# Proteomic Analysis of Paraffin-Embedded Intestines from *Schistosoma mansoni* Infection in Mice: Highlighting Molecular Players During Acute Schistosomiasis

**DOI:** 10.3390/proteomes14030039

**Published:** 2026-07-29

**Authors:** Lara Geralda Magela dos Santos Vieira, Ana Flávia Pinho Souza, Camilo Elber Vital, Dávila Regina Pacheco Silva, Flávia de Souza Marques, Gustavo Gonçalves Silva, Paula Melo de Abreu Vieira, R Alan Wilson, William Castro-Borges

**Affiliations:** 1 Programa de Pós-Graduação em Ciências Biológicas, Núcleo de Pesquisas em Ciências Biológicas, Instituto de Ciências Exatas e Biológicas, Universidade Federal de Ouro Preto, Ouro Preto 35402-136, Minas Gerais, Brazil; 2 Núcleo de Pesquisas em Ciências Biológicas, Instituto de Ciências Exatas e Biológicas, Universidade Federal de Ouro Preto, Ouro Preto 35402-136, Minas Gerais, Brazil; 3 Programa de Pós-Graduação em Biotecnologia, Núcleo de Pesquisas em Ciências Biológicas, Instituto de Ciências Exatas e Biológicas, Universidade Federal de Ouro Preto, Ouro Preto 35402-136, Minas Gerais, Brazil; 4 Departamento de Ciências Biológicas, Instituto de Ciências Exatas e Biológicas, Universidade Federal de Ouro Preto, Ouro Preto 35402-136, Minas Gerais, Brazil; 5 Department of Biology, York Biomedical Research Institute, University of York, York YO10 5DD, UK; alan.wilson@york.ac.uk

**Keywords:** *Schistosoma mansoni*, proteomic analysis, paraffin-embedded, intestines

## Abstract

Background: Adult *Schistosoma mansoni* parasites inhabit the hepatic portal system of the vertebrate host, their deposited eggs causing granulomatous pathology in both the intestines and liver. In the intestines, egg secretions drive inflammatory processes involved in extravasation to the gut lumen. Methods: Here, we investigate parasite-host interactions in a mouse model during the acute phase of a patent infection at 5 and 7 weeks, using parallel histological and proteomic analysis of paraffin-embedded ileal tissues. Results: Histology at week 7 showed infected animals had more inflammation and longer villi than at week 5, as well as more Goblet cells reflecting enhanced mucus production. LC-MS/MS analysis of deparaffinized ileal sections, subjected to in-solution tryptic digestion, revealed a total of 1615 protein groups. Differentially abundant proteins were found early at week 5, coinciding with the onset of egg deposition. A contrasting scenario, dominated by upregulation of protein components from the innate and adaptive immune systems, was seen at week 7; at this point, egg migration and excretion are underway. Among the proteins were mast cell proteases, fibrinogens, arginase-1, and molecules associated with extracellular matrix remodeling. Conclusions: Our findings reflect intestinal proteome changes likely participating in *S. mansoni* egg passage from the vascular bed to the intestinal lumen.

## 1. Introduction

Schistosomiasis is a disease caused by helminths of the genus *Schistosoma*, currently affecting more than 250 million people worldwide [1,2]. It is endemic in 78 countries, with three major species parasitising humans: *S. mansoni*, *S. haematobium*, and *S. japonicum*. Apart from *S. haematobium*, the other two species are responsible for the intestinal forms of the disease [3].

Once residence is established in the hepatic portal system, paired *S. mansoni* adults traverse the extensive venous and capillary beds that drain the blood supply from the intestines. From the superior mesenteric vein, they can reach the whole extent of the small intestine, plus the ascending and transverse colon. Alternatively, via the inferior mesenteric vein, they can reach the distributaries from the descending and sigmoid colon and the rectal venous plexus [4]. It needs to be emphasized that the adult worms are confined within this closed vascular network. For transmission to continue, the eggs laid by females must reach the gut lumen to be voided in feces. Those that do not are the source of the pathology, which is the hallmark of schistosomiasis. In humans, the available vascular bed drains an estimated area of 9 m^2^ of intestines, circular folds (plicae), and villi [5], providing extensive opportunities for developed schistosome eggs to find their way to the gut lumen. Moreover, secreted angiogenic factors may lead to a further extension of the capillary bed around deposited eggs [6].

The complexity of events associated with egg translocation has been poorly explored at the molecular level [7]. Nevertheless, a common belief is that they rely on the participation of both parasite and host factors acting coordinately to favor egg exit, whilst minimizing extensive tissue damage for the host. Although major gaps are yet to be filled, egg attachment is believed to occur by an altered composition of the endothelial surface with increased abundance of adhesion molecules, including ICAM-1, VCAM-1, and E-selectin, allowing for enhanced extracellular matrix interaction to take place, a process likely assisted by plasma and platelet-derived molecules [8,9]. Through stimulation of Rho/ROCK signaling in vascular endothelial cells, major alterations in the actin cytoskeleton and gap junctions lead to egg encapsulation in the vascular wall [10]. Once the endothelial barrier is traversed, the eggs encounter a multi-layered intestinal wall through which they migrate, surrounded by an inflammatory focus composed of Th2 cells, eosinophils, and alternatively activated macrophages [7]. Critical to the attraction of this cellular response is the assumed role played by egg secretions, including IPSE/α-1, omega-1, kappa-5, and the more recently discovered MEG-2 and -3 families of proteins [11,12,13]. Mastocytosis is another hallmark of intestinal schistosomiasis in the mouse model of the disease [14], but the role played by mast cell granules in egg extravasation has long been neglected. In 2010, it was observed that knocking out mast cell protease 1 expression did not compromise egg arrival in the gut lumen [15]. Enveloped by a cellular granuloma, eggs require around 6 days to complete their journey towards the gut lining, at which point, by unknown mechanisms, they escape to the gut lumen. The now fully developed miracidia are then free to hatch once they encounter suitable conditions in the external aquatic environment [16].

In this study, we have employed a shotgun proteomic approach based on paraffin-embedded intestinal tissues to provide an inventory of molecular signatures associated with this egg migration process in the mouse laboratory model of *S. mansoni* parasitism. Tissue sections for histopathological analysis and proteomics were obtained at the 5th and 7th weeks post-infection, representing the onset of egg laying and the peak of the acute phase of disease, respectively. Label-free quantitative analysis revealed differentially abundant proteins at these two time points, adding to the inventory of molecules linked to extracellular matrix and tissue remodeling required for egg passage. In parallel, it provided new avenues to understand not only this key aspect of the host–parasite interaction but, equally relevant, it allowed for inferences on the molecular scenarios associated with disrupting the integrity in the intestinal barrier, and the local and systemic effects this may trigger.

## 2. Materials and Methods

Animal experimentation was conducted in accordance with the Brazilian Federal Legislation (Arouca Law nº 11,794). All procedures involving mice were approved by the Local Ethics Committee on the Use of Animals (CEUA) from Universidade Federal de Ouro Preto (UFOP)–MG, under protocol nº 2017-34 approved on 18 January 2018.

### 2.1. Establishment of the Infection and Tissue Sampling

Twenty female mice (C57BL/6 strain) aged 4 weeks were provided by the local Animal Science Center. The infection was carried out using *Schistosoma mansoni* cercariae (Luiz Evangelista (LE) strain), provided by the Helminthology and Medical Malacology Laboratory at René Rachou Institute (Fundação Oswaldo Cruz, Belo Horizonte, Minas Gerais). Animals were housed in ventilated racks at the Animal Science Center/UFOP for up to 49 days, with water, chow, and wood shavings bedding replaced weekly.

Initially, the animals were anesthetized with a combination of 10% ketamine hydrochloride at 80 mg/kg (Dopalen—Sespo Indústria e Comércio Ltda., Jacareí, São Paulo, Brazil) and 2% xylazine hydrochloride at 10 mg/kg (Anasedan—Sespo Indústria e Comércio Ltda., Jacareí, São Paulo, Brazil), administered intraperitoneally. After sedation was confirmed, the tails were immersed in a suspension containing 200 cercariae/animal for 30 min. Following recovery from anesthesia, the animals were allocated in groups of 5 animals/cage and cared for weekly with replacement of sawdust, water, and food. At 5 or 7 weeks post-infection, the animals were euthanized using an anesthetic overdose, a combination of 10% Ketamine Hydrochloride at 240 mg/kg (Dopalen) and 2% Xylazine Hydrochloride at 30 mg/kg (Anasedan), administered intraperitoneally.

After incision of the abdominal region, the spleen was removed and weighed for evaluation of splenomegaly. The final third portion of the small intestine (ileum) was excised and slit longitudinally to remove the fecal content and expose the epithelium. The intestines were then washed with ice-cold RPMI medium pH 7.4 (Sigma-Aldrich, St. Louis, MO, USA) and immediately fixed in a solution of 20% methanol/80% dimethyl sulfoxide (DMSO), (Synth, Diadema, SP, Brazil) with changes in the fixative solution every 24 h for four days. Fixed intestines from control and infected animals were stored at −20 °C until further analyses.

### 2.2. Preparation of Intestine Sheets

#### 2.2.1. Hematoxylin and Eosin Analysis

Fixed intestines from control and infected animals at 5 and 7 weeks were subjected to histological analyses. Initially, the fixed samples were processed and sequentially dehydrated by immersion in alcoholic solutions (Synth, Diadema, SP, Brazil) of 70%, 80%, 90%, 95%, and 99.9% for 35 min; the last one was carried out three times. Then, the tissues were subjected to two independent xylene solutions (Synth, Diadema, SP, Brazil) for 15 and 13 min, respectively. Finally, the samples were immersed in liquid paraffin (Synth, Diadema, SP, Brazil/ Êxodo Científica, Sumaré, SP, Brazil). After cooling, the tissues were kept at room temperature until their inclusion in paraffin blocks.

Before inclusion, the paraffin was melted in an oven at 70 °C and filtered. The intestine samples were placed in metal molds containing liquid paraffin, identified, and subsequently refrigerated. Once the paraffin blocks were completely cooled, they were removed from the molds, and the samples were stored in a refrigerator at 4 °C until sectioning using a rotary microtome (Leica RM2235, Leica Biosystems, Nussloch, Germany). Tissue sections for histology were cut at 4 μm thickness; those intended for proteomic analyses at 10 μm. Sections were collected on glass slides, and those selected for microscopy were stained with Hematoxylin/Eosin (HE) solution. After the last washing step in running water, the slides were dried in an oven at 56 °C and finally mounted for microscopy using Entellan (Sigma-Aldrich, St. Louis, MO, USA).

#### 2.2.2. Morphometric Analysis of Intestine Tissue

This analysis consisted of the measurement of villus size and evaluation of the inflammatory infiltrate, based on the number of detected cell nuclei. These were quantified in 30 random fields per slide, visualized under a 40× objective, totaling a covered area equal to 30 × 217,540 μm^2^, using an optical microscope (Leica DM5000 B; Leica Microsystems, Wetzlar, Germany) coupled to a microscope camera (Leica DFC340 FX; Leica Microsystems, Wetzlar, Germany). Villi size was evaluated using ImageJ software (V. 1.8.0) through 20 readings/slide, visualized under a 20× objective, measuring the distance from the basal region to the apex of the villi.

#### 2.2.3. Assessment of Mucin Distribution

Histological sections of intestine from five animals for each group were deparaffinized in xylene (Synth, Diadema, SP, Brazil) for one hour and subsequently rehydrated through a descending ethanol series (Synth, Diadema, SP, Brazil) (absolute I and II, 90%, 80%, and 70%), with five-minute immersion in each solution. The slides were washed in running and distilled water to ensure complete removal of residues and then stained with Alcian Blue solution pH 2.7) (CI 74240; Dinâmica Química Contemporânea Ltda., Indaiatuba, SP, Brazil) for 30 min to visualize mucins. After rinsing in running water for ten minutes, counterstaining was performed using a 0.1% safranin solution (P.A.; Dinâmica Química Contemporânea Ltda., Indaiatuba, SP, Brazil) for one minute. Finally, the slides were washed again in running water and processed for permanent mounting.

#### 2.2.4. Morphometric Analysis of Mucins

Morphometric analysis consisted of measuring the area of tissue stained in blue. For this purpose, 20 images per slide were captured using a 40× objective on a light microscope (Leica DM5000 B; Leica Microsystems, Wetzlar, Germany) coupled to a ZEISS digital camera (Carl Zeiss AxioCam MRc; Carl Zeiss, Oberkochen, Germany) resulting in a total analyzed area of 20 × 217,540 µm^2^. The acquired images were processed using Leica Application Suite software (Version 2.4.0 R1) for the detection and quantification of the blue-stained area. 

### 2.3. Deparaffinization of Embedded Tissues and in Solution Digestion with Rapigest™

Out of the 20 paraffin-embedded intestine samples from control and infected C57BL/6 mice prepared for histology, 10 were also selected for mass spectrometry-based shotgun proteomic analysis. These encompassed three biological replicates from weeks 5 and 7, plus 4 additional samples representing control, non-infected animals, maintained during the 5 to 7 weeks interval.

Preparation of paraffin-embedded intestines for shotgun analysis followed the methodology of Coscia et al. [17], with some modifications. Briefly, deparaffinization consisted of eight steps. Initially, the paraffin blocks were melted in an oven at 60 °C for 20 min, followed by two immersions in xylene solution (Synth, Diadema, SP, Brazil) for 10 min. Tissues were then washed twice for 5 min in 99% ethanol, twice in 96% ethanol for 5 min, plus an additional wash for 5 min in 70% ethanol (Synth, Diadema, SP, Brazil), followed by running tap water for 10 min before submersion twice for 5 min in ultrapure LC-MS grade water (LiChrosolv^®^, Merck KGaA (Darmstadt, Germany)). Finally, deparaffinized tissue sections were scraped from the glass slides, a process facilitated by adding 5–10 µL of ultrapure water on top of them, before pipetting for transfer to 1.5 mL microtubes (Axygen Scientific, Union City, CA, USA). The recovered tissue sections were then dried in the Speedvac evaporator (RVC 2-18 CD Plus, Christ) for 120 min.

Protein extraction from 10 µm tissue sections was performed, using 100 µL of lysis solution composed of 0.2% RapiGest surfactant (Waters, Wilmslow, UK) solubilized in 50 mM ammonium bicarbonate (Ambic—(Sigma-Aldrich, St. Louis, MO, USA)). Subsequently, the samples were vortex-homogenized for 1 min. Spin-centrifuged samples were then incubated at 95 °C for 30 min in a heat block (Thermoblock incubator and timer IT-2002 13, Bio Plus, Seoul, Republic of Korea). Sonication steps were employed in a bioreactor (Unique) for 10 min, followed by incubation for 120 min at 80 °C and, finally, an additional sonication step for 10 min.

Extracted proteins were reduced and alkylated before digestion with Trypsin (Sequencing grade modified trypsin; Promega, Madison, WI, USA). They were reduced using 10 µL 5 mM dithiothreitol (DTT) (Sigma-Aldrich, St. Louis, MO, USA), incubated at 60 °C for 30 min in a heat block. Alkylation was performed using 15 mM Iodoacetamide (IAA) (Sigma-Aldrich, St. Louis, MO, USA) at room temperature for 45 min. Reduced and alkylated proteins were diluted by the addition of 110 µL 50 mM Ambic solution. For protein digestion, Trypsin (Sequencing grade modified trypsin; Promega, Madison, WI, USA) was added in a 1:25 ratio (trypsin: protein), followed by gentle homogenization and incubation at 37 °C for 16 h in a heat block. Trypsinolysis was stopped by the addition of 1% (*v*/*v*) trifluoroacetic acid (TFA) (Sigma-Aldrich, St. Louis, MO, USA), which resulted in pH ≤ 2, before incubation for 45 min at 37 °C for surfactant hydrolysis. Samples were centrifuged at 20,000× *g* for 15 min at 7 °C (Hettich Zentrifugen Mikro 200R). The supernatants containing the tryptic peptides were transferred to new tubes and subjected to a solid phase extraction process, using C18-E mini-columns (Strata, 55 µm, 70 A). Peptides eluted from the reversed-phase cartridge columns were dried by evaporation and kept at −20 °C until mass spectrometric analysis.

### 2.4. Mass Spectrometry

Peptide yield after digestion of the samples was estimated by measuring absorbance at 214 and 230 nm using a UV-1800 UV-Vis spectrophotometer (Shimadzu Corporation, Kyoto, Kyoto, Japan). Approximately 400 ng of peptides from each sample were applied to the Dionex UltiMate 3000 UHPLC platform (Thermo Scientific, Bremen, Germany). Peptides were targeted to the Acclaim PepMap100 C18 NanoTrap Column (100 μm i.d. × 2 cm, 5 μm, 100 Å; Thermo Scientific). Peptides retained in the pre-column were eluted for 3 min in 3.8% MeCN/0.1% TFA, at a flow rate of 5 µL/min, before their separation by reversed-phase nano liquid chromatography using an Easy Acclaim PepMap100 C18 column (75 μm i.d. × 10 cm, 2 μm, 100 Å; Thermo Scientific). This was achieved under an acetonitrile gradient through a combination of solutions A (0.1% formic acid) and B (80% MeCN/0.1% formic acid), at a flow rate of 0.300 µL/min, as follows: 3.8% B for 2 min (column equilibration); then 3.8–40% B until completing 110 min. Then, the acetonitrile gradient increased to 85% B in 121 min, and system conditioning with 3.8% B for up to 140 min. After UHPLC separation, peptides were directed to a Q-Exactive mass spectrometer (Thermo Scientific), through electrospray ionization using a Nanospray Flex Ion Source (Thermo Scientific), operated at +3.45 kV. The inlet capillary temperature was maintained at 250 °C, and the S-lens RF level was set to 50. Data-dependent acquisition (DDA) was programmed in Top12 selection mode. Precursor ion spectra (MS) were acquired in a *m/z* window of 300–2000, at a resolution of 70,000 and a maximum injection time of 120 ms for 1 × 10^6^ ions. Up to 12 most abundant ions per each MS spectrum were selected and isolated in a 2.0 *m/z* window for dissociation by High energy Collision Dissociation (HCD). Selected ions passed the criteria of exhibiting charge states ≥ +2 to ≤+4 and counting 5 × 10^5^ ions in up to 120 ms injection (threshold 1.0 × 10^5^). The activation of precursor ions occurred with normalized collision energy at 28–30 V, and product ion spectra (MS/MS) were acquired at 35,000 resolution and dynamic exclusion time of 40 ms.

### 2.5. Data Analysis

The histological data were subjected to a data normality analysis using the Shapiro–Wilk test. Parametric data were analyzed using one-way ANOVA and Tukey’s post hoc tests; data with a *p*-value ≤ 0.05 were considered statistically significant. All analyses were performed using the GraphPad Prism v8.0 program (San Diego, CA, USA). Values were expressed as mean ± standard deviation.

Mass spectral data were interrogated against the *Mus musculus* and *S. mansoni* proteome databases (55,398 and 10,770 sequences, respectively) downloaded from the UniProt website https://www.uniprot.org/ (accessed 10 October 2024 using PEAKS Studio (Bioinformatics Solutions Inc., Waterloo, ON, Canada)). Spectral data obtained were interrogated using the following search criteria: False Discovery Rate (FDR) set to 1%, −10logP = 13 (meaning *p*-value ≤ 0.05), and considering only proteins identified with at least one unique peptide for *M. musculus* proteins and 2 unique peptides for *S. mansoni* identities. Under these search parameters, FDR was shown to be 1% for Peptide-Spectrum Matches. The Area Under Curve values for each identified protein were used as input to build a Principal Component Analysis (PCA) plot. For label-free quantitative analysis, no predefined fold-change cutoff was applied; differential abundance was determined using FDR-adjusted statistical testing for protein −10logP = 13 (*p*-value ≤ 0.05) using ANOVA significance method, considering proteins identified with at least 1 unique peptide. Analysis of the compositional data was carried out using STRING tools (https://string-db.org/), to construct protein interaction networks and annotation of the enriched pathways. STRING confidence was set to 0.9 meaning highest score. A protein–protein interaction network was constructed using the totality of entries related to the compositional data. Pathway enrichment analysis was based on Reactome database terms (Supplementary Data S3). Protein network clustering was performed using the Markov Cluster Algorithm (MCL) with the inflation parameter set to 2. The SRplot https://www.bioinformatics.com.cn/en (Bioinformatics, Shanghai, China; accessed 10 October 2024) was used to construct the Venn diagram and the PCA plot.

## 3. Results and Discussion

### 3.1. Establishment of Murine Schistosomiasis Is Corroborated by Hallmarks of the Intestinal Disease

**Figure 1:**
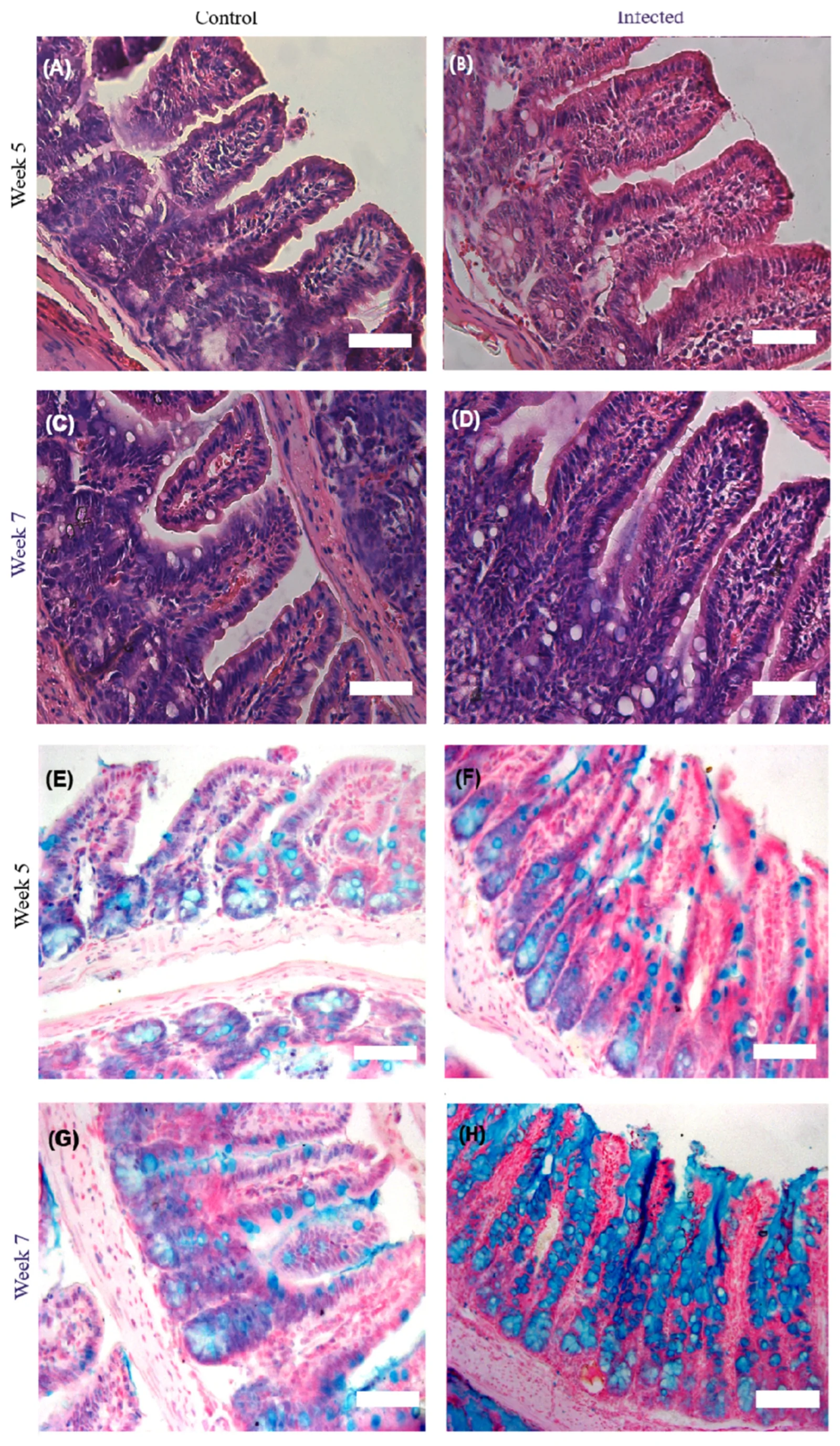
Intestinal histology during murine schistosomiasis. Hematoxylin and eosin (H&E) staining in panels (**A**–**D**) reveals a marked increase in leucocyte infiltration in individuals infected for 7 weeks. In addition, notable alterations in villus morphology are observed, with increased villus size in the 7-week infected group. Panels (**E**–**H**) demonstrate pronounced Goblet cell hyperplasia, culminating in enhanced mucin secretion at week 7, as revealed by Alcian Blue staining. All fields were acquired at 40× magnification. White scale bars indicate 100 μm.

**Figure 2:**
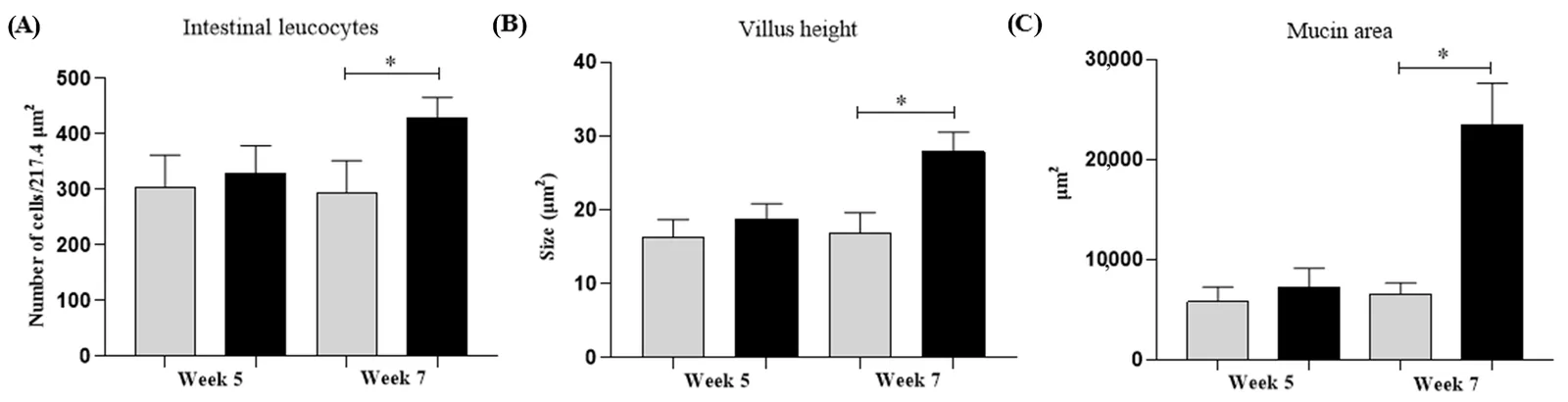
Quantification of the number of leucocytes, villus height, and mucin-stained area. (**A**) Quantitative analysis of leucocyte numbers demonstrates significant intestinal inflammation in animals infected for 7 weeks. (**B**) Inflammation was accompanied by increased villus size. (**C**) Mucin production was also enhanced at the same time point. Gray bars represent the non- infected groups and black bars represent the respective infected groups. The data are represented using the mean and standard deviation. Asterisk (*) indicates *p* < 0.05.

Confirmation of murine infection was primarily assessed by evaluation of splenomegaly, as it constitutes a hallmark of *S. mansoni* infection. As shown in Supplementary Figure S1, spleen weight/body weight ratios for infected animals at weeks 5 and 7 increased significantly compared to control animals. Moreover, in this study, our main goal was to evaluate the proteome associated with intestinal schistosomiasis in the murine model of the disease. For this purpose, demonstrating intestinal disease was also of major relevance. Accordingly, intestinal samples from weeks 5 and 7 post-infection were collected, paraffin-embedded, and processed for microscopy and morphometric analyses. Week 5 marks the onset of egg laying by *S. mansoni* adult paired worms, whereas week 7 represents the peak of the acute phase [18]. Microscopic analyses of the intestines confirmed the successful establishment of the infection at both time points (Figure 1 and Figure S2). At week 5, the number of leucocytes did not differ significantly from the control group (Figure 2). The main tissue alterations related to inflammation were associated with the intestines from infected animals at week 7, where the number of inflammatory cells increased significantly (Figure 1 and Figure 2A). Of note, a significant increase in villus size was also observed at week 5 in infected animals, becoming more pronounced at week 7 (Figure 1 and Figure 2B). The increase in villus size was also accompanied by a notable increase in Goblet cells, leading to a large area of alcian-blue-stained mucins (Figure 2C). Therefore, pronounced inflammation at week 7 and hyperplasia of Goblet cells with enhanced mucus production clearly demonstrated known hallmarks of intestinal disease caused by *S. mansoni* infection [19,20].

### 3.2. The Proteomes of Paraffin-Embedded Intestines from Murine Schistosomiasis Allowed for Discrimination Between Disease States

**Figure 3:**
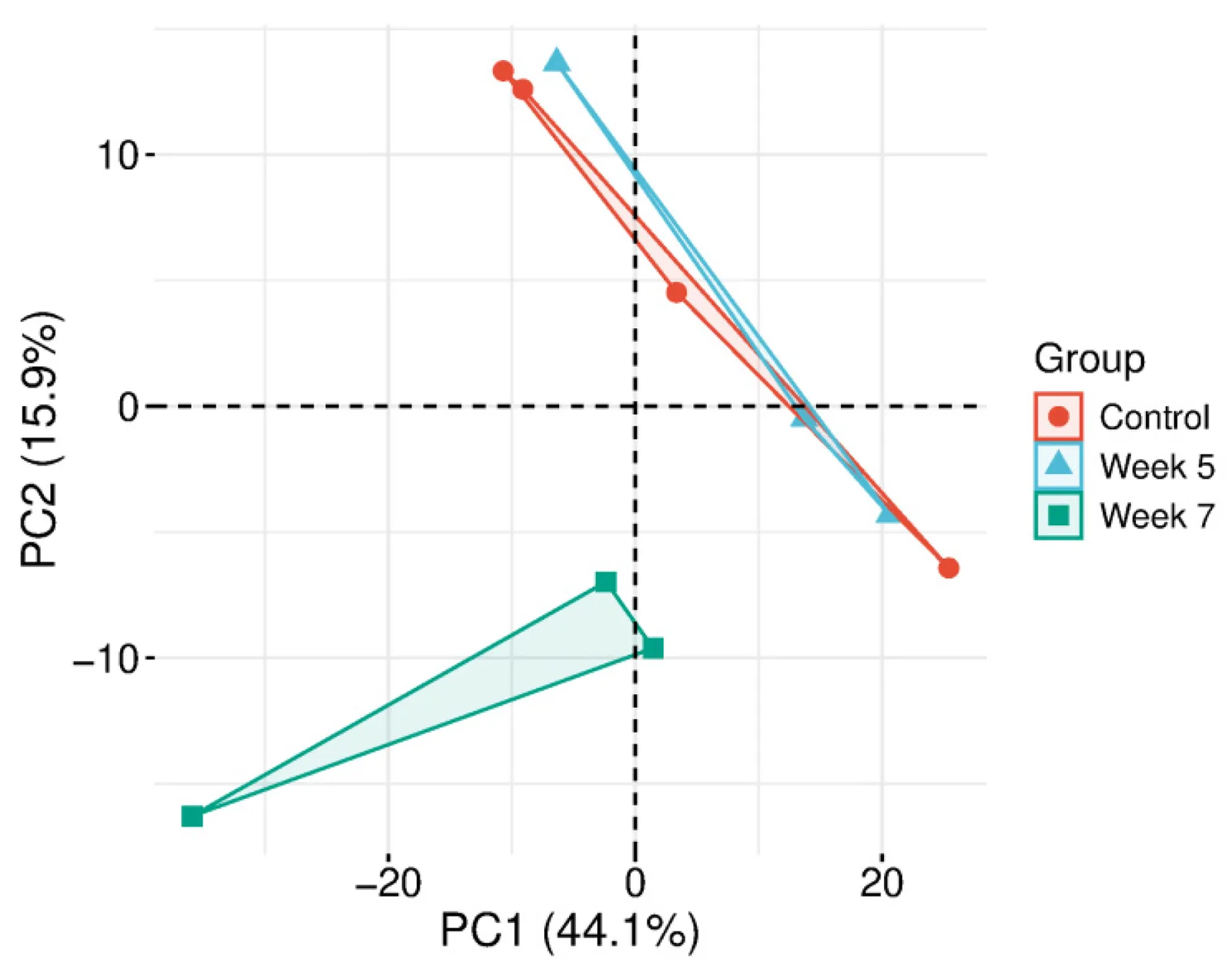
Principal component analysis of intestinal tissue samples representative of control, week 5, and week 7 infected mice. PCA summarizes the proteomic profiles of the experimental groups. Notably, the control group and the 5-week infected group exhibit highly similar proteomic signatures, whereas the 7-week infected group displays a pronounced divergence in protein composition relative to the other two groups.

Deparaffinization of paraffin-embedded intestines, protein extraction, and trypsin digestion followed by mass spectrometric analyses of the resulting peptides, from control and infected tissue samples, collectively allowed identification of 1615 protein groups (Supplementary Data S1 and S2). The relative contribution of constituents, provided by the recorded Area Under Curve (AUC), revealed that only 30 protein groups accounted for over 50% of the total ion signal (Supplementary Figure S3A). These were mainly represented by highly abundant components of the intestinal muscle layers and extracellular matrix, such as actin, myosins, keratins, and collagens. Adding these 30 to 370 protein groups achieved around 90% of the total ion signal. Most of the identified proteins contributed only approximately 10% to the cumulative abundance. By converting AUC to a logarithmic scale, a dynamic range of abundance encompassing five orders of magnitude was noted between the components associated with the most intense ion signal in comparison to that with the least (Supplementary Figure S3B). Principal component analysis (PCA) based on the total number of protein groups separately identified in each sample revealed complete discrimination between animals exhibiting altered inflammatory states. As shown in Figure 3, control and week 5 samples were closely related, whereas week 7 samples markedly segregated in the PCA plot. The composition of the three samples, representing the peak of the acute phase (week 7), altogether explained 44% of the observed variance.

**Figure 4:**
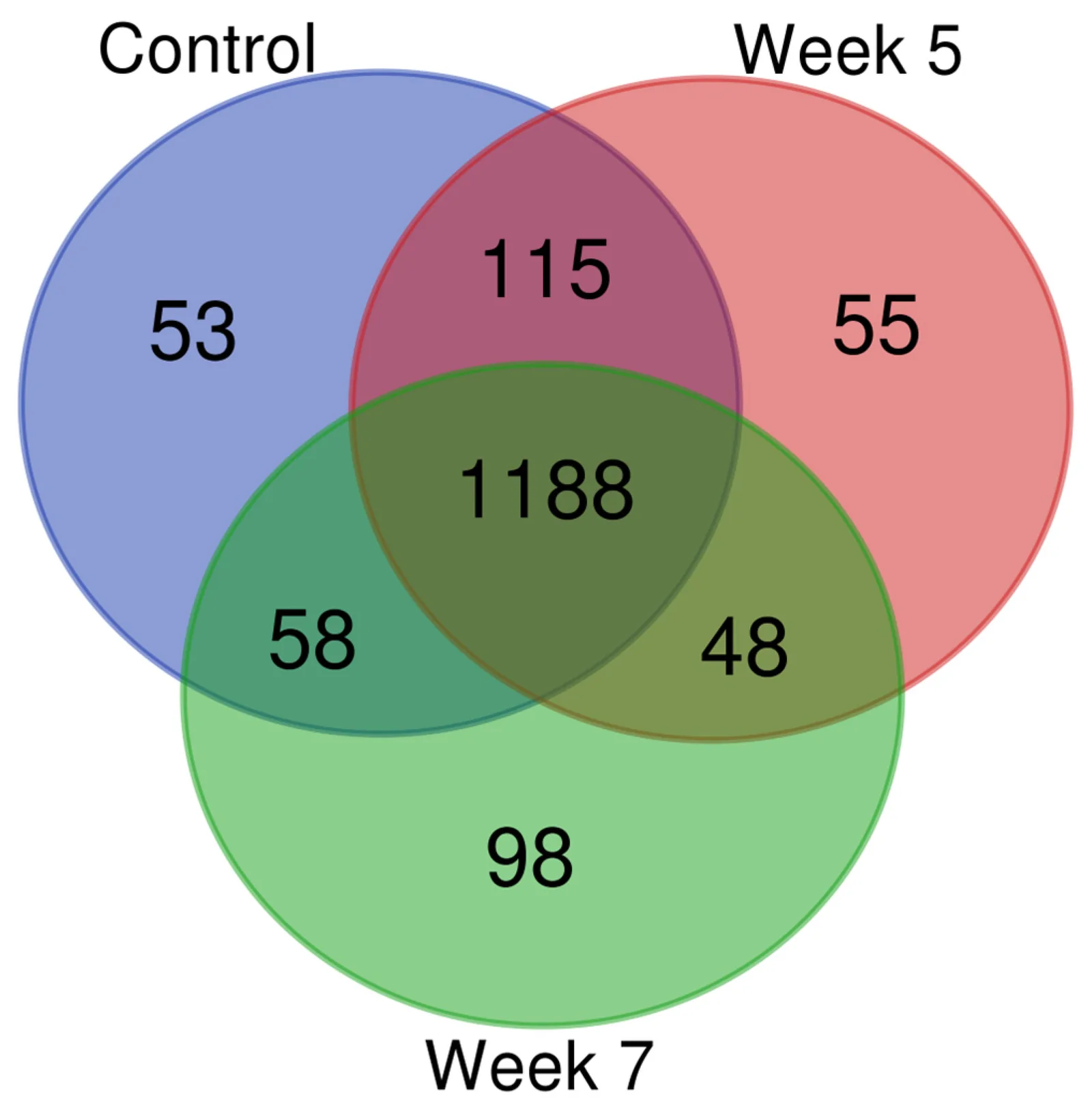
Venn diagram illustrating shared and unique protein groups identified in intestinal samples from control and infected animals at different experimental time points. The majority of the identified proteome (74%) is shared across all groups. Among uniquely identified proteins, the 7-week infected group exhibited the highest number of exclusive proteins, whereas the control group displayed the lowest number of unique identifications.

Overall, approximately 74% (1188 protein groups) of the identified proteome is shared among control and infected samples (Figure 4). In addition, 115 were common to the control and infected groups at week 5, 58 were identified only in the control and infected groups at week 7, and 48 were shared identities at weeks 5 and 7. Concerning the protein groups uniquely identified at each time point, 53 were from the control group, 55 were associated with the infected group at week 5, and almost double (98) were only detected at week 7.

To gain insights into the molecular diversity associated with the obtained intestinal proteome, a STRING-derived protein network was generated using its built-in highest confidence score, with exclusion of non-interacting protein groups (Supplementary Figure S4). Inspection of this interactome allowed us to pinpoint major overrepresented biological processes. Among them are those composed of housekeeping proteins involved in cellular respiration, energy metabolism, protein synthesis, heat shock response, and nuclear metabolism. Moreover, various proteasome subunits, building blocks of the 20S catalytic core and its regulatory particles, in addition to components of the ubiquitination system, were prominent features of the network. Extracellular matrix organization represented by collagens and integrins, in parallel to protein groups involved in cell signaling associated with cytoskeletal remodeling, provided molecular evidence for key processes related to inflammation.

**Figure 5:**
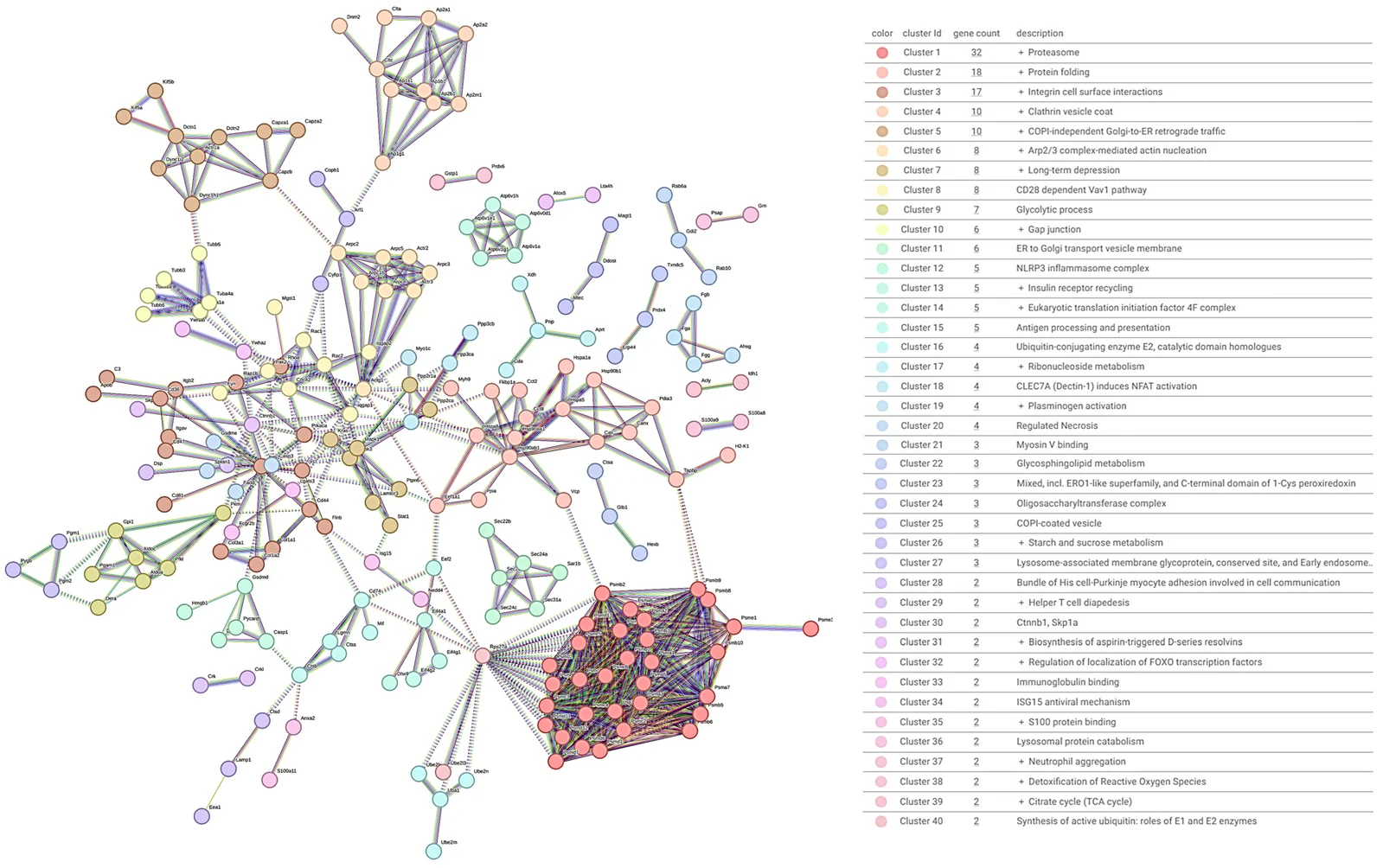
Protein–protein interaction network of 296 proteins identified on compositional analysis related to the term immune system. The network was generated using STRING and clustered using the Markov Cluster Algorithm (MCL). Colored nodes on the right describe the different protein clusters identified by the MCL. Also available at: https://string-db.org/cgi/network?taskId=brkO9sFCTB0H&sessionId=bDZNfzCaexpw (accessed on 30 June 2026).

In fact, enrichment analyses using the Reactome database, underpinned by STRING, indicated the terms metabolism, transcription, translation, immune system, and neutrophil degranulation among the top leading processes, as judged by their significantly low FDR (Table 1). When the 296 components associated with the term immune system were displayed in a second clustered network, a much clearer scenario for inflammation-related processes could be observed (Figure 5). Again, inspection of this secondary interactome containing 40 protein clusters revealed proteasome subunits highly connected with a member of the PA28-gamma regulatory particle (Psme3), pointing to immunoproteasome complex formation. Not surprisingly, proteasome subunits also appeared, interacting with members related to antigen presentation in the context of MHC-class I and II. Members of the Actin-related protein 2/3 complex were identified, linking them to cytoskeletal remodeling, a mandatory process for cell motility [21]. Of note, five V-type proton ATPase subunits were identified, illustrating phagocytosis-related endosome acidification [22]. This emerging picture of cellular activation also requires the recruitment of molecules involved in quality control and stability for proteins. These processes are illustrated by a cluster of heat shock members, including Hsp90 alpha and beta subunits, Hspa8 and Hspa9, in addition to proteins involved in endoplasmic reticulum-assisted folding, such as thioredoxin, calreticulin, and calnexin.

Key components of the extracellular matrix were also identified alongside known mediators of cell–cell interaction. These were represented by integrins and collagens highly connected with their binding partners known to be located at the cell surface [23]; among them CD44, CD36, and CD47. More convincing molecular evidence of the inflammatory foci revealed by this proteomic approach were S100-A8 and S100-A9 proteins, known to mediate neutrophil chemotaxis, adhesion, and enhancement of their microbicidal activity through stimulation of phagocytosis [24]. In this microenvironment, inflammatory regulated cell death (or pyroptosis) is possibly a required process, and the identification of two lipid-binding gasdermin-family members (C2 and C3) points to this ultimate fate for attracted leucocytes [25].

The aforementioned interpretation of the biological processes, underpinning these two interactomes, is statistically validated by the STRING enrichment analysis tool. In decreasing order of relevance, Table 1 expands the diversity of pathways composing the proteome of the intestines during the acute phase of murine schistosomiasis.

Supplementary Table S1 also provided compelling evidence that inflammation-related biological processes highlighted in the interactomes were primarily associated with the intestinal proteome at week 7. Inspection of uniquely identified proteins for each investigated group clearly showed that, in contrast to control and week 5, week 7 samples exhibited a repertoire of protein groups related to distinct aspects of the innate and adaptive immune systems. Two other enzymes, arachidonate-5-lipoxygenase (ALOX5) and polyunsaturated fatty acid lipoxygenase (ALOX15), involved in the synthesis of lipid mediators during inflammation [26,27,28], further corroborate our findings.

Under the more stringent criteria used to assess *S. mansoni* hits, we were able to assign 4 identified proteins (Supplementary Data S1, Table S2—highlighted in gray). The remaining 72 identities, representing housekeeping proteins, appeared indiscriminately in both control and infected animal-derived samples, attesting to their high amino acid conservation in *M. musculus* and *S. mansoni*, two evolutionarily distant bilaterian species. Inspection of the MS2 spectra for the identified peptides revealed their low abundance, as judged by their fragmentation pattern. Among the 4 retrieved hits, 1 associated with two of the three week 7 infected samples is annotated as a major egg antigen (P12812). Its presence in the preparation at week 7 merits consideration. This molecule is among the most abundant soluble egg antigens, as judged by 2D-gel-based analysis [29]. Given its intracellular nature, it is not trivial to anticipate a role for this protein in egg migration. However, identification of this parasite antigen in the ileum tissue reinforces its capability to trigger humoral immune response, strengthening its potential as an *S. mansoni*-specific tissue biomarker of infection.

### 3.3. Differential Abundance of Proteins in the Intestines at 5 and 7 Weeks After S. mansoni Infection

**Figure 6:**
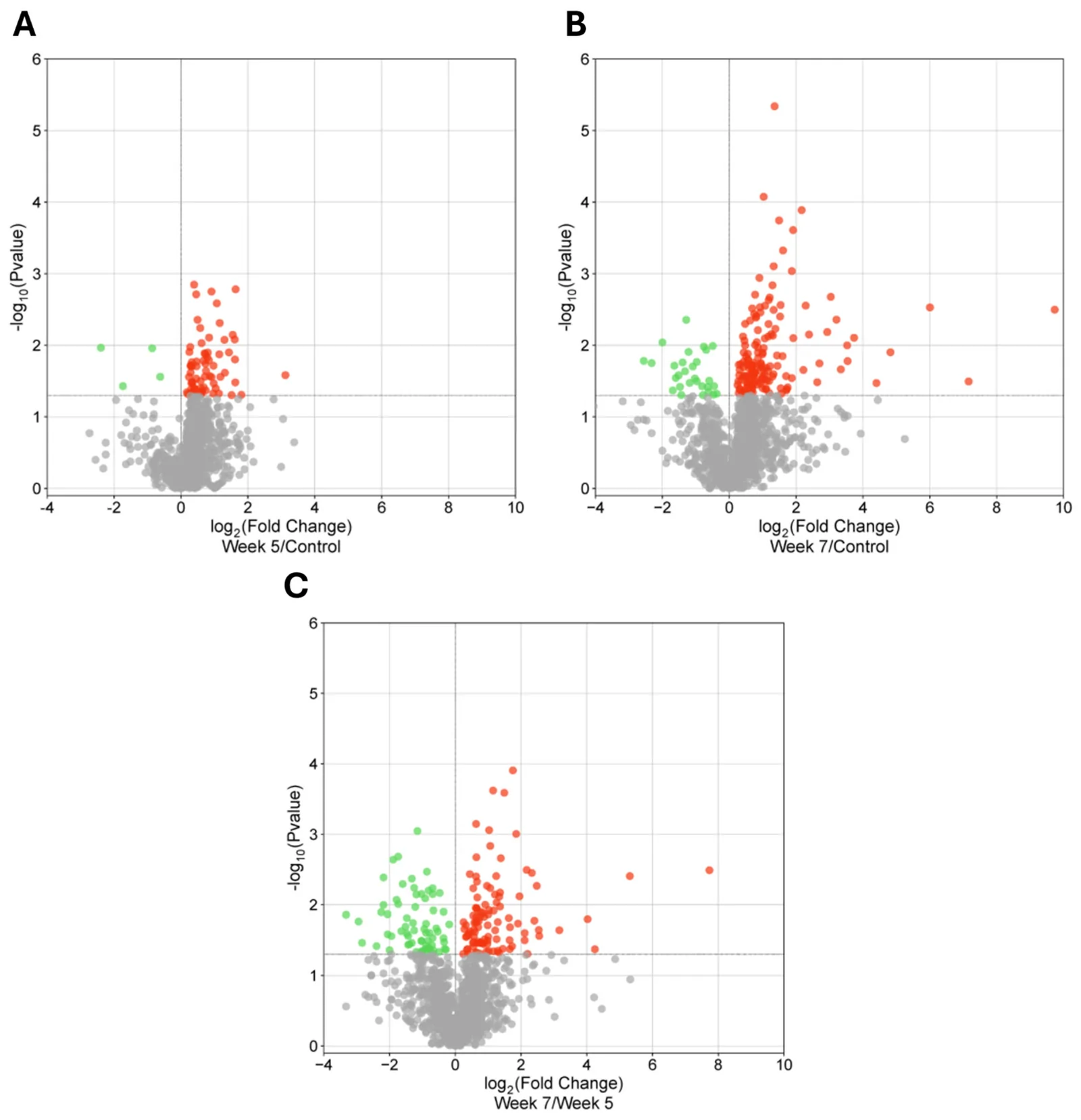
Volcano plots showing the set of differentially abundant proteins used for quantitative analyses in the intestine after 5 and 7 weeks of *S. mansoni* infection. (**A**) At week 5 post-infection, the quantitative profile revealed that 68 proteins displayed higher abundance relative to controls. (**B**) A more pronounced pattern was observed at week 7, in which 150 proteins increased in abundance, suggesting sustained proteomic alterations over the course of infection. (**C**) Comparative analysis between infected animals at 5 and 7 weeks further confirmed this trend, with most proteins exhibiting increased abundance at the later time point, reflecting progressive modulation of the intestinal proteome during infection. Red dots indicate increased abundance, whereas green dots indicate decreased abundance. Grey dots indicate not statistically significant proteins.

Label-free quantification for proteins commonly identified in the three conditions (infected at weeks 5 and 7 plus non-infected controls) confirmed observations made for the compositional analysis and complemented the repertoire of molecules differentially abundant during the acute phase of intestinal schistosomiasis (Table 2 and Table S2A–G). At week 5, 68 proteins exhibited higher abundance, among them enzymes involved in redox homeostasis, intracellular signaling, cytoskeletal remodeling, and extracellular matrix organization (Figure 6A, Table 1 and Table S2A–G). Of note, constituents of the extracellular matrix laminins (subunits beta-1 and alpha-4), destrin, and tenascin, required for cell adhesion and migration [30,31,32], and immunoglobulin heavy and light chains provided early evidence that tissue inflammation had been initiated, paralleling the onset of egg laying by sexually mature adult worms. It might be of relevance to stress that calpain 13 and cytoskeletal constituents were identified with a comparatively higher protein coverage. Whether this is linked to intense cytoskeletal remodeling intracellularly and/or at cell–cell junctions and, therefore, these molecules were more exposed to trypsin digestion during sample preparation, is a subject for future investigations.

Two weeks later (week 7) the inflammatory phenotype of the intestines was emphasized by 150 proteins exhibiting increased abundance (Figure 6).

These included a series of known circulating acute phase proteins and tissue resident proteins whose functions could be attributed to distinct steps involved in egg extravasation to the gut lumen [16]. Fibrinogen alpha, beta, and gamma chains, von Willebrand factor, and platelet-activation factor acetylhydrolase are a testament to the reported hemostatic perturbation possibly linked to the egg’s adherence to vascular endothelia, indicating cell damage and recruitment of immune responses [8,33,34]. Increased abundance of immunoglobulin heavy and light chains, alpha-2-HS-glycoprotein, hemopexin, and serotransferrin was detected, and these are known biomarkers of acute phase inflammation, previously reported by our group to be also elevated in the sera, using a shotgun proteomic approach [35]. Tissue markers of response to injury were again illustrated by increased abundance of extracellular matrix constituents, among them tenascin, integrin-alpha 5, galectin-1, and a pentraxin family member known to promote and facilitate leucocyte motility [36,37]. In addition, two single-pass transmembrane proteins (low-affinity Fc receptor for IgG and macrophage mannose receptor) reinforced phagocytosis-related processes at week 7. Of note, polyadenylate binding protein and serine/threonine protein phosphatase gamma catalytic subunit, both identified with various peptides, point to a cellular environment dominated by enhanced transcription and intracellular signaling, respectively (Table 2 and Table S2).

The marked presence of mast cells and eosinophils in the inflamed intestines was evident, with mast cell proteases (MCP)-1 and -2, in addition to Eosinophil-associated ribonuclease 6 (Ear6), exhibiting the highest positive fold change, relative to controls, at week 7. The dominance of these two leucocyte populations was further confirmed by the identification of MCP-4 and Eosinophil-cationic protein 2, respectively, in the list of exclusive proteins found in infected animals. Detection of three distinct mast cell proteases during the acute phase of *S. mansoni* infection suggests redundancy of proteolytic activity by mast cells and should stimulate revisiting their role in the process of egg transit to the gut lumen. This perhaps explains why knocking out only MCP-1 does not significantly compromise such a complex process [15]. Apart from mast cells and eosinophils, a variety of effector molecules with increased abundance in infected animals at week 7 emphasize the key role performed by macrophages in egg extravasation [16,38]. In particular, arginase-1, significantly displaying higher abundance at week 7, is a known marker of macrophages in the context of an *S. mansoni* infection, where it was shown to suppress rather than induce Th2-dependent inflammation and fibrosis [39]. Moreover, a macrophage mannose receptor, identified only in the infected animals, suggests that it may represent a binding partner for mannose-bearing glycoproteins of parasitic origin such as the abundantly secreted egg protein, IPSE-1 [40,41]. This could illustrate an initiation point for leucocyte activation and recruitment for granuloma formation under the influence of egg-secreted proteins.

Not surprisingly, in a scenario dominated by inflammation, proteins linked to stress response and remodeling of the actin cytoskeleton are mandatory. Among them, cytoglobin, known to detoxify reactive oxygen species through superoxide dismutase activity and protect cells against oxidative stress [42,43,44], exhibited >10 positive fold change at week 7. Of particular relevance to its possible role in *S. mansoni* infection is its reported ability to regulate nitric oxide (NO) decay in smooth muscle cells and therefore exert a critical role in the modulation of the vascular tone [45]. Comparable levels of abundance were seen for BAG family molecular chaperone regulator 3, another protein known to interact with HSPs to regulate their function and promote autophagy in response to inflammatory stimuli [46,47,48]. Higher abundance for tubulin, moesin, coronin-1, profilin-1, and a component of the Arp2/3 complex, known to mediate the formation of branched actin networks in the cytoplasm, providing the force for cell motility [49,50], represented molecular signatures of major cytoskeletal reorganization during this immune response induced by egg antigens. In this regard, increased abundance of legumain, a lysosomal protease, alongside various constituents of intracellular vesicular trafficking points to intense MHC class II antigen presentation, a crucial step for priming CD4^+^ T-cell activation and shaping Th-polarized immune responses characteristic of helminth-induced granulomas [51,52]. Concurrently, increased abundance for proteasome core and regulatory subunits also indicates enhanced intracellular protein turnover and MHC class I antigen presentation to Type I CD8^+^ T-cells as a result of exposure to *S. mansoni* egg-soluble antigens [53,54].

In the category of leucocyte effectors, apart from the differentially abundant mast cell proteases and eosinophil (Ear6) protein, four S100 molecules were found to be differentially abundant. In particular, protein S100-A9, a major calcium-binding protein of neutrophils and monocytes known to display a variety of functions related to immune modulation and transendothelial migration of phagocytes [55], occupied the 5th position among the most abundant proteins, relative to controls, at week 7. Of note, it is reported to facilitate leucocyte arachidonic acid trafficking and metabolism, and activate the neutrophilic NADPH-oxidase [56]. Corroborating this finding, increased abundance was detected for cytosolic phospholipase A2, which catalyzes the release of arachidonic acid, the precursor of pro-inflammatory lipid mediators, at the C-2 position from membrane glycerophospholipids [57]. In parallel, increased abundance of a downstream enzyme, polyunsaturated fatty acid lipoxygenase ALOX15, points to its involvement in the synthesis of arachidonic-derived lipid mediators, in particular lipoxins and resolvins, possibly as a relevant mechanism to prevent exacerbation of tissue damage induced by inflammation [58,59]. Another set of proteins with increased abundance (e.g., caspase 6 and the apoptosis inhibitor 5) signals programmed cell death and its modulation in the inflamed intestines. Moreover, detection of two gasdermins (C2 and C3), the final effectors responsible for cell death by pyroptosis [60], exclusively at week 7 in the infected animals, further corroborates inflammasome activation in response to *Schistosoma* sp. parasitism [61].

In order to provide a clearer picture of the biological processes that are initiated at week 5 and progress up to week 7, we interrogated the mass spectral data of week 7 samples against those from week 5. As shown in Figure 6C, 103 proteins significantly displayed positive fold change. Among them are ones listed as highly enriched in the infected condition when compared to their respective controls, strengthening our previous findings. The five leading proteins were MCP-1, MCP-2, protein S100-A9, ALOX15, and cytoglobin, in decreasing order of fold enrichment. As stated above, these molecules point to enhanced mastocytosis and inflammation, paralleling the activation of cell defense mechanisms counteracting exacerbated tissue damage. Again, this scenario was backed up by a sustained and increased abundance of retinol-binding protein, likely illustrating the tight link between vitamin A metabolism and gut health and integrity [62,63,64]. The remaining proteins exhibiting increased abundance are major constituents of the cytoskeleton and its modulators, in addition to extracellular matrix components known to act as a complex network, allowing for enhanced cell–cell interaction and motility to take place (Table 2 and Table S2G).

Finally, in the category termed mucosal defense, a higher abundance of mucin-2 variants at week 7 is in agreement with the increased number of mucus-producing Goblet cells observed in the histological findings, previously interpreted as a mechanism to facilitate egg exit from the mucosa [19]. Unique peptides assigned to each mucin variant confirmed their co-occurrence, with mucin-2 (Q80Z19) possibly forming the loosely attached outer mucus layer once secreted, due to its higher molecular mass and extensive O-glycosylation sites, favoring host microbiota entrapment and interactions [65]. Detection of defensin-alpha 30, exclusively in infected animals, provides evidence of a compensatory mechanism to limit bacterial invasion upon rupture of the epithelial barrier, further controlling systemic consequences to the host.

One particular aspect that poses a challenge for formulating hypotheses from shotgun proteomic approaches, based on analysis of whole tissues, is the cellular heterogeneity from which the proteins originated. For those differentially abundant proteins to which we could confidently assign cellular origin, the displayed scenario recapitulated degranulation of mast cells with their encoding proteases dominating the extracellular milieu and expanded the repertoire of signature proteins produced by eosinophils and neutrophils. It also corroborated the M2-like phenotype of macrophages in the intestinal tissue microenvironment during *S. mansoni* infection. The enzymatic machinery for the production of lipid species, through eicosanoid metabolism, suggests a balance between pro-inflammatory (leukotrienes) and anti-inflammatory (resolvins) mediators. Moreover, this study revealed the extracellular compartment as a dynamic platform possibly undergoing constant remodeling to accommodate and to facilitate egg passage. This type of information is not extractable from RNA-seq approaches or cell culture models, as they require cell isolation, disconnected from their microenvironment.

These proteomic findings reinforce the concept that liver and intestinal granulomas provoked by schistosome eggs are distinct cellular and molecular matrices. In the intestines, they do not normally represent the ultimate fate for trapped eggs but rather self-resolving inflammatory foci, with coordinated cellular and molecular processes primed to guarantee their arrival at the gut lumen, whilst preventing extensive damage to host tissue. This pioneering investigation, displaying the temporal proteome alterations observed at weeks 5 and 7, allowed appreciation of the initial tissue events associated with the onset of egg laying and their exacerbation at the peak of the acute phase. Moreover, it opened up novel possibilities for immunolocalization studies, cytometry analysis, and enzymatic assays to further attest the participation of the key highlighted molecules during egg transit to the gut lumen.

## 4. Conclusions

Although the cellular architecture defining the composition of hepatic and intestinal granulomas during *S. mansoni* infection is quite well established, protein identities governing cell function remain a topic largely open for exploration. In this investigation, we believe we have contributed by adding molecules at play, covering distinct steps involved in egg extravasation from the vascular bed to the intestinal lumen. Differential abundance of host constituents was observed early at the onset of egg laying (week 5), before histological changes were evident, implying tissue organization to serve as a site for attraction of an intense inflammatory infiltrate as mature eggs increase in numbers and their secretions accumulate. A variety of identified leucocyte effectors are testimonials of the proposed cellular content of intestinal granulomas, stimulating novel investigations and revisiting the role performed by host cells and their proteomic response to antigenic stimuli. In parallel, relevant molecular processes were revealed to be associated with dampening of tissue damage, spanning chaperone and redox responses, generation of lipid-resolving mediators, and epithelial barrier protection to limit systemic inflammation.

## Figures and Tables

**Table 1:** Top enriched biological pathways identified by functional enrichment analysis using the Reactome database.

Top Leading Enriched Pathways		
Reactome Pathways	Count in network	FDR value
Metabolism	388 of 1754	2.47 × 10^−78^
Metabolism of RNA	183 of 599	2.46 × 10^−50^
L13a-mediated translational silencing of Ceruloplasmin expression	90 of 109	1.01 × 10^−49^
GTP hydrolysis and joining of the 60S ribosomal subunit	90 of 110	1.01 × 10^−49^
Cap-dependent Translation Initiation	92 of 117	1.01 × 10^−49^
Innate Immune System	228 of 945	5.14 × 10^−49^
Metabolism of proteins	307 of 1616	4.23 × 10^−48^
Formation of a pool of free 40S subunits	83 of 99	1.53 × 10^−46^
Translation	108 of 225	1.42 × 10^−43^
Immune System	296 of 1615	1.51 × 10^−43^
Neutrophil degranulation	158 of 526	5.58 × 10^−43^
Nonsense Mediated Decay (NMD) independent of the Exon Junction Complex (EJC)	75 of 92	1.68 × 10^−41^

**Table 2:** Consolidated table of identities—differentially abundant proteins at week 5 and week 7 plus proteins uniquely identified at each time point. (**Cont**) Control. (**w 5**) week 5. (**w 7**) week 7.

Uniprot ID	Description	Cont × w 5	Cont × w 7	w 5 × w 7	w 5	w 7	w 5 and w 7
		Fold			Exclusive		
*Plasma proteins/Acute phase response*							
P29699	Alpha-2-HS-glycoprotein		3.4				
P06728	Apolipoprotein A-IV	2.5					
G3X9T8	Ceruloplasmin						√
Q8BH61	Coagulation factor XIII A chain					√	
P01027	Complement C3	2.0					
E9PV24	Fibrinogen alpha chain		13.3	5.0			
Q8K0E8	Fibrinogen beta chain		9.2				
Q8VCM7	Fibrinogen gamma chain		3.8	2.4			
Q91X72	Hemopexin	2.7	2.7				
P01820	Ig heavy chain V region PJ14					√	
P03976	Ig kappa chain V-II region 17S29.1						√
P01631	Ig kappa chain V-II region 26-10		5.2				
A0A0A6YXW6	Immunoglobulin heavy constant alpha (Fragment)		2.3				
A0A0A6YWR2	Immunoglobulin heavy constant gamma 1 (G1m marker)			5.3			
P01872	Immunoglobulin heavy constant mu			2.2			
A0A075B5V1	Immunoglobulin heavy variable 1–31	2.9					
A0A075B5R5	Immunoglobulin heavy variable 4–1 (Fragment)					√	
A0A075B5R2	Immunoglobulin heavy variable 7–3 (Fragment)					√	
P01837	Immunoglobulin kappa constant	2.5					
A0A075B5N7	Immunoglobulin kappa variable 6–13			3.9			
D3Z7E6	Platelet-activating factor acetylhydrolase isoform 1b subunit 3		2.5				
Q921I1	Serotransferrin	1.8	2.3				
Q99KC8	von Willebrand factor A domain-containing protein 5A		3.7	3.6			
*Extracellular matrix*							
Q3U8S1	CD44 antigen					√	
A2AX52	Collagen alpha-4(VI) chain	1.6					
A0A087WS56	Fibronectin			3.1			
P16045	Galectin-1		2.9	2.7			
P11688	Integrin alpha-5		2.3				
P09055	Integrin beta-1	1.2					
P97927	Laminin subunit alpha-4	2.1					
P02469	Laminin subunit beta-1	2.2					
D3YYJ7	Pentraxin family member		1.6				
Q80YX1	Tenascin	1.5	4.7	2.6			
*Cytoskeletal remodeling/Focal adhesion*							
D3YXG6	Arp2/3 complex 34 kDa subunit			1.5			
O88456	Calpain small subunit 1		2.5				
Q3UW68	Calpain-13					√	
O55111	Desmoglein-2	1.5					
Q9R0P5	Destrin	1.3					
Q62418	Drebrin-like protein			1.9			
Q61554	Fibrillin-1			1.3			
Q80X90	Filamin-B			1.5			
P26041	Moesin		1.7	2.5			
Q9ET54	Palladin			1.6			
Q61233	Plastin-2		3.3				
P62962	Profilin-1		1.7	1.6			
P26039	Talin-1			1.5			
Q7TMM9	Tubulin beta-2A chain						√
Q922F4	Tubulin beta-6 chain		11.6				
P20152	Vimentin		1.6				
*Leucocyte effectors (mast cells. eosinophils and macrophages)*							
Q61176	Arginase-1		7.6	5.6			
O35744	Chitinase-like protein 3					√	
Q923L7	Ear6 protein		143.1				
P97425	Eosinophil cationic protein 2					√	
H3BJE0	Granulin (Fragment)					√	
O89017	Legumain		6.5	4.3			
O09159	Lysosomal alpha-mannosidase					√	
Q61830	Macrophage mannose receptor 1					√	
P11034	Mast cell protease 1		852.3	212.4			
P15119	Mast cell protease 2		64.2	39.7			
Q3UN88	Mast cell protease 4					√	
A2AQ99	NAD(P)H oxidase (H_2_O_2_-forming)						√
O08692	Neutrophilic granule protein					√	
P50543	Protein S100-A11		2.6	2.1			
P27005	Protein S100-A8					√	
P31725	Protein S100-A9		21.2	19.0			
P97816	Protein S100-G						√
P21981	Protein-glutamine gamma-glutamyltransferase 2		1.9				
*Stress response and Intracellular signaling*							
Q91VR5	ATP-dependent RNA helicase DDX1	2.0					
Q9JLV1	BAG family molecular chaperone regulator 3		11.5				
Q8BT60	Copine-3	1.7					
Q9CX80	Cytoglobin		10.1	5.9			
Q9Z0E6	Guanylate-binding protein 2	2.9					
P30412	Peptidyl-prolyl cis-trans isomerase C					√	
Q61656	Probable ATP-dependent RNA helicase DDX5			1.2			
Q05144	Ras-related C3 botulinum toxin substrate 2					√	
P61028	Ras-related protein Rab-8B						√
Q8CIN4	Serine/threonine-protein kinase PAK 2				√		
P41241	Tyrosine-protein kinase CSK	1.8					
*Regulation of inflammation*							
E9Q5I9	26S proteasome non-ATPase regulatory subunit 13		8.2				
O35841	Apoptosis inhibitor 5		3.1				
E9Q6H6	Arachidonate 5-lipoxygenase					√	
P34914	Bifunctional epoxide hydrolase 2		2.2				
P23953	Carboxylesterase 1C	2.2					
O08738	Caspase-6		2.6	2.4			
P27784	C-C motif chemokine 6					√	
A0A140LJL0	CD81 antigen (Fragment)					√	
Q64GA5	Cytosolic phospholipase A2 gamma		3.7	3.2			
A0A0B4J1G1	Fc receptor IgG low affinity IIb					√	
Q2KHK6	Gasdermin-C2					√	
Q8CB12	Gasdermin-C3					√	
P14438	H-2 class II histocompatibility antigen A-U alpha chain	1.8					
Q3TJI8	Hydroxysteroid 11-beta dehydrogenase 1					√	
Q9D8C4	Interferon-induced 35 kDa protein homolog						√
P39654	Polyunsaturated fatty acid lipoxygenase ALOX15		28.3	16.3			
P56812	Programmed cell death protein 5	2.3					
Q62433	Protein NDRG1						√
Q08652	Retinol-binding protein 2		6.2	4.3			
Q60854	Serpin B6	1.4					
P19324	Serpin H1		2.8	2.6			
Q8C5G6	Toll interacting protein						√
*Mucosal response*							
E9QPZ2	Defensin alpha 30					√	
E9Q9C6	Mucin 2		2.2				
Q80Z19	Mucin-2		1.9				

## Data Availability

Protein identification and quantification data are provided in the accompanying CSV files. Raw data are available via the PRIDE Archive https://www.ebi.ac.uk/pride/login, Project accession: PXD079583—Token: 0ozhoftjmQQs (accessed on 15 July 2026).

## Supplementary Materials

The following supporting information can be downloaded at: https://www.mdpi.com/article/10.3390/proteomes14030039/s1, S1—Supplementary Data S1: Mass spectral data from *Mus musculus* and *Schistosoma mansoni*; S2—Supplementary Data S2: Mass spectral data from control and infected samples; S3—Supplementary Data S3: Reactome Pathway; Supplementary Figure S1: Evaluation of spleen weight ratio to body weight at weeks 5 and 7; Supplementary Figure S2: Overview of Intestinal tissue histology from *S. mansoni* infected animals; Supplementary Figure S3A,B: Cumulative abundance of intestinal proteome; Supplementary Figure S4: STRING access; Supplementary Table S1: Compositional analysis of the intestinal proteomes during *S. mansoni* infection and Reactome-based pathway enrichment; Supplementary Table S2: Label free quantitation-*S. mansoni* infected intestine proteome.

## References

[1] World Health Organization (2022). WHO Guideline on Control and Elimination of Human Schistosomiasis.

[2] Tavakoli Pirzaman A., Sepidarkish M., Alizadeh F., Al-Obidy S., Ebrahimi P., Kianifard N., Sheikhi Nooshabadi M., Jafari Tadi M., Zolfaghari Dehkharghani M., Mousavi S. (2024). Prevalence of Human *Schistosoma mansoni* Infection in Endemic Regions (2010–2024): A Systematic Review and Meta-Analysis. eClinicalMedicine.

[3] Ponzo E., Midiri A., Manno A., Pastorello M., Biondo C., Mancuso G. (2024). Insights into the Epidemiology, Pathogenesis, and Differential Diagnosis of Schistosomiasis. Eur. J. Microbiol. Immunol..

[4] Nation C.S., Da’dara A.A., Marchant J.K., Skelly P.J. (2020). Schistosome Migration in the Definitive Host. PLoS Negl. Trop. Dis..

[5] Helander H.F., Fändriks L. (2014). Surface Area of the Digestive Tract-Revisited. Scand. J. Gastroenterol..

[6] Loeffler D.A., Lundy S.K., Singh K.P., Gerard H.C., Hudson A.P., Boros D.L. (2002). Soluble Egg Antigens from *Schistosoma mansoni* Induce Angiogenesis-Related Processes by up-Regulating Vascular Endothelial Growth Factor in Human Endothelial Cells. J. Infect. Dis..

[7] Costain A.H., MacDonald A.S., Smits H.H. (2018). Schistosome Egg Migration: Mechanisms, Pathogenesis and Host Immune Responses. Front. Immunol..

[8] Mebius M.M., van Genderen P.J.J., Urbanus R.T., Tielens A.G.M., de Groot P.G., van Hellemond J.J. (2013). Interference with the Host Haemostatic System by Schistosomes. PLoS Pathog..

[9] Dewalick S., Hensbergen P.J., Bexkens M.L., Grosserichter-Wagener C., Hokke C.H., Deelder A.M., de Groot P.G., Tielens A.G.M., van Hellemond J.J. (2014). Binding of von Willebrand Factor and Plasma Proteins to the Eggshell of *Schistosoma mansoni*. Int. J. Parasitol..

[10] Yeh Y.-T., Skinner D.E., Criado-Hidalgo E., Chen N.S., Garcia-De Herreros A., El-Sakkary N., Liu L., Zhang S., Kandasamy A., Chien S. (2022). Biomechanical Interactions of *Schistosoma mansoni* Eggs with Vascular Endothelial Cells Facilitate Egg Extravasation. PLoS Pathog..

[11] DeMarco R., Mathieson W., Manuel S.J., Dillon G.P., Curwen R.S., Ashton P.D., Ivens A.C., Berriman M., Verjovski-Almeida S., Wilson R.A. (2010). Protein Variation in Blood-Dwelling Schistosome Worms Generated by Differential Splicing of Micro-Exon Gene Transcripts. Genome Res..

[12] Dunne D.W., Jones F.M., Doenhoff M.J. (1991). The Purification, Characterization, Serological Activity and Hepatotoxic Properties of Two Cationic Glycoproteins (alpha 1 and Omega 1) from *Schistosoma mansoni* Eggs. Parasitology.

[13] Schramm G., Hamilton J.V., Balog C.I.A., Wuhrer M., Gronow A., Beckmann S., Wippersteg V., Grevelding C.G., Goldmann T., Weber E. (2009). Molecular Characterisation of Kappa-5, a Major Antigenic Glycoprotein from *Schistosoma mansoni* Eggs. Mol. Biochem. Parasitol..

[14] Miller H.R., Newlands G.F., McKellar A., Inglis L., Coulson P.S., Wilson R.A. (1994). Hepatic Recruitment of Mast Cells Occurs in Rats but Not Mice Infected with *Schistosoma mansoni*. Parasite Immunol..

[15] Rychter J.W., Van Nassauw L., Brown J.K., Van Marck E., Knight P.A., Miller H.R.P., Kroese A.B.A., Timmermans J.-P. (2010). Impairment of Intestinal Barrier and Secretory Function as Well as Egg Excretion during Intestinal Schistosomiasis Occur Independently of Mouse Mast Cell Protease-1. Parasite Immunol..

[16] Schwartz C., Fallon P.G. (2018). Schistosoma “Eggs-Iting” the Host: Granuloma Formation and Egg Excretion. Front. Immunol..

[17] Coscia F., Doll S., Bech J.M., Schweizer L., Mund A., Lengyel E., Lindebjerg J., Madsen G.I., Moreira J.M., Mann M. (2020). A Streamlined Mass Spectrometry-Based Proteomics Workflow for Large-Scale FFPE Tissue Analysis. J. Pathol..

[18] Pearce E.J., MacDonald A.S. (2002). The Immunobiology of Schistosomiasis. Nat. Rev. Immunol..

[19] Marillier R.G., Michels C., Smith E.M., Fick L.C.E., Leeto M., Dewals B., Horsnell W.G.C., Brombacher F. (2008). IL-4/IL-13 Independent Goblet Cell Hyperplasia in Experimental Helminth Infections. BMC Immunol..

[20] Cheever A.W., Lenzi J.A., Lenzi H.L., Andrade Z.A. (2002). Experimental Models of *Schistosoma mansoni* Infection. Mem. Inst. Oswaldo Cruz.

[21] Xing J., Wang Y., Liang Y., Li J., Yao Y., Li J., Zhang K. (2025). The Role of Actin Cytoskeleton Component Arp2/3 in Inflammatory Response. Front. Cell Dev. Biol..

[22] Sun-Wada G.-H., Tabata H., Kawamura N., Aoyama M., Wada Y. (2009). Direct Recruitment of H+-ATPase from Lysosomes for Phagosomal Acidification. J. Cell Sci..

[23] Krieglstein C.F., Cerwinka W.H., Sprague A.G., Laroux F.S., Grisham M.B., Koteliansky V.E., Senninger N., Granger D.N., de Fougerolles A.R. (2002). Collagen-Binding Integrin alpha1beta1 Regulates Intestinal Inflammation in Experimental Colitis. J. Clin. Investig..

[24] Ryckman C., Vandal K., Rouleau P., Talbot M., Tessier P.A. (2003). Proinflammatory Activities of S100: Proteins S100A8, S100A9, and S100A8/A9 Induce Neutrophil Chemotaxis and Adhesion. J. Immunol..

[25] Shi J., Zhao Y., Wang K., Shi X., Wang Y., Huang H., Zhuang Y., Cai T., Wang F., Shao F. (2015). Cleavage of GSDMD by Inflammatory Caspases Determines Pyroptotic Cell Death. Nature.

[26] Jupp J., Hillier K., Elliott D.H., Fine D.R., Bateman A.C., Johnson P.A., Cazaly A.M., Penrose J.F., Sampson A.P. (2007). Colonic Expression of Leukotriene-Pathway Enzymes in Inflammatory Bowel Diseases. Inflamm. Bowel Dis..

[27] Cuzzocrea S., Rossi A., Mazzon E., Di Paola R., Genovese T., Muià C., Caputi A.P., Sautebin L. (2005). 5-Lipoxygenase Modulates Colitis through the Regulation of Adhesion Molecule Expression and Neutrophil Migration. Lab. Investig..

[28] Singh N.K., Rao G.N. (2019). Emerging Role of 12/15-Lipoxygenase (ALOX15) in Human Pathologies. Prog. Lipid Res..

[29] Silva-Moraes V., Shollenberger L.M., Castro-Borges W., Rabello A.L.T., Harn D.A., Medeiros L.C.S., de Jesus Jeremias W., Siqueira L.M.V., Pereira C.S.S., Pedrosa M.L.C. (2019). Serological Proteomic Screening and Evaluation of a Recombinant Egg Antigen for the Diagnosis of Low-Intensity *Schistosoma mansoni* Infections in Endemic Area in Brazil. PLoS Negl. Trop. Dis..

[30] Simon-Assmann P., Lefebvre O., Bellissent-Waydelich A., Olsen J., Orian-Rousseau V., de Arcangelis A. (1998). The Laminins: Role in Intestinal Morphogenesis and Differentiation. Ann. N. Y. Acad. Sci..

[31] Sorokin L. (2010). The Impact of the Extracellular Matrix on Inflammation. Nat. Rev. Immunol..

[32] Spenlé C., Hussenet T., Lacroute J., Lefebvre O., Kedinger M., Orend G., Simon-Assmann P. (2012). Dysregulation of Laminins in Intestinal Inflammation. Pathol. Biol..

[33] Greenman J.H., Moss L., Chakraborty S., Whitehead B.J., Palmfeldt J., Nejsum P., Hewitson J.P., Hitchcock I.S. (2025). Chronic Murine Schistosomiasis Causes Aberrant Hemostasis. Exp. Hematol..

[34] Tanabe M. (2003). Haemostatic Abnormalities in Hepatosplenic Schistosomiasis Mansoni. Parasitol. Int..

[35] Gonçalves-Silva G., Vieira L.G.M.D.S., Cosenza-Contreras M., Souza A.F.P., Costa D.C., Castro-Borges W. (2022). Profiling the Serum Proteome during *Schistosoma mansoni* Infection in the BALB/c Mice: A Focus on the Altered Lipid Metabolism as a Key Modulator of Host-Parasite Interactions. Front. Immunol..

[36] Deban L., Bottazzi B., Garlanda C., de la Torre Y.M., Mantovani A. (2009). Pentraxins: Multifunctional Proteins at the Interface of Innate Immunity and Inflammation. Biofactors.

[37] Agrawal A., Singh P.P., Bottazzi B., Garlanda C., Mantovani A. (2009). Pattern Recognition by Pentraxins. Adv. Exp. Med. Biol..

[38] Weinstock J.V., Boros D.L. (1983). Organ-Dependent Differences in Composition and Function Observed in Hepatic and Intestinal Granulomas Isolated from Mice with Schistosomiasis Mansoni. J. Immunol..

[39] Pesce J.T., Ramalingam T.R., Mentink-Kane M.M., Wilson M.S., El Kasmi K.C., Smith A.M., Thompson R.W., Cheever A.W., Murray P.J., Wynn T.A. (2009). Arginase-1-Expressing Macrophages Suppress Th2 Cytokine-Driven Inflammation and Fibrosis. PLoS Pathog..

[40] Schramm G., Gronow A., Knobloch J., Wippersteg V., Grevelding C.G., Galle J., Fuller H., Stanley R.G., Chiodini P.L., Haas H. (2006). IPSE/alpha-1: A Major Immunogenic Component Secreted from *Schistosoma mansoni* Eggs. Mol. Biochem. Parasitol..

[41] Mathieson W., Wilson R.A. (2010). A Comparative Proteomic Study of the Undeveloped and Developed *Schistosoma mansoni* Egg and Its Contents: The Miracidium, Hatch Fluid and Secretions. Int. J. Parasitol..

[42] Halligan K.E., Jourd’heuil F.L., Jourd’heuil D. (2009). Cytoglobin Is Expressed in the Vasculature and Regulates Cell Respiration and Proliferation via Nitric Oxide Dioxygenation. J. Biol. Chem..

[43] Liu X., El-Mahdy M.A., Boslett J., Varadharaj S., Hemann C., Abdelghany T.M., Ismail R.S., Little S.C., Zhou D., Thuy L.T.T. (2017). Cytoglobin Regulates Blood Pressure and Vascular Tone through Nitric Oxide Metabolism in the Vascular Wall. Nat. Commun..

[44] Zweier J.L., Hemann C., Kundu T., Ewees M.G., Khaleel S.A., Samouilov A., Ilangovan G., El-Mahdy M.A. (2021). Cytoglobin Has Potent Superoxide Dismutase Function. Proc. Natl. Acad. Sci. USA.

[45] Ilangovan G., Khaleel S.A., Kundu T., Hemann C., El-Mahdy M.A., Zweier J.L. (2021). Defining the Reducing System of the NO Dioxygenase Cytoglobin in Vascular Smooth Muscle Cells and Its Critical Role in Regulating Cellular NO Decay. J. Biol. Chem..

[46] Arndt V., Dick N., Tawo R., Dreiseidler M., Wenzel D., Hesse M., Fürst D.O., Saftig P., Saint R., Fleischmann B.K. (2010). Chaperone-Assisted Selective Autophagy Is Essential for Muscle Maintenance. Curr. Biol..

[47] Bravo-San Pedro J.M., Kroemer G., Galluzzi L. (2017). Autophagy and Inflammation. Cold Spring Harb. Perspect. Biol..

[48] Kaushik S., Cuervo A.M. (2018). The Coming of Age of Chaperone-Mediated Autophagy. Nat. Rev. Mol. Cell Biol..

[49] Mullins R.D., Heuser J.A., Pollard T.D. (1998). The Interaction of Arp2/3 Complex with Actin: Nucleation, High Affinity Pointed End Capping, and Formation of Branching Networks of Filaments. Proc. Natl. Acad. Sci. USA.

[50] Lechuga S., Ivanov A.I. (2021). Actin Cytoskeleton Dynamics during Mucosal Inflammation: A View from Broken Epithelial Barriers. Curr. Opin. Physiol..

[51] Manoury B. (2013). Proteases: Essential Actors in Processing Antigens and Intracellular Toll-like Receptors. Front. Immunol..

[52] Delamarre L., Couture R., Mellman I., Trombetta E.S. (2006). Enhancing Immunogenicity by Limiting Susceptibility to Lysosomal Proteolysis. J. Exp. Med..

[53] Pedras-Vasconcelos J.A., Pearce E.J. (1996). Type 1 CD8+ T Cell Responses during Infection with the Helminth *Schistosoma mansoni*. J. Immunol..

[54] Pancré V., Delacre M., Herno J., Auriault C. (1999). Schistosomal Egg Antigen-Responsive CD8 T-Cell Population in *Schistosoma mansoni*-Infected BALB/c Mice. Immunology.

[55] Vogl T., Ludwig S., Goebeler M., Strey A., Thorey I.S., Reichelt R., Foell D., Gerke V., Manitz M.P., Nacken W. (2004). MRP8 and MRP14 Control Microtubule Reorganization during Transendothelial Migration of Phagocytes. Blood.

[56] Wang S., Song R., Wang Z., Jing Z., Wang S., Ma J. (2018). S100A8/A9 in Inflammation. Front. Immunol..

[57] Dennis E.A., Norris P.C. (2015). Eicosanoid Storm in Infection and Inflammation. Nat. Rev. Immunol..

[58] Schebb N.H., Kühn H., Kahnt A.S., Rund K.M., O’Donnell V.B., Flamand N., Peters-Golden M., Jakobsson P.-J., Weylandt K.H., Rohwer N. (2022). Formation, Signaling and Occurrence of Specialized Pro-Resolving Lipid Mediators-What Is the Evidence so Far?. Front. Pharmacol..

[59] Tian R., Zuo X., Jaoude J., Mao F., Colby J., Shureiqi I. (2017). ALOX15 as a Suppressor of Inflammation and Cancer: Lost in the Link. Prostaglandins Other Lipid Mediat..

[60] Ding J., Wang K., Liu W., She Y., Sun Q., Shi J., Sun H., Wang D.-C., Shao F. (2016). Pore-Forming Activity and Structural Autoinhibition of the Gasdermin Family. Nature.

[61] Lu Y.-Q., Zhong S., Meng N., Fan Y.-P., Tang W.-X. (2017). NLRP3 Inflammasome Activation Results in Liver Inflammation and Fibrosis in Mice Infected with Schistosoma Japonicum in a Syk-Dependent Manner. Sci. Rep..

[62] Bakdash G., Vogelpoel L.T.C., van Capel T.M.M., Kapsenberg M.L., de Jong E.C. (2015). Retinoic Acid Primes Human Dendritic Cells to Induce Gut-Homing, IL-10-Producing Regulatory T Cells. Mucosal Immunol..

[63] Kang S.G., Lim H.W., Andrisani O.M., Broxmeyer H.E., Kim C.H. (2007). Vitamin A Metabolites Induce Gut-Homing FoxP3+ Regulatory T Cells. J. Immunol..

[64] Cassani B., Villablanca E.J., De Calisto J., Wang S., Mora J.R. (2012). Vitamin A and Immune Regulation: Role of Retinoic Acid in Gut-Associated Dendritic Cell Education, Immune Protection and Tolerance. Mol. Asp. Med..

[65] Johansson M.E.V., Larsson J.M.H., Hansson G.C. (2011). The Two Mucus Layers of Colon Are Organized by the MUC2 Mucin, Whereas the Outer Layer Is a Legislator of Host-Microbial Interactions. Proc. Natl. Acad. Sci. USA.

