# Supplementary material for: Proteomic Analysis of Paraffin-Embedded Intestines from Schistosoma mansoni Infection in Mice: Highlighting Molecular Players During Acute Schistosomiasis
Source: Proteomes. 2026 Jul 29;14(3):39. doi: 10.3390/proteomes14030039 (PMC13510969; doi:10.3390/proteomes14030039)
Supplement: Supplementary file 1 [file proteomes-14-00039-s001.zip › S2 - Supplementary Data S2 - Mass spectral data from control and infected samples.html]

proteins


Summary

  

# 1. Notes

# 2. Result Statistics

**Figure 1.**
False discovery rate (FDR) curve. X axis is the number of peptide-spectrum matches (PSM) being kept. Y axis is the corresponding FDR.

  


  

**Figure 2.**
PSM score distribution.  **(a)**
Distribution of PEAKS peptide score;  **(b)**
Scatterplot of PEAKS peptide score versus precursor mass error.

**(a)**

**(b)**

**Figure 3.**
De novo result validation. Distribution of residue local confidence:  **(a)**
Residues in de novo sequences validated by confident database peptide assignment;  **(b)**
Residues in "de novo only" sequences.

**(a)**

**(b)**

**Table 1.**
Statistics of data.

|  |  |
| --- | --- |
| # of MS scans | 265728 |
| # of MS/MS scans | 208260 |


**Table 2.**
Result filtration parameters.

|  |  |
| --- | --- |
| Peptide -10lgP | ≥22.2 |
| Peptide Ascore | ≥0 |
| Protein -10lgP | ≥39 |
| Proteins unique peptides | ≥1 |
| De novo ALC Score | ≥50% |


**Table 3.**
Statistics of filtered result.

|  |  |
| --- | --- |
| Peptide-Spectrum Matches | 85493 |
| Peptide sequences | 10697 |
| Protein groups | 1615 |
| Proteins | 2399 |
| Proteins (#Unique Peptides) | 1298 (>2); 407 (=2); 694 (=1); |
| FDR (Peptide-Spectrum Matches) | 1.0% |
| FDR (Peptide Sequences) | 5.0% |
| FDR (Protein) | 5.2% |
| De Novo Only Spectra | 63569 |

**Table 4.**
PTM profile.

| Name | ∆Mass | Position | #PSM | -10lgP | Area | AScore || Oxidation | 15.99 | M | 11634 | 144.30 | 8.51E6 | 1000.00 |
| Carbamidomethyl | 57.02 | C | 6125 | 157.78 | 7.78E6 | 1000.00 |

# 3. Experiment Control

**Figure 4.**
Precursor mass error of peptide-spectrum matches (PSM) in filtered result.  **(a)**
Distribution of precursor mass error in ppm;  **(b)**
Scatterplot of precursor m/z versus precursor mass error in ppm.

**(a)**

**(b)**

**Table 5.**
Number of identified peptides in each sample by the number of missed cleavages

|  |  |  |  |  |  |
| --- | --- | --- | --- | --- | --- |
| Missed Cleavages | 0 | 1 | 2 | 3 | 4+ |
| 10 | 951 | 116 | 11 | 0 | 0 |
| 01 | 871 | 122 | 5 | 0 | 0 |
| 02 | 1477 | 177 | 13 | 0 | 0 |
| 03 | 624 | 51 | 3 | 0 | 0 |
| 04 | 549 | 67 | 7 | 0 | 0 |
| 05 | 2098 | 221 | 15 | 0 | 0 |
| 06 | 835 | 72 | 2 | 0 | 0 |
| 07 | 342 | 30 | 1 | 0 | 0 |
| 08 | 1313 | 195 | 14 | 0 | 0 |
| 09 | 445 | 66 | 4 | 0 | 0 |

# 4. Other Information

**Table 6.**
Search parameters.

Search Engine Name: PEAKS  
Parent Mass Error Tolerance: 10.0 ppm  
Fragment Mass Error Tolerance: 0.1 Da  
Precursor Mass Search Type: monoisotopic  
Enzyme: Trypsin  
Max Missed Cleavages: 2  
Non-specific Cleavage: none  
Fixed Modifications:   
  Carbamidomethylation: 57.02  
Variable Modifications:   
  Oxidation (M): 15.99  
Max Variable PTM Per Peptide: 3  
Database: Mus musculus 10 10 24 54743  
Taxon: All  
Searched Entry: 54743  
FDR Estimation: Enabled  
Merge Options: no merge  
Precursor Options: corrected  
Charge Options: no correction  
Filter Quality: >0.65  
Process: true

**Table 7.**
Instrument parameters.

Fractions: ANa\_10.raw, Ana\_01.raw, Ana\_02.raw, Ana\_03.raw, Ana\_  
04.raw, Ana\_05.raw, Ana\_06.raw, Ana\_07.raw, Ana\_08.raw, Ana\_0  
9.raw  
Ion Source: ESI(nano-spray)  
Fragmentation Mode: high energy CID (y and b ions)  
MS Scan Mode: FT-ICR/Orbitrap  
MS/MS Scan Mode: FT-ICR/Orbitrap

  

Protein List

  

Protein Accession Contains:

Protein Description Contains:

Protein Sample Area >=

Protein Ptm Contains:

| Protein Group | Protein ID | Accession | -10lgP | Coverage (%) | Coverage (%) 10 | Coverage (%) 01 | Coverage (%) 02 | Coverage (%) 03 | Coverage (%) 04 | Coverage (%) 05 | Coverage (%) 06 | Coverage (%) 07 | Coverage (%) 08 | Coverage (%) 09 | Area 10 | Area 01 | Area 02 | Area 03 | Area 04 | Area 05 | Area 06 | Area 07 | Area 08 | Area 09 | #Peptides | #Unique | #Spec 10 | #Spec 01 | #Spec 02 | #Spec 03 | #Spec 04 | #Spec 05 | #Spec 06 | #Spec 07 | #Spec 08 | #Spec 09 | PTM | Avg. Mass | Description |
| --- | --- | --- | --- | --- | --- | --- | --- | --- | --- | --- | --- | --- | --- | --- | --- | --- | --- | --- | --- | --- | --- | --- | --- | --- | --- | --- | --- | --- | --- | --- | --- | --- | --- | --- | --- | --- | --- | --- | --- |
| 2 | 17 | A0A338P6K2\|A0A338P6K2\_MOUSE | 645.12 | 63 | 54 | 54 | 55 | 53 | 55 | 61 | 54 | 56 | 55 | 51 | 9.83E7 | 1.3E8 | 9.42E7 | 2.56E7 | 6.12E7 | 1.02E8 | 4.05E7 | 5.99E7 | 1.62E8 | 7.56E7 | 145 | 1 | 306 | 325 | 295 | 219 | 285 | 298 | 213 | 315 | 334 | 254 | Y | 227868 | Myosin-11 OS=Mus musculus OX=10090 GN=Myh11 PE=1 SV=1 |
| 6 | 16 | A0A2R8VHF9\|A0A2R8VHF9\_MOUSE | 642.11 | 63 | 54 | 54 | 54 | 53 | 56 | 61 | 54 | 56 | 55 | 50 | 1.09E7 | 0E0 | 2.63E7 | 9.6E6 | 1.79E7 | 3.99E7 | 1.23E7 | 1.34E7 | 1.55E7 | 5.91E6 | 143 | 1 | 300 | 319 | 288 | 215 | 279 | 292 | 209 | 308 | 329 | 248 | Y | 223355 | Myosin-11 OS=Mus musculus OX=10090 GN=Myh11 PE=1 SV=1 |
| 10 | 25 | B7FAU9\|B7FAU9\_MOUSE | 580.89 | 58 | 45 | 44 | 43 | 43 | 44 | 53 | 41 | 42 | 47 | 41 | 1.86E9 | 1.7E9 | 1.72E9 | 6.15E8 | 1.29E9 | 1.96E9 | 8.99E8 | 9.38E8 | 2.43E9 | 1.34E9 | 117 | 84 | 153 | 144 | 141 | 121 | 136 | 169 | 129 | 136 | 175 | 139 | Y | 280498 | Filamin, alpha OS=Mus musculus OX=10090 GN=Flna PE=1 SV=1 |
| 10 | 27 | sp\|Q8BTM8\|FLNA\_MOUSE | 580.89 | 58 | 45 | 44 | 43 | 43 | 43 | 53 | 41 | 42 | 47 | 41 | 1.86E9 | 1.7E9 | 1.72E9 | 6.15E8 | 1.29E9 | 1.96E9 | 8.99E8 | 9.38E8 | 2.43E9 | 1.34E9 | 117 | 84 | 153 | 144 | 141 | 121 | 136 | 169 | 129 | 136 | 175 | 139 | Y | 281219 | Filamin-A OS=Mus musculus OX=10090 GN=Flna PE=1 SV=5 |
| 16 | 36 | sp\|Q8VDD5\|MYH9\_MOUSE | 522.77 | 49 | 32 | 26 | 29 | 29 | 30 | 34 | 34 | 31 | 40 | 33 | 4.93E8 | 3.53E8 | 3.41E8 | 2.04E8 | 3.38E8 | 3.5E8 | 2.67E8 | 2.44E8 | 8.8E8 | 4.72E8 | 94 | 67 | 103 | 83 | 87 | 77 | 88 | 92 | 90 | 100 | 121 | 97 | Y | 226370 | Myosin-9 OS=Mus musculus OX=10090 GN=Myh9 PE=1 SV=4 |
| 9 | 2 | sp\|P07724\|ALBU\_MOUSE | 522.19 | 82 | 78 | 77 | 80 | 79 | 80 | 80 | 80 | 79 | 78 | 75 | 3.69E9 | 6.88E9 | 7.06E9 | 2.64E9 | 3.49E9 | 6.34E9 | 3.77E9 | 4.14E9 | 3.13E9 | 3.43E9 | 47 | 47 | 166 | 216 | 243 | 179 | 187 | 190 | 167 | 229 | 145 | 160 | Y | 68693 | Albumin OS=Mus musculus OX=10090 GN=Alb PE=1 SV=3 |
| 23 | 56 | J3QQ16\|J3QQ16\_MOUSE | 512.28 | 37 | 24 | 27 | 28 | 21 | 24 | 29 | 23 | 25 | 24 | 22 | 4.55E8 | 6E8 | 6.19E8 | 2.46E8 | 4.65E8 | 6.58E8 | 3.42E8 | 3.49E8 | 6.18E8 | 3.73E8 | 82 | 79 | 67 | 71 | 74 | 59 | 67 | 82 | 63 | 66 | 65 | 55 | Y | 288693 | Collagen, type VI, alpha 3 OS=Mus musculus OX=10090 GN=Col6a3 PE=1 SV=1 |
| 23 | 57 | A0A087WS16\|A0A087WS16\_MOUSE | 512.28 | 37 | 24 | 27 | 28 | 21 | 24 | 29 | 23 | 25 | 24 | 22 | 4.55E8 | 6E8 | 6.19E8 | 2.46E8 | 4.65E8 | 6.58E8 | 3.42E8 | 3.49E8 | 6.18E8 | 3.73E8 | 82 | 79 | 67 | 71 | 74 | 59 | 67 | 82 | 63 | 66 | 65 | 55 | Y | 288691 | Collagen, type VI, alpha 3 OS=Mus musculus OX=10090 GN=Col6a3 PE=1 SV=1 |
| 17 | 14 | sp\|P31001\|DESM\_MOUSE | 459.10 | 68 | 68 | 66 | 60 | 58 | 60 | 67 | 65 | 62 | 65 | 67 | 2.92E9 | 1.69E9 | 1.61E9 | 6.51E8 | 1.57E9 | 1.64E9 | 9.45E8 | 1.03E9 | 3.06E9 | 2.14E9 | 36 | 29 | 127 | 88 | 85 | 61 | 85 | 76 | 68 | 100 | 134 | 115 | Y | 53498 | Desmin OS=Mus musculus OX=10090 GN=Des PE=1 SV=3 |
| 58 | 59 | sp\|P26039\|TLN1\_MOUSE | 457.85 | 32 | 19 | 15 | 16 | 13 | 16 | 22 | 15 | 17 | 19 | 18 | 1.45E8 | 1.11E8 | 1.34E8 | 4.25E7 | 9.36E7 | 1.41E8 | 6.6E7 | 7.15E7 | 2.68E8 | 1.61E8 | 59 | 53 | 41 | 30 | 38 | 28 | 34 | 48 | 30 | 36 | 40 | 37 | Y | 269819 | Talin-1 OS=Mus musculus OX=10090 GN=Tln1 PE=1 SV=2 |
| 5 | 5 | sp\|P62737\|ACTA\_MOUSE | 445.86 | 78 | 72 | 75 | 69 | 73 | 67 | 77 | 77 | 68 | 75 | 66 | 1.31E8 | 2.18E8 | 1.99E8 | 8.09E7 | 1.55E8 | 2.32E8 | 1.01E8 | 1.17E8 | 2.79E8 | 1.3E8 | 30 | 1 | 343 | 279 | 277 | 229 | 284 | 244 | 202 | 311 | 338 | 276 | Y | 42009 | Actin, aortic smooth muscle OS=Mus musculus OX=10090 GN=Acta2 PE=1 SV=1 |
| 1 | 9 | sp\|P63260\|ACTG\_MOUSE | 432.42 | 74 | 68 | 73 | 74 | 69 | 70 | 66 | 73 | 71 | 71 | 71 | 5.25E9 | 4.73E9 | 3.81E9 | 1.49E9 | 2.84E9 | 3.17E9 | 2.11E9 | 2.4E9 | 8.87E9 | 3.85E9 | 29 | 3 | 370 | 291 | 277 | 254 | 292 | 235 | 206 | 315 | 369 | 312 | Y | 41793 | Actin, cytoplasmic 2 OS=Mus musculus OX=10090 GN=Actg1 PE=1 SV=1 |
| 1 | 10 | sp\|P60710\|ACTB\_MOUSE | 432.42 | 74 | 68 | 73 | 74 | 69 | 70 | 66 | 73 | 71 | 71 | 71 | 5.25E9 | 4.73E9 | 3.81E9 | 1.49E9 | 2.84E9 | 3.17E9 | 2.11E9 | 2.4E9 | 8.87E9 | 3.85E9 | 29 | 3 | 370 | 291 | 277 | 254 | 292 | 235 | 206 | 315 | 369 | 312 | Y | 41737 | Actin, cytoplasmic 1 OS=Mus musculus OX=10090 GN=Actb PE=1 SV=1 |
| 30 | 6 | sp\|P56480\|ATPB\_MOUSE | 429.12 | 73 | 70 | 65 | 61 | 64 | 60 | 68 | 58 | 63 | 61 | 64 | 8.51E8 | 9.75E8 | 9.42E8 | 3.61E8 | 6.85E8 | 8.81E8 | 5.46E8 | 4.95E8 | 1.32E9 | 6.82E8 | 27 | 27 | 66 | 68 | 58 | 47 | 54 | 57 | 42 | 57 | 72 | 56 | Y | 56301 | ATP synthase subunit beta, mitochondrial OS=Mus musculus OX=10090 GN=Atp5f1b PE=1 SV=2 |
| 71 | 60 | sp\|Q68FD5\|CLH1\_MOUSE | 426.19 | 33 | 20 | 21 | 16 | 18 | 19 | 18 | 19 | 19 | 25 | 22 | 2.07E8 | 1.21E8 | 1.21E8 | 6.43E7 | 1.17E8 | 1.67E8 | 1.21E8 | 9.32E7 | 2.69E8 | 1.66E8 | 46 | 46 | 35 | 32 | 28 | 28 | 35 | 31 | 32 | 34 | 41 | 36 | Y | 191555 | Clathrin heavy chain 1 OS=Mus musculus OX=10090 GN=Cltc PE=1 SV=3 |
| 71 | 61 | Q5SXR6\|Q5SXR6\_MOUSE | 426.19 | 33 | 20 | 21 | 16 | 18 | 19 | 18 | 19 | 19 | 25 | 22 | 2.07E8 | 1.21E8 | 1.21E8 | 6.43E7 | 1.17E8 | 1.67E8 | 1.21E8 | 9.32E7 | 2.69E8 | 1.66E8 | 46 | 46 | 35 | 32 | 28 | 28 | 35 | 31 | 32 | 34 | 41 | 36 | Y | 191985 | Clathrin heavy chain OS=Mus musculus OX=10090 GN=Cltc PE=1 SV=1 |
| 7 | 3 | sp\|P68134\|ACTS\_MOUSE | 424.25 | 78 | 66 | 69 | 69 | 67 | 62 | 71 | 71 | 63 | 69 | 60 |  |  | 1.24E6 |  |  |  |  |  |  |  | 29 | 1 | 328 | 272 | 270 | 229 | 278 | 236 | 197 | 302 | 319 | 272 | Y | 42051 | Actin, alpha skeletal muscle OS=Mus musculus OX=10090 GN=Acta1 PE=1 SV=1 |
| 25 | 40 | sp\|P63017\|HSP7C\_MOUSE | 418.91 | 51 | 40 | 43 | 45 | 47 | 46 | 43 | 45 | 43 | 41 | 45 | 5.76E8 | 5.65E8 | 4.91E8 | 2.55E8 | 4.63E8 | 3.62E8 | 2.71E8 | 3.11E8 | 8.68E8 | 4.5E8 | 35 | 20 | 64 | 70 | 67 | 54 | 64 | 56 | 52 | 66 | 72 | 67 | Y | 70871 | Heat shock cognate 71 kDa protein OS=Mus musculus OX=10090 GN=Hspa8 PE=1 SV=1 |
| 18 | 1 | sp\|P19001\|K1C19\_MOUSE | 417.41 | 87 | 78 | 72 | 69 | 73 | 77 | 77 | 78 | 75 | 77 | 78 | 1.95E9 | 2.65E9 | 2.16E9 | 9.73E8 | 2.24E9 | 2.52E9 | 1.52E9 | 1.31E9 | 2.14E9 | 1.65E9 | 40 | 30 | 95 | 103 | 84 | 74 | 104 | 86 | 81 | 90 | 108 | 86 | Y | 44542 | Keratin, type I cytoskeletal 19 OS=Mus musculus OX=10090 GN=Krt19 PE=1 SV=1 |
| 33 | 51 | sp\|Q6URW6\|MYH14\_MOUSE | 413.57 | 35 | 14 | 18 | 18 | 16 | 20 | 26 | 24 | 17 | 13 | 15 | 7.01E7 | 1.75E8 | 1.25E8 | 8.26E7 | 1.54E8 | 1.86E8 | 1.31E8 | 6.62E7 | 9.94E7 | 7.41E7 | 67 | 50 | 42 | 54 | 56 | 47 | 56 | 65 | 61 | 50 | 42 | 43 | Y | 228584 | Myosin-14 OS=Mus musculus OX=10090 GN=Myh14 PE=1 SV=1 |
| 37 | 94 | sp\|P99024\|TBB5\_MOUSE | 413.00 | 63 | 53 | 49 | 53 | 53 | 50 | 53 | 53 | 51 | 63 | 53 | 1.33E8 | 1.1E8 | 3.85E7 | 1.01E7 | 5.11E7 | 8.16E7 | 4.58E7 | 2.03E7 | 1.89E8 | 1.13E8 | 20 | 4 | 57 | 57 | 50 | 44 | 52 | 46 | 39 | 52 | 74 | 58 | Y | 49671 | Tubulin beta-5 chain OS=Mus musculus OX=10090 GN=Tubb5 PE=1 SV=1 |
| 34 | 23 | sp\|Q7TPR4\|ACTN1\_MOUSE | 409.98 | 60 | 39 | 39 | 41 | 39 | 36 | 48 | 40 | 38 | 46 | 36 |  |  |  |  |  |  |  |  | 5.04E6 |  | 45 | 1 | 57 | 57 | 59 | 42 | 53 | 58 | 44 | 50 | 63 | 56 | Y | 103068 | Alpha-actinin-1 OS=Mus musculus OX=10090 GN=Actn1 PE=1 SV=1 |
| 38 | 35 | sp\|P11499\|HS90B\_MOUSE | 409.93 | 51 | 37 | 40 | 40 | 44 | 43 | 44 | 40 | 38 | 39 | 33 | 3.03E8 | 4.03E8 | 4.17E8 | 2.87E8 | 3.55E8 | 3.55E8 | 3.09E8 | 3.02E8 | 4.77E8 | 2.79E8 | 38 | 22 | 46 | 55 | 56 | 51 | 53 | 41 | 47 | 58 | 54 | 45 | Y | 83281 | Heat shock protein HSP 90-beta OS=Mus musculus OX=10090 GN=Hsp90ab1 PE=1 SV=3 |
| 31 | 21 | A1BN54\|A1BN54\_MOUSE | 409.85 | 60 | 40 | 40 | 43 | 40 | 37 | 49 | 41 | 40 | 46 | 37 | 1.15E7 | 1.1E7 | 1.01E7 | 3.46E6 | 8.11E6 | 1.13E7 | 5.18E6 | 6.14E6 | 1.06E7 | 7.04E6 | 45 | 1 | 58 | 58 | 60 | 43 | 54 | 59 | 45 | 51 | 63 | 57 | Y | 102720 | Alpha-actinin-1 OS=Mus musculus OX=10090 GN=Actn1 PE=1 SV=1 |
| 41 | 95 | sp\|P68372\|TBB4B\_MOUSE | 409.09 | 63 | 53 | 53 | 53 | 53 | 53 | 50 | 53 | 53 | 63 | 53 | 3.84E7 | 3.93E7 | 3.34E7 | 1.72E7 | 3.01E7 | 3.22E7 | 2.38E7 | 2.17E7 | 6.64E7 | 3.83E7 | 20 | 1 | 55 | 55 | 45 | 40 | 49 | 46 | 38 | 49 | 71 | 53 | Y | 49831 | Tubulin beta-4B chain OS=Mus musculus OX=10090 GN=Tubb4b PE=1 SV=1 |
| 70 | 74 | sp\|Q9QXS1\|PLEC\_MOUSE | 407.55 | 17 | 9 | 6 | 4 | 3 | 5 | 8 | 6 | 3 | 10 | 8 | 1.37E8 | 1.35E8 | 4.8E7 | 2.11E7 | 2.07E8 | 3.18E8 | 2.19E8 | 2.25E7 | 2.48E8 | 1.09E8 | 79 | 74 | 42 | 28 | 18 | 17 | 25 | 39 | 29 | 16 | 46 | 37 | Y | 534193 | Plectin OS=Mus musculus OX=10090 GN=Plec PE=1 SV=3 |
| 46 | 22 | sp\|Q03265\|ATPA\_MOUSE | 404.25 | 57 | 46 | 41 | 48 | 49 | 48 | 47 | 44 | 49 | 43 | 43 | 7.29E8 | 9.54E8 | 7.46E8 | 3.23E8 | 6.2E8 | 7.06E8 | 4.75E8 | 5.24E8 | 8.39E8 | 6.2E8 | 26 | 25 | 47 | 51 | 46 | 43 | 47 | 44 | 42 | 53 | 61 | 45 | Y | 59753 | ATP synthase subunit alpha, mitochondrial OS=Mus musculus OX=10090 GN=Atp5f1a PE=1 SV=1 |
| 35 | 29 | sp\|P57780\|ACTN4\_MOUSE | 394.96 | 54 | 38 | 40 | 34 | 39 | 41 | 38 | 41 | 38 | 41 | 35 | 1.59E8 | 1.73E8 | 1.27E8 | 6.83E7 | 1.25E8 | 1.24E8 | 9.53E7 | 7.54E7 | 2.07E8 | 9.97E7 | 44 | 23 | 58 | 57 | 52 | 43 | 58 | 50 | 47 | 50 | 61 | 50 | Y | 104977 | Alpha-actinin-4 OS=Mus musculus OX=10090 GN=Actn4 PE=1 SV=1 |
| 13 | 13 | sp\|P11679\|K2C8\_MOUSE | 394.56 | 69 | 68 | 68 | 60 | 67 | 69 | 65 | 63 | 69 | 64 | 64 | 3.18E9 | 4.07E9 | 2.99E9 | 1.76E9 | 3.35E9 | 3.81E9 | 2.49E9 | 1.7E9 | 4.68E9 | 2.27E9 | 36 | 28 | 141 | 136 | 99 | 93 | 126 | 116 | 96 | 132 | 143 | 115 | Y | 54565 | Keratin, type II cytoskeletal 8 OS=Mus musculus OX=10090 GN=Krt8 PE=1 SV=4 |
| 101 | 66 | sp\|P16546\|SPTN1\_MOUSE | 390.54 | 22 | 6 | 11 | 10 | 10 | 13 | 15 | 12 | 10 | 10 | 7 | 3.5E7 | 1.01E8 | 6.81E7 | 3.1E7 | 8.01E7 | 1.14E8 | 5.3E7 | 3.13E7 | 1.24E8 | 5.08E7 | 45 | 45 | 13 | 25 | 21 | 20 | 28 | 34 | 28 | 19 | 23 | 17 | N | 284596 | Spectrin alpha chain, non-erythrocytic 1 OS=Mus musculus OX=10090 GN=Sptan1 PE=1 SV=4 |
| 101 | 67 | A3KGU7\|A3KGU7\_MOUSE | 390.54 | 22 | 6 | 11 | 10 | 10 | 13 | 15 | 12 | 10 | 10 | 7 | 3.5E7 | 1.01E8 | 6.81E7 | 3.1E7 | 8.01E7 | 1.14E8 | 5.3E7 | 3.13E7 | 1.24E8 | 5.08E7 | 45 | 45 | 13 | 25 | 21 | 20 | 28 | 34 | 28 | 19 | 23 | 17 | N | 285151 | Spectrin alpha, non-erythrocytic 1 OS=Mus musculus OX=10090 GN=Sptan1 PE=1 SV=1 |
| 101 | 68 | A3KGU9\|A3KGU9\_MOUSE | 390.54 | 22 | 6 | 11 | 10 | 9 | 13 | 15 | 12 | 10 | 10 | 7 | 3.5E7 | 1.01E8 | 6.81E7 | 3.1E7 | 8.01E7 | 1.14E8 | 5.3E7 | 3.13E7 | 1.24E8 | 5.08E7 | 45 | 45 | 13 | 25 | 21 | 20 | 28 | 34 | 28 | 19 | 23 | 17 | N | 287602 | Spectrin alpha, non-erythrocytic 1 OS=Mus musculus OX=10090 GN=Sptan1 PE=1 SV=2 |
| 101 | 72 | A3KGU5\|A3KGU5\_MOUSE | 390.54 | 22 | 6 | 11 | 10 | 10 | 13 | 15 | 12 | 10 | 11 | 7 | 3.5E7 | 1.01E8 | 6.81E7 | 3.1E7 | 8.01E7 | 1.14E8 | 5.3E7 | 3.13E7 | 1.24E8 | 5.08E7 | 45 | 45 | 13 | 25 | 21 | 20 | 28 | 34 | 28 | 19 | 23 | 17 | N | 282893 | Spectrin alpha, non-erythrocytic 1 OS=Mus musculus OX=10090 GN=Sptan1 PE=1 SV=1 |
| 101 | 73 | E9Q447\|E9Q447\_MOUSE | 390.54 | 22 | 6 | 11 | 10 | 10 | 13 | 15 | 12 | 10 | 10 | 7 | 3.5E7 | 1.01E8 | 6.81E7 | 3.1E7 | 8.01E7 | 1.14E8 | 5.3E7 | 3.13E7 | 1.24E8 | 5.08E7 | 45 | 45 | 13 | 25 | 21 | 20 | 28 | 34 | 28 | 19 | 23 | 17 | N | 285344 | Spectrin alpha, non-erythrocytic 1 OS=Mus musculus OX=10090 GN=Sptan1 PE=1 SV=1 |
| 44 | 45 | sp\|P27773\|PDIA3\_MOUSE | 390.05 | 49 | 41 | 43 | 42 | 44 | 44 | 46 | 44 | 42 | 44 | 42 | 8.6E8 | 9.54E8 | 9E8 | 2.86E8 | 8.26E8 | 6.51E8 | 5.11E8 | 5.57E8 | 9.26E8 | 7.36E8 | 26 | 26 | 50 | 50 | 48 | 39 | 53 | 48 | 49 | 51 | 57 | 45 | Y | 56678 | Protein disulfide-isomerase A3 OS=Mus musculus OX=10090 GN=Pdia3 PE=1 SV=2 |
| 52 | 53 | sp\|Q8VDN2\|AT1A1\_MOUSE | 389.77 | 40 | 28 | 31 | 31 | 27 | 30 | 34 | 32 | 34 | 34 | 25 | 2.22E8 | 3.2E8 | 2.68E8 | 1.43E8 | 2.13E8 | 2.66E8 | 1.67E8 | 1.39E8 | 2.98E8 | 1.93E8 | 45 | 28 | 34 | 47 | 42 | 37 | 42 | 44 | 43 | 42 | 39 | 34 | Y | 112982 | Sodium/potassium-transporting ATPase subunit alpha-1 OS=Mus musculus OX=10090 GN=Atp1a1 PE=1 SV=1 |
| 51 | 146 | sp\|Q7TMM9\|TBB2A\_MOUSE | 388.99 | 51 | 42 | 42 | 42 | 42 | 42 | 42 | 44 | 42 | 49 | 42 |  |  |  |  |  |  | 6.53E4 |  | 7.79E6 |  | 17 | 2 | 46 | 44 | 38 | 35 | 41 | 39 | 32 | 43 | 60 | 47 | Y | 49907 | Tubulin beta-2A chain OS=Mus musculus OX=10090 GN=Tubb2a PE=1 SV=1 |
| 78 | 41 | sp\|P40142\|TKT\_MOUSE | 383.50 | 49 | 35 | 45 | 40 | 44 | 42 | 47 | 45 | 44 | 47 | 43 | 3.74E8 | 3.84E8 | 3.65E8 | 1.76E8 | 2.96E8 | 3.58E8 | 2.83E8 | 1.93E8 | 6.22E8 | 3.79E8 | 27 | 27 | 26 | 29 | 30 | 27 | 29 | 32 | 36 | 29 | 47 | 29 | Y | 67631 | Transketolase OS=Mus musculus OX=10090 GN=Tkt PE=1 SV=1 |
| 47 | 140 | sp\|P10126\|EF1A1\_MOUSE | 383.40 | 56 | 44 | 43 | 44 | 38 | 39 | 49 | 49 | 35 | 38 | 40 | 6.44E8 | 7.38E8 | 6.96E8 | 4.05E8 | 6.25E8 | 6.13E8 | 5.03E8 | 4.6E8 | 1.54E9 | 7.41E8 | 19 | 10 | 48 | 51 | 43 | 33 | 46 | 36 | 40 | 47 | 60 | 46 | Y | 50114 | Elongation factor 1-alpha 1 OS=Mus musculus OX=10090 GN=Eef1a1 PE=1 SV=3 |
| 86 | 33 | sp\|Q64727\|VINC\_MOUSE | 377.37 | 47 | 26 | 28 | 27 | 23 | 28 | 28 | 27 | 28 | 32 | 26 | 1.7E8 | 1.77E8 | 1.82E8 | 7.78E7 | 1.54E8 | 1.9E8 | 1E8 | 8.59E7 | 2.8E8 | 1.82E8 | 46 | 45 | 25 | 30 | 27 | 24 | 29 | 27 | 26 | 27 | 31 | 28 | Y | 116717 | Vinculin OS=Mus musculus OX=10090 GN=Vcl PE=1 SV=4 |
| 57 | 79 | sp\|Q6PDN3\|MYLK\_MOUSE | 374.61 | 17 | 11 | 14 | 13 | 14 | 15 | 16 | 14 | 12 | 12 | 12 | 3.8E8 | 7.14E8 | 5.84E8 | 2.02E8 | 4.58E8 | 6.35E8 | 3.12E8 | 2.83E8 | 5.23E8 | 3.12E8 | 32 | 32 | 31 | 44 | 42 | 34 | 42 | 44 | 32 | 40 | 37 | 29 | Y | 212924 | Myosin light chain kinase, smooth muscle OS=Mus musculus OX=10090 GN=Mylk PE=1 SV=3 |
| 57 | 81 | B1B1A8\|B1B1A8\_MOUSE | 374.61 | 17 | 11 | 14 | 13 | 14 | 15 | 16 | 13 | 12 | 12 | 12 | 3.8E8 | 7.14E8 | 5.84E8 | 2.02E8 | 4.58E8 | 6.35E8 | 3.12E8 | 2.83E8 | 5.23E8 | 3.12E8 | 32 | 32 | 31 | 44 | 42 | 34 | 42 | 44 | 32 | 40 | 37 | 29 | Y | 213608 | Myosin light chain kinase, smooth muscle OS=Mus musculus OX=10090 GN=Mylk PE=1 SV=1 |
| 22 | 944 | Q91VB8\|Q91VB8\_MOUSE | 372.64 | 92 | 63 | 92 | 72 | 72 | 92 | 72 | 92 | 72 | 64 | 84 | 2.05E9 | 4.26E9 | 4.28E9 | 1.61E9 | 2.84E9 | 3.56E9 | 1.88E9 | 4.31E9 | 2.41E9 | 1.41E9 | 10 | 10 | 68 | 88 | 94 | 52 | 74 | 68 | 49 | 123 | 44 | 47 | Y | 15112 | Alpha globin 1 OS=Mus musculus OX=10090 GN=Hba-a1 PE=1 SV=1 |
| 133 | 86 | sp\|Q9JHU4\|DYHC1\_MOUSE | 372.59 | 10 | 6 | 4 | 4 | 5 | 6 | 5 | 5 | 4 | 4 | 4 | 3.34E7 | 2.86E7 | 3.55E7 | 2.33E7 | 3.37E7 | 4.54E7 | 2.49E7 | 1.69E7 | 5.73E7 | 3.14E7 | 43 | 41 | 21 | 16 | 16 | 20 | 22 | 20 | 19 | 15 | 19 | 16 | Y | 532050 | Cytoplasmic dynein 1 heavy chain 1 OS=Mus musculus OX=10090 GN=Dync1h1 PE=1 SV=2 |
| 159 | 76 | A0A087WS56\|A0A087WS56\_MOUSE | 372.25 | 22 | 17 | 3 | 7 | 5 | 4 | 9 | 4 | 4 | 17 | 16 | 1.63E8 | 3.58E7 | 5.98E7 | 1.82E7 | 2.73E7 | 6.22E7 | 2.34E7 | 2.2E7 | 3.65E8 | 1.97E8 | 38 | 38 | 29 | 7 | 13 | 10 | 7 | 17 | 9 | 7 | 36 | 33 | Y | 249550 | Fibronectin OS=Mus musculus OX=10090 GN=Fn1 PE=1 SV=1 |
| 159 | 78 | Q3UHL6\|Q3UHL6\_MOUSE | 372.25 | 21 | 16 | 3 | 7 | 5 | 4 | 8 | 4 | 4 | 17 | 16 | 1.63E8 | 3.58E7 | 5.98E7 | 1.82E7 | 2.73E7 | 6.22E7 | 2.34E7 | 2.2E7 | 3.65E8 | 1.97E8 | 38 | 38 | 29 | 7 | 13 | 10 | 7 | 17 | 9 | 7 | 36 | 33 | Y | 260126 | Fibronectin OS=Mus musculus OX=10090 GN=Fn1 PE=1 SV=1 |
| 159 | 82 | A0A087WR50\|A0A087WR50\_MOUSE | 372.25 | 21 | 16 | 3 | 7 | 5 | 4 | 8 | 4 | 4 | 17 | 15 | 1.63E8 | 3.58E7 | 5.98E7 | 1.82E7 | 2.73E7 | 6.22E7 | 2.34E7 | 2.2E7 | 3.65E8 | 1.97E8 | 38 | 38 | 29 | 7 | 13 | 10 | 7 | 17 | 9 | 7 | 36 | 33 | Y | 262840 | Fibronectin OS=Mus musculus OX=10090 GN=Fn1 PE=1 SV=1 |
| 95 | 31 | sp\|P14824\|ANXA6\_MOUSE | 370.98 | 51 | 31 | 29 | 31 | 24 | 27 | 33 | 30 | 37 | 38 | 30 | 1.58E8 | 1.63E8 | 1.66E8 | 5.88E7 | 1.51E8 | 1.8E8 | 6.63E7 | 8.37E7 | 2.5E8 | 1.63E8 | 30 | 28 | 27 | 24 | 26 | 21 | 27 | 26 | 22 | 28 | 28 | 29 | Y | 75885 | Annexin A6 OS=Mus musculus OX=10090 GN=Anxa6 PE=1 SV=3 |
| 90 | 62 | sp\|Q8C196\|CPSM\_MOUSE | 368.17 | 31 | 18 | 17 | 17 | 16 | 14 | 23 | 21 | 16 | 12 | 13 | 1.45E8 | 1.81E8 | 2.15E8 | 1.08E8 | 1.35E8 | 2.48E8 | 1.44E8 | 1.01E8 | 1.03E8 | 1.29E8 | 46 | 44 | 27 | 31 | 33 | 25 | 26 | 36 | 29 | 26 | 21 | 20 | Y | 164617 | Carbamoyl-phosphate synthase [ammonia], mitochondrial OS=Mus musculus OX=10090 GN=Cps1 PE=1 SV=2 |
| 39 | 37 | sp\|P20029\|BIP\_MOUSE | 365.33 | 53 | 42 | 42 | 46 | 43 | 42 | 42 | 48 | 42 | 42 | 39 | 5.86E8 | 5.24E8 | 6.32E8 | 2.66E8 | 4.55E8 | 3.18E8 | 4.07E8 | 3.04E8 | 7.61E8 | 4.34E8 | 33 | 29 | 55 | 52 | 58 | 46 | 47 | 41 | 51 | 55 | 58 | 48 | Y | 72422 | Endoplasmic reticulum chaperone BiP OS=Mus musculus OX=10090 GN=Hspa5 PE=1 SV=3 |
| 79 | 24 | sp\|P52480\|KPYM\_MOUSE | 363.15 | 58 | 39 | 39 | 39 | 43 | 41 | 37 | 39 | 37 | 44 | 45 | 4.29E8 | 2.44E8 | 3.25E8 | 1.15E8 | 2.17E8 | 1.91E8 | 1.43E8 | 1.58E8 | 5.7E8 | 4.81E8 | 29 | 27 | 29 | 28 | 27 | 28 | 30 | 27 | 25 | 27 | 43 | 36 | Y | 57845 | Pyruvate kinase PKM OS=Mus musculus OX=10090 GN=Pkm PE=1 SV=4 |
| 76 | 116 | sp\|Q04447\|KCRB\_MOUSE | 361.96 | 69 | 51 | 54 | 52 | 45 | 66 | 66 | 61 | 52 | 53 | 38 | 1.98E8 | 3.09E8 | 3.54E8 | 9.04E7 | 2.89E8 | 4.99E8 | 1.98E8 | 1.9E8 | 4.96E8 | 1.85E8 | 21 | 20 | 30 | 33 | 30 | 25 | 34 | 41 | 26 | 31 | 32 | 22 | Y | 42713 | Creatine kinase B-type OS=Mus musculus OX=10090 GN=Ckb PE=1 SV=1 |
| 42 | 96 | sp\|P09103\|PDIA1\_MOUSE | 358.52 | 58 | 47 | 50 | 44 | 51 | 46 | 52 | 48 | 42 | 44 | 47 | 5.74E8 | 7.95E8 | 9.06E8 | 4.08E8 | 5.72E8 | 6E8 | 5.19E8 | 4.39E8 | 6.66E8 | 5.58E8 | 31 | 29 | 40 | 51 | 52 | 55 | 47 | 47 | 50 | 53 | 50 | 42 | Y | 57059 | Protein disulfide-isomerase OS=Mus musculus OX=10090 GN=P4hb PE=1 SV=2 |
| 26 | 11 | sp\|P20152\|VIME\_MOUSE | 358.39 | 73 | 64 | 49 | 57 | 53 | 61 | 55 | 57 | 57 | 61 | 62 | 1.27E9 | 5.33E8 | 5.09E8 | 1.86E8 | 4.04E8 | 5.01E8 | 5.04E8 | 3.15E8 | 1.41E9 | 1.12E9 | 35 | 26 | 86 | 54 | 51 | 45 | 59 | 49 | 47 | 60 | 82 | 76 | Y | 53688 | Vimentin OS=Mus musculus OX=10090 GN=Vim PE=1 SV=3 |
| 67 | 50 | sp\|P08113\|ENPL\_MOUSE | 357.91 | 42 | 22 | 34 | 32 | 26 | 33 | 33 | 36 | 28 | 26 | 25 | 3.38E8 | 4.89E8 | 4.81E8 | 2.26E8 | 4.21E8 | 4.09E8 | 4.3E8 | 3.02E8 | 3.86E8 | 3.7E8 | 35 | 32 | 27 | 35 | 33 | 30 | 36 | 33 | 38 | 31 | 30 | 24 | Y | 92476 | Endoplasmin OS=Mus musculus OX=10090 GN=Hsp90b1 PE=1 SV=2 |
| 81 | 26 | sp\|Q9CZS1\|AL1B1\_MOUSE | 355.39 | 56 | 50 | 51 | 42 | 42 | 47 | 45 | 42 | 46 | 39 | 31 | 3.09E8 | 4.43E8 | 3.52E8 | 1.81E8 | 2.52E8 | 3.53E8 | 2.09E8 | 1.76E8 | 4.51E8 | 1.96E8 | 26 | 22 | 32 | 36 | 31 | 27 | 28 | 27 | 25 | 25 | 33 | 24 | Y | 57553 | Aldehyde dehydrogenase X, mitochondrial OS=Mus musculus OX=10090 GN=Aldh1b1 PE=1 SV=1 |
| 77 | 30 | sp\|Q921I1\|TRFE\_MOUSE | 351.92 | 58 | 43 | 30 | 40 | 32 | 36 | 46 | 43 | 45 | 39 | 44 | 2.98E8 | 1.3E8 | 2.12E8 | 8.38E7 | 1.14E8 | 3.09E8 | 1.47E8 | 1.74E8 | 2.27E8 | 3.12E8 | 35 | 25 | 41 | 22 | 31 | 21 | 24 | 37 | 33 | 34 | 31 | 44 | Y | 76724 | Serotransferrin OS=Mus musculus OX=10090 GN=Tf PE=1 SV=1 |
| 61 | 34 | sp\|P58252\|EF2\_MOUSE | 351.52 | 47 | 33 | 32 | 31 | 28 | 29 | 36 | 32 | 29 | 38 | 27 | 3.47E8 | 3.13E8 | 4.11E8 | 2.12E8 | 3.09E8 | 3.14E8 | 2.61E8 | 2.04E8 | 4.73E8 | 3.36E8 | 34 | 33 | 41 | 33 | 35 | 30 | 35 | 36 | 35 | 31 | 45 | 36 | Y | 95314 | Elongation factor 2 OS=Mus musculus OX=10090 GN=Eef2 PE=1 SV=2 |
| 74 | 92 | sp\|P08249\|MDHM\_MOUSE | 350.43 | 66 | 56 | 50 | 60 | 62 | 61 | 62 | 61 | 61 | 58 | 52 | 5.99E8 | 7.46E8 | 5.81E8 | 3.47E8 | 4.17E8 | 5.85E8 | 4.18E8 | 3.87E8 | 1.07E9 | 3.95E8 | 21 | 21 | 32 | 35 | 32 | 32 | 32 | 29 | 31 | 37 | 39 | 26 | Y | 35611 | Malate dehydrogenase, mitochondrial OS=Mus musculus OX=10090 GN=Mdh2 PE=1 SV=3 |
| 15 | 508 | A8DUK4\|A8DUK4\_MOUSE | 347.93 | 95 | 95 | 95 | 95 | 95 | 95 | 95 | 95 | 95 | 79 | 84 | 9.84E8 | 1.9E9 | 2.37E9 | 5.36E8 | 1.36E9 | 1.47E9 | 8.42E8 | 1.59E9 | 8.88E8 | 6.17E8 | 14 | 5 | 91 | 126 | 153 | 75 | 108 | 95 | 70 | 151 | 64 | 63 | Y | 15748 | Beta-globin OS=Mus musculus OX=10090 GN=Hbb-bs PE=1 SV=1 |
| 126 | 85 | sp\|Q8VHX6\|FLNC\_MOUSE | 347.25 | 16 | 10 | 8 | 5 | 6 | 6 | 6 | 6 | 6 | 10 | 7 | 5.12E7 | 2.64E7 | 1.98E7 | 1.18E7 | 2.06E7 | 3.73E7 | 2.68E7 | 1.13E7 | 6.38E7 | 3.32E7 | 35 | 25 | 25 | 19 | 15 | 18 | 16 | 17 | 19 | 18 | 26 | 20 | Y | 291117 | Filamin-C OS=Mus musculus OX=10090 GN=Flnc PE=1 SV=3 |
| 117 | 48 | sp\|Q01853\|TERA\_MOUSE | 346.65 | 45 | 26 | 26 | 23 | 33 | 35 | 31 | 29 | 24 | 32 | 20 | 1.07E8 | 1.31E8 | 1.4E8 | 8.58E7 | 1.32E8 | 1.29E8 | 7.81E7 | 5.06E7 | 2.23E8 | 9.88E7 | 33 | 33 | 21 | 20 | 21 | 25 | 27 | 26 | 20 | 16 | 31 | 17 | Y | 89322 | Transitional endoplasmic reticulum ATPase OS=Mus musculus OX=10090 GN=Vcp PE=1 SV=4 |
| 75 | 8 | sp\|P17182\|ENOA\_MOUSE | 346.27 | 72 | 44 | 34 | 49 | 38 | 42 | 42 | 54 | 48 | 66 | 47 | 4.67E8 | 3.28E8 | 3.91E8 | 1.41E8 | 2.22E8 | 3.13E8 | 2.14E8 | 1.84E8 | 6.5E8 | 4.98E8 | 25 | 18 | 35 | 22 | 32 | 25 | 33 | 26 | 32 | 36 | 43 | 36 | Y | 47141 | Alpha-enolase OS=Mus musculus OX=10090 GN=Eno1 PE=1 SV=3 |
| 63 | 43 | sp\|Q62468\|VILI\_MOUSE | 346.08 | 48 | 32 | 32 | 32 | 31 | 32 | 37 | 39 | 26 | 35 | 27 | 4.25E8 | 6.39E8 | 4.79E8 | 1.91E8 | 3.68E8 | 5.41E8 | 3.98E8 | 1.9E8 | 4.81E8 | 3.66E8 | 37 | 36 | 30 | 40 | 37 | 32 | 33 | 40 | 41 | 26 | 43 | 31 | Y | 92775 | Villin-1 OS=Mus musculus OX=10090 GN=Vil1 PE=1 SV=3 |
| 127 | 90 | B1B0C7\|B1B0C7\_MOUSE | 345.89 | 10 | 6 | 6 | 4 | 3 | 4 | 6 | 3 | 5 | 5 | 5 | 1.05E8 | 1.01E8 | 7.4E7 | 2.37E7 | 4.1E7 | 1.13E8 | 2.97E7 | 5.5E7 | 7.45E7 | 6.91E7 | 37 | 37 | 28 | 25 | 18 | 14 | 16 | 26 | 14 | 22 | 24 | 24 | Y | 469027 | Perlecan (heparan sulfate proteoglycan 2) OS=Mus musculus OX=10090 GN=Hspg2 PE=1 SV=1 |
| 127 | 91 | E9PZ16\|E9PZ16\_MOUSE | 345.89 | 10 | 6 | 6 | 4 | 3 | 4 | 6 | 3 | 5 | 5 | 5 | 1.05E8 | 1.01E8 | 7.4E7 | 2.37E7 | 4.1E7 | 1.13E8 | 2.97E7 | 5.5E7 | 7.45E7 | 6.91E7 | 37 | 37 | 28 | 25 | 18 | 14 | 16 | 26 | 14 | 22 | 24 | 24 | Y | 469789 | Perlecan (heparan sulfate proteoglycan 2) OS=Mus musculus OX=10090 GN=Hspg2 PE=1 SV=1 |
| 167 | 44 | sp\|Q8BMS1\|ECHA\_MOUSE | 345.47 | 44 | 25 | 26 | 21 | 20 | 24 | 34 | 19 | 18 | 33 | 25 | 7.53E7 | 4.95E7 | 6.79E7 | 1.66E7 | 5.61E7 | 8.54E7 | 4.31E7 | 2.69E7 | 1.3E8 | 5.71E7 | 25 | 25 | 14 | 15 | 15 | 13 | 17 | 20 | 12 | 11 | 22 | 16 | Y | 82670 | Trifunctional enzyme subunit alpha, mitochondrial OS=Mus musculus OX=10090 GN=Hadha PE=1 SV=1 |
| 59 | 100 | sp\|P68369\|TBA1A\_MOUSE | 344.12 | 55 | 41 | 39 | 36 | 45 | 48 | 37 | 45 | 30 | 44 | 48 |  |  |  |  |  |  |  |  | 4.81E6 | 7.09E5 | 20 | 1 | 45 | 39 | 32 | 37 | 34 | 32 | 33 | 33 | 49 | 43 | Y | 50136 | Tubulin alpha-1A chain OS=Mus musculus OX=10090 GN=Tuba1a PE=1 SV=1 |
| 254 | 222 | A2ACG7\|A2ACG7\_MOUSE | 343.06 | 33 | 22 | 30 | 15 | 27 | 21 | 24 | 17 | 22 | 26 | 16 | 3.74E7 | 5.3E7 | 2.14E7 | 2.23E7 | 2.35E7 | 5.32E7 | 3.18E7 | 2.29E7 | 6.76E7 | 4.08E7 | 16 | 16 | 9 | 13 | 7 | 13 | 10 | 14 | 10 | 11 | 12 | 7 | Y | 67502 | Dolichyl-diphosphooligosaccharide--protein glycosyltransferase subunit 2 OS=Mus musculus OX=10090 GN=Rpn2 PE=1 SV=1 |
| 62 | 46 | sp\|P07901\|HS90A\_MOUSE | 342.90 | 41 | 30 | 32 | 35 | 34 | 30 | 32 | 29 | 29 | 27 | 33 | 8.16E7 | 1.17E8 | 1.45E8 | 4.91E7 | 1E8 | 1.19E8 | 7.61E7 | 7.49E7 | 1.33E8 | 9.49E7 | 31 | 17 | 31 | 42 | 45 | 38 | 36 | 32 | 31 | 36 | 34 | 37 | Y | 84788 | Heat shock protein HSP 90-alpha OS=Mus musculus OX=10090 GN=Hsp90aa1 PE=1 SV=4 |
| 125 | 58 | sp\|Q99KI0\|ACON\_MOUSE | 342.83 | 38 | 28 | 30 | 28 | 26 | 31 | 33 | 29 | 29 | 26 | 29 | 1.5E8 | 2.39E8 | 1.88E8 | 6.57E7 | 1.52E8 | 2.39E8 | 1.1E8 | 1.19E8 | 1.52E8 | 9.02E7 | 26 | 26 | 20 | 25 | 20 | 17 | 23 | 26 | 20 | 22 | 21 | 19 | Y | 85464 | Aconitate hydratase, mitochondrial OS=Mus musculus OX=10090 GN=Aco2 PE=1 SV=1 |
| 94 | 64 | sp\|P97449\|AMPN\_MOUSE | 339.88 | 33 | 19 | 28 | 28 | 21 | 23 | 23 | 28 | 22 | 19 | 19 | 1.23E8 | 3.52E8 | 3.06E8 | 1.43E8 | 2.13E8 | 3.26E8 | 2.22E8 | 1.73E8 | 1.99E8 | 9.85E7 | 29 | 29 | 16 | 40 | 28 | 25 | 26 | 34 | 31 | 25 | 20 | 18 | Y | 109651 | Aminopeptidase N OS=Mus musculus OX=10090 GN=Anpep PE=1 SV=4 |
| 91 | 93 | sp\|P07356\|ANXA2\_MOUSE | 337.64 | 65 | 45 | 53 | 46 | 45 | 52 | 60 | 48 | 47 | 56 | 52 | 3.38E8 | 4.01E8 | 3.08E8 | 1.42E8 | 3.01E8 | 3.64E8 | 2.4E8 | 1.96E8 | 6.05E8 | 3.12E8 | 25 | 25 | 21 | 30 | 21 | 21 | 29 | 30 | 24 | 23 | 46 | 29 | Y | 38676 | Annexin A2 OS=Mus musculus OX=10090 GN=Anxa2 PE=1 SV=2 |
| 115 | 171 | sp\|Q9R100\|CAD17\_MOUSE | 336.16 | 25 | 20 | 23 | 20 | 25 | 25 | 23 | 23 | 20 | 21 | 18 | 1.58E8 | 2.74E8 | 2.33E8 | 1.41E8 | 1.94E8 | 1.94E8 | 1.68E8 | 9.29E7 | 2.64E8 | 1.3E8 | 17 | 17 | 21 | 26 | 24 | 22 | 26 | 22 | 24 | 20 | 22 | 22 | N | 91645 | Cadherin-17 OS=Mus musculus OX=10090 GN=Cdh17 PE=1 SV=1 |
| 36 | 135 | sp\|Q61879\|MYH10\_MOUSE | 335.54 | 15 | 10 | 8 | 11 | 9 | 10 | 10 | 10 | 10 | 10 | 8 | 1.34E7 | 2.17E7 | 1.95E7 | 8.42E6 | 2.03E7 | 2E7 | 1.01E7 | 1.27E7 | 3.6E6 |  | 30 | 4 | 50 | 52 | 54 | 44 | 49 | 49 | 45 | 61 | 51 | 44 | Y | 228994 | Myosin-10 OS=Mus musculus OX=10090 GN=Myh10 PE=1 SV=2 |
| 36 | 144 | Q5SV64\|Q5SV64\_MOUSE | 335.54 | 14 | 10 | 8 | 11 | 9 | 9 | 10 | 10 | 10 | 10 | 8 | 1.34E7 | 2.17E7 | 1.95E7 | 8.42E6 | 2.03E7 | 2E7 | 1.01E7 | 1.27E7 | 3.6E6 |  | 30 | 4 | 50 | 52 | 54 | 44 | 49 | 49 | 45 | 61 | 51 | 44 | Y | 232470 | Myosin, heavy polypeptide 10, non-muscle OS=Mus musculus OX=10090 GN=Myh10 PE=1 SV=1 |
| 36 | 145 | Q3UH59\|Q3UH59\_MOUSE | 335.54 | 14 | 10 | 8 | 11 | 9 | 9 | 10 | 10 | 10 | 10 | 8 | 1.34E7 | 2.17E7 | 1.95E7 | 8.42E6 | 2.03E7 | 2E7 | 1.01E7 | 1.27E7 | 3.6E6 |  | 30 | 4 | 50 | 52 | 54 | 44 | 49 | 49 | 45 | 61 | 51 | 44 | Y | 233447 | Myosin, heavy polypeptide 10, non-muscle OS=Mus musculus OX=10090 GN=Myh10 PE=1 SV=1 |
| 168 | 104 | sp\|P21981\|TGM2\_MOUSE | 335.38 | 34 | 23 | 16 | 16 | 17 | 23 | 19 | 20 | 19 | 29 | 27 | 1.51E8 | 7.64E7 | 7.57E7 | 3.36E7 | 8.38E7 | 8.16E7 | 6.79E7 | 5.29E7 | 3.43E8 | 1.93E8 | 22 | 21 | 18 | 13 | 11 | 10 | 16 | 12 | 13 | 12 | 25 | 22 | Y | 77061 | Protein-glutamine gamma-glutamyltransferase 2 OS=Mus musculus OX=10090 GN=Tgm2 PE=1 SV=4 |
| 146 | 109 | sp\|Q62261\|SPTB2\_MOUSE | 335.28 | 17 | 8 | 8 | 6 | 6 | 8 | 11 | 9 | 7 | 6 | 5 | 3.4E7 | 5.34E7 | 2.78E7 | 2.08E7 | 6.36E7 | 5.59E7 | 3.64E7 | 1.62E7 | 5.1E7 | 1.51E7 | 37 | 35 | 17 | 17 | 12 | 15 | 20 | 24 | 21 | 15 | 15 | 11 | Y | 274221 | Spectrin beta chain, non-erythrocytic 1 OS=Mus musculus OX=10090 GN=Sptbn1 PE=1 SV=2 |
| 98 | 228 | sp\|P51881\|ADT2\_MOUSE | 332.27 | 57 | 38 | 52 | 43 | 44 | 49 | 47 | 46 | 46 | 48 | 45 | 7.87E7 | 1.43E8 | 1.04E8 | 5.17E7 | 7.59E7 | 9.62E7 | 6.13E7 | 5.69E7 | 2.29E8 | 1.11E8 | 20 | 8 | 22 | 31 | 28 | 21 | 27 | 22 | 20 | 22 | 29 | 25 | Y | 32931 | ADP/ATP translocase 2 OS=Mus musculus OX=10090 GN=Slc25a5 PE=1 SV=3 |
| 124 | 87 | E9Q9C6\|E9Q9C6\_MOUSE | 330.68 | 13 | 7 | 7 | 6 | 4 | 5 | 8 | 8 | 5 | 10 | 7 | 1.93E8 | 1.77E8 | 1.39E8 | 3.88E7 | 7.53E7 | 1.57E8 | 1.1E8 | 5.51E7 | 3.35E8 | 2.33E8 | 28 | 27 | 25 | 22 | 18 | 14 | 17 | 24 | 22 | 16 | 31 | 25 | Y | 275239 | Fc fragment of IgG binding protein OS=Mus musculus OX=10090 GN=Fcgbp PE=1 SV=1 |
| 87 | 97 | sp\|P68368\|TBA4A\_MOUSE | 329.41 | 55 | 50 | 43 | 40 | 45 | 48 | 40 | 45 | 34 | 47 | 49 | 7.11E6 | 5.06E6 | 9.89E6 | 4.36E6 | 8.61E6 | 1.29E7 | 5.84E6 | 6.56E6 | 1.18E7 | 6.07E6 | 20 | 4 | 38 | 31 | 22 | 26 | 26 | 27 | 27 | 21 | 37 | 34 | Y | 49924 | Tubulin alpha-4A chain OS=Mus musculus OX=10090 GN=Tuba4a PE=1 SV=1 |
| 87 | 103 | A0A0A0MQA5\|A0A0A0MQA5\_MOUSE | 329.41 | 52 | 47 | 40 | 37 | 42 | 45 | 38 | 42 | 32 | 44 | 46 | 7.11E6 | 5.06E6 | 9.89E6 | 4.36E6 | 8.61E6 | 1.29E7 | 5.84E6 | 6.56E6 | 1.18E7 | 6.07E6 | 20 | 4 | 38 | 31 | 22 | 26 | 26 | 27 | 27 | 21 | 37 | 34 | Y | 52912 | Tubulin alpha chain (Fragment) OS=Mus musculus OX=10090 GN=Tuba4a PE=1 SV=1 |
| 181 | 65 | sp\|Q70IV5\|SYNEM\_MOUSE | 329.27 | 29 | 13 | 9 | 10 | 10 | 11 | 19 | 10 | 11 | 9 | 5 | 1.07E8 | 3.61E7 | 4.85E7 | 2.27E7 | 5.26E7 | 1.17E8 | 2.84E7 | 3.08E7 | 6.01E7 | 2.16E7 | 39 | 39 | 16 | 14 | 14 | 14 | 16 | 25 | 12 | 14 | 13 | 8 | Y | 173208 | Synemin OS=Mus musculus OX=10090 GN=Synm PE=1 SV=2 |
| 111 | 111 | sp\|P97429\|ANXA4\_MOUSE | 328.95 | 60 | 51 | 56 | 55 | 55 | 54 | 51 | 60 | 51 | 52 | 47 | 2.09E8 | 3.85E8 | 4.21E8 | 1.57E8 | 2.2E8 | 2.84E8 | 2.64E8 | 1.67E8 | 3.71E8 | 1.93E8 | 19 | 17 | 20 | 28 | 29 | 23 | 23 | 21 | 30 | 21 | 26 | 16 | Y | 35916 | Annexin A4 OS=Mus musculus OX=10090 GN=Anxa4 PE=1 SV=4 |
| 80 | 294 | sp\|P16858\|G3P\_MOUSE | 327.82 | 54 | 54 | 52 | 43 | 42 | 44 | 42 | 41 | 50 | 53 | 53 | 7.61E6 | 5.24E6 |  |  | 1.5E6 |  |  | 1.69E5 | 2.67E7 | 1.04E7 | 14 | 1 | 42 | 32 | 27 | 16 | 23 | 26 | 20 | 29 | 46 | 39 | Y | 35810 | Glyceraldehyde-3-phosphate dehydrogenase OS=Mus musculus OX=10090 GN=Gapdh PE=1 SV=2 |
| 80 | 295 | A0A0A0MQF6\|A0A0A0MQF6\_MOUSE | 327.82 | 50 | 50 | 48 | 40 | 39 | 40 | 39 | 38 | 46 | 49 | 49 | 7.61E6 | 5.24E6 |  |  | 1.5E6 |  |  | 1.69E5 | 2.67E7 | 1.04E7 | 14 | 1 | 42 | 32 | 27 | 16 | 23 | 26 | 20 | 29 | 46 | 39 | Y | 38653 | Glyceraldehyde-3-phosphate dehydrogenase OS=Mus musculus OX=10090 GN=Gapdh PE=1 SV=1 |
| 121 | 196 | sp\|P29758\|OAT\_MOUSE | 326.11 | 46 | 22 | 32 | 41 | 34 | 43 | 35 | 30 | 34 | 30 | 34 | 9.67E7 | 2.81E8 | 3.31E8 | 9.63E7 | 1.49E8 | 2.49E8 | 1.16E8 | 1.25E8 | 1.84E8 | 1.23E8 | 18 | 17 | 14 | 25 | 26 | 21 | 24 | 22 | 20 | 22 | 18 | 20 | Y | 48355 | Ornithine aminotransferase, mitochondrial OS=Mus musculus OX=10090 GN=Oat PE=1 SV=1 |
| 141 | 39 | sp\|P48678\|LMNA\_MOUSE | 325.67 | 46 | 26 | 33 | 28 | 30 | 29 | 32 | 33 | 24 | 29 | 23 | 1.9E8 | 2.61E8 | 1.94E8 | 8.82E7 | 1.28E8 | 1.81E8 | 1.86E8 | 7.5E7 | 2.38E8 | 1.38E8 | 32 | 31 | 16 | 22 | 17 | 19 | 18 | 21 | 20 | 15 | 23 | 15 | Y | 74238 | Prelamin-A/C OS=Mus musculus OX=10090 GN=Lmna PE=1 SV=2 |
| 261 | 134 | sp\|Q64GA5\|PA24C\_MOUSE | 325.46 | 38 | 34 | 0 | 0 | 0 | 0 | 0 | 0 | 2 | 27 | 27 | 5.55E8 |  |  |  |  |  |  | 5.37E5 | 4.81E8 | 1.86E8 | 22 | 22 | 38 | 0 | 0 | 0 | 0 | 0 | 0 | 1 | 34 | 22 | Y | 67882 | Cytosolic phospholipase A2 gamma OS=Mus musculus OX=10090 GN=Pla2g4c PE=1 SV=1 |
| 138 | 156 | sp\|P09411\|PGK1\_MOUSE | 324.48 | 53 | 45 | 35 | 35 | 35 | 29 | 46 | 28 | 37 | 36 | 41 | 1.51E8 | 9.38E7 | 1.25E8 | 7.34E7 | 9.7E7 | 8.97E7 | 8.46E7 | 6.4E7 | 3.27E8 | 1.56E8 | 20 | 14 | 23 | 15 | 18 | 17 | 18 | 20 | 14 | 20 | 26 | 24 | Y | 44550 | Phosphoglycerate kinase 1 OS=Mus musculus OX=10090 GN=Pgk1 PE=1 SV=4 |
| 272 | 54 | sp\|P49290\|PERE\_MOUSE | 324.38 | 40 | 25 | 2 | 3 | 4 | 0 | 3 | 0 | 4 | 37 | 27 | 1.5E8 | 4.89E6 | 1.93E6 | 2.17E6 |  | 5.02E6 |  | 3.34E6 | 4.11E8 | 3.11E8 | 27 | 26 | 22 | 1 | 2 | 2 | 0 | 2 | 0 | 2 | 37 | 28 | Y | 81380 | Eosinophil peroxidase OS=Mus musculus OX=10090 GN=Epx PE=1 SV=2 |
| 129 | 269 | sp\|P48962\|ADT1\_MOUSE | 322.60 | 52 | 39 | 42 | 45 | 39 | 47 | 43 | 45 | 42 | 34 | 40 | 2.58E7 | 4.29E7 | 6.03E7 | 2.97E7 | 4E7 | 5.64E7 | 3.64E7 | 3.64E7 | 1.42E7 | 1.91E7 | 17 | 5 | 18 | 22 | 26 | 17 | 23 | 19 | 17 | 20 | 18 | 19 | Y | 32904 | ADP/ATP translocase 1 OS=Mus musculus OX=10090 GN=Slc25a4 PE=1 SV=4 |
| 270 | 383 | sp\|Q00896\|A1AT3\_MOUSE | 319.25 | 37 | 17 | 15 | 29 | 34 | 29 | 33 | 34 | 26 | 9 | 13 | 3.55E6 | 1.57E6 | 4.07E6 | 1.99E6 | 1.92E6 | 4.01E6 | 4.64E6 | 3.35E6 |  |  | 13 | 3 | 8 | 7 | 12 | 16 | 11 | 10 | 12 | 11 | 3 | 5 | Y | 45823 | Alpha-1-antitrypsin 1-3 OS=Mus musculus OX=10090 GN=Serpina1c PE=1 SV=2 |
| 131 | 49 | sp\|Q3V0K9\|PLSI\_MOUSE | 318.86 | 45 | 22 | 37 | 38 | 25 | 34 | 40 | 37 | 28 | 33 | 26 | 4.38E7 | 1.8E8 | 1.03E8 | 4.73E7 | 1.13E8 | 1.54E8 | 1.01E8 | 3.85E7 | 1.01E8 | 4.75E7 | 24 | 21 | 14 | 25 | 24 | 17 | 22 | 25 | 23 | 18 | 22 | 14 | Y | 70408 | Plastin-1 OS=Mus musculus OX=10090 GN=Pls1 PE=1 SV=1 |
| 96 | 52 | E9QA15\|E9QA15\_MOUSE | 316.85 | 32 | 29 | 21 | 23 | 23 | 24 | 25 | 23 | 24 | 24 | 27 | 3.74E8 | 2.33E8 | 2.46E8 | 1.19E8 | 1.56E8 | 2.96E8 | 1.25E8 | 1.4E8 | 3.77E8 | 4.04E8 | 35 | 32 | 32 | 20 | 25 | 25 | 20 | 25 | 18 | 20 | 33 | 32 | Y | 89274 | Caldesmon 1 OS=Mus musculus OX=10090 GN=Cald1 PE=1 SV=1 |
| 85 | 143 | sp\|P17156\|HSP72\_MOUSE | 314.52 | 31 | 16 | 13 | 20 | 18 | 19 | 25 | 21 | 19 | 13 | 19 | 7.59E5 |  | 2.85E6 |  | 9.67E5 | 1.28E7 | 2.64E6 | 1.79E6 | 4.64E6 | 2.14E6 | 22 | 6 | 29 | 27 | 33 | 22 | 30 | 31 | 24 | 35 | 32 | 30 | Y | 69642 | Heat shock-related 70 kDa protein 2 OS=Mus musculus OX=10090 GN=Hspa2 PE=1 SV=2 |
| 108 | 338 | sp\|Q9ERD7\|TBB3\_MOUSE | 313.14 | 36 | 28 | 23 | 26 | 26 | 26 | 26 | 26 | 27 | 31 | 26 |  |  |  |  |  |  |  | 1.83E6 | 2.21E7 |  | 13 | 1 | 26 | 24 | 23 | 18 | 24 | 23 | 19 | 25 | 36 | 22 | Y | 50419 | Tubulin beta-3 chain OS=Mus musculus OX=10090 GN=Tubb3 PE=1 SV=1 |
| 12 | 422 | sp\|Q8BFZ3\|ACTBL\_MOUSE | 313.11 | 34 | 23 | 30 | 26 | 29 | 28 | 30 | 31 | 26 | 24 | 24 |  | 2.08E6 |  | 5.77E6 | 2.57E6 | 2.83E6 | 3.17E4 |  |  |  | 16 | 2 | 175 | 139 | 132 | 120 | 138 | 119 | 104 | 149 | 167 | 144 | Y | 42004 | Beta-actin-like protein 2 OS=Mus musculus OX=10090 GN=Actbl2 PE=1 SV=1 |
| 292 | 417 | sp\|P22599\|A1AT2\_MOUSE | 312.52 | 39 | 13 | 15 | 33 | 34 | 29 | 35 | 32 | 28 | 19 | 13 | 3.25E5 | 0E0 | 3.23E6 | 7.67E5 | 5.07E6 | 4.58E6 | 1.56E6 | 2.11E6 | 4.02E6 | 7.11E5 | 12 | 3 | 5 | 6 | 10 | 13 | 10 | 11 | 10 | 11 | 6 | 4 | Y | 45975 | Alpha-1-antitrypsin 1-2 OS=Mus musculus OX=10090 GN=Serpina1b PE=1 SV=2 |
| 166 | 55 | sp\|O88329\|MYO1A\_MOUSE | 310.24 | 32 | 10 | 19 | 15 | 14 | 13 | 25 | 14 | 10 | 17 | 16 | 3.86E7 | 6.67E7 | 5.73E7 | 1.96E7 | 4.23E7 | 8.23E7 | 4.54E7 | 2.09E7 | 1.13E8 | 4.89E7 | 27 | 22 | 12 | 18 | 14 | 12 | 13 | 23 | 14 | 11 | 21 | 15 | Y | 118695 | Unconventional myosin-Ia OS=Mus musculus OX=10090 GN=Myo1a PE=2 SV=2 |
| 247 | 626 | sp\|P14206\|RSSA\_MOUSE | 308.65 | 44 | 39 | 34 | 29 | 44 | 35 | 39 | 38 | 35 | 40 | 36 | 1.35E8 | 1.3E8 | 1.13E8 | 6.1E7 | 1E8 | 1.01E8 | 6.68E7 | 7.18E7 | 1.44E8 | 1.09E8 | 10 | 10 | 10 | 10 | 9 | 14 | 9 | 12 | 11 | 10 | 13 | 11 | Y | 32838 | Small ribosomal subunit protein uS2 OS=Mus musculus OX=10090 GN=Rpsa PE=1 SV=4 |
| 163 | 185 | sp\|Q61598\|GDIB\_MOUSE | 307.13 | 47 | 33 | 39 | 30 | 28 | 31 | 33 | 28 | 36 | 34 | 33 | 7.95E7 | 9.56E7 | 1.39E8 | 4.21E7 | 8.1E7 | 1.47E8 | 5.82E7 | 7.34E7 | 2.07E8 | 1.3E8 | 17 | 17 | 18 | 15 | 16 | 13 | 14 | 18 | 13 | 17 | 19 | 16 | Y | 50537 | Rab GDP dissociation inhibitor beta OS=Mus musculus OX=10090 GN=Gdi2 PE=1 SV=1 |
| 264 | 213 | A0A7N9VRC4\|A0A7N9VRC4\_MOUSE | 306.63 | 6 | 4 | 5 | 3 | 3 | 4 | 5 | 3 | 3 | 4 | 3 | 3.92E7 | 5.11E7 | 1.56E7 | 1.69E7 | 4.35E7 | 4.32E7 | 2.14E7 | 1.51E7 | 4.29E7 | 3.41E7 | 19 | 15 | 10 | 14 | 7 | 8 | 9 | 12 | 9 | 7 | 9 | 8 | Y | 381693 | Epiplakin 1 OS=Mus musculus OX=10090 GN=Eppk1 PE=1 SV=1 |
| 264 | 254 | sp\|Q8R0W0\|EPIPL\_MOUSE | 306.63 | 3 | 2 | 3 | 2 | 2 | 2 | 2 | 2 | 2 | 2 | 2 | 3.92E7 | 5.11E7 | 1.56E7 | 1.69E7 | 4.35E7 | 4.32E7 | 2.14E7 | 1.51E7 | 4.29E7 | 3.41E7 | 19 | 15 | 10 | 14 | 7 | 8 | 9 | 12 | 9 | 7 | 9 | 8 | Y | 724747 | Epiplakin OS=Mus musculus OX=10090 GN=Eppk1 PE=1 SV=2 |
| 54 | 246 | sp\|P63101\|1433Z\_MOUSE | 305.87 | 62 | 53 | 53 | 56 | 49 | 57 | 56 | 57 | 56 | 48 | 53 | 3.54E8 | 4.58E8 | 4.02E8 | 1.87E8 | 3.71E8 | 4.4E8 | 2.15E8 | 1.92E8 | 6.96E8 | 4.3E8 | 16 | 10 | 45 | 44 | 39 | 29 | 37 | 33 | 29 | 36 | 60 | 43 | Y | 27771 | 14-3-3 protein zeta/delta OS=Mus musculus OX=10090 GN=Ywhaz PE=1 SV=1 |
| 171 | 356 | sp\|P17751\|TPIS\_MOUSE | 304.74 | 61 | 52 | 43 | 45 | 54 | 61 | 44 | 57 | 61 | 49 | 54 | 2.48E8 | 2.31E8 | 2.36E8 | 1.32E8 | 1.71E8 | 2.35E8 | 1.56E8 | 1.03E8 | 3.6E8 | 1.77E8 | 12 | 12 | 14 | 13 | 14 | 18 | 17 | 15 | 18 | 18 | 18 | 14 | Y | 26713 | Triosephosphate isomerase OS=Mus musculus OX=10090 GN=Tpi1 PE=1 SV=5 |
| 50 | 202 | A2AIM4\|A2AIM4\_MOUSE | 304.38 | 55 | 43 | 37 | 45 | 45 | 41 | 47 | 49 | 43 | 39 | 38 | 2.96E8 | 2.99E8 | 2.16E8 | 9.11E7 | 2.15E8 | 3.45E8 | 1.18E8 | 1.82E8 | 2.72E8 | 2.19E8 | 23 | 11 | 44 | 36 | 40 | 34 | 43 | 44 | 38 | 47 | 41 | 35 | Y | 32994 | Tropomyosin beta chain OS=Mus musculus OX=10090 GN=Tpm2 PE=1 SV=1 |
| 337 | 187 | sp\|Q9CWJ9\|PUR9\_MOUSE | 303.51 | 34 | 14 | 20 | 18 | 17 | 16 | 25 | 18 | 24 | 20 | 15 | 1.82E7 | 1.34E7 | 3.7E7 | 9.9E6 | 1.24E7 | 1.82E7 | 7.2E6 | 1.26E7 | 2.81E7 | 7.73E6 | 17 | 17 | 6 | 9 | 8 | 7 | 6 | 10 | 8 | 9 | 8 | 7 | Y | 64217 | Bifunctional purine biosynthesis protein ATIC OS=Mus musculus OX=10090 GN=Atic PE=1 SV=2 |
| 21 | 625 | sp\|P02088\|HBB1\_MOUSE | 303.17 | 89 | 80 | 80 | 89 | 71 | 89 | 80 | 89 | 89 | 54 | 69 | 5.16E7 | 1.02E8 | 1.32E8 | 3.66E7 | 7.58E7 | 8.22E7 | 2.88E7 | 8.19E7 |  | 3.5E7 | 11 | 2 | 72 | 94 | 118 | 61 | 79 | 74 | 55 | 117 | 52 | 51 | Y | 15840 | Hemoglobin subunit beta-1 OS=Mus musculus OX=10090 GN=Hbb-b1 PE=1 SV=2 |
| 158 | 147 | sp\|Q04857\|CO6A1\_MOUSE | 301.12 | 20 | 13 | 18 | 16 | 11 | 12 | 14 | 16 | 11 | 16 | 10 | 1.3E8 | 1.69E8 | 1.97E8 | 6.88E7 | 1.04E8 | 1.77E8 | 9.46E7 | 9.56E7 | 1.58E8 | 1.02E8 | 17 | 17 | 19 | 21 | 21 | 13 | 15 | 18 | 17 | 16 | 21 | 14 | Y | 108489 | Collagen alpha-1(VI) chain OS=Mus musculus OX=10090 GN=Col6a1 PE=1 SV=1 |
| 215 | 238 | sp\|Q9D1A2\|CNDP2\_MOUSE | 300.83 | 44 | 25 | 34 | 30 | 31 | 28 | 36 | 34 | 34 | 34 | 28 | 7.05E7 | 9.29E7 | 9.06E7 | 3.95E7 | 5.36E7 | 1.21E8 | 6.16E7 | 2.7E7 | 1.54E8 | 5.96E7 | 15 | 15 | 10 | 15 | 12 | 12 | 12 | 18 | 12 | 10 | 15 | 12 | Y | 52767 | Cytosolic non-specific dipeptidase OS=Mus musculus OX=10090 GN=Cndp2 PE=1 SV=1 |
| 142 | 208 | sp\|Q02788\|CO6A2\_MOUSE | 300.17 | 18 | 15 | 14 | 15 | 14 | 15 | 15 | 13 | 15 | 15 | 13 | 2.15E8 | 2.56E8 | 2.35E8 | 9.27E7 | 2.27E8 | 3.04E8 | 1.4E8 | 1.5E8 | 2.59E8 | 1.83E8 | 20 | 20 | 20 | 20 | 20 | 19 | 22 | 21 | 18 | 18 | 20 | 17 | Y | 110334 | Collagen alpha-2(VI) chain OS=Mus musculus OX=10090 GN=Col6a2 PE=1 SV=3 |
| 226 | 250 | sp\|Q8BK48\|EST2E\_MOUSE | 300.03 | 33 | 15 | 17 | 21 | 18 | 18 | 29 | 24 | 17 | 24 | 12 | 3.95E7 | 6.03E7 | 7.4E7 | 1.07E7 | 3.34E7 | 6.65E7 | 5.38E7 | 1.87E7 | 1E8 | 2.75E7 | 15 | 10 | 12 | 14 | 15 | 8 | 11 | 16 | 13 | 8 | 17 | 8 | Y | 62318 | Pyrethroid hydrolase Ces2e OS=Mus musculus OX=10090 GN=Ces2e PE=1 SV=1 |
| 132 | 102 | sp\|P63038\|CH60\_MOUSE | 299.88 | 45 | 29 | 32 | 28 | 38 | 31 | 38 | 34 | 27 | 30 | 28 | 1.71E8 | 2.49E8 | 1.85E8 | 1.08E8 | 1.75E8 | 2.35E8 | 1.65E8 | 1.09E8 | 1.91E8 | 1.32E8 | 23 | 22 | 18 | 22 | 20 | 20 | 19 | 22 | 19 | 13 | 23 | 20 | Y | 60956 | 60 kDa heat shock protein, mitochondrial OS=Mus musculus OX=10090 GN=Hspd1 PE=1 SV=1 |
| 140 | 75 | sp\|O08601\|MTP\_MOUSE | 299.57 | 25 | 17 | 22 | 24 | 20 | 17 | 20 | 21 | 17 | 17 | 20 | 5.09E7 | 1.49E8 | 1.52E8 | 5.7E7 | 4.56E7 | 9.77E7 | 8.36E7 | 3.53E7 | 8.8E7 | 7.6E7 | 20 | 20 | 14 | 20 | 24 | 19 | 16 | 19 | 23 | 15 | 14 | 16 | Y | 99099 | Microsomal triglyceride transfer protein large subunit OS=Mus musculus OX=10090 GN=Mttp PE=1 SV=2 |
| 287 | 452 | sp\|Q00897\|A1AT4\_MOUSE | 298.51 | 34 | 10 | 17 | 29 | 27 | 22 | 28 | 34 | 21 | 6 | 8 | 2.93E6 | 1.12E7 | 1.09E7 | 2.64E6 | 6.58E6 | 8.33E6 | 4.3E6 | 6.69E6 |  |  | 12 | 2 | 5 | 7 | 12 | 13 | 10 | 10 | 11 | 10 | 3 | 4 | Y | 45998 | Alpha-1-antitrypsin 1-4 OS=Mus musculus OX=10090 GN=Serpina1d PE=1 SV=1 |
| 190 | 125 | sp\|Q02053\|UBA1\_MOUSE | 297.88 | 23 | 11 | 14 | 11 | 8 | 10 | 15 | 13 | 12 | 13 | 16 | 3.22E7 | 6.74E7 | 6.45E7 | 2.93E7 | 5.89E7 | 7.29E7 | 4.14E7 | 3.64E7 | 7.65E7 | 7.87E7 | 20 | 19 | 12 | 15 | 10 | 10 | 12 | 15 | 13 | 13 | 15 | 17 | Y | 117809 | Ubiquitin-like modifier-activating enzyme 1 OS=Mus musculus OX=10090 GN=Uba1 PE=1 SV=1 |
| 110 | 181 | sp\|P26040\|EZRI\_MOUSE | 297.44 | 32 | 20 | 19 | 23 | 20 | 30 | 26 | 25 | 21 | 22 | 23 | 3.73E7 | 5.15E7 | 4.17E7 | 3.79E7 | 9.89E7 | 1.1E8 | 5.61E7 | 4.09E7 | 7.33E7 | 4.73E7 | 26 | 16 | 22 | 21 | 20 | 22 | 34 | 20 | 22 | 19 | 26 | 23 | Y | 69407 | Ezrin OS=Mus musculus OX=10090 GN=Ezr PE=1 SV=3 |
| 315 | 780 | A0A0A6YXW6\|A0A0A6YXW6\_MOUSE | 297.12 | 30 | 8 | 16 | 16 | 18 | 18 | 18 | 20 | 22 | 25 | 8 | 6.69E7 | 5.93E7 | 5.14E7 | 1.99E7 | 4.42E7 | 4.16E7 | 3.88E7 | 4.16E7 | 1.64E8 | 4.91E7 | 9 | 3 | 7 | 9 | 6 | 7 | 9 | 7 | 10 | 10 | 12 | 8 | Y | 42078 | Immunoglobulin heavy constant alpha (Fragment) OS=Mus musculus OX=10090 GN=Igha PE=1 SV=1 |
| 252 | 247 | sp\|Q9WUM4\|COR1C\_MOUSE | 296.34 | 35 | 19 | 25 | 15 | 17 | 27 | 30 | 27 | 19 | 25 | 13 | 5.94E7 | 7.04E7 | 5.01E7 | 2.12E7 | 5.72E7 | 1.03E8 | 4.57E7 | 3.48E7 | 6.86E7 | 2.46E7 | 14 | 14 | 9 | 10 | 8 | 8 | 13 | 14 | 11 | 9 | 9 | 8 | Y | 53121 | Coronin-1C OS=Mus musculus OX=10090 GN=Coro1c PE=1 SV=2 |
| 326 | 605 | sp\|Q9DBJ1\|PGAM1\_MOUSE | 295.96 | 55 | 37 | 42 | 44 | 36 | 44 | 33 | 44 | 29 | 39 | 37 | 4.98E7 | 4.45E7 | 1.08E8 | 1.63E7 | 7.85E7 | 5.82E7 | 6.44E7 | 2.59E7 | 2.13E8 | 9.87E7 | 10 | 10 | 8 | 9 | 10 | 6 | 8 | 6 | 9 | 5 | 11 | 10 | Y | 28832 | Phosphoglycerate mutase 1 OS=Mus musculus OX=10090 GN=Pgam1 PE=1 SV=3 |
| 120 | 496 | sp\|Q922F4\|TBB6\_MOUSE | 295.46 | 28 | 24 | 17 | 20 | 20 | 20 | 20 | 20 | 19 | 23 | 22 | 2.96E6 |  |  |  |  |  |  | 8.5E5 | 4.27E6 | 2.28E6 | 11 | 2 | 25 | 21 | 21 | 17 | 22 | 21 | 18 | 24 | 33 | 22 | Y | 50090 | Tubulin beta-6 chain OS=Mus musculus OX=10090 GN=Tubb6 PE=1 SV=1 |
| 224 | 124 | sp\|P16406\|AMPE\_MOUSE | 294.33 | 24 | 12 | 15 | 11 | 13 | 10 | 16 | 12 | 9 | 20 | 10 | 1.65E7 | 9.46E7 | 1.92E8 | 1.62E7 | 2.86E7 | 8.99E7 | 4.38E7 | 1.2E7 | 9.15E7 | 2.98E7 | 21 | 21 | 9 | 16 | 10 | 11 | 8 | 14 | 12 | 7 | 17 | 10 | Y | 107956 | Glutamyl aminopeptidase OS=Mus musculus OX=10090 GN=Enpep PE=1 SV=1 |
| 97 | 272 | sp\|O88569\|ROA2\_MOUSE | 294.18 | 44 | 36 | 36 | 39 | 39 | 39 | 39 | 41 | 43 | 39 | 36 | 3.5E8 | 3.69E8 | 3.66E8 | 1.33E8 | 2.56E8 | 2.72E8 | 2.03E8 | 2.12E8 | 5.05E8 | 3.2E8 | 14 | 11 | 25 | 25 | 26 | 24 | 25 | 21 | 27 | 31 | 30 | 26 | Y | 37403 | Heterogeneous nuclear ribonucleoproteins A2/B1 OS=Mus musculus OX=10090 GN=Hnrnpa2b1 PE=1 SV=2 |
| 148 | 157 | sp\|P14211\|CALR\_MOUSE | 294.00 | 56 | 37 | 30 | 33 | 32 | 38 | 41 | 40 | 41 | 34 | 25 | 2.17E8 | 2.5E8 | 3.06E8 | 1.47E8 | 2.45E8 | 2.24E8 | 2.05E8 | 1.89E8 | 2.48E8 | 1.88E8 | 21 | 21 | 18 | 15 | 18 | 18 | 20 | 18 | 22 | 23 | 15 | 13 | Y | 47995 | Calreticulin OS=Mus musculus OX=10090 GN=Calr PE=1 SV=1 |
| 335 | 128 | A0A0U1RNJ1\|A0A0U1RNJ1\_MOUSE | 293.71 | 11 | 4 | 7 | 1 | 4 | 3 | 6 | 3 | 3 | 3 | 1 | 1.25E7 | 4.53E7 | 2.43E6 | 1.09E7 | 8.96E6 | 2.49E7 | 1.53E7 | 4.03E6 | 1.43E7 | 3.49E6 | 22 | 21 | 7 | 15 | 2 | 8 | 5 | 9 | 5 | 5 | 7 | 3 | Y | 272226 | Fatty acid synthase OS=Mus musculus OX=10090 GN=Fasn PE=1 SV=1 |
| 335 | 129 | sp\|P19096\|FAS\_MOUSE | 293.71 | 11 | 4 | 7 | 1 | 4 | 3 | 6 | 3 | 3 | 3 | 1 | 1.25E7 | 4.53E7 | 2.43E6 | 1.09E7 | 8.96E6 | 2.49E7 | 1.53E7 | 4.03E6 | 1.43E7 | 3.49E6 | 22 | 21 | 7 | 15 | 2 | 8 | 5 | 9 | 5 | 5 | 7 | 3 | Y | 272426 | Fatty acid synthase OS=Mus musculus OX=10090 GN=Fasn PE=1 SV=2 |
| 245 | 154 | sp\|Q62167\|DDX3X\_MOUSE | 292.47 | 30 | 18 | 16 | 14 | 16 | 13 | 16 | 16 | 10 | 26 | 17 | 5.05E7 | 4.58E7 | 5.13E7 | 2.71E7 | 2.29E7 | 3.36E7 | 2.63E7 | 1.5E7 | 9.61E7 | 4.84E7 | 17 | 16 | 11 | 11 | 10 | 11 | 8 | 8 | 10 | 5 | 17 | 12 | Y | 73102 | ATP-dependent RNA helicase DDX3X OS=Mus musculus OX=10090 GN=Ddx3x PE=1 SV=3 |
| 211 | 112 | sp\|Q8BZF8\|PGM5\_MOUSE | 292.33 | 43 | 19 | 26 | 17 | 22 | 20 | 33 | 23 | 21 | 17 | 16 | 6.26E7 | 9.76E7 | 9.83E7 | 4.12E7 | 8.74E7 | 1.11E8 | 7.14E7 | 6.04E7 | 5.82E7 | 4.26E7 | 21 | 19 | 9 | 13 | 11 | 12 | 12 | 16 | 14 | 12 | 10 | 9 | Y | 62220 | Phosphoglucomutase-like protein 5 OS=Mus musculus OX=10090 GN=Pgm5 PE=1 SV=2 |
| 169 | 582 | sp\|P18760\|COF1\_MOUSE | 292.11 | 66 | 47 | 47 | 52 | 66 | 49 | 55 | 62 | 37 | 52 | 56 | 1.36E8 | 1.52E8 | 1.53E8 | 8.33E7 | 1.27E8 | 1.41E8 | 7.9E7 | 9.15E7 | 3.16E8 | 1.82E8 | 11 | 9 | 16 | 17 | 13 | 16 | 14 | 12 | 13 | 15 | 20 | 19 | Y | 18560 | Cofilin-1 OS=Mus musculus OX=10090 GN=Cfl1 PE=1 SV=3 |
| 217 | 182 | Z4YJV4\|Z4YJV4\_MOUSE | 291.93 | 20 | 7 | 10 | 10 | 14 | 11 | 14 | 14 | 11 | 11 | 7 | 2.17E7 | 7.91E7 | 9.67E7 | 3.46E7 | 5.15E7 | 5.11E7 | 5.67E7 | 3.14E7 | 3.75E7 | 5.36E7 | 20 | 20 | 6 | 13 | 13 | 14 | 11 | 14 | 15 | 13 | 11 | 7 | Y | 116027 | 2-oxoglutarate dehydrogenase complex component E1 OS=Mus musculus OX=10090 GN=Ogdh PE=1 SV=1 |
| 217 | 183 | sp\|Q60597\|ODO1\_MOUSE | 291.93 | 20 | 7 | 10 | 10 | 14 | 11 | 13 | 14 | 11 | 11 | 7 | 2.17E7 | 7.91E7 | 9.67E7 | 3.46E7 | 5.15E7 | 5.11E7 | 5.67E7 | 3.14E7 | 3.75E7 | 5.36E7 | 20 | 20 | 6 | 13 | 13 | 14 | 11 | 14 | 15 | 13 | 11 | 7 | Y | 116449 | 2-oxoglutarate dehydrogenase complex component E1 OS=Mus musculus OX=10090 GN=Ogdh PE=1 SV=3 |
| 109 | 436 | A0A140LHI8\|A0A140LHI8\_MOUSE | 291.79 | 43 | 27 | 38 | 33 | 33 | 33 | 38 | 38 | 38 | 38 | 33 | 3.34E8 | 5.64E8 | 5.63E8 | 2.8E8 | 4.48E8 | 5.29E8 | 4E8 | 2.92E8 | 6.8E8 | 3.95E8 | 12 | 10 | 19 | 26 | 25 | 22 | 22 | 21 | 22 | 23 | 26 | 21 | Y | 33944 | Galectin OS=Mus musculus OX=10090 GN=Lgals4 PE=1 SV=1 |
| 109 | 438 | sp\|Q8K419\|LEG4\_MOUSE | 291.79 | 40 | 25 | 35 | 31 | 31 | 31 | 35 | 35 | 35 | 35 | 31 | 3.34E8 | 5.64E8 | 5.63E8 | 2.8E8 | 4.48E8 | 5.29E8 | 4E8 | 2.92E8 | 6.8E8 | 3.95E8 | 12 | 10 | 19 | 26 | 25 | 22 | 22 | 21 | 22 | 23 | 26 | 21 | Y | 36372 | Galectin-4 OS=Mus musculus OX=10090 GN=Lgals4 PE=1 SV=2 |
| 324 | 167 | sp\|P01872\|IGHM\_MOUSE | 291.14 | 42 | 33 | 0 | 0 | 3 | 0 | 3 | 6 | 9 | 39 | 27 | 2.98E8 |  |  | 6.8E5 |  | 7.2E5 | 9.5E5 | 3.01E6 | 5.99E8 | 1.5E8 | 17 | 17 | 21 | 0 | 0 | 1 | 0 | 1 | 2 | 3 | 32 | 16 | Y | 49972 | Immunoglobulin heavy constant mu OS=Mus musculus OX=10090 GN=Ighm PE=1 SV=2 |
| 324 | 168 | A0A075B5P6\|A0A075B5P6\_MOUSE | 291.14 | 42 | 33 | 0 | 0 | 3 | 0 | 3 | 6 | 9 | 39 | 27 | 2.98E8 |  |  | 6.8E5 |  | 7.2E5 | 9.5E5 | 3.01E6 | 5.99E8 | 1.5E8 | 17 | 17 | 21 | 0 | 0 | 1 | 0 | 1 | 2 | 3 | 32 | 16 | Y | 50063 | Immunoglobulin heavy constant mu (Fragment) OS=Mus musculus OX=10090 GN=Ighm PE=1 SV=1 |
| 324 | 169 | A0A075B6A0\|A0A075B6A0\_MOUSE | 291.14 | 40 | 31 | 0 | 0 | 3 | 0 | 3 | 5 | 9 | 38 | 26 | 2.98E8 |  |  | 6.8E5 |  | 7.2E5 | 9.5E5 | 3.01E6 | 5.99E8 | 1.5E8 | 17 | 17 | 21 | 0 | 0 | 1 | 0 | 1 | 2 | 3 | 32 | 16 | Y | 52618 | Immunoglobulin heavy constant mu (Fragment) OS=Mus musculus OX=10090 GN=Ighm PE=1 SV=2 |
| 249 | 126 | sp\|Q9JKF1\|IQGA1\_MOUSE | 291.12 | 19 | 8 | 6 | 10 | 8 | 10 | 7 | 8 | 8 | 10 | 9 | 2.86E7 | 2.36E7 | 2.68E7 | 1.08E7 | 2.75E7 | 3.04E7 | 1.43E7 | 1.11E7 | 6.96E7 | 6.16E7 | 25 | 24 | 10 | 7 | 13 | 9 | 12 | 9 | 10 | 10 | 12 | 12 | Y | 188741 | Ras GTPase-activating-like protein IQGAP1 OS=Mus musculus OX=10090 GN=Iqgap1 PE=1 SV=2 |
| 122 | 141 | sp\|P05784\|K1C18\_MOUSE | 290.85 | 50 | 37 | 30 | 43 | 24 | 37 | 31 | 28 | 25 | 35 | 35 | 1.89E8 | 1.4E8 | 1.46E8 | 5.65E7 | 1.23E8 | 1.31E8 | 8.24E7 | 6.81E7 | 2.93E8 | 1.56E8 | 21 | 18 | 23 | 18 | 25 | 18 | 21 | 14 | 18 | 16 | 24 | 21 | Y | 47538 | Keratin, type I cytoskeletal 18 OS=Mus musculus OX=10090 GN=Krt18 PE=1 SV=5 |
| 293 | 201 | sp\|Q9JKR6\|HYOU1\_MOUSE | 290.21 | 21 | 8 | 9 | 7 | 9 | 7 | 10 | 18 | 11 | 10 | 4 | 2.41E7 | 1.94E7 | 1.08E7 | 1.22E7 | 2.41E7 | 2.59E7 | 6.93E7 | 2.05E7 | 2.59E7 | 2.1E7 | 18 | 18 | 8 | 7 | 6 | 10 | 6 | 8 | 16 | 9 | 7 | 4 | Y | 111181 | Hypoxia up-regulated protein 1 OS=Mus musculus OX=10090 GN=Hyou1 PE=1 SV=1 |
| 151 | 151 | sp\|P47738\|ALDH2\_MOUSE | 289.58 | 43 | 31 | 25 | 36 | 36 | 38 | 34 | 29 | 36 | 36 | 24 | 1.25E8 | 1.01E8 | 1.4E8 | 6.06E7 | 9.75E7 | 1.15E8 | 5.07E7 | 6.31E7 | 1.75E8 | 8.89E7 | 17 | 16 | 18 | 15 | 19 | 19 | 18 | 18 | 12 | 18 | 22 | 12 | Y | 56538 | Aldehyde dehydrogenase, mitochondrial OS=Mus musculus OX=10090 GN=Aldh2 PE=1 SV=1 |
| 191 | 105 | sp\|Q9EQK5\|MVP\_MOUSE | 289.35 | 30 | 20 | 21 | 16 | 19 | 15 | 19 | 21 | 15 | 17 | 19 | 9.61E7 | 7.94E7 | 9.13E7 | 3.44E7 | 5.52E7 | 7.42E7 | 7.4E7 | 4.21E7 | 1.45E8 | 6.37E7 | 22 | 22 | 16 | 17 | 12 | 14 | 12 | 14 | 15 | 10 | 13 | 17 | Y | 95924 | Major vault protein OS=Mus musculus OX=10090 GN=Mvp PE=1 SV=4 |
| 191 | 106 | E9Q3X0\|E9Q3X0\_MOUSE | 289.35 | 30 | 20 | 21 | 15 | 19 | 15 | 19 | 21 | 15 | 16 | 19 | 9.61E7 | 7.94E7 | 9.13E7 | 3.44E7 | 5.52E7 | 7.42E7 | 7.4E7 | 4.21E7 | 1.45E8 | 6.37E7 | 22 | 22 | 16 | 17 | 12 | 14 | 12 | 14 | 15 | 10 | 13 | 17 | Y | 96857 | Major vault protein OS=Mus musculus OX=10090 GN=Mvp PE=1 SV=1 |
| 243 | 163 | sp\|P40124\|CAP1\_MOUSE | 288.95 | 47 | 22 | 24 | 27 | 22 | 28 | 26 | 28 | 15 | 31 | 37 | 3.81E7 | 3.07E7 | 2.42E7 | 1E7 | 2.89E7 | 3.29E7 | 1.06E8 | 2.04E7 | 6.34E7 | 5.12E7 | 17 | 17 | 8 | 10 | 9 | 7 | 11 | 9 | 12 | 6 | 12 | 14 | Y | 51565 | Adenylyl cyclase-associated protein 1 OS=Mus musculus OX=10090 GN=Cap1 PE=1 SV=4 |
| 267 | 864 | sp\|Q9EPB4\|ASC\_MOUSE | 288.94 | 58 | 51 | 58 | 43 | 44 | 58 | 50 | 44 | 51 | 51 | 43 | 6.48E7 | 1.17E8 | 9.78E7 | 2.74E7 | 6.65E7 | 5.82E7 | 7.37E7 | 5.1E7 | 1.35E8 | 4.86E7 | 8 | 8 | 9 | 11 | 9 | 8 | 12 | 11 | 10 | 11 | 11 | 8 | Y | 21459 | Apoptosis-associated speck-like protein containing a CARD OS=Mus musculus OX=10090 GN=Pycard PE=1 SV=1 |
| 187 | 209 | sp\|Q91X72\|HEMO\_MOUSE | 288.27 | 39 | 33 | 11 | 25 | 20 | 15 | 31 | 37 | 28 | 23 | 25 | 2.01E8 | 1.55E7 | 3.83E7 | 2.31E7 | 1.84E7 | 1.35E8 | 6.39E7 | 8.48E7 | 7.12E7 | 8.98E7 | 19 | 19 | 23 | 6 | 10 | 8 | 7 | 21 | 19 | 22 | 10 | 16 | Y | 51318 | Hemopexin OS=Mus musculus OX=10090 GN=Hpx PE=1 SV=2 |
| 160 | 184 | sp\|Q9D154\|ILEUA\_MOUSE | 287.33 | 47 | 33 | 32 | 33 | 37 | 37 | 35 | 41 | 33 | 35 | 30 | 2.22E8 | 2.57E8 | 2.12E8 | 1.08E8 | 1.84E8 | 2.09E8 | 1.22E8 | 1.05E8 | 4.03E8 | 1.58E8 | 15 | 14 | 17 | 20 | 15 | 17 | 16 | 14 | 16 | 13 | 26 | 15 | Y | 42575 | Leukocyte elastase inhibitor A OS=Mus musculus OX=10090 GN=Serpinb1a PE=1 SV=1 |
| 105 | 117 | sp\|Q9D312\|K1C20\_MOUSE | 287.30 | 55 | 41 | 48 | 44 | 38 | 47 | 42 | 45 | 32 | 44 | 46 | 1.68E8 | 3.96E8 | 2.48E8 | 1.76E8 | 2.85E8 | 4.16E8 | 2.65E8 | 1.5E8 | 2.69E8 | 1.68E8 | 24 | 21 | 20 | 25 | 21 | 20 | 26 | 27 | 25 | 19 | 28 | 22 | Y | 49034 | Keratin, type I cytoskeletal 20 OS=Mus musculus OX=10090 GN=Krt20 PE=1 SV=1 |
| 405 | 491 | A0A0R4J0I1\|A0A0R4J0I1\_MOUSE | 287.03 | 36 | 5 | 19 | 30 | 28 | 28 | 11 | 30 | 19 | 11 | 11 | 2.96E6 | 1.04E7 | 3.85E6 | 7.22E6 | 5.6E6 | 8.67E6 | 2.59E6 | 1.13E7 | 5.12E6 |  | 10 | 3 | 2 | 6 | 10 | 9 | 10 | 4 | 9 | 9 | 3 | 3 | Y | 46673 | Serine (or cysteine) peptidase inhibitor, clade A, member 3K OS=Mus musculus OX=10090 GN=Serpina3k PE=1 SV=1 |
| 225 | 150 | sp\|Q61233\|PLSL\_MOUSE | 286.86 | 32 | 17 | 13 | 17 | 9 | 15 | 15 | 16 | 21 | 21 | 26 | 4.39E7 | 1.32E7 | 2.53E7 | 4.83E6 | 1.66E7 | 1.11E7 | 1.27E7 | 1.81E7 | 8.62E7 | 5.62E7 | 17 | 12 | 11 | 10 | 11 | 6 | 12 | 9 | 10 | 14 | 16 | 15 | Y | 70149 | Plastin-2 OS=Mus musculus OX=10090 GN=Lcp1 PE=1 SV=4 |
| 246 | 161 | sp\|P29341\|PABP1\_MOUSE | 286.59 | 32 | 19 | 15 | 14 | 13 | 20 | 17 | 19 | 18 | 24 | 16 | 7.55E6 | 7.31E6 | 1.15E7 | 4.2E6 | 1.16E7 | 6.36E6 | 5.59E6 | 5.77E6 | 4.37E6 | 5.47E6 | 17 | 3 | 11 | 9 | 8 | 10 | 11 | 9 | 11 | 10 | 14 | 10 | Y | 70671 | Polyadenylate-binding protein 1 OS=Mus musculus OX=10090 GN=Pabpc1 PE=1 SV=2 |
| 175 | 71 | sp\|O55143\|AT2A2\_MOUSE | 286.12 | 24 | 12 | 10 | 16 | 16 | 19 | 17 | 16 | 19 | 14 | 10 | 3.26E7 | 5.1E7 | 7.96E7 | 2.65E7 | 4.73E7 | 6.39E7 | 4.01E7 | 3.94E7 | 5.54E7 | 3.41E7 | 21 | 13 | 11 | 10 | 18 | 13 | 19 | 18 | 16 | 18 | 13 | 8 | Y | 114858 | Sarcoplasmic/endoplasmic reticulum calcium ATPase 2 OS=Mus musculus OX=10090 GN=Atp2a2 PE=1 SV=2 |
| 317 | 225 | sp\|Q8BH64\|EHD2\_MOUSE | 284.74 | 33 | 12 | 21 | 19 | 16 | 19 | 16 | 13 | 22 | 14 | 10 | 2.68E7 | 2.98E7 | 2.06E7 | 7.75E6 | 1.49E7 | 2.49E7 | 1.28E7 | 1.65E7 | 4.91E7 | 2.86E7 | 18 | 16 | 6 | 10 | 10 | 7 | 8 | 9 | 8 | 10 | 6 | 6 | Y | 61175 | EH domain-containing protein 2 OS=Mus musculus OX=10090 GN=Ehd2 PE=1 SV=1 |
| 84 | 420 | D3Z6I8\|D3Z6I8\_MOUSE | 284.67 | 47 | 33 | 28 | 27 | 37 | 40 | 40 | 40 | 40 | 33 | 30 | 4.21E7 | 4.36E7 |  | 1.43E7 | 3.38E7 | 2.56E7 | 2.23E7 | 2.83E7 | 6.54E7 | 4.06E7 | 19 | 1 | 29 | 22 | 24 | 25 | 33 | 29 | 29 | 35 | 28 | 22 | Y | 28723 | Tropomyosin 3, gamma OS=Mus musculus OX=10090 GN=Tpm3 PE=1 SV=1 |
| 84 | 421 | E9Q7Q3\|E9Q7Q3\_MOUSE | 284.67 | 47 | 33 | 28 | 27 | 37 | 40 | 40 | 40 | 40 | 33 | 30 | 4.21E7 | 4.36E7 |  | 1.43E7 | 3.38E7 | 2.56E7 | 2.23E7 | 2.83E7 | 6.54E7 | 4.06E7 | 19 | 1 | 29 | 22 | 24 | 25 | 33 | 29 | 29 | 35 | 28 | 22 | Y | 28734 | Tropomyosin 3, gamma OS=Mus musculus OX=10090 GN=Tpm3 PE=1 SV=1 |
| 137 | 191 | sp\|P26041\|MOES\_MOUSE | 284.64 | 37 | 23 | 16 | 23 | 16 | 22 | 20 | 17 | 19 | 26 | 21 | 6.39E7 | 9.81E6 | 3.75E7 | 5.97E6 | 4.18E7 | 2.51E7 | 1.57E7 | 2.56E7 | 7.16E7 | 3.84E7 | 26 | 15 | 23 | 16 | 22 | 16 | 22 | 14 | 16 | 17 | 25 | 21 | Y | 67767 | Moesin OS=Mus musculus OX=10090 GN=Msn PE=1 SV=3 |
| 298 | 136 | sp\|Q61838\|PZP\_MOUSE | 284.30 | 18 | 6 | 8 | 10 | 17 | 9 | 10 | 11 | 9 | 2 | 5 | 3.51E7 | 2.79E7 | 6.67E7 | 3.98E7 | 3.51E7 | 5.99E7 | 3.81E7 | 3.1E7 | 1.36E7 | 1.89E7 | 16 | 16 | 7 | 7 | 11 | 17 | 8 | 9 | 11 | 9 | 1 | 5 | Y | 165852 | Pregnancy zone protein OS=Mus musculus OX=10090 GN=Pzp PE=1 SV=3 |
| 143 | 137 | E9Q616\|E9Q616\_MOUSE | 283.46 | 7 | 2 | 2 | 2 | 2 | 3 | 3 | 1 | 2 | 3 | 3 | 1.94E8 | 1.09E8 | 8.06E7 | 5.89E7 | 8.23E7 | 1.03E8 | 5.73E7 | 5.58E7 | 2.64E8 | 2.18E8 | 34 | 34 | 15 | 12 | 12 | 12 | 15 | 17 | 9 | 10 | 25 | 23 | Y | 604264 | AHNAK nucleoprotein OS=Mus musculus OX=10090 GN=Ahnak PE=1 SV=1 |
| 398 | 403 | sp\|O89053\|COR1A\_MOUSE | 282.85 | 31 | 14 | 14 | 21 | 19 | 16 | 14 | 11 | 24 | 23 | 18 | 4.89E7 | 3.48E7 | 6.68E7 | 2.98E7 | 3.28E7 | 2.22E7 | 3.97E7 | 4.89E7 | 1.24E8 | 3.16E7 | 12 | 12 | 4 | 4 | 7 | 7 | 5 | 5 | 5 | 9 | 8 | 6 | Y | 50989 | Coronin-1A OS=Mus musculus OX=10090 GN=Coro1a PE=1 SV=5 |
| 73 | 624 | sp\|Q9CQ19\|MYL9\_MOUSE | 282.41 | 80 | 62 | 63 | 62 | 62 | 62 | 80 | 62 | 62 | 62 | 62 | 3.57E8 | 3.43E8 | 4.02E8 | 1.68E8 | 3.32E8 | 4.19E8 | 2.05E8 | 2.58E8 | 4.76E8 | 2.04E8 | 10 | 5 | 40 | 39 | 29 | 21 | 29 | 35 | 20 | 36 | 36 | 29 | Y | 19854 | Myosin regulatory light polypeptide 9 OS=Mus musculus OX=10090 GN=Myl9 PE=1 SV=3 |
| 504 | 709 | sp\|P07759\|SPA3K\_MOUSE | 282.03 | 32 | 6 | 17 | 27 | 31 | 24 | 15 | 25 | 13 | 3 | 6 | 2.75E5 | 8.87E5 | 8.51E5 | 1.16E6 | 4.7E5 | 1.9E6 | 2.24E6 |  |  |  | 9 | 2 | 1 | 4 | 8 | 9 | 8 | 4 | 7 | 7 | 1 | 2 | Y | 46880 | Serine protease inhibitor A3K OS=Mus musculus OX=10090 GN=Serpina3k PE=1 SV=2 |
| 201 | 131 | sp\|Q78PY7\|SND1\_MOUSE | 281.23 | 30 | 14 | 13 | 13 | 15 | 19 | 13 | 15 | 19 | 14 | 14 | 5.85E7 | 3.17E7 | 4.62E7 | 2.74E7 | 4.05E7 | 2.56E7 | 2.66E7 | 4.14E7 | 4.06E7 | 3.2E7 | 24 | 24 | 13 | 9 | 11 | 13 | 16 | 9 | 12 | 15 | 11 | 11 | Y | 102088 | Staphylococcal nuclease domain-containing protein 1 OS=Mus musculus OX=10090 GN=Snd1 PE=1 SV=1 |
| 107 | 551 | sp\|Q9CQV8\|1433B\_MOUSE | 280.28 | 42 | 39 | 39 | 37 | 36 | 34 | 39 | 31 | 38 | 35 | 39 | 8.4E7 | 9.11E7 | 7.16E7 | 2.33E7 | 5.67E7 | 6.14E7 | 3.45E7 | 3.8E7 | 9.12E7 | 6.74E7 | 12 | 4 | 28 | 28 | 23 | 19 | 21 | 22 | 19 | 27 | 26 | 28 | Y | 28086 | 14-3-3 protein beta/alpha OS=Mus musculus OX=10090 GN=Ywhab PE=1 SV=3 |
| 199 | 162 | sp\|Q80Z19\|MUC2\_MOUSE | 279.97 | 7 | 5 | 2 | 1 | 2 | 2 | 3 | 2 | 2 | 5 | 3 | 1.88E8 | 3.47E7 | 1.92E7 | 1.31E7 | 3.09E7 | 6.42E7 | 2.04E7 | 3.05E7 | 2.25E8 | 1.4E8 | 30 | 30 | 24 | 7 | 6 | 8 | 7 | 14 | 10 | 8 | 25 | 17 | Y | 479291 | Mucin-2 OS=Mus musculus OX=10090 GN=Muc2 PE=1 SV=3 |
| 130 | 123 | sp\|P09405\|NUCL\_MOUSE | 279.36 | 31 | 21 | 16 | 27 | 25 | 25 | 25 | 25 | 27 | 22 | 21 | 1.22E8 | 9.35E7 | 1.64E8 | 1.04E8 | 1.3E8 | 1.02E8 | 1.06E8 | 1.18E8 | 2.23E8 | 1.38E8 | 22 | 22 | 18 | 14 | 21 | 23 | 21 | 18 | 18 | 24 | 19 | 18 | Y | 76723 | Nucleolin OS=Mus musculus OX=10090 GN=Ncl PE=1 SV=2 |
| 164 | 164 | B2M1R6\|B2M1R6\_MOUSE | 279.18 | 45 | 37 | 33 | 30 | 33 | 32 | 32 | 24 | 30 | 40 | 35 | 2.3E8 | 1.9E8 | 1.85E8 | 9.28E7 | 1.77E8 | 1.64E8 | 1.17E8 | 1.15E8 | 2.63E8 | 1.59E8 | 17 | 17 | 19 | 14 | 17 | 16 | 17 | 16 | 11 | 15 | 18 | 18 | Y | 48562 | Heterogeneous nuclear ribonucleoprotein K OS=Mus musculus OX=10090 GN=Hnrnpk PE=1 SV=1 |
| 164 | 165 | sp\|P61979\|HNRPK\_MOUSE | 279.18 | 43 | 35 | 31 | 29 | 31 | 30 | 31 | 22 | 29 | 38 | 33 | 2.3E8 | 1.9E8 | 1.85E8 | 9.28E7 | 1.77E8 | 1.64E8 | 1.17E8 | 1.15E8 | 2.63E8 | 1.59E8 | 17 | 17 | 19 | 14 | 17 | 16 | 17 | 16 | 11 | 15 | 18 | 18 | Y | 50976 | Heterogeneous nuclear ribonucleoprotein K OS=Mus musculus OX=10090 GN=Hnrnpk PE=1 SV=1 |
| 170 | 200 | sp\|P35564\|CALX\_MOUSE | 278.81 | 29 | 13 | 21 | 18 | 22 | 23 | 19 | 16 | 19 | 18 | 17 | 9.12E7 | 1.33E8 | 1.52E8 | 6.11E7 | 1.35E8 | 1.3E8 | 9.53E7 | 7.33E7 | 1.33E8 | 6.97E7 | 17 | 17 | 12 | 17 | 16 | 15 | 18 | 15 | 14 | 13 | 14 | 13 | Y | 67278 | Calnexin OS=Mus musculus OX=10090 GN=Canx PE=1 SV=1 |
| 193 | 271 | sp\|Q9CZU6\|CISY\_MOUSE | 278.68 | 39 | 27 | 29 | 24 | 34 | 29 | 32 | 39 | 31 | 27 | 18 | 1.32E8 | 2E8 | 7.39E7 | 6.54E7 | 9.17E7 | 1.55E8 | 9.69E7 | 4.54E7 | 1.66E8 | 9.28E7 | 13 | 13 | 14 | 15 | 11 | 17 | 15 | 15 | 16 | 14 | 15 | 11 | Y | 51737 | Citrate synthase, mitochondrial OS=Mus musculus OX=10090 GN=Cs PE=1 SV=1 |
| 150 | 107 | sp\|Q9D7Z6\|CLCA1\_MOUSE | 278.50 | 35 | 31 | 14 | 10 | 3 | 9 | 15 | 9 | 13 | 28 | 21 | 5.75E8 | 7.03E7 | 2.28E7 | 5.53E6 | 2.92E7 | 7.7E7 | 2.72E7 | 3.23E7 | 4.26E8 | 2.25E8 | 29 | 29 | 40 | 14 | 12 | 3 | 11 | 16 | 10 | 13 | 31 | 23 | Y | 100071 | Calcium-activated chloride channel regulator 1 OS=Mus musculus OX=10090 GN=Clca1 PE=1 SV=2 |
| 161 | 118 | sp\|P08003\|PDIA4\_MOUSE | 277.68 | 48 | 18 | 18 | 21 | 24 | 21 | 24 | 24 | 29 | 25 | 23 | 1.01E8 | 1.07E8 | 1.21E8 | 6.4E7 | 1.03E8 | 1.2E8 | 7.57E7 | 8.24E7 | 2.16E8 | 1.04E8 | 32 | 32 | 13 | 14 | 16 | 17 | 15 | 18 | 19 | 22 | 20 | 14 | Y | 71982 | Protein disulfide-isomerase A4 OS=Mus musculus OX=10090 GN=Pdia4 PE=1 SV=3 |
| 161 | 119 | A0A0R4J0Z1\|A0A0R4J0Z1\_MOUSE | 277.68 | 47 | 18 | 18 | 21 | 24 | 20 | 23 | 24 | 28 | 25 | 23 | 1.01E8 | 1.07E8 | 1.21E8 | 6.4E7 | 1.03E8 | 1.2E8 | 7.57E7 | 8.24E7 | 2.16E8 | 1.04E8 | 32 | 32 | 13 | 14 | 16 | 17 | 15 | 18 | 19 | 22 | 20 | 14 | Y | 72370 | Protein disulfide-isomerase A4 OS=Mus musculus OX=10090 GN=Pdia4 PE=1 SV=1 |
| 260 | 349 | sp\|Q8VIJ6\|SFPQ\_MOUSE | 277.41 | 20 | 11 | 10 | 15 | 12 | 14 | 19 | 15 | 15 | 11 | 14 | 6.83E7 | 4.46E7 | 7.66E7 | 3.7E7 | 7.29E7 | 6.91E7 | 5.1E7 | 5.86E7 | 4.82E7 | 5.32E7 | 13 | 12 | 9 | 7 | 10 | 9 | 11 | 13 | 11 | 11 | 8 | 9 | Y | 75442 | Splicing factor, proline- and glutamine-rich OS=Mus musculus OX=10090 GN=Sfpq PE=1 SV=1 |
| 260 | 351 | A0A991ENV6\|A0A991ENV6\_MOUSE | 277.41 | 19 | 11 | 9 | 14 | 11 | 14 | 18 | 15 | 15 | 11 | 13 | 6.83E7 | 4.46E7 | 7.66E7 | 3.7E7 | 7.29E7 | 6.91E7 | 5.1E7 | 5.86E7 | 4.82E7 | 5.32E7 | 13 | 12 | 9 | 7 | 10 | 9 | 11 | 13 | 11 | 11 | 8 | 9 | Y | 77877 | Splicing factor proline/glutamine rich (polypyrimidine tract binding protein associated) OS=Mus musculus OX=10090 GN=Sfpq PE=1 SV=1 |
| 153 | 408 | sp\|Q9R0P5\|DEST\_MOUSE | 276.93 | 75 | 52 | 54 | 60 | 56 | 73 | 60 | 53 | 55 | 34 | 64 | 3.16E8 | 3.36E8 | 3.03E8 | 1.42E8 | 2.62E8 | 3.5E8 | 1.49E8 | 1.32E8 | 2.65E8 | 2.51E8 | 14 | 13 | 14 | 21 | 16 | 14 | 22 | 19 | 12 | 14 | 16 | 18 | Y | 18522 | Destrin OS=Mus musculus OX=10090 GN=Dstn PE=1 SV=3 |
| 104 | 329 | sp\|P62259\|1433E\_MOUSE | 276.67 | 56 | 38 | 42 | 46 | 35 | 36 | 42 | 47 | 38 | 36 | 40 | 1.23E8 | 1.55E8 | 1.62E8 | 6.22E7 | 1.35E8 | 1.3E8 | 1.22E8 | 9.48E7 | 2.07E8 | 1.69E8 | 14 | 12 | 27 | 32 | 21 | 15 | 21 | 20 | 21 | 23 | 29 | 29 | Y | 29174 | 14-3-3 protein epsilon OS=Mus musculus OX=10090 GN=Ywhae PE=1 SV=1 |
| 381 | 178 | sp\|Q9Z1Q9\|SYVC\_MOUSE | 276.49 | 15 | 3 | 6 | 8 | 5 | 7 | 8 | 11 | 6 | 5 | 2 | 9.33E6 | 1.21E7 | 1.35E7 | 3.17E7 | 1.34E7 | 1.46E7 | 2.41E7 | 1.48E7 | 1.62E7 | 2.75E6 | 16 | 16 | 3 | 5 | 7 | 6 | 7 | 8 | 11 | 8 | 5 | 2 | Y | 140215 | Valine--tRNA ligase OS=Mus musculus OX=10090 GN=Vars1 PE=1 SV=1 |
| 102 | 316 | sp\|P68254\|1433T\_MOUSE | 276.29 | 53 | 45 | 47 | 50 | 31 | 34 | 30 | 41 | 33 | 43 | 50 | 6.93E7 | 6.86E7 | 6.94E7 | 2.36E7 | 5.08E7 | 4.69E7 | 3.76E7 | 3.45E7 | 9.88E7 | 6.97E7 | 16 | 8 | 30 | 28 | 25 | 17 | 20 | 20 | 22 | 25 | 28 | 30 | Y | 27778 | 14-3-3 protein theta OS=Mus musculus OX=10090 GN=Ywhaq PE=1 SV=1 |
| 173 | 644 | sp\|Q01149\|CO1A2\_MOUSE | 275.97 | 8 | 8 | 4 | 5 | 5 | 5 | 5 | 5 | 5 | 4 | 4 | 3.27E8 | 7.48E8 | 1.25E9 | 5.57E8 | 8.61E8 | 8.97E8 | 4.44E8 | 6.05E8 | 2.48E8 | 4.5E8 | 9 | 9 | 15 | 16 | 16 | 16 | 20 | 14 | 15 | 24 | 9 | 12 | N | 129557 | Collagen alpha-2(I) chain OS=Mus musculus OX=10090 GN=Col1a2 PE=1 SV=2 |
| 280 | 219 | sp\|Q9DB77\|QCR2\_MOUSE | 274.79 | 41 | 20 | 24 | 30 | 24 | 25 | 28 | 20 | 24 | 25 | 17 | 4.63E7 | 1.11E8 | 7.08E7 | 2.27E7 | 6.35E7 | 6.2E7 | 3.95E7 | 3.23E7 | 9.71E7 | 3.61E7 | 15 | 15 | 7 | 10 | 12 | 8 | 11 | 9 | 8 | 10 | 10 | 6 | Y | 48235 | Cytochrome b-c1 complex subunit 2, mitochondrial OS=Mus musculus OX=10090 GN=Uqcrc2 PE=1 SV=1 |
| 303 | 221 | sp\|P82198\|BGH3\_MOUSE | 274.72 | 30 | 13 | 16 | 13 | 12 | 17 | 23 | 16 | 20 | 9 | 8 | 3.86E7 | 8.47E7 | 6.15E7 | 1.95E7 | 6.81E7 | 7.72E7 | 3.41E7 | 5.31E7 | 4.75E7 | 2.97E7 | 15 | 14 | 7 | 10 | 8 | 7 | 9 | 11 | 9 | 11 | 5 | 5 | Y | 74597 | Transforming growth factor-beta-induced protein ig-h3 OS=Mus musculus OX=10090 GN=Tgfbi PE=1 SV=1 |
| 237 | 374 | sp\|P30275\|KCRU\_MOUSE | 274.50 | 39 | 24 | 27 | 37 | 20 | 22 | 35 | 25 | 27 | 32 | 24 | 8.36E7 | 7.8E7 | 1.17E8 | 3.35E7 | 4.56E7 | 8.61E7 | 4.68E7 | 3.82E7 | 1.04E8 | 8.42E7 | 12 | 7 | 12 | 9 | 14 | 9 | 8 | 12 | 12 | 11 | 15 | 9 | Y | 47004 | Creatine kinase U-type, mitochondrial OS=Mus musculus OX=10090 GN=Ckmt1 PE=1 SV=1 |
| 196 | 158 | A2AVJ7\|A2AVJ7\_MOUSE | 274.04 | 15 | 7 | 6 | 8 | 11 | 10 | 9 | 11 | 9 | 6 | 7 | 8.42E7 | 5.62E7 | 7.53E7 | 5.67E7 | 8.97E7 | 8.4E7 | 9.01E7 | 4.47E7 | 7.87E7 | 7.06E7 | 21 | 21 | 12 | 9 | 12 | 15 | 15 | 13 | 16 | 14 | 10 | 10 | Y | 158395 | Ribosome binding protein 1 OS=Mus musculus OX=10090 GN=Rrbp1 PE=1 SV=1 |
| 196 | 159 | sp\|Q99PL5\|RRBP1\_MOUSE | 274.04 | 13 | 6 | 5 | 7 | 10 | 9 | 8 | 10 | 9 | 6 | 6 | 8.42E7 | 5.62E7 | 7.53E7 | 5.67E7 | 8.97E7 | 8.4E7 | 9.01E7 | 4.47E7 | 7.87E7 | 7.06E7 | 21 | 21 | 12 | 9 | 12 | 15 | 15 | 13 | 16 | 14 | 10 | 10 | Y | 172878 | Ribosome-binding protein 1 OS=Mus musculus OX=10090 GN=Rrbp1 PE=1 SV=2 |
| 118 | 333 | sp\|P61982\|1433G\_MOUSE | 272.87 | 49 | 36 | 36 | 38 | 40 | 36 | 43 | 40 | 42 | 31 | 36 | 4.94E7 | 6.22E7 | 4.71E7 | 1.64E7 | 2.87E7 | 4.71E7 | 2.32E7 | 2.6E7 | 5.2E7 | 4.23E7 | 12 | 6 | 23 | 24 | 21 | 19 | 20 | 22 | 19 | 26 | 25 | 23 | Y | 28303 | 14-3-3 protein gamma OS=Mus musculus OX=10090 GN=Ywhag PE=1 SV=2 |
| 352 | 638 | sp\|P15119\|MCPT2\_MOUSE | 271.75 | 47 | 41 | 4 | 0 | 0 | 0 | 0 | 0 | 0 | 39 | 47 | 5.85E8 | 2.52E5 |  |  |  |  |  |  | 5.96E8 | 5.95E8 | 10 | 9 | 25 | 1 | 0 | 0 | 0 | 0 | 0 | 0 | 23 | 25 | Y | 26732 | Mast cell protease 2 OS=Mus musculus OX=10090 GN=Mcpt2 PE=2 SV=2 |
| 361 | 593 | sp\|P17225\|PTBP1\_MOUSE | 270.91 | 24 | 13 | 10 | 13 | 16 | 15 | 15 | 11 | 15 | 13 | 17 | 3.34E7 | 3.03E7 | 3.2E7 | 1.76E7 | 3.11E7 | 2.7E7 | 2E7 | 9.11E6 | 8.27E7 | 4.22E7 | 11 | 8 | 5 | 5 | 7 | 8 | 6 | 8 | 7 | 7 | 7 | 7 | N | 59322 | Polypyrimidine tract-binding protein 1 OS=Mus musculus OX=10090 GN=Ptbp1 PE=1 SV=3 |
| 361 | 597 | E9PZ39\|E9PZ39\_MOUSE | 270.91 | 23 | 12 | 10 | 12 | 15 | 14 | 14 | 11 | 15 | 12 | 17 | 3.34E7 | 3.03E7 | 3.2E7 | 1.76E7 | 3.11E7 | 2.7E7 | 2E7 | 9.11E6 | 8.27E7 | 4.22E7 | 11 | 8 | 5 | 5 | 7 | 8 | 6 | 8 | 7 | 7 | 7 | 7 | N | 63085 | Polypyrimidine tract binding protein 1 OS=Mus musculus OX=10090 GN=Ptbp1 PE=1 SV=2 |
| 152 | 127 | sp\|Q80X90\|FLNB\_MOUSE | 270.70 | 15 | 8 | 6 | 4 | 5 | 3 | 6 | 5 | 4 | 11 | 7 | 7.09E7 | 1.27E8 | 2.54E7 | 1.22E7 | 2.02E7 | 3.17E7 | 1.25E7 | 1.32E7 | 1.04E8 | 6.31E7 | 35 | 29 | 21 | 14 | 12 | 14 | 9 | 16 | 13 | 11 | 27 | 18 | Y | 277822 | Filamin-B OS=Mus musculus OX=10090 GN=Flnb PE=1 SV=3 |
| 219 | 746 | sp\|P97315\|CSRP1\_MOUSE | 269.72 | 64 | 51 | 51 | 37 | 37 | 47 | 54 | 46 | 37 | 59 | 46 | 2.39E8 | 1.98E8 | 1.65E8 | 6.28E7 | 1.27E8 | 2.02E8 | 9.04E7 | 1.11E8 | 3.48E8 | 1.77E8 | 10 | 10 | 15 | 15 | 9 | 8 | 11 | 10 | 8 | 9 | 21 | 14 | Y | 20583 | Cysteine and glycine-rich protein 1 OS=Mus musculus OX=10090 GN=Csrp1 PE=1 SV=3 |
| 206 | 331 | sp\|Q6PIC6\|AT1A3\_MOUSE | 269.10 | 15 | 10 | 11 | 14 | 8 | 11 | 14 | 13 | 11 | 13 | 7 |  |  |  |  | 8.26E5 |  |  |  |  |  | 15 | 1 | 10 | 14 | 15 | 10 | 12 | 15 | 12 | 12 | 12 | 7 | Y | 111691 | Sodium/potassium-transporting ATPase subunit alpha-3 OS=Mus musculus OX=10090 GN=Atp1a3 PE=1 SV=1 |
| 206 | 332 | A0A0G2JGX4\|A0A0G2JGX4\_MOUSE | 269.10 | 15 | 10 | 11 | 13 | 8 | 11 | 13 | 13 | 11 | 13 | 7 |  |  |  |  | 8.26E5 |  |  |  |  |  | 15 | 1 | 10 | 14 | 15 | 10 | 12 | 15 | 12 | 12 | 12 | 7 | Y | 112988 | Sodium/potassium-transporting ATPase subunit alpha OS=Mus musculus OX=10090 GN=Atp1a3 PE=1 SV=1 |
| 206 | 337 | Q8VCE0\|Q8VCE0\_MOUSE | 269.10 | 15 | 9 | 10 | 13 | 7 | 10 | 13 | 13 | 11 | 12 | 7 |  |  |  |  | 8.26E5 |  |  |  |  |  | 15 | 1 | 10 | 14 | 15 | 10 | 12 | 15 | 12 | 12 | 12 | 7 | Y | 115969 | Sodium/potassium-transporting ATPase subunit alpha OS=Mus musculus OX=10090 GN=Atp1a3 PE=1 SV=1 |
| 330 | 115 | sp\|P26231\|CTNA1\_MOUSE | 268.54 | 32 | 9 | 13 | 11 | 12 | 14 | 18 | 10 | 9 | 19 | 10 | 1.22E7 | 1.71E7 | 1.53E7 | 1.04E7 | 1.76E7 | 1.27E7 | 1.29E7 | 6.13E6 | 4.61E7 | 1.7E7 | 19 | 16 | 7 | 8 | 7 | 6 | 9 | 10 | 7 | 6 | 12 | 7 | Y | 100106 | Catenin alpha-1 OS=Mus musculus OX=10090 GN=Ctnna1 PE=1 SV=1 |
| 68 | 203 | sp\|P37804\|TAGL\_MOUSE | 267.80 | 85 | 61 | 48 | 58 | 59 | 66 | 65 | 60 | 59 | 49 | 60 | 1.38E9 | 1.08E9 | 8.87E8 | 4.21E8 | 8.5E8 | 1.08E9 | 5.91E8 | 6.79E8 | 1.63E9 | 8.64E8 | 18 | 18 | 45 | 31 | 24 | 28 | 29 | 34 | 26 | 33 | 50 | 36 | Y | 22576 | Transgelin OS=Mus musculus OX=10090 GN=Tagln PE=1 SV=3 |
| 212 | 379 | sp\|P97351\|RS3A\_MOUSE | 267.79 | 39 | 33 | 23 | 39 | 39 | 39 | 39 | 39 | 36 | 19 | 28 | 1.33E8 | 1.11E8 | 1.57E8 | 1.16E8 | 1.43E8 | 1.39E8 | 8.78E7 | 7.94E7 | 8.88E7 | 8.78E7 | 11 | 11 | 12 | 10 | 12 | 15 | 16 | 12 | 16 | 17 | 7 | 8 | Y | 29885 | Small ribosomal subunit protein eS1 OS=Mus musculus OX=10090 GN=Rps3a PE=1 SV=3 |
| 271 | 233 | sp\|P97807\|FUMH\_MOUSE | 267.70 | 34 | 18 | 21 | 23 | 22 | 27 | 17 | 20 | 20 | 15 | 10 | 2.77E7 | 3.24E7 | 4.12E7 | 2.06E7 | 3.86E7 | 3.98E7 | 3.55E7 | 2.41E7 | 4.32E7 | 2.31E7 | 16 | 16 | 8 | 9 | 10 | 11 | 12 | 9 | 11 | 9 | 8 | 6 | Y | 54357 | Fumarate hydratase, mitochondrial OS=Mus musculus OX=10090 GN=Fh PE=1 SV=3 |
| 116 | 223 | sp\|P06151\|LDHA\_MOUSE | 267.63 | 49 | 35 | 31 | 31 | 26 | 32 | 40 | 35 | 30 | 44 | 30 | 4.32E8 | 3.28E8 | 3.89E8 | 1.57E8 | 2.8E8 | 3.18E8 | 2.4E8 | 2.03E8 | 6.02E8 | 3.85E8 | 17 | 15 | 23 | 20 | 21 | 20 | 20 | 22 | 20 | 19 | 30 | 24 | Y | 36499 | L-lactate dehydrogenase A chain OS=Mus musculus OX=10090 GN=Ldha PE=1 SV=3 |
| 116 | 229 | A0A1B0GSX0\|A0A1B0GSX0\_MOUSE | 267.63 | 45 | 32 | 29 | 29 | 24 | 29 | 37 | 32 | 28 | 41 | 27 | 4.32E8 | 3.28E8 | 3.89E8 | 1.57E8 | 2.8E8 | 3.18E8 | 2.4E8 | 2.03E8 | 6.02E8 | 3.85E8 | 17 | 15 | 23 | 20 | 21 | 20 | 20 | 22 | 20 | 19 | 30 | 24 | Y | 39758 | L-lactate dehydrogenase OS=Mus musculus OX=10090 GN=Ldha PE=1 SV=1 |
| 302 | 211 | A0A087WPL5\|A0A087WPL5\_MOUSE | 267.04 | 14 | 5 | 6 | 5 | 4 | 7 | 7 | 4 | 4 | 8 | 6 | 2.35E7 | 2.58E7 | 2.58E7 | 7.9E6 | 2.77E7 | 1.96E7 | 8.85E6 | 9.24E6 | 4.42E7 | 1.69E7 | 17 | 16 | 7 | 8 | 9 | 6 | 10 | 12 | 7 | 6 | 10 | 9 | Y | 149618 | RNA helicase OS=Mus musculus OX=10090 GN=Dhx9 PE=1 SV=1 |
| 302 | 212 | E9QNN1\|E9QNN1\_MOUSE | 267.04 | 14 | 5 | 6 | 5 | 4 | 7 | 7 | 4 | 4 | 8 | 6 | 2.35E7 | 2.58E7 | 2.58E7 | 7.9E6 | 2.77E7 | 1.96E7 | 8.85E6 | 9.24E6 | 4.42E7 | 1.69E7 | 17 | 16 | 7 | 8 | 9 | 6 | 10 | 12 | 7 | 6 | 10 | 9 | Y | 149689 | RNA helicase OS=Mus musculus OX=10090 GN=Dhx9 PE=1 SV=1 |
| 65 | 1206 | F8WGM8\|F8WGM8\_MOUSE | 266.96 | 88 | 70 | 76 | 79 | 76 | 85 | 50 | 76 | 76 | 70 | 85 |  |  |  |  | 3.2E5 |  |  |  |  | 1.63E7 | 8 | 1 | 51 | 38 | 26 | 21 | 27 | 22 | 22 | 34 | 57 | 41 | Y | 11884 | Actin, cytoplasmic 2 (Fragment) OS=Mus musculus OX=10090 GN=Actg1 PE=1 SV=1 |
| 346 | 220 | sp\|Q80YX1\|TENA\_MOUSE | 266.66 | 9 | 5 | 2 | 2 | 2 | 3 | 4 | 4 | 3 | 6 | 4 | 7.45E7 | 7.93E6 | 1.22E7 | 9.76E6 | 1.01E7 | 2.59E7 | 1.81E7 | 1.56E7 | 1.2E8 | 6.29E7 | 18 | 18 | 11 | 3 | 4 | 5 | 5 | 7 | 8 | 6 | 14 | 10 | Y | 231805 | Tenascin OS=Mus musculus OX=10090 GN=Tnc PE=1 SV=1 |
| 255 | 315 | sp\|Q61425\|HCDH\_MOUSE | 265.75 | 58 | 16 | 28 | 27 | 28 | 39 | 28 | 48 | 16 | 41 | 30 | 4.31E7 | 7.98E7 | 7.7E7 | 3.59E7 | 5.27E7 | 8.75E7 | 6.8E7 | 2.51E7 | 9.82E7 | 6.7E7 | 15 | 15 | 6 | 10 | 10 | 8 | 11 | 10 | 10 | 5 | 11 | 8 | Y | 34464 | Hydroxyacyl-coenzyme A dehydrogenase, mitochondrial OS=Mus musculus OX=10090 GN=Hadh PE=1 SV=2 |
| 281 | 783 | sp\|Q99PT1\|GDIR1\_MOUSE | 265.04 | 52 | 49 | 49 | 42 | 51 | 51 | 42 | 36 | 44 | 49 | 39 | 8.14E7 | 9.43E7 | 7.35E7 | 3.9E7 | 7.69E7 | 6.02E7 | 3.81E7 | 5.1E7 | 1.8E8 | 7.63E7 | 10 | 10 | 9 | 10 | 8 | 9 | 11 | 8 | 7 | 9 | 14 | 10 | Y | 23407 | Rho GDP-dissociation inhibitor 1 OS=Mus musculus OX=10090 GN=Arhgdia PE=1 SV=3 |
| 203 | 174 | A0A571BF69\|A0A571BF69\_MOUSE | 264.85 | 7 | 2 | 4 | 4 | 4 | 2 | 5 | 3 | 2 | 4 | 3 | 3.38E7 | 8.77E7 | 9E7 | 4.21E7 | 3.48E7 | 6.8E7 | 4.48E7 | 2.34E7 | 4.81E7 | 5.41E7 | 23 | 20 | 9 | 17 | 18 | 16 | 8 | 18 | 12 | 10 | 14 | 10 | Y | 413024 | Maltase-glucoamylase OS=Mus musculus OX=10090 GN=Mgam PE=1 SV=1 |
| 348 | 276 | sp\|Q8BHN3\|GANAB\_MOUSE | 263.52 | 18 | 10 | 9 | 6 | 10 | 8 | 11 | 14 | 9 | 3 | 6 | 3.28E7 | 1.96E7 | 3.82E7 | 1.62E7 | 2.54E7 | 3.24E7 | 3.05E7 | 1.57E7 | 1.16E7 | 2.06E7 | 16 | 16 | 8 | 7 | 5 | 9 | 8 | 9 | 12 | 7 | 3 | 5 | N | 106911 | Neutral alpha-glucosidase AB OS=Mus musculus OX=10090 GN=Ganab PE=1 SV=1 |
| 156 | 234 | sp\|P48036\|ANXA5\_MOUSE | 262.65 | 46 | 37 | 37 | 39 | 40 | 39 | 40 | 39 | 39 | 39 | 41 | 1.38E8 | 1.66E8 | 1.91E8 | 6.01E7 | 1.28E8 | 1.86E8 | 1.02E8 | 8.48E7 | 2.74E8 | 1.43E8 | 14 | 14 | 16 | 17 | 17 | 15 | 17 | 17 | 17 | 18 | 20 | 16 | Y | 35752 | Annexin A5 OS=Mus musculus OX=10090 GN=Anxa5 PE=1 SV=1 |
| 186 | 122 | sp\|Q91YQ5\|RPN1\_MOUSE | 262.29 | 35 | 19 | 25 | 17 | 29 | 25 | 26 | 28 | 26 | 20 | 22 | 8.74E7 | 7.79E7 | 5.62E7 | 5.19E7 | 9.44E7 | 9.22E7 | 7.63E7 | 5.74E7 | 9.71E7 | 6.95E7 | 19 | 19 | 13 | 14 | 10 | 18 | 16 | 15 | 18 | 15 | 13 | 13 | N | 68528 | Dolichyl-diphosphooligosaccharide--protein glycosyltransferase subunit 1 OS=Mus musculus OX=10090 GN=Rpn1 PE=1 SV=1 |
| 60 | 950 | sp\|Q60605\|MYL6\_MOUSE | 262.26 | 56 | 56 | 56 | 48 | 48 | 48 | 56 | 48 | 48 | 48 | 56 | 6.6E8 | 6.55E8 | 1.09E9 | 3.16E8 | 7.2E8 | 6.56E8 | 4.44E8 | 5.32E8 | 1.25E9 | 6.28E8 | 8 | 7 | 47 | 41 | 37 | 24 | 36 | 38 | 22 | 45 | 44 | 36 | Y | 16930 | Myosin light polypeptide 6 OS=Mus musculus OX=10090 GN=Myl6 PE=1 SV=3 |
| 60 | 951 | A0A1W2P6F6\|A0A1W2P6F6\_MOUSE | 262.26 | 55 | 55 | 55 | 47 | 47 | 47 | 55 | 47 | 47 | 47 | 55 | 6.6E8 | 6.55E8 | 1.09E9 | 3.16E8 | 7.2E8 | 6.56E8 | 4.44E8 | 5.32E8 | 1.25E9 | 6.28E8 | 8 | 7 | 47 | 41 | 37 | 24 | 36 | 38 | 22 | 45 | 44 | 36 | Y | 17005 | Myosin, light polypeptide 6, alkali, smooth muscle and non-muscle OS=Mus musculus OX=10090 GN=Myl6 PE=1 SV=1 |
| 60 | 953 | A0A1W2P7Q9\|A0A1W2P7Q9\_MOUSE | 262.26 | 53 | 53 | 53 | 46 | 46 | 46 | 53 | 46 | 46 | 46 | 53 | 6.6E8 | 6.55E8 | 1.09E9 | 3.16E8 | 7.2E8 | 6.56E8 | 4.44E8 | 5.32E8 | 1.25E9 | 6.28E8 | 8 | 7 | 47 | 41 | 37 | 24 | 36 | 38 | 22 | 45 | 44 | 36 | Y | 17673 | Myosin, light polypeptide 6, alkali, smooth muscle and non-muscle OS=Mus musculus OX=10090 GN=Myl6 PE=1 SV=1 |
| 221 | 288 | sp\|P13020\|GELS\_MOUSE | 262.20 | 19 | 7 | 12 | 13 | 18 | 14 | 15 | 15 | 14 | 12 | 7 | 3.94E7 | 7.86E7 | 8.78E7 | 3.03E7 | 8.06E7 | 8.98E7 | 6.85E7 | 5.79E7 | 4.11E7 | 3.34E7 | 16 | 11 | 6 | 13 | 12 | 13 | 13 | 14 | 13 | 11 | 10 | 7 | Y | 85942 | Gelsolin OS=Mus musculus OX=10090 GN=Gsn PE=1 SV=3 |
| 176 | 418 | sp\|Q9WVA4\|TAGL2\_MOUSE | 262.17 | 68 | 64 | 52 | 55 | 55 | 62 | 56 | 58 | 58 | 51 | 50 | 2.4E8 | 1.8E8 | 1.89E8 | 8.26E7 | 1.32E8 | 2.07E8 | 8.08E7 | 9.25E7 | 2.85E8 | 1.61E8 | 12 | 12 | 18 | 14 | 14 | 11 | 14 | 16 | 12 | 19 | 22 | 15 | Y | 22395 | Transgelin-2 OS=Mus musculus OX=10090 GN=Tagln2 PE=1 SV=4 |
| 214 | 226 | sp\|Q9DCD0\|6PGD\_MOUSE | 262.15 | 32 | 25 | 18 | 24 | 22 | 26 | 23 | 22 | 19 | 27 | 24 | 1.19E8 | 6.59E7 | 1.24E8 | 6.31E7 | 9.21E7 | 7.18E7 | 5.94E7 | 4.92E7 | 1.58E8 | 1.03E8 | 15 | 15 | 16 | 7 | 10 | 11 | 11 | 11 | 14 | 9 | 14 | 14 | Y | 53247 | 6-phosphogluconate dehydrogenase, decarboxylating OS=Mus musculus OX=10090 GN=Pgd PE=1 SV=3 |
| 239 | 372 | sp\|Q9JI91\|ACTN2\_MOUSE | 262.15 | 14 | 8 | 11 | 9 | 11 | 9 | 11 | 11 | 11 | 7 | 9 |  | 4.17E5 |  |  |  |  |  |  |  |  | 13 | 1 | 8 | 12 | 13 | 12 | 10 | 13 | 13 | 11 | 10 | 9 | Y | 103834 | Alpha-actinin-2 OS=Mus musculus OX=10090 GN=Actn2 PE=1 SV=2 |
| 265 | 367 | sp\|Q61937\|NPM\_MOUSE | 262.05 | 46 | 34 | 30 | 35 | 27 | 30 | 26 | 34 | 31 | 24 | 28 | 9.22E7 | 8.81E7 | 1.1E8 | 3.96E7 | 5.78E7 | 7.88E7 | 7.07E7 | 5.84E7 | 1.13E8 | 7.95E7 | 12 | 12 | 9 | 9 | 9 | 8 | 10 | 9 | 10 | 9 | 11 | 8 | Y | 32560 | Nucleophosmin OS=Mus musculus OX=10090 GN=Npm1 PE=1 SV=1 |
| 263 | 138 | F8VQJ3\|F8VQJ3\_MOUSE | 261.59 | 14 | 3 | 7 | 5 | 8 | 4 | 6 | 7 | 7 | 4 | 3 |  |  | 6.41E6 | 3.82E6 | 1.25E7 |  |  | 6.47E5 |  |  | 20 | 2 | 6 | 12 | 10 | 13 | 7 | 9 | 11 | 9 | 6 | 5 | Y | 177187 | Laminin, gamma 1 OS=Mus musculus OX=10090 GN=Lamc1 PE=1 SV=1 |
| 213 | 391 | sp\|P12970\|RL7A\_MOUSE | 261.05 | 45 | 26 | 28 | 31 | 26 | 26 | 32 | 33 | 35 | 39 | 26 | 1.1E8 | 1.47E8 | 1.56E8 | 6.06E7 | 1.16E8 | 1.11E8 | 7.56E7 | 8.2E7 | 1.39E8 | 1.2E8 | 17 | 17 | 11 | 11 | 14 | 11 | 11 | 14 | 13 | 14 | 15 | 10 | Y | 29977 | Large ribosomal subunit protein eL8 OS=Mus musculus OX=10090 GN=Rpl7a PE=1 SV=2 |
| 205 | 312 | E9PXX7\|E9PXX7\_MOUSE | 260.77 | 42 | 25 | 25 | 29 | 32 | 27 | 31 | 36 | 36 | 34 | 34 | 5.88E7 | 1.18E8 | 8.64E7 | 3.68E7 | 8.36E7 | 6.3E7 | 5.85E7 | 7.14E7 | 1.14E8 | 8.69E7 | 13 | 12 | 11 | 12 | 14 | 14 | 13 | 11 | 13 | 16 | 13 | 15 | Y | 38532 | protein disulfide-isomerase OS=Mus musculus OX=10090 GN=Txndc5 PE=1 SV=1 |
| 205 | 313 | A0A0R4J1Y7\|A0A0R4J1Y7\_MOUSE | 260.77 | 45 | 27 | 27 | 31 | 34 | 29 | 33 | 38 | 38 | 37 | 36 | 5.88E7 | 1.18E8 | 8.64E7 | 3.68E7 | 8.36E7 | 6.3E7 | 5.85E7 | 7.14E7 | 1.14E8 | 8.69E7 | 13 | 12 | 11 | 12 | 14 | 14 | 13 | 11 | 13 | 16 | 13 | 15 | Y | 36084 | protein disulfide-isomerase OS=Mus musculus OX=10090 GN=Txndc5 PE=1 SV=1 |
| 205 | 317 | sp\|Q91W90\|TXND5\_MOUSE | 260.77 | 35 | 21 | 21 | 24 | 26 | 23 | 26 | 29 | 29 | 28 | 28 | 5.88E7 | 1.18E8 | 8.64E7 | 3.68E7 | 8.36E7 | 6.3E7 | 5.85E7 | 7.14E7 | 1.14E8 | 8.69E7 | 13 | 12 | 11 | 12 | 14 | 14 | 13 | 11 | 13 | 16 | 13 | 15 | Y | 46416 | Thioredoxin domain-containing protein 5 OS=Mus musculus OX=10090 GN=Txndc5 PE=1 SV=2 |
| 306 | 340 | sp\|Q7M758\|NALDL\_MOUSE | 260.49 | 25 | 10 | 19 | 13 | 16 | 11 | 21 | 14 | 8 | 15 | 9 | 2.12E7 | 1E8 | 3.67E7 | 3.37E7 | 2.31E7 | 1.01E8 | 3.56E7 | 1.91E7 | 8.86E7 | 1.56E7 | 13 | 13 | 5 | 13 | 9 | 11 | 7 | 11 | 9 | 4 | 10 | 4 | Y | 80513 | Aminopeptidase NAALADL1 OS=Mus musculus OX=10090 GN=Naaladl1 PE=2 SV=1 |
| 318 | 282 | sp\|P80314\|TCPB\_MOUSE | 260.11 | 32 | 18 | 18 | 18 | 18 | 17 | 17 | 18 | 20 | 20 | 14 | 1.2E7 | 2.38E7 | 2.57E7 | 1.56E7 | 2.6E7 | 3.29E7 | 1.27E7 | 1.73E7 | 4E7 | 2.41E7 | 16 | 16 | 6 | 9 | 9 | 8 | 8 | 8 | 8 | 8 | 9 | 7 | Y | 57477 | T-complex protein 1 subunit beta OS=Mus musculus OX=10090 GN=Cct2 PE=1 SV=4 |
| 278 | 189 | F8VQM5\|F8VQM5\_MOUSE | 260.04 | 11 | 5 | 5 | 6 | 6 | 3 | 5 | 3 | 3 | 9 | 4 | 2.09E7 | 2.51E7 | 4.06E7 | 2.14E7 | 6.11E6 | 1.99E7 | 1.01E7 | 5.5E6 | 7.71E7 | 2.17E7 | 20 | 17 | 8 | 10 | 12 | 11 | 5 | 8 | 5 | 5 | 17 | 8 | Y | 208983 | Sucrase isomaltase OS=Mus musculus OX=10090 GN=Sis PE=1 SV=1 |
| 601 | 253 | sp\|P01027\|CO3\_MOUSE | 259.27 | 11 | 8 | 1 | 5 | 2 | 1 | 3 | 2 | 2 | 2 | 3 | 3.82E7 | 2.77E6 | 6.39E6 | 3.93E6 | 1.79E6 | 6.04E6 | 1.48E6 | 1.51E6 | 3.33E6 | 2.84E6 | 15 | 15 | 11 | 1 | 7 | 3 | 2 | 3 | 2 | 3 | 2 | 3 | Y | 186483 | Complement C3 OS=Mus musculus OX=10090 GN=C3 PE=1 SV=3 |
| 112 | 262 | sp\|P68510\|1433F\_MOUSE | 258.86 | 57 | 37 | 37 | 50 | 41 | 44 | 53 | 37 | 39 | 35 | 40 | 5.8E8 | 6.54E8 | 6.13E8 | 4.07E7 | 4.59E8 | 4.66E8 | 3.01E8 | 3.74E8 | 6.28E8 | 4.41E7 | 14 | 8 | 24 | 24 | 23 | 19 | 21 | 23 | 18 | 24 | 26 | 24 | Y | 28212 | 14-3-3 protein eta OS=Mus musculus OX=10090 GN=Ywhah PE=1 SV=2 |
| 274 | 471 | Q3TML0\|Q3TML0\_MOUSE | 258.68 | 32 | 23 | 22 | 17 | 22 | 24 | 22 | 25 | 22 | 22 | 22 | 8.22E7 | 6.03E7 | 1.21E8 | 5.88E7 | 1.06E8 | 1E8 | 8.47E7 | 7.43E7 | 1.99E8 | 8.94E7 | 10 | 10 | 9 | 7 | 10 | 10 | 11 | 9 | 10 | 9 | 11 | 8 | Y | 48689 | Protein disulfide-isomerase A6 OS=Mus musculus OX=10090 GN=Pdia6 PE=1 SV=1 |
| 234 | 300 | sp\|Q9D8E6\|RL4\_MOUSE | 257.75 | 38 | 26 | 18 | 23 | 26 | 22 | 25 | 29 | 26 | 29 | 24 | 1.03E8 | 8.03E7 | 9.52E7 | 5.75E7 | 1.06E8 | 1.08E8 | 9.15E7 | 4.81E7 | 1.32E8 | 1.21E8 | 17 | 17 | 12 | 7 | 10 | 10 | 10 | 12 | 13 | 14 | 14 | 12 | Y | 47154 | Large ribosomal subunit protein uL4 OS=Mus musculus OX=10090 GN=Rpl4 PE=1 SV=3 |
| 399 | 399 | sp\|Q8K2B3\|SDHA\_MOUSE | 257.72 | 21 | 12 | 12 | 14 | 17 | 8 | 17 | 14 | 15 | 11 | 8 | 3.69E7 | 6.07E7 | 3.91E7 | 1.99E7 | 2.03E7 | 4.72E7 | 2.79E7 | 3.08E7 | 8.18E6 | 2.19E7 | 10 | 10 | 6 | 7 | 6 | 8 | 4 | 8 | 6 | 7 | 4 | 4 | Y | 72585 | Succinate dehydrogenase [ubiquinone] flavoprotein subunit, mitochondrial OS=Mus musculus OX=10090 GN=Sdha PE=1 SV=1 |
| 353 | 440 | sp\|Q8BFW7\|LPP\_MOUSE | 257.21 | 28 | 23 | 22 | 19 | 16 | 14 | 21 | 16 | 12 | 15 | 23 | 1.01E8 | 6.32E7 | 4.37E7 | 2.4E7 | 4.35E7 | 5.56E7 | 2.13E7 | 2.13E7 | 7.9E7 | 7.47E7 | 11 | 11 | 9 | 8 | 6 | 8 | 7 | 8 | 6 | 5 | 6 | 9 | Y | 65891 | Lipoma-preferred partner homolog OS=Mus musculus OX=10090 GN=Lpp PE=1 SV=1 |
| 72 | 326 | sp\|P35700\|PRDX1\_MOUSE | 257.15 | 64 | 64 | 63 | 64 | 61 | 64 | 64 | 64 | 64 | 61 | 56 | 7.11E8 | 6.86E8 | 7.16E8 | 3.05E8 | 5.67E8 | 5.16E8 | 4.38E8 | 4.18E8 | 9.53E8 | 6.1E8 | 14 | 11 | 38 | 35 | 33 | 26 | 30 | 22 | 27 | 33 | 42 | 35 | Y | 22176 | Peroxiredoxin-1 OS=Mus musculus OX=10090 GN=Prdx1 PE=1 SV=1 |
| 295 | 1093 | sp\|Q99JW5\|EPCAM\_MOUSE | 255.99 | 29 | 16 | 18 | 18 | 18 | 21 | 29 | 18 | 18 | 26 | 18 | 6.56E7 | 1.28E8 | 1.28E8 | 7.68E7 | 9.54E7 | 1.39E8 | 6.99E7 | 4.85E7 | 1.97E8 | 6.57E7 | 8 | 8 | 7 | 8 | 11 | 9 | 11 | 10 | 10 | 6 | 10 | 8 | Y | 35019 | Epithelial cell adhesion molecule OS=Mus musculus OX=10090 GN=Epcam PE=1 SV=1 |
| 408 | 328 | sp\|P24527\|LKHA4\_MOUSE | 255.27 | 23 | 11 | 10 | 9 | 10 | 9 | 15 | 15 | 10 | 11 | 7 | 2.49E7 | 3.19E7 | 2.96E7 | 1.12E7 | 6.16E6 | 3.11E7 | 2.43E7 | 8.13E6 | 4.92E7 | 2.37E7 | 12 | 12 | 7 | 7 | 6 | 5 | 4 | 8 | 7 | 5 | 8 | 4 | Y | 69051 | Leukotriene A-4 hydrolase OS=Mus musculus OX=10090 GN=Lta4h PE=1 SV=4 |
| 349 | 693 | sp\|P97372\|PSME2\_MOUSE | 254.60 | 36 | 26 | 26 | 25 | 33 | 30 | 33 | 36 | 22 | 16 | 31 | 3.49E7 | 3.41E7 | 2.63E7 | 2.39E7 | 2.27E7 | 5.87E7 | 4.63E7 | 7.89E6 | 1.54E7 | 4.34E7 | 10 | 9 | 8 | 8 | 6 | 8 | 8 | 8 | 11 | 5 | 4 | 9 | Y | 27057 | Proteasome activator complex subunit 2 OS=Mus musculus OX=10090 GN=Psme2 PE=1 SV=4 |
| 446 | 235 | sp\|P51432\|PLCB3\_MOUSE | 254.48 | 13 | 4 | 5 | 5 | 4 | 5 | 6 | 8 | 4 | 9 | 2 | 8.86E6 | 1.74E7 | 2.21E7 | 6.15E6 | 2.09E7 | 1.55E7 | 1.88E7 | 5.35E6 | 3.11E7 | 1.09E7 | 15 | 15 | 3 | 6 | 6 | 5 | 6 | 6 | 8 | 4 | 9 | 3 | Y | 139487 | 1-phosphatidylinositol 4,5-bisphosphate phosphodiesterase beta-3 OS=Mus musculus OX=10090 GN=Plcb3 PE=1 SV=2 |
| 416 | 311 | sp\|P10107\|ANXA1\_MOUSE | 253.78 | 43 | 21 | 13 | 17 | 8 | 3 | 21 | 11 | 14 | 32 | 25 | 6.88E7 | 2.09E7 | 2.12E7 | 3.22E6 | 5.15E6 | 1.46E7 | 6.55E6 | 7.1E6 | 1.19E8 | 7.58E7 | 13 | 13 | 8 | 4 | 5 | 2 | 1 | 6 | 3 | 4 | 13 | 8 | Y | 38734 | Annexin A1 OS=Mus musculus OX=10090 GN=Anxa1 PE=1 SV=2 |
| 240 | 175 | sp\|Q61656\|DDX5\_MOUSE | 253.34 | 29 | 17 | 12 | 18 | 17 | 20 | 19 | 21 | 18 | 22 | 20 | 1.54E7 | 2E7 | 2.27E7 | 1.29E7 | 2.46E7 | 2.26E7 | 1.77E7 | 1.69E7 | 4.79E7 | 2.91E7 | 18 | 12 | 9 | 6 | 12 | 10 | 12 | 11 | 12 | 11 | 14 | 12 | Y | 69290 | Probable ATP-dependent RNA helicase DDX5 OS=Mus musculus OX=10090 GN=Ddx5 PE=1 SV=2 |
| 240 | 176 | Q8BTS0\|Q8BTS0\_MOUSE | 253.34 | 29 | 17 | 12 | 18 | 17 | 20 | 19 | 20 | 18 | 22 | 20 | 1.54E7 | 2E7 | 2.27E7 | 1.29E7 | 2.46E7 | 2.26E7 | 1.77E7 | 1.69E7 | 4.79E7 | 2.91E7 | 18 | 12 | 9 | 6 | 12 | 10 | 12 | 11 | 12 | 11 | 14 | 12 | Y | 69266 | Probable ATP-dependent RNA helicase DDX5 OS=Mus musculus OX=10090 GN=Ddx5 PE=1 SV=1 |
| 470 | 348 | sp\|Q61176\|ARGI1\_MOUSE | 253.32 | 50 | 36 | 0 | 0 | 0 | 0 | 0 | 0 | 0 | 50 | 41 | 1.34E8 |  |  |  |  |  |  |  | 1.72E8 | 1.69E8 | 13 | 13 | 14 | 0 | 0 | 0 | 0 | 0 | 0 | 0 | 19 | 19 | Y | 34808 | Arginase-1 OS=Mus musculus OX=10090 GN=Arg1 PE=1 SV=1 |
| 106 | 619 | sp\|P17742\|PPIA\_MOUSE | 251.79 | 58 | 51 | 45 | 51 | 49 | 51 | 43 | 45 | 49 | 49 | 51 | 4.31E8 | 3.93E8 | 3.73E8 | 1.86E8 | 3.71E8 | 3.35E8 | 2.26E8 | 2.85E8 | 7.4E8 | 3.27E8 | 12 | 11 | 28 | 25 | 22 | 18 | 23 | 16 | 16 | 24 | 34 | 27 | Y | 17971 | Peptidyl-prolyl cis-trans isomerase A OS=Mus musculus OX=10090 GN=Ppia PE=1 SV=2 |
| 192 | 206 | sp\|Q61696\|HS71A\_MOUSE | 251.66 | 28 | 21 | 6 | 12 | 10 | 10 | 13 | 16 | 12 | 18 | 14 | 3.07E7 | 8.27E6 | 6.26E6 | 7.39E5 | 8.8E6 | 1.15E7 | 1.28E7 | 2.54E6 | 5.67E6 | 8.08E6 | 16 | 7 | 19 | 10 | 14 | 11 | 14 | 15 | 12 | 14 | 15 | 15 | Y | 70079 | Heat shock 70 kDa protein 1A OS=Mus musculus OX=10090 GN=Hspa1a PE=1 SV=2 |
| 192 | 207 | sp\|P17879\|HS71B\_MOUSE | 251.66 | 28 | 21 | 6 | 12 | 10 | 10 | 13 | 16 | 12 | 18 | 14 | 3.07E7 | 8.27E6 | 6.26E6 | 7.39E5 | 8.8E6 | 1.15E7 | 1.28E7 | 2.54E6 | 5.67E6 | 8.08E6 | 16 | 7 | 19 | 10 | 14 | 11 | 14 | 15 | 12 | 14 | 15 | 15 | Y | 70176 | Heat shock 70 kDa protein 1B OS=Mus musculus OX=10090 GN=Hspa1b PE=1 SV=3 |
| 48 | 1143 | sp\|P51162\|FABP6\_MOUSE | 250.99 | 66 | 45 | 66 | 45 | 45 | 45 | 66 | 45 | 45 | 45 | 45 | 1.27E9 | 3.86E9 | 8E8 | 8.84E8 | 2.65E9 | 3.08E9 | 1.29E9 | 1.04E9 | 3.77E9 | 1.42E9 | 8 | 8 | 36 | 73 | 20 | 30 | 61 | 62 | 30 | 36 | 61 | 39 | Y | 14486 | Gastrotropin OS=Mus musculus OX=10090 GN=Fabp6 PE=2 SV=2 |
| 123 | 751 | sp\|P62962\|PROF1\_MOUSE | 250.87 | 72 | 66 | 66 | 66 | 65 | 66 | 66 | 66 | 57 | 72 | 66 | 4E8 | 3.28E8 | 3.84E8 | 8.77E7 | 2.5E8 | 2.91E8 | 1.94E8 | 2E8 | 7.94E8 | 3.77E8 | 9 | 9 | 28 | 19 | 19 | 18 | 20 | 21 | 16 | 21 | 29 | 25 | Y | 14957 | Profilin-1 OS=Mus musculus OX=10090 GN=Pfn1 PE=1 SV=2 |
| 136 | 947 | sp\|Q3THE2\|ML12B\_MOUSE | 250.82 | 51 | 51 | 51 | 45 | 51 | 51 | 51 | 51 | 51 | 51 | 51 | 4.35E7 | 4.47E7 | 2.72E7 | 2.21E7 | 4.76E7 | 4.59E7 | 2.64E7 | 2.6E7 | 9.35E7 | 4.46E7 | 7 | 2 | 25 | 21 | 16 | 13 | 16 | 23 | 14 | 20 | 23 | 16 | Y | 19779 | Myosin regulatory light chain 12B OS=Mus musculus OX=10090 GN=Myl12b PE=1 SV=2 |
| 185 | 258 | sp\|Q8VEK3\|HNRPU\_MOUSE | 250.41 | 20 | 13 | 13 | 16 | 11 | 19 | 16 | 14 | 14 | 13 | 14 | 1.26E8 | 1.36E8 | 1.28E8 | 4.23E7 | 1.05E8 | 1.2E8 | 6.07E7 | 6.32E7 | 1.98E8 | 1.24E8 | 14 | 14 | 12 | 14 | 13 | 10 | 15 | 13 | 11 | 10 | 14 | 13 | Y | 87918 | Heterogeneous nuclear ribonucleoprotein U OS=Mus musculus OX=10090 GN=Hnrnpu PE=1 SV=1 |
| 459 | 587 | sp\|Q921U8\|SMTN\_MOUSE | 250.40 | 11 | 5 | 6 | 7 | 7 | 10 | 10 | 10 | 10 | 4 | 3 | 1.24E7 | 1.37E7 | 2.15E7 | 8.82E6 | 2.81E7 | 3.6E7 | 1.72E7 | 1.86E7 | 1.68E7 | 3.86E6 | 8 | 8 | 3 | 4 | 6 | 6 | 8 | 8 | 7 | 7 | 3 | 2 | Y | 100289 | Smoothelin OS=Mus musculus OX=10090 GN=Smtn PE=1 SV=2 |
| 459 | 588 | D3Z3Q3\|D3Z3Q3\_MOUSE | 250.40 | 11 | 5 | 6 | 7 | 7 | 9 | 9 | 10 | 10 | 4 | 3 | 1.24E7 | 1.37E7 | 2.15E7 | 8.82E6 | 2.81E7 | 3.6E7 | 1.72E7 | 1.86E7 | 1.68E7 | 3.86E6 | 8 | 8 | 3 | 4 | 6 | 6 | 8 | 8 | 7 | 7 | 3 | 2 | Y | 103040 | Smoothelin OS=Mus musculus OX=10090 GN=Smtn PE=1 SV=1 |
| 288 | 913 | sp\|P62141\|PP1B\_MOUSE | 249.98 | 29 | 21 | 21 | 24 | 24 | 24 | 29 | 24 | 24 | 18 | 18 | 3.82E6 | 4.53E6 | 3.22E6 | 8.97E5 | 4.96E6 | 5.76E6 | 1.6E6 | 1.95E6 |  |  | 8 | 2 | 7 | 9 | 10 | 9 | 11 | 14 | 10 | 8 | 7 | 7 | Y | 37187 | Serine/threonine-protein phosphatase PP1-beta catalytic subunit OS=Mus musculus OX=10090 GN=Ppp1cb PE=1 SV=3 |
| 179 | 153 | sp\|P38647\|GRP75\_MOUSE | 249.76 | 29 | 17 | 17 | 21 | 20 | 20 | 19 | 20 | 16 | 16 | 15 | 7.18E7 | 8.78E7 | 8.68E7 | 6.26E7 | 8.4E7 | 5.99E7 | 5.7E7 | 4.99E7 | 9.62E7 | 6.95E7 | 19 | 16 | 13 | 12 | 19 | 20 | 17 | 14 | 18 | 14 | 12 | 12 | Y | 73461 | Stress-70 protein, mitochondrial OS=Mus musculus OX=10090 GN=Hspa9 PE=1 SV=3 |
| 341 | 376 | sp\|Q8BFR5\|EFTU\_MOUSE | 249.07 | 30 | 12 | 22 | 21 | 17 | 25 | 23 | 22 | 27 | 18 | 18 | 2.09E7 | 4.1E7 | 3.65E7 | 7.64E6 | 3.34E7 | 2.43E7 | 3.89E7 | 2.77E7 | 5.84E7 | 3.17E7 | 11 | 11 | 5 | 9 | 8 | 6 | 10 | 9 | 9 | 10 | 6 | 6 | Y | 49508 | Elongation factor Tu, mitochondrial OS=Mus musculus OX=10090 GN=Tufm PE=1 SV=1 |
| 222 | 274 | sp\|P60843\|IF4A1\_MOUSE | 248.87 | 39 | 27 | 22 | 25 | 24 | 27 | 27 | 25 | 25 | 28 | 21 | 7.21E7 | 3.79E7 | 6.79E7 | 3.11E7 | 3.89E7 | 5.82E7 | 3.13E7 | 3.97E7 | 1.1E8 | 4.5E7 | 17 | 15 | 12 | 8 | 12 | 12 | 12 | 13 | 13 | 12 | 13 | 9 | Y | 46154 | Eukaryotic initiation factor 4A-I OS=Mus musculus OX=10090 GN=Eif4a1 PE=1 SV=1 |
| 396 | 305 | Q3U2G2\|Q3U2G2\_MOUSE | 247.87 | 16 | 8 | 7 | 10 | 10 | 7 | 11 | 7 | 9 | 7 | 8 | 3.65E7 | 1.56E7 | 3.08E7 | 1.58E7 | 7.27E6 | 2.8E7 | 9.2E6 | 1.78E7 | 4.14E7 | 2.59E7 | 12 | 11 | 7 | 5 | 8 | 7 | 5 | 9 | 5 | 7 | 5 | 5 | Y | 94209 | Heat shock 70 kDa protein 4 OS=Mus musculus OX=10090 GN=Hspa4 PE=1 SV=1 |
| 178 | 193 | sp\|P05064\|ALDOA\_MOUSE | 247.80 | 51 | 36 | 35 | 26 | 22 | 29 | 37 | 31 | 32 | 37 | 35 | 1.44E7 | 2.61E6 |  |  |  | 3.13E6 |  | 2.32E6 | 4.27E7 | 3.36E6 | 17 | 2 | 23 | 13 | 13 | 8 | 13 | 15 | 15 | 12 | 18 | 16 | Y | 39356 | Fructose-bisphosphate aldolase A OS=Mus musculus OX=10090 GN=Aldoa PE=1 SV=2 |
| 178 | 195 | A6ZI44\|A6ZI44\_MOUSE | 247.80 | 44 | 32 | 31 | 22 | 19 | 25 | 32 | 27 | 28 | 32 | 31 | 1.44E7 | 2.61E6 |  |  |  | 3.13E6 |  | 2.32E6 | 4.27E7 | 3.36E6 | 17 | 2 | 23 | 13 | 13 | 8 | 13 | 15 | 15 | 12 | 18 | 16 | Y | 45120 | Fructose-bisphosphate aldolase OS=Mus musculus OX=10090 GN=Aldoa PE=1 SV=1 |
| 236 | 468 | sp\|P14869\|RLA0\_MOUSE | 247.65 | 44 | 28 | 23 | 21 | 31 | 37 | 31 | 33 | 33 | 33 | 33 | 1.42E8 | 1.19E8 | 1.18E8 | 8.09E7 | 1.2E8 | 9.93E7 | 8.58E7 | 8.41E7 | 2.21E8 | 1.19E8 | 12 | 12 | 11 | 9 | 8 | 12 | 13 | 9 | 10 | 13 | 15 | 12 | Y | 34216 | Large ribosomal subunit protein uL10 OS=Mus musculus OX=10090 GN=Rplp0 PE=1 SV=3 |
| 128 | 148 | sp\|P62908\|RS3\_MOUSE | 247.47 | 79 | 66 | 73 | 70 | 52 | 72 | 63 | 65 | 60 | 55 | 59 | 1.96E8 | 2.45E8 | 2.33E8 | 1.08E8 | 2.11E8 | 1.78E8 | 1.52E8 | 1.47E8 | 3.45E8 | 1.66E8 | 18 | 17 | 23 | 23 | 20 | 18 | 26 | 20 | 18 | 21 | 21 | 18 | Y | 26674 | Small ribosomal subunit protein uS3 OS=Mus musculus OX=10090 GN=Rps3 PE=1 SV=1 |
| 14 | 573 | sp\|Q6ZWY9\|H2B1C\_MOUSE | 247.36 | 69 | 51 | 51 | 51 | 63 | 54 | 51 | 54 | 54 | 36 | 51 | 1.06E9 | 1.16E9 | 1.2E9 | 4.48E8 | 9.32E8 | 7.93E8 | 6.2E8 | 7.35E8 | 1.55E9 | 9.99E8 | 10 | 3 | 116 | 110 | 111 | 95 | 113 | 104 | 98 | 129 | 113 | 116 | Y | 13906 | Histone H2B type 1-C/E/G OS=Mus musculus OX=10090 GN=H2bc4 PE=1 SV=3 |
| 14 | 574 | sp\|Q64478\|H2B1H\_MOUSE | 247.36 | 69 | 51 | 51 | 51 | 63 | 54 | 51 | 54 | 54 | 36 | 51 | 1.06E9 | 1.16E9 | 1.2E9 | 4.48E8 | 9.32E8 | 7.93E8 | 6.2E8 | 7.35E8 | 1.55E9 | 9.99E8 | 10 | 3 | 116 | 110 | 111 | 95 | 113 | 104 | 98 | 129 | 113 | 116 | Y | 13920 | Histone H2B type 1-H OS=Mus musculus OX=10090 GN=H2bc9 PE=1 SV=3 |
| 14 | 575 | sp\|P10853\|H2B1F\_MOUSE | 247.36 | 69 | 51 | 51 | 51 | 63 | 54 | 51 | 54 | 54 | 36 | 51 | 1.06E9 | 1.16E9 | 1.2E9 | 4.48E8 | 9.32E8 | 7.93E8 | 6.2E8 | 7.35E8 | 1.55E9 | 9.99E8 | 10 | 3 | 116 | 110 | 111 | 95 | 113 | 104 | 98 | 129 | 113 | 116 | Y | 13936 | Histone H2B type 1-F/J/L OS=Mus musculus OX=10090 GN=H2bc7 PE=1 SV=2 |
| 325 | 546 | sp\|Q8QZT1\|THIL\_MOUSE | 247.04 | 32 | 17 | 17 | 16 | 13 | 18 | 18 | 22 | 14 | 14 | 12 | 3.15E7 | 4.82E7 | 3.57E7 | 1.66E7 | 2.57E7 | 4.39E7 | 1.94E7 | 1.15E7 | 4.91E7 | 1.9E7 | 11 | 11 | 8 | 9 | 7 | 6 | 9 | 10 | 10 | 7 | 8 | 5 | Y | 44816 | Acetyl-CoA acetyltransferase, mitochondrial OS=Mus musculus OX=10090 GN=Acat1 PE=1 SV=1 |
| 279 | 255 | sp\|Q8CI94\|PYGB\_MOUSE | 246.77 | 19 | 9 | 12 | 8 | 7 | 11 | 12 | 10 | 11 | 11 | 10 | 1.74E7 | 1.91E7 | 1.81E7 | 1.41E6 | 9.3E6 | 1.63E7 | 9.79E6 | 6.69E6 | 1.99E7 | 1.84E7 | 18 | 13 | 9 | 10 | 8 | 6 | 10 | 11 | 9 | 9 | 9 | 8 | N | 96730 | Glycogen phosphorylase, brain form OS=Mus musculus OX=10090 GN=Pygb PE=1 SV=3 |
| 147 | 959 | sp\|O88312\|AGR2\_MOUSE | 246.12 | 51 | 26 | 47 | 26 | 51 | 51 | 51 | 51 | 41 | 27 | 27 | 3.49E8 | 3.75E8 | 3.44E8 | 1.54E8 | 3.26E8 | 3.06E8 | 2.2E8 | 2.27E8 | 2.81E8 | 2.52E8 | 8 | 8 | 17 | 19 | 17 | 17 | 22 | 20 | 17 | 19 | 15 | 15 | Y | 19920 | Anterior gradient protein 2 homolog OS=Mus musculus OX=10090 GN=Agr2 PE=1 SV=1 |
| 174 | 375 | sp\|P14148\|RL7\_MOUSE | 245.89 | 47 | 29 | 36 | 29 | 39 | 32 | 38 | 36 | 29 | 36 | 43 | 1.4E8 | 1.3E8 | 1.54E8 | 8.92E7 | 1.22E8 | 1.35E8 | 9.52E7 | 9.12E7 | 2.76E8 | 1.43E8 | 14 | 13 | 12 | 16 | 14 | 13 | 14 | 14 | 14 | 13 | 16 | 15 | Y | 31420 | Large ribosomal subunit protein uL30 OS=Mus musculus OX=10090 GN=Rpl7 PE=1 SV=2 |
| 395 | 694 | sp\|Q9Z2X1\|HNRPF\_MOUSE | 245.55 | 26 | 11 | 14 | 14 | 20 | 23 | 9 | 18 | 16 | 18 | 10 | 4.13E6 | 2.65E7 | 2.98E7 | 2.15E7 | 5.01E7 | 3.98E7 | 1.9E7 | 2.3E7 | 5.45E7 | 1.79E7 | 10 | 8 | 4 | 6 | 6 | 10 | 9 | 4 | 7 | 6 | 8 | 4 | Y | 45730 | Heterogeneous nuclear ribonucleoprotein F OS=Mus musculus OX=10090 GN=Hnrnpf PE=1 SV=3 |
| 436 | 377 | sp\|Q99JY9\|ARP3\_MOUSE | 245.53 | 41 | 9 | 26 | 25 | 14 | 15 | 27 | 20 | 15 | 25 | 19 | 2.13E7 | 3.24E7 | 2.6E7 | 1.06E7 | 2.18E7 | 5.15E7 | 1.81E7 | 1.47E7 | 4.68E7 | 2.93E7 | 12 | 12 | 2 | 7 | 7 | 5 | 3 | 8 | 6 | 5 | 6 | 5 | Y | 47357 | Actin-related protein 3 OS=Mus musculus OX=10090 GN=Actr3 PE=1 SV=3 |
| 379 | 299 | sp\|P28271\|ACOHC\_MOUSE | 245.18 | 19 | 9 | 11 | 9 | 9 | 8 | 10 | 5 | 6 | 6 | 4 | 1.82E7 | 2.84E7 | 2.27E7 | 9.4E6 | 2.66E7 | 2.9E7 | 6.82E6 | 5.65E6 | 2.06E7 | 1.33E7 | 12 | 12 | 6 | 7 | 8 | 7 | 7 | 10 | 4 | 4 | 5 | 4 | Y | 98126 | Cytoplasmic aconitate hydratase OS=Mus musculus OX=10090 GN=Aco1 PE=1 SV=3 |
| 238 | 334 | sp\|P14152\|MDHC\_MOUSE | 245.03 | 44 | 20 | 24 | 20 | 21 | 20 | 30 | 26 | 23 | 26 | 11 | 1.29E8 | 1.99E8 | 2.44E8 | 7.86E7 | 1.56E8 | 1.68E8 | 1.32E8 | 6.53E7 | 2.35E8 | 9.66E7 | 14 | 14 | 12 | 14 | 11 | 10 | 12 | 11 | 10 | 10 | 11 | 6 | Y | 36511 | Malate dehydrogenase, cytoplasmic OS=Mus musculus OX=10090 GN=Mdh1 PE=1 SV=3 |
| 238 | 336 | A0A5F8MPN8\|A0A5F8MPN8\_MOUSE | 245.03 | 41 | 19 | 23 | 19 | 19 | 19 | 28 | 24 | 22 | 24 | 11 | 1.29E8 | 1.99E8 | 2.44E8 | 7.86E7 | 1.56E8 | 1.68E8 | 1.32E8 | 6.53E7 | 2.35E8 | 9.66E7 | 14 | 14 | 12 | 14 | 11 | 10 | 12 | 11 | 10 | 10 | 11 | 6 | Y | 39005 | Malate dehydrogenase OS=Mus musculus OX=10090 GN=Mdh1 PE=1 SV=1 |
| 235 | 549 | A0A0J9YUZ4\|A0A0J9YUZ4\_MOUSE | 244.97 | 51 | 28 | 36 | 36 | 41 | 40 | 40 | 33 | 48 | 39 | 36 | 1.29E8 | 1.39E8 | 1.61E8 | 4.33E7 | 1.1E8 | 1.05E8 | 4.4E7 | 8.72E7 | 2.02E8 | 9.84E7 | 13 | 11 | 11 | 13 | 12 | 10 | 13 | 12 | 10 | 11 | 14 | 11 | Y | 24234 | High mobility group protein B1 (Fragment) OS=Mus musculus OX=10090 GN=Hmgb1 PE=1 SV=1 |
| 235 | 550 | sp\|P63158\|HMGB1\_MOUSE | 244.97 | 50 | 28 | 35 | 35 | 40 | 39 | 39 | 32 | 47 | 39 | 35 | 1.29E8 | 1.39E8 | 1.61E8 | 4.33E7 | 1.1E8 | 1.05E8 | 4.4E7 | 8.72E7 | 2.02E8 | 9.84E7 | 13 | 11 | 11 | 13 | 12 | 10 | 13 | 12 | 10 | 11 | 14 | 11 | Y | 24894 | High mobility group protein B1 OS=Mus musculus OX=10090 GN=Hmgb1 PE=1 SV=2 |
| 316 | 974 | sp\|P63087\|PP1G\_MOUSE | 244.80 | 25 | 19 | 19 | 22 | 22 | 22 | 22 | 22 | 22 | 22 | 19 |  |  |  |  |  |  |  |  | 1.52E6 |  | 7 | 1 | 6 | 8 | 9 | 8 | 10 | 12 | 9 | 7 | 8 | 7 | Y | 36984 | Serine/threonine-protein phosphatase PP1-gamma catalytic subunit OS=Mus musculus OX=10090 GN=Ppp1cc PE=1 SV=1 |
| 165 | 562 | A2AL12\|A2AL12\_MOUSE | 244.42 | 39 | 24 | 29 | 26 | 34 | 31 | 31 | 31 | 29 | 24 | 29 | 1.65E8 | 1.93E8 | 2.02E8 | 8.28E7 | 1.6E8 | 1.61E8 | 1.19E8 | 1.18E8 | 1.45E8 | 1.54E8 | 13 | 10 | 14 | 14 | 18 | 15 | 18 | 16 | 19 | 17 | 16 | 16 | Y | 34476 | Heterogeneous nuclear ribonucleoprotein A3 OS=Mus musculus OX=10090 GN=Hnrnpa3 PE=1 SV=1 |
| 165 | 568 | sp\|Q8BG05\|ROA3\_MOUSE | 244.42 | 32 | 20 | 24 | 22 | 28 | 26 | 26 | 26 | 24 | 20 | 24 | 1.65E8 | 1.93E8 | 2.02E8 | 8.28E7 | 1.6E8 | 1.61E8 | 1.19E8 | 1.18E8 | 1.45E8 | 1.54E8 | 13 | 10 | 14 | 14 | 18 | 15 | 18 | 16 | 19 | 17 | 16 | 16 | Y | 39652 | Heterogeneous nuclear ribonucleoprotein A3 OS=Mus musculus OX=10090 GN=Hnrnpa3 PE=1 SV=1 |
| 321 | 324 | sp\|Q8BWT1\|THIM\_MOUSE | 243.88 | 42 | 21 | 27 | 29 | 19 | 20 | 24 | 15 | 28 | 25 | 21 | 3.94E7 | 6.44E7 | 4.68E7 | 1.33E7 | 3.12E7 | 4.36E7 | 1.42E7 | 1.78E7 | 6.66E7 | 3.61E7 | 13 | 13 | 7 | 9 | 10 | 6 | 7 | 8 | 6 | 8 | 10 | 7 | Y | 41830 | 3-ketoacyl-CoA thiolase, mitochondrial OS=Mus musculus OX=10090 GN=Acaa2 PE=1 SV=3 |
| 230 | 197 | sp\|P14733\|LMNB1\_MOUSE | 243.24 | 37 | 14 | 15 | 18 | 14 | 19 | 26 | 13 | 25 | 11 | 18 | 4.31E7 | 5.62E7 | 6.02E7 | 2.35E7 | 5.41E7 | 7.04E7 | 2.61E7 | 4.9E7 | 4.13E7 | 5.57E7 | 22 | 17 | 10 | 9 | 11 | 10 | 11 | 16 | 8 | 17 | 8 | 11 | Y | 66786 | Lamin-B1 OS=Mus musculus OX=10090 GN=Lmnb1 PE=1 SV=3 |
| 328 | 1027 | sp\|P62137\|PP1A\_MOUSE | 242.60 | 24 | 20 | 20 | 23 | 24 | 23 | 24 | 23 | 23 | 20 | 20 | 2.13E6 | 1.27E6 | 1.49E6 | 7.77E5 | 4.94E5 | 1.34E6 | 1.08E6 | 8.18E5 | 3.12E6 | 2.75E6 | 7 | 2 | 6 | 8 | 9 | 8 | 9 | 12 | 9 | 7 | 7 | 7 | Y | 37540 | Serine/threonine-protein phosphatase PP1-alpha catalytic subunit OS=Mus musculus OX=10090 GN=Ppp1ca PE=1 SV=1 |
| 194 | 918 | sp\|P11034\|MCPT1\_MOUSE | 242.59 | 40 | 40 | 0 | 0 | 0 | 0 | 18 | 0 | 9 | 40 | 40 | 1.42E9 |  |  |  |  | 5.47E6 |  | 1E6 | 1.35E9 | 1.07E9 | 8 | 6 | 51 | 0 | 0 | 0 | 0 | 3 | 0 | 1 | 48 | 41 | Y | 27013 | Mast cell protease 1 OS=Mus musculus OX=10090 GN=Mcpt1 PE=1 SV=2 |
| 360 | 579 | sp\|Q8K0C9\|GMDS\_MOUSE | 242.11 | 30 | 22 | 20 | 22 | 20 | 22 | 24 | 22 | 18 | 19 | 15 | 6.24E7 | 5.26E7 | 5.49E7 | 1.64E7 | 5.98E7 | 5.65E7 | 3.38E7 | 2.28E7 | 4.58E7 | 4.63E7 | 11 | 11 | 9 | 8 | 7 | 6 | 7 | 9 | 7 | 6 | 6 | 6 | Y | 41985 | GDP-mannose 4,6 dehydratase OS=Mus musculus OX=10090 GN=Gmds PE=1 SV=1 |
| 283 | 475 | sp\|Q60932\|VDAC1\_MOUSE | 241.83 | 44 | 20 | 24 | 22 | 18 | 25 | 30 | 24 | 22 | 35 | 33 | 5.21E7 | 5.76E7 | 6.49E7 | 3.17E7 | 5.66E7 | 6.67E7 | 3.02E7 | 1.58E7 | 6.86E7 | 4.14E7 | 10 | 8 | 8 | 11 | 10 | 7 | 9 | 10 | 7 | 7 | 12 | 10 | Y | 32351 | Non-selective voltage-gated ion channel VDAC1 OS=Mus musculus OX=10090 GN=Vdac1 PE=1 SV=3 |
| 691 | 820 | sp\|Q61029\|LAP2B\_MOUSE | 241.52 | 23 | 4 | 11 | 11 | 8 | 15 | 10 | 16 | 15 | 10 | 12 | 7.3E5 | 5.1E6 | 1.17E7 | 5.04E6 | 1.52E7 | 7.4E6 | 1.39E7 | 1.06E7 | 2.97E7 | 9.89E6 | 7 | 7 | 1 | 3 | 3 | 2 | 4 | 3 | 5 | 4 | 4 | 3 | Y | 50373 | Lamina-associated polypeptide 2, isoforms beta/delta/epsilon/gamma OS=Mus musculus OX=10090 GN=Tmpo PE=1 SV=4 |
| 282 | 239 | Q3UE92\|Q3UE92\_MOUSE | 241.19 | 23 | 17 | 22 | 12 | 15 | 14 | 13 | 20 | 14 | 15 | 10 | 2.84E7 | 6.38E7 | 2.35E7 | 1.95E7 | 1.55E7 | 3.86E7 | 3.74E7 | 1.31E7 | 4.1E7 | 1.48E7 | 14 | 13 | 10 | 14 | 6 | 9 | 8 | 7 | 12 | 9 | 8 | 6 | Y | 74560 | X-prolyl aminopeptidase (aminopeptidase P) 1, soluble OS=Mus musculus OX=10090 GN=Xpnpep1 PE=1 SV=1 |
| 282 | 241 | S4R1I3\|S4R1I3\_MOUSE | 241.19 | 24 | 18 | 23 | 13 | 16 | 14 | 14 | 21 | 14 | 15 | 11 | 2.84E7 | 6.38E7 | 2.35E7 | 1.95E7 | 1.55E7 | 3.86E7 | 3.74E7 | 1.31E7 | 4.1E7 | 1.48E7 | 14 | 13 | 10 | 14 | 6 | 9 | 8 | 7 | 12 | 9 | 8 | 6 | Y | 70990 | X-prolyl aminopeptidase (aminopeptidase P) 1, soluble OS=Mus musculus OX=10090 GN=Xpnpep1 PE=1 SV=1 |
| 415 | 474 | sp\|P68040\|RACK1\_MOUSE | 240.89 | 39 | 15 | 15 | 18 | 18 | 14 | 15 | 24 | 32 | 15 | 25 | 4.86E7 | 5.99E7 | 6.67E7 | 3.44E7 | 5.76E7 | 4.39E7 | 3.79E7 | 3.04E7 | 2.14E7 | 5.42E7 | 11 | 10 | 4 | 5 | 5 | 6 | 4 | 4 | 8 | 10 | 4 | 7 | Y | 35077 | Small ribosomal subunit protein RACK1 OS=Mus musculus OX=10090 GN=Rack1 PE=1 SV=3 |
| 465 | 287 | sp\|Q02248\|CTNB1\_MOUSE | 240.82 | 22 | 7 | 9 | 7 | 8 | 10 | 12 | 9 | 5 | 10 | 5 | 5.58E6 | 1.47E7 | 6.96E6 | 6.92E6 | 1.36E7 | 1.43E7 | 1.7E7 | 7.5E6 | 1.54E7 | 6.53E6 | 14 | 12 | 4 | 5 | 4 | 5 | 8 | 7 | 6 | 3 | 6 | 4 | Y | 85471 | Catenin beta-1 OS=Mus musculus OX=10090 GN=Ctnnb1 PE=1 SV=1 |
| 184 | 554 | sp\|Q6IRU2\|TPM4\_MOUSE | 240.77 | 39 | 30 | 26 | 23 | 26 | 21 | 25 | 31 | 28 | 26 | 29 | 4.77E7 | 3.24E7 | 2.94E7 | 8.09E6 | 2.22E7 | 2.14E7 | 3.78E7 | 2.45E7 | 8.03E7 | 5.24E7 | 14 | 8 | 13 | 11 | 11 | 13 | 15 | 16 | 18 | 18 | 12 | 13 | N | 28468 | Tropomyosin alpha-4 chain OS=Mus musculus OX=10090 GN=Tpm4 PE=1 SV=3 |
| 355 | 194 | sp\|Q9DBR7\|MYPT1\_MOUSE | 240.28 | 20 | 5 | 5 | 7 | 9 | 7 | 10 | 6 | 6 | 3 | 1 | 3.27E7 | 1.68E7 | 3.51E7 | 2.41E7 | 3.33E7 | 5.1E7 | 1.9E7 | 2.21E7 | 1.38E7 | 1.2E7 | 19 | 17 | 6 | 6 | 9 | 10 | 8 | 10 | 6 | 7 | 4 | 3 | N | 114996 | Protein phosphatase 1 regulatory subunit 12A OS=Mus musculus OX=10090 GN=Ppp1r12a PE=1 SV=2 |
| 373 | 368 | sp\|Q9CZ13\|QCR1\_MOUSE | 240.24 | 28 | 12 | 15 | 11 | 18 | 13 | 14 | 17 | 9 | 23 | 8 | 3.66E7 | 8.7E7 | 5.32E7 | 3.29E7 | 5.37E7 | 7.22E7 | 2.78E7 | 2.2E7 | 7.84E7 | 1.92E7 | 12 | 12 | 7 | 7 | 5 | 8 | 7 | 7 | 7 | 4 | 11 | 5 | Y | 52852 | Cytochrome b-c1 complex subunit 1, mitochondrial OS=Mus musculus OX=10090 GN=Uqcrc1 PE=1 SV=2 |
| 149 | 304 | sp\|Q08091\|CNN1\_MOUSE | 239.32 | 53 | 34 | 42 | 47 | 33 | 47 | 36 | 38 | 36 | 34 | 27 | 3.1E8 | 2.69E8 | 3.24E8 | 1.1E8 | 2.57E8 | 3.71E8 | 1.8E8 | 2.05E8 | 4.48E8 | 1.73E8 | 16 | 13 | 20 | 17 | 17 | 14 | 18 | 18 | 16 | 16 | 24 | 15 | Y | 33356 | Calponin-1 OS=Mus musculus OX=10090 GN=Cnn1 PE=1 SV=1 |
| 276 | 386 | sp\|P10649\|GSTM1\_MOUSE | 239.20 | 51 | 33 | 44 | 45 | 36 | 38 | 45 | 35 | 39 | 38 | 39 | 3.69E7 | 8.22E7 | 7.46E7 | 2.44E7 | 5.03E7 | 7.53E7 | 3E7 | 4.06E7 | 3.12E7 | 2.77E7 | 12 | 8 | 7 | 13 | 13 | 7 | 9 | 12 | 8 | 10 | 8 | 8 | Y | 25970 | Glutathione S-transferase Mu 1 OS=Mus musculus OX=10090 GN=Gstm1 PE=1 SV=2 |
| 276 | 389 | A2AE89\|A2AE89\_MOUSE | 239.20 | 45 | 30 | 39 | 40 | 32 | 34 | 40 | 31 | 34 | 34 | 34 | 3.69E7 | 8.22E7 | 7.46E7 | 2.44E7 | 5.03E7 | 7.53E7 | 3E7 | 4.06E7 | 3.12E7 | 2.77E7 | 12 | 8 | 7 | 13 | 13 | 7 | 9 | 12 | 8 | 10 | 8 | 8 | Y | 28556 | Glutathione S-transferase OS=Mus musculus OX=10090 GN=Gstm1 PE=1 SV=2 |
| 177 | 458 | sp\|P16627\|HS71L\_MOUSE | 239.19 | 21 | 13 | 9 | 9 | 9 | 7 | 9 | 9 | 11 | 12 | 10 |  | 0E0 |  |  |  |  |  |  |  |  | 11 | 1 | 17 | 15 | 17 | 11 | 16 | 15 | 10 | 18 | 18 | 16 | Y | 70637 | Heat shock 70 kDa protein 1-like OS=Mus musculus OX=10090 GN=Hspa1l PE=1 SV=4 |
| 322 | 296 | sp\|P70441\|NHRF1\_MOUSE | 238.81 | 40 | 25 | 29 | 18 | 21 | 21 | 29 | 28 | 21 | 21 | 23 | 8.46E7 | 1.5E8 | 4.65E7 | 2.49E7 | 4.93E7 | 1.01E8 | 6.22E7 | 2.8E7 | 6.46E7 | 6.24E7 | 13 | 13 | 10 | 12 | 5 | 7 | 8 | 10 | 8 | 6 | 7 | 8 | N | 38600 | Na(+)/H(+) exchange regulatory cofactor NHE-RF1 OS=Mus musculus OX=10090 GN=Nherf1 PE=1 SV=3 |
| 208 | 314 | sp\|Q64133\|AOFA\_MOUSE | 238.77 | 24 | 13 | 15 | 11 | 13 | 12 | 15 | 15 | 12 | 20 | 15 | 3.74E7 | 7.79E7 | 4.42E7 | 1.99E7 | 4.3E7 | 6.48E7 | 3.44E7 | 1.84E7 | 8.73E7 | 2.9E7 | 12 | 10 | 11 | 17 | 13 | 10 | 11 | 16 | 14 | 11 | 15 | 11 | N | 59602 | Amine oxidase [flavin-containing] A OS=Mus musculus OX=10090 GN=Maoa PE=1 SV=3 |
| 378 | 243 | sp\|Q99LC5\|ETFA\_MOUSE | 237.99 | 62 | 24 | 21 | 28 | 25 | 19 | 42 | 21 | 28 | 29 | 22 | 3.97E7 | 5.53E7 | 4.33E7 | 1.43E7 | 2.89E7 | 4.27E7 | 1.77E7 | 2.64E7 | 4.08E7 | 3.43E7 | 15 | 15 | 7 | 7 | 7 | 7 | 6 | 10 | 5 | 7 | 8 | 6 | Y | 35009 | Electron transfer flavoprotein subunit alpha, mitochondrial OS=Mus musculus OX=10090 GN=Etfa PE=1 SV=2 |
| 253 | 761 | sp\|O88310\|ITL1A\_MOUSE | 237.92 | 30 | 13 | 26 | 19 | 22 | 21 | 19 | 27 | 12 | 13 | 19 | 1.65E8 | 1.3E8 | 1.32E8 | 5.46E7 | 1.09E8 | 6.77E7 | 9.62E7 | 5.14E7 | 1.51E8 | 9.46E7 | 9 | 9 | 13 | 13 | 10 | 9 | 11 | 8 | 12 | 8 | 13 | 9 | N | 34953 | Intelectin-1a OS=Mus musculus OX=10090 GN=Itln1 PE=1 SV=1 |
| 11 | 1196 | sp\|P62806\|H4\_MOUSE | 237.07 | 57 | 51 | 56 | 51 | 51 | 56 | 51 | 51 | 51 | 53 | 52 | 6.62E9 | 6.73E9 | 8.89E9 | 3.3E9 | 4.27E9 | 5.48E9 | 3.95E9 | 5.1E9 | 1.08E10 | 6E9 | 10 | 10 | 145 | 151 | 158 | 135 | 155 | 126 | 126 | 170 | 152 | 161 | Y | 11367 | Histone H4 OS=Mus musculus OX=10090 GN=H4c1 PE=1 SV=2 |
| 250 | 170 | sp\|Q9D0E1\|HNRPM\_MOUSE | 236.94 | 26 | 11 | 8 | 19 | 19 | 16 | 19 | 17 | 16 | 12 | 10 | 2.55E7 | 2.66E7 | 5.3E7 | 2.73E7 | 4.61E7 | 5.04E7 | 4.03E7 | 2.96E7 | 5.38E7 | 2.62E7 | 17 | 16 | 7 | 5 | 12 | 13 | 10 | 13 | 14 | 13 | 7 | 6 | Y | 77649 | Heterogeneous nuclear ribonucleoprotein M OS=Mus musculus OX=10090 GN=Hnrnpm PE=1 SV=3 |
| 605 | 589 | sp\|P23953\|EST1C\_MOUSE | 236.89 | 24 | 8 | 9 | 15 | 11 | 13 | 14 | 9 | 12 | 0 | 3 | 7.41E6 | 4.22E6 | 5.29E6 | 2.56E6 | 2.33E6 | 1.17E7 | 6.51E6 | 5.55E6 |  | 1.37E6 | 9 | 7 | 4 | 4 | 5 | 5 | 4 | 6 | 5 | 5 | 0 | 2 | Y | 61056 | Carboxylesterase 1C OS=Mus musculus OX=10090 GN=Ces1c PE=1 SV=4 |
| 354 | 447 | sp\|O54724\|CAVN1\_MOUSE | 236.85 | 30 | 23 | 14 | 20 | 22 | 23 | 20 | 25 | 21 | 16 | 12 | 5.68E7 | 4.69E7 | 6.83E7 | 4.37E7 | 5.51E7 | 7.69E7 | 3.85E7 | 3.12E7 | 5.12E7 | 3.33E7 | 13 | 12 | 8 | 7 | 8 | 9 | 7 | 7 | 9 | 6 | 9 | 6 | Y | 43954 | Caveolae-associated protein 1 OS=Mus musculus OX=10090 GN=Cavin1 PE=1 SV=1 |
| 364 | 263 | sp\|Q62009\|POSTN\_MOUSE | 236.70 | 20 | 11 | 11 | 10 | 6 | 13 | 15 | 6 | 10 | 9 | 7 | 2.2E7 | 3.99E7 | 3.33E7 | 1.39E7 | 3.13E7 | 3.07E7 | 8.53E6 | 1.28E7 | 4.06E7 | 1.68E7 | 14 | 13 | 7 | 8 | 7 | 5 | 9 | 9 | 4 | 7 | 6 | 6 | Y | 93144 | Periostin OS=Mus musculus OX=10090 GN=Postn PE=1 SV=2 |
| 100 | 630 | sp\|Q9CQW5\|LEG2\_MOUSE | 236.47 | 82 | 62 | 62 | 62 | 66 | 62 | 62 | 62 | 66 | 77 | 62 | 8.69E8 | 5.85E8 | 5.82E8 | 2.72E8 | 3.74E8 | 4.23E8 | 3E8 | 3.13E8 | 5.78E8 | 3.27E8 | 9 | 9 | 24 | 26 | 28 | 24 | 26 | 25 | 20 | 25 | 30 | 24 | Y | 14880 | Galectin-2 OS=Mus musculus OX=10090 GN=Lgals2 PE=1 SV=1 |
| 450 | 609 | sp\|O35129\|PHB2\_MOUSE | 236.18 | 36 | 11 | 28 | 28 | 16 | 29 | 22 | 29 | 19 | 17 | 25 | 2.77E7 | 4.2E7 | 3.31E7 | 1.61E7 | 2.83E7 | 3.07E7 | 2.47E7 | 1.97E7 | 2.26E7 | 3.76E7 | 10 | 9 | 3 | 7 | 6 | 4 | 6 | 7 | 6 | 5 | 4 | 5 | N | 33296 | Prohibitin-2 OS=Mus musculus OX=10090 GN=Phb2 PE=1 SV=1 |
| 299 | 230 | sp\|P26443\|DHE3\_MOUSE | 236.10 | 36 | 14 | 19 | 20 | 14 | 13 | 22 | 10 | 14 | 18 | 15 | 4.47E7 | 6.83E7 | 7.85E7 | 3.1E7 | 3.88E7 | 6.57E7 | 3.49E7 | 4.12E7 | 6.26E7 | 3.58E7 | 17 | 17 | 8 | 10 | 11 | 7 | 7 | 11 | 5 | 8 | 9 | 7 | Y | 61337 | Glutamate dehydrogenase 1, mitochondrial OS=Mus musculus OX=10090 GN=Glud1 PE=1 SV=1 |
| 367 | 610 | sp\|Q9D6R2\|IDH3A\_MOUSE | 235.68 | 27 | 19 | 25 | 27 | 24 | 22 | 25 | 16 | 22 | 25 | 23 | 3.64E7 | 5.92E7 | 5.39E7 | 1.67E7 | 3.17E7 | 3.93E7 | 2.25E7 | 2.2E7 | 5.9E7 | 3.1E7 | 9 | 9 | 7 | 7 | 8 | 7 | 6 | 8 | 6 | 7 | 7 | 7 | Y | 39639 | Isocitrate dehydrogenase [NAD] subunit alpha, mitochondrial OS=Mus musculus OX=10090 GN=Idh3a PE=1 SV=1 |
| 367 | 617 | A0A1L1STE6\|A0A1L1STE6\_MOUSE | 235.68 | 26 | 18 | 24 | 26 | 23 | 21 | 23 | 15 | 21 | 24 | 22 | 3.64E7 | 5.92E7 | 5.39E7 | 1.67E7 | 3.17E7 | 3.93E7 | 2.25E7 | 2.2E7 | 5.9E7 | 3.1E7 | 9 | 9 | 7 | 7 | 8 | 7 | 6 | 8 | 6 | 7 | 7 | 7 | Y | 41520 | Isocitrate dehydrogenase [NAD] subunit, mitochondrial OS=Mus musculus OX=10090 GN=Idh3a PE=1 SV=1 |
| 294 | 319 | sp\|P54071\|IDHP\_MOUSE | 235.62 | 29 | 15 | 18 | 25 | 16 | 18 | 25 | 16 | 14 | 15 | 13 | 2.1E7 | 3.86E7 | 8.27E7 | 2.53E7 | 2.83E7 | 6.09E7 | 2.86E7 | 1.52E7 | 2.54E7 | 2.02E7 | 14 | 13 | 6 | 10 | 13 | 8 | 9 | 13 | 9 | 8 | 7 | 5 | N | 50906 | Isocitrate dehydrogenase [NADP], mitochondrial OS=Mus musculus OX=10090 GN=Idh2 PE=1 SV=3 |
| 119 | 224 | sp\|Q9DCV7\|K2C7\_MOUSE | 235.52 | 39 | 27 | 22 | 23 | 19 | 19 | 20 | 24 | 25 | 21 | 28 | 2.52E7 | 4.85E7 | 4.15E7 | 1.43E7 | 2.45E7 | 4.64E7 | 3.08E7 | 1.6E7 | 6.54E7 | 3.88E7 | 19 | 13 | 26 | 20 | 19 | 15 | 21 | 20 | 20 | 26 | 28 | 23 | Y | 50709 | Keratin, type II cytoskeletal 7 OS=Mus musculus OX=10090 GN=Krt7 PE=1 SV=1 |
| 943 | 1467 | sp\|P97425\|ECP2\_MOUSE | 234.92 | 50 | 26 | 0 | 0 | 0 | 0 | 0 | 0 | 0 | 39 | 50 | 2.76E7 |  |  |  |  |  |  |  | 2.31E7 | 3.97E7 | 5 | 3 | 6 | 0 | 0 | 0 | 0 | 0 | 0 | 0 | 7 | 5 | Y | 17620 | Eosinophil cationic protein 2 OS=Mus musculus OX=10090 GN=Ear2 PE=2 SV=1 |
| 162 | 310 | sp\|P47911\|RL6\_MOUSE | 234.73 | 42 | 34 | 36 | 41 | 40 | 36 | 39 | 37 | 36 | 35 | 40 | 1.49E8 | 2.06E8 | 2.61E8 | 1.24E8 | 1.63E8 | 1.75E8 | 1.59E8 | 1.3E8 | 2.58E8 | 1.89E8 | 14 | 14 | 19 | 15 | 18 | 18 | 16 | 16 | 16 | 18 | 18 | 15 | N | 33510 | Large ribosomal subunit protein eL6 OS=Mus musculus OX=10090 GN=Rpl6 PE=1 SV=3 |
| 256 | 318 | sp\|O88844\|IDHC\_MOUSE | 234.57 | 29 | 14 | 24 | 19 | 23 | 23 | 23 | 22 | 18 | 20 | 18 | 7.26E7 | 9.14E7 | 8.1E7 | 3.47E7 | 5.67E7 | 7.32E7 | 3.58E7 | 2.75E7 | 7.61E7 | 7.49E7 | 12 | 11 | 8 | 13 | 11 | 10 | 10 | 10 | 10 | 9 | 9 | 10 | Y | 46674 | Isocitrate dehydrogenase [NADP] cytoplasmic OS=Mus musculus OX=10090 GN=Idh1 PE=1 SV=2 |
| 296 | 555 | sp\|Q9DB20\|ATPO\_MOUSE | 234.08 | 55 | 41 | 44 | 36 | 41 | 41 | 49 | 37 | 52 | 41 | 31 | 4.79E7 | 6.93E7 | 6.45E7 | 6.74E7 | 4.38E7 | 5.18E7 | 1.08E8 | 2.83E7 | 9.07E7 | 3.58E7 | 10 | 10 | 9 | 11 | 9 | 7 | 8 | 9 | 8 | 10 | 9 | 7 | Y | 23364 | ATP synthase subunit O, mitochondrial OS=Mus musculus OX=10090 GN=Atp5po PE=1 SV=1 |
| 334 | 307 | sp\|P47856\|GFPT1\_MOUSE | 233.75 | 21 | 10 | 13 | 14 | 14 | 16 | 16 | 14 | 10 | 6 | 8 | 1.68E7 | 1.46E7 | 2.22E7 | 1.39E7 | 2E7 | 1.98E7 | 1.1E7 | 1.08E7 | 1.55E7 | 3.84E6 | 13 | 9 | 7 | 8 | 10 | 10 | 10 | 10 | 9 | 6 | 4 | 5 | Y | 78539 | Glutamine--fructose-6-phosphate aminotransferase [isomerizing] 1 OS=Mus musculus OX=10090 GN=Gfpt1 PE=1 SV=3 |
| 180 | 249 | sp\|Q91Y97\|ALDOB\_MOUSE | 233.60 | 39 | 29 | 32 | 33 | 33 | 35 | 35 | 35 | 19 | 27 | 27 | 1.41E8 | 2.28E8 | 1.96E8 | 7.22E7 | 9.88E7 | 1.82E8 | 1.1E8 | 4.22E7 | 2.18E8 | 1.19E8 | 15 | 14 | 14 | 19 | 16 | 14 | 14 | 17 | 16 | 8 | 15 | 15 | Y | 39507 | Fructose-bisphosphate aldolase B OS=Mus musculus OX=10090 GN=Aldob PE=1 SV=3 |
| 557 | 481 | sp\|Q9QZE5\|COPG1\_MOUSE | 232.62 | 14 | 3 | 9 | 7 | 3 | 3 | 8 | 8 | 3 | 12 | 7 | 1.28E6 | 5.95E6 | 3.08E6 | 2.05E6 | 6.94E5 | 3.07E6 | 4.94E6 | 5.83E5 | 1.71E7 | 7.39E6 | 10 | 8 | 2 | 6 | 5 | 3 | 2 | 5 | 5 | 2 | 8 | 5 | Y | 97513 | Coatomer subunit gamma-1 OS=Mus musculus OX=10090 GN=Copg1 PE=1 SV=1 |
| 319 | 301 | sp\|Q8JZR0\|ACSL5\_MOUSE | 232.48 | 22 | 11 | 18 | 17 | 12 | 10 | 17 | 19 | 6 | 8 | 6 | 1.12E7 | 3.43E7 | 3.52E7 | 1.52E7 | 1.28E7 | 3.21E7 | 2.78E7 | 8.93E6 | 1.31E7 | 3.93E5 | 12 | 10 | 6 | 11 | 10 | 9 | 6 | 12 | 12 | 4 | 5 | 3 | Y | 76206 | Long-chain-fatty-acid--CoA ligase 5 OS=Mus musculus OX=10090 GN=Acsl5 PE=1 SV=1 |
| 420 | 401 | G3UZI2\|G3UZI2\_MOUSE | 232.34 | 21 | 12 | 9 | 14 | 14 | 13 | 12 | 15 | 10 | 13 | 10 | 2.28E7 | 7.04E6 | 1.54E7 | 9.72E6 | 1.16E7 | 1.14E7 | 1.64E7 | 7.9E6 | 2.32E7 | 1.57E7 | 10 | 7 | 5 | 4 | 6 | 6 | 6 | 5 | 7 | 4 | 5 | 4 | N | 58752 | Synaptotagmin binding, cytoplasmic RNA interacting protein OS=Mus musculus OX=10090 GN=Syncrip PE=1 SV=1 |
| 420 | 404 | A0A0R4J259\|A0A0R4J259\_MOUSE | 232.34 | 20 | 11 | 9 | 13 | 13 | 12 | 11 | 14 | 9 | 12 | 9 | 2.28E7 | 7.04E6 | 1.54E7 | 9.72E6 | 1.16E7 | 1.14E7 | 1.64E7 | 7.9E6 | 2.32E7 | 1.57E7 | 10 | 7 | 5 | 4 | 6 | 6 | 6 | 5 | 7 | 4 | 5 | 4 | N | 62544 | Synaptotagmin binding, cytoplasmic RNA interacting protein OS=Mus musculus OX=10090 GN=Syncrip PE=1 SV=1 |
| 549 | 407 | sp\|P02469\|LAMB1\_MOUSE | 232.34 | 8 | 5 | 3 | 2 | 0 | 2 | 4 | 3 | 2 | 2 | 2 | 8.7E6 | 3.19E6 | 5.46E6 | 0E0 | 2.59E6 | 8.85E6 | 5.02E6 | 3.43E6 | 3.6E6 | 5.27E6 | 13 | 13 | 7 | 4 | 4 | 1 | 4 | 7 | 3 | 3 | 2 | 3 | Y | 197089 | Laminin subunit beta-1 OS=Mus musculus OX=10090 GN=Lamb1 PE=1 SV=3 |
| 549 | 409 | E9QN70\|E9QN70\_MOUSE | 232.34 | 7 | 5 | 3 | 2 | 0 | 2 | 4 | 2 | 2 | 2 | 2 | 8.7E6 | 3.19E6 | 5.46E6 | 0E0 | 2.59E6 | 8.85E6 | 5.02E6 | 3.43E6 | 3.6E6 | 5.27E6 | 13 | 13 | 7 | 4 | 4 | 1 | 4 | 7 | 3 | 3 | 2 | 3 | Y | 202440 | Laminin B1 OS=Mus musculus OX=10090 GN=Lamb1 PE=1 SV=1 |
| 560 | 413 | sp\|O70423\|AOC3\_MOUSE | 232.28 | 20 | 8 | 9 | 5 | 3 | 3 | 6 | 5 | 4 | 7 | 5 | 1.15E7 | 2.87E7 | 1.23E7 | 7.06E6 | 1.77E6 | 1.89E7 | 1.58E6 | 7.56E5 | 7.5E6 | 1.81E7 | 13 | 12 | 4 | 5 | 4 | 2 | 3 | 6 | 2 | 2 | 4 | 4 | Y | 84534 | Amine oxidase [copper-containing] 3 OS=Mus musculus OX=10090 GN=Aoc3 PE=1 SV=3 |
| 451 | 380 | sp\|Q9WUA3\|PFKAP\_MOUSE | 232.18 | 22 | 9 | 9 | 5 | 8 | 9 | 13 | 11 | 4 | 10 | 9 | 2.58E7 | 2.07E7 | 2E7 | 1.61E6 | 1.68E7 | 1.54E7 | 1.05E7 | 4.65E5 | 4.1E7 | 1.94E7 | 13 | 11 | 6 | 5 | 4 | 4 | 5 | 8 | 7 | 3 | 7 | 6 | Y | 85455 | ATP-dependent 6-phosphofructokinase, platelet type OS=Mus musculus OX=10090 GN=Pfkp PE=1 SV=1 |
| 451 | 381 | Q8C605\|Q8C605\_MOUSE | 232.18 | 22 | 9 | 9 | 5 | 8 | 9 | 13 | 11 | 4 | 10 | 9 | 2.58E7 | 2.07E7 | 2E7 | 1.61E6 | 1.68E7 | 1.54E7 | 1.05E7 | 4.65E5 | 4.1E7 | 1.94E7 | 13 | 11 | 6 | 5 | 4 | 4 | 5 | 8 | 7 | 3 | 7 | 6 | Y | 85547 | ATP-dependent 6-phosphofructokinase OS=Mus musculus OX=10090 GN=Pfkp PE=1 SV=1 |
| 512 | 522 | Q3UJQ9\|Q3UJQ9\_MOUSE | 231.50 | 29 | 16 | 16 | 10 | 2 | 13 | 18 | 14 | 11 | 17 | 13 | 1.49E7 | 1.04E7 | 1.25E7 | 4.81E5 | 1.09E7 | 2.16E7 | 6.29E6 | 4.95E6 | 1.71E7 | 9.21E6 | 10 | 10 | 5 | 4 | 4 | 1 | 5 | 8 | 5 | 5 | 5 | 4 | Y | 52278 | Succinyl-CoA:3-ketoacid-coenzyme A transferase OS=Mus musculus OX=10090 GN=Oxct1 PE=1 SV=1 |
| 512 | 528 | sp\|Q9D0K2\|SCOT1\_MOUSE | 231.50 | 27 | 15 | 15 | 9 | 2 | 12 | 17 | 13 | 10 | 16 | 12 | 1.49E7 | 1.04E7 | 1.25E7 | 4.81E5 | 1.09E7 | 2.16E7 | 6.29E6 | 4.95E6 | 1.71E7 | 9.21E6 | 10 | 10 | 5 | 4 | 4 | 1 | 5 | 8 | 5 | 5 | 5 | 4 | Y | 55989 | Succinyl-CoA:3-ketoacid coenzyme A transferase 1, mitochondrial OS=Mus musculus OX=10090 GN=Oxct1 PE=1 SV=1 |
| 368 | 353 | sp\|Q99JG3\|ANX13\_MOUSE | 231.07 | 43 | 11 | 19 | 35 | 43 | 11 | 27 | 30 | 13 | 20 | 15 | 8.8E6 | 1.72E7 | 4.18E7 | 1.99E7 | 6.02E6 | 2.03E7 | 1.39E7 | 2.32E6 | 2.75E7 | 1.29E7 | 11 | 11 | 4 | 6 | 12 | 12 | 3 | 7 | 10 | 4 | 6 | 6 | Y | 35922 | Annexin A13 OS=Mus musculus OX=10090 GN=Anxa13 PE=1 SV=3 |
| 469 | 392 | sp\|P19324\|SERPH\_MOUSE | 230.64 | 33 | 19 | 13 | 10 | 10 | 12 | 12 | 10 | 6 | 23 | 21 | 3.67E7 | 3.73E7 | 1.67E7 | 8.76E6 | 8.72E6 | 8.84E6 | 9.38E6 | 5.56E6 | 6.9E7 | 3.38E7 | 11 | 11 | 6 | 4 | 3 | 3 | 4 | 4 | 4 | 2 | 10 | 9 | Y | 46534 | Serpin H1 OS=Mus musculus OX=10090 GN=Serpinh1 PE=1 SV=3 |
| 227 | 632 | sp\|P49312\|ROA1\_MOUSE | 230.36 | 31 | 22 | 20 | 21 | 26 | 26 | 26 | 26 | 20 | 28 | 22 | 4.14E7 | 4.27E7 | 3.57E7 | 3.12E7 | 4.89E7 | 4.25E7 | 3.24E7 | 1.95E7 | 7.06E7 | 4.62E7 | 10 | 6 | 9 | 11 | 13 | 13 | 15 | 12 | 14 | 10 | 13 | 11 | N | 34196 | Heterogeneous nuclear ribonucleoprotein A1 OS=Mus musculus OX=10090 GN=Hnrnpa1 PE=1 SV=2 |
| 227 | 636 | Q5EBP8\|Q5EBP8\_MOUSE | 230.36 | 26 | 19 | 17 | 18 | 22 | 22 | 22 | 22 | 17 | 24 | 19 | 4.14E7 | 4.27E7 | 3.57E7 | 3.12E7 | 4.89E7 | 4.25E7 | 3.24E7 | 1.95E7 | 7.06E7 | 4.62E7 | 10 | 6 | 9 | 11 | 13 | 13 | 15 | 12 | 14 | 10 | 13 | 11 | N | 38834 | Heterogeneous nuclear ribonucleoprotein A1 OS=Mus musculus OX=10090 GN=Hnrnpa1 PE=1 SV=1 |
| 139 | 371 | sp\|O70456\|1433S\_MOUSE | 230.32 | 47 | 21 | 31 | 36 | 33 | 32 | 40 | 28 | 22 | 23 | 27 | 1.3E7 | 3.25E7 | 3.08E7 | 1.16E7 | 3.01E7 | 4.21E7 | 1.77E7 | 4.72E6 | 2.72E7 | 1.83E7 | 12 | 7 | 19 | 22 | 19 | 15 | 18 | 21 | 15 | 19 | 23 | 21 | Y | 27706 | 14-3-3 protein sigma OS=Mus musculus OX=10090 GN=Sfn PE=1 SV=2 |
| 530 | 833 | H3BLJ9\|H3BLJ9\_MOUSE | 229.32 | 40 | 29 | 33 | 25 | 27 | 17 | 23 | 21 | 17 | 37 | 7 | 1.69E7 | 1.77E7 | 1.46E7 | 8.68E6 | 9.93E6 | 1.82E7 | 9.79E6 | 5.62E6 | 3.96E7 | 2.46E6 | 8 | 8 | 6 | 6 | 4 | 5 | 3 | 4 | 4 | 3 | 8 | 1 | Y | 28554 | S-formylglutathione hydrolase OS=Mus musculus OX=10090 GN=Esd PE=1 SV=1 |
| 530 | 834 | H3BJL6\|H3BJL6\_MOUSE | 229.32 | 39 | 28 | 32 | 25 | 27 | 16 | 23 | 20 | 16 | 36 | 7 | 1.69E7 | 1.77E7 | 1.46E7 | 8.68E6 | 9.93E6 | 1.82E7 | 9.79E6 | 5.62E6 | 3.96E7 | 2.46E6 | 8 | 8 | 6 | 6 | 4 | 5 | 3 | 4 | 4 | 3 | 8 | 1 | Y | 29347 | S-formylglutathione hydrolase OS=Mus musculus OX=10090 GN=Esd PE=1 SV=1 |
| 530 | 835 | sp\|Q9R0P3\|ESTD\_MOUSE | 229.32 | 37 | 27 | 30 | 23 | 25 | 15 | 21 | 19 | 15 | 34 | 6 | 1.69E7 | 1.77E7 | 1.46E7 | 8.68E6 | 9.93E6 | 1.82E7 | 9.79E6 | 5.62E6 | 3.96E7 | 2.46E6 | 8 | 8 | 6 | 6 | 4 | 5 | 3 | 4 | 4 | 3 | 8 | 1 | Y | 31320 | S-formylglutathione hydrolase OS=Mus musculus OX=10090 GN=Esd PE=1 SV=1 |
| 530 | 836 | H3BKH6\|H3BKH6\_MOUSE | 229.32 | 35 | 25 | 29 | 22 | 24 | 15 | 20 | 18 | 15 | 32 | 6 | 1.69E7 | 1.77E7 | 1.46E7 | 8.68E6 | 9.93E6 | 1.82E7 | 9.79E6 | 5.62E6 | 3.96E7 | 2.46E6 | 8 | 8 | 6 | 6 | 4 | 5 | 3 | 4 | 4 | 3 | 8 | 1 | Y | 32829 | S-formylglutathione hydrolase OS=Mus musculus OX=10090 GN=Esd PE=1 SV=1 |
| 248 | 1067 | sp\|P11087\|CO1A1\_MOUSE | 229.22 | 6 | 5 | 4 | 2 | 4 | 4 | 3 | 4 | 4 | 5 | 4 | 5.2E8 | 6.02E8 | 5.49E8 | 2.8E8 | 6.3E8 | 5.38E8 | 3.64E8 | 4.37E8 | 5.46E8 | 3.79E8 | 7 | 7 | 10 | 9 | 9 | 11 | 12 | 10 | 11 | 13 | 12 | 10 | Y | 138033 | Collagen alpha-1(I) chain OS=Mus musculus OX=10090 GN=Col1a1 PE=1 SV=4 |
| 646 | 839 | Q91YZ8\|Q91YZ8\_MOUSE | 229.19 | 16 | 7 | 10 | 2 | 7 | 8 | 7 | 12 | 8 | 11 | 6 |  | 6.59E5 | 6.93E5 | 1.55E6 | 7.97E5 | 2.65E5 |  | 1.08E5 | 1.16E6 |  | 7 | 2 | 2 | 4 | 1 | 4 | 3 | 3 | 4 | 3 | 4 | 3 | Y | 67853 | Polyadenylate-binding protein OS=Mus musculus OX=10090 GN=Pabpc4 PE=1 SV=1 |
| 646 | 840 | A3KFU5\|A3KFU5\_MOUSE | 229.19 | 16 | 6 | 9 | 2 | 7 | 8 | 7 | 11 | 8 | 10 | 6 |  | 6.59E5 | 6.93E5 | 1.55E6 | 7.97E5 | 2.65E5 |  | 1.08E5 | 1.16E6 |  | 7 | 2 | 2 | 4 | 1 | 4 | 3 | 3 | 4 | 3 | 4 | 3 | Y | 69430 | Polyadenylate-binding protein OS=Mus musculus OX=10090 GN=Pabpc4 PE=1 SV=1 |
| 646 | 841 | A3KFU8\|A3KFU8\_MOUSE | 229.19 | 15 | 6 | 9 | 2 | 7 | 8 | 7 | 11 | 8 | 10 | 6 |  | 6.59E5 | 6.93E5 | 1.55E6 | 7.97E5 | 2.65E5 |  | 1.08E5 | 1.16E6 |  | 7 | 2 | 2 | 4 | 1 | 4 | 3 | 3 | 4 | 3 | 4 | 3 | Y | 70665 | Polyadenylate-binding protein OS=Mus musculus OX=10090 GN=Pabpc4 PE=1 SV=1 |
| 646 | 842 | Q6PHQ9\|Q6PHQ9\_MOUSE | 229.19 | 15 | 6 | 9 | 2 | 6 | 8 | 7 | 11 | 8 | 10 | 6 |  | 6.59E5 | 6.93E5 | 1.55E6 | 7.97E5 | 2.65E5 |  | 1.08E5 | 1.16E6 |  | 7 | 2 | 2 | 4 | 1 | 4 | 3 | 3 | 4 | 3 | 4 | 3 | Y | 72242 | Polyadenylate-binding protein OS=Mus musculus OX=10090 GN=Pabpc4 PE=1 SV=1 |
| 333 | 539 | sp\|O08709\|PRDX6\_MOUSE | 228.70 | 46 | 38 | 38 | 23 | 28 | 30 | 27 | 38 | 28 | 28 | 31 | 5.97E7 | 5.78E7 | 5.63E7 | 2.16E7 | 4.68E7 | 3.27E7 | 3.25E7 | 3.47E7 | 8.35E7 | 6.36E7 | 10 | 10 | 10 | 9 | 6 | 7 | 8 | 6 | 7 | 10 | 9 | 8 | Y | 24871 | Peroxiredoxin-6 OS=Mus musculus OX=10090 GN=Prdx6 PE=1 SV=3 |
| 333 | 540 | Q6GT24\|Q6GT24\_MOUSE | 228.70 | 46 | 38 | 38 | 23 | 28 | 30 | 27 | 38 | 28 | 28 | 31 | 5.97E7 | 5.78E7 | 5.63E7 | 2.16E7 | 4.68E7 | 3.27E7 | 3.25E7 | 3.47E7 | 8.35E7 | 6.36E7 | 10 | 10 | 10 | 9 | 6 | 7 | 8 | 6 | 7 | 10 | 9 | 8 | Y | 24827 | Peroxiredoxin-6 OS=Mus musculus OX=10090 GN=Prdx6 PE=1 SV=1 |
| 266 | 364 | sp\|Q61554\|FBN1\_MOUSE | 228.64 | 7 | 3 | 2 | 4 | 2 | 2 | 4 | 2 | 2 | 4 | 3 | 4.64E7 | 2.95E7 | 5.15E7 | 1.55E7 | 2.35E7 | 4.13E7 | 1.84E7 | 1.13E7 | 6.58E7 | 4.39E7 | 22 | 22 | 12 | 7 | 13 | 7 | 8 | 12 | 7 | 6 | 11 | 9 | Y | 312298 | Fibrillin-1 OS=Mus musculus OX=10090 GN=Fbn1 PE=1 SV=2 |
| 27 | 969 | A0A1W2P768\|A0A1W2P768\_MOUSE | 228.44 | 41 | 23 | 38 | 41 | 23 | 41 | 41 | 23 | 23 | 41 | 41 |  | 5.14E6 | 5.7E6 |  | 8.58E6 | 2.66E7 |  |  | 1.97E7 | 1.03E7 | 9 | 1 | 57 | 53 | 69 | 51 | 49 | 45 | 45 | 64 | 62 | 53 | Y | 20247 | H3 clustered histone 14 OS=Mus musculus OX=10090 GN=H3c14 PE=1 SV=1 |
| 307 | 760 | sp\|P62082\|RS7\_MOUSE | 227.99 | 45 | 30 | 20 | 38 | 35 | 28 | 34 | 39 | 24 | 29 | 24 | 7.9E7 | 9.89E7 | 1.51E8 | 5.76E7 | 6.92E7 | 1.05E8 | 7.7E7 | 4.75E7 | 8.04E7 | 8.52E7 | 9 | 9 | 6 | 4 | 8 | 8 | 7 | 9 | 9 | 8 | 7 | 8 | N | 22127 | Small ribosomal subunit protein eS7 OS=Mus musculus OX=10090 GN=Rps7 PE=1 SV=1 |
| 244 | 571 | G3UXZ5\|G3UXZ5\_MOUSE | 227.93 | 44 | 37 | 30 | 31 | 35 | 32 | 34 | 30 | 28 | 32 | 30 | 5.1E7 | 3.91E7 | 3.91E7 | 2.06E7 | 3.65E7 | 3.64E7 | 2.84E7 | 2.54E7 | 7.09E7 | 3.24E7 | 12 | 11 | 12 | 10 | 12 | 11 | 10 | 10 | 9 | 8 | 10 | 10 | N | 27411 | Proteasome activator complex subunit 1 (Fragment) OS=Mus musculus OX=10090 GN=Psme1 PE=1 SV=1 |
| 244 | 577 | sp\|P97371\|PSME1\_MOUSE | 227.93 | 42 | 35 | 29 | 29 | 33 | 31 | 32 | 29 | 27 | 31 | 29 | 5.1E7 | 3.91E7 | 3.91E7 | 2.06E7 | 3.65E7 | 3.64E7 | 2.84E7 | 2.54E7 | 7.09E7 | 3.24E7 | 12 | 11 | 12 | 10 | 12 | 11 | 10 | 10 | 9 | 8 | 10 | 10 | N | 28673 | Proteasome activator complex subunit 1 OS=Mus musculus OX=10090 GN=Psme1 PE=1 SV=2 |
| 377 | 320 | sp\|Q91VD9\|NDUS1\_MOUSE | 227.70 | 20 | 14 | 11 | 10 | 11 | 13 | 15 | 10 | 13 | 7 | 4 | 2.43E7 | 3.06E7 | 1.65E7 | 1.01E7 | 1.85E7 | 2.37E7 | 1.07E7 | 1.83E7 | 2.51E7 | 9.96E6 | 12 | 12 | 8 | 7 | 6 | 7 | 8 | 9 | 6 | 8 | 4 | 3 | Y | 79777 | NADH-ubiquinone oxidoreductase 75 kDa subunit, mitochondrial OS=Mus musculus OX=10090 GN=Ndufs1 PE=1 SV=2 |
| 592 | 869 | sp\|Q9D0M3\|CY1\_MOUSE | 227.66 | 28 | 8 | 16 | 12 | 18 | 12 | 21 | 13 | 12 | 18 | 7 | 1.18E7 | 2.16E7 | 1.23E7 | 4.11E6 | 9.42E6 | 1.69E7 | 1.15E7 | 5.68E6 | 2.07E7 | 7.54E6 | 8 | 8 | 4 | 5 | 3 | 4 | 3 | 6 | 5 | 3 | 5 | 3 | Y | 35328 | Cytochrome c1, heme protein, mitochondrial OS=Mus musculus OX=10090 GN=Cyc1 PE=1 SV=1 |
| 486 | 1183 | sp\|Q00898\|A1AT5\_MOUSE | 227.31 | 17 | 13 | 11 | 8 | 10 | 13 | 11 | 13 | 10 | 11 | 9 | 2.64E6 |  |  |  |  |  |  |  |  |  | 7 | 1 | 5 | 5 | 3 | 6 | 5 | 5 | 6 | 5 | 4 | 3 | Y | 45891 | Alpha-1-antitrypsin 1-5 OS=Mus musculus OX=10090 GN=Serpina1e PE=1 SV=1 |
| 312 | 227 | sp\|P42932\|TCPQ\_MOUSE | 227.06 | 27 | 13 | 13 | 17 | 18 | 16 | 14 | 16 | 12 | 18 | 15 | 2.41E7 | 2.81E7 | 3.91E7 | 2.11E7 | 3.32E7 | 2.46E7 | 2.65E7 | 1.6E7 | 4.95E7 | 3.9E7 | 14 | 14 | 6 | 6 | 8 | 10 | 8 | 7 | 8 | 6 | 9 | 7 | Y | 59556 | T-complex protein 1 subunit theta OS=Mus musculus OX=10090 GN=Cct8 PE=1 SV=3 |
| 257 | 251 | sp\|P06745\|G6PI\_MOUSE | 226.64 | 28 | 22 | 16 | 12 | 15 | 15 | 17 | 16 | 16 | 23 | 21 | 1.25E8 | 5.66E7 | 7.69E7 | 4.33E7 | 6.13E7 | 7.71E7 | 8.82E7 | 5.67E7 | 1.29E8 | 1.13E8 | 15 | 15 | 14 | 10 | 7 | 11 | 10 | 10 | 10 | 10 | 12 | 13 | Y | 62767 | Glucose-6-phosphate isomerase OS=Mus musculus OX=10090 GN=Gpi PE=1 SV=4 |
| 400 | 518 | Q5SW88\|Q5SW88\_MOUSE | 225.67 | 50 | 15 | 28 | 30 | 40 | 43 | 19 | 38 | 33 | 18 | 24 | 4.09E6 | 1.27E7 | 4.11E7 | 1.74E7 | 2.74E7 | 3.41E7 | 2.16E7 | 7.31E6 | 5.82E7 | 2.69E7 | 11 | 4 | 4 | 8 | 7 | 10 | 10 | 5 | 7 | 6 | 4 | 5 | Y | 22372 | Ras-related protein Rab-1A OS=Mus musculus OX=10090 GN=Rab1a PE=1 SV=1 |
| 400 | 519 | sp\|P62821\|RAB1A\_MOUSE | 225.67 | 49 | 15 | 27 | 30 | 40 | 42 | 19 | 37 | 33 | 18 | 23 | 4.09E6 | 1.27E7 | 4.11E7 | 1.74E7 | 2.74E7 | 3.41E7 | 2.16E7 | 7.31E6 | 5.82E7 | 2.69E7 | 11 | 4 | 4 | 8 | 7 | 10 | 10 | 5 | 7 | 6 | 4 | 5 | Y | 22678 | Ras-related protein Rab-1A OS=Mus musculus OX=10090 GN=Rab1a PE=1 SV=3 |
| 268 | 1223 | sp\|P17897\|LYZ1\_MOUSE | 225.41 | 49 | 24 | 44 | 37 | 49 | 49 | 49 | 49 | 49 | 28 | 40 | 6.37E7 | 1.1E8 | 1.48E8 | 6.97E7 | 1.36E8 | 5.68E7 | 8.96E7 | 9.88E7 | 1.18E8 | 5.76E7 | 6 | 6 | 7 | 10 | 9 | 11 | 9 | 8 | 9 | 14 | 7 | 7 | Y | 16794 | Lysozyme C-1 OS=Mus musculus OX=10090 GN=Lyz1 PE=1 SV=1 |
| 275 | 308 | sp\|Q9D8N0\|EF1G\_MOUSE | 225.10 | 31 | 20 | 15 | 21 | 21 | 20 | 15 | 19 | 22 | 25 | 11 | 5.31E7 | 2.8E7 | 5.36E7 | 3.38E7 | 3.9E7 | 4.61E7 | 3.36E7 | 4.57E7 | 9.88E7 | 4.68E7 | 16 | 16 | 11 | 6 | 10 | 10 | 9 | 7 | 9 | 10 | 16 | 8 | Y | 50061 | Elongation factor 1-gamma OS=Mus musculus OX=10090 GN=Eef1g PE=1 SV=3 |
| 286 | 382 | sp\|P05202\|AATM\_MOUSE | 225.09 | 31 | 18 | 24 | 27 | 22 | 26 | 27 | 20 | 25 | 24 | 13 | 4.43E7 | 4.86E7 | 6.22E7 | 2.3E7 | 4.74E7 | 5.24E7 | 2.07E7 | 3.22E7 | 6.33E7 | 2.19E7 | 12 | 12 | 7 | 8 | 10 | 7 | 11 | 11 | 8 | 9 | 10 | 5 | Y | 47411 | Aspartate aminotransferase, mitochondrial OS=Mus musculus OX=10090 GN=Got2 PE=1 SV=1 |
| 385 | 352 | sp\|P09055\|ITB1\_MOUSE | 224.64 | 16 | 8 | 8 | 6 | 9 | 7 | 14 | 6 | 8 | 8 | 11 | 3.85E7 | 4.25E7 | 2.92E7 | 1.37E7 | 2.8E7 | 4.57E7 | 1.46E7 | 2.2E7 | 8.72E7 | 3.82E7 | 12 | 12 | 6 | 6 | 4 | 8 | 6 | 11 | 5 | 7 | 8 | 8 | Y | 88231 | Integrin beta-1 OS=Mus musculus OX=10090 GN=Itgb1 PE=1 SV=1 |
| 456 | 702 | sp\|P35979\|RL12\_MOUSE | 224.40 | 45 | 23 | 31 | 22 | 38 | 27 | 29 | 33 | 24 | 24 | 20 | 3.75E7 | 4.69E7 | 3.3E7 | 3.48E7 | 5.22E7 | 2.66E7 | 4.65E7 | 2.66E7 | 4.51E7 | 3.09E7 | 9 | 9 | 7 | 6 | 4 | 7 | 6 | 5 | 6 | 4 | 5 | 6 | Y | 17805 | Large ribosomal subunit protein uL11 OS=Mus musculus OX=10090 GN=Rpl12 PE=1 SV=2 |
| 198 | 387 | sp\|P62702\|RS4X\_MOUSE | 224.32 | 43 | 30 | 35 | 36 | 25 | 35 | 33 | 27 | 33 | 28 | 31 | 1.22E8 | 1.34E8 | 1.9E8 | 6.38E7 | 1.52E8 | 1.05E8 | 1.03E8 | 9.56E7 | 1.21E8 | 8.63E7 | 16 | 16 | 12 | 12 | 13 | 10 | 15 | 13 | 12 | 14 | 11 | 11 | Y | 29598 | Small ribosomal subunit protein eS4 OS=Mus musculus OX=10090 GN=Rps4x PE=1 SV=2 |
| 492 | 501 | sp\|Q9EQH2\|ERAP1\_MOUSE | 224.16 | 13 | 10 | 5 | 10 | 6 | 7 | 9 | 7 | 6 | 5 | 3 | 1E7 | 9.27E6 | 2.57E7 | 6.63E6 | 1.45E7 | 1.63E7 | 1.5E7 | 8.58E6 | 3.84E6 | 2.47E6 | 10 | 10 | 7 | 4 | 7 | 4 | 5 | 6 | 5 | 4 | 3 | 2 | Y | 106599 | Endoplasmic reticulum aminopeptidase 1 OS=Mus musculus OX=10090 GN=Erap1 PE=1 SV=2 |
| 529 | 355 | sp\|Q76MZ3\|2AAA\_MOUSE | 224.07 | 23 | 7 | 5 | 10 | 7 | 13 | 16 | 10 | 4 | 6 | 3 | 2.59E7 | 1.28E7 | 1.13E7 | 7.28E6 | 2.64E7 | 1.23E7 | 1.72E7 | 4.66E6 | 2.69E6 | 1.87E7 | 12 | 8 | 3 | 2 | 5 | 4 | 7 | 8 | 5 | 2 | 2 | 2 | Y | 65323 | Serine/threonine-protein phosphatase 2A 65 kDa regulatory subunit A alpha isoform OS=Mus musculus OX=10090 GN=Ppp2r1a PE=1 SV=3 |
| 304 | 297 | Q52KG9\|Q52KG9\_MOUSE | 223.98 | 32 | 12 | 17 | 17 | 19 | 18 | 17 | 13 | 18 | 22 | 17 | 4.7E7 | 3.62E7 | 4.55E7 | 2.26E7 | 2.23E7 | 3.07E7 | 2.53E7 | 3.42E7 | 6.8E7 | 3.04E7 | 16 | 16 | 7 | 7 | 8 | 9 | 8 | 8 | 6 | 9 | 13 | 8 | Y | 58076 | T-complex protein 1 subunit zeta OS=Mus musculus OX=10090 GN=Cct6a PE=1 SV=1 |
| 304 | 298 | sp\|P80317\|TCPZ\_MOUSE | 223.98 | 32 | 12 | 17 | 17 | 19 | 18 | 17 | 13 | 18 | 22 | 17 | 4.7E7 | 3.62E7 | 4.55E7 | 2.26E7 | 2.23E7 | 3.07E7 | 2.53E7 | 3.42E7 | 6.8E7 | 3.04E7 | 16 | 16 | 7 | 7 | 8 | 9 | 8 | 8 | 6 | 9 | 13 | 8 | Y | 58004 | T-complex protein 1 subunit zeta OS=Mus musculus OX=10090 GN=Cct6a PE=1 SV=3 |
| 188 | 484 | E9PZF0\|E9PZF0\_MOUSE | 223.90 | 43 | 39 | 28 | 32 | 39 | 36 | 35 | 32 | 32 | 40 | 39 | 6.25E7 | 7.29E7 | 6.14E7 | 3.57E7 | 5.12E7 | 6.02E7 | 3.5E7 | 3.32E7 | 1.34E8 | 8.04E7 | 11 | 4 | 14 | 14 | 13 | 15 | 14 | 15 | 12 | 12 | 17 | 17 | Y | 30200 | Nucleoside diphosphate kinase OS=Mus musculus OX=10090 GN=Nme1nme2 PE=3 SV=1 |
| 388 | 1190 | sp\|P99029\|PRDX5\_MOUSE | 223.72 | 40 | 33 | 25 | 33 | 25 | 33 | 33 | 33 | 25 | 40 | 33 | 4.07E7 | 4.17E7 | 3.66E7 | 1.69E7 | 2.92E7 | 3.52E7 | 1.78E7 | 1.52E7 | 1.15E8 | 5.15E7 | 7 | 7 | 7 | 7 | 6 | 6 | 6 | 7 | 7 | 5 | 9 | 8 | Y | 21897 | Peroxiredoxin-5, mitochondrial OS=Mus musculus OX=10090 GN=Prdx5 PE=1 SV=2 |
| 390 | 509 | sp\|Q9CY58\|SERB1\_MOUSE | 223.30 | 29 | 17 | 13 | 21 | 19 | 17 | 15 | 15 | 17 | 9 | 14 | 4.57E7 | 2.5E7 | 3.69E7 | 1.23E7 | 3.27E7 | 1.55E7 | 1.13E7 | 1.99E7 | 5.31E7 | 3.02E7 | 10 | 10 | 6 | 5 | 7 | 9 | 7 | 5 | 7 | 7 | 5 | 5 | Y | 44714 | SERPINE1 mRNA-binding protein 1 OS=Mus musculus OX=10090 GN=Serbp1 PE=1 SV=2 |
| 702 | 662 | sp\|P21447\|MDR1A\_MOUSE | 223.23 | 8 | 5 | 6 | 0 | 1 | 4 | 5 | 0 | 0 | 7 | 2 | 3E6 | 7.71E6 |  | 4.2E5 | 4.94E6 | 7.47E6 |  |  | 2.72E7 | 1.81E6 | 8 | 7 | 3 | 6 | 0 | 1 | 3 | 4 | 0 | 0 | 6 | 2 | N | 140646 | ATP-dependent translocase ABCB1 OS=Mus musculus OX=10090 GN=Abcb1a PE=1 SV=3 |
| 394 | 732 | sp\|Q9CZM2\|RL15\_MOUSE | 222.90 | 41 | 27 | 24 | 32 | 35 | 27 | 26 | 23 | 26 | 27 | 24 | 1.02E8 | 6.25E7 | 1.11E8 | 5.67E7 | 5.87E7 | 5.27E7 | 4.8E7 | 4.69E7 | 8.59E7 | 7.28E7 | 9 | 9 | 9 | 5 | 9 | 9 | 6 | 5 | 5 | 5 | 7 | 5 | N | 24146 | Large ribosomal subunit protein eL15 OS=Mus musculus OX=10090 GN=Rpl15 PE=1 SV=4 |
| 597 | 723 | sp\|Q9D1Q6\|ERP44\_MOUSE | 222.21 | 26 | 22 | 24 | 10 | 13 | 5 | 9 | 11 | 11 | 13 | 15 | 1.7E7 | 1.9E7 | 5.91E6 | 2.37E6 | 3.62E6 | 4.34E6 | 5.05E6 | 7.95E6 | 1.97E7 | 8.04E6 | 8 | 8 | 7 | 8 | 3 | 3 | 3 | 2 | 3 | 3 | 4 | 5 | Y | 46853 | Endoplasmic reticulum resident protein 44 OS=Mus musculus OX=10090 GN=Erp44 PE=1 SV=1 |
| 401 | 360 | sp\|Q9R112\|SQOR\_MOUSE | 221.74 | 31 | 19 | 22 | 10 | 10 | 21 | 23 | 14 | 16 | 22 | 14 | 1.43E7 | 1.57E7 | 5.9E6 | 2.77E6 | 8.72E6 | 2.49E7 | 7.35E6 | 1.18E7 | 3.06E7 | 6.18E6 | 13 | 8 | 7 | 8 | 3 | 4 | 8 | 9 | 5 | 6 | 9 | 5 | Y | 50282 | Sulfide:quinone oxidoreductase, mitochondrial OS=Mus musculus OX=10090 GN=Sqor PE=1 SV=3 |
| 329 | 1828 | sp\|P99027\|RLA2\_MOUSE | 221.66 | 71 | 27 | 56 | 27 | 57 | 69 | 56 | 69 | 56 | 70 | 27 | 5.91E7 | 7.46E7 | 7.42E7 | 3.03E7 | 7.5E7 | 5.01E7 | 3.87E7 | 4.25E7 | 1.19E8 | 6.36E7 | 6 | 6 | 7 | 9 | 7 | 8 | 11 | 6 | 8 | 10 | 10 | 7 | Y | 11651 | Large ribosomal subunit protein P2 OS=Mus musculus OX=10090 GN=Rplp2 PE=1 SV=3 |
| 362 | 530 | sp\|Q9DCX2\|ATP5H\_MOUSE | 221.34 | 80 | 54 | 44 | 73 | 51 | 65 | 71 | 52 | 47 | 32 | 44 | 3.01E7 | 6.57E7 | 8.71E7 | 2.98E7 | 4.26E7 | 6.12E7 | 2.93E7 | 2.32E7 | 4.56E7 | 2E7 | 10 | 10 | 8 | 6 | 9 | 7 | 9 | 10 | 7 | 5 | 5 | 6 | Y | 18749 | ATP synthase subunit d, mitochondrial OS=Mus musculus OX=10090 GN=Atp5pd PE=1 SV=3 |
| 443 | 1092 | sp\|P19157\|GSTP1\_MOUSE | 221.02 | 47 | 15 | 35 | 35 | 34 | 35 | 35 | 38 | 26 | 35 | 22 | 2.37E7 | 4.93E7 | 4.2E7 | 1.84E7 | 2.48E7 | 4.53E7 | 1.78E7 | 1.6E7 | 7.21E7 | 3.53E7 | 6 | 6 | 3 | 8 | 6 | 5 | 6 | 8 | 6 | 5 | 7 | 5 | Y | 23609 | Glutathione S-transferase P 1 OS=Mus musculus OX=10090 GN=Gstp1 PE=1 SV=2 |
| 183 | 281 | sp\|Q00623\|APOA1\_MOUSE | 220.81 | 48 | 45 | 37 | 39 | 38 | 34 | 38 | 41 | 40 | 39 | 43 | 2.49E8 | 1.46E8 | 2.4E8 | 7.5E7 | 8.4E7 | 1.44E8 | 1.75E8 | 1.08E8 | 1.93E8 | 1.64E8 | 15 | 15 | 18 | 11 | 15 | 11 | 11 | 13 | 15 | 13 | 16 | 15 | Y | 30616 | Apolipoprotein A-I OS=Mus musculus OX=10090 GN=Apoa1 PE=1 SV=2 |
| 233 | 335 | sp\|P25444\|RS2\_MOUSE | 220.66 | 43 | 28 | 33 | 28 | 26 | 26 | 33 | 24 | 37 | 27 | 26 | 9.68E7 | 1.01E8 | 1.1E8 | 5.47E7 | 8.36E7 | 1.1E8 | 7.01E7 | 8.14E7 | 1.21E8 | 1.24E8 | 13 | 13 | 10 | 14 | 10 | 10 | 8 | 12 | 10 | 15 | 12 | 10 | Y | 31231 | Small ribosomal subunit protein uS5 OS=Mus musculus OX=10090 GN=Rps2 PE=1 SV=3 |
| 202 | 342 | sp\|O09131\|GSTO1\_MOUSE | 220.51 | 50 | 23 | 35 | 28 | 31 | 32 | 43 | 41 | 27 | 28 | 31 | 8.22E7 | 1.15E8 | 1.01E8 | 4.92E7 | 7.66E7 | 8.32E7 | 6.83E7 | 4.57E7 | 1.06E8 | 8.9E7 | 13 | 13 | 12 | 15 | 12 | 11 | 14 | 13 | 16 | 11 | 11 | 13 | Y | 27498 | Glutathione S-transferase omega-1 OS=Mus musculus OX=10090 GN=Gsto1 PE=1 SV=2 |
| 579 | 453 | sp\|Q8VC30\|TKFC\_MOUSE | 220.16 | 27 | 10 | 24 | 14 | 13 | 8 | 13 | 8 | 2 | 11 | 6 | 6.81E6 | 4.12E7 | 2.31E7 | 3.43E6 | 7.74E6 | 1.01E7 | 6.7E6 | 2.13E6 | 1.61E7 | 1.34E6 | 9 | 9 | 3 | 8 | 5 | 4 | 3 | 5 | 3 | 1 | 4 | 2 | Y | 59691 | Triokinase/FMN cyclase OS=Mus musculus OX=10090 GN=Tkfc PE=1 SV=1 |
| 365 | 344 | sp\|P67778\|PHB1\_MOUSE | 219.80 | 54 | 19 | 14 | 27 | 32 | 43 | 39 | 39 | 32 | 29 | 20 | 2.19E7 | 2.28E7 | 4.9E7 | 2.04E7 | 2.99E7 | 4.23E7 | 3.38E7 | 1.88E7 | 3.92E7 | 2.93E7 | 12 | 12 | 4 | 4 | 7 | 7 | 9 | 9 | 11 | 8 | 7 | 6 | N | 29820 | Prohibitin 1 OS=Mus musculus OX=10090 GN=Phb1 PE=1 SV=1 |
| 674 | 523 | E9Q174\|E9Q174\_MOUSE | 219.31 | 10 | 1 | 4 | 2 | 2 | 3 | 3 | 4 | 1 | 2 | 0 | 1.9E6 | 1.54E7 | 5.08E6 | 1.31E6 | 2.28E6 | 5.67E6 | 6.08E6 | 0E0 | 2.89E6 |  | 13 | 12 | 1 | 5 | 3 | 2 | 3 | 4 | 4 | 1 | 2 | 0 | Y | 148145 | Unconventional myosin-VI OS=Mus musculus OX=10090 GN=Myo6 PE=1 SV=1 |
| 674 | 524 | V9GX76\|V9GX76\_MOUSE | 219.31 | 10 | 1 | 4 | 2 | 2 | 3 | 3 | 4 | 1 | 2 | 0 | 1.9E6 | 1.54E7 | 5.08E6 | 1.31E6 | 2.28E6 | 5.67E6 | 6.08E6 | 0E0 | 2.89E6 |  | 13 | 12 | 1 | 5 | 3 | 2 | 3 | 4 | 4 | 1 | 2 | 0 | Y | 149229 | Unconventional myosin-VI OS=Mus musculus OX=10090 GN=Myo6 PE=1 SV=1 |
| 387 | 435 | sp\|Q9JII6\|AK1A1\_MOUSE | 219.26 | 34 | 12 | 20 | 20 | 17 | 15 | 21 | 20 | 18 | 30 | 19 | 2.34E7 | 4.4E7 | 5.7E7 | 2.42E7 | 1.9E7 | 4.71E7 | 3.05E7 | 1.21E7 | 1.15E8 | 5.03E7 | 11 | 11 | 4 | 6 | 8 | 6 | 5 | 7 | 6 | 5 | 10 | 7 | Y | 36587 | Aldo-keto reductase family 1 member A1 OS=Mus musculus OX=10090 GN=Akr1a1 PE=1 SV=3 |
| 706 | 778 | sp\|Q99LC3\|NDUAA\_MOUSE | 219.02 | 28 | 17 | 14 | 13 | 11 | 9 | 15 | 11 | 12 | 14 | 12 | 7.64E6 | 1.1E7 | 9.49E6 | 4.43E6 | 1.57E6 | 7.53E6 | 5.07E6 | 4.44E6 | 1.51E7 | 9.43E6 | 7 | 7 | 3 | 3 | 3 | 4 | 2 | 4 | 3 | 3 | 4 | 3 | N | 40603 | NADH dehydrogenase [ubiquinone] 1 alpha subcomplex subunit 10, mitochondrial OS=Mus musculus OX=10090 GN=Ndufa10 PE=1 SV=1 |
| 532 | 1379 | sp\|P63276\|RS17\_MOUSE | 219.00 | 53 | 39 | 41 | 39 | 39 | 43 | 39 | 39 | 32 | 48 | 41 | 1.05E7 | 1.09E7 | 3.18E7 | 9.81E6 | 1.1E7 | 3.63E7 | 2.29E7 | 6.88E6 | 7.33E7 | 2.17E7 | 7 | 6 | 4 | 3 | 5 | 5 | 4 | 5 | 4 | 5 | 8 | 4 | Y | 15524 | Small ribosomal subunit protein eS17 OS=Mus musculus OX=10090 GN=Rps17 PE=1 SV=2 |
| 668 | 1147 | sp\|P29699\|FETUA\_MOUSE | 218.84 | 30 | 19 | 19 | 15 | 17 | 7 | 17 | 8 | 7 | 28 | 22 | 2.63E7 | 8.16E6 | 1.4E7 | 7.05E6 | 7.46E6 | 1.65E7 | 1.26E7 | 9.27E6 | 8.68E6 | 2.01E7 | 6 | 6 | 4 | 3 | 3 | 4 | 2 | 4 | 3 | 2 | 5 | 4 | Y | 37326 | Alpha-2-HS-glycoprotein OS=Mus musculus OX=10090 GN=Ahsg PE=1 SV=1 |
| 103 | 1152 | sp\|P15864\|H12\_MOUSE | 218.82 | 32 | 27 | 27 | 27 | 26 | 32 | 27 | 27 | 27 | 27 | 27 | 8.04E7 | 6.61E7 | 8.48E7 | 2.93E7 | 6.93E7 | 5.55E7 | 4.83E7 | 4E7 | 4.83E7 | 1.17E8 | 9 | 3 | 23 | 22 | 27 | 19 | 24 | 17 | 24 | 29 | 26 | 21 | N | 21267 | Histone H1.2 OS=Mus musculus OX=10090 GN=H1-2 PE=1 SV=2 |
| 229 | 740 | sp\|Q61171\|PRDX2\_MOUSE | 218.29 | 42 | 30 | 36 | 42 | 39 | 38 | 36 | 38 | 35 | 26 | 22 | 4.46E7 | 1.15E8 | 1.02E8 | 5.27E7 | 9.02E7 | 1.17E8 | 7.06E7 | 4.77E7 | 1.3E8 | 9.35E7 | 9 | 8 | 12 | 14 | 16 | 11 | 13 | 10 | 10 | 12 | 11 | 9 | N | 21779 | Peroxiredoxin-2 OS=Mus musculus OX=10090 GN=Prdx2 PE=1 SV=3 |
| 172 | 1033 | sp\|P0DP28\|CALM3\_MOUSE | 218.20 | 58 | 50 | 48 | 24 | 35 | 35 | 24 | 50 | 24 | 45 | 39 | 8.62E7 | 1.37E8 | 1.44E8 | 7.05E7 | 1.25E8 | 1.23E8 | 8.48E7 | 6.63E7 | 1.8E8 | 7.55E7 | 10 | 7 | 15 | 17 | 14 | 16 | 19 | 13 | 15 | 13 | 14 | 16 | Y | 16838 | Calmodulin-3 OS=Mus musculus OX=10090 GN=Calm3 PE=1 SV=1 |
| 172 | 1034 | sp\|P0DP26\|CALM1\_MOUSE | 218.20 | 58 | 50 | 48 | 24 | 35 | 35 | 24 | 50 | 24 | 45 | 39 | 8.62E7 | 1.37E8 | 1.44E8 | 7.05E7 | 1.25E8 | 1.23E8 | 8.48E7 | 6.63E7 | 1.8E8 | 7.55E7 | 10 | 7 | 15 | 17 | 14 | 16 | 19 | 13 | 15 | 13 | 14 | 16 | Y | 16838 | Calmodulin-1 OS=Mus musculus OX=10090 GN=Calm1 PE=1 SV=1 |
| 172 | 1035 | sp\|P0DP27\|CALM2\_MOUSE | 218.20 | 58 | 50 | 48 | 24 | 35 | 35 | 24 | 50 | 24 | 45 | 39 | 8.62E7 | 1.37E8 | 1.44E8 | 7.05E7 | 1.25E8 | 1.23E8 | 8.48E7 | 6.63E7 | 1.8E8 | 7.55E7 | 10 | 7 | 15 | 17 | 14 | 16 | 19 | 13 | 15 | 13 | 14 | 16 | Y | 16838 | Calmodulin-2 OS=Mus musculus OX=10090 GN=Calm2 PE=1 SV=1 |
| 445 | 385 | sp\|Q6ZQ38\|CAND1\_MOUSE | 217.86 | 11 | 3 | 7 | 7 | 2 | 6 | 9 | 6 | 4 | 3 | 7 | 3.61E6 | 7.18E6 | 7.14E6 | 1.46E6 | 5.57E6 | 7.82E6 | 2.91E6 | 2.6E6 | 3.13E6 | 6.06E6 | 12 | 11 | 4 | 7 | 6 | 2 | 6 | 9 | 5 | 5 | 3 | 7 | N | 136331 | Cullin-associated NEDD8-dissociated protein 1 OS=Mus musculus OX=10090 GN=Cand1 PE=1 SV=2 |
| 421 | 543 | sp\|P32921\|SYWC\_MOUSE | 217.61 | 24 | 17 | 5 | 14 | 15 | 10 | 12 | 14 | 19 | 16 | 15 | 1.77E7 | 1.58E7 | 2.5E7 | 1.23E7 | 1.54E7 | 1.79E7 | 6.45E6 | 1.12E7 | 4.08E7 | 2.71E7 | 10 | 9 | 7 | 3 | 6 | 7 | 5 | 5 | 6 | 7 | 6 | 8 | Y | 54358 | Tryptophan--tRNA ligase, cytoplasmic OS=Mus musculus OX=10090 GN=Wars1 PE=1 SV=2 |
| 441 | 432 | sp\|Q91WG0\|EST2C\_MOUSE | 217.57 | 23 | 12 | 10 | 9 | 8 | 8 | 15 | 3 | 5 | 13 | 6 | 7.36E6 | 3.85E6 | 2.67E6 | 9.8E5 | 1.51E6 | 6.21E6 |  | 8.56E5 | 1.86E7 | 3.63E6 | 12 | 6 | 7 | 6 | 5 | 4 | 5 | 9 | 3 | 2 | 9 | 4 | Y | 62470 | Acylcarnitine hydrolase OS=Mus musculus OX=10090 GN=Ces2c PE=1 SV=1 |
| 890 | 620 | sp\|Q2KHK6\|GSDC2\_MOUSE | 217.47 | 21 | 19 | 0 | 0 | 0 | 0 | 0 | 0 | 0 | 19 | 14 | 9.53E6 |  |  |  |  |  |  |  | 1.96E7 | 3.54E6 | 10 | 5 | 8 | 0 | 0 | 0 | 0 | 0 | 0 | 0 | 8 | 6 | Y | 53952 | Gasdermin-C2 OS=Mus musculus OX=10090 GN=Gsdmc2 PE=1 SV=2 |
| 384 | 339 | sp\|Q9ET54\|PALLD\_MOUSE | 216.89 | 11 | 7 | 4 | 3 | 3 | 3 | 8 | 3 | 2 | 5 | 2 | 2.52E7 | 2.97E7 | 3.14E7 | 7.98E6 | 2.17E7 | 4.27E7 | 1.61E7 | 9.34E6 | 1.76E7 | 1.13E7 | 14 | 14 | 9 | 8 | 8 | 6 | 5 | 10 | 6 | 3 | 4 | 4 | Y | 152131 | Palladin OS=Mus musculus OX=10090 GN=Palld PE=1 SV=2 |
| 480 | 529 | sp\|P80316\|TCPE\_MOUSE | 216.72 | 18 | 12 | 6 | 10 | 12 | 11 | 11 | 12 | 6 | 11 | 7 | 2.75E7 | 3.3E7 | 1.47E7 | 8.41E6 | 2E7 | 5.35E6 | 2.44E7 | 1.11E7 | 2.54E7 | 2.06E7 | 9 | 9 | 6 | 5 | 5 | 5 | 5 | 4 | 6 | 4 | 5 | 5 | Y | 59624 | T-complex protein 1 subunit epsilon OS=Mus musculus OX=10090 GN=Cct5 PE=1 SV=1 |
| 588 | 476 | sp\|Q8QZR3\|EST2A\_MOUSE | 216.71 | 23 | 2 | 7 | 5 | 9 | 12 | 11 | 6 | 5 | 14 | 8 |  | 3.69E6 |  | 1.38E6 | 5.07E6 | 4.24E6 | 1.63E6 |  | 8.33E6 | 3.02E6 | 11 | 6 | 1 | 4 | 2 | 4 | 6 | 6 | 3 | 2 | 7 | 3 | Y | 61940 | Pyrethroid hydrolase Ces2a OS=Mus musculus OX=10090 GN=Ces2a PE=1 SV=1 |
| 336 | 266 | sp\|Q8CIE6\|COPA\_MOUSE | 216.19 | 13 | 6 | 6 | 7 | 3 | 4 | 5 | 9 | 5 | 5 | 7 | 2.22E7 | 2.59E7 | 1.96E7 | 5.54E6 | 1.15E7 | 1.57E7 | 1.47E7 | 8.26E6 | 3.06E7 | 2.55E7 | 15 | 15 | 7 | 8 | 10 | 5 | 6 | 7 | 9 | 6 | 6 | 8 | Y | 138432 | Coatomer subunit alpha OS=Mus musculus OX=10090 GN=Copa PE=1 SV=2 |
| 336 | 267 | F8WHL2\|F8WHL2\_MOUSE | 216.19 | 13 | 6 | 6 | 7 | 3 | 4 | 5 | 9 | 5 | 5 | 7 | 2.22E7 | 2.59E7 | 1.96E7 | 5.54E6 | 1.15E7 | 1.57E7 | 1.47E7 | 8.26E6 | 3.06E7 | 2.55E7 | 15 | 15 | 7 | 8 | 10 | 5 | 6 | 7 | 9 | 6 | 6 | 8 | Y | 139410 | Coatomer subunit alpha OS=Mus musculus OX=10090 GN=Copa PE=1 SV=1 |
| 332 | 1812 | sp\|P01837\|IGKC\_MOUSE | 216.16 | 64 | 56 | 43 | 50 | 50 | 50 | 64 | 64 | 64 | 33 | 64 | 1.11E8 | 2.81E7 | 7.06E7 | 4.44E7 | 3.97E7 | 1.99E8 | 1.3E8 | 9.51E7 | 1.69E7 | 9.4E7 | 5 | 5 | 8 | 4 | 7 | 7 | 8 | 11 | 10 | 7 | 9 | 9 | Y | 11934 | Immunoglobulin kappa constant OS=Mus musculus OX=10090 GN=Igkc PE=1 SV=2 |
| 345 | 537 | sp\|Q8VEM8\|S25A3\_MOUSE | 216.02 | 33 | 18 | 25 | 24 | 25 | 29 | 27 | 20 | 18 | 17 | 15 | 7.12E7 | 1.42E8 | 1.21E8 | 4.87E7 | 8.18E7 | 1.09E8 | 5.08E7 | 4.88E7 | 1.06E8 | 6.48E7 | 10 | 10 | 6 | 7 | 8 | 9 | 9 | 9 | 6 | 6 | 8 | 5 | Y | 39632 | Solute carrier family 25 member 3 OS=Mus musculus OX=10090 GN=Slc25a3 PE=1 SV=1 |
| 678 | 938 | sp\|Q7M6Y3\|PICAL\_MOUSE | 215.90 | 14 | 9 | 6 | 2 | 5 | 3 | 10 | 6 | 8 | 13 | 6 | 5.43E6 | 9.78E6 | 5.09E6 | 1.33E6 | 5.29E6 | 1.27E7 | 7.37E6 | 5.55E6 | 2.72E7 | 6.31E6 | 6 | 6 | 3 | 3 | 1 | 2 | 2 | 4 | 3 | 4 | 5 | 2 | Y | 71543 | Phosphatidylinositol-binding clathrin assembly protein OS=Mus musculus OX=10090 GN=Picalm PE=1 SV=1 |
| 452 | 1494 | sp\|Q8K0C5\|ZG16\_MOUSE | 215.56 | 49 | 20 | 29 | 40 | 44 | 49 | 46 | 38 | 44 | 49 | 40 | 2.83E7 | 5.81E7 | 4.67E7 | 2.37E7 | 4.53E7 | 5.05E7 | 3.43E7 | 3.42E7 | 1.4E8 | 3.09E7 | 5 | 5 | 2 | 6 | 5 | 6 | 6 | 7 | 5 | 7 | 10 | 4 | N | 18210 | Zymogen granule membrane protein 16 OS=Mus musculus OX=10090 GN=Zg16 PE=1 SV=1 |
| 320 | 542 | sp\|P14094\|AT1B1\_MOUSE | 215.39 | 35 | 24 | 30 | 27 | 22 | 30 | 22 | 14 | 18 | 22 | 22 | 7.67E7 | 1.27E8 | 9.27E7 | 4.46E7 | 8.07E7 | 9E7 | 4.62E7 | 4.49E7 | 8.54E7 | 5.94E7 | 10 | 10 | 8 | 10 | 8 | 9 | 10 | 8 | 6 | 7 | 8 | 7 | Y | 35195 | Sodium/potassium-transporting ATPase subunit beta-1 OS=Mus musculus OX=10090 GN=Atp1b1 PE=1 SV=1 |
| 568 | 1136 | sp\|P70695\|F16P2\_MOUSE | 215.31 | 24 | 9 | 15 | 15 | 9 | 15 | 24 | 15 | 15 | 19 | 9 | 1.12E7 | 3.67E7 | 3.29E7 | 7.92E6 | 1.77E7 | 3.43E7 | 1.75E7 | 9.54E6 | 3.43E7 | 1.57E7 | 6 | 6 | 3 | 4 | 4 | 2 | 4 | 7 | 4 | 4 | 6 | 3 | Y | 36947 | Fructose-1,6-bisphosphatase isozyme 2 OS=Mus musculus OX=10090 GN=Fbp2 PE=1 SV=2 |
| 305 | 278 | sp\|O88342\|WDR1\_MOUSE | 214.79 | 27 | 3 | 14 | 9 | 15 | 17 | 12 | 14 | 16 | 17 | 7 | 2.14E7 | 8.46E7 | 5.15E7 | 5.24E7 | 9.66E7 | 8.8E7 | 8.27E7 | 7.65E7 | 1.06E8 | 7E7 | 17 | 17 | 3 | 9 | 6 | 9 | 10 | 8 | 9 | 9 | 13 | 5 | Y | 66407 | WD repeat-containing protein 1 OS=Mus musculus OX=10090 GN=Wdr1 PE=1 SV=3 |
| 223 | 291 | sp\|P27659\|RL3\_MOUSE | 214.58 | 36 | 16 | 16 | 21 | 29 | 27 | 21 | 25 | 26 | 14 | 15 | 7.22E7 | 7.66E7 | 1.19E8 | 6.07E7 | 1.35E8 | 1.25E8 | 9.69E7 | 8.12E7 | 6.51E7 | 6.44E7 | 17 | 17 | 8 | 8 | 12 | 15 | 16 | 12 | 14 | 13 | 6 | 9 | Y | 46110 | Large ribosomal subunit protein uL3 OS=Mus musculus OX=10090 GN=Rpl3 PE=1 SV=3 |
| 413 | 412 | G5E839\|G5E839\_MOUSE | 214.35 | 25 | 16 | 16 | 12 | 11 | 14 | 15 | 13 | 7 | 9 | 14 | 2.99E7 | 3.08E7 | 4.64E7 | 1.77E7 | 3.19E7 | 3.87E7 | 2.27E7 | 1.4E7 | 4.18E7 | 3.12E7 | 11 | 11 | 6 | 7 | 7 | 6 | 7 | 8 | 6 | 3 | 5 | 8 | Y | 54862 | T-complex protein 1 subunit delta OS=Mus musculus OX=10090 GN=Cct4 PE=1 SV=1 |
| 413 | 414 | sp\|P80315\|TCPD\_MOUSE | 214.35 | 24 | 15 | 15 | 11 | 11 | 13 | 14 | 12 | 6 | 8 | 14 | 2.99E7 | 3.08E7 | 4.64E7 | 1.77E7 | 3.19E7 | 3.87E7 | 2.27E7 | 1.4E7 | 4.18E7 | 3.12E7 | 11 | 11 | 6 | 7 | 7 | 6 | 7 | 8 | 6 | 3 | 5 | 8 | Y | 58066 | T-complex protein 1 subunit delta OS=Mus musculus OX=10090 GN=Cct4 PE=1 SV=3 |
| 195 | 450 | sp\|P24369\|PPIB\_MOUSE | 213.58 | 57 | 28 | 36 | 30 | 34 | 30 | 29 | 36 | 45 | 29 | 34 | 1.82E8 | 1.2E8 | 1.23E8 | 6.08E7 | 9.49E7 | 9.51E7 | 1.3E8 | 1.2E8 | 1.56E8 | 1.56E8 | 12 | 11 | 13 | 15 | 14 | 13 | 12 | 10 | 13 | 18 | 13 | 15 | Y | 23713 | Peptidyl-prolyl cis-trans isomerase B OS=Mus musculus OX=10090 GN=Ppib PE=1 SV=2 |
| 550 | 584 | sp\|Q9JIF7\|COPB\_MOUSE | 213.36 | 11 | 2 | 5 | 3 | 4 | 4 | 5 | 9 | 2 | 2 | 4 | 6.92E6 | 1.21E7 | 7.22E6 | 4.49E6 | 8.67E6 | 1.24E7 | 9.55E6 | 2.54E6 | 8.25E6 | 9.1E6 | 11 | 11 | 2 | 5 | 4 | 5 | 4 | 4 | 8 | 2 | 2 | 4 | Y | 107066 | Coatomer subunit beta OS=Mus musculus OX=10090 GN=Copb1 PE=1 SV=1 |
| 412 | 787 | sp\|P70333\|HNRH2\_MOUSE | 213.33 | 23 | 16 | 11 | 14 | 15 | 14 | 4 | 17 | 12 | 11 | 7 | 1.08E6 |  |  |  |  |  |  |  |  |  | 10 | 1 | 6 | 5 | 7 | 7 | 7 | 2 | 7 | 5 | 11 | 4 | Y | 49280 | Heterogeneous nuclear ribonucleoprotein H2 OS=Mus musculus OX=10090 GN=Hnrnph2 PE=1 SV=1 |
| 371 | 343 | A0A0N4SV00\|A0A0N4SV00\_MOUSE | 213.27 | 27 | 9 | 18 | 14 | 8 | 22 | 18 | 21 | 15 | 16 | 13 | 1.39E7 | 2.96E7 | 1.82E7 | 2.79E6 | 2.81E7 | 2.13E7 | 1.45E7 | 1.41E7 | 2.95E7 | 1.84E7 | 12 | 12 | 4 | 8 | 6 | 4 | 9 | 7 | 10 | 7 | 7 | 6 | Y | 55058 | T-complex protein 1 subunit eta OS=Mus musculus OX=10090 GN=Cct7 PE=1 SV=1 |
| 371 | 346 | sp\|P80313\|TCPH\_MOUSE | 213.27 | 25 | 8 | 17 | 13 | 8 | 20 | 16 | 19 | 14 | 15 | 12 | 1.39E7 | 2.96E7 | 1.82E7 | 2.79E6 | 2.81E7 | 2.13E7 | 1.45E7 | 1.41E7 | 2.95E7 | 1.84E7 | 12 | 12 | 4 | 8 | 6 | 4 | 9 | 7 | 10 | 7 | 7 | 6 | Y | 59652 | T-complex protein 1 subunit eta OS=Mus musculus OX=10090 GN=Cct7 PE=1 SV=1 |
| 488 | 388 | sp\|P23116\|EIF3A\_MOUSE | 213.13 | 11 | 4 | 3 | 3 | 5 | 5 | 4 | 6 | 2 | 3 | 2 | 6.04E6 | 5.45E8 | 4.54E6 | 4.67E6 | 8.23E6 | 8.91E6 | 1.22E7 | 2.76E6 | 1.7E7 | 6.76E6 | 14 | 14 | 4 | 3 | 3 | 6 | 5 | 4 | 6 | 3 | 4 | 3 | Y | 161936 | Eukaryotic translation initiation factor 3 subunit A OS=Mus musculus OX=10090 GN=Eif3a PE=1 SV=5 |
| 374 | 742 | A0A0R4IZW5\|A0A0R4IZW5\_MOUSE | 212.95 | 9 | 6 | 7 | 6 | 5 | 5 | 5 | 8 | 5 | 2 | 7 | 2.96E7 | 2.82E7 | 1.67E7 | 1.29E7 | 3.18E7 | 3.25E7 | 2.62E7 | 1.35E7 | 2.84E7 | 2.82E7 | 7 | 7 | 5 | 7 | 6 | 5 | 5 | 6 | 7 | 4 | 1 | 6 | Y | 98284 | Cadherin-1 OS=Mus musculus OX=10090 GN=Cdh1 PE=1 SV=1 |
| 374 | 743 | sp\|P09803\|CADH1\_MOUSE | 212.95 | 9 | 6 | 7 | 6 | 5 | 5 | 5 | 8 | 5 | 2 | 7 | 2.96E7 | 2.82E7 | 1.67E7 | 1.29E7 | 3.18E7 | 3.25E7 | 2.62E7 | 1.35E7 | 2.84E7 | 2.82E7 | 7 | 7 | 5 | 7 | 6 | 5 | 5 | 6 | 7 | 4 | 1 | 6 | Y | 98256 | Cadherin-1 OS=Mus musculus OX=10090 GN=Cdh1 PE=1 SV=1 |
| 331 | 358 | Q543K9\|Q543K9\_MOUSE | 212.76 | 51 | 26 | 24 | 21 | 26 | 31 | 28 | 29 | 17 | 31 | 41 | 2.74E7 | 2.02E7 | 4.64E7 | 1.94E7 | 4.11E7 | 6.05E7 | 4.91E7 | 3.88E7 | 6.18E7 | 3.83E7 | 12 | 12 | 9 | 6 | 7 | 7 | 8 | 9 | 8 | 6 | 9 | 10 | Y | 32263 | Purine nucleoside phosphorylase OS=Mus musculus OX=10090 GN=Pnp PE=1 SV=1 |
| 331 | 359 | sp\|P23492\|PNPH\_MOUSE | 212.76 | 51 | 26 | 24 | 21 | 26 | 31 | 28 | 29 | 17 | 31 | 41 | 2.74E7 | 2.02E7 | 4.64E7 | 1.94E7 | 4.11E7 | 6.05E7 | 4.91E7 | 3.88E7 | 6.18E7 | 3.83E7 | 12 | 12 | 9 | 6 | 7 | 7 | 8 | 9 | 8 | 6 | 9 | 10 | Y | 32277 | Purine nucleoside phosphorylase OS=Mus musculus OX=10090 GN=Pnp PE=1 SV=2 |
| 323 | 650 | sp\|P51885\|LUM\_MOUSE | 212.51 | 27 | 18 | 16 | 20 | 17 | 22 | 20 | 19 | 21 | 18 | 17 | 5.81E7 | 5.03E7 | 5.33E7 | 2.96E7 | 5.04E7 | 6.18E7 | 3.77E7 | 4.45E7 | 5.73E7 | 2.06E7 | 10 | 9 | 8 | 7 | 8 | 6 | 9 | 9 | 10 | 11 | 7 | 6 | Y | 38265 | Lumican OS=Mus musculus OX=10090 GN=Lum PE=1 SV=2 |
| 430 | 558 | A0A494B945\|A0A494B945\_MOUSE | 212.40 | 41 | 12 | 27 | 21 | 25 | 28 | 12 | 30 | 26 | 19 | 19 | 1.27E7 | 1.51E7 | 1.04E7 | 3.03E6 | 4.67E6 | 1E7 | 4.62E6 | 1.49E6 | 2.07E7 | 2.51E6 | 10 | 2 | 4 | 7 | 6 | 7 | 8 | 5 | 6 | 7 | 4 | 5 | Y | 28254 | small monomeric GTPase (Fragment) OS=Mus musculus OX=10090 GN=Rab1b PE=1 SV=1 |
| 403 | 755 | sp\|P14602\|HSPB1\_MOUSE | 212.12 | 49 | 44 | 23 | 17 | 9 | 22 | 25 | 22 | 14 | 44 | 21 | 6.03E7 | 2.69E7 | 3.32E7 | 1.45E7 | 4.37E7 | 6.04E7 | 2.84E7 | 3.12E7 | 1.17E8 | 4.81E7 | 9 | 9 | 11 | 4 | 5 | 3 | 5 | 6 | 5 | 5 | 10 | 6 | N | 23014 | Heat shock protein beta-1 OS=Mus musculus OX=10090 GN=Hspb1 PE=1 SV=3 |
| 314 | 774 | sp\|P62242\|RS8\_MOUSE | 212.10 | 40 | 32 | 32 | 21 | 32 | 31 | 32 | 32 | 25 | 33 | 32 | 6.26E7 | 1.01E8 | 6.66E7 | 5.07E7 | 5.94E7 | 7.68E7 | 6.09E7 | 6.46E7 | 1.5E8 | 1E8 | 8 | 8 | 7 | 9 | 6 | 11 | 10 | 7 | 8 | 9 | 9 | 7 | Y | 24205 | Small ribosomal subunit protein eS8 OS=Mus musculus OX=10090 GN=Rps8 PE=1 SV=2 |
| 553 | 784 | sp\|Q8BMF4\|ODP2\_MOUSE | 211.52 | 14 | 9 | 9 | 10 | 10 | 9 | 11 | 9 | 6 | 9 | 9 | 1.11E7 | 1.84E7 | 1.89E7 | 3.86E6 | 1.2E7 | 2.27E7 | 4.93E6 | 1.07E7 | 2.05E7 | 8.76E6 | 7 | 7 | 4 | 4 | 5 | 6 | 4 | 6 | 4 | 3 | 4 | 4 | Y | 67942 | Dihydrolipoyllysine-residue acetyltransferase component of pyruvate dehydrogenase complex, mitochondrial OS=Mus musculus OX=10090 GN=Dlat PE=1 SV=2 |
| 703 | 857 | F6TVP2\|F6TVP2\_MOUSE | 211.36 | 15 | 7 | 2 | 4 | 8 | 6 | 7 | 10 | 7 | 12 | 9 | 8.63E6 | 2.14E6 | 3.14E6 | 2.5E6 | 2.47E6 | 6.44E6 | 5.13E6 | 4.67E6 | 2.7E7 | 1.6E7 | 7 | 7 | 3 | 1 | 1 | 3 | 2 | 3 | 3 | 3 | 5 | 4 | N | 72866 | Deoxynucleoside triphosphate triphosphohydrolase SAMHD1 (Fragment) OS=Mus musculus OX=10090 GN=Samhd1 PE=1 SV=1 |
| 703 | 858 | sp\|Q60710\|SAMH1\_MOUSE | 211.36 | 15 | 7 | 2 | 4 | 7 | 6 | 7 | 10 | 7 | 12 | 9 | 8.63E6 | 2.14E6 | 3.14E6 | 2.5E6 | 2.47E6 | 6.44E6 | 5.13E6 | 4.67E6 | 2.7E7 | 1.6E7 | 7 | 7 | 3 | 1 | 1 | 3 | 2 | 3 | 3 | 3 | 5 | 4 | N | 75893 | Deoxynucleoside triphosphate triphosphohydrolase SAMHD1 OS=Mus musculus OX=10090 GN=Samhd1 PE=1 SV=3 |
| 417 | 482 | sp\|Q99KE1\|MAOM\_MOUSE | 211.11 | 20 | 6 | 12 | 12 | 8 | 18 | 13 | 13 | 11 | 6 | 8 | 9.85E6 | 5.12E7 | 4.39E7 | 1.02E7 | 2.38E7 | 2.97E7 | 2.05E7 | 9.91E6 | 1.8E7 | 1.76E7 | 10 | 10 | 4 | 7 | 7 | 5 | 10 | 7 | 7 | 6 | 3 | 5 | Y | 65799 | NAD-dependent malic enzyme, mitochondrial OS=Mus musculus OX=10090 GN=Me2 PE=1 SV=1 |
| 273 | 782 | sp\|P35980\|RL18\_MOUSE | 210.14 | 40 | 32 | 32 | 28 | 33 | 28 | 30 | 40 | 36 | 36 | 26 | 9.78E7 | 1.11E8 | 1.29E8 | 7.33E7 | 9.42E7 | 1E8 | 8.81E7 | 7.15E7 | 1.8E8 | 7.53E7 | 7 | 7 | 9 | 10 | 9 | 8 | 8 | 7 | 9 | 9 | 12 | 6 | N | 21645 | Large ribosomal subunit protein eL18 OS=Mus musculus OX=10090 GN=Rpl18 PE=1 SV=3 |
| 273 | 847 | A0A1B0GQU8\|A0A1B0GQU8\_MOUSE | 210.14 | 48 | 38 | 38 | 33 | 39 | 33 | 35 | 48 | 42 | 42 | 30 | 9.78E7 | 1.11E8 | 1.29E8 | 7.33E7 | 9.42E7 | 1E8 | 8.81E7 | 7.15E7 | 1.8E8 | 7.53E7 | 7 | 7 | 9 | 10 | 9 | 8 | 8 | 7 | 9 | 9 | 12 | 6 | N | 18101 | Ribosomal protein L18 OS=Mus musculus OX=10090 GN=Rpl18 PE=1 SV=1 |
| 423 | 741 | sp\|P63325\|RS10\_MOUSE | 210.00 | 49 | 24 | 24 | 34 | 33 | 35 | 33 | 33 | 33 | 27 | 24 | 2.93E7 | 8.54E6 | 4.45E6 | 5.5E6 | 2.71E7 | 2.24E7 | 2.07E7 | 6.1E6 | 3.54E7 | 2.34E7 | 9 | 2 | 4 | 3 | 6 | 7 | 10 | 6 | 7 | 6 | 7 | 5 | N | 18916 | Small ribosomal subunit protein eS10 OS=Mus musculus OX=10090 GN=Rps10 PE=1 SV=1 |
| 565 | 602 | sp\|Q11136\|PEPD\_MOUSE | 209.27 | 18 | 6 | 9 | 13 | 9 | 4 | 11 | 6 | 4 | 5 | 8 | 2.2E7 | 3.76E7 | 5.64E7 | 1.94E7 | 1.35E7 | 2.61E7 | 1.63E7 | 1.21E7 | 4.5E7 | 1.33E7 | 9 | 9 | 3 | 4 | 7 | 4 | 2 | 6 | 3 | 2 | 3 | 4 | Y | 55029 | Xaa-Pro dipeptidase OS=Mus musculus OX=10090 GN=Pepd PE=1 SV=3 |
| 507 | 613 | sp\|Q6ZQM8\|UD17\_MOUSE | 209.23 | 22 | 11 | 18 | 7 | 5 | 5 | 12 | 9 | 6 | 14 | 14 | 3.36E6 | 1.35E7 | 1.06E7 | 3.6E5 | 6.21E6 | 3.36E6 | 3.31E6 | 1.75E6 | 3.68E6 | 9.11E6 | 8 | 5 | 4 | 8 | 3 | 2 | 2 | 6 | 4 | 3 | 7 | 7 | Y | 59758 | UDP-glucuronosyltransferase 1A7 OS=Mus musculus OX=10090 GN=Ugt1a7 PE=1 SV=1 |
| 586 | 1204 | sp\|Q9DCT2\|NDUS3\_MOUSE | 209.20 | 27 | 10 | 25 | 22 | 13 | 8 | 27 | 24 | 10 | 15 | 15 | 1.61E7 | 2.6E7 | 2.22E7 | 9.03E6 | 1.23E7 | 2.29E7 | 1.31E7 | 8.13E6 | 2.99E7 | 1.4E7 | 7 | 7 | 3 | 7 | 6 | 4 | 2 | 6 | 5 | 2 | 3 | 3 | N | 30149 | NADH dehydrogenase [ubiquinone] iron-sulfur protein 3, mitochondrial OS=Mus musculus OX=10090 GN=Ndufs3 PE=1 SV=2 |
| 311 | 232 | sp\|P80318\|TCPG\_MOUSE | 208.80 | 34 | 17 | 13 | 20 | 11 | 21 | 23 | 13 | 19 | 12 | 11 | 1.9E7 | 1.99E7 | 4.3E7 | 1E7 | 2.95E7 | 5.11E7 | 1.55E7 | 1.72E7 | 3.51E7 | 1.95E7 | 19 | 19 | 8 | 6 | 11 | 6 | 12 | 11 | 7 | 9 | 6 | 6 | Y | 60630 | T-complex protein 1 subunit gamma OS=Mus musculus OX=10090 GN=Cct3 PE=1 SV=1 |
| 518 | 395 | G3X9V2\|G3X9V2\_MOUSE | 208.66 | 16 | 1 | 5 | 5 | 5 | 5 | 6 | 5 | 4 | 8 | 2 | 7.16E5 | 6.38E6 | 8.8E6 | 8.98E6 | 2.41E6 | 1.83E7 | 1.13E7 | 2.43E6 | 3.84E7 | 6.64E6 | 15 | 15 | 1 | 4 | 4 | 5 | 4 | 5 | 5 | 3 | 8 | 2 | Y | 104104 | Catenin delta 1 OS=Mus musculus OX=10090 GN=Ctnnd1 PE=1 SV=1 |
| 518 | 396 | sp\|P30999\|CTND1\_MOUSE | 208.66 | 16 | 1 | 5 | 5 | 5 | 5 | 6 | 5 | 4 | 8 | 2 | 7.16E5 | 6.38E6 | 8.8E6 | 8.98E6 | 2.41E6 | 1.83E7 | 1.13E7 | 2.43E6 | 3.84E7 | 6.64E6 | 15 | 15 | 1 | 4 | 4 | 5 | 4 | 5 | 5 | 3 | 8 | 2 | Y | 104925 | Catenin delta-1 OS=Mus musculus OX=10090 GN=Ctnnd1 PE=1 SV=2 |
| 518 | 397 | E9Q8Z6\|E9Q8Z6\_MOUSE | 208.66 | 15 | 1 | 5 | 5 | 5 | 5 | 6 | 5 | 4 | 8 | 2 | 7.16E5 | 6.38E6 | 8.8E6 | 8.98E6 | 2.41E6 | 1.83E7 | 1.13E7 | 2.43E6 | 3.84E7 | 6.64E6 | 15 | 15 | 1 | 4 | 4 | 5 | 4 | 5 | 5 | 3 | 8 | 2 | Y | 107354 | Catenin delta 1 OS=Mus musculus OX=10090 GN=Ctnnd1 PE=1 SV=1 |
| 518 | 398 | E9Q8Z8\|E9Q8Z8\_MOUSE | 208.66 | 15 | 1 | 5 | 5 | 5 | 5 | 6 | 5 | 4 | 8 | 2 | 7.16E5 | 6.38E6 | 8.8E6 | 8.98E6 | 2.41E6 | 1.83E7 | 1.13E7 | 2.43E6 | 3.84E7 | 6.64E6 | 15 | 15 | 1 | 4 | 4 | 5 | 4 | 5 | 5 | 3 | 8 | 2 | Y | 108175 | Catenin delta 1 OS=Mus musculus OX=10090 GN=Ctnnd1 PE=1 SV=1 |
| 426 | 309 | sp\|Q60604\|SCIN\_MOUSE | 208.62 | 20 | 17 | 5 | 3 | 3 | 4 | 6 | 4 | 3 | 12 | 7 | 8.11E7 | 2.16E7 | 1.5E7 | 2.14E6 | 1.62E7 | 4.04E7 | 8.02E6 | 1.1E7 | 6.88E7 | 1.92E7 | 13 | 13 | 12 | 4 | 2 | 2 | 3 | 4 | 3 | 2 | 10 | 5 | Y | 80254 | Scinderin OS=Mus musculus OX=10090 GN=Scin PE=1 SV=3 |
| 350 | 290 | sp\|Q9CPY7\|AMPL\_MOUSE | 208.35 | 32 | 11 | 11 | 21 | 19 | 14 | 24 | 26 | 17 | 13 | 15 | 1.36E7 | 1.36E7 | 2.45E7 | 1.84E7 | 6.49E7 | 8.27E7 | 3.86E7 | 1.61E7 | 2.77E7 | 1.02E7 | 13 | 13 | 4 | 5 | 9 | 8 | 6 | 9 | 10 | 7 | 6 | 5 | Y | 56141 | Cytosol aminopeptidase OS=Mus musculus OX=10090 GN=Lap3 PE=1 SV=3 |
| 228 | 286 | sp\|Q93092\|TALDO\_MOUSE | 208.10 | 42 | 18 | 22 | 30 | 24 | 28 | 31 | 26 | 27 | 26 | 26 | 9.54E7 | 1.18E8 | 1.49E8 | 5.42E7 | 1.17E8 | 1.28E8 | 7.72E7 | 7.31E7 | 1.58E8 | 1.18E8 | 17 | 17 | 7 | 8 | 12 | 9 | 11 | 13 | 10 | 10 | 12 | 12 | Y | 37387 | Transaldolase OS=Mus musculus OX=10090 GN=Taldo1 PE=1 SV=2 |
| 228 | 292 | A0A1B0GR11\|A0A1B0GR11\_MOUSE | 208.10 | 37 | 16 | 19 | 27 | 21 | 25 | 27 | 23 | 24 | 23 | 23 | 9.54E7 | 1.18E8 | 1.49E8 | 5.42E7 | 1.17E8 | 1.28E8 | 7.72E7 | 7.31E7 | 1.58E8 | 1.18E8 | 17 | 17 | 7 | 8 | 12 | 9 | 11 | 13 | 10 | 10 | 12 | 12 | Y | 42151 | Transaldolase OS=Mus musculus OX=10090 GN=Taldo1 PE=1 SV=1 |
| 501 | 745 | Q62029\|Q62029\_MOUSE | 207.82 | 15 | 6 | 5 | 6 | 7 | 5 | 8 | 8 | 5 | 10 | 9 |  |  | 1.56E6 |  |  |  |  |  |  |  | 8 | 1 | 4 | 4 | 4 | 5 | 4 | 4 | 5 | 3 | 7 | 6 | Y | 69061 | Polyadenylate-binding protein OS=Mus musculus OX=10090 GN=Pabpc2 PE=1 SV=1 |
| 687 | 362 | sp\|Q3UQ44\|IQGA2\_MOUSE | 207.73 | 8 | 1 | 3 | 1 | 1 | 2 | 4 | 3 | 2 | 2 | 1 | 3.54E6 | 8.02E6 | 1.88E6 | 1.5E6 | 2.33E6 | 5.3E6 | 5.26E6 | 1.74E6 | 1.96E6 | 3.72E6 | 11 | 11 | 2 | 4 | 2 | 2 | 3 | 5 | 5 | 3 | 2 | 2 | Y | 180527 | Ras GTPase-activating-like protein IQGAP2 OS=Mus musculus OX=10090 GN=Iqgap2 PE=1 SV=2 |
| 419 | 724 | sp\|Q00519\|XDH\_MOUSE | 207.70 | 8 | 5 | 3 | 5 | 1 | 3 | 7 | 6 | 2 | 4 | 5 | 2.19E7 | 1.02E7 | 1.79E7 | 8.91E5 | 8.9E6 | 1.79E7 | 1.03E7 | 4.38E6 | 2.87E7 | 2.04E7 | 9 | 9 | 7 | 4 | 9 | 2 | 4 | 9 | 8 | 3 | 6 | 8 | Y | 146562 | Xanthine dehydrogenase/oxidase OS=Mus musculus OX=10090 GN=Xdh PE=1 SV=5 |
| 483 | 1101 | sp\|Q99020\|ROAA\_MOUSE | 207.35 | 24 | 18 | 11 | 18 | 24 | 21 | 14 | 9 | 18 | 14 | 18 | 3.57E7 | 2.81E7 | 3.15E7 | 2.1E7 | 3.97E7 | 2.19E7 | 6.42E6 | 2.07E7 | 5.95E7 | 3.2E7 | 7 | 6 | 6 | 6 | 7 | 8 | 6 | 3 | 2 | 5 | 4 | 5 | Y | 30831 | Heterogeneous nuclear ribonucleoprotein A/B OS=Mus musculus OX=10090 GN=Hnrnpab PE=1 SV=1 |
| 483 | 1103 | Q80XR6\|Q80XR6\_MOUSE | 207.35 | 22 | 16 | 10 | 16 | 22 | 19 | 13 | 9 | 16 | 13 | 16 | 3.57E7 | 2.81E7 | 3.15E7 | 2.1E7 | 3.97E7 | 2.19E7 | 6.42E6 | 2.07E7 | 5.95E7 | 3.2E7 | 7 | 6 | 6 | 6 | 7 | 8 | 6 | 3 | 2 | 5 | 4 | 5 | Y | 33816 | Heterogeneous nuclear ribonucleoprotein A/B OS=Mus musculus OX=10090 GN=Hnrnpab PE=1 SV=1 |
| 483 | 1106 | Q20BD0\|Q20BD0\_MOUSE | 207.35 | 20 | 15 | 10 | 15 | 20 | 18 | 12 | 8 | 15 | 12 | 15 | 3.57E7 | 2.81E7 | 3.15E7 | 2.1E7 | 3.97E7 | 2.19E7 | 6.42E6 | 2.07E7 | 5.95E7 | 3.2E7 | 7 | 6 | 6 | 6 | 7 | 8 | 6 | 3 | 2 | 5 | 4 | 5 | Y | 36211 | Heterogeneous nuclear ribonucleoprotein A/B OS=Mus musculus OX=10090 GN=Hnrnpab PE=1 SV=1 |
| 372 | 683 | sp\|P51150\|RAB7A\_MOUSE | 207.04 | 44 | 25 | 29 | 21 | 31 | 31 | 28 | 28 | 18 | 28 | 29 | 3.11E7 | 2.63E7 | 2.38E7 | 1.76E7 | 2.46E7 | 2.2E7 | 1.88E7 | 1.14E7 | 5.43E7 | 2.34E7 | 9 | 9 | 7 | 7 | 5 | 8 | 8 | 7 | 8 | 5 | 6 | 6 | N | 23490 | Ras-related protein Rab-7a OS=Mus musculus OX=10090 GN=Rab7a PE=1 SV=2 |
| 558 | 785 | E9Q1G8\|E9Q1G8\_MOUSE | 206.49 | 21 | 4 | 7 | 2 | 16 | 15 | 12 | 12 | 12 | 9 | 4 | 1.82E6 | 3.61E6 |  | 8.79E6 | 9.53E6 | 1.07E7 | 8.93E6 | 8.47E6 | 6.16E6 | 1.24E6 | 8 | 7 | 3 | 3 | 2 | 7 | 6 | 5 | 4 | 5 | 4 | 3 | Y | 50649 | Septin OS=Mus musculus OX=10090 GN=Septin7 PE=1 SV=2 |
| 609 | 1132 | sp\|P49722\|PSA2\_MOUSE | 206.35 | 35 | 15 | 12 | 20 | 20 | 11 | 20 | 20 | 9 | 32 | 26 | 2.23E7 | 2.09E7 | 1.3E7 | 2.15E7 | 1.18E7 | 1.5E7 | 8.82E6 | 6.15E6 | 3.1E7 | 2.46E7 | 7 | 7 | 4 | 4 | 3 | 4 | 2 | 3 | 3 | 2 | 7 | 5 | Y | 25927 | Proteasome subunit alpha type-2 OS=Mus musculus OX=10090 GN=Psma2 PE=1 SV=3 |
| 356 | 517 | Q9D4E6\|Q9D4E6\_MOUSE | 206.05 | 18 | 10 | 6 | 9 | 11 | 10 | 10 | 9 | 10 | 12 | 11 | 7.7E6 |  |  |  |  |  |  |  |  |  | 11 | 1 | 7 | 5 | 6 | 9 | 7 | 6 | 7 | 7 | 9 | 8 | Y | 71001 | Polyadenylate-binding protein OS=Mus musculus OX=10090 GN=Pabpc6 PE=1 SV=1 |
| 89 | 812 | sp\|P62983\|RS27A\_MOUSE | 205.83 | 50 | 34 | 44 | 47 | 35 | 31 | 35 | 31 | 31 | 50 | 42 | 7.95E8 | 7.92E8 | 5.27E8 | 3.8E8 | 5.66E8 | 4.29E8 | 3.32E8 | 3.67E8 | 7.2E8 | 6.86E8 | 8 | 8 | 35 | 29 | 27 | 27 | 28 | 19 | 26 | 31 | 23 | 25 | Y | 17951 | Ubiquitin-ribosomal protein eS31 fusion protein OS=Mus musculus OX=10090 GN=Rps27a PE=1 SV=2 |
| 701 | 600 | sp\|Q9ERG0\|LIMA1\_MOUSE | 205.15 | 13 | 5 | 6 | 4 | 4 | 5 | 5 | 3 | 2 | 4 | 3 | 2.16E7 | 6.77E6 | 2.11E6 | 1.36E6 | 6.24E6 | 4.99E6 | 7.01E5 | 0E0 | 1.95E7 | 3.05E6 | 9 | 9 | 5 | 3 | 2 | 2 | 3 | 3 | 2 | 1 | 4 | 2 | N | 84060 | LIM domain and actin-binding protein 1 OS=Mus musculus OX=10090 GN=Lima1 PE=1 SV=3 |
| 114 | 1831 | sp\|P43274\|H14\_MOUSE | 205.12 | 23 | 23 | 23 | 23 | 22 | 22 | 23 | 23 | 23 | 23 | 23 | 2.74E8 | 3.01E8 | 2.63E8 | 1.01E8 | 2.43E8 | 2.19E8 | 1.61E8 | 1.81E8 | 4.37E8 | 3E8 | 7 | 3 | 22 | 22 | 25 | 21 | 23 | 17 | 20 | 28 | 28 | 18 | N | 21977 | Histone H1.4 OS=Mus musculus OX=10090 GN=H1-4 PE=1 SV=2 |
| 539 | 433 | D3YVV9\|D3YVV9\_MOUSE | 205.07 | 12 | 8 | 4 | 6 | 5 | 3 | 6 | 5 | 7 | 8 | 3 | 1.1E7 | 7.47E6 | 1.09E7 | 3.12E6 | 4.4E6 | 5.85E6 | 4.17E6 | 4.87E6 | 1.46E7 | 1.95E6 | 10 | 10 | 6 | 3 | 5 | 5 | 3 | 5 | 4 | 6 | 5 | 2 | Y | 129630 | Synaptopodin 2 OS=Mus musculus OX=10090 GN=Synpo2 PE=1 SV=1 |
| 539 | 434 | E9Q1U2\|E9Q1U2\_MOUSE | 205.07 | 11 | 7 | 4 | 5 | 5 | 3 | 6 | 5 | 7 | 7 | 3 | 1.1E7 | 7.47E6 | 1.09E7 | 3.12E6 | 4.4E6 | 5.85E6 | 4.17E6 | 4.87E6 | 1.46E7 | 1.95E6 | 10 | 10 | 6 | 3 | 5 | 5 | 3 | 5 | 4 | 6 | 5 | 2 | Y | 135757 | Synaptopodin 2 OS=Mus musculus OX=10090 GN=Synpo2 PE=1 SV=1 |
| 397 | 393 | sp\|Q8BP47\|SYNC\_MOUSE | 204.95 | 23 | 6 | 9 | 11 | 12 | 11 | 12 | 18 | 9 | 7 | 10 | 1.78E7 | 2.27E7 | 2.41E7 | 1.07E7 | 1.74E7 | 1.57E7 | 2.04E7 | 1.25E7 | 2.93E7 | 2.45E7 | 13 | 13 | 4 | 5 | 6 | 7 | 6 | 6 | 9 | 5 | 5 | 5 | Y | 64279 | Asparagine--tRNA ligase, cytoplasmic OS=Mus musculus OX=10090 GN=Nars1 PE=1 SV=2 |
| 397 | 394 | A0A498WGK2\|A0A498WGK2\_MOUSE | 204.95 | 23 | 6 | 9 | 11 | 12 | 11 | 12 | 18 | 9 | 7 | 10 | 1.78E7 | 2.27E7 | 2.41E7 | 1.07E7 | 1.74E7 | 1.57E7 | 2.04E7 | 1.25E7 | 2.93E7 | 2.45E7 | 13 | 13 | 4 | 5 | 6 | 7 | 6 | 6 | 9 | 5 | 5 | 5 | Y | 64208 | Asparagine--tRNA ligase, cytoplasmic OS=Mus musculus OX=10090 GN=Nars1 PE=1 SV=1 |
| 269 | 488 | sp\|P47962\|RL5\_MOUSE | 204.72 | 34 | 18 | 24 | 18 | 27 | 26 | 24 | 28 | 19 | 27 | 25 | 6.83E7 | 3.04E8 | 8.68E7 | 6.54E7 | 9.06E7 | 8.67E7 | 8.85E7 | 4.13E7 | 2.72E7 | 1.7E8 | 11 | 11 | 7 | 8 | 6 | 9 | 9 | 10 | 10 | 8 | 8 | 10 | Y | 34401 | Large ribosomal subunit protein uL18 OS=Mus musculus OX=10090 GN=Rpl5 PE=1 SV=3 |
| 29 | 1048 | sp\|P02301\|H3C\_MOUSE | 204.49 | 54 | 31 | 26 | 54 | 31 | 54 | 54 | 31 | 31 | 54 | 54 |  |  | 4.66E5 |  | 2.18E6 | 3.79E6 |  |  | 3.2E6 | 9.19E6 | 9 | 1 | 57 | 50 | 68 | 51 | 49 | 44 | 45 | 64 | 58 | 49 | Y | 15315 | Histone H3.3C OS=Mus musculus OX=10090 GN=H3-5 PE=3 SV=3 |
| 29 | 1049 | sp\|P84244\|H33\_MOUSE | 204.49 | 54 | 31 | 26 | 54 | 31 | 54 | 54 | 31 | 31 | 54 | 54 |  |  | 4.66E5 |  | 2.18E6 | 3.79E6 |  |  | 3.2E6 | 9.19E6 | 9 | 1 | 57 | 50 | 68 | 51 | 49 | 44 | 45 | 64 | 58 | 49 | Y | 15328 | Histone H3.3 OS=Mus musculus OX=10090 GN=H3-3a PE=1 SV=2 |
| 625 | 955 | sp\|O70209\|PDLI3\_MOUSE | 204.19 | 33 | 14 | 19 | 9 | 15 | 33 | 24 | 14 | 24 | 20 | 9 | 7.53E6 | 1.35E7 | 7.43E6 | 4.7E6 | 1.58E7 | 1.74E7 | 3.93E6 | 1.19E7 | 3.74E7 | 6.28E6 | 7 | 7 | 3 | 4 | 2 | 3 | 7 | 5 | 3 | 5 | 5 | 2 | Y | 34300 | PDZ and LIM domain protein 3 OS=Mus musculus OX=10090 GN=Pdlim3 PE=1 SV=1 |
| 113 | 1722 | sp\|P43277\|H13\_MOUSE | 204.04 | 23 | 23 | 23 | 23 | 22 | 22 | 23 | 23 | 23 | 23 | 23 | 4.4E7 | 2.93E7 | 3.02E7 | 8.79E6 | 2.06E7 | 1.86E7 | 1.82E7 | 8.62E6 | 5.79E7 | 7.28E7 | 7 | 1 | 21 | 21 | 26 | 18 | 21 | 16 | 22 | 27 | 26 | 21 | N | 22100 | Histone H1.3 OS=Mus musculus OX=10090 GN=H1-3 PE=1 SV=2 |
| 737 | 557 | sp\|Q9JIK5\|DDX21\_MOUSE | 203.99 | 13 | 3 | 1 | 5 | 5 | 3 | 1 | 5 | 1 | 6 | 8 | 3.35E6 | 3.57E5 | 2.47E6 | 1.75E6 | 1.25E6 | 1.21E6 | 2.82E6 | 1.41E5 | 1.35E7 | 4.31E6 | 9 | 8 | 1 | 1 | 3 | 3 | 2 | 1 | 3 | 1 | 6 | 5 | N | 93551 | Nucleolar RNA helicase 2 OS=Mus musculus OX=10090 GN=Ddx21 PE=1 SV=3 |
| 259 | 1163 | sp\|P62897\|CYC\_MOUSE | 203.43 | 60 | 50 | 54 | 50 | 49 | 54 | 55 | 49 | 54 | 49 | 49 | 7.88E7 | 9.17E7 | 3.61E7 | 3.41E7 | 6.54E7 | 8.39E7 | 3.44E7 | 4.34E7 | 8.15E7 | 4.59E7 | 9 | 9 | 12 | 12 | 9 | 8 | 13 | 12 | 9 | 12 | 11 | 7 | Y | 11605 | Cytochrome c, somatic OS=Mus musculus OX=10090 GN=Cycs PE=1 SV=2 |
| 432 | 1271 | sp\|P47754\|CAZA2\_MOUSE | 203.25 | 26 | 24 | 23 | 23 | 24 | 16 | 24 | 26 | 19 | 24 | 26 | 3.45E7 | 3.18E7 | 3.01E7 | 1.88E7 | 1.44E7 | 3.87E7 | 2.27E7 | 1.81E7 | 7.39E7 | 4.47E7 | 6 | 4 | 6 | 6 | 6 | 6 | 4 | 6 | 7 | 5 | 6 | 7 | N | 32967 | F-actin-capping protein subunit alpha-2 OS=Mus musculus OX=10090 GN=Capza2 PE=1 SV=3 |
| 610 | 679 | sp\|P11983\|TCPA\_MOUSE | 203.07 | 16 | 10 | 4 | 4 | 5 | 4 | 6 | 5 | 4 | 9 | 5 | 1.82E7 | 1.1E7 | 1.03E7 | 1.29E7 | 1.45E7 | 1.75E7 | 8.3E6 | 5.77E6 | 2.17E7 | 1.91E7 | 8 | 8 | 5 | 2 | 2 | 5 | 3 | 4 | 4 | 2 | 5 | 4 | Y | 60449 | T-complex protein 1 subunit alpha OS=Mus musculus OX=10090 GN=Tcp1 PE=1 SV=3 |
| 464 | 511 | sp\|Q8JZQ5\|AOC1\_MOUSE | 202.75 | 14 | 5 | 11 | 7 | 7 | 6 | 7 | 8 | 6 | 5 | 6 | 6.13E6 | 4.43E7 | 2.84E7 | 9.54E6 | 1.69E8 | 3.05E7 | 1.75E7 | 7.79E6 | 1.86E7 | 1.35E7 | 10 | 10 | 3 | 9 | 5 | 6 | 4 | 6 | 6 | 5 | 4 | 4 | Y | 85424 | Diamine oxidase [copper-containing] OS=Mus musculus OX=10090 GN=Aoc1 PE=2 SV=1 |
| 464 | 513 | E9PYN9\|E9PYN9\_MOUSE | 202.75 | 14 | 5 | 11 | 7 | 7 | 6 | 7 | 8 | 6 | 5 | 6 | 6.13E6 | 4.43E7 | 2.84E7 | 9.54E6 | 1.69E8 | 3.05E7 | 1.75E7 | 7.79E6 | 1.86E7 | 1.35E7 | 10 | 10 | 3 | 9 | 5 | 6 | 4 | 6 | 6 | 5 | 4 | 4 | Y | 86023 | Amine oxidase OS=Mus musculus OX=10090 GN=Aoc1 PE=1 SV=1 |
| 380 | 754 | sp\|Q9WV27\|AT1A4\_MOUSE | 202.32 | 9 | 5 | 6 | 6 | 3 | 4 | 7 | 6 | 4 | 6 | 3 | 3.34E7 |  |  | 1.82E6 |  |  |  |  |  |  | 10 | 2 | 5 | 8 | 7 | 5 | 5 | 7 | 6 | 4 | 5 | 3 | Y | 114887 | Sodium/potassium-transporting ATPase subunit alpha-4 OS=Mus musculus OX=10090 GN=Atp1a4 PE=1 SV=3 |
| 514 | 580 | sp\|Q91V92\|ACLY\_MOUSE | 202.04 | 10 | 7 | 6 | 3 | 4 | 4 | 8 | 5 | 2 | 4 | 4 | 1.47E7 | 1.33E7 | 3.65E6 | 4.77E6 | 8.12E6 | 2.4E7 | 1.24E7 | 1.12E7 | 5.16E6 | 3.27E6 | 9 | 9 | 6 | 6 | 3 | 4 | 4 | 7 | 5 | 3 | 3 | 3 | Y | 119728 | ATP-citrate synthase OS=Mus musculus OX=10090 GN=Acly PE=1 SV=1 |
| 514 | 581 | Q3V117\|Q3V117\_MOUSE | 202.04 | 10 | 7 | 6 | 3 | 4 | 4 | 8 | 5 | 2 | 4 | 4 | 1.47E7 | 1.33E7 | 3.65E6 | 4.77E6 | 8.12E6 | 2.4E7 | 1.24E7 | 1.12E7 | 5.16E6 | 3.27E6 | 9 | 9 | 6 | 6 | 3 | 4 | 4 | 7 | 5 | 3 | 3 | 3 | Y | 120795 | ATP-citrate synthase OS=Mus musculus OX=10090 GN=Acly PE=1 SV=1 |
| 562 | 899 | sp\|Q921F2\|TADBP\_MOUSE | 202.00 | 22 | 13 | 9 | 8 | 18 | 16 | 21 | 21 | 16 | 6 | 13 | 1.71E7 | 5.28E6 | 1.5E7 | 9.62E6 | 2.21E7 | 2.43E7 | 1.85E7 | 1.13E7 | 2.76E6 | 1.74E7 | 8 | 7 | 4 | 2 | 3 | 6 | 5 | 6 | 6 | 5 | 2 | 4 | Y | 44548 | TAR DNA-binding protein 43 OS=Mus musculus OX=10090 GN=Tardbp PE=1 SV=1 |
| 391 | 838 | sp\|Q9CR68\|UCRI\_MOUSE | 201.51 | 31 | 23 | 26 | 23 | 23 | 19 | 31 | 28 | 28 | 22 | 20 | 1.29E8 | 6.83E7 | 5.81E7 | 3.71E7 | 3.39E7 | 1.36E8 | 1.13E8 | 4.57E7 | 5.25E7 | 9.8E7 | 8 | 8 | 6 | 6 | 5 | 7 | 7 | 7 | 8 | 8 | 5 | 6 | N | 29368 | Cytochrome b-c1 complex subunit Rieske, mitochondrial OS=Mus musculus OX=10090 GN=Uqcrfs1 PE=1 SV=1 |
| 566 | 874 | sp\|Q00PI9\|HNRL2\_MOUSE | 201.44 | 10 | 4 | 5 | 7 | 6 | 6 | 5 | 6 | 3 | 3 | 2 | 7.42E6 | 7.38E6 | 1.46E7 | 5.71E6 | 1.31E7 | 9.73E6 | 1.37E7 | 2.71E6 | 4.91E6 | 3.89E6 | 7 | 6 | 3 | 4 | 4 | 3 | 4 | 4 | 4 | 2 | 3 | 2 | Y | 84940 | Heterogeneous nuclear ribonucleoprotein U-like protein 2 OS=Mus musculus OX=10090 GN=Hnrnpul2 PE=1 SV=2 |
| 590 | 532 | F7AAP4\|F7AAP4\_MOUSE | 200.86 | 9 | 1 | 2 | 3 | 4 | 5 | 4 | 3 | 2 | 0 | 1 | 2.26E7 | 5.25E7 | 4.32E6 | 2.02E6 | 4.11E6 | 5.57E6 | 9.81E5 | 6.04E5 |  | 4.69E6 | 11 | 6 | 1 | 2 | 4 | 4 | 5 | 4 | 4 | 2 | 0 | 1 | Y | 128562 | Calcium-transporting ATPase OS=Mus musculus OX=10090 GN=Atp2b4 PE=1 SV=1 |
| 590 | 533 | E9Q828\|E9Q828\_MOUSE | 200.86 | 9 | 1 | 2 | 3 | 4 | 5 | 4 | 3 | 2 | 0 | 1 | 2.26E7 | 5.25E7 | 4.32E6 | 2.02E6 | 4.11E6 | 5.57E6 | 9.81E5 | 6.04E5 |  | 4.69E6 | 11 | 6 | 1 | 2 | 4 | 4 | 5 | 4 | 4 | 2 | 0 | 1 | Y | 129256 | Calcium-transporting ATPase OS=Mus musculus OX=10090 GN=Atp2b4 PE=1 SV=1 |
| 590 | 535 | sp\|Q6Q477\|AT2B4\_MOUSE | 200.86 | 9 | 1 | 2 | 3 | 4 | 5 | 4 | 3 | 2 | 0 | 1 | 2.26E7 | 5.25E7 | 4.32E6 | 2.02E6 | 4.11E6 | 5.57E6 | 9.81E5 | 6.04E5 |  | 4.69E6 | 11 | 6 | 1 | 2 | 4 | 4 | 5 | 4 | 4 | 2 | 0 | 1 | Y | 133068 | Plasma membrane calcium-transporting ATPase 4 OS=Mus musculus OX=10090 GN=Atp2b4 PE=1 SV=1 |
| 472 | 759 | sp\|Q9Z1Q5\|CLIC1\_MOUSE | 200.66 | 45 | 22 | 20 | 15 | 24 | 21 | 22 | 24 | 24 | 38 | 22 | 5.51E7 | 3.63E7 | 3.03E7 | 1.59E7 | 2.3E7 | 2.53E7 | 1.58E7 | 2E7 | 7.71E7 | 6.31E7 | 8 | 7 | 6 | 5 | 5 | 5 | 4 | 5 | 5 | 5 | 7 | 6 | Y | 27013 | Chloride intracellular channel protein 1 OS=Mus musculus OX=10090 GN=Clic1 PE=1 SV=3 |
| 289 | 748 | sp\|P15532\|NDKA\_MOUSE | 200.38 | 61 | 44 | 41 | 30 | 53 | 47 | 40 | 40 | 30 | 55 | 44 | 8.75E6 | 7.5E6 |  | 8.71E5 | 1.93E6 | 3.05E6 | 1.74E6 |  | 1.57E7 | 9.92E6 | 9 | 2 | 10 | 9 | 7 | 10 | 9 | 9 | 8 | 6 | 12 | 10 | Y | 17208 | Nucleoside diphosphate kinase A OS=Mus musculus OX=10090 GN=Nme1 PE=1 SV=1 |
| 527 | 965 | sp\|Q8VCT3\|AMPB\_MOUSE | 200.28 | 13 | 9 | 11 | 9 | 13 | 11 | 11 | 13 | 7 | 7 | 7 | 2.18E7 | 3.44E7 | 3.05E7 | 1.15E7 | 2.39E7 | 2.87E7 | 2.05E7 | 9.43E6 | 2.69E7 | 2.05E7 | 6 | 6 | 4 | 5 | 4 | 6 | 5 | 5 | 6 | 4 | 3 | 3 | Y | 72416 | Aminopeptidase B OS=Mus musculus OX=10090 GN=Rnpep PE=1 SV=2 |
| 454 | 1842 | sp\|P63028\|TCTP\_MOUSE | 200.15 | 28 | 15 | 14 | 21 | 28 | 28 | 22 | 28 | 21 | 28 | 21 | 2.64E7 | 3.82E7 | 2.81E7 | 2.28E7 | 4.14E7 | 3.85E7 | 3.11E7 | 2.34E7 | 4.25E7 | 3.43E7 | 5 | 5 | 2 | 4 | 5 | 8 | 6 | 6 | 8 | 6 | 8 | 5 | Y | 19462 | Translationally-controlled tumor protein OS=Mus musculus OX=10090 GN=Tpt1 PE=1 SV=1 |
| 435 | 451 | sp\|Q60598\|SRC8\_MOUSE | 200.15 | 21 | 8 | 5 | 5 | 9 | 12 | 15 | 13 | 13 | 4 | 13 | 1.01E7 | 1.5E7 | 8.13E6 | 5.21E6 | 1.33E7 | 3.4E7 | 1.42E7 | 7.28E6 | 2.02E7 | 2.75E7 | 12 | 12 | 5 | 4 | 3 | 6 | 7 | 10 | 8 | 7 | 2 | 8 | Y | 61250 | Src substrate cortactin OS=Mus musculus OX=10090 GN=Cttn PE=1 SV=2 |
| 363 | 520 | sp\|Q9DCW4\|ETFB\_MOUSE | 199.93 | 43 | 31 | 31 | 23 | 22 | 30 | 31 | 38 | 29 | 27 | 23 | 2.15E7 | 2.45E7 | 2.3E7 | 5.57E6 | 1.63E7 | 1.86E7 | 1.27E7 | 5.96E6 | 3.79E7 | 1.8E7 | 10 | 3 | 7 | 8 | 6 | 7 | 8 | 7 | 9 | 7 | 8 | 6 | Y | 27623 | Electron transfer flavoprotein subunit beta OS=Mus musculus OX=10090 GN=Etfb PE=1 SV=3 |
| 580 | 1409 | sp\|P62889\|RL30\_MOUSE | 199.76 | 58 | 48 | 48 | 48 | 41 | 48 | 37 | 41 | 41 | 31 | 58 | 4.73E7 | 5.09E7 | 4.03E7 | 5.49E6 | 4.23E7 | 3.25E7 | 6.94E6 | 5.58E6 | 2.29E7 | 4.3E7 | 5 | 5 | 4 | 4 | 4 | 3 | 4 | 3 | 5 | 4 | 5 | 5 | Y | 12784 | Large ribosomal subunit protein eL30 OS=Mus musculus OX=10090 GN=Rpl30 PE=1 SV=2 |
| 613 | 629 | sp\|Q9JMH6\|TRXR1\_MOUSE | 199.65 | 21 | 10 | 11 | 10 | 10 | 6 | 6 | 14 | 4 | 8 | 7 | 7.55E6 | 1.39E7 | 8.99E6 | 4.21E6 | 5.12E6 | 4.01E6 | 9.03E6 | 5.08E5 | 6.03E6 | 4.46E6 | 9 | 9 | 4 | 5 | 4 | 4 | 2 | 2 | 5 | 1 | 3 | 3 | Y | 67084 | Thioredoxin reductase 1, cytoplasmic OS=Mus musculus OX=10090 GN=Txnrd1 PE=1 SV=3 |
| 570 | 1214 | sp\|P68037\|UB2L3\_MOUSE | 199.49 | 45 | 24 | 30 | 30 | 36 | 45 | 34 | 34 | 34 | 30 | 10 | 1.85E7 | 3.19E7 | 2.02E7 | 1.72E7 | 4.2E7 | 3.04E7 | 1.6E7 | 1.96E7 | 4.14E7 | 5.64E6 | 6 | 6 | 4 | 4 | 3 | 5 | 6 | 4 | 5 | 4 | 4 | 1 | N | 17862 | Ubiquitin-conjugating enzyme E2 L3 OS=Mus musculus OX=10090 GN=Ube2l3 PE=1 SV=1 |
| 732 | 941 | sp\|Q9CQ60\|6PGL\_MOUSE | 199.47 | 40 | 12 | 25 | 22 | 5 | 21 | 15 | 9 | 27 | 15 | 15 | 4.29E6 | 1.14E7 | 1.04E7 | 1.66E6 | 1.04E7 | 5.31E6 | 3.05E6 | 7.17E6 | 1.67E7 | 6.56E6 | 7 | 7 | 3 | 4 | 3 | 1 | 4 | 3 | 2 | 5 | 3 | 3 | Y | 27254 | 6-phosphogluconolactonase OS=Mus musculus OX=10090 GN=Pgls PE=1 SV=1 |
| 569 | 1138 | sp\|O70251\|EF1B\_MOUSE | 199.44 | 28 | 11 | 11 | 16 | 13 | 17 | 16 | 14 | 19 | 14 | 14 | 4.15E7 | 3.83E7 | 4.03E7 | 9.41E6 | 3.3E7 | 1.83E7 | 2.22E7 | 2.07E7 | 6.87E7 | 3.19E7 | 6 | 6 | 3 | 3 | 5 | 3 | 5 | 5 | 4 | 5 | 4 | 5 | Y | 24694 | Elongation factor 1-beta OS=Mus musculus OX=10090 GN=Eef1b PE=1 SV=5 |
| 498 | 357 | sp\|Q11011\|PSA\_MOUSE | 199.36 | 14 | 5 | 6 | 6 | 7 | 5 | 7 | 7 | 4 | 6 | 2 | 4.45E6 | 5.86E6 | 5.29E6 | 5.16E6 | 4.16E6 | 9.02E6 | 1.08E7 | 3.95E6 | 5.76E6 | 2.02E6 | 13 | 13 | 4 | 5 | 6 | 6 | 4 | 6 | 6 | 4 | 5 | 2 | N | 103325 | Puromycin-sensitive aminopeptidase OS=Mus musculus OX=10090 GN=Npepps PE=1 SV=2 |
| 595 | 1004 | sp\|Q8BW75\|AOFB\_MOUSE | 199.06 | 15 | 4 | 4 | 12 | 9 | 11 | 15 | 8 | 9 | 4 | 4 | 4.74E5 | 6.97E6 | 1.99E7 | 7.98E6 | 8.02E6 | 2.66E7 | 3.38E6 | 7.07E6 | 4.63E6 | 2.64E6 | 6 | 5 | 2 | 4 | 6 | 5 | 5 | 7 | 4 | 3 | 2 | 2 | N | 58558 | Amine oxidase [flavin-containing] B OS=Mus musculus OX=10090 GN=Maob PE=1 SV=4 |
| 458 | 779 | G3UX26\|G3UX26\_MOUSE | 198.14 | 37 | 22 | 22 | 18 | 20 | 28 | 27 | 25 | 13 | 20 | 25 | 1.89E7 | 2.62E7 | 2.7E7 | 1.12E7 | 1.99E7 | 2.76E7 | 2.06E7 | 1.26E7 | 4.1E7 | 1.91E7 | 8 | 7 | 5 | 6 | 5 | 5 | 6 | 7 | 5 | 4 | 5 | 7 | Y | 30446 | Voltage-dependent anion-selective channel protein 2 OS=Mus musculus OX=10090 GN=Vdac2 PE=1 SV=1 |
| 458 | 781 | sp\|Q60930\|VDAC2\_MOUSE | 198.14 | 36 | 21 | 21 | 17 | 19 | 27 | 26 | 24 | 13 | 19 | 24 | 1.89E7 | 2.62E7 | 2.7E7 | 1.12E7 | 1.99E7 | 2.76E7 | 2.06E7 | 1.26E7 | 4.1E7 | 1.91E7 | 8 | 7 | 5 | 6 | 5 | 5 | 6 | 7 | 5 | 4 | 5 | 7 | Y | 31733 | Voltage-dependent anion-selective channel protein 2 OS=Mus musculus OX=10090 GN=Vdac2 PE=1 SV=2 |
| 478 | 733 | sp\|P70168\|IMB1\_MOUSE | 197.85 | 12 | 6 | 7 | 8 | 7 | 7 | 7 | 5 | 7 | 6 | 8 | 1.12E7 | 1.7E7 | 2.24E7 | 9.4E6 | 1.97E7 | 1.09E7 | 1.5E7 | 1.92E7 | 3.45E7 | 2.47E7 | 8 | 8 | 5 | 5 | 6 | 6 | 6 | 5 | 5 | 4 | 6 | 6 | Y | 97184 | Importin subunit beta-1 OS=Mus musculus OX=10090 GN=Kpnb1 PE=1 SV=2 |
| 300 | 3177 | sp\|P26350\|PTMA\_MOUSE | 197.62 | 26 | 26 | 26 | 26 | 25 | 25 | 25 | 26 | 14 | 26 | 26 | 7.35E7 | 1.11E8 | 9.82E7 | 2.04E7 | 3.56E7 | 7.96E7 | 6.51E7 | 3.18E7 | 1.08E8 | 4.23E7 | 5 | 5 | 11 | 14 | 11 | 6 | 7 | 7 | 12 | 6 | 7 | 8 | N | 12254 | Prothymosin alpha OS=Mus musculus OX=10090 GN=Ptma PE=1 SV=2 |
| 28 | 1047 | sp\|P68433\|H31\_MOUSE | 197.46 | 54 | 31 | 50 | 31 | 31 | 31 | 54 | 54 | 31 | 54 | 54 |  | 2.68E5 |  |  |  | 8.22E5 | 5.31E5 |  | 6.18E6 | 1.76E7 | 9 | 1 | 57 | 51 | 67 | 51 | 48 | 43 | 46 | 64 | 58 | 50 | Y | 15404 | Histone H3.1 OS=Mus musculus OX=10090 GN=H3c1 PE=1 SV=2 |
| 497 | 904 | D3YYJ7\|D3YYJ7\_MOUSE | 196.71 | 40 | 34 | 22 | 26 | 23 | 26 | 19 | 32 | 32 | 22 | 18 | 5.61E7 | 1.95E7 | 5.83E7 | 1.35E7 | 3.66E7 | 9.58E6 | 3.29E7 | 2.14E7 | 3.01E7 | 3.9E7 | 7 | 7 | 7 | 4 | 7 | 4 | 5 | 3 | 6 | 7 | 4 | 4 | N | 24549 | Pentraxin family member OS=Mus musculus OX=10090 GN=Mptx2 PE=1 SV=1 |
| 258 | 1248 | sp\|P55050\|FABPI\_MOUSE | 196.59 | 48 | 33 | 33 | 38 | 39 | 39 | 38 | 42 | 39 | 33 | 33 | 2.33E8 | 4.69E8 | 3.6E8 | 1.65E8 | 1.15E8 | 2.98E8 | 1.91E8 | 6.73E7 | 2.3E8 | 2.73E8 | 8 | 8 | 9 | 14 | 8 | 11 | 6 | 10 | 10 | 7 | 13 | 11 | Y | 15126 | Fatty acid-binding protein, intestinal OS=Mus musculus OX=10090 GN=Fabp2 PE=1 SV=2 |
| 591 | 442 | sp\|A2AX52\|CO6A4\_MOUSE | 196.25 | 5 | 0 | 2 | 1 | 2 | 1 | 2 | 3 | 3 | 1 | 0 |  | 6.8E6 | 8.36E6 | 2.21E6 | 3.44E6 | 1.13E7 | 8.19E6 | 5.67E6 | 3.36E6 |  | 12 | 12 | 0 | 5 | 4 | 5 | 3 | 5 | 7 | 6 | 2 | 0 | Y | 250795 | Collagen alpha-4(VI) chain OS=Mus musculus OX=10090 GN=Col6a4 PE=1 SV=2 |
| 637 | 830 | sp\|Q9Z2W0\|DNPEP\_MOUSE | 196.01 | 20 | 10 | 10 | 11 | 6 | 5 | 11 | 7 | 7 | 11 | 7 | 1.31E7 | 1.64E7 | 9.97E6 | 1.94E7 | 5.89E6 | 1.42E7 | 9.88E6 | 7.97E6 | 2.07E7 | 1.32E7 | 7 | 7 | 5 | 3 | 4 | 3 | 2 | 4 | 3 | 3 | 4 | 5 | Y | 52207 | Aspartyl aminopeptidase OS=Mus musculus OX=10090 GN=Dnpep PE=1 SV=2 |
| 637 | 831 | Q3TVK3\|Q3TVK3\_MOUSE | 196.01 | 20 | 10 | 10 | 11 | 6 | 5 | 11 | 7 | 7 | 11 | 7 | 1.31E7 | 1.64E7 | 9.97E6 | 1.94E7 | 5.89E6 | 1.42E7 | 9.88E6 | 7.97E6 | 2.07E7 | 1.32E7 | 7 | 7 | 5 | 3 | 4 | 3 | 2 | 4 | 3 | 3 | 4 | 5 | Y | 52466 | Aspartyl aminopeptidase OS=Mus musculus OX=10090 GN=Dnpep PE=1 SV=1 |
| 577 | 669 | sp\|O55029\|COPB2\_MOUSE | 195.95 | 9 | 3 | 6 | 4 | 5 | 4 | 3 | 3 | 5 | 3 | 5 | 1.11E7 | 1.31E7 | 1.1E7 | 5.14E6 | 1.14E7 | 1.14E7 | 6.06E6 | 7.92E6 | 1.69E7 | 1.21E7 | 7 | 7 | 3 | 4 | 3 | 4 | 4 | 3 | 3 | 4 | 3 | 5 | Y | 102449 | Coatomer subunit beta' OS=Mus musculus OX=10090 GN=Copb2 PE=1 SV=2 |
| 832 | 1284 | sp\|Q8R086\|SUOX\_MOUSE | 195.73 | 13 | 3 | 7 | 3 | 3 | 8 | 9 | 3 | 8 | 3 | 2 | 1.41E7 | 2.35E7 | 7.71E6 | 4.31E6 | 1.87E7 | 2.39E7 | 8.42E6 | 7.78E6 | 2.14E7 | 4.1E6 | 5 | 5 | 2 | 3 | 2 | 2 | 3 | 4 | 2 | 3 | 2 | 1 | Y | 60756 | Sulfite oxidase, mitochondrial OS=Mus musculus OX=10090 GN=Suox PE=1 SV=2 |
| 522 | 1133 | sp\|P16015\|CAH3\_MOUSE | 195.55 | 33 | 28 | 28 | 9 | 33 | 0 | 23 | 22 | 7 | 17 | 28 | 4.66E7 | 8.78E7 | 4.05E6 | 1.19E7 |  | 4.38E7 | 1.24E7 | 9.94E4 | 1.83E7 | 3.42E7 | 7 | 7 | 6 | 9 | 2 | 7 | 0 | 7 | 4 | 1 | 3 | 7 | Y | 29366 | Carbonic anhydrase 3 OS=Mus musculus OX=10090 GN=Ca3 PE=1 SV=3 |
| 561 | 564 | sp\|Q3UPL0\|SC31A\_MOUSE | 195.42 | 10 | 2 | 4 | 4 | 3 | 4 | 4 | 4 | 1 | 3 | 4 | 4.64E6 | 1.18E7 | 7.47E6 | 1.11E6 | 3.96E6 | 1.59E7 | 4.37E6 | 1.14E6 | 1.35E7 | 6.43E6 | 11 | 11 | 2 | 4 | 5 | 3 | 4 | 5 | 4 | 1 | 4 | 4 | Y | 133569 | Protein transport protein Sec31A OS=Mus musculus OX=10090 GN=Sec31a PE=1 SV=2 |
| 731 | 775 | sp\|Q91VI7\|RINI\_MOUSE | 195.11 | 24 | 10 | 6 | 3 | 9 | 2 | 10 | 5 | 10 | 16 | 16 | 2.72E6 | 3.93E6 | 1.81E6 | 4.2E6 | 5.89E6 | 4.88E6 | 6.06E6 | 3.97E6 | 2.55E7 | 1.03E7 | 8 | 8 | 3 | 2 | 1 | 3 | 1 | 3 | 2 | 3 | 5 | 5 | Y | 49817 | Ribonuclease inhibitor OS=Mus musculus OX=10090 GN=Rnh1 PE=1 SV=1 |
| 731 | 776 | A0A1B0GSG5\|A0A1B0GSG5\_MOUSE | 195.11 | 22 | 9 | 5 | 3 | 8 | 2 | 9 | 5 | 9 | 15 | 15 | 2.72E6 | 3.93E6 | 1.81E6 | 4.2E6 | 5.89E6 | 4.88E6 | 6.06E6 | 3.97E6 | 2.55E7 | 1.03E7 | 8 | 8 | 3 | 2 | 1 | 3 | 1 | 3 | 2 | 3 | 5 | 5 | Y | 53926 | Ribonuclease inhibitor OS=Mus musculus OX=10090 GN=Rnh1 PE=1 SV=1 |
| 145 | 1022 | sp\|Q922U2\|K2C5\_MOUSE | 195.09 | 9 | 8 | 8 | 8 | 8 | 8 | 8 | 9 | 8 | 7 | 6 |  |  |  |  |  |  | 3.98E6 |  |  |  | 7 | 1 | 20 | 18 | 16 | 16 | 19 | 19 | 17 | 22 | 20 | 18 | N | 61767 | Keratin, type II cytoskeletal 5 OS=Mus musculus OX=10090 GN=Krt5 PE=1 SV=1 |
| 846 | 658 | Q3U4W8\|Q3U4W8\_MOUSE | 194.69 | 13 | 5 | 5 | 5 | 3 | 6 | 7 | 3 | 1 | 3 | 1 | 8.87E6 | 4.03E6 | 7.21E6 | 7.86E5 | 4.68E6 | 7.42E6 | 5.79E5 | 4.11E5 | 2.02E6 | 5.92E6 | 8 | 8 | 4 | 2 | 2 | 2 | 3 | 4 | 2 | 1 | 2 | 1 | N | 93355 | Ubiquitin carboxyl-terminal hydrolase OS=Mus musculus OX=10090 GN=Usp5 PE=1 SV=1 |
| 846 | 660 | sp\|P56399\|UBP5\_MOUSE | 194.69 | 13 | 5 | 4 | 4 | 3 | 6 | 7 | 3 | 1 | 3 | 1 | 8.87E6 | 4.03E6 | 7.21E6 | 7.86E5 | 4.68E6 | 7.42E6 | 5.79E5 | 4.11E5 | 2.02E6 | 5.92E6 | 8 | 8 | 4 | 2 | 2 | 2 | 3 | 4 | 2 | 1 | 2 | 1 | N | 95833 | Ubiquitin carboxyl-terminal hydrolase 5 OS=Mus musculus OX=10090 GN=Usp5 PE=1 SV=1 |
| 207 | 1833 | sp\|P43275\|H11\_MOUSE | 194.53 | 29 | 19 | 19 | 29 | 19 | 19 | 19 | 19 | 19 | 29 | 29 | 7.31E7 | 1.15E8 | 1.49E8 | 6.48E7 | 1.15E8 | 1.16E8 | 7.92E7 | 1.01E8 | 6.01E7 | 1.3E8 | 6 | 4 | 12 | 10 | 15 | 11 | 13 | 10 | 14 | 15 | 14 | 10 | N | 21785 | Histone H1.1 OS=Mus musculus OX=10090 GN=H1-1 PE=1 SV=2 |
| 393 | 823 | sp\|P62754\|RS6\_MOUSE | 194.38 | 27 | 7 | 19 | 16 | 18 | 24 | 17 | 26 | 26 | 16 | 15 | 3.8E7 | 7.45E7 | 3.34E7 | 5.35E7 | 8.52E7 | 8.1E7 | 8.11E7 | 6.8E7 | 7.05E7 | 6.95E7 | 8 | 8 | 3 | 7 | 5 | 7 | 7 | 7 | 9 | 9 | 5 | 7 | Y | 28681 | Small ribosomal subunit protein eS6 OS=Mus musculus OX=10090 GN=Rps6 PE=1 SV=1 |
| 462 | 885 | sp\|Q9D2G2\|ODO2\_MOUSE | 193.37 | 17 | 8 | 13 | 17 | 9 | 13 | 11 | 13 | 17 | 6 | 6 | 1.83E7 | 2.4E7 | 3.4E7 | 1.23E7 | 3.18E7 | 3.36E7 | 2.62E7 | 2.16E7 | 1.12E7 | 1.32E7 | 7 | 7 | 4 | 5 | 7 | 5 | 7 | 6 | 7 | 8 | 3 | 3 | Y | 48995 | Dihydrolipoyllysine-residue succinyltransferase component of 2-oxoglutarate dehydrogenase complex, mitochondrial OS=Mus musculus OX=10090 GN=Dlst PE=1 SV=1 |
| 634 | 429 | sp\|P32067\|LA\_MOUSE | 193.30 | 24 | 7 | 6 | 5 | 10 | 3 | 11 | 6 | 6 | 14 | 12 | 6.05E6 | 3.82E6 | 3.76E6 | 3.55E6 | 1.78E6 | 5.61E6 | 4.12E6 | 2.01E5 | 1.44E7 | 6.23E6 | 10 | 10 | 3 | 2 | 2 | 4 | 1 | 4 | 2 | 2 | 7 | 5 | Y | 47756 | Lupus La protein homolog OS=Mus musculus OX=10090 GN=Ssb PE=1 SV=1 |
| 622 | 865 | sp\|Q63836\|SBP2\_MOUSE | 193.09 | 17 | 4 | 12 | 11 | 9 | 11 | 9 | 11 | 11 | 11 | 4 | 1.21E7 | 2.36E7 | 2.06E7 | 5.74E6 | 1.52E7 | 2.11E7 | 1.05E7 | 1.1E7 | 1.18E7 | 9.8E6 | 7 | 7 | 2 | 6 | 4 | 4 | 4 | 4 | 4 | 4 | 4 | 2 | Y | 52610 | Selenium-binding protein 2 OS=Mus musculus OX=10090 GN=Selenbp2 PE=1 SV=2 |
| 622 | 866 | sp\|P17563\|SBP1\_MOUSE | 193.09 | 17 | 4 | 12 | 11 | 9 | 11 | 9 | 11 | 11 | 11 | 4 | 1.21E7 | 2.36E7 | 2.06E7 | 5.74E6 | 1.52E7 | 2.11E7 | 1.05E7 | 1.1E7 | 1.18E7 | 9.8E6 | 7 | 7 | 2 | 6 | 4 | 4 | 4 | 4 | 4 | 4 | 4 | 2 | Y | 52514 | Methanethiol oxidase OS=Mus musculus OX=10090 GN=Selenbp1 PE=1 SV=2 |
| 612 | 982 | sp\|P18572\|BASI\_MOUSE | 192.56 | 20 | 11 | 9 | 13 | 14 | 13 | 9 | 11 | 11 | 9 | 11 | 2.79E7 | 1.74E7 | 2.56E7 | 6.83E6 | 1.44E7 | 2.04E7 | 1.51E7 | 1.2E7 | 3.09E7 | 1.92E7 | 7 | 7 | 4 | 3 | 4 | 5 | 4 | 3 | 4 | 4 | 3 | 4 | Y | 42445 | Basigin OS=Mus musculus OX=10090 GN=Bsg PE=1 SV=2 |
| 339 | 634 | F6ZV59\|F6ZV59\_MOUSE | 192.48 | 46 | 34 | 25 | 26 | 26 | 26 | 30 | 21 | 26 | 32 | 40 | 2.99E7 | 1.47E7 | 2.94E7 | 1.6E7 | 3.21E7 | 3.47E7 | 1.94E7 | 1.99E7 | 5.88E7 | 3.85E7 | 9 | 7 | 9 | 7 | 8 | 8 | 9 | 8 | 5 | 8 | 9 | 9 | Y | 24730 | Heterogeneous nuclear ribonucleoprotein D (Fragment) OS=Mus musculus OX=10090 GN=Hnrnpd PE=1 SV=1 |
| 339 | 637 | G5E8G0\|G5E8G0\_MOUSE | 192.48 | 34 | 25 | 19 | 20 | 20 | 20 | 23 | 16 | 20 | 24 | 30 | 2.99E7 | 1.47E7 | 2.94E7 | 1.6E7 | 3.21E7 | 3.47E7 | 1.94E7 | 1.99E7 | 5.88E7 | 3.85E7 | 9 | 7 | 9 | 7 | 8 | 8 | 9 | 8 | 5 | 8 | 9 | 9 | Y | 30608 | Heterogeneous nuclear ribonucleoprotein D OS=Mus musculus OX=10090 GN=Hnrnpd PE=1 SV=1 |
| 339 | 639 | G3X9W0\|G3X9W0\_MOUSE | 192.48 | 29 | 22 | 16 | 17 | 17 | 17 | 19 | 14 | 17 | 21 | 26 | 2.99E7 | 1.47E7 | 2.94E7 | 1.6E7 | 3.21E7 | 3.47E7 | 1.94E7 | 1.99E7 | 5.88E7 | 3.85E7 | 9 | 7 | 9 | 7 | 8 | 8 | 9 | 8 | 5 | 8 | 9 | 9 | Y | 36208 | Heterogeneous nuclear ribonucleoprotein D OS=Mus musculus OX=10090 GN=Hnrnpd PE=1 SV=1 |
| 339 | 640 | sp\|Q60668\|HNRPD\_MOUSE | 192.48 | 28 | 21 | 15 | 16 | 16 | 16 | 18 | 13 | 16 | 20 | 25 | 2.99E7 | 1.47E7 | 2.94E7 | 1.6E7 | 3.21E7 | 3.47E7 | 1.94E7 | 1.99E7 | 5.88E7 | 3.85E7 | 9 | 7 | 9 | 7 | 8 | 8 | 9 | 8 | 5 | 8 | 9 | 9 | Y | 38354 | Heterogeneous nuclear ribonucleoprotein D0 OS=Mus musculus OX=10090 GN=Hnrnpd PE=1 SV=2 |
| 772 | 868 | sp\|Q8VCT4\|EST1D\_MOUSE | 191.89 | 18 | 6 | 8 | 2 | 7 | 2 | 14 | 7 | 7 | 2 | 7 | 6.21E5 | 1.07E6 |  | 3.22E6 |  | 9.15E6 | 2.88E6 | 2.55E6 | 2.1E6 | 3.55E6 | 7 | 5 | 3 | 4 | 1 | 3 | 1 | 5 | 3 | 3 | 1 | 3 | N | 61788 | Carboxylesterase 1D OS=Mus musculus OX=10090 GN=Ces1d PE=1 SV=1 |
| 677 | 687 | sp\|Q61599\|GDIR2\_MOUSE | 191.05 | 62 | 29 | 4 | 4 | 10 | 24 | 0 | 10 | 26 | 34 | 22 | 1.59E7 | 5.59E6 | 6.14E6 | 1.35E6 | 5.14E6 |  | 1.39E6 | 2.87E6 | 1.91E7 | 2.77E7 | 9 | 9 | 5 | 2 | 2 | 2 | 3 | 0 | 1 | 4 | 5 | 6 | Y | 22851 | Rho GDP-dissociation inhibitor 2 OS=Mus musculus OX=10090 GN=Arhgdib PE=1 SV=3 |
| 309 | 303 | sp\|Q99JY0\|ECHB\_MOUSE | 191.03 | 32 | 20 | 18 | 22 | 17 | 11 | 21 | 19 | 18 | 19 | 20 | 7.47E7 | 4.6E7 | 9.87E7 | 2.51E7 | 2.89E7 | 1.05E8 | 4.06E7 | 3.56E7 | 1.04E8 | 6.17E7 | 13 | 13 | 9 | 7 | 9 | 9 | 5 | 10 | 10 | 8 | 9 | 9 | Y | 51386 | Trifunctional enzyme subunit beta, mitochondrial OS=Mus musculus OX=10090 GN=Hadhb PE=1 SV=1 |
| 476 | 363 | sp\|Q8CAQ8\|MIC60\_MOUSE | 190.93 | 16 | 7 | 5 | 6 | 10 | 7 | 7 | 4 | 4 | 4 | 5 | 8.38E6 | 2.05E7 | 2.82E6 | 5.17E6 | 3.76E6 | 1.82E7 |  | 3.25E5 | 9.65E6 | 1.18E7 | 12 | 5 | 5 | 4 | 5 | 7 | 6 | 7 | 3 | 3 | 3 | 4 | Y | 83900 | MICOS complex subunit Mic60 OS=Mus musculus OX=10090 GN=Immt PE=1 SV=1 |
| 494 | 598 | sp\|P62717\|RL18A\_MOUSE | 190.91 | 49 | 16 | 11 | 21 | 30 | 35 | 35 | 26 | 23 | 30 | 17 | 2.98E7 | 1.84E7 | 4.37E7 | 3.38E7 | 4.47E7 | 4.37E7 | 3.31E7 | 2.17E7 | 4.4E7 | 3.5E7 | 9 | 9 | 3 | 2 | 5 | 5 | 7 | 7 | 6 | 5 | 5 | 4 | Y | 20732 | Large ribosomal subunit protein eL20 OS=Mus musculus OX=10090 GN=Rpl18a PE=1 SV=1 |
| 494 | 652 | A0A1D5RLW5\|A0A1D5RLW5\_MOUSE | 190.91 | 59 | 20 | 13 | 25 | 36 | 42 | 42 | 31 | 27 | 36 | 20 | 2.98E7 | 1.84E7 | 4.37E7 | 3.38E7 | 4.47E7 | 4.37E7 | 3.31E7 | 2.17E7 | 4.4E7 | 3.5E7 | 9 | 9 | 3 | 2 | 5 | 5 | 7 | 7 | 6 | 5 | 5 | 4 | Y | 17447 | 60S ribosomal protein L18a OS=Mus musculus OX=10090 GN=Rpl18a PE=1 SV=1 |
| 216 | 1836 | sp\|P43276\|H15\_MOUSE | 190.68 | 22 | 22 | 15 | 22 | 22 | 22 | 22 | 22 | 22 | 22 | 22 | 3.21E8 | 2.73E8 | 2.97E8 | 1.18E8 | 2.73E8 | 2.05E8 | 1.74E8 | 1.86E8 | 5.37E8 | 2.91E8 | 6 | 6 | 17 | 12 | 12 | 12 | 12 | 9 | 10 | 14 | 14 | 15 | N | 22576 | Histone H1.5 OS=Mus musculus OX=10090 GN=H1-5 PE=1 SV=2 |
| 540 | 649 | sp\|Q5XJY5\|COPD\_MOUSE | 190.58 | 17 | 7 | 4 | 13 | 8 | 8 | 12 | 8 | 6 | 9 | 9 | 9.84E6 | 8.13E6 | 2.49E7 | 9.34E6 | 1.18E7 | 2.08E7 | 1.39E7 | 3.62E6 | 3.3E7 | 1.43E7 | 8 | 8 | 3 | 3 | 7 | 4 | 4 | 6 | 4 | 3 | 5 | 4 | Y | 57230 | Coatomer subunit delta OS=Mus musculus OX=10090 GN=Arcn1 PE=1 SV=2 |
| 410 | 1753 | sp\|P62264\|RS14\_MOUSE | 190.49 | 36 | 36 | 36 | 36 | 30 | 36 | 36 | 36 | 36 | 36 | 36 | 1.12E8 | 1.17E8 | 1.12E8 | 5.22E7 | 1.05E8 | 8.03E7 | 7.1E7 | 6.69E7 | 1.42E8 | 9.4E7 | 4 | 4 | 6 | 6 | 6 | 5 | 8 | 7 | 6 | 7 | 7 | 7 | Y | 16273 | Small ribosomal subunit protein uS11 OS=Mus musculus OX=10090 GN=Rps14 PE=1 SV=3 |
| 418 | 464 | sp\|P28654\|PGS2\_MOUSE | 190.46 | 27 | 14 | 20 | 19 | 14 | 17 | 16 | 14 | 12 | 7 | 5 | 9.3E6 | 4.4E7 | 4.25E7 | 2E7 | 2.15E7 | 4.6E7 | 2.25E7 | 1.5E7 | 1.17E7 | 2.05E6 | 10 | 8 | 5 | 10 | 9 | 6 | 6 | 8 | 6 | 5 | 2 | 2 | Y | 39809 | Decorin OS=Mus musculus OX=10090 GN=Dcn PE=1 SV=1 |
| 359 | 988 | sp\|Q9CZX8\|RS19\_MOUSE | 190.46 | 44 | 32 | 28 | 38 | 33 | 33 | 32 | 36 | 39 | 37 | 39 | 8.98E7 | 6.91E7 | 1.32E8 | 5.64E7 | 1E8 | 1.06E8 | 6.96E7 | 7.2E7 | 1.18E8 | 6.66E7 | 9 | 9 | 7 | 4 | 8 | 7 | 9 | 7 | 7 | 7 | 10 | 7 | N | 16085 | Small ribosomal subunit protein eS19 OS=Mus musculus OX=10090 GN=Rps19 PE=1 SV=3 |
| 697 | 1452 | sp\|Q921H8\|THIKA\_MOUSE | 190.26 | 17 | 8 | 8 | 8 | 8 | 8 | 8 | 10 | 8 | 15 | 11 | 1.09E7 | 1.51E7 | 1.18E7 | 2.18E6 | 4.99E6 | 1.06E7 | 4.82E6 | 3.85E6 | 1.9E7 | 9.59E6 | 6 | 6 | 2 | 3 | 4 | 2 | 3 | 4 | 4 | 3 | 5 | 3 | N | 43953 | 3-ketoacyl-CoA thiolase A, peroxisomal OS=Mus musculus OX=10090 GN=Acaa1a PE=1 SV=1 |
| 831 | 1137 | sp\|Q9DCS9\|NDUBA\_MOUSE | 190.18 | 39 | 19 | 11 | 19 | 28 | 24 | 24 | 30 | 13 | 5 | 3 | 1.16E6 | 9.67E5 | 1.07E6 | 1.59E6 | 4.06E6 | 7.7E6 | 5.32E6 | 3.47E6 | 7.05E5 |  | 6 | 5 | 2 | 1 | 2 | 4 | 3 | 3 | 4 | 2 | 1 | 1 | Y | 21024 | NADH dehydrogenase [ubiquinone] 1 beta subcomplex subunit 10 OS=Mus musculus OX=10090 GN=Ndufb10 PE=1 SV=3 |
| 662 | 444 | sp\|Q9Z110\|P5CS\_MOUSE | 190.13 | 14 | 5 | 5 | 7 | 7 | 2 | 5 | 8 | 2 | 9 | 0 | 4.68E6 | 3.28E6 | 1.6E7 | 4.55E6 | 2.11E6 | 9.64E6 | 7.89E6 | 5.54E5 | 1.44E7 |  | 10 | 10 | 3 | 3 | 5 | 5 | 2 | 4 | 6 | 1 | 5 | 0 | Y | 87266 | Delta-1-pyrroline-5-carboxylate synthase OS=Mus musculus OX=10090 GN=Aldh18a1 PE=1 SV=2 |
| 500 | 800 | sp\|P15626\|GSTM2\_MOUSE | 190.08 | 37 | 26 | 25 | 26 | 18 | 21 | 23 | 16 | 17 | 22 | 24 |  |  |  |  |  |  |  |  | 1.97E6 |  | 8 | 1 | 5 | 5 | 7 | 4 | 4 | 6 | 3 | 4 | 5 | 5 | Y | 25717 | Glutathione S-transferase Mu 2 OS=Mus musculus OX=10090 GN=Gstm2 PE=1 SV=2 |
| 457 | 666 | sp\|P16125\|LDHB\_MOUSE | 190.01 | 27 | 15 | 18 | 11 | 10 | 19 | 24 | 10 | 11 | 8 | 5 | 6.55E6 | 1.98E7 | 1.39E7 | 2.9E6 | 1.56E7 | 3.08E7 | 8.72E6 | 7.4E6 | 3.99E6 |  | 9 | 7 | 6 | 7 | 5 | 4 | 7 | 8 | 4 | 5 | 5 | 3 | Y | 36572 | L-lactate dehydrogenase B chain OS=Mus musculus OX=10090 GN=Ldhb PE=1 SV=2 |
| 457 | 667 | A0A6I8MX27\|A0A6I8MX27\_MOUSE | 190.01 | 27 | 15 | 17 | 11 | 10 | 18 | 23 | 10 | 11 | 8 | 5 | 6.55E6 | 1.98E7 | 1.39E7 | 2.9E6 | 1.56E7 | 3.08E7 | 8.72E6 | 7.4E6 | 3.99E6 |  | 9 | 7 | 6 | 7 | 5 | 4 | 7 | 8 | 4 | 5 | 5 | 3 | Y | 37357 | L-lactate dehydrogenase OS=Mus musculus OX=10090 GN=Ldhb PE=1 SV=1 |
| 642 | 441 | sp\|Q8CGC7\|SYEP\_MOUSE | 189.95 | 9 | 2 | 4 | 1 | 0 | 3 | 1 | 5 | 3 | 4 | 3 | 1.14E7 | 8.17E6 | 1.96E6 |  | 3.66E6 | 3.31E6 | 4.82E6 | 5.69E6 | 3.6E6 | 3.33E6 | 11 | 11 | 2 | 5 | 1 | 0 | 3 | 1 | 5 | 4 | 4 | 3 | Y | 170078 | Bifunctional glutamate/proline--tRNA ligase OS=Mus musculus OX=10090 GN=Eprs1 PE=1 SV=4 |
| 827 | 796 | B1ATS5\|B1ATS5\_MOUSE | 189.67 | 9 | 5 | 3 | 2 | 2 | 5 | 1 | 1 | 2 | 2 | 3 | 4.34E5 |  |  | 9.03E5 | 5.25E6 |  | 1.04E6 |  |  |  | 8 | 3 | 3 | 2 | 2 | 2 | 4 | 1 | 1 | 2 | 2 | 2 | Y | 111709 | Calcium-transporting ATPase OS=Mus musculus OX=10090 GN=Atp2a3 PE=1 SV=1 |
| 827 | 797 | E9Q559\|E9Q559\_MOUSE | 189.67 | 9 | 5 | 3 | 2 | 2 | 5 | 1 | 1 | 2 | 2 | 3 | 4.34E5 |  |  | 9.03E5 | 5.25E6 |  | 1.04E6 |  |  |  | 8 | 3 | 3 | 2 | 2 | 2 | 4 | 1 | 1 | 2 | 2 | 2 | Y | 112504 | Calcium-transporting ATPase OS=Mus musculus OX=10090 GN=Atp2a3 PE=1 SV=1 |
| 528 | 548 | sp\|Q922B2\|SYDC\_MOUSE | 189.67 | 18 | 6 | 5 | 11 | 8 | 11 | 9 | 7 | 5 | 13 | 10 | 1.39E7 | 8.72E6 | 1.12E7 | 8.49E6 | 1.66E7 | 1.29E7 | 6.32E6 | 5.94E6 | 2.4E7 | 1.44E7 | 8 | 8 | 3 | 3 | 5 | 4 | 6 | 4 | 4 | 3 | 6 | 5 | Y | 57147 | Aspartate--tRNA ligase, cytoplasmic OS=Mus musculus OX=10090 GN=Dars1 PE=1 SV=2 |
| 487 | 837 | sp\|O35381\|AN32A\_MOUSE | 189.66 | 32 | 14 | 21 | 20 | 13 | 21 | 21 | 24 | 24 | 19 | 14 | 1.97E7 | 2.27E7 | 1.78E7 | 1.97E6 | 2.1E7 | 1.25E7 | 4.32E6 | 1.18E7 | 5.85E6 | 1.52E7 | 8 | 5 | 3 | 5 | 5 | 3 | 6 | 5 | 7 | 6 | 4 | 5 | Y | 28538 | Acidic leucine-rich nuclear phosphoprotein 32 family member A OS=Mus musculus OX=10090 GN=Anp32a PE=1 SV=1 |
| 477 | 686 | sp\|P24270\|CATA\_MOUSE | 189.63 | 19 | 7 | 11 | 8 | 14 | 10 | 10 | 8 | 10 | 10 | 13 | 1.25E7 | 2.59E7 | 1.09E7 | 1.94E7 | 2.15E7 | 3.11E7 | 1.94E7 | 1.07E7 | 2.92E7 | 3.43E7 | 9 | 9 | 3 | 5 | 3 | 6 | 4 | 4 | 3 | 4 | 5 | 6 | N | 59795 | Catalase OS=Mus musculus OX=10090 GN=Cat PE=1 SV=4 |
| 347 | 910 | sp\|P62918\|RL8\_MOUSE | 189.40 | 28 | 19 | 22 | 16 | 16 | 19 | 22 | 22 | 22 | 19 | 19 | 1.48E8 | 1.74E8 | 1.61E8 | 7.77E7 | 1.37E8 | 1.34E8 | 9.97E7 | 1.07E8 | 1.6E8 | 1.45E8 | 8 | 8 | 7 | 7 | 5 | 5 | 6 | 7 | 8 | 9 | 7 | 8 | N | 28025 | Large ribosomal subunit protein uL2 OS=Mus musculus OX=10090 GN=Rpl8 PE=1 SV=2 |
| 576 | 855 | sp\|Q3U9G9\|LBR\_MOUSE | 189.14 | 12 | 7 | 7 | 9 | 5 | 9 | 7 | 2 | 6 | 7 | 9 | 6.99E6 | 1.09E7 | 2.92E7 | 1.3E6 | 1.05E7 | 8.17E6 | 3.55E6 | 1.46E7 | 1.47E7 | 1.14E7 | 7 | 7 | 3 | 3 | 5 | 2 | 5 | 3 | 2 | 5 | 5 | 5 | N | 71440 | Delta(14)-sterol reductase LBR OS=Mus musculus OX=10090 GN=Lbr PE=1 SV=2 |
| 1134 | 691 | sp\|O35744\|CHIL3\_MOUSE | 188.90 | 25 | 7 | 0 | 0 | 0 | 0 | 0 | 0 | 0 | 19 | 10 | 7.74E6 |  |  |  |  |  |  |  | 3.33E7 | 1.3E7 | 8 | 8 | 2 | 0 | 0 | 0 | 0 | 0 | 0 | 0 | 6 | 3 | N | 44458 | Chitinase-like protein 3 OS=Mus musculus OX=10090 GN=Chil3 PE=1 SV=2 |
| 564 | 685 | sp\|O08749\|DLDH\_MOUSE | 188.89 | 20 | 8 | 14 | 14 | 11 | 8 | 11 | 10 | 10 | 11 | 6 | 7E6 | 1.71E7 | 9.39E6 | 1.43E6 | 9.45E6 | 2.68E7 | 2.94E6 | 4.1E6 | 1.5E7 | 5.33E6 | 8 | 8 | 3 | 5 | 5 | 4 | 3 | 4 | 3 | 3 | 4 | 2 | Y | 54272 | Dihydrolipoyl dehydrogenase, mitochondrial OS=Mus musculus OX=10090 GN=Dld PE=1 SV=2 |
| 575 | 512 | A0A1C7CYU3\|A0A1C7CYU3\_MOUSE | 188.41 | 22 | 6 | 7 | 11 | 5 | 6 | 12 | 18 | 10 | 3 | 9 | 4.12E6 | 1.11E7 | 9.3E6 | 2.67E6 | 7.32E6 | 1.28E7 | 1.71E7 | 5.2E6 | 2.43E6 | 9.25E6 | 9 | 8 | 3 | 4 | 4 | 2 | 3 | 5 | 8 | 4 | 2 | 4 | Y | 52997 | Nucleobindin-1 OS=Mus musculus OX=10090 GN=Nucb1 PE=1 SV=1 |
| 582 | 875 | sp\|P08752\|GNAI2\_MOUSE | 188.36 | 24 | 9 | 10 | 10 | 7 | 16 | 5 | 5 | 14 | 14 | 10 | 5.13E5 | 2.25E6 | 2.46E6 | 6.78E5 | 2.17E6 |  |  | 2.74E6 | 1.06E7 | 1.77E6 | 7 | 4 | 4 | 4 | 3 | 3 | 6 | 3 | 3 | 6 | 4 | 3 | Y | 40489 | Guanine nucleotide-binding protein G(i) subunit alpha-2 OS=Mus musculus OX=10090 GN=Gnai2 PE=1 SV=5 |
| 471 | 767 | A0A2R8VI25\|A0A2R8VI25\_MOUSE | 187.88 | 32 | 23 | 23 | 16 | 21 | 19 | 19 | 16 | 32 | 23 | 23 | 1.97E7 | 1.51E7 | 1.39E7 | 7.24E6 | 1.35E7 | 1.39E7 | 1.11E7 | 8.76E6 | 3.05E7 | 2.15E7 | 8 | 4 | 6 | 6 | 4 | 5 | 5 | 5 | 4 | 8 | 5 | 7 | Y | 33299 | Poly(rC) binding protein 2 OS=Mus musculus OX=10090 GN=Pcbp2 PE=1 SV=1 |
| 471 | 768 | Q3TT81\|Q3TT81\_MOUSE | 187.88 | 31 | 23 | 23 | 16 | 20 | 18 | 18 | 16 | 31 | 23 | 23 | 1.97E7 | 1.51E7 | 1.39E7 | 7.24E6 | 1.35E7 | 1.39E7 | 1.11E7 | 8.76E6 | 3.05E7 | 2.15E7 | 8 | 4 | 6 | 6 | 4 | 5 | 5 | 5 | 4 | 8 | 5 | 7 | Y | 33926 | Poly(rC) binding protein 2 OS=Mus musculus OX=10090 GN=Pcbp2 PE=1 SV=1 |
| 471 | 769 | A0A2R8VHP9\|A0A2R8VHP9\_MOUSE | 187.88 | 30 | 22 | 22 | 15 | 19 | 18 | 18 | 15 | 30 | 22 | 22 | 1.97E7 | 1.51E7 | 1.39E7 | 7.24E6 | 1.35E7 | 1.39E7 | 1.11E7 | 8.76E6 | 3.05E7 | 2.15E7 | 8 | 4 | 6 | 6 | 4 | 5 | 5 | 5 | 4 | 8 | 5 | 7 | Y | 35347 | Poly(rC) binding protein 2 OS=Mus musculus OX=10090 GN=Pcbp2 PE=1 SV=1 |
| 471 | 770 | B2M1R7\|B2M1R7\_MOUSE | 187.88 | 28 | 20 | 20 | 14 | 18 | 16 | 16 | 14 | 28 | 20 | 20 | 1.97E7 | 1.51E7 | 1.39E7 | 7.24E6 | 1.35E7 | 1.39E7 | 1.11E7 | 8.76E6 | 3.05E7 | 2.15E7 | 8 | 4 | 6 | 6 | 4 | 5 | 5 | 5 | 4 | 8 | 5 | 7 | Y | 38151 | Poly(RC) binding protein 2 OS=Mus musculus OX=10090 GN=Pcbp2 PE=1 SV=1 |
| 471 | 771 | sp\|Q61990\|PCBP2\_MOUSE | 187.88 | 28 | 20 | 20 | 14 | 18 | 16 | 16 | 14 | 28 | 20 | 20 | 1.97E7 | 1.51E7 | 1.39E7 | 7.24E6 | 1.35E7 | 1.39E7 | 1.11E7 | 8.76E6 | 3.05E7 | 2.15E7 | 8 | 4 | 6 | 6 | 4 | 5 | 5 | 5 | 4 | 8 | 5 | 7 | Y | 38222 | Poly(rC)-binding protein 2 OS=Mus musculus OX=10090 GN=Pcbp2 PE=1 SV=1 |
| 471 | 772 | A0AAQ4VMQ7\|A0AAQ4VMQ7\_MOUSE | 187.88 | 21 | 15 | 15 | 11 | 14 | 13 | 13 | 11 | 21 | 16 | 15 | 1.97E7 | 1.51E7 | 1.39E7 | 7.24E6 | 1.35E7 | 1.39E7 | 1.11E7 | 8.76E6 | 3.05E7 | 2.15E7 | 8 | 4 | 6 | 6 | 4 | 5 | 5 | 5 | 4 | 8 | 5 | 7 | Y | 49152 | Poly(rC) binding protein 2 OS=Mus musculus OX=10090 GN=Pcbp2 PE=4 SV=1 |
| 407 | 544 | sp\|P50580\|PA2G4\_MOUSE | 187.82 | 26 | 13 | 8 | 18 | 18 | 11 | 18 | 18 | 13 | 19 | 14 | 2.76E7 | 2.25E7 | 4.6E7 | 2.23E7 | 2.24E7 | 4.74E7 | 4.03E7 | 2.45E7 | 4.91E7 | 3.38E7 | 10 | 10 | 5 | 3 | 9 | 7 | 4 | 7 | 8 | 5 | 7 | 6 | Y | 43699 | Proliferation-associated protein 2G4 OS=Mus musculus OX=10090 GN=Pa2g4 PE=1 SV=3 |
| 602 | 852 | sp\|Q8CIB5\|FERM2\_MOUSE | 187.47 | 13 | 8 | 9 | 9 | 5 | 8 | 8 | 6 | 6 | 4 | 6 | 2.2E7 | 2.49E7 | 1.48E7 | 2.5E6 | 5.57E6 | 1.21E7 | 3.11E6 | 1.73E7 | 1.12E7 | 3.79E6 | 7 | 7 | 4 | 5 | 5 | 3 | 4 | 4 | 3 | 4 | 2 | 3 | Y | 77800 | Fermitin family homolog 2 OS=Mus musculus OX=10090 GN=Fermt2 PE=1 SV=1 |
| 534 | 1538 | sp\|P49817\|CAV1\_MOUSE | 187.38 | 33 | 20 | 29 | 30 | 24 | 31 | 28 | 24 | 28 | 30 | 17 | 1.33E7 | 4.39E7 | 2.06E7 | 1.73E7 | 4.08E7 | 4.01E7 | 2.18E7 | 2.4E7 | 4.54E7 | 1.01E7 | 6 | 6 | 2 | 6 | 6 | 4 | 6 | 5 | 4 | 5 | 4 | 2 | N | 20539 | Caveolin-1 OS=Mus musculus OX=10090 GN=Cav1 PE=1 SV=1 |
| 448 | 1020 | sp\|P30681\|HMGB2\_MOUSE | 186.96 | 31 | 21 | 24 | 28 | 28 | 16 | 21 | 21 | 21 | 31 | 18 | 2.73E7 | 3.07E7 | 6.25E7 | 2.47E7 | 4.25E7 | 3.92E7 | 2.8E7 | 2.61E7 | 9.04E7 | 4.48E6 | 9 | 7 | 4 | 4 | 7 | 7 | 5 | 6 | 5 | 4 | 10 | 3 | Y | 24162 | High mobility group protein B2 OS=Mus musculus OX=10090 GN=Hmgb2 PE=1 SV=3 |
| 521 | 506 | sp\|Q501J6\|DDX17\_MOUSE | 186.82 | 14 | 9 | 5 | 9 | 6 | 7 | 9 | 6 | 9 | 8 | 7 | 1.32E6 | 0E0 | 1.22E6 | 2.59E6 | 2.21E6 | 9.15E5 |  | 2.02E6 | 6.55E7 |  | 10 | 4 | 5 | 3 | 5 | 4 | 4 | 5 | 3 | 5 | 6 | 4 | Y | 72400 | Probable ATP-dependent RNA helicase DDX17 OS=Mus musculus OX=10090 GN=Ddx17 PE=1 SV=1 |
| 521 | 507 | Q3U741\|Q3U741\_MOUSE | 186.82 | 14 | 9 | 5 | 9 | 6 | 7 | 9 | 6 | 9 | 8 | 7 | 1.32E6 | 0E0 | 1.22E6 | 2.59E6 | 2.21E6 | 9.15E5 |  | 2.02E6 | 6.55E7 |  | 10 | 4 | 5 | 3 | 5 | 4 | 4 | 5 | 3 | 5 | 6 | 4 | Y | 72585 | RNA helicase OS=Mus musculus OX=10090 GN=Ddx17 PE=1 SV=1 |
| 632 | 1053 | sp\|Q8BG95\|MYPT2\_MOUSE | 186.65 | 6 | 1 | 5 | 4 | 4 | 3 | 6 | 4 | 4 | 6 | 3 | 2.08E6 | 1.01E7 | 9.78E6 | 3.79E6 | 5.09E6 | 1.5E7 | 4.73E6 | 5.8E6 | 7.71E6 | 2.56E6 | 5 | 4 | 1 | 3 | 3 | 3 | 2 | 4 | 3 | 3 | 5 | 2 | N | 109049 | Protein phosphatase 1 regulatory subunit 12B OS=Mus musculus OX=10090 GN=Ppp1r12b PE=1 SV=2 |
| 632 | 1054 | A6H644\|A6H644\_MOUSE | 186.65 | 6 | 1 | 4 | 4 | 4 | 3 | 6 | 4 | 4 | 6 | 3 | 2.08E6 | 1.01E7 | 9.78E6 | 3.79E6 | 5.09E6 | 1.5E7 | 4.73E6 | 5.8E6 | 7.71E6 | 2.56E6 | 5 | 4 | 1 | 3 | 3 | 3 | 2 | 4 | 3 | 3 | 5 | 2 | N | 110972 | Protein phosphatase 1 regulatory subunit OS=Mus musculus OX=10090 GN=Ppp1r12b PE=1 SV=1 |
| 747 | 1332 | sp\|P47791\|GSHR\_MOUSE | 186.61 | 17 | 11 | 12 | 9 | 8 | 2 | 12 | 12 | 15 | 12 | 2 | 1.52E7 | 2.17E7 | 1.53E7 | 8.1E6 | 7.64E6 | 1.34E7 | 9.31E6 | 1.01E7 | 2.97E7 | 1.04E7 | 5 | 5 | 3 | 3 | 3 | 3 | 1 | 3 | 4 | 4 | 3 | 1 | Y | 53663 | Glutathione reductase, mitochondrial OS=Mus musculus OX=10090 GN=Gsr PE=1 SV=3 |
| 653 | 1385 | sp\|P62960\|YBOX1\_MOUSE | 186.33 | 21 | 11 | 11 | 13 | 11 | 17 | 11 | 19 | 8 | 7 | 13 | 1.22E7 | 1.34E7 | 2.82E6 | 5.86E6 | 1.65E7 | 1.5E7 | 1.44E7 | 2.37E6 | 7E6 | 9.93E6 | 7 | 5 | 2 | 2 | 3 | 2 | 6 | 3 | 5 | 2 | 2 | 4 | N | 35730 | Y-box-binding protein 1 OS=Mus musculus OX=10090 GN=Ybx1 PE=1 SV=3 |
| 721 | 578 | sp\|Q62318\|TIF1B\_MOUSE | 186.16 | 11 | 4 | 2 | 3 | 1 | 5 | 3 | 3 | 0 | 6 | 4 | 6.34E6 | 5.01E6 | 3.89E6 | 1.27E6 | 5.48E6 | 6.94E6 | 1.71E6 |  | 1.62E7 | 8.9E6 | 9 | 9 | 3 | 2 | 3 | 1 | 4 | 3 | 2 | 0 | 4 | 4 | Y | 88847 | Transcription intermediary factor 1-beta OS=Mus musculus OX=10090 GN=Trim28 PE=1 SV=3 |
| 491 | 490 | E9Q509\|E9Q509\_MOUSE | 185.77 | 22 | 16 | 9 | 7 | 9 | 9 | 3 | 13 | 5 | 11 | 9 | 1.04E7 | 4.81E6 | 7.7E6 | 3.08E6 | 3.35E6 | 2.77E6 | 7.94E6 | 2.42E5 | 1.26E7 | 5.79E6 | 11 | 9 | 8 | 4 | 5 | 4 | 4 | 2 | 7 | 3 | 7 | 7 | N | 58821 | Pyruvate kinase OS=Mus musculus OX=10090 GN=Pklr PE=1 SV=1 |
| 491 | 498 | G3X925\|G3X925\_MOUSE | 185.77 | 21 | 16 | 9 | 6 | 8 | 8 | 3 | 13 | 5 | 10 | 9 | 1.04E7 | 4.81E6 | 7.7E6 | 3.08E6 | 3.35E6 | 2.77E6 | 7.94E6 | 2.42E5 | 1.26E7 | 5.79E6 | 11 | 9 | 8 | 4 | 5 | 4 | 4 | 2 | 7 | 3 | 7 | 7 | N | 62228 | Pyruvate kinase OS=Mus musculus OX=10090 GN=Pklr PE=1 SV=1 |
| 491 | 499 | sp\|P53657\|KPYR\_MOUSE | 185.77 | 21 | 16 | 9 | 6 | 8 | 8 | 3 | 13 | 5 | 10 | 9 | 1.04E7 | 4.81E6 | 7.7E6 | 3.08E6 | 3.35E6 | 2.77E6 | 7.94E6 | 2.42E5 | 1.26E7 | 5.79E6 | 11 | 9 | 8 | 4 | 5 | 4 | 4 | 2 | 7 | 3 | 7 | 7 | N | 62309 | Pyruvate kinase PKLR OS=Mus musculus OX=10090 GN=Pklr PE=1 SV=1 |
| 551 | 894 | sp\|Q8R429\|AT2A1\_MOUSE | 185.47 | 9 | 5 | 4 | 2 | 3 | 5 | 4 | 5 | 5 | 5 | 1 |  |  |  |  |  | 5.44E6 |  |  | 2.02E5 |  | 7 | 1 | 4 | 3 | 4 | 2 | 5 | 5 | 4 | 4 | 4 | 1 | Y | 109425 | Sarcoplasmic/endoplasmic reticulum calcium ATPase 1 OS=Mus musculus OX=10090 GN=Atp2a1 PE=1 SV=1 |
| 628 | 1186 | D3Z7X7\|D3Z7X7\_MOUSE | 184.87 | 35 | 21 | 22 | 25 | 24 | 24 | 26 | 18 | 19 | 21 | 16 | 1.21E7 | 2.67E7 | 9.99E6 | 6.74E6 | 1.23E7 | 2.37E7 | 9.37E6 | 2.26E6 | 2.79E7 | 1.06E7 | 7 | 7 | 4 | 4 | 4 | 4 | 4 | 5 | 3 | 4 | 4 | 3 | N | 22562 | Tumor protein D52 (Fragment) OS=Mus musculus OX=10090 GN=Tpd52 PE=1 SV=1 |
| 628 | 1236 | D3Z637\|D3Z637\_MOUSE | 184.87 | 44 | 27 | 28 | 31 | 31 | 31 | 33 | 23 | 24 | 27 | 20 | 1.21E7 | 2.67E7 | 9.99E6 | 6.74E6 | 1.23E7 | 2.37E7 | 9.37E6 | 2.26E6 | 2.79E7 | 1.06E7 | 7 | 7 | 4 | 4 | 4 | 4 | 4 | 5 | 3 | 4 | 4 | 3 | N | 17603 | Tumor protein D52 OS=Mus musculus OX=10090 GN=Tpd52 PE=1 SV=1 |
| 628 | 1243 | D3Z125\|D3Z125\_MOUSE | 184.87 | 40 | 24 | 25 | 28 | 28 | 28 | 30 | 21 | 22 | 24 | 18 | 1.21E7 | 2.67E7 | 9.99E6 | 6.74E6 | 1.23E7 | 2.37E7 | 9.37E6 | 2.26E6 | 2.79E7 | 1.06E7 | 7 | 7 | 4 | 4 | 4 | 4 | 4 | 5 | 3 | 4 | 4 | 3 | N | 19417 | Tumor protein D52 (Fragment) OS=Mus musculus OX=10090 GN=Tpd52 PE=1 SV=1 |
| 628 | 1245 | E9PUA7\|E9PUA7\_MOUSE | 184.87 | 37 | 22 | 23 | 26 | 26 | 26 | 28 | 19 | 20 | 22 | 17 | 1.21E7 | 2.67E7 | 9.99E6 | 6.74E6 | 1.23E7 | 2.37E7 | 9.37E6 | 2.26E6 | 2.79E7 | 1.06E7 | 7 | 7 | 4 | 4 | 4 | 4 | 4 | 5 | 3 | 4 | 4 | 3 | N | 21134 | Tumor protein D52 OS=Mus musculus OX=10090 GN=Tpd52 PE=1 SV=1 |
| 628 | 1249 | sp\|Q62393\|TPD52\_MOUSE | 184.87 | 32 | 19 | 20 | 23 | 22 | 22 | 24 | 17 | 17 | 19 | 15 | 1.21E7 | 2.67E7 | 9.99E6 | 6.74E6 | 1.23E7 | 2.37E7 | 9.37E6 | 2.26E6 | 2.79E7 | 1.06E7 | 7 | 7 | 4 | 4 | 4 | 4 | 4 | 5 | 3 | 4 | 4 | 3 | N | 24313 | Tumor protein D52 OS=Mus musculus OX=10090 GN=Tpd52 PE=1 SV=2 |
| 628 | 1250 | F8WHQ1\|F8WHQ1\_MOUSE | 184.87 | 29 | 17 | 18 | 21 | 20 | 20 | 22 | 15 | 16 | 17 | 13 | 1.21E7 | 2.67E7 | 9.99E6 | 6.74E6 | 1.23E7 | 2.37E7 | 9.37E6 | 2.26E6 | 2.79E7 | 1.06E7 | 7 | 7 | 4 | 4 | 4 | 4 | 4 | 5 | 3 | 4 | 4 | 3 | N | 26927 | Tumor protein D52 OS=Mus musculus OX=10090 GN=Tpd52 PE=1 SV=1 |
| 482 | 592 | sp\|Q922Q8\|LRC59\_MOUSE | 184.84 | 32 | 11 | 12 | 25 | 14 | 18 | 20 | 26 | 14 | 10 | 15 | 3.31E7 | 1.95E7 | 4.57E7 | 2.2E7 | 3.24E7 | 2.37E7 | 3.08E7 | 1.85E7 | 4.7E7 | 3.17E7 | 8 | 7 | 4 | 4 | 7 | 4 | 5 | 5 | 6 | 5 | 4 | 6 | Y | 34877 | Leucine-rich repeat-containing protein 59 OS=Mus musculus OX=10090 GN=Lrrc59 PE=1 SV=1 |
| 620 | 494 | sp\|Q922D8\|C1TC\_MOUSE | 184.83 | 11 | 3 | 4 | 5 | 6 | 4 | 4 | 7 | 3 | 4 | 3 | 6.58E6 | 1.47E7 | 1.71E7 | 3.82E6 | 6.03E6 | 1.07E7 | 1.34E7 | 1.46E6 | 1.94E7 | 3.18E6 | 10 | 10 | 3 | 4 | 4 | 5 | 3 | 4 | 6 | 2 | 3 | 2 | N | 101200 | C-1-tetrahydrofolate synthase, cytoplasmic OS=Mus musculus OX=10090 GN=Mthfd1 PE=1 SV=4 |
| 736 | 912 | sp\|Q9WTI7\|MYO1C\_MOUSE | 184.40 | 8 | 1 | 3 | 2 | 3 | 4 | 3 | 2 | 4 | 5 | 2 | 2.49E7 | 3.94E6 | 1.52E6 | 2.39E6 | 2.16E6 | 3.01E6 | 9.6E6 | 9.22E6 | 5.67E6 | 2.08E6 | 7 | 6 | 1 | 2 | 1 | 2 | 3 | 2 | 2 | 3 | 4 | 2 | N | 121944 | Unconventional myosin-Ic OS=Mus musculus OX=10090 GN=Myo1c PE=1 SV=2 |
| 499 | 1077 | sp\|Q9DCN2\|NB5R3\_MOUSE | 184.22 | 26 | 23 | 23 | 14 | 14 | 11 | 23 | 11 | 7 | 20 | 15 | 4.02E7 | 2.56E7 | 2.47E7 | 6.69E6 | 1.22E7 | 2.01E7 | 1.1E7 | 7.8E6 | 2.97E7 | 2.51E7 | 6 | 6 | 6 | 5 | 4 | 3 | 3 | 5 | 3 | 2 | 4 | 4 | Y | 34128 | NADH-cytochrome b5 reductase 3 OS=Mus musculus OX=10090 GN=Cyb5r3 PE=1 SV=3 |
| 499 | 1078 | F2Z456\|F2Z456\_MOUSE | 184.22 | 25 | 22 | 22 | 13 | 14 | 11 | 22 | 11 | 7 | 19 | 14 | 4.02E7 | 2.56E7 | 2.47E7 | 6.69E6 | 1.22E7 | 2.01E7 | 1.1E7 | 7.8E6 | 2.97E7 | 2.51E7 | 6 | 6 | 6 | 5 | 4 | 3 | 3 | 5 | 3 | 2 | 4 | 4 | Y | 34929 | NADH-cytochrome b5 reductase OS=Mus musculus OX=10090 GN=Cyb5r3 PE=1 SV=1 |
| 425 | 411 | sp\|Q8VDJ3\|VIGLN\_MOUSE | 184.03 | 11 | 6 | 5 | 4 | 6 | 4 | 6 | 2 | 1 | 6 | 2 | 9.5E6 | 1.39E7 | 1.74E7 | 1.05E7 | 1.43E7 | 2.06E7 | 3.62E6 | 4.98E6 | 2.74E7 | 4.88E6 | 13 | 13 | 7 | 5 | 5 | 6 | 5 | 7 | 3 | 2 | 6 | 2 | Y | 141742 | Vigilin OS=Mus musculus OX=10090 GN=Hdlbp PE=1 SV=1 |
| 603 | 1134 | sp\|Q63886\|UD11\_MOUSE | 184.00 | 13 | 8 | 13 | 5 | 7 | 2 | 10 | 6 | 6 | 13 | 9 | 4.45E5 | 4.55E6 | 9.33E5 | 3.03E5 | 0E0 | 2.29E5 | 5.43E5 | 5.72E5 | 6.26E6 | 9.04E5 | 5 | 2 | 3 | 6 | 2 | 3 | 1 | 5 | 3 | 3 | 6 | 4 | Y | 60047 | UDP-glucuronosyltransferase 1A1 OS=Mus musculus OX=10090 GN=Ugt1a1 PE=1 SV=2 |
| 696 | 1160 | E9Q1S3\|E9Q1S3\_MOUSE | 184.00 | 8 | 5 | 3 | 4 | 5 | 5 | 3 | 7 | 5 | 7 | 5 | 6.26E6 | 6.7E6 | 1.06E7 | 1.69E6 | 3.77E6 | 1.88E6 | 3.56E6 | 2.8E6 | 1.97E6 | 3.63E6 | 5 | 4 | 3 | 2 | 3 | 3 | 3 | 2 | 5 | 3 | 4 | 3 | Y | 82956 | Protein transport protein SEC23 OS=Mus musculus OX=10090 GN=Sec23a PE=1 SV=1 |
| 696 | 1161 | sp\|Q01405\|SC23A\_MOUSE | 184.00 | 8 | 5 | 3 | 4 | 5 | 5 | 3 | 7 | 5 | 7 | 5 | 6.26E6 | 6.7E6 | 1.06E7 | 1.69E6 | 3.77E6 | 1.88E6 | 3.56E6 | 2.8E6 | 1.97E6 | 3.63E6 | 5 | 4 | 3 | 2 | 3 | 3 | 3 | 2 | 5 | 3 | 4 | 3 | Y | 86162 | Protein transport protein Sec23A OS=Mus musculus OX=10090 GN=Sec23a PE=1 SV=2 |
| 567 | 370 | sp\|Q5SYD0\|MYO1D\_MOUSE | 183.17 | 14 | 1 | 3 | 3 | 5 | 4 | 5 | 9 | 3 | 5 | 2 | 1.17E6 | 5.55E6 | 3.64E6 | 2.13E6 | 7.46E6 | 8.39E6 | 1.69E7 | 3.03E6 | 8.94E6 | 5.12E6 | 15 | 15 | 1 | 3 | 3 | 5 | 4 | 6 | 9 | 3 | 4 | 2 | Y | 116081 | Unconventional myosin-Id OS=Mus musculus OX=10090 GN=Myo1d PE=1 SV=1 |
| 810 | 738 | sp\|Q8BMK4\|CKAP4\_MOUSE | 183.08 | 14 | 7 | 0 | 0 | 4 | 3 | 5 | 4 | 5 | 9 | 3 | 2.27E6 |  |  | 2.27E6 | 1.97E6 | 2.15E6 | 3.29E6 | 2.5E6 | 1.15E7 | 1.18E6 | 7 | 7 | 3 | 0 | 0 | 3 | 2 | 2 | 3 | 2 | 5 | 1 | N | 63692 | Cytoskeleton-associated protein 4 OS=Mus musculus OX=10090 GN=Ckap4 PE=1 SV=2 |
| 231 | 921 | sp\|P14131\|RS16\_MOUSE | 183.08 | 53 | 36 | 33 | 38 | 53 | 42 | 42 | 52 | 47 | 30 | 33 | 1.26E8 | 9.98E7 | 1.59E8 | 8.4E7 | 1.04E8 | 9.96E7 | 9.58E7 | 7.98E7 | 1.73E8 | 1.04E8 | 10 | 10 | 13 | 11 | 12 | 14 | 12 | 8 | 11 | 15 | 11 | 11 | N | 16445 | Small ribosomal subunit protein uS9 OS=Mus musculus OX=10090 GN=Rps16 PE=1 SV=4 |
| 776 | 561 | sp\|Q60864\|STIP1\_MOUSE | 183.06 | 20 | 0 | 0 | 8 | 12 | 8 | 7 | 8 | 7 | 3 | 0 |  |  | 2.35E6 | 7.11E6 | 1.43E7 | 1.11E7 | 3.27E6 | 2.19E6 | 4.79E6 |  | 11 | 11 | 0 | 0 | 3 | 7 | 5 | 4 | 3 | 3 | 3 | 0 | Y | 62582 | Stress-induced-phosphoprotein 1 OS=Mus musculus OX=10090 GN=Stip1 PE=1 SV=1 |
| 708 | 1003 | sp\|Q91ZJ5\|UGPA\_MOUSE | 183.02 | 15 | 7 | 4 | 11 | 9 | 7 | 9 | 5 | 5 | 9 | 7 | 1.09E7 | 7.15E6 | 1.12E7 | 7.14E6 | 1.07E7 | 1.28E7 | 3.81E6 | 4.5E6 | 2.39E7 | 8.2E6 | 6 | 6 | 3 | 2 | 4 | 4 | 3 | 3 | 2 | 2 | 4 | 3 | N | 56979 | UTP--glucose-1-phosphate uridylyltransferase OS=Mus musculus OX=10090 GN=Ugp2 PE=1 SV=3 |
| 616 | 1310 | A0A0R4J1E2\|A0A0R4J1E2\_MOUSE | 182.94 | 11 | 5 | 11 | 7 | 7 | 6 | 5 | 4 | 7 | 6 | 7 | 2.18E7 | 4.64E7 | 2.6E7 | 1.16E7 | 1.8E7 | 7.2E6 | 5.66E6 | 9.52E6 | 5.13E7 | 1.84E7 | 5 | 5 | 4 | 6 | 5 | 3 | 4 | 3 | 1 | 3 | 4 | 4 | Y | 72930 | Elongation factor 1-delta OS=Mus musculus OX=10090 GN=Eef1d PE=1 SV=1 |
| 616 | 1353 | E9QN08\|E9QN08\_MOUSE | 182.94 | 29 | 14 | 29 | 19 | 20 | 15 | 14 | 10 | 20 | 15 | 19 | 2.18E7 | 4.64E7 | 2.6E7 | 1.16E7 | 1.8E7 | 7.2E6 | 5.66E6 | 9.52E6 | 5.13E7 | 1.84E7 | 5 | 5 | 4 | 6 | 5 | 3 | 4 | 3 | 1 | 3 | 4 | 4 | Y | 27218 | Elongation factor 1-delta (Fragment) OS=Mus musculus OX=10090 GN=Eef1d PE=1 SV=1 |
| 616 | 1354 | sp\|P57776\|EF1D\_MOUSE | 182.94 | 25 | 12 | 25 | 16 | 17 | 13 | 12 | 9 | 17 | 13 | 16 | 2.18E7 | 4.64E7 | 2.6E7 | 1.16E7 | 1.8E7 | 7.2E6 | 5.66E6 | 9.52E6 | 5.13E7 | 1.84E7 | 5 | 5 | 4 | 6 | 5 | 3 | 4 | 3 | 1 | 3 | 4 | 4 | Y | 31293 | Elongation factor 1-delta OS=Mus musculus OX=10090 GN=Eef1d PE=1 SV=3 |
| 616 | 1355 | Q80T06\|Q80T06\_MOUSE | 182.94 | 25 | 12 | 25 | 16 | 17 | 13 | 12 | 9 | 17 | 13 | 16 | 2.18E7 | 4.64E7 | 2.6E7 | 1.16E7 | 1.8E7 | 7.2E6 | 5.66E6 | 9.52E6 | 5.13E7 | 1.84E7 | 5 | 5 | 4 | 6 | 5 | 3 | 4 | 3 | 1 | 3 | 4 | 4 | Y | 31292 | Elongation factor 1-delta OS=Mus musculus OX=10090 GN=Eef1d PE=1 SV=1 |
| 1185 | 1302 | sp\|P17892\|LIPR2\_MOUSE | 182.71 | 13 | 13 | 0 | 0 | 0 | 0 | 0 | 0 | 0 | 6 | 4 | 4.1E7 |  |  |  |  |  |  |  | 6.76E6 | 4.45E6 | 5 | 5 | 6 | 0 | 0 | 0 | 0 | 0 | 0 | 0 | 3 | 2 | Y | 54017 | Pancreatic lipase-related protein 2 OS=Mus musculus OX=10090 GN=Pnliprp2 PE=1 SV=2 |
| 641 | 873 | A0A0N4SVP8\|A0A0N4SVP8\_MOUSE | 182.52 | 17 | 7 | 7 | 7 | 9 | 11 | 10 | 11 | 9 | 7 | 9 | 3.24E6 | 2.74E6 | 3.15E6 | 2.97E6 | 3.86E6 | 2.45E6 | 2.87E6 | 1.44E6 | 5.1E7 | 2.9E6 | 6 | 4 | 3 | 2 | 2 | 3 | 4 | 3 | 4 | 3 | 3 | 3 | N | 46959 | Eukaryotic initiation factor 4A-III OS=Mus musculus OX=10090 GN=Eif4a3l2 PE=3 SV=1 |
| 659 | 954 | sp\|Q9R111\|GUAD\_MOUSE | 182.09 | 14 | 4 | 4 | 9 | 9 | 9 | 9 | 9 | 5 | 7 | 11 | 9.8E6 | 9.29E6 | 1.81E7 | 1.77E7 | 8.53E6 | 3.64E7 | 8.42E6 | 4.47E6 | 1.58E7 | 3.84E7 | 6 | 6 | 2 | 2 | 4 | 4 | 4 | 4 | 4 | 2 | 3 | 5 | N | 51013 | Guanine deaminase OS=Mus musculus OX=10090 GN=Gda PE=1 SV=1 |
| 310 | 727 | sp\|P61161\|ARP2\_MOUSE | 182.08 | 20 | 17 | 11 | 14 | 9 | 14 | 17 | 14 | 12 | 16 | 17 | 3.68E7 | 2.09E7 | 2.11E7 | 9.58E6 | 1.92E7 | 2.36E7 | 2.31E7 | 2E7 | 5.41E7 | 2.09E7 | 8 | 7 | 8 | 6 | 8 | 9 | 10 | 9 | 6 | 5 | 15 | 7 | Y | 44761 | Actin-related protein 2 OS=Mus musculus OX=10090 GN=Actr2 PE=1 SV=1 |
| 232 | 848 | sp\|P15331\|PERI\_MOUSE | 182.03 | 16 | 7 | 12 | 9 | 5 | 10 | 3 | 5 | 5 | 5 | 5 | 3.54E6 | 5.8E6 | 2.33E6 |  | 2.62E6 |  |  |  |  |  | 9 | 4 | 16 | 13 | 10 | 8 | 12 | 9 | 9 | 12 | 13 | 11 | Y | 54268 | Peripherin OS=Mus musculus OX=10090 GN=Prph PE=1 SV=2 |
| 232 | 849 | G5E846\|G5E846\_MOUSE | 182.03 | 16 | 7 | 12 | 9 | 5 | 10 | 3 | 5 | 5 | 5 | 5 | 3.54E6 | 5.8E6 | 2.33E6 |  | 2.62E6 |  |  |  |  |  | 9 | 4 | 16 | 13 | 10 | 8 | 12 | 9 | 9 | 12 | 13 | 11 | Y | 54298 | Peripherin OS=Mus musculus OX=10090 GN=Prph PE=1 SV=1 |
| 392 | 813 | sp\|P62827\|RAN\_MOUSE | 182.03 | 37 | 20 | 18 | 21 | 31 | 24 | 21 | 25 | 20 | 26 | 21 | 6.2E7 | 5.64E7 | 5.5E7 | 5.18E7 | 6.87E7 | 5.04E7 | 6.16E7 | 4.93E7 | 1.07E8 | 6.21E7 | 8 | 8 | 5 | 6 | 5 | 8 | 8 | 6 | 8 | 7 | 8 | 6 | Y | 24423 | GTP-binding nuclear protein Ran OS=Mus musculus OX=10090 GN=Ran PE=1 SV=3 |
| 689 | 877 | sp\|Q02257\|PLAK\_MOUSE | 181.94 | 12 | 1 | 3 | 2 | 4 | 4 | 9 | 4 | 3 | 5 | 2 | 4.65E6 | 7.81E6 | 3.37E6 | 2.59E6 | 3.2E6 | 7.82E6 | 1.03E7 | 5.23E5 | 3.86E6 | 1.62E6 | 7 | 5 | 2 | 3 | 1 | 2 | 3 | 5 | 2 | 2 | 3 | 2 | Y | 81801 | Junction plakoglobin OS=Mus musculus OX=10090 GN=Jup PE=1 SV=3 |
| 427 | 416 | sp\|Q00612\|G6PD1\_MOUSE | 181.83 | 24 | 15 | 11 | 9 | 8 | 4 | 13 | 10 | 6 | 17 | 18 | 3.34E7 | 9.34E6 | 5.43E6 | 5.49E6 | 4.75E6 | 1.21E7 | 4.5E6 | 4.88E6 | 4.02E7 | 3.67E7 | 11 | 11 | 6 | 4 | 4 | 4 | 2 | 5 | 4 | 3 | 7 | 7 | N | 59263 | Glucose-6-phosphate 1-dehydrogenase X OS=Mus musculus OX=10090 GN=G6pdx PE=1 SV=3 |
| 433 | 1328 | A0A494BB86\|A0A494BB86\_MOUSE | 181.77 | 23 | 16 | 10 | 10 | 19 | 11 | 11 | 14 | 5 | 10 | 10 | 2.05E6 |  |  | 2.76E6 | 2.45E6 | 1.89E6 |  |  |  |  | 6 | 2 | 6 | 6 | 6 | 10 | 8 | 5 | 4 | 2 | 11 | 4 | Y | 37669 | Alpha-centractin OS=Mus musculus OX=10090 GN=Actr1a PE=1 SV=1 |
| 433 | 1331 | sp\|P61164\|ACTZ\_MOUSE | 181.77 | 20 | 14 | 9 | 9 | 16 | 10 | 10 | 12 | 4 | 9 | 9 | 2.05E6 |  |  | 2.76E6 | 2.45E6 | 1.89E6 |  |  |  |  | 6 | 2 | 6 | 6 | 6 | 10 | 8 | 5 | 4 | 2 | 11 | 4 | Y | 42614 | Alpha-centractin OS=Mus musculus OX=10090 GN=Actr1a PE=1 SV=1 |
| 503 | 623 | sp\|Q3TJD7\|PDLI7\_MOUSE | 181.70 | 20 | 10 | 14 | 14 | 11 | 9 | 14 | 11 | 12 | 12 | 7 | 1.96E7 | 3.38E7 | 2.96E7 | 9.45E6 | 1.14E7 | 2.72E7 | 1.59E7 | 1.21E7 | 3.78E7 | 1.07E7 | 9 | 9 | 4 | 6 | 6 | 5 | 4 | 6 | 5 | 5 | 5 | 3 | Y | 50119 | PDZ and LIM domain protein 7 OS=Mus musculus OX=10090 GN=Pdlim7 PE=1 SV=1 |
| 484 | 1210 | sp\|O70400\|PDLI1\_MOUSE | 181.63 | 19 | 9 | 16 | 9 | 16 | 16 | 16 | 12 | 12 | 19 | 12 | 1.77E7 | 3.27E7 | 1.89E7 | 1.67E7 | 3.63E7 | 3.85E7 | 2.33E7 | 1.89E7 | 5.63E7 | 1.59E7 | 6 | 6 | 3 | 6 | 4 | 5 | 6 | 7 | 4 | 4 | 8 | 3 | Y | 35774 | PDZ and LIM domain protein 1 OS=Mus musculus OX=10090 GN=Pdlim1 PE=1 SV=4 |
| 927 | 895 | sp\|Q99K41\|EMIL1\_MOUSE | 181.02 | 8 | 5 | 1 | 1 | 0 | 0 | 1 | 1 | 1 | 8 | 2 | 4.89E6 | 1.15E6 | 1.08E6 |  |  | 1.36E6 | 3.9E5 | 8.08E5 | 1.93E7 | 1.04E7 | 6 | 6 | 3 | 1 | 1 | 0 | 0 | 1 | 1 | 1 | 5 | 2 | N | 107585 | EMILIN-1 OS=Mus musculus OX=10090 GN=Emilin1 PE=1 SV=1 |
| 733 | 1460 | A0A0R3P9C8\|A0A0R3P9C8\_MOUSE | 180.73 | 25 | 17 | 20 | 12 | 17 | 21 | 17 | 17 | 7 | 12 | 12 | 6.8E6 | 1.07E7 | 3.56E6 | 3.93E6 | 1.02E7 | 1.61E7 | 3.42E6 | 2.48E6 | 1.91E7 | 7.09E6 | 5 | 5 | 3 | 5 | 2 | 3 | 4 | 4 | 4 | 1 | 2 | 2 | Y | 42122 | NADH dehydrogenase [ubiquinone] 1 alpha subcomplex subunit 9, mitochondrial OS=Mus musculus OX=10090 GN=Ndufa9 PE=1 SV=1 |
| 733 | 1461 | sp\|Q9DC69\|NDUA9\_MOUSE | 180.73 | 24 | 17 | 20 | 12 | 17 | 21 | 17 | 17 | 7 | 12 | 12 | 6.8E6 | 1.07E7 | 3.56E6 | 3.93E6 | 1.02E7 | 1.61E7 | 3.42E6 | 2.48E6 | 1.91E7 | 7.09E6 | 5 | 5 | 3 | 5 | 2 | 3 | 4 | 4 | 4 | 1 | 2 | 2 | Y | 42525 | NADH dehydrogenase [ubiquinone] 1 alpha subcomplex subunit 9, mitochondrial OS=Mus musculus OX=10090 GN=Ndufa9 PE=1 SV=2 |
| 389 | 384 | sp\|P50247\|SAHH\_MOUSE | 180.68 | 28 | 9 | 12 | 12 | 9 | 8 | 9 | 9 | 7 | 20 | 14 | 1.38E8 | 1.76E8 | 1.82E8 | 8.73E6 | 1.65E7 | 1.38E7 | 1.03E7 | 2.89E7 | 2.54E8 | 2.12E7 | 12 | 11 | 4 | 5 | 7 | 4 | 3 | 4 | 4 | 4 | 9 | 6 | Y | 47688 | Adenosylhomocysteinase OS=Mus musculus OX=10090 GN=Ahcy PE=1 SV=3 |
| 679 | 1126 | sp\|P62192\|PRS4\_MOUSE | 180.42 | 18 | 11 | 10 | 11 | 7 | 5 | 2 | 16 | 13 | 5 | 8 | 3.6E6 | 5.75E6 | 5.48E6 | 2.93E6 | 4.68E6 | 3.94E6 | 4.77E6 | 4.39E6 | 9.31E5 | 6.41E6 | 6 | 5 | 3 | 3 | 4 | 2 | 2 | 1 | 5 | 4 | 1 | 3 | Y | 49185 | 26S proteasome regulatory subunit 4 OS=Mus musculus OX=10090 GN=Psmc1 PE=1 SV=1 |
| 675 | 563 | sp\|Q99MZ6\|MYO7B\_MOUSE | 180.06 | 5 | 0 | 4 | 1 | 2 | 0 | 2 | 2 | 1 | 1 | 1 | 1.3E6 | 1.98E7 | 8.74E4 | 1.71E6 |  | 4.31E7 | 7.85E6 | 6.59E6 | 6.37E7 | 1.43E7 | 9 | 9 | 1 | 7 | 1 | 3 | 0 | 4 | 3 | 1 | 3 | 2 | Y | 240856 | Unconventional myosin-VIIb OS=Mus musculus OX=10090 GN=Myo7b PE=1 SV=3 |
| 635 | 678 | A0A5F8MPE1\|A0A5F8MPE1\_MOUSE | 180.03 | 9 | 4 | 7 | 3 | 5 | 0 | 4 | 4 | 3 | 3 | 2 | 2.78E7 | 1.93E7 | 3.1E6 | 3.45E6 |  | 3.6E6 | 4.24E6 | 3.25E6 | 3.6E6 | 2.31E7 | 8 | 8 | 3 | 6 | 3 | 4 | 0 | 3 | 4 | 3 | 3 | 3 | Y | 116535 | Erythrocyte membrane protein band 4.1 like 3 OS=Mus musculus OX=10090 GN=Epb41l3 PE=1 SV=1 |
| 593 | 906 | sp\|P46978\|STT3A\_MOUSE | 179.84 | 10 | 2 | 2 | 4 | 9 | 8 | 7 | 10 | 7 | 2 | 1 | 9.13E6 | 1.06E7 | 1.43E7 | 1.39E7 | 2.99E7 | 2.84E7 | 2.31E7 | 2.04E7 | 1.02E7 | 3.03E6 | 7 | 7 | 2 | 2 | 3 | 6 | 6 | 5 | 7 | 5 | 2 | 1 | N | 80598 | Dolichyl-diphosphooligosaccharide--protein glycosyltransferase subunit STT3A OS=Mus musculus OX=10090 GN=Stt3a PE=1 SV=1 |
| 898 | 810 | sp\|Q8BH59\|S2512\_MOUSE | 179.70 | 14 | 8 | 7 | 6 | 1 | 4 | 12 | 4 | 4 | 3 | 1 | 4.24E6 | 2.67E6 | 4.3E6 | 0E0 | 1.49E6 | 4.13E6 | 4.82E5 | 5.62E5 | 2.56E5 | 9.17E5 | 7 | 7 | 3 | 3 | 2 | 1 | 2 | 6 | 1 | 1 | 1 | 1 | N | 74570 | Electrogenic aspartate/glutamate antiporter SLC25A12, mitochondrial OS=Mus musculus OX=10090 GN=Slc25a12 PE=1 SV=1 |
| 523 | 1298 | sp\|P84078\|ARF1\_MOUSE | 179.61 | 35 | 28 | 28 | 22 | 25 | 17 | 29 | 29 | 22 | 28 | 22 | 2.66E7 | 3.24E7 | 1.66E7 | 7.13E5 |  | 2.53E7 | 2.58E7 | 1.93E7 | 1.77E7 | 2.44E7 | 5 | 2 | 6 | 6 | 4 | 4 | 3 | 5 | 5 | 4 | 5 | 4 | Y | 20697 | ADP-ribosylation factor 1 OS=Mus musculus OX=10090 GN=Arf1 PE=1 SV=2 |
| 523 | 1299 | sp\|P61205\|ARF3\_MOUSE | 179.61 | 35 | 28 | 28 | 22 | 25 | 17 | 29 | 29 | 22 | 28 | 22 | 2.66E7 | 3.24E7 | 1.66E7 | 7.13E5 |  | 2.53E7 | 2.58E7 | 1.93E7 | 1.77E7 | 2.44E7 | 5 | 2 | 6 | 6 | 4 | 4 | 3 | 5 | 5 | 4 | 5 | 4 | Y | 20601 | ADP-ribosylation factor 3 OS=Mus musculus OX=10090 GN=Arf3 PE=2 SV=2 |
| 519 | 611 | sp\|Q9Z2I8\|SUCB2\_MOUSE | 179.44 | 19 | 10 | 12 | 8 | 4 | 15 | 13 | 9 | 6 | 11 | 11 | 1.57E7 | 2.27E7 | 1.31E7 | 3.15E5 | 1.26E7 | 1.18E7 | 1.32E6 | 1.38E6 | 1.48E7 | 8.95E6 | 8 | 8 | 4 | 5 | 3 | 1 | 6 | 5 | 3 | 2 | 4 | 4 | Y | 46840 | Succinate--CoA ligase [GDP-forming] subunit beta, mitochondrial OS=Mus musculus OX=10090 GN=Suclg2 PE=1 SV=3 |
| 463 | 863 | A0A0R4J083\|A0A0R4J083\_MOUSE | 179.16 | 17 | 12 | 12 | 9 | 11 | 11 | 12 | 12 | 15 | 10 | 11 | 4.2E6 |  | 4.86E5 |  |  | 9.79E5 | 7.62E5 | 2.41E6 | 2.26E6 | 5.6E6 | 8 | 2 | 6 | 6 | 4 | 4 | 4 | 5 | 5 | 6 | 6 | 6 | Y | 47897 | Long-chain specific acyl-CoA dehydrogenase, mitochondrial OS=Mus musculus OX=10090 GN=Acadl PE=1 SV=1 |
| 1059 | 1167 | Q8CE80\|Q8CE80\_MOUSE | 179.12 | 11 | 2 | 2 | 2 | 0 | 5 | 5 | 8 | 0 | 4 | 1 | 2.51E5 | 7.2E5 | 7.08E5 |  | 3.35E6 | 4.41E6 | 3.34E6 |  | 5.08E6 | 5.94E4 | 5 | 5 | 1 | 1 | 1 | 0 | 2 | 2 | 3 | 0 | 3 | 1 | N | 81441 | Calpastatin OS=Mus musculus OX=10090 GN=Cast PE=1 SV=1 |
| 1059 | 1169 | sp\|P51125\|ICAL\_MOUSE | 179.12 | 10 | 2 | 2 | 2 | 0 | 4 | 4 | 7 | 0 | 4 | 1 | 2.51E5 | 7.2E5 | 7.08E5 |  | 3.35E6 | 4.41E6 | 3.34E6 |  | 5.08E6 | 5.94E4 | 5 | 5 | 1 | 1 | 1 | 0 | 2 | 2 | 3 | 0 | 3 | 1 | N | 84922 | Calpastatin OS=Mus musculus OX=10090 GN=Cast PE=1 SV=2 |
| 461 | 725 | sp\|P97384\|ANX11\_MOUSE | 179.09 | 16 | 6 | 10 | 6 | 11 | 9 | 11 | 11 | 9 | 13 | 10 | 1.22E7 | 2.76E7 | 1.43E7 | 1.09E7 | 1.99E7 | 1.29E7 | 1.62E7 | 1.05E7 | 3.48E7 | 1.57E7 | 8 | 7 | 3 | 5 | 4 | 7 | 6 | 6 | 7 | 6 | 7 | 5 | Y | 54079 | Annexin A11 OS=Mus musculus OX=10090 GN=Anxa11 PE=1 SV=2 |
| 440 | 570 | sp\|O54734\|OST48\_MOUSE | 178.75 | 25 | 7 | 12 | 11 | 11 | 11 | 17 | 23 | 2 | 16 | 15 | 7.07E6 | 2.06E7 | 1.73E8 | 1.83E7 | 1.53E7 | 3.6E7 | 2.44E7 | 4.11E5 | 1.97E7 | 1.49E8 | 9 | 9 | 3 | 6 | 5 | 5 | 5 | 8 | 9 | 1 | 7 | 7 | N | 49028 | Dolichyl-diphosphooligosaccharide--protein glycosyltransferase 48 kDa subunit OS=Mus musculus OX=10090 GN=Ddost PE=1 SV=2 |
| 770 | 985 | sp\|Q9CZD3\|GARS\_MOUSE | 178.23 | 10 | 3 | 3 | 6 | 7 | 3 | 4 | 5 | 5 | 2 | 6 | 9.01E6 | 8.3E6 | 1.22E7 | 6.07E6 | 6.19E6 | 9.3E6 | 1.1E7 | 5.36E6 | 1.16E7 | 5.46E7 | 6 | 6 | 2 | 2 | 3 | 4 | 2 | 3 | 2 | 2 | 1 | 3 | N | 81878 | Glycine--tRNA ligase OS=Mus musculus OX=10090 GN=Gars1 PE=1 SV=1 |
| 284 | 1118 | sp\|P97461\|RS5\_MOUSE | 178.19 | 31 | 16 | 16 | 24 | 24 | 24 | 20 | 20 | 26 | 26 | 16 | 8.07E7 | 6.87E7 | 1.04E8 | 3.24E7 | 8.74E7 | 8.38E7 | 6.38E7 | 5.6E7 | 1.53E8 | 9.39E7 | 8 | 8 | 7 | 8 | 13 | 6 | 9 | 7 | 7 | 10 | 14 | 12 | Y | 22876 | Small ribosomal subunit protein uS7 OS=Mus musculus OX=10090 GN=Rps5 PE=1 SV=4 |
| 695 | 560 | sp\|Q61847\|MEP1B\_MOUSE | 178.06 | 16 | 3 | 5 | 4 | 7 | 3 | 8 | 5 | 2 | 4 | 3 | 6.91E6 | 1.7E7 | 8.65E6 | 8.17E6 | 1.01E7 | 1.05E7 | 8.25E6 | 2.94E6 | 1.03E7 | 5.72E6 | 9 | 9 | 2 | 4 | 3 | 5 | 2 | 4 | 3 | 1 | 4 | 3 | Y | 79500 | Meprin A subunit beta OS=Mus musculus OX=10090 GN=Mep1b PE=1 SV=2 |
| 369 | 486 | sp\|Q60854\|SPB6\_MOUSE | 178.04 | 31 | 16 | 7 | 15 | 13 | 19 | 21 | 15 | 12 | 18 | 5 | 3.64E7 | 4.08E7 | 4.05E7 | 2.87E7 | 4.62E7 | 5.49E7 | 3.73E7 | 2.47E7 | 3.37E7 | 1.15E7 | 10 | 10 | 7 | 5 | 6 | 7 | 8 | 7 | 6 | 4 | 7 | 3 | Y | 42599 | Serpin B6 OS=Mus musculus OX=10090 GN=Serpinb6 PE=1 SV=1 |
| 369 | 487 | F8WIV2\|F8WIV2\_MOUSE | 178.04 | 30 | 15 | 7 | 15 | 13 | 18 | 20 | 14 | 12 | 17 | 5 | 3.64E7 | 4.08E7 | 4.05E7 | 2.87E7 | 4.62E7 | 5.49E7 | 3.73E7 | 2.47E7 | 3.37E7 | 1.15E7 | 10 | 10 | 7 | 5 | 6 | 7 | 8 | 7 | 6 | 4 | 7 | 3 | Y | 44774 | Serpin B6 OS=Mus musculus OX=10090 GN=Serpinb6a PE=1 SV=1 |
| 656 | 1030 | sp\|Q9D051\|ODPB\_MOUSE | 177.50 | 23 | 16 | 16 | 4 | 12 | 7 | 7 | 11 | 11 | 11 | 7 | 1.43E7 | 1.62E7 | 2.39E5 | 1.41E6 | 2.96E6 | 2.39E6 | 4.68E6 | 3.23E6 | 2.48E7 | 4E6 | 6 | 6 | 6 | 5 | 1 | 4 | 2 | 2 | 4 | 3 | 4 | 3 | Y | 38937 | Pyruvate dehydrogenase E1 component subunit beta, mitochondrial OS=Mus musculus OX=10090 GN=Pdhb PE=1 SV=1 |
| 533 | 752 | sp\|P60335\|PCBP1\_MOUSE | 177.37 | 33 | 13 | 13 | 11 | 11 | 13 | 15 | 11 | 26 | 16 | 13 | 1.8E7 | 2.25E7 | 1.41E7 | 1.21E6 | 1.4E7 | 1.61E7 | 2.48E6 | 1.58E7 | 4.5E7 | 1.69E7 | 9 | 5 | 5 | 5 | 4 | 3 | 5 | 5 | 3 | 7 | 5 | 5 | Y | 37498 | Poly(rC)-binding protein 1 OS=Mus musculus OX=10090 GN=Pcbp1 PE=1 SV=1 |
| 313 | 559 | sp\|P47963\|RL13\_MOUSE | 177.33 | 43 | 26 | 30 | 27 | 27 | 37 | 30 | 27 | 25 | 30 | 21 | 1.06E8 | 1.22E8 | 1.24E8 | 6.96E7 | 1.18E8 | 1.16E8 | 8.51E7 | 8.46E7 | 1.45E8 | 8.46E7 | 10 | 10 | 9 | 9 | 8 | 7 | 9 | 8 | 7 | 7 | 9 | 7 | Y | 24305 | Large ribosomal subunit protein eL13 OS=Mus musculus OX=10090 GN=Rpl13 PE=1 SV=3 |
| 376 | 1200 | sp\|P56391\|CX6B1\_MOUSE | 177.23 | 70 | 52 | 52 | 62 | 70 | 60 | 70 | 60 | 60 | 40 | 60 | 7.75E7 | 1.12E8 | 1.11E8 | 7.11E7 | 1.06E8 | 1.52E8 | 7.61E7 | 5.72E7 | 7.48E7 | 5.94E7 | 6 | 6 | 6 | 7 | 9 | 8 | 8 | 8 | 6 | 9 | 4 | 6 | Y | 10071 | Cytochrome c oxidase subunit 6B1 OS=Mus musculus OX=10090 GN=Cox6b1 PE=1 SV=2 |
| 1096 | 1319 | sp\|Q9R0H0\|ACOX1\_MOUSE | 176.91 | 10 | 0 | 0 | 5 | 3 | 5 | 9 | 2 | 2 | 0 | 1 |  |  | 3.84E6 | 1.27E6 | 2.51E6 | 8.32E6 | 7.77E5 | 7.15E5 |  | 4.22E5 | 5 | 5 | 0 | 0 | 2 | 2 | 3 | 4 | 1 | 1 | 0 | 1 | Y | 74649 | Peroxisomal acyl-coenzyme A oxidase 1 OS=Mus musculus OX=10090 GN=Acox1 PE=1 SV=5 |
| 218 | 1300 | sp\|Q6IFZ6\|K2C1B\_MOUSE | 176.66 | 8 | 3 | 3 | 6 | 5 | 5 | 6 | 8 | 8 | 3 | 3 |  |  |  |  | 3.4E7 | 3.99E7 | 2.41E7 | 2.1E7 |  |  | 6 | 1 | 12 | 11 | 12 | 8 | 12 | 13 | 12 | 17 | 15 | 11 | N | 61359 | Keratin, type II cytoskeletal 1b OS=Mus musculus OX=10090 GN=Krt77 PE=1 SV=1 |
| 645 | 710 | sp\|O35639\|ANXA3\_MOUSE | 176.52 | 25 | 15 | 5 | 6 | 3 | 11 | 10 | 6 | 6 | 9 | 8 | 5.22E6 | 3.71E6 | 1.2E6 |  | 3.07E6 | 1.24E6 | 3.27E5 | 2.66E5 | 4.46E6 | 3.99E6 | 8 | 6 | 4 | 2 | 3 | 2 | 5 | 4 | 3 | 3 | 4 | 3 | N | 36384 | Annexin A3 OS=Mus musculus OX=10090 GN=Anxa3 PE=1 SV=4 |
| 725 | 981 | sp\|Q3U7R1\|ESYT1\_MOUSE | 176.45 | 7 | 5 | 3 | 5 | 3 | 4 | 3 | 3 | 3 | 4 | 3 | 7.63E6 | 5.22E6 | 9.75E6 | 3.24E6 | 5.49E6 | 6.99E6 | 4.33E6 | 1.85E6 | 6.63E6 | 5.16E6 | 6 | 6 | 4 | 2 | 4 | 3 | 4 | 2 | 3 | 2 | 4 | 2 | N | 121553 | Extended synaptotagmin-1 OS=Mus musculus OX=10090 GN=Esyt1 PE=1 SV=2 |
| 618 | 606 | sp\|P32261\|ANT3\_MOUSE | 175.93 | 21 | 0 | 9 | 8 | 10 | 14 | 13 | 17 | 9 | 0 | 2 |  | 1.72E8 | 1.2E7 | 4.54E6 | 3.05E7 | 1.1E8 | 1.1E8 | 6.55E6 |  | 8.86E5 | 9 | 9 | 0 | 5 | 3 | 4 | 6 | 6 | 8 | 4 | 0 | 1 | Y | 52004 | Antithrombin-III OS=Mus musculus OX=10090 GN=Serpinc1 PE=1 SV=1 |
| 807 | 480 | sp\|Q8R0Y6\|AL1L1\_MOUSE | 175.86 | 13 | 3 | 3 | 0 | 2 | 3 | 4 | 2 | 1 | 3 | 6 | 1.4E6 | 3.14E6 |  | 5.4E5 | 1.62E6 | 5.17E6 | 3.58E6 | 1.01E6 | 5.7E6 | 2.97E6 | 9 | 9 | 2 | 2 | 0 | 1 | 2 | 3 | 2 | 1 | 2 | 4 | N | 98709 | Cytosolic 10-formyltetrahydrofolate dehydrogenase OS=Mus musculus OX=10090 GN=Aldh1l1 PE=1 SV=1 |
| 382 | 791 | Q8C2Q8\|Q8C2Q8\_MOUSE | 175.82 | 29 | 22 | 21 | 17 | 22 | 19 | 21 | 18 | 23 | 21 | 19 | 2.84E7 | 4.67E7 | 3.92E7 | 1.94E7 | 2.08E7 | 3.56E7 | 2.75E7 | 1.8E7 | 4.51E7 | 3.38E7 | 8 | 8 | 6 | 7 | 6 | 7 | 6 | 6 | 6 | 8 | 7 | 6 | Y | 30256 | ATP synthase subunit gamma OS=Mus musculus OX=10090 GN=Atp5f1c PE=1 SV=1 |
| 382 | 794 | sp\|Q91VR2\|ATPG\_MOUSE | 175.82 | 27 | 20 | 19 | 16 | 20 | 17 | 19 | 17 | 21 | 19 | 17 | 2.84E7 | 4.67E7 | 3.92E7 | 1.94E7 | 2.08E7 | 3.56E7 | 2.75E7 | 1.8E7 | 4.51E7 | 3.38E7 | 8 | 8 | 6 | 7 | 6 | 7 | 6 | 6 | 6 | 8 | 7 | 6 | Y | 32886 | ATP synthase subunit gamma, mitochondrial OS=Mus musculus OX=10090 GN=Atp5f1c PE=1 SV=1 |
| 517 | 1291 | sp\|Q9CQN1\|TRAP1\_MOUSE | 175.60 | 8 | 3 | 6 | 3 | 3 | 3 | 7 | 3 | 3 | 4 | 4 | 1.07E6 | 4.53E6 | 2.52E6 | 3.44E5 | 2.56E6 | 3.67E6 | 1.28E6 | 8.85E5 | 8.69E5 | 6.26E5 | 5 | 4 | 4 | 7 | 5 | 3 | 5 | 5 | 3 | 5 | 5 | 6 | N | 80209 | Heat shock protein 75 kDa, mitochondrial OS=Mus musculus OX=10090 GN=Trap1 PE=1 SV=1 |
| 740 | 957 | sp\|Q9CZ44\|NSF1C\_MOUSE | 175.49 | 21 | 2 | 5 | 7 | 10 | 4 | 9 | 2 | 6 | 12 | 12 | 2.37E6 | 6.1E6 | 8.42E6 | 2.77E6 | 4.49E6 | 9.06E6 | 9.05E5 | 7.63E5 | 1.85E7 | 9.42E6 | 7 | 7 | 1 | 2 | 3 | 4 | 1 | 4 | 1 | 2 | 4 | 4 | N | 40710 | NSFL1 cofactor p47 OS=Mus musculus OX=10090 GN=Nsfl1c PE=1 SV=1 |
| 241 | 472 | sp\|Q6ZWN5\|RS9\_MOUSE | 175.21 | 54 | 21 | 25 | 35 | 35 | 45 | 41 | 42 | 43 | 31 | 36 | 6.92E7 | 1.11E8 | 1.42E8 | 9.37E7 | 1.39E8 | 1.11E8 | 1.24E8 | 1.13E8 | 1.7E8 | 1.31E8 | 15 | 14 | 6 | 7 | 10 | 10 | 12 | 11 | 14 | 11 | 9 | 11 | Y | 22591 | Small ribosomal subunit protein uS4 OS=Mus musculus OX=10090 GN=Rps9 PE=1 SV=3 |
| 779 | 934 | sp\|Q07417\|ACADS\_MOUSE | 174.93 | 24 | 14 | 3 | 8 | 6 | 8 | 11 | 6 | 13 | 14 | 8 | 9.56E6 | 2.11E6 | 2.61E6 | 5.26E5 | 2.92E6 | 8.04E6 | 2.06E6 | 4.49E6 | 1.71E7 | 5.71E6 | 6 | 6 | 4 | 1 | 2 | 2 | 2 | 3 | 2 | 3 | 4 | 2 | Y | 44890 | Short-chain specific acyl-CoA dehydrogenase, mitochondrial OS=Mus musculus OX=10090 GN=Acads PE=1 SV=2 |
| 774 | 569 | A0A0J9YUS5\|A0A0J9YUS5\_MOUSE | 174.89 | 7 | 2 | 3 | 1 | 2 | 2 | 2 | 2 | 2 | 3 | 1 | 2.48E6 | 1.76E7 | 3.45E6 | 1.6E6 | 4.19E6 | 4.38E6 | 9.52E5 | 4.77E5 | 7.85E6 | 3.87E6 | 9 | 9 | 2 | 4 | 2 | 3 | 2 | 3 | 2 | 2 | 3 | 1 | Y | 145312 | Eukaryotic translation initiation factor 4, gamma 1 OS=Mus musculus OX=10090 GN=Eif4g1 PE=1 SV=1 |
| 774 | 583 | E9PVC6\|E9PVC6\_MOUSE | 174.89 | 6 | 2 | 2 | 1 | 2 | 1 | 2 | 1 | 1 | 2 | 1 | 2.48E6 | 1.76E7 | 3.45E6 | 1.6E6 | 4.19E6 | 4.38E6 | 9.52E5 | 4.77E5 | 7.85E6 | 3.87E6 | 9 | 9 | 2 | 4 | 2 | 3 | 2 | 3 | 2 | 2 | 3 | 1 | Y | 171283 | Eukaryotic translation initiation factor 4, gamma 1 OS=Mus musculus OX=10090 GN=Eif4g1 PE=1 SV=1 |
| 774 | 585 | E9Q9E1\|E9Q9E1\_MOUSE | 174.89 | 6 | 2 | 2 | 1 | 2 | 1 | 2 | 1 | 1 | 2 | 1 | 2.48E6 | 1.76E7 | 3.45E6 | 1.6E6 | 4.19E6 | 4.38E6 | 9.52E5 | 4.77E5 | 7.85E6 | 3.87E6 | 9 | 9 | 2 | 4 | 2 | 3 | 2 | 3 | 2 | 2 | 3 | 1 | Y | 175330 | Eukaryotic translation initiation factor 4, gamma 1 OS=Mus musculus OX=10090 GN=Eif4g1 PE=1 SV=1 |
| 774 | 586 | sp\|Q6NZJ6\|IF4G1\_MOUSE | 174.89 | 6 | 2 | 2 | 1 | 2 | 1 | 2 | 1 | 1 | 2 | 1 | 2.48E6 | 1.76E7 | 3.45E6 | 1.6E6 | 4.19E6 | 4.38E6 | 9.52E5 | 4.77E5 | 7.85E6 | 3.87E6 | 9 | 9 | 2 | 4 | 2 | 3 | 2 | 3 | 2 | 2 | 3 | 1 | Y | 176076 | Eukaryotic translation initiation factor 4 gamma 1 OS=Mus musculus OX=10090 GN=Eif4g1 PE=1 SV=1 |
| 626 | 1074 | sp\|P07310\|KCRM\_MOUSE | 174.72 | 16 | 10 | 8 | 9 | 12 | 10 | 9 | 9 | 10 | 4 | 8 | 6.51E6 | 9.57E6 | 6.73E6 | 6.99E6 | 6.48E6 | 1.13E7 | 5.36E6 | 6.36E6 | 3.53E6 | 5.75E6 | 6 | 5 | 4 | 3 | 4 | 5 | 4 | 3 | 4 | 4 | 2 | 3 | N | 43045 | Creatine kinase M-type OS=Mus musculus OX=10090 GN=Ckm PE=1 SV=1 |
| 555 | 720 | sp\|P45952\|ACADM\_MOUSE | 174.70 | 19 | 5 | 12 | 14 | 15 | 15 | 16 | 8 | 7 | 12 | 7 | 6.19E6 | 1.17E7 | 1.83E7 | 2.74E6 | 1.52E7 | 1.85E7 | 5.12E6 | 2.46E6 | 1.92E7 | 8.9E6 | 8 | 7 | 3 | 4 | 5 | 6 | 6 | 7 | 3 | 3 | 4 | 3 | N | 46481 | Medium-chain specific acyl-CoA dehydrogenase, mitochondrial OS=Mus musculus OX=10090 GN=Acadm PE=1 SV=1 |
| 251 | 594 | sp\|P62270\|RS18\_MOUSE | 174.65 | 53 | 40 | 40 | 38 | 41 | 43 | 49 | 41 | 35 | 34 | 39 | 1.72E8 | 1.4E8 | 1.73E8 | 9.33E7 | 1.36E8 | 1.57E8 | 1.29E8 | 9.6E7 | 1.21E8 | 1.62E8 | 11 | 11 | 9 | 9 | 9 | 10 | 11 | 13 | 11 | 10 | 9 | 10 | Y | 17719 | Small ribosomal subunit protein uS13 OS=Mus musculus OX=10090 GN=Rps18 PE=1 SV=3 |
| 664 | 706 | A2AMW0\|A2AMW0\_MOUSE | 174.60 | 30 | 9 | 16 | 16 | 8 | 7 | 21 | 5 | 10 | 10 | 13 | 1.62E6 | 1.72E7 | 2.09E7 | 9.97E6 | 1.51E7 | 2.4E7 | 4.37E6 | 5E6 | 3.78E7 | 1.2E7 | 9 | 9 | 2 | 4 | 4 | 2 | 2 | 5 | 1 | 2 | 4 | 4 | Y | 29295 | F-actin-capping protein subunit beta OS=Mus musculus OX=10090 GN=Capzb PE=1 SV=1 |
| 672 | 2426 | sp\|Q9CQX2\|CYB5B\_MOUSE | 174.42 | 36 | 36 | 36 | 36 | 32 | 36 | 32 | 32 | 32 | 32 | 36 | 7.12E7 | 6.19E7 | 7.26E7 | 1.13E7 | 2.67E7 | 5.66E7 | 1.82E7 | 1.49E7 | 5.49E7 | 4.7E7 | 3 | 3 | 4 | 3 | 4 | 4 | 4 | 3 | 2 | 3 | 2 | 4 | N | 16318 | Cytochrome b5 type B OS=Mus musculus OX=10090 GN=Cyb5b PE=1 SV=1 |
| 197 | 1437 | sp\|Q8VED5\|K2C79\_MOUSE | 174.18 | 8 | 5 | 7 | 8 | 7 | 7 | 8 | 8 | 8 | 3 | 5 |  | 9.49E5 | 1.12E6 | 9.57E5 | 7.32E5 | 2.97E6 | 1.24E6 | 2.73E6 |  |  | 6 | 1 | 13 | 13 | 13 | 10 | 14 | 15 | 13 | 18 | 15 | 12 | N | 57552 | Keratin, type II cytoskeletal 79 OS=Mus musculus OX=10090 GN=Krt79 PE=1 SV=2 |
| 578 | 608 | Q8C845\|Q8C845\_MOUSE | 174.03 | 37 | 11 | 18 | 4 | 13 | 10 | 15 | 20 | 22 | 21 | 19 | 8.43E6 | 1.32E7 | 4.44E6 | 4.85E6 | 8.44E6 | 1.35E7 | 6.32E6 | 6.97E6 | 1.81E7 | 1.06E7 | 9 | 9 | 2 | 5 | 1 | 3 | 3 | 4 | 4 | 5 | 4 | 4 | N | 26800 | EF hand domain containing 2 OS=Mus musculus OX=10090 GN=Efhd2 PE=1 SV=1 |
| 455 | 330 | sp\|Q9JLJ2\|AL9A1\_MOUSE | 173.97 | 30 | 4 | 18 | 10 | 13 | 12 | 11 | 13 | 13 | 21 | 7 | 1.04E7 | 5.92E7 | 5.68E7 | 2.57E7 | 3.86E7 | 4.92E7 | 4.59E7 | 2.86E7 | 2.5E7 | 1.07E7 | 13 | 13 | 2 | 8 | 5 | 6 | 5 | 5 | 7 | 6 | 8 | 3 | Y | 53515 | 4-trimethylaminobutyraldehyde dehydrogenase OS=Mus musculus OX=10090 GN=Aldh9a1 PE=1 SV=1 |
| 366 | 618 | sp\|Q9Z204\|HNRPC\_MOUSE | 173.66 | 27 | 10 | 16 | 24 | 12 | 17 | 19 | 12 | 9 | 10 | 18 | 3.2E7 | 3.28E7 | 5.47E7 | 6.77E6 | 4.49E7 | 3.57E7 | 2.48E7 | 2.01E7 | 3.01E7 | 4.37E7 | 10 | 10 | 6 | 6 | 10 | 5 | 8 | 7 | 5 | 5 | 4 | 8 | Y | 34385 | Heterogeneous nuclear ribonucleoproteins C1/C2 OS=Mus musculus OX=10090 GN=Hnrnpc PE=1 SV=1 |
| 308 | 1450 | sp\|P00405\|COX2\_MOUSE | 173.27 | 26 | 16 | 16 | 23 | 19 | 26 | 19 | 19 | 19 | 23 | 13 | 9.02E7 | 9.3E7 | 6.11E7 | 5.57E7 | 1.31E8 | 1.54E8 | 9.39E7 | 9.66E7 | 1.6E8 | 4.47E7 | 5 | 5 | 10 | 9 | 8 | 7 | 9 | 11 | 8 | 7 | 11 | 6 | Y | 25976 | Cytochrome c oxidase subunit 2 OS=Mus musculus OX=10090 GN=Mtco2 PE=1 SV=1 |
| 617 | 922 | sp\|P08122\|CO4A2\_MOUSE | 172.86 | 6 | 4 | 1 | 1 | 1 | 1 | 1 | 1 | 1 | 4 | 2 | 2.74E7 | 2.04E7 | 2.59E7 | 9.99E6 | 1.27E7 | 1.81E7 | 1.19E7 | 1.13E7 | 3.78E7 | 1.29E7 | 7 | 6 | 4 | 3 | 4 | 3 | 2 | 3 | 3 | 3 | 6 | 4 | Y | 167324 | Collagen alpha-2(IV) chain OS=Mus musculus OX=10090 GN=Col4a2 PE=1 SV=4 |
| 630 | 1490 | sp\|P04117\|FABP4\_MOUSE | 172.55 | 43 | 42 | 32 | 26 | 32 | 16 | 27 | 31 | 16 | 14 | 32 | 9.53E7 | 1.07E8 | 2.97E7 | 3.06E7 | 1.06E7 | 4.82E7 | 3.24E7 | 1.62E7 | 3.04E7 | 4.79E7 | 6 | 6 | 6 | 6 | 3 | 5 | 2 | 4 | 4 | 3 | 2 | 4 | Y | 14650 | Fatty acid-binding protein, adipocyte OS=Mus musculus OX=10090 GN=Fabp4 PE=1 SV=3 |
| 338 | 801 | sp\|Q64433\|CH10\_MOUSE | 172.47 | 73 | 29 | 29 | 43 | 64 | 62 | 67 | 67 | 53 | 39 | 39 | 4.54E7 | 5.53E7 | 7.78E7 | 4.79E7 | 7.54E7 | 9.25E7 | 7.18E7 | 6.19E7 | 1.06E8 | 6.39E7 | 9 | 8 | 7 | 7 | 7 | 7 | 10 | 9 | 8 | 9 | 8 | 7 | Y | 10963 | 10 kDa heat shock protein, mitochondrial OS=Mus musculus OX=10090 GN=Hspe1 PE=1 SV=2 |
| 724 | 1130 | Q6XLQ8\|Q6XLQ8\_MOUSE | 172.38 | 21 | 13 | 7 | 7 | 11 | 15 | 9 | 3 | 5 | 3 | 10 | 2.96E7 | 1.04E7 | 1.01E7 | 4.69E6 | 1.14E7 | 1.89E7 | 6.49E6 | 9.12E6 | 1.35E7 | 1.76E7 | 5 | 5 | 5 | 2 | 3 | 2 | 4 | 4 | 2 | 3 | 2 | 3 | N | 37118 | Calumenin OS=Mus musculus OX=10090 GN=Calu PE=1 SV=1 |
| 724 | 1157 | sp\|O35887\|CALU\_MOUSE | 172.38 | 21 | 13 | 7 | 7 | 11 | 15 | 9 | 3 | 5 | 3 | 10 | 2.96E7 | 1.04E7 | 1.01E7 | 4.69E6 | 1.14E7 | 1.89E7 | 6.49E6 | 9.12E6 | 1.35E7 | 1.76E7 | 5 | 5 | 5 | 2 | 3 | 2 | 4 | 4 | 2 | 3 | 2 | 3 | N | 37064 | Calumenin OS=Mus musculus OX=10090 GN=Calu PE=1 SV=1 |
| 546 | 698 | sp\|P55258\|RAB8A\_MOUSE | 171.71 | 40 | 14 | 31 | 21 | 24 | 24 | 23 | 21 | 15 | 19 | 14 | 7.59E5 | 7.73E6 | 6.02E5 | 2.73E5 | 1.74E6 | 5.47E6 | 2.96E6 | 9.02E5 | 2.71E6 | 2.33E6 | 9 | 4 | 3 | 6 | 4 | 5 | 6 | 5 | 4 | 3 | 3 | 3 | Y | 23668 | Ras-related protein Rab-8A OS=Mus musculus OX=10090 GN=Rab8a PE=1 SV=2 |
| 666 | 1069 | Q8R1A8\|Q8R1A8\_MOUSE | 171.60 | 20 | 8 | 10 | 12 | 16 | 8 | 16 | 16 | 3 | 3 | 2 | 1.23E7 | 1.98E7 | 1.76E7 | 1.73E7 | 1.06E7 | 1.87E7 | 1.99E7 | 4.69E6 | 5.53E6 | 3.35E6 | 6 | 6 | 3 | 3 | 4 | 5 | 3 | 5 | 5 | 2 | 1 | 1 | Y | 39365 | Ornithine transcarbamylase, mitochondrial OS=Mus musculus OX=10090 GN=Otc PE=1 SV=1 |
| 666 | 1070 | sp\|P11725\|OTC\_MOUSE | 171.60 | 19 | 8 | 10 | 12 | 16 | 8 | 16 | 16 | 3 | 3 | 2 | 1.23E7 | 1.98E7 | 1.76E7 | 1.73E7 | 1.06E7 | 1.87E7 | 1.99E7 | 4.69E6 | 5.53E6 | 3.35E6 | 6 | 6 | 3 | 3 | 4 | 5 | 3 | 5 | 5 | 2 | 1 | 1 | Y | 39765 | Ornithine transcarbamylase, mitochondrial OS=Mus musculus OX=10090 GN=Otc PE=1 SV=1 |
| 735 | 943 | sp\|Q99PV0\|PRP8\_MOUSE | 171.41 | 4 | 1 | 0 | 2 | 1 | 1 | 0 | 3 | 1 | 1 | 1 | 1.02E6 | 1.83E6 | 2.1E8 | 1.47E6 | 1.6E8 |  | 4.03E6 | 1.17E8 | 9.39E5 | 1.24E8 | 7 | 7 | 1 | 1 | 3 | 2 | 3 | 0 | 4 | 2 | 1 | 2 | Y | 273615 | Pre-mRNA-processing-splicing factor 8 OS=Mus musculus OX=10090 GN=Prpf8 PE=1 SV=2 |
| 370 | 701 | A6ZI47\|A6ZI47\_MOUSE | 171.29 | 19 | 13 | 16 | 11 | 14 | 16 | 16 | 16 | 14 | 12 | 10 |  | 4.53E6 |  |  |  |  |  |  | 6.37E6 |  | 9 | 1 | 8 | 7 | 6 | 5 | 7 | 7 | 8 | 6 | 6 | 6 | N | 39371 | Fructose-bisphosphate aldolase OS=Mus musculus OX=10090 GN=Aldoart2 PE=1 SV=1 |
| 429 | 829 | sp\|P61358\|RL27\_MOUSE | 171.27 | 49 | 39 | 39 | 43 | 44 | 33 | 39 | 39 | 39 | 35 | 35 | 5.77E7 | 4.93E7 | 4.1E7 | 2.48E7 | 3.39E7 | 3.33E7 | 2.79E7 | 3.52E7 | 7.73E7 | 3.51E7 | 8 | 8 | 6 | 6 | 6 | 7 | 4 | 5 | 6 | 7 | 8 | 5 | Y | 15798 | Large ribosomal subunit protein eL27 OS=Mus musculus OX=10090 GN=Rpl27 PE=1 SV=2 |
| 717 | 1350 | sp\|P53994\|RAB2A\_MOUSE | 170.86 | 30 | 11 | 11 | 11 | 17 | 17 | 30 | 19 | 17 | 13 | 24 | 1.71E7 | 1.9E7 | 1.73E7 | 9.36E6 | 1.4E7 | 1.9E7 | 5.64E6 | 8.76E6 | 1.33E7 | 1.58E7 | 5 | 3 | 3 | 2 | 2 | 3 | 3 | 5 | 3 | 3 | 3 | 4 | N | 23548 | Ras-related protein Rab-2A OS=Mus musculus OX=10090 GN=Rab2a PE=1 SV=1 |
| 508 | 708 | sp\|Q9D819\|IPYR\_MOUSE | 170.46 | 32 | 17 | 14 | 21 | 25 | 19 | 11 | 20 | 6 | 12 | 9 | 1.28E7 | 2.02E7 | 3.98E7 | 1.04E7 | 2.49E7 | 1.81E7 | 2.38E7 | 1.21E7 | 1.3E7 | 2.13E7 | 8 | 8 | 4 | 4 | 7 | 7 | 6 | 4 | 6 | 2 | 3 | 3 | Y | 32667 | Inorganic pyrophosphatase OS=Mus musculus OX=10090 GN=Ppa1 PE=1 SV=1 |
| 537 | 872 | sp\|P70670\|NACAM\_MOUSE | 170.46 | 4 | 2 | 2 | 2 | 2 | 1 | 2 | 2 | 2 | 2 | 2 | 2.23E7 | 2.91E7 | 1.97E7 | 1.15E7 | 1.52E7 | 2.52E7 | 6.59E6 | 1.37E7 | 2.99E7 | 1.79E7 | 7 | 7 | 3 | 3 | 3 | 4 | 2 | 4 | 4 | 3 | 3 | 3 | Y | 220497 | Nascent polypeptide-associated complex subunit alpha, muscle-specific form OS=Mus musculus OX=10090 GN=Naca PE=1 SV=2 |
| 870 | 879 | sp\|Q91VR5\|DDX1\_MOUSE | 169.75 | 14 | 2 | 4 | 4 | 5 | 2 | 10 | 4 | 6 | 0 | 5 | 5.7E5 | 5.79E6 | 3.25E6 | 1.52E6 | 1.37E6 | 4.91E6 | 4.86E5 | 2.32E6 |  | 1.36E6 | 7 | 7 | 1 | 2 | 2 | 2 | 1 | 4 | 2 | 3 | 0 | 2 | N | 82500 | ATP-dependent RNA helicase DDX1 OS=Mus musculus OX=10090 GN=Ddx1 PE=1 SV=1 |
| 1064 | 2073 | sp\|Q8BK64\|AHSA1\_MOUSE | 169.50 | 13 | 10 | 7 | 7 | 9 | 10 | 7 | 7 | 10 | 9 | 7 | 1.09E7 | 7.82E6 | 6.49E6 | 6.64E6 | 9.23E6 | 9.58E6 | 1.04E7 | 5.75E6 | 1.51E7 | 5.07E6 | 3 | 3 | 2 | 1 | 1 | 2 | 2 | 1 | 1 | 2 | 2 | 1 | N | 38117 | Activator of 90 kDa heat shock protein ATPase homolog 1 OS=Mus musculus OX=10090 GN=Ahsa1 PE=1 SV=2 |
| 290 | 572 | sp\|P19253\|RL13A\_MOUSE | 168.90 | 47 | 29 | 32 | 30 | 31 | 26 | 11 | 33 | 25 | 15 | 23 | 6.25E7 | 8.58E7 | 9.15E7 | 5.63E7 | 7.22E7 | 2.69E7 | 7.23E7 | 3E7 | 5.43E7 | 3.35E7 | 13 | 13 | 9 | 9 | 9 | 10 | 9 | 3 | 10 | 7 | 6 | 7 | Y | 23464 | Large ribosomal subunit protein uL13 OS=Mus musculus OX=10090 GN=Rpl13a PE=1 SV=4 |
| 1097 | 1572 | sp\|P26516\|PSMD7\_MOUSE | 168.70 | 19 | 0 | 0 | 0 | 16 | 3 | 5 | 13 | 13 | 16 | 5 |  |  |  | 2.86E6 | 1.93E6 | 3.66E5 | 1.74E6 | 1.07E6 | 6.15E6 | 9.11E5 | 4 | 4 | 0 | 0 | 0 | 3 | 1 | 1 | 2 | 2 | 3 | 1 | N | 36540 | 26S proteasome non-ATPase regulatory subunit 7 OS=Mus musculus OX=10090 GN=Psmd7 PE=1 SV=2 |
| 836 | 1948 | sp\|Q80XN0\|BDH\_MOUSE | 168.67 | 11 | 8 | 11 | 8 | 0 | 11 | 9 | 6 | 9 | 5 | 11 | 1.64E7 | 1.5E7 | 1.43E7 |  | 1.03E7 | 4.72E6 | 6.06E5 | 4.07E6 | 1.32E6 | 9.34E6 | 3 | 3 | 3 | 4 | 2 | 0 | 3 | 2 | 1 | 2 | 3 | 4 | Y | 38299 | D-beta-hydroxybutyrate dehydrogenase, mitochondrial OS=Mus musculus OX=10090 GN=Bdh1 PE=1 SV=2 |
| 956 | 635 | sp\|Q99KK7\|DPP3\_MOUSE | 168.60 | 14 | 1 | 2 | 0 | 1 | 0 | 4 | 2 | 4 | 1 | 7 | 6.12E5 | 3.69E6 |  | 1.9E6 |  | 1.53E6 | 8.73E4 | 1.52E6 | 1.38E6 | 5.63E6 | 8 | 8 | 1 | 2 | 0 | 2 | 0 | 2 | 1 | 3 | 1 | 4 | Y | 82898 | Dipeptidyl peptidase 3 OS=Mus musculus OX=10090 GN=Dpp3 PE=1 SV=2 |
| 581 | 802 | sp\|P51660\|DHB4\_MOUSE | 168.34 | 12 | 8 | 6 | 6 | 4 | 5 | 9 | 3 | 3 | 7 | 6 | 2.22E7 | 2.64E7 | 1.86E7 | 4.87E6 | 3.81E6 | 1.94E7 | 2.37E6 | 2.11E6 | 1.74E7 | 3.96E7 | 8 | 8 | 5 | 4 | 4 | 3 | 3 | 6 | 2 | 2 | 5 | 4 | N | 79482 | Peroxisomal multifunctional enzyme type 2 OS=Mus musculus OX=10090 GN=Hsd17b4 PE=1 SV=3 |
| 1067 | 1289 | sp\|Q6A0A9\|F120A\_MOUSE | 168.12 | 6 | 0 | 1 | 2 | 4 | 3 | 3 | 2 | 0 | 1 | 1 |  | 9.65E5 | 3.28E5 | 7.59E5 | 5.26E5 | 1.12E6 | 1.68E6 |  | 0E0 | 1.14E6 | 5 | 5 | 0 | 1 | 1 | 3 | 2 | 2 | 3 | 0 | 1 | 1 | N | 121646 | Constitutive coactivator of PPAR-gamma-like protein 1 OS=Mus musculus OX=10090 GN=FAM120A PE=1 SV=2 |
| 135 | 1293 | sp\|Q6IFX2\|K1C42\_MOUSE | 168.09 | 10 | 10 | 10 | 10 | 10 | 10 | 10 | 10 | 10 | 10 | 10 | 1.01E8 | 1.42E8 | 9.7E7 | 5.64E7 | 1.04E8 | 1.12E8 | 1.05E8 | 5.55E7 | 2.17E8 | 1.02E8 | 6 | 1 | 19 | 20 | 19 | 15 | 23 | 16 | 19 | 19 | 28 | 18 | N | 50133 | Keratin, type I cytoskeletal 42 OS=Mus musculus OX=10090 GN=Krt42 PE=1 SV=1 |
| 327 | 1324 | Q921R2\|Q921R2\_MOUSE | 168.09 | 39 | 16 | 25 | 31 | 38 | 39 | 23 | 31 | 32 | 31 | 31 | 7.9E7 | 7.9E7 | 1.33E8 | 3.71E7 | 1.09E8 | 1.04E8 | 7.75E7 | 4.15E7 | 1.98E8 | 1.09E8 | 6 | 6 | 9 | 9 | 8 | 8 | 11 | 5 | 6 | 9 | 9 | 9 | N | 16142 | Small ribosomal subunit protein uS15 OS=Mus musculus OX=10090 GN=Rps13 PE=1 SV=1 |
| 327 | 1325 | sp\|P62301\|RS13\_MOUSE | 168.09 | 36 | 15 | 23 | 29 | 35 | 36 | 21 | 29 | 30 | 29 | 29 | 7.9E7 | 7.9E7 | 1.33E8 | 3.71E7 | 1.09E8 | 1.04E8 | 7.75E7 | 4.15E7 | 1.98E8 | 1.09E8 | 6 | 6 | 9 | 9 | 8 | 8 | 11 | 5 | 6 | 9 | 9 | 9 | N | 17222 | Small ribosomal subunit protein uS15 OS=Mus musculus OX=10090 GN=Rps13 PE=1 SV=2 |
| 509 | 1153 | sp\|P62751\|RL23A\_MOUSE | 167.94 | 37 | 21 | 21 | 26 | 26 | 24 | 24 | 24 | 26 | 33 | 27 | 4.5E7 | 4.13E7 | 3.3E7 | 1.77E7 | 4.36E7 | 3.45E7 | 2.61E7 | 2.49E7 | 9.13E7 | 4.39E7 | 8 | 8 | 5 | 3 | 4 | 5 | 4 | 4 | 4 | 4 | 8 | 7 | Y | 17695 | Large ribosomal subunit protein uL23 OS=Mus musculus OX=10090 GN=Rpl23a PE=1 SV=1 |
| 953 | 1433 | E9Q740\|E9Q740\_MOUSE | 167.58 | 8 | 0 | 2 | 2 | 1 | 7 | 3 | 6 | 0 | 0 | 0 |  | 1.21E6 | 1.51E6 | 1.16E6 | 1.36E6 | 7.02E5 | 1.67E6 |  |  |  | 4 | 4 | 0 | 1 | 1 | 1 | 3 | 1 | 3 | 0 | 0 | 0 | N | 67929 | Signal recognition particle subunit SRP72 OS=Mus musculus OX=10090 GN=Srp72 PE=1 SV=1 |
| 953 | 1439 | F8VQC1\|F8VQC1\_MOUSE | 167.58 | 7 | 0 | 2 | 2 | 1 | 6 | 3 | 6 | 0 | 0 | 0 |  | 1.21E6 | 1.51E6 | 1.16E6 | 1.36E6 | 7.02E5 | 1.67E6 |  |  |  | 4 | 4 | 0 | 1 | 1 | 1 | 3 | 1 | 3 | 0 | 0 | 0 | N | 74657 | Signal recognition particle subunit SRP72 OS=Mus musculus OX=10090 GN=Srp72 PE=1 SV=1 |
| 734 | 1520 | sp\|E9Q7G0\|NUMA1\_MOUSE | 167.26 | 3 | 1 | 1 | 1 | 1 | 0 | 1 | 1 | 0 | 2 | 1 | 2.04E7 | 1.85E6 | 1.58E6 | 9.62E5 | 6.43E6 | 1.48E6 | 1.05E6 |  | 1.73E7 | 8.75E6 | 7 | 7 | 3 | 2 | 1 | 2 | 1 | 1 | 1 | 0 | 3 | 2 | N | 235628 | Nuclear mitotic apparatus protein 1 OS=Mus musculus OX=10090 GN=Numa1 PE=1 SV=1 |
| 690 | 1205 | sp\|Q3UZZ6\|ST1D1\_MOUSE | 167.20 | 20 | 10 | 11 | 8 | 12 | 11 | 16 | 13 | 5 | 8 | 5 | 3.69E6 | 4.01E7 | 4.31E6 | 9.29E6 | 9.64E6 | 1.73E7 | 1.22E7 | 1.07E6 | 1.05E7 | 3.03E5 | 6 | 6 | 3 | 4 | 2 | 4 | 3 | 4 | 4 | 1 | 2 | 1 | N | 35083 | Sulfotransferase 1 family member D1 OS=Mus musculus OX=10090 GN=Sult1d1 PE=1 SV=1 |
| 818 | 1038 | sp\|Q8BG32\|PSD11\_MOUSE | 166.84 | 19 | 14 | 3 | 3 | 6 | 5 | 14 | 14 | 5 | 3 | 14 | 2.67E6 | 6.39E5 | 1.81E6 | 8.1E5 | 9.26E6 | 6.89E6 | 5.63E7 | 6.17E7 | 7.27E6 | 5.47E6 | 6 | 6 | 4 | 1 | 1 | 2 | 2 | 4 | 4 | 2 | 1 | 4 | N | 47437 | 26S proteasome non-ATPase regulatory subunit 11 OS=Mus musculus OX=10090 GN=Psmd11 PE=1 SV=3 |
| 584 | 1100 | sp\|Q9Z2M7\|PMM2\_MOUSE | 166.79 | 27 | 21 | 7 | 17 | 16 | 17 | 17 | 16 | 17 | 13 | 11 | 3.12E7 | 1.81E7 | 2.26E7 | 1.19E7 | 3.12E7 | 1.93E7 | 2.42E7 | 1.09E7 | 3.06E7 | 1.21E7 | 7 | 7 | 6 | 2 | 4 | 4 | 5 | 4 | 4 | 4 | 3 | 3 | N | 27657 | Phosphomannomutase 2 OS=Mus musculus OX=10090 GN=Pmm2 PE=1 SV=1 |
| 531 | 1194 | sp\|P16045\|LEG1\_MOUSE | 166.74 | 51 | 51 | 23 | 23 | 23 | 12 | 19 | 23 | 19 | 51 | 32 | 8.78E7 | 3.95E7 | 2.86E7 | 9.39E6 | 1.59E7 | 2.83E7 | 2.01E7 | 9.8E6 | 1.47E8 | 6.7E7 | 6 | 6 | 9 | 3 | 4 | 4 | 2 | 3 | 3 | 3 | 10 | 5 | Y | 14866 | Galectin-1 OS=Mus musculus OX=10090 GN=Lgals1 PE=1 SV=3 |
| 515 | 867 | sp\|P61027\|RAB10\_MOUSE | 166.59 | 38 | 12 | 20 | 20 | 26 | 29 | 16 | 26 | 20 | 17 | 17 | 1.05E7 | 1.43E7 | 1.09E7 | 7.29E6 | 1.97E7 | 1.55E7 | 1.3E7 | 9.6E6 | 2.18E7 | 1.87E7 | 8 | 5 | 2 | 4 | 4 | 6 | 8 | 3 | 5 | 5 | 4 | 4 | Y | 22541 | Ras-related protein Rab-10 OS=Mus musculus OX=10090 GN=Rab10 PE=1 SV=1 |
| 825 | 886 | A2AFS0\|A2AFS0\_MOUSE | 166.51 | 27 | 12 | 7 | 7 | 7 | 9 | 9 | 7 | 7 | 15 | 2 | 4.57E6 | 2.46E6 | 2.09E6 | 1.8E6 | 2.84E6 | 6.8E5 | 2.9E6 | 1.02E6 | 7.94E6 | 5.2E5 | 7 | 7 | 4 | 2 | 2 | 2 | 3 | 2 | 2 | 2 | 4 | 1 | Y | 40164 | serine--tRNA ligase (Fragment) OS=Mus musculus OX=10090 GN=Sars1 PE=1 SV=1 |
| 825 | 896 | sp\|P26638\|SYSC\_MOUSE | 166.51 | 18 | 9 | 5 | 5 | 5 | 6 | 6 | 4 | 4 | 10 | 2 | 4.57E6 | 2.46E6 | 2.09E6 | 1.8E6 | 2.84E6 | 6.8E5 | 2.9E6 | 1.02E6 | 7.94E6 | 5.2E5 | 7 | 7 | 4 | 2 | 2 | 2 | 3 | 2 | 2 | 2 | 4 | 1 | Y | 58389 | Serine--tRNA ligase, cytoplasmic OS=Mus musculus OX=10090 GN=Sars1 PE=1 SV=3 |
| 825 | 897 | Q8C483\|Q8C483\_MOUSE | 166.51 | 18 | 8 | 4 | 4 | 4 | 6 | 6 | 4 | 4 | 10 | 1 | 4.57E6 | 2.46E6 | 2.09E6 | 1.8E6 | 2.84E6 | 6.8E5 | 2.9E6 | 1.02E6 | 7.94E6 | 5.2E5 | 7 | 7 | 4 | 2 | 2 | 2 | 3 | 2 | 2 | 2 | 4 | 1 | Y | 61167 | serine--tRNA ligase OS=Mus musculus OX=10090 GN=Sars1 PE=1 SV=1 |
| 794 | 887 | sp\|P14685\|PSMD3\_MOUSE | 166.49 | 14 | 0 | 8 | 6 | 6 | 2 | 6 | 5 | 4 | 8 | 7 |  | 5.7E6 | 4.5E6 | 2.53E6 | 8.44E5 | 7.73E6 | 4.74E6 | 1.19E6 | 1.65E7 | 7.06E6 | 6 | 6 | 0 | 3 | 3 | 3 | 1 | 3 | 2 | 1 | 3 | 2 | Y | 60718 | 26S proteasome non-ATPase regulatory subunit 3 OS=Mus musculus OX=10090 GN=Psmd3 PE=1 SV=3 |
| 489 | 962 | sp\|Q61792\|LASP1\_MOUSE | 165.80 | 23 | 14 | 14 | 11 | 8 | 8 | 14 | 19 | 16 | 13 | 11 | 6.93E7 | 5.64E7 | 1.64E7 | 6.36E6 | 2.45E7 | 3.23E7 | 3.61E7 | 1.65E7 | 4E7 | 4.77E7 | 7 | 7 | 6 | 4 | 3 | 2 | 3 | 7 | 6 | 5 | 4 | 5 | Y | 29994 | LIM and SH3 domain protein 1 OS=Mus musculus OX=10090 GN=Lasp1 PE=1 SV=1 |
| 489 | 1014 | A2A6H0\|A2A6H0\_MOUSE | 165.80 | 32 | 20 | 19 | 15 | 11 | 11 | 20 | 27 | 22 | 18 | 16 | 6.93E7 | 5.64E7 | 1.64E7 | 6.36E6 | 2.45E7 | 3.23E7 | 3.61E7 | 1.65E7 | 4E7 | 4.77E7 | 7 | 7 | 6 | 4 | 3 | 2 | 3 | 7 | 6 | 5 | 4 | 5 | Y | 21288 | LIM and SH3 protein 1 (Fragment) OS=Mus musculus OX=10090 GN=Lasp1 PE=1 SV=1 |
| 489 | 1052 | A2A6G9\|A2A6G9\_MOUSE | 165.80 | 47 | 29 | 28 | 22 | 16 | 16 | 29 | 39 | 32 | 26 | 23 | 6.93E7 | 5.64E7 | 1.64E7 | 6.36E6 | 2.45E7 | 3.23E7 | 3.61E7 | 1.65E7 | 4E7 | 4.77E7 | 7 | 7 | 6 | 4 | 3 | 2 | 3 | 7 | 6 | 5 | 4 | 5 | Y | 15153 | LIM and SH3 protein 1 (Fragment) OS=Mus musculus OX=10090 GN=Lasp1 PE=1 SV=1 |
| 644 | 853 | A0AAQ4VMY7\|A0AAQ4VMY7\_MOUSE | 165.69 | 10 | 8 | 8 | 3 | 4 | 7 | 6 | 4 | 6 | 4 | 7 | 6.14E7 | 6.33E7 | 6.5E7 | 3.39E5 | 1.47E7 | 1.67E7 | 8.84E6 | 1.07E7 |  | 5.4E7 | 6 | 4 | 5 | 5 | 2 | 2 | 4 | 3 | 2 | 3 | 2 | 4 | Y | 73191 | Dihydropyrimidinase-like 2 OS=Mus musculus OX=10090 GN=Dpysl2 PE=4 SV=1 |
| 644 | 881 | sp\|O08553\|DPYL2\_MOUSE | 165.69 | 12 | 9 | 9 | 4 | 5 | 9 | 7 | 5 | 7 | 4 | 8 | 6.14E7 | 6.33E7 | 6.5E7 | 3.39E5 | 1.47E7 | 1.67E7 | 8.84E6 | 1.07E7 |  | 5.4E7 | 6 | 4 | 5 | 5 | 2 | 2 | 4 | 3 | 2 | 3 | 2 | 4 | Y | 62278 | Dihydropyrimidinase-related protein 2 OS=Mus musculus OX=10090 GN=Dpysl2 PE=1 SV=2 |
| 977 | 1600 | sp\|P97927\|LAMA4\_MOUSE | 165.69 | 3 | 0 | 1 | 2 | 1 | 0 | 2 | 2 | 2 | 1 | 1 | 2.43E6 | 3.28E6 | 3.71E6 | 2.73E6 | 2.54E6 | 4.81E6 | 3.16E6 | 4.87E6 | 2.63E6 | 1.26E6 | 4 | 4 | 1 | 1 | 2 | 2 | 1 | 2 | 2 | 3 | 1 | 1 | N | 201818 | Laminin subunit alpha-4 OS=Mus musculus OX=10090 GN=Lama4 PE=1 SV=2 |
| 651 | 1578 | A6PWS5\|A6PWS5\_MOUSE | 165.63 | 18 | 6 | 14 | 14 | 18 | 14 | 14 | 14 | 14 | 10 | 10 |  |  |  | 1.58E6 |  |  |  |  |  |  | 5 | 1 | 1 | 5 | 3 | 4 | 5 | 5 | 4 | 3 | 2 | 2 | N | 28060 | Gelsolin (Fragment) OS=Mus musculus OX=10090 GN=Gsn PE=1 SV=1 |
| 520 | 633 | sp\|Q9D0I9\|SYRC\_MOUSE | 165.50 | 13 | 4 | 6 | 10 | 6 | 4 | 5 | 8 | 5 | 4 | 7 | 7.32E6 | 1.24E7 | 1.82E7 | 6.21E6 | 9.4E6 | 6.8E6 | 9.12E6 | 6.23E6 | 6.87E6 | 1.01E7 | 10 | 10 | 3 | 4 | 7 | 4 | 3 | 4 | 6 | 4 | 3 | 5 | Y | 75674 | Arginine--tRNA ligase, cytoplasmic OS=Mus musculus OX=10090 GN=Rars1 PE=1 SV=2 |
| 887 | 1371 | sp\|P21278\|GNA11\_MOUSE | 165.28 | 19 | 3 | 11 | 6 | 6 | 0 | 16 | 4 | 0 | 13 | 3 |  | 9.03E6 | 2.16E6 | 3.17E5 |  | 6.89E6 | 3.49E5 |  | 2.02E7 |  | 5 | 4 | 1 | 3 | 2 | 2 | 0 | 4 | 1 | 0 | 5 | 1 | N | 42024 | Guanine nucleotide-binding protein subunit alpha-11 OS=Mus musculus OX=10090 GN=Gna11 PE=1 SV=1 |
| 884 | 1017 | sp\|Q61001\|LAMA5\_MOUSE | 165.10 | 2 | 0 | 1 | 1 | 0 | 0 | 2 | 1 | 0 | 1 | 1 | 2.09E5 | 1.05E6 | 1.78E6 |  | 3.85E5 | 3.67E6 | 1.2E6 | 3.86E5 | 5.67E6 | 5.66E5 | 6 | 6 | 1 | 2 | 2 | 0 | 1 | 4 | 4 | 1 | 3 | 2 | Y | 404056 | Laminin subunit alpha-5 OS=Mus musculus OX=10090 GN=Lama5 PE=1 SV=4 |
| 554 | 888 | A2A547\|A2A547\_MOUSE | 164.80 | 35 | 15 | 12 | 16 | 11 | 15 | 12 | 11 | 15 | 20 | 19 | 9.27E7 | 1.02E8 | 9.65E7 | 2.9E7 | 5.1E7 | 5.73E7 | 4.1E7 | 5.37E7 | 7.83E7 | 8.97E7 | 8 | 7 | 4 | 4 | 5 | 3 | 4 | 3 | 3 | 5 | 4 | 5 | Y | 23248 | Ribosomal protein L19 OS=Mus musculus OX=10090 GN=Rpl19 PE=1 SV=1 |
| 554 | 889 | sp\|P84099\|RL19\_MOUSE | 164.80 | 35 | 15 | 12 | 16 | 11 | 15 | 12 | 11 | 15 | 20 | 18 | 9.27E7 | 1.02E8 | 9.65E7 | 2.9E7 | 5.1E7 | 5.73E7 | 4.1E7 | 5.37E7 | 7.83E7 | 8.97E7 | 8 | 7 | 4 | 4 | 5 | 3 | 4 | 3 | 3 | 5 | 4 | 5 | Y | 23466 | Large ribosomal subunit protein eL19 OS=Mus musculus OX=10090 GN=Rpl19 PE=1 SV=1 |
| 623 | 878 | Q8VHM5\|Q8VHM5\_MOUSE | 164.50 | 10 | 7 | 5 | 6 | 6 | 6 | 6 | 7 | 7 | 5 | 4 | 7.28E6 | 2.1E6 | 6.07E6 | 2.21E6 | 1.97E6 | 5.57E6 | 1.62E6 | 4.72E6 | 7.93E6 | 3.7E6 | 6 | 3 | 4 | 3 | 3 | 3 | 3 | 3 | 4 | 4 | 3 | 2 | N | 70888 | Heterogeneous nuclear ribonucleoprotein R OS=Mus musculus OX=10090 GN=Hnrnpr PE=1 SV=1 |
| 623 | 903 | F7B5B5\|F7B5B5\_MOUSE | 164.50 | 12 | 9 | 6 | 7 | 7 | 7 | 7 | 9 | 9 | 6 | 5 | 7.28E6 | 2.1E6 | 6.07E6 | 2.21E6 | 1.97E6 | 5.57E6 | 1.62E6 | 4.72E6 | 7.93E6 | 3.7E6 | 6 | 3 | 4 | 3 | 3 | 3 | 3 | 3 | 4 | 4 | 3 | 2 | N | 59626 | Heterogeneous nuclear ribonucleoprotein R OS=Mus musculus OX=10090 GN=Hnrnpr PE=1 SV=1 |
| 899 | 1031 | B1ATI0\|B1ATI0\_MOUSE | 164.47 | 14 | 5 | 7 | 2 | 5 | 7 | 3 | 4 | 6 | 6 | 0 | 2.56E6 | 1.16E6 | 1.15E6 | 1.19E6 | 2.32E6 | 9.52E5 | 1.4E6 | 1.16E6 | 5.06E6 |  | 6 | 6 | 2 | 3 | 1 | 2 | 3 | 1 | 2 | 2 | 2 | 0 | Y | 59277 | Aldehyde dehydrogenase OS=Mus musculus OX=10090 GN=Aldh3a2 PE=1 SV=1 |
| 899 | 1059 | sp\|P47740\|AL3A2\_MOUSE | 164.47 | 15 | 5 | 8 | 2 | 5 | 8 | 3 | 5 | 6 | 7 | 0 | 2.56E6 | 1.16E6 | 1.15E6 | 1.19E6 | 2.32E6 | 9.52E5 | 1.4E6 | 1.16E6 | 5.06E6 |  | 6 | 6 | 2 | 3 | 1 | 2 | 3 | 1 | 2 | 2 | 2 | 0 | Y | 53971 | Aldehyde dehydrogenase family 3 member A2 OS=Mus musculus OX=10090 GN=Aldh3a2 PE=1 SV=2 |
| 899 | 1060 | B1AV77\|B1AV77\_MOUSE | 164.47 | 15 | 5 | 8 | 2 | 5 | 8 | 3 | 5 | 6 | 7 | 0 | 2.56E6 | 1.16E6 | 1.15E6 | 1.19E6 | 2.32E6 | 9.52E5 | 1.4E6 | 1.16E6 | 5.06E6 |  | 6 | 6 | 2 | 3 | 1 | 2 | 3 | 1 | 2 | 2 | 2 | 0 | Y | 54330 | Aldehyde dehydrogenase OS=Mus musculus OX=10090 GN=Aldh3a2 PE=1 SV=1 |
| 467 | 971 | sp\|Q9CR57\|RL14\_MOUSE | 164.37 | 32 | 20 | 19 | 20 | 24 | 21 | 11 | 20 | 17 | 20 | 17 | 7.01E7 | 5.63E7 | 8.24E7 | 4.46E7 | 2.31E7 | 1.15E7 | 6.92E7 | 3.94E7 | 1.01E8 | 5.38E7 | 8 | 8 | 5 | 4 | 4 | 6 | 5 | 2 | 5 | 4 | 6 | 4 | Y | 23564 | Large ribosomal subunit protein eL14 OS=Mus musculus OX=10090 GN=Rpl14 PE=1 SV=3 |
| 358 | 1848 | sp\|P62855\|RS26\_MOUSE | 164.13 | 40 | 34 | 34 | 40 | 40 | 40 | 27 | 40 | 40 | 34 | 34 | 8.13E7 | 1.15E8 | 1.86E8 | 6.95E7 | 1.02E8 | 8.94E7 | 1.16E8 | 7.08E7 | 6.85E7 | 1.19E8 | 4 | 4 | 7 | 6 | 9 | 5 | 9 | 4 | 6 | 7 | 9 | 8 | Y | 13015 | Small ribosomal subunit protein eS26 OS=Mus musculus OX=10090 GN=Rps26 PE=1 SV=3 |
| 909 | 1002 | sp\|Q9CYZ2\|TPD54\_MOUSE | 163.68 | 42 | 12 | 0 | 9 | 9 | 4 | 14 | 10 | 4 | 27 | 4 | 4.09E6 |  | 2.54E6 | 5.08E5 | 2.84E6 | 4.88E6 | 2.45E6 | 1.51E6 | 9.64E6 | 1.11E6 | 7 | 7 | 3 | 0 | 2 | 2 | 1 | 2 | 2 | 1 | 4 | 1 | Y | 24043 | Tumor protein D54 OS=Mus musculus OX=10090 GN=Tpd52l2 PE=1 SV=1 |
| 909 | 1015 | Q3TAI4\|Q3TAI4\_MOUSE | 163.68 | 45 | 13 | 0 | 9 | 10 | 4 | 15 | 11 | 4 | 29 | 4 | 4.09E6 |  | 2.54E6 | 5.08E5 | 2.84E6 | 4.88E6 | 2.45E6 | 1.51E6 | 9.64E6 | 1.11E6 | 7 | 7 | 3 | 0 | 2 | 2 | 1 | 2 | 2 | 1 | 4 | 1 | Y | 22466 | Tumor protein D52-like 2 OS=Mus musculus OX=10090 GN=Tpd52l2 PE=1 SV=1 |
| 909 | 1016 | A2AUD5\|A2AUD5\_MOUSE | 163.68 | 40 | 12 | 0 | 8 | 9 | 4 | 13 | 10 | 4 | 26 | 4 | 4.09E6 |  | 2.54E6 | 5.08E5 | 2.84E6 | 4.88E6 | 2.45E6 | 1.51E6 | 9.64E6 | 1.11E6 | 7 | 7 | 3 | 0 | 2 | 2 | 1 | 2 | 2 | 1 | 4 | 1 | Y | 25067 | Tumor protein D52-like 2 OS=Mus musculus OX=10090 GN=Tpd52l2 PE=1 SV=1 |
| 845 | 684 | sp\|Q61768\|KINH\_MOUSE | 163.68 | 10 | 3 | 2 | 5 | 3 | 1 | 6 | 5 | 0 | 2 | 1 | 9.16E5 | 7.15E5 | 8.39E5 | 1.57E5 |  | 1.2E6 | 1.02E6 |  | 4.5E5 | 4.12E5 | 8 | 3 | 2 | 1 | 3 | 3 | 1 | 4 | 3 | 0 | 2 | 1 | Y | 109551 | Kinesin-1 heavy chain OS=Mus musculus OX=10090 GN=Kif5b PE=1 SV=3 |
| 856 | 1382 | sp\|P63085\|MK01\_MOUSE | 163.47 | 17 | 5 | 9 | 17 | 5 | 3 | 13 | 5 | 3 | 5 | 5 | 7.37E6 | 6.12E6 | 1.24E7 | 4.69E6 | 5.95E6 | 8.67E6 | 6.07E6 | 1.66E6 | 1.71E7 | 1.08E7 | 4 | 3 | 2 | 3 | 4 | 2 | 1 | 3 | 2 | 1 | 2 | 2 | Y | 41276 | Mitogen-activated protein kinase 1 OS=Mus musculus OX=10090 GN=Mapk1 PE=1 SV=3 |
| 874 | 1396 | sp\|P54775\|PRS6B\_MOUSE | 163.29 | 15 | 8 | 6 | 4 | 3 | 9 | 3 | 0 | 9 | 13 | 6 | 4.46E5 | 3.09E6 | 3.12E5 | 5.88E5 | 3.46E6 | 7.97E5 |  | 3.09E6 | 6.3E6 | 2.81E6 | 5 | 5 | 2 | 2 | 1 | 1 | 4 | 1 | 0 | 3 | 4 | 2 | N | 47408 | 26S proteasome regulatory subunit 6B OS=Mus musculus OX=10090 GN=Psmc4 PE=1 SV=2 |
| 874 | 1462 | A0A140LIZ5\|A0A140LIZ5\_MOUSE | 163.29 | 16 | 8 | 7 | 5 | 4 | 9 | 4 | 0 | 9 | 14 | 7 | 4.46E5 | 3.09E6 | 3.12E5 | 5.88E5 | 3.46E6 | 7.97E5 |  | 3.09E6 | 6.3E6 | 2.81E6 | 5 | 5 | 2 | 2 | 1 | 1 | 4 | 1 | 0 | 3 | 4 | 2 | N | 43550 | 26S proteasome regulatory subunit 6B OS=Mus musculus OX=10090 GN=Psmc4 PE=1 SV=1 |
| 826 | 1318 | E9QNA7\|E9QNA7\_MOUSE | 163.12 | 10 | 1 | 7 | 6 | 2 | 3 | 6 | 4 | 2 | 6 | 6 | 0E0 | 7.44E6 | 7.24E6 | 3.97E5 | 3.45E6 | 4.32E6 | 9.85E5 | 7.46E5 | 1.03E7 | 5.64E6 | 5 | 5 | 1 | 4 | 3 | 1 | 2 | 3 | 2 | 1 | 3 | 3 | N | 82875 | Sorbin and SH3 domain containing 1 OS=Mus musculus OX=10090 GN=Sorbs1 PE=1 SV=1 |
| 826 | 1321 | A0A668KL65\|A0A668KL65\_MOUSE | 163.12 | 7 | 1 | 5 | 4 | 2 | 2 | 4 | 3 | 2 | 5 | 4 | 0E0 | 7.44E6 | 7.24E6 | 3.97E5 | 3.45E6 | 4.32E6 | 9.85E5 | 7.46E5 | 1.03E7 | 5.64E6 | 5 | 5 | 1 | 4 | 3 | 1 | 2 | 3 | 2 | 1 | 3 | 3 | N | 111915 | Sorbin and SH3 domain containing 1 (Fragment) OS=Mus musculus OX=10090 GN=Sorbs1 PE=1 SV=1 |
| 1383 | 1719 | A0A075B5R5\|A0A075B5R5\_MOUSE | 163.02 | 53 | 53 | 0 | 0 | 0 | 0 | 0 | 0 | 0 | 34 | 23 | 2.49E7 |  |  |  |  |  |  |  | 8.28E6 | 1.96E6 | 4 | 3 | 4 | 0 | 0 | 0 | 0 | 0 | 0 | 0 | 3 | 2 | Y | 12874 | Immunoglobulin heavy variable 4-1 (Fragment) OS=Mus musculus OX=10090 GN=Ighv4-1 PE=1 SV=7 |
| 583 | 1712 | F6TQW2\|F6TQW2\_MOUSE | 163.01 | 17 | 12 | 7 | 12 | 12 | 12 | 12 | 12 | 12 | 14 | 17 | 9.08E6 | 7.67E5 | 1.36E7 | 3.79E7 | 9.08E6 | 3.79E7 | 3.54E7 | 2.62E7 | 1.28E7 | 2.06E7 | 4 | 4 | 3 | 1 | 4 | 5 | 4 | 5 | 6 | 5 | 3 | 5 | Y | 44214 | Immunoglobulin heavy constant gamma 2C (Fragment) OS=Mus musculus OX=10090 GN=Ighg2c PE=1 SV=2 |
| 583 | 1851 | A0A0A6YY53\|A0A0A6YY53\_MOUSE | 163.01 | 21 | 15 | 8 | 15 | 15 | 15 | 15 | 15 | 15 | 17 | 21 | 9.08E6 | 7.67E5 | 1.36E7 | 3.79E7 | 9.08E6 | 3.79E7 | 3.54E7 | 2.62E7 | 1.28E7 | 2.06E7 | 4 | 4 | 3 | 1 | 4 | 5 | 4 | 5 | 6 | 5 | 3 | 5 | Y | 36527 | Immunoglobulin heavy constant gamma 2C (Fragment) OS=Mus musculus OX=10090 GN=Ighg2c PE=1 SV=1 |
| 707 | 968 | sp\|Q9EQ06\|DHB11\_MOUSE | 163.00 | 27 | 6 | 21 | 3 | 15 | 9 | 9 | 24 | 3 | 21 | 15 | 3.55E6 | 1.56E7 | 3.31E6 | 4.82E6 | 2.26E6 | 4.4E6 | 1.14E7 | 7.71E5 | 2.31E7 | 1.23E7 | 6 | 6 | 2 | 5 | 1 | 4 | 2 | 2 | 5 | 1 | 4 | 3 | Y | 32881 | Estradiol 17-beta-dehydrogenase 11 OS=Mus musculus OX=10090 GN=Hsd17b11 PE=1 SV=1 |
| 460 | 489 | sp\|P24549\|AL1A1\_MOUSE | 162.24 | 28 | 6 | 14 | 17 | 8 | 11 | 17 | 8 | 12 | 10 | 11 | 6.87E6 | 1.93E7 | 1.45E7 | 4.37E6 | 1.49E7 | 2.27E7 | 7.36E6 | 8.83E6 | 1.61E7 | 1.88E7 | 10 | 9 | 3 | 6 | 7 | 4 | 5 | 6 | 4 | 5 | 6 | 5 | Y | 54468 | Aldehyde dehydrogenase 1A1 OS=Mus musculus OX=10090 GN=Aldh1a1 PE=1 SV=5 |
| 757 | 1018 | sp\|Q99NB9\|SF3B1\_MOUSE | 162.04 | 6 | 5 | 2 | 2 | 3 | 3 | 3 | 2 | 2 | 5 | 3 | 4.13E6 | 3.15E6 | 2.76E6 | 2.1E7 | 1.93E6 | 2.57E6 | 1.44E6 | 1.13E6 | 6.07E6 | 4.52E6 | 6 | 6 | 4 | 2 | 2 | 3 | 3 | 3 | 2 | 2 | 5 | 3 | N | 145816 | Splicing factor 3B subunit 1 OS=Mus musculus OX=10090 GN=Sf3b1 PE=1 SV=1 |
| 757 | 1019 | G5E866\|G5E866\_MOUSE | 162.04 | 6 | 5 | 2 | 2 | 3 | 3 | 3 | 2 | 2 | 5 | 3 | 4.13E6 | 3.15E6 | 2.76E6 | 2.1E7 | 1.93E6 | 2.57E6 | 1.44E6 | 1.13E6 | 6.07E6 | 4.52E6 | 6 | 6 | 4 | 2 | 2 | 3 | 3 | 3 | 2 | 2 | 5 | 3 | N | 145830 | Splicing factor 3b, subunit 1 OS=Mus musculus OX=10090 GN=Sf3b1 PE=1 SV=1 |
| 649 | 923 | sp\|Q9CQA3\|SDHB\_MOUSE | 161.93 | 23 | 8 | 6 | 12 | 17 | 9 | 11 | 12 | 5 | 9 | 8 | 7.93E6 | 1.74E7 | 3.33E7 | 1.41E7 | 1.41E7 | 2.65E7 | 6.99E6 | 2.03E6 | 1.44E7 | 6.81E6 | 7 | 7 | 3 | 2 | 4 | 5 | 4 | 4 | 3 | 1 | 2 | 3 | Y | 31814 | Succinate dehydrogenase [ubiquinone] iron-sulfur subunit, mitochondrial OS=Mus musculus OX=10090 GN=Sdhb PE=1 SV=1 |
| 998 | 2317 | Q923L6\|Q923L6\_MOUSE | 161.60 | 25 | 20 | 0 | 0 | 0 | 0 | 0 | 0 | 0 | 20 | 25 | 2.12E7 |  |  |  |  |  |  |  | 6.36E7 | 3.78E7 | 3 | 1 | 5 | 0 | 0 | 0 | 0 | 0 | 0 | 0 | 7 | 4 | Y | 17630 | Eosinophil-associated ribonuclease 10 OS=Mus musculus OX=10090 GN=Ear10 PE=3 SV=1 |
| 847 | 1041 | sp\|Q91VM5\|RMXL1\_MOUSE | 161.50 | 16 | 9 | 12 | 9 | 4 | 9 | 7 | 6 | 4 | 9 | 0 | 1.39E7 | 9E6 | 8.18E6 | 2.59E6 | 9.66E6 | 8.63E6 | 4.49E6 | 1.24E6 | 1.47E7 |  | 6 | 6 | 3 | 4 | 3 | 1 | 3 | 2 | 2 | 1 | 3 | 0 | N | 42162 | RNA binding motif protein, X-linked-like-1 OS=Mus musculus OX=10090 GN=Rbmxl1 PE=2 SV=1 |
| 847 | 1042 | A0A2I3BRL8\|A0A2I3BRL8\_MOUSE | 161.50 | 16 | 9 | 12 | 9 | 4 | 9 | 7 | 6 | 4 | 9 | 0 | 1.39E7 | 9E6 | 8.18E6 | 2.59E6 | 9.66E6 | 8.63E6 | 4.49E6 | 1.24E6 | 1.47E7 |  | 6 | 6 | 3 | 4 | 3 | 1 | 3 | 2 | 2 | 1 | 3 | 0 | N | 42179 | Predicted gene 7324 OS=Mus musculus OX=10090 GN=Gm7324 PE=4 SV=1 |
| 709 | 1075 | A0A0A6YWR2\|A0A0A6YWR2\_MOUSE | 161.37 | 22 | 13 | 0 | 0 | 0 | 0 | 10 | 8 | 3 | 20 | 13 | 8.38E7 |  |  |  |  | 5.19E6 | 9.65E6 | 1.16E7 | 8.18E7 | 8.67E7 | 6 | 6 | 7 | 0 | 0 | 0 | 0 | 3 | 3 | 1 | 10 | 7 | Y | 43434 | Immunoglobulin heavy constant gamma 1 (G1m marker) (Fragment) OS=Mus musculus OX=10090 GN=Ighg1 PE=1 SV=1 |
| 709 | 1127 | A0A075B5P4\|A0A075B5P4\_MOUSE | 161.37 | 27 | 15 | 0 | 0 | 0 | 0 | 12 | 10 | 4 | 24 | 15 | 8.38E7 |  |  |  |  | 5.19E6 | 9.65E6 | 1.16E7 | 8.18E7 | 8.67E7 | 6 | 6 | 7 | 0 | 0 | 0 | 0 | 3 | 3 | 1 | 10 | 7 | Y | 35752 | Immunoglobulin heavy constant gamma 1 (G1m marker) (Fragment) OS=Mus musculus OX=10090 GN=Ighg1 PE=1 SV=1 |
| 758 | 443 | sp\|Q9D0R2\|SYTC\_MOUSE | 161.05 | 15 | 10 | 2 | 5 | 4 | 4 | 3 | 5 | 2 | 6 | 3 | 6.62E6 | 6.82E5 | 2.16E6 | 1.32E6 | 4.93E6 | 2.11E6 | 6.49E6 | 7.09E5 | 7.88E6 | 1.55E6 | 9 | 9 | 5 | 1 | 3 | 3 | 3 | 2 | 3 | 1 | 3 | 1 | Y | 83356 | Threonine--tRNA ligase 1, cytoplasmic OS=Mus musculus OX=10090 GN=Tars1 PE=1 SV=2 |
| 868 | 1270 | sp\|Q62000\|MIME\_MOUSE | 160.93 | 20 | 0 | 3 | 14 | 8 | 11 | 18 | 8 | 6 | 0 | 0 |  | 4.3E6 | 1.22E7 | 4.88E6 | 4.88E6 | 2.15E7 | 8.45E6 | 7.13E6 |  |  | 6 | 5 | 0 | 1 | 4 | 3 | 3 | 5 | 3 | 2 | 0 | 0 | Y | 34012 | Mimecan OS=Mus musculus OX=10090 GN=Ogn PE=1 SV=1 |
| 669 | 1480 | sp\|O35955\|PSB10\_MOUSE | 160.77 | 23 | 7 | 11 | 11 | 23 | 19 | 15 | 15 | 15 | 15 | 11 | 2.17E6 | 3.71E6 | 4.56E6 | 1.02E7 | 1.94E7 | 1.66E7 | 9.22E6 | 7.31E6 | 1.14E7 | 3.59E6 | 5 | 5 | 1 | 3 | 2 | 5 | 5 | 3 | 4 | 4 | 4 | 2 | N | 29063 | Proteasome subunit beta type-10 OS=Mus musculus OX=10090 GN=Psmb10 PE=1 SV=1 |
| 1056 | 1898 | sp\|Q9CQE8\|RTRAF\_MOUSE | 160.72 | 18 | 8 | 4 | 7 | 0 | 3 | 0 | 7 | 3 | 4 | 4 | 2.39E6 | 1.52E6 | 1.44E6 |  | 5.96E6 |  | 6.74E6 | 1.71E6 | 2.33E6 | 8.28E6 | 4 | 4 | 1 | 1 | 2 | 0 | 1 | 0 | 2 | 1 | 1 | 1 | N | 28152 | RNA transcription, translation and transport factor protein OS=Mus musculus OX=10090 GN=RTRAF PE=1 SV=1 |
| 850 | 1135 | sp\|Q99KC8\|VMA5A\_MOUSE | 160.48 | 9 | 3 | 4 | 3 | 1 | 0 | 3 | 5 | 3 | 6 | 4 | 1.85E7 | 2.87E6 | 1.16E6 | 2.64E6 |  | 6.01E6 | 2.54E6 | 4.57E7 | 8.06E6 | 1.39E7 | 6 | 6 | 2 | 3 | 2 | 1 | 0 | 2 | 3 | 2 | 4 | 3 | Y | 87143 | von Willebrand factor A domain-containing protein 5A OS=Mus musculus OX=10090 GN=Vwa5a PE=1 SV=2 |
| 986 | 1386 | sp\|Q91V76\|CK054\_MOUSE | 160.39 | 20 | 5 | 5 | 7 | 9 | 7 | 20 | 0 | 5 | 5 | 5 | 4.78E5 | 5.22E6 | 4.53E6 | 2.78E6 | 3.67E6 | 1.44E7 |  | 5.78E5 | 1.96E6 | 8.9E5 | 5 | 5 | 1 | 1 | 2 | 2 | 2 | 6 | 0 | 1 | 1 | 1 | Y | 34996 | Ester hydrolase C11orf54 homolog OS=Mus musculus OX=10090 PE=1 SV=1 |
| 986 | 1442 | A0A1L1SSF8\|A0A1L1SSF8\_MOUSE | 160.39 | 23 | 6 | 6 | 8 | 10 | 8 | 23 | 0 | 6 | 6 | 6 | 4.78E5 | 5.22E6 | 4.53E6 | 2.78E6 | 3.67E6 | 1.44E7 |  | 5.78E5 | 1.96E6 | 8.9E5 | 5 | 5 | 1 | 1 | 2 | 2 | 2 | 6 | 0 | 1 | 1 | 1 | Y | 31335 | RIKEN cDNA 4931406C07 gene OS=Mus musculus OX=10090 GN=4931406C07Rik PE=1 SV=1 |
| 937 | 739 | sp\|Q61292\|LAMB2\_MOUSE | 160.30 | 5 | 0 | 1 | 1 | 2 | 2 | 2 | 0 | 2 | 0 | 0 |  | 2.22E6 | 2.64E6 | 2.89E6 | 1.86E6 | 3.25E6 |  | 2.26E6 |  |  | 8 | 8 | 0 | 2 | 2 | 5 | 3 | 3 | 0 | 3 | 0 | 0 | Y | 196578 | Laminin subunit beta-2 OS=Mus musculus OX=10090 GN=Lamb2 PE=1 SV=2 |
| 513 | 671 | Q3T9X3\|Q3T9X3\_MOUSE | 160.23 | 12 | 4 | 8 | 2 | 6 | 3 | 6 | 7 | 3 | 6 | 5 | 2.67E7 | 3.29E7 | 2.98E6 | 2.98E6 | 4.38E6 | 7.58E6 | 4.81E6 | 3.12E6 | 1.39E7 | 3.12E7 | 10 | 10 | 4 | 6 | 2 | 4 | 3 | 5 | 5 | 3 | 5 | 5 | N | 97195 | dynamin GTPase OS=Mus musculus OX=10090 GN=Dnm2 PE=1 SV=1 |
| 513 | 673 | sp\|P39054\|DYN2\_MOUSE | 160.23 | 11 | 4 | 8 | 2 | 6 | 3 | 6 | 7 | 3 | 6 | 5 | 2.67E7 | 3.29E7 | 2.98E6 | 2.98E6 | 4.38E6 | 7.58E6 | 4.81E6 | 3.12E6 | 1.39E7 | 3.12E7 | 10 | 10 | 4 | 6 | 2 | 4 | 3 | 5 | 5 | 3 | 5 | 5 | N | 98145 | Dynamin-2 OS=Mus musculus OX=10090 GN=Dnm2 PE=1 SV=2 |
| 705 | 1586 | Q80ZP8\|Q80ZP8\_MOUSE | 160.21 | 27 | 10 | 15 | 24 | 15 | 19 | 9 | 9 | 15 | 10 | 10 | 1.26E7 | 1.76E7 | 3.62E7 | 6.89E6 | 3.31E7 | 7.21E6 | 8.26E6 | 1.16E7 | 2.07E7 | 4.54E6 | 5 | 5 | 2 | 3 | 4 | 3 | 4 | 1 | 1 | 3 | 2 | 1 | Y | 19012 | Armet protein OS=Mus musculus OX=10090 GN=Manf PE=1 SV=1 |
| 705 | 1587 | sp\|Q9CXI5\|MANF\_MOUSE | 160.21 | 25 | 9 | 14 | 22 | 14 | 17 | 8 | 8 | 14 | 9 | 9 | 1.26E7 | 1.76E7 | 3.62E7 | 6.89E6 | 3.31E7 | 7.21E6 | 8.26E6 | 1.16E7 | 2.07E7 | 4.54E6 | 5 | 5 | 2 | 3 | 4 | 3 | 4 | 1 | 1 | 3 | 2 | 1 | Y | 20388 | Mesencephalic astrocyte-derived neurotrophic factor OS=Mus musculus OX=10090 GN=Manf PE=1 SV=2 |
| 780 | 1086 | sp\|Q62418\|DBNL\_MOUSE | 160.13 | 14 | 11 | 5 | 5 | 5 | 10 | 8 | 8 | 5 | 8 | 3 | 1.49E7 | 3.3E6 | 1.85E6 | 1.12E6 | 5.09E6 | 3.23E6 | 1.13E6 | 4.8E5 | 2.01E7 | 8.24E5 | 5 | 5 | 4 | 2 | 1 | 1 | 3 | 2 | 2 | 1 | 3 | 1 | Y | 48700 | Drebrin-like protein OS=Mus musculus OX=10090 GN=Dbnl PE=1 SV=2 |
| 906 | 1773 | sp\|P97823\|LYPA1\_MOUSE | 159.98 | 19 | 6 | 6 | 17 | 12 | 12 | 12 | 14 | 12 | 10 | 10 | 2.22E6 | 7.5E6 | 6.44E7 | 7.49E6 | 1.57E7 | 1.75E7 | 1.31E7 | 1.15E7 | 9.54E7 | 4.86E7 | 4 | 3 | 1 | 1 | 3 | 2 | 2 | 2 | 3 | 2 | 2 | 2 | Y | 24688 | Acyl-protein thioesterase 1 OS=Mus musculus OX=10090 GN=Lypla1 PE=1 SV=1 |
| 738 | 907 | sp\|P05201\|AATC\_MOUSE | 159.90 | 21 | 10 | 5 | 10 | 7 | 8 | 13 | 9 | 5 | 8 | 9 | 5.24E6 | 2.64E6 | 6.16E6 | 2.46E6 | 2.8E6 | 5.63E6 | 3.32E6 | 1.25E6 | 9.92E6 | 4.62E6 | 7 | 7 | 3 | 2 | 3 | 2 | 2 | 4 | 4 | 1 | 3 | 3 | N | 46248 | Aspartate aminotransferase, cytoplasmic OS=Mus musculus OX=10090 GN=Got1 PE=1 SV=3 |
| 573 | 1839 | sp\|P84084\|ARF5\_MOUSE | 159.87 | 29 | 29 | 29 | 23 | 23 | 23 | 23 | 23 | 23 | 23 | 23 | 1.78E7 | 1.82E7 | 7.7E6 | 3.78E6 | 7.27E6 | 6.24E6 | 3.43E6 | 2.14E6 |  | 1.37E7 | 4 | 1 | 5 | 6 | 4 | 4 | 4 | 4 | 4 | 4 | 4 | 4 | Y | 20530 | ADP-ribosylation factor 5 OS=Mus musculus OX=10090 GN=Arf5 PE=1 SV=2 |
| 761 | 1098 | sp\|Q8K310\|MATR3\_MOUSE | 159.86 | 11 | 6 | 6 | 5 | 5 | 5 | 8 | 4 | 2 | 8 | 4 | 5.67E6 | 5.28E6 | 8.14E6 | 1.2E6 | 2.74E6 | 6.99E6 | 2.29E6 | 1.47E6 | 9.08E6 | 4.26E6 | 6 | 6 | 3 | 3 | 3 | 3 | 3 | 4 | 2 | 1 | 4 | 2 | N | 94630 | Matrin-3 OS=Mus musculus OX=10090 GN=Matr3 PE=1 SV=1 |
| 808 | 1179 | sp\|Q61301\|CTNA2\_MOUSE | 159.77 | 7 | 1 | 5 | 1 | 1 | 3 | 2 | 2 | 1 | 3 | 3 |  | 2.61E6 |  |  |  |  | 1.27E7 |  |  | 2.22E7 | 5 | 2 | 1 | 3 | 1 | 1 | 3 | 2 | 2 | 1 | 2 | 3 | Y | 105286 | Catenin alpha-2 OS=Mus musculus OX=10090 GN=Ctnna2 PE=1 SV=3 |
| 808 | 1180 | E0CXB9\|E0CXB9\_MOUSE | 159.77 | 6 | 1 | 5 | 1 | 1 | 3 | 2 | 2 | 1 | 3 | 3 |  | 2.61E6 |  |  |  |  | 1.27E7 |  |  | 2.22E7 | 5 | 2 | 1 | 3 | 1 | 1 | 3 | 2 | 2 | 1 | 2 | 3 | Y | 106730 | Catenin alpha-2 OS=Mus musculus OX=10090 GN=Ctnna2 PE=1 SV=1 |
| 516 | 1832 | A2BE93\|A2BE93\_MOUSE | 159.69 | 22 | 22 | 22 | 22 | 22 | 22 | 22 | 22 | 22 | 22 | 22 | 2.88E7 | 3.38E7 | 3.67E7 | 1.67E7 | 2.85E7 | 3.01E7 | 2.2E7 | 2.19E7 | 4.68E7 | 3.24E7 | 4 | 4 | 5 | 6 | 5 | 4 | 5 | 4 | 5 | 4 | 6 | 4 | N | 24924 | SET nuclear oncogene (Fragment) OS=Mus musculus OX=10090 GN=Set PE=1 SV=1 |
| 516 | 1835 | sp\|Q9EQU5\|SET\_MOUSE | 159.69 | 17 | 17 | 17 | 17 | 17 | 17 | 17 | 17 | 17 | 17 | 17 | 2.88E7 | 3.38E7 | 3.67E7 | 1.67E7 | 2.85E7 | 3.01E7 | 2.2E7 | 2.19E7 | 4.68E7 | 3.24E7 | 4 | 4 | 5 | 6 | 5 | 4 | 5 | 4 | 5 | 4 | 6 | 4 | N | 33378 | Protein SET OS=Mus musculus OX=10090 GN=Set PE=1 SV=1 |
| 434 | 1483 | sp\|P59999\|ARPC4\_MOUSE | 159.52 | 28 | 23 | 28 | 23 | 23 | 28 | 28 | 28 | 23 | 23 | 28 | 5.12E7 | 6.81E7 | 3.83E7 | 2.16E7 | 4.28E7 | 3.9E7 | 2.89E7 | 1.86E7 | 9.24E7 | 6.18E7 | 5 | 5 | 5 | 7 | 5 | 5 | 7 | 7 | 8 | 6 | 5 | 7 | N | 19667 | Actin-related protein 2/3 complex subunit 4 OS=Mus musculus OX=10090 GN=Arpc4 PE=1 SV=3 |
| 444 | 1513 | sp\|P12787\|COX5A\_MOUSE | 159.48 | 36 | 36 | 36 | 32 | 21 | 36 | 22 | 21 | 21 | 36 | 32 | 6.44E7 | 8.09E7 | 7.54E7 | 1.69E7 | 6.17E7 | 6.48E7 | 4.91E7 | 4.77E7 | 1.06E8 | 6.22E7 | 5 | 4 | 7 | 6 | 7 | 5 | 6 | 4 | 4 | 6 | 7 | 6 | Y | 16101 | Cytochrome c oxidase subunit 5A, mitochondrial OS=Mus musculus OX=10090 GN=Cox5a PE=1 SV=2 |
| 710 | 1384 | sp\|Q60972\|RBBP4\_MOUSE | 158.98 | 12 | 7 | 5 | 7 | 12 | 7 | 7 | 7 | 7 | 5 | 5 | 8.87E6 | 4.42E6 | 8.55E6 | 7.43E6 | 4.72E6 | 4.88E6 | 5.44E6 | 3.34E6 | 5.28E6 | 2.34E6 | 5 | 5 | 3 | 2 | 3 | 5 | 3 | 3 | 3 | 3 | 2 | 2 | N | 47656 | Histone-binding protein RBBP4 OS=Mus musculus OX=10090 GN=Rbbp4 PE=1 SV=5 |
| 627 | 735 | sp\|Q9WUM5\|SUCA\_MOUSE | 158.89 | 25 | 10 | 18 | 16 | 11 | 8 | 9 | 12 | 8 | 13 | 10 | 6.17E6 | 1.62E7 | 1.82E7 | 7.47E6 | 2.84E6 | 1.16E7 | 7.92E6 | 6.86E6 | 1.96E7 | 4.8E6 | 8 | 7 | 3 | 7 | 5 | 3 | 2 | 3 | 4 | 2 | 5 | 3 | Y | 36155 | Succinate--CoA ligase [ADP/GDP-forming] subunit alpha, mitochondrial OS=Mus musculus OX=10090 GN=Suclg1 PE=1 SV=4 |
| 792 | 1065 | E9PX70\|E9PX70\_MOUSE | 158.48 | 2 | 0 | 1 | 0 | 1 | 2 | 1 | 1 | 2 | 1 | 1 | 1.31E6 | 2.84E6 | 2.09E6 | 7.43E5 | 4.01E6 | 2.95E6 | 1.63E6 | 1.43E6 | 3.51E6 | 8.49E5 | 5 | 5 | 1 | 3 | 1 | 2 | 4 | 2 | 2 | 4 | 3 | 2 | N | 333731 | Collagen, type XII, alpha 1 OS=Mus musculus OX=10090 GN=Col12a1 PE=1 SV=1 |
| 792 | 1066 | sp\|Q60847\|COCA1\_MOUSE | 158.48 | 2 | 0 | 1 | 0 | 1 | 2 | 1 | 1 | 2 | 1 | 1 | 1.31E6 | 2.84E6 | 2.09E6 | 7.43E5 | 4.01E6 | 2.95E6 | 1.63E6 | 1.43E6 | 3.51E6 | 8.49E5 | 5 | 5 | 1 | 3 | 1 | 2 | 4 | 2 | 2 | 4 | 3 | 2 | N | 340215 | Collagen alpha-1(XII) chain OS=Mus musculus OX=10090 GN=Col12a1 PE=2 SV=3 |
| 640 | 3785 | Q9D809\|Q9D809\_MOUSE | 158.28 | 23 | 12 | 12 | 23 | 23 | 23 | 23 | 23 | 23 | 12 | 12 | 6.89E6 | 2.94E7 | 2.12E7 | 1.25E7 | 2.45E7 | 1.89E7 | 1.07E7 | 1.17E7 | 3.41E7 | 1.75E7 | 2 | 2 | 3 | 3 | 4 | 5 | 4 | 4 | 4 | 4 | 4 | 3 | N | 12123 | RIKEN cDNA 2200002D01 gene OS=Mus musculus OX=10090 GN=2200002D01Rik PE=1 SV=1 |
| 1286 | 1802 | sp\|P28825\|MEP1A\_MOUSE | 158.11 | 7 | 0 | 3 | 1 | 3 | 1 | 7 | 3 | 1 | 0 | 0 |  | 1.35E6 |  | 6.53E5 | 2.57E6 | 5.47E6 | 1.67E6 | 5.85E5 |  |  | 3 | 3 | 0 | 1 | 1 | 1 | 1 | 3 | 1 | 1 | 0 | 0 | N | 84231 | Meprin A subunit alpha OS=Mus musculus OX=10090 GN=Mep1a PE=1 SV=4 |
| 1286 | 1803 | A0A0R4J043\|A0A0R4J043\_MOUSE | 158.11 | 6 | 0 | 2 | 1 | 2 | 1 | 6 | 2 | 1 | 0 | 0 |  | 1.35E6 |  | 6.53E5 | 2.57E6 | 5.47E6 | 1.67E6 | 5.85E5 |  |  | 3 | 3 | 0 | 1 | 1 | 1 | 1 | 3 | 1 | 1 | 0 | 0 | N | 85665 | Meprin A subunit OS=Mus musculus OX=10090 GN=Mep1a PE=1 SV=1 |
| 773 | 898 | sp\|Q99MN1\|SYK\_MOUSE | 158.07 | 12 | 5 | 1 | 7 | 1 | 3 | 10 | 4 | 4 | 3 | 1 | 3.26E6 | 2.17E6 | 8.21E6 | 3.55E5 | 3.37E6 | 1.28E7 | 2.62E6 | 3.28E6 | 1.07E6 | 1.59E6 | 6 | 6 | 2 | 1 | 3 | 1 | 2 | 6 | 3 | 3 | 2 | 1 | N | 67840 | Lysine--tRNA ligase OS=Mus musculus OX=10090 GN=Kars1 PE=1 SV=1 |
| 773 | 908 | Q8R2P8\|Q8R2P8\_MOUSE | 158.07 | 12 | 5 | 1 | 7 | 1 | 2 | 10 | 4 | 4 | 3 | 1 | 3.26E6 | 2.17E6 | 8.21E6 | 3.55E5 | 3.37E6 | 1.28E7 | 2.62E6 | 3.28E6 | 1.07E6 | 1.59E6 | 6 | 6 | 2 | 1 | 3 | 1 | 2 | 6 | 3 | 3 | 2 | 1 | N | 71291 | Lysine--tRNA ligase OS=Mus musculus OX=10090 GN=Kars1 PE=1 SV=1 |
| 750 | 1096 | sp\|Q64442\|DHSO\_MOUSE | 157.97 | 18 | 9 | 10 | 12 | 10 | 11 | 10 | 7 | 3 | 3 | 10 | 8.91E6 | 8.65E6 | 2.08E7 | 5.17E6 | 1.22E7 | 1.25E7 | 5.78E6 | 5.17E5 | 1.07E7 | 1.05E7 | 7 | 7 | 3 | 3 | 5 | 3 | 4 | 3 | 2 | 1 | 1 | 3 | Y | 38249 | Sorbitol dehydrogenase OS=Mus musculus OX=10090 GN=Sord PE=1 SV=3 |
| 1073 | 1423 | sp\|P39654\|LOX15\_MOUSE | 157.95 | 9 | 5 | 0 | 0 | 0 | 0 | 0 | 0 | 0 | 9 | 8 | 7.06E6 |  |  |  |  |  |  |  | 2.23E7 | 1.74E7 | 5 | 5 | 4 | 0 | 0 | 0 | 0 | 0 | 0 | 0 | 6 | 5 | N | 75445 | Polyunsaturated fatty acid lipoxygenase ALOX15 OS=Mus musculus OX=10090 GN=Alox15 PE=1 SV=4 |
| 988 | 1421 | F8WIR1\|F8WIR1\_MOUSE | 157.59 | 19 | 11 | 2 | 2 | 2 | 2 | 0 | 2 | 8 | 15 | 17 | 3.73E6 | 6.5E5 | 0E0 | 3.27E5 | 8.08E5 |  | 3.82E5 | 1.43E6 | 9.45E6 | 6.48E6 | 5 | 5 | 3 | 1 | 1 | 1 | 1 | 0 | 1 | 2 | 4 | 4 | Y | 44215 | Cathepsin D OS=Mus musculus OX=10090 GN=Ctsd PE=1 SV=2 |
| 988 | 1422 | sp\|P18242\|CATD\_MOUSE | 157.59 | 19 | 11 | 2 | 2 | 2 | 2 | 0 | 2 | 8 | 14 | 17 | 3.73E6 | 6.5E5 | 0E0 | 3.27E5 | 8.08E5 |  | 3.82E5 | 1.43E6 | 9.45E6 | 6.48E6 | 5 | 5 | 3 | 1 | 1 | 1 | 1 | 0 | 1 | 2 | 4 | 4 | Y | 44954 | Cathepsin D OS=Mus musculus OX=10090 GN=Ctsd PE=1 SV=1 |
| 988 | 1470 | A0A1B0GT66\|A0A1B0GT66\_MOUSE | 157.59 | 28 | 17 | 3 | 3 | 4 | 3 | 0 | 4 | 12 | 22 | 25 | 3.73E6 | 6.5E5 | 0E0 | 3.27E5 | 8.08E5 |  | 3.82E5 | 1.43E6 | 9.45E6 | 6.48E6 | 5 | 5 | 3 | 1 | 1 | 1 | 1 | 0 | 1 | 2 | 4 | 4 | Y | 29418 | Cathepsin D (Fragment) OS=Mus musculus OX=10090 GN=Gm49369 PE=1 SV=1 |
| 988 | 1471 | F6Y6L6\|F6Y6L6\_MOUSE | 157.59 | 28 | 17 | 3 | 3 | 4 | 3 | 0 | 4 | 12 | 21 | 24 | 3.73E6 | 6.5E5 | 0E0 | 3.27E5 | 8.08E5 |  | 3.82E5 | 1.43E6 | 9.45E6 | 6.48E6 | 5 | 5 | 3 | 1 | 1 | 1 | 1 | 0 | 1 | 2 | 4 | 4 | Y | 29961 | Cathepsin D (Fragment) OS=Mus musculus OX=10090 GN=Gm49369 PE=1 SV=1 |
| 1010 | 1181 | sp\|P63330\|PP2AA\_MOUSE | 157.51 | 18 | 4 | 8 | 6 | 4 | 11 | 6 | 8 | 4 | 13 | 0 | 7.32E6 | 7.72E6 | 1.09E7 | 3.76E6 | 1.37E7 | 1.34E7 | 4.01E6 | 1.84E6 | 1.61E7 |  | 5 | 5 | 1 | 2 | 2 | 1 | 3 | 2 | 2 | 1 | 3 | 0 | Y | 35608 | Serine/threonine-protein phosphatase 2A catalytic subunit alpha isoform OS=Mus musculus OX=10090 GN=Ppp2ca PE=1 SV=1 |
| 1010 | 1306 | sp\|P62715\|PP2AB\_MOUSE | 157.51 | 18 | 4 | 8 | 6 | 4 | 11 | 6 | 8 | 4 | 13 | 0 | 7.32E6 | 7.72E6 | 1.09E7 | 3.76E6 | 1.37E7 | 1.34E7 | 4.01E6 | 1.84E6 | 1.61E7 |  | 5 | 5 | 1 | 2 | 2 | 1 | 3 | 2 | 2 | 1 | 3 | 0 | Y | 35575 | Serine/threonine-protein phosphatase 2A catalytic subunit beta isoform OS=Mus musculus OX=10090 GN=Ppp2cb PE=1 SV=1 |
| 782 | 1046 | sp\|O35350\|CAN1\_MOUSE | 157.44 | 8 | 6 | 6 | 1 | 3 | 1 | 3 | 4 | 2 | 6 | 6 | 1.36E6 | 8.66E6 | 3.75E6 | 2.14E6 | 3.64E6 | 3.61E6 | 4.48E6 | 2.64E5 | 1.19E7 | 1.03E7 | 6 | 6 | 4 | 4 | 1 | 2 | 1 | 2 | 3 | 1 | 4 | 4 | N | 82106 | Calpain-1 catalytic subunit OS=Mus musculus OX=10090 GN=Capn1 PE=1 SV=1 |
| 32 | 1893 | A0A0N4SV66\|A0A0N4SV66\_MOUSE | 157.28 | 29 | 28 | 28 | 29 | 29 | 29 | 29 | 28 | 28 | 28 | 28 | 3.95E9 | 4.92E8 | 3.65E9 | 1.45E8 | 2.98E9 | 6.76E8 | 1.71E9 | 2.14E9 | 5.85E9 | 3.33E9 | 4 | 2 | 55 | 43 | 59 | 56 | 64 | 55 | 57 | 62 | 50 | 49 | N | 13660 | Histone H2A OS=Mus musculus OX=10090 GN=H2aj PE=1 SV=1 |
| 32 | 1895 | sp\|Q8R1M2\|H2AJ\_MOUSE | 157.28 | 28 | 27 | 27 | 28 | 28 | 28 | 28 | 27 | 27 | 27 | 27 | 3.95E9 | 4.92E8 | 3.65E9 | 1.45E8 | 2.98E9 | 6.76E8 | 1.71E9 | 2.14E9 | 5.85E9 | 3.33E9 | 4 | 2 | 55 | 43 | 59 | 56 | 64 | 55 | 57 | 62 | 50 | 49 | N | 14045 | Histone H2A.J OS=Mus musculus OX=10090 GN=H2aj PE=1 SV=1 |
| 1152 | 2644 | sp\|O89017\|LGMN\_MOUSE | 157.14 | 7 | 7 | 4 | 4 | 4 | 4 | 4 | 0 | 4 | 7 | 7 | 1.49E7 | 1.78E6 | 4.54E6 | 2.1E6 | 4.01E6 | 4.58E6 |  | 2.88E6 | 3.05E7 | 1.77E7 | 2 | 2 | 2 | 1 | 1 | 1 | 1 | 1 | 0 | 1 | 2 | 2 | Y | 49373 | Legumain OS=Mus musculus OX=10090 GN=Lgmn PE=1 SV=1 |
| 357 | 1357 | sp\|Q9DBP5\|KCY\_MOUSE | 156.74 | 33 | 16 | 10 | 16 | 16 | 16 | 17 | 27 | 11 | 23 | 16 | 1.28E8 | 2.13E7 | 3.39E7 | 1.46E7 | 2.82E7 | 2.31E7 | 1.88E7 | 1.46E7 | 1.6E8 | 3.84E7 | 5 | 5 | 9 | 6 | 8 | 6 | 8 | 6 | 7 | 6 | 10 | 9 | Y | 22165 | UMP-CMP kinase OS=Mus musculus OX=10090 GN=Cmpk1 PE=1 SV=1 |
| 357 | 1358 | A0A0R4J093\|A0A0R4J093\_MOUSE | 156.74 | 29 | 14 | 8 | 14 | 14 | 14 | 15 | 23 | 9 | 20 | 14 | 1.28E8 | 2.13E7 | 3.39E7 | 1.46E7 | 2.82E7 | 2.31E7 | 1.88E7 | 1.46E7 | 1.6E8 | 3.84E7 | 5 | 5 | 9 | 6 | 8 | 6 | 8 | 6 | 7 | 6 | 10 | 9 | Y | 25715 | UMP-CMP kinase OS=Mus musculus OX=10090 GN=Cmpk1 PE=1 SV=1 |
| 606 | 497 | sp\|Q9WU78\|PDC6I\_MOUSE | 156.69 | 13 | 1 | 3 | 3 | 2 | 8 | 7 | 5 | 3 | 6 | 3 | 4.31E6 | 8.46E6 | 6.5E6 | 7.07E5 | 9.65E6 | 8.87E6 | 5.76E6 | 2.43E6 | 5.31E6 | 2.41E6 | 10 | 10 | 1 | 3 | 3 | 2 | 7 | 6 | 4 | 2 | 4 | 2 | Y | 96024 | Programmed cell death 6-interacting protein OS=Mus musculus OX=10090 GN=Pdcd6ip PE=1 SV=3 |
| 812 | 1224 | sp\|P31428\|DPEP1\_MOUSE | 156.65 | 17 | 10 | 5 | 2 | 7 | 8 | 17 | 5 | 3 | 7 | 5 | 2.21E6 | 4.38E6 | 2.55E6 | 1.27E6 | 1.81E6 | 1.62E7 | 1.8E6 | 4.86E5 | 1.28E7 | 3.83E6 | 6 | 6 | 3 | 2 | 1 | 2 | 2 | 6 | 2 | 1 | 3 | 2 | Y | 45722 | Dipeptidase 1 OS=Mus musculus OX=10090 GN=Dpep1 PE=1 SV=2 |
| 958 | 1117 | sp\|Q7TSV4\|PGM2\_MOUSE | 156.58 | 10 | 5 | 5 | 3 | 5 | 2 | 5 | 5 | 2 | 3 | 3 | 1.49E6 | 2.12E6 | 1.33E5 | 2.89E6 | 3.14E6 | 7.5E6 | 4.5E6 | 2.22E6 | 2.51E6 | 1.61E6 | 5 | 5 | 2 | 2 | 1 | 2 | 1 | 2 | 2 | 1 | 2 | 1 | N | 68748 | Phosphopentomutase OS=Mus musculus OX=10090 GN=Pgm2 PE=1 SV=1 |
| 838 | 1841 | sp\|P70296\|PEBP1\_MOUSE | 156.47 | 33 | 12 | 19 | 19 | 12 | 12 | 19 | 12 | 12 | 26 | 12 | 1.21E7 | 2.12E7 | 1.79E7 | 4.46E6 | 1.15E7 | 1.69E7 | 9.99E6 | 9.8E6 | 1.65E7 | 1.04E7 | 4 | 4 | 2 | 3 | 3 | 2 | 2 | 3 | 2 | 2 | 3 | 2 | N | 20830 | Phosphatidylethanolamine-binding protein 1 OS=Mus musculus OX=10090 GN=Pebp1 PE=1 SV=3 |
| 744 | 1449 | sp\|Q62465\|VAT1\_MOUSE | 156.38 | 16 | 12 | 12 | 12 | 4 | 8 | 8 | 4 | 2 | 12 | 8 | 1.33E7 | 1.03E7 | 8.91E6 | 2.78E6 | 4.27E6 | 6.9E6 | 4.47E6 | 2.55E5 | 2.24E7 | 5.51E6 | 5 | 5 | 4 | 4 | 4 | 2 | 3 | 3 | 2 | 1 | 4 | 3 | Y | 43097 | Synaptic vesicle membrane protein VAT-1 homolog OS=Mus musculus OX=10090 GN=Vat1 PE=1 SV=3 |
| 844 | 989 | E9Q1Y3\|E9Q1Y3\_MOUSE | 156.17 | 2 | 1 | 0 | 1 | 1 | 1 | 1 | 1 | 0 | 1 | 0 | 1.1E7 | 6.04E5 | 3.16E6 | 7.65E5 | 3.91E5 | 1.04E6 | 3.04E6 | 4.32E4 | 2.54E7 | 1.19E6 | 7 | 7 | 2 | 1 | 2 | 2 | 2 | 2 | 3 | 1 | 4 | 1 | Y | 503910 | Apolipoprotein B (Fragment) OS=Mus musculus OX=10090 GN=Apob PE=1 SV=1 |
| 844 | 991 | sp\|E9Q414\|APOB\_MOUSE | 156.17 | 2 | 1 | 0 | 1 | 1 | 1 | 1 | 1 | 0 | 1 | 0 | 1.1E7 | 6.04E5 | 3.16E6 | 7.65E5 | 3.91E5 | 1.04E6 | 3.04E6 | 4.32E4 | 2.54E7 | 1.19E6 | 7 | 7 | 2 | 1 | 2 | 2 | 2 | 2 | 3 | 1 | 4 | 1 | Y | 509437 | Apolipoprotein B-100 OS=Mus musculus OX=10090 GN=Apob PE=1 SV=1 |
| 829 | 845 | sp\|Q99L45\|IF2B\_MOUSE | 156.05 | 27 | 3 | 8 | 5 | 5 | 13 | 16 | 18 | 6 | 16 | 11 | 1.77E6 | 4.95E6 | 2.64E5 | 5.85E5 | 9.12E6 | 1.44E7 | 7.71E6 | 1.91E6 | 1.74E7 | 3.25E6 | 7 | 7 | 1 | 2 | 1 | 1 | 3 | 4 | 4 | 1 | 4 | 3 | Y | 38092 | Eukaryotic translation initiation factor 2 subunit 2 OS=Mus musculus OX=10090 GN=Eif2s2 PE=1 SV=1 |
| 493 | 1044 | sp\|P32020\|SCP2\_MOUSE | 155.89 | 10 | 7 | 7 | 5 | 7 | 7 | 9 | 7 | 6 | 7 | 5 | 2.78E7 | 3.71E7 | 2.58E7 | 1.19E7 | 2.21E7 | 3.54E7 | 1.34E7 | 1.1E7 | 7.55E7 | 2.77E7 | 7 | 7 | 5 | 4 | 5 | 5 | 5 | 6 | 4 | 4 | 5 | 5 | Y | 59126 | Sterol carrier protein 2 OS=Mus musculus OX=10090 GN=Scp2 PE=1 SV=3 |
| 771 | 672 | sp\|Q6PB66\|LPPRC\_MOUSE | 155.81 | 6 | 0 | 2 | 3 | 4 | 2 | 3 | 3 | 1 | 2 | 1 |  | 2.3E6 | 3.18E6 | 3.79E6 | 2.88E6 | 8.51E6 | 5.24E6 | 3.39E5 | 3.72E6 | 3.88E6 | 8 | 8 | 0 | 2 | 3 | 5 | 2 | 4 | 3 | 1 | 3 | 1 | Y | 156614 | Leucine-rich PPR motif-containing protein, mitochondrial OS=Mus musculus OX=10090 GN=Lrpprc PE=1 SV=2 |
| 753 | 552 | sp\|E9Q557\|DESP\_MOUSE | 155.69 | 6 | 1 | 0 | 0 | 0 | 1 | 1 | 1 | 0 | 3 | 1 | 8.53E5 | 4.11E5 |  | 3.54E6 | 5.47E6 | 7.08E6 | 4.82E6 | 1.86E6 | 2.86E7 | 2.16E6 | 17 | 16 | 2 | 2 | 1 | 1 | 2 | 3 | 4 | 1 | 7 | 2 | Y | 332913 | Desmoplakin OS=Mus musculus OX=10090 GN=Dsp PE=1 SV=1 |
| 563 | 1911 | A0A3B2WDD2\|A0A3B2WDD2\_MOUSE | 155.55 | 22 | 15 | 15 | 15 | 15 | 22 | 15 | 22 | 8 | 15 | 15 | 4.45E7 | 4.12E7 | 4.92E7 | 2.53E7 | 5.51E7 | 3.64E7 | 4.03E7 | 2.76E7 | 1.15E7 | 4.2E7 | 5 | 5 | 4 | 4 | 4 | 3 | 6 | 4 | 6 | 2 | 4 | 4 | Y | 21637 | Ribosomal protein OS=Mus musculus OX=10090 GN=Rpl10a PE=1 SV=1 |
| 563 | 1912 | A0A3B2WBL1\|A0A3B2WBL1\_MOUSE | 155.55 | 19 | 13 | 13 | 13 | 13 | 19 | 13 | 19 | 7 | 13 | 13 | 4.45E7 | 4.12E7 | 4.92E7 | 2.53E7 | 5.51E7 | 3.64E7 | 4.03E7 | 2.76E7 | 1.15E7 | 4.2E7 | 5 | 5 | 4 | 4 | 4 | 3 | 6 | 4 | 6 | 2 | 4 | 4 | Y | 24744 | Ribosomal protein OS=Mus musculus OX=10090 GN=Rpl10a PE=1 SV=1 |
| 563 | 1913 | Q5XJF6\|Q5XJF6\_MOUSE | 155.55 | 19 | 13 | 13 | 13 | 13 | 19 | 13 | 19 | 7 | 13 | 13 | 4.45E7 | 4.12E7 | 4.92E7 | 2.53E7 | 5.51E7 | 3.64E7 | 4.03E7 | 2.76E7 | 1.15E7 | 4.2E7 | 5 | 5 | 4 | 4 | 4 | 3 | 6 | 4 | 6 | 2 | 4 | 4 | Y | 24831 | Ribosomal protein OS=Mus musculus OX=10090 GN=Rpl10a PE=1 SV=1 |
| 563 | 1914 | sp\|P53026\|RL10A\_MOUSE | 155.55 | 19 | 13 | 13 | 13 | 13 | 19 | 13 | 19 | 7 | 13 | 13 | 4.45E7 | 4.12E7 | 4.92E7 | 2.53E7 | 5.51E7 | 3.64E7 | 4.03E7 | 2.76E7 | 1.15E7 | 4.2E7 | 5 | 5 | 4 | 4 | 4 | 3 | 6 | 4 | 6 | 2 | 4 | 4 | Y | 24916 | Large ribosomal subunit protein uL1 OS=Mus musculus OX=10090 GN=Rpl10a PE=1 SV=3 |
| 713 | 961 | sp\|Q9Z2U0\|PSA7\_MOUSE | 155.37 | 34 | 15 | 9 | 17 | 25 | 16 | 14 | 9 | 8 | 12 | 8 | 1.16E7 | 7.02E6 | 3.81E6 | 6.39E6 | 9.31E6 | 1.14E7 | 7.38E6 | 3.59E6 | 2.44E7 | 1.55E7 | 7 | 6 | 3 | 2 | 3 | 5 | 4 | 3 | 2 | 2 | 3 | 2 | Y | 27855 | Proteasome subunit alpha type-7 OS=Mus musculus OX=10090 GN=Psma7 PE=1 SV=1 |
| 431 | 1131 | sp\|P19783\|COX41\_MOUSE | 155.33 | 33 | 17 | 19 | 19 | 20 | 30 | 25 | 25 | 19 | 30 | 16 | 5.53E7 | 7.65E7 | 6.69E7 | 3.42E7 | 7.29E7 | 6.85E7 | 5.13E7 | 4.4E7 | 1.06E8 | 4.79E7 | 6 | 6 | 4 | 7 | 5 | 4 | 7 | 5 | 5 | 5 | 7 | 5 | Y | 19530 | Cytochrome c oxidase subunit 4 isoform 1, mitochondrial OS=Mus musculus OX=10090 GN=Cox4i1 PE=1 SV=2 |
| 704 | 1662 | sp\|Q9WV55\|VAPA\_MOUSE | 155.27 | 19 | 16 | 19 | 9 | 5 | 12 | 12 | 9 | 9 | 19 | 9 | 1.44E7 | 1.97E7 | 4.25E6 |  | 5.82E6 | 1.16E7 | 8.03E5 | 8.75E5 | 2.98E7 | 1.43E6 | 4 | 3 | 4 | 5 | 2 | 1 | 3 | 3 | 2 | 2 | 5 | 2 | Y | 27855 | Vesicle-associated membrane protein-associated protein A OS=Mus musculus OX=10090 GN=Vapa PE=1 SV=2 |
| 861 | 1837 | G5E850\|G5E850\_MOUSE | 155.15 | 56 | 44 | 44 | 31 | 35 | 31 | 43 | 31 | 31 | 44 | 31 | 1.98E7 | 3.32E7 | 1.81E7 | 1.43E7 | 1.47E7 | 1.85E7 | 1.24E7 | 9.62E6 | 3.19E7 | 1.33E7 | 4 | 4 | 3 | 3 | 2 | 2 | 2 | 3 | 2 | 2 | 3 | 2 | N | 11142 | Cytochrome b5 OS=Mus musculus OX=10090 GN=Cyb5a PE=1 SV=1 |
| 861 | 1838 | sp\|P56395\|CYB5\_MOUSE | 155.15 | 41 | 32 | 32 | 22 | 25 | 22 | 31 | 22 | 22 | 32 | 22 | 1.98E7 | 3.32E7 | 1.81E7 | 1.43E7 | 1.47E7 | 1.85E7 | 1.24E7 | 9.62E6 | 3.19E7 | 1.33E7 | 4 | 4 | 3 | 3 | 2 | 2 | 2 | 3 | 2 | 2 | 3 | 2 | N | 15241 | Cytochrome b5 OS=Mus musculus OX=10090 GN=Cyb5a PE=1 SV=2 |
| 621 | 1863 | A0A0N4SVQ1\|A0A0N4SVQ1\_MOUSE | 155.12 | 78 | 45 | 61 | 61 | 61 | 61 | 41 | 78 | 78 | 45 | 45 | 1.88E7 | 7.25E7 | 4.98E7 | 2.92E7 | 2.55E7 | 3.96E7 | 3.27E7 | 4.3E7 | 5.43E7 | 1.57E7 | 4 | 4 | 3 | 4 | 5 | 4 | 4 | 3 | 5 | 5 | 3 | 3 | N | 5877 | Ndufa4, mitochondrial complex associated OS=Mus musculus OX=10090 GN=Ndufa4 PE=1 SV=1 |
| 621 | 1869 | sp\|Q62425\|NDUA4\_MOUSE | 155.12 | 46 | 27 | 37 | 37 | 37 | 37 | 24 | 46 | 46 | 27 | 27 | 1.88E7 | 7.25E7 | 4.98E7 | 2.92E7 | 2.55E7 | 3.96E7 | 3.27E7 | 4.3E7 | 5.43E7 | 1.57E7 | 4 | 4 | 3 | 4 | 5 | 4 | 4 | 3 | 5 | 5 | 3 | 3 | N | 9327 | Cytochrome c oxidase subunit NDUFA4 OS=Mus musculus OX=10090 GN=Ndufa4 PE=1 SV=2 |
| 777 | 821 | sp\|P17427\|AP2A2\_MOUSE | 154.97 | 9 | 4 | 2 | 3 | 2 | 3 | 2 | 6 | 1 | 4 | 4 | 2.9E5 | 0E0 | 5.74E5 | 6.45E5 | 3E5 |  | 1.17E6 | 3.41E5 | 8.45E6 | 1.88E6 | 7 | 5 | 3 | 2 | 2 | 2 | 2 | 1 | 4 | 1 | 3 | 3 | Y | 104016 | AP-2 complex subunit alpha-2 OS=Mus musculus OX=10090 GN=Ap2a2 PE=1 SV=2 |
| 756 | 2031 | sp\|Q9Z0X1\|AIFM1\_MOUSE | 154.92 | 5 | 2 | 2 | 2 | 5 | 4 | 4 | 4 | 2 | 2 | 2 | 1.66E6 | 2.66E6 | 1.54E6 | 3.7E6 | 3.18E6 | 5.88E6 | 3.01E6 | 2.82E5 | 9.94E5 | 1.39E6 | 4 | 4 | 3 | 4 | 3 | 3 | 3 | 3 | 3 | 1 | 1 | 3 | N | 66766 | Apoptosis-inducing factor 1, mitochondrial OS=Mus musculus OX=10090 GN=Aifm1 PE=1 SV=1 |
| 804 | 1553 | sp\|P14483\|HB2A\_MOUSE | 154.87 | 16 | 3 | 8 | 8 | 11 | 8 | 11 | 10 | 14 | 0 | 3 | 2.22E6 | 1.04E7 | 1.81E7 | 7.71E6 | 1.98E7 | 2.49E7 | 9.53E6 | 1.84E7 |  | 4.05E6 | 5 | 5 | 1 | 2 | 3 | 3 | 3 | 4 | 3 | 5 | 0 | 2 | Y | 30128 | H-2 class II histocompatibility antigen, A beta chain OS=Mus musculus OX=10090 GN=H2-Ab1 PE=1 SV=1 |
| 959 | 1557 | sp\|Q91YR1\|TWF1\_MOUSE | 154.79 | 14 | 5 | 8 | 5 | 0 | 5 | 8 | 8 | 0 | 14 | 8 | 6.93E5 | 2.79E6 | 4.63E5 |  | 2.11E5 | 3.66E6 | 8.01E5 |  | 2.22E7 | 6.51E5 | 4 | 4 | 1 | 2 | 1 | 0 | 1 | 2 | 2 | 0 | 5 | 2 | N | 40079 | Twinfilin-1 OS=Mus musculus OX=10090 GN=Twf1 PE=1 SV=2 |
| 1130 | 1654 | sp\|Q99K30\|ES8L2\_MOUSE | 154.79 | 6 | 2 | 2 | 0 | 4 | 4 | 4 | 2 | 2 | 0 | 2 | 5.17E5 | 1.52E6 |  | 9.71E5 | 1.65E6 | 6.13E6 | 2.04E5 | 2.16E5 |  | 2E5 | 3 | 3 | 1 | 1 | 0 | 2 | 2 | 2 | 1 | 1 | 0 | 1 | N | 82229 | Epidermal growth factor receptor kinase substrate 8-like protein 2 OS=Mus musculus OX=10090 GN=Eps8l2 PE=1 SV=1 |
| 933 | 1201 | sp\|P27546\|MAP4\_MOUSE | 154.71 | 5 | 2 | 2 | 2 | 3 | 2 | 2 | 2 | 2 | 0 | 1 | 1.29E6 | 8.17E5 | 1.52E6 | 5.28E5 | 7.65E5 | 2.13E6 | 4.83E5 | 1.12E6 |  | 8.08E5 | 5 | 3 | 2 | 2 | 3 | 3 | 3 | 2 | 2 | 2 | 0 | 1 | Y | 117429 | Microtubule-associated protein 4 OS=Mus musculus OX=10090 GN=Map4 PE=1 SV=3 |
| 442 | 3021 | sp\|P08207\|S10AA\_MOUSE | 154.32 | 28 | 28 | 28 | 28 | 28 | 28 | 28 | 28 | 28 | 28 | 28 | 6.96E7 | 1.02E8 | 6.07E7 | 3.3E7 | 4.02E7 | 8.94E7 | 2.24E7 | 1.89E7 | 6.23E7 | 4.99E7 | 4 | 4 | 6 | 5 | 6 | 5 | 7 | 8 | 5 | 4 | 6 | 4 | N | 11186 | Protein S100-A10 OS=Mus musculus OX=10090 GN=S100a10 PE=1 SV=2 |
| 813 | 819 | sp\|Q8VDM4\|PSMD2\_MOUSE | 154.16 | 8 | 3 | 3 | 1 | 4 | 0 | 3 | 6 | 3 | 2 | 5 | 2.28E6 | 1.56E6 | 3.82E6 | 2.85E6 |  | 5.18E6 | 9.98E6 | 2.49E6 | 4.36E6 | 7.68E6 | 7 | 7 | 2 | 2 | 1 | 3 | 0 | 2 | 6 | 2 | 2 | 4 | Y | 100203 | 26S proteasome non-ATPase regulatory subunit 2 OS=Mus musculus OX=10090 GN=Psmd2 PE=1 SV=1 |
| 1063 | 1112 | Q5SVG5\|Q5SVG5\_MOUSE | 153.85 | 9 | 3 | 3 | 0 | 3 | 1 | 3 | 4 | 3 | 3 | 1 | 2.76E6 | 3.32E6 |  | 3.13E5 |  | 1.89E6 | 1.19E6 | 3.5E5 | 4.23E5 |  | 5 | 3 | 1 | 1 | 0 | 2 | 1 | 1 | 2 | 1 | 2 | 1 | Y | 101193 | AP complex subunit beta OS=Mus musculus OX=10090 GN=Ap1b1 PE=1 SV=1 |
| 1063 | 1113 | Q5SVG4\|Q5SVG4\_MOUSE | 153.85 | 9 | 3 | 3 | 0 | 2 | 1 | 3 | 4 | 3 | 3 | 1 | 2.76E6 | 3.32E6 |  | 3.13E5 |  | 1.89E6 | 1.19E6 | 3.5E5 | 4.23E5 |  | 5 | 3 | 1 | 1 | 0 | 2 | 1 | 1 | 2 | 1 | 2 | 1 | Y | 101898 | AP complex subunit beta OS=Mus musculus OX=10090 GN=Ap1b1 PE=1 SV=1 |
| 1063 | 1115 | sp\|O35643\|AP1B1\_MOUSE | 153.85 | 9 | 3 | 3 | 0 | 2 | 1 | 3 | 4 | 3 | 3 | 1 | 2.76E6 | 3.32E6 |  | 3.13E5 |  | 1.89E6 | 1.19E6 | 3.5E5 | 4.23E5 |  | 5 | 3 | 1 | 1 | 0 | 2 | 1 | 1 | 2 | 1 | 2 | 1 | Y | 103935 | AP-1 complex subunit beta-1 OS=Mus musculus OX=10090 GN=Ap1b1 PE=1 SV=2 |
| 809 | 1107 | sp\|P10493\|NID1\_MOUSE | 153.75 | 4 | 1 | 3 | 1 | 2 | 2 | 2 | 2 | 2 | 1 | 2 | 3.31E6 | 2.21E7 | 8.21E6 | 3.58E6 | 5.67E6 | 1.07E7 | 4.53E6 | 5.42E6 | 8.25E6 | 4.89E6 | 5 | 5 | 1 | 3 | 2 | 2 | 3 | 4 | 2 | 3 | 1 | 2 | Y | 136538 | Nidogen-1 OS=Mus musculus OX=10090 GN=Nid1 PE=1 SV=2 |
| 743 | 1356 | sp\|P08228\|SODC\_MOUSE | 153.73 | 37 | 32 | 32 | 7 | 15 | 16 | 7 | 15 | 12 | 32 | 25 | 2.31E7 | 4.85E7 | 1.48E7 | 1.37E7 | 1.56E7 | 1.54E7 | 2.09E7 | 1.31E7 | 6.3E7 | 1.85E7 | 5 | 5 | 4 | 4 | 1 | 3 | 3 | 1 | 4 | 2 | 5 | 3 | Y | 15943 | Superoxide dismutase [Cu-Zn] OS=Mus musculus OX=10090 GN=Sod1 PE=1 SV=2 |
| 1066 | 1029 | sp\|Q8BMJ2\|SYLC\_MOUSE | 153.62 | 6 | 1 | 1 | 1 | 0 | 1 | 2 | 2 | 1 | 2 | 1 | 8.66E5 | 0E0 | 6.01E5 |  | 4.29E7 | 1.04E6 | 3.52E7 |  | 2.37E6 | 9.46E5 | 6 | 6 | 1 | 1 | 1 | 0 | 1 | 2 | 3 | 1 | 2 | 1 | Y | 134192 | Leucine--tRNA ligase, cytoplasmic OS=Mus musculus OX=10090 GN=Lars1 PE=1 SV=2 |
| 719 | 824 | F8WIH0\|F8WIH0\_MOUSE | 153.11 | 9 | 5 | 7 | 6 | 5 | 4 | 3 | 5 | 4 | 1 | 4 | 6.31E6 | 1.35E7 | 9.94E6 | 4.13E6 | 4.36E6 | 2.73E6 | 1.17E7 | 3.53E6 | 3.83E6 | 3.29E6 | 6 | 6 | 3 | 4 | 4 | 3 | 2 | 2 | 3 | 2 | 1 | 3 | N | 79447 | Sarcolemma associated protein (Fragment) OS=Mus musculus OX=10090 GN=Slmap PE=1 SV=1 |
| 719 | 826 | D3Z7V3\|D3Z7V3\_MOUSE | 153.11 | 8 | 4 | 6 | 6 | 5 | 3 | 3 | 5 | 3 | 1 | 4 | 6.31E6 | 1.35E7 | 9.94E6 | 4.13E6 | 4.36E6 | 2.73E6 | 1.17E7 | 3.53E6 | 3.83E6 | 3.29E6 | 6 | 6 | 3 | 4 | 4 | 3 | 2 | 2 | 3 | 2 | 1 | 3 | N | 90721 | Sarcolemma associated protein OS=Mus musculus OX=10090 GN=Slmap PE=1 SV=1 |
| 719 | 827 | H7BX64\|H7BX64\_MOUSE | 153.11 | 8 | 4 | 6 | 5 | 4 | 3 | 3 | 4 | 3 | 1 | 4 | 6.31E6 | 1.35E7 | 9.94E6 | 4.13E6 | 4.36E6 | 2.73E6 | 1.17E7 | 3.53E6 | 3.83E6 | 3.29E6 | 6 | 6 | 3 | 4 | 4 | 3 | 2 | 2 | 3 | 2 | 1 | 3 | N | 95090 | Sarcolemma associated protein OS=Mus musculus OX=10090 GN=Slmap PE=1 SV=1 |
| 719 | 828 | sp\|Q3URD3\|SLMAP\_MOUSE | 153.11 | 8 | 4 | 5 | 5 | 4 | 3 | 3 | 4 | 3 | 1 | 4 | 6.31E6 | 1.35E7 | 9.94E6 | 4.13E6 | 4.36E6 | 2.73E6 | 1.17E7 | 3.53E6 | 3.83E6 | 3.29E6 | 6 | 6 | 3 | 4 | 4 | 3 | 2 | 2 | 3 | 2 | 1 | 3 | N | 96933 | Sarcolemmal membrane-associated protein OS=Mus musculus OX=10090 GN=Slmap PE=1 SV=2 |
| 611 | 762 | A2AUR7\|A2AUR7\_MOUSE | 153.00 | 32 | 19 | 16 | 21 | 6 | 12 | 23 | 0 | 23 | 22 | 13 | 1.05E7 | 5.85E6 | 5.74E6 | 1.78E6 | 2.2E6 | 8.7E6 |  | 5.72E6 | 1.28E7 | 2.75E6 | 8 | 8 | 5 | 3 | 5 | 2 | 4 | 6 | 0 | 6 | 6 | 2 | Y | 29543 | Ras suppressor protein 1 OS=Mus musculus OX=10090 GN=Rsu1 PE=1 SV=1 |
| 611 | 765 | Q9D031\|Q9D031\_MOUSE | 153.00 | 30 | 18 | 15 | 20 | 6 | 12 | 22 | 0 | 21 | 21 | 13 | 1.05E7 | 5.85E6 | 5.74E6 | 1.78E6 | 2.2E6 | 8.7E6 |  | 5.72E6 | 1.28E7 | 2.75E6 | 8 | 8 | 5 | 3 | 5 | 2 | 4 | 6 | 0 | 6 | 6 | 2 | Y | 31461 | Ras suppressor protein 1 OS=Mus musculus OX=10090 GN=Rsu1 PE=1 SV=1 |
| 611 | 766 | sp\|Q01730\|RSU1\_MOUSE | 153.00 | 30 | 18 | 15 | 20 | 6 | 12 | 22 | 0 | 21 | 21 | 13 | 1.05E7 | 5.85E6 | 5.74E6 | 1.78E6 | 2.2E6 | 8.7E6 |  | 5.72E6 | 1.28E7 | 2.75E6 | 8 | 8 | 5 | 3 | 5 | 2 | 4 | 6 | 0 | 6 | 6 | 2 | Y | 31550 | Ras suppressor protein 1 OS=Mus musculus OX=10090 GN=Rsu1 PE=1 SV=3 |
| 424 | 595 | D3YXG6\|D3YXG6\_MOUSE | 152.74 | 35 | 18 | 16 | 18 | 12 | 21 | 25 | 15 | 23 | 21 | 16 | 2.97E7 | 2.49E7 | 3.26E7 | 8.58E6 | 2.33E7 | 1.87E7 | 1.2E7 | 2.39E7 | 3.98E7 | 1.87E7 | 9 | 9 | 6 | 5 | 6 | 4 | 6 | 6 | 5 | 7 | 8 | 5 | Y | 32513 | Arp2/3 complex 34 kDa subunit OS=Mus musculus OX=10090 GN=Arpc2 PE=1 SV=1 |
| 424 | 599 | sp\|Q9CVB6\|ARPC2\_MOUSE | 152.74 | 33 | 17 | 15 | 17 | 11 | 20 | 23 | 14 | 22 | 20 | 15 | 2.97E7 | 2.49E7 | 3.26E7 | 8.58E6 | 2.33E7 | 1.87E7 | 1.2E7 | 2.39E7 | 3.98E7 | 1.87E7 | 9 | 9 | 6 | 5 | 6 | 4 | 6 | 6 | 5 | 7 | 8 | 5 | Y | 34357 | Actin-related protein 2/3 complex subunit 2 OS=Mus musculus OX=10090 GN=Arpc2 PE=1 SV=3 |
| 839 | 1876 | sp\|P62830\|RL23\_MOUSE | 152.62 | 33 | 27 | 13 | 0 | 21 | 21 | 21 | 26 | 21 | 20 | 27 | 5.6E7 | 4.89E7 |  | 2.32E7 | 3.86E7 | 2.38E7 | 8.95E6 | 3.13E7 | 4.58E7 | 4.85E7 | 4 | 4 | 3 | 2 | 0 | 4 | 2 | 3 | 3 | 2 | 3 | 3 | Y | 14865 | Large ribosomal subunit protein uL14 OS=Mus musculus OX=10090 GN=Rpl23 PE=1 SV=1 |
| 495 | 1388 | sp\|P62267\|RS23\_MOUSE | 152.39 | 34 | 22 | 30 | 14 | 22 | 30 | 30 | 30 | 30 | 22 | 27 | 4.36E7 | 5.83E7 | 4.66E7 | 2.42E7 | 3.85E7 | 3.55E7 | 2.45E7 | 1.76E7 | 3.35E7 | 3.51E7 | 7 | 7 | 5 | 6 | 4 | 5 | 5 | 5 | 4 | 4 | 4 | 6 | N | 15808 | Small ribosomal subunit protein uS12 OS=Mus musculus OX=10090 GN=Rps23 PE=1 SV=3 |
| 619 | 1448 | sp\|P02463\|CO4A1\_MOUSE | 152.37 | 3 | 2 | 2 | 1 | 1 | 1 | 3 | 1 | 1 | 1 | 1 | 2.99E7 | 1.81E7 | 2.23E7 | 4.4E6 | 6.96E6 | 3.11E7 | 1.08E7 | 1.06E7 | 1.5E7 | 2.41E7 | 4 | 4 | 5 | 3 | 3 | 3 | 2 | 6 | 3 | 4 | 4 | 3 | Y | 160679 | Collagen alpha-1(IV) chain OS=Mus musculus OX=10090 GN=Col4a1 PE=1 SV=4 |
| 479 | 659 | sp\|P21619\|LMNB2\_MOUSE | 152.04 | 14 | 4 | 6 | 7 | 4 | 6 | 10 | 5 | 10 | 4 | 8 |  | 1.19E6 | 2.51E6 |  | 1.38E6 | 2.39E6 | 1.24E6 | 9.3E5 | 8.39E5 | 2.9E5 | 9 | 4 | 4 | 4 | 5 | 3 | 4 | 8 | 4 | 7 | 2 | 6 | Y | 67318 | Lamin-B2 OS=Mus musculus OX=10090 GN=Lmnb2 PE=1 SV=2 |
| 479 | 661 | A0A0R4J0Q5\|A0A0R4J0Q5\_MOUSE | 152.04 | 14 | 4 | 6 | 7 | 4 | 6 | 10 | 5 | 10 | 3 | 8 |  | 1.19E6 | 2.51E6 |  | 1.38E6 | 2.39E6 | 1.24E6 | 9.3E5 | 8.39E5 | 2.9E5 | 9 | 4 | 4 | 4 | 5 | 3 | 4 | 8 | 4 | 7 | 2 | 6 | Y | 69085 | Lamin B2 OS=Mus musculus OX=10090 GN=Lmnb2 PE=1 SV=1 |
| 572 | 1305 | sp\|Q9CPR4\|RL17\_MOUSE | 152.03 | 35 | 18 | 16 | 22 | 22 | 22 | 22 | 27 | 27 | 18 | 13 | 4.49E7 | 4.55E7 | 5.14E7 | 2.24E7 | 4.5E7 | 3.86E7 | 5.51E7 | 4.34E7 | 3.73E7 | 3.94E7 | 6 | 6 | 4 | 4 | 4 | 5 | 4 | 4 | 5 | 6 | 4 | 3 | Y | 21397 | Large ribosomal subunit protein uL22 OS=Mus musculus OX=10090 GN=Rpl17 PE=1 SV=4 |
| 869 | 1644 | sp\|P17426\|AP2A1\_MOUSE | 151.29 | 4 | 4 | 2 | 3 | 2 | 3 | 3 | 4 | 0 | 3 | 3 | 4.2E5 | 1.61E6 | 6.38E5 | 1.32E5 | 7.44E5 | 4.97E5 | 4.22E5 |  | 8.9E5 |  | 3 | 1 | 3 | 2 | 2 | 2 | 2 | 2 | 3 | 0 | 2 | 2 | N | 107664 | AP-2 complex subunit alpha-1 OS=Mus musculus OX=10090 GN=Ap2a1 PE=1 SV=1 |
| 995 | 1885 | sp\|O09049\|REG3G\_MOUSE | 151.26 | 32 | 0 | 0 | 21 | 21 | 32 | 3 | 17 | 11 | 13 | 3 |  |  | 1.26E6 | 1.31E7 | 1.06E7 |  | 5.64E6 | 4.95E5 | 1.29E6 |  | 4 | 3 | 0 | 0 | 2 | 3 | 5 | 1 | 3 | 2 | 1 | 1 | Y | 19307 | Regenerating islet-derived protein 3-gamma OS=Mus musculus OX=10090 GN=Reg3g PE=1 SV=1 |
| 714 | 1159 | A0A1B0GS70\|A0A1B0GS70\_MOUSE | 151.16 | 23 | 16 | 16 | 8 | 15 | 4 | 7 | 6 | 9 | 16 | 10 | 1.28E7 | 2.02E7 | 4.86E6 | 2.87E7 | 2.8E6 | 1.04E7 | 6.53E5 | 1.59E6 | 3.94E7 | 1.43E7 | 6 | 5 | 4 | 4 | 2 | 4 | 1 | 2 | 2 | 3 | 5 | 2 | N | 26496 | Proteasome subunit alpha 1 OS=Mus musculus OX=10090 GN=Psma1 PE=1 SV=1 |
| 714 | 1165 | sp\|Q9R1P4\|PSA1\_MOUSE | 151.16 | 21 | 14 | 14 | 7 | 13 | 4 | 6 | 6 | 8 | 15 | 9 | 1.28E7 | 2.02E7 | 4.86E6 | 2.87E7 | 2.8E6 | 1.04E7 | 6.53E5 | 1.59E6 | 3.94E7 | 1.43E7 | 6 | 5 | 4 | 4 | 2 | 4 | 1 | 2 | 2 | 3 | 5 | 2 | N | 29547 | Proteasome subunit alpha type-1 OS=Mus musculus OX=10090 GN=Psma1 PE=1 SV=1 |
| 485 | 1256 | sp\|Q9QUI0\|RHOA\_MOUSE | 150.98 | 33 | 13 | 13 | 13 | 16 | 17 | 10 | 10 | 17 | 13 | 17 | 3.36E7 | 3.12E7 | 3.51E7 | 7.32E6 | 2.36E7 | 3.06E7 | 1.84E7 | 1.65E7 | 1.3E7 | 2.62E7 | 6 | 6 | 4 | 5 | 5 | 4 | 5 | 4 | 4 | 4 | 4 | 6 | Y | 21782 | Transforming protein RhoA OS=Mus musculus OX=10090 GN=Rhoa PE=1 SV=1 |
| 923 | 1252 | sp\|Q64511\|TOP2B\_MOUSE | 150.93 | 4 | 1 | 2 | 0 | 1 | 0 | 3 | 2 | 1 | 2 | 1 | 6.08E5 | 1.13E6 |  | 1.47E6 |  | 7.1E6 | 1.68E6 | 7.98E5 | 6.22E6 | 5.4E5 | 7 | 5 | 2 | 3 | 0 | 2 | 1 | 3 | 2 | 1 | 3 | 1 | Y | 181908 | DNA topoisomerase 2-beta OS=Mus musculus OX=10090 GN=Top2b PE=1 SV=2 |
| 760 | 2302 | sp\|Q3TMQ6\|ANG4\_MOUSE | 150.68 | 31 | 31 | 7 | 14 | 6 | 0 | 7 | 7 | 13 | 31 | 31 | 2.03E8 | 3.49E7 | 2.06E7 | 1.76E6 |  | 5.92E6 | 1.79E6 | 6.98E6 | 6.59E7 | 8.18E7 | 4 | 4 | 8 | 2 | 3 | 1 | 0 | 1 | 1 | 2 | 5 | 6 | Y | 16425 | Angiogenin-4 OS=Mus musculus OX=10090 GN=Ang4 PE=1 SV=1 |
| 681 | 1788 | Q50HX3\|Q50HX3\_MOUSE | 150.33 | 21 | 12 | 13 | 17 | 14 | 21 | 16 | 21 | 21 | 13 | 17 | 1.86E7 | 1.05E6 | 2.85E7 | 1.06E7 | 1.53E7 | 3.45E7 | 1.11E7 | 1.24E7 | 1.25E7 | 8.77E6 | 4 | 3 | 2 | 2 | 3 | 3 | 5 | 3 | 5 | 4 | 2 | 3 | N | 21889 | RAB14 protein variant OS=Mus musculus OX=10090 GN=Rab14 PE=1 SV=1 |
| 681 | 1789 | sp\|Q91V41\|RAB14\_MOUSE | 150.33 | 20 | 11 | 12 | 16 | 13 | 20 | 14 | 20 | 20 | 12 | 15 | 1.86E7 | 1.05E6 | 2.85E7 | 1.06E7 | 1.53E7 | 3.45E7 | 1.11E7 | 1.24E7 | 1.25E7 | 8.77E6 | 4 | 3 | 2 | 2 | 3 | 3 | 5 | 3 | 5 | 4 | 2 | 3 | N | 23897 | Ras-related protein Rab-14 OS=Mus musculus OX=10090 GN=Rab14 PE=1 SV=3 |
| 688 | 689 | D3Z158\|D3Z158\_MOUSE | 150.29 | 11 | 1 | 4 | 6 | 5 | 7 | 3 | 4 | 4 | 3 | 4 | 2.59E6 | 6.18E6 | 6.67E6 | 3.86E6 | 1.22E7 | 5.83E5 | 5.43E6 | 9.16E5 | 4.44E6 | 3.58E6 | 8 | 8 | 1 | 3 | 4 | 4 | 5 | 2 | 3 | 3 | 2 | 3 | N | 85055 | glutamine--tRNA ligase OS=Mus musculus OX=10090 GN=Qars1 PE=1 SV=2 |
| 688 | 690 | sp\|Q8BML9\|SYQ\_MOUSE | 150.29 | 11 | 1 | 4 | 5 | 5 | 6 | 3 | 4 | 4 | 3 | 4 | 2.59E6 | 6.18E6 | 6.67E6 | 3.86E6 | 1.22E7 | 5.83E5 | 5.43E6 | 9.16E5 | 4.44E6 | 3.58E6 | 8 | 8 | 1 | 3 | 4 | 4 | 5 | 2 | 3 | 3 | 2 | 3 | N | 87677 | Glutamine--tRNA ligase OS=Mus musculus OX=10090 GN=Qars1 PE=1 SV=1 |
| 262 | 1810 | sp\|Q9Z2K1\|K1C16\_MOUSE | 150.15 | 6 | 5 | 6 | 6 | 6 | 6 | 6 | 6 | 3 | 6 | 6 | 1.23E7 | 1.58E7 | 1.73E7 | 9.52E6 | 1.64E7 | 1.71E7 | 1.19E7 |  | 8.85E6 | 1.14E7 | 4 | 1 | 9 | 11 | 11 | 8 | 12 | 8 | 12 | 9 | 14 | 9 | N | 51606 | Keratin, type I cytoskeletal 16 OS=Mus musculus OX=10090 GN=Krt16 PE=1 SV=3 |
| 785 | 1151 | sp\|P45376\|ALDR\_MOUSE | 150.02 | 16 | 4 | 7 | 7 | 7 | 4 | 10 | 4 | 6 | 9 | 9 | 9.75E6 | 1.66E7 | 1.43E7 | 2.86E6 | 1.05E7 | 1.69E7 | 6.45E6 | 4.27E6 | 5.04E6 | 1.73E7 | 6 | 5 | 2 | 2 | 3 | 2 | 2 | 3 | 1 | 2 | 3 | 4 | N | 35732 | Aldo-keto reductase family 1 member B1 OS=Mus musculus OX=10090 GN=Akr1b1 PE=1 SV=3 |
| 449 | 2065 | sp\|P62843\|RS15\_MOUSE | 149.63 | 26 | 17 | 17 | 17 | 22 | 22 | 22 | 17 | 22 | 17 | 21 | 6.25E7 | 6.31E7 | 6.3E7 | 2.91E7 | 3.61E7 | 5.23E7 | 3.42E7 | 2.89E7 | 5.12E7 | 6.22E7 | 4 | 4 | 6 | 5 | 4 | 6 | 6 | 7 | 5 | 5 | 5 | 5 | Y | 17040 | Small ribosomal subunit protein uS19 OS=Mus musculus OX=10090 GN=Rps15 PE=1 SV=2 |
| 881 | 1849 | sp\|P50543\|S10AB\_MOUSE | 149.56 | 48 | 37 | 23 | 0 | 7 | 23 | 23 | 23 | 7 | 35 | 48 | 3.78E7 | 1.92E7 |  | 1.25E7 | 1.7E7 | 1.87E7 | 1.63E7 | 1.37E7 | 5.46E7 | 4.55E7 | 4 | 4 | 3 | 2 | 0 | 1 | 2 | 2 | 2 | 2 | 4 | 5 | Y | 11083 | Protein S100-A11 OS=Mus musculus OX=10090 GN=S100a11 PE=1 SV=1 |
| 1038 | 1667 | sp\|Q9DBM2\|ECHP\_MOUSE | 149.55 | 8 | 4 | 2 | 2 | 2 | 2 | 8 | 3 | 2 | 3 | 2 | 1.03E6 | 8.44E5 | 4.78E6 | 5.81E5 | 4.18E5 | 5.12E6 | 2.18E6 | 4.15E5 | 3.11E6 | 5.2E5 | 4 | 4 | 2 | 1 | 1 | 1 | 1 | 4 | 2 | 1 | 2 | 1 | Y | 78302 | Peroxisomal bifunctional enzyme OS=Mus musculus OX=10090 GN=Ehhadh PE=1 SV=4 |
| 886 | 998 | sp\|P11688\|ITA5\_MOUSE | 149.28 | 6 | 4 | 1 | 0 | 1 | 1 | 2 | 2 | 1 | 3 | 4 | 1.04E7 | 1.68E6 |  | 9.86E5 | 9.79E5 | 7.16E6 | 2.68E6 | 9.78E5 | 1.45E7 | 1.33E7 | 6 | 6 | 4 | 1 | 0 | 1 | 1 | 2 | 2 | 1 | 3 | 5 | N | 115043 | Integrin alpha-5 OS=Mus musculus OX=10090 GN=Itga5 PE=1 SV=3 |
| 763 | 1816 | sp\|Q99JI6\|RAP1B\_MOUSE | 149.23 | 21 | 12 | 11 | 15 | 14 | 21 | 18 | 14 | 18 | 18 | 18 | 1.15E7 | 1.56E7 | 1.44E7 | 1.03E7 | 2.2E7 | 2.47E7 | 1.18E7 | 1.23E7 | 3.37E7 | 2.27E7 | 4 | 3 | 2 | 2 | 3 | 3 | 4 | 3 | 3 | 3 | 3 | 3 | N | 20825 | Ras-related protein Rap-1b OS=Mus musculus OX=10090 GN=Rap1b PE=1 SV=2 |
| 763 | 1826 | sp\|P62835\|RAP1A\_MOUSE | 149.23 | 21 | 12 | 11 | 15 | 14 | 21 | 18 | 14 | 18 | 18 | 18 | 1.15E7 | 1.56E7 | 1.44E7 | 1.03E7 | 2.2E7 | 2.47E7 | 1.18E7 | 1.23E7 | 3.37E7 | 2.27E7 | 4 | 3 | 2 | 2 | 3 | 3 | 4 | 3 | 3 | 3 | 3 | 3 | N | 20987 | Ras-related protein Rap-1A OS=Mus musculus OX=10090 GN=Rap1a PE=1 SV=1 |
| 490 | 1149 | sp\|Q6PDM2\|SRSF1\_MOUSE | 149.16 | 24 | 17 | 17 | 17 | 12 | 21 | 17 | 21 | 21 | 17 | 17 | 3.29E7 | 3.96E7 | 4.3E7 | 9.61E6 | 3.67E7 | 2.96E7 | 2E7 | 2.35E7 | 2.75E7 | 2.61E7 | 6 | 6 | 4 | 4 | 4 | 4 | 6 | 4 | 5 | 5 | 4 | 4 | N | 27745 | Serine/arginine-rich splicing factor 1 OS=Mus musculus OX=10090 GN=Srsf1 PE=1 SV=3 |
| 490 | 1150 | H7BX95\|H7BX95\_MOUSE | 149.16 | 24 | 17 | 17 | 17 | 12 | 21 | 17 | 20 | 20 | 17 | 17 | 3.29E7 | 3.96E7 | 4.3E7 | 9.61E6 | 3.67E7 | 2.96E7 | 2E7 | 2.35E7 | 2.75E7 | 2.61E7 | 6 | 6 | 4 | 4 | 4 | 4 | 6 | 4 | 5 | 5 | 4 | 4 | N | 28329 | Serine/arginine-rich splicing factor 1 OS=Mus musculus OX=10090 GN=Srsf1 PE=1 SV=1 |
| 624 | 729 | sp\|Q9WV32\|ARC1B\_MOUSE | 149.12 | 24 | 5 | 2 | 0 | 7 | 9 | 7 | 17 | 9 | 12 | 2 | 1.75E7 | 3.48E6 |  | 1.78E7 | 3.53E7 | 2.68E7 | 2.61E7 | 3.12E6 | 1.86E7 | 1.5E7 | 9 | 9 | 3 | 1 | 0 | 3 | 4 | 3 | 6 | 3 | 4 | 2 | N | 41064 | Actin-related protein 2/3 complex subunit 1B OS=Mus musculus OX=10090 GN=Arpc1b PE=1 SV=4 |
| 624 | 730 | Q91Z25\|Q91Z25\_MOUSE | 149.12 | 24 | 5 | 2 | 0 | 7 | 9 | 7 | 17 | 9 | 12 | 2 | 1.75E7 | 3.48E6 |  | 1.78E7 | 3.53E7 | 2.68E7 | 2.61E7 | 3.12E6 | 1.86E7 | 1.5E7 | 9 | 9 | 3 | 1 | 0 | 3 | 4 | 3 | 6 | 3 | 4 | 2 | N | 41498 | Actin-related protein 2/3 complex subunit OS=Mus musculus OX=10090 GN=Arpc1b PE=1 SV=1 |
| 475 | 1542 | sp\|P62900\|RL31\_MOUSE | 148.93 | 34 | 25 | 25 | 25 | 25 | 29 | 24 | 25 | 25 | 25 | 25 | 8.47E7 | 8.69E7 | 7.81E7 | 3.58E7 | 7.8E7 | 7.82E7 | 6.51E7 | 5.22E7 | 7.3E7 | 7.45E7 | 5 | 5 | 5 | 6 | 6 | 5 | 7 | 4 | 5 | 6 | 5 | 6 | N | 14463 | Large ribosomal subunit protein eL31 OS=Mus musculus OX=10090 GN=Rpl31 PE=1 SV=1 |
| 475 | 1543 | A0A0A6YX26\|A0A0A6YX26\_MOUSE | 148.93 | 33 | 24 | 24 | 24 | 24 | 28 | 23 | 24 | 24 | 24 | 24 | 8.47E7 | 8.69E7 | 7.81E7 | 3.58E7 | 7.8E7 | 7.82E7 | 6.51E7 | 5.22E7 | 7.3E7 | 7.45E7 | 5 | 5 | 5 | 6 | 6 | 5 | 7 | 4 | 5 | 6 | 5 | 6 | N | 14997 | Large ribosomal subunit protein eL31 OS=Mus musculus OX=10090 GN=Rpl31 PE=1 SV=1 |
| 908 | 2066 | sp\|P07309\|TTHY\_MOUSE | 148.81 | 33 | 10 | 17 | 33 | 22 | 22 | 16 | 16 | 22 | 17 | 10 | 1.89E6 | 1.5E7 | 2.68E7 | 8.05E6 | 1.68E7 | 1.46E6 | 2.62E6 | 1.4E7 | 8.49E6 | 8.27E5 | 3 | 3 | 1 | 2 | 3 | 2 | 2 | 1 | 2 | 2 | 2 | 1 | N | 15776 | Transthyretin OS=Mus musculus OX=10090 GN=Ttr PE=1 SV=1 |
| 907 | 1874 | sp\|Q9WTX5\|SKP1\_MOUSE | 148.65 | 20 | 7 | 7 | 15 | 12 | 12 | 12 | 20 | 12 | 7 | 7 | 2.98E6 | 4.04E6 | 6.42E6 | 5.49E5 | 3.96E6 | 3.32E6 | 5.79E6 | 2.67E6 | 5.16E6 | 2.99E6 | 4 | 3 | 1 | 1 | 2 | 2 | 2 | 2 | 4 | 2 | 1 | 1 | N | 18672 | S-phase kinase-associated protein 1 OS=Mus musculus OX=10090 GN=Skp1 PE=1 SV=3 |
| 411 | 1108 | sp\|P62281\|RS11\_MOUSE | 148.60 | 38 | 22 | 16 | 22 | 27 | 16 | 22 | 27 | 27 | 33 | 33 | 4.62E7 | 3.68E7 | 7.04E7 | 4.68E7 | 4.29E7 | 5.65E7 | 7.01E7 | 5.32E7 | 8.26E7 | 5.71E7 | 7 | 7 | 4 | 4 | 5 | 7 | 4 | 5 | 8 | 7 | 8 | 6 | Y | 18431 | Small ribosomal subunit protein uS17 OS=Mus musculus OX=10090 GN=Rps11 PE=1 SV=3 |
| 789 | 1116 | sp\|Q9WUK2\|IF4H\_MOUSE | 148.57 | 26 | 5 | 8 | 8 | 17 | 14 | 18 | 12 | 5 | 14 | 0 | 3.14E6 | 2.72E7 | 2.58E7 | 7.86E6 | 2.28E7 | 1.55E7 | 2.15E7 | 8.96E5 | 1.14E7 |  | 7 | 7 | 1 | 3 | 2 | 4 | 3 | 4 | 3 | 1 | 4 | 0 | Y | 27341 | Eukaryotic translation initiation factor 4H OS=Mus musculus OX=10090 GN=Eif4h PE=1 SV=3 |
| 729 | 2144 | sp\|Q9CQR2\|RS21\_MOUSE | 148.41 | 40 | 29 | 29 | 29 | 40 | 40 | 40 | 29 | 29 | 40 | 40 | 2.03E7 | 1.84E7 | 2.61E7 | 1.3E7 | 2.76E7 | 2.06E7 | 1.42E7 | 1.2E7 | 2.48E7 | 1.15E7 | 3 | 3 | 3 | 3 | 3 | 3 | 4 | 4 | 2 | 3 | 3 | 3 | Y | 9141 | Small ribosomal subunit protein eS21 OS=Mus musculus OX=10090 GN=Rps21 PE=1 SV=1 |
| 1437 | 2147 | sp\|O88456\|CPNS1\_MOUSE | 148.19 | 18 | 6 | 0 | 0 | 0 | 0 | 15 | 15 | 0 | 9 | 13 | 2.73E6 |  |  |  |  | 8.54E6 | 5.35E6 |  | 1.34E7 | 6.37E6 | 3 | 3 | 1 | 0 | 0 | 0 | 0 | 2 | 2 | 0 | 1 | 2 | Y | 28406 | Calpain small subunit 1 OS=Mus musculus OX=10090 GN=Capns1 PE=1 SV=2 |
| 994 | 1783 | sp\|Q99P72\|RTN4\_MOUSE | 148.04 | 6 | 1 | 1 | 1 | 2 | 2 | 2 | 1 | 1 | 2 | 4 | 1.22E7 | 7.91E6 | 2.12E6 | 4.1E6 | 8.63E6 | 1.11E7 | 4.21E6 | 1.64E6 | 2.11E7 | 1.15E7 | 4 | 4 | 2 | 1 | 2 | 2 | 2 | 2 | 1 | 1 | 2 | 3 | N | 126613 | Reticulon-4 OS=Mus musculus OX=10090 GN=Rtn4 PE=1 SV=2 |
| 402 | 1323 | sp\|P14115\|RL27A\_MOUSE | 148.03 | 34 | 21 | 21 | 24 | 28 | 16 | 24 | 28 | 28 | 28 | 24 | 7.34E7 | 7.14E7 | 7.82E7 | 4.14E7 | 4.7E7 | 5.83E7 | 4.84E7 | 5.38E7 | 1.24E8 | 8.19E7 | 6 | 6 | 7 | 6 | 7 | 7 | 5 | 5 | 6 | 7 | 7 | 8 | Y | 16605 | Large ribosomal subunit protein uL15 OS=Mus musculus OX=10090 GN=Rpl27a PE=1 SV=5 |
| 474 | 1171 | D3YZX3\|D3YZX3\_MOUSE | 148.00 | 23 | 13 | 9 | 15 | 11 | 15 | 23 | 13 | 9 | 7 | 13 | 5.7E6 |  | 4.87E6 | 2.04E6 | 3.78E6 | 4.83E6 | 2.81E6 |  | 1.05E7 | 6.32E6 | 6 | 1 | 5 | 4 | 6 | 5 | 7 | 7 | 6 | 5 | 2 | 5 | Y | 32408 | Guanine nucleotide binding protein (G protein), beta 2 OS=Mus musculus OX=10090 GN=Gnb2 PE=1 SV=1 |
| 474 | 1176 | sp\|P62880\|GBB2\_MOUSE | 148.00 | 20 | 11 | 8 | 13 | 9 | 13 | 20 | 11 | 8 | 6 | 11 | 5.7E6 |  | 4.87E6 | 2.04E6 | 3.78E6 | 4.83E6 | 2.81E6 |  | 1.05E7 | 6.32E6 | 6 | 1 | 5 | 4 | 6 | 5 | 7 | 7 | 6 | 5 | 2 | 5 | Y | 37331 | Guanine nucleotide-binding protein G(I)/G(S)/G(T) subunit beta-2 OS=Mus musculus OX=10090 GN=Gnb2 PE=1 SV=3 |
| 474 | 1185 | E9QKR0\|E9QKR0\_MOUSE | 148.00 | 18 | 10 | 7 | 12 | 8 | 12 | 18 | 10 | 7 | 6 | 10 | 5.7E6 |  | 4.87E6 | 2.04E6 | 3.78E6 | 4.83E6 | 2.81E6 |  | 1.05E7 | 6.32E6 | 6 | 1 | 5 | 4 | 6 | 5 | 7 | 7 | 6 | 5 | 2 | 5 | Y | 41409 | Guanine nucleotide binding protein (G protein), beta 2 OS=Mus musculus OX=10090 GN=Gnb2 PE=1 SV=1 |
| 939 | 1394 | E9Q3V6\|E9Q3V6\_MOUSE | 147.95 | 21 | 11 | 10 | 5 | 2 | 9 | 16 | 7 | 13 | 5 | 5 | 3.23E6 | 1.96E6 | 1.05E6 | 1.81E6 | 6.23E6 | 3.03E6 | 4.22E6 | 3.73E6 | 2.37E6 | 1.47E6 | 5 | 5 | 2 | 2 | 1 | 1 | 3 | 3 | 2 | 3 | 1 | 1 | N | 36978 | Septin-2 OS=Mus musculus OX=10090 GN=Septin2 PE=1 SV=1 |
| 939 | 1397 | sp\|P42208\|SEPT2\_MOUSE | 147.95 | 18 | 10 | 9 | 4 | 2 | 8 | 14 | 6 | 11 | 4 | 4 | 3.23E6 | 1.96E6 | 1.05E6 | 1.81E6 | 6.23E6 | 3.03E6 | 4.22E6 | 3.73E6 | 2.37E6 | 1.47E6 | 5 | 5 | 2 | 2 | 1 | 1 | 3 | 3 | 2 | 3 | 1 | 1 | N | 41526 | Septin-2 OS=Mus musculus OX=10090 GN=Septin2 PE=1 SV=2 |
| 835 | 1128 | E9Q0U7\|E9Q0U7\_MOUSE | 147.72 | 8 | 3 | 2 | 6 | 6 | 3 | 5 | 6 | 6 | 0 | 0 | 2.22E6 | 4.85E6 | 8.83E6 | 6.17E6 | 4.37E6 | 7.43E6 | 6.16E6 | 6.21E6 |  |  | 6 | 5 | 2 | 1 | 4 | 4 | 2 | 3 | 4 | 4 | 0 | 0 | Y | 91678 | Heat shock 105kDa/110kDa protein 1 OS=Mus musculus OX=10090 GN=Hsph1 PE=1 SV=1 |
| 835 | 1129 | sp\|Q61699\|HS105\_MOUSE | 147.72 | 8 | 3 | 2 | 6 | 6 | 3 | 5 | 6 | 5 | 0 | 0 | 2.22E6 | 4.85E6 | 8.83E6 | 6.17E6 | 4.37E6 | 7.43E6 | 6.16E6 | 6.21E6 |  |  | 6 | 5 | 2 | 1 | 4 | 4 | 2 | 3 | 4 | 4 | 0 | 0 | Y | 96407 | Heat shock protein 105 kDa OS=Mus musculus OX=10090 GN=Hsph1 PE=1 SV=2 |
| 720 | 1095 | G3UVV4\|G3UVV4\_MOUSE | 147.65 | 7 | 2 | 4 | 3 | 5 | 4 | 1 | 2 | 1 | 3 | 3 | 1.01E6 | 2.01E5 | 2.68E5 | 1.98E5 | 3.51E5 |  |  |  |  | 1.99E6 | 7 | 3 | 1 | 3 | 3 | 4 | 4 | 1 | 2 | 1 | 3 | 3 | Y | 101873 | Hexokinase-1 OS=Mus musculus OX=10090 GN=Hk1 PE=1 SV=1 |
| 720 | 1111 | sp\|P17710\|HXK1\_MOUSE | 147.65 | 6 | 2 | 3 | 3 | 4 | 4 | 1 | 2 | 1 | 2 | 3 | 1.01E6 | 2.01E5 | 2.68E5 | 1.98E5 | 3.51E5 |  |  |  |  | 1.99E6 | 7 | 3 | 1 | 3 | 3 | 4 | 4 | 1 | 2 | 1 | 3 | 3 | Y | 108303 | Hexokinase-1 OS=Mus musculus OX=10090 GN=Hk1 PE=1 SV=3 |
| 800 | 1689 | sp\|Q9CZ30\|OLA1\_MOUSE | 147.65 | 11 | 11 | 7 | 8 | 0 | 8 | 8 | 7 | 8 | 7 | 11 | 1.57E7 | 4.72E6 | 1.09E7 |  | 8.5E6 | 1.06E7 | 5.02E6 | 3.75E6 | 9.21E6 | 1.1E7 | 4 | 4 | 4 | 2 | 3 | 0 | 3 | 3 | 2 | 3 | 2 | 4 | N | 44730 | Obg-like ATPase 1 OS=Mus musculus OX=10090 GN=Ola1 PE=1 SV=1 |
| 685 | 1519 | F8WJ41\|F8WJ41\_MOUSE | 147.64 | 43 | 43 | 26 | 20 | 29 | 29 | 34 | 34 | 29 | 13 | 43 | 2.93E7 | 3.44E7 | 2.41E7 | 2.35E7 | 3.56E7 | 3.26E7 | 2.76E7 | 2.69E7 | 1.01E7 | 3.57E7 | 5 | 5 | 5 | 3 | 2 | 4 | 3 | 4 | 4 | 3 | 1 | 5 | Y | 12311 | 40S ribosomal protein S15a (Fragment) OS=Mus musculus OX=10090 GN=Rps15a PE=1 SV=1 |
| 685 | 1524 | sp\|P62245\|RS15A\_MOUSE | 147.64 | 35 | 35 | 22 | 17 | 24 | 24 | 28 | 28 | 24 | 11 | 35 | 2.93E7 | 3.44E7 | 2.41E7 | 2.35E7 | 3.56E7 | 3.26E7 | 2.76E7 | 2.69E7 | 1.01E7 | 3.57E7 | 5 | 5 | 5 | 3 | 2 | 4 | 3 | 4 | 4 | 3 | 1 | 5 | Y | 14840 | Small ribosomal subunit protein uS8 OS=Mus musculus OX=10090 GN=Rps15a PE=1 SV=2 |
| 755 | 1317 | sp\|Q64521\|GPDM\_MOUSE | 147.60 | 6 | 3 | 4 | 1 | 5 | 4 | 3 | 3 | 1 | 4 | 4 | 3.34E6 | 6.62E6 | 3.39E6 | 4.05E6 | 4.31E6 | 4.46E6 | 3.79E6 | 2.32E6 | 8.35E6 | 3.04E6 | 4 | 4 | 2 | 3 | 1 | 3 | 3 | 3 | 2 | 1 | 3 | 3 | N | 80954 | Glycerol-3-phosphate dehydrogenase, mitochondrial OS=Mus musculus OX=10090 GN=Gpd2 PE=1 SV=2 |
| 755 | 1322 | A2AQR0\|A2AQR0\_MOUSE | 147.60 | 6 | 3 | 4 | 1 | 5 | 4 | 3 | 3 | 1 | 4 | 4 | 3.34E6 | 6.62E6 | 3.39E6 | 4.05E6 | 4.31E6 | 4.46E6 | 3.79E6 | 2.32E6 | 8.35E6 | 3.04E6 | 4 | 4 | 2 | 3 | 1 | 3 | 3 | 3 | 2 | 1 | 3 | 3 | N | 82830 | Glycerol-3-phosphate dehydrogenase OS=Mus musculus OX=10090 GN=Gpd2 PE=1 SV=1 |
| 759 | 1087 | sp\|Q07076\|ANXA7\_MOUSE | 147.39 | 13 | 3 | 4 | 10 | 8 | 8 | 8 | 2 | 10 | 3 | 2 | 1.36E6 | 1.81E6 | 1.4E7 | 3.84E6 | 1.13E7 | 5.65E6 | 2.68E6 | 5.59E6 | 2.41E6 | 2.24E6 | 6 | 5 | 1 | 2 | 5 | 4 | 4 | 3 | 2 | 5 | 2 | 1 | N | 49925 | Annexin A7 OS=Mus musculus OX=10090 GN=Anxa7 PE=1 SV=2 |
| 759 | 1088 | A0A2C9F2D2\|A0A2C9F2D2\_MOUSE | 147.39 | 12 | 3 | 4 | 9 | 7 | 8 | 7 | 2 | 9 | 3 | 2 | 1.36E6 | 1.81E6 | 1.4E7 | 3.84E6 | 1.13E7 | 5.65E6 | 2.68E6 | 5.59E6 | 2.41E6 | 2.24E6 | 6 | 5 | 1 | 2 | 5 | 4 | 4 | 3 | 2 | 5 | 2 | 1 | N | 52271 | Annexin OS=Mus musculus OX=10090 GN=Anxa7 PE=1 SV=1 |
| 599 | 1857 | sp\|P61750\|ARF4\_MOUSE | 147.38 | 27 | 27 | 27 | 21 | 17 | 21 | 21 | 17 | 17 | 27 | 27 | 2.22E7 | 2.13E7 | 2.14E7 | 6.78E6 | 2.03E7 | 2.02E7 | 9.63E6 | 8.69E6 | 1.63E7 | 2.2E7 | 4 | 2 | 5 | 5 | 4 | 3 | 4 | 4 | 3 | 3 | 5 | 5 | Y | 20397 | ADP-ribosylation factor 4 OS=Mus musculus OX=10090 GN=Arf4 PE=1 SV=2 |
| 648 | 1361 | sp\|Q9WUB3\|PYGM\_MOUSE | 147.19 | 6 | 2 | 4 | 3 | 4 | 4 | 5 | 3 | 4 | 3 | 2 |  |  |  |  |  | 1.48E6 |  |  |  |  | 6 | 1 | 2 | 4 | 3 | 4 | 4 | 4 | 3 | 4 | 3 | 2 | N | 97286 | Glycogen phosphorylase, muscle form OS=Mus musculus OX=10090 GN=Pygm PE=1 SV=3 |
| 670 | 1191 | sp\|Q9QUM9\|PSA6\_MOUSE | 147.03 | 30 | 21 | 15 | 4 | 17 | 20 | 20 | 21 | 9 | 17 | 17 | 6.51E6 | 1.07E7 | 1.57E6 | 2.43E6 | 1.05E7 | 1.03E7 | 6.13E6 | 5.54E6 | 1.46E7 | 9.34E6 | 6 | 6 | 4 | 3 | 1 | 3 | 4 | 4 | 4 | 2 | 5 | 3 | Y | 27372 | Proteasome subunit alpha type-6 OS=Mus musculus OX=10090 GN=Psma6 PE=1 SV=1 |
| 979 | 1933 | I7HPW8\|I7HPW8\_MOUSE | 146.80 | 1 | 0 | 1 | 0 | 0 | 1 | 1 | 1 | 1 | 0 | 0 | 2.44E6 | 5.47E6 | 2.38E6 |  | 3.48E6 | 5.87E6 | 3.26E6 | 1.53E6 | 3.31E6 | 1.22E6 | 3 | 3 | 1 | 2 | 1 | 0 | 3 | 3 | 2 | 2 | 1 | 1 | N | 332970 | Myosin XVB OS=Mus musculus OX=10090 GN=Myo15b PE=1 SV=2 |
| 979 | 1993 | A0A1Y7VNG3\|A0A1Y7VNG3\_MOUSE | 146.80 | 3 | 1 | 2 | 1 | 0 | 2 | 3 | 2 | 2 | 1 | 1 | 2.44E6 | 5.47E6 | 2.38E6 |  | 3.48E6 | 5.87E6 | 3.26E6 | 1.53E6 | 3.31E6 | 1.22E6 | 3 | 3 | 1 | 2 | 1 | 0 | 3 | 3 | 2 | 2 | 1 | 1 | N | 178303 | Myosin XVB OS=Mus musculus OX=10090 GN=Myo15b PE=1 SV=1 |
| 466 | 773 | sp\|P41105\|RL28\_MOUSE | 146.36 | 48 | 28 | 19 | 19 | 28 | 33 | 25 | 29 | 25 | 37 | 23 | 9.84E7 | 6.73E7 | 8.58E7 | 4.54E7 | 7.91E7 | 6.33E7 | 7.59E7 | 3.58E7 | 4.57E7 | 7.61E7 | 9 | 9 | 7 | 3 | 4 | 6 | 6 | 4 | 6 | 4 | 6 | 5 | Y | 15733 | Large ribosomal subunit protein eL28 OS=Mus musculus OX=10090 GN=Rpl28 PE=1 SV=2 |
| 1069 | 1692 | sp\|Q920E5\|FPPS\_MOUSE | 146.31 | 13 | 4 | 4 | 4 | 9 | 7 | 4 | 8 | 7 | 4 | 4 | 3.43E6 | 6E6 | 1.23E6 | 2.19E7 | 3.64E6 | 2.25E6 | 3.18E6 | 1.75E6 | 4.8E6 | 2.74E6 | 4 | 4 | 1 | 1 | 1 | 3 | 2 | 1 | 2 | 2 | 1 | 1 | N | 40582 | Farnesyl pyrophosphate synthase OS=Mus musculus OX=10090 GN=Fdps PE=1 SV=1 |
| 980 | 1583 | sp\|P62334\|PRS10\_MOUSE | 146.30 | 13 | 2 | 2 | 4 | 7 | 4 | 7 | 7 | 4 | 10 | 0 | 1.13E6 | 1.02E6 | 1.44E6 | 1.6E6 | 1.18E6 | 6.88E6 | 7.29E5 | 3.21E5 | 1.48E7 |  | 5 | 5 | 1 | 1 | 1 | 2 | 1 | 2 | 2 | 1 | 3 | 0 | N | 44173 | 26S proteasome regulatory subunit 10B OS=Mus musculus OX=10090 GN=Psmc6 PE=1 SV=1 |
| 866 | 2214 | E9PV44\|E9PV44\_MOUSE | 146.29 | 39 | 0 | 0 | 28 | 9 | 39 | 28 | 24 | 24 | 9 | 0 |  |  | 2.31E7 | 5.66E6 | 1.16E7 | 2.24E7 | 1.09E7 | 8.32E6 | 7.04E6 |  | 5 | 5 | 0 | 0 | 4 | 1 | 6 | 6 | 2 | 2 | 1 | 0 | N | 8789 | ATPase inhibitor, mitochondrial OS=Mus musculus OX=10090 GN=Atp5if1 PE=1 SV=1 |
| 866 | 2215 | sp\|O35143\|ATIF1\_MOUSE | 146.29 | 27 | 0 | 0 | 20 | 7 | 27 | 20 | 17 | 17 | 7 | 0 |  |  | 2.31E7 | 5.66E6 | 1.16E7 | 2.24E7 | 1.09E7 | 8.32E6 | 7.04E6 |  | 5 | 5 | 0 | 0 | 4 | 1 | 6 | 6 | 2 | 2 | 1 | 0 | N | 12159 | ATPase inhibitor, mitochondrial OS=Mus musculus OX=10090 GN=Atp5if1 PE=1 SV=2 |
| 775 | 1057 | Q9D9P1\|Q9D9P1\_MOUSE | 146.23 | 35 | 0 | 13 | 11 | 17 | 6 | 20 | 30 | 17 | 15 | 6 |  | 1.27E7 | 1.47E7 | 8.6E6 | 2.99E6 | 1.47E7 | 1.27E7 | 6.64E6 | 5.5E6 | 3.17E6 | 6 | 6 | 0 | 2 | 2 | 4 | 1 | 3 | 5 | 3 | 2 | 1 | N | 20376 | Coiled-coil-helix-coiled-coil-helix domain containing 3 OS=Mus musculus OX=10090 GN=Chchd3 PE=1 SV=1 |
| 775 | 1058 | D3Z0L4\|D3Z0L4\_MOUSE | 146.23 | 32 | 0 | 12 | 10 | 16 | 6 | 18 | 28 | 16 | 14 | 6 |  | 1.27E7 | 1.47E7 | 8.6E6 | 2.99E6 | 1.47E7 | 1.27E7 | 6.64E6 | 5.5E6 | 3.17E6 | 6 | 6 | 0 | 2 | 2 | 4 | 1 | 3 | 5 | 3 | 2 | 1 | N | 22452 | Coiled-coil-helix-coiled-coil-helix domain containing 3 (Fragment) OS=Mus musculus OX=10090 GN=Chchd3 PE=1 SV=1 |
| 775 | 1063 | sp\|Q9CRB9\|MIC19\_MOUSE | 146.23 | 27 | 0 | 10 | 9 | 13 | 5 | 15 | 23 | 13 | 11 | 5 |  | 1.27E7 | 1.47E7 | 8.6E6 | 2.99E6 | 1.47E7 | 1.27E7 | 6.64E6 | 5.5E6 | 3.17E6 | 6 | 6 | 0 | 2 | 2 | 4 | 1 | 3 | 5 | 3 | 2 | 1 | N | 26335 | MICOS complex subunit Mic19 OS=Mus musculus OX=10090 GN=Chchd3 PE=1 SV=1 |
| 893 | 1296 | sp\|P58021\|TM9S2\_MOUSE | 146.20 | 8 | 1 | 4 | 0 | 6 | 1 | 3 | 5 | 2 | 1 | 2 | 1.04E7 | 1.48E7 |  | 1.21E7 | 3.89E6 | 4.08E6 | 1.32E7 | 7.41E6 | 5.81E6 | 1.36E7 | 5 | 5 | 1 | 3 | 0 | 5 | 1 | 2 | 3 | 3 | 1 | 2 | Y | 75330 | Transmembrane 9 superfamily member 2 OS=Mus musculus OX=10090 GN=Tm9sf2 PE=1 SV=1 |
| 545 | 1942 | sp\|P19536\|COX5B\_MOUSE | 146.17 | 30 | 16 | 24 | 30 | 22 | 22 | 22 | 22 | 22 | 24 | 16 | 4.05E7 | 4.78E7 | 3.67E7 | 2.87E7 | 5.67E7 | 6.64E7 | 3.74E7 | 4.51E7 | 3E7 | 2.99E7 | 4 | 4 | 2 | 4 | 5 | 5 | 5 | 5 | 4 | 5 | 4 | 3 | Y | 13813 | Cytochrome c oxidase subunit 5B, mitochondrial OS=Mus musculus OX=10090 GN=Cox5b PE=1 SV=1 |
| 545 | 1943 | Q9D881\|Q9D881\_MOUSE | 146.17 | 30 | 16 | 24 | 30 | 22 | 22 | 22 | 22 | 22 | 24 | 16 | 4.05E7 | 4.78E7 | 3.67E7 | 2.87E7 | 5.67E7 | 6.64E7 | 3.74E7 | 4.51E7 | 3E7 | 2.99E7 | 4 | 4 | 2 | 4 | 5 | 5 | 5 | 5 | 4 | 5 | 4 | 3 | Y | 13847 | Cytochrome c oxidase subunit 5B, mitochondrial OS=Mus musculus OX=10090 GN=Cox5b-ps PE=1 SV=1 |
| 682 | 1168 | sp\|P01899\|HA11\_MOUSE | 146.15 | 19 | 9 | 5 | 6 | 14 | 12 | 8 | 14 | 8 | 12 | 5 | 3.69E6 |  | 1.1E6 | 2.75E6 | 4E6 |  | 1.55E6 |  | 9.88E6 |  | 6 | 3 | 3 | 2 | 2 | 6 | 4 | 3 | 5 | 3 | 4 | 2 | N | 40836 | H-2 class I histocompatibility antigen, D-B alpha chain OS=Mus musculus OX=10090 GN=H2-D1 PE=1 SV=2 |
| 682 | 1170 | G3UZP7\|G3UZP7\_MOUSE | 146.15 | 18 | 9 | 5 | 6 | 14 | 11 | 8 | 14 | 8 | 12 | 5 | 3.69E6 |  | 1.1E6 | 2.75E6 | 4E6 |  | 1.55E6 |  | 9.88E6 |  | 6 | 3 | 3 | 2 | 2 | 6 | 4 | 3 | 5 | 3 | 4 | 2 | N | 42780 | Histocompatibility 2, D region locus 1 OS=Mus musculus OX=10090 GN=H2-D1 PE=1 SV=2 |
| 1070 | 2016 | sp\|P46935\|NEDD4\_MOUSE | 146.05 | 5 | 1 | 4 | 1 | 3 | 1 | 1 | 3 | 1 | 1 | 3 | 3.28E6 | 2.44E6 | 1.81E6 | 5.93E5 | 1.73E6 | 2.38E6 | 1.56E6 | 6.13E5 | 4.11E6 | 2.51E6 | 3 | 2 | 2 | 2 | 1 | 2 | 1 | 1 | 2 | 1 | 1 | 2 | N | 102706 | E3 ubiquitin-protein ligase NEDD4 OS=Mus musculus OX=10090 GN=Nedd4 PE=1 SV=3 |
| 851 | 2610 | sp\|P53810\|PIPNA\_MOUSE | 145.89 | 11 | 11 | 2 | 11 | 11 | 11 | 11 | 8 | 11 | 8 | 11 | 7.39E6 | 4.93E6 | 8.67E6 | 5.18E6 | 9.7E6 | 7.36E6 | 3.24E5 | 7.93E6 | 1.06E7 | 1.18E7 | 2 | 2 | 3 | 1 | 2 | 3 | 2 | 3 | 1 | 3 | 1 | 2 | N | 31893 | Phosphatidylinositol transfer protein alpha isoform OS=Mus musculus OX=10090 GN=Pitpna PE=1 SV=2 |
| 667 | 1125 | sp\|Q99LX0\|PARK7\_MOUSE | 145.81 | 37 | 16 | 5 | 9 | 29 | 16 | 21 | 33 | 7 | 20 | 13 | 6.98E6 | 7.46E6 | 1.69E7 | 9.63E6 | 1.32E7 | 2.39E7 | 1.56E7 | 0E0 | 1.94E7 | 1.64E7 | 6 | 6 | 3 | 1 | 2 | 4 | 3 | 4 | 5 | 1 | 4 | 3 | Y | 20021 | Parkinson disease protein 7 homolog OS=Mus musculus OX=10090 GN=Park7 PE=1 SV=1 |
| 1290 | 1297 | sp\|P28352\|APEX1\_MOUSE | 145.73 | 25 | 5 | 0 | 5 | 6 | 5 | 11 | 0 | 0 | 5 | 8 | 4.26E5 |  | 5.93E5 | 7.53E5 | 3.68E5 | 2.3E6 |  |  | 1.32E6 | 3.34E6 | 5 | 5 | 1 | 0 | 1 | 1 | 1 | 2 | 0 | 0 | 1 | 2 | Y | 35490 | DNA repair nuclease/redox regulator APEX1 OS=Mus musculus OX=10090 GN=Apex1 PE=1 SV=2 |
| 1308 | 1827 | sp\|O08691\|ARGI2\_MOUSE | 145.47 | 14 | 4 | 9 | 12 | 0 | 2 | 9 | 4 | 0 | 0 | 0 | 2.12E6 | 6.07E6 | 6.73E6 |  | 2.09E6 | 2.4E6 | 5.03E5 |  |  |  | 4 | 4 | 1 | 2 | 3 | 0 | 1 | 2 | 1 | 0 | 0 | 0 | N | 38878 | Arginase-2, mitochondrial OS=Mus musculus OX=10090 GN=Arg2 PE=1 SV=1 |
| 406 | 844 | sp\|Q9Z1N5\|DX39B\_MOUSE | 145.18 | 15 | 11 | 4 | 11 | 7 | 11 | 11 | 9 | 13 | 11 | 11 |  |  |  |  |  |  |  | 8.01E5 |  |  | 7 | 1 | 6 | 3 | 7 | 3 | 7 | 7 | 6 | 7 | 8 | 8 | N | 49035 | Spliceosome RNA helicase Ddx39b OS=Mus musculus OX=10090 GN=Ddx39b PE=1 SV=1 |
| 604 | 882 | sp\|P54227\|STMN1\_MOUSE | 145.12 | 42 | 19 | 15 | 11 | 32 | 26 | 19 | 36 | 30 | 0 | 5 | 1.25E7 | 2.85E6 | 1.45E7 | 6.03E6 | 1.83E7 | 9.96E6 | 1.45E7 | 7.19E6 |  | 2.64E6 | 8 | 8 | 3 | 2 | 2 | 5 | 6 | 3 | 6 | 6 | 0 | 1 | N | 17274 | Stathmin OS=Mus musculus OX=10090 GN=Stmn1 PE=1 SV=2 |
| 657 | 1402 | H7BWZ3\|H7BWZ3\_MOUSE | 144.84 | 28 | 18 | 18 | 17 | 18 | 22 | 22 | 18 | 18 | 24 | 22 | 1.85E6 | 2.83E6 | 4.74E6 | 6.66E5 | 3.46E6 | 7.4E5 | 2.93E6 | 4.76E5 | 1.17E7 | 4.03E6 | 5 | 1 | 3 | 3 | 3 | 3 | 4 | 4 | 3 | 3 | 5 | 4 | Y | 19638 | Actin-related protein 2/3 complex subunit 3 OS=Mus musculus OX=10090 GN=Arpc3 PE=1 SV=1 |
| 657 | 1405 | sp\|Q9JM76\|ARPC3\_MOUSE | 144.84 | 27 | 17 | 17 | 16 | 17 | 21 | 21 | 17 | 17 | 23 | 21 | 1.85E6 | 2.83E6 | 4.74E6 | 6.66E5 | 3.46E6 | 7.4E5 | 2.93E6 | 4.76E5 | 1.17E7 | 4.03E6 | 5 | 1 | 3 | 3 | 3 | 3 | 4 | 4 | 3 | 3 | 5 | 4 | Y | 20525 | Actin-related protein 2/3 complex subunit 3 OS=Mus musculus OX=10090 GN=Arpc3 PE=1 SV=3 |
| 525 | 1177 | sp\|P62874\|GBB1\_MOUSE | 144.78 | 20 | 8 | 8 | 9 | 9 | 9 | 16 | 11 | 8 | 3 | 8 |  |  |  | 2.16E6 |  |  | 3.59E6 |  |  |  | 6 | 1 | 4 | 4 | 5 | 5 | 6 | 6 | 6 | 5 | 1 | 4 | Y | 37377 | Guanine nucleotide-binding protein G(I)/G(S)/G(T) subunit beta-1 OS=Mus musculus OX=10090 GN=Gnb1 PE=1 SV=3 |
| 978 | 1198 | sp\|Q8JZQ9\|EIF3B\_MOUSE | 144.66 | 7 | 0 | 0 | 3 | 2 | 3 | 2 | 3 | 4 | 3 | 1 |  |  | 4.46E6 | 1.42E6 | 1.53E6 | 3.81E6 | 7.05E5 | 3.09E6 | 7.06E6 | 8.41E5 | 5 | 5 | 0 | 0 | 2 | 2 | 2 | 2 | 2 | 3 | 2 | 1 | N | 91370 | Eukaryotic translation initiation factor 3 subunit B OS=Mus musculus OX=10090 GN=Eif3b PE=1 SV=1 |
| 828 | 935 | sp\|Q9EQP2\|EHD4\_MOUSE | 144.31 | 12 | 1 | 3 | 4 | 4 | 2 | 11 | 7 | 2 | 2 | 3 |  | 4.89E6 | 5.49E6 | 2.94E6 | 3.34E6 | 8.56E6 | 3.57E6 | 7.9E5 | 3.36E6 | 1.52E6 | 7 | 5 | 1 | 2 | 3 | 2 | 2 | 6 | 4 | 1 | 1 | 2 | N | 61481 | EH domain-containing protein 4 OS=Mus musculus OX=10090 GN=Ehd4 PE=1 SV=1 |
| 510 | 1279 | sp\|O55142\|RL35A\_MOUSE | 144.25 | 45 | 21 | 15 | 27 | 32 | 38 | 38 | 32 | 32 | 21 | 27 | 2.81E7 | 2.54E7 | 6.44E7 | 4.02E7 | 7.06E7 | 4.44E7 | 4.33E7 | 4.1E7 | 3.47E7 | 4.22E7 | 6 | 6 | 3 | 2 | 4 | 5 | 7 | 6 | 6 | 7 | 3 | 4 | Y | 12554 | Large ribosomal subunit protein eL33 OS=Mus musculus OX=10090 GN=Rpl35a PE=1 SV=2 |
| 1213 | 1804 | sp\|A6H584\|CO6A5\_MOUSE | 144.13 | 1 | 1 | 1 | 1 | 0 | 0 | 0 | 1 | 0 | 1 | 0 | 1.89E6 | 2.89E5 | 2.74E5 | 1.68E6 |  | 2.49E6 | 1.38E5 |  | 1.14E6 |  | 3 | 3 | 2 | 1 | 1 | 1 | 0 | 1 | 1 | 0 | 1 | 0 | N | 289572 | Collagen alpha-5(VI) chain OS=Mus musculus OX=10090 GN=Col6a5 PE=1 SV=4 |
| 1213 | 1805 | A0A140T8W1\|A0A140T8W1\_MOUSE | 144.13 | 1 | 1 | 1 | 1 | 0 | 0 | 0 | 1 | 0 | 1 | 0 | 1.89E6 | 2.89E5 | 2.74E5 | 1.68E6 |  | 2.49E6 | 1.38E5 |  | 1.14E6 |  | 3 | 3 | 2 | 1 | 1 | 1 | 0 | 1 | 1 | 0 | 1 | 0 | N | 289613 | Collagen, type VI, alpha 5 OS=Mus musculus OX=10090 GN=Col6a5 PE=1 SV=1 |
| 726 | 1707 | sp\|P42125\|ECI1\_MOUSE | 143.90 | 18 | 15 | 10 | 10 | 10 | 15 | 12 | 18 | 15 | 10 | 10 | 5.45E6 | 4.11E6 | 1.13E7 | 3.04E6 | 8.24E6 | 2.21E7 | 1.42E7 | 2E6 | 1.66E7 | 4.57E6 | 4 | 4 | 4 | 2 | 2 | 2 | 3 | 3 | 4 | 3 | 2 | 2 | N | 32250 | Enoyl-CoA delta isomerase 1, mitochondrial OS=Mus musculus OX=10090 GN=Eci1 PE=1 SV=2 |
| 726 | 1771 | A0A452J8A5\|A0A452J8A5\_MOUSE | 143.90 | 18 | 15 | 10 | 10 | 10 | 15 | 13 | 18 | 15 | 10 | 10 | 5.45E6 | 4.11E6 | 1.13E7 | 3.04E6 | 8.24E6 | 2.21E7 | 1.42E7 | 2E6 | 1.66E7 | 4.57E6 | 4 | 4 | 4 | 2 | 2 | 2 | 3 | 3 | 4 | 3 | 2 | 2 | N | 32023 | Enoyl-Coenzyme A delta isomerase 1 OS=Mus musculus OX=10090 GN=Eci1 PE=1 SV=1 |
| 726 | 1897 | A0A3Q4EC00\|A0A3Q4EC00\_MOUSE | 143.90 | 21 | 18 | 11 | 11 | 11 | 18 | 15 | 21 | 18 | 11 | 11 | 5.45E6 | 4.11E6 | 1.13E7 | 3.04E6 | 8.24E6 | 2.21E7 | 1.42E7 | 2E6 | 1.66E7 | 4.57E6 | 4 | 4 | 4 | 2 | 2 | 2 | 3 | 3 | 4 | 3 | 2 | 2 | N | 27842 | Enoyl-Coenzyme A delta isomerase 1 OS=Mus musculus OX=10090 GN=Eci1 PE=1 SV=1 |
| 1145 | 1009 | sp\|O70475\|UGDH\_MOUSE | 143.85 | 13 | 0 | 2 | 0 | 3 | 3 | 4 | 2 | 0 | 6 | 5 |  | 4.16E6 |  | 1.34E6 | 3.14E6 | 4.06E6 | 3.08E5 |  | 7.56E6 | 5.81E6 | 6 | 6 | 0 | 1 | 0 | 2 | 1 | 2 | 1 | 0 | 2 | 3 | Y | 54832 | UDP-glucose 6-dehydrogenase OS=Mus musculus OX=10090 GN=Ugdh PE=1 SV=1 |
| 879 | 2790 | sp\|O35658\|C1QBP\_MOUSE | 143.60 | 10 | 10 | 5 | 5 | 5 | 10 | 5 | 5 | 10 | 5 | 10 | 1.23E6 | 7.96E6 | 8.09E5 | 4.64E6 | 1.99E6 | 8.95E6 | 7.1E5 | 1.93E6 | 5.25E6 | 4.15E6 | 3 | 3 | 2 | 2 | 1 | 3 | 2 | 2 | 3 | 3 | 2 | 2 | N | 31013 | Complement component 1 Q subcomponent-binding protein, mitochondrial OS=Mus musculus OX=10090 GN=C1qbp PE=1 SV=1 |
| 351 | 734 | I7HLV2\|I7HLV2\_MOUSE | 143.56 | 38 | 22 | 19 | 29 | 27 | 32 | 31 | 19 | 16 | 23 | 26 | 4.69E7 | 4.57E7 | 7.38E7 | 3.67E7 | 6.62E7 | 6.36E7 | 3.74E7 | 3.47E7 | 7.38E7 | 5.48E7 | 9 | 9 | 6 | 5 | 9 | 7 | 9 | 10 | 5 | 5 | 8 | 8 | Y | 23072 | Large ribosomal subunit protein uL16 (Fragment) OS=Mus musculus OX=10090 GN=Rpl10 PE=1 SV=1 |
| 351 | 737 | sp\|Q6ZWV3\|RL10\_MOUSE | 143.56 | 36 | 21 | 18 | 27 | 25 | 30 | 29 | 18 | 15 | 22 | 25 | 4.69E7 | 4.57E7 | 7.38E7 | 3.67E7 | 6.62E7 | 6.36E7 | 3.74E7 | 3.47E7 | 7.38E7 | 5.48E7 | 9 | 9 | 6 | 5 | 9 | 7 | 9 | 10 | 5 | 5 | 8 | 8 | Y | 24604 | Large ribosomal subunit protein uL16 OS=Mus musculus OX=10090 GN=Rpl10 PE=1 SV=3 |
| 414 | 1605 | sp\|Q9CX86\|ROA0\_MOUSE | 143.17 | 20 | 5 | 11 | 9 | 12 | 17 | 12 | 12 | 15 | 11 | 11 | 6.65E6 | 1.41E7 | 9.13E6 | 2.92E6 | 1.15E7 | 5.82E6 | 7.84E6 | 4.4E6 | 2.73E7 | 1.32E7 | 6 | 4 | 5 | 6 | 6 | 7 | 9 | 6 | 7 | 7 | 6 | 6 | N | 30530 | Heterogeneous nuclear ribonucleoprotein A0 OS=Mus musculus OX=10090 GN=Hnrnpa0 PE=1 SV=1 |
| 1183 | 856 | sp\|Q9WTP7\|KAD3\_MOUSE | 143.03 | 31 | 0 | 6 | 11 | 3 | 0 | 15 | 0 | 0 | 0 | 7 |  | 1.27E6 | 5.73E6 | 6.8E6 |  | 1.26E7 |  |  |  | 8.69E5 | 7 | 7 | 0 | 1 | 2 | 1 | 0 | 4 | 0 | 0 | 0 | 1 | Y | 25426 | GTP:AMP phosphotransferase AK3, mitochondrial OS=Mus musculus OX=10090 GN=Ak3 PE=1 SV=3 |
| 615 | 2321 | A0A0A0MQM0\|A0A0A0MQM0\_MOUSE | 142.90 | 21 | 21 | 16 | 16 | 13 | 16 | 11 | 16 | 21 | 16 | 16 | 1.88E7 | 3.98E7 | 4.26E7 | 2.22E7 | 7.83E6 | 3.59E7 | 3.99E7 | 2.4E7 | 2.31E7 | 5.45E7 | 4 | 4 | 4 | 4 | 3 | 3 | 4 | 2 | 4 | 5 | 4 | 4 | N | 16303 | Eukaryotic translation initiation factor 5A (Fragment) OS=Mus musculus OX=10090 GN=Eif5a PE=1 SV=1 |
| 615 | 2324 | sp\|P63242\|IF5A1\_MOUSE | 142.90 | 21 | 21 | 16 | 16 | 13 | 16 | 10 | 16 | 21 | 16 | 16 | 1.88E7 | 3.98E7 | 4.26E7 | 2.22E7 | 7.83E6 | 3.59E7 | 3.99E7 | 2.4E7 | 2.31E7 | 5.45E7 | 4 | 4 | 4 | 4 | 3 | 3 | 4 | 2 | 4 | 5 | 4 | 4 | N | 16832 | Eukaryotic translation initiation factor 5A-1 OS=Mus musculus OX=10090 GN=Eif5a PE=1 SV=2 |
| 342 | 1696 | A0A2R8VHP3\|A0A2R8VHP3\_MOUSE | 142.77 | 7 | 5 | 5 | 5 | 7 | 5 | 5 | 5 | 5 | 5 | 3 |  |  |  | 1.7E6 |  |  |  |  |  |  | 4 | 1 | 8 | 8 | 7 | 7 | 9 | 6 | 6 | 11 | 9 | 7 | N | 57920 | Predicted pseudogene 5478 OS=Mus musculus OX=10090 GN=Gm5478 PE=1 SV=1 |
| 544 | 1678 | A0A3Q4EH04\|A0A3Q4EH04\_MOUSE | 142.59 | 31 | 23 | 23 | 26 | 26 | 26 | 23 | 31 | 31 | 23 | 16 | 5.66E6 | 6.37E6 | 3.95E6 | 3.84E6 | 8.03E6 | 7.33E6 | 9.77E6 | 6.67E6 | 1.23E7 | 3.31E6 | 4 | 3 | 3 | 4 | 4 | 4 | 5 | 5 | 6 | 6 | 5 | 3 | N | 15763 | Serine and arginine-rich splicing factor 7 OS=Mus musculus OX=10090 GN=Srsf7 PE=1 SV=1 |
| 544 | 1679 | A0A3Q4L335\|A0A3Q4L335\_MOUSE | 142.59 | 20 | 14 | 14 | 16 | 16 | 16 | 14 | 20 | 20 | 14 | 10 | 5.66E6 | 6.37E6 | 3.95E6 | 3.84E6 | 8.03E6 | 7.33E6 | 9.77E6 | 6.67E6 | 1.23E7 | 3.31E6 | 4 | 3 | 3 | 4 | 4 | 4 | 5 | 5 | 6 | 6 | 5 | 3 | N | 24827 | Serine and arginine-rich splicing factor 7 OS=Mus musculus OX=10090 GN=Srsf7 PE=1 SV=1 |
| 544 | 1681 | A0A3Q4L393\|A0A3Q4L393\_MOUSE | 142.59 | 18 | 13 | 13 | 15 | 15 | 15 | 13 | 18 | 18 | 13 | 9 | 5.66E6 | 6.37E6 | 3.95E6 | 3.84E6 | 8.03E6 | 7.33E6 | 9.77E6 | 6.67E6 | 1.23E7 | 3.31E6 | 4 | 3 | 3 | 4 | 4 | 4 | 5 | 5 | 6 | 6 | 5 | 3 | N | 26939 | Serine and arginine-rich splicing factor 7 OS=Mus musculus OX=10090 GN=Srsf7 PE=1 SV=1 |
| 544 | 1682 | sp\|Q8BL97\|SRSF7\_MOUSE | 142.59 | 16 | 12 | 12 | 13 | 13 | 13 | 12 | 16 | 16 | 12 | 8 | 5.66E6 | 6.37E6 | 3.95E6 | 3.84E6 | 8.03E6 | 7.33E6 | 9.77E6 | 6.67E6 | 1.23E7 | 3.31E6 | 4 | 3 | 3 | 4 | 4 | 4 | 5 | 5 | 6 | 6 | 5 | 3 | N | 30818 | Serine/arginine-rich splicing factor 7 OS=Mus musculus OX=10090 GN=Srsf7 PE=1 SV=1 |
| 571 | 1784 | sp\|P45591\|COF2\_MOUSE | 142.07 | 28 | 7 | 19 | 19 | 11 | 11 | 7 | 11 | 7 | 7 | 11 |  | 1.34E6 | 1.99E6 |  |  |  | 6.63E5 |  |  |  | 4 | 2 | 5 | 5 | 3 | 3 | 3 | 2 | 3 | 4 | 4 | 6 | N | 18710 | Cofilin-2 OS=Mus musculus OX=10090 GN=Cfl2 PE=1 SV=1 |
| 1241 | 2187 | sp\|P26645\|MARCS\_MOUSE | 141.77 | 17 | 10 | 0 | 6 | 6 | 6 | 10 | 6 | 6 | 0 | 12 | 1.61E7 |  | 4.5E5 | 1.49E6 | 2.58E6 | 3.54E6 | 2.48E6 | 1.45E6 |  | 6.21E6 | 3 | 3 | 2 | 0 | 1 | 1 | 1 | 2 | 1 | 1 | 0 | 2 | N | 29661 | Myristoylated alanine-rich C-kinase substrate OS=Mus musculus OX=10090 GN=Marcks PE=1 SV=2 |
| 1366 | 2142 | sp\|P21614\|VTDB\_MOUSE | 141.73 | 9 | 0 | 5 | 5 | 5 | 0 | 5 | 9 | 0 | 5 | 0 |  | 7.42E5 | 1.85E6 | 3.34E5 |  | 1.07E6 | 1.85E6 |  | 1.01E6 |  | 3 | 3 | 0 | 1 | 2 | 1 | 0 | 1 | 3 | 0 | 1 | 0 | N | 53600 | Vitamin D-binding protein OS=Mus musculus OX=10090 GN=Gc PE=1 SV=2 |
| 1039 | 1825 | sp\|P52825\|CPT2\_MOUSE | 141.67 | 8 | 3 | 7 | 1 | 1 | 0 | 3 | 2 | 0 | 1 | 3 | 3.52E6 | 4.2E6 | 1.75E6 | 4.34E5 |  | 2.58E6 | 2.1E6 |  | 2.11E6 | 1.35E6 | 4 | 4 | 2 | 3 | 1 | 1 | 0 | 2 | 2 | 0 | 1 | 2 | N | 73981 | Carnitine O-palmitoyltransferase 2, mitochondrial OS=Mus musculus OX=10090 GN=Cpt2 PE=1 SV=2 |
| 841 | 1391 | Q8CFQ9\|Q8CFQ9\_MOUSE | 141.61 | 10 | 3 | 2 | 3 | 4 | 6 | 6 | 8 | 2 | 7 | 6 | 1E7 | 9.47E6 | 3.22E6 |  | 1.4E7 | 1.08E7 | 1.38E6 |  | 8.63E6 | 1.13E7 | 6 | 3 | 2 | 1 | 1 | 2 | 3 | 3 | 4 | 1 | 4 | 3 | Y | 52602 | Fused in sarcoma OS=Mus musculus OX=10090 GN=Fus PE=1 SV=1 |
| 841 | 1392 | sp\|P56959\|FUS\_MOUSE | 141.61 | 10 | 3 | 2 | 3 | 4 | 6 | 6 | 8 | 2 | 7 | 6 | 1E7 | 9.47E6 | 3.22E6 |  | 1.4E7 | 1.08E7 | 1.38E6 |  | 8.63E6 | 1.13E7 | 6 | 3 | 2 | 1 | 1 | 2 | 3 | 3 | 4 | 1 | 4 | 3 | Y | 52673 | RNA-binding protein FUS OS=Mus musculus OX=10090 GN=Fus PE=1 SV=1 |
| 340 | 1674 | sp\|P04104\|K2C1\_MOUSE | 141.39 | 6 | 4 | 2 | 6 | 6 | 2 | 4 | 6 | 6 | 2 | 2 | 6.54E6 |  | 7.62E6 | 3.01E6 |  | 3E6 | 2.94E6 | 5.28E6 |  |  | 4 | 1 | 8 | 7 | 8 | 6 | 8 | 6 | 7 | 12 | 8 | 7 | N | 65606 | Keratin, type II cytoskeletal 1 OS=Mus musculus OX=10090 GN=Krt1 PE=1 SV=4 |
| 1140 | 1155 | sp\|Q8K0E8\|FIBB\_MOUSE | 141.27 | 13 | 10 | 0 | 4 | 2 | 0 | 0 | 0 | 0 | 1 | 5 | 3.38E7 |  | 7.59E5 | 4.22E5 |  |  |  |  | 3.89E6 | 6.8E6 | 6 | 6 | 5 | 0 | 1 | 1 | 0 | 0 | 0 | 0 | 1 | 2 | Y | 54753 | Fibrinogen beta chain OS=Mus musculus OX=10090 GN=Fgb PE=1 SV=1 |
| 848 | 1883 | sp\|Q8BKC5\|IPO5\_MOUSE | 141.25 | 4 | 4 | 4 | 3 | 4 | 3 | 3 | 1 | 3 | 3 | 2 | 2.47E6 | 6.58E6 | 4.52E6 | 2.22E6 | 3.09E6 | 2.17E6 | 1.62E6 | 2.11E6 | 7.34E6 | 2.64E6 | 3 | 3 | 4 | 3 | 2 | 3 | 2 | 2 | 1 | 2 | 2 | 2 | Y | 123591 | Importin-5 OS=Mus musculus OX=10090 GN=Ipo5 PE=1 SV=3 |
| 189 | 1444 | E9Q1Z0\|E9Q1Z0\_MOUSE | 141.22 | 8 | 6 | 6 | 6 | 8 | 6 | 6 | 6 | 6 | 5 | 6 |  |  |  | 9.21E6 |  |  |  |  |  |  | 5 | 1 | 14 | 14 | 12 | 11 | 16 | 12 | 11 | 17 | 13 | 14 | Y | 58224 | Keratin 90 OS=Mus musculus OX=10090 GN=Krt90 PE=1 SV=1 |
| 727 | 1745 | sp\|Q9CQ62\|DECR\_MOUSE | 141.21 | 13 | 3 | 4 | 7 | 4 | 4 | 7 | 13 | 7 | 7 | 7 | 3.29E6 | 3.38E6 | 4.83E6 | 1.8E5 | 2.3E6 | 6.65E6 | 3.75E7 | 3.09E6 | 7.92E6 | 4.17E6 | 5 | 5 | 1 | 2 | 2 | 1 | 2 | 3 | 5 | 2 | 2 | 3 | N | 36214 | 2,4-dienoyl-CoA reductase [(3E)-enoyl-CoA-producing], mitochondrial OS=Mus musculus OX=10090 GN=Decr1 PE=1 SV=1 |
| 925 | 914 | B1AR28\|B1AR28\_MOUSE | 141.20 | 12 | 2 | 2 | 3 | 1 | 1 | 5 | 3 | 2 | 4 | 2 | 1.22E6 | 7.31E5 | 3.11E6 | 1.59E6 | 1.07E6 | 1.11E7 | 3.66E5 | 2.63E6 | 5.86E6 | 1.06E6 | 6 | 6 | 1 | 1 | 2 | 1 | 1 | 3 | 1 | 1 | 2 | 1 | N | 68551 | Very long-chain specific acyl-CoA dehydrogenase, mitochondrial OS=Mus musculus OX=10090 GN=Acadvl PE=1 SV=1 |
| 925 | 916 | sp\|P50544\|ACADV\_MOUSE | 141.20 | 11 | 2 | 2 | 3 | 1 | 1 | 5 | 3 | 2 | 4 | 2 | 1.22E6 | 7.31E5 | 3.11E6 | 1.59E6 | 1.07E6 | 1.11E7 | 3.66E5 | 2.63E6 | 5.86E6 | 1.06E6 | 6 | 6 | 1 | 1 | 2 | 1 | 1 | 3 | 1 | 1 | 2 | 1 | N | 70876 | Very long-chain specific acyl-CoA dehydrogenase, mitochondrial OS=Mus musculus OX=10090 GN=Acadvl PE=1 SV=3 |
| 473 | 1590 | E9PZ00\|E9PZ00\_MOUSE | 141.18 | 8 | 8 | 8 | 8 | 7 | 7 | 7 | 8 | 7 | 8 | 8 | 2.1E7 | 4.27E7 | 3.78E7 | 4.61E6 | 2.3E7 | 1.97E7 | 2.61E7 | 1.17E7 | 6.38E7 | 4.85E7 | 4 | 4 | 5 | 6 | 6 | 3 | 5 | 4 | 6 | 4 | 8 | 6 | Y | 60672 | Prosaposin OS=Mus musculus OX=10090 GN=Psap PE=1 SV=1 |
| 473 | 1591 | Q8BFQ1\|Q8BFQ1\_MOUSE | 141.18 | 8 | 8 | 8 | 8 | 6 | 6 | 6 | 8 | 6 | 8 | 8 | 2.1E7 | 4.27E7 | 3.78E7 | 4.61E6 | 2.3E7 | 1.97E7 | 2.61E7 | 1.17E7 | 6.38E7 | 4.85E7 | 4 | 4 | 5 | 6 | 6 | 3 | 5 | 4 | 6 | 4 | 8 | 6 | Y | 61051 | Prosaposin OS=Mus musculus OX=10090 GN=Psap PE=1 SV=1 |
| 473 | 1592 | J3QPG5\|J3QPG5\_MOUSE | 141.18 | 8 | 8 | 8 | 8 | 6 | 6 | 6 | 8 | 6 | 8 | 8 | 2.1E7 | 4.27E7 | 3.78E7 | 4.61E6 | 2.3E7 | 1.97E7 | 2.61E7 | 1.17E7 | 6.38E7 | 4.85E7 | 4 | 4 | 5 | 6 | 6 | 3 | 5 | 4 | 6 | 4 | 8 | 6 | Y | 61363 | Prosaposin OS=Mus musculus OX=10090 GN=Psap PE=1 SV=1 |
| 473 | 1593 | sp\|Q61207\|SAP\_MOUSE | 141.18 | 8 | 8 | 8 | 8 | 6 | 6 | 6 | 8 | 6 | 8 | 8 | 2.1E7 | 4.27E7 | 3.78E7 | 4.61E6 | 2.3E7 | 1.97E7 | 2.61E7 | 1.17E7 | 6.38E7 | 4.85E7 | 4 | 4 | 5 | 6 | 6 | 3 | 5 | 4 | 6 | 4 | 8 | 6 | Y | 61422 | Prosaposin OS=Mus musculus OX=10090 GN=Psap PE=1 SV=2 |
| 608 | 1406 | Q5RKN9\|Q5RKN9\_MOUSE | 141.11 | 22 | 14 | 12 | 12 | 3 | 10 | 14 | 22 | 14 | 15 | 10 | 1.89E7 | 3.57E6 | 1.75E7 |  | 7.97E6 | 1.85E7 | 7.35E6 | 5.64E6 | 1.01E7 | 3.24E6 | 5 | 3 | 5 | 3 | 4 | 1 | 2 | 4 | 5 | 3 | 3 | 3 | N | 32954 | F-actin-capping protein subunit alpha OS=Mus musculus OX=10090 GN=Capza1 PE=1 SV=1 |
| 608 | 1407 | sp\|P47753\|CAZA1\_MOUSE | 141.11 | 22 | 14 | 12 | 12 | 3 | 10 | 14 | 22 | 14 | 15 | 10 | 1.89E7 | 3.57E6 | 1.75E7 |  | 7.97E6 | 1.85E7 | 7.35E6 | 5.64E6 | 1.01E7 | 3.24E6 | 5 | 3 | 5 | 3 | 4 | 1 | 2 | 4 | 5 | 3 | 3 | 3 | N | 32940 | F-actin-capping protein subunit alpha-1 OS=Mus musculus OX=10090 GN=Capza1 PE=1 SV=4 |
| 745 | 1039 | A0A3B2WCD8\|A0A3B2WCD8\_MOUSE | 141.08 | 9 | 3 | 3 | 3 | 7 | 4 | 3 | 7 | 1 | 3 | 5 | 4.58E6 | 4.08E6 | 4.69E6 | 5.91E6 | 4.54E6 | 1.75E6 | 7.79E6 | 8.6E5 | 6.28E6 | 1.35E7 | 6 | 6 | 2 | 2 | 2 | 5 | 3 | 2 | 6 | 1 | 2 | 4 | N | 74253 | KH-type splicing regulatory protein OS=Mus musculus OX=10090 GN=Khsrp PE=1 SV=1 |
| 745 | 1040 | sp\|Q3U0V1\|FUBP2\_MOUSE | 141.08 | 8 | 3 | 3 | 3 | 7 | 4 | 3 | 7 | 1 | 3 | 5 | 4.58E6 | 4.08E6 | 4.69E6 | 5.91E6 | 4.54E6 | 1.75E6 | 7.79E6 | 8.6E5 | 6.28E6 | 1.35E7 | 6 | 6 | 2 | 2 | 2 | 5 | 3 | 2 | 6 | 1 | 2 | 4 | N | 76776 | Far upstream element-binding protein 2 OS=Mus musculus OX=10090 GN=Khsrp PE=1 SV=2 |
| 1154 | 1829 | Q923L7\|Q923L7\_MOUSE | 140.97 | 46 | 28 | 0 | 0 | 0 | 0 | 0 | 0 | 0 | 28 | 46 | 4.44E7 |  |  |  |  |  |  |  | 1.01E8 | 5.23E7 | 4 | 4 | 4 | 0 | 0 | 0 | 0 | 0 | 0 | 0 | 5 | 4 | Y | 17357 | Ear6 protein OS=Mus musculus OX=10090 GN=Ear6 PE=1 SV=1 |
| 1029 | 1073 | sp\|Q91YT0\|NDUV1\_MOUSE | 140.92 | 17 | 2 | 12 | 2 | 2 | 2 | 2 | 8 | 2 | 4 | 3 | 3.58E6 | 1.06E7 | 2.87E6 | 3.99E6 | 5.36E6 | 2.83E6 | 6.74E6 | 1.67E6 | 3.87E6 | 2.25E6 | 6 | 6 | 1 | 3 | 1 | 1 | 1 | 1 | 3 | 1 | 2 | 2 | Y | 50834 | NADH dehydrogenase [ubiquinone] flavoprotein 1, mitochondrial OS=Mus musculus OX=10090 GN=Ndufv1 PE=1 SV=1 |
| 1029 | 1084 | D3YUM1\|D3YUM1\_MOUSE | 140.92 | 18 | 2 | 12 | 2 | 2 | 2 | 2 | 9 | 2 | 4 | 3 | 3.58E6 | 1.06E7 | 2.87E6 | 3.99E6 | 5.36E6 | 2.83E6 | 6.74E6 | 1.67E6 | 3.87E6 | 2.25E6 | 6 | 6 | 1 | 3 | 1 | 1 | 1 | 1 | 3 | 1 | 2 | 2 | Y | 49913 | NADH dehydrogenase [ubiquinone] flavoprotein 1, mitochondrial OS=Mus musculus OX=10090 GN=Ndufv1 PE=1 SV=1 |
| 383 | 1230 | sp\|P61255\|RL26\_MOUSE | 140.92 | 33 | 17 | 21 | 24 | 23 | 33 | 29 | 24 | 29 | 22 | 33 | 8.01E7 | 8.04E7 | 1.37E8 | 5.52E7 | 1.11E8 | 8.8E7 | 6.69E7 | 5.94E7 | 9.96E7 | 1.33E8 | 7 | 7 | 5 | 6 | 8 | 7 | 8 | 6 | 5 | 7 | 6 | 9 | N | 17258 | Large ribosomal subunit protein uL24 OS=Mus musculus OX=10090 GN=Rpl26 PE=1 SV=1 |
| 790 | 1287 | A0A494BAT0\|A0A494BAT0\_MOUSE | 140.59 | 15 | 8 | 5 | 3 | 5 | 9 | 9 | 13 | 5 | 9 | 8 | 2.31E6 |  |  |  | 8.38E5 | 6.4E5 | 9.69E5 |  | 4.57E5 | 1.49E6 | 5 | 3 | 3 | 2 | 1 | 3 | 3 | 3 | 4 | 2 | 3 | 3 | Y | 39969 | Histocompatibility 2, K1, K region OS=Mus musculus OX=10090 GN=H2-K1 PE=1 SV=1 |
| 790 | 1288 | Q3TH01\|Q3TH01\_MOUSE | 140.59 | 15 | 8 | 5 | 2 | 5 | 9 | 9 | 12 | 5 | 9 | 8 | 2.31E6 |  |  |  | 8.38E5 | 6.4E5 | 9.69E5 |  | 4.57E5 | 1.49E6 | 5 | 3 | 3 | 2 | 1 | 3 | 3 | 3 | 4 | 2 | 3 | 3 | Y | 40286 | Histocompatibility 2, K1, K region OS=Mus musculus OX=10090 GN=H2-K1 PE=1 SV=1 |
| 790 | 1290 | sp\|P01901\|HA1B\_MOUSE | 140.59 | 15 | 8 | 5 | 2 | 5 | 9 | 8 | 12 | 5 | 9 | 8 | 2.31E6 |  |  |  | 8.38E5 | 6.4E5 | 9.69E5 |  | 4.57E5 | 1.49E6 | 5 | 3 | 3 | 2 | 1 | 3 | 3 | 3 | 4 | 2 | 3 | 3 | Y | 41302 | H-2 class I histocompatibility antigen, K-B alpha chain OS=Mus musculus OX=10090 GN=H2-K1 PE=1 SV=1 |
| 754 | 692 | A0A0U1RP81\|A0A0U1RP81\_MOUSE | 140.51 | 15 | 3 | 2 | 8 | 8 | 6 | 6 | 6 | 6 | 2 | 3 |  |  |  |  |  |  |  | 5.44E5 |  |  | 8 | 1 | 2 | 1 | 4 | 4 | 3 | 3 | 3 | 3 | 1 | 2 | Y | 53717 | MICOS complex subunit MIC60 OS=Mus musculus OX=10090 GN=Immt PE=1 SV=1 |
| 650 | 1565 | sp\|O08795\|GLU2B\_MOUSE | 140.26 | 8 | 4 | 6 | 8 | 8 | 6 | 8 | 8 | 8 | 4 | 8 | 1.59E7 | 2.27E7 | 2.41E7 | 1.03E7 | 1.89E7 | 2.07E7 | 7.56E6 | 1.44E7 | 3.02E6 | 1.6E7 | 4 | 4 | 2 | 3 | 4 | 4 | 4 | 4 | 4 | 4 | 2 | 4 | N | 58793 | Glucosidase 2 subunit beta OS=Mus musculus OX=10090 GN=Prkcsh PE=1 SV=1 |
| 767 | 1158 | sp\|Q922Q1\|MARC2\_MOUSE | 140.14 | 17 | 11 | 9 | 11 | 4 | 9 | 10 | 2 | 9 | 0 | 7 | 2.13E7 | 9.03E6 | 2.57E7 | 6.28E5 | 3.12E6 | 1.64E7 | 2.47E6 | 1.1E7 |  | 1.8E7 | 7 | 7 | 4 | 3 | 4 | 2 | 3 | 4 | 1 | 3 | 0 | 3 | Y | 38194 | Mitochondrial amidoxime reducing component 2 OS=Mus musculus OX=10090 GN=Mtarc2 PE=1 SV=1 |
| 987 | 2084 | A0A494BA97\|A0A494BA97\_MOUSE | 139.66 | 21 | 6 | 17 | 9 | 0 | 21 | 21 | 17 | 0 | 21 | 9 | 1.92E6 | 4.23E6 | 4.74E6 |  | 3.86E6 | 4.85E6 | 1.82E6 |  | 2.01E7 | 8.21E6 | 3 | 3 | 1 | 2 | 2 | 0 | 3 | 3 | 2 | 0 | 3 | 2 | N | 23394 | Osteoclast-stimulating factor 1 OS=Mus musculus OX=10090 GN=Ostf1 PE=1 SV=1 |
| 987 | 2085 | sp\|Q62422\|OSTF1\_MOUSE | 139.66 | 21 | 6 | 17 | 9 | 0 | 21 | 21 | 17 | 0 | 21 | 9 | 1.92E6 | 4.23E6 | 4.74E6 |  | 3.86E6 | 4.85E6 | 1.82E6 |  | 2.01E7 | 8.21E6 | 3 | 3 | 1 | 2 | 2 | 0 | 3 | 3 | 2 | 0 | 3 | 2 | N | 23783 | Osteoclast-stimulating factor 1 OS=Mus musculus OX=10090 GN=Ostf1 PE=1 SV=2 |
| 481 | 1345 | sp\|Q63918\|CAVN2\_MOUSE | 139.54 | 13 | 4 | 6 | 7 | 7 | 9 | 11 | 9 | 6 | 4 | 6 | 4.5E8 | 5.01E8 | 1.08E7 | 3.86E6 | 3.82E7 | 2.44E7 | 3.35E8 | 3.91E8 | 3.95E8 | 3.59E8 | 5 | 5 | 4 | 4 | 3 | 3 | 4 | 4 | 3 | 4 | 2 | 4 | N | 46764 | Caveolae-associated protein 2 OS=Mus musculus OX=10090 GN=Cavin2 PE=1 SV=3 |
| 997 | 2239 | sp\|P10605\|CATB\_MOUSE | 139.43 | 13 | 13 | 8 | 4 | 4 | 4 | 8 | 4 | 4 | 4 | 13 | 1.12E7 | 6.89E6 | 2.9E6 | 1.12E6 | 2.33E6 | 4.52E6 | 1.9E6 | 1.81E6 | 2.87E6 | 6.79E6 | 3 | 3 | 3 | 3 | 1 | 1 | 1 | 2 | 1 | 1 | 1 | 4 | Y | 37280 | Cathepsin B OS=Mus musculus OX=10090 GN=Ctsb PE=1 SV=2 |
| 742 | 1036 | sp\|P35486\|ODPA\_MOUSE | 139.29 | 14 | 6 | 10 | 4 | 8 | 6 | 10 | 3 | 8 | 6 | 6 | 4.15E6 | 2.09E7 | 1.99E7 | 4.71E6 | 2.5E6 | 1.64E7 | 3.95E6 | 8.91E6 | 1.55E7 | 6.7E6 | 6 | 6 | 2 | 4 | 2 | 3 | 2 | 5 | 1 | 4 | 3 | 2 | Y | 43232 | Pyruvate dehydrogenase E1 component subunit alpha, somatic form, mitochondrial OS=Mus musculus OX=10090 GN=Pdha1 PE=1 SV=1 |
| 1226 | 1928 | sp\|O35841\|API5\_MOUSE | 139.18 | 8 | 3 | 0 | 4 | 0 | 0 | 4 | 4 | 0 | 6 | 8 | 0E0 |  | 2.83E5 |  |  | 1.63E6 | 3.76E5 |  | 3.23E6 | 2.49E6 | 3 | 3 | 1 | 0 | 1 | 0 | 0 | 1 | 1 | 0 | 2 | 3 | N | 56785 | Apoptosis inhibitor 5 OS=Mus musculus OX=10090 GN=Api5 PE=1 SV=2 |
| 784 | 1492 | sp\|P20108\|PRDX3\_MOUSE | 138.90 | 20 | 3 | 6 | 13 | 11 | 13 | 7 | 18 | 6 | 10 | 10 | 2.85E6 | 1.34E7 | 5.9E6 | 7.93E6 | 5.26E6 | 4.88E7 | 3.15E6 | 9.79E6 | 1.73E7 | 1.26E7 | 5 | 5 | 1 | 2 | 3 | 3 | 3 | 3 | 4 | 2 | 3 | 3 | N | 28127 | Thioredoxin-dependent peroxide reductase, mitochondrial OS=Mus musculus OX=10090 GN=Prdx3 PE=1 SV=1 |
| 880 | 1257 | sp\|Q5FWK3\|RHG01\_MOUSE | 138.36 | 19 | 9 | 0 | 8 | 9 | 7 | 2 | 15 | 8 | 7 | 5 | 4.64E6 |  | 9.52E6 | 2.43E6 | 6.77E6 | 4.8E6 | 7.02E6 | 5.03E6 | 8.79E6 | 3.76E6 | 6 | 6 | 2 | 0 | 3 | 2 | 2 | 1 | 5 | 3 | 2 | 1 | Y | 50411 | Rho GTPase-activating protein 1 OS=Mus musculus OX=10090 GN=Arhgap1 PE=1 SV=1 |
| 880 | 1268 | A2AH25\|A2AH25\_MOUSE | 138.36 | 17 | 9 | 0 | 8 | 8 | 6 | 2 | 13 | 7 | 6 | 5 | 4.64E6 |  | 9.52E6 | 2.43E6 | 6.77E6 | 4.8E6 | 7.02E6 | 5.03E6 | 8.79E6 | 3.76E6 | 6 | 6 | 2 | 0 | 3 | 2 | 2 | 1 | 5 | 3 | 2 | 1 | Y | 54437 | Rho GTPase activating protein 1 OS=Mus musculus OX=10090 GN=Arhgap1 PE=1 SV=1 |
| 849 | 653 | sp\|Q9Z2I9\|SUCB1\_MOUSE | 138.14 | 21 | 0 | 3 | 3 | 11 | 4 | 10 | 4 | 2 | 5 | 4 |  | 7.15E6 | 4.57E6 | 3.38E6 | 4.22E6 | 1.27E7 | 9.29E6 | 4.36E5 | 7.27E6 | 3.6E6 | 9 | 9 | 0 | 2 | 2 | 4 | 2 | 5 | 2 | 1 | 3 | 2 | Y | 50114 | Succinate--CoA ligase [ADP-forming] subunit beta, mitochondrial OS=Mus musculus OX=10090 GN=Sucla2 PE=1 SV=2 |
| 1364 | 2564 | sp\|Q9Z0E6\|GBP2\_MOUSE | 137.84 | 5 | 3 | 0 | 3 | 3 | 3 | 5 | 3 | 0 | 3 | 0 | 3.18E6 |  | 1.16E6 | 8.93E5 | 7.66E5 | 2.81E6 | 2.1E6 |  | 0E0 |  | 2 | 2 | 1 | 0 | 1 | 1 | 1 | 2 | 1 | 0 | 1 | 0 | N | 66740 | Guanylate-binding protein 2 OS=Mus musculus OX=10090 GN=Gbp2 PE=1 SV=1 |
| 864 | 942 | sp\|Q9D279\|MISP\_MOUSE | 137.82 | 12 | 1 | 8 | 2 | 5 | 3 | 4 | 3 | 1 | 4 | 1 | 6.7E7 | 1.99E7 | 4.3E6 | 5.28E6 | 6.74E6 | 3.12E6 | 6.99E6 | 1.12E6 | 2.84E6 | 2.74E6 | 7 | 7 | 1 | 4 | 1 | 3 | 2 | 2 | 2 | 1 | 2 | 1 | N | 72281 | Mitotic interactor and substrate of PLK1 OS=Mus musculus OX=10090 GN=Misp PE=1 SV=1 |
| 647 | 1979 | sp\|P99028\|QCR6\_MOUSE | 137.71 | 37 | 20 | 28 | 20 | 29 | 37 | 28 | 29 | 29 | 28 | 20 | 5.17E6 | 4.23E7 | 3.14E7 | 6.05E6 | 3.15E7 | 3.18E7 | 2.05E7 | 2.09E7 | 4.6E7 | 1.97E7 | 3 | 3 | 3 | 4 | 2 | 3 | 6 | 3 | 4 | 4 | 3 | 2 | Y | 10435 | Cytochrome b-c1 complex subunit 6, mitochondrial OS=Mus musculus OX=10090 GN=Uqcrh PE=1 SV=2 |
| 547 | 2045 | sp\|P10639\|THIO\_MOUSE | 137.70 | 31 | 12 | 21 | 31 | 21 | 31 | 31 | 21 | 31 | 12 | 21 | 2E8 | 3.37E8 | 1.8E8 | 1.73E8 | 1.71E8 | 3.03E8 | 2.38E8 | 2.46E8 | 4.24E8 | 3.35E8 | 3 | 3 | 2 | 3 | 7 | 5 | 5 | 4 | 3 | 7 | 2 | 4 | N | 11675 | Thioredoxin OS=Mus musculus OX=10090 GN=Txn PE=1 SV=3 |
| 797 | 909 | Q9JLI0\|Q9JLI0\_MOUSE | 137.70 | 26 | 3 | 12 | 9 | 9 | 3 | 17 | 10 | 10 | 8 | 8 |  |  | 9.57E5 |  |  | 2.82E6 |  |  | 4.04E6 |  | 7 | 1 | 1 | 3 | 2 | 2 | 1 | 5 | 2 | 2 | 2 | 3 | N | 37064 | Aldo-keto reductase a OS=Mus musculus OX=10090 GN=Akr1c12 PE=1 SV=1 |
| 932 | 1648 | sp\|Q8BVA4\|LMOD1\_MOUSE | 137.57 | 7 | 5 | 6 | 5 | 3 | 6 | 5 | 3 | 5 | 5 | 3 | 1.09E7 | 1.22E7 | 6.7E6 | 2.52E6 | 4.33E6 | 9.7E6 | 3.87E6 | 4.85E6 | 1.78E7 | 6.78E6 | 4 | 4 | 2 | 3 | 2 | 1 | 3 | 2 | 1 | 2 | 2 | 1 | N | 66310 | Leiomodin-1 OS=Mus musculus OX=10090 GN=Lmod1 PE=1 SV=1 |
| 932 | 1649 | F8VPR1\|F8VPR1\_MOUSE | 137.57 | 7 | 5 | 6 | 5 | 3 | 6 | 5 | 3 | 5 | 5 | 3 | 1.09E7 | 1.22E7 | 6.7E6 | 2.52E6 | 4.33E6 | 9.7E6 | 3.87E6 | 4.85E6 | 1.78E7 | 6.78E6 | 4 | 4 | 2 | 3 | 2 | 1 | 3 | 2 | 1 | 2 | 2 | 1 | N | 66268 | Leiomodin 1 (smooth muscle) OS=Mus musculus OX=10090 GN=Lmod1 PE=1 SV=1 |
| 1251 | 2695 | sp\|Q80W94\|MOGT2\_MOUSE | 137.29 | 11 | 4 | 4 | 4 | 4 | 4 | 4 | 11 | 4 | 4 | 0 | 1.51E6 | 4.48E6 | 3.38E6 | 1.05E6 | 1.45E6 | 3.73E6 | 3.23E6 | 1.24E6 | 1.7E6 |  | 2 | 2 | 1 | 1 | 1 | 1 | 1 | 1 | 2 | 1 | 1 | 0 | N | 38591 | 2-acylglycerol O-acyltransferase 2 OS=Mus musculus OX=10090 GN=Mogat2 PE=1 SV=1 |
| 764 | 2532 | sp\|P60766\|CDC42\_MOUSE | 137.11 | 20 | 9 | 15 | 14 | 14 | 14 | 9 | 9 | 14 | 9 | 14 | 1.25E6 | 2.07E7 | 2.9E7 | 1.07E7 | 3.35E7 | 2.48E7 | 9.18E6 | 1.37E7 | 5.81E7 | 4.14E7 | 3 | 3 | 1 | 2 | 3 | 3 | 3 | 2 | 2 | 4 | 4 | 3 | Y | 21259 | Cell division control protein 42 homolog OS=Mus musculus OX=10090 GN=Cdc42 PE=1 SV=2 |
| 781 | 1447 | sp\|Q9QWG7\|ST1B1\_MOUSE | 136.72 | 19 | 9 | 12 | 15 | 10 | 6 | 9 | 13 | 6 | 9 | 3 | 2.2E7 | 2.6E7 | 1.75E7 | 6.37E6 | 1.1E7 | 1.51E7 | 1.14E7 | 8.52E6 | 6.91E6 | 2.09E7 | 5 | 5 | 2 | 3 | 4 | 3 | 2 | 4 | 4 | 2 | 2 | 1 | Y | 34901 | Sulfotransferase 1B1 OS=Mus musculus OX=10090 GN=Sult1b1 PE=1 SV=2 |
| 686 | 2293 | sp\|Q62186\|SSRD\_MOUSE | 136.61 | 20 | 12 | 20 | 12 | 12 | 20 | 12 | 20 | 12 | 20 | 12 | 2.41E7 | 2.97E7 | 2.8E7 | 7.72E6 | 2.12E7 | 2E7 | 1.77E7 | 1.33E7 | 3.41E7 | 2.24E7 | 3 | 3 | 3 | 4 | 3 | 3 | 4 | 3 | 4 | 3 | 3 | 4 | Y | 18937 | Translocon-associated protein subunit delta OS=Mus musculus OX=10090 GN=Ssr4 PE=1 SV=1 |
| 686 | 2294 | Q9D8L3\|Q9D8L3\_MOUSE | 136.61 | 20 | 12 | 20 | 12 | 12 | 20 | 12 | 20 | 12 | 20 | 12 | 2.41E7 | 2.97E7 | 2.8E7 | 7.72E6 | 2.12E7 | 2E7 | 1.77E7 | 1.33E7 | 3.41E7 | 2.24E7 | 3 | 3 | 3 | 4 | 3 | 3 | 4 | 3 | 4 | 3 | 3 | 4 | Y | 19008 | Translocon-associated protein subunit delta OS=Mus musculus OX=10090 GN=Ssr4 PE=1 SV=1 |
| 699 | 1221 | sp\|P61089\|UBE2N\_MOUSE | 136.27 | 39 | 24 | 14 | 29 | 29 | 29 | 7 | 22 | 24 | 22 | 16 | 4.18E7 | 3.61E7 | 4.18E7 | 2.29E7 | 3.91E7 | 2.24E7 | 2.43E7 | 2.12E7 | 1.38E7 | 2.03E7 | 6 | 6 | 3 | 2 | 4 | 4 | 4 | 1 | 3 | 4 | 4 | 2 | Y | 17138 | Ubiquitin-conjugating enzyme E2 N OS=Mus musculus OX=10090 GN=Ube2n PE=1 SV=1 |
| 1535 | 4394 | sp\|P27005\|S10A8\_MOUSE | 136.19 | 22 | 22 | 0 | 0 | 0 | 0 | 0 | 0 | 0 | 22 | 22 | 1.34E7 |  |  |  |  |  |  |  | 3.08E7 | 1.21E7 | 1 | 1 | 3 | 0 | 0 | 0 | 0 | 0 | 0 | 0 | 2 | 2 | Y | 10295 | Protein S100-A8 OS=Mus musculus OX=10090 GN=S100a8 PE=1 SV=3 |
| 692 | 1547 | Q8C253\|Q8C253\_MOUSE | 136.13 | 19 | 7 | 6 | 6 | 7 | 9 | 6 | 6 | 4 | 11 | 16 | 1.19E7 | 5.64E6 | 6.9E6 | 2.83E6 | 2.38E7 | 1.15E7 | 8.15E6 | 2.71E6 | 4.05E7 | 2.58E7 | 6 | 6 | 2 | 2 | 2 | 2 | 5 | 2 | 2 | 1 | 5 | 7 | Y | 27415 | Galectin OS=Mus musculus OX=10090 GN=Lgals3 PE=1 SV=1 |
| 692 | 1548 | sp\|P16110\|LEG3\_MOUSE | 136.13 | 19 | 7 | 6 | 6 | 7 | 9 | 6 | 6 | 4 | 11 | 16 | 1.19E7 | 5.64E6 | 6.9E6 | 2.83E6 | 2.38E7 | 1.15E7 | 8.15E6 | 2.71E6 | 4.05E7 | 2.58E7 | 6 | 6 | 2 | 2 | 2 | 2 | 5 | 2 | 2 | 1 | 5 | 7 | Y | 27515 | Galectin-3 OS=Mus musculus OX=10090 GN=Lgals3 PE=1 SV=3 |
| 801 | 1985 | A0A0N4SVT3\|A0A0N4SVT3\_MOUSE | 135.96 | 63 | 35 | 52 | 52 | 52 | 42 | 42 | 25 | 42 | 28 | 17 | 3.61E7 | 7.23E7 | 2.34E7 | 1.8E7 | 1.07E7 | 1.69E7 | 5.69E5 | 5.37E6 | 3.14E7 | 9.28E6 | 4 | 4 | 2 | 4 | 3 | 4 | 2 | 2 | 1 | 2 | 2 | 1 | Y | 7197 | Guanine nucleotide-binding protein subunit gamma (Fragment) OS=Mus musculus OX=10090 GN=Gng12 PE=1 SV=1 |
| 801 | 1986 | sp\|Q9DAS9\|GBG12\_MOUSE | 135.96 | 57 | 32 | 47 | 47 | 47 | 38 | 38 | 22 | 38 | 25 | 15 | 3.61E7 | 7.23E7 | 2.34E7 | 1.8E7 | 1.07E7 | 1.69E7 | 5.69E5 | 5.37E6 | 3.14E7 | 9.28E6 | 4 | 4 | 2 | 4 | 3 | 4 | 2 | 2 | 1 | 2 | 2 | 1 | Y | 7997 | Guanine nucleotide-binding protein G(I)/G(S)/G(O) subunit gamma-12 OS=Mus musculus OX=10090 GN=Gng12 PE=1 SV=3 |
| 801 | 1991 | A0A0N4SW28\|A0A0N4SW28\_MOUSE | 135.96 | 49 | 28 | 41 | 41 | 41 | 33 | 33 | 19 | 33 | 22 | 13 | 3.61E7 | 7.23E7 | 2.34E7 | 1.8E7 | 1.07E7 | 1.69E7 | 5.69E5 | 5.37E6 | 3.14E7 | 9.28E6 | 4 | 4 | 2 | 4 | 3 | 4 | 2 | 2 | 1 | 2 | 2 | 1 | Y | 9182 | Guanine nucleotide-binding protein subunit gamma OS=Mus musculus OX=10090 GN=Gng12 PE=1 SV=1 |
| 786 | 1330 | sp\|O09061\|PSB1\_MOUSE | 135.77 | 22 | 6 | 4 | 14 | 10 | 10 | 10 | 13 | 18 | 11 | 14 | 6E6 | 6.6E6 | 9.97E6 | 1.34E7 | 5.33E6 | 7.2E6 | 1.8E7 | 1.51E7 | 1.73E7 | 1.45E7 | 6 | 6 | 1 | 1 | 3 | 3 | 2 | 2 | 3 | 4 | 3 | 3 | N | 26372 | Proteasome subunit beta type-1 OS=Mus musculus OX=10090 GN=Psmb1 PE=1 SV=1 |
| 877 | 1148 | sp\|P19639\|GSTM3\_MOUSE | 135.57 | 26 | 9 | 22 | 6 | 4 | 4 | 6 | 4 | 0 | 9 | 12 |  | 4.09E6 |  |  |  |  |  |  |  |  | 6 | 3 | 2 | 5 | 2 | 1 | 1 | 2 | 1 | 0 | 3 | 3 | Y | 25702 | Glutathione S-transferase Mu 3 OS=Mus musculus OX=10090 GN=Gstm3 PE=1 SV=2 |
| 930 | 930 | sp\|Q6P5E4\|UGGG1\_MOUSE | 135.54 | 4 | 1 | 1 | 1 | 1 | 1 | 2 | 1 | 1 | 1 | 1 | 2.58E6 | 2.69E6 | 3.33E6 | 1.84E6 | 7.22E5 | 3.54E6 | 2.57E6 | 1.58E6 | 7.42E5 | 2.59E6 | 6 | 6 | 1 | 1 | 2 | 2 | 2 | 2 | 2 | 1 | 1 | 2 | N | 176433 | UDP-glucose:glycoprotein glucosyltransferase 1 OS=Mus musculus OX=10090 GN=Uggt1 PE=1 SV=4 |
| 1169 | 1779 | sp\|Q99KP3\|CRYL1\_MOUSE | 135.45 | 14 | 0 | 6 | 0 | 3 | 0 | 6 | 5 | 0 | 0 | 0 |  | 9.9E6 |  | 3.68E5 |  | 4.5E6 | 6.91E5 |  |  |  | 3 | 3 | 0 | 1 | 0 | 1 | 0 | 1 | 1 | 0 | 0 | 0 | N | 35209 | Lambda-crystallin homolog OS=Mus musculus OX=10090 GN=Cryl1 PE=1 SV=3 |
| 716 | 1055 | sp\|P61620\|S61A1\_MOUSE | 135.42 | 18 | 0 | 8 | 6 | 13 | 13 | 12 | 11 | 9 | 1 | 6 |  | 1.26E7 | 2.9E7 | 1.99E7 | 2.41E7 | 1.55E7 | 1.67E7 | 8.05E6 | 3.45E6 | 2.31E6 | 7 | 7 | 0 | 3 | 3 | 7 | 5 | 4 | 4 | 3 | 1 | 2 | Y | 52265 | Protein transport protein Sec61 subunit alpha isoform 1 OS=Mus musculus OX=10090 GN=Sec61a1 PE=1 SV=2 |
| 938 | 1089 | sp\|Q7TPV4\|MBB1A\_MOUSE | 135.41 | 5 | 1 | 0 | 3 | 2 | 3 | 2 | 1 | 3 | 1 | 1 | 1.3E6 |  | 7.67E6 | 2.34E6 | 4.46E6 | 3.35E6 | 0E0 | 7.46E6 | 1.7E6 | 3.1E5 | 6 | 6 | 1 | 0 | 4 | 3 | 3 | 2 | 1 | 3 | 2 | 1 | N | 152036 | Myb-binding protein 1A OS=Mus musculus OX=10090 GN=Mybbp1a PE=1 SV=2 |
| 1044 | 2489 | sp\|Q8CG76\|ARK72\_MOUSE | 135.05 | 7 | 3 | 7 | 7 | 7 | 4 | 4 | 4 | 4 | 7 | 3 | 3.61E6 | 1.31E7 | 7.42E6 | 5.1E6 | 4.1E6 | 5.57E6 | 2.88E6 | 3.64E6 | 1.57E7 | 2.33E6 | 2 | 2 | 1 | 2 | 2 | 2 | 1 | 1 | 1 | 1 | 2 | 1 | N | 40612 | Aflatoxin B1 aldehyde reductase member 2 OS=Mus musculus OX=10090 GN=Akr7a2 PE=1 SV=3 |
| 676 | 1365 | A2AFW6\|A2AFW6\_MOUSE | 135.01 | 16 | 3 | 6 | 5 | 8 | 5 | 5 | 8 | 8 | 11 | 5 | 3.29E6 | 2.02E7 | 1.15E7 | 7.87E6 | 7.37E6 | 9.61E6 | 8.49E6 | 8.76E6 | 2.03E7 | 5.74E6 | 5 | 5 | 1 | 3 | 3 | 4 | 3 | 3 | 4 | 4 | 4 | 2 | Y | 32345 | Mitochondrial carrier 2 OS=Mus musculus OX=10090 GN=Mtch2 PE=1 SV=1 |
| 676 | 1368 | sp\|Q791V5\|MTCH2\_MOUSE | 135.01 | 16 | 3 | 6 | 5 | 8 | 5 | 5 | 8 | 8 | 11 | 5 | 3.29E6 | 2.02E7 | 1.15E7 | 7.87E6 | 7.37E6 | 9.61E6 | 8.49E6 | 8.76E6 | 2.03E7 | 5.74E6 | 5 | 5 | 1 | 3 | 3 | 4 | 3 | 3 | 4 | 4 | 4 | 2 | Y | 33499 | Mitochondrial carrier homolog 2 OS=Mus musculus OX=10090 GN=Mtch2 PE=1 SV=1 |
| 676 | 1370 | Q9D050\|Q9D050\_MOUSE | 135.01 | 15 | 3 | 5 | 5 | 7 | 5 | 5 | 7 | 7 | 11 | 5 | 3.29E6 | 2.02E7 | 1.15E7 | 7.87E6 | 7.37E6 | 9.61E6 | 8.49E6 | 8.76E6 | 2.03E7 | 5.74E6 | 5 | 5 | 1 | 3 | 3 | 4 | 3 | 3 | 4 | 4 | 4 | 2 | Y | 34554 | Mitochondrial carrier 2 OS=Mus musculus OX=10090 GN=Mtch2 PE=1 SV=1 |
| 638 | 1493 | sp\|P19467\|MUC13\_MOUSE | 134.90 | 11 | 4 | 4 | 4 | 9 | 6 | 5 | 5 | 6 | 8 | 8 | 9.4E6 | 1.3E7 | 9.24E6 | 7.97E6 | 1.4E7 | 8.15E6 | 9.76E6 | 7.05E6 | 2.41E7 | 1.51E7 | 5 | 5 | 3 | 3 | 2 | 4 | 3 | 2 | 3 | 3 | 5 | 5 | N | 58701 | Mucin-13 OS=Mus musculus OX=10090 GN=Muc13 PE=2 SV=1 |
| 798 | 1032 | sp\|P30416\|FKBP4\_MOUSE | 134.66 | 14 | 3 | 12 | 5 | 8 | 5 | 5 | 5 | 5 | 0 | 0 | 6.44E5 | 2.37E7 | 8.94E6 | 6.53E6 | 1.12E7 | 6.14E6 | 3.51E6 | 2.55E6 |  |  | 7 | 7 | 1 | 6 | 2 | 4 | 2 | 2 | 2 | 2 | 0 | 0 | N | 51572 | Peptidyl-prolyl cis-trans isomerase FKBP4 OS=Mus musculus OX=10090 GN=Fkbp4 PE=1 SV=5 |
| 749 | 1723 | sp\|Q9JJU8\|SH3L1\_MOUSE | 134.27 | 45 | 29 | 20 | 29 | 18 | 29 | 29 | 45 | 29 | 20 | 29 | 1.18E7 | 8.66E6 | 1.38E7 | 4.02E6 | 1.04E7 | 1.43E7 | 9.51E6 | 7.1E6 | 2.22E7 | 2.7E7 | 4 | 4 | 3 | 2 | 3 | 2 | 3 | 4 | 4 | 3 | 2 | 4 | N | 12811 | Adapter Sh3bgrl OS=Mus musculus OX=10090 GN=Sh3bgrl PE=1 SV=1 |
| 949 | 1971 | sp\|Q99LY9\|NDUS5\_MOUSE | 134.20 | 39 | 21 | 21 | 21 | 9 | 21 | 21 | 9 | 16 | 11 | 21 | 2.36E6 | 4.46E6 | 1.01E7 | 8.5E5 | 4.25E6 | 7.78E6 | 4.48E6 | 1.34E6 | 5.91E6 | 5.62E6 | 4 | 4 | 3 | 2 | 3 | 1 | 2 | 2 | 1 | 2 | 1 | 3 | Y | 12648 | NADH dehydrogenase [ubiquinone] iron-sulfur protein 5 OS=Mus musculus OX=10090 GN=Ndufs5 PE=1 SV=3 |
| 723 | 952 | sp\|Q8R081\|HNRPL\_MOUSE | 134.16 | 12 | 3 | 3 | 7 | 2 | 6 | 7 | 1 | 2 | 5 | 3 | 2.96E7 | 2.79E7 | 1.39E7 | 4.38E6 | 2.53E7 | 1.75E8 | 1.49E6 | 3.83E6 | 2.31E7 | 2.41E7 | 7 | 7 | 3 | 3 | 4 | 1 | 4 | 5 | 1 | 1 | 3 | 2 | Y | 63964 | Heterogeneous nuclear ribonucleoprotein L OS=Mus musculus OX=10090 GN=Hnrnpl PE=1 SV=2 |
| 636 | 726 | sp\|P46638\|RB11B\_MOUSE | 133.92 | 34 | 7 | 16 | 16 | 12 | 15 | 20 | 11 | 9 | 16 | 17 | 1.24E7 | 1.48E7 | 2.82E7 | 1.18E7 | 2.79E7 | 3.19E7 | 1.86E7 | 1.2E7 | 1.38E7 | 2.36E7 | 8 | 8 | 2 | 4 | 4 | 3 | 4 | 5 | 3 | 2 | 4 | 4 | N | 24489 | Ras-related protein Rab-11B OS=Mus musculus OX=10090 GN=Rab11b PE=1 SV=3 |
| 1201 | 1940 | A0A1W2P729\|A0A1W2P729\_MOUSE | 133.92 | 39 | 0 | 28 | 9 | 9 | 9 | 9 | 9 | 9 | 11 | 0 |  | 7.3E6 | 4.19E6 | 6.81E5 | 2.97E6 | 4.75E6 | 1.28E6 | 1.67E6 | 4.95E6 |  | 4 | 4 | 0 | 3 | 1 | 1 | 1 | 1 | 1 | 1 | 1 | 0 | Y | 15316 | Canopy FGF signaling regulator 2 (Fragment) OS=Mus musculus OX=10090 GN=Cnpy2 PE=1 SV=1 |
| 1201 | 1941 | sp\|Q9QXT0\|CNPY2\_MOUSE | 133.92 | 29 | 0 | 21 | 7 | 7 | 7 | 7 | 7 | 7 | 8 | 0 |  | 7.3E6 | 4.19E6 | 6.81E5 | 2.97E6 | 4.75E6 | 1.28E6 | 1.67E6 | 4.95E6 |  | 4 | 4 | 0 | 3 | 1 | 1 | 1 | 1 | 1 | 1 | 1 | 0 | Y | 20767 | Protein canopy homolog 2 OS=Mus musculus OX=10090 GN=Cnpy2 PE=1 SV=1 |
| 658 | 1425 | sp\|P57759\|ERP29\_MOUSE | 133.77 | 19 | 16 | 7 | 16 | 12 | 16 | 16 | 16 | 11 | 19 | 11 | 1.04E7 | 4.05E6 | 1.24E7 | 5.07E6 | 8.29E6 | 8.68E6 | 5.51E6 | 4.33E6 | 2.21E7 | 4.88E6 | 5 | 5 | 4 | 2 | 4 | 3 | 4 | 4 | 4 | 3 | 5 | 3 | N | 28823 | Endoplasmic reticulum resident protein 29 OS=Mus musculus OX=10090 GN=Erp29 PE=1 SV=2 |
| 1006 | 980 | sp\|P57016\|LAD1\_MOUSE | 133.73 | 13 | 5 | 7 | 3 | 5 | 4 | 4 | 2 | 0 | 5 | 2 | 1.42E6 | 9.21E6 | 4.49E6 | 9.43E5 | 1.78E6 | 3.4E6 | 6.85E5 |  | 4.7E6 | 1.34E6 | 7 | 7 | 2 | 3 | 2 | 2 | 2 | 2 | 1 | 0 | 2 | 1 | N | 58864 | Ladinin-1 OS=Mus musculus OX=10090 GN=Lad1 PE=1 SV=1 |
| 819 | 2064 | sp\|Q9CY50\|SSRA\_MOUSE | 133.54 | 12 | 9 | 9 | 9 | 9 | 9 | 5 | 4 | 9 | 12 | 12 | 1.26E7 | 1.53E7 | 1.28E7 | 5.67E6 | 1.07E7 | 5.41E6 | 5.44E6 | 6.72E6 | 2.62E7 | 1.31E7 | 3 | 3 | 2 | 3 | 2 | 2 | 2 | 2 | 1 | 3 | 3 | 3 | N | 32065 | Translocon-associated protein subunit alpha OS=Mus musculus OX=10090 GN=Ssr1 PE=1 SV=1 |
| 819 | 2067 | A0A286YCT4\|A0A286YCT4\_MOUSE | 133.54 | 11 | 9 | 9 | 9 | 9 | 9 | 5 | 4 | 9 | 11 | 11 | 1.26E7 | 1.53E7 | 1.28E7 | 5.67E6 | 1.07E7 | 5.41E6 | 5.44E6 | 6.72E6 | 2.62E7 | 1.31E7 | 3 | 3 | 2 | 3 | 2 | 2 | 2 | 2 | 1 | 3 | 3 | 3 | N | 33678 | Translocon-associated protein subunit alpha OS=Mus musculus OX=10090 GN=Ssr1 PE=1 SV=1 |
| 819 | 2074 | A0A286YCG8\|A0A286YCG8\_MOUSE | 133.54 | 11 | 8 | 8 | 8 | 8 | 8 | 5 | 3 | 8 | 11 | 11 | 1.26E7 | 1.53E7 | 1.28E7 | 5.67E6 | 1.07E7 | 5.41E6 | 5.44E6 | 6.72E6 | 2.62E7 | 1.31E7 | 3 | 3 | 2 | 3 | 2 | 2 | 2 | 2 | 1 | 3 | 3 | 3 | N | 35558 | Translocon-associated protein subunit alpha OS=Mus musculus OX=10090 GN=Ssr1 PE=1 SV=1 |
| 935 | 1080 | Q99LB4\|Q99LB4\_MOUSE | 133.50 | 18 | 3 | 3 | 3 | 3 | 7 | 3 | 7 | 7 | 15 | 9 | 1.93E6 | 5.38E6 | 6.67E6 | 2.26E6 | 2.9E6 | 4.07E6 | 3.31E6 | 4.7E6 | 3.21E7 | 3.75E6 | 5 | 5 | 1 | 1 | 1 | 1 | 2 | 1 | 2 | 2 | 5 | 2 | Y | 38769 | Capping actin protein, gelsolin like OS=Mus musculus OX=10090 GN=Capg PE=1 SV=1 |
| 1060 | 1675 | sp\|Q91WK2\|EIF3H\_MOUSE | 132.99 | 14 | 8 | 5 | 8 | 0 | 3 | 5 | 11 | 3 | 9 | 9 | 4.14E6 | 4.12E6 | 4.21E6 |  | 1.31E6 | 1.92E6 | 3.08E6 | 1.23E6 | 3.37E6 | 3.05E6 | 4 | 4 | 2 | 1 | 2 | 0 | 1 | 1 | 3 | 1 | 2 | 2 | N | 39832 | Eukaryotic translation initiation factor 3 subunit H OS=Mus musculus OX=10090 GN=Eif3h PE=1 SV=1 |
| 1291 | 1702 | sp\|P61222\|ABCE1\_MOUSE | 132.99 | 8 | 2 | 0 | 3 | 3 | 4 | 0 | 0 | 3 | 4 | 0 | 6.61E5 |  | 3.53E5 | 6E5 | 1.07E6 |  |  | 9.35E5 | 1.67E7 |  | 4 | 3 | 1 | 0 | 2 | 1 | 2 | 0 | 0 | 1 | 2 | 0 | Y | 67314 | ATP-binding cassette sub-family E member 1 OS=Mus musculus OX=10090 GN=Abce1 PE=1 SV=1 |
| 982 | 1551 | A0A0R4J0D3\|A0A0R4J0D3\_MOUSE | 132.96 | 6 | 1 | 2 | 1 | 2 | 4 | 1 | 4 | 1 | 1 | 0 | 7.08E5 | 2.29E6 | 2.29E6 | 1.79E6 | 3.68E6 | 2.9E6 | 3.36E6 | 1.84E6 | 2.13E6 |  | 5 | 5 | 1 | 2 | 1 | 2 | 4 | 1 | 3 | 1 | 1 | 0 | Y | 93315 | dolichyl-diphosphooligosaccharide--protein glycotransferase OS=Mus musculus OX=10090 GN=Stt3b PE=1 SV=1 |
| 982 | 1552 | sp\|Q3TDQ1\|STT3B\_MOUSE | 132.96 | 6 | 1 | 2 | 1 | 2 | 4 | 1 | 4 | 1 | 1 | 0 | 7.08E5 | 2.29E6 | 2.29E6 | 1.79E6 | 3.68E6 | 2.9E6 | 3.36E6 | 1.84E6 | 2.13E6 |  | 5 | 5 | 1 | 2 | 1 | 2 | 4 | 1 | 3 | 1 | 1 | 0 | Y | 93246 | Dolichyl-diphosphooligosaccharide--protein glycosyltransferase subunit STT3B OS=Mus musculus OX=10090 GN=Stt3b PE=1 SV=2 |
| 596 | 1013 | sp\|Q9QZQ8\|H2AY\_MOUSE | 132.23 | 23 | 3 | 12 | 17 | 14 | 17 | 17 | 14 | 13 | 13 | 12 | 3.28E6 | 2.47E7 | 3.6E7 | 1.09E7 | 2.96E7 | 2.79E7 | 1.8E7 | 1.1E7 | 1.94E7 | 1.7E7 | 6 | 6 | 1 | 3 | 6 | 4 | 5 | 5 | 4 | 3 | 3 | 3 | N | 39290 | Core histone macro-H2A.1 OS=Mus musculus OX=10090 GN=Macroh2a1 PE=1 SV=4 |
| 1032 | 1417 | sp\|Q8BJ64\|CHDH\_MOUSE | 131.89 | 7 | 2 | 2 | 2 | 0 | 5 | 7 | 3 | 2 | 2 | 0 | 1.17E6 | 1.89E6 | 1.73E6 |  | 2.51E6 | 7.02E6 | 3.07E6 | 1.22E6 | 1.32E6 |  | 4 | 4 | 1 | 1 | 1 | 0 | 3 | 4 | 2 | 1 | 1 | 0 | N | 66415 | Choline dehydrogenase, mitochondrial OS=Mus musculus OX=10090 GN=Chdh PE=1 SV=1 |
| 700 | 1541 | sp\|Q06185\|ATP5I\_MOUSE | 131.87 | 69 | 0 | 61 | 28 | 44 | 61 | 52 | 61 | 61 | 61 | 35 |  | 4.1E7 | 1.82E7 | 5.26E6 | 3.06E7 | 1.37E7 | 1.96E7 | 2.33E7 | 4.7E7 | 1.38E7 | 5 | 4 | 0 | 4 | 3 | 3 | 5 | 4 | 4 | 4 | 4 | 2 | Y | 8236 | ATP synthase subunit e, mitochondrial OS=Mus musculus OX=10090 GN=Atp5me PE=1 SV=2 |
| 1614 | 2454 | sp\|P07146\|TRY2\_MOUSE | 131.85 | 15 | 4 | 0 | 8 | 7 | 0 | 0 | 0 | 4 | 0 | 0 | 4.55E6 |  | 5.54E6 | 2.69E6 |  |  |  | 4.29E6 |  |  | 3 | 2 | 1 | 0 | 1 | 2 | 0 | 0 | 0 | 1 | 0 | 0 | Y | 26204 | Anionic trypsin-2 OS=Mus musculus OX=10090 GN=Prss2 PE=1 SV=1 |
| 556 | 2409 | D3YX03\|D3YX03\_MOUSE | 131.69 | 27 | 10 | 27 | 27 | 16 | 26 | 16 | 27 | 27 | 16 | 10 |  | 1.82E6 | 1.61E6 |  | 1.35E6 |  | 7.96E5 | 9.64E5 |  |  | 4 | 1 | 2 | 6 | 7 | 4 | 5 | 3 | 5 | 5 | 3 | 1 | Y | 12799 | Defensin, alpha, 42 OS=Mus musculus OX=10090 GN=Defa42 PE=1 SV=1 |
| 795 | 1619 | sp\|Q64464\|CP3AD\_MOUSE | 131.65 | 10 | 6 | 8 | 6 | 6 | 8 | 6 | 9 | 5 | 8 | 10 | 2.99E6 | 1.45E6 | 5E6 | 2.78E6 | 3.8E6 | 6.15E6 | 6.05E6 | 1.1E6 | 6.23E6 | 7.44E6 | 4 | 4 | 3 | 3 | 2 | 2 | 3 | 2 | 3 | 2 | 3 | 4 | N | 57492 | Cytochrome P450 3A13 OS=Mus musculus OX=10090 GN=Cyp3a13 PE=1 SV=1 |
| 683 | 1589 | sp\|P35230\|REG3B\_MOUSE | 131.50 | 26 | 5 | 9 | 9 | 22 | 26 | 26 | 26 | 26 | 0 | 3 | 4.21E6 | 2.02E7 | 3.94E7 | 5.07E7 | 1.08E8 | 2.91E7 | 6.46E7 | 3.82E7 |  |  | 4 | 3 | 1 | 2 | 2 | 3 | 6 | 5 | 5 | 4 | 0 | 1 | Y | 19476 | Regenerating islet-derived protein 3-beta OS=Mus musculus OX=10090 GN=Reg3b PE=1 SV=1 |
| 917 | 2015 | sp\|Q8K157\|GALM\_MOUSE | 131.48 | 13 | 8 | 8 | 4 | 4 | 8 | 8 | 13 | 8 | 8 | 4 | 2.66E6 | 5.15E6 | 3.35E6 | 9.96E5 | 3.12E6 | 4.77E6 | 2.55E6 | 9.02E5 | 5.25E6 | 3.73E5 | 3 | 3 | 2 | 2 | 2 | 1 | 2 | 2 | 3 | 2 | 2 | 1 | N | 37799 | Galactose mutarotase OS=Mus musculus OX=10090 GN=Galm PE=1 SV=1 |
| 1018 | 1585 | sp\|Q9R0Q7\|TEBP\_MOUSE | 131.39 | 27 | 0 | 16 | 12 | 12 | 5 | 5 | 5 | 8 | 14 | 11 |  | 4E6 | 6.58E6 | 1.51E6 | 3.99E6 | 4.13E6 | 3.86E6 | 8.3E5 | 1.17E7 | 8.47E6 | 4 | 4 | 0 | 2 | 2 | 2 | 1 | 1 | 1 | 1 | 2 | 2 | Y | 18721 | Prostaglandin E synthase 3 OS=Mus musculus OX=10090 GN=Ptges3 PE=1 SV=1 |
| 1050 | 2561 | sp\|P70372\|ELAV1\_MOUSE | 131.33 | 7 | 4 | 0 | 4 | 7 | 4 | 4 | 4 | 7 | 7 | 7 | 3.32E6 |  | 8.28E6 | 4.35E6 | 5.25E6 | 4.94E6 | 2.24E6 | 2.1E6 | 1.32E7 | 1.11E7 | 2 | 2 | 2 | 0 | 1 | 2 | 1 | 1 | 1 | 2 | 3 | 2 | N | 36169 | ELAV-like protein 1 OS=Mus musculus OX=10090 GN=Elavl1 PE=1 SV=2 |
| 854 | 1064 | sp\|Q8VC28\|AK1CD\_MOUSE | 131.17 | 26 | 3 | 12 | 3 | 9 | 3 | 17 | 10 | 10 | 2 | 8 |  |  |  |  |  | 1.03E6 |  |  |  |  | 7 | 1 | 1 | 3 | 1 | 2 | 1 | 5 | 2 | 2 | 1 | 3 | N | 37058 | Aldo-keto reductase family 1 member C13 OS=Mus musculus OX=10090 GN=Akr1c13 PE=1 SV=2 |
| 607 | 1340 | sp\|Q8R1B4\|EIF3C\_MOUSE | 131.11 | 5 | 4 | 4 | 2 | 3 | 4 | 3 | 4 | 4 | 3 | 4 | 1.16E7 | 6.14E7 | 5.65E6 | 3.57E6 | 2.69E7 | 3.39E7 | 4.21E7 | 2.88E7 | 1.95E7 | 2.35E7 | 6 | 6 | 4 | 5 | 2 | 3 | 4 | 3 | 5 | 4 | 3 | 5 | N | 105531 | Eukaryotic translation initiation factor 3 subunit C OS=Mus musculus OX=10090 GN=Eif3c PE=1 SV=1 |
| 778 | 1594 | Q3TT92\|Q3TT92\_MOUSE | 131.02 | 7 | 5 | 5 | 0 | 4 | 4 | 4 | 3 | 3 | 5 | 5 | 1.2E6 | 1.29E6 |  | 0E0 |  | 1.16E6 |  |  | 3.41E6 | 9.98E5 | 4 | 2 | 3 | 3 | 0 | 2 | 2 | 2 | 1 | 1 | 3 | 3 | Y | 61780 | Dihydropyrimidinase-like 3 OS=Mus musculus OX=10090 GN=Dpysl3 PE=1 SV=1 |
| 778 | 1599 | E9PWE8\|E9PWE8\_MOUSE | 131.02 | 6 | 5 | 5 | 0 | 4 | 4 | 3 | 2 | 2 | 5 | 5 | 1.2E6 | 1.29E6 |  | 0E0 |  | 1.16E6 |  |  | 3.41E6 | 9.98E5 | 4 | 2 | 3 | 3 | 0 | 2 | 2 | 2 | 1 | 1 | 3 | 3 | Y | 73884 | Dihydropyrimidinase-like 3 OS=Mus musculus OX=10090 GN=Dpysl3 PE=1 SV=1 |
| 778 | 1601 | sp\|Q62188\|DPYL3\_MOUSE | 131.02 | 7 | 5 | 5 | 0 | 4 | 4 | 4 | 3 | 3 | 5 | 5 | 1.2E6 | 1.29E6 |  | 0E0 |  | 1.16E6 |  |  | 3.41E6 | 9.98E5 | 4 | 2 | 3 | 3 | 0 | 2 | 2 | 2 | 1 | 1 | 3 | 3 | Y | 61936 | Dihydropyrimidinase-related protein 3 OS=Mus musculus OX=10090 GN=Dpysl3 PE=1 SV=1 |
| 942 | 1877 | sp\|P61804\|DAD1\_MOUSE | 130.71 | 35 | 19 | 9 | 11 | 35 | 19 | 19 | 18 | 19 | 19 | 19 | 8.25E6 | 1.28E7 | 3.34E6 | 1.19E7 | 3.46E6 | 1.16E7 | 5.1E6 | 1.58E7 | 1.94E7 | 1.15E6 | 4 | 4 | 2 | 1 | 1 | 4 | 2 | 2 | 2 | 2 | 2 | 2 | N | 12497 | Dolichyl-diphosphooligosaccharide--protein glycosyltransferase subunit DAD1 OS=Mus musculus OX=10090 GN=Dad1 PE=1 SV=3 |
| 629 | 1314 | sp\|P63323\|RS12\_MOUSE | 130.67 | 39 | 14 | 14 | 28 | 6 | 6 | 6 | 14 | 14 | 39 | 14 | 2.1E7 | 2.3E7 | 3.05E7 | 9.39E6 | 1.39E7 | 1.28E7 | 1.53E7 | 1.59E7 | 4.58E7 | 2.15E7 | 5 | 5 | 3 | 3 | 6 | 2 | 2 | 2 | 3 | 4 | 6 | 4 | Y | 14515 | Small ribosomal subunit protein eS12 OS=Mus musculus OX=10090 GN=Rps12 PE=1 SV=3 |
| 629 | 1315 | F7AEH4\|F7AEH4\_MOUSE | 130.67 | 38 | 13 | 13 | 27 | 6 | 6 | 6 | 13 | 13 | 38 | 13 | 2.1E7 | 2.3E7 | 3.05E7 | 9.39E6 | 1.39E7 | 1.28E7 | 1.53E7 | 1.59E7 | 4.58E7 | 2.15E7 | 5 | 5 | 3 | 3 | 6 | 2 | 2 | 2 | 3 | 4 | 6 | 4 | Y | 15181 | 40S ribosomal protein S12 OS=Mus musculus OX=10090 GN=Rps12 PE=1 SV=1 |
| 629 | 1316 | A0A1W2P7A1\|A0A1W2P7A1\_MOUSE | 130.67 | 35 | 12 | 12 | 25 | 5 | 5 | 5 | 12 | 12 | 35 | 12 | 2.1E7 | 2.3E7 | 3.05E7 | 9.39E6 | 1.39E7 | 1.28E7 | 1.53E7 | 1.59E7 | 4.58E7 | 2.15E7 | 5 | 5 | 3 | 3 | 6 | 2 | 2 | 2 | 3 | 4 | 6 | 4 | Y | 15979 | 40S ribosomal protein S12 OS=Mus musculus OX=10090 GN=Rps12 PE=1 SV=1 |
| 680 | 1175 | sp\|O70435\|PSA3\_MOUSE | 130.43 | 22 | 3 | 6 | 6 | 9 | 15 | 8 | 6 | 6 | 0 | 11 | 3.71E6 | 6.83E6 | 7.7E6 | 7.82E6 | 2.67E7 | 1.79E7 | 1.39E7 | 5.99E6 |  | 1.81E7 | 8 | 8 | 1 | 2 | 4 | 4 | 6 | 5 | 4 | 2 | 0 | 4 | Y | 28405 | Proteasome subunit alpha type-3 OS=Mus musculus OX=10090 GN=Psma3 PE=1 SV=3 |
| 862 | 1081 | sp\|Q9WVK4\|EHD1\_MOUSE | 130.38 | 11 | 5 | 5 | 3 | 0 | 8 | 7 | 5 | 5 | 5 | 3 | 2.57E6 | 1.45E6 | 7.29E5 |  | 1.39E6 | 4.5E6 | 9.76E5 | 2E6 | 2.84E6 | 2.3E6 | 6 | 4 | 2 | 2 | 2 | 0 | 4 | 3 | 2 | 2 | 2 | 2 | N | 60603 | EH domain-containing protein 1 OS=Mus musculus OX=10090 GN=Ehd1 PE=1 SV=1 |
| 1205 | 2372 | sp\|Q9DCH4\|EIF3F\_MOUSE | 130.25 | 11 | 6 | 5 | 4 | 4 | 2 | 2 | 4 | 6 | 5 | 2 | 5.7E6 | 0E0 | 3.35E5 | 3.27E5 | 1.12E6 | 2.63E6 | 3.72E5 | 5.84E5 | 1.12E6 | 6.12E5 | 3 | 3 | 2 | 1 | 1 | 1 | 1 | 1 | 1 | 2 | 1 | 1 | N | 37984 | Eukaryotic translation initiation factor 3 subunit F OS=Mus musculus OX=10090 GN=Eif3f PE=1 SV=2 |
| 1179 | 2089 | sp\|Q9D662\|SC23B\_MOUSE | 130.12 | 5 | 0 | 3 | 1 | 2 | 2 | 0 | 2 | 3 | 2 | 5 |  | 0E0 | 7.27E6 |  |  |  |  | 1.39E6 |  | 3.61E6 | 3 | 2 | 0 | 2 | 1 | 1 | 1 | 0 | 1 | 2 | 1 | 3 | Y | 86437 | Protein transport protein Sec23B OS=Mus musculus OX=10090 GN=Sec23b PE=1 SV=1 |
| 799 | 1380 | sp\|Q8BP67\|RL24\_MOUSE | 130.03 | 30 | 15 | 15 | 15 | 19 | 15 | 16 | 18 | 15 | 15 | 15 | 1.43E7 | 3.36E6 | 1.32E7 | 1.38E7 | 1.18E7 | 1.91E7 | 2.63E7 | 4.74E6 | 2.31E7 | 1.17E7 | 5 | 5 | 3 | 2 | 2 | 3 | 2 | 3 | 4 | 2 | 3 | 3 | Y | 17779 | Large ribosomal subunit protein eL24 OS=Mus musculus OX=10090 GN=Rpl24 PE=1 SV=2 |
| 587 | 2337 | sp\|P84104\|SRSF3\_MOUSE | 130.00 | 19 | 14 | 14 | 9 | 19 | 13 | 13 | 19 | 19 | 14 | 14 | 1.04E7 | 1.29E7 |  | 9.03E6 | 2.03E6 | 2.69E6 | 1.25E7 | 1.08E7 | 2.44E7 | 1.18E7 | 3 | 2 | 3 | 4 | 2 | 5 | 4 | 4 | 5 | 5 | 6 | 4 | Y | 19330 | Serine/arginine-rich splicing factor 3 OS=Mus musculus OX=10090 GN=Srsf3 PE=1 SV=1 |
| 711 | 1349 | Q8K1K2\|Q8K1K2\_MOUSE | 129.84 | 14 | 6 | 3 | 10 | 10 | 10 | 4 | 7 | 6 | 10 | 10 | 5.7E6 | 9.69E6 | 4.57E6 | 9.35E6 | 8.98E6 | 2.67E6 | 2.88E6 | 4.63E6 | 1.7E7 | 8.26E6 | 4 | 3 | 2 | 1 | 3 | 3 | 3 | 1 | 2 | 2 | 3 | 3 | N | 38752 | 26S proteasome regulatory subunit 8 OS=Mus musculus OX=10090 GN=Psmc5 PE=1 SV=1 |
| 711 | 1351 | sp\|P62196\|PRS8\_MOUSE | 129.84 | 12 | 5 | 2 | 8 | 9 | 9 | 3 | 6 | 5 | 9 | 8 | 5.7E6 | 9.69E6 | 4.57E6 | 9.35E6 | 8.98E6 | 2.67E6 | 2.88E6 | 4.63E6 | 1.7E7 | 8.26E6 | 4 | 3 | 2 | 1 | 3 | 3 | 3 | 1 | 2 | 2 | 3 | 3 | N | 45626 | 26S proteasome regulatory subunit 8 OS=Mus musculus OX=10090 GN=Psmc5 PE=1 SV=1 |
| 1015 | 1957 | F7AA45\|F7AA45\_MOUSE | 129.69 | 12 | 4 | 9 | 4 | 4 | 9 | 9 | 9 | 9 | 6 | 4 | 2.07E6 | 4.53E6 | 3.32E6 | 6.55E5 | 3.06E6 | 1.79E6 | 2.42E6 | 6.7E5 | 7.4E6 | 2.82E6 | 3 | 3 | 1 | 2 | 1 | 1 | 2 | 2 | 2 | 2 | 2 | 1 | N | 33947 | RNA binding motif protein 39 (Fragment) OS=Mus musculus OX=10090 GN=Rbm39 PE=1 SV=1 |
| 1015 | 1961 | E9Q8F0\|E9Q8F0\_MOUSE | 129.69 | 9 | 3 | 7 | 3 | 3 | 7 | 7 | 7 | 7 | 5 | 3 | 2.07E6 | 4.53E6 | 3.32E6 | 6.55E5 | 3.06E6 | 1.79E6 | 2.42E6 | 6.7E5 | 7.4E6 | 2.82E6 | 3 | 3 | 1 | 2 | 1 | 1 | 2 | 2 | 2 | 2 | 2 | 1 | N | 47051 | RNA binding motif protein 39 OS=Mus musculus OX=10090 GN=Rbm39 PE=1 SV=1 |
| 1015 | 1964 | sp\|Q8VH51\|RBM39\_MOUSE | 129.69 | 7 | 2 | 5 | 2 | 2 | 5 | 5 | 5 | 5 | 4 | 2 | 2.07E6 | 4.53E6 | 3.32E6 | 6.55E5 | 3.06E6 | 1.79E6 | 2.42E6 | 6.7E5 | 7.4E6 | 2.82E6 | 3 | 3 | 1 | 2 | 1 | 1 | 2 | 2 | 2 | 2 | 2 | 1 | N | 59407 | RNA-binding protein 39 OS=Mus musculus OX=10090 GN=Rbm39 PE=1 SV=2 |
| 511 | 2422 | sp\|P60867\|RS20\_MOUSE | 129.62 | 24 | 14 | 24 | 24 | 24 | 24 | 24 | 24 | 24 | 24 | 24 | 4.55E7 | 4.77E7 | 4.83E7 | 2.49E7 | 3.79E7 | 3.5E7 | 3.47E7 | 3.58E7 | 3.69E7 | 3.66E7 | 3 | 2 | 3 | 5 | 5 | 5 | 4 | 5 | 5 | 5 | 4 | 4 | N | 13373 | Small ribosomal subunit protein uS10 OS=Mus musculus OX=10090 GN=Rps20 PE=1 SV=1 |
| 966 | 1412 | sp\|O70570\|PIGR\_MOUSE | 129.27 | 8 | 1 | 0 | 3 | 7 | 3 | 0 | 5 | 7 | 0 | 1 | 5.05E6 |  | 9.18E6 | 4.28E6 | 5.21E6 |  | 5.43E6 | 4.98E6 |  | 4.01E6 | 5 | 5 | 1 | 0 | 2 | 4 | 2 | 0 | 3 | 4 | 0 | 1 | Y | 84999 | Polymeric immunoglobulin receptor OS=Mus musculus OX=10090 GN=Pigr PE=1 SV=1 |
| 524 | 1755 | sp\|Q62093\|SRSF2\_MOUSE | 129.07 | 15 | 8 | 12 | 12 | 12 | 8 | 12 | 12 | 15 | 8 | 12 | 1.97E7 | 4.18E7 | 3.65E7 | 1.15E7 | 2.16E7 | 3.58E7 | 2.37E7 | 2.13E7 | 2.49E7 | 3E7 | 4 | 4 | 3 | 6 | 6 | 4 | 3 | 5 | 5 | 4 | 5 | 3 | N | 25476 | Serine/arginine-rich splicing factor 2 OS=Mus musculus OX=10090 GN=Srsf2 PE=1 SV=4 |
| 878 | 2049 | sp\|Q60737\|CSK21\_MOUSE | 128.85 | 11 | 5 | 5 | 7 | 5 | 5 | 6 | 5 | 3 | 8 | 3 | 4.99E5 | 1.85E6 | 2.28E6 | 1.09E6 | 1.56E6 | 3.39E6 | 4.86E5 | 1.79E6 | 2.77E6 | 2.35E6 | 5 | 4 | 2 | 2 | 3 | 2 | 2 | 3 | 2 | 1 | 4 | 1 | N | 45134 | Casein kinase II subunit alpha OS=Mus musculus OX=10090 GN=Csnk2a1 PE=1 SV=2 |
| 548 | 1769 | sp\|Q60931\|VDAC3\_MOUSE | 128.79 | 19 | 6 | 10 | 8 | 10 | 8 | 19 | 8 | 8 | 8 | 10 |  | 6.94E6 | 3.89E6 | 9.02E6 | 6E6 | 9.44E6 | 2.24E5 | 1.86E6 | 8.9E6 | 4.4E6 | 5 | 3 | 3 | 5 | 6 | 4 | 5 | 6 | 3 | 3 | 5 | 5 | Y | 30753 | Voltage-dependent anion-selective channel protein 3 OS=Mus musculus OX=10090 GN=Vdac3 PE=1 SV=1 |
| 741 | 1512 | sp\|Q05144\|RAC2\_MOUSE | 128.62 | 24 | 16 | 9 | 9 | 4 | 0 | 9 | 9 | 9 | 24 | 21 | 6.56E6 |  |  |  |  |  |  |  | 1.26E7 | 8.98E6 | 5 | 3 | 4 | 3 | 3 | 1 | 0 | 2 | 2 | 3 | 6 | 5 | Y | 21441 | Ras-related C3 botulinum toxin substrate 2 OS=Mus musculus OX=10090 GN=Rac2 PE=1 SV=1 |
| 1221 | 2082 | sp\|Q8VCW8\|ACSF2\_MOUSE | 128.58 | 7 | 0 | 5 | 3 | 0 | 3 | 7 | 3 | 3 | 2 | 2 |  | 4.73E6 | 2.62E6 |  | 1.3E6 | 1.04E7 | 2.42E6 | 1.13E6 | 2.76E6 | 1.72E5 | 3 | 3 | 0 | 2 | 1 | 0 | 1 | 3 | 1 | 1 | 1 | 1 | N | 67951 | Medium-chain acyl-CoA ligase ACSF2, mitochondrial OS=Mus musculus OX=10090 GN=Acsf2 PE=1 SV=1 |
| 1234 | 2080 | sp\|P97821\|CATC\_MOUSE | 128.53 | 8 | 3 | 4 | 3 | 1 | 1 | 1 | 0 | 0 | 7 | 0 | 5.99E7 | 2.28E4 | 1.55E7 | 3.12E5 | 2.06E5 | 4.39E5 |  |  | 1.24E7 |  | 3 | 3 | 1 | 1 | 1 | 1 | 1 | 1 | 0 | 0 | 2 | 0 | Y | 52376 | Dipeptidyl peptidase 1 OS=Mus musculus OX=10090 GN=Ctsc PE=1 SV=1 |
| 92 | 1865 | sp\|P0C0S6\|H2AZ\_MOUSE | 128.48 | 31 | 20 | 31 | 31 | 31 | 20 | 20 | 20 | 20 | 20 | 20 | 4.29E7 | 6.13E7 | 4.88E7 | 2.32E7 | 3.62E7 | 3.82E7 | 2.86E7 | 3.25E7 | 8.23E7 | 3.94E7 | 4 | 2 | 23 | 22 | 31 | 29 | 38 | 20 | 25 | 36 | 22 | 21 | N | 13553 | Histone H2A.Z OS=Mus musculus OX=10090 GN=H2az1 PE=1 SV=2 |
| 92 | 1866 | sp\|Q3THW5\|H2AV\_MOUSE | 128.48 | 31 | 20 | 31 | 31 | 31 | 20 | 20 | 20 | 20 | 20 | 20 | 4.29E7 | 6.13E7 | 4.88E7 | 2.32E7 | 3.62E7 | 3.82E7 | 2.86E7 | 3.25E7 | 8.23E7 | 3.94E7 | 4 | 2 | 23 | 22 | 31 | 29 | 38 | 20 | 25 | 36 | 22 | 21 | N | 13509 | Histone H2A.V OS=Mus musculus OX=10090 GN=H2az2 PE=1 SV=3 |
| 855 | 1313 | sp\|P10852\|4F2\_MOUSE | 128.10 | 11 | 8 | 8 | 6 | 2 | 2 | 6 | 6 | 8 | 5 | 5 | 4.91E6 | 8.99E6 | 1.73E6 | 3.5E6 | 4.14E6 | 3.04E6 | 4.33E6 | 3.99E6 | 5.02E6 | 1.91E6 | 5 | 5 | 3 | 3 | 2 | 1 | 1 | 3 | 3 | 3 | 2 | 2 | N | 58337 | Amino acid transporter heavy chain SLC3A2 OS=Mus musculus OX=10090 GN=Slc3a2 PE=1 SV=1 |
| 945 | 2472 | A0A0N4SUV7\|A0A0N4SUV7\_MOUSE | 128.10 | 20 | 8 | 8 | 11 | 20 | 20 | 17 | 20 | 9 | 8 | 8 | 9.32E5 | 6.96E5 | 1.16E6 | 1.65E6 | 2.94E6 | 2.58E6 | 1.76E6 | 1.36E6 | 1.18E6 | 9.12E5 | 3 | 2 | 1 | 1 | 2 | 3 | 3 | 2 | 3 | 1 | 1 | 1 | N | 16894 | deoxyribose-phosphate aldolase (Fragment) OS=Mus musculus OX=10090 GN=Dera PE=1 SV=1 |
| 945 | 2589 | sp\|Q91YP3\|DEOC\_MOUSE | 128.10 | 10 | 4 | 4 | 5 | 10 | 10 | 8 | 10 | 4 | 4 | 4 | 9.32E5 | 6.96E5 | 1.16E6 | 1.65E6 | 2.94E6 | 2.58E6 | 1.76E6 | 1.36E6 | 1.18E6 | 9.12E5 | 3 | 2 | 1 | 1 | 2 | 3 | 3 | 2 | 3 | 1 | 1 | 1 | N | 34975 | Deoxyribose-phosphate aldolase OS=Mus musculus OX=10090 GN=Dera PE=1 SV=1 |
| 1057 | 2394 | Q921K2\|Q921K2\_MOUSE | 128.09 | 2 | 1 | 0 | 2 | 1 | 2 | 2 | 1 | 1 | 0 | 0 | 5.06E5 |  | 1.18E6 | 9.34E5 | 6.61E5 | 1.42E6 | 4.22E5 | 3.49E5 |  |  | 2 | 2 | 1 | 0 | 2 | 1 | 2 | 2 | 1 | 1 | 0 | 0 | N | 112721 | Poly [ADP-ribose] polymerase OS=Mus musculus OX=10090 GN=Parp1 PE=1 SV=1 |
| 1232 | 1734 | sp\|Q3THS6\|METK2\_MOUSE | 128.03 | 11 | 4 | 0 | 8 | 2 | 0 | 2 | 2 | 3 | 4 | 4 | 1.75E6 |  | 2.84E6 | 1.4E6 |  | 2.88E6 | 1.09E6 | 1.2E5 | 2.75E6 | 6.88E5 | 4 | 4 | 1 | 0 | 3 | 1 | 0 | 1 | 1 | 1 | 1 | 1 | N | 43689 | S-adenosylmethionine synthase isoform type-2 OS=Mus musculus OX=10090 GN=Mat2a PE=1 SV=2 |
| 1021 | 1613 | sp\|P14438\|HA2U\_MOUSE | 127.99 | 26 | 4 | 0 | 7 | 4 | 16 | 11 | 18 | 17 | 7 | 4 | 4.04E6 |  | 5.76E6 | 2.15E6 | 4.71E6 | 1.09E7 | 5.48E6 | 6.34E6 | 8.81E5 | 3.49E6 | 5 | 5 | 1 | 0 | 1 | 1 | 3 | 2 | 3 | 3 | 1 | 1 | Y | 25136 | H-2 class II histocompatibility antigen, A-U alpha chain (Fragment) OS=Mus musculus OX=10090 GN=H2-Aa PE=1 SV=1 |
| 1021 | 1615 | sp\|P14434\|HA2B\_MOUSE | 127.99 | 23 | 3 | 0 | 7 | 3 | 14 | 10 | 16 | 15 | 7 | 3 | 4.04E6 |  | 5.76E6 | 2.15E6 | 4.71E6 | 1.09E7 | 5.48E6 | 6.34E6 | 8.81E5 | 3.49E6 | 5 | 5 | 1 | 0 | 1 | 1 | 3 | 2 | 3 | 3 | 1 | 1 | Y | 28093 | H-2 class II histocompatibility antigen, A-B alpha chain OS=Mus musculus OX=10090 GN=H2-Aa PE=1 SV=2 |
| 1019 | 1595 | sp\|Q9WUA2\|SYFB\_MOUSE | 127.87 | 8 | 0 | 2 | 4 | 6 | 5 | 4 | 4 | 5 | 2 | 0 |  | 6.49E5 | 1.53E6 | 8.75E5 | 1.53E6 | 9.51E5 | 1.52E6 | 3.7E6 | 1.96E6 |  | 5 | 5 | 0 | 1 | 2 | 3 | 3 | 2 | 2 | 3 | 1 | 0 | Y | 65697 | Phenylalanine--tRNA ligase beta subunit OS=Mus musculus OX=10090 GN=Farsb PE=1 SV=2 |
| 1247 | 1793 | sp\|Q9EPC1\|PARVA\_MOUSE | 127.86 | 11 | 0 | 2 | 2 | 0 | 2 | 6 | 9 | 4 | 0 | 0 |  | 3.81E6 | 5.52E6 |  | 5.3E6 | 9.33E6 | 3.97E6 | 7.31E6 |  |  | 4 | 4 | 0 | 1 | 1 | 0 | 1 | 2 | 3 | 2 | 0 | 0 | N | 42330 | Alpha-parvin OS=Mus musculus OX=10090 GN=Parva PE=1 SV=1 |
| 1176 | 2041 | Q3UW98\|Q3UW98\_MOUSE | 127.56 | 4 | 0 | 0 | 0 | 2 | 4 | 3 | 4 | 2 | 2 | 0 |  |  |  | 4.65E6 | 4.5E6 | 4.17E5 | 5.94E6 | 2.62E5 | 2.2E6 |  | 3 | 3 | 0 | 0 | 0 | 2 | 3 | 2 | 3 | 1 | 1 | 0 | N | 102165 | Chloride channel accessory 4B OS=Mus musculus OX=10090 GN=Clca4b PE=1 SV=1 |
| 1055 | 1387 | sp\|P06728\|APOA4\_MOUSE | 127.40 | 12 | 0 | 0 | 3 | 7 | 0 | 5 | 10 | 9 | 3 | 8 |  |  | 1.39E6 | 5.36E5 |  | 6.64E5 | 1.61E6 | 1.43E7 | 7.18E6 | 1.3E6 | 4 | 4 | 0 | 0 | 1 | 2 | 0 | 1 | 3 | 3 | 1 | 2 | N | 45029 | Apolipoprotein A-IV OS=Mus musculus OX=10090 GN=Apoa4 PE=1 SV=3 |
| 594 | 846 | sp\|P28653\|PGS1\_MOUSE | 127.24 | 19 | 8 | 8 | 5 | 10 | 8 | 8 | 12 | 5 | 3 | 5 | 9.29E6 | 1.64E7 | 1.49E7 | 3.08E6 | 1.45E7 | 1.79E7 | 1.01E7 | 8.36E6 | 2.57E7 | 1.61E7 | 7 | 6 | 4 | 4 | 3 | 4 | 5 | 4 | 6 | 2 | 2 | 3 | N | 41639 | Biglycan OS=Mus musculus OX=10090 GN=Bgn PE=1 SV=1 |
| 1121 | 1729 | Q99N15\|Q99N15\_MOUSE | 127.18 | 29 | 3 | 3 | 10 | 0 | 13 | 10 | 7 | 10 | 3 | 3 | 3.8E6 | 5.92E6 | 6.35E6 |  | 4.93E7 | 8.56E6 | 4.95E5 | 9.35E5 | 4.36E6 | 4.56E6 | 5 | 5 | 1 | 1 | 2 | 0 | 3 | 2 | 1 | 1 | 1 | 1 | Y | 27274 | 17beta-hydroxysteroid dehydrogenase type 10/short chain L-3-hydroxyacyl-CoA dehydrogenase OS=Mus musculus OX=10090 GN=Hsd17b10 PE=1 SV=1 |
| 1121 | 1730 | A2AFQ2\|A2AFQ2\_MOUSE | 127.18 | 28 | 3 | 3 | 9 | 0 | 12 | 10 | 7 | 9 | 3 | 3 | 3.8E6 | 5.92E6 | 6.35E6 |  | 4.93E7 | 8.56E6 | 4.95E5 | 9.35E5 | 4.36E6 | 4.56E6 | 5 | 5 | 1 | 1 | 2 | 0 | 3 | 2 | 1 | 1 | 1 | 1 | Y | 28374 | Hydroxysteroid (17-beta) dehydrogenase 10 OS=Mus musculus OX=10090 GN=Hsd17b10 PE=1 SV=1 |
| 768 | 1312 | sp\|P61028\|RAB8B\_MOUSE | 127.16 | 24 | 14 | 9 | 14 | 14 | 14 | 3 | 15 | 9 | 12 | 9 | 6.47E5 |  |  |  |  |  | 3.05E5 |  | 1.12E6 |  | 5 | 1 | 3 | 2 | 3 | 3 | 4 | 2 | 3 | 2 | 2 | 2 | N | 23603 | Ras-related protein Rab-8B OS=Mus musculus OX=10090 GN=Rab8b PE=1 SV=1 |
| 220 | 3905 | sp\|Q8C1P2\|DFA21\_MOUSE | 126.86 | 23 | 23 | 23 | 23 | 23 | 23 | 23 | 23 | 23 | 23 | 23 | 3.29E8 | 5.23E8 | 4.75E8 | 2.4E8 | 2.48E8 | 2.58E8 | 1.91E8 | 2.06E8 | 5.31E8 | 2.67E8 | 2 | 2 | 12 | 18 | 20 | 13 | 15 | 9 | 9 | 13 | 8 | 8 | Y | 10507 | Alpha-defensin 21 OS=Mus musculus OX=10090 GN=Defa21 PE=3 SV=1 |
| 220 | 3906 | W4VSN6\|W4VSN6\_MOUSE | 126.86 | 23 | 23 | 23 | 23 | 23 | 23 | 23 | 23 | 23 | 23 | 23 | 3.29E8 | 5.23E8 | 4.75E8 | 2.4E8 | 2.48E8 | 2.58E8 | 1.91E8 | 2.06E8 | 5.31E8 | 2.67E8 | 2 | 2 | 12 | 18 | 20 | 13 | 15 | 9 | 9 | 13 | 8 | 8 | Y | 10535 | Defensin, alpha, 22 OS=Mus musculus OX=10090 GN=Defa22 PE=1 SV=1 |
| 220 | 3907 | sp\|Q8C1N8\|DFA22\_MOUSE | 126.86 | 23 | 23 | 23 | 23 | 23 | 23 | 23 | 23 | 23 | 23 | 23 | 3.29E8 | 5.23E8 | 4.75E8 | 2.4E8 | 2.48E8 | 2.58E8 | 1.91E8 | 2.06E8 | 5.31E8 | 2.67E8 | 2 | 2 | 12 | 18 | 20 | 13 | 15 | 9 | 9 | 13 | 8 | 8 | Y | 10548 | Alpha-defensin 22 OS=Mus musculus OX=10090 GN=Defa22 PE=3 SV=1 |
| 971 | 2428 | sp\|Q9Z0G9\|CLD3\_MOUSE | 126.48 | 15 | 6 | 12 | 6 | 12 | 15 | 10 | 12 | 12 | 12 | 12 | 4.82E6 | 1.77E7 | 6.13E6 | 7.55E6 | 1.75E7 | 1.52E7 | 1E7 | 5.5E6 | 1.34E7 | 5.08E6 | 3 | 3 | 1 | 2 | 1 | 2 | 3 | 2 | 2 | 2 | 2 | 2 | N | 23285 | Claudin-3 OS=Mus musculus OX=10090 GN=Cldn3 PE=1 SV=1 |
| 830 | 1294 | Q924B0\|Q924B0\_MOUSE | 126.29 | 23 | 16 | 12 | 7 | 12 | 7 | 16 | 0 | 16 | 16 | 16 | 3.97E6 | 4.06E6 | 3.38E6 | 1.15E6 | 2.97E6 | 4.25E6 |  | 3.59E6 | 7.77E6 | 2.98E6 | 5 | 5 | 3 | 2 | 2 | 2 | 2 | 3 | 0 | 3 | 3 | 3 | N | 30429 | Inositol-1-monophosphatase OS=Mus musculus OX=10090 GN=Impa1 PE=1 SV=1 |
| 805 | 1569 | sp\|Q9Z2U1\|PSA5\_MOUSE | 126.24 | 22 | 13 | 10 | 10 | 5 | 10 | 0 | 14 | 10 | 17 | 14 | 1.04E7 | 2.87E7 | 2.84E7 |  | 2.21E7 |  | 1.54E7 | 1.54E7 | 2.69E7 | 3.2E7 | 5 | 5 | 2 | 2 | 2 | 1 | 2 | 0 | 3 | 2 | 4 | 3 | Y | 26411 | Proteasome subunit alpha type-5 OS=Mus musculus OX=10090 GN=Psma5 PE=1 SV=1 |
| 888 | 1265 | sp\|Q99MR6\|SRRT\_MOUSE | 126.22 | 6 | 1 | 1 | 3 | 1 | 3 | 4 | 3 | 4 | 1 | 1 | 3.11E6 | 4.26E6 | 3.11E6 |  |  | 4.58E6 | 2.16E6 | 2.33E6 | 8.88E5 | 2.92E6 | 5 | 3 | 1 | 1 | 2 | 1 | 2 | 3 | 2 | 3 | 1 | 1 | N | 100452 | Serrate RNA effector molecule homolog OS=Mus musculus OX=10090 GN=Srrt PE=1 SV=1 |
| 751 | 1489 | A0A0R4J111\|A0A0R4J111\_MOUSE | 126.14 | 27 | 9 | 10 | 6 | 19 | 16 | 10 | 20 | 10 | 9 | 6 | 1.11E7 | 1.22E7 | 9.05E6 | 6.2E6 | 7.6E6 | 5.04E5 | 7.89E6 | 6.04E6 | 1.58E7 | 8.29E6 | 5 | 5 | 3 | 2 | 2 | 4 | 3 | 2 | 5 | 3 | 2 | 2 | N | 21929 | Glutathione peroxidase OS=Mus musculus OX=10090 GN=Gpx2 PE=1 SV=1 |
| 816 | 1445 | sp\|Q8QZY1\|EIF3L\_MOUSE | 125.96 | 8 | 0 | 3 | 1 | 5 | 5 | 5 | 7 | 2 | 3 | 2 |  | 6.31E7 | 9.49E5 | 5.29E6 | 2.44E6 | 9.66E6 | 1.03E7 | 1.3E6 | 4.99E7 | 5.52E7 | 5 | 5 | 0 | 2 | 1 | 3 | 3 | 3 | 4 | 2 | 2 | 1 | N | 66613 | Eukaryotic translation initiation factor 3 subunit L OS=Mus musculus OX=10090 GN=Eif3l PE=1 SV=1 |
| 788 | 1785 | sp\|P35278\|RAB5C\_MOUSE | 125.86 | 21 | 16 | 5 | 16 | 16 | 16 | 16 | 16 | 16 | 16 | 16 | 1.44E7 | 7.81E6 | 1.97E7 | 8.78E6 | 1.89E7 | 1.57E7 | 1.11E7 | 6.48E6 | 1.57E7 | 1.87E7 | 4 | 4 | 3 | 1 | 3 | 3 | 3 | 3 | 3 | 3 | 3 | 3 | N | 23412 | Ras-related protein Rab-5C OS=Mus musculus OX=10090 GN=Rab5c PE=1 SV=2 |
| 788 | 1786 | Q8C266\|Q8C266\_MOUSE | 125.86 | 20 | 15 | 5 | 15 | 15 | 15 | 15 | 15 | 15 | 15 | 15 | 1.44E7 | 7.81E6 | 1.97E7 | 8.78E6 | 1.89E7 | 1.57E7 | 1.11E7 | 6.48E6 | 1.57E7 | 1.87E7 | 4 | 4 | 3 | 1 | 3 | 3 | 3 | 3 | 3 | 3 | 3 | 3 | N | 25352 | small monomeric GTPase OS=Mus musculus OX=10090 GN=Rab5c PE=1 SV=1 |
| 1027 | 2686 | sp\|P23198\|CBX3\_MOUSE | 125.75 | 16 | 8 | 16 | 8 | 8 | 8 | 0 | 8 | 8 | 8 | 8 | 6E6 | 6.38E6 | 8.35E6 | 2.68E6 | 6.6E6 |  | 3.8E6 | 5.09E6 | 8.69E6 | 1.06E7 | 3 | 3 | 1 | 2 | 1 | 2 | 1 | 0 | 1 | 1 | 1 | 3 | Y | 20855 | Chromobox protein homolog 3 OS=Mus musculus OX=10090 GN=Cbx3 PE=1 SV=2 |
| 1027 | 2687 | Q9DCC5\|Q9DCC5\_MOUSE | 125.75 | 16 | 8 | 16 | 8 | 8 | 8 | 0 | 8 | 8 | 8 | 8 | 6E6 | 6.38E6 | 8.35E6 | 2.68E6 | 6.6E6 |  | 3.8E6 | 5.09E6 | 8.69E6 | 1.06E7 | 3 | 3 | 1 | 2 | 1 | 2 | 1 | 0 | 1 | 1 | 1 | 3 | Y | 20811 | Cbx3 protein OS=Mus musculus OX=10090 GN=Cbx3 PE=1 SV=1 |
| 536 | 1697 | A0A1L1SQA8\|A0A1L1SQA8\_MOUSE | 125.75 | 32 | 30 | 20 | 30 | 30 | 30 | 30 | 30 | 30 | 32 | 20 | 7.66E7 | 4.92E7 | 8.7E7 | 4.28E7 | 7.09E7 | 5.64E7 | 5.15E7 | 5.37E7 | 1.28E8 | 4.66E7 | 4 | 4 | 4 | 3 | 3 | 5 | 4 | 4 | 5 | 7 | 7 | 3 | N | 10309 | 40S ribosomal protein S25 OS=Mus musculus OX=10090 GN=Rps25 PE=1 SV=1 |
| 536 | 1700 | sp\|P62852\|RS25\_MOUSE | 125.75 | 24 | 22 | 15 | 22 | 22 | 22 | 22 | 22 | 22 | 24 | 15 | 7.66E7 | 4.92E7 | 8.7E7 | 4.28E7 | 7.09E7 | 5.64E7 | 5.15E7 | 5.37E7 | 1.28E8 | 4.66E7 | 4 | 4 | 4 | 3 | 3 | 5 | 4 | 4 | 5 | 7 | 7 | 3 | N | 13742 | Small ribosomal subunit protein eS25 OS=Mus musculus OX=10090 GN=Rps25 PE=1 SV=1 |
| 1194 | 1737 | Q3TF41\|Q3TF41\_MOUSE | 125.72 | 13 | 3 | 3 | 3 | 9 | 3 | 6 | 5 | 3 | 3 | 0 |  | 1.02E6 | 3.82E5 | 5.16E5 | 6.2E5 | 2.39E6 | 4.74E5 | 1.13E6 |  |  | 4 | 3 | 1 | 1 | 1 | 3 | 1 | 2 | 1 | 1 | 1 | 0 | N | 42733 | Nucleosome assembly protein 1-like 1 OS=Mus musculus OX=10090 GN=Nap1l1 PE=1 SV=1 |
| 1194 | 1738 | Q8BSH9\|Q8BSH9\_MOUSE | 125.72 | 13 | 3 | 3 | 3 | 8 | 3 | 6 | 4 | 3 | 3 | 0 |  | 1.02E6 | 3.82E5 | 5.16E5 | 6.2E5 | 2.39E6 | 4.74E5 | 1.13E6 |  |  | 4 | 3 | 1 | 1 | 1 | 3 | 1 | 2 | 1 | 1 | 1 | 0 | N | 44598 | Nucleosome assembly protein 1-like 1 OS=Mus musculus OX=10090 GN=Nap1l1 PE=1 SV=1 |
| 1194 | 1739 | sp\|P28656\|NP1L1\_MOUSE | 125.72 | 13 | 3 | 3 | 3 | 8 | 3 | 6 | 4 | 3 | 3 | 0 |  | 1.02E6 | 3.82E5 | 5.16E5 | 6.2E5 | 2.39E6 | 4.74E5 | 1.13E6 |  |  | 4 | 3 | 1 | 1 | 1 | 3 | 1 | 2 | 1 | 1 | 1 | 0 | N | 45345 | Nucleosome assembly protein 1-like 1 OS=Mus musculus OX=10090 GN=Nap1l1 PE=1 SV=2 |
| 1031 | 1664 | sp\|Q8BT60\|CPNE3\_MOUSE | 125.24 | 9 | 2 | 4 | 0 | 6 | 2 | 0 | 2 | 2 | 3 | 2 | 7.89E6 | 7.28E6 |  | 8.52E6 | 1.74E6 |  | 4.1E6 | 3.66E6 | 1.49E6 | 2.44E6 | 4 | 4 | 1 | 2 | 0 | 3 | 1 | 0 | 1 | 1 | 1 | 1 | Y | 59585 | Copine-3 OS=Mus musculus OX=10090 GN=Cpne3 PE=1 SV=2 |
| 1300 | 920 | A0A6I8MWZ2\|A0A6I8MWZ2\_MOUSE | 125.24 | 5 | 2 | 1 | 0 | 0 | 0 | 1 | 1 | 0 | 0 | 0 |  |  |  |  |  |  | 1.1E7 |  |  |  | 7 | 1 | 2 | 2 | 0 | 1 | 1 | 1 | 1 | 0 | 0 | 0 | Y | 197192 | Tensin 1 OS=Mus musculus OX=10090 GN=Tns1 PE=1 SV=1 |
| 344 | 1767 | B1AZS9\|B1AZS9\_MOUSE | 125.23 | 16 | 8 | 11 | 13 | 8 | 8 | 13 | 13 | 13 | 8 | 13 |  | 4.34E6 | 6.06E6 |  |  | 6.5E6 | 1.06E7 | 4.68E6 |  | 1.09E6 | 4 | 2 | 9 | 9 | 7 | 5 | 7 | 4 | 7 | 7 | 11 | 11 | N | 26391 | Peroxiredoxin 4 (Fragment) OS=Mus musculus OX=10090 GN=Prdx4 PE=1 SV=1 |
| 344 | 1768 | sp\|O08807\|PRDX4\_MOUSE | 125.23 | 13 | 7 | 9 | 11 | 7 | 7 | 11 | 11 | 11 | 7 | 11 |  | 4.34E6 | 6.06E6 |  |  | 6.5E6 | 1.06E7 | 4.68E6 |  | 1.09E6 | 4 | 2 | 9 | 9 | 7 | 5 | 7 | 4 | 7 | 7 | 11 | 11 | N | 31053 | Peroxiredoxin-4 OS=Mus musculus OX=10090 GN=Prdx4 PE=1 SV=1 |
| 837 | 1560 | A0A0G2JES3\|A0A0G2JES3\_MOUSE | 125.13 | 28 | 10 | 10 | 10 | 15 | 20 | 23 | 13 | 20 | 14 | 4 | 3.69E6 | 1.43E7 | 1.49E7 | 1.22E7 | 1.22E7 | 1.81E7 | 1.56E7 | 3.73E6 | 6.04E6 |  | 5 | 5 | 2 | 2 | 2 | 3 | 3 | 4 | 3 | 3 | 2 | 1 | Y | 21741 | Large ribosomal subunit protein uL6 (Fragment) OS=Mus musculus OX=10090 GN=Rpl9 PE=1 SV=1 |
| 837 | 1562 | sp\|P51410\|RL9\_MOUSE | 125.13 | 28 | 10 | 10 | 10 | 15 | 20 | 23 | 13 | 20 | 14 | 4 | 3.69E6 | 1.43E7 | 1.49E7 | 1.22E7 | 1.22E7 | 1.81E7 | 1.56E7 | 3.73E6 | 6.04E6 |  | 5 | 5 | 2 | 2 | 2 | 3 | 3 | 4 | 3 | 3 | 2 | 1 | Y | 21881 | Large ribosomal subunit protein uL6 OS=Mus musculus OX=10090 GN=Rpl9 PE=1 SV=2 |
| 1186 | 1766 | sp\|Q9QYJ0\|DNJA2\_MOUSE | 125.13 | 13 | 5 | 5 | 3 | 2 | 0 | 8 | 0 | 2 | 0 | 3 | 3.72E6 | 4.84E6 | 1.48E6 | 2.56E6 |  | 5.57E6 |  | 7.68E5 |  | 1.18E6 | 4 | 4 | 3 | 2 | 1 | 1 | 0 | 2 | 0 | 1 | 0 | 1 | N | 45746 | DnaJ homolog subfamily A member 2 OS=Mus musculus OX=10090 GN=Dnaja2 PE=1 SV=1 |
| 787 | 1922 | sp\|Q9Z0S1\|BPNT1\_MOUSE | 125.06 | 13 | 9 | 7 | 9 | 5 | 5 | 7 | 2 | 5 | 10 | 10 | 1.12E7 | 1.34E7 | 1.22E7 | 4.31E6 | 5.42E6 | 9.31E6 | 4.34E6 | 3.02E6 | 1.34E7 | 1.21E7 | 4 | 4 | 3 | 3 | 4 | 2 | 2 | 2 | 1 | 2 | 4 | 3 | N | 33196 | 3'(2'),5'-bisphosphate nucleotidase 1 OS=Mus musculus OX=10090 GN=Bpnt1 PE=1 SV=2 |
| 787 | 1927 | A0A1B0GRV0\|A0A1B0GRV0\_MOUSE | 125.06 | 12 | 9 | 7 | 9 | 5 | 5 | 7 | 2 | 5 | 10 | 10 | 1.12E7 | 1.34E7 | 1.22E7 | 4.31E6 | 5.42E6 | 9.31E6 | 4.34E6 | 3.02E6 | 1.34E7 | 1.21E7 | 4 | 4 | 3 | 3 | 4 | 2 | 2 | 2 | 1 | 2 | 4 | 3 | N | 34974 | 3'(2'),5'-bisphosphate nucleotidase 1 OS=Mus musculus OX=10090 GN=Bpnt1 PE=1 SV=1 |
| 1450 | 1791 | sp\|Q7TNV0\|DEK\_MOUSE | 124.82 | 9 | 0 | 3 | 3 | 0 | 4 | 0 | 0 | 6 | 0 | 3 |  | 1.43E6 | 1.25E6 |  | 3.09E5 |  |  | 2.93E6 |  | 2.11E6 | 3 | 3 | 0 | 1 | 1 | 0 | 1 | 0 | 0 | 2 | 0 | 1 | N | 43159 | Protein DEK OS=Mus musculus OX=10090 GN=Dek PE=1 SV=1 |
| 811 | 1432 | E0CXN5\|E0CXN5\_MOUSE | 124.66 | 13 | 4 | 8 | 8 | 4 | 6 | 7 | 8 | 4 | 8 | 6 | 2.97E6 | 4.91E6 | 5.83E6 | 1.37E6 | 8.12E6 | 9.45E6 | 1.35E6 | 4.94E5 | 2.64E6 | 1.23E7 | 4 | 4 | 2 | 3 | 3 | 2 | 2 | 3 | 2 | 2 | 2 | 2 | Y | 35231 | Glycerol-3-phosphate dehydrogenase [NAD(+)] OS=Mus musculus OX=10090 GN=Gpd1 PE=1 SV=1 |
| 811 | 1435 | sp\|P13707\|GPDA\_MOUSE | 124.66 | 12 | 4 | 8 | 8 | 4 | 6 | 6 | 8 | 4 | 8 | 6 | 2.97E6 | 4.91E6 | 5.83E6 | 1.37E6 | 8.12E6 | 9.45E6 | 1.35E6 | 4.94E5 | 2.64E6 | 1.23E7 | 4 | 4 | 2 | 3 | 3 | 2 | 2 | 3 | 2 | 2 | 2 | 2 | Y | 37573 | Glycerol-3-phosphate dehydrogenase [NAD(+)], cytoplasmic OS=Mus musculus OX=10090 GN=Gpd1 PE=1 SV=3 |
| 1095 | 1110 | sp\|Q9EQH3\|VPS35\_MOUSE | 124.40 | 7 | 0 | 3 | 0 | 4 | 0 | 0 | 1 | 1 | 4 | 2 |  | 3.11E6 |  | 8.89E5 |  |  | 3.86E5 | 4.37E5 | 7.26E6 | 7.27E5 | 5 | 5 | 0 | 2 | 0 | 3 | 0 | 0 | 1 | 1 | 3 | 1 | N | 91713 | Vacuolar protein sorting-associated protein 35 OS=Mus musculus OX=10090 GN=Vps35 PE=1 SV=1 |
| 872 | 1529 | sp\|Q8VCM7\|FIBG\_MOUSE | 124.35 | 11 | 6 | 2 | 2 | 2 | 4 | 2 | 2 | 4 | 11 | 4 | 4.07E7 | 3.32E6 | 2.56E6 | 5.51E5 | 3.12E6 | 6.2E6 | 3.2E6 | 3.55E6 | 2.74E7 | 1.65E7 | 4 | 4 | 4 | 1 | 1 | 1 | 2 | 1 | 1 | 2 | 5 | 3 | Y | 49391 | Fibrinogen gamma chain OS=Mus musculus OX=10090 GN=Fgg PE=1 SV=1 |
| 872 | 1530 | Q3UER8\|Q3UER8\_MOUSE | 124.35 | 11 | 6 | 2 | 2 | 2 | 4 | 2 | 2 | 4 | 11 | 4 | 4.07E7 | 3.32E6 | 2.56E6 | 5.51E5 | 3.12E6 | 6.2E6 | 3.2E6 | 3.55E6 | 2.74E7 | 1.65E7 | 4 | 4 | 4 | 1 | 1 | 1 | 2 | 1 | 1 | 2 | 5 | 3 | Y | 50349 | Fibrinogen gamma chain OS=Mus musculus OX=10090 GN=Fgg PE=1 SV=1 |
| 242 | 2401 | Q8C1N9\|Q8C1N9\_MOUSE | 124.09 | 25 | 25 | 25 | 25 | 25 | 25 | 25 | 25 | 25 | 25 | 25 | 1.6E8 | 8.53E8 | 8.4E8 | 3.91E8 | 5.76E8 | 6.06E8 | 4.91E8 | 2.32E8 | 5.78E8 | 1.73E8 | 3 | 3 | 10 | 12 | 12 | 9 | 11 | 9 | 9 | 12 | 10 | 11 | Y | 10575 | Defensin, alpha, 2 OS=Mus musculus OX=10090 GN=Defa2 PE=2 SV=1 |
| 242 | 2402 | sp\|Q45VN2\|DFA20\_MOUSE | 124.09 | 25 | 25 | 25 | 25 | 25 | 25 | 25 | 25 | 25 | 25 | 25 | 1.6E8 | 8.53E8 | 8.4E8 | 3.91E8 | 5.76E8 | 6.06E8 | 4.91E8 | 2.32E8 | 5.78E8 | 1.73E8 | 3 | 3 | 10 | 12 | 12 | 9 | 11 | 9 | 9 | 12 | 10 | 11 | Y | 10601 | Alpha-defensin 20 OS=Mus musculus OX=10090 GN=Defa20 PE=3 SV=2 |
| 1103 | 1259 | Q99K94\|Q99K94\_MOUSE | 124.01 | 10 | 2 | 0 | 0 | 2 | 3 | 2 | 7 | 4 | 0 | 2 | 9.23E5 |  |  | 8.3E5 | 1.62E6 | 1.22E6 | 1.62E6 | 2.65E6 |  | 8.85E5 | 6 | 6 | 1 | 0 | 0 | 1 | 2 | 1 | 4 | 3 | 0 | 1 | N | 83107 | Signal transducer and activator of transcription OS=Mus musculus OX=10090 GN=Stat1 PE=1 SV=1 |
| 1103 | 1260 | sp\|P42225\|STAT1\_MOUSE | 124.01 | 9 | 2 | 0 | 0 | 2 | 3 | 2 | 7 | 4 | 0 | 2 | 9.23E5 |  |  | 8.3E5 | 1.62E6 | 1.22E6 | 1.62E6 | 2.65E6 |  | 8.85E5 | 6 | 6 | 1 | 0 | 0 | 1 | 2 | 1 | 4 | 3 | 0 | 1 | N | 87197 | Signal transducer and activator of transcription 1 OS=Mus musculus OX=10090 GN=Stat1 PE=1 SV=1 |
| 1103 | 1261 | Q8C3V4\|Q8C3V4\_MOUSE | 124.01 | 9 | 2 | 0 | 0 | 2 | 3 | 2 | 7 | 4 | 0 | 2 | 9.23E5 |  |  | 8.3E5 | 1.62E6 | 1.22E6 | 1.62E6 | 2.65E6 |  | 8.85E5 | 6 | 6 | 1 | 0 | 0 | 1 | 2 | 1 | 4 | 3 | 0 | 1 | N | 87315 | Signal transducer and activator of transcription OS=Mus musculus OX=10090 GN=Stat1 PE=1 SV=1 |
| 1103 | 1262 | A0A087WSP5\|A0A087WSP5\_MOUSE | 124.01 | 9 | 2 | 0 | 0 | 2 | 3 | 2 | 7 | 4 | 0 | 2 | 9.23E5 |  |  | 8.3E5 | 1.62E6 | 1.22E6 | 1.62E6 | 2.65E6 |  | 8.85E5 | 6 | 6 | 1 | 0 | 0 | 1 | 2 | 1 | 4 | 3 | 0 | 1 | N | 88033 | Signal transducer and activator of transcription OS=Mus musculus OX=10090 GN=Stat1 PE=1 SV=1 |
| 1225 | 1573 | sp\|Q9CPV4\|GLOD4\_MOUSE | 123.81 | 14 | 0 | 5 | 6 | 0 | 0 | 0 | 9 | 0 | 6 | 6 |  | 0E0 | 1.74E6 |  |  |  | 1.08E6 |  | 2.23E6 | 1.07E6 | 3 | 3 | 0 | 1 | 1 | 0 | 0 | 0 | 2 | 0 | 1 | 1 | N | 33317 | Glyoxalase domain-containing protein 4 OS=Mus musculus OX=10090 GN=Glod4 PE=1 SV=1 |
| 1071 | 1295 | sp\|P50516\|VATA\_MOUSE | 123.75 | 11 | 4 | 3 | 2 | 0 | 1 | 1 | 1 | 1 | 7 | 3 | 1.75E7 | 8.02E5 | 4.3E5 |  | 1.5E7 | 1.4E7 | 1.18E7 | 1.08E7 | 4.65E6 | 5.69E6 | 6 | 6 | 2 | 1 | 1 | 0 | 1 | 1 | 1 | 1 | 3 | 2 | Y | 68326 | V-type proton ATPase catalytic subunit A OS=Mus musculus OX=10090 GN=Atp6v1a PE=1 SV=2 |
| 963 | 2282 | A0A1L1SVK0\|A0A1L1SVK0\_MOUSE | 123.69 | 18 | 14 | 9 | 14 | 5 | 14 | 14 | 14 | 14 | 14 | 9 | 2.09E6 | 2.52E6 | 2.68E6 | 9.37E5 | 3.67E6 | 2.1E6 | 1.72E6 | 1.89E6 | 3.46E6 | 3.66E6 | 3 | 3 | 2 | 2 | 2 | 1 | 2 | 2 | 2 | 2 | 2 | 2 | N | 22056 | 1-alkyl-2-acetylglycerophosphocholine esterase (Fragment) OS=Mus musculus OX=10090 GN=Pafah1b2 PE=1 SV=1 |
| 963 | 2284 | sp\|Q61206\|PA1B2\_MOUSE | 123.69 | 16 | 12 | 7 | 12 | 4 | 12 | 12 | 12 | 12 | 12 | 7 | 2.09E6 | 2.52E6 | 2.68E6 | 9.37E5 | 3.67E6 | 2.1E6 | 1.72E6 | 1.89E6 | 3.46E6 | 3.66E6 | 3 | 3 | 2 | 2 | 2 | 1 | 2 | 2 | 2 | 2 | 2 | 2 | N | 25581 | Platelet-activating factor acetylhydrolase IB subunit alpha2 OS=Mus musculus OX=10090 GN=Pafah1b2 PE=1 SV=2 |
| 842 | 2468 | sp\|P31725\|S10A9\_MOUSE | 123.53 | 27 | 19 | 0 | 12 | 12 | 20 | 0 | 12 | 12 | 19 | 19 | 3.21E7 |  | 1.65E6 | 2.64E6 | 1.82E6 |  | 4.86E5 | 3.86E6 | 8.25E7 | 5.42E7 | 3 | 3 | 4 | 0 | 1 | 1 | 2 | 0 | 1 | 2 | 8 | 6 | Y | 13049 | Protein S100-A9 OS=Mus musculus OX=10090 GN=S100a9 PE=1 SV=3 |
| 895 | 3550 | sp\|Q9D0J8\|PTMS\_MOUSE | 123.32 | 23 | 23 | 23 | 23 | 23 | 11 | 23 | 23 | 12 | 0 | 23 | 2.15E7 | 3.27E7 | 3.42E7 | 1.53E7 | 1.66E7 | 1.84E7 | 2.3E7 | 6.83E6 |  | 2.22E7 | 2 | 2 | 2 | 3 | 3 | 3 | 1 | 3 | 3 | 2 | 0 | 2 | N | 11430 | Parathymosin OS=Mus musculus OX=10090 GN=Ptms PE=1 SV=3 |
| 895 | 3551 | A0AA74KUT7\|A0AA74KUT7\_MOUSE | 123.32 | 14 | 14 | 14 | 14 | 14 | 6 | 14 | 14 | 7 | 0 | 14 | 2.15E7 | 3.27E7 | 3.42E7 | 1.53E7 | 1.66E7 | 1.84E7 | 2.3E7 | 6.83E6 |  | 2.22E7 | 2 | 2 | 2 | 3 | 3 | 3 | 1 | 3 | 3 | 2 | 0 | 2 | N | 18909 | Parathymosin OS=Mus musculus OX=10090 GN=Ptms PE=1 SV=1 |
| 928 | 2562 | sp\|P55012\|S12A2\_MOUSE | 123.30 | 3 | 2 | 2 | 2 | 2 | 2 | 2 | 3 | 2 | 2 | 2 | 1.21E6 | 6.35E5 | 2.41E6 | 5.26E5 | 1.14E6 | 3.7E6 | 4.62E6 | 4.68E5 | 3.14E6 | 2.97E6 | 2 | 2 | 2 | 1 | 2 | 2 | 1 | 2 | 3 | 1 | 1 | 2 | N | 131033 | Solute carrier family 12 member 2 OS=Mus musculus OX=10090 GN=Slc12a2 PE=1 SV=2 |
| 928 | 2563 | E9QM38\|E9QM38\_MOUSE | 123.30 | 3 | 2 | 2 | 2 | 2 | 2 | 2 | 3 | 2 | 2 | 2 | 1.21E6 | 6.35E5 | 2.41E6 | 5.26E5 | 1.14E6 | 3.7E6 | 4.62E6 | 4.68E5 | 3.14E6 | 2.97E6 | 2 | 2 | 2 | 1 | 2 | 2 | 1 | 2 | 3 | 1 | 1 | 2 | N | 130668 | Solute carrier family 12, member 2 OS=Mus musculus OX=10090 GN=Slc12a2 PE=1 SV=1 |
| 526 | 777 | sp\|Q9WUM3\|COR1B\_MOUSE | 123.27 | 16 | 8 | 7 | 6 | 5 | 8 | 9 | 8 | 5 | 4 | 4 | 3.06E7 | 3.43E7 | 5.48E7 | 1.22E7 | 2.6E7 | 2.47E7 | 2.65E7 | 1.31E7 | 8.56E6 | 2.38E7 | 9 | 9 | 5 | 4 | 4 | 3 | 5 | 5 | 5 | 3 | 2 | 3 | Y | 53912 | Coronin-1B OS=Mus musculus OX=10090 GN=Coro1b PE=1 SV=1 |
| 671 | 1873 | sp\|P10922\|H10\_MOUSE | 122.88 | 21 | 15 | 14 | 15 | 15 | 16 | 21 | 15 | 15 | 11 | 16 | 2.68E7 | 1.19E8 | 7.92E7 | 3.01E7 | 2.28E7 | 6.56E7 | 4.22E7 | 3.85E7 | 3.46E7 | 2.65E7 | 4 | 4 | 3 | 4 | 4 | 3 | 3 | 5 | 4 | 4 | 2 | 3 | Y | 20861 | Histone H1.0 OS=Mus musculus OX=10090 GN=H1-0 PE=2 SV=4 |
| 901 | 1544 | sp\|P28843\|DPP4\_MOUSE | 122.76 | 6 | 0 | 5 | 5 | 1 | 5 | 5 | 2 | 5 | 0 | 1 |  | 1.94E7 | 1.22E7 | 2.55E6 | 9.8E6 | 9.68E6 | 6.64E6 | 9.97E6 |  | 7.58E6 | 4 | 4 | 0 | 3 | 3 | 1 | 3 | 3 | 2 | 3 | 0 | 1 | Y | 87437 | Dipeptidyl peptidase 4 OS=Mus musculus OX=10090 GN=Dpp4 PE=1 SV=3 |
| 985 | 1373 | sp\|O55111\|DSG2\_MOUSE | 122.68 | 5 | 2 | 2 | 1 | 1 | 2 | 1 | 4 | 1 | 2 | 0 | 1.21E7 | 5.32E6 | 3.92E5 | 1.83E6 | 4.34E6 | 3.19E6 | 4.55E6 | 1.57E6 | 6.39E6 |  | 5 | 5 | 2 | 2 | 1 | 1 | 2 | 1 | 3 | 1 | 2 | 0 | N | 122385 | Desmoglein-2 OS=Mus musculus OX=10090 GN=Dsg2 PE=1 SV=3 |
| 957 | 2055 | sp\|P83882\|RL36A\_MOUSE | 122.46 | 26 | 17 | 18 | 16 | 9 | 16 | 9 | 8 | 8 | 16 | 8 | 1.72E7 | 2.03E7 | 2.73E7 | 8.11E6 | 2.1E7 | 3.59E6 | 6.47E6 | 6.56E6 | 1.2E7 | 1.35E7 | 5 | 5 | 2 | 3 | 2 | 2 | 2 | 1 | 1 | 1 | 2 | 1 | Y | 12441 | Large ribosomal subunit protein eL42 OS=Mus musculus OX=10090 GN=Rpl36a PE=1 SV=2 |
| 913 | 1539 | sp\|Q6ZQI3\|MLEC\_MOUSE | 122.07 | 15 | 7 | 7 | 7 | 8 | 7 | 12 | 7 | 4 | 4 | 4 | 5.96E6 | 7.35E6 | 2.76E6 | 2.58E6 | 3.47E6 | 9.07E6 | 2.41E6 | 1.43E6 | 3.49E6 | 2.66E6 | 5 | 5 | 2 | 2 | 2 | 2 | 2 | 4 | 2 | 1 | 1 | 1 | N | 32342 | Malectin OS=Mus musculus OX=10090 GN=Mlec PE=1 SV=2 |
| 718 | 2458 | sp\|Q9D3D9\|ATPD\_MOUSE | 121.87 | 17 | 14 | 17 | 17 | 17 | 17 | 17 | 17 | 14 | 8 | 17 | 5.62E7 | 6.11E7 | 6.26E7 | 1.86E7 | 4.74E7 | 5.38E7 | 3.65E7 | 3.67E7 | 4.88E6 | 5.11E7 | 3 | 2 | 3 | 3 | 3 | 3 | 3 | 4 | 3 | 3 | 2 | 3 | N | 17600 | ATP synthase subunit delta, mitochondrial OS=Mus musculus OX=10090 GN=Atp5f1d PE=1 SV=1 |
| 1115 | 3663 | sp\|Q8R2Y8\|PTH2\_MOUSE | 121.82 | 20 | 20 | 12 | 20 | 8 | 8 | 20 | 20 | 8 | 20 | 0 | 2.13E6 | 4.77E5 | 1.89E6 | 3.92E5 | 2.92E5 | 1.77E6 | 2.95E6 | 5.59E5 | 7.12E6 |  | 2 | 2 | 2 | 1 | 2 | 1 | 1 | 2 | 2 | 1 | 2 | 0 | N | 19527 | Peptidyl-tRNA hydrolase 2, mitochondrial OS=Mus musculus OX=10090 GN=Ptrh2 PE=1 SV=1 |
| 918 | 2303 | sp\|Q9CQM5\|TXD17\_MOUSE | 121.82 | 27 | 15 | 15 | 15 | 15 | 15 | 15 | 15 | 27 | 27 | 8 | 8.94E6 | 8.53E6 | 8.67E6 | 2.72E6 | 8.75E6 | 6.92E6 | 4.63E6 | 3.82E6 | 1.42E7 | 3.04E6 | 3 | 3 | 2 | 2 | 2 | 2 | 2 | 2 | 2 | 3 | 3 | 1 | Y | 14015 | Thioredoxin domain-containing protein 17 OS=Mus musculus OX=10090 GN=Txndc17 PE=1 SV=1 |
| 663 | 884 | sp\|O55222\|ILK\_MOUSE | 121.78 | 16 | 4 | 5 | 10 | 5 | 5 | 9 | 9 | 3 | 3 | 7 | 7.92E6 | 1.16E6 | 7.41E6 | 2.34E6 | 2.51E6 | 1.1E7 | 1.78E6 | 3.66E5 | 8.05E5 | 7.31E6 | 7 | 7 | 2 | 2 | 5 | 2 | 2 | 4 | 4 | 1 | 1 | 3 | Y | 51373 | Integrin-linked protein kinase OS=Mus musculus OX=10090 GN=Ilk PE=1 SV=2 |
| 1040 | 2225 | sp\|O55022\|PGRC1\_MOUSE | 121.64 | 16 | 11 | 11 | 11 | 11 | 12 | 7 | 4 | 0 | 11 | 11 | 1.97E7 | 2.15E7 | 1.97E7 | 6.08E6 | 9.55E5 | 8.34E6 | 6.22E6 |  | 1.66E7 | 1.32E7 | 3 | 2 | 2 | 2 | 2 | 2 | 2 | 1 | 1 | 0 | 2 | 2 | Y | 21694 | Membrane-associated progesterone receptor component 1 OS=Mus musculus OX=10090 GN=Pgrmc1 PE=1 SV=4 |
| 739 | 2190 | Q9CQB4\|Q9CQB4\_MOUSE | 121.44 | 27 | 15 | 27 | 27 | 10 | 10 | 27 | 10 | 22 | 27 | 27 | 2.38E7 | 4.1E7 | 4.03E7 | 4.89E6 | 9.1E6 | 2.22E7 | 3E6 | 8.92E6 | 2.86E7 | 2.6E7 | 3 | 3 | 2 | 4 | 4 | 1 | 1 | 4 | 1 | 2 | 3 | 3 | N | 13561 | Cytochrome b-c1 complex subunit 7 OS=Mus musculus OX=10090 GN=Uqcrb PE=1 SV=1 |
| 739 | 2191 | sp\|Q9D855\|QCR7\_MOUSE | 121.44 | 27 | 15 | 27 | 27 | 10 | 10 | 27 | 10 | 22 | 27 | 27 | 2.38E7 | 4.1E7 | 4.03E7 | 4.89E6 | 9.1E6 | 2.22E7 | 3E6 | 8.92E6 | 2.86E7 | 2.6E7 | 3 | 3 | 2 | 4 | 4 | 1 | 1 | 4 | 1 | 2 | 3 | 3 | N | 13527 | Cytochrome b-c1 complex subunit 7 OS=Mus musculus OX=10090 GN=Uqcrb PE=1 SV=3 |
| 1619 | 3702 | A0A2K6EDJ7\|A0A2K6EDJ7\_MOUSE | 121.44 | 3 | 0 | 0 | 0 | 3 | 2 | 2 | 2 | 2 | 0 | 0 |  |  |  | 1.64E6 | 7.25E5 | 1.01E6 | 9.15E5 | 6.37E5 |  |  | 2 | 2 | 0 | 0 | 0 | 2 | 1 | 1 | 1 | 1 | 0 | 0 | N | 100324 | Inter alpha-trypsin inhibitor, heavy chain 4 OS=Mus musculus OX=10090 GN=Itih4 PE=1 SV=1 |
| 1619 | 3703 | E9Q5L2\|E9Q5L2\_MOUSE | 121.44 | 3 | 0 | 0 | 0 | 3 | 2 | 2 | 2 | 2 | 0 | 0 |  |  |  | 1.64E6 | 7.25E5 | 1.01E6 | 9.15E5 | 6.37E5 |  |  | 2 | 2 | 0 | 0 | 0 | 2 | 1 | 1 | 1 | 1 | 0 | 0 | N | 102823 | Inter alpha-trypsin inhibitor, heavy chain 4 OS=Mus musculus OX=10090 GN=Itih4 PE=1 SV=1 |
| 1619 | 3704 | E9PVD2\|E9PVD2\_MOUSE | 121.44 | 3 | 0 | 0 | 0 | 3 | 2 | 2 | 2 | 2 | 0 | 0 |  |  |  | 1.64E6 | 7.25E5 | 1.01E6 | 9.15E5 | 6.37E5 |  |  | 2 | 2 | 0 | 0 | 0 | 2 | 1 | 1 | 1 | 1 | 0 | 0 | N | 104588 | Inter alpha-trypsin inhibitor, heavy chain 4 OS=Mus musculus OX=10090 GN=Itih4 PE=1 SV=1 |
| 1619 | 3705 | sp\|A6X935\|ITIH4\_MOUSE | 121.44 | 3 | 0 | 0 | 0 | 3 | 2 | 2 | 2 | 2 | 0 | 0 |  |  |  | 1.64E6 | 7.25E5 | 1.01E6 | 9.15E5 | 6.37E5 |  |  | 2 | 2 | 0 | 0 | 0 | 2 | 1 | 1 | 1 | 1 | 0 | 0 | N | 104660 | Inter alpha-trypsin inhibitor, heavy chain 4 OS=Mus musculus OX=10090 GN=Itih4 PE=1 SV=2 |
| 386 | 2199 | sp\|Q3TTY5\|K22E\_MOUSE | 121.39 | 4 | 2 | 2 | 2 | 2 | 2 | 2 | 2 | 4 | 2 | 2 |  |  |  |  |  |  |  | 1.38E6 |  |  | 3 | 1 | 7 | 7 | 6 | 4 | 8 | 5 | 5 | 11 | 8 | 7 | N | 70923 | Keratin, type II cytoskeletal 2 epidermal OS=Mus musculus OX=10090 GN=Krt2 PE=1 SV=1 |
| 1081 | 3816 | A0A0N4SW94\|A0A0N4SW94\_MOUSE | 121.38 | 33 | 33 | 33 | 21 | 33 | 21 | 21 | 21 | 21 | 33 | 33 | 1.05E7 | 1.09E7 | 7.92E6 | 7.34E6 | 5.12E6 | 6.42E6 | 4.6E6 | 4.22E6 | 1.2E7 | 9.71E6 | 2 | 2 | 2 | 2 | 1 | 2 | 1 | 1 | 1 | 1 | 2 | 2 | N | 8640 | Myeloid-associated differentiation marker (Fragment) OS=Mus musculus OX=10090 GN=Myadm PE=1 SV=1 |
| 1081 | 3826 | sp\|O35682\|MYADM\_MOUSE | 121.38 | 8 | 8 | 8 | 5 | 8 | 5 | 5 | 5 | 5 | 8 | 8 | 1.05E7 | 1.09E7 | 7.92E6 | 7.34E6 | 5.12E6 | 6.42E6 | 4.6E6 | 4.22E6 | 1.2E7 | 9.71E6 | 2 | 2 | 2 | 2 | 1 | 2 | 1 | 1 | 1 | 1 | 2 | 2 | N | 35285 | Myeloid-associated differentiation marker OS=Mus musculus OX=10090 GN=Myadm PE=1 SV=2 |
| 1150 | 3469 | sp\|Q99JX4\|EIF3M\_MOUSE | 121.33 | 5 | 2 | 2 | 3 | 3 | 3 | 3 | 3 | 3 | 0 | 0 | 2.23E6 | 2.65E6 | 1.06E5 | 7.23E5 | 1.13E6 | 3.96E5 | 1.26E5 | 4.01E5 |  |  | 3 | 3 | 1 | 1 | 1 | 3 | 2 | 2 | 1 | 2 | 0 | 0 | N | 42517 | Eukaryotic translation initiation factor 3 subunit M OS=Mus musculus OX=10090 GN=Eif3m PE=1 SV=1 |
| 1631 | 3856 | sp\|P61922\|GABT\_MOUSE | 121.28 | 5 | 0 | 3 | 0 | 0 | 3 | 5 | 3 | 0 | 3 | 0 |  | 1.52E6 |  |  | 9.6E4 | 3.57E6 | 3.79E5 |  | 1.57E6 |  | 2 | 2 | 0 | 1 | 0 | 0 | 1 | 2 | 1 | 0 | 1 | 0 | N | 56452 | 4-aminobutyrate aminotransferase, mitochondrial OS=Mus musculus OX=10090 GN=Abat PE=1 SV=1 |
| 1089 | 2169 | A0A668KLV9\|A0A668KLV9\_MOUSE | 121.22 | 2 | 1 | 1 | 0 | 0 | 1 | 2 | 1 | 0 | 1 | 0 | 2.23E5 | 1.99E5 |  |  | 1.71E5 | 7.7E5 | 3.94E5 |  | 6.4E5 |  | 2 | 2 | 1 | 1 | 0 | 0 | 1 | 2 | 1 | 0 | 1 | 0 | N | 172559 | A kinase anchor protein 12 OS=Mus musculus OX=10090 GN=Akap12 PE=1 SV=1 |
| 1089 | 2170 | A0A668KLD3\|A0A668KLD3\_MOUSE | 121.22 | 2 | 1 | 1 | 0 | 0 | 1 | 2 | 1 | 0 | 1 | 0 | 2.23E5 | 1.99E5 |  |  | 1.71E5 | 7.7E5 | 3.94E5 |  | 6.4E5 |  | 2 | 2 | 1 | 1 | 0 | 0 | 1 | 2 | 1 | 0 | 1 | 0 | N | 172794 | A kinase (PRKA) anchor protein (Gravin) 12, isoform CRA\_a OS=Mus musculus OX=10090 GN=Akap12 PE=1 SV=1 |
| 1089 | 2172 | sp\|Q9WTQ5\|AKA12\_MOUSE | 121.22 | 2 | 1 | 1 | 0 | 0 | 1 | 2 | 1 | 0 | 1 | 0 | 2.23E5 | 1.99E5 |  |  | 1.71E5 | 7.7E5 | 3.94E5 |  | 6.4E5 |  | 2 | 2 | 1 | 1 | 0 | 0 | 1 | 2 | 1 | 0 | 1 | 0 | N | 180694 | A-kinase anchor protein 12 OS=Mus musculus OX=10090 GN=Akap12 PE=1 SV=1 |
| 502 | 2710 | sp\|Q8CI43\|MYL6B\_MOUSE | 121.17 | 10 | 10 | 10 | 10 | 10 | 10 | 10 | 10 | 10 | 10 | 10 | 0E0 | 1.35E7 | 1.48E7 | 5.89E6 | 9.59E6 | 1.4E7 | 8.83E6 | 5.74E6 | 1.38E7 | 7.49E6 | 2 | 1 | 5 | 5 | 3 | 6 | 5 | 6 | 4 | 5 | 6 | 3 | N | 22749 | Myosin light chain 6B OS=Mus musculus OX=10090 GN=Myl6b PE=1 SV=1 |
| 437 | 2044 | A2A513\|A2A513\_MOUSE | 121.17 | 5 | 3 | 3 | 5 | 5 | 1 | 3 | 3 | 5 | 1 | 1 | 2.79E6 | 2.7E6 | 1.27E7 | 1.04E7 |  | 3.26E6 | 2.13E6 | 1.86E7 |  |  | 3 | 2 | 6 | 6 | 8 | 7 | 6 | 3 | 6 | 8 | 5 | 4 | N | 57041 | Keratin, type I cytoskeletal 10 OS=Mus musculus OX=10090 GN=Krt10 PE=1 SV=1 |
| 437 | 2046 | sp\|P02535\|K1C10\_MOUSE | 121.17 | 5 | 3 | 3 | 5 | 5 | 1 | 3 | 3 | 5 | 1 | 1 | 2.79E6 | 2.7E6 | 1.27E7 | 1.04E7 |  | 3.26E6 | 2.13E6 | 1.86E7 |  |  | 3 | 2 | 6 | 6 | 8 | 7 | 6 | 3 | 6 | 8 | 5 | 4 | N | 57770 | Keratin, type I cytoskeletal 10 OS=Mus musculus OX=10090 GN=Krt10 PE=1 SV=3 |
| 863 | 2182 | Q149K3\|Q149K3\_MOUSE | 121.14 | 8 | 3 | 5 | 3 | 3 | 3 | 5 | 3 | 0 | 8 | 3 |  |  |  |  |  |  |  |  | 7.98E5 |  | 3 | 1 | 3 | 3 | 2 | 1 | 2 | 3 | 2 | 0 | 4 | 2 | N | 48884 | 2310038E17Rik protein OS=Mus musculus OX=10090 GN=Ces2f PE=1 SV=1 |
| 863 | 2196 | Q08ED5\|Q08ED5\_MOUSE | 121.14 | 6 | 2 | 4 | 2 | 2 | 2 | 4 | 2 | 0 | 6 | 2 |  |  |  |  |  |  |  |  | 7.98E5 |  | 3 | 1 | 3 | 3 | 2 | 1 | 2 | 3 | 2 | 0 | 4 | 2 | N | 62708 | Carboxylic ester hydrolase OS=Mus musculus OX=10090 GN=Ces2f PE=1 SV=1 |
| 867 | 2739 | A0A286YEB7\|A0A286YEB7\_MOUSE | 121.12 | 23 | 13 | 13 | 23 | 23 | 23 | 23 | 23 | 23 | 13 | 13 | 8.36E6 | 4.49E6 | 6.82E6 | 3.68E6 | 8.71E6 | 6.47E6 | 3.13E6 | 2.16E6 | 1.59E7 | 8.32E6 | 2 | 2 | 2 | 1 | 2 | 3 | 3 | 3 | 2 | 2 | 2 | 2 | Y | 13605 | 40S ribosomal protein S24 OS=Mus musculus OX=10090 GN=Rps24 PE=1 SV=1 |
| 867 | 2805 | sp\|P62849\|RS24\_MOUSE | 121.12 | 20 | 11 | 11 | 20 | 20 | 20 | 20 | 20 | 20 | 11 | 11 | 8.36E6 | 4.49E6 | 6.82E6 | 3.68E6 | 8.71E6 | 6.47E6 | 3.13E6 | 2.16E6 | 1.59E7 | 8.32E6 | 2 | 2 | 2 | 1 | 2 | 3 | 3 | 3 | 2 | 2 | 2 | 2 | Y | 15423 | Small ribosomal subunit protein eS24 OS=Mus musculus OX=10090 GN=Rps24 PE=1 SV=1 |
| 961 | 2002 | sp\|P70404\|IDHG1\_MOUSE | 121.06 | 10 | 8 | 8 | 8 | 6 | 10 | 8 | 6 | 6 | 6 | 2 | 3.83E6 | 3.68E6 | 5.72E6 | 1.21E6 | 7.44E6 | 1.16E7 | 4.32E6 | 1.21E6 | 8.84E6 | 2.73E6 | 3 | 3 | 2 | 2 | 2 | 1 | 3 | 2 | 1 | 1 | 1 | 1 | N | 42785 | Isocitrate dehydrogenase [NAD] subunit gamma 1, mitochondrial OS=Mus musculus OX=10090 GN=Idh3g PE=1 SV=1 |
| 277 | 1453 | sp\|Q3UV17\|K22O\_MOUSE | 120.78 | 7 | 4 | 4 | 4 | 5 | 4 | 5 | 5 | 5 | 4 | 4 |  |  |  | 2.64E6 |  | 5.63E6 | 5.12E6 | 2.85E6 |  |  | 5 | 1 | 8 | 9 | 8 | 6 | 11 | 8 | 8 | 13 | 10 | 8 | Y | 62845 | Keratin, type II cytoskeletal 2 oral OS=Mus musculus OX=10090 GN=Krt76 PE=1 SV=1 |
| 1020 | 2242 | sp\|P09671\|SODM\_MOUSE | 120.67 | 13 | 3 | 3 | 13 | 6 | 9 | 9 | 6 | 9 | 0 | 3 | 4.88E6 | 4.55E6 | 6.82E6 | 3.62E5 | 9.76E6 | 1.29E7 | 1.83E6 | 3.77E6 |  | 6.53E6 | 3 | 3 | 1 | 1 | 3 | 1 | 2 | 2 | 1 | 2 | 0 | 1 | N | 24603 | Superoxide dismutase [Mn], mitochondrial OS=Mus musculus OX=10090 GN=Sod2 PE=1 SV=3 |
| 712 | 1526 | sp\|P63001\|RAC1\_MOUSE | 120.54 | 24 | 11 | 9 | 9 | 7 | 7 | 17 | 9 | 9 | 17 | 24 | 9.69E5 |  |  | 1.53E6 | 7.68E5 | 1.77E6 |  |  | 1.57E7 | 1.08E7 | 5 | 3 | 3 | 3 | 3 | 2 | 1 | 3 | 2 | 3 | 5 | 6 | Y | 21450 | Ras-related C3 botulinum toxin substrate 1 OS=Mus musculus OX=10090 GN=Rac1 PE=1 SV=1 |
| 712 | 1531 | Q3TLP8\|Q3TLP8\_MOUSE | 120.54 | 22 | 10 | 9 | 9 | 7 | 7 | 15 | 9 | 9 | 16 | 22 | 9.69E5 |  |  | 1.53E6 | 7.68E5 | 1.77E6 |  |  | 1.57E7 | 1.08E7 | 5 | 3 | 3 | 3 | 3 | 2 | 1 | 3 | 2 | 3 | 5 | 6 | Y | 23432 | Ras-related C3 botulinum toxin substrate 1 OS=Mus musculus OX=10090 GN=Rac1 PE=1 SV=1 |
| 1075 | 1777 | sp\|Q8BFZ9\|ERLN2\_MOUSE | 120.20 | 12 | 4 | 3 | 4 | 4 | 4 | 6 | 9 | 6 | 4 | 4 | 1.55E6 | 2.26E6 | 1.52E6 | 1.35E6 | 3.16E6 | 4.53E6 | 4.04E6 | 2.12E6 | 4.04E6 | 2.6E6 | 4 | 4 | 1 | 1 | 1 | 1 | 1 | 2 | 3 | 2 | 1 | 1 | Y | 37873 | Erlin-2 OS=Mus musculus OX=10090 GN=Erlin2 PE=1 SV=1 |
| 99 | 2375 | sp\|P27661\|H2AX\_MOUSE | 120.20 | 27 | 27 | 27 | 27 | 27 | 27 | 27 | 27 | 27 | 27 | 27 | 2.14E7 | 2.77E7 | 1.25E7 | 6.05E6 | 3.51E6 | 3.33E7 | 1.51E7 | 8.81E6 | 7.31E7 | 4.06E7 | 3 | 1 | 23 | 20 | 29 | 27 | 37 | 21 | 26 | 34 | 22 | 21 | N | 15143 | Histone H2AX OS=Mus musculus OX=10090 GN=H2ax PE=1 SV=2 |
| 722 | 929 | sp\|Q99K48\|NONO\_MOUSE | 120.16 | 15 | 9 | 7 | 8 | 3 | 7 | 2 | 3 | 2 | 6 | 6 | 1.53E7 | 3.62E6 | 1.25E7 | 2.04E6 | 7.97E6 |  | 6.99E5 |  | 7.01E6 | 4.74E6 | 7 | 6 | 5 | 3 | 3 | 2 | 3 | 1 | 2 | 1 | 4 | 3 | Y | 54541 | Non-POU domain-containing octamer-binding protein OS=Mus musculus OX=10090 GN=Nono PE=1 SV=3 |
| 969 | 1718 | sp\|Q99J77\|SIAS\_MOUSE | 120.14 | 12 | 5 | 4 | 8 | 0 | 4 | 5 | 5 | 3 | 4 | 2 | 8.06E6 | 3.26E5 | 9.48E6 |  | 3.55E6 | 7.15E6 | 3.54E6 | 1.82E6 | 1.25E7 | 8.42E6 | 5 | 5 | 2 | 1 | 3 | 0 | 1 | 2 | 2 | 1 | 3 | 1 | N | 40024 | Sialic acid synthase OS=Mus musculus OX=10090 GN=Nans PE=1 SV=1 |
| 911 | 2052 | sp\|Q6WVG3\|KCD12\_MOUSE | 119.88 | 9 | 7 | 7 | 7 | 7 | 7 | 7 | 7 | 9 | 4 | 7 | 1.19E7 | 4.1E6 | 2.64E6 | 1.91E6 | 7.52E6 | 4.1E6 | 3.58E6 | 1.4E6 | 1.37E7 | 5.6E6 | 3 | 3 | 2 | 2 | 2 | 2 | 2 | 2 | 2 | 3 | 1 | 2 | N | 35892 | BTB/POZ domain-containing protein KCTD12 OS=Mus musculus OX=10090 GN=Kctd12 PE=1 SV=1 |
| 911 | 2053 | A0A0R4J2B2\|A0A0R4J2B2\_MOUSE | 119.88 | 9 | 7 | 7 | 7 | 7 | 7 | 7 | 7 | 9 | 4 | 7 | 1.19E7 | 4.1E6 | 2.64E6 | 1.91E6 | 7.52E6 | 4.1E6 | 3.58E6 | 1.4E6 | 1.37E7 | 5.6E6 | 3 | 3 | 2 | 2 | 2 | 2 | 2 | 2 | 2 | 3 | 1 | 2 | N | 35888 | Potassium channel tetramerisation domain containing 12 OS=Mus musculus OX=10090 GN=Kctd12 PE=1 SV=1 |
| 996 | 2114 | sp\|Q9D8V7\|SC11C\_MOUSE | 119.80 | 20 | 10 | 10 | 20 | 16 | 9 | 16 | 20 | 16 | 0 | 10 | 3.34E5 | 1.58E6 | 5.5E6 | 1.69E6 | 2.95E6 | 5.23E6 | 5.55E6 | 3E6 |  | 4.38E5 | 3 | 3 | 1 | 1 | 3 | 2 | 2 | 2 | 4 | 2 | 0 | 1 | N | 21660 | Signal peptidase complex catalytic subunit SEC11C OS=Mus musculus OX=10090 GN=Sec11c PE=1 SV=3 |
| 996 | 2115 | D3Z5H3\|D3Z5H3\_MOUSE | 119.80 | 18 | 10 | 10 | 18 | 15 | 9 | 15 | 18 | 15 | 0 | 10 | 3.34E5 | 1.58E6 | 5.5E6 | 1.69E6 | 2.95E6 | 5.23E6 | 5.55E6 | 3E6 |  | 4.38E5 | 3 | 3 | 1 | 1 | 3 | 2 | 2 | 2 | 4 | 2 | 0 | 1 | N | 23266 | Signal peptidase complex catalytic subunit SEC11 OS=Mus musculus OX=10090 GN=Sec11c PE=1 SV=2 |
| 1046 | 1717 | Q8BQ46\|Q8BQ46\_MOUSE | 119.53 | 7 | 1 | 0 | 0 | 4 | 2 | 4 | 7 | 2 | 5 | 2 |  |  |  |  |  |  | 2.58E5 |  |  |  | 4 | 1 | 1 | 0 | 0 | 2 | 1 | 2 | 4 | 1 | 3 | 1 | N | 58601 | TAF15 RNA polymerase II, TATA box binding protein (TBP)-associated factor OS=Mus musculus OX=10090 GN=Taf15 PE=1 SV=1 |
| 1527 | 2636 | sp\|Q9CXS4\|CENPV\_MOUSE | 119.45 | 10 | 0 | 0 | 5 | 5 | 0 | 5 | 10 | 5 | 0 | 0 |  |  | 4.75E6 | 5.37E5 |  | 4.64E6 | 2.68E6 | 2.25E6 |  |  | 2 | 2 | 0 | 0 | 1 | 1 | 0 | 1 | 2 | 1 | 0 | 0 | N | 27541 | Centromere protein V OS=Mus musculus OX=10090 GN=Cenpv PE=1 SV=2 |
| 746 | 1320 | sp\|Q6P069\|SORCN\_MOUSE | 119.37 | 24 | 9 | 4 | 17 | 20 | 17 | 20 | 17 | 14 | 8 | 6 | 7.91E6 | 6.23E6 | 6.06E6 | 5.1E6 | 7.44E6 | 1.27E7 | 5.01E6 | 1.01E7 | 1.29E7 | 4.46E6 | 5 | 5 | 2 | 1 | 3 | 4 | 3 | 4 | 3 | 3 | 2 | 1 | Y | 21627 | Sorcin OS=Mus musculus OX=10090 GN=Sri PE=1 SV=1 |
| 1233 | 1989 | sp\|Q9JKA5\|GPA33\_MOUSE | 119.31 | 9 | 0 | 0 | 0 | 2 | 2 | 4 | 2 | 2 | 5 | 0 |  |  |  | 4.19E6 | 5.25E6 | 1.51E7 | 7.36E6 | 3.42E6 | 9.05E5 |  | 3 | 3 | 0 | 0 | 0 | 1 | 1 | 2 | 1 | 1 | 1 | 0 | Y | 35692 | Cell surface A33 antigen OS=Mus musculus OX=10090 GN=Gpa33 PE=2 SV=2 |
| 1233 | 1990 | A0A0R4J209\|A0A0R4J209\_MOUSE | 119.31 | 9 | 0 | 0 | 0 | 2 | 2 | 4 | 2 | 2 | 5 | 0 |  |  |  | 4.19E6 | 5.25E6 | 1.51E7 | 7.36E6 | 3.42E6 | 9.05E5 |  | 3 | 3 | 0 | 0 | 0 | 1 | 1 | 2 | 1 | 1 | 1 | 0 | Y | 35756 | Glycoprotein A33 transmembrane OS=Mus musculus OX=10090 GN=Gpa33 PE=1 SV=1 |
| 1458 | 3842 | Q3LFS9\|Q3LFS9\_MOUSE | 119.23 | 8 | 0 | 5 | 0 | 8 | 8 | 5 | 8 | 0 | 0 | 0 |  | 1.13E6 |  | 6.22E5 | 2.92E6 | 9.9E5 | 1.38E6 |  |  |  | 2 | 2 | 0 | 1 | 0 | 2 | 2 | 1 | 2 | 0 | 0 | 0 | Y | 37241 | CEA cell adhesion molecule 1 OS=Mus musculus OX=10090 GN=Ceacam1 PE=1 SV=2 |
| 1458 | 3844 | Q925P3\|Q925P3\_MOUSE | 119.23 | 5 | 0 | 3 | 0 | 5 | 5 | 3 | 5 | 0 | 0 | 0 |  | 1.13E6 |  | 6.22E5 | 2.92E6 | 9.9E5 | 1.38E6 |  |  |  | 2 | 2 | 0 | 1 | 0 | 2 | 2 | 1 | 2 | 0 | 0 | 0 | Y | 56986 | CEA cell adhesion molecule 1 OS=Mus musculus OX=10090 GN=Ceacam1 PE=1 SV=1 |
| 1458 | 3845 | sp\|P31809\|CEAM1\_MOUSE | 119.23 | 5 | 0 | 3 | 0 | 5 | 5 | 3 | 5 | 0 | 0 | 0 |  | 1.13E6 |  | 6.22E5 | 2.92E6 | 9.9E5 | 1.38E6 |  |  |  | 2 | 2 | 0 | 1 | 0 | 2 | 2 | 1 | 2 | 0 | 0 | 0 | Y | 57016 | Carcinoembryonic antigen-related cell adhesion molecule 1 OS=Mus musculus OX=10090 GN=Ceacam1 PE=1 SV=1 |
| 1447 | 3490 | sp\|P56812\|PDCD5\_MOUSE | 119.23 | 20 | 0 | 0 | 10 | 10 | 10 | 20 | 10 | 20 | 0 | 0 |  |  | 5.51E5 | 2.95E5 | 7.54E5 | 1.59E6 | 4.92E5 | 6.2E5 |  |  | 2 | 2 | 0 | 0 | 1 | 1 | 1 | 2 | 1 | 2 | 0 | 0 | N | 14275 | Programmed cell death protein 5 OS=Mus musculus OX=10090 GN=Pdcd5 PE=1 SV=3 |
| 931 | 2245 | sp\|Q62351\|TFR1\_MOUSE | 118.99 | 5 | 4 | 1 | 4 | 2 | 0 | 4 | 2 | 1 | 2 | 3 | 6.92E5 |  | 5.98E6 | 2.02E6 |  | 5.13E6 | 1.23E6 |  | 7.06E6 | 3.88E6 | 4 | 3 | 3 | 1 | 3 | 2 | 0 | 3 | 2 | 1 | 1 | 2 | N | 85731 | Transferrin receptor protein 1 OS=Mus musculus OX=10090 GN=Tfrc PE=1 SV=1 |
| 2157 | 3788 | sp\|Q91VH2\|SNX9\_MOUSE | 118.89 | 5 | 0 | 0 | 0 | 0 | 3 | 5 | 0 | 0 | 0 | 0 |  |  |  |  | 6.58E5 | 1.37E6 |  |  |  |  | 2 | 2 | 0 | 0 | 0 | 0 | 1 | 2 | 0 | 0 | 0 | 0 | Y | 66546 | Sorting nexin-9 OS=Mus musculus OX=10090 GN=Snx9 PE=1 SV=1 |
| 762 | 1234 | sp\|P29452\|CASP1\_MOUSE | 118.59 | 13 | 7 | 7 | 5 | 7 | 2 | 7 | 2 | 2 | 9 | 7 | 4.01E6 | 1.07E7 | 4.08E6 | 3.6E6 | 2.9E6 | 9.02E6 | 7.68E5 | 2.47E6 | 1.27E7 | 6.11E6 | 6 | 6 | 3 | 3 | 2 | 4 | 1 | 3 | 1 | 1 | 4 | 3 | Y | 45641 | Caspase-1 OS=Mus musculus OX=10090 GN=Casp1 PE=1 SV=1 |
| 1162 | 2548 | sp\|Q8JZU2\|TXTP\_MOUSE | 118.15 | 10 | 0 | 4 | 7 | 4 | 4 | 4 | 6 | 4 | 4 | 4 |  | 5.23E5 | 2.68E6 | 5.87E5 | 6.36E5 | 1.89E6 | 1.12E7 | 2.61E5 | 1.91E6 | 4.07E5 | 3 | 3 | 0 | 1 | 2 | 1 | 1 | 1 | 2 | 1 | 1 | 1 | N | 33932 | Tricarboxylate transport protein, mitochondrial OS=Mus musculus OX=10090 GN=Slc25a1 PE=1 SV=1 |
| 1377 | 2195 | sp\|P60122\|RUVB1\_MOUSE | 118.06 | 9 | 0 | 0 | 0 | 5 | 0 | 3 | 5 | 6 | 2 | 2 |  |  |  | 9.01E5 |  | 8.46E5 | 2.44E5 | 8.09E5 | 1.51E6 | 1.23E6 | 3 | 3 | 0 | 0 | 0 | 2 | 0 | 1 | 2 | 2 | 1 | 1 | N | 50214 | RuvB-like 1 OS=Mus musculus OX=10090 GN=Ruvbl1 PE=1 SV=1 |
| 859 | 2027 | sp\|Q61878\|PRG2\_MOUSE | 118.04 | 20 | 9 | 5 | 5 | 5 | 0 | 5 | 0 | 0 | 17 | 11 | 7.94E7 | 1.35E6 | 5.64E6 | 1.36E6 |  | 2.1E6 |  |  | 2.7E8 | 1.54E8 | 4 | 4 | 4 | 1 | 1 | 1 | 0 | 1 | 0 | 0 | 8 | 5 | Y | 24255 | Bone marrow proteoglycan OS=Mus musculus OX=10090 GN=Prg2 PE=1 SV=1 |
| 865 | 1807 | sp\|Q9CXW4\|RL11\_MOUSE | 117.59 | 20 | 4 | 4 | 12 | 12 | 15 | 12 | 12 | 17 | 13 | 4 | 2.84E7 | 2.66E7 | 3.46E7 | 2.03E7 | 3.13E7 | 2.73E7 | 2.17E7 | 2.6E7 | 3.47E7 | 2.7E7 | 4 | 4 | 1 | 1 | 2 | 3 | 3 | 2 | 2 | 3 | 2 | 1 | N | 20252 | Large ribosomal subunit protein uL5 OS=Mus musculus OX=10090 GN=Rpl11 PE=1 SV=4 |
| 991 | 2645 | sp\|P16675\|PPGB\_MOUSE | 117.23 | 6 | 3 | 3 | 3 | 3 | 3 | 3 | 0 | 0 | 5 | 4 | 1.21E6 | 3.75E6 | 2.81E6 | 7.6E5 | 1.16E6 | 2.69E6 |  |  | 6.04E6 | 1.48E7 | 3 | 3 | 2 | 2 | 2 | 1 | 1 | 1 | 0 | 0 | 3 | 3 | Y | 53844 | Lysosomal protective protein OS=Mus musculus OX=10090 GN=Ctsa PE=1 SV=1 |
| 991 | 2658 | G3X8T3\|G3X8T3\_MOUSE | 117.23 | 5 | 2 | 2 | 2 | 2 | 2 | 2 | 0 | 0 | 4 | 3 | 1.21E6 | 3.75E6 | 2.81E6 | 7.6E5 | 1.16E6 | 2.69E6 |  |  | 6.04E6 | 1.48E7 | 3 | 3 | 2 | 2 | 2 | 1 | 1 | 1 | 0 | 0 | 3 | 3 | Y | 55726 | Carboxypeptidase OS=Mus musculus OX=10090 GN=Ctsa PE=1 SV=1 |
| 919 | 2791 | sp\|O54962\|BAF\_MOUSE | 117.17 | 35 | 35 | 35 | 35 | 27 | 35 | 35 | 35 | 35 | 35 | 27 | 1.35E7 | 1.81E7 | 1.79E7 | 1.51E6 | 2.09E7 | 8.91E6 | 5.27E6 | 1.52E7 | 2.48E7 | 1.01E7 | 3 | 3 | 3 | 2 | 2 | 1 | 2 | 2 | 3 | 2 | 2 | 1 | Y | 10103 | Barrier-to-autointegration factor OS=Mus musculus OX=10090 GN=Banf1 PE=1 SV=1 |
| 1025 | 2131 | sp\|P97855\|G3BP1\_MOUSE | 117.17 | 7 | 2 | 5 | 5 | 5 | 5 | 5 | 5 | 5 | 0 | 2 | 1.01E7 | 1.51E7 | 1.76E7 | 2.15E6 | 1.55E7 | 1.28E7 | 8.99E6 | 4.81E6 |  | 6.62E6 | 3 | 3 | 1 | 2 | 2 | 2 | 2 | 2 | 2 | 2 | 0 | 1 | N | 51829 | Ras GTPase-activating protein-binding protein 1 OS=Mus musculus OX=10090 GN=G3bp1 PE=1 SV=1 |
| 900 | 1443 | sp\|Q99KK2\|NEUA\_MOUSE | 117.15 | 12 | 6 | 5 | 3 | 0 | 4 | 4 | 6 | 3 | 9 | 3 | 2.4E6 | 2.66E7 | 2.92E5 |  | 1.64E6 | 2.37E6 | 1.29E6 | 4.34E5 | 7.42E6 | 7.36E5 | 5 | 5 | 2 | 2 | 1 | 0 | 2 | 2 | 2 | 1 | 4 | 1 | N | 48058 | N-acylneuraminate cytidylyltransferase OS=Mus musculus OX=10090 GN=Cmas PE=1 SV=2 |
| 1137 | 1346 | F8WHM5\|F8WHM5\_MOUSE | 116.73 | 5 | 1 | 0 | 1 | 0 | 2 | 0 | 1 | 0 | 3 | 1 | 1.77E6 |  | 3.46E5 |  | 4.86E6 |  | 5.13E5 |  | 4.03E6 | 4.27E5 | 5 | 5 | 2 | 0 | 1 | 0 | 2 | 0 | 1 | 0 | 4 | 1 | Y | 132351 | Golgi apparatus protein 1 (Fragment) OS=Mus musculus OX=10090 GN=Glg1 PE=1 SV=1 |
| 1137 | 1347 | sp\|Q61543\|GSLG1\_MOUSE | 116.73 | 5 | 1 | 0 | 1 | 0 | 2 | 0 | 1 | 0 | 3 | 1 | 1.77E6 |  | 3.46E5 |  | 4.86E6 |  | 5.13E5 |  | 4.03E6 | 4.27E5 | 5 | 5 | 2 | 0 | 1 | 0 | 2 | 0 | 1 | 0 | 4 | 1 | Y | 133734 | Golgi apparatus protein 1 OS=Mus musculus OX=10090 GN=Glg1 PE=1 SV=1 |
| 654 | 928 | sp\|Q9EST5\|AN32B\_MOUSE | 116.70 | 28 | 0 | 6 | 15 | 18 | 13 | 13 | 16 | 9 | 10 | 3 |  |  | 6.99E5 | 5.4E5 | 2.5E6 | 1.56E6 | 9.36E5 |  | 1.74E6 |  | 7 | 4 | 0 | 2 | 3 | 4 | 4 | 3 | 5 | 3 | 3 | 1 | Y | 31079 | Acidic leucine-rich nuclear phosphoprotein 32 family member B OS=Mus musculus OX=10090 GN=Anp32b PE=1 SV=1 |
| 1141 | 1521 | E9Q1K3\|E9Q1K3\_MOUSE | 116.61 | 9 | 0 | 3 | 4 | 0 | 2 | 5 | 2 | 0 | 0 | 0 |  | 6.21E5 | 6.75E6 |  | 8.66E6 | 3.39E6 | 4.11E5 |  |  |  | 4 | 4 | 0 | 1 | 2 | 0 | 1 | 2 | 1 | 0 | 0 | 0 | N | 72926 | Adducin 1 OS=Mus musculus OX=10090 GN=Add1 PE=1 SV=1 |
| 1141 | 1522 | F8WHZ9\|F8WHZ9\_MOUSE | 116.61 | 9 | 0 | 3 | 4 | 0 | 2 | 5 | 2 | 0 | 0 | 0 |  | 6.21E5 | 6.75E6 |  | 8.66E6 | 3.39E6 | 4.11E5 |  |  |  | 4 | 4 | 0 | 1 | 2 | 0 | 1 | 2 | 1 | 0 | 0 | 0 | N | 73164 | Adducin 1 OS=Mus musculus OX=10090 GN=Add1 PE=1 SV=1 |
| 1141 | 1523 | F8WGR0\|F8WGR0\_MOUSE | 116.61 | 9 | 0 | 3 | 4 | 0 | 2 | 5 | 2 | 0 | 0 | 0 |  | 6.21E5 | 6.75E6 |  | 8.66E6 | 3.39E6 | 4.11E5 |  |  |  | 4 | 4 | 0 | 1 | 2 | 0 | 1 | 2 | 1 | 0 | 0 | 0 | N | 73235 | Adducin 1 OS=Mus musculus OX=10090 GN=Add1 PE=1 SV=1 |
| 1141 | 1528 | sp\|Q9QYC0\|ADDA\_MOUSE | 116.61 | 8 | 0 | 2 | 3 | 0 | 1 | 4 | 2 | 0 | 0 | 0 |  | 6.21E5 | 6.75E6 |  | 8.66E6 | 3.39E6 | 4.11E5 |  |  |  | 4 | 4 | 0 | 1 | 2 | 0 | 1 | 2 | 1 | 0 | 0 | 0 | N | 80647 | Alpha-adducin OS=Mus musculus OX=10090 GN=Add1 PE=1 SV=2 |
| 1496 | 2751 | sp\|Q91XV3\|BASP1\_MOUSE | 116.60 | 12 | 12 | 0 | 6 | 6 | 0 | 0 | 6 | 6 | 6 | 0 | 2.85E6 |  | 1.03E6 | 8.7E5 |  |  | 1.75E6 | 9.3E5 | 0E0 |  | 2 | 2 | 2 | 0 | 1 | 1 | 0 | 0 | 1 | 1 | 1 | 0 | N | 22087 | Brain acid soluble protein 1 OS=Mus musculus OX=10090 GN=Basp1 PE=1 SV=3 |
| 1702 | 1747 | sp\|P28665\|MUG1\_MOUSE | 116.43 | 3 | 0 | 0 | 3 | 0 | 0 | 0 | 0 | 1 | 0 | 0 |  |  | 2.97E6 |  | 9.79E5 |  |  | 7.79E5 |  |  | 4 | 4 | 0 | 0 | 3 | 0 | 1 | 0 | 0 | 1 | 0 | 0 | Y | 165297 | Murinoglobulin-1 OS=Mus musculus OX=10090 GN=Mug1 PE=1 SV=3 |
| 853 | 1637 | D3Z041\|D3Z041\_MOUSE | 116.37 | 6 | 4 | 3 | 3 | 3 | 3 | 3 | 3 | 2 | 5 | 3 | 8.02E5 |  |  |  |  |  |  |  | 1.94E6 |  | 4 | 2 | 3 | 3 | 2 | 2 | 2 | 3 | 2 | 1 | 3 | 2 | N | 78035 | Long-chain-fatty-acid--CoA ligase OS=Mus musculus OX=10090 GN=Acsl1 PE=1 SV=1 |
| 853 | 1638 | sp\|P41216\|ACSL1\_MOUSE | 116.37 | 6 | 4 | 3 | 3 | 3 | 3 | 3 | 3 | 2 | 5 | 3 | 8.02E5 |  |  |  |  |  |  |  | 1.94E6 |  | 4 | 2 | 3 | 3 | 2 | 2 | 2 | 3 | 2 | 1 | 3 | 2 | N | 77952 | Long-chain-fatty-acid--CoA ligase 1 OS=Mus musculus OX=10090 GN=Acsl1 PE=1 SV=2 |
| 983 | 1336 | Q497W9\|Q497W9\_MOUSE | 116.24 | 6 | 2 | 0 | 2 | 2 | 4 | 3 | 3 | 0 | 2 | 2 | 6E6 |  | 7.7E6 | 2.44E6 | 5.86E6 | 6.54E6 | 5.98E6 |  | 3.8E6 | 3.59E6 | 5 | 5 | 2 | 0 | 1 | 1 | 3 | 2 | 2 | 0 | 2 | 1 | Y | 79971 | RNA helicase OS=Mus musculus OX=10090 GN=Dhx15 PE=1 SV=1 |
| 983 | 1342 | sp\|O35286\|DHX15\_MOUSE | 116.24 | 6 | 2 | 0 | 2 | 2 | 4 | 3 | 2 | 0 | 2 | 2 | 6E6 |  | 7.7E6 | 2.44E6 | 5.86E6 | 6.54E6 | 5.98E6 |  | 3.8E6 | 3.59E6 | 5 | 5 | 2 | 0 | 1 | 1 | 3 | 2 | 2 | 0 | 2 | 1 | Y | 91007 | ATP-dependent RNA helicase DHX15 OS=Mus musculus OX=10090 GN=Dhx15 PE=1 SV=2 |
| 1228 | 1884 | sp\|Q60829\|PPR1B\_MOUSE | 116.21 | 30 | 8 | 0 | 0 | 8 | 13 | 8 | 8 | 6 | 0 | 0 | 3.95E6 |  |  | 2.54E6 | 1.25E6 | 4.85E6 | 6.39E6 | 4.07E5 |  |  | 4 | 4 | 1 | 0 | 0 | 1 | 2 | 2 | 2 | 1 | 0 | 0 | Y | 21781 | Protein phosphatase 1 regulatory subunit 1B OS=Mus musculus OX=10090 GN=Ppp1r1b PE=1 SV=2 |
| 589 | 1886 | sp\|Q9D1R9\|RL34\_MOUSE | 115.94 | 29 | 21 | 21 | 21 | 29 | 28 | 21 | 23 | 29 | 21 | 21 | 4.96E7 | 6.05E7 | 5.92E7 | 1.95E7 | 5.48E7 | 5.06E7 | 3.61E7 | 4.46E7 | 5.66E7 | 4.77E7 | 5 | 5 | 4 | 3 | 3 | 4 | 5 | 3 | 4 | 6 | 4 | 5 | Y | 13293 | Large ribosomal subunit protein eL34 OS=Mus musculus OX=10090 GN=Rpl34 PE=1 SV=2 |
| 538 | 1303 | sp\|Q9Z1W8\|AT12A\_MOUSE | 115.60 | 5 | 4 | 4 | 3 | 1 | 2 | 4 | 3 | 2 | 3 | 2 | 1.14E7 |  |  |  |  | 1.16E6 |  |  |  |  | 7 | 2 | 4 | 6 | 5 | 3 | 3 | 5 | 4 | 3 | 3 | 2 | Y | 114726 | Potassium-transporting ATPase alpha chain 2 OS=Mus musculus OX=10090 GN=Atp12a PE=1 SV=3 |
| 1430 | 2839 | sp\|Q8K183\|PDXK\_MOUSE | 115.41 | 7 | 7 | 7 | 0 | 0 | 0 | 7 | 7 | 0 | 7 | 0 | 1.24E6 | 4.22E6 |  |  |  | 1.91E6 | 9.84E5 |  | 5.33E6 |  | 1 | 1 | 1 | 2 | 0 | 0 | 0 | 1 | 1 | 0 | 1 | 0 | N | 35015 | Pyridoxal kinase OS=Mus musculus OX=10090 GN=Pdxk PE=1 SV=1 |
| 946 | 1558 | sp\|P11352\|GPX1\_MOUSE | 115.14 | 28 | 8 | 15 | 23 | 14 | 5 | 14 | 5 | 18 | 8 | 14 | 1.91E6 | 5.11E6 | 4.07E6 | 3.6E6 | 3.2E6 | 2.02E6 | 7.41E5 | 3.51E6 | 1.55E6 | 3.52E6 | 5 | 5 | 1 | 3 | 4 | 2 | 1 | 2 | 1 | 3 | 1 | 2 | Y | 22329 | Glutathione peroxidase 1 OS=Mus musculus OX=10090 GN=Gpx1 PE=1 SV=2 |
| 1219 | 2694 | sp\|P08121\|CO3A1\_MOUSE | 114.68 | 1 | 1 | 0 | 0 | 1 | 0 | 1 | 0 | 0 | 1 | 1 | 9.91E6 |  |  | 3.84E6 | 1.58E6 | 1.24E7 | 5.35E5 |  | 1.02E7 | 1.13E7 | 2 | 2 | 1 | 0 | 0 | 1 | 1 | 1 | 1 | 0 | 1 | 1 | N | 138944 | Collagen alpha-1(III) chain OS=Mus musculus OX=10090 GN=Col3a1 PE=1 SV=4 |
| 1101 | 1534 | A2AN84\|A2AN84\_MOUSE | 114.66 | 10 | 6 | 2 | 3 | 3 | 3 | 3 | 6 | 0 | 3 | 0 | 1.57E6 | 8.67E5 | 8.87E5 | 2.12E5 | 8.66E5 | 3.28E5 | 1.29E6 |  | 1.72E6 |  | 4 | 4 | 2 | 1 | 1 | 1 | 1 | 1 | 2 | 0 | 1 | 0 | Y | 50536 | 55 kDa erythrocyte membrane protein OS=Mus musculus OX=10090 GN=Mpp1 PE=1 SV=1 |
| 1101 | 1606 | sp\|P70290\|EM55\_MOUSE | 114.66 | 10 | 6 | 2 | 3 | 3 | 3 | 3 | 6 | 0 | 3 | 0 | 1.57E6 | 8.67E5 | 8.87E5 | 2.12E5 | 8.66E5 | 3.28E5 | 1.29E6 |  | 1.72E6 |  | 4 | 4 | 2 | 1 | 1 | 1 | 1 | 1 | 2 | 0 | 1 | 0 | Y | 52227 | 55 kDa erythrocyte membrane protein OS=Mus musculus OX=10090 GN=Mpp1 PE=1 SV=1 |
| 1101 | 1636 | B7ZCL8\|B7ZCL8\_MOUSE | 114.66 | 11 | 6 | 2 | 3 | 3 | 3 | 3 | 6 | 0 | 3 | 0 | 1.57E6 | 8.67E5 | 8.87E5 | 2.12E5 | 8.66E5 | 3.28E5 | 1.29E6 |  | 1.72E6 |  | 4 | 4 | 2 | 1 | 1 | 1 | 1 | 1 | 2 | 0 | 1 | 0 | Y | 49784 | Membrane protein, palmitoylated OS=Mus musculus OX=10090 GN=Mpp1 PE=1 SV=1 |
| 453 | 3834 | G3UYV7\|G3UYV7\_MOUSE | 114.07 | 38 | 38 | 38 | 38 | 38 | 38 | 38 | 38 | 38 | 38 | 38 | 7.89E7 | 7.2E7 | 6.03E7 | 4.18E7 | 6.66E7 | 4.89E7 | 4.48E7 | 3.77E7 | 1.04E8 | 4.9E7 | 2 | 2 | 8 | 5 | 6 | 6 | 7 | 4 | 5 | 7 | 6 | 4 | Y | 6344 | Small ribosomal subunit protein eS28 (Fragment) OS=Mus musculus OX=10090 GN=Rps28 PE=1 SV=1 |
| 453 | 3837 | sp\|P62858\|RS28\_MOUSE | 114.07 | 30 | 30 | 30 | 30 | 30 | 30 | 30 | 30 | 30 | 30 | 30 | 7.89E7 | 7.2E7 | 6.03E7 | 4.18E7 | 6.66E7 | 4.89E7 | 4.48E7 | 3.77E7 | 1.04E8 | 4.9E7 | 2 | 2 | 8 | 5 | 6 | 6 | 7 | 4 | 5 | 7 | 6 | 4 | Y | 7841 | Small ribosomal subunit protein eS28 OS=Mus musculus OX=10090 GN=Rps28 PE=1 SV=1 |
| 1112 | 1580 | sp\|P12815\|PDCD6\_MOUSE | 113.92 | 21 | 10 | 10 | 5 | 7 | 7 | 0 | 7 | 10 | 0 | 6 | 2.3E6 | 4.29E6 | 6.92E5 | 5.85E5 | 5.42E5 |  | 4.63E5 | 3.04E6 |  | 7.91E5 | 4 | 4 | 2 | 2 | 1 | 1 | 1 | 0 | 1 | 2 | 0 | 1 | N | 21867 | Programmed cell death protein 6 OS=Mus musculus OX=10090 GN=Pdcd6 PE=1 SV=2 |
| 1068 | 1507 | sp\|P12382\|PFKAL\_MOUSE | 113.66 | 7 | 1 | 1 | 1 | 1 | 3 | 3 | 1 | 1 | 4 | 1 | 2.27E5 |  |  |  | 9.3E5 | 1.2E6 |  |  |  |  | 4 | 2 | 1 | 1 | 1 | 1 | 2 | 2 | 1 | 1 | 2 | 1 | Y | 85360 | ATP-dependent 6-phosphofructokinase, liver type OS=Mus musculus OX=10090 GN=Pfkl PE=1 SV=4 |
| 1113 | 2397 | sp\|P08030\|APT\_MOUSE | 113.56 | 20 | 13 | 13 | 13 | 7 | 0 | 7 | 7 | 6 | 14 | 13 | 1.38E7 | 9.47E6 | 3.08E6 | 1.93E5 |  | 4.45E6 | 1.31E6 | 1.75E6 | 8.29E6 | 1.1E7 | 3 | 3 | 2 | 2 | 2 | 1 | 0 | 1 | 1 | 1 | 2 | 2 | N | 19724 | Adenine phosphoribosyltransferase OS=Mus musculus OX=10090 GN=Aprt PE=1 SV=2 |
| 1026 | 2474 | sp\|Q64310\|SURF4\_MOUSE | 113.24 | 15 | 7 | 10 | 0 | 4 | 0 | 10 | 5 | 7 | 7 | 5 | 4.82E5 | 7.32E6 |  | 4.7E6 |  | 7.7E6 | 7.89E5 | 6.83E5 | 2.36E6 | 7.45E5 | 3 | 3 | 1 | 2 | 0 | 1 | 0 | 3 | 1 | 1 | 1 | 1 | Y | 30381 | Surfeit locus protein 4 OS=Mus musculus OX=10090 GN=Surf4 PE=1 SV=1 |
| 652 | 1735 | A0A0G2JDV8\|A0A0G2JDV8\_MOUSE | 113.20 | 12 | 4 | 10 | 6 | 10 | 6 | 6 | 9 | 6 | 6 | 10 | 2.56E7 | 3.04E7 | 2.29E7 | 9.53E6 | 1.79E7 | 2.28E7 | 1.47E7 | 1.56E7 | 3.33E7 | 5.79E6 | 5 | 2 | 1 | 6 | 2 | 3 | 3 | 4 | 5 | 2 | 4 | 3 | N | 31395 | Calponin OS=Mus musculus OX=10090 GN=Cnn3 PE=1 SV=1 |
| 652 | 1740 | sp\|Q9DAW9\|CNN3\_MOUSE | 113.20 | 11 | 3 | 8 | 5 | 8 | 5 | 5 | 8 | 5 | 5 | 8 | 2.56E7 | 3.04E7 | 2.29E7 | 9.53E6 | 1.79E7 | 2.28E7 | 1.47E7 | 1.56E7 | 3.33E7 | 5.79E6 | 5 | 2 | 1 | 6 | 2 | 3 | 3 | 4 | 5 | 2 | 4 | 3 | N | 36429 | Calponin-3 OS=Mus musculus OX=10090 GN=Cnn3 PE=1 SV=1 |
| 1305 | 3800 | sp\|P62317\|SMD2\_MOUSE | 113.07 | 24 | 8 | 15 | 8 | 24 | 8 | 8 | 8 | 0 | 0 | 8 | 1.29E7 | 1.71E6 | 1.31E7 | 5.32E6 | 9.09E6 | 4.53E6 | 2.21E6 |  |  | 7.98E6 | 2 | 2 | 1 | 1 | 1 | 2 | 1 | 1 | 1 | 0 | 0 | 1 | Y | 13527 | Small nuclear ribonucleoprotein Sm D2 OS=Mus musculus OX=10090 GN=Snrpd2 PE=1 SV=1 |
| 1242 | 2551 | sp\|Q921M7\|CYRIB\_MOUSE | 113.06 | 9 | 0 | 0 | 4 | 4 | 5 | 5 | 9 | 9 | 5 | 5 |  |  | 2.48E5 | 2.68E5 | 5.31E5 | 1.46E6 | 1.54E6 | 1.89E6 | 1.64E6 | 8.61E5 | 2 | 2 | 0 | 0 | 1 | 1 | 1 | 1 | 2 | 2 | 1 | 1 | N | 36776 | CYFIP-related Rac1 interactor B OS=Mus musculus OX=10090 GN=Cyrib PE=1 SV=1 |
| 1198 | 2526 | E9Q1J7\|E9Q1J7\_MOUSE | 112.60 | 7 | 2 | 2 | 4 | 0 | 0 | 0 | 0 | 0 | 5 | 2 | 1.97E6 | 2E6 | 9.84E5 |  |  |  |  |  | 5.77E6 | 1.54E6 | 3 | 3 | 2 | 2 | 2 | 0 | 0 | 0 | 0 | 0 | 3 | 2 | N | 54614 | Propionyl-CoA carboxylase beta chain, mitochondrial OS=Mus musculus OX=10090 GN=Pccb PE=1 SV=1 |
| 1198 | 2527 | sp\|Q99MN9\|PCCB\_MOUSE | 112.60 | 7 | 2 | 2 | 4 | 0 | 0 | 0 | 0 | 0 | 5 | 2 | 1.97E6 | 2E6 | 9.84E5 |  |  |  |  |  | 5.77E6 | 1.54E6 | 3 | 3 | 2 | 2 | 2 | 0 | 0 | 0 | 0 | 0 | 3 | 2 | N | 58409 | Propionyl-CoA carboxylase beta chain, mitochondrial OS=Mus musculus OX=10090 GN=Pccb PE=1 SV=2 |
| 1318 | 2220 | sp\|E9PV24\|FIBA\_MOUSE | 112.27 | 4 | 3 | 0 | 0 | 0 | 1 | 1 | 1 | 1 | 1 | 4 | 3.07E6 |  |  |  | 3.63E5 | 7.71E5 | 2.77E5 | 6.27E5 | 4.11E6 | 7.36E6 | 3 | 3 | 2 | 0 | 0 | 0 | 1 | 1 | 1 | 1 | 1 | 3 | N | 87429 | Fibrinogen alpha chain OS=Mus musculus OX=10090 GN=Fga PE=1 SV=1 |
| 1171 | 2149 | A0A494B9K4\|A0A494B9K4\_MOUSE | 112.10 | 5 | 0 | 0 | 0 | 2 | 1 | 4 | 2 | 2 | 2 | 0 |  |  |  | 5.1E5 | 2.18E6 | 1.21E6 | 8.04E5 | 6.54E5 | 4.89E5 |  | 3 | 3 | 0 | 0 | 0 | 1 | 1 | 2 | 1 | 1 | 1 | 0 | N | 78318 | Adducin 3 OS=Mus musculus OX=10090 GN=Add3 PE=1 SV=1 |
| 1171 | 2150 | sp\|Q9QYB5\|ADDG\_MOUSE | 112.10 | 5 | 0 | 0 | 0 | 2 | 1 | 4 | 2 | 2 | 2 | 0 |  |  |  | 5.1E5 | 2.18E6 | 1.21E6 | 8.04E5 | 6.54E5 | 4.89E5 |  | 3 | 3 | 0 | 0 | 0 | 1 | 1 | 2 | 1 | 1 | 1 | 0 | N | 78777 | Gamma-adducin OS=Mus musculus OX=10090 GN=Add3 PE=1 SV=2 |
| 1695 | 1956 | sp\|Q8CGN5\|PLIN1\_MOUSE | 112.10 | 8 | 0 | 8 | 0 | 0 | 0 | 2 | 0 | 0 | 0 | 0 |  | 6.12E6 |  |  |  | 1.27E6 |  |  |  |  | 3 | 3 | 0 | 3 | 0 | 0 | 0 | 1 | 0 | 0 | 0 | 0 | N | 55596 | Perilipin-1 OS=Mus musculus OX=10090 GN=Plin1 PE=1 SV=2 |
| 843 | 5227 | sp\|Q9JJI8\|RL38\_MOUSE | 112.06 | 19 | 17 | 17 | 17 | 17 | 19 | 19 | 19 | 17 | 17 | 17 | 1.09E7 | 3.14E6 | 1.36E7 | 1.07E6 | 6.2E6 | 8.43E6 | 6.14E6 | 1.37E6 | 2.5E7 | 1.55E7 | 4 | 3 | 2 | 1 | 2 | 3 | 4 | 3 | 3 | 2 | 2 | 2 | N | 8204 | Large ribosomal subunit protein eL38 OS=Mus musculus OX=10090 GN=Rpl38 PE=1 SV=3 |
| 916 | 2437 | D3YWF6\|D3YWF6\_MOUSE | 111.98 | 18 | 6 | 6 | 6 | 10 | 10 | 10 | 18 | 18 | 6 | 10 | 6.43E5 | 2.15E6 | 1.04E6 | 9.56E5 | 1.76E6 | 6.07E6 | 5.2E6 | 4.98E6 | 3.44E6 | 1.99E6 | 3 | 3 | 1 | 2 | 1 | 3 | 2 | 2 | 3 | 3 | 1 | 2 | N | 28037 | ubiquitinyl hydrolase 1 OS=Mus musculus OX=10090 GN=Otub1 PE=1 SV=1 |
| 916 | 2438 | sp\|Q7TQI3\|OTUB1\_MOUSE | 111.98 | 16 | 6 | 6 | 6 | 9 | 9 | 9 | 16 | 16 | 6 | 9 | 6.43E5 | 2.15E6 | 1.04E6 | 9.56E5 | 1.76E6 | 6.07E6 | 5.2E6 | 4.98E6 | 3.44E6 | 1.99E6 | 3 | 3 | 1 | 2 | 1 | 3 | 2 | 2 | 3 | 3 | 1 | 2 | N | 31270 | Ubiquitin thioesterase OTUB1 OS=Mus musculus OX=10090 GN=Otub1 PE=1 SV=2 |
| 896 | 1574 | sp\|P51855\|GSHB\_MOUSE | 111.53 | 9 | 5 | 4 | 5 | 2 | 4 | 5 | 2 | 5 | 5 | 7 | 3.97E6 | 1.39E6 | 2.15E6 | 8.68E5 | 1.2E6 | 2.63E6 | 1.81E6 | 2.91E6 | 1.27E7 | 3.9E6 | 4 | 4 | 2 | 2 | 2 | 1 | 2 | 2 | 1 | 2 | 3 | 3 | N | 52247 | Glutathione synthetase OS=Mus musculus OX=10090 GN=Gss PE=1 SV=1 |
| 1449 | 1764 | sp\|P01867\|IGG2B\_MOUSE | 111.33 | 13 | 4 | 0 | 0 | 2 | 0 | 0 | 0 | 7 | 8 | 4 | 2.36E6 |  |  | 2.07E6 |  |  |  | 2.13E6 | 1.03E7 | 3.56E5 | 4 | 4 | 1 | 0 | 0 | 1 | 0 | 0 | 0 | 2 | 3 | 1 | Y | 44259 | Immunoglobulin heavy constant gamma 2B OS=Mus musculus OX=10090 GN=Ighg2b PE=1 SV=3 |
| 1449 | 1765 | A0A0A6YVP0\|A0A0A6YVP0\_MOUSE | 111.33 | 13 | 4 | 0 | 0 | 2 | 0 | 0 | 0 | 7 | 8 | 4 | 2.36E6 |  |  | 2.07E6 |  |  |  | 2.13E6 | 1.03E7 | 3.56E5 | 4 | 4 | 1 | 0 | 0 | 1 | 0 | 0 | 0 | 2 | 3 | 1 | Y | 44359 | Immunoglobulin heavy constant gamma 2B (Fragment) OS=Mus musculus OX=10090 GN=Ighg2b PE=1 SV=2 |
| 1449 | 1929 | A0A075B5P3\|A0A075B5P3\_MOUSE | 111.33 | 16 | 5 | 0 | 0 | 3 | 0 | 0 | 0 | 9 | 10 | 5 | 2.36E6 |  |  | 2.07E6 |  |  |  | 2.13E6 | 1.03E7 | 3.56E5 | 4 | 4 | 1 | 0 | 0 | 1 | 0 | 0 | 0 | 2 | 3 | 1 | Y | 36687 | Immunoglobulin heavy constant gamma 2B (Fragment) OS=Mus musculus OX=10090 GN=Ighg2b PE=1 SV=1 |
| 1181 | 3939 | sp\|P40240\|CD9\_MOUSE | 111.21 | 14 | 3 | 14 | 3 | 14 | 3 | 3 | 3 | 14 | 3 | 0 | 4.96E6 | 8.21E6 | 6.7E6 | 2.88E6 | 2.1E5 | 2.45E6 | 3.15E6 | 4.91E6 | 7.61E6 |  | 2 | 2 | 1 | 2 | 1 | 2 | 1 | 1 | 1 | 2 | 1 | 0 | Y | 25258 | CD9 antigen OS=Mus musculus OX=10090 GN=Cd9 PE=1 SV=2 |
| 1565 | 1974 | E9QAS4\|E9QAS4\_MOUSE | 110.99 | 2 | 1 | 0 | 1 | 0 | 1 | 1 | 0 | 1 | 0 | 0 | 7.84E5 |  | 4.21E5 |  | 3.09E5 | 3.72E5 |  | 5.5E5 |  |  | 3 | 3 | 1 | 0 | 1 | 0 | 1 | 1 | 0 | 1 | 0 | 0 | Y | 216369 | DNA helicase OS=Mus musculus OX=10090 GN=Chd4 PE=1 SV=1 |
| 1565 | 1975 | sp\|Q6PDQ2\|CHD4\_MOUSE | 110.99 | 2 | 1 | 0 | 1 | 0 | 1 | 1 | 0 | 1 | 0 | 0 | 7.84E5 |  | 4.21E5 |  | 3.09E5 | 3.72E5 |  | 5.5E5 |  |  | 3 | 3 | 1 | 0 | 1 | 0 | 1 | 1 | 0 | 1 | 0 | 0 | Y | 217749 | Chromodomain-helicase-DNA-binding protein 4 OS=Mus musculus OX=10090 GN=Chd4 PE=1 SV=1 |
| 1565 | 1976 | E9QAS5\|E9QAS5\_MOUSE | 110.99 | 2 | 1 | 0 | 1 | 0 | 1 | 1 | 0 | 1 | 0 | 0 | 7.84E5 |  | 4.21E5 |  | 3.09E5 | 3.72E5 |  | 5.5E5 |  |  | 3 | 3 | 1 | 0 | 1 | 0 | 1 | 1 | 0 | 1 | 0 | 0 | Y | 218632 | DNA helicase OS=Mus musculus OX=10090 GN=Chd4 PE=1 SV=1 |
| 1136 | 2690 | B9EHJ3\|B9EHJ3\_MOUSE | 110.76 | 1 | 1 | 1 | 1 | 1 | 1 | 1 | 1 | 1 | 0 | 1 | 9.56E5 | 1.88E6 | 1.6E6 | 6.59E5 | 8.94E5 | 2.17E6 | 1.16E6 | 1.15E6 |  | 1.32E6 | 2 | 1 | 1 | 1 | 2 | 1 | 1 | 1 | 1 | 1 | 0 | 1 | N | 188851 | Tight junction protein 1 OS=Mus musculus OX=10090 GN=Tjp1 PE=1 SV=1 |
| 1136 | 2691 | sp\|P39447\|ZO1\_MOUSE | 110.76 | 1 | 1 | 1 | 1 | 1 | 1 | 1 | 1 | 1 | 0 | 1 | 9.56E5 | 1.88E6 | 1.6E6 | 6.59E5 | 8.94E5 | 2.17E6 | 1.16E6 | 1.15E6 |  | 1.32E6 | 2 | 1 | 1 | 1 | 2 | 1 | 1 | 1 | 1 | 1 | 0 | 1 | N | 194740 | Tight junction protein ZO-1 OS=Mus musculus OX=10090 GN=Tjp1 PE=1 SV=2 |
| 885 | 2145 | sp\|Q3TTY0\|PLB1\_MOUSE | 110.72 | 2 | 1 | 2 | 0 | 0 | 0 | 1 | 2 | 0 | 0 | 0 | 9.12E7 | 1.03E7 |  |  |  | 4.76E5 | 6.84E6 |  | 1.16E7 |  | 3 | 3 | 2 | 4 | 0 | 0 | 0 | 1 | 3 | 0 | 1 | 0 | N | 164540 | Phospholipase B1, membrane-associated OS=Mus musculus OX=10090 GN=Plb1 PE=1 SV=2 |
| 972 | 2557 | A0A075B5V1\|A0A075B5V1\_MOUSE | 110.52 | 32 | 24 | 0 | 7 | 14 | 21 | 21 | 7 | 21 | 14 | 7 | 5.67E6 |  | 2.83E7 | 1.44E6 | 3.69E7 | 7.52E6 | 1.8E7 | 1.39E7 | 3.29E6 | 6.17E6 | 3 | 3 | 2 | 0 | 1 | 1 | 2 | 2 | 1 | 2 | 1 | 1 | Y | 10966 | Immunoglobulin heavy variable 1-31 OS=Mus musculus OX=10090 GN=Ighv1-31 PE=4 SV=1 |
| 972 | 2558 | A0A075B5V2\|A0A075B5V2\_MOUSE | 110.52 | 26 | 21 | 0 | 6 | 12 | 18 | 18 | 6 | 18 | 12 | 6 | 5.67E6 |  | 2.83E7 | 1.44E6 | 3.69E7 | 7.52E6 | 1.8E7 | 1.39E7 | 3.29E6 | 6.17E6 | 3 | 3 | 2 | 0 | 1 | 1 | 2 | 2 | 1 | 2 | 1 | 1 | Y | 13032 | Immunoglobulin heavy variable 1-34 (Fragment) OS=Mus musculus OX=10090 GN=Ighv1-34 PE=1 SV=1 |
| 972 | 2559 | A0A075B5V0\|A0A075B5V0\_MOUSE | 110.52 | 26 | 21 | 0 | 6 | 12 | 18 | 18 | 6 | 18 | 12 | 6 | 5.67E6 |  | 2.83E7 | 1.44E6 | 3.69E7 | 7.52E6 | 1.8E7 | 1.39E7 | 3.29E6 | 6.17E6 | 3 | 3 | 2 | 0 | 1 | 1 | 2 | 2 | 1 | 2 | 1 | 1 | Y | 12911 | Immunoglobulin heavy variable 1-26 (Fragment) OS=Mus musculus OX=10090 GN=Ighv1-26 PE=4 SV=1 |
| 972 | 2560 | A0A0A6YWK5\|A0A0A6YWK5\_MOUSE | 110.52 | 26 | 21 | 0 | 6 | 12 | 18 | 18 | 6 | 18 | 12 | 6 | 5.67E6 |  | 2.83E7 | 1.44E6 | 3.69E7 | 7.52E6 | 1.8E7 | 1.39E7 | 3.29E6 | 6.17E6 | 3 | 3 | 2 | 0 | 1 | 1 | 2 | 2 | 1 | 2 | 1 | 1 | Y | 13115 | Immunoglobulin heavy variable 1-31 (Fragment) OS=Mus musculus OX=10090 GN=Ighv1-31 PE=4 SV=1 |
| 1665 | 1978 | sp\|Q6PDI5\|ECM29\_MOUSE | 110.44 | 2 | 0 | 0 | 0 | 1 | 0 | 2 | 1 | 0 | 0 | 0 |  |  |  | 1.23E5 |  | 6.41E5 | 3.56E5 |  |  |  | 3 | 3 | 0 | 0 | 0 | 1 | 0 | 2 | 2 | 0 | 0 | 0 | N | 203913 | Proteasome adapter and scaffold protein ECM29 OS=Mus musculus OX=10090 GN=Ecpas PE=1 SV=4 |
| 967 | 1900 | A0A494B955\|A0A494B955\_MOUSE | 110.35 | 16 | 6 | 6 | 6 | 6 | 13 | 8 | 0 | 6 | 12 | 8 | 1.89E6 | 3.43E6 | 2.99E6 | 3.05E6 | 3.73E6 | 2.81E6 |  | 2.43E6 | 8.33E6 | 3.33E6 | 4 | 4 | 1 | 2 | 2 | 2 | 3 | 2 | 0 | 2 | 3 | 2 | Y | 29238 | Thioredoxin-like 1 OS=Mus musculus OX=10090 GN=Txnl1 PE=1 SV=1 |
| 967 | 1901 | sp\|Q8CDN6\|TXNL1\_MOUSE | 110.35 | 15 | 5 | 6 | 6 | 6 | 11 | 8 | 0 | 6 | 11 | 8 | 1.89E6 | 3.43E6 | 2.99E6 | 3.05E6 | 3.73E6 | 2.81E6 |  | 2.43E6 | 8.33E6 | 3.33E6 | 4 | 4 | 1 | 2 | 2 | 2 | 3 | 2 | 0 | 2 | 3 | 2 | Y | 32237 | Thioredoxin-like protein 1 OS=Mus musculus OX=10090 GN=Txnl1 PE=1 SV=3 |
| 967 | 1902 | A0A498WGD8\|A0A498WGD8\_MOUSE | 110.35 | 14 | 5 | 5 | 5 | 5 | 11 | 8 | 0 | 5 | 11 | 8 | 1.89E6 | 3.43E6 | 2.99E6 | 3.05E6 | 3.73E6 | 2.81E6 |  | 2.43E6 | 8.33E6 | 3.33E6 | 4 | 4 | 1 | 2 | 2 | 2 | 3 | 2 | 0 | 2 | 3 | 2 | Y | 32715 | Thioredoxin-like 1 OS=Mus musculus OX=10090 GN=Txnl1 PE=1 SV=1 |
| 944 | 1643 | sp\|Q6PHN9\|RAB35\_MOUSE | 110.08 | 26 | 0 | 13 | 11 | 21 | 11 | 0 | 5 | 8 | 10 | 8 |  | 1.55E6 |  | 6.05E4 |  |  |  |  | 4.87E6 |  | 5 | 2 | 0 | 3 | 2 | 4 | 3 | 0 | 1 | 2 | 2 | 2 | Y | 23025 | Ras-related protein Rab-35 OS=Mus musculus OX=10090 GN=Rab35 PE=1 SV=1 |
| 1132 | 1184 | sp\|P33175\|KIF5A\_MOUSE | 109.67 | 7 | 2 | 0 | 1 | 2 | 1 | 3 | 1 | 0 | 1 | 0 | 2.09E7 |  |  |  |  |  |  |  |  |  | 6 | 1 | 2 | 0 | 1 | 2 | 1 | 2 | 1 | 0 | 1 | 0 | Y | 117019 | Kinesin heavy chain isoform 5A OS=Mus musculus OX=10090 GN=Kif5a PE=1 SV=3 |
| 817 | 3182 | sp\|P63254\|CRIP1\_MOUSE | 109.62 | 25 | 9 | 9 | 9 | 21 | 25 | 25 | 21 | 21 | 13 | 0 | 5.62E7 | 1.05E8 | 9.28E7 | 6.12E7 | 1.19E8 | 9.7E7 | 8.6E7 | 5.3E7 | 1.09E7 |  | 3 | 3 | 1 | 1 | 2 | 4 | 5 | 3 | 3 | 3 | 3 | 0 | Y | 8550 | Cysteine-rich protein 1 OS=Mus musculus OX=10090 GN=Crip1 PE=1 SV=2 |
| 817 | 3183 | A0A0G2JEK2\|A0A0G2JEK2\_MOUSE | 109.62 | 15 | 5 | 5 | 5 | 12 | 15 | 15 | 12 | 12 | 8 | 0 | 5.62E7 | 1.05E8 | 9.28E7 | 6.12E7 | 1.19E8 | 9.7E7 | 8.6E7 | 5.3E7 | 1.09E7 |  | 3 | 3 | 1 | 1 | 2 | 4 | 5 | 3 | 3 | 3 | 3 | 0 | Y | 13582 | Cysteine-rich protein 1 OS=Mus musculus OX=10090 GN=Crip1 PE=1 SV=1 |
| 585 | 1144 | sp\|Q9CQI6\|COTL1\_MOUSE | 109.37 | 32 | 10 | 10 | 20 | 22 | 16 | 9 | 21 | 27 | 20 | 20 | 2.24E7 | 2.03E7 | 4.39E7 | 3.27E7 | 2.42E7 | 1.39E7 | 3.3E7 | 2.96E7 | 7.53E7 | 5.75E7 | 6 | 6 | 2 | 2 | 4 | 4 | 3 | 2 | 4 | 6 | 5 | 5 | Y | 15944 | Coactosin-like protein OS=Mus musculus OX=10090 GN=Cotl1 PE=1 SV=3 |
| 1393 | 4218 | sp\|Q9CRY7\|GDPD1\_MOUSE | 109.30 | 5 | 5 | 5 | 5 | 0 | 5 | 5 | 5 | 5 | 5 | 0 | 2.49E6 | 3.16E6 | 4.89E6 |  | 1.88E6 | 3.02E6 | 3.16E6 | 3.44E6 | 1.73E6 |  | 1 | 1 | 1 | 1 | 1 | 0 | 1 | 1 | 1 | 1 | 1 | 0 | N | 35867 | Lysophospholipase D GDPD1 OS=Mus musculus OX=10090 GN=Gdpd1 PE=1 SV=1 |
| 791 | 2379 | sp\|Q9Z130\|HNRDL\_MOUSE | 109.29 | 9 | 6 | 6 | 6 | 6 | 6 | 3 | 3 | 6 | 5 | 6 |  |  |  |  |  |  |  |  | 1.73E6 |  | 3 | 1 | 3 | 3 | 4 | 4 | 4 | 2 | 1 | 3 | 2 | 2 | Y | 33559 | Heterogeneous nuclear ribonucleoprotein D-like OS=Mus musculus OX=10090 GN=Hnrnpdl PE=1 SV=1 |
| 791 | 2383 | F6VQH5\|F6VQH5\_MOUSE | 109.29 | 8 | 6 | 6 | 6 | 6 | 6 | 2 | 2 | 6 | 5 | 6 |  |  |  |  |  |  |  |  | 1.73E6 |  | 3 | 1 | 3 | 3 | 4 | 4 | 4 | 2 | 1 | 3 | 2 | 2 | Y | 35654 | Heterogeneous nuclear ribonucleoprotein D-like (Fragment) OS=Mus musculus OX=10090 GN=Hnrnpdl PE=1 SV=1 |
| 791 | 2386 | D3YTQ3\|D3YTQ3\_MOUSE | 109.29 | 6 | 4 | 4 | 4 | 4 | 4 | 2 | 2 | 4 | 4 | 4 |  |  |  |  |  |  |  |  | 1.73E6 |  | 3 | 1 | 3 | 3 | 4 | 4 | 4 | 2 | 1 | 3 | 2 | 2 | Y | 46270 | Heterogeneous nuclear ribonucleoprotein D-like OS=Mus musculus OX=10090 GN=Hnrnpdl PE=1 SV=1 |
| 802 | 2061 | sp\|Q9R1P0\|PSA4\_MOUSE | 109.26 | 10 | 10 | 7 | 10 | 10 | 7 | 7 | 10 | 7 | 7 | 7 | 2.18E7 | 1.71E7 | 2.53E7 | 1.35E7 | 7.72E6 | 1.08E7 | 1.58E7 | 7.05E6 | 2.97E7 | 1.77E7 | 3 | 3 | 3 | 2 | 3 | 3 | 2 | 2 | 3 | 2 | 3 | 2 | N | 29471 | Proteasome subunit alpha type-4 OS=Mus musculus OX=10090 GN=Psma4 PE=1 SV=1 |
| 730 | 2414 | sp\|P67984\|RL22\_MOUSE | 109.12 | 23 | 19 | 23 | 23 | 23 | 19 | 23 | 19 | 23 | 23 | 23 | 3.91E7 | 5.37E7 | 5.37E7 | 2.72E7 | 3.05E7 | 3.92E7 | 2.23E7 | 2.72E7 | 9.29E7 | 4.36E7 | 3 | 3 | 2 | 3 | 5 | 3 | 3 | 3 | 2 | 3 | 3 | 3 | N | 14759 | Large ribosomal subunit protein eL22 OS=Mus musculus OX=10090 GN=Rpl22 PE=1 SV=2 |
| 1100 | 1285 | sp\|Q8CB12\|GSDC3\_MOUSE | 109.09 | 12 | 12 | 0 | 0 | 0 | 0 | 0 | 0 | 0 | 7 | 7 | 2.93E5 |  |  |  |  |  |  |  |  |  | 6 | 1 | 6 | 0 | 0 | 0 | 0 | 0 | 0 | 0 | 4 | 4 | Y | 54188 | Gasdermin-C3 OS=Mus musculus OX=10090 GN=Gsdmc3 PE=2 SV=2 |
| 1155 | 1910 | sp\|P42669\|PURA\_MOUSE | 109.03 | 11 | 3 | 5 | 0 | 6 | 6 | 6 | 6 | 0 | 0 | 3 | 6E5 | 3.96E6 |  | 4.87E6 | 3.86E6 | 3.58E6 | 3.51E6 |  |  | 1.53E6 | 4 | 3 | 1 | 2 | 0 | 2 | 2 | 2 | 2 | 0 | 0 | 1 | N | 34884 | Transcriptional activator protein Pur-alpha OS=Mus musculus OX=10090 GN=Pura PE=1 SV=1 |
| 1728 | 3820 | E9Q5H2\|E9Q5H2\_MOUSE | 108.98 | 26 | 0 | 0 | 0 | 15 | 11 | 0 | 11 | 0 | 0 | 15 |  |  |  | 2.84E5 | 1.1E6 |  | 4.28E5 |  |  | 1.52E6 | 2 | 2 | 0 | 0 | 0 | 1 | 1 | 0 | 1 | 0 | 0 | 1 | Y | 12078 | Acidic leucine-rich nuclear phosphoprotein 32 family member (Fragment) OS=Mus musculus OX=10090 GN=Anp32e PE=1 SV=1 |
| 1728 | 3821 | E9PZF5\|E9PZF5\_MOUSE | 108.98 | 16 | 0 | 0 | 0 | 9 | 7 | 0 | 7 | 0 | 0 | 9 |  |  |  | 2.84E5 | 1.1E6 |  | 4.28E5 |  |  | 1.52E6 | 2 | 2 | 0 | 0 | 0 | 1 | 1 | 0 | 1 | 0 | 0 | 1 | Y | 19234 | Acidic leucine-rich nuclear phosphoprotein 32 family member (Fragment) OS=Mus musculus OX=10090 GN=Anp32e PE=1 SV=1 |
| 1728 | 3822 | sp\|P97822\|AN32E\_MOUSE | 108.98 | 11 | 0 | 0 | 0 | 6 | 5 | 0 | 5 | 0 | 0 | 6 |  |  |  | 2.84E5 | 1.1E6 |  | 4.28E5 |  |  | 1.52E6 | 2 | 2 | 0 | 0 | 0 | 1 | 1 | 0 | 1 | 0 | 0 | 1 | Y | 29622 | Acidic leucine-rich nuclear phosphoprotein 32 family member E OS=Mus musculus OX=10090 GN=Anp32e PE=1 SV=2 |
| 1195 | 2232 | sp\|Q923D2\|BLVRB\_MOUSE | 108.28 | 17 | 8 | 8 | 17 | 0 | 8 | 8 | 4 | 8 | 8 | 5 | 1.03E6 | 4.05E6 | 1.42E7 |  | 2.67E6 | 6.88E5 | 1.06E6 | 2.99E6 | 8.9E6 | 2.85E6 | 3 | 3 | 1 | 1 | 3 | 0 | 1 | 1 | 1 | 1 | 1 | 1 | N | 22197 | Flavin reductase (NADPH) OS=Mus musculus OX=10090 GN=Blvrb PE=1 SV=3 |
| 1195 | 2348 | E9PZC3\|E9PZC3\_MOUSE | 108.28 | 15 | 7 | 7 | 15 | 0 | 7 | 7 | 4 | 7 | 7 | 4 | 1.03E6 | 4.05E6 | 1.42E7 |  | 2.67E6 | 6.88E5 | 1.06E6 | 2.99E6 | 8.9E6 | 2.85E6 | 3 | 3 | 1 | 1 | 3 | 0 | 1 | 1 | 1 | 1 | 1 | 1 | N | 26647 | Biliverdin reductase B OS=Mus musculus OX=10090 GN=Blvrb PE=1 SV=1 |
| 1250 | 2588 | sp\|P10833\|RRAS\_MOUSE | 108.18 | 18 | 6 | 12 | 0 | 11 | 6 | 6 | 6 | 0 | 6 | 6 | 8.26E6 | 4.88E6 |  | 5.47E6 | 4.6E6 | 2.73E7 | 2.93E6 |  | 3.17E7 | 1.93E7 | 3 | 3 | 1 | 2 | 0 | 2 | 1 | 1 | 1 | 0 | 1 | 1 | Y | 23764 | Ras-related protein R-Ras OS=Mus musculus OX=10090 GN=Rras PE=1 SV=1 |
| 1102 | 2722 | sp\|Q60749\|KHDR1\_MOUSE | 108.17 | 5 | 0 | 0 | 2 | 2 | 5 | 2 | 5 | 5 | 3 | 0 |  |  | 2.13E6 | 2.45E6 | 5.94E6 | 5.53E6 | 2.54E6 | 8.62E5 | 1.4E6 |  | 2 | 2 | 0 | 0 | 1 | 2 | 3 | 2 | 2 | 2 | 1 | 0 | N | 48371 | KH domain-containing, RNA-binding, signal transduction-associated protein 1 OS=Mus musculus OX=10090 GN=Khdrbs1 PE=1 SV=2 |
| 684 | 2443 | A0A0G2JDW7\|A0A0G2JDW7\_MOUSE | 108.01 | 39 | 39 | 23 | 23 | 23 | 39 | 23 | 23 | 23 | 29 | 23 | 1.15E7 | 1.17E7 | 1.42E7 | 6.13E6 | 1.21E7 | 6.12E6 | 9.73E6 | 8.94E6 | 1.14E7 | 1.23E7 | 3 | 1 | 4 | 3 | 3 | 3 | 4 | 3 | 3 | 3 | 3 | 3 | Y | 9233 | 40S ribosomal protein S27 (Fragment) OS=Mus musculus OX=10090 GN=Rps27 PE=1 SV=1 |
| 684 | 2444 | sp\|Q6ZWU9\|RS27\_MOUSE | 108.01 | 38 | 38 | 23 | 23 | 23 | 38 | 23 | 23 | 23 | 29 | 23 | 1.15E7 | 1.17E7 | 1.42E7 | 6.13E6 | 1.21E7 | 6.12E6 | 9.73E6 | 8.94E6 | 1.14E7 | 1.23E7 | 3 | 1 | 4 | 3 | 3 | 3 | 4 | 3 | 3 | 3 | 3 | 3 | Y | 9461 | Small ribosomal subunit protein eS27 OS=Mus musculus OX=10090 GN=Rps27 PE=1 SV=3 |
| 1033 | 1676 | sp\|Q3UJB0\|SF3B2\_MOUSE | 107.50 | 4 | 2 | 0 | 3 | 1 | 1 | 3 | 2 | 3 | 2 | 2 | 7.72E5 |  | 2.99E6 | 3.82E5 | 5.95E5 | 9.22E5 | 8.96E5 | 9.49E5 | 1.7E5 | 1.92E6 | 3 | 3 | 1 | 0 | 2 | 1 | 1 | 2 | 1 | 2 | 1 | 1 | N | 98199 | Splicing factor 3B subunit 2 OS=Mus musculus OX=10090 GN=Sf3b2 PE=2 SV=1 |
| 1188 | 2057 | sp\|P97447\|FHL1\_MOUSE | 107.43 | 14 | 5 | 0 | 4 | 0 | 10 | 4 | 10 | 0 | 10 | 4 | 6.06E5 |  | 1.31E7 |  | 9.59E6 | 9.53E6 | 7.28E6 |  | 3.08E6 | 1.09E7 | 3 | 3 | 1 | 0 | 1 | 0 | 2 | 1 | 2 | 0 | 2 | 1 | Y | 31889 | Four and a half LIM domains protein 1 OS=Mus musculus OX=10090 GN=Fhl1 PE=1 SV=3 |
| 1188 | 2058 | A2AEX8\|A2AEX8\_MOUSE | 107.43 | 14 | 4 | 0 | 4 | 0 | 9 | 4 | 9 | 0 | 9 | 4 | 6.06E5 |  | 1.31E7 |  | 9.59E6 | 9.53E6 | 7.28E6 |  | 3.08E6 | 1.09E7 | 3 | 3 | 1 | 0 | 1 | 0 | 2 | 1 | 2 | 0 | 2 | 1 | Y | 33564 | Four and a half LIM domains 1 OS=Mus musculus OX=10090 GN=Fhl1 PE=1 SV=1 |
| 1188 | 2059 | A2AEX6\|A2AEX6\_MOUSE | 107.43 | 13 | 4 | 0 | 4 | 0 | 9 | 4 | 9 | 0 | 9 | 4 | 6.06E5 |  | 1.31E7 |  | 9.59E6 | 9.53E6 | 7.28E6 |  | 3.08E6 | 1.09E7 | 3 | 3 | 1 | 0 | 1 | 0 | 2 | 1 | 2 | 0 | 2 | 1 | Y | 35081 | Four and a half LIM domains 1 OS=Mus musculus OX=10090 GN=Fhl1 PE=1 SV=1 |
| 1374 | 1944 | sp\|O08677\|KNG1\_MOUSE | 107.32 | 5 | 0 | 0 | 3 | 0 | 2 | 3 | 0 | 3 | 0 | 2 |  |  | 3.49E6 |  | 2.82E5 | 3E6 |  | 5.49E5 |  | 4.08E5 | 3 | 3 | 0 | 0 | 2 | 0 | 1 | 2 | 0 | 2 | 0 | 1 | Y | 73102 | Kininogen-1 OS=Mus musculus OX=10090 GN=Kng1 PE=1 SV=1 |
| 1374 | 1945 | A0A0R4J038\|A0A0R4J038\_MOUSE | 107.32 | 5 | 0 | 0 | 3 | 0 | 2 | 3 | 0 | 3 | 0 | 2 |  |  | 3.49E6 |  | 2.82E5 | 3E6 |  | 5.49E5 |  | 4.08E5 | 3 | 3 | 0 | 0 | 2 | 0 | 1 | 2 | 0 | 2 | 0 | 1 | Y | 73101 | Kininogen 1 OS=Mus musculus OX=10090 GN=Kng1 PE=1 SV=1 |
| 1374 | 1972 | D3YTY9\|D3YTY9\_MOUSE | 107.32 | 7 | 0 | 0 | 5 | 0 | 2 | 5 | 0 | 5 | 0 | 2 |  |  | 3.49E6 |  | 2.82E5 | 3E6 |  | 5.49E5 |  | 4.08E5 | 3 | 3 | 0 | 0 | 2 | 0 | 1 | 2 | 0 | 2 | 0 | 1 | Y | 53205 | Kininogen 1 OS=Mus musculus OX=10090 GN=Kng1 PE=1 SV=1 |
| 660 | 2752 | F8WHP8\|F8WHP8\_MOUSE | 107.17 | 16 | 16 | 16 | 16 | 16 | 16 | 16 | 16 | 16 | 16 | 16 | 3.95E7 | 4.71E7 | 5.44E7 | 2.19E7 | 3.59E7 | 3.94E7 | 2.75E7 | 2.44E7 | 6.26E7 | 3.1E7 | 1 | 1 | 3 | 4 | 4 | 3 | 2 | 2 | 2 | 4 | 4 | 4 | N | 9006 | ATP synthase subunit f, mitochondrial OS=Mus musculus OX=10090 GN=Atp5mf PE=1 SV=1 |
| 660 | 2758 | sp\|P56135\|ATPK\_MOUSE | 107.17 | 14 | 14 | 14 | 14 | 14 | 14 | 14 | 14 | 14 | 14 | 14 | 3.95E7 | 4.71E7 | 5.44E7 | 2.19E7 | 3.59E7 | 3.94E7 | 2.75E7 | 2.44E7 | 6.26E7 | 3.1E7 | 1 | 1 | 3 | 4 | 4 | 3 | 2 | 2 | 2 | 4 | 4 | 4 | N | 10344 | ATP synthase subunit f, mitochondrial OS=Mus musculus OX=10090 GN=Atp5mf PE=1 SV=3 |
| 1817 | 3083 | sp\|Q61081\|CDC37\_MOUSE | 107.15 | 6 | 6 | 6 | 0 | 0 | 6 | 0 | 6 | 0 | 0 | 0 | 2.13E6 | 2.03E6 |  |  | 2.28E6 |  | 1.73E6 |  |  |  | 1 | 1 | 1 | 1 | 0 | 0 | 1 | 0 | 1 | 0 | 0 | 0 | N | 44593 | Hsp90 co-chaperone Cdc37 OS=Mus musculus OX=10090 GN=Cdc37 PE=1 SV=1 |
| 950 | 2583 | sp\|O55135\|IF6\_MOUSE | 107.09 | 16 | 14 | 4 | 7 | 14 | 14 | 14 | 14 | 14 | 4 | 14 | 5.3E6 | 1.18E6 | 2.36E6 | 1.8E6 | 2.47E6 | 5.39E6 | 3.7E6 | 2.95E6 | 1.73E6 | 5.62E6 | 3 | 3 | 2 | 1 | 2 | 2 | 2 | 2 | 2 | 2 | 1 | 2 | N | 26511 | Eukaryotic translation initiation factor 6 OS=Mus musculus OX=10090 GN=Eif6 PE=1 SV=2 |
| 1184 | 3194 | A0A0J9YUI1\|A0A0J9YUI1\_MOUSE | 107.05 | 19 | 11 | 0 | 11 | 11 | 19 | 9 | 19 | 19 | 0 | 19 | 3.17E6 |  | 4.19E6 | 2.08E6 | 4.26E6 | 1.23E6 | 4.21E6 | 3.58E6 |  | 4.21E6 | 2 | 2 | 1 | 0 | 1 | 1 | 2 | 1 | 2 | 2 | 0 | 2 | N | 15810 | E2 ubiquitin-conjugating enzyme OS=Mus musculus OX=10090 GN=Ube2k PE=1 SV=1 |
| 1184 | 3195 | sp\|P61087\|UBE2K\_MOUSE | 107.05 | 14 | 8 | 0 | 8 | 8 | 14 | 6 | 14 | 14 | 0 | 14 | 3.17E6 |  | 4.19E6 | 2.08E6 | 4.26E6 | 1.23E6 | 4.21E6 | 3.58E6 |  | 4.21E6 | 2 | 2 | 1 | 0 | 1 | 1 | 2 | 1 | 2 | 2 | 0 | 2 | N | 22407 | Ubiquitin-conjugating enzyme E2 K OS=Mus musculus OX=10090 GN=Ube2k PE=1 SV=3 |
| 1184 | 3222 | D3Z4U3\|D3Z4U3\_MOUSE | 107.05 | 17 | 10 | 0 | 10 | 10 | 17 | 8 | 17 | 17 | 0 | 17 | 3.17E6 |  | 4.19E6 | 2.08E6 | 4.26E6 | 1.23E6 | 4.21E6 | 3.58E6 |  | 4.21E6 | 2 | 2 | 1 | 0 | 1 | 1 | 2 | 1 | 2 | 2 | 0 | 2 | N | 17447 | E2 ubiquitin-conjugating enzyme OS=Mus musculus OX=10090 GN=Ube2k PE=1 SV=1 |
| 910 | 1561 | sp\|P46471\|PRS7\_MOUSE | 107.01 | 11 | 10 | 5 | 3 | 3 | 5 | 4 | 3 | 3 | 9 | 5 | 6.77E6 | 1.08E6 | 1.55E6 | 0E0 | 2.3E6 | 2.97E6 | 2.65E6 | 4.07E5 | 1.15E7 | 1.98E6 | 5 | 5 | 4 | 2 | 1 | 1 | 2 | 2 | 1 | 1 | 4 | 2 | N | 48648 | 26S proteasome regulatory subunit 7 OS=Mus musculus OX=10090 GN=Psmc2 PE=1 SV=5 |
| 910 | 1567 | Q8BVQ9\|Q8BVQ9\_MOUSE | 107.01 | 10 | 9 | 5 | 3 | 3 | 5 | 4 | 3 | 3 | 8 | 5 | 6.77E6 | 1.08E6 | 1.55E6 | 0E0 | 2.3E6 | 2.97E6 | 2.65E6 | 4.07E5 | 1.15E7 | 1.98E6 | 5 | 5 | 4 | 2 | 1 | 1 | 2 | 2 | 1 | 1 | 4 | 2 | N | 52867 | 26S proteasome regulatory subunit 7 OS=Mus musculus OX=10090 GN=Psmc2 PE=1 SV=1 |
| 1079 | 2566 | sp\|Q9Z1Z0\|USO1\_MOUSE | 106.87 | 3 | 1 | 1 | 1 | 2 | 3 | 2 | 3 | 0 | 0 | 0 | 1.63E6 | 8.25E5 |  | 7.34E5 | 2.18E6 | 2.5E6 | 2.19E6 |  |  |  | 3 | 2 | 1 | 1 | 1 | 2 | 3 | 2 | 3 | 0 | 0 | 0 | N | 106983 | General vesicular transport factor p115 OS=Mus musculus OX=10090 GN=Uso1 PE=1 SV=2 |
| 1490 | 2586 | sp\|Q9CRB6\|TPPP3\_MOUSE | 106.75 | 14 | 0 | 9 | 5 | 0 | 5 | 5 | 0 | 0 | 0 | 0 |  | 1.85E6 | 1.68E6 |  | 3.11E6 | 9.28E5 |  |  |  |  | 2 | 2 | 0 | 1 | 1 | 0 | 1 | 1 | 0 | 0 | 0 | 0 | Y | 18965 | Tubulin polymerization-promoting protein family member 3 OS=Mus musculus OX=10090 GN=Tppp3 PE=1 SV=1 |
| 1123 | 3846 | Z4YKT6\|Z4YKT6\_MOUSE | 106.70 | 8 | 5 | 5 | 8 | 3 | 3 | 8 | 8 | 3 | 8 | 3 | 4.61E5 | 1.55E5 | 2.31E6 | 4.71E5 | 7.76E5 | 7.1E5 | 1.09E6 | 1.78E5 | 2.55E6 | 4.34E5 | 2 | 2 | 1 | 1 | 2 | 1 | 1 | 2 | 2 | 1 | 2 | 1 | N | 33225 | Dehydrogenase/reductase SDR family member 7B OS=Mus musculus OX=10090 GN=Dhrs7b PE=1 SV=1 |
| 1123 | 3847 | sp\|Q99J47\|DRS7B\_MOUSE | 106.70 | 7 | 5 | 5 | 7 | 3 | 3 | 7 | 7 | 3 | 7 | 3 | 4.61E5 | 1.55E5 | 2.31E6 | 4.71E5 | 7.76E5 | 7.1E5 | 1.09E6 | 1.78E5 | 2.55E6 | 4.34E5 | 2 | 2 | 1 | 1 | 2 | 1 | 1 | 2 | 2 | 1 | 2 | 1 | N | 34987 | Dehydrogenase/reductase SDR family member 7B OS=Mus musculus OX=10090 GN=Dhrs7b PE=1 SV=1 |
| 1078 | 2902 | sp\|Q9D0S9\|HINT2\_MOUSE | 106.49 | 21 | 9 | 21 | 12 | 21 | 21 | 21 | 21 | 21 | 0 | 12 | 1.53E6 | 1.68E6 | 5.16E5 | 1.67E6 | 2.2E6 | 3.49E6 | 1.09E6 | 1.82E6 |  | 5.91E5 | 2 | 2 | 1 | 2 | 1 | 2 | 2 | 2 | 2 | 2 | 0 | 1 | N | 17320 | Adenosine 5'-monophosphoramidase HINT2 OS=Mus musculus OX=10090 GN=Hint2 PE=1 SV=1 |
| 1444 | 2314 | E9Q6R3\|E9Q6R3\_MOUSE | 106.27 | 20 | 8 | 0 | 11 | 0 | 6 | 6 | 5 | 0 | 0 | 6 | 4.44E5 |  | 8.76E6 |  | 3.37E6 | 7.45E6 | 1.58E6 |  |  | 7.66E6 | 3 | 3 | 1 | 0 | 2 | 0 | 1 | 1 | 1 | 0 | 0 | 1 | N | 18923 | SEC22 homolog B, vesicle trafficking protein OS=Mus musculus OX=10090 GN=Sec22b PE=1 SV=1 |
| 1444 | 2315 | sp\|O08547\|SC22B\_MOUSE | 106.27 | 15 | 7 | 0 | 9 | 0 | 5 | 5 | 4 | 0 | 0 | 5 | 4.44E5 |  | 8.76E6 |  | 3.37E6 | 7.45E6 | 1.58E6 |  |  | 7.66E6 | 3 | 3 | 1 | 0 | 2 | 0 | 1 | 1 | 1 | 0 | 0 | 1 | N | 24741 | Vesicle-trafficking protein SEC22b OS=Mus musculus OX=10090 GN=Sec22b PE=1 SV=3 |
| 1024 | 1706 | sp\|P97864\|CASP7\_MOUSE | 106.26 | 11 | 3 | 9 | 9 | 3 | 7 | 7 | 7 | 0 | 3 | 0 | 2.85E6 | 4.49E6 | 8.86E6 | 1.45E6 | 4.77E6 | 5.07E6 | 2.5E6 |  | 1.78E6 |  | 4 | 4 | 1 | 3 | 3 | 1 | 2 | 2 | 2 | 0 | 1 | 0 | N | 34061 | Caspase-7 OS=Mus musculus OX=10090 GN=Casp7 PE=1 SV=2 |
| 1884 | 2203 | Q3TQP6\|Q3TQP6\_MOUSE | 106.17 | 7 | 5 | 0 | 0 | 0 | 0 | 0 | 0 | 0 | 2 | 0 | 5.76E6 |  |  |  |  |  |  |  | 2.08E6 |  | 3 | 3 | 2 | 0 | 0 | 0 | 0 | 0 | 0 | 0 | 1 | 0 | Y | 61481 | Malic enzyme OS=Mus musculus OX=10090 GN=Me1 PE=1 SV=1 |
| 1884 | 2204 | sp\|P06801\|MAOX\_MOUSE | 106.17 | 6 | 5 | 0 | 0 | 0 | 0 | 0 | 0 | 0 | 2 | 0 | 5.76E6 |  |  |  |  |  |  |  | 2.08E6 |  | 3 | 3 | 2 | 0 | 0 | 0 | 0 | 0 | 0 | 0 | 1 | 0 | Y | 63954 | NADP-dependent malic enzyme OS=Mus musculus OX=10090 GN=Me1 PE=1 SV=2 |
| 1042 | 3292 | sp\|P01592\|IGJ\_MOUSE | 106.13 | 14 | 14 | 9 | 9 | 0 | 14 | 14 | 14 | 14 | 14 | 14 | 3.34E6 | 1.47E6 | 6.89E5 |  | 4.13E6 | 4.34E6 | 3.19E6 | 2.61E6 | 2.15E7 | 5.63E6 | 2 | 2 | 2 | 1 | 1 | 0 | 2 | 2 | 2 | 2 | 2 | 2 | N | 18014 | Immunoglobulin J chain OS=Mus musculus OX=10090 GN=Jchain PE=1 SV=4 |
| 1187 | 1819 | A0A0A6YX73\|A0A0A6YX73\_MOUSE | 106.03 | 11 | 3 | 0 | 5 | 6 | 5 | 5 | 6 | 0 | 0 | 3 | 1.53E6 |  | 5.4E6 | 1.84E6 | 4.2E6 | 4.22E6 | 4.76E6 |  |  | 2.21E6 | 4 | 4 | 1 | 0 | 2 | 2 | 2 | 2 | 2 | 0 | 0 | 1 | Y | 43166 | cAMP-dependent protein kinase type II-alpha regulatory subunit OS=Mus musculus OX=10090 GN=Prkar2a PE=1 SV=1 |
| 1187 | 1820 | Q8K1M3\|Q8K1M3\_MOUSE | 106.03 | 10 | 3 | 0 | 5 | 5 | 5 | 5 | 6 | 0 | 0 | 3 | 1.53E6 |  | 5.4E6 | 1.84E6 | 4.2E6 | 4.22E6 | 4.76E6 |  |  | 2.21E6 | 4 | 4 | 1 | 0 | 2 | 2 | 2 | 2 | 2 | 0 | 0 | 1 | Y | 45589 | cAMP-dependent protein kinase type II-alpha regulatory subunit OS=Mus musculus OX=10090 GN=Prkar2a PE=1 SV=1 |
| 1245 | 2892 | sp\|Q9QZD8\|DIC\_MOUSE | 105.92 | 11 | 0 | 3 | 11 | 11 | 3 | 11 | 3 | 3 | 8 | 0 |  | 4.65E6 | 1.87E6 | 1.39E6 |  | 3.86E6 | 1.54E6 | 0E0 | 2.01E6 |  | 2 | 2 | 0 | 1 | 2 | 2 | 1 | 2 | 1 | 1 | 1 | 0 | N | 31715 | Mitochondrial dicarboxylate carrier OS=Mus musculus OX=10090 GN=Slc25a10 PE=1 SV=2 |
| 1542 | 4184 | Q8JZR2\|Q8JZR2\_MOUSE | 105.56 | 8 | 0 | 8 | 8 | 8 | 0 | 8 | 8 | 0 | 0 | 0 |  | 1.77E6 | 1.07E6 | 3.32E5 |  | 9.72E5 | 6.86E5 |  |  |  | 1 | 1 | 0 | 1 | 1 | 1 | 0 | 1 | 1 | 0 | 0 | 0 | N | 22890 | Crk protein OS=Mus musculus OX=10090 GN=Crk PE=1 SV=1 |
| 1542 | 4185 | F7D232\|F7D232\_MOUSE | 105.56 | 8 | 0 | 8 | 8 | 8 | 0 | 8 | 8 | 0 | 0 | 0 |  | 1.77E6 | 1.07E6 | 3.32E5 |  | 9.72E5 | 6.86E5 |  |  |  | 1 | 1 | 0 | 1 | 1 | 1 | 0 | 1 | 1 | 0 | 0 | 0 | N | 24673 | V-crk avian sarcoma virus CT10 oncogene homolog (Fragment) OS=Mus musculus OX=10090 GN=Crk PE=1 SV=1 |
| 1542 | 4187 | Q5ND50\|Q5ND50\_MOUSE | 105.56 | 7 | 0 | 7 | 7 | 7 | 0 | 7 | 7 | 0 | 0 | 0 |  | 1.77E6 | 1.07E6 | 3.32E5 |  | 9.72E5 | 6.86E5 |  |  |  | 1 | 1 | 0 | 1 | 1 | 1 | 0 | 1 | 1 | 0 | 0 | 0 | N | 29341 | V-crk avian sarcoma virus CT10 oncogene homolog OS=Mus musculus OX=10090 GN=Crk PE=1 SV=2 |
| 1542 | 4188 | sp\|Q64010\|CRK\_MOUSE | 105.56 | 6 | 0 | 6 | 6 | 6 | 0 | 6 | 6 | 0 | 0 | 0 |  | 1.77E6 | 1.07E6 | 3.32E5 |  | 9.72E5 | 6.86E5 |  |  |  | 1 | 1 | 0 | 1 | 1 | 1 | 0 | 1 | 1 | 0 | 0 | 0 | N | 33815 | Adapter molecule crk OS=Mus musculus OX=10090 GN=Crk PE=1 SV=1 |
| 1240 | 2078 | sp\|P61290\|PSME3\_MOUSE | 105.52 | 15 | 12 | 0 | 0 | 6 | 0 | 6 | 6 | 6 | 3 | 0 | 2.03E6 |  |  | 1.12E6 |  | 4.18E5 | 5.66E5 | 2.81E5 | 1.42E6 |  | 3 | 3 | 2 | 0 | 0 | 2 | 0 | 1 | 1 | 1 | 1 | 0 | N | 29506 | Proteasome activator complex subunit 3 OS=Mus musculus OX=10090 GN=Psme3 PE=1 SV=1 |
| 698 | 2249 | sp\|Q9WTP6\|KAD2\_MOUSE | 105.45 | 15 | 5 | 15 | 15 | 10 | 15 | 15 | 15 | 15 | 10 | 10 | 1.65E6 | 1.18E7 | 2.02E7 | 6.85E6 | 1.53E7 | 1.8E7 | 1.29E7 | 1.04E7 | 9.38E6 | 2.88E6 | 3 | 3 | 1 | 3 | 4 | 3 | 6 | 5 | 3 | 3 | 3 | 2 | Y | 26469 | Adenylate kinase 2, mitochondrial OS=Mus musculus OX=10090 GN=Ak2 PE=1 SV=5 |
| 1231 | 1280 | sp\|Q9CZN7\|GLYM\_MOUSE | 105.38 | 10 | 2 | 0 | 5 | 0 | 2 | 0 | 0 | 0 | 8 | 4 | 0E0 |  | 3.57E6 |  | 2.33E6 |  |  |  | 8.51E6 | 5.02E6 | 5 | 4 | 1 | 0 | 2 | 0 | 1 | 0 | 0 | 0 | 4 | 2 | Y | 55759 | Serine hydroxymethyltransferase, mitochondrial OS=Mus musculus OX=10090 GN=Shmt2 PE=1 SV=1 |
| 574 | 2522 | Q9D848\|Q9D848\_MOUSE | 105.24 | 20 | 15 | 16 | 16 | 20 | 15 | 16 | 20 | 16 | 16 | 10 | 3.12E6 |  |  | 2.2E6 |  |  | 3.58E6 |  |  |  | 4 | 1 | 3 | 5 | 6 | 5 | 4 | 3 | 5 | 4 | 3 | 1 | Y | 12784 | CRS1C-3 OS=Mus musculus OX=10090 GN=Defa39 PE=1 SV=1 |
| 2061 | 2160 | G3X8T9\|G3X8T9\_MOUSE | 105.24 | 8 | 3 | 0 | 0 | 3 | 0 | 0 | 0 | 0 | 0 | 2 |  |  |  |  |  |  |  |  |  | 4.78E5 | 3 | 1 | 1 | 0 | 0 | 1 | 0 | 0 | 0 | 0 | 0 | 1 | N | 46766 | Serine (or cysteine) peptidase inhibitor, clade A, member 3N OS=Mus musculus OX=10090 GN=Serpina3n PE=1 SV=1 |
| 2061 | 2188 | sp\|Q91WP6\|SPA3N\_MOUSE | 105.24 | 8 | 3 | 0 | 0 | 3 | 0 | 0 | 0 | 0 | 0 | 2 |  |  |  |  |  |  |  |  |  | 4.78E5 | 3 | 1 | 1 | 0 | 0 | 1 | 0 | 0 | 0 | 0 | 0 | 1 | N | 46718 | Serine protease inhibitor A3N OS=Mus musculus OX=10090 GN=Serpina3n PE=1 SV=1 |
| 1147 | 2036 | sp\|Q99JI4\|PSMD6\_MOUSE | 105.23 | 9 | 4 | 4 | 4 | 0 | 5 | 7 | 3 | 0 | 4 | 4 | 7.95E5 | 2.53E6 | 3.8E6 |  | 7.24E6 | 1.8E6 | 3.13E5 |  | 2.13E6 | 1.69E6 | 3 | 3 | 1 | 1 | 1 | 0 | 2 | 2 | 1 | 0 | 1 | 1 | N | 45536 | 26S proteasome non-ATPase regulatory subunit 6 OS=Mus musculus OX=10090 GN=Psmd6 PE=1 SV=1 |
| 1167 | 2108 | A0A3B2WBH9\|A0A3B2WBH9\_MOUSE | 105.21 | 3 | 0 | 0 | 0 | 1 | 1 | 0 | 2 | 0 | 0 | 0 |  |  |  | 2.7E4 | 3.74E5 |  | 2.1E6 |  |  |  | 4 | 3 | 0 | 0 | 0 | 1 | 2 | 0 | 2 | 0 | 0 | 0 | N | 131106 | Tight junction protein 2 OS=Mus musculus OX=10090 GN=Tjp2 PE=1 SV=1 |
| 1167 | 2109 | sp\|Q9Z0U1\|ZO2\_MOUSE | 105.21 | 3 | 0 | 0 | 0 | 1 | 1 | 0 | 2 | 0 | 0 | 0 |  |  |  | 2.7E4 | 3.74E5 |  | 2.1E6 |  |  |  | 4 | 3 | 0 | 0 | 0 | 1 | 2 | 0 | 2 | 0 | 0 | 0 | N | 131280 | Tight junction protein ZO-2 OS=Mus musculus OX=10090 GN=Tjp2 PE=1 SV=2 |
| 1167 | 2110 | A0A3B2WCN9\|A0A3B2WCN9\_MOUSE | 105.21 | 3 | 0 | 0 | 0 | 1 | 1 | 0 | 2 | 0 | 0 | 0 |  |  |  | 2.7E4 | 3.74E5 |  | 2.1E6 |  |  |  | 4 | 3 | 0 | 0 | 0 | 1 | 2 | 0 | 2 | 0 | 0 | 0 | N | 133871 | Tight junction protein 2 OS=Mus musculus OX=10090 GN=Tjp2 PE=1 SV=1 |
| 769 | 2021 | sp\|Q9DC51\|GNAI3\_MOUSE | 105.18 | 9 | 5 | 5 | 3 | 5 | 7 | 5 | 5 | 5 | 5 | 5 |  |  |  | 1.65E6 |  |  |  |  |  |  | 4 | 1 | 3 | 3 | 1 | 3 | 4 | 3 | 3 | 4 | 2 | 2 | Y | 40538 | Guanine nucleotide-binding protein G(i) subunit alpha-3 OS=Mus musculus OX=10090 GN=Gnai3 PE=1 SV=3 |
| 715 | 1966 | sp\|Q9CPQ1\|COX6C\_MOUSE | 105.08 | 38 | 18 | 9 | 20 | 29 | 38 | 20 | 38 | 29 | 29 | 9 | 5.5E7 | 3.92E7 | 7.94E7 | 4.68E7 | 9E7 | 6.25E7 | 6.44E7 | 4.15E7 | 5.88E7 | 3.05E7 | 4 | 4 | 2 | 1 | 2 | 3 | 4 | 2 | 5 | 4 | 3 | 1 | Y | 8469 | Cytochrome c oxidase subunit 6C OS=Mus musculus OX=10090 GN=Cox6c PE=1 SV=3 |
| 748 | 1935 | sp\|Q9CZY3\|UB2V1\_MOUSE | 104.91 | 22 | 11 | 18 | 6 | 17 | 22 | 10 | 10 | 10 | 18 | 11 | 8.37E6 | 2.69E7 | 1.23E7 | 7.28E6 | 2.01E7 | 1.3E7 | 1.13E7 | 7.17E6 | 2.45E7 | 1.26E7 | 4 | 4 | 2 | 3 | 2 | 3 | 5 | 2 | 3 | 2 | 4 | 3 | N | 16355 | Ubiquitin-conjugating enzyme E2 variant 1 OS=Mus musculus OX=10090 GN=Ube2v1 PE=1 SV=1 |
| 748 | 1951 | B7ZBY7\|B7ZBY7\_MOUSE | 104.91 | 18 | 9 | 15 | 5 | 14 | 18 | 9 | 9 | 9 | 15 | 9 | 8.37E6 | 2.69E7 | 1.23E7 | 7.28E6 | 2.01E7 | 1.3E7 | 1.13E7 | 7.17E6 | 2.45E7 | 1.26E7 | 4 | 4 | 2 | 3 | 2 | 3 | 5 | 2 | 3 | 2 | 4 | 3 | N | 19339 | Ubiquitin-conjugating enzyme E2 variant 1 OS=Mus musculus OX=10090 GN=Ube2v1 PE=4 SV=1 |
| 748 | 1995 | E9PY39\|E9PY39\_MOUSE | 104.91 | 9 | 4 | 7 | 2 | 7 | 9 | 4 | 4 | 4 | 7 | 4 | 8.37E6 | 2.69E7 | 1.23E7 | 7.28E6 | 2.01E7 | 1.3E7 | 1.13E7 | 7.17E6 | 2.45E7 | 1.26E7 | 4 | 4 | 2 | 3 | 2 | 3 | 5 | 2 | 3 | 2 | 4 | 3 | N | 42096 | Predicted gene 20431 OS=Mus musculus OX=10090 GN=Gm20431 PE=3 SV=1 |
| 962 | 1430 | sp\|Q61335\|BAP31\_MOUSE | 104.88 | 20 | 9 | 0 | 11 | 13 | 5 | 11 | 7 | 7 | 0 | 0 | 4.11E6 |  | 2.18E7 | 2.4E7 | 7.32E6 | 2.3E7 | 8.94E6 | 7.19E6 |  |  | 6 | 5 | 2 | 0 | 3 | 4 | 2 | 3 | 2 | 2 | 0 | 0 | Y | 27957 | B-cell receptor-associated protein 31 OS=Mus musculus OX=10090 GN=Bcap31 PE=1 SV=4 |
| 1303 | 2608 | E9QJW0\|E9QJW0\_MOUSE | 104.88 | 13 | 6 | 6 | 6 | 0 | 6 | 6 | 6 | 0 | 13 | 6 | 7.73E6 | 1.13E7 | 5.93E6 |  | 5.03E6 | 6.7E6 | 1.37E6 |  | 1.5E7 | 9.98E6 | 2 | 2 | 1 | 1 | 1 | 0 | 1 | 1 | 1 | 0 | 2 | 1 | Y | 25793 | Microsomal glutathione S-transferase 1 OS=Mus musculus OX=10090 GN=Mgst1 PE=1 SV=1 |
| 1303 | 2624 | sp\|Q91VS7\|MGST1\_MOUSE | 104.88 | 19 | 8 | 8 | 8 | 0 | 8 | 8 | 8 | 0 | 19 | 8 | 7.73E6 | 1.13E7 | 5.93E6 |  | 5.03E6 | 6.7E6 | 1.37E6 |  | 1.5E7 | 9.98E6 | 2 | 2 | 1 | 1 | 1 | 0 | 1 | 1 | 1 | 0 | 2 | 1 | Y | 17552 | Microsomal glutathione S-transferase 1 OS=Mus musculus OX=10090 GN=Mgst1 PE=1 SV=3 |
| 765 | 4075 | sp\|P47955\|RLA1\_MOUSE | 104.88 | 19 | 14 | 14 | 14 | 14 | 14 | 19 | 14 | 14 | 14 | 19 | 5.66E7 | 6.03E5 | 2.22E7 | 3.48E7 | 4.98E7 | 7.32E7 | 5.5E7 | 3.9E7 | 1.03E8 | 7.21E6 | 2 | 2 | 3 | 1 | 1 | 1 | 2 | 2 | 1 | 3 | 3 | 2 | N | 11475 | Large ribosomal subunit protein P1 OS=Mus musculus OX=10090 GN=Rplp1 PE=1 SV=1 |
| 915 | 2412 | sp\|Q9D1D4\|TMEDA\_MOUSE | 104.68 | 15 | 11 | 3 | 11 | 11 | 15 | 11 | 3 | 11 | 7 | 3 | 1.13E7 | 1.26E7 | 8.24E6 | 6.17E6 | 1.16E7 | 1.14E7 | 8.56E6 | 8.36E6 | 5.31E6 | 1.12E7 | 4 | 4 | 2 | 1 | 2 | 2 | 3 | 2 | 1 | 2 | 2 | 1 | Y | 24911 | Transmembrane emp24 domain-containing protein 10 OS=Mus musculus OX=10090 GN=Tmed10 PE=1 SV=1 |
| 1157 | 2647 | sp\|Q3UMR5\|MCU\_MOUSE | 104.31 | 8 | 3 | 8 | 0 | 0 | 5 | 8 | 8 | 5 | 0 | 3 | 2.85E6 | 7.51E6 |  |  | 4.39E6 | 9.62E6 | 6.43E6 | 3.28E6 |  | 2.07E6 | 2 | 2 | 1 | 2 | 0 | 0 | 1 | 2 | 2 | 1 | 0 | 1 | Y | 39682 | Calcium uniporter protein, mitochondrial OS=Mus musculus OX=10090 GN=Mcu PE=1 SV=2 |
| 1518 | 2553 | sp\|Q8K2I3\|FMO2\_MOUSE | 104.27 | 6 | 4 | 5 | 4 | 0 | 4 | 4 | 0 | 0 | 0 | 1 | 3.89E5 | 3.65E6 | 0E0 |  | 6.43E5 | 3.58E5 |  |  |  | 1.16E6 | 3 | 3 | 1 | 2 | 1 | 0 | 1 | 1 | 0 | 0 | 0 | 1 | N | 60975 | Dimethylaniline monooxygenase [N-oxide-forming] 2 OS=Mus musculus OX=10090 GN=Fmo2 PE=1 SV=3 |
| 1745 | 2091 | sp\|O08692\|NGP\_MOUSE | 104.24 | 17 | 0 | 0 | 0 | 0 | 0 | 0 | 0 | 0 | 13 | 10 |  |  |  |  |  |  |  |  | 2.44E6 | 5.01E6 | 3 | 3 | 0 | 0 | 0 | 0 | 0 | 0 | 0 | 0 | 2 | 2 | Y | 19332 | Neutrophilic granule protein OS=Mus musculus OX=10090 GN=Ngp PE=1 SV=1 |
| 1301 | 1333 | sp\|Q8QZR5\|ALAT1\_MOUSE | 104.22 | 11 | 0 | 3 | 0 | 2 | 4 | 2 | 2 | 0 | 1 | 0 |  | 5.9E6 |  | 2.07E5 | 5.29E5 | 8.61E5 | 1.93E5 |  | 1E6 |  | 6 | 5 | 0 | 2 | 0 | 1 | 2 | 1 | 1 | 0 | 1 | 0 | N | 55143 | Alanine aminotransferase 1 OS=Mus musculus OX=10090 GN=Gpt PE=1 SV=3 |
| 1311 | 2684 | sp\|Q9JKB3\|YBOX3\_MOUSE | 104.13 | 10 | 0 | 0 | 3 | 0 | 1 | 0 | 3 | 0 | 7 | 3 |  |  |  |  |  |  |  |  | 1.23E6 |  | 3 | 1 | 0 | 0 | 1 | 0 | 1 | 0 | 1 | 0 | 2 | 2 | N | 38814 | Y-box-binding protein 3 OS=Mus musculus OX=10090 GN=Ybx3 PE=1 SV=2 |
| 1516 | 2183 | Q3TJI8\|Q3TJI8\_MOUSE | 104.04 | 12 | 5 | 0 | 0 | 0 | 0 | 0 | 0 | 0 | 9 | 12 | 3.15E6 |  |  |  |  |  |  |  | 9.67E6 | 7.35E6 | 3 | 3 | 1 | 0 | 0 | 0 | 0 | 0 | 0 | 0 | 3 | 3 | N | 28690 | 11-beta-hydroxysteroid dehydrogenase 1 OS=Mus musculus OX=10090 GN=Hsd11b1 PE=1 SV=1 |
| 1516 | 2184 | sp\|P50172\|DHI1\_MOUSE | 104.04 | 11 | 4 | 0 | 0 | 0 | 0 | 0 | 0 | 0 | 8 | 11 | 3.15E6 |  |  |  |  |  |  |  | 9.67E6 | 7.35E6 | 3 | 3 | 1 | 0 | 0 | 0 | 0 | 0 | 0 | 0 | 3 | 3 | N | 32364 | 11-beta-hydroxysteroid dehydrogenase 1 OS=Mus musculus OX=10090 GN=Hsd11b1 PE=1 SV=3 |
| 1109 | 1665 | E9Q1V9\|E9Q1V9\_MOUSE | 103.92 | 10 | 5 | 5 | 0 | 0 | 6 | 4 | 5 | 2 | 6 | 0 |  |  |  |  | 1.64E6 |  | 1.53E6 |  |  |  | 4 | 1 | 2 | 2 | 0 | 0 | 2 | 1 | 2 | 1 | 2 | 0 | N | 55293 | Calcium/calmodulin-dependent protein kinase type II subunit delta OS=Mus musculus OX=10090 GN=Camk2d PE=1 SV=2 |
| 1109 | 1666 | sp\|Q6PHZ2\|KCC2D\_MOUSE | 103.92 | 9 | 5 | 5 | 0 | 0 | 6 | 4 | 4 | 2 | 6 | 0 |  |  |  |  | 1.64E6 |  | 1.53E6 |  |  |  | 4 | 1 | 2 | 2 | 0 | 0 | 2 | 1 | 2 | 1 | 2 | 0 | N | 56369 | Calcium/calmodulin-dependent protein kinase type II subunit delta OS=Mus musculus OX=10090 GN=Camk2d PE=1 SV=1 |
| 1109 | 1669 | E9Q1W0\|E9Q1W0\_MOUSE | 103.92 | 9 | 5 | 5 | 0 | 0 | 6 | 4 | 4 | 2 | 6 | 0 |  |  |  |  | 1.64E6 |  | 1.53E6 |  |  |  | 4 | 1 | 2 | 2 | 0 | 0 | 2 | 1 | 2 | 1 | 2 | 0 | N | 57749 | calcium/calmodulin-dependent protein kinase OS=Mus musculus OX=10090 GN=Camk2d PE=1 SV=2 |
| 1109 | 1671 | A0A0G2JGS4\|A0A0G2JGS4\_MOUSE | 103.92 | 9 | 5 | 5 | 0 | 0 | 5 | 4 | 4 | 2 | 6 | 0 |  |  |  |  | 1.64E6 |  | 1.53E6 |  |  |  | 4 | 1 | 2 | 2 | 0 | 0 | 2 | 1 | 2 | 1 | 2 | 0 | N | 60004 | Calcium/calmodulin-dependent protein kinase type II subunit delta OS=Mus musculus OX=10090 GN=Camk2d PE=1 SV=1 |
| 1109 | 1672 | E9Q1T1\|E9Q1T1\_MOUSE | 103.92 | 9 | 5 | 5 | 0 | 0 | 5 | 4 | 4 | 2 | 6 | 0 |  |  |  |  | 1.64E6 |  | 1.53E6 |  |  |  | 4 | 1 | 2 | 2 | 0 | 0 | 2 | 1 | 2 | 1 | 2 | 0 | N | 60005 | calcium/calmodulin-dependent protein kinase OS=Mus musculus OX=10090 GN=Camk2d PE=1 SV=1 |
| 1023 | 2171 | Q8K154\|Q8K154\_MOUSE | 103.56 | 6 | 2 | 2 | 2 | 4 | 0 | 4 | 4 | 0 | 2 | 0 | 2.84E6 | 9.01E6 | 5.54E6 | 4.71E6 |  | 1.14E7 | 3.55E6 |  | 8.52E5 |  | 3 | 3 | 1 | 2 | 1 | 2 | 0 | 2 | 2 | 0 | 1 | 0 | N | 60877 | UDP-glucuronosyltransferase OS=Mus musculus OX=10090 GN=Ugt2b34 PE=1 SV=1 |
| 2549 | 5170 | A0A140LJL0\|A0A140LJL0\_MOUSE | 103.50 | 19 | 19 | 0 | 0 | 0 | 0 | 0 | 0 | 0 | 0 | 19 | 4.61E6 |  |  |  |  |  |  |  |  | 2.5E6 | 1 | 1 | 1 | 0 | 0 | 0 | 0 | 0 | 0 | 0 | 0 | 1 | Y | 13176 | CD81 antigen (Fragment) OS=Mus musculus OX=10090 GN=Cd81 PE=1 SV=1 |
| 2549 | 5174 | sp\|P35762\|CD81\_MOUSE | 103.50 | 10 | 10 | 0 | 0 | 0 | 0 | 0 | 0 | 0 | 0 | 10 | 4.61E6 |  |  |  |  |  |  |  |  | 2.5E6 | 1 | 1 | 1 | 0 | 0 | 0 | 0 | 0 | 0 | 0 | 0 | 1 | Y | 25815 | CD81 antigen OS=Mus musculus OX=10090 GN=Cd81 PE=1 SV=2 |
| 904 | 1404 | sp\|Q68FL4\|SAHH3\_MOUSE | 103.45 | 7 | 0 | 1 | 1 | 4 | 2 | 6 | 5 | 2 | 0 | 1 |  | 3.84E6 |  | 1.67E6 |  | 2.58E6 | 2.49E6 |  |  | 2.6E6 | 5 | 1 | 0 | 1 | 1 | 3 | 1 | 4 | 4 | 1 | 0 | 1 | N | 66899 | Putative adenosylhomocysteinase 3 OS=Mus musculus OX=10090 GN=Ahcyl2 PE=1 SV=1 |
| 904 | 1420 | H3BKT5\|H3BKT5\_MOUSE | 103.45 | 11 | 0 | 2 | 2 | 7 | 3 | 10 | 8 | 3 | 0 | 2 |  | 3.84E6 |  | 1.67E6 |  | 2.58E6 | 2.49E6 |  |  | 2.6E6 | 5 | 1 | 0 | 1 | 1 | 3 | 1 | 4 | 4 | 1 | 0 | 1 | N | 45055 | S-adenosylhomocysteine hydrolase-like 2 OS=Mus musculus OX=10090 GN=Ahcyl2 PE=1 SV=1 |
| 904 | 1426 | F8WI65\|F8WI65\_MOUSE | 103.45 | 9 | 0 | 2 | 2 | 5 | 2 | 8 | 6 | 2 | 0 | 2 |  | 3.84E6 |  | 1.67E6 |  | 2.58E6 | 2.49E6 |  |  | 2.6E6 | 5 | 1 | 0 | 1 | 1 | 3 | 1 | 4 | 4 | 1 | 0 | 1 | N | 56944 | Adenosylhomocysteinase 3 isoform 4 OS=Mus musculus OX=10090 GN=Ahcyl2 PE=1 SV=1 |
| 904 | 1429 | F8WGT1\|F8WGT1\_MOUSE | 103.45 | 7 | 0 | 1 | 1 | 4 | 2 | 6 | 5 | 2 | 0 | 1 |  | 3.84E6 |  | 1.67E6 |  | 2.58E6 | 2.49E6 |  |  | 2.6E6 | 5 | 1 | 0 | 1 | 1 | 3 | 1 | 4 | 4 | 1 | 0 | 1 | N | 66771 | S-adenosylhomocysteine hydrolase-like 2 OS=Mus musculus OX=10090 GN=Ahcyl2 PE=1 SV=1 |
| 1293 | 1887 | sp\|Q921M3\|SF3B3\_MOUSE | 103.38 | 4 | 1 | 1 | 1 | 1 | 1 | 0 | 1 | 1 | 1 | 0 | 1.32E6 | 5.08E5 | 2.64E5 | 4.06E5 | 1.48E6 |  | 6.44E5 | 5.51E5 | 2.21E6 |  | 4 | 4 | 1 | 1 | 1 | 1 | 1 | 0 | 1 | 1 | 1 | 0 | N | 135550 | Splicing factor 3B subunit 3 OS=Mus musculus OX=10090 GN=Sf3b3 PE=1 SV=1 |
| 934 | 1456 | sp\|P54728\|RD23B\_MOUSE | 103.32 | 11 | 2 | 6 | 5 | 2 | 6 | 2 | 2 | 3 | 9 | 5 | 1.99E6 | 1.8E6 | 1.23E7 | 5.55E5 | 4.81E5 | 4.34E5 | 1.05E6 |  | 1.34E7 | 7.02E6 | 5 | 5 | 1 | 2 | 2 | 1 | 2 | 1 | 1 | 1 | 4 | 2 | Y | 43513 | UV excision repair protein RAD23 homolog B OS=Mus musculus OX=10090 GN=Rad23b PE=1 SV=2 |
| 1686 | 2517 | G3UZ26\|G3UZ26\_MOUSE | 103.10 | 6 | 0 | 0 | 3 | 0 | 0 | 0 | 3 | 0 | 6 | 0 |  |  |  |  |  |  | 3.37E4 |  | 2.28E6 |  | 2 | 1 | 0 | 0 | 1 | 0 | 0 | 0 | 1 | 0 | 2 | 0 | Y | 48453 | Serine hydroxymethyltransferase (Fragment) OS=Mus musculus OX=10090 GN=Shmt1 PE=1 SV=1 |
| 1686 | 2518 | sp\|P50431\|GLYC\_MOUSE | 103.10 | 5 | 0 | 0 | 3 | 0 | 0 | 0 | 3 | 0 | 5 | 0 |  |  |  |  |  |  | 3.37E4 |  | 2.28E6 |  | 2 | 1 | 0 | 0 | 1 | 0 | 0 | 0 | 1 | 0 | 2 | 0 | Y | 52601 | Serine hydroxymethyltransferase, cytosolic OS=Mus musculus OX=10090 GN=Shmt1 PE=1 SV=3 |
| 1686 | 2599 | G3UYY1\|G3UYY1\_MOUSE | 103.10 | 6 | 0 | 0 | 3 | 0 | 0 | 0 | 3 | 0 | 6 | 0 |  |  |  |  |  |  | 3.37E4 |  | 2.28E6 |  | 2 | 1 | 0 | 0 | 1 | 0 | 0 | 0 | 1 | 0 | 2 | 0 | Y | 48532 | Serine hydroxymethyltransferase (Fragment) OS=Mus musculus OX=10090 GN=Shmt1 PE=1 SV=1 |
| 960 | 1660 | sp\|Q9D0F9\|PGM1\_MOUSE | 103.07 | 7 | 2 | 4 | 4 | 4 | 2 | 4 | 0 | 4 | 5 | 2 |  | 3.46E6 | 2.64E6 | 2.19E6 |  | 3.66E6 |  | 1.84E6 | 1.34E7 |  | 4 | 3 | 1 | 2 | 2 | 2 | 1 | 2 | 0 | 2 | 3 | 1 | N | 61418 | Phosphoglucomutase-1 OS=Mus musculus OX=10090 GN=Pgm1 PE=1 SV=4 |
| 1139 | 1687 | sp\|O88685\|PRS6A\_MOUSE | 102.95 | 9 | 5 | 2 | 2 | 2 | 2 | 2 | 2 | 0 | 6 | 2 | 3.84E6 | 3.85E6 | 4.54E6 | 1.42E6 | 4.08E6 | 3.98E6 | 2.6E6 |  | 5.84E6 | 5.36E6 | 3 | 3 | 2 | 1 | 1 | 1 | 1 | 1 | 1 | 0 | 2 | 1 | Y | 49549 | 26S proteasome regulatory subunit 6A OS=Mus musculus OX=10090 GN=Psmc3 PE=1 SV=2 |
| 1139 | 1720 | B7ZCF1\|B7ZCF1\_MOUSE | 102.95 | 8 | 5 | 2 | 2 | 2 | 2 | 2 | 2 | 0 | 6 | 2 | 3.84E6 | 3.85E6 | 4.54E6 | 1.42E6 | 4.08E6 | 3.98E6 | 2.6E6 |  | 5.84E6 | 5.36E6 | 3 | 3 | 2 | 1 | 1 | 1 | 1 | 1 | 1 | 0 | 2 | 1 | Y | 50396 | Proteasome (prosome, macropain) 26S subunit, ATPase 3 OS=Mus musculus OX=10090 GN=Psmc3 PE=1 SV=1 |
| 1139 | 1721 | A2AGN7\|A2AGN7\_MOUSE | 102.95 | 10 | 6 | 2 | 2 | 2 | 2 | 2 | 2 | 0 | 6 | 2 | 3.84E6 | 3.85E6 | 4.54E6 | 1.42E6 | 4.08E6 | 3.98E6 | 2.6E6 |  | 5.84E6 | 5.36E6 | 3 | 3 | 2 | 1 | 1 | 1 | 1 | 1 | 1 | 0 | 2 | 1 | Y | 44669 | Proteasome (prosome, macropain) 26S subunit, ATPase 3 OS=Mus musculus OX=10090 GN=Psmc3 PE=1 SV=1 |
| 912 | 3798 | sp\|Q78IK2\|ATPMK\_MOUSE | 102.83 | 43 | 43 | 43 | 43 | 43 | 43 | 43 | 43 | 43 | 43 | 43 | 1.1E7 | 1.63E7 | 1.19E7 | 6.69E6 | 1.27E7 | 1.59E7 | 5.76E6 | 3.92E6 | 1.05E7 | 9.81E6 | 2 | 2 | 2 | 2 | 2 | 2 | 2 | 2 | 3 | 2 | 2 | 2 | N | 6382 | ATP synthase membrane subunit K, mitochondrial OS=Mus musculus OX=10090 GN=Atp5mk PE=1 SV=1 |
| 1144 | 2274 | sp\|Q9Z2I0\|LETM1\_MOUSE | 102.51 | 3 | 1 | 3 | 3 | 2 | 2 | 2 | 2 | 2 | 0 | 3 | 1.42E7 | 1.05E7 | 6.82E6 | 1.2E6 | 5.02E5 | 2.54E5 | 4.38E5 | 9.12E5 |  | 1.77E7 | 2 | 2 | 1 | 2 | 2 | 1 | 1 | 1 | 1 | 1 | 0 | 2 | N | 82989 | Mitochondrial proton/calcium exchanger protein OS=Mus musculus OX=10090 GN=Letm1 PE=1 SV=1 |
| 1098 | 2283 | Q3U1U4\|Q3U1U4\_MOUSE | 102.27 | 3 | 1 | 0 | 1 | 0 | 0 | 0 | 0 | 0 | 2 | 2 | 0E0 |  |  |  |  |  |  |  | 3.95E6 | 1.64E6 | 4 | 2 | 1 | 0 | 1 | 1 | 1 | 0 | 1 | 0 | 2 | 2 | Y | 135944 | Predicted gene, 49368 OS=Mus musculus OX=10090 GN=Gm49368 PE=1 SV=1 |
| 1014 | 1431 | sp\|Q80SW1\|SAHH2\_MOUSE | 102.26 | 8 | 0 | 0 | 2 | 4 | 2 | 6 | 5 | 2 | 2 | 0 |  |  |  |  |  |  |  |  | 3.37E6 |  | 5 | 1 | 0 | 0 | 1 | 2 | 1 | 3 | 3 | 1 | 1 | 0 | N | 58952 | S-adenosylhomocysteine hydrolase-like protein 1 OS=Mus musculus OX=10090 GN=Ahcyl1 PE=1 SV=1 |
| 1034 | 1536 | sp\|G5E829\|AT2B1\_MOUSE | 102.26 | 5 | 0 | 0 | 0 | 1 | 1 | 2 | 2 | 1 | 0 | 0 |  |  |  |  |  | 1.1E6 | 1.32E6 |  |  |  | 7 | 2 | 0 | 0 | 0 | 1 | 2 | 2 | 3 | 1 | 0 | 0 | N | 134747 | Plasma membrane calcium-transporting ATPase 1 OS=Mus musculus OX=10090 GN=Atp2b1 PE=1 SV=1 |
| 1143 | 1794 | sp\|Q09200\|B4GN1\_MOUSE | 102.19 | 8 | 2 | 4 | 2 | 4 | 4 | 2 | 4 | 2 | 0 | 2 | 6.09E5 | 3.56E6 | 6.93E5 | 8.52E5 | 1.51E6 | 1.44E6 | 5E5 | 3.02E5 |  | 0E0 | 4 | 4 | 1 | 2 | 1 | 2 | 2 | 1 | 2 | 1 | 0 | 1 | N | 59212 | Beta-1,4 N-acetylgalactosaminyltransferase 1 OS=Mus musculus OX=10090 GN=B4galnt1 PE=1 SV=1 |
| 993 | 1329 | sp\|Q63844\|MK03\_MOUSE | 102.15 | 16 | 4 | 8 | 8 | 4 | 9 | 7 | 4 | 0 | 2 | 4 | 4.31E6 | 5.04E6 | 7.39E5 | 5.44E5 | 5.26E6 | 3.9E6 | 1.21E6 |  | 1.04E6 | 3.03E6 | 5 | 4 | 1 | 2 | 2 | 1 | 3 | 2 | 1 | 0 | 1 | 1 | Y | 43067 | Mitogen-activated protein kinase 3 OS=Mus musculus OX=10090 GN=Mapk3 PE=1 SV=5 |
| 1507 | 2238 | sp\|Q9DBG5\|PLIN3\_MOUSE | 102.12 | 9 | 2 | 2 | 2 | 0 | 0 | 3 | 0 | 0 | 6 | 2 | 4.34E6 | 6.82E6 | 2.32E6 |  |  | 8.5E5 |  |  | 5.42E6 | 4.4E6 | 3 | 3 | 1 | 1 | 1 | 0 | 0 | 1 | 0 | 0 | 2 | 1 | Y | 47262 | Perilipin-3 OS=Mus musculus OX=10090 GN=Plin3 PE=1 SV=1 |
| 1530 | 3347 | sp\|Q91WT8\|RBM47\_MOUSE | 102.07 | 4 | 0 | 0 | 3 | 0 | 3 | 3 | 3 | 3 | 1 | 0 |  |  | 1.37E6 |  | 7.4E5 | 1.79E6 | 1.18E6 | 8.66E5 |  |  | 2 | 1 | 0 | 0 | 1 | 0 | 1 | 1 | 1 | 1 | 1 | 0 | N | 64062 | RNA-binding protein 47 OS=Mus musculus OX=10090 GN=Rbm47 PE=1 SV=1 |
| 1530 | 4212 | D3Z113\|D3Z113\_MOUSE | 102.07 | 16 | 0 | 0 | 13 | 0 | 13 | 13 | 13 | 13 | 3 | 0 |  |  | 1.37E6 |  | 7.4E5 | 1.79E6 | 1.18E6 | 8.66E5 |  |  | 2 | 1 | 0 | 0 | 1 | 0 | 1 | 1 | 1 | 1 | 1 | 0 | N | 17455 | RNA binding motif protein 47 (Fragment) OS=Mus musculus OX=10090 GN=Rbm47 PE=1 SV=1 |
| 1530 | 4398 | D3YXZ5\|D3YXZ5\_MOUSE | 102.07 | 5 | 0 | 0 | 4 | 0 | 4 | 4 | 4 | 4 | 1 | 0 |  |  | 1.37E6 |  | 7.4E5 | 1.79E6 | 1.18E6 | 8.66E5 |  |  | 2 | 1 | 0 | 0 | 1 | 0 | 1 | 1 | 1 | 1 | 1 | 0 | N | 56869 | RNA-binding protein 47 OS=Mus musculus OX=10090 GN=Rbm47 PE=1 SV=1 |
| 1074 | 1710 | sp\|Q8BGQ7\|SYAC\_MOUSE | 102.05 | 5 | 3 | 1 | 0 | 0 | 2 | 0 | 1 | 2 | 1 | 2 | 2.22E6 | 6.61E5 |  |  | 1.05E6 |  | 4.86E6 | 5.45E6 | 7.79E5 | 8.79E6 | 4 | 4 | 2 | 1 | 0 | 0 | 2 | 0 | 1 | 2 | 1 | 2 | N | 106908 | Alanine--tRNA ligase, cytoplasmic OS=Mus musculus OX=10090 GN=Aars1 PE=1 SV=1 |
| 1009 | 1304 | sp\|Q80UG5\|SEPT9\_MOUSE | 102.03 | 9 | 2 | 3 | 0 | 2 | 2 | 2 | 6 | 2 | 3 | 2 | 2.61E6 | 2.06E6 |  | 7.15E5 | 2.59E6 | 0E0 | 4.56E6 | 6.63E5 | 7.51E6 | 5.94E5 | 6 | 5 | 1 | 2 | 0 | 2 | 1 | 1 | 4 | 1 | 2 | 1 | N | 65575 | Septin-9 OS=Mus musculus OX=10090 GN=Septin9 PE=1 SV=1 |
| 1009 | 1308 | A2A6U3\|A2A6U3\_MOUSE | 102.03 | 9 | 2 | 3 | 0 | 2 | 2 | 2 | 6 | 2 | 3 | 2 | 2.61E6 | 2.06E6 |  | 7.15E5 | 2.59E6 | 0E0 | 4.56E6 | 6.63E5 | 7.51E6 | 5.94E5 | 6 | 5 | 1 | 2 | 0 | 2 | 1 | 1 | 4 | 1 | 2 | 1 | N | 63773 | Septin 9 OS=Mus musculus OX=10090 GN=Septin9 PE=1 SV=1 |
| 1725 | 2922 | sp\|Q9JM14\|NT5C\_MOUSE | 101.80 | 17 | 0 | 0 | 0 | 0 | 0 | 0 | 17 | 0 | 17 | 10 |  |  |  |  |  |  | 1.49E6 |  | 5.43E6 | 2.85E5 | 2 | 2 | 0 | 0 | 0 | 0 | 0 | 0 | 2 | 0 | 2 | 1 | Y | 23076 | 5'(3')-deoxyribonucleotidase, cytosolic type OS=Mus musculus OX=10090 GN=Nt5c PE=1 SV=1 |
| 1725 | 3792 | A2A9X5\|A2A9X5\_MOUSE | 101.80 | 18 | 0 | 0 | 0 | 0 | 0 | 0 | 18 | 0 | 18 | 11 |  |  |  |  |  |  | 1.49E6 |  | 5.43E6 | 2.85E5 | 2 | 2 | 0 | 0 | 0 | 0 | 0 | 0 | 2 | 0 | 2 | 1 | Y | 21939 | 5',3'-nucleotidase, cytosolic OS=Mus musculus OX=10090 GN=Nt5c PE=1 SV=1 |
| 834 | 2445 | sp\|Q6ZWY3\|RS27L\_MOUSE | 101.56 | 38 | 38 | 10 | 23 | 10 | 38 | 23 | 10 | 10 | 29 | 23 | 9.64E6 |  | 8.08E6 |  | 3.41E5 | 4.65E6 |  |  | 2.05E7 | 9.07E6 | 3 | 1 | 4 | 1 | 3 | 1 | 3 | 3 | 1 | 1 | 3 | 2 | Y | 9477 | Small ribosomal subunit protein eS27-like OS=Mus musculus OX=10090 GN=Rps27l PE=1 SV=3 |
| 1151 | 2039 | E9Q715\|E9Q715\_MOUSE | 101.55 | 13 | 6 | 0 | 3 | 7 | 3 | 6 | 6 | 11 | 6 | 0 | 4.08E5 |  | 5.25E6 | 4.58E6 | 6.34E6 | 1.06E7 | 9.09E5 | 5.58E6 | 1.62E6 |  | 4 | 3 | 1 | 0 | 1 | 3 | 1 | 2 | 1 | 3 | 1 | 0 | N | 32647 | RNA-binding protein Luc7-like OS=Mus musculus OX=10090 GN=Luc7l2 PE=1 SV=1 |
| 1151 | 2056 | Q05CX5\|Q05CX5\_MOUSE | 101.55 | 10 | 4 | 0 | 2 | 6 | 2 | 4 | 4 | 9 | 4 | 0 | 4.08E5 |  | 5.25E6 | 4.58E6 | 6.34E6 | 1.06E7 | 9.09E5 | 5.58E6 | 1.62E6 |  | 4 | 3 | 1 | 0 | 1 | 3 | 1 | 2 | 1 | 3 | 1 | 0 | N | 40618 | RNA-binding protein Luc7-like OS=Mus musculus OX=10090 GN=Luc7l2 PE=1 SV=1 |
| 1151 | 2072 | sp\|Q7TNC4\|LC7L2\_MOUSE | 101.55 | 9 | 4 | 0 | 2 | 5 | 2 | 4 | 4 | 8 | 4 | 0 | 4.08E5 |  | 5.25E6 | 4.58E6 | 6.34E6 | 1.06E7 | 9.09E5 | 5.58E6 | 1.62E6 |  | 4 | 3 | 1 | 0 | 1 | 3 | 1 | 2 | 1 | 3 | 1 | 0 | N | 46583 | Putative RNA-binding protein Luc7-like 2 OS=Mus musculus OX=10090 GN=Luc7l2 PE=1 SV=1 |
| 1605 | 2506 | sp\|Q922H2\|PDK3\_MOUSE | 101.48 | 6 | 0 | 4 | 6 | 0 | 0 | 4 | 0 | 0 | 0 | 0 |  | 8.84E5 | 2.62E6 |  |  | 4.06E5 |  |  |  |  | 2 | 2 | 0 | 1 | 2 | 0 | 0 | 1 | 0 | 0 | 0 | 0 | N | 47923 | [Pyruvate dehydrogenase (acetyl-transferring)] kinase isozyme 3, mitochondrial OS=Mus musculus OX=10090 GN=Pdk3 PE=1 SV=1 |
| 815 | 2510 | sp\|P47964\|RL36\_MOUSE | 101.42 | 29 | 20 | 10 | 18 | 10 | 20 | 10 | 10 | 10 | 20 | 20 | 2.31E7 | 2.4E7 | 3.08E7 | 1.27E7 | 2.36E7 | 1.81E7 | 1.68E7 | 1.55E7 | 3.85E7 | 2.45E7 | 3 | 3 | 3 | 1 | 3 | 1 | 3 | 1 | 1 | 1 | 4 | 3 | Y | 12254 | Large ribosomal subunit protein eL36 OS=Mus musculus OX=10090 GN=Rpl36 PE=1 SV=3 |
| 873 | 1617 | G3UZ01\|G3UZ01\_MOUSE | 101.05 | 9 | 2 | 1 | 0 | 6 | 4 | 3 | 4 | 4 | 2 | 2 |  |  |  | 1.83E6 | 3.92E5 | 9.98E5 | 5.79E5 |  |  |  | 5 | 2 | 1 | 1 | 0 | 3 | 2 | 2 | 2 | 2 | 1 | 1 | N | 49633 | Polypyrimidine tract binding protein 3 OS=Mus musculus OX=10090 GN=Ptbp3 PE=1 SV=1 |
| 873 | 1635 | sp\|Q8BHD7\|PTBP3\_MOUSE | 101.05 | 8 | 1 | 1 | 0 | 5 | 4 | 3 | 4 | 3 | 1 | 1 |  |  |  | 1.83E6 | 3.92E5 | 9.98E5 | 5.79E5 |  |  |  | 5 | 2 | 1 | 1 | 0 | 3 | 2 | 2 | 2 | 2 | 1 | 1 | N | 56701 | Polypyrimidine tract-binding protein 3 OS=Mus musculus OX=10090 GN=Ptbp3 PE=1 SV=1 |
| 873 | 1639 | G8JL74\|G8JL74\_MOUSE | 101.05 | 7 | 1 | 1 | 0 | 5 | 3 | 3 | 3 | 3 | 1 | 1 |  |  |  | 1.83E6 | 3.92E5 | 9.98E5 | 5.79E5 |  |  |  | 5 | 2 | 1 | 1 | 0 | 3 | 2 | 2 | 2 | 2 | 1 | 1 | N | 59465 | Polypyrimidine tract binding protein 3 OS=Mus musculus OX=10090 GN=Ptbp3 PE=1 SV=1 |
| 873 | 1641 | G3UXA6\|G3UXA6\_MOUSE | 101.05 | 7 | 1 | 1 | 0 | 5 | 3 | 3 | 3 | 3 | 1 | 1 |  |  |  | 1.83E6 | 3.92E5 | 9.98E5 | 5.79E5 |  |  |  | 5 | 2 | 1 | 1 | 0 | 3 | 2 | 2 | 2 | 2 | 1 | 1 | N | 59785 | Polypyrimidine tract binding protein 3 OS=Mus musculus OX=10090 GN=Ptbp3 PE=1 SV=1 |
| 1175 | 3171 | E9PWY9\|E9PWY9\_MOUSE | 100.83 | 7 | 3 | 3 | 4 | 7 | 3 | 7 | 3 | 4 | 0 | 3 | 1.07E6 | 1.25E6 | 1.92E6 | 7.28E5 | 8.25E5 | 2.46E6 | 1.78E6 | 2.08E6 |  | 9.54E5 | 2 | 2 | 1 | 1 | 1 | 2 | 1 | 2 | 2 | 1 | 0 | 1 | Y | 57471 | Phenylalanine--tRNA ligase alpha subunit OS=Mus musculus OX=10090 GN=Farsa PE=1 SV=1 |
| 1175 | 3172 | sp\|Q8C0C7\|SYFA\_MOUSE | 100.83 | 6 | 3 | 3 | 4 | 6 | 3 | 6 | 3 | 4 | 0 | 3 | 1.07E6 | 1.25E6 | 1.92E6 | 7.28E5 | 8.25E5 | 2.46E6 | 1.78E6 | 2.08E6 |  | 9.54E5 | 2 | 2 | 1 | 1 | 1 | 2 | 1 | 2 | 2 | 1 | 0 | 1 | Y | 57599 | Phenylalanine--tRNA ligase alpha subunit OS=Mus musculus OX=10090 GN=Farsa PE=1 SV=1 |
| 693 | 1742 | sp\|Q9ERS2\|NDUAD\_MOUSE | 100.79 | 30 | 23 | 30 | 23 | 13 | 21 | 30 | 15 | 23 | 22 | 15 | 9.65E6 | 1.61E7 | 1.06E7 | 3.73E6 | 9.55E6 | 1.47E7 | 5.24E6 | 5.22E6 | 1.54E7 | 6.82E6 | 4 | 4 | 3 | 4 | 3 | 2 | 3 | 5 | 2 | 3 | 3 | 2 | Y | 16860 | NADH dehydrogenase [ubiquinone] 1 alpha subcomplex subunit 13 OS=Mus musculus OX=10090 GN=Ndufa13 PE=1 SV=3 |
| 796 | 1774 | sp\|Q9R233\|TPSN\_MOUSE | 100.54 | 7 | 5 | 3 | 3 | 5 | 7 | 5 | 5 | 7 | 3 | 2 | 4.46E6 | 7.96E5 | 1.65E6 | 8.77E5 | 4.59E6 | 1.08E6 | 5E6 | 2.27E6 | 6.03E6 | 1.98E6 | 3 | 3 | 3 | 1 | 2 | 2 | 3 | 3 | 3 | 3 | 2 | 1 | Y | 49736 | Tapasin OS=Mus musculus OX=10090 GN=Tapbp PE=1 SV=2 |
| 796 | 1775 | Q3TCU5\|Q3TCU5\_MOUSE | 100.54 | 7 | 5 | 3 | 3 | 5 | 7 | 5 | 5 | 7 | 3 | 2 | 4.46E6 | 7.96E5 | 1.65E6 | 8.77E5 | 4.59E6 | 1.08E6 | 5E6 | 2.27E6 | 6.03E6 | 1.98E6 | 3 | 3 | 3 | 1 | 2 | 2 | 3 | 3 | 3 | 3 | 2 | 1 | Y | 49836 | Tapasin OS=Mus musculus OX=10090 GN=Tapbp PE=1 SV=1 |
| 1005 | 2336 | sp\|E9PVA8\|GCN1\_MOUSE | 100.09 | 1 | 1 | 1 | 0 | 0 | 0 | 0 | 1 | 0 | 1 | 1 | 4.15E5 | 7.06E5 |  |  | 3.07E5 |  | 1.27E6 |  | 5.86E5 | 1.19E6 | 2 | 2 | 2 | 2 | 0 | 0 | 1 | 0 | 2 | 0 | 1 | 2 | N | 293018 | Stalled ribosome sensor GCN1 OS=Mus musculus OX=10090 GN=Gcn1 PE=1 SV=1 |
| 1506 | 2068 | Q3ULG5\|Q3ULG5\_MOUSE | 99.97 | 4 | 0 | 2 | 2 | 1 | 0 | 0 | 0 | 2 | 0 | 2 |  | 2.95E6 | 1.86E6 | 2.05E5 |  |  |  | 1.71E6 |  | 1.44E6 | 3 | 3 | 0 | 1 | 1 | 1 | 0 | 0 | 0 | 1 | 0 | 1 | N | 89784 | DNA replication licensing factor MCM6 OS=Mus musculus OX=10090 GN=Mcm6 PE=1 SV=1 |
| 1506 | 2069 | sp\|P97311\|MCM6\_MOUSE | 99.97 | 4 | 0 | 1 | 2 | 1 | 0 | 0 | 0 | 2 | 0 | 2 |  | 2.95E6 | 1.86E6 | 2.05E5 |  |  |  | 1.71E6 |  | 1.44E6 | 3 | 3 | 0 | 1 | 1 | 1 | 0 | 0 | 0 | 1 | 0 | 1 | N | 92867 | DNA replication licensing factor MCM6 OS=Mus musculus OX=10090 GN=Mcm6 PE=1 SV=1 |
| 1629 | 2816 | A0A2R8VI30\|A0A2R8VI30\_MOUSE | 99.70 | 6 | 0 | 0 | 0 | 5 | 0 | 0 | 6 | 0 | 5 | 5 |  |  |  | 3.94E5 |  |  | 7.83E5 |  | 2.42E6 | 7.38E5 | 2 | 2 | 0 | 0 | 0 | 1 | 0 | 0 | 1 | 0 | 1 | 1 | Y | 29345 | Leukocyte surface antigen CD47 OS=Mus musculus OX=10090 GN=Cd47 PE=1 SV=1 |
| 1629 | 2817 | sp\|Q61735\|CD47\_MOUSE | 99.70 | 6 | 0 | 0 | 0 | 5 | 0 | 0 | 6 | 0 | 5 | 5 |  |  |  | 3.94E5 |  |  | 7.83E5 |  | 2.42E6 | 7.38E5 | 2 | 2 | 0 | 0 | 0 | 1 | 0 | 0 | 1 | 0 | 1 | 1 | Y | 33098 | Leukocyte surface antigen CD47 OS=Mus musculus OX=10090 GN=Cd47 PE=1 SV=2 |
| 1629 | 2818 | A0A2R8VK70\|A0A2R8VK70\_MOUSE | 99.70 | 5 | 0 | 0 | 0 | 4 | 0 | 0 | 5 | 0 | 4 | 4 |  |  |  | 3.94E5 |  |  | 7.83E5 |  | 2.42E6 | 7.38E5 | 2 | 2 | 0 | 0 | 0 | 1 | 0 | 0 | 1 | 0 | 1 | 1 | Y | 35077 | Leukocyte surface antigen CD47 OS=Mus musculus OX=10090 GN=Cd47 PE=1 SV=1 |
| 1122 | 1959 | sp\|Q9R1P1\|PSB3\_MOUSE | 99.61 | 27 | 11 | 8 | 8 | 17 | 8 | 8 | 8 | 0 | 15 | 8 | 1.19E7 | 8.17E6 | 1.81E6 | 1.74E6 | 8.68E5 | 1.59E6 | 2.56E6 |  | 1.47E7 | 3.18E6 | 4 | 4 | 2 | 2 | 1 | 2 | 1 | 1 | 1 | 0 | 3 | 1 | Y | 22965 | Proteasome subunit beta type-3 OS=Mus musculus OX=10090 GN=Psmb3 PE=1 SV=1 |
| 1248 | 1796 | sp\|P46460\|NSF\_MOUSE | 99.61 | 6 | 2 | 2 | 2 | 0 | 0 | 0 | 0 | 1 | 3 | 3 | 3.79E5 | 5.99E5 | 5.41E5 |  |  |  |  | 1.79E6 | 2.6E6 | 8.8E5 | 4 | 4 | 1 | 1 | 1 | 0 | 0 | 0 | 0 | 1 | 2 | 2 | N | 82613 | Vesicle-fusing ATPase OS=Mus musculus OX=10090 GN=Nsf PE=1 SV=2 |
| 614 | 1555 | sp\|P61514\|RL37A\_MOUSE | 99.47 | 52 | 25 | 25 | 18 | 25 | 25 | 33 | 46 | 25 | 10 | 25 | 2.34E7 | 5.89E6 | 2.06E7 | 2.03E7 | 3.22E7 | 4.08E7 | 2.39E7 | 2.09E7 | 1.59E7 | 3.2E7 | 5 | 5 | 4 | 3 | 3 | 4 | 4 | 5 | 5 | 4 | 2 | 4 | Y | 10275 | Large ribosomal subunit protein eL43 OS=Mus musculus OX=10090 GN=Rpl37a PE=1 SV=2 |
| 1294 | 2123 | sp\|Q9ESX5\|DKC1\_MOUSE | 99.30 | 10 | 8 | 4 | 4 | 0 | 4 | 4 | 4 | 2 | 4 | 4 | 2.32E6 | 6.49E5 | 1.51E6 |  | 4.75E5 | 6.82E5 | 1.36E6 | 0E0 | 1.58E6 | 9E5 | 3 | 3 | 2 | 1 | 1 | 0 | 1 | 1 | 1 | 1 | 1 | 1 | N | 57402 | H/ACA ribonucleoprotein complex subunit DKC1 OS=Mus musculus OX=10090 GN=Dkc1 PE=1 SV=4 |
| 2110 | 2868 | sp\|O35098\|DPYL4\_MOUSE | 99.26 | 3 | 0 | 0 | 3 | 0 | 0 | 0 | 0 | 0 | 0 | 0 |  |  | 6.2E5 |  |  |  |  |  |  |  | 1 | 1 | 0 | 0 | 1 | 0 | 0 | 0 | 0 | 0 | 0 | 0 | Y | 61962 | Dihydropyrimidinase-related protein 4 OS=Mus musculus OX=10090 GN=Dpysl4 PE=1 SV=1 |
| 2110 | 2869 | Q3TMU8\|Q3TMU8\_MOUSE | 99.26 | 3 | 0 | 0 | 3 | 0 | 0 | 0 | 0 | 0 | 0 | 0 |  |  | 6.2E5 |  |  |  |  |  |  |  | 1 | 1 | 0 | 0 | 1 | 0 | 0 | 0 | 0 | 0 | 0 | 0 | Y | 61928 | Dihydropyrimidinase-like 4 OS=Mus musculus OX=10090 GN=Dpysl4 PE=1 SV=1 |
| 2110 | 2870 | A0A0R4J1N0\|A0A0R4J1N0\_MOUSE | 99.26 | 3 | 0 | 0 | 3 | 0 | 0 | 0 | 0 | 0 | 0 | 0 |  |  | 6.2E5 |  |  |  |  |  |  |  | 1 | 1 | 0 | 0 | 1 | 0 | 0 | 0 | 0 | 0 | 0 | 0 | Y | 64303 | Dihydropyrimidinase-like 4 OS=Mus musculus OX=10090 GN=Dpysl4 PE=1 SV=1 |
| 1459 | 3923 | D3YYP5\|D3YYP5\_MOUSE | 99.06 | 4 | 0 | 0 | 0 | 2 | 4 | 4 | 4 | 0 | 0 | 0 |  |  |  | 1.49E5 | 8.54E5 | 3.68E6 | 4.51E5 |  |  |  | 2 | 2 | 0 | 0 | 0 | 1 | 2 | 3 | 2 | 0 | 0 | 0 | N | 44278 | Gasdermin E OS=Mus musculus OX=10090 GN=Gsdme PE=1 SV=2 |
| 1459 | 3925 | E9Q5V3\|E9Q5V3\_MOUSE | 99.06 | 4 | 0 | 0 | 0 | 2 | 4 | 4 | 4 | 0 | 0 | 0 |  |  |  | 1.49E5 | 8.54E5 | 3.68E6 | 4.51E5 |  |  |  | 2 | 2 | 0 | 0 | 0 | 1 | 2 | 3 | 2 | 0 | 0 | 0 | N | 46840 | Gasdermin E OS=Mus musculus OX=10090 GN=Gsdme PE=1 SV=1 |
| 1459 | 3938 | sp\|Q9Z2D3\|GSDME\_MOUSE | 99.06 | 3 | 0 | 0 | 0 | 2 | 3 | 3 | 3 | 0 | 0 | 0 |  |  |  | 1.49E5 | 8.54E5 | 3.68E6 | 4.51E5 |  |  |  | 2 | 2 | 0 | 0 | 0 | 1 | 2 | 3 | 2 | 0 | 0 | 0 | N | 56631 | Gasdermin-E OS=Mus musculus OX=10090 GN=Gsdme PE=1 SV=1 |
| 1244 | 2735 | A2A864\|A2A864\_MOUSE | 98.85 | 1 | 0 | 1 | 1 | 1 | 1 | 1 | 1 | 1 | 0 | 0 |  | 1.88E6 | 6.22E5 | 0E0 | 5.36E5 | 1.34E6 | 6.01E5 | 7.03E5 |  |  | 2 | 2 | 0 | 2 | 1 | 1 | 1 | 2 | 1 | 2 | 0 | 0 | N | 199865 | Integrin beta OS=Mus musculus OX=10090 GN=Itgb4 PE=1 SV=1 |
| 1244 | 2736 | sp\|A2A863\|ITB4\_MOUSE | 98.85 | 1 | 0 | 1 | 1 | 1 | 1 | 1 | 1 | 1 | 0 | 0 |  | 1.88E6 | 6.22E5 | 0E0 | 5.36E5 | 1.34E6 | 6.01E5 | 7.03E5 |  |  | 2 | 2 | 0 | 2 | 1 | 1 | 1 | 2 | 1 | 2 | 0 | 0 | N | 201649 | Integrin beta-4 OS=Mus musculus OX=10090 GN=Itgb4 PE=1 SV=1 |
| 1190 | 2186 | sp\|Q91VK1\|5MP1\_MOUSE | 98.56 | 9 | 2 | 7 | 0 | 0 | 2 | 2 | 2 | 0 | 5 | 7 | 3.38E5 | 1.5E6 |  |  | 4.53E5 | 1.54E5 | 4.44E5 |  | 1.37E6 | 3.57E5 | 3 | 3 | 1 | 3 | 0 | 0 | 1 | 1 | 1 | 0 | 3 | 2 | N | 48063 | eIF5-mimic protein 1 OS=Mus musculus OX=10090 GN=Bzw2 PE=1 SV=1 |
| 1236 | 3186 | sp\|E9Q7P9\|CDHR2\_MOUSE | 98.28 | 2 | 1 | 1 | 1 | 1 | 1 | 1 | 2 | 1 | 1 | 0 | 2.61E6 | 2.8E6 | 2.53E6 | 9.08E5 | 2.6E6 | 2.93E6 | 4.29E6 | 3.61E5 | 3.25E6 |  | 2 | 2 | 1 | 1 | 1 | 1 | 1 | 1 | 2 | 1 | 1 | 0 | N | 142633 | Cadherin-related family member 2 OS=Mus musculus OX=10090 GN=Cdhr2 PE=1 SV=1 |
| 639 | 1988 | sp\|Q6ZWV7\|RL35\_MOUSE | 98.08 | 27 | 15 | 15 | 27 | 20 | 26 | 21 | 26 | 8 | 21 | 21 | 2.86E7 | 2.14E7 | 6.25E7 | 1.75E7 | 5.1E7 | 4.32E7 | 4.1E7 | 1.44E7 | 3.9E7 | 4.86E7 | 5 | 3 | 3 | 2 | 5 | 3 | 4 | 3 | 5 | 1 | 3 | 4 | N | 14553 | Large ribosomal subunit protein uL29 OS=Mus musculus OX=10090 GN=Rpl35 PE=1 SV=1 |
| 1508 | 2876 | Q7TQE2\|Q7TQE2\_MOUSE | 97.95 | 6 | 0 | 2 | 0 | 0 | 2 | 4 | 0 | 0 | 6 | 6 |  | 1.19E6 |  |  | 1.12E6 | 1.01E6 |  |  | 1.13E7 | 3.61E6 | 2 | 2 | 0 | 1 | 0 | 0 | 1 | 1 | 0 | 0 | 2 | 2 | N | 57027 | Zyxin OS=Mus musculus OX=10090 GN=Zyx PE=1 SV=1 |
| 1508 | 2877 | sp\|Q62523\|ZYX\_MOUSE | 97.95 | 5 | 0 | 2 | 0 | 0 | 2 | 3 | 0 | 0 | 5 | 5 |  | 1.19E6 |  |  | 1.12E6 | 1.01E6 |  |  | 1.13E7 | 3.61E6 | 2 | 2 | 0 | 1 | 0 | 0 | 1 | 1 | 0 | 0 | 2 | 2 | N | 60546 | Zyxin OS=Mus musculus OX=10090 GN=Zyx PE=1 SV=2 |
| 2474 | 3453 | sp\|P62774\|MTPN\_MOUSE | 97.94 | 14 | 0 | 0 | 0 | 0 | 0 | 0 | 0 | 0 | 14 | 0 |  |  |  |  |  |  |  |  | 4.7E6 |  | 1 | 1 | 0 | 0 | 0 | 0 | 0 | 0 | 0 | 0 | 1 | 0 | N | 12861 | Myotrophin OS=Mus musculus OX=10090 GN=Mtpn PE=1 SV=2 |
| 1696 | 2708 | sp\|Q3U487\|HECD3\_MOUSE | 97.82 | 3 | 0 | 2 | 0 | 0 | 0 | 0 | 2 | 0 | 2 | 0 |  | 1.9E5 |  |  |  |  | 8.93E5 |  | 0E0 |  | 2 | 2 | 0 | 1 | 0 | 0 | 0 | 0 | 1 | 0 | 1 | 0 | N | 97347 | E3 ubiquitin-protein ligase HECTD3 OS=Mus musculus OX=10090 GN=Hectd3 PE=1 SV=2 |
| 1080 | 3815 | G3UYJ7\|G3UYJ7\_MOUSE | 97.62 | 10 | 4 | 4 | 10 | 4 | 4 | 4 | 4 | 4 | 4 | 0 | 3.56E6 | 9.15E5 | 1.05E7 | 5.09E6 | 3.17E6 | 2.06E6 | 1.66E6 | 1.43E6 | 1.69E6 |  | 2 | 2 | 1 | 1 | 3 | 2 | 2 | 1 | 2 | 2 | 1 | 0 | N | 28642 | glutathione transferase (Fragment) OS=Mus musculus OX=10090 GN=Gm20441 PE=3 SV=1 |
| 1359 | 2236 | sp\|Q99K01\|PDXD1\_MOUSE | 97.51 | 4 | 0 | 2 | 0 | 0 | 3 | 2 | 3 | 0 | 3 | 0 |  | 2.28E6 |  |  | 4.8E6 | 1.43E6 | 3.29E6 |  | 6.83E6 |  | 3 | 3 | 0 | 1 | 0 | 0 | 2 | 1 | 2 | 0 | 2 | 0 | N | 87336 | Pyridoxal-dependent decarboxylase domain-containing protein 1 OS=Mus musculus OX=10090 GN=Pdxdc1 PE=1 SV=2 |
| 1359 | 2237 | A0A0R4J034\|A0A0R4J034\_MOUSE | 97.51 | 4 | 0 | 2 | 0 | 0 | 3 | 2 | 3 | 0 | 3 | 0 |  | 2.28E6 |  |  | 4.8E6 | 1.43E6 | 3.29E6 |  | 6.83E6 |  | 3 | 3 | 0 | 1 | 0 | 0 | 2 | 1 | 2 | 0 | 2 | 0 | N | 87480 | Pyridoxal-dependent decarboxylase domain containing 1 OS=Mus musculus OX=10090 GN=Pdxdc1 PE=1 SV=1 |
| 2482 | 4607 | A0A2R8VHH9\|A0A2R8VHH9\_MOUSE | 97.46 | 9 | 0 | 9 | 0 | 0 | 0 | 0 | 0 | 0 | 0 | 9 |  | 1.16E6 |  |  |  |  |  |  |  | 1.26E5 | 1 | 1 | 0 | 1 | 0 | 0 | 0 | 0 | 0 | 0 | 0 | 1 | N | 24662 | Alpha-galactosidase (Fragment) OS=Mus musculus OX=10090 GN=Naga PE=1 SV=1 |
| 2482 | 4608 | A0A2R8VHC0\|A0A2R8VHC0\_MOUSE | 97.46 | 8 | 0 | 8 | 0 | 0 | 0 | 0 | 0 | 0 | 0 | 8 |  | 1.16E6 |  |  |  |  |  |  |  | 1.26E5 | 1 | 1 | 0 | 1 | 0 | 0 | 0 | 0 | 0 | 0 | 0 | 1 | N | 26983 | Alpha-galactosidase (Fragment) OS=Mus musculus OX=10090 GN=Naga PE=1 SV=1 |
| 2482 | 4609 | Q3UZX5\|Q3UZX5\_MOUSE | 97.46 | 7 | 0 | 7 | 0 | 0 | 0 | 0 | 0 | 0 | 0 | 7 |  | 1.16E6 |  |  |  |  |  |  |  | 1.26E5 | 1 | 1 | 0 | 1 | 0 | 0 | 0 | 0 | 0 | 0 | 0 | 1 | N | 32090 | Alpha-galactosidase OS=Mus musculus OX=10090 GN=Naga PE=1 SV=1 |
| 2482 | 4610 | sp\|Q9QWR8\|NAGAB\_MOUSE | 97.46 | 5 | 0 | 5 | 0 | 0 | 0 | 0 | 0 | 0 | 0 | 5 |  | 1.16E6 |  |  |  |  |  |  |  | 1.26E5 | 1 | 1 | 0 | 1 | 0 | 0 | 0 | 0 | 0 | 0 | 0 | 1 | N | 47235 | Alpha-N-acetylgalactosaminidase OS=Mus musculus OX=10090 GN=Naga PE=1 SV=2 |
| 1439 | 2113 | sp\|O08529\|CAN2\_MOUSE | 97.44 | 4 | 0 | 0 | 0 | 2 | 2 | 2 | 1 | 3 | 0 | 3 |  |  |  | 7.62E5 | 8.22E5 | 2.95E6 | 1.43E5 | 4.9E5 |  | 3.58E6 | 3 | 3 | 0 | 0 | 0 | 1 | 1 | 1 | 1 | 2 | 0 | 2 | N | 79872 | Calpain-2 catalytic subunit OS=Mus musculus OX=10090 GN=Capn2 PE=1 SV=4 |
| 1203 | 2306 | A0A087WP24\|A0A087WP24\_MOUSE | 97.17 | 22 | 0 | 15 | 14 | 6 | 0 | 14 | 14 | 0 | 6 | 0 |  | 8.5E6 | 5.27E6 | 1.28E6 |  | 3.55E6 | 2.07E6 |  | 1.27E6 |  | 3 | 3 | 0 | 2 | 2 | 1 | 0 | 2 | 2 | 0 | 1 | 0 | N | 14948 | Putative protein-lysine deacylase ABHD14B (Fragment) OS=Mus musculus OX=10090 GN=Abhd14b PE=1 SV=1 |
| 1203 | 2307 | A0A087WRJ2\|A0A087WRJ2\_MOUSE | 97.17 | 21 | 0 | 15 | 13 | 6 | 0 | 13 | 13 | 0 | 6 | 0 |  | 8.5E6 | 5.27E6 | 1.28E6 |  | 3.55E6 | 2.07E6 |  | 1.27E6 |  | 3 | 3 | 0 | 2 | 2 | 1 | 0 | 2 | 2 | 0 | 1 | 0 | N | 15896 | Putative protein-lysine deacylase ABHD14B (Fragment) OS=Mus musculus OX=10090 GN=Abhd14b PE=1 SV=1 |
| 1203 | 2308 | A0A087WPF8\|A0A087WPF8\_MOUSE | 97.17 | 18 | 0 | 13 | 11 | 5 | 0 | 11 | 11 | 0 | 5 | 0 |  | 8.5E6 | 5.27E6 | 1.28E6 |  | 3.55E6 | 2.07E6 |  | 1.27E6 |  | 3 | 3 | 0 | 2 | 2 | 1 | 0 | 2 | 2 | 0 | 1 | 0 | N | 18398 | Putative protein-lysine deacylase ABHD14B (Fragment) OS=Mus musculus OX=10090 GN=Abhd14b PE=1 SV=1 |
| 1203 | 2309 | A0A087WSR2\|A0A087WSR2\_MOUSE | 97.17 | 16 | 0 | 11 | 10 | 5 | 0 | 10 | 10 | 0 | 5 | 0 |  | 8.5E6 | 5.27E6 | 1.28E6 |  | 3.55E6 | 2.07E6 |  | 1.27E6 |  | 3 | 3 | 0 | 2 | 2 | 1 | 0 | 2 | 2 | 0 | 1 | 0 | N | 20630 | Putative protein-lysine deacylase ABHD14B (Fragment) OS=Mus musculus OX=10090 GN=Abhd14b PE=1 SV=1 |
| 1203 | 2310 | E9QN99\|E9QN99\_MOUSE | 97.17 | 15 | 0 | 10 | 10 | 4 | 0 | 10 | 10 | 0 | 4 | 0 |  | 8.5E6 | 5.27E6 | 1.28E6 |  | 3.55E6 | 2.07E6 |  | 1.27E6 |  | 3 | 3 | 0 | 2 | 2 | 1 | 0 | 2 | 2 | 0 | 1 | 0 | N | 22451 | Putative protein-lysine deacylase ABHD14B OS=Mus musculus OX=10090 GN=Abhd14b PE=1 SV=1 |
| 1441 | 2940 | sp\|Q9DAR7\|DCPS\_MOUSE | 97.14 | 9 | 0 | 3 | 9 | 9 | 3 | 0 | 0 | 9 | 0 | 0 |  | 2.15E5 | 1.26E6 | 4.53E5 | 4.99E5 |  |  | 4.55E5 |  |  | 2 | 2 | 0 | 1 | 2 | 2 | 1 | 0 | 0 | 2 | 0 | 0 | N | 38988 | m7GpppX diphosphatase OS=Mus musculus OX=10090 GN=Dcps PE=1 SV=1 |
| 1488 | 2960 | sp\|P61924\|COPZ1\_MOUSE | 97.07 | 16 | 0 | 0 | 6 | 6 | 16 | 6 | 6 | 6 | 0 | 0 |  |  | 3.49E6 | 1.55E6 | 4.22E6 | 3.23E6 | 3.01E6 | 2.45E6 |  |  | 2 | 2 | 0 | 0 | 1 | 1 | 2 | 1 | 1 | 1 | 0 | 0 | N | 20198 | Coatomer subunit zeta-1 OS=Mus musculus OX=10090 GN=Copz1 PE=1 SV=1 |
| 1618 | 3608 | sp\|Q9CQS8\|SC61B\_MOUSE | 97.03 | 26 | 0 | 0 | 10 | 26 | 0 | 10 | 0 | 0 | 10 | 10 |  |  | 1.85E7 | 1.09E7 |  | 1.41E7 |  |  | 2.38E7 | 1.48E7 | 2 | 2 | 0 | 0 | 1 | 2 | 0 | 1 | 0 | 0 | 1 | 1 | N | 9958 | Protein transport protein Sec61 subunit beta OS=Mus musculus OX=10090 GN=Sec61b PE=1 SV=3 |
| 1265 | 5168 | B1AR46\|B1AR46\_MOUSE | 96.97 | 19 | 19 | 19 | 19 | 19 | 19 | 19 | 0 | 19 | 19 | 0 | 1E6 | 7.53E6 | 4.56E6 | 6.13E5 | 2.96E6 | 1.46E6 |  | 4.83E5 | 5.43E6 |  | 1 | 1 | 1 | 2 | 1 | 2 | 1 | 2 | 0 | 1 | 1 | 0 | Y | 8885 | Claudin (Fragment) OS=Mus musculus OX=10090 GN=Cldn7 PE=1 SV=1 |
| 1265 | 5175 | sp\|Q9Z261\|CLD7\_MOUSE | 96.97 | 8 | 8 | 8 | 8 | 8 | 8 | 8 | 0 | 8 | 8 | 0 | 1E6 | 7.53E6 | 4.56E6 | 6.13E5 | 2.96E6 | 1.46E6 |  | 4.83E5 | 5.43E6 |  | 1 | 1 | 1 | 2 | 1 | 2 | 1 | 2 | 0 | 1 | 1 | 0 | Y | 22359 | Claudin-7 OS=Mus musculus OX=10090 GN=Cldn7 PE=1 SV=1 |
| 1114 | 3313 | sp\|P50518\|VATE1\_MOUSE | 96.96 | 10 | 6 | 6 | 6 | 6 | 10 | 6 | 6 | 4 | 6 | 6 | 2.91E6 | 6.57E5 | 2.84E5 | 1.89E5 | 4.24E6 | 9.64E5 | 6.08E5 | 1.59E6 | 1.82E6 | 2.73E6 | 2 | 2 | 2 | 1 | 2 | 1 | 2 | 1 | 1 | 1 | 1 | 2 | N | 26157 | V-type proton ATPase subunit E 1 OS=Mus musculus OX=10090 GN=Atp6v1e1 PE=1 SV=2 |
| 1801 | 2795 | sp\|Q99JY3\|GIMA4\_MOUSE | 96.85 | 8 | 0 | 0 | 2 | 6 | 6 | 0 | 0 | 6 | 0 | 0 |  |  | 2.89E6 | 7.76E5 | 1.46E6 |  |  | 9.52E4 |  |  | 2 | 2 | 0 | 0 | 1 | 1 | 1 | 0 | 0 | 1 | 0 | 0 | N | 38044 | GTPase IMAP family member 4 OS=Mus musculus OX=10090 GN=Gimap4 PE=1 SV=2 |
| 1621 | 4010 | sp\|O35295\|PURB\_MOUSE | 96.74 | 7 | 0 | 2 | 6 | 0 | 6 | 6 | 0 | 0 | 0 | 6 |  |  | 6.52E6 |  | 1.14E6 | 1.26E6 |  |  |  | 7.15E6 | 2 | 1 | 0 | 1 | 1 | 0 | 1 | 1 | 0 | 0 | 0 | 1 | N | 33901 | Transcriptional regulator protein Pur-beta OS=Mus musculus OX=10090 GN=Purb PE=1 SV=3 |
| 1065 | 2847 | G3X9U9\|G3X9U9\_MOUSE | 96.67 | 17 | 17 | 17 | 8 | 16 | 8 | 8 | 16 | 8 | 8 | 17 | 6.03E6 | 6E6 | 3.55E6 | 1.76E6 | 4.06E6 | 6.03E6 | 2.09E6 | 1.34E6 | 5.91E6 | 3.36E6 | 3 | 3 | 2 | 2 | 1 | 2 | 1 | 1 | 2 | 1 | 1 | 2 | N | 16265 | Mitochondrial fission 1 protein OS=Mus musculus OX=10090 GN=Fis1 PE=1 SV=1 |
| 1065 | 2848 | sp\|Q9CQ92\|FIS1\_MOUSE | 96.67 | 16 | 16 | 16 | 7 | 15 | 7 | 7 | 15 | 7 | 7 | 16 | 6.03E6 | 6E6 | 3.55E6 | 1.76E6 | 4.06E6 | 6.03E6 | 2.09E6 | 1.34E6 | 5.91E6 | 3.36E6 | 3 | 3 | 2 | 2 | 1 | 2 | 1 | 1 | 2 | 1 | 1 | 2 | N | 17009 | Mitochondrial fission 1 protein OS=Mus musculus OX=10090 GN=Fis1 PE=1 SV=1 |
| 852 | 1930 | sp\|O89086\|RBM3\_MOUSE | 96.56 | 33 | 16 | 19 | 7 | 12 | 21 | 16 | 16 | 16 | 21 | 16 | 1.39E7 | 1.06E7 | 2.74E6 | 2.74E6 | 1.2E7 | 5.87E6 | 5.32E6 | 4.07E6 | 1.59E7 | 1.5E7 | 4 | 4 | 2 | 2 | 1 | 2 | 3 | 2 | 2 | 2 | 3 | 3 | Y | 16605 | RNA-binding protein 3 OS=Mus musculus OX=10090 GN=Rbm3 PE=1 SV=1 |
| 1329 | 3909 | sp\|Q8C166\|CPNE1\_MOUSE | 96.46 | 3 | 0 | 2 | 2 | 0 | 2 | 2 | 2 | 0 | 3 | 3 |  | 1.31E6 | 1.37E6 |  | 6.26E5 | 6.12E5 | 9.11E5 |  | 1.03E6 | 7.42E5 | 2 | 2 | 0 | 1 | 1 | 0 | 1 | 1 | 1 | 0 | 2 | 2 | N | 58887 | Copine-1 OS=Mus musculus OX=10090 GN=Cpne1 PE=1 SV=1 |
| 1142 | 2525 | Q8BH80\|Q8BH80\_MOUSE | 96.40 | 9 | 5 | 5 | 5 | 9 | 5 | 5 | 5 | 5 | 5 | 5 |  |  |  | 4.41E5 |  |  |  |  |  |  | 2 | 1 | 2 | 1 | 1 | 2 | 1 | 1 | 1 | 1 | 1 | 1 | Y | 26918 | Vesicle-associated membrane protein, associated protein B and C OS=Mus musculus OX=10090 GN=Vapb PE=1 SV=1 |
| 1142 | 2575 | sp\|Q9QY76\|VAPB\_MOUSE | 96.40 | 9 | 5 | 5 | 5 | 9 | 5 | 5 | 5 | 5 | 5 | 5 |  |  |  | 4.41E5 |  |  |  |  |  |  | 2 | 1 | 2 | 1 | 1 | 2 | 1 | 1 | 1 | 1 | 1 | 1 | Y | 26946 | Vesicle-associated membrane protein-associated protein B OS=Mus musculus OX=10090 GN=Vapb PE=1 SV=3 |
| 1202 | 2157 | sp\|Q9QYB1\|CLIC4\_MOUSE | 96.23 | 10 | 7 | 5 | 2 | 3 | 0 | 2 | 2 | 0 | 5 | 7 | 2.06E6 | 3.12E6 |  |  |  |  |  |  | 4.09E6 | 1.84E6 | 3 | 1 | 2 | 1 | 1 | 1 | 0 | 1 | 1 | 0 | 2 | 2 | Y | 28729 | Chloride intracellular channel protein 4 OS=Mus musculus OX=10090 GN=Clic4 PE=1 SV=3 |
| 1105 | 2717 | G3UXX3\|G3UXX3\_MOUSE | 95.98 | 11 | 11 | 11 | 11 | 0 | 0 | 11 | 7 | 0 | 11 | 11 | 6.18E6 | 6.76E6 | 7.24E6 |  |  | 7E6 | 7.74E5 |  | 1.65E7 | 5.29E6 | 2 | 2 | 2 | 2 | 2 | 0 | 0 | 2 | 1 | 0 | 2 | 2 | N | 23357 | Sepiapterin reductase OS=Mus musculus OX=10090 GN=Spr PE=1 SV=1 |
| 1105 | 2729 | sp\|Q64105\|SPRE\_MOUSE | 95.98 | 9 | 9 | 9 | 9 | 0 | 0 | 9 | 6 | 0 | 9 | 9 | 6.18E6 | 6.76E6 | 7.24E6 |  |  | 7E6 | 7.74E5 |  | 1.65E7 | 5.29E6 | 2 | 2 | 2 | 2 | 2 | 0 | 0 | 2 | 1 | 0 | 2 | 2 | N | 27883 | Sepiapterin reductase OS=Mus musculus OX=10090 GN=Spr PE=1 SV=1 |
| 1105 | 2730 | Q91XH5\|Q91XH5\_MOUSE | 95.98 | 9 | 9 | 9 | 9 | 0 | 0 | 9 | 6 | 0 | 9 | 9 | 6.18E6 | 6.76E6 | 7.24E6 |  |  | 7E6 | 7.74E5 |  | 1.65E7 | 5.29E6 | 2 | 2 | 2 | 2 | 2 | 0 | 0 | 2 | 1 | 0 | 2 | 2 | N | 27928 | Sepiapterin reductase OS=Mus musculus OX=10090 GN=Spr PE=1 SV=1 |
| 1004 | 1535 | sp\|P05063\|ALDOC\_MOUSE | 95.96 | 16 | 2 | 2 | 2 | 0 | 2 | 6 | 2 | 2 | 4 | 9 |  |  |  |  |  | 2.23E6 |  |  | 4.29E6 |  | 4 | 2 | 1 | 1 | 2 | 0 | 1 | 2 | 2 | 2 | 2 | 2 | Y | 39395 | Fructose-bisphosphate aldolase C OS=Mus musculus OX=10090 GN=Aldoc PE=1 SV=4 |
| 1322 | 3431 | sp\|Q6P1F6\|2ABA\_MOUSE | 95.89 | 3 | 0 | 3 | 3 | 3 | 3 | 3 | 3 | 0 | 3 | 3 |  | 2E6 | 4.25E3 | 2.9E5 | 1.14E6 | 6.64E5 | 2.08E5 |  | 2.14E5 | 1.84E6 | 1 | 1 | 0 | 1 | 1 | 1 | 1 | 1 | 1 | 0 | 1 | 1 | N | 51692 | Serine/threonine-protein phosphatase 2A 55 kDa regulatory subunit B alpha isoform OS=Mus musculus OX=10090 GN=Ppp2r2a PE=1 SV=1 |
| 1161 | 2369 | A0A498WGK6\|A0A498WGK6\_MOUSE | 95.72 | 5 | 1 | 0 | 0 | 4 | 2 | 4 | 5 | 3 | 0 | 3 | 7.72E5 |  |  | 1.39E6 | 9.05E5 | 1.49E6 | 2.19E6 | 2.22E6 |  | 9.88E5 | 3 | 3 | 1 | 0 | 0 | 2 | 1 | 2 | 3 | 2 | 0 | 2 | Y | 67508 | Transmembrane 9 superfamily member OS=Mus musculus OX=10090 GN=Tm9sf3 PE=1 SV=1 |
| 1161 | 2370 | sp\|Q9ET30\|TM9S3\_MOUSE | 95.72 | 5 | 1 | 0 | 0 | 4 | 2 | 4 | 5 | 3 | 0 | 3 | 7.72E5 |  |  | 1.39E6 | 9.05E5 | 1.49E6 | 2.19E6 | 2.22E6 |  | 9.88E5 | 3 | 3 | 1 | 0 | 0 | 2 | 1 | 2 | 3 | 2 | 0 | 2 | Y | 67545 | Transmembrane 9 superfamily member 3 OS=Mus musculus OX=10090 GN=Tm9sf3 PE=1 SV=1 |
| 1148 | 2051 | sp\|P16460\|ASSY\_MOUSE | 95.69 | 9 | 2 | 0 | 2 | 0 | 2 | 8 | 4 | 4 | 4 | 6 | 1.13E7 |  | 8.24E6 |  | 9.07E6 | 1.22E7 | 1.54E6 | 1.86E6 | 4.85E6 | 9.22E6 | 4 | 4 | 1 | 0 | 1 | 0 | 1 | 3 | 1 | 1 | 2 | 2 | Y | 46585 | Argininosuccinate synthase OS=Mus musculus OX=10090 GN=Ass1 PE=1 SV=1 |
| 920 | 3807 | sp\|Q9CQC9\|SAR1B\_MOUSE | 95.41 | 11 | 6 | 6 | 11 | 11 | 11 | 11 | 11 | 11 | 11 | 6 | 3.26E6 | 5.96E6 | 2.09E7 | 6.86E6 | 1.76E7 | 1.75E7 | 1.63E7 | 1.01E7 | 1.26E7 | 1.29E6 | 2 | 2 | 1 | 1 | 3 | 3 | 3 | 2 | 3 | 2 | 2 | 1 | N | 22382 | Small COPII coat GTPase SAR1B OS=Mus musculus OX=10090 GN=Sar1b PE=1 SV=1 |
| 1048 | 2212 | sp\|Q3UGC7\|EI3JA\_MOUSE | 95.27 | 11 | 8 | 8 | 3 | 0 | 8 | 3 | 3 | 0 | 4 | 11 | 2.73E6 | 2.32E6 | 2.15E6 |  | 1.84E6 | 1E6 | 1.37E6 |  | 3.51E6 | 4.76E6 | 3 | 3 | 2 | 2 | 1 | 0 | 2 | 1 | 1 | 0 | 1 | 3 | N | 29344 | Eukaryotic translation initiation factor 3 subunit J-A OS=Mus musculus OX=10090 GN=Eif3j1 PE=2 SV=1 |
| 1048 | 2213 | sp\|Q66JS6\|EI3JB\_MOUSE | 95.27 | 11 | 8 | 8 | 3 | 0 | 8 | 3 | 3 | 0 | 4 | 11 | 2.73E6 | 2.32E6 | 2.15E6 |  | 1.84E6 | 1E6 | 1.37E6 |  | 3.51E6 | 4.76E6 | 3 | 3 | 2 | 2 | 1 | 0 | 2 | 1 | 1 | 0 | 1 | 3 | N | 29486 | Eukaryotic translation initiation factor 3 subunit J-B OS=Mus musculus OX=10090 GN=Eif3j2 PE=1 SV=1 |
| 1456 | 2903 | sp\|Q9D1H9\|MFAP4\_MOUSE | 95.25 | 9 | 0 | 0 | 0 | 7 | 9 | 9 | 7 | 7 | 0 | 0 |  |  |  | 6.75E5 | 3.85E6 | 7.62E6 | 1.27E6 | 9.21E5 |  |  | 2 | 2 | 0 | 0 | 0 | 1 | 2 | 2 | 1 | 1 | 0 | 0 | N | 28938 | Microfibril-associated glycoprotein 4 OS=Mus musculus OX=10090 GN=Mfap4 PE=1 SV=1 |
| 1260 | 3839 | Q3UN88\|Q3UN88\_MOUSE | 95.22 | 10 | 5 | 0 | 0 | 0 | 0 | 0 | 0 | 0 | 5 | 10 |  |  |  |  |  |  |  |  |  | 1.22E6 | 2 | 1 | 3 | 0 | 0 | 0 | 0 | 0 | 0 | 0 | 3 | 4 | N | 27197 | Mast cell protease 4 OS=Mus musculus OX=10090 GN=Mcpt4 PE=1 SV=1 |
| 1022 | 2075 | sp\|P46412\|GPX3\_MOUSE | 95.16 | 17 | 8 | 10 | 10 | 3 | 3 | 10 | 8 | 8 | 14 | 14 | 6.8E5 | 1.45E6 | 1.67E6 | 7.36E5 | 1.3E6 | 3.42E6 | 2.93E6 | 9.61E5 | 4.03E6 | 1.1E6 | 3 | 3 | 1 | 2 | 2 | 1 | 1 | 2 | 1 | 1 | 2 | 2 | Y | 25424 | Glutathione peroxidase 3 OS=Mus musculus OX=10090 GN=Gpx3 PE=1 SV=2 |
| 1172 | 2862 | sp\|P62911\|RL32\_MOUSE | 95.13 | 10 | 10 | 10 | 0 | 10 | 10 | 0 | 10 | 10 | 10 | 10 | 8.5E6 | 9.16E6 |  | 6.11E6 | 6.1E6 |  | 1.34E7 | 1.44E7 | 1.23E7 | 9.36E6 | 1 | 1 | 1 | 1 | 0 | 1 | 1 | 0 | 2 | 2 | 2 | 1 | Y | 15860 | Large ribosomal subunit protein eL32 OS=Mus musculus OX=10090 GN=Rpl32 PE=1 SV=2 |
| 914 | 2154 | sp\|O09106\|HDAC1\_MOUSE | 95.01 | 7 | 4 | 1 | 4 | 4 | 6 | 6 | 4 | 4 | 4 | 0 | 4.66E6 | 2.3E6 | 3.22E6 | 2E6 | 4.28E6 | 3.92E6 | 1.67E6 | 3.61E6 | 5.06E6 |  | 4 | 4 | 2 | 1 | 2 | 2 | 3 | 3 | 2 | 2 | 2 | 0 | Y | 55075 | Histone deacetylase 1 OS=Mus musculus OX=10090 GN=Hdac1 PE=1 SV=1 |
| 1249 | 2185 | sp\|Q9D6J6\|NDUV2\_MOUSE | 94.97 | 17 | 4 | 0 | 4 | 4 | 4 | 12 | 4 | 4 | 4 | 8 | 6.89E6 |  | 8.59E6 | 3.66E6 | 3.61E6 | 8.99E6 | 4.68E6 | 1.27E6 | 3.41E6 | 7.54E6 | 3 | 3 | 1 | 0 | 1 | 1 | 1 | 2 | 1 | 1 | 1 | 2 | N | 27285 | NADH dehydrogenase [ubiquinone] flavoprotein 2, mitochondrial OS=Mus musculus OX=10090 GN=Ndufv2 PE=1 SV=2 |
| 1047 | 1894 | sp\|Q62376\|RU17\_MOUSE | 94.96 | 6 | 2 | 2 | 2 | 4 | 6 | 2 | 4 | 4 | 0 | 2 | 1.39E6 | 4.89E5 | 9.82E5 | 1.71E6 | 5.17E6 | 3.1E6 | 9.07E5 | 1.98E6 |  | 1.76E5 | 3 | 3 | 1 | 1 | 1 | 2 | 3 | 1 | 2 | 2 | 0 | 1 | N | 51992 | U1 small nuclear ribonucleoprotein 70 kDa OS=Mus musculus OX=10090 GN=Snrnp70 PE=1 SV=2 |
| 1312 | 2772 | A0A494B952\|A0A494B952\_MOUSE | 94.88 | 13 | 5 | 5 | 5 | 0 | 5 | 5 | 5 | 0 | 5 | 13 | 7.96E5 | 4.61E6 | 4.43E6 |  | 3.9E5 | 3.41E6 | 5.85E5 |  | 1.88E6 | 3.51E6 | 2 | 2 | 1 | 1 | 1 | 0 | 1 | 1 | 1 | 0 | 1 | 2 | Y | 27514 | Isochorismatase domain-containing protein 1 OS=Mus musculus OX=10090 GN=Isoc1 PE=1 SV=1 |
| 1312 | 2773 | sp\|Q91V64\|ISOC1\_MOUSE | 94.88 | 11 | 5 | 5 | 5 | 0 | 5 | 5 | 5 | 0 | 5 | 11 | 7.96E5 | 4.61E6 | 4.43E6 |  | 3.9E5 | 3.41E6 | 5.85E5 |  | 1.88E6 | 3.51E6 | 2 | 2 | 1 | 1 | 1 | 0 | 1 | 1 | 1 | 0 | 1 | 2 | Y | 32033 | Isochorismatase domain-containing protein 1 OS=Mus musculus OX=10090 GN=Isoc1 PE=1 SV=1 |
| 2107 | 2124 | sp\|Q61739\|ITA6\_MOUSE | 94.60 | 3 | 0 | 0 | 0 | 0 | 0 | 1 | 1 | 0 | 1 | 0 |  |  |  |  |  | 3.32E5 | 0E0 |  | 6.17E5 |  | 3 | 3 | 0 | 0 | 0 | 0 | 0 | 1 | 1 | 0 | 1 | 0 | N | 122159 | Integrin alpha-6 OS=Mus musculus OX=10090 GN=Itga6 PE=1 SV=3 |
| 2107 | 2125 | A0A5F8MPB5\|A0A5F8MPB5\_MOUSE | 94.60 | 3 | 0 | 0 | 0 | 0 | 0 | 1 | 1 | 0 | 1 | 0 |  |  |  |  |  | 3.32E5 | 0E0 |  | 6.17E5 |  | 3 | 3 | 0 | 0 | 0 | 0 | 0 | 1 | 1 | 0 | 1 | 0 | N | 126723 | Integrin alpha 6 OS=Mus musculus OX=10090 GN=Itga6 PE=1 SV=1 |
| 2107 | 2158 | F6VSK8\|F6VSK8\_MOUSE | 94.60 | 6 | 0 | 0 | 0 | 0 | 0 | 1 | 2 | 0 | 2 | 0 |  |  |  |  |  | 3.32E5 | 0E0 |  | 6.17E5 |  | 3 | 3 | 0 | 0 | 0 | 0 | 0 | 1 | 1 | 0 | 1 | 0 | N | 75074 | Integrin alpha 6 (Fragment) OS=Mus musculus OX=10090 GN=Itga6 PE=1 SV=1 |
| 2161 | 3913 | D3YU17\|D3YU17\_MOUSE | 94.33 | 4 | 0 | 0 | 1 | 1 | 0 | 2 | 0 | 0 | 0 | 0 |  |  | 4.93E5 | 2.19E5 |  | 7.56E5 |  |  |  |  | 2 | 2 | 0 | 0 | 1 | 1 | 0 | 1 | 0 | 0 | 0 | 0 | N | 62780 | Nicalin OS=Mus musculus OX=10090 GN=Ncln PE=1 SV=1 |
| 2161 | 3915 | sp\|Q8VCM8\|NCLN\_MOUSE | 94.33 | 4 | 0 | 0 | 1 | 1 | 0 | 2 | 0 | 0 | 0 | 0 |  |  | 4.93E5 | 2.19E5 |  | 7.56E5 |  |  |  |  | 2 | 2 | 0 | 0 | 1 | 1 | 0 | 1 | 0 | 0 | 0 | 0 | N | 62908 | BOS complex subunit NCLN OS=Mus musculus OX=10090 GN=Ncln PE=1 SV=2 |
| 1454 | 1973 | sp\|Q8BXK9\|CLIC5\_MOUSE | 94.15 | 18 | 2 | 0 | 2 | 3 | 0 | 10 | 7 | 0 | 0 | 2 |  |  |  |  |  | 1.3E6 | 7.63E5 |  |  |  | 4 | 2 | 1 | 0 | 1 | 1 | 0 | 2 | 2 | 0 | 0 | 1 | Y | 28287 | Chloride intracellular channel protein 5 OS=Mus musculus OX=10090 GN=Clic5 PE=1 SV=1 |
| 1454 | 2005 | A0A668KLB7\|A0A668KLB7\_MOUSE | 94.15 | 9 | 1 | 0 | 1 | 2 | 0 | 5 | 4 | 0 | 0 | 1 |  |  |  |  |  | 1.3E6 | 7.63E5 |  |  |  | 4 | 2 | 1 | 0 | 1 | 1 | 0 | 2 | 2 | 0 | 0 | 1 | Y | 54150 | Chloride intracellular channel protein OS=Mus musculus OX=10090 GN=Clic5 PE=1 SV=1 |
| 897 | 2387 | G3UZ34\|G3UZ34\_MOUSE | 93.94 | 3 | 1 | 2 | 2 | 1 | 1 | 1 | 1 | 1 | 1 | 1 |  | 4.21E6 | 2.66E5 |  |  |  |  |  |  |  | 3 | 2 | 2 | 3 | 3 | 2 | 2 | 2 | 2 | 2 | 2 | 1 | N | 108339 | 116 kDa U5 small nuclear ribonucleoprotein component OS=Mus musculus OX=10090 GN=Eftud2 PE=1 SV=1 |
| 897 | 2388 | sp\|O08810\|U5S1\_MOUSE | 93.94 | 3 | 1 | 2 | 2 | 1 | 1 | 1 | 1 | 1 | 1 | 1 |  | 4.21E6 | 2.66E5 |  |  |  |  |  |  |  | 3 | 2 | 2 | 3 | 3 | 2 | 2 | 2 | 2 | 2 | 2 | 1 | N | 109361 | 116 kDa U5 small nuclear ribonucleoprotein component OS=Mus musculus OX=10090 GN=Eftud2 PE=1 SV=1 |
| 897 | 2389 | A2AH85\|A2AH85\_MOUSE | 93.94 | 3 | 1 | 2 | 2 | 1 | 1 | 1 | 1 | 1 | 1 | 1 |  | 4.21E6 | 2.66E5 |  |  |  |  |  |  |  | 3 | 2 | 2 | 3 | 3 | 2 | 2 | 2 | 2 | 2 | 2 | 1 | N | 109492 | 116 kDa U5 small nuclear ribonucleoprotein component OS=Mus musculus OX=10090 GN=Eftud2 PE=1 SV=1 |
| 1299 | 4579 | sp\|Q9D8I3\|GLOD5\_MOUSE | 93.83 | 9 | 9 | 9 | 9 | 9 | 9 | 9 | 9 | 9 | 9 | 9 | 4.43E6 | 7.94E6 | 8.88E6 | 2.71E6 | 3.93E6 | 6.48E6 | 2.57E6 | 2.64E5 | 1.09E7 | 5.79E6 | 1 | 1 | 1 | 1 | 1 | 1 | 1 | 1 | 1 | 1 | 1 | 1 | N | 16595 | Glyoxalase domain-containing protein 5 OS=Mus musculus OX=10090 GN=Glod5 PE=1 SV=1 |
| 1061 | 2094 | Q542V3\|Q542V3\_MOUSE | 93.82 | 6 | 2 | 2 | 1 | 1 | 3 | 1 | 6 | 6 | 0 | 1 |  |  |  |  |  |  | 1.11E6 | 3.12E5 |  |  | 3 | 1 | 1 | 1 | 1 | 1 | 2 | 1 | 3 | 3 | 0 | 1 | N | 56249 | Serine and arginine-rich splicing factor 4 OS=Mus musculus OX=10090 GN=Srsf4 PE=1 SV=1 |
| 1061 | 2111 | sp\|Q8VE97\|SRSF4\_MOUSE | 93.82 | 6 | 2 | 2 | 1 | 1 | 3 | 1 | 6 | 6 | 0 | 1 |  |  |  |  |  |  | 1.11E6 | 3.12E5 |  |  | 3 | 1 | 1 | 1 | 1 | 1 | 2 | 1 | 3 | 3 | 0 | 1 | N | 55979 | Serine/arginine-rich splicing factor 4 OS=Mus musculus OX=10090 GN=Srsf4 PE=2 SV=1 |
| 1111 | 3827 | sp\|Q8R3G9\|TSN8\_MOUSE | 93.74 | 10 | 6 | 10 | 0 | 10 | 0 | 0 | 10 | 4 | 6 | 6 | 8.46E6 | 1.59E7 |  | 3.75E6 |  |  | 4.69E6 | 1.19E6 | 1.66E7 | 8.65E6 | 2 | 2 | 1 | 3 | 0 | 2 | 0 | 0 | 2 | 1 | 1 | 2 | Y | 25582 | Tetraspanin-8 OS=Mus musculus OX=10090 GN=Tspan8 PE=1 SV=1 |
| 1428 | 1487 | sp\|Q6GQT9\|NOMO1\_MOUSE | 93.69 | 5 | 0 | 0 | 1 | 2 | 0 | 0 | 2 | 1 | 1 | 1 |  |  | 7.69E5 | 1.16E7 |  |  | 1.66E6 | 1.97E5 | 1.1E6 | 1.28E6 | 5 | 5 | 0 | 0 | 1 | 2 | 0 | 0 | 2 | 1 | 1 | 1 | N | 133420 | BOS complex subunit NOMO1 OS=Mus musculus OX=10090 GN=Nomo1 PE=1 SV=1 |
| 1189 | 2116 | sp\|Q99LF4\|RTCB\_MOUSE | 93.55 | 9 | 2 | 2 | 0 | 2 | 2 | 2 | 2 | 5 | 2 | 4 | 3.18E6 | 2.05E6 |  | 6.96E5 | 4.4E5 | 2.85E6 | 1.8E6 | 2.18E6 | 3.24E6 | 3.04E6 | 4 | 4 | 1 | 1 | 0 | 1 | 1 | 1 | 1 | 2 | 1 | 2 | Y | 55249 | RNA-splicing ligase RtcB homolog OS=Mus musculus OX=10090 GN=Rtcb PE=1 SV=1 |
| 1193 | 3056 | sp\|P62315\|SMD1\_MOUSE | 93.45 | 28 | 0 | 17 | 28 | 11 | 28 | 11 | 28 | 0 | 28 | 17 |  | 5.42E5 | 2.65E6 | 5.07E5 | 2.81E6 | 1.57E6 | 1.75E6 |  | 2.63E6 | 1.09E6 | 2 | 2 | 0 | 1 | 2 | 1 | 2 | 1 | 2 | 0 | 2 | 1 | N | 13282 | Small nuclear ribonucleoprotein Sm D1 OS=Mus musculus OX=10090 GN=Snrpd1 PE=1 SV=1 |
| 1041 | 2446 | sp\|Q6ZWX6\|IF2A\_MOUSE | 93.41 | 6 | 6 | 4 | 6 | 0 | 4 | 6 | 0 | 4 | 4 | 6 | 1.38E7 | 1.06E7 | 1.26E7 |  | 1.34E7 | 1.47E7 |  | 9.53E6 | 3.63E6 | 1.17E7 | 2 | 2 | 2 | 1 | 2 | 0 | 1 | 2 | 0 | 1 | 1 | 2 | N | 36108 | Eukaryotic translation initiation factor 2 subunit 1 OS=Mus musculus OX=10090 GN=Eif2s1 PE=1 SV=3 |
| 1256 | 2465 | sp\|Q91V01\|MBOA5\_MOUSE | 93.37 | 6 | 0 | 2 | 2 | 2 | 2 | 2 | 2 | 2 | 6 | 0 |  | 1.07E6 | 8.03E5 | 1.95E5 | 3.35E5 | 1.88E5 | 7.63E5 | 5.48E5 | 1.06E7 |  | 3 | 3 | 0 | 1 | 1 | 1 | 1 | 1 | 1 | 1 | 4 | 0 | N | 56147 | Lysophospholipid acyltransferase 5 OS=Mus musculus OX=10090 GN=Lpcat3 PE=1 SV=1 |
| 1200 | 3671 | sp\|Q91V12\|BACH\_MOUSE | 93.34 | 6 | 3 | 3 | 3 | 3 | 3 | 3 | 3 | 3 | 6 | 3 | 8.61E6 | 1.69E6 | 4.36E6 | 1.98E6 | 6.06E6 | 5.99E6 | 2.42E6 | 1.77E6 | 1.41E7 | 6.49E6 | 2 | 2 | 1 | 1 | 1 | 1 | 1 | 1 | 1 | 1 | 3 | 1 | N | 42537 | Cytosolic acyl coenzyme A thioester hydrolase OS=Mus musculus OX=10090 GN=Acot7 PE=1 SV=2 |
| 1200 | 3672 | E9PYH2\|E9PYH2\_MOUSE | 93.34 | 6 | 3 | 3 | 3 | 3 | 3 | 3 | 3 | 3 | 6 | 3 | 8.61E6 | 1.69E6 | 4.36E6 | 1.98E6 | 6.06E6 | 5.99E6 | 2.42E6 | 1.77E6 | 1.41E7 | 6.49E6 | 2 | 2 | 1 | 1 | 1 | 1 | 1 | 1 | 1 | 1 | 3 | 1 | N | 42826 | Acyl-CoA thioesterase 7 OS=Mus musculus OX=10090 GN=Acot7 PE=1 SV=1 |
| 1385 | 2469 | sp\|O70492\|SNX3\_MOUSE | 93.23 | 17 | 7 | 0 | 0 | 0 | 0 | 7 | 0 | 0 | 17 | 7 | 2.46E6 |  |  |  |  | 1.12E6 |  |  | 9.25E6 | 2.36E6 | 3 | 2 | 1 | 0 | 0 | 0 | 0 | 1 | 0 | 0 | 4 | 1 | N | 18757 | Sorting nexin-3 OS=Mus musculus OX=10090 GN=Snx3 PE=1 SV=3 |
| 1385 | 2470 | Q78ZM0\|Q78ZM0\_MOUSE | 93.23 | 17 | 7 | 0 | 0 | 0 | 0 | 7 | 0 | 0 | 17 | 7 | 2.46E6 |  |  |  |  | 1.12E6 |  |  | 9.25E6 | 2.36E6 | 3 | 2 | 1 | 0 | 0 | 0 | 0 | 1 | 0 | 0 | 4 | 1 | N | 18762 | Sorting nexin-3 OS=Mus musculus OX=10090 GN=Snx3 PE=1 SV=1 |
| 876 | 3320 | sp\|P27601\|GNA13\_MOUSE | 93.16 | 6 | 6 | 3 | 6 | 6 | 3 | 6 | 6 | 6 | 6 | 3 | 8.97E5 |  | 5.04E5 | 2.19E5 |  | 7.71E5 | 3.44E5 | 1.66E5 | 1.14E6 |  | 2 | 1 | 3 | 1 | 2 | 3 | 2 | 3 | 3 | 3 | 2 | 1 | N | 44055 | Guanine nucleotide-binding protein subunit alpha-13 OS=Mus musculus OX=10090 GN=Gna13 PE=1 SV=1 |
| 876 | 3501 | Q9D034\|Q9D034\_MOUSE | 93.16 | 12 | 12 | 6 | 12 | 12 | 6 | 12 | 12 | 12 | 12 | 6 | 8.97E5 |  | 5.04E5 | 2.19E5 |  | 7.71E5 | 3.44E5 | 1.66E5 | 1.14E6 |  | 2 | 1 | 3 | 1 | 2 | 3 | 2 | 3 | 3 | 3 | 2 | 1 | N | 19937 | Guanine nucleotide binding protein, alpha 13 OS=Mus musculus OX=10090 GN=Gna13 PE=1 SV=1 |
| 2469 | 3065 | A0A0A0MQG7\|A0A0A0MQG7\_MOUSE | 92.87 | 18 | 0 | 0 | 0 | 0 | 0 | 0 | 18 | 0 | 0 | 0 |  |  |  |  |  |  | 2.46E5 |  |  |  | 1 | 1 | 0 | 0 | 0 | 0 | 0 | 0 | 1 | 0 | 0 | 0 | N | 13024 | Uroporphyrinogen decarboxylase (Fragment) OS=Mus musculus OX=10090 GN=Urod PE=1 SV=1 |
| 1630 | 3823 | sp\|Q60692\|PSB6\_MOUSE | 92.86 | 9 | 0 | 0 | 4 | 4 | 4 | 4 | 4 | 0 | 5 | 0 |  |  | 5.14E6 | 1.9E6 | 6.71E6 | 6.17E6 | 3.37E6 |  | 4.81E6 |  | 2 | 2 | 0 | 0 | 1 | 1 | 1 | 1 | 1 | 0 | 1 | 0 | N | 25379 | Proteasome subunit beta type-6 OS=Mus musculus OX=10090 GN=Psmb6 PE=1 SV=3 |
| 941 | 2473 | sp\|P34022\|RANG\_MOUSE | 92.77 | 13 | 5 | 10 | 0 | 10 | 5 | 9 | 10 | 5 | 13 | 10 | 6.67E6 | 8.75E6 |  | 4.75E6 | 4.39E6 | 1.29E7 | 8.13E6 | 1.92E6 | 2.2E7 | 8.05E6 | 3 | 3 | 1 | 2 | 0 | 2 | 1 | 2 | 2 | 1 | 4 | 2 | N | 23596 | Ran-specific GTPase-activating protein OS=Mus musculus OX=10090 GN=Ranbp1 PE=1 SV=2 |
| 1654 | 2932 | sp\|Q9QY30\|ABCBB\_MOUSE | 92.68 | 1 | 0 | 0 | 0 | 0 | 0 | 1 | 0 | 0 | 1 | 0 |  |  |  |  |  | 2.16E6 |  |  | 7.07E6 |  | 1 | 1 | 0 | 0 | 0 | 0 | 0 | 1 | 0 | 0 | 1 | 0 | N | 146748 | Bile salt export pump OS=Mus musculus OX=10090 GN=Abcb11 PE=1 SV=2 |
| 824 | 2637 | sp\|A2AQP0\|MYH7B\_MOUSE | 92.67 | 1 | 1 | 1 | 1 | 1 | 0 | 1 | 1 | 1 | 0 | 0 |  |  | 1.29E7 |  |  |  |  |  |  |  | 4 | 1 | 3 | 3 | 3 | 2 | 1 | 3 | 2 | 3 | 1 | 1 | N | 221495 | Myosin-7B OS=Mus musculus OX=10090 GN=Myh7b PE=3 SV=1 |
| 947 | 1962 | Q3TWV4\|Q3TWV4\_MOUSE | 92.46 | 7 | 2 | 5 | 3 | 2 | 4 | 2 | 2 | 4 | 5 | 4 | 3.35E6 | 6.61E6 | 8.56E6 | 1.06E6 | 2.86E6 | 2.42E6 | 3.44E6 | 3.49E6 | 7.9E6 | 3.94E6 | 4 | 4 | 1 | 3 | 2 | 1 | 2 | 1 | 1 | 2 | 3 | 2 | N | 49389 | AP-2 complex subunit mu OS=Mus musculus OX=10090 GN=Ap2m1 PE=1 SV=1 |
| 947 | 1963 | sp\|P84091\|AP2M1\_MOUSE | 92.46 | 7 | 2 | 5 | 3 | 2 | 4 | 2 | 2 | 4 | 5 | 4 | 3.35E6 | 6.61E6 | 8.56E6 | 1.06E6 | 2.86E6 | 2.42E6 | 3.44E6 | 3.49E6 | 7.9E6 | 3.94E6 | 4 | 4 | 1 | 3 | 2 | 1 | 2 | 1 | 1 | 2 | 3 | 2 | N | 49655 | AP-2 complex subunit mu OS=Mus musculus OX=10090 GN=Ap2m1 PE=1 SV=1 |
| 871 | 2252 | A0A0J9YTY0\|A0A0J9YTY0\_MOUSE | 92.44 | 6 | 2 | 2 | 2 | 4 | 2 | 2 | 2 | 2 | 5 | 5 |  |  |  |  |  |  |  |  | 2.44E6 | 9.16E5 | 3 | 1 | 2 | 2 | 2 | 3 | 2 | 2 | 1 | 2 | 3 | 3 | N | 48979 | Septin OS=Mus musculus OX=10090 GN=Septin11 PE=1 SV=1 |
| 871 | 2253 | sp\|Q8C1B7\|SEP11\_MOUSE | 92.44 | 6 | 2 | 2 | 2 | 3 | 2 | 2 | 2 | 2 | 5 | 5 |  |  |  |  |  |  |  |  | 2.44E6 | 9.16E5 | 3 | 1 | 2 | 2 | 2 | 3 | 2 | 2 | 1 | 2 | 3 | 3 | N | 49695 | Septin-11 OS=Mus musculus OX=10090 GN=Septin11 PE=1 SV=4 |
| 871 | 2254 | A0A0J9YUL3\|A0A0J9YUL3\_MOUSE | 92.44 | 6 | 2 | 2 | 2 | 3 | 2 | 2 | 2 | 2 | 5 | 5 |  |  |  |  |  |  |  |  | 2.44E6 | 9.16E5 | 3 | 1 | 2 | 2 | 2 | 3 | 2 | 2 | 1 | 2 | 3 | 3 | N | 49782 | Septin OS=Mus musculus OX=10090 GN=Septin11 PE=1 SV=1 |
| 1149 | 2206 | sp\|P35550\|FBRL\_MOUSE | 92.34 | 10 | 6 | 6 | 0 | 3 | 3 | 0 | 0 | 3 | 0 | 8 | 7.88E6 | 8.06E6 |  | 3.21E6 | 4E6 |  |  | 3.65E6 |  | 4.69E6 | 3 | 3 | 2 | 2 | 0 | 2 | 1 | 0 | 0 | 1 | 0 | 2 | N | 34307 | rRNA 2'-O-methyltransferase fibrillarin OS=Mus musculus OX=10090 GN=Fbl PE=1 SV=2 |
| 1580 | 2705 | sp\|Q8R0X7\|SGPL1\_MOUSE | 92.20 | 5 | 0 | 2 | 2 | 5 | 0 | 0 | 2 | 0 | 0 | 0 |  | 1.9E5 | 6.02E5 | 6.49E5 |  |  | 7.05E5 |  |  |  | 2 | 2 | 0 | 1 | 1 | 2 | 0 | 0 | 1 | 0 | 0 | 0 | N | 63677 | Sphingosine-1-phosphate lyase 1 OS=Mus musculus OX=10090 GN=Sgpl1 PE=1 SV=1 |
| 1375 | 2612 | sp\|P22892\|AP1G1\_MOUSE | 92.12 | 4 | 1 | 1 | 0 | 0 | 1 | 1 | 0 | 1 | 1 | 1 | 1.58E6 | 1.8E5 |  |  | 1.25E5 | 1.7E6 |  | 3.27E5 | 9.35E5 | 5.05E5 | 3 | 3 | 2 | 1 | 0 | 0 | 1 | 2 | 0 | 1 | 1 | 1 | N | 91350 | AP-1 complex subunit gamma-1 OS=Mus musculus OX=10090 GN=Ap1g1 PE=1 SV=3 |
| 1375 | 2613 | Q8CBB7\|Q8CBB7\_MOUSE | 92.12 | 4 | 1 | 1 | 0 | 0 | 1 | 1 | 0 | 1 | 1 | 1 | 1.58E6 | 1.8E5 |  |  | 1.25E5 | 1.7E6 |  | 3.27E5 | 9.35E5 | 5.05E5 | 3 | 3 | 2 | 1 | 0 | 0 | 1 | 2 | 0 | 1 | 1 | 1 | N | 91722 | AP-1 complex subunit gamma OS=Mus musculus OX=10090 GN=Ap1g1 PE=1 SV=1 |
| 1452 | 2153 | sp\|Q09199\|B4GN2\_MOUSE | 91.93 | 6 | 0 | 2 | 0 | 3 | 0 | 3 | 0 | 0 | 0 | 0 |  | 1.91E6 |  | 1.16E6 |  | 5.6E5 |  |  |  |  | 3 | 3 | 0 | 1 | 0 | 2 | 0 | 1 | 0 | 0 | 0 | 0 | Y | 58317 | Beta-1,4 N-acetylgalactosaminyltransferase 2 OS=Mus musculus OX=10090 GN=B4galnt2 PE=2 SV=1 |
| 1036 | 2435 | sp\|Q9JLQ0\|CD2AP\_MOUSE | 91.88 | 5 | 2 | 2 | 2 | 5 | 4 | 4 | 2 | 4 | 2 | 0 | 8.3E5 | 4.59E5 | 3.8E5 | 1.75E6 | 2.21E6 | 1.67E6 | 7.68E5 | 7.89E5 | 1.05E6 |  | 3 | 3 | 1 | 1 | 1 | 3 | 2 | 2 | 1 | 2 | 1 | 0 | N | 70450 | CD2-associated protein OS=Mus musculus OX=10090 GN=Cd2ap PE=1 SV=3 |
| 1036 | 2511 | A0A3B2W812\|A0A3B2W812\_MOUSE | 91.88 | 6 | 2 | 2 | 2 | 6 | 4 | 4 | 2 | 4 | 2 | 0 | 8.3E5 | 4.59E5 | 3.8E5 | 1.75E6 | 2.21E6 | 1.67E6 | 7.68E5 | 7.89E5 | 1.05E6 |  | 3 | 3 | 1 | 1 | 1 | 3 | 2 | 2 | 1 | 2 | 1 | 0 | N | 58394 | CD2-associated protein OS=Mus musculus OX=10090 GN=Cd2ap PE=1 SV=1 |
| 1901 | 2341 | sp\|Q80UU9\|PGRC2\_MOUSE | 91.81 | 14 | 6 | 0 | 0 | 0 | 4 | 4 | 0 | 0 | 0 | 6 | 1.4E6 |  |  |  |  | 1.19E6 |  |  |  | 1.11E6 | 3 | 2 | 1 | 0 | 0 | 0 | 1 | 1 | 0 | 0 | 0 | 1 | Y | 23334 | Membrane-associated progesterone receptor component 2 OS=Mus musculus OX=10090 GN=Pgrmc2 PE=1 SV=2 |
| 1117 | 3883 | sp\|P45878\|FKBP2\_MOUSE | 91.49 | 14 | 14 | 14 | 14 | 9 | 9 | 9 | 9 | 9 | 9 | 14 | 7.24E6 | 7.91E6 | 6.73E6 | 1.77E6 | 2.12E6 | 3.6E6 | 3.62E6 | 2.87E6 | 7.35E6 | 5.18E6 | 2 | 2 | 2 | 2 | 2 | 1 | 1 | 1 | 1 | 1 | 1 | 2 | Y | 15344 | Peptidyl-prolyl cis-trans isomerase FKBP2 OS=Mus musculus OX=10090 GN=Fkbp2 PE=1 SV=1 |
| 1539 | 1936 | sp\|Q8R180\|ERO1A\_MOUSE | 91.35 | 12 | 0 | 3 | 0 | 0 | 0 | 0 | 6 | 0 | 6 | 0 |  | 1.71E5 |  |  |  |  | 1.06E6 |  | 2.5E6 |  | 4 | 4 | 0 | 1 | 0 | 0 | 0 | 0 | 3 | 0 | 2 | 0 | N | 54085 | ERO1-like protein alpha OS=Mus musculus OX=10090 GN=Ero1a PE=1 SV=2 |
| 1369 | 2063 | B7ZNL2\|B7ZNL2\_MOUSE | 91.35 | 10 | 3 | 0 | 3 | 3 | 0 | 0 | 5 | 3 | 3 | 3 |  |  | 2.28E5 |  |  |  | 3.4E5 | 3.39E5 |  | 3.07E5 | 3 | 2 | 1 | 0 | 1 | 1 | 0 | 0 | 1 | 1 | 1 | 1 | Y | 43935 | Nap1l4 protein OS=Mus musculus OX=10090 GN=Nap1l4 PE=1 SV=1 |
| 1049 | 2492 | sp\|P00920\|CAH2\_MOUSE | 91.33 | 14 | 6 | 6 | 14 | 8 | 8 | 6 | 8 | 8 | 0 | 0 | 3.89E5 | 1.34E6 | 1.61E7 | 2.42E6 | 2.97E6 | 1.31E6 | 2.12E6 | 2.81E6 |  |  | 3 | 3 | 1 | 1 | 3 | 2 | 2 | 1 | 2 | 2 | 0 | 0 | Y | 29033 | Carbonic anhydrase 2 OS=Mus musculus OX=10090 GN=Ca2 PE=1 SV=4 |
| 1387 | 2591 | sp\|Q8CFV9\|RIFK\_MOUSE | 91.30 | 18 | 0 | 14 | 0 | 0 | 5 | 14 | 0 | 0 | 18 | 14 |  | 1.05E6 |  |  | 1.3E6 | 2.11E6 |  |  | 3.56E6 | 1.39E6 | 2 | 2 | 0 | 1 | 0 | 0 | 1 | 1 | 0 | 0 | 2 | 1 | Y | 17437 | Riboflavin kinase OS=Mus musculus OX=10090 GN=Rfk PE=1 SV=2 |
| 905 | 1757 | sp\|Q91W97\|HKDC1\_MOUSE | 91.12 | 3 | 1 | 3 | 2 | 2 | 1 | 1 | 2 | 0 | 2 | 2 | 4.66E6 | 4.3E6 | 3.3E6 | 1.73E6 |  |  | 8.4E5 |  |  | 1.26E6 | 3 | 1 | 1 | 3 | 2 | 2 | 1 | 1 | 2 | 0 | 2 | 2 | N | 102259 | Hexokinase HKDC1 OS=Mus musculus OX=10090 GN=Hkdc1 PE=2 SV=1 |
| 1296 | 3300 | F8WJK8\|F8WJK8\_MOUSE | 91.10 | 6 | 3 | 6 | 0 | 4 | 4 | 4 | 4 | 4 | 3 | 3 | 5.85E6 | 7.57E6 |  | 2.06E6 | 9.94E5 | 3.58E6 | 1.7E6 | 1.66E6 | 0E0 | 3.6E6 | 2 | 2 | 1 | 2 | 0 | 1 | 1 | 1 | 1 | 1 | 1 | 1 | N | 40539 | Suppression of tumorigenicity 13 OS=Mus musculus OX=10090 GN=St13 PE=1 SV=1 |
| 1296 | 3301 | sp\|Q99L47\|F10A1\_MOUSE | 91.10 | 6 | 3 | 6 | 0 | 4 | 4 | 4 | 4 | 4 | 3 | 3 | 5.85E6 | 7.57E6 |  | 2.06E6 | 9.94E5 | 3.58E6 | 1.7E6 | 1.66E6 | 0E0 | 3.6E6 | 2 | 2 | 1 | 2 | 0 | 1 | 1 | 1 | 1 | 1 | 1 | 1 | N | 41656 | Hsc70-interacting protein OS=Mus musculus OX=10090 GN=St13 PE=1 SV=1 |
| 1319 | 2226 | sp\|Q6P9R2\|OXSR1\_MOUSE | 91.07 | 6 | 1 | 0 | 2 | 1 | 2 | 3 | 3 | 4 | 0 | 2 | 7.65E5 |  | 1.25E7 | 9.01E5 | 7.7E5 | 6.45E5 | 2.14E6 | 1E6 |  | 8.77E6 | 3 | 3 | 1 | 0 | 1 | 1 | 1 | 1 | 2 | 2 | 0 | 1 | N | 58214 | Serine/threonine-protein kinase OSR1 OS=Mus musculus OX=10090 GN=Oxsr1 PE=1 SV=1 |
| 1204 | 2343 | sp\|Q8BWY3\|ERF1\_MOUSE | 90.97 | 7 | 5 | 2 | 0 | 5 | 0 | 2 | 2 | 0 | 5 | 5 | 3.98E6 | 2.49E6 |  | 1.28E6 |  | 1.73E6 | 1.57E6 |  | 4.87E6 | 3.55E6 | 3 | 3 | 2 | 1 | 0 | 2 | 0 | 1 | 1 | 0 | 2 | 2 | N | 49031 | Eukaryotic peptide chain release factor subunit 1 OS=Mus musculus OX=10090 GN=Etf1 PE=1 SV=4 |
| 1451 | 2112 | sp\|Q9CY64\|BIEA\_MOUSE | 90.93 | 12 | 5 | 3 | 0 | 3 | 3 | 3 | 0 | 0 | 7 | 0 | 4.84E5 | 4.48E6 |  | 1.22E6 | 1.41E6 | 1.85E6 |  |  | 2.09E6 |  | 3 | 3 | 1 | 1 | 0 | 1 | 1 | 1 | 0 | 0 | 2 | 0 | N | 33525 | Biliverdin reductase A OS=Mus musculus OX=10090 GN=Blvra PE=1 SV=1 |
| 1741 | 2669 | A2AA71\|A2AA71\_MOUSE | 90.73 | 2 | 1 | 0 | 0 | 0 | 1 | 0 | 1 | 0 | 0 | 1 | 7.84E5 |  |  |  | 5.35E5 |  | 3.61E5 |  |  | 9.46E5 | 2 | 2 | 1 | 0 | 0 | 0 | 1 | 0 | 1 | 0 | 0 | 1 | N | 118654 | SEC24 homolog A, COPII coat complex component OS=Mus musculus OX=10090 GN=Sec24a PE=1 SV=1 |
| 1741 | 2670 | sp\|Q3U2P1\|SC24A\_MOUSE | 90.73 | 2 | 1 | 0 | 0 | 0 | 1 | 0 | 1 | 0 | 0 | 1 | 7.84E5 |  |  |  | 5.35E5 |  | 3.61E5 |  |  | 9.46E5 | 2 | 2 | 1 | 0 | 0 | 0 | 1 | 0 | 1 | 0 | 0 | 1 | N | 118782 | Protein transport protein Sec24A OS=Mus musculus OX=10090 GN=Sec24a PE=1 SV=1 |
| 1257 | 2480 | Q9CPX4\|Q9CPX4\_MOUSE | 90.73 | 20 | 13 | 9 | 0 | 13 | 9 | 9 | 16 | 9 | 9 | 0 | 4.76E6 | 5.4E5 |  | 2.91E5 | 8.11E5 | 4.03E5 | 9.47E5 | 1.57E6 | 4.19E6 |  | 3 | 3 | 2 | 1 | 0 | 2 | 1 | 1 | 2 | 1 | 1 | 0 | N | 20772 | Ferritin OS=Mus musculus OX=10090 GN=Ftl1 PE=1 SV=1 |
| 1257 | 2481 | sp\|P29391\|FRIL1\_MOUSE | 90.73 | 20 | 13 | 9 | 0 | 13 | 9 | 9 | 16 | 9 | 9 | 0 | 4.76E6 | 5.4E5 |  | 2.91E5 | 8.11E5 | 4.03E5 | 9.47E5 | 1.57E6 | 4.19E6 |  | 3 | 3 | 2 | 1 | 0 | 2 | 1 | 1 | 2 | 1 | 1 | 0 | N | 20802 | Ferritin light chain 1 OS=Mus musculus OX=10090 GN=Ftl1 PE=1 SV=2 |
| 1227 | 1663 | sp\|Q9DBG3\|AP2B1\_MOUSE | 90.71 | 6 | 1 | 0 | 2 | 1 | 1 | 0 | 1 | 0 | 2 | 2 | 2.06E6 |  | 7.03E5 |  |  |  |  |  | 2.7E6 | 7.01E5 | 4 | 2 | 1 | 0 | 1 | 1 | 1 | 0 | 1 | 0 | 2 | 2 | Y | 104583 | AP-2 complex subunit beta OS=Mus musculus OX=10090 GN=Ap2b1 PE=1 SV=1 |
| 1386 | 2475 | sp\|Q99JR1\|SFXN1\_MOUSE | 90.56 | 9 | 0 | 7 | 0 | 2 | 2 | 2 | 2 | 2 | 4 | 0 |  | 4.58E6 |  | 3.1E5 | 2.6E6 | 4.47E5 | 3.88E5 | 4.32E5 | 2.44E6 |  | 3 | 3 | 0 | 2 | 0 | 1 | 2 | 1 | 1 | 1 | 1 | 0 | N | 35649 | Sideroflexin-1 OS=Mus musculus OX=10090 GN=Sfxn1 PE=1 SV=3 |
| 1723 | 2602 | E9PV85\|E9PV85\_MOUSE | 90.49 | 9 | 0 | 0 | 9 | 4 | 0 | 0 | 0 | 0 | 4 | 0 |  |  | 3.84E6 | 2.2E5 |  |  |  |  | 8.95E5 |  | 2 | 2 | 0 | 0 | 2 | 1 | 0 | 0 | 0 | 0 | 1 | 0 | N | 28033 | alpha-amylase (Fragment) OS=Mus musculus OX=10090 GN=Amy2a1 PE=1 SV=1 |
| 1723 | 2606 | sp\|P00688\|AM2A5\_MOUSE | 90.49 | 4 | 0 | 0 | 4 | 2 | 0 | 0 | 0 | 0 | 2 | 0 |  |  | 3.84E6 | 2.2E5 |  |  |  |  | 8.95E5 |  | 2 | 2 | 0 | 0 | 2 | 1 | 0 | 0 | 0 | 0 | 1 | 0 | N | 57318 | Pancreatic alpha-amylase 2a5 OS=Mus musculus OX=10090 GN=Amy2a5 PE=1 SV=2 |
| 1107 | 2429 | sp\|Q8VCN5\|CGL\_MOUSE | 90.38 | 7 | 3 | 3 | 3 | 3 | 3 | 5 | 5 | 3 | 3 | 3 | 1.09E6 | 7.54E6 | 5.11E6 | 9.4E5 | 9.72E5 | 1.3E7 | 2.21E6 | 1.48E6 | 7.07E6 | 3.51E6 | 3 | 3 | 1 | 1 | 1 | 1 | 1 | 2 | 2 | 1 | 1 | 1 | N | 43567 | Cystathionine gamma-lyase OS=Mus musculus OX=10090 GN=Cth PE=1 SV=1 |
| 1196 | 2320 | sp\|P20060\|HEXB\_MOUSE | 90.23 | 6 | 0 | 0 | 2 | 2 | 2 | 4 | 0 | 2 | 4 | 0 |  |  | 1.91E6 | 8.57E5 | 1.4E6 | 3.19E6 |  | 1.01E6 | 9.44E6 |  | 3 | 3 | 0 | 0 | 1 | 1 | 1 | 2 | 0 | 1 | 2 | 0 | N | 61116 | Beta-hexosaminidase subunit beta OS=Mus musculus OX=10090 GN=Hexb PE=1 SV=2 |
| 1572 | 2875 | sp\|Q3UIR3\|DTX3L\_MOUSE | 90.05 | 2 | 0 | 0 | 0 | 0 | 0 | 2 | 2 | 0 | 0 | 0 |  |  |  |  |  | 1.97E6 | 9.12E5 |  |  |  | 1 | 1 | 0 | 0 | 0 | 0 | 0 | 2 | 1 | 0 | 0 | 0 | N | 83044 | E3 ubiquitin-protein ligase DTX3L OS=Mus musculus OX=10090 GN=Dtx3l PE=1 SV=1 |
| 1603 | 2811 | sp\|P04441\|HG2A\_MOUSE | 89.95 | 5 | 5 | 0 | 5 | 0 | 0 | 0 | 0 | 5 | 0 | 0 | 3.83E5 |  | 1.98E5 |  |  |  |  | 1.09E5 |  |  | 1 | 1 | 1 | 0 | 1 | 0 | 0 | 0 | 0 | 1 | 0 | 0 | N | 31557 | H-2 class II histocompatibility antigen gamma chain OS=Mus musculus OX=10090 GN=Cd74 PE=1 SV=3 |
| 2158 | 3802 | A0A1L1SSA3\|A0A1L1SSA3\_MOUSE | 89.92 | 30 | 16 | 0 | 14 | 0 | 0 | 0 | 0 | 0 | 14 | 0 | 1.2E6 |  | 8.23E5 |  |  |  |  |  | 5.27E6 |  | 2 | 2 | 1 | 0 | 1 | 0 | 0 | 0 | 0 | 0 | 1 | 0 | N | 11358 | Eukaryotic translation initiation factor 1B (Fragment) OS=Mus musculus OX=10090 GN=Eif1b PE=1 SV=1 |
| 2158 | 3803 | J3QPZ8\|J3QPZ8\_MOUSE | 89.92 | 27 | 15 | 0 | 13 | 0 | 0 | 0 | 0 | 0 | 13 | 0 | 1.2E6 |  | 8.23E5 |  |  |  |  |  | 5.27E6 |  | 2 | 2 | 1 | 0 | 1 | 0 | 0 | 0 | 0 | 0 | 1 | 0 | N | 12100 | Eukaryotic translation initiation factor 1 (Fragment) OS=Mus musculus OX=10090 GN=Eif1 PE=4 SV=1 |
| 2158 | 3804 | sp\|P48024\|EIF1\_MOUSE | 89.92 | 27 | 14 | 0 | 12 | 0 | 0 | 0 | 0 | 0 | 12 | 0 | 1.2E6 |  | 8.23E5 |  |  |  |  |  | 5.27E6 |  | 2 | 2 | 1 | 0 | 1 | 0 | 0 | 0 | 0 | 0 | 1 | 0 | N | 12747 | Eukaryotic translation initiation factor 1 OS=Mus musculus OX=10090 GN=Eif1 PE=1 SV=2 |
| 2158 | 3805 | sp\|Q9CXU9\|EIF1B\_MOUSE | 89.92 | 27 | 14 | 0 | 12 | 0 | 0 | 0 | 0 | 0 | 12 | 0 | 1.2E6 |  | 8.23E5 |  |  |  |  |  | 5.27E6 |  | 2 | 2 | 1 | 0 | 1 | 0 | 0 | 0 | 0 | 0 | 1 | 0 | N | 12824 | Eukaryotic translation initiation factor 1b OS=Mus musculus OX=10090 GN=Eif1b PE=1 SV=2 |
| 1600 | 2118 | A0A0G2JF72\|A0A0G2JF72\_MOUSE | 89.84 | 5 | 2 | 2 | 0 | 3 | 0 | 2 | 0 | 0 | 2 | 0 | 9.67E5 | 7.73E5 |  | 1.27E6 |  | 1.7E6 |  |  | 2.41E5 |  | 3 | 3 | 1 | 1 | 0 | 2 | 0 | 1 | 0 | 0 | 1 | 0 | N | 74483 | Cold shock domain containing E1, RNA binding OS=Mus musculus OX=10090 GN=Csde1 PE=1 SV=1 |
| 1600 | 2121 | Q8JZN2\|Q8JZN2\_MOUSE | 89.84 | 4 | 2 | 2 | 0 | 2 | 0 | 1 | 0 | 0 | 2 | 0 | 9.67E5 | 7.73E5 |  | 1.27E6 |  | 1.7E6 |  |  | 2.41E5 |  | 3 | 3 | 1 | 1 | 0 | 2 | 0 | 1 | 0 | 0 | 1 | 0 | N | 85653 | Cold shock domain containing E1, RNA binding OS=Mus musculus OX=10090 GN=Csde1 PE=1 SV=1 |
| 1600 | 2122 | sp\|Q91W50\|CSDE1\_MOUSE | 89.84 | 4 | 2 | 2 | 0 | 2 | 0 | 1 | 0 | 0 | 2 | 0 | 9.67E5 | 7.73E5 |  | 1.27E6 |  | 1.7E6 |  |  | 2.41E5 |  | 3 | 3 | 1 | 1 | 0 | 2 | 0 | 1 | 0 | 0 | 1 | 0 | N | 88791 | Cold shock domain-containing protein E1 OS=Mus musculus OX=10090 GN=Csde1 PE=1 SV=1 |
| 1013 | 2524 | sp\|Q9D8W5\|PSD12\_MOUSE | 89.81 | 4 | 3 | 3 | 3 | 4 | 4 | 4 | 3 | 4 | 3 | 3 | 3.69E6 | 3.08E6 | 3.03E6 | 2.12E6 | 4.52E6 | 5.58E6 | 2.11E6 | 2.91E6 | 5.61E6 | 3.06E6 | 2 | 2 | 1 | 1 | 1 | 2 | 2 | 2 | 1 | 2 | 1 | 1 | N | 52895 | 26S proteasome non-ATPase regulatory subunit 12 OS=Mus musculus OX=10090 GN=Psmd12 PE=1 SV=4 |
| 1120 | 1655 | A0A2I3BQL9\|A0A2I3BQL9\_MOUSE | 89.80 | 8 | 1 | 3 | 0 | 1 | 0 | 3 | 4 | 4 | 2 | 3 | 2.61E6 | 3.18E6 |  | 9.17E5 |  | 3.55E5 | 3.07E6 | 3.86E6 | 3.31E6 | 1.95E6 | 4 | 4 | 1 | 2 | 0 | 1 | 0 | 1 | 2 | 2 | 1 | 2 | N | 66056 | phosphoenolpyruvate carboxykinase (GTP) (Fragment) OS=Mus musculus OX=10090 GN=Pck2 PE=1 SV=1 |
| 1120 | 1656 | A0A2I3BS39\|A0A2I3BS39\_MOUSE | 89.80 | 8 | 1 | 3 | 0 | 1 | 0 | 3 | 4 | 4 | 2 | 3 | 2.61E6 | 3.18E6 |  | 9.17E5 |  | 3.55E5 | 3.07E6 | 3.86E6 | 3.31E6 | 1.95E6 | 4 | 4 | 1 | 2 | 0 | 1 | 0 | 1 | 2 | 2 | 1 | 2 | N | 66910 | phosphoenolpyruvate carboxykinase (GTP) OS=Mus musculus OX=10090 GN=Pck2 PE=1 SV=1 |
| 1120 | 1658 | sp\|Q8BH04\|PCKGM\_MOUSE | 89.80 | 7 | 1 | 3 | 0 | 1 | 0 | 2 | 4 | 4 | 2 | 3 | 2.61E6 | 3.18E6 |  | 9.17E5 |  | 3.55E5 | 3.07E6 | 3.86E6 | 3.31E6 | 1.95E6 | 4 | 4 | 1 | 2 | 0 | 1 | 0 | 1 | 2 | 2 | 1 | 2 | N | 70528 | Phosphoenolpyruvate carboxykinase [GTP], mitochondrial OS=Mus musculus OX=10090 GN=Pck2 PE=1 SV=1 |
| 1120 | 1659 | A0A0R4J0G0\|A0A0R4J0G0\_MOUSE | 89.80 | 7 | 1 | 3 | 0 | 1 | 0 | 2 | 4 | 4 | 2 | 3 | 2.61E6 | 3.18E6 |  | 9.17E5 |  | 3.55E5 | 3.07E6 | 3.86E6 | 3.31E6 | 1.95E6 | 4 | 4 | 1 | 2 | 0 | 1 | 0 | 1 | 2 | 2 | 1 | 2 | N | 73418 | phosphoenolpyruvate carboxykinase (GTP) OS=Mus musculus OX=10090 GN=Pck2 PE=1 SV=1 |
| 1146 | 1628 | sp\|O08585\|CLCA\_MOUSE | 89.78 | 14 | 7 | 4 | 4 | 4 | 7 | 4 | 4 | 4 | 4 | 4 | 5.21E6 | 6.48E4 | 3.73E6 | 4.71E6 | 4.68E6 | 8.58E6 | 3.29E6 | 2.2E6 | 7.48E5 | 4.4E6 | 4 | 4 | 2 | 1 | 1 | 1 | 2 | 1 | 1 | 1 | 1 | 1 | Y | 25604 | Clathrin light chain A OS=Mus musculus OX=10090 GN=Clta PE=1 SV=2 |
| 1146 | 1629 | B1AWD8\|B1AWD8\_MOUSE | 89.78 | 14 | 7 | 4 | 4 | 4 | 7 | 4 | 4 | 4 | 4 | 4 | 5.21E6 | 6.48E4 | 3.73E6 | 4.71E6 | 4.68E6 | 8.58E6 | 3.29E6 | 2.2E6 | 7.48E5 | 4.4E6 | 4 | 4 | 2 | 1 | 1 | 1 | 2 | 1 | 1 | 1 | 1 | 1 | Y | 25661 | Clathrin light chain OS=Mus musculus OX=10090 GN=Clta PE=1 SV=1 |
| 1146 | 1630 | B1AWD9\|B1AWD9\_MOUSE | 89.78 | 13 | 7 | 4 | 4 | 4 | 6 | 4 | 4 | 4 | 4 | 4 | 5.21E6 | 6.48E4 | 3.73E6 | 4.71E6 | 4.68E6 | 8.58E6 | 3.29E6 | 2.2E6 | 7.48E5 | 4.4E6 | 4 | 4 | 2 | 1 | 1 | 1 | 2 | 1 | 1 | 1 | 1 | 1 | Y | 27062 | Clathrin light chain OS=Mus musculus OX=10090 GN=Clta PE=1 SV=1 |
| 1146 | 1684 | B1AWE0\|B1AWE0\_MOUSE | 89.78 | 15 | 8 | 4 | 4 | 4 | 7 | 4 | 4 | 4 | 4 | 4 | 5.21E6 | 6.48E4 | 3.73E6 | 4.71E6 | 4.68E6 | 8.58E6 | 3.29E6 | 2.2E6 | 7.48E5 | 4.4E6 | 4 | 4 | 2 | 1 | 1 | 1 | 2 | 1 | 1 | 1 | 1 | 1 | Y | 23479 | Clathrin light chain OS=Mus musculus OX=10090 GN=Clta PE=1 SV=1 |
| 1146 | 1685 | Q6PFA2\|Q6PFA2\_MOUSE | 89.78 | 15 | 8 | 4 | 4 | 4 | 7 | 4 | 4 | 4 | 4 | 4 | 5.21E6 | 6.48E4 | 3.73E6 | 4.71E6 | 4.68E6 | 8.58E6 | 3.29E6 | 2.2E6 | 7.48E5 | 4.4E6 | 4 | 4 | 2 | 1 | 1 | 1 | 2 | 1 | 1 | 1 | 1 | 1 | Y | 23633 | Clathrin light chain OS=Mus musculus OX=10090 GN=Clta PE=1 SV=1 |
| 1146 | 1686 | B1AWE1\|B1AWE1\_MOUSE | 89.78 | 14 | 7 | 4 | 4 | 4 | 7 | 4 | 4 | 4 | 4 | 4 | 5.21E6 | 6.48E4 | 3.73E6 | 4.71E6 | 4.68E6 | 8.58E6 | 3.29E6 | 2.2E6 | 7.48E5 | 4.4E6 | 4 | 4 | 2 | 1 | 1 | 1 | 2 | 1 | 1 | 1 | 1 | 1 | Y | 24964 | Clathrin light chain OS=Mus musculus OX=10090 GN=Clta PE=1 SV=1 |
| 1252 | 3248 | sp\|P55096\|ABCD3\_MOUSE | 89.68 | 3 | 2 | 3 | 2 | 2 | 2 | 2 | 2 | 2 | 2 | 2 | 3.14E6 | 7.04E6 | 9.82E5 | 1.98E6 | 3.15E6 | 5.46E6 | 4.08E6 | 2.12E6 | 4.1E6 | 2.66E6 | 2 | 2 | 1 | 2 | 1 | 1 | 1 | 1 | 1 | 1 | 1 | 1 | N | 75475 | ATP-binding cassette sub-family D member 3 OS=Mus musculus OX=10090 GN=Abcd3 PE=1 SV=2 |
| 1370 | 2590 | sp\|O55234\|PSB5\_MOUSE | 89.65 | 10 | 3 | 3 | 3 | 0 | 0 | 3 | 3 | 0 | 7 | 3 | 6.56E6 | 6.75E6 | 4.76E6 |  |  | 1.21E6 | 1.43E6 |  | 2.97E6 | 5.94E6 | 3 | 2 | 1 | 1 | 1 | 0 | 0 | 1 | 1 | 0 | 2 | 1 | N | 28532 | Proteasome subunit beta type-5 OS=Mus musculus OX=10090 GN=Psmb5 PE=1 SV=3 |
| 1072 | 1923 | sp\|Q9D0F3\|LMAN1\_MOUSE | 89.61 | 6 | 1 | 1 | 1 | 0 | 3 | 3 | 5 | 4 | 1 | 1 | 2.62E6 | 2.32E6 | 3.16E6 |  | 6.51E6 | 2.15E6 | 1.19E7 | 9.08E6 | 2.14E6 | 2.4E6 | 4 | 4 | 1 | 1 | 1 | 0 | 2 | 2 | 3 | 2 | 1 | 1 | N | 57789 | Protein ERGIC-53 OS=Mus musculus OX=10090 GN=Lman1 PE=1 SV=1 |
| 1984 | 2740 | sp\|P29351\|PTN6\_MOUSE | 89.50 | 5 | 0 | 0 | 0 | 0 | 2 | 0 | 3 | 2 | 0 | 0 |  |  |  |  | 8.36E5 |  | 7.98E5 | 4.6E5 |  |  | 2 | 2 | 0 | 0 | 0 | 0 | 1 | 0 | 1 | 1 | 0 | 0 | N | 67559 | Tyrosine-protein phosphatase non-receptor type 6 OS=Mus musculus OX=10090 GN=Ptpn6 PE=1 SV=2 |
| 1001 | 3850 | sp\|Q9Z1D1\|EIF3G\_MOUSE | 89.37 | 8 | 5 | 8 | 8 | 8 | 8 | 8 | 8 | 8 | 5 | 8 | 1.73E6 | 7.71E6 | 8.17E6 | 4.71E6 | 6.1E6 | 6.76E6 | 6.65E6 | 3.23E6 | 2.76E6 | 5.2E6 | 2 | 2 | 1 | 2 | 2 | 2 | 2 | 2 | 2 | 2 | 1 | 2 | N | 35638 | Eukaryotic translation initiation factor 3 subunit G OS=Mus musculus OX=10090 GN=Eif3g PE=1 SV=2 |
| 1656 | 1947 | sp\|Q6P4T2\|U520\_MOUSE | 89.26 | 2 | 0 | 0 | 0 | 1 | 0 | 0 | 0 | 0 | 0 | 0 | 3.62E6 |  |  | 5.9E5 |  |  |  |  |  |  | 3 | 3 | 1 | 0 | 0 | 2 | 0 | 0 | 0 | 0 | 0 | 0 | N | 244544 | U5 small nuclear ribonucleoprotein 200 kDa helicase OS=Mus musculus OX=10090 GN=Snrnp200 PE=1 SV=1 |
| 1106 | 2104 | sp\|Q9R1P3\|PSB2\_MOUSE | 88.80 | 16 | 12 | 0 | 0 | 9 | 4 | 9 | 4 | 9 | 9 | 4 | 2.56E6 |  |  | 4.3E6 | 4.85E6 | 6.52E6 | 2.56E6 | 4.8E6 | 7.17E6 | 2.88E6 | 3 | 3 | 2 | 0 | 0 | 2 | 1 | 2 | 1 | 2 | 2 | 1 | Y | 22906 | Proteasome subunit beta type-2 OS=Mus musculus OX=10090 GN=Psmb2 PE=1 SV=1 |
| 1017 | 3026 | sp\|Q99JB2\|STML2\_MOUSE | 88.61 | 7 | 7 | 4 | 7 | 7 | 7 | 7 | 7 | 7 | 4 | 3 | 5.18E6 | 2.35E6 | 5.28E6 | 2.07E6 | 3.8E6 | 3.55E6 | 3.91E6 | 1.48E6 | 4.55E6 | 2.86E6 | 2 | 2 | 2 | 1 | 2 | 2 | 2 | 2 | 2 | 2 | 1 | 1 | N | 38385 | Stomatin-like protein 2, mitochondrial OS=Mus musculus OX=10090 GN=Stoml2 PE=1 SV=1 |
| 1304 | 2712 | sp\|Q5EBG6\|HSPB6\_MOUSE | 88.60 | 15 | 0 | 0 | 0 | 8 | 7 | 7 | 8 | 8 | 0 | 0 |  |  |  | 1.58E6 | 1.15E6 | 2.23E6 | 3.85E6 | 2.3E6 |  |  | 2 | 2 | 0 | 0 | 0 | 1 | 1 | 1 | 1 | 1 | 0 | 0 | N | 17521 | Heat shock protein beta-6 OS=Mus musculus OX=10090 GN=Hspb6 PE=1 SV=1 |
| 1271 | 2638 | D3YUW7\|D3YUW7\_MOUSE | 88.41 | 2 | 0 | 2 | 1 | 0 | 1 | 0 | 1 | 0 | 1 | 1 |  | 5.53E5 | 2.55E5 |  | 5.59E5 |  | 4.66E5 |  | 1.37E6 | 5.72E4 | 2 | 2 | 0 | 2 | 1 | 0 | 1 | 0 | 1 | 0 | 1 | 1 | N | 135613 | Cingulin OS=Mus musculus OX=10090 GN=Cgn PE=1 SV=2 |
| 1271 | 2639 | E9QMC1\|E9QMC1\_MOUSE | 88.41 | 2 | 0 | 2 | 1 | 0 | 1 | 0 | 1 | 0 | 1 | 1 |  | 5.53E5 | 2.55E5 |  | 5.59E5 |  | 4.66E5 |  | 1.37E6 | 5.72E4 | 2 | 2 | 0 | 2 | 1 | 0 | 1 | 0 | 1 | 0 | 1 | 1 | N | 136415 | Cingulin OS=Mus musculus OX=10090 GN=Cgn PE=1 SV=2 |
| 1271 | 2640 | sp\|P59242\|CING\_MOUSE | 88.41 | 2 | 0 | 2 | 1 | 0 | 1 | 0 | 1 | 0 | 1 | 1 |  | 5.53E5 | 2.55E5 |  | 5.59E5 |  | 4.66E5 |  | 1.37E6 | 5.72E4 | 2 | 2 | 0 | 2 | 1 | 0 | 1 | 0 | 1 | 0 | 1 | 1 | N | 136447 | Cingulin OS=Mus musculus OX=10090 GN=Cgn PE=1 SV=1 |
| 1271 | 2641 | A0A5F8MPT6\|A0A5F8MPT6\_MOUSE | 88.41 | 2 | 0 | 2 | 1 | 0 | 1 | 0 | 1 | 0 | 1 | 1 |  | 5.53E5 | 2.55E5 |  | 5.59E5 |  | 4.66E5 |  | 1.37E6 | 5.72E4 | 2 | 2 | 0 | 2 | 1 | 0 | 1 | 0 | 1 | 0 | 1 | 1 | N | 136546 | Cingulin OS=Mus musculus OX=10090 GN=Cgn PE=1 SV=1 |
| 1178 | 1752 | sp\|Q9CWK8\|SNX2\_MOUSE | 88.40 | 8 | 2 | 0 | 0 | 2 | 0 | 2 | 0 | 4 | 4 | 2 | 1.24E6 |  |  |  |  | 9.04E5 |  | 5.18E5 | 2.67E6 |  | 4 | 2 | 1 | 0 | 0 | 1 | 0 | 1 | 0 | 2 | 2 | 1 | N | 58471 | Sorting nexin-2 OS=Mus musculus OX=10090 GN=Snx2 PE=1 SV=2 |
| 891 | 2754 | sp\|Q6R0H7\|GNAS1\_MOUSE | 88.10 | 2 | 1 | 1 | 2 | 1 | 2 | 2 | 1 | 1 | 1 | 1 |  |  | 1.15E6 |  | 1.48E6 | 7.11E5 |  |  |  |  | 2 | 1 | 2 | 1 | 2 | 2 | 3 | 3 | 2 | 2 | 1 | 1 | N | 121505 | Guanine nucleotide-binding protein G(s) subunit alpha isoforms XLas OS=Mus musculus OX=10090 GN=Gnas PE=1 SV=1 |
| 1124 | 3848 | Q8R3V2\|Q8R3V2\_MOUSE | 87.96 | 8 | 8 | 8 | 3 | 3 | 3 | 3 | 3 | 3 | 4 | 8 | 1.65E6 | 3.29E6 | 3.49E5 |  | 5.74E5 | 3E6 | 2.4E6 | 1.28E6 | 3.95E6 | 1.92E6 | 2 | 2 | 2 | 2 | 1 | 1 | 1 | 1 | 1 | 1 | 2 | 2 | N | 31098 | Aminoacyl tRNA synthetase complex-interacting multifunctional protein 2 OS=Mus musculus OX=10090 GN=Aimp2 PE=1 SV=1 |
| 1124 | 3851 | sp\|Q8R010\|AIMP2\_MOUSE | 87.96 | 7 | 7 | 7 | 3 | 3 | 3 | 3 | 3 | 3 | 4 | 7 | 1.65E6 | 3.29E6 | 3.49E5 |  | 5.74E5 | 3E6 | 2.4E6 | 1.28E6 | 3.95E6 | 1.92E6 | 2 | 2 | 2 | 2 | 1 | 1 | 1 | 1 | 1 | 1 | 2 | 2 | N | 35378 | Aminoacyl tRNA synthase complex-interacting multifunctional protein 2 OS=Mus musculus OX=10090 GN=Aimp2 PE=1 SV=2 |
| 1499 | 2233 | A2AAN2\|A2AAN2\_MOUSE | 87.94 | 5 | 2 | 0 | 2 | 1 | 3 | 0 | 2 | 2 | 0 | 0 | 1.13E6 |  | 1.23E5 | 5.23E5 | 4.11E5 |  | 4.78E5 | 5.15E5 |  |  | 3 | 3 | 1 | 0 | 1 | 1 | 2 | 0 | 1 | 1 | 0 | 0 | N | 65953 | Signal recognition particle subunit SRP68 OS=Mus musculus OX=10090 GN=Srp68 PE=1 SV=1 |
| 1499 | 2234 | sp\|Q8BMA6\|SRP68\_MOUSE | 87.94 | 5 | 1 | 0 | 2 | 1 | 3 | 0 | 2 | 2 | 0 | 0 | 1.13E6 |  | 1.23E5 | 5.23E5 | 4.11E5 |  | 4.78E5 | 5.15E5 |  |  | 3 | 3 | 1 | 0 | 1 | 1 | 2 | 0 | 1 | 1 | 0 | 0 | N | 70574 | Signal recognition particle subunit SRP68 OS=Mus musculus OX=10090 GN=Srp68 PE=1 SV=2 |
| 1384 | 2141 | sp\|Q9Z2H7\|GIPC2\_MOUSE | 87.93 | 10 | 10 | 8 | 4 | 4 | 4 | 0 | 0 | 0 | 0 | 0 | 5.4E6 | 1.12E6 | 0E0 | 2.52E5 | 5.8E5 |  |  |  |  |  | 3 | 3 | 3 | 2 | 1 | 1 | 1 | 0 | 0 | 0 | 0 | 0 | Y | 34118 | PDZ domain-containing protein GIPC2 OS=Mus musculus OX=10090 GN=Gipc2 PE=1 SV=1 |
| 1008 | 2520 | F6V6T4\|F6V6T4\_MOUSE | 87.85 | 15 | 9 | 7 | 5 | 5 | 5 | 9 | 5 | 0 | 12 | 5 | 8.79E6 | 3.89E5 | 3.85E6 | 1.55E6 | 8.99E5 | 7.69E6 | 2.4E6 |  | 7.87E6 | 2.36E6 | 3 | 3 | 2 | 1 | 1 | 1 | 1 | 2 | 2 | 0 | 2 | 1 | Y | 20033 | Transmembrane p24 trafficking protein 2 (Fragment) OS=Mus musculus OX=10090 GN=Tmed2 PE=1 SV=1 |
| 1008 | 2523 | sp\|Q9R0Q3\|TMED2\_MOUSE | 87.85 | 13 | 7 | 6 | 4 | 4 | 4 | 7 | 4 | 0 | 10 | 4 | 8.79E6 | 3.89E5 | 3.85E6 | 1.55E6 | 8.99E5 | 7.69E6 | 2.4E6 |  | 7.87E6 | 2.36E6 | 3 | 3 | 2 | 1 | 1 | 1 | 1 | 2 | 2 | 0 | 2 | 1 | Y | 22705 | Transmembrane emp24 domain-containing protein 2 OS=Mus musculus OX=10090 GN=Tmed2 PE=1 SV=1 |
| 1104 | 2020 | E9QAI5\|E9QAI5\_MOUSE | 87.80 | 1 | 0 | 0 | 1 | 1 | 0 | 0 | 0 | 1 | 1 | 0 |  |  |  | 2.7E5 |  |  |  |  |  |  | 3 | 1 | 1 | 1 | 2 | 1 | 1 | 1 | 0 | 2 | 3 | 1 | N | 236247 | Carbamoyl-phosphate synthetase 2, aspartate transcarbamylase, and dihydroorotase OS=Mus musculus OX=10090 GN=Cad PE=1 SV=1 |
| 1104 | 2022 | G3UWN2\|G3UWN2\_MOUSE | 87.80 | 1 | 0 | 0 | 1 | 1 | 0 | 0 | 0 | 1 | 1 | 0 |  |  |  | 2.7E5 |  |  |  |  |  |  | 3 | 1 | 1 | 1 | 2 | 1 | 1 | 1 | 0 | 2 | 3 | 1 | N | 236993 | Carbamoyl-phosphate synthetase 2, aspartate transcarbamylase, and dihydroorotase OS=Mus musculus OX=10090 GN=Cad PE=1 SV=1 |
| 1104 | 2025 | sp\|B2RQC6\|PYR1\_MOUSE | 87.80 | 1 | 0 | 0 | 1 | 0 | 0 | 0 | 0 | 1 | 1 | 0 |  |  |  | 2.7E5 |  |  |  |  |  |  | 3 | 1 | 1 | 1 | 2 | 1 | 1 | 1 | 0 | 2 | 3 | 1 | N | 243236 | Multifunctional protein CAD OS=Mus musculus OX=10090 GN=Cad PE=1 SV=1 |
| 1932 | 5179 | sp\|Q3TW96\|UAP1L\_MOUSE | 87.78 | 3 | 3 | 3 | 0 | 0 | 0 | 0 | 0 | 0 | 3 | 3 | 1.61E5 | 3.42E5 |  |  |  |  |  |  | 6E5 | 3.92E5 | 1 | 1 | 1 | 1 | 0 | 0 | 0 | 0 | 0 | 0 | 1 | 1 | N | 56614 | UDP-N-acetylhexosamine pyrophosphorylase-like protein 1 OS=Mus musculus OX=10090 GN=Uap1l1 PE=1 SV=1 |
| 889 | 3072 | E9Q0F0\|E9Q0F0\_MOUSE | 87.50 | 2 | 2 | 2 | 2 | 2 | 1 | 2 | 2 | 2 | 0 | 1 | 5.37E6 | 7.75E5 | 4.32E6 | 9.43E5 |  | 2.04E6 | 0E0 | 4.54E6 |  |  | 2 | 1 | 2 | 2 | 2 | 3 | 2 | 3 | 3 | 3 | 0 | 1 | N | 112265 | Keratin 78 OS=Mus musculus OX=10090 GN=Krt78 PE=1 SV=1 |
| 1158 | 3366 | sp\|P47915\|RL29\_MOUSE | 87.17 | 14 | 7 | 7 | 0 | 7 | 14 | 7 | 14 | 7 | 7 | 7 | 1.85E7 | 1.87E7 |  | 1.07E7 | 4.37E7 | 1.62E7 | 2.42E7 | 1.62E7 | 2E7 | 1.9E7 | 2 | 2 | 1 | 1 | 0 | 1 | 2 | 1 | 2 | 1 | 2 | 1 | N | 17587 | Large ribosomal subunit protein eL29 OS=Mus musculus OX=10090 GN=Rpl29 PE=1 SV=2 |
| 2113 | 4132 | sp\|Q9DC50\|OCTC\_MOUSE | 87.14 | 4 | 0 | 0 | 0 | 0 | 0 | 3 | 1 | 0 | 0 | 0 |  |  |  |  |  | 7.72E5 | 0E0 |  |  |  | 2 | 2 | 0 | 0 | 0 | 0 | 0 | 1 | 1 | 0 | 0 | 0 | N | 70264 | Peroxisomal carnitine O-octanoyltransferase OS=Mus musculus OX=10090 GN=Crot PE=1 SV=1 |
| 1928 | 3884 | D6RJ80\|D6RJ80\_MOUSE | 87.10 | 28 | 0 | 14 | 13 | 0 | 13 | 13 | 0 | 0 | 0 | 0 |  | 8.07E5 | 6.18E5 |  | 1.36E5 | 7.43E5 |  |  |  |  | 2 | 2 | 0 | 1 | 1 | 0 | 1 | 1 | 0 | 0 | 0 | 0 | N | 9196 | Solute carrier family 6 (neurotransmitter transporter), member 19 OS=Mus musculus OX=10090 GN=Slc6a19 PE=1 SV=1 |
| 1928 | 3886 | sp\|Q9D687\|S6A19\_MOUSE | 87.10 | 4 | 0 | 2 | 2 | 0 | 2 | 2 | 0 | 0 | 0 | 0 |  | 8.07E5 | 6.18E5 |  | 1.36E5 | 7.43E5 |  |  |  |  | 2 | 2 | 0 | 1 | 1 | 0 | 1 | 1 | 0 | 0 | 0 | 0 | N | 71367 | Sodium-dependent neutral amino acid transporter B(0)AT1 OS=Mus musculus OX=10090 GN=Slc6a19 PE=1 SV=1 |
| 1350 | 1994 | Q80UE5\|Q80UE5\_MOUSE | 87.04 | 4 | 1 | 1 | 1 | 1 | 0 | 2 | 0 | 0 | 1 | 0 | 6.76E6 | 4.55E7 | 5.12E6 | 2.12E5 |  | 7.61E5 |  |  | 5.71E7 |  | 3 | 3 | 1 | 1 | 1 | 1 | 0 | 1 | 0 | 0 | 1 | 0 | N | 101814 | Epb4.1l2 protein OS=Mus musculus OX=10090 GN=Epb41l2 PE=1 SV=1 |
| 1350 | 1996 | sp\|O70318\|E41L2\_MOUSE | 87.04 | 4 | 1 | 1 | 1 | 1 | 0 | 2 | 0 | 0 | 1 | 0 | 6.76E6 | 4.55E7 | 5.12E6 | 2.12E5 |  | 7.61E5 |  |  | 5.71E7 |  | 3 | 3 | 1 | 1 | 1 | 1 | 0 | 1 | 0 | 0 | 1 | 0 | N | 109940 | Band 4.1-like protein 2 OS=Mus musculus OX=10090 GN=Epb41l2 PE=1 SV=2 |
| 1133 | 3333 | sp\|Q91YI0\|ARLY\_MOUSE | 86.99 | 7 | 4 | 3 | 0 | 4 | 4 | 4 | 4 | 4 | 4 | 4 | 8.84E5 | 9.57E5 |  | 7.76E5 | 1.85E6 | 3.81E6 | 4.48E5 | 8.82E5 | 5.04E6 | 5.34E5 | 2 | 2 | 1 | 1 | 0 | 1 | 1 | 1 | 1 | 1 | 1 | 1 | Y | 51739 | Argininosuccinate lyase OS=Mus musculus OX=10090 GN=Asl PE=1 SV=1 |
| 1133 | 3408 | E0CY49\|E0CY49\_MOUSE | 86.99 | 12 | 8 | 5 | 0 | 8 | 8 | 8 | 8 | 8 | 8 | 8 | 8.84E5 | 9.57E5 |  | 7.76E5 | 1.85E6 | 3.81E6 | 4.48E5 | 8.82E5 | 5.04E6 | 5.34E5 | 2 | 2 | 1 | 1 | 0 | 1 | 1 | 1 | 1 | 1 | 1 | 1 | Y | 29478 | Argininosuccinate lyase (Fragment) OS=Mus musculus OX=10090 GN=Asl PE=1 SV=1 |
| 1259 | 2512 | sp\|Q6P8X1\|SNX6\_MOUSE | 86.88 | 6 | 3 | 3 | 2 | 3 | 3 | 4 | 3 | 1 | 3 | 3 | 2.34E5 | 7.85E5 | 5.07E6 | 3.61E5 | 3.88E5 | 7.86E5 | 4.58E5 |  | 1.83E6 | 4.88E5 | 3 | 2 | 1 | 1 | 1 | 1 | 1 | 2 | 1 | 1 | 1 | 1 | N | 46649 | Sorting nexin-6 OS=Mus musculus OX=10090 GN=Snx6 PE=1 SV=2 |
| 2159 | 3817 | D3Z7F1\|D3Z7F1\_MOUSE | 86.87 | 7 | 0 | 3 | 0 | 0 | 0 | 0 | 0 | 0 | 0 | 7 |  | 2.82E6 |  |  |  |  |  |  |  | 1.74E6 | 2 | 2 | 0 | 1 | 0 | 0 | 0 | 0 | 0 | 0 | 0 | 2 | N | 49792 | Cytochrome P450 OS=Mus musculus OX=10090 GN=Cyp2s1 PE=1 SV=1 |
| 2159 | 3818 | sp\|Q9DBX6\|CP2S1\_MOUSE | 86.87 | 6 | 0 | 3 | 0 | 0 | 0 | 0 | 0 | 0 | 0 | 6 |  | 2.82E6 |  |  |  |  |  |  |  | 1.74E6 | 2 | 2 | 0 | 1 | 0 | 0 | 0 | 0 | 0 | 0 | 0 | 2 | N | 55632 | Cytochrome P450 2S1 OS=Mus musculus OX=10090 GN=Cyp2s1 PE=1 SV=1 |
| 882 | 2054 | sp\|G3UW82\|MYH2\_MOUSE | 86.74 | 1 | 0 | 0 | 0 | 0 | 0 | 0 | 1 | 1 | 0 | 0 |  |  |  |  |  |  |  | 2.03E5 |  |  | 3 | 1 | 2 | 2 | 1 | 1 | 1 | 2 | 2 | 3 | 0 | 0 | N | 223217 | Myosin-2 OS=Mus musculus OX=10090 GN=Myh2 PE=1 SV=1 |
| 970 | 2378 | E9Q6P2\|E9Q6P2\_MOUSE | 86.49 | 6 | 4 | 4 | 4 | 4 | 4 | 4 | 2 | 2 | 6 | 4 | 2.28E6 | 4.68E6 | 4.13E6 | 2.53E6 |  | 3.55E6 |  | 6.37E5 | 3.86E6 | 7.09E5 | 3 | 1 | 2 | 2 | 2 | 2 | 2 | 2 | 1 | 1 | 3 | 2 | N | 56098 | Serine/threonine-protein phosphatase OS=Mus musculus OX=10090 GN=Ppp3cb PE=1 SV=1 |
| 970 | 2380 | G3X8U7\|G3X8U7\_MOUSE | 86.49 | 6 | 4 | 3 | 3 | 4 | 4 | 3 | 2 | 2 | 6 | 3 | 2.28E6 | 4.68E6 | 4.13E6 | 2.53E6 |  | 3.55E6 |  | 6.37E5 | 3.86E6 | 7.09E5 | 3 | 1 | 2 | 2 | 2 | 2 | 2 | 2 | 1 | 1 | 3 | 2 | N | 58131 | Serine/threonine-protein phosphatase OS=Mus musculus OX=10090 GN=Ppp3cb PE=1 SV=1 |
| 970 | 2381 | E0CZ78\|E0CZ78\_MOUSE | 86.49 | 6 | 4 | 3 | 3 | 4 | 4 | 3 | 2 | 2 | 6 | 3 | 2.28E6 | 4.68E6 | 4.13E6 | 2.53E6 |  | 3.55E6 |  | 6.37E5 | 3.86E6 | 7.09E5 | 3 | 1 | 2 | 2 | 2 | 2 | 2 | 2 | 1 | 1 | 3 | 2 | N | 59074 | Serine/threonine-protein phosphatase OS=Mus musculus OX=10090 GN=Ppp3cb PE=1 SV=1 |
| 970 | 2382 | sp\|P48453\|PP2BB\_MOUSE | 86.49 | 6 | 4 | 3 | 3 | 4 | 4 | 3 | 2 | 2 | 6 | 3 | 2.28E6 | 4.68E6 | 4.13E6 | 2.53E6 |  | 3.55E6 |  | 6.37E5 | 3.86E6 | 7.09E5 | 3 | 1 | 2 | 2 | 2 | 2 | 2 | 2 | 1 | 1 | 3 | 2 | N | 59174 | Serine/threonine-protein phosphatase 2B catalytic subunit beta isoform OS=Mus musculus OX=10090 GN=Ppp3cb PE=1 SV=2 |
| 2154 | 2593 | sp\|Q9DB05\|SNAA\_MOUSE | 86.47 | 9 | 5 | 0 | 0 | 0 | 0 | 0 | 4 | 0 | 0 | 0 | 3.57E5 |  |  |  |  |  | 4.01E5 |  |  |  | 2 | 2 | 1 | 0 | 0 | 0 | 0 | 0 | 1 | 0 | 0 | 0 | N | 33190 | Alpha-soluble NSF attachment protein OS=Mus musculus OX=10090 GN=Napa PE=1 SV=1 |
| 1316 | 4622 | sp\|Q921L3\|TMCO1\_MOUSE | 86.39 | 8 | 8 | 8 | 8 | 0 | 8 | 8 | 8 | 8 | 8 | 8 | 7.28E5 | 9.6E5 | 6.93E5 |  | 1.11E6 | 5.67E6 | 1.01E6 | 2.29E6 | 5.7E6 | 4.18E5 | 1 | 1 | 1 | 1 | 1 | 0 | 1 | 1 | 1 | 1 | 1 | 1 | N | 21175 | Calcium load-activated calcium channel OS=Mus musculus OX=10090 GN=Tmco1 PE=1 SV=1 |
| 1316 | 4623 | A0A0A6YVS2\|A0A0A6YVS2\_MOUSE | 86.39 | 8 | 8 | 8 | 8 | 0 | 8 | 8 | 8 | 8 | 8 | 8 | 7.28E5 | 9.6E5 | 6.93E5 |  | 1.11E6 | 5.67E6 | 1.01E6 | 2.29E6 | 5.7E6 | 4.18E5 | 1 | 1 | 1 | 1 | 1 | 0 | 1 | 1 | 1 | 1 | 1 | 1 | N | 22402 | Calcium load-activated calcium channel (Fragment) OS=Mus musculus OX=10090 GN=Tmco1 PE=1 SV=1 |
| 1243 | 2576 | sp\|P48758\|CBR1\_MOUSE | 86.10 | 14 | 3 | 3 | 11 | 3 | 0 | 0 | 3 | 0 | 3 | 3 | 7.21E6 | 8.41E6 | 6.25E5 | 1.39E6 |  |  | 5.55E6 |  | 6.51E6 | 7.24E6 | 2 | 2 | 1 | 1 | 1 | 1 | 0 | 0 | 1 | 0 | 1 | 1 | N | 30641 | Carbonyl reductase [NADPH] 1 OS=Mus musculus OX=10090 GN=Cbr1 PE=1 SV=3 |
| 1886 | 2596 | B7ZWL1\|B7ZWL1\_MOUSE | 86.07 | 1 | 0 | 0 | 0 | 0 | 0 | 0 | 0 | 0 | 0 | 0 |  |  |  |  | 1.78E5 |  | 3.14E5 |  |  |  | 2 | 2 | 0 | 0 | 0 | 0 | 1 | 0 | 1 | 0 | 0 | 0 | N | 260955 | CCR4-NOT transcription complex, subunit 1 OS=Mus musculus OX=10090 GN=Cnot1 PE=1 SV=1 |
| 1886 | 2597 | A0A1D5RMJ8\|A0A1D5RMJ8\_MOUSE | 86.07 | 1 | 0 | 0 | 0 | 0 | 0 | 0 | 0 | 0 | 0 | 0 |  |  |  |  | 1.78E5 |  | 3.14E5 |  |  |  | 2 | 2 | 0 | 0 | 0 | 0 | 1 | 0 | 1 | 0 | 0 | 0 | N | 266118 | CCR4-NOT transcription complex, subunit 1 OS=Mus musculus OX=10090 GN=Cnot1 PE=1 SV=1 |
| 1886 | 2598 | sp\|Q6ZQ08\|CNOT1\_MOUSE | 86.07 | 1 | 0 | 0 | 0 | 0 | 0 | 0 | 0 | 0 | 0 | 0 |  |  |  |  | 1.78E5 |  | 3.14E5 |  |  |  | 2 | 2 | 0 | 0 | 0 | 0 | 1 | 0 | 1 | 0 | 0 | 0 | N | 266805 | CCR4-NOT transcription complex subunit 1 OS=Mus musculus OX=10090 GN=Cnot1 PE=1 SV=2 |
| 1746 | 3814 | sp\|Q3UM45\|PP1R7\_MOUSE | 86.03 | 8 | 0 | 0 | 0 | 8 | 0 | 0 | 0 | 0 | 8 | 3 |  |  |  | 6.94E5 |  |  |  |  | 2.71E6 | 4.41E5 | 2 | 2 | 0 | 0 | 0 | 2 | 0 | 0 | 0 | 0 | 2 | 1 | N | 41292 | Protein phosphatase 1 regulatory subunit 7 OS=Mus musculus OX=10090 GN=Ppp1r7 PE=1 SV=2 |
| 1729 | 4323 | sp\|O88587\|COMT\_MOUSE | 85.93 | 6 | 0 | 0 | 6 | 0 | 0 | 0 | 0 | 0 | 0 | 0 |  |  | 1.51E6 |  |  |  |  |  |  |  | 1 | 1 | 0 | 0 | 2 | 0 | 0 | 0 | 0 | 0 | 0 | 0 | Y | 29486 | Catechol O-methyltransferase OS=Mus musculus OX=10090 GN=Comt PE=1 SV=2 |
| 1093 | 2895 | sp\|P58389\|PTPA\_MOUSE | 85.87 | 7 | 3 | 7 | 3 | 3 | 3 | 3 | 3 | 3 | 7 | 7 | 1.45E6 | 3.52E6 | 1.57E6 | 1.85E5 | 5.96E5 | 5.92E5 | 3.33E5 | 1.63E5 | 2.9E6 | 1.45E6 | 2 | 2 | 1 | 2 | 1 | 1 | 1 | 1 | 1 | 1 | 2 | 2 | Y | 36710 | Serine/threonine-protein phosphatase 2A activator OS=Mus musculus OX=10090 GN=Ptpa PE=1 SV=1 |
| 989 | 2162 | sp\|Q9CXZ1\|NDUS4\_MOUSE | 85.75 | 19 | 9 | 14 | 14 | 5 | 19 | 11 | 5 | 5 | 14 | 14 | 9.38E5 | 6.97E6 | 5.04E6 | 5.96E5 | 4.22E6 | 5.56E6 | 1.92E6 | 1.6E6 | 5.49E6 | 2.83E6 | 3 | 3 | 1 | 2 | 2 | 1 | 3 | 2 | 1 | 1 | 2 | 2 | N | 19785 | NADH dehydrogenase [ubiquinone] iron-sulfur protein 4, mitochondrial OS=Mus musculus OX=10090 GN=Ndufs4 PE=1 SV=3 |
| 989 | 2163 | E9QPX3\|E9QPX3\_MOUSE | 85.75 | 19 | 9 | 14 | 14 | 5 | 19 | 11 | 5 | 5 | 14 | 14 | 9.38E5 | 6.97E6 | 5.04E6 | 5.96E5 | 4.22E6 | 5.56E6 | 1.92E6 | 1.6E6 | 5.49E6 | 2.83E6 | 3 | 3 | 1 | 2 | 2 | 1 | 3 | 2 | 1 | 1 | 2 | 2 | N | 19803 | NADH dehydrogenase [ubiquinone] iron-sulfur protein 4, mitochondrial OS=Mus musculus OX=10090 GN=Ndufs4 PE=1 SV=1 |
| 1110 | 3316 | sp\|P30412\|PPIC\_MOUSE | 85.70 | 9 | 9 | 3 | 3 | 3 | 3 | 0 | 3 | 3 | 6 | 9 | 2.87E6 |  |  |  |  |  |  |  | 3.78E5 | 1.91E6 | 2 | 1 | 2 | 1 | 2 | 1 | 1 | 0 | 1 | 1 | 1 | 4 | N | 22794 | Peptidyl-prolyl cis-trans isomerase C OS=Mus musculus OX=10090 GN=Ppic PE=1 SV=1 |
| 1292 | 1778 | sp\|Q9CQC6\|5MP2\_MOUSE | 85.66 | 9 | 0 | 0 | 3 | 5 | 0 | 0 | 2 | 2 | 0 | 2 |  |  | 6.82E5 | 1.16E6 |  |  | 1.7E6 | 8.37E5 |  | 3.65E6 | 4 | 4 | 0 | 0 | 1 | 3 | 0 | 0 | 1 | 1 | 0 | 1 | N | 48043 | eIF5-mimic protein 2 OS=Mus musculus OX=10090 GN=Bzw1 PE=1 SV=1 |
| 1292 | 1781 | A0A087WQS2\|A0A087WQS2\_MOUSE | 85.66 | 8 | 0 | 0 | 2 | 4 | 0 | 0 | 2 | 2 | 0 | 2 |  |  | 6.82E5 | 1.16E6 |  |  | 1.7E6 | 8.37E5 |  | 3.65E6 | 4 | 4 | 0 | 0 | 1 | 3 | 0 | 0 | 1 | 1 | 0 | 1 | N | 51210 | Basic leucine zipper and W2 domains 1 OS=Mus musculus OX=10090 GN=Bzw1 PE=1 SV=1 |
| 1519 | 2633 | sp\|Q8VE37\|RCC1\_MOUSE | 85.33 | 5 | 3 | 0 | 5 | 0 | 3 | 0 | 0 | 0 | 3 | 0 | 4.7E6 |  | 3.43E6 |  | 2.35E6 |  |  |  | 2.35E6 |  | 2 | 2 | 1 | 0 | 2 | 0 | 1 | 0 | 0 | 0 | 1 | 0 | Y | 44931 | Regulator of chromosome condensation OS=Mus musculus OX=10090 GN=Rcc1 PE=1 SV=1 |
| 1519 | 2634 | Q6PFB2\|Q6PFB2\_MOUSE | 85.33 | 5 | 3 | 0 | 5 | 0 | 3 | 0 | 0 | 0 | 3 | 0 | 4.7E6 |  | 3.43E6 |  | 2.35E6 |  |  |  | 2.35E6 |  | 2 | 2 | 1 | 0 | 2 | 0 | 1 | 0 | 0 | 0 | 1 | 0 | Y | 46375 | Regulator of chromosome condensation OS=Mus musculus OX=10090 GN=Rcc1 PE=1 SV=1 |
| 1302 | 2099 | A2BFF8\|A2BFF8\_MOUSE | 85.22 | 5 | 3 | 2 | 0 | 2 | 2 | 0 | 2 | 2 | 1 | 3 | 5.96E6 | 0E0 |  | 2.31E6 | 3.5E6 |  | 3.78E6 | 1.8E6 | 6.17E6 | 8.51E6 | 3 | 3 | 2 | 1 | 0 | 1 | 1 | 0 | 1 | 1 | 1 | 2 | N | 68266 | Cytoplasmic dynein 1 intermediate chain 2 OS=Mus musculus OX=10090 GN=Dync1i2 PE=1 SV=1 |
| 1302 | 2100 | sp\|O88487\|DC1I2\_MOUSE | 85.22 | 5 | 3 | 2 | 0 | 2 | 2 | 0 | 2 | 2 | 1 | 3 | 5.96E6 | 0E0 |  | 2.31E6 | 3.5E6 |  | 3.78E6 | 1.8E6 | 6.17E6 | 8.51E6 | 3 | 3 | 2 | 1 | 0 | 1 | 1 | 0 | 1 | 1 | 1 | 2 | N | 68394 | Cytoplasmic dynein 1 intermediate chain 2 OS=Mus musculus OX=10090 GN=Dync1i2 PE=1 SV=1 |
| 1302 | 2101 | Q3TPJ8\|Q3TPJ8\_MOUSE | 85.22 | 5 | 3 | 2 | 0 | 2 | 2 | 0 | 2 | 2 | 1 | 3 | 5.96E6 | 0E0 |  | 2.31E6 | 3.5E6 |  | 3.78E6 | 1.8E6 | 6.17E6 | 8.51E6 | 3 | 3 | 2 | 1 | 0 | 1 | 1 | 0 | 1 | 1 | 1 | 2 | N | 70613 | Cytoplasmic dynein 1 intermediate chain 2 OS=Mus musculus OX=10090 GN=Dync1i2 PE=1 SV=1 |
| 1302 | 2102 | A2BFF5\|A2BFF5\_MOUSE | 85.22 | 5 | 3 | 2 | 0 | 2 | 2 | 0 | 2 | 2 | 1 | 3 | 5.96E6 | 0E0 |  | 2.31E6 | 3.5E6 |  | 3.78E6 | 1.8E6 | 6.17E6 | 8.51E6 | 3 | 3 | 2 | 1 | 0 | 1 | 1 | 0 | 1 | 1 | 1 | 2 | N | 71296 | Cytoplasmic dynein 1 intermediate chain 2 OS=Mus musculus OX=10090 GN=Dync1i2 PE=1 SV=1 |
| 1302 | 2103 | A2BFF9\|A2BFF9\_MOUSE | 85.22 | 5 | 3 | 2 | 0 | 2 | 2 | 0 | 2 | 2 | 1 | 3 | 5.96E6 | 0E0 |  | 2.31E6 | 3.5E6 |  | 3.78E6 | 1.8E6 | 6.17E6 | 8.51E6 | 3 | 3 | 2 | 1 | 0 | 1 | 1 | 0 | 1 | 1 | 1 | 2 | N | 71424 | Cytoplasmic dynein 1 intermediate chain 2 OS=Mus musculus OX=10090 GN=Dync1i2 PE=1 SV=1 |
| 858 | 3145 | sp\|Q9CPT4\|MYDGF\_MOUSE | 85.16 | 12 | 7 | 12 | 12 | 12 | 12 | 12 | 12 | 12 | 12 | 12 | 0E0 | 6.32E6 | 3.28E6 | 2.41E6 | 1.66E6 | 1.47E6 | 1.34E6 | 1.99E6 | 5.18E6 | 1.75E6 | 2 | 2 | 1 | 3 | 2 | 2 | 2 | 2 | 2 | 3 | 3 | 2 | N | 17982 | Myeloid-derived growth factor OS=Mus musculus OX=10090 GN=Mydgf PE=1 SV=1 |
| 1295 | 3118 | Q80XR5\|Q80XR5\_MOUSE | 85.14 | 4 | 0 | 2 | 4 | 2 | 2 | 2 | 4 | 2 | 0 | 2 |  | 6.11E5 | 1.8E6 | 1.49E6 | 2.05E6 | 8.36E5 | 1.85E6 | 4.39E5 |  | 9.14E5 | 2 | 2 | 0 | 1 | 2 | 1 | 1 | 1 | 2 | 1 | 0 | 1 | N | 53121 | Splicing factor U2AF subunit OS=Mus musculus OX=10090 GN=U2af2 PE=1 SV=1 |
| 1295 | 3119 | sp\|P26369\|U2AF2\_MOUSE | 85.14 | 4 | 0 | 2 | 4 | 2 | 2 | 2 | 4 | 2 | 0 | 2 |  | 6.11E5 | 1.8E6 | 1.49E6 | 2.05E6 | 8.36E5 | 1.85E6 | 4.39E5 |  | 9.14E5 | 2 | 2 | 0 | 1 | 2 | 1 | 1 | 1 | 2 | 1 | 0 | 1 | N | 53517 | Splicing factor U2AF 65 kDa subunit OS=Mus musculus OX=10090 GN=U2af2 PE=1 SV=3 |
| 2093 | 3207 | sp\|Q91V61\|SFXN3\_MOUSE | 85.06 | 9 | 9 | 0 | 0 | 0 | 0 | 9 | 0 | 0 | 0 | 0 | 9.25E5 |  |  |  |  | 4.76E5 |  |  |  |  | 1 | 1 | 1 | 0 | 0 | 0 | 0 | 1 | 0 | 0 | 0 | 0 | N | 35406 | Sideroflexin-3 OS=Mus musculus OX=10090 GN=Sfxn3 PE=1 SV=1 |
| 1261 | 3890 | sp\|Q9CX80\|CYGB\_MOUSE | 84.75 | 9 | 5 | 5 | 5 | 5 | 0 | 5 | 5 | 5 | 9 | 9 | 2.68E6 | 4.55E5 | 7.79E5 | 4.01E5 |  | 1.07E6 | 2.06E5 | 1.76E5 | 8.54E6 | 2.5E6 | 2 | 2 | 1 | 1 | 1 | 1 | 0 | 1 | 1 | 1 | 2 | 2 | N | 21466 | Cytoglobin OS=Mus musculus OX=10090 GN=Cygb PE=1 SV=1 |
| 1628 | 2173 | sp\|Q9CR35\|CTRB1\_MOUSE | 84.70 | 13 | 6 | 0 | 13 | 0 | 0 | 0 | 0 | 0 | 0 | 0 | 1.88E6 |  | 9.54E6 |  |  |  |  |  |  |  | 3 | 3 | 1 | 0 | 3 | 0 | 0 | 0 | 0 | 0 | 0 | 0 | Y | 27822 | Chymotrypsinogen B OS=Mus musculus OX=10090 GN=Ctrb1 PE=1 SV=1 |
| 2067 | 3386 | A0A0A6YX18\|A0A0A6YX18\_MOUSE | 84.68 | 5 | 5 | 0 | 0 | 0 | 0 | 5 | 0 | 0 | 0 | 0 | 1.13E6 |  |  |  |  | 4.31E5 |  |  |  |  | 1 | 1 | 1 | 0 | 0 | 0 | 0 | 1 | 0 | 0 | 0 | 0 | N | 54109 | V-type proton ATPase subunit H OS=Mus musculus OX=10090 GN=Atp6v1h PE=1 SV=1 |
| 2067 | 3394 | sp\|Q8BVE3\|VATH\_MOUSE | 84.68 | 5 | 5 | 0 | 0 | 0 | 0 | 5 | 0 | 0 | 0 | 0 | 1.13E6 |  |  |  |  | 4.31E5 |  |  |  |  | 1 | 1 | 1 | 0 | 0 | 0 | 0 | 1 | 0 | 0 | 0 | 0 | N | 55855 | V-type proton ATPase subunit H OS=Mus musculus OX=10090 GN=Atp6v1h PE=1 SV=1 |
| 2067 | 3441 | A0A0A6YWP6\|A0A0A6YWP6\_MOUSE | 84.68 | 5 | 5 | 0 | 0 | 0 | 0 | 5 | 0 | 0 | 0 | 0 | 1.13E6 |  |  |  |  | 4.31E5 |  |  |  |  | 1 | 1 | 1 | 0 | 0 | 0 | 0 | 1 | 0 | 0 | 0 | 0 | N | 50811 | V-type proton ATPase subunit H OS=Mus musculus OX=10090 GN=Atp6v1h PE=1 SV=1 |
| 1182 | 2675 | sp\|Q80UM7\|MOGS\_MOUSE | 84.68 | 3 | 1 | 1 | 1 | 1 | 1 | 1 | 1 | 1 | 1 | 3 | 6.32E5 | 2.48E5 | 3.37E5 | 1.94E5 | 2.19E5 | 3.86E5 | 4.32E5 | 5.24E5 | 1.38E6 | 1.02E6 | 2 | 2 | 1 | 1 | 1 | 1 | 1 | 1 | 1 | 1 | 1 | 2 | N | 91831 | Mannosyl-oligosaccharide glucosidase OS=Mus musculus OX=10090 GN=Mogs PE=1 SV=1 |
| 974 | 3897 | sp\|Q8R1I1\|QCR9\_MOUSE | 84.58 | 38 | 11 | 38 | 38 | 38 | 38 | 11 | 38 | 38 | 38 | 38 | 3.46E6 | 3.12E6 | 1.88E7 | 7.74E6 | 1.34E7 | 5.64E6 | 9.99E6 | 8.41E6 | 1.48E7 | 8.94E6 | 2 | 2 | 1 | 2 | 2 | 2 | 2 | 1 | 2 | 2 | 2 | 2 | N | 7446 | Cytochrome b-c1 complex subunit 9 OS=Mus musculus OX=10090 GN=Uqcr10 PE=1 SV=1 |
| 1462 | 5201 | D3Z6V2\|D3Z6V2\_MOUSE | 84.52 | 10 | 10 | 10 | 10 | 0 | 0 | 10 | 10 | 0 | 10 | 10 | 3.49E5 | 1.1E6 | 1.22E6 |  |  | 1.87E6 | 1.44E6 |  | 3.7E5 | 1.39E6 | 1 | 1 | 1 | 1 | 2 | 0 | 0 | 1 | 1 | 0 | 1 | 1 | N | 14005 | Receptor expression-enhancing protein OS=Mus musculus OX=10090 GN=Reep6 PE=1 SV=1 |
| 1462 | 5207 | sp\|Q9JM62\|REEP6\_MOUSE | 84.52 | 6 | 6 | 6 | 6 | 0 | 0 | 6 | 6 | 0 | 6 | 6 | 3.49E5 | 1.1E6 | 1.22E6 |  |  | 1.87E6 | 1.44E6 |  | 3.7E5 | 1.39E6 | 1 | 1 | 1 | 1 | 2 | 0 | 0 | 1 | 1 | 0 | 1 | 1 | N | 22204 | Receptor expression-enhancing protein 6 OS=Mus musculus OX=10090 GN=Reep6 PE=1 SV=1 |
| 1740 | 2657 | A0A075B5R2\|A0A075B5R2\_MOUSE | 84.47 | 20 | 8 | 0 | 0 | 0 | 0 | 0 | 0 | 0 | 20 | 0 | 7.11E5 |  |  |  |  |  |  |  | 1.3E7 |  | 2 | 2 | 1 | 0 | 0 | 0 | 0 | 0 | 0 | 0 | 3 | 0 | Y | 13457 | Immunoglobulin heavy variable 7-3 (Fragment) OS=Mus musculus OX=10090 GN=Ighv7-3 PE=1 SV=2 |
| 936 | 1726 | sp\|Q9EP69\|SAC1\_MOUSE | 84.41 | 7 | 2 | 3 | 2 | 2 | 3 | 6 | 2 | 2 | 3 | 2 | 7.12E5 | 3.88E6 | 6.18E5 | 2.4E5 | 6.44E6 | 1.1E7 | 6.42E5 | 7.36E5 | 3.77E6 | 1.49E6 | 5 | 5 | 1 | 2 | 1 | 1 | 2 | 4 | 1 | 1 | 2 | 1 | N | 66944 | Phosphatidylinositol-3-phosphatase SAC1 OS=Mus musculus OX=10090 GN=Sacm1l PE=1 SV=1 |
| 936 | 1728 | A0A5F8MPK9\|A0A5F8MPK9\_MOUSE | 84.41 | 7 | 2 | 3 | 2 | 2 | 3 | 6 | 2 | 2 | 3 | 2 | 7.12E5 | 3.88E6 | 6.18E5 | 2.4E5 | 6.44E6 | 1.1E7 | 6.42E5 | 7.36E5 | 3.77E6 | 1.49E6 | 5 | 5 | 1 | 2 | 1 | 1 | 2 | 4 | 1 | 1 | 2 | 1 | N | 69492 | Phosphatidylinositol-3-phosphatase SAC1 OS=Mus musculus OX=10090 GN=Sacm1l PE=1 SV=1 |
| 2124 | 2984 | sp\|Q9DCJ5\|NDUA8\_MOUSE | 84.29 | 15 | 0 | 0 | 0 | 0 | 7 | 0 | 8 | 0 | 0 | 0 |  |  |  |  | 1.85E5 |  | 9.29E5 |  |  |  | 2 | 2 | 0 | 0 | 0 | 0 | 1 | 0 | 1 | 0 | 0 | 0 | Y | 19992 | NADH dehydrogenase [ubiquinone] 1 alpha subcomplex subunit 8 OS=Mus musculus OX=10090 GN=Ndufa8 PE=1 SV=3 |
| 984 | 2965 | sp\|P35279\|RAB6A\_MOUSE | 83.95 | 11 | 5 | 11 | 11 | 11 | 11 | 5 | 11 | 5 | 11 | 5 | 1.06E6 | 2.13E6 | 2.01E6 | 7.91E5 | 1.38E6 | 2.25E6 | 1.97E6 |  | 1.65E6 |  | 2 | 1 | 1 | 2 | 2 | 2 | 3 | 1 | 2 | 1 | 2 | 1 | N | 23590 | Ras-related protein Rab-6A OS=Mus musculus OX=10090 GN=Rab6a PE=1 SV=4 |
| 984 | 3542 | sp\|P61294\|RAB6B\_MOUSE | 83.95 | 11 | 5 | 11 | 11 | 11 | 11 | 5 | 11 | 5 | 11 | 5 | 1.06E6 | 2.13E6 | 2.01E6 | 7.91E5 | 1.38E6 | 2.25E6 | 1.97E6 |  | 1.65E6 |  | 2 | 1 | 1 | 2 | 2 | 2 | 3 | 1 | 2 | 1 | 2 | 1 | N | 23462 | Ras-related protein Rab-6B OS=Mus musculus OX=10090 GN=Rab6b PE=1 SV=1 |
| 1282 | 1704 | sp\|O35326\|SRSF5\_MOUSE | 83.80 | 14 | 3 | 8 | 3 | 3 | 3 | 0 | 7 | 3 | 0 | 0 |  | 2.75E6 | 3.11E6 | 1.01E6 |  |  | 3.41E5 |  |  |  | 4 | 3 | 1 | 2 | 1 | 1 | 1 | 0 | 2 | 1 | 0 | 0 | N | 30891 | Serine/arginine-rich splicing factor 5 OS=Mus musculus OX=10090 GN=Srsf5 PE=1 SV=2 |
| 1282 | 1705 | Q9D8S5\|Q9D8S5\_MOUSE | 83.80 | 14 | 3 | 8 | 3 | 3 | 3 | 0 | 7 | 3 | 0 | 0 |  | 2.75E6 | 3.11E6 | 1.01E6 |  |  | 3.41E5 |  |  |  | 4 | 3 | 1 | 2 | 1 | 1 | 1 | 0 | 2 | 1 | 0 | 0 | N | 30978 | Serine and arginine-rich splicing factor 5 OS=Mus musculus OX=10090 GN=Srsf5 PE=1 SV=1 |
| 1748 | 5182 | sp\|Q62395\|TFF3\_MOUSE | 83.63 | 20 | 20 | 20 | 0 | 0 | 0 | 0 | 0 | 0 | 20 | 20 | 1.94E6 | 5.96E5 |  |  |  |  |  |  | 4.61E6 | 0E0 | 1 | 1 | 1 | 1 | 0 | 0 | 0 | 0 | 0 | 0 | 2 | 1 | Y | 8808 | Trefoil factor 3 OS=Mus musculus OX=10090 GN=Tff3 PE=1 SV=2 |
| 1153 | 1741 | sp\|Q99JW2\|ACY1\_MOUSE | 83.43 | 9 | 0 | 2 | 5 | 7 | 5 | 5 | 3 | 0 | 2 | 0 |  | 1.4E6 | 1.46E7 | 2.47E6 | 1.09E7 | 1.41E7 | 7.67E6 |  | 1.04E6 |  | 4 | 4 | 0 | 1 | 2 | 3 | 2 | 2 | 1 | 0 | 1 | 0 | N | 45781 | Aminoacylase-1 OS=Mus musculus OX=10090 GN=Acy1 PE=1 SV=1 |
| 1153 | 1916 | A0A1L1SS90\|A0A1L1SS90\_MOUSE | 83.43 | 10 | 0 | 2 | 5 | 8 | 5 | 5 | 3 | 0 | 2 | 0 |  | 1.4E6 | 1.46E7 | 2.47E6 | 1.09E7 | 1.41E7 | 7.67E6 |  | 1.04E6 |  | 4 | 4 | 0 | 1 | 2 | 3 | 2 | 2 | 1 | 0 | 1 | 0 | N | 42068 | N-acyl-aliphatic-L-amino acid amidohydrolase OS=Mus musculus OX=10090 GN=Acy1 PE=1 SV=1 |
| 1153 | 1917 | A0A0R4J050\|A0A0R4J050\_MOUSE | 83.43 | 9 | 0 | 2 | 5 | 7 | 5 | 5 | 3 | 0 | 2 | 0 |  | 1.4E6 | 1.46E7 | 2.47E6 | 1.09E7 | 1.41E7 | 7.67E6 |  | 1.04E6 |  | 4 | 4 | 0 | 1 | 2 | 3 | 2 | 2 | 1 | 0 | 1 | 0 | N | 45795 | N-acyl-aliphatic-L-amino acid amidohydrolase OS=Mus musculus OX=10090 GN=Acy1 PE=1 SV=1 |
| 1043 | 1968 | sp\|P51859\|HDGF\_MOUSE | 83.08 | 19 | 4 | 8 | 8 | 12 | 4 | 4 | 4 | 8 | 11 | 4 | 3.65E6 | 7.08E6 | 9.68E6 | 4.18E6 | 1.33E6 | 3.36E6 | 2.34E6 | 4.24E6 | 1.02E7 | 2.3E6 | 4 | 4 | 1 | 2 | 2 | 3 | 1 | 1 | 1 | 2 | 2 | 1 | Y | 26269 | Hepatoma-derived growth factor OS=Mus musculus OX=10090 GN=Hdgf PE=1 SV=2 |
| 1043 | 1970 | A0AA74KSY4\|A0AA74KSY4\_MOUSE | 83.08 | 14 | 3 | 6 | 6 | 9 | 3 | 3 | 3 | 6 | 8 | 3 | 3.65E6 | 7.08E6 | 9.68E6 | 4.18E6 | 1.33E6 | 3.36E6 | 2.34E6 | 4.24E6 | 1.02E7 | 2.3E6 | 4 | 4 | 1 | 2 | 2 | 3 | 1 | 1 | 1 | 2 | 2 | 1 | Y | 34537 | Heparin binding growth factor OS=Mus musculus OX=10090 GN=Hdgf PE=1 SV=1 |
| 1591 | 4032 | sp\|Q60590\|A1AG1\_MOUSE | 82.89 | 12 | 7 | 0 | 0 | 7 | 0 | 0 | 7 | 7 | 4 | 0 | 4.8E5 |  |  | 6.68E5 |  |  | 1.05E6 | 1.07E6 | 6.17E6 |  | 2 | 2 | 1 | 0 | 0 | 1 | 0 | 0 | 1 | 1 | 1 | 0 | N | 23895 | Alpha-1-acid glycoprotein 1 OS=Mus musculus OX=10090 GN=Orm1 PE=1 SV=1 |
| 2091 | 2192 | sp\|Q05920\|PYC\_MOUSE | 82.77 | 3 | 0 | 2 | 0 | 0 | 0 | 0 | 0 | 0 | 1 | 0 |  | 3.63E6 |  |  |  |  |  |  | 4.89E5 |  | 3 | 3 | 0 | 2 | 0 | 0 | 0 | 0 | 0 | 0 | 1 | 0 | N | 129684 | Pyruvate carboxylase, mitochondrial OS=Mus musculus OX=10090 GN=Pc PE=1 SV=1 |
| 2091 | 2193 | G5E8R3\|G5E8R3\_MOUSE | 82.77 | 3 | 0 | 2 | 0 | 0 | 0 | 0 | 0 | 0 | 1 | 0 |  | 3.63E6 |  |  |  |  |  |  | 4.89E5 |  | 3 | 3 | 0 | 2 | 0 | 0 | 0 | 0 | 0 | 0 | 1 | 0 | N | 129701 | Pyruvate carboxylase OS=Mus musculus OX=10090 GN=Pcx PE=1 SV=1 |
| 2091 | 2194 | E9QPD7\|E9QPD7\_MOUSE | 82.77 | 3 | 0 | 2 | 0 | 0 | 0 | 0 | 0 | 0 | 1 | 0 |  | 3.63E6 |  |  |  |  |  |  | 4.89E5 |  | 3 | 3 | 0 | 2 | 0 | 0 | 0 | 0 | 0 | 0 | 1 | 0 | N | 129832 | Pyruvate carboxylase OS=Mus musculus OX=10090 GN=Pcx PE=1 SV=1 |
| 1324 | 3953 | sp\|O08583\|THOC4\_MOUSE | 82.66 | 7 | 0 | 4 | 0 | 3 | 7 | 0 | 3 | 4 | 4 | 0 |  | 2.13E6 |  | 8.97E5 | 4.08E6 |  | 1.33E6 | 3.22E6 | 1.01E6 |  | 2 | 2 | 0 | 1 | 0 | 1 | 2 | 0 | 1 | 1 | 1 | 0 | N | 26940 | THO complex subunit 4 OS=Mus musculus OX=10090 GN=Alyref PE=1 SV=3 |
| 1604 | 2164 | A2AU61\|A2AU61\_MOUSE | 82.66 | 15 | 6 | 0 | 0 | 4 | 0 | 0 | 5 | 0 | 0 | 6 | 9.75E5 |  |  | 1.59E6 |  |  | 5.74E5 |  |  | 4.14E5 | 3 | 3 | 1 | 0 | 0 | 1 | 0 | 0 | 1 | 0 | 0 | 1 | N | 23120 | HnRNP-associated with lethal yellow (Fragment) OS=Mus musculus OX=10090 GN=Raly PE=1 SV=1 |
| 1604 | 2165 | A2AU62\|A2AU62\_MOUSE | 82.66 | 11 | 4 | 0 | 0 | 3 | 0 | 0 | 4 | 0 | 0 | 4 | 9.75E5 |  |  | 1.59E6 |  |  | 5.74E5 |  |  | 4.14E5 | 3 | 3 | 1 | 0 | 0 | 1 | 0 | 0 | 1 | 0 | 0 | 1 | N | 31690 | HnRNP-associated with lethal yellow (Fragment) OS=Mus musculus OX=10090 GN=Raly PE=1 SV=1 |
| 1604 | 2166 | sp\|Q64012\|RALY\_MOUSE | 82.66 | 10 | 4 | 0 | 0 | 3 | 0 | 0 | 4 | 0 | 0 | 4 | 9.75E5 |  |  | 1.59E6 |  |  | 5.74E5 |  |  | 4.14E5 | 3 | 3 | 1 | 0 | 0 | 1 | 0 | 0 | 1 | 0 | 0 | 1 | N | 33188 | RNA-binding protein Raly OS=Mus musculus OX=10090 GN=Raly PE=1 SV=3 |
| 1238 | 1987 | sp\|Q9D8U8\|SNX5\_MOUSE | 82.65 | 7 | 3 | 0 | 3 | 3 | 0 | 1 | 3 | 4 | 3 | 2 | 1.63E6 |  | 8.81E5 | 4.4E5 |  |  | 6.5E5 | 8.29E5 | 3.22E6 | 4.48E5 | 3 | 2 | 1 | 0 | 1 | 1 | 0 | 1 | 1 | 2 | 1 | 1 | N | 46797 | Sorting nexin-5 OS=Mus musculus OX=10090 GN=Snx5 PE=1 SV=1 |
| 1495 | 2436 | sp\|Q3UMT1\|PP12C\_MOUSE | 82.51 | 3 | 0 | 0 | 0 | 0 | 1 | 2 | 0 | 0 | 1 | 0 |  |  |  |  | 8.05E5 | 0E0 |  |  | 4.18E6 |  | 2 | 2 | 0 | 0 | 0 | 0 | 1 | 1 | 0 | 0 | 1 | 0 | N | 84685 | Protein phosphatase 1 regulatory subunit 12C OS=Mus musculus OX=10090 GN=Ppp1r12c PE=1 SV=1 |
| 1713 | 2677 | E9PYT3\|E9PYT3\_MOUSE | 82.46 | 4 | 0 | 0 | 0 | 0 | 0 | 0 | 0 | 0 | 2 | 2 |  |  |  |  |  |  |  |  | 2.64E6 | 2.18E6 | 2 | 2 | 0 | 0 | 0 | 0 | 0 | 0 | 0 | 0 | 2 | 1 | N | 60232 | Atlastin GTPase 3 OS=Mus musculus OX=10090 GN=Atl3 PE=1 SV=1 |
| 1713 | 2678 | sp\|Q91YH5\|ATLA3\_MOUSE | 82.46 | 4 | 0 | 0 | 0 | 0 | 0 | 0 | 0 | 0 | 2 | 2 |  |  |  |  |  |  |  |  | 2.64E6 | 2.18E6 | 2 | 2 | 0 | 0 | 0 | 0 | 0 | 0 | 0 | 0 | 2 | 1 | N | 60575 | Atlastin-3 OS=Mus musculus OX=10090 GN=Atl3 PE=1 SV=1 |
| 1197 | 2488 | sp\|P63328\|PP2BA\_MOUSE | 82.35 | 6 | 4 | 2 | 2 | 2 | 4 | 2 | 2 | 0 | 4 | 2 | 9.2E5 |  |  |  |  |  |  |  |  |  | 3 | 1 | 2 | 1 | 1 | 1 | 2 | 1 | 1 | 0 | 2 | 1 | N | 58644 | Protein phosphatase 3 catalytic subunit alpha OS=Mus musculus OX=10090 GN=Ppp3ca PE=1 SV=1 |
| 1007 | 3985 | B1AVK5\|B1AVK5\_MOUSE | 82.35 | 1 | 1 | 0 | 1 | 1 | 0 | 1 | 0 | 0 | 1 | 1 | 8.01E7 |  | 1.27E8 | 7.81E6 |  | 1.09E8 |  |  | 1.41E7 | 6.3E7 | 2 | 1 | 2 | 0 | 1 | 1 | 0 | 1 | 0 | 0 | 2 | 2 | Y | 164033 | Collagen, type IV, alpha 6 OS=Mus musculus OX=10090 GN=Col4a6 PE=1 SV=1 |
| 999 | 2451 | sp\|P00493\|HPRT\_MOUSE | 82.14 | 15 | 12 | 7 | 12 | 7 | 12 | 5 | 8 | 12 | 12 | 12 | 7.45E6 | 2.32E6 | 7.73E6 | 2.17E5 | 4.61E6 | 3.86E5 | 2.24E6 | 1.56E6 | 1.3E7 | 4.99E6 | 3 | 3 | 2 | 1 | 2 | 1 | 2 | 1 | 2 | 2 | 2 | 2 | Y | 24570 | Hypoxanthine-guanine phosphoribosyltransferase OS=Mus musculus OX=10090 GN=Hprt1 PE=1 SV=3 |
| 1275 | 1806 | sp\|Q91WQ3\|SYYC\_MOUSE | 82.00 | 7 | 0 | 2 | 0 | 2 | 0 | 3 | 4 | 2 | 0 | 0 |  | 1.08E6 |  | 2.73E5 |  | 4.56E6 | 2.13E6 | 7.01E5 |  |  | 4 | 4 | 0 | 1 | 0 | 1 | 0 | 2 | 2 | 1 | 0 | 0 | N | 59105 | Tyrosine--tRNA ligase, cytoplasmic OS=Mus musculus OX=10090 GN=Yars1 PE=1 SV=3 |
| 1275 | 1808 | A2A7S7\|A2A7S7\_MOUSE | 82.00 | 7 | 0 | 2 | 0 | 2 | 0 | 3 | 3 | 2 | 0 | 0 |  | 1.08E6 |  | 2.73E5 |  | 4.56E6 | 2.13E6 | 7.01E5 |  |  | 4 | 4 | 0 | 1 | 0 | 1 | 0 | 2 | 2 | 1 | 0 | 0 | N | 63002 | Tyrosine--tRNA ligase OS=Mus musculus OX=10090 GN=Yars1 PE=1 SV=1 |
| 1461 | 4017 | sp\|P00416\|COX3\_MOUSE | 81.96 | 8 | 5 | 5 | 2 | 0 | 2 | 2 | 2 | 0 | 5 | 0 | 6.05E5 | 1.67E6 | 4.62E6 |  | 2.63E6 | 2.9E6 | 2.27E6 |  | 9.68E5 |  | 2 | 2 | 1 | 1 | 1 | 0 | 1 | 1 | 1 | 0 | 1 | 0 | N | 29923 | Cytochrome c oxidase subunit 3 OS=Mus musculus OX=10090 GN=mt-Co3 PE=1 SV=2 |
| 990 | 2221 | sp\|Q9EPU0\|RENT1\_MOUSE | 81.92 | 4 | 0 | 0 | 0 | 0 | 2 | 3 | 1 | 0 | 0 | 1 |  |  |  |  | 1.16E6 | 1.24E6 | 1.2E7 |  |  | 2.68E5 | 4 | 4 | 0 | 0 | 0 | 0 | 2 | 3 | 1 | 0 | 0 | 1 | N | 123967 | Regulator of nonsense transcripts 1 OS=Mus musculus OX=10090 GN=Upf1 PE=1 SV=2 |
| 1424 | 2501 | F6QHD1\|F6QHD1\_MOUSE | 81.88 | 5 | 3 | 3 | 0 | 0 | 3 | 0 | 2 | 0 | 0 | 3 | 4.34E6 | 4.62E6 |  |  | 1.99E6 |  | 1.76E6 |  |  | 3.25E6 | 2 | 2 | 1 | 1 | 0 | 0 | 1 | 0 | 1 | 0 | 0 | 1 | N | 43388 | Metadherin (Fragment) OS=Mus musculus OX=10090 GN=Mtdh PE=1 SV=2 |
| 1424 | 2502 | sp\|Q80WJ7\|LYRIC\_MOUSE | 81.88 | 3 | 2 | 2 | 0 | 0 | 2 | 0 | 2 | 0 | 0 | 2 | 4.34E6 | 4.62E6 |  |  | 1.99E6 |  | 1.76E6 |  |  | 3.25E6 | 2 | 2 | 1 | 1 | 0 | 0 | 1 | 0 | 1 | 0 | 0 | 1 | N | 63846 | Protein LYRIC OS=Mus musculus OX=10090 GN=Mtdh PE=1 SV=1 |
| 1116 | 3689 | G3UZW8\|G3UZW8\_MOUSE | 81.70 | 7 | 7 | 7 | 0 | 4 | 4 | 4 | 4 | 7 | 4 | 7 | 1.18E7 | 1.1E7 |  | 2.03E6 | 3.25E6 | 2.92E6 | 2.39E6 | 4.79E6 | 6.05E6 | 8.81E6 | 2 | 2 | 2 | 2 | 0 | 1 | 1 | 1 | 1 | 2 | 1 | 2 | N | 28092 | Proteasome subunit beta (Fragment) OS=Mus musculus OX=10090 GN=Psmb8 PE=1 SV=3 |
| 1116 | 3690 | sp\|P28063\|PSB8\_MOUSE | 81.70 | 7 | 7 | 7 | 0 | 4 | 4 | 4 | 4 | 7 | 4 | 7 | 1.18E7 | 1.1E7 |  | 2.03E6 | 3.25E6 | 2.92E6 | 2.39E6 | 4.79E6 | 6.05E6 | 8.81E6 | 2 | 2 | 2 | 2 | 0 | 1 | 1 | 1 | 1 | 2 | 1 | 2 | N | 30260 | Proteasome subunit beta type-8 OS=Mus musculus OX=10090 GN=Psmb8 PE=1 SV=2 |
| 1712 | 2155 | Q5SUH7\|Q5SUH7\_MOUSE | 81.68 | 5 | 0 | 2 | 0 | 0 | 0 | 1 | 0 | 0 | 2 | 2 |  | 2.64E6 |  |  |  | 9.95E5 |  |  | 2.35E6 | 1.99E6 | 3 | 3 | 0 | 1 | 0 | 0 | 0 | 1 | 0 | 0 | 1 | 1 | N | 67731 | Clathrin interactor 1 OS=Mus musculus OX=10090 GN=Clint1 PE=1 SV=1 |
| 1712 | 2161 | sp\|Q99KN9\|EPN4\_MOUSE | 81.68 | 5 | 0 | 2 | 0 | 0 | 0 | 1 | 0 | 0 | 2 | 2 |  | 2.64E6 |  |  |  | 9.95E5 |  |  | 2.35E6 | 1.99E6 | 3 | 3 | 0 | 1 | 0 | 0 | 0 | 1 | 0 | 0 | 1 | 1 | N | 68513 | Clathrin interactor 1 OS=Mus musculus OX=10090 GN=Clint1 PE=1 SV=2 |
| 1712 | 2176 | Q5SUH6\|Q5SUH6\_MOUSE | 81.68 | 5 | 0 | 2 | 0 | 0 | 0 | 1 | 0 | 0 | 2 | 2 |  | 2.64E6 |  |  |  | 9.95E5 |  |  | 2.35E6 | 1.99E6 | 3 | 3 | 0 | 1 | 0 | 0 | 0 | 1 | 0 | 0 | 1 | 1 | N | 69759 | Clathrin interactor 1 OS=Mus musculus OX=10090 GN=Clint1 PE=1 SV=1 |
| 2762 | 4087 | sp\|Q9DC61\|MPPA\_MOUSE | 81.49 | 3 | 0 | 0 | 0 | 0 | 0 | 3 | 0 | 0 | 0 | 0 |  |  |  |  |  | 2.71E5 |  |  |  |  | 1 | 1 | 0 | 0 | 0 | 0 | 0 | 1 | 0 | 0 | 0 | 0 | N | 58279 | Mitochondrial-processing peptidase subunit alpha OS=Mus musculus OX=10090 GN=Pmpca PE=1 SV=1 |
| 2762 | 5138 | A2AIW9\|A2AIW9\_MOUSE | 81.49 | 4 | 0 | 0 | 0 | 0 | 0 | 4 | 0 | 0 | 0 | 0 |  |  |  |  |  | 2.71E5 |  |  |  |  | 1 | 1 | 0 | 0 | 0 | 0 | 0 | 1 | 0 | 0 | 0 | 0 | N | 48455 | Mitochondrial-processing peptidase subunit alpha OS=Mus musculus OX=10090 GN=Pmpca PE=1 SV=1 |
| 1156 | 2509 | sp\|Q9CQ54\|NDUC2\_MOUSE | 81.46 | 24 | 0 | 11 | 18 | 11 | 17 | 0 | 17 | 11 | 11 | 11 |  | 1.24E6 | 4.84E6 | 1.95E6 | 6.71E6 |  | 8.23E6 | 6.09E5 | 6.79E6 | 2.36E6 | 3 | 3 | 0 | 1 | 2 | 1 | 2 | 0 | 2 | 1 | 1 | 1 | N | 14164 | NADH dehydrogenase [ubiquinone] 1 subunit C2 OS=Mus musculus OX=10090 GN=Ndufc2 PE=1 SV=1 |
| 1594 | 2095 | sp\|Q60865\|CAPR1\_MOUSE | 81.23 | 4 | 0 | 0 | 1 | 3 | 0 | 0 | 2 | 0 | 2 | 0 |  |  | 4.11E6 | 3.71E6 |  |  | 0E0 |  | 1.53E6 |  | 3 | 3 | 0 | 0 | 1 | 2 | 0 | 0 | 1 | 0 | 1 | 0 | N | 78169 | Caprin-1 OS=Mus musculus OX=10090 GN=Caprin1 PE=1 SV=2 |
| 1206 | 3854 | sp\|P17665\|COX7C\_MOUSE | 81.13 | 29 | 14 | 14 | 14 | 14 | 29 | 14 | 14 | 14 | 14 | 14 | 2.03E7 | 2.68E7 | 2.32E7 | 1.56E7 | 1.84E7 | 1.34E7 | 8.1E6 | 9.94E6 | 2.53E7 | 2.01E7 | 2 | 2 | 1 | 2 | 1 | 1 | 2 | 1 | 1 | 1 | 1 | 1 | N | 7333 | Cytochrome c oxidase subunit 7C, mitochondrial OS=Mus musculus OX=10090 GN=Cox7c PE=1 SV=1 |
| 1262 | 3893 | E9Q7G1\|E9Q7G1\_MOUSE | 80.87 | 12 | 7 | 7 | 7 | 7 | 7 | 7 | 7 | 7 | 7 | 12 | 1.44E6 | 2.04E6 | 1.58E6 | 5.06E5 | 5.93E5 | 1.07E6 | 9.48E5 | 2.25E5 | 3.18E6 | 9.92E5 | 2 | 2 | 1 | 1 | 1 | 1 | 1 | 1 | 1 | 1 | 1 | 2 | Y | 21276 | Transmembrane p24 trafficking protein 7 OS=Mus musculus OX=10090 GN=Tmed7 PE=1 SV=1 |
| 1262 | 3894 | D3YZZ5\|D3YZZ5\_MOUSE | 80.87 | 10 | 6 | 6 | 6 | 6 | 6 | 6 | 6 | 6 | 6 | 10 | 1.44E6 | 2.04E6 | 1.58E6 | 5.06E5 | 5.93E5 | 1.07E6 | 9.48E5 | 2.25E5 | 3.18E6 | 9.92E5 | 2 | 2 | 1 | 1 | 1 | 1 | 1 | 1 | 1 | 1 | 1 | 2 | Y | 25201 | Transmembrane p24 trafficking protein 7 OS=Mus musculus OX=10090 GN=Tmed7 PE=1 SV=1 |
| 1526 | 2487 | sp\|Q91Z53\|GRHPR\_MOUSE | 80.79 | 12 | 0 | 3 | 4 | 0 | 4 | 4 | 0 | 5 | 7 | 0 |  | 2.82E6 | 0E0 |  | 9.27E5 | 8.76E5 |  | 3.08E5 | 2.89E6 |  | 3 | 3 | 0 | 1 | 1 | 0 | 1 | 1 | 0 | 1 | 2 | 0 | Y | 35329 | Glyoxylate reductase/hydroxypyruvate reductase OS=Mus musculus OX=10090 GN=Grhpr PE=1 SV=1 |
| 2450 | 4933 | sp\|Q9ESB3\|HRG\_MOUSE | 80.74 | 3 | 0 | 0 | 0 | 0 | 3 | 0 | 3 | 0 | 0 | 0 |  |  |  |  | 0E0 |  | 2.21E5 |  |  |  | 1 | 1 | 0 | 0 | 0 | 0 | 1 | 0 | 1 | 0 | 0 | 0 | N | 59163 | Histidine-rich glycoprotein OS=Mus musculus OX=10090 GN=Hrg PE=1 SV=2 |
| 2450 | 4934 | A0A0R4J039\|A0A0R4J039\_MOUSE | 80.74 | 3 | 0 | 0 | 0 | 0 | 3 | 0 | 3 | 0 | 0 | 0 |  |  |  |  | 0E0 |  | 2.21E5 |  |  |  | 1 | 1 | 0 | 0 | 0 | 0 | 1 | 0 | 1 | 0 | 0 | 0 | N | 60439 | Histidine-rich glycoprotein OS=Mus musculus OX=10090 GN=Hrg PE=1 SV=1 |
| 2450 | 5192 | A0A338P6H8\|A0A338P6H8\_MOUSE | 80.74 | 8 | 0 | 0 | 0 | 0 | 8 | 0 | 8 | 0 | 0 | 0 |  |  |  |  | 0E0 |  | 2.21E5 |  |  |  | 1 | 1 | 0 | 0 | 0 | 0 | 1 | 0 | 1 | 0 | 0 | 0 | N | 22257 | Histidine-rich glycoprotein OS=Mus musculus OX=10090 GN=Hrg PE=1 SV=1 |
| 1321 | 3413 | sp\|Q6P5F9\|XPO1\_MOUSE | 80.71 | 3 | 1 | 1 | 1 | 1 | 1 | 3 | 1 | 1 | 0 | 1 | 1.39E6 | 1.85E6 | 1.57E6 | 5.61E5 | 1.41E6 | 4.14E6 | 9.78E5 | 9.15E5 |  | 1.34E6 | 2 | 2 | 1 | 1 | 1 | 1 | 1 | 2 | 1 | 1 | 0 | 1 | Y | 123093 | Exportin-1 OS=Mus musculus OX=10090 GN=Xpo1 PE=1 SV=1 |
| 1380 | 2898 | sp\|Q61160\|FADD\_MOUSE | 80.66 | 8 | 8 | 8 | 0 | 0 | 8 | 0 | 8 | 0 | 8 | 8 | 2.22E6 | 7.1E5 |  |  | 6.82E5 |  | 6.45E5 |  | 1.3E6 | 3.42E5 | 1 | 1 | 2 | 1 | 0 | 0 | 2 | 0 | 1 | 0 | 1 | 1 | N | 22960 | FAS-associated death domain protein OS=Mus musculus OX=10090 GN=Fadd PE=1 SV=1 |
| 1263 | 3940 | sp\|Q9Z2Y8\|PLPHP\_MOUSE | 80.46 | 8 | 5 | 8 | 5 | 5 | 5 | 5 | 5 | 5 | 5 | 5 |  | 1.34E6 |  |  |  |  |  |  |  |  | 2 | 1 | 1 | 2 | 1 | 1 | 1 | 1 | 1 | 1 | 1 | 1 | N | 30049 | Pyridoxal phosphate homeostasis protein OS=Mus musculus OX=10090 GN=Plpbp PE=1 SV=1 |
| 1263 | 3941 | A0A1B0GRP7\|A0A1B0GRP7\_MOUSE | 80.46 | 7 | 4 | 7 | 4 | 4 | 4 | 4 | 4 | 4 | 4 | 4 |  | 1.34E6 |  |  |  |  |  |  |  |  | 2 | 1 | 1 | 2 | 1 | 1 | 1 | 1 | 1 | 1 | 1 | 1 | N | 36180 | Pyridoxal phosphate binding protein (Fragment) OS=Mus musculus OX=10090 GN=Plpbp PE=1 SV=1 |
| 1902 | 2706 | sp\|Q9JK48\|SHLB1\_MOUSE | 80.39 | 4 | 4 | 0 | 0 | 0 | 0 | 0 | 0 | 0 | 0 | 0 | 1.09E6 |  |  |  |  |  |  |  |  |  | 1 | 1 | 1 | 0 | 0 | 0 | 0 | 0 | 0 | 0 | 0 | 0 | N | 40855 | Endophilin-B1 OS=Mus musculus OX=10090 GN=Sh3glb1 PE=1 SV=1 |
| 1902 | 2707 | A0A0G2JEC4\|A0A0G2JEC4\_MOUSE | 80.39 | 4 | 4 | 0 | 0 | 0 | 0 | 0 | 0 | 0 | 0 | 0 | 1.09E6 |  |  |  |  |  |  |  |  |  | 1 | 1 | 1 | 0 | 0 | 0 | 0 | 0 | 0 | 0 | 0 | 0 | N | 44303 | Endophilin-B1 OS=Mus musculus OX=10090 GN=Sh3glb1 PE=1 SV=1 |
| 860 | 3181 | sp\|P62320\|SMD3\_MOUSE | 80.35 | 15 | 15 | 15 | 15 | 15 | 15 | 15 | 8 | 15 | 7 | 15 | 1.64E7 | 4.64E6 | 1.56E7 | 6.87E6 | 1.15E7 | 8.98E6 | 2.46E6 | 8.28E6 | 1.63E7 | 1.39E7 | 2 | 2 | 3 | 2 | 2 | 2 | 2 | 3 | 1 | 2 | 2 | 3 | Y | 13916 | Small nuclear ribonucleoprotein Sm D3 OS=Mus musculus OX=10090 GN=Snrpd3 PE=1 SV=1 |
| 1831 | 2760 | sp\|Q61830\|MRC1\_MOUSE | 80.31 | 2 | 1 | 0 | 0 | 0 | 0 | 0 | 0 | 0 | 1 | 1 | 4.91E5 |  |  |  |  |  |  |  | 6.46E5 | 7.37E5 | 2 | 2 | 1 | 0 | 0 | 0 | 0 | 0 | 0 | 0 | 1 | 1 | Y | 164981 | Macrophage mannose receptor 1 OS=Mus musculus OX=10090 GN=Mrc1 PE=1 SV=2 |
| 1199 | 2821 | sp\|P81117\|NUCB2\_MOUSE | 80.28 | 4 | 2 | 2 | 2 | 2 | 2 | 4 | 2 | 2 | 2 | 2 | 3.9E5 | 1.97E6 | 7.43E5 | 6.84E5 | 1.02E6 | 1.74E6 | 2.06E6 | 1.48E6 | 1.23E6 | 6.46E5 | 2 | 1 | 1 | 1 | 1 | 1 | 1 | 2 | 1 | 1 | 1 | 1 | N | 50305 | Nucleobindin-2 OS=Mus musculus OX=10090 GN=Nucb2 PE=1 SV=2 |
| 1199 | 2822 | Q3UKN6\|Q3UKN6\_MOUSE | 80.28 | 4 | 2 | 2 | 2 | 2 | 2 | 4 | 2 | 2 | 2 | 2 | 3.9E5 | 1.97E6 | 7.43E5 | 6.84E5 | 1.02E6 | 1.74E6 | 2.06E6 | 1.48E6 | 1.23E6 | 6.46E5 | 2 | 1 | 1 | 1 | 1 | 1 | 1 | 2 | 1 | 1 | 1 | 1 | N | 50354 | Nucleobindin 2 OS=Mus musculus OX=10090 GN=Nucb2 PE=1 SV=1 |
| 1453 | 2626 | A0A1W2P7G2\|A0A1W2P7G2\_MOUSE | 80.27 | 15 | 0 | 0 | 7 | 7 | 7 | 7 | 0 | 7 | 15 | 0 |  |  | 2.35E6 | 1.1E6 | 2.42E6 | 1.78E6 |  | 2.36E6 | 1.47E7 |  | 2 | 2 | 0 | 0 | 1 | 1 | 1 | 1 | 0 | 1 | 2 | 0 | N | 14114 | SAP domain containing ribonucleoprotein OS=Mus musculus OX=10090 GN=Sarnp PE=1 SV=1 |
| 1453 | 2627 | sp\|Q9D1J3\|SARNP\_MOUSE | 80.27 | 9 | 0 | 0 | 4 | 4 | 4 | 4 | 0 | 4 | 9 | 0 |  |  | 2.35E6 | 1.1E6 | 2.42E6 | 1.78E6 |  | 2.36E6 | 1.47E7 |  | 2 | 2 | 0 | 0 | 1 | 1 | 1 | 1 | 0 | 1 | 2 | 0 | N | 23533 | SAP domain-containing ribonucleoprotein OS=Mus musculus OX=10090 GN=Sarnp PE=1 SV=3 |
| 1668 | 2819 | sp\|Q9R190\|MTA2\_MOUSE | 80.17 | 3 | 3 | 0 | 0 | 0 | 0 | 0 | 3 | 3 | 0 | 0 | 1.37E6 |  |  |  |  |  | 9.72E5 | 6E5 |  |  | 1 | 1 | 1 | 0 | 0 | 0 | 0 | 0 | 1 | 1 | 0 | 0 | N | 75030 | Metastasis-associated protein MTA2 OS=Mus musculus OX=10090 GN=Mta2 PE=1 SV=1 |
| 1511 | 4815 | sp\|P03921\|NU5M\_MOUSE | 80.16 | 3 | 0 | 3 | 3 | 3 | 3 | 0 | 0 | 0 | 3 | 3 |  | 2.45E6 | 1.32E6 | 3.3E5 | 1.33E6 |  |  |  | 2.86E6 | 1.97E6 | 1 | 1 | 0 | 1 | 1 | 1 | 1 | 0 | 0 | 0 | 1 | 1 | N | 68475 | NADH-ubiquinone oxidoreductase chain 5 OS=Mus musculus OX=10090 GN=Mtnd5 PE=1 SV=3 |
| 1632 | 3946 | sp\|P56389\|CDD\_MOUSE | 80.13 | 21 | 9 | 0 | 9 | 9 | 0 | 0 | 0 | 0 | 21 | 9 | 5.49E5 |  | 8.27E5 | 1.82E5 |  |  |  |  | 1.99E6 | 3.36E5 | 2 | 2 | 1 | 0 | 1 | 1 | 0 | 0 | 0 | 0 | 2 | 1 | N | 16131 | Cytidine deaminase OS=Mus musculus OX=10090 GN=Cda PE=1 SV=2 |
| 1455 | 2222 | A2AM65\|A2AM65\_MOUSE | 80.08 | 10 | 4 | 0 | 0 | 0 | 3 | 4 | 7 | 0 | 7 | 4 | 1.58E2 |  |  |  | 8.46E5 | 6.22E6 | 3.92E6 |  | 2.37E6 | 6.3E6 | 3 | 3 | 1 | 0 | 0 | 0 | 1 | 1 | 2 | 0 | 2 | 1 | N | 32539 | Heterochromatin protein 1-binding protein 3 (Fragment) OS=Mus musculus OX=10090 GN=Hp1bp3 PE=1 SV=1 |
| 1455 | 2227 | Z4YKA3\|Z4YKA3\_MOUSE | 80.08 | 5 | 2 | 0 | 0 | 0 | 2 | 2 | 4 | 0 | 4 | 2 | 1.58E2 |  |  |  | 8.46E5 | 6.22E6 | 3.92E6 |  | 2.37E6 | 6.3E6 | 3 | 3 | 1 | 0 | 0 | 0 | 1 | 1 | 2 | 0 | 2 | 1 | N | 56825 | Heterochromatin protein 1-binding protein 3 OS=Mus musculus OX=10090 GN=Hp1bp3 PE=1 SV=1 |
| 1455 | 2229 | Z4YKB8\|Z4YKB8\_MOUSE | 80.08 | 5 | 2 | 0 | 0 | 0 | 2 | 2 | 4 | 0 | 3 | 2 | 1.58E2 |  |  |  | 8.46E5 | 6.22E6 | 3.92E6 |  | 2.37E6 | 6.3E6 | 3 | 3 | 1 | 0 | 0 | 0 | 1 | 1 | 2 | 0 | 2 | 1 | N | 60851 | Heterochromatin protein 1-binding protein 3 OS=Mus musculus OX=10090 GN=Hp1bp3 PE=1 SV=1 |
| 1455 | 2230 | sp\|Q3TEA8\|HP1B3\_MOUSE | 80.08 | 5 | 2 | 0 | 0 | 0 | 2 | 2 | 4 | 0 | 3 | 2 | 1.58E2 |  |  |  | 8.46E5 | 6.22E6 | 3.92E6 |  | 2.37E6 | 6.3E6 | 3 | 3 | 1 | 0 | 0 | 0 | 1 | 1 | 2 | 0 | 2 | 1 | N | 60867 | Heterochromatin protein 1-binding protein 3 OS=Mus musculus OX=10090 GN=Hp1bp3 PE=1 SV=1 |
| 2162 | 3920 | sp\|Q9CPP6\|NDUA5\_MOUSE | 79.96 | 25 | 0 | 0 | 0 | 25 | 0 | 0 | 8 | 0 | 0 | 0 |  |  |  | 2.34E6 |  |  | 5.36E5 |  |  |  | 2 | 2 | 0 | 0 | 0 | 2 | 0 | 0 | 1 | 0 | 0 | 0 | N | 13360 | NADH dehydrogenase [ubiquinone] 1 alpha subcomplex subunit 5 OS=Mus musculus OX=10090 GN=Ndufa5 PE=1 SV=3 |
| 1283 | 2543 | sp\|Q91XA2\|GOLM1\_MOUSE | 79.89 | 5 | 2 | 0 | 0 | 5 | 4 | 0 | 2 | 0 | 4 | 0 | 1.33E7 |  |  | 9.58E6 | 1.26E6 |  | 9.32E6 |  | 3.27E6 |  | 2 | 2 | 1 | 0 | 0 | 2 | 1 | 0 | 1 | 0 | 1 | 0 | N | 44326 | Golgi membrane protein 1 OS=Mus musculus OX=10090 GN=Golm1 PE=1 SV=2 |
| 1443 | 2007 | Q05DV1\|Q05DV1\_MOUSE | 79.79 | 5 | 0 | 0 | 1 | 1 | 0 | 0 | 1 | 0 | 2 | 3 |  |  | 5.04E6 | 1.95E6 |  |  | 1.33E6 |  | 1.61E6 | 4.33E6 | 3 | 3 | 0 | 0 | 1 | 1 | 0 | 0 | 1 | 0 | 1 | 2 | Y | 72931 | NADPH--hemoprotein reductase OS=Mus musculus OX=10090 GN=Por PE=1 SV=1 |
| 1443 | 2011 | sp\|P37040\|NCPR\_MOUSE | 79.79 | 5 | 0 | 0 | 1 | 1 | 0 | 0 | 1 | 0 | 2 | 3 |  |  | 5.04E6 | 1.95E6 |  |  | 1.33E6 |  | 1.61E6 | 4.33E6 | 3 | 3 | 0 | 0 | 1 | 1 | 0 | 0 | 1 | 0 | 1 | 2 | Y | 77044 | NADPH--cytochrome P450 reductase OS=Mus musculus OX=10090 GN=Por PE=1 SV=2 |
| 1625 | 5041 | sp\|Q9CQ65\|MTAP\_MOUSE | 79.78 | 6 | 0 | 6 | 0 | 6 | 6 | 6 | 0 | 0 | 6 | 6 |  | 1.13E6 |  | 2.4E5 | 0E0 | 9.06E5 |  |  | 1.24E6 | 3.63E5 | 1 | 1 | 0 | 1 | 0 | 1 | 1 | 1 | 0 | 0 | 1 | 1 | N | 31062 | S-methyl-5'-thioadenosine phosphorylase OS=Mus musculus OX=10090 GN=Mtap PE=1 SV=1 |
| 1159 | 3901 | sp\|Q64674\|SPEE\_MOUSE | 79.78 | 6 | 3 | 3 | 6 | 3 | 3 | 3 | 6 | 6 | 3 | 3 | 1.95E6 | 1.43E6 | 9.83E5 | 1.05E6 | 2.07E6 | 1.99E6 | 1.79E6 | 1.31E6 | 5.77E6 | 1.47E6 | 2 | 2 | 1 | 1 | 2 | 1 | 1 | 1 | 2 | 2 | 1 | 1 | N | 33995 | Spermidine synthase OS=Mus musculus OX=10090 GN=Srm PE=1 SV=1 |
| 2006 | 2765 | A0A0R4J0I9\|A0A0R4J0I9\_MOUSE | 79.76 | 0 | 0 | 0 | 0 | 0 | 0 | 0 | 0 | 0 | 0 | 0 |  |  |  |  |  |  |  |  | 5.52E5 |  | 1 | 1 | 0 | 0 | 0 | 0 | 0 | 0 | 0 | 0 | 1 | 0 | N | 504775 | Low density lipoprotein receptor-related protein 1 OS=Mus musculus OX=10090 GN=Lrp1 PE=1 SV=1 |
| 2006 | 2766 | sp\|Q91ZX7\|LRP1\_MOUSE | 79.76 | 0 | 0 | 0 | 0 | 0 | 0 | 0 | 0 | 0 | 0 | 0 |  |  |  |  |  |  |  |  | 5.52E5 |  | 1 | 1 | 0 | 0 | 0 | 0 | 0 | 0 | 0 | 0 | 1 | 0 | N | 504745 | Prolow-density lipoprotein receptor-related protein 1 OS=Mus musculus OX=10090 GN=Lrp1 PE=1 SV=1 |
| 2126 | 3348 | sp\|O70310\|NMT1\_MOUSE | 79.58 | 5 | 0 | 0 | 0 | 0 | 3 | 3 | 2 | 0 | 0 | 0 |  |  |  |  | 3.68E5 | 1.56E6 | 2.04E6 |  |  |  | 2 | 2 | 0 | 0 | 0 | 0 | 1 | 1 | 1 | 0 | 0 | 0 | Y | 56888 | Glycylpeptide N-tetradecanoyltransferase 1 OS=Mus musculus OX=10090 GN=Nmt1 PE=1 SV=1 |
| 1314 | 3101 | A0A286YDA2\|A0A286YDA2\_MOUSE | 79.26 | 3 | 2 | 2 | 3 | 2 | 2 | 2 | 2 | 0 | 2 | 2 | 1.46E6 | 1.45E6 | 1.86E6 | 7.52E5 | 2.38E5 | 4.48E5 | 6.13E5 |  | 2.57E6 | 3.38E5 | 2 | 2 | 1 | 1 | 2 | 1 | 1 | 1 | 1 | 0 | 1 | 1 | N | 73428 | Nucleolar and coiled-body phosphoprotein 1 OS=Mus musculus OX=10090 GN=Nolc1 PE=1 SV=1 |
| 1314 | 3102 | A0A286YDT3\|A0A286YDT3\_MOUSE | 79.26 | 3 | 2 | 2 | 3 | 2 | 2 | 2 | 2 | 0 | 2 | 2 | 1.46E6 | 1.45E6 | 1.86E6 | 7.52E5 | 2.38E5 | 4.48E5 | 6.13E5 |  | 2.57E6 | 3.38E5 | 2 | 2 | 1 | 1 | 2 | 1 | 1 | 1 | 1 | 0 | 1 | 1 | N | 73570 | Nucleolar and coiled-body phosphoprotein 1 OS=Mus musculus OX=10090 GN=Nolc1 PE=1 SV=1 |
| 1314 | 3103 | A0A286YDV7\|A0A286YDV7\_MOUSE | 79.26 | 3 | 2 | 2 | 3 | 2 | 2 | 2 | 2 | 0 | 2 | 2 | 1.46E6 | 1.45E6 | 1.86E6 | 7.52E5 | 2.38E5 | 4.48E5 | 6.13E5 |  | 2.57E6 | 3.38E5 | 2 | 2 | 1 | 1 | 2 | 1 | 1 | 1 | 1 | 0 | 1 | 1 | N | 73686 | Nucleolar and coiled-body phosphoprotein 1 (Fragment) OS=Mus musculus OX=10090 GN=Nolc1 PE=1 SV=1 |
| 1314 | 3104 | sp\|E9Q5C9\|NOLC1\_MOUSE | 79.26 | 3 | 2 | 2 | 3 | 2 | 2 | 2 | 2 | 0 | 2 | 2 | 1.46E6 | 1.45E6 | 1.86E6 | 7.52E5 | 2.38E5 | 4.48E5 | 6.13E5 |  | 2.57E6 | 3.38E5 | 2 | 2 | 1 | 1 | 2 | 1 | 1 | 1 | 1 | 0 | 1 | 1 | N | 73698 | Nucleolar and coiled-body phosphoprotein 1 OS=Mus musculus OX=10090 GN=Nolc1 PE=1 SV=1 |
| 1330 | 3944 | sp\|Q9CQZ5\|NDUA6\_MOUSE | 79.17 | 15 | 0 | 0 | 15 | 8 | 8 | 15 | 6 | 0 | 8 | 0 |  |  | 4.5E6 | 1.42E6 | 3.04E6 | 5.59E6 | 1.3E6 |  | 3.79E6 |  | 2 | 2 | 0 | 0 | 2 | 1 | 1 | 2 | 1 | 0 | 1 | 0 | N | 15283 | NADH dehydrogenase [ubiquinone] 1 alpha subcomplex subunit 6 OS=Mus musculus OX=10090 GN=Ndufa6 PE=1 SV=1 |
| 1749 | 5219 | F8WHU8\|F8WHU8\_MOUSE | 79.02 | 12 | 0 | 0 | 12 | 12 | 0 | 12 | 12 | 0 | 0 | 0 |  |  | 1.17E6 | 1.03E6 |  | 1.09E6 | 7.22E5 |  |  |  | 1 | 1 | 0 | 0 | 1 | 2 | 0 | 1 | 1 | 0 | 0 | 0 | N | 11444 | SRA stem-loop interacting RNA binding protein (Fragment) OS=Mus musculus OX=10090 GN=Slirp PE=1 SV=1 |
| 1749 | 5222 | sp\|Q9D8T7\|SLIRP\_MOUSE | 79.02 | 11 | 0 | 0 | 11 | 11 | 0 | 11 | 11 | 0 | 0 | 0 |  |  | 1.17E6 | 1.03E6 |  | 1.09E6 | 7.22E5 |  |  |  | 1 | 1 | 0 | 0 | 1 | 2 | 0 | 1 | 1 | 0 | 0 | 0 | N | 12605 | SRA stem-loop-interacting RNA-binding protein, mitochondrial OS=Mus musculus OX=10090 GN=Slirp PE=1 SV=2 |
| 1378 | 2550 | sp\|Q99LP6\|GRPE1\_MOUSE | 79.00 | 9 | 0 | 0 | 4 | 4 | 4 | 4 | 0 | 0 | 9 | 5 |  |  | 6.81E6 | 2.69E6 | 7.18E6 | 1.18E7 |  |  | 5.3E6 | 1.47E6 | 2 | 2 | 0 | 0 | 1 | 1 | 1 | 1 | 0 | 0 | 2 | 1 | N | 24307 | GrpE protein homolog 1, mitochondrial OS=Mus musculus OX=10090 GN=Grpel1 PE=1 SV=1 |
| 1363 | 2267 | G3X9T8\|G3X9T8\_MOUSE | 78.94 | 3 | 1 | 0 | 0 | 0 | 0 | 2 | 1 | 0 | 1 | 0 | 9.76E5 |  |  |  |  | 1.61E6 | 7.4E5 |  | 2.06E5 |  | 3 | 3 | 1 | 0 | 0 | 0 | 0 | 2 | 1 | 0 | 1 | 0 | Y | 121080 | ferroxidase OS=Mus musculus OX=10090 GN=Cp PE=1 SV=1 |
| 1363 | 2268 | sp\|Q61147\|CERU\_MOUSE | 78.94 | 3 | 1 | 0 | 0 | 0 | 0 | 2 | 1 | 0 | 1 | 0 | 9.76E5 |  |  |  |  | 1.61E6 | 7.4E5 |  | 2.06E5 |  | 3 | 3 | 1 | 0 | 0 | 0 | 0 | 2 | 1 | 0 | 1 | 0 | Y | 121151 | Ceruloplasmin OS=Mus musculus OX=10090 GN=Cp PE=1 SV=2 |
| 1363 | 2269 | G3X8Q5\|G3X8Q5\_MOUSE | 78.94 | 3 | 1 | 0 | 0 | 0 | 0 | 2 | 1 | 0 | 1 | 0 | 9.76E5 |  |  |  |  | 1.61E6 | 7.4E5 |  | 2.06E5 |  | 3 | 3 | 1 | 0 | 0 | 0 | 0 | 2 | 1 | 0 | 1 | 0 | Y | 124140 | ferroxidase OS=Mus musculus OX=10090 GN=Cp PE=1 SV=1 |
| 1363 | 2270 | E9PZD8\|E9PZD8\_MOUSE | 78.94 | 3 | 1 | 0 | 0 | 0 | 0 | 2 | 1 | 0 | 1 | 0 | 9.76E5 |  |  |  |  | 1.61E6 | 7.4E5 |  | 2.06E5 |  | 3 | 3 | 1 | 0 | 0 | 0 | 0 | 2 | 1 | 0 | 1 | 0 | Y | 124211 | ferroxidase OS=Mus musculus OX=10090 GN=Cp PE=1 SV=1 |
| 1037 | 3281 | sp\|Q569Z6\|TR150\_MOUSE | 78.75 | 2 | 1 | 1 | 1 | 1 | 1 | 2 | 1 | 1 | 1 | 1 |  |  |  |  |  | 1.31E6 |  |  |  |  | 2 | 1 | 1 | 2 | 1 | 2 | 2 | 2 | 2 | 1 | 1 | 1 | N | 108178 | Thyroid hormone receptor-associated protein 3 OS=Mus musculus OX=10090 GN=Thrap3 PE=1 SV=1 |
| 1052 | 2863 | sp\|Q80YP0\|CDK3\_MOUSE | 78.57 | 7 | 0 | 7 | 3 | 3 | 0 | 3 | 3 | 7 | 3 | 0 |  | 2.14E5 |  |  |  |  |  | 8.19E5 |  |  | 2 | 1 | 0 | 2 | 1 | 2 | 0 | 2 | 2 | 3 | 1 | 0 | N | 34075 | Cyclin-dependent kinase 3 OS=Mus musculus OX=10090 GN=Cdk3 PE=1 SV=3 |
| 1118 | 3916 | B0R1E3\|B0R1E3\_MOUSE | 78.56 | 18 | 18 | 18 | 18 | 12 | 6 | 12 | 12 | 12 | 12 | 18 | 2.21E7 | 2.89E7 | 2.42E7 | 5.98E6 | 8.26E6 | 1.27E7 | 8.16E6 | 8.8E6 | 2.29E7 | 2.3E7 | 2 | 2 | 2 | 2 | 2 | 1 | 1 | 1 | 1 | 1 | 1 | 2 | N | 13439 | Adenosine 5'-monophosphoramidase HINT1 OS=Mus musculus OX=10090 GN=Hint1 PE=1 SV=1 |
| 1118 | 3917 | sp\|P70349\|HINT1\_MOUSE | 78.56 | 17 | 17 | 17 | 17 | 11 | 6 | 11 | 11 | 11 | 11 | 17 | 2.21E7 | 2.89E7 | 2.42E7 | 5.98E6 | 8.26E6 | 1.27E7 | 8.16E6 | 8.8E6 | 2.29E7 | 2.3E7 | 2 | 2 | 2 | 2 | 2 | 1 | 1 | 1 | 1 | 1 | 1 | 2 | N | 13777 | Adenosine 5'-monophosphoramidase HINT1 OS=Mus musculus OX=10090 GN=Hint1 PE=1 SV=3 |
| 1715 | 3093 | sp\|Q9R1T4\|SEPT6\_MOUSE | 78.56 | 3 | 0 | 2 | 0 | 1 | 0 | 2 | 2 | 0 | 0 | 0 |  | 2.5E5 |  |  |  | 4.55E5 | 3.26E5 |  |  |  | 2 | 1 | 0 | 1 | 0 | 1 | 0 | 1 | 1 | 0 | 0 | 0 | N | 49620 | Septin-6 OS=Mus musculus OX=10090 GN=Septin6 PE=1 SV=4 |
| 1391 | 4903 | sp\|P63024\|VAMP3\_MOUSE | 78.55 | 17 | 17 | 17 | 0 | 17 | 17 | 17 | 17 | 17 | 17 | 17 | 1.48E6 | 3.79E6 |  | 2.31E6 | 3.81E6 | 4.32E6 | 3.31E6 | 3.21E6 | 4.48E5 | 4.6E6 | 1 | 1 | 1 | 1 | 0 | 1 | 1 | 1 | 1 | 1 | 1 | 1 | N | 11480 | Vesicle-associated membrane protein 3 OS=Mus musculus OX=10090 GN=Vamp3 PE=1 SV=1 |
| 1391 | 5204 | sp\|P63044\|VAMP2\_MOUSE | 78.55 | 15 | 15 | 15 | 0 | 15 | 15 | 15 | 15 | 15 | 15 | 15 | 1.48E6 | 3.79E6 |  | 2.31E6 | 3.81E6 | 4.32E6 | 3.31E6 | 3.21E6 | 4.48E5 | 4.6E6 | 1 | 1 | 1 | 1 | 0 | 1 | 1 | 1 | 1 | 1 | 1 | 1 | N | 12691 | Vesicle-associated membrane protein 2 OS=Mus musculus OX=10090 GN=Vamp2 PE=1 SV=2 |
| 1391 | 5205 | B0QZN5\|B0QZN5\_MOUSE | 78.55 | 10 | 10 | 10 | 0 | 10 | 10 | 10 | 10 | 10 | 10 | 10 | 1.48E6 | 3.79E6 |  | 2.31E6 | 3.81E6 | 4.32E6 | 3.31E6 | 3.21E6 | 4.48E5 | 4.6E6 | 1 | 1 | 1 | 1 | 0 | 1 | 1 | 1 | 1 | 1 | 1 | 1 | N | 17862 | Vesicle-associated membrane protein 2 OS=Mus musculus OX=10090 GN=Vamp2 PE=1 SV=1 |
| 1616 | 2906 | sp\|P97742\|CPT1A\_MOUSE | 78.45 | 3 | 0 | 0 | 1 | 0 | 0 | 2 | 1 | 0 | 2 | 2 |  |  | 3.48E5 |  |  | 2.84E5 | 7.12E5 |  | 7.1E5 | 3E5 | 2 | 2 | 0 | 0 | 1 | 0 | 0 | 1 | 1 | 0 | 1 | 1 | N | 88252 | Carnitine O-palmitoyltransferase 1, liver isoform OS=Mus musculus OX=10090 GN=Cpt1a PE=1 SV=4 |
| 2401 | 3044 | sp\|Q91VM9\|IPYR2\_MOUSE | 78.44 | 6 | 0 | 0 | 2 | 0 | 0 | 4 | 0 | 0 | 0 | 0 |  |  | 3.03E6 |  |  | 4.78E5 |  |  |  |  | 2 | 2 | 0 | 0 | 1 | 0 | 0 | 1 | 0 | 0 | 0 | 0 | N | 38115 | Inorganic pyrophosphatase 2, mitochondrial OS=Mus musculus OX=10090 GN=Ppa2 PE=1 SV=1 |
| 1253 | 2425 | sp\|Q9CQ69\|QCR8\_MOUSE | 78.41 | 33 | 24 | 9 | 0 | 9 | 0 | 9 | 9 | 17 | 24 | 9 | 1.29E6 | 2.11E6 |  | 1.55E6 |  | 2.49E6 | 1.77E6 | 5.88E6 | 2.55E6 | 1.09E6 | 3 | 3 | 2 | 1 | 0 | 1 | 0 | 1 | 1 | 2 | 2 | 1 | N | 9768 | Cytochrome b-c1 complex subunit 8 OS=Mus musculus OX=10090 GN=Uqcrq PE=1 SV=3 |
| 992 | 4536 | sp\|Q9CPQ8\|ATP5L\_MOUSE | 78.33 | 18 | 18 | 18 | 18 | 18 | 18 | 18 | 18 | 18 | 18 | 18 | 6.25E5 | 2.07E6 | 4.01E6 | 2.04E6 | 8.68E5 | 1.83E6 | 2.69E6 | 2.17E6 | 1.53E7 | 3.68E4 | 1 | 1 | 1 | 2 | 3 | 3 | 1 | 2 | 2 | 1 | 2 | 1 | N | 11425 | ATP synthase subunit g, mitochondrial OS=Mus musculus OX=10090 GN=Atp5mg PE=1 SV=1 |
| 1502 | 3307 | sp\|Q8VHF2\|CDHR5\_MOUSE | 78.32 | 2 | 0 | 2 | 2 | 0 | 0 | 2 | 2 | 0 | 2 | 0 |  | 1.22E6 | 3.29E5 |  |  | 5.86E5 | 3.63E5 |  | 1.71E6 |  | 1 | 1 | 0 | 1 | 1 | 0 | 0 | 1 | 1 | 0 | 1 | 0 | N | 88208 | Cadherin-related family member 5 OS=Mus musculus OX=10090 GN=Cdhr5 PE=1 SV=1 |
| 1502 | 3308 | A0PJK7\|A0PJK7\_MOUSE | 78.32 | 2 | 0 | 2 | 2 | 0 | 0 | 2 | 2 | 0 | 2 | 0 |  | 1.22E6 | 3.29E5 |  |  | 5.86E5 | 3.63E5 |  | 1.71E6 |  | 1 | 1 | 0 | 1 | 1 | 0 | 0 | 1 | 1 | 0 | 1 | 0 | N | 88227 | Cadherin-related family member 5 OS=Mus musculus OX=10090 GN=Cdhr5 PE=1 SV=1 |
| 1546 | 5226 | sp\|Q78XF5\|OSTC\_MOUSE | 78.26 | 8 | 0 | 0 | 8 | 0 | 8 | 8 | 8 | 8 | 8 | 8 |  |  | 1.95E6 |  | 4.11E5 | 1.61E6 | 8.78E5 | 6.5E5 | 1.25E6 | 4.37E5 | 1 | 1 | 0 | 0 | 1 | 0 | 1 | 1 | 1 | 1 | 1 | 1 | Y | 16815 | Oligosaccharyltransferase complex subunit OSTC OS=Mus musculus OX=10090 GN=Ostc PE=1 SV=1 |
| 1313 | 2985 | sp\|P54320\|ELN\_MOUSE | 78.16 | 3 | 2 | 3 | 3 | 2 | 0 | 0 | 3 | 2 | 0 | 2 | 4.88E6 | 9.76E6 | 9.6E6 | 2.2E6 |  |  | 4.56E6 | 3.41E6 |  | 4.17E6 | 2 | 2 | 1 | 2 | 2 | 1 | 0 | 0 | 2 | 1 | 0 | 1 | N | 71938 | Elastin OS=Mus musculus OX=10090 GN=Eln PE=1 SV=2 |
| 1352 | 2718 | sp\|P21279\|GNAQ\_MOUSE | 78.12 | 5 | 3 | 3 | 3 | 3 | 0 | 5 | 0 | 0 | 3 | 3 |  |  |  |  |  | 1.05E6 |  |  |  |  | 2 | 1 | 1 | 1 | 1 | 1 | 0 | 2 | 0 | 0 | 1 | 1 | N | 42158 | Guanine nucleotide-binding protein G(q) subunit alpha OS=Mus musculus OX=10090 GN=Gnaq PE=1 SV=4 |
| 3434 | 5190 | A0A2I3BRK6\|A0A2I3BRK6\_MOUSE | 77.94 | 18 | 0 | 0 | 0 | 0 | 0 | 0 | 0 | 0 | 18 | 0 |  |  |  |  |  |  |  |  | 8.55E5 |  | 1 | 1 | 0 | 0 | 0 | 0 | 0 | 0 | 0 | 0 | 1 | 0 | N | 10984 | Transcription and mRNA export factor ENY2 OS=Mus musculus OX=10090 GN=Eny2 PE=1 SV=1 |
| 3434 | 5191 | sp\|Q9JIX0\|ENY2\_MOUSE | 77.94 | 17 | 0 | 0 | 0 | 0 | 0 | 0 | 0 | 0 | 17 | 0 |  |  |  |  |  |  |  |  | 8.55E5 |  | 1 | 1 | 0 | 0 | 0 | 0 | 0 | 0 | 0 | 0 | 1 | 0 | N | 11529 | Transcription and mRNA export factor ENY2 OS=Mus musculus OX=10090 GN=Eny2 PE=1 SV=1 |
| 2949 | 4489 | Q5NCU3\|Q5NCU3\_MOUSE | 77.86 | 9 | 0 | 0 | 0 | 0 | 0 | 0 | 0 | 0 | 9 | 0 |  |  |  |  |  |  |  |  | 4.46E5 |  | 1 | 1 | 0 | 0 | 0 | 0 | 0 | 0 | 0 | 0 | 1 | 0 | Y | 19096 | SPARC (Fragment) OS=Mus musculus OX=10090 GN=Sparc PE=1 SV=1 |
| 2949 | 4490 | Q5NCU4\|Q5NCU4\_MOUSE | 77.86 | 5 | 0 | 0 | 0 | 0 | 0 | 0 | 0 | 0 | 5 | 0 |  |  |  |  |  |  |  |  | 4.46E5 |  | 1 | 1 | 0 | 0 | 0 | 0 | 0 | 0 | 0 | 0 | 1 | 0 | Y | 34322 | SPARC OS=Mus musculus OX=10090 GN=Sparc PE=1 SV=1 |
| 2949 | 4491 | sp\|P07214\|SPRC\_MOUSE | 77.86 | 5 | 0 | 0 | 0 | 0 | 0 | 0 | 0 | 0 | 5 | 0 |  |  |  |  |  |  |  |  | 4.46E5 |  | 1 | 1 | 0 | 0 | 0 | 0 | 0 | 0 | 0 | 0 | 1 | 0 | Y | 34450 | SPARC OS=Mus musculus OX=10090 GN=Sparc PE=1 SV=1 |
| 2949 | 4492 | A0A1L1SSH9\|A0A1L1SSH9\_MOUSE | 77.86 | 5 | 0 | 0 | 0 | 0 | 0 | 0 | 0 | 0 | 5 | 0 |  |  |  |  |  |  |  |  | 4.46E5 |  | 1 | 1 | 0 | 0 | 0 | 0 | 0 | 0 | 0 | 0 | 1 | 0 | Y | 37837 | SPARC OS=Mus musculus OX=10090 GN=Sparc PE=1 SV=1 |
| 1716 | 3178 | A0A0R4J195\|A0A0R4J195\_MOUSE | 77.57 | 5 | 5 | 0 | 0 | 0 | 5 | 5 | 5 | 0 | 0 | 0 | 0E0 |  |  |  | 6.53E5 | 3.03E5 | 3.06E5 |  |  |  | 1 | 1 | 1 | 0 | 0 | 0 | 1 | 1 | 1 | 0 | 0 | 0 | N | 36813 | Regulation of nuclear pre-mRNA domain-containing protein OS=Mus musculus OX=10090 GN=Rprd1b PE=1 SV=1 |
| 1716 | 3179 | sp\|Q9CSU0\|RPR1B\_MOUSE | 77.57 | 5 | 5 | 0 | 0 | 0 | 5 | 5 | 5 | 0 | 0 | 0 | 0E0 |  |  |  | 6.53E5 | 3.03E5 | 3.06E5 |  |  |  | 1 | 1 | 1 | 0 | 0 | 0 | 1 | 1 | 1 | 0 | 0 | 0 | N | 36884 | Regulation of nuclear pre-mRNA domain-containing protein 1B OS=Mus musculus OX=10090 GN=Rprd1b PE=1 SV=2 |
| 2515 | 4719 | sp\|Q7TQD2\|TPPP\_MOUSE | 77.41 | 7 | 0 | 7 | 0 | 0 | 0 | 7 | 0 | 0 | 0 | 0 |  | 7.46E4 |  |  |  | 1.45E6 |  |  |  |  | 1 | 1 | 0 | 1 | 0 | 0 | 0 | 1 | 0 | 0 | 0 | 0 | N | 23575 | Tubulin polymerization-promoting protein OS=Mus musculus OX=10090 GN=Tppp PE=1 SV=1 |
| 1521 | 3861 | A0AAA9WUU2\|A0AAA9WUU2\_MOUSE | 77.27 | 27 | 0 | 0 | 27 | 0 | 27 | 15 | 0 | 12 | 0 | 15 |  |  | 3.34E6 |  | 4.48E6 | 3.17E6 |  | 2.36E5 |  | 1.21E6 | 2 | 2 | 0 | 0 | 2 | 0 | 2 | 1 | 0 | 1 | 0 | 1 | N | 9582 | SUMO-conjugating enzyme UBC9 OS=Mus musculus OX=10090 GN=Ube2i PE=1 SV=1 |
| 1521 | 3862 | G3UYP0\|G3UYP0\_MOUSE | 77.27 | 22 | 0 | 0 | 22 | 0 | 22 | 13 | 0 | 10 | 0 | 13 |  |  | 3.34E6 |  | 4.48E6 | 3.17E6 |  | 2.36E5 |  | 1.21E6 | 2 | 2 | 0 | 0 | 2 | 0 | 2 | 1 | 0 | 1 | 0 | 1 | N | 11696 | SUMO-conjugating enzyme UBC9 (Fragment) OS=Mus musculus OX=10090 GN=Ube2i PE=1 SV=1 |
| 1521 | 3864 | A0AAA9WVZ3\|A0AAA9WVZ3\_MOUSE | 77.27 | 19 | 0 | 0 | 19 | 0 | 19 | 11 | 0 | 8 | 0 | 11 |  |  | 3.34E6 |  | 4.48E6 | 3.17E6 |  | 2.36E5 |  | 1.21E6 | 2 | 2 | 0 | 0 | 2 | 0 | 2 | 1 | 0 | 1 | 0 | 1 | N | 13690 | SUMO-conjugating enzyme UBC9 OS=Mus musculus OX=10090 GN=Ube2i PE=1 SV=1 |
| 1521 | 3865 | A0AAA9WWE8\|A0AAA9WWE8\_MOUSE | 77.27 | 17 | 0 | 0 | 17 | 0 | 17 | 9 | 0 | 7 | 0 | 9 |  |  | 3.34E6 |  | 4.48E6 | 3.17E6 |  | 2.36E5 |  | 1.21E6 | 2 | 2 | 0 | 0 | 2 | 0 | 2 | 1 | 0 | 1 | 0 | 1 | N | 15516 | SUMO-conjugating enzyme UBC9 OS=Mus musculus OX=10090 GN=Ube2i PE=1 SV=1 |
| 1521 | 3866 | A0AAA9WWE2\|A0AAA9WWE2\_MOUSE | 77.27 | 16 | 0 | 0 | 16 | 0 | 16 | 9 | 0 | 7 | 0 | 9 |  |  | 3.34E6 |  | 4.48E6 | 3.17E6 |  | 2.36E5 |  | 1.21E6 | 2 | 2 | 0 | 0 | 2 | 0 | 2 | 1 | 0 | 1 | 0 | 1 | N | 16106 | SUMO-conjugating enzyme UBC9 OS=Mus musculus OX=10090 GN=Ube2i PE=1 SV=1 |
| 1521 | 3868 | sp\|P63280\|UBC9\_MOUSE | 77.27 | 15 | 0 | 0 | 15 | 0 | 15 | 8 | 0 | 6 | 0 | 8 |  |  | 3.34E6 |  | 4.48E6 | 3.17E6 |  | 2.36E5 |  | 1.21E6 | 2 | 2 | 0 | 0 | 2 | 0 | 2 | 1 | 0 | 1 | 0 | 1 | N | 18007 | SUMO-conjugating enzyme UBC9 OS=Mus musculus OX=10090 GN=Ube2i PE=1 SV=1 |
| 1521 | 3869 | A0AAA9WWE5\|A0AAA9WWE5\_MOUSE | 77.27 | 13 | 0 | 0 | 13 | 0 | 13 | 7 | 0 | 6 | 0 | 7 |  |  | 3.34E6 |  | 4.48E6 | 3.17E6 |  | 2.36E5 |  | 1.21E6 | 2 | 2 | 0 | 0 | 2 | 0 | 2 | 1 | 0 | 1 | 0 | 1 | N | 20364 | SUMO-conjugating enzyme UBC9 OS=Mus musculus OX=10090 GN=Ube2i PE=1 SV=1 |
| 1521 | 3870 | A0AAA9WW15\|A0AAA9WW15\_MOUSE | 77.27 | 13 | 0 | 0 | 13 | 0 | 13 | 7 | 0 | 6 | 0 | 7 |  |  | 3.34E6 |  | 4.48E6 | 3.17E6 |  | 2.36E5 |  | 1.21E6 | 2 | 2 | 0 | 0 | 2 | 0 | 2 | 1 | 0 | 1 | 0 | 1 | N | 20225 | SUMO-conjugating enzyme UBC9 OS=Mus musculus OX=10090 GN=Ube2i PE=1 SV=1 |
| 1521 | 3871 | A0AAA9WVY9\|A0AAA9WVY9\_MOUSE | 77.27 | 13 | 0 | 0 | 13 | 0 | 13 | 7 | 0 | 5 | 0 | 7 |  |  | 3.34E6 |  | 4.48E6 | 3.17E6 |  | 2.36E5 |  | 1.21E6 | 2 | 2 | 0 | 0 | 2 | 0 | 2 | 1 | 0 | 1 | 0 | 1 | N | 20757 | SUMO-conjugating enzyme UBC9 OS=Mus musculus OX=10090 GN=Ube2i PE=1 SV=1 |
| 1328 | 5117 | sp\|Q9EPL8\|IPO7\_MOUSE | 77.18 | 1 | 1 | 1 | 1 | 1 | 1 | 1 | 1 | 1 | 1 | 1 | 1.12E6 | 8.08E5 | 2.94E6 | 7.49E5 | 1.6E6 | 1.78E6 | 2.06E6 | 1.4E6 | 2.25E6 | 1.77E6 | 1 | 1 | 1 | 1 | 1 | 1 | 1 | 1 | 1 | 1 | 1 | 1 | N | 119486 | Importin-7 OS=Mus musculus OX=10090 GN=Ipo7 PE=1 SV=2 |
| 1287 | 2966 | sp\|Q08652\|RET2\_MOUSE | 76.90 | 14 | 14 | 0 | 0 | 0 | 0 | 0 | 0 | 0 | 14 | 14 | 3.88E7 |  |  |  |  |  |  |  | 4.29E7 | 5.11E7 | 2 | 2 | 3 | 0 | 0 | 0 | 0 | 0 | 0 | 0 | 3 | 3 | N | 15610 | Retinol-binding protein 2 OS=Mus musculus OX=10090 GN=Rbp2 PE=2 SV=2 |
| 1927 | 3855 | sp\|Q99MQ4\|ASPN\_MOUSE | 76.85 | 7 | 0 | 3 | 5 | 3 | 0 | 5 | 0 | 0 | 0 | 0 |  | 5.94E5 | 1.38E6 | 0E0 |  | 2.86E6 |  |  |  |  | 2 | 2 | 0 | 1 | 1 | 1 | 0 | 1 | 0 | 0 | 0 | 0 | N | 42573 | Asporin OS=Mus musculus OX=10090 GN=Aspn PE=1 SV=1 |
| 1924 | 2391 | sp\|Q3UW68\|CAN13\_MOUSE | 76.82 | 4 | 4 | 0 | 0 | 0 | 0 | 0 | 0 | 0 | 0 | 1 | 9.03E6 |  |  |  |  |  |  |  |  | 0E0 | 3 | 3 | 3 | 0 | 0 | 0 | 0 | 0 | 0 | 0 | 0 | 1 | N | 76049 | Calpain-13 OS=Mus musculus OX=10090 GN=Capn13 PE=2 SV=1 |
| 2550 | 5197 | A0A1B0GRG3\|A0A1B0GRG3\_MOUSE | 76.76 | 10 | 0 | 10 | 0 | 0 | 0 | 0 | 0 | 0 | 10 | 0 |  | 3.86E6 |  |  |  |  |  |  | 1.04E6 |  | 1 | 1 | 0 | 1 | 0 | 0 | 0 | 0 | 0 | 0 | 1 | 0 | N | 16784 | CD151 antigen (Fragment) OS=Mus musculus OX=10090 GN=Cd151 PE=1 SV=1 |
| 2550 | 5199 | A0A1B0GSK8\|A0A1B0GSK8\_MOUSE | 76.76 | 7 | 0 | 7 | 0 | 0 | 0 | 0 | 0 | 0 | 7 | 0 |  | 3.86E6 |  |  |  |  |  |  | 1.04E6 |  | 1 | 1 | 0 | 1 | 0 | 0 | 0 | 0 | 0 | 0 | 1 | 0 | N | 22575 | Tetraspanin (Fragment) OS=Mus musculus OX=10090 GN=Cd151 PE=1 SV=1 |
| 2550 | 5200 | sp\|O35566\|CD151\_MOUSE | 76.76 | 6 | 0 | 6 | 0 | 0 | 0 | 0 | 0 | 0 | 6 | 0 |  | 3.86E6 |  |  |  |  |  |  | 1.04E6 |  | 1 | 1 | 0 | 1 | 0 | 0 | 0 | 0 | 0 | 0 | 1 | 0 | N | 28246 | CD151 antigen OS=Mus musculus OX=10090 GN=Cd151 PE=1 SV=2 |
| 2371 | 2824 | sp\|Q99JF8\|PSIP1\_MOUSE | 76.48 | 5 | 0 | 2 | 0 | 0 | 0 | 0 | 3 | 0 | 0 | 0 |  | 1.33E6 |  |  |  |  | 5.87E5 |  |  |  | 2 | 2 | 0 | 1 | 0 | 0 | 0 | 0 | 1 | 0 | 0 | 0 | N | 59697 | PC4 and SFRS1-interacting protein OS=Mus musculus OX=10090 GN=Psip1 PE=1 SV=1 |
| 1726 | 3212 | sp\|Q9D6Y7\|MSRA\_MOUSE | 76.41 | 9 | 4 | 4 | 4 | 0 | 0 | 4 | 0 | 0 | 5 | 0 | 3.78E6 | 5.64E6 | 4.54E6 |  |  | 3.96E6 |  |  | 3.03E6 |  | 2 | 2 | 1 | 1 | 1 | 0 | 0 | 1 | 0 | 0 | 1 | 0 | N | 25988 | Mitochondrial peptide methionine sulfoxide reductase OS=Mus musculus OX=10090 GN=Msra PE=1 SV=1 |
| 1192 | 2565 | sp\|Q9JHJ0\|TMOD3\_MOUSE | 76.39 | 5 | 0 | 0 | 2 | 5 | 2 | 2 | 5 | 0 | 3 | 3 |  |  | 5.68E5 | 1.4E6 | 2.73E6 | 1.93E6 | 1.29E6 |  | 1.79E6 | 0E0 | 2 | 2 | 0 | 0 | 1 | 2 | 1 | 1 | 2 | 0 | 1 | 1 | N | 39503 | Tropomodulin-3 OS=Mus musculus OX=10090 GN=Tmod3 PE=1 SV=1 |
| 2100 | 3726 | sp\|Q8K0C4\|CP51A\_MOUSE | 76.24 | 3 | 3 | 0 | 0 | 0 | 0 | 3 | 0 | 0 | 3 | 0 | 1.34E6 |  |  |  |  | 5.71E5 |  |  | 1.8E6 |  | 1 | 1 | 1 | 0 | 0 | 0 | 0 | 1 | 0 | 0 | 1 | 0 | N | 56776 | Lanosterol 14-alpha demethylase OS=Mus musculus OX=10090 GN=Cyp51a1 PE=1 SV=1 |
| 955 | 2537 | sp\|Q02566\|MYH6\_MOUSE | 76.23 | 1 | 0 | 0 | 1 | 1 | 1 | 1 | 1 | 1 | 0 | 0 |  |  | 0E0 |  |  |  |  |  |  |  | 3 | 1 | 2 | 2 | 2 | 2 | 2 | 3 | 2 | 4 | 0 | 0 | N | 223563 | Myosin-6 OS=Mus musculus OX=10090 GN=Myh6 PE=1 SV=2 |
| 1875 | 3198 | sp\|Q61881\|MCM7\_MOUSE | 76.14 | 2 | 0 | 0 | 0 | 2 | 0 | 2 | 0 | 0 | 2 | 0 |  |  |  | 5.06E5 |  | 1.01E6 |  |  | 1.45E6 |  | 1 | 1 | 0 | 0 | 0 | 1 | 0 | 1 | 0 | 0 | 1 | 0 | N | 81211 | DNA replication licensing factor MCM7 OS=Mus musculus OX=10090 GN=Mcm7 PE=1 SV=1 |
| 1875 | 4393 | D3Z6N3\|D3Z6N3\_MOUSE | 76.14 | 4 | 0 | 0 | 0 | 4 | 0 | 4 | 0 | 0 | 4 | 0 |  |  |  | 5.06E5 |  | 1.01E6 |  |  | 1.45E6 |  | 1 | 1 | 0 | 0 | 0 | 1 | 0 | 1 | 0 | 0 | 1 | 0 | N | 44610 | DNA replication licensing factor MCM7 OS=Mus musculus OX=10090 GN=Mcm7 PE=1 SV=1 |
| 1108 | 2661 | Q9CXS2\|Q9CXS2\_MOUSE | 76.03 | 10 | 0 | 7 | 7 | 7 | 10 | 0 | 10 | 7 | 7 | 7 |  |  |  |  | 7.55E5 |  | 4.41E5 |  |  |  | 2 | 1 | 0 | 1 | 1 | 1 | 3 | 0 | 2 | 1 | 1 | 1 | N | 19434 | RAB3C, member RAS oncogene family OS=Mus musculus OX=10090 GN=Rab3c PE=1 SV=1 |
| 1108 | 2713 | D3YWL1\|D3YWL1\_MOUSE | 76.03 | 16 | 0 | 10 | 10 | 10 | 16 | 0 | 16 | 10 | 10 | 10 |  |  |  |  | 7.55E5 |  | 4.41E5 |  |  |  | 2 | 1 | 0 | 1 | 1 | 1 | 3 | 0 | 2 | 1 | 1 | 1 | N | 12054 | RAB3D, member RAS oncogene family (Fragment) OS=Mus musculus OX=10090 GN=Rab3d PE=1 SV=1 |
| 1108 | 2714 | D3YW33\|D3YW33\_MOUSE | 76.03 | 12 | 0 | 8 | 8 | 8 | 12 | 0 | 12 | 8 | 8 | 8 |  |  |  |  | 7.55E5 |  | 4.41E5 |  |  |  | 2 | 1 | 0 | 1 | 1 | 1 | 3 | 0 | 2 | 1 | 1 | 1 | N | 15697 | RAB3D, member RAS oncogene family OS=Mus musculus OX=10090 GN=Rab3d PE=1 SV=1 |
| 1541 | 4088 | sp\|Q9D8W7\|OCAD2\_MOUSE | 75.64 | 12 | 8 | 8 | 3 | 8 | 0 | 0 | 8 | 0 | 8 | 8 | 1.96E6 | 0E0 | 4.78E5 | 8.7E5 |  |  | 9.65E5 |  | 4.85E5 | 1.13E6 | 2 | 2 | 1 | 1 | 1 | 1 | 0 | 0 | 1 | 0 | 1 | 1 | N | 16926 | OCIA domain-containing protein 2 OS=Mus musculus OX=10090 GN=Ociad2 PE=1 SV=1 |
| 2470 | 3067 | D3Z315\|D3Z315\_MOUSE | 75.53 | 10 | 0 | 0 | 10 | 0 | 0 | 0 | 0 | 0 | 0 | 0 |  |  | 5.05E5 |  |  |  |  |  |  |  | 1 | 1 | 0 | 0 | 1 | 0 | 0 | 0 | 0 | 0 | 0 | 0 | N | 25370 | Coatomer subunit epsilon (Fragment) OS=Mus musculus OX=10090 GN=Cope PE=1 SV=1 |
| 2470 | 3311 | E9Q6I5\|E9Q6I5\_MOUSE | 75.53 | 20 | 0 | 0 | 20 | 0 | 0 | 0 | 0 | 0 | 0 | 0 |  |  | 5.05E5 |  |  |  |  |  |  |  | 1 | 1 | 0 | 0 | 1 | 0 | 0 | 0 | 0 | 0 | 0 | 0 | N | 12111 | Coatomer subunit epsilon OS=Mus musculus OX=10090 GN=Cope PE=1 SV=1 |
| 2470 | 3312 | sp\|O89079\|COPE\_MOUSE | 75.53 | 7 | 0 | 0 | 7 | 0 | 0 | 0 | 0 | 0 | 0 | 0 |  |  | 5.05E5 |  |  |  |  |  |  |  | 1 | 1 | 0 | 0 | 1 | 0 | 0 | 0 | 0 | 0 | 0 | 0 | N | 34567 | Coatomer subunit epsilon OS=Mus musculus OX=10090 GN=Cope PE=1 SV=3 |
| 1866 | 3240 | A0A1Y7VJZ2\|A0A1Y7VJZ2\_MOUSE | 75.34 | 6 | 6 | 0 | 0 | 6 | 0 | 6 | 0 | 0 | 0 | 0 | 2.03E5 |  |  | 2.22E5 |  | 9.63E5 |  |  |  |  | 1 | 1 | 1 | 0 | 0 | 1 | 0 | 1 | 0 | 0 | 0 | 0 | N | 24205 | maleylacetoacetate isomerase OS=Mus musculus OX=10090 GN=Gstz1 PE=1 SV=1 |
| 1866 | 3241 | sp\|Q9WVL0\|MAAI\_MOUSE | 75.34 | 6 | 6 | 0 | 0 | 6 | 0 | 6 | 0 | 0 | 0 | 0 | 2.03E5 |  |  | 2.22E5 |  | 9.63E5 |  |  |  |  | 1 | 1 | 1 | 0 | 0 | 1 | 0 | 1 | 0 | 0 | 0 | 0 | N | 24275 | Maleylacetoacetate isomerase OS=Mus musculus OX=10090 GN=Gstz1 PE=1 SV=1 |
| 1309 | 2536 | sp\|Q8BFY9\|TNPO1\_MOUSE | 75.28 | 3 | 0 | 1 | 1 | 1 | 1 | 2 | 0 | 0 | 1 | 0 |  | 1.53E5 | 3.92E5 | 4.51E5 | 5.31E5 | 2.16E6 |  |  | 1.18E6 |  | 3 | 3 | 0 | 1 | 1 | 1 | 1 | 2 | 0 | 0 | 1 | 0 | N | 102357 | Transportin-1 OS=Mus musculus OX=10090 GN=Tnpo1 PE=1 SV=2 |
| 1544 | 5208 | sp\|Q99LT0\|DPY30\_MOUSE | 74.99 | 16 | 0 | 16 | 16 | 0 | 0 | 16 | 16 | 16 | 16 | 16 |  | 2.1E5 | 2.56E5 |  |  | 3.53E5 | 2.45E5 | 1.79E5 | 1.62E6 | 1.05E6 | 1 | 1 | 0 | 1 | 1 | 0 | 0 | 1 | 1 | 1 | 1 | 1 | N | 11213 | Protein dpy-30 homolog OS=Mus musculus OX=10090 GN=Dpy30 PE=1 SV=1 |
| 1888 | 2959 | sp\|Q9D967\|MGDP1\_MOUSE | 74.73 | 11 | 0 | 0 | 0 | 11 | 0 | 11 | 11 | 0 | 0 | 0 |  |  |  | 6.05E5 |  | 5.43E5 | 2.76E5 |  |  |  | 1 | 1 | 0 | 0 | 0 | 1 | 0 | 1 | 1 | 0 | 0 | 0 | N | 18582 | Magnesium-dependent phosphatase 1 OS=Mus musculus OX=10090 GN=Mdp1 PE=1 SV=1 |
| 948 | 2530 | sp\|P97450\|ATP5J\_MOUSE | 74.68 | 24 | 16 | 18 | 16 | 9 | 9 | 16 | 9 | 9 | 9 | 0 | 1.44E7 | 3.21E7 | 1.49E7 | 5.21E6 | 1.1E7 | 1.86E7 | 4.93E6 | 5.74E6 | 9.48E5 |  | 3 | 3 | 2 | 3 | 2 | 1 | 2 | 2 | 1 | 2 | 1 | 0 | Y | 12496 | ATP synthase-coupling factor 6, mitochondrial OS=Mus musculus OX=10090 GN=Atp5pf PE=1 SV=1 |
| 964 | 2406 | sp\|Q3TWW8\|SRSF6\_MOUSE | 74.54 | 7 | 5 | 5 | 2 | 2 | 7 | 5 | 7 | 5 | 0 | 2 | 2.96E6 | 4.02E6 |  |  | 1.16E6 | 5.66E5 | 2.82E6 |  |  |  | 3 | 1 | 2 | 2 | 1 | 1 | 3 | 2 | 4 | 2 | 0 | 1 | N | 39025 | Serine/arginine-rich splicing factor 6 OS=Mus musculus OX=10090 GN=Srsf6 PE=1 SV=1 |
| 1714 | 2891 | sp\|Q8R050\|ERF3A\_MOUSE | 74.40 | 3 | 2 | 0 | 0 | 2 | 0 | 0 | 0 | 0 | 0 | 2 | 5.22E6 |  |  | 1.02E7 |  |  |  |  |  | 2.99E6 | 2 | 2 | 1 | 0 | 0 | 1 | 0 | 0 | 0 | 0 | 0 | 1 | N | 68626 | Eukaryotic peptide chain release factor GTP-binding subunit ERF3A OS=Mus musculus OX=10090 GN=Gspt1 PE=1 SV=2 |
| 2109 | 2806 | sp\|Q8VDP4\|CCAR2\_MOUSE | 74.39 | 3 | 0 | 0 | 0 | 0 | 1 | 0 | 0 | 2 | 0 | 0 |  |  |  |  | 0E0 |  |  | 4.98E5 |  |  | 2 | 2 | 0 | 0 | 0 | 0 | 1 | 0 | 0 | 1 | 0 | 0 | N | 103002 | Cell cycle and apoptosis regulator protein 2 OS=Mus musculus OX=10090 GN=Ccar2 PE=1 SV=2 |
| 3115 | 4839 | sp\|P61967\|AP1S1\_MOUSE | 74.35 | 10 | 10 | 0 | 0 | 0 | 0 | 0 | 0 | 0 | 0 | 0 | 4.28E5 |  |  |  |  |  |  |  |  |  | 1 | 1 | 1 | 0 | 0 | 0 | 0 | 0 | 0 | 0 | 0 | 0 | N | 18733 | AP-1 complex subunit sigma-1A OS=Mus musculus OX=10090 GN=Ap1s1 PE=1 SV=1 |
| 823 | 2515 | sp\|Q5SX39\|MYH4\_MOUSE | 74.34 | 1 | 0 | 0 | 0 | 0 | 0 | 0 | 0 | 1 | 0 | 0 |  |  |  |  |  |  |  | 3.69E6 |  |  | 2 | 1 | 2 | 2 | 1 | 1 | 1 | 2 | 1 | 3 | 0 | 0 | N | 222857 | Myosin-4 OS=Mus musculus OX=10090 GN=Myh4 PE=1 SV=1 |
| 2160 | 3887 | sp\|P00329\|ADH1\_MOUSE | 74.29 | 7 | 4 | 0 | 0 | 0 | 0 | 2 | 0 | 0 | 0 | 0 | 6.89E5 |  |  |  |  | 5.74E5 |  |  |  |  | 2 | 2 | 1 | 0 | 0 | 0 | 0 | 1 | 0 | 0 | 0 | 0 | N | 39771 | Alcohol dehydrogenase 1 OS=Mus musculus OX=10090 GN=Adh1 PE=1 SV=2 |
| 1609 | 3880 | sp\|Q99JI1\|MSTN1\_MOUSE | 74.23 | 32 | 32 | 13 | 13 | 0 | 13 | 0 | 0 | 0 | 18 | 0 | 4.15E6 | 4.11E6 | 7.73E5 |  | 2.89E5 |  |  |  | 1.13E6 |  | 2 | 2 | 2 | 1 | 1 | 0 | 1 | 0 | 0 | 0 | 1 | 0 | N | 8941 | Musculoskeletal embryonic nuclear protein 1 OS=Mus musculus OX=10090 GN=Mustn1 PE=1 SV=1 |
| 1929 | 4054 | A0A2R8VK72\|A0A2R8VK72\_MOUSE | 74.19 | 12 | 0 | 8 | 0 | 8 | 0 | 0 | 5 | 8 | 0 | 0 |  | 6.8E5 |  | 2.49E5 |  |  | 1.37E6 | 1.14E5 |  |  | 2 | 2 | 0 | 1 | 0 | 1 | 0 | 0 | 1 | 1 | 0 | 0 | N | 22494 | Thiol methyltransferase 1A1 OS=Mus musculus OX=10090 GN=Tmt1a PE=1 SV=1 |
| 1929 | 4056 | Q8C6B0\|Q8C6B0\_MOUSE | 74.19 | 10 | 0 | 6 | 0 | 6 | 0 | 0 | 4 | 6 | 0 | 0 |  | 6.8E5 |  | 2.49E5 |  |  | 1.37E6 | 1.14E5 |  |  | 2 | 2 | 0 | 1 | 0 | 1 | 0 | 0 | 1 | 1 | 0 | 0 | N | 28096 | Methyltransferase like 7A1 OS=Mus musculus OX=10090 GN=Tmt1a PE=1 SV=1 |
| 1320 | 2482 | sp\|Q9D8T2\|GSDMD\_MOUSE | 74.19 | 6 | 6 | 2 | 0 | 2 | 0 | 2 | 2 | 2 | 4 | 0 | 5.65E6 | 2.09E6 |  | 6.7E5 |  | 1.74E6 | 2.62E5 | 7.78E5 | 6.88E6 |  | 3 | 3 | 3 | 1 | 0 | 1 | 0 | 1 | 1 | 1 | 2 | 0 | N | 53238 | Gasdermin-D OS=Mus musculus OX=10090 GN=Gsdmd PE=1 SV=1 |
| 1323 | 3443 | D3YWT0\|D3YWT0\_MOUSE | 74.11 | 12 | 5 | 5 | 12 | 12 | 5 | 5 | 0 | 0 | 0 | 0 | 1.29E6 | 1.54E6 | 3.42E6 | 3.53E5 | 4.35E5 | 1.49E6 |  |  |  |  | 2 | 2 | 1 | 1 | 2 | 2 | 1 | 1 | 0 | 0 | 0 | 0 | Y | 19569 | Signal peptidase complex catalytic subunit SEC11 OS=Mus musculus OX=10090 GN=Sec11a PE=1 SV=1 |
| 1323 | 3444 | sp\|Q9R0P6\|SC11A\_MOUSE | 74.11 | 11 | 5 | 5 | 11 | 11 | 5 | 5 | 0 | 0 | 0 | 0 | 1.29E6 | 1.54E6 | 3.42E6 | 3.53E5 | 4.35E5 | 1.49E6 |  |  |  |  | 2 | 2 | 1 | 1 | 2 | 2 | 1 | 1 | 0 | 0 | 0 | 0 | Y | 20626 | Signal peptidase complex catalytic subunit SEC11A OS=Mus musculus OX=10090 GN=Sec11a PE=1 SV=1 |
| 1323 | 3445 | D3Z569\|D3Z569\_MOUSE | 74.11 | 11 | 5 | 5 | 11 | 11 | 5 | 5 | 0 | 0 | 0 | 0 | 1.29E6 | 1.54E6 | 3.42E6 | 3.53E5 | 4.35E5 | 1.49E6 |  |  |  |  | 2 | 2 | 1 | 1 | 2 | 2 | 1 | 1 | 0 | 0 | 0 | 0 | Y | 21868 | Signal peptidase complex catalytic subunit SEC11 OS=Mus musculus OX=10090 GN=Sec11a PE=1 SV=1 |
| 1323 | 3447 | D3YTS1\|D3YTS1\_MOUSE | 74.11 | 8 | 4 | 4 | 8 | 8 | 4 | 4 | 0 | 0 | 0 | 0 | 1.29E6 | 1.54E6 | 3.42E6 | 3.53E5 | 4.35E5 | 1.49E6 |  |  |  |  | 2 | 2 | 1 | 1 | 2 | 2 | 1 | 1 | 0 | 0 | 0 | 0 | Y | 27931 | Signal peptidase complex catalytic subunit SEC11 OS=Mus musculus OX=10090 GN=Sec11a PE=1 SV=1 |
| 973 | 3863 | sp\|Q9CR61\|NDUB7\_MOUSE | 74.10 | 17 | 9 | 17 | 17 | 17 | 7 | 7 | 7 | 7 | 7 | 9 | 1.92E6 | 2.07E6 | 2.74E6 | 6.39E5 | 1.56E6 | 2.48E6 | 1.72E6 | 1.73E6 | 2.37E6 | 7.57E5 | 2 | 2 | 1 | 3 | 3 | 2 | 1 | 1 | 1 | 1 | 1 | 1 | Y | 16331 | NADH dehydrogenase [ubiquinone] 1 beta subcomplex subunit 7 OS=Mus musculus OX=10090 GN=Ndufb7 PE=1 SV=3 |
| 2939 | 4003 | D3YYP8\|D3YYP8\_MOUSE | 73.95 | 13 | 13 | 0 | 0 | 0 | 0 | 0 | 0 | 0 | 0 | 0 | 9.71E5 |  |  |  |  |  |  |  |  |  | 1 | 1 | 1 | 0 | 0 | 0 | 0 | 0 | 0 | 0 | 0 | 0 | N | 14786 | Integrin beta (Fragment) OS=Mus musculus OX=10090 GN=Itgb2 PE=1 SV=1 |
| 2939 | 4004 | M0QWA7\|M0QWA7\_MOUSE | 73.95 | 4 | 4 | 0 | 0 | 0 | 0 | 0 | 0 | 0 | 0 | 0 | 9.71E5 |  |  |  |  |  |  |  |  |  | 1 | 1 | 1 | 0 | 0 | 0 | 0 | 0 | 0 | 0 | 0 | 0 | N | 53043 | Integrin beta OS=Mus musculus OX=10090 GN=Itgb2 PE=1 SV=1 |
| 2939 | 4005 | Q542I8\|Q542I8\_MOUSE | 73.95 | 2 | 2 | 0 | 0 | 0 | 0 | 0 | 0 | 0 | 0 | 0 | 9.71E5 |  |  |  |  |  |  |  |  |  | 1 | 1 | 1 | 0 | 0 | 0 | 0 | 0 | 0 | 0 | 0 | 0 | N | 84894 | Integrin beta OS=Mus musculus OX=10090 GN=Itgb2 PE=1 SV=1 |
| 2939 | 4006 | sp\|P11835\|ITB2\_MOUSE | 73.95 | 2 | 2 | 0 | 0 | 0 | 0 | 0 | 0 | 0 | 0 | 0 | 9.71E5 |  |  |  |  |  |  |  |  |  | 1 | 1 | 1 | 0 | 0 | 0 | 0 | 0 | 0 | 0 | 0 | 0 | N | 85026 | Integrin beta-2 OS=Mus musculus OX=10090 GN=Itgb2 PE=1 SV=2 |
| 783 | 2799 | A3KGR9\|A3KGR9\_MOUSE | 73.81 | 6 | 3 | 6 | 6 | 6 | 3 | 6 | 6 | 6 | 6 | 3 | 8.06E5 | 1.73E6 | 5.09E6 | 2.79E6 | 3.68E6 | 7.21E5 | 3.3E6 | 6.16E5 | 1.74E6 | 2.36E6 | 2 | 2 | 1 | 2 | 4 | 3 | 2 | 2 | 3 | 2 | 2 | 2 | Y | 38175 | Histocompatibility 13 (Fragment) OS=Mus musculus OX=10090 GN=H13 PE=1 SV=1 |
| 783 | 2800 | sp\|Q9D8V0\|HM13\_MOUSE | 73.81 | 6 | 3 | 6 | 6 | 6 | 3 | 6 | 6 | 6 | 6 | 3 | 8.06E5 | 1.73E6 | 5.09E6 | 2.79E6 | 3.68E6 | 7.21E5 | 3.3E6 | 6.16E5 | 1.74E6 | 2.36E6 | 2 | 2 | 1 | 2 | 4 | 3 | 2 | 2 | 3 | 2 | 2 | 2 | Y | 41748 | Minor histocompatibility antigen H13 OS=Mus musculus OX=10090 GN=Hm13 PE=1 SV=1 |
| 1492 | 2979 | E9QND8\|E9QND8\_MOUSE | 73.75 | 4 | 0 | 4 | 0 | 0 | 0 | 0 | 4 | 0 | 4 | 4 |  | 1.34E6 |  |  |  |  | 1.11E6 |  | 2.11E6 | 1.05E6 | 1 | 1 | 0 | 1 | 0 | 0 | 0 | 0 | 1 | 0 | 1 | 1 | N | 46876 | Atlastin GTPase 2 OS=Mus musculus OX=10090 GN=Atl2 PE=1 SV=1 |
| 1492 | 2993 | sp\|Q6PA06\|ATLA2\_MOUSE | 73.75 | 3 | 0 | 3 | 0 | 0 | 0 | 0 | 3 | 0 | 3 | 3 |  | 1.34E6 |  |  |  |  | 1.11E6 |  | 2.11E6 | 1.05E6 | 1 | 1 | 0 | 1 | 0 | 0 | 0 | 0 | 1 | 0 | 1 | 1 | N | 66224 | Atlastin-2 OS=Mus musculus OX=10090 GN=Atl2 PE=1 SV=1 |
| 1590 | 2925 | sp\|P70302\|STIM1\_MOUSE | 73.60 | 4 | 1 | 2 | 0 | 0 | 2 | 0 | 4 | 2 | 0 | 0 | 5.95E5 | 7.97E5 |  |  | 4.98E5 |  | 6.59E5 | 2.75E5 |  |  | 2 | 2 | 1 | 1 | 0 | 0 | 1 | 0 | 2 | 1 | 0 | 0 | N | 77567 | Stromal interaction molecule 1 OS=Mus musculus OX=10090 GN=Stim1 PE=1 SV=2 |
| 1590 | 2926 | A0A1B0GRA5\|A0A1B0GRA5\_MOUSE | 73.60 | 3 | 1 | 2 | 0 | 0 | 2 | 0 | 3 | 2 | 0 | 0 | 5.95E5 | 7.97E5 |  |  | 4.98E5 |  | 6.59E5 | 2.75E5 |  |  | 2 | 2 | 1 | 1 | 0 | 0 | 1 | 0 | 2 | 1 | 0 | 0 | N | 88931 | Stromal interaction molecule 1 OS=Mus musculus OX=10090 GN=Stim1 PE=1 SV=1 |
| 1590 | 3227 | A0A1B0GR78\|A0A1B0GR78\_MOUSE | 73.60 | 4 | 2 | 3 | 0 | 0 | 3 | 0 | 4 | 3 | 0 | 0 | 5.95E5 | 7.97E5 |  |  | 4.98E5 |  | 6.59E5 | 2.75E5 |  |  | 2 | 2 | 1 | 1 | 0 | 0 | 1 | 0 | 2 | 1 | 0 | 0 | N | 62195 | Stromal interaction molecule 1 OS=Mus musculus OX=10090 GN=Stim1 PE=1 SV=1 |
| 1035 | 2570 | Z4YJR7\|Z4YJR7\_MOUSE | 73.53 | 2 | 1 | 2 | 1 | 1 | 0 | 1 | 0 | 1 | 1 | 1 | 6.8E5 | 1.54E6 | 9.38E5 | 1.47E5 |  | 3.01E6 |  | 2.83E5 | 8.98E5 | 1.67E5 | 2 | 2 | 1 | 3 | 1 | 1 | 0 | 2 | 0 | 1 | 2 | 1 | N | 121432 | Sorbin and SH3 domain containing 2 OS=Mus musculus OX=10090 GN=Sorbs2 PE=1 SV=1 |
| 1035 | 2571 | A0A0R4J1T1\|A0A0R4J1T1\_MOUSE | 73.53 | 2 | 1 | 2 | 1 | 1 | 0 | 1 | 0 | 1 | 1 | 1 | 6.8E5 | 1.54E6 | 9.38E5 | 1.47E5 |  | 3.01E6 |  | 2.83E5 | 8.98E5 | 1.67E5 | 2 | 2 | 1 | 3 | 1 | 1 | 0 | 2 | 0 | 1 | 2 | 1 | N | 127731 | Sorbin and SH3 domain containing 2 (Fragment) OS=Mus musculus OX=10090 GN=Sorbs2 PE=1 SV=1 |
| 1035 | 2572 | sp\|Q3UTJ2\|SRBS2\_MOUSE | 73.53 | 2 | 1 | 2 | 1 | 1 | 0 | 1 | 0 | 1 | 1 | 1 | 6.8E5 | 1.54E6 | 9.38E5 | 1.47E5 |  | 3.01E6 |  | 2.83E5 | 8.98E5 | 1.67E5 | 2 | 2 | 1 | 3 | 1 | 1 | 0 | 2 | 0 | 1 | 2 | 1 | N | 132349 | Sorbin and SH3 domain-containing protein 2 OS=Mus musculus OX=10090 GN=Sorbs2 PE=1 SV=2 |
| 1035 | 2573 | B9EKP8\|B9EKP8\_MOUSE | 73.53 | 2 | 1 | 2 | 1 | 1 | 0 | 1 | 0 | 1 | 1 | 1 | 6.8E5 | 1.54E6 | 9.38E5 | 1.47E5 |  | 3.01E6 |  | 2.83E5 | 8.98E5 | 1.67E5 | 2 | 2 | 1 | 3 | 1 | 1 | 0 | 2 | 0 | 1 | 2 | 1 | N | 136197 | Sorbin and SH3 domain containing 2 OS=Mus musculus OX=10090 GN=Sorbs2 PE=1 SV=1 |
| 1035 | 2605 | B7ZWM6\|B7ZWM6\_MOUSE | 73.53 | 2 | 1 | 2 | 1 | 1 | 0 | 1 | 0 | 1 | 1 | 1 | 6.8E5 | 1.54E6 | 9.38E5 | 1.47E5 |  | 3.01E6 |  | 2.83E5 | 8.98E5 | 1.67E5 | 2 | 2 | 1 | 3 | 1 | 1 | 0 | 2 | 0 | 1 | 2 | 1 | N | 134243 | Sorbin and SH3 domain containing 2 OS=Mus musculus OX=10090 GN=Sorbs2 PE=1 SV=1 |
| 1035 | 2614 | B2RXQ9\|B2RXQ9\_MOUSE | 73.53 | 3 | 1 | 3 | 2 | 2 | 0 | 2 | 0 | 2 | 2 | 2 | 6.8E5 | 1.54E6 | 9.38E5 | 1.47E5 |  | 3.01E6 |  | 2.83E5 | 8.98E5 | 1.67E5 | 2 | 2 | 1 | 3 | 1 | 1 | 0 | 2 | 0 | 1 | 2 | 1 | N | 72404 | Sorbin and SH3 domain containing 2 OS=Mus musculus OX=10090 GN=Sorbs2 PE=1 SV=1 |
| 1035 | 2615 | D3Z080\|D3Z080\_MOUSE | 73.53 | 3 | 1 | 3 | 2 | 2 | 0 | 2 | 0 | 2 | 2 | 2 | 6.8E5 | 1.54E6 | 9.38E5 | 1.47E5 |  | 3.01E6 |  | 2.83E5 | 8.98E5 | 1.67E5 | 2 | 2 | 1 | 3 | 1 | 1 | 0 | 2 | 0 | 1 | 2 | 1 | N | 78155 | Sorbin and SH3 domain containing 2 OS=Mus musculus OX=10090 GN=Sorbs2 PE=1 SV=1 |
| 1035 | 2682 | A0A0R4J1W0\|A0A0R4J1W0\_MOUSE | 73.53 | 3 | 1 | 3 | 2 | 2 | 0 | 2 | 0 | 2 | 2 | 2 | 6.8E5 | 1.54E6 | 9.38E5 | 1.47E5 |  | 3.01E6 |  | 2.83E5 | 8.98E5 | 1.67E5 | 2 | 2 | 1 | 3 | 1 | 1 | 0 | 2 | 0 | 1 | 2 | 1 | N | 69576 | Sorbin and SH3 domain containing 2 OS=Mus musculus OX=10090 GN=Sorbs2 PE=1 SV=1 |
| 1125 | 3859 | sp\|P31786\|ACBP\_MOUSE | 73.51 | 39 | 18 | 18 | 0 | 18 | 18 | 18 | 18 | 18 | 39 | 0 | 1.43E6 | 3.31E6 |  | 7.87E5 | 2.39E6 | 3.37E6 | 2.62E6 | 1E6 | 1.28E7 |  | 2 | 2 | 1 | 1 | 0 | 1 | 1 | 2 | 2 | 1 | 3 | 0 | Y | 10000 | Acyl-CoA-binding protein OS=Mus musculus OX=10090 GN=Dbi PE=1 SV=2 |
| 1125 | 3860 | Q4VWZ5\|Q4VWZ5\_MOUSE | 73.51 | 25 | 12 | 12 | 0 | 12 | 12 | 12 | 12 | 12 | 25 | 0 | 1.43E6 | 3.31E6 |  | 7.87E5 | 2.39E6 | 3.37E6 | 2.62E6 | 1E6 | 1.28E7 |  | 2 | 2 | 1 | 1 | 0 | 1 | 1 | 2 | 2 | 1 | 3 | 0 | Y | 15229 | Acyl-CoA-binding protein OS=Mus musculus OX=10090 GN=Dbi PE=1 SV=1 |
| 1306 | 4046 | sp\|P05132\|KAPCA\_MOUSE | 73.49 | 3 | 3 | 3 | 3 | 3 | 3 | 3 | 0 | 3 | 0 | 3 | 5.61E5 | 1.61E5 | 1.57E6 | 3.03E5 | 5.93E5 | 5.54E5 |  | 2.84E5 |  | 5.73E5 | 1 | 1 | 1 | 1 | 1 | 1 | 1 | 1 | 0 | 1 | 0 | 1 | N | 40571 | cAMP-dependent protein kinase catalytic subunit alpha OS=Mus musculus OX=10090 GN=Prkaca PE=1 SV=3 |
| 1491 | 2780 | A0A0R4J119\|A0A0R4J119\_MOUSE | 73.46 | 2 | 0 | 1 | 1 | 0 | 0 | 1 | 1 | 0 | 1 | 0 |  | 8.86E5 | 8.02E5 |  |  | 1.24E6 | 1.99E5 |  | 2.05E6 |  | 2 | 2 | 0 | 1 | 1 | 0 | 0 | 1 | 1 | 0 | 1 | 0 | N | 144917 | Cytoplasmic FMR1-interacting protein OS=Mus musculus OX=10090 GN=Cyfip1 PE=1 SV=1 |
| 1491 | 2781 | sp\|Q7TMB8\|CYFP1\_MOUSE | 73.46 | 2 | 0 | 1 | 1 | 0 | 0 | 1 | 1 | 0 | 1 | 0 |  | 8.86E5 | 8.02E5 |  |  | 1.24E6 | 1.99E5 |  | 2.05E6 |  | 2 | 2 | 0 | 1 | 1 | 0 | 0 | 1 | 1 | 0 | 1 | 0 | N | 145241 | Cytoplasmic FMR1-interacting protein 1 OS=Mus musculus OX=10090 GN=Cyfip1 PE=1 SV=1 |
| 1705 | 3086 | F7CBP1\|F7CBP1\_MOUSE | 73.34 | 2 | 0 | 1 | 0 | 0 | 1 | 1 | 2 | 0 | 0 | 0 |  | 2.73E5 |  |  | 4.2E5 | 2.6E5 | 1.77E6 |  |  |  | 2 | 2 | 0 | 1 | 0 | 0 | 1 | 1 | 2 | 0 | 0 | 0 | N | 97889 | Eukaryotic translation initiation factor 4 gamma 2 OS=Mus musculus OX=10090 GN=Eif4g2 PE=1 SV=1 |
| 1705 | 3087 | G3XA17\|G3XA17\_MOUSE | 73.34 | 2 | 0 | 1 | 0 | 0 | 1 | 1 | 2 | 0 | 0 | 0 |  | 2.73E5 |  |  | 4.2E5 | 2.6E5 | 1.77E6 |  |  |  | 2 | 2 | 0 | 1 | 0 | 0 | 1 | 1 | 2 | 0 | 0 | 0 | N | 102100 | Eukaryotic translation initiation factor 4 gamma 2 OS=Mus musculus OX=10090 GN=Eif4g2 PE=1 SV=1 |
| 1705 | 3088 | sp\|Q62448\|IF4G2\_MOUSE | 73.34 | 2 | 0 | 1 | 0 | 0 | 1 | 1 | 2 | 0 | 0 | 0 |  | 2.73E5 |  |  | 4.2E5 | 2.6E5 | 1.77E6 |  |  |  | 2 | 2 | 0 | 1 | 0 | 0 | 1 | 1 | 2 | 0 | 0 | 0 | N | 102105 | Eukaryotic translation initiation factor 4 gamma 2 OS=Mus musculus OX=10090 GN=Eif4g2 PE=1 SV=2 |
| 2376 | 3393 | sp\|Q8CHR6\|DPYD\_MOUSE | 73.26 | 1 | 0 | 0 | 0 | 0 | 0 | 1 | 0 | 0 | 0 | 0 |  |  |  |  |  | 1.85E6 |  |  |  |  | 1 | 1 | 0 | 0 | 0 | 0 | 0 | 2 | 0 | 0 | 0 | 0 | N | 111253 | Dihydropyrimidine dehydrogenase [NADP(+)] OS=Mus musculus OX=10090 GN=Dpyd PE=1 SV=1 |
| 2376 | 5213 | A0A0G2JF48\|A0A0G2JF48\_MOUSE | 73.26 | 18 | 0 | 0 | 0 | 0 | 0 | 18 | 0 | 0 | 0 | 0 |  |  |  |  |  | 1.85E6 |  |  |  |  | 1 | 1 | 0 | 0 | 0 | 0 | 0 | 2 | 0 | 0 | 0 | 0 | N | 8789 | Dihydrothymine dehydrogenase OS=Mus musculus OX=10090 GN=Dpyd PE=1 SV=1 |
| 1012 | 3036 | E9PX29\|E9PX29\_MOUSE | 73.25 | 1 | 0 | 0 | 0 | 0 | 0 | 1 | 0 | 0 | 0 | 0 |  |  |  |  |  | 2.34E6 |  |  |  |  | 2 | 1 | 1 | 1 | 1 | 2 | 2 | 3 | 1 | 2 | 1 | 1 | N | 288822 | Spectrin beta chain OS=Mus musculus OX=10090 GN=Sptbn4 PE=1 SV=1 |
| 1160 | 3973 | sp\|P34884\|MIF\_MOUSE | 73.25 | 16 | 6 | 6 | 16 | 16 | 6 | 6 | 6 | 16 | 6 | 0 | 2.11E7 | 1.41E7 | 1.74E7 | 6.78E6 | 1.12E7 | 1.33E7 | 8.61E6 | 1.01E7 | 1.94E7 |  | 2 | 2 | 1 | 1 | 2 | 2 | 1 | 1 | 1 | 2 | 1 | 0 | Y | 12504 | Macrophage migration inhibitory factor OS=Mus musculus OX=10090 GN=Mif PE=1 SV=2 |
| 1697 | 2901 | sp\|Q64737\|PUR2\_MOUSE | 73.13 | 2 | 0 | 1 | 0 | 0 | 1 | 0 | 0 | 0 | 1 | 1 |  | 2.03E6 |  |  | 4.99E5 |  |  |  | 2.32E6 | 2.94E5 | 2 | 2 | 0 | 1 | 0 | 0 | 1 | 0 | 0 | 0 | 1 | 1 | Y | 107503 | Trifunctional purine biosynthetic protein adenosine-3 OS=Mus musculus OX=10090 GN=Gart PE=1 SV=3 |
| 1595 | 3775 | sp\|Q9R069\|BCAM\_MOUSE | 72.85 | 2 | 2 | 2 | 0 | 0 | 2 | 2 | 2 | 0 | 0 | 0 | 6.6E5 | 1.03E6 |  |  | 7.76E5 | 9.77E5 | 3.81E5 |  |  |  | 1 | 1 | 1 | 1 | 0 | 0 | 1 | 1 | 1 | 0 | 0 | 0 | N | 67670 | Basal cell adhesion molecule OS=Mus musculus OX=10090 GN=Bcam PE=1 SV=1 |
| 902 | 2494 | A0A494BAF7\|A0A494BAF7\_MOUSE | 72.85 | 19 | 12 | 19 | 19 | 0 | 12 | 12 | 12 | 0 | 12 | 7 | 1.09E6 | 9.01E6 | 1.17E7 |  | 8.74E6 | 8.29E6 | 4.77E6 |  | 9.5E6 | 1.16E6 | 3 | 3 | 2 | 3 | 3 | 0 | 2 | 2 | 2 | 0 | 2 | 1 | Y | 15455 | lactoylglutathione lyase OS=Mus musculus OX=10090 GN=Glo1 PE=1 SV=1 |
| 902 | 2495 | A0A494BBE7\|A0A494BBE7\_MOUSE | 72.85 | 17 | 11 | 17 | 17 | 0 | 11 | 11 | 11 | 0 | 11 | 6 | 1.09E6 | 9.01E6 | 1.17E7 |  | 8.74E6 | 8.29E6 | 4.77E6 |  | 9.5E6 | 1.16E6 | 3 | 3 | 2 | 3 | 3 | 0 | 2 | 2 | 2 | 0 | 2 | 1 | Y | 16912 | Glyoxalase 1 OS=Mus musculus OX=10090 GN=Glo1 PE=1 SV=1 |
| 902 | 2498 | sp\|Q9CPU0\|LGUL\_MOUSE | 72.85 | 14 | 9 | 14 | 14 | 0 | 9 | 9 | 9 | 0 | 9 | 5 | 1.09E6 | 9.01E6 | 1.17E7 |  | 8.74E6 | 8.29E6 | 4.77E6 |  | 9.5E6 | 1.16E6 | 3 | 3 | 2 | 3 | 3 | 0 | 2 | 2 | 2 | 0 | 2 | 1 | Y | 20810 | Lactoylglutathione lyase OS=Mus musculus OX=10090 GN=Glo1 PE=1 SV=3 |
| 1637 | 5405 | A0A1Y7VM65\|A0A1Y7VM65\_MOUSE | 72.72 | 14 | 0 | 13 | 0 | 13 | 0 | 13 | 14 | 0 | 0 | 13 |  | 5.58E6 |  | 1.39E6 |  | 4.95E6 | 4.53E6 |  |  | 2.48E6 | 2 | 2 | 0 | 1 | 0 | 1 | 0 | 1 | 2 | 0 | 0 | 1 | N | 9387 | Glutaredoxin-1 OS=Mus musculus OX=10090 GN=Glrx PE=1 SV=1 |
| 1637 | 5408 | sp\|Q9QUH0\|GLRX1\_MOUSE | 72.72 | 11 | 0 | 10 | 0 | 10 | 0 | 10 | 11 | 0 | 0 | 10 |  | 5.58E6 |  | 1.39E6 |  | 4.95E6 | 4.53E6 |  |  | 2.48E6 | 2 | 2 | 0 | 1 | 0 | 1 | 0 | 1 | 2 | 0 | 0 | 1 | N | 11871 | Glutaredoxin-1 OS=Mus musculus OX=10090 GN=Glrx PE=1 SV=3 |
| 1440 | 2763 | Q5SX46\|Q5SX46\_MOUSE | 72.72 | 9 | 0 | 9 | 4 | 5 | 0 | 0 | 5 | 0 | 0 | 4 |  | 2.2E6 | 1.51E6 | 3.92E5 |  |  | 1.96E5 |  |  | 8.92E5 | 2 | 2 | 0 | 2 | 1 | 1 | 0 | 0 | 1 | 0 | 0 | 1 | N | 20815 | Mitochondrial 2-oxoglutarate/malate carrier protein (Fragment) OS=Mus musculus OX=10090 GN=Slc25a11 PE=1 SV=1 |
| 1440 | 2764 | sp\|Q9CR62\|M2OM\_MOUSE | 72.72 | 6 | 0 | 6 | 3 | 3 | 0 | 0 | 3 | 0 | 0 | 3 |  | 2.2E6 | 1.51E6 | 3.92E5 |  |  | 1.96E5 |  |  | 8.92E5 | 2 | 2 | 0 | 2 | 1 | 1 | 0 | 0 | 1 | 0 | 0 | 1 | N | 34155 | Mitochondrial 2-oxoglutarate/malate carrier protein OS=Mus musculus OX=10090 GN=Slc25a11 PE=1 SV=3 |
| 2000 | 2745 | sp\|Q01320\|TOP2A\_MOUSE | 72.67 | 1 | 0 | 1 | 0 | 1 | 0 | 0 | 0 | 1 | 0 | 0 |  |  |  | 3.19E5 |  |  |  | 8.62E4 |  |  | 2 | 1 | 0 | 1 | 0 | 1 | 0 | 0 | 0 | 1 | 0 | 0 | N | 172789 | DNA topoisomerase 2-alpha OS=Mus musculus OX=10090 GN=Top2a PE=1 SV=2 |
| 2063 | 2850 | sp\|Q62348\|TSN\_MOUSE | 72.28 | 10 | 0 | 0 | 0 | 0 | 0 | 10 | 0 | 0 | 0 | 0 |  |  |  |  |  | 1.32E6 |  |  |  |  | 2 | 2 | 0 | 0 | 0 | 0 | 0 | 2 | 0 | 0 | 0 | 0 | N | 26201 | Translin OS=Mus musculus OX=10090 GN=Tsn PE=1 SV=1 |
| 1277 | 3048 | sp\|P63037\|DNJA1\_MOUSE | 72.26 | 5 | 3 | 3 | 3 | 3 | 0 | 5 | 3 | 0 | 0 | 3 | 1.12E6 | 2.03E6 | 2.18E6 | 7.57E5 |  | 1.69E6 | 7.54E5 |  |  | 1.05E6 | 2 | 1 | 1 | 2 | 2 | 1 | 0 | 2 | 1 | 0 | 0 | 1 | N | 44868 | DnaJ homolog subfamily A member 1 OS=Mus musculus OX=10090 GN=Dnaja1 PE=1 SV=1 |
| 1239 | 2255 | sp\|Q04750\|TOP1\_MOUSE | 72.26 | 5 | 0 | 0 | 3 | 2 | 3 | 0 | 2 | 2 | 1 | 2 |  |  | 2.13E6 | 5.1E5 | 7.92E5 |  | 8.86E5 | 9.73E5 | 1.03E6 | 1.15E6 | 3 | 3 | 0 | 0 | 2 | 1 | 2 | 0 | 1 | 1 | 1 | 1 | N | 90876 | DNA topoisomerase 1 OS=Mus musculus OX=10090 GN=Top1 PE=1 SV=2 |
| 1925 | 2540 | sp\|P62869\|ELOB\_MOUSE | 72.18 | 21 | 0 | 0 | 0 | 0 | 0 | 15 | 0 | 0 | 6 | 8 |  |  |  |  |  | 6.85E6 |  |  | 2.66E6 | 7.54E5 | 3 | 3 | 0 | 0 | 0 | 0 | 0 | 2 | 0 | 0 | 1 | 1 | N | 13170 | Elongin-B OS=Mus musculus OX=10090 GN=Elob PE=1 SV=1 |
| 1477 | 2779 | J3QNW0\|J3QNW0\_MOUSE | 72.16 | 1 | 0 | 0 | 1 | 0 | 0 | 0 | 0 | 0 | 0 | 0 |  |  | 4.09E5 |  |  |  |  |  |  |  | 1 | 1 | 0 | 0 | 1 | 0 | 0 | 0 | 0 | 0 | 0 | 0 | N | 169956 | DNA (cytosine-5)-methyltransferase OS=Mus musculus OX=10090 GN=Dnmt1 PE=1 SV=1 |
| 1477 | 2782 | sp\|P13864\|DNMT1\_MOUSE | 72.16 | 1 | 0 | 0 | 1 | 0 | 0 | 0 | 0 | 0 | 0 | 0 |  |  | 4.09E5 |  |  |  |  |  |  |  | 1 | 1 | 0 | 0 | 1 | 0 | 0 | 0 | 0 | 0 | 0 | 0 | N | 183188 | DNA (cytosine-5)-methyltransferase 1 OS=Mus musculus OX=10090 GN=Dnmt1 PE=1 SV=5 |
| 1540 | 4076 | sp\|P54823\|DDX6\_MOUSE | 72.13 | 4 | 2 | 0 | 2 | 2 | 0 | 2 | 2 | 0 | 2 | 2 | 2.41E6 |  | 3.92E6 | 1.21E6 |  | 2.16E6 | 6.27E5 |  | 2.17E6 | 7.36E5 | 2 | 2 | 1 | 0 | 1 | 1 | 0 | 1 | 1 | 0 | 1 | 1 | Y | 54192 | Probable ATP-dependent RNA helicase DDX6 OS=Mus musculus OX=10090 GN=Ddx6 PE=1 SV=1 |
| 1174 | 2683 | sp\|Q8VCC2\|EST1\_MOUSE | 71.87 | 3 | 2 | 2 | 2 | 2 | 2 | 3 | 2 | 2 | 0 | 2 |  |  |  |  |  | 1E6 |  |  |  |  | 2 | 1 | 1 | 1 | 1 | 1 | 1 | 2 | 1 | 1 | 0 | 1 | N | 62680 | Liver carboxylesterase 1 OS=Mus musculus OX=10090 GN=Ces1 PE=1 SV=1 |
| 1397 | 5364 | A0A494BBE3\|A0A494BBE3\_MOUSE | 71.79 | 10 | 10 | 10 | 10 | 10 | 0 | 10 | 10 | 10 | 10 | 10 | 1.13E7 | 9.52E6 | 5.77E6 | 1.94E6 |  | 5.13E6 | 2.29E6 | 1.88E6 | 1.05E7 | 6.81E6 | 1 | 1 | 1 | 1 | 1 | 1 | 0 | 1 | 1 | 1 | 1 | 1 | N | 11535 | Receptor accessory protein 5 (Fragment) OS=Mus musculus OX=10090 GN=Reep5 PE=1 SV=1 |
| 1397 | 5393 | sp\|Q60870\|REEP5\_MOUSE | 71.79 | 5 | 5 | 5 | 5 | 5 | 0 | 5 | 5 | 5 | 5 | 5 | 1.13E7 | 9.52E6 | 5.77E6 | 1.94E6 |  | 5.13E6 | 2.29E6 | 1.88E6 | 1.05E7 | 6.81E6 | 1 | 1 | 1 | 1 | 1 | 1 | 0 | 1 | 1 | 1 | 1 | 1 | N | 21051 | Receptor expression-enhancing protein 5 OS=Mus musculus OX=10090 GN=Reep5 PE=1 SV=1 |
| 1397 | 5396 | G3X8R0\|G3X8R0\_MOUSE | 71.79 | 5 | 5 | 5 | 5 | 5 | 0 | 5 | 5 | 5 | 5 | 5 | 1.13E7 | 9.52E6 | 5.77E6 | 1.94E6 |  | 5.13E6 | 2.29E6 | 1.88E6 | 1.05E7 | 6.81E6 | 1 | 1 | 1 | 1 | 1 | 1 | 0 | 1 | 1 | 1 | 1 | 1 | N | 21447 | Receptor expression-enhancing protein OS=Mus musculus OX=10090 GN=Reep5 PE=1 SV=1 |
| 1545 | 5214 | sp\|Q6ZWM4\|LSM8\_MOUSE | 71.56 | 17 | 0 | 17 | 17 | 17 | 0 | 17 | 17 | 17 | 17 | 0 |  | 2.65E5 | 1.99E6 | 6.89E5 |  | 1.05E6 | 3.74E5 | 3.9E5 | 1.56E6 |  | 1 | 1 | 0 | 1 | 1 | 1 | 0 | 1 | 1 | 1 | 1 | 0 | N | 10403 | U6 snRNA-associated Sm-like protein LSm8 OS=Mus musculus OX=10090 GN=Lsm8 PE=1 SV=3 |
| 1545 | 5215 | A0A0N4SUZ3\|A0A0N4SUZ3\_MOUSE | 71.56 | 16 | 0 | 16 | 16 | 16 | 0 | 16 | 16 | 16 | 16 | 0 |  | 2.65E5 | 1.99E6 | 6.89E5 |  | 1.05E6 | 3.74E5 | 3.9E5 | 1.56E6 |  | 1 | 1 | 0 | 1 | 1 | 1 | 0 | 1 | 1 | 1 | 1 | 0 | N | 11039 | U6 snRNA-associated Sm-like protein LSm8 OS=Mus musculus OX=10090 GN=Lsm8 PE=1 SV=1 |
| 1743 | 4149 | D3YZ05\|D3YZ05\_MOUSE | 71.48 | 13 | 0 | 0 | 0 | 0 | 0 | 13 | 0 | 0 | 0 | 13 |  |  |  |  |  | 3.8E5 |  |  |  | 3.13E5 | 1 | 1 | 0 | 0 | 0 | 0 | 0 | 1 | 0 | 0 | 0 | 1 | Y | 14898 | Translation initiation factor eIF2B subunit alpha (Fragment) OS=Mus musculus OX=10090 GN=Eif2b1 PE=1 SV=1 |
| 1743 | 4152 | D3Z151\|D3Z151\_MOUSE | 71.48 | 9 | 0 | 0 | 0 | 0 | 0 | 9 | 0 | 0 | 0 | 9 |  |  |  |  |  | 3.8E5 |  |  |  | 3.13E5 | 1 | 1 | 0 | 0 | 0 | 0 | 0 | 1 | 0 | 0 | 0 | 1 | Y | 20551 | Translation initiation factor eIF2B subunit alpha (Fragment) OS=Mus musculus OX=10090 GN=Eif2b1 PE=1 SV=8 |
| 1743 | 4172 | sp\|Q99LC8\|EI2BA\_MOUSE | 71.48 | 6 | 0 | 0 | 0 | 0 | 0 | 6 | 0 | 0 | 0 | 6 |  |  |  |  |  | 3.8E5 |  |  |  | 3.13E5 | 1 | 1 | 0 | 0 | 0 | 0 | 0 | 1 | 0 | 0 | 0 | 1 | Y | 33816 | Translation initiation factor eIF2B subunit alpha OS=Mus musculus OX=10090 GN=Eif2b1 PE=1 SV=1 |
| 1615 | 2885 | F7CDT0\|F7CDT0\_MOUSE | 71.43 | 10 | 0 | 0 | 0 | 6 | 6 | 10 | 0 | 0 | 6 | 0 |  |  |  | 1.51E6 | 8.9E5 | 3E6 |  |  | 2.31E6 |  | 2 | 2 | 0 | 0 | 0 | 1 | 1 | 2 | 0 | 0 | 1 | 0 | N | 19824 | E2 NEDD8-conjugating enzyme (Fragment) OS=Mus musculus OX=10090 GN=Ube2m PE=1 SV=1 |
| 1615 | 2886 | F6WMC0\|F6WMC0\_MOUSE | 71.43 | 10 | 0 | 0 | 0 | 6 | 6 | 10 | 0 | 0 | 6 | 0 |  |  |  | 1.51E6 | 8.9E5 | 3E6 |  |  | 2.31E6 |  | 2 | 2 | 0 | 0 | 0 | 1 | 1 | 2 | 0 | 0 | 1 | 0 | N | 19973 | E2 ubiquitin-conjugating enzyme (Fragment) OS=Mus musculus OX=10090 GN=Ube2m PE=1 SV=1 |
| 1615 | 2889 | sp\|P61082\|UBC12\_MOUSE | 71.43 | 9 | 0 | 0 | 0 | 6 | 6 | 9 | 0 | 0 | 6 | 0 |  |  |  | 1.51E6 | 8.9E5 | 3E6 |  |  | 2.31E6 |  | 2 | 2 | 0 | 0 | 0 | 1 | 1 | 2 | 0 | 0 | 1 | 0 | N | 20900 | NEDD8-conjugating enzyme Ubc12 OS=Mus musculus OX=10090 GN=Ube2m PE=1 SV=1 |
| 1422 | 1921 | sp\|Q8BMD8\|SCMC1\_MOUSE | 70.77 | 7 | 0 | 0 | 2 | 0 | 2 | 3 | 0 | 0 | 3 | 0 |  |  | 6.07E6 |  | 2.4E6 | 1.11E7 |  |  | 5.14E6 |  | 4 | 4 | 0 | 0 | 1 | 0 | 1 | 2 | 0 | 0 | 2 | 0 | Y | 52902 | Mitochondrial adenyl nucleotide antiporter SLC25A24 OS=Mus musculus OX=10090 GN=Slc25a24 PE=1 SV=1 |
| 2551 | 5225 | sp\|Q9CPU4\|MGST3\_MOUSE | 70.72 | 8 | 0 | 0 | 8 | 8 | 0 | 0 | 0 | 0 | 0 | 0 |  |  | 8.31E5 | 2.78E5 |  |  |  |  |  |  | 1 | 1 | 0 | 0 | 1 | 1 | 0 | 0 | 0 | 0 | 0 | 0 | N | 16958 | Glutathione S-transferase 3, mitochondrial OS=Mus musculus OX=10090 GN=Mgst3 PE=1 SV=1 |
| 1173 | 2181 | E9Q397\|E9Q397\_MOUSE | 70.66 | 1 | 0 | 0 | 0 | 1 | 1 | 1 | 0 | 0 | 0 | 0 |  |  | 5.27E6 | 6.35E5 |  | 3.56E6 |  |  | 2.22E6 |  | 3 | 1 | 0 | 0 | 1 | 2 | 3 | 3 | 0 | 0 | 1 | 0 | N | 246259 | Spectrin beta chain OS=Mus musculus OX=10090 GN=Sptb PE=1 SV=1 |
| 1173 | 2207 | Q3UGX2\|Q3UGX2\_MOUSE | 70.66 | 1 | 0 | 0 | 0 | 1 | 1 | 1 | 0 | 0 | 0 | 0 |  |  | 5.27E6 | 6.35E5 |  | 3.56E6 |  |  | 2.22E6 |  | 3 | 1 | 0 | 0 | 1 | 2 | 3 | 3 | 0 | 0 | 1 | 0 | N | 268089 | Spectrin beta chain OS=Mus musculus OX=10090 GN=Sptb PE=1 SV=1 |
| 1173 | 2228 | sp\|P15508\|SPTB1\_MOUSE | 70.66 | 1 | 0 | 0 | 0 | 1 | 1 | 1 | 0 | 0 | 0 | 0 |  |  | 5.27E6 | 6.35E5 |  | 3.56E6 |  |  | 2.22E6 |  | 3 | 1 | 0 | 0 | 1 | 2 | 3 | 3 | 0 | 0 | 1 | 0 | N | 245248 | Spectrin beta chain, erythrocytic OS=Mus musculus OX=10090 GN=Sptb PE=1 SV=4 |
| 1381 | 4240 | sp\|Q9DBX3\|SUSD2\_MOUSE | 70.66 | 1 | 1 | 1 | 1 | 0 | 0 | 1 | 0 | 0 | 0 | 1 | 8.24E5 | 2.48E5 | 6.56E5 |  |  | 6.29E5 |  |  |  | 7.07E5 | 1 | 1 | 2 | 1 | 2 | 0 | 0 | 2 | 0 | 0 | 0 | 2 | N | 90641 | Sushi domain-containing protein 2 OS=Mus musculus OX=10090 GN=Susd2 PE=1 SV=1 |
| 1912 | 4837 | A0A0M3HEP8\|A0A0M3HEP8\_MOUSE | 70.41 | 8 | 0 | 0 | 8 | 0 | 8 | 8 | 0 | 0 | 8 | 0 |  |  | 4.57E5 |  | 4.47E5 | 5.68E5 |  |  | 2.33E5 |  | 1 | 1 | 0 | 0 | 1 | 0 | 1 | 1 | 0 | 0 | 1 | 0 | N | 14664 | vitamin-K-epoxide reductase (warfarin-sensitive) OS=Mus musculus OX=10090 GN=Vkorc1l1 PE=1 SV=1 |
| 1912 | 4841 | sp\|Q6TEK5\|VKORL\_MOUSE | 70.41 | 6 | 0 | 0 | 6 | 0 | 6 | 6 | 0 | 0 | 6 | 0 |  |  | 4.57E5 |  | 4.47E5 | 5.68E5 |  |  | 2.33E5 |  | 1 | 1 | 0 | 0 | 1 | 0 | 1 | 1 | 0 | 0 | 1 | 0 | N | 19779 | Vitamin K epoxide reductase complex subunit 1-like protein 1 OS=Mus musculus OX=10090 GN=Vkorc1l1 PE=1 SV=1 |
| 1327 | 5070 | sp\|Q9DCF9\|SSRG\_MOUSE | 70.37 | 8 | 8 | 8 | 8 | 8 | 8 | 8 | 8 | 8 | 8 | 8 | 9.62E5 | 1.95E6 | 1.62E6 | 8.7E5 | 1.28E6 | 2.56E5 | 1.2E6 | 1.31E6 | 3.06E6 | 2.51E6 | 1 | 1 | 1 | 1 | 1 | 1 | 1 | 1 | 1 | 1 | 1 | 1 | N | 21064 | Translocon-associated protein subunit gamma OS=Mus musculus OX=10090 GN=Ssr3 PE=1 SV=1 |
| 1523 | 4846 | sp\|O70370\|CATS\_MOUSE | 70.35 | 4 | 4 | 4 | 4 | 4 | 0 | 0 | 0 | 4 | 4 | 4 | 4.74E6 | 6.08E6 | 1.27E6 | 5.66E5 |  |  |  | 1.74E6 | 1.13E7 | 5.37E6 | 1 | 1 | 1 | 1 | 1 | 1 | 0 | 0 | 0 | 1 | 1 | 1 | N | 38475 | Cathepsin S OS=Mus musculus OX=10090 GN=Ctss PE=1 SV=2 |
| 1523 | 4847 | F6WR04\|F6WR04\_MOUSE | 70.35 | 4 | 4 | 4 | 4 | 4 | 0 | 0 | 0 | 4 | 4 | 4 | 4.74E6 | 6.08E6 | 1.27E6 | 5.66E5 |  |  |  | 1.74E6 | 1.13E7 | 5.37E6 | 1 | 1 | 1 | 1 | 1 | 1 | 0 | 0 | 0 | 1 | 1 | 1 | N | 38631 | Cathepsin S OS=Mus musculus OX=10090 GN=Ctss PE=1 SV=1 |
| 1887 | 2666 | sp\|Q9WVE8\|PACN2\_MOUSE | 70.27 | 4 | 0 | 2 | 0 | 0 | 2 | 2 | 0 | 0 | 0 | 0 |  | 6.22E6 |  |  | 4.87E5 | 3.01E6 |  |  |  |  | 2 | 2 | 0 | 1 | 0 | 0 | 1 | 1 | 0 | 0 | 0 | 0 | N | 55833 | Protein kinase C and casein kinase substrate in neurons protein 2 OS=Mus musculus OX=10090 GN=Pacsin2 PE=1 SV=1 |
| 1264 | 4224 | sp\|P01631\|KV2A7\_MOUSE | 70.25 | 12 | 12 | 12 | 12 | 12 | 12 | 12 | 12 | 12 | 0 | 12 | 1.4E7 | 2.74E6 | 4.76E6 | 6.47E5 | 4.98E5 | 6.11E6 | 3.95E4 | 6.84E6 |  | 8.88E6 | 1 | 1 | 1 | 1 | 1 | 1 | 1 | 1 | 1 | 1 | 0 | 1 | N | 12273 | Ig kappa chain V-II region 26-10 OS=Mus musculus OX=10090 PE=1 SV=1 |
| 1264 | 4227 | A0A0B4J1I0\|A0A0B4J1I0\_MOUSE | 70.25 | 11 | 11 | 11 | 11 | 11 | 11 | 11 | 11 | 11 | 0 | 11 | 1.4E7 | 2.74E6 | 4.76E6 | 6.47E5 | 4.98E5 | 6.11E6 | 3.95E4 | 6.84E6 |  | 8.88E6 | 1 | 1 | 1 | 1 | 1 | 1 | 1 | 1 | 1 | 1 | 0 | 1 | N | 13079 | Immunoglobulin kappa variable 1-110 (Fragment) OS=Mus musculus OX=10090 GN=Igkv1-110 PE=1 SV=5 |
| 1264 | 4228 | A0A140T8M0\|A0A140T8M0\_MOUSE | 70.25 | 11 | 11 | 11 | 11 | 11 | 11 | 11 | 11 | 11 | 0 | 11 | 1.4E7 | 2.74E6 | 4.76E6 | 6.47E5 | 4.98E5 | 6.11E6 | 3.95E4 | 6.84E6 |  | 8.88E6 | 1 | 1 | 1 | 1 | 1 | 1 | 1 | 1 | 1 | 1 | 0 | 1 | N | 13117 | Immunoglobulin kappa variable 1-117 (Fragment) OS=Mus musculus OX=10090 GN=Igkv1-117 PE=1 SV=2 |
| 1529 | 2941 | F7BX26\|F7BX26\_MOUSE | 70.17 | 2 | 2 | 2 | 0 | 0 | 0 | 0 | 0 | 0 | 2 | 2 | 1.23E6 | 1.46E6 |  |  |  |  |  |  | 1.1E6 | 2.27E6 | 1 | 1 | 1 | 1 | 0 | 0 | 0 | 0 | 0 | 0 | 1 | 1 | N | 54324 | Serine/threonine-protein phosphatase (Fragment) OS=Mus musculus OX=10090 GN=Ppp5c PE=1 SV=1 |
| 1529 | 2942 | sp\|Q60676\|PPP5\_MOUSE | 70.17 | 2 | 2 | 2 | 0 | 0 | 0 | 0 | 0 | 0 | 2 | 2 | 1.23E6 | 1.46E6 |  |  |  |  |  |  | 1.1E6 | 2.27E6 | 1 | 1 | 1 | 1 | 0 | 0 | 0 | 0 | 0 | 0 | 1 | 1 | N | 56877 | Serine/threonine-protein phosphatase 5 OS=Mus musculus OX=10090 GN=Ppp5c PE=1 SV=3 |
| 1326 | 4204 | sp\|O70591\|PFD2\_MOUSE | 70.12 | 12 | 0 | 0 | 0 | 9 | 9 | 12 | 9 | 9 | 0 | 0 |  |  |  | 0E0 | 1.77E6 | 1.44E6 | 6.46E5 | 7.55E5 |  |  | 2 | 1 | 0 | 0 | 0 | 1 | 1 | 2 | 1 | 1 | 0 | 0 | N | 16534 | Prefoldin subunit 2 OS=Mus musculus OX=10090 GN=Pfdn2 PE=1 SV=2 |
| 1930 | 4275 | sp\|Q9DCM0\|ETHE1\_MOUSE | 70.11 | 4 | 4 | 0 | 0 | 0 | 0 | 0 | 0 | 0 | 4 | 0 | 1.4E6 |  |  |  |  |  |  |  | 1.27E6 |  | 1 | 1 | 1 | 0 | 0 | 0 | 0 | 0 | 0 | 0 | 1 | 0 | N | 27739 | Persulfide dioxygenase ETHE1, mitochondrial OS=Mus musculus OX=10090 GN=Ethe1 PE=1 SV=2 |
| 968 | 2547 | sp\|Q9QZD9\|EIF3I\_MOUSE | 70.09 | 10 | 7 | 7 | 7 | 4 | 7 | 7 | 10 | 7 | 4 | 4 | 3.87E6 | 3.49E6 | 4.7E6 | 9.41E5 | 3.22E6 | 2.16E6 | 3E6 | 1.9E6 | 2.7E6 | 2.2E6 | 3 | 3 | 2 | 2 | 2 | 1 | 2 | 2 | 3 | 2 | 1 | 1 | Y | 36461 | Eukaryotic translation initiation factor 3 subunit I OS=Mus musculus OX=10090 GN=Eif3i PE=1 SV=1 |
| 822 | 5338 | A0A494B9Z0\|A0A494B9Z0\_MOUSE | 70.02 | 16 | 16 | 16 | 16 | 16 | 16 | 16 | 16 | 16 | 16 | 16 | 2.38E7 | 2.65E7 | 2.9E7 | 1.46E7 | 2.65E7 | 1.91E7 | 1.88E7 | 1.62E7 | 5E7 | 1.59E7 | 1 | 1 | 3 | 3 | 2 | 2 | 2 | 2 | 2 | 4 | 4 | 2 | N | 7006 | 40S ribosomal protein S30 OS=Mus musculus OX=10090 GN=Fau PE=1 SV=1 |
| 822 | 5361 | A0A494BBA8\|A0A494BBA8\_MOUSE | 70.02 | 10 | 10 | 10 | 10 | 10 | 10 | 10 | 10 | 10 | 10 | 10 | 2.38E7 | 2.65E7 | 2.9E7 | 1.46E7 | 2.65E7 | 1.91E7 | 1.88E7 | 1.62E7 | 5E7 | 1.59E7 | 1 | 1 | 3 | 3 | 2 | 2 | 2 | 2 | 2 | 4 | 4 | 2 | N | 10871 | FAU ubiquitin like and ribosomal protein S30 fusion OS=Mus musculus OX=10090 GN=Fau PE=1 SV=1 |
| 822 | 5384 | sp\|P62862\|RS30\_MOUSE | 70.02 | 8 | 8 | 8 | 8 | 8 | 8 | 8 | 8 | 8 | 8 | 8 | 2.38E7 | 2.65E7 | 2.9E7 | 1.46E7 | 2.65E7 | 1.91E7 | 1.88E7 | 1.62E7 | 5E7 | 1.59E7 | 1 | 1 | 3 | 3 | 2 | 2 | 2 | 2 | 2 | 4 | 4 | 2 | N | 14416 | Ubiquitin-like FUBI-ribosomal protein eS30 fusion protein OS=Mus musculus OX=10090 GN=Fau PE=1 SV=2 |
| 1388 | 3520 | sp\|P70460\|VASP\_MOUSE | 69.80 | 6 | 2 | 2 | 2 | 2 | 0 | 0 | 2 | 2 | 6 | 2 | 9.97E6 | 7.49E6 | 6.91E6 | 3.19E6 |  |  | 5.86E6 | 4.49E6 | 9.74E6 | 1.39E7 | 2 | 2 | 1 | 1 | 1 | 1 | 0 | 0 | 1 | 1 | 2 | 1 | N | 39667 | Vasodilator-stimulated phosphoprotein OS=Mus musculus OX=10090 GN=Vasp PE=1 SV=4 |
| 1362 | 2076 | sp\|Q3U1J4\|DDB1\_MOUSE | 69.67 | 2 | 1 | 0 | 1 | 1 | 1 | 0 | 0 | 0 | 1 | 1 | 8.74E6 |  | 2.64E5 | 1.29E5 | 0E0 |  |  |  | 4.89E6 | 8.72E6 | 3 | 3 | 1 | 0 | 1 | 1 | 1 | 0 | 0 | 0 | 1 | 1 | N | 126853 | DNA damage-binding protein 1 OS=Mus musculus OX=10090 GN=Ddb1 PE=1 SV=2 |
| 1617 | 3454 | sp\|Q9CQE1\|NPS3B\_MOUSE | 69.62 | 8 | 0 | 0 | 4 | 0 | 4 | 4 | 4 | 0 | 4 | 4 |  |  | 5.84E5 |  | 4.75E5 | 3.7E5 | 2.8E5 |  | 9.66E5 | 6.54E5 | 2 | 2 | 0 | 0 | 1 | 0 | 1 | 1 | 1 | 0 | 1 | 1 | N | 28308 | Protein NipSnap homolog 3B OS=Mus musculus OX=10090 GN=Nipsnap3b PE=1 SV=1 |
| 1617 | 3958 | B1AWZ5\|B1AWZ5\_MOUSE | 69.62 | 10 | 0 | 0 | 6 | 0 | 6 | 6 | 5 | 0 | 6 | 6 |  |  | 5.84E5 |  | 4.75E5 | 3.7E5 | 2.8E5 |  | 9.66E5 | 6.54E5 | 2 | 2 | 0 | 0 | 1 | 0 | 1 | 1 | 1 | 0 | 1 | 1 | N | 22887 | Nipsnap homolog 3B OS=Mus musculus OX=10090 GN=Nipsnap3b PE=1 SV=1 |
| 2098 | 3652 | sp\|P23780\|BGAL\_MOUSE | 69.57 | 2 | 0 | 0 | 0 | 2 | 0 | 0 | 2 | 2 | 0 | 0 |  |  |  | 8.15E5 |  |  | 1.31E6 | 1.1E6 |  |  | 1 | 1 | 0 | 0 | 0 | 1 | 0 | 0 | 1 | 1 | 0 | 0 | N | 73121 | Beta-galactosidase OS=Mus musculus OX=10090 GN=Glb1 PE=1 SV=1 |
| 2098 | 5256 | A0A1L1SSJ7\|A0A1L1SSJ7\_MOUSE | 69.57 | 4 | 0 | 0 | 0 | 4 | 0 | 0 | 4 | 4 | 0 | 0 |  |  |  | 8.15E5 |  |  | 1.31E6 | 1.1E6 |  |  | 1 | 1 | 0 | 0 | 0 | 1 | 0 | 0 | 1 | 1 | 0 | 0 | N | 32929 | Galactosidase, beta 1 OS=Mus musculus OX=10090 GN=Glb1 PE=1 SV=1 |
| 1191 | 2464 | sp\|P34928\|APOC1\_MOUSE | 69.54 | 28 | 0 | 18 | 10 | 0 | 10 | 20 | 20 | 10 | 0 | 0 |  | 5.81E6 | 1.74E5 |  | 1.12E6 | 6.11E6 | 4.02E6 | 4.23E5 |  |  | 3 | 3 | 0 | 2 | 1 | 0 | 1 | 2 | 2 | 1 | 0 | 0 | N | 9696 | Apolipoprotein C-I OS=Mus musculus OX=10090 GN=Apoc1 PE=1 SV=1 |
| 1460 | 3964 | H3BKN0\|H3BKN0\_MOUSE | 69.30 | 4 | 0 | 2 | 2 | 2 | 2 | 0 | 2 | 2 | 4 | 0 |  | 2.1E5 | 7.23E5 | 9.01E4 | 8.61E5 |  | 2.77E5 | 3.18E5 | 2.19E6 |  | 2 | 2 | 0 | 1 | 1 | 1 | 1 | 0 | 1 | 1 | 2 | 0 | N | 81374 | tRNA (cytosine(34)-C(5))-methyltransferase OS=Mus musculus OX=10090 GN=Nsun2 PE=1 SV=1 |
| 1460 | 3965 | sp\|Q1HFZ0\|NSUN2\_MOUSE | 69.30 | 4 | 0 | 1 | 1 | 1 | 1 | 0 | 1 | 1 | 4 | 0 |  | 2.1E5 | 7.23E5 | 9.01E4 | 8.61E5 |  | 2.77E5 | 3.18E5 | 2.19E6 |  | 2 | 2 | 0 | 1 | 1 | 1 | 1 | 0 | 1 | 1 | 2 | 0 | N | 85452 | RNA cytosine C(5)-methyltransferase NSUN2 OS=Mus musculus OX=10090 GN=Nsun2 PE=1 SV=2 |
| 3436 | 5327 | sp\|Q9WVQ5\|MTNB\_MOUSE | 69.29 | 5 | 5 | 0 | 0 | 0 | 0 | 0 | 0 | 0 | 0 | 0 | 1.26E5 |  |  |  |  |  |  |  |  |  | 1 | 1 | 1 | 0 | 0 | 0 | 0 | 0 | 0 | 0 | 0 | 0 | N | 26949 | Methylthioribulose-1-phosphate dehydratase OS=Mus musculus OX=10090 GN=Apip PE=1 SV=1 |
| 1613 | 1999 | Q6ZWQ5\|Q6ZWQ5\_MOUSE | 69.26 | 17 | 0 | 0 | 0 | 0 | 0 | 0 | 0 | 0 | 10 | 7 |  |  |  |  |  |  |  |  | 1.73E6 | 8.62E5 | 3 | 2 | 0 | 0 | 0 | 0 | 0 | 0 | 0 | 0 | 2 | 1 | N | 18885 | Sorting nexin 12 OS=Mus musculus OX=10090 GN=Snx12 PE=1 SV=1 |
| 1613 | 2003 | Q3TGS7\|Q3TGS7\_MOUSE | 69.26 | 16 | 0 | 0 | 0 | 0 | 0 | 0 | 0 | 0 | 10 | 7 |  |  |  |  |  |  |  |  | 1.73E6 | 8.62E5 | 3 | 2 | 0 | 0 | 0 | 0 | 0 | 0 | 0 | 0 | 2 | 1 | N | 19246 | Sorting nexin 12 OS=Mus musculus OX=10090 GN=Snx12 PE=1 SV=1 |
| 1613 | 2004 | Q3V2H3\|Q3V2H3\_MOUSE | 69.26 | 16 | 0 | 0 | 0 | 0 | 0 | 0 | 0 | 0 | 9 | 6 |  |  |  |  |  |  |  |  | 1.73E6 | 8.62E5 | 3 | 2 | 0 | 0 | 0 | 0 | 0 | 0 | 0 | 0 | 2 | 1 | N | 19808 | Sorting nexin 12 OS=Mus musculus OX=10090 GN=Snx12 PE=1 SV=1 |
| 2078 | 2969 | sp\|Q8K224\|NAT10\_MOUSE | 69.21 | 2 | 0 | 0 | 2 | 0 | 0 | 0 | 0 | 0 | 2 | 0 |  |  | 5.33E5 |  |  |  |  |  | 4.42E5 |  | 1 | 1 | 0 | 0 | 1 | 0 | 0 | 0 | 0 | 0 | 1 | 0 | N | 115418 | RNA cytidine acetyltransferase OS=Mus musculus OX=10090 GN=Nat10 PE=1 SV=1 |
| 1517 | 2466 | sp\|Q9JK53\|PRELP\_MOUSE | 69.18 | 7 | 3 | 2 | 0 | 0 | 0 | 2 | 0 | 0 | 2 | 2 | 3.78E6 | 5.79E5 |  |  |  | 5.92E5 |  |  | 1.11E6 | 4.01E5 | 3 | 3 | 1 | 1 | 0 | 0 | 0 | 1 | 0 | 0 | 1 | 1 | Y | 43293 | Prolargin OS=Mus musculus OX=10090 GN=Prelp PE=1 SV=2 |
| 2076 | 2668 | D3Z568\|D3Z568\_MOUSE | 68.80 | 15 | 0 | 0 | 0 | 0 | 0 | 15 | 0 | 0 | 0 | 0 |  |  |  |  |  | 7.37E5 |  |  |  |  | 2 | 2 | 0 | 0 | 0 | 0 | 0 | 2 | 0 | 0 | 0 | 0 | N | 14038 | NADH dehydrogenase [ubiquinone] 1 beta subcomplex subunit 5, mitochondrial OS=Mus musculus OX=10090 GN=Ndufb5 PE=1 SV=1 |
| 2076 | 2672 | D3YX99\|D3YX99\_MOUSE | 68.80 | 10 | 0 | 0 | 0 | 0 | 0 | 10 | 0 | 0 | 0 | 0 |  |  |  |  |  | 7.37E5 |  |  |  |  | 2 | 2 | 0 | 0 | 0 | 0 | 0 | 2 | 0 | 0 | 0 | 0 | N | 19957 | NADH dehydrogenase [ubiquinone] 1 beta subcomplex subunit 5, mitochondrial (Fragment) OS=Mus musculus OX=10090 GN=Ndufb5 PE=1 SV=1 |
| 2076 | 2674 | sp\|Q9CQH3\|NDUB5\_MOUSE | 68.80 | 10 | 0 | 0 | 0 | 0 | 0 | 10 | 0 | 0 | 0 | 0 |  |  |  |  |  | 7.37E5 |  |  |  |  | 2 | 2 | 0 | 0 | 0 | 0 | 0 | 2 | 0 | 0 | 0 | 0 | N | 21710 | NADH dehydrogenase [ubiquinone] 1 beta subcomplex subunit 5, mitochondrial OS=Mus musculus OX=10090 GN=Ndufb5 PE=1 SV=1 |
| 1082 | 3959 | sp\|Q9WUU7\|CATZ\_MOUSE | 68.80 | 6 | 6 | 6 | 2 | 3 | 2 | 3 | 6 | 6 | 3 | 6 | 1.64E7 | 6.58E6 | 4.86E6 | 4.77E6 | 4.1E6 | 9.56E6 | 1.12E7 | 8.42E6 | 7.22E5 | 1.45E7 | 2 | 2 | 2 | 2 | 1 | 1 | 1 | 1 | 2 | 2 | 1 | 2 | N | 33996 | Cathepsin Z OS=Mus musculus OX=10090 GN=Ctsz PE=1 SV=1 |
| 1687 | 2918 | Q3TMN1\|Q3TMN1\_MOUSE | 68.80 | 5 | 5 | 5 | 0 | 0 | 5 | 5 | 0 | 0 | 0 | 0 | 1.85E6 | 2.79E6 |  |  | 1.37E6 | 1.64E6 |  |  |  |  | 1 | 1 | 1 | 1 | 0 | 0 | 1 | 1 | 0 | 0 | 0 | 0 | N | 50527 | Methionine aminopeptidase 2 OS=Mus musculus OX=10090 GN=Metap2 PE=1 SV=1 |
| 1687 | 2919 | sp\|O08663\|MAP2\_MOUSE | 68.80 | 5 | 5 | 5 | 0 | 0 | 5 | 5 | 0 | 0 | 0 | 0 | 1.85E6 | 2.79E6 |  |  | 1.37E6 | 1.64E6 |  |  |  |  | 1 | 1 | 1 | 1 | 0 | 0 | 1 | 1 | 0 | 0 | 0 | 0 | N | 52922 | Methionine aminopeptidase 2 OS=Mus musculus OX=10090 GN=Metap2 PE=1 SV=1 |
| 1687 | 2920 | Q3UI33\|Q3UI33\_MOUSE | 68.80 | 5 | 5 | 5 | 0 | 0 | 5 | 5 | 0 | 0 | 0 | 0 | 1.85E6 | 2.79E6 |  |  | 1.37E6 | 1.64E6 |  |  |  |  | 1 | 1 | 1 | 1 | 0 | 0 | 1 | 1 | 0 | 0 | 0 | 0 | N | 54117 | Methionine aminopeptidase 2 OS=Mus musculus OX=10090 GN=Metap2 PE=1 SV=1 |
| 1687 | 3266 | M0QWM9\|M0QWM9\_MOUSE | 68.80 | 10 | 10 | 10 | 0 | 0 | 10 | 10 | 0 | 0 | 0 | 0 | 1.85E6 | 2.79E6 |  |  | 1.37E6 | 1.64E6 |  |  |  |  | 1 | 1 | 1 | 1 | 0 | 0 | 1 | 1 | 0 | 0 | 0 | 0 | N | 26710 | Methionine aminopeptidase 2 (Fragment) OS=Mus musculus OX=10090 GN=Metap2 PE=1 SV=1 |
| 1606 | 3097 | A0A0B4J1G1\|A0A0B4J1G1\_MOUSE | 68.70 | 7 | 3 | 0 | 0 | 0 | 0 | 0 | 0 | 0 | 3 | 7 | 1.22E6 |  |  |  |  |  |  |  | 1.25E6 | 4.32E6 | 2 | 2 | 2 | 0 | 0 | 0 | 0 | 0 | 0 | 0 | 1 | 2 | N | 32977 | Fc receptor, IgG, low affinity IIb OS=Mus musculus OX=10090 GN=Fcgr2b PE=1 SV=1 |
| 1606 | 3387 | E9Q415\|E9Q415\_MOUSE | 68.70 | 6 | 3 | 0 | 0 | 0 | 0 | 0 | 0 | 0 | 3 | 6 | 1.22E6 |  |  |  |  |  |  |  | 1.25E6 | 4.32E6 | 2 | 2 | 2 | 0 | 0 | 0 | 0 | 0 | 0 | 0 | 1 | 2 | N | 35050 | Fc receptor, IgG, low affinity IIb OS=Mus musculus OX=10090 GN=Fcgr2b PE=1 SV=1 |
| 1606 | 3388 | sp\|P08101\|FCGR2\_MOUSE | 68.70 | 6 | 3 | 0 | 0 | 0 | 0 | 0 | 0 | 0 | 3 | 6 | 1.22E6 |  |  |  |  |  |  |  | 1.25E6 | 4.32E6 | 2 | 2 | 2 | 0 | 0 | 0 | 0 | 0 | 0 | 0 | 1 | 2 | N | 36695 | Low affinity immunoglobulin gamma Fc region receptor II OS=Mus musculus OX=10090 GN=Fcgr2 PE=1 SV=2 |
| 1606 | 3389 | A0A0B4J1E6\|A0A0B4J1E6\_MOUSE | 68.70 | 6 | 3 | 0 | 0 | 0 | 0 | 0 | 0 | 0 | 3 | 6 | 1.22E6 |  |  |  |  |  |  |  | 1.25E6 | 4.32E6 | 2 | 2 | 2 | 0 | 0 | 0 | 0 | 0 | 0 | 0 | 1 | 2 | N | 37791 | Fc receptor, IgG, low affinity IIb OS=Mus musculus OX=10090 GN=Fcgr2b PE=1 SV=1 |
| 1926 | 2720 | sp\|Q8K2C9\|HACD3\_MOUSE | 68.65 | 7 | 0 | 0 | 1 | 6 | 0 | 0 | 0 | 0 | 0 | 0 |  |  |  | 9.87E5 |  |  |  |  |  |  | 3 | 2 | 0 | 0 | 1 | 2 | 0 | 0 | 0 | 0 | 0 | 0 | N | 43131 | Very-long-chain (3R)-3-hydroxyacyl-CoA dehydratase 3 OS=Mus musculus OX=10090 GN=Hacd3 PE=1 SV=2 |
| 1358 | 2128 | sp\|Q9EQ20\|MMSA\_MOUSE | 68.63 | 5 | 0 | 0 | 2 | 0 | 2 | 4 | 2 | 0 | 1 | 0 |  |  | 2.08E6 |  | 1.48E6 | 4.86E6 | 5.2E5 |  | 1.01E5 |  | 3 | 3 | 0 | 0 | 1 | 0 | 1 | 2 | 1 | 0 | 1 | 0 | N | 57916 | Methylmalonate-semialdehyde/malonate-semialdehyde dehydrogenase [acylating], mitochondrial OS=Mus musculus OX=10090 GN=Aldh6a1 PE=1 SV=1 |
| 2166 | 5233 | sp\|P62627\|DLRB1\_MOUSE | 68.61 | 12 | 0 | 0 | 0 | 0 | 12 | 12 | 12 | 0 | 0 | 0 |  |  |  |  | 2.79E5 | 5.64E5 | 9.9E5 |  |  |  | 1 | 1 | 0 | 0 | 0 | 0 | 1 | 1 | 1 | 0 | 0 | 0 | N | 10990 | Dynein light chain roadblock-type 1 OS=Mus musculus OX=10090 GN=Dynlrb1 PE=1 SV=3 |
| 2166 | 5234 | A2AVR9\|A2AVR9\_MOUSE | 68.61 | 12 | 0 | 0 | 0 | 0 | 12 | 12 | 12 | 0 | 0 | 0 |  |  |  |  | 2.79E5 | 5.64E5 | 9.9E5 |  |  |  | 1 | 1 | 0 | 0 | 0 | 0 | 1 | 1 | 1 | 0 | 0 | 0 | N | 11928 | Dynein light chain roadblock OS=Mus musculus OX=10090 GN=Dynlrb1 PE=1 SV=1 |
| 1750 | 5228 | A2AUM6\|A2AUM6\_MOUSE | 68.36 | 14 | 0 | 0 | 14 | 0 | 14 | 14 | 14 | 14 | 0 | 0 |  |  | 1.57E6 |  | 1.99E6 | 2.2E6 | 3.56E5 | 1.01E6 |  |  | 1 | 1 | 0 | 0 | 1 | 0 | 1 | 1 | 1 | 1 | 0 | 0 | N | 11234 | Signal recognition particle 14 kDa protein OS=Mus musculus OX=10090 GN=Srp14 PE=1 SV=1 |
| 1750 | 5229 | sp\|P16254\|SRP14\_MOUSE | 68.36 | 13 | 0 | 0 | 13 | 0 | 13 | 13 | 13 | 13 | 0 | 0 |  |  | 1.57E6 |  | 1.99E6 | 2.2E6 | 3.56E5 | 1.01E6 |  |  | 1 | 1 | 0 | 0 | 1 | 0 | 1 | 1 | 1 | 1 | 0 | 0 | N | 12510 | Signal recognition particle 14 kDa protein OS=Mus musculus OX=10090 GN=Srp14 PE=1 SV=1 |
| 1865 | 3066 | sp\|O54774\|AP3D1\_MOUSE | 68.21 | 3 | 0 | 0 | 0 | 1 | 0 | 0 | 1 | 0 | 0 | 2 |  |  |  | 3.31E5 |  |  | 3.78E5 |  |  | 2.8E5 | 2 | 2 | 0 | 0 | 0 | 1 | 0 | 0 | 1 | 0 | 0 | 1 | N | 135081 | AP-3 complex subunit delta-1 OS=Mus musculus OX=10090 GN=Ap3d1 PE=1 SV=1 |
| 1601 | 2508 | sp\|P03930\|ATP8\_MOUSE | 68.18 | 43 | 0 | 9 | 0 | 19 | 0 | 0 | 28 | 0 | 15 | 0 |  | 1.73E6 |  | 3.76E6 |  |  | 1.95E6 |  | 5.74E6 |  | 3 | 3 | 0 | 1 | 0 | 1 | 0 | 0 | 2 | 0 | 1 | 0 | N | 7766 | ATP synthase protein 8 OS=Mus musculus OX=10090 GN=Mtatp8 PE=1 SV=1 |
| 1738 | 2619 | F8VPQ6\|F8VPQ6\_MOUSE | 67.80 | 4 | 0 | 2 | 0 | 0 | 0 | 2 | 2 | 0 | 2 | 0 |  | 1.07E6 |  |  |  | 3.36E6 | 2.21E6 |  | 5.14E6 |  | 2 | 2 | 0 | 1 | 0 | 0 | 0 | 1 | 1 | 0 | 1 | 0 | N | 59532 | Alkaline phosphatase OS=Mus musculus OX=10090 GN=Alpi PE=1 SV=1 |
| 2271 | 4096 | sp\|Q99J27\|ACATN\_MOUSE | 67.66 | 2 | 0 | 0 | 0 | 0 | 2 | 0 | 2 | 0 | 0 | 0 |  |  |  |  | 1.35E5 |  | 4.23E5 |  |  |  | 1 | 1 | 0 | 0 | 0 | 0 | 1 | 0 | 1 | 0 | 0 | 0 | N | 61075 | Acetyl-coenzyme A transporter 1 OS=Mus musculus OX=10090 GN=Slc33a1 PE=1 SV=1 |
| 1177 | 2135 | sp\|Q8BH95\|ECHM\_MOUSE | 67.51 | 14 | 0 | 0 | 7 | 0 | 0 | 10 | 3 | 10 | 0 | 0 |  |  | 5.91E6 |  |  | 6.28E6 | 2.77E6 | 3.29E6 |  |  | 3 | 3 | 0 | 0 | 2 | 0 | 0 | 2 | 1 | 2 | 0 | 0 | Y | 31474 | Enoyl-CoA hydratase, mitochondrial OS=Mus musculus OX=10090 GN=Echs1 PE=1 SV=1 |
| 2511 | 4453 | B1AT83\|B1AT83\_MOUSE | 67.49 | 7 | 0 | 0 | 7 | 0 | 0 | 0 | 7 | 0 | 0 | 0 |  |  | 1.17E6 |  |  |  | 1.4E6 |  |  |  | 1 | 1 | 0 | 0 | 1 | 0 | 0 | 0 | 1 | 0 | 0 | 0 | N | 21678 | Phosphoribosyl pyrophosphate synthetase-associated protein 1 (Fragment) OS=Mus musculus OX=10090 GN=Prpsap1 PE=1 SV=1 |
| 2511 | 4455 | B1AT84\|B1AT84\_MOUSE | 67.49 | 4 | 0 | 0 | 4 | 0 | 0 | 0 | 4 | 0 | 0 | 0 |  |  | 1.17E6 |  |  |  | 1.4E6 |  |  |  | 1 | 1 | 0 | 0 | 1 | 0 | 0 | 0 | 1 | 0 | 0 | 0 | N | 38682 | Phosphoribosyl pyrophosphate synthetase-associated protein 1 OS=Mus musculus OX=10090 GN=Prpsap1 PE=1 SV=2 |
| 2511 | 4456 | sp\|Q9D0M1\|KPRA\_MOUSE | 67.49 | 4 | 0 | 0 | 4 | 0 | 0 | 0 | 4 | 0 | 0 | 0 |  |  | 1.17E6 |  |  |  | 1.4E6 |  |  |  | 1 | 1 | 0 | 0 | 1 | 0 | 0 | 0 | 1 | 0 | 0 | 0 | N | 39432 | Phosphoribosyl pyrophosphate synthase-associated protein 1 OS=Mus musculus OX=10090 GN=Prpsap1 PE=1 SV=1 |
| 2511 | 4459 | B1AT82\|B1AT82\_MOUSE | 67.49 | 4 | 0 | 0 | 4 | 0 | 0 | 0 | 4 | 0 | 0 | 0 |  |  | 1.17E6 |  |  |  | 1.4E6 |  |  |  | 1 | 1 | 0 | 0 | 1 | 0 | 0 | 0 | 1 | 0 | 0 | 0 | N | 42468 | Phosphoribosyl pyrophosphate synthetase-associated protein 1 OS=Mus musculus OX=10090 GN=Prpsap1 PE=1 SV=1 |
| 1332 | 5240 | sp\|P53986\|MOT1\_MOUSE | 67.44 | 3 | 3 | 3 | 3 | 3 | 3 | 3 | 3 | 3 | 3 | 3 | 1.22E6 | 3.58E6 | 3.21E6 | 9.58E5 | 2.3E6 | 2.98E6 | 1.96E6 | 1.33E6 | 9.42E5 | 1.62E6 | 1 | 1 | 1 | 1 | 1 | 1 | 1 | 1 | 1 | 1 | 1 | 1 | N | 53267 | Monocarboxylate transporter 1 OS=Mus musculus OX=10090 GN=Slc16a1 PE=1 SV=1 |
| 1586 | 2986 | sp\|Q08943\|SSRP1\_MOUSE | 67.42 | 3 | 0 | 2 | 2 | 2 | 0 | 0 | 0 | 0 | 0 | 3 |  | 7.53E5 | 1.26E6 | 3.9E5 |  |  |  |  |  | 1.1E7 | 2 | 2 | 0 | 1 | 1 | 1 | 0 | 0 | 0 | 0 | 0 | 2 | N | 80860 | FACT complex subunit SSRP1 OS=Mus musculus OX=10090 GN=Ssrp1 PE=1 SV=2 |
| 1520 | 2680 | sp\|Q9D8B3\|CHM4B\_MOUSE | 67.39 | 11 | 0 | 6 | 4 | 0 | 0 | 0 | 0 | 4 | 0 | 0 |  | 5.69E5 | 1.49E6 |  |  |  |  | 9.1E5 |  |  | 2 | 2 | 0 | 1 | 1 | 0 | 0 | 0 | 0 | 1 | 0 | 0 | Y | 24936 | Charged multivesicular body protein 4b OS=Mus musculus OX=10090 GN=Chmp4b PE=1 SV=2 |
| 2064 | 3250 | sp\|P57784\|RU2A\_MOUSE | 67.33 | 5 | 5 | 5 | 0 | 0 | 0 | 0 | 0 | 0 | 0 | 0 | 3.2E5 | 1.48E6 |  |  |  |  |  |  |  |  | 1 | 1 | 1 | 1 | 0 | 0 | 0 | 0 | 0 | 0 | 0 | 0 | N | 28357 | U2 small nuclear ribonucleoprotein A' OS=Mus musculus OX=10090 GN=Snrpa1 PE=1 SV=2 |
| 2378 | 3510 | E9Q6H6\|E9Q6H6\_MOUSE | 67.29 | 2 | 2 | 0 | 0 | 0 | 0 | 0 | 0 | 0 | 0 | 0 | 1.62E6 |  |  |  |  |  |  |  |  |  | 1 | 1 | 1 | 0 | 0 | 0 | 0 | 0 | 0 | 0 | 0 | 0 | N | 74636 | Arachidonate 5-lipoxygenase OS=Mus musculus OX=10090 GN=Alox5 PE=1 SV=1 |
| 2378 | 3511 | sp\|P48999\|LOX5\_MOUSE | 67.29 | 2 | 2 | 0 | 0 | 0 | 0 | 0 | 0 | 0 | 0 | 0 | 1.62E6 |  |  |  |  |  |  |  |  |  | 1 | 1 | 1 | 0 | 0 | 0 | 0 | 0 | 0 | 0 | 0 | 0 | N | 77967 | Polyunsaturated fatty acid 5-lipoxygenase OS=Mus musculus OX=10090 GN=Alox5 PE=1 SV=3 |
| 2111 | 3572 | sp\|P03911\|NU4M\_MOUSE | 67.16 | 6 | 0 | 0 | 0 | 0 | 0 | 4 | 2 | 0 | 4 | 0 |  |  |  |  |  | 6.08E5 | 1.16E5 |  | 5.51E5 |  | 2 | 2 | 0 | 0 | 0 | 0 | 0 | 1 | 1 | 0 | 1 | 0 | Y | 51882 | NADH-ubiquinone oxidoreductase chain 4 OS=Mus musculus OX=10090 GN=Mtnd4 PE=1 SV=1 |
| 1333 | 5253 | B0R091\|B0R091\_MOUSE | 67.11 | 6 | 6 | 6 | 6 | 6 | 6 | 6 | 6 | 6 | 6 | 6 | 7.3E5 | 1.74E5 | 4.49E5 | 2.18E5 | 4.31E5 |  | 8.15E5 | 2.79E5 | 2.85E6 | 7.92E5 | 1 | 1 | 1 | 1 | 1 | 1 | 1 | 1 | 1 | 1 | 1 | 1 | N | 21998 | Calcineurin B homologous protein 1 OS=Mus musculus OX=10090 GN=Chp1 PE=1 SV=1 |
| 1333 | 5254 | sp\|P61022\|CHP1\_MOUSE | 67.11 | 6 | 6 | 6 | 6 | 6 | 6 | 6 | 6 | 6 | 6 | 6 | 7.3E5 | 1.74E5 | 4.49E5 | 2.18E5 | 4.31E5 |  | 8.15E5 | 2.79E5 | 2.85E6 | 7.92E5 | 1 | 1 | 1 | 1 | 1 | 1 | 1 | 1 | 1 | 1 | 1 | 1 | N | 22432 | Calcineurin B homologous protein 1 OS=Mus musculus OX=10090 GN=Chp1 PE=1 SV=2 |
| 2163 | 3982 | sp\|Q9CXR1\|DHRS7\_MOUSE | 67.06 | 9 | 0 | 0 | 0 | 0 | 0 | 9 | 0 | 0 | 0 | 0 |  |  |  |  |  | 2.71E6 |  |  |  |  | 2 | 2 | 0 | 0 | 0 | 0 | 0 | 2 | 0 | 0 | 0 | 0 | N | 38168 | Dehydrogenase/reductase SDR family member 7 OS=Mus musculus OX=10090 GN=Dhrs7 PE=1 SV=2 |
| 2163 | 3983 | A0A1Y7VN19\|A0A1Y7VN19\_MOUSE | 67.06 | 8 | 0 | 0 | 0 | 0 | 0 | 8 | 0 | 0 | 0 | 0 |  |  |  |  |  | 2.71E6 |  |  |  |  | 2 | 2 | 0 | 0 | 0 | 0 | 0 | 2 | 0 | 0 | 0 | 0 | N | 40708 | Dehydrogenase/reductase 7 (Fragment) OS=Mus musculus OX=10090 GN=Dhrs7 PE=1 SV=1 |
| 1727 | 3607 | sp\|Q9D2V7\|CORO7\_MOUSE | 66.52 | 1 | 0 | 0 | 1 | 0 | 0 | 1 | 0 | 0 | 1 | 0 |  |  | 1.09E6 |  |  | 1.05E6 |  |  | 2.6E6 |  | 1 | 1 | 0 | 0 | 1 | 0 | 0 | 1 | 0 | 0 | 2 | 0 | N | 100812 | Coronin-7 OS=Mus musculus OX=10090 GN=Coro7 PE=1 SV=2 |
| 1515 | 2180 | sp\|Q8C2Q3\|RBM14\_MOUSE | 66.48 | 4 | 0 | 2 | 0 | 0 | 0 | 1 | 1 | 0 | 0 | 0 |  | 5.48E6 |  |  |  | 1.13E6 | 3.12E5 |  |  |  | 3 | 3 | 0 | 1 | 0 | 0 | 0 | 1 | 1 | 0 | 0 | 0 | N | 69449 | RNA-binding protein 14 OS=Mus musculus OX=10090 GN=Rbm14 PE=1 SV=1 |
| 2553 | 5270 | Q3U8S1\|Q3U8S1\_MOUSE | 66.38 | 3 | 3 | 0 | 0 | 0 | 0 | 0 | 0 | 0 | 3 | 0 | 1.11E6 |  |  |  |  |  |  |  | 1.87E6 |  | 1 | 1 | 1 | 0 | 0 | 0 | 0 | 0 | 0 | 0 | 1 | 0 | N | 40263 | CD44 antigen OS=Mus musculus OX=10090 GN=Cd44 PE=1 SV=1 |
| 2553 | 5277 | A2APM5\|A2APM5\_MOUSE | 66.38 | 3 | 3 | 0 | 0 | 0 | 0 | 0 | 0 | 0 | 3 | 0 | 1.11E6 |  |  |  |  |  |  |  | 1.87E6 |  | 1 | 1 | 1 | 0 | 0 | 0 | 0 | 0 | 0 | 0 | 1 | 0 | N | 47418 | CD44 antigen OS=Mus musculus OX=10090 GN=Cd44 PE=1 SV=1 |
| 2553 | 5278 | A2APM3\|A2APM3\_MOUSE | 66.38 | 3 | 3 | 0 | 0 | 0 | 0 | 0 | 0 | 0 | 3 | 0 | 1.11E6 |  |  |  |  |  |  |  | 1.87E6 |  | 1 | 1 | 1 | 0 | 0 | 0 | 0 | 0 | 0 | 0 | 1 | 0 | N | 50707 | CD44 antigen OS=Mus musculus OX=10090 GN=Cd44 PE=1 SV=1 |
| 2553 | 5283 | A2APM4\|A2APM4\_MOUSE | 66.38 | 2 | 2 | 0 | 0 | 0 | 0 | 0 | 0 | 0 | 2 | 0 | 1.11E6 |  |  |  |  |  |  |  | 1.87E6 |  | 1 | 1 | 1 | 0 | 0 | 0 | 0 | 0 | 0 | 0 | 1 | 0 | N | 54262 | CD44 antigen OS=Mus musculus OX=10090 GN=Cd44 PE=1 SV=1 |
| 2553 | 5289 | E9QKM8\|E9QKM8\_MOUSE | 66.38 | 2 | 2 | 0 | 0 | 0 | 0 | 0 | 0 | 0 | 2 | 0 | 1.11E6 |  |  |  |  |  |  |  | 1.87E6 |  | 1 | 1 | 1 | 0 | 0 | 0 | 0 | 0 | 0 | 0 | 1 | 0 | N | 63007 | CD44 antigen OS=Mus musculus OX=10090 GN=Cd44 PE=1 SV=1 |
| 2553 | 5290 | Q80X37\|Q80X37\_MOUSE | 66.38 | 2 | 2 | 0 | 0 | 0 | 0 | 0 | 0 | 0 | 2 | 0 | 1.11E6 |  |  |  |  |  |  |  | 1.87E6 |  | 1 | 1 | 1 | 0 | 0 | 0 | 0 | 0 | 0 | 0 | 1 | 0 | N | 63264 | CD44 antigen OS=Mus musculus OX=10090 GN=Cd44 PE=1 SV=1 |
| 2553 | 5295 | A2APM1\|A2APM1\_MOUSE | 66.38 | 2 | 2 | 0 | 0 | 0 | 0 | 0 | 0 | 0 | 2 | 0 | 1.11E6 |  |  |  |  |  |  |  | 1.87E6 |  | 1 | 1 | 1 | 0 | 0 | 0 | 0 | 0 | 0 | 0 | 1 | 0 | N | 72088 | CD44 antigen OS=Mus musculus OX=10090 GN=Cd44 PE=1 SV=1 |
| 2553 | 5309 | sp\|P15379\|CD44\_MOUSE | 66.38 | 2 | 2 | 0 | 0 | 0 | 0 | 0 | 0 | 0 | 2 | 0 | 1.11E6 |  |  |  |  |  |  |  | 1.87E6 |  | 1 | 1 | 1 | 0 | 0 | 0 | 0 | 0 | 0 | 0 | 1 | 0 | N | 85617 | CD44 antigen OS=Mus musculus OX=10090 GN=Cd44 PE=1 SV=3 |
| 2553 | 5311 | A2APM2\|A2APM2\_MOUSE | 66.38 | 2 | 2 | 0 | 0 | 0 | 0 | 0 | 0 | 0 | 2 | 0 | 1.11E6 |  |  |  |  |  |  |  | 1.87E6 |  | 1 | 1 | 1 | 0 | 0 | 0 | 0 | 0 | 0 | 0 | 1 | 0 | N | 85838 | CD44 antigen OS=Mus musculus OX=10090 GN=Cd44 PE=1 SV=1 |
| 1536 | 4561 | sp\|P26883\|FKB1A\_MOUSE | 66.24 | 12 | 0 | 0 | 12 | 12 | 12 | 12 | 12 | 12 | 12 | 0 |  |  | 1.04E7 | 3.16E6 | 9.31E6 | 1.02E7 | 8.72E6 | 1.04E7 | 3.02E7 |  | 1 | 1 | 0 | 0 | 1 | 1 | 1 | 1 | 1 | 1 | 1 | 0 | N | 11923 | Peptidyl-prolyl cis-trans isomerase FKBP1A OS=Mus musculus OX=10090 GN=Fkbp1a PE=1 SV=2 |
| 2445 | 4309 | D3Z4J1\|D3Z4J1\_MOUSE | 66.15 | 9 | 0 | 0 | 9 | 0 | 0 | 9 | 0 | 0 | 0 | 0 |  |  | 2.94E5 |  |  | 9.17E5 |  |  |  |  | 1 | 1 | 0 | 0 | 1 | 0 | 0 | 1 | 0 | 0 | 0 | 0 | N | 19364 | DAZ associated protein 1 (Fragment) OS=Mus musculus OX=10090 GN=Dazap1 PE=1 SV=1 |
| 2445 | 4312 | Q3UGB5\|Q3UGB5\_MOUSE | 66.15 | 4 | 0 | 0 | 4 | 0 | 0 | 4 | 0 | 0 | 0 | 0 |  |  | 2.94E5 |  |  | 9.17E5 |  |  |  |  | 1 | 1 | 0 | 0 | 1 | 0 | 0 | 1 | 0 | 0 | 0 | 0 | N | 43157 | DAZ associated protein 1 OS=Mus musculus OX=10090 GN=Dazap1 PE=1 SV=1 |
| 2445 | 4324 | sp\|Q9JII5\|DAZP1\_MOUSE | 66.15 | 4 | 0 | 0 | 4 | 0 | 0 | 4 | 0 | 0 | 0 | 0 |  |  | 2.94E5 |  |  | 9.17E5 |  |  |  |  | 1 | 1 | 0 | 0 | 1 | 0 | 0 | 1 | 0 | 0 | 0 | 0 | N | 43214 | DAZ-associated protein 1 OS=Mus musculus OX=10090 GN=Dazap1 PE=1 SV=2 |
| 1208 | 5463 | B0V3P6\|B0V3P6\_MOUSE | 66.09 | 5 | 5 | 5 | 5 | 5 | 5 | 5 | 5 | 5 | 0 | 5 | 4.55E6 | 1.84E6 | 3.43E6 | 2.22E6 | 2.91E6 | 3.62E6 | 1.88E6 | 1.67E6 |  | 3.38E6 | 1 | 1 | 2 | 1 | 2 | 2 | 1 | 1 | 1 | 1 | 0 | 1 | N | 18067 | BCLl2-like 15 OS=Mus musculus OX=10090 GN=Bcl2l15 PE=1 SV=1 |
| 1208 | 5465 | sp\|Q08ED0\|B2L15\_MOUSE | 66.09 | 5 | 5 | 5 | 5 | 5 | 5 | 5 | 5 | 5 | 0 | 5 | 4.55E6 | 1.84E6 | 3.43E6 | 2.22E6 | 2.91E6 | 3.62E6 | 1.88E6 | 1.67E6 |  | 3.38E6 | 1 | 1 | 2 | 1 | 2 | 2 | 1 | 1 | 1 | 1 | 0 | 1 | N | 18300 | Bcl-2-like protein 15 OS=Mus musculus OX=10090 GN=Bcl2l15 PE=2 SV=2 |
| 1394 | 5235 | D5MCW4\|D5MCW4\_MOUSE | 66.00 | 9 | 9 | 9 | 9 | 0 | 9 | 9 | 0 | 9 | 9 | 0 | 1.66E6 | 1.31E6 | 1.47E6 |  | 2.22E6 | 2.31E6 |  | 8.57E5 | 1.77E6 |  | 1 | 1 | 1 | 1 | 1 | 0 | 1 | 2 | 0 | 2 | 1 | 0 | N | 16453 | CutA divalent cation tolerance homolog OS=Mus musculus OX=10090 GN=Cuta PE=1 SV=1 |
| 1394 | 5236 | sp\|Q9CQ89\|CUTA\_MOUSE | 66.00 | 8 | 8 | 8 | 8 | 0 | 8 | 8 | 0 | 8 | 8 | 0 | 1.66E6 | 1.31E6 | 1.47E6 |  | 2.22E6 | 2.31E6 |  | 8.57E5 | 1.77E6 |  | 1 | 1 | 1 | 1 | 1 | 0 | 1 | 2 | 0 | 2 | 1 | 0 | N | 18865 | Protein CutA OS=Mus musculus OX=10090 GN=Cuta PE=1 SV=3 |
| 3302 | 5020 | sp\|Q8CHP8\|PGP\_MOUSE | 65.88 | 5 | 0 | 0 | 0 | 0 | 0 | 5 | 0 | 0 | 0 | 0 |  |  |  |  |  | 3.84E5 |  |  |  |  | 1 | 1 | 0 | 0 | 0 | 0 | 0 | 1 | 0 | 0 | 0 | 0 | N | 34541 | Glycerol-3-phosphate phosphatase OS=Mus musculus OX=10090 GN=Pgp PE=1 SV=1 |
| 2108 | 2673 | sp\|Q8R059\|GALE\_MOUSE | 65.58 | 9 | 0 | 0 | 0 | 3 | 0 | 0 | 0 | 0 | 6 | 0 |  |  |  | 2.17E5 |  |  |  |  | 5.79E6 |  | 2 | 2 | 0 | 0 | 0 | 1 | 0 | 0 | 0 | 0 | 1 | 0 | Y | 38225 | UDP-glucose 4-epimerase OS=Mus musculus OX=10090 GN=Gale PE=1 SV=1 |
| 1914 | 5245 | sp\|P03976\|KV2A5\_MOUSE | 65.51 | 12 | 12 | 0 | 0 | 0 | 0 | 12 | 0 | 0 | 12 | 12 | 2.53E7 |  |  |  |  | 1.09E6 |  |  | 2.2E7 | 6.66E6 | 1 | 1 | 1 | 0 | 0 | 0 | 0 | 1 | 0 | 0 | 1 | 1 | N | 12390 | Ig kappa chain V-II region 17S29.1 OS=Mus musculus OX=10090 PE=1 SV=1 |
| 1914 | 5246 | A0A075B5K6\|A0A075B5K6\_MOUSE | 65.51 | 11 | 11 | 0 | 0 | 0 | 0 | 11 | 0 | 0 | 11 | 11 | 2.53E7 |  |  |  |  | 1.09E6 |  |  | 2.2E7 | 6.66E6 | 1 | 1 | 1 | 0 | 0 | 0 | 0 | 1 | 0 | 0 | 1 | 1 | N | 13057 | Immunoglobulin kappa variable 2-109 (Fragment) OS=Mus musculus OX=10090 GN=Igkv2-109 PE=4 SV=4 |
| 543 | 2949 | Q6IFZ8\|Q6IFZ8\_MOUSE | 65.46 | 3 | 1 | 3 | 1 | 1 | 1 | 1 | 1 | 1 | 1 | 1 |  | 1.89E5 |  |  |  |  |  |  |  |  | 2 | 1 | 5 | 6 | 4 | 2 | 5 | 3 | 3 | 6 | 6 | 5 | N | 60228 | Predicted gene 5414 OS=Mus musculus OX=10090 GN=Gm5414 PE=1 SV=1 |
| 1747 | 3966 | sp\|P28076\|PSB9\_MOUSE | 65.41 | 9 | 4 | 0 | 0 | 5 | 5 | 0 | 5 | 0 | 4 | 0 | 6.16E6 |  |  | 3.2E6 | 7.8E6 |  | 4.72E6 |  | 4.75E6 |  | 2 | 2 | 1 | 0 | 0 | 1 | 1 | 0 | 1 | 0 | 1 | 0 | N | 23397 | Proteasome subunit beta type-9 OS=Mus musculus OX=10090 GN=Psmb9 PE=1 SV=1 |
| 1747 | 3967 | A0A0R4J256\|A0A0R4J256\_MOUSE | 65.41 | 9 | 4 | 0 | 0 | 5 | 5 | 0 | 5 | 0 | 4 | 0 | 6.16E6 |  |  | 3.2E6 | 7.8E6 |  | 4.72E6 |  | 4.75E6 |  | 2 | 2 | 1 | 0 | 0 | 1 | 1 | 0 | 1 | 0 | 1 | 0 | N | 23325 | Proteasome subunit beta OS=Mus musculus OX=10090 GN=Psmb9 PE=1 SV=1 |
| 3093 | 3561 | A0A0R4J1Z3\|A0A0R4J1Z3\_MOUSE | 65.19 | 5 | 0 | 0 | 0 | 0 | 0 | 0 | 5 | 0 | 0 | 0 |  |  |  |  |  |  | 1.27E6 |  |  |  | 1 | 1 | 0 | 0 | 0 | 0 | 0 | 0 | 1 | 0 | 0 | 0 | N | 27932 | Transmembrane protein 33 OS=Mus musculus OX=10090 GN=Tmem33 PE=1 SV=1 |
| 3093 | 3562 | sp\|Q9CR67\|TMM33\_MOUSE | 65.19 | 5 | 0 | 0 | 0 | 0 | 0 | 0 | 5 | 0 | 0 | 0 |  |  |  |  |  |  | 1.27E6 |  |  |  | 1 | 1 | 0 | 0 | 0 | 0 | 0 | 0 | 1 | 0 | 0 | 0 | N | 28031 | Transmembrane protein 33 OS=Mus musculus OX=10090 GN=Tmem33 PE=1 SV=1 |
| 3093 | 4991 | Q9CZM3\|Q9CZM3\_MOUSE | 65.19 | 10 | 0 | 0 | 0 | 0 | 0 | 0 | 10 | 0 | 0 | 0 |  |  |  |  |  |  | 1.27E6 |  |  |  | 1 | 1 | 0 | 0 | 0 | 0 | 0 | 0 | 1 | 0 | 0 | 0 | N | 14049 | Transmembrane protein 33 OS=Mus musculus OX=10090 GN=Tmem33 PE=1 SV=1 |
| 1751 | 5252 | A0A1B0GTA0\|A0A1B0GTA0\_MOUSE | 65.12 | 22 | 0 | 22 | 22 | 22 | 0 | 22 | 0 | 22 | 0 | 0 |  | 6.47E5 | 1.4E6 | 7.54E5 |  | 1.56E6 |  | 7.98E5 |  |  | 1 | 1 | 0 | 1 | 1 | 1 | 0 | 1 | 0 | 1 | 0 | 0 | N | 5725 | L-fucose mutarotase (Fragment) OS=Mus musculus OX=10090 GN=Fuom PE=1 SV=1 |
| 1751 | 5258 | D3YWS7\|D3YWS7\_MOUSE | 65.12 | 9 | 0 | 9 | 9 | 9 | 0 | 9 | 0 | 9 | 0 | 0 |  | 6.47E5 | 1.4E6 | 7.54E5 |  | 1.56E6 |  | 7.98E5 |  |  | 1 | 1 | 0 | 1 | 1 | 1 | 0 | 1 | 0 | 1 | 0 | 0 | N | 14790 | L-fucose mutarotase OS=Mus musculus OX=10090 GN=Fuom PE=1 SV=1 |
| 1751 | 5259 | sp\|Q8R2K1\|FUCM\_MOUSE | 65.12 | 8 | 0 | 8 | 8 | 8 | 0 | 8 | 0 | 8 | 0 | 0 |  | 6.47E5 | 1.4E6 | 7.54E5 |  | 1.56E6 |  | 7.98E5 |  |  | 1 | 1 | 0 | 1 | 1 | 1 | 0 | 1 | 0 | 1 | 0 | 0 | N | 16805 | Fucose mutarotase OS=Mus musculus OX=10090 GN=Fuom PE=1 SV=1 |
| 1751 | 5260 | F6SJM7\|F6SJM7\_MOUSE | 65.12 | 8 | 0 | 8 | 8 | 8 | 0 | 8 | 0 | 8 | 0 | 0 |  | 6.47E5 | 1.4E6 | 7.54E5 |  | 1.56E6 |  | 7.98E5 |  |  | 1 | 1 | 0 | 1 | 1 | 1 | 0 | 1 | 0 | 1 | 0 | 0 | N | 17359 | L-fucose mutarotase (Fragment) OS=Mus musculus OX=10090 GN=Fuom PE=1 SV=1 |
| 2167 | 5243 | Q8C5G6\|Q8C5G6\_MOUSE | 65.06 | 6 | 0 | 0 | 0 | 0 | 0 | 6 | 0 | 6 | 6 | 0 |  |  |  |  |  | 8.67E5 |  | 1.36E6 | 3.17E6 |  | 1 | 1 | 0 | 0 | 0 | 0 | 0 | 1 | 0 | 1 | 1 | 0 | N | 24540 | Toll interacting protein OS=Mus musculus OX=10090 GN=Tollip PE=1 SV=1 |
| 2167 | 5244 | sp\|Q9QZ06\|TOLIP\_MOUSE | 65.06 | 5 | 0 | 0 | 0 | 0 | 0 | 5 | 0 | 5 | 5 | 0 |  |  |  |  |  | 8.67E5 |  | 1.36E6 | 3.17E6 |  | 1 | 1 | 0 | 0 | 0 | 0 | 0 | 1 | 0 | 1 | 1 | 0 | N | 30345 | Toll-interacting protein OS=Mus musculus OX=10090 GN=Tollip PE=1 SV=1 |
| 1371 | 3038 | sp\|P24547\|IMDH2\_MOUSE | 64.77 | 4 | 2 | 0 | 0 | 0 | 4 | 2 | 2 | 2 | 2 | 2 | 1.52E5 |  |  |  | 2.14E6 | 1.24E6 | 9.86E5 | 1.09E6 | 1.41E6 | 0E0 | 2 | 2 | 1 | 0 | 0 | 0 | 2 | 1 | 1 | 1 | 1 | 1 | N | 55815 | Inosine-5'-monophosphate dehydrogenase 2 OS=Mus musculus OX=10090 GN=Impdh2 PE=1 SV=2 |
| 1371 | 3077 | A0A0A6YY72\|A0A0A6YY72\_MOUSE | 64.77 | 7 | 3 | 0 | 0 | 0 | 7 | 4 | 4 | 4 | 3 | 3 | 1.52E5 |  |  |  | 2.14E6 | 1.24E6 | 9.86E5 | 1.09E6 | 1.41E6 | 0E0 | 2 | 2 | 1 | 0 | 0 | 0 | 2 | 1 | 1 | 1 | 1 | 1 | N | 34129 | Inosine monophosphate dehydrogenase 2 (Fragment) OS=Mus musculus OX=10090 GN=Impdh2 PE=1 SV=1 |
| 1552 | 2929 | sp\|Q8R2Y2\|MUC18\_MOUSE | 64.65 | 2 | 0 | 2 | 0 | 0 | 0 | 2 | 0 | 0 | 0 | 2 |  | 2.07E6 |  |  |  | 2.93E5 |  |  |  | 1.67E6 | 1 | 1 | 0 | 1 | 0 | 0 | 0 | 1 | 0 | 0 | 0 | 1 | N | 71546 | Cell surface glycoprotein MUC18 OS=Mus musculus OX=10090 GN=Mcam PE=1 SV=1 |
| 820 | 3972 | E9QPZ2\|E9QPZ2\_MOUSE | 64.03 | 18 | 8 | 8 | 8 | 8 | 8 | 8 | 8 | 8 | 18 | 8 |  |  |  |  |  |  |  |  | 5.09E6 |  | 2 | 1 | 3 | 3 | 2 | 3 | 1 | 1 | 1 | 4 | 6 | 2 | Y | 10361 | Defensin, alpha, 30 OS=Mus musculus OX=10090 GN=Defa30 PE=1 SV=1 |
| 1876 | 3577 | sp\|O09159\|MA2B1\_MOUSE | 64.00 | 2 | 0 | 0 | 0 | 0 | 0 | 0 | 0 | 0 | 2 | 1 |  |  |  |  |  |  |  |  | 2.25E7 | 1.53E7 | 2 | 2 | 0 | 0 | 0 | 0 | 0 | 0 | 0 | 0 | 2 | 1 | N | 114648 | Lysosomal alpha-mannosidase OS=Mus musculus OX=10090 GN=Man2b1 PE=1 SV=4 |
| 1325 | 4013 | sp\|P00158\|CYB\_MOUSE | 63.99 | 4 | 2 | 4 | 2 | 0 | 2 | 2 | 0 | 2 | 4 | 0 | 1.76E6 | 2.28E6 | 9.21E5 |  | 5.36E5 | 5.72E5 |  | 2.37E5 | 1.12E6 |  | 2 | 2 | 1 | 2 | 1 | 0 | 1 | 1 | 0 | 1 | 2 | 0 | N | 43210 | Cytochrome b OS=Mus musculus OX=10090 GN=Mt-Cyb PE=1 SV=1 |
| 1331 | 3990 | sp\|Q9WVH9\|FBLN5\_MOUSE | 63.94 | 4 | 2 | 2 | 2 | 0 | 2 | 2 | 4 | 2 | 2 | 2 | 6.47E5 | 4.63E5 | 2.36E6 |  | 1.06E6 | 2.56E6 | 5.03E6 | 1.17E6 | 8.5E5 | 2.11E5 | 2 | 2 | 1 | 1 | 1 | 0 | 1 | 1 | 2 | 1 | 1 | 1 | Y | 50193 | Fibulin-5 OS=Mus musculus OX=10090 GN=Fbln5 PE=1 SV=1 |
| 1331 | 3992 | A0A1Y7VJW9\|A0A1Y7VJW9\_MOUSE | 63.94 | 3 | 2 | 2 | 2 | 0 | 2 | 2 | 3 | 2 | 2 | 2 | 6.47E5 | 4.63E5 | 2.36E6 |  | 1.06E6 | 2.56E6 | 5.03E6 | 1.17E6 | 8.5E5 | 2.11E5 | 2 | 2 | 1 | 1 | 1 | 0 | 1 | 1 | 2 | 1 | 1 | 1 | Y | 51769 | Fibulin-5 OS=Mus musculus OX=10090 GN=Fbln5 PE=1 SV=1 |
| 1410 | 2631 | F7ACR9\|F7ACR9\_MOUSE | 63.89 | 1 | 0 | 0 | 0 | 0 | 0 | 0 | 0 | 0 | 0 | 0 |  | 3.98E6 |  |  |  |  |  |  | 3.12E6 |  | 4 | 2 | 0 | 1 | 0 | 1 | 1 | 0 | 0 | 0 | 1 | 0 | N | 608246 | Microtubule-actin crosslinking factor 1 (Fragment) OS=Mus musculus OX=10090 GN=Macf1 PE=1 SV=1 |
| 1821 | 2649 | A0A286YDI8\|A0A286YDI8\_MOUSE | 63.62 | 3 | 0 | 0 | 1 | 0 | 0 | 0 | 2 | 0 | 0 | 0 |  |  | 5.15E5 |  |  |  | 3.54E5 |  |  |  | 2 | 2 | 0 | 0 | 1 | 0 | 0 | 0 | 1 | 0 | 0 | 0 | N | 111613 | SEC24 homolog C, COPII coat complex component OS=Mus musculus OX=10090 GN=Sec24c PE=1 SV=1 |
| 1821 | 2650 | G3X972\|G3X972\_MOUSE | 63.62 | 2 | 0 | 0 | 1 | 0 | 0 | 0 | 1 | 0 | 0 | 0 |  |  | 5.15E5 |  |  |  | 3.54E5 |  |  |  | 2 | 2 | 0 | 0 | 1 | 0 | 0 | 0 | 1 | 0 | 0 | 0 | N | 118581 | SEC24 homolog C, COPII coat complex component OS=Mus musculus OX=10090 GN=Sec24c PE=1 SV=1 |
| 1266 | 5518 | E9Q1D5\|E9Q1D5\_MOUSE | 63.58 | 2 | 0 | 2 | 2 | 2 | 2 | 2 | 2 | 2 | 2 | 2 |  | 2.94E6 | 3.37E6 | 2.11E5 | 4.04E6 | 5.28E6 | 2.12E6 | 7.43E5 | 2.48E6 | 5.24E5 | 1 | 1 | 0 | 1 | 2 | 1 | 2 | 1 | 1 | 1 | 1 | 1 | N | 46006 | Transforming growth factor beta-1-induced transcript 1 protein OS=Mus musculus OX=10090 GN=Tgfb1i1 PE=1 SV=1 |
| 1266 | 5520 | sp\|Q62219\|TGFI1\_MOUSE | 63.58 | 2 | 0 | 2 | 2 | 2 | 2 | 2 | 2 | 2 | 2 | 2 |  | 2.94E6 | 3.37E6 | 2.11E5 | 4.04E6 | 5.28E6 | 2.12E6 | 7.43E5 | 2.48E6 | 5.24E5 | 1 | 1 | 0 | 1 | 2 | 1 | 2 | 1 | 1 | 1 | 1 | 1 | N | 50101 | Transforming growth factor beta-1-induced transcript 1 protein OS=Mus musculus OX=10090 GN=Tgfb1i1 PE=1 SV=2 |
| 1000 | 2499 | sp\|P14069\|S10A6\_MOUSE | 63.52 | 25 | 17 | 9 | 9 | 9 | 9 | 9 | 0 | 9 | 9 | 16 | 8.68E7 | 4.51E7 | 3.83E7 | 1.78E7 | 2.89E7 | 3.82E7 |  | 2.27E7 | 1.83E8 | 3.26E6 | 3 | 2 | 3 | 1 | 1 | 1 | 1 | 1 | 0 | 1 | 3 | 2 | Y | 10051 | Protein S100-A6 OS=Mus musculus OX=10090 GN=S100a6 PE=1 SV=3 |
| 2552 | 5250 | sp\|Q9DBG9\|TX1B3\_MOUSE | 63.44 | 14 | 14 | 0 | 0 | 0 | 0 | 0 | 0 | 0 | 14 | 0 | 8.94E5 |  |  |  |  |  |  |  | 2.46E6 |  | 1 | 1 | 1 | 0 | 0 | 0 | 0 | 0 | 0 | 0 | 1 | 0 | N | 13723 | Tax1-binding protein 3 OS=Mus musculus OX=10090 GN=Tax1bp3 PE=1 SV=1 |
| 3295 | 4798 | sp\|Q3UJU9\|RMD3\_MOUSE | 63.43 | 3 | 0 | 0 | 0 | 0 | 0 | 3 | 0 | 0 | 0 | 0 |  |  |  |  |  | 1.16E6 |  |  |  |  | 1 | 1 | 0 | 0 | 0 | 0 | 0 | 1 | 0 | 0 | 0 | 0 | N | 52029 | Regulator of microtubule dynamics protein 3 OS=Mus musculus OX=10090 GN=Rmdn3 PE=1 SV=2 |
| 1336 | 5413 | A0A1W2P7X0\|A0A1W2P7X0\_MOUSE | 63.43 | 16 | 16 | 16 | 16 | 16 | 16 | 16 | 16 | 16 | 16 | 16 | 3.22E6 | 3.64E6 | 3.8E6 | 2.77E5 | 3.46E6 | 4.35E6 | 2.26E6 | 3E6 | 7.58E6 | 3.48E6 | 1 | 1 | 1 | 1 | 1 | 1 | 1 | 1 | 1 | 1 | 1 | 1 | N | 6437 | Costars family protein ABRACL (Fragment) OS=Mus musculus OX=10090 GN=Abracl PE=1 SV=1 |
| 1336 | 5421 | E9QMV2\|E9QMV2\_MOUSE | 63.43 | 11 | 11 | 11 | 11 | 11 | 11 | 11 | 11 | 11 | 11 | 11 | 3.22E6 | 3.64E6 | 3.8E6 | 2.77E5 | 3.46E6 | 4.35E6 | 2.26E6 | 3E6 | 7.58E6 | 3.48E6 | 1 | 1 | 1 | 1 | 1 | 1 | 1 | 1 | 1 | 1 | 1 | 1 | N | 9058 | Costars family protein ABRACL OS=Mus musculus OX=10090 GN=Abracl PE=1 SV=1 |
| 1336 | 5422 | sp\|Q4KML4\|ABRAL\_MOUSE | 63.43 | 11 | 11 | 11 | 11 | 11 | 11 | 11 | 11 | 11 | 11 | 11 | 3.22E6 | 3.64E6 | 3.8E6 | 2.77E5 | 3.46E6 | 4.35E6 | 2.26E6 | 3E6 | 7.58E6 | 3.48E6 | 1 | 1 | 1 | 1 | 1 | 1 | 1 | 1 | 1 | 1 | 1 | 1 | N | 9030 | Costars family protein ABRACL OS=Mus musculus OX=10090 GN=Abracl PE=1 SV=1 |
| 1903 | 2719 | sp\|Q8BH24\|TM9S4\_MOUSE | 63.42 | 4 | 0 | 3 | 0 | 0 | 3 | 0 | 0 | 0 | 1 | 0 |  | 4.93E5 |  |  | 6.25E5 |  |  |  | 4.3E5 |  | 2 | 2 | 0 | 1 | 0 | 0 | 1 | 0 | 0 | 0 | 1 | 0 | N | 74693 | Transmembrane 9 superfamily member 4 OS=Mus musculus OX=10090 GN=Tm9sf4 PE=1 SV=1 |
| 2155 | 3095 | sp\|Q07797\|LG3BP\_MOUSE | 63.28 | 4 | 0 | 0 | 0 | 0 | 0 | 0 | 0 | 0 | 4 | 0 |  |  |  |  |  |  |  |  | 4.88E6 |  | 2 | 2 | 0 | 0 | 0 | 0 | 0 | 0 | 0 | 0 | 2 | 0 | Y | 64491 | Galectin-3-binding protein OS=Mus musculus OX=10090 GN=Lgals3bp PE=1 SV=1 |
| 2125 | 3291 | sp\|Q99LJ1\|FUCO\_MOUSE | 63.28 | 6 | 2 | 0 | 0 | 0 | 0 | 4 | 2 | 0 | 0 | 0 | 4.86E5 |  |  |  |  | 7.79E5 | 0E0 |  |  |  | 2 | 2 | 1 | 0 | 0 | 0 | 0 | 1 | 1 | 0 | 0 | 0 | N | 52281 | Tissue alpha-L-fucosidase OS=Mus musculus OX=10090 GN=Fuca1 PE=1 SV=1 |
| 2165 | 4467 | A0A338P7D2\|A0A338P7D2\_MOUSE | 63.26 | 4 | 0 | 0 | 4 | 0 | 0 | 4 | 0 | 0 | 0 | 0 |  |  | 0E0 |  |  | 3.99E5 |  |  |  |  | 1 | 1 | 0 | 0 | 1 | 0 | 0 | 1 | 0 | 0 | 0 | 0 | N | 28332 | Sorting nexin 4 OS=Mus musculus OX=10090 GN=Snx4 PE=1 SV=1 |
| 2165 | 4468 | sp\|Q91YJ2\|SNX4\_MOUSE | 63.26 | 2 | 0 | 0 | 2 | 0 | 0 | 2 | 0 | 0 | 0 | 0 |  |  | 0E0 |  |  | 3.99E5 |  |  |  |  | 1 | 1 | 0 | 0 | 1 | 0 | 0 | 1 | 0 | 0 | 0 | 0 | N | 51778 | Sorting nexin-4 OS=Mus musculus OX=10090 GN=Snx4 PE=1 SV=1 |
| 2096 | 3456 | sp\|P01820\|HVM44\_MOUSE | 63.08 | 10 | 0 | 0 | 0 | 0 | 0 | 0 | 0 | 0 | 10 | 10 |  |  |  |  |  |  |  |  | 5.37E6 | 6.66E6 | 1 | 1 | 0 | 0 | 0 | 0 | 0 | 0 | 0 | 0 | 1 | 1 | Y | 12447 | Ig heavy chain V region PJ14 OS=Mus musculus OX=10090 PE=1 SV=1 |
| 2096 | 3457 | A0A075B697\|A0A075B697\_MOUSE | 63.08 | 9 | 0 | 0 | 0 | 0 | 0 | 0 | 0 | 0 | 9 | 9 |  |  |  |  |  |  |  |  | 5.37E6 | 6.66E6 | 1 | 1 | 0 | 0 | 0 | 0 | 0 | 0 | 0 | 0 | 1 | 1 | Y | 12481 | Immunoglobulin heavy variable 2-9-1 (Fragment) OS=Mus musculus OX=10090 GN=Ighv2-9-1 PE=1 SV=1 |
| 2096 | 4171 | sp\|P01819\|HVM43\_MOUSE | 63.08 | 8 | 0 | 0 | 0 | 0 | 0 | 0 | 0 | 0 | 8 | 8 |  |  |  |  |  |  |  |  | 5.37E6 | 6.66E6 | 1 | 1 | 0 | 0 | 0 | 0 | 0 | 0 | 0 | 0 | 1 | 1 | Y | 15759 | Ig heavy chain V region MOPC 141 OS=Mus musculus OX=10090 PE=4 SV=1 |
| 1395 | 5263 | Q8VCR4\|Q8VCR4\_MOUSE | 63.04 | 12 | 12 | 12 | 0 | 12 | 12 | 12 | 12 | 12 | 12 | 12 | 2.97E6 | 2.14E6 |  | 4.43E5 | 8.84E5 | 7.26E5 | 4.48E5 | 4.61E5 | 6.88E4 | 1.7E6 | 1 | 1 | 1 | 1 | 0 | 1 | 1 | 1 | 1 | 1 | 1 | 1 | N | 12208 | 0610038D11Rik protein OS=Mus musculus OX=10090 GN=Trmt112 PE=1 SV=1 |
| 1395 | 5264 | sp\|Q9DCG9\|TR112\_MOUSE | 63.04 | 10 | 10 | 10 | 0 | 10 | 10 | 10 | 10 | 10 | 10 | 10 | 2.97E6 | 2.14E6 |  | 4.43E5 | 8.84E5 | 7.26E5 | 4.48E5 | 4.61E5 | 6.88E4 | 1.7E6 | 1 | 1 | 1 | 1 | 0 | 1 | 1 | 1 | 1 | 1 | 1 | 1 | N | 14141 | Multifunctional methyltransferase subunit TRM112-like protein OS=Mus musculus OX=10090 GN=Trmt112 PE=1 SV=1 |
| 1737 | 2592 | sp\|Q9WVJ2\|PSD13\_MOUSE | 62.97 | 7 | 2 | 0 | 2 | 0 | 0 | 0 | 0 | 0 | 2 | 5 | 2.34E6 |  | 8.36E5 |  |  |  |  |  | 1.54E6 | 1.94E6 | 3 | 3 | 1 | 0 | 1 | 0 | 0 | 0 | 0 | 0 | 1 | 2 | N | 42809 | 26S proteasome non-ATPase regulatory subunit 13 OS=Mus musculus OX=10090 GN=Psmd13 PE=1 SV=1 |
| 1737 | 2595 | E9Q5I9\|E9Q5I9\_MOUSE | 62.97 | 8 | 2 | 0 | 2 | 0 | 0 | 0 | 0 | 0 | 2 | 6 | 2.34E6 |  | 8.36E5 |  |  |  |  |  | 1.54E6 | 1.94E6 | 3 | 3 | 1 | 0 | 1 | 0 | 0 | 0 | 0 | 0 | 1 | 2 | N | 39695 | 26S proteasome non-ATPase regulatory subunit 13 OS=Mus musculus OX=10090 GN=Psmd13 PE=1 SV=1 |
| 1298 | 3709 | B2RUJ2\|B2RUJ2\_MOUSE | 62.94 | 1 | 1 | 1 | 1 | 1 | 1 | 1 | 1 | 1 | 1 | 0 | 1.26E6 | 1.11E6 | 3.41E5 | 3.46E5 | 5.74E5 | 1.61E6 | 7.61E5 | 8.86E5 | 3.21E5 |  | 1 | 1 | 1 | 1 | 1 | 1 | 1 | 1 | 1 | 1 | 1 | 0 | N | 145095 | Erbb2 interacting protein OS=Mus musculus OX=10090 GN=Erbin PE=1 SV=1 |
| 3435 | 5279 | sp\|Q64339\|ISG15\_MOUSE | 62.85 | 11 | 0 | 0 | 0 | 0 | 0 | 0 | 11 | 0 | 0 | 0 |  |  |  |  |  |  | 9.61E5 |  |  |  | 1 | 1 | 0 | 0 | 0 | 0 | 0 | 0 | 1 | 0 | 0 | 0 | N | 17898 | Ubiquitin-like protein ISG15 OS=Mus musculus OX=10090 GN=Isg15 PE=1 SV=4 |
| 2471 | 3111 | B1AQR8\|B1AQR8\_MOUSE | 62.81 | 3 | 0 | 0 | 0 | 0 | 0 | 0 | 0 | 0 | 3 | 0 |  |  |  |  |  |  |  |  | 5.05E6 |  | 1 | 1 | 0 | 0 | 0 | 0 | 0 | 0 | 0 | 0 | 1 | 0 | Y | 39908 | Galectin OS=Mus musculus OX=10090 GN=Lgals9 PE=1 SV=1 |
| 2471 | 3112 | G3X9T7\|G3X9T7\_MOUSE | 62.81 | 3 | 0 | 0 | 0 | 0 | 0 | 0 | 0 | 0 | 3 | 0 |  |  |  |  |  |  |  |  | 5.05E6 |  | 1 | 1 | 0 | 0 | 0 | 0 | 0 | 0 | 0 | 0 | 1 | 0 | Y | 40022 | Galectin OS=Mus musculus OX=10090 GN=Lgals9 PE=1 SV=1 |
| 2471 | 3113 | sp\|O08573\|LEG9\_MOUSE | 62.81 | 3 | 0 | 0 | 0 | 0 | 0 | 0 | 0 | 0 | 3 | 0 |  |  |  |  |  |  |  |  | 5.05E6 |  | 1 | 1 | 0 | 0 | 0 | 0 | 0 | 0 | 0 | 0 | 1 | 0 | Y | 40036 | Galectin-9 OS=Mus musculus OX=10090 GN=Lgals9 PE=1 SV=1 |
| 2099 | 3716 | sp\|Q9JIP7\|S15A1\_MOUSE | 62.77 | 3 | 0 | 2 | 0 | 0 | 0 | 0 | 1 | 0 | 0 | 0 |  | 4E5 |  |  |  |  | 9.13E5 |  |  |  | 2 | 2 | 0 | 1 | 0 | 0 | 0 | 0 | 1 | 0 | 0 | 0 | Y | 78560 | Solute carrier family 15 member 1 OS=Mus musculus OX=10090 GN=Slc15a1 PE=1 SV=2 |
| 2374 | 3290 | sp\|P54276\|MSH6\_MOUSE | 62.75 | 1 | 0 | 0 | 0 | 0 | 1 | 0 | 0 | 0 | 0 | 0 |  |  |  |  | 4.14E4 |  |  |  |  |  | 1 | 1 | 0 | 0 | 0 | 0 | 1 | 0 | 0 | 0 | 0 | 0 | N | 151083 | DNA mismatch repair protein Msh6 OS=Mus musculus OX=10090 GN=Msh6 PE=1 SV=3 |
| 1855 | 2750 | sp\|P49718\|MCM5\_MOUSE | 62.28 | 2 | 0 | 0 | 0 | 0 | 0 | 0 | 0 | 2 | 0 | 2 |  |  |  |  |  |  |  | 8.56E5 |  | 9.73E5 | 1 | 1 | 0 | 0 | 0 | 0 | 0 | 0 | 0 | 1 | 0 | 1 | N | 82407 | DNA replication licensing factor MCM5 OS=Mus musculus OX=10090 GN=Mcm5 PE=1 SV=2 |
| 1045 | 3626 | A0A668KL51\|A0A668KL51\_MOUSE | 62.10 | 5 | 5 | 5 | 5 | 5 | 5 | 5 | 2 | 0 | 5 | 2 | 9.76E6 | 1.45E7 | 1E7 | 3.25E6 | 7.92E6 | 8.79E6 | 3.11E6 |  | 1.62E7 | 4.55E6 | 2 | 2 | 2 | 2 | 2 | 2 | 2 | 2 | 1 | 0 | 2 | 1 | N | 41700 | Isocitrate dehydrogenase [NAD] subunit, mitochondrial OS=Mus musculus OX=10090 GN=Idh3b PE=1 SV=1 |
| 1045 | 3627 | Q91VA7\|Q91VA7\_MOUSE | 62.10 | 5 | 5 | 5 | 5 | 5 | 5 | 5 | 2 | 0 | 5 | 2 | 9.76E6 | 1.45E7 | 1E7 | 3.25E6 | 7.92E6 | 8.79E6 | 3.11E6 |  | 1.62E7 | 4.55E6 | 2 | 2 | 2 | 2 | 2 | 2 | 2 | 2 | 1 | 0 | 2 | 1 | N | 42195 | Isocitrate dehydrogenase [NAD] subunit, mitochondrial OS=Mus musculus OX=10090 GN=Idh3b PE=1 SV=1 |
| 3207 | 4616 | sp\|Q9CR51\|VATG1\_MOUSE | 62.05 | 9 | 0 | 9 | 0 | 0 | 0 | 0 | 0 | 0 | 0 | 0 |  | 2.79E6 |  |  |  |  |  |  |  |  | 1 | 1 | 0 | 1 | 0 | 0 | 0 | 0 | 0 | 0 | 0 | 0 | N | 13724 | V-type proton ATPase subunit G 1 OS=Mus musculus OX=10090 GN=Atp6v1g1 PE=1 SV=3 |
| 821 | 4022 | sp\|P50706\|DEFA8\_MOUSE | 61.96 | 21 | 9 | 9 | 9 | 9 | 9 | 9 | 9 | 9 | 21 | 9 |  |  |  |  |  |  |  |  | 1.52E6 |  | 2 | 1 | 3 | 3 | 2 | 3 | 1 | 1 | 1 | 4 | 6 | 2 | Y | 9226 | Alpha-defensin 8 (Fragment) OS=Mus musculus OX=10090 GN=Defa8 PE=2 SV=2 |
| 821 | 4027 | sp\|P28310\|DEFA3\_MOUSE | 61.96 | 18 | 8 | 8 | 8 | 8 | 8 | 8 | 8 | 8 | 18 | 8 |  |  |  |  |  |  |  |  | 1.52E6 |  | 2 | 1 | 3 | 3 | 2 | 3 | 1 | 1 | 1 | 4 | 6 | 2 | Y | 10528 | Alpha-defensin 3 OS=Mus musculus OX=10090 GN=Defa3 PE=1 SV=2 |
| 821 | 4028 | sp\|P28309\|DEFA2\_MOUSE | 61.96 | 18 | 8 | 8 | 8 | 8 | 8 | 8 | 8 | 8 | 18 | 8 |  |  |  |  |  |  |  |  | 1.52E6 |  | 2 | 1 | 3 | 3 | 2 | 3 | 1 | 1 | 1 | 4 | 6 | 2 | Y | 10560 | Alpha-defensin 2 OS=Mus musculus OX=10090 GN=Defa2 PE=1 SV=2 |
| 821 | 4029 | sp\|P50705\|DEFA7\_MOUSE | 61.96 | 18 | 8 | 8 | 8 | 8 | 8 | 8 | 8 | 8 | 18 | 8 |  |  |  |  |  |  |  |  | 1.52E6 |  | 2 | 1 | 3 | 3 | 2 | 3 | 1 | 1 | 1 | 4 | 6 | 2 | Y | 10496 | Alpha-defensin 7 OS=Mus musculus OX=10090 GN=Defa7 PE=2 SV=2 |
| 821 | 4030 | sp\|P50711\|DFA13\_MOUSE | 61.96 | 18 | 8 | 8 | 8 | 8 | 8 | 8 | 8 | 8 | 18 | 8 |  |  |  |  |  |  |  |  | 1.52E6 |  | 2 | 1 | 3 | 3 | 2 | 3 | 1 | 1 | 1 | 4 | 6 | 2 | Y | 10565 | Alpha-defensin 13 OS=Mus musculus OX=10090 GN=Defa13 PE=2 SV=2 |
| 821 | 4031 | sp\|P50714\|DFA16\_MOUSE | 61.96 | 18 | 8 | 8 | 8 | 8 | 8 | 8 | 8 | 8 | 18 | 8 |  |  |  |  |  |  |  |  | 1.52E6 |  | 2 | 1 | 3 | 3 | 2 | 3 | 1 | 1 | 1 | 4 | 6 | 2 | Y | 10430 | Alpha-defensin 16 OS=Mus musculus OX=10090 GN=Defa16 PE=2 SV=2 |
| 2489 | 5067 | V9GXB6\|V9GXB6\_MOUSE | 61.93 | 4 | 0 | 0 | 4 | 0 | 0 | 0 | 4 | 0 | 0 | 0 |  |  | 9.27E5 |  |  |  | 7.5E5 |  |  |  | 1 | 1 | 0 | 0 | 1 | 0 | 0 | 0 | 1 | 0 | 0 | 0 | Y | 35346 | Heterogeneous nuclear ribonucleoprotein L-like OS=Mus musculus OX=10090 GN=Hnrnpll PE=1 SV=1 |
| 2489 | 5068 | sp\|Q921F4\|HNRLL\_MOUSE | 61.93 | 2 | 0 | 0 | 2 | 0 | 0 | 0 | 2 | 0 | 0 | 0 |  |  | 9.27E5 |  |  |  | 7.5E5 |  |  |  | 1 | 1 | 0 | 0 | 1 | 0 | 0 | 0 | 1 | 0 | 0 | 0 | Y | 64125 | Heterogeneous nuclear ribonucleoprotein L-like OS=Mus musculus OX=10090 GN=Hnrnpll PE=1 SV=3 |
| 1623 | 4602 | sp\|P41241\|CSK\_MOUSE | 61.92 | 3 | 0 | 3 | 3 | 0 | 0 | 3 | 0 | 3 | 3 | 3 |  | 9.26E5 | 6.7E5 |  |  | 1.04E6 |  | 7.56E5 | 1.17E6 | 1.17E6 | 1 | 1 | 0 | 1 | 1 | 0 | 0 | 1 | 0 | 1 | 1 | 1 | N | 50716 | Tyrosine-protein kinase CSK OS=Mus musculus OX=10090 GN=Csk PE=1 SV=2 |
| 1623 | 5286 | A0A1L1SR46\|A0A1L1SR46\_MOUSE | 61.92 | 12 | 0 | 12 | 12 | 0 | 0 | 12 | 0 | 12 | 12 | 12 |  | 9.26E5 | 6.7E5 |  |  | 1.04E6 |  | 7.56E5 | 1.17E6 | 1.17E6 | 1 | 1 | 0 | 1 | 1 | 0 | 0 | 1 | 0 | 1 | 1 | 1 | N | 12122 | C-src tyrosine kinase (Fragment) OS=Mus musculus OX=10090 GN=Csk PE=1 SV=1 |
| 1623 | 5287 | A0A1L1STA1\|A0A1L1STA1\_MOUSE | 61.92 | 9 | 0 | 9 | 9 | 0 | 0 | 9 | 0 | 9 | 9 | 9 |  | 9.26E5 | 6.7E5 |  |  | 1.04E6 |  | 7.56E5 | 1.17E6 | 1.17E6 | 1 | 1 | 0 | 1 | 1 | 0 | 0 | 1 | 0 | 1 | 1 | 1 | N | 15657 | C-src tyrosine kinase (Fragment) OS=Mus musculus OX=10090 GN=Csk PE=1 SV=1 |
| 1392 | 4050 | A0A1Y7VKT5\|A0A1Y7VKT5\_MOUSE | 61.91 | 53 | 0 | 25 | 25 | 0 | 25 | 25 | 28 | 53 | 25 | 25 |  | 9.32E5 | 7.43E5 |  | 8.06E5 | 3.99E5 | 3.62E5 | 9.05E5 | 9.23E5 | 8.72E5 | 2 | 2 | 0 | 1 | 1 | 0 | 1 | 1 | 1 | 2 | 1 | 1 | N | 4299 | Eukaryotic translation initiation factor 5 (Fragment) OS=Mus musculus OX=10090 GN=Eif5 PE=1 SV=1 |
| 1392 | 4051 | Q8BVV6\|Q8BVV6\_MOUSE | 61.91 | 5 | 0 | 2 | 2 | 0 | 2 | 2 | 3 | 5 | 2 | 2 |  | 9.32E5 | 7.43E5 |  | 8.06E5 | 3.99E5 | 3.62E5 | 9.05E5 | 9.23E5 | 8.72E5 | 2 | 2 | 0 | 1 | 1 | 0 | 1 | 1 | 1 | 2 | 1 | 1 | N | 42587 | Eukaryotic translation initiation factor 5 OS=Mus musculus OX=10090 GN=Eif5 PE=1 SV=1 |
| 1392 | 4052 | sp\|P59325\|IF5\_MOUSE | 61.91 | 4 | 0 | 2 | 2 | 0 | 2 | 2 | 2 | 4 | 2 | 2 |  | 9.32E5 | 7.43E5 |  | 8.06E5 | 3.99E5 | 3.62E5 | 9.05E5 | 9.23E5 | 8.72E5 | 2 | 2 | 0 | 1 | 1 | 0 | 1 | 1 | 1 | 2 | 1 | 1 | N | 48968 | Eukaryotic translation initiation factor 5 OS=Mus musculus OX=10090 GN=Eif5 PE=1 SV=1 |
| 1522 | 4480 | sp\|P11438\|LAMP1\_MOUSE | 61.82 | 3 | 3 | 0 | 3 | 0 | 0 | 3 | 3 | 3 | 3 | 3 | 7.23E5 |  | 5.94E6 |  |  | 6.07E6 | 2.45E6 | 3.86E6 | 4.61E6 | 2.17E6 | 1 | 1 | 1 | 0 | 1 | 0 | 0 | 1 | 1 | 1 | 1 | 1 | Y | 43865 | Lysosome-associated membrane glycoprotein 1 OS=Mus musculus OX=10090 GN=Lamp1 PE=1 SV=2 |
| 1620 | 3962 | sp\|Q9QZZ6\|DERM\_MOUSE | 61.76 | 9 | 0 | 0 | 0 | 9 | 0 | 0 | 9 | 0 | 4 | 4 |  |  |  | 4.7E6 |  |  | 3.92E6 |  | 2.01E6 | 8.26E5 | 2 | 2 | 0 | 0 | 0 | 2 | 0 | 0 | 2 | 0 | 1 | 1 | N | 23995 | Dermatopontin OS=Mus musculus OX=10090 GN=Dpt PE=1 SV=1 |
| 2112 | 3742 | sp\|Q8CIM7\|CP2DQ\_MOUSE | 61.59 | 2 | 0 | 2 | 0 | 0 | 0 | 2 | 0 | 0 | 2 | 0 |  | 9.26E5 |  |  |  | 4.03E5 |  |  | 1.76E6 |  | 1 | 1 | 0 | 1 | 0 | 0 | 0 | 1 | 0 | 0 | 1 | 0 | N | 56976 | Cytochrome P450 2D26 OS=Mus musculus OX=10090 GN=Cyp2d26 PE=1 SV=1 |
| 2112 | 3743 | A0A0R4IZY2\|A0A0R4IZY2\_MOUSE | 61.59 | 2 | 0 | 2 | 0 | 0 | 0 | 2 | 0 | 0 | 2 | 0 |  | 9.26E5 |  |  |  | 4.03E5 |  |  | 1.76E6 |  | 1 | 1 | 0 | 1 | 0 | 0 | 0 | 1 | 0 | 0 | 1 | 0 | N | 56950 | Cytochrome P450 OS=Mus musculus OX=10090 GN=Cyp2d26 PE=1 SV=1 |
| 2112 | 4771 | Q9JKY7\|Q9JKY7\_MOUSE | 61.59 | 2 | 0 | 2 | 0 | 0 | 0 | 2 | 0 | 0 | 2 | 0 |  | 9.26E5 |  |  |  | 4.03E5 |  |  | 1.76E6 |  | 1 | 1 | 0 | 1 | 0 | 0 | 0 | 1 | 0 | 0 | 1 | 0 | N | 56468 | Cytochrome P450 OS=Mus musculus OX=10090 GN=Cyp2d22 PE=1 SV=1 |
| 2546 | 4239 | D3YX34\|D3YX34\_MOUSE | 61.59 | 2 | 0 | 0 | 1 | 0 | 0 | 1 | 0 | 0 | 0 | 0 |  |  | 1.29E6 |  |  |  |  |  |  |  | 2 | 1 | 0 | 0 | 1 | 0 | 0 | 1 | 0 | 0 | 0 | 0 | N | 126821 | Dynactin 1 OS=Mus musculus OX=10090 GN=Dctn1 PE=1 SV=1 |
| 2546 | 4250 | E9Q586\|E9Q586\_MOUSE | 61.59 | 1 | 0 | 0 | 1 | 0 | 0 | 0 | 0 | 0 | 0 | 0 |  |  | 1.29E6 |  |  |  |  |  |  |  | 2 | 1 | 0 | 0 | 1 | 0 | 0 | 1 | 0 | 0 | 0 | 0 | N | 136848 | Dynactin subunit 1 OS=Mus musculus OX=10090 GN=Dctn1 PE=1 SV=1 |
| 2546 | 4254 | E9Q3M3\|E9Q3M3\_MOUSE | 61.59 | 1 | 0 | 0 | 1 | 0 | 0 | 0 | 0 | 0 | 0 | 0 |  |  | 1.29E6 |  |  |  |  |  |  |  | 2 | 1 | 0 | 0 | 1 | 0 | 0 | 1 | 0 | 0 | 0 | 0 | N | 139761 | Dynactin subunit 1 OS=Mus musculus OX=10090 GN=Dctn1 PE=1 SV=1 |
| 2546 | 4255 | sp\|O08788\|DCTN1\_MOUSE | 61.59 | 1 | 0 | 0 | 1 | 0 | 0 | 0 | 0 | 0 | 0 | 0 |  |  | 1.29E6 |  |  |  |  |  |  |  | 2 | 1 | 0 | 0 | 1 | 0 | 0 | 1 | 0 | 0 | 0 | 0 | N | 141675 | Dynactin subunit 1 OS=Mus musculus OX=10090 GN=Dctn1 PE=1 SV=3 |
| 1379 | 2835 | sp\|Q9WV54\|ASAH1\_MOUSE | 61.58 | 5 | 0 | 3 | 3 | 0 | 5 | 3 | 3 | 0 | 3 | 3 |  | 1.13E6 | 4.28E5 |  | 1.1E6 | 1.31E6 | 3.96E5 |  | 1.54E5 | 3.15E5 | 2 | 2 | 0 | 1 | 1 | 0 | 2 | 1 | 1 | 0 | 1 | 1 | Y | 44670 | Acid ceramidase OS=Mus musculus OX=10090 GN=Asah1 PE=1 SV=1 |
| 1885 | 2460 | A2AQ99\|A2AQ99\_MOUSE | 61.57 | 2 | 1 | 0 | 0 | 0 | 0 | 0 | 0 | 0 | 1 | 0 | 1.5E6 |  |  |  |  |  |  | 5.65E5 | 9.36E6 |  | 3 | 3 | 1 | 0 | 0 | 0 | 0 | 0 | 0 | 1 | 2 | 0 | N | 171625 | NAD(P)H oxidase (H2O2-forming) OS=Mus musculus OX=10090 GN=Duox2 PE=1 SV=1 |
| 1885 | 2462 | A0A494BAW1\|A0A494BAW1\_MOUSE | 61.57 | 2 | 1 | 0 | 0 | 0 | 0 | 0 | 0 | 0 | 1 | 0 | 1.5E6 |  |  |  |  |  |  | 5.65E5 | 9.36E6 |  | 3 | 3 | 1 | 0 | 0 | 0 | 0 | 0 | 0 | 1 | 2 | 0 | N | 174546 | NAD(P)H oxidase (H2O2-forming) OS=Mus musculus OX=10090 GN=Duox2 PE=1 SV=1 |
| 3306 | 5069 | sp\|Q5SUF2\|LC7L3\_MOUSE | 61.57 | 4 | 0 | 0 | 0 | 0 | 0 | 0 | 0 | 4 | 0 | 0 |  |  |  |  |  |  |  | 1.57E5 |  |  | 1 | 1 | 0 | 0 | 0 | 0 | 0 | 0 | 0 | 1 | 0 | 0 | N | 51450 | Luc7-like protein 3 OS=Mus musculus OX=10090 GN=Luc7l3 PE=1 SV=1 |
| 1822 | 2825 | sp\|E9Q634\|MYO1E\_MOUSE | 61.34 | 1 | 0 | 0 | 1 | 0 | 0 | 0 | 0 | 0 | 1 | 1 |  |  |  |  |  |  |  |  | 0E0 | 6.17E6 | 2 | 1 | 0 | 0 | 1 | 0 | 0 | 0 | 0 | 0 | 1 | 1 | Y | 126818 | Unconventional myosin-Ie OS=Mus musculus OX=10090 GN=Myo1e PE=1 SV=1 |
| 2050 | 2924 | sp\|Q9QUR6\|PPCE\_MOUSE | 61.30 | 4 | 2 | 0 | 0 | 0 | 0 | 0 | 0 | 0 | 4 | 0 | 0E0 |  |  |  |  |  |  |  | 1.26E6 |  | 2 | 2 | 1 | 0 | 0 | 0 | 0 | 0 | 0 | 0 | 2 | 0 | N | 80752 | Prolyl endopeptidase OS=Mus musculus OX=10090 GN=Prep PE=1 SV=1 |
| 1216 | 2981 | A0A668KMA1\|A0A668KMA1\_MOUSE | 61.28 | 1 | 0 | 0 | 0 | 0 | 1 | 0 | 0 | 0 | 0 | 0 |  |  | 1.96E7 | 2.67E6 | 1.64E8 |  |  |  |  | 4.34E6 | 2 | 1 | 0 | 0 | 1 | 1 | 2 | 1 | 0 | 0 | 0 | 1 | N | 266495 | non-specific serine/threonine protein kinase OS=Mus musculus OX=10090 GN=Mast4 PE=1 SV=1 |
| 3303 | 5028 | H3BJE0\|H3BJE0\_MOUSE | 61.24 | 5 | 0 | 0 | 0 | 0 | 0 | 0 | 0 | 0 | 5 | 0 |  |  |  |  |  |  |  |  | 3.06E6 |  | 1 | 1 | 0 | 0 | 0 | 0 | 0 | 0 | 0 | 0 | 1 | 0 | Y | 27083 | Granulin (Fragment) OS=Mus musculus OX=10090 GN=Grn PE=1 SV=1 |
| 3303 | 5030 | sp\|P28798\|GRN\_MOUSE | 61.24 | 2 | 0 | 0 | 0 | 0 | 0 | 0 | 0 | 0 | 2 | 0 |  |  |  |  |  |  |  |  | 3.06E6 |  | 1 | 1 | 0 | 0 | 0 | 0 | 0 | 0 | 0 | 0 | 1 | 0 | Y | 63458 | Progranulin OS=Mus musculus OX=10090 GN=Grn PE=1 SV=2 |
| 3303 | 5031 | Q3U9N4\|Q3U9N4\_MOUSE | 61.24 | 2 | 0 | 0 | 0 | 0 | 0 | 0 | 0 | 0 | 2 | 0 |  |  |  |  |  |  |  |  | 3.06E6 |  | 1 | 1 | 0 | 0 | 0 | 0 | 0 | 0 | 0 | 0 | 1 | 0 | Y | 65018 | Granulin OS=Mus musculus OX=10090 GN=Grn PE=1 SV=1 |
| 1933 | 5306 | A0A0G2JFB7\|A0A0G2JFB7\_MOUSE | 61.21 | 8 | 0 | 8 | 8 | 8 | 0 | 8 | 0 | 0 | 0 | 0 |  | 2.95E6 | 3.36E5 | 2.36E5 |  | 1.74E6 |  |  |  |  | 1 | 1 | 0 | 1 | 1 | 1 | 0 | 1 | 0 | 0 | 0 | 0 | N | 15700 | Platelet glycoprotein 4 (Fragment) OS=Mus musculus OX=10090 GN=Cd36 PE=1 SV=1 |
| 1933 | 5334 | sp\|Q08857\|CD36\_MOUSE | 61.21 | 3 | 0 | 3 | 3 | 3 | 0 | 3 | 0 | 0 | 0 | 0 |  | 2.95E6 | 3.36E5 | 2.36E5 |  | 1.74E6 |  |  |  |  | 1 | 1 | 0 | 1 | 1 | 1 | 0 | 1 | 0 | 0 | 0 | 0 | N | 52698 | Platelet glycoprotein 4 OS=Mus musculus OX=10090 GN=Cd36 PE=1 SV=2 |
| 1547 | 5336 | A0A1Y7VKP8\|A0A1Y7VKP8\_MOUSE | 61.08 | 11 | 0 | 11 | 11 | 11 | 0 | 11 | 0 | 11 | 11 | 0 |  | 1.58E6 | 2.42E6 | 2.98E6 |  | 4.22E6 |  | 6.05E5 | 3.84E6 |  | 1 | 1 | 0 | 1 | 1 | 2 | 0 | 1 | 0 | 1 | 1 | 0 | N | 10908 | NADH:ubiquinone oxidoreductase core subunit S6 OS=Mus musculus OX=10090 GN=Ndufs6 PE=1 SV=1 |
| 1547 | 5343 | sp\|P52503\|NDUS6\_MOUSE | 61.08 | 9 | 0 | 9 | 9 | 9 | 0 | 9 | 0 | 9 | 9 | 0 |  | 1.58E6 | 2.42E6 | 2.98E6 |  | 4.22E6 |  | 6.05E5 | 3.84E6 |  | 1 | 1 | 0 | 1 | 1 | 2 | 0 | 1 | 0 | 1 | 1 | 0 | N | 13020 | NADH dehydrogenase [ubiquinone] iron-sulfur protein 6, mitochondrial OS=Mus musculus OX=10090 GN=Ndufs6 PE=1 SV=2 |
| 1547 | 5344 | A0A1Y7VM38\|A0A1Y7VM38\_MOUSE | 61.08 | 9 | 0 | 9 | 9 | 9 | 0 | 9 | 0 | 9 | 9 | 0 |  | 1.58E6 | 2.42E6 | 2.98E6 |  | 4.22E6 |  | 6.05E5 | 3.84E6 |  | 1 | 1 | 0 | 1 | 1 | 2 | 0 | 1 | 0 | 1 | 1 | 0 | N | 14092 | NADH dehydrogenase [ubiquinone] iron-sulfur protein 6, mitochondrial OS=Mus musculus OX=10090 GN=Ndufs6 PE=1 SV=1 |
| 1494 | 4281 | A0A1B0GRX1\|A0A1B0GRX1\_MOUSE | 61.08 | 8 | 0 | 0 | 0 | 8 | 0 | 0 | 0 | 0 | 8 | 0 |  |  |  | 1.4E6 |  |  |  |  | 4.35E6 |  | 1 | 1 | 0 | 0 | 0 | 1 | 0 | 0 | 0 | 0 | 1 | 0 | N | 14835 | Caspase 3 OS=Mus musculus OX=10090 GN=Casp3 PE=1 SV=1 |
| 1494 | 4284 | sp\|P70677\|CASP3\_MOUSE | 61.08 | 4 | 0 | 0 | 0 | 4 | 0 | 0 | 0 | 0 | 4 | 0 |  |  |  | 1.4E6 |  |  |  |  | 4.35E6 |  | 1 | 1 | 0 | 0 | 0 | 1 | 0 | 0 | 0 | 0 | 1 | 0 | N | 31475 | Caspase-3 OS=Mus musculus OX=10090 GN=Casp3 PE=1 SV=1 |
| 2062 | 2737 | sp\|P38060\|HMGCL\_MOUSE | 60.67 | 4 | 0 | 0 | 0 | 0 | 0 | 0 | 0 | 0 | 0 | 4 |  |  |  |  |  |  |  |  |  | 4.77E5 | 1 | 1 | 0 | 0 | 0 | 0 | 0 | 0 | 0 | 0 | 0 | 1 | N | 34239 | Hydroxymethylglutaryl-CoA lyase, mitochondrial OS=Mus musculus OX=10090 GN=Hmgcl PE=1 SV=2 |
| 1532 | 3718 | sp\|P32883\|RASK\_MOUSE | 60.64 | 12 | 0 | 0 | 6 | 0 | 6 | 12 | 6 | 0 | 0 | 6 |  |  | 1.47E6 |  | 1.09E6 | 1.58E6 | 8.35E5 |  |  | 1.19E6 | 2 | 2 | 0 | 0 | 1 | 0 | 1 | 2 | 1 | 0 | 0 | 1 | N | 21656 | GTPase KRas OS=Mus musculus OX=10090 GN=Kras PE=1 SV=1 |
| 1532 | 3954 | A0A0G2JE25\|A0A0G2JE25\_MOUSE | 60.64 | 15 | 0 | 0 | 7 | 0 | 7 | 15 | 7 | 0 | 0 | 7 |  |  | 1.47E6 |  | 1.09E6 | 1.58E6 | 8.35E5 |  |  | 1.19E6 | 2 | 2 | 0 | 0 | 1 | 0 | 1 | 2 | 1 | 0 | 0 | 1 | N | 16835 | GTPase NRas (Fragment) OS=Mus musculus OX=10090 GN=Nras PE=1 SV=1 |
| 1532 | 3955 | A0A0G2JGP4\|A0A0G2JGP4\_MOUSE | 60.64 | 13 | 0 | 0 | 6 | 0 | 6 | 13 | 6 | 0 | 0 | 6 |  |  | 1.47E6 |  | 1.09E6 | 1.58E6 | 8.35E5 |  |  | 1.19E6 | 2 | 2 | 0 | 0 | 1 | 0 | 1 | 2 | 1 | 0 | 0 | 1 | N | 20586 | GTPase NRas (Fragment) OS=Mus musculus OX=10090 GN=Nras PE=1 SV=1 |
| 1532 | 3956 | sp\|P08556\|RASN\_MOUSE | 60.64 | 12 | 0 | 0 | 6 | 0 | 6 | 12 | 6 | 0 | 0 | 6 |  |  | 1.47E6 |  | 1.09E6 | 1.58E6 | 8.35E5 |  |  | 1.19E6 | 2 | 2 | 0 | 0 | 1 | 0 | 1 | 2 | 1 | 0 | 0 | 1 | N | 21199 | GTPase NRas OS=Mus musculus OX=10090 GN=Nras PE=1 SV=1 |
| 1532 | 3957 | Q9D091\|Q9D091\_MOUSE | 60.64 | 12 | 0 | 0 | 6 | 0 | 6 | 12 | 6 | 0 | 0 | 6 |  |  | 1.47E6 |  | 1.09E6 | 1.58E6 | 8.35E5 |  |  | 1.19E6 | 2 | 2 | 0 | 0 | 1 | 0 | 1 | 2 | 1 | 0 | 0 | 1 | N | 21243 | GTPase NRas OS=Mus musculus OX=10090 GN=Nras PE=1 SV=1 |
| 2168 | 5281 | sp\|Q9JKB1\|UCHL3\_MOUSE | 60.58 | 7 | 0 | 0 | 0 | 0 | 7 | 0 | 7 | 0 | 7 | 0 |  |  |  |  | 3.63E5 |  | 5.15E5 |  | 2.09E6 |  | 1 | 1 | 0 | 0 | 0 | 0 | 1 | 0 | 1 | 0 | 1 | 0 | N | 26152 | Ubiquitin carboxyl-terminal hydrolase isozyme L3 OS=Mus musculus OX=10090 GN=Uchl3 PE=1 SV=2 |
| 2168 | 5282 | sp\|P58321\|UCHL4\_MOUSE | 60.58 | 7 | 0 | 0 | 0 | 0 | 7 | 0 | 7 | 0 | 7 | 0 |  |  |  |  | 3.63E5 |  | 5.15E5 |  | 2.09E6 |  | 1 | 1 | 0 | 0 | 0 | 0 | 1 | 0 | 1 | 0 | 1 | 0 | N | 26450 | Ubiquitin carboxyl-terminal hydrolase isozyme L4 OS=Mus musculus OX=10090 GN=Uchl4 PE=2 SV=1 |
| 1288 | 3950 | A0A140T8M5\|A0A140T8M5\_MOUSE | 60.54 | 14 | 6 | 0 | 8 | 0 | 0 | 14 | 8 | 8 | 0 | 0 | 1E7 |  |  |  |  | 3.63E6 |  |  |  |  | 2 | 1 | 1 | 0 | 1 | 0 | 0 | 2 | 2 | 1 | 0 | 0 | Y | 12757 | Immunoglobulin kappa variable 6-15 (Fragment) OS=Mus musculus OX=10090 GN=Igkv6-15 PE=1 SV=2 |
| 1505 | 3997 | A0A140LJF4\|A0A140LJF4\_MOUSE | 60.17 | 6 | 6 | 0 | 6 | 6 | 0 | 6 | 0 | 6 | 6 | 6 | 1.42E6 |  | 1.09E6 | 1.19E6 |  | 5.49E5 |  | 9.69E5 | 8.35E5 | 8.06E5 | 1 | 1 | 1 | 0 | 1 | 1 | 0 | 1 | 0 | 1 | 1 | 1 | N | 22451 | Protein arginine N-methyltransferase 1 (Fragment) OS=Mus musculus OX=10090 GN=Prmt1 PE=1 SV=1 |
| 1505 | 3998 | A0A140LHF7\|A0A140LHF7\_MOUSE | 60.17 | 6 | 6 | 0 | 6 | 6 | 0 | 6 | 0 | 6 | 6 | 6 | 1.42E6 |  | 1.09E6 | 1.19E6 |  | 5.49E5 |  | 9.69E5 | 8.35E5 | 8.06E5 | 1 | 1 | 1 | 0 | 1 | 1 | 0 | 1 | 0 | 1 | 1 | 1 | N | 24419 | Protein arginine N-methyltransferase 1 OS=Mus musculus OX=10090 GN=Prmt1 PE=1 SV=1 |
| 1505 | 3999 | A0A171KXD3\|A0A171KXD3\_MOUSE | 60.17 | 4 | 4 | 0 | 4 | 4 | 0 | 4 | 0 | 4 | 4 | 4 | 1.42E6 |  | 1.09E6 | 1.19E6 |  | 5.49E5 |  | 9.69E5 | 8.35E5 | 8.06E5 | 1 | 1 | 1 | 0 | 1 | 1 | 0 | 1 | 0 | 1 | 1 | 1 | N | 36567 | type I protein arginine methyltransferase OS=Mus musculus OX=10090 GN=Prmt1 PE=1 SV=1 |
| 1505 | 4000 | sp\|Q9JIF0\|ANM1\_MOUSE | 60.17 | 3 | 3 | 0 | 3 | 3 | 0 | 3 | 0 | 3 | 3 | 3 | 1.42E6 |  | 1.09E6 | 1.19E6 |  | 5.49E5 |  | 9.69E5 | 8.35E5 | 8.06E5 | 1 | 1 | 1 | 0 | 1 | 1 | 0 | 1 | 0 | 1 | 1 | 1 | N | 42436 | Protein arginine N-methyltransferase 1 OS=Mus musculus OX=10090 GN=Prmt1 PE=1 SV=1 |
| 2142 | 5074 | sp\|Q78IK4\|MIC27\_MOUSE | 60.14 | 6 | 0 | 0 | 0 | 0 | 0 | 6 | 0 | 6 | 6 | 0 |  |  |  |  |  | 1.42E6 |  | 3.68E5 | 4.24E5 |  | 1 | 1 | 0 | 0 | 0 | 0 | 0 | 1 | 0 | 1 | 1 | 0 | Y | 29261 | MICOS complex subunit Mic27 OS=Mus musculus OX=10090 GN=Apool PE=1 SV=1 |
| 2142 | 5328 | B1AV14\|B1AV14\_MOUSE | 60.14 | 8 | 0 | 0 | 0 | 0 | 0 | 8 | 0 | 8 | 8 | 0 |  |  |  |  |  | 1.42E6 |  | 3.68E5 | 4.24E5 |  | 1 | 1 | 0 | 0 | 0 | 0 | 0 | 1 | 0 | 1 | 1 | 0 | Y | 21063 | MICOS complex subunit OS=Mus musculus OX=10090 GN=Apool PE=1 SV=1 |
| 1892 | 3662 | sp\|Q99KP6\|PRP19\_MOUSE | 60.02 | 4 | 0 | 2 | 0 | 0 | 0 | 2 | 0 | 0 | 0 | 4 |  | 6.14E6 |  |  |  | 6.55E6 |  |  |  | 6.79E6 | 2 | 2 | 0 | 1 | 0 | 0 | 0 | 1 | 0 | 0 | 0 | 2 | N | 55239 | Pre-mRNA-processing factor 19 OS=Mus musculus OX=10090 GN=Prpf19 PE=1 SV=1 |
| 1280 | 2893 | sp\|Q9CY27\|TECR\_MOUSE | 59.90 | 6 | 3 | 3 | 0 | 0 | 3 | 3 | 3 | 3 | 3 | 6 | 1.5E6 | 1.14E6 |  |  | 7.45E5 | 1.3E6 | 3.17E5 | 3.83E5 | 1.59E6 | 3.01E6 | 2 | 2 | 1 | 1 | 0 | 0 | 1 | 1 | 1 | 1 | 1 | 2 | Y | 36090 | Very-long-chain enoyl-CoA reductase OS=Mus musculus OX=10090 GN=Tecr PE=1 SV=1 |
| 1280 | 2894 | Q52L67\|Q52L67\_MOUSE | 59.90 | 5 | 2 | 2 | 0 | 0 | 2 | 2 | 2 | 2 | 2 | 5 | 1.5E6 | 1.14E6 |  |  | 7.45E5 | 1.3E6 | 3.17E5 | 3.83E5 | 1.59E6 | 3.01E6 | 2 | 2 | 1 | 1 | 0 | 0 | 1 | 1 | 1 | 1 | 1 | 2 | Y | 41896 | Gpsn2 protein OS=Mus musculus OX=10090 GN=Tecr PE=1 SV=1 |
| 1280 | 2904 | G3UWE1\|G3UWE1\_MOUSE | 59.90 | 6 | 3 | 3 | 0 | 0 | 3 | 3 | 3 | 3 | 3 | 6 | 1.5E6 | 1.14E6 |  |  | 7.45E5 | 1.3E6 | 3.17E5 | 3.83E5 | 1.59E6 | 3.01E6 | 2 | 2 | 1 | 1 | 0 | 0 | 1 | 1 | 1 | 1 | 1 | 2 | Y | 34244 | Trans-2,3-enoyl-CoA reductase OS=Mus musculus OX=10090 GN=Tecr PE=1 SV=1 |
| 1280 | 3032 | A0A5F8MQC8\|A0A5F8MQC8\_MOUSE | 59.90 | 5 | 2 | 2 | 0 | 0 | 2 | 2 | 2 | 2 | 2 | 5 | 1.5E6 | 1.14E6 |  |  | 7.45E5 | 1.3E6 | 3.17E5 | 3.83E5 | 1.59E6 | 3.01E6 | 2 | 2 | 1 | 1 | 0 | 0 | 1 | 1 | 1 | 1 | 1 | 2 | Y | 39689 | very-long-chain enoyl-CoA reductase OS=Mus musculus OX=10090 GN=Tecr PE=1 SV=1 |
| 965 | 4559 | G3UZX6\|G3UZX6\_MOUSE | 59.85 | 26 | 26 | 0 | 26 | 26 | 26 | 26 | 26 | 26 | 26 | 26 | 9.87E6 |  | 9.11E6 | 4.76E6 | 9.81E6 | 8.73E6 | 6.27E6 | 6.35E6 | 1.29E7 | 8.02E6 | 1 | 1 | 2 | 0 | 1 | 2 | 2 | 3 | 2 | 2 | 1 | 2 | N | 5382 | Small ubiquitin-like modifier 3 (Fragment) OS=Mus musculus OX=10090 GN=Sumo3 PE=4 SV=1 |
| 965 | 5307 | H7BWX9\|H7BWX9\_MOUSE | 59.85 | 23 | 23 | 0 | 23 | 23 | 23 | 23 | 23 | 23 | 23 | 23 | 9.87E6 |  | 9.11E6 | 4.76E6 | 9.81E6 | 8.73E6 | 6.27E6 | 6.35E6 | 1.29E7 | 8.02E6 | 1 | 1 | 2 | 0 | 1 | 2 | 2 | 3 | 2 | 2 | 1 | 2 | N | 6014 | Small ubiquitin-like modifier 2 OS=Mus musculus OX=10090 GN=Sumo2 PE=1 SV=1 |
| 965 | 5310 | D3Z794\|D3Z794\_MOUSE | 59.85 | 17 | 17 | 0 | 17 | 17 | 17 | 17 | 17 | 17 | 17 | 17 | 9.87E6 |  | 9.11E6 | 4.76E6 | 9.81E6 | 8.73E6 | 6.27E6 | 6.35E6 | 1.29E7 | 8.02E6 | 1 | 1 | 2 | 0 | 1 | 2 | 2 | 3 | 2 | 2 | 1 | 2 | N | 8111 | Small ubiquitin-like modifier 2 OS=Mus musculus OX=10090 GN=Sumo2 PE=1 SV=1 |
| 965 | 5312 | G3UZ60\|G3UZ60\_MOUSE | 59.85 | 16 | 16 | 0 | 16 | 16 | 16 | 16 | 16 | 16 | 16 | 16 | 9.87E6 |  | 9.11E6 | 4.76E6 | 9.81E6 | 8.73E6 | 6.27E6 | 6.35E6 | 1.29E7 | 8.02E6 | 1 | 1 | 2 | 0 | 1 | 2 | 2 | 3 | 2 | 2 | 1 | 2 | N | 8602 | Predicted gene, 49325 OS=Mus musculus OX=10090 GN=Gm49325 PE=4 SV=1 |
| 965 | 5313 | G3UWI9\|G3UWI9\_MOUSE | 59.85 | 15 | 15 | 0 | 15 | 15 | 15 | 15 | 15 | 15 | 15 | 15 | 9.87E6 |  | 9.11E6 | 4.76E6 | 9.81E6 | 8.73E6 | 6.27E6 | 6.35E6 | 1.29E7 | 8.02E6 | 1 | 1 | 2 | 0 | 1 | 2 | 2 | 3 | 2 | 2 | 1 | 2 | N | 9373 | Small ubiquitin-like modifier 3 OS=Mus musculus OX=10090 GN=Sumo3 PE=3 SV=1 |
| 965 | 5314 | sp\|P61957\|SUMO2\_MOUSE | 59.85 | 13 | 13 | 0 | 13 | 13 | 13 | 13 | 13 | 13 | 13 | 13 | 9.87E6 |  | 9.11E6 | 4.76E6 | 9.81E6 | 8.73E6 | 6.27E6 | 6.35E6 | 1.29E7 | 8.02E6 | 1 | 1 | 2 | 0 | 1 | 2 | 2 | 3 | 2 | 2 | 1 | 2 | N | 10871 | Small ubiquitin-related modifier 2 OS=Mus musculus OX=10090 GN=Sumo2 PE=1 SV=1 |
| 965 | 5315 | G3UWX9\|G3UWX9\_MOUSE | 59.85 | 12 | 12 | 0 | 12 | 12 | 12 | 12 | 12 | 12 | 12 | 12 | 9.87E6 |  | 9.11E6 | 4.76E6 | 9.81E6 | 8.73E6 | 6.27E6 | 6.35E6 | 1.29E7 | 8.02E6 | 1 | 1 | 2 | 0 | 1 | 2 | 2 | 3 | 2 | 2 | 1 | 2 | N | 10736 | Small ubiquitin-like modifier 3 OS=Mus musculus OX=10090 GN=Sumo3 PE=3 SV=1 |
| 965 | 5317 | sp\|Q9Z172\|SUMO3\_MOUSE | 59.85 | 11 | 11 | 0 | 11 | 11 | 11 | 11 | 11 | 11 | 11 | 11 | 9.87E6 |  | 9.11E6 | 4.76E6 | 9.81E6 | 8.73E6 | 6.27E6 | 6.35E6 | 1.29E7 | 8.02E6 | 1 | 1 | 2 | 0 | 1 | 2 | 2 | 3 | 2 | 2 | 1 | 2 | N | 12430 | Small ubiquitin-related modifier 3 OS=Mus musculus OX=10090 GN=Sumo3 PE=1 SV=1 |
| 965 | 5318 | G3UZA7\|G3UZA7\_MOUSE | 59.85 | 11 | 11 | 0 | 11 | 11 | 11 | 11 | 11 | 11 | 11 | 11 | 9.87E6 |  | 9.11E6 | 4.76E6 | 9.81E6 | 8.73E6 | 6.27E6 | 6.35E6 | 1.29E7 | 8.02E6 | 1 | 1 | 2 | 0 | 1 | 2 | 2 | 3 | 2 | 2 | 1 | 2 | N | 12713 | Small ubiquitin-related modifier OS=Mus musculus OX=10090 GN=Sumo3 PE=1 SV=1 |
| 1396 | 5362 | sp\|Q8BPB0\|MOB1B\_MOUSE | 59.67 | 5 | 0 | 5 | 5 | 5 | 5 | 5 | 5 | 5 | 5 | 5 |  | 1.42E6 | 2.04E6 | 1.48E6 | 1.53E6 | 2.59E6 | 1.25E6 | 5.02E5 | 3.46E6 | 1.47E6 | 1 | 1 | 0 | 1 | 1 | 1 | 1 | 1 | 1 | 1 | 1 | 1 | N | 25091 | MOB kinase activator 1B OS=Mus musculus OX=10090 GN=Mob1b PE=1 SV=3 |
| 1396 | 5363 | sp\|Q921Y0\|MOB1A\_MOUSE | 59.67 | 5 | 0 | 5 | 5 | 5 | 5 | 5 | 5 | 5 | 5 | 5 |  | 1.42E6 | 2.04E6 | 1.48E6 | 1.53E6 | 2.59E6 | 1.25E6 | 5.02E5 | 3.46E6 | 1.47E6 | 1 | 1 | 0 | 1 | 1 | 1 | 1 | 1 | 1 | 1 | 1 | 1 | N | 25080 | MOB kinase activator 1A OS=Mus musculus OX=10090 GN=Mob1a PE=2 SV=3 |
| 1396 | 5367 | Q3UDM0\|Q3UDM0\_MOUSE | 59.67 | 5 | 0 | 5 | 5 | 5 | 5 | 5 | 5 | 5 | 5 | 5 |  | 1.42E6 | 2.04E6 | 1.48E6 | 1.53E6 | 2.59E6 | 1.25E6 | 5.02E5 | 3.46E6 | 1.47E6 | 1 | 1 | 0 | 1 | 1 | 1 | 1 | 1 | 1 | 1 | 1 | 1 | N | 25609 | MOB kinase activator 1B OS=Mus musculus OX=10090 GN=Mob1b PE=1 SV=1 |
| 1543 | 4248 | sp\|Q8BMJ3\|IF1AX\_MOUSE | 59.67 | 8 | 0 | 0 | 0 | 8 | 8 | 8 | 8 | 0 | 0 | 0 |  |  |  | 1.37E6 | 3.48E6 | 2.2E6 | 1.98E6 |  |  |  | 1 | 1 | 0 | 0 | 0 | 1 | 1 | 1 | 1 | 0 | 0 | 0 | N | 16460 | Eukaryotic translation initiation factor 1A, X-chromosomal OS=Mus musculus OX=10090 GN=Eif1ax PE=2 SV=3 |
| 1543 | 4249 | sp\|Q60872\|IF1A\_MOUSE | 59.67 | 8 | 0 | 0 | 0 | 8 | 8 | 8 | 8 | 0 | 0 | 0 |  |  |  | 1.37E6 | 3.48E6 | 2.2E6 | 1.98E6 |  |  |  | 1 | 1 | 0 | 0 | 0 | 1 | 1 | 1 | 1 | 0 | 0 | 0 | N | 16502 | Eukaryotic translation initiation factor 1A OS=Mus musculus OX=10090 GN=Eif1a PE=2 SV=3 |
| 1543 | 4311 | A0A494BB95\|A0A494BB95\_MOUSE | 59.67 | 4 | 0 | 0 | 0 | 4 | 4 | 4 | 4 | 0 | 0 | 0 |  |  |  | 1.37E6 | 3.48E6 | 2.2E6 | 1.98E6 |  |  |  | 1 | 1 | 0 | 0 | 0 | 1 | 1 | 1 | 1 | 0 | 0 | 0 | N | 28613 | Eukaryotic translation initiation factor 4C OS=Mus musculus OX=10090 GN=Eif1a PE=3 SV=1 |
| 3437 | 5339 | sp\|Q9D7S7\|RL22L\_MOUSE | 59.43 | 20 | 0 | 0 | 0 | 0 | 0 | 0 | 20 | 0 | 0 | 0 |  |  |  |  |  |  | 1.33E6 |  |  |  | 1 | 1 | 0 | 0 | 0 | 0 | 0 | 0 | 1 | 0 | 0 | 0 | N | 14467 | Ribosomal protein eL22-like 1 OS=Mus musculus OX=10090 GN=Rpl22l1 PE=1 SV=1 |
| 2138 | 4905 | sp\|O08738\|CASP6\_MOUSE | 59.40 | 4 | 0 | 0 | 0 | 0 | 0 | 4 | 0 | 0 | 4 | 4 |  |  |  |  |  | 3.4E5 |  |  | 4.66E5 | 8.59E5 | 1 | 1 | 0 | 0 | 0 | 0 | 0 | 1 | 0 | 0 | 1 | 1 | N | 31595 | Caspase-6 OS=Mus musculus OX=10090 GN=Casp6 PE=1 SV=1 |
| 1602 | 2700 | sp\|P97300\|NPTN\_MOUSE | 59.33 | 2 | 0 | 0 | 0 | 0 | 0 | 2 | 2 | 0 | 0 | 0 |  |  |  |  |  | 3.68E6 | 2.33E6 |  |  |  | 1 | 1 | 0 | 0 | 0 | 0 | 0 | 1 | 1 | 0 | 0 | 0 | N | 44373 | Neuroplastin OS=Mus musculus OX=10090 GN=Nptn PE=1 SV=3 |
| 1163 | 5342 | E9Q0W8\|E9Q0W8\_MOUSE | 59.32 | 21 | 21 | 21 | 21 | 21 | 21 | 21 | 21 | 21 | 21 | 21 | 2.19E6 | 4.97E6 | 7.25E6 | 4.96E6 | 5.51E6 | 7.72E5 | 7.87E6 | 7.16E6 | 1.34E7 | 3.96E6 | 1 | 1 | 2 | 1 | 1 | 1 | 1 | 1 | 2 | 1 | 2 | 1 | Y | 6159 | Small nuclear ribonucleoprotein E OS=Mus musculus OX=10090 GN=Snrpe PE=1 SV=1 |
| 1163 | 5347 | E9Q4F4\|E9Q4F4\_MOUSE | 59.32 | 12 | 12 | 12 | 12 | 12 | 12 | 12 | 12 | 12 | 12 | 12 | 2.19E6 | 4.97E6 | 7.25E6 | 4.96E6 | 5.51E6 | 7.72E5 | 7.87E6 | 7.16E6 | 1.34E7 | 3.96E6 | 1 | 1 | 2 | 1 | 1 | 1 | 1 | 1 | 2 | 1 | 2 | 1 | Y | 10873 | Small nuclear ribonucleoprotein E OS=Mus musculus OX=10090 GN=Snrpe PE=1 SV=1 |
| 1163 | 5348 | sp\|P62305\|RUXE\_MOUSE | 59.32 | 12 | 12 | 12 | 12 | 12 | 12 | 12 | 12 | 12 | 12 | 12 | 2.19E6 | 4.97E6 | 7.25E6 | 4.96E6 | 5.51E6 | 7.72E5 | 7.87E6 | 7.16E6 | 1.34E7 | 3.96E6 | 1 | 1 | 2 | 1 | 1 | 1 | 1 | 1 | 2 | 1 | 2 | 1 | Y | 10804 | Small nuclear ribonucleoprotein E OS=Mus musculus OX=10090 GN=Snrpe PE=1 SV=1 |
| 1258 | 2503 | sp\|Q9DC70\|NDUS7\_MOUSE | 59.31 | 10 | 0 | 10 | 10 | 3 | 0 | 3 | 0 | 3 | 0 | 0 |  | 1.45E7 | 1.45E7 | 1.35E6 |  | 2.85E6 |  | 1.61E6 |  |  | 3 | 3 | 0 | 3 | 3 | 1 | 0 | 1 | 0 | 1 | 0 | 0 | N | 24683 | NADH dehydrogenase [ubiquinone] iron-sulfur protein 7, mitochondrial OS=Mus musculus OX=10090 GN=Ndufs7 PE=1 SV=1 |
| 1667 | 2768 | sp\|O35691\|PININ\_MOUSE | 59.25 | 1 | 0 | 0 | 0 | 0 | 0 | 0 | 0 | 0 | 0 | 1 |  |  |  |  |  |  |  |  |  | 1.55E6 | 1 | 1 | 0 | 0 | 0 | 0 | 0 | 0 | 0 | 0 | 0 | 1 | N | 82436 | Pinin OS=Mus musculus OX=10090 GN=Pnn PE=1 SV=4 |
| 1667 | 2769 | Q3TUQ5\|Q3TUQ5\_MOUSE | 59.25 | 1 | 0 | 0 | 0 | 0 | 0 | 0 | 0 | 0 | 0 | 1 |  |  |  |  |  |  |  |  |  | 1.55E6 | 1 | 1 | 0 | 0 | 0 | 0 | 0 | 0 | 0 | 0 | 0 | 1 | N | 82505 | Pinin OS=Mus musculus OX=10090 GN=Pnn PE=1 SV=1 |
| 2554 | 5349 | sp\|Q9D554\|SF3A3\_MOUSE | 59.06 | 2 | 2 | 0 | 0 | 0 | 0 | 0 | 0 | 0 | 0 | 2 | 1.94E5 |  |  |  |  |  |  |  |  | 3.82E5 | 1 | 1 | 1 | 0 | 0 | 0 | 0 | 0 | 0 | 0 | 0 | 1 | N | 58842 | Splicing factor 3A subunit 3 OS=Mus musculus OX=10090 GN=Sf3a3 PE=1 SV=2 |
| 3210 | 4804 | sp\|P50295\|ARY2\_MOUSE | 58.95 | 4 | 0 | 0 | 4 | 0 | 0 | 0 | 0 | 0 | 0 | 0 |  |  | 1.24E5 |  |  |  |  |  |  |  | 1 | 1 | 0 | 0 | 1 | 0 | 0 | 0 | 0 | 0 | 0 | 0 | N | 33701 | Arylamine N-acetyltransferase 2 OS=Mus musculus OX=10090 GN=Nat2 PE=1 SV=1 |
| 1597 | 4057 | sp\|A2BH40\|ARI1A\_MOUSE | 58.94 | 1 | 0 | 0 | 1 | 0 | 0 | 0 | 0 | 0 | 0 | 1 |  |  | 0E0 |  |  |  |  | 6.22E5 |  |  | 2 | 2 | 0 | 0 | 1 | 0 | 0 | 0 | 0 | 1 | 0 | 1 | N | 242089 | AT-rich interactive domain-containing protein 1A OS=Mus musculus OX=10090 GN=Arid1a PE=1 SV=1 |
| 1597 | 4058 | E9QAQ7\|E9QAQ7\_MOUSE | 58.94 | 1 | 0 | 0 | 1 | 0 | 0 | 0 | 0 | 0 | 0 | 1 |  |  | 0E0 |  |  |  |  | 6.22E5 |  |  | 2 | 2 | 0 | 0 | 1 | 0 | 0 | 0 | 0 | 1 | 0 | 1 | N | 242671 | AT-rich interaction domain 1A OS=Mus musculus OX=10090 GN=Arid1a PE=1 SV=1 |
| 1633 | 4157 | sp\|Q9DB15\|RM12\_MOUSE | 58.93 | 4 | 0 | 0 | 0 | 4 | 0 | 4 | 4 | 0 | 0 | 0 |  |  |  | 2.76E5 |  | 1.25E6 | 6.42E5 |  |  |  | 1 | 1 | 0 | 0 | 0 | 1 | 0 | 1 | 1 | 0 | 0 | 0 | N | 21708 | Large ribosomal subunit protein bL12m OS=Mus musculus OX=10090 GN=Mrpl12 PE=1 SV=2 |
| 2128 | 3530 | sp\|P97872\|FMO5\_MOUSE | 58.92 | 3 | 0 | 0 | 0 | 0 | 0 | 3 | 2 | 0 | 0 | 0 |  |  |  |  |  | 4.81E6 | 2.19E6 |  |  |  | 2 | 2 | 0 | 0 | 0 | 0 | 0 | 2 | 1 | 0 | 0 | 0 | N | 60001 | Flavin-containing monooxygenase 5 OS=Mus musculus OX=10090 GN=Fmo5 PE=1 SV=4 |
| 2170 | 5337 | sp\|O35114\|SCRB2\_MOUSE | 58.84 | 3 | 0 | 3 | 0 | 3 | 0 | 0 | 0 | 0 | 0 | 3 |  | 8.98E5 |  | 2.44E5 |  |  |  |  |  | 2.13E5 | 1 | 1 | 0 | 1 | 0 | 1 | 0 | 0 | 0 | 0 | 0 | 1 | Y | 54044 | Lysosome membrane protein 2 OS=Mus musculus OX=10090 GN=Scarb2 PE=1 SV=3 |
| 1489 | 3996 | sp\|P60229\|EIF3E\_MOUSE | 58.83 | 3 | 0 | 3 | 3 | 3 | 0 | 3 | 3 | 0 | 0 | 3 |  | 5.37E5 | 2.48E5 | 1.24E5 |  | 8.14E5 | 5.49E5 |  |  | 4.99E5 | 1 | 1 | 0 | 1 | 1 | 1 | 0 | 2 | 1 | 0 | 0 | 1 | N | 52221 | Eukaryotic translation initiation factor 3 subunit E OS=Mus musculus OX=10090 GN=Eif3e PE=1 SV=1 |
| 2300 | 3224 | sp\|Q3TXS7\|PSMD1\_MOUSE | 58.65 | 2 | 0 | 0 | 0 | 0 | 2 | 0 | 0 | 0 | 0 | 2 |  |  |  |  | 5.51E5 |  |  |  |  | 1.43E6 | 1 | 1 | 0 | 0 | 0 | 0 | 1 | 0 | 0 | 0 | 0 | 1 | N | 105730 | 26S proteasome non-ATPase regulatory subunit 1 OS=Mus musculus OX=10090 GN=Psmd1 PE=1 SV=1 |
| 2300 | 5335 | J3QN38\|J3QN38\_MOUSE | 58.65 | 19 | 0 | 0 | 0 | 0 | 19 | 0 | 0 | 0 | 0 | 19 |  |  |  |  | 5.51E5 |  |  |  |  | 1.43E6 | 1 | 1 | 0 | 0 | 0 | 0 | 1 | 0 | 0 | 0 | 0 | 1 | N | 11238 | Predicted gene 21972 (Fragment) OS=Mus musculus OX=10090 GN=Gm21972 PE=4 SV=1 |
| 2129 | 4072 | sp\|Q9D172\|GAL3A\_MOUSE | 58.48 | 7 | 0 | 0 | 0 | 0 | 0 | 3 | 0 | 4 | 0 | 0 |  |  |  |  |  | 5.47E6 |  | 1.16E6 |  |  | 2 | 2 | 0 | 0 | 0 | 0 | 0 | 1 | 0 | 1 | 0 | 0 | N | 28090 | Glutamine amidotransferase-like class 1 domain-containing protein 3, mitochondrial OS=Mus musculus OX=10090 GN=Gatd3 PE=1 SV=1 |
| 2509 | 4181 | sp\|Q99J45\|NRBP\_MOUSE | 58.41 | 3 | 3 | 0 | 0 | 0 | 0 | 0 | 0 | 0 | 0 | 0 | 1.12E6 |  |  |  |  |  |  |  |  |  | 1 | 1 | 1 | 0 | 0 | 0 | 0 | 0 | 0 | 0 | 0 | 0 | N | 59866 | Nuclear receptor-binding protein OS=Mus musculus OX=10090 GN=Nrbp1 PE=1 SV=1 |
| 2509 | 4182 | D3YUV1\|D3YUV1\_MOUSE | 58.41 | 3 | 3 | 0 | 0 | 0 | 0 | 0 | 0 | 0 | 0 | 0 | 1.12E6 |  |  |  |  |  |  |  |  |  | 1 | 1 | 1 | 0 | 0 | 0 | 0 | 0 | 0 | 0 | 0 | 0 | N | 60851 | Nuclear receptor binding protein 1 OS=Mus musculus OX=10090 GN=Nrbp1 PE=1 SV=1 |
| 2432 | 2801 | sp\|Q61166\|MARE1\_MOUSE | 58.38 | 5 | 0 | 0 | 2 | 0 | 0 | 0 | 3 | 0 | 0 | 0 |  |  | 1.92E6 |  |  |  | 3.68E5 |  |  |  | 2 | 2 | 0 | 0 | 1 | 0 | 0 | 0 | 1 | 0 | 0 | 0 | N | 30016 | Microtubule-associated protein RP/EB family member 1 OS=Mus musculus OX=10090 GN=Mapre1 PE=1 SV=3 |
| 2169 | 5331 | sp\|Q62433\|NDRG1\_MOUSE | 58.28 | 4 | 0 | 0 | 0 | 0 | 0 | 4 | 4 | 0 | 4 | 0 |  |  |  |  |  | 1.19E6 | 3.34E5 |  | 3.16E6 |  | 1 | 1 | 0 | 0 | 0 | 0 | 0 | 1 | 1 | 0 | 1 | 0 | N | 43009 | Protein NDRG1 OS=Mus musculus OX=10090 GN=Ndrg1 PE=1 SV=1 |
| 2141 | 5049 | sp\|Q9JMG1\|EDF1\_MOUSE | 58.26 | 6 | 0 | 0 | 0 | 0 | 6 | 0 | 6 | 6 | 0 | 0 |  |  |  |  | 5.88E5 |  | 2.67E5 | 2.52E5 |  |  | 1 | 1 | 0 | 0 | 0 | 0 | 1 | 0 | 1 | 1 | 0 | 0 | N | 16369 | Endothelial differentiation-related factor 1 OS=Mus musculus OX=10090 GN=Edf1 PE=1 SV=1 |
| 1211 | 2804 | A0A1D5RLM8\|A0A1D5RLM8\_MOUSE | 58.26 | 0 | 0 | 0 | 0 | 0 | 0 | 0 | 0 | 0 | 0 | 0 |  |  |  |  | 4.33E7 | 6.37E6 |  |  |  |  | 2 | 2 | 0 | 0 | 0 | 0 | 2 | 2 | 0 | 0 | 0 | 0 | N | 654216 | Predicted gene 11639 OS=Mus musculus OX=10090 GN=Efcab3 PE=1 SV=1 |
| 3291 | 4547 | sp\|P57716\|NICA\_MOUSE | 58.18 | 2 | 0 | 0 | 0 | 0 | 0 | 0 | 0 | 0 | 2 | 0 |  |  |  |  |  |  |  |  | 2.04E6 |  | 1 | 1 | 0 | 0 | 0 | 0 | 0 | 0 | 0 | 0 | 1 | 0 | N | 78492 | Nicastrin OS=Mus musculus OX=10090 GN=Ncstn PE=1 SV=3 |
| 1334 | 5350 | H7BWZ1\|H7BWZ1\_MOUSE | 58.14 | 26 | 26 | 26 | 26 | 26 | 26 | 26 | 26 | 26 | 26 | 26 | 2.36E6 | 5.94E6 | 4.51E6 |  | 2.6E6 | 1.87E6 | 2.96E6 | 1.97E6 | 3.39E6 | 2.55E6 | 1 | 1 | 1 | 1 | 1 | 1 | 1 | 1 | 1 | 1 | 1 | 1 | N | 6142 | Ubiquitin-fold modifier 1 OS=Mus musculus OX=10090 GN=Ufm1 PE=1 SV=1 |
| 1334 | 5351 | D3YW97\|D3YW97\_MOUSE | 58.14 | 18 | 18 | 18 | 18 | 18 | 18 | 18 | 18 | 18 | 18 | 18 | 2.36E6 | 5.94E6 | 4.51E6 |  | 2.6E6 | 1.87E6 | 2.96E6 | 1.97E6 | 3.39E6 | 2.55E6 | 1 | 1 | 1 | 1 | 1 | 1 | 1 | 1 | 1 | 1 | 1 | 1 | N | 8770 | Ubiquitin-fold modifier 1 OS=Mus musculus OX=10090 GN=Ufm1 PE=1 SV=1 |
| 1334 | 5352 | sp\|P61961\|UFM1\_MOUSE | 58.14 | 18 | 18 | 18 | 18 | 18 | 18 | 18 | 18 | 18 | 18 | 18 | 2.36E6 | 5.94E6 | 4.51E6 |  | 2.6E6 | 1.87E6 | 2.96E6 | 1.97E6 | 3.39E6 | 2.55E6 | 1 | 1 | 1 | 1 | 1 | 1 | 1 | 1 | 1 | 1 | 1 | 1 | N | 9118 | Ubiquitin-fold modifier 1 OS=Mus musculus OX=10090 GN=Ufm1 PE=1 SV=1 |
| 1289 | 4335 | sp\|P11031\|TCP4\_MOUSE | 58.06 | 9 | 9 | 9 | 9 | 9 | 9 | 9 | 9 | 9 | 0 | 9 | 1.01E7 | 9.79E6 | 1.29E7 | 4.13E6 | 1.01E7 | 7.33E6 | 6.56E6 | 6.61E6 |  | 7.85E6 | 1 | 1 | 1 | 1 | 1 | 1 | 1 | 1 | 1 | 1 | 0 | 1 | N | 14427 | Activated RNA polymerase II transcriptional coactivator p15 OS=Mus musculus OX=10090 GN=Sub1 PE=1 SV=3 |
| 2145 | 5458 | sp\|Q8CGA3\|LAT4\_MOUSE | 58.05 | 2 | 0 | 0 | 0 | 2 | 0 | 0 | 2 | 2 | 0 | 0 |  |  |  | 2.14E5 |  |  | 1.76E5 | 2.89E5 |  |  | 1 | 1 | 0 | 0 | 0 | 1 | 0 | 0 | 1 | 1 | 0 | 0 | N | 62430 | Large neutral amino acids transporter small subunit 4 OS=Mus musculus OX=10090 GN=Slc43a2 PE=1 SV=1 |
| 1622 | 4175 | sp\|Q9DB16\|CB39L\_MOUSE | 57.62 | 3 | 0 | 0 | 3 | 0 | 0 | 3 | 0 | 3 | 3 | 3 |  |  | 1.37E6 |  |  | 5.08E5 |  | 2.66E5 | 2.93E6 | 1.33E6 | 1 | 1 | 0 | 0 | 1 | 0 | 0 | 1 | 0 | 1 | 1 | 1 | N | 39106 | Calcium-binding protein 39-like OS=Mus musculus OX=10090 GN=Cab39l PE=1 SV=3 |
| 1724 | 2838 | sp\|Q8JZQ2\|AFG32\_MOUSE | 57.61 | 1 | 0 | 0 | 0 | 0 | 0 | 1 | 0 | 0 | 1 | 0 |  |  |  |  |  | 4.16E6 |  |  | 1.08E6 |  | 1 | 1 | 0 | 0 | 0 | 0 | 0 | 1 | 0 | 0 | 1 | 0 | N | 89519 | Mitochondrial inner membrane m-AAA protease component AFG3L2 OS=Mus musculus OX=10090 GN=Afg3l2 PE=1 SV=1 |
| 2556 | 5360 | Q6PBC0\|Q6PBC0\_MOUSE | 57.58 | 9 | 0 | 0 | 0 | 0 | 9 | 9 | 0 | 0 | 0 | 0 |  |  |  |  | 0E0 | 1.46E6 |  |  |  |  | 1 | 1 | 0 | 0 | 0 | 0 | 1 | 1 | 0 | 0 | 0 | 0 | N | 14931 | phosphatidate cytidylyltransferase OS=Mus musculus OX=10090 GN=Cds2 PE=1 SV=1 |
| 2556 | 5376 | A2AMQ5\|A2AMQ5\_MOUSE | 57.58 | 3 | 0 | 0 | 0 | 0 | 3 | 3 | 0 | 0 | 0 | 0 |  |  |  |  | 0E0 | 1.46E6 |  |  |  |  | 1 | 1 | 0 | 0 | 0 | 0 | 1 | 1 | 0 | 0 | 0 | 0 | N | 49294 | Phosphatidate cytidylyltransferase OS=Mus musculus OX=10090 GN=Cds2 PE=1 SV=1 |
| 2556 | 5377 | sp\|Q99L43\|CDS2\_MOUSE | 57.58 | 3 | 0 | 0 | 0 | 0 | 3 | 3 | 0 | 0 | 0 | 0 |  |  |  |  | 0E0 | 1.46E6 |  |  |  |  | 1 | 1 | 0 | 0 | 0 | 0 | 1 | 1 | 0 | 0 | 0 | 0 | N | 51314 | Phosphatidate cytidylyltransferase 2 OS=Mus musculus OX=10090 GN=Cds2 PE=1 SV=1 |
| 3442 | 5519 | H3BJ97\|H3BJ97\_MOUSE | 57.57 | 2 | 0 | 0 | 0 | 0 | 0 | 2 | 0 | 0 | 0 | 0 |  |  |  |  |  | 0E0 |  |  |  |  | 1 | 1 | 0 | 0 | 0 | 0 | 0 | 1 | 0 | 0 | 0 | 0 | N | 49277 | Tubulointerstitial nephritis antigen-like 1 OS=Mus musculus OX=10090 GN=Tinagl1 PE=1 SV=1 |
| 3442 | 5527 | sp\|Q99JR5\|TINAL\_MOUSE | 57.57 | 2 | 0 | 0 | 0 | 0 | 0 | 2 | 0 | 0 | 0 | 0 |  |  |  |  |  | 0E0 |  |  |  |  | 1 | 1 | 0 | 0 | 0 | 0 | 0 | 1 | 0 | 0 | 0 | 0 | N | 52665 | Tubulointerstitial nephritis antigen-like OS=Mus musculus OX=10090 GN=Tinagl1 PE=1 SV=1 |
| 1335 | 5388 | sp\|P48771\|CX7A2\_MOUSE | 57.20 | 12 | 12 | 12 | 12 | 12 | 12 | 12 | 12 | 12 | 12 | 12 | 1.93E7 | 2.67E7 | 2.82E7 | 1.23E7 | 2.1E7 | 2.35E7 | 1.9E7 | 1.5E7 | 2.76E7 | 1.82E7 | 1 | 1 | 1 | 1 | 1 | 1 | 1 | 1 | 1 | 1 | 1 | 1 | N | 9291 | Cytochrome c oxidase subunit 7A2, mitochondrial OS=Mus musculus OX=10090 GN=Cox7a2 PE=1 SV=2 |
| 2075 | 2607 | E9PYI8\|E9PYI8\_MOUSE | 57.15 | 6 | 0 | 0 | 0 | 0 | 0 | 0 | 0 | 0 | 6 | 0 |  |  |  |  |  |  |  |  | 3.61E6 |  | 2 | 2 | 0 | 0 | 0 | 0 | 0 | 0 | 0 | 0 | 2 | 0 | N | 52319 | Ubiquitin carboxyl-terminal hydrolase OS=Mus musculus OX=10090 GN=Usp14 PE=1 SV=1 |
| 2075 | 2609 | sp\|Q9JMA1\|UBP14\_MOUSE | 57.15 | 5 | 0 | 0 | 0 | 0 | 0 | 0 | 0 | 0 | 5 | 0 |  |  |  |  |  |  |  |  | 3.61E6 |  | 2 | 2 | 0 | 0 | 0 | 0 | 0 | 0 | 0 | 0 | 2 | 0 | N | 56002 | Ubiquitin carboxyl-terminal hydrolase 14 OS=Mus musculus OX=10090 GN=Usp14 PE=1 SV=3 |
| 3439 | 5370 | A0A2R8VHE5\|A0A2R8VHE5\_MOUSE | 57.04 | 24 | 24 | 0 | 0 | 0 | 0 | 0 | 0 | 0 | 0 | 0 | 4.07E5 |  |  |  |  |  |  |  |  |  | 1 | 1 | 1 | 0 | 0 | 0 | 0 | 0 | 0 | 0 | 0 | 0 | N | 6316 | Mercaptopyruvate sulfurtransferase (Fragment) OS=Mus musculus OX=10090 GN=Mpst PE=1 SV=1 |
| 3439 | 5372 | A0A2R8VHT1\|A0A2R8VHT1\_MOUSE | 57.04 | 8 | 8 | 0 | 0 | 0 | 0 | 0 | 0 | 0 | 0 | 0 | 4.07E5 |  |  |  |  |  |  |  |  |  | 1 | 1 | 1 | 0 | 0 | 0 | 0 | 0 | 0 | 0 | 0 | 0 | N | 18977 | Mercaptopyruvate sulfurtransferase (Fragment) OS=Mus musculus OX=10090 GN=Mpst PE=1 SV=1 |
| 3439 | 5373 | sp\|Q99J99\|THTM\_MOUSE | 57.04 | 4 | 4 | 0 | 0 | 0 | 0 | 0 | 0 | 0 | 0 | 0 | 4.07E5 |  |  |  |  |  |  |  |  |  | 1 | 1 | 1 | 0 | 0 | 0 | 0 | 0 | 0 | 0 | 0 | 0 | N | 33097 | 3-mercaptopyruvate sulfurtransferase OS=Mus musculus OX=10090 GN=Mpst PE=1 SV=4 |
| 3438 | 5355 | sp\|P27784\|CCL6\_MOUSE | 56.82 | 11 | 0 | 0 | 0 | 0 | 0 | 0 | 0 | 0 | 11 | 0 |  |  |  |  |  |  |  |  | 1.55E6 |  | 1 | 1 | 0 | 0 | 0 | 0 | 0 | 0 | 0 | 0 | 1 | 0 | Y | 12984 | C-C motif chemokine 6 OS=Mus musculus OX=10090 GN=Ccl6 PE=1 SV=1 |
| 2171 | 5365 | sp\|Q4FZG7\|TI8AB\_MOUSE | 56.70 | 11 | 0 | 11 | 0 | 0 | 0 | 11 | 0 | 0 | 0 | 11 |  | 4.63E5 |  |  |  | 8.19E5 |  |  |  | 1.28E6 | 1 | 1 | 0 | 1 | 0 | 0 | 0 | 1 | 0 | 0 | 0 | 1 | N | 11283 | Putative mitochondrial import inner membrane translocase subunit Tim8 A-B OS=Mus musculus OX=10090 GN=Timm8a2 PE=3 SV=1 |
| 2171 | 5366 | sp\|Q9WVA2\|TIM8A\_MOUSE | 56.70 | 11 | 0 | 11 | 0 | 0 | 0 | 11 | 0 | 0 | 0 | 11 |  | 4.63E5 |  |  |  | 8.19E5 |  |  |  | 1.28E6 | 1 | 1 | 0 | 1 | 0 | 0 | 0 | 1 | 0 | 0 | 0 | 1 | N | 11042 | Mitochondrial import inner membrane translocase subunit Tim8 A OS=Mus musculus OX=10090 GN=Timm8a1 PE=1 SV=1 |
| 3288 | 4401 | sp\|Q99KV1\|DJB11\_MOUSE | 56.27 | 4 | 0 | 0 | 0 | 0 | 0 | 0 | 0 | 0 | 4 | 0 |  |  |  |  |  |  |  |  | 3.71E6 |  | 1 | 1 | 0 | 0 | 0 | 0 | 0 | 0 | 0 | 0 | 1 | 0 | N | 40555 | DnaJ homolog subfamily B member 11 OS=Mus musculus OX=10090 GN=Dnajb11 PE=1 SV=1 |
| 3294 | 4713 | sp\|Q9D2R0\|AACS\_MOUSE | 56.26 | 1 | 0 | 0 | 0 | 0 | 0 | 0 | 0 | 0 | 1 | 0 |  |  |  |  |  |  |  |  | 3.59E5 |  | 1 | 1 | 0 | 0 | 0 | 0 | 0 | 0 | 0 | 0 | 1 | 0 | N | 75200 | Acetoacetyl-CoA synthetase OS=Mus musculus OX=10090 GN=Aacs PE=1 SV=1 |
| 1752 | 5391 | sp\|Q60766\|IRGM1\_MOUSE | 56.24 | 3 | 0 | 3 | 0 | 0 | 3 | 3 | 3 | 0 | 0 | 3 |  | 2.03E5 |  |  | 5.31E5 | 4.59E5 | 2.6E5 |  |  | 5.78E5 | 1 | 1 | 0 | 1 | 0 | 0 | 1 | 1 | 1 | 0 | 0 | 1 | N | 46552 | Immunity-related GTPase family M protein 1 OS=Mus musculus OX=10090 GN=Irgm1 PE=1 SV=1 |
| 1051 | 4315 | sp\|O88444\|ADCY1\_MOUSE | 56.16 | 1 | 0 | 0 | 0 | 0 | 0 | 0 | 0 | 1 | 0 | 0 |  |  |  |  |  |  |  | 3.72E6 |  |  | 2 | 1 | 1 | 0 | 0 | 3 | 3 | 1 | 4 | 2 | 1 | 0 | N | 123373 | Adenylate cyclase type 1 OS=Mus musculus OX=10090 GN=Adcy1 PE=1 SV=2 |
| 1915 | 5443 | S4R2J8\|S4R2J8\_MOUSE | 56.05 | 2 | 2 | 0 | 0 | 0 | 0 | 0 | 0 | 0 | 2 | 2 | 3.1E5 |  |  |  |  |  |  |  | 1.17E6 | 1.99E5 | 1 | 1 | 1 | 0 | 0 | 0 | 0 | 0 | 0 | 0 | 2 | 1 | N | 50892 | Bridging integrator 2 OS=Mus musculus OX=10090 GN=Bin2 PE=1 SV=1 |
| 1915 | 5445 | S4R1B8\|S4R1B8\_MOUSE | 56.05 | 2 | 2 | 0 | 0 | 0 | 0 | 0 | 0 | 0 | 2 | 2 | 3.1E5 |  |  |  |  |  |  |  | 1.17E6 | 1.99E5 | 1 | 1 | 1 | 0 | 0 | 0 | 0 | 0 | 0 | 0 | 2 | 1 | N | 51582 | Bridging integrator 2 OS=Mus musculus OX=10090 GN=Bin2 PE=1 SV=1 |
| 1915 | 5449 | sp\|D3Z6Q9\|BIN2\_MOUSE | 56.05 | 2 | 2 | 0 | 0 | 0 | 0 | 0 | 0 | 0 | 2 | 2 | 3.1E5 |  |  |  |  |  |  |  | 1.17E6 | 1.99E5 | 1 | 1 | 1 | 0 | 0 | 0 | 0 | 0 | 0 | 0 | 2 | 1 | N | 52554 | Bridging integrator 2 OS=Mus musculus OX=10090 GN=Bin2 PE=1 SV=1 |
| 1915 | 5452 | S4R270\|S4R270\_MOUSE | 56.05 | 2 | 2 | 0 | 0 | 0 | 0 | 0 | 0 | 0 | 2 | 2 | 3.1E5 |  |  |  |  |  |  |  | 1.17E6 | 1.99E5 | 1 | 1 | 1 | 0 | 0 | 0 | 0 | 0 | 0 | 0 | 2 | 1 | N | 54767 | Bridging integrator 2 OS=Mus musculus OX=10090 GN=Bin2 PE=1 SV=1 |
| 1915 | 5454 | A0A5F8MQ05\|A0A5F8MQ05\_MOUSE | 56.05 | 2 | 2 | 0 | 0 | 0 | 0 | 0 | 0 | 0 | 2 | 2 | 3.1E5 |  |  |  |  |  |  |  | 1.17E6 | 1.99E5 | 1 | 1 | 1 | 0 | 0 | 0 | 0 | 0 | 0 | 0 | 2 | 1 | N | 55808 | Bridging integrator 2 OS=Mus musculus OX=10090 GN=Bin2 PE=1 SV=1 |
| 2127 | 3353 | Z4YKM2\|Z4YKM2\_MOUSE | 56.05 | 15 | 0 | 6 | 0 | 0 | 0 | 9 | 0 | 0 | 0 | 0 |  | 5.96E6 |  |  |  | 1.1E6 |  |  |  |  | 2 | 2 | 0 | 1 | 0 | 0 | 0 | 1 | 0 | 0 | 0 | 0 | N | 13155 | CDGSH iron sulfur domain 3 OS=Mus musculus OX=10090 GN=Cisd3 PE=1 SV=1 |
| 2127 | 3354 | B2RWE3\|B2RWE3\_MOUSE | 56.05 | 13 | 0 | 5 | 0 | 0 | 0 | 7 | 0 | 0 | 0 | 0 |  | 5.96E6 |  |  |  | 1.1E6 |  |  |  |  | 2 | 2 | 0 | 1 | 0 | 0 | 0 | 1 | 0 | 0 | 0 | 0 | N | 14994 | CDGSH iron sulfur domain 3 OS=Mus musculus OX=10090 GN=Cisd3 PE=1 SV=1 |
| 2127 | 3355 | sp\|B1AR13\|CISD3\_MOUSE | 56.05 | 12 | 0 | 5 | 0 | 0 | 0 | 7 | 0 | 0 | 0 | 0 |  | 5.96E6 |  |  |  | 1.1E6 |  |  |  |  | 2 | 2 | 0 | 1 | 0 | 0 | 0 | 1 | 0 | 0 | 0 | 0 | N | 15676 | CDGSH iron-sulfur domain-containing protein 3, mitochondrial OS=Mus musculus OX=10090 GN=Cisd3 PE=1 SV=1 |
| 1868 | 3460 | sp\|Q8BPY9\|FIGL1\_MOUSE | 55.97 | 2 | 0 | 2 | 0 | 0 | 2 | 0 | 0 | 0 | 0 | 2 |  | 1.3E6 |  |  | 7.26E5 |  |  |  |  | 6.14E5 | 1 | 1 | 0 | 1 | 0 | 0 | 1 | 0 | 0 | 0 | 0 | 1 | N | 74851 | Fidgetin-like protein 1 OS=Mus musculus OX=10090 GN=Fignl1 PE=1 SV=1 |
| 1868 | 4168 | sp\|Q8VEJ9\|VPS4A\_MOUSE | 55.97 | 3 | 0 | 3 | 0 | 0 | 3 | 0 | 0 | 0 | 0 | 3 |  | 1.3E6 |  |  | 7.26E5 |  |  |  |  | 6.14E5 | 1 | 1 | 0 | 1 | 0 | 0 | 1 | 0 | 0 | 0 | 0 | 1 | N | 48907 | Vacuolar protein sorting-associated protein 4A OS=Mus musculus OX=10090 GN=Vps4a PE=1 SV=1 |
| 1868 | 4508 | sp\|P46467\|VPS4B\_MOUSE | 55.97 | 3 | 0 | 3 | 0 | 0 | 3 | 0 | 0 | 0 | 0 | 3 |  | 1.3E6 |  |  | 7.26E5 |  |  |  |  | 6.14E5 | 1 | 1 | 0 | 1 | 0 | 0 | 1 | 0 | 0 | 0 | 0 | 1 | N | 49419 | Vacuolar protein sorting-associated protein 4B OS=Mus musculus OX=10090 GN=Vps4b PE=1 SV=2 |
| 2555 | 5358 | sp\|Q9D7N3\|RT09\_MOUSE | 55.75 | 3 | 0 | 0 | 0 | 0 | 3 | 0 | 0 | 0 | 3 | 0 |  |  |  |  | 4.56E5 |  |  |  | 6.31E5 |  | 1 | 1 | 0 | 0 | 0 | 0 | 1 | 0 | 0 | 0 | 1 | 0 | N | 44929 | Small ribosomal subunit protein uS9m OS=Mus musculus OX=10090 GN=Mrps9 PE=1 SV=3 |
| 1935 | 5385 | sp\|Q9CXE7\|TMED5\_MOUSE | 55.69 | 5 | 0 | 0 | 0 | 5 | 5 | 0 | 5 | 5 | 0 | 0 |  |  |  | 2.21E5 | 6.8E5 |  | 5.9E5 | 1.65E5 |  |  | 1 | 1 | 0 | 0 | 0 | 1 | 1 | 0 | 1 | 1 | 0 | 0 | N | 26172 | Transmembrane emp24 domain-containing protein 5 OS=Mus musculus OX=10090 GN=Tmed5 PE=1 SV=1 |
| 2130 | 4085 | sp\|Q63850\|NUP62\_MOUSE | 55.62 | 3 | 0 | 0 | 0 | 0 | 0 | 0 | 0 | 0 | 3 | 3 |  |  |  |  |  |  |  |  | 3.37E6 | 0E0 | 1 | 1 | 0 | 0 | 0 | 0 | 0 | 0 | 0 | 0 | 1 | 1 | N | 53255 | Nuclear pore glycoprotein p62 OS=Mus musculus OX=10090 GN=Nup62 PE=1 SV=2 |
| 1310 | 2555 | sp\|Q9ES97\|RTN3\_MOUSE | 55.59 | 3 | 1 | 1 | 1 | 2 | 0 | 0 | 2 | 1 | 1 | 1 | 2.42E6 | 3.07E6 | 2.22E6 | 2.69E6 |  |  | 5.05E6 | 2.22E6 | 4.54E6 | 3.52E6 | 3 | 3 | 1 | 1 | 1 | 2 | 0 | 0 | 2 | 1 | 1 | 1 | N | 103879 | Reticulon-3 OS=Mus musculus OX=10090 GN=Rtn3 PE=1 SV=2 |
| 1934 | 5357 | sp\|P28667\|MRP\_MOUSE | 55.54 | 8 | 8 | 8 | 0 | 0 | 0 | 0 | 0 | 0 | 8 | 8 | 2.88E6 | 1.32E6 |  |  |  |  |  |  | 4.75E6 | 2.51E6 | 1 | 1 | 1 | 1 | 0 | 0 | 0 | 0 | 0 | 0 | 1 | 1 | N | 20165 | MARCKS-related protein OS=Mus musculus OX=10090 GN=Marcksl1 PE=1 SV=2 |
| 1538 | 5392 | A0A140LJ59\|A0A140LJ59\_MOUSE | 55.54 | 21 | 0 | 21 | 21 | 21 | 21 | 21 | 21 | 21 | 0 | 0 |  | 4.56E6 | 2.56E6 | 1.2E6 | 1.06E6 | 1.74E6 | 6.74E5 | 8.4E5 |  |  | 1 | 1 | 0 | 1 | 1 | 1 | 1 | 1 | 1 | 1 | 0 | 0 | N | 6088 | Eukaryotic translation initiation factor 3, subunit K OS=Mus musculus OX=10090 GN=Eif3k PE=1 SV=1 |
| 1538 | 5400 | sp\|Q9DBZ5\|EIF3K\_MOUSE | 55.54 | 5 | 0 | 5 | 5 | 5 | 5 | 5 | 5 | 5 | 0 | 0 |  | 4.56E6 | 2.56E6 | 1.2E6 | 1.06E6 | 1.74E6 | 6.74E5 | 8.4E5 |  |  | 1 | 1 | 0 | 1 | 1 | 1 | 1 | 1 | 1 | 1 | 0 | 0 | N | 25087 | Eukaryotic translation initiation factor 3 subunit K OS=Mus musculus OX=10090 GN=Eif3k PE=1 SV=1 |
| 1717 | 3476 | sp\|Q9Z2H5\|E41L1\_MOUSE | 55.50 | 1 | 0 | 0 | 1 | 0 | 0 | 1 | 0 | 0 | 0 | 1 |  |  | 5.84E6 |  |  | 5.04E6 |  |  |  | 3.05E6 | 1 | 1 | 0 | 0 | 1 | 0 | 0 | 1 | 0 | 0 | 0 | 1 | N | 98315 | Band 4.1-like protein 1 OS=Mus musculus OX=10090 GN=Epb41l1 PE=1 SV=2 |
| 1717 | 3477 | A2AUK5\|A2AUK5\_MOUSE | 55.50 | 1 | 0 | 0 | 1 | 0 | 0 | 1 | 0 | 0 | 0 | 1 |  |  | 5.84E6 |  |  | 5.04E6 |  |  |  | 3.05E6 | 1 | 1 | 0 | 0 | 1 | 0 | 0 | 1 | 0 | 0 | 0 | 1 | N | 98315 | Erythrocyte membrane protein band 4.1 like 1 OS=Mus musculus OX=10090 GN=Epb41l1 PE=1 SV=1 |
| 1717 | 3479 | E9PV14\|E9PV14\_MOUSE | 55.50 | 1 | 0 | 0 | 1 | 0 | 0 | 1 | 0 | 0 | 0 | 1 |  |  | 5.84E6 |  |  | 5.04E6 |  |  |  | 3.05E6 | 1 | 1 | 0 | 0 | 1 | 0 | 0 | 1 | 0 | 0 | 0 | 1 | N | 169891 | Erythrocyte membrane protein band 4.1 like 1 OS=Mus musculus OX=10090 GN=Epb41l1 PE=1 SV=2 |
| 1607 | 3485 | sp\|Q9CYH2\|PXL2A\_MOUSE | 55.42 | 5 | 0 | 0 | 5 | 5 | 5 | 5 | 5 | 0 | 0 | 5 |  |  | 2.31E6 | 4.48E5 | 4.33E5 | 4.53E5 | 7.71E5 |  |  | 5.36E5 | 1 | 1 | 0 | 0 | 1 | 1 | 1 | 1 | 1 | 0 | 0 | 1 | N | 24395 | Peroxiredoxin-like 2A OS=Mus musculus OX=10090 GN=Prxl2a PE=1 SV=2 |
| 1607 | 3487 | Q3U125\|Q3U125\_MOUSE | 55.42 | 5 | 0 | 0 | 5 | 5 | 5 | 5 | 5 | 0 | 0 | 5 |  |  | 2.31E6 | 4.48E5 | 4.33E5 | 4.53E5 | 7.71E5 |  |  | 5.36E5 | 1 | 1 | 0 | 0 | 1 | 1 | 1 | 1 | 1 | 0 | 0 | 1 | N | 25682 | Peroxiredoxin-like 2A OS=Mus musculus OX=10090 GN=Prxl2a PE=1 SV=1 |
| 2944 | 4259 | M0QWP1\|M0QWP1\_MOUSE | 55.32 | 1 | 0 | 0 | 1 | 0 | 0 | 0 | 0 | 0 | 0 | 0 |  |  | 4.42E5 |  |  |  |  |  |  |  | 1 | 1 | 0 | 0 | 1 | 0 | 0 | 0 | 0 | 0 | 0 | 0 | N | 216647 | Agrin OS=Mus musculus OX=10090 GN=Agrn PE=1 SV=1 |
| 2944 | 4655 | Z4YJS5\|Z4YJS5\_MOUSE | 55.32 | 1 | 0 | 0 | 1 | 0 | 0 | 0 | 0 | 0 | 0 | 0 |  |  | 4.42E5 |  |  |  |  |  |  |  | 1 | 1 | 0 | 0 | 1 | 0 | 0 | 0 | 0 | 0 | 0 | 0 | N | 121883 | Follistatin OS=Mus musculus OX=10090 GN=Agrn PE=1 SV=1 |
| 2944 | 4658 | Z4YK85\|Z4YK85\_MOUSE | 55.32 | 1 | 0 | 0 | 1 | 0 | 0 | 0 | 0 | 0 | 0 | 0 |  |  | 4.42E5 |  |  |  |  |  |  |  | 1 | 1 | 0 | 0 | 1 | 0 | 0 | 0 | 0 | 0 | 0 | 0 | N | 204952 | Agrin OS=Mus musculus OX=10090 GN=Agrn PE=1 SV=1 |
| 2944 | 4660 | sp\|A2ASQ1\|AGRIN\_MOUSE | 55.32 | 1 | 0 | 0 | 1 | 0 | 0 | 0 | 0 | 0 | 0 | 0 |  |  | 4.42E5 |  |  |  |  |  |  |  | 1 | 1 | 0 | 0 | 1 | 0 | 0 | 0 | 0 | 0 | 0 | 0 | N | 207537 | Agrin OS=Mus musculus OX=10090 GN=Agrn PE=1 SV=1 |
| 2116 | 4576 | D3YTR5\|D3YTR5\_MOUSE | 55.11 | 6 | 6 | 0 | 0 | 0 | 0 | 6 | 0 | 0 | 6 | 0 | 1.01E6 |  |  |  |  | 3.39E5 |  |  | 1.75E6 |  | 1 | 1 | 1 | 0 | 0 | 0 | 0 | 1 | 0 | 0 | 1 | 0 | N | 29070 | Methyl-CpG binding domain protein 3 OS=Mus musculus OX=10090 GN=Mbd3 PE=1 SV=1 |
| 2116 | 4577 | D3YTR4\|D3YTR4\_MOUSE | 55.11 | 5 | 5 | 0 | 0 | 0 | 0 | 5 | 0 | 0 | 5 | 0 | 1.01E6 |  |  |  |  | 3.39E5 |  |  | 1.75E6 |  | 1 | 1 | 1 | 0 | 0 | 0 | 0 | 1 | 0 | 0 | 1 | 0 | N | 31276 | Methyl-CpG binding domain protein 3 OS=Mus musculus OX=10090 GN=Mbd3 PE=1 SV=1 |
| 2116 | 4578 | sp\|Q9Z2D8\|MBD3\_MOUSE | 55.11 | 5 | 5 | 0 | 0 | 0 | 0 | 5 | 0 | 0 | 5 | 0 | 1.01E6 |  |  |  |  | 3.39E5 |  |  | 1.75E6 |  | 1 | 1 | 1 | 0 | 0 | 0 | 0 | 1 | 0 | 0 | 1 | 0 | N | 32168 | Methyl-CpG-binding domain protein 3 OS=Mus musculus OX=10090 GN=Mbd3 PE=1 SV=1 |
| 2116 | 4805 | F6WZH1\|F6WZH1\_MOUSE | 55.11 | 8 | 8 | 0 | 0 | 0 | 0 | 8 | 0 | 0 | 8 | 0 | 1.01E6 |  |  |  |  | 3.39E5 |  |  | 1.75E6 |  | 1 | 1 | 1 | 0 | 0 | 0 | 0 | 1 | 0 | 0 | 1 | 0 | N | 20564 | Methyl-CpG binding domain protein 3 (Fragment) OS=Mus musculus OX=10090 GN=Mbd3 PE=1 SV=1 |
| 2544 | 4215 | D3Z0B9\|D3Z0B9\_MOUSE | 55.07 | 2 | 0 | 0 | 0 | 0 | 0 | 2 | 0 | 0 | 0 | 0 |  |  |  |  |  | 2.14E5 |  |  |  |  | 1 | 1 | 0 | 0 | 0 | 0 | 0 | 1 | 0 | 0 | 0 | 0 | N | 79489 | Aldehyde dehydrogenase family 16 member A1 OS=Mus musculus OX=10090 GN=Aldh16a1 PE=1 SV=3 |
| 2544 | 4216 | A0A1B0GSU0\|A0A1B0GSU0\_MOUSE | 55.07 | 2 | 0 | 0 | 0 | 0 | 0 | 2 | 0 | 0 | 0 | 0 |  |  |  |  |  | 2.14E5 |  |  |  |  | 1 | 1 | 0 | 0 | 0 | 0 | 0 | 1 | 0 | 0 | 0 | 0 | N | 84798 | Aldehyde dehydrogenase family 16 member A1 OS=Mus musculus OX=10090 GN=Aldh16a1 PE=1 SV=1 |
| 2544 | 4217 | sp\|Q571I9\|A16A1\_MOUSE | 55.07 | 2 | 0 | 0 | 0 | 0 | 0 | 2 | 0 | 0 | 0 | 0 |  |  |  |  |  | 2.14E5 |  |  |  |  | 1 | 1 | 0 | 0 | 0 | 0 | 0 | 1 | 0 | 0 | 0 | 0 | N | 84756 | Aldehyde dehydrogenase family 16 member A1 OS=Mus musculus OX=10090 GN=Aldh16a1 PE=1 SV=2 |
| 1389 | 4665 | Q3UA72\|Q3UA72\_MOUSE | 55.05 | 13 | 13 | 13 | 0 | 13 | 13 | 13 | 13 | 13 | 13 | 0 | 1.13E7 | 9.54E6 |  | 3.75E6 | 6.65E6 | 5.99E6 | 5.29E6 | 4.95E6 | 2.47E7 |  | 1 | 1 | 1 | 1 | 0 | 1 | 1 | 1 | 1 | 1 | 2 | 0 | N | 9696 | Actin-related protein 2/3 complex subunit 5 OS=Mus musculus OX=10090 GN=Arpc5 PE=1 SV=1 |
| 1389 | 4666 | sp\|Q9CPW4\|ARPC5\_MOUSE | 55.05 | 8 | 8 | 8 | 0 | 8 | 8 | 8 | 8 | 8 | 8 | 0 | 1.13E7 | 9.54E6 |  | 3.75E6 | 6.65E6 | 5.99E6 | 5.29E6 | 4.95E6 | 2.47E7 |  | 1 | 1 | 1 | 1 | 0 | 1 | 1 | 1 | 1 | 1 | 2 | 0 | N | 16288 | Actin-related protein 2/3 complex subunit 5 OS=Mus musculus OX=10090 GN=Arpc5 PE=1 SV=3 |
| 3113 | 4793 | A0A494B985\|A0A494B985\_MOUSE | 55.03 | 7 | 0 | 0 | 7 | 0 | 0 | 0 | 0 | 0 | 0 | 0 |  |  | 1.17E6 |  |  |  |  |  |  |  | 1 | 1 | 0 | 0 | 1 | 0 | 0 | 0 | 0 | 0 | 0 | 0 | N | 30492 | Arsenite methyltransferase OS=Mus musculus OX=10090 GN=As3mt PE=1 SV=1 |
| 3113 | 4794 | sp\|Q91WU5\|AS3MT\_MOUSE | 55.03 | 5 | 0 | 0 | 5 | 0 | 0 | 0 | 0 | 0 | 0 | 0 |  |  | 1.17E6 |  |  |  |  |  |  |  | 1 | 1 | 0 | 0 | 1 | 0 | 0 | 0 | 0 | 0 | 0 | 0 | N | 41794 | Arsenite methyltransferase OS=Mus musculus OX=10090 GN=As3mt PE=1 SV=2 |
| 1337 | 5437 | D3Z3Y2\|D3Z3Y2\_MOUSE | 54.93 | 12 | 12 | 12 | 12 | 12 | 12 | 12 | 12 | 12 | 12 | 12 | 7.52E6 | 1.57E7 | 1.45E7 | 6.04E6 | 5.82E6 | 9.18E6 | 8.03E6 | 3.44E6 | 6.46E6 | 6.95E6 | 1 | 1 | 1 | 1 | 1 | 1 | 1 | 1 | 1 | 1 | 1 | 1 | N | 8251 | Small integral membrane protein 24 OS=Mus musculus OX=10090 GN=Smim24 PE=1 SV=1 |
| 1337 | 5460 | sp\|Q0VG18\|SIM24\_MOUSE | 54.93 | 8 | 8 | 8 | 8 | 8 | 8 | 8 | 8 | 8 | 8 | 8 | 7.52E6 | 1.57E7 | 1.45E7 | 6.04E6 | 5.82E6 | 9.18E6 | 8.03E6 | 3.44E6 | 6.46E6 | 6.95E6 | 1 | 1 | 1 | 1 | 1 | 1 | 1 | 1 | 1 | 1 | 1 | 1 | N | 13547 | Small integral membrane protein 24 OS=Mus musculus OX=10090 GN=Smim24 PE=1 SV=1 |
| 2558 | 5416 | sp\|Q8K4L4\|POF1B\_MOUSE | 54.68 | 2 | 0 | 0 | 0 | 0 | 2 | 0 | 0 | 0 | 0 | 2 |  |  |  |  | 3.12E5 |  |  |  |  | 2.66E5 | 1 | 1 | 0 | 0 | 0 | 0 | 1 | 0 | 0 | 0 | 0 | 1 | N | 67785 | Protein POF1B OS=Mus musculus OX=10090 GN=Pof1b PE=2 SV=3 |
| 1445 | 3141 | sp\|Q9CZU3\|MTREX\_MOUSE | 54.65 | 3 | 1 | 1 | 3 | 0 | 1 | 1 | 2 | 0 | 1 | 0 | 4.71E6 | 5.04E6 | 7.35E6 |  | 6.51E6 | 7.6E6 | 3.69E5 |  | 4.37E6 |  | 2 | 2 | 1 | 1 | 2 | 0 | 1 | 1 | 1 | 0 | 1 | 0 | Y | 117636 | Exosome RNA helicase MTR4 OS=Mus musculus OX=10090 GN=Mtrex PE=1 SV=1 |
| 1537 | 4748 | sp\|Q9JLV1\|BAG3\_MOUSE | 54.55 | 2 | 2 | 0 | 2 | 2 | 0 | 2 | 0 | 2 | 2 | 2 | 1.22E6 |  | 2.66E5 | 2.41E5 |  | 2.58E6 |  | 9.69E5 | 1.86E6 | 1.65E6 | 1 | 1 | 1 | 0 | 1 | 1 | 0 | 1 | 0 | 1 | 1 | 1 | N | 61860 | BAG family molecular chaperone regulator 3 OS=Mus musculus OX=10090 GN=Bag3 PE=1 SV=2 |
| 2082 | 3921 | Q921G9\|Q921G9\_MOUSE | 54.54 | 2 | 0 | 2 | 0 | 0 | 2 | 0 | 0 | 0 | 0 | 2 |  | 9.51E5 |  |  | 2.05E5 |  |  |  |  | 2.98E5 | 1 | 1 | 0 | 1 | 0 | 0 | 1 | 0 | 0 | 0 | 0 | 1 | N | 99169 | Tight junction protein 3 OS=Mus musculus OX=10090 GN=Tjp3 PE=1 SV=1 |
| 2082 | 3922 | sp\|Q9QXY1\|ZO3\_MOUSE | 54.54 | 2 | 0 | 2 | 0 | 0 | 2 | 0 | 0 | 0 | 0 | 2 |  | 9.51E5 |  |  | 2.05E5 |  |  |  |  | 2.98E5 | 1 | 1 | 0 | 1 | 0 | 0 | 1 | 0 | 0 | 0 | 0 | 1 | N | 99324 | Tight junction protein ZO-3 OS=Mus musculus OX=10090 GN=Tjp3 PE=1 SV=1 |
| 2136 | 4768 | Q6P8Q0\|Q6P8Q0\_MOUSE | 54.44 | 5 | 0 | 0 | 5 | 0 | 5 | 0 | 0 | 0 | 5 | 0 |  |  | 2.95E5 |  | 7.41E5 |  |  |  | 3.66E6 |  | 1 | 1 | 0 | 0 | 1 | 0 | 1 | 0 | 0 | 0 | 1 | 0 | N | 25582 | Glutathione S-transferase OS=Mus musculus OX=10090 GN=Gsta13 PE=2 SV=1 |
| 2136 | 4769 | sp\|P13745\|GSTA1\_MOUSE | 54.44 | 5 | 0 | 0 | 5 | 0 | 5 | 0 | 0 | 0 | 5 | 0 |  |  | 2.95E5 |  | 7.41E5 |  |  |  | 3.66E6 |  | 1 | 1 | 0 | 0 | 1 | 0 | 1 | 0 | 0 | 0 | 1 | 0 | N | 25608 | Glutathione S-transferase A1 OS=Mus musculus OX=10090 GN=Gsta1 PE=1 SV=2 |
| 2136 | 5390 | A0A087WQI6\|A0A087WQI6\_MOUSE | 54.44 | 8 | 0 | 0 | 8 | 0 | 8 | 0 | 0 | 0 | 8 | 0 |  |  | 2.95E5 |  | 7.41E5 |  |  |  | 3.66E6 |  | 1 | 1 | 0 | 0 | 1 | 0 | 1 | 0 | 0 | 0 | 1 | 0 | N | 15369 | Glutathione S-transferase (Fragment) OS=Mus musculus OX=10090 GN=Gsta3 PE=1 SV=1 |
| 2136 | 5394 | D3Z6A6\|D3Z6A6\_MOUSE | 54.44 | 6 | 0 | 0 | 6 | 0 | 6 | 0 | 0 | 0 | 6 | 0 |  |  | 2.95E5 |  | 7.41E5 |  |  |  | 3.66E6 |  | 1 | 1 | 0 | 0 | 1 | 0 | 1 | 0 | 0 | 0 | 1 | 0 | N | 20581 | Glutathione S-transferase (Fragment) OS=Mus musculus OX=10090 GN=Gsta2 PE=3 SV=1 |
| 2136 | 5395 | D3YZV3\|D3YZV3\_MOUSE | 54.44 | 6 | 0 | 0 | 6 | 0 | 6 | 0 | 0 | 0 | 6 | 0 |  |  | 2.95E5 |  | 7.41E5 |  |  |  | 3.66E6 |  | 1 | 1 | 0 | 0 | 1 | 0 | 1 | 0 | 0 | 0 | 1 | 0 | N | 20936 | Glutathione S-transferase (Fragment) OS=Mus musculus OX=10090 GN=Gsta2 PE=1 SV=1 |
| 2136 | 5397 | sp\|P30115\|GSTA3\_MOUSE | 54.44 | 5 | 0 | 0 | 5 | 0 | 5 | 0 | 0 | 0 | 5 | 0 |  |  | 2.95E5 |  | 7.41E5 |  |  |  | 3.66E6 |  | 1 | 1 | 0 | 0 | 1 | 0 | 1 | 0 | 0 | 0 | 1 | 0 | N | 25361 | Glutathione S-transferase A3 OS=Mus musculus OX=10090 GN=Gsta3 PE=1 SV=2 |
| 2136 | 5398 | sp\|P10648\|GSTA2\_MOUSE | 54.44 | 5 | 0 | 0 | 5 | 0 | 5 | 0 | 0 | 0 | 5 | 0 |  |  | 2.95E5 |  | 7.41E5 |  |  |  | 3.66E6 |  | 1 | 1 | 0 | 0 | 1 | 0 | 1 | 0 | 0 | 0 | 1 | 0 | N | 25542 | Glutathione S-transferase A2 OS=Mus musculus OX=10090 GN=Gsta2 PE=1 SV=3 |
| 2468 | 3040 | sp\|Q3V3R1\|C1TM\_MOUSE | 54.41 | 2 | 0 | 0 | 1 | 0 | 0 | 0 | 0 | 0 | 0 | 1 |  |  | 1.14E5 |  |  |  |  |  |  | 0E0 | 2 | 2 | 0 | 0 | 1 | 0 | 0 | 0 | 0 | 0 | 0 | 1 | N | 105729 | Monofunctional C1-tetrahydrofolate synthase, mitochondrial OS=Mus musculus OX=10090 GN=Mthfd1l PE=1 SV=2 |
| 1435 | 3492 | E9Q242\|E9Q242\_MOUSE | 54.16 | 2 | 0 | 0 | 2 | 2 | 2 | 2 | 2 | 2 | 2 | 0 |  |  | 2.28E6 | 7E5 | 2.31E6 | 2.4E6 | 1.57E6 | 1.49E6 | 3.91E6 |  | 1 | 1 | 0 | 0 | 1 | 1 | 1 | 1 | 1 | 1 | 1 | 0 | N | 53130 | Adenylosuccinate lyase OS=Mus musculus OX=10090 GN=Adsl PE=1 SV=1 |
| 1435 | 3493 | sp\|P54822\|PUR8\_MOUSE | 54.16 | 2 | 0 | 0 | 2 | 2 | 2 | 2 | 2 | 2 | 2 | 0 |  |  | 2.28E6 | 7E5 | 2.31E6 | 2.4E6 | 1.57E6 | 1.49E6 | 3.91E6 |  | 1 | 1 | 0 | 0 | 1 | 1 | 1 | 1 | 1 | 1 | 1 | 0 | N | 54866 | Adenylosuccinate lyase OS=Mus musculus OX=10090 GN=Adsl PE=1 SV=2 |
| 840 | 4633 | A0A2I3BQA5\|A0A2I3BQA5\_MOUSE | 54.09 | 1 | 0 | 0 | 1 | 0 | 0 | 0 | 0 | 0 | 0 | 0 |  |  | 1.9E6 |  |  |  |  |  |  |  | 2 | 1 | 2 | 2 | 3 | 2 | 2 | 2 | 2 | 3 | 2 | 2 | N | 147895 | Leucine-rich repeats and guanylate kinase domain containing OS=Mus musculus OX=10090 GN=Lrguk PE=1 SV=1 |
| 3011 | 4061 | A0A075B5P5\|A0A075B5P5\_MOUSE | 53.82 | 5 | 0 | 0 | 0 | 0 | 0 | 0 | 0 | 0 | 0 | 5 |  |  |  |  |  |  |  |  |  | 1.1E6 | 1 | 1 | 0 | 0 | 0 | 0 | 0 | 0 | 0 | 0 | 0 | 1 | N | 36317 | Immunoglobulin heavy constant gamma 3 (Fragment) OS=Mus musculus OX=10090 GN=Ighg3 PE=4 SV=1 |
| 3011 | 4062 | sp\|P03987\|IGHG3\_MOUSE | 53.82 | 4 | 0 | 0 | 0 | 0 | 0 | 0 | 0 | 0 | 0 | 4 |  |  |  |  |  |  |  |  |  | 1.1E6 | 1 | 1 | 0 | 0 | 0 | 0 | 0 | 0 | 0 | 0 | 0 | 1 | N | 43929 | Ig gamma-3 chain C region OS=Mus musculus OX=10090 PE=1 SV=2 |
| 3011 | 4063 | A0A1Y7VJN6\|A0A1Y7VJN6\_MOUSE | 53.82 | 4 | 0 | 0 | 0 | 0 | 0 | 0 | 0 | 0 | 0 | 4 |  |  |  |  |  |  |  |  |  | 1.1E6 | 1 | 1 | 0 | 0 | 0 | 0 | 0 | 0 | 0 | 0 | 0 | 1 | N | 44018 | Immunoglobulin heavy constant gamma 3 (Fragment) OS=Mus musculus OX=10090 GN=Ighg3 PE=4 SV=1 |
| 1509 | 3681 | sp\|P32233\|DRG1\_MOUSE | 53.62 | 3 | 3 | 0 | 3 | 3 | 3 | 0 | 3 | 3 | 3 | 0 | 7.12E5 |  | 8.52E5 | 1.4E5 | 5.38E5 |  | 4.71E5 | 5.27E5 | 8.32E5 |  | 1 | 1 | 1 | 0 | 1 | 1 | 1 | 0 | 1 | 1 | 1 | 0 | N | 40512 | Developmentally-regulated GTP-binding protein 1 OS=Mus musculus OX=10090 GN=Drg1 PE=1 SV=1 |
| 1909 | 4208 | sp\|Q9D8C4\|IN35\_MOUSE | 53.62 | 4 | 0 | 0 | 0 | 0 | 0 | 0 | 0 | 0 | 4 | 0 |  |  |  |  |  |  |  |  | 1.58E6 |  | 1 | 1 | 0 | 0 | 0 | 0 | 0 | 0 | 0 | 0 | 1 | 0 | N | 31876 | Interferon-induced 35 kDa protein homolog OS=Mus musculus OX=10090 GN=Ifi35 PE=1 SV=3 |
| 1342 | 2611 | sp\|E9PVX6\|KI67\_MOUSE | 53.12 | 0 | 0 | 0 | 0 | 0 | 0 | 0 | 0 | 0 | 0 | 0 |  |  |  |  |  | 2.08E6 |  |  |  | 3.33E6 | 1 | 1 | 0 | 0 | 0 | 0 | 0 | 1 | 0 | 0 | 0 | 1 | N | 350864 | Proliferation marker protein Ki-67 OS=Mus musculus OX=10090 GN=Mki67 PE=1 SV=1 |
| 1815 | 2776 | sp\|Q6P8I4\|PCNP\_MOUSE | 53.09 | 12 | 0 | 0 | 6 | 0 | 0 | 6 | 6 | 6 | 0 | 0 |  |  | 3.22E5 |  |  | 2.31E5 | 2.94E5 | 9.48E4 |  |  | 2 | 2 | 0 | 0 | 1 | 0 | 0 | 1 | 1 | 1 | 0 | 0 | N | 18963 | PEST proteolytic signal-containing nuclear protein OS=Mus musculus OX=10090 GN=Pcnp PE=1 SV=1 |
| 1815 | 2807 | Q3TM89\|Q3TM89\_MOUSE | 53.09 | 15 | 0 | 0 | 7 | 0 | 0 | 7 | 7 | 7 | 0 | 0 |  |  | 3.22E5 |  |  | 2.31E5 | 2.94E5 | 9.48E4 |  |  | 2 | 2 | 0 | 0 | 1 | 0 | 0 | 1 | 1 | 1 | 0 | 0 | N | 15562 | PEST proteolytic signal-containing nuclear protein OS=Mus musculus OX=10090 GN=Pcnp PE=1 SV=1 |
| 1851 | 2441 | sp\|Q8CGF7\|TCRG1\_MOUSE | 53.02 | 3 | 0 | 0 | 0 | 0 | 1 | 0 | 1 | 0 | 1 | 0 |  |  |  |  | 0E0 |  | 5.76E5 |  | 0E0 |  | 3 | 3 | 0 | 0 | 0 | 0 | 1 | 0 | 1 | 0 | 1 | 0 | N | 123788 | Transcription elongation regulator 1 OS=Mus musculus OX=10090 GN=Tcerg1 PE=1 SV=2 |
| 2066 | 3298 | sp\|Q9JIL4\|NHRF3\_MOUSE | 52.97 | 5 | 0 | 0 | 0 | 2 | 0 | 3 | 3 | 0 | 0 | 0 |  |  |  | 2.01E6 |  | 6.3E5 | 2.02E5 |  |  |  | 2 | 2 | 0 | 0 | 0 | 1 | 0 | 1 | 1 | 0 | 0 | 0 | N | 56499 | Na(+)/H(+) exchange regulatory cofactor NHE-RF3 OS=Mus musculus OX=10090 GN=Pdzk1 PE=1 SV=1 |
| 2066 | 3299 | A0A0R4J1V0\|A0A0R4J1V0\_MOUSE | 52.97 | 5 | 0 | 0 | 0 | 2 | 0 | 3 | 3 | 0 | 0 | 0 |  |  |  | 2.01E6 |  | 6.3E5 | 2.02E5 |  |  |  | 2 | 2 | 0 | 0 | 0 | 1 | 0 | 1 | 1 | 0 | 0 | 0 | N | 56498 | PDZ domain containing 1 OS=Mus musculus OX=10090 GN=Pdzk1 PE=1 SV=1 |
| 2560 | 5424 | sp\|Q9D880\|TIM50\_MOUSE | 52.91 | 3 | 0 | 0 | 0 | 0 | 0 | 3 | 3 | 0 | 0 | 0 |  |  |  |  |  | 4.38E5 | 1.82E5 |  |  |  | 1 | 1 | 0 | 0 | 0 | 0 | 0 | 1 | 1 | 0 | 0 | 0 | N | 39776 | Mitochondrial import inner membrane translocase subunit TIM50 OS=Mus musculus OX=10090 GN=Timm50 PE=1 SV=1 |
| 1742 | 3632 | sp\|Q9DCC4\|P5CR3\_MOUSE | 52.80 | 7 | 0 | 4 | 4 | 0 | 0 | 4 | 0 | 3 | 0 | 0 |  | 2.43E6 | 1.72E6 |  |  | 8.76E5 |  | 8.93E5 |  |  | 2 | 2 | 0 | 1 | 1 | 0 | 0 | 1 | 0 | 1 | 0 | 0 | N | 28721 | Pyrroline-5-carboxylate reductase 3 OS=Mus musculus OX=10090 GN=Pycr3 PE=1 SV=2 |
| 1797 | 2861 | sp\|Q8BH61\|F13A\_MOUSE | 52.66 | 2 | 2 | 0 | 0 | 0 | 0 | 0 | 0 | 0 | 0 | 2 | 2.4E5 |  |  |  |  |  |  |  |  | 1.83E5 | 1 | 1 | 1 | 0 | 0 | 0 | 0 | 0 | 0 | 0 | 0 | 1 | N | 83207 | Coagulation factor XIII A chain OS=Mus musculus OX=10090 GN=F13a1 PE=1 SV=3 |
| 2545 | 4229 | sp\|Q3TZZ7\|ESYT2\_MOUSE | 52.43 | 2 | 0 | 0 | 0 | 0 | 0 | 2 | 0 | 0 | 0 | 0 |  |  |  |  |  | 2.58E5 |  |  |  |  | 1 | 1 | 0 | 0 | 0 | 0 | 0 | 1 | 0 | 0 | 0 | 0 | N | 94139 | Extended synaptotagmin-2 OS=Mus musculus OX=10090 GN=Esyt2 PE=1 SV=1 |
| 1382 | 4772 | sp\|Q91VE0\|S27A4\_MOUSE | 52.22 | 1 | 1 | 1 | 1 | 1 | 1 | 1 | 1 | 0 | 1 | 1 | 3.28E5 | 1.63E6 | 2.45E6 | 6.37E5 | 9.76E5 | 1.03E6 | 1.18E6 |  | 2.67E6 | 1.02E6 | 1 | 1 | 1 | 1 | 1 | 1 | 1 | 1 | 1 | 0 | 1 | 1 | N | 72319 | Long-chain fatty acid transport protein 4 OS=Mus musculus OX=10090 GN=Slc27a4 PE=1 SV=1 |
| 2559 | 5420 | K4DI63\|K4DI63\_MOUSE | 52.15 | 12 | 0 | 0 | 0 | 0 | 12 | 12 | 0 | 0 | 0 | 0 |  |  |  |  | 3.09E5 | 2.89E5 |  |  |  |  | 1 | 1 | 0 | 0 | 0 | 0 | 1 | 1 | 0 | 0 | 0 | 0 | N | 11021 | Cellular repressor of E1A-stimulated genes 1 OS=Mus musculus OX=10090 GN=Creg1 PE=1 SV=1 |
| 2559 | 5426 | sp\|O88668\|CREG1\_MOUSE | 52.15 | 5 | 0 | 0 | 0 | 0 | 5 | 5 | 0 | 0 | 0 | 0 |  |  |  |  | 3.09E5 | 2.89E5 |  |  |  |  | 1 | 1 | 0 | 0 | 0 | 0 | 1 | 1 | 0 | 0 | 0 | 0 | N | 24452 | Protein CREG1 OS=Mus musculus OX=10090 GN=Creg1 PE=1 SV=1 |
| 2321 | 3252 | A2AKI5\|A2AKI5\_MOUSE | 52.07 | 1 | 0 | 0 | 0 | 0 | 0 | 0 | 0 | 0 | 1 | 0 |  |  |  |  |  |  |  |  | 3.81E6 |  | 1 | 1 | 0 | 0 | 0 | 0 | 0 | 0 | 0 | 0 | 1 | 0 | N | 111512 | Integrin alpha V OS=Mus musculus OX=10090 GN=Itgav PE=1 SV=1 |
| 2321 | 3253 | sp\|P43406\|ITAV\_MOUSE | 52.07 | 1 | 0 | 0 | 0 | 0 | 0 | 0 | 0 | 0 | 1 | 0 |  |  |  |  |  |  |  |  | 3.81E6 |  | 1 | 1 | 0 | 0 | 0 | 0 | 0 | 0 | 0 | 0 | 1 | 0 | N | 115360 | Integrin alpha-V OS=Mus musculus OX=10090 GN=Itgav PE=1 SV=2 |
| 2321 | 4412 | F6W6Q6\|F6W6Q6\_MOUSE | 52.07 | 8 | 0 | 0 | 0 | 0 | 0 | 0 | 0 | 0 | 8 | 0 |  |  |  |  |  |  |  |  | 3.81E6 |  | 1 | 1 | 0 | 0 | 0 | 0 | 0 | 0 | 0 | 0 | 1 | 0 | N | 13799 | Integrin alpha V (Fragment) OS=Mus musculus OX=10090 GN=Itgav PE=1 SV=1 |
| 2557 | 5412 | sp\|Q9Z0J0\|NPC2\_MOUSE | 51.68 | 8 | 0 | 0 | 0 | 0 | 0 | 0 | 8 | 0 | 0 | 8 |  |  |  |  |  |  | 2.62E5 |  |  | 6.19E5 | 1 | 1 | 0 | 0 | 0 | 0 | 0 | 0 | 1 | 0 | 0 | 1 | Y | 16442 | NPC intracellular cholesterol transporter 2 OS=Mus musculus OX=10090 GN=Npc2 PE=1 SV=1 |
| 3293 | 4587 | sp\|Q8C3K6\|SC5A1\_MOUSE | 51.64 | 2 | 0 | 0 | 0 | 2 | 0 | 0 | 0 | 0 | 0 | 0 |  |  |  | 3.15E5 |  |  |  |  |  |  | 1 | 1 | 0 | 0 | 0 | 1 | 0 | 0 | 0 | 0 | 0 | 0 | N | 73449 | Sodium/glucose cotransporter 1 OS=Mus musculus OX=10090 GN=Slc5a1 PE=1 SV=1 |
| 3293 | 4588 | Q9QXI6\|Q9QXI6\_MOUSE | 51.64 | 2 | 0 | 0 | 0 | 2 | 0 | 0 | 0 | 0 | 0 | 0 |  |  |  | 3.15E5 |  |  |  |  |  |  | 1 | 1 | 0 | 0 | 0 | 1 | 0 | 0 | 0 | 0 | 0 | 0 | N | 73318 | Sodium/glucose cotransporter 1 OS=Mus musculus OX=10090 GN=Slc5a1 PE=1 SV=1 |
| 1398 | 5474 | sp\|Q6ZWQ7\|SPCS3\_MOUSE | 51.31 | 5 | 5 | 5 | 5 | 5 | 5 | 5 | 5 | 5 | 0 | 5 | 8.72E6 | 8.45E6 | 1.2E7 | 5.21E6 | 8.78E6 | 8.02E6 | 8.17E6 | 6.07E6 |  | 6.86E6 | 1 | 1 | 1 | 1 | 1 | 1 | 1 | 1 | 1 | 1 | 0 | 1 | N | 20313 | Signal peptidase complex subunit 3 OS=Mus musculus OX=10090 GN=Spcs3 PE=1 SV=1 |
| 1611 | 5455 | sp\|Q9D3P8\|PLRKT\_MOUSE | 50.99 | 7 | 7 | 7 | 7 | 0 | 0 | 7 | 0 | 0 | 7 | 7 | 1.55E6 | 8.23E5 | 7.95E5 |  |  | 6.54E5 |  |  | 3.49E5 | 5.93E5 | 1 | 1 | 1 | 1 | 1 | 0 | 0 | 1 | 0 | 0 | 1 | 1 | N | 17261 | Plasminogen receptor (KT) OS=Mus musculus OX=10090 GN=Plgrkt PE=1 SV=1 |
| 3440 | 5428 | A0A1B0GT97\|A0A1B0GT97\_MOUSE | 50.87 | 8 | 0 | 0 | 8 | 0 | 0 | 0 | 0 | 0 | 0 | 0 |  |  | 2.5E6 |  |  |  |  |  |  |  | 1 | 1 | 0 | 0 | 1 | 0 | 0 | 0 | 0 | 0 | 0 | 0 | N | 15901 | ER lumen protein-retaining receptor OS=Mus musculus OX=10090 GN=Kdelr1 PE=1 SV=1 |
| 3440 | 5429 | A0A1B0GQW0\|A0A1B0GQW0\_MOUSE | 50.87 | 7 | 0 | 0 | 7 | 0 | 0 | 0 | 0 | 0 | 0 | 0 |  |  | 2.5E6 |  |  |  |  |  |  |  | 1 | 1 | 0 | 0 | 1 | 0 | 0 | 0 | 0 | 0 | 0 | 0 | N | 17505 | ER lumen protein-retaining receptor OS=Mus musculus OX=10090 GN=Kdelr1 PE=1 SV=1 |
| 3440 | 5431 | sp\|Q9CQM2\|ERD22\_MOUSE | 50.87 | 5 | 0 | 0 | 5 | 0 | 0 | 0 | 0 | 0 | 0 | 0 |  |  | 2.5E6 |  |  |  |  |  |  |  | 1 | 1 | 0 | 0 | 1 | 0 | 0 | 0 | 0 | 0 | 0 | 0 | N | 24454 | ER lumen protein-retaining receptor 2 OS=Mus musculus OX=10090 GN=Kdelr2 PE=1 SV=1 |
| 3440 | 5432 | sp\|Q99JH8\|ERD21\_MOUSE | 50.87 | 5 | 0 | 0 | 5 | 0 | 0 | 0 | 0 | 0 | 0 | 0 |  |  | 2.5E6 |  |  |  |  |  |  |  | 1 | 1 | 0 | 0 | 1 | 0 | 0 | 0 | 0 | 0 | 0 | 0 | N | 24560 | ER lumen protein-retaining receptor 1 OS=Mus musculus OX=10090 GN=Kdelr1 PE=1 SV=1 |
| 2599 | 3035 | B1AU75\|B1AU75\_MOUSE | 50.73 | 1 | 0 | 1 | 0 | 0 | 0 | 0 | 0 | 0 | 0 | 0 |  | 6.71E5 |  |  |  |  |  |  |  |  | 1 | 1 | 0 | 1 | 0 | 0 | 0 | 0 | 0 | 0 | 0 | 0 | N | 84013 | Nuclear autoantigenic sperm protein OS=Mus musculus OX=10090 GN=Nasp PE=1 SV=1 |
| 2599 | 3043 | sp\|Q99MD9\|NASP\_MOUSE | 50.73 | 1 | 0 | 1 | 0 | 0 | 0 | 0 | 0 | 0 | 0 | 0 |  | 6.71E5 |  |  |  |  |  |  |  |  | 1 | 1 | 0 | 1 | 0 | 0 | 0 | 0 | 0 | 0 | 0 | 0 | N | 83954 | Nuclear autoantigenic sperm protein OS=Mus musculus OX=10090 GN=Nasp PE=1 SV=2 |
| 2599 | 3449 | B1AU76\|B1AU76\_MOUSE | 50.73 | 2 | 0 | 2 | 0 | 0 | 0 | 0 | 0 | 0 | 0 | 0 |  | 6.71E5 |  |  |  |  |  |  |  |  | 1 | 1 | 0 | 1 | 0 | 0 | 0 | 0 | 0 | 0 | 0 | 0 | N | 48766 | Nuclear autoantigenic sperm protein OS=Mus musculus OX=10090 GN=Nasp PE=1 SV=1 |
| 2140 | 5045 | sp\|Q8JZN5\|ACAD9\_MOUSE | 50.66 | 2 | 0 | 0 | 2 | 2 | 0 | 0 | 2 | 0 | 0 | 0 |  |  | 9.62E5 | 4.47E5 |  |  | 4.04E5 |  |  |  | 1 | 1 | 0 | 0 | 1 | 1 | 0 | 0 | 1 | 0 | 0 | 0 | N | 68722 | Complex I assembly factor ACAD9, mitochondrial OS=Mus musculus OX=10090 GN=Acad9 PE=1 SV=2 |
| 2140 | 5419 | A0A0G2JF25\|A0A0G2JF25\_MOUSE | 50.66 | 9 | 0 | 0 | 9 | 9 | 0 | 0 | 9 | 0 | 0 | 0 |  |  | 9.62E5 | 4.47E5 |  |  | 4.04E5 |  |  |  | 1 | 1 | 0 | 0 | 1 | 1 | 0 | 0 | 1 | 0 | 0 | 0 | N | 17659 | Acyl-Coenzyme A dehydrogenase family, member 9 (Fragment) OS=Mus musculus OX=10090 GN=Acad9 PE=1 SV=1 |
| 954 | 3205 | sp\|Q4U2R1\|HERC2\_MOUSE | 50.65 | 0 | 0 | 0 | 0 | 0 | 0 | 0 | 0 | 0 | 0 | 0 | 1.4E6 |  |  |  |  | 7.41E6 |  |  | 2.4E7 |  | 2 | 1 | 2 | 1 | 1 | 1 | 0 | 2 | 1 | 1 | 1 | 1 | N | 527462 | E3 ubiquitin-protein ligase HERC2 OS=Mus musculus OX=10090 GN=Herc2 PE=1 SV=3 |
| 1446 | 3373 | sp\|Q9CQY5\|MAGT1\_MOUSE | 50.51 | 3 | 0 | 3 | 3 | 3 | 3 | 3 | 0 | 3 | 3 | 3 |  | 2.97E6 | 3.77E6 | 1.72E6 | 2.99E6 | 3.33E6 |  | 2.39E6 | 1.96E6 | 3E6 | 1 | 1 | 0 | 1 | 1 | 1 | 1 | 1 | 0 | 1 | 1 | 1 | N | 37970 | Magnesium transporter protein 1 OS=Mus musculus OX=10090 GN=Magt1 PE=1 SV=1 |
| 1446 | 3398 | A2ADH1\|A2ADH1\_MOUSE | 50.51 | 2 | 0 | 2 | 2 | 2 | 2 | 2 | 0 | 2 | 2 | 2 |  | 2.97E6 | 3.77E6 | 1.72E6 | 2.99E6 | 3.33E6 |  | 2.39E6 | 1.96E6 | 3E6 | 1 | 1 | 0 | 1 | 1 | 1 | 1 | 1 | 0 | 1 | 1 | 1 | N | 41638 | Magnesium transporter protein 1 OS=Mus musculus OX=10090 GN=Magt1 PE=1 SV=1 |
| 3098 | 4362 | A0A0H2UH17\|A0A0H2UH17\_MOUSE | 50.46 | 1 | 0 | 0 | 0 | 0 | 0 | 1 | 0 | 0 | 0 | 0 |  |  |  |  |  | 2.42E5 |  |  |  |  | 1 | 1 | 0 | 0 | 0 | 0 | 0 | 1 | 0 | 0 | 0 | 0 | N | 103946 | Ubiquitin-associated protein 2-like OS=Mus musculus OX=10090 GN=Ubap2l PE=1 SV=1 |
| 3098 | 4364 | A0A0G2JDV6\|A0A0G2JDV6\_MOUSE | 50.46 | 1 | 0 | 0 | 0 | 0 | 0 | 1 | 0 | 0 | 0 | 0 |  |  |  |  |  | 2.42E5 |  |  |  |  | 1 | 1 | 0 | 0 | 0 | 0 | 0 | 1 | 0 | 0 | 0 | 0 | N | 112421 | Ubiquitin-associated protein 2-like OS=Mus musculus OX=10090 GN=Ubap2l PE=1 SV=1 |
| 3098 | 4366 | sp\|Q80X50\|UBP2L\_MOUSE | 50.46 | 1 | 0 | 0 | 0 | 0 | 0 | 1 | 0 | 0 | 0 | 0 |  |  |  |  |  | 2.42E5 |  |  |  |  | 1 | 1 | 0 | 0 | 0 | 0 | 0 | 1 | 0 | 0 | 0 | 0 | N | 116799 | Ubiquitin-associated protein 2-like OS=Mus musculus OX=10090 GN=Ubap2l PE=1 SV=1 |
| 1753 | 5438 | A2AP78\|A2AP78\_MOUSE | 50.39 | 8 | 0 | 8 | 8 | 0 | 8 | 8 | 0 | 8 | 0 | 0 |  | 7.15E5 | 5.19E5 |  | 0E0 | 1.89E6 |  | 1.88E6 |  |  | 1 | 1 | 0 | 1 | 1 | 0 | 1 | 1 | 0 | 1 | 0 | 0 | N | 18311 | High mobility group box 3 (Fragment) OS=Mus musculus OX=10090 GN=Hmgb3 PE=1 SV=1 |
| 1753 | 5439 | sp\|O54879\|HMGB3\_MOUSE | 50.39 | 6 | 0 | 6 | 6 | 0 | 6 | 6 | 0 | 6 | 0 | 0 |  | 7.15E5 | 5.19E5 |  | 0E0 | 1.89E6 |  | 1.88E6 |  |  | 1 | 1 | 0 | 1 | 1 | 0 | 1 | 1 | 0 | 1 | 0 | 0 | N | 23010 | High mobility group protein B3 OS=Mus musculus OX=10090 GN=Hmgb3 PE=1 SV=3 |
| 2417 | 4667 | sp\|P55095\|GLUC\_MOUSE | 50.26 | 6 | 0 | 0 | 0 | 0 | 6 | 0 | 6 | 0 | 0 | 0 |  |  |  |  | 7.68E5 |  | 2.63E5 |  |  |  | 1 | 1 | 0 | 0 | 0 | 0 | 1 | 0 | 1 | 0 | 0 | 0 | N | 20906 | Pro-glucagon OS=Mus musculus OX=10090 GN=Gcg PE=1 SV=1 |
| 1916 | 5488 | sp\|Q6ZWY8\|TYB10\_MOUSE | 50.20 | 18 | 0 | 0 | 0 | 18 | 18 | 0 | 0 | 18 | 0 | 0 |  |  |  | 4.66E5 | 4.82E6 |  |  | 7.1E5 |  |  | 1 | 1 | 0 | 0 | 0 | 1 | 2 | 0 | 0 | 1 | 0 | 0 | N | 5026 | Thymosin beta-10 OS=Mus musculus OX=10090 GN=Tmsb10 PE=1 SV=3 |
| 2510 | 4326 | sp\|P59017\|B2L13\_MOUSE | 50.03 | 3 | 0 | 0 | 0 | 0 | 0 | 0 | 0 | 0 | 0 | 3 |  |  |  |  |  |  |  |  |  | 5.62E5 | 1 | 1 | 0 | 0 | 0 | 0 | 0 | 0 | 0 | 0 | 0 | 1 | N | 46719 | Bcl-2-like protein 13 OS=Mus musculus OX=10090 GN=Bcl2l13 PE=1 SV=2 |
| 2484 | 4744 | sp\|Q8BND5\|QSOX1\_MOUSE | 49.91 | 1 | 1 | 0 | 0 | 0 | 0 | 1 | 0 | 0 | 0 | 0 | 0E0 |  |  |  |  | 1.74E5 |  |  |  |  | 1 | 1 | 1 | 0 | 0 | 0 | 0 | 1 | 0 | 0 | 0 | 0 | N | 82785 | Sulfhydryl oxidase 1 OS=Mus musculus OX=10090 GN=Qsox1 PE=1 SV=1 |
| 1895 | 4106 | sp\|Q6IRU5\|CLCB\_MOUSE | 49.91 | 8 | 0 | 0 | 0 | 8 | 4 | 0 | 4 | 0 | 0 | 0 |  |  |  | 4.14E6 | 6.23E6 |  | 4.91E6 |  |  |  | 2 | 2 | 0 | 0 | 0 | 2 | 1 | 0 | 1 | 0 | 0 | 0 | Y | 25172 | Clathrin light chain B OS=Mus musculus OX=10090 GN=Cltb PE=1 SV=1 |
| 3441 | 5440 | sp\|Q91WD9\|SEGN\_MOUSE | 49.48 | 5 | 0 | 0 | 0 | 0 | 5 | 0 | 0 | 0 | 0 | 0 |  |  |  |  | 1.55E5 |  |  |  |  |  | 1 | 1 | 0 | 0 | 0 | 0 | 1 | 0 | 0 | 0 | 0 | 0 | N | 32146 | Secretagogin OS=Mus musculus OX=10090 GN=Scgn PE=1 SV=1 |
| 2518 | 4813 | D3Z7E6\|D3Z7E6\_MOUSE | 49.34 | 5 | 5 | 0 | 0 | 0 | 0 | 0 | 0 | 0 | 0 | 5 | 3.07E6 |  |  |  |  |  |  |  |  | 3.22E5 | 1 | 1 | 1 | 0 | 0 | 0 | 0 | 0 | 0 | 0 | 0 | 1 | N | 20149 | Platelet-activating factor acetylhydrolase IB subunit alpha1 OS=Mus musculus OX=10090 GN=Pafah1b3 PE=1 SV=1 |
| 2518 | 4814 | sp\|Q61205\|PA1B3\_MOUSE | 49.34 | 4 | 4 | 0 | 0 | 0 | 0 | 0 | 0 | 0 | 0 | 4 | 3.07E6 |  |  |  |  |  |  |  |  | 3.22E5 | 1 | 1 | 1 | 0 | 0 | 0 | 0 | 0 | 0 | 0 | 0 | 1 | N | 25853 | Platelet-activating factor acetylhydrolase IB subunit alpha1 OS=Mus musculus OX=10090 GN=Pafah1b3 PE=1 SV=1 |
| 2518 | 5475 | D3Z2X5\|D3Z2X5\_MOUSE | 49.34 | 6 | 6 | 0 | 0 | 0 | 0 | 0 | 0 | 0 | 0 | 6 | 3.07E6 |  |  |  |  |  |  |  |  | 3.22E5 | 1 | 1 | 1 | 0 | 0 | 0 | 0 | 0 | 0 | 0 | 0 | 1 | N | 15893 | 1-alkyl-2-acetylglycerophosphocholine esterase (Fragment) OS=Mus musculus OX=10090 GN=Pafah1b3 PE=1 SV=1 |
| 3289 | 4503 | sp\|Q8BW96\|KCC1D\_MOUSE | 49.33 | 3 | 0 | 0 | 0 | 0 | 0 | 0 | 0 | 0 | 3 | 0 |  |  |  |  |  |  |  |  | 2.45E6 |  | 1 | 1 | 0 | 0 | 0 | 0 | 0 | 0 | 0 | 0 | 1 | 0 | N | 42919 | Calcium/calmodulin-dependent protein kinase type 1D OS=Mus musculus OX=10090 GN=Camk1d PE=1 SV=2 |
| 2361 | 5457 | sp\|Q9Z1Z2\|STRAP\_MOUSE | 49.31 | 3 | 0 | 0 | 0 | 3 | 0 | 0 | 3 | 0 | 0 | 0 |  |  |  | 1.15E6 |  |  | 3.79E5 |  |  |  | 1 | 1 | 0 | 0 | 0 | 1 | 0 | 0 | 1 | 0 | 0 | 0 | N | 38442 | Serine-threonine kinase receptor-associated protein OS=Mus musculus OX=10090 GN=Strap PE=1 SV=2 |
| 1587 | 3263 | sp\|Q4VAA2\|CDV3\_MOUSE | 49.31 | 6 | 3 | 3 | 0 | 0 | 0 | 4 | 0 | 0 | 0 | 0 | 4.16E6 | 4.64E6 |  |  |  | 4.39E6 |  |  |  |  | 2 | 2 | 1 | 1 | 0 | 0 | 0 | 1 | 0 | 0 | 0 | 0 | Y | 29729 | Protein CDV3 OS=Mus musculus OX=10090 GN=Cdv3 PE=1 SV=2 |
| 3031 | 4917 | A0A0U1RPF2\|A0A0U1RPF2\_MOUSE | 49.24 | 4 | 0 | 0 | 0 | 4 | 0 | 0 | 0 | 0 | 0 | 0 |  |  |  | 1.78E5 |  |  |  |  |  |  | 1 | 1 | 0 | 0 | 0 | 1 | 0 | 0 | 0 | 0 | 0 | 0 | N | 29582 | HSPA (heat shock 70kDa) binding protein, cytoplasmic cochaperone 1 (Fragment) OS=Mus musculus OX=10090 GN=Hspbp1 PE=1 SV=1 |
| 3031 | 4918 | sp\|Q99P31\|HPBP1\_MOUSE | 49.24 | 3 | 0 | 0 | 0 | 3 | 0 | 0 | 0 | 0 | 0 | 0 |  |  |  | 1.78E5 |  |  |  |  |  |  | 1 | 1 | 0 | 0 | 0 | 1 | 0 | 0 | 0 | 0 | 0 | 0 | N | 39167 | Hsp70-binding protein 1 OS=Mus musculus OX=10090 GN=Hspbp1 PE=1 SV=1 |
| 2071 | 4676 | sp\|Q8BU14\|SEC62\_MOUSE | 49.21 | 3 | 3 | 0 | 0 | 0 | 0 | 0 | 0 | 0 | 3 | 0 | 9.14E5 |  |  |  |  |  |  |  | 1.3E6 |  | 1 | 1 | 1 | 0 | 0 | 0 | 0 | 0 | 0 | 0 | 1 | 0 | N | 45581 | Translocation protein SEC62 OS=Mus musculus OX=10090 GN=Sec62 PE=1 SV=1 |
| 2561 | 5453 | sp\|O88653\|LTOR3\_MOUSE | 49.12 | 9 | 0 | 0 | 0 | 9 | 0 | 0 | 9 | 0 | 0 | 0 |  |  |  | 3.07E5 |  |  | 1.38E6 |  |  |  | 1 | 1 | 0 | 0 | 0 | 1 | 0 | 0 | 1 | 0 | 0 | 0 | N | 13553 | Ragulator complex protein LAMTOR3 OS=Mus musculus OX=10090 GN=Lamtor3 PE=1 SV=1 |
| 2026 | 2914 | E9Q6A6\|E9Q6A6\_MOUSE | 48.98 | 1 | 0 | 0 | 0 | 0 | 0 | 0 | 0 | 0 | 0 | 0 |  | 2.18E7 |  |  | 4E6 |  |  |  |  |  | 2 | 2 | 0 | 1 | 0 | 0 | 1 | 0 | 0 | 0 | 0 | 0 | Y | 246422 | Collagen, type VI, alpha 6 OS=Mus musculus OX=10090 GN=Col6a6 PE=1 SV=2 |
| 2026 | 2915 | sp\|Q8C6K9\|CO6A6\_MOUSE | 48.98 | 1 | 0 | 0 | 0 | 0 | 0 | 0 | 0 | 0 | 0 | 0 |  | 2.18E7 |  |  | 4E6 |  |  |  |  |  | 2 | 2 | 0 | 1 | 0 | 0 | 1 | 0 | 0 | 0 | 0 | 0 | Y | 246320 | Collagen alpha-6(VI) chain OS=Mus musculus OX=10090 GN=Col6a6 PE=1 SV=2 |
| 1963 | 3005 | Q8BWW3\|Q8BWW3\_MOUSE | 48.75 | 3 | 0 | 0 | 0 | 0 | 3 | 0 | 0 | 0 | 3 | 0 |  |  |  |  | 1.11E6 |  |  |  | 3.62E6 |  | 1 | 1 | 0 | 0 | 0 | 0 | 1 | 0 | 0 | 0 | 1 | 0 | N | 54989 | Phosphoacetylglucosamine mutase OS=Mus musculus OX=10090 GN=Pgm3 PE=1 SV=1 |
| 1963 | 3006 | sp\|Q9CYR6\|AGM1\_MOUSE | 48.75 | 2 | 0 | 0 | 0 | 0 | 2 | 0 | 0 | 0 | 2 | 0 |  |  |  |  | 1.11E6 |  |  |  | 3.62E6 |  | 1 | 1 | 0 | 0 | 0 | 0 | 1 | 0 | 0 | 0 | 1 | 0 | N | 59453 | Phosphoacetylglucosamine mutase OS=Mus musculus OX=10090 GN=Pgm3 PE=1 SV=1 |
| 1911 | 4777 | sp\|Q99JX7\|NXF1\_MOUSE | 48.62 | 2 | 2 | 0 | 0 | 2 | 0 | 0 | 2 | 0 | 0 | 2 | 6.43E5 |  |  | 1.99E5 |  |  | 1.44E5 |  |  | 2.82E5 | 1 | 1 | 1 | 0 | 0 | 1 | 0 | 0 | 1 | 0 | 0 | 1 | N | 70300 | Nuclear RNA export factor 1 OS=Mus musculus OX=10090 GN=Nxf1 PE=1 SV=3 |
| 2444 | 4286 | sp\|Q10738\|MMP7\_MOUSE | 48.41 | 4 | 0 | 0 | 0 | 0 | 0 | 0 | 0 | 0 | 4 | 0 |  |  |  |  |  |  |  |  | 5.56E6 |  | 1 | 1 | 0 | 0 | 0 | 0 | 0 | 0 | 0 | 0 | 1 | 0 | N | 29755 | Matrilysin OS=Mus musculus OX=10090 GN=Mmp7 PE=1 SV=1 |
| 2444 | 4287 | Q3UN27\|Q3UN27\_MOUSE | 48.41 | 4 | 0 | 0 | 0 | 0 | 0 | 0 | 0 | 0 | 4 | 0 |  |  |  |  |  |  |  |  | 5.56E6 |  | 1 | 1 | 0 | 0 | 0 | 0 | 0 | 0 | 0 | 0 | 1 | 0 | N | 30028 | Matrix metallopeptidase 7 OS=Mus musculus OX=10090 GN=Mmp7 PE=1 SV=1 |
| 2562 | 5461 | sp\|P97352\|S10AD\_MOUSE | 48.21 | 11 | 0 | 0 | 0 | 11 | 11 | 0 | 0 | 0 | 0 | 0 |  |  |  | 1.26E6 | 1.45E6 |  |  |  |  |  | 1 | 1 | 0 | 0 | 0 | 1 | 1 | 0 | 0 | 0 | 0 | 0 | N | 11158 | Protein S100-A13 OS=Mus musculus OX=10090 GN=S100a13 PE=1 SV=1 |
| 2562 | 5462 | A0A0A0MQ90\|A0A0A0MQ90\_MOUSE | 48.21 | 7 | 0 | 0 | 0 | 7 | 7 | 0 | 0 | 0 | 0 | 0 |  |  |  | 1.26E6 | 1.45E6 |  |  |  |  |  | 1 | 1 | 0 | 0 | 0 | 1 | 1 | 0 | 0 | 0 | 0 | 0 | N | 17744 | Protein S100-A13 OS=Mus musculus OX=10090 GN=S100a13 PE=1 SV=1 |
| 1906 | 3505 | sp\|Q05816\|FABP5\_MOUSE | 48.20 | 7 | 7 | 0 | 0 | 7 | 0 | 0 | 0 | 0 | 0 | 7 | 1.49E6 |  |  | 8.34E5 |  |  |  |  |  | 4.65E6 | 1 | 1 | 1 | 0 | 0 | 1 | 0 | 0 | 0 | 0 | 0 | 1 | N | 15137 | Fatty acid-binding protein 5 OS=Mus musculus OX=10090 GN=Fabp5 PE=1 SV=3 |
| 1638 | 5469 | D3YZX5\|D3YZX5\_MOUSE | 48.04 | 9 | 0 | 0 | 0 | 9 | 9 | 9 | 9 | 0 | 0 | 9 |  |  |  | 2.64E5 | 4.77E5 | 1.23E6 | 1.95E5 |  |  | 2.25E5 | 1 | 1 | 0 | 0 | 0 | 1 | 1 | 1 | 1 | 0 | 0 | 1 | N | 12280 | ATP binding cassette subfamily G member 2 (Junior blood group) (Fragment) OS=Mus musculus OX=10090 GN=Abcg2 PE=1 SV=1 |
| 1638 | 5470 | A0A0N4SWA2\|A0A0N4SWA2\_MOUSE | 48.04 | 6 | 0 | 0 | 0 | 6 | 6 | 6 | 6 | 0 | 0 | 6 |  |  |  | 2.64E5 | 4.77E5 | 1.23E6 | 1.95E5 |  |  | 2.25E5 | 1 | 1 | 0 | 0 | 0 | 1 | 1 | 1 | 1 | 0 | 0 | 1 | N | 17399 | ATP binding cassette subfamily G member 2 (Junior blood group) (Fragment) OS=Mus musculus OX=10090 GN=Abcg2 PE=1 SV=1 |
| 1638 | 5471 | D3Z150\|D3Z150\_MOUSE | 48.04 | 6 | 0 | 0 | 0 | 6 | 6 | 6 | 6 | 0 | 0 | 6 |  |  |  | 2.64E5 | 4.77E5 | 1.23E6 | 1.95E5 |  |  | 2.25E5 | 1 | 1 | 0 | 0 | 0 | 1 | 1 | 1 | 1 | 0 | 0 | 1 | N | 18745 | ATP binding cassette subfamily G member 2 (Junior blood group) (Fragment) OS=Mus musculus OX=10090 GN=Abcg2 PE=1 SV=1 |
| 1638 | 5491 | S4R2E1\|S4R2E1\_MOUSE | 48.04 | 2 | 0 | 0 | 0 | 2 | 2 | 2 | 2 | 0 | 0 | 2 |  |  |  | 2.64E5 | 4.77E5 | 1.23E6 | 1.95E5 |  |  | 2.25E5 | 1 | 1 | 0 | 0 | 0 | 1 | 1 | 1 | 1 | 0 | 0 | 1 | N | 63608 | Broad substrate specificity ATP-binding cassette transporter ABCG2 OS=Mus musculus OX=10090 GN=Abcg2 PE=1 SV=1 |
| 1638 | 5498 | A0A0R4J0B6\|A0A0R4J0B6\_MOUSE | 48.04 | 2 | 0 | 0 | 0 | 2 | 2 | 2 | 2 | 0 | 0 | 2 |  |  |  | 2.64E5 | 4.77E5 | 1.23E6 | 1.95E5 |  |  | 2.25E5 | 1 | 1 | 0 | 0 | 0 | 1 | 1 | 1 | 1 | 0 | 0 | 1 | N | 73022 | Broad substrate specificity ATP-binding cassette transporter ABCG2 OS=Mus musculus OX=10090 GN=Abcg2 PE=1 SV=1 |
| 1638 | 5499 | sp\|Q7TMS5\|ABCG2\_MOUSE | 48.04 | 2 | 0 | 0 | 0 | 2 | 2 | 2 | 2 | 0 | 0 | 2 |  |  |  | 2.64E5 | 4.77E5 | 1.23E6 | 1.95E5 |  |  | 2.25E5 | 1 | 1 | 0 | 0 | 0 | 1 | 1 | 1 | 1 | 0 | 0 | 1 | N | 72978 | Broad substrate specificity ATP-binding cassette transporter ABCG2 OS=Mus musculus OX=10090 GN=Abcg2 PE=1 SV=1 |
| 2506 | 3089 | A0A0G2JG00\|A0A0G2JG00\_MOUSE | 47.99 | 2 | 0 | 0 | 0 | 0 | 0 | 0 | 0 | 0 | 2 | 0 |  |  |  |  |  |  |  |  | 1.92E6 |  | 1 | 1 | 0 | 0 | 0 | 0 | 0 | 0 | 0 | 0 | 1 | 0 | N | 54845 | Far upstream element (FUSE) binding protein 1 OS=Mus musculus OX=10090 GN=Fubp1 PE=1 SV=1 |
| 2156 | 3335 | sp\|Q91WC3\|ACSL6\_MOUSE | 47.75 | 1 | 0 | 0 | 0 | 1 | 0 | 0 | 0 | 0 | 0 | 0 |  |  |  | 4.06E5 |  |  |  |  |  |  | 1 | 1 | 0 | 0 | 0 | 1 | 0 | 0 | 0 | 0 | 0 | 0 | N | 78017 | Long-chain-fatty-acid--CoA ligase 6 OS=Mus musculus OX=10090 GN=Acsl6 PE=1 SV=1 |
| 2092 | 3125 | sp\|Q8BU30\|SYIC\_MOUSE | 47.75 | 1 | 0 | 1 | 0 | 0 | 1 | 0 | 0 | 0 | 0 | 0 |  | 1.97E5 |  |  | 5.69E5 |  |  |  |  |  | 2 | 1 | 0 | 1 | 0 | 0 | 1 | 0 | 1 | 0 | 0 | 0 | N | 144270 | Isoleucine--tRNA ligase, cytoplasmic OS=Mus musculus OX=10090 GN=Iars1 PE=1 SV=2 |
| 3292 | 4586 | sp\|Q9CS42\|PRPS2\_MOUSE | 47.66 | 4 | 0 | 0 | 0 | 4 | 0 | 0 | 0 | 0 | 0 | 0 |  |  |  | 0E0 |  |  |  |  |  |  | 1 | 1 | 0 | 0 | 0 | 1 | 0 | 0 | 0 | 0 | 0 | 0 | Y | 34786 | Ribose-phosphate pyrophosphokinase 2 OS=Mus musculus OX=10090 GN=Prps2 PE=1 SV=4 |
| 3292 | 4844 | G3UXL2\|G3UXL2\_MOUSE | 47.66 | 4 | 0 | 0 | 0 | 4 | 0 | 0 | 0 | 0 | 0 | 0 |  |  |  | 0E0 |  |  |  |  |  |  | 1 | 1 | 0 | 0 | 0 | 1 | 0 | 0 | 0 | 0 | 0 | 0 | Y | 34824 | ribose-phosphate diphosphokinase OS=Mus musculus OX=10090 GN=Prps1l3 PE=3 SV=1 |
| 3292 | 4845 | sp\|Q9D7G0\|PRPS1\_MOUSE | 47.66 | 4 | 0 | 0 | 0 | 4 | 0 | 0 | 0 | 0 | 0 | 0 |  |  |  | 0E0 |  |  |  |  |  |  | 1 | 1 | 0 | 0 | 0 | 1 | 0 | 0 | 0 | 0 | 0 | 0 | Y | 34834 | Ribose-phosphate pyrophosphokinase 1 OS=Mus musculus OX=10090 GN=Prps1 PE=1 SV=4 |
| 1558 | 2802 | sp\|P21440\|MDR3\_MOUSE | 47.43 | 1 | 1 | 1 | 0 | 0 | 0 | 0 | 0 | 0 | 0 | 0 | 5.37E5 |  |  |  |  |  |  |  |  |  | 2 | 1 | 1 | 1 | 0 | 0 | 0 | 0 | 0 | 0 | 0 | 0 | N | 140377 | Phosphatidylcholine translocator ABCB4 OS=Mus musculus OX=10090 GN=Abcb4 PE=1 SV=2 |
| 2823 | 3614 | sp\|Q8BK72\|RT27\_MOUSE | 47.40 | 3 | 0 | 0 | 0 | 0 | 0 | 0 | 3 | 0 | 0 | 0 |  |  |  |  |  |  | 1.23E5 |  |  |  | 1 | 1 | 0 | 0 | 0 | 0 | 0 | 0 | 1 | 0 | 0 | 0 | N | 47779 | Small ribosomal subunit protein mS27 OS=Mus musculus OX=10090 GN=Mrps27 PE=1 SV=2 |
| 2097 | 3521 | sp\|P97379\|G3BP2\_MOUSE | 47.16 | 3 | 0 | 0 | 3 | 0 | 3 | 3 | 0 | 0 | 0 | 0 |  |  | 1.13E6 |  | 2.04E6 | 9.65E5 |  |  |  |  | 1 | 1 | 0 | 0 | 1 | 0 | 1 | 1 | 0 | 0 | 0 | 0 | N | 54088 | Ras GTPase-activating protein-binding protein 2 OS=Mus musculus OX=10090 GN=G3bp2 PE=1 SV=2 |
| 1707 | 3384 | sp\|Q9QZS0\|CO4A3\_MOUSE | 46.95 | 1 | 0 | 0 | 0 | 1 | 1 | 0 | 1 | 1 | 0 | 0 |  |  |  | 1.58E7 | 3.65E7 |  | 2.09E7 | 5.08E6 |  |  | 1 | 1 | 0 | 0 | 0 | 1 | 1 | 0 | 1 | 1 | 0 | 0 | N | 161726 | Collagen alpha-3(IV) chain OS=Mus musculus OX=10090 GN=Col4a3 PE=1 SV=2 |
| 1588 | 4505 | D3YW09\|D3YW09\_MOUSE | 46.68 | 5 | 0 | 0 | 0 | 5 | 5 | 5 | 5 | 5 | 0 | 0 |  |  |  | 2.17E6 | 3.67E6 | 3.03E6 | 3.26E6 | 1.59E6 |  |  | 1 | 1 | 0 | 0 | 0 | 1 | 1 | 1 | 1 | 1 | 0 | 0 | N | 22135 | Splicing factor 3a, subunit 2 OS=Mus musculus OX=10090 GN=Sf3a2 PE=1 SV=1 |
| 1588 | 4526 | G3UVU2\|G3UVU2\_MOUSE | 46.68 | 2 | 0 | 0 | 0 | 2 | 2 | 2 | 2 | 2 | 0 | 0 |  |  |  | 2.17E6 | 3.67E6 | 3.03E6 | 3.26E6 | 1.59E6 |  |  | 1 | 1 | 0 | 0 | 0 | 1 | 1 | 1 | 1 | 1 | 0 | 0 | N | 51279 | Splicing factor 3a, subunit 2 OS=Mus musculus OX=10090 GN=Sf3a2 PE=1 SV=1 |
| 2095 | 3430 | sp\|Q8K389\|CK5P2\_MOUSE | 46.61 | 0 | 0 | 0 | 0 | 0 | 0 | 0 | 0 | 0 | 0 | 0 |  |  | 3.53E6 |  |  | 3.53E6 |  |  |  |  | 1 | 1 | 0 | 0 | 1 | 0 | 0 | 1 | 0 | 0 | 0 | 0 | N | 205943 | CDK5 regulatory subunit-associated protein 2 OS=Mus musculus OX=10090 GN=Cdk5rap2 PE=1 SV=3 |
| 1639 | 5531 | sp\|Q9D7S0\|LYPD8\_MOUSE | 46.57 | 4 | 4 | 0 | 4 | 0 | 4 | 4 | 4 | 4 | 0 | 0 | 5.85E6 |  | 1.72E6 |  | 2.8E6 | 1.83E6 | 2.05E6 | 8.49E5 |  |  | 1 | 1 | 1 | 0 | 1 | 0 | 1 | 1 | 1 | 1 | 0 | 0 | Y | 27524 | Ly6/PLAUR domain-containing protein 8 OS=Mus musculus OX=10090 GN=Lypd8 PE=1 SV=1 |
| 1317 | 5490 | A0A338P6G6\|A0A338P6G6\_MOUSE | 46.53 | 10 | 0 | 0 | 10 | 10 | 10 | 10 | 10 | 10 | 10 | 10 |  |  | 1.38E6 | 4.71E6 | 1.85E6 | 1.91E6 | 0E0 | 6.03E6 | 8.85E6 | 1.65E6 | 1 | 1 | 0 | 0 | 1 | 1 | 1 | 1 | 1 | 1 | 1 | 1 | N | 9423 | High mobility group protein HMG-I/HMG-Y OS=Mus musculus OX=10090 GN=Hmga1 PE=1 SV=1 |
| 1317 | 5492 | A0A384DV79\|A0A384DV79\_MOUSE | 46.53 | 9 | 0 | 0 | 9 | 9 | 9 | 9 | 9 | 9 | 9 | 9 |  |  | 1.38E6 | 4.71E6 | 1.85E6 | 1.91E6 | 0E0 | 6.03E6 | 8.85E6 | 1.65E6 | 1 | 1 | 0 | 0 | 1 | 1 | 1 | 1 | 1 | 1 | 1 | 1 | N | 10488 | High mobility group protein HMG-I/HMG-Y OS=Mus musculus OX=10090 GN=Hmga1 PE=1 SV=1 |
| 1317 | 5495 | Q3TE85\|Q3TE85\_MOUSE | 46.53 | 8 | 0 | 0 | 8 | 8 | 8 | 8 | 8 | 8 | 8 | 8 |  |  | 1.38E6 | 4.71E6 | 1.85E6 | 1.91E6 | 0E0 | 6.03E6 | 8.85E6 | 1.65E6 | 1 | 1 | 0 | 0 | 1 | 1 | 1 | 1 | 1 | 1 | 1 | 1 | N | 11485 | High mobility group protein HMG-I/HMG-Y OS=Mus musculus OX=10090 GN=Hmga1 PE=1 SV=1 |
| 1317 | 5500 | sp\|P17095\|HMGA1\_MOUSE | 46.53 | 8 | 0 | 0 | 8 | 8 | 8 | 8 | 8 | 8 | 8 | 8 |  |  | 1.38E6 | 4.71E6 | 1.85E6 | 1.91E6 | 0E0 | 6.03E6 | 8.85E6 | 1.65E6 | 1 | 1 | 0 | 0 | 1 | 1 | 1 | 1 | 1 | 1 | 1 | 1 | N | 11614 | High mobility group protein HMG-I/HMG-Y OS=Mus musculus OX=10090 GN=Hmga1 PE=1 SV=4 |
| 1317 | 5503 | A0A338P6E8\|A0A338P6E8\_MOUSE | 46.53 | 8 | 0 | 0 | 8 | 8 | 8 | 8 | 8 | 8 | 8 | 8 |  |  | 1.38E6 | 4.71E6 | 1.85E6 | 1.91E6 | 0E0 | 6.03E6 | 8.85E6 | 1.65E6 | 1 | 1 | 0 | 0 | 1 | 1 | 1 | 1 | 1 | 1 | 1 | 1 | N | 12043 | High mobility group protein HMG-I/HMG-Y OS=Mus musculus OX=10090 GN=Hmga1 PE=1 SV=1 |
| 1317 | 5506 | A0A338P6F6\|A0A338P6F6\_MOUSE | 46.53 | 8 | 0 | 0 | 8 | 8 | 8 | 8 | 8 | 8 | 8 | 8 |  |  | 1.38E6 | 4.71E6 | 1.85E6 | 1.91E6 | 0E0 | 6.03E6 | 8.85E6 | 1.65E6 | 1 | 1 | 0 | 0 | 1 | 1 | 1 | 1 | 1 | 1 | 1 | 1 | N | 13027 | High mobility group protein HMG-I/HMG-Y OS=Mus musculus OX=10090 GN=Hmga1 PE=1 SV=1 |
| 2435 | 3682 | sp\|Q9JIX8\|ACINU\_MOUSE | 46.47 | 1 | 0 | 0 | 0 | 1 | 0 | 0 | 0 | 0 | 0 | 0 |  |  |  | 1.42E5 |  |  |  |  |  |  | 1 | 1 | 0 | 0 | 0 | 1 | 0 | 0 | 0 | 0 | 0 | 0 | N | 150719 | Apoptotic chromatin condensation inducer in the nucleus OS=Mus musculus OX=10090 GN=Acin1 PE=1 SV=3 |
| 926 | 2697 | A0A1B0GRM4\|A0A1B0GRM4\_MOUSE | 46.41 | 2 | 0 | 0 | 2 | 0 | 1 | 0 | 0 | 0 | 0 | 0 |  |  | 2.73E7 |  | 1.75E7 |  |  |  |  |  | 2 | 2 | 0 | 0 | 2 | 0 | 1 | 0 | 0 | 0 | 0 | 0 | N | 115164 | Liprin-alpha-3 (Fragment) OS=Mus musculus OX=10090 GN=Ppfia3 PE=1 SV=1 |
| 1548 | 5479 | sp\|P62311\|LSM3\_MOUSE | 46.40 | 12 | 0 | 0 | 12 | 12 | 12 | 12 | 12 | 12 | 0 | 12 |  |  | 1.44E6 | 5.39E5 | 4.59E5 | 9.01E5 | 3.12E5 | 8.98E5 |  | 7.65E5 | 1 | 1 | 0 | 0 | 1 | 1 | 1 | 1 | 1 | 1 | 0 | 1 | N | 11845 | U6 snRNA-associated Sm-like protein LSm3 OS=Mus musculus OX=10090 GN=Lsm3 PE=1 SV=2 |
| 1338 | 5482 | E9PVP3\|E9PVP3\_MOUSE | 46.37 | 9 | 0 | 9 | 9 | 9 | 9 | 9 | 9 | 9 | 9 | 9 |  | 1.04E6 | 1.95E6 | 1.78E6 | 1.07E6 | 2.51E6 | 1.73E6 | 1.64E6 | 2.19E5 | 2.58E6 | 1 | 1 | 0 | 1 | 1 | 1 | 1 | 1 | 1 | 1 | 1 | 1 | N | 12838 | Mannose-P-dolichol utilization defect 1 OS=Mus musculus OX=10090 GN=Mpdu1 PE=1 SV=1 |
| 1338 | 5484 | Q5F2B1\|Q5F2B1\_MOUSE | 46.37 | 7 | 0 | 7 | 7 | 7 | 7 | 7 | 7 | 7 | 7 | 7 |  | 1.04E6 | 1.95E6 | 1.78E6 | 1.07E6 | 2.51E6 | 1.73E6 | 1.64E6 | 2.19E5 | 2.58E6 | 1 | 1 | 0 | 1 | 1 | 1 | 1 | 1 | 1 | 1 | 1 | 1 | N | 16094 | Mannose-P-dolichol utilization defect 1 (Fragment) OS=Mus musculus OX=10090 GN=Mpdu1 PE=1 SV=8 |
| 1338 | 5485 | F6XX36\|F6XX36\_MOUSE | 46.37 | 6 | 0 | 6 | 6 | 6 | 6 | 6 | 6 | 6 | 6 | 6 |  | 1.04E6 | 1.95E6 | 1.78E6 | 1.07E6 | 2.51E6 | 1.73E6 | 1.64E6 | 2.19E5 | 2.58E6 | 1 | 1 | 0 | 1 | 1 | 1 | 1 | 1 | 1 | 1 | 1 | 1 | N | 21599 | Mannose-P-dolichol utilization defect 1 (Fragment) OS=Mus musculus OX=10090 GN=Mpdu1 PE=1 SV=1 |
| 1338 | 5486 | sp\|Q9R0Q9\|MPU1\_MOUSE | 46.37 | 4 | 0 | 4 | 4 | 4 | 4 | 4 | 4 | 4 | 4 | 4 |  | 1.04E6 | 1.95E6 | 1.78E6 | 1.07E6 | 2.51E6 | 1.73E6 | 1.64E6 | 2.19E5 | 2.58E6 | 1 | 1 | 0 | 1 | 1 | 1 | 1 | 1 | 1 | 1 | 1 | 1 | N | 26498 | Mannose-P-dolichol utilization defect 1 protein OS=Mus musculus OX=10090 GN=Mpdu1 PE=1 SV=1 |
| 1338 | 5487 | Q8R0J2\|Q8R0J2\_MOUSE | 46.37 | 4 | 0 | 4 | 4 | 4 | 4 | 4 | 4 | 4 | 4 | 4 |  | 1.04E6 | 1.95E6 | 1.78E6 | 1.07E6 | 2.51E6 | 1.73E6 | 1.64E6 | 2.19E5 | 2.58E6 | 1 | 1 | 0 | 1 | 1 | 1 | 1 | 1 | 1 | 1 | 1 | 1 | N | 26438 | Mannose-P-dolichol utilization defect 1 protein OS=Mus musculus OX=10090 GN=Mpdu1 PE=1 SV=1 |
| 2543 | 4083 | sp\|Q99KJ8\|DCTN2\_MOUSE | 46.18 | 6 | 3 | 0 | 0 | 0 | 0 | 2 | 0 | 0 | 0 | 0 | 5.17E5 |  |  |  |  | 4.81E5 |  |  |  |  | 2 | 2 | 1 | 0 | 0 | 0 | 0 | 1 | 0 | 0 | 0 | 0 | N | 44117 | Dynactin subunit 2 OS=Mus musculus OX=10090 GN=Dctn2 PE=1 SV=3 |
| 2206 | 4404 | sp\|Q8R1F1\|NIBA2\_MOUSE | 46.07 | 1 | 0 | 0 | 0 | 0 | 0 | 0 | 0 | 0 | 1 | 1 |  |  |  |  |  |  |  |  | 9.41E4 | 8.62E5 | 1 | 1 | 0 | 0 | 0 | 0 | 0 | 0 | 0 | 0 | 1 | 1 | N | 84819 | Protein Niban 2 OS=Mus musculus OX=10090 GN=Niban2 PE=1 SV=2 |
| 1087 | 3016 | A0A1L1STC6\|A0A1L1STC6\_MOUSE | 45.95 | 0 | 0 | 0 | 0 | 0 | 0 | 0 | 0 | 0 | 0 | 0 |  |  |  |  |  |  | 7.65E6 |  |  |  | 1 | 1 | 0 | 0 | 0 | 0 | 0 | 0 | 1 | 0 | 0 | 0 | N | 1009943 | Spectrin repeat containing, nuclear envelope 1 OS=Mus musculus OX=10090 GN=Syne1 PE=1 SV=1 |
| 1087 | 3017 | sp\|Q6ZWR6\|SYNE1\_MOUSE | 45.95 | 0 | 0 | 0 | 0 | 0 | 0 | 0 | 0 | 0 | 0 | 0 |  |  |  |  |  |  | 7.65E6 |  |  |  | 1 | 1 | 0 | 0 | 0 | 0 | 0 | 0 | 1 | 0 | 0 | 0 | N | 1009946 | Nesprin-1 OS=Mus musculus OX=10090 GN=Syne1 PE=1 SV=2 |
| 2372 | 2976 | sp\|Q61245\|COBA1\_MOUSE | 45.78 | 0 | 0 | 0 | 0 | 0 | 0 | 0 | 0 | 0 | 0 | 0 |  |  |  |  |  |  |  |  | 1.28E7 |  | 1 | 1 | 0 | 0 | 0 | 0 | 0 | 0 | 0 | 0 | 1 | 0 | N | 181031 | Collagen alpha-1(XI) chain OS=Mus musculus OX=10090 GN=Col11a1 PE=1 SV=2 |
| 2563 | 5496 | sp\|P97816\|S100G\_MOUSE | 45.70 | 15 | 0 | 0 | 0 | 0 | 0 | 15 | 0 | 0 | 15 | 0 |  |  |  |  |  | 1.98E6 |  |  | 1.69E6 |  | 1 | 1 | 0 | 0 | 0 | 0 | 0 | 1 | 0 | 0 | 1 | 0 | N | 8970 | Protein S100-G OS=Mus musculus OX=10090 GN=S100g PE=1 SV=3 |
| 1634 | 4279 | sp\|Q9WTL7\|LYPA2\_MOUSE | 45.70 | 6 | 0 | 0 | 6 | 2 | 2 | 0 | 3 | 0 | 0 | 0 |  |  | 8.6E5 |  |  |  | 2.57E6 |  |  |  | 2 | 1 | 0 | 0 | 2 | 1 | 1 | 0 | 1 | 0 | 0 | 0 | N | 24794 | Acyl-protein thioesterase 2 OS=Mus musculus OX=10090 GN=Lypla2 PE=1 SV=1 |
| 1307 | 5004 | sp\|Q9CQR4\|ACO13\_MOUSE | 45.60 | 7 | 0 | 7 | 7 | 7 | 7 | 7 | 7 | 7 | 7 | 7 |  | 2.31E6 | 2.97E6 | 1.29E6 | 2.15E6 | 2.75E6 | 1.74E6 | 1.23E6 | 2.69E6 | 1.87E6 | 1 | 1 | 0 | 1 | 1 | 1 | 1 | 1 | 1 | 1 | 1 | 1 | N | 15183 | Acyl-coenzyme A thioesterase 13 OS=Mus musculus OX=10090 GN=Acot13 PE=1 SV=1 |
| 1463 | 5483 | sp\|O09111\|NDUBB\_MOUSE | 45.44 | 7 | 0 | 0 | 7 | 7 | 7 | 7 | 7 | 7 | 7 | 7 |  |  | 1.35E4 | 1.05E7 | 6.53E6 | 8.71E6 | 7.03E6 | 4.68E6 | 5.36E6 | 7.88E6 | 1 | 1 | 0 | 0 | 1 | 1 | 1 | 1 | 1 | 1 | 1 | 1 | N | 17444 | NADH dehydrogenase [ubiquinone] 1 beta subcomplex subunit 11, mitochondrial OS=Mus musculus OX=10090 GN=Ndufb11 PE=1 SV=2 |
| 3206 | 4495 | A0A2U3TZ67\|A0A2U3TZ67\_MOUSE | 45.37 | 4 | 0 | 0 | 0 | 0 | 0 | 4 | 0 | 0 | 0 | 0 |  |  |  |  |  | 5.74E5 |  |  |  |  | 1 | 1 | 0 | 0 | 0 | 0 | 0 | 1 | 0 | 0 | 0 | 0 | N | 65395 | Dynamin-1-like protein OS=Mus musculus OX=10090 GN=Dnm1l PE=1 SV=1 |
| 3206 | 4497 | sp\|Q8K1M6\|DNM1L\_MOUSE | 45.37 | 3 | 0 | 0 | 0 | 0 | 0 | 3 | 0 | 0 | 0 | 0 |  |  |  |  |  | 5.74E5 |  |  |  |  | 1 | 1 | 0 | 0 | 0 | 0 | 0 | 1 | 0 | 0 | 0 | 0 | N | 81825 | Dynamin-1-like protein OS=Mus musculus OX=10090 GN=Dnm1l PE=1 SV=3 |
| 2524 | 5512 | A0A1B0GRI0\|A0A1B0GRI0\_MOUSE | 45.19 | 8 | 8 | 0 | 0 | 0 | 0 | 0 | 0 | 0 | 0 | 8 | 3.05E6 |  |  |  |  |  |  |  |  | 3.34E6 | 1 | 1 | 1 | 0 | 0 | 0 | 0 | 0 | 0 | 0 | 0 | 1 | N | 13194 | Reticulocalbin 3, EF-hand calcium binding domain (Fragment) OS=Mus musculus OX=10090 GN=Rcn3 PE=1 SV=1 |
| 2524 | 5532 | A0A1B0GR19\|A0A1B0GR19\_MOUSE | 45.19 | 3 | 3 | 0 | 0 | 0 | 0 | 0 | 0 | 0 | 0 | 3 | 3.05E6 |  |  |  |  |  |  |  |  | 3.34E6 | 1 | 1 | 1 | 0 | 0 | 0 | 0 | 0 | 0 | 0 | 0 | 1 | N | 31843 | Reticulocalbin 3, EF-hand calcium binding domain (Fragment) OS=Mus musculus OX=10090 GN=Rcn3 PE=1 SV=1 |
| 2524 | 5533 | A0A1B0GR86\|A0A1B0GR86\_MOUSE | 45.19 | 3 | 3 | 0 | 0 | 0 | 0 | 0 | 0 | 0 | 0 | 3 | 3.05E6 |  |  |  |  |  |  |  |  | 3.34E6 | 1 | 1 | 1 | 0 | 0 | 0 | 0 | 0 | 0 | 0 | 0 | 1 | N | 32524 | Reticulocalbin 3, EF-hand calcium binding domain (Fragment) OS=Mus musculus OX=10090 GN=Rcn3 PE=1 SV=1 |
| 2524 | 5536 | sp\|Q8BH97\|RCN3\_MOUSE | 45.19 | 3 | 3 | 0 | 0 | 0 | 0 | 0 | 0 | 0 | 0 | 3 | 3.05E6 |  |  |  |  |  |  |  |  | 3.34E6 | 1 | 1 | 1 | 0 | 0 | 0 | 0 | 0 | 0 | 0 | 0 | 1 | N | 38002 | Reticulocalbin-3 OS=Mus musculus OX=10090 GN=Rcn3 PE=1 SV=1 |
| 3296 | 4908 | A0A338P6Q0\|A0A338P6Q0\_MOUSE | 45.12 | 16 | 0 | 0 | 16 | 0 | 0 | 0 | 0 | 0 | 0 | 0 |  |  | 3.37E5 |  |  |  |  |  |  |  | 1 | 1 | 0 | 0 | 1 | 0 | 0 | 0 | 0 | 0 | 0 | 0 | N | 12435 | V-crk avian sarcoma virus CT10 oncogene homolog-like OS=Mus musculus OX=10090 GN=Crkl PE=1 SV=1 |
| 3296 | 4909 | A0A338P675\|A0A338P675\_MOUSE | 45.12 | 11 | 0 | 0 | 11 | 0 | 0 | 0 | 0 | 0 | 0 | 0 |  |  | 3.37E5 |  |  |  |  |  |  |  | 1 | 1 | 0 | 0 | 1 | 0 | 0 | 0 | 0 | 0 | 0 | 0 | N | 16816 | V-crk avian sarcoma virus CT10 oncogene homolog-like (Fragment) OS=Mus musculus OX=10090 GN=Crkl PE=1 SV=1 |
| 3296 | 4910 | sp\|P47941\|CRKL\_MOUSE | 45.12 | 6 | 0 | 0 | 6 | 0 | 0 | 0 | 0 | 0 | 0 | 0 |  |  | 3.37E5 |  |  |  |  |  |  |  | 1 | 1 | 0 | 0 | 1 | 0 | 0 | 0 | 0 | 0 | 0 | 0 | N | 33830 | Crk-like protein OS=Mus musculus OX=10090 GN=Crkl PE=1 SV=2 |
| 2516 | 4757 | Q8CCP6\|Q8CCP6\_MOUSE | 44.88 | 1 | 0 | 0 | 0 | 0 | 0 | 1 | 0 | 0 | 0 | 0 |  |  |  |  |  | 2.07E6 |  |  |  |  | 1 | 1 | 0 | 0 | 0 | 0 | 0 | 1 | 0 | 0 | 0 | 0 | N | 73091 | Zfp108 protein OS=Mus musculus OX=10090 GN=Zfp108 PE=2 SV=1 |
| 2516 | 4884 | Q8R1N3\|Q8R1N3\_MOUSE | 44.88 | 2 | 0 | 0 | 0 | 0 | 0 | 2 | 0 | 0 | 0 | 0 |  |  |  |  |  | 2.07E6 |  |  |  |  | 1 | 1 | 0 | 0 | 0 | 0 | 0 | 1 | 0 | 0 | 0 | 0 | N | 67518 | Predicted gene, OTTMUSG00000010657 OS=Mus musculus OX=10090 GN=Zfp991 PE=2 SV=2 |
| 2516 | 5008 | S4R299\|S4R299\_MOUSE | 44.88 | 0 | 0 | 0 | 0 | 0 | 0 | 0 | 0 | 0 | 0 | 0 |  |  |  |  |  | 2.07E6 |  |  |  |  | 1 | 1 | 0 | 0 | 0 | 0 | 0 | 1 | 0 | 0 | 0 | 0 | N | 202866 | Zinc finger protein 236 OS=Mus musculus OX=10090 GN=Zfp236 PE=4 SV=1 |
| 2516 | 5040 | Q8C393\|Q8C393\_MOUSE | 44.88 | 2 | 0 | 0 | 0 | 0 | 0 | 2 | 0 | 0 | 0 | 0 |  |  |  |  |  | 2.07E6 |  |  |  |  | 1 | 1 | 0 | 0 | 0 | 0 | 0 | 1 | 0 | 0 | 0 | 0 | N | 65340 | Zinc finger, imprinted 1 OS=Mus musculus OX=10090 GN=Zim1 PE=2 SV=1 |
| 2516 | 5046 | Q91W94\|Q91W94\_MOUSE | 44.88 | 2 | 0 | 0 | 0 | 0 | 0 | 2 | 0 | 0 | 0 | 0 |  |  |  |  |  | 2.07E6 |  |  |  |  | 1 | 1 | 0 | 0 | 0 | 0 | 0 | 1 | 0 | 0 | 0 | 0 | N | 44946 | Zfp71-rs1 protein OS=Mus musculus OX=10090 GN=Zfp65 PE=2 SV=1 |
| 2516 | 5066 | sp\|Q6GQR8\|ZN329\_MOUSE | 44.88 | 2 | 0 | 0 | 0 | 0 | 0 | 2 | 0 | 0 | 0 | 0 |  |  |  |  |  | 2.07E6 |  |  |  |  | 1 | 1 | 0 | 0 | 0 | 0 | 0 | 1 | 0 | 0 | 0 | 0 | N | 59832 | Zinc finger protein 329 OS=Mus musculus OX=10090 GN=Znf329 PE=1 SV=2 |
| 2516 | 5582 | Q91VM8\|Q91VM8\_MOUSE | 44.88 | 2 | 0 | 0 | 0 | 0 | 0 | 2 | 0 | 0 | 0 | 0 |  |  |  |  |  | 2.07E6 |  |  |  |  | 1 | 1 | 0 | 0 | 0 | 0 | 0 | 1 | 0 | 0 | 0 | 0 | N | 62669 | Zinc finger protein 959 OS=Mus musculus OX=10090 GN=Zfp959 PE=2 SV=1 |
| 2516 | 5583 | Q8K2A4\|Q8K2A4\_MOUSE | 44.88 | 2 | 0 | 0 | 0 | 0 | 0 | 2 | 0 | 0 | 0 | 0 |  |  |  |  |  | 2.07E6 |  |  |  |  | 1 | 1 | 0 | 0 | 0 | 0 | 0 | 1 | 0 | 0 | 0 | 0 | N | 63039 | Zinc finger protein 87 OS=Mus musculus OX=10090 GN=Zfp87 PE=2 SV=1 |
| 2516 | 5584 | Q8K0G9\|Q8K0G9\_MOUSE | 44.88 | 2 | 0 | 0 | 0 | 0 | 0 | 2 | 0 | 0 | 0 | 0 |  |  |  |  |  | 2.07E6 |  |  |  |  | 1 | 1 | 0 | 0 | 0 | 0 | 0 | 1 | 0 | 0 | 0 | 0 | N | 62408 | Zinc finger protein 119b OS=Mus musculus OX=10090 GN=Zfp119b PE=2 SV=1 |
| 2516 | 5585 | Q9JIC0\|Q9JIC0\_MOUSE | 44.88 | 2 | 0 | 0 | 0 | 0 | 0 | 2 | 0 | 0 | 0 | 0 |  |  |  |  |  | 2.07E6 |  |  |  |  | 1 | 1 | 0 | 0 | 0 | 0 | 0 | 1 | 0 | 0 | 0 | 0 | N | 63027 | KRAB zinc finger protein OS=Mus musculus OX=10090 GN=Zfp119a PE=2 SV=1 |
| 2516 | 5590 | Q9R166\|Q9R166\_MOUSE | 44.88 | 1 | 0 | 0 | 0 | 0 | 0 | 1 | 0 | 0 | 0 | 0 |  |  |  |  |  | 2.07E6 |  |  |  |  | 1 | 1 | 0 | 0 | 0 | 0 | 0 | 1 | 0 | 0 | 0 | 0 | N | 73456 | Zinc finger protein 109 OS=Mus musculus OX=10090 GN=Zfp109 PE=2 SV=1 |
| 2516 | 5594 | E9QAG4\|E9QAG4\_MOUSE | 44.88 | 1 | 0 | 0 | 0 | 0 | 0 | 1 | 0 | 0 | 0 | 0 |  |  |  |  |  | 2.07E6 |  |  |  |  | 1 | 1 | 0 | 0 | 0 | 0 | 0 | 1 | 0 | 0 | 0 | 0 | N | 78473 | RIKEN cDNA 4930522L14 gene OS=Mus musculus OX=10090 GN=4930522L14Rik PE=4 SV=1 |
| 2516 | 5598 | Q8BJ45\|Q8BJ45\_MOUSE | 44.88 | 1 | 0 | 0 | 0 | 0 | 0 | 1 | 0 | 0 | 0 | 0 |  |  |  |  |  | 2.07E6 |  |  |  |  | 1 | 1 | 0 | 0 | 0 | 0 | 0 | 1 | 0 | 0 | 0 | 0 | N | 85411 | Zinc finger protein 52 OS=Mus musculus OX=10090 GN=Zfp52 PE=2 SV=1 |
| 2131 | 4352 | sp\|P61211\|ARL1\_MOUSE | 44.53 | 6 | 0 | 6 | 6 | 0 | 0 | 0 | 0 | 0 | 0 | 0 |  | 8.51E5 | 9.34E5 |  |  |  |  |  |  |  | 1 | 1 | 0 | 1 | 1 | 0 | 0 | 0 | 0 | 0 | 0 | 0 | N | 20412 | ADP-ribosylation factor-like protein 1 OS=Mus musculus OX=10090 GN=Arl1 PE=1 SV=1 |
| 3301 | 5017 | sp\|Q9DCG6\|PBLD1\_MOUSE | 44.30 | 4 | 0 | 0 | 0 | 0 | 0 | 0 | 0 | 0 | 4 | 0 |  |  |  |  |  |  |  |  | 2.59E5 |  | 1 | 1 | 0 | 0 | 0 | 0 | 0 | 0 | 0 | 0 | 1 | 0 | N | 32048 | Phenazine biosynthesis-like domain-containing protein 1 OS=Mus musculus OX=10090 GN=Pbld1 PE=1 SV=2 |
| 3301 | 5018 | K3W4L7\|K3W4L7\_MOUSE | 44.30 | 4 | 0 | 0 | 0 | 0 | 0 | 0 | 0 | 0 | 4 | 0 |  |  |  |  |  |  |  |  | 2.59E5 |  | 1 | 1 | 0 | 0 | 0 | 0 | 0 | 0 | 0 | 0 | 1 | 0 | N | 32190 | Phenazine biosynthesis-like protein domain containing 1 OS=Mus musculus OX=10090 GN=Pbld1 PE=1 SV=1 |
| 3301 | 5497 | A0A1W2P7N2\|A0A1W2P7N2\_MOUSE | 44.30 | 9 | 0 | 0 | 0 | 0 | 0 | 0 | 0 | 0 | 9 | 0 |  |  |  |  |  |  |  |  | 2.59E5 |  | 1 | 1 | 0 | 0 | 0 | 0 | 0 | 0 | 0 | 0 | 1 | 0 | N | 14765 | Phenazine biosynthesis-like protein domain containing 1 (Fragment) OS=Mus musculus OX=10090 GN=Pbld1 PE=1 SV=1 |
| 3301 | 5501 | sp\|Q9CXN7\|PBLD2\_MOUSE | 44.30 | 4 | 0 | 0 | 0 | 0 | 0 | 0 | 0 | 0 | 4 | 0 |  |  |  |  |  |  |  |  | 2.59E5 |  | 1 | 1 | 0 | 0 | 0 | 0 | 0 | 0 | 0 | 0 | 1 | 0 | N | 31983 | Phenazine biosynthesis-like domain-containing protein 2 OS=Mus musculus OX=10090 GN=Pbld2 PE=1 SV=1 |
| 2133 | 4629 | sp\|Q8BK30\|NDUV3\_MOUSE | 44.16 | 11 | 0 | 0 | 0 | 11 | 0 | 0 | 11 | 11 | 0 | 0 |  |  |  | 8.54E5 |  |  | 7.14E5 | 7.83E5 |  |  | 1 | 1 | 0 | 0 | 0 | 1 | 0 | 0 | 1 | 1 | 0 | 0 | N | 11813 | NADH dehydrogenase [ubiquinone] flavoprotein 3, mitochondrial OS=Mus musculus OX=10090 GN=Ndufv3 PE=1 SV=1 |
| 3300 | 5014 | A0A2I3BRG8\|A0A2I3BRG8\_MOUSE | 44.10 | 7 | 0 | 0 | 0 | 0 | 0 | 0 | 0 | 0 | 0 | 7 |  |  |  |  |  |  |  |  |  | 5.17E6 | 1 | 1 | 0 | 0 | 0 | 0 | 0 | 0 | 0 | 0 | 0 | 1 | N | 19344 | Dehydrogenase/reductase 4 (Fragment) OS=Mus musculus OX=10090 GN=Dhrs4 PE=1 SV=1 |
| 3300 | 5015 | sp\|Q99LB2\|DHRS4\_MOUSE | 44.10 | 5 | 0 | 0 | 0 | 0 | 0 | 0 | 0 | 0 | 0 | 5 |  |  |  |  |  |  |  |  |  | 5.17E6 | 1 | 1 | 0 | 0 | 0 | 0 | 0 | 0 | 0 | 0 | 0 | 1 | N | 29885 | Dehydrogenase/reductase SDR family member 4 OS=Mus musculus OX=10090 GN=Dhrs4 PE=1 SV=3 |
| 3300 | 5508 | A0A2I3BQ25\|A0A2I3BQ25\_MOUSE | 44.10 | 10 | 0 | 0 | 0 | 0 | 0 | 0 | 0 | 0 | 0 | 10 |  |  |  |  |  |  |  |  |  | 5.17E6 | 1 | 1 | 0 | 0 | 0 | 0 | 0 | 0 | 0 | 0 | 0 | 1 | N | 13189 | Dehydrogenase/reductase 4 (Fragment) OS=Mus musculus OX=10090 GN=Dhrs4 PE=1 SV=1 |
| 3300 | 5509 | A0A2I3BPY3\|A0A2I3BPY3\_MOUSE | 44.10 | 7 | 0 | 0 | 0 | 0 | 0 | 0 | 0 | 0 | 0 | 7 |  |  |  |  |  |  |  |  |  | 5.17E6 | 1 | 1 | 0 | 0 | 0 | 0 | 0 | 0 | 0 | 0 | 0 | 1 | N | 21220 | Dehydrogenase/reductase 4 (Fragment) OS=Mus musculus OX=10090 GN=Dhrs4 PE=1 SV=1 |
| 3300 | 5510 | Q9D2U3\|Q9D2U3\_MOUSE | 44.10 | 6 | 0 | 0 | 0 | 0 | 0 | 0 | 0 | 0 | 0 | 6 |  |  |  |  |  |  |  |  |  | 5.17E6 | 1 | 1 | 0 | 0 | 0 | 0 | 0 | 0 | 0 | 0 | 0 | 1 | N | 23178 | Dehydrogenase/reductase 4 OS=Mus musculus OX=10090 GN=Dhrs4 PE=1 SV=1 |
| 1582 | 2917 | sp\|Q6P542\|ABCF1\_MOUSE | 44.07 | 2 | 0 | 1 | 0 | 1 | 0 | 0 | 0 | 0 | 0 | 1 |  | 3.77E5 |  | 1.77E5 |  |  |  |  |  | 3.21E5 | 2 | 2 | 0 | 1 | 0 | 1 | 0 | 0 | 0 | 0 | 0 | 1 | N | 94945 | ATP-binding cassette sub-family F member 1 OS=Mus musculus OX=10090 GN=Abcf1 PE=1 SV=1 |
| 1372 | 3115 | sp\|Q8CIN4\|PAK2\_MOUSE | 43.73 | 3 | 2 | 0 | 0 | 0 | 0 | 2 | 2 | 2 | 0 | 0 | 2.46E6 |  |  |  |  | 1.56E6 | 2.61E6 | 1.4E6 |  |  | 2 | 2 | 1 | 0 | 0 | 0 | 0 | 1 | 1 | 1 | 0 | 0 | Y | 57930 | Serine/threonine-protein kinase PAK 2 OS=Mus musculus OX=10090 GN=Pak2 PE=1 SV=1 |
| 3443 | 5539 | G3UYN6\|G3UYN6\_MOUSE | 43.42 | 14 | 0 | 14 | 0 | 0 | 0 | 0 | 0 | 0 | 0 | 0 |  | 1.16E6 |  |  |  |  |  |  |  |  | 1 | 1 | 0 | 1 | 0 | 0 | 0 | 0 | 0 | 0 | 0 | 0 | N | 9634 | StAR related lipid transfer domain containing 10 (Fragment) OS=Mus musculus OX=10090 GN=Stard10 PE=1 SV=1 |
| 3443 | 5540 | G3UYM0\|G3UYM0\_MOUSE | 43.42 | 8 | 0 | 8 | 0 | 0 | 0 | 0 | 0 | 0 | 0 | 0 |  | 1.16E6 |  |  |  |  |  |  |  |  | 1 | 1 | 0 | 1 | 0 | 0 | 0 | 0 | 0 | 0 | 0 | 0 | N | 17301 | StAR related lipid transfer domain containing 10 (Fragment) OS=Mus musculus OX=10090 GN=Stard10 PE=1 SV=1 |
| 3443 | 5541 | G3UY59\|G3UY59\_MOUSE | 43.42 | 8 | 0 | 8 | 0 | 0 | 0 | 0 | 0 | 0 | 0 | 0 |  | 1.16E6 |  |  |  |  |  |  |  |  | 1 | 1 | 0 | 1 | 0 | 0 | 0 | 0 | 0 | 0 | 0 | 0 | N | 18422 | StAR related lipid transfer domain containing 10 OS=Mus musculus OX=10090 GN=Stard10 PE=1 SV=1 |
| 3443 | 5542 | G3UW37\|G3UW37\_MOUSE | 43.42 | 5 | 0 | 5 | 0 | 0 | 0 | 0 | 0 | 0 | 0 | 0 |  | 1.16E6 |  |  |  |  |  |  |  |  | 1 | 1 | 0 | 1 | 0 | 0 | 0 | 0 | 0 | 0 | 0 | 0 | N | 29731 | StAR related lipid transfer domain containing 10 (Fragment) OS=Mus musculus OX=10090 GN=Stard10 PE=1 SV=1 |
| 3443 | 5543 | sp\|Q9JMD3\|STA10\_MOUSE | 43.42 | 4 | 0 | 4 | 0 | 0 | 0 | 0 | 0 | 0 | 0 | 0 |  | 1.16E6 |  |  |  |  |  |  |  |  | 1 | 1 | 0 | 1 | 0 | 0 | 0 | 0 | 0 | 0 | 0 | 0 | N | 32951 | START domain-containing protein 10 OS=Mus musculus OX=10090 GN=Stard10 PE=1 SV=1 |
| 3443 | 5544 | E9PVP0\|E9PVP0\_MOUSE | 43.42 | 3 | 0 | 3 | 0 | 0 | 0 | 0 | 0 | 0 | 0 | 0 |  | 1.16E6 |  |  |  |  |  |  |  |  | 1 | 1 | 0 | 1 | 0 | 0 | 0 | 0 | 0 | 0 | 0 | 0 | N | 40116 | StAR related lipid transfer domain containing 10 OS=Mus musculus OX=10090 GN=Stard10 PE=1 SV=1 |
| 3009 | 3736 | sp\|Q6DID3\|SCAF8\_MOUSE | 43.41 | 1 | 0 | 0 | 0 | 1 | 0 | 0 | 0 | 0 | 0 | 0 |  |  |  | 3.82E5 |  |  |  |  |  |  | 1 | 1 | 0 | 0 | 0 | 1 | 0 | 0 | 0 | 0 | 0 | 0 | N | 139572 | SR-related and CTD-associated factor 8 OS=Mus musculus OX=10090 GN=Scaf8 PE=1 SV=1 |
| 2115 | 4570 | sp\|Q8JZK9\|HMCS1\_MOUSE | 43.39 | 2 | 2 | 2 | 0 | 0 | 2 | 0 | 0 | 0 | 0 | 0 | 3.58E5 | 0E0 |  |  | 1.52E6 |  |  |  |  |  | 1 | 1 | 1 | 1 | 0 | 0 | 1 | 0 | 0 | 0 | 0 | 0 | N | 57569 | Hydroxymethylglutaryl-CoA synthase, cytoplasmic OS=Mus musculus OX=10090 GN=Hmgcs1 PE=1 SV=1 |
| 3444 | 5547 | A0A3B2W450\|A0A3B2W450\_MOUSE | 43.25 | 6 | 0 | 0 | 0 | 0 | 6 | 0 | 0 | 0 | 0 | 0 |  |  |  |  | 1.97E6 |  |  |  |  |  | 1 | 1 | 0 | 0 | 0 | 0 | 1 | 0 | 0 | 0 | 0 | 0 | N | 17042 | U1 small nuclear ribonucleoprotein C OS=Mus musculus OX=10090 GN=Snrpc PE=1 SV=1 |
| 3444 | 5548 | sp\|Q62241\|RU1C\_MOUSE | 43.25 | 6 | 0 | 0 | 0 | 0 | 6 | 0 | 0 | 0 | 0 | 0 |  |  |  |  | 1.97E6 |  |  |  |  |  | 1 | 1 | 0 | 0 | 0 | 0 | 1 | 0 | 0 | 0 | 0 | 0 | N | 17364 | U1 small nuclear ribonucleoprotein C OS=Mus musculus OX=10090 GN=Snrpc PE=1 SV=1 |
| 3444 | 5553 | A0A3B2WCR6\|A0A3B2WCR6\_MOUSE | 43.25 | 5 | 0 | 0 | 0 | 0 | 5 | 0 | 0 | 0 | 0 | 0 |  |  |  |  | 1.97E6 |  |  |  |  |  | 1 | 1 | 0 | 0 | 0 | 0 | 1 | 0 | 0 | 0 | 0 | 0 | N | 19709 | U1 small nuclear ribonucleoprotein C OS=Mus musculus OX=10090 GN=Snrpc PE=1 SV=1 |
| 1429 | 2826 | sp\|Q7TT50\|MRCKB\_MOUSE | 43.02 | 1 | 0 | 0 | 0 | 0 | 0 | 1 | 1 | 0 | 0 | 0 |  |  |  |  |  | 4.14E6 | 2.9E6 |  |  |  | 1 | 1 | 0 | 0 | 0 | 0 | 0 | 1 | 1 | 0 | 0 | 0 | N | 194751 | Serine/threonine-protein kinase MRCK beta OS=Mus musculus OX=10090 GN=Cdc42bpb PE=1 SV=2 |
| 3445 | 5554 | D3YWT1\|D3YWT1\_MOUSE | 43.00 | 4 | 0 | 0 | 0 | 0 | 0 | 4 | 0 | 0 | 0 | 0 |  |  |  |  |  | 0E0 |  |  |  |  | 1 | 1 | 0 | 0 | 0 | 0 | 0 | 1 | 0 | 0 | 0 | 0 | N | 35182 | Heterogeneous nuclear ribonucleoprotein H3 OS=Mus musculus OX=10090 GN=Hnrnph3 PE=1 SV=1 |
| 3445 | 5555 | D3Z3N4\|D3Z3N4\_MOUSE | 43.00 | 3 | 0 | 0 | 0 | 0 | 0 | 3 | 0 | 0 | 0 | 0 |  |  |  |  |  | 0E0 |  |  |  |  | 1 | 1 | 0 | 0 | 0 | 0 | 0 | 1 | 0 | 0 | 0 | 0 | N | 36869 | Heterogeneous nuclear ribonucleoprotein H3 OS=Mus musculus OX=10090 GN=Hnrnph3 PE=1 SV=1 |
| 2433 | 3228 | sp\|Q6NZR5\|SKI2\_MOUSE | 42.41 | 1 | 1 | 0 | 0 | 0 | 0 | 0 | 0 | 0 | 0 | 0 | 7.58E5 |  |  |  |  |  |  |  |  |  | 1 | 1 | 1 | 0 | 0 | 0 | 0 | 0 | 0 | 0 | 0 | 0 | N | 137524 | Superkiller complex protein 2 OS=Mus musculus OX=10090 GN=Skic2 PE=2 SV=1 |
| 2402 | 3362 | S4R2B0\|S4R2B0\_MOUSE | 42.25 | 3 | 0 | 0 | 0 | 0 | 0 | 3 | 3 | 0 | 0 | 0 |  |  |  |  |  | 2.38E5 | 2.52E5 |  |  |  | 1 | 1 | 0 | 0 | 0 | 0 | 0 | 1 | 1 | 0 | 0 | 0 | N | 71007 | RNA binding motif protein 25 (Fragment) OS=Mus musculus OX=10090 GN=Rbm25 PE=1 SV=1 |
| 2402 | 3364 | sp\|B2RY56\|RBM25\_MOUSE | 42.25 | 2 | 0 | 0 | 0 | 0 | 0 | 2 | 2 | 0 | 0 | 0 |  |  |  |  |  | 2.38E5 | 2.52E5 |  |  |  | 1 | 1 | 0 | 0 | 0 | 0 | 0 | 1 | 1 | 0 | 0 | 0 | N | 99552 | RNA-binding protein 25 OS=Mus musculus OX=10090 GN=Rbm25 PE=1 SV=2 |
| 3446 | 5556 | A0A2I3BR94\|A0A2I3BR94\_MOUSE | 42.21 | 17 | 0 | 0 | 0 | 0 | 0 | 0 | 17 | 0 | 0 | 0 |  |  |  |  |  |  | 4.65E5 |  |  |  | 1 | 1 | 0 | 0 | 0 | 0 | 0 | 0 | 1 | 0 | 0 | 0 | N | 15916 | Glia maturation factor beta (Fragment) OS=Mus musculus OX=10090 GN=Gmfb PE=1 SV=1 |
| 3446 | 5557 | sp\|Q9CQI3\|GMFB\_MOUSE | 42.21 | 16 | 0 | 0 | 0 | 0 | 0 | 0 | 16 | 0 | 0 | 0 |  |  |  |  |  |  | 4.65E5 |  |  |  | 1 | 1 | 0 | 0 | 0 | 0 | 0 | 0 | 1 | 0 | 0 | 0 | N | 16723 | Glia maturation factor beta OS=Mus musculus OX=10090 GN=Gmfb PE=1 SV=3 |
| 3446 | 5558 | A0A2I3BPS1\|A0A2I3BPS1\_MOUSE | 42.21 | 15 | 0 | 0 | 0 | 0 | 0 | 0 | 15 | 0 | 0 | 0 |  |  |  |  |  |  | 4.65E5 |  |  |  | 1 | 1 | 0 | 0 | 0 | 0 | 0 | 0 | 1 | 0 | 0 | 0 | N | 18377 | Glia maturation factor beta (Fragment) OS=Mus musculus OX=10090 GN=Gmfb PE=1 SV=1 |
| 2137 | 4878 | F6ZSB7\|F6ZSB7\_MOUSE | 42.15 | 6 | 6 | 0 | 0 | 0 | 0 | 0 | 0 | 0 | 6 | 6 | 8.91E5 |  |  |  |  |  |  |  | 1.21E6 | 8.11E5 | 1 | 1 | 1 | 0 | 0 | 0 | 0 | 0 | 0 | 0 | 1 | 1 | N | 15845 | 3-phosphoglycerate dehydrogenase (Fragment) OS=Mus musculus OX=10090 GN=Phgdh PE=1 SV=1 |
| 3448 | 5575 | sp\|Q8R2Y0\|ABHD6\_MOUSE | 42.12 | 4 | 0 | 0 | 0 | 0 | 0 | 4 | 0 | 0 | 0 | 0 |  |  |  |  |  | 5.94E5 |  |  |  |  | 1 | 1 | 0 | 0 | 0 | 0 | 0 | 1 | 0 | 0 | 0 | 0 | N | 38205 | Monoacylglycerol lipase ABHD6 OS=Mus musculus OX=10090 GN=Abhd6 PE=1 SV=1 |
| 2146 | 5549 | sp\|Q9D8B4\|NDUAB\_MOUSE | 42.12 | 8 | 0 | 8 | 0 | 8 | 0 | 0 | 0 | 0 | 8 | 0 |  | 2.58E5 |  | 7.48E5 |  |  |  |  | 1.52E6 |  | 1 | 1 | 0 | 1 | 0 | 1 | 0 | 0 | 0 | 0 | 1 | 0 | N | 14982 | NADH dehydrogenase [ubiquinone] 1 alpha subcomplex subunit 11 OS=Mus musculus OX=10090 GN=Ndufa11 PE=1 SV=2 |
| 2146 | 5550 | G5E814\|G5E814\_MOUSE | 42.12 | 8 | 0 | 8 | 0 | 8 | 0 | 0 | 0 | 0 | 8 | 0 |  | 2.58E5 |  | 7.48E5 |  |  |  |  | 1.52E6 |  | 1 | 1 | 0 | 1 | 0 | 1 | 0 | 0 | 0 | 0 | 1 | 0 | N | 15115 | NADH dehydrogenase [ubiquinone] 1 alpha subcomplex subunit 11 OS=Mus musculus OX=10090 GN=Ndufa11 PE=1 SV=1 |
| 2520 | 4965 | A2AI62\|A2AI62\_MOUSE | 42.00 | 2 | 0 | 0 | 0 | 0 | 0 | 2 | 0 | 0 | 2 | 0 |  |  |  |  |  | 0E0 |  |  |  |  | 1 | 1 | 0 | 0 | 0 | 0 | 0 | 1 | 0 | 0 | 1 | 0 | N | 95137 | Hephaestin OS=Mus musculus OX=10090 GN=Heph PE=1 SV=1 |
| 2443 | 4234 | sp\|Q19LI2\|A1BG\_MOUSE | 41.98 | 2 | 0 | 0 | 0 | 0 | 0 | 2 | 0 | 0 | 0 | 0 |  |  |  |  |  | 6.09E5 |  |  |  |  | 1 | 1 | 0 | 0 | 0 | 0 | 0 | 1 | 0 | 0 | 0 | 0 | N | 56554 | Alpha-1B-glycoprotein OS=Mus musculus OX=10090 GN=A1bg PE=1 SV=1 |
| 1917 | 5561 | D3YVS7\|D3YVS7\_MOUSE | 41.90 | 12 | 0 | 0 | 0 | 12 | 12 | 12 | 12 | 0 | 0 | 0 |  |  |  | 1.09E6 | 2.53E6 | 2.28E6 | 1.62E6 |  |  |  | 1 | 1 | 0 | 0 | 0 | 1 | 1 | 1 | 1 | 0 | 0 | 0 | N | 9165 | Cysteine-rich and transmembrane domain-containing protein 1 (Fragment) OS=Mus musculus OX=10090 GN=Cystm1 PE=1 SV=1 |
| 1917 | 5562 | sp\|Q8K353\|CYTM1\_MOUSE | 41.90 | 10 | 0 | 0 | 0 | 10 | 10 | 10 | 10 | 0 | 0 | 0 |  |  |  | 1.09E6 | 2.53E6 | 2.28E6 | 1.62E6 |  |  |  | 1 | 1 | 0 | 0 | 0 | 1 | 1 | 1 | 1 | 0 | 0 | 0 | N | 11402 | Cysteine-rich and transmembrane domain-containing protein 1 OS=Mus musculus OX=10090 GN=Cystm1 PE=1 SV=1 |
| 1936 | 5559 | F6V084\|F6V084\_MOUSE | 41.85 | 10 | 0 | 0 | 0 | 0 | 10 | 10 | 10 | 10 | 0 | 0 |  |  |  |  | 1.52E6 | 9.95E5 | 7.84E5 | 8.58E5 |  |  | 1 | 1 | 0 | 0 | 0 | 0 | 1 | 1 | 1 | 1 | 0 | 0 | N | 14462 | Thioredoxin-related transmembrane protein 1 (Fragment) OS=Mus musculus OX=10090 GN=Tmx1 PE=1 SV=1 |
| 1936 | 5560 | sp\|Q8VBT0\|TMX1\_MOUSE | 41.85 | 4 | 0 | 0 | 0 | 0 | 4 | 4 | 4 | 4 | 0 | 0 |  |  |  |  | 1.52E6 | 9.95E5 | 7.84E5 | 8.58E5 |  |  | 1 | 1 | 0 | 0 | 0 | 0 | 1 | 1 | 1 | 1 | 0 | 0 | N | 31396 | Thioredoxin-related transmembrane protein 1 OS=Mus musculus OX=10090 GN=Tmx1 PE=1 SV=1 |
| 1897 | 4785 | Z4YN97\|Z4YN97\_MOUSE | 41.70 | 13 | 0 | 0 | 13 | 0 | 13 | 13 | 13 | 0 | 0 | 0 |  |  | 2.88E6 |  | 6.21E6 | 7.02E6 | 3.03E6 |  |  |  | 1 | 1 | 0 | 0 | 1 | 0 | 1 | 1 | 1 | 0 | 0 | 0 | N | 9541 | nucleoside-triphosphate--adenylate kinase (Fragment) OS=Mus musculus OX=10090 GN=Ak1 PE=1 SV=1 |
| 1897 | 4786 | sp\|Q9R0Y5\|KAD1\_MOUSE | 41.70 | 6 | 0 | 0 | 6 | 0 | 6 | 6 | 6 | 0 | 0 | 0 |  |  | 2.88E6 |  | 6.21E6 | 7.02E6 | 3.03E6 |  |  |  | 1 | 1 | 0 | 0 | 1 | 0 | 1 | 1 | 1 | 0 | 0 | 0 | N | 21540 | Adenylate kinase isoenzyme 1 OS=Mus musculus OX=10090 GN=Ak1 PE=1 SV=1 |
| 1792 | 2982 | sp\|P28481\|CO2A1\_MOUSE | 41.67 | 1 | 0 | 1 | 0 | 0 | 0 | 0 | 0 | 0 | 0 | 0 |  | 6.89E6 |  |  |  |  |  |  |  |  | 1 | 1 | 0 | 1 | 0 | 0 | 0 | 0 | 0 | 0 | 0 | 0 | N | 141974 | Collagen alpha-1(II) chain OS=Mus musculus OX=10090 GN=Col2a1 PE=1 SV=2 |
| 2164 | 4251 | sp\|Q99MJ9\|DDX50\_MOUSE | 41.60 | 2 | 1 | 0 | 0 | 0 | 0 | 0 | 0 | 0 | 0 | 1 | 8.08E4 |  |  |  |  |  |  |  |  |  | 2 | 1 | 1 | 0 | 0 | 0 | 0 | 0 | 0 | 0 | 0 | 1 | N | 82176 | ATP-dependent RNA helicase DDX50 OS=Mus musculus OX=10090 GN=Ddx50 PE=2 SV=1 |
| 3449 | 5576 | sp\|Q80WK2\|OSTB\_MOUSE | 41.58 | 7 | 0 | 0 | 0 | 0 | 7 | 0 | 0 | 0 | 0 | 0 |  |  |  |  | 2.34E6 |  |  |  |  |  | 1 | 1 | 0 | 0 | 0 | 0 | 1 | 0 | 0 | 0 | 0 | 0 | N | 14684 | Organic solute transporter subunit beta OS=Mus musculus OX=10090 GN=Slc51b PE=1 SV=1 |
| 2173 | 5577 | D3YYX0\|D3YYX0\_MOUSE | 41.23 | 19 | 0 | 0 | 0 | 0 | 0 | 19 | 0 | 0 | 0 | 0 |  |  |  |  |  | 1.64E5 |  |  |  |  | 1 | 1 | 0 | 0 | 0 | 0 | 0 | 1 | 0 | 0 | 0 | 0 | N | 9075 | Thioredoxin-related transmembrane protein 2 (Fragment) OS=Mus musculus OX=10090 GN=Tmx2 PE=1 SV=1 |
| 2173 | 5578 | D3Z2J6\|D3Z2J6\_MOUSE | 41.23 | 6 | 0 | 0 | 0 | 0 | 0 | 6 | 0 | 0 | 0 | 0 |  |  |  |  |  | 1.64E5 |  |  |  |  | 1 | 1 | 0 | 0 | 0 | 0 | 0 | 1 | 0 | 0 | 0 | 0 | N | 29591 | Thioredoxin-related transmembrane protein 2 OS=Mus musculus OX=10090 GN=Tmx2 PE=1 SV=1 |
| 2173 | 5579 | sp\|Q9D710\|TMX2\_MOUSE | 41.23 | 5 | 0 | 0 | 0 | 0 | 0 | 5 | 0 | 0 | 0 | 0 |  |  |  |  |  | 1.64E5 |  |  |  |  | 1 | 1 | 0 | 0 | 0 | 0 | 0 | 1 | 0 | 0 | 0 | 0 | N | 33943 | Thioredoxin-related transmembrane protein 2 OS=Mus musculus OX=10090 GN=Tmx2 PE=1 SV=1 |
| 3451 | 5603 | D3Z3S1\|D3Z3S1\_MOUSE | 41.22 | 3 | 0 | 0 | 0 | 0 | 0 | 0 | 3 | 0 | 0 | 0 |  |  |  |  |  |  | 0E0 |  |  |  | 1 | 1 | 0 | 0 | 0 | 0 | 0 | 0 | 1 | 0 | 0 | 0 | N | 37965 | Prolactin regulatory element-binding protein OS=Mus musculus OX=10090 GN=Preb PE=1 SV=1 |
| 3451 | 5606 | sp\|Q9WUQ2\|SEC12\_MOUSE | 41.22 | 2 | 0 | 0 | 0 | 0 | 0 | 0 | 2 | 0 | 0 | 0 |  |  |  |  |  |  | 0E0 |  |  |  | 1 | 1 | 0 | 0 | 0 | 0 | 0 | 0 | 1 | 0 | 0 | 0 | N | 45437 | Guanine nucleotide-exchange factor SEC12 OS=Mus musculus OX=10090 GN=Preb PE=1 SV=1 |
| 1918 | 5566 | sp\|Q99NB1\|ACS2L\_MOUSE | 41.17 | 2 | 0 | 2 | 0 | 2 | 0 | 0 | 0 | 0 | 0 | 2 |  | 2.75E6 |  | 1.17E6 |  |  |  |  |  | 2.22E6 | 1 | 1 | 0 | 1 | 0 | 1 | 0 | 0 | 0 | 0 | 0 | 1 | N | 74623 | Acetyl-coenzyme A synthetase 2-like, mitochondrial OS=Mus musculus OX=10090 GN=Acss1 PE=1 SV=1 |
| 3447 | 5567 | sp\|P22907\|HEM3\_MOUSE | 41.12 | 6 | 0 | 0 | 0 | 0 | 0 | 0 | 0 | 0 | 6 | 0 |  |  |  |  |  |  |  |  | 2.61E5 |  | 1 | 1 | 0 | 0 | 0 | 0 | 0 | 0 | 0 | 0 | 1 | 0 | N | 39344 | Porphobilinogen deaminase OS=Mus musculus OX=10090 GN=Hmbs PE=1 SV=2 |
| 1938 | 5587 | A0A087WRY3\|A0A087WRY3\_MOUSE | 41.03 | 4 | 0 | 0 | 0 | 4 | 0 | 4 | 4 | 4 | 0 | 0 |  |  |  | 3.7E6 |  | 7.58E6 | 3.22E6 | 4.84E6 |  |  | 1 | 1 | 0 | 0 | 0 | 1 | 0 | 1 | 1 | 1 | 0 | 0 | N | 26184 | Nuclear casein kinase and cyclin-dependent kinase substrate 1 OS=Mus musculus OX=10090 GN=Nucks1 PE=1 SV=1 |
| 1938 | 5588 | sp\|Q80XU3\|NUCKS\_MOUSE | 41.03 | 4 | 0 | 0 | 0 | 4 | 0 | 4 | 4 | 4 | 0 | 0 |  |  |  | 3.7E6 |  | 7.58E6 | 3.22E6 | 4.84E6 |  |  | 1 | 1 | 0 | 0 | 0 | 1 | 0 | 1 | 1 | 1 | 0 | 0 | N | 26313 | Nuclear ubiquitous casein and cyclin-dependent kinase substrate 1 OS=Mus musculus OX=10090 GN=Nucks1 PE=1 SV=1 |
| 2132 | 4516 | sp\|Q9DBC7\|KAP0\_MOUSE | 40.92 | 3 | 0 | 3 | 0 | 0 | 0 | 0 | 0 | 0 | 3 | 0 |  | 8.4E5 |  |  |  |  |  |  | 1.35E6 |  | 1 | 1 | 0 | 1 | 0 | 0 | 0 | 0 | 0 | 0 | 1 | 0 | N | 43185 | cAMP-dependent protein kinase type I-alpha regulatory subunit OS=Mus musculus OX=10090 GN=Prkar1a PE=1 SV=3 |
| 2172 | 5573 | sp\|Q8C0E2\|VP26B\_MOUSE | 40.61 | 3 | 0 | 3 | 0 | 0 | 0 | 0 | 0 | 0 | 3 | 3 |  | 3.76E6 |  |  |  |  |  |  | 2.46E6 | 1.81E6 | 1 | 1 | 0 | 1 | 0 | 0 | 0 | 0 | 0 | 0 | 1 | 1 | N | 39125 | Vacuolar protein sorting-associated protein 26B OS=Mus musculus OX=10090 GN=Vps26b PE=1 SV=1 |
| 1991 | 3259 | sp\|E9Q555\|RN213\_MOUSE | 40.59 | 0 | 0 | 0 | 0 | 0 | 0 | 0 | 0 | 0 | 0 | 0 |  |  |  |  | 4.88E5 |  |  |  |  |  | 1 | 1 | 0 | 0 | 0 | 0 | 1 | 0 | 0 | 0 | 0 | 0 | N | 584265 | E3 ubiquitin-protein ligase RNF213 OS=Mus musculus OX=10090 GN=Rnf213 PE=1 SV=3 |
| 1991 | 3260 | A0A171EBL2\|A0A171EBL2\_MOUSE | 40.59 | 0 | 0 | 0 | 0 | 0 | 0 | 0 | 0 | 0 | 0 | 0 |  |  |  |  | 4.88E5 |  |  |  |  |  | 1 | 1 | 0 | 0 | 0 | 0 | 1 | 0 | 0 | 0 | 0 | 0 | N | 584635 | Ring finger protein 213 OS=Mus musculus OX=10090 GN=Rnf213 PE=1 SV=1 |
| 1991 | 3261 | A0A8I4P9C4\|A0A8I4P9C4\_MOUSE | 40.59 | 0 | 0 | 0 | 0 | 0 | 0 | 0 | 0 | 0 | 0 | 0 |  |  |  |  | 4.88E5 |  |  |  |  |  | 1 | 1 | 0 | 0 | 0 | 0 | 1 | 0 | 0 | 0 | 0 | 0 | N | 584791 | Ring finger protein 213 OS=Mus musculus OX=10090 GN=Rnf213 PE=1 SV=1 |
| 1985 | 3034 | sp\|A2AN08\|UBR4\_MOUSE | 40.59 | 0 | 0 | 0 | 0 | 0 | 0 | 0 | 0 | 0 | 0 | 0 |  |  |  |  |  |  |  |  | 2.04E5 |  | 1 | 1 | 0 | 0 | 0 | 0 | 0 | 0 | 0 | 0 | 1 | 0 | N | 572298 | E3 ubiquitin-protein ligase UBR4 OS=Mus musculus OX=10090 GN=Ubr4 PE=1 SV=1 |
| 1937 | 5580 | A0A1L1SRY4\|A0A1L1SRY4\_MOUSE | 40.49 | 4 | 4 | 0 | 0 | 0 | 0 | 4 | 4 | 0 | 0 | 0 | 5.54E5 |  |  |  |  | 1.07E6 | 2.32E5 |  |  |  | 1 | 1 | 1 | 0 | 0 | 0 | 0 | 1 | 1 | 0 | 0 | 0 | N | 50146 | Predicted gene, 38431 (Fragment) OS=Mus musculus OX=10090 GN=Gm38431 PE=1 SV=1 |
| 1937 | 5581 | sp\|Q9CW46\|RAVR1\_MOUSE | 40.49 | 3 | 3 | 0 | 0 | 0 | 0 | 3 | 3 | 0 | 0 | 0 | 5.54E5 |  |  |  |  | 1.07E6 | 2.32E5 |  |  |  | 1 | 1 | 1 | 0 | 0 | 0 | 0 | 1 | 1 | 0 | 0 | 0 | N | 79382 | Ribonucleoprotein PTB-binding 1 OS=Mus musculus OX=10090 GN=Raver1 PE=1 SV=2 |
| 2565 | 5618 | sp\|P56528\|CD38\_MOUSE | 40.32 | 3 | 0 | 0 | 0 | 0 | 0 | 3 | 0 | 0 | 0 | 0 |  |  |  |  |  | 2.98E5 |  |  |  |  | 1 | 1 | 0 | 0 | 0 | 0 | 0 | 1 | 0 | 0 | 0 | 0 | N | 34408 | ADP-ribosyl cyclase/cyclic ADP-ribose hydrolase 1 OS=Mus musculus OX=10090 GN=Cd38 PE=1 SV=2 |
| 1431 | 2872 | sp\|Q80U35\|ARHGH\_MOUSE | 40.26 | 0 | 0 | 0 | 0 | 0 | 0 | 0 | 0 | 0 | 0 | 0 |  |  | 7.74E6 |  |  | 9.25E6 |  |  |  | 3.22E6 | 1 | 1 | 0 | 0 | 1 | 0 | 0 | 1 | 0 | 0 | 0 | 1 | N | 221669 | Rho guanine nucleotide exchange factor 17 OS=Mus musculus OX=10090 GN=Arhgef17 PE=1 SV=2 |
| 1434 | 3232 | sp\|Q5XG71\|UTP20\_MOUSE | 40.18 | 0 | 0 | 0 | 0 | 0 | 0 | 0 | 0 | 0 | 0 | 0 |  |  |  | 7.85E5 |  |  |  |  |  |  | 1 | 1 | 0 | 0 | 0 | 1 | 0 | 0 | 0 | 0 | 0 | 0 | N | 317744 | Small subunit processome component 20 homolog OS=Mus musculus OX=10090 GN=Utp20 PE=1 SV=2 |
| 2564 | 5589 | sp\|Q8BGB7\|ENOPH\_MOUSE | 40.13 | 5 | 0 | 0 | 0 | 0 | 0 | 0 | 0 | 0 | 5 | 5 |  |  |  |  |  |  |  |  | 7.45E5 | 1.95E5 | 1 | 1 | 0 | 0 | 0 | 0 | 0 | 0 | 0 | 0 | 1 | 1 | N | 28600 | Enolase-phosphatase E1 OS=Mus musculus OX=10090 GN=Enoph1 PE=1 SV=1 |
| 2472 | 3403 | sp\|P51863\|VA0D1\_MOUSE | 40.02 | 3 | 0 | 0 | 0 | 0 | 0 | 0 | 0 | 0 | 0 | 3 |  |  |  |  |  |  |  |  |  | 2.1E6 | 1 | 1 | 0 | 0 | 0 | 0 | 0 | 0 | 0 | 0 | 0 | 1 | N | 40301 | V-type proton ATPase subunit d 1 OS=Mus musculus OX=10090 GN=Atp6v0d1 PE=1 SV=2 |
| 3452 | 5613 | A0A2R8W753\|A0A2R8W753\_MOUSE | 39.96 | 8 | 0 | 0 | 8 | 0 | 0 | 0 | 0 | 0 | 0 | 0 |  |  | 3.27E6 |  |  |  |  |  |  |  | 1 | 1 | 0 | 0 | 1 | 0 | 0 | 0 | 0 | 0 | 0 | 0 | N | 15373 | RAN GTPase activating protein 1 (Fragment) OS=Mus musculus OX=10090 GN=Rangap1 PE=1 SV=1 |
| 3452 | 5614 | A0A2R8VHK9\|A0A2R8VHK9\_MOUSE | 39.96 | 5 | 0 | 0 | 5 | 0 | 0 | 0 | 0 | 0 | 0 | 0 |  |  | 3.27E6 |  |  |  |  |  |  |  | 1 | 1 | 0 | 0 | 1 | 0 | 0 | 0 | 0 | 0 | 0 | 0 | N | 27117 | RAN GTPase activating protein 1 (Fragment) OS=Mus musculus OX=10090 GN=Rangap1 PE=1 SV=1 |
| 3452 | 5616 | sp\|P46061\|RAGP1\_MOUSE | 39.96 | 2 | 0 | 0 | 2 | 0 | 0 | 0 | 0 | 0 | 0 | 0 |  |  | 3.27E6 |  |  |  |  |  |  |  | 1 | 1 | 0 | 0 | 1 | 0 | 0 | 0 | 0 | 0 | 0 | 0 | N | 63531 | Ran GTPase-activating protein 1 OS=Mus musculus OX=10090 GN=Rangap1 PE=1 SV=2 |
| 1237 | 3735 | sp\|Q3UQ84\|SYTM\_MOUSE | 39.67 | 1 | 0 | 1 | 0 | 0 | 0 | 0 | 0 | 0 | 0 | 0 |  | 3.43E6 |  |  |  |  |  |  |  |  | 1 | 1 | 0 | 1 | 0 | 0 | 0 | 0 | 0 | 0 | 0 | 0 | N | 81700 | Threonine--tRNA ligase, mitochondrial OS=Mus musculus OX=10090 GN=Tars2 PE=1 SV=1 |
| 1237 | 3761 | Q3TP97\|Q3TP97\_MOUSE | 39.67 | 2 | 0 | 2 | 0 | 0 | 0 | 0 | 0 | 0 | 0 | 0 |  | 3.43E6 |  |  |  |  |  |  |  |  | 1 | 1 | 0 | 1 | 0 | 0 | 0 | 0 | 0 | 0 | 0 | 0 | N | 47813 | Threonyl-tRNA synthetase 2, mitochondrial (putative) OS=Mus musculus OX=10090 GN=Tars2 PE=1 SV=1 |
| 1964 | 2896 | E9Q3P4\|E9Q3P4\_MOUSE | 39.61 | 1 | 0 | 0 | 0 | 0 | 0 | 0 | 0 | 0 | 0 | 0 | 1.33E7 |  |  |  |  |  | 2.08E5 |  |  |  | 2 | 2 | 1 | 0 | 0 | 0 | 0 | 0 | 1 | 0 | 0 | 0 | N | 342472 | Centromere protein F OS=Mus musculus OX=10090 GN=Cenpf PE=1 SV=1 |
| 3450 | 5602 | sp\|P63054\|PCP4\_MOUSE | 39.50 | 16 | 0 | 0 | 0 | 0 | 0 | 0 | 16 | 0 | 0 | 0 |  |  |  |  |  |  | 4.24E6 |  |  |  | 1 | 1 | 0 | 0 | 0 | 0 | 0 | 0 | 1 | 0 | 0 | 0 | N | 6807 | Calmodulin regulator protein PCP4 OS=Mus musculus OX=10090 GN=Pcp4 PE=3 SV=2 |
| 3450 | 5604 | D3YXH0\|D3YXH0\_MOUSE | 39.50 | 3 | 0 | 0 | 0 | 0 | 0 | 0 | 3 | 0 | 0 | 0 |  |  |  |  |  |  | 4.24E6 |  |  |  | 1 | 1 | 0 | 0 | 0 | 0 | 0 | 0 | 1 | 0 | 0 | 0 | N | 32628 | Predicted gene, 49948 OS=Mus musculus OX=10090 GN=Gm49948 PE=4 SV=1 |
| total 2399 proteins |  |  |  |  |  |  |  |  |  |  |  |  |  |  |  |  |  |  |  |  |  |  |  |  |  |  |  |  |  |  |  |  |  |  |  |  |  |  |  |
| --- | --- | --- | --- | --- | --- | --- | --- | --- | --- | --- | --- | --- | --- | --- | --- | --- | --- | --- | --- | --- | --- | --- | --- | --- | --- | --- | --- | --- | --- | --- | --- | --- | --- | --- | --- | --- | --- | --- | --- |

  

A0A338P6K2|A0A338P6K2\_MOUSE

back to list

  

| Protein Coverage
| Supporting Peptides
| Best Unique PSM
|

Protein Coverage:

Supporting Peptides:

| Peptide | Uniq | -10lgP | Mass | Length | ppm | m/z | z | RT | Fraction | Scan | Source File | Area 10 | Area 01 | Area 02 | Area 03 | Area 04 | Area 05 | Area 06 | Area 07 | Area 08 | Area 09 | #Spec | #Spec 10 | #Spec 01 | #Spec 02 | #Spec 03 | #Spec 04 | #Spec 05 | #Spec 06 | #Spec 07 | #Spec 08 | #Spec 09 | Start | End | PTM |
| --- | --- | --- | --- | --- | --- | --- | --- | --- | --- | --- | --- | --- | --- | --- | --- | --- | --- | --- | --- | --- | --- | --- | --- | --- | --- | --- | --- | --- | --- | --- | --- | --- | --- | --- | --- |
| R.KLEGDASDFHEQIADLQAQIAELK.M | N | 159.90 | 2668.3342 | 24 | -0.7 | 890.4514 | 3 | 124.42 | 3 | 34168 | Ana\_02.raw | 6.47E7 | 1.55E8 | 1.33E8 | 1.05E7 | 1.64E8 | 2.73E8 | 1.13E8 | 5.72E7 | 1.76E8 | 7.7E7 | 55 | 5 | 6 | 8 | 4 | 6 | 5 | 3 | 8 | 5 | 5 | 1066 | 1089 |  |
| K.DVASLGSQLQDTQELLQEETR.Q | N | 149.74 | 2359.1504 | 21 | 0.6 | 1180.5831 | 2 | 121.54 | 3 | 33431 | Ana\_02.raw | 7.72E7 | 3.61E7 | 1.47E8 | 4.55E7 | 9.2E7 | 1.65E8 | 6.84E7 | 3.28E7 | 2.37E8 | 8.75E7 | 56 | 4 | 7 | 5 | 5 | 6 | 4 | 5 | 7 | 6 | 7 | 1316 | 1336 |  |
| R.DLGEELEALKTELEDTLDSTATQQELR.A | N | 138.52 | 3046.4829 | 27 | 7.3 | 1016.5090 | 3 | 138.44 | 9 | 38224 | Ana\_08.raw | 3.23E6 | 6.46E6 | 1.63E6 |  | 1.78E6 | 8.62E6 | 2.99E5 |  | 1.03E7 | 9.25E6 | 12 | 1 | 2 | 1 | 0 | 1 | 3 | 1 | 0 | 1 | 2 | 1150 | 1176 |  |
| K.HTQAVEELTEQLEQFK.R | N | 133.51 | 1928.9479 | 16 | 2.4 | 965.4835 | 2 | 109.11 | 6 | 30645 | Ana\_05.raw | 5.81E7 | 8.76E7 | 1.83E7 | 1.72E7 | 7.48E7 | 1.09E8 | 4.92E7 | 1.34E7 | 1.06E8 | 4.94E6 | 53 | 4 | 6 | 5 | 3 | 6 | 6 | 3 | 9 | 6 | 5 | 1208 | 1223 |  |
| R.HEM(+15.99)PPHIYAIADTAYR.S | N | 129.92 | 1899.8937 | 16 | 1.7 | 634.3063 | 3 | 63.28 | 3 | 18023 | Ana\_02.raw | 5.99E7 | 8.34E7 | 7.48E7 | 1.53E7 | 5.08E7 | 5.82E7 | 3.66E7 | 4.15E7 | 9.56E7 | 4.68E7 | 22 | 3 | 3 | 2 | 1 | 2 | 2 | 2 | 2 | 3 | 2 | 148 | 163 | Oxidation (M) |
| R.KQADLEKEELAEELASSLSGR.N | N | 127.02 | 2302.1650 | 21 | -0.5 | 768.3953 | 3 | 97.95 | 3 | 27359 | Ana\_02.raw | 6.13E7 | 6.62E7 | 9.15E7 | 2.64E6 | 2.89E7 | 4.03E7 | 2.23E7 | 9.37E6 | 1.26E8 | 5.89E7 | 27 | 4 | 2 | 3 | 3 | 2 | 1 | 3 | 4 | 3 | 2 | 1711 | 1731 |  |
| R.KATLQAEQLSNELATER.S | N | 123.88 | 1900.9854 | 17 | 2.6 | 951.5024 | 2 | 69.14 | 3 | 19657 | Ana\_02.raw | 5.33E7 | 7.5E7 | 5.97E7 | 1.77E7 | 3.76E7 | 7.17E7 | 2.38E7 | 4.11E7 | 9.24E7 | 4.28E7 | 30 | 4 | 3 | 2 | 2 | 3 | 3 | 2 | 4 | 4 | 3 | 1768 | 1784 |  |
| R.LQQELDDLVVDLDNQR.Q | N | 119.49 | 1911.9537 | 16 | 0.7 | 638.3256 | 3 | 114.73 | 3 | 31696 | Ana\_02.raw | 2.19E8 | 3.6E7 | 6.58E7 | 7.99E7 | 1.62E8 | 2.16E8 | 8.92E7 | 1.4E8 | 2.63E8 | 1.07E8 | 63 | 7 | 6 | 7 | 5 | 5 | 6 | 4 | 8 | 8 | 7 | 1432 | 1447 |  |
| K.HTQAVEELTEQLEQFKR.A | N | 118.81 | 2085.0491 | 17 | 5.3 | 696.0273 | 3 | 97.61 | 2 | 27502 | Ana\_01.raw | 6.29E7 | 1.46E7 | 3.45E6 | 4.86E6 | 2.96E6 | 2.97E7 | 9.91E5 | 5.07E6 | 6.08E7 | 2.67E7 | 23 | 3 | 2 | 2 | 2 | 3 | 2 | 2 | 1 | 3 | 3 | 1208 | 1224 |  |
| K.ATLQAEQLSNELATER.S | N | 116.90 | 1772.8904 | 16 | 3.7 | 887.4557 | 2 | 82.32 | 6 | 23502 | Ana\_05.raw | 6.3E7 | 9.03E7 | 3.97E7 | 2.43E7 | 4.89E7 | 7.85E7 | 2.99E7 | 4.17E7 | 1.04E8 | 3.9E7 | 33 | 5 | 4 | 2 | 3 | 3 | 3 | 3 | 3 | 4 | 3 | 1769 | 1784 |  |
| K.TQLEELEDELQATEDAK.L | N | 116.11 | 1960.9113 | 17 | 1.2 | 981.4641 | 2 | 112.32 | 3 | 31061 | Ana\_02.raw | 1.36E8 | 2.22E8 | 1.17E8 | 7.57E7 | 1.05E8 | 1.7E8 | 8.31E7 | 4.83E7 | 2.7E8 | 1.45E8 | 68 | 10 | 10 | 5 | 5 | 5 | 4 | 5 | 9 | 8 | 7 | 1553 | 1569 |  |
| K.LEGDASDFHEQIADLQAQIAELK.M | N | 115.03 | 2540.2393 | 23 | 4.4 | 847.7574 | 3 | 129.25 | 9 | 35796 | Ana\_08.raw | 1.95E7 | 2.72E7 | 1.16E7 | 9.86E6 | 1.69E7 | 3.3E7 | 1.42E7 | 1.24E7 | 2.81E7 | 1.71E7 | 11 | 2 | 1 | 1 | 1 | 1 | 1 | 1 | 1 | 1 | 1 | 1067 | 1089 |  |
| K.VEDM(+15.99)AELTC(+57.02)LNEASVLHNLR.E | N | 114.91 | 2329.1042 | 20 | 0.2 | 777.3755 | 3 | 98.97 | 3 | 27632 | Ana\_02.raw | 1.71E7 | 6.41E6 | 3.37E7 | 8.86E5 | 7.27E6 | 1.13E7 | 1.52E6 | 2.99E6 | 9.78E7 | 2.58E7 | 21 | 3 | 2 | 3 | 1 | 2 | 3 | 1 | 1 | 4 | 1 | 87 | 106 | Oxidation (M); Carbamidomethylation |
| K.QADLEKEELAEELASSLSGR.N | N | 114.68 | 2174.0703 | 20 | 2.5 | 725.6992 | 3 | 109.89 | 6 | 30848 | Ana\_05.raw | 1.17E7 | 2.12E7 | 2.44E7 | 7.14E6 | 1.99E7 | 4.01E7 | 1.12E7 | 1.33E7 | 3.31E7 | 2.17E6 | 20 | 1 | 2 | 3 | 3 | 2 | 3 | 1 | 2 | 2 | 1 | 1712 | 1731 |  |
| R.ELEGHISDLQEDLDSER.A | N | 114.66 | 1983.9021 | 17 | -0.4 | 992.9579 | 2 | 81.47 | 3 | 23007 | Ana\_02.raw | 8.87E7 | 1.12E7 | 6.26E7 | 1.25E7 | 5.39E7 | 5.99E7 | 7.4E6 | 1.47E7 | 6.39E7 | 7.53E7 | 27 | 4 | 1 | 3 | 1 | 4 | 3 | 3 | 3 | 3 | 2 | 1122 | 1138 |  |
| K.NM(+15.99)DPLNDNVTSLLNASSDK.F | N | 114.43 | 2062.9478 | 19 | 0.4 | 1032.4816 | 2 | 97.63 | 3 | 27276 | Ana\_02.raw | 2.75E7 | 2.95E7 | 3.52E7 | 5.79E6 | 3.04E6 | 2.52E7 | 3.66E6 | 8.43E6 | 5.22E7 | 1.38E7 | 26 | 3 | 4 | 3 | 2 | 2 | 3 | 2 | 3 | 2 | 2 | 602 | 620 | Oxidation (M) |
| K.SLEADLM(+15.99)QLQEDLAAAER.A | N | 112.51 | 2017.9625 | 18 | 4.2 | 1009.9927 | 2 | 134.02 | 7 | 36928 | Ana\_06.raw | 5.3E7 | 7.73E7 | 6.11E7 | 3.12E7 | 4.73E7 | 1.56E8 | 3.76E7 | 1.93E7 | 1.1E8 | 6.31E7 | 87 | 8 | 16 | 13 | 2 | 6 | 8 | 3 | 7 | 14 | 10 | 1691 | 1708 | Oxidation (M) |
| K.M(+15.99)AQQM(+15.99)LDLEEQLEEEEAAR.Q | N | 110.76 | 2294.0042 | 19 | 0.3 | 1148.0096 | 2 | 91.09 | 3 | 25554 | Ana\_02.raw | 1.39E7 | 4.96E6 | 9.72E6 | 8.79E5 | 1.01E7 | 4.57E6 |  | 1.82E6 | 8.38E6 |  | 17 | 3 | 2 | 2 | 2 | 2 | 2 | 0 | 1 | 3 | 0 | 955 | 973 | Oxidation (M) |
| R.VSDLTTNLAEEEEK.A | N | 110.12 | 1576.7468 | 14 | 2.8 | 789.3829 | 2 | 63.60 | 6 | 18249 | Ana\_05.raw | 1.31E8 | 1.92E8 | 1.43E8 | 4.08E7 | 8.46E7 | 1.1E8 | 5.46E7 | 8.41E7 | 1.61E8 | 8.77E7 | 41 | 5 | 6 | 5 | 3 | 3 | 3 | 3 | 4 | 5 | 4 | 1015 | 1028 |  |
| K.TELEDTLDSTATQQELR.A | N | 108.59 | 1948.9225 | 17 | 1.1 | 975.4695 | 2 | 80.55 | 3 | 22763 | Ana\_02.raw | 8.58E5 | 1.09E7 | 1.61E6 | 5.1E5 |  | 6.53E6 |  | 2.14E5 | 7.34E6 | 2.52E6 | 8 | 1 | 1 | 1 | 1 | 0 | 1 | 0 | 1 | 1 | 1 | 1160 | 1176 |  |
| K.DSSITQGPSFAYGELEK.Q | Y | 107.12 | 1827.8527 | 17 | 2.3 | 914.9357 | 2 | 91.62 | 6 | 26019 | Ana\_05.raw | 9.83E7 | 1.3E8 | 9.42E7 | 2.56E7 | 6.12E7 | 1.02E8 | 4.05E7 | 5.99E7 | 1.62E8 | 7.56E7 | 41 | 4 | 4 | 6 | 2 | 5 | 4 | 2 | 6 | 4 | 4 | 207 | 223 |  |
| R.ALETQM(+15.99)EEM(+15.99)KTQLEELEDELQATEDAK.L | N | 105.94 | 3183.4321 | 27 | -0.2 | 1062.1511 | 3 | 114.45 | 3 | 31624 | Ana\_02.raw | 4.63E7 | 1.55E7 | 8.02E6 | 2.59E6 | 1.6E7 | 1.91E7 | 6.25E6 | 2.76E7 | 3.93E7 | 4.9E5 | 19 | 2 | 2 | 2 | 1 | 2 | 2 | 2 | 3 | 2 | 1 | 1543 | 1569 | Oxidation (M) |
| K.SLEADLMQLQEDLAAAER.A | N | 104.96 | 2001.9677 | 18 | 1.9 | 1001.9930 | 2 | 134.96 | 9 | 37315 | Ana\_08.raw |  | 5E5 |  |  |  | 1.9E6 |  |  | 1.95E6 | 3.44E6 | 5 | 0 | 1 | 0 | 0 | 0 | 1 | 0 | 0 | 1 | 2 | 1691 | 1708 |  |
| K.IRELEGHISDLQEDLDSER.A | N | 104.93 | 2253.0872 | 19 | 6.2 | 752.0410 | 3 | 83.44 | 2 | 23659 | Ana\_01.raw | 2.67E6 | 1.81E6 |  |  |  | 1.64E6 |  |  | 7.52E6 | 5.57E6 | 5 | 1 | 1 | 0 | 0 | 0 | 1 | 0 | 0 | 1 | 1 | 1120 | 1138 |  |
| K.TLLQEQLQAETELYAEAEEM(+15.99)R.V | N | 104.54 | 2510.1846 | 21 | 1.2 | 837.7365 | 3 | 125.64 | 6 | 34949 | Ana\_05.raw | 1.91E7 | 8.37E7 | 2.26E7 | 3.25E6 | 7.91E7 | 1.57E8 | 1.63E7 | 4.21E7 | 1.21E8 | 6.55E6 | 56 | 5 | 6 | 9 | 4 | 5 | 7 | 2 | 7 | 8 | 3 | 897 | 917 | Oxidation (M) |
| K.KLEDDILVM(+15.99)DDQNSK.L | N | 103.26 | 1777.8403 | 15 | 4.0 | 889.9310 | 2 | 60.00 | 6 | 17162 | Ana\_05.raw | 2.48E7 | 1E7 | 6.06E6 | 1.61E6 | 3.71E6 | 8.41E6 | 3.45E6 | 3.93E6 | 3.85E7 | 1.29E7 | 13 | 2 | 1 | 1 | 1 | 1 | 1 | 1 | 1 | 2 | 2 | 989 | 1003 | Oxidation (M) |
| K.LQQLFNHTMFILEQEEYQR.E | N | 102.05 | 2466.2002 | 19 | 4.9 | 823.0780 | 3 | 118.36 | 6 | 33058 | Ana\_05.raw |  |  |  |  |  | 4.77E6 |  |  |  |  | 1 | 0 | 0 | 0 | 0 | 0 | 1 | 0 | 0 | 0 | 0 | 490 | 508 |  |
| K.M(+15.99)AQQMLDLEEQLEEEEAAR.Q | N | 101.73 | 2278.0093 | 19 | 2.6 | 1140.0149 | 2 | 105.76 | 9 | 29767 | Ana\_08.raw |  |  |  |  |  |  |  |  | 2.69E6 |  | 2 | 0 | 0 | 0 | 0 | 0 | 0 | 0 | 0 | 2 | 0 | 955 | 973 | Oxidation (M) |
| R.LEEEEDRGQQLQAER.K | N | 99.43 | 1828.8551 | 15 | 6.1 | 915.4404 | 2 | 38.08 | 1 | 10426 | ANa\_10.raw | 3.25E8 | 3.81E8 | 2.77E8 | 1E8 | 2.38E8 | 3.24E8 | 1.51E8 | 2.13E8 | 1.92E8 | 2.08E8 | 52 | 6 | 8 | 4 | 4 | 4 | 5 | 4 | 6 | 7 | 4 | 938 | 952 |  |
| K.EELAEELASSLSGR.N | N | 98.34 | 1489.7260 | 14 | 2.0 | 745.8718 | 2 | 111.24 | 6 | 31198 | Ana\_05.raw | 7.14E5 | 7.56E6 | 7.3E6 | 6.42E6 | 7.57E6 | 1.48E7 | 6.18E6 | 8.38E6 | 1.9E6 | 5.32E6 | 17 | 1 | 1 | 1 | 1 | 3 | 3 | 2 | 3 | 1 | 1 | 1718 | 1731 |  |
| K.QLLQANPILEAFGNAK.T | N | 97.96 | 1725.9413 | 16 | 2.9 | 863.9804 | 2 | 125.10 | 6 | 34810 | Ana\_05.raw | 1.57E7 | 1.55E7 | 4.42E7 | 4.06E6 | 2.63E7 | 7.1E7 | 2.22E7 | 4.41E7 | 7.06E7 | 1.03E7 | 45 | 6 | 4 | 8 | 4 | 4 | 4 | 3 | 5 | 4 | 3 | 224 | 239 |  |
| K.VEDMAELTC(+57.02)LNEASVLHNLR.E | N | 96.90 | 2313.1094 | 20 | 0.7 | 772.0443 | 3 | 115.12 | 6 | 32207 | Ana\_05.raw | 1.11E6 | 1.57E7 |  |  |  | 8.58E6 |  | 9.72E5 |  | 1.54E7 | 5 | 1 | 1 | 0 | 0 | 0 | 1 | 0 | 1 | 0 | 1 | 87 | 106 | Carbamidomethylation |
| K.LQQLFNHTM(+15.99)FILEQEEYQR.E | N | 96.51 | 2482.1951 | 19 | 2.7 | 828.4079 | 3 | 103.10 | 5 | 28829 | Ana\_04.raw | 6.34E6 |  | 2.18E7 |  | 2.15E7 | 2.26E7 | 1.24E6 | 8.44E4 | 2.31E7 |  | 8 | 1 | 0 | 1 | 0 | 1 | 1 | 1 | 1 | 2 | 0 | 490 | 508 | Oxidation (M) |
| K.KQELEEILHEM(+15.99)EAR.L | N | 95.61 | 1769.8617 | 14 | 2.9 | 590.9629 | 3 | 102.05 | 6 | 28795 | Ana\_05.raw | 4.13E7 | 3.89E7 | 3.62E6 | 8.18E6 | 8.32E5 | 2.86E7 | 2.01E6 | 1.61E7 | 4.11E7 | 1.93E7 | 20 | 3 | 2 | 1 | 2 | 1 | 2 | 1 | 2 | 4 | 2 | 924 | 937 | Oxidation (M) |
| R.HVSTLNIQLSDSK.K | N | 95.47 | 1440.7572 | 13 | 5.1 | 721.3895 | 2 | 56.36 | 5 | 15999 | Ana\_04.raw | 1.49E7 | 2.59E7 | 2.13E7 | 7.07E6 | 1.18E7 | 1.67E7 | 8.24E6 | 1.25E7 | 1.19E7 | 1.3E7 | 21 | 2 | 3 | 2 | 2 | 2 | 2 | 2 | 2 | 2 | 2 | 1372 | 1384 |  |
| R.ALEEALEAKEELER.T | N | 94.95 | 1628.8257 | 14 | 0.6 | 815.4206 | 2 | 69.89 | 9 | 19947 | Ana\_08.raw | 2.64E7 | 1.3E7 | 9.85E6 | 4.02E6 | 1.01E7 | 1.87E7 | 4.91E6 | 6.41E6 | 1.08E7 | 1.45E7 | 21 | 3 | 2 | 2 | 2 | 2 | 2 | 2 | 2 | 2 | 2 | 1498 | 1511 |  |
| R.EDQSILC(+57.02)TGESGAGK.T | N | 94.65 | 1550.6882 | 15 | 0.4 | 776.3517 | 2 | 46.91 | 3 | 13245 | Ana\_02.raw | 6.73E7 | 9.75E7 | 5.91E7 | 2.44E7 | 4.17E7 | 6.67E7 | 3.22E7 | 4.18E7 | 1.11E8 | 4.88E7 | 27 | 4 | 5 | 2 | 2 | 3 | 2 | 1 | 3 | 3 | 2 | 170 | 184 | Carbamidomethylation |
| R.TFHIFYYLLAGAK.E | N | 94.59 | 1542.8235 | 13 | 9.1 | 515.2864 | 3 | 127.05 | 6 | 35318 | Ana\_05.raw |  | 3.12E6 | 2.45E6 | 4.13E6 | 7.52E6 | 1.54E7 | 7.86E6 | 6.87E6 |  | 1.71E6 | 15 | 0 | 1 | 2 | 2 | 2 | 2 | 2 | 2 | 0 | 2 | 287 | 299 |  |
| K.IAQLEEQVEQEAR.E | N | 94.51 | 1541.7686 | 13 | 3.8 | 771.8945 | 2 | 69.28 | 1 | 19636 | ANa\_10.raw | 1.56E8 | 1.81E8 | 1.31E8 | 4.51E7 | 1.09E8 | 1.24E8 | 5.75E7 | 9.21E7 | 2.09E8 | 1.37E8 | 41 | 4 | 5 | 4 | 2 | 4 | 5 | 2 | 5 | 6 | 4 | 1830 | 1842 |  |
| K.LQNEVESVTGM(+15.99)LNEAEGK.A | N | 93.53 | 1962.9204 | 18 | 3.1 | 982.4705 | 2 | 84.53 | 1 | 23809 | ANa\_10.raw | 1.35E7 | 3.64E7 | 1.13E7 |  | 1.66E6 | 3.19E6 | 3.06E5 |  | 5.83E7 | 2.91E6 | 21 | 2 | 6 | 2 | 0 | 1 | 2 | 1 | 0 | 4 | 3 | 1292 | 1309 | Oxidation (M) |
| R.TGVLAHLEEER.D | N | 92.10 | 1252.6411 | 11 | 2.1 | 627.3292 | 2 | 57.41 | 3 | 16300 | Ana\_02.raw | 1.44E8 | 2.05E8 | 1.82E8 | 5.64E7 | 1.17E8 | 1.42E8 | 7.9E7 | 8.68E7 | 2.48E8 | 1.16E8 | 63 | 7 | 9 | 8 | 4 | 6 | 4 | 3 | 9 | 8 | 5 | 779 | 789 |  |
| K.VDYNASAWLTK.N | N | 91.97 | 1266.6244 | 11 | 2.5 | 634.3210 | 2 | 84.38 | 6 | 24059 | Ana\_05.raw | 2.32E7 | 2.4E7 | 2E7 | 1.18E7 | 2.27E7 | 3.62E7 | 1.28E7 | 1.94E7 | 5.66E7 | 1.45E7 | 17 | 2 | 2 | 1 | 2 | 2 | 1 | 1 | 2 | 3 | 1 | 591 | 601 |  |
| R.VSDLTTNLAEEEEKAK.N | N | 91.90 | 1775.8788 | 16 | 3.3 | 888.9496 | 2 | 64.62 | 6 | 18560 | Ana\_05.raw | 5.28E7 | 4.52E7 | 1.6E6 |  | 3.46E7 | 4.49E7 | 1.7E7 | 2.47E7 | 6.96E7 | 4.04E7 | 17 | 3 | 2 | 1 | 0 | 2 | 2 | 1 | 2 | 2 | 2 | 1015 | 1030 |  |
| K.VVSSVLQLGNIVFK.K | N | 91.84 | 1501.8867 | 14 | 1.1 | 751.9515 | 2 | 125.10 | 3 | 34346 | Ana\_02.raw | 8.1E6 | 2.52E7 | 3.71E7 | 2.53E7 | 4.43E7 | 8.21E7 | 7.4E6 | 3.99E7 | 8.05E7 | 4.18E7 | 40 | 4 | 5 | 7 | 3 | 4 | 3 | 2 | 3 | 6 | 3 | 356 | 369 |  |
| K.LDAFLVLEQLR.C | N | 91.07 | 1315.7500 | 11 | 3.3 | 658.8845 | 2 | 130.52 | 6 | 36264 | Ana\_05.raw | 1.24E7 | 4.75E7 | 2.28E7 | 2.23E7 | 4.47E7 | 6.74E7 | 3.85E7 | 2.66E7 | 3.2E7 | 2.15E7 | 57 | 2 | 10 | 6 | 4 | 11 | 8 | 4 | 5 | 3 | 4 | 697 | 707 |  |
| R.SHEAQVQEMR.Q | N | 90.82 | 1213.5509 | 10 | 4.0 | 607.7852 | 2 | 22.42 | 9 | 5433 | Ana\_08.raw | 2.07E7 | 9.7E6 |  |  |  |  |  |  | 1.56E7 | 1.03E7 | 8 | 2 | 2 | 0 | 0 | 0 | 0 | 0 | 0 | 2 | 2 | 1196 | 1205 |  |
| K.VVSSVLQLGNIVFKK.E | N | 90.15 | 1629.9817 | 15 | 5.1 | 816.0023 | 2 | 113.24 | 6 | 31713 | Ana\_05.raw | 9.4E7 | 6.04E7 | 1.37E8 | 4.71E7 | 1.23E8 | 1.61E8 | 9.34E7 | 7.37E7 | 1.4E8 | 3.6E7 | 74 | 7 | 9 | 8 | 7 | 8 | 8 | 6 | 7 | 6 | 8 | 356 | 370 |  |
| K.RQLEEAEEESQR.I | N | 89.47 | 1502.6960 | 12 | 5.5 | 752.3594 | 2 | 24.69 | 9 | 6199 | Ana\_08.raw | 1.58E7 | 2.24E7 | 7.07E6 | 7.31E6 | 4.29E6 | 7.84E6 | 2.12E6 | 9.29E6 | 1.43E7 | 7.57E6 | 16 | 2 | 2 | 1 | 2 | 1 | 1 | 1 | 2 | 2 | 2 | 1891 | 1902 |  |
| K.NM(+15.99)DPLNDNVTSLLNASSDKFVADLWK.D | N | 88.92 | 2922.4070 | 26 | 3.2 | 975.1461 | 3 | 132.73 | 6 | 36897 | Ana\_05.raw |  |  |  |  |  | 2.3E6 |  |  |  |  | 1 | 0 | 0 | 0 | 0 | 0 | 1 | 0 | 0 | 0 | 0 | 602 | 627 | Oxidation (M) |
| R.SHEAQVQEM(+15.99)R.Q | N | 87.74 | 1229.5459 | 10 | 1.7 | 615.7812 | 2 | 17.04 | 5 | 3993 | Ana\_04.raw | 7.94E6 |  | 4.97E6 | 3.62E7 | 7.45E7 | 1.64E8 | 6.63E7 | 7.6E7 | 5.62E6 | 3.98E6 | 14 | 1 | 0 | 1 | 2 | 2 | 2 | 2 | 2 | 1 | 1 | 1196 | 1205 | Oxidation (M) |
| R.NSLQDQLDEEM(+15.99)EAK.Q | N | 84.86 | 1664.7200 | 14 | 1.5 | 833.3685 | 2 | 58.98 | 3 | 16774 | Ana\_02.raw | 6.22E7 | 8.5E7 | 5.25E7 | 1.77E7 | 4.14E7 | 5.79E7 | 2.9E7 | 3.86E7 | 9.22E7 | 3.81E7 | 30 | 3 | 3 | 2 | 2 | 3 | 4 | 3 | 4 | 3 | 3 | 1353 | 1366 | Oxidation (M) |
| R.RGNEASFVPSR.R | N | 84.31 | 1218.6105 | 11 | 4.2 | 407.2125 | 3 | 36.30 | 6 | 10012 | Ana\_05.raw | 3.24E7 | 3.48E7 | 2.17E7 | 1.49E7 | 2.58E7 | 3.73E7 | 1.12E7 | 1.04E7 | 2.68E7 | 1.74E7 | 14 | 1 | 1 | 1 | 2 | 2 | 2 | 2 | 1 | 1 | 1 | 1937 | 1947 |  |
| R.ELDEATESNEAM(+15.99)GR.E | N | 84.11 | 1566.6467 | 14 | -0.4 | 784.3303 | 2 | 32.74 | 3 | 8938 | Ana\_02.raw | 1.98E8 | 2.47E8 | 2.41E8 | 7.86E7 | 1.72E8 | 2.32E8 | 1.23E8 | 1.52E8 | 1.99E8 | 1.23E8 | 31 | 3 | 2 | 3 | 2 | 5 | 3 | 3 | 4 | 3 | 3 | 1913 | 1926 | Oxidation (M) |
| K.VC(+57.02)HLVGINVTDFTR.A | N | 83.20 | 1629.8297 | 14 | 4.7 | 544.2864 | 3 | 89.32 | 2 | 25270 | Ana\_01.raw |  | 2.95E6 | 1.38E6 |  | 1.27E6 | 7.6E6 | 8.52E5 | 1.61E6 | 5.91E6 | 1.8E6 | 11 | 0 | 2 | 1 | 1 | 1 | 1 | 1 | 1 | 2 | 1 | 388 | 401 | Carbamidomethylation |
| R.YEILAANAIPK.G | N | 82.90 | 1201.6707 | 11 | 3.9 | 601.8450 | 2 | 81.05 | 6 | 23160 | Ana\_05.raw | 6E7 | 9.81E7 | 1.02E8 | 3.65E7 | 6.62E7 | 7.76E7 | 3.29E7 | 5.77E7 | 1.45E8 | 5.75E7 | 35 | 5 | 5 | 4 | 2 | 3 | 1 | 2 | 3 | 6 | 4 | 735 | 745 |  |
| K.NFM(+15.99)NSPM(+15.99)AQADWVAK.K | N | 82.41 | 1740.7600 | 15 | -0.9 | 871.3865 | 2 | 64.52 | 3 | 18376 | Ana\_02.raw | 1.91E7 | 2.93E7 | 2.1E7 | 8.31E6 | 1.69E7 | 2.65E7 | 2.58E6 | 1.86E7 | 2.81E7 | 6.03E6 | 17 | 2 | 2 | 2 | 1 | 2 | 2 | 1 | 2 | 2 | 1 | 19 | 33 | Oxidation (M) |
| K.KLEVQLQDLQSK.C | N | 82.13 | 1427.7983 | 12 | 2.1 | 714.9080 | 2 | 61.63 | 3 | 17556 | Ana\_02.raw | 1.85E7 | 1.77E7 | 1.49E7 | 1.37E6 | 1.07E7 | 1.53E7 | 4.11E6 | 9.1E6 | 1.19E7 | 1.11E7 | 19 | 2 | 2 | 2 | 1 | 2 | 2 | 1 | 2 | 3 | 2 | 1263 | 1274 |  |
| R.QLEEAEEESQR.I | N | 81.80 | 1346.5950 | 11 | 1.4 | 674.3057 | 2 | 27.38 | 3 | 7315 | Ana\_02.raw | 4.18E7 | 4.82E7 | 5.09E7 | 2.22E7 | 3.65E7 | 5.49E7 | 1.72E7 | 2.64E7 | 5.42E6 | 5.41E6 | 18 | 2 | 2 | 2 | 2 | 2 | 2 | 1 | 2 | 2 | 1 | 1892 | 1902 |  |
| K.KEEELQAALAR.L | N | 81.72 | 1256.6724 | 11 | 3.0 | 629.3453 | 2 | 48.00 | 6 | 13598 | Ana\_05.raw | 1.52E8 | 1.91E8 | 1.81E8 | 6.35E7 | 1.28E8 | 2.09E8 | 9.44E7 | 1.29E8 | 1.4E8 | 1.62E8 | 43 | 5 | 4 | 4 | 4 | 4 | 5 | 3 | 5 | 5 | 4 | 1095 | 1105 |  |
| K.AEM(+15.99)EDLVSSKDDVGK.N | N | 81.11 | 1637.7454 | 15 | 1.3 | 819.8810 | 2 | 38.88 | 1 | 10671 | ANa\_10.raw | 1.64E7 | 1.55E7 | 2.05E7 | 6.42E6 | 1.13E7 | 3.13E7 | 1.42E7 | 1.22E7 | 1.42E7 | 9.48E6 | 23 | 2 | 2 | 2 | 2 | 1 | 3 | 3 | 2 | 4 | 2 | 1518 | 1532 | Oxidation (M) |
| K.ALELDPNLYR.I | N | 81.07 | 1202.6295 | 10 | 2.4 | 602.3235 | 2 | 85.18 | 6 | 24273 | Ana\_05.raw | 9.75E7 | 1.34E8 | 1.19E8 | 4.21E7 | 8.95E7 | 1.03E8 | 5.04E7 | 5.85E7 | 2.02E8 | 6.89E7 | 54 | 5 | 7 | 7 | 5 | 5 | 5 | 4 | 6 | 6 | 4 | 760 | 769 |  |
| R.ELDEATESNEAMGR.E | N | 80.15 | 1550.6519 | 14 | 3.8 | 776.3361 | 2 | 45.47 | 1 | 12685 | ANa\_10.raw | 9.05E6 | 4.54E6 |  |  |  |  |  |  |  | 5.99E6 | 4 | 1 | 2 | 0 | 0 | 0 | 0 | 0 | 0 | 0 | 1 | 1913 | 1926 |  |
| K.LQDFASTIEVM(+15.99)EEGK.K | N | 79.47 | 1711.7975 | 15 | 2.9 | 856.9085 | 2 | 83.87 | 6 | 23920 | Ana\_05.raw | 1.04E7 | 1.24E7 | 6.18E6 | 1.05E6 | 6.27E6 | 8.8E6 | 6.52E5 | 3.79E6 | 1.75E7 | 5.67E6 | 17 | 2 | 2 | 1 | 1 | 2 | 1 | 1 | 2 | 3 | 2 | 1387 | 1401 | Oxidation (M) |
| K.VIQYLAVVASSHK.G | N | 79.27 | 1413.7980 | 13 | 2.5 | 707.9080 | 2 | 75.00 | 6 | 21515 | Ana\_05.raw | 4.3E6 | 7.02E6 | 2.63E6 | 1.24E6 | 9.33E5 | 4.14E6 |  | 2.72E6 | 6.77E6 | 3.02E6 | 9 | 1 | 1 | 1 | 1 | 1 | 1 | 0 | 1 | 1 | 1 | 191 | 203 |  |
| K.LQNEVESVTGMLNEAEGK.A | N | 79.09 | 1946.9255 | 18 | 3.2 | 974.4732 | 2 | 106.89 | 9 | 30065 | Ana\_08.raw |  |  |  |  |  |  |  |  | 1.51E7 |  | 2 | 0 | 0 | 0 | 0 | 0 | 0 | 0 | 0 | 2 | 0 | 1292 | 1309 |  |
| R.NTDQASM(+15.99)PDNTAAQK.V | N | 77.94 | 1606.6893 | 15 | 0.7 | 804.3525 | 2 | 17.95 | 5 | 4299 | Ana\_04.raw |  |  |  | 1.79E7 | 4.46E7 | 2.32E6 | 2.28E7 | 2.37E7 |  |  | 7 | 0 | 0 | 0 | 1 | 2 | 1 | 1 | 2 | 0 | 0 | 373 | 387 | Oxidation (M) |
| K.ATYERLFRWILSR.V | N | 77.91 | 1709.9365 | 13 | 3.3 | 428.4928 | 4 | 125.23 | 5 | 34534 | Ana\_04.raw |  |  |  | 2.73E5 | 1.72E6 | 3.4E6 | 6.6E5 |  |  | 7.61E5 | 6 | 0 | 0 | 0 | 1 | 2 | 1 | 1 | 0 | 0 | 1 | 434 | 446 |  |
| R.QLHEYETELEDER.K | N | 77.63 | 1689.7482 | 13 | 2.2 | 845.8832 | 2 | 59.45 | 1 | 16806 | ANa\_10.raw | 3.06E6 | 2.99E6 |  |  |  |  |  |  | 4.11E6 |  | 3 | 1 | 1 | 0 | 0 | 0 | 0 | 0 | 0 | 1 | 0 | 1604 | 1616 |  |
| K.TEFSIIHYAGK.V | N | 77.58 | 1264.6451 | 11 | 3.9 | 633.3323 | 2 | 69.33 | 2 | 19759 | Ana\_01.raw | 3.43E7 | 5.88E7 | 5.55E7 | 1.24E7 | 3.91E7 | 6.24E7 | 1.54E7 | 2.08E7 | 3.31E7 | 4.55E7 | 26 | 2 | 4 | 2 | 2 | 3 | 3 | 2 | 3 | 2 | 3 | 580 | 590 |  |
| K.ENADLAGELR.V | N | 77.43 | 1086.5305 | 10 | 1.0 | 544.2731 | 2 | 49.24 | 3 | 13919 | Ana\_02.raw | 1.78E8 | 2.26E8 | 2.02E8 | 7.09E7 | 1.46E8 | 1.82E8 | 9.26E7 | 1.29E8 | 1.9E8 | 1.21E8 | 51 | 5 | 4 | 5 | 4 | 4 | 5 | 4 | 8 | 8 | 4 | 1239 | 1248 |  |
| R.GNEASFVPSR.R | N | 77.39 | 1062.5094 | 10 | 3.3 | 532.2637 | 2 | 45.10 | 6 | 12725 | Ana\_05.raw | 6.77E7 | 9.42E7 | 8.77E7 | 3.18E7 | 7.18E7 | 8.84E7 | 4.75E7 | 5.02E7 | 6.49E7 | 5.93E7 | 20 | 3 | 2 | 2 | 2 | 3 | 2 | 2 | 2 | 1 | 1 | 1938 | 1947 |  |
| K.LEDDILVM(+15.99)DDQNSK.L | N | 76.67 | 1649.7454 | 14 | 3.7 | 825.8830 | 2 | 65.35 | 6 | 18763 | Ana\_05.raw | 2.38E6 | 1.16E6 | 4.12E6 |  |  | 1.92E6 | 8.79E5 | 2.58E6 | 6.03E6 | 5.36E5 | 8 | 1 | 1 | 1 | 0 | 0 | 1 | 1 | 1 | 1 | 1 | 990 | 1003 | Oxidation (M) |
| K.M(+15.99)AEQYKEQAEK.G | N | 76.03 | 1369.6183 | 11 | 3.8 | 685.8190 | 2 | 19.83 | 1 | 4708 | ANa\_10.raw | 3.81E6 | 3.25E5 | 1.87E6 |  | 1.35E6 | 9.38E6 | 3.39E6 | 1.18E6 |  | 4.9E6 | 12 | 2 | 1 | 1 | 0 | 1 | 2 | 2 | 1 | 0 | 2 | 1871 | 1881 | Oxidation (M) |
| K.KQELEEILHEMEAR.L | N | 75.82 | 1753.8668 | 14 | 3.7 | 585.6317 | 3 | 100.26 | 1 | 28046 | ANa\_10.raw | 3.76E6 |  |  |  |  |  |  |  |  |  | 1 | 1 | 0 | 0 | 0 | 0 | 0 | 0 | 0 | 0 | 0 | 924 | 937 |  |
| K.KFDQLLAEEK.N | N | 75.17 | 1219.6448 | 10 | 2.0 | 610.8309 | 2 | 53.25 | 3 | 15096 | Ana\_02.raw | 7E7 | 8.1E7 | 7.96E7 | 3.21E7 | 6.01E7 | 7.54E7 | 3.96E7 | 3.99E7 | 1.26E8 | 5.97E7 | 37 | 4 | 4 | 3 | 3 | 4 | 4 | 4 | 3 | 4 | 4 | 1459 | 1468 |  |
| R.VIENTDGSEEEM(+15.99)DAR.D | N | 74.67 | 1709.7050 | 15 | 3.8 | 855.8630 | 2 | 32.83 | 6 | 8983 | Ana\_05.raw | 1.29E7 | 1.6E7 | 9.72E6 | 3.72E6 |  | 1.2E7 | 1.07E6 | 7.3E6 | 8.07E6 | 7.46E6 | 9 | 1 | 1 | 1 | 1 | 0 | 1 | 1 | 1 | 1 | 1 | 1954 | 1968 | Oxidation (M) |
| K.EM(+15.99)EGLSQQYEEK.A | N | 73.31 | 1485.6293 | 12 | 5.4 | 743.8259 | 2 | 39.52 | 6 | 11003 | Ana\_05.raw | 4.6E7 | 4.93E7 |  | 1.53E7 | 3.56E7 | 5.38E7 | 2.44E7 | 3.27E7 | 3.98E7 | 2.76E7 | 19 | 3 | 2 | 0 | 1 | 1 | 3 | 1 | 3 | 3 | 2 | 1407 | 1418 | Oxidation (M) |
| K.HESM(+15.99)ISELEVR.L | N | 73.11 | 1344.6344 | 11 | 3.1 | 673.3265 | 2 | 51.34 | 7 | 14439 | Ana\_06.raw | 1.11E7 | 2.22E7 | 2.73E7 | 6.07E6 | 1.61E7 | 2.3E7 | 1.2E7 | 1.7E7 |  | 3.86E6 | 24 | 2 | 2 | 3 | 3 | 3 | 3 | 3 | 4 | 0 | 1 | 1039 | 1049 | Oxidation (M) |
| R.SGKLDAFLVLEQLR.C | N | 71.83 | 1587.8984 | 14 | 1.4 | 530.3075 | 3 | 121.03 | 6 | 33756 | Ana\_05.raw |  | 7.43E5 |  |  |  | 3.98E5 | 2.34E5 |  |  |  | 3 | 0 | 1 | 0 | 0 | 0 | 1 | 1 | 0 | 0 | 0 | 694 | 707 |  |
| K.LQDFASTIEVMEEGK.K | N | 71.66 | 1695.8025 | 15 | 4.7 | 848.9125 | 2 | 110.63 | 10 | 30552 | Ana\_09.raw |  |  |  |  |  | 8.17E5 |  |  | 1.6E7 | 2.73E7 | 3 | 0 | 0 | 0 | 0 | 0 | 1 | 0 | 0 | 1 | 1 | 1387 | 1401 |  |
| K.FDQLLAEEK.N | N | 71.45 | 1091.5498 | 9 | 2.9 | 546.7838 | 2 | 58.18 | 6 | 16636 | Ana\_05.raw | 3.53E7 | 3.9E7 | 4.13E7 | 1.48E7 | 2.94E7 | 4.16E7 | 2.35E7 | 2.34E7 | 5.36E7 | 2.71E7 | 11 | 1 | 1 | 1 | 1 | 1 | 1 | 1 | 1 | 2 | 1 | 1460 | 1468 |  |
| K.M(+15.99)TESSLPSASK.T | N | 68.97 | 1152.5332 | 11 | 3.5 | 577.2759 | 2 | 27.47 | 6 | 7335 | Ana\_05.raw | 3.72E7 | 1.32E8 | 0 | 6.96E7 | 1.23E8 | 2.09E8 | 1E8 | 3.2E7 | 1.31E8 |  | 15 | 1 | 1 | 1 | 1 | 2 | 2 | 2 | 3 | 2 | 0 | 641 | 651 | Oxidation (M) |
| R.ALEEALEAK.E | N | 68.87 | 972.5127 | 9 | 1.7 | 487.2645 | 2 | 44.90 | 3 | 12627 | Ana\_02.raw | 1.13E8 | 1.41E8 | 1.47E8 | 5.37E7 | 1.14E8 | 1.46E8 | 7.47E7 | 8.94E7 | 1.33E8 | 9.07E7 | 38 | 4 | 4 | 5 | 4 | 4 | 3 | 2 | 5 | 3 | 4 | 1498 | 1506 |  |
| K.DLELQADSAIK.G | N | 67.66 | 1201.6190 | 11 | 5.4 | 601.8200 | 2 | 68.82 | 6 | 19744 | Ana\_05.raw | 2.42E7 | 3.1E7 | 2.32E7 | 1.38E7 | 2.23E7 | 2.9E7 | 1.87E7 | 1.52E7 | 2.51E7 | 1.91E7 | 19 | 3 | 2 | 3 | 2 | 1 | 2 | 1 | 1 | 3 | 1 | 1635 | 1645 |  |
| R.LDEEIAQK.N | N | 67.52 | 944.4814 | 8 | 2.9 | 473.2494 | 2 | 30.85 | 6 | 8388 | Ana\_05.raw | 2.45E8 | 3.03E8 | 2.14E8 | 9.41E7 | 1.58E8 | 2.46E8 | 1.16E8 | 1.39E8 | 2.26E8 | 1.46E8 | 26 | 4 | 4 | 4 | 2 | 1 | 2 | 1 | 2 | 3 | 3 | 1106 | 1113 |  |
| K.EQADFAIEALAK.A | N | 67.21 | 1304.6611 | 12 | 3.3 | 653.3400 | 2 | 90.14 | 6 | 25623 | Ana\_05.raw | 1.03E8 | 1.07E8 | 1.05E8 | 3.27E7 | 6.53E7 | 7.22E7 | 4.35E7 | 5.16E7 | 1.8E8 | 8.13E7 | 42 | 4 | 5 | 4 | 3 | 4 | 4 | 4 | 3 | 6 | 5 | 422 | 433 |  |
| K.DKTEFSIIHYAGK.V | N | 67.14 | 1507.7671 | 13 | 8.5 | 754.8972 | 2 | 62.66 | 9 | 17847 | Ana\_08.raw | 1.68E7 | 2.15E7 | 1.33E7 |  | 1.24E7 | 1.86E7 | 9.2E6 | 7.06E6 | 3.79E7 | 1.81E7 | 11 | 1 | 2 | 1 | 0 | 1 | 1 | 1 | 1 | 2 | 1 | 578 | 590 |  |
| K.EMEGLSQQYEEK.A | N | 65.04 | 1469.6344 | 12 | 4.1 | 735.8275 | 2 | 48.70 | 9 | 13645 | Ana\_08.raw | 9.07E6 | 5.63E6 |  |  |  | 3.47E6 |  | 1.3E6 | 1.04E7 | 5.86E6 | 6 | 1 | 1 | 0 | 0 | 0 | 1 | 0 | 1 | 1 | 1 | 1407 | 1418 |  |
| R.ASRDEIFATSK.E | N | 64.33 | 1223.6146 | 11 | 2.1 | 408.8797 | 3 | 37.66 | 4 | 10135 | Ana\_03.raw | 4.88E7 | 4.31E7 | 3.25E7 | 1.32E7 | 3.05E7 | 5.66E7 | 2.76E7 | 2.51E7 | 3.12E7 | 3.75E7 | 20 | 2 | 2 | 2 | 2 | 2 | 2 | 2 | 2 | 1 | 3 | 1673 | 1683 |  |
| K.SKHESM(+15.99)ISELEVR.L | N | 64.09 | 1559.7614 | 13 | 3.0 | 780.8903 | 2 | 36.07 | 9 | 9742 | Ana\_08.raw | 2.8E7 | 1.49E7 | 3.39E7 |  | 1.14E7 | 1.37E7 | 9.81E6 | 2.43E5 | 1.09E7 | 2.06E7 | 21 | 4 | 2 | 3 | 0 | 2 | 1 | 1 | 1 | 4 | 3 | 1037 | 1049 | Oxidation (M) |
| K.LEVQLQDLQSK.C | N | 63.87 | 1299.7034 | 11 | 6.0 | 650.8629 | 2 | 72.30 | 1 | 20480 | ANa\_10.raw | 2.99E6 |  | 0 |  | 1.53E5 | 6.67E5 |  |  | 7.88E6 |  | 5 | 1 | 0 | 1 | 0 | 1 | 1 | 0 | 0 | 1 | 0 | 1264 | 1274 |  |
| K.AAAYDKLEK.T | N | 63.81 | 1007.5287 | 9 | 3.7 | 504.7735 | 2 | 27.80 | 6 | 7440 | Ana\_05.raw | 2.1E7 | 6.67E7 | 5.29E7 | 1.76E7 | 3.87E7 | 5.75E7 | 1.53E7 | 2.51E7 | 5.22E7 | 1.3E7 | 22 | 2 | 4 | 3 | 2 | 2 | 2 | 1 | 2 | 2 | 2 | 1419 | 1427 |  |
| R.DEIFATSK.E | N | 62.67 | 909.4443 | 8 | 3.5 | 455.7310 | 2 | 41.18 | 6 | 11518 | Ana\_05.raw | 9.85E7 | 1.52E8 | 1.93E8 | 6.81E7 | 1.33E8 | 1.54E8 | 9.23E7 | 1.05E8 | 4.64E7 | 9.51E7 | 36 | 3 | 5 | 3 | 3 | 3 | 3 | 3 | 5 | 4 | 4 | 1676 | 1683 |  |
| K.YLPIYSEK.I | N | 62.49 | 1011.5276 | 8 | 0.6 | 506.7714 | 2 | 56.72 | 3 | 16097 | Ana\_02.raw | 8.27E7 | 1.14E8 | 1.08E8 | 3.84E7 | 6.9E7 | 1.01E8 | 5.2E7 | 5.61E7 | 8.68E7 | 6.12E7 | 20 | 2 | 2 | 2 | 2 | 3 | 2 | 2 | 2 | 1 | 2 | 130 | 137 |  |
| R.DLGEELEALK.T | N | 61.89 | 1115.5709 | 10 | 3.2 | 558.7945 | 2 | 97.87 | 6 | 27687 | Ana\_05.raw | 4.23E7 | 3.48E7 | 5.12E7 | 1.32E7 | 3.52E7 | 3.93E7 | 1.91E7 | 2.39E7 | 5.83E7 | 2.24E7 | 27 | 3 | 2 | 3 | 2 | 3 | 2 | 2 | 4 | 4 | 2 | 1150 | 1159 |  |
| K.VTVGKDDIQK.M | N | 61.69 | 1101.6029 | 10 | 2.9 | 551.8103 | 2 | 24.00 | 1 | 6101 | ANa\_10.raw | 3.64E7 | 3.47E7 | 2.67E7 | 5.07E6 | 3.3E7 | 4.49E7 |  | 1.7E7 | 2.3E7 | 1.93E7 | 14 | 2 | 1 | 1 | 2 | 3 | 2 | 0 | 1 | 1 | 1 | 69 | 78 |  |
| K.AEM(+15.99)EDLVSSK.D | N | 61.23 | 1123.5067 | 10 | 4.8 | 562.7633 | 2 | 29.53 | 6 | 7982 | Ana\_05.raw | 3.16E7 | 4.16E7 | 4.86E7 | 1.55E7 | 2.66E7 | 4.49E7 | 2.35E7 | 2.5E7 | 2.4E7 | 2.72E6 | 13 | 2 | 1 | 1 | 2 | 1 | 2 | 1 | 1 | 1 | 1 | 1518 | 1527 | Oxidation (M) |
| R.IVGLDQM(+15.99)AK.M | N | 60.65 | 989.5215 | 9 | 3.7 | 495.7699 | 2 | 39.85 | 6 | 11107 | Ana\_05.raw | 7.81E7 | 8.5E7 | 1.04E8 | 3.38E7 | 7.27E7 | 1.06E8 | 5.61E7 | 6.28E7 | 6.13E7 | 4.6E7 | 16 | 2 | 1 | 1 | 1 | 2 | 2 | 2 | 2 | 2 | 1 | 632 | 640 | Oxidation (M) |
| K.EVLLQVEDERK.M | N | 60.42 | 1356.7249 | 11 | 6.5 | 679.3741 | 2 | 55.18 | 7 | 15549 | Ana\_06.raw | 1.76E7 | 2.17E7 |  |  | 1.67E7 | 2.38E7 | 3.12E6 |  | 1.74E7 |  | 6 | 1 | 1 | 0 | 0 | 1 | 1 | 1 | 0 | 1 | 0 | 1860 | 1870 |  |
| R.IVFQEFR.Q | N | 60.19 | 937.5021 | 7 | 2.1 | 469.7593 | 2 | 70.64 | 3 | 20071 | Ana\_02.raw | 1.78E8 | 2.55E8 | 2.31E8 | 9.57E7 | 1.71E8 | 2.24E8 | 1.16E8 | 1.52E8 | 2.58E8 | 1.28E8 | 82 | 7 | 9 | 10 | 6 | 9 | 8 | 8 | 10 | 8 | 7 | 726 | 732 |  |
| K.MTESSLPSASK.T | N | 60.00 | 1136.5383 | 11 | 6.6 | 569.2802 | 2 | 36.23 | 4 | 9704 | Ana\_03.raw |  |  |  | 1.8E6 |  |  | 2.51E6 |  |  |  | 2 | 0 | 0 | 0 | 1 | 0 | 0 | 1 | 0 | 0 | 0 | 641 | 651 |  |
| K.LKEVLLQVEDER.K | N | 58.91 | 1469.8090 | 12 | 5.0 | 490.9460 | 3 | 70.48 | 6 | 20225 | Ana\_05.raw |  |  |  |  |  | 2.53E6 |  | 2.73E6 | 1.18E6 |  | 3 | 0 | 0 | 0 | 0 | 0 | 1 | 0 | 1 | 1 | 0 | 1858 | 1869 |  |
| K.AETELKELEQK.H | N | 58.85 | 1316.6824 | 11 | 3.4 | 659.3507 | 2 | 45.14 | 1 | 12588 | ANa\_10.raw | 2.46E6 |  |  |  |  | 3.57E6 |  |  | 7.89E5 | 1.06E6 | 4 | 1 | 0 | 0 | 0 | 0 | 1 | 0 | 0 | 1 | 1 | 878 | 888 |  |
| K.FVADLWK.D | N | 58.69 | 877.4697 | 7 | 2.6 | 439.7433 | 2 | 82.66 | 6 | 23594 | Ana\_05.raw | 1.56E7 | 2.33E7 | 1.6E7 |  | 1.36E7 | 3.18E7 | 1.01E7 | 1.88E7 | 4.32E7 | 9.24E6 | 14 | 2 | 2 | 1 | 0 | 1 | 1 | 1 | 1 | 4 | 1 | 621 | 627 |  |
| K.EVLLQVEDER.K | N | 57.24 | 1228.6299 | 10 | 5.1 | 615.3254 | 2 | 67.38 | 7 | 19119 | Ana\_06.raw |  | 0 | 2.7E7 | 1.08E7 | 1.43E7 | 2.41E7 | 1.16E7 | 1.13E7 |  | 1.55E7 | 8 | 0 | 1 | 1 | 1 | 1 | 1 | 1 | 1 | 0 | 1 | 1860 | 1869 |  |
| R.NTTPNFVR.C | N | 57.07 | 947.4825 | 8 | 0.2 | 474.7486 | 2 | 36.48 | 3 | 10067 | Ana\_02.raw | 1.77E8 | 2.17E8 | 2.07E8 | 8.12E7 | 1.61E8 | 1.99E8 | 1.07E8 | 1.42E8 | 7.84E7 | 1.2E8 | 25 | 3 | 2 | 2 | 1 | 3 | 2 | 1 | 5 | 3 | 3 | 677 | 684 |  |
| R.NC(+57.02)AAYLK.L | N | 56.53 | 838.4007 | 7 | 3.5 | 420.2091 | 2 | 28.49 | 6 | 7650 | Ana\_05.raw | 3.89E7 |  | 4.95E7 | 2.34E7 | 4.81E7 | 7.1E7 | 3.25E7 | 2.9E7 | 3.32E7 | 3.28E7 | 11 | 1 | 0 | 1 | 2 | 1 | 1 | 1 | 2 | 1 | 1 | 829 | 835 | Carbamidomethylation |
| K.KVIQYLAVVASSHK.G | N | 56.05 | 1541.8929 | 14 | 2.4 | 514.9728 | 3 | 71.36 | 4 | 19853 | Ana\_03.raw |  |  |  | 1.99E6 |  |  |  |  |  |  | 1 | 0 | 0 | 0 | 1 | 0 | 0 | 0 | 0 | 0 | 0 | 190 | 203 |  |
| K.STVAALEAK.I | N | 55.97 | 888.4916 | 9 | 2.7 | 445.2543 | 2 | 33.22 | 6 | 9095 | Ana\_05.raw | 1.06E8 | 1.39E8 | 1.32E8 | 3.91E7 | 9.22E7 | 5.75E7 | 7.49E7 | 6.4E7 | 1.1E8 | 8.86E7 | 16 | 2 | 2 | 2 | 1 | 2 | 1 | 1 | 1 | 2 | 2 | 1821 | 1829 |  |
| R.IAQLEEELEEEQGNM(+15.99)EAM(+15.99)SDR.V | N | 52.63 | 2482.0476 | 21 | 3.9 | 1242.0359 | 2 | 80.25 | 9 | 22852 | Ana\_08.raw |  |  | 3.98E6 |  | 4.21E6 | 3.08E6 |  |  | 6.27E6 |  | 5 | 0 | 0 | 1 | 0 | 1 | 1 | 0 | 0 | 2 | 0 | 1745 | 1765 | Oxidation (M) |
| K.QGFEAASIKEEK.G | N | 52.32 | 1335.6670 | 12 | 5.8 | 668.8447 | 2 | 39.33 | 1 | 10811 | ANa\_10.raw | 2.45E6 | 2.42E6 |  |  |  | 1.47E7 |  |  | 1.13E6 |  | 5 | 1 | 1 | 0 | 0 | 0 | 2 | 0 | 0 | 1 | 0 | 43 | 54 |  |
| R.QLHEYETELEDERK.Q | N | 51.81 | 1817.8431 | 14 | 0.7 | 606.9554 | 3 | 53.37 | 6 | 15217 | Ana\_05.raw |  |  |  |  |  | 7.26E6 |  | 3.06E6 | 5.79E6 |  | 3 | 0 | 0 | 0 | 0 | 0 | 1 | 0 | 1 | 1 | 0 | 1604 | 1617 |  |
| R.KM(+15.99)AEQYKEQAEK.G | N | 51.68 | 1497.7133 | 12 | -0.1 | 500.2450 | 3 | 17.57 | 6 | 4122 | Ana\_05.raw | 3.16E6 | 1.02E6 |  |  |  | 2.59E6 |  |  |  |  | 4 | 2 | 1 | 0 | 0 | 0 | 1 | 0 | 0 | 0 | 0 | 1870 | 1881 | Oxidation (M) |
| R.C(+57.02)IIPNHEK.R | N | 51.44 | 1009.5015 | 8 | 3.8 | 505.7599 | 2 | 28.00 | 5 | 7477 | Ana\_04.raw | 3.6E6 | 5.27E6 | 2.95E6 |  | 3.18E6 | 3.44E6 | 0 | 1.91E6 |  |  | 7 | 1 | 1 | 1 | 0 | 1 | 1 | 1 | 1 | 0 | 0 | 685 | 692 | Carbamidomethylation |
| K.ALDEETR.S | N | 51.25 | 832.3926 | 7 | 0.1 | 417.2036 | 2 | 20.23 | 5 | 5059 | Ana\_04.raw |  |  |  | 3.38E6 | 5.94E7 |  | 7.01E6 | 5.73E6 |  |  | 4 | 0 | 0 | 0 | 1 | 1 | 0 | 1 | 1 | 0 | 0 | 1189 | 1195 |  |
| K.RQQQLTAM(+15.99)K.V | N | 51.23 | 1118.5867 | 9 | 3.8 | 560.3027 | 2 | 17.54 | 6 | 4110 | Ana\_05.raw |  |  |  | 3.15E6 | 9.88E6 | 3.99E6 | 6.24E6 | 6.76E6 |  |  | 5 | 0 | 0 | 0 | 1 | 1 | 1 | 1 | 1 | 0 | 0 | 816 | 824 | Oxidation (M) |
| K.LQEVEGAVK.A | N | 50.94 | 971.5287 | 9 | 1.0 | 486.7722 | 2 | 30.03 | 3 | 8136 | Ana\_02.raw |  | 1.73E8 | 1.48E8 |  | 1.14E8 | 1.6E8 |  | 8.18E7 | 1.21E8 |  | 7 | 0 | 2 | 1 | 0 | 1 | 1 | 0 | 1 | 1 | 0 | 1808 | 1816 |  |
| K.KALDEETR.S | N | 50.77 | 960.4876 | 8 | 1.9 | 321.1704 | 3 | 18.14 | 4 | 4322 | Ana\_03.raw |  |  |  | 3.51E5 | 5.68E6 |  | 1.02E6 |  |  |  | 3 | 0 | 0 | 0 | 1 | 1 | 0 | 1 | 0 | 0 | 0 | 1188 | 1195 |  |
| R.GQQLQAER.K | N | 50.71 | 928.4726 | 8 | 2.1 | 465.2446 | 2 | 19.39 | 5 | 4780 | Ana\_04.raw |  |  |  | 7.53E6 | 2.49E7 |  | 3.31E6 | 6.58E6 |  |  | 4 | 0 | 0 | 0 | 1 | 1 | 0 | 1 | 1 | 0 | 0 | 945 | 952 |  |
| K.LVWVPSEK.Q | N | 48.98 | 956.5331 | 8 | 3.1 | 479.2753 | 2 | 66.05 | 6 | 18966 | Ana\_05.raw | 2.38E7 | 3.38E7 | 3.02E7 | 2.56E7 | 1.44E7 | 2.88E7 |  |  | 2.32E7 | 0 | 9 | 2 | 1 | 1 | 1 | 1 | 1 | 0 | 0 | 1 | 1 | 35 | 42 |  |
| K.LRNWQWWRLFTK.V | N | 48.91 | 1732.9314 | 12 | 3.0 | 578.6528 | 3 | 117.69 | 6 | 32880 | Ana\_05.raw |  |  |  |  |  | 1.07E7 |  |  |  |  | 2 | 0 | 0 | 0 | 0 | 0 | 2 | 0 | 0 | 0 | 0 | 836 | 847 |  |
| K.AEMEDLVSSK.D | N | 48.32 | 1107.5117 | 10 | 4.4 | 554.7656 | 2 | 51.76 | 1 | 14541 | ANa\_10.raw | 3.59E6 |  |  |  |  |  |  |  |  | 1.86E6 | 2 | 1 | 0 | 0 | 0 | 0 | 0 | 0 | 0 | 0 | 1 | 1518 | 1527 |  |
| R.LEVNM(+15.99)QALK.G | N | 47.90 | 1060.5587 | 9 | 1.8 | 531.2876 | 2 | 45.29 | 4 | 12401 | Ana\_03.raw | 8.81E6 | 5.8E7 | 5.85E7 | 1.59E7 |  | 5.61E7 | 2.48E7 | 3.04E7 | 6.41E7 | 9.94E6 | 11 | 1 | 2 | 1 | 1 | 0 | 2 | 1 | 1 | 1 | 1 | 1572 | 1580 | Oxidation (M) |
| K.NVHELEK.S | N | 46.85 | 867.4450 | 7 | 2.4 | 434.7308 | 2 | 18.44 | 6 | 4415 | Ana\_05.raw |  |  |  | 4.05E7 | 8.23E7 | 8.16E7 | 6.58E7 | 1.74E8 |  |  | 6 | 0 | 0 | 0 | 1 | 1 | 1 | 1 | 2 | 0 | 0 | 1533 | 1539 |  |
| K.KLVWVPSEK.Q | N | 46.63 | 1084.6281 | 9 | 2.7 | 543.3228 | 2 | 57.34 | 6 | 16386 | Ana\_05.raw |  |  | 5.44E6 | 1.75E6 |  | 6.46E6 | 2.56E6 |  | 9.29E6 | 1.51E6 | 6 | 0 | 0 | 1 | 1 | 0 | 1 | 1 | 0 | 1 | 1 | 34 | 42 |  |
| R.ALETQM(+15.99)EEM(+15.99)K.T | N | 45.79 | 1240.5315 | 10 | 1.8 | 621.2741 | 2 | 20.29 | 3 | 5032 | Ana\_02.raw | 6.37E6 |  | 0 | 1.64E7 | 2.08E7 | 2.65E7 | 2.22E7 | 2.91E7 |  | 7.92E6 | 9 | 1 | 0 | 1 | 1 | 1 | 1 | 1 | 2 | 0 | 1 | 1543 | 1552 | Oxidation (M) |
| K.ITDVIM(+15.99)AFQAM(+15.99)C(+57.02)R.G | N | 45.02 | 1586.7255 | 13 | 0.4 | 794.3704 | 2 | 88.57 | 3 | 24886 | Ana\_02.raw |  | 4.35E6 | 2.13E6 | 8.01E5 | 1.87E6 | 1.11E6 | 8.56E5 | 2.41E6 | 6.3E6 |  | 10 | 0 | 1 | 1 | 1 | 2 | 1 | 2 | 1 | 1 | 0 | 793 | 805 | Oxidation (M); Carbamidomethylation |
| K.KLEGDLK.D | N | 43.92 | 801.4596 | 7 | 2.3 | 401.7380 | 2 | 20.25 | 6 | 5002 | Ana\_05.raw | 7.05E7 | 6.06E7 | 5.96E7 | 2.41E7 | 4.21E7 | 8.36E7 | 6.49E6 | 4.29E7 |  |  | 12 | 2 | 2 | 1 | 1 | 1 | 2 | 1 | 2 | 0 | 0 | 1628 | 1634 |  |
| R.QRYEILAANAIPK.G | N | 43.83 | 1485.8303 | 13 | 0.4 | 496.2842 | 3 | 68.44 | 9 | 19529 | Ana\_08.raw |  |  |  |  |  |  |  |  | 1.14E6 |  | 1 | 0 | 0 | 0 | 0 | 0 | 0 | 0 | 0 | 1 | 0 | 733 | 745 |  |
| K.KKLEGDLK.D | N | 43.81 | 929.5546 | 8 | 1.4 | 465.7852 | 2 | 17.40 | 3 | 4067 | Ana\_02.raw | 7.47E6 | 4.25E6 | 1.5E7 | 5.12E6 | 1.18E7 | 2.36E7 | 4.88E6 | 4.9E6 |  | 1.14E7 | 14 | 1 | 1 | 2 | 2 | 2 | 2 | 1 | 1 | 0 | 2 | 1627 | 1634 |  |
| K.VKPLLQVTR.Q | N | 43.60 | 1052.6705 | 9 | 4.3 | 351.8990 | 3 | 47.58 | 8 | 13112 | Ana\_07.raw | 5.04E7 | 3.17E7 | 7.22E7 | 2.28E7 | 5.31E7 | 7.4E7 | 3.29E7 | 3.78E7 | 6.72E7 | 4.05E7 | 26 | 2 | 1 | 3 | 3 | 4 | 3 | 3 | 3 | 2 | 2 | 848 | 856 |  |
| R.QGFPNR.I | N | 43.46 | 717.3558 | 6 | 0.9 | 359.6855 | 2 | 22.62 | 3 | 5802 | Ana\_02.raw |  | 4.83E6 | 1.13E7 | 7.76E6 |  | 1.82E7 | 7.99E6 | 1.36E7 |  |  | 6 | 0 | 1 | 1 | 1 | 0 | 1 | 1 | 1 | 0 | 0 | 720 | 725 |  |
| R.EQEVTVLK.K | N | 42.54 | 944.5178 | 8 | 3.3 | 473.2678 | 2 | 38.27 | 6 | 10622 | Ana\_05.raw | 9.95E7 | 1.45E8 | 1.44E8 | 5E7 | 1.07E8 | 1.42E8 | 7.48E7 | 8.02E7 | 1.07E8 |  | 18 | 1 | 2 | 1 | 2 | 2 | 2 | 2 | 3 | 3 | 0 | 1180 | 1187 |  |
| R.KLLEER.V | N | 41.76 | 786.4599 | 6 | 4.8 | 394.2391 | 2 | 21.71 | 9 | 5194 | Ana\_08.raw | 4.77E7 | 7.84E7 | 7.3E7 | 2.37E7 |  |  |  | 3.46E7 | 4.24E7 | 3.86E7 | 7 | 1 | 1 | 1 | 1 | 0 | 0 | 0 | 1 | 1 | 1 | 1009 | 1014 |  |
| R.KLQAQMK.D | N | 41.48 | 845.4793 | 7 | 4.2 | 423.7487 | 2 | 18.45 | 8 | 4358 | Ana\_07.raw |  |  |  | 1.72E5 |  |  |  | 5.1E5 |  |  | 2 | 0 | 0 | 0 | 1 | 0 | 0 | 0 | 1 | 0 | 0 | 1656 | 1662 |  |
| R.EQEVTVLKK.A | N | 40.98 | 1072.6128 | 9 | 2.6 | 537.3151 | 2 | 25.53 | 3 | 6730 | Ana\_02.raw | 1.36E7 |  | 1.48E7 | 4.06E6 | 7.84E6 | 1.33E7 |  | 4.73E6 | 2.23E7 | 6.69E6 | 11 | 1 | 0 | 2 | 2 | 1 | 1 | 0 | 1 | 2 | 1 | 1180 | 1188 |  |
| K.FLFVDK.N | N | 39.11 | 767.4218 | 6 | 5.0 | 384.7201 | 2 | 70.11 | 2 | 19973 | Ana\_01.raw | 5.15E7 | 7.78E7 | 8.54E7 | 3.15E7 | 5.75E7 | 8.31E7 | 3.65E7 | 4.73E7 | 8.82E7 | 4.85E7 | 34 | 3 | 4 | 6 | 3 | 6 | 2 | 1 | 1 | 6 | 2 | 13 | 18 |  |
| R.TVGQLYK.E | N | 38.67 | 807.4490 | 7 | 2.6 | 404.7328 | 2 | 33.40 | 4 | 8890 | Ana\_03.raw | 3.54E6 | 1.11E8 | 1.2E8 | 4.55E7 | 8.98E7 | 1.13E8 | 7.15E7 | 6.55E7 | 1.02E8 | 6.97E7 | 17 | 1 | 1 | 2 | 1 | 3 | 2 | 2 | 3 | 1 | 1 | 659 | 665 |  |
| K.LQDFASTIEVM(+15.99)EEGKK.R | N | 36.24 | 1839.8923 | 16 | 3.1 | 614.3066 | 3 | 72.20 | 1 | 20454 | ANa\_10.raw | 2.71E6 | 1.62E6 |  |  |  | 3.17E6 |  |  |  |  | 3 | 1 | 1 | 0 | 0 | 0 | 1 | 0 | 0 | 0 | 0 | 1387 | 1402 | Oxidation (M) |
| R.SKLQEVEGAVK.A | N | 36.17 | 1186.6558 | 11 | 4.0 | 594.3375 | 2 | 42.97 | 2 | 11976 | Ana\_01.raw | 3.35E6 | 1.64E6 |  |  |  |  |  |  |  |  | 2 | 1 | 1 | 0 | 0 | 0 | 0 | 0 | 0 | 0 | 0 | 1806 | 1816 |  |
| K.REQEVTVLK.K | N | 35.82 | 1100.6189 | 9 | 2.3 | 551.3180 | 2 | 27.06 | 3 | 7215 | Ana\_02.raw |  |  | 1.85E7 | 9.94E6 | 2.04E7 | 1.69E7 | 1.39E7 |  |  |  | 5 | 0 | 0 | 1 | 1 | 1 | 1 | 1 | 0 | 0 | 0 | 1179 | 1187 |  |
| R.ALETQM(+15.99)EEMK.T | N | 35.77 | 1224.5366 | 10 | 1.5 | 613.2765 | 2 | 30.52 | 1 | 8174 | ANa\_10.raw | 5.5E6 |  |  |  |  |  |  |  | 2.04E6 |  | 2 | 1 | 0 | 0 | 0 | 0 | 0 | 0 | 0 | 1 | 0 | 1543 | 1552 | Oxidation (M) |
| K.ALSLAR.A | N | 35.77 | 629.3860 | 6 | 2.7 | 315.7011 | 2 | 31.28 | 6 | 8518 | Ana\_05.raw |  |  |  |  |  | 3.8E7 | 2.13E7 | 1.28E7 |  | 1.54E7 | 4 | 0 | 0 | 0 | 0 | 0 | 1 | 1 | 1 | 0 | 1 | 1492 | 1497 |  |
| K.QGFEAASIK.E | N | 34.98 | 949.4869 | 9 | 3.7 | 475.7525 | 2 | 45.37 | 4 | 12425 | Ana\_03.raw |  |  |  | 2.94E6 |  |  |  |  |  |  | 1 | 0 | 0 | 0 | 1 | 0 | 0 | 0 | 0 | 0 | 0 | 43 | 51 |  |
| R.QLVSNLEK.K | N | 33.88 | 929.5182 | 8 | 5.3 | 465.7688 | 2 | 35.36 | 9 | 9523 | Ana\_08.raw | 1.19E7 |  |  |  |  |  |  |  | 7.42E6 |  | 2 | 1 | 0 | 0 | 0 | 0 | 0 | 0 | 0 | 1 | 0 | 1448 | 1455 |  |
| K.LLEER.V | N | 33.24 | 658.3650 | 5 | 3.3 | 330.1909 | 2 | 23.06 | 6 | 5936 | Ana\_05.raw |  |  |  |  | 2.63E7 | 3.88E7 | 3.76E6 | 1.26E7 |  |  | 4 | 0 | 0 | 0 | 0 | 1 | 1 | 1 | 1 | 0 | 0 | 1010 | 1014 |  |
| K.IVDMYK.G | N | 33.02 | 767.3887 | 6 | 2.9 | 384.7028 | 2 | 33.55 | 9 | 8954 | Ana\_08.raw |  |  |  |  |  |  |  |  | 4.01E6 |  | 1 | 0 | 0 | 0 | 0 | 0 | 0 | 0 | 0 | 1 | 0 | 138 | 143 |  |
| K.REQEVTVLKK.A | N | 32.76 | 1228.7139 | 10 | 2.7 | 410.5797 | 3 | 22.61 | 1 | 5639 | ANa\_10.raw | 1.64E6 |  |  |  |  |  |  |  |  |  | 1 | 1 | 0 | 0 | 0 | 0 | 0 | 0 | 0 | 0 | 0 | 1179 | 1188 |  |
| R.WILSR.V | N | 32.69 | 673.3911 | 5 | 0.3 | 337.7029 | 2 | 55.30 | 3 | 15697 | Ana\_02.raw |  |  | 6.82E6 |  |  |  |  |  |  |  | 1 | 0 | 0 | 1 | 0 | 0 | 0 | 0 | 0 | 0 | 0 | 442 | 446 |  |
| K.LM(+15.99)TTLR.N | N | 32.45 | 749.4106 | 6 | 4.9 | 375.7144 | 2 | 26.68 | 2 | 6975 | Ana\_01.raw | 4.02E7 | 2.78E7 | 3.66E7 |  |  |  |  | 1.52E7 | 3.01E7 |  | 6 | 2 | 1 | 1 | 0 | 0 | 0 | 0 | 1 | 1 | 0 | 671 | 676 | Oxidation (M) |
| R.NWQWWR.L | N | 29.97 | 974.4511 | 6 | 5.4 | 488.2355 | 2 | 98.83 | 8 | 27494 | Ana\_07.raw |  |  |  |  |  |  |  | 8.23E5 |  |  | 1 | 0 | 0 | 0 | 0 | 0 | 0 | 0 | 1 | 0 | 0 | 838 | 843 |  |
| R.AILTPR.I | N | 29.60 | 669.4174 | 6 | 3.2 | 335.7170 | 2 | 26.50 | 9 | 6805 | Ana\_08.raw | 3.95E7 | 5.44E7 | 6.82E7 | 2.88E7 | 4.05E7 | 7.21E7 | 3.34E7 | 3.05E7 | 6.5E6 | 2.28E7 | 14 | 1 | 2 | 1 | 2 | 1 | 2 | 2 | 1 | 1 | 1 | 402 | 407 |  |
| K.IVDM(+15.99)YK.G | N | 29.48 | 783.3837 | 6 | 4.2 | 392.7007 | 2 | 26.31 | 8 | 6743 | Ana\_07.raw | 1.41E7 |  | 9.05E7 |  |  | 3.86E7 | 5.33E7 | 1.8E7 |  |  | 6 | 1 | 0 | 1 | 0 | 0 | 1 | 2 | 1 | 0 | 0 | 138 | 143 | Oxidation (M) |
| K.LKEVLLQVEDERK.M | N | 28.26 | 1597.9038 | 13 | 3.8 | 533.6439 | 3 | 59.69 | 5 | 16966 | Ana\_04.raw |  |  |  |  | 1.51E6 |  |  |  |  |  | 1 | 0 | 0 | 0 | 0 | 1 | 0 | 0 | 0 | 0 | 0 | 1858 | 1870 |  |
| R.QEEEM(+15.99)QAKEEEM(+15.99)QK.I | N | 27.83 | 1797.7396 | 14 | 1.2 | 600.2545 | 3 | 21.92 | 2 | 5386 | Ana\_01.raw |  | 2.06E6 |  |  |  | 2.88E6 |  |  |  |  | 2 | 0 | 1 | 0 | 0 | 0 | 1 | 0 | 0 | 0 | 0 | 857 | 870 | Oxidation (M) |
| R.SM(+15.99)LQDR.E | N | 26.72 | 764.3487 | 6 | 4.0 | 383.1832 | 2 | 24.74 | 1 | 6348 | ANa\_10.raw | 2.06E6 |  |  |  |  |  |  |  |  |  | 1 | 1 | 0 | 0 | 0 | 0 | 0 | 0 | 0 | 0 | 0 | 164 | 169 | Oxidation (M) |
| K.LQLEK.V | N | 26.40 | 629.3748 | 5 | 5.2 | 315.6963 | 2 | 24.82 | 10 | 6436 | Ana\_09.raw |  |  | 5.13E7 |  |  |  |  |  |  | 3.11E7 | 2 | 0 | 0 | 1 | 0 | 0 | 0 | 0 | 0 | 0 | 1 | 976 | 980 |  |
| K.LMTTLR.N | N | 23.92 | 733.4156 | 6 | 6.6 | 367.7175 | 2 | 34.22 | 9 | 9164 | Ana\_08.raw |  |  |  |  |  |  |  |  | 2.23E6 |  | 1 | 0 | 0 | 0 | 0 | 0 | 0 | 0 | 0 | 1 | 0 | 671 | 676 |  |
| K.LRNWQWWR.L | N | 23.70 | 1243.6362 | 8 | 2.7 | 415.5538 | 3 | 88.09 | 6 | 25077 | Ana\_05.raw |  |  |  |  |  | 1.89E6 |  |  |  |  | 1 | 0 | 0 | 0 | 0 | 0 | 1 | 0 | 0 | 0 | 0 | 836 | 843 |  |
| K.QAC(+57.02)ILM(+15.99)IK.A | N | 22.53 | 991.5195 | 8 | 2.7 | 496.7683 | 2 | 59.24 | 6 | 16946 | Ana\_05.raw |  |  |  |  |  | 2.13E6 |  |  |  |  | 1 | 0 | 0 | 0 | 0 | 0 | 1 | 0 | 0 | 0 | 0 | 752 | 759 | Carbamidomethylation; Oxidation (M) |
| total 160 peptides |  |  |  |  |  |  |  |  |  |  |  |  |  |  |  |  |  |  |  |  |  |  |  |  |  |  |  |  |  |  |  |  |  |  |  |
| --- | --- | --- | --- | --- | --- | --- | --- | --- | --- | --- | --- | --- | --- | --- | --- | --- | --- | --- | --- | --- | --- | --- | --- | --- | --- | --- | --- | --- | --- | --- | --- | --- | --- | --- | --- |

Best Unique PSM (Scan F6:26019, m/z=914.9357, z=2, RT=91.62, ppm=2.3):

A0A2R8VHF9|A0A2R8VHF9\_MOUSE

back to list

  

| Protein Coverage
| Supporting Peptides
| Best Unique PSM
|

Protein Coverage:

Supporting Peptides:

| Peptide | Uniq | -10lgP | Mass | Length | ppm | m/z | z | RT | Fraction | Scan | Source File | Area 10 | Area 01 | Area 02 | Area 03 | Area 04 | Area 05 | Area 06 | Area 07 | Area 08 | Area 09 | #Spec | #Spec 10 | #Spec 01 | #Spec 02 | #Spec 03 | #Spec 04 | #Spec 05 | #Spec 06 | #Spec 07 | #Spec 08 | #Spec 09 | Start | End | PTM |
| --- | --- | --- | --- | --- | --- | --- | --- | --- | --- | --- | --- | --- | --- | --- | --- | --- | --- | --- | --- | --- | --- | --- | --- | --- | --- | --- | --- | --- | --- | --- | --- | --- | --- | --- | --- |
| R.KLEGDASDFHEQIADLQAQIAELK.M | N | 159.90 | 2668.3342 | 24 | -0.7 | 890.4514 | 3 | 124.42 | 3 | 34168 | Ana\_02.raw | 6.47E7 | 1.55E8 | 1.33E8 | 1.05E7 | 1.64E8 | 2.73E8 | 1.13E8 | 5.72E7 | 1.76E8 | 7.7E7 | 55 | 5 | 6 | 8 | 4 | 6 | 5 | 3 | 8 | 5 | 5 | 1059 | 1082 |  |
| K.DVASLGSQLQDTQELLQEETR.Q | N | 149.74 | 2359.1504 | 21 | 0.6 | 1180.5831 | 2 | 121.54 | 3 | 33431 | Ana\_02.raw | 7.72E7 | 3.61E7 | 1.47E8 | 4.55E7 | 9.2E7 | 1.65E8 | 6.84E7 | 3.28E7 | 2.37E8 | 8.75E7 | 56 | 4 | 7 | 5 | 5 | 6 | 4 | 5 | 7 | 6 | 7 | 1309 | 1329 |  |
| R.DLGEELEALKTELEDTLDSTATQQELR.A | N | 138.52 | 3046.4829 | 27 | 7.3 | 1016.5090 | 3 | 138.44 | 9 | 38224 | Ana\_08.raw | 3.23E6 | 6.46E6 | 1.63E6 |  | 1.78E6 | 8.62E6 | 2.99E5 |  | 1.03E7 | 9.25E6 | 12 | 1 | 2 | 1 | 0 | 1 | 3 | 1 | 0 | 1 | 2 | 1143 | 1169 |  |
| K.HTQAVEELTEQLEQFK.R | N | 133.51 | 1928.9479 | 16 | 2.4 | 965.4835 | 2 | 109.11 | 6 | 30645 | Ana\_05.raw | 5.81E7 | 8.76E7 | 1.83E7 | 1.72E7 | 7.48E7 | 1.09E8 | 4.92E7 | 1.34E7 | 1.06E8 | 4.94E6 | 53 | 4 | 6 | 5 | 3 | 6 | 6 | 3 | 9 | 6 | 5 | 1201 | 1216 |  |
| R.HEM(+15.99)PPHIYAIADTAYR.S | N | 129.92 | 1899.8937 | 16 | 1.7 | 634.3063 | 3 | 63.28 | 3 | 18023 | Ana\_02.raw | 5.99E7 | 8.34E7 | 7.48E7 | 1.53E7 | 5.08E7 | 5.82E7 | 3.66E7 | 4.15E7 | 9.56E7 | 4.68E7 | 22 | 3 | 3 | 2 | 1 | 2 | 2 | 2 | 2 | 3 | 2 | 148 | 163 | Oxidation (M) |
| R.KQADLEKEELAEELASSLSGR.N | N | 127.02 | 2302.1650 | 21 | -0.5 | 768.3953 | 3 | 97.95 | 3 | 27359 | Ana\_02.raw | 6.13E7 | 6.62E7 | 9.15E7 | 2.64E6 | 2.89E7 | 4.03E7 | 2.23E7 | 9.37E6 | 1.26E8 | 5.89E7 | 27 | 4 | 2 | 3 | 3 | 2 | 1 | 3 | 4 | 3 | 2 | 1704 | 1724 |  |
| R.KATLQAEQLSNELATER.S | N | 123.88 | 1900.9854 | 17 | 2.6 | 951.5024 | 2 | 69.14 | 3 | 19657 | Ana\_02.raw | 5.33E7 | 7.5E7 | 5.97E7 | 1.77E7 | 3.76E7 | 7.17E7 | 2.38E7 | 4.11E7 | 9.24E7 | 4.28E7 | 30 | 4 | 3 | 2 | 2 | 3 | 3 | 2 | 4 | 4 | 3 | 1761 | 1777 |  |
| R.LQQELDDLVVDLDNQR.Q | N | 119.49 | 1911.9537 | 16 | 0.7 | 638.3256 | 3 | 114.73 | 3 | 31696 | Ana\_02.raw | 2.19E8 | 3.6E7 | 6.58E7 | 7.99E7 | 1.62E8 | 2.16E8 | 8.92E7 | 1.4E8 | 2.63E8 | 1.07E8 | 63 | 7 | 6 | 7 | 5 | 5 | 6 | 4 | 8 | 8 | 7 | 1425 | 1440 |  |
| K.HTQAVEELTEQLEQFKR.A | N | 118.81 | 2085.0491 | 17 | 5.3 | 696.0273 | 3 | 97.61 | 2 | 27502 | Ana\_01.raw | 6.29E7 | 1.46E7 | 3.45E6 | 4.86E6 | 2.96E6 | 2.97E7 | 9.91E5 | 5.07E6 | 6.08E7 | 2.67E7 | 23 | 3 | 2 | 2 | 2 | 3 | 2 | 2 | 1 | 3 | 3 | 1201 | 1217 |  |
| K.ATLQAEQLSNELATER.S | N | 116.90 | 1772.8904 | 16 | 3.7 | 887.4557 | 2 | 82.32 | 6 | 23502 | Ana\_05.raw | 6.3E7 | 9.03E7 | 3.97E7 | 2.43E7 | 4.89E7 | 7.85E7 | 2.99E7 | 4.17E7 | 1.04E8 | 3.9E7 | 33 | 5 | 4 | 2 | 3 | 3 | 3 | 3 | 3 | 4 | 3 | 1762 | 1777 |  |
| K.TQLEELEDELQATEDAK.L | N | 116.11 | 1960.9113 | 17 | 1.2 | 981.4641 | 2 | 112.32 | 3 | 31061 | Ana\_02.raw | 1.36E8 | 2.22E8 | 1.17E8 | 7.57E7 | 1.05E8 | 1.7E8 | 8.31E7 | 4.83E7 | 2.7E8 | 1.45E8 | 68 | 10 | 10 | 5 | 5 | 5 | 4 | 5 | 9 | 8 | 7 | 1546 | 1562 |  |
| K.LEGDASDFHEQIADLQAQIAELK.M | N | 115.03 | 2540.2393 | 23 | 4.4 | 847.7574 | 3 | 129.25 | 9 | 35796 | Ana\_08.raw | 1.95E7 | 2.72E7 | 1.16E7 | 9.86E6 | 1.69E7 | 3.3E7 | 1.42E7 | 1.24E7 | 2.81E7 | 1.71E7 | 11 | 2 | 1 | 1 | 1 | 1 | 1 | 1 | 1 | 1 | 1 | 1060 | 1082 |  |
| K.VEDM(+15.99)AELTC(+57.02)LNEASVLHNLR.E | N | 114.91 | 2329.1042 | 20 | 0.2 | 777.3755 | 3 | 98.97 | 3 | 27632 | Ana\_02.raw | 1.71E7 | 6.41E6 | 3.37E7 | 8.86E5 | 7.27E6 | 1.13E7 | 1.52E6 | 2.99E6 | 9.78E7 | 2.58E7 | 21 | 3 | 2 | 3 | 1 | 2 | 3 | 1 | 1 | 4 | 1 | 87 | 106 | Oxidation (M); Carbamidomethylation |
| K.QADLEKEELAEELASSLSGR.N | N | 114.68 | 2174.0703 | 20 | 2.5 | 725.6992 | 3 | 109.89 | 6 | 30848 | Ana\_05.raw | 1.17E7 | 2.12E7 | 2.44E7 | 7.14E6 | 1.99E7 | 4.01E7 | 1.12E7 | 1.33E7 | 3.31E7 | 2.17E6 | 20 | 1 | 2 | 3 | 3 | 2 | 3 | 1 | 2 | 2 | 1 | 1705 | 1724 |  |
| R.ELEGHISDLQEDLDSER.A | N | 114.66 | 1983.9021 | 17 | -0.4 | 992.9579 | 2 | 81.47 | 3 | 23007 | Ana\_02.raw | 8.87E7 | 1.12E7 | 6.26E7 | 1.25E7 | 5.39E7 | 5.99E7 | 7.4E6 | 1.47E7 | 6.39E7 | 7.53E7 | 27 | 4 | 1 | 3 | 1 | 4 | 3 | 3 | 3 | 3 | 2 | 1115 | 1131 |  |
| K.NM(+15.99)DPLNDNVTSLLNASSDK.F | N | 114.43 | 2062.9478 | 19 | 0.4 | 1032.4816 | 2 | 97.63 | 3 | 27276 | Ana\_02.raw | 2.75E7 | 2.95E7 | 3.52E7 | 5.79E6 | 3.04E6 | 2.52E7 | 3.66E6 | 8.43E6 | 5.22E7 | 1.38E7 | 26 | 3 | 4 | 3 | 2 | 2 | 3 | 2 | 3 | 2 | 2 | 595 | 613 | Oxidation (M) |
| K.SLEADLM(+15.99)QLQEDLAAAER.A | N | 112.51 | 2017.9625 | 18 | 4.2 | 1009.9927 | 2 | 134.02 | 7 | 36928 | Ana\_06.raw | 5.3E7 | 7.73E7 | 6.11E7 | 3.12E7 | 4.73E7 | 1.56E8 | 3.76E7 | 1.93E7 | 1.1E8 | 6.31E7 | 87 | 8 | 16 | 13 | 2 | 6 | 8 | 3 | 7 | 14 | 10 | 1684 | 1701 | Oxidation (M) |
| K.M(+15.99)AQQM(+15.99)LDLEEQLEEEEAAR.Q | N | 110.76 | 2294.0042 | 19 | 0.3 | 1148.0096 | 2 | 91.09 | 3 | 25554 | Ana\_02.raw | 1.39E7 | 4.96E6 | 9.72E6 | 8.79E5 | 1.01E7 | 4.57E6 |  | 1.82E6 | 8.38E6 |  | 17 | 3 | 2 | 2 | 2 | 2 | 2 | 0 | 1 | 3 | 0 | 948 | 966 | Oxidation (M) |
| R.VSDLTTNLAEEEEK.A | N | 110.12 | 1576.7468 | 14 | 2.8 | 789.3829 | 2 | 63.60 | 6 | 18249 | Ana\_05.raw | 1.31E8 | 1.92E8 | 1.43E8 | 4.08E7 | 8.46E7 | 1.1E8 | 5.46E7 | 8.41E7 | 1.61E8 | 8.77E7 | 41 | 5 | 6 | 5 | 3 | 3 | 3 | 3 | 4 | 5 | 4 | 1008 | 1021 |  |
| K.TELEDTLDSTATQQELR.A | N | 108.59 | 1948.9225 | 17 | 1.1 | 975.4695 | 2 | 80.55 | 3 | 22763 | Ana\_02.raw | 8.58E5 | 1.09E7 | 1.61E6 | 5.1E5 |  | 6.53E6 |  | 2.14E5 | 7.34E6 | 2.52E6 | 8 | 1 | 1 | 1 | 1 | 0 | 1 | 0 | 1 | 1 | 1 | 1153 | 1169 |  |
| R.ALETQM(+15.99)EEM(+15.99)KTQLEELEDELQATEDAK.L | N | 105.94 | 3183.4321 | 27 | -0.2 | 1062.1511 | 3 | 114.45 | 3 | 31624 | Ana\_02.raw | 4.63E7 | 1.55E7 | 8.02E6 | 2.59E6 | 1.6E7 | 1.91E7 | 6.25E6 | 2.76E7 | 3.93E7 | 4.9E5 | 19 | 2 | 2 | 2 | 1 | 2 | 2 | 2 | 3 | 2 | 1 | 1536 | 1562 | Oxidation (M) |
| K.SLEADLMQLQEDLAAAER.A | N | 104.96 | 2001.9677 | 18 | 1.9 | 1001.9930 | 2 | 134.96 | 9 | 37315 | Ana\_08.raw |  | 5E5 |  |  |  | 1.9E6 |  |  | 1.95E6 | 3.44E6 | 5 | 0 | 1 | 0 | 0 | 0 | 1 | 0 | 0 | 1 | 2 | 1684 | 1701 |  |
| K.IRELEGHISDLQEDLDSER.A | N | 104.93 | 2253.0872 | 19 | 6.2 | 752.0410 | 3 | 83.44 | 2 | 23659 | Ana\_01.raw | 2.67E6 | 1.81E6 |  |  |  | 1.64E6 |  |  | 7.52E6 | 5.57E6 | 5 | 1 | 1 | 0 | 0 | 0 | 1 | 0 | 0 | 1 | 1 | 1113 | 1131 |  |
| K.TLLQEQLQAETELYAEAEEM(+15.99)R.V | N | 104.54 | 2510.1846 | 21 | 1.2 | 837.7365 | 3 | 125.64 | 6 | 34949 | Ana\_05.raw | 1.91E7 | 8.37E7 | 2.26E7 | 3.25E6 | 7.91E7 | 1.57E8 | 1.63E7 | 4.21E7 | 1.21E8 | 6.55E6 | 56 | 5 | 6 | 9 | 4 | 5 | 7 | 2 | 7 | 8 | 3 | 890 | 910 | Oxidation (M) |
| K.KLEDDILVM(+15.99)DDQNSK.L | N | 103.26 | 1777.8403 | 15 | 4.0 | 889.9310 | 2 | 60.00 | 6 | 17162 | Ana\_05.raw | 2.48E7 | 1E7 | 6.06E6 | 1.61E6 | 3.71E6 | 8.41E6 | 3.45E6 | 3.93E6 | 3.85E7 | 1.29E7 | 13 | 2 | 1 | 1 | 1 | 1 | 1 | 1 | 1 | 2 | 2 | 982 | 996 | Oxidation (M) |
| K.LQQLFNHTMFILEQEEYQR.E | N | 102.05 | 2466.2002 | 19 | 4.9 | 823.0780 | 3 | 118.36 | 6 | 33058 | Ana\_05.raw |  |  |  |  |  | 4.77E6 |  |  |  |  | 1 | 0 | 0 | 0 | 0 | 0 | 1 | 0 | 0 | 0 | 0 | 483 | 501 |  |
| K.M(+15.99)AQQMLDLEEQLEEEEAAR.Q | N | 101.73 | 2278.0093 | 19 | 2.6 | 1140.0149 | 2 | 105.76 | 9 | 29767 | Ana\_08.raw |  |  |  |  |  |  |  |  | 2.69E6 |  | 2 | 0 | 0 | 0 | 0 | 0 | 0 | 0 | 0 | 2 | 0 | 948 | 966 | Oxidation (M) |
| R.LEEEEDRGQQLQAER.K | N | 99.43 | 1828.8551 | 15 | 6.1 | 915.4404 | 2 | 38.08 | 1 | 10426 | ANa\_10.raw | 3.25E8 | 3.81E8 | 2.77E8 | 1E8 | 2.38E8 | 3.24E8 | 1.51E8 | 2.13E8 | 1.92E8 | 2.08E8 | 52 | 6 | 8 | 4 | 4 | 4 | 5 | 4 | 6 | 7 | 4 | 931 | 945 |  |
| K.EELAEELASSLSGR.N | N | 98.34 | 1489.7260 | 14 | 2.0 | 745.8718 | 2 | 111.24 | 6 | 31198 | Ana\_05.raw | 7.14E5 | 7.56E6 | 7.3E6 | 6.42E6 | 7.57E6 | 1.48E7 | 6.18E6 | 8.38E6 | 1.9E6 | 5.32E6 | 17 | 1 | 1 | 1 | 1 | 3 | 3 | 2 | 3 | 1 | 1 | 1711 | 1724 |  |
| K.QLLQANPILEAFGNAK.T | N | 97.96 | 1725.9413 | 16 | 2.9 | 863.9804 | 2 | 125.10 | 6 | 34810 | Ana\_05.raw | 1.57E7 | 1.55E7 | 4.42E7 | 4.06E6 | 2.63E7 | 7.1E7 | 2.22E7 | 4.41E7 | 7.06E7 | 1.03E7 | 45 | 6 | 4 | 8 | 4 | 4 | 4 | 3 | 5 | 4 | 3 | 217 | 232 |  |
| K.VEDMAELTC(+57.02)LNEASVLHNLR.E | N | 96.90 | 2313.1094 | 20 | 0.7 | 772.0443 | 3 | 115.12 | 6 | 32207 | Ana\_05.raw | 1.11E6 | 1.57E7 |  |  |  | 8.58E6 |  | 9.72E5 |  | 1.54E7 | 5 | 1 | 1 | 0 | 0 | 0 | 1 | 0 | 1 | 0 | 1 | 87 | 106 | Carbamidomethylation |
| K.LQQLFNHTM(+15.99)FILEQEEYQR.E | N | 96.51 | 2482.1951 | 19 | 2.7 | 828.4079 | 3 | 103.10 | 5 | 28829 | Ana\_04.raw | 6.34E6 |  | 2.18E7 |  | 2.15E7 | 2.26E7 | 1.24E6 | 8.44E4 | 2.31E7 |  | 8 | 1 | 0 | 1 | 0 | 1 | 1 | 1 | 1 | 2 | 0 | 483 | 501 | Oxidation (M) |
| K.KQELEEILHEM(+15.99)EAR.L | N | 95.61 | 1769.8617 | 14 | 2.9 | 590.9629 | 3 | 102.05 | 6 | 28795 | Ana\_05.raw | 4.13E7 | 3.89E7 | 3.62E6 | 8.18E6 | 8.32E5 | 2.86E7 | 2.01E6 | 1.61E7 | 4.11E7 | 1.93E7 | 20 | 3 | 2 | 1 | 2 | 1 | 2 | 1 | 2 | 4 | 2 | 917 | 930 | Oxidation (M) |
| R.HVSTLNIQLSDSK.K | N | 95.47 | 1440.7572 | 13 | 5.1 | 721.3895 | 2 | 56.36 | 5 | 15999 | Ana\_04.raw | 1.49E7 | 2.59E7 | 2.13E7 | 7.07E6 | 1.18E7 | 1.67E7 | 8.24E6 | 1.25E7 | 1.19E7 | 1.3E7 | 21 | 2 | 3 | 2 | 2 | 2 | 2 | 2 | 2 | 2 | 2 | 1365 | 1377 |  |
| R.ALEEALEAKEELER.T | N | 94.95 | 1628.8257 | 14 | 0.6 | 815.4206 | 2 | 69.89 | 9 | 19947 | Ana\_08.raw | 2.64E7 | 1.3E7 | 9.85E6 | 4.02E6 | 1.01E7 | 1.87E7 | 4.91E6 | 6.41E6 | 1.08E7 | 1.45E7 | 21 | 3 | 2 | 2 | 2 | 2 | 2 | 2 | 2 | 2 | 2 | 1491 | 1504 |  |
| R.EDQSILC(+57.02)TGESGAGK.T | N | 94.65 | 1550.6882 | 15 | 0.4 | 776.3517 | 2 | 46.91 | 3 | 13245 | Ana\_02.raw | 6.73E7 | 9.75E7 | 5.91E7 | 2.44E7 | 4.17E7 | 6.67E7 | 3.22E7 | 4.18E7 | 1.11E8 | 4.88E7 | 27 | 4 | 5 | 2 | 2 | 3 | 2 | 1 | 3 | 3 | 2 | 170 | 184 | Carbamidomethylation |
| R.TFHIFYYLLAGAK.E | N | 94.59 | 1542.8235 | 13 | 9.1 | 515.2864 | 3 | 127.05 | 6 | 35318 | Ana\_05.raw |  | 3.12E6 | 2.45E6 | 4.13E6 | 7.52E6 | 1.54E7 | 7.86E6 | 6.87E6 |  | 1.71E6 | 15 | 0 | 1 | 2 | 2 | 2 | 2 | 2 | 2 | 0 | 2 | 280 | 292 |  |
| K.IAQLEEQVEQEAR.E | N | 94.51 | 1541.7686 | 13 | 3.8 | 771.8945 | 2 | 69.28 | 1 | 19636 | ANa\_10.raw | 1.56E8 | 1.81E8 | 1.31E8 | 4.51E7 | 1.09E8 | 1.24E8 | 5.75E7 | 9.21E7 | 2.09E8 | 1.37E8 | 41 | 4 | 5 | 4 | 2 | 4 | 5 | 2 | 5 | 6 | 4 | 1823 | 1835 |  |
| K.LQNEVESVTGM(+15.99)LNEAEGK.A | N | 93.53 | 1962.9204 | 18 | 3.1 | 982.4705 | 2 | 84.53 | 1 | 23809 | ANa\_10.raw | 1.35E7 | 3.64E7 | 1.13E7 |  | 1.66E6 | 3.19E6 | 3.06E5 |  | 5.83E7 | 2.91E6 | 21 | 2 | 6 | 2 | 0 | 1 | 2 | 1 | 0 | 4 | 3 | 1285 | 1302 | Oxidation (M) |
| R.TGVLAHLEEER.D | N | 92.10 | 1252.6411 | 11 | 2.1 | 627.3292 | 2 | 57.41 | 3 | 16300 | Ana\_02.raw | 1.44E8 | 2.05E8 | 1.82E8 | 5.64E7 | 1.17E8 | 1.42E8 | 7.9E7 | 8.68E7 | 2.48E8 | 1.16E8 | 63 | 7 | 9 | 8 | 4 | 6 | 4 | 3 | 9 | 8 | 5 | 772 | 782 |  |
| K.VDYNASAWLTK.N | N | 91.97 | 1266.6244 | 11 | 2.5 | 634.3210 | 2 | 84.38 | 6 | 24059 | Ana\_05.raw | 2.32E7 | 2.4E7 | 2E7 | 1.18E7 | 2.27E7 | 3.62E7 | 1.28E7 | 1.94E7 | 5.66E7 | 1.45E7 | 17 | 2 | 2 | 1 | 2 | 2 | 1 | 1 | 2 | 3 | 1 | 584 | 594 |  |
| R.VSDLTTNLAEEEEKAK.N | N | 91.90 | 1775.8788 | 16 | 3.3 | 888.9496 | 2 | 64.62 | 6 | 18560 | Ana\_05.raw | 5.28E7 | 4.52E7 | 1.6E6 |  | 3.46E7 | 4.49E7 | 1.7E7 | 2.47E7 | 6.96E7 | 4.04E7 | 17 | 3 | 2 | 1 | 0 | 2 | 2 | 1 | 2 | 2 | 2 | 1008 | 1023 |  |
| K.VVSSVLQLGNIVFK.K | N | 91.84 | 1501.8867 | 14 | 1.1 | 751.9515 | 2 | 125.10 | 3 | 34346 | Ana\_02.raw | 8.1E6 | 2.52E7 | 3.71E7 | 2.53E7 | 4.43E7 | 8.21E7 | 7.4E6 | 3.99E7 | 8.05E7 | 4.18E7 | 40 | 4 | 5 | 7 | 3 | 4 | 3 | 2 | 3 | 6 | 3 | 349 | 362 |  |
| K.LDAFLVLEQLR.C | N | 91.07 | 1315.7500 | 11 | 3.3 | 658.8845 | 2 | 130.52 | 6 | 36264 | Ana\_05.raw | 1.24E7 | 4.75E7 | 2.28E7 | 2.23E7 | 4.47E7 | 6.74E7 | 3.85E7 | 2.66E7 | 3.2E7 | 2.15E7 | 57 | 2 | 10 | 6 | 4 | 11 | 8 | 4 | 5 | 3 | 4 | 690 | 700 |  |
| R.SHEAQVQEMR.Q | N | 90.82 | 1213.5509 | 10 | 4.0 | 607.7852 | 2 | 22.42 | 9 | 5433 | Ana\_08.raw | 2.07E7 | 9.7E6 |  |  |  |  |  |  | 1.56E7 | 1.03E7 | 8 | 2 | 2 | 0 | 0 | 0 | 0 | 0 | 0 | 2 | 2 | 1189 | 1198 |  |
| K.VVSSVLQLGNIVFKK.E | N | 90.15 | 1629.9817 | 15 | 5.1 | 816.0023 | 2 | 113.24 | 6 | 31713 | Ana\_05.raw | 9.4E7 | 6.04E7 | 1.37E8 | 4.71E7 | 1.23E8 | 1.61E8 | 9.34E7 | 7.37E7 | 1.4E8 | 3.6E7 | 74 | 7 | 9 | 8 | 7 | 8 | 8 | 6 | 7 | 6 | 8 | 349 | 363 |  |
| K.RQLEEAEEESQR.I | N | 89.47 | 1502.6960 | 12 | 5.5 | 752.3594 | 2 | 24.69 | 9 | 6199 | Ana\_08.raw | 1.58E7 | 2.24E7 | 7.07E6 | 7.31E6 | 4.29E6 | 7.84E6 | 2.12E6 | 9.29E6 | 1.43E7 | 7.57E6 | 16 | 2 | 2 | 1 | 2 | 1 | 1 | 1 | 2 | 2 | 2 | 1884 | 1895 |  |
| K.NM(+15.99)DPLNDNVTSLLNASSDKFVADLWK.D | N | 88.92 | 2922.4070 | 26 | 3.2 | 975.1461 | 3 | 132.73 | 6 | 36897 | Ana\_05.raw |  |  |  |  |  | 2.3E6 |  |  |  |  | 1 | 0 | 0 | 0 | 0 | 0 | 1 | 0 | 0 | 0 | 0 | 595 | 620 | Oxidation (M) |
| R.SHEAQVQEM(+15.99)R.Q | N | 87.74 | 1229.5459 | 10 | 1.7 | 615.7812 | 2 | 17.04 | 5 | 3993 | Ana\_04.raw | 7.94E6 |  | 4.97E6 | 3.62E7 | 7.45E7 | 1.64E8 | 6.63E7 | 7.6E7 | 5.62E6 | 3.98E6 | 14 | 1 | 0 | 1 | 2 | 2 | 2 | 2 | 2 | 1 | 1 | 1189 | 1198 | Oxidation (M) |
| R.NSLQDQLDEEM(+15.99)EAK.Q | N | 84.86 | 1664.7200 | 14 | 1.5 | 833.3685 | 2 | 58.98 | 3 | 16774 | Ana\_02.raw | 6.22E7 | 8.5E7 | 5.25E7 | 1.77E7 | 4.14E7 | 5.79E7 | 2.9E7 | 3.86E7 | 9.22E7 | 3.81E7 | 30 | 3 | 3 | 2 | 2 | 3 | 4 | 3 | 4 | 3 | 3 | 1346 | 1359 | Oxidation (M) |
| R.ELDEATESNEAM(+15.99)GR.E | N | 84.11 | 1566.6467 | 14 | -0.4 | 784.3303 | 2 | 32.74 | 3 | 8938 | Ana\_02.raw | 1.98E8 | 2.47E8 | 2.41E8 | 7.86E7 | 1.72E8 | 2.32E8 | 1.23E8 | 1.52E8 | 1.99E8 | 1.23E8 | 31 | 3 | 2 | 3 | 2 | 5 | 3 | 3 | 4 | 3 | 3 | 1906 | 1919 | Oxidation (M) |
| K.VC(+57.02)HLVGINVTDFTR.A | N | 83.20 | 1629.8297 | 14 | 4.7 | 544.2864 | 3 | 89.32 | 2 | 25270 | Ana\_01.raw |  | 2.95E6 | 1.38E6 |  | 1.27E6 | 7.6E6 | 8.52E5 | 1.61E6 | 5.91E6 | 1.8E6 | 11 | 0 | 2 | 1 | 1 | 1 | 1 | 1 | 1 | 2 | 1 | 381 | 394 | Carbamidomethylation |
| R.YEILAANAIPK.G | N | 82.90 | 1201.6707 | 11 | 3.9 | 601.8450 | 2 | 81.05 | 6 | 23160 | Ana\_05.raw | 6E7 | 9.81E7 | 1.02E8 | 3.65E7 | 6.62E7 | 7.76E7 | 3.29E7 | 5.77E7 | 1.45E8 | 5.75E7 | 35 | 5 | 5 | 4 | 2 | 3 | 1 | 2 | 3 | 6 | 4 | 728 | 738 |  |
| K.NFM(+15.99)NSPM(+15.99)AQADWVAK.K | N | 82.41 | 1740.7600 | 15 | -0.9 | 871.3865 | 2 | 64.52 | 3 | 18376 | Ana\_02.raw | 1.91E7 | 2.93E7 | 2.1E7 | 8.31E6 | 1.69E7 | 2.65E7 | 2.58E6 | 1.86E7 | 2.81E7 | 6.03E6 | 17 | 2 | 2 | 2 | 1 | 2 | 2 | 1 | 2 | 2 | 1 | 19 | 33 | Oxidation (M) |
| K.KLEVQLQDLQSK.C | N | 82.13 | 1427.7983 | 12 | 2.1 | 714.9080 | 2 | 61.63 | 3 | 17556 | Ana\_02.raw | 1.85E7 | 1.77E7 | 1.49E7 | 1.37E6 | 1.07E7 | 1.53E7 | 4.11E6 | 9.1E6 | 1.19E7 | 1.11E7 | 19 | 2 | 2 | 2 | 1 | 2 | 2 | 1 | 2 | 3 | 2 | 1256 | 1267 |  |
| R.QLEEAEEESQR.I | N | 81.80 | 1346.5950 | 11 | 1.4 | 674.3057 | 2 | 27.38 | 3 | 7315 | Ana\_02.raw | 4.18E7 | 4.82E7 | 5.09E7 | 2.22E7 | 3.65E7 | 5.49E7 | 1.72E7 | 2.64E7 | 5.42E6 | 5.41E6 | 18 | 2 | 2 | 2 | 2 | 2 | 2 | 1 | 2 | 2 | 1 | 1885 | 1895 |  |
| K.KEEELQAALAR.L | N | 81.72 | 1256.6724 | 11 | 3.0 | 629.3453 | 2 | 48.00 | 6 | 13598 | Ana\_05.raw | 1.52E8 | 1.91E8 | 1.81E8 | 6.35E7 | 1.28E8 | 2.09E8 | 9.44E7 | 1.29E8 | 1.4E8 | 1.62E8 | 43 | 5 | 4 | 4 | 4 | 4 | 5 | 3 | 5 | 5 | 4 | 1088 | 1098 |  |
| K.AEM(+15.99)EDLVSSKDDVGK.N | N | 81.11 | 1637.7454 | 15 | 1.3 | 819.8810 | 2 | 38.88 | 1 | 10671 | ANa\_10.raw | 1.64E7 | 1.55E7 | 2.05E7 | 6.42E6 | 1.13E7 | 3.13E7 | 1.42E7 | 1.22E7 | 1.42E7 | 9.48E6 | 23 | 2 | 2 | 2 | 2 | 1 | 3 | 3 | 2 | 4 | 2 | 1511 | 1525 | Oxidation (M) |
| K.ALELDPNLYR.I | N | 81.07 | 1202.6295 | 10 | 2.4 | 602.3235 | 2 | 85.18 | 6 | 24273 | Ana\_05.raw | 9.75E7 | 1.34E8 | 1.19E8 | 4.21E7 | 8.95E7 | 1.03E8 | 5.04E7 | 5.85E7 | 2.02E8 | 6.89E7 | 54 | 5 | 7 | 7 | 5 | 5 | 5 | 4 | 6 | 6 | 4 | 753 | 762 |  |
| R.ELDEATESNEAMGR.E | N | 80.15 | 1550.6519 | 14 | 3.8 | 776.3361 | 2 | 45.47 | 1 | 12685 | ANa\_10.raw | 9.05E6 | 4.54E6 |  |  |  |  |  |  |  | 5.99E6 | 4 | 1 | 2 | 0 | 0 | 0 | 0 | 0 | 0 | 0 | 1 | 1906 | 1919 |  |
| K.LQDFASTIEVM(+15.99)EEGK.K | N | 79.47 | 1711.7975 | 15 | 2.9 | 856.9085 | 2 | 83.87 | 6 | 23920 | Ana\_05.raw | 1.04E7 | 1.24E7 | 6.18E6 | 1.05E6 | 6.27E6 | 8.8E6 | 6.52E5 | 3.79E6 | 1.75E7 | 5.67E6 | 17 | 2 | 2 | 1 | 1 | 2 | 1 | 1 | 2 | 3 | 2 | 1380 | 1394 | Oxidation (M) |
| K.VIQYLAVVASSHK.G | N | 79.27 | 1413.7980 | 13 | 2.5 | 707.9080 | 2 | 75.00 | 6 | 21515 | Ana\_05.raw | 4.3E6 | 7.02E6 | 2.63E6 | 1.24E6 | 9.33E5 | 4.14E6 |  | 2.72E6 | 6.77E6 | 3.02E6 | 9 | 1 | 1 | 1 | 1 | 1 | 1 | 0 | 1 | 1 | 1 | 191 | 203 |  |
| K.LQNEVESVTGMLNEAEGK.A | N | 79.09 | 1946.9255 | 18 | 3.2 | 974.4732 | 2 | 106.89 | 9 | 30065 | Ana\_08.raw |  |  |  |  |  |  |  |  | 1.51E7 |  | 2 | 0 | 0 | 0 | 0 | 0 | 0 | 0 | 0 | 2 | 0 | 1285 | 1302 |  |
| R.NTDQASM(+15.99)PDNTAAQK.V | N | 77.94 | 1606.6893 | 15 | 0.7 | 804.3525 | 2 | 17.95 | 5 | 4299 | Ana\_04.raw |  |  |  | 1.79E7 | 4.46E7 | 2.32E6 | 2.28E7 | 2.37E7 |  |  | 7 | 0 | 0 | 0 | 1 | 2 | 1 | 1 | 2 | 0 | 0 | 366 | 380 | Oxidation (M) |
| K.ATYERLFRWILSR.V | N | 77.91 | 1709.9365 | 13 | 3.3 | 428.4928 | 4 | 125.23 | 5 | 34534 | Ana\_04.raw |  |  |  | 2.73E5 | 1.72E6 | 3.4E6 | 6.6E5 |  |  | 7.61E5 | 6 | 0 | 0 | 0 | 1 | 2 | 1 | 1 | 0 | 0 | 1 | 427 | 439 |  |
| R.QLHEYETELEDER.K | N | 77.63 | 1689.7482 | 13 | 2.2 | 845.8832 | 2 | 59.45 | 1 | 16806 | ANa\_10.raw | 3.06E6 | 2.99E6 |  |  |  |  |  |  | 4.11E6 |  | 3 | 1 | 1 | 0 | 0 | 0 | 0 | 0 | 0 | 1 | 0 | 1597 | 1609 |  |
| K.TEFSIIHYAGK.V | N | 77.58 | 1264.6451 | 11 | 3.9 | 633.3323 | 2 | 69.33 | 2 | 19759 | Ana\_01.raw | 3.43E7 | 5.88E7 | 5.55E7 | 1.24E7 | 3.91E7 | 6.24E7 | 1.54E7 | 2.08E7 | 3.31E7 | 4.55E7 | 26 | 2 | 4 | 2 | 2 | 3 | 3 | 2 | 3 | 2 | 3 | 573 | 583 |  |
| K.ENADLAGELR.V | N | 77.43 | 1086.5305 | 10 | 1.0 | 544.2731 | 2 | 49.24 | 3 | 13919 | Ana\_02.raw | 1.78E8 | 2.26E8 | 2.02E8 | 7.09E7 | 1.46E8 | 1.82E8 | 9.26E7 | 1.29E8 | 1.9E8 | 1.21E8 | 51 | 5 | 4 | 5 | 4 | 4 | 5 | 4 | 8 | 8 | 4 | 1232 | 1241 |  |
| K.LEDDILVM(+15.99)DDQNSK.L | N | 76.67 | 1649.7454 | 14 | 3.7 | 825.8830 | 2 | 65.35 | 6 | 18763 | Ana\_05.raw | 2.38E6 | 1.16E6 | 4.12E6 |  |  | 1.92E6 | 8.79E5 | 2.58E6 | 6.03E6 | 5.36E5 | 8 | 1 | 1 | 1 | 0 | 0 | 1 | 1 | 1 | 1 | 1 | 983 | 996 | Oxidation (M) |
| K.M(+15.99)AEQYKEQAEK.G | N | 76.03 | 1369.6183 | 11 | 3.8 | 685.8190 | 2 | 19.83 | 1 | 4708 | ANa\_10.raw | 3.81E6 | 3.25E5 | 1.87E6 |  | 1.35E6 | 9.38E6 | 3.39E6 | 1.18E6 |  | 4.9E6 | 12 | 2 | 1 | 1 | 0 | 1 | 2 | 2 | 1 | 0 | 2 | 1864 | 1874 | Oxidation (M) |
| K.KQELEEILHEMEAR.L | N | 75.82 | 1753.8668 | 14 | 3.7 | 585.6317 | 3 | 100.26 | 1 | 28046 | ANa\_10.raw | 3.76E6 |  |  |  |  |  |  |  |  |  | 1 | 1 | 0 | 0 | 0 | 0 | 0 | 0 | 0 | 0 | 0 | 917 | 930 |  |
| K.KFDQLLAEEK.N | N | 75.17 | 1219.6448 | 10 | 2.0 | 610.8309 | 2 | 53.25 | 3 | 15096 | Ana\_02.raw | 7E7 | 8.1E7 | 7.96E7 | 3.21E7 | 6.01E7 | 7.54E7 | 3.96E7 | 3.99E7 | 1.26E8 | 5.97E7 | 37 | 4 | 4 | 3 | 3 | 4 | 4 | 4 | 3 | 4 | 4 | 1452 | 1461 |  |
| K.EM(+15.99)EGLSQQYEEK.A | N | 73.31 | 1485.6293 | 12 | 5.4 | 743.8259 | 2 | 39.52 | 6 | 11003 | Ana\_05.raw | 4.6E7 | 4.93E7 |  | 1.53E7 | 3.56E7 | 5.38E7 | 2.44E7 | 3.27E7 | 3.98E7 | 2.76E7 | 19 | 3 | 2 | 0 | 1 | 1 | 3 | 1 | 3 | 3 | 2 | 1400 | 1411 | Oxidation (M) |
| K.HESM(+15.99)ISELEVR.L | N | 73.11 | 1344.6344 | 11 | 3.1 | 673.3265 | 2 | 51.34 | 7 | 14439 | Ana\_06.raw | 1.11E7 | 2.22E7 | 2.73E7 | 6.07E6 | 1.61E7 | 2.3E7 | 1.2E7 | 1.7E7 |  | 3.86E6 | 24 | 2 | 2 | 3 | 3 | 3 | 3 | 3 | 4 | 0 | 1 | 1032 | 1042 | Oxidation (M) |
| R.SGKLDAFLVLEQLR.C | N | 71.83 | 1587.8984 | 14 | 1.4 | 530.3075 | 3 | 121.03 | 6 | 33756 | Ana\_05.raw |  | 7.43E5 |  |  |  | 3.98E5 | 2.34E5 |  |  |  | 3 | 0 | 1 | 0 | 0 | 0 | 1 | 1 | 0 | 0 | 0 | 687 | 700 |  |
| K.LQDFASTIEVMEEGK.K | N | 71.66 | 1695.8025 | 15 | 4.7 | 848.9125 | 2 | 110.63 | 10 | 30552 | Ana\_09.raw |  |  |  |  |  | 8.17E5 |  |  | 1.6E7 | 2.73E7 | 3 | 0 | 0 | 0 | 0 | 0 | 1 | 0 | 0 | 1 | 1 | 1380 | 1394 |  |
| K.FDQLLAEEK.N | N | 71.45 | 1091.5498 | 9 | 2.9 | 546.7838 | 2 | 58.18 | 6 | 16636 | Ana\_05.raw | 3.53E7 | 3.9E7 | 4.13E7 | 1.48E7 | 2.94E7 | 4.16E7 | 2.35E7 | 2.34E7 | 5.36E7 | 2.71E7 | 11 | 1 | 1 | 1 | 1 | 1 | 1 | 1 | 1 | 2 | 1 | 1453 | 1461 |  |
| K.M(+15.99)TESSLPSASK.T | N | 68.97 | 1152.5332 | 11 | 3.5 | 577.2759 | 2 | 27.47 | 6 | 7335 | Ana\_05.raw | 3.72E7 | 1.32E8 | 0 | 6.96E7 | 1.23E8 | 2.09E8 | 1E8 | 3.2E7 | 1.31E8 |  | 15 | 1 | 1 | 1 | 1 | 2 | 2 | 2 | 3 | 2 | 0 | 634 | 644 | Oxidation (M) |
| R.ALEEALEAK.E | N | 68.87 | 972.5127 | 9 | 1.7 | 487.2645 | 2 | 44.90 | 3 | 12627 | Ana\_02.raw | 1.13E8 | 1.41E8 | 1.47E8 | 5.37E7 | 1.14E8 | 1.46E8 | 7.47E7 | 8.94E7 | 1.33E8 | 9.07E7 | 38 | 4 | 4 | 5 | 4 | 4 | 3 | 2 | 5 | 3 | 4 | 1491 | 1499 |  |
| K.DLELQADSAIK.G | N | 67.66 | 1201.6190 | 11 | 5.4 | 601.8200 | 2 | 68.82 | 6 | 19744 | Ana\_05.raw | 2.42E7 | 3.1E7 | 2.32E7 | 1.38E7 | 2.23E7 | 2.9E7 | 1.87E7 | 1.52E7 | 2.51E7 | 1.91E7 | 19 | 3 | 2 | 3 | 2 | 1 | 2 | 1 | 1 | 3 | 1 | 1628 | 1638 |  |
| R.LDEEIAQK.N | N | 67.52 | 944.4814 | 8 | 2.9 | 473.2494 | 2 | 30.85 | 6 | 8388 | Ana\_05.raw | 2.45E8 | 3.03E8 | 2.14E8 | 9.41E7 | 1.58E8 | 2.46E8 | 1.16E8 | 1.39E8 | 2.26E8 | 1.46E8 | 26 | 4 | 4 | 4 | 2 | 1 | 2 | 1 | 2 | 3 | 3 | 1099 | 1106 |  |
| K.EQADFAIEALAK.A | N | 67.21 | 1304.6611 | 12 | 3.3 | 653.3400 | 2 | 90.14 | 6 | 25623 | Ana\_05.raw | 1.03E8 | 1.07E8 | 1.05E8 | 3.27E7 | 6.53E7 | 7.22E7 | 4.35E7 | 5.16E7 | 1.8E8 | 8.13E7 | 42 | 4 | 5 | 4 | 3 | 4 | 4 | 4 | 3 | 6 | 5 | 415 | 426 |  |
| K.DKTEFSIIHYAGK.V | N | 67.14 | 1507.7671 | 13 | 8.5 | 754.8972 | 2 | 62.66 | 9 | 17847 | Ana\_08.raw | 1.68E7 | 2.15E7 | 1.33E7 |  | 1.24E7 | 1.86E7 | 9.2E6 | 7.06E6 | 3.79E7 | 1.81E7 | 11 | 1 | 2 | 1 | 0 | 1 | 1 | 1 | 1 | 2 | 1 | 571 | 583 |  |
| K.EMEGLSQQYEEK.A | N | 65.04 | 1469.6344 | 12 | 4.1 | 735.8275 | 2 | 48.70 | 9 | 13645 | Ana\_08.raw | 9.07E6 | 5.63E6 |  |  |  | 3.47E6 |  | 1.3E6 | 1.04E7 | 5.86E6 | 6 | 1 | 1 | 0 | 0 | 0 | 1 | 0 | 1 | 1 | 1 | 1400 | 1411 |  |
| R.ASRDEIFATSK.E | N | 64.33 | 1223.6146 | 11 | 2.1 | 408.8797 | 3 | 37.66 | 4 | 10135 | Ana\_03.raw | 4.88E7 | 4.31E7 | 3.25E7 | 1.32E7 | 3.05E7 | 5.66E7 | 2.76E7 | 2.51E7 | 3.12E7 | 3.75E7 | 20 | 2 | 2 | 2 | 2 | 2 | 2 | 2 | 2 | 1 | 3 | 1666 | 1676 |  |
| K.SKHESM(+15.99)ISELEVR.L | N | 64.09 | 1559.7614 | 13 | 3.0 | 780.8903 | 2 | 36.07 | 9 | 9742 | Ana\_08.raw | 2.8E7 | 1.49E7 | 3.39E7 |  | 1.14E7 | 1.37E7 | 9.81E6 | 2.43E5 | 1.09E7 | 2.06E7 | 21 | 4 | 2 | 3 | 0 | 2 | 1 | 1 | 1 | 4 | 3 | 1030 | 1042 | Oxidation (M) |
| K.LEVQLQDLQSK.C | N | 63.87 | 1299.7034 | 11 | 6.0 | 650.8629 | 2 | 72.30 | 1 | 20480 | ANa\_10.raw | 2.99E6 |  | 0 |  | 1.53E5 | 6.67E5 |  |  | 7.88E6 |  | 5 | 1 | 0 | 1 | 0 | 1 | 1 | 0 | 0 | 1 | 0 | 1257 | 1267 |  |
| K.AAAYDKLEK.T | N | 63.81 | 1007.5287 | 9 | 3.7 | 504.7735 | 2 | 27.80 | 6 | 7440 | Ana\_05.raw | 2.1E7 | 6.67E7 | 5.29E7 | 1.76E7 | 3.87E7 | 5.75E7 | 1.53E7 | 2.51E7 | 5.22E7 | 1.3E7 | 22 | 2 | 4 | 3 | 2 | 2 | 2 | 1 | 2 | 2 | 2 | 1412 | 1420 |  |
| R.DEIFATSK.E | N | 62.67 | 909.4443 | 8 | 3.5 | 455.7310 | 2 | 41.18 | 6 | 11518 | Ana\_05.raw | 9.85E7 | 1.52E8 | 1.93E8 | 6.81E7 | 1.33E8 | 1.54E8 | 9.23E7 | 1.05E8 | 4.64E7 | 9.51E7 | 36 | 3 | 5 | 3 | 3 | 3 | 3 | 3 | 5 | 4 | 4 | 1669 | 1676 |  |
| K.YLPIYSEK.I | N | 62.49 | 1011.5276 | 8 | 0.6 | 506.7714 | 2 | 56.72 | 3 | 16097 | Ana\_02.raw | 8.27E7 | 1.14E8 | 1.08E8 | 3.84E7 | 6.9E7 | 1.01E8 | 5.2E7 | 5.61E7 | 8.68E7 | 6.12E7 | 20 | 2 | 2 | 2 | 2 | 3 | 2 | 2 | 2 | 1 | 2 | 130 | 137 |  |
| R.DLGEELEALK.T | N | 61.89 | 1115.5709 | 10 | 3.2 | 558.7945 | 2 | 97.87 | 6 | 27687 | Ana\_05.raw | 4.23E7 | 3.48E7 | 5.12E7 | 1.32E7 | 3.52E7 | 3.93E7 | 1.91E7 | 2.39E7 | 5.83E7 | 2.24E7 | 27 | 3 | 2 | 3 | 2 | 3 | 2 | 2 | 4 | 4 | 2 | 1143 | 1152 |  |
| K.VTVGKDDIQK.M | N | 61.69 | 1101.6029 | 10 | 2.9 | 551.8103 | 2 | 24.00 | 1 | 6101 | ANa\_10.raw | 3.64E7 | 3.47E7 | 2.67E7 | 5.07E6 | 3.3E7 | 4.49E7 |  | 1.7E7 | 2.3E7 | 1.93E7 | 14 | 2 | 1 | 1 | 2 | 3 | 2 | 0 | 1 | 1 | 1 | 69 | 78 |  |
| K.AEM(+15.99)EDLVSSK.D | N | 61.23 | 1123.5067 | 10 | 4.8 | 562.7633 | 2 | 29.53 | 6 | 7982 | Ana\_05.raw | 3.16E7 | 4.16E7 | 4.86E7 | 1.55E7 | 2.66E7 | 4.49E7 | 2.35E7 | 2.5E7 | 2.4E7 | 2.72E6 | 13 | 2 | 1 | 1 | 2 | 1 | 2 | 1 | 1 | 1 | 1 | 1511 | 1520 | Oxidation (M) |
| R.IVGLDQM(+15.99)AK.M | N | 60.65 | 989.5215 | 9 | 3.7 | 495.7699 | 2 | 39.85 | 6 | 11107 | Ana\_05.raw | 7.81E7 | 8.5E7 | 1.04E8 | 3.38E7 | 7.27E7 | 1.06E8 | 5.61E7 | 6.28E7 | 6.13E7 | 4.6E7 | 16 | 2 | 1 | 1 | 1 | 2 | 2 | 2 | 2 | 2 | 1 | 625 | 633 | Oxidation (M) |
| K.EVLLQVEDERK.M | N | 60.42 | 1356.7249 | 11 | 6.5 | 679.3741 | 2 | 55.18 | 7 | 15549 | Ana\_06.raw | 1.76E7 | 2.17E7 |  |  | 1.67E7 | 2.38E7 | 3.12E6 |  | 1.74E7 |  | 6 | 1 | 1 | 0 | 0 | 1 | 1 | 1 | 0 | 1 | 0 | 1853 | 1863 |  |
| R.IVFQEFR.Q | N | 60.19 | 937.5021 | 7 | 2.1 | 469.7593 | 2 | 70.64 | 3 | 20071 | Ana\_02.raw | 1.78E8 | 2.55E8 | 2.31E8 | 9.57E7 | 1.71E8 | 2.24E8 | 1.16E8 | 1.52E8 | 2.58E8 | 1.28E8 | 82 | 7 | 9 | 10 | 6 | 9 | 8 | 8 | 10 | 8 | 7 | 719 | 725 |  |
| K.MTESSLPSASK.T | N | 60.00 | 1136.5383 | 11 | 6.6 | 569.2802 | 2 | 36.23 | 4 | 9704 | Ana\_03.raw |  |  |  | 1.8E6 |  |  | 2.51E6 |  |  |  | 2 | 0 | 0 | 0 | 1 | 0 | 0 | 1 | 0 | 0 | 0 | 634 | 644 |  |
| K.LKEVLLQVEDER.K | N | 58.91 | 1469.8090 | 12 | 5.0 | 490.9460 | 3 | 70.48 | 6 | 20225 | Ana\_05.raw |  |  |  |  |  | 2.53E6 |  | 2.73E6 | 1.18E6 |  | 3 | 0 | 0 | 0 | 0 | 0 | 1 | 0 | 1 | 1 | 0 | 1851 | 1862 |  |
| K.AETELKELEQK.H | N | 58.85 | 1316.6824 | 11 | 3.4 | 659.3507 | 2 | 45.14 | 1 | 12588 | ANa\_10.raw | 2.46E6 |  |  |  |  | 3.57E6 |  |  | 7.89E5 | 1.06E6 | 4 | 1 | 0 | 0 | 0 | 0 | 1 | 0 | 0 | 1 | 1 | 871 | 881 |  |
| K.LRGPPPQETSQ | Y | 58.73 | 1208.6149 | 11 | 4.5 | 605.3174 | 2 | 25.00 | 6 | 6554 | Ana\_05.raw | 1.09E7 | 0 | 2.63E7 | 9.6E6 | 1.79E7 | 3.99E7 | 1.23E7 | 1.34E7 | 1.55E7 | 5.91E6 | 19 | 2 | 1 | 3 | 2 | 3 | 2 | 2 | 2 | 1 | 1 | 1928 | 1938 |  |
| K.FVADLWK.D | N | 58.69 | 877.4697 | 7 | 2.6 | 439.7433 | 2 | 82.66 | 6 | 23594 | Ana\_05.raw | 1.56E7 | 2.33E7 | 1.6E7 |  | 1.36E7 | 3.18E7 | 1.01E7 | 1.88E7 | 4.32E7 | 9.24E6 | 14 | 2 | 2 | 1 | 0 | 1 | 1 | 1 | 1 | 4 | 1 | 614 | 620 |  |
| K.EVLLQVEDER.K | N | 57.24 | 1228.6299 | 10 | 5.1 | 615.3254 | 2 | 67.38 | 7 | 19119 | Ana\_06.raw |  | 0 | 2.7E7 | 1.08E7 | 1.43E7 | 2.41E7 | 1.16E7 | 1.13E7 |  | 1.55E7 | 8 | 0 | 1 | 1 | 1 | 1 | 1 | 1 | 1 | 0 | 1 | 1853 | 1862 |  |
| R.NTTPNFVR.C | N | 57.07 | 947.4825 | 8 | 0.2 | 474.7486 | 2 | 36.48 | 3 | 10067 | Ana\_02.raw | 1.77E8 | 2.17E8 | 2.07E8 | 8.12E7 | 1.61E8 | 1.99E8 | 1.07E8 | 1.42E8 | 7.84E7 | 1.2E8 | 25 | 3 | 2 | 2 | 1 | 3 | 2 | 1 | 5 | 3 | 3 | 670 | 677 |  |
| R.NC(+57.02)AAYLK.L | N | 56.53 | 838.4007 | 7 | 3.5 | 420.2091 | 2 | 28.49 | 6 | 7650 | Ana\_05.raw | 3.89E7 |  | 4.95E7 | 2.34E7 | 4.81E7 | 7.1E7 | 3.25E7 | 2.9E7 | 3.32E7 | 3.28E7 | 11 | 1 | 0 | 1 | 2 | 1 | 1 | 1 | 2 | 1 | 1 | 822 | 828 | Carbamidomethylation |
| K.KVIQYLAVVASSHK.G | N | 56.05 | 1541.8929 | 14 | 2.4 | 514.9728 | 3 | 71.36 | 4 | 19853 | Ana\_03.raw |  |  |  | 1.99E6 |  |  |  |  |  |  | 1 | 0 | 0 | 0 | 1 | 0 | 0 | 0 | 0 | 0 | 0 | 190 | 203 |  |
| K.STVAALEAK.I | N | 55.97 | 888.4916 | 9 | 2.7 | 445.2543 | 2 | 33.22 | 6 | 9095 | Ana\_05.raw | 1.06E8 | 1.39E8 | 1.32E8 | 3.91E7 | 9.22E7 | 5.75E7 | 7.49E7 | 6.4E7 | 1.1E8 | 8.86E7 | 16 | 2 | 2 | 2 | 1 | 2 | 1 | 1 | 1 | 2 | 2 | 1814 | 1822 |  |
| R.IAQLEEELEEEQGNM(+15.99)EAM(+15.99)SDR.V | N | 52.63 | 2482.0476 | 21 | 3.9 | 1242.0359 | 2 | 80.25 | 9 | 22852 | Ana\_08.raw |  |  | 3.98E6 |  | 4.21E6 | 3.08E6 |  |  | 6.27E6 |  | 5 | 0 | 0 | 1 | 0 | 1 | 1 | 0 | 0 | 2 | 0 | 1738 | 1758 | Oxidation (M) |
| K.QGFEAASIKEEK.G | N | 52.32 | 1335.6670 | 12 | 5.8 | 668.8447 | 2 | 39.33 | 1 | 10811 | ANa\_10.raw | 2.45E6 | 2.42E6 |  |  |  | 1.47E7 |  |  | 1.13E6 |  | 5 | 1 | 1 | 0 | 0 | 0 | 2 | 0 | 0 | 1 | 0 | 43 | 54 |  |
| R.QLHEYETELEDERK.Q | N | 51.81 | 1817.8431 | 14 | 0.7 | 606.9554 | 3 | 53.37 | 6 | 15217 | Ana\_05.raw |  |  |  |  |  | 7.26E6 |  | 3.06E6 | 5.79E6 |  | 3 | 0 | 0 | 0 | 0 | 0 | 1 | 0 | 1 | 1 | 0 | 1597 | 1610 |  |
| R.KM(+15.99)AEQYKEQAEK.G | N | 51.68 | 1497.7133 | 12 | -0.1 | 500.2450 | 3 | 17.57 | 6 | 4122 | Ana\_05.raw | 3.16E6 | 1.02E6 |  |  |  | 2.59E6 |  |  |  |  | 4 | 2 | 1 | 0 | 0 | 0 | 1 | 0 | 0 | 0 | 0 | 1863 | 1874 | Oxidation (M) |
| R.C(+57.02)IIPNHEK.R | N | 51.44 | 1009.5015 | 8 | 3.8 | 505.7599 | 2 | 28.00 | 5 | 7477 | Ana\_04.raw | 3.6E6 | 5.27E6 | 2.95E6 |  | 3.18E6 | 3.44E6 | 0 | 1.91E6 |  |  | 7 | 1 | 1 | 1 | 0 | 1 | 1 | 1 | 1 | 0 | 0 | 678 | 685 | Carbamidomethylation |
| K.ALDEETR.S | N | 51.25 | 832.3926 | 7 | 0.1 | 417.2036 | 2 | 20.23 | 5 | 5059 | Ana\_04.raw |  |  |  | 3.38E6 | 5.94E7 |  | 7.01E6 | 5.73E6 |  |  | 4 | 0 | 0 | 0 | 1 | 1 | 0 | 1 | 1 | 0 | 0 | 1182 | 1188 |  |
| K.RQQQLTAM(+15.99)K.V | N | 51.23 | 1118.5867 | 9 | 3.8 | 560.3027 | 2 | 17.54 | 6 | 4110 | Ana\_05.raw |  |  |  | 3.15E6 | 9.88E6 | 3.99E6 | 6.24E6 | 6.76E6 |  |  | 5 | 0 | 0 | 0 | 1 | 1 | 1 | 1 | 1 | 0 | 0 | 809 | 817 | Oxidation (M) |
| K.LQEVEGAVK.A | N | 50.94 | 971.5287 | 9 | 1.0 | 486.7722 | 2 | 30.03 | 3 | 8136 | Ana\_02.raw |  | 1.73E8 | 1.48E8 |  | 1.14E8 | 1.6E8 |  | 8.18E7 | 1.21E8 |  | 7 | 0 | 2 | 1 | 0 | 1 | 1 | 0 | 1 | 1 | 0 | 1801 | 1809 |  |
| K.KALDEETR.S | N | 50.77 | 960.4876 | 8 | 1.9 | 321.1704 | 3 | 18.14 | 4 | 4322 | Ana\_03.raw |  |  |  | 3.51E5 | 5.68E6 |  | 1.02E6 |  |  |  | 3 | 0 | 0 | 0 | 1 | 1 | 0 | 1 | 0 | 0 | 0 | 1181 | 1188 |  |
| R.GQQLQAER.K | N | 50.71 | 928.4726 | 8 | 2.1 | 465.2446 | 2 | 19.39 | 5 | 4780 | Ana\_04.raw |  |  |  | 7.53E6 | 2.49E7 |  | 3.31E6 | 6.58E6 |  |  | 4 | 0 | 0 | 0 | 1 | 1 | 0 | 1 | 1 | 0 | 0 | 938 | 945 |  |
| K.LVWVPSEK.Q | N | 48.98 | 956.5331 | 8 | 3.1 | 479.2753 | 2 | 66.05 | 6 | 18966 | Ana\_05.raw | 2.38E7 | 3.38E7 | 3.02E7 | 2.56E7 | 1.44E7 | 2.88E7 |  |  | 2.32E7 | 0 | 9 | 2 | 1 | 1 | 1 | 1 | 1 | 0 | 0 | 1 | 1 | 35 | 42 |  |
| K.LRNWQWWRLFTK.V | N | 48.91 | 1732.9314 | 12 | 3.0 | 578.6528 | 3 | 117.69 | 6 | 32880 | Ana\_05.raw |  |  |  |  |  | 1.07E7 |  |  |  |  | 2 | 0 | 0 | 0 | 0 | 0 | 2 | 0 | 0 | 0 | 0 | 829 | 840 |  |
| K.AEMEDLVSSK.D | N | 48.32 | 1107.5117 | 10 | 4.4 | 554.7656 | 2 | 51.76 | 1 | 14541 | ANa\_10.raw | 3.59E6 |  |  |  |  |  |  |  |  | 1.86E6 | 2 | 1 | 0 | 0 | 0 | 0 | 0 | 0 | 0 | 0 | 1 | 1511 | 1520 |  |
| R.LEVNM(+15.99)QALK.G | N | 47.90 | 1060.5587 | 9 | 1.8 | 531.2876 | 2 | 45.29 | 4 | 12401 | Ana\_03.raw | 8.81E6 | 5.8E7 | 5.85E7 | 1.59E7 |  | 5.61E7 | 2.48E7 | 3.04E7 | 6.41E7 | 9.94E6 | 11 | 1 | 2 | 1 | 1 | 0 | 2 | 1 | 1 | 1 | 1 | 1565 | 1573 | Oxidation (M) |
| K.DSSITGELEK.Q | N | 47.89 | 1077.5189 | 10 | 6.5 | 539.7703 | 2 | 49.96 | 1 | 14018 | ANa\_10.raw | 1.04E7 | 5.59E6 |  | 2.42E6 | 9E5 | 5.7E6 | 4.29E6 | 3.73E5 | 1.41E7 |  | 8 | 1 | 1 | 0 | 1 | 1 | 1 | 1 | 1 | 1 | 0 | 207 | 216 |  |
| K.NVHELEK.S | N | 46.85 | 867.4450 | 7 | 2.4 | 434.7308 | 2 | 18.44 | 6 | 4415 | Ana\_05.raw |  |  |  | 4.05E7 | 8.23E7 | 8.16E7 | 6.58E7 | 1.74E8 |  |  | 6 | 0 | 0 | 0 | 1 | 1 | 1 | 1 | 2 | 0 | 0 | 1526 | 1532 |  |
| K.KLVWVPSEK.Q | N | 46.63 | 1084.6281 | 9 | 2.7 | 543.3228 | 2 | 57.34 | 6 | 16386 | Ana\_05.raw |  |  | 5.44E6 | 1.75E6 |  | 6.46E6 | 2.56E6 |  | 9.29E6 | 1.51E6 | 6 | 0 | 0 | 1 | 1 | 0 | 1 | 1 | 0 | 1 | 1 | 34 | 42 |  |
| R.ALETQM(+15.99)EEM(+15.99)K.T | N | 45.79 | 1240.5315 | 10 | 1.8 | 621.2741 | 2 | 20.29 | 3 | 5032 | Ana\_02.raw | 6.37E6 |  | 0 | 1.64E7 | 2.08E7 | 2.65E7 | 2.22E7 | 2.91E7 |  | 7.92E6 | 9 | 1 | 0 | 1 | 1 | 1 | 1 | 1 | 2 | 0 | 1 | 1536 | 1545 | Oxidation (M) |
| K.ITDVIM(+15.99)AFQAM(+15.99)C(+57.02)R.G | N | 45.02 | 1586.7255 | 13 | 0.4 | 794.3704 | 2 | 88.57 | 3 | 24886 | Ana\_02.raw |  | 4.35E6 | 2.13E6 | 8.01E5 | 1.87E6 | 1.11E6 | 8.56E5 | 2.41E6 | 6.3E6 |  | 10 | 0 | 1 | 1 | 1 | 2 | 1 | 2 | 1 | 1 | 0 | 786 | 798 | Oxidation (M); Carbamidomethylation |
| K.KLEGDLK.D | N | 43.92 | 801.4596 | 7 | 2.3 | 401.7380 | 2 | 20.25 | 6 | 5002 | Ana\_05.raw | 7.05E7 | 6.06E7 | 5.96E7 | 2.41E7 | 4.21E7 | 8.36E7 | 6.49E6 | 4.29E7 |  |  | 12 | 2 | 2 | 1 | 1 | 1 | 2 | 1 | 2 | 0 | 0 | 1621 | 1627 |  |
| R.QRYEILAANAIPK.G | N | 43.83 | 1485.8303 | 13 | 0.4 | 496.2842 | 3 | 68.44 | 9 | 19529 | Ana\_08.raw |  |  |  |  |  |  |  |  | 1.14E6 |  | 1 | 0 | 0 | 0 | 0 | 0 | 0 | 0 | 0 | 1 | 0 | 726 | 738 |  |
| K.KKLEGDLK.D | N | 43.81 | 929.5546 | 8 | 1.4 | 465.7852 | 2 | 17.40 | 3 | 4067 | Ana\_02.raw | 7.47E6 | 4.25E6 | 1.5E7 | 5.12E6 | 1.18E7 | 2.36E7 | 4.88E6 | 4.9E6 |  | 1.14E7 | 14 | 1 | 1 | 2 | 2 | 2 | 2 | 1 | 1 | 0 | 2 | 1620 | 1627 |  |
| K.VKPLLQVTR.Q | N | 43.60 | 1052.6705 | 9 | 4.3 | 351.8990 | 3 | 47.58 | 8 | 13112 | Ana\_07.raw | 5.04E7 | 3.17E7 | 7.22E7 | 2.28E7 | 5.31E7 | 7.4E7 | 3.29E7 | 3.78E7 | 6.72E7 | 4.05E7 | 26 | 2 | 1 | 3 | 3 | 4 | 3 | 3 | 3 | 2 | 2 | 841 | 849 |  |
| R.QGFPNR.I | N | 43.46 | 717.3558 | 6 | 0.9 | 359.6855 | 2 | 22.62 | 3 | 5802 | Ana\_02.raw |  | 4.83E6 | 1.13E7 | 7.76E6 |  | 1.82E7 | 7.99E6 | 1.36E7 |  |  | 6 | 0 | 1 | 1 | 1 | 0 | 1 | 1 | 1 | 0 | 0 | 713 | 718 |  |
| R.EQEVTVLK.K | N | 42.54 | 944.5178 | 8 | 3.3 | 473.2678 | 2 | 38.27 | 6 | 10622 | Ana\_05.raw | 9.95E7 | 1.45E8 | 1.44E8 | 5E7 | 1.07E8 | 1.42E8 | 7.48E7 | 8.02E7 | 1.07E8 |  | 18 | 1 | 2 | 1 | 2 | 2 | 2 | 2 | 3 | 3 | 0 | 1173 | 1180 |  |
| R.KLLEER.V | N | 41.76 | 786.4599 | 6 | 4.8 | 394.2391 | 2 | 21.71 | 9 | 5194 | Ana\_08.raw | 4.77E7 | 7.84E7 | 7.3E7 | 2.37E7 |  |  |  | 3.46E7 | 4.24E7 | 3.86E7 | 7 | 1 | 1 | 1 | 1 | 0 | 0 | 0 | 1 | 1 | 1 | 1002 | 1007 |  |
| R.KLQAQMK.D | N | 41.48 | 845.4793 | 7 | 4.2 | 423.7487 | 2 | 18.45 | 8 | 4358 | Ana\_07.raw |  |  |  | 1.72E5 |  |  |  | 5.1E5 |  |  | 2 | 0 | 0 | 0 | 1 | 0 | 0 | 0 | 1 | 0 | 0 | 1649 | 1655 |  |
| R.EQEVTVLKK.A | N | 40.98 | 1072.6128 | 9 | 2.6 | 537.3151 | 2 | 25.53 | 3 | 6730 | Ana\_02.raw | 1.36E7 |  | 1.48E7 | 4.06E6 | 7.84E6 | 1.33E7 |  | 4.73E6 | 2.23E7 | 6.69E6 | 11 | 1 | 0 | 2 | 2 | 1 | 1 | 0 | 1 | 2 | 1 | 1173 | 1181 |  |
| K.FLFVDK.N | N | 39.11 | 767.4218 | 6 | 5.0 | 384.7201 | 2 | 70.11 | 2 | 19973 | Ana\_01.raw | 5.15E7 | 7.78E7 | 8.54E7 | 3.15E7 | 5.75E7 | 8.31E7 | 3.65E7 | 4.73E7 | 8.82E7 | 4.85E7 | 34 | 3 | 4 | 6 | 3 | 6 | 2 | 1 | 1 | 6 | 2 | 13 | 18 |  |
| R.TVGQLYK.E | N | 38.67 | 807.4490 | 7 | 2.6 | 404.7328 | 2 | 33.40 | 4 | 8890 | Ana\_03.raw | 3.54E6 | 1.11E8 | 1.2E8 | 4.55E7 | 8.98E7 | 1.13E8 | 7.15E7 | 6.55E7 | 1.02E8 | 6.97E7 | 17 | 1 | 1 | 2 | 1 | 3 | 2 | 2 | 3 | 1 | 1 | 652 | 658 |  |
| K.LQDFASTIEVM(+15.99)EEGKK.R | N | 36.24 | 1839.8923 | 16 | 3.1 | 614.3066 | 3 | 72.20 | 1 | 20454 | ANa\_10.raw | 2.71E6 | 1.62E6 |  |  |  | 3.17E6 |  |  |  |  | 3 | 1 | 1 | 0 | 0 | 0 | 1 | 0 | 0 | 0 | 0 | 1380 | 1395 | Oxidation (M) |
| R.SKLQEVEGAVK.A | N | 36.17 | 1186.6558 | 11 | 4.0 | 594.3375 | 2 | 42.97 | 2 | 11976 | Ana\_01.raw | 3.35E6 | 1.64E6 |  |  |  |  |  |  |  |  | 2 | 1 | 1 | 0 | 0 | 0 | 0 | 0 | 0 | 0 | 0 | 1799 | 1809 |  |
| K.REQEVTVLK.K | N | 35.82 | 1100.6189 | 9 | 2.3 | 551.3180 | 2 | 27.06 | 3 | 7215 | Ana\_02.raw |  |  | 1.85E7 | 9.94E6 | 2.04E7 | 1.69E7 | 1.39E7 |  |  |  | 5 | 0 | 0 | 1 | 1 | 1 | 1 | 1 | 0 | 0 | 0 | 1172 | 1180 |  |
| R.ALETQM(+15.99)EEMK.T | N | 35.77 | 1224.5366 | 10 | 1.5 | 613.2765 | 2 | 30.52 | 1 | 8174 | ANa\_10.raw | 5.5E6 |  |  |  |  |  |  |  | 2.04E6 |  | 2 | 1 | 0 | 0 | 0 | 0 | 0 | 0 | 0 | 1 | 0 | 1536 | 1545 | Oxidation (M) |
| K.ALSLAR.A | N | 35.77 | 629.3860 | 6 | 2.7 | 315.7011 | 2 | 31.28 | 6 | 8518 | Ana\_05.raw |  |  |  |  |  | 3.8E7 | 2.13E7 | 1.28E7 |  | 1.54E7 | 4 | 0 | 0 | 0 | 0 | 0 | 1 | 1 | 1 | 0 | 1 | 1485 | 1490 |  |
| K.QGFEAASIK.E | N | 34.98 | 949.4869 | 9 | 3.7 | 475.7525 | 2 | 45.37 | 4 | 12425 | Ana\_03.raw |  |  |  | 2.94E6 |  |  |  |  |  |  | 1 | 0 | 0 | 0 | 1 | 0 | 0 | 0 | 0 | 0 | 0 | 43 | 51 |  |
| R.QLVSNLEK.K | N | 33.88 | 929.5182 | 8 | 5.3 | 465.7688 | 2 | 35.36 | 9 | 9523 | Ana\_08.raw | 1.19E7 |  |  |  |  |  |  |  | 7.42E6 |  | 2 | 1 | 0 | 0 | 0 | 0 | 0 | 0 | 0 | 1 | 0 | 1441 | 1448 |  |
| K.LLEER.V | N | 33.24 | 658.3650 | 5 | 3.3 | 330.1909 | 2 | 23.06 | 6 | 5936 | Ana\_05.raw |  |  |  |  | 2.63E7 | 3.88E7 | 3.76E6 | 1.26E7 |  |  | 4 | 0 | 0 | 0 | 0 | 1 | 1 | 1 | 1 | 0 | 0 | 1003 | 1007 |  |
| K.IVDMYK.G | N | 33.02 | 767.3887 | 6 | 2.9 | 384.7028 | 2 | 33.55 | 9 | 8954 | Ana\_08.raw |  |  |  |  |  |  |  |  | 4.01E6 |  | 1 | 0 | 0 | 0 | 0 | 0 | 0 | 0 | 0 | 1 | 0 | 138 | 143 |  |
| K.REQEVTVLKK.A | N | 32.76 | 1228.7139 | 10 | 2.7 | 410.5797 | 3 | 22.61 | 1 | 5639 | ANa\_10.raw | 1.64E6 |  |  |  |  |  |  |  |  |  | 1 | 1 | 0 | 0 | 0 | 0 | 0 | 0 | 0 | 0 | 0 | 1172 | 1181 |  |
| R.WILSR.V | N | 32.69 | 673.3911 | 5 | 0.3 | 337.7029 | 2 | 55.30 | 3 | 15697 | Ana\_02.raw |  |  | 6.82E6 |  |  |  |  |  |  |  | 1 | 0 | 0 | 1 | 0 | 0 | 0 | 0 | 0 | 0 | 0 | 435 | 439 |  |
| K.LM(+15.99)TTLR.N | N | 32.45 | 749.4106 | 6 | 4.9 | 375.7144 | 2 | 26.68 | 2 | 6975 | Ana\_01.raw | 4.02E7 | 2.78E7 | 3.66E7 |  |  |  |  | 1.52E7 | 3.01E7 |  | 6 | 2 | 1 | 1 | 0 | 0 | 0 | 0 | 1 | 1 | 0 | 664 | 669 | Oxidation (M) |
| R.NWQWWR.L | N | 29.97 | 974.4511 | 6 | 5.4 | 488.2355 | 2 | 98.83 | 8 | 27494 | Ana\_07.raw |  |  |  |  |  |  |  | 8.23E5 |  |  | 1 | 0 | 0 | 0 | 0 | 0 | 0 | 0 | 1 | 0 | 0 | 831 | 836 |  |
| R.AILTPR.I | N | 29.60 | 669.4174 | 6 | 3.2 | 335.7170 | 2 | 26.50 | 9 | 6805 | Ana\_08.raw | 3.95E7 | 5.44E7 | 6.82E7 | 2.88E7 | 4.05E7 | 7.21E7 | 3.34E7 | 3.05E7 | 6.5E6 | 2.28E7 | 14 | 1 | 2 | 1 | 2 | 1 | 2 | 2 | 1 | 1 | 1 | 395 | 400 |  |
| K.IVDM(+15.99)YK.G | N | 29.48 | 783.3837 | 6 | 4.2 | 392.7007 | 2 | 26.31 | 8 | 6743 | Ana\_07.raw | 1.41E7 |  | 9.05E7 |  |  | 3.86E7 | 5.33E7 | 1.8E7 |  |  | 6 | 1 | 0 | 1 | 0 | 0 | 1 | 2 | 1 | 0 | 0 | 138 | 143 | Oxidation (M) |
| K.LKEVLLQVEDERK.M | N | 28.26 | 1597.9038 | 13 | 3.8 | 533.6439 | 3 | 59.69 | 5 | 16966 | Ana\_04.raw |  |  |  |  | 1.51E6 |  |  |  |  |  | 1 | 0 | 0 | 0 | 0 | 1 | 0 | 0 | 0 | 0 | 0 | 1851 | 1863 |  |
| R.QEEEM(+15.99)QAKEEEM(+15.99)QK.I | N | 27.83 | 1797.7396 | 14 | 1.2 | 600.2545 | 3 | 21.92 | 2 | 5386 | Ana\_01.raw |  | 2.06E6 |  |  |  | 2.88E6 |  |  |  |  | 2 | 0 | 1 | 0 | 0 | 0 | 1 | 0 | 0 | 0 | 0 | 850 | 863 | Oxidation (M) |
| R.SM(+15.99)LQDR.E | N | 26.72 | 764.3487 | 6 | 4.0 | 383.1832 | 2 | 24.74 | 1 | 6348 | ANa\_10.raw | 2.06E6 |  |  |  |  |  |  |  |  |  | 1 | 1 | 0 | 0 | 0 | 0 | 0 | 0 | 0 | 0 | 0 | 164 | 169 | Oxidation (M) |
| K.LQLEK.V | N | 26.40 | 629.3748 | 5 | 5.2 | 315.6963 | 2 | 24.82 | 10 | 6436 | Ana\_09.raw |  |  | 5.13E7 |  |  |  |  |  |  | 3.11E7 | 2 | 0 | 0 | 1 | 0 | 0 | 0 | 0 | 0 | 0 | 1 | 969 | 973 |  |
| K.LMTTLR.N | N | 23.92 | 733.4156 | 6 | 6.6 | 367.7175 | 2 | 34.22 | 9 | 9164 | Ana\_08.raw |  |  |  |  |  |  |  |  | 2.23E6 |  | 1 | 0 | 0 | 0 | 0 | 0 | 0 | 0 | 0 | 1 | 0 | 664 | 669 |  |
| K.LRNWQWWR.L | N | 23.70 | 1243.6362 | 8 | 2.7 | 415.5538 | 3 | 88.09 | 6 | 25077 | Ana\_05.raw |  |  |  |  |  | 1.89E6 |  |  |  |  | 1 | 0 | 0 | 0 | 0 | 0 | 1 | 0 | 0 | 0 | 0 | 829 | 836 |  |
| K.QAC(+57.02)ILM(+15.99)IK.A | N | 22.53 | 991.5195 | 8 | 2.7 | 496.7683 | 2 | 59.24 | 6 | 16946 | Ana\_05.raw |  |  |  |  |  | 2.13E6 |  |  |  |  | 1 | 0 | 0 | 0 | 0 | 0 | 1 | 0 | 0 | 0 | 0 | 745 | 752 | Carbamidomethylation; Oxidation (M) |
| total 158 peptides |  |  |  |  |  |  |  |  |  |  |  |  |  |  |  |  |  |  |  |  |  |  |  |  |  |  |  |  |  |  |  |  |  |  |  |
| --- | --- | --- | --- | --- | --- | --- | --- | --- | --- | --- | --- | --- | --- | --- | --- | --- | --- | --- | --- | --- | --- | --- | --- | --- | --- | --- | --- | --- | --- | --- | --- | --- | --- | --- | --- |

Best Unique PSM (Scan F6:6554, m/z=605.3174, z=2, RT=25.00, ppm=4.5):

B7FAU9|B7FAU9\_MOUSE

back to list

  

| Protein Coverage
| Supporting Peptides
| Best Unique PSM
|

Protein Coverage:

Supporting Peptides:

| Peptide | Uniq | -10lgP | Mass | Length | ppm | m/z | z | RT | Fraction | Scan | Source File | Area 10 | Area 01 | Area 02 | Area 03 | Area 04 | Area 05 | Area 06 | Area 07 | Area 08 | Area 09 | #Spec | #Spec 10 | #Spec 01 | #Spec 02 | #Spec 03 | #Spec 04 | #Spec 05 | #Spec 06 | #Spec 07 | #Spec 08 | #Spec 09 | Start | End | PTM |
| --- | --- | --- | --- | --- | --- | --- | --- | --- | --- | --- | --- | --- | --- | --- | --- | --- | --- | --- | --- | --- | --- | --- | --- | --- | --- | --- | --- | --- | --- | --- | --- | --- | --- | --- | --- |
| K.FNEEHIPDSPFVVPVASPSGDAR.R | Y | 139.48 | 2466.1814 | 23 | 0.0 | 823.0677 | 3 | 99.61 | 3 | 27795 | Ana\_02.raw | 1.37E8 | 1.17E8 | 9.82E7 | 2.86E7 | 7.2E7 | 9.35E7 | 3.56E7 | 6.1E7 | 2.24E8 | 7.34E7 | 46 | 6 | 5 | 6 | 3 | 4 | 5 | 2 | 6 | 5 | 4 | 2303 | 2325 |  |
| R.FGGEHVPNSPFQVTALAGDQPTVQTPLR.S | Y | 133.91 | 2962.4937 | 28 | 1.8 | 988.5070 | 3 | 107.99 | 6 | 30360 | Ana\_05.raw | 5.11E7 | 5.03E7 | 2.37E7 | 7.57E5 | 3.46E7 | 1.16E7 | 8.58E6 | 1.04E7 | 9.52E7 |  | 16 | 2 | 2 | 1 | 1 | 4 | 1 | 1 | 2 | 2 | 0 | 1718 | 1745 |  |
| K.GLVEPVDVVDNADGTQTVNYVPSR.E | Y | 131.40 | 2543.2502 | 24 | 1.4 | 1272.6342 | 2 | 96.61 | 9 | 27341 | Ana\_08.raw | 1.35E7 | 1.15E7 |  | 6.07E5 |  | 2.06E6 |  | 9.97E5 | 3.54E7 | 5.42E6 | 8 | 1 | 1 | 0 | 1 | 0 | 1 | 0 | 1 | 2 | 1 | 1492 | 1515 |  |
| K.VGSAADIPINISETDLSLLTATVVPPSGR.E | Y | 123.12 | 2892.5444 | 29 | 2.6 | 1447.2832 | 2 | 134.53 | 8 | 36883 | Ana\_07.raw | 2.83E7 | 1.53E7 | 2.23E7 | 7.35E6 | 2.04E7 | 9.31E6 | 2.06E7 | 1.2E7 | 4.77E7 | 9.59E6 | 19 | 2 | 2 | 2 | 2 | 2 | 2 | 2 | 2 | 2 | 1 | 1957 | 1985 |  |
| K.LQVEPAVDTSGVQC(+57.02)YGPGIEGQGVFR.E | Y | 122.99 | 2762.3333 | 26 | 2.1 | 1382.1768 | 2 | 102.98 | 9 | 29031 | Ana\_08.raw |  | 1.3E6 | 1.92E6 |  | 1.09E6 | 2.71E5 |  |  | 1.31E6 |  | 5 | 0 | 1 | 1 | 0 | 1 | 1 | 0 | 0 | 1 | 0 | 1247 | 1272 | Carbamidomethylation |
| R.ALGALVDSC(+57.02)APGLC(+57.02)PDWDSWDASKPVNNAR.E | N | 120.06 | 3241.4919 | 30 | 0.7 | 1081.5054 | 3 | 115.02 | 6 | 32179 | Ana\_05.raw | 5.49E6 | 8.67E6 |  | 4.05E6 |  | 4.4E6 |  | 2.32E6 | 2.02E7 | 3.12E6 | 7 | 1 | 1 | 0 | 1 | 0 | 1 | 0 | 1 | 1 | 1 | 197 | 226 | Carbamidomethylation |
| K.GQHVPGSPFQFTVGPLGEGGAHK.V | N | 114.28 | 2303.1445 | 23 | 3.8 | 576.7956 | 4 | 90.06 | 1 | 25304 | ANa\_10.raw | 1.86E7 | 5.87E6 | 2.37E7 | 6.44E6 | 1.54E7 | 1.46E7 |  | 1.38E7 | 3.15E7 | 4.2E6 | 18 | 3 | 2 | 2 | 1 | 2 | 1 | 0 | 3 | 2 | 2 | 2210 | 2232 |  |
| K.VATVPQHATSGPGPADVSK.V | Y | 111.55 | 1817.9271 | 19 | 1.7 | 909.9724 | 2 | 37.79 | 3 | 10475 | Ana\_02.raw | 7.81E7 | 7.96E7 | 7.66E7 | 3.2E7 | 6.38E7 | 8.55E7 | 4.17E7 | 5.37E7 | 4.96E7 | 4.5E7 | 26 | 2 | 2 | 2 | 3 | 2 | 3 | 2 | 3 | 4 | 3 | 2533 | 2551 |  |
| R.VHGPGIQSGTTNKPNK.F | Y | 111.27 | 1633.8536 | 16 | 4.2 | 817.9376 | 2 | 21.30 | 1 | 5200 | ANa\_10.raw | 5.24E7 | 8.68E6 | 4.32E7 | 1.64E7 | 3.43E7 | 2.76E7 | 1.91E7 | 8.36E6 | 6.77E7 | 3.64E7 | 32 | 3 | 3 | 3 | 2 | 3 | 3 | 3 | 2 | 6 | 4 | 1360 | 1375 |  |
| R.YWPQEAGEYAVHVLC(+57.02)NSEDIR.L | Y | 110.26 | 2535.1489 | 21 | 1.3 | 846.0580 | 3 | 126.34 | 3 | 34660 | Ana\_02.raw | 7.99E6 | 2.9E6 | 1.6E7 | 4.94E6 | 2.05E7 | 3.32E7 | 1.21E7 | 1.1E7 | 6.95E7 | 3.12E7 | 18 | 1 | 1 | 2 | 2 | 1 | 2 | 2 | 2 | 3 | 2 | 635 | 655 | Carbamidomethylation |
| R.VSGQGLHEGHTFEPAEFIIDTR.D | Y | 109.44 | 2439.1819 | 22 | 4.9 | 814.0719 | 3 | 90.76 | 9 | 25750 | Ana\_08.raw |  | 1.07E7 | 3E7 | 2.36E6 | 1.95E6 | 8.97E6 | 8.56E6 | 5.2E6 | 3.78E7 | 3.3E6 | 15 | 1 | 1 | 2 | 1 | 1 | 2 | 2 | 1 | 2 | 2 | 2044 | 2065 |  |
| K.AFGPGLQGGNAGSPAR.F | Y | 107.88 | 1455.7218 | 16 | 1.5 | 728.8693 | 2 | 49.35 | 3 | 13951 | Ana\_02.raw | 7.43E7 | 6E7 | 4.77E7 | 1.96E7 | 4.07E7 | 4.94E7 | 2.6E7 | 3.27E7 | 7.3E7 | 4.25E7 | 23 | 4 | 2 | 2 | 2 | 2 | 2 | 1 | 2 | 3 | 3 | 1072 | 1087 |  |
| K.THEAEIVEGENHTYC(+57.02)IR.F | Y | 106.99 | 2056.9272 | 17 | -0.8 | 686.6491 | 3 | 53.53 | 3 | 15177 | Ana\_02.raw |  | 9.13E6 | 7.94E6 | 2.84E6 | 6.02E6 | 1.47E7 |  | 8.03E6 |  | 9.21E6 | 9 | 0 | 1 | 1 | 1 | 1 | 2 | 0 | 2 | 0 | 1 | 2177 | 2193 | Carbamidomethylation |
| K.SADFVVEAIGDDVGTLGFSVEGPSQAK.I | Y | 105.96 | 2694.3022 | 27 | 3.8 | 899.1115 | 3 | 132.39 | 6 | 36801 | Ana\_05.raw | 2E6 |  | 5.34E6 |  |  | 8.92E6 | 2.91E6 |  |  |  | 4 | 1 | 0 | 1 | 0 | 0 | 1 | 1 | 0 | 0 | 0 | 594 | 620 |  |
| R.SPYTVTVGQAC(+57.02)NPAAC(+57.02)R.A | N | 105.52 | 1850.8403 | 17 | 4.0 | 926.4312 | 2 | 64.97 | 8 | 18225 | Ana\_07.raw | 1.38E7 | 1.71E7 | 1.32E7 | 4.49E6 | 1.27E7 | 1.88E7 | 5.1E6 | 6.29E6 | 2E7 | 1.11E7 | 15 | 1 | 1 | 2 | 1 | 2 | 3 | 1 | 1 | 2 | 1 | 468 | 484 | Carbamidomethylation |
| R.EEGPYEVEVTYDGVPVPGSPFPLEAVAPTKPSK.V | Y | 105.25 | 3484.7288 | 33 | 6.7 | 1162.5913 | 3 | 123.64 | 1 | 34044 | ANa\_10.raw | 5.45E6 | 1.29E7 | 5.51E6 | 1.25E6 | 1.97E6 | 4.39E6 | 9.58E6 | 1.43E6 | 1.53E7 |  | 11 | 2 | 2 | 1 | 1 | 1 | 1 | 1 | 1 | 1 | 0 | 1037 | 1069 |  |
| K.DAGEGLLAVQITDPEGKPK.K | Y | 101.62 | 1937.0105 | 19 | -2.2 | 969.5104 | 2 | 87.12 | 6 | 24812 | Ana\_05.raw | 3.98E5 | 2.92E6 | 1.93E6 |  | 8.43E5 | 6.08E6 | 6.7E5 |  | 5.13E6 | 5.61E5 | 11 | 1 | 2 | 1 | 0 | 2 | 2 | 1 | 0 | 1 | 1 | 1574 | 1592 |  |
| K.ASGPGLNTTGVPASLPVEFTIDAK.D | Y | 98.70 | 2341.2166 | 24 | 2.1 | 1171.6180 | 2 | 116.37 | 6 | 32531 | Ana\_05.raw | 2.29E6 | 6.26E6 | 4.49E6 | 6.45E5 | 6.06E5 | 1.03E7 | 3.35E6 | 7.44E5 | 4.34E6 |  | 12 | 1 | 2 | 2 | 1 | 1 | 1 | 2 | 1 | 1 | 0 | 1550 | 1573 |  |
| K.SPFSVGVSPSLDLSK.I | Y | 97.50 | 1518.7930 | 15 | -1.0 | 760.4030 | 2 | 93.39 | 3 | 26157 | Ana\_02.raw | 2.6E7 | 1.94E7 | 2.18E7 | 6.89E5 | 2.24E6 | 1.93E7 | 9.75E6 | 1.14E7 | 5.31E7 | 1.67E7 | 19 | 2 | 1 | 2 | 2 | 2 | 2 | 2 | 2 | 3 | 1 | 959 | 973 |  |
| R.GAGTGGLGLAVEGPSEAK.M | Y | 95.14 | 1569.7998 | 18 | 6.7 | 785.9124 | 2 | 66.11 | 1 | 18749 | ANa\_10.raw | 4.07E7 | 4.44E7 | 3.04E7 | 1.35E7 | 2.16E7 | 3.58E7 | 1.7E7 | 2.14E7 | 6.26E7 | 2.88E7 | 20 | 2 | 2 | 2 | 2 | 2 | 2 | 2 | 2 | 3 | 1 | 1382 | 1399 |  |
| K.VHSPSGALEEC(+57.02)YVTEIDQDK.Y | Y | 95.02 | 2276.0266 | 20 | -0.9 | 759.6821 | 3 | 83.27 | 6 | 23761 | Ana\_05.raw |  |  |  | 3.18E6 | 8.03E5 | 7.51E6 |  | 1.9E6 | 8.01E6 |  | 5 | 0 | 0 | 0 | 1 | 1 | 1 | 0 | 1 | 1 | 0 | 2360 | 2379 | Carbamidomethylation |
| K.HTAM(+15.99)VSWGGVSIPNSPFR.V | Y | 94.90 | 1957.9469 | 18 | 3.8 | 653.6587 | 3 | 100.25 | 1 | 28042 | ANa\_10.raw | 2.42E6 | 2.68E6 |  |  |  |  |  |  |  |  | 2 | 1 | 1 | 0 | 0 | 0 | 0 | 0 | 0 | 0 | 0 | 743 | 760 | Oxidation (M) |
| R.EGSYSISVLYGEEEVPR.S | Y | 93.11 | 1912.9054 | 17 | 4.4 | 957.4642 | 2 | 98.50 | 6 | 27854 | Ana\_05.raw |  | 1.87E6 |  |  | 1.04E6 | 9.47E5 |  | 3.67E5 | 1.16E7 | 2.82E6 | 6 | 0 | 1 | 0 | 0 | 1 | 1 | 0 | 1 | 1 | 1 | 1516 | 1532 |  |
| K.AHVAPC(+57.02)FDASK.V | Y | 92.51 | 1201.5549 | 11 | 4.1 | 601.7872 | 2 | 33.88 | 6 | 9295 | Ana\_05.raw | 7.71E6 | 1.8E7 | 2.17E7 | 4.06E6 | 7.18E6 | 2.13E7 | 6.29E6 | 1.37E7 | 2.07E7 | 5.62E6 | 13 | 1 | 1 | 2 | 1 | 1 | 2 | 1 | 2 | 1 | 1 | 1152 | 1162 | Carbamidomethylation |
| R.EAGAGGLAIAVEGPSK.A | Y | 92.40 | 1425.7463 | 16 | 1.4 | 713.8814 | 2 | 64.99 | 3 | 18515 | Ana\_02.raw | 2.9E7 | 2.75E7 | 1.93E7 | 7.62E6 | 1.55E7 | 2.18E7 | 1.17E7 | 1.4E7 | 2.38E7 | 1.87E7 | 17 | 2 | 2 | 2 | 2 | 1 | 1 | 1 | 2 | 2 | 2 | 2257 | 2272 |  |
| K.VEPGLGADNSVVR.F | Y | 92.07 | 1311.6782 | 13 | 2.8 | 656.8482 | 2 | 53.15 | 6 | 15152 | Ana\_05.raw | 4.62E7 | 4.52E7 | 4.39E7 | 1.5E7 | 3.06E7 | 4.22E7 | 2.17E7 | 2.51E7 | 6.31E7 | 3.27E7 | 17 | 2 | 2 | 1 | 2 | 1 | 1 | 1 | 1 | 3 | 3 | 1020 | 1032 |  |
| R.TFSVWYVPEVTGTHK.V | N | 91.78 | 1749.8726 | 15 | 3.2 | 584.3000 | 3 | 98.33 | 3 | 27460 | Ana\_02.raw | 5.77E6 | 2.61E7 | 3.86E7 | 1.01E7 | 1.77E7 | 3.11E7 | 1.2E7 | 2.04E7 | 4.72E7 | 1.08E7 | 25 | 2 | 2 | 2 | 3 | 2 | 3 | 3 | 3 | 4 | 1 | 341 | 355 |  |
| R.LVSNHSLHETSSVFVDSLTK.V | Y | 91.24 | 2199.1172 | 20 | 5.1 | 734.0501 | 3 | 76.34 | 9 | 21751 | Ana\_08.raw |  |  |  |  |  |  |  |  | 1.01E7 |  | 2 | 0 | 0 | 0 | 0 | 0 | 0 | 0 | 0 | 2 | 0 | 2513 | 2532 |  |
| K.IPEISIQDMTAQVTSPSGK.T | Y | 91.12 | 2001.0088 | 19 | 0.4 | 1001.5121 | 2 | 106.39 | 9 | 29930 | Ana\_08.raw |  |  |  |  |  |  |  |  | 0 |  | 1 | 0 | 0 | 0 | 0 | 0 | 0 | 0 | 0 | 1 | 0 | 2158 | 2176 |  |
| K.YNDQHIPGSPFTAR.V | Y | 90.75 | 1601.7587 | 14 | 1.5 | 534.9276 | 3 | 60.11 | 3 | 17104 | Ana\_02.raw | 8.29E6 | 8.48E6 | 3.67E7 | 1.47E7 | 2.58E7 | 3.81E7 | 2.15E7 | 2.49E7 | 1.05E7 | 2.41E7 | 15 | 1 | 1 | 2 | 1 | 1 | 2 | 2 | 2 | 1 | 2 | 1930 | 1943 |  |
| R.ANLPQSFQVDTSK.A | Y | 90.56 | 1433.7151 | 13 | 4.2 | 717.8679 | 2 | 66.05 | 6 | 18965 | Ana\_05.raw | 3.11E7 | 3.16E7 | 2.64E7 | 1.09E7 | 2.01E7 | 2.65E7 | 1.42E7 | 1.72E7 | 4.69E7 | 2.47E7 | 19 | 2 | 1 | 2 | 1 | 2 | 2 | 2 | 2 | 3 | 2 | 1465 | 1477 |  |
| K.IEC(+57.02)DDKGDGSC(+57.02)DVR.Y | N | 89.31 | 1624.6458 | 14 | 0.4 | 813.3305 | 2 | 21.87 | 3 | 5563 | Ana\_02.raw | 1.78E7 | 2.73E7 | 2.13E7 | 1.72E6 | 7E6 | 2.34E7 | 5.65E6 | 1.04E7 | 2.12E7 | 1.83E7 | 19 | 2 | 2 | 2 | 1 | 2 | 2 | 2 | 2 | 2 | 2 | 621 | 634 | Carbamidomethylation |
| R.QM(+15.99)QLENVSVALEFLDR.E | N | 88.37 | 1906.9458 | 16 | 6.6 | 954.4865 | 2 | 134.38 | 6 | 37359 | Ana\_05.raw | 1.04E6 |  |  |  | 1.01E6 | 2.46E6 | 7.85E5 |  | 3.11E6 | 1.53E6 | 6 | 1 | 0 | 0 | 0 | 1 | 1 | 1 | 0 | 1 | 1 | 101 | 116 | Oxidation (M) |
| K.YGGPYHIGGSPFK.A | Y | 88.27 | 1378.6670 | 13 | 1.0 | 690.3415 | 2 | 65.13 | 3 | 18556 | Ana\_02.raw | 3.7E7 | 4.07E7 | 2.69E7 | 1.37E7 | 2.24E7 | 3.34E7 | 1.87E7 | 1.89E7 | 5.82E7 | 3.04E7 | 22 | 3 | 3 | 2 | 2 | 2 | 2 | 2 | 2 | 2 | 2 | 2493 | 2505 |  |
| R.VQVQDNEGC(+57.02)SVEATVK.D | Y | 88.24 | 1761.8203 | 16 | 2.6 | 881.9197 | 2 | 52.02 | 6 | 14809 | Ana\_05.raw |  | 2.2E6 |  |  |  | 1.16E6 |  |  | 2.56E6 | 9.53E5 | 4 | 0 | 1 | 0 | 0 | 0 | 1 | 0 | 0 | 1 | 1 | 709 | 724 | Carbamidomethylation |
| R.VNVGAGSHPNK.V | Y | 87.83 | 1078.5519 | 11 | 1.6 | 540.2841 | 2 | 17.76 | 3 | 4187 | Ana\_02.raw | 0 | 1.46E7 | 5.25E7 | 1.83E7 | 2.95E7 | 5.89E7 | 2.88E7 | 2.6E7 |  | 2.75E7 | 20 | 2 | 2 | 2 | 2 | 3 | 2 | 2 | 3 | 0 | 2 | 761 | 771 |  |
| K.TGVAVNKPAEFTVDAK.H | Y | 87.61 | 1645.8674 | 16 | 2.8 | 549.6313 | 3 | 58.81 | 6 | 16817 | Ana\_05.raw | 4.53E7 | 4.35E7 | 3.45E7 | 1.26E7 | 2.34E7 | 3.96E7 | 1.82E7 | 1.94E7 | 4.94E7 | 2.55E7 | 19 | 2 | 3 | 2 | 1 | 2 | 2 | 1 | 2 | 3 | 1 | 685 | 700 |  |
| K.VTAQGPGLEPSGNIANK.T | N | 87.54 | 1651.8529 | 17 | 4.7 | 826.9376 | 2 | 51.30 | 5 | 14510 | Ana\_04.raw | 4.78E7 | 4.9E7 | 3.49E7 | 1.17E7 | 2.62E7 | 3.38E7 | 1.79E7 | 2.08E7 | 6.6E7 | 3.05E7 | 20 | 2 | 3 | 1 | 2 | 1 | 2 | 2 | 2 | 3 | 2 | 384 | 400 |  |
| R.AEVGVPAEFGIWTR.E | Y | 87.07 | 1530.7831 | 14 | 0.9 | 766.3995 | 2 | 109.33 | 3 | 30299 | Ana\_02.raw | 4.68E7 | 3.5E7 | 2.77E7 | 1.59E7 | 2.09E7 | 3.53E7 | 1.54E7 | 2.6E7 | 8.83E7 | 2.37E7 | 27 | 4 | 3 | 3 | 1 | 3 | 2 | 2 | 3 | 4 | 2 | 2243 | 2256 |  |
| R.IANLQTDLSDGLR.L | N | 86.97 | 1414.7416 | 13 | 1.5 | 708.3792 | 2 | 82.32 | 3 | 23230 | Ana\_02.raw | 2.35E7 | 2.69E7 | 6.27E6 | 1.22E6 | 1.38E7 | 1.5E7 | 6.62E6 | 7.33E6 | 4.62E7 | 1.86E7 | 16 | 2 | 3 | 1 | 1 | 1 | 1 | 1 | 1 | 3 | 2 | 64 | 76 |  |
| K.C(+57.02)APGVVGPTEADIDFDIIR.N | Y | 86.50 | 2043.9935 | 19 | 4.0 | 1023.0082 | 2 | 130.36 | 6 | 36218 | Ana\_05.raw |  |  |  |  |  | 1.58E6 |  |  |  |  | 1 | 0 | 0 | 0 | 0 | 0 | 1 | 0 | 0 | 0 | 0 | 810 | 828 | Carbamidomethylation |
| R.LLGWIQNKLPQLPITNFSR.D | N | 86.36 | 2237.2683 | 19 | 5.1 | 746.7672 | 3 | 128.97 | 9 | 35721 | Ana\_08.raw | 2.56E7 | 2.46E6 | 8.41E6 | 8.11E6 | 1.22E7 | 2.5E7 | 1.11E7 | 3.39E6 | 2.78E7 | 4.32E6 | 11 | 2 | 1 | 1 | 1 | 1 | 1 | 1 | 1 | 1 | 1 | 172 | 190 |  |
| R.DVDIIDHHDNTYTVK.Y | Y | 86.12 | 1783.8376 | 15 | 1.5 | 892.9274 | 2 | 56.10 | 9 | 15890 | Ana\_08.raw | 2.03E7 | 2.45E7 | 5.25E5 |  |  | 2.8E7 | 8.42E6 |  | 1.68E6 | 1.84E7 | 11 | 2 | 2 | 1 | 0 | 0 | 2 | 1 | 0 | 1 | 2 | 922 | 936 |  |
| K.YGGQPVPNFPSK.L | Y | 85.98 | 1289.6404 | 12 | 0.9 | 645.8281 | 2 | 58.25 | 3 | 16553 | Ana\_02.raw | 6.79E7 | 5.74E7 | 5.65E7 | 2.23E7 | 4.31E7 | 5.2E7 | 2.78E7 | 3.32E7 | 9.61E7 | 4.91E7 | 21 | 3 | 1 | 1 | 1 | 3 | 2 | 2 | 1 | 3 | 4 | 1235 | 1246 |  |
| K.SPFEVYVDK.S | N | 85.26 | 1082.5283 | 9 | 4.8 | 542.2740 | 2 | 70.04 | 6 | 20098 | Ana\_05.raw | 3.22E7 | 2.75E7 | 2.74E7 | 1.33E7 | 2.47E7 | 2.66E7 | 1.48E7 | 1.81E7 | 3.94E7 | 3.32E7 | 12 | 1 | 1 | 1 | 1 | 1 | 1 | 1 | 2 | 1 | 2 | 368 | 376 |  |
| R.AYGPGIEPTGNM(+15.99)VK.K | N | 85.02 | 1448.6969 | 14 | 2.4 | 725.3575 | 2 | 51.20 | 6 | 14570 | Ana\_05.raw | 6.44E7 | 5.77E7 | 4.94E7 | 1.74E7 | 3.83E7 | 4.92E7 | 2.94E7 | 3.05E7 | 7.8E7 | 3.88E7 | 17 | 2 | 1 | 2 | 2 | 1 | 2 | 2 | 2 | 1 | 2 | 286 | 299 | Oxidation (M) |
| R.EATTEFSVDAR.A | Y | 82.42 | 1224.5623 | 11 | 2.2 | 613.2897 | 2 | 51.40 | 6 | 14630 | Ana\_05.raw | 1.1E8 | 9.94E7 | 8.84E7 | 3.14E7 | 6.94E7 | 8.07E7 | 4.9E7 | 5.66E7 | 1.13E8 | 7.42E7 | 35 | 4 | 4 | 2 | 3 | 3 | 4 | 2 | 3 | 5 | 5 | 1273 | 1283 |  |
| K.VDVGKDQEFTVK.S | Y | 81.31 | 1363.6982 | 12 | 1.8 | 682.8576 | 2 | 46.28 | 3 | 13048 | Ana\_02.raw | 1.79E7 | 2.32E7 | 2.36E7 | 8.04E6 | 1.86E7 | 2.74E7 | 1.38E7 | 1.33E7 | 9.91E6 |  | 16 | 1 | 2 | 2 | 2 | 2 | 2 | 2 | 2 | 1 | 0 | 983 | 994 |  |
| K.VPVHDVTDASK.V | Y | 81.10 | 1166.5931 | 11 | 5.8 | 584.3073 | 2 | 23.71 | 9 | 5873 | Ana\_08.raw | 7.71E7 |  | 4.83E7 | 2.18E7 | 3.45E7 | 1.07E8 | 2.67E7 | 3.96E7 | 3.77E7 | 7.69E7 | 15 | 2 | 0 | 2 | 1 | 1 | 2 | 2 | 2 | 1 | 2 | 1440 | 1450 |  |
| R.VKVEPSHDASK.V | Y | 79.82 | 1195.6196 | 11 | 5.3 | 598.8203 | 2 | 16.67 | 7 | 3844 | Ana\_06.raw |  |  | 1.83E7 | 6.96E6 | 1.87E7 | 3E7 | 1.21E7 | 1.22E7 |  | 2.06E6 | 14 | 0 | 0 | 2 | 2 | 2 | 2 | 2 | 2 | 0 | 2 | 864 | 874 |  |
| R.VTYC(+57.02)PTEPGNYIINIK.F | Y | 79.69 | 1880.9342 | 16 | 6.5 | 941.4805 | 2 | 97.14 | 6 | 27491 | Ana\_05.raw | 1.52E7 | 2.87E6 | 2.62E6 | 2.33E6 | 4.68E6 | 8.29E6 | 3.07E5 |  | 1.85E7 | 2.13E6 | 9 | 1 | 1 | 1 | 1 | 1 | 1 | 1 | 0 | 1 | 1 | 2096 | 2111 | Carbamidomethylation |
| R.VTYTPM(+15.99)APGSYLISIK.Y | Y | 78.99 | 1755.9116 | 16 | 5.4 | 878.9678 | 2 | 97.20 | 9 | 27501 | Ana\_08.raw | 3.39E7 | 3.83E7 | 4.85E7 | 9.47E6 | 1.83E7 | 2.57E7 | 1.51E7 |  | 5.73E7 | 1.8E7 | 10 | 1 | 1 | 1 | 1 | 1 | 1 | 1 | 0 | 2 | 1 | 2477 | 2492 | Oxidation (M) |
| R.LYSVSYLLK.D | Y | 78.68 | 1084.6168 | 9 | 1.4 | 543.3165 | 2 | 92.08 | 3 | 25816 | Ana\_02.raw |  |  | 1.54E7 | 3.33E6 | 7.79E6 | 1.24E7 | 5.56E6 | 5.21E6 |  |  | 6 | 0 | 0 | 1 | 1 | 1 | 1 | 1 | 1 | 0 | 0 | 2605 | 2613 |  |
| K.FADQHVPGSPFSVK.V | Y | 78.15 | 1514.7517 | 14 | 0.5 | 758.3835 | 2 | 68.70 | 6 | 19712 | Ana\_05.raw | 2.11E7 | 1.75E7 | 1.34E7 | 9.79E6 | 2.38E7 | 2.19E7 | 1.27E7 | 6.81E5 | 3.47E7 | 9.01E6 | 17 | 1 | 2 | 1 | 1 | 2 | 2 | 2 | 1 | 3 | 2 | 2112 | 2125 |  |
| K.WGDEHIPGSPYR.I | Y | 77.67 | 1412.6472 | 12 | 9.9 | 707.3379 | 2 | 60.92 | 9 | 17330 | Ana\_08.raw | 2E7 | 1.84E7 | 1.93E7 | 6.31E6 | 1.47E7 | 1.87E7 | 8.56E6 | 1.18E7 | 3.52E7 | 2.88E6 | 19 | 2 | 2 | 3 | 1 | 2 | 2 | 2 | 2 | 2 | 1 | 2624 | 2635 |  |
| K.DAGEGGLSLAIEGPSK.A | Y | 77.43 | 1499.7467 | 16 | 0.9 | 750.8813 | 2 | 77.41 | 3 | 21919 | Ana\_02.raw | 9.65E5 | 4.4E6 | 2.51E6 | 3.34E5 | 1.11E6 | 3.79E6 | 1.62E6 | 2.05E6 | 8.73E6 | 4.92E6 | 12 | 1 | 2 | 1 | 1 | 1 | 1 | 1 | 1 | 2 | 1 | 1884 | 1899 |  |
| K.DKGEYTLVVK.W | Y | 77.09 | 1150.6233 | 10 | 2.3 | 576.3203 | 2 | 49.49 | 4 | 13632 | Ana\_03.raw | 1.62E7 | 1.86E7 | 1.99E7 | 5.86E6 | 1.39E7 | 2.06E7 | 1.95E6 |  | 1.09E7 | 1.45E7 | 13 | 1 | 2 | 2 | 1 | 2 | 2 | 1 | 0 | 1 | 1 | 2614 | 2623 |  |
| K.ETGEHLVHVK.K | Y | 75.80 | 1147.5985 | 10 | 5.2 | 574.8095 | 2 | 23.76 | 6 | 6161 | Ana\_05.raw | 3.4E7 | 1.66E7 | 2.74E7 | 1.04E7 | 3.95E6 | 5.06E6 | 1.06E7 | 9.41E6 | 3.22E7 | 3.27E7 | 20 | 2 | 2 | 2 | 2 | 2 | 2 | 2 | 2 | 2 | 2 | 2007 | 2016 |  |
| R.RLTVSSLQESGLK.V | Y | 74.70 | 1416.7936 | 13 | 3.1 | 473.2733 | 3 | 57.56 | 3 | 16343 | Ana\_02.raw | 1.06E6 | 3.14E6 | 8.46E6 |  | 4.15E6 | 6.6E6 |  | 9.38E5 | 2.37E6 |  | 9 | 1 | 1 | 2 | 0 | 1 | 2 | 0 | 1 | 1 | 0 | 2326 | 2338 |  |
| R.EAM(+15.99)QQADDWLGIPQVITPEEIVDPNVDEHSVM(+15.99)TYLSQFPK.A | N | 73.70 | 4601.1616 | 40 | 9.1 | 1534.7417 | 3 | 134.80 | 6 | 37472 | Ana\_05.raw | 2.66E6 |  |  |  |  | 3.98E6 |  |  |  |  | 2 | 1 | 0 | 0 | 0 | 0 | 1 | 0 | 0 | 0 | 0 | 227 | 266 | Oxidation (M) |
| R.YSPSEAGLHEM(+15.99)DIR.Y | Y | 72.35 | 1619.7250 | 14 | 6.2 | 810.8748 | 2 | 50.49 | 2 | 14237 | Ana\_01.raw | 4.13E7 | 3.43E7 | 4.19E7 | 1.29E7 | 5.57E6 | 3.34E7 | 1.98E7 | 3.28E6 | 5.9E7 | 2.81E7 | 18 | 2 | 2 | 2 | 2 | 1 | 2 | 2 | 1 | 2 | 2 | 1824 | 1837 | Oxidation (M) |
| R.LTVSSLQESGLK.V | Y | 71.71 | 1260.6925 | 12 | 2.7 | 631.3552 | 2 | 68.27 | 6 | 19602 | Ana\_05.raw | 1.18E6 | 2.19E7 | 1.22E7 | 6.72E6 | 1.53E7 | 1.55E7 | 9.17E6 | 8.88E6 | 2.61E7 | 1.54E7 | 14 | 1 | 2 | 1 | 1 | 2 | 2 | 1 | 2 | 1 | 1 | 2327 | 2338 |  |
| K.HNQRPTFR.Q | N | 71.56 | 1054.5420 | 8 | 0.3 | 352.5214 | 3 | 18.34 | 3 | 4378 | Ana\_02.raw |  | 2.29E7 | 2.51E7 | 7.69E5 | 1.93E7 | 2.79E7 | 1.13E7 | 7.21E6 | 3.53E7 | 2.7E7 | 15 | 0 | 1 | 2 | 1 | 2 | 2 | 2 | 1 | 2 | 2 | 93 | 100 |  |
| K.GTVEPQLEAR.G | N | 71.47 | 1098.5669 | 10 | 2.4 | 550.2921 | 2 | 41.93 | 4 | 11411 | Ana\_03.raw | 9.02E7 | 8.35E7 | 8.01E7 | 2.85E7 | 5.68E7 | 7.98E7 | 4.06E7 | 5.31E7 | 8.54E7 | 6.27E7 | 24 | 3 | 3 | 1 | 2 | 2 | 2 | 2 | 4 | 3 | 2 | 428 | 437 |  |
| K.VAQPSITDNKDGTVTVR.Y | Y | 70.77 | 1799.9377 | 17 | 3.2 | 600.9885 | 3 | 46.28 | 3 | 13050 | Ana\_02.raw | 1.54E7 |  | 9.85E6 | 2.99E6 | 6.65E6 | 1.36E7 |  | 5.1E6 | 2.32E7 | 2.94E6 | 11 | 2 | 0 | 1 | 1 | 1 | 2 | 0 | 1 | 2 | 1 | 1807 | 1823 |  |
| K.QRLLGWIQNKLPQLPITNFSR.D | N | 69.78 | 2521.4280 | 21 | 4.4 | 631.3671 | 4 | 128.48 | 8 | 35238 | Ana\_07.raw |  |  |  |  |  |  |  | 1.78E6 |  |  | 1 | 0 | 0 | 0 | 0 | 0 | 0 | 0 | 1 | 0 | 0 | 170 | 190 |  |
| K.YGGDEIPFSPYR.V | Y | 69.54 | 1399.6407 | 12 | -2.5 | 700.8259 | 2 | 85.21 | 3 | 24009 | Ana\_02.raw | 6.05E7 | 4.02E7 | 3.53E7 | 5.38E6 | 2.41E7 | 5.7E7 |  | 2.85E7 | 9.89E7 | 3.33E7 | 19 | 4 | 2 | 1 | 1 | 2 | 2 | 0 | 2 | 3 | 2 | 1622 | 1633 |  |
| R.LIALLEVLSQK.K | N | 69.25 | 1225.7645 | 11 | 3.9 | 613.8919 | 2 | 129.98 | 7 | 35763 | Ana\_06.raw | 4.91E6 | 1.99E6 | 1.02E7 | 4.83E6 | 8.34E6 | 9.14E6 | 6.96E6 | 2.18E6 | 1.08E7 | 5.95E6 | 10 | 1 | 1 | 1 | 1 | 1 | 1 | 1 | 1 | 1 | 1 | 77 | 87 |  |
| R.DAGYGGLSLSIEGPSK.V | Y | 68.41 | 1549.7623 | 16 | 4.4 | 775.8918 | 2 | 88.04 | 9 | 25008 | Ana\_08.raw |  |  |  |  |  |  |  |  | 1.4E6 |  | 1 | 0 | 0 | 0 | 0 | 0 | 0 | 0 | 0 | 1 | 0 | 2066 | 2081 |  |
| R.SAGQGEVLVYVEDPAGHQEEAK.V | N | 67.66 | 2312.0920 | 22 | 4.7 | 771.7083 | 3 | 75.59 | 10 | 21357 | Ana\_09.raw | 1.86E7 |  | 6.01E6 | 4.52E6 | 8.46E6 | 1.71E7 |  | 1.05E7 |  | 7.76E6 | 8 | 1 | 0 | 1 | 1 | 1 | 2 | 0 | 1 | 0 | 1 | 310 | 331 |  |
| K.LDVQFSGLAK.G | Y | 65.87 | 1076.5865 | 10 | 2.8 | 539.3021 | 2 | 77.93 | 6 | 22307 | Ana\_05.raw | 2.64E6 | 6.6E6 | 3.95E6 | 1.96E6 | 3.92E6 | 5.27E6 | 2.78E6 | 2.95E6 | 8.86E6 | 3.53E6 | 11 | 1 | 2 | 1 | 1 | 1 | 1 | 1 | 1 | 1 | 1 | 907 | 916 |  |
| K.AEISFEDR.K | Y | 65.39 | 965.4454 | 8 | 4.4 | 483.7321 | 2 | 48.28 | 2 | 13595 | Ana\_01.raw | 5.17E7 | 5.43E7 | 4.9E7 | 2.05E7 | 3.57E7 | 4.74E7 | 2.7E7 | 2.84E7 | 6.74E7 | 3.77E7 | 18 | 3 | 1 | 2 | 1 | 1 | 2 | 1 | 3 | 3 | 1 | 2273 | 2280 |  |
| K.DLAEDAPWK.K | N | 65.01 | 1043.4923 | 9 | 3.5 | 522.7552 | 2 | 72.72 | 6 | 20864 | Ana\_05.raw | 1.24E7 | 7.6E6 | 1.07E7 | 6.87E6 | 8.48E6 | 1.44E7 | 7.45E6 |  | 1.14E7 | 5.52E6 | 13 | 1 | 1 | 2 | 2 | 1 | 2 | 2 | 0 | 1 | 1 | 34 | 42 |  |
| K.VAQPSITDNK.D | Y | 64.71 | 1071.5560 | 10 | 4.9 | 536.7879 | 2 | 30.54 | 6 | 8294 | Ana\_05.raw | 3.18E7 | 3.51E7 | 1.63E7 | 1.12E7 | 1.82E7 | 2.62E7 | 1.39E7 |  | 3.33E7 | 2.44E7 | 9 | 1 | 1 | 1 | 1 | 1 | 1 | 1 | 0 | 1 | 1 | 1807 | 1816 |  |
| K.M(+15.99)DC(+57.02)QEC(+57.02)PEGYR.V | Y | 64.46 | 1459.5166 | 11 | 1.7 | 730.7668 | 2 | 32.71 | 4 | 8696 | Ana\_03.raw | 3.7E6 | 5.25E6 | 2.74E6 | 1.25E6 | 2.53E6 | 3.63E6 | 1.38E6 | 2.49E6 | 3E6 | 2.53E6 | 10 | 1 | 1 | 1 | 1 | 1 | 1 | 1 | 1 | 1 | 1 | 2466 | 2476 | Oxidation (M); Carbamidomethylation |
| K.KTHIQDNHDGTYTVAYVPDVPGR.Y | Y | 63.39 | 2582.2512 | 23 | 3.4 | 646.5723 | 4 | 64.17 | 2 | 18280 | Ana\_01.raw |  | 3.31E6 |  |  |  |  |  |  |  |  | 1 | 0 | 1 | 0 | 0 | 0 | 0 | 0 | 0 | 0 | 0 | 1593 | 1615 |  |
| R.TPC(+57.02)EEILVK.H | Y | 63.17 | 1087.5583 | 9 | 5.1 | 544.7892 | 2 | 54.53 | 6 | 15556 | Ana\_05.raw |  |  |  | 5.06E6 |  | 8.44E6 |  |  |  | 1.11E7 | 3 | 0 | 0 | 0 | 1 | 0 | 1 | 0 | 0 | 0 | 1 | 2591 | 2599 | Carbamidomethylation |
| K.MDC(+57.02)QEC(+57.02)PEGYR.V | Y | 62.83 | 1443.5217 | 11 | 3.8 | 722.7709 | 2 | 38.45 | 2 | 10577 | Ana\_01.raw | 1.55E6 | 1.37E6 |  |  |  |  |  |  |  |  | 2 | 1 | 1 | 0 | 0 | 0 | 0 | 0 | 0 | 0 | 0 | 2466 | 2476 | Carbamidomethylation |
| R.EAPQDFHPDR.V | Y | 62.71 | 1210.5366 | 10 | 1.7 | 606.2766 | 2 | 29.24 | 3 | 7898 | Ana\_02.raw | 6.57E7 | 5.93E7 | 2.89E7 |  | 5.72E7 | 7.66E7 | 3.31E7 | 2.59E7 | 5.94E7 | 3.86E7 | 18 | 3 | 2 | 1 | 0 | 3 | 2 | 2 | 1 | 2 | 2 | 665 | 674 |  |
| K.LPQLPITNFSR.D | N | 60.80 | 1284.7190 | 11 | 0.7 | 643.3672 | 2 | 97.59 | 2 | 27496 | Ana\_01.raw | 2.55E7 | 1.06E7 | 6.2E6 | 5.04E5 |  | 1.92E6 | 3.92E5 | 2.74E5 | 2.02E7 | 1.08E7 | 9 | 1 | 1 | 1 | 1 | 0 | 1 | 1 | 1 | 1 | 1 | 180 | 190 |  |
| K.IVSPSGAAVPC(+57.02)K.V | Y | 60.31 | 1184.6223 | 12 | 4.0 | 593.3208 | 2 | 43.18 | 6 | 12135 | Ana\_05.raw | 4.13E7 | 3.95E7 | 3.62E7 | 5.84E6 | 2.67E7 | 3.65E7 | 1.84E7 | 2.26E7 | 9.49E6 | 3.48E7 | 16 | 2 | 1 | 1 | 1 | 1 | 2 | 2 | 2 | 2 | 2 | 1008 | 1019 | Carbamidomethylation |
| K.VLPTHDASK.V | Y | 60.00 | 966.5134 | 9 | 1.9 | 323.1790 | 3 | 18.21 | 3 | 4334 | Ana\_02.raw | 3.29E6 | 9.56E5 | 4.19E7 | 1.59E7 | 2.97E7 | 5.65E7 | 2.19E7 | 1.71E7 |  | 1.17E7 | 22 | 1 | 1 | 3 | 3 | 2 | 4 | 3 | 2 | 0 | 3 | 1539 | 1547 |  |
| K.KGEITGEVR.M | Y | 58.95 | 987.5349 | 9 | 2.0 | 330.1862 | 3 | 21.78 | 6 | 5517 | Ana\_05.raw | 7.1E7 | 6.19E7 | 4.95E7 | 2.66E6 | 3E7 | 9.91E6 | 1.84E7 | 2.27E7 | 5.22E6 | 5.23E7 | 17 | 2 | 2 | 2 | 1 | 2 | 2 | 2 | 2 | 1 | 1 | 1793 | 1801 |  |
| R.ALTQTGGPHVK.A | Y | 58.75 | 1107.6036 | 11 | 1.4 | 554.8099 | 2 | 23.84 | 4 | 6051 | Ana\_03.raw |  | 3.03E7 | 2.5E7 | 8.31E6 | 1.65E7 | 1E7 | 6.36E6 | 4.74E6 | 4.2E7 | 2.1E7 | 11 | 0 | 2 | 1 | 1 | 1 | 1 | 1 | 1 | 2 | 1 | 1284 | 1294 |  |
| R.LIALLEVLSQKK.M | N | 58.22 | 1353.8595 | 12 | 3.2 | 452.2952 | 3 | 118.02 | 7 | 32626 | Ana\_06.raw | 3.02E6 | 6.32E6 | 4.03E6 | 1.24E6 | 6.98E6 | 1.09E7 | 2.78E6 | 5.23E6 | 3.58E6 | 5.53E6 | 19 | 1 | 2 | 1 | 2 | 2 | 3 | 3 | 2 | 1 | 2 | 77 | 88 |  |
| K.VYGPGVAK.T | Y | 57.94 | 789.4385 | 8 | 4.5 | 395.7283 | 2 | 26.67 | 5 | 7062 | Ana\_04.raw | 1.41E7 | 4.5E7 | 4.09E7 | 1.74E7 | 2.27E7 | 4.05E7 | 2.36E7 | 2.21E7 | 3.77E7 | 1.82E7 | 10 | 1 | 1 | 1 | 1 | 1 | 1 | 1 | 1 | 1 | 1 | 774 | 781 |  |
| R.AEFTVETR.S | N | 57.51 | 951.4661 | 8 | 6.1 | 476.7432 | 2 | 41.85 | 1 | 11585 | ANa\_10.raw | 6.37E7 | 5.87E7 | 5.92E7 | 1.91E7 |  | 5.15E7 | 3E7 | 3.42E7 | 2.18E7 | 4.17E7 | 18 | 2 | 2 | 2 | 2 | 1 | 2 | 2 | 1 | 2 | 2 | 302 | 309 |  |
| K.VTVLFAGQHIAK.S | N | 57.09 | 1282.7397 | 12 | 3.4 | 642.3793 | 2 | 65.73 | 4 | 18317 | Ana\_03.raw | 2E7 | 2.04E7 | 1.8E7 | 6.77E6 | 1.47E7 | 1.39E7 | 6.82E6 | 7.14E6 | 3.11E7 | 1.56E7 | 18 | 2 | 2 | 2 | 2 | 2 | 2 | 1 | 1 | 2 | 2 | 356 | 367 |  |
| R.AWGPGLEGGIVGK.S | Y | 56.61 | 1239.6611 | 13 | 2.7 | 620.8395 | 2 | 82.65 | 4 | 22929 | Ana\_03.raw |  | 1.53E7 | 1.31E7 | 4.93E6 | 9.64E6 | 1.89E7 | 5.25E6 | 1.21E7 | 4.2E7 | 9.23E6 | 10 | 0 | 1 | 1 | 1 | 1 | 1 | 1 | 1 | 2 | 1 | 581 | 593 |  |
| R.C(+57.02)GQSAAVASPGGSIDSR.D | Y | 55.27 | 1618.7369 | 17 | -4.2 | 810.3723 | 2 | 58.72 | 7 | 16598 | Ana\_06.raw |  |  |  | 8.41E5 |  |  | 7.63E5 | 1.2E6 |  |  | 3 | 0 | 0 | 0 | 1 | 0 | 0 | 1 | 1 | 0 | 0 | 8 | 24 | Carbamidomethylation |
| R.NDNDTFTVK.Y | N | 54.27 | 1052.4774 | 9 | 5.4 | 527.2488 | 2 | 34.22 | 2 | 9284 | Ana\_01.raw | 2.84E7 | 2.74E7 |  |  |  |  |  |  | 8.6E6 | 1.49E7 | 5 | 1 | 1 | 0 | 0 | 0 | 0 | 0 | 0 | 2 | 1 | 829 | 837 |  |
| K.IQQNTFTR.W | N | 53.76 | 1006.5196 | 8 | 2.0 | 504.2681 | 2 | 28.64 | 3 | 7713 | Ana\_02.raw |  |  | 4.24E7 | 1.74E7 |  |  |  | 2.58E7 | 5.6E7 | 4.3E7 | 6 | 0 | 0 | 1 | 1 | 0 | 0 | 0 | 1 | 1 | 2 | 44 | 51 |  |
| R.VTYTPMAPGSYLISIK.Y | Y | 53.27 | 1739.9167 | 16 | 3.9 | 870.9691 | 2 | 110.15 | 1 | 30577 | ANa\_10.raw | 2.81E5 |  |  |  |  |  |  |  |  |  | 1 | 1 | 0 | 0 | 0 | 0 | 0 | 0 | 0 | 0 | 0 | 2477 | 2492 |  |
| R.VKETADFK.V | N | 53.19 | 936.4916 | 8 | 3.3 | 313.1722 | 3 | 21.84 | 6 | 5538 | Ana\_05.raw | 1.93E7 | 1.5E7 | 1.57E7 | 4.97E6 | 1.04E7 | 1.25E7 | 6.49E6 | 5.96E6 | 3.22E6 |  | 13 | 2 | 1 | 2 | 2 | 1 | 2 | 1 | 1 | 1 | 0 | 497 | 504 |  |
| K.AGNNM(+15.99)LLVGVHGPR.T | Y | 53.10 | 1449.7511 | 14 | 2.3 | 484.2588 | 3 | 53.10 | 5 | 15040 | Ana\_04.raw | 1.33E7 | 1.12E7 | 1.31E7 | 4.73E6 | 5.84E6 | 1.06E7 | 7.23E6 | 6.61E6 | 8.23E6 | 8.3E6 | 11 | 1 | 1 | 1 | 1 | 1 | 1 | 1 | 1 | 2 | 1 | 2577 | 2590 | Oxidation (M) |
| K.VKAEGPGLNR.T | Y | 52.29 | 1039.5774 | 10 | 4.6 | 347.5347 | 3 | 22.90 | 1 | 5733 | ANa\_10.raw | 4.2E6 |  | 7.13E5 |  |  |  |  |  | 7.16E6 |  | 5 | 2 | 0 | 1 | 0 | 0 | 0 | 0 | 0 | 2 | 0 | 875 | 884 |  |
| K.RAEFTVETR.S | N | 52.22 | 1107.5673 | 9 | 5.0 | 554.7937 | 2 | 32.78 | 10 | 8897 | Ana\_09.raw |  |  | 1.49E7 |  |  |  | 9.16E6 |  |  | 1.99E7 | 3 | 0 | 0 | 1 | 0 | 0 | 0 | 1 | 0 | 0 | 1 | 301 | 309 |  |
| R.AGGPGLER.A | N | 50.66 | 755.3926 | 8 | 2.6 | 378.7045 | 2 | 21.77 | 6 | 5512 | Ana\_05.raw |  |  |  | 2.88E7 | 4.09E7 | 1.25E7 | 3.55E7 | 3.18E7 |  |  | 5 | 0 | 0 | 0 | 1 | 1 | 1 | 1 | 1 | 0 | 0 | 2235 | 2242 |  |
| R.LSPFM(+15.99)ADIR.E | Y | 49.59 | 1064.5325 | 9 | 4.3 | 533.2758 | 2 | 68.11 | 2 | 19409 | Ana\_01.raw | 3.13E7 | 2.29E7 | 1.93E7 | 7.33E6 | 2.04E7 | 2.01E7 | 1.13E7 |  | 4.28E7 | 2.45E7 | 11 | 1 | 1 | 1 | 1 | 2 | 1 | 1 | 0 | 2 | 1 | 656 | 664 | Oxidation (M) |
| K.AGVAPLQVK.V | Y | 48.52 | 881.5334 | 9 | 4.8 | 441.7761 | 2 | 39.07 | 9 | 10685 | Ana\_08.raw | 3.17E7 | 2.88E7 | 1.35E7 | 1.31E7 | 6.56E6 | 1.76E7 | 1.5E7 | 1.42E7 | 6.2E6 | 2.44E7 | 14 | 2 | 2 | 1 | 2 | 1 | 1 | 1 | 1 | 2 | 1 | 1478 | 1486 |  |
| K.AEGPGLNR.T | Y | 47.22 | 812.4141 | 8 | 8.1 | 407.2176 | 2 | 21.64 | 5 | 5514 | Ana\_04.raw |  |  | 3.63E7 | 2.47E7 | 3.11E7 | 6.91E7 | 6.09E6 | 1.93E6 |  |  | 6 | 0 | 0 | 1 | 1 | 1 | 1 | 1 | 1 | 0 | 0 | 877 | 884 |  |
| R.ENGIYLIDVK.F | Y | 46.84 | 1162.6233 | 10 | 9.5 | 582.3245 | 2 | 83.90 | 2 | 23786 | Ana\_01.raw | 2.39E5 | 1.1E6 |  |  | 0 |  |  |  | 2.78E6 | 2.03E5 | 7 | 1 | 1 | 1 | 0 | 1 | 1 | 0 | 0 | 1 | 1 | 2388 | 2397 |  |
| R.KHNQRPTFR.Q | N | 46.51 | 1182.6370 | 9 | 4.1 | 395.2212 | 3 | 17.94 | 10 | 4130 | Ana\_09.raw |  | 1.24E7 | 1.1E7 |  |  | 1.25E7 |  | 4.02E6 | 1.24E7 | 9.28E6 | 10 | 0 | 2 | 2 | 0 | 0 | 1 | 0 | 1 | 2 | 2 | 92 | 100 |  |
| K.FTVETR.G | N | 45.35 | 751.3864 | 6 | 2.0 | 376.7012 | 2 | 29.33 | 3 | 7925 | Ana\_02.raw | 4.96E7 | 4.71E7 | 4.24E7 | 1.96E7 | 3.51E7 | 4.5E7 | 2.76E7 | 2.49E7 | 4.87E7 | 3.52E7 | 15 | 2 | 1 | 1 | 2 | 1 | 2 | 2 | 2 | 1 | 1 | 1376 | 1381 |  |
| K.GKLDVQFSGLAK.G | Y | 43.29 | 1261.7030 | 12 | 2.8 | 631.8605 | 2 | 65.13 | 3 | 18557 | Ana\_02.raw | 6.11E5 | 1.73E7 | 5.05E5 |  | 1.07E7 |  |  |  | 1.18E7 |  | 5 | 1 | 1 | 1 | 0 | 1 | 0 | 0 | 0 | 1 | 0 | 905 | 916 |  |
| R.TGVELGKPTHFTVNAK.T | Y | 43.25 | 1697.9100 | 16 | 2.8 | 425.4860 | 4 | 54.93 | 6 | 15679 | Ana\_05.raw |  |  |  |  |  | 2.66E6 |  |  | 1.18E7 |  | 2 | 0 | 0 | 0 | 0 | 0 | 1 | 0 | 0 | 1 | 0 | 885 | 900 |  |
| R.FVPAEM(+15.99)GM(+15.99)HTVSVK.Y | Y | 42.43 | 1563.7426 | 14 | 3.6 | 782.8813 | 2 | 42.50 | 6 | 11929 | Ana\_05.raw | 3.41E7 |  |  | 2.64E6 | 2.66E7 | 3.54E7 | 4.36E6 | 2.96E7 |  |  | 7 | 1 | 0 | 0 | 1 | 1 | 2 | 1 | 1 | 0 | 0 | 2194 | 2207 | Oxidation (M) |
| K.LVSIDSK.A | N | 40.94 | 760.4330 | 7 | 5.1 | 381.2257 | 2 | 29.51 | 1 | 7861 | ANa\_10.raw | 6.01E7 | 6.64E7 | 5.26E7 |  |  | 4.66E7 | 2.86E7 | 3.95E7 | 4.72E7 | 3.77E7 | 8 | 1 | 1 | 1 | 0 | 0 | 1 | 1 | 1 | 1 | 1 | 121 | 127 |  |
| K.LKPGAPLRPK.L | N | 40.18 | 1075.6865 | 10 | 1.3 | 538.8513 | 2 | 22.02 | 3 | 5610 | Ana\_02.raw | 5.59E7 | 2.09E7 | 3.94E7 | 2.4E6 | 1.78E7 | 1.45E7 | 1.12E6 | 9.17E5 | 4.58E7 | 4.15E7 | 15 | 2 | 1 | 2 | 1 | 2 | 1 | 1 | 1 | 2 | 2 | 269 | 278 |  |
| K.DQEFTVK.S | Y | 39.21 | 865.4181 | 7 | 3.9 | 433.7180 | 2 | 32.47 | 9 | 8621 | Ana\_08.raw | 7.4E7 | 8.29E7 | 8.26E7 | 4.02E7 | 6.74E7 | 6.49E7 | 4.8E7 | 5.13E7 | 5.34E7 | 8.12E7 | 11 | 1 | 1 | 1 | 1 | 2 | 1 | 1 | 1 | 1 | 1 | 988 | 994 |  |
| K.GEITGEVR.M | N | 38.99 | 859.4399 | 8 | 4.0 | 430.7290 | 2 | 26.51 | 2 | 6920 | Ana\_01.raw |  | 1.32E7 |  | 5.95E6 |  | 6.47E6 | 1.14E7 |  |  |  | 6 | 0 | 1 | 0 | 1 | 0 | 1 | 3 | 0 | 0 | 0 | 1794 | 1801 |  |
| R.FTIDTK.G | Y | 38.40 | 723.3803 | 6 | 4.0 | 362.6989 | 2 | 38.48 | 6 | 10684 | Ana\_05.raw | 2.27E7 | 2.21E7 | 2.44E7 | 9.1E6 | 1.93E7 | 2.35E7 | 1.3E7 | 1.53E7 |  | 1.54E7 | 18 | 2 | 2 | 2 | 2 | 2 | 2 | 2 | 2 | 0 | 2 | 1088 | 1093 |  |
| K.YVIC(+57.02)VR.F | Y | 37.62 | 808.4265 | 6 | -0.5 | 405.2203 | 2 | 51.13 | 4 | 14114 | Ana\_03.raw |  |  | 6.42E6 | 3.37E6 | 5.84E6 | 8.21E6 |  |  |  | 5.6E6 | 5 | 0 | 0 | 1 | 1 | 1 | 1 | 0 | 0 | 0 | 1 | 1712 | 1717 | Carbamidomethylation |
| R.WC(+57.02)NEHLK.C | N | 37.58 | 985.4440 | 7 | 3.6 | 329.4898 | 3 | 24.09 | 9 | 6000 | Ana\_08.raw |  |  |  |  |  |  |  |  | 6.53E6 |  | 2 | 0 | 0 | 0 | 0 | 0 | 0 | 0 | 0 | 2 | 0 | 52 | 58 | Carbamidomethylation |
| R.SPFEVK.V | Y | 35.30 | 705.3697 | 6 | 4.0 | 353.6935 | 2 | 37.23 | 7 | 10157 | Ana\_06.raw | 3.45E7 |  |  |  |  | 3.74E7 | 2.44E7 |  | 4.68E7 |  | 4 | 1 | 0 | 0 | 0 | 0 | 1 | 1 | 0 | 1 | 0 | 564 | 569 |  |
| R.EEPC(+57.02)LLK.R | Y | 33.68 | 887.4422 | 7 | 1.3 | 444.7289 | 2 | 43.46 | 6 | 12224 | Ana\_05.raw | 1.36E6 | 1.1E7 |  |  |  | 1.06E7 |  |  | 5.66E6 | 8.12E6 | 5 | 1 | 1 | 0 | 0 | 0 | 1 | 0 | 0 | 1 | 1 | 1986 | 1992 | Carbamidomethylation |
| R.VKESITR.R | Y | 29.01 | 831.4814 | 7 | 3.6 | 416.7495 | 2 | 19.07 | 7 | 4598 | Ana\_06.raw |  |  |  |  |  | 3.03E6 | 1.26E6 |  |  |  | 2 | 0 | 0 | 0 | 0 | 0 | 1 | 1 | 0 | 0 | 0 | 2132 | 2138 |  |
| K.YIPVQQGPVGVNVTYGGDHIPK.S | Y | 27.93 | 2337.2117 | 22 | 1.0 | 780.0786 | 3 | 85.93 | 7 | 24193 | Ana\_06.raw |  |  |  |  |  | 2.95E6 | 8.77E5 |  |  |  | 2 | 0 | 0 | 0 | 0 | 0 | 1 | 1 | 0 | 0 | 0 | 937 | 958 |  |
| R.YTILIK.Y | Y | 26.84 | 749.4687 | 6 | 5.7 | 375.7438 | 2 | 53.99 | 9 | 15274 | Ana\_08.raw |  |  |  |  |  |  |  |  | 2.42E7 |  | 1 | 0 | 0 | 0 | 0 | 0 | 0 | 0 | 0 | 1 | 0 | 1616 | 1621 |  |
| total 119 peptides |  |  |  |  |  |  |  |  |  |  |  |  |  |  |  |  |  |  |  |  |  |  |  |  |  |  |  |  |  |  |  |  |  |  |  |
| --- | --- | --- | --- | --- | --- | --- | --- | --- | --- | --- | --- | --- | --- | --- | --- | --- | --- | --- | --- | --- | --- | --- | --- | --- | --- | --- | --- | --- | --- | --- | --- | --- | --- | --- | --- |

Best Unique PSM (Scan F3:27795, m/z=823.0677, z=3, RT=99.61, ppm=0.0):

sp|Q8BTM8|FLNA\_MOUSE

back to list

  

| Protein Coverage
| Supporting Peptides
| Best Unique PSM
|

Protein Coverage:

Supporting Peptides:

| Peptide | Uniq | -10lgP | Mass | Length | ppm | m/z | z | RT | Fraction | Scan | Source File | Area 10 | Area 01 | Area 02 | Area 03 | Area 04 | Area 05 | Area 06 | Area 07 | Area 08 | Area 09 | #Spec | #Spec 10 | #Spec 01 | #Spec 02 | #Spec 03 | #Spec 04 | #Spec 05 | #Spec 06 | #Spec 07 | #Spec 08 | #Spec 09 | Start | End | PTM |
| --- | --- | --- | --- | --- | --- | --- | --- | --- | --- | --- | --- | --- | --- | --- | --- | --- | --- | --- | --- | --- | --- | --- | --- | --- | --- | --- | --- | --- | --- | --- | --- | --- | --- | --- | --- |
| K.FNEEHIPDSPFVVPVASPSGDAR.R | Y | 139.48 | 2466.1814 | 23 | 0.0 | 823.0677 | 3 | 99.61 | 3 | 27795 | Ana\_02.raw | 1.37E8 | 1.17E8 | 9.82E7 | 2.86E7 | 7.2E7 | 9.35E7 | 3.56E7 | 6.1E7 | 2.24E8 | 7.34E7 | 46 | 6 | 5 | 6 | 3 | 4 | 5 | 2 | 6 | 5 | 4 | 2311 | 2333 |  |
| R.FGGEHVPNSPFQVTALAGDQPTVQTPLR.S | Y | 133.91 | 2962.4937 | 28 | 1.8 | 988.5070 | 3 | 107.99 | 6 | 30360 | Ana\_05.raw | 5.11E7 | 5.03E7 | 2.37E7 | 7.57E5 | 3.46E7 | 1.16E7 | 8.58E6 | 1.04E7 | 9.52E7 |  | 16 | 2 | 2 | 1 | 1 | 4 | 1 | 1 | 2 | 2 | 0 | 1726 | 1753 |  |
| K.GLVEPVDVVDNADGTQTVNYVPSR.E | Y | 131.40 | 2543.2502 | 24 | 1.4 | 1272.6342 | 2 | 96.61 | 9 | 27341 | Ana\_08.raw | 1.35E7 | 1.15E7 |  | 6.07E5 |  | 2.06E6 |  | 9.97E5 | 3.54E7 | 5.42E6 | 8 | 1 | 1 | 0 | 1 | 0 | 1 | 0 | 1 | 2 | 1 | 1492 | 1515 |  |
| K.VGSAADIPINISETDLSLLTATVVPPSGR.E | Y | 123.12 | 2892.5444 | 29 | 2.6 | 1447.2832 | 2 | 134.53 | 8 | 36883 | Ana\_07.raw | 2.83E7 | 1.53E7 | 2.23E7 | 7.35E6 | 2.04E7 | 9.31E6 | 2.06E7 | 1.2E7 | 4.77E7 | 9.59E6 | 19 | 2 | 2 | 2 | 2 | 2 | 2 | 2 | 2 | 2 | 1 | 1965 | 1993 |  |
| K.LQVEPAVDTSGVQC(+57.02)YGPGIEGQGVFR.E | Y | 122.99 | 2762.3333 | 26 | 2.1 | 1382.1768 | 2 | 102.98 | 9 | 29031 | Ana\_08.raw |  | 1.3E6 | 1.92E6 |  | 1.09E6 | 2.71E5 |  |  | 1.31E6 |  | 5 | 0 | 1 | 1 | 0 | 1 | 1 | 0 | 0 | 1 | 0 | 1247 | 1272 | Carbamidomethylation |
| R.ALGALVDSC(+57.02)APGLC(+57.02)PDWDSWDASKPVNNAR.E | N | 120.06 | 3241.4919 | 30 | 0.7 | 1081.5054 | 3 | 115.02 | 6 | 32179 | Ana\_05.raw | 5.49E6 | 8.67E6 |  | 4.05E6 |  | 4.4E6 |  | 2.32E6 | 2.02E7 | 3.12E6 | 7 | 1 | 1 | 0 | 1 | 0 | 1 | 0 | 1 | 1 | 1 | 197 | 226 | Carbamidomethylation |
| K.GQHVPGSPFQFTVGPLGEGGAHK.V | N | 114.28 | 2303.1445 | 23 | 3.8 | 576.7956 | 4 | 90.06 | 1 | 25304 | ANa\_10.raw | 1.86E7 | 5.87E6 | 2.37E7 | 6.44E6 | 1.54E7 | 1.46E7 |  | 1.38E7 | 3.15E7 | 4.2E6 | 18 | 3 | 2 | 2 | 1 | 2 | 1 | 0 | 3 | 2 | 2 | 2218 | 2240 |  |
| K.VATVPQHATSGPGPADVSK.V | Y | 111.55 | 1817.9271 | 19 | 1.7 | 909.9724 | 2 | 37.79 | 3 | 10475 | Ana\_02.raw | 7.81E7 | 7.96E7 | 7.66E7 | 3.2E7 | 6.38E7 | 8.55E7 | 4.17E7 | 5.37E7 | 4.96E7 | 4.5E7 | 26 | 2 | 2 | 2 | 3 | 2 | 3 | 2 | 3 | 4 | 3 | 2541 | 2559 |  |
| R.VHGPGIQSGTTNKPNK.F | Y | 111.27 | 1633.8536 | 16 | 4.2 | 817.9376 | 2 | 21.30 | 1 | 5200 | ANa\_10.raw | 5.24E7 | 8.68E6 | 4.32E7 | 1.64E7 | 3.43E7 | 2.76E7 | 1.91E7 | 8.36E6 | 6.77E7 | 3.64E7 | 32 | 3 | 3 | 3 | 2 | 3 | 3 | 3 | 2 | 6 | 4 | 1360 | 1375 |  |
| R.YWPQEAGEYAVHVLC(+57.02)NSEDIR.L | Y | 110.26 | 2535.1489 | 21 | 1.3 | 846.0580 | 3 | 126.34 | 3 | 34660 | Ana\_02.raw | 7.99E6 | 2.9E6 | 1.6E7 | 4.94E6 | 2.05E7 | 3.32E7 | 1.21E7 | 1.1E7 | 6.95E7 | 3.12E7 | 18 | 1 | 1 | 2 | 2 | 1 | 2 | 2 | 2 | 3 | 2 | 635 | 655 | Carbamidomethylation |
| R.VSGQGLHEGHTFEPAEFIIDTR.D | Y | 109.44 | 2439.1819 | 22 | 4.9 | 814.0719 | 3 | 90.76 | 9 | 25750 | Ana\_08.raw |  | 1.07E7 | 3E7 | 2.36E6 | 1.95E6 | 8.97E6 | 8.56E6 | 5.2E6 | 3.78E7 | 3.3E6 | 15 | 1 | 1 | 2 | 1 | 1 | 2 | 2 | 1 | 2 | 2 | 2052 | 2073 |  |
| K.AFGPGLQGGNAGSPAR.F | Y | 107.88 | 1455.7218 | 16 | 1.5 | 728.8693 | 2 | 49.35 | 3 | 13951 | Ana\_02.raw | 7.43E7 | 6E7 | 4.77E7 | 1.96E7 | 4.07E7 | 4.94E7 | 2.6E7 | 3.27E7 | 7.3E7 | 4.25E7 | 23 | 4 | 2 | 2 | 2 | 2 | 2 | 1 | 2 | 3 | 3 | 1072 | 1087 |  |
| K.THEAEIVEGENHTYC(+57.02)IR.F | Y | 106.99 | 2056.9272 | 17 | -0.8 | 686.6491 | 3 | 53.53 | 3 | 15177 | Ana\_02.raw |  | 9.13E6 | 7.94E6 | 2.84E6 | 6.02E6 | 1.47E7 |  | 8.03E6 |  | 9.21E6 | 9 | 0 | 1 | 1 | 1 | 1 | 2 | 0 | 2 | 0 | 1 | 2185 | 2201 | Carbamidomethylation |
| K.SADFVVEAIGDDVGTLGFSVEGPSQAK.I | Y | 105.96 | 2694.3022 | 27 | 3.8 | 899.1115 | 3 | 132.39 | 6 | 36801 | Ana\_05.raw | 2E6 |  | 5.34E6 |  |  | 8.92E6 | 2.91E6 |  |  |  | 4 | 1 | 0 | 1 | 0 | 0 | 1 | 1 | 0 | 0 | 0 | 594 | 620 |  |
| R.SPYTVTVGQAC(+57.02)NPAAC(+57.02)R.A | N | 105.52 | 1850.8403 | 17 | 4.0 | 926.4312 | 2 | 64.97 | 8 | 18225 | Ana\_07.raw | 1.38E7 | 1.71E7 | 1.32E7 | 4.49E6 | 1.27E7 | 1.88E7 | 5.1E6 | 6.29E6 | 2E7 | 1.11E7 | 15 | 1 | 1 | 2 | 1 | 2 | 3 | 1 | 1 | 2 | 1 | 468 | 484 | Carbamidomethylation |
| R.EEGPYEVEVTYDGVPVPGSPFPLEAVAPTKPSK.V | Y | 105.25 | 3484.7288 | 33 | 6.7 | 1162.5913 | 3 | 123.64 | 1 | 34044 | ANa\_10.raw | 5.45E6 | 1.29E7 | 5.51E6 | 1.25E6 | 1.97E6 | 4.39E6 | 9.58E6 | 1.43E6 | 1.53E7 |  | 11 | 2 | 2 | 1 | 1 | 1 | 1 | 1 | 1 | 1 | 0 | 1037 | 1069 |  |
| K.DAGEGLLAVQITDPEGKPK.K | Y | 101.62 | 1937.0105 | 19 | -2.2 | 969.5104 | 2 | 87.12 | 6 | 24812 | Ana\_05.raw | 3.98E5 | 2.92E6 | 1.93E6 |  | 8.43E5 | 6.08E6 | 6.7E5 |  | 5.13E6 | 5.61E5 | 11 | 1 | 2 | 1 | 0 | 2 | 2 | 1 | 0 | 1 | 1 | 1574 | 1592 |  |
| K.ASGPGLNTTGVPASLPVEFTIDAK.D | Y | 98.70 | 2341.2166 | 24 | 2.1 | 1171.6180 | 2 | 116.37 | 6 | 32531 | Ana\_05.raw | 2.29E6 | 6.26E6 | 4.49E6 | 6.45E5 | 6.06E5 | 1.03E7 | 3.35E6 | 7.44E5 | 4.34E6 |  | 12 | 1 | 2 | 2 | 1 | 1 | 1 | 2 | 1 | 1 | 0 | 1550 | 1573 |  |
| K.SPFSVGVSPSLDLSK.I | Y | 97.50 | 1518.7930 | 15 | -1.0 | 760.4030 | 2 | 93.39 | 3 | 26157 | Ana\_02.raw | 2.6E7 | 1.94E7 | 2.18E7 | 6.89E5 | 2.24E6 | 1.93E7 | 9.75E6 | 1.14E7 | 5.31E7 | 1.67E7 | 19 | 2 | 1 | 2 | 2 | 2 | 2 | 2 | 2 | 3 | 1 | 959 | 973 |  |
| R.GAGTGGLGLAVEGPSEAK.M | Y | 95.14 | 1569.7998 | 18 | 6.7 | 785.9124 | 2 | 66.11 | 1 | 18749 | ANa\_10.raw | 4.07E7 | 4.44E7 | 3.04E7 | 1.35E7 | 2.16E7 | 3.58E7 | 1.7E7 | 2.14E7 | 6.26E7 | 2.88E7 | 20 | 2 | 2 | 2 | 2 | 2 | 2 | 2 | 2 | 3 | 1 | 1382 | 1399 |  |
| K.VHSPSGALEEC(+57.02)YVTEIDQDK.Y | Y | 95.02 | 2276.0266 | 20 | -0.9 | 759.6821 | 3 | 83.27 | 6 | 23761 | Ana\_05.raw |  |  |  | 3.18E6 | 8.03E5 | 7.51E6 |  | 1.9E6 | 8.01E6 |  | 5 | 0 | 0 | 0 | 1 | 1 | 1 | 0 | 1 | 1 | 0 | 2368 | 2387 | Carbamidomethylation |
| K.HTAM(+15.99)VSWGGVSIPNSPFR.V | Y | 94.90 | 1957.9469 | 18 | 3.8 | 653.6587 | 3 | 100.25 | 1 | 28042 | ANa\_10.raw | 2.42E6 | 2.68E6 |  |  |  |  |  |  |  |  | 2 | 1 | 1 | 0 | 0 | 0 | 0 | 0 | 0 | 0 | 0 | 743 | 760 | Oxidation (M) |
| R.EGSYSISVLYGEEEVPR.S | Y | 93.11 | 1912.9054 | 17 | 4.4 | 957.4642 | 2 | 98.50 | 6 | 27854 | Ana\_05.raw |  | 1.87E6 |  |  | 1.04E6 | 9.47E5 |  | 3.67E5 | 1.16E7 | 2.82E6 | 6 | 0 | 1 | 0 | 0 | 1 | 1 | 0 | 1 | 1 | 1 | 1516 | 1532 |  |
| K.AHVAPC(+57.02)FDASK.V | Y | 92.51 | 1201.5549 | 11 | 4.1 | 601.7872 | 2 | 33.88 | 6 | 9295 | Ana\_05.raw | 7.71E6 | 1.8E7 | 2.17E7 | 4.06E6 | 7.18E6 | 2.13E7 | 6.29E6 | 1.37E7 | 2.07E7 | 5.62E6 | 13 | 1 | 1 | 2 | 1 | 1 | 2 | 1 | 2 | 1 | 1 | 1152 | 1162 | Carbamidomethylation |
| R.EAGAGGLAIAVEGPSK.A | Y | 92.40 | 1425.7463 | 16 | 1.4 | 713.8814 | 2 | 64.99 | 3 | 18515 | Ana\_02.raw | 2.9E7 | 2.75E7 | 1.93E7 | 7.62E6 | 1.55E7 | 2.18E7 | 1.17E7 | 1.4E7 | 2.38E7 | 1.87E7 | 17 | 2 | 2 | 2 | 2 | 1 | 1 | 1 | 2 | 2 | 2 | 2265 | 2280 |  |
| K.VEPGLGADNSVVR.F | Y | 92.07 | 1311.6782 | 13 | 2.8 | 656.8482 | 2 | 53.15 | 6 | 15152 | Ana\_05.raw | 4.62E7 | 4.52E7 | 4.39E7 | 1.5E7 | 3.06E7 | 4.22E7 | 2.17E7 | 2.51E7 | 6.31E7 | 3.27E7 | 17 | 2 | 2 | 1 | 2 | 1 | 1 | 1 | 1 | 3 | 3 | 1020 | 1032 |  |
| R.TFSVWYVPEVTGTHK.V | N | 91.78 | 1749.8726 | 15 | 3.2 | 584.3000 | 3 | 98.33 | 3 | 27460 | Ana\_02.raw | 5.77E6 | 2.61E7 | 3.86E7 | 1.01E7 | 1.77E7 | 3.11E7 | 1.2E7 | 2.04E7 | 4.72E7 | 1.08E7 | 25 | 2 | 2 | 2 | 3 | 2 | 3 | 3 | 3 | 4 | 1 | 341 | 355 |  |
| R.LVSNHSLHETSSVFVDSLTK.V | Y | 91.24 | 2199.1172 | 20 | 5.1 | 734.0501 | 3 | 76.34 | 9 | 21751 | Ana\_08.raw |  |  |  |  |  |  |  |  | 1.01E7 |  | 2 | 0 | 0 | 0 | 0 | 0 | 0 | 0 | 0 | 2 | 0 | 2521 | 2540 |  |
| K.IPEISIQDMTAQVTSPSGK.T | Y | 91.12 | 2001.0088 | 19 | 0.4 | 1001.5121 | 2 | 106.39 | 9 | 29930 | Ana\_08.raw |  |  |  |  |  |  |  |  | 0 |  | 1 | 0 | 0 | 0 | 0 | 0 | 0 | 0 | 0 | 1 | 0 | 2166 | 2184 |  |
| K.YNDQHIPGSPFTAR.V | Y | 90.75 | 1601.7587 | 14 | 1.5 | 534.9276 | 3 | 60.11 | 3 | 17104 | Ana\_02.raw | 8.29E6 | 8.48E6 | 3.67E7 | 1.47E7 | 2.58E7 | 3.81E7 | 2.15E7 | 2.49E7 | 1.05E7 | 2.41E7 | 15 | 1 | 1 | 2 | 1 | 1 | 2 | 2 | 2 | 1 | 2 | 1938 | 1951 |  |
| R.ANLPQSFQVDTSK.A | Y | 90.56 | 1433.7151 | 13 | 4.2 | 717.8679 | 2 | 66.05 | 6 | 18965 | Ana\_05.raw | 3.11E7 | 3.16E7 | 2.64E7 | 1.09E7 | 2.01E7 | 2.65E7 | 1.42E7 | 1.72E7 | 4.69E7 | 2.47E7 | 19 | 2 | 1 | 2 | 1 | 2 | 2 | 2 | 2 | 3 | 2 | 1465 | 1477 |  |
| K.IEC(+57.02)DDKGDGSC(+57.02)DVR.Y | N | 89.31 | 1624.6458 | 14 | 0.4 | 813.3305 | 2 | 21.87 | 3 | 5563 | Ana\_02.raw | 1.78E7 | 2.73E7 | 2.13E7 | 1.72E6 | 7E6 | 2.34E7 | 5.65E6 | 1.04E7 | 2.12E7 | 1.83E7 | 19 | 2 | 2 | 2 | 1 | 2 | 2 | 2 | 2 | 2 | 2 | 621 | 634 | Carbamidomethylation |
| R.QM(+15.99)QLENVSVALEFLDR.E | N | 88.37 | 1906.9458 | 16 | 6.6 | 954.4865 | 2 | 134.38 | 6 | 37359 | Ana\_05.raw | 1.04E6 |  |  |  | 1.01E6 | 2.46E6 | 7.85E5 |  | 3.11E6 | 1.53E6 | 6 | 1 | 0 | 0 | 0 | 1 | 1 | 1 | 0 | 1 | 1 | 101 | 116 | Oxidation (M) |
| K.YGGPYHIGGSPFK.A | Y | 88.27 | 1378.6670 | 13 | 1.0 | 690.3415 | 2 | 65.13 | 3 | 18556 | Ana\_02.raw | 3.7E7 | 4.07E7 | 2.69E7 | 1.37E7 | 2.24E7 | 3.34E7 | 1.87E7 | 1.89E7 | 5.82E7 | 3.04E7 | 22 | 3 | 3 | 2 | 2 | 2 | 2 | 2 | 2 | 2 | 2 | 2501 | 2513 |  |
| R.VQVQDNEGC(+57.02)SVEATVK.D | Y | 88.24 | 1761.8203 | 16 | 2.6 | 881.9197 | 2 | 52.02 | 6 | 14809 | Ana\_05.raw |  | 2.2E6 |  |  |  | 1.16E6 |  |  | 2.56E6 | 9.53E5 | 4 | 0 | 1 | 0 | 0 | 0 | 1 | 0 | 0 | 1 | 1 | 709 | 724 | Carbamidomethylation |
| R.VNVGAGSHPNK.V | Y | 87.83 | 1078.5519 | 11 | 1.6 | 540.2841 | 2 | 17.76 | 3 | 4187 | Ana\_02.raw | 0 | 1.46E7 | 5.25E7 | 1.83E7 | 2.95E7 | 5.89E7 | 2.88E7 | 2.6E7 |  | 2.75E7 | 20 | 2 | 2 | 2 | 2 | 3 | 2 | 2 | 3 | 0 | 2 | 761 | 771 |  |
| K.TGVAVNKPAEFTVDAK.H | Y | 87.61 | 1645.8674 | 16 | 2.8 | 549.6313 | 3 | 58.81 | 6 | 16817 | Ana\_05.raw | 4.53E7 | 4.35E7 | 3.45E7 | 1.26E7 | 2.34E7 | 3.96E7 | 1.82E7 | 1.94E7 | 4.94E7 | 2.55E7 | 19 | 2 | 3 | 2 | 1 | 2 | 2 | 1 | 2 | 3 | 1 | 685 | 700 |  |
| K.VTAQGPGLEPSGNIANK.T | N | 87.54 | 1651.8529 | 17 | 4.7 | 826.9376 | 2 | 51.30 | 5 | 14510 | Ana\_04.raw | 4.78E7 | 4.9E7 | 3.49E7 | 1.17E7 | 2.62E7 | 3.38E7 | 1.79E7 | 2.08E7 | 6.6E7 | 3.05E7 | 20 | 2 | 3 | 1 | 2 | 1 | 2 | 2 | 2 | 3 | 2 | 384 | 400 |  |
| R.AEVGVPAEFGIWTR.E | Y | 87.07 | 1530.7831 | 14 | 0.9 | 766.3995 | 2 | 109.33 | 3 | 30299 | Ana\_02.raw | 4.68E7 | 3.5E7 | 2.77E7 | 1.59E7 | 2.09E7 | 3.53E7 | 1.54E7 | 2.6E7 | 8.83E7 | 2.37E7 | 27 | 4 | 3 | 3 | 1 | 3 | 2 | 2 | 3 | 4 | 2 | 2251 | 2264 |  |
| R.IANLQTDLSDGLR.L | N | 86.97 | 1414.7416 | 13 | 1.5 | 708.3792 | 2 | 82.32 | 3 | 23230 | Ana\_02.raw | 2.35E7 | 2.69E7 | 6.27E6 | 1.22E6 | 1.38E7 | 1.5E7 | 6.62E6 | 7.33E6 | 4.62E7 | 1.86E7 | 16 | 2 | 3 | 1 | 1 | 1 | 1 | 1 | 1 | 3 | 2 | 64 | 76 |  |
| K.C(+57.02)APGVVGPTEADIDFDIIR.N | Y | 86.50 | 2043.9935 | 19 | 4.0 | 1023.0082 | 2 | 130.36 | 6 | 36218 | Ana\_05.raw |  |  |  |  |  | 1.58E6 |  |  |  |  | 1 | 0 | 0 | 0 | 0 | 0 | 1 | 0 | 0 | 0 | 0 | 810 | 828 | Carbamidomethylation |
| R.LLGWIQNKLPQLPITNFSR.D | N | 86.36 | 2237.2683 | 19 | 5.1 | 746.7672 | 3 | 128.97 | 9 | 35721 | Ana\_08.raw | 2.56E7 | 2.46E6 | 8.41E6 | 8.11E6 | 1.22E7 | 2.5E7 | 1.11E7 | 3.39E6 | 2.78E7 | 4.32E6 | 11 | 2 | 1 | 1 | 1 | 1 | 1 | 1 | 1 | 1 | 1 | 172 | 190 |  |
| R.DVDIIDHHDNTYTVK.Y | Y | 86.12 | 1783.8376 | 15 | 1.5 | 892.9274 | 2 | 56.10 | 9 | 15890 | Ana\_08.raw | 2.03E7 | 2.45E7 | 5.25E5 |  |  | 2.8E7 | 8.42E6 |  | 1.68E6 | 1.84E7 | 11 | 2 | 2 | 1 | 0 | 0 | 2 | 1 | 0 | 1 | 2 | 922 | 936 |  |
| K.YGGQPVPNFPSK.L | Y | 85.98 | 1289.6404 | 12 | 0.9 | 645.8281 | 2 | 58.25 | 3 | 16553 | Ana\_02.raw | 6.79E7 | 5.74E7 | 5.65E7 | 2.23E7 | 4.31E7 | 5.2E7 | 2.78E7 | 3.32E7 | 9.61E7 | 4.91E7 | 21 | 3 | 1 | 1 | 1 | 3 | 2 | 2 | 1 | 3 | 4 | 1235 | 1246 |  |
| K.SPFEVYVDK.S | N | 85.26 | 1082.5283 | 9 | 4.8 | 542.2740 | 2 | 70.04 | 6 | 20098 | Ana\_05.raw | 3.22E7 | 2.75E7 | 2.74E7 | 1.33E7 | 2.47E7 | 2.66E7 | 1.48E7 | 1.81E7 | 3.94E7 | 3.32E7 | 12 | 1 | 1 | 1 | 1 | 1 | 1 | 1 | 2 | 1 | 2 | 368 | 376 |  |
| R.AYGPGIEPTGNM(+15.99)VK.K | N | 85.02 | 1448.6969 | 14 | 2.4 | 725.3575 | 2 | 51.20 | 6 | 14570 | Ana\_05.raw | 6.44E7 | 5.77E7 | 4.94E7 | 1.74E7 | 3.83E7 | 4.92E7 | 2.94E7 | 3.05E7 | 7.8E7 | 3.88E7 | 17 | 2 | 1 | 2 | 2 | 1 | 2 | 2 | 2 | 1 | 2 | 286 | 299 | Oxidation (M) |
| R.EATTEFSVDAR.A | Y | 82.42 | 1224.5623 | 11 | 2.2 | 613.2897 | 2 | 51.40 | 6 | 14630 | Ana\_05.raw | 1.1E8 | 9.94E7 | 8.84E7 | 3.14E7 | 6.94E7 | 8.07E7 | 4.9E7 | 5.66E7 | 1.13E8 | 7.42E7 | 35 | 4 | 4 | 2 | 3 | 3 | 4 | 2 | 3 | 5 | 5 | 1273 | 1283 |  |
| K.VDVGKDQEFTVK.S | Y | 81.31 | 1363.6982 | 12 | 1.8 | 682.8576 | 2 | 46.28 | 3 | 13048 | Ana\_02.raw | 1.79E7 | 2.32E7 | 2.36E7 | 8.04E6 | 1.86E7 | 2.74E7 | 1.38E7 | 1.33E7 | 9.91E6 |  | 16 | 1 | 2 | 2 | 2 | 2 | 2 | 2 | 2 | 1 | 0 | 983 | 994 |  |
| K.VPVHDVTDASK.V | Y | 81.10 | 1166.5931 | 11 | 5.8 | 584.3073 | 2 | 23.71 | 9 | 5873 | Ana\_08.raw | 7.71E7 |  | 4.83E7 | 2.18E7 | 3.45E7 | 1.07E8 | 2.67E7 | 3.96E7 | 3.77E7 | 7.69E7 | 15 | 2 | 0 | 2 | 1 | 1 | 2 | 2 | 2 | 1 | 2 | 1440 | 1450 |  |
| R.VKVEPSHDASK.V | Y | 79.82 | 1195.6196 | 11 | 5.3 | 598.8203 | 2 | 16.67 | 7 | 3844 | Ana\_06.raw |  |  | 1.83E7 | 6.96E6 | 1.87E7 | 3E7 | 1.21E7 | 1.22E7 |  | 2.06E6 | 14 | 0 | 0 | 2 | 2 | 2 | 2 | 2 | 2 | 0 | 2 | 864 | 874 |  |
| R.VTYC(+57.02)PTEPGNYIINIK.F | Y | 79.69 | 1880.9342 | 16 | 6.5 | 941.4805 | 2 | 97.14 | 6 | 27491 | Ana\_05.raw | 1.52E7 | 2.87E6 | 2.62E6 | 2.33E6 | 4.68E6 | 8.29E6 | 3.07E5 |  | 1.85E7 | 2.13E6 | 9 | 1 | 1 | 1 | 1 | 1 | 1 | 1 | 0 | 1 | 1 | 2104 | 2119 | Carbamidomethylation |
| R.VTYTPM(+15.99)APGSYLISIK.Y | Y | 78.99 | 1755.9116 | 16 | 5.4 | 878.9678 | 2 | 97.20 | 9 | 27501 | Ana\_08.raw | 3.39E7 | 3.83E7 | 4.85E7 | 9.47E6 | 1.83E7 | 2.57E7 | 1.51E7 |  | 5.73E7 | 1.8E7 | 10 | 1 | 1 | 1 | 1 | 1 | 1 | 1 | 0 | 2 | 1 | 2485 | 2500 | Oxidation (M) |
| R.LYSVSYLLK.D | Y | 78.68 | 1084.6168 | 9 | 1.4 | 543.3165 | 2 | 92.08 | 3 | 25816 | Ana\_02.raw |  |  | 1.54E7 | 3.33E6 | 7.79E6 | 1.24E7 | 5.56E6 | 5.21E6 |  |  | 6 | 0 | 0 | 1 | 1 | 1 | 1 | 1 | 1 | 0 | 0 | 2613 | 2621 |  |
| K.FADQHVPGSPFSVK.V | Y | 78.15 | 1514.7517 | 14 | 0.5 | 758.3835 | 2 | 68.70 | 6 | 19712 | Ana\_05.raw | 2.11E7 | 1.75E7 | 1.34E7 | 9.79E6 | 2.38E7 | 2.19E7 | 1.27E7 | 6.81E5 | 3.47E7 | 9.01E6 | 17 | 1 | 2 | 1 | 1 | 2 | 2 | 2 | 1 | 3 | 2 | 2120 | 2133 |  |
| K.WGDEHIPGSPYR.I | Y | 77.67 | 1412.6472 | 12 | 9.9 | 707.3379 | 2 | 60.92 | 9 | 17330 | Ana\_08.raw | 2E7 | 1.84E7 | 1.93E7 | 6.31E6 | 1.47E7 | 1.87E7 | 8.56E6 | 1.18E7 | 3.52E7 | 2.88E6 | 19 | 2 | 2 | 3 | 1 | 2 | 2 | 2 | 2 | 2 | 1 | 2632 | 2643 |  |
| K.DAGEGGLSLAIEGPSK.A | Y | 77.43 | 1499.7467 | 16 | 0.9 | 750.8813 | 2 | 77.41 | 3 | 21919 | Ana\_02.raw | 9.65E5 | 4.4E6 | 2.51E6 | 3.34E5 | 1.11E6 | 3.79E6 | 1.62E6 | 2.05E6 | 8.73E6 | 4.92E6 | 12 | 1 | 2 | 1 | 1 | 1 | 1 | 1 | 1 | 2 | 1 | 1892 | 1907 |  |
| K.DKGEYTLVVK.W | Y | 77.09 | 1150.6233 | 10 | 2.3 | 576.3203 | 2 | 49.49 | 4 | 13632 | Ana\_03.raw | 1.62E7 | 1.86E7 | 1.99E7 | 5.86E6 | 1.39E7 | 2.06E7 | 1.95E6 |  | 1.09E7 | 1.45E7 | 13 | 1 | 2 | 2 | 1 | 2 | 2 | 1 | 0 | 1 | 1 | 2622 | 2631 |  |
| K.ETGEHLVHVK.K | Y | 75.80 | 1147.5985 | 10 | 5.2 | 574.8095 | 2 | 23.76 | 6 | 6161 | Ana\_05.raw | 3.4E7 | 1.66E7 | 2.74E7 | 1.04E7 | 3.95E6 | 5.06E6 | 1.06E7 | 9.41E6 | 3.22E7 | 3.27E7 | 20 | 2 | 2 | 2 | 2 | 2 | 2 | 2 | 2 | 2 | 2 | 2015 | 2024 |  |
| R.RLTVSSLQESGLK.V | Y | 74.70 | 1416.7936 | 13 | 3.1 | 473.2733 | 3 | 57.56 | 3 | 16343 | Ana\_02.raw | 1.06E6 | 3.14E6 | 8.46E6 |  | 4.15E6 | 6.6E6 |  | 9.38E5 | 2.37E6 |  | 9 | 1 | 1 | 2 | 0 | 1 | 2 | 0 | 1 | 1 | 0 | 2334 | 2346 |  |
| R.EAM(+15.99)QQADDWLGIPQVITPEEIVDPNVDEHSVM(+15.99)TYLSQFPK.A | N | 73.70 | 4601.1616 | 40 | 9.1 | 1534.7417 | 3 | 134.80 | 6 | 37472 | Ana\_05.raw | 2.66E6 |  |  |  |  | 3.98E6 |  |  |  |  | 2 | 1 | 0 | 0 | 0 | 0 | 1 | 0 | 0 | 0 | 0 | 227 | 266 | Oxidation (M) |
| R.YSPSEAGLHEM(+15.99)DIR.Y | Y | 72.35 | 1619.7250 | 14 | 6.2 | 810.8748 | 2 | 50.49 | 2 | 14237 | Ana\_01.raw | 4.13E7 | 3.43E7 | 4.19E7 | 1.29E7 | 5.57E6 | 3.34E7 | 1.98E7 | 3.28E6 | 5.9E7 | 2.81E7 | 18 | 2 | 2 | 2 | 2 | 1 | 2 | 2 | 1 | 2 | 2 | 1832 | 1845 | Oxidation (M) |
| R.LTVSSLQESGLK.V | Y | 71.71 | 1260.6925 | 12 | 2.7 | 631.3552 | 2 | 68.27 | 6 | 19602 | Ana\_05.raw | 1.18E6 | 2.19E7 | 1.22E7 | 6.72E6 | 1.53E7 | 1.55E7 | 9.17E6 | 8.88E6 | 2.61E7 | 1.54E7 | 14 | 1 | 2 | 1 | 1 | 2 | 2 | 1 | 2 | 1 | 1 | 2335 | 2346 |  |
| K.HNQRPTFR.Q | N | 71.56 | 1054.5420 | 8 | 0.3 | 352.5214 | 3 | 18.34 | 3 | 4378 | Ana\_02.raw |  | 2.29E7 | 2.51E7 | 7.69E5 | 1.93E7 | 2.79E7 | 1.13E7 | 7.21E6 | 3.53E7 | 2.7E7 | 15 | 0 | 1 | 2 | 1 | 2 | 2 | 2 | 1 | 2 | 2 | 93 | 100 |  |
| K.GTVEPQLEAR.G | N | 71.47 | 1098.5669 | 10 | 2.4 | 550.2921 | 2 | 41.93 | 4 | 11411 | Ana\_03.raw | 9.02E7 | 8.35E7 | 8.01E7 | 2.85E7 | 5.68E7 | 7.98E7 | 4.06E7 | 5.31E7 | 8.54E7 | 6.27E7 | 24 | 3 | 3 | 1 | 2 | 2 | 2 | 2 | 4 | 3 | 2 | 428 | 437 |  |
| K.VAQPSITDNKDGTVTVR.Y | Y | 70.77 | 1799.9377 | 17 | 3.2 | 600.9885 | 3 | 46.28 | 3 | 13050 | Ana\_02.raw | 1.54E7 |  | 9.85E6 | 2.99E6 | 6.65E6 | 1.36E7 |  | 5.1E6 | 2.32E7 | 2.94E6 | 11 | 2 | 0 | 1 | 1 | 1 | 2 | 0 | 1 | 2 | 1 | 1815 | 1831 |  |
| K.QRLLGWIQNKLPQLPITNFSR.D | N | 69.78 | 2521.4280 | 21 | 4.4 | 631.3671 | 4 | 128.48 | 8 | 35238 | Ana\_07.raw |  |  |  |  |  |  |  | 1.78E6 |  |  | 1 | 0 | 0 | 0 | 0 | 0 | 0 | 0 | 1 | 0 | 0 | 170 | 190 |  |
| K.YGGDEIPFSPYR.V | Y | 69.54 | 1399.6407 | 12 | -2.5 | 700.8259 | 2 | 85.21 | 3 | 24009 | Ana\_02.raw | 6.05E7 | 4.02E7 | 3.53E7 | 5.38E6 | 2.41E7 | 5.7E7 |  | 2.85E7 | 9.89E7 | 3.33E7 | 19 | 4 | 2 | 1 | 1 | 2 | 2 | 0 | 2 | 3 | 2 | 1622 | 1633 |  |
| R.LIALLEVLSQK.K | N | 69.25 | 1225.7645 | 11 | 3.9 | 613.8919 | 2 | 129.98 | 7 | 35763 | Ana\_06.raw | 4.91E6 | 1.99E6 | 1.02E7 | 4.83E6 | 8.34E6 | 9.14E6 | 6.96E6 | 2.18E6 | 1.08E7 | 5.95E6 | 10 | 1 | 1 | 1 | 1 | 1 | 1 | 1 | 1 | 1 | 1 | 77 | 87 |  |
| R.DAGYGGLSLSIEGPSK.V | Y | 68.41 | 1549.7623 | 16 | 4.4 | 775.8918 | 2 | 88.04 | 9 | 25008 | Ana\_08.raw |  |  |  |  |  |  |  |  | 1.4E6 |  | 1 | 0 | 0 | 0 | 0 | 0 | 0 | 0 | 0 | 1 | 0 | 2074 | 2089 |  |
| R.SAGQGEVLVYVEDPAGHQEEAK.V | N | 67.66 | 2312.0920 | 22 | 4.7 | 771.7083 | 3 | 75.59 | 10 | 21357 | Ana\_09.raw | 1.86E7 |  | 6.01E6 | 4.52E6 | 8.46E6 | 1.71E7 |  | 1.05E7 |  | 7.76E6 | 8 | 1 | 0 | 1 | 1 | 1 | 2 | 0 | 1 | 0 | 1 | 310 | 331 |  |
| K.LDVQFSGLAK.G | Y | 65.87 | 1076.5865 | 10 | 2.8 | 539.3021 | 2 | 77.93 | 6 | 22307 | Ana\_05.raw | 2.64E6 | 6.6E6 | 3.95E6 | 1.96E6 | 3.92E6 | 5.27E6 | 2.78E6 | 2.95E6 | 8.86E6 | 3.53E6 | 11 | 1 | 2 | 1 | 1 | 1 | 1 | 1 | 1 | 1 | 1 | 907 | 916 |  |
| K.AEISFEDR.K | Y | 65.39 | 965.4454 | 8 | 4.4 | 483.7321 | 2 | 48.28 | 2 | 13595 | Ana\_01.raw | 5.17E7 | 5.43E7 | 4.9E7 | 2.05E7 | 3.57E7 | 4.74E7 | 2.7E7 | 2.84E7 | 6.74E7 | 3.77E7 | 18 | 3 | 1 | 2 | 1 | 1 | 2 | 1 | 3 | 3 | 1 | 2281 | 2288 |  |
| K.DLAEDAPWK.K | N | 65.01 | 1043.4923 | 9 | 3.5 | 522.7552 | 2 | 72.72 | 6 | 20864 | Ana\_05.raw | 1.24E7 | 7.6E6 | 1.07E7 | 6.87E6 | 8.48E6 | 1.44E7 | 7.45E6 |  | 1.14E7 | 5.52E6 | 13 | 1 | 1 | 2 | 2 | 1 | 2 | 2 | 0 | 1 | 1 | 34 | 42 |  |
| K.VAQPSITDNK.D | Y | 64.71 | 1071.5560 | 10 | 4.9 | 536.7879 | 2 | 30.54 | 6 | 8294 | Ana\_05.raw | 3.18E7 | 3.51E7 | 1.63E7 | 1.12E7 | 1.82E7 | 2.62E7 | 1.39E7 |  | 3.33E7 | 2.44E7 | 9 | 1 | 1 | 1 | 1 | 1 | 1 | 1 | 0 | 1 | 1 | 1815 | 1824 |  |
| K.M(+15.99)DC(+57.02)QEC(+57.02)PEGYR.V | Y | 64.46 | 1459.5166 | 11 | 1.7 | 730.7668 | 2 | 32.71 | 4 | 8696 | Ana\_03.raw | 3.7E6 | 5.25E6 | 2.74E6 | 1.25E6 | 2.53E6 | 3.63E6 | 1.38E6 | 2.49E6 | 3E6 | 2.53E6 | 10 | 1 | 1 | 1 | 1 | 1 | 1 | 1 | 1 | 1 | 1 | 2474 | 2484 | Oxidation (M); Carbamidomethylation |
| K.KTHIQDNHDGTYTVAYVPDVPGR.Y | Y | 63.39 | 2582.2512 | 23 | 3.4 | 646.5723 | 4 | 64.17 | 2 | 18280 | Ana\_01.raw |  | 3.31E6 |  |  |  |  |  |  |  |  | 1 | 0 | 1 | 0 | 0 | 0 | 0 | 0 | 0 | 0 | 0 | 1593 | 1615 |  |
| R.TPC(+57.02)EEILVK.H | Y | 63.17 | 1087.5583 | 9 | 5.1 | 544.7892 | 2 | 54.53 | 6 | 15556 | Ana\_05.raw |  |  |  | 5.06E6 |  | 8.44E6 |  |  |  | 1.11E7 | 3 | 0 | 0 | 0 | 1 | 0 | 1 | 0 | 0 | 0 | 1 | 2599 | 2607 | Carbamidomethylation |
| K.MDC(+57.02)QEC(+57.02)PEGYR.V | Y | 62.83 | 1443.5217 | 11 | 3.8 | 722.7709 | 2 | 38.45 | 2 | 10577 | Ana\_01.raw | 1.55E6 | 1.37E6 |  |  |  |  |  |  |  |  | 2 | 1 | 1 | 0 | 0 | 0 | 0 | 0 | 0 | 0 | 0 | 2474 | 2484 | Carbamidomethylation |
| R.EAPQDFHPDR.V | Y | 62.71 | 1210.5366 | 10 | 1.7 | 606.2766 | 2 | 29.24 | 3 | 7898 | Ana\_02.raw | 6.57E7 | 5.93E7 | 2.89E7 |  | 5.72E7 | 7.66E7 | 3.31E7 | 2.59E7 | 5.94E7 | 3.86E7 | 18 | 3 | 2 | 1 | 0 | 3 | 2 | 2 | 1 | 2 | 2 | 665 | 674 |  |
| K.LPQLPITNFSR.D | N | 60.80 | 1284.7190 | 11 | 0.7 | 643.3672 | 2 | 97.59 | 2 | 27496 | Ana\_01.raw | 2.55E7 | 1.06E7 | 6.2E6 | 5.04E5 |  | 1.92E6 | 3.92E5 | 2.74E5 | 2.02E7 | 1.08E7 | 9 | 1 | 1 | 1 | 1 | 0 | 1 | 1 | 1 | 1 | 1 | 180 | 190 |  |
| K.IVSPSGAAVPC(+57.02)K.V | Y | 60.31 | 1184.6223 | 12 | 4.0 | 593.3208 | 2 | 43.18 | 6 | 12135 | Ana\_05.raw | 4.13E7 | 3.95E7 | 3.62E7 | 5.84E6 | 2.67E7 | 3.65E7 | 1.84E7 | 2.26E7 | 9.49E6 | 3.48E7 | 16 | 2 | 1 | 1 | 1 | 1 | 2 | 2 | 2 | 2 | 2 | 1008 | 1019 | Carbamidomethylation |
| K.VLPTHDASK.V | Y | 60.00 | 966.5134 | 9 | 1.9 | 323.1790 | 3 | 18.21 | 3 | 4334 | Ana\_02.raw | 3.29E6 | 9.56E5 | 4.19E7 | 1.59E7 | 2.97E7 | 5.65E7 | 2.19E7 | 1.71E7 |  | 1.17E7 | 22 | 1 | 1 | 3 | 3 | 2 | 4 | 3 | 2 | 0 | 3 | 1539 | 1547 |  |
| K.KGEITGEVR.M | Y | 58.95 | 987.5349 | 9 | 2.0 | 330.1862 | 3 | 21.78 | 6 | 5517 | Ana\_05.raw | 7.1E7 | 6.19E7 | 4.95E7 | 2.66E6 | 3E7 | 9.91E6 | 1.84E7 | 2.27E7 | 5.22E6 | 5.23E7 | 17 | 2 | 2 | 2 | 1 | 2 | 2 | 2 | 2 | 1 | 1 | 1801 | 1809 |  |
| R.ALTQTGGPHVK.A | Y | 58.75 | 1107.6036 | 11 | 1.4 | 554.8099 | 2 | 23.84 | 4 | 6051 | Ana\_03.raw |  | 3.03E7 | 2.5E7 | 8.31E6 | 1.65E7 | 1E7 | 6.36E6 | 4.74E6 | 4.2E7 | 2.1E7 | 11 | 0 | 2 | 1 | 1 | 1 | 1 | 1 | 1 | 2 | 1 | 1284 | 1294 |  |
| R.LIALLEVLSQKK.M | N | 58.22 | 1353.8595 | 12 | 3.2 | 452.2952 | 3 | 118.02 | 7 | 32626 | Ana\_06.raw | 3.02E6 | 6.32E6 | 4.03E6 | 1.24E6 | 6.98E6 | 1.09E7 | 2.78E6 | 5.23E6 | 3.58E6 | 5.53E6 | 19 | 1 | 2 | 1 | 2 | 2 | 3 | 3 | 2 | 1 | 2 | 77 | 88 |  |
| K.VYGPGVAK.T | Y | 57.94 | 789.4385 | 8 | 4.5 | 395.7283 | 2 | 26.67 | 5 | 7062 | Ana\_04.raw | 1.41E7 | 4.5E7 | 4.09E7 | 1.74E7 | 2.27E7 | 4.05E7 | 2.36E7 | 2.21E7 | 3.77E7 | 1.82E7 | 10 | 1 | 1 | 1 | 1 | 1 | 1 | 1 | 1 | 1 | 1 | 774 | 781 |  |
| R.AEFTVETR.S | N | 57.51 | 951.4661 | 8 | 6.1 | 476.7432 | 2 | 41.85 | 1 | 11585 | ANa\_10.raw | 6.37E7 | 5.87E7 | 5.92E7 | 1.91E7 |  | 5.15E7 | 3E7 | 3.42E7 | 2.18E7 | 4.17E7 | 18 | 2 | 2 | 2 | 2 | 1 | 2 | 2 | 1 | 2 | 2 | 302 | 309 |  |
| K.VTVLFAGQHIAK.S | N | 57.09 | 1282.7397 | 12 | 3.4 | 642.3793 | 2 | 65.73 | 4 | 18317 | Ana\_03.raw | 2E7 | 2.04E7 | 1.8E7 | 6.77E6 | 1.47E7 | 1.39E7 | 6.82E6 | 7.14E6 | 3.11E7 | 1.56E7 | 18 | 2 | 2 | 2 | 2 | 2 | 2 | 1 | 1 | 2 | 2 | 356 | 367 |  |
| R.AWGPGLEGGIVGK.S | Y | 56.61 | 1239.6611 | 13 | 2.7 | 620.8395 | 2 | 82.65 | 4 | 22929 | Ana\_03.raw |  | 1.53E7 | 1.31E7 | 4.93E6 | 9.64E6 | 1.89E7 | 5.25E6 | 1.21E7 | 4.2E7 | 9.23E6 | 10 | 0 | 1 | 1 | 1 | 1 | 1 | 1 | 1 | 2 | 1 | 581 | 593 |  |
| R.C(+57.02)GQSAAVASPGGSIDSR.D | Y | 55.27 | 1618.7369 | 17 | -4.2 | 810.3723 | 2 | 58.72 | 7 | 16598 | Ana\_06.raw |  |  |  | 8.41E5 |  |  | 7.63E5 | 1.2E6 |  |  | 3 | 0 | 0 | 0 | 1 | 0 | 0 | 1 | 1 | 0 | 0 | 8 | 24 | Carbamidomethylation |
| R.NDNDTFTVK.Y | N | 54.27 | 1052.4774 | 9 | 5.4 | 527.2488 | 2 | 34.22 | 2 | 9284 | Ana\_01.raw | 2.84E7 | 2.74E7 |  |  |  |  |  |  | 8.6E6 | 1.49E7 | 5 | 1 | 1 | 0 | 0 | 0 | 0 | 0 | 0 | 2 | 1 | 829 | 837 |  |
| K.IQQNTFTR.W | N | 53.76 | 1006.5196 | 8 | 2.0 | 504.2681 | 2 | 28.64 | 3 | 7713 | Ana\_02.raw |  |  | 4.24E7 | 1.74E7 |  |  |  | 2.58E7 | 5.6E7 | 4.3E7 | 6 | 0 | 0 | 1 | 1 | 0 | 0 | 0 | 1 | 1 | 2 | 44 | 51 |  |
| R.VTYTPMAPGSYLISIK.Y | Y | 53.27 | 1739.9167 | 16 | 3.9 | 870.9691 | 2 | 110.15 | 1 | 30577 | ANa\_10.raw | 2.81E5 |  |  |  |  |  |  |  |  |  | 1 | 1 | 0 | 0 | 0 | 0 | 0 | 0 | 0 | 0 | 0 | 2485 | 2500 |  |
| R.VKETADFK.V | N | 53.19 | 936.4916 | 8 | 3.3 | 313.1722 | 3 | 21.84 | 6 | 5538 | Ana\_05.raw | 1.93E7 | 1.5E7 | 1.57E7 | 4.97E6 | 1.04E7 | 1.25E7 | 6.49E6 | 5.96E6 | 3.22E6 |  | 13 | 2 | 1 | 2 | 2 | 1 | 2 | 1 | 1 | 1 | 0 | 497 | 504 |  |
| K.AGNNM(+15.99)LLVGVHGPR.T | Y | 53.10 | 1449.7511 | 14 | 2.3 | 484.2588 | 3 | 53.10 | 5 | 15040 | Ana\_04.raw | 1.33E7 | 1.12E7 | 1.31E7 | 4.73E6 | 5.84E6 | 1.06E7 | 7.23E6 | 6.61E6 | 8.23E6 | 8.3E6 | 11 | 1 | 1 | 1 | 1 | 1 | 1 | 1 | 1 | 2 | 1 | 2585 | 2598 | Oxidation (M) |
| K.VKAEGPGLNR.T | Y | 52.29 | 1039.5774 | 10 | 4.6 | 347.5347 | 3 | 22.90 | 1 | 5733 | ANa\_10.raw | 4.2E6 |  | 7.13E5 |  |  |  |  |  | 7.16E6 |  | 5 | 2 | 0 | 1 | 0 | 0 | 0 | 0 | 0 | 2 | 0 | 875 | 884 |  |
| K.RAEFTVETR.S | N | 52.22 | 1107.5673 | 9 | 5.0 | 554.7937 | 2 | 32.78 | 10 | 8897 | Ana\_09.raw |  |  | 1.49E7 |  |  |  | 9.16E6 |  |  | 1.99E7 | 3 | 0 | 0 | 1 | 0 | 0 | 0 | 1 | 0 | 0 | 1 | 301 | 309 |  |
| R.AGGPGLER.A | N | 50.66 | 755.3926 | 8 | 2.6 | 378.7045 | 2 | 21.77 | 6 | 5512 | Ana\_05.raw |  |  |  | 2.88E7 | 4.09E7 | 1.25E7 | 3.55E7 | 3.18E7 |  |  | 5 | 0 | 0 | 0 | 1 | 1 | 1 | 1 | 1 | 0 | 0 | 2243 | 2250 |  |
| R.LSPFM(+15.99)ADIR.E | Y | 49.59 | 1064.5325 | 9 | 4.3 | 533.2758 | 2 | 68.11 | 2 | 19409 | Ana\_01.raw | 3.13E7 | 2.29E7 | 1.93E7 | 7.33E6 | 2.04E7 | 2.01E7 | 1.13E7 |  | 4.28E7 | 2.45E7 | 11 | 1 | 1 | 1 | 1 | 2 | 1 | 1 | 0 | 2 | 1 | 656 | 664 | Oxidation (M) |
| K.AGVAPLQVK.V | Y | 48.52 | 881.5334 | 9 | 4.8 | 441.7761 | 2 | 39.07 | 9 | 10685 | Ana\_08.raw | 3.17E7 | 2.88E7 | 1.35E7 | 1.31E7 | 6.56E6 | 1.76E7 | 1.5E7 | 1.42E7 | 6.2E6 | 2.44E7 | 14 | 2 | 2 | 1 | 2 | 1 | 1 | 1 | 1 | 2 | 1 | 1478 | 1486 |  |
| K.AEGPGLNR.T | Y | 47.22 | 812.4141 | 8 | 8.1 | 407.2176 | 2 | 21.64 | 5 | 5514 | Ana\_04.raw |  |  | 3.63E7 | 2.47E7 | 3.11E7 | 6.91E7 | 6.09E6 | 1.93E6 |  |  | 6 | 0 | 0 | 1 | 1 | 1 | 1 | 1 | 1 | 0 | 0 | 877 | 884 |  |
| R.ENGIYLIDVK.F | Y | 46.84 | 1162.6233 | 10 | 9.5 | 582.3245 | 2 | 83.90 | 2 | 23786 | Ana\_01.raw | 2.39E5 | 1.1E6 |  |  | 0 |  |  |  | 2.78E6 | 2.03E5 | 7 | 1 | 1 | 1 | 0 | 1 | 1 | 0 | 0 | 1 | 1 | 2396 | 2405 |  |
| R.KHNQRPTFR.Q | N | 46.51 | 1182.6370 | 9 | 4.1 | 395.2212 | 3 | 17.94 | 10 | 4130 | Ana\_09.raw |  | 1.24E7 | 1.1E7 |  |  | 1.25E7 |  | 4.02E6 | 1.24E7 | 9.28E6 | 10 | 0 | 2 | 2 | 0 | 0 | 1 | 0 | 1 | 2 | 2 | 92 | 100 |  |
| K.FTVETR.G | N | 45.35 | 751.3864 | 6 | 2.0 | 376.7012 | 2 | 29.33 | 3 | 7925 | Ana\_02.raw | 4.96E7 | 4.71E7 | 4.24E7 | 1.96E7 | 3.51E7 | 4.5E7 | 2.76E7 | 2.49E7 | 4.87E7 | 3.52E7 | 15 | 2 | 1 | 1 | 2 | 1 | 2 | 2 | 2 | 1 | 1 | 1376 | 1381 |  |
| K.GKLDVQFSGLAK.G | Y | 43.29 | 1261.7030 | 12 | 2.8 | 631.8605 | 2 | 65.13 | 3 | 18557 | Ana\_02.raw | 6.11E5 | 1.73E7 | 5.05E5 |  | 1.07E7 |  |  |  | 1.18E7 |  | 5 | 1 | 1 | 1 | 0 | 1 | 0 | 0 | 0 | 1 | 0 | 905 | 916 |  |
| R.TGVELGKPTHFTVNAK.T | Y | 43.25 | 1697.9100 | 16 | 2.8 | 425.4860 | 4 | 54.93 | 6 | 15679 | Ana\_05.raw |  |  |  |  |  | 2.66E6 |  |  | 1.18E7 |  | 2 | 0 | 0 | 0 | 0 | 0 | 1 | 0 | 0 | 1 | 0 | 885 | 900 |  |
| R.FVPAEM(+15.99)GM(+15.99)HTVSVK.Y | Y | 42.43 | 1563.7426 | 14 | 3.6 | 782.8813 | 2 | 42.50 | 6 | 11929 | Ana\_05.raw | 3.41E7 |  |  | 2.64E6 | 2.66E7 | 3.54E7 | 4.36E6 | 2.96E7 |  |  | 7 | 1 | 0 | 0 | 1 | 1 | 2 | 1 | 1 | 0 | 0 | 2202 | 2215 | Oxidation (M) |
| K.LVSIDSK.A | N | 40.94 | 760.4330 | 7 | 5.1 | 381.2257 | 2 | 29.51 | 1 | 7861 | ANa\_10.raw | 6.01E7 | 6.64E7 | 5.26E7 |  |  | 4.66E7 | 2.86E7 | 3.95E7 | 4.72E7 | 3.77E7 | 8 | 1 | 1 | 1 | 0 | 0 | 1 | 1 | 1 | 1 | 1 | 121 | 127 |  |
| K.LKPGAPLRPK.L | N | 40.18 | 1075.6865 | 10 | 1.3 | 538.8513 | 2 | 22.02 | 3 | 5610 | Ana\_02.raw | 5.59E7 | 2.09E7 | 3.94E7 | 2.4E6 | 1.78E7 | 1.45E7 | 1.12E6 | 9.17E5 | 4.58E7 | 4.15E7 | 15 | 2 | 1 | 2 | 1 | 2 | 1 | 1 | 1 | 2 | 2 | 269 | 278 |  |
| K.DQEFTVK.S | Y | 39.21 | 865.4181 | 7 | 3.9 | 433.7180 | 2 | 32.47 | 9 | 8621 | Ana\_08.raw | 7.4E7 | 8.29E7 | 8.26E7 | 4.02E7 | 6.74E7 | 6.49E7 | 4.8E7 | 5.13E7 | 5.34E7 | 8.12E7 | 11 | 1 | 1 | 1 | 1 | 2 | 1 | 1 | 1 | 1 | 1 | 988 | 994 |  |
| K.GEITGEVR.M | N | 38.99 | 859.4399 | 8 | 4.0 | 430.7290 | 2 | 26.51 | 2 | 6920 | Ana\_01.raw |  | 1.32E7 |  | 5.95E6 |  | 6.47E6 | 1.14E7 |  |  |  | 6 | 0 | 1 | 0 | 1 | 0 | 1 | 3 | 0 | 0 | 0 | 1802 | 1809 |  |
| R.FTIDTK.G | Y | 38.40 | 723.3803 | 6 | 4.0 | 362.6989 | 2 | 38.48 | 6 | 10684 | Ana\_05.raw | 2.27E7 | 2.21E7 | 2.44E7 | 9.1E6 | 1.93E7 | 2.35E7 | 1.3E7 | 1.53E7 |  | 1.54E7 | 18 | 2 | 2 | 2 | 2 | 2 | 2 | 2 | 2 | 0 | 2 | 1088 | 1093 |  |
| K.YVIC(+57.02)VR.F | Y | 37.62 | 808.4265 | 6 | -0.5 | 405.2203 | 2 | 51.13 | 4 | 14114 | Ana\_03.raw |  |  | 6.42E6 | 3.37E6 | 5.84E6 | 8.21E6 |  |  |  | 5.6E6 | 5 | 0 | 0 | 1 | 1 | 1 | 1 | 0 | 0 | 0 | 1 | 1720 | 1725 | Carbamidomethylation |
| R.WC(+57.02)NEHLK.C | N | 37.58 | 985.4440 | 7 | 3.6 | 329.4898 | 3 | 24.09 | 9 | 6000 | Ana\_08.raw |  |  |  |  |  |  |  |  | 6.53E6 |  | 2 | 0 | 0 | 0 | 0 | 0 | 0 | 0 | 0 | 2 | 0 | 52 | 58 | Carbamidomethylation |
| R.SPFEVK.V | Y | 35.30 | 705.3697 | 6 | 4.0 | 353.6935 | 2 | 37.23 | 7 | 10157 | Ana\_06.raw | 3.45E7 |  |  |  |  | 3.74E7 | 2.44E7 |  | 4.68E7 |  | 4 | 1 | 0 | 0 | 0 | 0 | 1 | 1 | 0 | 1 | 0 | 564 | 569 |  |
| R.EEPC(+57.02)LLK.R | Y | 33.68 | 887.4422 | 7 | 1.3 | 444.7289 | 2 | 43.46 | 6 | 12224 | Ana\_05.raw | 1.36E6 | 1.1E7 |  |  |  | 1.06E7 |  |  | 5.66E6 | 8.12E6 | 5 | 1 | 1 | 0 | 0 | 0 | 1 | 0 | 0 | 1 | 1 | 1994 | 2000 | Carbamidomethylation |
| R.VKESITR.R | Y | 29.01 | 831.4814 | 7 | 3.6 | 416.7495 | 2 | 19.07 | 7 | 4598 | Ana\_06.raw |  |  |  |  |  | 3.03E6 | 1.26E6 |  |  |  | 2 | 0 | 0 | 0 | 0 | 0 | 1 | 1 | 0 | 0 | 0 | 2140 | 2146 |  |
| K.YIPVQQGPVGVNVTYGGDHIPK.S | Y | 27.93 | 2337.2117 | 22 | 1.0 | 780.0786 | 3 | 85.93 | 7 | 24193 | Ana\_06.raw |  |  |  |  |  | 2.95E6 | 8.77E5 |  |  |  | 2 | 0 | 0 | 0 | 0 | 0 | 1 | 1 | 0 | 0 | 0 | 937 | 958 |  |
| R.YTILIK.Y | Y | 26.84 | 749.4687 | 6 | 5.7 | 375.7438 | 2 | 53.99 | 9 | 15274 | Ana\_08.raw |  |  |  |  |  |  |  |  | 2.42E7 |  | 1 | 0 | 0 | 0 | 0 | 0 | 0 | 0 | 0 | 1 | 0 | 1616 | 1621 |  |
| total 119 peptides |  |  |  |  |  |  |  |  |  |  |  |  |  |  |  |  |  |  |  |  |  |  |  |  |  |  |  |  |  |  |  |  |  |  |  |
| --- | --- | --- | --- | --- | --- | --- | --- | --- | --- | --- | --- | --- | --- | --- | --- | --- | --- | --- | --- | --- | --- | --- | --- | --- | --- | --- | --- | --- | --- | --- | --- | --- | --- | --- | --- |

Best Unique PSM (Scan F3:27795, m/z=823.0677, z=3, RT=99.61, ppm=0.0):

sp|Q8VDD5|MYH9\_MOUSE

back to list

  

| Protein Coverage
| Supporting Peptides
| Best Unique PSM
|

Protein Coverage:

Supporting Peptides:

| Peptide | Uniq | -10lgP | Mass | Length | ppm | m/z | z | RT | Fraction | Scan | Source File | Area 10 | Area 01 | Area 02 | Area 03 | Area 04 | Area 05 | Area 06 | Area 07 | Area 08 | Area 09 | #Spec | #Spec 10 | #Spec 01 | #Spec 02 | #Spec 03 | #Spec 04 | #Spec 05 | #Spec 06 | #Spec 07 | #Spec 08 | #Spec 09 | Start | End | PTM |
| --- | --- | --- | --- | --- | --- | --- | --- | --- | --- | --- | --- | --- | --- | --- | --- | --- | --- | --- | --- | --- | --- | --- | --- | --- | --- | --- | --- | --- | --- | --- | --- | --- | --- | --- | --- |
| K.DFSALESQLQDTQELLQEENR.Q | Y | 149.25 | 2492.1667 | 21 | 1.3 | 1247.0923 | 2 | 129.90 | 3 | 35591 | Ana\_02.raw | 2.08E7 | 8.19E6 | 1.12E7 | 3.31E6 | 1.12E7 | 1.25E7 | 7.65E6 | 8.4E6 | 3.38E7 | 1.53E7 | 19 | 2 | 1 | 2 | 2 | 2 | 2 | 2 | 2 | 2 | 2 | 1302 | 1322 |  |
| R.IAQLEEELEEEQGNTELINDR.L | Y | 125.34 | 2471.1663 | 21 | 5.2 | 1236.5968 | 2 | 100.86 | 9 | 28475 | Ana\_08.raw |  |  |  |  |  | 5.13E5 |  |  | 1.14E7 |  | 3 | 0 | 0 | 0 | 0 | 0 | 1 | 0 | 0 | 2 | 0 | 1731 | 1751 |  |
| R.KLEGDSTDLSDQIAELQAQIAELK.M | Y | 122.12 | 2614.3337 | 24 | 6.8 | 872.4578 | 3 | 131.72 | 1 | 36209 | ANa\_10.raw | 1.39E7 | 1.09E7 | 9.43E6 | 3.83E6 |  |  | 6.13E6 | 6.58E6 | 1.97E7 | 1.06E7 | 8 | 1 | 1 | 1 | 1 | 0 | 0 | 1 | 1 | 1 | 1 | 1052 | 1075 |  |
| K.TQLEELEDELQATEDAK.L | N | 116.11 | 1960.9113 | 17 | 1.2 | 981.4641 | 2 | 112.32 | 3 | 31061 | Ana\_02.raw | 1.36E8 | 2.22E8 | 1.17E8 | 7.57E7 | 1.05E8 | 1.7E8 | 8.31E7 | 4.83E7 | 2.7E8 | 1.45E8 | 68 | 10 | 10 | 5 | 5 | 5 | 4 | 5 | 9 | 8 | 7 | 1539 | 1555 |  |
| K.VSHLLGINVTDFTR.G | Y | 106.47 | 1570.8467 | 14 | 1.3 | 524.6235 | 3 | 95.70 | 3 | 26772 | Ana\_02.raw | 1.94E7 | 1.07E6 | 1.98E6 | 1.98E6 | 4.16E6 | 8.61E5 | 1.84E6 |  | 2.23E7 | 4.35E6 | 14 | 2 | 1 | 2 | 1 | 2 | 1 | 2 | 0 | 2 | 1 | 374 | 387 |  |
| K.IRELETQISELQEDLESER.A | Y | 104.94 | 2316.1445 | 19 | 4.0 | 773.0585 | 3 | 116.41 | 6 | 32541 | Ana\_05.raw | 1.89E6 | 3.72E6 |  |  | 1.16E6 | 0 | 2.27E6 |  | 9E6 |  | 6 | 1 | 1 | 0 | 0 | 1 | 1 | 1 | 0 | 1 | 0 | 1106 | 1124 |  |
| R.LQQELDDLLVDLDHQR.Q | Y | 103.01 | 1948.9854 | 16 | 0.9 | 975.5008 | 2 | 122.05 | 6 | 34023 | Ana\_05.raw | 8.45E6 | 4.05E7 | 4.41E7 | 1.74E7 | 1.43E7 | 3.33E7 | 2.57E7 | 2.64E7 | 8.57E7 | 5.08E7 | 31 | 4 | 2 | 3 | 2 | 1 | 3 | 3 | 5 | 4 | 4 | 1418 | 1433 |  |
| K.LQQLFNHTMFILEQEEYQR.E | N | 102.05 | 2466.2002 | 19 | 4.9 | 823.0780 | 3 | 118.36 | 6 | 33058 | Ana\_05.raw |  |  |  |  |  | 4.77E6 |  |  |  |  | 1 | 0 | 0 | 0 | 0 | 0 | 1 | 0 | 0 | 0 | 0 | 476 | 494 |  |
| K.LQVELDSVTGLLSQSDSK.S | Y | 101.56 | 1917.9895 | 18 | -1.6 | 960.0005 | 2 | 113.56 | 3 | 31390 | Ana\_02.raw | 2.6E6 | 2.39E6 | 1.56E6 | 2.33E6 |  | 5.53E6 | 1.25E6 | 5.97E5 | 1.44E7 | 2.21E6 | 9 | 1 | 1 | 1 | 1 | 0 | 1 | 1 | 1 | 1 | 1 | 1278 | 1295 |  |
| R.DLGEELEALKTELEDTLDSTAAQQELR.S | Y | 101.55 | 3016.4724 | 27 | 2.8 | 1006.5009 | 3 | 138.56 | 6 | 38465 | Ana\_05.raw |  |  |  |  |  | 1.2E6 |  |  |  |  | 1 | 0 | 0 | 0 | 0 | 0 | 1 | 0 | 0 | 0 | 0 | 1136 | 1162 |  |
| R.ELETQISELQEDLESER.A | Y | 98.63 | 2046.9592 | 17 | 3.9 | 1024.4908 | 2 | 116.32 | 9 | 32507 | Ana\_08.raw | 1.9E7 | 2.75E7 | 1.37E7 | 2.98E6 | 1.73E7 | 1.69E7 | 9.67E6 | 3.3E6 | 5.48E7 | 1.7E7 | 24 | 3 | 3 | 3 | 2 | 2 | 2 | 2 | 3 | 3 | 1 | 1108 | 1124 |  |
| R.QLLQANPILEAFGNAK.T | N | 97.96 | 1725.9413 | 16 | 2.9 | 863.9804 | 2 | 125.10 | 6 | 34810 | Ana\_05.raw | 1.57E7 | 1.55E7 | 4.42E7 | 4.06E6 | 2.63E7 | 7.1E7 | 2.22E7 | 4.41E7 | 7.06E7 | 1.03E7 | 45 | 6 | 4 | 8 | 4 | 4 | 4 | 3 | 5 | 4 | 3 | 210 | 225 |  |
| K.LQQLFNHTM(+15.99)FILEQEEYQR.E | N | 96.51 | 2482.1951 | 19 | 2.7 | 828.4079 | 3 | 103.10 | 5 | 28829 | Ana\_04.raw | 6.34E6 |  | 2.18E7 |  | 2.15E7 | 2.26E7 | 1.24E6 | 8.44E4 | 2.31E7 |  | 8 | 1 | 0 | 1 | 0 | 1 | 1 | 1 | 1 | 2 | 0 | 476 | 494 | Oxidation (M) |
| R.TFHIFYYLLSGAGEHLK.T | Y | 96.06 | 1995.0254 | 17 | 4.0 | 499.7656 | 4 | 128.25 | 7 | 35291 | Ana\_06.raw |  |  |  | 2.88E6 | 4.22E6 | 4.21E6 | 4.16E5 | 1.02E6 |  |  | 7 | 0 | 0 | 0 | 2 | 2 | 1 | 1 | 1 | 0 | 0 | 273 | 289 |  |
| R.EDQSILC(+57.02)TGESGAGK.T | N | 94.65 | 1550.6882 | 15 | 0.4 | 776.3517 | 2 | 46.91 | 3 | 13245 | Ana\_02.raw | 6.73E7 | 9.75E7 | 5.91E7 | 2.44E7 | 4.17E7 | 6.67E7 | 3.22E7 | 4.18E7 | 1.11E8 | 4.88E7 | 27 | 4 | 5 | 2 | 2 | 3 | 2 | 1 | 3 | 3 | 2 | 166 | 180 | Carbamidomethylation |
| K.ANLQIDQINTDLNLER.S | Y | 93.32 | 1868.9592 | 16 | 8.7 | 935.4950 | 2 | 97.64 | 9 | 27623 | Ana\_08.raw | 9.43E5 |  |  |  | 0 | 1.19E6 |  |  | 4.97E6 | 1.9E5 | 6 | 1 | 0 | 0 | 1 | 1 | 1 | 0 | 0 | 1 | 1 | 1755 | 1770 |  |
| K.NM(+15.99)DPLNDNIATLLHQSSDK.F | Y | 88.55 | 2141.0059 | 19 | 4.2 | 714.6789 | 3 | 96.58 | 9 | 27334 | Ana\_08.raw | 3.71E6 |  |  |  | 1.38E5 | 2.13E6 | 9.95E5 | 8.58E5 | 1.51E7 | 5.88E5 | 7 | 1 | 0 | 0 | 0 | 1 | 1 | 1 | 1 | 1 | 1 | 588 | 606 | Oxidation (M) |
| R.ALEQQVEEM(+15.99)KTQLEELEDELQATEDAK.L | Y | 85.26 | 3162.4761 | 27 | 6.0 | 1055.1722 | 3 | 127.70 | 1 | 35112 | ANa\_10.raw | 1.4E6 |  |  |  |  |  |  |  |  |  | 1 | 1 | 0 | 0 | 0 | 0 | 0 | 0 | 0 | 0 | 0 | 1529 | 1555 | Oxidation (M) |
| K.NFINNPLAQADWAAK.K | Y | 84.92 | 1671.8369 | 15 | 3.9 | 836.9290 | 2 | 106.53 | 6 | 29985 | Ana\_05.raw | 1.15E7 | 9.22E6 | 1.84E6 |  | 9.89E5 | 7.17E6 | 1.8E6 | 2.97E5 | 3.44E7 | 9.05E6 | 10 | 1 | 1 | 1 | 0 | 1 | 1 | 1 | 1 | 2 | 1 | 15 | 29 |  |
| K.LDPHLVLDQLR.C | N | 84.87 | 1317.7405 | 11 | 1.6 | 440.2548 | 3 | 92.61 | 3 | 25955 | Ana\_02.raw | 2.14E6 | 4.21E6 | 2.49E6 | 1.52E6 | 7.25E6 | 1.77E6 | 4.2E6 | 3.71E6 | 3.64E7 | 1.88E7 | 23 | 2 | 3 | 3 | 1 | 2 | 2 | 2 | 2 | 3 | 3 | 683 | 693 |  |
| R.IIGLDQVAGM(+15.99)SETALPGAFK.T | Y | 81.97 | 2033.0503 | 20 | 6.7 | 1017.5392 | 2 | 112.17 | 1 | 31091 | ANa\_10.raw | 3.51E7 | 2.96E6 | 3.41E6 | 4.15E6 | 1.52E7 | 1.97E6 | 1.17E7 | 1.25E7 | 4.69E7 | 1.12E7 | 28 | 2 | 2 | 3 | 3 | 2 | 2 | 3 | 3 | 4 | 4 | 618 | 637 | Oxidation (M) |
| K.KEEELQAALAR.V | N | 81.72 | 1256.6724 | 11 | 3.0 | 629.3453 | 2 | 48.00 | 6 | 13598 | Ana\_05.raw | 1.52E8 | 1.91E8 | 1.81E8 | 6.35E7 | 1.28E8 | 2.09E8 | 9.44E7 | 1.29E8 | 1.4E8 | 1.62E8 | 43 | 5 | 4 | 4 | 4 | 4 | 5 | 3 | 5 | 5 | 4 | 1081 | 1091 |  |
| K.NLPIYSEEIVEM(+15.99)YK.G | Y | 81.37 | 1742.8436 | 14 | 3.3 | 872.4320 | 2 | 103.73 | 6 | 29239 | Ana\_05.raw | 1.42E7 | 1.25E7 | 1.3E7 |  | 9.37E6 | 9.53E6 | 1.02E6 | 4.16E6 | 2.95E7 |  | 10 | 2 | 1 | 1 | 0 | 2 | 1 | 1 | 1 | 1 | 0 | 126 | 139 | Oxidation (M) |
| K.DLEAHIDTANK.N | Y | 80.85 | 1225.5939 | 11 | 2.1 | 613.8055 | 2 | 36.82 | 3 | 10169 | Ana\_02.raw | 1.29E7 | 1.23E7 | 8.31E6 | 5.57E6 | 9.44E6 | 6.56E6 | 4.31E6 | 5.67E6 | 1.94E6 | 1.03E7 | 11 | 1 | 1 | 1 | 2 | 1 | 1 | 1 | 1 | 1 | 1 | 1621 | 1631 |  |
| K.TDLLLEPYNK.Y | Y | 78.90 | 1204.6339 | 10 | 2.0 | 603.3254 | 2 | 75.48 | 6 | 21638 | Ana\_05.raw | 1.23E7 | 1.26E6 | 7.68E6 | 3.13E6 | 8.09E6 | 6.74E6 | 4.17E6 | 1.67E6 | 2.78E7 | 1.3E7 | 12 | 2 | 1 | 1 | 1 | 1 | 1 | 1 | 1 | 1 | 2 | 290 | 299 |  |
| R.ELEDATETADAM(+15.99)NR.E | Y | 78.70 | 1580.6624 | 14 | 1.4 | 791.3395 | 2 | 36.90 | 3 | 10193 | Ana\_02.raw | 2.86E7 | 2.09E7 | 1.69E7 | 5.64E6 | 1.75E7 | 1.24E7 | 9.53E6 | 1.01E7 | 1.13E7 | 1.66E7 | 10 | 1 | 1 | 1 | 1 | 1 | 1 | 1 | 1 | 1 | 1 | 1899 | 1912 | Oxidation (M) |
| R.QLEEAEEEAQR.A | Y | 78.18 | 1330.6001 | 11 | 2.5 | 666.3090 | 2 | 32.72 | 9 | 8696 | Ana\_08.raw | 6.8E6 |  | 1.58E6 | 1.41E6 | 5.24E5 |  | 5.72E5 | 3.72E6 | 7.44E6 | 2.15E6 | 8 | 1 | 0 | 1 | 1 | 1 | 0 | 1 | 1 | 1 | 1 | 1878 | 1888 |  |
| R.NTDQASM(+15.99)PDNTAAQK.V | N | 77.94 | 1606.6893 | 15 | 0.7 | 804.3525 | 2 | 17.95 | 5 | 4299 | Ana\_04.raw |  |  |  | 1.79E7 | 4.46E7 | 2.32E6 | 2.28E7 | 2.37E7 |  |  | 7 | 0 | 0 | 0 | 1 | 2 | 1 | 1 | 2 | 0 | 0 | 359 | 373 | Oxidation (M) |
| K.IAQLEEQLDNETK.E | Y | 77.00 | 1529.7573 | 13 | -6.4 | 765.8810 | 2 | 63.93 | 3 | 18207 | Ana\_02.raw | 1.36E7 | 1.18E7 | 8.97E6 |  | 5.88E6 | 7E6 | 6.55E5 | 3.34E6 | 2.85E7 | 1.3E7 | 9 | 1 | 1 | 1 | 0 | 1 | 1 | 1 | 1 | 1 | 1 | 1816 | 1828 |  |
| R.AGVLAHLEEER.D | N | 75.88 | 1222.6306 | 11 | 4.9 | 612.3256 | 2 | 56.76 | 1 | 16028 | ANa\_10.raw | 1.1E7 |  | 8.62E6 | 8.53E6 | 4.85E6 | 1.4E7 | 3.79E6 | 9.81E6 | 2.4E7 | 1.23E7 | 14 | 1 | 0 | 2 | 2 | 1 | 2 | 1 | 2 | 1 | 2 | 765 | 775 |  |
| K.KFDQLLAEEK.T | N | 75.17 | 1219.6448 | 10 | 2.0 | 610.8309 | 2 | 53.25 | 3 | 15096 | Ana\_02.raw | 7E7 | 8.1E7 | 7.96E7 | 3.21E7 | 6.01E7 | 7.54E7 | 3.96E7 | 3.99E7 | 1.26E8 | 5.97E7 | 37 | 4 | 4 | 3 | 3 | 4 | 4 | 4 | 3 | 4 | 4 | 1445 | 1454 |  |
| K.VEDM(+15.99)AELTC(+57.02)LNEASVLHNLK.E | N | 73.50 | 2301.0981 | 20 | -0.6 | 768.0396 | 3 | 95.93 | 9 | 27155 | Ana\_08.raw |  |  |  |  |  |  |  |  | 7.55E6 |  | 1 | 0 | 0 | 0 | 0 | 0 | 0 | 0 | 0 | 1 | 0 | 83 | 102 | Oxidation (M); Carbamidomethylation |
| R.NAEQFKDQADK.A | Y | 73.30 | 1292.5996 | 11 | 4.7 | 647.3101 | 2 | 22.30 | 9 | 5388 | Ana\_08.raw | 1.42E7 | 5.39E6 | 4.69E6 |  | 3.05E6 | 5.41E6 | 1.7E6 | 1.1E6 | 1.97E7 | 5.26E6 | 11 | 2 | 1 | 1 | 0 | 1 | 1 | 1 | 1 | 2 | 1 | 1857 | 1867 |  |
| R.VAEFTTNLM(+15.99)EEEEK.S | Y | 73.15 | 1684.7501 | 14 | -0.6 | 843.3818 | 2 | 65.44 | 3 | 18643 | Ana\_02.raw | 1.79E7 | 1.19E7 | 1.18E7 | 5.17E6 | 1.02E7 | 9.79E6 | 7.43E6 | 7.38E6 | 2.12E7 | 1.26E7 | 19 | 2 | 2 | 2 | 1 | 2 | 2 | 2 | 2 | 2 | 2 | 1001 | 1014 | Oxidation (M) |
| R.IIGLDQVAGMSETALPGAFK.T | Y | 72.61 | 2017.0554 | 20 | 6.0 | 1009.5410 | 2 | 128.09 | 1 | 35217 | ANa\_10.raw | 5.69E6 |  |  |  |  |  |  |  | 4.53E6 | 1.44E6 | 3 | 1 | 0 | 0 | 0 | 0 | 0 | 0 | 0 | 1 | 1 | 618 | 637 |  |
| K.FDQLLAEEK.T | N | 71.45 | 1091.5498 | 9 | 2.9 | 546.7838 | 2 | 58.18 | 6 | 16636 | Ana\_05.raw | 3.53E7 | 3.9E7 | 4.13E7 | 1.48E7 | 2.94E7 | 4.16E7 | 2.35E7 | 2.34E7 | 5.36E7 | 2.71E7 | 11 | 1 | 1 | 1 | 1 | 1 | 1 | 1 | 1 | 2 | 1 | 1446 | 1454 |  |
| K.SM(+15.99)EAEM(+15.99)IQLQEELAAAER.A | Y | 70.76 | 2079.9453 | 18 | 9.1 | 1040.9894 | 2 | 91.98 | 9 | 26085 | Ana\_08.raw |  |  |  |  |  |  |  |  | 3.83E5 |  | 1 | 0 | 0 | 0 | 0 | 0 | 0 | 0 | 0 | 1 | 0 | 1677 | 1694 | Oxidation (M) |
| K.HSQAVEELADQLEQTK.R | Y | 70.44 | 1824.8854 | 16 | 4.3 | 609.3050 | 3 | 80.47 | 9 | 22911 | Ana\_08.raw |  | 1.62E6 |  |  |  |  |  |  | 1.42E6 | 2.67E6 | 3 | 0 | 1 | 0 | 0 | 0 | 0 | 0 | 0 | 1 | 1 | 1194 | 1209 |  |
| R.VISGVLQLGNIAFK.K | Y | 70.41 | 1457.8606 | 14 | 2.4 | 729.9393 | 2 | 118.06 | 6 | 32977 | Ana\_05.raw | 1.09E7 | 6.83E6 | 7.8E6 |  | 4.59E6 | 6.26E6 | 4.19E6 |  | 1.78E7 | 1.07E7 | 9 | 1 | 1 | 1 | 0 | 1 | 1 | 1 | 0 | 1 | 2 | 342 | 355 |  |
| K.THEAQIQEM(+15.99)R.Q | Y | 69.57 | 1257.5771 | 10 | 4.5 | 420.2015 | 3 | 20.86 | 9 | 4905 | Ana\_08.raw | 5.26E7 | 1.73E7 |  |  |  |  |  |  | 1.05E7 | 3.28E7 | 6 | 2 | 1 | 0 | 0 | 0 | 0 | 0 | 0 | 1 | 2 | 1182 | 1191 | Oxidation (M) |
| K.ALELDSNLYR.I | Y | 67.37 | 1192.6088 | 10 | 6.1 | 597.3153 | 2 | 75.62 | 6 | 21676 | Ana\_05.raw |  |  |  |  |  | 8.03E6 | 1.77E6 |  |  |  | 2 | 0 | 0 | 0 | 0 | 0 | 1 | 1 | 0 | 0 | 0 | 746 | 755 |  |
| K.EQADFAIEALAK.A | N | 67.21 | 1304.6611 | 12 | 3.3 | 653.3400 | 2 | 90.14 | 6 | 25623 | Ana\_05.raw | 1.03E8 | 1.07E8 | 1.05E8 | 3.27E7 | 6.53E7 | 7.22E7 | 4.35E7 | 5.16E7 | 1.8E8 | 8.13E7 | 42 | 4 | 5 | 4 | 3 | 4 | 4 | 4 | 3 | 6 | 5 | 408 | 419 |  |
| R.DELADEIANSSGK.G | Y | 65.92 | 1347.6154 | 13 | 6.8 | 674.8195 | 2 | 63.49 | 1 | 18002 | ANa\_10.raw | 3.1E6 |  | 2.93E6 | 2.39E6 | 2.67E6 |  | 2.15E6 |  | 1.25E7 | 1.96E6 | 7 | 1 | 0 | 1 | 1 | 1 | 0 | 1 | 0 | 1 | 1 | 1704 | 1716 |  |
| K.NLPIYSEEIVEMYK.G | Y | 65.43 | 1726.8488 | 14 | 2.2 | 864.4336 | 2 | 113.42 | 9 | 31754 | Ana\_08.raw |  |  |  |  |  |  |  |  | 4.11E6 |  | 1 | 0 | 0 | 0 | 0 | 0 | 0 | 0 | 0 | 1 | 0 | 126 | 139 |  |
| R.HEM(+15.99)PPHIYAITDTAYR.S | Y | 64.78 | 1929.9043 | 16 | 6.7 | 644.3130 | 3 | 59.49 | 9 | 16908 | Ana\_08.raw | 4.73E6 |  |  |  |  |  |  |  | 7.13E6 | 5.22E6 | 3 | 1 | 0 | 0 | 0 | 0 | 0 | 0 | 0 | 1 | 1 | 144 | 159 | Oxidation (M) |
| R.RGDLPFVVTR.R | Y | 63.70 | 1158.6509 | 10 | -0.5 | 387.2240 | 3 | 63.04 | 3 | 17953 | Ana\_02.raw | 1.66E7 | 1.44E7 | 1.6E7 | 1.16E6 | 1.37E7 | 9.06E6 | 7.39E6 | 8.83E6 | 2.82E7 | 2.17E7 | 23 | 2 | 3 | 3 | 1 | 2 | 2 | 2 | 2 | 3 | 3 | 1923 | 1932 |  |
| R.DLGEELEALK.T | N | 61.89 | 1115.5709 | 10 | 3.2 | 558.7945 | 2 | 97.87 | 6 | 27687 | Ana\_05.raw | 4.23E7 | 3.48E7 | 5.12E7 | 1.32E7 | 3.52E7 | 3.93E7 | 1.91E7 | 2.39E7 | 5.83E7 | 2.24E7 | 27 | 3 | 2 | 3 | 2 | 3 | 2 | 2 | 4 | 4 | 2 | 1136 | 1145 |  |
| R.HEDELLAK.E | Y | 60.60 | 953.4818 | 8 | 1.1 | 477.7487 | 2 | 23.33 | 3 | 6029 | Ana\_02.raw | 1.25E7 |  | 2.37E7 | 1.1E7 | 1.5E7 | 2.09E7 | 1.49E7 | 1.16E7 | 2.98E7 | 2.74E7 | 9 | 1 | 0 | 1 | 1 | 1 | 1 | 1 | 1 | 1 | 1 | 843 | 850 |  |
| R.VVFQEFR.Q | Y | 60.05 | 923.4865 | 7 | 4.1 | 462.7524 | 2 | 63.53 | 1 | 18010 | ANa\_10.raw | 3.45E7 | 2.63E7 | 3.07E7 | 1.54E7 | 2.31E7 | 2.09E7 | 1.86E7 | 1.62E7 | 5E7 | 3.68E7 | 21 | 2 | 3 | 2 | 1 | 2 | 2 | 2 | 2 | 3 | 2 | 712 | 718 |  |
| K.RQLEEAEEEAQR.A | Y | 60.01 | 1486.7012 | 12 | 4.7 | 744.3613 | 2 | 30.06 | 9 | 7893 | Ana\_08.raw |  |  |  |  |  |  |  |  | 2.87E6 |  | 1 | 0 | 0 | 0 | 0 | 0 | 0 | 0 | 0 | 1 | 0 | 1877 | 1888 |  |
| K.KVEAQLQELQVK.F | Y | 59.08 | 1411.8035 | 12 | 5.2 | 706.9127 | 2 | 52.25 | 9 | 14738 | Ana\_08.raw |  |  |  |  |  |  |  |  | 2.5E6 |  | 1 | 0 | 0 | 0 | 0 | 0 | 0 | 0 | 0 | 1 | 0 | 1249 | 1260 |  |
| R.EM(+15.99)EAELEDERK.Q | Y | 57.47 | 1393.6030 | 11 | 2.6 | 697.8106 | 2 | 25.53 | 1 | 6597 | ANa\_10.raw | 2.45E6 | 3.08E6 | 9.22E6 | 2.07E6 | 6.34E6 |  | 5.42E6 | 3.79E6 | 5.93E6 | 2.45E6 | 13 | 1 | 1 | 2 | 1 | 2 | 0 | 2 | 2 | 1 | 1 | 1593 | 1603 | Oxidation (M) |
| R.EM(+15.99)EAELEDER.K | Y | 56.28 | 1265.5082 | 10 | 4.8 | 633.7644 | 2 | 35.76 | 7 | 9725 | Ana\_06.raw | 1.12E7 | 4.2E6 | 5.71E6 | 2.37E6 | 6.53E6 | 4.43E6 | 6.01E6 | 2.95E6 | 3.04E6 | 7.41E6 | 12 | 1 | 1 | 1 | 1 | 1 | 1 | 2 | 2 | 1 | 1 | 1593 | 1602 | Oxidation (M) |
| R.EQLEEEEEAKR.N | Y | 55.12 | 1388.6418 | 11 | 3.4 | 463.8895 | 3 | 29.65 | 7 | 7863 | Ana\_06.raw |  | 2.99E7 |  | 4.78E7 | 9.12E6 | 2.23E7 | 1.72E7 | 1.49E7 | 5.2E7 |  | 10 | 0 | 1 | 0 | 2 | 1 | 1 | 2 | 1 | 2 | 0 | 1343 | 1353 |  |
| R.VISGVLQLGNIAFKK.E | Y | 54.49 | 1585.9556 | 15 | 5.3 | 529.6619 | 3 | 103.45 | 6 | 29165 | Ana\_05.raw |  |  |  | 3.44E5 | 2.49E6 | 1.64E6 | 8.62E5 | 2.02E5 | 2.16E6 |  | 6 | 0 | 0 | 0 | 1 | 1 | 1 | 1 | 1 | 1 | 0 | 342 | 356 |  |
| R.EEILAQAK.E | Y | 53.24 | 900.4916 | 8 | 3.2 | 451.2545 | 2 | 33.33 | 6 | 9124 | Ana\_05.raw | 2.31E7 | 2.02E7 |  | 8.66E6 | 2.03E7 | 1.55E7 | 1.46E7 | 1.21E7 | 1.61E7 | 2.31E7 | 10 | 2 | 1 | 0 | 1 | 1 | 1 | 1 | 1 | 1 | 1 | 1662 | 1669 |  |
| R.C(+57.02)IIPNHEK.K | N | 51.44 | 1009.5015 | 8 | 3.8 | 505.7599 | 2 | 28.00 | 5 | 7477 | Ana\_04.raw | 3.6E6 | 5.27E6 | 2.95E6 |  | 3.18E6 | 3.44E6 | 0 | 1.91E6 |  |  | 7 | 1 | 1 | 1 | 0 | 1 | 1 | 1 | 1 | 0 | 0 | 671 | 678 | Carbamidomethylation |
| K.RQQQLTAM(+15.99)K.V | N | 51.23 | 1118.5867 | 9 | 3.8 | 560.3027 | 2 | 17.54 | 6 | 4110 | Ana\_05.raw |  |  |  | 3.15E6 | 9.88E6 | 3.99E6 | 6.24E6 | 6.76E6 |  |  | 5 | 0 | 0 | 0 | 1 | 1 | 1 | 1 | 1 | 0 | 0 | 802 | 810 | Oxidation (M) |
| K.ASIAALEAK.I | Y | 51.00 | 872.4967 | 9 | 1.5 | 437.2563 | 2 | 42.27 | 3 | 11845 | Ana\_02.raw | 1.31E7 | 9.35E6 | 1.35E7 | 5.28E6 | 1.06E7 |  | 7.94E6 | 6.44E6 | 2.05E7 | 1.58E7 | 9 | 1 | 1 | 1 | 1 | 1 | 0 | 1 | 1 | 1 | 1 | 1807 | 1815 |  |
| K.VEAQLQELQVK.F | Y | 50.61 | 1283.7085 | 11 | 6.6 | 642.8658 | 2 | 64.33 | 1 | 18234 | ANa\_10.raw | 4.3E6 |  |  | 8.38E5 | 1.45E6 | 7.05E5 | 1.68E6 |  |  | 5.03E6 | 6 | 1 | 0 | 0 | 1 | 1 | 1 | 1 | 0 | 0 | 1 | 1250 | 1260 |  |
| K.VIQYLAHVASSHK.S | N | 49.69 | 1451.7885 | 13 | 1.2 | 484.9373 | 3 | 48.09 | 3 | 13589 | Ana\_02.raw | 2.84E6 |  | 3.39E6 |  |  |  |  |  |  | 4.06E6 | 3 | 1 | 0 | 1 | 0 | 0 | 0 | 0 | 0 | 0 | 1 | 187 | 199 |  |
| R.LRNWQWWRLFTK.V | N | 48.91 | 1732.9314 | 12 | 3.0 | 578.6528 | 3 | 117.69 | 6 | 32880 | Ana\_05.raw |  |  |  |  |  | 1.07E7 |  |  |  |  | 2 | 0 | 0 | 0 | 0 | 0 | 2 | 0 | 0 | 0 | 0 | 822 | 833 |  |
| R.ALEEAM(+15.99)EQK.A | Y | 47.39 | 1063.4856 | 9 | 3.2 | 532.7518 | 2 | 21.37 | 7 | 5332 | Ana\_06.raw |  |  |  | 6.49E6 |  |  | 1.16E7 | 1.01E7 |  |  | 3 | 0 | 0 | 0 | 1 | 0 | 0 | 1 | 1 | 0 | 0 | 1484 | 1492 | Oxidation (M) |
| K.SVHELEK.S | Y | 47.37 | 840.4341 | 7 | 1.7 | 421.2250 | 2 | 17.99 | 4 | 4279 | Ana\_03.raw |  |  |  | 4.88E6 |  |  |  |  |  |  | 1 | 0 | 0 | 0 | 1 | 0 | 0 | 0 | 0 | 0 | 0 | 1519 | 1525 |  |
| R.NC(+57.02)AAYLR.L | Y | 47.08 | 866.4069 | 7 | 1.6 | 434.2114 | 2 | 31.34 | 6 | 8540 | Ana\_05.raw | 1.13E7 | 8.55E6 | 1.01E7 | 6.19E6 | 1.09E7 | 4.4E6 | 4.45E6 | 6.58E6 | 1.2E7 | 1.26E7 | 10 | 1 | 1 | 1 | 1 | 1 | 1 | 1 | 1 | 1 | 1 | 815 | 821 | Carbamidomethylation |
| K.LQEM(+15.99)ESAVK.S | Y | 46.49 | 1049.5063 | 9 | 5.8 | 525.7635 | 2 | 22.88 | 2 | 5710 | Ana\_01.raw |  | 6.99E6 | 1.63E7 | 6.81E6 | 4.83E6 | 1.44E7 | 3.78E6 |  |  | 1.79E7 | 7 | 0 | 1 | 1 | 1 | 1 | 1 | 1 | 0 | 0 | 1 | 1794 | 1802 | Oxidation (M) |
| K.VAAYDKLEK.T | Y | 45.16 | 1035.5601 | 9 | 4.1 | 518.7894 | 2 | 27.79 | 9 | 7218 | Ana\_08.raw |  |  |  |  |  |  |  |  | 6.87E6 | 1.01E7 | 3 | 0 | 0 | 0 | 0 | 0 | 0 | 0 | 0 | 1 | 2 | 1405 | 1413 |  |
| K.HEAM(+15.99)ITDLEER.L | Y | 43.79 | 1358.6136 | 11 | 6.5 | 680.3185 | 2 | 42.92 | 9 | 11877 | Ana\_08.raw |  |  |  |  |  |  |  |  | 2.34E6 | 0 | 2 | 0 | 0 | 0 | 0 | 0 | 0 | 0 | 0 | 1 | 1 | 1025 | 1035 | Oxidation (M) |
| R.QGFPNR.V | N | 43.46 | 717.3558 | 6 | 0.9 | 359.6855 | 2 | 22.62 | 3 | 5802 | Ana\_02.raw |  | 4.83E6 | 1.13E7 | 7.76E6 |  | 1.82E7 | 7.99E6 | 1.36E7 |  |  | 6 | 0 | 1 | 1 | 1 | 0 | 1 | 1 | 1 | 0 | 0 | 706 | 711 |  |
| R.NTNPNFVR.C | N | 43.08 | 960.4777 | 8 | 5.1 | 481.2486 | 2 | 29.96 | 10 | 8058 | Ana\_09.raw | 4.19E7 |  | 2.88E7 | 1.24E7 |  |  | 1.83E7 | 1.6E7 | 4.87E7 | 3.61E7 | 8 | 1 | 0 | 1 | 1 | 0 | 0 | 1 | 2 | 1 | 1 | 663 | 670 |  |
| K.LEGDSTDLSDQIAELQAQIAELK.M | Y | 43.06 | 2486.2388 | 23 | 4.6 | 829.7573 | 3 | 133.87 | 1 | 36803 | ANa\_10.raw | 1E6 |  |  |  |  |  |  |  |  |  | 1 | 1 | 0 | 0 | 0 | 0 | 0 | 0 | 0 | 0 | 0 | 1053 | 1075 |  |
| R.GDLPFVVTR.R | Y | 42.85 | 1002.5498 | 9 | -0.4 | 502.2820 | 2 | 80.91 | 4 | 22461 | Ana\_03.raw | 3.53E5 | 1.92E5 |  | 1.2E6 | 1.77E6 | 9.47E5 | 1.13E6 | 7.46E5 | 6.85E6 | 2.44E5 | 9 | 1 | 1 | 0 | 1 | 1 | 1 | 1 | 1 | 1 | 1 | 1924 | 1932 |  |
| K.DLEGLSQR.L | Y | 42.69 | 916.4614 | 8 | 4.6 | 459.2401 | 2 | 43.31 | 6 | 12177 | Ana\_05.raw |  |  | 1.72E7 | 7.67E6 | 1.39E7 | 1.42E7 |  | 1.16E7 | 2.5E7 | 0 | 8 | 0 | 0 | 1 | 2 | 1 | 1 | 0 | 1 | 1 | 1 | 1393 | 1400 |  |
| K.ADFC(+57.02)IIHYAGK.V | N | 42.52 | 1293.6176 | 11 | 0.7 | 432.2134 | 3 | 70.59 | 3 | 20057 | Ana\_02.raw | 5.04E5 | 1.28E6 | 6.1E5 | 8.67E5 | 3.82E5 | 9.18E5 | 4.85E5 |  | 0 | 1.79E6 | 9 | 1 | 1 | 1 | 1 | 1 | 1 | 1 | 0 | 1 | 1 | 566 | 576 | Carbamidomethylation |
| R.GELANEVK.A | Y | 42.10 | 858.4446 | 8 | 2.8 | 430.2308 | 2 | 28.18 | 7 | 7412 | Ana\_06.raw | 1.08E7 |  |  |  | 2.85E7 | 2.16E7 | 1.61E7 | 1.38E7 | 9.27E6 |  | 6 | 1 | 0 | 0 | 0 | 1 | 1 | 1 | 1 | 1 | 0 | 1227 | 1234 |  |
| R.KLQAQMK.D | N | 41.48 | 845.4793 | 7 | 4.2 | 423.7487 | 2 | 18.45 | 8 | 4358 | Ana\_07.raw |  |  |  | 1.72E5 |  |  |  | 5.1E5 |  |  | 2 | 0 | 0 | 0 | 1 | 0 | 0 | 0 | 1 | 0 | 0 | 1642 | 1648 |  |
| R.YEILTPNSIPK.G | Y | 40.95 | 1273.6918 | 11 | 5.0 | 637.8563 | 2 | 79.95 | 10 | 22527 | Ana\_09.raw |  |  |  |  |  | 6.15E5 |  |  | 3.57E6 | 9.96E5 | 3 | 0 | 0 | 0 | 0 | 0 | 1 | 0 | 0 | 1 | 1 | 721 | 731 |  |
| K.LVWVPSSK.N | Y | 40.93 | 914.5225 | 8 | 7.3 | 458.2719 | 2 | 57.50 | 9 | 16304 | Ana\_08.raw |  |  |  |  |  |  |  |  | 4.14E6 |  | 1 | 0 | 0 | 0 | 0 | 0 | 0 | 0 | 0 | 1 | 0 | 31 | 38 |  |
| R.EQEVSILK.K | Y | 39.61 | 944.5178 | 8 | 4.6 | 473.2684 | 2 | 48.88 | 8 | 13492 | Ana\_07.raw |  |  | 1.88E7 | 7.04E6 | 1.39E7 | 1.09E7 | 9.91E6 | 1.09E7 |  |  | 6 | 0 | 0 | 1 | 1 | 1 | 1 | 1 | 1 | 0 | 0 | 1166 | 1173 |  |
| K.GALALEEK.R | Y | 39.12 | 829.4545 | 8 | 5.9 | 415.7369 | 2 | 35.57 | 10 | 9702 | Ana\_09.raw |  | 1.43E7 |  |  | 1.23E7 | 1.28E7 | 1.07E7 | 1E7 | 1.82E7 | 1.71E7 | 7 | 0 | 1 | 0 | 0 | 1 | 1 | 1 | 1 | 1 | 1 | 1717 | 1724 |  |
| R.KKLEM(+15.99)DLK.D | Y | 38.86 | 1019.5685 | 8 | 5.4 | 510.7943 | 2 | 19.30 | 1 | 4532 | ANa\_10.raw | 0 |  |  |  |  |  |  |  |  |  | 1 | 1 | 0 | 0 | 0 | 0 | 0 | 0 | 0 | 0 | 0 | 1613 | 1620 | Oxidation (M) |
| R.IM(+15.99)GIPEDEQM(+15.99)GLLR.V | Y | 38.74 | 1632.7852 | 14 | 5.7 | 817.4045 | 2 | 74.93 | 9 | 21353 | Ana\_08.raw |  | 5.01E6 | 1.71E6 |  |  |  |  |  | 1.29E6 | 3.04E6 | 4 | 0 | 1 | 1 | 0 | 0 | 0 | 0 | 0 | 1 | 1 | 328 | 341 | Oxidation (M) |
| R.TVGQLYK.E | N | 38.67 | 807.4490 | 7 | 2.6 | 404.7328 | 2 | 33.40 | 4 | 8890 | Ana\_03.raw | 3.54E6 | 1.11E8 | 1.2E8 | 4.55E7 | 8.98E7 | 1.13E8 | 7.15E7 | 6.55E7 | 1.02E8 | 6.97E7 | 17 | 1 | 1 | 2 | 1 | 3 | 2 | 2 | 3 | 1 | 1 | 645 | 651 |  |
| R.TEM(+15.99)EDLM(+15.99)SSK.D | Y | 38.03 | 1201.4843 | 10 | 4.9 | 601.7523 | 2 | 22.60 | 8 | 5627 | Ana\_07.raw |  |  |  | 3.32E6 |  |  |  | 4.34E6 |  |  | 2 | 0 | 0 | 0 | 1 | 0 | 0 | 0 | 1 | 0 | 0 | 1504 | 1513 | Oxidation (M) |
| K.YLYVDK.N | Y | 36.99 | 799.4116 | 6 | 4.6 | 400.7149 | 2 | 39.51 | 10 | 10909 | Ana\_09.raw | 1.17E7 |  |  | 3.58E6 |  | 9.86E6 | 6.73E6 |  | 9.18E6 | 1.24E7 | 6 | 1 | 0 | 0 | 1 | 0 | 1 | 1 | 0 | 1 | 1 | 9 | 14 |  |
| K.DVLLQVEDERR.N | Y | 36.08 | 1370.7153 | 11 | 4.8 | 457.9146 | 3 | 60.91 | 9 | 17326 | Ana\_08.raw |  |  |  |  |  |  |  |  | 1.01E6 |  | 1 | 0 | 0 | 0 | 0 | 0 | 0 | 0 | 0 | 1 | 0 | 1846 | 1856 |  |
| K.ALSLAR.A | N | 35.77 | 629.3860 | 6 | 2.7 | 315.7011 | 2 | 31.28 | 6 | 8518 | Ana\_05.raw |  |  |  |  |  | 3.8E7 | 2.13E7 | 1.28E7 |  | 1.54E7 | 4 | 0 | 0 | 0 | 0 | 0 | 1 | 1 | 1 | 0 | 1 | 1478 | 1483 |  |
| K.LKDVLLQVEDERR.N | Y | 34.22 | 1611.8944 | 13 | 6.3 | 538.3088 | 3 | 61.69 | 9 | 17550 | Ana\_08.raw |  |  |  |  |  |  |  |  | 1.02E6 |  | 1 | 0 | 0 | 0 | 0 | 0 | 0 | 0 | 0 | 1 | 0 | 1844 | 1856 |  |
| K.VKPLLNSIR.H | Y | 33.45 | 1038.6549 | 9 | 5.8 | 347.2276 | 3 | 41.00 | 9 | 11295 | Ana\_08.raw |  |  |  |  |  |  |  |  | 2.08E6 |  | 1 | 0 | 0 | 0 | 0 | 0 | 0 | 0 | 0 | 1 | 0 | 834 | 842 |  |
| R.LTEM(+15.99)ETM(+15.99)QSQLM(+15.99)AEK.L | Y | 33.22 | 1816.7893 | 15 | 4.2 | 909.4058 | 2 | 39.06 | 9 | 10683 | Ana\_08.raw |  |  |  |  |  |  | 4.68E5 |  | 1.83E6 |  | 2 | 0 | 0 | 0 | 0 | 0 | 0 | 1 | 0 | 1 | 0 | 868 | 882 | Oxidation (M) |
| K.KLEM(+15.99)DLK.D | Y | 33.13 | 891.4735 | 7 | 2.8 | 446.7453 | 2 | 21.93 | 9 | 5265 | Ana\_08.raw | 9.02E5 | 1.83E6 |  |  |  | 2.55E6 |  |  | 3.7E6 | 6.2E6 | 5 | 1 | 1 | 0 | 0 | 0 | 1 | 0 | 0 | 1 | 1 | 1614 | 1620 | Oxidation (M) |
| R.NWQWWR.L | N | 29.97 | 974.4511 | 6 | 5.4 | 488.2355 | 2 | 98.83 | 8 | 27494 | Ana\_07.raw |  |  |  |  |  |  |  | 8.23E5 |  |  | 1 | 0 | 0 | 0 | 0 | 0 | 0 | 0 | 1 | 0 | 0 | 824 | 829 |  |
| K.LM(+15.99)ATLR.N | N | 29.79 | 719.4000 | 6 | 2.6 | 360.7082 | 2 | 25.75 | 9 | 6555 | Ana\_08.raw |  |  |  |  |  |  |  |  | 6.95E6 |  | 1 | 0 | 0 | 0 | 0 | 0 | 0 | 0 | 0 | 1 | 0 | 657 | 662 | Oxidation (M) |
| K.LSLSTK.L | Y | 28.92 | 647.3854 | 6 | 4.1 | 324.7013 | 2 | 27.29 | 10 | 7228 | Ana\_09.raw | 2.77E6 |  |  |  | 3.02E6 | 2.9E6 |  | 1.65E6 |  | 2.36E6 | 5 | 1 | 0 | 0 | 0 | 1 | 1 | 0 | 1 | 0 | 1 | 1325 | 1330 |  |
| K.LQLEK.V | N | 26.40 | 629.3748 | 5 | 5.2 | 315.6963 | 2 | 24.82 | 10 | 6436 | Ana\_09.raw |  |  | 5.13E7 |  |  |  |  |  |  | 3.11E7 | 2 | 0 | 0 | 1 | 0 | 0 | 0 | 0 | 0 | 0 | 1 | 962 | 966 |  |
| R.ALEQQVEEM(+15.99)K.T | Y | 26.07 | 1219.5754 | 10 | 3.1 | 610.7969 | 2 | 34.52 | 3 | 9469 | Ana\_02.raw |  |  | 7.75E6 |  |  |  |  |  |  |  | 1 | 0 | 0 | 1 | 0 | 0 | 0 | 0 | 0 | 0 | 0 | 1529 | 1538 | Oxidation (M) |
| R.LRNWQWWR.L | N | 23.70 | 1243.6362 | 8 | 2.7 | 415.5538 | 3 | 88.09 | 6 | 25077 | Ana\_05.raw |  |  |  |  |  | 1.89E6 |  |  |  |  | 1 | 0 | 0 | 0 | 0 | 0 | 1 | 0 | 0 | 0 | 0 | 822 | 829 |  |
| total 97 peptides |  |  |  |  |  |  |  |  |  |  |  |  |  |  |  |  |  |  |  |  |  |  |  |  |  |  |  |  |  |  |  |  |  |  |  |
| --- | --- | --- | --- | --- | --- | --- | --- | --- | --- | --- | --- | --- | --- | --- | --- | --- | --- | --- | --- | --- | --- | --- | --- | --- | --- | --- | --- | --- | --- | --- | --- | --- | --- | --- | --- |

Best Unique PSM (Scan F3:35591, m/z=1247.0923, z=2, RT=129.90, ppm=1.3):

sp|P07724|ALBU\_MOUSE

back to list

  

| Protein Coverage
| Supporting Peptides
| Best Unique PSM
|

Protein Coverage:

Supporting Peptides:

| Peptide | Uniq | -10lgP | Mass | Length | ppm | m/z | z | RT | Fraction | Scan | Source File | Area 10 | Area 01 | Area 02 | Area 03 | Area 04 | Area 05 | Area 06 | Area 07 | Area 08 | Area 09 | #Spec | #Spec 10 | #Spec 01 | #Spec 02 | #Spec 03 | #Spec 04 | #Spec 05 | #Spec 06 | #Spec 07 | #Spec 08 | #Spec 09 | Start | End | PTM |
| --- | --- | --- | --- | --- | --- | --- | --- | --- | --- | --- | --- | --- | --- | --- | --- | --- | --- | --- | --- | --- | --- | --- | --- | --- | --- | --- | --- | --- | --- | --- | --- | --- | --- | --- | --- |
| R.RHPYFYAPELLYYAEQYNEILTQC(+57.02)C(+57.02)AEADKESC(+57.02)LTPK.L | Y | 150.88 | 4570.0918 | 37 | 1.3 | 1143.5317 | 4 | 131.93 | 5 | 36311 | Ana\_04.raw |  | 2.28E6 | 1.42E7 | 8.97E6 | 1.58E7 | 2.83E7 | 2.2E7 |  |  |  | 6 | 0 | 1 | 1 | 1 | 1 | 1 | 1 | 0 | 0 | 0 | 169 | 205 | Carbamidomethylation |
| R.HPYFYAPELLYYAEQYNEILTQC(+57.02)C(+57.02)AEADKESC(+57.02)LTPK.L | Y | 146.91 | 4413.9907 | 36 | 0.9 | 1104.5060 | 4 | 133.33 | 3 | 36506 | Ana\_02.raw | 7.02E6 | 1.03E7 | 3.29E7 | 2.28E6 | 2.44E7 | 2.28E7 | 8.45E6 | 2.83E6 | 8.19E6 | 5.17E6 | 12 | 1 | 1 | 2 | 1 | 2 | 1 | 1 | 1 | 1 | 1 | 170 | 205 | Carbamidomethylation |
| K.AHC(+57.02)LSEVEHDTM(+15.99)PADLPAIAADFVEDQEVC(+57.02)K.N | Y | 139.54 | 3512.5535 | 31 | 1.1 | 1171.8597 | 3 | 123.20 | 3 | 33852 | Ana\_02.raw | 4.67E7 | 5.12E7 | 2.09E8 | 1.96E7 | 2.58E7 | 2.59E8 | 9.93E7 | 1.8E8 | 9.48E7 | 1.06E8 | 57 | 5 | 7 | 8 | 4 | 8 | 6 | 5 | 6 | 3 | 5 | 311 | 341 | Carbamidomethylation; Oxidation (M) |
| K.AADKDTC(+57.02)FSTEGPNLVTR.C | Y | 120.92 | 1980.9210 | 18 | 0.9 | 991.4686 | 2 | 61.01 | 3 | 17369 | Ana\_02.raw | 2.18E8 | 3.79E8 | 3.8E8 | 1.5E8 | 2.06E8 | 2.84E8 | 1.79E8 | 2.48E8 | 2.21E8 | 2.01E8 | 76 | 8 | 10 | 9 | 6 | 7 | 6 | 6 | 8 | 8 | 8 | 585 | 602 | Carbamidomethylation |
| R.RPC(+57.02)FSALTVDETYVPK.E | Y | 119.56 | 1881.9294 | 16 | 2.1 | 628.3184 | 3 | 84.95 | 3 | 23940 | Ana\_02.raw | 9.11E7 | 1.68E8 | 1.86E8 | 7.98E7 | 3.38E7 | 1.77E8 | 1.11E8 | 1.22E8 | 1.38E8 | 8.76E7 | 68 | 6 | 9 | 8 | 6 | 6 | 7 | 6 | 8 | 5 | 7 | 509 | 524 | Carbamidomethylation |
| K.APQVSTPTLVEAAR.N | Y | 116.16 | 1438.7780 | 14 | 0.7 | 720.3968 | 2 | 64.01 | 3 | 18232 | Ana\_02.raw | 2.73E8 | 4.61E8 | 4.13E8 | 1.27E8 | 1.26E7 | 2.8E8 | 1.82E8 | 2.45E8 | 2.99E8 | 2.39E8 | 76 | 7 | 10 | 9 | 4 | 7 | 8 | 5 | 10 | 8 | 8 | 439 | 452 |  |
| K.C(+57.02)C(+57.02)AEANPPAC(+57.02)YGTVLAEFQPLVEEPK.N | Y | 110.89 | 2949.3347 | 26 | -0.9 | 984.1179 | 3 | 128.11 | 3 | 35112 | Ana\_02.raw | 1.5E7 | 3.57E7 | 2.51E7 | 1.95E7 | 4.16E7 | 4.09E7 | 3.85E7 | 3.63E7 | 2.05E7 | 1.42E6 | 30 | 1 | 4 | 2 | 5 | 4 | 3 | 4 | 4 | 2 | 1 | 384 | 409 | Carbamidomethylation |
| R.RHPYFYAPELLYYAEQYNEILTQC(+57.02)C(+57.02)AEADK.E | Y | 110.22 | 3754.7070 | 30 | 1.3 | 939.6852 | 4 | 132.45 | 5 | 36446 | Ana\_04.raw |  |  | 5.69E6 | 5.58E6 | 6.01E6 | 7.61E6 | 4.91E6 |  |  |  | 5 | 0 | 0 | 1 | 1 | 1 | 1 | 1 | 0 | 0 | 0 | 169 | 198 | Carbamidomethylation |
| K.LGEYGFQNAILVR.Y | Y | 106.95 | 1478.7881 | 13 | 4.6 | 740.4047 | 2 | 98.49 | 1 | 27574 | ANa\_10.raw | 2.49E8 | 4.34E8 | 3.39E8 | 1.48E8 | 2.02E8 | 3.18E8 | 1.75E8 | 2.14E8 | 3.46E8 | 2.44E8 | 84 | 7 | 10 | 13 | 8 | 7 | 8 | 6 | 11 | 7 | 7 | 422 | 434 |  |
| R.LPC(+57.02)VEDYLSAILNR.V | Y | 104.03 | 1661.8446 | 14 | -4.3 | 831.9260 | 2 | 140.08 | 7 | 38461 | Ana\_06.raw | 2.18E8 | 4.96E8 | 6.39E8 | 3.53E7 | 5.49E7 | 5.61E8 | 2.78E8 | 1.3E8 | 1.99E8 | 2.42E8 | 271 | 28 | 34 | 34 | 23 | 25 | 27 | 22 | 23 | 28 | 27 | 470 | 483 | Carbamidomethylation |
| K.TVM(+15.99)DDFAQFLDTC(+57.02)C(+57.02)K.A | Y | 103.37 | 1865.7634 | 15 | -0.1 | 933.8889 | 2 | 110.72 | 3 | 30653 | Ana\_02.raw | 4.9E7 | 1.3E8 | 1.2E8 | 3.14E7 | 5.64E7 | 6.13E7 | 1.6E7 | 6.13E7 | 6.58E7 | 2.04E7 | 69 | 7 | 9 | 10 | 6 | 7 | 7 | 6 | 8 | 5 | 4 | 570 | 584 | Oxidation (M); Carbamidomethylation |
| K.AETFTFHSDIC(+57.02)TLPEK.E | Y | 100.89 | 1894.8771 | 16 | 4.0 | 948.4496 | 2 | 82.51 | 2 | 23399 | Ana\_01.raw | 4.33E7 | 8.43E7 | 6.67E7 | 5.26E7 | 5.47E7 | 8.85E7 | 4.84E7 | 7.3E7 | 4.81E7 | 4.24E7 | 37 | 3 | 5 | 4 | 3 | 3 | 4 | 3 | 6 | 3 | 3 | 528 | 543 | Carbamidomethylation |
| K.DVFLGTFLYEYSR.R | Y | 100.87 | 1608.7823 | 13 | 3.5 | 805.4012 | 2 | 131.11 | 6 | 36427 | Ana\_05.raw | 4.11E7 | 5.79E6 | 1.26E8 | 1.21E8 | 9.5E7 | 8.46E7 | 1.04E8 | 7.93E7 | 3.38E7 | 4.92E7 | 86 | 3 | 7 | 14 | 14 | 15 | 4 | 5 | 12 | 5 | 7 | 348 | 360 |  |
| R.LSQTFPNADFAEITK.L | Y | 100.29 | 1680.8358 | 15 | 1.1 | 841.4261 | 2 | 95.76 | 3 | 26788 | Ana\_02.raw | 4.1E8 | 7.45E8 | 6.46E8 | 1.73E8 | 3.04E8 | 4.55E8 | 2.92E8 | 3.66E8 | 4.12E8 | 3.26E8 | 85 | 8 | 12 | 13 | 6 | 8 | 6 | 6 | 12 | 6 | 8 | 243 | 257 |  |
| K.ENPTTFM(+15.99)GHYLHEVAR.R | Y | 99.38 | 1916.8839 | 16 | 2.5 | 480.2294 | 4 | 61.52 | 6 | 17626 | Ana\_05.raw | 8.9E7 | 1.11E8 | 8.81E7 | 3.59E7 | 3.05E7 | 1.09E8 | 6E7 | 7.69E7 | 3.97E7 | 4.09E7 | 71 | 10 | 7 | 5 | 7 | 5 | 10 | 7 | 9 | 5 | 6 | 153 | 168 | Oxidation (M) |
| K.KAHC(+57.02)LSEVEHDTM(+15.99)PADLPAIAADFVEDQEVC(+57.02)K.N | Y | 96.72 | 3640.6482 | 32 | 2.2 | 911.1713 | 4 | 118.72 | 6 | 33156 | Ana\_05.raw |  | 1.06E7 |  | 1.92E6 | 1.58E6 | 2.44E7 | 4.14E6 | 4.82E6 |  | 7.2E6 | 10 | 0 | 2 | 0 | 1 | 1 | 2 | 2 | 1 | 0 | 1 | 310 | 341 | Carbamidomethylation; Oxidation (M) |
| K.SLHTLFGDKLC(+57.02)AIPNLR.E | Y | 95.36 | 1954.0458 | 17 | 1.7 | 489.5196 | 4 | 102.43 | 6 | 28897 | Ana\_05.raw |  | 2.3E6 | 0 | 1.59E6 | 2.38E6 | 1.38E7 | 3.95E5 |  | 1.84E6 |  | 9 | 0 | 2 | 1 | 1 | 1 | 2 | 1 | 0 | 1 | 0 | 89 | 105 | Carbamidomethylation |
| K.TVMDDFAQFLDTC(+57.02)C(+57.02)K.A | Y | 95.25 | 1849.7684 | 15 | 1.1 | 925.8925 | 2 | 128.97 | 3 | 35338 | Ana\_02.raw | 1.68E7 | 2.29E7 | 4.22E6 | 7.08E6 | 2.26E6 | 3.11E7 | 7.08E6 | 6.7E6 | 2.67E7 | 2.35E7 | 18 | 2 | 2 | 1 | 2 | 1 | 2 | 2 | 2 | 2 | 2 | 570 | 584 | Carbamidomethylation |
| K.DTC(+57.02)FSTEGPNLVTR.C | Y | 94.78 | 1595.7250 | 14 | -0.5 | 798.8694 | 2 | 70.00 | 3 | 19896 | Ana\_02.raw | 7.37E6 | 1.61E7 | 9.92E6 | 2.42E6 | 5.26E6 | 1.1E7 | 4.7E6 | 6.24E6 | 2.98E6 | 3.4E6 | 10 | 1 | 1 | 1 | 1 | 1 | 1 | 1 | 1 | 1 | 1 | 589 | 602 | Carbamidomethylation |
| K.EC(+57.02)C(+57.02)HGDLLEC(+57.02)ADDRAELAK.Y | Y | 94.12 | 2260.9512 | 19 | 5.2 | 754.6616 | 3 | 67.06 | 7 | 19029 | Ana\_06.raw | 6.51E6 | 2.94E7 | 1.62E7 | 9.01E6 | 3.37E6 | 2.03E7 | 1.03E7 | 8.92E6 | 1.09E7 | 7.77E6 | 17 | 1 | 2 | 2 | 2 | 1 | 2 | 2 | 1 | 2 | 2 | 268 | 286 | Carbamidomethylation |
| K.EC(+57.02)C(+57.02)HGDLLEC(+57.02)ADDR.A | Y | 92.39 | 1748.6553 | 14 | 1.6 | 875.3363 | 2 | 51.51 | 4 | 14228 | Ana\_03.raw | 2.37E7 | 6.56E7 | 7.62E7 | 2.45E7 | 4.28E7 | 6.7E7 | 4.04E7 | 4.15E7 | 2.47E7 | 2.79E7 | 19 | 2 | 2 | 2 | 2 | 2 | 2 | 2 | 2 | 1 | 2 | 268 | 281 | Carbamidomethylation |
| K.GLVLIAFSQYLQK.C | Y | 90.76 | 1478.8496 | 13 | 3.4 | 740.4346 | 2 | 129.67 | 3 | 35526 | Ana\_02.raw | 1.55E8 | 1.78E8 | 3.58E8 | 3.66E8 | 2.28E8 | 2.96E8 | 2.87E8 | 2.46E8 | 1.28E8 | 1.59E8 | 146 | 13 | 14 | 20 | 18 | 13 | 13 | 15 | 18 | 10 | 12 | 45 | 57 |  |
| R.RHPDYSVSLLLR.L | Y | 90.61 | 1454.7993 | 12 | 6.1 | 485.9434 | 3 | 71.88 | 2 | 20475 | Ana\_01.raw | 3.29E7 | 7.43E7 | 5.13E7 | 3.66E7 | 4.25E7 | 5.08E7 | 3.01E7 | 3.7E7 | 3.89E7 | 2.49E7 | 28 | 3 | 3 | 4 | 2 | 3 | 3 | 3 | 2 | 2 | 3 | 361 | 372 |  |
| K.AETFTFHSDIC(+57.02)TLPEKEK.Q | Y | 90.01 | 2152.0146 | 18 | 2.8 | 718.3475 | 3 | 72.70 | 3 | 20648 | Ana\_02.raw | 4.67E6 | 2.53E6 | 1.75E7 | 2.52E7 | 1.84E7 | 3.45E7 | 3.29E7 | 2.55E7 |  |  | 14 | 1 | 1 | 2 | 2 | 2 | 2 | 2 | 2 | 0 | 0 | 528 | 545 | Carbamidomethylation |
| K.YM(+15.99)C(+57.02)ENQATISSK.L | Y | 87.94 | 1446.6119 | 12 | 4.5 | 724.3165 | 2 | 31.18 | 6 | 8488 | Ana\_05.raw | 5.68E7 | 8.79E7 | 2.91E7 | 2.54E7 | 3.51E7 | 7.05E7 | 3.65E7 | 5.68E7 | 1.56E7 | 3.77E7 | 33 | 3 | 5 | 3 | 3 | 2 | 3 | 3 | 5 | 3 | 3 | 287 | 298 | Oxidation (M); Carbamidomethylation |
| K.LVQEVTDFAK.T | Y | 83.71 | 1148.6077 | 10 | 3.6 | 575.3132 | 2 | 66.60 | 6 | 19122 | Ana\_05.raw | 1.91E8 | 3.41E8 | 3.13E8 | 1.42E8 | 1.66E8 | 2.14E8 | 1.59E8 | 1.95E8 | 2.33E8 | 1.98E8 | 68 | 6 | 8 | 9 | 7 | 8 | 6 | 5 | 9 | 5 | 5 | 66 | 75 |  |
| R.ENYGELADC(+57.02)C(+57.02)TK.Q | Y | 81.52 | 1458.5756 | 12 | 1.2 | 730.2960 | 2 | 47.34 | 3 | 13372 | Ana\_02.raw | 9.42E7 | 2.22E8 | 1.95E8 | 6.34E7 | 1.16E8 | 1.9E8 | 1.04E8 | 1.26E8 | 8.92E7 | 8.38E7 | 34 | 3 | 5 | 4 | 4 | 3 | 3 | 2 | 6 | 2 | 2 | 106 | 117 | Carbamidomethylation |
| K.SLHTLFGDK.L | Y | 81.07 | 1016.5291 | 9 | 3.0 | 509.2733 | 2 | 53.91 | 6 | 15375 | Ana\_05.raw | 1.72E7 |  | 1.16E8 | 3.29E7 | 4.69E7 | 9.86E7 | 6.2E7 | 6.09E7 | 2.22E7 | 3.38E7 | 25 | 1 | 0 | 4 | 3 | 3 | 4 | 3 | 3 | 2 | 2 | 89 | 97 |  |
| K.YMC(+57.02)ENQATISSK.L | Y | 79.60 | 1430.6169 | 12 | 0.7 | 716.3162 | 2 | 21.34 | 3 | 5386 | Ana\_02.raw | 2.38E7 | 2.55E7 | 4.39E6 | 3.88E6 | 5.48E6 | 1.84E7 | 4.4E6 | 1.03E7 | 1.09E7 | 2.25E7 | 15 | 1 | 2 | 1 | 2 | 1 | 2 | 2 | 1 | 1 | 2 | 287 | 298 | Carbamidomethylation |
| K.TC(+57.02)VADESAANC(+57.02)DK.S | Y | 78.90 | 1439.5657 | 13 | 3.1 | 720.7924 | 2 | 25.73 | 6 | 6794 | Ana\_05.raw | 9.18E7 | 2.21E8 | 2.57E8 | 8.04E7 | 1.26E8 | 2.26E8 | 1.25E8 | 1.24E8 | 4.92E7 | 9.89E6 | 39 | 3 | 4 | 5 | 3 | 4 | 5 | 4 | 5 | 2 | 4 | 76 | 88 | Carbamidomethylation |
| R.HPDYSVSLLLR.L | Y | 78.72 | 1298.6982 | 11 | -0.2 | 433.9066 | 3 | 82.57 | 3 | 23297 | Ana\_02.raw | 1.33E6 | 6.93E6 | 2.47E6 | 2.07E6 | 3.77E6 | 3.33E6 | 2.66E6 | 4.07E5 |  |  | 14 | 1 | 2 | 2 | 2 | 2 | 2 | 2 | 1 | 0 | 0 | 362 | 372 |  |
| R.YNDLGEQHFK.G | Y | 73.28 | 1249.5728 | 10 | 1.3 | 625.7945 | 2 | 37.67 | 3 | 10437 | Ana\_02.raw | 1.82E8 | 3.29E8 | 3.65E8 | 1.02E8 | 1.98E8 | 3.08E8 | 1.91E8 | 1.99E8 | 6.9E7 | 1.7E8 | 39 | 4 | 5 | 6 | 2 | 4 | 3 | 3 | 7 | 2 | 3 | 35 | 44 |  |
| K.TPVSEHVTK.C | Y | 70.61 | 996.5240 | 9 | 1.6 | 499.2701 | 2 | 19.01 | 3 | 4608 | Ana\_02.raw | 3.54E8 | 5.3E8 | 5.35E8 | 1.55E8 | 1.57E8 | 4.74E8 | 2.08E8 | 5.65E7 | 3.2E6 | 3.32E8 | 34 | 3 | 3 | 7 | 3 | 3 | 2 | 3 | 4 | 2 | 4 | 491 | 499 |  |
| K.C(+57.02)C(+57.02)TLPEDQR.L | Y | 70.57 | 1177.4856 | 9 | 4.7 | 589.7529 | 2 | 29.50 | 6 | 7970 | Ana\_05.raw | 1.2E7 |  |  | 1.04E7 | 1.79E7 | 2.64E7 | 1.57E7 | 1.97E7 | 2.46E6 |  | 7 | 1 | 0 | 0 | 1 | 1 | 1 | 1 | 1 | 1 | 0 | 461 | 469 | Carbamidomethylation |
| K.C(+57.02)C(+57.02)SGSLVER.R | Y | 69.69 | 1066.4536 | 9 | 3.3 | 534.2358 | 2 | 26.73 | 5 | 7082 | Ana\_04.raw |  | 1.65E7 | 2.22E7 | 9.67E6 | 1.69E7 |  |  | 1.94E7 | 6.46E6 |  | 6 | 0 | 1 | 1 | 1 | 1 | 0 | 0 | 1 | 1 | 0 | 500 | 508 | Carbamidomethylation |
| K.DDNPSLPPFERPEAEAM(+15.99)C(+57.02)TSFK.E | Y | 67.54 | 2553.1152 | 22 | 4.8 | 852.0498 | 3 | 85.82 | 2 | 24314 | Ana\_01.raw | 7.15E7 | 1.58E8 | 1.57E8 | 4.08E7 | 8.1E7 | 1.01E8 | 8.22E7 | 1.31E8 | 7.8E7 | 3.95E7 | 40 | 3 | 5 | 5 | 3 | 4 | 4 | 4 | 6 | 3 | 3 | 131 | 152 | Oxidation (M); Carbamidomethylation |
| K.KYEATLEK.C | Y | 66.89 | 980.5178 | 8 | 1.7 | 491.2670 | 2 | 21.86 | 3 | 5558 | Ana\_02.raw | 1.13E7 | 2.85E8 | 3.56E8 | 1.09E8 | 1.18E8 | 2.84E8 | 5.46E7 | 1.53E8 | 1.38E8 | 1.61E8 | 38 | 4 | 3 | 6 | 4 | 3 | 4 | 1 | 5 | 3 | 5 | 376 | 383 |  |
| K.KQTALAELVK.H | Y | 61.75 | 1099.6600 | 10 | 1.6 | 367.5612 | 3 | 50.06 | 6 | 14243 | Ana\_05.raw | 1.85E7 | 3.53E7 | 4.34E7 | 1.19E7 | 2.06E7 | 4.2E7 | 2.43E7 | 1.77E7 | 1E7 | 1.62E7 | 20 | 2 | 2 | 2 | 3 | 1 | 2 | 3 | 2 | 2 | 1 | 549 | 558 |  |
| K.DDNPSLPPFERPEAEAMC(+57.02)TSFK.E | Y | 61.21 | 2537.1201 | 22 | 2.1 | 846.7158 | 3 | 98.97 | 6 | 27978 | Ana\_05.raw | 1.04E7 | 5.03E6 |  |  |  | 1.2E6 |  | 1.26E6 | 3.8E6 |  | 5 | 1 | 1 | 0 | 0 | 0 | 1 | 0 | 1 | 1 | 0 | 131 | 152 | Carbamidomethylation |
| K.ALVSSVR.Q | Y | 61.08 | 730.4337 | 7 | 3.1 | 366.2253 | 2 | 28.99 | 6 | 7811 | Ana\_05.raw | 9.89E7 | 1.68E8 | 1.75E8 | 5.85E7 | 1.05E8 | 1.7E8 | 1.06E8 | 1.07E8 | 4.88E7 | 8.32E7 | 21 | 3 | 2 | 2 | 2 | 3 | 2 | 2 | 3 | 1 | 1 | 213 | 219 |  |
| K.LQTC(+57.02)C(+57.02)DKPLLK.K | Y | 60.92 | 1374.7000 | 11 | 2.8 | 688.3592 | 2 | 39.06 | 6 | 10857 | Ana\_05.raw | 6.55E6 | 6.47E7 | 8E7 | 1.99E7 | 3.73E7 | 6.95E7 | 7.2E6 | 3.12E7 | 1.36E7 | 2.39E7 | 18 | 1 | 2 | 2 | 2 | 2 | 2 | 1 | 2 | 2 | 2 | 299 | 309 | Carbamidomethylation |
| K.TNC(+57.02)DLYEK.L | Y | 59.78 | 1041.4437 | 8 | 3.5 | 521.7310 | 2 | 28.18 | 6 | 7557 | Ana\_05.raw | 1.55E8 | 2.98E8 | 3.31E8 | 1.09E8 | 2.04E8 | 2.6E8 | 1.72E8 | 1.77E8 | 3.19E7 | 6.4E7 | 17 | 1 | 1 | 3 | 1 | 1 | 2 | 2 | 4 | 1 | 1 | 414 | 421 | Carbamidomethylation |
| R.NEC(+57.02)FLQHK.D | Y | 57.18 | 1074.4917 | 8 | 4.2 | 538.2554 | 2 | 30.30 | 6 | 8220 | Ana\_05.raw | 5.12E7 | 8.84E7 | 5.18E7 | 3.76E7 | 6.8E7 | 1.12E8 | 6.3E7 | 7.44E7 | 4.48E6 | 5.28E7 | 20 | 3 | 2 | 1 | 2 | 2 | 2 | 2 | 3 | 1 | 2 | 123 | 130 | Carbamidomethylation |
| K.LC(+57.02)AIPNLR.E | Y | 55.96 | 955.5273 | 8 | 3.4 | 478.7726 | 2 | 66.00 | 5 | 18820 | Ana\_04.raw | 4.72E7 | 1.09E8 | 1.11E8 | 2.65E7 | 5.17E7 | 6.57E7 | 4.81E7 | 7.45E7 | 9.19E7 | 8.06E7 | 21 | 1 | 3 | 3 | 1 | 1 | 2 | 3 | 3 | 2 | 2 | 98 | 105 | Carbamidomethylation |
| K.HKPKATAEQLK.T | Y | 54.40 | 1249.7142 | 11 | 3.3 | 417.5801 | 3 | 16.18 | 6 | 3676 | Ana\_05.raw |  |  | 6.75E5 |  |  | 3.26E5 | 2.14E5 |  |  |  | 4 | 0 | 0 | 2 | 0 | 0 | 1 | 1 | 0 | 0 | 0 | 559 | 569 |  |
| K.YEATLEK.C | Y | 53.49 | 852.4229 | 7 | 0.8 | 427.2190 | 2 | 27.25 | 3 | 7275 | Ana\_02.raw | 3.81E7 | 7.17E7 | 6.25E7 | 2.39E7 | 3.8E7 | 5.77E7 | 4E7 | 3.46E7 |  | 3.77E7 | 9 | 1 | 1 | 1 | 1 | 1 | 1 | 1 | 1 | 0 | 1 | 377 | 383 |  |
| K.LATDLTK.V | Y | 49.01 | 760.4330 | 7 | -1.2 | 381.2233 | 2 | 28.30 | 2 | 7487 | Ana\_01.raw | 1.09E8 | 2.38E8 | 2.34E8 | 8.15E7 | 1.36E8 | 2.23E8 | 1.48E8 | 1.79E8 | 4.4E7 | 9.3E7 | 21 | 1 | 3 | 3 | 1 | 1 | 2 | 2 | 5 | 2 | 1 | 258 | 264 |  |
| R.VC(+57.02)LLHEK.T | Y | 47.41 | 897.4742 | 7 | 2.7 | 449.7456 | 2 | 28.91 | 6 | 7786 | Ana\_05.raw | 2.79E7 | 6.46E7 | 7.52E7 |  | 5.47E7 | 7.77E7 | 4.12E7 | 5.32E7 | 5.51E6 | 3.27E7 | 15 | 1 | 1 | 3 | 1 | 2 | 2 | 1 | 2 | 1 | 1 | 484 | 490 | Carbamidomethylation |
| K.QTALAELVK.H | Y | 41.40 | 971.5651 | 9 | 3.2 | 486.7914 | 2 | 66.65 | 6 | 19141 | Ana\_05.raw |  |  | 1.8E7 | 8.68E6 | 1.11E7 | 2.34E7 | 1.53E7 | 2.58E7 |  |  | 9 | 0 | 0 | 1 | 1 | 2 | 2 | 1 | 2 | 0 | 0 | 550 | 558 |  |
| K.AWAVAR.L | Y | 36.08 | 672.3707 | 6 | 3.7 | 337.1939 | 2 | 36.53 | 7 | 9942 | Ana\_06.raw | 1.82E7 |  | 4.19E7 |  | 2.21E7 | 4.07E7 | 2.51E7 |  |  |  | 6 | 1 | 0 | 1 | 0 | 1 | 2 | 1 | 0 | 0 | 0 | 237 | 242 |  |
| total 50 peptides |  |  |  |  |  |  |  |  |  |  |  |  |  |  |  |  |  |  |  |  |  |  |  |  |  |  |  |  |  |  |  |  |  |  |  |
| --- | --- | --- | --- | --- | --- | --- | --- | --- | --- | --- | --- | --- | --- | --- | --- | --- | --- | --- | --- | --- | --- | --- | --- | --- | --- | --- | --- | --- | --- | --- | --- | --- | --- | --- | --- |

Best Unique PSM (Scan F5:36311, m/z=1143.5317, z=4, RT=131.93, ppm=1.3):

J3QQ16|J3QQ16\_MOUSE

back to list

  

| Protein Coverage
| Supporting Peptides
| Best Unique PSM
|

Protein Coverage:

Supporting Peptides:

| Peptide | Uniq | -10lgP | Mass | Length | ppm | m/z | z | RT | Fraction | Scan | Source File | Area 10 | Area 01 | Area 02 | Area 03 | Area 04 | Area 05 | Area 06 | Area 07 | Area 08 | Area 09 | #Spec | #Spec 10 | #Spec 01 | #Spec 02 | #Spec 03 | #Spec 04 | #Spec 05 | #Spec 06 | #Spec 07 | #Spec 08 | #Spec 09 | Start | End | PTM |
| --- | --- | --- | --- | --- | --- | --- | --- | --- | --- | --- | --- | --- | --- | --- | --- | --- | --- | --- | --- | --- | --- | --- | --- | --- | --- | --- | --- | --- | --- | --- | --- | --- | --- | --- | --- |
| R.YPPSVVESDAADIVFLIDSSDAVKPDGIAHIR.D | Y | 152.52 | 3395.7249 | 32 | 3.2 | 849.9412 | 4 | 133.75 | 6 | 37183 | Ana\_05.raw | 1.64E6 | 5.72E6 | 2.75E6 |  |  | 4.27E6 | 2.02E6 | 9.68E5 | 3.97E6 | 2.89E6 | 8 | 1 | 1 | 1 | 0 | 0 | 1 | 1 | 1 | 1 | 1 | 815 | 846 |  |
| R.VGSVQELSELSETVLETLHDAM(+15.99)HETLC(+57.02)PGVTDVSK.A | Y | 132.56 | 3825.8289 | 35 | 1.0 | 957.4655 | 4 | 135.76 | 3 | 37137 | Ana\_02.raw | 8.57E6 | 9.6E6 | 1.86E6 |  | 2.82E6 | 1.25E6 |  | 4.8E6 | 1.11E7 |  | 10 | 1 | 2 | 1 | 0 | 1 | 2 | 0 | 1 | 2 | 0 | 1189 | 1223 | Oxidation (M); Carbamidomethylation |
| R.IGVVQFSNDVFPEFYLK.T | Y | 115.60 | 2001.0247 | 17 | 3.1 | 1001.5227 | 2 | 131.84 | 6 | 36641 | Ana\_05.raw | 8.08E6 | 1.13E7 | 1.04E7 | 2.91E6 | 8.69E6 | 1.52E7 |  | 8.11E6 | 1.97E7 | 1.15E7 | 9 | 1 | 1 | 1 | 1 | 1 | 1 | 0 | 1 | 1 | 1 | 865 | 881 |  |
| R.LNLLDLDYELAEQLDNIAEK.A | Y | 106.05 | 2331.1846 | 20 | 0.6 | 1166.6002 | 2 | 137.69 | 6 | 38241 | Ana\_05.raw |  |  |  |  |  | 1.19E6 |  |  |  |  | 2 | 0 | 0 | 0 | 0 | 0 | 2 | 0 | 0 | 0 | 0 | 1398 | 1417 |  |
| R.LTLLGGPTPNTGAALEFVLR.N | Y | 104.49 | 2039.1415 | 20 | 2.2 | 1020.5803 | 2 | 131.30 | 6 | 36482 | Ana\_05.raw | 4.12E6 | 1.91E6 | 1.85E5 |  | 2.44E6 | 4.58E6 | 2.53E6 | 1.66E6 | 7.63E6 |  | 14 | 2 | 1 | 1 | 0 | 2 | 2 | 2 | 2 | 2 | 0 | 488 | 507 |  |
| R.VGLVQYNSDPTDEFFLR.D | Y | 102.38 | 1998.9686 | 17 | 1.5 | 1000.4930 | 2 | 114.78 | 3 | 31710 | Ana\_02.raw | 3.37E6 | 1.27E7 | 8.66E6 | 1.44E5 | 1.11E6 | 1.24E7 |  | 1.14E6 | 4.52E6 | 5.65E6 | 14 | 3 | 2 | 2 | 1 | 1 | 2 | 0 | 1 | 1 | 1 | 1068 | 1084 |  |
| K.ISLSPEYVYSVSTFR.E | Y | 99.24 | 1746.8828 | 15 | -0.4 | 874.4484 | 2 | 114.37 | 6 | 32002 | Ana\_05.raw | 9.96E6 | 2.87E7 | 1.42E7 | 1.08E7 | 1.3E7 | 1.87E7 | 1.37E7 | 2.65E6 | 2.96E7 | 1.42E7 | 28 | 3 | 4 | 2 | 3 | 3 | 2 | 3 | 3 | 4 | 1 | 771 | 785 |  |
| R.IAEGVPQLLIVLTAEPSGDDVR.G | Y | 97.77 | 2291.2373 | 22 | 3.4 | 764.7557 | 3 | 132.81 | 7 | 36587 | Ana\_06.raw | 2.93E6 | 2.13E6 | 3.61E6 | 1.18E6 | 2.33E6 | 5.14E6 | 2.4E6 |  | 3.6E6 |  | 12 | 2 | 1 | 2 | 1 | 1 | 2 | 2 | 0 | 1 | 0 | 518 | 539 |  |
| K.SALLDSIQNLQVALTSK.Q | Y | 96.91 | 1799.9993 | 17 | 1.6 | 901.0084 | 2 | 130.56 | 3 | 35764 | Ana\_02.raw | 1.44E7 | 1.22E7 | 1.54E7 | 4.4E6 | 8.86E6 | 1.58E7 | 7.63E6 | 7.62E6 | 1.92E7 | 2.67E6 | 10 | 1 | 1 | 1 | 1 | 1 | 1 | 1 | 1 | 1 | 1 | 1857 | 1873 |  |
| K.LLTPITTLTSQQIHQILASTR.Y | Y | 94.82 | 2334.3271 | 21 | 1.4 | 779.1174 | 3 | 129.21 | 7 | 35553 | Ana\_06.raw | 2.06E7 |  | 0 |  |  |  | 3.15E6 | 6.31E6 | 3.45E7 | 1.4E7 | 6 | 1 | 0 | 1 | 0 | 0 | 0 | 1 | 1 | 1 | 1 | 794 | 814 |  |
| K.IGDLQSQIVSLLK.A | Y | 92.46 | 1412.8239 | 13 | 3.6 | 707.4218 | 2 | 127.31 | 6 | 35384 | Ana\_05.raw | 6.51E6 | 9.84E6 | 2.04E7 | 1.29E7 | 2.59E7 | 3.59E7 | 3.72E6 | 7.15E5 | 4.58E7 | 2.65E7 | 21 | 3 | 3 | 4 | 1 | 2 | 2 | 1 | 1 | 2 | 2 | 394 | 406 |  |
| R.IEDNVQQFLVLLVAGR.S | Y | 91.43 | 1813.0098 | 16 | 5.6 | 907.5173 | 2 | 136.72 | 7 | 37643 | Ana\_06.raw |  | 2.81E5 |  | 1.46E5 |  | 1.44E6 | 4.35E5 | 7.5E5 |  |  | 7 | 0 | 1 | 0 | 1 | 0 | 2 | 2 | 1 | 0 | 0 | 328 | 343 |  |
| K.SDDEVDDSAVELK.Q | Y | 91.02 | 1420.6205 | 13 | 1.5 | 711.3186 | 2 | 52.19 | 3 | 14783 | Ana\_02.raw | 1.11E7 | 1.83E7 | 1.3E7 | 5.33E6 | 1.07E7 | 1.15E7 | 8.05E6 | 8.08E6 | 1.89E7 | 1.24E7 | 13 | 1 | 2 | 1 | 1 | 2 | 1 | 1 | 1 | 1 | 2 | 738 | 750 |  |
| R.LLPSFVSSENAFYLPPDLR.K | Y | 90.86 | 2164.1204 | 19 | 5.5 | 1083.0734 | 2 | 130.86 | 1 | 35971 | ANa\_10.raw | 1.04E6 |  |  |  |  |  |  |  |  |  | 1 | 1 | 0 | 0 | 0 | 0 | 0 | 0 | 0 | 0 | 0 | 2205 | 2223 |  |
| K.NANPSELEQIVLSPAFILAAESLPK.I | Y | 89.82 | 2650.4216 | 25 | 3.0 | 884.4838 | 3 | 138.72 | 9 | 38295 | Ana\_08.raw |  | 1.36E6 | 2.75E6 |  | 1.85E6 | 3.83E6 | 2.57E6 | 7E5 | 7.96E6 | 8.87E5 | 11 | 0 | 1 | 2 | 0 | 1 | 1 | 2 | 1 | 2 | 1 | 369 | 393 |  |
| R.TLTGTTEESKR.D | Y | 86.95 | 1221.6201 | 11 | 2.2 | 611.8187 | 2 | 20.19 | 7 | 4954 | Ana\_06.raw |  | 1.35E7 | 9.29E6 | 7.36E6 |  | 2.15E7 | 1.14E7 | 7.67E6 | 3.05E6 | 7.47E6 | 13 | 0 | 1 | 1 | 2 | 0 | 2 | 2 | 2 | 2 | 1 | 218 | 228 |  |
| K.ALNLGYALDYALR.N | Y | 85.48 | 1451.7772 | 13 | 4.7 | 726.8993 | 2 | 125.58 | 6 | 34932 | Ana\_05.raw | 1.45E6 | 1.94E6 | 4.28E5 | 3.64E5 | 4.86E6 | 1.08E7 | 4.6E6 | 1.96E6 | 1.45E7 | 1.89E6 | 15 | 2 | 2 | 1 | 1 | 2 | 2 | 1 | 1 | 2 | 1 | 305 | 317 |  |
| R.FASEIVDTVYEDGDSIR.V | Y | 85.44 | 1914.8846 | 17 | 0.5 | 958.4501 | 2 | 95.08 | 6 | 26950 | Ana\_05.raw |  | 1.01E7 | 6.05E5 |  | 2.03E6 | 4.84E6 | 2.53E6 |  | 3.96E6 | 3.41E5 | 11 | 0 | 1 | 1 | 0 | 1 | 3 | 2 | 0 | 2 | 1 | 1051 | 1067 |  |
| R.VSVM(+15.99)ANTPSGPVEAFDFAEYQPELFEK.F | Y | 83.06 | 3017.4004 | 27 | -0.3 | 1006.8071 | 3 | 130.01 | 6 | 36117 | Ana\_05.raw |  |  |  |  |  | 4.9E6 |  |  |  |  | 2 | 0 | 0 | 0 | 0 | 0 | 2 | 0 | 0 | 0 | 0 | 1277 | 1303 | Oxidation (M) |
| K.LSDAGITPLFLTSQEDR.Q | Y | 82.10 | 1861.9421 | 17 | 10.0 | 931.9876 | 2 | 107.93 | 6 | 30347 | Ana\_05.raw |  | 2.23E7 | 1.12E7 | 5.61E6 | 4.66E6 | 1.16E7 | 7.24E6 |  | 3.03E6 | 7.4E5 | 11 | 0 | 2 | 1 | 1 | 2 | 2 | 1 | 0 | 1 | 1 | 1923 | 1939 |  |
| R.VAIAQFSDDVR.L | Y | 82.06 | 1219.6196 | 11 | 4.9 | 610.8201 | 2 | 70.21 | 2 | 20000 | Ana\_01.raw | 1.94E7 | 2.5E7 | 2.26E7 | 9.16E6 | 1.62E7 | 2.26E7 | 1.17E7 | 1.37E7 | 2.89E7 | 8.41E5 | 12 | 1 | 1 | 1 | 2 | 2 | 1 | 1 | 1 | 1 | 1 | 267 | 277 |  |
| R.GGPGQPGFEGEQGTR.G | Y | 81.43 | 1472.6643 | 15 | 2.9 | 737.3416 | 2 | 38.53 | 6 | 10702 | Ana\_05.raw | 1.85E7 | 1.93E7 | 1.58E7 | 6.15E6 | 1.38E7 | 1.66E7 | 9.29E6 | 1.13E7 | 8.14E6 | 1.13E7 | 10 | 1 | 1 | 1 | 1 | 1 | 1 | 1 | 1 | 1 | 1 | 1603 | 1617 |  |
| K.SLDEVSQPAQELK.R | Y | 79.32 | 1442.7252 | 13 | 5.1 | 722.3736 | 2 | 57.11 | 3 | 16212 | Ana\_02.raw |  | 6.78E6 | 7.17E6 | 2.33E6 | 5.11E6 | 6.88E6 | 4.41E6 | 3.8E6 |  | 6.52E6 | 8 | 0 | 1 | 1 | 1 | 1 | 1 | 1 | 1 | 0 | 1 | 153 | 165 |  |
| R.ELGTIQQVISER.V | Y | 79.27 | 1371.7357 | 12 | 4.9 | 686.8785 | 2 | 86.16 | 6 | 24543 | Ana\_05.raw | 4.83E6 | 1.97E7 | 1.82E7 | 5.9E6 | 1.17E7 | 1.65E7 | 9.79E6 | 1.08E7 | 2.64E7 | 1.05E7 | 10 | 1 | 1 | 1 | 1 | 1 | 1 | 1 | 1 | 1 | 1 | 583 | 594 |  |
| R.VVESLDVGPDR.V | Y | 78.80 | 1184.6036 | 11 | 2.6 | 593.3106 | 2 | 52.32 | 3 | 14818 | Ana\_02.raw | 1.72E7 | 2.43E7 | 2.86E7 | 8.76E6 | 1.89E7 | 2.5E7 | 1.61E7 | 1.56E7 | 2.66E7 | 2.13E7 | 18 | 1 | 1 | 2 | 1 | 2 | 2 | 2 | 3 | 2 | 2 | 444 | 454 |  |
| R.VAVIQFSEDSK.M | Y | 76.30 | 1221.6240 | 11 | 0.5 | 611.8196 | 2 | 61.42 | 3 | 17495 | Ana\_02.raw | 7.43E6 | 1.17E7 | 1.15E7 | 4.99E6 | 8.24E6 |  |  | 7.23E6 | 7.98E6 |  | 8 | 1 | 2 | 1 | 1 | 1 | 0 | 0 | 1 | 1 | 0 | 661 | 671 |  |
| R.SSDAVAGPASSLK.Q | Y | 75.69 | 1188.5986 | 13 | 6.2 | 595.3103 | 2 | 39.04 | 1 | 10719 | ANa\_10.raw | 2.34E7 | 2.85E7 | 2.65E7 | 1.09E7 | 2.13E7 | 2.7E7 | 1.68E7 | 1.77E7 | 8.49E5 | 1.7E7 | 17 | 2 | 1 | 2 | 2 | 2 | 2 | 2 | 2 | 1 | 1 | 344 | 356 |  |
| K.GGSQVYIGNALEYVLKNIFQRPLGSR.I | Y | 75.68 | 2878.5452 | 26 | 5.7 | 720.6476 | 4 | 137.55 | 2 | 38012 | Ana\_01.raw |  | 2.3E6 |  |  |  | 5.94E5 |  | 2.21E5 |  | 2.1E6 | 4 | 0 | 1 | 0 | 0 | 0 | 1 | 0 | 1 | 0 | 1 | 696 | 721 |  |
| R.IEDGVPQHLVLFLGGK.S | Y | 74.98 | 1720.9512 | 16 | 0.8 | 861.4836 | 2 | 111.47 | 6 | 31260 | Ana\_05.raw | 2.54E6 | 7.63E5 | 2.43E6 | 1.28E6 | 1.6E5 | 1.45E7 | 2.42E6 | 3.28E6 | 1.24E7 | 2.44E6 | 22 | 2 | 2 | 1 | 3 | 1 | 4 | 2 | 2 | 2 | 3 | 926 | 941 |  |
| R.HTDQEELVK.I | Y | 74.86 | 1097.5353 | 9 | 5.9 | 549.7781 | 2 | 21.30 | 3 | 5372 | Ana\_02.raw |  |  | 2.39E7 | 9.52E6 | 8.1E6 | 2.72E7 | 1.02E7 | 2.49E6 | 6.7E6 | 1.74E7 | 8 | 0 | 0 | 1 | 1 | 1 | 1 | 1 | 1 | 1 | 1 | 762 | 770 |  |
| R.VPQIAFVITGGK.S | Y | 74.79 | 1228.7179 | 12 | -0.8 | 615.3657 | 2 | 95.01 | 3 | 26588 | Ana\_02.raw | 1.05E7 | 5.95E6 | 7.32E6 | 3.09E5 | 4.76E6 | 7.28E6 | 3.28E6 | 2.55E6 | 1.12E7 | 5.77E6 | 14 | 2 | 2 | 1 | 1 | 1 | 2 | 1 | 1 | 1 | 2 | 1133 | 1144 |  |
| R.EELSSLKPILTPSTGAGVGSK.K | Y | 73.85 | 2070.1208 | 21 | 3.8 | 691.0502 | 3 | 82.43 | 6 | 23532 | Ana\_05.raw | 5.2E6 |  | 4.51E6 |  |  | 6.19E6 | 9.64E5 | 3.37E6 | 1.13E7 |  | 6 | 1 | 0 | 1 | 0 | 0 | 1 | 1 | 1 | 1 | 0 | 601 | 621 |  |
| R.ISC(+57.02)SGNQLPTVR.V | Y | 70.40 | 1330.6664 | 12 | 4.2 | 666.3433 | 2 | 48.04 | 9 | 13445 | Ana\_08.raw |  | 4.92E6 | 9.38E5 |  |  | 9.83E5 |  |  | 8.43E6 | 1.71E6 | 5 | 0 | 1 | 1 | 0 | 0 | 1 | 0 | 0 | 1 | 1 | 1265 | 1276 | Carbamidomethylation |
| K.SVEDAQDVSLALTQK.G | Y | 69.77 | 1602.8101 | 15 | 4.4 | 802.4158 | 2 | 76.68 | 6 | 21959 | Ana\_05.raw |  | 2.23E6 | 1.06E6 | 0 | 7.42E5 | 1.94E6 | 1.17E6 | 1.71E6 |  |  | 7 | 0 | 1 | 1 | 1 | 1 | 1 | 1 | 1 | 0 | 0 | 1145 | 1159 |  |
| R.NILTSSTGSR.I | Y | 69.10 | 1034.5356 | 10 | 3.4 | 518.2769 | 2 | 36.10 | 3 | 9947 | Ana\_02.raw |  |  | 1.81E7 | 2.58E6 | 1.35E7 |  | 1.05E7 |  |  | 8.99E6 | 5 | 0 | 0 | 1 | 1 | 1 | 0 | 1 | 0 | 0 | 1 | 508 | 517 |  |
| R.NNLFTSSAGHR.A | Y | 66.48 | 1202.5792 | 11 | 1.9 | 602.2980 | 2 | 33.20 | 1 | 8952 | ANa\_10.raw | 4.6E6 | 6.01E6 | 5.2E6 | 4.12E6 |  |  |  |  | 9.04E6 |  | 6 | 1 | 1 | 1 | 1 | 0 | 0 | 0 | 0 | 2 | 0 | 126 | 136 |  |
| R.NAGPEFQYIR.T | Y | 66.32 | 1193.5829 | 10 | 3.6 | 597.8008 | 2 | 68.10 | 6 | 19555 | Ana\_05.raw | 2.72E7 | 3.79E7 | 2.22E7 | 1.03E7 | 2.03E7 | 2.31E7 | 1.57E7 | 2.06E7 | 2.47E7 | 1.85E7 | 11 | 1 | 1 | 1 | 1 | 1 | 1 | 1 | 1 | 1 | 2 | 633 | 642 |  |
| R.IIEELDVKPDGTR.V | Y | 66.23 | 1483.7882 | 13 | 0.6 | 742.9018 | 2 | 57.24 | 3 | 16250 | Ana\_02.raw | 1.93E6 | 1.07E6 | 1.12E7 | 3.26E6 | 7.83E6 | 7.78E6 | 3.98E6 | 4.84E6 | 6.02E6 |  | 10 | 1 | 1 | 2 | 1 | 1 | 1 | 1 | 1 | 1 | 0 | 254 | 266 |  |
| R.VALVQYSDR.T | Y | 64.77 | 1049.5505 | 9 | 4.5 | 525.7849 | 2 | 48.76 | 1 | 13663 | ANa\_10.raw | 1.78E7 | 1.95E7 | 2.58E7 | 7.84E6 | 1.55E7 |  | 1.35E7 | 1.11E7 | 2.16E7 | 1.45E7 | 12 | 1 | 2 | 2 | 1 | 1 | 0 | 1 | 1 | 1 | 2 | 457 | 465 |  |
| R.NHFVPEAGSR.L | Y | 64.36 | 1112.5363 | 10 | 2.8 | 557.2770 | 2 | 27.50 | 2 | 7240 | Ana\_01.raw | 1.62E7 | 2.01E7 | 1.75E7 | 7.22E6 | 1.31E7 | 1.55E7 | 1.1E7 | 9.47E6 | 1.07E7 | 1.13E7 | 11 | 1 | 1 | 1 | 1 | 1 | 1 | 1 | 2 | 1 | 1 | 1119 | 1128 |  |
| R.IEEGVPQFLVLISSGK.S | Y | 64.29 | 1714.9504 | 16 | 4.0 | 572.6597 | 3 | 132.21 | 7 | 36414 | Ana\_06.raw | 4.36E6 | 4.29E6 |  | 3.41E6 | 7.63E6 | 1.46E6 | 1.06E7 | 6.12E6 | 1.2E7 | 7.22E6 | 11 | 1 | 1 | 1 | 1 | 1 | 1 | 2 | 1 | 1 | 1 | 722 | 737 |  |
| R.DQNVFVSQK.G | Y | 64.18 | 1063.5298 | 9 | 4.8 | 532.7747 | 2 | 38.53 | 6 | 10700 | Ana\_05.raw | 1.72E7 | 1.74E7 | 1.88E7 | 7.43E6 | 1.66E7 | 1.96E7 | 1.2E7 | 1.45E7 | 1.37E7 | 1.41E7 | 12 | 1 | 1 | 1 | 1 | 2 | 1 | 1 | 1 | 1 | 2 | 1238 | 1246 |  |
| R.GETGDDGRDGVGSEGR.R | Y | 62.82 | 1562.6556 | 16 | 4.0 | 521.8946 | 3 | 21.25 | 1 | 5184 | ANa\_10.raw | 3.3E6 | 0 | 1.34E6 |  |  |  |  |  |  | 2.08E6 | 4 | 1 | 1 | 1 | 0 | 0 | 0 | 0 | 0 | 0 | 1 | 1681 | 1696 |  |
| R.TDLQTITNDPR.L | Y | 61.60 | 1272.6310 | 11 | -0.7 | 637.3223 | 2 | 49.49 | 3 | 13993 | Ana\_02.raw | 1.31E7 | 1.89E7 | 1.36E7 | 5.18E6 | 8.93E6 |  | 7.04E6 | 7.64E6 |  | 9.21E6 | 8 | 1 | 1 | 1 | 1 | 1 | 0 | 1 | 1 | 0 | 1 | 971 | 981 |  |
| K.VAVFFSNKPTR.A | Y | 59.61 | 1264.6927 | 11 | 2.0 | 422.5724 | 3 | 53.65 | 6 | 15297 | Ana\_05.raw | 4.27E6 | 4.16E6 | 4.55E6 | 4.77E5 | 1.97E6 | 5.19E6 | 3.14E6 |  |  | 3.29E6 | 8 | 1 | 1 | 1 | 1 | 1 | 1 | 1 | 0 | 0 | 1 | 1901 | 1911 |  |
| K.IASNSATAFR.V | Y | 59.24 | 1036.5302 | 10 | 4.3 | 519.2746 | 2 | 35.24 | 5 | 9662 | Ana\_04.raw | 1.99E7 | 3.19E7 | 2.4E7 |  | 1.75E7 | 2.49E7 | 1.67E7 | 1.57E7 | 1.15E7 | 1.77E7 | 12 | 2 | 2 | 1 | 0 | 1 | 1 | 1 | 2 | 1 | 1 | 1179 | 1188 |  |
| R.GDSVDQC(+57.02)ALIQSIR.D | Y | 58.80 | 1560.7566 | 14 | 5.8 | 781.3901 | 2 | 80.80 | 9 | 23007 | Ana\_08.raw | 9.96E5 | 2.33E6 |  |  |  |  |  |  | 3.99E6 |  | 4 | 2 | 1 | 0 | 0 | 0 | 0 | 0 | 0 | 1 | 0 | 1761 | 1774 | Carbamidomethylation |
| K.VFAVGVR.N | Y | 58.29 | 746.4438 | 7 | 1.8 | 374.2299 | 2 | 48.31 | 6 | 13696 | Ana\_05.raw | 8.77E6 | 1.03E7 | 1.22E7 |  | 8.72E6 | 1.24E7 | 7.99E6 | 6.71E6 | 1.25E7 | 1.11E7 | 10 | 1 | 1 | 1 | 0 | 1 | 1 | 1 | 1 | 2 | 1 | 1163 | 1169 |  |
| R.ELPNIEER.V | Y | 57.56 | 998.5032 | 8 | 1.6 | 500.2597 | 2 | 51.56 | 6 | 14675 | Ana\_05.raw | 1.95E7 | 2.06E7 | 1.99E7 | 7.88E6 | 1.63E7 | 1.8E7 | 1.18E7 | 1.21E7 | 2.42E7 |  | 18 | 2 | 2 | 2 | 2 | 2 | 2 | 2 | 2 | 2 | 0 | 991 | 998 |  |
| K.SQSSVLEAIR.R | Y | 57.50 | 1088.5825 | 10 | 4.2 | 545.3008 | 2 | 63.57 | 7 | 18023 | Ana\_06.raw | 9.54E6 | 1.27E7 | 1.75E7 | 6.76E6 | 1.55E7 | 1.27E7 | 9.48E6 | 9.48E6 | 1.77E7 | 2.69E6 | 10 | 1 | 1 | 1 | 1 | 1 | 1 | 1 | 1 | 1 | 1 | 885 | 894 |  |
| R.GVVPFIFQAK.N | Y | 55.98 | 1104.6331 | 10 | 3.5 | 553.3257 | 2 | 102.03 | 6 | 28790 | Ana\_05.raw | 8.47E6 |  | 5.87E6 | 3.07E5 | 5.67E6 | 8.49E6 | 9.6E5 | 3.91E6 | 1.24E7 | 6.58E6 | 13 | 2 | 0 | 2 | 1 | 1 | 2 | 1 | 2 | 1 | 1 | 359 | 368 |  |
| R.DSFQEVLR.F | Y | 54.85 | 992.4927 | 8 | 2.5 | 497.2549 | 2 | 64.69 | 6 | 18579 | Ana\_05.raw | 7.85E6 | 1.09E7 | 1.27E7 | 6.27E6 | 1.03E7 | 1.08E7 | 7.49E6 | 8.79E6 |  | 7.55E6 | 11 | 2 | 2 | 1 | 1 | 1 | 1 | 1 | 1 | 0 | 1 | 1043 | 1050 |  |
| K.AEILNLVK.K | Y | 53.09 | 898.5487 | 8 | 0.9 | 450.2820 | 2 | 73.55 | 6 | 21099 | Ana\_05.raw | 9.24E6 | 1E7 | 8.09E6 | 3.75E6 | 6.62E6 | 8.33E6 | 4.12E6 | 5.12E6 | 1.01E7 | 6.05E6 | 11 | 2 | 1 | 1 | 1 | 1 | 1 | 1 | 1 | 1 | 1 | 289 | 296 |  |
| R.GPIGSIGPK.G | Y | 52.23 | 824.4756 | 9 | 2.8 | 413.2462 | 2 | 39.12 | 2 | 10782 | Ana\_01.raw | 5.95E6 | 6.19E6 | 7.4E6 | 1.38E6 |  |  |  | 3.43E6 |  |  | 5 | 1 | 1 | 1 | 1 | 0 | 0 | 0 | 1 | 0 | 0 | 1434 | 1442 |  |
| R.TLTGTTEESK.R | Y | 51.90 | 1065.5189 | 10 | 1.0 | 533.7673 | 2 | 21.27 | 8 | 5220 | Ana\_07.raw |  |  |  | 1.04E6 |  |  | 1.14E6 | 1.58E6 |  |  | 3 | 0 | 0 | 0 | 1 | 0 | 0 | 1 | 1 | 0 | 0 | 218 | 227 |  |
| K.DVVFLIDGSR.N | Y | 49.26 | 1119.5924 | 10 | 4.6 | 560.8060 | 2 | 93.08 | 6 | 26410 | Ana\_05.raw |  |  |  |  | 1.45E5 | 3.4E5 |  |  |  |  | 2 | 0 | 0 | 0 | 0 | 1 | 1 | 0 | 0 | 0 | 0 | 623 | 632 |  |
| R.ALEFVAR.N | Y | 49.09 | 804.4493 | 7 | 4.5 | 403.2338 | 2 | 51.78 | 2 | 14621 | Ana\_01.raw | 2.15E7 | 1.77E7 | 1.87E7 | 8.9E6 | 1.41E7 | 1.75E7 | 1.52E7 | 1.24E7 | 2.65E7 | 2.04E7 | 16 | 1 | 1 | 2 | 2 | 2 | 2 | 2 | 2 | 1 | 1 | 909 | 915 |  |
| K.GISGEDGYR.G | Y | 49.07 | 952.4250 | 9 | 3.0 | 477.2212 | 2 | 27.14 | 3 | 7241 | Ana\_02.raw |  |  | 9.62E6 |  |  |  |  | 4.46E6 |  |  | 3 | 0 | 0 | 1 | 0 | 0 | 0 | 0 | 2 | 0 | 0 | 1443 | 1451 |  |
| K.DEVQNAVR.R | Y | 48.53 | 929.4567 | 8 | 2.5 | 465.7368 | 2 | 26.41 | 8 | 6775 | Ana\_07.raw | 4.77E6 | 2.8E6 | 2.21E7 | 1.01E7 | 1.36E7 | 2.47E7 |  | 1.15E7 |  |  | 7 | 1 | 1 | 1 | 1 | 1 | 1 | 0 | 1 | 0 | 0 | 683 | 690 |  |
| R.NIDSEEVGK.I | Y | 47.38 | 989.4666 | 9 | 1.5 | 495.7413 | 2 | 26.25 | 4 | 6770 | Ana\_03.raw |  |  |  | 1.23E7 |  | 2.9E7 |  |  |  | 0 | 3 | 0 | 0 | 0 | 1 | 0 | 1 | 0 | 0 | 0 | 1 | 1170 | 1178 |  |
| R.TLIER.I | N | 45.14 | 630.3701 | 5 | 1.7 | 316.1928 | 2 | 23.18 | 3 | 5981 | Ana\_02.raw |  |  | 8.6E6 |  |  |  |  |  |  |  | 1 | 0 | 0 | 1 | 0 | 0 | 0 | 0 | 0 | 0 | 0 | 643 | 647 |  |
| R.VGIEHLLR.N | Y | 45.02 | 935.5552 | 8 | -0.4 | 468.7847 | 2 | 55.32 | 3 | 15702 | Ana\_02.raw | 2.7E6 | 3.77E6 | 2.77E6 | 8.48E5 |  | 3.72E6 | 1.89E6 | 1.75E6 | 2.11E6 | 1.95E6 | 9 | 1 | 1 | 1 | 1 | 0 | 1 | 1 | 1 | 1 | 1 | 1111 | 1118 |  |
| R.SGFPLLK.D | Y | 44.22 | 760.4483 | 7 | 1.0 | 381.2318 | 2 | 68.19 | 3 | 19389 | Ana\_02.raw |  | 1.67E6 | 3.79E6 | 3.3E6 | 5.89E6 | 6.59E6 |  | 5.06E6 | 2.7E6 |  | 9 | 0 | 1 | 2 | 1 | 1 | 1 | 0 | 1 | 2 | 0 | 432 | 438 |  |
| K.VDIILNR.I | Y | 43.50 | 841.5021 | 7 | 3.8 | 421.7599 | 2 | 55.75 | 2 | 15815 | Ana\_01.raw | 7.52E6 | 9.92E6 | 1.1E7 | 5.47E6 | 8.39E6 | 1.09E7 | 6.07E6 | 7.15E6 |  | 5.59E6 | 9 | 1 | 1 | 1 | 1 | 1 | 1 | 1 | 1 | 0 | 1 | 1252 | 1258 |  |
| R.DLTDFLK.N | Y | 43.25 | 850.4436 | 7 | 0.7 | 426.2294 | 2 | 94.47 | 3 | 26446 | Ana\_02.raw | 6.02E5 |  | 7.7E5 |  |  | 1.52E6 |  |  | 2.14E6 | 1.91E5 | 5 | 1 | 0 | 1 | 0 | 0 | 1 | 0 | 0 | 1 | 1 | 1963 | 1969 |  |
| K.M(+15.99)EFPLNAHFSK.D | Y | 42.25 | 1335.6282 | 11 | 3.3 | 446.2181 | 3 | 65.06 | 2 | 18544 | Ana\_01.raw |  | 4.58E6 |  |  |  |  |  |  |  |  | 1 | 0 | 1 | 0 | 0 | 0 | 0 | 0 | 0 | 0 | 0 | 672 | 682 | Oxidation (M) |
| K.LLDFLSR.R | Y | 41.90 | 862.4912 | 7 | 3.9 | 432.2546 | 2 | 87.99 | 9 | 24995 | Ana\_08.raw |  |  |  |  |  |  |  |  | 1.05E6 |  | 1 | 0 | 0 | 0 | 0 | 0 | 0 | 0 | 0 | 1 | 0 | 2088 | 2094 |  |
| R.GFM(+15.99)YDRPLR.L | Y | 41.20 | 1169.5652 | 9 | -0.5 | 390.8622 | 3 | 46.72 | 3 | 13190 | Ana\_02.raw | 4.43E6 |  | 2.98E6 | 1.4E6 | 2.85E6 | 4.53E6 |  |  | 3.88E6 |  | 6 | 1 | 0 | 1 | 1 | 1 | 1 | 0 | 0 | 1 | 0 | 1389 | 1397 | Oxidation (M) |
| R.VGSVQELSELSETVLETLHDAMHETLC(+57.02)PGVTDVSK.A | Y | 37.50 | 3809.8340 | 35 | 3.8 | 953.4694 | 4 | 140.46 | 9 | 38720 | Ana\_08.raw |  |  |  |  |  |  |  |  | 3.78E6 |  | 1 | 0 | 0 | 0 | 0 | 0 | 0 | 0 | 0 | 1 | 0 | 1189 | 1223 | Carbamidomethylation |
| R.DAITAVR.K | Y | 37.07 | 744.4130 | 7 | 3.4 | 373.2150 | 2 | 30.65 | 6 | 8330 | Ana\_05.raw |  |  |  |  | 5.08E6 | 8.53E6 | 5.37E6 | 9.26E6 |  |  | 4 | 0 | 0 | 0 | 0 | 1 | 1 | 1 | 1 | 0 | 0 | 99 | 105 |  |
| R.LVFTVR.E | Y | 35.95 | 733.4486 | 6 | 3.7 | 367.7329 | 2 | 61.87 | 7 | 17513 | Ana\_06.raw | 3.24E6 |  |  | 2.58E6 | 4.17E6 | 6.21E6 | 3.63E6 | 3.65E6 |  |  | 6 | 1 | 0 | 0 | 1 | 1 | 1 | 1 | 1 | 0 | 0 | 982 | 987 |  |
| R.GYPGDEGGPGER.G | Y | 34.59 | 1189.4999 | 12 | 7.6 | 595.7617 | 2 | 30.08 | 10 | 8097 | Ana\_09.raw |  |  |  |  |  |  |  |  |  | 2.92E6 | 1 | 0 | 0 | 0 | 0 | 0 | 0 | 0 | 0 | 0 | 1 | 1452 | 1463 |  |
| R.GPSVVLK.Q | Y | 33.87 | 698.4326 | 7 | 5.0 | 350.2253 | 2 | 29.28 | 2 | 7794 | Ana\_01.raw |  | 1.05E7 |  |  | 7.43E6 | 6.3E6 |  | 6.23E6 |  |  | 4 | 0 | 1 | 0 | 0 | 1 | 1 | 0 | 1 | 0 | 0 | 540 | 546 |  |
| R.LNIGPSK.V | Y | 33.76 | 727.4228 | 7 | 0.6 | 364.7189 | 2 | 28.81 | 3 | 7766 | Ana\_02.raw | 7.44E6 |  | 6.42E6 |  | 4.74E6 |  |  |  | 5.82E6 | 3.34E6 | 5 | 1 | 0 | 1 | 0 | 1 | 0 | 0 | 0 | 1 | 1 | 856 | 862 |  |
| R.DFVSR.I | N | 32.72 | 622.3074 | 5 | 2.3 | 312.1617 | 2 | 25.06 | 5 | 6571 | Ana\_04.raw |  |  |  |  | 2.71E6 |  |  |  |  |  | 1 | 0 | 0 | 0 | 0 | 1 | 0 | 0 | 0 | 0 | 0 | 847 | 851 |  |
| K.AC(+57.02)C(+57.02)GVPC(+57.02)K.C | Y | 31.85 | 950.3773 | 8 | 1.1 | 476.1964 | 2 | 21.53 | 7 | 5389 | Ana\_06.raw |  |  |  |  |  |  | 1.81E6 |  |  |  | 1 | 0 | 0 | 0 | 0 | 0 | 0 | 1 | 0 | 0 | 0 | 1418 | 1425 | Carbamidomethylation |
| K.DFVQR.V | Y | 31.52 | 663.3340 | 5 | 4.8 | 332.6759 | 2 | 25.05 | 6 | 6571 | Ana\_05.raw |  |  |  | 3.46E6 | 2.43E6 | 6.71E6 |  |  |  |  | 3 | 0 | 0 | 0 | 1 | 1 | 1 | 0 | 0 | 0 | 0 | 439 | 443 |  |
| R.VANLER.L | Y | 31.48 | 700.3868 | 6 | 0.8 | 351.2010 | 2 | 20.17 | 5 | 5040 | Ana\_04.raw |  |  |  | 2.89E6 | 1.17E7 |  | 3.01E6 |  |  |  | 3 | 0 | 0 | 0 | 1 | 1 | 0 | 1 | 0 | 0 | 0 | 1375 | 1380 |  |
| R.RDLTDFLK.N | Y | 30.00 | 1006.5447 | 8 | 2.5 | 504.2809 | 2 | 76.55 | 6 | 21926 | Ana\_05.raw |  |  |  |  |  | 1.3E6 |  |  |  |  | 1 | 0 | 0 | 0 | 0 | 0 | 1 | 0 | 0 | 0 | 0 | 1962 | 1969 |  |
| R.LM(+15.99)HLEFGR.G | Y | 28.81 | 1017.5066 | 8 | 3.4 | 340.1773 | 3 | 48.45 | 6 | 13739 | Ana\_05.raw |  |  |  |  |  | 3.41E6 |  |  |  |  | 1 | 0 | 0 | 0 | 0 | 0 | 1 | 0 | 0 | 0 | 0 | 1381 | 1388 | Oxidation (M) |
| R.SGFLM(+15.99)R.K | Y | 27.77 | 725.3530 | 6 | 2.7 | 363.6848 | 2 | 38.19 | 4 | 10292 | Ana\_03.raw |  |  | 9.17E6 | 4.76E6 |  |  |  |  |  |  | 2 | 0 | 0 | 1 | 1 | 0 | 0 | 0 | 0 | 0 | 0 | 1894 | 1899 | Oxidation (M) |
| R.QIIDAINK.V | Y | 22.76 | 913.5233 | 8 | 6.1 | 457.7717 | 2 | 52.75 | 2 | 14912 | Ana\_01.raw |  | 1.77E6 |  |  |  |  |  |  |  |  | 1 | 0 | 1 | 0 | 0 | 0 | 0 | 0 | 0 | 0 | 0 | 1091 | 1098 |  |
| R.NIFVR.S | N | 22.75 | 647.3755 | 5 | 2.9 | 324.6960 | 2 | 40.26 | 5 | 11188 | Ana\_04.raw |  |  |  |  | 2.22E6 |  |  |  |  |  | 1 | 0 | 0 | 0 | 0 | 1 | 0 | 0 | 0 | 0 | 0 | 318 | 322 |  |
| total 83 peptides |  |  |  |  |  |  |  |  |  |  |  |  |  |  |  |  |  |  |  |  |  |  |  |  |  |  |  |  |  |  |  |  |  |  |  |
| --- | --- | --- | --- | --- | --- | --- | --- | --- | --- | --- | --- | --- | --- | --- | --- | --- | --- | --- | --- | --- | --- | --- | --- | --- | --- | --- | --- | --- | --- | --- | --- | --- | --- | --- | --- |

Best Unique PSM (Scan F6:37183, m/z=849.9412, z=4, RT=133.75, ppm=3.2):

A0A087WS16|A0A087WS16\_MOUSE

back to list

  

| Protein Coverage
| Supporting Peptides
| Best Unique PSM
|

Protein Coverage:

Supporting Peptides:

| Peptide | Uniq | -10lgP | Mass | Length | ppm | m/z | z | RT | Fraction | Scan | Source File | Area 10 | Area 01 | Area 02 | Area 03 | Area 04 | Area 05 | Area 06 | Area 07 | Area 08 | Area 09 | #Spec | #Spec 10 | #Spec 01 | #Spec 02 | #Spec 03 | #Spec 04 | #Spec 05 | #Spec 06 | #Spec 07 | #Spec 08 | #Spec 09 | Start | End | PTM |
| --- | --- | --- | --- | --- | --- | --- | --- | --- | --- | --- | --- | --- | --- | --- | --- | --- | --- | --- | --- | --- | --- | --- | --- | --- | --- | --- | --- | --- | --- | --- | --- | --- | --- | --- | --- |
| R.YPPSVVESDAADIVFLIDSSDAVKPDGIAHIR.D | Y | 152.52 | 3395.7249 | 32 | 3.2 | 849.9412 | 4 | 133.75 | 6 | 37183 | Ana\_05.raw | 1.64E6 | 5.72E6 | 2.75E6 |  |  | 4.27E6 | 2.02E6 | 9.68E5 | 3.97E6 | 2.89E6 | 8 | 1 | 1 | 1 | 0 | 0 | 1 | 1 | 1 | 1 | 1 | 815 | 846 |  |
| R.VGSVQELSELSETVLETLHDAM(+15.99)HETLC(+57.02)PGVTDVSK.A | Y | 132.56 | 3825.8289 | 35 | 1.0 | 957.4655 | 4 | 135.76 | 3 | 37137 | Ana\_02.raw | 8.57E6 | 9.6E6 | 1.86E6 |  | 2.82E6 | 1.25E6 |  | 4.8E6 | 1.11E7 |  | 10 | 1 | 2 | 1 | 0 | 1 | 2 | 0 | 1 | 2 | 0 | 1189 | 1223 | Oxidation (M); Carbamidomethylation |
| R.IGVVQFSNDVFPEFYLK.T | Y | 115.60 | 2001.0247 | 17 | 3.1 | 1001.5227 | 2 | 131.84 | 6 | 36641 | Ana\_05.raw | 8.08E6 | 1.13E7 | 1.04E7 | 2.91E6 | 8.69E6 | 1.52E7 |  | 8.11E6 | 1.97E7 | 1.15E7 | 9 | 1 | 1 | 1 | 1 | 1 | 1 | 0 | 1 | 1 | 1 | 865 | 881 |  |
| R.LNLLDLDYELAEQLDNIAEK.A | Y | 106.05 | 2331.1846 | 20 | 0.6 | 1166.6002 | 2 | 137.69 | 6 | 38241 | Ana\_05.raw |  |  |  |  |  | 1.19E6 |  |  |  |  | 2 | 0 | 0 | 0 | 0 | 0 | 2 | 0 | 0 | 0 | 0 | 1398 | 1417 |  |
| R.LTLLGGPTPNTGAALEFVLR.N | Y | 104.49 | 2039.1415 | 20 | 2.2 | 1020.5803 | 2 | 131.30 | 6 | 36482 | Ana\_05.raw | 4.12E6 | 1.91E6 | 1.85E5 |  | 2.44E6 | 4.58E6 | 2.53E6 | 1.66E6 | 7.63E6 |  | 14 | 2 | 1 | 1 | 0 | 2 | 2 | 2 | 2 | 2 | 0 | 488 | 507 |  |
| R.VGLVQYNSDPTDEFFLR.D | Y | 102.38 | 1998.9686 | 17 | 1.5 | 1000.4930 | 2 | 114.78 | 3 | 31710 | Ana\_02.raw | 3.37E6 | 1.27E7 | 8.66E6 | 1.44E5 | 1.11E6 | 1.24E7 |  | 1.14E6 | 4.52E6 | 5.65E6 | 14 | 3 | 2 | 2 | 1 | 1 | 2 | 0 | 1 | 1 | 1 | 1068 | 1084 |  |
| K.ISLSPEYVYSVSTFR.E | Y | 99.24 | 1746.8828 | 15 | -0.4 | 874.4484 | 2 | 114.37 | 6 | 32002 | Ana\_05.raw | 9.96E6 | 2.87E7 | 1.42E7 | 1.08E7 | 1.3E7 | 1.87E7 | 1.37E7 | 2.65E6 | 2.96E7 | 1.42E7 | 28 | 3 | 4 | 2 | 3 | 3 | 2 | 3 | 3 | 4 | 1 | 771 | 785 |  |
| R.IAEGVPQLLIVLTAEPSGDDVR.G | Y | 97.77 | 2291.2373 | 22 | 3.4 | 764.7557 | 3 | 132.81 | 7 | 36587 | Ana\_06.raw | 2.93E6 | 2.13E6 | 3.61E6 | 1.18E6 | 2.33E6 | 5.14E6 | 2.4E6 |  | 3.6E6 |  | 12 | 2 | 1 | 2 | 1 | 1 | 2 | 2 | 0 | 1 | 0 | 518 | 539 |  |
| K.SALLDSIQNLQVALTSK.Q | Y | 96.91 | 1799.9993 | 17 | 1.6 | 901.0084 | 2 | 130.56 | 3 | 35764 | Ana\_02.raw | 1.44E7 | 1.22E7 | 1.54E7 | 4.4E6 | 8.86E6 | 1.58E7 | 7.63E6 | 7.62E6 | 1.92E7 | 2.67E6 | 10 | 1 | 1 | 1 | 1 | 1 | 1 | 1 | 1 | 1 | 1 | 1857 | 1873 |  |
| K.LLTPITTLTSQQIHQILASTR.Y | Y | 94.82 | 2334.3271 | 21 | 1.4 | 779.1174 | 3 | 129.21 | 7 | 35553 | Ana\_06.raw | 2.06E7 |  | 0 |  |  |  | 3.15E6 | 6.31E6 | 3.45E7 | 1.4E7 | 6 | 1 | 0 | 1 | 0 | 0 | 0 | 1 | 1 | 1 | 1 | 794 | 814 |  |
| K.IGDLQSQIVSLLK.A | Y | 92.46 | 1412.8239 | 13 | 3.6 | 707.4218 | 2 | 127.31 | 6 | 35384 | Ana\_05.raw | 6.51E6 | 9.84E6 | 2.04E7 | 1.29E7 | 2.59E7 | 3.59E7 | 3.72E6 | 7.15E5 | 4.58E7 | 2.65E7 | 21 | 3 | 3 | 4 | 1 | 2 | 2 | 1 | 1 | 2 | 2 | 394 | 406 |  |
| R.IEDNVQQFLVLLVAGR.S | Y | 91.43 | 1813.0098 | 16 | 5.6 | 907.5173 | 2 | 136.72 | 7 | 37643 | Ana\_06.raw |  | 2.81E5 |  | 1.46E5 |  | 1.44E6 | 4.35E5 | 7.5E5 |  |  | 7 | 0 | 1 | 0 | 1 | 0 | 2 | 2 | 1 | 0 | 0 | 328 | 343 |  |
| K.SDDEVDDSAVELK.Q | Y | 91.02 | 1420.6205 | 13 | 1.5 | 711.3186 | 2 | 52.19 | 3 | 14783 | Ana\_02.raw | 1.11E7 | 1.83E7 | 1.3E7 | 5.33E6 | 1.07E7 | 1.15E7 | 8.05E6 | 8.08E6 | 1.89E7 | 1.24E7 | 13 | 1 | 2 | 1 | 1 | 2 | 1 | 1 | 1 | 1 | 2 | 738 | 750 |  |
| R.LLPSFVSSENAFYLPPDLR.K | Y | 90.86 | 2164.1204 | 19 | 5.5 | 1083.0734 | 2 | 130.86 | 1 | 35971 | ANa\_10.raw | 1.04E6 |  |  |  |  |  |  |  |  |  | 1 | 1 | 0 | 0 | 0 | 0 | 0 | 0 | 0 | 0 | 0 | 2205 | 2223 |  |
| K.NANPSELEQIVLSPAFILAAESLPK.I | Y | 89.82 | 2650.4216 | 25 | 3.0 | 884.4838 | 3 | 138.72 | 9 | 38295 | Ana\_08.raw |  | 1.36E6 | 2.75E6 |  | 1.85E6 | 3.83E6 | 2.57E6 | 7E5 | 7.96E6 | 8.87E5 | 11 | 0 | 1 | 2 | 0 | 1 | 1 | 2 | 1 | 2 | 1 | 369 | 393 |  |
| R.TLTGTTEESKR.D | Y | 86.95 | 1221.6201 | 11 | 2.2 | 611.8187 | 2 | 20.19 | 7 | 4954 | Ana\_06.raw |  | 1.35E7 | 9.29E6 | 7.36E6 |  | 2.15E7 | 1.14E7 | 7.67E6 | 3.05E6 | 7.47E6 | 13 | 0 | 1 | 1 | 2 | 0 | 2 | 2 | 2 | 2 | 1 | 218 | 228 |  |
| K.ALNLGYALDYALR.N | Y | 85.48 | 1451.7772 | 13 | 4.7 | 726.8993 | 2 | 125.58 | 6 | 34932 | Ana\_05.raw | 1.45E6 | 1.94E6 | 4.28E5 | 3.64E5 | 4.86E6 | 1.08E7 | 4.6E6 | 1.96E6 | 1.45E7 | 1.89E6 | 15 | 2 | 2 | 1 | 1 | 2 | 2 | 1 | 1 | 2 | 1 | 305 | 317 |  |
| R.FASEIVDTVYEDGDSIR.V | Y | 85.44 | 1914.8846 | 17 | 0.5 | 958.4501 | 2 | 95.08 | 6 | 26950 | Ana\_05.raw |  | 1.01E7 | 6.05E5 |  | 2.03E6 | 4.84E6 | 2.53E6 |  | 3.96E6 | 3.41E5 | 11 | 0 | 1 | 1 | 0 | 1 | 3 | 2 | 0 | 2 | 1 | 1051 | 1067 |  |
| R.VSVM(+15.99)ANTPSGPVEAFDFAEYQPELFEK.F | Y | 83.06 | 3017.4004 | 27 | -0.3 | 1006.8071 | 3 | 130.01 | 6 | 36117 | Ana\_05.raw |  |  |  |  |  | 4.9E6 |  |  |  |  | 2 | 0 | 0 | 0 | 0 | 0 | 2 | 0 | 0 | 0 | 0 | 1277 | 1303 | Oxidation (M) |
| K.LSDAGITPLFLTSQEDR.Q | Y | 82.10 | 1861.9421 | 17 | 10.0 | 931.9876 | 2 | 107.93 | 6 | 30347 | Ana\_05.raw |  | 2.23E7 | 1.12E7 | 5.61E6 | 4.66E6 | 1.16E7 | 7.24E6 |  | 3.03E6 | 7.4E5 | 11 | 0 | 2 | 1 | 1 | 2 | 2 | 1 | 0 | 1 | 1 | 1923 | 1939 |  |
| R.VAIAQFSDDVR.L | Y | 82.06 | 1219.6196 | 11 | 4.9 | 610.8201 | 2 | 70.21 | 2 | 20000 | Ana\_01.raw | 1.94E7 | 2.5E7 | 2.26E7 | 9.16E6 | 1.62E7 | 2.26E7 | 1.17E7 | 1.37E7 | 2.89E7 | 8.41E5 | 12 | 1 | 1 | 1 | 2 | 2 | 1 | 1 | 1 | 1 | 1 | 267 | 277 |  |
| R.GGPGQPGFEGEQGTR.G | Y | 81.43 | 1472.6643 | 15 | 2.9 | 737.3416 | 2 | 38.53 | 6 | 10702 | Ana\_05.raw | 1.85E7 | 1.93E7 | 1.58E7 | 6.15E6 | 1.38E7 | 1.66E7 | 9.29E6 | 1.13E7 | 8.14E6 | 1.13E7 | 10 | 1 | 1 | 1 | 1 | 1 | 1 | 1 | 1 | 1 | 1 | 1603 | 1617 |  |
| K.SLDEVSQPAQELK.R | Y | 79.32 | 1442.7252 | 13 | 5.1 | 722.3736 | 2 | 57.11 | 3 | 16212 | Ana\_02.raw |  | 6.78E6 | 7.17E6 | 2.33E6 | 5.11E6 | 6.88E6 | 4.41E6 | 3.8E6 |  | 6.52E6 | 8 | 0 | 1 | 1 | 1 | 1 | 1 | 1 | 1 | 0 | 1 | 153 | 165 |  |
| R.ELGTIQQVISER.V | Y | 79.27 | 1371.7357 | 12 | 4.9 | 686.8785 | 2 | 86.16 | 6 | 24543 | Ana\_05.raw | 4.83E6 | 1.97E7 | 1.82E7 | 5.9E6 | 1.17E7 | 1.65E7 | 9.79E6 | 1.08E7 | 2.64E7 | 1.05E7 | 10 | 1 | 1 | 1 | 1 | 1 | 1 | 1 | 1 | 1 | 1 | 583 | 594 |  |
| R.VVESLDVGPDR.V | Y | 78.80 | 1184.6036 | 11 | 2.6 | 593.3106 | 2 | 52.32 | 3 | 14818 | Ana\_02.raw | 1.72E7 | 2.43E7 | 2.86E7 | 8.76E6 | 1.89E7 | 2.5E7 | 1.61E7 | 1.56E7 | 2.66E7 | 2.13E7 | 18 | 1 | 1 | 2 | 1 | 2 | 2 | 2 | 3 | 2 | 2 | 444 | 454 |  |
| R.VAVIQFSEDSK.M | Y | 76.30 | 1221.6240 | 11 | 0.5 | 611.8196 | 2 | 61.42 | 3 | 17495 | Ana\_02.raw | 7.43E6 | 1.17E7 | 1.15E7 | 4.99E6 | 8.24E6 |  |  | 7.23E6 | 7.98E6 |  | 8 | 1 | 2 | 1 | 1 | 1 | 0 | 0 | 1 | 1 | 0 | 661 | 671 |  |
| R.SSDAVAGPASSLK.Q | Y | 75.69 | 1188.5986 | 13 | 6.2 | 595.3103 | 2 | 39.04 | 1 | 10719 | ANa\_10.raw | 2.34E7 | 2.85E7 | 2.65E7 | 1.09E7 | 2.13E7 | 2.7E7 | 1.68E7 | 1.77E7 | 8.49E5 | 1.7E7 | 17 | 2 | 1 | 2 | 2 | 2 | 2 | 2 | 2 | 1 | 1 | 344 | 356 |  |
| K.GGSQVYIGNALEYVLKNIFQRPLGSR.I | Y | 75.68 | 2878.5452 | 26 | 5.7 | 720.6476 | 4 | 137.55 | 2 | 38012 | Ana\_01.raw |  | 2.3E6 |  |  |  | 5.94E5 |  | 2.21E5 |  | 2.1E6 | 4 | 0 | 1 | 0 | 0 | 0 | 1 | 0 | 1 | 0 | 1 | 696 | 721 |  |
| R.IEDGVPQHLVLFLGGK.S | Y | 74.98 | 1720.9512 | 16 | 0.8 | 861.4836 | 2 | 111.47 | 6 | 31260 | Ana\_05.raw | 2.54E6 | 7.63E5 | 2.43E6 | 1.28E6 | 1.6E5 | 1.45E7 | 2.42E6 | 3.28E6 | 1.24E7 | 2.44E6 | 22 | 2 | 2 | 1 | 3 | 1 | 4 | 2 | 2 | 2 | 3 | 926 | 941 |  |
| R.HTDQEELVK.I | Y | 74.86 | 1097.5353 | 9 | 5.9 | 549.7781 | 2 | 21.30 | 3 | 5372 | Ana\_02.raw |  |  | 2.39E7 | 9.52E6 | 8.1E6 | 2.72E7 | 1.02E7 | 2.49E6 | 6.7E6 | 1.74E7 | 8 | 0 | 0 | 1 | 1 | 1 | 1 | 1 | 1 | 1 | 1 | 762 | 770 |  |
| R.VPQIAFVITGGK.S | Y | 74.79 | 1228.7179 | 12 | -0.8 | 615.3657 | 2 | 95.01 | 3 | 26588 | Ana\_02.raw | 1.05E7 | 5.95E6 | 7.32E6 | 3.09E5 | 4.76E6 | 7.28E6 | 3.28E6 | 2.55E6 | 1.12E7 | 5.77E6 | 14 | 2 | 2 | 1 | 1 | 1 | 2 | 1 | 1 | 1 | 2 | 1133 | 1144 |  |
| R.EELSSLKPILTPSTGAGVGSK.K | Y | 73.85 | 2070.1208 | 21 | 3.8 | 691.0502 | 3 | 82.43 | 6 | 23532 | Ana\_05.raw | 5.2E6 |  | 4.51E6 |  |  | 6.19E6 | 9.64E5 | 3.37E6 | 1.13E7 |  | 6 | 1 | 0 | 1 | 0 | 0 | 1 | 1 | 1 | 1 | 0 | 601 | 621 |  |
| R.ISC(+57.02)SGNQLPTVR.V | Y | 70.40 | 1330.6664 | 12 | 4.2 | 666.3433 | 2 | 48.04 | 9 | 13445 | Ana\_08.raw |  | 4.92E6 | 9.38E5 |  |  | 9.83E5 |  |  | 8.43E6 | 1.71E6 | 5 | 0 | 1 | 1 | 0 | 0 | 1 | 0 | 0 | 1 | 1 | 1265 | 1276 | Carbamidomethylation |
| K.SVEDAQDVSLALTQK.G | Y | 69.77 | 1602.8101 | 15 | 4.4 | 802.4158 | 2 | 76.68 | 6 | 21959 | Ana\_05.raw |  | 2.23E6 | 1.06E6 | 0 | 7.42E5 | 1.94E6 | 1.17E6 | 1.71E6 |  |  | 7 | 0 | 1 | 1 | 1 | 1 | 1 | 1 | 1 | 0 | 0 | 1145 | 1159 |  |
| R.NILTSSTGSR.I | Y | 69.10 | 1034.5356 | 10 | 3.4 | 518.2769 | 2 | 36.10 | 3 | 9947 | Ana\_02.raw |  |  | 1.81E7 | 2.58E6 | 1.35E7 |  | 1.05E7 |  |  | 8.99E6 | 5 | 0 | 0 | 1 | 1 | 1 | 0 | 1 | 0 | 0 | 1 | 508 | 517 |  |
| R.NNLFTSSAGHR.A | Y | 66.48 | 1202.5792 | 11 | 1.9 | 602.2980 | 2 | 33.20 | 1 | 8952 | ANa\_10.raw | 4.6E6 | 6.01E6 | 5.2E6 | 4.12E6 |  |  |  |  | 9.04E6 |  | 6 | 1 | 1 | 1 | 1 | 0 | 0 | 0 | 0 | 2 | 0 | 126 | 136 |  |
| R.NAGPEFQYIR.T | Y | 66.32 | 1193.5829 | 10 | 3.6 | 597.8008 | 2 | 68.10 | 6 | 19555 | Ana\_05.raw | 2.72E7 | 3.79E7 | 2.22E7 | 1.03E7 | 2.03E7 | 2.31E7 | 1.57E7 | 2.06E7 | 2.47E7 | 1.85E7 | 11 | 1 | 1 | 1 | 1 | 1 | 1 | 1 | 1 | 1 | 2 | 633 | 642 |  |
| R.IIEELDVKPDGTR.V | Y | 66.23 | 1483.7882 | 13 | 0.6 | 742.9018 | 2 | 57.24 | 3 | 16250 | Ana\_02.raw | 1.93E6 | 1.07E6 | 1.12E7 | 3.26E6 | 7.83E6 | 7.78E6 | 3.98E6 | 4.84E6 | 6.02E6 |  | 10 | 1 | 1 | 2 | 1 | 1 | 1 | 1 | 1 | 1 | 0 | 254 | 266 |  |
| R.VALVQYSDR.T | Y | 64.77 | 1049.5505 | 9 | 4.5 | 525.7849 | 2 | 48.76 | 1 | 13663 | ANa\_10.raw | 1.78E7 | 1.95E7 | 2.58E7 | 7.84E6 | 1.55E7 |  | 1.35E7 | 1.11E7 | 2.16E7 | 1.45E7 | 12 | 1 | 2 | 2 | 1 | 1 | 0 | 1 | 1 | 1 | 2 | 457 | 465 |  |
| R.NHFVPEAGSR.L | Y | 64.36 | 1112.5363 | 10 | 2.8 | 557.2770 | 2 | 27.50 | 2 | 7240 | Ana\_01.raw | 1.62E7 | 2.01E7 | 1.75E7 | 7.22E6 | 1.31E7 | 1.55E7 | 1.1E7 | 9.47E6 | 1.07E7 | 1.13E7 | 11 | 1 | 1 | 1 | 1 | 1 | 1 | 1 | 2 | 1 | 1 | 1119 | 1128 |  |
| R.IEEGVPQFLVLISSGK.S | Y | 64.29 | 1714.9504 | 16 | 4.0 | 572.6597 | 3 | 132.21 | 7 | 36414 | Ana\_06.raw | 4.36E6 | 4.29E6 |  | 3.41E6 | 7.63E6 | 1.46E6 | 1.06E7 | 6.12E6 | 1.2E7 | 7.22E6 | 11 | 1 | 1 | 1 | 1 | 1 | 1 | 2 | 1 | 1 | 1 | 722 | 737 |  |
| R.DQNVFVSQK.G | Y | 64.18 | 1063.5298 | 9 | 4.8 | 532.7747 | 2 | 38.53 | 6 | 10700 | Ana\_05.raw | 1.72E7 | 1.74E7 | 1.88E7 | 7.43E6 | 1.66E7 | 1.96E7 | 1.2E7 | 1.45E7 | 1.37E7 | 1.41E7 | 12 | 1 | 1 | 1 | 1 | 2 | 1 | 1 | 1 | 1 | 2 | 1238 | 1246 |  |
| R.GETGDDGRDGVGSEGR.R | Y | 62.82 | 1562.6556 | 16 | 4.0 | 521.8946 | 3 | 21.25 | 1 | 5184 | ANa\_10.raw | 3.3E6 | 0 | 1.34E6 |  |  |  |  |  |  | 2.08E6 | 4 | 1 | 1 | 1 | 0 | 0 | 0 | 0 | 0 | 0 | 1 | 1681 | 1696 |  |
| R.TDLQTITNDPR.L | Y | 61.60 | 1272.6310 | 11 | -0.7 | 637.3223 | 2 | 49.49 | 3 | 13993 | Ana\_02.raw | 1.31E7 | 1.89E7 | 1.36E7 | 5.18E6 | 8.93E6 |  | 7.04E6 | 7.64E6 |  | 9.21E6 | 8 | 1 | 1 | 1 | 1 | 1 | 0 | 1 | 1 | 0 | 1 | 971 | 981 |  |
| K.VAVFFSNKPTR.A | Y | 59.61 | 1264.6927 | 11 | 2.0 | 422.5724 | 3 | 53.65 | 6 | 15297 | Ana\_05.raw | 4.27E6 | 4.16E6 | 4.55E6 | 4.77E5 | 1.97E6 | 5.19E6 | 3.14E6 |  |  | 3.29E6 | 8 | 1 | 1 | 1 | 1 | 1 | 1 | 1 | 0 | 0 | 1 | 1901 | 1911 |  |
| K.IASNSATAFR.V | Y | 59.24 | 1036.5302 | 10 | 4.3 | 519.2746 | 2 | 35.24 | 5 | 9662 | Ana\_04.raw | 1.99E7 | 3.19E7 | 2.4E7 |  | 1.75E7 | 2.49E7 | 1.67E7 | 1.57E7 | 1.15E7 | 1.77E7 | 12 | 2 | 2 | 1 | 0 | 1 | 1 | 1 | 2 | 1 | 1 | 1179 | 1188 |  |
| R.GDSVDQC(+57.02)ALIQSIR.D | Y | 58.80 | 1560.7566 | 14 | 5.8 | 781.3901 | 2 | 80.80 | 9 | 23007 | Ana\_08.raw | 9.96E5 | 2.33E6 |  |  |  |  |  |  | 3.99E6 |  | 4 | 2 | 1 | 0 | 0 | 0 | 0 | 0 | 0 | 1 | 0 | 1761 | 1774 | Carbamidomethylation |
| K.VFAVGVR.N | Y | 58.29 | 746.4438 | 7 | 1.8 | 374.2299 | 2 | 48.31 | 6 | 13696 | Ana\_05.raw | 8.77E6 | 1.03E7 | 1.22E7 |  | 8.72E6 | 1.24E7 | 7.99E6 | 6.71E6 | 1.25E7 | 1.11E7 | 10 | 1 | 1 | 1 | 0 | 1 | 1 | 1 | 1 | 2 | 1 | 1163 | 1169 |  |
| R.ELPNIEER.V | Y | 57.56 | 998.5032 | 8 | 1.6 | 500.2597 | 2 | 51.56 | 6 | 14675 | Ana\_05.raw | 1.95E7 | 2.06E7 | 1.99E7 | 7.88E6 | 1.63E7 | 1.8E7 | 1.18E7 | 1.21E7 | 2.42E7 |  | 18 | 2 | 2 | 2 | 2 | 2 | 2 | 2 | 2 | 2 | 0 | 991 | 998 |  |
| K.SQSSVLEAIR.R | Y | 57.50 | 1088.5825 | 10 | 4.2 | 545.3008 | 2 | 63.57 | 7 | 18023 | Ana\_06.raw | 9.54E6 | 1.27E7 | 1.75E7 | 6.76E6 | 1.55E7 | 1.27E7 | 9.48E6 | 9.48E6 | 1.77E7 | 2.69E6 | 10 | 1 | 1 | 1 | 1 | 1 | 1 | 1 | 1 | 1 | 1 | 885 | 894 |  |
| R.GVVPFIFQAK.N | Y | 55.98 | 1104.6331 | 10 | 3.5 | 553.3257 | 2 | 102.03 | 6 | 28790 | Ana\_05.raw | 8.47E6 |  | 5.87E6 | 3.07E5 | 5.67E6 | 8.49E6 | 9.6E5 | 3.91E6 | 1.24E7 | 6.58E6 | 13 | 2 | 0 | 2 | 1 | 1 | 2 | 1 | 2 | 1 | 1 | 359 | 368 |  |
| R.DSFQEVLR.F | Y | 54.85 | 992.4927 | 8 | 2.5 | 497.2549 | 2 | 64.69 | 6 | 18579 | Ana\_05.raw | 7.85E6 | 1.09E7 | 1.27E7 | 6.27E6 | 1.03E7 | 1.08E7 | 7.49E6 | 8.79E6 |  | 7.55E6 | 11 | 2 | 2 | 1 | 1 | 1 | 1 | 1 | 1 | 0 | 1 | 1043 | 1050 |  |
| K.AEILNLVK.K | Y | 53.09 | 898.5487 | 8 | 0.9 | 450.2820 | 2 | 73.55 | 6 | 21099 | Ana\_05.raw | 9.24E6 | 1E7 | 8.09E6 | 3.75E6 | 6.62E6 | 8.33E6 | 4.12E6 | 5.12E6 | 1.01E7 | 6.05E6 | 11 | 2 | 1 | 1 | 1 | 1 | 1 | 1 | 1 | 1 | 1 | 289 | 296 |  |
| R.GPIGSIGPK.G | Y | 52.23 | 824.4756 | 9 | 2.8 | 413.2462 | 2 | 39.12 | 2 | 10782 | Ana\_01.raw | 5.95E6 | 6.19E6 | 7.4E6 | 1.38E6 |  |  |  | 3.43E6 |  |  | 5 | 1 | 1 | 1 | 1 | 0 | 0 | 0 | 1 | 0 | 0 | 1434 | 1442 |  |
| R.TLTGTTEESK.R | Y | 51.90 | 1065.5189 | 10 | 1.0 | 533.7673 | 2 | 21.27 | 8 | 5220 | Ana\_07.raw |  |  |  | 1.04E6 |  |  | 1.14E6 | 1.58E6 |  |  | 3 | 0 | 0 | 0 | 1 | 0 | 0 | 1 | 1 | 0 | 0 | 218 | 227 |  |
| K.DVVFLIDGSR.N | Y | 49.26 | 1119.5924 | 10 | 4.6 | 560.8060 | 2 | 93.08 | 6 | 26410 | Ana\_05.raw |  |  |  |  | 1.45E5 | 3.4E5 |  |  |  |  | 2 | 0 | 0 | 0 | 0 | 1 | 1 | 0 | 0 | 0 | 0 | 623 | 632 |  |
| R.ALEFVAR.N | Y | 49.09 | 804.4493 | 7 | 4.5 | 403.2338 | 2 | 51.78 | 2 | 14621 | Ana\_01.raw | 2.15E7 | 1.77E7 | 1.87E7 | 8.9E6 | 1.41E7 | 1.75E7 | 1.52E7 | 1.24E7 | 2.65E7 | 2.04E7 | 16 | 1 | 1 | 2 | 2 | 2 | 2 | 2 | 2 | 1 | 1 | 909 | 915 |  |
| K.GISGEDGYR.G | Y | 49.07 | 952.4250 | 9 | 3.0 | 477.2212 | 2 | 27.14 | 3 | 7241 | Ana\_02.raw |  |  | 9.62E6 |  |  |  |  | 4.46E6 |  |  | 3 | 0 | 0 | 1 | 0 | 0 | 0 | 0 | 2 | 0 | 0 | 1443 | 1451 |  |
| K.DEVQNAVR.R | Y | 48.53 | 929.4567 | 8 | 2.5 | 465.7368 | 2 | 26.41 | 8 | 6775 | Ana\_07.raw | 4.77E6 | 2.8E6 | 2.21E7 | 1.01E7 | 1.36E7 | 2.47E7 |  | 1.15E7 |  |  | 7 | 1 | 1 | 1 | 1 | 1 | 1 | 0 | 1 | 0 | 0 | 683 | 690 |  |
| R.NIDSEEVGK.I | Y | 47.38 | 989.4666 | 9 | 1.5 | 495.7413 | 2 | 26.25 | 4 | 6770 | Ana\_03.raw |  |  |  | 1.23E7 |  | 2.9E7 |  |  |  | 0 | 3 | 0 | 0 | 0 | 1 | 0 | 1 | 0 | 0 | 0 | 1 | 1170 | 1178 |  |
| R.TLIER.I | N | 45.14 | 630.3701 | 5 | 1.7 | 316.1928 | 2 | 23.18 | 3 | 5981 | Ana\_02.raw |  |  | 8.6E6 |  |  |  |  |  |  |  | 1 | 0 | 0 | 1 | 0 | 0 | 0 | 0 | 0 | 0 | 0 | 643 | 647 |  |
| R.VGIEHLLR.N | Y | 45.02 | 935.5552 | 8 | -0.4 | 468.7847 | 2 | 55.32 | 3 | 15702 | Ana\_02.raw | 2.7E6 | 3.77E6 | 2.77E6 | 8.48E5 |  | 3.72E6 | 1.89E6 | 1.75E6 | 2.11E6 | 1.95E6 | 9 | 1 | 1 | 1 | 1 | 0 | 1 | 1 | 1 | 1 | 1 | 1111 | 1118 |  |
| R.SGFPLLK.D | Y | 44.22 | 760.4483 | 7 | 1.0 | 381.2318 | 2 | 68.19 | 3 | 19389 | Ana\_02.raw |  | 1.67E6 | 3.79E6 | 3.3E6 | 5.89E6 | 6.59E6 |  | 5.06E6 | 2.7E6 |  | 9 | 0 | 1 | 2 | 1 | 1 | 1 | 0 | 1 | 2 | 0 | 432 | 438 |  |
| K.VDIILNR.I | Y | 43.50 | 841.5021 | 7 | 3.8 | 421.7599 | 2 | 55.75 | 2 | 15815 | Ana\_01.raw | 7.52E6 | 9.92E6 | 1.1E7 | 5.47E6 | 8.39E6 | 1.09E7 | 6.07E6 | 7.15E6 |  | 5.59E6 | 9 | 1 | 1 | 1 | 1 | 1 | 1 | 1 | 1 | 0 | 1 | 1252 | 1258 |  |
| R.DLTDFLK.N | Y | 43.25 | 850.4436 | 7 | 0.7 | 426.2294 | 2 | 94.47 | 3 | 26446 | Ana\_02.raw | 6.02E5 |  | 7.7E5 |  |  | 1.52E6 |  |  | 2.14E6 | 1.91E5 | 5 | 1 | 0 | 1 | 0 | 0 | 1 | 0 | 0 | 1 | 1 | 1963 | 1969 |  |
| K.M(+15.99)EFPLNAHFSK.D | Y | 42.25 | 1335.6282 | 11 | 3.3 | 446.2181 | 3 | 65.06 | 2 | 18544 | Ana\_01.raw |  | 4.58E6 |  |  |  |  |  |  |  |  | 1 | 0 | 1 | 0 | 0 | 0 | 0 | 0 | 0 | 0 | 0 | 672 | 682 | Oxidation (M) |
| K.LLDFLSR.R | Y | 41.90 | 862.4912 | 7 | 3.9 | 432.2546 | 2 | 87.99 | 9 | 24995 | Ana\_08.raw |  |  |  |  |  |  |  |  | 1.05E6 |  | 1 | 0 | 0 | 0 | 0 | 0 | 0 | 0 | 0 | 1 | 0 | 2088 | 2094 |  |
| R.GFM(+15.99)YDRPLR.L | Y | 41.20 | 1169.5652 | 9 | -0.5 | 390.8622 | 3 | 46.72 | 3 | 13190 | Ana\_02.raw | 4.43E6 |  | 2.98E6 | 1.4E6 | 2.85E6 | 4.53E6 |  |  | 3.88E6 |  | 6 | 1 | 0 | 1 | 1 | 1 | 1 | 0 | 0 | 1 | 0 | 1389 | 1397 | Oxidation (M) |
| R.VGSVQELSELSETVLETLHDAMHETLC(+57.02)PGVTDVSK.A | Y | 37.50 | 3809.8340 | 35 | 3.8 | 953.4694 | 4 | 140.46 | 9 | 38720 | Ana\_08.raw |  |  |  |  |  |  |  |  | 3.78E6 |  | 1 | 0 | 0 | 0 | 0 | 0 | 0 | 0 | 0 | 1 | 0 | 1189 | 1223 | Carbamidomethylation |
| R.DAITAVR.K | Y | 37.07 | 744.4130 | 7 | 3.4 | 373.2150 | 2 | 30.65 | 6 | 8330 | Ana\_05.raw |  |  |  |  | 5.08E6 | 8.53E6 | 5.37E6 | 9.26E6 |  |  | 4 | 0 | 0 | 0 | 0 | 1 | 1 | 1 | 1 | 0 | 0 | 99 | 105 |  |
| R.LVFTVR.E | Y | 35.95 | 733.4486 | 6 | 3.7 | 367.7329 | 2 | 61.87 | 7 | 17513 | Ana\_06.raw | 3.24E6 |  |  | 2.58E6 | 4.17E6 | 6.21E6 | 3.63E6 | 3.65E6 |  |  | 6 | 1 | 0 | 0 | 1 | 1 | 1 | 1 | 1 | 0 | 0 | 982 | 987 |  |
| R.GYPGDEGGPGER.G | Y | 34.59 | 1189.4999 | 12 | 7.6 | 595.7617 | 2 | 30.08 | 10 | 8097 | Ana\_09.raw |  |  |  |  |  |  |  |  |  | 2.92E6 | 1 | 0 | 0 | 0 | 0 | 0 | 0 | 0 | 0 | 0 | 1 | 1452 | 1463 |  |
| R.GPSVVLK.Q | Y | 33.87 | 698.4326 | 7 | 5.0 | 350.2253 | 2 | 29.28 | 2 | 7794 | Ana\_01.raw |  | 1.05E7 |  |  | 7.43E6 | 6.3E6 |  | 6.23E6 |  |  | 4 | 0 | 1 | 0 | 0 | 1 | 1 | 0 | 1 | 0 | 0 | 540 | 546 |  |
| R.LNIGPSK.V | Y | 33.76 | 727.4228 | 7 | 0.6 | 364.7189 | 2 | 28.81 | 3 | 7766 | Ana\_02.raw | 7.44E6 |  | 6.42E6 |  | 4.74E6 |  |  |  | 5.82E6 | 3.34E6 | 5 | 1 | 0 | 1 | 0 | 1 | 0 | 0 | 0 | 1 | 1 | 856 | 862 |  |
| R.DFVSR.I | N | 32.72 | 622.3074 | 5 | 2.3 | 312.1617 | 2 | 25.06 | 5 | 6571 | Ana\_04.raw |  |  |  |  | 2.71E6 |  |  |  |  |  | 1 | 0 | 0 | 0 | 0 | 1 | 0 | 0 | 0 | 0 | 0 | 847 | 851 |  |
| K.AC(+57.02)C(+57.02)GVPC(+57.02)K.C | Y | 31.85 | 950.3773 | 8 | 1.1 | 476.1964 | 2 | 21.53 | 7 | 5389 | Ana\_06.raw |  |  |  |  |  |  | 1.81E6 |  |  |  | 1 | 0 | 0 | 0 | 0 | 0 | 0 | 1 | 0 | 0 | 0 | 1418 | 1425 | Carbamidomethylation |
| K.DFVQR.V | Y | 31.52 | 663.3340 | 5 | 4.8 | 332.6759 | 2 | 25.05 | 6 | 6571 | Ana\_05.raw |  |  |  | 3.46E6 | 2.43E6 | 6.71E6 |  |  |  |  | 3 | 0 | 0 | 0 | 1 | 1 | 1 | 0 | 0 | 0 | 0 | 439 | 443 |  |
| R.VANLER.L | Y | 31.48 | 700.3868 | 6 | 0.8 | 351.2010 | 2 | 20.17 | 5 | 5040 | Ana\_04.raw |  |  |  | 2.89E6 | 1.17E7 |  | 3.01E6 |  |  |  | 3 | 0 | 0 | 0 | 1 | 1 | 0 | 1 | 0 | 0 | 0 | 1375 | 1380 |  |
| R.RDLTDFLK.N | Y | 30.00 | 1006.5447 | 8 | 2.5 | 504.2809 | 2 | 76.55 | 6 | 21926 | Ana\_05.raw |  |  |  |  |  | 1.3E6 |  |  |  |  | 1 | 0 | 0 | 0 | 0 | 0 | 1 | 0 | 0 | 0 | 0 | 1962 | 1969 |  |
| R.LM(+15.99)HLEFGR.G | Y | 28.81 | 1017.5066 | 8 | 3.4 | 340.1773 | 3 | 48.45 | 6 | 13739 | Ana\_05.raw |  |  |  |  |  | 3.41E6 |  |  |  |  | 1 | 0 | 0 | 0 | 0 | 0 | 1 | 0 | 0 | 0 | 0 | 1381 | 1388 | Oxidation (M) |
| R.SGFLM(+15.99)R.K | Y | 27.77 | 725.3530 | 6 | 2.7 | 363.6848 | 2 | 38.19 | 4 | 10292 | Ana\_03.raw |  |  | 9.17E6 | 4.76E6 |  |  |  |  |  |  | 2 | 0 | 0 | 1 | 1 | 0 | 0 | 0 | 0 | 0 | 0 | 1894 | 1899 | Oxidation (M) |
| R.QIIDAINK.V | Y | 22.76 | 913.5233 | 8 | 6.1 | 457.7717 | 2 | 52.75 | 2 | 14912 | Ana\_01.raw |  | 1.77E6 |  |  |  |  |  |  |  |  | 1 | 0 | 1 | 0 | 0 | 0 | 0 | 0 | 0 | 0 | 0 | 1091 | 1098 |  |
| R.NIFVR.S | N | 22.75 | 647.3755 | 5 | 2.9 | 324.6960 | 2 | 40.26 | 5 | 11188 | Ana\_04.raw |  |  |  |  | 2.22E6 |  |  |  |  |  | 1 | 0 | 0 | 0 | 0 | 1 | 0 | 0 | 0 | 0 | 0 | 318 | 322 |  |
| total 83 peptides |  |  |  |  |  |  |  |  |  |  |  |  |  |  |  |  |  |  |  |  |  |  |  |  |  |  |  |  |  |  |  |  |  |  |  |
| --- | --- | --- | --- | --- | --- | --- | --- | --- | --- | --- | --- | --- | --- | --- | --- | --- | --- | --- | --- | --- | --- | --- | --- | --- | --- | --- | --- | --- | --- | --- | --- | --- | --- | --- | --- |

Best Unique PSM (Scan F6:37183, m/z=849.9412, z=4, RT=133.75, ppm=3.2):

sp|P31001|DESM\_MOUSE

back to list

  

| Protein Coverage
| Supporting Peptides
| Best Unique PSM
|

Protein Coverage:

Supporting Peptides:

| Peptide | Uniq | -10lgP | Mass | Length | ppm | m/z | z | RT | Fraction | Scan | Source File | Area 10 | Area 01 | Area 02 | Area 03 | Area 04 | Area 05 | Area 06 | Area 07 | Area 08 | Area 09 | #Spec | #Spec 10 | #Spec 01 | #Spec 02 | #Spec 03 | #Spec 04 | #Spec 05 | #Spec 06 | #Spec 07 | #Spec 08 | #Spec 09 | Start | End | PTM |
| --- | --- | --- | --- | --- | --- | --- | --- | --- | --- | --- | --- | --- | --- | --- | --- | --- | --- | --- | --- | --- | --- | --- | --- | --- | --- | --- | --- | --- | --- | --- | --- | --- | --- | --- | --- |
| R.APSYGAGELLDFSLADAVNQEFLATR.T | Y | 165.70 | 2754.3501 | 26 | 3.6 | 1378.1873 | 2 | 135.03 | 6 | 37541 | Ana\_05.raw | 1.59E7 | 1.21E7 | 2.96E6 | 1.38E6 | 7.19E6 | 2.58E7 | 4.36E6 | 5.6E6 | 4.03E7 | 1.63E7 | 27 | 2 | 3 | 1 | 2 | 2 | 2 | 2 | 2 | 8 | 3 | 79 | 104 |  |
| R.TFGGAPGFSLGSPLSSPVFPR.A | Y | 139.90 | 2077.0632 | 21 | 5.1 | 1039.5442 | 2 | 126.73 | 1 | 34856 | ANa\_10.raw | 1.85E8 | 1.71E8 | 1.43E8 | 3.22E7 | 2.68E8 | 1.16E8 | 6.69E7 | 9.26E7 | 7.28E8 | 1.68E8 | 93 | 16 | 8 | 9 | 3 | 9 | 5 | 4 | 9 | 16 | 14 | 17 | 37 |  |
| R.RTFGGAPGFSLGSPLSSPVFPR.A | Y | 105.04 | 2233.1643 | 22 | -1.2 | 745.3945 | 3 | 111.97 | 3 | 30970 | Ana\_02.raw |  |  | 2.5E6 | 5.14E6 | 1.62E6 | 7.8E6 | 5.23E6 | 1.34E6 |  |  | 6 | 0 | 0 | 1 | 1 | 1 | 1 | 1 | 1 | 0 | 0 | 16 | 37 |  |
| R.FLEQQNAALAAEVNR.L | Y | 104.78 | 1672.8533 | 15 | 3.3 | 837.4366 | 2 | 77.60 | 4 | 21569 | Ana\_03.raw | 9.83E7 | 6.35E7 | 2.98E7 | 1.1E7 | 2.49E7 | 3.36E7 | 1.74E7 | 2.15E7 | 1.17E8 | 4.81E7 | 34 | 5 | 5 | 2 | 2 | 2 | 2 | 2 | 3 | 7 | 4 | 127 | 141 |  |
| R.HQIQSYTC(+57.02)EIDALK.G | Y | 100.13 | 1704.8141 | 14 | 2.8 | 853.4167 | 2 | 67.95 | 9 | 19392 | Ana\_08.raw | 7.25E6 | 8.64E6 | 2.48E6 |  | 1.78E6 | 1.08E7 | 2.19E6 | 6.78E5 | 2.46E7 | 4.96E6 | 13 | 1 | 1 | 2 | 0 | 2 | 2 | 1 | 1 | 2 | 1 | 325 | 338 | Carbamidomethylation |
| R.INLPIQTFSALNFR.E | Y | 93.75 | 1632.8987 | 14 | -0.2 | 817.4565 | 2 | 128.11 | 3 | 35111 | Ana\_02.raw | 2.98E8 | 6.63E6 | 1.33E8 | 6.5E6 | 1.19E8 | 2.92E7 | 4.06E7 | 1.34E7 | 3.91E8 | 1.98E8 | 60 | 15 | 4 | 6 | 2 | 5 | 2 | 2 | 3 | 10 | 11 | 415 | 428 |  |
| R.RIESLNEEIAFLK.K | Y | 91.20 | 1560.8511 | 13 | 0.7 | 521.2913 | 3 | 97.28 | 6 | 27530 | Ana\_05.raw | 6.92E7 | 1.03E7 | 6.9E6 | 1.18E6 | 1.35E7 | 1.41E7 | 7.49E6 | 3.5E6 | 7.14E7 | 1.31E7 | 29 | 5 | 2 | 2 | 2 | 2 | 3 | 2 | 3 | 4 | 4 | 227 | 239 |  |
| R.FASEANGYQDNIAR.L | Y | 89.73 | 1554.7063 | 14 | 4.5 | 778.3640 | 2 | 52.13 | 1 | 14653 | ANa\_10.raw | 2.6E7 | 1.03E7 |  |  |  | 1.04E7 | 7.7E6 |  | 2.06E7 | 1E7 | 7 | 1 | 1 | 0 | 0 | 0 | 1 | 1 | 0 | 1 | 2 | 355 | 368 |  |
| R.IESLNEEIAFLK.K | Y | 88.49 | 1404.7500 | 12 | 1.5 | 703.3834 | 2 | 106.49 | 3 | 29569 | Ana\_02.raw | 1.27E8 | 9.24E7 | 7.08E7 | 2.21E7 | 6.46E7 | 6.44E7 | 4.29E7 | 8.21E6 | 2.22E8 | 7.74E7 | 38 | 5 | 5 | 3 | 2 | 3 | 3 | 3 | 4 | 5 | 5 | 228 | 239 |  |
| R.TSGGAGGLGSLR.S | Y | 84.28 | 1031.5359 | 12 | 3.9 | 516.7772 | 2 | 41.48 | 6 | 11611 | Ana\_05.raw | 2.68E8 | 1.81E8 | 1.3E8 | 6.41E7 | 1.4E8 | 1.45E8 | 8.57E7 | 1.08E8 | 6.48E5 | 1.9E8 | 25 | 4 | 2 | 2 | 2 | 3 | 3 | 2 | 3 | 1 | 3 | 59 | 70 |  |
| R.EEAENNLAAFR.A | Y | 83.90 | 1262.5891 | 11 | 0.9 | 632.3024 | 2 | 57.02 | 3 | 16186 | Ana\_02.raw | 2.79E8 | 1.52E8 | 1.46E8 | 5.79E7 | 1.16E8 | 1.41E8 | 8.55E7 | 7.97E7 | 2.57E8 | 1.87E8 | 36 | 3 | 3 | 4 | 3 | 3 | 4 | 4 | 4 | 4 | 4 | 201 | 211 |  |
| R.KLLEGEESR.I | N | 81.04 | 1059.5560 | 9 | 8.7 | 354.1957 | 3 | 23.79 | 6 | 6172 | Ana\_05.raw | 1.35E8 | 7.36E7 | 8.47E7 | 3.98E7 | 3.65E7 | 5.87E7 | 4.47E7 | 2.25E7 | 1.49E8 | 2.57E7 | 25 | 5 | 2 | 1 | 1 | 2 | 2 | 2 | 2 | 5 | 3 | 406 | 414 |  |
| K.M(+15.99)ALDVEIATYR.K | Y | 76.36 | 1296.6383 | 11 | 8.4 | 649.3318 | 2 | 79.88 | 6 | 22840 | Ana\_05.raw | 1.43E6 |  |  | 1.53E6 |  | 1.65E5 | 7.46E5 | 7.93E5 | 2.08E7 | 4.2E6 | 9 | 1 | 0 | 0 | 1 | 0 | 1 | 1 | 1 | 2 | 2 | 395 | 405 | Oxidation (M) |
| R.VAELYEEEM(+15.99)R.E | Y | 74.25 | 1283.5703 | 10 | 3.2 | 642.7945 | 2 | 49.26 | 7 | 13797 | Ana\_06.raw | 1.57E8 | 1.14E8 | 2.2E6 | 4.33E7 | 9.24E7 | 1.04E8 | 6.82E7 | 7.89E7 | 1.61E7 | 1.1E8 | 33 | 4 | 4 | 3 | 2 | 3 | 3 | 2 | 3 | 4 | 5 | 150 | 159 | Oxidation (M) |
| R.TNEKVELQELNDR.F | N | 73.46 | 1586.7900 | 13 | 7.9 | 794.4086 | 2 | 48.77 | 9 | 13667 | Ana\_08.raw | 1.95E7 | 6.68E6 | 4.32E6 |  | 2.98E6 | 7.99E6 | 2.95E6 |  | 3.01E7 | 1.28E7 | 10 | 1 | 1 | 2 | 0 | 1 | 1 | 1 | 0 | 2 | 1 | 105 | 117 |  |
| R.DNLIDDLQR.L | Y | 72.08 | 1100.5461 | 9 | 5.1 | 551.2831 | 2 | 80.36 | 1 | 22683 | ANa\_10.raw | 1.1E8 | 8.11E7 | 7.51E7 | 2.59E7 | 5.47E7 | 5.88E7 | 2.94E7 | 4.01E7 | 1.8E8 | 9.04E7 | 53 | 8 | 4 | 6 | 3 | 6 | 3 | 5 | 7 | 4 | 7 | 180 | 188 |  |
| K.VHEEEIR.E | Y | 71.77 | 910.4508 | 7 | 0.6 | 456.2330 | 2 | 19.38 | 4 | 4704 | Ana\_03.raw | 4.17E7 | 1.77E7 |  | 3.05E7 | 5.29E7 |  |  | 4.32E7 |  | 8.92E7 | 7 | 1 | 1 | 0 | 1 | 1 | 0 | 0 | 2 | 0 | 1 | 241 | 247 |  |
| K.KVHEEEIR.E | Y | 71.51 | 1038.5458 | 8 | 1.9 | 520.2811 | 2 | 17.41 | 3 | 4069 | Ana\_02.raw | 2.92E7 | 1.52E7 | 2.85E7 | 1.09E7 | 2.95E7 | 3.49E7 | 2.33E7 | 1.65E7 |  | 3.92E7 | 16 | 2 | 1 | 2 | 2 | 2 | 1 | 2 | 2 | 0 | 2 | 240 | 247 |  |
| K.LLEGEESR.I | N | 70.89 | 931.4611 | 8 | 2.9 | 466.7392 | 2 | 26.90 | 6 | 7157 | Ana\_05.raw | 1.04E8 | 5.04E8 | 4.04E8 | 2.11E8 | 3.45E8 | 4.18E8 | 2.77E8 | 2.42E8 | 3.67E8 | 3.25E8 | 45 | 6 | 4 | 4 | 4 | 4 | 4 | 4 | 6 | 5 | 4 | 407 | 414 |  |
| R.ADVDAATLAR.I | Y | 69.66 | 1001.5142 | 10 | 3.6 | 501.7662 | 2 | 42.42 | 6 | 11903 | Ana\_05.raw | 3.59E8 | 2.26E8 | 2.08E8 | 8.68E7 | 1.83E8 | 1.93E8 | 1.27E8 | 1.47E8 | 9.51E7 | 2.57E8 | 42 | 4 | 4 | 4 | 3 | 3 | 2 | 4 | 7 | 6 | 5 | 212 | 221 |  |
| R.DGEVVSEATQQQHEVL | Y | 68.56 | 1767.8275 | 16 | 5.3 | 884.9257 | 2 | 69.70 | 10 | 19736 | Ana\_09.raw | 1.2E8 | 5.65E7 | 4.69E7 | 2.24E7 | 4.79E7 | 5.29E7 | 2.92E7 | 4.22E7 | 1.77E8 | 8.72E7 | 40 | 4 | 3 | 5 | 2 | 3 | 4 | 3 | 4 | 8 | 4 | 454 | 469 |  |
| K.VELQELNDR.F | N | 68.56 | 1114.5618 | 9 | 1.7 | 558.2891 | 2 | 53.93 | 6 | 15380 | Ana\_05.raw | 1.87E8 | 1.42E8 | 1.14E8 | 4.2E7 | 9.84E7 | 1.18E8 | 6.37E7 | 8.36E7 | 2.32E8 | 1.36E8 | 49 | 5 | 5 | 5 | 3 | 6 | 3 | 3 | 5 | 10 | 4 | 109 | 117 |  |
| R.VAELYEEEMR.E | Y | 68.36 | 1267.5754 | 10 | 3.3 | 634.7971 | 2 | 61.19 | 1 | 17334 | ANa\_10.raw | 8.19E6 | 8.85E5 |  |  |  |  |  |  | 8.58E6 | 5.5E6 | 4 | 1 | 1 | 0 | 0 | 0 | 0 | 0 | 0 | 1 | 1 | 150 | 159 |  |
| R.EYQDLLNVK.M | N | 65.67 | 1120.5764 | 9 | 3.7 | 561.2975 | 2 | 72.28 | 6 | 20741 | Ana\_05.raw | 1.18E8 | 9.27E7 | 7.55E7 | 3.42E7 | 5.46E7 | 8.33E7 | 3.64E7 | 6.08E7 | 2.11E8 | 9.03E7 | 38 | 6 | 4 | 3 | 3 | 2 | 4 | 1 | 6 | 4 | 5 | 386 | 394 |  |
| K.VSDLTQAANK.N | Y | 64.04 | 1045.5404 | 10 | 3.6 | 523.7794 | 2 | 25.74 | 6 | 6796 | Ana\_05.raw | 1.5E8 | 7.99E7 | 1.13E8 | 4.08E7 | 2.34E7 | 1.05E8 | 5.78E7 | 5.5E7 | 1.17E8 | 1.09E8 | 27 | 4 | 2 | 2 | 2 | 2 | 2 | 3 | 5 | 3 | 2 | 299 | 308 |  |
| R.RQVEVLTNQR.A | Y | 63.57 | 1241.6840 | 10 | 2.9 | 621.8511 | 2 | 29.18 | 6 | 7872 | Ana\_05.raw | 2.01E7 | 1.21E7 | 1.64E7 |  | 8.49E6 | 1.53E7 | 4.85E6 | 9.69E6 | 1.93E7 |  | 13 | 1 | 1 | 2 | 0 | 2 | 2 | 1 | 2 | 2 | 0 | 163 | 172 |  |
| R.VDVERDNLIDDLQR.L | Y | 62.67 | 1698.8536 | 14 | 4.2 | 567.2942 | 3 | 81.99 | 2 | 23262 | Ana\_01.raw | 3.97E6 | 5.17E5 |  |  |  |  |  |  | 8.7E6 |  | 6 | 2 | 1 | 0 | 0 | 0 | 0 | 0 | 0 | 3 | 0 | 175 | 188 |  |
| K.NISEAEEWYK.S | Y | 62.47 | 1267.5720 | 10 | 2.6 | 634.7949 | 2 | 70.53 | 5 | 20097 | Ana\_04.raw | 5.42E7 | 2.43E7 | 2.88E7 | 1.43E7 | 2.89E7 | 2.95E7 | 1.61E7 | 1.91E7 | 6.26E7 | 3.83E7 | 15 | 1 | 1 | 2 | 1 | 2 | 2 | 1 | 1 | 2 | 2 | 287 | 296 |  |
| R.HLKDEMAR.H | Y | 59.51 | 998.4967 | 8 | 3.5 | 333.8407 | 3 | 19.22 | 10 | 4548 | Ana\_09.raw | 2.53E7 | 5.74E6 |  | 6.75E5 |  | 2.33E6 |  |  | 1.12E7 | 1.33E7 | 10 | 2 | 2 | 0 | 1 | 0 | 1 | 0 | 0 | 2 | 2 | 375 | 382 |  |
| R.AQYETIAAK.N | Y | 59.36 | 993.5131 | 9 | 3.3 | 497.7654 | 2 | 33.46 | 6 | 9164 | Ana\_05.raw | 1.73E8 | 1.87E8 | 1.5E8 | 6.61E7 | 1.33E8 | 1.76E8 | 9.57E7 | 1.13E8 | 1.67E8 | 1.43E8 | 19 | 1 | 2 | 2 | 3 | 2 | 2 | 2 | 2 | 2 | 1 | 278 | 286 |  |
| K.LQEEIQLR.E | Y | 58.64 | 1027.5662 | 8 | 1.2 | 514.7910 | 2 | 52.99 | 3 | 15015 | Ana\_02.raw | 7.09E7 | 5.22E7 | 5.41E7 | 2.54E7 | 4.72E7 | 5.15E7 | 2.91E7 | 3.9E7 | 9.14E7 | 6.58E7 | 22 | 2 | 2 | 2 | 2 | 2 | 2 | 2 | 3 | 2 | 3 | 193 | 200 |  |
| R.QVEVLTNQR.A | Y | 55.58 | 1085.5829 | 9 | 4.6 | 543.8012 | 2 | 37.90 | 8 | 10206 | Ana\_07.raw | 3.21E7 |  | 2.23E7 |  | 2.03E7 | 2.67E7 | 1.38E7 | 2E7 | 3.23E7 | 1.17E7 | 12 | 1 | 0 | 2 | 0 | 2 | 2 | 1 | 1 | 1 | 2 | 164 | 172 |  |
| R.VYQVSR.T | Y | 54.02 | 750.4024 | 6 | 3.9 | 376.2099 | 2 | 23.27 | 2 | 5844 | Ana\_01.raw | 3.39E7 | 1.17E7 | 9.13E7 | 4.23E7 | 7.58E6 | 9.44E7 | 2.79E7 | 1.1E7 |  | 5.95E7 | 11 | 1 | 1 | 1 | 2 | 1 | 1 | 1 | 2 | 0 | 1 | 53 | 58 |  |
| R.FANYIEK.V | Y | 53.33 | 883.4439 | 7 | 4.0 | 442.7310 | 2 | 42.18 | 9 | 11654 | Ana\_08.raw | 1.53E8 | 1.02E8 | 1.05E8 | 3.81E7 | 8.18E7 | 9.74E7 | 5.61E7 | 5.99E7 | 1.69E8 | 1.08E8 | 22 | 3 | 3 | 2 | 2 | 2 | 2 | 1 | 2 | 2 | 3 | 118 | 124 |  |
| R.HLKDEM(+15.99)AR.H | Y | 43.18 | 1014.4916 | 8 | 4.7 | 508.2555 | 2 | 20.11 | 9 | 4662 | Ana\_08.raw |  |  |  |  |  |  |  |  | 6.81E6 |  | 2 | 0 | 0 | 0 | 0 | 0 | 0 | 0 | 0 | 2 | 0 | 375 | 382 | Oxidation (M) |
| R.IESLNEEIAFLKK.V | Y | 42.20 | 1532.8450 | 13 | 3.5 | 511.9574 | 3 | 91.35 | 9 | 25906 | Ana\_08.raw |  |  |  |  |  |  |  |  | 1.96E6 |  | 1 | 0 | 0 | 0 | 0 | 0 | 0 | 0 | 0 | 1 | 0 | 228 | 240 |  |
| R.LEEEIR.H | N | 37.84 | 787.4075 | 6 | 3.6 | 394.7125 | 2 | 26.67 | 9 | 6857 | Ana\_08.raw | 2.45E8 | 1.88E8 | 1.4E8 | 6.36E7 | 1.19E8 | 1.56E8 | 8.53E7 | 8.93E7 | 1.82E8 | 1.83E8 | 26 | 3 | 3 | 2 | 2 | 3 | 3 | 2 | 3 | 2 | 3 | 369 | 374 |  |
| R.IDLER.R | N | 35.02 | 644.3493 | 5 | 4.6 | 323.1834 | 2 | 33.12 | 7 | 8934 | Ana\_06.raw | 2.18E7 | 4.86E7 | 1.51E7 | 2.77E7 | 3.2E7 | 1.31E7 | 3.61E7 | 8.73E6 | 2.45E7 | 2.04E7 | 14 | 1 | 2 | 1 | 2 | 2 | 1 | 2 | 1 | 1 | 1 | 222 | 226 |  |
| total 38 peptides |  |  |  |  |  |  |  |  |  |  |  |  |  |  |  |  |  |  |  |  |  |  |  |  |  |  |  |  |  |  |  |  |  |  |  |
| --- | --- | --- | --- | --- | --- | --- | --- | --- | --- | --- | --- | --- | --- | --- | --- | --- | --- | --- | --- | --- | --- | --- | --- | --- | --- | --- | --- | --- | --- | --- | --- | --- | --- | --- | --- |

Best Unique PSM (Scan F6:37541, m/z=1378.1873, z=2, RT=135.03, ppm=3.6):

sp|P26039|TLN1\_MOUSE

back to list

  

| Protein Coverage
| Supporting Peptides
| Best Unique PSM
|

Protein Coverage:

Supporting Peptides:

| Peptide | Uniq | -10lgP | Mass | Length | ppm | m/z | z | RT | Fraction | Scan | Source File | Area 10 | Area 01 | Area 02 | Area 03 | Area 04 | Area 05 | Area 06 | Area 07 | Area 08 | Area 09 | #Spec | #Spec 10 | #Spec 01 | #Spec 02 | #Spec 03 | #Spec 04 | #Spec 05 | #Spec 06 | #Spec 07 | #Spec 08 | #Spec 09 | Start | End | PTM |
| --- | --- | --- | --- | --- | --- | --- | --- | --- | --- | --- | --- | --- | --- | --- | --- | --- | --- | --- | --- | --- | --- | --- | --- | --- | --- | --- | --- | --- | --- | --- | --- | --- | --- | --- | --- |
| R.GVAALTSDPAVQAIVLDTASDVLDK.A | Y | 136.08 | 2468.3010 | 25 | -0.5 | 1235.1572 | 2 | 134.23 | 3 | 36742 | Ana\_02.raw | 3.65E6 | 3.01E5 | 5.04E6 | 5.44E5 | 1.82E6 | 4.16E6 |  |  | 1.4E7 | 4.95E6 | 11 | 1 | 1 | 2 | 1 | 1 | 1 | 0 | 0 | 2 | 2 | 1137 | 1161 |  |
| R.EGISQEALHTQM(+15.99)LTAVQEISHLIEPLASAAR.A | Y | 119.55 | 3358.7190 | 31 | 3.8 | 840.6902 | 4 | 133.46 | 6 | 37102 | Ana\_05.raw | 3.48E6 | 3.25E6 |  | 3.7E5 | 5.4E6 | 4.63E6 |  | 3.86E6 | 8.43E6 | 3.43E6 | 8 | 1 | 1 | 0 | 1 | 1 | 1 | 0 | 1 | 1 | 1 | 1691 | 1721 | Oxidation (M) |
| R.GVGAAATAVTQALNELLQHVKAHATGAGPAGR.Y | Y | 114.02 | 3036.6216 | 32 | 1.7 | 760.1639 | 4 | 133.46 | 6 | 37103 | Ana\_05.raw | 4.33E6 |  |  |  | 2.16E6 | 3.34E6 | 1.66E6 | 2.17E6 |  | 3.93E6 | 6 | 1 | 0 | 0 | 0 | 1 | 1 | 1 | 1 | 0 | 1 | 766 | 797 |  |
| R.LNEAAAGLNQAATELVQASR.G | Y | 109.35 | 2026.0443 | 20 | 1.4 | 1014.0309 | 2 | 108.27 | 3 | 30028 | Ana\_02.raw | 3.55E6 | 3.48E6 | 4.75E6 | 3.1E6 | 2.85E6 | 2.42E6 | 1.92E6 | 2.53E5 | 1.05E7 | 3.06E6 | 17 | 2 | 2 | 2 | 2 | 1 | 2 | 2 | 1 | 1 | 2 | 1242 | 1261 |  |
| K.GAAAHPDSEEQQQR.L | Y | 102.39 | 1522.6760 | 14 | 2.9 | 762.3475 | 2 | 17.24 | 6 | 4010 | Ana\_05.raw |  |  |  | 1.72E6 | 3.51E6 | 3.7E6 | 1.89E6 | 2.69E6 |  |  | 9 | 0 | 0 | 0 | 2 | 2 | 2 | 1 | 2 | 0 | 0 | 876 | 889 |  |
| R.AATAPLLEAVDNLSAFASNPEFSSVPAQISPEGR.A | Y | 99.29 | 3455.7207 | 34 | 6.5 | 1152.9216 | 3 | 136.31 | 9 | 37675 | Ana\_08.raw |  |  |  |  |  | 4.99E6 | 1.58E6 |  | 5.72E6 |  | 5 | 0 | 0 | 0 | 0 | 0 | 2 | 1 | 0 | 2 | 0 | 1560 | 1593 |  |
| R.GVGAAATAVTQALNELLQHVK.A | Y | 97.71 | 2090.1484 | 21 | 4.4 | 1046.0861 | 2 | 136.28 | 6 | 37875 | Ana\_05.raw | 4.59E5 | 5.56E6 | 6.4E6 |  | 4.35E6 | 2.14E6 | 1.74E6 | 7.6E5 | 2.04E7 | 9.22E6 | 16 | 2 | 1 | 1 | 0 | 3 | 2 | 2 | 1 | 3 | 1 | 766 | 786 |  |
| K.TLAESALQLLYTAK.E | Y | 96.96 | 1520.8450 | 14 | 2.9 | 761.4319 | 2 | 127.67 | 3 | 34997 | Ana\_02.raw | 1.76E7 |  | 1.39E7 | 5.58E6 | 1.16E7 | 8.56E6 | 7.9E6 | 2.57E6 | 2.47E7 | 1.45E7 | 9 | 1 | 0 | 1 | 1 | 1 | 1 | 1 | 1 | 1 | 1 | 1767 | 1780 |  |
| K.GTEWVDPEDPTVIAENELLGAAAAIEAAAK.K | Y | 94.03 | 3050.5083 | 30 | 2.0 | 1017.8454 | 3 | 139.84 | 9 | 38573 | Ana\_08.raw | 6.03E5 |  |  |  |  | 1.11E6 |  |  | 4.27E6 |  | 3 | 1 | 0 | 0 | 0 | 0 | 1 | 0 | 0 | 1 | 0 | 2292 | 2321 |  |
| K.EADESLNFEEQILEAAK.S | Y | 93.84 | 1934.9109 | 17 | 1.8 | 968.4644 | 2 | 119.71 | 1 | 33021 | ANa\_10.raw | 8.91E6 |  | 1.64E6 | 8.56E5 | 2.68E5 | 2.89E6 | 2.01E6 | 2.11E6 | 1.24E7 | 6.45E6 | 9 | 1 | 0 | 1 | 1 | 1 | 1 | 1 | 1 | 1 | 1 | 2334 | 2350 |  |
| K.LLGEIAQGNENYAGIAAR.D | Y | 89.13 | 1858.9536 | 18 | 1.0 | 930.4850 | 2 | 82.88 | 9 | 23582 | Ana\_08.raw |  | 5.9E5 |  |  |  |  |  |  | 3.24E6 | 6.46E5 | 3 | 0 | 1 | 0 | 0 | 0 | 0 | 0 | 0 | 1 | 1 | 1105 | 1122 |  |
| K.LAQAAQSSVATITR.L | Y | 88.38 | 1415.7732 | 14 | 2.6 | 708.8957 | 2 | 51.83 | 5 | 14670 | Ana\_04.raw | 4.48E6 | 4.08E6 | 3.36E6 |  | 2.08E6 | 2.48E6 | 1.23E6 | 1.53E6 | 8.8E6 | 4.32E6 | 9 | 1 | 1 | 1 | 0 | 1 | 1 | 1 | 1 | 1 | 1 | 2044 | 2057 |  |
| R.DLDQASLAAVSQQLAPR.E | Y | 87.69 | 1781.9271 | 17 | 5.3 | 891.9755 | 2 | 105.38 | 1 | 29357 | ANa\_10.raw | 1.44E6 | 5.13E5 | 1.32E6 |  |  | 7.08E5 |  |  | 1.03E6 | 1.21E6 | 8 | 2 | 1 | 1 | 0 | 0 | 2 | 0 | 0 | 1 | 1 | 1674 | 1690 |  |
| K.VVAPTISSPVC(+57.02)QEQLVEAGR.L | Y | 82.10 | 2139.0994 | 20 | 1.0 | 1070.5580 | 2 | 85.30 | 9 | 24264 | Ana\_08.raw |  |  |  |  |  |  |  |  | 0 |  | 1 | 0 | 0 | 0 | 0 | 0 | 0 | 0 | 0 | 1 | 0 | 722 | 741 | Carbamidomethylation |
| R.ILAQATSDLVNAIK.A | Y | 80.76 | 1455.8297 | 14 | -0.6 | 728.9217 | 2 | 97.60 | 3 | 27268 | Ana\_02.raw | 9.69E6 | 2.34E6 | 9.38E5 |  | 1.63E6 | 3.19E5 | 1.86E6 | 2E6 | 1.87E7 | 1.04E7 | 9 | 1 | 1 | 1 | 0 | 1 | 1 | 1 | 1 | 1 | 1 | 828 | 841 |  |
| K.ADAEGESDLENSR.K | Y | 80.26 | 1391.5801 | 13 | 2.0 | 696.7987 | 2 | 33.64 | 6 | 9220 | Ana\_05.raw | 6.18E6 | 5.69E6 | 4.46E6 | 1.98E6 | 3.79E6 | 4.61E6 | 3E6 | 2.49E6 | 6.28E6 | 5.46E6 | 10 | 1 | 1 | 1 | 1 | 1 | 1 | 1 | 1 | 1 | 1 | 842 | 854 |  |
| K.M(+15.99)VGGIAQIIAAQEEM(+15.99)LR.K | Y | 77.34 | 1860.9437 | 17 | 4.9 | 621.3249 | 3 | 123.12 | 7 | 33953 | Ana\_06.raw | 3.25E5 | 7.47E5 |  |  |  |  | 2.37E5 |  |  | 0 | 4 | 1 | 1 | 0 | 0 | 0 | 0 | 1 | 0 | 0 | 1 | 2494 | 2510 | Oxidation (M) |
| R.EGISQEALHTQMLTAVQEISHLIEPLASAAR.A | Y | 76.40 | 3342.7241 | 31 | 5.3 | 836.6927 | 4 | 138.27 | 9 | 38181 | Ana\_08.raw |  |  |  |  |  |  |  |  | 9.28E5 |  | 1 | 0 | 0 | 0 | 0 | 0 | 0 | 0 | 0 | 1 | 0 | 1691 | 1721 |  |
| K.GLAGAVSELLR.S | Y | 73.28 | 1084.6240 | 11 | 4.4 | 543.3217 | 2 | 105.58 | 6 | 29733 | Ana\_05.raw | 7.08E6 | 1.38E7 | 1.1E7 | 3.06E6 | 5.18E6 | 1.01E7 | 5.56E6 | 4.91E6 | 1.9E7 | 1.65E7 | 18 | 2 | 2 | 1 | 2 | 2 | 2 | 2 | 2 | 2 | 1 | 614 | 624 |  |
| K.AVAEQIPLLVQGVR.G | Y | 71.96 | 1491.8773 | 14 | 1.7 | 746.9472 | 2 | 98.46 | 3 | 27496 | Ana\_02.raw | 2.35E6 | 2.08E6 | 3.45E6 | 2.53E5 |  | 1.65E6 | 1.34E6 | 2.1E6 | 1.73E7 | 2.49E6 | 11 | 1 | 1 | 1 | 1 | 0 | 1 | 1 | 1 | 2 | 2 | 958 | 971 |  |
| K.AAAFEDQENETVVVK.E | Y | 71.76 | 1648.7944 | 15 | 0.7 | 825.4051 | 2 | 61.87 | 4 | 17207 | Ana\_03.raw | 5.38E6 | 5.02E6 | 3.84E6 | 1.43E6 | 2.25E6 | 2.62E6 |  | 3.74E5 | 3.03E6 | 4.6E6 | 9 | 1 | 1 | 1 | 1 | 1 | 1 | 0 | 1 | 1 | 1 | 2477 | 2491 |  |
| K.ALSTDPASPNLK.S | Y | 71.19 | 1212.6350 | 12 | 5.4 | 607.3281 | 2 | 45.09 | 1 | 12573 | ANa\_10.raw | 3.91E6 | 3.19E6 | 3.45E6 | 1.1E6 | 2.74E6 | 2.83E6 | 1.47E6 | 1.76E6 | 4.93E6 |  | 9 | 1 | 1 | 1 | 1 | 1 | 1 | 1 | 1 | 1 | 0 | 1321 | 1332 |  |
| K.EVIQEWSLTNIK.R | Y | 71.17 | 1458.7719 | 12 | 1.9 | 730.3946 | 2 | 98.49 | 6 | 27853 | Ana\_05.raw |  |  |  |  |  | 0 |  |  |  |  | 1 | 0 | 0 | 0 | 0 | 0 | 1 | 0 | 0 | 0 | 0 | 346 | 357 |  |
| K.VLVQNAAGSQEK.L | Y | 70.81 | 1242.6567 | 12 | 3.4 | 622.3378 | 2 | 27.38 | 5 | 7287 | Ana\_04.raw | 1.13E7 | 1.2E7 | 8.12E6 | 3.15E6 | 6.6E6 | 8.27E6 | 4.85E6 | 4.7E6 | 6.88E6 | 1.05E7 | 10 | 1 | 1 | 1 | 1 | 1 | 1 | 1 | 1 | 1 | 1 | 2032 | 2043 |  |
| K.FFYSDQNVDSR.D | N | 69.38 | 1376.5996 | 11 | 2.0 | 689.3085 | 2 | 58.81 | 3 | 16721 | Ana\_02.raw | 6.01E6 | 9.54E6 | 5.91E6 | 2.58E6 | 5.03E6 | 5.48E6 | 2.72E6 | 3.34E6 | 8.57E6 | 4.8E6 | 10 | 1 | 1 | 1 | 1 | 1 | 1 | 1 | 1 | 1 | 1 | 197 | 207 |  |
| K.ASGHPGDPESQQR.L | Y | 68.70 | 1364.6068 | 13 | 3.0 | 683.3127 | 2 | 17.61 | 6 | 4135 | Ana\_05.raw |  |  |  | 4.19E5 | 1.12E6 | 4.49E6 |  |  |  |  | 4 | 0 | 0 | 0 | 1 | 1 | 2 | 0 | 0 | 0 | 0 | 1172 | 1184 |  |
| R.EQGVEEHETLLLR.R | Y | 68.13 | 1551.7893 | 13 | 6.6 | 518.2738 | 3 | 62.40 | 6 | 17894 | Ana\_05.raw | 7.26E6 | 5.76E6 | 5.32E6 |  | 4.11E6 | 5.32E6 |  | 3.26E6 | 9.62E6 | 4.5E6 | 8 | 1 | 1 | 1 | 0 | 1 | 1 | 0 | 1 | 1 | 1 | 182 | 194 |  |
| K.TSTPEDFIR.M | Y | 66.04 | 1064.5138 | 9 | 1.7 | 533.2651 | 2 | 63.74 | 7 | 18076 | Ana\_06.raw | 1.15E7 | 1.04E7 | 1.06E7 |  | 7.51E6 | 1.02E7 | 5.46E6 | 5.43E6 | 1.31E7 | 9.51E6 | 9 | 1 | 1 | 1 | 0 | 1 | 1 | 1 | 1 | 1 | 1 | 2169 | 2177 |  |
| R.M(+15.99)ATNAAAQNAIK.K | Y | 65.33 | 1218.6027 | 12 | 4.5 | 610.3113 | 2 | 25.41 | 6 | 6694 | Ana\_05.raw | 2E6 |  | 3.41E6 |  |  | 3.26E6 |  | 1.43E6 |  |  | 4 | 1 | 0 | 1 | 0 | 0 | 1 | 0 | 1 | 0 | 0 | 899 | 910 | Oxidation (M) |
| K.EAAFHPEVAPDVR.L | Y | 64.15 | 1436.7047 | 13 | 5.2 | 479.9113 | 3 | 58.03 | 8 | 16189 | Ana\_07.raw | 7.34E6 | 7.36E6 | 5.24E5 | 3.76E6 | 6.31E6 | 7.68E6 | 5.1E6 | 4.45E6 | 1.4E7 | 7.34E6 | 10 | 1 | 1 | 1 | 1 | 1 | 1 | 1 | 1 | 1 | 1 | 2221 | 2233 |  |
| K.NLGTALAELR.T | Y | 62.81 | 1056.5928 | 10 | 3.2 | 529.3054 | 2 | 87.02 | 6 | 24784 | Ana\_05.raw | 4.72E6 | 6.42E6 | 6.71E6 | 2.05E6 | 3.45E6 | 5.88E6 | 3.39E6 | 3.63E6 | 1.29E7 | 3.62E6 | 11 | 2 | 1 | 1 | 1 | 1 | 1 | 1 | 1 | 1 | 1 | 1026 | 1035 |  |
| K.ALDGDFTEENR.A | Y | 62.66 | 1265.5524 | 11 | 2.0 | 633.7847 | 2 | 48.54 | 6 | 13766 | Ana\_05.raw | 2.33E6 |  | 2.62E6 | 2.83E6 |  | 4.48E6 | 2.04E6 | 2E6 |  | 3.11E6 | 7 | 1 | 0 | 1 | 1 | 0 | 1 | 1 | 1 | 0 | 1 | 1545 | 1555 |  |
| K.EVANSTANLVK.T | N | 57.95 | 1144.6088 | 11 | 2.5 | 573.3131 | 2 | 38.43 | 6 | 10669 | Ana\_05.raw | 4.72E6 | 4.56E6 | 4.51E6 | 1.53E6 | 3.37E6 | 4.28E6 | 2.82E6 | 2.81E6 |  | 4.16E6 | 9 | 1 | 1 | 1 | 1 | 1 | 1 | 1 | 1 | 0 | 1 | 1531 | 1541 |  |
| K.ALGDLISATK.A | Y | 54.11 | 987.5601 | 10 | 2.2 | 494.7884 | 2 | 67.94 | 3 | 19320 | Ana\_02.raw | 3.64E6 | 2.38E6 | 2.63E6 | 6.39E5 | 2.07E6 | 2.02E6 | 1.17E6 | 1.49E6 | 7.3E6 | 3.46E6 | 10 | 1 | 1 | 1 | 1 | 1 | 1 | 1 | 1 | 1 | 1 | 2090 | 2099 |  |
| K.AVTQALNR.C | Y | 53.02 | 871.4875 | 8 | 4.4 | 436.7530 | 2 | 24.59 | 9 | 6162 | Ana\_08.raw |  |  | 9.64E6 | 4.93E6 |  | 8.96E6 | 5.29E6 | 4.52E6 | 1.28E7 | 8.26E6 | 8 | 0 | 0 | 1 | 1 | 1 | 1 | 1 | 1 | 1 | 1 | 1191 | 1198 |  |
| K.ASAGPQPLLVQSC(+57.02)K.A | Y | 52.61 | 1454.7551 | 14 | 4.7 | 728.3882 | 2 | 50.40 | 9 | 14175 | Ana\_08.raw |  |  |  |  |  |  |  |  | 2.63E6 |  | 1 | 0 | 0 | 0 | 0 | 0 | 0 | 0 | 0 | 1 | 0 | 944 | 957 | Carbamidomethylation |
| R.IGITNHDEYSLVR.E | Y | 52.18 | 1515.7681 | 13 | 0.3 | 506.2635 | 3 | 68.64 | 8 | 19277 | Ana\_07.raw |  | 3.58E5 | 7.61E5 |  | 1.06E6 | 2.68E6 |  | 1.62E6 | 1.84E6 | 2.33E6 | 7 | 0 | 1 | 1 | 0 | 1 | 1 | 0 | 1 | 1 | 1 | 119 | 131 |  |
| K.AVASAAAALVLK.A | Y | 50.94 | 1083.6652 | 12 | 2.0 | 542.8409 | 2 | 76.10 | 3 | 21561 | Ana\_02.raw | 2.14E6 |  | 1.38E6 | 6.82E5 |  |  |  |  |  | 1.85E6 | 4 | 1 | 0 | 1 | 1 | 0 | 0 | 0 | 0 | 0 | 1 | 674 | 685 |  |
| R.GTPQDLAR.A | Y | 50.57 | 856.4402 | 8 | 3.5 | 429.2289 | 2 | 25.20 | 2 | 6491 | Ana\_01.raw | 1.54E6 | 9.55E6 |  |  |  |  |  | 4.54E6 |  | 4.69E6 | 4 | 1 | 1 | 0 | 0 | 0 | 0 | 0 | 1 | 0 | 1 | 1262 | 1269 |  |
| K.ILADATAK.M | Y | 48.46 | 801.4596 | 8 | 4.3 | 401.7388 | 2 | 23.97 | 2 | 6079 | Ana\_01.raw |  | 6.03E6 | 7.61E6 |  |  |  |  |  |  |  | 2 | 0 | 1 | 1 | 0 | 0 | 0 | 0 | 0 | 0 | 0 | 862 | 869 |  |
| K.SQLAAAAR.A | Y | 48.31 | 786.4348 | 8 | 1.2 | 394.2251 | 2 | 21.15 | 7 | 5260 | Ana\_06.raw |  |  |  | 3.25E6 |  |  | 3.96E6 | 3.15E6 |  |  | 3 | 0 | 0 | 0 | 1 | 0 | 0 | 1 | 1 | 0 | 0 | 1333 | 1340 |  |
| K.VLGEAM(+15.99)TGISQNAK.N | Y | 48.17 | 1433.7184 | 14 | 4.0 | 717.8693 | 2 | 43.07 | 10 | 11988 | Ana\_09.raw |  |  |  |  |  |  |  |  |  | 6.4E5 | 1 | 0 | 0 | 0 | 0 | 0 | 0 | 0 | 0 | 0 | 1 | 1402 | 1415 | Oxidation (M) |
| K.TYGVSFFLVK.E | N | 48.17 | 1159.6277 | 10 | 3.9 | 580.8234 | 2 | 111.46 | 8 | 30792 | Ana\_07.raw |  |  |  |  |  | 9.89E5 |  | 8.38E5 |  |  | 2 | 0 | 0 | 0 | 0 | 0 | 1 | 0 | 1 | 0 | 0 | 307 | 316 |  |
| R.VAGSVTELIQAAEAM(+15.99)K.G | Y | 47.54 | 1632.8392 | 16 | 4.5 | 817.4306 | 2 | 107.88 | 5 | 30071 | Ana\_04.raw |  |  |  |  | 1.5E6 | 3.58E5 | 1.49E5 |  |  |  | 3 | 0 | 0 | 0 | 0 | 1 | 1 | 1 | 0 | 0 | 0 | 2276 | 2291 | Oxidation (M) |
| K.KLEQLKPR.A | N | 46.07 | 1010.6236 | 8 | 2.8 | 506.3205 | 2 | 21.60 | 9 | 5160 | Ana\_08.raw | 7.54E6 | 4.09E6 | 6.14E6 | 1.8E6 | 3.18E6 | 4.36E6 | 3.05E6 | 1.22E6 | 7.03E6 | 5.84E6 | 13 | 2 | 1 | 2 | 1 | 1 | 1 | 1 | 1 | 2 | 1 | 2322 | 2329 |  |
| R.LQAAGNAVK.R | N | 44.93 | 870.4923 | 9 | 4.2 | 436.2553 | 2 | 20.95 | 6 | 5240 | Ana\_05.raw |  |  |  | 2.7E6 | 4.2E6 | 9.83E6 |  | 4.99E6 |  |  | 4 | 0 | 0 | 0 | 1 | 1 | 1 | 0 | 1 | 0 | 0 | 2456 | 2464 |  |
| R.ALEATTEHIR.Q | Y | 43.25 | 1139.5935 | 10 | -0.3 | 380.8717 | 3 | 30.15 | 3 | 8171 | Ana\_02.raw |  |  | 6.69E6 |  |  | 5.13E6 |  |  |  |  | 3 | 0 | 0 | 2 | 0 | 0 | 1 | 0 | 0 | 0 | 0 | 2145 | 2154 |  |
| K.VM(+15.99)VTNVTSLLK.T | N | 40.89 | 1219.6846 | 11 | 3.3 | 610.8516 | 2 | 83.10 | 9 | 23645 | Ana\_08.raw | 6.84E5 | 5.23E5 | 6.65E5 |  |  | 1.03E6 |  |  | 4.57E6 |  | 5 | 1 | 1 | 1 | 0 | 0 | 1 | 0 | 0 | 1 | 0 | 2120 | 2130 | Oxidation (M) |
| K.AGFLDLK.D | Y | 37.74 | 762.4276 | 7 | 0.2 | 382.2211 | 2 | 70.28 | 3 | 19974 | Ana\_02.raw | 4.84E5 |  | 1.39E6 | 7.89E5 | 9.67E5 | 6.48E5 |  | 9.29E5 | 2.01E6 |  | 7 | 1 | 0 | 1 | 1 | 1 | 1 | 0 | 1 | 1 | 0 | 257 | 263 |  |
| R.ALHYGR.E | Y | 37.67 | 715.3765 | 6 | 3.5 | 358.6968 | 2 | 20.56 | 6 | 5101 | Ana\_05.raw |  |  |  |  | 3.07E6 | 3.8E6 |  |  |  |  | 2 | 0 | 0 | 0 | 0 | 1 | 1 | 0 | 0 | 0 | 0 | 2236 | 2241 |  |
| K.ASSLIEEAK.K | Y | 37.06 | 946.4971 | 9 | 7.0 | 474.2591 | 2 | 36.28 | 10 | 9922 | Ana\_09.raw | 4.85E6 |  |  |  |  |  |  |  |  | 4.63E6 | 2 | 1 | 0 | 0 | 0 | 0 | 0 | 0 | 0 | 0 | 1 | 1162 | 1170 |  |
| K.ISIGNVVK.T | Y | 36.07 | 828.5069 | 8 | 4.4 | 415.2625 | 2 | 55.45 | 10 | 15657 | Ana\_09.raw |  |  |  |  |  |  |  |  |  | 4.71E6 | 1 | 0 | 0 | 0 | 0 | 0 | 0 | 0 | 0 | 0 | 1 | 8 | 15 |  |
| K.RQFVQSAK.E | Y | 35.75 | 962.5297 | 8 | 1.4 | 482.2728 | 2 | 19.99 | 1 | 4763 | ANa\_10.raw | 1.31E6 |  | 8.28E5 |  |  |  |  |  |  |  | 2 | 1 | 0 | 1 | 0 | 0 | 0 | 0 | 0 | 0 | 0 | 1523 | 1530 |  |
| K.VGDDPAVWQLK.N | Y | 33.44 | 1226.6295 | 11 | 2.8 | 614.3237 | 2 | 73.81 | 9 | 21046 | Ana\_08.raw |  |  |  |  |  |  |  |  | 1.34E6 |  | 1 | 0 | 0 | 0 | 0 | 0 | 0 | 0 | 0 | 1 | 0 | 2105 | 2115 |  |
| R.KIFQAHK.N | Y | 32.57 | 870.5076 | 7 | 0.8 | 436.2614 | 2 | 17.46 | 3 | 4087 | Ana\_02.raw | 9.67E5 |  | 7.26E5 |  |  |  |  |  |  | 5.51E5 | 3 | 1 | 0 | 1 | 0 | 0 | 0 | 0 | 0 | 0 | 1 | 278 | 284 |  |
| K.HTSALC(+57.02)NSC(+57.02)R.L | Y | 30.75 | 1204.5077 | 10 | 2.6 | 603.2627 | 2 | 18.47 | 8 | 4365 | Ana\_07.raw |  |  |  |  |  |  |  | 3.17E5 |  |  | 1 | 0 | 0 | 0 | 0 | 0 | 0 | 0 | 1 | 0 | 0 | 1501 | 1510 | Carbamidomethylation |
| R.DVDNALR.A | Y | 27.06 | 801.3981 | 7 | 1.2 | 401.7068 | 2 | 26.55 | 3 | 7052 | Ana\_02.raw |  |  | 1.11E7 |  |  | 4.18E6 |  |  |  |  | 2 | 0 | 0 | 1 | 0 | 0 | 1 | 0 | 0 | 0 | 0 | 1208 | 1214 |  |
| R.YDQATDTILTVTENIFSSM(+15.99)GDAGEM(+15.99)VR.Q | Y | 26.81 | 2995.3428 | 27 | 0.9 | 999.4558 | 3 | 134.38 | 7 | 37026 | Ana\_06.raw |  |  |  |  |  |  | 1.2E6 |  |  |  | 1 | 0 | 0 | 0 | 0 | 0 | 0 | 1 | 0 | 0 | 0 | 798 | 824 | Oxidation (M) |
| R.QELAVFC(+57.02)SPEPPAK.T | Y | 26.79 | 1571.7654 | 14 | 5.4 | 786.8942 | 2 | 97.66 | 2 | 27515 | Ana\_01.raw |  | 1.25E5 |  |  |  |  |  |  |  |  | 1 | 0 | 1 | 0 | 0 | 0 | 0 | 0 | 0 | 0 | 0 | 2155 | 2168 | Carbamidomethylation |
| K.VGAIPANALDDGQWSQGLISAAR.M | Y | 25.84 | 2309.1763 | 23 | 2.3 | 770.7344 | 3 | 117.97 | 8 | 32490 | Ana\_07.raw |  |  |  |  |  |  |  | 4.29E5 |  |  | 1 | 0 | 0 | 0 | 0 | 0 | 0 | 0 | 1 | 0 | 0 | 2376 | 2398 |  |
| total 60 peptides |  |  |  |  |  |  |  |  |  |  |  |  |  |  |  |  |  |  |  |  |  |  |  |  |  |  |  |  |  |  |  |  |  |  |  |
| --- | --- | --- | --- | --- | --- | --- | --- | --- | --- | --- | --- | --- | --- | --- | --- | --- | --- | --- | --- | --- | --- | --- | --- | --- | --- | --- | --- | --- | --- | --- | --- | --- | --- | --- | --- |

Best Unique PSM (Scan F3:36742, m/z=1235.1572, z=2, RT=134.23, ppm=-0.5):

sp|P62737|ACTA\_MOUSE

back to list

  

| Protein Coverage
| Supporting Peptides
| Best Unique PSM
|

Protein Coverage:

Supporting Peptides:

| Peptide | Uniq | -10lgP | Mass | Length | ppm | m/z | z | RT | Fraction | Scan | Source File | Area 10 | Area 01 | Area 02 | Area 03 | Area 04 | Area 05 | Area 06 | Area 07 | Area 08 | Area 09 | #Spec | #Spec 10 | #Spec 01 | #Spec 02 | #Spec 03 | #Spec 04 | #Spec 05 | #Spec 06 | #Spec 07 | #Spec 08 | #Spec 09 | Start | End | PTM |
| --- | --- | --- | --- | --- | --- | --- | --- | --- | --- | --- | --- | --- | --- | --- | --- | --- | --- | --- | --- | --- | --- | --- | --- | --- | --- | --- | --- | --- | --- | --- | --- | --- | --- | --- | --- |
| R.VAPEEHPTLLTEAPLNPK.A | N | 151.97 | 1955.0364 | 18 | 1.1 | 978.5266 | 2 | 73.32 | 3 | 20810 | Ana\_02.raw | 2.45E9 | 2.5E9 | 1.77E9 | 7.47E8 | 1.61E9 | 1.6E9 | 9.26E8 | 1.38E9 | 4.04E9 | 1.56E9 | 127 | 13 | 17 | 11 | 9 | 14 | 13 | 9 | 13 | 11 | 17 | 98 | 115 |  |
| K.SYELPDGQVITIGNER.F | N | 136.16 | 1789.8846 | 16 | 0.5 | 895.9501 | 2 | 101.19 | 3 | 28209 | Ana\_02.raw | 2.56E9 | 2.49E9 | 2.2E9 | 5.02E8 | 1.2E9 | 1.61E9 | 8.03E8 | 1.08E9 | 4.17E9 | 2.27E9 | 445 | 51 | 48 | 45 | 42 | 44 | 40 | 40 | 47 | 46 | 42 | 241 | 256 |  |
| R.KDLYANNVLSGGTTM(+15.99)YPGIADR.M | N | 132.74 | 2371.1477 | 22 | 5.7 | 791.3943 | 3 | 83.46 | 1 | 23524 | ANa\_10.raw | 7.77E7 | 6.77E7 | 5.46E7 | 2.44E7 | 5.59E7 | 7.19E7 | 2.69E7 | 5.49E7 | 1.65E8 | 3.63E7 | 29 | 3 | 3 | 3 | 3 | 3 | 3 | 2 | 3 | 4 | 2 | 293 | 314 | Oxidation (M) |
| K.DLYANNVLSGGTTM(+15.99)YPGIADR.M | N | 119.21 | 2243.0527 | 21 | 2.5 | 1122.5365 | 2 | 98.06 | 7 | 27431 | Ana\_06.raw | 4.24E8 | 3.71E8 | 2.28E8 | 7.65E5 | 1.13E8 | 1.58E8 | 4.13E7 | 1.04E8 | 6.79E8 | 1.15E8 | 57 | 11 | 6 | 6 | 1 | 5 | 3 | 3 | 5 | 11 | 6 | 294 | 314 | Oxidation (M) |
| K.DLYANNVLSGGTTMYPGIADR.M | N | 117.02 | 2227.0579 | 21 | 7.1 | 1114.5441 | 2 | 105.61 | 9 | 29725 | Ana\_08.raw | 5.21E6 |  |  |  |  |  |  |  | 1.4E7 |  | 5 | 2 | 0 | 0 | 0 | 0 | 0 | 0 | 0 | 3 | 0 | 294 | 314 |  |
| K.LC(+57.02)YVALDFENEMATAASSSSLEK.S | N | 111.03 | 2535.1509 | 23 | 5.6 | 1268.5898 | 2 | 129.15 | 1 | 35504 | ANa\_10.raw | 3.4E6 |  |  |  |  |  |  |  |  | 3.91E6 | 2 | 1 | 0 | 0 | 0 | 0 | 0 | 0 | 0 | 0 | 1 | 218 | 240 | Carbamidomethylation |
| R.TTGIVLDSGDGVTHNVPIYEGYALPHAIMR.L | N | 110.75 | 3195.6023 | 30 | 6.6 | 1066.2151 | 3 | 112.07 | 9 | 31405 | Ana\_08.raw | 6.3E6 |  |  |  |  | 3.6E6 |  |  | 1.67E7 |  | 3 | 1 | 0 | 0 | 0 | 0 | 1 | 0 | 0 | 1 | 0 | 150 | 179 |  |
| K.LC(+57.02)YVALDFENEM(+15.99)ATAASSSSLEK.S | N | 101.77 | 2551.1458 | 23 | 2.6 | 1276.5835 | 2 | 103.36 | 9 | 29127 | Ana\_08.raw | 3.65E6 | 6.44E6 | 9.4E6 | 4.23E5 |  | 1.01E7 | 2.94E6 |  | 3.63E7 | 3.17E6 | 14 | 2 | 2 | 2 | 1 | 0 | 2 | 2 | 0 | 2 | 1 | 218 | 240 | Carbamidomethylation; Oxidation (M) |
| R.TTGIVLDSGDGVTHNVPIYEGYALPHAIM(+15.99)R.L | N | 98.15 | 3211.5972 | 30 | 0.6 | 1071.5403 | 3 | 103.74 | 3 | 28873 | Ana\_02.raw | 1.38E8 | 8.64E7 | 2.27E8 | 8.5E6 | 8.89E7 | 3.16E8 | 1.03E8 | 1.09E8 | 4.11E8 | 2.12E8 | 72 | 10 | 5 | 6 | 4 | 5 | 8 | 4 | 9 | 14 | 7 | 150 | 179 | Oxidation (M) |
| K.IWHHSFYNELR.V | Y | 94.96 | 1500.7262 | 11 | 3.5 | 501.2511 | 3 | 60.94 | 6 | 17452 | Ana\_05.raw | 1.31E8 | 2.18E8 | 1.99E8 | 8.09E7 | 1.55E8 | 2.32E8 | 1.01E8 | 1.17E8 | 2.79E8 | 1.3E8 | 45 | 3 | 4 | 4 | 3 | 5 | 6 | 4 | 6 | 7 | 3 | 87 | 97 |  |
| K.DSYVGDEAQSKR.G | N | 93.37 | 1353.6160 | 12 | 3.6 | 677.8177 | 2 | 27.36 | 1 | 7192 | ANa\_10.raw | 9.62E7 | 3.55E7 | 6.96E7 | 1.04E7 | 3.31E7 | 5.26E7 | 2.56E7 | 1.83E7 | 1.05E8 | 4.34E7 | 30 | 4 | 2 | 4 | 2 | 3 | 3 | 3 | 3 | 3 | 3 | 53 | 64 |  |
| K.QEYDEAGPSIVHR.K | N | 91.09 | 1499.7004 | 13 | 3.8 | 500.9093 | 3 | 48.57 | 6 | 13776 | Ana\_05.raw | 7.68E7 | 6.13E7 | 9.07E7 | 3.96E7 | 8.61E7 | 1.01E8 | 4.95E7 | 8.99E7 | 5.06E7 | 4.33E6 | 35 | 3 | 3 | 3 | 3 | 5 | 4 | 3 | 7 | 3 | 1 | 362 | 374 |  |
| K.DSYVGDEAQSK.R | N | 89.89 | 1197.5149 | 11 | 1.9 | 599.7659 | 2 | 26.18 | 3 | 6939 | Ana\_02.raw | 2.5E9 | 2.55E9 | 2.09E9 | 9.4E8 | 1.67E9 | 2.04E9 | 1.22E9 | 1.3E9 | 1.9E9 | 2.02E9 | 140 | 13 | 12 | 15 | 13 | 15 | 15 | 13 | 18 | 11 | 15 | 53 | 63 |  |
| R.KDLYANNVLSGGTTMYPGIADR.M | N | 89.03 | 2355.1528 | 22 | 3.6 | 786.0610 | 3 | 92.11 | 1 | 25855 | ANa\_10.raw | 3.05E5 |  |  |  |  |  |  |  | 2.35E6 |  | 2 | 1 | 0 | 0 | 0 | 0 | 0 | 0 | 0 | 1 | 0 | 293 | 314 |  |
| K.YPIEHGIITNWDDM(+15.99)EK.I | N | 87.01 | 1975.8986 | 16 | 4.7 | 988.9612 | 2 | 75.68 | 9 | 21564 | Ana\_08.raw | 3.19E8 | 2.43E8 | 2.13E8 | 1.25E8 | 2.7E8 | 2.71E8 | 1.07E8 | 2.43E8 | 8.47E8 | 1.87E8 | 63 | 9 | 6 | 7 | 4 | 6 | 3 | 4 | 7 | 13 | 4 | 71 | 86 | Oxidation (M) |
| R.GYSFVTTAER.E | N | 84.49 | 1129.5404 | 10 | 1.5 | 565.7783 | 2 | 57.04 | 3 | 16191 | Ana\_02.raw | 1.61E9 | 1.67E9 | 1.48E9 | 5.34E8 | 1.06E9 | 1.19E9 | 7.36E8 | 9.1E8 | 1.96E9 | 1.22E9 | 186 | 21 | 21 | 21 | 15 | 20 | 18 | 14 | 24 | 17 | 15 | 199 | 208 |  |
| R.DLTDYLM(+15.99)KILTER.G | N | 84.48 | 1625.8334 | 13 | -0.5 | 813.9235 | 2 | 131.53 | 3 | 36031 | Ana\_02.raw | 2.95E6 | 3.96E6 | 7.7E6 | 1.52E6 | 5.67E6 | 8.87E6 | 6.41E6 | 2.09E6 | 2.69E6 | 6.16E6 | 17 | 2 | 1 | 2 | 1 | 2 | 2 | 2 | 2 | 1 | 2 | 186 | 198 | Oxidation (M) |
| R.HQGVMVGM(+15.99)GQK.D | N | 84.12 | 1186.5587 | 11 | 1.7 | 594.2877 | 2 | 16.20 | 3 | 3687 | Ana\_02.raw | 8.78E6 | 2.67E7 | 5.55E6 | 4.81E6 | 4.46E6 | 2.99E7 | 5.97E6 | 1.31E7 | 1.79E7 | 2.08E7 | 21 | 2 | 2 | 2 | 2 | 1 | 3 | 3 | 2 | 1 | 3 | 42 | 52 | Oxidation (M) |
| K.EITALAPSTM(+15.99)K.I | N | 81.55 | 1176.6060 | 11 | 3.3 | 589.3122 | 2 | 45.25 | 6 | 12771 | Ana\_05.raw | 1.46E9 | 1.19E9 | 1.22E9 | 4.08E8 | 9.94E8 | 1.04E9 | 6.43E8 | 8.39E8 | 2.22E9 | 8.74E8 | 142 | 18 | 15 | 12 | 10 | 13 | 11 | 10 | 18 | 18 | 17 | 318 | 328 | Oxidation (M) |
| K.YPIEHGIITNWDDMEK.I | N | 80.26 | 1959.9037 | 16 | 2.8 | 980.9619 | 2 | 90.08 | 1 | 25309 | ANa\_10.raw | 3.61E6 |  |  |  |  |  |  |  | 2.49E7 | 7.39E6 | 4 | 2 | 0 | 0 | 0 | 0 | 0 | 0 | 0 | 1 | 1 | 71 | 86 |  |
| K.AGFAGDDAPR.A | N | 76.24 | 975.4410 | 10 | 4.8 | 488.7301 | 2 | 31.38 | 1 | 8414 | ANa\_10.raw | 3.67E9 | 4.81E9 | 3.7E9 | 1.86E9 | 3.52E9 | 3.73E9 | 2.42E9 | 2.63E9 | 6.06E9 | 4.43E9 | 286 | 36 | 29 | 31 | 28 | 26 | 21 | 16 | 35 | 35 | 29 | 21 | 30 |  |
| R.AVFPSIVGRPR.H | N | 74.48 | 1197.6981 | 11 | 5.3 | 599.8595 | 2 | 62.78 | 2 | 17880 | Ana\_01.raw | 1.6E9 | 1.34E9 | 1.32E9 | 8.13E8 | 1.16E9 | 1.21E9 | 8.12E8 | 8.11E8 | 2.43E9 | 1.42E9 | 261 | 32 | 21 | 25 | 23 | 36 | 23 | 17 | 28 | 32 | 24 | 31 | 41 |  |
| R.HQGVM(+15.99)VGMGQK.D | N | 69.21 | 1186.5587 | 11 | 1.9 | 594.2878 | 2 | 15.71 | 5 | 3571 | Ana\_04.raw | 1.62E7 | 9.07E6 |  |  | 1.39E6 | 1.35E7 | 8.82E5 | 7.09E6 | 1.92E6 | 1.76E7 | 12 | 1 | 1 | 0 | 0 | 1 | 2 | 2 | 2 | 1 | 2 | 42 | 52 | Oxidation (M) |
| K.EITALAPSTMK.I | N | 67.15 | 1160.6111 | 11 | -0.1 | 581.3127 | 2 | 30.48 | 9 | 8027 | Ana\_08.raw | 6.1E7 | 2.19E7 |  | 5.22E5 |  |  | 1.96E6 |  | 1.1E8 | 4.16E7 | 14 | 4 | 1 | 0 | 1 | 0 | 0 | 1 | 0 | 5 | 2 | 318 | 328 |  |
| R.HQGVM(+15.99)VGM(+15.99)GQK.D | N | 65.50 | 1202.5536 | 11 | 7.8 | 602.2888 | 2 | 21.12 | 1 | 5142 | ANa\_10.raw | 3.51E8 | 1.68E8 | 6.85E8 | 2.25E8 | 4.15E8 | 7.33E8 | 4.21E8 | 3.56E8 | 9.25E7 | 3.14E8 | 59 | 8 | 4 | 5 | 7 | 6 | 4 | 5 | 8 | 6 | 6 | 42 | 52 | Oxidation (M) |
| R.C(+57.02)PETLFQPSFIGM(+15.99)ESAGIHETTYNSIMK.C | N | 60.09 | 3203.4614 | 28 | 4.5 | 1068.8325 | 3 | 117.97 | 1 | 32577 | ANa\_10.raw | 1.9E6 |  |  |  |  |  |  |  |  |  | 1 | 1 | 0 | 0 | 0 | 0 | 0 | 0 | 0 | 0 | 0 | 259 | 286 | Carbamidomethylation; Oxidation (M) |
| R.DLTDYLM(+15.99)K.I | N | 58.74 | 1013.4739 | 8 | 3.9 | 507.7462 | 2 | 92.37 | 6 | 26220 | Ana\_05.raw | 8.4E8 | 6.93E8 | 6.22E8 | 2.72E8 | 4.42E8 | 4.88E8 | 3.63E8 | 5.27E8 | 1.32E9 | 5.32E8 | 291 | 38 | 34 | 28 | 21 | 30 | 22 | 17 | 33 | 35 | 33 | 186 | 193 | Oxidation (M) |
| R.DLTDYLMK.I | N | 58.29 | 997.4790 | 8 | 4.0 | 499.7487 | 2 | 92.24 | 1 | 25887 | ANa\_10.raw | 3.85E7 | 6.67E6 | 1.26E5 | 5.05E5 |  | 6.74E6 | 2.41E5 | 3.81E6 | 2.57E7 | 2.99E6 | 21 | 5 | 2 | 1 | 1 | 0 | 2 | 1 | 1 | 5 | 3 | 186 | 193 |  |
| K.IIAPPER.K | N | 57.46 | 794.4650 | 7 | 1.7 | 398.2404 | 2 | 33.34 | 3 | 9119 | Ana\_02.raw | 3.97E9 | 4.12E9 | 3.95E9 | 1.66E9 | 3.05E9 | 3.75E9 | 2.4E9 | 2.4E9 | 4.68E9 | 3.63E9 | 283 | 36 | 29 | 33 | 22 | 29 | 22 | 16 | 34 | 31 | 31 | 331 | 337 |  |
| R.C(+57.02)PETLFQPSFIGM(+15.99)ESAGIHETTYNSIM(+15.99)K.C | N | 56.25 | 3219.4563 | 28 | 3.5 | 1074.1631 | 3 | 106.43 | 9 | 29941 | Ana\_08.raw | 9.39E6 | 7.29E6 |  | 2.94E6 | 1.83E7 | 6.25E6 | 1E6 | 1.05E7 | 1.17E7 |  | 10 | 1 | 2 | 0 | 1 | 2 | 1 | 1 | 1 | 1 | 0 | 259 | 286 | Carbamidomethylation; Oxidation (M) |
| R.LDLAGRDLTDYLM(+15.99)K.I | N | 51.33 | 1638.8286 | 14 | 3.2 | 820.4242 | 2 | 95.36 | 9 | 26994 | Ana\_08.raw | 9.63E6 |  |  |  |  |  |  |  | 1.04E7 |  | 3 | 1 | 0 | 0 | 0 | 0 | 0 | 0 | 0 | 2 | 0 | 180 | 193 | Oxidation (M) |
| K.IIAPPERK.Y | N | 46.97 | 922.5599 | 8 | 3.9 | 462.2891 | 2 | 23.58 | 9 | 5830 | Ana\_08.raw | 8.34E7 | 2.85E7 | 2.04E7 |  | 1.06E7 | 3.16E7 | 5.24E6 | 4.71E6 | 8.32E7 | 2.96E7 | 18 | 3 | 2 | 2 | 0 | 2 | 2 | 1 | 1 | 3 | 2 | 331 | 338 |  |
| K.C(+57.02)DIDIR.K | N | 45.41 | 790.3643 | 6 | 3.2 | 396.1907 | 2 | 36.67 | 6 | 10134 | Ana\_05.raw |  |  |  | 1.65E7 | 3.9E7 | 5.72E7 | 2.82E7 | 3.43E7 |  |  | 5 | 0 | 0 | 0 | 1 | 1 | 1 | 1 | 1 | 0 | 0 | 287 | 292 | Carbamidomethylation |
| K.IKIIAPPER.K | N | 42.43 | 1035.6440 | 9 | 0.5 | 346.2221 | 3 | 44.96 | 3 | 12647 | Ana\_02.raw | 3.02E6 |  | 7.18E6 | 2.01E6 | 2.08E6 | 3.03E6 | 1.58E6 |  | 4.82E6 | 2.45E6 | 12 | 1 | 0 | 2 | 2 | 1 | 2 | 1 | 0 | 2 | 1 | 329 | 337 |  |
| K.ILTER.G | N | 39.01 | 630.3701 | 5 | 3.4 | 316.1934 | 2 | 20.56 | 6 | 5102 | Ana\_05.raw | 2.01E7 |  |  | 2.76E6 | 1.13E7 | 9.27E6 | 2.65E8 | 1.7E8 | 7.79E6 |  | 14 | 1 | 0 | 0 | 3 | 3 | 1 | 4 | 1 | 1 | 0 | 194 | 198 |  |
| R.EIVRDIK.E | N | 36.72 | 871.5127 | 7 | 6.3 | 436.7664 | 2 | 23.86 | 9 | 5929 | Ana\_08.raw |  | 3.4E6 | 3.11E6 |  |  | 3.2E6 | 5.84E5 |  | 3.56E6 | 3.27E6 | 6 | 0 | 1 | 1 | 0 | 0 | 1 | 1 | 0 | 1 | 1 | 209 | 215 |  |
| R.LDLAGR.D | N | 35.64 | 643.3653 | 6 | -0.6 | 322.6897 | 2 | 30.23 | 3 | 8192 | Ana\_02.raw | 7.61E8 | 7.49E8 | 8.47E8 | 3.81E8 | 6.81E8 | 6.9E7 | 4E5 | 1.11E2 | 9.23E8 | 6.75E8 | 38 | 3 | 3 | 4 | 6 | 6 | 3 | 1 | 1 | 9 | 2 | 180 | 185 |  |
| K.RGILTLK.Y | N | 29.35 | 799.5280 | 7 | 4.0 | 400.7729 | 2 | 41.36 | 6 | 11572 | Ana\_05.raw |  |  |  |  |  | 1.41E8 |  | 9.14E7 |  |  | 2 | 0 | 0 | 0 | 0 | 0 | 1 | 0 | 1 | 0 | 0 | 64 | 70 |  |
| R.M(+15.99)QKEITALAPSTM(+15.99)K.I | N | 25.56 | 1579.7949 | 14 | 0.7 | 527.6060 | 3 | 38.99 | 3 | 10840 | Ana\_02.raw |  |  | 2.42E6 |  |  |  |  |  |  |  | 1 | 0 | 0 | 1 | 0 | 0 | 0 | 0 | 0 | 0 | 0 | 315 | 328 | Oxidation (M) |
| R.C(+57.02)PETLFQPSFIGMESAGIHETTYNSIM(+15.99)K.C | N | 25.32 | 3203.4614 | 28 | -1.1 | 1602.7362 | 2 | 126.87 | 2 | 35153 | Ana\_01.raw |  | 4.47E5 |  |  |  |  |  |  |  |  | 1 | 0 | 1 | 0 | 0 | 0 | 0 | 0 | 0 | 0 | 0 | 259 | 286 | Carbamidomethylation; Oxidation (M) |
| R.GILTLK.Y | N | 25.30 | 643.4268 | 6 | 5.7 | 322.7225 | 2 | 51.01 | 9 | 14360 | Ana\_08.raw |  | 1.76E8 | 1.95E8 |  |  | 1.81E8 | 1.2E8 |  | 2.57E2 |  | 6 | 0 | 2 | 1 | 0 | 0 | 1 | 1 | 0 | 1 | 0 | 65 | 70 |  |
| total 41 peptides |  |  |  |  |  |  |  |  |  |  |  |  |  |  |  |  |  |  |  |  |  |  |  |  |  |  |  |  |  |  |  |  |  |  |  |
| --- | --- | --- | --- | --- | --- | --- | --- | --- | --- | --- | --- | --- | --- | --- | --- | --- | --- | --- | --- | --- | --- | --- | --- | --- | --- | --- | --- | --- | --- | --- | --- | --- | --- | --- | --- |

Best Unique PSM (Scan F6:17452, m/z=501.2511, z=3, RT=60.94, ppm=3.5):

sp|P63260|ACTG\_MOUSE

back to list

  

| Protein Coverage
| Supporting Peptides
| Best Unique PSM
|

Protein Coverage:

Supporting Peptides:

| Peptide | Uniq | -10lgP | Mass | Length | ppm | m/z | z | RT | Fraction | Scan | Source File | Area 10 | Area 01 | Area 02 | Area 03 | Area 04 | Area 05 | Area 06 | Area 07 | Area 08 | Area 09 | #Spec | #Spec 10 | #Spec 01 | #Spec 02 | #Spec 03 | #Spec 04 | #Spec 05 | #Spec 06 | #Spec 07 | #Spec 08 | #Spec 09 | Start | End | PTM |
| --- | --- | --- | --- | --- | --- | --- | --- | --- | --- | --- | --- | --- | --- | --- | --- | --- | --- | --- | --- | --- | --- | --- | --- | --- | --- | --- | --- | --- | --- | --- | --- | --- | --- | --- | --- |
| K.SYELPDGQVITIGNER.F | N | 136.16 | 1789.8846 | 16 | 0.5 | 895.9501 | 2 | 101.19 | 3 | 28209 | Ana\_02.raw | 2.56E9 | 2.49E9 | 2.2E9 | 5.02E8 | 1.2E9 | 1.61E9 | 8.03E8 | 1.08E9 | 4.17E9 | 2.27E9 | 445 | 51 | 48 | 45 | 42 | 44 | 40 | 40 | 47 | 46 | 42 | 239 | 254 |  |
| R.TTGIVM(+15.99)DSGDGVTHTVPIYEGYALPHAILR.L | N | 134.68 | 3198.6018 | 30 | 0.6 | 1067.2085 | 3 | 109.44 | 6 | 30732 | Ana\_05.raw | 5.15E8 | 1.48E8 | 1.08E8 | 2.77E7 | 1.97E8 | 1.61E8 | 1.12E8 | 4.1E7 | 1.33E9 | 1.87E8 | 94 | 17 | 6 | 5 | 8 | 10 | 7 | 7 | 11 | 15 | 8 | 148 | 177 | Oxidation (M) |
| R.VAPEEHPVLLTEAPLNPK.A | Y | 131.47 | 1953.0570 | 18 | 0.4 | 977.5361 | 2 | 76.06 | 3 | 21552 | Ana\_02.raw | 3.07E9 | 2.82E9 | 2.13E9 | 8.06E8 | 1.46E9 | 1.76E9 | 1.08E9 | 1.35E9 | 5.91E9 | 1.83E9 | 228 | 30 | 24 | 22 | 22 | 24 | 15 | 15 | 21 | 30 | 25 | 96 | 113 |  |
| R.TTGIVMDSGDGVTHTVPIYEGYALPHAILR.L | N | 130.29 | 3182.6069 | 30 | 5.3 | 1061.8818 | 3 | 113.09 | 9 | 31668 | Ana\_08.raw | 1.04E8 | 4.47E7 | 3.97E6 | 1.57E7 | 0 | 4.85E7 | 5.93E6 | 1.32E7 | 2.9E8 | 1.9E8 | 37 | 6 | 3 | 1 | 3 | 1 | 3 | 1 | 3 | 8 | 8 | 148 | 177 |  |
| K.LC(+57.02)YVALDFEQEMATAASSSSLEK.S | Y | 122.70 | 2549.1665 | 23 | 4.0 | 1275.5956 | 2 | 130.62 | 1 | 35904 | ANa\_10.raw | 2.63E7 | 1.91E7 |  | 7.19E5 | 2.61E6 | 1.12E7 | 2.92E6 | 6.76E6 | 2.16E7 | 3.03E7 | 14 | 2 | 2 | 0 | 1 | 1 | 2 | 1 | 2 | 1 | 2 | 216 | 238 | Carbamidomethylation |
| R.KDLYANTVLSGGTTM(+15.99)YPGIADR.M | N | 120.48 | 2358.1526 | 22 | 3.9 | 787.0612 | 3 | 83.50 | 9 | 23760 | Ana\_08.raw | 4.51E7 | 4.59E7 | 4.16E7 | 1.5E7 | 3.13E7 | 2.96E7 | 2.16E7 | 2.39E7 | 1.12E8 | 2.15E7 | 19 | 2 | 4 | 1 | 1 | 1 | 1 | 1 | 2 | 5 | 1 | 291 | 312 | Oxidation (M) |
| K.DLYANTVLSGGTTM(+15.99)YPGIADR.M | N | 117.82 | 2230.0576 | 21 | 3.4 | 1116.0399 | 2 | 100.01 | 1 | 27979 | ANa\_10.raw | 3.93E8 | 2.93E8 | 1.86E8 | 4.46E7 | 6.34E7 | 1.11E8 | 5.83E7 | 5.32E7 | 1.47E9 | 1.98E8 | 84 | 17 | 11 | 7 | 2 | 5 | 4 | 4 | 5 | 16 | 13 | 292 | 312 | Oxidation (M) |
| K.DLYANTVLSGGTTMYPGIADR.M | N | 116.82 | 2214.0627 | 21 | 5.3 | 1108.0446 | 2 | 108.94 | 9 | 30607 | Ana\_08.raw | 7.32E6 | 1.81E6 |  |  |  | 1.9E5 |  |  | 5.45E7 | 8.18E6 | 9 | 1 | 1 | 0 | 0 | 0 | 1 | 0 | 0 | 5 | 1 | 292 | 312 |  |
| K.LC(+57.02)YVALDFEQEM(+15.99)ATAASSSSLEK.S | Y | 104.80 | 2565.1614 | 23 | 3.0 | 1283.5918 | 2 | 106.82 | 9 | 30047 | Ana\_08.raw | 7.31E6 | 3.56E6 | 4.04E6 | 2.12E6 | 8.73E5 | 1.42E6 |  | 2.53E6 | 1.57E7 | 2.2E6 | 19 | 2 | 1 | 2 | 2 | 1 | 1 | 0 | 3 | 6 | 1 | 216 | 238 | Carbamidomethylation; Oxidation (M) |
| K.IWHHTFYNELR.V | N | 103.41 | 1514.7418 | 11 | 1.7 | 505.9221 | 3 | 58.21 | 3 | 16543 | Ana\_02.raw | 1.86E8 | 2.44E8 | 2.11E8 | 9.2E7 | 1.75E8 | 1.74E8 | 1.18E8 | 1.31E8 | 3.7E8 | 2.13E8 | 57 | 5 | 6 | 5 | 7 | 7 | 4 | 4 | 5 | 7 | 7 | 85 | 95 |  |
| K.QEYDESGPSIVHR.K | N | 100.31 | 1515.6953 | 13 | 4.7 | 758.8585 | 2 | 44.88 | 1 | 12508 | ANa\_10.raw | 2.15E8 | 5.17E7 | 4.08E7 | 9.64E7 | 5.34E7 | 1.9E8 | 1.2E8 | 2.14E8 | 1.41E8 | 7.44E7 | 41 | 5 | 3 | 3 | 5 | 4 | 4 | 3 | 6 | 5 | 3 | 360 | 372 |  |
| K.DSYVGDEAQSKR.G | N | 93.37 | 1353.6160 | 12 | 3.6 | 677.8177 | 2 | 27.36 | 1 | 7192 | ANa\_10.raw | 9.62E7 | 3.55E7 | 6.96E7 | 1.04E7 | 3.31E7 | 5.26E7 | 2.56E7 | 1.83E7 | 1.05E8 | 4.34E7 | 30 | 4 | 2 | 4 | 2 | 3 | 3 | 3 | 3 | 3 | 3 | 51 | 62 |  |
| K.DSYVGDEAQSK.R | N | 89.89 | 1197.5149 | 11 | 1.9 | 599.7659 | 2 | 26.18 | 3 | 6939 | Ana\_02.raw | 2.5E9 | 2.55E9 | 2.09E9 | 9.4E8 | 1.67E9 | 2.04E9 | 1.22E9 | 1.3E9 | 1.9E9 | 2.02E9 | 140 | 13 | 12 | 15 | 13 | 15 | 15 | 13 | 18 | 11 | 15 | 51 | 61 |  |
| R.GYSFTTTAER.E | Y | 87.08 | 1131.5197 | 10 | 0.2 | 566.7672 | 2 | 47.24 | 3 | 13342 | Ana\_02.raw | 2.15E9 | 1.89E9 | 1.68E9 | 6.85E8 | 1.37E9 | 1.4E9 | 1.03E9 | 1.04E9 | 2.92E9 | 1.99E9 | 170 | 20 | 18 | 15 | 17 | 17 | 11 | 12 | 19 | 19 | 22 | 197 | 206 |  |
| R.DLTDYLM(+15.99)KILTER.G | N | 84.48 | 1625.8334 | 13 | -0.5 | 813.9235 | 2 | 131.53 | 3 | 36031 | Ana\_02.raw | 2.95E6 | 3.96E6 | 7.7E6 | 1.52E6 | 5.67E6 | 8.87E6 | 6.41E6 | 2.09E6 | 2.69E6 | 6.16E6 | 17 | 2 | 1 | 2 | 1 | 2 | 2 | 2 | 2 | 1 | 2 | 184 | 196 | Oxidation (M) |
| R.HQGVMVGM(+15.99)GQK.D | N | 84.12 | 1186.5587 | 11 | 1.7 | 594.2877 | 2 | 16.20 | 3 | 3687 | Ana\_02.raw | 8.78E6 | 2.67E7 | 5.55E6 | 4.81E6 | 4.46E6 | 2.99E7 | 5.97E6 | 1.31E7 | 1.79E7 | 2.08E7 | 21 | 2 | 2 | 2 | 2 | 1 | 3 | 3 | 2 | 1 | 3 | 40 | 50 | Oxidation (M) |
| K.EITALAPSTM(+15.99)K.I | N | 81.55 | 1176.6060 | 11 | 3.3 | 589.3122 | 2 | 45.25 | 6 | 12771 | Ana\_05.raw | 1.46E9 | 1.19E9 | 1.22E9 | 4.08E8 | 9.94E8 | 1.04E9 | 6.43E8 | 8.39E8 | 2.22E9 | 8.74E8 | 142 | 18 | 15 | 12 | 10 | 13 | 11 | 10 | 18 | 18 | 17 | 316 | 326 | Oxidation (M) |
| K.AGFAGDDAPR.A | N | 76.24 | 975.4410 | 10 | 4.8 | 488.7301 | 2 | 31.38 | 1 | 8414 | ANa\_10.raw | 3.67E9 | 4.81E9 | 3.7E9 | 1.86E9 | 3.52E9 | 3.73E9 | 2.42E9 | 2.63E9 | 6.06E9 | 4.43E9 | 286 | 36 | 29 | 31 | 28 | 26 | 21 | 16 | 35 | 35 | 29 | 19 | 28 |  |
| R.AVFPSIVGRPR.H | N | 74.48 | 1197.6981 | 11 | 5.3 | 599.8595 | 2 | 62.78 | 2 | 17880 | Ana\_01.raw | 1.6E9 | 1.34E9 | 1.32E9 | 8.13E8 | 1.16E9 | 1.21E9 | 8.12E8 | 8.11E8 | 2.43E9 | 1.42E9 | 261 | 32 | 21 | 25 | 23 | 36 | 23 | 17 | 28 | 32 | 24 | 29 | 39 |  |
| R.C(+57.02)PEALFQPSFLGM(+15.99)ESC(+57.02)GIHETTFNSIMK.C | N | 70.79 | 3246.4495 | 28 | 4.5 | 1083.1620 | 3 | 123.56 | 9 | 34350 | Ana\_08.raw | 3.93E6 |  |  |  |  |  |  |  | 4.59E6 |  | 2 | 1 | 0 | 0 | 0 | 0 | 0 | 0 | 0 | 1 | 0 | 257 | 284 | Carbamidomethylation; Oxidation (M) |
| R.KDLYANTVLSGGTTMYPGIADR.M | N | 69.63 | 2342.1577 | 22 | 3.1 | 781.7289 | 3 | 94.52 | 1 | 26498 | ANa\_10.raw | 3.49E5 |  |  |  |  |  |  |  |  |  | 1 | 1 | 0 | 0 | 0 | 0 | 0 | 0 | 0 | 0 | 0 | 291 | 312 |  |
| R.HQGVM(+15.99)VGMGQK.D | N | 69.21 | 1186.5587 | 11 | 1.9 | 594.2878 | 2 | 15.71 | 5 | 3571 | Ana\_04.raw | 1.62E7 | 9.07E6 |  |  | 1.39E6 | 1.35E7 | 8.82E5 | 7.09E6 | 1.92E6 | 1.76E7 | 12 | 1 | 1 | 0 | 0 | 1 | 2 | 2 | 2 | 1 | 2 | 40 | 50 | Oxidation (M) |
| R.C(+57.02)PEALFQPSFLGM(+15.99)ESC(+57.02)GIHETTFNSIM(+15.99)K.C | N | 68.96 | 3262.4443 | 28 | 4.0 | 1088.4930 | 3 | 115.42 | 2 | 32132 | Ana\_01.raw | 2.15E7 | 3.46E7 | 4.02E6 | 6.18E6 | 6.21E6 |  | 1.48E7 | 2.62E7 | 3.67E7 | 1.41E7 | 12 | 1 | 2 | 1 | 1 | 1 | 0 | 2 | 1 | 1 | 2 | 257 | 284 | Carbamidomethylation; Oxidation (M) |
| K.EITALAPSTMK.I | N | 67.15 | 1160.6111 | 11 | -0.1 | 581.3127 | 2 | 30.48 | 9 | 8027 | Ana\_08.raw | 6.1E7 | 2.19E7 |  | 5.22E5 |  |  | 1.96E6 |  | 1.1E8 | 4.16E7 | 14 | 4 | 1 | 0 | 1 | 0 | 0 | 1 | 0 | 5 | 2 | 316 | 326 |  |
| R.HQGVM(+15.99)VGM(+15.99)GQK.D | N | 65.50 | 1202.5536 | 11 | 7.8 | 602.2888 | 2 | 21.12 | 1 | 5142 | ANa\_10.raw | 3.51E8 | 1.68E8 | 6.85E8 | 2.25E8 | 4.15E8 | 7.33E8 | 4.21E8 | 3.56E8 | 9.25E7 | 3.14E8 | 59 | 8 | 4 | 5 | 7 | 6 | 4 | 5 | 8 | 6 | 6 | 40 | 50 | Oxidation (M) |
| R.C(+57.02)PEALFQPSFLGMESC(+57.02)GIHETTFNSIM(+15.99)K.C | N | 61.68 | 3246.4495 | 28 | 3.4 | 1083.1608 | 3 | 122.40 | 9 | 34055 | Ana\_08.raw | 9.92E5 |  |  |  | 2.9E6 |  |  |  | 4.5E6 |  | 3 | 1 | 0 | 0 | 0 | 1 | 0 | 0 | 0 | 1 | 0 | 257 | 284 | Carbamidomethylation; Oxidation (M) |
| R.DLTDYLM(+15.99)K.I | N | 58.74 | 1013.4739 | 8 | 3.9 | 507.7462 | 2 | 92.37 | 6 | 26220 | Ana\_05.raw | 8.4E8 | 6.93E8 | 6.22E8 | 2.72E8 | 4.42E8 | 4.88E8 | 3.63E8 | 5.27E8 | 1.32E9 | 5.32E8 | 291 | 38 | 34 | 28 | 21 | 30 | 22 | 17 | 33 | 35 | 33 | 184 | 191 | Oxidation (M) |
| R.DLTDYLMK.I | N | 58.29 | 997.4790 | 8 | 4.0 | 499.7487 | 2 | 92.24 | 1 | 25887 | ANa\_10.raw | 3.85E7 | 6.67E6 | 1.26E5 | 5.05E5 |  | 6.74E6 | 2.41E5 | 3.81E6 | 2.57E7 | 2.99E6 | 21 | 5 | 2 | 1 | 1 | 0 | 2 | 1 | 1 | 5 | 3 | 184 | 191 |  |
| K.IIAPPER.K | N | 57.46 | 794.4650 | 7 | 1.7 | 398.2404 | 2 | 33.34 | 3 | 9119 | Ana\_02.raw | 3.97E9 | 4.12E9 | 3.95E9 | 1.66E9 | 3.05E9 | 3.75E9 | 2.4E9 | 2.4E9 | 4.68E9 | 3.63E9 | 283 | 36 | 29 | 33 | 22 | 29 | 22 | 16 | 34 | 31 | 31 | 329 | 335 |  |
| R.LDLAGRDLTDYLM(+15.99)K.I | N | 51.33 | 1638.8286 | 14 | 3.2 | 820.4242 | 2 | 95.36 | 9 | 26994 | Ana\_08.raw | 9.63E6 |  |  |  |  |  |  |  | 1.04E7 |  | 3 | 1 | 0 | 0 | 0 | 0 | 0 | 0 | 0 | 2 | 0 | 178 | 191 | Oxidation (M) |
| K.IIAPPERK.Y | N | 46.97 | 922.5599 | 8 | 3.9 | 462.2891 | 2 | 23.58 | 9 | 5830 | Ana\_08.raw | 8.34E7 | 2.85E7 | 2.04E7 |  | 1.06E7 | 3.16E7 | 5.24E6 | 4.71E6 | 8.32E7 | 2.96E7 | 18 | 3 | 2 | 2 | 0 | 2 | 2 | 1 | 1 | 3 | 2 | 329 | 336 |  |
| K.C(+57.02)DVDIR.K | N | 43.98 | 776.3487 | 6 | 4.4 | 389.1833 | 2 | 28.80 | 8 | 7498 | Ana\_07.raw |  | 4.69E7 | 4.42E7 | 2.63E7 | 5.45E7 | 4.38E7 | 3.39E7 | 3.7E7 |  | 9.42E6 | 9 | 0 | 1 | 1 | 1 | 1 | 1 | 1 | 2 | 0 | 1 | 285 | 290 | Carbamidomethylation |
| K.IKIIAPPER.K | N | 42.43 | 1035.6440 | 9 | 0.5 | 346.2221 | 3 | 44.96 | 3 | 12647 | Ana\_02.raw | 3.02E6 |  | 7.18E6 | 2.01E6 | 2.08E6 | 3.03E6 | 1.58E6 |  | 4.82E6 | 2.45E6 | 12 | 1 | 0 | 2 | 2 | 1 | 2 | 1 | 0 | 2 | 1 | 327 | 335 |  |
| K.ILTER.G | N | 39.01 | 630.3701 | 5 | 3.4 | 316.1934 | 2 | 20.56 | 6 | 5102 | Ana\_05.raw | 2.01E7 |  |  | 2.76E6 | 1.13E7 | 9.27E6 | 2.65E8 | 1.7E8 | 7.79E6 |  | 14 | 1 | 0 | 0 | 3 | 3 | 1 | 4 | 1 | 1 | 0 | 192 | 196 |  |
| R.EIVRDIK.E | N | 36.72 | 871.5127 | 7 | 6.3 | 436.7664 | 2 | 23.86 | 9 | 5929 | Ana\_08.raw |  | 3.4E6 | 3.11E6 |  |  | 3.2E6 | 5.84E5 |  | 3.56E6 | 3.27E6 | 6 | 0 | 1 | 1 | 0 | 0 | 1 | 1 | 0 | 1 | 1 | 207 | 213 |  |
| R.LDLAGR.D | N | 35.64 | 643.3653 | 6 | -0.6 | 322.6897 | 2 | 30.23 | 3 | 8192 | Ana\_02.raw | 7.61E8 | 7.49E8 | 8.47E8 | 3.81E8 | 6.81E8 | 6.9E7 | 4E5 | 1.11E2 | 9.23E8 | 6.75E8 | 38 | 3 | 3 | 4 | 6 | 6 | 3 | 1 | 1 | 9 | 2 | 178 | 183 |  |
| K.RGILTLK.Y | N | 29.35 | 799.5280 | 7 | 4.0 | 400.7729 | 2 | 41.36 | 6 | 11572 | Ana\_05.raw |  |  |  |  |  | 1.41E8 |  | 9.14E7 |  |  | 2 | 0 | 0 | 0 | 0 | 0 | 1 | 0 | 1 | 0 | 0 | 62 | 68 |  |
| R.M(+15.99)QKEITALAPSTM(+15.99)K.I | N | 25.56 | 1579.7949 | 14 | 0.7 | 527.6060 | 3 | 38.99 | 3 | 10840 | Ana\_02.raw |  |  | 2.42E6 |  |  |  |  |  |  |  | 1 | 0 | 0 | 1 | 0 | 0 | 0 | 0 | 0 | 0 | 0 | 313 | 326 | Oxidation (M) |
| R.GILTLK.Y | N | 25.30 | 643.4268 | 6 | 5.7 | 322.7225 | 2 | 51.01 | 9 | 14360 | Ana\_08.raw |  | 1.76E8 | 1.95E8 |  |  | 1.81E8 | 1.2E8 |  | 2.57E2 |  | 6 | 0 | 2 | 1 | 0 | 0 | 1 | 1 | 0 | 1 | 0 | 63 | 68 |  |
| total 39 peptides |  |  |  |  |  |  |  |  |  |  |  |  |  |  |  |  |  |  |  |  |  |  |  |  |  |  |  |  |  |  |  |  |  |  |  |
| --- | --- | --- | --- | --- | --- | --- | --- | --- | --- | --- | --- | --- | --- | --- | --- | --- | --- | --- | --- | --- | --- | --- | --- | --- | --- | --- | --- | --- | --- | --- | --- | --- | --- | --- | --- |

Best Unique PSM (Scan F3:21552, m/z=977.5361, z=2, RT=76.06, ppm=0.4):

sp|P60710|ACTB\_MOUSE

back to list

  

| Protein Coverage
| Supporting Peptides
| Best Unique PSM
|

Protein Coverage:

Supporting Peptides:

| Peptide | Uniq | -10lgP | Mass | Length | ppm | m/z | z | RT | Fraction | Scan | Source File | Area 10 | Area 01 | Area 02 | Area 03 | Area 04 | Area 05 | Area 06 | Area 07 | Area 08 | Area 09 | #Spec | #Spec 10 | #Spec 01 | #Spec 02 | #Spec 03 | #Spec 04 | #Spec 05 | #Spec 06 | #Spec 07 | #Spec 08 | #Spec 09 | Start | End | PTM |
| --- | --- | --- | --- | --- | --- | --- | --- | --- | --- | --- | --- | --- | --- | --- | --- | --- | --- | --- | --- | --- | --- | --- | --- | --- | --- | --- | --- | --- | --- | --- | --- | --- | --- | --- | --- |
| K.SYELPDGQVITIGNER.F | N | 136.16 | 1789.8846 | 16 | 0.5 | 895.9501 | 2 | 101.19 | 3 | 28209 | Ana\_02.raw | 2.56E9 | 2.49E9 | 2.2E9 | 5.02E8 | 1.2E9 | 1.61E9 | 8.03E8 | 1.08E9 | 4.17E9 | 2.27E9 | 445 | 51 | 48 | 45 | 42 | 44 | 40 | 40 | 47 | 46 | 42 | 239 | 254 |  |
| R.TTGIVM(+15.99)DSGDGVTHTVPIYEGYALPHAILR.L | N | 134.68 | 3198.6018 | 30 | 0.6 | 1067.2085 | 3 | 109.44 | 6 | 30732 | Ana\_05.raw | 5.15E8 | 1.48E8 | 1.08E8 | 2.77E7 | 1.97E8 | 1.61E8 | 1.12E8 | 4.1E7 | 1.33E9 | 1.87E8 | 94 | 17 | 6 | 5 | 8 | 10 | 7 | 7 | 11 | 15 | 8 | 148 | 177 | Oxidation (M) |
| R.VAPEEHPVLLTEAPLNPK.A | Y | 131.47 | 1953.0570 | 18 | 0.4 | 977.5361 | 2 | 76.06 | 3 | 21552 | Ana\_02.raw | 3.07E9 | 2.82E9 | 2.13E9 | 8.06E8 | 1.46E9 | 1.76E9 | 1.08E9 | 1.35E9 | 5.91E9 | 1.83E9 | 228 | 30 | 24 | 22 | 22 | 24 | 15 | 15 | 21 | 30 | 25 | 96 | 113 |  |
| R.TTGIVMDSGDGVTHTVPIYEGYALPHAILR.L | N | 130.29 | 3182.6069 | 30 | 5.3 | 1061.8818 | 3 | 113.09 | 9 | 31668 | Ana\_08.raw | 1.04E8 | 4.47E7 | 3.97E6 | 1.57E7 | 0 | 4.85E7 | 5.93E6 | 1.32E7 | 2.9E8 | 1.9E8 | 37 | 6 | 3 | 1 | 3 | 1 | 3 | 1 | 3 | 8 | 8 | 148 | 177 |  |
| K.LC(+57.02)YVALDFEQEMATAASSSSLEK.S | Y | 122.70 | 2549.1665 | 23 | 4.0 | 1275.5956 | 2 | 130.62 | 1 | 35904 | ANa\_10.raw | 2.63E7 | 1.91E7 |  | 7.19E5 | 2.61E6 | 1.12E7 | 2.92E6 | 6.76E6 | 2.16E7 | 3.03E7 | 14 | 2 | 2 | 0 | 1 | 1 | 2 | 1 | 2 | 1 | 2 | 216 | 238 | Carbamidomethylation |
| R.KDLYANTVLSGGTTM(+15.99)YPGIADR.M | N | 120.48 | 2358.1526 | 22 | 3.9 | 787.0612 | 3 | 83.50 | 9 | 23760 | Ana\_08.raw | 4.51E7 | 4.59E7 | 4.16E7 | 1.5E7 | 3.13E7 | 2.96E7 | 2.16E7 | 2.39E7 | 1.12E8 | 2.15E7 | 19 | 2 | 4 | 1 | 1 | 1 | 1 | 1 | 2 | 5 | 1 | 291 | 312 | Oxidation (M) |
| K.DLYANTVLSGGTTM(+15.99)YPGIADR.M | N | 117.82 | 2230.0576 | 21 | 3.4 | 1116.0399 | 2 | 100.01 | 1 | 27979 | ANa\_10.raw | 3.93E8 | 2.93E8 | 1.86E8 | 4.46E7 | 6.34E7 | 1.11E8 | 5.83E7 | 5.32E7 | 1.47E9 | 1.98E8 | 84 | 17 | 11 | 7 | 2 | 5 | 4 | 4 | 5 | 16 | 13 | 292 | 312 | Oxidation (M) |
| K.DLYANTVLSGGTTMYPGIADR.M | N | 116.82 | 2214.0627 | 21 | 5.3 | 1108.0446 | 2 | 108.94 | 9 | 30607 | Ana\_08.raw | 7.32E6 | 1.81E6 |  |  |  | 1.9E5 |  |  | 5.45E7 | 8.18E6 | 9 | 1 | 1 | 0 | 0 | 0 | 1 | 0 | 0 | 5 | 1 | 292 | 312 |  |
| K.LC(+57.02)YVALDFEQEM(+15.99)ATAASSSSLEK.S | Y | 104.80 | 2565.1614 | 23 | 3.0 | 1283.5918 | 2 | 106.82 | 9 | 30047 | Ana\_08.raw | 7.31E6 | 3.56E6 | 4.04E6 | 2.12E6 | 8.73E5 | 1.42E6 |  | 2.53E6 | 1.57E7 | 2.2E6 | 19 | 2 | 1 | 2 | 2 | 1 | 1 | 0 | 3 | 6 | 1 | 216 | 238 | Carbamidomethylation; Oxidation (M) |
| K.IWHHTFYNELR.V | N | 103.41 | 1514.7418 | 11 | 1.7 | 505.9221 | 3 | 58.21 | 3 | 16543 | Ana\_02.raw | 1.86E8 | 2.44E8 | 2.11E8 | 9.2E7 | 1.75E8 | 1.74E8 | 1.18E8 | 1.31E8 | 3.7E8 | 2.13E8 | 57 | 5 | 6 | 5 | 7 | 7 | 4 | 4 | 5 | 7 | 7 | 85 | 95 |  |
| K.QEYDESGPSIVHR.K | N | 100.31 | 1515.6953 | 13 | 4.7 | 758.8585 | 2 | 44.88 | 1 | 12508 | ANa\_10.raw | 2.15E8 | 5.17E7 | 4.08E7 | 9.64E7 | 5.34E7 | 1.9E8 | 1.2E8 | 2.14E8 | 1.41E8 | 7.44E7 | 41 | 5 | 3 | 3 | 5 | 4 | 4 | 3 | 6 | 5 | 3 | 360 | 372 |  |
| K.DSYVGDEAQSKR.G | N | 93.37 | 1353.6160 | 12 | 3.6 | 677.8177 | 2 | 27.36 | 1 | 7192 | ANa\_10.raw | 9.62E7 | 3.55E7 | 6.96E7 | 1.04E7 | 3.31E7 | 5.26E7 | 2.56E7 | 1.83E7 | 1.05E8 | 4.34E7 | 30 | 4 | 2 | 4 | 2 | 3 | 3 | 3 | 3 | 3 | 3 | 51 | 62 |  |
| K.DSYVGDEAQSK.R | N | 89.89 | 1197.5149 | 11 | 1.9 | 599.7659 | 2 | 26.18 | 3 | 6939 | Ana\_02.raw | 2.5E9 | 2.55E9 | 2.09E9 | 9.4E8 | 1.67E9 | 2.04E9 | 1.22E9 | 1.3E9 | 1.9E9 | 2.02E9 | 140 | 13 | 12 | 15 | 13 | 15 | 15 | 13 | 18 | 11 | 15 | 51 | 61 |  |
| R.GYSFTTTAER.E | Y | 87.08 | 1131.5197 | 10 | 0.2 | 566.7672 | 2 | 47.24 | 3 | 13342 | Ana\_02.raw | 2.15E9 | 1.89E9 | 1.68E9 | 6.85E8 | 1.37E9 | 1.4E9 | 1.03E9 | 1.04E9 | 2.92E9 | 1.99E9 | 170 | 20 | 18 | 15 | 17 | 17 | 11 | 12 | 19 | 19 | 22 | 197 | 206 |  |
| R.DLTDYLM(+15.99)KILTER.G | N | 84.48 | 1625.8334 | 13 | -0.5 | 813.9235 | 2 | 131.53 | 3 | 36031 | Ana\_02.raw | 2.95E6 | 3.96E6 | 7.7E6 | 1.52E6 | 5.67E6 | 8.87E6 | 6.41E6 | 2.09E6 | 2.69E6 | 6.16E6 | 17 | 2 | 1 | 2 | 1 | 2 | 2 | 2 | 2 | 1 | 2 | 184 | 196 | Oxidation (M) |
| R.HQGVMVGM(+15.99)GQK.D | N | 84.12 | 1186.5587 | 11 | 1.7 | 594.2877 | 2 | 16.20 | 3 | 3687 | Ana\_02.raw | 8.78E6 | 2.67E7 | 5.55E6 | 4.81E6 | 4.46E6 | 2.99E7 | 5.97E6 | 1.31E7 | 1.79E7 | 2.08E7 | 21 | 2 | 2 | 2 | 2 | 1 | 3 | 3 | 2 | 1 | 3 | 40 | 50 | Oxidation (M) |
| K.EITALAPSTM(+15.99)K.I | N | 81.55 | 1176.6060 | 11 | 3.3 | 589.3122 | 2 | 45.25 | 6 | 12771 | Ana\_05.raw | 1.46E9 | 1.19E9 | 1.22E9 | 4.08E8 | 9.94E8 | 1.04E9 | 6.43E8 | 8.39E8 | 2.22E9 | 8.74E8 | 142 | 18 | 15 | 12 | 10 | 13 | 11 | 10 | 18 | 18 | 17 | 316 | 326 | Oxidation (M) |
| K.AGFAGDDAPR.A | N | 76.24 | 975.4410 | 10 | 4.8 | 488.7301 | 2 | 31.38 | 1 | 8414 | ANa\_10.raw | 3.67E9 | 4.81E9 | 3.7E9 | 1.86E9 | 3.52E9 | 3.73E9 | 2.42E9 | 2.63E9 | 6.06E9 | 4.43E9 | 286 | 36 | 29 | 31 | 28 | 26 | 21 | 16 | 35 | 35 | 29 | 19 | 28 |  |
| R.AVFPSIVGRPR.H | N | 74.48 | 1197.6981 | 11 | 5.3 | 599.8595 | 2 | 62.78 | 2 | 17880 | Ana\_01.raw | 1.6E9 | 1.34E9 | 1.32E9 | 8.13E8 | 1.16E9 | 1.21E9 | 8.12E8 | 8.11E8 | 2.43E9 | 1.42E9 | 261 | 32 | 21 | 25 | 23 | 36 | 23 | 17 | 28 | 32 | 24 | 29 | 39 |  |
| R.C(+57.02)PEALFQPSFLGM(+15.99)ESC(+57.02)GIHETTFNSIMK.C | N | 70.79 | 3246.4495 | 28 | 4.5 | 1083.1620 | 3 | 123.56 | 9 | 34350 | Ana\_08.raw | 3.93E6 |  |  |  |  |  |  |  | 4.59E6 |  | 2 | 1 | 0 | 0 | 0 | 0 | 0 | 0 | 0 | 1 | 0 | 257 | 284 | Carbamidomethylation; Oxidation (M) |
| R.KDLYANTVLSGGTTMYPGIADR.M | N | 69.63 | 2342.1577 | 22 | 3.1 | 781.7289 | 3 | 94.52 | 1 | 26498 | ANa\_10.raw | 3.49E5 |  |  |  |  |  |  |  |  |  | 1 | 1 | 0 | 0 | 0 | 0 | 0 | 0 | 0 | 0 | 0 | 291 | 312 |  |
| R.HQGVM(+15.99)VGMGQK.D | N | 69.21 | 1186.5587 | 11 | 1.9 | 594.2878 | 2 | 15.71 | 5 | 3571 | Ana\_04.raw | 1.62E7 | 9.07E6 |  |  | 1.39E6 | 1.35E7 | 8.82E5 | 7.09E6 | 1.92E6 | 1.76E7 | 12 | 1 | 1 | 0 | 0 | 1 | 2 | 2 | 2 | 1 | 2 | 40 | 50 | Oxidation (M) |
| R.C(+57.02)PEALFQPSFLGM(+15.99)ESC(+57.02)GIHETTFNSIM(+15.99)K.C | N | 68.96 | 3262.4443 | 28 | 4.0 | 1088.4930 | 3 | 115.42 | 2 | 32132 | Ana\_01.raw | 2.15E7 | 3.46E7 | 4.02E6 | 6.18E6 | 6.21E6 |  | 1.48E7 | 2.62E7 | 3.67E7 | 1.41E7 | 12 | 1 | 2 | 1 | 1 | 1 | 0 | 2 | 1 | 1 | 2 | 257 | 284 | Carbamidomethylation; Oxidation (M) |
| K.EITALAPSTMK.I | N | 67.15 | 1160.6111 | 11 | -0.1 | 581.3127 | 2 | 30.48 | 9 | 8027 | Ana\_08.raw | 6.1E7 | 2.19E7 |  | 5.22E5 |  |  | 1.96E6 |  | 1.1E8 | 4.16E7 | 14 | 4 | 1 | 0 | 1 | 0 | 0 | 1 | 0 | 5 | 2 | 316 | 326 |  |
| R.HQGVM(+15.99)VGM(+15.99)GQK.D | N | 65.50 | 1202.5536 | 11 | 7.8 | 602.2888 | 2 | 21.12 | 1 | 5142 | ANa\_10.raw | 3.51E8 | 1.68E8 | 6.85E8 | 2.25E8 | 4.15E8 | 7.33E8 | 4.21E8 | 3.56E8 | 9.25E7 | 3.14E8 | 59 | 8 | 4 | 5 | 7 | 6 | 4 | 5 | 8 | 6 | 6 | 40 | 50 | Oxidation (M) |
| R.C(+57.02)PEALFQPSFLGMESC(+57.02)GIHETTFNSIM(+15.99)K.C | N | 61.68 | 3246.4495 | 28 | 3.4 | 1083.1608 | 3 | 122.40 | 9 | 34055 | Ana\_08.raw | 9.92E5 |  |  |  | 2.9E6 |  |  |  | 4.5E6 |  | 3 | 1 | 0 | 0 | 0 | 1 | 0 | 0 | 0 | 1 | 0 | 257 | 284 | Carbamidomethylation; Oxidation (M) |
| R.DLTDYLM(+15.99)K.I | N | 58.74 | 1013.4739 | 8 | 3.9 | 507.7462 | 2 | 92.37 | 6 | 26220 | Ana\_05.raw | 8.4E8 | 6.93E8 | 6.22E8 | 2.72E8 | 4.42E8 | 4.88E8 | 3.63E8 | 5.27E8 | 1.32E9 | 5.32E8 | 291 | 38 | 34 | 28 | 21 | 30 | 22 | 17 | 33 | 35 | 33 | 184 | 191 | Oxidation (M) |
| R.DLTDYLMK.I | N | 58.29 | 997.4790 | 8 | 4.0 | 499.7487 | 2 | 92.24 | 1 | 25887 | ANa\_10.raw | 3.85E7 | 6.67E6 | 1.26E5 | 5.05E5 |  | 6.74E6 | 2.41E5 | 3.81E6 | 2.57E7 | 2.99E6 | 21 | 5 | 2 | 1 | 1 | 0 | 2 | 1 | 1 | 5 | 3 | 184 | 191 |  |
| K.IIAPPER.K | N | 57.46 | 794.4650 | 7 | 1.7 | 398.2404 | 2 | 33.34 | 3 | 9119 | Ana\_02.raw | 3.97E9 | 4.12E9 | 3.95E9 | 1.66E9 | 3.05E9 | 3.75E9 | 2.4E9 | 2.4E9 | 4.68E9 | 3.63E9 | 283 | 36 | 29 | 33 | 22 | 29 | 22 | 16 | 34 | 31 | 31 | 329 | 335 |  |
| R.LDLAGRDLTDYLM(+15.99)K.I | N | 51.33 | 1638.8286 | 14 | 3.2 | 820.4242 | 2 | 95.36 | 9 | 26994 | Ana\_08.raw | 9.63E6 |  |  |  |  |  |  |  | 1.04E7 |  | 3 | 1 | 0 | 0 | 0 | 0 | 0 | 0 | 0 | 2 | 0 | 178 | 191 | Oxidation (M) |
| K.IIAPPERK.Y | N | 46.97 | 922.5599 | 8 | 3.9 | 462.2891 | 2 | 23.58 | 9 | 5830 | Ana\_08.raw | 8.34E7 | 2.85E7 | 2.04E7 |  | 1.06E7 | 3.16E7 | 5.24E6 | 4.71E6 | 8.32E7 | 2.96E7 | 18 | 3 | 2 | 2 | 0 | 2 | 2 | 1 | 1 | 3 | 2 | 329 | 336 |  |
| K.C(+57.02)DVDIR.K | N | 43.98 | 776.3487 | 6 | 4.4 | 389.1833 | 2 | 28.80 | 8 | 7498 | Ana\_07.raw |  | 4.69E7 | 4.42E7 | 2.63E7 | 5.45E7 | 4.38E7 | 3.39E7 | 3.7E7 |  | 9.42E6 | 9 | 0 | 1 | 1 | 1 | 1 | 1 | 1 | 2 | 0 | 1 | 285 | 290 | Carbamidomethylation |
| K.IKIIAPPER.K | N | 42.43 | 1035.6440 | 9 | 0.5 | 346.2221 | 3 | 44.96 | 3 | 12647 | Ana\_02.raw | 3.02E6 |  | 7.18E6 | 2.01E6 | 2.08E6 | 3.03E6 | 1.58E6 |  | 4.82E6 | 2.45E6 | 12 | 1 | 0 | 2 | 2 | 1 | 2 | 1 | 0 | 2 | 1 | 327 | 335 |  |
| K.ILTER.G | N | 39.01 | 630.3701 | 5 | 3.4 | 316.1934 | 2 | 20.56 | 6 | 5102 | Ana\_05.raw | 2.01E7 |  |  | 2.76E6 | 1.13E7 | 9.27E6 | 2.65E8 | 1.7E8 | 7.79E6 |  | 14 | 1 | 0 | 0 | 3 | 3 | 1 | 4 | 1 | 1 | 0 | 192 | 196 |  |
| R.EIVRDIK.E | N | 36.72 | 871.5127 | 7 | 6.3 | 436.7664 | 2 | 23.86 | 9 | 5929 | Ana\_08.raw |  | 3.4E6 | 3.11E6 |  |  | 3.2E6 | 5.84E5 |  | 3.56E6 | 3.27E6 | 6 | 0 | 1 | 1 | 0 | 0 | 1 | 1 | 0 | 1 | 1 | 207 | 213 |  |
| R.LDLAGR.D | N | 35.64 | 643.3653 | 6 | -0.6 | 322.6897 | 2 | 30.23 | 3 | 8192 | Ana\_02.raw | 7.61E8 | 7.49E8 | 8.47E8 | 3.81E8 | 6.81E8 | 6.9E7 | 4E5 | 1.11E2 | 9.23E8 | 6.75E8 | 38 | 3 | 3 | 4 | 6 | 6 | 3 | 1 | 1 | 9 | 2 | 178 | 183 |  |
| K.RGILTLK.Y | N | 29.35 | 799.5280 | 7 | 4.0 | 400.7729 | 2 | 41.36 | 6 | 11572 | Ana\_05.raw |  |  |  |  |  | 1.41E8 |  | 9.14E7 |  |  | 2 | 0 | 0 | 0 | 0 | 0 | 1 | 0 | 1 | 0 | 0 | 62 | 68 |  |
| R.M(+15.99)QKEITALAPSTM(+15.99)K.I | N | 25.56 | 1579.7949 | 14 | 0.7 | 527.6060 | 3 | 38.99 | 3 | 10840 | Ana\_02.raw |  |  | 2.42E6 |  |  |  |  |  |  |  | 1 | 0 | 0 | 1 | 0 | 0 | 0 | 0 | 0 | 0 | 0 | 313 | 326 | Oxidation (M) |
| R.GILTLK.Y | N | 25.30 | 643.4268 | 6 | 5.7 | 322.7225 | 2 | 51.01 | 9 | 14360 | Ana\_08.raw |  | 1.76E8 | 1.95E8 |  |  | 1.81E8 | 1.2E8 |  | 2.57E2 |  | 6 | 0 | 2 | 1 | 0 | 0 | 1 | 1 | 0 | 1 | 0 | 63 | 68 |  |
| total 39 peptides |  |  |  |  |  |  |  |  |  |  |  |  |  |  |  |  |  |  |  |  |  |  |  |  |  |  |  |  |  |  |  |  |  |  |  |
| --- | --- | --- | --- | --- | --- | --- | --- | --- | --- | --- | --- | --- | --- | --- | --- | --- | --- | --- | --- | --- | --- | --- | --- | --- | --- | --- | --- | --- | --- | --- | --- | --- | --- | --- | --- |

Best Unique PSM (Scan F3:21552, m/z=977.5361, z=2, RT=76.06, ppm=0.4):

sp|P56480|ATPB\_MOUSE

back to list

  

| Protein Coverage
| Supporting Peptides
| Best Unique PSM
|

Protein Coverage:

Supporting Peptides:

| Peptide | Uniq | -10lgP | Mass | Length | ppm | m/z | z | RT | Fraction | Scan | Source File | Area 10 | Area 01 | Area 02 | Area 03 | Area 04 | Area 05 | Area 06 | Area 07 | Area 08 | Area 09 | #Spec | #Spec 10 | #Spec 01 | #Spec 02 | #Spec 03 | #Spec 04 | #Spec 05 | #Spec 06 | #Spec 07 | #Spec 08 | #Spec 09 | Start | End | PTM |
| --- | --- | --- | --- | --- | --- | --- | --- | --- | --- | --- | --- | --- | --- | --- | --- | --- | --- | --- | --- | --- | --- | --- | --- | --- | --- | --- | --- | --- | --- | --- | --- | --- | --- | --- | --- |
| K.KGSITSVQAIYVPADDLTDPAPATTFAHLDATTVLSR.A | Y | 140.71 | 3841.9736 | 37 | 4.5 | 961.5050 | 4 | 124.11 | 6 | 34559 | Ana\_05.raw | 6.14E6 |  |  | 1.4E6 | 6.69E6 | 1.66E7 | 3.13E6 | 4.88E6 |  |  | 6 | 1 | 0 | 0 | 1 | 1 | 1 | 1 | 1 | 0 | 0 | 351 | 387 |  |
| K.SLQDIIAILGM(+15.99)DELSEEDKLTVSR.A | Y | 128.37 | 2690.3684 | 24 | 3.0 | 897.7994 | 3 | 134.04 | 3 | 36692 | Ana\_02.raw | 1.15E7 | 5.49E6 | 1.3E7 | 4.09E6 | 9.73E6 | 2.27E7 | 8.59E6 | 3.26E6 | 2.03E7 | 1.51E7 | 10 | 1 | 1 | 1 | 1 | 1 | 1 | 1 | 1 | 1 | 1 | 433 | 456 | Oxidation (M) |
| K.GSITSVQAIYVPADDLTDPAPATTFAHLDATTVLSR.A | Y | 118.29 | 3713.8787 | 36 | 3.9 | 1238.9717 | 3 | 129.14 | 6 | 35871 | Ana\_05.raw | 8.37E6 | 1.03E7 | 1.7E6 |  | 6.7E6 | 8.97E6 | 4.88E6 | 4.1E6 |  | 4.35E6 | 8 | 1 | 1 | 1 | 0 | 1 | 1 | 1 | 1 | 0 | 1 | 352 | 387 |  |
| R.FLSQPFQVAEVFTGHM(+15.99)GK.L | Y | 112.75 | 2037.9982 | 18 | 1.2 | 680.3408 | 3 | 112.60 | 3 | 31137 | Ana\_02.raw | 9.97E7 | 1.24E8 | 1.22E8 | 6.43E7 | 7.67E7 | 1.05E8 | 6.33E7 | 7.38E7 | 1.54E8 | 6.68E7 | 45 | 6 | 5 | 5 | 4 | 5 | 4 | 3 | 5 | 5 | 3 | 463 | 480 | Oxidation (M) |
| R.FTQAGSEVSALLGR.I | Y | 110.40 | 1434.7467 | 14 | 3.4 | 718.3831 | 2 | 93.58 | 6 | 26549 | Ana\_05.raw | 8.78E7 | 7.61E7 | 7.02E7 | 6.26E6 | 5.66E7 | 4.61E7 | 2.5E7 | 2.65E7 | 1.55E8 | 2.91E7 | 45 | 6 | 7 | 5 | 2 | 4 | 3 | 2 | 3 | 7 | 6 | 311 | 324 |  |
| R.AIAELGIYPAVDPLDSTSR.I | Y | 110.17 | 1987.0261 | 19 | 4.1 | 994.5244 | 2 | 114.68 | 7 | 31745 | Ana\_06.raw | 3.89E7 | 4.04E7 | 4.04E7 | 2.21E7 | 3.18E7 | 4.15E7 | 3.58E7 | 1.34E7 | 7.59E7 | 3.73E7 | 38 | 5 | 4 | 4 | 3 | 3 | 4 | 3 | 4 | 5 | 3 | 388 | 406 |  |
| R.DQEGQDVLLFIDNIFR.F | Y | 104.54 | 1920.9581 | 16 | 4.9 | 961.4911 | 2 | 139.68 | 6 | 38753 | Ana\_05.raw |  |  |  |  |  | 4.14E5 |  |  | 7.77E5 | 4.79E5 | 4 | 0 | 0 | 0 | 0 | 0 | 2 | 0 | 0 | 1 | 1 | 295 | 310 |  |
| R.VALTGLTVAEYFR.D | Y | 97.00 | 1438.7820 | 13 | 0.7 | 720.3987 | 2 | 117.03 | 3 | 32294 | Ana\_02.raw | 4.49E7 | 7.25E7 | 4.87E7 | 3.42E7 | 2.99E7 | 5.26E7 | 3.82E7 | 2.18E7 | 6.19E7 | 4.72E7 | 37 | 3 | 5 | 4 | 4 | 4 | 3 | 2 | 4 | 4 | 4 | 282 | 294 |  |
| R.IM(+15.99)DPNIVGNEHYDVAR.G | Y | 95.88 | 1857.8679 | 16 | -1.6 | 929.9398 | 2 | 61.69 | 3 | 17575 | Ana\_02.raw | 3.45E7 | 3.69E7 | 4.06E7 | 1.37E7 | 2.81E7 | 2.53E7 | 1.69E7 | 1.86E7 | 5.55E7 | 2.57E7 | 16 | 2 | 2 | 2 | 1 | 2 | 2 | 1 | 1 | 2 | 1 | 407 | 422 | Oxidation (M) |
| K.GFQQILAGEYDHLPEQAFYM(+15.99)VGPIEEAVAK.A | Y | 94.96 | 3365.6277 | 30 | 0.3 | 1122.8835 | 3 | 131.13 | 3 | 35923 | Ana\_02.raw | 3.37E7 | 4.08E7 | 2.9E7 | 5.74E6 | 2.28E7 | 5.13E7 | 2.26E7 | 1.85E7 | 4.23E7 | 2.48E7 | 11 | 1 | 1 | 1 | 1 | 1 | 2 | 1 | 1 | 1 | 1 | 490 | 519 | Oxidation (M) |
| R.LVLEVAQHLGESTVR.T | Y | 93.76 | 1649.9100 | 15 | 0.7 | 825.9629 | 2 | 85.70 | 6 | 24414 | Ana\_05.raw | 1.93E7 | 4.27E7 | 1.59E7 | 3.62E6 | 1.78E7 | 2.24E7 | 1.05E7 | 4.63E6 | 7.36E7 | 3.21E7 | 25 | 3 | 3 | 2 | 2 | 2 | 2 | 2 | 2 | 4 | 3 | 95 | 109 |  |
| K.TVLIMELINNVAK.A | Y | 93.03 | 1456.8323 | 13 | 1.7 | 729.4247 | 2 | 132.65 | 3 | 36330 | Ana\_02.raw | 3.78E5 | 1.16E7 | 1.02E7 | 6.6E6 | 2.08E6 | 3.38E6 | 8.07E6 | 2.96E6 | 1.31E7 | 4.4E6 | 10 | 1 | 1 | 1 | 1 | 1 | 1 | 1 | 1 | 1 | 1 | 213 | 225 |  |
| R.FLSQPFQVAEVFTGHMGK.L | Y | 90.76 | 2022.0033 | 18 | 6.0 | 675.0125 | 3 | 125.42 | 6 | 34891 | Ana\_05.raw | 3.88E6 | 1.29E6 |  |  |  | 1.4E6 |  |  | 1.93E7 | 1.35E7 | 9 | 2 | 1 | 0 | 0 | 0 | 1 | 0 | 1 | 2 | 2 | 463 | 480 |  |
| K.AHGGYSVFAGVGER.T | Y | 90.26 | 1405.6738 | 14 | 5.2 | 703.8478 | 2 | 63.73 | 7 | 18070 | Ana\_06.raw | 3.03E7 | 3.32E7 | 3.09E7 | 1.3E7 | 2.19E7 | 2.83E7 | 1.8E7 | 1.69E7 | 4.23E7 | 2.57E7 | 28 | 3 | 3 | 3 | 3 | 3 | 3 | 3 | 2 | 2 | 3 | 226 | 239 |  |
| K.TVLIM(+15.99)ELINNVAK.A | Y | 89.89 | 1472.8273 | 13 | 4.3 | 737.4241 | 2 | 124.67 | 1 | 34303 | ANa\_10.raw | 1.4E7 | 1.29E7 | 4.65E7 | 8.26E6 | 4.39E7 | 4.86E7 | 3.84E7 | 3.24E7 | 6.51E7 | 2.35E7 | 41 | 3 | 6 | 5 | 3 | 6 | 3 | 3 | 5 | 4 | 3 | 213 | 225 | Oxidation (M) |
| R.IM(+15.99)NVIGEPIDER.G | Y | 80.53 | 1400.6969 | 12 | 3.8 | 701.3584 | 2 | 67.17 | 7 | 19061 | Ana\_06.raw | 4.95E7 | 5.27E7 | 5.34E7 | 1.96E7 | 3.8E7 | 3.67E7 | 2.79E7 | 3.05E7 | 6.89E7 | 4.07E7 | 23 | 3 | 2 | 2 | 2 | 3 | 2 | 2 | 2 | 2 | 3 | 144 | 155 | Oxidation (M) |
| K.VALVYGQM(+15.99)NEPPGAR.A | Y | 80.35 | 1616.7981 | 15 | 2.3 | 809.4082 | 2 | 60.07 | 7 | 16989 | Ana\_06.raw | 3.48E7 | 4.36E7 | 4.24E7 | 1.17E7 | 2.57E7 | 2.21E7 | 1.95E7 | 2.68E7 | 4.9E7 | 2.17E7 | 45 | 4 | 5 | 5 | 4 | 4 | 4 | 4 | 5 | 5 | 5 | 265 | 279 | Oxidation (M) |
| K.VVDLLAPYAK.G | Y | 80.07 | 1087.6277 | 10 | 4.5 | 544.8235 | 2 | 85.80 | 6 | 24442 | Ana\_05.raw | 5.6E7 | 6.24E7 | 5.98E7 | 2.48E7 | 4.3E7 | 4.36E7 | 3.17E7 | 2.88E7 | 8.73E7 | 4.64E7 | 43 | 5 | 5 | 5 | 4 | 4 | 2 | 2 | 5 | 6 | 5 | 189 | 198 |  |
| R.EGNDLYHEMIESGVINLK.D | Y | 77.63 | 2059.9885 | 18 | 3.0 | 687.6722 | 3 | 115.77 | 2 | 32227 | Ana\_01.raw | 8.35E6 | 6.33E6 |  | 4.68E5 |  | 2.75E6 |  | 1.82E6 |  | 2.96E6 | 6 | 1 | 1 | 0 | 1 | 0 | 1 | 0 | 1 | 0 | 1 | 242 | 259 |  |
| K.IPVGPETLGR.I | Y | 74.09 | 1037.5869 | 10 | 6.0 | 519.8038 | 2 | 58.59 | 1 | 16555 | ANa\_10.raw | 9.87E7 | 1.27E8 | 9.82E7 | 4.24E7 | 7.73E7 | 8.31E7 | 6.09E7 | 5.3E7 | 1.35E8 | 8.48E7 | 23 | 2 | 3 | 3 | 1 | 1 | 2 | 1 | 4 | 4 | 2 | 134 | 143 |  |
| R.TIAM(+15.99)DGTEGLVR.G | Y | 73.26 | 1277.6285 | 12 | 2.8 | 639.8233 | 2 | 53.46 | 6 | 15244 | Ana\_05.raw | 2.15E7 | 3.57E7 | 3.84E7 | 1.76E7 | 2.74E7 | 4.98E7 | 7.82E6 | 3.96E7 | 5.69E7 | 1.36E7 | 20 | 2 | 4 | 1 | 2 | 1 | 2 | 1 | 2 | 4 | 1 | 110 | 121 | Oxidation (M) |
| K.SLQDIIAILGM(+15.99)DELSEEDK.L | Y | 63.33 | 2134.0352 | 19 | 3.9 | 1068.0291 | 2 | 134.12 | 6 | 37287 | Ana\_05.raw |  |  |  |  | 4.59E5 | 1.36E6 | 2.19E6 |  |  |  | 4 | 0 | 0 | 0 | 0 | 1 | 1 | 2 | 0 | 0 | 0 | 433 | 451 | Oxidation (M) |
| K.VLDSGAPIKIPVGPETLGR.I | Y | 62.28 | 1918.0887 | 19 | 5.1 | 640.3735 | 3 | 94.10 | 10 | 26300 | Ana\_09.raw | 7.96E6 | 9.07E5 |  | 2.77E5 | 1.77E6 | 1.92E6 | 1.16E6 |  | 1.76E7 | 2.4E6 | 9 | 1 | 1 | 0 | 1 | 1 | 1 | 1 | 0 | 2 | 1 | 125 | 143 |  |
| R.IPSAVGYQPTLATDM(+15.99)GTM(+15.99)QER.I | Y | 60.56 | 2297.0667 | 21 | 3.1 | 1149.5442 | 2 | 72.56 | 1 | 20557 | ANa\_10.raw | 1.68E7 | 7.53E6 | 1E7 | 9.91E5 | 6.69E6 | 1.28E7 |  | 3.11E6 | 7.49E6 |  | 12 | 2 | 1 | 2 | 1 | 1 | 2 | 0 | 1 | 2 | 0 | 325 | 345 | Oxidation (M) |
| K.IGLFGGAGVGK.T | Y | 59.22 | 974.5549 | 11 | 3.9 | 488.2866 | 2 | 73.19 | 6 | 20995 | Ana\_05.raw | 5.01E7 | 6.15E7 | 4.7E7 | 2.14E7 | 4.12E7 | 3.59E7 | 2.52E7 | 3.13E7 | 6.67E7 | 3.49E7 | 22 | 3 | 3 | 2 | 2 | 2 | 2 | 1 | 3 | 3 | 1 | 202 | 212 |  |
| K.VLDSGAPIK.I | Y | 52.47 | 898.5123 | 9 | 3.2 | 450.2649 | 2 | 37.71 | 6 | 10443 | Ana\_05.raw | 2.26E7 | 2.41E7 | 6.74E7 | 3.21E7 | 5.85E7 | 6.26E7 | 4.16E7 | 3.83E7 | 1.23E7 | 4.1E7 | 15 | 1 | 1 | 1 | 2 | 1 | 2 | 2 | 2 | 1 | 2 | 125 | 133 |  |
| R.IMDPNIVGNEHYDVAR.G | Y | 37.71 | 1841.8730 | 16 | 5.8 | 614.9685 | 3 | 68.71 | 9 | 19600 | Ana\_08.raw |  |  |  |  |  |  |  |  | 2.35E6 |  | 1 | 0 | 0 | 0 | 0 | 0 | 0 | 0 | 0 | 1 | 0 | 407 | 422 |  |
| R.EGNDLYHEM(+15.99)IESGVINLK.D | Y | 34.27 | 2075.9834 | 18 | 5.7 | 693.0057 | 3 | 86.50 | 1 | 24355 | ANa\_10.raw | 1.94E6 |  |  |  |  |  |  |  | 1.71E6 |  | 2 | 1 | 0 | 0 | 0 | 0 | 0 | 0 | 0 | 1 | 0 | 242 | 259 | Oxidation (M) |
| MLSLVGR.V | Y | 33.54 | 774.4422 | 7 | 0.9 | 388.2287 | 2 | 73.89 | 2 | 21036 | Ana\_01.raw | 1.52E7 | 6.04E6 | 1.47E7 | 5.57E6 | 1.06E7 | 1.14E7 | 6.75E6 |  | 1.28E7 | 1.22E7 | 11 | 1 | 1 | 2 | 1 | 1 | 2 | 1 | 0 | 1 | 1 | 1 | 7 |  |
| K.ILQDYK.S | Y | 32.30 | 778.4225 | 6 | 3.7 | 390.2199 | 2 | 28.83 | 10 | 7709 | Ana\_09.raw | 3.3E7 | 3.94E7 | 4.15E7 |  |  | 4.21E7 | 2.81E7 |  | 2.64E7 | 3.13E7 | 7 | 1 | 1 | 1 | 0 | 0 | 1 | 1 | 0 | 1 | 1 | 427 | 432 |  |
| K.QFAPIHAEAPEFIEMSVEQEILVTGIK.V | Y | 22.73 | 3025.5469 | 27 | -2.1 | 1513.7775 | 2 | 136.20 | 1 | 37418 | ANa\_10.raw | 8.76E5 |  |  |  |  |  |  |  |  |  | 1 | 1 | 0 | 0 | 0 | 0 | 0 | 0 | 0 | 0 | 0 | 162 | 188 |  |
| total 31 peptides |  |  |  |  |  |  |  |  |  |  |  |  |  |  |  |  |  |  |  |  |  |  |  |  |  |  |  |  |  |  |  |  |  |  |  |
| --- | --- | --- | --- | --- | --- | --- | --- | --- | --- | --- | --- | --- | --- | --- | --- | --- | --- | --- | --- | --- | --- | --- | --- | --- | --- | --- | --- | --- | --- | --- | --- | --- | --- | --- | --- |

Best Unique PSM (Scan F6:34559, m/z=961.5050, z=4, RT=124.11, ppm=4.5):

sp|Q68FD5|CLH1\_MOUSE

back to list

  

| Protein Coverage
| Supporting Peptides
| Best Unique PSM
|

Protein Coverage:

Supporting Peptides:

| Peptide | Uniq | -10lgP | Mass | Length | ppm | m/z | z | RT | Fraction | Scan | Source File | Area 10 | Area 01 | Area 02 | Area 03 | Area 04 | Area 05 | Area 06 | Area 07 | Area 08 | Area 09 | #Spec | #Spec 10 | #Spec 01 | #Spec 02 | #Spec 03 | #Spec 04 | #Spec 05 | #Spec 06 | #Spec 07 | #Spec 08 | #Spec 09 | Start | End | PTM |
| --- | --- | --- | --- | --- | --- | --- | --- | --- | --- | --- | --- | --- | --- | --- | --- | --- | --- | --- | --- | --- | --- | --- | --- | --- | --- | --- | --- | --- | --- | --- | --- | --- | --- | --- | --- |
| R.KFDVNTSAVQVLIEHIGNLDR.A | Y | 126.67 | 2367.2546 | 21 | 1.9 | 790.0936 | 3 | 131.24 | 6 | 36466 | Ana\_05.raw | 3.37E6 | 6.46E6 | 6.9E6 | 5.13E6 | 9.09E6 | 1.13E7 | 9.23E6 | 6.88E6 | 5.69E6 | 1.16E7 | 19 | 2 | 2 | 2 | 2 | 2 | 2 | 2 | 2 | 1 | 2 | 1074 | 1094 |  |
| R.RPLIDQVVQTALSETQDPEEVSVTVK.A | Y | 124.91 | 2880.5081 | 26 | 2.9 | 961.1794 | 3 | 130.09 | 1 | 35757 | ANa\_10.raw | 8.5E6 | 1.9E6 |  |  |  | 6.76E6 | 3.23E6 |  | 1.07E7 | 6.07E6 | 6 | 1 | 1 | 0 | 0 | 0 | 1 | 1 | 0 | 1 | 1 | 968 | 993 |  |
| K.SVNESLNNLFITEEDYQALR.T | Y | 116.21 | 2354.1389 | 20 | 3.6 | 1178.0809 | 2 | 121.23 | 9 | 33761 | Ana\_08.raw |  | 5.83E6 |  | 6.83E5 |  |  |  |  | 4.54E6 | 1.62E6 | 6 | 0 | 2 | 0 | 1 | 0 | 0 | 0 | 0 | 1 | 2 | 1462 | 1481 |  |
| R.LPVVIGGLLDVDC(+57.02)SEDVIK.N | Y | 107.13 | 2040.0813 | 19 | 4.8 | 1021.0528 | 2 | 128.70 | 9 | 35652 | Ana\_08.raw | 6.46E5 | 1.42E6 |  | 6.12E5 |  | 0 |  |  | 3.64E6 | 1.62E6 | 6 | 1 | 1 | 0 | 1 | 0 | 1 | 0 | 0 | 1 | 1 | 812 | 830 | Carbamidomethylation |
| R.ISGETIFVTAPHEATAGIIGVNR.K | Y | 100.29 | 2352.2437 | 23 | 4.6 | 785.0921 | 3 | 100.62 | 9 | 28410 | Ana\_08.raw |  |  |  | 4.19E5 | 2.88E6 |  | 1.87E6 | 6.46E5 | 3.02E6 |  | 5 | 0 | 0 | 0 | 1 | 1 | 0 | 1 | 1 | 1 | 0 | 298 | 320 |  |
| K.AFM(+15.99)TADLPNELIELLEK.I | Y | 95.51 | 1962.0020 | 17 | 2.7 | 982.0109 | 2 | 135.71 | 6 | 37720 | Ana\_05.raw | 4.72E6 | 1.57E6 | 3.14E6 |  | 1.5E6 | 4.32E6 | 4.55E6 | 2.91E6 | 1.02E7 | 2.13E6 | 12 | 1 | 1 | 1 | 0 | 1 | 2 | 2 | 1 | 2 | 1 | 994 | 1010 | Oxidation (M) |
| R.TSIDAYDNFDNISLAQR.L | Y | 90.38 | 1941.9067 | 17 | 3.8 | 971.9643 | 2 | 96.76 | 9 | 27385 | Ana\_08.raw |  |  |  |  |  |  |  |  | 4.57E6 | 3.42E5 | 2 | 0 | 0 | 0 | 0 | 0 | 0 | 0 | 0 | 1 | 1 | 1482 | 1498 |  |
| R.ALEHFTDLYDIK.R | Y | 87.76 | 1463.7296 | 12 | 5.4 | 732.8760 | 2 | 88.19 | 1 | 24808 | ANa\_10.raw | 7.29E6 | 1.81E6 | 4.37E6 |  | 5.02E6 | 3.53E6 | 2.74E6 | 1.26E6 | 1.61E7 | 4.92E6 | 12 | 2 | 1 | 1 | 0 | 2 | 1 | 1 | 1 | 2 | 1 | 626 | 637 |  |
| R.LASTLVHLGEYQAAVDGAR.K | Y | 83.74 | 1970.0221 | 19 | 4.9 | 657.6845 | 3 | 85.25 | 9 | 24252 | Ana\_08.raw | 1.64E6 | 1.45E6 |  |  | 3.18E4 |  |  |  | 5.41E6 | 2.97E6 | 5 | 1 | 1 | 0 | 0 | 1 | 0 | 0 | 0 | 1 | 1 | 1227 | 1245 |  |
| K.KVGYTPDWIFLLR.N | Y | 80.20 | 1606.8871 | 13 | 4.1 | 536.6385 | 3 | 126.69 | 6 | 35228 | Ana\_05.raw |  | 1.78E5 |  |  | 1.1E6 | 1.22E6 | 2.62E6 |  |  |  | 4 | 0 | 1 | 0 | 0 | 1 | 1 | 1 | 0 | 0 | 0 | 507 | 519 |  |
| K.VGYTPDWIFLLR.N | Y | 78.53 | 1478.7921 | 12 | 5.8 | 740.4076 | 2 | 132.97 | 6 | 36965 | Ana\_05.raw |  | 5.14E6 |  | 2.01E6 | 3.03E6 | 4.08E6 |  | 1.19E6 |  | 2.01E6 | 6 | 0 | 1 | 0 | 1 | 1 | 1 | 0 | 1 | 0 | 1 | 508 | 519 |  |
| K.LHIIEVGTPPTGNQPFPK.K | Y | 77.43 | 1944.0469 | 18 | 4.6 | 649.0259 | 3 | 81.47 | 9 | 23192 | Ana\_08.raw | 3.51E5 |  |  | 1.86E6 | 1.59E5 |  | 1.03E6 | 4.32E5 | 1.99E6 |  | 6 | 1 | 0 | 0 | 1 | 1 | 0 | 1 | 1 | 1 | 0 | 228 | 245 |  |
| R.GQFSTDELVAEVEK.R | Y | 77.10 | 1550.7463 | 14 | 5.9 | 776.3850 | 2 | 96.54 | 6 | 27333 | Ana\_05.raw | 6.5E6 | 3.55E6 | 1.19E6 | 4.57E5 | 2.56E6 | 4.1E6 | 1.22E6 | 8.19E5 | 8.49E6 | 5.58E5 | 10 | 1 | 1 | 1 | 1 | 1 | 1 | 1 | 1 | 1 | 1 | 838 | 851 |  |
| K.LLLPWLEAR.I | Y | 73.10 | 1109.6597 | 9 | 3.5 | 555.8391 | 2 | 124.31 | 6 | 34612 | Ana\_05.raw | 3.11E7 | 6.86E6 | 1.55E7 | 3.73E6 | 2.4E7 | 2.5E7 | 1.16E7 | 6.99E6 | 4.1E7 | 2.59E7 | 35 | 4 | 3 | 4 | 2 | 5 | 3 | 2 | 4 | 4 | 4 | 857 | 865 |  |
| R.KDPELWGSVLLESNPYR.R | Y | 73.02 | 2002.0159 | 17 | 8.3 | 668.3514 | 3 | 110.95 | 9 | 31124 | Ana\_08.raw |  | 9.29E5 |  |  |  |  |  |  | 1.99E6 |  | 2 | 0 | 1 | 0 | 0 | 0 | 0 | 0 | 0 | 1 | 0 | 951 | 967 |  |
| K.NNRPSEGPLQTR.L | Y | 72.84 | 1367.6906 | 12 | -1.1 | 684.8518 | 2 | 24.70 | 3 | 6465 | Ana\_02.raw | 8.67E6 | 7.43E5 | 3.11E6 |  |  | 2.45E6 | 3.57E6 | 3.27E6 | 7.91E6 | 3.55E6 | 10 | 2 | 1 | 1 | 0 | 0 | 1 | 1 | 2 | 1 | 1 | 572 | 583 |  |
| R.GQFSTDELVAEVEKR.N | Y | 72.46 | 1706.8474 | 15 | 4.6 | 569.9590 | 3 | 91.41 | 9 | 25925 | Ana\_08.raw |  | 3.03E5 | 1.25E6 |  | 2.39E6 | 2.47E6 | 2.29E6 | 7.6E5 | 8.78E6 | 5.03E6 | 8 | 0 | 1 | 1 | 0 | 1 | 1 | 1 | 1 | 1 | 1 | 838 | 852 |  |
| R.RPISADSAIM(+15.99)NPASK.V | Y | 72.06 | 1572.7930 | 15 | 4.8 | 525.2741 | 3 | 40.54 | 1 | 11180 | ANa\_10.raw | 1.17E7 | 1.33E6 | 9.46E6 |  |  |  |  |  | 4.29E6 | 1.47E6 | 7 | 2 | 1 | 1 | 0 | 0 | 0 | 0 | 0 | 2 | 1 | 64 | 78 | Oxidation (M) |
| R.IHEGC(+57.02)EEPATHNALAK.I | Y | 69.67 | 1775.8260 | 16 | 5.8 | 592.9527 | 3 | 25.88 | 9 | 6599 | Ana\_08.raw |  |  |  |  |  |  |  |  | 3.31E6 |  | 1 | 0 | 0 | 0 | 0 | 0 | 0 | 0 | 0 | 1 | 0 | 866 | 881 | Carbamidomethylation |
| K.HDVVFLITK.Y | Y | 69.67 | 1070.6124 | 9 | 3.5 | 536.3154 | 2 | 68.62 | 6 | 19693 | Ana\_05.raw |  | 8.16E6 |  | 9.4E5 | 3.74E6 | 2.55E6 | 2.66E6 | 1.88E6 |  |  | 6 | 0 | 1 | 0 | 1 | 1 | 1 | 1 | 1 | 0 | 0 | 270 | 278 |  |
| R.KFNALFAQGNYSEAAK.V | Y | 68.37 | 1757.8737 | 16 | 4.5 | 586.9678 | 3 | 70.90 | 5 | 20205 | Ana\_04.raw |  |  | 1.26E6 |  | 1.72E6 | 2.39E6 | 4.84E5 | 2.24E6 | 3.82E6 |  | 6 | 0 | 0 | 1 | 0 | 1 | 1 | 1 | 1 | 1 | 0 | 367 | 382 |  |
| K.IYIDSNNNPER.F | Y | 66.43 | 1333.6262 | 11 | 6.4 | 667.8246 | 2 | 42.37 | 6 | 11887 | Ana\_05.raw | 1.17E7 | 1.1E7 | 8.26E6 | 1.99E6 | 8.17E6 | 9.66E6 | 7.02E6 | 5.26E6 | 1.35E7 | 8.65E6 | 11 | 1 | 1 | 1 | 1 | 1 | 1 | 1 | 1 | 2 | 1 | 882 | 892 |  |
| R.NNLAGAEELFAR.K | Y | 65.55 | 1303.6520 | 12 | 3.4 | 652.8354 | 2 | 88.06 | 6 | 25069 | Ana\_05.raw | 1.39E7 | 1.42E7 | 1.38E7 | 6.82E6 | 1.13E7 | 1.08E7 | 9.6E6 | 8.14E6 | 2.55E7 | 9.64E6 | 10 | 1 | 1 | 1 | 1 | 1 | 1 | 1 | 1 | 1 | 1 | 355 | 366 |  |
| K.VIQC(+57.02)FAETGQVQK.I | Y | 62.42 | 1506.7500 | 13 | 7.9 | 754.3882 | 2 | 51.26 | 9 | 14439 | Ana\_08.raw | 2.9E6 | 3.49E6 | 4.04E5 | 3.1E5 | 1.61E6 |  | 1.06E6 | 9.47E5 | 7.08E6 | 3.47E6 | 9 | 1 | 1 | 1 | 1 | 1 | 0 | 1 | 1 | 1 | 1 | 488 | 500 | Carbamidomethylation |
| K.IVLDNSVFSEHR.N | Y | 62.14 | 1414.7205 | 12 | 3.2 | 708.3698 | 2 | 73.72 | 8 | 20696 | Ana\_07.raw | 9.48E5 | 1.33E6 |  |  | 1.06E6 | 2.01E6 | 7.36E5 | 1.18E6 |  | 1.83E6 | 7 | 1 | 1 | 0 | 0 | 1 | 1 | 1 | 1 | 0 | 1 | 1011 | 1022 |  |
| K.VSQPIEGHAASFAQFK.M | Y | 61.31 | 1715.8630 | 16 | 4.7 | 572.9643 | 3 | 67.76 | 2 | 19309 | Ana\_01.raw | 1.36E7 | 8.64E6 | 5.35E6 |  |  |  |  | 5.99E6 |  | 1.28E7 | 6 | 2 | 1 | 1 | 0 | 0 | 0 | 0 | 1 | 0 | 1 | 190 | 205 |  |
| R.ESNC(+57.02)YDPER.V | Y | 59.87 | 1168.4454 | 9 | 6.0 | 585.2335 | 2 | 26.98 | 6 | 7179 | Ana\_05.raw | 3.05E6 |  |  | 2.27E6 |  | 3.96E6 | 1.51E6 |  |  | 1.64E6 | 5 | 1 | 0 | 0 | 1 | 0 | 1 | 1 | 0 | 0 | 1 | 750 | 758 | Carbamidomethylation |
| R.NLQNLLILTAIK.A | Y | 59.77 | 1352.8391 | 12 | 4.1 | 677.4296 | 2 | 126.96 | 6 | 35294 | Ana\_05.raw | 8.59E6 | 2.11E6 | 2.39E6 | 3.37E6 | 9.41E6 | 1.72E6 | 9.56E6 | 6.14E5 | 4.85E6 | 6.01E6 | 11 | 1 | 1 | 2 | 1 | 1 | 1 | 1 | 1 | 1 | 1 | 1023 | 1034 |  |
| K.HELIEFR.R | Y | 59.00 | 942.4923 | 7 | 3.2 | 472.2549 | 2 | 55.38 | 6 | 15808 | Ana\_05.raw |  |  |  | 2.89E6 |  | 7.92E6 | 4.63E6 |  |  |  | 3 | 0 | 0 | 0 | 1 | 0 | 1 | 1 | 0 | 0 | 0 | 1502 | 1508 |  |
| R.AHIAQLC(+57.02)EK.A | Y | 57.84 | 1068.5386 | 9 | 4.7 | 535.2791 | 2 | 24.48 | 9 | 6125 | Ana\_08.raw |  |  |  |  |  |  |  |  | 3.97E6 | 1.35E6 | 2 | 0 | 0 | 0 | 0 | 0 | 0 | 0 | 0 | 1 | 1 | 611 | 619 | Carbamidomethylation |
| K.AFMTADLPNELIELLEK.I | Y | 54.92 | 1946.0070 | 17 | 5.9 | 974.0165 | 2 | 137.63 | 10 | 37493 | Ana\_09.raw |  |  |  |  |  |  |  |  | 7.73E5 | 7.42E5 | 2 | 0 | 0 | 0 | 0 | 0 | 0 | 0 | 0 | 1 | 1 | 994 | 1010 |  |
| K.LLYNNVSNFGR.L | Y | 54.34 | 1295.6622 | 11 | 4.8 | 648.8415 | 2 | 71.46 | 9 | 20397 | Ana\_08.raw | 6.15E6 | 4.18E5 |  | 1.72E6 | 0 | 1.17E6 |  | 9.46E5 | 9.79E6 | 4.81E6 | 9 | 1 | 1 | 1 | 1 | 1 | 1 | 0 | 1 | 1 | 1 | 1216 | 1226 |  |
| K.TLQIFNIEM(+15.99)K.S | Y | 53.51 | 1251.6533 | 10 | 1.4 | 626.8348 | 2 | 88.66 | 3 | 24909 | Ana\_02.raw | 9.37E5 | 2.21E6 | 2.39E6 | 7.11E5 | 4.99E5 | 2.77E6 | 2.24E6 | 2.09E6 | 6.55E5 | 1.22E6 | 11 | 1 | 1 | 1 | 1 | 1 | 1 | 2 | 1 | 1 | 1 | 87 | 96 | Oxidation (M) |
| K.DAM(+15.99)QYASESK.D | Y | 52.98 | 1144.4706 | 10 | 3.0 | 573.2443 | 2 | 21.39 | 3 | 5402 | Ana\_02.raw |  |  | 3.87E6 | 2.83E6 | 2.28E6 | 5.68E6 | 2.36E6 | 2.71E6 |  |  | 6 | 0 | 0 | 1 | 1 | 1 | 1 | 1 | 1 | 0 | 0 | 1536 | 1545 | Oxidation (M) |
| R.ENPYYDSR.V | Y | 49.85 | 1042.4355 | 8 | 5.8 | 522.2281 | 2 | 30.75 | 10 | 8300 | Ana\_09.raw | 1.25E7 | 1.4E7 | 1.11E7 | 7.07E6 | 1.28E7 | 1.46E7 | 1.02E7 | 7.68E6 | 1.98E7 | 1.59E7 | 10 | 1 | 1 | 1 | 1 | 1 | 1 | 1 | 1 | 1 | 1 | 896 | 903 |  |
| R.AYEFAER.C | Y | 47.79 | 884.4028 | 7 | 4.0 | 443.2104 | 2 | 43.12 | 4 | 11765 | Ana\_03.raw | 1.92E7 |  | 1.68E7 | 7.09E6 | 1.44E7 | 1.49E7 | 1.16E7 | 1E7 |  |  | 7 | 1 | 0 | 1 | 1 | 1 | 1 | 1 | 1 | 0 | 0 | 1095 | 1101 |  |
| R.IAAYLFK.G | Y | 46.81 | 824.4796 | 7 | 4.0 | 413.2487 | 2 | 66.95 | 10 | 18980 | Ana\_09.raw | 3.68E6 | 3.7E6 |  |  |  |  |  | 1.26E6 | 4.94E6 | 2.85E6 | 5 | 1 | 1 | 0 | 0 | 0 | 0 | 0 | 1 | 1 | 1 | 1510 | 1516 |  |
| K.VDKLDASESLR.K | Y | 45.70 | 1231.6407 | 11 | 6.1 | 411.5567 | 3 | 36.41 | 9 | 9855 | Ana\_08.raw |  |  |  |  |  |  |  |  | 3.34E6 |  | 2 | 0 | 0 | 0 | 0 | 0 | 0 | 0 | 0 | 2 | 0 | 1610 | 1620 |  |
| R.LKLLLPWLEAR.I | Y | 45.64 | 1350.8387 | 11 | 3.3 | 451.2884 | 3 | 124.82 | 5 | 34426 | Ana\_04.raw |  |  |  | 2.33E5 | 7.68E5 |  | 1.32E5 |  |  |  | 3 | 0 | 0 | 0 | 1 | 1 | 0 | 1 | 0 | 0 | 0 | 855 | 865 |  |
| K.EAIDSYIK.A | Y | 44.95 | 937.4756 | 8 | 5.5 | 469.7477 | 2 | 55.00 | 2 | 15592 | Ana\_01.raw | 5.94E6 | 5.43E6 | 5.83E6 | 2.34E6 | 5.03E6 | 4.37E6 |  | 2.68E6 | 9.61E6 | 6.37E6 | 9 | 1 | 1 | 1 | 1 | 1 | 1 | 0 | 1 | 1 | 1 | 1123 | 1130 |  |
| K.YIQAAC(+57.02)K.T | Y | 43.01 | 852.4164 | 7 | 1.5 | 427.2161 | 2 | 21.21 | 7 | 5279 | Ana\_06.raw |  |  |  | 1.73E6 | 2.84E6 |  | 1.94E6 | 1.68E6 |  |  | 4 | 0 | 0 | 0 | 1 | 1 | 0 | 1 | 1 | 0 | 0 | 731 | 737 | Carbamidomethylation |
| K.WLLLTGISAQQNR.V | Y | 42.39 | 1498.8256 | 13 | 1.8 | 750.4214 | 2 | 107.90 | 4 | 29545 | Ana\_03.raw |  |  |  | 2.58E5 |  |  |  |  |  |  | 1 | 0 | 0 | 0 | 1 | 0 | 0 | 0 | 0 | 0 | 0 | 164 | 176 |  |
| R.AVNYFSK.V | Y | 40.46 | 827.4177 | 7 | 5.6 | 414.7184 | 2 | 38.94 | 10 | 10734 | Ana\_09.raw | 8.79E6 | 7.02E6 |  |  |  |  |  |  | 6.95E6 | 8.37E6 | 4 | 1 | 1 | 0 | 0 | 0 | 0 | 0 | 0 | 1 | 1 | 1435 | 1441 |  |
| K.EVC(+57.02)FAC(+57.02)VDGK.E | Y | 40.37 | 1183.5001 | 10 | 2.8 | 592.7590 | 2 | 56.35 | 5 | 15996 | Ana\_04.raw |  |  | 8.43E5 |  | 8.09E5 |  |  |  |  |  | 2 | 0 | 0 | 1 | 0 | 1 | 0 | 0 | 0 | 0 | 0 | 1255 | 1264 | Carbamidomethylation |
| R.VM(+15.99)EYINR.L | Y | 38.44 | 939.4484 | 7 | 4.5 | 470.7336 | 2 | 32.95 | 7 | 8884 | Ana\_06.raw | 6.73E6 |  |  | 3.99E6 | 7.98E6 | 1.37E7 | 7.76E6 | 1.1E7 | 8.31E6 | 7.3E6 | 9 | 1 | 0 | 0 | 1 | 1 | 1 | 1 | 2 | 1 | 1 | 1040 | 1046 | Oxidation (M) |
| K.AGLLQR.A | Y | 29.76 | 656.3969 | 6 | 4.2 | 329.2071 | 2 | 28.52 | 10 | 7613 | Ana\_09.raw | 3.49E6 |  | 3.28E6 | 2.81E6 | 3.36E6 | 3.8E6 | 3.13E6 | 1.72E6 | 3.91E6 | 3.62E6 | 9 | 1 | 0 | 1 | 1 | 1 | 1 | 1 | 1 | 1 | 1 | 620 | 625 |  |
| R.VVGAM(+15.99)QLYSVDR.K | Y | 22.59 | 1352.6758 | 12 | 3.1 | 677.3473 | 2 | 60.19 | 3 | 17126 | Ana\_02.raw |  |  | 8.57E5 |  |  |  |  |  |  |  | 1 | 0 | 0 | 1 | 0 | 0 | 0 | 0 | 0 | 0 | 0 | 177 | 188 | Oxidation (M) |
| total 47 peptides |  |  |  |  |  |  |  |  |  |  |  |  |  |  |  |  |  |  |  |  |  |  |  |  |  |  |  |  |  |  |  |  |  |  |  |
| --- | --- | --- | --- | --- | --- | --- | --- | --- | --- | --- | --- | --- | --- | --- | --- | --- | --- | --- | --- | --- | --- | --- | --- | --- | --- | --- | --- | --- | --- | --- | --- | --- | --- | --- | --- |

Best Unique PSM (Scan F6:36466, m/z=790.0936, z=3, RT=131.24, ppm=1.9):

Q5SXR6|Q5SXR6\_MOUSE

back to list

  

| Protein Coverage
| Supporting Peptides
| Best Unique PSM
|

Protein Coverage:

Supporting Peptides:

| Peptide | Uniq | -10lgP | Mass | Length | ppm | m/z | z | RT | Fraction | Scan | Source File | Area 10 | Area 01 | Area 02 | Area 03 | Area 04 | Area 05 | Area 06 | Area 07 | Area 08 | Area 09 | #Spec | #Spec 10 | #Spec 01 | #Spec 02 | #Spec 03 | #Spec 04 | #Spec 05 | #Spec 06 | #Spec 07 | #Spec 08 | #Spec 09 | Start | End | PTM |
| --- | --- | --- | --- | --- | --- | --- | --- | --- | --- | --- | --- | --- | --- | --- | --- | --- | --- | --- | --- | --- | --- | --- | --- | --- | --- | --- | --- | --- | --- | --- | --- | --- | --- | --- | --- |
| R.KFDVNTSAVQVLIEHIGNLDR.A | Y | 126.67 | 2367.2546 | 21 | 1.9 | 790.0936 | 3 | 131.24 | 6 | 36466 | Ana\_05.raw | 3.37E6 | 6.46E6 | 6.9E6 | 5.13E6 | 9.09E6 | 1.13E7 | 9.23E6 | 6.88E6 | 5.69E6 | 1.16E7 | 19 | 2 | 2 | 2 | 2 | 2 | 2 | 2 | 2 | 1 | 2 | 1078 | 1098 |  |
| R.RPLIDQVVQTALSETQDPEEVSVTVK.A | Y | 124.91 | 2880.5081 | 26 | 2.9 | 961.1794 | 3 | 130.09 | 1 | 35757 | ANa\_10.raw | 8.5E6 | 1.9E6 |  |  |  | 6.76E6 | 3.23E6 |  | 1.07E7 | 6.07E6 | 6 | 1 | 1 | 0 | 0 | 0 | 1 | 1 | 0 | 1 | 1 | 972 | 997 |  |
| K.SVNESLNNLFITEEDYQALR.T | Y | 116.21 | 2354.1389 | 20 | 3.6 | 1178.0809 | 2 | 121.23 | 9 | 33761 | Ana\_08.raw |  | 5.83E6 |  | 6.83E5 |  |  |  |  | 4.54E6 | 1.62E6 | 6 | 0 | 2 | 0 | 1 | 0 | 0 | 0 | 0 | 1 | 2 | 1466 | 1485 |  |
| R.LPVVIGGLLDVDC(+57.02)SEDVIK.N | Y | 107.13 | 2040.0813 | 19 | 4.8 | 1021.0528 | 2 | 128.70 | 9 | 35652 | Ana\_08.raw | 6.46E5 | 1.42E6 |  | 6.12E5 |  | 0 |  |  | 3.64E6 | 1.62E6 | 6 | 1 | 1 | 0 | 1 | 0 | 1 | 0 | 0 | 1 | 1 | 816 | 834 | Carbamidomethylation |
| R.ISGETIFVTAPHEATAGIIGVNR.K | Y | 100.29 | 2352.2437 | 23 | 4.6 | 785.0921 | 3 | 100.62 | 9 | 28410 | Ana\_08.raw |  |  |  | 4.19E5 | 2.88E6 |  | 1.87E6 | 6.46E5 | 3.02E6 |  | 5 | 0 | 0 | 0 | 1 | 1 | 0 | 1 | 1 | 1 | 0 | 302 | 324 |  |
| K.AFM(+15.99)TADLPNELIELLEK.I | Y | 95.51 | 1962.0020 | 17 | 2.7 | 982.0109 | 2 | 135.71 | 6 | 37720 | Ana\_05.raw | 4.72E6 | 1.57E6 | 3.14E6 |  | 1.5E6 | 4.32E6 | 4.55E6 | 2.91E6 | 1.02E7 | 2.13E6 | 12 | 1 | 1 | 1 | 0 | 1 | 2 | 2 | 1 | 2 | 1 | 998 | 1014 | Oxidation (M) |
| R.TSIDAYDNFDNISLAQR.L | Y | 90.38 | 1941.9067 | 17 | 3.8 | 971.9643 | 2 | 96.76 | 9 | 27385 | Ana\_08.raw |  |  |  |  |  |  |  |  | 4.57E6 | 3.42E5 | 2 | 0 | 0 | 0 | 0 | 0 | 0 | 0 | 0 | 1 | 1 | 1486 | 1502 |  |
| R.ALEHFTDLYDIK.R | Y | 87.76 | 1463.7296 | 12 | 5.4 | 732.8760 | 2 | 88.19 | 1 | 24808 | ANa\_10.raw | 7.29E6 | 1.81E6 | 4.37E6 |  | 5.02E6 | 3.53E6 | 2.74E6 | 1.26E6 | 1.61E7 | 4.92E6 | 12 | 2 | 1 | 1 | 0 | 2 | 1 | 1 | 1 | 2 | 1 | 630 | 641 |  |
| R.LASTLVHLGEYQAAVDGAR.K | Y | 83.74 | 1970.0221 | 19 | 4.9 | 657.6845 | 3 | 85.25 | 9 | 24252 | Ana\_08.raw | 1.64E6 | 1.45E6 |  |  | 3.18E4 |  |  |  | 5.41E6 | 2.97E6 | 5 | 1 | 1 | 0 | 0 | 1 | 0 | 0 | 0 | 1 | 1 | 1231 | 1249 |  |
| K.KVGYTPDWIFLLR.N | Y | 80.20 | 1606.8871 | 13 | 4.1 | 536.6385 | 3 | 126.69 | 6 | 35228 | Ana\_05.raw |  | 1.78E5 |  |  | 1.1E6 | 1.22E6 | 2.62E6 |  |  |  | 4 | 0 | 1 | 0 | 0 | 1 | 1 | 1 | 0 | 0 | 0 | 511 | 523 |  |
| K.VGYTPDWIFLLR.N | Y | 78.53 | 1478.7921 | 12 | 5.8 | 740.4076 | 2 | 132.97 | 6 | 36965 | Ana\_05.raw |  | 5.14E6 |  | 2.01E6 | 3.03E6 | 4.08E6 |  | 1.19E6 |  | 2.01E6 | 6 | 0 | 1 | 0 | 1 | 1 | 1 | 0 | 1 | 0 | 1 | 512 | 523 |  |
| K.LHIIEVGTPPTGNQPFPK.K | Y | 77.43 | 1944.0469 | 18 | 4.6 | 649.0259 | 3 | 81.47 | 9 | 23192 | Ana\_08.raw | 3.51E5 |  |  | 1.86E6 | 1.59E5 |  | 1.03E6 | 4.32E5 | 1.99E6 |  | 6 | 1 | 0 | 0 | 1 | 1 | 0 | 1 | 1 | 1 | 0 | 232 | 249 |  |
| R.GQFSTDELVAEVEK.R | Y | 77.10 | 1550.7463 | 14 | 5.9 | 776.3850 | 2 | 96.54 | 6 | 27333 | Ana\_05.raw | 6.5E6 | 3.55E6 | 1.19E6 | 4.57E5 | 2.56E6 | 4.1E6 | 1.22E6 | 8.19E5 | 8.49E6 | 5.58E5 | 10 | 1 | 1 | 1 | 1 | 1 | 1 | 1 | 1 | 1 | 1 | 842 | 855 |  |
| K.LLLPWLEAR.I | Y | 73.10 | 1109.6597 | 9 | 3.5 | 555.8391 | 2 | 124.31 | 6 | 34612 | Ana\_05.raw | 3.11E7 | 6.86E6 | 1.55E7 | 3.73E6 | 2.4E7 | 2.5E7 | 1.16E7 | 6.99E6 | 4.1E7 | 2.59E7 | 35 | 4 | 3 | 4 | 2 | 5 | 3 | 2 | 4 | 4 | 4 | 861 | 869 |  |
| R.KDPELWGSVLLESNPYR.R | Y | 73.02 | 2002.0159 | 17 | 8.3 | 668.3514 | 3 | 110.95 | 9 | 31124 | Ana\_08.raw |  | 9.29E5 |  |  |  |  |  |  | 1.99E6 |  | 2 | 0 | 1 | 0 | 0 | 0 | 0 | 0 | 0 | 1 | 0 | 955 | 971 |  |
| K.NNRPSEGPLQTR.L | Y | 72.84 | 1367.6906 | 12 | -1.1 | 684.8518 | 2 | 24.70 | 3 | 6465 | Ana\_02.raw | 8.67E6 | 7.43E5 | 3.11E6 |  |  | 2.45E6 | 3.57E6 | 3.27E6 | 7.91E6 | 3.55E6 | 10 | 2 | 1 | 1 | 0 | 0 | 1 | 1 | 2 | 1 | 1 | 576 | 587 |  |
| R.GQFSTDELVAEVEKR.N | Y | 72.46 | 1706.8474 | 15 | 4.6 | 569.9590 | 3 | 91.41 | 9 | 25925 | Ana\_08.raw |  | 3.03E5 | 1.25E6 |  | 2.39E6 | 2.47E6 | 2.29E6 | 7.6E5 | 8.78E6 | 5.03E6 | 8 | 0 | 1 | 1 | 0 | 1 | 1 | 1 | 1 | 1 | 1 | 842 | 856 |  |
| R.RPISADSAIM(+15.99)NPASK.V | Y | 72.06 | 1572.7930 | 15 | 4.8 | 525.2741 | 3 | 40.54 | 1 | 11180 | ANa\_10.raw | 1.17E7 | 1.33E6 | 9.46E6 |  |  |  |  |  | 4.29E6 | 1.47E6 | 7 | 2 | 1 | 1 | 0 | 0 | 0 | 0 | 0 | 2 | 1 | 64 | 78 | Oxidation (M) |
| R.IHEGC(+57.02)EEPATHNALAK.I | Y | 69.67 | 1775.8260 | 16 | 5.8 | 592.9527 | 3 | 25.88 | 9 | 6599 | Ana\_08.raw |  |  |  |  |  |  |  |  | 3.31E6 |  | 1 | 0 | 0 | 0 | 0 | 0 | 0 | 0 | 0 | 1 | 0 | 870 | 885 | Carbamidomethylation |
| K.HDVVFLITK.Y | Y | 69.67 | 1070.6124 | 9 | 3.5 | 536.3154 | 2 | 68.62 | 6 | 19693 | Ana\_05.raw |  | 8.16E6 |  | 9.4E5 | 3.74E6 | 2.55E6 | 2.66E6 | 1.88E6 |  |  | 6 | 0 | 1 | 0 | 1 | 1 | 1 | 1 | 1 | 0 | 0 | 274 | 282 |  |
| R.KFNALFAQGNYSEAAK.V | Y | 68.37 | 1757.8737 | 16 | 4.5 | 586.9678 | 3 | 70.90 | 5 | 20205 | Ana\_04.raw |  |  | 1.26E6 |  | 1.72E6 | 2.39E6 | 4.84E5 | 2.24E6 | 3.82E6 |  | 6 | 0 | 0 | 1 | 0 | 1 | 1 | 1 | 1 | 1 | 0 | 371 | 386 |  |
| K.IYIDSNNNPER.F | Y | 66.43 | 1333.6262 | 11 | 6.4 | 667.8246 | 2 | 42.37 | 6 | 11887 | Ana\_05.raw | 1.17E7 | 1.1E7 | 8.26E6 | 1.99E6 | 8.17E6 | 9.66E6 | 7.02E6 | 5.26E6 | 1.35E7 | 8.65E6 | 11 | 1 | 1 | 1 | 1 | 1 | 1 | 1 | 1 | 2 | 1 | 886 | 896 |  |
| R.NNLAGAEELFAR.K | Y | 65.55 | 1303.6520 | 12 | 3.4 | 652.8354 | 2 | 88.06 | 6 | 25069 | Ana\_05.raw | 1.39E7 | 1.42E7 | 1.38E7 | 6.82E6 | 1.13E7 | 1.08E7 | 9.6E6 | 8.14E6 | 2.55E7 | 9.64E6 | 10 | 1 | 1 | 1 | 1 | 1 | 1 | 1 | 1 | 1 | 1 | 359 | 370 |  |
| K.VIQC(+57.02)FAETGQVQK.I | Y | 62.42 | 1506.7500 | 13 | 7.9 | 754.3882 | 2 | 51.26 | 9 | 14439 | Ana\_08.raw | 2.9E6 | 3.49E6 | 4.04E5 | 3.1E5 | 1.61E6 |  | 1.06E6 | 9.47E5 | 7.08E6 | 3.47E6 | 9 | 1 | 1 | 1 | 1 | 1 | 0 | 1 | 1 | 1 | 1 | 492 | 504 | Carbamidomethylation |
| K.IVLDNSVFSEHR.N | Y | 62.14 | 1414.7205 | 12 | 3.2 | 708.3698 | 2 | 73.72 | 8 | 20696 | Ana\_07.raw | 9.48E5 | 1.33E6 |  |  | 1.06E6 | 2.01E6 | 7.36E5 | 1.18E6 |  | 1.83E6 | 7 | 1 | 1 | 0 | 0 | 1 | 1 | 1 | 1 | 0 | 1 | 1015 | 1026 |  |
| K.VSQPIEGHAASFAQFK.M | Y | 61.31 | 1715.8630 | 16 | 4.7 | 572.9643 | 3 | 67.76 | 2 | 19309 | Ana\_01.raw | 1.36E7 | 8.64E6 | 5.35E6 |  |  |  |  | 5.99E6 |  | 1.28E7 | 6 | 2 | 1 | 1 | 0 | 0 | 0 | 0 | 1 | 0 | 1 | 194 | 209 |  |
| R.ESNC(+57.02)YDPER.V | Y | 59.87 | 1168.4454 | 9 | 6.0 | 585.2335 | 2 | 26.98 | 6 | 7179 | Ana\_05.raw | 3.05E6 |  |  | 2.27E6 |  | 3.96E6 | 1.51E6 |  |  | 1.64E6 | 5 | 1 | 0 | 0 | 1 | 0 | 1 | 1 | 0 | 0 | 1 | 754 | 762 | Carbamidomethylation |
| R.NLQNLLILTAIK.A | Y | 59.77 | 1352.8391 | 12 | 4.1 | 677.4296 | 2 | 126.96 | 6 | 35294 | Ana\_05.raw | 8.59E6 | 2.11E6 | 2.39E6 | 3.37E6 | 9.41E6 | 1.72E6 | 9.56E6 | 6.14E5 | 4.85E6 | 6.01E6 | 11 | 1 | 1 | 2 | 1 | 1 | 1 | 1 | 1 | 1 | 1 | 1027 | 1038 |  |
| K.HELIEFR.R | Y | 59.00 | 942.4923 | 7 | 3.2 | 472.2549 | 2 | 55.38 | 6 | 15808 | Ana\_05.raw |  |  |  | 2.89E6 |  | 7.92E6 | 4.63E6 |  |  |  | 3 | 0 | 0 | 0 | 1 | 0 | 1 | 1 | 0 | 0 | 0 | 1506 | 1512 |  |
| R.AHIAQLC(+57.02)EK.A | Y | 57.84 | 1068.5386 | 9 | 4.7 | 535.2791 | 2 | 24.48 | 9 | 6125 | Ana\_08.raw |  |  |  |  |  |  |  |  | 3.97E6 | 1.35E6 | 2 | 0 | 0 | 0 | 0 | 0 | 0 | 0 | 0 | 1 | 1 | 615 | 623 | Carbamidomethylation |
| K.AFMTADLPNELIELLEK.I | Y | 54.92 | 1946.0070 | 17 | 5.9 | 974.0165 | 2 | 137.63 | 10 | 37493 | Ana\_09.raw |  |  |  |  |  |  |  |  | 7.73E5 | 7.42E5 | 2 | 0 | 0 | 0 | 0 | 0 | 0 | 0 | 0 | 1 | 1 | 998 | 1014 |  |
| K.LLYNNVSNFGR.L | Y | 54.34 | 1295.6622 | 11 | 4.8 | 648.8415 | 2 | 71.46 | 9 | 20397 | Ana\_08.raw | 6.15E6 | 4.18E5 |  | 1.72E6 | 0 | 1.17E6 |  | 9.46E5 | 9.79E6 | 4.81E6 | 9 | 1 | 1 | 1 | 1 | 1 | 1 | 0 | 1 | 1 | 1 | 1220 | 1230 |  |
| K.TLQIFNIEM(+15.99)K.S | Y | 53.51 | 1251.6533 | 10 | 1.4 | 626.8348 | 2 | 88.66 | 3 | 24909 | Ana\_02.raw | 9.37E5 | 2.21E6 | 2.39E6 | 7.11E5 | 4.99E5 | 2.77E6 | 2.24E6 | 2.09E6 | 6.55E5 | 1.22E6 | 11 | 1 | 1 | 1 | 1 | 1 | 1 | 2 | 1 | 1 | 1 | 91 | 100 | Oxidation (M) |
| K.DAM(+15.99)QYASESK.D | Y | 52.98 | 1144.4706 | 10 | 3.0 | 573.2443 | 2 | 21.39 | 3 | 5402 | Ana\_02.raw |  |  | 3.87E6 | 2.83E6 | 2.28E6 | 5.68E6 | 2.36E6 | 2.71E6 |  |  | 6 | 0 | 0 | 1 | 1 | 1 | 1 | 1 | 1 | 0 | 0 | 1540 | 1549 | Oxidation (M) |
| R.ENPYYDSR.V | Y | 49.85 | 1042.4355 | 8 | 5.8 | 522.2281 | 2 | 30.75 | 10 | 8300 | Ana\_09.raw | 1.25E7 | 1.4E7 | 1.11E7 | 7.07E6 | 1.28E7 | 1.46E7 | 1.02E7 | 7.68E6 | 1.98E7 | 1.59E7 | 10 | 1 | 1 | 1 | 1 | 1 | 1 | 1 | 1 | 1 | 1 | 900 | 907 |  |
| R.AYEFAER.C | Y | 47.79 | 884.4028 | 7 | 4.0 | 443.2104 | 2 | 43.12 | 4 | 11765 | Ana\_03.raw | 1.92E7 |  | 1.68E7 | 7.09E6 | 1.44E7 | 1.49E7 | 1.16E7 | 1E7 |  |  | 7 | 1 | 0 | 1 | 1 | 1 | 1 | 1 | 1 | 0 | 0 | 1099 | 1105 |  |
| R.IAAYLFK.G | Y | 46.81 | 824.4796 | 7 | 4.0 | 413.2487 | 2 | 66.95 | 10 | 18980 | Ana\_09.raw | 3.68E6 | 3.7E6 |  |  |  |  |  | 1.26E6 | 4.94E6 | 2.85E6 | 5 | 1 | 1 | 0 | 0 | 0 | 0 | 0 | 1 | 1 | 1 | 1514 | 1520 |  |
| K.VDKLDASESLR.K | Y | 45.70 | 1231.6407 | 11 | 6.1 | 411.5567 | 3 | 36.41 | 9 | 9855 | Ana\_08.raw |  |  |  |  |  |  |  |  | 3.34E6 |  | 2 | 0 | 0 | 0 | 0 | 0 | 0 | 0 | 0 | 2 | 0 | 1614 | 1624 |  |
| R.LKLLLPWLEAR.I | Y | 45.64 | 1350.8387 | 11 | 3.3 | 451.2884 | 3 | 124.82 | 5 | 34426 | Ana\_04.raw |  |  |  | 2.33E5 | 7.68E5 |  | 1.32E5 |  |  |  | 3 | 0 | 0 | 0 | 1 | 1 | 0 | 1 | 0 | 0 | 0 | 859 | 869 |  |
| K.EAIDSYIK.A | Y | 44.95 | 937.4756 | 8 | 5.5 | 469.7477 | 2 | 55.00 | 2 | 15592 | Ana\_01.raw | 5.94E6 | 5.43E6 | 5.83E6 | 2.34E6 | 5.03E6 | 4.37E6 |  | 2.68E6 | 9.61E6 | 6.37E6 | 9 | 1 | 1 | 1 | 1 | 1 | 1 | 0 | 1 | 1 | 1 | 1127 | 1134 |  |
| K.YIQAAC(+57.02)K.T | Y | 43.01 | 852.4164 | 7 | 1.5 | 427.2161 | 2 | 21.21 | 7 | 5279 | Ana\_06.raw |  |  |  | 1.73E6 | 2.84E6 |  | 1.94E6 | 1.68E6 |  |  | 4 | 0 | 0 | 0 | 1 | 1 | 0 | 1 | 1 | 0 | 0 | 735 | 741 | Carbamidomethylation |
| K.WLLLTGISAQQNR.V | Y | 42.39 | 1498.8256 | 13 | 1.8 | 750.4214 | 2 | 107.90 | 4 | 29545 | Ana\_03.raw |  |  |  | 2.58E5 |  |  |  |  |  |  | 1 | 0 | 0 | 0 | 1 | 0 | 0 | 0 | 0 | 0 | 0 | 168 | 180 |  |
| R.AVNYFSK.V | Y | 40.46 | 827.4177 | 7 | 5.6 | 414.7184 | 2 | 38.94 | 10 | 10734 | Ana\_09.raw | 8.79E6 | 7.02E6 |  |  |  |  |  |  | 6.95E6 | 8.37E6 | 4 | 1 | 1 | 0 | 0 | 0 | 0 | 0 | 0 | 1 | 1 | 1439 | 1445 |  |
| K.EVC(+57.02)FAC(+57.02)VDGK.E | Y | 40.37 | 1183.5001 | 10 | 2.8 | 592.7590 | 2 | 56.35 | 5 | 15996 | Ana\_04.raw |  |  | 8.43E5 |  | 8.09E5 |  |  |  |  |  | 2 | 0 | 0 | 1 | 0 | 1 | 0 | 0 | 0 | 0 | 0 | 1259 | 1268 | Carbamidomethylation |
| R.VM(+15.99)EYINR.L | Y | 38.44 | 939.4484 | 7 | 4.5 | 470.7336 | 2 | 32.95 | 7 | 8884 | Ana\_06.raw | 6.73E6 |  |  | 3.99E6 | 7.98E6 | 1.37E7 | 7.76E6 | 1.1E7 | 8.31E6 | 7.3E6 | 9 | 1 | 0 | 0 | 1 | 1 | 1 | 1 | 2 | 1 | 1 | 1044 | 1050 | Oxidation (M) |
| K.AGLLQR.A | Y | 29.76 | 656.3969 | 6 | 4.2 | 329.2071 | 2 | 28.52 | 10 | 7613 | Ana\_09.raw | 3.49E6 |  | 3.28E6 | 2.81E6 | 3.36E6 | 3.8E6 | 3.13E6 | 1.72E6 | 3.91E6 | 3.62E6 | 9 | 1 | 0 | 1 | 1 | 1 | 1 | 1 | 1 | 1 | 1 | 624 | 629 |  |
| R.VVGAM(+15.99)QLYSVDR.K | Y | 22.59 | 1352.6758 | 12 | 3.1 | 677.3473 | 2 | 60.19 | 3 | 17126 | Ana\_02.raw |  |  | 8.57E5 |  |  |  |  |  |  |  | 1 | 0 | 0 | 1 | 0 | 0 | 0 | 0 | 0 | 0 | 0 | 181 | 192 | Oxidation (M) |
| total 47 peptides |  |  |  |  |  |  |  |  |  |  |  |  |  |  |  |  |  |  |  |  |  |  |  |  |  |  |  |  |  |  |  |  |  |  |  |
| --- | --- | --- | --- | --- | --- | --- | --- | --- | --- | --- | --- | --- | --- | --- | --- | --- | --- | --- | --- | --- | --- | --- | --- | --- | --- | --- | --- | --- | --- | --- | --- | --- | --- | --- | --- |

Best Unique PSM (Scan F6:36466, m/z=790.0936, z=3, RT=131.24, ppm=1.9):

sp|P68134|ACTS\_MOUSE

back to list

  

| Protein Coverage
| Supporting Peptides
| Best Unique PSM
|

Protein Coverage:

Supporting Peptides:

| Peptide | Uniq | -10lgP | Mass | Length | ppm | m/z | z | RT | Fraction | Scan | Source File | Area 10 | Area 01 | Area 02 | Area 03 | Area 04 | Area 05 | Area 06 | Area 07 | Area 08 | Area 09 | #Spec | #Spec 10 | #Spec 01 | #Spec 02 | #Spec 03 | #Spec 04 | #Spec 05 | #Spec 06 | #Spec 07 | #Spec 08 | #Spec 09 | Start | End | PTM |
| --- | --- | --- | --- | --- | --- | --- | --- | --- | --- | --- | --- | --- | --- | --- | --- | --- | --- | --- | --- | --- | --- | --- | --- | --- | --- | --- | --- | --- | --- | --- | --- | --- | --- | --- | --- |
| R.VAPEEHPTLLTEAPLNPK.A | N | 151.97 | 1955.0364 | 18 | 1.1 | 978.5266 | 2 | 73.32 | 3 | 20810 | Ana\_02.raw | 2.45E9 | 2.5E9 | 1.77E9 | 7.47E8 | 1.61E9 | 1.6E9 | 9.26E8 | 1.38E9 | 4.04E9 | 1.56E9 | 127 | 13 | 17 | 11 | 9 | 14 | 13 | 9 | 13 | 11 | 17 | 98 | 115 |  |
| K.SYELPDGQVITIGNER.F | N | 136.16 | 1789.8846 | 16 | 0.5 | 895.9501 | 2 | 101.19 | 3 | 28209 | Ana\_02.raw | 2.56E9 | 2.49E9 | 2.2E9 | 5.02E8 | 1.2E9 | 1.61E9 | 8.03E8 | 1.08E9 | 4.17E9 | 2.27E9 | 445 | 51 | 48 | 45 | 42 | 44 | 40 | 40 | 47 | 46 | 42 | 241 | 256 |  |
| K.LC(+57.02)YVALDFENEMATAASSSSLEK.S | N | 111.03 | 2535.1509 | 23 | 5.6 | 1268.5898 | 2 | 129.15 | 1 | 35504 | ANa\_10.raw | 3.4E6 |  |  |  |  |  |  |  |  | 3.91E6 | 2 | 1 | 0 | 0 | 0 | 0 | 0 | 0 | 0 | 0 | 1 | 218 | 240 | Carbamidomethylation |
| R.TTGIVLDSGDGVTHNVPIYEGYALPHAIMR.L | N | 110.75 | 3195.6023 | 30 | 6.6 | 1066.2151 | 3 | 112.07 | 9 | 31405 | Ana\_08.raw | 6.3E6 |  |  |  |  | 3.6E6 |  |  | 1.67E7 |  | 3 | 1 | 0 | 0 | 0 | 0 | 1 | 0 | 0 | 1 | 0 | 150 | 179 |  |
| K.IWHHTFYNELR.V | N | 103.41 | 1514.7418 | 11 | 1.7 | 505.9221 | 3 | 58.21 | 3 | 16543 | Ana\_02.raw | 1.86E8 | 2.44E8 | 2.11E8 | 9.2E7 | 1.75E8 | 1.74E8 | 1.18E8 | 1.31E8 | 3.7E8 | 2.13E8 | 57 | 5 | 6 | 5 | 7 | 7 | 4 | 4 | 5 | 7 | 7 | 87 | 97 |  |
| K.LC(+57.02)YVALDFENEM(+15.99)ATAASSSSLEK.S | N | 101.77 | 2551.1458 | 23 | 2.6 | 1276.5835 | 2 | 103.36 | 9 | 29127 | Ana\_08.raw | 3.65E6 | 6.44E6 | 9.4E6 | 4.23E5 |  | 1.01E7 | 2.94E6 |  | 3.63E7 | 3.17E6 | 14 | 2 | 2 | 2 | 1 | 0 | 2 | 2 | 0 | 2 | 1 | 218 | 240 | Carbamidomethylation; Oxidation (M) |
| R.TTGIVLDSGDGVTHNVPIYEGYALPHAIM(+15.99)R.L | N | 98.15 | 3211.5972 | 30 | 0.6 | 1071.5403 | 3 | 103.74 | 3 | 28873 | Ana\_02.raw | 1.38E8 | 8.64E7 | 2.27E8 | 8.5E6 | 8.89E7 | 3.16E8 | 1.03E8 | 1.09E8 | 4.11E8 | 2.12E8 | 72 | 10 | 5 | 6 | 4 | 5 | 8 | 4 | 9 | 14 | 7 | 150 | 179 | Oxidation (M) |
| K.DSYVGDEAQSKR.G | N | 93.37 | 1353.6160 | 12 | 3.6 | 677.8177 | 2 | 27.36 | 1 | 7192 | ANa\_10.raw | 9.62E7 | 3.55E7 | 6.96E7 | 1.04E7 | 3.31E7 | 5.26E7 | 2.56E7 | 1.83E7 | 1.05E8 | 4.34E7 | 30 | 4 | 2 | 4 | 2 | 3 | 3 | 3 | 3 | 3 | 3 | 53 | 64 |  |
| K.QEYDEAGPSIVHR.K | N | 91.09 | 1499.7004 | 13 | 3.8 | 500.9093 | 3 | 48.57 | 6 | 13776 | Ana\_05.raw | 7.68E7 | 6.13E7 | 9.07E7 | 3.96E7 | 8.61E7 | 1.01E8 | 4.95E7 | 8.99E7 | 5.06E7 | 4.33E6 | 35 | 3 | 3 | 3 | 3 | 5 | 4 | 3 | 7 | 3 | 1 | 362 | 374 |  |
| K.DSYVGDEAQSK.R | N | 89.89 | 1197.5149 | 11 | 1.9 | 599.7659 | 2 | 26.18 | 3 | 6939 | Ana\_02.raw | 2.5E9 | 2.55E9 | 2.09E9 | 9.4E8 | 1.67E9 | 2.04E9 | 1.22E9 | 1.3E9 | 1.9E9 | 2.02E9 | 140 | 13 | 12 | 15 | 13 | 15 | 15 | 13 | 18 | 11 | 15 | 53 | 63 |  |
| K.YPIEHGIITNWDDM(+15.99)EK.I | N | 87.01 | 1975.8986 | 16 | 4.7 | 988.9612 | 2 | 75.68 | 9 | 21564 | Ana\_08.raw | 3.19E8 | 2.43E8 | 2.13E8 | 1.25E8 | 2.7E8 | 2.71E8 | 1.07E8 | 2.43E8 | 8.47E8 | 1.87E8 | 63 | 9 | 6 | 7 | 4 | 6 | 3 | 4 | 7 | 13 | 4 | 71 | 86 | Oxidation (M) |
| R.GYSFVTTAER.E | N | 84.49 | 1129.5404 | 10 | 1.5 | 565.7783 | 2 | 57.04 | 3 | 16191 | Ana\_02.raw | 1.61E9 | 1.67E9 | 1.48E9 | 5.34E8 | 1.06E9 | 1.19E9 | 7.36E8 | 9.1E8 | 1.96E9 | 1.22E9 | 186 | 21 | 21 | 21 | 15 | 20 | 18 | 14 | 24 | 17 | 15 | 199 | 208 |  |
| R.DLTDYLM(+15.99)KILTER.G | N | 84.48 | 1625.8334 | 13 | -0.5 | 813.9235 | 2 | 131.53 | 3 | 36031 | Ana\_02.raw | 2.95E6 | 3.96E6 | 7.7E6 | 1.52E6 | 5.67E6 | 8.87E6 | 6.41E6 | 2.09E6 | 2.69E6 | 6.16E6 | 17 | 2 | 1 | 2 | 1 | 2 | 2 | 2 | 2 | 1 | 2 | 186 | 198 | Oxidation (M) |
| R.HQGVMVGM(+15.99)GQK.D | N | 84.12 | 1186.5587 | 11 | 1.7 | 594.2877 | 2 | 16.20 | 3 | 3687 | Ana\_02.raw | 8.78E6 | 2.67E7 | 5.55E6 | 4.81E6 | 4.46E6 | 2.99E7 | 5.97E6 | 1.31E7 | 1.79E7 | 2.08E7 | 21 | 2 | 2 | 2 | 2 | 1 | 3 | 3 | 2 | 1 | 3 | 42 | 52 | Oxidation (M) |
| K.EITALAPSTM(+15.99)K.I | N | 81.55 | 1176.6060 | 11 | 3.3 | 589.3122 | 2 | 45.25 | 6 | 12771 | Ana\_05.raw | 1.46E9 | 1.19E9 | 1.22E9 | 4.08E8 | 9.94E8 | 1.04E9 | 6.43E8 | 8.39E8 | 2.22E9 | 8.74E8 | 142 | 18 | 15 | 12 | 10 | 13 | 11 | 10 | 18 | 18 | 17 | 318 | 328 | Oxidation (M) |
| K.YPIEHGIITNWDDMEK.I | N | 80.26 | 1959.9037 | 16 | 2.8 | 980.9619 | 2 | 90.08 | 1 | 25309 | ANa\_10.raw | 3.61E6 |  |  |  |  |  |  |  | 2.49E7 | 7.39E6 | 4 | 2 | 0 | 0 | 0 | 0 | 0 | 0 | 0 | 1 | 1 | 71 | 86 |  |
| K.AGFAGDDAPR.A | N | 76.24 | 975.4410 | 10 | 4.8 | 488.7301 | 2 | 31.38 | 1 | 8414 | ANa\_10.raw | 3.67E9 | 4.81E9 | 3.7E9 | 1.86E9 | 3.52E9 | 3.73E9 | 2.42E9 | 2.63E9 | 6.06E9 | 4.43E9 | 286 | 36 | 29 | 31 | 28 | 26 | 21 | 16 | 35 | 35 | 29 | 21 | 30 |  |
| R.AVFPSIVGRPR.H | N | 74.48 | 1197.6981 | 11 | 5.3 | 599.8595 | 2 | 62.78 | 2 | 17880 | Ana\_01.raw | 1.6E9 | 1.34E9 | 1.32E9 | 8.13E8 | 1.16E9 | 1.21E9 | 8.12E8 | 8.11E8 | 2.43E9 | 1.42E9 | 261 | 32 | 21 | 25 | 23 | 36 | 23 | 17 | 28 | 32 | 24 | 31 | 41 |  |
| R.HQGVM(+15.99)VGMGQK.D | N | 69.21 | 1186.5587 | 11 | 1.9 | 594.2878 | 2 | 15.71 | 5 | 3571 | Ana\_04.raw | 1.62E7 | 9.07E6 |  |  | 1.39E6 | 1.35E7 | 8.82E5 | 7.09E6 | 1.92E6 | 1.76E7 | 12 | 1 | 1 | 0 | 0 | 1 | 2 | 2 | 2 | 1 | 2 | 42 | 52 | Oxidation (M) |
| K.EITALAPSTMK.I | N | 67.15 | 1160.6111 | 11 | -0.1 | 581.3127 | 2 | 30.48 | 9 | 8027 | Ana\_08.raw | 6.1E7 | 2.19E7 |  | 5.22E5 |  |  | 1.96E6 |  | 1.1E8 | 4.16E7 | 14 | 4 | 1 | 0 | 1 | 0 | 0 | 1 | 0 | 5 | 2 | 318 | 328 |  |
| R.HQGVM(+15.99)VGM(+15.99)GQK.D | N | 65.50 | 1202.5536 | 11 | 7.8 | 602.2888 | 2 | 21.12 | 1 | 5142 | ANa\_10.raw | 3.51E8 | 1.68E8 | 6.85E8 | 2.25E8 | 4.15E8 | 7.33E8 | 4.21E8 | 3.56E8 | 9.25E7 | 3.14E8 | 59 | 8 | 4 | 5 | 7 | 6 | 4 | 5 | 8 | 6 | 6 | 42 | 52 | Oxidation (M) |
| R.C(+57.02)PETLFQPSFIGM(+15.99)ESAGIHETTYNSIMK.C | N | 60.09 | 3203.4614 | 28 | 4.5 | 1068.8325 | 3 | 117.97 | 1 | 32577 | ANa\_10.raw | 1.9E6 |  |  |  |  |  |  |  |  |  | 1 | 1 | 0 | 0 | 0 | 0 | 0 | 0 | 0 | 0 | 0 | 259 | 286 | Carbamidomethylation; Oxidation (M) |
| R.DLTDYLM(+15.99)K.I | N | 58.74 | 1013.4739 | 8 | 3.9 | 507.7462 | 2 | 92.37 | 6 | 26220 | Ana\_05.raw | 8.4E8 | 6.93E8 | 6.22E8 | 2.72E8 | 4.42E8 | 4.88E8 | 3.63E8 | 5.27E8 | 1.32E9 | 5.32E8 | 291 | 38 | 34 | 28 | 21 | 30 | 22 | 17 | 33 | 35 | 33 | 186 | 193 | Oxidation (M) |
| R.DLTDYLMK.I | N | 58.29 | 997.4790 | 8 | 4.0 | 499.7487 | 2 | 92.24 | 1 | 25887 | ANa\_10.raw | 3.85E7 | 6.67E6 | 1.26E5 | 5.05E5 |  | 6.74E6 | 2.41E5 | 3.81E6 | 2.57E7 | 2.99E6 | 21 | 5 | 2 | 1 | 1 | 0 | 2 | 1 | 1 | 5 | 3 | 186 | 193 |  |
| R.KDLYANNVM(+15.99)SGGTTM(+15.99)YPGIADR.M | Y | 58.01 | 2405.0991 | 22 | 9.6 | 802.7147 | 3 | 84.08 | 3 | 23710 | Ana\_02.raw |  |  | 1.24E6 |  |  |  |  |  |  |  | 1 | 0 | 0 | 1 | 0 | 0 | 0 | 0 | 0 | 0 | 0 | 293 | 314 | Oxidation (M) |
| K.IIAPPER.K | N | 57.46 | 794.4650 | 7 | 1.7 | 398.2404 | 2 | 33.34 | 3 | 9119 | Ana\_02.raw | 3.97E9 | 4.12E9 | 3.95E9 | 1.66E9 | 3.05E9 | 3.75E9 | 2.4E9 | 2.4E9 | 4.68E9 | 3.63E9 | 283 | 36 | 29 | 33 | 22 | 29 | 22 | 16 | 34 | 31 | 31 | 331 | 337 |  |
| R.C(+57.02)PETLFQPSFIGM(+15.99)ESAGIHETTYNSIM(+15.99)K.C | N | 56.25 | 3219.4563 | 28 | 3.5 | 1074.1631 | 3 | 106.43 | 9 | 29941 | Ana\_08.raw | 9.39E6 | 7.29E6 |  | 2.94E6 | 1.83E7 | 6.25E6 | 1E6 | 1.05E7 | 1.17E7 |  | 10 | 1 | 2 | 0 | 1 | 2 | 1 | 1 | 1 | 1 | 0 | 259 | 286 | Carbamidomethylation; Oxidation (M) |
| R.LDLAGRDLTDYLM(+15.99)K.I | N | 51.33 | 1638.8286 | 14 | 3.2 | 820.4242 | 2 | 95.36 | 9 | 26994 | Ana\_08.raw | 9.63E6 |  |  |  |  |  |  |  | 1.04E7 |  | 3 | 1 | 0 | 0 | 0 | 0 | 0 | 0 | 0 | 2 | 0 | 180 | 193 | Oxidation (M) |
| K.IIAPPERK.Y | N | 46.97 | 922.5599 | 8 | 3.9 | 462.2891 | 2 | 23.58 | 9 | 5830 | Ana\_08.raw | 8.34E7 | 2.85E7 | 2.04E7 |  | 1.06E7 | 3.16E7 | 5.24E6 | 4.71E6 | 8.32E7 | 2.96E7 | 18 | 3 | 2 | 2 | 0 | 2 | 2 | 1 | 1 | 3 | 2 | 331 | 338 |  |
| K.C(+57.02)DIDIR.K | N | 45.41 | 790.3643 | 6 | 3.2 | 396.1907 | 2 | 36.67 | 6 | 10134 | Ana\_05.raw |  |  |  | 1.65E7 | 3.9E7 | 5.72E7 | 2.82E7 | 3.43E7 |  |  | 5 | 0 | 0 | 0 | 1 | 1 | 1 | 1 | 1 | 0 | 0 | 287 | 292 | Carbamidomethylation |
| K.IKIIAPPER.K | N | 42.43 | 1035.6440 | 9 | 0.5 | 346.2221 | 3 | 44.96 | 3 | 12647 | Ana\_02.raw | 3.02E6 |  | 7.18E6 | 2.01E6 | 2.08E6 | 3.03E6 | 1.58E6 |  | 4.82E6 | 2.45E6 | 12 | 1 | 0 | 2 | 2 | 1 | 2 | 1 | 0 | 2 | 1 | 329 | 337 |  |
| K.ILTER.G | N | 39.01 | 630.3701 | 5 | 3.4 | 316.1934 | 2 | 20.56 | 6 | 5102 | Ana\_05.raw | 2.01E7 |  |  | 2.76E6 | 1.13E7 | 9.27E6 | 2.65E8 | 1.7E8 | 7.79E6 |  | 14 | 1 | 0 | 0 | 3 | 3 | 1 | 4 | 1 | 1 | 0 | 194 | 198 |  |
| R.EIVRDIK.E | N | 36.72 | 871.5127 | 7 | 6.3 | 436.7664 | 2 | 23.86 | 9 | 5929 | Ana\_08.raw |  | 3.4E6 | 3.11E6 |  |  | 3.2E6 | 5.84E5 |  | 3.56E6 | 3.27E6 | 6 | 0 | 1 | 1 | 0 | 0 | 1 | 1 | 0 | 1 | 1 | 209 | 215 |  |
| R.LDLAGR.D | N | 35.64 | 643.3653 | 6 | -0.6 | 322.6897 | 2 | 30.23 | 3 | 8192 | Ana\_02.raw | 7.61E8 | 7.49E8 | 8.47E8 | 3.81E8 | 6.81E8 | 6.9E7 | 4E5 | 1.11E2 | 9.23E8 | 6.75E8 | 38 | 3 | 3 | 4 | 6 | 6 | 3 | 1 | 1 | 9 | 2 | 180 | 185 |  |
| K.RGILTLK.Y | N | 29.35 | 799.5280 | 7 | 4.0 | 400.7729 | 2 | 41.36 | 6 | 11572 | Ana\_05.raw |  |  |  |  |  | 1.41E8 |  | 9.14E7 |  |  | 2 | 0 | 0 | 0 | 0 | 0 | 1 | 0 | 1 | 0 | 0 | 64 | 70 |  |
| R.M(+15.99)QKEITALAPSTM(+15.99)K.I | N | 25.56 | 1579.7949 | 14 | 0.7 | 527.6060 | 3 | 38.99 | 3 | 10840 | Ana\_02.raw |  |  | 2.42E6 |  |  |  |  |  |  |  | 1 | 0 | 0 | 1 | 0 | 0 | 0 | 0 | 0 | 0 | 0 | 315 | 328 | Oxidation (M) |
| R.C(+57.02)PETLFQPSFIGMESAGIHETTYNSIM(+15.99)K.C | N | 25.32 | 3203.4614 | 28 | -1.1 | 1602.7362 | 2 | 126.87 | 2 | 35153 | Ana\_01.raw |  | 4.47E5 |  |  |  |  |  |  |  |  | 1 | 0 | 1 | 0 | 0 | 0 | 0 | 0 | 0 | 0 | 0 | 259 | 286 | Carbamidomethylation; Oxidation (M) |
| R.GILTLK.Y | N | 25.30 | 643.4268 | 6 | 5.7 | 322.7225 | 2 | 51.01 | 9 | 14360 | Ana\_08.raw |  | 1.76E8 | 1.95E8 |  |  | 1.81E8 | 1.2E8 |  | 2.57E2 |  | 6 | 0 | 2 | 1 | 0 | 0 | 1 | 1 | 0 | 1 | 0 | 65 | 70 |  |
| total 38 peptides |  |  |  |  |  |  |  |  |  |  |  |  |  |  |  |  |  |  |  |  |  |  |  |  |  |  |  |  |  |  |  |  |  |  |  |
| --- | --- | --- | --- | --- | --- | --- | --- | --- | --- | --- | --- | --- | --- | --- | --- | --- | --- | --- | --- | --- | --- | --- | --- | --- | --- | --- | --- | --- | --- | --- | --- | --- | --- | --- | --- |

Best Unique PSM (Scan F3:23710, m/z=802.7147, z=3, RT=84.08, ppm=9.6):

sp|P63017|HSP7C\_MOUSE

back to list

  

| Protein Coverage
| Supporting Peptides
| Best Unique PSM
|

Protein Coverage:

Supporting Peptides:

| Peptide | Uniq | -10lgP | Mass | Length | ppm | m/z | z | RT | Fraction | Scan | Source File | Area 10 | Area 01 | Area 02 | Area 03 | Area 04 | Area 05 | Area 06 | Area 07 | Area 08 | Area 09 | #Spec | #Spec 10 | #Spec 01 | #Spec 02 | #Spec 03 | #Spec 04 | #Spec 05 | #Spec 06 | #Spec 07 | #Spec 08 | #Spec 09 | Start | End | PTM |
| --- | --- | --- | --- | --- | --- | --- | --- | --- | --- | --- | --- | --- | --- | --- | --- | --- | --- | --- | --- | --- | --- | --- | --- | --- | --- | --- | --- | --- | --- | --- | --- | --- | --- | --- | --- |
| K.SINPDEAVAYGAAVQAAILSGDK.S | Y | 145.50 | 2259.1382 | 23 | 2.9 | 1130.5796 | 2 | 130.48 | 3 | 35742 | Ana\_02.raw | 7.21E7 | 8.44E7 | 8.5E7 | 1.81E7 | 6.94E7 | 4.21E7 | 4.07E7 | 2.46E7 | 9.58E7 | 6.76E7 | 31 | 4 | 5 | 4 | 2 | 3 | 2 | 2 | 2 | 3 | 4 | 362 | 384 |  |
| K.STAGDTHLGGEDFDNR.M | N | 112.04 | 1690.7183 | 16 | 4.9 | 846.3705 | 2 | 45.17 | 2 | 12649 | Ana\_01.raw | 1.15E8 | 1.44E8 | 1.35E8 | 6.12E7 | 1.07E8 | 1E8 | 7.94E7 | 8.64E7 | 1.4E8 | 9.77E7 | 40 | 4 | 6 | 4 | 3 | 4 | 3 | 3 | 4 | 6 | 3 | 221 | 236 |  |
| K.NQTAEKEEFEHQQK.E | Y | 105.17 | 1744.8016 | 14 | 1.6 | 873.4095 | 2 | 21.33 | 3 | 5383 | Ana\_02.raw | 5.23E7 | 2.41E7 | 5.95E7 | 2.7E7 | 9.45E6 | 2.57E7 | 1.01E7 | 1.93E7 | 7.42E7 | 4.6E7 | 25 | 3 | 2 | 3 | 2 | 2 | 1 | 3 | 3 | 3 | 3 | 584 | 597 |  |
| R.TTPSYVAFTDTER.L | N | 99.69 | 1486.6940 | 13 | 5.3 | 744.3582 | 2 | 69.89 | 1 | 19802 | ANa\_10.raw | 1.62E8 | 1.76E8 | 1.5E8 | 6.46E7 | 1.23E8 | 1.06E8 | 7.24E7 | 8.59E7 | 2.27E8 | 1.42E8 | 51 | 5 | 5 | 6 | 3 | 7 | 6 | 2 | 6 | 6 | 5 | 37 | 49 |  |
| R.IINEPTAAAIAYGLDK.K | N | 98.74 | 1658.8879 | 16 | 2.1 | 830.4530 | 2 | 98.36 | 6 | 27817 | Ana\_05.raw | 1.5E8 | 1.38E8 | 1.16E8 | 1.71E7 | 1.84E7 | 6.76E7 | 5.7E7 | 5.77E7 | 1.85E8 | 9.55E7 | 49 | 5 | 6 | 6 | 2 | 5 | 4 | 3 | 6 | 7 | 5 | 172 | 187 |  |
| R.IINEPTAAAIAYGLDKK.V | N | 95.42 | 1786.9828 | 17 | 0.5 | 596.6685 | 3 | 86.45 | 3 | 24333 | Ana\_02.raw | 2.7E7 | 7.31E5 | 1.63E7 | 5.34E6 | 1.79E7 | 1.63E7 | 1.26E7 | 8.11E6 | 5.92E7 | 2.04E7 | 17 | 2 | 1 | 1 | 1 | 1 | 2 | 2 | 2 | 3 | 2 | 172 | 188 |  |
| K.SQIHDIVLVGGSTR.I | Y | 95.41 | 1480.7998 | 14 | 1.0 | 494.6077 | 3 | 59.59 | 3 | 16954 | Ana\_02.raw | 2.85E7 | 4.09E7 | 2.44E7 | 1.04E7 | 2.06E7 | 1.98E7 | 3.64E6 |  | 6.48E7 | 3.29E7 | 13 | 2 | 2 | 1 | 1 | 1 | 1 | 1 | 0 | 2 | 2 | 329 | 342 |  |
| K.NQVAM(+15.99)NPTNTVFDAK.R | Y | 94.56 | 1664.7828 | 15 | 5.9 | 833.4036 | 2 | 58.68 | 1 | 16583 | ANa\_10.raw | 4.66E7 | 4.92E7 | 4.56E7 | 1.89E7 | 3.69E7 | 3.54E7 | 2.75E7 | 2.71E7 | 6.53E7 | 2.98E7 | 23 | 3 | 3 | 2 | 2 | 2 | 2 | 2 | 2 | 3 | 2 | 57 | 71 | Oxidation (M) |
| K.TVTNAVVTVPAYFNDSQR.Q | Y | 94.38 | 1980.9905 | 18 | 3.7 | 991.5062 | 2 | 90.95 | 9 | 25803 | Ana\_08.raw | 2E7 | 1.53E7 | 1.8E7 | 7.66E6 | 9.42E6 | 1.13E7 | 1.14E7 | 1.07E7 | 3.78E7 | 7.63E6 | 26 | 3 | 4 | 2 | 3 | 2 | 3 | 2 | 3 | 2 | 2 | 138 | 155 |  |
| K.VEIIANDQGNR.T | N | 92.33 | 1227.6207 | 11 | 5.5 | 614.8210 | 2 | 37.88 | 1 | 10363 | ANa\_10.raw | 1.27E8 | 1.28E8 | 1.27E8 | 5.59E7 | 1.2E8 | 1.04E8 | 8.8E7 | 8.5E7 | 1.27E8 | 1.01E8 | 31 | 3 | 3 | 3 | 3 | 3 | 3 | 3 | 4 | 3 | 3 | 26 | 36 |  |
| K.DAGTIAGLNVLR.I | Y | 88.09 | 1198.6670 | 12 | 3.1 | 600.3427 | 2 | 89.33 | 6 | 25409 | Ana\_05.raw | 6.82E7 | 8.75E7 | 8.04E7 | 2.29E7 | 5.71E7 | 4.3E7 | 3.03E7 | 3.19E7 | 1.38E8 | 6.99E7 | 48 | 5 | 6 | 7 | 2 | 5 | 5 | 2 | 4 | 7 | 5 | 160 | 171 |  |
| R.M(+15.99)VNHFIAEFK.R | Y | 87.16 | 1250.6117 | 10 | 5.0 | 626.3162 | 2 | 61.69 | 3 | 17573 | Ana\_02.raw | 2.99E7 | 1.26E7 | 3.33E7 | 2.55E7 | 4.54E7 | 3.65E7 |  | 3.41E7 | 4.6E7 | 1.13E7 | 17 | 2 | 1 | 2 | 2 | 2 | 2 | 0 | 2 | 2 | 2 | 237 | 246 | Oxidation (M) |
| R.FEELNADLFR.G | N | 87.12 | 1252.6088 | 10 | 2.3 | 627.3131 | 2 | 97.91 | 6 | 27699 | Ana\_05.raw | 5.57E7 | 4.44E7 | 6.22E7 | 2.2E7 | 4.03E7 | 3.82E7 | 2.75E7 | 2.99E7 | 7.88E7 | 4.35E7 | 39 | 5 | 4 | 5 | 2 | 4 | 2 | 3 | 5 | 4 | 5 | 302 | 311 |  |
| K.SFYPEEVSSM(+15.99)VLTK.M | Y | 82.36 | 1631.7753 | 14 | -2.2 | 816.8931 | 2 | 89.20 | 3 | 25055 | Ana\_02.raw | 4.48E7 | 4.81E7 | 3.68E7 | 1.49E7 | 2.89E7 | 2.59E7 | 2.03E7 | 2.67E7 | 8.24E7 | 1.97E7 | 37 | 4 | 4 | 4 | 4 | 3 | 3 | 4 | 4 | 4 | 3 | 113 | 126 | Oxidation (M) |
| K.HWPFM(+15.99)VVNDAGRPK.V | Y | 78.80 | 1668.8195 | 14 | 7.3 | 557.2845 | 3 | 62.17 | 9 | 17701 | Ana\_08.raw |  |  | 1.85E6 | 1.29E6 |  |  | 6.4E6 | 2.45E6 | 1.23E7 | 3.83E6 | 8 | 0 | 0 | 1 | 1 | 0 | 0 | 2 | 2 | 1 | 1 | 89 | 102 | Oxidation (M) |
| R.ARFEELNADLFR.G | N | 78.55 | 1479.7469 | 12 | 0.6 | 494.2566 | 3 | 86.00 | 9 | 24462 | Ana\_08.raw | 3.8E5 |  |  | 4.24E5 |  | 2.1E6 | 1.72E6 | 1.02E6 | 1.11E7 | 1.35E6 | 8 | 1 | 0 | 0 | 1 | 0 | 1 | 1 | 1 | 2 | 1 | 300 | 311 |  |
| R.FDDAVVQSDM(+15.99)K.H | Y | 66.82 | 1269.5547 | 11 | 3.2 | 635.7867 | 2 | 42.36 | 1 | 11741 | ANa\_10.raw | 7.85E6 | 1.21E7 | 1.03E7 | 8.91E5 | 8.73E6 | 7.81E6 | 7.37E6 | 6.06E6 |  | 6.45E6 | 9 | 1 | 1 | 1 | 1 | 1 | 1 | 1 | 1 | 0 | 1 | 78 | 88 | Oxidation (M) |
| K.FELTGIPPAPR.G | Y | 64.46 | 1196.6553 | 11 | 8.5 | 599.3400 | 2 | 84.73 | 7 | 23881 | Ana\_06.raw | 5.37E6 | 1.16E7 | 5.77E6 | 8.61E6 | 1.27E7 | 1.13E7 | 5.55E6 | 6.23E6 | 2.49E7 | 4.85E6 | 53 | 4 | 6 | 6 | 6 | 5 | 5 | 5 | 4 | 7 | 5 | 459 | 469 |  |
| R.RFDDAVVQSDM(+15.99)K.H | Y | 62.78 | 1425.6558 | 12 | 2.7 | 476.2271 | 3 | 40.03 | 4 | 10838 | Ana\_03.raw | 1.99E7 | 2.26E7 | 2.14E7 | 1.85E7 | 3.86E7 | 1.53E7 | 3.23E7 | 3.36E7 | 2.16E7 | 1.47E7 | 16 | 1 | 2 | 1 | 2 | 2 | 1 | 2 | 2 | 1 | 2 | 77 | 88 | Oxidation (M) |
| K.M(+15.99)KEIAEAYLGK.T | Y | 59.10 | 1267.6482 | 11 | 8.4 | 634.8367 | 2 | 44.60 | 9 | 12407 | Ana\_08.raw |  | 1.15E6 |  |  | 3.98E6 |  | 4.47E6 |  | 3.07E6 |  | 4 | 0 | 1 | 0 | 0 | 1 | 0 | 1 | 0 | 1 | 0 | 127 | 137 | Oxidation (M) |
| K.SFYPEEVSSMVLTK.M | Y | 58.68 | 1615.7803 | 14 | 6.1 | 808.9023 | 2 | 103.88 | 9 | 29269 | Ana\_08.raw |  |  |  |  |  |  |  |  | 1.32E7 |  | 1 | 0 | 0 | 0 | 0 | 0 | 0 | 0 | 0 | 1 | 0 | 113 | 126 |  |
| K.C(+57.02)NEIISWLDK.N | Y | 57.03 | 1276.6122 | 10 | 3.0 | 639.3153 | 2 | 131.94 | 5 | 36314 | Ana\_04.raw |  | 0 |  |  | 4.33E5 | 6.88E5 | 7.96E4 | 3.57E5 |  |  | 5 | 0 | 1 | 0 | 0 | 1 | 1 | 1 | 1 | 0 | 0 | 574 | 583 | Carbamidomethylation |
| K.EIAEAYLGK.T | Y | 55.78 | 992.5178 | 9 | 4.6 | 497.2685 | 2 | 56.73 | 8 | 15809 | Ana\_07.raw | 3.92E7 |  | 4.84E7 | 1.33E7 |  |  |  | 2.89E7 | 6.21E7 |  | 5 | 1 | 0 | 1 | 1 | 0 | 0 | 0 | 1 | 1 | 0 | 129 | 137 |  |
| K.ITITNDK.G | N | 55.47 | 803.4388 | 7 | 4.4 | 402.7285 | 2 | 24.39 | 1 | 6231 | ANa\_10.raw | 1.08E7 |  |  | 2.48E7 | 4.24E7 | 4.79E7 | 3.85E7 | 2.79E7 |  | 3.03E7 | 7 | 1 | 0 | 0 | 1 | 1 | 1 | 1 | 1 | 0 | 1 | 501 | 507 |  |
| K.VC(+57.02)NPIITK.L | Y | 55.45 | 943.5161 | 8 | 1.7 | 472.7661 | 2 | 41.13 | 4 | 11170 | Ana\_03.raw | 4.46E7 | 3.53E7 | 1.05E7 | 1.93E7 | 3.41E7 |  |  |  | 3.5E7 | 3.66E7 | 7 | 1 | 1 | 1 | 1 | 1 | 0 | 0 | 0 | 1 | 1 | 602 | 609 | Carbamidomethylation |
| K.NSLESYAFNM(+15.99)K.A | Y | 53.52 | 1318.5863 | 11 | 4.1 | 660.3031 | 2 | 69.27 | 6 | 19874 | Ana\_05.raw | 1.24E7 | 9.93E6 | 9.61E6 | 4.81E6 | 1.19E7 | 9.04E6 | 7.83E6 | 6.82E6 | 1.53E6 | 8.33E6 | 10 | 1 | 1 | 1 | 1 | 1 | 1 | 1 | 1 | 1 | 1 | 540 | 550 | Oxidation (M) |
| K.LLQDFFNGK.E | N | 50.24 | 1080.5603 | 9 | 2.1 | 541.2886 | 2 | 91.11 | 8 | 25457 | Ana\_07.raw |  | 8.07E6 | 9.57E6 | 3.38E6 | 6.29E6 | 1.15E6 | 5.38E6 | 3.94E6 |  | 5.69E6 | 8 | 0 | 1 | 1 | 1 | 1 | 1 | 1 | 1 | 0 | 1 | 349 | 357 |  |
| K.EEFEHQQK.E | Y | 43.56 | 1073.4778 | 8 | 1.7 | 537.7471 | 2 | 17.87 | 5 | 4273 | Ana\_04.raw |  |  |  |  | 1.18E6 |  |  |  |  |  | 1 | 0 | 0 | 0 | 0 | 1 | 0 | 0 | 0 | 0 | 0 | 590 | 597 |  |
| R.LSKEDIER.M | Y | 43.34 | 988.5189 | 8 | 9.1 | 495.2712 | 2 | 26.20 | 10 | 6873 | Ana\_09.raw |  | 2.01E7 |  |  |  | 1.78E6 |  |  |  | 9.82E6 | 5 | 0 | 2 | 0 | 0 | 0 | 1 | 0 | 0 | 0 | 2 | 510 | 517 |  |
| R.GTLDPVEK.A | Y | 43.32 | 857.4494 | 8 | 3.7 | 429.7336 | 2 | 33.45 | 8 | 8905 | Ana\_07.raw | 8.32E7 | 9.01E7 |  | 4.3E7 | 7.39E7 | 7.5E7 | 6.29E7 | 5.22E7 | 8.98E7 | 7.73E7 | 11 | 1 | 1 | 1 | 1 | 2 | 1 | 1 | 1 | 1 | 1 | 312 | 319 |  |
| K.VQVEYK.G | N | 40.81 | 764.4069 | 6 | 1.4 | 383.2112 | 2 | 24.93 | 3 | 6541 | Ana\_02.raw | 3.23E7 | 4.68E7 | 5.56E7 | 2.79E7 | 4.3E7 | 5.09E7 | 3.53E7 | 2.75E7 |  | 3.07E7 | 10 | 1 | 1 | 1 | 1 | 1 | 1 | 1 | 2 | 0 | 1 | 103 | 108 |  |
| K.RNTTIPTK.Q | N | 40.62 | 929.5294 | 8 | 1.0 | 465.7724 | 2 | 18.51 | 3 | 4438 | Ana\_02.raw |  |  | 3.47E6 | 1.52E6 | 2.62E6 | 3.09E6 | 2.5E6 | 1.67E6 |  | 1.8E6 | 7 | 0 | 0 | 1 | 1 | 1 | 1 | 1 | 1 | 0 | 1 | 416 | 423 |  |
| R.MVNHFIAEFK.R | Y | 36.97 | 1234.6168 | 10 | 4.9 | 618.3187 | 2 | 69.95 | 10 | 19810 | Ana\_09.raw |  |  |  |  |  | 9.64E5 |  |  |  | 3.09E6 | 3 | 0 | 0 | 0 | 0 | 0 | 1 | 0 | 0 | 0 | 2 | 237 | 246 |  |
| K.DNNLLGK.F | N | 35.26 | 772.4079 | 7 | 3.2 | 387.2125 | 2 | 30.19 | 5 | 8155 | Ana\_04.raw |  |  | 3.03E7 |  | 2.51E7 |  | 1.95E7 |  |  |  | 3 | 0 | 0 | 1 | 0 | 1 | 0 | 1 | 0 | 0 | 0 | 452 | 458 |  |
| R.LIGDAAK.N | N | 28.78 | 686.3962 | 7 | 1.5 | 344.2059 | 2 | 21.83 | 4 | 5454 | Ana\_03.raw |  |  |  | 3.57E7 |  |  |  |  |  |  | 1 | 0 | 0 | 0 | 1 | 0 | 0 | 0 | 0 | 0 | 0 | 50 | 56 |  |
| K.RLIGR.R | N | 26.60 | 613.4023 | 5 | 0.6 | 307.7086 | 2 | 18.67 | 4 | 4488 | Ana\_03.raw |  |  |  | 4.8E6 |  |  |  |  |  |  | 1 | 0 | 0 | 0 | 1 | 0 | 0 | 0 | 0 | 0 | 0 | 72 | 76 |  |
| K.NQVAMNPTNTVFDAK.R | Y | 25.04 | 1648.7878 | 15 | 1.8 | 825.4026 | 2 | 71.42 | 1 | 20230 | ANa\_10.raw | 1.48E6 |  |  |  |  |  |  |  |  |  | 1 | 1 | 0 | 0 | 0 | 0 | 0 | 0 | 0 | 0 | 0 | 57 | 71 |  |
| R.NTTIPTK.Q | N | 23.26 | 773.4283 | 7 | 0.8 | 387.7218 | 2 | 21.30 | 4 | 5291 | Ana\_03.raw |  |  |  | 3.64E7 |  |  |  |  |  |  | 1 | 0 | 0 | 0 | 1 | 0 | 0 | 0 | 0 | 0 | 0 | 417 | 423 |  |
| total 38 peptides |  |  |  |  |  |  |  |  |  |  |  |  |  |  |  |  |  |  |  |  |  |  |  |  |  |  |  |  |  |  |  |  |  |  |  |
| --- | --- | --- | --- | --- | --- | --- | --- | --- | --- | --- | --- | --- | --- | --- | --- | --- | --- | --- | --- | --- | --- | --- | --- | --- | --- | --- | --- | --- | --- | --- | --- | --- | --- | --- | --- |

Best Unique PSM (Scan F3:35742, m/z=1130.5796, z=2, RT=130.48, ppm=2.9):

sp|P19001|K1C19\_MOUSE

back to list

  

| Protein Coverage
| Supporting Peptides
| Best Unique PSM
|

Protein Coverage:

Supporting Peptides:

| Peptide | Uniq | -10lgP | Mass | Length | ppm | m/z | z | RT | Fraction | Scan | Source File | Area 10 | Area 01 | Area 02 | Area 03 | Area 04 | Area 05 | Area 06 | Area 07 | Area 08 | Area 09 | #Spec | #Spec 10 | #Spec 01 | #Spec 02 | #Spec 03 | #Spec 04 | #Spec 05 | #Spec 06 | #Spec 07 | #Spec 08 | #Spec 09 | Start | End | PTM |
| --- | --- | --- | --- | --- | --- | --- | --- | --- | --- | --- | --- | --- | --- | --- | --- | --- | --- | --- | --- | --- | --- | --- | --- | --- | --- | --- | --- | --- | --- | --- | --- | --- | --- | --- | --- |
| R.TDLEM(+15.99)QIESLKEELAYLK.K | Y | 122.56 | 2168.0923 | 18 | 1.8 | 1085.0554 | 2 | 131.70 | 3 | 36080 | Ana\_02.raw | 6.36E7 | 2.57E7 | 1.37E7 | 4.9E6 | 2.14E7 | 2.17E7 | 1.72E7 | 1.1E7 | 3.19E7 | 1.08E7 | 51 | 3 | 7 | 8 | 2 | 9 | 3 | 1 | 7 | 6 | 5 | 201 | 218 | Oxidation (M) |
| R.YGVQLSQIQSVISGFEAQLSDVR.A | Y | 119.77 | 2523.2969 | 23 | 3.4 | 842.1091 | 3 | 138.78 | 9 | 38309 | Ana\_08.raw | 7.32E6 | 4.05E6 | 2.71E6 |  | 9.77E6 | 3.45E7 | 2.59E6 | 1.42E6 | 2.85E7 | 1.93E7 | 18 | 2 | 3 | 1 | 0 | 2 | 2 | 2 | 2 | 2 | 2 | 334 | 356 |  |
| R.SLLEGQEAHYNNLPTPK.A | Y | 118.17 | 1909.9534 | 17 | 2.7 | 955.9866 | 2 | 68.37 | 3 | 19441 | Ana\_02.raw | 8.49E7 | 8.98E7 | 5.82E7 | 3.76E7 | 6.6E7 | 7.67E7 | 5.03E7 | 4.23E7 | 1.55E8 | 5.7E7 | 29 | 4 | 4 | 2 | 2 | 3 | 3 | 3 | 3 | 4 | 1 | 385 | 401 |  |
| R.TDLEM(+15.99)QIESLKEELAYLKK.N | Y | 103.97 | 2296.1873 | 19 | 3.6 | 575.0562 | 4 | 128.49 | 6 | 35695 | Ana\_05.raw | 2.75E7 | 2.2E6 |  |  | 1.15E7 | 2.92E7 | 5.09E6 |  | 2.72E7 | 1.75E7 | 13 | 2 | 2 | 0 | 0 | 2 | 2 | 2 | 0 | 1 | 2 | 201 | 219 | Oxidation (M) |
| R.IEELNTQVAVHSEQIQISK.T | Y | 103.01 | 2165.1328 | 19 | 4.1 | 1083.5781 | 2 | 73.75 | 9 | 21031 | Ana\_08.raw | 6.53E5 |  |  |  |  |  |  |  | 8.82E6 | 0 | 4 | 1 | 0 | 0 | 0 | 0 | 0 | 0 | 0 | 2 | 1 | 278 | 296 |  |
| R.TDLEMQIESLKEELAYLK.K | Y | 98.60 | 2152.0972 | 18 | 4.7 | 718.3763 | 3 | 131.95 | 6 | 36676 | Ana\_05.raw |  | 7.66E6 |  | 2.45E6 |  | 6.42E6 | 1.77E6 |  | 7.95E6 | 8.31E6 | 8 | 0 | 1 | 0 | 1 | 0 | 1 | 1 | 0 | 2 | 2 | 201 | 218 |  |
| R.TLQGLEIELQSQLSM(+15.99)K.A | N | 97.22 | 1832.9553 | 16 | 2.5 | 611.9939 | 3 | 122.92 | 6 | 34244 | Ana\_05.raw | 2.46E7 | 1.07E8 | 1.14E8 | 2.48E7 | 7.6E7 | 1.16E8 | 7.14E7 | 4.17E7 | 1.4E8 | 6.1E7 | 38 | 3 | 4 | 4 | 4 | 4 | 4 | 4 | 3 | 4 | 4 | 305 | 320 | Oxidation (M) |
| R.GGSFSGTLAVSDGLLSGNEK.I | Y | 96.84 | 1894.9272 | 20 | 6.2 | 948.4768 | 2 | 97.53 | 2 | 27482 | Ana\_01.raw | 1.87E7 | 1.68E7 |  | 1E6 | 8.87E6 | 1.56E6 | 3.62E6 | 3.1E5 | 3.85E7 | 7.66E6 | 14 | 3 | 1 | 0 | 1 | 1 | 2 | 1 | 1 | 3 | 1 | 65 | 84 |  |
| R.FVTSSSGSYGGVR.G | Y | 92.91 | 1302.6204 | 13 | 3.5 | 652.3198 | 2 | 47.29 | 6 | 13380 | Ana\_05.raw | 3.22E8 | 4.13E8 | 2.51E8 | 1.3E8 | 3E8 | 3.09E8 | 2.03E8 | 1.78E8 | 4.2E8 | 2.51E8 | 51 | 5 | 7 | 3 | 3 | 6 | 5 | 4 | 7 | 6 | 5 | 52 | 64 |  |
| K.SRLEQEIATYR.S | Y | 91.62 | 1364.7048 | 11 | 5.6 | 683.3635 | 2 | 61.29 | 1 | 17362 | ANa\_10.raw | 7.29E7 | 8.38E7 | 5.35E7 | 2.48E7 | 5.32E7 | 5.95E7 | 3.66E7 | 3.45E7 | 1.23E8 | 5.4E7 | 38 | 3 | 4 | 4 | 4 | 4 | 4 | 4 | 4 | 5 | 2 | 374 | 384 |  |
| R.QTSAM(+15.99)SSFGGTGGGSVR.I | Y | 91.05 | 1601.7103 | 17 | 3.9 | 801.8656 | 2 | 37.65 | 6 | 10424 | Ana\_05.raw | 4.25E7 | 5.72E7 | 4.17E7 | 2.67E7 | 6.31E7 | 6.22E7 | 4.16E7 | 3.87E7 | 1.11E7 | 1.27E7 | 32 | 4 | 3 | 3 | 3 | 4 | 5 | 3 | 3 | 2 | 2 | 8 | 24 | Oxidation (M) |
| K.NHEEEITALR.S | Y | 90.24 | 1210.5942 | 10 | 0.7 | 606.3048 | 2 | 38.17 | 3 | 10595 | Ana\_02.raw | 1.39E8 | 1.63E8 | 1.41E8 | 6.52E7 | 1.44E8 | 1.47E8 | 9.61E7 | 8.3E7 | 4.52E7 | 1.27E8 | 35 | 4 | 4 | 3 | 2 | 4 | 3 | 3 | 3 | 5 | 4 | 220 | 229 |  |
| K.AALEGTLAETEAR.Y | Y | 87.11 | 1330.6729 | 13 | 1.1 | 666.3444 | 2 | 55.76 | 3 | 15828 | Ana\_02.raw | 1.81E8 | 2.95E8 | 2.11E8 | 7.03E7 | 1.59E8 | 2.14E8 | 1.29E8 | 1.22E8 | 3.06E8 | 1.83E8 | 45 | 6 | 5 | 4 | 3 | 5 | 4 | 3 | 4 | 5 | 6 | 321 | 333 |  |
| R.TKFETEHALR.L | Y | 80.06 | 1230.6356 | 10 | 3.1 | 616.3270 | 2 | 27.97 | 6 | 7494 | Ana\_05.raw | 1.21E8 | 3.06E8 | 2.2E8 | 1.13E8 | 2.06E8 | 2.35E8 | 1.43E8 | 1.24E8 | 1.05E8 | 1.66E8 | 46 | 4 | 5 | 4 | 5 | 5 | 3 | 4 | 6 | 6 | 4 | 170 | 179 |  |
| R.APSIHGGSGGR.G | Y | 74.03 | 994.4944 | 11 | 4.3 | 498.2566 | 2 | 17.35 | 5 | 4096 | Ana\_04.raw |  |  |  | 5E7 | 1.46E8 | 8.34E7 | 9.11E7 | 5.82E7 |  |  | 13 | 0 | 0 | 0 | 2 | 4 | 2 | 2 | 3 | 0 | 0 | 33 | 43 |  |
| K.DAEATYLAR.I | Y | 72.50 | 1008.4876 | 9 | 3.2 | 505.2527 | 2 | 45.40 | 6 | 12818 | Ana\_05.raw | 1.91E8 | 2.21E8 | 1.92E8 | 8.96E7 | 1.93E8 | 2.22E8 | 1.44E8 | 1.26E8 | 2.35E8 | 1.67E8 | 41 | 4 | 5 | 3 | 3 | 4 | 5 | 5 | 3 | 3 | 6 | 269 | 277 |  |
| K.KNHEEEITALR.S | Y | 70.39 | 1338.6891 | 11 | 4.9 | 447.2392 | 3 | 29.31 | 9 | 7677 | Ana\_08.raw | 8.7E6 | 1.04E7 | 1.33E6 | 6.23E5 | 1.05E7 | 8.12E6 | 7.49E6 | 3.64E6 | 1.95E7 | 8.07E6 | 10 | 1 | 1 | 1 | 1 | 1 | 1 | 1 | 1 | 1 | 1 | 219 | 229 |  |
| R.LEQEIATYR.S | N | 69.83 | 1121.5717 | 9 | 7.2 | 561.7971 | 2 | 47.45 | 1 | 13287 | ANa\_10.raw | 1.25E8 | 2.24E8 | 1.66E8 | 7.19E7 | 1.72E8 | 1.74E8 | 1.25E8 | 1.03E8 | 2.2E8 | 1.15E8 | 31 | 3 | 4 | 1 | 1 | 3 | 3 | 4 | 3 | 6 | 3 | 376 | 384 |  |
| R.TDLEM(+15.99)QIESLK.E | Y | 69.56 | 1321.6436 | 11 | 0.4 | 661.8293 | 2 | 89.25 | 3 | 25069 | Ana\_02.raw |  |  | 5.72E6 |  |  | 3.6E6 | 3.78E5 |  |  |  | 3 | 0 | 0 | 1 | 0 | 0 | 1 | 1 | 0 | 0 | 0 | 201 | 211 | Oxidation (M) |
| K.ITM(+15.99)QNLNDR.L | N | 68.69 | 1119.5343 | 9 | 3.9 | 560.7766 | 2 | 28.28 | 7 | 7444 | Ana\_06.raw | 7.98E7 | 1.25E8 | 1.19E8 | 6.08E7 | 1.19E8 | 1.53E8 | 8.61E7 | 6.1E7 | 3.75E7 | 2.42E7 | 19 | 2 | 2 | 2 | 2 | 2 | 3 | 1 | 1 | 2 | 2 | 85 | 93 | Oxidation (M) |
| R.DYNHYFK.T | Y | 67.57 | 985.4293 | 7 | 3.0 | 493.7234 | 2 | 46.05 | 6 | 13011 | Ana\_05.raw | 6.3E7 | 8.12E7 | 9.85E7 | 3.23E7 | 8.93E7 | 7.91E7 | 4.85E7 | 3.88E7 | 8.09E7 | 6.16E7 | 18 | 1 | 1 | 3 | 2 | 3 | 2 | 2 | 2 | 1 | 1 | 129 | 135 |  |
| K.ILGATIDNSK.I | Y | 66.18 | 1030.5658 | 10 | 4.6 | 516.2925 | 2 | 45.54 | 7 | 12672 | Ana\_06.raw | 1.06E8 | 1.51E8 | 1.19E8 | 5.39E7 | 1.15E8 | 1.27E8 | 8.39E7 | 7.63E7 | 4.06E7 | 8.8E7 | 31 | 4 | 6 | 3 | 2 | 3 | 2 | 2 | 3 | 4 | 2 | 144 | 153 |  |
| R.ALEQANGELEVK.I | Y | 65.34 | 1299.6670 | 12 | 6.5 | 650.8450 | 2 | 49.44 | 1 | 13864 | ANa\_10.raw | 2.13E7 | 2.23E7 |  | 5.5E6 |  | 1.68E7 | 1.43E7 | 7.07E6 | 3.14E7 | 1.09E7 | 11 | 2 | 2 | 0 | 1 | 0 | 1 | 1 | 1 | 2 | 1 | 103 | 114 |  |
| R.VLDELTLAR.T | N | 65.07 | 1028.5865 | 9 | 4.4 | 515.3028 | 2 | 74.87 | 7 | 21218 | Ana\_06.raw | 1.82E8 | 2.53E8 | 1.62E8 | 7.94E7 | 1.55E8 | 1.49E8 | 9.25E7 | 1.09E8 | 3.03E8 | 1.34E8 | 49 | 6 | 5 | 6 | 4 | 6 | 3 | 3 | 5 | 6 | 5 | 192 | 200 |  |
| K.IVLQIDNAR.L | N | 63.88 | 1040.5978 | 9 | 4.1 | 521.3083 | 2 | 60.33 | 6 | 17263 | Ana\_05.raw | 1.45E8 | 1.35E8 | 6.92E6 | 5.18E7 | 1.12E8 | 9.67E7 | 6E7 |  | 9.07E7 | 9.46E7 | 17 | 2 | 1 | 1 | 2 | 1 | 1 | 2 | 0 | 3 | 4 | 154 | 162 |  |
| R.IGSGGVFR.A | Y | 61.94 | 791.4290 | 8 | 2.7 | 396.7228 | 2 | 40.26 | 6 | 11237 | Ana\_05.raw | 1.47E8 | 1.96E8 | 1.26E8 | 7.61E7 | 1.55E8 | 1.78E8 | 1.21E8 | 9.55E7 |  | 1.07E8 | 12 | 1 | 1 | 1 | 2 | 1 | 1 | 2 | 2 | 0 | 1 | 25 | 32 |  |
| R.KDAEATYLAR.I | Y | 60.40 | 1136.5825 | 10 | 5.7 | 569.3018 | 2 | 33.94 | 6 | 9314 | Ana\_05.raw |  | 3E6 |  |  |  | 0 |  |  |  |  | 4 | 0 | 1 | 1 | 0 | 0 | 1 | 0 | 1 | 0 | 0 | 268 | 277 |  |
| K.FETEHALR.L | Y | 60.34 | 1001.4930 | 8 | 3.6 | 501.7556 | 2 | 26.95 | 9 | 6949 | Ana\_08.raw | 1.97E7 | 5.95E7 | 6.48E7 | 2.39E7 | 4.96E7 | 6.29E7 | 1.81E7 | 3.1E7 | 4.94E7 | 3.55E7 | 18 | 1 | 2 | 2 | 2 | 2 | 2 | 1 | 2 | 2 | 2 | 172 | 179 |  |
| R.LSVEADINGLR.R | Y | 59.56 | 1185.6353 | 11 | 4.6 | 593.8276 | 2 | 78.93 | 10 | 22250 | Ana\_09.raw | 3.42E6 | 2.54E7 | 2.77E7 | 1.48E5 | 1.36E7 | 1.76E7 | 1.32E7 | 1.1E7 | 1.91E7 | 4.51E6 | 15 | 1 | 1 | 2 | 1 | 2 | 2 | 2 | 1 | 2 | 1 | 180 | 190 |  |
| R.LAADDFR.T | N | 57.33 | 806.3922 | 7 | 4.2 | 404.2051 | 2 | 42.34 | 2 | 11777 | Ana\_01.raw | 2.24E8 | 2.78E8 | 2.22E8 | 1.32E8 | 2.61E8 | 2.64E8 | 1.84E8 | 1.78E8 | 2.86E8 | 1.96E8 | 49 | 5 | 5 | 6 | 5 | 6 | 2 | 5 | 6 | 5 | 4 | 163 | 169 |  |
| R.SQYEIMAEK.N | Y | 55.76 | 1097.5063 | 9 | 3.2 | 549.7622 | 2 | 46.59 | 2 | 13088 | Ana\_01.raw | 7.15E6 | 2.4E5 |  |  |  | 5.08E6 |  |  |  | 7.22E6 | 4 | 1 | 1 | 0 | 0 | 0 | 1 | 0 | 0 | 0 | 1 | 257 | 265 |  |
| K.TEVTDLR.R | Y | 55.32 | 832.4290 | 7 | 5.6 | 417.2241 | 2 | 32.09 | 2 | 8647 | Ana\_01.raw | 1.34E8 | 2.18E8 | 1.76E8 | 9.25E7 | 1.81E8 | 1.79E8 | 1.31E8 | 1.04E8 | 1.46E8 | 1.22E8 | 31 | 4 | 5 | 3 | 3 | 4 | 2 | 2 | 3 | 3 | 2 | 297 | 303 |  |
| R.SQYEIM(+15.99)AEK.N | Y | 54.39 | 1113.5012 | 9 | 4.4 | 557.7603 | 2 | 35.80 | 6 | 9863 | Ana\_05.raw | 1.52E8 | 1.9E8 | 1.41E8 | 5.84E7 | 1.09E8 | 1.57E8 | 1.04E8 | 9.82E7 | 1.53E8 | 1.11E8 | 33 | 5 | 3 | 3 | 2 | 3 | 3 | 3 | 3 | 4 | 4 | 257 | 265 | Oxidation (M) |
| R.TLQGLEIELQSQLSMK.A | N | 52.70 | 1816.9604 | 16 | 1.6 | 909.4890 | 2 | 127.27 | 9 | 35288 | Ana\_08.raw |  |  |  |  |  |  |  |  | 0 |  | 1 | 0 | 0 | 0 | 0 | 0 | 0 | 0 | 0 | 1 | 0 | 305 | 320 |  |
| R.LASYLDK.V | N | 49.65 | 808.4330 | 7 | 0.3 | 405.2239 | 2 | 36.28 | 3 | 10002 | Ana\_02.raw | 1.07E8 | 1.23E8 | 1.07E8 | 4.67E7 | 1.05E8 | 1.26E8 | 7.91E7 | 7.01E7 | 1.06E8 | 8.02E7 | 17 | 3 | 2 | 1 | 2 | 1 | 1 | 2 | 2 | 2 | 1 | 94 | 100 |  |
| K.TEVTDLRR.T | Y | 48.94 | 988.5302 | 8 | 4.8 | 495.2747 | 2 | 26.57 | 1 | 6929 | ANa\_10.raw | 8.93E6 |  | 3.34E7 | 9.01E6 | 5.71E7 | 4.82E7 |  | 2.12E7 | 5.36E7 |  | 7 | 1 | 0 | 1 | 1 | 1 | 1 | 0 | 1 | 1 | 0 | 297 | 304 |  |
| R.GVSVSSTR.F | Y | 45.36 | 791.4137 | 8 | 3.0 | 396.7153 | 2 | 19.22 | 5 | 4727 | Ana\_04.raw |  |  |  | 3.53E6 | 4.97E7 |  | 2.06E6 | 1.29E6 |  |  | 4 | 0 | 0 | 0 | 1 | 1 | 0 | 1 | 1 | 0 | 0 | 44 | 51 |  |
| K.TIEDLR.D | N | 40.52 | 745.3970 | 6 | 5.8 | 373.7079 | 2 | 32.91 | 2 | 8896 | Ana\_01.raw | 9.61E7 | 1.47E8 | 1.08E8 | 5.7E7 | 1.19E8 | 1.05E8 | 7.36E7 | 6.93E7 | 1.2E8 | 8.41E7 | 20 | 2 | 3 | 1 | 1 | 2 | 1 | 1 | 3 | 4 | 2 | 136 | 141 |  |
| K.TIEDLRDK.I | Y | 37.64 | 988.5189 | 8 | 3.3 | 495.2684 | 2 | 28.53 | 5 | 7645 | Ana\_04.raw |  | 9.96E6 | 1.72E8 | 1.08E7 | 8.61E6 | 1.4E8 | 6.05E6 |  | 7.55E6 | 8.08E6 | 12 | 0 | 1 | 2 | 2 | 2 | 2 | 1 | 0 | 1 | 1 | 136 | 143 |  |
| K.EELAYLK.K | N | 37.32 | 864.4592 | 7 | 1.0 | 433.2373 | 2 | 53.31 | 6 | 15201 | Ana\_05.raw | 2.33E6 | 5.1E6 | 4.4E6 | 3.95E5 | 4.54E6 | 5.67E6 | 5.16E6 |  | 4.69E6 |  | 8 | 1 | 1 | 1 | 1 | 1 | 1 | 1 | 0 | 1 | 0 | 212 | 218 |  |
| M.TSYSYR.Q | Y | 33.16 | 775.3500 | 6 | 4.7 | 388.6841 | 2 | 23.81 | 10 | 6101 | Ana\_09.raw |  |  | 4.03E6 |  | 3.38E6 |  |  |  |  | 2.58E6 | 3 | 0 | 0 | 1 | 0 | 1 | 0 | 0 | 0 | 0 | 1 | 2 | 7 |  |
| K.QLMDIK.S | Y | 31.54 | 746.3997 | 6 | -8.0 | 374.2041 | 2 | 37.75 | 1 | 10321 | ANa\_10.raw | 1.37E6 |  |  |  |  |  |  |  |  |  | 1 | 1 | 0 | 0 | 0 | 0 | 0 | 0 | 0 | 0 | 0 | 368 | 373 |  |
| K.ILSEMR.S | N | 30.39 | 747.3949 | 6 | 2.3 | 374.7056 | 2 | 29.71 | 9 | 7791 | Ana\_08.raw |  |  |  |  |  |  |  |  | 3.76E6 |  | 1 | 0 | 0 | 0 | 0 | 0 | 0 | 0 | 0 | 1 | 0 | 251 | 256 |  |
| K.ILSEM(+15.99)R.S | N | 27.54 | 763.3898 | 6 | 1.7 | 382.7029 | 2 | 20.48 | 3 | 5099 | Ana\_02.raw |  |  | 2.76E7 | 2.52E7 | 4.34E7 | 4.55E7 | 4.24E7 |  |  |  | 5 | 0 | 0 | 1 | 1 | 1 | 1 | 1 | 0 | 0 | 0 | 251 | 256 | Oxidation (M) |
| K.QLM(+15.99)DIK.S | Y | 22.95 | 762.3946 | 6 | -0.1 | 382.2045 | 2 | 22.61 | 3 | 5796 | Ana\_02.raw |  |  | 6.12E6 |  |  |  |  |  |  |  | 1 | 0 | 0 | 1 | 0 | 0 | 0 | 0 | 0 | 0 | 0 | 368 | 373 | Oxidation (M) |
| total 45 peptides |  |  |  |  |  |  |  |  |  |  |  |  |  |  |  |  |  |  |  |  |  |  |  |  |  |  |  |  |  |  |  |  |  |  |  |
| --- | --- | --- | --- | --- | --- | --- | --- | --- | --- | --- | --- | --- | --- | --- | --- | --- | --- | --- | --- | --- | --- | --- | --- | --- | --- | --- | --- | --- | --- | --- | --- | --- | --- | --- | --- |

Best Unique PSM (Scan F3:36080, m/z=1085.0554, z=2, RT=131.70, ppm=1.8):

sp|Q6URW6|MYH14\_MOUSE

back to list

  

| Protein Coverage
| Supporting Peptides
| Best Unique PSM
|

Protein Coverage:

Supporting Peptides:

| Peptide | Uniq | -10lgP | Mass | Length | ppm | m/z | z | RT | Fraction | Scan | Source File | Area 10 | Area 01 | Area 02 | Area 03 | Area 04 | Area 05 | Area 06 | Area 07 | Area 08 | Area 09 | #Spec | #Spec 10 | #Spec 01 | #Spec 02 | #Spec 03 | #Spec 04 | #Spec 05 | #Spec 06 | #Spec 07 | #Spec 08 | #Spec 09 | Start | End | PTM |
| --- | --- | --- | --- | --- | --- | --- | --- | --- | --- | --- | --- | --- | --- | --- | --- | --- | --- | --- | --- | --- | --- | --- | --- | --- | --- | --- | --- | --- | --- | --- | --- | --- | --- | --- | --- |
| R.DLGEELEALRGELEDTLDSTNAQQELR.S | Y | 119.66 | 3043.4580 | 27 | 4.6 | 1015.4980 | 3 | 136.05 | 6 | 37812 | Ana\_05.raw |  |  |  |  |  | 1E6 |  |  |  |  | 1 | 0 | 0 | 0 | 0 | 0 | 1 | 0 | 0 | 0 | 0 | 1164 | 1190 |  |
| K.VASRPGPVPEAAQSFLYAPR.T | Y | 99.91 | 2112.1116 | 20 | 1.9 | 705.0458 | 3 | 90.99 | 6 | 25851 | Ana\_05.raw |  |  |  |  |  | 4.92E5 |  |  |  |  | 1 | 0 | 0 | 0 | 0 | 0 | 1 | 0 | 0 | 0 | 0 | 13 | 32 |  |
| R.QLLQANPILEAFGNAK.T | N | 97.96 | 1725.9413 | 16 | 2.9 | 863.9804 | 2 | 125.10 | 6 | 34810 | Ana\_05.raw | 1.57E7 | 1.55E7 | 4.42E7 | 4.06E6 | 2.63E7 | 7.1E7 | 2.22E7 | 4.41E7 | 7.06E7 | 1.03E7 | 45 | 6 | 4 | 8 | 4 | 4 | 4 | 3 | 5 | 4 | 3 | 241 | 256 |  |
| K.AQTSAAGQGKEEAVK.Q | Y | 95.67 | 1473.7423 | 15 | 2.4 | 737.8802 | 2 | 18.65 | 6 | 4486 | Ana\_05.raw | 1.86E6 | 2.2E6 | 9.81E5 |  | 3.47E5 | 1.88E6 |  |  |  | 1.21E6 | 10 | 2 | 2 | 2 | 0 | 1 | 2 | 0 | 0 | 0 | 1 | 1652 | 1666 |  |
| R.EDQSILC(+57.02)TGESGAGK.T | N | 94.65 | 1550.6882 | 15 | 0.4 | 776.3517 | 2 | 46.91 | 3 | 13245 | Ana\_02.raw | 6.73E7 | 9.75E7 | 5.91E7 | 2.44E7 | 4.17E7 | 6.67E7 | 3.22E7 | 4.18E7 | 1.11E8 | 4.88E7 | 27 | 4 | 5 | 2 | 2 | 3 | 2 | 1 | 3 | 3 | 2 | 186 | 200 | Carbamidomethylation |
| R.EAQAGLAEAQEDLEAER.V | Y | 92.87 | 1828.8439 | 17 | -0.8 | 915.4285 | 2 | 79.02 | 2 | 22435 | Ana\_01.raw |  | 4.02E6 |  |  | 4.92E6 | 5.66E6 | 1.19E6 | 2.03E6 | 7.9E6 | 3.72E6 | 7 | 0 | 1 | 0 | 0 | 1 | 1 | 1 | 1 | 1 | 1 | 1136 | 1152 |  |
| K.DEC(+57.02)SFHIFYQLLGGAGEQLK.A | Y | 88.25 | 2311.0942 | 20 | 3.0 | 771.3743 | 3 | 131.97 | 6 | 36681 | Ana\_05.raw |  |  |  |  |  | 2.03E6 | 1.06E6 |  |  |  | 2 | 0 | 0 | 0 | 0 | 0 | 1 | 1 | 0 | 0 | 0 | 301 | 320 | Carbamidomethylation |
| R.LAEFSSQAAEEEEK.V | Y | 85.84 | 1566.7048 | 14 | -0.8 | 784.3591 | 2 | 55.10 | 4 | 15256 | Ana\_03.raw |  | 4.65E6 | 2.69E6 | 1.83E6 | 3.27E6 | 4.14E6 | 2.71E6 | 1.75E6 | 4.46E6 |  | 8 | 0 | 1 | 1 | 1 | 1 | 1 | 1 | 1 | 1 | 0 | 1029 | 1042 |  |
| R.AQAELESVSTALSEAESK.A | Y | 80.41 | 1848.8953 | 18 | 6.1 | 925.4605 | 2 | 97.38 | 1 | 27274 | ANa\_10.raw | 3.22E6 | 2.08E6 | 1.52E6 |  |  | 9.48E5 |  |  | 9.25E5 | 3.16E5 | 6 | 1 | 1 | 1 | 0 | 0 | 1 | 0 | 0 | 1 | 1 | 1306 | 1323 |  |
| K.EDELQAALLR.A | Y | 76.88 | 1156.6088 | 10 | 3.7 | 579.3138 | 2 | 82.26 | 6 | 23485 | Ana\_05.raw |  | 1.55E6 | 5.55E5 | 4.41E5 |  | 1.82E6 | 7.39E5 | 4.61E5 |  | 3.66E5 | 7 | 0 | 1 | 1 | 1 | 0 | 1 | 1 | 1 | 0 | 1 | 1110 | 1119 |  |
| K.ELSSAESQLHDTQELLQEETR.A | Y | 76.62 | 2442.1509 | 21 | 1.1 | 815.0585 | 3 | 98.76 | 5 | 27685 | Ana\_04.raw |  |  |  |  | 1.75E6 |  | 8.54E5 |  |  |  | 2 | 0 | 0 | 0 | 0 | 1 | 0 | 1 | 0 | 0 | 0 | 1330 | 1350 |  |
| K.DVEGIVGLEQVSSLGDGPPGGRPR.R | Y | 76.38 | 2390.2190 | 24 | 4.6 | 797.7506 | 3 | 104.15 | 6 | 29350 | Ana\_05.raw | 2.21E5 |  |  |  |  | 4.86E5 |  |  |  |  | 2 | 1 | 0 | 0 | 0 | 0 | 1 | 0 | 0 | 0 | 0 | 644 | 667 |  |
| R.NTDQATM(+15.99)PDNTAAQK.L | Y | 75.73 | 1620.7050 | 15 | 1.0 | 811.3606 | 2 | 20.07 | 6 | 4947 | Ana\_05.raw |  |  |  |  | 9.43E5 | 8.37E5 | 1.23E6 |  |  |  | 3 | 0 | 0 | 0 | 0 | 1 | 1 | 1 | 0 | 0 | 0 | 389 | 403 | Oxidation (M) |
| R.AEAELC(+57.02)SEAEETR.A | Y | 75.60 | 1493.6304 | 13 | 5.9 | 747.8268 | 2 | 44.73 | 2 | 12515 | Ana\_01.raw |  | 1.14E7 |  |  | 5.69E6 | 4.47E6 |  |  |  |  | 3 | 0 | 1 | 0 | 0 | 1 | 1 | 0 | 0 | 0 | 0 | 919 | 931 | Carbamidomethylation |
| R.KFDQLLAEEK.A | N | 75.17 | 1219.6448 | 10 | 2.0 | 610.8309 | 2 | 53.25 | 3 | 15096 | Ana\_02.raw | 7E7 | 8.1E7 | 7.96E7 | 3.21E7 | 6.01E7 | 7.54E7 | 3.96E7 | 3.99E7 | 1.26E8 | 5.97E7 | 37 | 4 | 4 | 3 | 3 | 4 | 4 | 4 | 3 | 4 | 4 | 1473 | 1482 |  |
| R.KQELELVVTELEAR.V | Y | 74.24 | 1655.9093 | 14 | 4.3 | 552.9794 | 3 | 106.47 | 7 | 29640 | Ana\_06.raw |  |  |  |  |  | 5.03E5 | 2.01E5 |  |  |  | 2 | 0 | 0 | 0 | 0 | 0 | 1 | 1 | 0 | 0 | 0 | 938 | 951 |  |
| R.TQVTELEDELTAAEDAK.L | Y | 73.90 | 1861.8793 | 17 | 4.0 | 931.9506 | 2 | 108.25 | 4 | 29639 | Ana\_03.raw |  | 1.72E6 |  | 5.95E5 |  | 2.65E5 |  | 4.42E5 |  |  | 6 | 0 | 2 | 0 | 2 | 0 | 1 | 0 | 1 | 0 | 0 | 1567 | 1583 |  |
| R.KAAEQAASDLR.T | Y | 72.59 | 1158.5992 | 11 | 4.9 | 580.3098 | 2 | 22.58 | 1 | 5627 | ANa\_10.raw | 2.29E6 | 3.31E6 | 1.7E6 | 9.38E5 | 7.26E5 | 3.09E6 | 1.29E6 |  | 1.64E6 | 1.04E6 | 9 | 1 | 1 | 1 | 1 | 1 | 1 | 1 | 0 | 1 | 1 | 1556 | 1566 |  |
| K.FDQLLAEEK.A | N | 71.45 | 1091.5498 | 9 | 2.9 | 546.7838 | 2 | 58.18 | 6 | 16636 | Ana\_05.raw | 3.53E7 | 3.9E7 | 4.13E7 | 1.48E7 | 2.94E7 | 4.16E7 | 2.35E7 | 2.34E7 | 5.36E7 | 2.71E7 | 11 | 1 | 1 | 1 | 1 | 1 | 1 | 1 | 1 | 2 | 1 | 1474 | 1482 |  |
| R.LLGLGVTDFSR.A | Y | 71.02 | 1176.6503 | 11 | 3.0 | 589.3342 | 2 | 97.96 | 6 | 27713 | Ana\_05.raw |  | 4.02E6 | 1.82E6 | 8.08E5 | 1.22E4 | 3.57E5 | 2.88E5 | 8.33E5 |  | 3.5E6 | 8 | 0 | 1 | 1 | 1 | 1 | 1 | 1 | 1 | 0 | 1 | 407 | 417 |  |
| R.AGVLAQLEEER.D | Y | 69.65 | 1213.6302 | 11 | 9.0 | 607.8279 | 2 | 80.00 | 6 | 22872 | Ana\_05.raw | 7.07E6 | 1.2E7 | 3.13E6 | 2.83E6 | 1.03E7 | 1.16E7 | 4.25E6 | 4.37E6 | 2.53E6 | 1.48E6 | 12 | 1 | 1 | 1 | 1 | 1 | 2 | 2 | 1 | 1 | 1 | 793 | 803 |  |
| R.ELFQETLESLR.V | Y | 69.39 | 1363.6982 | 11 | 4.5 | 682.8594 | 2 | 106.74 | 6 | 30039 | Ana\_05.raw | 6.65E6 | 2.45E6 | 1.17E7 | 3.17E6 | 3.86E6 | 1.32E7 | 7.05E6 | 5.97E6 | 2.09E7 | 7.93E5 | 14 | 1 | 1 | 2 | 1 | 1 | 2 | 1 | 1 | 2 | 2 | 347 | 357 |  |
| K.EQADFALEALAK.A | N | 67.21 | 1304.6611 | 12 | 3.3 | 653.3400 | 2 | 90.14 | 6 | 25623 | Ana\_05.raw | 1.03E8 | 1.07E8 | 1.05E8 | 3.27E7 | 6.53E7 | 7.22E7 | 4.35E7 | 5.16E7 | 1.8E8 | 8.13E7 | 42 | 4 | 5 | 4 | 3 | 4 | 4 | 4 | 3 | 6 | 5 | 438 | 449 |  |
| R.AEELLAQLGR.K | Y | 66.39 | 1098.6033 | 10 | 2.9 | 550.3105 | 2 | 78.37 | 6 | 22427 | Ana\_05.raw |  | 2.92E6 | 1.75E6 | 1.3E6 | 1.27E6 | 1.43E6 | 2.07E6 | 1.16E6 | 2.67E6 |  | 8 | 0 | 1 | 1 | 1 | 1 | 1 | 1 | 1 | 1 | 0 | 1099 | 1108 |  |
| R.ELEDVTESAESM(+15.99)NR.E | Y | 66.37 | 1624.6886 | 14 | 1.2 | 813.3525 | 2 | 49.78 | 6 | 14159 | Ana\_05.raw | 2.22E6 | 5.71E6 | 7.26E5 | 6.49E5 |  | 2.54E6 | 2.67E6 | 1.95E6 | 2.74E6 |  | 8 | 1 | 1 | 1 | 1 | 0 | 1 | 1 | 1 | 1 | 0 | 1927 | 1940 | Oxidation (M) |
| R.RQEEEAAVLEAGEEAR.R | Y | 64.83 | 1785.8492 | 16 | 0.0 | 596.2903 | 3 | 59.26 | 5 | 16839 | Ana\_04.raw |  |  | 6.61E5 |  | 6.28E6 |  |  |  |  |  | 2 | 0 | 0 | 1 | 0 | 1 | 0 | 0 | 0 | 0 | 0 | 1400 | 1415 |  |
| R.RLESQLQEVQGR.S | Y | 64.71 | 1441.7637 | 12 | 8.9 | 721.8955 | 2 | 39.88 | 9 | 10936 | Ana\_08.raw |  |  |  |  |  |  |  |  | 9.63E5 |  | 1 | 0 | 0 | 0 | 0 | 0 | 0 | 0 | 0 | 1 | 0 | 1277 | 1288 |  |
| R.LAEFSSQAAEEEEKVK.S | Y | 62.64 | 1793.8683 | 16 | 3.1 | 598.9652 | 3 | 60.42 | 1 | 17101 | ANa\_10.raw | 3.08E6 | 5.09E6 | 2.76E6 | 1.2E6 | 1.82E6 | 3.81E6 | 2.71E6 | 6.87E5 | 4.74E6 | 3.16E6 | 10 | 1 | 1 | 1 | 1 | 1 | 1 | 1 | 1 | 1 | 1 | 1029 | 1044 |  |
| R.DLGEELEALR.G | Y | 61.84 | 1143.5771 | 10 | 4.7 | 572.7985 | 2 | 101.01 | 6 | 28524 | Ana\_05.raw |  | 1.1E6 | 3.85E6 | 4.18E5 | 3E5 | 1.75E6 | 2.76E6 | 1.69E6 | 1.95E6 | 1.66E6 | 9 | 0 | 1 | 1 | 1 | 1 | 1 | 1 | 1 | 1 | 1 | 1164 | 1173 |  |
| K.VQELQQQSAR.E | Y | 61.43 | 1185.6102 | 10 | 4.8 | 593.8152 | 2 | 24.21 | 6 | 6300 | Ana\_05.raw |  |  | 4.39E6 | 3.22E6 | 5.62E6 | 8.44E6 | 4.68E6 | 2.98E6 |  |  | 6 | 0 | 0 | 1 | 1 | 1 | 1 | 1 | 1 | 0 | 0 | 885 | 894 |  |
| R.DAEVERDEER.K | Y | 59.75 | 1246.5425 | 10 | 3.3 | 624.2806 | 2 | 19.32 | 4 | 4686 | Ana\_03.raw |  |  |  | 2.63E6 | 4.09E6 |  | 6.5E6 | 8.06E5 |  |  | 5 | 0 | 0 | 0 | 1 | 1 | 0 | 2 | 1 | 0 | 0 | 1621 | 1630 |  |
| R.AELEALLSSK.D | Y | 58.54 | 1059.5812 | 10 | 2.5 | 530.7992 | 2 | 82.29 | 7 | 23235 | Ana\_06.raw |  |  |  | 1.78E6 | 3.03E6 | 3.26E6 | 1.62E6 | 1.48E6 | 4.03E6 |  | 6 | 0 | 0 | 0 | 1 | 1 | 1 | 1 | 1 | 1 | 0 | 1532 | 1541 |  |
| R.ALEAEAAGLR.E | Y | 58.18 | 999.5349 | 10 | 3.2 | 500.7763 | 2 | 48.86 | 5 | 13800 | Ana\_04.raw | 7.94E6 | 1.34E7 | 1.1E7 | 5.97E6 | 1.11E7 | 1.31E7 | 1.05E7 | 6.86E6 | 7.05E6 |  | 9 | 1 | 1 | 1 | 1 | 1 | 1 | 1 | 1 | 1 | 0 | 1361 | 1370 |  |
| K.LAQAEEQLEQESR.E | Y | 56.94 | 1529.7322 | 13 | -0.7 | 765.8729 | 2 | 51.90 | 4 | 14344 | Ana\_03.raw |  |  |  | 6.85E6 |  |  | 2.86E6 |  |  |  | 2 | 0 | 0 | 0 | 1 | 0 | 0 | 1 | 0 | 0 | 0 | 1844 | 1856 |  |
| R.NC(+57.02)AAYLK.L | N | 56.53 | 838.4007 | 7 | 3.5 | 420.2091 | 2 | 28.49 | 6 | 7650 | Ana\_05.raw | 3.89E7 |  | 4.95E7 | 2.34E7 | 4.81E7 | 7.1E7 | 3.25E7 | 2.9E7 | 3.32E7 | 3.28E7 | 11 | 1 | 0 | 1 | 2 | 1 | 1 | 1 | 2 | 1 | 1 | 843 | 849 | Carbamidomethylation |
| R.LQEELAASDR.A | Y | 53.25 | 1130.5568 | 10 | 0.5 | 566.2859 | 2 | 35.41 | 3 | 9739 | Ana\_02.raw |  | 1.69E7 | 1.47E7 | 3.81E6 | 8.65E6 |  | 9.68E6 | 8.65E6 | 3.87E6 | 1.02E7 | 8 | 0 | 1 | 1 | 1 | 1 | 0 | 1 | 1 | 1 | 1 | 1713 | 1722 |  |
| R.QLEEAEEEASR.A | Y | 52.66 | 1289.5735 | 11 | 2.6 | 645.7957 | 2 | 35.77 | 7 | 9728 | Ana\_06.raw |  |  |  |  |  | 1.85E5 | 4.42E5 | 5.03E4 |  |  | 4 | 0 | 0 | 0 | 0 | 0 | 1 | 2 | 1 | 0 | 0 | 1906 | 1916 |  |
| R.ALEEEQEAR.E | Y | 52.46 | 1073.4989 | 9 | 5.7 | 537.7598 | 2 | 23.77 | 2 | 6017 | Ana\_01.raw | 1.02E7 | 7.56E6 | 1.33E7 | 7.95E6 |  | 1.22E7 | 5.98E6 | 6.49E6 |  | 9.2E6 | 8 | 1 | 1 | 1 | 1 | 0 | 1 | 1 | 1 | 0 | 1 | 1512 | 1520 |  |
| R.LGEEDAGAR.A | Y | 50.23 | 916.4250 | 9 | 3.0 | 459.2212 | 2 | 22.41 | 7 | 5668 | Ana\_06.raw |  |  |  | 8.92E6 | 1.5E7 | 1.78E7 | 9.89E6 | 7.28E6 |  |  | 5 | 0 | 0 | 0 | 1 | 1 | 1 | 1 | 1 | 0 | 0 | 1822 | 1830 |  |
| R.EQM(+15.99)EEEVVAR.E | Y | 49.35 | 1234.5499 | 10 | -2.1 | 618.2809 | 2 | 27.80 | 3 | 7450 | Ana\_02.raw | 2.23E6 |  | 4.48E6 |  | 3.6E6 | 5.06E6 | 1.09E6 | 1.15E6 |  |  | 6 | 1 | 0 | 1 | 0 | 1 | 1 | 1 | 1 | 0 | 0 | 1371 | 1380 | Oxidation (M) |
| K.AAEQAASDLR.T | Y | 48.17 | 1030.5043 | 10 | 2.9 | 516.2609 | 2 | 24.65 | 9 | 6186 | Ana\_08.raw | 5.59E6 | 7.29E6 | 1.3E7 | 1.07E7 | 1.11E7 | 1.25E7 | 7.13E6 |  | 1.22E7 | 9.92E6 | 9 | 1 | 1 | 1 | 1 | 1 | 1 | 1 | 0 | 1 | 1 | 1557 | 1566 |  |
| R.VAQLEEER.T | Y | 48.05 | 972.4876 | 8 | 4.9 | 487.2534 | 2 | 26.92 | 2 | 7055 | Ana\_01.raw |  | 2.44E7 | 8.21E6 | 7.53E6 | 1.32E7 | 1.61E7 | 1.17E7 | 6.13E6 | 1.34E7 | 1.18E7 | 9 | 0 | 1 | 1 | 1 | 1 | 1 | 1 | 1 | 1 | 1 | 903 | 910 |  |
| R.EQEVTELKK.A | Y | 48.05 | 1102.5869 | 9 | 2.1 | 552.3019 | 2 | 22.18 | 3 | 5664 | Ana\_02.raw | 8E6 | 6.32E6 | 3.29E6 |  |  | 3.11E6 |  |  |  | 3.57E6 | 6 | 1 | 2 | 1 | 0 | 0 | 1 | 0 | 0 | 0 | 1 | 1194 | 1202 |  |
| K.M(+15.99)IQALELDPNLYR.V | Y | 47.82 | 1590.8075 | 13 | 2.0 | 796.4126 | 2 | 99.97 | 6 | 28246 | Ana\_05.raw | 2.54E6 |  | 4.54E5 |  |  | 7.65E5 | 4.72E5 |  |  | 3.51E5 | 5 | 1 | 0 | 1 | 0 | 0 | 1 | 1 | 0 | 0 | 1 | 771 | 783 | Oxidation (M) |
| R.AHEVSM(+15.99)QELR.Q | Y | 47.57 | 1214.5714 | 10 | 4.1 | 608.2955 | 2 | 23.51 | 2 | 5929 | Ana\_01.raw |  | 1.62E6 | 2.51E6 | 7.83E5 | 3.43E6 | 1.48E6 | 9.86E5 |  |  | 1.34E6 | 7 | 0 | 1 | 1 | 1 | 1 | 1 | 1 | 0 | 0 | 1 | 1210 | 1219 | Oxidation (M) |
| R.VLGLLPEEITAM(+15.99)LR.T | Y | 44.37 | 1569.8800 | 14 | 2.4 | 785.9492 | 2 | 127.19 | 4 | 34550 | Ana\_03.raw |  |  |  | 1.04E6 | 3.16E5 |  |  |  |  |  | 2 | 0 | 0 | 0 | 1 | 1 | 0 | 0 | 0 | 0 | 0 | 358 | 371 | Oxidation (M) |
| R.EAETLTQR.L | Y | 44.35 | 946.4719 | 8 | 8.9 | 474.2475 | 2 | 24.11 | 7 | 6177 | Ana\_06.raw |  |  |  |  |  |  | 3.23E6 |  |  |  | 1 | 0 | 0 | 0 | 0 | 0 | 0 | 1 | 0 | 0 | 0 | 1421 | 1428 |  |
| R.EVGELQGR.V | Y | 44.22 | 886.4508 | 8 | 3.4 | 444.2342 | 2 | 25.76 | 6 | 6801 | Ana\_05.raw | 2.66E6 | 1.05E7 |  |  | 1.22E7 | 1.04E7 | 7.24E6 |  |  | 5.38E6 | 6 | 1 | 1 | 0 | 0 | 1 | 1 | 1 | 0 | 0 | 1 | 895 | 902 |  |
| K.NVHELER.A | Y | 44.16 | 895.4512 | 7 | 3.2 | 448.7343 | 2 | 20.77 | 2 | 4994 | Ana\_01.raw |  | 5.13E6 | 5.82E6 |  | 5.52E6 |  |  |  |  |  | 3 | 0 | 1 | 1 | 0 | 1 | 0 | 0 | 0 | 0 | 0 | 1547 | 1553 |  |
| R.ILFQEFR.Q | Y | 43.75 | 951.5178 | 7 | 6.7 | 476.7693 | 2 | 83.79 | 6 | 23899 | Ana\_05.raw |  |  | 4.79E6 | 7.18E6 | 1.12E7 | 1.37E7 | 7.63E6 |  |  |  | 5 | 0 | 0 | 1 | 1 | 1 | 1 | 1 | 0 | 0 | 0 | 740 | 746 |  |
| K.VKPLLQVTR.Q | N | 43.60 | 1052.6705 | 9 | 4.3 | 351.8990 | 3 | 47.58 | 8 | 13112 | Ana\_07.raw | 5.04E7 | 3.17E7 | 7.22E7 | 2.28E7 | 5.31E7 | 7.4E7 | 3.29E7 | 3.78E7 | 6.72E7 | 4.05E7 | 26 | 2 | 1 | 3 | 3 | 4 | 3 | 3 | 3 | 2 | 2 | 862 | 870 |  |
| R.QGFPNR.I | N | 43.46 | 717.3558 | 6 | 0.9 | 359.6855 | 2 | 22.62 | 3 | 5802 | Ana\_02.raw |  | 4.83E6 | 1.13E7 | 7.76E6 |  | 1.82E7 | 7.99E6 | 1.36E7 |  |  | 6 | 0 | 1 | 1 | 1 | 0 | 1 | 1 | 1 | 0 | 0 | 734 | 739 |  |
| R.TVGQLYK.E | N | 38.67 | 807.4490 | 7 | 2.6 | 404.7328 | 2 | 33.40 | 4 | 8890 | Ana\_03.raw | 3.54E6 | 1.11E8 | 1.2E8 | 4.55E7 | 8.98E7 | 1.13E8 | 7.15E7 | 6.55E7 | 1.02E8 | 6.97E7 | 17 | 1 | 1 | 2 | 1 | 3 | 2 | 2 | 3 | 1 | 1 | 673 | 679 |  |
| R.LVLDQLR.C | Y | 37.63 | 855.5178 | 7 | 4.6 | 428.7681 | 2 | 62.09 | 9 | 17675 | Ana\_08.raw | 4.33E6 | 7.32E6 | 4.07E6 |  | 4.51E6 | 5.4E6 | 4.06E6 | 2.99E6 | 7.45E6 | 4.55E6 | 9 | 1 | 1 | 1 | 0 | 1 | 1 | 1 | 1 | 1 | 1 | 715 | 721 |  |
| K.NM(+15.99)DPLNDNVAALLHQSTDR.L | Y | 37.41 | 2139.0015 | 19 | 4.8 | 714.0112 | 3 | 100.63 | 7 | 28103 | Ana\_06.raw |  |  |  |  |  |  | 2.58E6 |  |  |  | 1 | 0 | 0 | 0 | 0 | 0 | 0 | 1 | 0 | 0 | 0 | 618 | 636 | Oxidation (M) |
| R.LTAEIWK.D | Y | 37.36 | 859.4803 | 7 | 2.1 | 430.7484 | 2 | 61.78 | 3 | 17602 | Ana\_02.raw |  | 1.64E6 | 8.99E5 |  |  |  |  |  |  |  | 2 | 0 | 1 | 1 | 0 | 0 | 0 | 0 | 0 | 0 | 0 | 637 | 643 |  |
| R.LLEER.L | N | 33.24 | 658.3650 | 5 | 3.3 | 330.1909 | 2 | 23.06 | 6 | 5936 | Ana\_05.raw |  |  |  |  | 2.63E7 | 3.88E7 | 3.76E6 | 1.26E7 |  |  | 4 | 0 | 0 | 0 | 0 | 1 | 1 | 1 | 1 | 0 | 0 | 1024 | 1028 |  |
| R.ALSLTR.A | Y | 33.02 | 659.3966 | 6 | 3.4 | 330.7067 | 2 | 34.74 | 7 | 9416 | Ana\_06.raw |  |  |  |  |  |  | 1.29E6 |  |  |  | 1 | 0 | 0 | 0 | 0 | 0 | 0 | 1 | 0 | 0 | 0 | 1506 | 1511 |  |
| R.NWQWWR.L | N | 29.97 | 974.4511 | 6 | 5.4 | 488.2355 | 2 | 98.83 | 8 | 27494 | Ana\_07.raw |  |  |  |  |  |  |  | 8.23E5 |  |  | 1 | 0 | 0 | 0 | 0 | 0 | 0 | 0 | 1 | 0 | 0 | 852 | 857 |  |
| R.ALLTPR.I | N | 29.60 | 669.4174 | 6 | 3.2 | 335.7170 | 2 | 26.50 | 9 | 6805 | Ana\_08.raw | 3.95E7 | 5.44E7 | 6.82E7 | 2.88E7 | 4.05E7 | 7.21E7 | 3.34E7 | 3.05E7 | 6.5E6 | 2.28E7 | 14 | 1 | 2 | 1 | 2 | 1 | 2 | 2 | 1 | 1 | 1 | 418 | 423 |  |
| K.LALGSR.V | N | 29.45 | 615.3704 | 6 | 2.5 | 308.6932 | 2 | 26.17 | 5 | 6907 | Ana\_04.raw |  |  |  |  | 1.5E6 |  |  |  |  |  | 1 | 0 | 0 | 0 | 0 | 1 | 0 | 0 | 0 | 0 | 0 | 1353 | 1358 |  |
| R.SM(+15.99)LQDR.E | N | 26.72 | 764.3487 | 6 | 4.0 | 383.1832 | 2 | 24.74 | 1 | 6348 | ANa\_10.raw | 2.06E6 |  |  |  |  |  |  |  |  |  | 1 | 1 | 0 | 0 | 0 | 0 | 0 | 0 | 0 | 0 | 0 | 180 | 185 | Oxidation (M) |
| R.LAEQLR.A | N | 26.64 | 728.4181 | 6 | 4.4 | 365.2179 | 2 | 26.49 | 10 | 6965 | Ana\_09.raw |  |  |  |  |  |  |  | 1.64E6 |  | 4.02E6 | 2 | 0 | 0 | 0 | 0 | 0 | 0 | 0 | 1 | 0 | 1 | 913 | 918 |  |
| K.LQLEK.V | N | 26.40 | 629.3748 | 5 | 5.2 | 315.6963 | 2 | 24.82 | 10 | 6436 | Ana\_09.raw |  |  | 5.13E7 |  |  |  |  |  |  | 3.11E7 | 2 | 0 | 0 | 1 | 0 | 0 | 0 | 0 | 0 | 0 | 1 | 990 | 994 |  |
| R.LESQLQEVQGR.S | Y | 25.90 | 1285.6626 | 11 | 5.0 | 643.8418 | 2 | 46.18 | 10 | 12923 | Ana\_09.raw |  |  |  |  |  |  |  |  |  | 5.69E5 | 1 | 0 | 0 | 0 | 0 | 0 | 0 | 0 | 0 | 0 | 1 | 1278 | 1288 |  |
| R.RLLEER.L | Y | 25.12 | 814.4661 | 6 | 2.3 | 408.2412 | 2 | 22.39 | 2 | 5541 | Ana\_01.raw |  | 8.21E6 |  |  |  |  |  |  |  |  | 1 | 0 | 1 | 0 | 0 | 0 | 0 | 0 | 0 | 0 | 0 | 1023 | 1028 |  |
| K.LRNWQWWR.L | N | 23.70 | 1243.6362 | 8 | 2.7 | 415.5538 | 3 | 88.09 | 6 | 25077 | Ana\_05.raw |  |  |  |  |  | 1.89E6 |  |  |  |  | 1 | 0 | 0 | 0 | 0 | 0 | 1 | 0 | 0 | 0 | 0 | 850 | 857 |  |
| total 67 peptides |  |  |  |  |  |  |  |  |  |  |  |  |  |  |  |  |  |  |  |  |  |  |  |  |  |  |  |  |  |  |  |  |  |  |  |
| --- | --- | --- | --- | --- | --- | --- | --- | --- | --- | --- | --- | --- | --- | --- | --- | --- | --- | --- | --- | --- | --- | --- | --- | --- | --- | --- | --- | --- | --- | --- | --- | --- | --- | --- | --- |

Best Unique PSM (Scan F6:37812, m/z=1015.4980, z=3, RT=136.05, ppm=4.6):

sp|P99024|TBB5\_MOUSE

back to list

  

| Protein Coverage
| Supporting Peptides
| Best Unique PSM
|

Protein Coverage:

Supporting Peptides:

| Peptide | Uniq | -10lgP | Mass | Length | ppm | m/z | z | RT | Fraction | Scan | Source File | Area 10 | Area 01 | Area 02 | Area 03 | Area 04 | Area 05 | Area 06 | Area 07 | Area 08 | Area 09 | #Spec | #Spec 10 | #Spec 01 | #Spec 02 | #Spec 03 | #Spec 04 | #Spec 05 | #Spec 06 | #Spec 07 | #Spec 08 | #Spec 09 | Start | End | PTM |
| --- | --- | --- | --- | --- | --- | --- | --- | --- | --- | --- | --- | --- | --- | --- | --- | --- | --- | --- | --- | --- | --- | --- | --- | --- | --- | --- | --- | --- | --- | --- | --- | --- | --- | --- | --- |
| R.SGPFGQIFRPDNFVFGQSGAGNNWAK.G | N | 151.86 | 2797.3359 | 26 | 0.6 | 933.4532 | 3 | 124.87 | 3 | 34288 | Ana\_02.raw | 1.28E7 | 3.44E7 | 9.58E7 | 5.21E6 | 1.06E8 | 1.1E8 | 2.56E7 | 4.47E7 | 2.04E8 | 2.41E7 | 20 | 2 | 1 | 2 | 2 | 2 | 2 | 2 | 2 | 3 | 2 | 78 | 103 |  |
| K.GHYTEGAELVDSVLDVVR.K | N | 135.92 | 1957.9745 | 18 | 3.6 | 979.9980 | 2 | 127.77 | 6 | 35505 | Ana\_05.raw | 2.36E7 | 5.44E7 | 1.6E8 | 4.71E7 | 1.26E8 | 1.66E8 | 6.78E7 | 6.96E7 | 3.65E8 | 1.97E8 | 32 | 2 | 4 | 5 | 2 | 4 | 2 | 1 | 5 | 4 | 3 | 104 | 121 |  |
| K.LTTPTYGDLNHLVSATMSGVTTC(+57.02)LR.F | N | 113.62 | 2707.3311 | 25 | 1.9 | 903.4526 | 3 | 131.13 | 6 | 36433 | Ana\_05.raw |  | 5.46E5 | 9.82E5 | 4.59E6 | 4.43E6 | 9.86E6 | 2.28E6 | 7.01E6 | 3.92E7 | 2.05E7 | 9 | 0 | 1 | 1 | 1 | 1 | 1 | 1 | 1 | 1 | 1 | 217 | 241 | Carbamidomethylation |
| K.NSSYFVEWIPNNVK.T | N | 110.81 | 1695.8257 | 14 | 0.6 | 848.9207 | 2 | 109.87 | 3 | 30437 | Ana\_02.raw | 3.45E7 | 3.29E7 | 2.2E7 | 1.6E7 | 2.71E7 | 1.96E7 | 1.72E7 | 1.87E7 | 6.7E7 | 2.73E7 | 21 | 2 | 3 | 2 | 1 | 4 | 1 | 3 | 1 | 3 | 1 | 337 | 350 |  |
| R.LHFFM(+15.99)PGFAPLTSR.G | N | 108.26 | 1635.8231 | 14 | 1.6 | 546.2825 | 3 | 100.14 | 3 | 27934 | Ana\_02.raw | 1.44E8 | 1.06E8 | 1.27E8 | 4.7E6 | 1.01E8 | 8.11E7 | 1.18E7 | 4.61E7 | 3.33E8 | 1.08E8 | 72 | 8 | 9 | 7 | 5 | 7 | 7 | 4 | 8 | 9 | 8 | 263 | 276 | Oxidation (M) |
| K.M(+15.99)AVTFIGNSTAIQELFK.R | Y | 106.98 | 1884.9655 | 17 | 2.6 | 943.4924 | 2 | 128.56 | 6 | 35718 | Ana\_05.raw | 3.52E6 |  | 4.34E6 | 1.33E6 |  | 3.31E6 | 2.24E6 | 2.77E6 | 7.7E6 | 2.84E6 | 8 | 1 | 0 | 1 | 1 | 0 | 1 | 1 | 1 | 1 | 1 | 363 | 379 | Oxidation (M) |
| K.FWEVISDEHGIDPTGTYHGDSDLQLDR.I | Y | 100.67 | 3101.4001 | 27 | 3.9 | 776.3604 | 4 | 103.61 | 9 | 29196 | Ana\_08.raw |  |  |  |  |  |  |  |  | 2.11E7 |  | 2 | 0 | 0 | 0 | 0 | 0 | 0 | 0 | 0 | 2 | 0 | 20 | 46 |  |
| R.ALTVPELTQQVFDAK.N | Y | 97.20 | 1658.8879 | 15 | 4.0 | 830.4545 | 2 | 115.85 | 6 | 32397 | Ana\_05.raw | 1.06E8 | 8.57E7 | 5.1E7 | 8.2E6 | 3.02E7 | 3.76E7 | 1.21E7 | 1.75E7 | 1.55E8 | 7.86E7 | 66 | 8 | 9 | 8 | 4 | 7 | 4 | 4 | 6 | 9 | 7 | 283 | 297 |  |
| R.LHFFMPGFAPLTSR.G | N | 97.12 | 1619.8282 | 14 | 6.0 | 540.9532 | 3 | 118.11 | 1 | 32615 | ANa\_10.raw | 3.14E7 | 1.19E7 |  | 8.19E5 | 3.08E5 | 5.07E6 |  | 2.64E5 | 3.8E7 | 2.23E7 | 17 | 3 | 3 | 0 | 1 | 1 | 1 | 0 | 1 | 3 | 4 | 263 | 276 |  |
| K.LTTPTYGDLNHLVSATM(+15.99)SGVTTC(+57.02)LR.F | N | 96.84 | 2723.3259 | 25 | 3.5 | 908.7858 | 3 | 116.60 | 2 | 32447 | Ana\_01.raw | 6.74E7 | 3.02E7 | 1.26E7 | 8.03E6 | 2E7 | 2.71E7 | 5.03E6 | 2.06E7 | 7.16E7 | 2.84E7 | 26 | 4 | 3 | 2 | 3 | 2 | 2 | 2 | 1 | 5 | 2 | 217 | 241 | Oxidation (M); Carbamidomethylation |
| K.EVDEQM(+15.99)LNVQNK.N | N | 82.36 | 1461.6770 | 12 | 4.6 | 731.8491 | 2 | 41.89 | 6 | 11733 | Ana\_05.raw | 4.47E7 | 4.06E7 | 4.89E7 | 1.69E7 | 3.72E7 | 3.72E7 | 2.65E7 | 3.63E7 | 4.7E7 | 3.59E7 | 25 | 2 | 2 | 3 | 2 | 3 | 2 | 2 | 4 | 2 | 3 | 325 | 336 | Oxidation (M) |
| R.FPGQLNADLR.K | N | 78.02 | 1129.5880 | 10 | 3.0 | 565.8030 | 2 | 69.05 | 6 | 19811 | Ana\_05.raw | 7.68E7 | 5.71E7 | 5.55E7 | 2.67E7 | 6.02E7 | 4.21E7 | 2.93E7 | 4.94E7 | 1.57E8 | 8.76E7 | 45 | 4 | 4 | 4 | 4 | 4 | 5 | 3 | 5 | 6 | 6 | 242 | 251 |  |
| K.LAVNM(+15.99)VPFPR.L | N | 76.83 | 1158.6219 | 10 | 4.1 | 580.3206 | 2 | 79.35 | 6 | 22695 | Ana\_05.raw | 8.22E7 | 5.68E7 | 5.35E7 | 2.41E7 | 4.94E7 | 5.03E7 | 3.23E7 | 3.95E7 | 1.48E8 | 5.62E7 | 48 | 6 | 5 | 4 | 4 | 4 | 4 | 4 | 6 | 7 | 4 | 253 | 262 | Oxidation (M) |
| R.AILVDLEPGTM(+15.99)DSVR.S | N | 73.85 | 1630.8236 | 15 | 3.2 | 816.4217 | 2 | 86.14 | 1 | 24257 | ANa\_10.raw | 1.6E7 | 1.38E7 | 6.82E5 | 2.16E6 | 7.97E6 | 2.12E6 | 5.93E6 | 7.31E6 | 3.21E7 | 1.06E7 | 13 | 2 | 1 | 1 | 1 | 1 | 1 | 1 | 1 | 2 | 2 | 63 | 77 | Oxidation (M) |
| R.IM(+15.99)NTFSVVPSPK.V | N | 72.89 | 1334.6904 | 12 | 2.0 | 668.3538 | 2 | 67.16 | 3 | 19097 | Ana\_02.raw | 4.81E7 | 3.48E7 | 2.62E7 | 1.36E7 | 3.4E7 | 2.79E7 | 2.17E7 | 2.24E7 | 6.59E7 | 3.08E7 | 21 | 2 | 3 | 2 | 2 | 2 | 2 | 2 | 2 | 3 | 1 | 163 | 174 | Oxidation (M) |
| R.ISVYYNEATGGK.Y | Y | 69.86 | 1300.6299 | 12 | 5.6 | 651.3259 | 2 | 53.95 | 1 | 15195 | ANa\_10.raw | 2.35E7 | 2.44E7 | 2.41E7 | 5.88E5 | 4.68E6 | 4.07E7 | 3.15E7 |  | 5.01E6 | 3.11E7 | 10 | 1 | 1 | 1 | 2 | 1 | 1 | 1 | 0 | 1 | 1 | 47 | 58 |  |
| K.LAVNMVPFPR.L | N | 69.21 | 1142.6270 | 10 | 5.4 | 572.3239 | 2 | 92.17 | 1 | 25869 | ANa\_10.raw | 6.59E6 |  |  |  |  | 1.83E5 |  |  | 1.45E6 |  | 3 | 1 | 0 | 0 | 0 | 0 | 1 | 0 | 0 | 1 | 0 | 253 | 262 |  |
| R.ISEQFTAM(+15.99)FR.R | N | 65.28 | 1244.5859 | 10 | 1.5 | 623.3012 | 2 | 70.89 | 3 | 20141 | Ana\_02.raw | 2.85E7 | 2.58E7 | 1.92E7 | 1E7 | 8.26E6 | 1.54E7 | 6.67E6 | 1.69E7 | 2.69E7 | 1.4E7 | 10 | 1 | 1 | 1 | 1 | 1 | 1 | 1 | 1 | 1 | 1 | 381 | 390 | Oxidation (M) |
| K.IREEYPDR.I | N | 65.00 | 1076.5250 | 8 | 2.5 | 539.2711 | 2 | 25.17 | 6 | 6612 | Ana\_05.raw | 8.6E7 | 1.16E8 | 1.13E8 | 5.23E7 | 7.83E7 | 1.03E8 | 7.1E7 | 6.1E7 | 5.8E7 | 1.33E8 | 27 | 3 | 2 | 2 | 3 | 2 | 2 | 3 | 3 | 4 | 3 | 155 | 162 |  |
| R.YLTVAAVFR.G | N | 63.70 | 1038.5862 | 9 | 3.5 | 520.3022 | 2 | 93.88 | 6 | 26627 | Ana\_05.raw | 7.84E6 | 9.68E6 | 1.04E7 | 5.49E6 | 8.6E6 | 8.9E6 | 7.22E6 | 3.63E6 | 1.87E7 | 1.03E7 | 15 | 2 | 2 | 1 | 1 | 2 | 1 | 1 | 1 | 2 | 2 | 310 | 318 |  |
| R.EIVHIQAGQC(+57.02)GNQIGAK.F | N | 61.23 | 1821.9155 | 17 | 6.6 | 911.9710 | 2 | 49.98 | 9 | 14043 | Ana\_08.raw |  |  |  |  |  |  |  |  | 4.97E6 |  | 2 | 0 | 0 | 0 | 0 | 0 | 0 | 0 | 0 | 2 | 0 | 3 | 19 | Carbamidomethylation |
| K.TAVC(+57.02)DIPPR.G | N | 54.26 | 1027.5121 | 9 | 1.9 | 514.7643 | 2 | 37.94 | 6 | 10515 | Ana\_05.raw | 1.32E7 | 1.04E7 | 1.14E7 | 1.69E6 | 9.99E6 | 4.43E7 | 1.78E7 | 6.51E6 | 1.8E7 | 5.15E7 | 32 | 3 | 3 | 3 | 3 | 3 | 4 | 3 | 2 | 2 | 6 | 351 | 359 | Carbamidomethylation |
| R.KLAVNM(+15.99)VPFPR.L | N | 49.45 | 1286.7169 | 11 | 2.9 | 429.9142 | 3 | 66.65 | 5 | 19005 | Ana\_04.raw |  |  |  | 2.22E6 | 4.19E6 | 8.25E6 |  | 2.66E6 | 3.2E6 |  | 5 | 0 | 0 | 0 | 1 | 1 | 1 | 0 | 1 | 1 | 0 | 252 | 262 | Oxidation (M) |
| total 23 peptides |  |  |  |  |  |  |  |  |  |  |  |  |  |  |  |  |  |  |  |  |  |  |  |  |  |  |  |  |  |  |  |  |  |  |  |
| --- | --- | --- | --- | --- | --- | --- | --- | --- | --- | --- | --- | --- | --- | --- | --- | --- | --- | --- | --- | --- | --- | --- | --- | --- | --- | --- | --- | --- | --- | --- | --- | --- | --- | --- | --- |

Best Unique PSM (Scan F6:35718, m/z=943.4924, z=2, RT=128.56, ppm=2.6):

sp|Q7TPR4|ACTN1\_MOUSE

back to list

  

| Protein Coverage
| Supporting Peptides
| Best Unique PSM
|

Protein Coverage:

Supporting Peptides:

| Peptide | Uniq | -10lgP | Mass | Length | ppm | m/z | z | RT | Fraction | Scan | Source File | Area 10 | Area 01 | Area 02 | Area 03 | Area 04 | Area 05 | Area 06 | Area 07 | Area 08 | Area 09 | #Spec | #Spec 10 | #Spec 01 | #Spec 02 | #Spec 03 | #Spec 04 | #Spec 05 | #Spec 06 | #Spec 07 | #Spec 08 | #Spec 09 | Start | End | PTM |
| --- | --- | --- | --- | --- | --- | --- | --- | --- | --- | --- | --- | --- | --- | --- | --- | --- | --- | --- | --- | --- | --- | --- | --- | --- | --- | --- | --- | --- | --- | --- | --- | --- | --- | --- | --- |
| R.VEQIAAIAQELNELDYYDSPSVNAR.C | N | 128.63 | 2807.3613 | 25 | 3.9 | 1404.6935 | 2 | 130.98 | 9 | 36254 | Ana\_08.raw | 4.88E6 | 6.61E6 |  | 2.21E6 |  | 7.27E6 | 3.62E6 | 4.18E6 | 1.38E7 |  | 8 | 1 | 1 | 0 | 1 | 0 | 1 | 1 | 1 | 2 | 0 | 451 | 475 |  |
| K.KHEAFESDLAAHQDR.V | N | 104.04 | 1752.8179 | 15 | 6.5 | 877.4219 | 2 | 32.17 | 2 | 8671 | Ana\_01.raw | 5.96E7 | 4.97E7 | 5.26E7 | 4.62E6 | 3.7E7 | 5.14E7 | 3.7E7 | 2.49E7 | 4.86E7 | 3.63E7 | 22 | 2 | 2 | 3 | 2 | 2 | 2 | 2 | 2 | 4 | 1 | 436 | 450 |  |
| K.DDPLTNLNTAFDVAER.F | N | 100.33 | 1789.8483 | 16 | 7.2 | 895.9379 | 2 | 115.61 | 6 | 32332 | Ana\_05.raw |  |  |  |  |  | 9.14E6 | 2.21E6 |  |  |  | 2 | 0 | 0 | 0 | 0 | 0 | 1 | 1 | 0 | 0 | 0 | 199 | 214 |  |
| R.RDQALTEEHAR.Q | N | 95.86 | 1324.6483 | 11 | 5.8 | 442.5593 | 3 | 19.76 | 1 | 4685 | ANa\_10.raw | 5.07E7 | 4.57E7 | 4.03E7 | 1.42E7 | 2.84E7 | 4.12E7 | 1.95E7 | 1.54E7 | 5.42E7 | 4.43E7 | 26 | 3 | 3 | 3 | 2 | 2 | 3 | 2 | 2 | 2 | 4 | 614 | 624 |  |
| K.LLETIDQLYLEYAK.R | N | 95.68 | 1710.9080 | 14 | 3.4 | 856.4642 | 2 | 128.84 | 3 | 35301 | Ana\_02.raw | 4.09E6 | 1.13E7 | 9.81E6 | 5.43E6 | 7.74E6 | 1.54E7 | 6.63E6 | 4.77E6 | 1.24E7 | 1.13E7 | 10 | 1 | 1 | 1 | 1 | 1 | 1 | 1 | 1 | 1 | 1 | 503 | 516 |  |
| K.HEAFESDLAAHQDR.V | N | 93.86 | 1624.7229 | 14 | 6.1 | 542.5849 | 3 | 43.62 | 8 | 11920 | Ana\_07.raw | 6.79E6 | 8.02E6 | 1.01E7 | 3.33E6 | 5.65E6 | 8.62E6 | 5.39E6 | 5.37E6 | 1.32E6 | 5.79E6 | 10 | 1 | 1 | 1 | 1 | 1 | 1 | 1 | 1 | 1 | 1 | 437 | 450 |  |
| K.GYEEWLLNEIR.R | N | 91.22 | 1420.6986 | 11 | 1.9 | 711.3579 | 2 | 123.41 | 7 | 34028 | Ana\_06.raw | 2.56E6 | 1.41E7 | 2.88E7 | 8.74E6 | 1.06E7 | 3.06E7 | 2.98E6 | 1.24E7 |  | 2.63E7 | 14 | 1 | 2 | 2 | 1 | 1 | 2 | 2 | 1 | 0 | 2 | 377 | 387 |  |
| R.VGWEQLLTTIAR.T | N | 86.75 | 1385.7667 | 12 | 4.9 | 693.8940 | 2 | 130.96 | 6 | 36386 | Ana\_05.raw | 6.62E7 | 7.2E7 | 8.58E7 | 4.24E7 | 7.11E7 | 7.69E7 | 3.11E7 | 5.19E7 | 9.16E7 | 5.85E7 | 83 | 11 | 9 | 10 | 4 | 12 | 4 | 6 | 9 | 8 | 10 | 715 | 726 |  |
| K.AIMTYVSSFYHAFSGAQK.A | N | 86.55 | 2006.9561 | 18 | 3.9 | 669.9952 | 3 | 128.25 | 1 | 35264 | ANa\_10.raw | 1.11E7 | 6.88E6 |  | 2.86E6 | 3.46E6 | 7.31E6 |  | 3.73E6 | 1.62E7 | 1.43E7 | 9 | 2 | 1 | 0 | 1 | 1 | 1 | 0 | 1 | 1 | 1 | 237 | 254 |  |
| K.LASDLLEWIR.R | N | 83.62 | 1214.6659 | 10 | 1.9 | 608.3414 | 2 | 124.08 | 3 | 34079 | Ana\_02.raw | 2.24E7 | 3.6E7 | 1.72E7 | 3.28E7 | 7.24E7 | 7.44E7 | 3.6E7 | 4.66E7 | 1E8 | 5.02E7 | 44 | 6 | 6 | 5 | 2 | 4 | 3 | 2 | 5 | 6 | 5 | 282 | 291 |  |
| R.KDDPLTNLNTAFDVAER.F | N | 83.37 | 1917.9431 | 17 | 1.8 | 640.3228 | 3 | 101.27 | 4 | 27837 | Ana\_03.raw | 3.37E6 |  |  | 5.42E5 |  |  |  |  | 1.28E7 | 6.99E5 | 4 | 1 | 0 | 0 | 1 | 0 | 0 | 0 | 0 | 1 | 1 | 198 | 214 |  |
| R.TINEVENQILTR.D | N | 83.00 | 1428.7572 | 12 | 6.3 | 715.3904 | 2 | 79.01 | 1 | 22311 | ANa\_10.raw | 6.74E6 | 2.24E7 | 2.21E7 | 8.27E6 | 1.79E7 | 1.93E7 | 8.77E6 | 1.06E7 | 6.01E7 | 2.35E7 | 12 | 1 | 1 | 1 | 1 | 2 | 2 | 1 | 1 | 1 | 1 | 727 | 738 |  |
| K.IDQLEC(+57.02)DHQLIQEALIFDNK.H | N | 82.36 | 2441.1897 | 20 | 5.3 | 814.7415 | 3 | 122.24 | 6 | 34071 | Ana\_05.raw | 3E6 |  | 6.97E6 | 2.39E6 | 5.22E5 | 7.02E6 | 6.79E5 | 2.44E6 | 4.55E6 | 1.58E6 | 9 | 1 | 0 | 1 | 1 | 1 | 1 | 1 | 1 | 1 | 1 | 685 | 704 | Carbamidomethylation |
| R.FAIQDISVEETSAK.E | N | 78.06 | 1536.7671 | 14 | 3.8 | 769.3937 | 2 | 82.14 | 9 | 23377 | Ana\_08.raw | 1.19E7 | 1.61E7 | 1.28E7 | 6.08E6 | 1.02E7 | 9.32E6 | 6.33E6 | 5.83E6 | 2.83E7 | 1.39E7 | 11 | 1 | 1 | 1 | 1 | 2 | 1 | 1 | 1 | 1 | 1 | 134 | 147 |  |
| K.AIM(+15.99)TYVSSFYHAFSGAQK.A | N | 77.57 | 2022.9509 | 18 | 0.5 | 675.3246 | 3 | 111.48 | 2 | 31114 | Ana\_01.raw | 1.77E7 | 1.16E7 | 2.14E7 | 1.36E7 | 1.42E7 | 3.33E6 | 1.45E7 | 3.65E6 | 1.39E7 | 7.21E5 | 19 | 2 | 2 | 2 | 2 | 2 | 2 | 2 | 2 | 2 | 1 | 237 | 254 | Oxidation (M) |
| K.HTNYNM(+15.99)EHIR.V | N | 77.30 | 1329.5884 | 10 | 2.4 | 444.2045 | 3 | 20.55 | 6 | 5100 | Ana\_05.raw | 1.17E7 | 1.23E7 | 1.41E7 |  | 7.36E5 | 1.26E7 |  | 7.01E5 | 2.51E6 | 8.74E6 | 13 | 2 | 2 | 2 | 0 | 1 | 2 | 0 | 1 | 1 | 2 | 705 | 714 | Oxidation (M) |
| K.AGTQIENIEEDFRDGLK.L | N | 73.48 | 1933.9381 | 17 | 4.7 | 645.6564 | 3 | 107.05 | 1 | 29783 | ANa\_10.raw | 2.96E6 | 6.38E5 | 1.03E6 | 8.41E5 | 1.03E6 | 1.52E6 | 1.41E6 | 2.63E5 | 5.57E6 | 8.92E5 | 11 | 1 | 1 | 1 | 1 | 1 | 1 | 1 | 1 | 2 | 1 | 48 | 64 |  |
| K.VLAVNQENEQLM(+15.99)EDYEK.L | N | 72.64 | 2066.9465 | 17 | 6.7 | 1034.4874 | 2 | 69.05 | 9 | 19697 | Ana\_08.raw |  | 3.63E6 | 1.89E6 |  |  |  |  |  | 2.76E6 |  | 3 | 0 | 1 | 1 | 0 | 0 | 0 | 0 | 0 | 1 | 0 | 265 | 281 | Oxidation (M) |
| R.LSNRPAFM(+15.99)PSEGR.M | N | 71.07 | 1476.7144 | 13 | 4.2 | 493.2475 | 3 | 37.09 | 1 | 10119 | ANa\_10.raw | 7.73E7 | 0 | 8.88E7 | 3.92E7 | 4.01E7 | 8.61E7 | 5.08E7 | 5.09E7 | 5.68E7 | 3.21E7 | 25 | 2 | 2 | 3 | 3 | 2 | 4 | 2 | 2 | 2 | 3 | 347 | 359 | Oxidation (M) |
| R.ELPPDQAEYC(+57.02)IAR.M | N | 70.16 | 1560.7242 | 13 | 3.7 | 781.3723 | 2 | 63.81 | 9 | 18193 | Ana\_08.raw | 1.4E7 | 2.47E7 | 4.32E6 | 3.57E5 | 8.53E6 | 8.27E6 | 0 | 4.3E6 | 1.55E7 | 1.96E7 | 35 | 5 | 5 | 2 | 1 | 4 | 3 | 1 | 3 | 6 | 5 | 851 | 863 | Carbamidomethylation |
| K.AGTQIENIEEDFR.D | N | 69.94 | 1520.7107 | 13 | 5.0 | 761.3664 | 2 | 90.74 | 2 | 25658 | Ana\_01.raw | 6.27E6 | 7.93E6 | 6.28E6 | 6.4E5 | 3.01E6 | 6.52E6 | 1.43E6 | 2.43E6 |  | 5.02E6 | 9 | 1 | 1 | 1 | 1 | 1 | 1 | 1 | 1 | 0 | 1 | 48 | 60 |  |
| R.ETADTDTADQVM(+15.99)ASFK.I | N | 68.34 | 1744.7461 | 16 | -3.1 | 873.3776 | 2 | 60.81 | 3 | 17310 | Ana\_02.raw | 1.13E7 | 1.44E6 | 1.01E7 |  | 7.47E6 |  | 5.03E6 | 6.38E6 | 1.47E7 | 6.2E6 | 9 | 1 | 1 | 1 | 0 | 1 | 0 | 1 | 2 | 1 | 1 | 819 | 834 | Oxidation (M) |
| R.DLLLDPAWEK.Q | N | 64.97 | 1198.6233 | 10 | 5.7 | 600.3223 | 2 | 100.06 | 2 | 28148 | Ana\_01.raw | 1.22E7 | 1.24E7 | 1.75E7 | 6E6 | 8.22E6 | 6.59E6 | 7.77E6 | 7.33E6 | 4.18E7 | 1.9E6 | 11 | 1 | 1 | 2 | 1 | 1 | 1 | 1 | 1 | 1 | 1 | 22 | 31 |  |
| K.GISQEQM(+15.99)NEFR.A | N | 64.05 | 1353.5983 | 11 | 4.7 | 677.8096 | 2 | 40.92 | 2 | 11339 | Ana\_01.raw | 1.89E7 | 1.6E7 | 1.59E7 | 4.85E6 |  | 1.55E7 | 7.74E6 | 9.67E6 | 2.24E7 | 1.26E7 | 9 | 1 | 1 | 1 | 1 | 0 | 1 | 1 | 1 | 1 | 1 | 742 | 752 | Oxidation (M) |
| R.LAILGIHNEVSK.I | N | 63.18 | 1292.7452 | 12 | 4.7 | 647.3829 | 2 | 66.22 | 2 | 18881 | Ana\_01.raw | 5.66E6 | 9.88E6 | 4.06E5 | 1.14E6 | 1.17E6 | 2.67E6 |  | 6.25E5 | 1.33E7 | 7.08E6 | 11 | 1 | 2 | 1 | 1 | 1 | 1 | 0 | 1 | 1 | 2 | 566 | 577 |  |
| K.ALDFIASK.G | N | 61.40 | 863.4752 | 8 | 2.2 | 432.7458 | 2 | 61.89 | 6 | 17737 | Ana\_05.raw | 1.98E7 | 2.27E7 | 2.33E7 | 1.06E7 | 1.77E7 | 1.93E7 | 1.47E7 | 1.41E7 | 3.63E7 | 1.65E7 | 17 | 2 | 1 | 2 | 2 | 1 | 2 | 2 | 1 | 3 | 1 | 96 | 103 |  |
| R.ISIEM(+15.99)HGTLEDQLSHLR.Q | N | 61.10 | 1993.9891 | 17 | 4.1 | 499.5066 | 4 | 83.49 | 9 | 23758 | Ana\_08.raw | 1.6E6 |  |  |  |  |  |  |  | 3.84E6 |  | 2 | 1 | 0 | 0 | 0 | 0 | 0 | 0 | 0 | 1 | 0 | 656 | 672 | Oxidation (M) |
| K.NYITEDELR.R | N | 60.72 | 1151.5459 | 9 | 4.1 | 576.7826 | 2 | 61.00 | 6 | 17471 | Ana\_05.raw |  | 2.46E7 | 2.29E7 |  |  | 1.72E7 |  |  |  |  | 3 | 0 | 1 | 1 | 0 | 0 | 1 | 0 | 0 | 0 | 0 | 841 | 849 |  |
| K.EGLLLWC(+57.02)QR.K | N | 60.29 | 1173.5964 | 9 | 4.7 | 587.8082 | 2 | 94.15 | 2 | 26589 | Ana\_01.raw |  | 4.64E6 |  |  |  |  |  |  | 1.06E7 | 1.1E6 | 3 | 0 | 1 | 0 | 0 | 0 | 0 | 0 | 0 | 1 | 1 | 148 | 156 | Carbamidomethylation |
| K.DYETATLSEIK.A | N | 59.88 | 1268.6135 | 11 | 5.4 | 635.3174 | 2 | 77.87 | 8 | 21841 | Ana\_07.raw |  | 2.55E6 | 1.63E6 |  | 1.14E6 |  | 1.61E6 | 6.91E5 | 2.86E6 |  | 6 | 0 | 1 | 1 | 0 | 1 | 0 | 1 | 1 | 1 | 0 | 421 | 431 |  |
| K.M(+15.99)LDAEDIVGTARPDEK.A | N | 59.22 | 1774.8407 | 16 | 3.7 | 592.6230 | 3 | 70.68 | 6 | 20281 | Ana\_05.raw | 1.12E7 | 9.12E6 | 1.13E7 | 4.18E6 | 9.64E6 | 1.06E7 | 6.08E6 | 6.54E6 | 1.04E7 | 7.54E6 | 13 | 2 | 2 | 1 | 1 | 1 | 2 | 1 | 1 | 1 | 1 | 221 | 236 | Oxidation (M) |
| R.DHSGTLGPEEFK.A | Y | 55.91 | 1315.6044 | 12 | 6.3 | 658.8136 | 2 | 45.13 | 9 | 12578 | Ana\_08.raw |  |  |  |  |  |  |  |  | 5.04E6 |  | 1 | 0 | 0 | 0 | 0 | 0 | 0 | 0 | 0 | 1 | 0 | 761 | 772 |  |
| R.ASFNHFDR.D | N | 55.40 | 992.4464 | 8 | 5.0 | 497.2330 | 2 | 33.69 | 6 | 9235 | Ana\_05.raw |  |  | 3.12E6 | 5.01E6 | 2.3E6 | 1.05E7 | 7.38E6 |  | 1.08E7 |  | 10 | 0 | 0 | 1 | 2 | 1 | 2 | 2 | 1 | 1 | 0 | 753 | 760 |  |
| R.HRPELIDYGK.L | N | 54.71 | 1226.6407 | 10 | 1.4 | 409.8881 | 3 | 35.40 | 3 | 9737 | Ana\_02.raw | 1.82E7 | 1.98E7 | 2.25E7 | 8.56E6 | 1.66E7 | 2.01E7 | 1.21E7 | 1.27E7 | 2.85E7 | 2.53E7 | 11 | 1 | 1 | 1 | 1 | 1 | 1 | 1 | 1 | 1 | 2 | 186 | 195 |  |
| K.ATLPDADKER.L | N | 53.61 | 1114.5618 | 10 | 2.4 | 558.2895 | 2 | 25.66 | 2 | 6644 | Ana\_01.raw |  | 1.04E6 |  |  |  | 8.16E6 |  |  | 9.01E6 |  | 3 | 0 | 1 | 0 | 0 | 0 | 1 | 0 | 0 | 1 | 0 | 556 | 565 |  |
| R.TIPWLENR.V | N | 52.70 | 1027.5450 | 8 | 6.5 | 514.7831 | 2 | 81.81 | 1 | 23083 | ANa\_10.raw | 4.77E6 | 5.25E6 | 3.2E6 | 3.29E6 | 4.65E6 | 7.81E6 | 2.61E6 | 3.83E6 | 1.2E7 | 2.56E6 | 10 | 1 | 1 | 1 | 1 | 1 | 1 | 1 | 1 | 1 | 1 | 293 | 300 |  |
| K.GISQEQMNEFR.A | N | 50.65 | 1337.6034 | 11 | 5.1 | 669.8124 | 2 | 58.94 | 10 | 16667 | Ana\_09.raw |  |  |  |  |  |  |  |  |  | 1.81E6 | 1 | 0 | 0 | 0 | 0 | 0 | 0 | 0 | 0 | 0 | 1 | 742 | 752 |  |
| K.IC(+57.02)DQWDNLGALTQK.R | N | 49.74 | 1660.7878 | 14 | 1.0 | 831.4020 | 2 | 92.31 | 3 | 25878 | Ana\_02.raw |  |  | 6.95E5 |  |  |  |  |  |  |  | 1 | 0 | 0 | 1 | 0 | 0 | 0 | 0 | 0 | 0 | 0 | 479 | 492 | Carbamidomethylation |
| R.VPENTM(+15.99)HAM(+15.99)QQK.L | N | 48.52 | 1444.6439 | 12 | 2.5 | 723.3311 | 2 | 16.42 | 5 | 3788 | Ana\_04.raw |  |  |  | 2.87E5 | 1.13E6 |  |  |  |  |  | 2 | 0 | 0 | 0 | 1 | 1 | 0 | 0 | 0 | 0 | 0 | 301 | 312 | Oxidation (M) |
| R.AAPFNNWM(+15.99)EGAM(+15.99)EDLQDTFIVHTIEEIQGLTTAHEQFK.A | N | 47.50 | 4393.0308 | 38 | 6.4 | 1099.2720 | 4 | 137.43 | 6 | 38175 | Ana\_05.raw |  |  |  |  |  | 1.13E6 |  |  |  |  | 1 | 0 | 0 | 0 | 0 | 0 | 1 | 0 | 0 | 0 | 0 | 518 | 555 | Oxidation (M) |
| R.FLDIPK.M | N | 46.94 | 731.4218 | 6 | 3.4 | 366.7194 | 2 | 61.25 | 9 | 17419 | Ana\_08.raw | 1.06E7 | 1.02E7 | 1.12E7 | 4.65E6 | 7.77E6 | 9.3E6 | 6.1E6 |  | 1.84E7 |  | 11 | 1 | 1 | 2 | 1 | 1 | 2 | 1 | 0 | 2 | 0 | 215 | 220 |  |
| K.SIVNYKPK.I | N | 45.23 | 947.5440 | 8 | 0.7 | 316.8555 | 3 | 28.31 | 3 | 7612 | Ana\_02.raw |  |  | 2.22E6 | 1.59E6 | 1E6 | 1.43E6 | 1.4E6 |  | 9.44E6 | 8.82E6 | 8 | 0 | 0 | 1 | 1 | 1 | 1 | 1 | 0 | 2 | 1 | 677 | 684 |  |
| R.LDHLAEK.F | N | 44.09 | 824.4392 | 7 | 2.3 | 413.2278 | 2 | 19.09 | 5 | 4680 | Ana\_04.raw |  |  |  |  | 1.46E7 | 2.65E7 | 1.21E7 | 1.15E7 |  |  | 4 | 0 | 0 | 0 | 0 | 1 | 1 | 1 | 1 | 0 | 0 | 392 | 398 |  |
| K.MEEIGR.I | N | 36.59 | 733.3428 | 6 | 4.4 | 367.6803 | 2 | 25.12 | 6 | 6593 | Ana\_05.raw |  |  |  |  |  | 4.69E6 |  | 2.55E6 |  | 3.65E6 | 3 | 0 | 0 | 0 | 0 | 0 | 1 | 0 | 1 | 0 | 1 | 650 | 655 |  |
| K.TFTAWC(+57.02)NSHLR.K | N | 36.01 | 1391.6405 | 11 | 2.5 | 464.8886 | 3 | 63.09 | 6 | 18092 | Ana\_05.raw |  |  |  |  |  | 5.73E5 |  |  |  |  | 1 | 0 | 0 | 0 | 0 | 0 | 1 | 0 | 0 | 0 | 0 | 36 | 46 | Carbamidomethylation |
| K.LEDFR.D | N | 34.55 | 678.3337 | 5 | 1.6 | 340.1747 | 2 | 32.77 | 4 | 8713 | Ana\_03.raw |  |  | 1.16E7 | 1.11E7 |  | 2.14E7 | 1.54E7 | 1.44E7 |  |  | 5 | 0 | 0 | 1 | 1 | 0 | 1 | 1 | 1 | 0 | 0 | 313 | 317 |  |
| K.ASIHEAWTDGK.E | N | 31.75 | 1213.5728 | 11 | 3.5 | 405.5329 | 3 | 41.70 | 6 | 11672 | Ana\_05.raw |  |  | 6.42E6 | 4.14E6 |  | 8.99E6 | 6.97E6 |  |  |  | 4 | 0 | 0 | 1 | 1 | 0 | 1 | 1 | 0 | 0 | 0 | 403 | 413 |  |
| total 47 peptides |  |  |  |  |  |  |  |  |  |  |  |  |  |  |  |  |  |  |  |  |  |  |  |  |  |  |  |  |  |  |  |  |  |  |  |
| --- | --- | --- | --- | --- | --- | --- | --- | --- | --- | --- | --- | --- | --- | --- | --- | --- | --- | --- | --- | --- | --- | --- | --- | --- | --- | --- | --- | --- | --- | --- | --- | --- | --- | --- | --- |

Best Unique PSM (Scan F9:12578, m/z=658.8136, z=2, RT=45.13, ppm=6.3):

sp|P11499|HS90B\_MOUSE

back to list

  

| Protein Coverage
| Supporting Peptides
| Best Unique PSM
|

Protein Coverage:

Supporting Peptides:

| Peptide | Uniq | -10lgP | Mass | Length | ppm | m/z | z | RT | Fraction | Scan | Source File | Area 10 | Area 01 | Area 02 | Area 03 | Area 04 | Area 05 | Area 06 | Area 07 | Area 08 | Area 09 | #Spec | #Spec 10 | #Spec 01 | #Spec 02 | #Spec 03 | #Spec 04 | #Spec 05 | #Spec 06 | #Spec 07 | #Spec 08 | #Spec 09 | Start | End | PTM |
| --- | --- | --- | --- | --- | --- | --- | --- | --- | --- | --- | --- | --- | --- | --- | --- | --- | --- | --- | --- | --- | --- | --- | --- | --- | --- | --- | --- | --- | --- | --- | --- | --- | --- | --- | --- |
| R.VFIM(+15.99)DSC(+57.02)DELIPEYLNFIR.G | Y | 130.13 | 2389.1333 | 19 | 3.8 | 1195.5785 | 2 | 133.35 | 6 | 37072 | Ana\_05.raw | 9.57E6 | 8.66E6 | 5.2E6 | 4.26E6 | 5.12E6 | 9.06E6 | 4.6E6 | 1.39E6 | 4.94E6 | 8.35E6 | 18 | 2 | 2 | 2 | 2 | 2 | 2 | 2 | 1 | 1 | 2 | 360 | 378 | Oxidation (M); Carbamidomethylation |
| K.HSQFIGYPITLYLEK.E | Y | 129.06 | 1807.9508 | 15 | 0.9 | 904.9835 | 2 | 112.22 | 3 | 31036 | Ana\_02.raw |  | 7.48E6 | 6.98E6 | 3.03E7 | 1.13E7 | 9.55E6 | 2.56E6 |  | 9.72E6 |  | 18 | 0 | 3 | 3 | 3 | 3 | 2 | 2 | 0 | 2 | 0 | 205 | 219 |  |
| R.NPDDITQEEYGEFYK.S | Y | 106.07 | 1846.7897 | 15 | 1.6 | 924.4036 | 2 | 83.57 | 6 | 23844 | Ana\_05.raw | 3.3E7 | 4.1E7 | 3.44E7 | 2.08E7 | 3.89E7 | 3.76E7 | 2.25E7 | 3.03E7 | 6.27E7 | 3.02E7 | 26 | 3 | 2 | 2 | 2 | 3 | 2 | 2 | 4 | 3 | 3 | 292 | 306 |  |
| R.GVVDSEDLPLNISR.E | N | 102.97 | 1512.7783 | 14 | 0.9 | 757.3971 | 2 | 86.49 | 3 | 24346 | Ana\_02.raw | 6.13E7 | 7.03E7 | 3.65E6 | 3.61E6 | 5.27E7 | 4.2E7 | 2.46E7 | 2.66E7 | 1.07E8 | 7.09E7 | 35 | 3 | 5 | 4 | 2 | 4 | 2 | 2 | 4 | 4 | 5 | 379 | 392 |  |
| K.HLEINPDHPIVETLR.Q | Y | 94.69 | 1781.9424 | 15 | 7.2 | 594.9924 | 3 | 72.81 | 6 | 20889 | Ana\_05.raw | 1.19E7 | 1.67E7 | 9.65E6 | 6.07E6 | 7.61E6 | 8.69E6 | 3.91E5 | 6.21E6 | 2.23E7 | 7.67E6 | 12 | 1 | 1 | 2 | 1 | 1 | 1 | 1 | 1 | 2 | 1 | 625 | 639 |  |
| R.YHTSQSGDEM(+15.99)TSLSEYVSR.M | Y | 90.90 | 2191.9329 | 19 | 5.3 | 731.6555 | 3 | 62.33 | 6 | 17871 | Ana\_05.raw | 8.83E6 | 1.87E7 | 1.49E7 | 9.77E6 |  | 1.42E7 | 1.26E7 | 1.61E7 | 1.81E7 | 7.18E6 | 9 | 1 | 1 | 1 | 1 | 0 | 1 | 1 | 1 | 1 | 1 | 457 | 475 | Oxidation (M) |
| R.ADHGEPIGR.G | Y | 82.92 | 950.4570 | 9 | 0.4 | 476.2360 | 2 | 18.58 | 4 | 4462 | Ana\_03.raw |  |  |  | 3.01E7 | 3.36E7 | 1.06E7 | 2.3E7 | 7.47E6 |  |  | 7 | 0 | 0 | 0 | 2 | 1 | 1 | 1 | 2 | 0 | 0 | 169 | 177 |  |
| K.LGIHEDSTNR.R | Y | 80.50 | 1140.5524 | 10 | 3.0 | 571.2852 | 2 | 26.14 | 7 | 6790 | Ana\_06.raw | 3.5E7 | 3.38E7 | 4.05E7 | 2.32E7 | 5.61E7 | 2.55E7 | 5.15E7 | 4.32E7 | 5.45E7 | 2.18E7 | 15 | 2 | 1 | 1 | 1 | 2 | 1 | 2 | 2 | 2 | 1 | 439 | 448 |  |
| K.IDIIPNPQER.T | Y | 80.33 | 1193.6404 | 10 | 2.1 | 597.8287 | 2 | 67.57 | 3 | 19215 | Ana\_02.raw | 5.15E7 | 5.49E7 | 5.76E7 | 3.06E7 | 5.63E7 | 4.15E7 | 3.44E7 | 3.73E7 | 7.21E7 | 5.44E7 | 33 | 3 | 2 | 3 | 3 | 3 | 3 | 3 | 5 | 4 | 4 | 73 | 82 |  |
| R.RAPFDLFENK.K | Y | 78.93 | 1235.6298 | 10 | 2.1 | 412.8847 | 3 | 80.47 | 3 | 22740 | Ana\_02.raw | 8.67E6 | 1.19E7 | 1.9E7 | 1.16E7 | 1.75E7 | 1.82E7 | 1.29E7 | 1.57E7 | 2.85E7 | 8.17E6 | 24 | 1 | 2 | 3 | 2 | 3 | 4 | 3 | 3 | 2 | 1 | 338 | 347 |  |
| R.TLTLVDTGIGM(+15.99)TK.A | N | 76.46 | 1364.7222 | 13 | 2.8 | 683.3703 | 2 | 75.39 | 6 | 21614 | Ana\_05.raw | 1.7E7 | 2.94E7 | 3.4E7 | 1.31E7 | 2.19E7 | 1.58E7 | 1.83E7 | 1.87E7 | 6.4E6 | 1.47E7 | 22 | 2 | 2 | 3 | 3 | 2 | 1 | 2 | 2 | 1 | 4 | 83 | 95 | Oxidation (M) |
| K.EGLELPEDEEEK.K | N | 76.27 | 1415.6304 | 12 | 4.3 | 708.8255 | 2 | 63.16 | 6 | 18113 | Ana\_05.raw | 1.47E7 | 2.12E7 | 2.24E7 | 9.03E6 |  | 1.3E7 | 1.23E7 | 9.36E6 | 1.85E7 |  | 9 | 1 | 1 | 2 | 1 | 0 | 1 | 1 | 1 | 1 | 0 | 539 | 550 |  |
| K.EDQTEYLEER.R | N | 73.56 | 1310.5626 | 10 | 2.1 | 656.2900 | 2 | 46.56 | 3 | 13135 | Ana\_02.raw | 8.17E7 | 1.16E8 | 9.68E7 | 5.07E7 | 8.64E7 | 7.77E7 | 6.62E7 | 7.11E7 | 9.8E7 | 7.47E7 | 27 | 3 | 3 | 2 | 4 | 3 | 1 | 3 | 3 | 2 | 3 | 187 | 196 |  |
| R.ELISNASDALDK.I | N | 71.73 | 1274.6354 | 12 | 0.7 | 638.3254 | 2 | 63.17 | 7 | 17908 | Ana\_06.raw | 5.3E7 | 6.22E7 | 6.1E7 | 2.33E7 | 3.85E7 | 3.66E7 | 3.47E7 | 3.57E7 | 8.97E7 | 4.28E7 | 22 | 3 | 2 | 2 | 2 | 2 | 1 | 3 | 3 | 3 | 1 | 42 | 53 |  |
| K.ADLINNLGTIAK.S | N | 71.56 | 1241.6979 | 12 | 2.5 | 621.8578 | 2 | 85.58 | 6 | 24379 | Ana\_05.raw | 9.14E6 | 4.85E7 | 4.8E7 | 1.77E7 | 3.55E7 | 3.13E7 | 2.49E7 | 2.43E7 | 6.64E7 | 2.83E7 | 23 | 2 | 3 | 3 | 1 | 3 | 1 | 1 | 3 | 4 | 2 | 96 | 107 |  |
| K.HNDDEQYAWESSAGGSFTVR.A | N | 71.42 | 2254.9514 | 20 | 1.3 | 752.6588 | 3 | 83.49 | 4 | 23153 | Ana\_03.raw |  |  |  | 5.26E5 |  |  |  |  |  |  | 1 | 0 | 0 | 0 | 1 | 0 | 0 | 0 | 0 | 0 | 0 | 149 | 168 |  |
| K.LGLGIDEDEVTAEEPSAAVPDEIPPLEGDEDASR.M | Y | 69.69 | 3534.6372 | 34 | 1.2 | 1179.2211 | 3 | 119.29 | 5 | 33012 | Ana\_04.raw |  |  |  |  | 2.42E6 | 5.64E5 |  |  |  |  | 2 | 0 | 0 | 0 | 0 | 1 | 1 | 0 | 0 | 0 | 0 | 686 | 719 |  |
| R.YESLTDPSK.L | N | 68.33 | 1038.4869 | 9 | 5.5 | 520.2536 | 2 | 35.77 | 1 | 9709 | ANa\_10.raw | 4.69E7 | 6.87E7 | 7.75E7 | 3.49E7 | 5.34E7 | 5.02E7 | 4.55E7 | 4.71E7 | 6.1E7 | 4.21E7 | 21 | 2 | 2 | 3 | 2 | 2 | 2 | 1 | 3 | 2 | 2 | 56 | 64 |  |
| K.EQVANSAFVER.V | Y | 68.00 | 1248.6099 | 11 | 5.8 | 625.3159 | 2 | 46.46 | 6 | 13129 | Ana\_05.raw | 4.52E7 | 5.09E7 | 5.55E7 | 2.38E7 | 4.24E7 | 4E7 | 3.42E7 | 3.76E7 | 3.17E7 | 3.97E7 | 15 | 1 | 1 | 2 | 1 | 1 | 2 | 2 | 3 | 1 | 1 | 492 | 502 |  |
| K.YIDQEELNK.T | N | 66.73 | 1150.5505 | 9 | 4.3 | 576.2850 | 2 | 41.44 | 6 | 11597 | Ana\_05.raw | 7.59E7 | 8.68E7 | 1.08E8 | 3.52E7 | 7.65E7 | 5.89E7 | 4.76E7 | 3.83E7 | 4.98E7 | 6.56E7 | 10 | 1 | 1 | 1 | 1 | 1 | 1 | 1 | 1 | 1 | 1 | 276 | 284 |  |
| K.SIYYITGESK.E | Y | 65.08 | 1159.5760 | 10 | 2.4 | 580.7967 | 2 | 59.89 | 4 | 16639 | Ana\_03.raw | 2.42E7 | 3.6E7 | 3.45E7 | 1.73E7 | 3.05E7 | 2.92E7 | 2.53E7 | 2.38E7 | 3.83E7 | 2.62E7 | 11 | 1 | 1 | 1 | 1 | 1 | 1 | 1 | 2 | 1 | 1 | 482 | 491 |  |
| K.EGLELPEDEEEKKK.M | N | 65.05 | 1671.8202 | 14 | 7.1 | 558.2846 | 3 | 42.33 | 3 | 11863 | Ana\_02.raw | 0 | 9.31E6 | 1.33E7 | 1.55E6 | 7.96E6 |  |  |  |  |  | 5 | 1 | 1 | 1 | 1 | 1 | 0 | 0 | 0 | 0 | 0 | 539 | 552 |  |
| R.APFDLFENK.K | Y | 64.99 | 1079.5287 | 9 | 4.2 | 540.7739 | 2 | 90.11 | 2 | 25487 | Ana\_01.raw |  | 8.08E6 |  |  |  |  |  |  | 1.24E7 | 5.55E6 | 4 | 0 | 2 | 0 | 0 | 0 | 0 | 0 | 0 | 1 | 1 | 339 | 347 |  |
| K.SLTNDWEDHLAVK.H | Y | 64.81 | 1526.7365 | 13 | 2.7 | 764.3776 | 2 | 76.98 | 9 | 21930 | Ana\_08.raw | 1.14E6 | 3.96E6 | 2.63E6 | 3.57E6 | 5.85E6 | 3.36E6 | 2.68E6 | 5.81E6 | 3.66E6 |  | 14 | 1 | 2 | 1 | 2 | 2 | 1 | 1 | 2 | 2 | 0 | 307 | 319 |  |
| R.ELISNASDALDKIR.Y | N | 58.86 | 1543.8206 | 14 | 5.5 | 515.6169 | 3 | 80.38 | 9 | 22886 | Ana\_08.raw |  |  |  |  |  |  |  |  | 2.43E6 |  | 1 | 0 | 0 | 0 | 0 | 0 | 0 | 0 | 0 | 1 | 0 | 42 | 55 |  |
| K.LGIHEDSTNRR.R | Y | 56.82 | 1296.6534 | 11 | 1.4 | 649.3349 | 2 | 22.20 | 2 | 5474 | Ana\_01.raw | 2.93E6 | 7.34E6 |  |  |  |  |  |  |  | 4.34E6 | 5 | 1 | 2 | 0 | 0 | 0 | 0 | 0 | 0 | 0 | 2 | 439 | 449 |  |
| R.ALLFIPR.R | Y | 49.76 | 828.5221 | 7 | 2.8 | 415.2695 | 2 | 87.44 | 6 | 24899 | Ana\_05.raw | 3.55E7 | 4.76E7 | 6.37E7 | 4.84E7 | 1.47E7 | 4.75E7 | 3.99E7 | 4.48E7 | 6.66E7 | 3.62E7 | 42 | 4 | 4 | 6 | 4 | 5 | 2 | 4 | 5 | 5 | 3 | 331 | 337 |  |
| K.FYEAFSK.N | Y | 49.31 | 890.4174 | 7 | 5.4 | 446.2184 | 2 | 52.07 | 2 | 14711 | Ana\_01.raw | 2.33E7 | 2.77E7 | 2.74E7 | 1.48E7 | 2.46E7 | 2.38E7 | 1.95E7 | 1.67E7 | 3.16E7 | 2.16E7 | 10 | 1 | 1 | 1 | 1 | 1 | 1 | 1 | 1 | 1 | 1 | 429 | 435 |  |
| K.TKPIWTR.N | N | 48.99 | 900.5181 | 7 | 0.7 | 301.1802 | 3 | 33.07 | 3 | 9038 | Ana\_02.raw | 1.67E7 | 2.38E7 | 5.34E5 | 5.35E6 |  |  |  |  |  | 1.51E7 | 5 | 1 | 1 | 1 | 1 | 0 | 0 | 0 | 0 | 0 | 1 | 285 | 291 |  |
| K.KHLEINPDHPIVETLR.Q | Y | 47.51 | 1910.0374 | 16 | 4.1 | 478.5186 | 4 | 62.39 | 6 | 17888 | Ana\_05.raw | 1.36E6 |  |  |  |  | 1.46E6 |  |  |  |  | 2 | 1 | 0 | 0 | 0 | 0 | 1 | 0 | 0 | 0 | 0 | 624 | 639 |  |
| R.DNSTM(+15.99)GYM(+15.99)M(+15.99)AK.K | Y | 45.90 | 1295.4833 | 11 | 3.4 | 648.7511 | 2 | 22.04 | 7 | 5549 | Ana\_06.raw |  |  |  | 3.79E6 | 5.85E6 |  | 5.62E6 | 5.5E6 |  |  | 4 | 0 | 0 | 0 | 1 | 1 | 0 | 1 | 1 | 0 | 0 | 613 | 623 | Oxidation (M) |
| K.EGLELPEDEEEKK.K | N | 44.93 | 1543.7252 | 13 | 0.3 | 772.8701 | 2 | 51.90 | 2 | 14657 | Ana\_01.raw |  | 1.38E7 |  | 0 |  |  | 2.61E6 |  | 7.3E6 |  | 5 | 0 | 2 | 0 | 1 | 0 | 0 | 1 | 0 | 1 | 0 | 539 | 551 |  |
| K.SLVSVTK.E | Y | 44.27 | 732.4381 | 7 | 0.9 | 367.2267 | 2 | 29.70 | 3 | 8042 | Ana\_02.raw | 1.06E7 | 1.46E7 | 1.96E7 | 9.01E6 |  | 1.6E7 |  | 1.04E7 | 1.45E7 |  | 7 | 1 | 1 | 1 | 1 | 0 | 1 | 0 | 1 | 1 | 0 | 532 | 538 |  |
| K.FENLC(+57.02)K.L | N | 42.97 | 809.3741 | 6 | 0.8 | 405.6947 | 2 | 28.27 | 3 | 7600 | Ana\_02.raw | 8.65E6 | 1.22E7 | 1.26E7 | 7.61E6 | 9.52E6 | 1.1E7 | 8.16E6 | 7.56E6 | 9.31E6 | 9.82E6 | 10 | 1 | 1 | 1 | 1 | 1 | 1 | 1 | 1 | 1 | 1 | 560 | 565 | Carbamidomethylation |
| R.LSELLR.Y | N | 42.95 | 729.4385 | 6 | 5.6 | 365.7285 | 2 | 43.93 | 2 | 12274 | Ana\_01.raw | 2.33E7 | 2.55E7 | 3.68E7 | 1.67E7 | 3.13E7 | 2.93E7 | 2.32E7 | 2.44E7 | 6.01E6 | 2.41E7 | 21 | 2 | 2 | 2 | 2 | 2 | 2 | 2 | 3 | 2 | 2 | 451 | 456 |  |
| K.AKFENLC(+57.02)K.L | Y | 34.90 | 1008.5062 | 8 | 6.3 | 505.2636 | 2 | 30.65 | 7 | 8170 | Ana\_06.raw |  |  |  |  | 1.93E6 |  | 5.65E5 |  | 5.6E6 |  | 3 | 0 | 0 | 0 | 0 | 1 | 0 | 1 | 0 | 1 | 0 | 558 | 565 | Carbamidomethylation |
| K.VVVITK.H | Y | 31.76 | 657.4425 | 6 | 4.2 | 329.7299 | 2 | 25.16 | 2 | 6478 | Ana\_01.raw |  | 1.4E7 | 2.54E7 |  |  | 1.84E7 | 1.64E7 |  |  | 7.63E6 | 5 | 0 | 1 | 1 | 0 | 0 | 1 | 1 | 0 | 0 | 1 | 143 | 148 |  |
| K.EIFLR.E | N | 28.74 | 676.3907 | 5 | 1.2 | 339.2031 | 2 | 46.69 | 3 | 13179 | Ana\_02.raw |  |  | 1.52E7 |  | 1.07E7 |  |  |  | 6.65E6 |  | 3 | 0 | 0 | 1 | 0 | 1 | 0 | 0 | 0 | 1 | 0 | 37 | 41 |  |
| total 38 peptides |  |  |  |  |  |  |  |  |  |  |  |  |  |  |  |  |  |  |  |  |  |  |  |  |  |  |  |  |  |  |  |  |  |  |  |
| --- | --- | --- | --- | --- | --- | --- | --- | --- | --- | --- | --- | --- | --- | --- | --- | --- | --- | --- | --- | --- | --- | --- | --- | --- | --- | --- | --- | --- | --- | --- | --- | --- | --- | --- | --- |

Best Unique PSM (Scan F6:37072, m/z=1195.5785, z=2, RT=133.35, ppm=3.8):

A1BN54|A1BN54\_MOUSE

back to list

  

| Protein Coverage
| Supporting Peptides
| Best Unique PSM
|

Protein Coverage:

Supporting Peptides:

| Peptide | Uniq | -10lgP | Mass | Length | ppm | m/z | z | RT | Fraction | Scan | Source File | Area 10 | Area 01 | Area 02 | Area 03 | Area 04 | Area 05 | Area 06 | Area 07 | Area 08 | Area 09 | #Spec | #Spec 10 | #Spec 01 | #Spec 02 | #Spec 03 | #Spec 04 | #Spec 05 | #Spec 06 | #Spec 07 | #Spec 08 | #Spec 09 | Start | End | PTM |
| --- | --- | --- | --- | --- | --- | --- | --- | --- | --- | --- | --- | --- | --- | --- | --- | --- | --- | --- | --- | --- | --- | --- | --- | --- | --- | --- | --- | --- | --- | --- | --- | --- | --- | --- | --- |
| R.VEQIAAIAQELNELDYYDSPSVNAR.C | N | 128.63 | 2807.3613 | 25 | 3.9 | 1404.6935 | 2 | 130.98 | 9 | 36254 | Ana\_08.raw | 4.88E6 | 6.61E6 |  | 2.21E6 |  | 7.27E6 | 3.62E6 | 4.18E6 | 1.38E7 |  | 8 | 1 | 1 | 0 | 1 | 0 | 1 | 1 | 1 | 2 | 0 | 451 | 475 |  |
| K.KHEAFESDLAAHQDR.V | N | 104.04 | 1752.8179 | 15 | 6.5 | 877.4219 | 2 | 32.17 | 2 | 8671 | Ana\_01.raw | 5.96E7 | 4.97E7 | 5.26E7 | 4.62E6 | 3.7E7 | 5.14E7 | 3.7E7 | 2.49E7 | 4.86E7 | 3.63E7 | 22 | 2 | 2 | 3 | 2 | 2 | 2 | 2 | 2 | 4 | 1 | 436 | 450 |  |
| K.DDPLTNLNTAFDVAER.F | N | 100.33 | 1789.8483 | 16 | 7.2 | 895.9379 | 2 | 115.61 | 6 | 32332 | Ana\_05.raw |  |  |  |  |  | 9.14E6 | 2.21E6 |  |  |  | 2 | 0 | 0 | 0 | 0 | 0 | 1 | 1 | 0 | 0 | 0 | 199 | 214 |  |
| R.RDQALTEEHAR.Q | N | 95.86 | 1324.6483 | 11 | 5.8 | 442.5593 | 3 | 19.76 | 1 | 4685 | ANa\_10.raw | 5.07E7 | 4.57E7 | 4.03E7 | 1.42E7 | 2.84E7 | 4.12E7 | 1.95E7 | 1.54E7 | 5.42E7 | 4.43E7 | 26 | 3 | 3 | 3 | 2 | 2 | 3 | 2 | 2 | 2 | 4 | 614 | 624 |  |
| K.LLETIDQLYLEYAK.R | N | 95.68 | 1710.9080 | 14 | 3.4 | 856.4642 | 2 | 128.84 | 3 | 35301 | Ana\_02.raw | 4.09E6 | 1.13E7 | 9.81E6 | 5.43E6 | 7.74E6 | 1.54E7 | 6.63E6 | 4.77E6 | 1.24E7 | 1.13E7 | 10 | 1 | 1 | 1 | 1 | 1 | 1 | 1 | 1 | 1 | 1 | 503 | 516 |  |
| K.HEAFESDLAAHQDR.V | N | 93.86 | 1624.7229 | 14 | 6.1 | 542.5849 | 3 | 43.62 | 8 | 11920 | Ana\_07.raw | 6.79E6 | 8.02E6 | 1.01E7 | 3.33E6 | 5.65E6 | 8.62E6 | 5.39E6 | 5.37E6 | 1.32E6 | 5.79E6 | 10 | 1 | 1 | 1 | 1 | 1 | 1 | 1 | 1 | 1 | 1 | 437 | 450 |  |
| K.GYEEWLLNEIR.R | N | 91.22 | 1420.6986 | 11 | 1.9 | 711.3579 | 2 | 123.41 | 7 | 34028 | Ana\_06.raw | 2.56E6 | 1.41E7 | 2.88E7 | 8.74E6 | 1.06E7 | 3.06E7 | 2.98E6 | 1.24E7 |  | 2.63E7 | 14 | 1 | 2 | 2 | 1 | 1 | 2 | 2 | 1 | 0 | 2 | 377 | 387 |  |
| R.VGWEQLLTTIAR.T | N | 86.75 | 1385.7667 | 12 | 4.9 | 693.8940 | 2 | 130.96 | 6 | 36386 | Ana\_05.raw | 6.62E7 | 7.2E7 | 8.58E7 | 4.24E7 | 7.11E7 | 7.69E7 | 3.11E7 | 5.19E7 | 9.16E7 | 5.85E7 | 83 | 11 | 9 | 10 | 4 | 12 | 4 | 6 | 9 | 8 | 10 | 715 | 726 |  |
| K.AIMTYVSSFYHAFSGAQK.A | N | 86.55 | 2006.9561 | 18 | 3.9 | 669.9952 | 3 | 128.25 | 1 | 35264 | ANa\_10.raw | 1.11E7 | 6.88E6 |  | 2.86E6 | 3.46E6 | 7.31E6 |  | 3.73E6 | 1.62E7 | 1.43E7 | 9 | 2 | 1 | 0 | 1 | 1 | 1 | 0 | 1 | 1 | 1 | 237 | 254 |  |
| K.LASDLLEWIR.R | N | 83.62 | 1214.6659 | 10 | 1.9 | 608.3414 | 2 | 124.08 | 3 | 34079 | Ana\_02.raw | 2.24E7 | 3.6E7 | 1.72E7 | 3.28E7 | 7.24E7 | 7.44E7 | 3.6E7 | 4.66E7 | 1E8 | 5.02E7 | 44 | 6 | 6 | 5 | 2 | 4 | 3 | 2 | 5 | 6 | 5 | 282 | 291 |  |
| R.KDDPLTNLNTAFDVAER.F | N | 83.37 | 1917.9431 | 17 | 1.8 | 640.3228 | 3 | 101.27 | 4 | 27837 | Ana\_03.raw | 3.37E6 |  |  | 5.42E5 |  |  |  |  | 1.28E7 | 6.99E5 | 4 | 1 | 0 | 0 | 1 | 0 | 0 | 0 | 0 | 1 | 1 | 198 | 214 |  |
| R.TINEVENQILTR.D | N | 83.00 | 1428.7572 | 12 | 6.3 | 715.3904 | 2 | 79.01 | 1 | 22311 | ANa\_10.raw | 6.74E6 | 2.24E7 | 2.21E7 | 8.27E6 | 1.79E7 | 1.93E7 | 8.77E6 | 1.06E7 | 6.01E7 | 2.35E7 | 12 | 1 | 1 | 1 | 1 | 2 | 2 | 1 | 1 | 1 | 1 | 727 | 738 |  |
| K.IDQLEC(+57.02)DHQLIQEALIFDNK.H | N | 82.36 | 2441.1897 | 20 | 5.3 | 814.7415 | 3 | 122.24 | 6 | 34071 | Ana\_05.raw | 3E6 |  | 6.97E6 | 2.39E6 | 5.22E5 | 7.02E6 | 6.79E5 | 2.44E6 | 4.55E6 | 1.58E6 | 9 | 1 | 0 | 1 | 1 | 1 | 1 | 1 | 1 | 1 | 1 | 685 | 704 | Carbamidomethylation |
| R.FAIQDISVEETSAK.E | N | 78.06 | 1536.7671 | 14 | 3.8 | 769.3937 | 2 | 82.14 | 9 | 23377 | Ana\_08.raw | 1.19E7 | 1.61E7 | 1.28E7 | 6.08E6 | 1.02E7 | 9.32E6 | 6.33E6 | 5.83E6 | 2.83E7 | 1.39E7 | 11 | 1 | 1 | 1 | 1 | 2 | 1 | 1 | 1 | 1 | 1 | 134 | 147 |  |
| K.AIM(+15.99)TYVSSFYHAFSGAQK.A | N | 77.57 | 2022.9509 | 18 | 0.5 | 675.3246 | 3 | 111.48 | 2 | 31114 | Ana\_01.raw | 1.77E7 | 1.16E7 | 2.14E7 | 1.36E7 | 1.42E7 | 3.33E6 | 1.45E7 | 3.65E6 | 1.39E7 | 7.21E5 | 19 | 2 | 2 | 2 | 2 | 2 | 2 | 2 | 2 | 2 | 1 | 237 | 254 | Oxidation (M) |
| K.HTNYNM(+15.99)EHIR.V | N | 77.30 | 1329.5884 | 10 | 2.4 | 444.2045 | 3 | 20.55 | 6 | 5100 | Ana\_05.raw | 1.17E7 | 1.23E7 | 1.41E7 |  | 7.36E5 | 1.26E7 |  | 7.01E5 | 2.51E6 | 8.74E6 | 13 | 2 | 2 | 2 | 0 | 1 | 2 | 0 | 1 | 1 | 2 | 705 | 714 | Oxidation (M) |
| K.AGTQIENIEEDFRDGLK.L | N | 73.48 | 1933.9381 | 17 | 4.7 | 645.6564 | 3 | 107.05 | 1 | 29783 | ANa\_10.raw | 2.96E6 | 6.38E5 | 1.03E6 | 8.41E5 | 1.03E6 | 1.52E6 | 1.41E6 | 2.63E5 | 5.57E6 | 8.92E5 | 11 | 1 | 1 | 1 | 1 | 1 | 1 | 1 | 1 | 2 | 1 | 48 | 64 |  |
| K.VLAVNQENEQLM(+15.99)EDYEK.L | N | 72.64 | 2066.9465 | 17 | 6.7 | 1034.4874 | 2 | 69.05 | 9 | 19697 | Ana\_08.raw |  | 3.63E6 | 1.89E6 |  |  |  |  |  | 2.76E6 |  | 3 | 0 | 1 | 1 | 0 | 0 | 0 | 0 | 0 | 1 | 0 | 265 | 281 | Oxidation (M) |
| R.LSNRPAFM(+15.99)PSEGR.M | N | 71.07 | 1476.7144 | 13 | 4.2 | 493.2475 | 3 | 37.09 | 1 | 10119 | ANa\_10.raw | 7.73E7 | 0 | 8.88E7 | 3.92E7 | 4.01E7 | 8.61E7 | 5.08E7 | 5.09E7 | 5.68E7 | 3.21E7 | 25 | 2 | 2 | 3 | 3 | 2 | 4 | 2 | 2 | 2 | 3 | 347 | 359 | Oxidation (M) |
| R.ELPPDQAEYC(+57.02)IAR.M | N | 70.16 | 1560.7242 | 13 | 3.7 | 781.3723 | 2 | 63.81 | 9 | 18193 | Ana\_08.raw | 1.4E7 | 2.47E7 | 4.32E6 | 3.57E5 | 8.53E6 | 8.27E6 | 0 | 4.3E6 | 1.55E7 | 1.96E7 | 35 | 5 | 5 | 2 | 1 | 4 | 3 | 1 | 3 | 6 | 5 | 846 | 858 | Carbamidomethylation |
| K.AGTQIENIEEDFR.D | N | 69.94 | 1520.7107 | 13 | 5.0 | 761.3664 | 2 | 90.74 | 2 | 25658 | Ana\_01.raw | 6.27E6 | 7.93E6 | 6.28E6 | 6.4E5 | 3.01E6 | 6.52E6 | 1.43E6 | 2.43E6 |  | 5.02E6 | 9 | 1 | 1 | 1 | 1 | 1 | 1 | 1 | 1 | 0 | 1 | 48 | 60 |  |
| R.ETADTDTADQVM(+15.99)ASFK.I | N | 68.34 | 1744.7461 | 16 | -3.1 | 873.3776 | 2 | 60.81 | 3 | 17310 | Ana\_02.raw | 1.13E7 | 1.44E6 | 1.01E7 |  | 7.47E6 |  | 5.03E6 | 6.38E6 | 1.47E7 | 6.2E6 | 9 | 1 | 1 | 1 | 0 | 1 | 0 | 1 | 2 | 1 | 1 | 814 | 829 | Oxidation (M) |
| R.DLLLDPAWEK.Q | N | 64.97 | 1198.6233 | 10 | 5.7 | 600.3223 | 2 | 100.06 | 2 | 28148 | Ana\_01.raw | 1.22E7 | 1.24E7 | 1.75E7 | 6E6 | 8.22E6 | 6.59E6 | 7.77E6 | 7.33E6 | 4.18E7 | 1.9E6 | 11 | 1 | 1 | 2 | 1 | 1 | 1 | 1 | 1 | 1 | 1 | 22 | 31 |  |
| K.GISQEQM(+15.99)NEFR.A | N | 64.05 | 1353.5983 | 11 | 4.7 | 677.8096 | 2 | 40.92 | 2 | 11339 | Ana\_01.raw | 1.89E7 | 1.6E7 | 1.59E7 | 4.85E6 |  | 1.55E7 | 7.74E6 | 9.67E6 | 2.24E7 | 1.26E7 | 9 | 1 | 1 | 1 | 1 | 0 | 1 | 1 | 1 | 1 | 1 | 742 | 752 | Oxidation (M) |
| R.LAILGIHNEVSK.I | N | 63.18 | 1292.7452 | 12 | 4.7 | 647.3829 | 2 | 66.22 | 2 | 18881 | Ana\_01.raw | 5.66E6 | 9.88E6 | 4.06E5 | 1.14E6 | 1.17E6 | 2.67E6 |  | 6.25E5 | 1.33E7 | 7.08E6 | 11 | 1 | 2 | 1 | 1 | 1 | 1 | 0 | 1 | 1 | 2 | 566 | 577 |  |
| K.ALDFIASK.G | N | 61.40 | 863.4752 | 8 | 2.2 | 432.7458 | 2 | 61.89 | 6 | 17737 | Ana\_05.raw | 1.98E7 | 2.27E7 | 2.33E7 | 1.06E7 | 1.77E7 | 1.93E7 | 1.47E7 | 1.41E7 | 3.63E7 | 1.65E7 | 17 | 2 | 1 | 2 | 2 | 1 | 2 | 2 | 1 | 3 | 1 | 96 | 103 |  |
| R.ISIEM(+15.99)HGTLEDQLSHLR.Q | N | 61.10 | 1993.9891 | 17 | 4.1 | 499.5066 | 4 | 83.49 | 9 | 23758 | Ana\_08.raw | 1.6E6 |  |  |  |  |  |  |  | 3.84E6 |  | 2 | 1 | 0 | 0 | 0 | 0 | 0 | 0 | 0 | 1 | 0 | 656 | 672 | Oxidation (M) |
| K.NYITEDELR.R | N | 60.72 | 1151.5459 | 9 | 4.1 | 576.7826 | 2 | 61.00 | 6 | 17471 | Ana\_05.raw |  | 2.46E7 | 2.29E7 |  |  | 1.72E7 |  |  |  |  | 3 | 0 | 1 | 1 | 0 | 0 | 1 | 0 | 0 | 0 | 0 | 836 | 844 |  |
| K.EGLLLWC(+57.02)QR.K | N | 60.29 | 1173.5964 | 9 | 4.7 | 587.8082 | 2 | 94.15 | 2 | 26589 | Ana\_01.raw |  | 4.64E6 |  |  |  |  |  |  | 1.06E7 | 1.1E6 | 3 | 0 | 1 | 0 | 0 | 0 | 0 | 0 | 0 | 1 | 1 | 148 | 156 | Carbamidomethylation |
| K.DYETATLSEIK.A | N | 59.88 | 1268.6135 | 11 | 5.4 | 635.3174 | 2 | 77.87 | 8 | 21841 | Ana\_07.raw |  | 2.55E6 | 1.63E6 |  | 1.14E6 |  | 1.61E6 | 6.91E5 | 2.86E6 |  | 6 | 0 | 1 | 1 | 0 | 1 | 0 | 1 | 1 | 1 | 0 | 421 | 431 |  |
| K.M(+15.99)LDAEDIVGTARPDEK.A | N | 59.22 | 1774.8407 | 16 | 3.7 | 592.6230 | 3 | 70.68 | 6 | 20281 | Ana\_05.raw | 1.12E7 | 9.12E6 | 1.13E7 | 4.18E6 | 9.64E6 | 1.06E7 | 6.08E6 | 6.54E6 | 1.04E7 | 7.54E6 | 13 | 2 | 2 | 1 | 1 | 1 | 2 | 1 | 1 | 1 | 1 | 221 | 236 | Oxidation (M) |
| R.ASFNHFDR.K | N | 55.40 | 992.4464 | 8 | 5.0 | 497.2330 | 2 | 33.69 | 6 | 9235 | Ana\_05.raw |  |  | 3.12E6 | 5.01E6 | 2.3E6 | 1.05E7 | 7.38E6 |  | 1.08E7 |  | 10 | 0 | 0 | 1 | 2 | 1 | 2 | 2 | 1 | 1 | 0 | 753 | 760 |  |
| R.HRPELIDYGK.L | N | 54.71 | 1226.6407 | 10 | 1.4 | 409.8881 | 3 | 35.40 | 3 | 9737 | Ana\_02.raw | 1.82E7 | 1.98E7 | 2.25E7 | 8.56E6 | 1.66E7 | 2.01E7 | 1.21E7 | 1.27E7 | 2.85E7 | 2.53E7 | 11 | 1 | 1 | 1 | 1 | 1 | 1 | 1 | 1 | 1 | 2 | 186 | 195 |  |
| K.ATLPDADKER.L | N | 53.61 | 1114.5618 | 10 | 2.4 | 558.2895 | 2 | 25.66 | 2 | 6644 | Ana\_01.raw |  | 1.04E6 |  |  |  | 8.16E6 |  |  | 9.01E6 |  | 3 | 0 | 1 | 0 | 0 | 0 | 1 | 0 | 0 | 1 | 0 | 556 | 565 |  |
| R.TIPWLENR.V | N | 52.70 | 1027.5450 | 8 | 6.5 | 514.7831 | 2 | 81.81 | 1 | 23083 | ANa\_10.raw | 4.77E6 | 5.25E6 | 3.2E6 | 3.29E6 | 4.65E6 | 7.81E6 | 2.61E6 | 3.83E6 | 1.2E7 | 2.56E6 | 10 | 1 | 1 | 1 | 1 | 1 | 1 | 1 | 1 | 1 | 1 | 293 | 300 |  |
| K.TGM(+15.99)M(+15.99)DTDDFR.A | Y | 51.82 | 1219.4485 | 10 | 6.3 | 610.7354 | 2 | 29.35 | 1 | 7813 | ANa\_10.raw | 1.15E7 | 1.1E7 | 1.01E7 | 3.46E6 | 8.11E6 | 1.13E7 | 5.18E6 | 6.14E6 | 1.06E7 | 7.04E6 | 10 | 1 | 1 | 1 | 1 | 1 | 1 | 1 | 1 | 1 | 1 | 763 | 772 | Oxidation (M) |
| K.GISQEQMNEFR.A | N | 50.65 | 1337.6034 | 11 | 5.1 | 669.8124 | 2 | 58.94 | 10 | 16667 | Ana\_09.raw |  |  |  |  |  |  |  |  |  | 1.81E6 | 1 | 0 | 0 | 0 | 0 | 0 | 0 | 0 | 0 | 0 | 1 | 742 | 752 |  |
| K.IC(+57.02)DQWDNLGALTQK.R | N | 49.74 | 1660.7878 | 14 | 1.0 | 831.4020 | 2 | 92.31 | 3 | 25878 | Ana\_02.raw |  |  | 6.95E5 |  |  |  |  |  |  |  | 1 | 0 | 0 | 1 | 0 | 0 | 0 | 0 | 0 | 0 | 0 | 479 | 492 | Carbamidomethylation |
| R.VPENTM(+15.99)HAM(+15.99)QQK.L | N | 48.52 | 1444.6439 | 12 | 2.5 | 723.3311 | 2 | 16.42 | 5 | 3788 | Ana\_04.raw |  |  |  | 2.87E5 | 1.13E6 |  |  |  |  |  | 2 | 0 | 0 | 0 | 1 | 1 | 0 | 0 | 0 | 0 | 0 | 301 | 312 | Oxidation (M) |
| R.AAPFNNWM(+15.99)EGAM(+15.99)EDLQDTFIVHTIEEIQGLTTAHEQFK.A | N | 47.50 | 4393.0308 | 38 | 6.4 | 1099.2720 | 4 | 137.43 | 6 | 38175 | Ana\_05.raw |  |  |  |  |  | 1.13E6 |  |  |  |  | 1 | 0 | 0 | 0 | 0 | 0 | 1 | 0 | 0 | 0 | 0 | 518 | 555 | Oxidation (M) |
| R.FLDIPK.M | N | 46.94 | 731.4218 | 6 | 3.4 | 366.7194 | 2 | 61.25 | 9 | 17419 | Ana\_08.raw | 1.06E7 | 1.02E7 | 1.12E7 | 4.65E6 | 7.77E6 | 9.3E6 | 6.1E6 |  | 1.84E7 |  | 11 | 1 | 1 | 2 | 1 | 1 | 2 | 1 | 0 | 2 | 0 | 215 | 220 |  |
| K.SIVNYKPK.I | N | 45.23 | 947.5440 | 8 | 0.7 | 316.8555 | 3 | 28.31 | 3 | 7612 | Ana\_02.raw |  |  | 2.22E6 | 1.59E6 | 1E6 | 1.43E6 | 1.4E6 |  | 9.44E6 | 8.82E6 | 8 | 0 | 0 | 1 | 1 | 1 | 1 | 1 | 0 | 2 | 1 | 677 | 684 |  |
| R.LDHLAEK.F | N | 44.09 | 824.4392 | 7 | 2.3 | 413.2278 | 2 | 19.09 | 5 | 4680 | Ana\_04.raw |  |  |  |  | 1.46E7 | 2.65E7 | 1.21E7 | 1.15E7 |  |  | 4 | 0 | 0 | 0 | 0 | 1 | 1 | 1 | 1 | 0 | 0 | 392 | 398 |  |
| K.MEEIGR.I | N | 36.59 | 733.3428 | 6 | 4.4 | 367.6803 | 2 | 25.12 | 6 | 6593 | Ana\_05.raw |  |  |  |  |  | 4.69E6 |  | 2.55E6 |  | 3.65E6 | 3 | 0 | 0 | 0 | 0 | 0 | 1 | 0 | 1 | 0 | 1 | 650 | 655 |  |
| K.TFTAWC(+57.02)NSHLR.K | N | 36.01 | 1391.6405 | 11 | 2.5 | 464.8886 | 3 | 63.09 | 6 | 18092 | Ana\_05.raw |  |  |  |  |  | 5.73E5 |  |  |  |  | 1 | 0 | 0 | 0 | 0 | 0 | 1 | 0 | 0 | 0 | 0 | 36 | 46 | Carbamidomethylation |
| K.LEDFR.D | N | 34.55 | 678.3337 | 5 | 1.6 | 340.1747 | 2 | 32.77 | 4 | 8713 | Ana\_03.raw |  |  | 1.16E7 | 1.11E7 |  | 2.14E7 | 1.54E7 | 1.44E7 |  |  | 5 | 0 | 0 | 1 | 1 | 0 | 1 | 1 | 1 | 0 | 0 | 313 | 317 |  |
| K.ASIHEAWTDGK.E | N | 31.75 | 1213.5728 | 11 | 3.5 | 405.5329 | 3 | 41.70 | 6 | 11672 | Ana\_05.raw |  |  | 6.42E6 | 4.14E6 |  | 8.99E6 | 6.97E6 |  |  |  | 4 | 0 | 0 | 1 | 1 | 0 | 1 | 1 | 0 | 0 | 0 | 403 | 413 |  |
| total 47 peptides |  |  |  |  |  |  |  |  |  |  |  |  |  |  |  |  |  |  |  |  |  |  |  |  |  |  |  |  |  |  |  |  |  |  |  |
| --- | --- | --- | --- | --- | --- | --- | --- | --- | --- | --- | --- | --- | --- | --- | --- | --- | --- | --- | --- | --- | --- | --- | --- | --- | --- | --- | --- | --- | --- | --- | --- | --- | --- | --- | --- |

Best Unique PSM (Scan F1:7813, m/z=610.7354, z=2, RT=29.35, ppm=6.3):

sp|P68372|TBB4B\_MOUSE

back to list

  

| Protein Coverage
| Supporting Peptides
| Best Unique PSM
|

Protein Coverage:

Supporting Peptides:

| Peptide | Uniq | -10lgP | Mass | Length | ppm | m/z | z | RT | Fraction | Scan | Source File | Area 10 | Area 01 | Area 02 | Area 03 | Area 04 | Area 05 | Area 06 | Area 07 | Area 08 | Area 09 | #Spec | #Spec 10 | #Spec 01 | #Spec 02 | #Spec 03 | #Spec 04 | #Spec 05 | #Spec 06 | #Spec 07 | #Spec 08 | #Spec 09 | Start | End | PTM |
| --- | --- | --- | --- | --- | --- | --- | --- | --- | --- | --- | --- | --- | --- | --- | --- | --- | --- | --- | --- | --- | --- | --- | --- | --- | --- | --- | --- | --- | --- | --- | --- | --- | --- | --- | --- |
| R.SGPFGQIFRPDNFVFGQSGAGNNWAK.G | N | 151.86 | 2797.3359 | 26 | 0.6 | 933.4532 | 3 | 124.87 | 3 | 34288 | Ana\_02.raw | 1.28E7 | 3.44E7 | 9.58E7 | 5.21E6 | 1.06E8 | 1.1E8 | 2.56E7 | 4.47E7 | 2.04E8 | 2.41E7 | 20 | 2 | 1 | 2 | 2 | 2 | 2 | 2 | 2 | 3 | 2 | 78 | 103 |  |
| K.GHYTEGAELVDSVLDVVR.K | N | 135.92 | 1957.9745 | 18 | 3.6 | 979.9980 | 2 | 127.77 | 6 | 35505 | Ana\_05.raw | 2.36E7 | 5.44E7 | 1.6E8 | 4.71E7 | 1.26E8 | 1.66E8 | 6.78E7 | 6.96E7 | 3.65E8 | 1.97E8 | 32 | 2 | 4 | 5 | 2 | 4 | 2 | 1 | 5 | 4 | 3 | 104 | 121 |  |
| K.LTTPTYGDLNHLVSATMSGVTTC(+57.02)LR.F | N | 113.62 | 2707.3311 | 25 | 1.9 | 903.4526 | 3 | 131.13 | 6 | 36433 | Ana\_05.raw |  | 5.46E5 | 9.82E5 | 4.59E6 | 4.43E6 | 9.86E6 | 2.28E6 | 7.01E6 | 3.92E7 | 2.05E7 | 9 | 0 | 1 | 1 | 1 | 1 | 1 | 1 | 1 | 1 | 1 | 217 | 241 | Carbamidomethylation |
| K.FWEVISDEHGIDPTGTYHGDSDLQLER.I | N | 111.17 | 3115.4158 | 27 | 1.9 | 779.8627 | 4 | 103.47 | 9 | 29159 | Ana\_08.raw |  |  |  |  |  |  |  |  | 3.85E7 |  | 1 | 0 | 0 | 0 | 0 | 0 | 0 | 0 | 0 | 1 | 0 | 20 | 46 |  |
| K.NSSYFVEWIPNNVK.T | N | 110.81 | 1695.8257 | 14 | 0.6 | 848.9207 | 2 | 109.87 | 3 | 30437 | Ana\_02.raw | 3.45E7 | 3.29E7 | 2.2E7 | 1.6E7 | 2.71E7 | 1.96E7 | 1.72E7 | 1.87E7 | 6.7E7 | 2.73E7 | 21 | 2 | 3 | 2 | 1 | 4 | 1 | 3 | 1 | 3 | 1 | 337 | 350 |  |
| R.LHFFM(+15.99)PGFAPLTSR.G | N | 108.26 | 1635.8231 | 14 | 1.6 | 546.2825 | 3 | 100.14 | 3 | 27934 | Ana\_02.raw | 1.44E8 | 1.06E8 | 1.27E8 | 4.7E6 | 1.01E8 | 8.11E7 | 1.18E7 | 4.61E7 | 3.33E8 | 1.08E8 | 72 | 8 | 9 | 7 | 5 | 7 | 7 | 4 | 8 | 9 | 8 | 263 | 276 | Oxidation (M) |
| R.LHFFMPGFAPLTSR.G | N | 97.12 | 1619.8282 | 14 | 6.0 | 540.9532 | 3 | 118.11 | 1 | 32615 | ANa\_10.raw | 3.14E7 | 1.19E7 |  | 8.19E5 | 3.08E5 | 5.07E6 |  | 2.64E5 | 3.8E7 | 2.23E7 | 17 | 3 | 3 | 0 | 1 | 1 | 1 | 0 | 1 | 3 | 4 | 263 | 276 |  |
| K.LTTPTYGDLNHLVSATM(+15.99)SGVTTC(+57.02)LR.F | N | 96.84 | 2723.3259 | 25 | 3.5 | 908.7858 | 3 | 116.60 | 2 | 32447 | Ana\_01.raw | 6.74E7 | 3.02E7 | 1.26E7 | 8.03E6 | 2E7 | 2.71E7 | 5.03E6 | 2.06E7 | 7.16E7 | 2.84E7 | 26 | 4 | 3 | 2 | 3 | 2 | 2 | 2 | 1 | 5 | 2 | 217 | 241 | Oxidation (M); Carbamidomethylation |
| R.INVYYNEATGGK.Y | Y | 83.59 | 1327.6407 | 12 | 4.8 | 664.8308 | 2 | 54.40 | 1 | 15327 | ANa\_10.raw | 3.84E7 | 3.93E7 | 3.34E7 | 1.72E7 | 3.01E7 | 3.22E7 | 2.38E7 | 2.17E7 | 6.64E7 | 3.83E7 | 11 | 1 | 2 | 1 | 1 | 1 | 1 | 1 | 1 | 1 | 1 | 47 | 58 |  |
| K.EVDEQM(+15.99)LNVQNK.N | N | 82.36 | 1461.6770 | 12 | 4.6 | 731.8491 | 2 | 41.89 | 6 | 11733 | Ana\_05.raw | 4.47E7 | 4.06E7 | 4.89E7 | 1.69E7 | 3.72E7 | 3.72E7 | 2.65E7 | 3.63E7 | 4.7E7 | 3.59E7 | 25 | 2 | 2 | 3 | 2 | 3 | 2 | 2 | 4 | 2 | 3 | 325 | 336 | Oxidation (M) |
| K.MSATFIGNSTAIQELFK.R | N | 82.10 | 1856.9342 | 17 | 4.7 | 929.4788 | 2 | 128.68 | 6 | 35752 | Ana\_05.raw | 2.9E6 | 3.03E6 |  |  |  | 1.87E6 |  | 1.08E6 | 4.78E6 |  | 5 | 1 | 1 | 0 | 0 | 0 | 1 | 0 | 1 | 1 | 0 | 363 | 379 |  |
| R.FPGQLNADLR.K | N | 78.02 | 1129.5880 | 10 | 3.0 | 565.8030 | 2 | 69.05 | 6 | 19811 | Ana\_05.raw | 7.68E7 | 5.71E7 | 5.55E7 | 2.67E7 | 6.02E7 | 4.21E7 | 2.93E7 | 4.94E7 | 1.57E8 | 8.76E7 | 45 | 4 | 4 | 4 | 4 | 4 | 5 | 3 | 5 | 6 | 6 | 242 | 251 |  |
| K.LAVNM(+15.99)VPFPR.L | N | 76.83 | 1158.6219 | 10 | 4.1 | 580.3206 | 2 | 79.35 | 6 | 22695 | Ana\_05.raw | 8.22E7 | 5.68E7 | 5.35E7 | 2.41E7 | 4.94E7 | 5.03E7 | 3.23E7 | 3.95E7 | 1.48E8 | 5.62E7 | 48 | 6 | 5 | 4 | 4 | 4 | 4 | 4 | 6 | 7 | 4 | 253 | 262 | Oxidation (M) |
| K.M(+15.99)SATFIGNSTAIQELFK.R | N | 76.67 | 1872.9291 | 17 | 5.1 | 937.4766 | 2 | 123.87 | 6 | 34493 | Ana\_05.raw | 5.15E6 | 2.88E6 | 9.44E6 | 3.34E5 | 1.38E6 | 1.15E7 | 5.53E6 |  | 2.21E7 | 3.6E6 | 11 | 2 | 1 | 1 | 1 | 1 | 1 | 1 | 0 | 2 | 1 | 363 | 379 | Oxidation (M) |
| R.IM(+15.99)NTFSVVPSPK.V | N | 72.89 | 1334.6904 | 12 | 2.0 | 668.3538 | 2 | 67.16 | 3 | 19097 | Ana\_02.raw | 4.81E7 | 3.48E7 | 2.62E7 | 1.36E7 | 3.4E7 | 2.79E7 | 2.17E7 | 2.24E7 | 6.59E7 | 3.08E7 | 21 | 2 | 3 | 2 | 2 | 2 | 2 | 2 | 2 | 3 | 1 | 163 | 174 | Oxidation (M) |
| R.ALTVPELTQQM(+15.99)FDAK.N | N | 71.14 | 1706.8549 | 15 | 4.9 | 854.4389 | 2 | 89.92 | 3 | 25244 | Ana\_02.raw | 3.6E7 | 2.33E7 | 1.45E7 | 4.92E6 | 1.46E7 | 1.85E7 | 9.93E6 | 1.33E7 | 7.58E7 | 9.88E6 | 26 | 4 | 2 | 3 | 1 | 2 | 3 | 3 | 1 | 5 | 2 | 283 | 297 | Oxidation (M) |
| K.LAVNMVPFPR.L | N | 69.21 | 1142.6270 | 10 | 5.4 | 572.3239 | 2 | 92.17 | 1 | 25869 | ANa\_10.raw | 6.59E6 |  |  |  |  | 1.83E5 |  |  | 1.45E6 |  | 3 | 1 | 0 | 0 | 0 | 0 | 1 | 0 | 0 | 1 | 0 | 253 | 262 |  |
| R.AVLVDLEPGTM(+15.99)DSVR.S | N | 67.26 | 1616.8080 | 15 | 4.5 | 809.4149 | 2 | 79.74 | 5 | 22629 | Ana\_04.raw | 1.39E7 | 1.65E7 | 1.58E7 | 7.18E6 | 1.33E7 |  | 5.23E6 | 6.6E6 | 3.5E7 | 9.08E6 | 13 | 2 | 2 | 1 | 1 | 2 | 0 | 1 | 1 | 2 | 1 | 63 | 77 | Oxidation (M) |
| R.ISEQFTAM(+15.99)FR.R | N | 65.28 | 1244.5859 | 10 | 1.5 | 623.3012 | 2 | 70.89 | 3 | 20141 | Ana\_02.raw | 2.85E7 | 2.58E7 | 1.92E7 | 1E7 | 8.26E6 | 1.54E7 | 6.67E6 | 1.69E7 | 2.69E7 | 1.4E7 | 10 | 1 | 1 | 1 | 1 | 1 | 1 | 1 | 1 | 1 | 1 | 381 | 390 | Oxidation (M) |
| K.IREEYPDR.I | N | 65.00 | 1076.5250 | 8 | 2.5 | 539.2711 | 2 | 25.17 | 6 | 6612 | Ana\_05.raw | 8.6E7 | 1.16E8 | 1.13E8 | 5.23E7 | 7.83E7 | 1.03E8 | 7.1E7 | 6.1E7 | 5.8E7 | 1.33E8 | 27 | 3 | 2 | 2 | 3 | 2 | 2 | 3 | 3 | 4 | 3 | 155 | 162 |  |
| R.YLTVAAVFR.G | N | 63.70 | 1038.5862 | 9 | 3.5 | 520.3022 | 2 | 93.88 | 6 | 26627 | Ana\_05.raw | 7.84E6 | 9.68E6 | 1.04E7 | 5.49E6 | 8.6E6 | 8.9E6 | 7.22E6 | 3.63E6 | 1.87E7 | 1.03E7 | 15 | 2 | 2 | 1 | 1 | 2 | 1 | 1 | 1 | 2 | 2 | 310 | 318 |  |
| R.EIVHLQAGQC(+57.02)GNQIGAK.F | N | 61.23 | 1821.9155 | 17 | 6.6 | 911.9710 | 2 | 49.98 | 9 | 14043 | Ana\_08.raw |  |  |  |  |  |  |  |  | 4.97E6 |  | 2 | 0 | 0 | 0 | 0 | 0 | 0 | 0 | 0 | 2 | 0 | 3 | 19 | Carbamidomethylation |
| K.TAVC(+57.02)DIPPR.G | N | 54.26 | 1027.5121 | 9 | 1.9 | 514.7643 | 2 | 37.94 | 6 | 10515 | Ana\_05.raw | 1.32E7 | 1.04E7 | 1.14E7 | 1.69E6 | 9.99E6 | 4.43E7 | 1.78E7 | 6.51E6 | 1.8E7 | 5.15E7 | 32 | 3 | 3 | 3 | 3 | 3 | 4 | 3 | 2 | 2 | 6 | 351 | 359 | Carbamidomethylation |
| R.ALTVPELTQQMFDAK.N | N | 51.60 | 1690.8600 | 15 | 9.7 | 846.4455 | 2 | 117.55 | 10 | 32318 | Ana\_09.raw |  | 3.09E6 |  |  |  | 1.51E6 |  | 3.44E5 |  | 2.19E6 | 4 | 0 | 1 | 0 | 0 | 0 | 1 | 0 | 1 | 0 | 1 | 283 | 297 |  |
| R.KLAVNM(+15.99)VPFPR.L | N | 49.45 | 1286.7169 | 11 | 2.9 | 429.9142 | 3 | 66.65 | 5 | 19005 | Ana\_04.raw |  |  |  | 2.22E6 | 4.19E6 | 8.25E6 |  | 2.66E6 | 3.2E6 |  | 5 | 0 | 0 | 0 | 1 | 1 | 1 | 0 | 1 | 1 | 0 | 252 | 262 | Oxidation (M) |
| total 25 peptides |  |  |  |  |  |  |  |  |  |  |  |  |  |  |  |  |  |  |  |  |  |  |  |  |  |  |  |  |  |  |  |  |  |  |  |
| --- | --- | --- | --- | --- | --- | --- | --- | --- | --- | --- | --- | --- | --- | --- | --- | --- | --- | --- | --- | --- | --- | --- | --- | --- | --- | --- | --- | --- | --- | --- | --- | --- | --- | --- | --- |

Best Unique PSM (Scan F1:15327, m/z=664.8308, z=2, RT=54.40, ppm=4.8):

sp|Q9QXS1|PLEC\_MOUSE

back to list

  

| Protein Coverage
| Supporting Peptides
| Best Unique PSM
|

Protein Coverage:

Supporting Peptides:

| Peptide | Uniq | -10lgP | Mass | Length | ppm | m/z | z | RT | Fraction | Scan | Source File | Area 10 | Area 01 | Area 02 | Area 03 | Area 04 | Area 05 | Area 06 | Area 07 | Area 08 | Area 09 | #Spec | #Spec 10 | #Spec 01 | #Spec 02 | #Spec 03 | #Spec 04 | #Spec 05 | #Spec 06 | #Spec 07 | #Spec 08 | #Spec 09 | Start | End | PTM |
| --- | --- | --- | --- | --- | --- | --- | --- | --- | --- | --- | --- | --- | --- | --- | --- | --- | --- | --- | --- | --- | --- | --- | --- | --- | --- | --- | --- | --- | --- | --- | --- | --- | --- | --- | --- |
| R.ALVPAAELLDSGVISHELYQQLQR.G | Y | 127.18 | 2649.4126 | 24 | 1.0 | 884.1457 | 3 | 131.56 | 3 | 36039 | Ana\_02.raw | 8.63E6 | 9.5E6 | 4.95E6 | 2.59E6 | 5.07E6 | 1.01E7 | 4.39E6 |  | 1.16E7 | 8.18E6 | 9 | 1 | 1 | 1 | 1 | 1 | 1 | 1 | 0 | 1 | 1 | 3123 | 3146 |  |
| K.GFFDPNTHENLTYLQLLER.C | N | 112.87 | 2306.1331 | 19 | 4.9 | 769.7220 | 3 | 126.30 | 2 | 34999 | Ana\_01.raw | 2.27E6 | 6.61E6 | 4.35E6 |  |  | 1.49E7 |  | 1.3E6 | 1.31E7 | 9.32E6 | 7 | 1 | 1 | 1 | 0 | 0 | 1 | 0 | 1 | 1 | 1 | 2996 | 3014 |  |
| R.DGHNLISLLEVLSGDSLPR.E | Y | 101.17 | 2034.0745 | 19 | 4.1 | 679.0349 | 3 | 136.71 | 7 | 37642 | Ana\_06.raw |  | 3.99E5 |  |  |  |  | 3.36E5 |  |  | 9.06E5 | 3 | 0 | 1 | 0 | 0 | 0 | 0 | 1 | 0 | 0 | 1 | 220 | 238 |  |
| R.AALAHSEIATTQAASTK.A | Y | 90.47 | 1669.8635 | 17 | 3.3 | 835.9418 | 2 | 34.31 | 9 | 9192 | Ana\_08.raw |  |  |  |  |  |  |  |  | 5.07E5 |  | 1 | 0 | 0 | 0 | 0 | 0 | 0 | 0 | 0 | 1 | 0 | 2752 | 2768 |  |
| R.QTNLENLDQAFSVAER.D | Y | 77.21 | 1833.8856 | 16 | 8.7 | 917.9581 | 2 | 119.49 | 6 | 33357 | Ana\_05.raw |  |  |  |  |  | 3.81E5 |  |  |  |  | 1 | 0 | 0 | 0 | 0 | 0 | 1 | 0 | 0 | 0 | 0 | 361 | 376 |  |
| R.LGFHLPLEVAYQR.G | Y | 76.79 | 1541.8354 | 13 | 5.5 | 514.9553 | 3 | 101.01 | 9 | 28517 | Ana\_08.raw |  |  | 8.88E5 |  |  | 3.53E5 |  |  | 5.45E5 |  | 3 | 0 | 0 | 1 | 0 | 0 | 1 | 0 | 0 | 1 | 0 | 3958 | 3970 |  |
| K.VLALPEPSPAAPTLR.S | Y | 75.37 | 1530.8770 | 15 | 3.0 | 766.4481 | 2 | 85.55 | 9 | 24333 | Ana\_08.raw | 5.19E6 | 1.29E6 |  |  |  | 1.56E6 |  |  | 1.11E7 | 4.13E6 | 5 | 1 | 1 | 0 | 0 | 0 | 1 | 0 | 0 | 1 | 1 | 1195 | 1209 |  |
| R.ESADPLSAWLQDAK.R | Y | 71.10 | 1529.7361 | 14 | -1.0 | 765.8746 | 2 | 117.52 | 6 | 32833 | Ana\_05.raw | 1.74E6 |  |  |  |  | 1.2E6 |  |  |  | 3.77E5 | 3 | 1 | 0 | 0 | 0 | 0 | 1 | 0 | 0 | 0 | 1 | 1359 | 1372 |  |
| R.RAAEEAEEAR.E | Y | 69.03 | 1130.5315 | 10 | 3.5 | 566.2750 | 2 | 17.29 | 6 | 4028 | Ana\_05.raw |  |  |  |  | 6.71E5 | 5.93E5 | 4.04E5 | 2.8E5 |  |  | 4 | 0 | 0 | 0 | 0 | 1 | 1 | 1 | 1 | 0 | 0 | 2233 | 2242 |  |
| R.LQHEATAATQK.R | Y | 68.24 | 1196.6150 | 11 | 3.0 | 599.3166 | 2 | 16.72 | 4 | 3876 | Ana\_03.raw |  |  |  | 1.26E6 |  | 2.46E6 |  | 8.41E5 |  |  | 4 | 0 | 0 | 0 | 2 | 0 | 1 | 0 | 1 | 0 | 0 | 1884 | 1894 |  |
| R.DSQDAGGFGPEDR.L | Y | 66.07 | 1349.5483 | 13 | 3.5 | 675.7838 | 2 | 39.82 | 9 | 10919 | Ana\_08.raw | 3.05E6 |  |  |  |  |  |  |  | 1.32E6 |  | 2 | 1 | 0 | 0 | 0 | 0 | 0 | 0 | 0 | 1 | 0 | 1084 | 1096 |  |
| R.LLEAAAQSSK.G | Y | 65.88 | 1016.5502 | 10 | 3.5 | 509.2841 | 2 | 27.57 | 6 | 7369 | Ana\_05.raw |  |  |  |  | 1.83E6 | 2.62E6 | 1.59E6 |  |  |  | 3 | 0 | 0 | 0 | 0 | 1 | 1 | 1 | 0 | 0 | 0 | 4607 | 4616 |  |
| K.ATVSAPFGK.F | Y | 64.27 | 876.4705 | 9 | 4.1 | 439.2443 | 2 | 44.24 | 6 | 12463 | Ana\_05.raw |  |  |  |  | 4.87E6 | 6.66E6 | 4.95E6 |  | 1.13E6 |  | 4 | 0 | 0 | 0 | 0 | 1 | 1 | 1 | 0 | 1 | 0 | 3052 | 3060 |  |
| K.EAQAVPATLQELEATK.A | Y | 64.04 | 1697.8835 | 16 | 3.6 | 849.9521 | 2 | 87.57 | 9 | 24879 | Ana\_08.raw |  |  |  |  |  | 3.43E5 |  |  | 1.98E6 |  | 2 | 0 | 0 | 0 | 0 | 0 | 1 | 0 | 0 | 1 | 0 | 1259 | 1274 |  |
| R.YSELTTLTSQYIK.F | Y | 63.03 | 1545.7926 | 13 | 0.9 | 773.9042 | 2 | 96.65 | 9 | 27354 | Ana\_08.raw | 1.05E6 |  |  |  |  |  |  |  | 4.78E6 | 1.15E6 | 3 | 1 | 0 | 0 | 0 | 0 | 0 | 0 | 0 | 1 | 1 | 1468 | 1480 |  |
| K.AQVEQELTTLR.L | Y | 62.37 | 1286.6830 | 11 | 5.4 | 644.3522 | 2 | 69.30 | 6 | 19883 | Ana\_05.raw | 3.64E6 | 3.92E6 | 1.29E6 | 7.32E5 | 2.67E6 | 1.74E6 | 1.58E6 |  |  | 3.34E6 | 8 | 1 | 1 | 1 | 1 | 1 | 1 | 1 | 0 | 0 | 1 | 2327 | 2337 |  |
| R.SQVEEELFSVR.V | Y | 61.44 | 1321.6514 | 11 | 3.5 | 661.8353 | 2 | 87.28 | 6 | 24854 | Ana\_05.raw | 3.38E6 |  |  | 3.22E5 | 8.35E5 | 2.79E6 | 1.79E6 | 8.99E5 | 9.14E6 | 2.67E6 | 8 | 1 | 0 | 0 | 1 | 1 | 1 | 1 | 1 | 1 | 1 | 2369 | 2379 |  |
| R.IISLETYNLFR.E | Y | 60.67 | 1367.7449 | 11 | 0.5 | 684.8801 | 2 | 118.50 | 7 | 32749 | Ana\_06.raw | 3.57E6 | 3.41E6 |  | 1.36E6 | 4.17E5 | 5.32E5 | 1.33E6 | 8.68E5 | 4.07E6 | 3.03E6 | 9 | 1 | 1 | 0 | 1 | 1 | 1 | 1 | 1 | 1 | 1 | 3801 | 3811 |  |
| R.SWSLVTFR.T | Y | 58.05 | 994.5236 | 8 | 2.7 | 498.2704 | 2 | 97.31 | 6 | 27538 | Ana\_05.raw |  |  |  |  |  | 4.16E5 |  |  |  |  | 1 | 0 | 0 | 0 | 0 | 0 | 1 | 0 | 0 | 0 | 0 | 1053 | 1060 |  |
| R.RLEEQAALHK.A | Y | 57.89 | 1193.6516 | 10 | 3.9 | 398.8927 | 3 | 22.60 | 9 | 5496 | Ana\_08.raw |  |  |  |  |  |  |  |  | 2.02E6 |  | 1 | 0 | 0 | 0 | 0 | 0 | 0 | 0 | 0 | 1 | 0 | 2024 | 2033 |  |
| R.APVPASELLDAK.I | Y | 57.63 | 1209.6604 | 12 | 5.6 | 605.8409 | 2 | 69.94 | 10 | 19807 | Ana\_09.raw |  |  |  |  | 6.15E6 |  | 2.67E6 |  | 1.04E7 | 6.75E6 | 4 | 0 | 0 | 0 | 0 | 1 | 0 | 1 | 0 | 1 | 1 | 3454 | 3465 |  |
| R.ILSDPSDDTK.G | N | 56.91 | 1089.5189 | 10 | 5.1 | 545.7695 | 2 | 28.12 | 3 | 7553 | Ana\_02.raw | 7.89E6 | 1.53E7 | 5.42E6 |  |  |  | 2.54E6 |  | 4.9E6 |  | 5 | 1 | 1 | 1 | 0 | 0 | 0 | 1 | 0 | 1 | 0 | 2986 | 2995 |  |
| R.REEAAVDAQQQK.R | Y | 56.33 | 1371.6742 | 12 | 5.0 | 686.8478 | 2 | 18.08 | 5 | 4340 | Ana\_04.raw |  |  |  |  | 5.97E5 | 1.13E6 |  |  |  | 4.66E5 | 3 | 0 | 0 | 0 | 0 | 1 | 1 | 0 | 0 | 0 | 1 | 1549 | 1560 |  |
| K.AGVGAPVTQVTLQSTQR.R | Y | 55.85 | 1711.9216 | 17 | 5.5 | 856.9728 | 2 | 62.98 | 9 | 17940 | Ana\_08.raw |  |  |  |  |  |  |  |  | 7.67E5 |  | 1 | 0 | 0 | 0 | 0 | 0 | 0 | 0 | 0 | 1 | 0 | 642 | 658 |  |
| R.LAEDEAFQR.R | Y | 54.65 | 1077.5090 | 9 | 7.2 | 539.7657 | 2 | 43.71 | 10 | 12188 | Ana\_09.raw | 5.08E6 |  |  | 1.76E6 | 7.82E6 |  | 4.83E6 |  |  | 5.23E6 | 5 | 1 | 0 | 0 | 1 | 1 | 0 | 1 | 0 | 0 | 1 | 2014 | 2022 |  |
| K.AQLEPVASPAK.K | Y | 53.81 | 1109.6080 | 11 | 4.0 | 555.8135 | 2 | 35.17 | 9 | 9467 | Ana\_08.raw | 2.54E6 | 2.22E6 |  |  |  |  |  |  | 2.35E6 | 2.52E6 | 4 | 1 | 1 | 0 | 0 | 0 | 0 | 0 | 0 | 1 | 1 | 1436 | 1446 |  |
| R.LTAEDLYEAR.I | Y | 52.86 | 1179.5771 | 10 | 2.7 | 590.7974 | 2 | 71.35 | 6 | 20475 | Ana\_05.raw |  | 2.29E5 | 1.7E6 |  | 2.46E6 | 2.39E6 |  |  | 4.48E6 | 5.54E5 | 6 | 0 | 1 | 1 | 0 | 1 | 1 | 0 | 0 | 1 | 1 | 3791 | 3800 |  |
| K.ELIPAEEALR.L | Y | 52.23 | 1139.6185 | 10 | 5.2 | 570.8195 | 2 | 69.98 | 9 | 19976 | Ana\_08.raw | 3.02E6 | 2.89E6 |  |  |  |  |  |  | 3.74E6 | 2.33E6 | 4 | 1 | 1 | 0 | 0 | 0 | 0 | 0 | 0 | 1 | 1 | 3933 | 3942 |  |
| R.LLFNDVQTLK.D | Y | 52.10 | 1189.6707 | 10 | 6.5 | 595.8465 | 2 | 84.30 | 9 | 23988 | Ana\_08.raw | 1.42E6 |  |  |  | 7.1E5 | 1.35E6 | 0 | 4.61E5 | 3.08E6 | 9.64E5 | 7 | 1 | 0 | 0 | 0 | 1 | 1 | 1 | 1 | 1 | 1 | 599 | 608 |  |
| R.SLAAEEEAAR.Q | Y | 50.51 | 1045.5039 | 10 | 4.6 | 523.7617 | 2 | 31.23 | 9 | 8246 | Ana\_08.raw |  |  |  |  |  |  |  |  | 9.67E6 | 1.15E7 | 2 | 0 | 0 | 0 | 0 | 0 | 0 | 0 | 0 | 1 | 1 | 2138 | 2147 |  |
| R.VPVDVAYK.R | Y | 50.22 | 889.4909 | 8 | 5.6 | 445.7552 | 2 | 43.42 | 1 | 12067 | ANa\_10.raw | 2.6E6 |  |  |  |  |  |  |  |  | 2.23E6 | 2 | 1 | 0 | 0 | 0 | 0 | 0 | 0 | 0 | 0 | 1 | 2968 | 2975 |  |
| R.LSVAAQEAAR.L | Y | 49.98 | 1014.5458 | 10 | 4.9 | 508.2827 | 2 | 34.89 | 7 | 9457 | Ana\_06.raw |  |  | 4.12E6 | 3.3E6 | 4.79E6 | 5.28E6 | 3.68E6 | 3.05E6 | 5.2E6 | 6.63E6 | 8 | 0 | 0 | 1 | 1 | 1 | 1 | 1 | 1 | 1 | 1 | 2428 | 2437 |  |
| R.AQAEAQQPVFNTLR.D | Y | 49.93 | 1571.8055 | 14 | 5.7 | 786.9146 | 2 | 64.57 | 10 | 18295 | Ana\_09.raw |  |  |  |  |  |  |  |  |  | 5.46E5 | 1 | 0 | 0 | 0 | 0 | 0 | 0 | 0 | 0 | 0 | 1 | 1282 | 1295 |  |
| R.SIQEELQHLR.Q | Y | 49.92 | 1251.6571 | 10 | 6.7 | 418.2291 | 3 | 68.06 | 9 | 19423 | Ana\_08.raw | 9.51E5 |  |  |  |  | 9.04E5 |  |  | 1.32E6 |  | 3 | 1 | 0 | 0 | 0 | 0 | 1 | 0 | 0 | 1 | 0 | 1562 | 1571 |  |
| R.GGAEGELQALR.A | Y | 48.78 | 1099.5621 | 11 | 6.5 | 550.7919 | 2 | 56.31 | 1 | 15889 | ANa\_10.raw | 4.04E6 | 3.33E6 |  |  | 1.14E6 |  |  |  | 6.27E6 |  | 4 | 1 | 1 | 0 | 0 | 1 | 0 | 0 | 0 | 1 | 0 | 1614 | 1624 |  |
| R.LQAEEVAQQK.S | Y | 48.30 | 1142.5931 | 10 | 6.4 | 572.3075 | 2 | 29.99 | 1 | 8003 | ANa\_10.raw | 3.62E6 | 4.9E6 |  |  |  |  |  |  |  | 4.58E6 | 3 | 1 | 1 | 0 | 0 | 0 | 0 | 0 | 0 | 0 | 1 | 1791 | 1800 |  |
| R.ALQALDELR.L | Y | 47.02 | 1027.5662 | 9 | -2.4 | 514.7891 | 2 | 71.91 | 6 | 20634 | Ana\_05.raw | 1.42E6 |  | 1.2E6 | 3.95E5 | 1.72E6 | 2.43E6 | 3.29E5 |  |  | 2.12E6 | 7 | 1 | 0 | 1 | 1 | 1 | 1 | 1 | 0 | 0 | 1 | 1679 | 1687 |  |
| R.LAAEQELIR.L | Y | 46.61 | 1041.5818 | 9 | 5.0 | 521.8008 | 2 | 52.68 | 9 | 14874 | Ana\_08.raw | 3.78E6 | 4.5E6 | 2.25E6 |  | 2.67E6 | 4.03E6 | 2.33E6 | 2.27E5 | 9.9E6 | 4.17E6 | 9 | 1 | 1 | 1 | 0 | 1 | 1 | 1 | 1 | 1 | 1 | 1854 | 1862 |  |
| R.EVAEADSVR.Q | Y | 46.53 | 974.4669 | 9 | 3.5 | 488.2424 | 2 | 23.72 | 6 | 6150 | Ana\_05.raw |  |  |  |  | 5.65E6 | 3.88E6 | 1.93E6 | 1.45E6 |  |  | 4 | 0 | 0 | 0 | 0 | 1 | 1 | 1 | 1 | 0 | 0 | 3153 | 3161 |  |
| R.AGLVGPELHEK.L | Y | 45.61 | 1148.6189 | 11 | 9.5 | 383.8839 | 3 | 37.83 | 9 | 10291 | Ana\_08.raw |  |  |  |  |  |  |  |  | 1.38E6 |  | 1 | 0 | 0 | 0 | 0 | 0 | 0 | 0 | 0 | 1 | 0 | 3228 | 3238 |  |
| R.VLADPSDDTK.G | Y | 44.67 | 1059.5084 | 10 | 3.7 | 530.7634 | 2 | 27.03 | 5 | 7172 | Ana\_04.raw |  |  | 3.11E6 | 1.13E6 | 2.54E6 |  |  | 2.59E6 | 5.37E6 |  | 5 | 0 | 0 | 1 | 1 | 1 | 0 | 0 | 1 | 1 | 0 | 3645 | 3654 |  |
| R.LSFSGLR.A | Y | 44.14 | 778.4337 | 7 | 5.8 | 390.2264 | 2 | 62.35 | 2 | 17757 | Ana\_01.raw |  | 1.11E6 |  |  | 1.08E6 | 1.33E6 |  |  | 2.27E6 | 1.57E6 | 5 | 0 | 1 | 0 | 0 | 1 | 1 | 0 | 0 | 1 | 1 | 3447 | 3453 |  |
| R.ELAEQELEK.Q | Y | 43.77 | 1087.5397 | 9 | -0.8 | 544.7767 | 2 | 44.07 | 7 | 12228 | Ana\_06.raw | 7.48E6 |  |  |  | 1.49E8 | 2.04E8 | 1.37E8 |  | 9.97E5 |  | 5 | 1 | 0 | 0 | 0 | 1 | 1 | 1 | 0 | 1 | 0 | 1833 | 1841 |  |
| K.SILDEELQR.L | Y | 43.52 | 1101.5665 | 9 | 3.3 | 551.7924 | 2 | 69.67 | 1 | 19739 | ANa\_10.raw | 1.26E6 |  |  |  |  | 5.52E5 |  |  | 3.85E6 |  | 3 | 1 | 0 | 0 | 0 | 0 | 1 | 0 | 0 | 1 | 0 | 2348 | 2356 |  |
| K.AQAEVEGLGK.G | Y | 43.41 | 1000.5189 | 10 | 6.0 | 501.2697 | 2 | 32.08 | 10 | 8680 | Ana\_09.raw | 2.75E6 | 4.77E6 | 1.28E6 |  |  | 4.67E6 | 3.76E6 |  | 1.86E6 | 1.03E6 | 7 | 1 | 1 | 1 | 0 | 0 | 1 | 1 | 0 | 1 | 1 | 1174 | 1183 |  |
| K.AHAFVVQQR.E | Y | 43.27 | 1054.5671 | 9 | 3.9 | 528.2929 | 2 | 25.05 | 9 | 6325 | Ana\_08.raw | 7.46E5 | 9.33E5 |  |  |  |  |  |  | 2.04E6 | 6.23E5 | 4 | 1 | 1 | 0 | 0 | 0 | 0 | 0 | 0 | 1 | 1 | 2198 | 2206 |  |
| R.SM(+15.99)VEEGTGLR.L | Y | 41.83 | 1093.5073 | 10 | 3.9 | 547.7631 | 2 | 28.77 | 1 | 7631 | ANa\_10.raw | 5.24E6 |  |  |  |  | 3.34E6 |  |  |  |  | 2 | 1 | 0 | 0 | 0 | 0 | 1 | 0 | 0 | 0 | 0 | 4597 | 4606 | Oxidation (M) |
| R.LQLETTER.Q | Y | 41.80 | 988.5189 | 8 | 5.8 | 495.2696 | 2 | 38.21 | 10 | 10508 | Ana\_09.raw | 2.45E6 |  |  |  |  |  |  |  | 2.82E6 | 2.85E6 | 3 | 1 | 0 | 0 | 0 | 0 | 0 | 0 | 0 | 1 | 1 | 1604 | 1611 |  |
| R.LTVDEAVR.A | Y | 41.70 | 901.4869 | 8 | 2.7 | 451.7519 | 2 | 44.40 | 4 | 12143 | Ana\_03.raw | 1.58E7 | 2.22E7 | 5.83E6 | 2.51E6 |  | 2.28E7 | 9.78E6 | 6.94E6 | 4.2E6 | 5.63E6 | 9 | 1 | 1 | 1 | 1 | 0 | 1 | 1 | 1 | 1 | 1 | 3220 | 3227 |  |
| R.NDESQLSPATR.G | Y | 41.53 | 1216.5684 | 11 | 4.3 | 609.2941 | 2 | 30.61 | 2 | 8211 | Ana\_01.raw | 6.31E6 | 1.71E6 |  |  |  |  |  |  |  |  | 2 | 1 | 1 | 0 | 0 | 0 | 0 | 0 | 0 | 0 | 0 | 722 | 732 |  |
| R.FLEEEAEK.M | Y | 40.31 | 993.4655 | 8 | 4.7 | 497.7423 | 2 | 37.08 | 2 | 10155 | Ana\_01.raw | 4.58E6 | 3.46E6 |  | 1.73E6 |  | 3.31E6 | 4.9E6 | 4.15E6 | 3.75E6 | 4.27E6 | 8 | 1 | 1 | 0 | 1 | 0 | 1 | 1 | 1 | 1 | 1 | 2410 | 2417 |  |
| R.EAIAELER.E | Y | 40.22 | 929.4818 | 8 | 3.8 | 465.7499 | 2 | 47.88 | 4 | 13155 | Ana\_03.raw |  |  |  | 8.98E5 |  |  | 8.55E5 |  |  |  | 2 | 0 | 0 | 0 | 1 | 0 | 0 | 1 | 0 | 0 | 0 | 2595 | 2602 |  |
| K.AQAELEAQELQR.R | Y | 40.19 | 1384.6946 | 12 | 7.7 | 693.3599 | 2 | 48.98 | 9 | 13731 | Ana\_08.raw |  |  |  |  |  |  |  |  | 5.78E6 |  | 1 | 0 | 0 | 0 | 0 | 0 | 0 | 0 | 0 | 1 | 0 | 1529 | 1540 |  |
| K.ALLEEIER.H | Y | 39.10 | 971.5287 | 8 | 4.4 | 486.7738 | 2 | 68.29 | 1 | 19355 | ANa\_10.raw | 2.5E6 |  |  |  |  |  |  |  | 4.17E6 | 2.92E6 | 3 | 1 | 0 | 0 | 0 | 0 | 0 | 0 | 0 | 1 | 1 | 1398 | 1405 |  |
| R.ELAEEAAR.L | Y | 38.16 | 887.4348 | 8 | 3.8 | 444.7263 | 2 | 23.18 | 10 | 5896 | Ana\_09.raw |  |  |  |  |  | 3.87E6 |  |  |  | 3.79E6 | 2 | 0 | 0 | 0 | 0 | 0 | 1 | 0 | 0 | 0 | 1 | 1943 | 1950 |  |
| R.IEEEIR.V | N | 37.84 | 787.4075 | 6 | 3.6 | 394.7125 | 2 | 26.67 | 9 | 6857 | Ana\_08.raw | 2.45E8 | 1.88E8 | 1.4E8 | 6.36E7 | 1.19E8 | 1.56E8 | 8.53E7 | 8.93E7 | 1.82E8 | 1.83E8 | 26 | 3 | 3 | 2 | 2 | 3 | 3 | 2 | 3 | 2 | 3 | 1595 | 1600 |  |
| R.LQAEEAER.R | Y | 37.31 | 944.4563 | 8 | 4.4 | 473.2375 | 2 | 21.39 | 6 | 5389 | Ana\_05.raw |  |  |  | 2.23E6 | 4.16E6 | 8.59E6 | 4.49E6 |  |  |  | 4 | 0 | 0 | 0 | 1 | 1 | 1 | 1 | 0 | 0 | 0 | 1688 | 1695 |  |
| K.LAAISEATR.L | Y | 37.13 | 930.5134 | 9 | -2.2 | 466.2630 | 2 | 40.54 | 7 | 11152 | Ana\_06.raw |  |  |  |  |  |  | 2.33E7 |  |  |  | 1 | 0 | 0 | 0 | 0 | 0 | 0 | 1 | 0 | 0 | 0 | 1984 | 1992 |  |
| R.QELEAELAK.V | Y | 34.64 | 1029.5342 | 9 | 2.3 | 515.7756 | 2 | 45.04 | 3 | 12674 | Ana\_02.raw | 8.25E6 | 1.14E7 | 1.93E7 |  |  |  |  |  |  |  | 3 | 1 | 1 | 1 | 0 | 0 | 0 | 0 | 0 | 0 | 0 | 1896 | 1904 |  |
| R.FPVTEAVNK.G | Y | 34.47 | 1003.5338 | 9 | 2.5 | 502.7754 | 2 | 46.64 | 6 | 13184 | Ana\_05.raw |  |  | 1.49E6 |  |  | 6.51E6 |  |  |  |  | 2 | 0 | 0 | 1 | 0 | 0 | 1 | 0 | 0 | 0 | 0 | 4474 | 4482 |  |
| K.GLVEDTLR.Q | Y | 34.04 | 901.4869 | 8 | -2.2 | 451.7497 | 2 | 54.26 | 1 | 15287 | ANa\_10.raw | 1.72E6 |  |  |  |  |  |  |  |  |  | 1 | 1 | 0 | 0 | 0 | 0 | 0 | 0 | 0 | 0 | 0 | 2056 | 2063 |  |
| R.TEYNLR.L | Y | 33.01 | 794.3923 | 6 | 5.1 | 398.2054 | 2 | 26.76 | 9 | 6891 | Ana\_08.raw |  |  |  |  |  |  |  |  | 2.97E6 |  | 1 | 0 | 0 | 0 | 0 | 0 | 0 | 0 | 0 | 1 | 0 | 634 | 639 |  |
| K.AEVTEAAR.Q | Y | 32.67 | 845.4243 | 8 | 8.8 | 423.7231 | 2 | 47.60 | 10 | 13357 | Ana\_09.raw | 5.99E6 |  |  |  |  |  |  |  |  | 7.27E6 | 2 | 1 | 0 | 0 | 0 | 0 | 0 | 0 | 0 | 0 | 1 | 2359 | 2366 |  |
| K.RQELEAELAK.V | Y | 32.33 | 1185.6353 | 10 | 5.5 | 593.8282 | 2 | 34.37 | 9 | 9212 | Ana\_08.raw |  | 8.04E5 |  |  |  |  |  |  | 1.27E6 |  | 2 | 0 | 1 | 0 | 0 | 0 | 0 | 0 | 0 | 1 | 0 | 1895 | 1904 |  |
| R.NLVDNITGQR.L | Y | 31.68 | 1128.5887 | 10 | -0.8 | 565.3012 | 2 | 68.06 | 7 | 19314 | Ana\_06.raw |  |  |  |  |  |  | 0 |  | 3.17E6 |  | 2 | 0 | 0 | 0 | 0 | 0 | 0 | 1 | 0 | 1 | 0 | 4445 | 4454 |  |
| R.VVIVDPETGK.E | Y | 31.38 | 1055.5862 | 10 | 3.9 | 528.8024 | 2 | 45.36 | 9 | 12647 | Ana\_08.raw |  |  |  |  |  |  |  |  | 8.55E5 |  | 1 | 0 | 0 | 0 | 0 | 0 | 0 | 0 | 0 | 1 | 0 | 4285 | 4294 |  |
| R.LTVNEAVK.E | Y | 31.32 | 872.4967 | 8 | 4.0 | 437.2574 | 2 | 32.13 | 9 | 8522 | Ana\_08.raw | 3.22E6 |  |  |  |  |  |  |  | 6.73E6 |  | 3 | 1 | 0 | 0 | 0 | 0 | 0 | 0 | 0 | 2 | 0 | 2892 | 2899 |  |
| R.RPELEDSTLR.Y | Y | 30.89 | 1214.6255 | 10 | 4.2 | 608.3226 | 2 | 39.33 | 6 | 10946 | Ana\_05.raw |  |  |  |  |  | 3.24E6 |  |  |  |  | 1 | 0 | 0 | 0 | 0 | 0 | 1 | 0 | 0 | 0 | 0 | 659 | 668 |  |
| R.VPGAQDGVR.A | Y | 30.64 | 897.4668 | 9 | 1.3 | 449.7413 | 2 | 23.08 | 1 | 5798 | ANa\_10.raw | 4.93E6 |  |  |  |  |  |  |  |  |  | 1 | 1 | 0 | 0 | 0 | 0 | 0 | 0 | 0 | 0 | 0 | 413 | 421 |  |
| K.LLSAER.A | N | 30.63 | 687.3915 | 6 | 4.5 | 344.7046 | 2 | 23.39 | 1 | 5905 | ANa\_10.raw | 1.15E7 | 1.14E7 | 2.09E7 |  |  |  | 1.01E6 |  |  |  | 4 | 1 | 1 | 1 | 0 | 0 | 0 | 1 | 0 | 0 | 0 | 2911 | 2916 |  |
| K.EQAELEAAR.Q | Y | 30.32 | 1015.4934 | 9 | 3.9 | 508.7560 | 2 | 29.16 | 7 | 7712 | Ana\_06.raw |  |  |  | 8.35E5 |  |  | 1.94E6 |  |  |  | 2 | 0 | 0 | 0 | 1 | 0 | 0 | 1 | 0 | 0 | 0 | 2108 | 2116 |  |
| R.RAQAEQAALK.Q | Y | 30.08 | 1084.5989 | 10 | 4.9 | 543.3094 | 2 | 19.42 | 10 | 4616 | Ana\_09.raw | 1.18E6 | 3.64E5 | 5.87E5 |  |  | 1.01E6 | 4.97E5 |  |  | 1.54E6 | 6 | 1 | 1 | 1 | 0 | 0 | 1 | 1 | 0 | 0 | 1 | 2294 | 2303 |  |
| R.KASESELER.Q | Y | 28.69 | 1047.5197 | 9 | 3.1 | 524.7687 | 2 | 19.72 | 10 | 4718 | Ana\_09.raw |  |  |  |  |  |  |  |  |  | 3.06E6 | 1 | 0 | 0 | 0 | 0 | 0 | 0 | 0 | 0 | 0 | 1 | 2045 | 2053 |  |
| R.TLKPEEQR.Q | Y | 28.26 | 999.5349 | 8 | 4.6 | 334.1871 | 3 | 18.92 | 8 | 4498 | Ana\_07.raw |  |  |  |  |  |  |  | 7.4E5 |  |  | 1 | 0 | 0 | 0 | 0 | 0 | 0 | 0 | 1 | 0 | 0 | 1061 | 1068 |  |
| R.DLGVTR.L | Y | 27.89 | 659.3602 | 6 | 4.0 | 330.6887 | 2 | 30.46 | 2 | 8166 | Ana\_01.raw |  | 4.24E7 |  |  |  |  |  |  | 4.18E7 |  | 2 | 0 | 1 | 0 | 0 | 0 | 0 | 0 | 0 | 1 | 0 | 377 | 382 |  |
| R.LAEVEAALEK.Q | Y | 27.69 | 1071.5812 | 10 | 7.4 | 536.8018 | 2 | 56.95 | 2 | 16173 | Ana\_01.raw | 3.81E6 | 9.34E6 |  |  |  |  |  |  | 4.72E7 |  | 3 | 1 | 1 | 0 | 0 | 0 | 0 | 0 | 0 | 1 | 0 | 1507 | 1516 |  |
| R.HPQGEQM(+15.99)YR.R | Y | 24.30 | 1160.5033 | 9 | 4.8 | 581.2617 | 2 | 17.83 | 6 | 4208 | Ana\_05.raw |  |  |  |  |  | 8.25E5 |  |  |  |  | 1 | 0 | 0 | 0 | 0 | 0 | 1 | 0 | 0 | 0 | 0 | 612 | 620 | Oxidation (M) |
| R.EAIAELEREK.E | Y | 24.04 | 1186.6193 | 10 | -3.3 | 594.3149 | 2 | 88.65 | 1 | 24934 | ANa\_10.raw | 5.42E5 |  |  |  |  |  |  |  |  |  | 1 | 1 | 0 | 0 | 0 | 0 | 0 | 0 | 0 | 0 | 0 | 2595 | 2604 |  |
| K.LVNIR.N | N | 22.39 | 613.3911 | 5 | 2.7 | 307.7037 | 2 | 25.08 | 5 | 6578 | Ana\_04.raw |  |  |  |  | 1.6E6 |  |  |  |  |  | 1 | 0 | 0 | 0 | 0 | 1 | 0 | 0 | 0 | 0 | 0 | 265 | 269 |  |
| total 79 peptides |  |  |  |  |  |  |  |  |  |  |  |  |  |  |  |  |  |  |  |  |  |  |  |  |  |  |  |  |  |  |  |  |  |  |  |
| --- | --- | --- | --- | --- | --- | --- | --- | --- | --- | --- | --- | --- | --- | --- | --- | --- | --- | --- | --- | --- | --- | --- | --- | --- | --- | --- | --- | --- | --- | --- | --- | --- | --- | --- | --- |

Best Unique PSM (Scan F3:36039, m/z=884.1457, z=3, RT=131.56, ppm=1.0):

sp|Q03265|ATPA\_MOUSE

back to list

  

| Protein Coverage
| Supporting Peptides
| Best Unique PSM
|

Protein Coverage:

Supporting Peptides:

| Peptide | Uniq | -10lgP | Mass | Length | ppm | m/z | z | RT | Fraction | Scan | Source File | Area 10 | Area 01 | Area 02 | Area 03 | Area 04 | Area 05 | Area 06 | Area 07 | Area 08 | Area 09 | #Spec | #Spec 10 | #Spec 01 | #Spec 02 | #Spec 03 | #Spec 04 | #Spec 05 | #Spec 06 | #Spec 07 | #Spec 08 | #Spec 09 | Start | End | PTM |
| --- | --- | --- | --- | --- | --- | --- | --- | --- | --- | --- | --- | --- | --- | --- | --- | --- | --- | --- | --- | --- | --- | --- | --- | --- | --- | --- | --- | --- | --- | --- | --- | --- | --- | --- | --- |
| R.EVAAFAQFGSDLDAATQQLLSR.G | Y | 138.30 | 2337.1599 | 22 | 0.8 | 1169.5881 | 2 | 134.65 | 3 | 36853 | Ana\_02.raw | 2.05E7 | 1.91E7 | 3.58E6 | 2.63E6 | 6.19E6 | 1.06E7 | 4.27E6 | 5.99E6 | 6.68E6 | 1.54E7 | 21 | 2 | 2 | 1 | 1 | 1 | 1 | 2 | 2 | 7 | 2 | 442 | 463 |  |
| K.FENAFLSHVISQHQSLLGNIR.S | Y | 136.20 | 2409.2554 | 21 | 1.0 | 603.3217 | 4 | 117.33 | 3 | 32369 | Ana\_02.raw | 5.6E7 | 8.43E7 | 8.28E7 | 2.42E7 | 3.44E7 | 6.55E7 | 1.11E7 | 2.98E7 | 9.66E7 | 5.83E7 | 30 | 2 | 3 | 4 | 4 | 4 | 2 | 2 | 2 | 4 | 3 | 507 | 527 |  |
| K.GM(+15.99)SLNLEPDNVGVVVFGNDK.L | Y | 112.08 | 2119.0256 | 20 | 4.0 | 1060.5243 | 2 | 108.11 | 3 | 29985 | Ana\_02.raw | 1.09E6 | 3.91E5 | 2.64E6 | 1.34E6 | 3.21E6 | 5.59E6 | 5.11E6 | 1.4E6 | 2.1E7 | 5.45E5 | 14 | 1 | 1 | 1 | 2 | 1 | 2 | 2 | 1 | 2 | 1 | 104 | 123 | Oxidation (M) |
| K.ITKFENAFLSHVISQHQSLLGNIR.S | Y | 111.74 | 2751.4819 | 24 | 4.3 | 688.8807 | 4 | 117.33 | 1 | 32408 | ANa\_10.raw | 1.08E7 | 3.4E7 | 2.18E7 | 1.69E7 | 1.97E7 | 2.93E7 | 1.6E7 | 1.53E7 | 2.53E6 | 2.39E6 | 16 | 2 | 1 | 1 | 2 | 2 | 2 | 2 | 2 | 1 | 1 | 504 | 527 |  |
| R.TGAIVDVPVGEELLGR.V | Y | 107.16 | 1623.8832 | 16 | 3.7 | 812.9518 | 2 | 108.00 | 6 | 30364 | Ana\_05.raw | 1.41E8 | 1.19E8 | 7.35E7 | 1.09E7 | 1.22E7 | 4.97E7 | 6.57E7 | 7.34E7 | 5.71E7 | 6.77E7 | 52 | 4 | 7 | 6 | 5 | 6 | 3 | 4 | 6 | 6 | 5 | 134 | 149 |  |
| R.EAYPGDVFYLHSR.L | Y | 101.28 | 1552.7310 | 13 | 4.4 | 518.5865 | 3 | 82.98 | 7 | 23420 | Ana\_06.raw | 4.86E7 | 6.46E7 | 6.35E7 | 3.22E7 | 5.03E7 | 5.11E7 | 3.08E7 | 3.89E7 | 1.11E8 | 4.89E7 | 46 | 5 | 5 | 4 | 4 | 5 | 4 | 3 | 5 | 6 | 5 | 335 | 347 |  |
| R.ILGADTSVDLEETGR.V | Y | 98.91 | 1574.7787 | 15 | 2.7 | 788.3987 | 2 | 72.98 | 3 | 20720 | Ana\_02.raw | 7.15E6 | 1.66E7 | 4.87E6 | 2.09E6 |  | 4.64E6 | 2.25E6 | 1.44E6 | 1.44E7 | 9.62E6 | 12 | 1 | 3 | 1 | 1 | 0 | 1 | 1 | 1 | 2 | 1 | 59 | 73 |  |
| K.TSIAIDTIINQK.R | Y | 81.04 | 1315.7347 | 12 | 5.3 | 658.8781 | 2 | 85.47 | 6 | 24350 | Ana\_05.raw | 7.92E6 | 1.02E7 | 7.82E6 | 2.58E6 | 5.41E6 | 5.99E6 | 1.12E4 | 1.38E6 | 1.74E7 | 8.26E6 | 10 | 1 | 1 | 1 | 1 | 1 | 1 | 1 | 1 | 1 | 1 | 219 | 230 |  |
| K.HALIIYDDLSK.Q | Y | 80.75 | 1286.6870 | 11 | 1.6 | 644.3518 | 2 | 74.14 | 4 | 20624 | Ana\_03.raw |  | 6.04E6 | 1.29E7 | 6.8E6 | 1.11E7 | 1.86E7 | 9.08E6 | 1E7 |  | 1E7 | 9 | 0 | 1 | 1 | 1 | 1 | 2 | 1 | 1 | 0 | 1 | 306 | 316 |  |
| K.TGTAEM(+15.99)SSILEER.I | Y | 78.65 | 1438.6610 | 13 | 3.7 | 720.3405 | 2 | 51.97 | 6 | 14795 | Ana\_05.raw | 5.91E7 | 6.23E7 | 5.37E7 | 2.26E7 | 4.24E7 | 4.27E7 | 3.09E7 | 3.62E7 | 7.9E7 | 3.13E7 | 31 | 4 | 4 | 2 | 3 | 2 | 3 | 2 | 4 | 4 | 3 | 46 | 58 | Oxidation (M) |
| K.GIRPAINVGLSVSR.V | Y | 74.65 | 1437.8416 | 14 | 2.8 | 719.9301 | 2 | 69.61 | 1 | 19724 | ANa\_10.raw | 9.83E5 |  |  |  |  |  |  |  | 4.5E6 |  | 2 | 1 | 0 | 0 | 0 | 0 | 0 | 0 | 0 | 1 | 0 | 403 | 416 |  |
| K.AVDSLVPIGR.G | Y | 74.34 | 1025.5869 | 10 | 4.1 | 513.8029 | 2 | 69.56 | 6 | 19955 | Ana\_05.raw | 6.13E7 | 7.74E7 | 7.17E7 | 3.44E7 | 6.27E7 | 5.44E7 | 4.38E7 | 4.73E7 | 9.16E7 | 6.45E7 | 39 | 4 | 5 | 4 | 3 | 3 | 3 | 3 | 4 | 5 | 5 | 195 | 204 |  |
| R.VVDALGNAIDGK.G | Y | 73.66 | 1170.6244 | 12 | 4.0 | 586.3218 | 2 | 61.08 | 1 | 17303 | ANa\_10.raw | 6.93E7 | 7.72E7 | 7.04E7 | 2.05E7 | 4.61E7 | 1.45E7 | 3.22E7 | 1.87E7 | 8.66E7 | 5.43E7 | 39 | 5 | 6 | 3 | 2 | 4 | 2 | 4 | 5 | 4 | 4 | 150 | 161 |  |
| R.GYLDKLEPSK.I | Y | 66.23 | 1148.6077 | 10 | 1.6 | 575.3120 | 2 | 57.61 | 5 | 16369 | Ana\_04.raw | 3.14E6 |  | 4.71E6 | 1.27E6 | 2.8E6 | 4.48E6 | 2.87E6 | 1.9E6 | 1.3E7 | 2.11E6 | 13 | 1 | 0 | 2 | 1 | 2 | 2 | 1 | 1 | 2 | 1 | 494 | 503 |  |
| R.NVQAEEM(+15.99)VEFSSGLK.G | Y | 65.38 | 1682.7821 | 15 | 3.5 | 842.4013 | 2 | 72.55 | 6 | 20818 | Ana\_05.raw |  |  | 6.07E5 | 7.03E5 | 5.65E5 | 3.63E5 |  | 7.18E5 |  |  | 5 | 0 | 0 | 1 | 1 | 1 | 1 | 0 | 1 | 0 | 0 | 89 | 103 | Oxidation (M) |
| R.VLSIGDGIAR.V | Y | 58.13 | 999.5713 | 10 | 3.8 | 500.7948 | 2 | 63.23 | 2 | 18010 | Ana\_01.raw | 7.66E7 | 9.05E7 | 8.78E7 | 2.93E7 | 6.08E7 | 5.86E7 | 4.02E7 | 4.28E7 | 1.17E8 | 6.56E7 | 37 | 5 | 4 | 4 | 2 | 3 | 3 | 3 | 4 | 5 | 4 | 74 | 83 |  |
| K.RLTDADAM(+15.99)K.Y | Y | 50.40 | 1035.5018 | 9 | 3.4 | 346.1757 | 3 | 18.29 | 6 | 4366 | Ana\_05.raw |  |  |  | 5.94E6 | 9.27E6 | 1.49E7 | 7.44E6 | 8E6 |  |  | 6 | 0 | 0 | 0 | 1 | 1 | 2 | 1 | 1 | 0 | 0 | 262 | 270 | Oxidation (M) |
| R.STVAQLVK.R | Y | 50.01 | 844.5018 | 8 | 2.5 | 423.2592 | 2 | 38.51 | 2 | 10594 | Ana\_01.raw |  | 1.11E8 |  | 2.5E7 | 9.97E7 | 9.99E7 | 7.69E7 | 6.8E7 | 2.32E5 | 6.38E7 | 10 | 0 | 1 | 0 | 1 | 1 | 2 | 1 | 2 | 1 | 1 | 254 | 261 |  |
| K.APGIIPR.I | Y | 47.69 | 722.4438 | 7 | 1.9 | 362.2299 | 2 | 40.07 | 6 | 11179 | Ana\_05.raw | 4.65E7 | 5.62E7 | 5.84E7 | 2.58E7 | 4.45E7 | 5.3E7 | 3.73E7 | 3.34E7 | 3.02E7 | 4.51E7 | 21 | 3 | 2 | 2 | 2 | 1 | 2 | 2 | 3 | 3 | 1 | 176 | 182 |  |
| R.ELIIGDR.Q | Y | 46.63 | 814.4548 | 7 | 3.9 | 408.2363 | 2 | 48.53 | 6 | 13763 | Ana\_05.raw | 5.42E7 | 5.85E7 | 5.72E7 | 2.42E7 | 4.31E7 | 4.84E7 | 3.57E7 | 4.12E7 | 4.1E6 | 4.96E7 | 19 | 2 | 2 | 3 | 1 | 2 | 2 | 2 | 1 | 2 | 2 | 208 | 214 |  |
| K.LELAQYR.E | Y | 41.85 | 891.4814 | 7 | 5.0 | 446.7502 | 2 | 51.68 | 1 | 14518 | ANa\_10.raw | 3.82E7 | 4.16E7 | 4.99E7 | 2.38E7 | 3.94E7 | 4.28E7 | 2.18E7 | 3.38E7 | 5.6E7 | 1.2E6 | 13 | 1 | 1 | 1 | 1 | 1 | 1 | 2 | 2 | 2 | 1 | 435 | 441 |  |
| K.LKEIVTNFLAGFEP | Y | 40.78 | 1576.8501 | 14 | 6.3 | 789.4373 | 2 | 130.22 | 2 | 36060 | Ana\_01.raw | 2.65E7 | 2.52E7 | 1.82E7 | 8.42E6 | 2.03E7 | 3.08E7 |  | 1.26E7 | 2.92E7 | 2.16E7 | 9 | 1 | 1 | 1 | 1 | 1 | 1 | 0 | 1 | 1 | 1 | 540 | 553 |  |
| K.QGQYSPMAIEEQVAVIYAGVR.G | Y | 38.45 | 2308.1521 | 21 | -8.4 | 770.3848 | 3 | 133.55 | 3 | 36566 | Ana\_02.raw | 6.59E3 |  | 0 |  |  |  |  |  |  |  | 2 | 1 | 0 | 1 | 0 | 0 | 0 | 0 | 0 | 0 | 0 | 473 | 493 |  |
| R.EPM(+15.99)QTGIK.A | Y | 37.87 | 918.4481 | 8 | 4.1 | 460.2332 | 2 | 18.86 | 5 | 4603 | Ana\_04.raw |  |  |  |  | 3.68E6 |  |  |  |  |  | 1 | 0 | 0 | 0 | 0 | 1 | 0 | 0 | 0 | 0 | 0 | 187 | 194 | Oxidation (M) |
| K.RFNDGTDEK.K | Y | 37.13 | 1080.4835 | 9 | 4.5 | 541.2515 | 2 | 17.62 | 5 | 4185 | Ana\_04.raw |  |  |  | 1.37E6 | 2.44E6 |  | 1.66E6 | 1.36E6 |  |  | 4 | 0 | 0 | 0 | 1 | 1 | 0 | 1 | 1 | 0 | 0 | 231 | 239 |  |
| R.LTELLK.Q | N | 33.58 | 715.4480 | 6 | 3.2 | 358.7324 | 2 | 47.10 | 7 | 13150 | Ana\_06.raw | 2.06E7 | 2.05E7 | 2.69E7 | 1.1E7 | 2.14E7 | 2.37E7 | 1.58E7 | 1.57E7 | 2.39E7 | 1.81E7 | 18 | 1 | 1 | 2 | 2 | 2 | 2 | 2 | 2 | 2 | 2 | 467 | 472 |  |
| total 26 peptides |  |  |  |  |  |  |  |  |  |  |  |  |  |  |  |  |  |  |  |  |  |  |  |  |  |  |  |  |  |  |  |  |  |  |  |
| --- | --- | --- | --- | --- | --- | --- | --- | --- | --- | --- | --- | --- | --- | --- | --- | --- | --- | --- | --- | --- | --- | --- | --- | --- | --- | --- | --- | --- | --- | --- | --- | --- | --- | --- | --- |

Best Unique PSM (Scan F3:36853, m/z=1169.5881, z=2, RT=134.65, ppm=0.8):

sp|P57780|ACTN4\_MOUSE

back to list

  

| Protein Coverage
| Supporting Peptides
| Best Unique PSM
|

Protein Coverage:

Supporting Peptides:

| Peptide | Uniq | -10lgP | Mass | Length | ppm | m/z | z | RT | Fraction | Scan | Source File | Area 10 | Area 01 | Area 02 | Area 03 | Area 04 | Area 05 | Area 06 | Area 07 | Area 08 | Area 09 | #Spec | #Spec 10 | #Spec 01 | #Spec 02 | #Spec 03 | #Spec 04 | #Spec 05 | #Spec 06 | #Spec 07 | #Spec 08 | #Spec 09 | Start | End | PTM |
| --- | --- | --- | --- | --- | --- | --- | --- | --- | --- | --- | --- | --- | --- | --- | --- | --- | --- | --- | --- | --- | --- | --- | --- | --- | --- | --- | --- | --- | --- | --- | --- | --- | --- | --- | --- |
| R.VEQIAAIAQELNELDYYDSHNVNTR.C | Y | 117.38 | 2904.3889 | 25 | 3.0 | 969.1398 | 3 | 128.96 | 7 | 35481 | Ana\_06.raw | 8.24E6 |  | 1.2E7 | 2.14E6 | 1.25E7 |  | 8.01E6 | 7.95E6 | 2.28E7 | 9.9E6 | 11 | 1 | 0 | 1 | 1 | 1 | 0 | 1 | 2 | 3 | 1 | 471 | 495 |  |
| R.SIVDYKPSLDLLEQQHQLIQEALIFDNK.H | Y | 104.72 | 3296.7292 | 28 | 4.2 | 825.1931 | 4 | 131.19 | 6 | 36450 | Ana\_05.raw | 3.14E6 | 7.34E6 |  |  | 5.38E6 | 8.03E6 | 7.63E6 | 3.12E6 | 7.48E6 | 3.43E6 | 9 | 1 | 1 | 0 | 0 | 1 | 1 | 2 | 1 | 1 | 1 | 697 | 724 |  |
| R.KHEAFESDLAAHQDR.V | N | 104.04 | 1752.8179 | 15 | 6.5 | 877.4219 | 2 | 32.17 | 2 | 8671 | Ana\_01.raw | 5.96E7 | 4.97E7 | 5.26E7 | 4.62E6 | 3.7E7 | 5.14E7 | 3.7E7 | 2.49E7 | 4.86E7 | 3.63E7 | 22 | 2 | 2 | 3 | 2 | 2 | 2 | 2 | 2 | 4 | 1 | 456 | 470 |  |
| R.M(+15.99)APYQGPDAAPGALDYK.S | Y | 95.80 | 1779.8137 | 17 | 0.9 | 890.9149 | 2 | 66.26 | 3 | 18852 | Ana\_02.raw | 1.19E7 | 1.1E7 | 2.14E6 | 3.77E6 |  | 8.44E6 | 6.42E6 |  | 1.43E7 | 7.84E6 | 9 | 1 | 1 | 1 | 1 | 0 | 1 | 1 | 0 | 2 | 1 | 884 | 900 | Oxidation (M) |
| K.HEAFESDLAAHQDR.V | N | 93.86 | 1624.7229 | 14 | 6.1 | 542.5849 | 3 | 43.62 | 8 | 11920 | Ana\_07.raw | 6.79E6 | 8.02E6 | 1.01E7 | 3.33E6 | 5.65E6 | 8.62E6 | 5.39E6 | 5.37E6 | 1.32E6 | 5.79E6 | 10 | 1 | 1 | 1 | 1 | 1 | 1 | 1 | 1 | 1 | 1 | 457 | 470 |  |
| K.GYEEWLLNEIR.R | N | 91.22 | 1420.6986 | 11 | 1.9 | 711.3579 | 2 | 123.41 | 7 | 34028 | Ana\_06.raw | 2.56E6 | 1.41E7 | 2.88E7 | 8.74E6 | 1.06E7 | 3.06E7 | 2.98E6 | 1.24E7 |  | 2.63E7 | 14 | 1 | 2 | 2 | 1 | 1 | 2 | 2 | 1 | 0 | 2 | 397 | 407 |  |
| K.LSGSNPYTTVTPQIINSK.W | Y | 87.11 | 1919.0000 | 18 | 2.3 | 960.5095 | 2 | 79.00 | 3 | 22351 | Ana\_02.raw |  |  | 5.63E5 |  | 6.75E5 | 1.33E6 | 9.09E5 |  | 4.23E5 |  | 5 | 0 | 0 | 1 | 0 | 1 | 1 | 1 | 0 | 1 | 0 | 606 | 623 |  |
| R.VGWEQLLTTIAR.T | N | 86.75 | 1385.7667 | 12 | 4.9 | 693.8940 | 2 | 130.96 | 6 | 36386 | Ana\_05.raw | 6.62E7 | 7.2E7 | 8.58E7 | 4.24E7 | 7.11E7 | 7.69E7 | 3.11E7 | 5.19E7 | 9.16E7 | 5.85E7 | 83 | 11 | 9 | 10 | 4 | 12 | 4 | 6 | 9 | 8 | 10 | 735 | 746 |  |
| K.AIMTYVSSFYHAFSGAQK.A | N | 86.55 | 2006.9561 | 18 | 3.9 | 669.9952 | 3 | 128.25 | 1 | 35264 | ANa\_10.raw | 1.11E7 | 6.88E6 |  | 2.86E6 | 3.46E6 | 7.31E6 |  | 3.73E6 | 1.62E7 | 1.43E7 | 9 | 2 | 1 | 0 | 1 | 1 | 1 | 0 | 1 | 1 | 1 | 257 | 274 |  |
| K.RDHALLEEQSK.Q | Y | 84.51 | 1324.6735 | 11 | 2.2 | 442.5661 | 3 | 21.19 | 3 | 5333 | Ana\_02.raw | 1.96E7 | 1.55E7 | 1.65E7 | 7.01E6 | 8.42E6 | 1.84E7 | 2.56E6 | 1.86E6 | 3.07E7 | 1.13E7 | 17 | 2 | 1 | 2 | 1 | 2 | 2 | 2 | 1 | 2 | 2 | 634 | 644 |  |
| R.LASDLLEWIR.R | N | 83.62 | 1214.6659 | 10 | 1.9 | 608.3414 | 2 | 124.08 | 3 | 34079 | Ana\_02.raw | 2.24E7 | 3.6E7 | 1.72E7 | 3.28E7 | 7.24E7 | 7.44E7 | 3.6E7 | 4.66E7 | 1E8 | 5.02E7 | 44 | 6 | 6 | 5 | 2 | 4 | 3 | 2 | 5 | 6 | 5 | 302 | 311 |  |
| R.ETTDTDTADQVIASFK.V | Y | 83.59 | 1740.8054 | 16 | 3.8 | 871.4133 | 2 | 95.23 | 1 | 26687 | ANa\_10.raw | 1.67E7 | 0 |  | 3.43E6 | 1.03E7 | 1.49E7 |  | 7.99E6 | 2.9E7 |  | 8 | 1 | 1 | 0 | 1 | 1 | 1 | 0 | 1 | 2 | 0 | 839 | 854 |  |
| R.TINEVENQILTR.D | N | 83.00 | 1428.7572 | 12 | 6.3 | 715.3904 | 2 | 79.01 | 1 | 22311 | ANa\_10.raw | 6.74E6 | 2.24E7 | 2.21E7 | 8.27E6 | 1.79E7 | 1.93E7 | 8.77E6 | 1.06E7 | 6.01E7 | 2.35E7 | 12 | 1 | 1 | 1 | 1 | 2 | 2 | 1 | 1 | 1 | 1 | 747 | 758 |  |
| R.FAIQDISVEETSAK.E | N | 78.06 | 1536.7671 | 14 | 3.8 | 769.3937 | 2 | 82.14 | 9 | 23377 | Ana\_08.raw | 1.19E7 | 1.61E7 | 1.28E7 | 6.08E6 | 1.02E7 | 9.32E6 | 6.33E6 | 5.83E6 | 2.83E7 | 1.39E7 | 11 | 1 | 1 | 1 | 1 | 2 | 1 | 1 | 1 | 1 | 1 | 154 | 167 |  |
| R.KDDPVTNLNNAFEVAEK.Y | Y | 77.84 | 1902.9323 | 17 | 6.2 | 635.3220 | 3 | 84.01 | 9 | 23909 | Ana\_08.raw |  |  |  |  |  |  |  |  | 6.28E6 |  | 1 | 0 | 0 | 0 | 0 | 0 | 0 | 0 | 0 | 1 | 0 | 218 | 234 |  |
| K.AIM(+15.99)TYVSSFYHAFSGAQK.A | N | 77.57 | 2022.9509 | 18 | 0.5 | 675.3246 | 3 | 111.48 | 2 | 31114 | Ana\_01.raw | 1.77E7 | 1.16E7 | 2.14E7 | 1.36E7 | 1.42E7 | 3.33E6 | 1.45E7 | 3.65E6 | 1.39E7 | 7.21E5 | 19 | 2 | 2 | 2 | 2 | 2 | 2 | 2 | 2 | 2 | 1 | 257 | 274 | Oxidation (M) |
| R.DHALLEEQSK.Q | Y | 76.33 | 1168.5724 | 10 | 7.3 | 585.2977 | 2 | 27.34 | 1 | 7185 | ANa\_10.raw | 7.39E5 | 2.65E6 |  |  |  |  |  |  |  |  | 2 | 1 | 1 | 0 | 0 | 0 | 0 | 0 | 0 | 0 | 0 | 635 | 644 |  |
| R.LSNRPAFM(+15.99)PSEGR.M | N | 71.07 | 1476.7144 | 13 | 4.2 | 493.2475 | 3 | 37.09 | 1 | 10119 | ANa\_10.raw | 7.73E7 | 0 | 8.88E7 | 3.92E7 | 4.01E7 | 8.61E7 | 5.08E7 | 5.09E7 | 5.68E7 | 3.21E7 | 25 | 2 | 2 | 3 | 3 | 2 | 4 | 2 | 2 | 2 | 3 | 367 | 379 | Oxidation (M) |
| R.ELPPDQAEYC(+57.02)IAR.M | N | 70.16 | 1560.7242 | 13 | 3.7 | 781.3723 | 2 | 63.81 | 9 | 18193 | Ana\_08.raw | 1.4E7 | 2.47E7 | 4.32E6 | 3.57E5 | 8.53E6 | 8.27E6 | 0 | 4.3E6 | 1.55E7 | 1.96E7 | 35 | 5 | 5 | 2 | 1 | 4 | 3 | 1 | 3 | 6 | 5 | 871 | 883 | Carbamidomethylation |
| K.NFITAEELR.R | Y | 66.76 | 1091.5610 | 9 | 3.8 | 546.7899 | 2 | 73.58 | 6 | 21108 | Ana\_05.raw | 2.04E7 | 2.42E7 | 1.73E7 | 7.64E6 | 1.46E7 | 1.23E7 | 1.05E7 | 8.93E6 | 2.97E7 | 1.63E7 | 12 | 2 | 1 | 1 | 1 | 1 | 1 | 2 | 1 | 1 | 1 | 861 | 869 |  |
| K.GISQEQM(+15.99)QEFR.A | Y | 66.51 | 1367.6139 | 11 | 5.2 | 684.8178 | 2 | 41.96 | 6 | 11755 | Ana\_05.raw | 1.51E7 | 1.56E7 | 1.53E7 | 6.41E6 | 1.38E7 | 1.39E7 | 1.09E7 | 1.11E7 | 5.22E6 | 9.93E6 | 11 | 2 | 1 | 1 | 1 | 1 | 1 | 1 | 1 | 1 | 1 | 762 | 772 | Oxidation (M) |
| R.DYETATLSDIK.A | Y | 66.20 | 1254.5979 | 11 | -0.6 | 628.3058 | 2 | 69.56 | 3 | 19775 | Ana\_02.raw | 1.15E7 | 1.48E7 | 7.78E6 | 5.89E6 | 8.6E6 | 8.27E6 | 6.85E6 | 7.24E6 |  | 7.91E6 | 9 | 1 | 1 | 1 | 1 | 1 | 1 | 1 | 1 | 0 | 1 | 441 | 451 |  |
| R.DLLLDPAWEK.Q | N | 64.97 | 1198.6233 | 10 | 5.7 | 600.3223 | 2 | 100.06 | 2 | 28148 | Ana\_01.raw | 1.22E7 | 1.24E7 | 1.75E7 | 6E6 | 8.22E6 | 6.59E6 | 7.77E6 | 7.33E6 | 4.18E7 | 1.9E6 | 11 | 1 | 1 | 2 | 1 | 1 | 1 | 1 | 1 | 1 | 1 | 42 | 51 |  |
| R.HRPELIEYDK.L | Y | 63.97 | 1298.6619 | 10 | 0.6 | 433.8948 | 3 | 39.42 | 4 | 10665 | Ana\_03.raw | 2.04E7 | 2.69E7 | 2.31E7 | 1.02E7 | 2.28E7 | 2.23E7 | 1.61E7 | 1.27E7 | 1.52E7 | 2.08E7 | 17 | 2 | 2 | 2 | 1 | 2 | 2 | 1 | 1 | 2 | 2 | 206 | 215 |  |
| K.DGLAFNALIHR.H | N | 62.11 | 1225.6567 | 11 | 0.7 | 409.5598 | 3 | 88.26 | 6 | 25125 | Ana\_05.raw | 6.16E5 | 3.47E6 | 1.42E6 | 1.3E6 | 3.03E6 | 3.87E6 | 1.48E6 | 9.86E5 | 1.43E6 | 1.37E6 | 14 | 1 | 1 | 1 | 2 | 2 | 2 | 1 | 2 | 1 | 1 | 195 | 205 |  |
| K.AGTQIENIDEDFR.D | Y | 61.75 | 1506.6951 | 13 | 2.7 | 754.3569 | 2 | 78.75 | 5 | 22360 | Ana\_04.raw | 2.32E6 | 2.92E6 |  | 5.95E5 | 1.59E6 | 3.1E6 |  | 7.47E5 | 4.04E6 |  | 7 | 1 | 1 | 0 | 1 | 1 | 1 | 0 | 1 | 1 | 0 | 68 | 80 |  |
| K.ALDFIASK.G | N | 61.40 | 863.4752 | 8 | 2.2 | 432.7458 | 2 | 61.89 | 6 | 17737 | Ana\_05.raw | 1.98E7 | 2.27E7 | 2.33E7 | 1.06E7 | 1.77E7 | 1.93E7 | 1.47E7 | 1.41E7 | 3.63E7 | 1.65E7 | 17 | 2 | 1 | 2 | 2 | 1 | 2 | 2 | 1 | 3 | 1 | 116 | 123 |  |
| K.EGLLLWC(+57.02)QR.K | N | 60.29 | 1173.5964 | 9 | 4.7 | 587.8082 | 2 | 94.15 | 2 | 26589 | Ana\_01.raw |  | 4.64E6 |  |  |  |  |  |  | 1.06E7 | 1.1E6 | 3 | 0 | 1 | 0 | 0 | 0 | 0 | 0 | 0 | 1 | 1 | 168 | 176 | Carbamidomethylation |
| R.TIPWLEDR.V | Y | 58.98 | 1028.5291 | 8 | 3.2 | 515.2734 | 2 | 86.22 | 1 | 24280 | ANa\_10.raw | 2.68E6 | 1.46E6 | 1.7E6 | 1.74E6 | 3.79E5 |  | 2.98E6 | 2.12E6 | 8.32E6 | 5.47E5 | 9 | 1 | 1 | 1 | 1 | 1 | 0 | 1 | 1 | 1 | 1 | 313 | 320 |  |
| K.HTNYTM(+15.99)EHIR.V | N | 56.35 | 1316.5931 | 10 | 2.4 | 439.8727 | 3 | 23.11 | 4 | 5838 | Ana\_03.raw |  | 2.97E7 |  | 1.95E7 |  |  | 6.33E6 | 3.24E6 |  |  | 4 | 0 | 1 | 0 | 1 | 0 | 0 | 1 | 1 | 0 | 0 | 725 | 734 | Oxidation (M) |
| K.STLPDADR.E | Y | 49.99 | 873.4192 | 8 | 0.4 | 437.7170 | 2 | 24.23 | 3 | 6314 | Ana\_02.raw |  | 4.58E6 | 1.11E7 | 3.53E6 | 0 | 4.62E6 | 4.72E6 | 3.21E6 |  | 7.3E6 | 8 | 0 | 1 | 1 | 1 | 1 | 1 | 1 | 1 | 0 | 1 | 576 | 583 |  |
| K.STLPDADRER.E | Y | 48.96 | 1158.5629 | 10 | 6.8 | 580.2927 | 2 | 24.80 | 2 | 6365 | Ana\_01.raw | 8.76E6 | 1.4E7 |  | 2.21E6 | 4.01E6 |  | 3.81E6 |  | 0 | 3.63E6 | 7 | 1 | 1 | 0 | 1 | 1 | 0 | 1 | 0 | 1 | 1 | 576 | 585 |  |
| K.AGTQIENIDEDFRDGLK.L | Y | 47.96 | 1919.9224 | 17 | 3.8 | 640.9838 | 3 | 90.04 | 2 | 25468 | Ana\_01.raw |  | 3.73E6 |  |  |  | 1.54E6 | 9.07E5 |  | 3.84E6 | 8.18E5 | 5 | 0 | 1 | 0 | 0 | 0 | 1 | 1 | 0 | 1 | 1 | 68 | 84 |  |
| K.LVSIGAEEIVDGNAK.M | Y | 46.61 | 1513.7987 | 15 | 1.4 | 757.9077 | 2 | 80.10 | 5 | 22728 | Ana\_04.raw |  |  |  |  | 0 |  |  |  |  |  | 1 | 0 | 0 | 0 | 0 | 1 | 0 | 0 | 0 | 0 | 0 | 127 | 141 |  |
| R.EAILAIHK.E | Y | 45.85 | 893.5334 | 8 | 3.9 | 447.7757 | 2 | 40.68 | 6 | 11371 | Ana\_05.raw |  | 7.03E6 | 7.68E6 | 3.41E6 | 6.89E6 | 7.3E6 | 5.48E6 | 4.07E6 |  |  | 7 | 0 | 1 | 1 | 1 | 1 | 1 | 1 | 1 | 0 | 0 | 586 | 593 |  |
| R.LDHLAEK.F | N | 44.09 | 824.4392 | 7 | 2.3 | 413.2278 | 2 | 19.09 | 5 | 4680 | Ana\_04.raw |  |  |  |  | 1.46E7 | 2.65E7 | 1.21E7 | 1.15E7 |  |  | 4 | 0 | 0 | 0 | 0 | 1 | 1 | 1 | 1 | 0 | 0 | 412 | 418 |  |
| K.M(+15.99)LDAEDIVNTARPDEK.A | Y | 42.98 | 1831.8622 | 16 | -0.2 | 611.6279 | 3 | 74.17 | 5 | 21127 | Ana\_04.raw | 6.88E6 | 2.66E6 |  | 4.16E6 | 4.47E6 |  |  | 4.39E6 | 1.77E7 |  | 6 | 1 | 1 | 0 | 1 | 1 | 0 | 0 | 1 | 1 | 0 | 241 | 256 | Oxidation (M) |
| R.QGDAEFNR.I | Y | 38.80 | 935.4097 | 8 | 1.5 | 468.7128 | 2 | 23.96 | 4 | 6087 | Ana\_03.raw |  |  |  | 4.51E5 |  |  |  |  |  |  | 1 | 0 | 0 | 0 | 1 | 0 | 0 | 0 | 0 | 0 | 0 | 807 | 814 |  |
| K.YLDIPK.M | N | 38.09 | 747.4166 | 6 | 3.3 | 374.7168 | 2 | 49.39 | 9 | 13857 | Ana\_08.raw | 5.29E6 | 5.26E6 | 5.22E6 |  | 3.24E6 |  | 4.37E6 |  | 8.75E6 |  | 6 | 1 | 1 | 1 | 0 | 1 | 0 | 1 | 0 | 1 | 0 | 235 | 240 |  |
| K.MEEIGR.I | N | 36.59 | 733.3428 | 6 | 4.4 | 367.6803 | 2 | 25.12 | 6 | 6593 | Ana\_05.raw |  |  |  |  |  | 4.69E6 |  | 2.55E6 |  | 3.65E6 | 3 | 0 | 0 | 0 | 0 | 0 | 1 | 0 | 1 | 0 | 1 | 670 | 675 |  |
| K.TFTAWC(+57.02)NSHLR.K | N | 36.01 | 1391.6405 | 11 | 2.5 | 464.8886 | 3 | 63.09 | 6 | 18092 | Ana\_05.raw |  |  |  |  |  | 5.73E5 |  |  |  |  | 1 | 0 | 0 | 0 | 0 | 0 | 1 | 0 | 0 | 0 | 0 | 56 | 66 | Carbamidomethylation |
| K.LEDFR.D | N | 34.55 | 678.3337 | 5 | 1.6 | 340.1747 | 2 | 32.77 | 4 | 8713 | Ana\_03.raw |  |  | 1.16E7 | 1.11E7 |  | 2.14E7 | 1.54E7 | 1.44E7 |  |  | 5 | 0 | 0 | 1 | 1 | 0 | 1 | 1 | 1 | 0 | 0 | 333 | 337 |  |
| K.ASIHEAWTDGK.E | N | 31.75 | 1213.5728 | 11 | 3.5 | 405.5329 | 3 | 41.70 | 6 | 11672 | Ana\_05.raw |  |  | 6.42E6 | 4.14E6 |  | 8.99E6 | 6.97E6 |  |  |  | 4 | 0 | 0 | 1 | 1 | 0 | 1 | 1 | 0 | 0 | 0 | 423 | 433 |  |
| K.VQQLVPK.R | Y | 31.00 | 810.4963 | 7 | 1.9 | 406.2562 | 2 | 28.50 | 3 | 7668 | Ana\_02.raw | 1.08E7 | 1.29E7 | 1.14E7 | 5.68E6 | 1.03E7 |  | 7.5E6 |  | 1.22E7 |  | 7 | 1 | 1 | 1 | 1 | 1 | 0 | 1 | 0 | 1 | 0 | 627 | 633 |  |
| K.VLAVNQENEHLM(+15.99)EDYER.L | Y | 25.73 | 2103.9531 | 17 | 8.6 | 702.3310 | 3 | 60.31 | 2 | 17148 | Ana\_01.raw |  | 5.46E6 |  |  |  |  |  |  |  |  | 1 | 0 | 1 | 0 | 0 | 0 | 0 | 0 | 0 | 0 | 0 | 285 | 301 | Oxidation (M) |
| total 45 peptides |  |  |  |  |  |  |  |  |  |  |  |  |  |  |  |  |  |  |  |  |  |  |  |  |  |  |  |  |  |  |  |  |  |  |  |
| --- | --- | --- | --- | --- | --- | --- | --- | --- | --- | --- | --- | --- | --- | --- | --- | --- | --- | --- | --- | --- | --- | --- | --- | --- | --- | --- | --- | --- | --- | --- | --- | --- | --- | --- | --- |

Best Unique PSM (Scan F7:35481, m/z=969.1398, z=3, RT=128.96, ppm=3.0):

sp|P11679|K2C8\_MOUSE

back to list

  

| Protein Coverage
| Supporting Peptides
| Best Unique PSM
|

Protein Coverage:

Supporting Peptides:

| Peptide | Uniq | -10lgP | Mass | Length | ppm | m/z | z | RT | Fraction | Scan | Source File | Area 10 | Area 01 | Area 02 | Area 03 | Area 04 | Area 05 | Area 06 | Area 07 | Area 08 | Area 09 | #Spec | #Spec 10 | #Spec 01 | #Spec 02 | #Spec 03 | #Spec 04 | #Spec 05 | #Spec 06 | #Spec 07 | #Spec 08 | #Spec 09 | Start | End | PTM |
| --- | --- | --- | --- | --- | --- | --- | --- | --- | --- | --- | --- | --- | --- | --- | --- | --- | --- | --- | --- | --- | --- | --- | --- | --- | --- | --- | --- | --- | --- | --- | --- | --- | --- | --- | --- |
| R.GSM(+15.99)GTGVGLGGFGGAGVGGITAVTVNQSLLSPLK.L | Y | 124.22 | 3074.6069 | 34 | 6.0 | 1538.3199 | 2 | 130.11 | 5 | 35809 | Ana\_04.raw | 9.92E7 | 9.49E7 | 4.65E7 | 2.51E7 | 6.54E7 | 9.95E7 | 5.24E7 | 3.42E7 | 1.04E8 | 4.96E7 | 24 | 3 | 4 | 2 | 1 | 2 | 3 | 2 | 2 | 2 | 3 | 50 | 83 | Oxidation (M) |
| K.LEAELGNMQGLVEDFK.N | Y | 109.09 | 1791.8712 | 16 | 5.2 | 896.9476 | 2 | 121.80 | 1 | 33562 | ANa\_10.raw | 5.85E7 | 3E7 |  | 1.93E5 |  | 5.1E6 | 1.62E6 | 1.31E7 | 6.51E7 | 2.82E7 | 12 | 2 | 1 | 0 | 1 | 0 | 1 | 1 | 1 | 3 | 2 | 167 | 182 |  |
| K.LKLEAELGNMQGLVEDFK.N | Y | 98.43 | 2033.0503 | 18 | 4.3 | 1017.5367 | 2 | 127.10 | 1 | 34955 | ANa\_10.raw | 3.8E6 | 1.57E6 |  | 1.49E6 |  | 5.19E6 |  |  |  | 2.65E6 | 6 | 2 | 1 | 0 | 1 | 0 | 1 | 0 | 0 | 0 | 1 | 165 | 182 |  |
| R.LEGLTDEINFLR.Q | Y | 98.18 | 1418.7405 | 12 | 2.9 | 710.3796 | 2 | 114.06 | 6 | 31925 | Ana\_05.raw | 6.2E8 | 8.78E8 | 6.77E8 | 3.76E8 | 6.3E8 | 7.01E8 | 4.29E8 | 9.27E7 | 1.31E9 | 4.86E8 | 186 | 23 | 20 | 20 | 11 | 21 | 19 | 14 | 20 | 21 | 17 | 220 | 231 |  |
| R.ASLEAAIADAEQR.G | Y | 97.88 | 1343.6681 | 13 | 1.5 | 672.8423 | 2 | 83.73 | 3 | 23611 | Ana\_02.raw | 4.89E8 | 3.35E8 | 2.38E8 | 1.21E8 | 2.4E8 | 2.48E8 | 2.99E8 | 1.52E8 | 3.97E8 | 1.97E8 | 65 | 7 | 9 | 5 | 6 | 6 | 5 | 6 | 7 | 7 | 7 | 335 | 347 |  |
| R.SNM(+15.99)DNM(+15.99)FESYINNLR.R | Y | 93.49 | 1878.7876 | 15 | 2.6 | 940.4035 | 2 | 102.75 | 6 | 28979 | Ana\_05.raw | 3.19E7 | 3.43E7 | 3.23E7 | 5.58E6 | 3.86E7 | 3E7 | 1.29E7 | 2.77E7 | 7.43E7 | 1.2E7 | 35 | 5 | 4 | 4 | 3 | 4 | 3 | 1 | 3 | 5 | 3 | 140 | 154 | Oxidation (M) |
| R.SNMDNMFESYINNLR.R | Y | 86.02 | 1846.7979 | 15 | 5.3 | 924.4111 | 2 | 128.50 | 1 | 35331 | ANa\_10.raw | 1.5E6 |  |  |  |  |  |  |  | 3.39E6 |  | 2 | 1 | 0 | 0 | 0 | 0 | 0 | 0 | 0 | 1 | 0 | 140 | 154 |  |
| R.SLDM(+15.99)DGIIAEVR.A | Y | 85.68 | 1333.6548 | 12 | 4.5 | 667.8376 | 2 | 78.81 | 1 | 22258 | ANa\_10.raw | 6.84E7 | 9.87E7 | 5.52E7 | 2.04E7 | 7.57E7 | 7.41E7 | 3.72E7 | 3.77E7 | 1.21E8 | 5.31E7 | 60 | 7 | 8 | 5 | 4 | 5 | 5 | 3 | 7 | 11 | 5 | 259 | 270 | Oxidation (M) |
| K.NKYEDEINKR.T | N | 84.60 | 1307.6470 | 10 | 4.8 | 654.8339 | 2 | 31.10 | 6 | 8464 | Ana\_05.raw | 1.89E8 | 2.18E8 | 1.22E8 | 7.44E7 | 1.25E8 | 1.57E8 | 1.13E8 | 8.46E7 | 2.33E8 | 1.6E8 | 44 | 5 | 4 | 4 | 3 | 3 | 6 | 4 | 4 | 7 | 4 | 183 | 192 |  |
| R.SNM(+15.99)DNMFESYINNLR.R | Y | 83.72 | 1862.7927 | 15 | 3.7 | 932.4070 | 2 | 117.22 | 1 | 32379 | ANa\_10.raw | 4.45E6 | 2.57E6 |  |  |  | 6.5E5 |  |  | 5.26E6 | 9.09E5 | 6 | 2 | 1 | 0 | 0 | 0 | 1 | 0 | 0 | 1 | 1 | 140 | 154 | Oxidation (M) |
| R.ELQSQISDTSVVLSM(+15.99)DNSR.S | Y | 81.96 | 2124.0005 | 19 | -1.1 | 1063.0063 | 2 | 78.21 | 5 | 22209 | Ana\_04.raw | 2.63E6 | 3.79E6 |  | 3.89E5 | 1.88E6 |  |  | 8.42E5 | 3.74E6 |  | 6 | 1 | 1 | 0 | 1 | 1 | 0 | 0 | 1 | 1 | 0 | 240 | 258 | Oxidation (M) |
| K.LEVDPNIQAVR.T | Y | 81.66 | 1252.6775 | 11 | 2.4 | 627.3475 | 2 | 68.29 | 6 | 19605 | Ana\_05.raw | 1.68E8 | 1.78E8 | 9.13E7 | 5.22E7 | 1.37E8 | 1.29E8 | 9.11E7 | 7.84E7 | 2.77E8 | 1.15E8 | 46 | 5 | 5 | 5 | 4 | 5 | 4 | 3 | 5 | 5 | 5 | 84 | 94 |  |
| R.TEM(+15.99)ENEFVLIK.K | Y | 81.05 | 1367.6642 | 11 | 6.8 | 684.8440 | 2 | 79.83 | 6 | 22825 | Ana\_05.raw | 1.85E8 | 2.32E8 | 1.57E8 | 9.55E7 | 1.92E8 | 1.95E8 | 9.2E7 | 1.25E8 | 3.62E8 | 1.42E8 | 52 | 6 | 7 | 4 | 4 | 6 | 4 | 3 | 7 | 6 | 5 | 193 | 203 | Oxidation (M) |
| R.KLLEGEESR.L | N | 81.04 | 1059.5560 | 9 | 8.7 | 354.1957 | 3 | 23.79 | 6 | 6172 | Ana\_05.raw | 1.35E8 | 7.36E7 | 8.47E7 | 3.98E7 | 3.65E7 | 5.87E7 | 4.47E7 | 2.25E7 | 1.49E8 | 2.57E7 | 25 | 5 | 2 | 1 | 1 | 2 | 2 | 2 | 2 | 5 | 3 | 399 | 407 |  |
| R.AQYEDIANR.S | N | 77.62 | 1078.5043 | 9 | 1.9 | 540.2604 | 2 | 36.42 | 3 | 10050 | Ana\_02.raw | 3.24E8 | 3.44E8 | 3.06E8 | 1.66E8 | 3.4E8 | 3.73E8 | 2.43E8 | 2.08E8 | 1.05E8 | 2.79E8 | 49 | 6 | 5 | 4 | 5 | 5 | 5 | 4 | 5 | 4 | 6 | 271 | 279 |  |
| K.YEDEINKR.T | N | 77.25 | 1065.5090 | 8 | 0.9 | 533.7623 | 2 | 21.18 | 3 | 5329 | Ana\_02.raw | 9.68E7 | 1.2E8 | 1.02E8 | 2.41E7 | 3.33E7 | 6.9E7 | 3.61E7 | 4.69E7 | 1.25E8 | 7.09E7 | 23 | 2 | 2 | 2 | 2 | 3 | 2 | 2 | 4 | 2 | 2 | 185 | 192 |  |
| K.YEELQTLAGK.H | Y | 76.89 | 1150.5869 | 10 | 2.1 | 576.3019 | 2 | 57.48 | 3 | 16320 | Ana\_02.raw | 1.23E8 | 1.7E8 | 1E8 | 5.36E7 | 1.12E8 | 1.29E8 | 8.33E7 | 6.51E7 | 2.05E8 | 1.1E8 | 32 | 3 | 5 | 2 | 2 | 4 | 4 | 3 | 3 | 3 | 3 | 292 | 301 |  |
| R.SLDMDGIIAEVR.A | Y | 73.81 | 1317.6598 | 12 | 2.3 | 659.8387 | 2 | 107.59 | 6 | 30261 | Ana\_05.raw | 5.47E5 | 1.3E6 |  | 1.48E5 |  | 0 |  |  | 7.19E6 |  | 8 | 1 | 1 | 0 | 1 | 1 | 1 | 0 | 0 | 3 | 0 | 259 | 270 |  |
| R.SRAEAETM(+15.99)YQIK.Y | Y | 71.39 | 1441.6871 | 12 | 6.4 | 481.5727 | 3 | 32.03 | 2 | 8627 | Ana\_01.raw | 7.51E7 | 9.21E7 | 4.82E7 | 2.26E7 | 3.95E7 | 8E7 | 4.52E7 | 3.66E7 | 8.98E7 | 4.38E7 | 32 | 3 | 4 | 2 | 3 | 3 | 4 | 3 | 5 | 3 | 2 | 280 | 291 | Oxidation (M) |
| K.LLEGEESR.L | N | 70.89 | 931.4611 | 8 | 2.9 | 466.7392 | 2 | 26.90 | 6 | 7157 | Ana\_05.raw | 1.04E8 | 5.04E8 | 4.04E8 | 2.11E8 | 3.45E8 | 4.18E8 | 2.77E8 | 2.42E8 | 3.67E8 | 3.25E8 | 45 | 6 | 4 | 4 | 4 | 4 | 4 | 4 | 6 | 5 | 4 | 400 | 407 |  |
| R.SFTSGPGAR.I | Y | 70.12 | 878.4246 | 9 | 2.1 | 440.2205 | 2 | 26.01 | 4 | 6698 | Ana\_03.raw | 4.86E7 | 3.51E8 | 2.84E8 | 1.69E8 | 3.22E8 | 3.71E8 | 2.58E8 | 1.68E8 | 2.22E8 | 1.66E8 | 46 | 3 | 5 | 4 | 6 | 5 | 5 | 5 | 4 | 3 | 6 | 24 | 32 |  |
| K.LAELEAALQR.A | Y | 69.01 | 1112.6189 | 10 | 3.6 | 557.3187 | 2 | 72.70 | 6 | 20858 | Ana\_05.raw | 1.25E8 | 1.83E8 | 9.36E7 | 5.89E7 | 1.08E8 | 1.31E8 | 6.94E7 | 7.2E7 | 2.1E8 | 8.93E7 | 39 | 5 | 5 | 4 | 3 | 4 | 4 | 3 | 4 | 4 | 3 | 359 | 368 |  |
| K.LVSESSDVVSK | Y | 68.25 | 1148.5924 | 11 | 5.2 | 575.3065 | 2 | 32.11 | 9 | 8516 | Ana\_08.raw | 2.88E8 | 4.06E8 | 3.43E8 | 1.38E8 | 3.14E8 | 3.69E8 | 2.46E8 | 1.63E8 | 3.95E8 | 2.7E8 | 12 | 1 | 1 | 1 | 1 | 1 | 1 | 2 | 1 | 2 | 1 | 480 | 490 |  |
| R.TKTEISEM(+15.99)NR.N | Y | 67.68 | 1223.5815 | 10 | 2.8 | 612.7997 | 2 | 18.48 | 4 | 4428 | Ana\_03.raw | 2.71E6 | 8.37E5 | 4.14E7 | 2.24E7 | 5.15E7 | 7.09E7 | 1.49E7 | 1.31E7 |  | 7.64E6 | 15 | 1 | 1 | 2 | 2 | 2 | 2 | 2 | 2 | 0 | 1 | 309 | 318 | Oxidation (M) |
| R.LESGM(+15.99)QNM(+15.99)SIHTK.T | Y | 65.07 | 1506.6807 | 13 | 1.0 | 754.3484 | 2 | 21.28 | 6 | 5355 | Ana\_05.raw | 9.79E7 | 1.54E8 | 9.08E7 | 9.18E6 | 8.05E6 | 2.1E7 | 8.37E7 | 6.63E7 | 9.91E7 | 6.52E6 | 21 | 2 | 2 | 2 | 1 | 2 | 2 | 2 | 4 | 3 | 1 | 408 | 420 | Oxidation (M) |
| R.SNMDNM(+15.99)FESYINNLR.R | Y | 65.04 | 1862.7927 | 15 | 2.2 | 932.4057 | 2 | 109.88 | 9 | 30852 | Ana\_08.raw | 1.72E6 | 0 |  |  |  |  |  |  | 2.59E6 | 4.75E5 | 4 | 1 | 1 | 0 | 0 | 0 | 0 | 0 | 0 | 1 | 1 | 140 | 154 | Oxidation (M) |
| K.DVDEAYMNK.V | Y | 62.22 | 1083.4542 | 9 | 5.9 | 542.7376 | 2 | 39.13 | 1 | 10746 | ANa\_10.raw | 1.48E7 | 8.99E6 |  |  |  | 5.71E6 |  | 3.38E6 | 2.39E6 | 1.11E7 | 6 | 1 | 1 | 0 | 0 | 0 | 1 | 0 | 1 | 1 | 1 | 205 | 213 |  |
| R.TEMENEFVLIK.K | Y | 60.62 | 1351.6693 | 11 | 2.4 | 676.8436 | 2 | 91.57 | 1 | 25713 | ANa\_10.raw | 6.18E6 | 4.58E6 |  |  |  | 3.1E6 | 5.03E5 |  | 2.03E7 | 6.17E6 | 7 | 1 | 1 | 0 | 0 | 0 | 1 | 1 | 0 | 1 | 2 | 193 | 203 |  |
| R.EYQELM(+15.99)NVK.L | N | 60.61 | 1168.5435 | 9 | 3.8 | 585.2812 | 2 | 43.66 | 6 | 12282 | Ana\_05.raw | 8.17E7 | 1.18E8 | 1.07E8 | 2.16E7 | 1.14E8 | 1.16E8 | 6.81E7 | 9.11E7 | 2.93E7 | 4.85E7 | 26 | 2 | 3 | 3 | 1 | 4 | 2 | 2 | 4 | 3 | 2 | 379 | 387 | Oxidation (M) |
| R.VGSSSSSFR.G | Y | 60.05 | 912.4301 | 9 | 2.4 | 457.2234 | 2 | 22.10 | 6 | 5626 | Ana\_05.raw | 2.89E7 | 1.23E7 | 2.09E8 | 1.65E8 | 2.67E8 | 4.28E8 | 1.98E8 | 4.57E7 |  | 5.7E7 | 20 | 1 | 1 | 1 | 3 | 4 | 2 | 4 | 3 | 0 | 1 | 41 | 49 |  |
| R.AEAETM(+15.99)YQIK.Y | Y | 58.43 | 1198.5540 | 10 | 3.0 | 600.2861 | 2 | 38.83 | 6 | 10793 | Ana\_05.raw | 1.27E8 | 1.76E8 | 1.37E8 | 7.12E7 | 1.39E8 | 1.27E8 | 1.03E8 | 1.06E8 | 1.02E8 | 7.87E7 | 26 | 2 | 3 | 2 | 2 | 3 | 2 | 2 | 3 | 5 | 2 | 282 | 291 | Oxidation (M) |
| R.QIHEEEIR.E | Y | 57.87 | 1052.5250 | 8 | 4.4 | 527.2721 | 2 | 23.38 | 9 | 5759 | Ana\_08.raw | 5.25E7 | 4.91E7 | 5.54E7 | 3.06E7 | 5.81E7 | 3.9E7 | 4.36E7 | 4.28E7 | 5.91E7 | 1.22E7 | 21 | 4 | 2 | 2 | 2 | 2 | 1 | 2 | 2 | 2 | 2 | 232 | 239 |  |
| K.DVDEAYM(+15.99)NK.V | Y | 57.71 | 1099.4492 | 9 | 3.9 | 550.7340 | 2 | 27.67 | 6 | 7399 | Ana\_05.raw | 5E7 | 6.1E7 | 5.97E7 | 3.33E7 | 5.73E7 | 6.28E7 | 4.42E7 | 4.12E7 |  | 1.67E7 | 21 | 3 | 1 | 2 | 3 | 2 | 3 | 2 | 3 | 0 | 2 | 205 | 213 | Oxidation (M) |
| K.WSLLQQQK.T | Y | 56.89 | 1029.5607 | 8 | 6.7 | 515.7911 | 2 | 62.28 | 9 | 17734 | Ana\_08.raw | 2.33E7 | 3.65E7 | 8.14E6 |  | 2.17E7 | 3.11E7 |  | 1.46E7 | 6.41E7 | 2.43E7 | 10 | 1 | 2 | 1 | 0 | 1 | 1 | 0 | 1 | 2 | 1 | 129 | 136 |  |
| R.LQAEIEALK.G | Y | 56.75 | 1013.5757 | 9 | 3.6 | 507.7970 | 2 | 61.66 | 6 | 17665 | Ana\_05.raw | 7.18E7 | 8.71E7 | 6.45E7 | 3.7E7 | 5.99E7 | 6.03E7 | 3.73E7 | 3.87E7 | 1.18E8 | 6.72E7 | 21 | 3 | 3 | 2 | 1 | 2 | 2 | 2 | 1 | 3 | 2 | 323 | 331 |  |
| K.FASFIDK.V | N | 53.69 | 826.4225 | 7 | 4.0 | 414.2202 | 2 | 59.07 | 6 | 16895 | Ana\_05.raw | 1.22E8 | 1.75E8 | 1.3E8 | 6.85E7 | 1.33E8 | 1.39E8 | 9.02E7 | 8.34E7 | 2.01E8 | 1.11E8 | 44 | 5 | 5 | 4 | 2 | 5 | 3 | 3 | 6 | 6 | 5 | 108 | 114 |  |
| R.ISSSSFSR.V | Y | 53.16 | 869.4243 | 8 | 2.9 | 435.7207 | 2 | 32.33 | 6 | 8843 | Ana\_05.raw | 2.69E8 | 3.24E8 | 1.54E8 | 1.27E8 | 2.65E8 | 2.78E8 | 2.04E8 | 1.36E8 | 3.14E8 | 1.89E8 | 39 | 5 | 4 | 2 | 3 | 5 | 3 | 4 | 4 | 5 | 4 | 33 | 40 |  |
| R.TKTEISEMNR.N | Y | 50.25 | 1207.5867 | 10 | 3.9 | 403.5378 | 3 | 24.47 | 9 | 6124 | Ana\_08.raw | 3.05E6 |  |  |  | 1.59E5 |  |  |  | 5.01E6 | 3.32E6 | 4 | 1 | 0 | 0 | 0 | 1 | 0 | 0 | 0 | 1 | 1 | 309 | 318 |  |
| R.RQLEALGQEK.L | Y | 49.96 | 1170.6356 | 10 | 3.9 | 586.3274 | 2 | 27.62 | 2 | 7279 | Ana\_01.raw | 9.33E6 | 1.37E7 | 4.41E6 | 2.14E6 | 5.21E6 | 6.17E6 | 3.18E6 | 1.49E6 | 8.87E6 | 8.36E6 | 10 | 1 | 1 | 1 | 1 | 1 | 1 | 1 | 1 | 1 | 1 | 155 | 164 |  |
| R.QLEALGQEK.L | Y | 49.86 | 1014.5345 | 9 | -3.7 | 508.2727 | 2 | 69.91 | 4 | 19454 | Ana\_03.raw | 2.58E7 | 3.48E7 | 1.07E6 | 2.13E7 | 3.83E7 |  | 2.97E7 | 6.28E7 | 1.01E7 | 1.18E7 | 13 | 1 | 3 | 1 | 1 | 2 | 0 | 1 | 2 | 1 | 1 | 156 | 164 |  |
| K.TEISEMNR.N | Y | 47.56 | 978.4440 | 8 | 2.9 | 490.2307 | 2 | 29.82 | 1 | 7952 | ANa\_10.raw | 6.94E6 |  |  |  |  |  |  |  | 1.9E7 |  | 2 | 1 | 0 | 0 | 0 | 0 | 0 | 0 | 0 | 1 | 0 | 311 | 318 |  |
| K.LEAELGNM(+15.99)QGLVEDFK.N | Y | 45.13 | 1807.8662 | 16 | 8.6 | 904.9481 | 2 | 72.04 | 9 | 20562 | Ana\_08.raw |  |  |  |  | 2.89E6 | 1.59E5 |  |  | 0 |  | 3 | 0 | 0 | 0 | 0 | 1 | 1 | 0 | 0 | 1 | 0 | 167 | 182 | Oxidation (M) |
| R.FLEQQNK.M | N | 42.78 | 905.4606 | 7 | 6.4 | 453.7405 | 2 | 25.02 | 7 | 6450 | Ana\_06.raw | 7.66E6 | 2.16E7 | 2.19E8 | 1.48E8 | 2.21E8 | 2.67E8 | 2.03E8 | 8.66E7 |  | 9.67E7 | 14 | 1 | 1 | 1 | 2 | 2 | 2 | 2 | 2 | 0 | 1 | 117 | 123 |  |
| K.TEISEM(+15.99)NR.N | Y | 42.56 | 994.4390 | 8 | 2.6 | 498.2280 | 2 | 20.10 | 4 | 4920 | Ana\_03.raw |  | 1.06E7 |  | 1.58E7 | 5.3E7 | 3.94E6 |  |  |  |  | 4 | 0 | 1 | 0 | 1 | 1 | 1 | 0 | 0 | 0 | 0 | 311 | 318 | Oxidation (M) |
| R.AEAETMYQIK.Y | Y | 38.87 | 1182.5591 | 10 | 9.0 | 592.2921 | 2 | 47.95 | 9 | 13420 | Ana\_08.raw |  | 1.17E6 |  |  |  | 1.25E6 |  |  | 1.75E6 | 5.47E6 | 4 | 0 | 1 | 0 | 0 | 0 | 1 | 0 | 0 | 1 | 1 | 282 | 291 |  |
| K.VELESR.L | Y | 38.86 | 731.3813 | 6 | 2.9 | 366.6990 | 2 | 22.66 | 6 | 5810 | Ana\_05.raw |  |  |  | 8.34E7 | 4.97E7 | 1.07E8 | 1.17E7 | 6.4E7 |  |  | 6 | 0 | 0 | 0 | 1 | 1 | 1 | 1 | 2 | 0 | 0 | 214 | 219 |  |
| R.LESGM(+15.99)QNMSIHTK.T | Y | 31.22 | 1490.6858 | 13 | 4.4 | 746.3535 | 2 | 28.76 | 9 | 7521 | Ana\_08.raw |  |  |  |  |  |  |  |  | 4.02E6 |  | 1 | 0 | 0 | 0 | 0 | 0 | 0 | 0 | 0 | 1 | 0 | 408 | 420 | Oxidation (M) |
| total 47 peptides |  |  |  |  |  |  |  |  |  |  |  |  |  |  |  |  |  |  |  |  |  |  |  |  |  |  |  |  |  |  |  |  |  |  |  |
| --- | --- | --- | --- | --- | --- | --- | --- | --- | --- | --- | --- | --- | --- | --- | --- | --- | --- | --- | --- | --- | --- | --- | --- | --- | --- | --- | --- | --- | --- | --- | --- | --- | --- | --- | --- |

Best Unique PSM (Scan F5:35809, m/z=1538.3199, z=2, RT=130.11, ppm=6.0):

sp|P16546|SPTN1\_MOUSE

back to list

  

| Protein Coverage
| Supporting Peptides
| Best Unique PSM
|

Protein Coverage:

Supporting Peptides:

| Peptide | Uniq | -10lgP | Mass | Length | ppm | m/z | z | RT | Fraction | Scan | Source File | Area 10 | Area 01 | Area 02 | Area 03 | Area 04 | Area 05 | Area 06 | Area 07 | Area 08 | Area 09 | #Spec | #Spec 10 | #Spec 01 | #Spec 02 | #Spec 03 | #Spec 04 | #Spec 05 | #Spec 06 | #Spec 07 | #Spec 08 | #Spec 09 | Start | End | PTM |
| --- | --- | --- | --- | --- | --- | --- | --- | --- | --- | --- | --- | --- | --- | --- | --- | --- | --- | --- | --- | --- | --- | --- | --- | --- | --- | --- | --- | --- | --- | --- | --- | --- | --- | --- | --- |
| K.ALINADELANDVAGAEALLDR.H | Y | 120.24 | 2153.0964 | 21 | 3.6 | 1077.5593 | 2 | 134.84 | 7 | 37152 | Ana\_06.raw |  |  |  | 4.95E5 | 8.34E5 | 2.58E6 | 1.17E6 | 4.41E5 | 3.91E6 | 1.89E6 | 13 | 0 | 0 | 0 | 2 | 2 | 2 | 2 | 1 | 2 | 2 | 382 | 402 |  |
| R.AGTFQAFEQFGQQLLAHGHYASPEIK.E | Y | 112.57 | 2874.4089 | 26 | 4.6 | 719.6128 | 4 | 130.51 | 7 | 35914 | Ana\_06.raw | 6.11E6 | 9.12E6 | 6.22E6 | 1.86E6 | 6.67E6 | 9.99E6 | 4.81E6 | 4.8E6 |  |  | 8 | 1 | 1 | 1 | 1 | 1 | 1 | 1 | 1 | 0 | 0 | 1392 | 1417 |  |
| K.IAALQAFADQLIAVDHYAK.G | Y | 107.82 | 2057.0945 | 19 | 1.2 | 686.7062 | 3 | 131.57 | 3 | 36043 | Ana\_02.raw |  | 7.45E6 | 4.7E6 | 4.22E6 | 3.85E6 | 9.01E6 | 7.59E6 | 2.57E6 | 8.07E6 | 6.1E6 | 9 | 0 | 1 | 1 | 1 | 1 | 1 | 1 | 1 | 1 | 1 | 1501 | 1519 |  |
| R.SSLSSAQADFNQLAELDR.Q | Y | 103.93 | 1950.9282 | 18 | 2.7 | 976.4741 | 2 | 110.76 | 6 | 31074 | Ana\_05.raw |  | 2.21E6 | 8.97E5 | 1.41E6 | 1.47E6 | 3.91E6 | 2.26E6 | 1.55E6 | 5.42E6 |  | 8 | 0 | 1 | 1 | 1 | 1 | 1 | 1 | 1 | 1 | 0 | 2138 | 2155 |  |
| R.AQLADSFHLQQFFR.D | Y | 98.18 | 1706.8528 | 14 | 3.5 | 569.9602 | 3 | 114.44 | 6 | 32019 | Ana\_05.raw | 4.07E5 | 1.37E6 | 1.45E6 | 2.71E6 | 5.03E5 | 5.45E6 | 1.2E6 | 1.15E6 | 6.99E5 | 1.05E6 | 12 | 1 | 1 | 1 | 1 | 1 | 2 | 2 | 1 | 1 | 1 | 567 | 580 |  |
| R.EAFLNTEDKGDSLDSVEALIK.K | Y | 77.66 | 2293.1326 | 21 | 2.0 | 765.3863 | 3 | 114.10 | 6 | 31934 | Ana\_05.raw |  | 4.87E6 | 1.56E6 |  | 3.53E6 | 3.8E6 |  |  |  |  | 4 | 0 | 1 | 1 | 0 | 1 | 1 | 0 | 0 | 0 | 0 | 1465 | 1485 |  |
| R.ELPTAFDYVEFTR.S | Y | 77.14 | 1586.7616 | 13 | 0.5 | 794.3885 | 2 | 116.65 | 7 | 32272 | Ana\_06.raw | 2.68E6 | 5.14E6 | 1.02E7 | 3.84E6 | 8.62E6 | 1.1E7 | 1.4E6 | 4.28E6 | 1.81E7 | 1.02E7 | 13 | 1 | 2 | 1 | 1 | 2 | 1 | 1 | 1 | 1 | 2 | 2455 | 2467 |  |
| K.LSDDNTIGQEEIQQR.L | Y | 75.78 | 1744.8228 | 15 | 5.4 | 873.4234 | 2 | 55.09 | 1 | 15534 | ANa\_10.raw | 9.83E5 | 1.32E6 |  |  |  | 9.3E5 | 3.4E5 |  | 1.81E6 |  | 5 | 1 | 1 | 0 | 0 | 0 | 1 | 1 | 0 | 1 | 0 | 1830 | 1844 |  |
| K.DVTGAEALLER.H | Y | 74.99 | 1172.6036 | 11 | 8.1 | 587.3138 | 2 | 80.00 | 6 | 22871 | Ana\_05.raw |  | 1.13E7 | 1.04E7 | 4.88E6 | 1.38E7 | 1.75E7 | 5.33E6 | 5.36E6 | 2.86E7 | 9.69E6 | 12 | 0 | 2 | 1 | 1 | 1 | 1 | 2 | 2 | 1 | 1 | 1370 | 1380 |  |
| R.LAALADQWQFLVQK.S | Y | 74.35 | 1629.8878 | 14 | 3.6 | 815.9541 | 2 | 119.85 | 6 | 33450 | Ana\_05.raw |  | 2.91E6 |  | 5.12E5 |  | 2.53E6 | 5.25E5 | 8.19E5 | 1.18E6 |  | 6 | 0 | 1 | 0 | 1 | 0 | 1 | 1 | 1 | 1 | 0 | 1633 | 1646 |  |
| R.SQLLGSAHEVQR.F | Y | 72.11 | 1323.6895 | 12 | 4.4 | 662.8549 | 2 | 37.98 | 6 | 10528 | Ana\_05.raw |  | 1.65E6 |  |  |  | 4.56E6 |  |  | 7.12E6 |  | 4 | 0 | 1 | 0 | 0 | 0 | 2 | 0 | 0 | 1 | 0 | 1226 | 1237 |  |
| R.KVEDLFLTFAK.K | Y | 71.27 | 1309.7281 | 11 | 3.5 | 437.5848 | 3 | 103.36 | 6 | 29141 | Ana\_05.raw |  |  |  |  |  | 7.25E5 | 1.3E5 |  |  |  | 3 | 0 | 0 | 0 | 0 | 0 | 2 | 1 | 0 | 0 | 0 | 2088 | 2098 |  |
| R.TALLELWELR.R | Y | 70.11 | 1242.6971 | 10 | 4.4 | 622.3586 | 2 | 127.41 | 6 | 35412 | Ana\_05.raw |  | 1.45E6 | 1.28E6 | 6.88E5 | 2.46E6 | 2.87E6 | 1.36E6 |  |  | 8.71E5 | 7 | 0 | 1 | 1 | 1 | 1 | 1 | 1 | 0 | 0 | 1 | 450 | 459 |  |
| K.VLETAEDIQER.R | Y | 67.11 | 1301.6462 | 11 | 4.5 | 651.8333 | 2 | 46.43 | 9 | 12965 | Ana\_08.raw |  |  |  |  |  |  |  |  | 8.83E6 |  | 1 | 0 | 0 | 0 | 0 | 0 | 0 | 0 | 0 | 1 | 0 | 8 | 18 |  |
| R.DLASVQALLR.K | Y | 63.40 | 1084.6240 | 10 | 4.5 | 543.3217 | 2 | 101.48 | 6 | 28648 | Ana\_05.raw |  |  |  | 3.97E5 | 1.04E6 | 3.35E6 | 5.08E5 |  |  |  | 4 | 0 | 0 | 0 | 1 | 1 | 1 | 1 | 0 | 0 | 0 | 286 | 295 |  |
| K.SSEEIESAFR.A | Y | 63.18 | 1153.5251 | 10 | 1.1 | 577.7705 | 2 | 58.57 | 6 | 16750 | Ana\_05.raw | 3.84E5 | 5.73E6 | 4.32E6 | 2.4E6 | 4.2E6 | 4.67E6 | 3.46E6 | 3.15E6 | 6.43E6 | 3.81E6 | 10 | 1 | 1 | 1 | 1 | 1 | 1 | 1 | 1 | 1 | 1 | 2405 | 2414 |  |
| K.HQAFEAELHANADR.I | Y | 62.68 | 1607.7440 | 14 | 3.6 | 536.9239 | 3 | 43.65 | 5 | 12219 | Ana\_04.raw |  |  |  |  | 2.24E6 |  |  |  |  |  | 1 | 0 | 0 | 0 | 0 | 1 | 0 | 0 | 0 | 0 | 0 | 1592 | 1605 |  |
| R.EKEPIAASTNR.G | Y | 62.53 | 1214.6255 | 11 | 8.2 | 608.3250 | 2 | 21.71 | 1 | 5341 | ANa\_10.raw | 2.8E6 | 8.85E6 | 5.62E6 | 5.64E5 | 1.7E6 | 2.35E6 | 1.34E6 | 1.25E6 | 6.68E6 | 2.14E6 | 14 | 1 | 2 | 2 | 1 | 1 | 2 | 1 | 1 | 2 | 1 | 802 | 812 |  |
| R.DLSSVQTLLTK.Q | Y | 60.58 | 1203.6710 | 11 | 4.2 | 602.8453 | 2 | 94.23 | 6 | 26719 | Ana\_05.raw |  |  |  |  | 1.11E6 | 2.59E6 | 5.01E5 | 3.99E5 |  |  | 4 | 0 | 0 | 0 | 0 | 1 | 1 | 1 | 1 | 0 | 0 | 2007 | 2017 |  |
| K.DLIGVQNLLK.K | Y | 59.31 | 1111.6600 | 10 | 4.2 | 556.8396 | 2 | 105.07 | 6 | 29600 | Ana\_05.raw | 8.22E5 |  | 2.01E6 | 2.13E5 | 3.2E5 | 3.49E6 | 7.93E5 | 2.39E5 | 1.03E6 |  | 8 | 1 | 0 | 1 | 1 | 1 | 1 | 1 | 1 | 1 | 0 | 815 | 824 |  |
| K.HQAFEAELSANQSR.I | Y | 55.43 | 1586.7437 | 14 | 3.9 | 529.9239 | 3 | 50.21 | 7 | 14088 | Ana\_06.raw |  |  |  |  |  |  | 5.38E5 |  |  |  | 1 | 0 | 0 | 0 | 0 | 0 | 0 | 1 | 0 | 0 | 0 | 614 | 627 |  |
| R.SLQQLAEER.S | Y | 54.70 | 1072.5513 | 9 | -0.1 | 537.2828 | 2 | 55.95 | 6 | 15974 | Ana\_05.raw | 3.87E6 | 7.11E6 | 2.68E6 | 2.14E6 |  | 4.99E5 |  | 0 | 8.68E6 | 3.27E6 | 8 | 1 | 1 | 1 | 1 | 0 | 1 | 0 | 1 | 1 | 1 | 1217 | 1225 |  |
| K.ITALDEFATK.L | Y | 52.53 | 1107.5812 | 10 | 6.8 | 554.8016 | 2 | 71.03 | 3 | 20183 | Ana\_02.raw | 8.72E6 | 1.03E7 | 9.54E5 | 7.67E5 |  |  |  |  |  | 3.91E6 | 5 | 1 | 1 | 1 | 1 | 0 | 0 | 0 | 0 | 0 | 1 | 523 | 532 |  |
| K.SADESGQALLAASHYASDEVR.E | Y | 51.46 | 2176.0032 | 21 | 4.9 | 726.3452 | 3 | 89.10 | 6 | 25348 | Ana\_05.raw |  |  |  |  |  | 4.5E5 |  | 9.67E5 |  |  | 2 | 0 | 0 | 0 | 0 | 0 | 1 | 0 | 1 | 0 | 0 | 419 | 439 |  |
| K.EAIVTSEELGQDLEHVEVLQK.K | Y | 51.20 | 2365.2012 | 21 | 8.4 | 789.4143 | 3 | 112.16 | 9 | 31429 | Ana\_08.raw |  |  |  |  |  |  |  |  | 1.49E6 |  | 1 | 0 | 0 | 0 | 0 | 0 | 0 | 0 | 0 | 1 | 0 | 169 | 189 |  |
| R.DLTGVQNLR.K | Y | 50.95 | 1014.5458 | 9 | 4.4 | 508.2824 | 2 | 55.53 | 2 | 15750 | Ana\_01.raw | 1.32E6 | 2.81E6 | 2.26E6 | 1.23E6 | 2.46E6 | 2.56E6 | 1.01E6 |  | 2.24E6 | 3.91E5 | 9 | 1 | 1 | 1 | 1 | 1 | 1 | 1 | 0 | 1 | 1 | 1795 | 1803 |  |
| K.GEIDAHEDSFK.S | Y | 50.70 | 1246.5465 | 11 | 2.0 | 624.2818 | 2 | 39.37 | 6 | 10959 | Ana\_05.raw |  |  |  |  | 2.81E6 | 1.72E6 |  |  |  |  | 2 | 0 | 0 | 0 | 0 | 1 | 1 | 0 | 0 | 0 | 0 | 408 | 418 |  |
| K.LSILSEER.T | Y | 48.99 | 945.5131 | 8 | 3.0 | 473.7652 | 2 | 57.61 | 6 | 16466 | Ana\_05.raw | 1.75E6 | 3.75E6 | 2.84E6 |  | 2.13E6 | 3.2E6 |  |  | 6.2E6 | 2.17E6 | 7 | 1 | 1 | 1 | 0 | 1 | 1 | 0 | 0 | 1 | 1 | 442 | 449 |  |
| R.RQQVLDR.Y | Y | 48.35 | 913.5093 | 7 | 1.8 | 457.7628 | 2 | 19.41 | 6 | 4737 | Ana\_05.raw |  |  | 1.13E6 | 7.94E5 | 1.07E6 | 1.53E6 | 1.5E6 | 4.2E5 |  | 1.76E6 | 7 | 0 | 0 | 1 | 1 | 1 | 1 | 1 | 1 | 0 | 1 | 19 | 25 |  |
| R.NTTGVTEEALK.E | Y | 47.55 | 1161.5878 | 11 | 1.8 | 581.8022 | 2 | 38.73 | 6 | 10762 | Ana\_05.raw | 4.16E6 | 5.61E6 |  |  | 3.7E6 | 4.29E6 | 1.08E6 | 2.26E6 |  |  | 6 | 1 | 1 | 0 | 0 | 1 | 1 | 1 | 1 | 0 | 0 | 2316 | 2326 |  |
| K.EELYQNLTR.E | Y | 45.15 | 1164.5775 | 9 | 4.8 | 583.2988 | 2 | 60.51 | 5 | 17214 | Ana\_04.raw |  |  |  |  | 2.51E6 |  |  |  |  |  | 1 | 0 | 0 | 0 | 0 | 1 | 0 | 0 | 0 | 0 | 0 | 2427 | 2435 |  |
| K.LGDSHDLQR.F | Y | 44.96 | 1039.5046 | 9 | 5.5 | 347.5107 | 3 | 23.51 | 2 | 5930 | Ana\_01.raw |  | 2.26E6 |  | 1.12E6 |  |  |  |  |  |  | 2 | 0 | 1 | 0 | 1 | 0 | 0 | 0 | 0 | 0 | 0 | 1335 | 1343 |  |
| K.LLVSSEDYGR.D | Y | 44.88 | 1137.5665 | 10 | 4.6 | 569.7932 | 2 | 53.75 | 5 | 15229 | Ana\_04.raw |  |  | 2.6E6 |  | 2.1E6 |  |  |  | 1.52E6 |  | 3 | 0 | 0 | 1 | 0 | 1 | 0 | 0 | 0 | 1 | 0 | 1785 | 1794 |  |
| R.GLVSSDELAK.D | Y | 44.23 | 1017.5342 | 10 | 6.0 | 509.7774 | 2 | 44.75 | 9 | 12455 | Ana\_08.raw |  |  |  |  |  |  |  |  | 3.61E6 |  | 1 | 0 | 0 | 0 | 0 | 0 | 0 | 0 | 0 | 1 | 0 | 1360 | 1369 |  |
| R.EANELQQWITEK.E | Y | 43.50 | 1487.7256 | 12 | 1.5 | 744.8712 | 2 | 97.66 | 8 | 27190 | Ana\_07.raw |  |  |  |  |  |  |  | 3.59E5 |  |  | 1 | 0 | 0 | 0 | 0 | 0 | 0 | 0 | 1 | 0 | 0 | 1099 | 1110 |  |
| R.FLADFR.D | Y | 42.71 | 767.3966 | 6 | -0.1 | 384.7055 | 2 | 62.23 | 3 | 17731 | Ana\_02.raw | 9.72E5 | 1.67E6 | 2.7E6 | 7.73E5 | 2.28E6 | 8.38E5 | 1.54E6 |  | 2.07E6 | 1.94E6 | 9 | 1 | 1 | 1 | 1 | 1 | 1 | 1 | 0 | 1 | 1 | 366 | 371 |  |
| K.KQEALVAR.Y | Y | 41.75 | 913.5345 | 8 | 1.6 | 457.7753 | 2 | 19.95 | 6 | 4912 | Ana\_05.raw |  |  |  |  |  | 3.7E6 |  |  |  |  | 1 | 0 | 0 | 0 | 0 | 0 | 1 | 0 | 0 | 0 | 0 | 758 | 765 |  |
| K.TATDEAYKDPSNLQGK.V | Y | 41.66 | 1736.8217 | 16 | 3.4 | 579.9498 | 3 | 39.67 | 5 | 11003 | Ana\_04.raw |  |  | 2.76E6 |  | 2.47E6 | 2.44E6 |  |  |  |  | 3 | 0 | 0 | 1 | 0 | 1 | 1 | 0 | 0 | 0 | 0 | 595 | 610 |  |
| K.KFDDFQK.D | Y | 39.22 | 926.4497 | 7 | 6.6 | 464.2352 | 2 | 29.61 | 2 | 7898 | Ana\_01.raw |  | 2E6 |  |  |  |  |  |  |  |  | 1 | 0 | 1 | 0 | 0 | 0 | 0 | 0 | 0 | 0 | 0 | 1132 | 1138 |  |
| R.EAHDAFR.S | Y | 38.78 | 844.3828 | 7 | 2.9 | 423.1999 | 2 | 18.52 | 5 | 4491 | Ana\_04.raw |  |  |  |  | 2.84E6 |  |  |  |  |  | 1 | 0 | 0 | 0 | 0 | 1 | 0 | 0 | 0 | 0 | 0 | 2131 | 2137 |  |
| R.FQFFQR.D | Y | 37.35 | 871.4340 | 6 | 3.9 | 436.7260 | 2 | 74.88 | 6 | 21483 | Ana\_05.raw |  | 2.22E6 | 1.53E6 |  | 3.39E6 | 1.05E6 | 1.74E6 | 1.26E6 |  | 1.64E6 | 7 | 0 | 1 | 1 | 0 | 1 | 1 | 1 | 1 | 0 | 1 | 46 | 51 |  |
| K.KHEAFETDFTVHK.D | Y | 25.96 | 1587.7681 | 13 | 3.9 | 397.9508 | 4 | 48.47 | 7 | 13557 | Ana\_06.raw |  |  |  |  |  |  | 9.07E5 |  |  |  | 1 | 0 | 0 | 0 | 0 | 0 | 0 | 1 | 0 | 0 | 0 | 1911 | 1923 |  |
| R.FLSDFR.D | Y | 25.64 | 783.3915 | 6 | 3.6 | 392.7044 | 2 | 55.68 | 9 | 15767 | Ana\_08.raw |  |  |  |  |  |  |  |  | 7.07E5 |  | 1 | 0 | 0 | 0 | 0 | 0 | 0 | 0 | 0 | 1 | 0 | 1344 | 1349 |  |
| R.EANQQQQFNR.N | Y | 25.43 | 1261.5800 | 10 | -4.6 | 631.7944 | 2 | 30.34 | 7 | 8074 | Ana\_06.raw |  |  |  |  |  |  | 4.22E6 |  |  |  | 1 | 0 | 0 | 0 | 0 | 0 | 0 | 1 | 0 | 0 | 0 | 676 | 685 |  |
| R.ELELQK.E | Y | 24.81 | 758.4174 | 6 | -8.9 | 380.2126 | 2 | 20.42 | 7 | 5030 | Ana\_06.raw |  |  |  |  |  |  | 7.73E6 |  |  |  | 1 | 0 | 0 | 0 | 0 | 0 | 0 | 1 | 0 | 0 | 0 | 2190 | 2195 |  |
| total 45 peptides |  |  |  |  |  |  |  |  |  |  |  |  |  |  |  |  |  |  |  |  |  |  |  |  |  |  |  |  |  |  |  |  |  |  |  |
| --- | --- | --- | --- | --- | --- | --- | --- | --- | --- | --- | --- | --- | --- | --- | --- | --- | --- | --- | --- | --- | --- | --- | --- | --- | --- | --- | --- | --- | --- | --- | --- | --- | --- | --- | --- |

Best Unique PSM (Scan F7:37152, m/z=1077.5593, z=2, RT=134.84, ppm=3.6):

A3KGU7|A3KGU7\_MOUSE

back to list

  

| Protein Coverage
| Supporting Peptides
| Best Unique PSM
|

Protein Coverage:

Supporting Peptides:

| Peptide | Uniq | -10lgP | Mass | Length | ppm | m/z | z | RT | Fraction | Scan | Source File | Area 10 | Area 01 | Area 02 | Area 03 | Area 04 | Area 05 | Area 06 | Area 07 | Area 08 | Area 09 | #Spec | #Spec 10 | #Spec 01 | #Spec 02 | #Spec 03 | #Spec 04 | #Spec 05 | #Spec 06 | #Spec 07 | #Spec 08 | #Spec 09 | Start | End | PTM |
| --- | --- | --- | --- | --- | --- | --- | --- | --- | --- | --- | --- | --- | --- | --- | --- | --- | --- | --- | --- | --- | --- | --- | --- | --- | --- | --- | --- | --- | --- | --- | --- | --- | --- | --- | --- |
| K.ALINADELANDVAGAEALLDR.H | Y | 120.24 | 2153.0964 | 21 | 3.6 | 1077.5593 | 2 | 134.84 | 7 | 37152 | Ana\_06.raw |  |  |  | 4.95E5 | 8.34E5 | 2.58E6 | 1.17E6 | 4.41E5 | 3.91E6 | 1.89E6 | 13 | 0 | 0 | 0 | 2 | 2 | 2 | 2 | 1 | 2 | 2 | 382 | 402 |  |
| R.AGTFQAFEQFGQQLLAHGHYASPEIK.E | Y | 112.57 | 2874.4089 | 26 | 4.6 | 719.6128 | 4 | 130.51 | 7 | 35914 | Ana\_06.raw | 6.11E6 | 9.12E6 | 6.22E6 | 1.86E6 | 6.67E6 | 9.99E6 | 4.81E6 | 4.8E6 |  |  | 8 | 1 | 1 | 1 | 1 | 1 | 1 | 1 | 1 | 0 | 0 | 1392 | 1417 |  |
| K.IAALQAFADQLIAVDHYAK.G | Y | 107.82 | 2057.0945 | 19 | 1.2 | 686.7062 | 3 | 131.57 | 3 | 36043 | Ana\_02.raw |  | 7.45E6 | 4.7E6 | 4.22E6 | 3.85E6 | 9.01E6 | 7.59E6 | 2.57E6 | 8.07E6 | 6.1E6 | 9 | 0 | 1 | 1 | 1 | 1 | 1 | 1 | 1 | 1 | 1 | 1501 | 1519 |  |
| R.SSLSSAQADFNQLAELDR.Q | Y | 103.93 | 1950.9282 | 18 | 2.7 | 976.4741 | 2 | 110.76 | 6 | 31074 | Ana\_05.raw |  | 2.21E6 | 8.97E5 | 1.41E6 | 1.47E6 | 3.91E6 | 2.26E6 | 1.55E6 | 5.42E6 |  | 8 | 0 | 1 | 1 | 1 | 1 | 1 | 1 | 1 | 1 | 0 | 2143 | 2160 |  |
| R.AQLADSFHLQQFFR.D | Y | 98.18 | 1706.8528 | 14 | 3.5 | 569.9602 | 3 | 114.44 | 6 | 32019 | Ana\_05.raw | 4.07E5 | 1.37E6 | 1.45E6 | 2.71E6 | 5.03E5 | 5.45E6 | 1.2E6 | 1.15E6 | 6.99E5 | 1.05E6 | 12 | 1 | 1 | 1 | 1 | 1 | 2 | 2 | 1 | 1 | 1 | 567 | 580 |  |
| R.EAFLNTEDKGDSLDSVEALIK.K | Y | 77.66 | 2293.1326 | 21 | 2.0 | 765.3863 | 3 | 114.10 | 6 | 31934 | Ana\_05.raw |  | 4.87E6 | 1.56E6 |  | 3.53E6 | 3.8E6 |  |  |  |  | 4 | 0 | 1 | 1 | 0 | 1 | 1 | 0 | 0 | 0 | 0 | 1465 | 1485 |  |
| R.ELPTAFDYVEFTR.S | Y | 77.14 | 1586.7616 | 13 | 0.5 | 794.3885 | 2 | 116.65 | 7 | 32272 | Ana\_06.raw | 2.68E6 | 5.14E6 | 1.02E7 | 3.84E6 | 8.62E6 | 1.1E7 | 1.4E6 | 4.28E6 | 1.81E7 | 1.02E7 | 13 | 1 | 2 | 1 | 1 | 2 | 1 | 1 | 1 | 1 | 2 | 2460 | 2472 |  |
| K.LSDDNTIGQEEIQQR.L | Y | 75.78 | 1744.8228 | 15 | 5.4 | 873.4234 | 2 | 55.09 | 1 | 15534 | ANa\_10.raw | 9.83E5 | 1.32E6 |  |  |  | 9.3E5 | 3.4E5 |  | 1.81E6 |  | 5 | 1 | 1 | 0 | 0 | 0 | 1 | 1 | 0 | 1 | 0 | 1835 | 1849 |  |
| K.DVTGAEALLER.H | Y | 74.99 | 1172.6036 | 11 | 8.1 | 587.3138 | 2 | 80.00 | 6 | 22871 | Ana\_05.raw |  | 1.13E7 | 1.04E7 | 4.88E6 | 1.38E7 | 1.75E7 | 5.33E6 | 5.36E6 | 2.86E7 | 9.69E6 | 12 | 0 | 2 | 1 | 1 | 1 | 1 | 2 | 2 | 1 | 1 | 1370 | 1380 |  |
| R.LAALADQWQFLVQK.S | Y | 74.35 | 1629.8878 | 14 | 3.6 | 815.9541 | 2 | 119.85 | 6 | 33450 | Ana\_05.raw |  | 2.91E6 |  | 5.12E5 |  | 2.53E6 | 5.25E5 | 8.19E5 | 1.18E6 |  | 6 | 0 | 1 | 0 | 1 | 0 | 1 | 1 | 1 | 1 | 0 | 1638 | 1651 |  |
| R.SQLLGSAHEVQR.F | Y | 72.11 | 1323.6895 | 12 | 4.4 | 662.8549 | 2 | 37.98 | 6 | 10528 | Ana\_05.raw |  | 1.65E6 |  |  |  | 4.56E6 |  |  | 7.12E6 |  | 4 | 0 | 1 | 0 | 0 | 0 | 2 | 0 | 0 | 1 | 0 | 1226 | 1237 |  |
| R.KVEDLFLTFAK.K | Y | 71.27 | 1309.7281 | 11 | 3.5 | 437.5848 | 3 | 103.36 | 6 | 29141 | Ana\_05.raw |  |  |  |  |  | 7.25E5 | 1.3E5 |  |  |  | 3 | 0 | 0 | 0 | 0 | 0 | 2 | 1 | 0 | 0 | 0 | 2093 | 2103 |  |
| R.TALLELWELR.R | Y | 70.11 | 1242.6971 | 10 | 4.4 | 622.3586 | 2 | 127.41 | 6 | 35412 | Ana\_05.raw |  | 1.45E6 | 1.28E6 | 6.88E5 | 2.46E6 | 2.87E6 | 1.36E6 |  |  | 8.71E5 | 7 | 0 | 1 | 1 | 1 | 1 | 1 | 1 | 0 | 0 | 1 | 450 | 459 |  |
| K.VLETAEDIQER.R | Y | 67.11 | 1301.6462 | 11 | 4.5 | 651.8333 | 2 | 46.43 | 9 | 12965 | Ana\_08.raw |  |  |  |  |  |  |  |  | 8.83E6 |  | 1 | 0 | 0 | 0 | 0 | 0 | 0 | 0 | 0 | 1 | 0 | 8 | 18 |  |
| R.DLASVQALLR.K | Y | 63.40 | 1084.6240 | 10 | 4.5 | 543.3217 | 2 | 101.48 | 6 | 28648 | Ana\_05.raw |  |  |  | 3.97E5 | 1.04E6 | 3.35E6 | 5.08E5 |  |  |  | 4 | 0 | 0 | 0 | 1 | 1 | 1 | 1 | 0 | 0 | 0 | 286 | 295 |  |
| K.SSEEIESAFR.A | Y | 63.18 | 1153.5251 | 10 | 1.1 | 577.7705 | 2 | 58.57 | 6 | 16750 | Ana\_05.raw | 3.84E5 | 5.73E6 | 4.32E6 | 2.4E6 | 4.2E6 | 4.67E6 | 3.46E6 | 3.15E6 | 6.43E6 | 3.81E6 | 10 | 1 | 1 | 1 | 1 | 1 | 1 | 1 | 1 | 1 | 1 | 2410 | 2419 |  |
| K.HQAFEAELHANADR.I | Y | 62.68 | 1607.7440 | 14 | 3.6 | 536.9239 | 3 | 43.65 | 5 | 12219 | Ana\_04.raw |  |  |  |  | 2.24E6 |  |  |  |  |  | 1 | 0 | 0 | 0 | 0 | 1 | 0 | 0 | 0 | 0 | 0 | 1597 | 1610 |  |
| R.EKEPIAASTNR.G | Y | 62.53 | 1214.6255 | 11 | 8.2 | 608.3250 | 2 | 21.71 | 1 | 5341 | ANa\_10.raw | 2.8E6 | 8.85E6 | 5.62E6 | 5.64E5 | 1.7E6 | 2.35E6 | 1.34E6 | 1.25E6 | 6.68E6 | 2.14E6 | 14 | 1 | 2 | 2 | 1 | 1 | 2 | 1 | 1 | 2 | 1 | 802 | 812 |  |
| R.DLSSVQTLLTK.Q | Y | 60.58 | 1203.6710 | 11 | 4.2 | 602.8453 | 2 | 94.23 | 6 | 26719 | Ana\_05.raw |  |  |  |  | 1.11E6 | 2.59E6 | 5.01E5 | 3.99E5 |  |  | 4 | 0 | 0 | 0 | 0 | 1 | 1 | 1 | 1 | 0 | 0 | 2012 | 2022 |  |
| K.DLIGVQNLLK.K | Y | 59.31 | 1111.6600 | 10 | 4.2 | 556.8396 | 2 | 105.07 | 6 | 29600 | Ana\_05.raw | 8.22E5 |  | 2.01E6 | 2.13E5 | 3.2E5 | 3.49E6 | 7.93E5 | 2.39E5 | 1.03E6 |  | 8 | 1 | 0 | 1 | 1 | 1 | 1 | 1 | 1 | 1 | 0 | 815 | 824 |  |
| K.HQAFEAELSANQSR.I | Y | 55.43 | 1586.7437 | 14 | 3.9 | 529.9239 | 3 | 50.21 | 7 | 14088 | Ana\_06.raw |  |  |  |  |  |  | 5.38E5 |  |  |  | 1 | 0 | 0 | 0 | 0 | 0 | 0 | 1 | 0 | 0 | 0 | 614 | 627 |  |
| R.SLQQLAEER.S | Y | 54.70 | 1072.5513 | 9 | -0.1 | 537.2828 | 2 | 55.95 | 6 | 15974 | Ana\_05.raw | 3.87E6 | 7.11E6 | 2.68E6 | 2.14E6 |  | 4.99E5 |  | 0 | 8.68E6 | 3.27E6 | 8 | 1 | 1 | 1 | 1 | 0 | 1 | 0 | 1 | 1 | 1 | 1217 | 1225 |  |
| K.ITALDEFATK.L | Y | 52.53 | 1107.5812 | 10 | 6.8 | 554.8016 | 2 | 71.03 | 3 | 20183 | Ana\_02.raw | 8.72E6 | 1.03E7 | 9.54E5 | 7.67E5 |  |  |  |  |  | 3.91E6 | 5 | 1 | 1 | 1 | 1 | 0 | 0 | 0 | 0 | 0 | 1 | 523 | 532 |  |
| K.SADESGQALLAASHYASDEVR.E | Y | 51.46 | 2176.0032 | 21 | 4.9 | 726.3452 | 3 | 89.10 | 6 | 25348 | Ana\_05.raw |  |  |  |  |  | 4.5E5 |  | 9.67E5 |  |  | 2 | 0 | 0 | 0 | 0 | 0 | 1 | 0 | 1 | 0 | 0 | 419 | 439 |  |
| K.EAIVTSEELGQDLEHVEVLQK.K | Y | 51.20 | 2365.2012 | 21 | 8.4 | 789.4143 | 3 | 112.16 | 9 | 31429 | Ana\_08.raw |  |  |  |  |  |  |  |  | 1.49E6 |  | 1 | 0 | 0 | 0 | 0 | 0 | 0 | 0 | 0 | 1 | 0 | 169 | 189 |  |
| R.DLTGVQNLR.K | Y | 50.95 | 1014.5458 | 9 | 4.4 | 508.2824 | 2 | 55.53 | 2 | 15750 | Ana\_01.raw | 1.32E6 | 2.81E6 | 2.26E6 | 1.23E6 | 2.46E6 | 2.56E6 | 1.01E6 |  | 2.24E6 | 3.91E5 | 9 | 1 | 1 | 1 | 1 | 1 | 1 | 1 | 0 | 1 | 1 | 1800 | 1808 |  |
| K.GEIDAHEDSFK.S | Y | 50.70 | 1246.5465 | 11 | 2.0 | 624.2818 | 2 | 39.37 | 6 | 10959 | Ana\_05.raw |  |  |  |  | 2.81E6 | 1.72E6 |  |  |  |  | 2 | 0 | 0 | 0 | 0 | 1 | 1 | 0 | 0 | 0 | 0 | 408 | 418 |  |
| K.LSILSEER.T | Y | 48.99 | 945.5131 | 8 | 3.0 | 473.7652 | 2 | 57.61 | 6 | 16466 | Ana\_05.raw | 1.75E6 | 3.75E6 | 2.84E6 |  | 2.13E6 | 3.2E6 |  |  | 6.2E6 | 2.17E6 | 7 | 1 | 1 | 1 | 0 | 1 | 1 | 0 | 0 | 1 | 1 | 442 | 449 |  |
| R.RQQVLDR.Y | Y | 48.35 | 913.5093 | 7 | 1.8 | 457.7628 | 2 | 19.41 | 6 | 4737 | Ana\_05.raw |  |  | 1.13E6 | 7.94E5 | 1.07E6 | 1.53E6 | 1.5E6 | 4.2E5 |  | 1.76E6 | 7 | 0 | 0 | 1 | 1 | 1 | 1 | 1 | 1 | 0 | 1 | 19 | 25 |  |
| R.NTTGVTEEALK.E | Y | 47.55 | 1161.5878 | 11 | 1.8 | 581.8022 | 2 | 38.73 | 6 | 10762 | Ana\_05.raw | 4.16E6 | 5.61E6 |  |  | 3.7E6 | 4.29E6 | 1.08E6 | 2.26E6 |  |  | 6 | 1 | 1 | 0 | 0 | 1 | 1 | 1 | 1 | 0 | 0 | 2321 | 2331 |  |
| K.EELYQNLTR.E | Y | 45.15 | 1164.5775 | 9 | 4.8 | 583.2988 | 2 | 60.51 | 5 | 17214 | Ana\_04.raw |  |  |  |  | 2.51E6 |  |  |  |  |  | 1 | 0 | 0 | 0 | 0 | 1 | 0 | 0 | 0 | 0 | 0 | 2432 | 2440 |  |
| K.LGDSHDLQR.F | Y | 44.96 | 1039.5046 | 9 | 5.5 | 347.5107 | 3 | 23.51 | 2 | 5930 | Ana\_01.raw |  | 2.26E6 |  | 1.12E6 |  |  |  |  |  |  | 2 | 0 | 1 | 0 | 1 | 0 | 0 | 0 | 0 | 0 | 0 | 1335 | 1343 |  |
| K.LLVSSEDYGR.D | Y | 44.88 | 1137.5665 | 10 | 4.6 | 569.7932 | 2 | 53.75 | 5 | 15229 | Ana\_04.raw |  |  | 2.6E6 |  | 2.1E6 |  |  |  | 1.52E6 |  | 3 | 0 | 0 | 1 | 0 | 1 | 0 | 0 | 0 | 1 | 0 | 1790 | 1799 |  |
| R.GLVSSDELAK.D | Y | 44.23 | 1017.5342 | 10 | 6.0 | 509.7774 | 2 | 44.75 | 9 | 12455 | Ana\_08.raw |  |  |  |  |  |  |  |  | 3.61E6 |  | 1 | 0 | 0 | 0 | 0 | 0 | 0 | 0 | 0 | 1 | 0 | 1360 | 1369 |  |
| R.EANELQQWITEK.E | Y | 43.50 | 1487.7256 | 12 | 1.5 | 744.8712 | 2 | 97.66 | 8 | 27190 | Ana\_07.raw |  |  |  |  |  |  |  | 3.59E5 |  |  | 1 | 0 | 0 | 0 | 0 | 0 | 0 | 0 | 1 | 0 | 0 | 1099 | 1110 |  |
| R.FLADFR.D | Y | 42.71 | 767.3966 | 6 | -0.1 | 384.7055 | 2 | 62.23 | 3 | 17731 | Ana\_02.raw | 9.72E5 | 1.67E6 | 2.7E6 | 7.73E5 | 2.28E6 | 8.38E5 | 1.54E6 |  | 2.07E6 | 1.94E6 | 9 | 1 | 1 | 1 | 1 | 1 | 1 | 1 | 0 | 1 | 1 | 366 | 371 |  |
| K.KQEALVAR.Y | Y | 41.75 | 913.5345 | 8 | 1.6 | 457.7753 | 2 | 19.95 | 6 | 4912 | Ana\_05.raw |  |  |  |  |  | 3.7E6 |  |  |  |  | 1 | 0 | 0 | 0 | 0 | 0 | 1 | 0 | 0 | 0 | 0 | 758 | 765 |  |
| K.TATDEAYKDPSNLQGK.V | Y | 41.66 | 1736.8217 | 16 | 3.4 | 579.9498 | 3 | 39.67 | 5 | 11003 | Ana\_04.raw |  |  | 2.76E6 |  | 2.47E6 | 2.44E6 |  |  |  |  | 3 | 0 | 0 | 1 | 0 | 1 | 1 | 0 | 0 | 0 | 0 | 595 | 610 |  |
| K.KFDDFQK.D | Y | 39.22 | 926.4497 | 7 | 6.6 | 464.2352 | 2 | 29.61 | 2 | 7898 | Ana\_01.raw |  | 2E6 |  |  |  |  |  |  |  |  | 1 | 0 | 1 | 0 | 0 | 0 | 0 | 0 | 0 | 0 | 0 | 1132 | 1138 |  |
| R.EAHDAFR.S | Y | 38.78 | 844.3828 | 7 | 2.9 | 423.1999 | 2 | 18.52 | 5 | 4491 | Ana\_04.raw |  |  |  |  | 2.84E6 |  |  |  |  |  | 1 | 0 | 0 | 0 | 0 | 1 | 0 | 0 | 0 | 0 | 0 | 2136 | 2142 |  |
| R.FQFFQR.D | Y | 37.35 | 871.4340 | 6 | 3.9 | 436.7260 | 2 | 74.88 | 6 | 21483 | Ana\_05.raw |  | 2.22E6 | 1.53E6 |  | 3.39E6 | 1.05E6 | 1.74E6 | 1.26E6 |  | 1.64E6 | 7 | 0 | 1 | 1 | 0 | 1 | 1 | 1 | 1 | 0 | 1 | 46 | 51 |  |
| K.KHEAFETDFTVHK.D | Y | 25.96 | 1587.7681 | 13 | 3.9 | 397.9508 | 4 | 48.47 | 7 | 13557 | Ana\_06.raw |  |  |  |  |  |  | 9.07E5 |  |  |  | 1 | 0 | 0 | 0 | 0 | 0 | 0 | 1 | 0 | 0 | 0 | 1916 | 1928 |  |
| R.FLSDFR.D | Y | 25.64 | 783.3915 | 6 | 3.6 | 392.7044 | 2 | 55.68 | 9 | 15767 | Ana\_08.raw |  |  |  |  |  |  |  |  | 7.07E5 |  | 1 | 0 | 0 | 0 | 0 | 0 | 0 | 0 | 0 | 1 | 0 | 1344 | 1349 |  |
| R.EANQQQQFNR.N | Y | 25.43 | 1261.5800 | 10 | -4.6 | 631.7944 | 2 | 30.34 | 7 | 8074 | Ana\_06.raw |  |  |  |  |  |  | 4.22E6 |  |  |  | 1 | 0 | 0 | 0 | 0 | 0 | 0 | 1 | 0 | 0 | 0 | 676 | 685 |  |
| R.ELELQK.E | Y | 24.81 | 758.4174 | 6 | -8.9 | 380.2126 | 2 | 20.42 | 7 | 5030 | Ana\_06.raw |  |  |  |  |  |  | 7.73E6 |  |  |  | 1 | 0 | 0 | 0 | 0 | 0 | 0 | 1 | 0 | 0 | 0 | 2195 | 2200 |  |
| total 45 peptides |  |  |  |  |  |  |  |  |  |  |  |  |  |  |  |  |  |  |  |  |  |  |  |  |  |  |  |  |  |  |  |  |  |  |  |
| --- | --- | --- | --- | --- | --- | --- | --- | --- | --- | --- | --- | --- | --- | --- | --- | --- | --- | --- | --- | --- | --- | --- | --- | --- | --- | --- | --- | --- | --- | --- | --- | --- | --- | --- | --- |

Best Unique PSM (Scan F7:37152, m/z=1077.5593, z=2, RT=134.84, ppm=3.6):

A3KGU9|A3KGU9\_MOUSE

back to list

  

| Protein Coverage
| Supporting Peptides
| Best Unique PSM
|

Protein Coverage:

Supporting Peptides:

| Peptide | Uniq | -10lgP | Mass | Length | ppm | m/z | z | RT | Fraction | Scan | Source File | Area 10 | Area 01 | Area 02 | Area 03 | Area 04 | Area 05 | Area 06 | Area 07 | Area 08 | Area 09 | #Spec | #Spec 10 | #Spec 01 | #Spec 02 | #Spec 03 | #Spec 04 | #Spec 05 | #Spec 06 | #Spec 07 | #Spec 08 | #Spec 09 | Start | End | PTM |
| --- | --- | --- | --- | --- | --- | --- | --- | --- | --- | --- | --- | --- | --- | --- | --- | --- | --- | --- | --- | --- | --- | --- | --- | --- | --- | --- | --- | --- | --- | --- | --- | --- | --- | --- | --- |
| K.ALINADELANDVAGAEALLDR.H | Y | 120.24 | 2153.0964 | 21 | 3.6 | 1077.5593 | 2 | 134.84 | 7 | 37152 | Ana\_06.raw |  |  |  | 4.95E5 | 8.34E5 | 2.58E6 | 1.17E6 | 4.41E5 | 3.91E6 | 1.89E6 | 13 | 0 | 0 | 0 | 2 | 2 | 2 | 2 | 1 | 2 | 2 | 382 | 402 |  |
| R.AGTFQAFEQFGQQLLAHGHYASPEIK.E | Y | 112.57 | 2874.4089 | 26 | 4.6 | 719.6128 | 4 | 130.51 | 7 | 35914 | Ana\_06.raw | 6.11E6 | 9.12E6 | 6.22E6 | 1.86E6 | 6.67E6 | 9.99E6 | 4.81E6 | 4.8E6 |  |  | 8 | 1 | 1 | 1 | 1 | 1 | 1 | 1 | 1 | 0 | 0 | 1392 | 1417 |  |
| K.IAALQAFADQLIAVDHYAK.G | Y | 107.82 | 2057.0945 | 19 | 1.2 | 686.7062 | 3 | 131.57 | 3 | 36043 | Ana\_02.raw |  | 7.45E6 | 4.7E6 | 4.22E6 | 3.85E6 | 9.01E6 | 7.59E6 | 2.57E6 | 8.07E6 | 6.1E6 | 9 | 0 | 1 | 1 | 1 | 1 | 1 | 1 | 1 | 1 | 1 | 1501 | 1519 |  |
| R.SSLSSAQADFNQLAELDR.Q | Y | 103.93 | 1950.9282 | 18 | 2.7 | 976.4741 | 2 | 110.76 | 6 | 31074 | Ana\_05.raw |  | 2.21E6 | 8.97E5 | 1.41E6 | 1.47E6 | 3.91E6 | 2.26E6 | 1.55E6 | 5.42E6 |  | 8 | 0 | 1 | 1 | 1 | 1 | 1 | 1 | 1 | 1 | 0 | 2143 | 2160 |  |
| R.AQLADSFHLQQFFR.D | Y | 98.18 | 1706.8528 | 14 | 3.5 | 569.9602 | 3 | 114.44 | 6 | 32019 | Ana\_05.raw | 4.07E5 | 1.37E6 | 1.45E6 | 2.71E6 | 5.03E5 | 5.45E6 | 1.2E6 | 1.15E6 | 6.99E5 | 1.05E6 | 12 | 1 | 1 | 1 | 1 | 1 | 2 | 2 | 1 | 1 | 1 | 567 | 580 |  |
| R.EAFLNTEDKGDSLDSVEALIK.K | Y | 77.66 | 2293.1326 | 21 | 2.0 | 765.3863 | 3 | 114.10 | 6 | 31934 | Ana\_05.raw |  | 4.87E6 | 1.56E6 |  | 3.53E6 | 3.8E6 |  |  |  |  | 4 | 0 | 1 | 1 | 0 | 1 | 1 | 0 | 0 | 0 | 0 | 1465 | 1485 |  |
| R.ELPTAFDYVEFTR.S | Y | 77.14 | 1586.7616 | 13 | 0.5 | 794.3885 | 2 | 116.65 | 7 | 32272 | Ana\_06.raw | 2.68E6 | 5.14E6 | 1.02E7 | 3.84E6 | 8.62E6 | 1.1E7 | 1.4E6 | 4.28E6 | 1.81E7 | 1.02E7 | 13 | 1 | 2 | 1 | 1 | 2 | 1 | 1 | 1 | 1 | 2 | 2481 | 2493 |  |
| K.LSDDNTIGQEEIQQR.L | Y | 75.78 | 1744.8228 | 15 | 5.4 | 873.4234 | 2 | 55.09 | 1 | 15534 | ANa\_10.raw | 9.83E5 | 1.32E6 |  |  |  | 9.3E5 | 3.4E5 |  | 1.81E6 |  | 5 | 1 | 1 | 0 | 0 | 0 | 1 | 1 | 0 | 1 | 0 | 1835 | 1849 |  |
| K.DVTGAEALLER.H | Y | 74.99 | 1172.6036 | 11 | 8.1 | 587.3138 | 2 | 80.00 | 6 | 22871 | Ana\_05.raw |  | 1.13E7 | 1.04E7 | 4.88E6 | 1.38E7 | 1.75E7 | 5.33E6 | 5.36E6 | 2.86E7 | 9.69E6 | 12 | 0 | 2 | 1 | 1 | 1 | 1 | 2 | 2 | 1 | 1 | 1370 | 1380 |  |
| R.LAALADQWQFLVQK.S | Y | 74.35 | 1629.8878 | 14 | 3.6 | 815.9541 | 2 | 119.85 | 6 | 33450 | Ana\_05.raw |  | 2.91E6 |  | 5.12E5 |  | 2.53E6 | 5.25E5 | 8.19E5 | 1.18E6 |  | 6 | 0 | 1 | 0 | 1 | 0 | 1 | 1 | 1 | 1 | 0 | 1638 | 1651 |  |
| R.SQLLGSAHEVQR.F | Y | 72.11 | 1323.6895 | 12 | 4.4 | 662.8549 | 2 | 37.98 | 6 | 10528 | Ana\_05.raw |  | 1.65E6 |  |  |  | 4.56E6 |  |  | 7.12E6 |  | 4 | 0 | 1 | 0 | 0 | 0 | 2 | 0 | 0 | 1 | 0 | 1226 | 1237 |  |
| R.KVEDLFLTFAK.K | Y | 71.27 | 1309.7281 | 11 | 3.5 | 437.5848 | 3 | 103.36 | 6 | 29141 | Ana\_05.raw |  |  |  |  |  | 7.25E5 | 1.3E5 |  |  |  | 3 | 0 | 0 | 0 | 0 | 0 | 2 | 1 | 0 | 0 | 0 | 2093 | 2103 |  |
| R.TALLELWELR.R | Y | 70.11 | 1242.6971 | 10 | 4.4 | 622.3586 | 2 | 127.41 | 6 | 35412 | Ana\_05.raw |  | 1.45E6 | 1.28E6 | 6.88E5 | 2.46E6 | 2.87E6 | 1.36E6 |  |  | 8.71E5 | 7 | 0 | 1 | 1 | 1 | 1 | 1 | 1 | 0 | 0 | 1 | 450 | 459 |  |
| K.VLETAEDIQER.R | Y | 67.11 | 1301.6462 | 11 | 4.5 | 651.8333 | 2 | 46.43 | 9 | 12965 | Ana\_08.raw |  |  |  |  |  |  |  |  | 8.83E6 |  | 1 | 0 | 0 | 0 | 0 | 0 | 0 | 0 | 0 | 1 | 0 | 8 | 18 |  |
| R.DLASVQALLR.K | Y | 63.40 | 1084.6240 | 10 | 4.5 | 543.3217 | 2 | 101.48 | 6 | 28648 | Ana\_05.raw |  |  |  | 3.97E5 | 1.04E6 | 3.35E6 | 5.08E5 |  |  |  | 4 | 0 | 0 | 0 | 1 | 1 | 1 | 1 | 0 | 0 | 0 | 286 | 295 |  |
| K.SSEEIESAFR.A | Y | 63.18 | 1153.5251 | 10 | 1.1 | 577.7705 | 2 | 58.57 | 6 | 16750 | Ana\_05.raw | 3.84E5 | 5.73E6 | 4.32E6 | 2.4E6 | 4.2E6 | 4.67E6 | 3.46E6 | 3.15E6 | 6.43E6 | 3.81E6 | 10 | 1 | 1 | 1 | 1 | 1 | 1 | 1 | 1 | 1 | 1 | 2431 | 2440 |  |
| K.HQAFEAELHANADR.I | Y | 62.68 | 1607.7440 | 14 | 3.6 | 536.9239 | 3 | 43.65 | 5 | 12219 | Ana\_04.raw |  |  |  |  | 2.24E6 |  |  |  |  |  | 1 | 0 | 0 | 0 | 0 | 1 | 0 | 0 | 0 | 0 | 0 | 1597 | 1610 |  |
| R.EKEPIAASTNR.G | Y | 62.53 | 1214.6255 | 11 | 8.2 | 608.3250 | 2 | 21.71 | 1 | 5341 | ANa\_10.raw | 2.8E6 | 8.85E6 | 5.62E6 | 5.64E5 | 1.7E6 | 2.35E6 | 1.34E6 | 1.25E6 | 6.68E6 | 2.14E6 | 14 | 1 | 2 | 2 | 1 | 1 | 2 | 1 | 1 | 2 | 1 | 802 | 812 |  |
| R.DLSSVQTLLTK.Q | Y | 60.58 | 1203.6710 | 11 | 4.2 | 602.8453 | 2 | 94.23 | 6 | 26719 | Ana\_05.raw |  |  |  |  | 1.11E6 | 2.59E6 | 5.01E5 | 3.99E5 |  |  | 4 | 0 | 0 | 0 | 0 | 1 | 1 | 1 | 1 | 0 | 0 | 2012 | 2022 |  |
| K.DLIGVQNLLK.K | Y | 59.31 | 1111.6600 | 10 | 4.2 | 556.8396 | 2 | 105.07 | 6 | 29600 | Ana\_05.raw | 8.22E5 |  | 2.01E6 | 2.13E5 | 3.2E5 | 3.49E6 | 7.93E5 | 2.39E5 | 1.03E6 |  | 8 | 1 | 0 | 1 | 1 | 1 | 1 | 1 | 1 | 1 | 0 | 815 | 824 |  |
| K.HQAFEAELSANQSR.I | Y | 55.43 | 1586.7437 | 14 | 3.9 | 529.9239 | 3 | 50.21 | 7 | 14088 | Ana\_06.raw |  |  |  |  |  |  | 5.38E5 |  |  |  | 1 | 0 | 0 | 0 | 0 | 0 | 0 | 1 | 0 | 0 | 0 | 614 | 627 |  |
| R.SLQQLAEER.S | Y | 54.70 | 1072.5513 | 9 | -0.1 | 537.2828 | 2 | 55.95 | 6 | 15974 | Ana\_05.raw | 3.87E6 | 7.11E6 | 2.68E6 | 2.14E6 |  | 4.99E5 |  | 0 | 8.68E6 | 3.27E6 | 8 | 1 | 1 | 1 | 1 | 0 | 1 | 0 | 1 | 1 | 1 | 1217 | 1225 |  |
| K.ITALDEFATK.L | Y | 52.53 | 1107.5812 | 10 | 6.8 | 554.8016 | 2 | 71.03 | 3 | 20183 | Ana\_02.raw | 8.72E6 | 1.03E7 | 9.54E5 | 7.67E5 |  |  |  |  |  | 3.91E6 | 5 | 1 | 1 | 1 | 1 | 0 | 0 | 0 | 0 | 0 | 1 | 523 | 532 |  |
| K.SADESGQALLAASHYASDEVR.E | Y | 51.46 | 2176.0032 | 21 | 4.9 | 726.3452 | 3 | 89.10 | 6 | 25348 | Ana\_05.raw |  |  |  |  |  | 4.5E5 |  | 9.67E5 |  |  | 2 | 0 | 0 | 0 | 0 | 0 | 1 | 0 | 1 | 0 | 0 | 419 | 439 |  |
| K.EAIVTSEELGQDLEHVEVLQK.K | Y | 51.20 | 2365.2012 | 21 | 8.4 | 789.4143 | 3 | 112.16 | 9 | 31429 | Ana\_08.raw |  |  |  |  |  |  |  |  | 1.49E6 |  | 1 | 0 | 0 | 0 | 0 | 0 | 0 | 0 | 0 | 1 | 0 | 169 | 189 |  |
| R.DLTGVQNLR.K | Y | 50.95 | 1014.5458 | 9 | 4.4 | 508.2824 | 2 | 55.53 | 2 | 15750 | Ana\_01.raw | 1.32E6 | 2.81E6 | 2.26E6 | 1.23E6 | 2.46E6 | 2.56E6 | 1.01E6 |  | 2.24E6 | 3.91E5 | 9 | 1 | 1 | 1 | 1 | 1 | 1 | 1 | 0 | 1 | 1 | 1800 | 1808 |  |
| K.GEIDAHEDSFK.S | Y | 50.70 | 1246.5465 | 11 | 2.0 | 624.2818 | 2 | 39.37 | 6 | 10959 | Ana\_05.raw |  |  |  |  | 2.81E6 | 1.72E6 |  |  |  |  | 2 | 0 | 0 | 0 | 0 | 1 | 1 | 0 | 0 | 0 | 0 | 408 | 418 |  |
| K.LSILSEER.T | Y | 48.99 | 945.5131 | 8 | 3.0 | 473.7652 | 2 | 57.61 | 6 | 16466 | Ana\_05.raw | 1.75E6 | 3.75E6 | 2.84E6 |  | 2.13E6 | 3.2E6 |  |  | 6.2E6 | 2.17E6 | 7 | 1 | 1 | 1 | 0 | 1 | 1 | 0 | 0 | 1 | 1 | 442 | 449 |  |
| R.RQQVLDR.Y | Y | 48.35 | 913.5093 | 7 | 1.8 | 457.7628 | 2 | 19.41 | 6 | 4737 | Ana\_05.raw |  |  | 1.13E6 | 7.94E5 | 1.07E6 | 1.53E6 | 1.5E6 | 4.2E5 |  | 1.76E6 | 7 | 0 | 0 | 1 | 1 | 1 | 1 | 1 | 1 | 0 | 1 | 19 | 25 |  |
| R.NTTGVTEEALK.E | Y | 47.55 | 1161.5878 | 11 | 1.8 | 581.8022 | 2 | 38.73 | 6 | 10762 | Ana\_05.raw | 4.16E6 | 5.61E6 |  |  | 3.7E6 | 4.29E6 | 1.08E6 | 2.26E6 |  |  | 6 | 1 | 1 | 0 | 0 | 1 | 1 | 1 | 1 | 0 | 0 | 2342 | 2352 |  |
| K.EELYQNLTR.E | Y | 45.15 | 1164.5775 | 9 | 4.8 | 583.2988 | 2 | 60.51 | 5 | 17214 | Ana\_04.raw |  |  |  |  | 2.51E6 |  |  |  |  |  | 1 | 0 | 0 | 0 | 0 | 1 | 0 | 0 | 0 | 0 | 0 | 2453 | 2461 |  |
| K.LGDSHDLQR.F | Y | 44.96 | 1039.5046 | 9 | 5.5 | 347.5107 | 3 | 23.51 | 2 | 5930 | Ana\_01.raw |  | 2.26E6 |  | 1.12E6 |  |  |  |  |  |  | 2 | 0 | 1 | 0 | 1 | 0 | 0 | 0 | 0 | 0 | 0 | 1335 | 1343 |  |
| K.LLVSSEDYGR.D | Y | 44.88 | 1137.5665 | 10 | 4.6 | 569.7932 | 2 | 53.75 | 5 | 15229 | Ana\_04.raw |  |  | 2.6E6 |  | 2.1E6 |  |  |  | 1.52E6 |  | 3 | 0 | 0 | 1 | 0 | 1 | 0 | 0 | 0 | 1 | 0 | 1790 | 1799 |  |
| R.GLVSSDELAK.D | Y | 44.23 | 1017.5342 | 10 | 6.0 | 509.7774 | 2 | 44.75 | 9 | 12455 | Ana\_08.raw |  |  |  |  |  |  |  |  | 3.61E6 |  | 1 | 0 | 0 | 0 | 0 | 0 | 0 | 0 | 0 | 1 | 0 | 1360 | 1369 |  |
| R.EANELQQWITEK.E | Y | 43.50 | 1487.7256 | 12 | 1.5 | 744.8712 | 2 | 97.66 | 8 | 27190 | Ana\_07.raw |  |  |  |  |  |  |  | 3.59E5 |  |  | 1 | 0 | 0 | 0 | 0 | 0 | 0 | 0 | 1 | 0 | 0 | 1099 | 1110 |  |
| R.FLADFR.D | Y | 42.71 | 767.3966 | 6 | -0.1 | 384.7055 | 2 | 62.23 | 3 | 17731 | Ana\_02.raw | 9.72E5 | 1.67E6 | 2.7E6 | 7.73E5 | 2.28E6 | 8.38E5 | 1.54E6 |  | 2.07E6 | 1.94E6 | 9 | 1 | 1 | 1 | 1 | 1 | 1 | 1 | 0 | 1 | 1 | 366 | 371 |  |
| K.KQEALVAR.Y | Y | 41.75 | 913.5345 | 8 | 1.6 | 457.7753 | 2 | 19.95 | 6 | 4912 | Ana\_05.raw |  |  |  |  |  | 3.7E6 |  |  |  |  | 1 | 0 | 0 | 0 | 0 | 0 | 1 | 0 | 0 | 0 | 0 | 758 | 765 |  |
| K.TATDEAYKDPSNLQGK.V | Y | 41.66 | 1736.8217 | 16 | 3.4 | 579.9498 | 3 | 39.67 | 5 | 11003 | Ana\_04.raw |  |  | 2.76E6 |  | 2.47E6 | 2.44E6 |  |  |  |  | 3 | 0 | 0 | 1 | 0 | 1 | 1 | 0 | 0 | 0 | 0 | 595 | 610 |  |
| K.KFDDFQK.D | Y | 39.22 | 926.4497 | 7 | 6.6 | 464.2352 | 2 | 29.61 | 2 | 7898 | Ana\_01.raw |  | 2E6 |  |  |  |  |  |  |  |  | 1 | 0 | 1 | 0 | 0 | 0 | 0 | 0 | 0 | 0 | 0 | 1132 | 1138 |  |
| R.EAHDAFR.S | Y | 38.78 | 844.3828 | 7 | 2.9 | 423.1999 | 2 | 18.52 | 5 | 4491 | Ana\_04.raw |  |  |  |  | 2.84E6 |  |  |  |  |  | 1 | 0 | 0 | 0 | 0 | 1 | 0 | 0 | 0 | 0 | 0 | 2136 | 2142 |  |
| R.FQFFQR.D | Y | 37.35 | 871.4340 | 6 | 3.9 | 436.7260 | 2 | 74.88 | 6 | 21483 | Ana\_05.raw |  | 2.22E6 | 1.53E6 |  | 3.39E6 | 1.05E6 | 1.74E6 | 1.26E6 |  | 1.64E6 | 7 | 0 | 1 | 1 | 0 | 1 | 1 | 1 | 1 | 0 | 1 | 46 | 51 |  |
| K.KHEAFETDFTVHK.D | Y | 25.96 | 1587.7681 | 13 | 3.9 | 397.9508 | 4 | 48.47 | 7 | 13557 | Ana\_06.raw |  |  |  |  |  |  | 9.07E5 |  |  |  | 1 | 0 | 0 | 0 | 0 | 0 | 0 | 1 | 0 | 0 | 0 | 1916 | 1928 |  |
| R.FLSDFR.D | Y | 25.64 | 783.3915 | 6 | 3.6 | 392.7044 | 2 | 55.68 | 9 | 15767 | Ana\_08.raw |  |  |  |  |  |  |  |  | 7.07E5 |  | 1 | 0 | 0 | 0 | 0 | 0 | 0 | 0 | 0 | 1 | 0 | 1344 | 1349 |  |
| R.EANQQQQFNR.N | Y | 25.43 | 1261.5800 | 10 | -4.6 | 631.7944 | 2 | 30.34 | 7 | 8074 | Ana\_06.raw |  |  |  |  |  |  | 4.22E6 |  |  |  | 1 | 0 | 0 | 0 | 0 | 0 | 0 | 1 | 0 | 0 | 0 | 676 | 685 |  |
| R.ELELQK.E | Y | 24.81 | 758.4174 | 6 | -8.9 | 380.2126 | 2 | 20.42 | 7 | 5030 | Ana\_06.raw |  |  |  |  |  |  | 7.73E6 |  |  |  | 1 | 0 | 0 | 0 | 0 | 0 | 0 | 1 | 0 | 0 | 0 | 2195 | 2200 |  |
| total 45 peptides |  |  |  |  |  |  |  |  |  |  |  |  |  |  |  |  |  |  |  |  |  |  |  |  |  |  |  |  |  |  |  |  |  |  |  |
| --- | --- | --- | --- | --- | --- | --- | --- | --- | --- | --- | --- | --- | --- | --- | --- | --- | --- | --- | --- | --- | --- | --- | --- | --- | --- | --- | --- | --- | --- | --- | --- | --- | --- | --- | --- |

Best Unique PSM (Scan F7:37152, m/z=1077.5593, z=2, RT=134.84, ppm=3.6):

A3KGU5|A3KGU5\_MOUSE

back to list

  

| Protein Coverage
| Supporting Peptides
| Best Unique PSM
|

Protein Coverage:

Supporting Peptides:

| Peptide | Uniq | -10lgP | Mass | Length | ppm | m/z | z | RT | Fraction | Scan | Source File | Area 10 | Area 01 | Area 02 | Area 03 | Area 04 | Area 05 | Area 06 | Area 07 | Area 08 | Area 09 | #Spec | #Spec 10 | #Spec 01 | #Spec 02 | #Spec 03 | #Spec 04 | #Spec 05 | #Spec 06 | #Spec 07 | #Spec 08 | #Spec 09 | Start | End | PTM |
| --- | --- | --- | --- | --- | --- | --- | --- | --- | --- | --- | --- | --- | --- | --- | --- | --- | --- | --- | --- | --- | --- | --- | --- | --- | --- | --- | --- | --- | --- | --- | --- | --- | --- | --- | --- |
| K.ALINADELANDVAGAEALLDR.H | Y | 120.24 | 2153.0964 | 21 | 3.6 | 1077.5593 | 2 | 134.84 | 7 | 37152 | Ana\_06.raw |  |  |  | 4.95E5 | 8.34E5 | 2.58E6 | 1.17E6 | 4.41E5 | 3.91E6 | 1.89E6 | 13 | 0 | 0 | 0 | 2 | 2 | 2 | 2 | 1 | 2 | 2 | 382 | 402 |  |
| R.AGTFQAFEQFGQQLLAHGHYASPEIK.E | Y | 112.57 | 2874.4089 | 26 | 4.6 | 719.6128 | 4 | 130.51 | 7 | 35914 | Ana\_06.raw | 6.11E6 | 9.12E6 | 6.22E6 | 1.86E6 | 6.67E6 | 9.99E6 | 4.81E6 | 4.8E6 |  |  | 8 | 1 | 1 | 1 | 1 | 1 | 1 | 1 | 1 | 0 | 0 | 1372 | 1397 |  |
| K.IAALQAFADQLIAVDHYAK.G | Y | 107.82 | 2057.0945 | 19 | 1.2 | 686.7062 | 3 | 131.57 | 3 | 36043 | Ana\_02.raw |  | 7.45E6 | 4.7E6 | 4.22E6 | 3.85E6 | 9.01E6 | 7.59E6 | 2.57E6 | 8.07E6 | 6.1E6 | 9 | 0 | 1 | 1 | 1 | 1 | 1 | 1 | 1 | 1 | 1 | 1481 | 1499 |  |
| R.SSLSSAQADFNQLAELDR.Q | Y | 103.93 | 1950.9282 | 18 | 2.7 | 976.4741 | 2 | 110.76 | 6 | 31074 | Ana\_05.raw |  | 2.21E6 | 8.97E5 | 1.41E6 | 1.47E6 | 3.91E6 | 2.26E6 | 1.55E6 | 5.42E6 |  | 8 | 0 | 1 | 1 | 1 | 1 | 1 | 1 | 1 | 1 | 0 | 2123 | 2140 |  |
| R.AQLADSFHLQQFFR.D | Y | 98.18 | 1706.8528 | 14 | 3.5 | 569.9602 | 3 | 114.44 | 6 | 32019 | Ana\_05.raw | 4.07E5 | 1.37E6 | 1.45E6 | 2.71E6 | 5.03E5 | 5.45E6 | 1.2E6 | 1.15E6 | 6.99E5 | 1.05E6 | 12 | 1 | 1 | 1 | 1 | 1 | 2 | 2 | 1 | 1 | 1 | 567 | 580 |  |
| R.EAFLNTEDKGDSLDSVEALIK.K | Y | 77.66 | 2293.1326 | 21 | 2.0 | 765.3863 | 3 | 114.10 | 6 | 31934 | Ana\_05.raw |  | 4.87E6 | 1.56E6 |  | 3.53E6 | 3.8E6 |  |  |  |  | 4 | 0 | 1 | 1 | 0 | 1 | 1 | 0 | 0 | 0 | 0 | 1445 | 1465 |  |
| R.ELPTAFDYVEFTR.S | Y | 77.14 | 1586.7616 | 13 | 0.5 | 794.3885 | 2 | 116.65 | 7 | 32272 | Ana\_06.raw | 2.68E6 | 5.14E6 | 1.02E7 | 3.84E6 | 8.62E6 | 1.1E7 | 1.4E6 | 4.28E6 | 1.81E7 | 1.02E7 | 13 | 1 | 2 | 1 | 1 | 2 | 1 | 1 | 1 | 1 | 2 | 2440 | 2452 |  |
| K.LSDDNTIGQEEIQQR.L | Y | 75.78 | 1744.8228 | 15 | 5.4 | 873.4234 | 2 | 55.09 | 1 | 15534 | ANa\_10.raw | 9.83E5 | 1.32E6 |  |  |  | 9.3E5 | 3.4E5 |  | 1.81E6 |  | 5 | 1 | 1 | 0 | 0 | 0 | 1 | 1 | 0 | 1 | 0 | 1815 | 1829 |  |
| K.DVTGAEALLER.H | Y | 74.99 | 1172.6036 | 11 | 8.1 | 587.3138 | 2 | 80.00 | 6 | 22871 | Ana\_05.raw |  | 1.13E7 | 1.04E7 | 4.88E6 | 1.38E7 | 1.75E7 | 5.33E6 | 5.36E6 | 2.86E7 | 9.69E6 | 12 | 0 | 2 | 1 | 1 | 1 | 1 | 2 | 2 | 1 | 1 | 1350 | 1360 |  |
| R.LAALADQWQFLVQK.S | Y | 74.35 | 1629.8878 | 14 | 3.6 | 815.9541 | 2 | 119.85 | 6 | 33450 | Ana\_05.raw |  | 2.91E6 |  | 5.12E5 |  | 2.53E6 | 5.25E5 | 8.19E5 | 1.18E6 |  | 6 | 0 | 1 | 0 | 1 | 0 | 1 | 1 | 1 | 1 | 0 | 1618 | 1631 |  |
| R.SQLLGSAHEVQR.F | Y | 72.11 | 1323.6895 | 12 | 4.4 | 662.8549 | 2 | 37.98 | 6 | 10528 | Ana\_05.raw |  | 1.65E6 |  |  |  | 4.56E6 |  |  | 7.12E6 |  | 4 | 0 | 1 | 0 | 0 | 0 | 2 | 0 | 0 | 1 | 0 | 1206 | 1217 |  |
| R.KVEDLFLTFAK.K | Y | 71.27 | 1309.7281 | 11 | 3.5 | 437.5848 | 3 | 103.36 | 6 | 29141 | Ana\_05.raw |  |  |  |  |  | 7.25E5 | 1.3E5 |  |  |  | 3 | 0 | 0 | 0 | 0 | 0 | 2 | 1 | 0 | 0 | 0 | 2073 | 2083 |  |
| R.TALLELWELR.R | Y | 70.11 | 1242.6971 | 10 | 4.4 | 622.3586 | 2 | 127.41 | 6 | 35412 | Ana\_05.raw |  | 1.45E6 | 1.28E6 | 6.88E5 | 2.46E6 | 2.87E6 | 1.36E6 |  |  | 8.71E5 | 7 | 0 | 1 | 1 | 1 | 1 | 1 | 1 | 0 | 0 | 1 | 450 | 459 |  |
| K.VLETAEDIQER.R | Y | 67.11 | 1301.6462 | 11 | 4.5 | 651.8333 | 2 | 46.43 | 9 | 12965 | Ana\_08.raw |  |  |  |  |  |  |  |  | 8.83E6 |  | 1 | 0 | 0 | 0 | 0 | 0 | 0 | 0 | 0 | 1 | 0 | 8 | 18 |  |
| R.DLASVQALLR.K | Y | 63.40 | 1084.6240 | 10 | 4.5 | 543.3217 | 2 | 101.48 | 6 | 28648 | Ana\_05.raw |  |  |  | 3.97E5 | 1.04E6 | 3.35E6 | 5.08E5 |  |  |  | 4 | 0 | 0 | 0 | 1 | 1 | 1 | 1 | 0 | 0 | 0 | 286 | 295 |  |
| K.SSEEIESAFR.A | Y | 63.18 | 1153.5251 | 10 | 1.1 | 577.7705 | 2 | 58.57 | 6 | 16750 | Ana\_05.raw | 3.84E5 | 5.73E6 | 4.32E6 | 2.4E6 | 4.2E6 | 4.67E6 | 3.46E6 | 3.15E6 | 6.43E6 | 3.81E6 | 10 | 1 | 1 | 1 | 1 | 1 | 1 | 1 | 1 | 1 | 1 | 2390 | 2399 |  |
| K.HQAFEAELHANADR.I | Y | 62.68 | 1607.7440 | 14 | 3.6 | 536.9239 | 3 | 43.65 | 5 | 12219 | Ana\_04.raw |  |  |  |  | 2.24E6 |  |  |  |  |  | 1 | 0 | 0 | 0 | 0 | 1 | 0 | 0 | 0 | 0 | 0 | 1577 | 1590 |  |
| R.EKEPIAASTNR.G | Y | 62.53 | 1214.6255 | 11 | 8.2 | 608.3250 | 2 | 21.71 | 1 | 5341 | ANa\_10.raw | 2.8E6 | 8.85E6 | 5.62E6 | 5.64E5 | 1.7E6 | 2.35E6 | 1.34E6 | 1.25E6 | 6.68E6 | 2.14E6 | 14 | 1 | 2 | 2 | 1 | 1 | 2 | 1 | 1 | 2 | 1 | 802 | 812 |  |
| R.DLSSVQTLLTK.Q | Y | 60.58 | 1203.6710 | 11 | 4.2 | 602.8453 | 2 | 94.23 | 6 | 26719 | Ana\_05.raw |  |  |  |  | 1.11E6 | 2.59E6 | 5.01E5 | 3.99E5 |  |  | 4 | 0 | 0 | 0 | 0 | 1 | 1 | 1 | 1 | 0 | 0 | 1992 | 2002 |  |
| K.DLIGVQNLLK.K | Y | 59.31 | 1111.6600 | 10 | 4.2 | 556.8396 | 2 | 105.07 | 6 | 29600 | Ana\_05.raw | 8.22E5 |  | 2.01E6 | 2.13E5 | 3.2E5 | 3.49E6 | 7.93E5 | 2.39E5 | 1.03E6 |  | 8 | 1 | 0 | 1 | 1 | 1 | 1 | 1 | 1 | 1 | 0 | 815 | 824 |  |
| K.HQAFEAELSANQSR.I | Y | 55.43 | 1586.7437 | 14 | 3.9 | 529.9239 | 3 | 50.21 | 7 | 14088 | Ana\_06.raw |  |  |  |  |  |  | 5.38E5 |  |  |  | 1 | 0 | 0 | 0 | 0 | 0 | 0 | 1 | 0 | 0 | 0 | 614 | 627 |  |
| R.SLQQLAEER.S | Y | 54.70 | 1072.5513 | 9 | -0.1 | 537.2828 | 2 | 55.95 | 6 | 15974 | Ana\_05.raw | 3.87E6 | 7.11E6 | 2.68E6 | 2.14E6 |  | 4.99E5 |  | 0 | 8.68E6 | 3.27E6 | 8 | 1 | 1 | 1 | 1 | 0 | 1 | 0 | 1 | 1 | 1 | 1197 | 1205 |  |
| K.ITALDEFATK.L | Y | 52.53 | 1107.5812 | 10 | 6.8 | 554.8016 | 2 | 71.03 | 3 | 20183 | Ana\_02.raw | 8.72E6 | 1.03E7 | 9.54E5 | 7.67E5 |  |  |  |  |  | 3.91E6 | 5 | 1 | 1 | 1 | 1 | 0 | 0 | 0 | 0 | 0 | 1 | 523 | 532 |  |
| K.SADESGQALLAASHYASDEVR.E | Y | 51.46 | 2176.0032 | 21 | 4.9 | 726.3452 | 3 | 89.10 | 6 | 25348 | Ana\_05.raw |  |  |  |  |  | 4.5E5 |  | 9.67E5 |  |  | 2 | 0 | 0 | 0 | 0 | 0 | 1 | 0 | 1 | 0 | 0 | 419 | 439 |  |
| K.EAIVTSEELGQDLEHVEVLQK.K | Y | 51.20 | 2365.2012 | 21 | 8.4 | 789.4143 | 3 | 112.16 | 9 | 31429 | Ana\_08.raw |  |  |  |  |  |  |  |  | 1.49E6 |  | 1 | 0 | 0 | 0 | 0 | 0 | 0 | 0 | 0 | 1 | 0 | 169 | 189 |  |
| R.DLTGVQNLR.K | Y | 50.95 | 1014.5458 | 9 | 4.4 | 508.2824 | 2 | 55.53 | 2 | 15750 | Ana\_01.raw | 1.32E6 | 2.81E6 | 2.26E6 | 1.23E6 | 2.46E6 | 2.56E6 | 1.01E6 |  | 2.24E6 | 3.91E5 | 9 | 1 | 1 | 1 | 1 | 1 | 1 | 1 | 0 | 1 | 1 | 1780 | 1788 |  |
| K.GEIDAHEDSFK.S | Y | 50.70 | 1246.5465 | 11 | 2.0 | 624.2818 | 2 | 39.37 | 6 | 10959 | Ana\_05.raw |  |  |  |  | 2.81E6 | 1.72E6 |  |  |  |  | 2 | 0 | 0 | 0 | 0 | 1 | 1 | 0 | 0 | 0 | 0 | 408 | 418 |  |
| K.LSILSEER.T | Y | 48.99 | 945.5131 | 8 | 3.0 | 473.7652 | 2 | 57.61 | 6 | 16466 | Ana\_05.raw | 1.75E6 | 3.75E6 | 2.84E6 |  | 2.13E6 | 3.2E6 |  |  | 6.2E6 | 2.17E6 | 7 | 1 | 1 | 1 | 0 | 1 | 1 | 0 | 0 | 1 | 1 | 442 | 449 |  |
| R.RQQVLDR.Y | Y | 48.35 | 913.5093 | 7 | 1.8 | 457.7628 | 2 | 19.41 | 6 | 4737 | Ana\_05.raw |  |  | 1.13E6 | 7.94E5 | 1.07E6 | 1.53E6 | 1.5E6 | 4.2E5 |  | 1.76E6 | 7 | 0 | 0 | 1 | 1 | 1 | 1 | 1 | 1 | 0 | 1 | 19 | 25 |  |
| R.NTTGVTEEALK.E | Y | 47.55 | 1161.5878 | 11 | 1.8 | 581.8022 | 2 | 38.73 | 6 | 10762 | Ana\_05.raw | 4.16E6 | 5.61E6 |  |  | 3.7E6 | 4.29E6 | 1.08E6 | 2.26E6 |  |  | 6 | 1 | 1 | 0 | 0 | 1 | 1 | 1 | 1 | 0 | 0 | 2301 | 2311 |  |
| K.EELYQNLTR.E | Y | 45.15 | 1164.5775 | 9 | 4.8 | 583.2988 | 2 | 60.51 | 5 | 17214 | Ana\_04.raw |  |  |  |  | 2.51E6 |  |  |  |  |  | 1 | 0 | 0 | 0 | 0 | 1 | 0 | 0 | 0 | 0 | 0 | 2412 | 2420 |  |
| K.LGDSHDLQR.F | Y | 44.96 | 1039.5046 | 9 | 5.5 | 347.5107 | 3 | 23.51 | 2 | 5930 | Ana\_01.raw |  | 2.26E6 |  | 1.12E6 |  |  |  |  |  |  | 2 | 0 | 1 | 0 | 1 | 0 | 0 | 0 | 0 | 0 | 0 | 1315 | 1323 |  |
| K.LLVSSEDYGR.D | Y | 44.88 | 1137.5665 | 10 | 4.6 | 569.7932 | 2 | 53.75 | 5 | 15229 | Ana\_04.raw |  |  | 2.6E6 |  | 2.1E6 |  |  |  | 1.52E6 |  | 3 | 0 | 0 | 1 | 0 | 1 | 0 | 0 | 0 | 1 | 0 | 1770 | 1779 |  |
| R.GLVSSDELAK.D | Y | 44.23 | 1017.5342 | 10 | 6.0 | 509.7774 | 2 | 44.75 | 9 | 12455 | Ana\_08.raw |  |  |  |  |  |  |  |  | 3.61E6 |  | 1 | 0 | 0 | 0 | 0 | 0 | 0 | 0 | 0 | 1 | 0 | 1340 | 1349 |  |
| R.EANELQQWITEK.E | Y | 43.50 | 1487.7256 | 12 | 1.5 | 744.8712 | 2 | 97.66 | 8 | 27190 | Ana\_07.raw |  |  |  |  |  |  |  | 3.59E5 |  |  | 1 | 0 | 0 | 0 | 0 | 0 | 0 | 0 | 1 | 0 | 0 | 1079 | 1090 |  |
| R.FLADFR.D | Y | 42.71 | 767.3966 | 6 | -0.1 | 384.7055 | 2 | 62.23 | 3 | 17731 | Ana\_02.raw | 9.72E5 | 1.67E6 | 2.7E6 | 7.73E5 | 2.28E6 | 8.38E5 | 1.54E6 |  | 2.07E6 | 1.94E6 | 9 | 1 | 1 | 1 | 1 | 1 | 1 | 1 | 0 | 1 | 1 | 366 | 371 |  |
| K.KQEALVAR.Y | Y | 41.75 | 913.5345 | 8 | 1.6 | 457.7753 | 2 | 19.95 | 6 | 4912 | Ana\_05.raw |  |  |  |  |  | 3.7E6 |  |  |  |  | 1 | 0 | 0 | 0 | 0 | 0 | 1 | 0 | 0 | 0 | 0 | 758 | 765 |  |
| K.TATDEAYKDPSNLQGK.V | Y | 41.66 | 1736.8217 | 16 | 3.4 | 579.9498 | 3 | 39.67 | 5 | 11003 | Ana\_04.raw |  |  | 2.76E6 |  | 2.47E6 | 2.44E6 |  |  |  |  | 3 | 0 | 0 | 1 | 0 | 1 | 1 | 0 | 0 | 0 | 0 | 595 | 610 |  |
| K.KFDDFQK.D | Y | 39.22 | 926.4497 | 7 | 6.6 | 464.2352 | 2 | 29.61 | 2 | 7898 | Ana\_01.raw |  | 2E6 |  |  |  |  |  |  |  |  | 1 | 0 | 1 | 0 | 0 | 0 | 0 | 0 | 0 | 0 | 0 | 1112 | 1118 |  |
| R.EAHDAFR.S | Y | 38.78 | 844.3828 | 7 | 2.9 | 423.1999 | 2 | 18.52 | 5 | 4491 | Ana\_04.raw |  |  |  |  | 2.84E6 |  |  |  |  |  | 1 | 0 | 0 | 0 | 0 | 1 | 0 | 0 | 0 | 0 | 0 | 2116 | 2122 |  |
| R.FQFFQR.D | Y | 37.35 | 871.4340 | 6 | 3.9 | 436.7260 | 2 | 74.88 | 6 | 21483 | Ana\_05.raw |  | 2.22E6 | 1.53E6 |  | 3.39E6 | 1.05E6 | 1.74E6 | 1.26E6 |  | 1.64E6 | 7 | 0 | 1 | 1 | 0 | 1 | 1 | 1 | 1 | 0 | 1 | 46 | 51 |  |
| K.KHEAFETDFTVHK.D | Y | 25.96 | 1587.7681 | 13 | 3.9 | 397.9508 | 4 | 48.47 | 7 | 13557 | Ana\_06.raw |  |  |  |  |  |  | 9.07E5 |  |  |  | 1 | 0 | 0 | 0 | 0 | 0 | 0 | 1 | 0 | 0 | 0 | 1896 | 1908 |  |
| R.FLSDFR.D | Y | 25.64 | 783.3915 | 6 | 3.6 | 392.7044 | 2 | 55.68 | 9 | 15767 | Ana\_08.raw |  |  |  |  |  |  |  |  | 7.07E5 |  | 1 | 0 | 0 | 0 | 0 | 0 | 0 | 0 | 0 | 1 | 0 | 1324 | 1329 |  |
| R.EANQQQQFNR.N | Y | 25.43 | 1261.5800 | 10 | -4.6 | 631.7944 | 2 | 30.34 | 7 | 8074 | Ana\_06.raw |  |  |  |  |  |  | 4.22E6 |  |  |  | 1 | 0 | 0 | 0 | 0 | 0 | 0 | 1 | 0 | 0 | 0 | 676 | 685 |  |
| R.ELELQK.E | Y | 24.81 | 758.4174 | 6 | -8.9 | 380.2126 | 2 | 20.42 | 7 | 5030 | Ana\_06.raw |  |  |  |  |  |  | 7.73E6 |  |  |  | 1 | 0 | 0 | 0 | 0 | 0 | 0 | 1 | 0 | 0 | 0 | 2175 | 2180 |  |
| total 45 peptides |  |  |  |  |  |  |  |  |  |  |  |  |  |  |  |  |  |  |  |  |  |  |  |  |  |  |  |  |  |  |  |  |  |  |  |
| --- | --- | --- | --- | --- | --- | --- | --- | --- | --- | --- | --- | --- | --- | --- | --- | --- | --- | --- | --- | --- | --- | --- | --- | --- | --- | --- | --- | --- | --- | --- | --- | --- | --- | --- | --- |

Best Unique PSM (Scan F7:37152, m/z=1077.5593, z=2, RT=134.84, ppm=3.6):

E9Q447|E9Q447\_MOUSE

back to list

  

| Protein Coverage
| Supporting Peptides
| Best Unique PSM
|

Protein Coverage:

Supporting Peptides:

| Peptide | Uniq | -10lgP | Mass | Length | ppm | m/z | z | RT | Fraction | Scan | Source File | Area 10 | Area 01 | Area 02 | Area 03 | Area 04 | Area 05 | Area 06 | Area 07 | Area 08 | Area 09 | #Spec | #Spec 10 | #Spec 01 | #Spec 02 | #Spec 03 | #Spec 04 | #Spec 05 | #Spec 06 | #Spec 07 | #Spec 08 | #Spec 09 | Start | End | PTM |
| --- | --- | --- | --- | --- | --- | --- | --- | --- | --- | --- | --- | --- | --- | --- | --- | --- | --- | --- | --- | --- | --- | --- | --- | --- | --- | --- | --- | --- | --- | --- | --- | --- | --- | --- | --- |
| K.ALINADELANDVAGAEALLDR.H | Y | 120.24 | 2153.0964 | 21 | 3.6 | 1077.5593 | 2 | 134.84 | 7 | 37152 | Ana\_06.raw |  |  |  | 4.95E5 | 8.34E5 | 2.58E6 | 1.17E6 | 4.41E5 | 3.91E6 | 1.89E6 | 13 | 0 | 0 | 0 | 2 | 2 | 2 | 2 | 1 | 2 | 2 | 382 | 402 |  |
| R.AGTFQAFEQFGQQLLAHGHYASPEIK.E | Y | 112.57 | 2874.4089 | 26 | 4.6 | 719.6128 | 4 | 130.51 | 7 | 35914 | Ana\_06.raw | 6.11E6 | 9.12E6 | 6.22E6 | 1.86E6 | 6.67E6 | 9.99E6 | 4.81E6 | 4.8E6 |  |  | 8 | 1 | 1 | 1 | 1 | 1 | 1 | 1 | 1 | 0 | 0 | 1372 | 1397 |  |
| K.IAALQAFADQLIAVDHYAK.G | Y | 107.82 | 2057.0945 | 19 | 1.2 | 686.7062 | 3 | 131.57 | 3 | 36043 | Ana\_02.raw |  | 7.45E6 | 4.7E6 | 4.22E6 | 3.85E6 | 9.01E6 | 7.59E6 | 2.57E6 | 8.07E6 | 6.1E6 | 9 | 0 | 1 | 1 | 1 | 1 | 1 | 1 | 1 | 1 | 1 | 1481 | 1499 |  |
| R.SSLSSAQADFNQLAELDR.Q | Y | 103.93 | 1950.9282 | 18 | 2.7 | 976.4741 | 2 | 110.76 | 6 | 31074 | Ana\_05.raw |  | 2.21E6 | 8.97E5 | 1.41E6 | 1.47E6 | 3.91E6 | 2.26E6 | 1.55E6 | 5.42E6 |  | 8 | 0 | 1 | 1 | 1 | 1 | 1 | 1 | 1 | 1 | 0 | 2123 | 2140 |  |
| R.AQLADSFHLQQFFR.D | Y | 98.18 | 1706.8528 | 14 | 3.5 | 569.9602 | 3 | 114.44 | 6 | 32019 | Ana\_05.raw | 4.07E5 | 1.37E6 | 1.45E6 | 2.71E6 | 5.03E5 | 5.45E6 | 1.2E6 | 1.15E6 | 6.99E5 | 1.05E6 | 12 | 1 | 1 | 1 | 1 | 1 | 2 | 2 | 1 | 1 | 1 | 567 | 580 |  |
| R.EAFLNTEDKGDSLDSVEALIK.K | Y | 77.66 | 2293.1326 | 21 | 2.0 | 765.3863 | 3 | 114.10 | 6 | 31934 | Ana\_05.raw |  | 4.87E6 | 1.56E6 |  | 3.53E6 | 3.8E6 |  |  |  |  | 4 | 0 | 1 | 1 | 0 | 1 | 1 | 0 | 0 | 0 | 0 | 1445 | 1465 |  |
| R.ELPTAFDYVEFTR.S | Y | 77.14 | 1586.7616 | 13 | 0.5 | 794.3885 | 2 | 116.65 | 7 | 32272 | Ana\_06.raw | 2.68E6 | 5.14E6 | 1.02E7 | 3.84E6 | 8.62E6 | 1.1E7 | 1.4E6 | 4.28E6 | 1.81E7 | 1.02E7 | 13 | 1 | 2 | 1 | 1 | 2 | 1 | 1 | 1 | 1 | 2 | 2461 | 2473 |  |
| K.LSDDNTIGQEEIQQR.L | Y | 75.78 | 1744.8228 | 15 | 5.4 | 873.4234 | 2 | 55.09 | 1 | 15534 | ANa\_10.raw | 9.83E5 | 1.32E6 |  |  |  | 9.3E5 | 3.4E5 |  | 1.81E6 |  | 5 | 1 | 1 | 0 | 0 | 0 | 1 | 1 | 0 | 1 | 0 | 1815 | 1829 |  |
| K.DVTGAEALLER.H | Y | 74.99 | 1172.6036 | 11 | 8.1 | 587.3138 | 2 | 80.00 | 6 | 22871 | Ana\_05.raw |  | 1.13E7 | 1.04E7 | 4.88E6 | 1.38E7 | 1.75E7 | 5.33E6 | 5.36E6 | 2.86E7 | 9.69E6 | 12 | 0 | 2 | 1 | 1 | 1 | 1 | 2 | 2 | 1 | 1 | 1350 | 1360 |  |
| R.LAALADQWQFLVQK.S | Y | 74.35 | 1629.8878 | 14 | 3.6 | 815.9541 | 2 | 119.85 | 6 | 33450 | Ana\_05.raw |  | 2.91E6 |  | 5.12E5 |  | 2.53E6 | 5.25E5 | 8.19E5 | 1.18E6 |  | 6 | 0 | 1 | 0 | 1 | 0 | 1 | 1 | 1 | 1 | 0 | 1618 | 1631 |  |
| R.SQLLGSAHEVQR.F | Y | 72.11 | 1323.6895 | 12 | 4.4 | 662.8549 | 2 | 37.98 | 6 | 10528 | Ana\_05.raw |  | 1.65E6 |  |  |  | 4.56E6 |  |  | 7.12E6 |  | 4 | 0 | 1 | 0 | 0 | 0 | 2 | 0 | 0 | 1 | 0 | 1206 | 1217 |  |
| R.KVEDLFLTFAK.K | Y | 71.27 | 1309.7281 | 11 | 3.5 | 437.5848 | 3 | 103.36 | 6 | 29141 | Ana\_05.raw |  |  |  |  |  | 7.25E5 | 1.3E5 |  |  |  | 3 | 0 | 0 | 0 | 0 | 0 | 2 | 1 | 0 | 0 | 0 | 2073 | 2083 |  |
| R.TALLELWELR.R | Y | 70.11 | 1242.6971 | 10 | 4.4 | 622.3586 | 2 | 127.41 | 6 | 35412 | Ana\_05.raw |  | 1.45E6 | 1.28E6 | 6.88E5 | 2.46E6 | 2.87E6 | 1.36E6 |  |  | 8.71E5 | 7 | 0 | 1 | 1 | 1 | 1 | 1 | 1 | 0 | 0 | 1 | 450 | 459 |  |
| K.VLETAEDIQER.R | Y | 67.11 | 1301.6462 | 11 | 4.5 | 651.8333 | 2 | 46.43 | 9 | 12965 | Ana\_08.raw |  |  |  |  |  |  |  |  | 8.83E6 |  | 1 | 0 | 0 | 0 | 0 | 0 | 0 | 0 | 0 | 1 | 0 | 8 | 18 |  |
| R.DLASVQALLR.K | Y | 63.40 | 1084.6240 | 10 | 4.5 | 543.3217 | 2 | 101.48 | 6 | 28648 | Ana\_05.raw |  |  |  | 3.97E5 | 1.04E6 | 3.35E6 | 5.08E5 |  |  |  | 4 | 0 | 0 | 0 | 1 | 1 | 1 | 1 | 0 | 0 | 0 | 286 | 295 |  |
| K.SSEEIESAFR.A | Y | 63.18 | 1153.5251 | 10 | 1.1 | 577.7705 | 2 | 58.57 | 6 | 16750 | Ana\_05.raw | 3.84E5 | 5.73E6 | 4.32E6 | 2.4E6 | 4.2E6 | 4.67E6 | 3.46E6 | 3.15E6 | 6.43E6 | 3.81E6 | 10 | 1 | 1 | 1 | 1 | 1 | 1 | 1 | 1 | 1 | 1 | 2411 | 2420 |  |
| K.HQAFEAELHANADR.I | Y | 62.68 | 1607.7440 | 14 | 3.6 | 536.9239 | 3 | 43.65 | 5 | 12219 | Ana\_04.raw |  |  |  |  | 2.24E6 |  |  |  |  |  | 1 | 0 | 0 | 0 | 0 | 1 | 0 | 0 | 0 | 0 | 0 | 1577 | 1590 |  |
| R.EKEPIAASTNR.G | Y | 62.53 | 1214.6255 | 11 | 8.2 | 608.3250 | 2 | 21.71 | 1 | 5341 | ANa\_10.raw | 2.8E6 | 8.85E6 | 5.62E6 | 5.64E5 | 1.7E6 | 2.35E6 | 1.34E6 | 1.25E6 | 6.68E6 | 2.14E6 | 14 | 1 | 2 | 2 | 1 | 1 | 2 | 1 | 1 | 2 | 1 | 802 | 812 |  |
| R.DLSSVQTLLTK.Q | Y | 60.58 | 1203.6710 | 11 | 4.2 | 602.8453 | 2 | 94.23 | 6 | 26719 | Ana\_05.raw |  |  |  |  | 1.11E6 | 2.59E6 | 5.01E5 | 3.99E5 |  |  | 4 | 0 | 0 | 0 | 0 | 1 | 1 | 1 | 1 | 0 | 0 | 1992 | 2002 |  |
| K.DLIGVQNLLK.K | Y | 59.31 | 1111.6600 | 10 | 4.2 | 556.8396 | 2 | 105.07 | 6 | 29600 | Ana\_05.raw | 8.22E5 |  | 2.01E6 | 2.13E5 | 3.2E5 | 3.49E6 | 7.93E5 | 2.39E5 | 1.03E6 |  | 8 | 1 | 0 | 1 | 1 | 1 | 1 | 1 | 1 | 1 | 0 | 815 | 824 |  |
| K.HQAFEAELSANQSR.I | Y | 55.43 | 1586.7437 | 14 | 3.9 | 529.9239 | 3 | 50.21 | 7 | 14088 | Ana\_06.raw |  |  |  |  |  |  | 5.38E5 |  |  |  | 1 | 0 | 0 | 0 | 0 | 0 | 0 | 1 | 0 | 0 | 0 | 614 | 627 |  |
| R.SLQQLAEER.S | Y | 54.70 | 1072.5513 | 9 | -0.1 | 537.2828 | 2 | 55.95 | 6 | 15974 | Ana\_05.raw | 3.87E6 | 7.11E6 | 2.68E6 | 2.14E6 |  | 4.99E5 |  | 0 | 8.68E6 | 3.27E6 | 8 | 1 | 1 | 1 | 1 | 0 | 1 | 0 | 1 | 1 | 1 | 1197 | 1205 |  |
| K.ITALDEFATK.L | Y | 52.53 | 1107.5812 | 10 | 6.8 | 554.8016 | 2 | 71.03 | 3 | 20183 | Ana\_02.raw | 8.72E6 | 1.03E7 | 9.54E5 | 7.67E5 |  |  |  |  |  | 3.91E6 | 5 | 1 | 1 | 1 | 1 | 0 | 0 | 0 | 0 | 0 | 1 | 523 | 532 |  |
| K.SADESGQALLAASHYASDEVR.E | Y | 51.46 | 2176.0032 | 21 | 4.9 | 726.3452 | 3 | 89.10 | 6 | 25348 | Ana\_05.raw |  |  |  |  |  | 4.5E5 |  | 9.67E5 |  |  | 2 | 0 | 0 | 0 | 0 | 0 | 1 | 0 | 1 | 0 | 0 | 419 | 439 |  |
| K.EAIVTSEELGQDLEHVEVLQK.K | Y | 51.20 | 2365.2012 | 21 | 8.4 | 789.4143 | 3 | 112.16 | 9 | 31429 | Ana\_08.raw |  |  |  |  |  |  |  |  | 1.49E6 |  | 1 | 0 | 0 | 0 | 0 | 0 | 0 | 0 | 0 | 1 | 0 | 169 | 189 |  |
| R.DLTGVQNLR.K | Y | 50.95 | 1014.5458 | 9 | 4.4 | 508.2824 | 2 | 55.53 | 2 | 15750 | Ana\_01.raw | 1.32E6 | 2.81E6 | 2.26E6 | 1.23E6 | 2.46E6 | 2.56E6 | 1.01E6 |  | 2.24E6 | 3.91E5 | 9 | 1 | 1 | 1 | 1 | 1 | 1 | 1 | 0 | 1 | 1 | 1780 | 1788 |  |
| K.GEIDAHEDSFK.S | Y | 50.70 | 1246.5465 | 11 | 2.0 | 624.2818 | 2 | 39.37 | 6 | 10959 | Ana\_05.raw |  |  |  |  | 2.81E6 | 1.72E6 |  |  |  |  | 2 | 0 | 0 | 0 | 0 | 1 | 1 | 0 | 0 | 0 | 0 | 408 | 418 |  |
| K.LSILSEER.T | Y | 48.99 | 945.5131 | 8 | 3.0 | 473.7652 | 2 | 57.61 | 6 | 16466 | Ana\_05.raw | 1.75E6 | 3.75E6 | 2.84E6 |  | 2.13E6 | 3.2E6 |  |  | 6.2E6 | 2.17E6 | 7 | 1 | 1 | 1 | 0 | 1 | 1 | 0 | 0 | 1 | 1 | 442 | 449 |  |
| R.RQQVLDR.Y | Y | 48.35 | 913.5093 | 7 | 1.8 | 457.7628 | 2 | 19.41 | 6 | 4737 | Ana\_05.raw |  |  | 1.13E6 | 7.94E5 | 1.07E6 | 1.53E6 | 1.5E6 | 4.2E5 |  | 1.76E6 | 7 | 0 | 0 | 1 | 1 | 1 | 1 | 1 | 1 | 0 | 1 | 19 | 25 |  |
| R.NTTGVTEEALK.E | Y | 47.55 | 1161.5878 | 11 | 1.8 | 581.8022 | 2 | 38.73 | 6 | 10762 | Ana\_05.raw | 4.16E6 | 5.61E6 |  |  | 3.7E6 | 4.29E6 | 1.08E6 | 2.26E6 |  |  | 6 | 1 | 1 | 0 | 0 | 1 | 1 | 1 | 1 | 0 | 0 | 2322 | 2332 |  |
| K.EELYQNLTR.E | Y | 45.15 | 1164.5775 | 9 | 4.8 | 583.2988 | 2 | 60.51 | 5 | 17214 | Ana\_04.raw |  |  |  |  | 2.51E6 |  |  |  |  |  | 1 | 0 | 0 | 0 | 0 | 1 | 0 | 0 | 0 | 0 | 0 | 2433 | 2441 |  |
| K.LGDSHDLQR.F | Y | 44.96 | 1039.5046 | 9 | 5.5 | 347.5107 | 3 | 23.51 | 2 | 5930 | Ana\_01.raw |  | 2.26E6 |  | 1.12E6 |  |  |  |  |  |  | 2 | 0 | 1 | 0 | 1 | 0 | 0 | 0 | 0 | 0 | 0 | 1315 | 1323 |  |
| K.LLVSSEDYGR.D | Y | 44.88 | 1137.5665 | 10 | 4.6 | 569.7932 | 2 | 53.75 | 5 | 15229 | Ana\_04.raw |  |  | 2.6E6 |  | 2.1E6 |  |  |  | 1.52E6 |  | 3 | 0 | 0 | 1 | 0 | 1 | 0 | 0 | 0 | 1 | 0 | 1770 | 1779 |  |
| R.GLVSSDELAK.D | Y | 44.23 | 1017.5342 | 10 | 6.0 | 509.7774 | 2 | 44.75 | 9 | 12455 | Ana\_08.raw |  |  |  |  |  |  |  |  | 3.61E6 |  | 1 | 0 | 0 | 0 | 0 | 0 | 0 | 0 | 0 | 1 | 0 | 1340 | 1349 |  |
| R.EANELQQWITEK.E | Y | 43.50 | 1487.7256 | 12 | 1.5 | 744.8712 | 2 | 97.66 | 8 | 27190 | Ana\_07.raw |  |  |  |  |  |  |  | 3.59E5 |  |  | 1 | 0 | 0 | 0 | 0 | 0 | 0 | 0 | 1 | 0 | 0 | 1079 | 1090 |  |
| R.FLADFR.D | Y | 42.71 | 767.3966 | 6 | -0.1 | 384.7055 | 2 | 62.23 | 3 | 17731 | Ana\_02.raw | 9.72E5 | 1.67E6 | 2.7E6 | 7.73E5 | 2.28E6 | 8.38E5 | 1.54E6 |  | 2.07E6 | 1.94E6 | 9 | 1 | 1 | 1 | 1 | 1 | 1 | 1 | 0 | 1 | 1 | 366 | 371 |  |
| K.KQEALVAR.Y | Y | 41.75 | 913.5345 | 8 | 1.6 | 457.7753 | 2 | 19.95 | 6 | 4912 | Ana\_05.raw |  |  |  |  |  | 3.7E6 |  |  |  |  | 1 | 0 | 0 | 0 | 0 | 0 | 1 | 0 | 0 | 0 | 0 | 758 | 765 |  |
| K.TATDEAYKDPSNLQGK.V | Y | 41.66 | 1736.8217 | 16 | 3.4 | 579.9498 | 3 | 39.67 | 5 | 11003 | Ana\_04.raw |  |  | 2.76E6 |  | 2.47E6 | 2.44E6 |  |  |  |  | 3 | 0 | 0 | 1 | 0 | 1 | 1 | 0 | 0 | 0 | 0 | 595 | 610 |  |
| K.KFDDFQK.D | Y | 39.22 | 926.4497 | 7 | 6.6 | 464.2352 | 2 | 29.61 | 2 | 7898 | Ana\_01.raw |  | 2E6 |  |  |  |  |  |  |  |  | 1 | 0 | 1 | 0 | 0 | 0 | 0 | 0 | 0 | 0 | 0 | 1112 | 1118 |  |
| R.EAHDAFR.S | Y | 38.78 | 844.3828 | 7 | 2.9 | 423.1999 | 2 | 18.52 | 5 | 4491 | Ana\_04.raw |  |  |  |  | 2.84E6 |  |  |  |  |  | 1 | 0 | 0 | 0 | 0 | 1 | 0 | 0 | 0 | 0 | 0 | 2116 | 2122 |  |
| R.FQFFQR.D | Y | 37.35 | 871.4340 | 6 | 3.9 | 436.7260 | 2 | 74.88 | 6 | 21483 | Ana\_05.raw |  | 2.22E6 | 1.53E6 |  | 3.39E6 | 1.05E6 | 1.74E6 | 1.26E6 |  | 1.64E6 | 7 | 0 | 1 | 1 | 0 | 1 | 1 | 1 | 1 | 0 | 1 | 46 | 51 |  |
| K.KHEAFETDFTVHK.D | Y | 25.96 | 1587.7681 | 13 | 3.9 | 397.9508 | 4 | 48.47 | 7 | 13557 | Ana\_06.raw |  |  |  |  |  |  | 9.07E5 |  |  |  | 1 | 0 | 0 | 0 | 0 | 0 | 0 | 1 | 0 | 0 | 0 | 1896 | 1908 |  |
| R.FLSDFR.D | Y | 25.64 | 783.3915 | 6 | 3.6 | 392.7044 | 2 | 55.68 | 9 | 15767 | Ana\_08.raw |  |  |  |  |  |  |  |  | 7.07E5 |  | 1 | 0 | 0 | 0 | 0 | 0 | 0 | 0 | 0 | 1 | 0 | 1324 | 1329 |  |
| R.EANQQQQFNR.N | Y | 25.43 | 1261.5800 | 10 | -4.6 | 631.7944 | 2 | 30.34 | 7 | 8074 | Ana\_06.raw |  |  |  |  |  |  | 4.22E6 |  |  |  | 1 | 0 | 0 | 0 | 0 | 0 | 0 | 1 | 0 | 0 | 0 | 676 | 685 |  |
| R.ELELQK.E | Y | 24.81 | 758.4174 | 6 | -8.9 | 380.2126 | 2 | 20.42 | 7 | 5030 | Ana\_06.raw |  |  |  |  |  |  | 7.73E6 |  |  |  | 1 | 0 | 0 | 0 | 0 | 0 | 0 | 1 | 0 | 0 | 0 | 2175 | 2180 |  |
| total 45 peptides |  |  |  |  |  |  |  |  |  |  |  |  |  |  |  |  |  |  |  |  |  |  |  |  |  |  |  |  |  |  |  |  |  |  |  |
| --- | --- | --- | --- | --- | --- | --- | --- | --- | --- | --- | --- | --- | --- | --- | --- | --- | --- | --- | --- | --- | --- | --- | --- | --- | --- | --- | --- | --- | --- | --- | --- | --- | --- | --- | --- |

Best Unique PSM (Scan F7:37152, m/z=1077.5593, z=2, RT=134.84, ppm=3.6):

sp|P27773|PDIA3\_MOUSE

back to list

  

| Protein Coverage
| Supporting Peptides
| Best Unique PSM
|

Protein Coverage:

Supporting Peptides:

| Peptide | Uniq | -10lgP | Mass | Length | ppm | m/z | z | RT | Fraction | Scan | Source File | Area 10 | Area 01 | Area 02 | Area 03 | Area 04 | Area 05 | Area 06 | Area 07 | Area 08 | Area 09 | #Spec | #Spec 10 | #Spec 01 | #Spec 02 | #Spec 03 | #Spec 04 | #Spec 05 | #Spec 06 | #Spec 07 | #Spec 08 | #Spec 09 | Start | End | PTM |
| --- | --- | --- | --- | --- | --- | --- | --- | --- | --- | --- | --- | --- | --- | --- | --- | --- | --- | --- | --- | --- | --- | --- | --- | --- | --- | --- | --- | --- | --- | --- | --- | --- | --- | --- | --- |
| K.TFSHELSDFGLESTTGEVPVVAIR.T | Y | 161.43 | 2590.2915 | 24 | 0.8 | 864.4385 | 3 | 115.34 | 3 | 31854 | Ana\_02.raw | 1.53E8 | 1.69E8 | 9.01E7 | 1.08E7 | 1.11E8 | 8.82E7 | 6.51E7 | 8.04E7 | 2.75E7 | 9.89E7 | 47 | 6 | 6 | 4 | 3 | 6 | 2 | 4 | 6 | 6 | 4 | 306 | 329 |  |
| K.SEPIPESNEGPVK.V | Y | 94.56 | 1381.6725 | 13 | 1.0 | 691.8442 | 2 | 43.19 | 3 | 12118 | Ana\_02.raw | 8.07E7 | 7.28E7 | 7.37E7 | 3.47E7 | 6.78E7 | 6.25E7 | 5.18E7 | 4.91E7 | 1.12E8 | 5.39E7 | 24 | 3 | 1 | 3 | 2 | 2 | 2 | 2 | 3 | 4 | 2 | 367 | 379 |  |
| K.ALEQFLQEYFDGNLK.R | Y | 91.74 | 1813.8885 | 15 | 2.3 | 907.9537 | 2 | 131.20 | 6 | 36453 | Ana\_05.raw | 8.58E6 | 2.91E7 | 1.88E7 | 1.13E7 | 1.9E7 | 2.72E7 | 2E7 | 3.76E6 | 2.33E7 | 2.64E7 | 34 | 2 | 4 | 3 | 4 | 3 | 4 | 4 | 2 | 3 | 5 | 348 | 362 |  |
| K.GFPTIYFSPANK.K | Y | 88.49 | 1340.6764 | 12 | 2.9 | 671.3474 | 2 | 96.65 | 6 | 27362 | Ana\_05.raw | 3.31E7 | 3.1E7 | 3.2E7 | 1.36E7 | 2.13E7 | 2.34E7 | 2E7 | 2.1E7 | 5.04E7 | 2.81E7 | 22 | 2 | 3 | 2 | 2 | 2 | 2 | 2 | 3 | 2 | 2 | 449 | 460 |  |
| K.ALEQFLQEYFDGNLKR.Y | Y | 86.83 | 1969.9897 | 16 | 4.2 | 657.6733 | 3 | 128.50 | 6 | 35699 | Ana\_05.raw | 4.92E6 | 3.94E6 | 3.87E6 | 3.14E6 | 1.03E7 | 9.4E6 | 7.29E6 | 5.32E6 | 1.14E7 | 8.64E6 | 15 | 1 | 2 | 1 | 1 | 2 | 2 | 2 | 1 | 2 | 1 | 348 | 363 |  |
| R.ELNDFISYLQR.E | Y | 85.79 | 1396.6986 | 11 | 0.8 | 699.3571 | 2 | 111.69 | 3 | 30898 | Ana\_02.raw | 5.29E7 | 6.69E7 | 5.77E7 | 3.13E7 | 5E7 | 4.78E7 | 4.66E7 | 1.4E7 | 8.81E7 | 5.48E7 | 31 | 3 | 4 | 4 | 2 | 3 | 2 | 3 | 4 | 4 | 2 | 472 | 482 |  |
| K.M(+15.99)DATANDVPSPYEVK.G | Y | 85.59 | 1651.7399 | 15 | 6.2 | 826.8823 | 2 | 59.44 | 2 | 16889 | Ana\_01.raw | 1.81E7 | 1.9E7 | 2.01E7 | 9.52E5 | 1.75E7 | 1.74E7 | 1.56E7 | 1.47E7 | 2.63E7 | 1.08E7 | 11 | 1 | 1 | 1 | 1 | 1 | 1 | 2 | 1 | 1 | 1 | 434 | 448 | Oxidation (M) |
| K.DASVVGFFR.D | Y | 80.38 | 996.5028 | 9 | 0.7 | 499.2590 | 2 | 89.06 | 3 | 25018 | Ana\_02.raw | 2.01E7 | 2.6E7 | 2.89E7 | 1.44E7 | 2.25E7 | 2.36E7 | 1.91E7 | 1.75E7 | 3.72E7 | 2.23E7 | 23 | 2 | 2 | 3 | 2 | 2 | 3 | 2 | 3 | 2 | 2 | 153 | 161 |  |
| R.DGEEAGAYDGPR.T | Y | 79.34 | 1235.5054 | 12 | 3.2 | 618.7620 | 2 | 38.59 | 7 | 10566 | Ana\_06.raw | 2.44E7 | 3.08E7 | 3E7 | 1.35E7 | 2.74E7 | 2.74E7 | 2.44E7 | 1.63E7 | 1.7E7 | 2.29E7 | 26 | 2 | 1 | 3 | 3 | 3 | 4 | 4 | 3 | 1 | 2 | 108 | 119 |  |
| R.EATNPPIIQEEKPK.K | Y | 78.32 | 1592.8409 | 14 | 0.8 | 797.4284 | 2 | 40.64 | 3 | 11344 | Ana\_02.raw | 5.65E7 | 6.55E7 | 5.46E7 | 1.47E7 | 5.32E7 | 4.1E7 | 3.78E7 | 3.28E7 | 1.74E7 | 3.1E7 | 23 | 2 | 3 | 3 | 1 | 3 | 2 | 2 | 3 | 3 | 1 | 483 | 496 |  |
| R.DLFSDGHSEFLK.A | Y | 77.97 | 1393.6514 | 12 | 3.3 | 697.8353 | 2 | 85.46 | 2 | 24214 | Ana\_01.raw | 3.75E7 | 3.43E7 | 3.39E7 | 1.05E7 | 2.41E7 | 3.07E7 | 2.62E7 | 2.67E7 | 5.74E7 | 2.58E7 | 42 | 5 | 4 | 3 | 3 | 4 | 4 | 4 | 5 | 6 | 4 | 162 | 173 |  |
| K.FISDKDASVVGFFR.D | Y | 77.83 | 1586.8092 | 14 | 0.6 | 529.9440 | 3 | 94.89 | 3 | 26552 | Ana\_02.raw | 1.97E7 | 1.2E7 | 1.62E6 | 5.97E6 | 1.14E7 | 1.26E7 | 8.51E6 | 6.79E6 | 2.06E7 | 1.48E7 | 21 | 3 | 2 | 1 | 1 | 3 | 2 | 2 | 2 | 3 | 2 | 148 | 161 |  |
| R.LAPEYEAAATR.L | Y | 75.48 | 1190.5931 | 11 | 1.5 | 596.3047 | 2 | 45.53 | 4 | 12473 | Ana\_03.raw | 2.31E8 | 2.64E8 | 2.87E8 | 4.99E7 | 2.29E8 | 8.83E7 | 6.65E7 | 1.76E8 | 2.95E8 | 2.13E8 | 32 | 4 | 3 | 4 | 2 | 3 | 2 | 2 | 4 | 4 | 4 | 63 | 73 |  |
| K.YGVSGYPTLK.I | Y | 73.03 | 1083.5601 | 10 | 3.0 | 542.7889 | 2 | 65.69 | 7 | 18641 | Ana\_06.raw | 1.28E7 | 2.46E7 | 2.34E7 | 1.1E7 | 1.99E7 | 1.78E7 | 1.37E7 | 1.31E7 | 3.71E7 | 2.29E7 | 18 | 1 | 2 | 2 | 2 | 2 | 2 | 2 | 2 | 2 | 1 | 95 | 104 |  |
| R.FAHTNIESLVK.E | Y | 70.59 | 1257.6718 | 11 | 2.8 | 629.8449 | 2 | 62.10 | 1 | 17595 | ANa\_10.raw | 2.31E7 | 2.44E7 | 2.36E7 | 7.84E6 | 1.75E7 | 1.06E7 | 1.23E7 | 9.86E6 | 4.02E7 | 2.1E7 | 17 | 2 | 2 | 2 | 1 | 2 | 1 | 2 | 1 | 2 | 2 | 184 | 194 |  |
| R.TADGIVSHLK.K | Y | 69.11 | 1039.5662 | 10 | 4.2 | 520.7925 | 2 | 41.82 | 5 | 11666 | Ana\_04.raw | 9.71E6 | 1.05E7 | 6.33E6 | 4.61E6 | 1.26E7 | 5.14E6 | 4.35E6 |  | 6.91E6 | 8.17E6 | 12 | 1 | 1 | 1 | 2 | 2 | 1 | 1 | 0 | 2 | 1 | 120 | 129 |  |
| K.IFRDGEEAGAYDGPR.T | Y | 66.08 | 1651.7590 | 15 | -0.8 | 551.5932 | 3 | 50.99 | 3 | 14432 | Ana\_02.raw | 1.7E7 | 3.06E6 | 1.56E7 | 6.05E6 | 1.36E7 | 1.43E7 | 9.2E6 | 7.79E6 | 3.17E6 | 1.54E7 | 20 | 3 | 2 | 3 | 1 | 2 | 3 | 1 | 1 | 1 | 3 | 105 | 119 |  |
| K.FVM(+15.99)QEEFSR.D | Y | 62.05 | 1187.5281 | 9 | 4.4 | 594.7739 | 2 | 50.98 | 7 | 14325 | Ana\_06.raw | 3.84E7 | 4.23E7 | 3.97E7 | 1.48E7 | 3.44E7 | 3.33E7 | 2.74E7 | 2.66E7 | 0 | 3.27E7 | 21 | 2 | 3 | 1 | 2 | 2 | 2 | 3 | 2 | 2 | 2 | 336 | 344 | Oxidation (M) |
| K.GFPTIYFSPANKK.L | Y | 61.16 | 1468.7714 | 13 | 2.0 | 490.5987 | 3 | 82.41 | 5 | 23356 | Ana\_04.raw | 5.87E6 | 6.95E6 | 4.66E6 | 1.29E6 | 6.2E6 | 7.58E6 | 9.03E5 | 3.75E6 | 1.27E7 | 4.09E6 | 13 | 2 | 2 | 1 | 1 | 1 | 1 | 1 | 1 | 2 | 1 | 449 | 461 |  |
| K.FVMQEEFSR.D | Y | 57.70 | 1171.5332 | 9 | 2.5 | 586.7753 | 2 | 70.99 | 6 | 20373 | Ana\_05.raw | 4.55E5 |  |  |  | 9.33E5 | 2.09E6 | 5.19E5 | 1.24E6 | 5.09E6 | 3.99E6 | 7 | 1 | 0 | 0 | 0 | 1 | 1 | 1 | 1 | 1 | 1 | 336 | 344 |  |
| R.TEEEFKK.F | Y | 48.21 | 909.4443 | 7 | 3.1 | 455.7309 | 2 | 18.66 | 7 | 4472 | Ana\_06.raw |  |  |  | 2.71E6 | 7.91E6 |  | 2.81E6 | 1.77E6 |  |  | 4 | 0 | 0 | 0 | 1 | 1 | 0 | 1 | 1 | 0 | 0 | 141 | 147 |  |
| K.LNFAVASR.K | Y | 47.97 | 876.4817 | 8 | 2.6 | 439.2493 | 2 | 54.70 | 6 | 15606 | Ana\_05.raw | 1.22E7 | 1.48E7 | 1.71E7 | 5.41E6 | 1.19E7 | 9.41E6 |  | 7.85E6 | 2.1E7 | 1.21E7 | 11 | 2 | 1 | 2 | 1 | 1 | 1 | 0 | 1 | 1 | 1 | 297 | 304 |  |
| K.QAGPASVPLR.T | Y | 45.28 | 994.5559 | 10 | 9.7 | 498.2901 | 2 | 40.18 | 2 | 11115 | Ana\_01.raw |  | 3.31E6 |  |  |  |  |  |  |  |  | 1 | 0 | 1 | 0 | 0 | 0 | 0 | 0 | 0 | 0 | 0 | 131 | 140 |  |
| K.DPNIVIAK.M | Y | 44.70 | 868.5018 | 8 | 3.7 | 435.2598 | 2 | 46.58 | 8 | 12809 | Ana\_07.raw |  |  | 3.73E7 | 1.75E7 | 3.38E7 | 3.25E7 | 2.67E7 | 2.02E7 | 5.82E6 |  | 7 | 0 | 0 | 1 | 1 | 1 | 1 | 1 | 1 | 1 | 0 | 426 | 433 |  |
| K.LSKDPNIVIAK.M | Y | 41.85 | 1196.7129 | 11 | 3.9 | 399.9131 | 3 | 42.99 | 5 | 12016 | Ana\_04.raw |  |  |  |  | 2.92E6 | 3.71E6 |  |  |  |  | 2 | 0 | 0 | 0 | 0 | 1 | 1 | 0 | 0 | 0 | 0 | 423 | 433 |  |
| K.MDATANDVPSPYEVK.G | Y | 39.00 | 1635.7450 | 15 | 2.2 | 818.8816 | 2 | 64.38 | 9 | 18357 | Ana\_08.raw |  |  |  |  |  |  |  |  | 2.27E6 |  | 1 | 0 | 0 | 0 | 0 | 0 | 0 | 0 | 0 | 1 | 0 | 434 | 448 |  |
| K.YKELGEK.L | Y | 38.19 | 865.4545 | 7 | 3.0 | 433.7358 | 2 | 19.93 | 6 | 4906 | Ana\_05.raw |  |  |  |  |  | 1.02E7 |  |  |  |  | 1 | 0 | 0 | 0 | 0 | 0 | 1 | 0 | 0 | 0 | 0 | 416 | 422 |  |
| K.GIVPLAK.V | Y | 31.85 | 696.4534 | 7 | 3.7 | 349.2353 | 2 | 47.97 | 7 | 13413 | Ana\_06.raw |  |  |  |  |  | 5.06E6 | 4.49E6 |  | 8.11E6 | 4.57E6 | 4 | 0 | 0 | 0 | 0 | 0 | 1 | 1 | 0 | 1 | 1 | 76 | 82 |  |
| total 28 peptides |  |  |  |  |  |  |  |  |  |  |  |  |  |  |  |  |  |  |  |  |  |  |  |  |  |  |  |  |  |  |  |  |  |  |  |
| --- | --- | --- | --- | --- | --- | --- | --- | --- | --- | --- | --- | --- | --- | --- | --- | --- | --- | --- | --- | --- | --- | --- | --- | --- | --- | --- | --- | --- | --- | --- | --- | --- | --- | --- | --- |

Best Unique PSM (Scan F3:31854, m/z=864.4385, z=3, RT=115.34, ppm=0.8):

sp|Q8VDN2|AT1A1\_MOUSE

back to list

  

| Protein Coverage
| Supporting Peptides
| Best Unique PSM
|

Protein Coverage:

Supporting Peptides:

| Peptide | Uniq | -10lgP | Mass | Length | ppm | m/z | z | RT | Fraction | Scan | Source File | Area 10 | Area 01 | Area 02 | Area 03 | Area 04 | Area 05 | Area 06 | Area 07 | Area 08 | Area 09 | #Spec | #Spec 10 | #Spec 01 | #Spec 02 | #Spec 03 | #Spec 04 | #Spec 05 | #Spec 06 | #Spec 07 | #Spec 08 | #Spec 09 | Start | End | PTM |
| --- | --- | --- | --- | --- | --- | --- | --- | --- | --- | --- | --- | --- | --- | --- | --- | --- | --- | --- | --- | --- | --- | --- | --- | --- | --- | --- | --- | --- | --- | --- | --- | --- | --- | --- | --- |
| K.QAADM(+15.99)ILLDDNFASIVTGVEEGR.L | N | 116.52 | 2479.1899 | 23 | -0.1 | 1240.6022 | 2 | 136.29 | 3 | 37274 | Ana\_02.raw |  |  | 4.04E5 |  |  | 1.41E6 | 4.04E5 |  | 8.62E5 |  | 4 | 0 | 0 | 1 | 0 | 0 | 1 | 1 | 0 | 1 | 0 | 744 | 766 | Oxidation (M) |
| R.SPDFTNENPLETR.N | Y | 105.43 | 1518.6951 | 13 | -0.3 | 760.3546 | 2 | 71.74 | 3 | 20380 | Ana\_02.raw | 4.09E7 | 5.17E7 | 3.85E7 | 2.01E7 | 3.5E7 | 3.42E7 | 2.27E7 | 2.23E7 | 5.96E7 | 2.11E7 | 25 | 2 | 3 | 3 | 3 | 2 | 2 | 2 | 4 | 2 | 2 | 228 | 240 |  |
| K.GVGIISEGNETVEDIAAR.L | N | 103.92 | 1828.9166 | 18 | -1.4 | 915.4643 | 2 | 92.92 | 3 | 26035 | Ana\_02.raw | 5.92E5 |  | 9E6 | 3.97E6 | 1.19E7 | 5.5E6 | 8.95E6 | 2.72E6 | 4.1E7 | 2.06E7 | 12 | 1 | 0 | 2 | 1 | 2 | 2 | 1 | 1 | 1 | 1 | 630 | 647 |  |
| K.QAADMILLDDNFASIVTGVEEGR.L | N | 97.47 | 2463.1951 | 23 | -5.5 | 1232.5980 | 2 | 136.98 | 2 | 37870 | Ana\_01.raw |  | 4.47E5 |  |  |  |  |  | 3.23E5 |  |  | 3 | 0 | 2 | 0 | 0 | 0 | 0 | 0 | 1 | 0 | 0 | 744 | 766 |  |
| K.EQPLDEELKDAFQNAYLELGGLGER.V | Y | 97.29 | 2833.3770 | 25 | 1.3 | 945.4675 | 3 | 135.22 | 3 | 37001 | Ana\_02.raw | 9.73E5 | 3.85E6 | 2.83E6 | 2.17E6 | 2.23E6 | 1.18E7 | 1.46E6 | 1.12E6 | 7.32E6 | 1.01E7 | 10 | 1 | 1 | 1 | 1 | 1 | 1 | 1 | 1 | 1 | 1 | 527 | 551 |  |
| K.DAFQNAYLELGGLGER.V | Y | 96.85 | 1751.8478 | 16 | 4.3 | 876.9349 | 2 | 120.51 | 7 | 33285 | Ana\_06.raw |  | 0 | 8.71E6 | 1.17E6 |  | 5.81E6 | 1.26E6 | 1.29E6 | 3.97E6 | 1.33E6 | 8 | 0 | 1 | 1 | 1 | 0 | 1 | 1 | 1 | 1 | 1 | 536 | 551 |  |
| R.AVFQANQENLPILK.R | Y | 96.12 | 1583.8671 | 14 | 0.1 | 792.9409 | 2 | 90.49 | 3 | 25394 | Ana\_02.raw | 2.36E7 | 3.17E7 | 7.32E6 | 9.73E6 | 1.6E7 | 1.97E7 | 1.24E7 | 1.04E7 | 3.74E7 | 1.9E7 | 21 | 2 | 4 | 1 | 1 | 2 | 2 | 2 | 2 | 2 | 3 | 431 | 444 |  |
| K.M(+15.99)SINAEDVVVGDLVEVK.G | Y | 89.14 | 1831.9237 | 17 | 6.3 | 916.9749 | 2 | 112.42 | 7 | 31153 | Ana\_06.raw | 2.21E6 | 2.82E5 | 2.36E6 | 8.87E6 | 3.32E6 | 1.21E7 | 2.64E6 | 1.8E7 | 6.12E6 | 2.05E6 | 20 | 1 | 1 | 2 | 3 | 3 | 3 | 3 | 2 | 1 | 1 | 178 | 194 | Oxidation (M) |
| K.VDNSSLTGESEPQTR.S | N | 88.21 | 1618.7434 | 15 | 5.6 | 810.3835 | 2 | 36.79 | 3 | 10160 | Ana\_02.raw | 2.9E7 | 4.65E7 | 3.68E7 | 1.67E7 | 2.97E7 | 3.29E7 | 2.3E7 | 2.3E7 | 2.12E7 | 1.78E7 | 24 | 2 | 3 | 2 | 3 | 2 | 2 | 2 | 3 | 3 | 2 | 213 | 227 |  |
| R.NIAFFSTNC(+57.02)VEGTAR.G | Y | 83.80 | 1685.7831 | 15 | -1.2 | 843.8978 | 2 | 90.70 | 4 | 25076 | Ana\_03.raw |  | 7.92E5 | 7.39E5 | 1.15E6 | 2.38E5 | 9.8E5 | 2.67E6 | 4.26E5 |  | 0 | 8 | 0 | 1 | 1 | 1 | 1 | 1 | 1 | 1 | 0 | 1 | 241 | 255 | Carbamidomethylation |
| K.DM(+15.99)TSEELDDILR.Y | Y | 82.47 | 1451.6450 | 12 | 2.3 | 726.8315 | 2 | 98.22 | 7 | 27474 | Ana\_06.raw | 9.54E6 | 1.14E6 |  | 2.7E5 | 8.35E5 | 7.15E6 | 4.83E6 | 1.1E6 | 6.19E6 |  | 8 | 1 | 1 | 0 | 1 | 1 | 1 | 1 | 1 | 1 | 0 | 672 | 683 | Oxidation (M) |
| K.YEPAAVSEHGDKK.G | Y | 82.07 | 1429.6837 | 13 | 5.1 | 715.8528 | 2 | 21.99 | 2 | 5407 | Ana\_01.raw | 1.09E7 | 1.78E7 | 1.03E7 |  | 8.05E6 | 1.07E7 | 5.59E6 | 7.41E5 | 1.04E7 | 1.06E7 | 17 | 2 | 2 | 2 | 0 | 2 | 2 | 2 | 1 | 2 | 2 | 10 | 22 |  |
| R.DKYEPAAVSEHGDKK.G | Y | 80.90 | 1672.8055 | 15 | 1.8 | 558.6101 | 3 | 21.96 | 3 | 5591 | Ana\_02.raw | 5.84E6 |  | 6.25E6 | 2.47E6 | 3.5E6 | 2.13E6 |  |  | 6.25E6 | 4.56E6 | 7 | 1 | 0 | 1 | 1 | 1 | 1 | 0 | 0 | 1 | 1 | 8 | 22 |  |
| K.IVEIPFNSTNK.Y | Y | 74.92 | 1260.6714 | 11 | 1.9 | 631.3442 | 2 | 71.81 | 6 | 20606 | Ana\_05.raw | 1.44E7 | 2.46E7 | 1.85E7 | 9.6E6 | 1.44E7 | 1.94E7 | 1.08E7 | 9.85E6 | 2.32E7 | 1.25E7 | 13 | 1 | 2 | 1 | 2 | 1 | 1 | 1 | 1 | 1 | 2 | 477 | 487 |  |
| R.DKYEPAAVSEHGDK.K | Y | 71.81 | 1544.7107 | 14 | 3.4 | 515.9126 | 3 | 28.53 | 6 | 7665 | Ana\_05.raw |  |  |  |  | 1.73E6 | 2.76E6 | 2.08E6 |  |  |  | 3 | 0 | 0 | 0 | 0 | 1 | 1 | 1 | 0 | 0 | 0 | 8 | 21 |  |
| R.LNIPVNQVNPR.D | Y | 68.83 | 1262.7095 | 11 | 2.5 | 632.3636 | 2 | 63.64 | 6 | 18262 | Ana\_05.raw | 2.32E7 | 3.86E7 | 3.46E7 | 1.26E7 | 2.2E7 | 2.18E7 | 1.5E7 | 1.41E7 | 4.23E7 | 2.37E7 | 17 | 2 | 2 | 2 | 1 | 1 | 2 | 2 | 2 | 1 | 2 | 648 | 658 |  |
| R.AVAGDASESALLK.C | Y | 68.44 | 1230.6455 | 13 | 0.6 | 616.3304 | 2 | 52.14 | 3 | 14766 | Ana\_02.raw | 2.27E7 | 3.68E7 | 3.33E7 | 1.51E7 | 2.54E7 | 2.55E7 | 1.97E7 | 1.34E7 | 4.47E7 | 2.3E7 | 20 | 2 | 2 | 2 | 2 | 3 | 1 | 3 | 1 | 2 | 2 | 446 | 458 |  |
| K.AC(+57.02)VVHGSDLK.D | N | 67.47 | 1084.5336 | 10 | 2.9 | 543.2756 | 2 | 22.87 | 3 | 5880 | Ana\_02.raw |  | 1.41E7 | 4.85E6 | 1.98E6 | 3.78E6 |  |  | 2.26E6 | 4.64E6 |  | 7 | 0 | 2 | 1 | 1 | 1 | 0 | 0 | 1 | 1 | 0 | 662 | 671 | Carbamidomethylation |
| R.YHTEIVFAR.T | Y | 66.48 | 1134.5822 | 9 | 2.3 | 379.2022 | 3 | 55.96 | 6 | 15979 | Ana\_05.raw | 2.26E6 | 1.13E7 | 1.67E7 | 8.81E6 | 1.2E7 | 2.64E6 | 1.08E7 | 2.43E6 | 2.3E6 | 2.88E6 | 16 | 1 | 2 | 2 | 2 | 2 | 1 | 3 | 1 | 1 | 1 | 684 | 692 |  |
| R.DGPNALTPPPTTPEWVK.F | N | 64.90 | 1818.9152 | 17 | 0.1 | 910.4649 | 2 | 89.07 | 4 | 24649 | Ana\_03.raw | 4.8E6 | 9.15E6 | 7.79E6 | 1.07E6 | 0 | 2.31E6 | 2.42E6 | 1E6 | 1.36E7 |  | 10 | 2 | 1 | 1 | 1 | 1 | 1 | 1 | 1 | 1 | 0 | 75 | 91 |  |
| K.YEPAAVSEHGDK.K | Y | 63.59 | 1301.5887 | 12 | 3.3 | 651.8038 | 2 | 25.50 | 2 | 6590 | Ana\_01.raw | 5.07E5 | 3.88E6 | 5.39E5 |  |  |  | 3.58E5 | 3.23E5 | 3.82E6 | 8.66E5 | 8 | 1 | 1 | 1 | 0 | 0 | 0 | 1 | 1 | 2 | 1 | 10 | 21 |  |
| K.LSLDELHR.K | Y | 61.35 | 981.5243 | 8 | 1.2 | 328.1824 | 3 | 59.36 | 4 | 16487 | Ana\_03.raw | 4.62E6 |  | 1.1E7 | 3.07E6 |  | 4.7E6 |  | 2.58E6 | 8.77E6 | 3.86E6 | 9 | 1 | 0 | 2 | 2 | 0 | 1 | 0 | 1 | 1 | 1 | 46 | 53 |  |
| K.NM(+15.99)VPQQALVIR.N | N | 60.74 | 1283.7020 | 11 | 0.4 | 642.8585 | 2 | 58.76 | 3 | 16708 | Ana\_02.raw | 1.55E5 |  | 1.57E7 | 4.59E6 | 6.22E6 | 6.02E6 | 5.14E6 |  | 5.17E6 | 3.65E6 | 8 | 1 | 0 | 1 | 1 | 1 | 1 | 1 | 0 | 1 | 1 | 163 | 173 | Oxidation (M) |
| K.TSATWFALSR.I | Y | 59.68 | 1138.5771 | 10 | 8.0 | 570.3004 | 2 | 89.66 | 7 | 25198 | Ana\_06.raw | 8.75E5 | 7.98E6 |  |  |  | 6.44E6 | 5.47E6 | 8.03E5 | 1.3E7 | 3.75E6 | 9 | 1 | 1 | 0 | 0 | 0 | 2 | 1 | 1 | 2 | 1 | 414 | 423 |  |
| R.GIVVYTGDR.T | Y | 58.61 | 978.5134 | 9 | 5.2 | 490.2665 | 2 | 46.48 | 2 | 13052 | Ana\_01.raw | 1.6E7 | 1.54E7 |  | 1.04E7 | 1.57E7 | 2.14E7 | 1.6E7 | 9E6 | 1.28E7 | 1.56E7 | 10 | 1 | 1 | 0 | 1 | 1 | 1 | 1 | 2 | 1 | 1 | 256 | 264 |  |
| K.YGTDLSR.G | Y | 54.61 | 810.3871 | 7 | 4.4 | 406.2026 | 2 | 28.61 | 6 | 7689 | Ana\_05.raw | 1.62E7 | 2.64E7 | 2.32E7 | 1.22E7 | 1.67E7 | 1.96E7 | 1.44E7 | 2.15E6 | 4.66E6 | 1.12E7 | 10 | 1 | 1 | 1 | 1 | 1 | 1 | 1 | 1 | 1 | 1 | 55 | 61 |  |
| K.ERDM(+15.99)DELKK.E | Y | 51.42 | 1178.5601 | 9 | 2.6 | 393.8616 | 3 | 16.97 | 6 | 3926 | Ana\_05.raw |  |  |  |  | 9.38E6 | 2.66E6 | 6.72E6 | 4.2E6 |  |  | 7 | 0 | 0 | 0 | 0 | 2 | 1 | 2 | 2 | 0 | 0 | 29 | 37 | Oxidation (M) |
| K.VIM(+15.99)VTGDHPITAK.A | N | 51.05 | 1396.7384 | 13 | 0.1 | 466.5868 | 3 | 41.98 | 3 | 11757 | Ana\_02.raw | 2.67E6 | 0 | 4.23E6 |  | 4.77E6 | 4.45E6 | 5.72E6 |  | 3.89E6 |  | 7 | 1 | 1 | 1 | 0 | 1 | 1 | 1 | 0 | 1 | 0 | 613 | 625 | Oxidation (M) |
| K.EQPLDEELK.D | Y | 49.13 | 1099.5397 | 9 | 5.2 | 550.7800 | 2 | 49.13 | 5 | 13886 | Ana\_04.raw |  |  |  | 4.47E6 | 6.28E6 |  | 4.97E6 | 4.2E6 |  |  | 4 | 0 | 0 | 0 | 1 | 1 | 0 | 1 | 1 | 0 | 0 | 527 | 535 |  |
| R.KYGTDLSR.G | Y | 48.76 | 938.4821 | 8 | 4.0 | 470.2502 | 2 | 23.38 | 2 | 5884 | Ana\_01.raw |  | 8.67E6 |  | 2.85E6 | 4.96E6 | 6.29E6 |  |  |  | 7.71E5 | 5 | 0 | 1 | 0 | 1 | 1 | 1 | 0 | 0 | 0 | 1 | 54 | 61 |  |
| R.AAVPDAVGK.C | N | 47.89 | 826.4548 | 9 | 4.5 | 414.2366 | 2 | 27.38 | 2 | 7204 | Ana\_01.raw | 3.26E7 | 4.39E7 | 3.93E7 | 0 | 2.68E7 | 3.32E7 | 2.31E7 | 1.68E7 | 2.21E7 | 2.67E7 | 11 | 1 | 1 | 1 | 1 | 1 | 2 | 1 | 1 | 1 | 1 | 597 | 605 |  |
| R.AAEILAR.D | N | 47.53 | 742.4337 | 7 | 5.8 | 372.2263 | 2 | 28.79 | 2 | 7637 | Ana\_01.raw |  | 3.18E7 |  |  |  |  |  |  |  |  | 1 | 0 | 1 | 0 | 0 | 0 | 0 | 0 | 0 | 0 | 0 | 68 | 74 |  |
| R.NSVFQQGM(+15.99)K.N | N | 47.02 | 1053.4913 | 9 | 4.4 | 527.7552 | 2 | 29.87 | 8 | 7831 | Ana\_07.raw |  |  | 1.13E7 |  | 8.04E6 | 9.6E6 | 7.62E6 | 5.16E6 | 5.39E6 |  | 6 | 0 | 0 | 1 | 0 | 1 | 1 | 1 | 1 | 1 | 0 | 942 | 950 | Oxidation (M) |
| K.YQLSIHK.N | Y | 43.26 | 887.4865 | 7 | 4.2 | 444.7523 | 2 | 39.30 | 6 | 10934 | Ana\_05.raw |  |  | 1.09E7 |  |  | 1.29E7 | 7.33E6 | 5.33E6 |  |  | 4 | 0 | 0 | 1 | 0 | 0 | 1 | 1 | 1 | 0 | 0 | 488 | 494 |  |
| K.LIIVEGC(+57.02)QR.Q | N | 42.89 | 1086.5856 | 9 | 3.1 | 544.3018 | 2 | 47.56 | 9 | 13306 | Ana\_08.raw | 6.42E6 | 1.11E7 | 8.3E6 |  |  | 0 | 4.71E6 | 9.29E6 | 1.19E7 | 6.32E6 | 8 | 1 | 1 | 1 | 0 | 0 | 1 | 1 | 1 | 1 | 1 | 699 | 707 | Carbamidomethylation |
| K.HLLVM(+15.99)K.G | N | 42.16 | 755.4364 | 6 | 3.0 | 378.7266 | 2 | 21.75 | 1 | 5355 | ANa\_10.raw | 8.95E6 |  |  |  |  |  |  |  | 1.24E7 |  | 2 | 1 | 0 | 0 | 0 | 0 | 0 | 0 | 0 | 1 | 0 | 503 | 508 | Oxidation (M) |
| R.LIFDNLK.K | N | 39.03 | 861.4960 | 7 | 4.3 | 431.7571 | 2 | 74.92 | 1 | 21210 | ANa\_10.raw | 6.2E6 | 1.18E7 | 1.32E7 | 6.49E6 | 7.88E6 | 9.52E6 | 8.58E6 | 6.65E6 | 1.7E6 | 6.39E6 | 11 | 1 | 2 | 1 | 1 | 1 | 1 | 1 | 1 | 1 | 1 | 767 | 773 |  |
| R.ETWDDR.W | Y | 36.80 | 820.3351 | 6 | 4.3 | 411.1766 | 2 | 27.60 | 2 | 7274 | Ana\_01.raw | 3.79E6 | 5.92E6 | 5.9E6 |  |  |  |  |  |  | 2.65E6 | 4 | 1 | 1 | 1 | 0 | 0 | 0 | 0 | 0 | 0 | 1 | 888 | 893 |  |
| R.LIFDNLKK.S | N | 36.64 | 989.5909 | 8 | 3.5 | 330.8721 | 3 | 58.00 | 3 | 16481 | Ana\_02.raw |  | 2.69E6 | 3.36E6 | 2.39E6 | 2.01E6 | 2.6E6 | 1.92E6 | 1.32E6 |  |  | 9 | 0 | 1 | 2 | 2 | 1 | 1 | 1 | 1 | 0 | 0 | 767 | 774 |  |
| R.IPADLR.I | Y | 31.81 | 683.3966 | 6 | 1.2 | 342.7060 | 2 | 32.52 | 4 | 8638 | Ana\_03.raw | 2.29E7 | 3.29E7 | 3.63E7 | 1.81E7 | 1.52E7 | 1.56E7 |  | 1.4E7 |  | 2.31E7 | 11 | 1 | 2 | 2 | 1 | 2 | 1 | 0 | 1 | 0 | 1 | 199 | 204 |  |
| R.RNSVFQQGM(+15.99)K.N | N | 30.93 | 1209.5924 | 10 | 4.3 | 605.8061 | 2 | 22.76 | 2 | 5670 | Ana\_01.raw |  | 4.93E5 |  |  |  |  |  |  |  |  | 1 | 0 | 1 | 0 | 0 | 0 | 0 | 0 | 0 | 0 | 0 | 941 | 950 | Oxidation (M) |
| R.RPGGWVEK.E | Y | 29.28 | 927.4926 | 8 | 3.1 | 464.7550 | 2 | 23.28 | 9 | 5727 | Ana\_08.raw |  |  |  |  |  |  |  |  | 5.2E6 |  | 1 | 0 | 0 | 0 | 0 | 0 | 0 | 0 | 0 | 1 | 0 | 1012 | 1019 |  |
| R.IAGLC(+57.02)NR.A | N | 27.91 | 802.4119 | 7 | 2.2 | 402.2141 | 2 | 29.15 | 5 | 7838 | Ana\_04.raw |  |  |  |  | 7.7E6 |  |  |  |  |  | 1 | 0 | 0 | 0 | 0 | 1 | 0 | 0 | 0 | 0 | 0 | 424 | 430 | Carbamidomethylation |
| K.IM(+15.99)ESFK.N | N | 24.32 | 769.3680 | 6 | 5.0 | 385.6932 | 2 | 27.55 | 2 | 7258 | Ana\_01.raw |  | 1.14E7 |  |  |  | 7.41E6 |  |  |  |  | 2 | 0 | 1 | 0 | 0 | 0 | 1 | 0 | 0 | 0 | 0 | 157 | 162 | Oxidation (M) |
| K.ADIGVAM(+15.99)GIVGSDVSK.Q | Y | 23.87 | 1533.7709 | 16 | 3.6 | 767.8954 | 2 | 68.85 | 9 | 19638 | Ana\_08.raw |  |  |  |  |  |  |  |  | 0 |  | 1 | 0 | 0 | 0 | 0 | 0 | 0 | 0 | 0 | 1 | 0 | 728 | 743 | Oxidation (M) |
| K.KADIGVAM(+15.99)GIVGSDVSK.Q | Y | 22.62 | 1661.8657 | 17 | 4.5 | 554.9650 | 3 | 64.07 | 8 | 17959 | Ana\_07.raw |  |  |  |  |  |  |  | 9.03E5 |  |  | 1 | 0 | 0 | 0 | 0 | 0 | 0 | 0 | 1 | 0 | 0 | 727 | 743 | Oxidation (M) |
| total 46 peptides |  |  |  |  |  |  |  |  |  |  |  |  |  |  |  |  |  |  |  |  |  |  |  |  |  |  |  |  |  |  |  |  |  |  |  |
| --- | --- | --- | --- | --- | --- | --- | --- | --- | --- | --- | --- | --- | --- | --- | --- | --- | --- | --- | --- | --- | --- | --- | --- | --- | --- | --- | --- | --- | --- | --- | --- | --- | --- | --- | --- |

Best Unique PSM (Scan F3:20380, m/z=760.3546, z=2, RT=71.74, ppm=-0.3):

sp|Q7TMM9|TBB2A\_MOUSE

back to list

  

| Protein Coverage
| Supporting Peptides
| Best Unique PSM
|

Protein Coverage:

Supporting Peptides:

| Peptide | Uniq | -10lgP | Mass | Length | ppm | m/z | z | RT | Fraction | Scan | Source File | Area 10 | Area 01 | Area 02 | Area 03 | Area 04 | Area 05 | Area 06 | Area 07 | Area 08 | Area 09 | #Spec | #Spec 10 | #Spec 01 | #Spec 02 | #Spec 03 | #Spec 04 | #Spec 05 | #Spec 06 | #Spec 07 | #Spec 08 | #Spec 09 | Start | End | PTM |
| --- | --- | --- | --- | --- | --- | --- | --- | --- | --- | --- | --- | --- | --- | --- | --- | --- | --- | --- | --- | --- | --- | --- | --- | --- | --- | --- | --- | --- | --- | --- | --- | --- | --- | --- | --- |
| R.SGPFGQIFRPDNFVFGQSGAGNNWAK.G | N | 151.86 | 2797.3359 | 26 | 0.6 | 933.4532 | 3 | 124.87 | 3 | 34288 | Ana\_02.raw | 1.28E7 | 3.44E7 | 9.58E7 | 5.21E6 | 1.06E8 | 1.1E8 | 2.56E7 | 4.47E7 | 2.04E8 | 2.41E7 | 20 | 2 | 1 | 2 | 2 | 2 | 2 | 2 | 2 | 3 | 2 | 78 | 103 |  |
| K.GHYTEGAELVDSVLDVVR.K | N | 135.92 | 1957.9745 | 18 | 3.6 | 979.9980 | 2 | 127.77 | 6 | 35505 | Ana\_05.raw | 2.36E7 | 5.44E7 | 1.6E8 | 4.71E7 | 1.26E8 | 1.66E8 | 6.78E7 | 6.96E7 | 3.65E8 | 1.97E8 | 32 | 2 | 4 | 5 | 2 | 4 | 2 | 1 | 5 | 4 | 3 | 104 | 121 |  |
| K.LTTPTYGDLNHLVSATMSGVTTC(+57.02)LR.F | N | 113.62 | 2707.3311 | 25 | 1.9 | 903.4526 | 3 | 131.13 | 6 | 36433 | Ana\_05.raw |  | 5.46E5 | 9.82E5 | 4.59E6 | 4.43E6 | 9.86E6 | 2.28E6 | 7.01E6 | 3.92E7 | 2.05E7 | 9 | 0 | 1 | 1 | 1 | 1 | 1 | 1 | 1 | 1 | 1 | 217 | 241 | Carbamidomethylation |
| K.NSSYFVEWIPNNVK.T | N | 110.81 | 1695.8257 | 14 | 0.6 | 848.9207 | 2 | 109.87 | 3 | 30437 | Ana\_02.raw | 3.45E7 | 3.29E7 | 2.2E7 | 1.6E7 | 2.71E7 | 1.96E7 | 1.72E7 | 1.87E7 | 6.7E7 | 2.73E7 | 21 | 2 | 3 | 2 | 1 | 4 | 1 | 3 | 1 | 3 | 1 | 337 | 350 |  |
| R.LHFFM(+15.99)PGFAPLTSR.G | N | 108.26 | 1635.8231 | 14 | 1.6 | 546.2825 | 3 | 100.14 | 3 | 27934 | Ana\_02.raw | 1.44E8 | 1.06E8 | 1.27E8 | 4.7E6 | 1.01E8 | 8.11E7 | 1.18E7 | 4.61E7 | 3.33E8 | 1.08E8 | 72 | 8 | 9 | 7 | 5 | 7 | 7 | 4 | 8 | 9 | 8 | 263 | 276 | Oxidation (M) |
| R.LHFFMPGFAPLTSR.G | N | 97.12 | 1619.8282 | 14 | 6.0 | 540.9532 | 3 | 118.11 | 1 | 32615 | ANa\_10.raw | 3.14E7 | 1.19E7 |  | 8.19E5 | 3.08E5 | 5.07E6 |  | 2.64E5 | 3.8E7 | 2.23E7 | 17 | 3 | 3 | 0 | 1 | 1 | 1 | 0 | 1 | 3 | 4 | 263 | 276 |  |
| K.LTTPTYGDLNHLVSATM(+15.99)SGVTTC(+57.02)LR.F | N | 96.84 | 2723.3259 | 25 | 3.5 | 908.7858 | 3 | 116.60 | 2 | 32447 | Ana\_01.raw | 6.74E7 | 3.02E7 | 1.26E7 | 8.03E6 | 2E7 | 2.71E7 | 5.03E6 | 2.06E7 | 7.16E7 | 2.84E7 | 26 | 4 | 3 | 2 | 3 | 2 | 2 | 2 | 1 | 5 | 2 | 217 | 241 | Oxidation (M); Carbamidomethylation |
| K.EVDEQM(+15.99)LNVQNK.N | N | 82.36 | 1461.6770 | 12 | 4.6 | 731.8491 | 2 | 41.89 | 6 | 11733 | Ana\_05.raw | 4.47E7 | 4.06E7 | 4.89E7 | 1.69E7 | 3.72E7 | 3.72E7 | 2.65E7 | 3.63E7 | 4.7E7 | 3.59E7 | 25 | 2 | 2 | 3 | 2 | 3 | 2 | 2 | 4 | 2 | 3 | 325 | 336 | Oxidation (M) |
| K.MSATFIGNSTAIQELFK.R | N | 82.10 | 1856.9342 | 17 | 4.7 | 929.4788 | 2 | 128.68 | 6 | 35752 | Ana\_05.raw | 2.9E6 | 3.03E6 |  |  |  | 1.87E6 |  | 1.08E6 | 4.78E6 |  | 5 | 1 | 1 | 0 | 0 | 0 | 1 | 0 | 1 | 1 | 0 | 363 | 379 |  |
| R.FPGQLNADLR.K | N | 78.02 | 1129.5880 | 10 | 3.0 | 565.8030 | 2 | 69.05 | 6 | 19811 | Ana\_05.raw | 7.68E7 | 5.71E7 | 5.55E7 | 2.67E7 | 6.02E7 | 4.21E7 | 2.93E7 | 4.94E7 | 1.57E8 | 8.76E7 | 45 | 4 | 4 | 4 | 4 | 4 | 5 | 3 | 5 | 6 | 6 | 242 | 251 |  |
| K.LAVNM(+15.99)VPFPR.L | N | 76.83 | 1158.6219 | 10 | 4.1 | 580.3206 | 2 | 79.35 | 6 | 22695 | Ana\_05.raw | 8.22E7 | 5.68E7 | 5.35E7 | 2.41E7 | 4.94E7 | 5.03E7 | 3.23E7 | 3.95E7 | 1.48E8 | 5.62E7 | 48 | 6 | 5 | 4 | 4 | 4 | 4 | 4 | 6 | 7 | 4 | 253 | 262 | Oxidation (M) |
| K.M(+15.99)SATFIGNSTAIQELFK.R | N | 76.67 | 1872.9291 | 17 | 5.1 | 937.4766 | 2 | 123.87 | 6 | 34493 | Ana\_05.raw | 5.15E6 | 2.88E6 | 9.44E6 | 3.34E5 | 1.38E6 | 1.15E7 | 5.53E6 |  | 2.21E7 | 3.6E6 | 11 | 2 | 1 | 1 | 1 | 1 | 1 | 1 | 0 | 2 | 1 | 363 | 379 | Oxidation (M) |
| R.AILVDLEPGTM(+15.99)DSVR.S | N | 73.85 | 1630.8236 | 15 | 3.2 | 816.4217 | 2 | 86.14 | 1 | 24257 | ANa\_10.raw | 1.6E7 | 1.38E7 | 6.82E5 | 2.16E6 | 7.97E6 | 2.12E6 | 5.93E6 | 7.31E6 | 3.21E7 | 1.06E7 | 13 | 2 | 1 | 1 | 1 | 1 | 1 | 1 | 1 | 2 | 2 | 63 | 77 | Oxidation (M) |
| R.INVYYNEAAGNK.Y | Y | 71.01 | 1354.6517 | 12 | 3.5 | 678.3355 | 2 | 48.78 | 9 | 13669 | Ana\_08.raw |  |  |  |  |  |  |  |  | 7.79E6 |  | 1 | 0 | 0 | 0 | 0 | 0 | 0 | 0 | 0 | 1 | 0 | 47 | 58 |  |
| K.LAVNMVPFPR.L | N | 69.21 | 1142.6270 | 10 | 5.4 | 572.3239 | 2 | 92.17 | 1 | 25869 | ANa\_10.raw | 6.59E6 |  |  |  |  | 1.83E5 |  |  | 1.45E6 |  | 3 | 1 | 0 | 0 | 0 | 0 | 1 | 0 | 0 | 1 | 0 | 253 | 262 |  |
| R.ISEQFTAM(+15.99)FR.R | N | 65.28 | 1244.5859 | 10 | 1.5 | 623.3012 | 2 | 70.89 | 3 | 20141 | Ana\_02.raw | 2.85E7 | 2.58E7 | 1.92E7 | 1E7 | 8.26E6 | 1.54E7 | 6.67E6 | 1.69E7 | 2.69E7 | 1.4E7 | 10 | 1 | 1 | 1 | 1 | 1 | 1 | 1 | 1 | 1 | 1 | 381 | 390 | Oxidation (M) |
| K.IREEYPDR.I | N | 65.00 | 1076.5250 | 8 | 2.5 | 539.2711 | 2 | 25.17 | 6 | 6612 | Ana\_05.raw | 8.6E7 | 1.16E8 | 1.13E8 | 5.23E7 | 7.83E7 | 1.03E8 | 7.1E7 | 6.1E7 | 5.8E7 | 1.33E8 | 27 | 3 | 2 | 2 | 3 | 2 | 2 | 3 | 3 | 4 | 3 | 155 | 162 |  |
| R.EIVHIQAGQC(+57.02)GNQIGAK.F | N | 61.23 | 1821.9155 | 17 | 6.6 | 911.9710 | 2 | 49.98 | 9 | 14043 | Ana\_08.raw |  |  |  |  |  |  |  |  | 4.97E6 |  | 2 | 0 | 0 | 0 | 0 | 0 | 0 | 0 | 0 | 2 | 0 | 3 | 19 | Carbamidomethylation |
| K.TAVC(+57.02)DIPPR.G | N | 54.26 | 1027.5121 | 9 | 1.9 | 514.7643 | 2 | 37.94 | 6 | 10515 | Ana\_05.raw | 1.32E7 | 1.04E7 | 1.14E7 | 1.69E6 | 9.99E6 | 4.43E7 | 1.78E7 | 6.51E6 | 1.8E7 | 5.15E7 | 32 | 3 | 3 | 3 | 3 | 3 | 4 | 3 | 2 | 2 | 6 | 351 | 359 | Carbamidomethylation |
| R.KLAVNM(+15.99)VPFPR.L | N | 49.45 | 1286.7169 | 11 | 2.9 | 429.9142 | 3 | 66.65 | 5 | 19005 | Ana\_04.raw |  |  |  | 2.22E6 | 4.19E6 | 8.25E6 |  | 2.66E6 | 3.2E6 |  | 5 | 0 | 0 | 0 | 1 | 1 | 1 | 0 | 1 | 1 | 0 | 252 | 262 | Oxidation (M) |
| R.YLTVAAIFR.G | Y | 47.97 | 1052.6018 | 9 | 5.0 | 527.3108 | 2 | 107.72 | 7 | 29962 | Ana\_06.raw |  |  |  |  |  |  | 6.53E4 |  |  |  | 1 | 0 | 0 | 0 | 0 | 0 | 0 | 1 | 0 | 0 | 0 | 310 | 318 |  |
| total 21 peptides |  |  |  |  |  |  |  |  |  |  |  |  |  |  |  |  |  |  |  |  |  |  |  |  |  |  |  |  |  |  |  |  |  |  |  |
| --- | --- | --- | --- | --- | --- | --- | --- | --- | --- | --- | --- | --- | --- | --- | --- | --- | --- | --- | --- | --- | --- | --- | --- | --- | --- | --- | --- | --- | --- | --- | --- | --- | --- | --- | --- |

Best Unique PSM (Scan F9:13669, m/z=678.3355, z=2, RT=48.78, ppm=3.5):

sp|P40142|TKT\_MOUSE

back to list

  

| Protein Coverage
| Supporting Peptides
| Best Unique PSM
|

Protein Coverage:

Supporting Peptides:

| Peptide | Uniq | -10lgP | Mass | Length | ppm | m/z | z | RT | Fraction | Scan | Source File | Area 10 | Area 01 | Area 02 | Area 03 | Area 04 | Area 05 | Area 06 | Area 07 | Area 08 | Area 09 | #Spec | #Spec 10 | #Spec 01 | #Spec 02 | #Spec 03 | #Spec 04 | #Spec 05 | #Spec 06 | #Spec 07 | #Spec 08 | #Spec 09 | Start | End | PTM |
| --- | --- | --- | --- | --- | --- | --- | --- | --- | --- | --- | --- | --- | --- | --- | --- | --- | --- | --- | --- | --- | --- | --- | --- | --- | --- | --- | --- | --- | --- | --- | --- | --- | --- | --- | --- |
| R.ILTVEDHYYEGGIGEAVSAAVVGEPGVTVTR.L | Y | 127.39 | 3187.6038 | 31 | 0.8 | 1063.5427 | 3 | 118.41 | 6 | 33071 | Ana\_05.raw |  | 1.62E7 |  | 2.79E6 | 1.36E6 | 7.64E6 | 1.88E6 | 1.81E6 | 3.79E6 | 4.99E6 | 8 | 0 | 1 | 0 | 1 | 1 | 1 | 1 | 1 | 1 | 1 | 556 | 586 |  |
| K.ILATPPQEDAPSVDIANIR.M | Y | 116.75 | 2019.0636 | 19 | -0.4 | 1010.5387 | 2 | 91.33 | 3 | 25620 | Ana\_02.raw | 3.12E7 | 4.23E7 | 1.19E7 | 1.63E6 | 1.01E7 | 2.57E6 | 4.07E6 | 3.37E6 | 7.03E7 | 9.44E5 | 22 | 2 | 4 | 2 | 1 | 2 | 2 | 2 | 2 | 4 | 1 | 284 | 302 |  |
| K.SKDDQVTVIGAGVTLHEALAAAESLK.K | Y | 116.44 | 2622.3865 | 26 | 2.0 | 656.6052 | 4 | 129.65 | 4 | 35215 | Ana\_03.raw | 1.91E7 | 9.75E6 | 4.65E6 | 5.06E6 | 5.92E6 | 9.07E6 | 5.8E6 | 4.59E6 | 1.51E7 | 1.12E7 | 13 | 2 | 1 | 2 | 1 | 1 | 1 | 1 | 1 | 1 | 2 | 498 | 523 |  |
| R.TSRPENAIIYSNNEDFQVGQAK.V | Y | 113.15 | 2480.1931 | 22 | 1.8 | 827.7398 | 3 | 73.06 | 5 | 20805 | Ana\_04.raw | 1.9E7 | 1.57E7 | 9.44E6 | 6.2E6 | 9.63E6 | 9.74E6 | 6.63E6 | 6.6E6 | 2.42E7 | 1.05E7 | 11 | 1 | 1 | 1 | 1 | 1 | 1 | 2 | 1 | 1 | 1 | 472 | 493 |  |
| K.SKDDQVTVIGAGVTLHEALAAAESLKK.D | Y | 108.92 | 2750.4814 | 27 | 2.7 | 688.6295 | 4 | 122.80 | 6 | 34215 | Ana\_05.raw | 1.73E6 | 2.95E5 | 4.81E5 | 5.69E5 | 1.82E6 | 1E6 | 3.37E6 | 2.22E6 | 1.59E6 | 1.09E7 | 12 | 2 | 1 | 1 | 1 | 1 | 1 | 1 | 1 | 2 | 1 | 498 | 524 |  |
| R.TVPFC(+57.02)STFAAFFTR.A | Y | 99.73 | 1650.7864 | 14 | 5.0 | 826.4046 | 2 | 131.73 | 6 | 36608 | Ana\_05.raw | 3.63E6 | 3.52E6 | 1.15E7 | 6.23E6 | 1.04E7 | 1.7E7 | 1.31E7 | 5.54E6 | 1.68E7 | 1.43E7 | 20 | 1 | 2 | 2 | 2 | 2 | 2 | 2 | 2 | 3 | 2 | 382 | 395 | Carbamidomethylation |
| K.NM(+15.99)AEQIIQEIYSQVQSK.K | Y | 98.96 | 2023.9884 | 17 | 1.1 | 675.6708 | 3 | 131.01 | 3 | 35888 | Ana\_02.raw | 9.8E6 | 7.82E6 | 1.07E7 | 7.21E5 | 6.65E6 | 1.25E7 | 9.4E6 | 7.22E6 | 1.22E7 | 5.53E6 | 15 | 1 | 1 | 2 | 1 | 1 | 2 | 2 | 2 | 2 | 1 | 265 | 281 | Oxidation (M) |
| R.SVPM(+15.99)STVFYPSDGVATEK.A | Y | 92.12 | 1929.9030 | 18 | 0.7 | 965.9595 | 2 | 73.74 | 3 | 20920 | Ana\_02.raw | 2.23E7 | 2.05E7 | 1.85E7 | 3.04E6 | 7.61E6 | 7.9E6 | 6.87E6 | 8.54E6 | 3.8E7 | 3.97E6 | 23 | 2 | 2 | 2 | 2 | 2 | 2 | 3 | 2 | 4 | 2 | 439 | 456 | Oxidation (M) |
| R.LGQSDPAPLQHQVDIYQK.R | Y | 90.19 | 2036.0327 | 18 | 4.8 | 679.6881 | 3 | 63.46 | 9 | 18085 | Ana\_08.raw |  | 1.19E7 | 8.49E6 | 5.42E6 | 8.6E6 | 1.15E7 | 1.87E6 | 8.02E6 | 2.18E7 | 1.26E7 | 9 | 0 | 1 | 1 | 1 | 1 | 1 | 1 | 1 | 1 | 1 | 187 | 204 |  |
| K.NMAEQIIQEIYSQVQSK.K | Y | 85.78 | 2007.9935 | 17 | 3.2 | 670.3406 | 3 | 136.16 | 1 | 37408 | ANa\_10.raw | 2.47E6 |  |  |  |  |  |  |  | 6.53E6 | 3.7E6 | 4 | 1 | 0 | 0 | 0 | 0 | 0 | 0 | 0 | 2 | 1 | 265 | 281 |  |
| K.LDNLVAIFDINR.L | Y | 83.52 | 1401.7616 | 12 | 0.5 | 701.8884 | 2 | 123.77 | 3 | 33998 | Ana\_02.raw | 3.76E6 | 2.56E6 | 2.04E6 | 3.31E6 | 1.32E7 | 5.8E5 | 1.06E7 | 8.85E6 | 1.71E7 | 1.22E7 | 17 | 2 | 2 | 2 | 1 | 2 | 1 | 2 | 1 | 2 | 2 | 175 | 186 |  |
| R.VLDPFTIKPLDR.K | Y | 66.66 | 1412.8027 | 12 | 4.8 | 471.9438 | 3 | 94.38 | 2 | 26651 | Ana\_01.raw | 3.96E7 | 3.75E7 | 3.76E7 | 1.33E7 | 2.12E7 | 3.25E7 | 1.97E7 | 1.61E7 | 7.02E7 | 4.34E7 | 23 | 2 | 2 | 2 | 1 | 2 | 2 | 1 | 3 | 5 | 3 | 531 | 542 |  |
| K.AVELAANTK.G | Y | 63.47 | 915.5025 | 9 | 4.5 | 458.7606 | 2 | 26.67 | 6 | 7084 | Ana\_05.raw | 1.11E7 |  | 2.99E7 | 1.49E7 | 2.3E7 | 3.36E7 | 1.92E7 | 1.84E7 |  |  | 7 | 1 | 0 | 1 | 1 | 1 | 1 | 1 | 1 | 0 | 0 | 457 | 465 |  |
| K.ISSDLDGHPVPK.Q | Y | 62.41 | 1263.6459 | 12 | 4.1 | 632.8328 | 2 | 35.32 | 9 | 9511 | Ana\_08.raw | 9.93E6 | 1.25E7 | 1.06E7 | 6E6 |  |  | 4.42E6 |  | 8.86E6 | 2.58E7 | 9 | 1 | 1 | 1 | 1 | 0 | 0 | 1 | 0 | 2 | 2 | 103 | 114 |  |
| K.HQPTAIIAK.T | Y | 52.46 | 977.5658 | 9 | 2.2 | 489.7913 | 2 | 22.40 | 3 | 5735 | Ana\_02.raw | 2.46E7 | 2.96E7 | 3.94E7 | 1.9E7 | 2.99E7 | 3.54E7 | 2.45E7 | 5.99E6 | 5.75E7 | 4.02E7 | 14 | 1 | 1 | 1 | 2 | 2 | 2 | 2 | 1 | 1 | 1 | 233 | 241 |  |
| R.IIALDGDTK.N | Y | 51.10 | 944.5178 | 9 | 3.1 | 473.2676 | 2 | 47.02 | 4 | 12902 | Ana\_03.raw | 6.03E7 | 5.32E7 | 5E7 | 2.2E7 | 3.91E7 | 3.69E7 | 3.06E7 | 2.32E7 | 7.01E7 | 5.72E7 | 20 | 2 | 1 | 3 | 2 | 3 | 2 | 2 | 1 | 2 | 2 | 335 | 343 |  |
| R.LAVSQVPR.S | Y | 49.87 | 868.5130 | 8 | 5.1 | 435.2660 | 2 | 40.80 | 2 | 11302 | Ana\_01.raw |  | 2.51E6 | 4.39E7 | 5.87E6 | 3.9E7 | 3.49E7 | 3.04E7 |  | 6.18E7 | 4.62E7 | 8 | 0 | 1 | 1 | 1 | 1 | 1 | 1 | 0 | 1 | 1 | 587 | 594 |  |
| K.NSTFSELFK.K | Y | 49.11 | 1071.5237 | 9 | 0.9 | 536.7696 | 2 | 88.25 | 4 | 24432 | Ana\_03.raw | 1.75E7 | 2.06E7 |  | 8.61E6 |  | 1.74E7 | 1.25E7 | 1.16E7 | 4.06E7 | 1.64E7 | 11 | 1 | 1 | 0 | 1 | 0 | 1 | 2 | 1 | 3 | 1 | 344 | 352 |  |
| R.M(+15.99)PTPPSYK.V | Y | 48.78 | 935.4423 | 8 | 5.2 | 468.7308 | 2 | 29.20 | 2 | 7767 | Ana\_01.raw | 3.01E7 | 2.74E7 | 1.89E7 | 2.66E7 | 1.91E7 | 2.45E7 | 3.52E7 | 3.7E7 | 2.91E6 | 2.23E7 | 16 | 1 | 1 | 1 | 3 | 1 | 2 | 3 | 2 | 1 | 1 | 303 | 310 | Oxidation (M) |
| R.KISSDLDGHPVPK.Q | Y | 46.72 | 1391.7408 | 13 | 4.6 | 464.9230 | 3 | 36.42 | 6 | 10052 | Ana\_05.raw |  |  |  |  |  | 2.07E6 |  | 8.78E5 |  |  | 2 | 0 | 0 | 0 | 0 | 0 | 1 | 0 | 1 | 0 | 0 | 102 | 114 |  |
| R.AFDQIR.M | Y | 46.19 | 748.3868 | 6 | 5.3 | 375.2026 | 2 | 34.93 | 1 | 9458 | ANa\_10.raw | 3.48E7 | 3.24E7 | 3.18E7 | 1.9E7 | 2.53E7 | 2.23E7 | 2.29E7 | 1.54E7 | 2.96E7 | 2.53E7 | 10 | 1 | 1 | 1 | 1 | 1 | 1 | 1 | 1 | 1 | 1 | 396 | 401 |  |
| K.LILDSAR.A | Y | 44.50 | 786.4599 | 7 | 5.9 | 394.2396 | 2 | 41.76 | 1 | 11557 | ANa\_10.raw | 2.82E7 | 2.58E7 |  |  | 1.24E7 | 1.48E7 | 8.63E6 |  | 7.77E6 |  | 6 | 1 | 1 | 0 | 0 | 1 | 1 | 1 | 0 | 1 | 0 | 544 | 550 |  |
| R.SGKPAELLK.M | Y | 44.49 | 941.5546 | 9 | 3.7 | 471.7863 | 2 | 24.37 | 9 | 6089 | Ana\_08.raw |  | 2.22E6 | 9.78E6 |  | 4.29E6 | 1.29E7 |  | 2.64E6 | 4.94E6 |  | 7 | 0 | 1 | 1 | 0 | 1 | 1 | 0 | 1 | 2 | 0 | 595 | 603 |  |
| R.MPTPPSYK.V | Y | 43.66 | 919.4473 | 8 | 5.1 | 460.7333 | 2 | 42.54 | 9 | 11764 | Ana\_08.raw |  |  |  |  |  |  |  |  | 3.13E6 |  | 1 | 0 | 0 | 0 | 0 | 0 | 0 | 0 | 0 | 1 | 0 | 303 | 310 |  |
| K.AYGLALAK.L | Y | 43.08 | 805.4697 | 8 | 5.2 | 403.7442 | 2 | 53.29 | 2 | 15074 | Ana\_01.raw | 5.28E6 | 5.86E6 | 5.19E6 | 2.25E6 | 2.63E6 | 3E6 | 3.2E6 | 2.25E6 | 1.31E7 | 6.1E6 | 10 | 1 | 1 | 1 | 1 | 1 | 1 | 1 | 1 | 1 | 1 | 320 | 327 |  |
| R.KLILDSAR.A | Y | 37.92 | 914.5549 | 8 | 0.4 | 458.2849 | 2 | 31.60 | 3 | 8609 | Ana\_02.raw |  |  | 5.36E6 |  | 4.56E6 | 4.54E6 | 3.26E6 |  | 3.5E6 |  | 5 | 0 | 0 | 1 | 0 | 1 | 1 | 1 | 0 | 1 | 0 | 543 | 550 |  |
| K.GIC(+57.02)FIR.T | Y | 36.61 | 764.4003 | 6 | 5.9 | 383.2097 | 2 | 58.71 | 10 | 16602 | Ana\_09.raw |  | 4.36E6 | 4.78E6 | 2.95E6 |  | 3.24E6 | 2.87E6 | 1.44E6 | 8.55E6 | 5.22E6 | 8 | 0 | 1 | 1 | 1 | 0 | 1 | 1 | 1 | 1 | 1 | 466 | 471 | Carbamidomethylation |
| R.KAYGLALAK.L | Y | 35.51 | 933.5647 | 9 | 4.3 | 467.7916 | 2 | 42.34 | 7 | 11710 | Ana\_06.raw |  |  |  |  |  |  | 2.12E6 | 1.44E6 |  |  | 2 | 0 | 0 | 0 | 0 | 0 | 0 | 1 | 1 | 0 | 0 | 319 | 327 |  |
| K.EAWHGKPLPK.N | Y | 26.14 | 1161.6294 | 10 | 3.8 | 388.2185 | 3 | 25.04 | 9 | 6319 | Ana\_08.raw |  |  |  |  |  |  |  |  | 1.16E7 |  | 1 | 0 | 0 | 0 | 0 | 0 | 0 | 0 | 0 | 1 | 0 | 255 | 264 |  |
| total 29 peptides |  |  |  |  |  |  |  |  |  |  |  |  |  |  |  |  |  |  |  |  |  |  |  |  |  |  |  |  |  |  |  |  |  |  |  |
| --- | --- | --- | --- | --- | --- | --- | --- | --- | --- | --- | --- | --- | --- | --- | --- | --- | --- | --- | --- | --- | --- | --- | --- | --- | --- | --- | --- | --- | --- | --- | --- | --- | --- | --- | --- |

Best Unique PSM (Scan F6:33071, m/z=1063.5427, z=3, RT=118.41, ppm=0.8):

sp|P10126|EF1A1\_MOUSE

back to list

  

| Protein Coverage
| Supporting Peptides
| Best Unique PSM
|

Protein Coverage:

Supporting Peptides:

| Peptide | Uniq | -10lgP | Mass | Length | ppm | m/z | z | RT | Fraction | Scan | Source File | Area 10 | Area 01 | Area 02 | Area 03 | Area 04 | Area 05 | Area 06 | Area 07 | Area 08 | Area 09 | #Spec | #Spec 10 | #Spec 01 | #Spec 02 | #Spec 03 | #Spec 04 | #Spec 05 | #Spec 06 | #Spec 07 | #Spec 08 | #Spec 09 | Start | End | PTM |
| --- | --- | --- | --- | --- | --- | --- | --- | --- | --- | --- | --- | --- | --- | --- | --- | --- | --- | --- | --- | --- | --- | --- | --- | --- | --- | --- | --- | --- | --- | --- | --- | --- | --- | --- | --- |
| R.KDGSASGTTLLEALDC(+57.02)ILPPTRPTDKPLR.L | Y | 141.45 | 3121.6440 | 29 | 1.1 | 781.4191 | 4 | 123.30 | 3 | 33877 | Ana\_02.raw | 1.76E7 | 3.45E7 | 6.59E7 | 4.98E7 | 1.03E8 | 1.2E8 | 9.16E7 | 6.52E7 | 2.38E8 | 1.12E8 | 43 | 5 | 6 | 4 | 2 | 3 | 4 | 4 | 5 | 6 | 4 | 219 | 247 | Carbamidomethylation |
| R.VETGVLKPGM(+15.99)VVTFAPVNVTTEVK.S | Y | 123.34 | 2530.3716 | 24 | -1.1 | 1266.1917 | 2 | 99.74 | 3 | 27828 | Ana\_02.raw | 4.04E7 | 1.03E8 | 4.83E7 | 4.19E6 | 3.78E7 | 2.95E7 | 8.35E6 | 8.21E6 | 3.97E8 | 9.18E7 | 47 | 3 | 7 | 6 | 3 | 6 | 4 | 3 | 4 | 6 | 5 | 267 | 290 | Oxidation (M) |
| K.NM(+15.99)ITGTSQADC(+57.02)AVLIVAAGVGEFEAGISK.N | N | 122.91 | 2924.4260 | 29 | 0.7 | 975.8167 | 3 | 136.80 | 6 | 38014 | Ana\_05.raw | 6.35E5 |  | 8.93E5 | 4.7E5 |  | 2.24E6 | 4.52E5 |  |  |  | 5 | 1 | 0 | 1 | 1 | 0 | 1 | 1 | 0 | 0 | 0 | 101 | 129 | Oxidation (M); Carbamidomethylation |
| K.DGSASGTTLLEALDC(+57.02)ILPPTRPTDKPLR.L | Y | 119.71 | 2993.5491 | 28 | 6.5 | 749.3994 | 4 | 130.76 | 7 | 35989 | Ana\_06.raw | 1.32E8 | 1.27E8 | 9.85E7 | 2.9E7 | 5.71E7 | 1.06E8 | 7.68E7 | 7.09E7 | 2.26E8 | 9.41E7 | 28 | 5 | 3 | 2 | 1 | 1 | 2 | 2 | 3 | 6 | 3 | 220 | 247 | Carbamidomethylation |
| K.SGDAAIVDM(+15.99)VPGKPM(+15.99)C(+57.02)VESFSDYPPLGR.F | Y | 117.01 | 3026.3823 | 28 | 4.8 | 1009.8062 | 3 | 99.59 | 1 | 27867 | ANa\_10.raw | 1.65E7 | 3.77E6 | 8.31E6 |  | 8.77E5 | 1.21E6 | 2.19E6 | 4.27E6 | 9.2E7 | 2.43E6 | 14 | 2 | 1 | 2 | 0 | 1 | 1 | 1 | 2 | 3 | 1 | 396 | 423 | Oxidation (M); Carbamidomethylation |
| R.VETGVLKPGMVVTFAPVNVTTEVK.S | Y | 110.47 | 2514.3767 | 24 | 6.0 | 1258.2031 | 2 | 107.83 | 9 | 30312 | Ana\_08.raw | 1.39E7 | 8.29E6 |  | 4.54E6 |  | 8.87E5 | 8.79E5 | 2.1E6 | 8.54E7 | 8.98E6 | 10 | 1 | 1 | 0 | 1 | 0 | 1 | 1 | 1 | 3 | 1 | 267 | 290 |  |
| K.YYVTIIDAPGHR.D | Y | 98.11 | 1403.7197 | 12 | 2.1 | 468.9149 | 3 | 67.61 | 3 | 19226 | Ana\_02.raw | 7.23E7 | 8.36E7 | 9.97E7 | 5.59E7 | 1.14E8 | 6.96E7 | 6.47E7 | 6.19E7 | 1.22E8 | 1.29E8 | 37 | 3 | 3 | 2 | 3 | 5 | 3 | 3 | 5 | 5 | 5 | 85 | 96 |  |
| K.STTTGHLIYK.C | N | 88.63 | 1119.5924 | 10 | 0.7 | 560.8039 | 2 | 28.40 | 3 | 7637 | Ana\_02.raw | 1.38E8 | 1.88E8 | 2.14E8 | 9.45E7 | 1.5E8 | 1.16E8 | 1.36E8 | 7.12E7 | 6.18E7 | 1.88E8 | 32 | 3 | 3 | 4 | 3 | 3 | 3 | 3 | 2 | 5 | 3 | 21 | 30 |  |
| K.M(+15.99)DSTEPPYSQK.R | Y | 82.58 | 1297.5496 | 11 | 1.4 | 649.7830 | 2 | 25.72 | 3 | 6792 | Ana\_02.raw | 1.03E8 | 1.32E8 | 1.29E8 | 6.7E7 | 1.05E8 | 8.93E7 | 8.17E7 | 8.69E7 | 7.49E7 | 4.08E7 | 32 | 2 | 4 | 3 | 3 | 4 | 3 | 4 | 4 | 3 | 2 | 155 | 165 | Oxidation (M) |
| R.EHALLAYTLGVK.Q | N | 82.06 | 1313.7343 | 12 | 1.0 | 438.9191 | 3 | 77.36 | 3 | 21905 | Ana\_02.raw | 5.26E6 | 3.72E6 | 4.64E6 | 2.82E6 | 5.59E6 | 4.35E6 | 3.01E6 |  | 6.02E6 | 4.1E6 | 12 | 1 | 1 | 1 | 1 | 1 | 1 | 2 | 0 | 2 | 2 | 135 | 146 |  |
| R.LPLQDVYK.I | N | 67.48 | 974.5436 | 8 | 5.0 | 488.2815 | 2 | 62.59 | 1 | 17742 | ANa\_10.raw | 2.01E8 | 1.85E8 | 1.64E8 | 7.17E7 | 1.33E8 | 1E8 | 8.03E7 | 8.38E7 | 3.21E8 | 1.9E8 | 36 | 4 | 4 | 4 | 2 | 4 | 3 | 2 | 3 | 6 | 4 | 248 | 255 |  |
| K.MDSTEPPYSQK.R | Y | 65.37 | 1281.5547 | 11 | 2.7 | 641.7864 | 2 | 31.60 | 9 | 8363 | Ana\_08.raw | 1.4E7 | 1.55E7 |  | 3.11E6 |  | 6.06E6 |  | 3.07E6 | 1.7E7 | 1.76E7 | 7 | 1 | 1 | 0 | 1 | 0 | 1 | 0 | 1 | 1 | 1 | 155 | 165 |  |
| K.THINIVVIGHVDSGK.S | N | 65.22 | 1587.8733 | 15 | 2.1 | 530.2995 | 3 | 60.78 | 2 | 17296 | Ana\_01.raw |  | 5.35E5 |  |  | 4.05E6 |  |  |  |  |  | 2 | 0 | 1 | 0 | 0 | 1 | 0 | 0 | 0 | 0 | 0 | 6 | 20 |  |
| K.IGGIGTVPVGR.V | N | 63.02 | 1024.6029 | 11 | 5.0 | 513.3113 | 2 | 57.49 | 1 | 16237 | ANa\_10.raw | 2.78E8 | 3.1E8 | 3.15E8 | 1.16E8 | 2.47E8 | 2.21E8 | 1.68E8 | 1.86E8 | 3.85E8 | 1.76E8 | 60 | 7 | 7 | 8 | 3 | 7 | 4 | 5 | 7 | 7 | 5 | 256 | 266 |  |
| K.EVSTYIK.K | Y | 58.49 | 838.4436 | 7 | 1.4 | 420.2296 | 2 | 33.51 | 3 | 9169 | Ana\_02.raw | 1.11E8 | 1.22E8 | 1.31E8 | 6.11E7 | 1.15E8 | 1.01E8 | 9.43E7 | 8.24E7 | 1.45E8 | 1.05E8 | 26 | 2 | 2 | 2 | 2 | 4 | 2 | 3 | 4 | 2 | 3 | 173 | 179 |  |
| K.RYEEIVK.E | Y | 50.25 | 935.5076 | 7 | 3.0 | 312.8441 | 3 | 25.51 | 2 | 6594 | Ana\_01.raw | 1.19E7 | 1.84E6 |  | 7.6E7 | 1.99E6 |  |  |  | 2.77E6 | 2.54E7 | 9 | 2 | 2 | 0 | 2 | 1 | 0 | 0 | 0 | 1 | 1 | 166 | 172 |  |
| K.IGYNPDTVAFVPISGWNGDNM(+15.99)LEPSANM(+15.99)PWFK.G | Y | 47.89 | 3598.6536 | 32 | 5.0 | 1200.5645 | 3 | 131.97 | 7 | 36341 | Ana\_06.raw |  |  |  |  |  | 3.43E6 | 3.32E6 |  |  |  | 2 | 0 | 0 | 0 | 0 | 0 | 1 | 1 | 0 | 0 | 0 | 181 | 212 | Oxidation (M) |
| R.YEEIVK.E | Y | 38.96 | 779.4065 | 6 | 3.6 | 390.7119 | 2 | 28.38 | 9 | 7401 | Ana\_08.raw | 1.11E8 | 1.06E8 | 1.15E8 | 5.39E7 | 9.12E7 | 8.55E7 | 7.87E7 | 7.56E7 | 1.39E8 | 1.13E8 | 24 | 3 | 2 | 2 | 2 | 3 | 1 | 3 | 3 | 2 | 3 | 167 | 172 |  |
| K.QLIVGVNK.M | N | 38.34 | 869.5334 | 8 | 5.6 | 435.7764 | 2 | 39.66 | 9 | 10867 | Ana\_08.raw | 1.73E7 | 1.45E7 | 2.07E7 | 1.11E7 | 1.99E7 | 1.91E7 | 1.6E7 | 1.74E7 | 1.34E7 | 8.62E6 | 15 | 2 | 1 | 1 | 2 | 2 | 1 | 2 | 2 | 1 | 1 | 147 | 154 |  |
| R.QTVAVGVIK.A | N | 33.40 | 913.5596 | 9 | 4.3 | 457.7891 | 2 | 44.30 | 10 | 12366 | Ana\_09.raw |  | 2.94E7 | 3.57E7 | 1.71E7 |  |  |  | 2.96E7 | 4.31E7 | 1.88E7 | 6 | 0 | 1 | 1 | 1 | 0 | 0 | 0 | 1 | 1 | 1 | 431 | 439 |  |
| R.TIEKFEK.E | N | 33.38 | 893.4858 | 7 | 4.1 | 447.7520 | 2 | 23.34 | 1 | 5887 | ANa\_10.raw | 3.29E6 | 9.7E5 |  |  |  |  |  |  |  | 2.53E6 | 3 | 1 | 1 | 0 | 0 | 0 | 0 | 0 | 0 | 0 | 1 | 38 | 44 |  |
| total 21 peptides |  |  |  |  |  |  |  |  |  |  |  |  |  |  |  |  |  |  |  |  |  |  |  |  |  |  |  |  |  |  |  |  |  |  |  |
| --- | --- | --- | --- | --- | --- | --- | --- | --- | --- | --- | --- | --- | --- | --- | --- | --- | --- | --- | --- | --- | --- | --- | --- | --- | --- | --- | --- | --- | --- | --- | --- | --- | --- | --- | --- |

Best Unique PSM (Scan F3:33877, m/z=781.4191, z=4, RT=123.30, ppm=1.1):

sp|Q64727|VINC\_MOUSE

back to list

  

| Protein Coverage
| Supporting Peptides
| Best Unique PSM
|

Protein Coverage:

Supporting Peptides:

| Peptide | Uniq | -10lgP | Mass | Length | ppm | m/z | z | RT | Fraction | Scan | Source File | Area 10 | Area 01 | Area 02 | Area 03 | Area 04 | Area 05 | Area 06 | Area 07 | Area 08 | Area 09 | #Spec | #Spec 10 | #Spec 01 | #Spec 02 | #Spec 03 | #Spec 04 | #Spec 05 | #Spec 06 | #Spec 07 | #Spec 08 | #Spec 09 | Start | End | PTM |
| --- | --- | --- | --- | --- | --- | --- | --- | --- | --- | --- | --- | --- | --- | --- | --- | --- | --- | --- | --- | --- | --- | --- | --- | --- | --- | --- | --- | --- | --- | --- | --- | --- | --- | --- | --- |
| R.GILSGTSDLLLTFDEAEVR.K | Y | 118.87 | 2035.0472 | 19 | 2.7 | 1018.5336 | 2 | 132.23 | 6 | 36757 | Ana\_05.raw |  | 1.94E6 | 1.61E6 |  | 1.32E6 | 2.72E6 | 1.29E6 | 6.22E5 | 2.83E6 |  | 7 | 0 | 1 | 1 | 0 | 1 | 1 | 1 | 1 | 1 | 0 | 114 | 132 |  |
| R.DPNASPGDAGEQAIR.Q | Y | 105.55 | 1496.6855 | 15 | 4.9 | 749.3537 | 2 | 39.33 | 3 | 10947 | Ana\_02.raw |  |  | 1.97E7 | 6.73E6 | 1.42E7 | 1.88E7 |  |  | 1.6E7 | 1.63E7 | 6 | 0 | 0 | 1 | 1 | 1 | 1 | 0 | 0 | 1 | 1 | 286 | 300 |  |
| R.TIESILEPVAQQISHLVIMHEEGEVDGK.A | Y | 105.47 | 3100.5750 | 28 | 5.3 | 776.1552 | 4 | 135.91 | 6 | 37772 | Ana\_05.raw |  | 7.49E5 |  |  |  | 1.43E6 |  |  | 6.7E6 | 2.14E6 | 4 | 0 | 1 | 0 | 0 | 0 | 1 | 0 | 0 | 1 | 1 | 8 | 35 |  |
| R.TIESILEPVAQQISHLVIM(+15.99)HEEGEVDGK.A | Y | 102.51 | 3116.5698 | 28 | 2.9 | 780.1520 | 4 | 134.95 | 9 | 37313 | Ana\_08.raw | 7.23E6 | 1.02E7 | 4.98E6 | 1.61E6 | 7.96E6 |  | 1.68E6 | 1.52E6 | 1.07E7 | 7.68E6 | 10 | 1 | 2 | 1 | 1 | 1 | 0 | 1 | 1 | 1 | 1 | 8 | 35 | Oxidation (M) |
| R.VLQLTSWDEDAWASK.D | Y | 88.98 | 1747.8417 | 15 | -0.8 | 874.9274 | 2 | 107.13 | 3 | 29730 | Ana\_02.raw | 2.41E6 | 5.35E6 | 1.35E6 |  | 6.27E5 |  | 3.47E5 |  | 1.46E7 |  | 6 | 1 | 1 | 1 | 0 | 1 | 0 | 1 | 0 | 1 | 0 | 247 | 261 |  |
| R.VDQLTAQLADLAAR.G | Y | 88.49 | 1483.7994 | 14 | 5.7 | 742.9112 | 2 | 101.97 | 5 | 28531 | Ana\_04.raw | 9.48E6 | 7.8E6 | 3.37E6 | 4.48E5 | 6.02E6 | 9.06E5 | 1.26E6 | 7.01E5 | 1.72E7 | 5.08E6 | 10 | 1 | 1 | 1 | 1 | 1 | 1 | 1 | 1 | 1 | 1 | 548 | 561 |  |
| K.AQQVSQGLDVLTAK.V | Y | 84.11 | 1456.7886 | 14 | 2.5 | 729.4034 | 2 | 71.50 | 6 | 20520 | Ana\_05.raw | 9.63E6 | 9.56E6 | 7.99E6 | 2.52E6 | 5.36E6 | 8.69E6 | 3.03E6 | 4.83E6 | 2.02E7 | 9.81E6 | 10 | 1 | 1 | 1 | 1 | 1 | 1 | 1 | 1 | 1 | 1 | 353 | 366 |  |
| K.LLAVAATAPPDAPNREEVFDER.A | Y | 81.83 | 2380.2021 | 22 | 7.0 | 794.4136 | 3 | 83.79 | 2 | 23755 | Ana\_01.raw | 3.08E6 | 3.88E6 |  |  | 5.05E6 | 6.25E6 | 2.11E6 | 1.01E6 | 1.89E7 | 3.94E6 | 8 | 1 | 1 | 0 | 0 | 1 | 1 | 1 | 1 | 1 | 1 | 608 | 629 |  |
| K.AIPDLTAPVAAVQAAVSNLVR.V | Y | 79.10 | 2075.1738 | 21 | 1.7 | 1038.5959 | 2 | 136.97 | 3 | 37443 | Ana\_02.raw | 1.16E7 | 1.53E6 | 1.22E6 |  |  | 4.75E5 |  | 9.7E5 | 6.92E6 | 5.54E6 | 15 | 2 | 1 | 1 | 0 | 0 | 1 | 0 | 2 | 5 | 3 | 36 | 56 |  |
| K.ETVQTTEDQILK.R | Y | 74.54 | 1403.7144 | 12 | 3.9 | 702.8672 | 2 | 61.90 | 6 | 17739 | Ana\_05.raw | 4.33E6 | 6.28E6 | 4.45E6 |  |  | 3.22E6 |  | 1.45E6 | 8.61E6 | 3.83E6 | 7 | 1 | 1 | 1 | 0 | 0 | 1 | 0 | 1 | 1 | 1 | 60 | 71 |  |
| R.AANFENHSGR.L | Y | 74.50 | 1101.4951 | 10 | 0.1 | 551.7549 | 2 | 18.54 | 3 | 4446 | Ana\_02.raw |  |  | 9.1E6 | 3.59E6 | 6.89E6 | 1.04E7 | 5.57E6 | 3.69E6 |  | 1.08E6 | 13 | 0 | 0 | 2 | 2 | 2 | 2 | 2 | 2 | 0 | 1 | 630 | 639 |  |
| R.QQELTHQEHR.V | Y | 73.65 | 1304.6222 | 10 | 3.2 | 653.3204 | 2 | 17.08 | 6 | 3959 | Ana\_05.raw |  |  | 8.78E5 | 4.92E5 | 1.4E6 | 1.65E6 | 8.24E5 | 1.15E6 |  |  | 9 | 0 | 0 | 1 | 1 | 2 | 2 | 1 | 2 | 0 | 0 | 179 | 188 |  |
| R.SLGEIAALTSK.L | Y | 69.82 | 1088.6077 | 11 | 2.2 | 545.3123 | 2 | 75.16 | 6 | 21554 | Ana\_05.raw | 2.2E6 | 2.69E6 |  | 7.53E5 | 8.12E6 | 8.61E6 | 5.19E6 | 5.39E6 | 3.95E6 |  | 8 | 1 | 1 | 0 | 1 | 1 | 1 | 1 | 1 | 1 | 0 | 434 | 444 |  |
| K.M(+15.99)SAEINEIIR.V | Y | 68.92 | 1190.5966 | 10 | 7.0 | 596.3097 | 2 | 62.36 | 1 | 17674 | ANa\_10.raw | 7.05E6 | 4.52E6 | 5.96E6 | 1.32E6 | 1.77E6 |  | 1.88E6 | 6.28E5 | 9.3E6 | 3.46E6 | 9 | 1 | 1 | 1 | 1 | 1 | 0 | 1 | 1 | 1 | 1 | 237 | 246 | Oxidation (M) |
| R.ELTPQVISAAR.I | Y | 68.21 | 1183.6560 | 11 | 1.4 | 592.8361 | 2 | 59.22 | 3 | 16843 | Ana\_02.raw | 1.54E7 | 1.5E7 | 1.49E7 | 5.72E6 | 9.54E6 | 1.35E7 | 6.85E6 | 7.86E6 | 2.61E7 | 1.19E7 | 10 | 1 | 1 | 1 | 1 | 1 | 1 | 1 | 1 | 1 | 1 | 670 | 680 |  |
| R.WIDNPTVDDR.G | Y | 65.25 | 1229.5676 | 10 | 0.1 | 615.7911 | 2 | 57.92 | 6 | 16558 | Ana\_05.raw | 1.71E6 | 2.26E6 | 5.16E5 | 9.37E5 | 2.15E6 | 7.36E6 | 3.51E6 | 2.27E6 | 1.21E5 | 4.22E6 | 10 | 1 | 1 | 1 | 1 | 1 | 1 | 1 | 1 | 1 | 1 | 503 | 512 |  |
| K.AVAGNISDPGLQK.S | Y | 63.74 | 1268.6724 | 13 | 6.3 | 635.3475 | 2 | 43.94 | 9 | 12196 | Ana\_08.raw | 1.05E7 | 9.4E6 | 1.02E7 | 2.9E6 | 6.63E6 | 8.96E6 | 4.38E6 | 5.36E6 | 4.43E6 | 8.81E6 | 10 | 1 | 1 | 1 | 1 | 1 | 1 | 1 | 1 | 1 | 1 | 803 | 815 |  |
| K.SLLDASEEAIKK.D | Y | 62.66 | 1302.7030 | 12 | 5.3 | 652.3622 | 2 | 65.44 | 2 | 18652 | Ana\_01.raw | 6.23E6 | 3.86E6 | 1.57E6 | 3.31E6 | 4.52E6 | 6.47E6 | 3.55E5 | 3.8E6 | 1.15E7 | 8.3E6 | 13 | 1 | 2 | 1 | 2 | 1 | 1 | 1 | 1 | 1 | 2 | 721 | 732 |  |
| R.ALASQLQDSLK.D | Y | 58.52 | 1172.6400 | 11 | 4.7 | 587.3300 | 2 | 68.40 | 4 | 19033 | Ana\_03.raw |  | 3.74E6 |  | 2.18E6 |  |  | 1.47E6 |  | 1.25E7 |  | 4 | 0 | 1 | 0 | 1 | 0 | 0 | 1 | 0 | 1 | 0 | 571 | 581 |  |
| K.SFLDSGYR.I | Y | 57.39 | 943.4399 | 8 | 3.3 | 472.7288 | 2 | 60.87 | 6 | 17432 | Ana\_05.raw | 9.24E6 | 9.55E6 | 1.06E7 | 7.83E6 | 1.07E7 | 1.56E7 |  |  | 4.42E6 | 7E6 | 9 | 1 | 1 | 2 | 1 | 1 | 1 | 0 | 0 | 1 | 1 | 816 | 823 |  |
| R.GLVAEGHR.L | Y | 52.43 | 837.4457 | 8 | 2.7 | 419.7312 | 2 | 19.82 | 6 | 4866 | Ana\_05.raw | 1.44E7 | 1.32E7 |  | 3.87E6 |  | 1.43E7 | 5.63E6 | 5.59E6 |  | 1.65E7 | 7 | 1 | 1 | 0 | 1 | 0 | 1 | 1 | 1 | 0 | 1 | 521 | 528 |  |
| K.GNDIIAAAK.R | Y | 51.27 | 871.4763 | 9 | 3.5 | 436.7469 | 2 | 34.08 | 6 | 9361 | Ana\_05.raw |  |  | 9.35E6 | 3.78E6 |  | 8.65E6 | 6.39E6 | 5.56E6 |  | 5.92E6 | 6 | 0 | 0 | 1 | 1 | 0 | 1 | 1 | 1 | 0 | 1 | 916 | 924 |  |
| R.TDAGFTLR.W | Y | 51.10 | 879.4450 | 8 | 2.5 | 440.7309 | 2 | 51.49 | 7 | 14483 | Ana\_06.raw |  | 1.9E7 | 1.74E7 | 9.91E6 | 2.19E7 |  | 1.49E7 |  | 6.7E6 | 1.14E7 | 7 | 0 | 1 | 1 | 1 | 1 | 0 | 1 | 0 | 1 | 1 | 1050 | 1057 |  |
| K.M(+15.99)TGLVDEAIDTK.S | Y | 50.84 | 1307.6278 | 12 | 3.0 | 654.8231 | 2 | 62.30 | 5 | 17749 | Ana\_04.raw |  |  |  |  | 3.24E6 | 0 |  |  | 1.18E6 |  | 3 | 0 | 0 | 0 | 0 | 1 | 1 | 0 | 0 | 1 | 0 | 709 | 720 | Oxidation (M) |
| K.STVEGIQASVK.T | Y | 49.31 | 1117.5979 | 11 | 7.2 | 559.8102 | 2 | 43.26 | 2 | 12067 | Ana\_01.raw |  | 1.2E7 |  |  |  |  |  | 7.26E6 |  |  | 2 | 0 | 1 | 0 | 0 | 0 | 0 | 0 | 1 | 0 | 0 | 656 | 666 |  |
| K.VGELC(+57.02)AGK.E | Y | 49.13 | 832.4113 | 8 | 4.1 | 417.2146 | 2 | 24.64 | 8 | 6240 | Ana\_07.raw |  |  | 8.12E6 |  |  | 1.86E6 |  | 1.72E6 |  |  | 3 | 0 | 0 | 1 | 0 | 0 | 1 | 0 | 1 | 0 | 0 | 309 | 316 | Carbamidomethylation |
| R.DYLIDGSR.G | Y | 48.30 | 937.4505 | 8 | 0.8 | 469.7329 | 2 | 48.58 | 3 | 13728 | Ana\_02.raw |  | 9.71E6 | 7.31E6 |  | 2.1E6 |  |  | 1.41E6 | 8.37E6 |  | 6 | 0 | 2 | 1 | 0 | 1 | 0 | 0 | 1 | 1 | 0 | 106 | 113 |  |
| K.AAVHLEGK.I | Y | 46.30 | 823.4552 | 8 | 1.7 | 412.7356 | 2 | 19.64 | 3 | 4809 | Ana\_02.raw |  |  | 7.27E6 | 2.2E6 | 4.71E6 | 6.83E6 | 2.72E6 | 2.7E6 |  | 5.01E6 | 7 | 0 | 0 | 1 | 1 | 1 | 1 | 1 | 1 | 0 | 1 | 489 | 496 |  |
| K.M(+15.99)LGQM(+15.99)TDQVADLR.A | Y | 46.19 | 1508.6963 | 13 | 6.9 | 755.3607 | 2 | 56.52 | 1 | 15954 | ANa\_10.raw | 2.55E6 |  |  |  |  |  |  |  |  |  | 2 | 2 | 0 | 0 | 0 | 0 | 0 | 0 | 0 | 0 | 0 | 327 | 339 | Oxidation (M) |
| K.SLLDASEEAIK.K | Y | 45.94 | 1174.6080 | 11 | 1.8 | 588.3124 | 2 | 74.96 | 2 | 21329 | Ana\_01.raw |  | 0 |  |  | 1.08E5 |  |  |  |  |  | 3 | 1 | 1 | 0 | 0 | 1 | 0 | 0 | 0 | 0 | 0 | 721 | 731 |  |
| R.IPTISTQLK.I | Y | 45.15 | 999.5964 | 9 | 6.1 | 500.8086 | 2 | 60.75 | 1 | 17199 | ANa\_10.raw | 5.6E6 | 4.89E6 |  |  |  |  |  |  |  | 4.2E6 | 3 | 1 | 1 | 0 | 0 | 0 | 0 | 0 | 0 | 0 | 1 | 988 | 996 |  |
| K.ELLPVLISAM(+15.99)K.I | Y | 45.13 | 1228.7101 | 11 | 6.1 | 615.3661 | 2 | 94.32 | 9 | 26717 | Ana\_08.raw |  |  |  |  |  |  |  |  | 1.29E6 |  | 1 | 0 | 0 | 0 | 0 | 0 | 0 | 0 | 0 | 1 | 0 | 200 | 210 | Oxidation (M) |
| K.TISPM(+15.99)VM(+15.99)DAK.A | Y | 45.05 | 1123.5254 | 10 | 2.5 | 562.7714 | 2 | 28.07 | 9 | 7304 | Ana\_08.raw | 1.16E7 |  |  |  |  |  |  |  | 1.39E7 |  | 2 | 1 | 0 | 0 | 0 | 0 | 0 | 0 | 0 | 1 | 0 | 793 | 802 | Oxidation (M) |
| R.ALASIDSK.L | Y | 44.73 | 803.4388 | 8 | -0.3 | 402.7266 | 2 | 26.44 | 3 | 7017 | Ana\_02.raw | 1.43E7 |  | 2.09E7 | 1.03E7 | 9.61E6 | 1.88E7 | 1.16E7 | 8.47E6 | 7.59E6 | 1.8E7 | 9 | 1 | 0 | 1 | 1 | 1 | 1 | 1 | 1 | 1 | 1 | 269 | 276 |  |
| K.NQGIEEALK.N | Y | 42.85 | 1000.5189 | 9 | 4.1 | 501.2688 | 2 | 43.76 | 5 | 12254 | Ana\_04.raw |  |  |  | 5.5E6 | 7.24E6 | 1.39E7 | 9.09E6 |  |  |  | 4 | 0 | 0 | 0 | 1 | 1 | 1 | 1 | 0 | 0 | 0 | 220 | 228 |  |
| R.GVGQAAIR.G | Y | 42.52 | 770.4399 | 8 | 4.3 | 386.2289 | 2 | 23.32 | 1 | 5882 | ANa\_10.raw | 1.37E7 |  |  |  |  |  |  |  |  |  | 1 | 1 | 0 | 0 | 0 | 0 | 0 | 0 | 0 | 0 | 0 | 513 | 520 |  |
| K.IAELC(+57.02)DDPK.E | Y | 38.79 | 1059.4906 | 9 | 1.7 | 530.7535 | 2 | 39.49 | 7 | 10835 | Ana\_06.raw |  | 4.33E6 |  | 1.85E6 | 2.9E6 |  | 3.27E6 |  |  | 3.02E6 | 5 | 0 | 1 | 0 | 1 | 1 | 0 | 1 | 0 | 0 | 1 | 418 | 426 | Carbamidomethylation |
| R.EEVFDER.A | Y | 37.94 | 922.4032 | 7 | 0.0 | 462.2089 | 2 | 38.94 | 6 | 10825 | Ana\_05.raw |  | 2.85E6 | 4.06E6 |  | 3.45E6 | 4.44E6 | 1.24E6 |  |  |  | 5 | 0 | 1 | 1 | 0 | 1 | 1 | 1 | 0 | 0 | 0 | 623 | 629 |  |
| R.LANVM(+15.99)M(+15.99)GPYR.Q | Y | 37.65 | 1182.5525 | 10 | 2.7 | 592.2851 | 2 | 42.76 | 8 | 11661 | Ana\_07.raw |  |  |  |  |  |  |  | 9.11E6 |  |  | 1 | 0 | 0 | 0 | 0 | 0 | 0 | 0 | 1 | 0 | 0 | 529 | 538 | Oxidation (M) |
| R.EILGTC(+57.02)K.M | Y | 35.76 | 819.4160 | 7 | 2.1 | 410.7161 | 2 | 27.13 | 5 | 7205 | Ana\_04.raw |  |  |  |  | 6.79E6 |  |  |  |  | 5.51E6 | 2 | 0 | 0 | 0 | 0 | 1 | 0 | 0 | 0 | 0 | 1 | 320 | 326 | Carbamidomethylation |
| R.DM(+15.99)PPAFIK.V | Y | 31.85 | 933.4630 | 8 | 2.1 | 467.7397 | 2 | 50.00 | 3 | 14144 | Ana\_02.raw | 3.33E6 | 7.8E6 | 3.63E6 |  |  |  |  |  | 9.84E6 |  | 4 | 1 | 1 | 1 | 0 | 0 | 0 | 0 | 0 | 1 | 0 | 73 | 80 | Oxidation (M) |
| R.NPGNQAAYEHFETM(+15.99)K.N | Y | 30.93 | 1751.7573 | 15 | 3.3 | 584.9283 | 3 | 46.63 | 1 | 13035 | ANa\_10.raw | 4.56E6 |  |  |  |  |  |  |  | 6.66E6 |  | 2 | 1 | 0 | 0 | 0 | 0 | 0 | 0 | 0 | 1 | 0 | 685 | 699 | Oxidation (M) |
| K.DEEFPEQK.A | Y | 29.64 | 1020.4399 | 8 | 5.1 | 511.2298 | 2 | 32.53 | 10 | 8821 | Ana\_09.raw |  |  |  |  |  |  |  |  |  | 9.46E6 | 1 | 0 | 0 | 0 | 0 | 0 | 0 | 0 | 0 | 0 | 1 | 882 | 889 |  |
| R.ILGAVAK.V | Y | 28.38 | 670.4377 | 7 | 5.1 | 336.2278 | 2 | 27.37 | 2 | 7200 | Ana\_01.raw |  | 5.41E6 | 5.4E6 |  |  | 5.91E6 | 3.43E6 | 2.77E6 |  | 4.15E6 | 6 | 0 | 1 | 1 | 0 | 0 | 1 | 1 | 1 | 0 | 1 | 824 | 830 |  |
| R.QILDEAGK.V | Y | 23.35 | 872.4603 | 8 | 2.2 | 437.2384 | 2 | 32.28 | 7 | 8676 | Ana\_06.raw |  |  |  |  |  |  | 2.49E6 |  |  |  | 1 | 0 | 0 | 0 | 0 | 0 | 0 | 1 | 0 | 0 | 0 | 301 | 308 |  |
| R.ILLVAK.R | N | 22.63 | 655.4632 | 6 | 2.6 | 328.7397 | 2 | 43.68 | 5 | 12230 | Ana\_04.raw |  |  |  | 1.33E6 | 2.22E6 |  |  |  |  |  | 2 | 0 | 0 | 0 | 1 | 1 | 0 | 0 | 0 | 0 | 0 | 763 | 768 |  |
| R.ARGQGASPVAMQK.A | Y | 22.22 | 1299.6718 | 13 | 3.0 | 650.8451 | 2 | 62.64 | 9 | 17840 | Ana\_08.raw |  |  |  |  |  |  |  |  | 2.93E7 |  | 1 | 0 | 0 | 0 | 0 | 0 | 0 | 0 | 0 | 1 | 0 | 340 | 352 |  |
| total 47 peptides |  |  |  |  |  |  |  |  |  |  |  |  |  |  |  |  |  |  |  |  |  |  |  |  |  |  |  |  |  |  |  |  |  |  |  |
| --- | --- | --- | --- | --- | --- | --- | --- | --- | --- | --- | --- | --- | --- | --- | --- | --- | --- | --- | --- | --- | --- | --- | --- | --- | --- | --- | --- | --- | --- | --- | --- | --- | --- | --- | --- |

Best Unique PSM (Scan F6:36757, m/z=1018.5336, z=2, RT=132.23, ppm=2.7):

sp|Q6PDN3|MYLK\_MOUSE

back to list

  

| Protein Coverage
| Supporting Peptides
| Best Unique PSM
|

Protein Coverage:

Supporting Peptides:

| Peptide | Uniq | -10lgP | Mass | Length | ppm | m/z | z | RT | Fraction | Scan | Source File | Area 10 | Area 01 | Area 02 | Area 03 | Area 04 | Area 05 | Area 06 | Area 07 | Area 08 | Area 09 | #Spec | #Spec 10 | #Spec 01 | #Spec 02 | #Spec 03 | #Spec 04 | #Spec 05 | #Spec 06 | #Spec 07 | #Spec 08 | #Spec 09 | Start | End | PTM |
| --- | --- | --- | --- | --- | --- | --- | --- | --- | --- | --- | --- | --- | --- | --- | --- | --- | --- | --- | --- | --- | --- | --- | --- | --- | --- | --- | --- | --- | --- | --- | --- | --- | --- | --- | --- |
| K.LESEDDVSQAFLEAVAEEKPHVKPYFSK.T | Y | 129.78 | 3191.5662 | 28 | 3.7 | 798.9018 | 4 | 115.30 | 9 | 32245 | Ana\_08.raw | 9.53E6 | 3.53E7 |  | 1.05E6 | 1.85E7 | 1.37E6 | 4.25E6 | 1.29E7 | 4.1E6 | 6.18E6 | 10 | 1 | 2 | 0 | 1 | 1 | 1 | 1 | 1 | 1 | 1 | 1808 | 1835 |  |
| R.STSFNVQDLLPDR.E | Y | 102.79 | 1490.7365 | 13 | 4.1 | 746.3785 | 2 | 107.31 | 6 | 30186 | Ana\_05.raw | 1.19E7 | 4.32E7 | 3.08E7 | 1.56E7 | 2.1E7 | 2.79E7 | 1.44E7 | 2.07E7 | 2.69E7 | 1.54E7 | 31 | 2 | 4 | 4 | 3 | 4 | 3 | 2 | 5 | 2 | 2 | 1408 | 1420 |  |
| K.ANIVM(+15.99)VLEIVSGGELFER.I | Y | 94.66 | 1991.0397 | 18 | 4.8 | 664.6904 | 3 | 137.81 | 6 | 38271 | Ana\_05.raw |  | 2.21E5 | 4.03E5 | 4.89E4 | 5.54E5 | 7.03E5 | 2.77E5 |  |  |  | 7 | 0 | 1 | 1 | 1 | 1 | 2 | 1 | 0 | 0 | 0 | 1554 | 1571 | Oxidation (M) |
| K.IEGYPDPEVVWFK.D | Y | 93.97 | 1577.7765 | 13 | 2.6 | 789.8976 | 2 | 115.48 | 6 | 32300 | Ana\_05.raw | 7.58E7 | 1.38E8 | 9.64E7 | 4.56E7 | 5.04E7 | 1.03E8 | 3.43E7 | 6.37E7 | 1.37E8 | 4.48E7 | 46 | 6 | 6 | 7 | 3 | 4 | 4 | 3 | 5 | 5 | 3 | 1854 | 1866 |  |
| K.SSLPPVLGTESDATVK.K | Y | 90.21 | 1599.8356 | 16 | 6.9 | 800.9306 | 2 | 72.85 | 1 | 20645 | ANa\_10.raw | 7.05E6 | 1.38E7 | 8.57E6 | 3.05E6 | 1.12E7 | 1.03E7 | 3.92E6 | 7.71E6 | 5.22E6 | 1.04E6 | 13 | 1 | 2 | 1 | 1 | 1 | 2 | 1 | 1 | 2 | 1 | 1230 | 1245 |  |
| K.RPESQGSAPVFK.E | Y | 84.30 | 1301.6727 | 12 | 2.7 | 434.8994 | 3 | 30.11 | 6 | 8163 | Ana\_05.raw | 1.08E7 | 2.07E7 | 3.41E7 | 1.04E7 | 1.97E7 | 3.42E7 | 9.2E6 | 1.77E7 | 1.09E7 | 6.2E6 | 16 | 1 | 1 | 2 | 2 | 2 | 2 | 2 | 2 | 1 | 1 | 1112 | 1123 |  |
| R.DLEVVEGSAAR.F | Y | 83.43 | 1144.5724 | 11 | 3.7 | 573.2956 | 2 | 51.38 | 7 | 14452 | Ana\_06.raw | 1.05E8 | 1.5E8 | 1.27E8 | 1.26E6 | 1.04E8 | 1.34E8 | 8.47E7 | 4.82E6 | 1.28E8 | 7.48E7 | 21 | 3 | 3 | 2 | 1 | 2 | 2 | 1 | 2 | 3 | 2 | 1839 | 1849 |  |
| K.SSTGSPTSPINAEK.L | Y | 82.20 | 1374.6626 | 14 | 3.5 | 688.3410 | 2 | 34.13 | 6 | 9376 | Ana\_05.raw | 1.64E7 | 3.41E7 | 8.51E5 | 9.87E6 | 1.49E7 | 3.31E7 | 1.52E7 | 1.45E7 | 1.6E7 | 2.41E6 | 10 | 1 | 1 | 1 | 1 | 1 | 1 | 1 | 1 | 1 | 1 | 1794 | 1807 |  |
| R.IIDEDFELTER.E | Y | 77.19 | 1378.6616 | 11 | 2.5 | 690.3398 | 2 | 78.75 | 3 | 22282 | Ana\_02.raw | 5.02E6 | 1.39E7 | 1.08E7 | 3.16E6 | 7.55E6 |  |  |  | 1.22E7 | 5.42E6 | 7 | 1 | 1 | 1 | 1 | 1 | 0 | 0 | 0 | 1 | 1 | 1572 | 1582 |  |
| K.VSDVYDIEER.L | Y | 76.84 | 1223.5669 | 10 | 6.4 | 612.7946 | 2 | 57.14 | 2 | 16225 | Ana\_01.raw | 2.05E7 | 3.08E7 | 3.23E7 | 1.29E7 | 2.3E7 | 3.17E7 | 1.7E7 | 1.8E7 | 2.53E7 | 1.69E7 | 17 | 1 | 2 | 1 | 2 | 2 | 1 | 2 | 2 | 3 | 1 | 1482 | 1491 |  |
| R.KSSTGSPTSPINAEK.L | Y | 76.29 | 1502.7576 | 15 | 2.3 | 752.3878 | 2 | 25.68 | 2 | 6650 | Ana\_01.raw | 1.69E6 | 3.23E6 |  |  |  | 3.62E6 |  | 3.37E5 | 3.56E6 | 9.22E5 | 6 | 1 | 1 | 0 | 0 | 0 | 1 | 0 | 1 | 1 | 1 | 1793 | 1807 |  |
| K.AAM(+15.99)PPQIIQFPEDQK.V | Y | 75.87 | 1727.8552 | 15 | 6.1 | 864.9401 | 2 | 80.81 | 9 | 23011 | Ana\_08.raw | 9.66E6 |  | 1.04E7 | 2.96E6 | 7.94E6 | 8.19E6 | 1.22E6 | 6.13E6 | 1.6E7 | 1.73E6 | 12 | 1 | 0 | 1 | 1 | 2 | 2 | 1 | 2 | 1 | 1 | 1256 | 1270 | Oxidation (M) |
| K.LQDVHVAEGEK.L | Y | 72.82 | 1223.6146 | 11 | 6.1 | 612.8183 | 2 | 31.48 | 7 | 8430 | Ana\_06.raw | 9.2E6 |  | 1.99E7 | 1.08E7 | 1.67E7 | 3.37E7 | 1.84E7 | 4.83E6 | 7.35E6 | 1.39E7 | 15 | 1 | 0 | 1 | 2 | 2 | 2 | 2 | 2 | 1 | 2 | 1126 | 1136 |  |
| K.LVQC(+57.02)VDAFEEK.A | Y | 72.53 | 1336.6333 | 11 | 1.4 | 669.3249 | 2 | 64.26 | 6 | 18452 | Ana\_05.raw |  | 5.95E6 | 8.26E6 | 3.05E6 | 4.97E6 | 8.22E6 | 3.53E6 | 4.08E6 |  | 3.07E6 | 8 | 0 | 1 | 1 | 1 | 1 | 1 | 1 | 1 | 0 | 1 | 1543 | 1553 | Carbamidomethylation |
| R.AGEPVELFGK.V | Y | 72.10 | 1045.5443 | 10 | 5.0 | 523.7820 | 2 | 71.85 | 7 | 20372 | Ana\_06.raw | 2.65E7 | 3.35E7 | 2.63E7 | 1.17E7 | 2.72E7 | 2.67E7 | 1.49E7 | 1.57E7 | 2.83E7 | 2.06E7 | 18 | 1 | 1 | 2 | 2 | 2 | 2 | 2 | 2 | 3 | 1 | 1273 | 1282 |  |
| R.TVTVNTEQK.V | Y | 67.23 | 1018.5295 | 9 | 2.0 | 510.2730 | 2 | 21.52 | 6 | 5430 | Ana\_05.raw |  |  | 6.39E6 | 1.27E7 | 2.11E7 | 1.21E7 | 1.76E7 | 1.61E7 |  |  | 6 | 0 | 0 | 1 | 1 | 1 | 1 | 1 | 1 | 0 | 0 | 1473 | 1481 |  |
| K.DFISNLLK.K | Y | 61.69 | 948.5280 | 8 | 5.1 | 475.2737 | 2 | 115.51 | 2 | 32157 | Ana\_01.raw | 1.7E7 | 3.05E7 | 2.99E7 | 1.3E7 | 2.08E7 | 3E7 | 1.34E7 | 1.76E7 | 2.42E7 | 1.34E7 | 31 | 3 | 5 | 4 | 2 | 4 | 2 | 2 | 4 | 3 | 2 | 1716 | 1723 |  |
| K.FIVLAQEGSR.F | Y | 57.46 | 1118.6084 | 10 | 0.2 | 560.3116 | 2 | 60.09 | 3 | 17098 | Ana\_02.raw | 1.16E7 | 2.13E7 | 1.9E7 | 7.23E6 | 1.36E7 | 1.92E7 | 1.02E7 | 1.21E7 | 1.64E7 | 9.68E6 | 16 | 1 | 1 | 2 | 2 | 2 | 2 | 2 | 2 | 1 | 1 | 1165 | 1174 |  |
| R.LSSM(+15.99)AM(+15.99)ISGLSGR.K | Y | 57.43 | 1340.6428 | 13 | 1.5 | 671.3297 | 2 | 53.32 | 4 | 14743 | Ana\_03.raw |  | 6.29E6 | 6.97E6 | 1.77E6 | 5.41E6 | 7.02E6 | 3.17E6 | 5.57E6 |  |  | 7 | 0 | 1 | 1 | 1 | 1 | 1 | 1 | 1 | 0 | 0 | 1780 | 1792 | Oxidation (M) |
| R.RLENAGSLK.V | Y | 55.66 | 986.5508 | 9 | 2.8 | 494.2841 | 2 | 21.98 | 6 | 5584 | Ana\_05.raw |  | 2.21E7 | 2.25E7 | 8.79E6 | 1.4E7 | 1.02E7 | 1.39E7 | 9.36E6 |  | 9.52E6 | 8 | 0 | 1 | 1 | 1 | 1 | 1 | 1 | 1 | 0 | 1 | 1633 | 1641 |  |
| K.VHSPQQVDFR.S | Y | 55.64 | 1211.6047 | 10 | 6.9 | 606.8138 | 2 | 36.88 | 2 | 10091 | Ana\_01.raw | 4.76E6 | 8.03E6 | 5.83E6 |  |  |  |  |  |  |  | 3 | 1 | 1 | 1 | 0 | 0 | 0 | 0 | 0 | 0 | 0 | 933 | 942 |  |
| R.KQIQESEHIK.V | Y | 50.41 | 1238.6619 | 10 | 4.2 | 620.3408 | 2 | 20.55 | 9 | 4802 | Ana\_08.raw |  | 2.26E6 | 2.52E5 |  |  | 1.24E6 |  |  | 1.11E6 |  | 5 | 0 | 1 | 1 | 0 | 0 | 1 | 0 | 0 | 2 | 0 | 1298 | 1307 |  |
| K.LIDFGLAR.R | Y | 50.24 | 903.5178 | 8 | 0.3 | 452.7663 | 2 | 80.72 | 3 | 22812 | Ana\_02.raw | 1.41E7 | 2.08E7 | 1.86E7 | 9.49E6 | 1.45E7 | 2.45E7 | 1.19E7 | 1.45E7 | 2.08E7 | 1.32E7 | 14 | 1 | 1 | 1 | 1 | 1 | 2 | 2 | 2 | 1 | 2 | 1625 | 1632 |  |
| R.KVHSPQQVDFR.S | Y | 49.07 | 1339.6997 | 11 | 5.5 | 447.5763 | 3 | 29.14 | 2 | 7749 | Ana\_01.raw |  | 4.73E6 |  |  |  | 3.3E6 | 2.65E6 |  |  |  | 3 | 0 | 1 | 0 | 0 | 0 | 1 | 1 | 0 | 0 | 0 | 932 | 942 |  |
| K.VAGTQPITC(+57.02)K.W | Y | 47.75 | 1073.5540 | 10 | 2.3 | 537.7855 | 2 | 25.63 | 6 | 6761 | Ana\_05.raw |  | 2.78E7 |  |  | 3.71E6 | 2.56E7 |  |  |  |  | 3 | 0 | 1 | 0 | 0 | 1 | 1 | 0 | 0 | 0 | 0 | 1283 | 1292 | Carbamidomethylation |
| K.SAETSKPAGKEEVK.E | Y | 47.30 | 1459.7518 | 14 | 3.8 | 487.5931 | 3 | 16.69 | 6 | 3833 | Ana\_05.raw |  |  |  |  |  | 6.59E5 |  |  |  |  | 1 | 0 | 0 | 0 | 0 | 0 | 1 | 0 | 0 | 0 | 0 | 1078 | 1091 |  |
| K.LTILAAR.Q | Y | 45.66 | 756.4857 | 7 | 5.0 | 379.2520 | 2 | 50.85 | 1 | 14272 | ANa\_10.raw | 7.78E6 | 1.46E7 | 1.37E7 | 5.36E6 | 9.59E6 | 1.35E7 |  |  | 1.14E7 | 6.57E6 | 15 | 2 | 2 | 2 | 2 | 2 | 2 | 0 | 0 | 1 | 2 | 1317 | 1323 |  |
| K.FGQVFR.L | Y | 45.01 | 752.3969 | 6 | 4.1 | 377.2073 | 2 | 48.14 | 6 | 13642 | Ana\_05.raw | 9.18E6 | 1.54E7 | 1.7E7 | 6.69E6 | 1.19E7 | 1.77E7 | 9.75E6 | 9.56E6 | 1.24E7 |  | 11 | 1 | 1 | 1 | 1 | 1 | 2 | 1 | 1 | 2 | 0 | 1497 | 1502 |  |
| R.LENAGSLK.V | Y | 44.09 | 830.4498 | 8 | 5.8 | 416.2346 | 2 | 24.20 | 2 | 6162 | Ana\_01.raw | 6.71E6 | 6.33E6 | 2.81E7 |  |  |  |  |  |  | 4.18E7 | 4 | 1 | 1 | 1 | 0 | 0 | 0 | 0 | 0 | 0 | 1 | 1634 | 1641 |  |
| K.AATPDFR.S | Y | 42.31 | 776.3817 | 7 | 2.2 | 389.1990 | 2 | 28.57 | 9 | 7462 | Ana\_08.raw |  |  |  |  |  |  |  |  | 7.93E6 | 4.38E6 | 2 | 0 | 0 | 0 | 0 | 0 | 0 | 0 | 0 | 1 | 1 | 963 | 969 |  |
| R.FSVSIEK.A | Y | 36.16 | 808.4330 | 7 | 5.6 | 405.2260 | 2 | 52.83 | 2 | 14933 | Ana\_01.raw |  | 1.16E7 | 1.04E7 | 5.86E6 | 1.11E7 | 1.34E7 | 8.39E6 | 7.56E6 | 7.85E6 |  | 8 | 0 | 1 | 1 | 1 | 1 | 1 | 1 | 1 | 1 | 0 | 1175 | 1181 |  |
| R.QISEGVEYIHK.Q | Y | 22.38 | 1301.6615 | 11 | 2.5 | 434.8955 | 3 | 47.70 | 5 | 13449 | Ana\_04.raw |  |  |  |  | 4.41E6 |  |  |  |  |  | 1 | 0 | 0 | 0 | 0 | 1 | 0 | 0 | 0 | 0 | 0 | 1590 | 1600 |  |
| total 32 peptides |  |  |  |  |  |  |  |  |  |  |  |  |  |  |  |  |  |  |  |  |  |  |  |  |  |  |  |  |  |  |  |  |  |  |  |
| --- | --- | --- | --- | --- | --- | --- | --- | --- | --- | --- | --- | --- | --- | --- | --- | --- | --- | --- | --- | --- | --- | --- | --- | --- | --- | --- | --- | --- | --- | --- | --- | --- | --- | --- | --- |

Best Unique PSM (Scan F9:32245, m/z=798.9018, z=4, RT=115.30, ppm=3.7):

B1B1A8|B1B1A8\_MOUSE

back to list

  

| Protein Coverage
| Supporting Peptides
| Best Unique PSM
|

Protein Coverage:

Supporting Peptides:

| Peptide | Uniq | -10lgP | Mass | Length | ppm | m/z | z | RT | Fraction | Scan | Source File | Area 10 | Area 01 | Area 02 | Area 03 | Area 04 | Area 05 | Area 06 | Area 07 | Area 08 | Area 09 | #Spec | #Spec 10 | #Spec 01 | #Spec 02 | #Spec 03 | #Spec 04 | #Spec 05 | #Spec 06 | #Spec 07 | #Spec 08 | #Spec 09 | Start | End | PTM |
| --- | --- | --- | --- | --- | --- | --- | --- | --- | --- | --- | --- | --- | --- | --- | --- | --- | --- | --- | --- | --- | --- | --- | --- | --- | --- | --- | --- | --- | --- | --- | --- | --- | --- | --- | --- |
| K.LESEDDVSQAFLEAVAEEKPHVKPYFSK.T | Y | 129.78 | 3191.5662 | 28 | 3.7 | 798.9018 | 4 | 115.30 | 9 | 32245 | Ana\_08.raw | 9.53E6 | 3.53E7 |  | 1.05E6 | 1.85E7 | 1.37E6 | 4.25E6 | 1.29E7 | 4.1E6 | 6.18E6 | 10 | 1 | 2 | 0 | 1 | 1 | 1 | 1 | 1 | 1 | 1 | 1817 | 1844 |  |
| R.STSFNVQDLLPDR.E | Y | 102.79 | 1490.7365 | 13 | 4.1 | 746.3785 | 2 | 107.31 | 6 | 30186 | Ana\_05.raw | 1.19E7 | 4.32E7 | 3.08E7 | 1.56E7 | 2.1E7 | 2.79E7 | 1.44E7 | 2.07E7 | 2.69E7 | 1.54E7 | 31 | 2 | 4 | 4 | 3 | 4 | 3 | 2 | 5 | 2 | 2 | 1417 | 1429 |  |
| K.ANIVM(+15.99)VLEIVSGGELFER.I | Y | 94.66 | 1991.0397 | 18 | 4.8 | 664.6904 | 3 | 137.81 | 6 | 38271 | Ana\_05.raw |  | 2.21E5 | 4.03E5 | 4.89E4 | 5.54E5 | 7.03E5 | 2.77E5 |  |  |  | 7 | 0 | 1 | 1 | 1 | 1 | 2 | 1 | 0 | 0 | 0 | 1563 | 1580 | Oxidation (M) |
| K.IEGYPDPEVVWFK.D | Y | 93.97 | 1577.7765 | 13 | 2.6 | 789.8976 | 2 | 115.48 | 6 | 32300 | Ana\_05.raw | 7.58E7 | 1.38E8 | 9.64E7 | 4.56E7 | 5.04E7 | 1.03E8 | 3.43E7 | 6.37E7 | 1.37E8 | 4.48E7 | 46 | 6 | 6 | 7 | 3 | 4 | 4 | 3 | 5 | 5 | 3 | 1863 | 1875 |  |
| K.SSLPPVLGTESDATVK.K | Y | 90.21 | 1599.8356 | 16 | 6.9 | 800.9306 | 2 | 72.85 | 1 | 20645 | ANa\_10.raw | 7.05E6 | 1.38E7 | 8.57E6 | 3.05E6 | 1.12E7 | 1.03E7 | 3.92E6 | 7.71E6 | 5.22E6 | 1.04E6 | 13 | 1 | 2 | 1 | 1 | 1 | 2 | 1 | 1 | 2 | 1 | 1239 | 1254 |  |
| K.RPESQGSAPVFK.E | Y | 84.30 | 1301.6727 | 12 | 2.7 | 434.8994 | 3 | 30.11 | 6 | 8163 | Ana\_05.raw | 1.08E7 | 2.07E7 | 3.41E7 | 1.04E7 | 1.97E7 | 3.42E7 | 9.2E6 | 1.77E7 | 1.09E7 | 6.2E6 | 16 | 1 | 1 | 2 | 2 | 2 | 2 | 2 | 2 | 1 | 1 | 1121 | 1132 |  |
| R.DLEVVEGSAAR.F | Y | 83.43 | 1144.5724 | 11 | 3.7 | 573.2956 | 2 | 51.38 | 7 | 14452 | Ana\_06.raw | 1.05E8 | 1.5E8 | 1.27E8 | 1.26E6 | 1.04E8 | 1.34E8 | 8.47E7 | 4.82E6 | 1.28E8 | 7.48E7 | 21 | 3 | 3 | 2 | 1 | 2 | 2 | 1 | 2 | 3 | 2 | 1848 | 1858 |  |
| K.SSTGSPTSPINAEK.L | Y | 82.20 | 1374.6626 | 14 | 3.5 | 688.3410 | 2 | 34.13 | 6 | 9376 | Ana\_05.raw | 1.64E7 | 3.41E7 | 8.51E5 | 9.87E6 | 1.49E7 | 3.31E7 | 1.52E7 | 1.45E7 | 1.6E7 | 2.41E6 | 10 | 1 | 1 | 1 | 1 | 1 | 1 | 1 | 1 | 1 | 1 | 1803 | 1816 |  |
| R.IIDEDFELTER.E | Y | 77.19 | 1378.6616 | 11 | 2.5 | 690.3398 | 2 | 78.75 | 3 | 22282 | Ana\_02.raw | 5.02E6 | 1.39E7 | 1.08E7 | 3.16E6 | 7.55E6 |  |  |  | 1.22E7 | 5.42E6 | 7 | 1 | 1 | 1 | 1 | 1 | 0 | 0 | 0 | 1 | 1 | 1581 | 1591 |  |
| K.VSDVYDIEER.L | Y | 76.84 | 1223.5669 | 10 | 6.4 | 612.7946 | 2 | 57.14 | 2 | 16225 | Ana\_01.raw | 2.05E7 | 3.08E7 | 3.23E7 | 1.29E7 | 2.3E7 | 3.17E7 | 1.7E7 | 1.8E7 | 2.53E7 | 1.69E7 | 17 | 1 | 2 | 1 | 2 | 2 | 1 | 2 | 2 | 3 | 1 | 1491 | 1500 |  |
| R.KSSTGSPTSPINAEK.L | Y | 76.29 | 1502.7576 | 15 | 2.3 | 752.3878 | 2 | 25.68 | 2 | 6650 | Ana\_01.raw | 1.69E6 | 3.23E6 |  |  |  | 3.62E6 |  | 3.37E5 | 3.56E6 | 9.22E5 | 6 | 1 | 1 | 0 | 0 | 0 | 1 | 0 | 1 | 1 | 1 | 1802 | 1816 |  |
| K.AAM(+15.99)PPQIIQFPEDQK.V | Y | 75.87 | 1727.8552 | 15 | 6.1 | 864.9401 | 2 | 80.81 | 9 | 23011 | Ana\_08.raw | 9.66E6 |  | 1.04E7 | 2.96E6 | 7.94E6 | 8.19E6 | 1.22E6 | 6.13E6 | 1.6E7 | 1.73E6 | 12 | 1 | 0 | 1 | 1 | 2 | 2 | 1 | 2 | 1 | 1 | 1265 | 1279 | Oxidation (M) |
| K.LQDVHVAEGEK.L | Y | 72.82 | 1223.6146 | 11 | 6.1 | 612.8183 | 2 | 31.48 | 7 | 8430 | Ana\_06.raw | 9.2E6 |  | 1.99E7 | 1.08E7 | 1.67E7 | 3.37E7 | 1.84E7 | 4.83E6 | 7.35E6 | 1.39E7 | 15 | 1 | 0 | 1 | 2 | 2 | 2 | 2 | 2 | 1 | 2 | 1135 | 1145 |  |
| K.LVQC(+57.02)VDAFEEK.A | Y | 72.53 | 1336.6333 | 11 | 1.4 | 669.3249 | 2 | 64.26 | 6 | 18452 | Ana\_05.raw |  | 5.95E6 | 8.26E6 | 3.05E6 | 4.97E6 | 8.22E6 | 3.53E6 | 4.08E6 |  | 3.07E6 | 8 | 0 | 1 | 1 | 1 | 1 | 1 | 1 | 1 | 0 | 1 | 1552 | 1562 | Carbamidomethylation |
| R.AGEPVELFGK.V | Y | 72.10 | 1045.5443 | 10 | 5.0 | 523.7820 | 2 | 71.85 | 7 | 20372 | Ana\_06.raw | 2.65E7 | 3.35E7 | 2.63E7 | 1.17E7 | 2.72E7 | 2.67E7 | 1.49E7 | 1.57E7 | 2.83E7 | 2.06E7 | 18 | 1 | 1 | 2 | 2 | 2 | 2 | 2 | 2 | 3 | 1 | 1282 | 1291 |  |
| R.TVTVNTEQK.V | Y | 67.23 | 1018.5295 | 9 | 2.0 | 510.2730 | 2 | 21.52 | 6 | 5430 | Ana\_05.raw |  |  | 6.39E6 | 1.27E7 | 2.11E7 | 1.21E7 | 1.76E7 | 1.61E7 |  |  | 6 | 0 | 0 | 1 | 1 | 1 | 1 | 1 | 1 | 0 | 0 | 1482 | 1490 |  |
| K.DFISNLLK.K | Y | 61.69 | 948.5280 | 8 | 5.1 | 475.2737 | 2 | 115.51 | 2 | 32157 | Ana\_01.raw | 1.7E7 | 3.05E7 | 2.99E7 | 1.3E7 | 2.08E7 | 3E7 | 1.34E7 | 1.76E7 | 2.42E7 | 1.34E7 | 31 | 3 | 5 | 4 | 2 | 4 | 2 | 2 | 4 | 3 | 2 | 1725 | 1732 |  |
| K.FIVLAQEGSR.F | Y | 57.46 | 1118.6084 | 10 | 0.2 | 560.3116 | 2 | 60.09 | 3 | 17098 | Ana\_02.raw | 1.16E7 | 2.13E7 | 1.9E7 | 7.23E6 | 1.36E7 | 1.92E7 | 1.02E7 | 1.21E7 | 1.64E7 | 9.68E6 | 16 | 1 | 1 | 2 | 2 | 2 | 2 | 2 | 2 | 1 | 1 | 1174 | 1183 |  |
| R.LSSM(+15.99)AM(+15.99)ISGLSGR.K | Y | 57.43 | 1340.6428 | 13 | 1.5 | 671.3297 | 2 | 53.32 | 4 | 14743 | Ana\_03.raw |  | 6.29E6 | 6.97E6 | 1.77E6 | 5.41E6 | 7.02E6 | 3.17E6 | 5.57E6 |  |  | 7 | 0 | 1 | 1 | 1 | 1 | 1 | 1 | 1 | 0 | 0 | 1789 | 1801 | Oxidation (M) |
| R.RLENAGSLK.V | Y | 55.66 | 986.5508 | 9 | 2.8 | 494.2841 | 2 | 21.98 | 6 | 5584 | Ana\_05.raw |  | 2.21E7 | 2.25E7 | 8.79E6 | 1.4E7 | 1.02E7 | 1.39E7 | 9.36E6 |  | 9.52E6 | 8 | 0 | 1 | 1 | 1 | 1 | 1 | 1 | 1 | 0 | 1 | 1642 | 1650 |  |
| K.VHSPQQVDFR.S | Y | 55.64 | 1211.6047 | 10 | 6.9 | 606.8138 | 2 | 36.88 | 2 | 10091 | Ana\_01.raw | 4.76E6 | 8.03E6 | 5.83E6 |  |  |  |  |  |  |  | 3 | 1 | 1 | 1 | 0 | 0 | 0 | 0 | 0 | 0 | 0 | 942 | 951 |  |
| R.KQIQESEHIK.V | Y | 50.41 | 1238.6619 | 10 | 4.2 | 620.3408 | 2 | 20.55 | 9 | 4802 | Ana\_08.raw |  | 2.26E6 | 2.52E5 |  |  | 1.24E6 |  |  | 1.11E6 |  | 5 | 0 | 1 | 1 | 0 | 0 | 1 | 0 | 0 | 2 | 0 | 1307 | 1316 |  |
| K.LIDFGLAR.R | Y | 50.24 | 903.5178 | 8 | 0.3 | 452.7663 | 2 | 80.72 | 3 | 22812 | Ana\_02.raw | 1.41E7 | 2.08E7 | 1.86E7 | 9.49E6 | 1.45E7 | 2.45E7 | 1.19E7 | 1.45E7 | 2.08E7 | 1.32E7 | 14 | 1 | 1 | 1 | 1 | 1 | 2 | 2 | 2 | 1 | 2 | 1634 | 1641 |  |
| R.KVHSPQQVDFR.S | Y | 49.07 | 1339.6997 | 11 | 5.5 | 447.5763 | 3 | 29.14 | 2 | 7749 | Ana\_01.raw |  | 4.73E6 |  |  |  | 3.3E6 | 2.65E6 |  |  |  | 3 | 0 | 1 | 0 | 0 | 0 | 1 | 1 | 0 | 0 | 0 | 941 | 951 |  |
| K.VAGTQPITC(+57.02)K.W | Y | 47.75 | 1073.5540 | 10 | 2.3 | 537.7855 | 2 | 25.63 | 6 | 6761 | Ana\_05.raw |  | 2.78E7 |  |  | 3.71E6 | 2.56E7 |  |  |  |  | 3 | 0 | 1 | 0 | 0 | 1 | 1 | 0 | 0 | 0 | 0 | 1292 | 1301 | Carbamidomethylation |
| K.SAETSKPAGKEEVK.E | Y | 47.30 | 1459.7518 | 14 | 3.8 | 487.5931 | 3 | 16.69 | 6 | 3833 | Ana\_05.raw |  |  |  |  |  | 6.59E5 |  |  |  |  | 1 | 0 | 0 | 0 | 0 | 0 | 1 | 0 | 0 | 0 | 0 | 1087 | 1100 |  |
| K.LTILAAR.Q | Y | 45.66 | 756.4857 | 7 | 5.0 | 379.2520 | 2 | 50.85 | 1 | 14272 | ANa\_10.raw | 7.78E6 | 1.46E7 | 1.37E7 | 5.36E6 | 9.59E6 | 1.35E7 |  |  | 1.14E7 | 6.57E6 | 15 | 2 | 2 | 2 | 2 | 2 | 2 | 0 | 0 | 1 | 2 | 1326 | 1332 |  |
| K.FGQVFR.L | Y | 45.01 | 752.3969 | 6 | 4.1 | 377.2073 | 2 | 48.14 | 6 | 13642 | Ana\_05.raw | 9.18E6 | 1.54E7 | 1.7E7 | 6.69E6 | 1.19E7 | 1.77E7 | 9.75E6 | 9.56E6 | 1.24E7 |  | 11 | 1 | 1 | 1 | 1 | 1 | 2 | 1 | 1 | 2 | 0 | 1506 | 1511 |  |
| R.LENAGSLK.V | Y | 44.09 | 830.4498 | 8 | 5.8 | 416.2346 | 2 | 24.20 | 2 | 6162 | Ana\_01.raw | 6.71E6 | 6.33E6 | 2.81E7 |  |  |  |  |  |  | 4.18E7 | 4 | 1 | 1 | 1 | 0 | 0 | 0 | 0 | 0 | 0 | 1 | 1643 | 1650 |  |
| K.AATPDFR.S | Y | 42.31 | 776.3817 | 7 | 2.2 | 389.1990 | 2 | 28.57 | 9 | 7462 | Ana\_08.raw |  |  |  |  |  |  |  |  | 7.93E6 | 4.38E6 | 2 | 0 | 0 | 0 | 0 | 0 | 0 | 0 | 0 | 1 | 1 | 972 | 978 |  |
| R.FSVSIEK.A | Y | 36.16 | 808.4330 | 7 | 5.6 | 405.2260 | 2 | 52.83 | 2 | 14933 | Ana\_01.raw |  | 1.16E7 | 1.04E7 | 5.86E6 | 1.11E7 | 1.34E7 | 8.39E6 | 7.56E6 | 7.85E6 |  | 8 | 0 | 1 | 1 | 1 | 1 | 1 | 1 | 1 | 1 | 0 | 1184 | 1190 |  |
| R.QISEGVEYIHK.Q | Y | 22.38 | 1301.6615 | 11 | 2.5 | 434.8955 | 3 | 47.70 | 5 | 13449 | Ana\_04.raw |  |  |  |  | 4.41E6 |  |  |  |  |  | 1 | 0 | 0 | 0 | 0 | 1 | 0 | 0 | 0 | 0 | 0 | 1599 | 1609 |  |
| total 32 peptides |  |  |  |  |  |  |  |  |  |  |  |  |  |  |  |  |  |  |  |  |  |  |  |  |  |  |  |  |  |  |  |  |  |  |  |
| --- | --- | --- | --- | --- | --- | --- | --- | --- | --- | --- | --- | --- | --- | --- | --- | --- | --- | --- | --- | --- | --- | --- | --- | --- | --- | --- | --- | --- | --- | --- | --- | --- | --- | --- | --- |

Best Unique PSM (Scan F9:32245, m/z=798.9018, z=4, RT=115.30, ppm=3.7):

Q91VB8|Q91VB8\_MOUSE

back to list

  

| Protein Coverage
| Supporting Peptides
| Best Unique PSM
|

Protein Coverage:

Supporting Peptides:

| Peptide | Uniq | -10lgP | Mass | Length | ppm | m/z | z | RT | Fraction | Scan | Source File | Area 10 | Area 01 | Area 02 | Area 03 | Area 04 | Area 05 | Area 06 | Area 07 | Area 08 | Area 09 | #Spec | #Spec 10 | #Spec 01 | #Spec 02 | #Spec 03 | #Spec 04 | #Spec 05 | #Spec 06 | #Spec 07 | #Spec 08 | #Spec 09 | Start | End | PTM |
| --- | --- | --- | --- | --- | --- | --- | --- | --- | --- | --- | --- | --- | --- | --- | --- | --- | --- | --- | --- | --- | --- | --- | --- | --- | --- | --- | --- | --- | --- | --- | --- | --- | --- | --- | --- |
| K.VADALANAAGHLDDLPGALSALSDLHAHK.L | Y | 161.60 | 2862.4624 | 29 | 1.1 | 955.1624 | 3 | 125.65 | 3 | 34485 | Ana\_02.raw | 7.6E7 | 5.97E8 | 1.2E9 | 2.64E8 | 5.36E8 | 9.86E8 | 6.62E7 | 1.01E9 | 5.09E8 | 2.5E8 | 187 | 18 | 21 | 29 | 13 | 23 | 20 | 10 | 33 | 10 | 10 | 63 | 91 |  |
| K.KVADALANAAGHLDDLPGALSALSDLHAHK.L | Y | 147.15 | 2990.5574 | 30 | 4.7 | 997.8644 | 3 | 126.98 | 7 | 34953 | Ana\_06.raw | 4.78E7 | 4.97E7 | 4.38E7 | 2.49E7 | 2.89E7 | 1.16E8 | 5.56E7 | 3.57E7 | 1.37E7 | 7.84E6 | 32 | 3 | 4 | 5 | 3 | 3 | 3 | 2 | 5 | 2 | 2 | 62 | 91 |  |
| K.IGGHGAEYGAEALER.M | Y | 135.77 | 1528.7269 | 15 | 0.8 | 765.3713 | 2 | 48.74 | 3 | 13776 | Ana\_02.raw | 9.05E8 | 1.43E9 | 1.66E9 | 5.13E8 | 1.09E9 | 1.26E9 | 7.42E8 | 1.29E9 | 9.01E8 | 5.87E8 | 163 | 16 | 23 | 19 | 12 | 16 | 16 | 11 | 28 | 11 | 11 | 18 | 32 |  |
| K.TYFPHFDVSHGSAQVK.G | Y | 118.06 | 1818.8689 | 16 | 0.2 | 910.4419 | 2 | 70.09 | 3 | 19921 | Ana\_02.raw | 2.56E8 | 5.34E8 | 4.64E8 | 1.56E8 | 2.91E8 | 5.06E8 | 1.98E8 | 4.9E8 | 3.29E8 | 1.54E8 | 93 | 10 | 11 | 13 | 4 | 10 | 8 | 5 | 18 | 8 | 6 | 42 | 57 |  |
| K.LLSHC(+57.02)LLVTLASHHPADFTPAVHASLDKFLASVSTVLTSK.Y | Y | 104.55 | 4283.2773 | 40 | 4.8 | 1071.8318 | 4 | 135.36 | 2 | 37451 | Ana\_01.raw |  | 3.15E6 |  |  | 4.75E6 |  | 9.95E5 |  |  | 3.03E6 | 4 | 0 | 1 | 0 | 0 | 1 | 0 | 1 | 0 | 0 | 1 | 101 | 140 | Carbamidomethylation |
| R.VDPVNFK.L | Y | 70.40 | 817.4333 | 7 | 0.9 | 409.7243 | 2 | 42.21 | 3 | 11828 | Ana\_02.raw | 2.79E8 | 4.92E8 | 8.8E8 | 2.33E8 | 5.01E8 | 5.29E8 | 3.2E8 | 5.48E8 | 2.19E8 | 1.76E8 | 67 | 6 | 8 | 8 | 6 | 6 | 6 | 7 | 12 | 4 | 4 | 94 | 100 |  |
| K.FLASVSTVLTSK.Y | Y | 66.13 | 1251.7074 | 12 | 2.7 | 626.8627 | 2 | 88.04 | 6 | 25064 | Ana\_05.raw |  | 6.34E6 | 1.46E7 | 2.57E6 | 2.74E6 | 5.2E6 | 3.11E6 | 4.71E6 | 2.98E6 | 1.19E6 | 10 | 0 | 2 | 1 | 1 | 1 | 1 | 1 | 1 | 1 | 1 | 129 | 140 |  |
| K.LRVDPVNFK.L | Y | 64.35 | 1086.6185 | 9 | 0.4 | 363.2136 | 3 | 55.65 | 3 | 15798 | Ana\_02.raw | 1.7E7 | 2.33E7 | 3.87E7 | 7.87E6 | 1.2E7 | 2.8E7 | 7.09E6 | 2.04E7 | 1.82E7 | 1.11E7 | 18 | 2 | 2 | 2 | 2 | 1 | 2 | 1 | 2 | 2 | 2 | 92 | 100 |  |
| R.M(+15.99)FASFPTTK.T | Y | 63.41 | 1044.4950 | 9 | 0.4 | 523.2550 | 2 | 55.09 | 3 | 15633 | Ana\_02.raw | 4.3E8 | 1.05E9 | 1.34E9 | 3.75E8 | 6.2E8 | 8.21E8 | 4.73E8 | 1.04E9 | 3.83E8 | 2.02E8 | 105 | 10 | 12 | 14 | 9 | 10 | 9 | 8 | 21 | 4 | 8 | 33 | 41 | Oxidation (M) |
| R.MFASFPTTK.T | Y | 63.39 | 1028.5001 | 9 | 3.7 | 515.2592 | 2 | 66.89 | 2 | 19061 | Ana\_01.raw | 3.41E7 | 5.54E7 | 8.87E6 | 1.57E7 | 2.06E7 | 3.91E7 | 1.03E7 | 1.81E7 | 3.84E7 | 1.59E7 | 15 | 2 | 2 | 1 | 1 | 1 | 2 | 1 | 1 | 2 | 2 | 33 | 41 |  |
| M.VLSGEDKSNIK.A | Y | 55.00 | 1188.6350 | 11 | 4.4 | 595.3274 | 2 | 22.76 | 2 | 5669 | Ana\_01.raw | 2.74E6 | 1.65E7 | 1.28E7 | 1.38E6 | 4.55E6 | 5.75E6 | 2.36E6 | 3.2E6 |  |  | 13 | 1 | 2 | 2 | 1 | 2 | 1 | 2 | 2 | 0 | 0 | 2 | 12 |  |
| total 11 peptides |  |  |  |  |  |  |  |  |  |  |  |  |  |  |  |  |  |  |  |  |  |  |  |  |  |  |  |  |  |  |  |  |  |  |  |
| --- | --- | --- | --- | --- | --- | --- | --- | --- | --- | --- | --- | --- | --- | --- | --- | --- | --- | --- | --- | --- | --- | --- | --- | --- | --- | --- | --- | --- | --- | --- | --- | --- | --- | --- | --- |

Best Unique PSM (Scan F3:34485, m/z=955.1624, z=3, RT=125.65, ppm=1.1):

sp|Q9JHU4|DYHC1\_MOUSE

back to list

  

| Protein Coverage
| Supporting Peptides
| Best Unique PSM
|

Protein Coverage:

Supporting Peptides:

| Peptide | Uniq | -10lgP | Mass | Length | ppm | m/z | z | RT | Fraction | Scan | Source File | Area 10 | Area 01 | Area 02 | Area 03 | Area 04 | Area 05 | Area 06 | Area 07 | Area 08 | Area 09 | #Spec | #Spec 10 | #Spec 01 | #Spec 02 | #Spec 03 | #Spec 04 | #Spec 05 | #Spec 06 | #Spec 07 | #Spec 08 | #Spec 09 | Start | End | PTM |
| --- | --- | --- | --- | --- | --- | --- | --- | --- | --- | --- | --- | --- | --- | --- | --- | --- | --- | --- | --- | --- | --- | --- | --- | --- | --- | --- | --- | --- | --- | --- | --- | --- | --- | --- | --- |
| R.FGNPLLVQDVESYDPVLNPVLNR.E | Y | 135.62 | 2597.3489 | 23 | 5.1 | 1299.6884 | 2 | 132.26 | 6 | 36763 | Ana\_05.raw | 2.81E6 |  |  | 7.96E5 | 7.54E5 | 1.38E6 | 1.83E6 |  | 8.63E6 |  | 7 | 1 | 0 | 0 | 1 | 1 | 1 | 1 | 0 | 2 | 0 | 3627 | 3649 |  |
| R.GIFEALRPLETLPVEGLIR.I | Y | 101.46 | 2122.2148 | 19 | 3.0 | 708.4144 | 3 | 133.24 | 6 | 37041 | Ana\_05.raw | 2.44E6 | 3.12E6 | 1.54E6 | 1.46E6 | 1.67E6 | 2.4E6 | 1.53E6 | 7.52E5 |  | 3.03E6 | 9 | 1 | 1 | 1 | 1 | 1 | 1 | 1 | 1 | 0 | 1 | 2803 | 2821 |  |
| R.ILDDDTIITTLENLK.R | Y | 98.01 | 1715.9193 | 15 | 2.4 | 858.9690 | 2 | 132.10 | 3 | 36184 | Ana\_02.raw | 2.12E6 | 2.62E6 | 1.94E6 | 6.11E5 | 1.72E6 | 1.25E6 | 1.31E6 | 8.13E5 | 3.26E6 | 2.69E6 | 10 | 1 | 1 | 1 | 1 | 1 | 1 | 1 | 1 | 1 | 1 | 3758 | 3772 |  |
| K.GTVGEPTYDAEFQHFLR.G | Y | 90.13 | 1965.9220 | 17 | 2.9 | 656.3165 | 3 | 97.58 | 3 | 27263 | Ana\_02.raw | 1.83E6 | 9.38E5 | 2.19E6 | 9.5E5 | 6.55E5 | 3.42E6 |  | 1.68E6 |  |  | 7 | 1 | 1 | 1 | 1 | 1 | 1 | 0 | 1 | 0 | 0 | 3892 | 3908 |  |
| R.ENFIPTIVNFSAEEISDAIR.E | N | 82.93 | 2264.1323 | 20 | 7.8 | 755.7239 | 3 | 136.62 | 6 | 37969 | Ana\_05.raw |  |  |  |  |  | 5.99E5 |  |  |  |  | 1 | 0 | 0 | 0 | 0 | 0 | 1 | 0 | 0 | 0 | 0 | 3343 | 3362 |  |
| R.HYLDFINHYANLFHEK.R | Y | 81.72 | 2059.9905 | 16 | 4.2 | 516.0071 | 4 | 116.29 | 7 | 32177 | Ana\_06.raw |  |  |  | 7.2E5 | 5.2E5 |  | 2.62E5 |  |  |  | 3 | 0 | 0 | 0 | 1 | 1 | 0 | 1 | 0 | 0 | 0 | 3173 | 3188 |  |
| K.LSLSNAISTVLPLTQLR.W | Y | 73.29 | 1825.0673 | 17 | 2.1 | 913.5428 | 2 | 133.46 | 4 | 36259 | Ana\_03.raw | 1.05E6 |  |  | 9.31E4 |  | 4.43E5 | 4.17E5 |  | 1.75E6 | 6.64E5 | 6 | 1 | 0 | 0 | 1 | 0 | 1 | 1 | 0 | 1 | 1 | 4573 | 4589 |  |
| K.VNFLPEIITLSK.E | Y | 67.53 | 1372.7965 | 12 | 3.8 | 687.4081 | 2 | 126.39 | 6 | 35146 | Ana\_05.raw | 3.58E5 | 1.35E6 | 3.39E5 | 9.98E5 | 2.32E6 | 4.49E6 | 1.41E6 | 1.44E6 | 1.59E6 |  | 9 | 1 | 1 | 1 | 1 | 1 | 1 | 1 | 1 | 1 | 0 | 735 | 746 |  |
| K.ASVVTLPVYLNFTR.A | Y | 61.30 | 1578.8770 | 14 | 1.7 | 790.4471 | 2 | 122.47 | 4 | 33337 | Ana\_03.raw | 6.71E5 | 1.98E6 | 6.31E5 | 3.43E5 | 1.21E6 | 1.18E6 | 9.92E5 | 4.11E5 | 1.27E6 |  | 9 | 1 | 1 | 1 | 1 | 1 | 1 | 1 | 1 | 1 | 0 | 4600 | 4613 |  |
| R.VQVALEELQDLK.G | Y | 61.28 | 1383.7609 | 12 | 4.0 | 692.8905 | 2 | 94.49 | 9 | 26766 | Ana\_08.raw |  |  |  |  |  |  |  |  | 2.41E6 |  | 1 | 0 | 0 | 0 | 0 | 0 | 0 | 0 | 0 | 1 | 0 | 1317 | 1328 |  |
| R.TSFLDDAFR.K | Y | 61.12 | 1070.5032 | 9 | -0.8 | 536.2584 | 2 | 88.01 | 3 | 24740 | Ana\_02.raw | 1.16E6 | 3.19E5 | 5.64E5 |  | 1.04E6 | 8.34E5 | 8.33E5 |  |  |  | 6 | 1 | 1 | 1 | 0 | 1 | 1 | 1 | 0 | 0 | 0 | 3610 | 3618 |  |
| R.IFVFEPPPGVK.A | Y | 59.18 | 1228.6855 | 11 | 1.1 | 615.3507 | 2 | 93.17 | 3 | 26102 | Ana\_02.raw | 4.62E6 | 4.94E6 | 1.41E6 | 2.25E6 |  | 3.5E6 | 3E6 |  | 5E6 | 5.01E6 | 9 | 1 | 1 | 1 | 1 | 0 | 1 | 1 | 0 | 2 | 1 | 4142 | 4152 |  |
| R.TEYLSNADER.L | Y | 58.98 | 1196.5309 | 10 | 6.4 | 599.2766 | 2 | 38.16 | 6 | 10586 | Ana\_05.raw |  |  |  | 2.67E6 | 3.36E6 | 4.1E6 | 4.01E6 | 2.71E6 |  |  | 5 | 0 | 0 | 0 | 1 | 1 | 1 | 1 | 1 | 0 | 0 | 3548 | 3557 |  |
| R.SLAM(+15.99)TKPDR.Q | Y | 58.78 | 1033.5226 | 9 | 2.7 | 345.5157 | 3 | 18.22 | 3 | 4340 | Ana\_02.raw | 1.37E6 | 4.25E5 | 1.3E6 | 5.13E5 | 1.18E6 |  | 9.06E5 | 7.31E5 |  | 1.06E6 | 8 | 1 | 1 | 1 | 1 | 1 | 0 | 1 | 1 | 0 | 1 | 2036 | 2044 | Oxidation (M) |
| R.SIPLDEGEDEAQR.R | Y | 55.98 | 1457.6633 | 13 | 5.8 | 729.8431 | 2 | 51.39 | 10 | 14461 | Ana\_09.raw |  |  |  |  |  |  |  |  | 2.66E6 | 1.99E6 | 2 | 0 | 0 | 0 | 0 | 0 | 0 | 0 | 0 | 1 | 1 | 2382 | 2394 |  |
| R.TFSSIPVSR.I | Y | 53.84 | 992.5291 | 9 | 6.2 | 497.2749 | 2 | 52.29 | 6 | 14890 | Ana\_05.raw | 1.46E6 | 7.6E5 | 3.35E6 | 1.55E6 | 3.39E6 | 3.8E6 | 2.37E6 | 2.04E6 | 6.16E6 |  | 9 | 1 | 1 | 1 | 1 | 1 | 1 | 1 | 1 | 1 | 0 | 4158 | 4166 |  |
| R.LGGSPFGPAGTGK.T | Y | 53.14 | 1144.5876 | 13 | 6.5 | 573.3048 | 2 | 51.90 | 10 | 14613 | Ana\_09.raw |  | 2.56E6 | 4.02E6 | 1.64E6 | 2.27E6 | 3.63E6 | 6.27E5 | 2.05E6 | 1.26E7 | 2.05E6 | 9 | 0 | 1 | 1 | 1 | 1 | 1 | 1 | 1 | 1 | 1 | 1898 | 1910 |  |
| K.SLLQALNEVK.G | Y | 49.69 | 1113.6394 | 10 | 4.1 | 557.8293 | 2 | 94.75 | 7 | 26556 | Ana\_06.raw | 6.18E5 | 1.44E6 |  | 2.15E5 | 6.76E5 | 1.21E6 | 3.17E5 | 1.96E5 | 2.47E6 | 6.71E5 | 9 | 1 | 1 | 0 | 1 | 1 | 1 | 1 | 1 | 1 | 1 | 3746 | 3755 |  |
| R.LVEDEERR.W | Y | 49.18 | 1044.5199 | 8 | 4.2 | 523.2694 | 2 | 20.08 | 10 | 4845 | Ana\_09.raw | 4.84E6 |  | 3.7E6 | 1.99E6 |  |  |  |  |  | 6.19E6 | 4 | 1 | 0 | 1 | 1 | 0 | 0 | 0 | 0 | 0 | 1 | 2835 | 2842 |  |
| K.IQGLTVEQAEAVVR.L | Y | 49.04 | 1511.8307 | 14 | 3.1 | 756.9250 | 2 | 87.58 | 1 | 24648 | ANa\_10.raw | 3.47E5 |  |  |  |  |  |  |  | 0 | 5.96E5 | 3 | 1 | 0 | 0 | 0 | 0 | 0 | 0 | 0 | 1 | 1 | 3922 | 3935 |  |
| R.IFTIESAR.V | Y | 48.52 | 935.5076 | 8 | 3.0 | 468.7625 | 2 | 58.81 | 6 | 16818 | Ana\_05.raw | 3.22E6 | 3.02E6 | 2.57E6 | 1.83E6 | 2.83E6 | 3.09E6 |  | 1.18E6 |  | 3E6 | 8 | 1 | 1 | 1 | 1 | 1 | 1 | 0 | 1 | 0 | 1 | 715 | 722 |  |
| K.DYIPVDQEELR.D | Y | 48.49 | 1375.6619 | 11 | 4.8 | 688.8415 | 2 | 73.95 | 6 | 21217 | Ana\_05.raw |  |  |  |  | 1.2E6 | 1.27E6 |  |  | 3.37E6 |  | 3 | 0 | 0 | 0 | 0 | 1 | 1 | 0 | 0 | 1 | 0 | 2878 | 2888 |  |
| K.KYEFGESDLR.S | Y | 46.56 | 1242.5880 | 10 | 3.9 | 622.3037 | 2 | 51.90 | 2 | 14656 | Ana\_01.raw |  | 1.9E6 |  |  |  |  |  |  |  | 1.1E6 | 2 | 0 | 1 | 0 | 0 | 0 | 0 | 0 | 0 | 0 | 1 | 4202 | 4211 |  |
| K.EALELTDTGLLSGSEER.V | Y | 46.21 | 1818.8846 | 17 | 9.5 | 910.4583 | 2 | 96.22 | 1 | 26956 | ANa\_10.raw | 1.36E6 |  |  |  |  |  |  |  |  |  | 1 | 1 | 0 | 0 | 0 | 0 | 0 | 0 | 0 | 0 | 0 | 1300 | 1316 |  |
| K.VAAPDVVVPTLDTVR.H | Y | 44.43 | 1550.8668 | 15 | -2.0 | 776.4391 | 2 | 91.80 | 3 | 25743 | Ana\_02.raw |  |  | 1.43E6 |  |  |  |  |  |  |  | 1 | 0 | 0 | 1 | 0 | 0 | 0 | 0 | 0 | 0 | 0 | 2560 | 2574 |  |
| R.ILDDDTIITTLENLKR.E | Y | 43.64 | 1872.0204 | 16 | 5.6 | 625.0176 | 3 | 128.36 | 6 | 35661 | Ana\_05.raw |  |  |  |  |  | 2.51E6 |  |  |  |  | 1 | 0 | 0 | 0 | 0 | 0 | 1 | 0 | 0 | 0 | 0 | 3758 | 3773 |  |
| K.AERPDVDEK.R | Y | 42.85 | 1057.5039 | 9 | 1.4 | 529.7599 | 2 | 19.18 | 6 | 4662 | Ana\_05.raw |  |  |  | 7.21E5 | 1.98E6 | 2.53E6 | 1.08E6 | 8.77E5 |  |  | 5 | 0 | 0 | 0 | 1 | 1 | 1 | 1 | 1 | 0 | 0 | 3717 | 3725 |  |
| R.FTQDTQPHYIYSPR.E | Y | 42.39 | 1751.8267 | 14 | 1.4 | 584.9503 | 3 | 58.99 | 7 | 16680 | Ana\_06.raw |  |  | 2.68E6 |  | 2.17E6 | 8.44E5 | 1.19E6 |  |  |  | 4 | 0 | 0 | 1 | 0 | 1 | 1 | 1 | 0 | 0 | 0 | 2782 | 2795 |  |
| K.YTGEDFDEDLR.T | Y | 39.91 | 1358.5626 | 11 | 4.1 | 680.2914 | 2 | 67.53 | 1 | 19140 | ANa\_10.raw | 2.67E5 |  |  |  |  |  |  |  |  |  | 1 | 1 | 0 | 0 | 0 | 0 | 0 | 0 | 0 | 0 | 0 | 2965 | 2975 |  |
| R.AATSPALFNR.C | Y | 39.06 | 1046.5509 | 10 | 2.9 | 524.2842 | 2 | 53.36 | 7 | 15019 | Ana\_06.raw |  |  |  | 9.86E5 |  |  | 5.79E5 |  |  |  | 2 | 0 | 0 | 0 | 1 | 0 | 0 | 1 | 0 | 0 | 0 | 3077 | 3086 |  |
| K.EFGPVVIDYGK.V | Y | 38.90 | 1222.6233 | 11 | 2.6 | 612.3205 | 2 | 86.31 | 8 | 24160 | Ana\_07.raw |  |  |  |  |  |  |  | 1.13E6 |  |  | 1 | 0 | 0 | 0 | 0 | 0 | 0 | 0 | 1 | 0 | 0 | 1099 | 1109 |  |
| K.EIVLSAGSTPK.I | Y | 38.76 | 1100.6077 | 11 | 5.7 | 551.3143 | 2 | 45.10 | 9 | 12568 | Ana\_08.raw |  |  | 4.79E6 |  | 0 |  |  |  | 7.63E5 | 6.14E5 | 4 | 0 | 0 | 1 | 0 | 1 | 0 | 0 | 0 | 1 | 1 | 3911 | 3921 |  |
| R.LLNTFLER.L | Y | 38.74 | 1004.5654 | 8 | 4.2 | 503.2921 | 2 | 77.92 | 10 | 21983 | Ana\_09.raw |  |  |  | 8.09E5 | 9.44E5 |  | 3.54E5 | 8.6E5 |  | 1.64E6 | 5 | 0 | 0 | 0 | 1 | 1 | 0 | 1 | 1 | 0 | 1 | 4262 | 4269 |  |
| K.VPVNLLR.A | Y | 35.40 | 809.5123 | 7 | 1.6 | 405.7641 | 2 | 55.99 | 1 | 15797 | ANa\_10.raw | 1.03E6 | 9.29E5 |  |  | 5.64E5 |  |  |  | 2.65E6 | 1.1E6 | 5 | 1 | 1 | 0 | 0 | 1 | 0 | 0 | 0 | 1 | 1 | 4132 | 4138 |  |
| K.ALGHQLGR.F | Y | 34.50 | 850.4773 | 8 | 3.8 | 426.2475 | 2 | 22.44 | 9 | 5438 | Ana\_08.raw |  |  |  |  |  |  |  |  | 2.7E6 |  | 1 | 0 | 0 | 0 | 0 | 0 | 0 | 0 | 0 | 1 | 0 | 1916 | 1923 |  |
| R.DLEASIAR.Y | N | 34.02 | 873.4556 | 8 | 3.5 | 437.7366 | 2 | 47.65 | 10 | 13374 | Ana\_09.raw | 3.91E6 | 4.89E6 |  |  |  |  |  |  |  | 3.05E6 | 3 | 1 | 1 | 0 | 0 | 0 | 0 | 0 | 0 | 0 | 1 | 3437 | 3444 |  |
| R.LVEAISR.D | Y | 29.34 | 786.4599 | 7 | 2.8 | 394.2383 | 2 | 33.96 | 6 | 9324 | Ana\_05.raw |  |  | 3.07E6 |  | 2.23E6 | 3.53E6 | 1.91E6 |  |  |  | 4 | 0 | 0 | 1 | 0 | 1 | 1 | 1 | 0 | 0 | 0 | 378 | 384 |  |
| R.VISFIR.Q | Y | 29.03 | 733.4486 | 6 | 5.5 | 367.7336 | 2 | 56.41 | 9 | 15979 | Ana\_08.raw |  |  |  |  |  |  |  |  | 0 |  | 1 | 0 | 0 | 0 | 0 | 0 | 0 | 0 | 0 | 1 | 0 | 2677 | 2682 |  |
| R.TLINELVK.G | Y | 28.25 | 928.5593 | 8 | -3.6 | 465.2852 | 2 | 80.16 | 8 | 22471 | Ana\_07.raw |  |  |  |  |  |  |  | 0 |  |  | 1 | 0 | 0 | 0 | 0 | 0 | 0 | 0 | 1 | 0 | 0 | 4448 | 4455 |  |
| K.LNTQEIFDDWAR.K | Y | 26.91 | 1506.7103 | 12 | 4.8 | 754.3661 | 2 | 113.51 | 1 | 31432 | ANa\_10.raw | 0 |  |  |  |  |  |  |  |  |  | 1 | 1 | 0 | 0 | 0 | 0 | 0 | 0 | 0 | 0 | 0 | 691 | 702 |  |
| R.IMFEVQDLK.Y | Y | 25.55 | 1121.5791 | 9 | -5.9 | 561.7935 | 2 | 70.09 | 1 | 19861 | ANa\_10.raw | 1.78E6 | 2.28E6 |  |  |  |  |  |  |  |  | 2 | 1 | 1 | 0 | 0 | 0 | 0 | 0 | 0 | 0 | 0 | 2339 | 2347 |  |
| R.LADLLGK.I | Y | 23.90 | 728.4432 | 7 | 2.6 | 365.2298 | 2 | 56.33 | 5 | 15991 | Ana\_04.raw |  |  |  |  | 1.06E6 |  |  |  |  |  | 1 | 0 | 0 | 0 | 0 | 1 | 0 | 0 | 0 | 0 | 0 | 1602 | 1608 |  |
| K.NEAVVK.D | Y | 22.27 | 658.3650 | 6 | 0.3 | 330.1899 | 2 | 24.24 | 4 | 6170 | Ana\_03.raw |  |  |  | 2.11E6 |  |  |  |  |  |  | 1 | 0 | 0 | 0 | 1 | 0 | 0 | 0 | 0 | 0 | 0 | 1440 | 1445 |  |
| total 43 peptides |  |  |  |  |  |  |  |  |  |  |  |  |  |  |  |  |  |  |  |  |  |  |  |  |  |  |  |  |  |  |  |  |  |  |  |
| --- | --- | --- | --- | --- | --- | --- | --- | --- | --- | --- | --- | --- | --- | --- | --- | --- | --- | --- | --- | --- | --- | --- | --- | --- | --- | --- | --- | --- | --- | --- | --- | --- | --- | --- | --- |

Best Unique PSM (Scan F6:36763, m/z=1299.6884, z=2, RT=132.26, ppm=5.1):

A0A087WS56|A0A087WS56\_MOUSE

back to list

  

| Protein Coverage
| Supporting Peptides
| Best Unique PSM
|

Protein Coverage:

Supporting Peptides:

| Peptide | Uniq | -10lgP | Mass | Length | ppm | m/z | z | RT | Fraction | Scan | Source File | Area 10 | Area 01 | Area 02 | Area 03 | Area 04 | Area 05 | Area 06 | Area 07 | Area 08 | Area 09 | #Spec | #Spec 10 | #Spec 01 | #Spec 02 | #Spec 03 | #Spec 04 | #Spec 05 | #Spec 06 | #Spec 07 | #Spec 08 | #Spec 09 | Start | End | PTM |
| --- | --- | --- | --- | --- | --- | --- | --- | --- | --- | --- | --- | --- | --- | --- | --- | --- | --- | --- | --- | --- | --- | --- | --- | --- | --- | --- | --- | --- | --- | --- | --- | --- | --- | --- | --- |
| R.NTFAEITGLSPGVTYLFK.V | Y | 117.62 | 1957.0197 | 18 | 4.8 | 979.5218 | 2 | 130.93 | 1 | 35991 | ANa\_10.raw | 1.71E7 | 5.47E6 | 4.55E6 | 1.66E6 | 3.39E6 | 6.49E6 | 3.83E6 | 2.37E6 | 2.53E7 | 1.31E7 | 10 | 1 | 1 | 1 | 1 | 1 | 1 | 1 | 1 | 1 | 1 | 958 | 975 |  |
| R.EESPPLIGQQATVSDIPR.D | Y | 100.51 | 1935.9901 | 18 | 4.9 | 969.0071 | 2 | 84.70 | 1 | 23859 | ANa\_10.raw | 3.41E6 |  | 0 |  |  |  |  |  | 7.46E6 | 2.77E6 | 4 | 1 | 0 | 1 | 0 | 0 | 0 | 0 | 0 | 1 | 1 | 1434 | 1451 |  |
| R.HALQSASAGSGSFTDVR.T | Y | 94.98 | 1689.8070 | 17 | 5.1 | 564.2792 | 3 | 43.82 | 9 | 12156 | Ana\_08.raw | 1.52E7 |  |  | 1.61E6 |  | 4.88E6 |  |  | 1.4E7 | 1.39E7 | 8 | 2 | 0 | 0 | 1 | 0 | 1 | 0 | 0 | 2 | 2 | 274 | 290 |  |
| R.VVTPLSPPTNLHLEANPDTGVLTVSWER.S | Y | 94.80 | 3041.5820 | 28 | 4.6 | 1014.8726 | 3 | 123.84 | 9 | 34421 | Ana\_08.raw | 1.96E6 |  |  |  |  |  |  |  | 1.29E7 | 1.45E6 | 3 | 1 | 0 | 0 | 0 | 0 | 0 | 0 | 0 | 1 | 1 | 1169 | 1196 |  |
| R.VTWAPPPSIELTNLLVR.Y | Y | 92.89 | 1905.0724 | 17 | 2.8 | 636.0332 | 3 | 131.32 | 9 | 36352 | Ana\_08.raw | 1.06E7 | 4.32E6 | 3.39E6 | 2.23E6 | 2.78E6 | 9.18E6 | 4.42E6 | 3.25E6 | 2.79E7 | 1.32E7 | 15 | 1 | 1 | 1 | 2 | 1 | 2 | 2 | 1 | 2 | 2 | 1284 | 1300 |  |
| R.VEVLPVSLPGEHGQR.L | Y | 92.67 | 1615.8682 | 15 | 4.9 | 539.6326 | 3 | 73.48 | 9 | 20959 | Ana\_08.raw | 7.38E6 |  | 1.06E6 | 5.73E5 |  | 2.67E6 |  |  | 2.62E7 | 7.44E6 | 7 | 1 | 0 | 1 | 1 | 0 | 1 | 0 | 0 | 2 | 1 | 938 | 952 |  |
| R.FLTTTPNSLLVSWQAPR.A | Y | 88.78 | 1930.0312 | 17 | 3.8 | 966.0266 | 2 | 116.92 | 9 | 32658 | Ana\_08.raw | 4.5E4 |  |  |  |  |  |  |  | 6.62E6 |  | 2 | 1 | 0 | 0 | 0 | 0 | 0 | 0 | 0 | 1 | 0 | 1910 | 1926 |  |
| R.HHAEHSVGRPR.Q | Y | 83.94 | 1281.6439 | 11 | 1.2 | 428.2224 | 3 | 14.86 | 3 | 3344 | Ana\_02.raw |  |  | 8.87E5 |  | 7.83E5 | 9.15E5 | 4.02E5 |  |  | 2.42E6 | 5 | 0 | 0 | 1 | 0 | 1 | 1 | 1 | 0 | 0 | 1 | 1391 | 1401 |  |
| R.SSPVIIDASTAIDAPSNLR.F | Y | 83.89 | 1926.0057 | 19 | 9.0 | 964.0188 | 2 | 96.73 | 1 | 27093 | ANa\_10.raw | 9.01E5 |  |  |  |  |  |  |  | 5.98E6 | 7.54E5 | 3 | 1 | 0 | 0 | 0 | 0 | 0 | 0 | 0 | 1 | 1 | 1891 | 1909 |  |
| K.LGVRPSQGGEAPR.E | Y | 79.62 | 1322.7054 | 13 | 3.6 | 441.9107 | 3 | 28.22 | 6 | 7569 | Ana\_05.raw | 3.06E7 | 1.33E7 | 7.94E6 | 3.68E6 | 6.85E6 | 3.74E6 | 5.18E6 | 4.99E6 | 3.25E7 | 2.93E7 | 14 | 2 | 2 | 1 | 1 | 1 | 1 | 1 | 1 | 2 | 2 | 1116 | 1128 |  |
| R.GVTYNIIVEALQNQR.R | Y | 79.16 | 1716.9159 | 15 | 1.2 | 859.4662 | 2 | 121.21 | 6 | 33804 | Ana\_05.raw | 1.37E6 |  |  |  |  | 7.85E5 |  |  | 8.16E5 | 9.27E5 | 5 | 1 | 0 | 0 | 0 | 0 | 1 | 0 | 0 | 1 | 2 | 2044 | 2058 |  |
| K.GLAFTDVDVDSIK.I | Y | 70.23 | 1378.6980 | 13 | 4.1 | 690.3591 | 2 | 95.79 | 1 | 26840 | ANa\_10.raw | 4.33E5 |  |  |  |  |  |  |  | 7.04E6 | 1.82E6 | 3 | 1 | 0 | 0 | 0 | 0 | 0 | 0 | 0 | 1 | 1 | 1636 | 1648 |  |
| K.YIVNVYQISEEGK.Q | Y | 69.41 | 1540.7773 | 13 | 2.0 | 771.3975 | 2 | 84.99 | 9 | 24182 | Ana\_08.raw |  |  |  |  |  |  |  |  | 2.89E6 |  | 1 | 0 | 0 | 0 | 0 | 0 | 0 | 0 | 0 | 1 | 0 | 785 | 797 |  |
| K.FSQVTPTSFTAQWIAPSVQLTGYR.V | Y | 68.66 | 2684.3599 | 24 | 1.8 | 895.7955 | 3 | 130.13 | 1 | 35767 | ANa\_10.raw | 3.74E6 |  |  |  |  |  |  |  |  | 1.53E6 | 2 | 1 | 0 | 0 | 0 | 0 | 0 | 0 | 0 | 0 | 1 | 1729 | 1752 |  |
| R.ITYGETGGNSPVQEFTVPGSK.S | Y | 68.33 | 2167.0432 | 21 | 2.4 | 1084.5315 | 2 | 84.79 | 9 | 24127 | Ana\_08.raw | 3.12E6 |  |  |  |  |  |  |  | 3.61E6 |  | 3 | 1 | 0 | 0 | 0 | 0 | 0 | 0 | 0 | 2 | 0 | 1479 | 1499 |  |
| R.STTPDITGYR.I | Y | 64.06 | 1109.5353 | 10 | 5.4 | 555.7779 | 2 | 47.08 | 9 | 13163 | Ana\_08.raw |  | 7.02E6 | 7.15E6 | 2.47E6 | 5.01E6 | 7.27E6 | 4.1E6 | 3.92E6 | 2.88E7 | 1.66E7 | 9 | 0 | 1 | 1 | 1 | 1 | 1 | 1 | 1 | 1 | 1 | 1197 | 1206 |  |
| R.TVLVTWTPPR.A | Y | 62.14 | 1168.6604 | 10 | 4.6 | 585.3401 | 2 | 80.93 | 1 | 22843 | ANa\_10.raw | 1.58E6 |  |  | 7.48E4 | 6.42E5 | 9.15E5 |  | 5.23E5 |  | 1.52E6 | 6 | 1 | 0 | 0 | 1 | 1 | 1 | 0 | 1 | 0 | 1 | 1011 | 1020 |  |
| R.TFYQIGDSWEK.F | Y | 62.12 | 1372.6299 | 11 | 4.9 | 687.3256 | 2 | 86.26 | 1 | 24291 | ANa\_10.raw | 3.72E6 |  |  |  |  | 1.92E6 |  |  | 9.15E6 | 1.29E6 | 4 | 1 | 0 | 0 | 0 | 0 | 1 | 0 | 0 | 1 | 1 | 568 | 578 |  |
| K.DDKESAPISDTVVPAVPPPTDLR.F | Y | 61.07 | 2418.2278 | 23 | 4.5 | 807.0868 | 3 | 90.50 | 1 | 25426 | ANa\_10.raw | 2.62E6 |  |  |  |  |  |  |  |  |  | 1 | 1 | 0 | 0 | 0 | 0 | 0 | 0 | 0 | 0 | 0 | 1251 | 1273 |  |
| R.TFYSC(+57.02)TTEGR.Q | Y | 55.80 | 1220.5132 | 10 | 5.5 | 611.2672 | 2 | 37.91 | 9 | 10316 | Ana\_08.raw |  |  |  |  |  |  |  |  | 8.25E6 | 1.25E7 | 2 | 0 | 0 | 0 | 0 | 0 | 0 | 0 | 0 | 1 | 1 | 370 | 379 | Carbamidomethylation |
| K.VGDTYERPK.D | Y | 55.80 | 1063.5298 | 9 | 4.3 | 532.7745 | 2 | 21.65 | 9 | 5173 | Ana\_08.raw |  |  | 3.04E6 |  |  |  |  | 2.03E6 | 5.28E6 | 5.6E6 | 4 | 0 | 0 | 1 | 0 | 0 | 0 | 0 | 1 | 1 | 1 | 109 | 117 |  |
| R.SYTITGLQPGTDYK.I | Y | 55.43 | 1542.7566 | 14 | 3.9 | 772.3886 | 2 | 73.40 | 10 | 20778 | Ana\_09.raw | 7.93E6 |  |  |  |  | 5.31E6 | 1.34E6 | 4.94E6 | 2.49E7 | 7.11E6 | 6 | 1 | 0 | 0 | 0 | 0 | 1 | 1 | 1 | 1 | 1 | 1866 | 1879 |  |
| R.VTYSSPEDGIR.E | Y | 53.92 | 1222.5830 | 11 | 4.7 | 612.3016 | 2 | 46.60 | 1 | 13025 | ANa\_10.raw | 1.33E7 |  | 0 | 5.14E6 | 7.88E6 |  |  |  | 1.44E7 |  | 6 | 1 | 0 | 1 | 1 | 1 | 0 | 0 | 0 | 1 | 1 | 1663 | 1673 |  |
| K.ATGVFTTLQPLR.S | Y | 52.78 | 1302.7296 | 12 | 5.1 | 652.3754 | 2 | 88.60 | 6 | 25213 | Ana\_05.raw |  |  | 2.22E7 | 8.14E5 |  | 3.91E6 | 2.42E6 |  | 1.81E7 | 7.22E6 | 6 | 0 | 0 | 1 | 1 | 0 | 1 | 1 | 0 | 1 | 1 | 1077 | 1088 |  |
| K.VFAVHQGR.E | Y | 51.62 | 912.4930 | 8 | 3.1 | 457.2552 | 2 | 23.27 | 9 | 5724 | Ana\_08.raw | 4.9E6 |  |  |  |  |  |  |  | 1.12E7 | 6.98E6 | 3 | 1 | 0 | 0 | 0 | 0 | 0 | 0 | 0 | 1 | 1 | 976 | 983 |  |
| K.IHLYTLNDNAR.S | Y | 49.75 | 1328.6837 | 11 | 6.2 | 443.9046 | 3 | 54.09 | 9 | 15305 | Ana\_08.raw |  |  |  |  |  |  |  |  | 4.77E6 |  | 1 | 0 | 0 | 0 | 0 | 0 | 0 | 0 | 0 | 1 | 0 | 1880 | 1890 |  |
| R.YQC(+57.02)YC(+57.02)YGR.G | Y | 48.91 | 1168.4430 | 8 | 4.5 | 585.2314 | 2 | 36.71 | 9 | 9944 | Ana\_08.raw | 2.96E6 |  |  |  |  |  |  |  | 3.52E6 | 3.96E6 | 3 | 1 | 0 | 0 | 0 | 0 | 0 | 0 | 0 | 1 | 1 | 585 | 592 | Carbamidomethylation |
| R.APITGYIIR.H | Y | 47.75 | 1002.5862 | 9 | 6.8 | 502.3038 | 2 | 72.23 | 6 | 20726 | Ana\_05.raw | 4.32E6 | 1.01E6 | 6.89E5 |  |  | 1.95E6 |  |  | 1.21E7 | 2.79E6 | 6 | 1 | 1 | 1 | 0 | 0 | 1 | 0 | 0 | 1 | 1 | 1382 | 1390 |  |
| K.YEKPGSPPR.E | Y | 47.33 | 1029.5243 | 9 | 2.1 | 515.7705 | 2 | 21.44 | 9 | 5104 | Ana\_08.raw | 8.25E6 |  | 5.39E6 |  |  | 5.96E6 |  |  | 2.09E7 | 8.14E6 | 6 | 1 | 0 | 1 | 0 | 0 | 1 | 0 | 0 | 2 | 1 | 1936 | 1944 |  |
| R.LTAGLTR.G | Y | 41.63 | 730.4337 | 7 | 2.6 | 366.2251 | 2 | 26.64 | 9 | 6850 | Ana\_08.raw |  |  |  |  |  |  |  |  | 1.91E7 | 1.01E7 | 2 | 0 | 0 | 0 | 0 | 0 | 0 | 0 | 0 | 1 | 1 | 1028 | 1034 |  |
| K.FVHGVR.Y | Y | 39.65 | 713.3973 | 6 | 4.7 | 357.7076 | 2 | 20.58 | 10 | 5011 | Ana\_09.raw |  | 4.69E6 | 3.45E6 |  |  | 3.72E6 | 1.7E6 |  | 1.8E6 | 1.27E7 | 6 | 0 | 1 | 1 | 0 | 0 | 1 | 1 | 0 | 1 | 1 | 579 | 584 |  |
| R.GEWAC(+57.02)IPYSQLR.D | Y | 38.57 | 1478.6976 | 12 | 6.2 | 740.3607 | 2 | 93.94 | 10 | 26257 | Ana\_09.raw |  |  |  |  |  |  |  |  |  | 2.44E6 | 1 | 0 | 0 | 0 | 0 | 0 | 0 | 0 | 0 | 0 | 1 | 504 | 515 | Carbamidomethylation |
| K.YEVSVYALK.D | Y | 37.51 | 1070.5648 | 9 | 3.5 | 536.2916 | 2 | 72.64 | 1 | 20582 | ANa\_10.raw | 2.02E6 |  |  |  |  |  |  |  |  |  | 1 | 1 | 0 | 0 | 0 | 0 | 0 | 0 | 0 | 0 | 0 | 1787 | 1795 |  |
| R.NLQPGSEYTVTLVAVK.G | Y | 36.44 | 1717.9250 | 16 | 1.4 | 859.9710 | 2 | 87.24 | 9 | 24790 | Ana\_08.raw | 3.17E5 |  |  |  |  |  |  |  | 2.14E6 |  | 2 | 1 | 0 | 0 | 0 | 0 | 0 | 0 | 0 | 1 | 0 | 1054 | 1069 |  |
| K.SEPLIGR.K | Y | 36.01 | 770.4286 | 7 | 5.0 | 386.2235 | 2 | 33.34 | 10 | 9058 | Ana\_09.raw | 8.16E6 |  |  |  |  | 2.54E6 |  |  |  | 6.91E6 | 3 | 1 | 0 | 0 | 0 | 0 | 1 | 0 | 0 | 0 | 1 | 1981 | 1987 |  |
| R.ITGYIIK.Y | Y | 35.76 | 806.4902 | 7 | 5.9 | 404.2547 | 2 | 46.62 | 9 | 13020 | Ana\_08.raw | 2.61E6 |  |  |  |  |  |  |  | 5.61E6 | 2.73E6 | 3 | 1 | 0 | 0 | 0 | 0 | 0 | 0 | 0 | 1 | 1 | 1929 | 1935 |  |
| R.IGDTWSK.K | Y | 35.03 | 805.3970 | 7 | 5.0 | 403.7078 | 2 | 32.35 | 1 | 8701 | ANa\_10.raw | 4.74E6 |  |  |  |  |  |  |  |  |  | 1 | 1 | 0 | 0 | 0 | 0 | 0 | 0 | 0 | 0 | 0 | 243 | 249 |  |
| K.EATIPGHLNSYTIK.G | Y | 27.24 | 1542.8042 | 14 | 8.1 | 772.4156 | 2 | 61.90 | 9 | 17617 | Ana\_08.raw |  |  |  |  |  |  |  |  | 1.59E6 |  | 1 | 0 | 0 | 0 | 0 | 0 | 0 | 0 | 0 | 1 | 0 | 656 | 669 |  |
| total 38 peptides |  |  |  |  |  |  |  |  |  |  |  |  |  |  |  |  |  |  |  |  |  |  |  |  |  |  |  |  |  |  |  |  |  |  |  |
| --- | --- | --- | --- | --- | --- | --- | --- | --- | --- | --- | --- | --- | --- | --- | --- | --- | --- | --- | --- | --- | --- | --- | --- | --- | --- | --- | --- | --- | --- | --- | --- | --- | --- | --- | --- |

Best Unique PSM (Scan F1:35991, m/z=979.5218, z=2, RT=130.93, ppm=4.8):

Q3UHL6|Q3UHL6\_MOUSE

back to list

  

| Protein Coverage
| Supporting Peptides
| Best Unique PSM
|

Protein Coverage:

Supporting Peptides:

| Peptide | Uniq | -10lgP | Mass | Length | ppm | m/z | z | RT | Fraction | Scan | Source File | Area 10 | Area 01 | Area 02 | Area 03 | Area 04 | Area 05 | Area 06 | Area 07 | Area 08 | Area 09 | #Spec | #Spec 10 | #Spec 01 | #Spec 02 | #Spec 03 | #Spec 04 | #Spec 05 | #Spec 06 | #Spec 07 | #Spec 08 | #Spec 09 | Start | End | PTM |
| --- | --- | --- | --- | --- | --- | --- | --- | --- | --- | --- | --- | --- | --- | --- | --- | --- | --- | --- | --- | --- | --- | --- | --- | --- | --- | --- | --- | --- | --- | --- | --- | --- | --- | --- | --- |
| R.NTFAEITGLSPGVTYLFK.V | Y | 117.62 | 1957.0197 | 18 | 4.8 | 979.5218 | 2 | 130.93 | 1 | 35991 | ANa\_10.raw | 1.71E7 | 5.47E6 | 4.55E6 | 1.66E6 | 3.39E6 | 6.49E6 | 3.83E6 | 2.37E6 | 2.53E7 | 1.31E7 | 10 | 1 | 1 | 1 | 1 | 1 | 1 | 1 | 1 | 1 | 1 | 958 | 975 |  |
| R.EESPPLIGQQATVSDIPR.D | Y | 100.51 | 1935.9901 | 18 | 4.9 | 969.0071 | 2 | 84.70 | 1 | 23859 | ANa\_10.raw | 3.41E6 |  | 0 |  |  |  |  |  | 7.46E6 | 2.77E6 | 4 | 1 | 0 | 1 | 0 | 0 | 0 | 0 | 0 | 1 | 1 | 1434 | 1451 |  |
| R.HALQSASAGSGSFTDVR.T | Y | 94.98 | 1689.8070 | 17 | 5.1 | 564.2792 | 3 | 43.82 | 9 | 12156 | Ana\_08.raw | 1.52E7 |  |  | 1.61E6 |  | 4.88E6 |  |  | 1.4E7 | 1.39E7 | 8 | 2 | 0 | 0 | 1 | 0 | 1 | 0 | 0 | 2 | 2 | 274 | 290 |  |
| R.VVTPLSPPTNLHLEANPDTGVLTVSWER.S | Y | 94.80 | 3041.5820 | 28 | 4.6 | 1014.8726 | 3 | 123.84 | 9 | 34421 | Ana\_08.raw | 1.96E6 |  |  |  |  |  |  |  | 1.29E7 | 1.45E6 | 3 | 1 | 0 | 0 | 0 | 0 | 0 | 0 | 0 | 1 | 1 | 1169 | 1196 |  |
| R.VTWAPPPSIELTNLLVR.Y | Y | 92.89 | 1905.0724 | 17 | 2.8 | 636.0332 | 3 | 131.32 | 9 | 36352 | Ana\_08.raw | 1.06E7 | 4.32E6 | 3.39E6 | 2.23E6 | 2.78E6 | 9.18E6 | 4.42E6 | 3.25E6 | 2.79E7 | 1.32E7 | 15 | 1 | 1 | 1 | 2 | 1 | 2 | 2 | 1 | 2 | 2 | 1284 | 1300 |  |
| R.VEVLPVSLPGEHGQR.L | Y | 92.67 | 1615.8682 | 15 | 4.9 | 539.6326 | 3 | 73.48 | 9 | 20959 | Ana\_08.raw | 7.38E6 |  | 1.06E6 | 5.73E5 |  | 2.67E6 |  |  | 2.62E7 | 7.44E6 | 7 | 1 | 0 | 1 | 1 | 0 | 1 | 0 | 0 | 2 | 1 | 938 | 952 |  |
| R.FLTTTPNSLLVSWQAPR.A | Y | 88.78 | 1930.0312 | 17 | 3.8 | 966.0266 | 2 | 116.92 | 9 | 32658 | Ana\_08.raw | 4.5E4 |  |  |  |  |  |  |  | 6.62E6 |  | 2 | 1 | 0 | 0 | 0 | 0 | 0 | 0 | 0 | 1 | 0 | 1910 | 1926 |  |
| R.HHAEHSVGRPR.Q | Y | 83.94 | 1281.6439 | 11 | 1.2 | 428.2224 | 3 | 14.86 | 3 | 3344 | Ana\_02.raw |  |  | 8.87E5 |  | 7.83E5 | 9.15E5 | 4.02E5 |  |  | 2.42E6 | 5 | 0 | 0 | 1 | 0 | 1 | 1 | 1 | 0 | 0 | 1 | 1391 | 1401 |  |
| R.SSPVIIDASTAIDAPSNLR.F | Y | 83.89 | 1926.0057 | 19 | 9.0 | 964.0188 | 2 | 96.73 | 1 | 27093 | ANa\_10.raw | 9.01E5 |  |  |  |  |  |  |  | 5.98E6 | 7.54E5 | 3 | 1 | 0 | 0 | 0 | 0 | 0 | 0 | 0 | 1 | 1 | 1891 | 1909 |  |
| K.LGVRPSQGGEAPR.E | Y | 79.62 | 1322.7054 | 13 | 3.6 | 441.9107 | 3 | 28.22 | 6 | 7569 | Ana\_05.raw | 3.06E7 | 1.33E7 | 7.94E6 | 3.68E6 | 6.85E6 | 3.74E6 | 5.18E6 | 4.99E6 | 3.25E7 | 2.93E7 | 14 | 2 | 2 | 1 | 1 | 1 | 1 | 1 | 1 | 2 | 2 | 1116 | 1128 |  |
| R.GVTYNIIVEALQNQR.R | Y | 79.16 | 1716.9159 | 15 | 1.2 | 859.4662 | 2 | 121.21 | 6 | 33804 | Ana\_05.raw | 1.37E6 |  |  |  |  | 7.85E5 |  |  | 8.16E5 | 9.27E5 | 5 | 1 | 0 | 0 | 0 | 0 | 1 | 0 | 0 | 1 | 2 | 2139 | 2153 |  |
| K.GLAFTDVDVDSIK.I | Y | 70.23 | 1378.6980 | 13 | 4.1 | 690.3591 | 2 | 95.79 | 1 | 26840 | ANa\_10.raw | 4.33E5 |  |  |  |  |  |  |  | 7.04E6 | 1.82E6 | 3 | 1 | 0 | 0 | 0 | 0 | 0 | 0 | 0 | 1 | 1 | 1636 | 1648 |  |
| K.YIVNVYQISEEGK.Q | Y | 69.41 | 1540.7773 | 13 | 2.0 | 771.3975 | 2 | 84.99 | 9 | 24182 | Ana\_08.raw |  |  |  |  |  |  |  |  | 2.89E6 |  | 1 | 0 | 0 | 0 | 0 | 0 | 0 | 0 | 0 | 1 | 0 | 785 | 797 |  |
| K.FSQVTPTSFTAQWIAPSVQLTGYR.V | Y | 68.66 | 2684.3599 | 24 | 1.8 | 895.7955 | 3 | 130.13 | 1 | 35767 | ANa\_10.raw | 3.74E6 |  |  |  |  |  |  |  |  | 1.53E6 | 2 | 1 | 0 | 0 | 0 | 0 | 0 | 0 | 0 | 0 | 1 | 1729 | 1752 |  |
| R.ITYGETGGNSPVQEFTVPGSK.S | Y | 68.33 | 2167.0432 | 21 | 2.4 | 1084.5315 | 2 | 84.79 | 9 | 24127 | Ana\_08.raw | 3.12E6 |  |  |  |  |  |  |  | 3.61E6 |  | 3 | 1 | 0 | 0 | 0 | 0 | 0 | 0 | 0 | 2 | 0 | 1479 | 1499 |  |
| R.STTPDITGYR.I | Y | 64.06 | 1109.5353 | 10 | 5.4 | 555.7779 | 2 | 47.08 | 9 | 13163 | Ana\_08.raw |  | 7.02E6 | 7.15E6 | 2.47E6 | 5.01E6 | 7.27E6 | 4.1E6 | 3.92E6 | 2.88E7 | 1.66E7 | 9 | 0 | 1 | 1 | 1 | 1 | 1 | 1 | 1 | 1 | 1 | 1197 | 1206 |  |
| R.TVLVTWTPPR.A | Y | 62.14 | 1168.6604 | 10 | 4.6 | 585.3401 | 2 | 80.93 | 1 | 22843 | ANa\_10.raw | 1.58E6 |  |  | 7.48E4 | 6.42E5 | 9.15E5 |  | 5.23E5 |  | 1.52E6 | 6 | 1 | 0 | 0 | 1 | 1 | 1 | 0 | 1 | 0 | 1 | 1011 | 1020 |  |
| R.TFYQIGDSWEK.F | Y | 62.12 | 1372.6299 | 11 | 4.9 | 687.3256 | 2 | 86.26 | 1 | 24291 | ANa\_10.raw | 3.72E6 |  |  |  |  | 1.92E6 |  |  | 9.15E6 | 1.29E6 | 4 | 1 | 0 | 0 | 0 | 0 | 1 | 0 | 0 | 1 | 1 | 568 | 578 |  |
| K.DDKESAPISDTVVPAVPPPTDLR.F | Y | 61.07 | 2418.2278 | 23 | 4.5 | 807.0868 | 3 | 90.50 | 1 | 25426 | ANa\_10.raw | 2.62E6 |  |  |  |  |  |  |  |  |  | 1 | 1 | 0 | 0 | 0 | 0 | 0 | 0 | 0 | 0 | 0 | 1251 | 1273 |  |
| R.TFYSC(+57.02)TTEGR.Q | Y | 55.80 | 1220.5132 | 10 | 5.5 | 611.2672 | 2 | 37.91 | 9 | 10316 | Ana\_08.raw |  |  |  |  |  |  |  |  | 8.25E6 | 1.25E7 | 2 | 0 | 0 | 0 | 0 | 0 | 0 | 0 | 0 | 1 | 1 | 370 | 379 | Carbamidomethylation |
| K.VGDTYERPK.D | Y | 55.80 | 1063.5298 | 9 | 4.3 | 532.7745 | 2 | 21.65 | 9 | 5173 | Ana\_08.raw |  |  | 3.04E6 |  |  |  |  | 2.03E6 | 5.28E6 | 5.6E6 | 4 | 0 | 0 | 1 | 0 | 0 | 0 | 0 | 1 | 1 | 1 | 109 | 117 |  |
| R.SYTITGLQPGTDYK.I | Y | 55.43 | 1542.7566 | 14 | 3.9 | 772.3886 | 2 | 73.40 | 10 | 20778 | Ana\_09.raw | 7.93E6 |  |  |  |  | 5.31E6 | 1.34E6 | 4.94E6 | 2.49E7 | 7.11E6 | 6 | 1 | 0 | 0 | 0 | 0 | 1 | 1 | 1 | 1 | 1 | 1866 | 1879 |  |
| R.VTYSSPEDGIR.E | Y | 53.92 | 1222.5830 | 11 | 4.7 | 612.3016 | 2 | 46.60 | 1 | 13025 | ANa\_10.raw | 1.33E7 |  | 0 | 5.14E6 | 7.88E6 |  |  |  | 1.44E7 |  | 6 | 1 | 0 | 1 | 1 | 1 | 0 | 0 | 0 | 1 | 1 | 1663 | 1673 |  |
| K.ATGVFTTLQPLR.S | Y | 52.78 | 1302.7296 | 12 | 5.1 | 652.3754 | 2 | 88.60 | 6 | 25213 | Ana\_05.raw |  |  | 2.22E7 | 8.14E5 |  | 3.91E6 | 2.42E6 |  | 1.81E7 | 7.22E6 | 6 | 0 | 0 | 1 | 1 | 0 | 1 | 1 | 0 | 1 | 1 | 1077 | 1088 |  |
| K.VFAVHQGR.E | Y | 51.62 | 912.4930 | 8 | 3.1 | 457.2552 | 2 | 23.27 | 9 | 5724 | Ana\_08.raw | 4.9E6 |  |  |  |  |  |  |  | 1.12E7 | 6.98E6 | 3 | 1 | 0 | 0 | 0 | 0 | 0 | 0 | 0 | 1 | 1 | 976 | 983 |  |
| K.IHLYTLNDNAR.S | Y | 49.75 | 1328.6837 | 11 | 6.2 | 443.9046 | 3 | 54.09 | 9 | 15305 | Ana\_08.raw |  |  |  |  |  |  |  |  | 4.77E6 |  | 1 | 0 | 0 | 0 | 0 | 0 | 0 | 0 | 0 | 1 | 0 | 1880 | 1890 |  |
| R.YQC(+57.02)YC(+57.02)YGR.G | Y | 48.91 | 1168.4430 | 8 | 4.5 | 585.2314 | 2 | 36.71 | 9 | 9944 | Ana\_08.raw | 2.96E6 |  |  |  |  |  |  |  | 3.52E6 | 3.96E6 | 3 | 1 | 0 | 0 | 0 | 0 | 0 | 0 | 0 | 1 | 1 | 585 | 592 | Carbamidomethylation |
| R.APITGYIIR.H | Y | 47.75 | 1002.5862 | 9 | 6.8 | 502.3038 | 2 | 72.23 | 6 | 20726 | Ana\_05.raw | 4.32E6 | 1.01E6 | 6.89E5 |  |  | 1.95E6 |  |  | 1.21E7 | 2.79E6 | 6 | 1 | 1 | 1 | 0 | 0 | 1 | 0 | 0 | 1 | 1 | 1382 | 1390 |  |
| K.YEKPGSPPR.E | Y | 47.33 | 1029.5243 | 9 | 2.1 | 515.7705 | 2 | 21.44 | 9 | 5104 | Ana\_08.raw | 8.25E6 |  | 5.39E6 |  |  | 5.96E6 |  |  | 2.09E7 | 8.14E6 | 6 | 1 | 0 | 1 | 0 | 0 | 1 | 0 | 0 | 2 | 1 | 1936 | 1944 |  |
| R.LTAGLTR.G | Y | 41.63 | 730.4337 | 7 | 2.6 | 366.2251 | 2 | 26.64 | 9 | 6850 | Ana\_08.raw |  |  |  |  |  |  |  |  | 1.91E7 | 1.01E7 | 2 | 0 | 0 | 0 | 0 | 0 | 0 | 0 | 0 | 1 | 1 | 1028 | 1034 |  |
| K.FVHGVR.Y | Y | 39.65 | 713.3973 | 6 | 4.7 | 357.7076 | 2 | 20.58 | 10 | 5011 | Ana\_09.raw |  | 4.69E6 | 3.45E6 |  |  | 3.72E6 | 1.7E6 |  | 1.8E6 | 1.27E7 | 6 | 0 | 1 | 1 | 0 | 0 | 1 | 1 | 0 | 1 | 1 | 579 | 584 |  |
| R.GEWAC(+57.02)IPYSQLR.D | Y | 38.57 | 1478.6976 | 12 | 6.2 | 740.3607 | 2 | 93.94 | 10 | 26257 | Ana\_09.raw |  |  |  |  |  |  |  |  |  | 2.44E6 | 1 | 0 | 0 | 0 | 0 | 0 | 0 | 0 | 0 | 0 | 1 | 504 | 515 | Carbamidomethylation |
| K.YEVSVYALK.D | Y | 37.51 | 1070.5648 | 9 | 3.5 | 536.2916 | 2 | 72.64 | 1 | 20582 | ANa\_10.raw | 2.02E6 |  |  |  |  |  |  |  |  |  | 1 | 1 | 0 | 0 | 0 | 0 | 0 | 0 | 0 | 0 | 0 | 1787 | 1795 |  |
| R.NLQPGSEYTVTLVAVK.G | Y | 36.44 | 1717.9250 | 16 | 1.4 | 859.9710 | 2 | 87.24 | 9 | 24790 | Ana\_08.raw | 3.17E5 |  |  |  |  |  |  |  | 2.14E6 |  | 2 | 1 | 0 | 0 | 0 | 0 | 0 | 0 | 0 | 1 | 0 | 1054 | 1069 |  |
| K.SEPLIGR.K | Y | 36.01 | 770.4286 | 7 | 5.0 | 386.2235 | 2 | 33.34 | 10 | 9058 | Ana\_09.raw | 8.16E6 |  |  |  |  | 2.54E6 |  |  |  | 6.91E6 | 3 | 1 | 0 | 0 | 0 | 0 | 1 | 0 | 0 | 0 | 1 | 1981 | 1987 |  |
| R.ITGYIIK.Y | Y | 35.76 | 806.4902 | 7 | 5.9 | 404.2547 | 2 | 46.62 | 9 | 13020 | Ana\_08.raw | 2.61E6 |  |  |  |  |  |  |  | 5.61E6 | 2.73E6 | 3 | 1 | 0 | 0 | 0 | 0 | 0 | 0 | 0 | 1 | 1 | 1929 | 1935 |  |
| R.IGDTWSK.K | Y | 35.03 | 805.3970 | 7 | 5.0 | 403.7078 | 2 | 32.35 | 1 | 8701 | ANa\_10.raw | 4.74E6 |  |  |  |  |  |  |  |  |  | 1 | 1 | 0 | 0 | 0 | 0 | 0 | 0 | 0 | 0 | 0 | 243 | 249 |  |
| K.EATIPGHLNSYTIK.G | Y | 27.24 | 1542.8042 | 14 | 8.1 | 772.4156 | 2 | 61.90 | 9 | 17617 | Ana\_08.raw |  |  |  |  |  |  |  |  | 1.59E6 |  | 1 | 0 | 0 | 0 | 0 | 0 | 0 | 0 | 0 | 1 | 0 | 656 | 669 |  |
| total 38 peptides |  |  |  |  |  |  |  |  |  |  |  |  |  |  |  |  |  |  |  |  |  |  |  |  |  |  |  |  |  |  |  |  |  |  |  |
| --- | --- | --- | --- | --- | --- | --- | --- | --- | --- | --- | --- | --- | --- | --- | --- | --- | --- | --- | --- | --- | --- | --- | --- | --- | --- | --- | --- | --- | --- | --- | --- | --- | --- | --- | --- |

Best Unique PSM (Scan F1:35991, m/z=979.5218, z=2, RT=130.93, ppm=4.8):

A0A087WR50|A0A087WR50\_MOUSE

back to list

  

| Protein Coverage
| Supporting Peptides
| Best Unique PSM
|

Protein Coverage:

Supporting Peptides:

| Peptide | Uniq | -10lgP | Mass | Length | ppm | m/z | z | RT | Fraction | Scan | Source File | Area 10 | Area 01 | Area 02 | Area 03 | Area 04 | Area 05 | Area 06 | Area 07 | Area 08 | Area 09 | #Spec | #Spec 10 | #Spec 01 | #Spec 02 | #Spec 03 | #Spec 04 | #Spec 05 | #Spec 06 | #Spec 07 | #Spec 08 | #Spec 09 | Start | End | PTM |
| --- | --- | --- | --- | --- | --- | --- | --- | --- | --- | --- | --- | --- | --- | --- | --- | --- | --- | --- | --- | --- | --- | --- | --- | --- | --- | --- | --- | --- | --- | --- | --- | --- | --- | --- | --- |
| R.NTFAEITGLSPGVTYLFK.V | Y | 117.62 | 1957.0197 | 18 | 4.8 | 979.5218 | 2 | 130.93 | 1 | 35991 | ANa\_10.raw | 1.71E7 | 5.47E6 | 4.55E6 | 1.66E6 | 3.39E6 | 6.49E6 | 3.83E6 | 2.37E6 | 2.53E7 | 1.31E7 | 10 | 1 | 1 | 1 | 1 | 1 | 1 | 1 | 1 | 1 | 1 | 958 | 975 |  |
| R.EESPPLIGQQATVSDIPR.D | Y | 100.51 | 1935.9901 | 18 | 4.9 | 969.0071 | 2 | 84.70 | 1 | 23859 | ANa\_10.raw | 3.41E6 |  | 0 |  |  |  |  |  | 7.46E6 | 2.77E6 | 4 | 1 | 0 | 1 | 0 | 0 | 0 | 0 | 0 | 1 | 1 | 1434 | 1451 |  |
| R.HALQSASAGSGSFTDVR.T | Y | 94.98 | 1689.8070 | 17 | 5.1 | 564.2792 | 3 | 43.82 | 9 | 12156 | Ana\_08.raw | 1.52E7 |  |  | 1.61E6 |  | 4.88E6 |  |  | 1.4E7 | 1.39E7 | 8 | 2 | 0 | 0 | 1 | 0 | 1 | 0 | 0 | 2 | 2 | 274 | 290 |  |
| R.VVTPLSPPTNLHLEANPDTGVLTVSWER.S | Y | 94.80 | 3041.5820 | 28 | 4.6 | 1014.8726 | 3 | 123.84 | 9 | 34421 | Ana\_08.raw | 1.96E6 |  |  |  |  |  |  |  | 1.29E7 | 1.45E6 | 3 | 1 | 0 | 0 | 0 | 0 | 0 | 0 | 0 | 1 | 1 | 1169 | 1196 |  |
| R.VTWAPPPSIELTNLLVR.Y | Y | 92.89 | 1905.0724 | 17 | 2.8 | 636.0332 | 3 | 131.32 | 9 | 36352 | Ana\_08.raw | 1.06E7 | 4.32E6 | 3.39E6 | 2.23E6 | 2.78E6 | 9.18E6 | 4.42E6 | 3.25E6 | 2.79E7 | 1.32E7 | 15 | 1 | 1 | 1 | 2 | 1 | 2 | 2 | 1 | 2 | 2 | 1284 | 1300 |  |
| R.VEVLPVSLPGEHGQR.L | Y | 92.67 | 1615.8682 | 15 | 4.9 | 539.6326 | 3 | 73.48 | 9 | 20959 | Ana\_08.raw | 7.38E6 |  | 1.06E6 | 5.73E5 |  | 2.67E6 |  |  | 2.62E7 | 7.44E6 | 7 | 1 | 0 | 1 | 1 | 0 | 1 | 0 | 0 | 2 | 1 | 938 | 952 |  |
| R.FLTTTPNSLLVSWQAPR.A | Y | 88.78 | 1930.0312 | 17 | 3.8 | 966.0266 | 2 | 116.92 | 9 | 32658 | Ana\_08.raw | 4.5E4 |  |  |  |  |  |  |  | 6.62E6 |  | 2 | 1 | 0 | 0 | 0 | 0 | 0 | 0 | 0 | 1 | 0 | 1910 | 1926 |  |
| R.HHAEHSVGRPR.Q | Y | 83.94 | 1281.6439 | 11 | 1.2 | 428.2224 | 3 | 14.86 | 3 | 3344 | Ana\_02.raw |  |  | 8.87E5 |  | 7.83E5 | 9.15E5 | 4.02E5 |  |  | 2.42E6 | 5 | 0 | 0 | 1 | 0 | 1 | 1 | 1 | 0 | 0 | 1 | 1391 | 1401 |  |
| R.SSPVIIDASTAIDAPSNLR.F | Y | 83.89 | 1926.0057 | 19 | 9.0 | 964.0188 | 2 | 96.73 | 1 | 27093 | ANa\_10.raw | 9.01E5 |  |  |  |  |  |  |  | 5.98E6 | 7.54E5 | 3 | 1 | 0 | 0 | 0 | 0 | 0 | 0 | 0 | 1 | 1 | 1891 | 1909 |  |
| K.LGVRPSQGGEAPR.E | Y | 79.62 | 1322.7054 | 13 | 3.6 | 441.9107 | 3 | 28.22 | 6 | 7569 | Ana\_05.raw | 3.06E7 | 1.33E7 | 7.94E6 | 3.68E6 | 6.85E6 | 3.74E6 | 5.18E6 | 4.99E6 | 3.25E7 | 2.93E7 | 14 | 2 | 2 | 1 | 1 | 1 | 1 | 1 | 1 | 2 | 2 | 1116 | 1128 |  |
| R.GVTYNIIVEALQNQR.R | Y | 79.16 | 1716.9159 | 15 | 1.2 | 859.4662 | 2 | 121.21 | 6 | 33804 | Ana\_05.raw | 1.37E6 |  |  |  |  | 7.85E5 |  |  | 8.16E5 | 9.27E5 | 5 | 1 | 0 | 0 | 0 | 0 | 1 | 0 | 0 | 1 | 2 | 2164 | 2178 |  |
| K.GLAFTDVDVDSIK.I | Y | 70.23 | 1378.6980 | 13 | 4.1 | 690.3591 | 2 | 95.79 | 1 | 26840 | ANa\_10.raw | 4.33E5 |  |  |  |  |  |  |  | 7.04E6 | 1.82E6 | 3 | 1 | 0 | 0 | 0 | 0 | 0 | 0 | 0 | 1 | 1 | 1636 | 1648 |  |
| K.YIVNVYQISEEGK.Q | Y | 69.41 | 1540.7773 | 13 | 2.0 | 771.3975 | 2 | 84.99 | 9 | 24182 | Ana\_08.raw |  |  |  |  |  |  |  |  | 2.89E6 |  | 1 | 0 | 0 | 0 | 0 | 0 | 0 | 0 | 0 | 1 | 0 | 785 | 797 |  |
| K.FSQVTPTSFTAQWIAPSVQLTGYR.V | Y | 68.66 | 2684.3599 | 24 | 1.8 | 895.7955 | 3 | 130.13 | 1 | 35767 | ANa\_10.raw | 3.74E6 |  |  |  |  |  |  |  |  | 1.53E6 | 2 | 1 | 0 | 0 | 0 | 0 | 0 | 0 | 0 | 0 | 1 | 1729 | 1752 |  |
| R.ITYGETGGNSPVQEFTVPGSK.S | Y | 68.33 | 2167.0432 | 21 | 2.4 | 1084.5315 | 2 | 84.79 | 9 | 24127 | Ana\_08.raw | 3.12E6 |  |  |  |  |  |  |  | 3.61E6 |  | 3 | 1 | 0 | 0 | 0 | 0 | 0 | 0 | 0 | 2 | 0 | 1479 | 1499 |  |
| R.STTPDITGYR.I | Y | 64.06 | 1109.5353 | 10 | 5.4 | 555.7779 | 2 | 47.08 | 9 | 13163 | Ana\_08.raw |  | 7.02E6 | 7.15E6 | 2.47E6 | 5.01E6 | 7.27E6 | 4.1E6 | 3.92E6 | 2.88E7 | 1.66E7 | 9 | 0 | 1 | 1 | 1 | 1 | 1 | 1 | 1 | 1 | 1 | 1197 | 1206 |  |
| R.TVLVTWTPPR.A | Y | 62.14 | 1168.6604 | 10 | 4.6 | 585.3401 | 2 | 80.93 | 1 | 22843 | ANa\_10.raw | 1.58E6 |  |  | 7.48E4 | 6.42E5 | 9.15E5 |  | 5.23E5 |  | 1.52E6 | 6 | 1 | 0 | 0 | 1 | 1 | 1 | 0 | 1 | 0 | 1 | 1011 | 1020 |  |
| R.TFYQIGDSWEK.F | Y | 62.12 | 1372.6299 | 11 | 4.9 | 687.3256 | 2 | 86.26 | 1 | 24291 | ANa\_10.raw | 3.72E6 |  |  |  |  | 1.92E6 |  |  | 9.15E6 | 1.29E6 | 4 | 1 | 0 | 0 | 0 | 0 | 1 | 0 | 0 | 1 | 1 | 568 | 578 |  |
| K.DDKESAPISDTVVPAVPPPTDLR.F | Y | 61.07 | 2418.2278 | 23 | 4.5 | 807.0868 | 3 | 90.50 | 1 | 25426 | ANa\_10.raw | 2.62E6 |  |  |  |  |  |  |  |  |  | 1 | 1 | 0 | 0 | 0 | 0 | 0 | 0 | 0 | 0 | 0 | 1251 | 1273 |  |
| R.TFYSC(+57.02)TTEGR.Q | Y | 55.80 | 1220.5132 | 10 | 5.5 | 611.2672 | 2 | 37.91 | 9 | 10316 | Ana\_08.raw |  |  |  |  |  |  |  |  | 8.25E6 | 1.25E7 | 2 | 0 | 0 | 0 | 0 | 0 | 0 | 0 | 0 | 1 | 1 | 370 | 379 | Carbamidomethylation |
| K.VGDTYERPK.D | Y | 55.80 | 1063.5298 | 9 | 4.3 | 532.7745 | 2 | 21.65 | 9 | 5173 | Ana\_08.raw |  |  | 3.04E6 |  |  |  |  | 2.03E6 | 5.28E6 | 5.6E6 | 4 | 0 | 0 | 1 | 0 | 0 | 0 | 0 | 1 | 1 | 1 | 109 | 117 |  |
| R.SYTITGLQPGTDYK.I | Y | 55.43 | 1542.7566 | 14 | 3.9 | 772.3886 | 2 | 73.40 | 10 | 20778 | Ana\_09.raw | 7.93E6 |  |  |  |  | 5.31E6 | 1.34E6 | 4.94E6 | 2.49E7 | 7.11E6 | 6 | 1 | 0 | 0 | 0 | 0 | 1 | 1 | 1 | 1 | 1 | 1866 | 1879 |  |
| R.VTYSSPEDGIR.E | Y | 53.92 | 1222.5830 | 11 | 4.7 | 612.3016 | 2 | 46.60 | 1 | 13025 | ANa\_10.raw | 1.33E7 |  | 0 | 5.14E6 | 7.88E6 |  |  |  | 1.44E7 |  | 6 | 1 | 0 | 1 | 1 | 1 | 0 | 0 | 0 | 1 | 1 | 1663 | 1673 |  |
| K.ATGVFTTLQPLR.S | Y | 52.78 | 1302.7296 | 12 | 5.1 | 652.3754 | 2 | 88.60 | 6 | 25213 | Ana\_05.raw |  |  | 2.22E7 | 8.14E5 |  | 3.91E6 | 2.42E6 |  | 1.81E7 | 7.22E6 | 6 | 0 | 0 | 1 | 1 | 0 | 1 | 1 | 0 | 1 | 1 | 1077 | 1088 |  |
| K.VFAVHQGR.E | Y | 51.62 | 912.4930 | 8 | 3.1 | 457.2552 | 2 | 23.27 | 9 | 5724 | Ana\_08.raw | 4.9E6 |  |  |  |  |  |  |  | 1.12E7 | 6.98E6 | 3 | 1 | 0 | 0 | 0 | 0 | 0 | 0 | 0 | 1 | 1 | 976 | 983 |  |
| K.IHLYTLNDNAR.S | Y | 49.75 | 1328.6837 | 11 | 6.2 | 443.9046 | 3 | 54.09 | 9 | 15305 | Ana\_08.raw |  |  |  |  |  |  |  |  | 4.77E6 |  | 1 | 0 | 0 | 0 | 0 | 0 | 0 | 0 | 0 | 1 | 0 | 1880 | 1890 |  |
| R.YQC(+57.02)YC(+57.02)YGR.G | Y | 48.91 | 1168.4430 | 8 | 4.5 | 585.2314 | 2 | 36.71 | 9 | 9944 | Ana\_08.raw | 2.96E6 |  |  |  |  |  |  |  | 3.52E6 | 3.96E6 | 3 | 1 | 0 | 0 | 0 | 0 | 0 | 0 | 0 | 1 | 1 | 585 | 592 | Carbamidomethylation |
| R.APITGYIIR.H | Y | 47.75 | 1002.5862 | 9 | 6.8 | 502.3038 | 2 | 72.23 | 6 | 20726 | Ana\_05.raw | 4.32E6 | 1.01E6 | 6.89E5 |  |  | 1.95E6 |  |  | 1.21E7 | 2.79E6 | 6 | 1 | 1 | 1 | 0 | 0 | 1 | 0 | 0 | 1 | 1 | 1382 | 1390 |  |
| K.YEKPGSPPR.E | Y | 47.33 | 1029.5243 | 9 | 2.1 | 515.7705 | 2 | 21.44 | 9 | 5104 | Ana\_08.raw | 8.25E6 |  | 5.39E6 |  |  | 5.96E6 |  |  | 2.09E7 | 8.14E6 | 6 | 1 | 0 | 1 | 0 | 0 | 1 | 0 | 0 | 2 | 1 | 1936 | 1944 |  |
| R.LTAGLTR.G | Y | 41.63 | 730.4337 | 7 | 2.6 | 366.2251 | 2 | 26.64 | 9 | 6850 | Ana\_08.raw |  |  |  |  |  |  |  |  | 1.91E7 | 1.01E7 | 2 | 0 | 0 | 0 | 0 | 0 | 0 | 0 | 0 | 1 | 1 | 1028 | 1034 |  |
| K.FVHGVR.Y | Y | 39.65 | 713.3973 | 6 | 4.7 | 357.7076 | 2 | 20.58 | 10 | 5011 | Ana\_09.raw |  | 4.69E6 | 3.45E6 |  |  | 3.72E6 | 1.7E6 |  | 1.8E6 | 1.27E7 | 6 | 0 | 1 | 1 | 0 | 0 | 1 | 1 | 0 | 1 | 1 | 579 | 584 |  |
| R.GEWAC(+57.02)IPYSQLR.D | Y | 38.57 | 1478.6976 | 12 | 6.2 | 740.3607 | 2 | 93.94 | 10 | 26257 | Ana\_09.raw |  |  |  |  |  |  |  |  |  | 2.44E6 | 1 | 0 | 0 | 0 | 0 | 0 | 0 | 0 | 0 | 0 | 1 | 504 | 515 | Carbamidomethylation |
| K.YEVSVYALK.D | Y | 37.51 | 1070.5648 | 9 | 3.5 | 536.2916 | 2 | 72.64 | 1 | 20582 | ANa\_10.raw | 2.02E6 |  |  |  |  |  |  |  |  |  | 1 | 1 | 0 | 0 | 0 | 0 | 0 | 0 | 0 | 0 | 0 | 1787 | 1795 |  |
| R.NLQPGSEYTVTLVAVK.G | Y | 36.44 | 1717.9250 | 16 | 1.4 | 859.9710 | 2 | 87.24 | 9 | 24790 | Ana\_08.raw | 3.17E5 |  |  |  |  |  |  |  | 2.14E6 |  | 2 | 1 | 0 | 0 | 0 | 0 | 0 | 0 | 0 | 1 | 0 | 1054 | 1069 |  |
| K.SEPLIGR.K | Y | 36.01 | 770.4286 | 7 | 5.0 | 386.2235 | 2 | 33.34 | 10 | 9058 | Ana\_09.raw | 8.16E6 |  |  |  |  | 2.54E6 |  |  |  | 6.91E6 | 3 | 1 | 0 | 0 | 0 | 0 | 1 | 0 | 0 | 0 | 1 | 1981 | 1987 |  |
| R.ITGYIIK.Y | Y | 35.76 | 806.4902 | 7 | 5.9 | 404.2547 | 2 | 46.62 | 9 | 13020 | Ana\_08.raw | 2.61E6 |  |  |  |  |  |  |  | 5.61E6 | 2.73E6 | 3 | 1 | 0 | 0 | 0 | 0 | 0 | 0 | 0 | 1 | 1 | 1929 | 1935 |  |
| R.IGDTWSK.K | Y | 35.03 | 805.3970 | 7 | 5.0 | 403.7078 | 2 | 32.35 | 1 | 8701 | ANa\_10.raw | 4.74E6 |  |  |  |  |  |  |  |  |  | 1 | 1 | 0 | 0 | 0 | 0 | 0 | 0 | 0 | 0 | 0 | 243 | 249 |  |
| K.EATIPGHLNSYTIK.G | Y | 27.24 | 1542.8042 | 14 | 8.1 | 772.4156 | 2 | 61.90 | 9 | 17617 | Ana\_08.raw |  |  |  |  |  |  |  |  | 1.59E6 |  | 1 | 0 | 0 | 0 | 0 | 0 | 0 | 0 | 0 | 1 | 0 | 656 | 669 |  |
| total 38 peptides |  |  |  |  |  |  |  |  |  |  |  |  |  |  |  |  |  |  |  |  |  |  |  |  |  |  |  |  |  |  |  |  |  |  |  |
| --- | --- | --- | --- | --- | --- | --- | --- | --- | --- | --- | --- | --- | --- | --- | --- | --- | --- | --- | --- | --- | --- | --- | --- | --- | --- | --- | --- | --- | --- | --- | --- | --- | --- | --- | --- |

Best Unique PSM (Scan F1:35991, m/z=979.5218, z=2, RT=130.93, ppm=4.8):

sp|P14824|ANXA6\_MOUSE

back to list

  

| Protein Coverage
| Supporting Peptides
| Best Unique PSM
|

Protein Coverage:

Supporting Peptides:

| Peptide | Uniq | -10lgP | Mass | Length | ppm | m/z | z | RT | Fraction | Scan | Source File | Area 10 | Area 01 | Area 02 | Area 03 | Area 04 | Area 05 | Area 06 | Area 07 | Area 08 | Area 09 | #Spec | #Spec 10 | #Spec 01 | #Spec 02 | #Spec 03 | #Spec 04 | #Spec 05 | #Spec 06 | #Spec 07 | #Spec 08 | #Spec 09 | Start | End | PTM |
| --- | --- | --- | --- | --- | --- | --- | --- | --- | --- | --- | --- | --- | --- | --- | --- | --- | --- | --- | --- | --- | --- | --- | --- | --- | --- | --- | --- | --- | --- | --- | --- | --- | --- | --- | --- |
| R.ENDDVVSEDLVQQDVQDLYEAGELK.W | Y | 128.66 | 2849.3091 | 25 | 3.6 | 1425.6670 | 2 | 130.80 | 6 | 36343 | Ana\_05.raw |  |  | 7.1E5 |  |  | 2.07E6 |  | 6.04E5 | 7.79E6 | 4.06E6 | 5 | 0 | 0 | 1 | 0 | 0 | 1 | 0 | 1 | 1 | 1 | 167 | 191 |  |
| R.DQAQEDAQVAAEILEIADTPSGDK.T | Y | 104.73 | 2513.1768 | 24 | 1.8 | 1257.5979 | 2 | 131.96 | 7 | 36336 | Ana\_06.raw | 1.98E6 |  |  |  |  | 1.07E6 | 9.88E5 |  |  |  | 3 | 1 | 0 | 0 | 0 | 0 | 1 | 1 | 0 | 0 | 0 | 517 | 540 |  |
| R.DLESDIIGDTSGHFQK.M | Y | 104.10 | 1760.8217 | 16 | -0.1 | 881.4180 | 2 | 102.48 | 6 | 28908 | Ana\_05.raw | 1.27E7 | 1.05E7 | 9.09E5 |  | 1.08E7 | 1.23E7 | 1.89E6 | 1.26E6 | 3.97E7 | 1.21E7 | 16 | 2 | 2 | 1 | 0 | 2 | 2 | 1 | 2 | 2 | 2 | 141 | 156 |  |
| K.GIGTDEATIIDIVTHR.S | Y | 95.82 | 1709.8948 | 16 | -0.2 | 855.9545 | 2 | 122.60 | 4 | 33368 | Ana\_03.raw | 9.04E6 | 3.35E7 | 3.32E7 | 1.47E7 | 1.88E7 | 3.06E7 | 5.1E6 | 1.97E7 | 4.38E7 | 2.67E7 | 31 | 3 | 3 | 3 | 3 | 3 | 3 | 2 | 4 | 3 | 4 | 378 | 393 |  |
| K.GFGSDKESILELITSR.S | Y | 87.95 | 1750.9100 | 16 | 6.6 | 584.6478 | 3 | 119.14 | 1 | 32875 | ANa\_10.raw | 8.77E6 | 1.14E7 | 1.27E7 | 1.85E6 | 9.93E6 | 1.37E7 | 2.61E6 | 7.25E6 | 1.67E7 | 7.86E6 | 18 | 2 | 1 | 1 | 1 | 2 | 2 | 2 | 3 | 2 | 2 | 35 | 50 |  |
| K.GTVC(+57.02)AANDFNPDADAK.A | Y | 82.94 | 1664.7100 | 16 | 2.3 | 833.3642 | 2 | 53.89 | 5 | 15268 | Ana\_04.raw | 4.15E6 | 3.93E6 |  | 3.36E5 | 2.15E6 | 2.77E6 | 1.27E6 | 1.61E6 | 6.09E6 | 3.36E6 | 9 | 1 | 1 | 0 | 1 | 1 | 1 | 1 | 1 | 1 | 1 | 355 | 370 | Carbamidomethylation |
| K.ESILELITSR.S | Y | 82.45 | 1159.6448 | 10 | 0.7 | 580.8301 | 2 | 125.33 | 6 | 34869 | Ana\_05.raw | 1.43E6 | 2.11E6 | 5.83E6 | 4.85E5 | 8.98E6 | 1.11E7 | 6.62E6 | 5.56E6 | 1.01E6 | 7.62E6 | 19 | 2 | 2 | 2 | 2 | 2 | 2 | 2 | 2 | 1 | 2 | 41 | 50 |  |
| K.DLIEDLKYELTGK.F | Y | 79.51 | 1535.8082 | 13 | 4.2 | 512.9455 | 3 | 130.16 | 3 | 35658 | Ana\_02.raw | 8.94E6 | 9.34E6 | 8.44E6 | 3.37E6 | 7.59E6 | 1.16E7 | 6.75E6 | 3.11E6 | 3.05E6 | 9.78E6 | 19 | 2 | 2 | 2 | 2 | 2 | 2 | 2 | 2 | 1 | 2 | 69 | 81 |  |
| K.WGTDEAQFIYILGNR.S | Y | 76.33 | 1781.8737 | 15 | 2.7 | 891.9465 | 2 | 127.44 | 8 | 34960 | Ana\_07.raw |  |  |  |  |  |  |  | 4.9E5 |  | 0 | 2 | 0 | 0 | 0 | 0 | 0 | 0 | 0 | 1 | 0 | 1 | 192 | 206 |  |
| R.GSVHDFPEFDANQDAEALYTAM(+15.99)K.G | Y | 75.13 | 2571.1223 | 23 | 4.2 | 858.0516 | 3 | 98.38 | 1 | 27544 | ANa\_10.raw | 3.12E6 | 9.9E5 |  |  |  |  |  | 1.61E6 | 3.35E6 |  | 4 | 1 | 1 | 0 | 0 | 0 | 0 | 0 | 1 | 1 | 0 | 12 | 34 | Oxidation (M) |
| K.SLEDALSSDTSGHFR.R | Y | 75.03 | 1620.7379 | 15 | 3.5 | 811.3791 | 2 | 78.40 | 7 | 22183 | Ana\_06.raw |  |  | 4.63E6 |  |  |  | 5.93E5 | 8.57E5 | 8.5E6 |  | 4 | 0 | 0 | 1 | 0 | 0 | 0 | 1 | 1 | 1 | 0 | 484 | 498 |  |
| K.DAFVAIVQSVK.N | Y | 74.22 | 1175.6550 | 11 | -1.5 | 588.8339 | 2 | 95.03 | 3 | 26593 | Ana\_02.raw | 8.13E6 | 5.48E6 | 7.44E6 | 2.28E6 | 9.32E5 | 5.53E6 | 3.35E6 | 3.7E6 | 1.17E7 | 6.93E6 | 13 | 2 | 1 | 1 | 1 | 1 | 1 | 1 | 1 | 2 | 2 | 588 | 598 |  |
| R.STPEYFAER.L | Y | 73.85 | 1098.4982 | 9 | 1.5 | 550.2572 | 2 | 47.17 | 3 | 13322 | Ana\_02.raw | 2.29E7 | 2.17E7 | 2.59E7 | 9.32E6 | 2.24E7 | 2.28E7 | 1.09E7 | 1.28E7 | 1.86E7 | 2.23E7 | 13 | 2 | 1 | 1 | 1 | 2 | 1 | 1 | 1 | 1 | 2 | 251 | 259 |  |
| R.SEIDLLNIR.R | Y | 73.65 | 1071.5924 | 9 | 5.2 | 536.8063 | 2 | 92.90 | 1 | 26066 | ANa\_10.raw | 1.61E7 | 1.32E6 | 1.63E7 | 4.83E6 | 1.18E7 | 9.94E6 | 2.02E6 | 4.79E6 | 2.19E7 | 1.07E7 | 14 | 1 | 1 | 2 | 1 | 2 | 1 | 1 | 2 | 1 | 2 | 630 | 638 |  |
| R.AINEAYKEDYHK.S | Y | 71.20 | 1479.6993 | 12 | 3.9 | 740.8599 | 2 | 29.37 | 2 | 7822 | Ana\_01.raw | 1.33E7 | 1.22E7 |  | 3.14E6 | 3.73E6 | 6.18E6 |  | 3.31E6 | 0 |  | 8 | 1 | 2 | 0 | 1 | 1 | 1 | 0 | 1 | 1 | 0 | 472 | 483 |  |
| K.TTGKPIEASIR.G | Y | 56.07 | 1171.6560 | 11 | 3.8 | 586.8375 | 2 | 27.65 | 3 | 7402 | Ana\_02.raw | 1.58E7 | 1.04E7 | 1.88E7 | 4.33E6 |  |  |  |  |  | 1.51E7 | 8 | 2 | 1 | 2 | 1 | 0 | 0 | 0 | 0 | 0 | 2 | 221 | 231 |  |
| K.TNYDIEHVIK.K | Y | 54.13 | 1230.6244 | 10 | 2.5 | 616.3210 | 2 | 57.62 | 9 | 16342 | Ana\_08.raw |  |  | 2.94E6 |  |  | 2.62E6 |  |  | 1.26E7 |  | 4 | 0 | 0 | 1 | 0 | 0 | 1 | 0 | 0 | 2 | 0 | 570 | 579 |  |
| K.DAISGVGTDEK.C | Y | 53.25 | 1090.5142 | 11 | 2.3 | 546.2656 | 2 | 34.33 | 4 | 9159 | Ana\_03.raw |  | 1.22E7 |  | 4.89E6 | 1.09E7 | 1.25E7 | 7.99E6 |  | 1.43E7 | 1.04E7 | 7 | 0 | 1 | 0 | 1 | 1 | 1 | 1 | 0 | 1 | 1 | 103 | 113 |  |
| K.TLIEILATR.T | Y | 51.12 | 1028.6229 | 9 | 4.0 | 515.3208 | 2 | 96.01 | 2 | 27080 | Ana\_01.raw | 5.82E6 | 4.81E6 | 4.84E6 | 7.67E5 | 2.42E6 | 4.42E6 | 1.5E5 | 8.07E5 | 8.69E6 | 4.72E6 | 10 | 1 | 1 | 1 | 1 | 1 | 1 | 1 | 1 | 1 | 1 | 457 | 465 |  |
| R.GELSGDFEK.L | Y | 49.88 | 980.4451 | 9 | 4.9 | 491.2322 | 2 | 43.68 | 6 | 12288 | Ana\_05.raw | 1.26E7 | 9.4E6 | 1.37E7 | 4.69E6 | 1.07E7 | 1.18E7 | 7.76E6 | 7.65E6 | 9.94E6 | 1.15E7 | 10 | 1 | 1 | 1 | 1 | 1 | 1 | 1 | 1 | 1 | 1 | 232 | 240 |  |
| K.DLIEDLK.Y | N | 49.65 | 844.4542 | 7 | 1.3 | 423.2349 | 2 | 75.59 | 4 | 21028 | Ana\_03.raw | 8.45E6 | 1.3E7 | 1.69E7 | 7.59E6 | 8.85E6 | 8.73E6 | 7.03E6 |  | 2.59E6 |  | 9 | 1 | 1 | 2 | 1 | 1 | 1 | 1 | 0 | 1 | 0 | 69 | 75 |  |
| R.LVFDEYLK.T | Y | 48.47 | 1025.5433 | 8 | 3.0 | 513.7805 | 2 | 91.78 | 6 | 26063 | Ana\_05.raw | 7.66E6 | 8.66E6 | 7.91E6 | 3.23E6 | 5.01E6 | 7.27E6 | 3.96E6 | 4.63E6 | 1.25E7 | 7.59E6 | 10 | 1 | 1 | 1 | 1 | 1 | 1 | 1 | 1 | 1 | 1 | 213 | 220 |  |
| R.SELDM(+15.99)LDIR.E | Y | 48.36 | 1106.5277 | 9 | 6.9 | 554.2750 | 2 | 72.05 | 2 | 20526 | Ana\_01.raw | 6.02E6 | 5.52E6 |  | 6.26E5 | 2.62E6 |  | 2.42E6 | 3.18E6 | 7.96E6 | 2.42E6 | 9 | 1 | 1 | 0 | 1 | 1 | 0 | 1 | 1 | 1 | 2 | 282 | 290 | Oxidation (M) |
| K.SEISGDLAR.L | Y | 47.13 | 946.4719 | 9 | 7.0 | 474.2466 | 2 | 34.43 | 9 | 9230 | Ana\_08.raw |  |  |  |  |  |  |  |  | 6.53E5 |  | 1 | 0 | 0 | 0 | 0 | 0 | 0 | 0 | 0 | 1 | 0 | 419 | 427 |  |
| K.SLHQAIEGDTSGDFM(+15.99)K.A | Y | 46.00 | 1750.7832 | 16 | 4.7 | 584.6044 | 3 | 55.27 | 8 | 15383 | Ana\_07.raw |  |  | 1.1E6 |  |  |  |  | 7.85E5 |  |  | 2 | 0 | 0 | 1 | 0 | 0 | 0 | 0 | 1 | 0 | 0 | 648 | 663 | Oxidation (M) |
| R.ILISLATGNR.E | Y | 37.86 | 1056.6292 | 10 | 0.2 | 529.3220 | 2 | 67.56 | 9 | 19283 | Ana\_08.raw |  |  | 6.77E5 |  |  | 8.35E5 |  |  | 1.23E6 |  | 3 | 0 | 0 | 1 | 0 | 0 | 1 | 0 | 0 | 1 | 0 | 500 | 509 |  |
| R.AINEAYK.E | Y | 36.50 | 807.4127 | 7 | 3.7 | 404.7151 | 2 | 24.11 | 6 | 6268 | Ana\_05.raw |  |  |  |  |  | 1.1E7 |  |  |  |  | 1 | 0 | 0 | 0 | 0 | 0 | 1 | 0 | 0 | 0 | 0 | 472 | 478 |  |
| K.NKPLFFADK.L | Y | 36.22 | 1078.5811 | 9 | 4.7 | 540.3004 | 2 | 55.85 | 7 | 15747 | Ana\_06.raw |  |  |  |  | 1.28E7 |  | 1.98E6 |  |  |  | 2 | 0 | 0 | 0 | 0 | 1 | 0 | 1 | 0 | 0 | 0 | 599 | 607 |  |
| R.SYPHLR.R | Y | 27.51 | 771.4028 | 6 | 2.8 | 386.7097 | 2 | 27.02 | 5 | 7169 | Ana\_04.raw |  |  |  |  | 9.6E6 |  |  |  |  |  | 1 | 0 | 0 | 0 | 0 | 1 | 0 | 0 | 0 | 0 | 0 | 555 | 560 |  |
| R.EFIEK.Y | N | 24.32 | 664.3431 | 5 | 0.7 | 333.1791 | 2 | 25.26 | 3 | 6644 | Ana\_02.raw |  |  | 7.6E6 | 1.48E6 | 2.66E5 |  |  |  |  |  | 3 | 0 | 0 | 1 | 1 | 1 | 0 | 0 | 0 | 0 | 0 | 640 | 644 |  |
| total 30 peptides |  |  |  |  |  |  |  |  |  |  |  |  |  |  |  |  |  |  |  |  |  |  |  |  |  |  |  |  |  |  |  |  |  |  |  |
| --- | --- | --- | --- | --- | --- | --- | --- | --- | --- | --- | --- | --- | --- | --- | --- | --- | --- | --- | --- | --- | --- | --- | --- | --- | --- | --- | --- | --- | --- | --- | --- | --- | --- | --- | --- |

Best Unique PSM (Scan F6:36343, m/z=1425.6670, z=2, RT=130.80, ppm=3.6):

sp|Q8C196|CPSM\_MOUSE

back to list

  

| Protein Coverage
| Supporting Peptides
| Best Unique PSM
|

Protein Coverage:

Supporting Peptides:

| Peptide | Uniq | -10lgP | Mass | Length | ppm | m/z | z | RT | Fraction | Scan | Source File | Area 10 | Area 01 | Area 02 | Area 03 | Area 04 | Area 05 | Area 06 | Area 07 | Area 08 | Area 09 | #Spec | #Spec 10 | #Spec 01 | #Spec 02 | #Spec 03 | #Spec 04 | #Spec 05 | #Spec 06 | #Spec 07 | #Spec 08 | #Spec 09 | Start | End | PTM |
| --- | --- | --- | --- | --- | --- | --- | --- | --- | --- | --- | --- | --- | --- | --- | --- | --- | --- | --- | --- | --- | --- | --- | --- | --- | --- | --- | --- | --- | --- | --- | --- | --- | --- | --- | --- |
| K.GYSFGHPSSVAGEVVFNTGLGGYPEALTDPAYK.G | Y | 120.65 | 3386.6094 | 33 | -1.8 | 1129.8750 | 3 | 124.14 | 2 | 34421 | Ana\_01.raw | 1.01E6 | 0 |  |  |  | 3.05E6 | 2.06E6 |  |  |  | 4 | 1 | 1 | 0 | 0 | 0 | 1 | 1 | 0 | 0 | 0 | 58 | 90 |  |
| K.EPLFGISTGNIITGLAAGAK.S | Y | 102.39 | 1929.0571 | 20 | 6.3 | 965.5419 | 2 | 129.53 | 6 | 35983 | Ana\_05.raw |  |  |  |  |  | 9.65E6 | 5.51E6 |  |  |  | 2 | 0 | 0 | 0 | 0 | 0 | 1 | 1 | 0 | 0 | 0 | 288 | 307 |  |
| R.TAVDSGIALLTNFQVTK.L | Y | 101.56 | 1776.9622 | 17 | -1.8 | 889.4868 | 2 | 122.41 | 3 | 33654 | Ana\_02.raw | 2.16E6 |  | 2.9E6 | 2.21E6 | 6.33E6 | 1.18E7 | 5.61E6 | 2.71E6 | 8.84E6 | 1.2E6 | 14 | 1 | 0 | 1 | 2 | 2 | 2 | 2 | 2 | 1 | 1 | 1455 | 1471 |  |
| K.AFAISGPFNVQFLVK.G | Y | 83.36 | 1636.8977 | 15 | 3.6 | 819.4591 | 2 | 129.92 | 3 | 35597 | Ana\_02.raw |  | 4.17E6 | 4.29E6 | 1.77E6 |  |  | 2.7E6 |  | 4.07E6 |  | 5 | 0 | 1 | 1 | 1 | 0 | 0 | 1 | 0 | 1 | 0 | 1233 | 1247 |  |
| K.TLGVDFIDVATK.V | Y | 80.80 | 1277.6866 | 12 | 3.7 | 639.8530 | 2 | 101.35 | 6 | 28614 | Ana\_05.raw | 2.69E6 |  | 7.52E6 | 2.21E5 | 2.72E6 | 3.43E6 | 5.18E5 | 8.49E5 |  | 4.77E6 | 8 | 1 | 0 | 1 | 1 | 1 | 1 | 1 | 1 | 0 | 1 | 1270 | 1281 |  |
| K.VPAIYGVDTR.M | Y | 78.06 | 1089.5818 | 10 | 1.9 | 545.7992 | 2 | 59.32 | 3 | 16873 | Ana\_02.raw | 8.81E6 |  | 9.23E6 | 6.85E6 |  | 9.34E6 | 4.52E6 | 8.6E6 |  |  | 6 | 1 | 0 | 1 | 1 | 0 | 1 | 1 | 1 | 0 | 0 | 158 | 167 |  |
| K.GLNSDSVTEETLR.K | Y | 77.27 | 1419.6841 | 13 | 4.3 | 710.8524 | 2 | 55.94 | 9 | 15844 | Ana\_08.raw |  | 5.43E6 |  | 1.63E6 |  | 3.8E6 | 2.4E6 | 6.84E5 | 7.17E6 |  | 6 | 0 | 1 | 0 | 1 | 0 | 1 | 1 | 1 | 1 | 0 | 893 | 905 |  |
| K.IEFEGQSVDFVDPNK.Q | Y | 76.13 | 1722.8101 | 15 | 0.3 | 862.4126 | 2 | 93.79 | 3 | 26266 | Ana\_02.raw |  |  | 4.21E6 |  |  |  |  | 7.38E5 | 5.21E6 |  | 3 | 0 | 0 | 1 | 0 | 0 | 0 | 0 | 1 | 1 | 0 | 183 | 197 |  |
| R.SIFSAVLDELK.V | Y | 75.17 | 1220.6652 | 11 | 2.7 | 611.3415 | 2 | 131.91 | 3 | 36137 | Ana\_02.raw | 1.14E7 | 1.64E7 | 1.28E7 | 7.17E6 | 1.36E7 | 1.86E7 | 1.03E7 | 4.67E6 | 4.82E5 | 1.26E7 | 23 | 3 | 6 | 5 | 1 | 3 | 1 | 1 | 1 | 1 | 1 | 1090 | 1100 |  |
| R.VSQEHPVVLTK.F | Y | 74.57 | 1235.6874 | 11 | 2.9 | 618.8528 | 2 | 31.55 | 6 | 8604 | Ana\_05.raw | 1.32E7 | 2.07E7 | 2.18E7 | 9.74E6 | 1.34E7 | 1.6E7 | 6E6 | 8.84E6 | 1.51E7 | 1.09E7 | 18 | 2 | 2 | 2 | 2 | 2 | 2 | 1 | 1 | 2 | 2 | 1158 | 1168 |  |
| K.RLPTLEQPIIPSDYVAIK.A | Y | 74.49 | 2052.1619 | 18 | 3.0 | 685.0633 | 3 | 106.43 | 6 | 29957 | Ana\_05.raw | 2.14E6 | 9.78E5 | 2.89E5 | 6.32E5 | 1.04E6 | 3.73E6 | 1.65E6 | 4.17E5 | 6.54E6 |  | 9 | 1 | 1 | 1 | 1 | 1 | 1 | 1 | 1 | 1 | 0 | 1292 | 1309 |  |
| R.LPTLEQPIIPSDYVAIK.A | Y | 70.60 | 1896.0608 | 17 | 3.4 | 949.0409 | 2 | 115.71 | 1 | 31990 | ANa\_10.raw | 9.51E6 | 9.48E5 | 2.17E6 | 5.58E5 | 4.04E6 | 8.26E6 | 1.97E6 | 3.77E6 | 1.15E7 | 8.38E6 | 10 | 1 | 1 | 1 | 1 | 1 | 1 | 1 | 1 | 1 | 1 | 1293 | 1309 |  |
| K.AFAM(+15.99)TNQILVER.S | Y | 70.51 | 1407.7180 | 12 | 4.0 | 704.8691 | 2 | 68.12 | 2 | 19415 | Ana\_01.raw | 5.49E6 | 5.4E6 | 5.55E6 | 1.98E6 | 4.93E6 | 4.37E6 | 3.08E6 | 3.28E6 |  | 4.41E6 | 9 | 1 | 1 | 1 | 1 | 1 | 1 | 1 | 1 | 0 | 1 | 613 | 624 | Oxidation (M) |
| R.FLGVAEQLHNEGFK.L | Y | 65.95 | 1587.8044 | 14 | 2.1 | 530.2766 | 3 | 84.50 | 6 | 24092 | Ana\_05.raw | 1.02E6 | 3.82E6 |  |  | 5.33E5 | 2.33E6 | 1.64E6 |  | 7.81E6 | 3.35E6 | 8 | 1 | 1 | 0 | 0 | 1 | 1 | 1 | 0 | 2 | 1 | 1374 | 1387 |  |
| K.IALGIPLPEIK.N | Y | 64.53 | 1162.7325 | 11 | 2.0 | 582.3747 | 2 | 113.41 | 6 | 31755 | Ana\_05.raw | 1.43E7 | 1.69E7 | 2.23E7 | 8.24E6 | 1.37E7 | 1.76E7 | 1.42E7 | 3.59E6 | 1.87E7 | 1.06E7 | 20 | 2 | 2 | 3 | 1 | 2 | 2 | 2 | 3 | 2 | 1 | 741 | 751 |  |
| K.FVHDNYVIR.R | Y | 61.31 | 1161.5930 | 9 | 3.8 | 388.2064 | 3 | 45.53 | 3 | 12828 | Ana\_02.raw | 3.18E6 | 1.05E7 | 7.22E6 | 3.77E6 | 4.47E6 | 5.64E6 | 4.77E6 |  |  | 2.39E6 | 9 | 1 | 2 | 1 | 1 | 1 | 1 | 1 | 0 | 0 | 1 | 1445 | 1453 |  |
| K.IAPSFAVESM(+15.99)EDALK.A | Y | 59.74 | 1622.7861 | 15 | 2.5 | 812.4024 | 2 | 80.40 | 6 | 22983 | Ana\_05.raw | 1.34E6 | 2.98E6 | 8.86E6 |  |  | 0 |  | 1.08E6 |  | 5.95E5 | 7 | 1 | 1 | 2 | 0 | 0 | 1 | 0 | 1 | 0 | 1 | 561 | 575 | Oxidation (M) |
| K.IM(+15.99)GTSPLQIDR.A | Y | 58.04 | 1245.6387 | 11 | -3.1 | 623.8247 | 2 | 58.03 | 6 | 16591 | Ana\_05.raw |  | 5.35E6 | 5.53E6 |  | 3.69E6 | 3.35E6 | 2.14E6 | 3.43E6 |  |  | 6 | 0 | 1 | 1 | 0 | 1 | 1 | 1 | 1 | 0 | 0 | 1075 | 1085 | Oxidation (M) |
| K.ELSEPSSTR.I | Y | 56.97 | 1004.4774 | 9 | 3.2 | 503.2476 | 2 | 22.20 | 3 | 5672 | Ana\_02.raw | 1.43E7 | 1.52E7 | 2.01E7 | 9.44E6 |  | 3.1E6 | 6.26E6 | 8.1E6 |  | 1.26E7 | 8 | 1 | 1 | 1 | 1 | 0 | 1 | 1 | 1 | 0 | 1 | 842 | 850 |  |
| K.IAPSFAVESMEDALK.A | Y | 56.39 | 1606.7913 | 15 | -2.8 | 804.4006 | 2 | 116.08 | 10 | 31929 | Ana\_09.raw |  |  |  |  |  |  |  |  |  | 1.5E6 | 1 | 0 | 0 | 0 | 0 | 0 | 0 | 0 | 0 | 0 | 1 | 561 | 575 |  |
| R.TFEESFQK.A | Y | 53.03 | 1014.4658 | 8 | 6.4 | 508.2434 | 2 | 45.72 | 2 | 12813 | Ana\_01.raw |  | 1.48E7 |  | 7.2E6 | 1.1E7 | 1.3E7 | 9.91E6 | 9.55E6 |  | 1.07E7 | 7 | 0 | 1 | 0 | 1 | 1 | 1 | 1 | 1 | 0 | 1 | 804 | 811 |  |
| R.GQNQPVLNITNR.Q | Y | 52.50 | 1352.7161 | 12 | 4.0 | 677.3680 | 2 | 56.77 | 7 | 16026 | Ana\_06.raw |  |  | 1.03E7 |  |  |  | 6.29E6 |  |  |  | 2 | 0 | 0 | 1 | 0 | 0 | 0 | 1 | 0 | 0 | 0 | 317 | 328 |  |
| R.TSVNVVR.H | Y | 50.07 | 773.4396 | 7 | 4.5 | 387.7288 | 2 | 24.25 | 2 | 6175 | Ana\_01.raw | 9.69E6 | 1.51E7 | 2.3E7 | 8.77E6 |  | 2.05E7 | 1.17E7 | 8.42E6 |  | 1.13E7 | 8 | 1 | 1 | 1 | 1 | 0 | 1 | 1 | 1 | 0 | 1 | 683 | 689 |  |
| R.KEPLFGISTGNIITGLAAGAK.S | Y | 48.49 | 2057.1521 | 21 | 5.4 | 686.7283 | 3 | 117.92 | 2 | 32805 | Ana\_01.raw |  | 2E6 |  |  |  |  |  |  |  |  | 1 | 0 | 1 | 0 | 0 | 0 | 0 | 0 | 0 | 0 | 0 | 287 | 307 |  |
| K.EIEYEVVR.D | N | 47.57 | 1035.5237 | 8 | 8.0 | 518.7733 | 2 | 51.55 | 9 | 14530 | Ana\_08.raw |  |  | 1.42E7 |  |  |  |  | 5.98E6 | 2.08E7 | 1.34E7 | 4 | 0 | 0 | 1 | 0 | 0 | 0 | 0 | 1 | 1 | 1 | 631 | 638 |  |
| R.RTSVNVVR.H | Y | 47.31 | 929.5406 | 8 | 7.9 | 465.7813 | 2 | 21.22 | 2 | 5146 | Ana\_01.raw |  | 1.41E6 | 1.5E6 |  |  |  |  |  | 0 |  | 3 | 0 | 1 | 1 | 0 | 0 | 0 | 0 | 0 | 1 | 0 | 682 | 689 |  |
| K.LNEINEK.I | Y | 44.96 | 858.4447 | 7 | 3.6 | 430.2312 | 2 | 22.27 | 6 | 5682 | Ana\_05.raw |  |  |  | 1.24E7 |  | 1.64E7 | 4.64E6 | 5.01E6 |  |  | 4 | 0 | 0 | 0 | 1 | 0 | 1 | 1 | 1 | 0 | 0 | 554 | 560 |  |
| R.DADPILR.C | Y | 44.80 | 798.4235 | 7 | 1.3 | 400.2195 | 2 | 36.88 | 3 | 10189 | Ana\_02.raw |  |  | 9.47E6 |  | 8.2E6 | 7.93E6 |  |  |  | 7.24E6 | 4 | 0 | 0 | 1 | 0 | 1 | 1 | 0 | 0 | 0 | 1 | 1320 | 1326 |  |
| R.SFPFVSK.T | N | 43.11 | 810.4276 | 7 | 6.1 | 406.2235 | 2 | 68.52 | 6 | 19664 | Ana\_05.raw | 2.58E6 | 5.3E6 | 5.65E6 |  | 2.88E6 | 3.13E6 |  | 2.1E6 | 9.21E6 |  | 8 | 1 | 1 | 1 | 0 | 1 | 1 | 0 | 1 | 2 | 0 | 1263 | 1269 |  |
| K.VVAVDC(+57.02)GIK.N | Y | 42.97 | 959.5110 | 9 | 1.9 | 480.7637 | 2 | 40.96 | 4 | 11120 | Ana\_03.raw |  | 7.26E6 |  | 2.52E6 |  |  |  |  |  |  | 2 | 0 | 1 | 0 | 1 | 0 | 0 | 0 | 0 | 0 | 0 | 220 | 228 | Carbamidomethylation |
| K.LFAEAVQK.S | Y | 40.54 | 904.5018 | 8 | 2.9 | 453.2595 | 2 | 43.67 | 6 | 12284 | Ana\_05.raw | 8.41E6 | 1.05E7 | 1.22E7 | 6.26E6 | 8.21E6 | 1.11E7 | 8.04E6 | 6.45E6 | 4.58E6 | 7.47E6 | 10 | 1 | 1 | 1 | 1 | 1 | 1 | 1 | 1 | 1 | 1 | 1472 | 1479 |  |
| K.TAHIVLEDGTK.M | Y | 39.30 | 1182.6244 | 11 | 7.3 | 395.2183 | 3 | 37.13 | 7 | 10129 | Ana\_06.raw |  |  |  |  | 2.82E6 |  | 4.37E6 |  |  |  | 2 | 0 | 0 | 0 | 0 | 1 | 0 | 1 | 0 | 0 | 0 | 45 | 55 |  |
| K.VM(+15.99)IGESIDEK.R | Y | 38.78 | 1135.5431 | 10 | 3.2 | 568.7806 | 2 | 48.65 | 7 | 13616 | Ana\_06.raw |  |  |  | 3.09E6 | 5.96E6 | 5.74E6 | 4.56E6 | 4.66E6 |  |  | 5 | 0 | 0 | 0 | 1 | 1 | 1 | 1 | 1 | 0 | 0 | 1282 | 1291 | Oxidation (M) |
| R.FLEEATR.V | Y | 37.93 | 864.4341 | 7 | 7.2 | 433.2274 | 2 | 33.92 | 9 | 9072 | Ana\_08.raw | 1.79E7 | 1.63E7 |  | 9.37E6 | 2.03E7 | 2.62E7 | 1.54E7 | 1.59E7 | 9E6 | 1.12E7 | 9 | 1 | 1 | 0 | 1 | 1 | 1 | 1 | 1 | 1 | 1 | 1151 | 1157 |  |
| K.AADTIGYPVM(+15.99)IR.S | Y | 34.87 | 1321.6700 | 12 | 2.6 | 661.8440 | 2 | 71.33 | 3 | 20266 | Ana\_02.raw | 6.26E6 |  | 5.09E6 | 1.67E6 | 4.45E6 | 4.07E6 | 2.65E6 |  | 3.44E6 |  | 7 | 1 | 0 | 1 | 1 | 1 | 1 | 1 | 0 | 1 | 0 | 576 | 587 | Oxidation (M) |
| K.QADAVYFLPITPQFVTEVIK.A | Y | 34.06 | 2278.2249 | 20 | 3.0 | 1140.1232 | 2 | 139.24 | 1 | 38179 | ANa\_10.raw | 3.24E5 |  |  |  |  | 7.94E5 |  |  |  |  | 2 | 1 | 0 | 0 | 0 | 0 | 1 | 0 | 0 | 0 | 0 | 478 | 497 |  |
| K.FVEGAR.E | Y | 33.22 | 677.3496 | 6 | 3.0 | 339.6831 | 2 | 20.06 | 5 | 5004 | Ana\_04.raw |  |  |  |  | 5.43E6 |  | 9.77E5 |  |  |  | 2 | 0 | 0 | 0 | 0 | 1 | 0 | 1 | 0 | 0 | 0 | 1169 | 1174 |  |
| K.KILESDRK.E | Y | 33.14 | 987.5712 | 8 | 4.0 | 330.1990 | 3 | 16.81 | 6 | 3873 | Ana\_05.raw |  |  |  |  |  | 7.59E5 |  |  |  |  | 2 | 0 | 0 | 0 | 0 | 0 | 2 | 0 | 0 | 0 | 0 | 280 | 287 |  |
| R.WDLDR.F | Y | 30.35 | 703.3289 | 5 | -0.7 | 352.6715 | 2 | 44.80 | 3 | 12597 | Ana\_02.raw |  |  | 0 |  |  |  |  |  |  |  | 1 | 0 | 0 | 1 | 0 | 0 | 0 | 0 | 0 | 0 | 0 | 776 | 780 |  |
| R.DELGLNK.Y | Y | 29.43 | 787.4075 | 7 | 1.9 | 394.7118 | 2 | 34.17 | 1 | 9237 | ANa\_10.raw | 7.58E6 |  | 1.88E7 |  |  | 1.1E7 |  |  |  | 7.61E6 | 4 | 1 | 0 | 1 | 0 | 0 | 1 | 0 | 0 | 0 | 1 | 113 | 119 |  |
| R.SVTGWK.E | Y | 28.12 | 676.3544 | 6 | 3.4 | 339.1856 | 2 | 28.64 | 6 | 7697 | Ana\_05.raw |  |  |  |  |  | 2.16E6 |  |  |  |  | 1 | 0 | 0 | 0 | 0 | 0 | 1 | 0 | 0 | 0 | 0 | 625 | 630 |  |
| K.SLGQWLQEEK.V | Y | 27.65 | 1216.6088 | 10 | -8.1 | 609.3067 | 2 | 66.33 | 8 | 18622 | Ana\_07.raw | 2.32E6 |  |  |  |  |  |  | 7.38E5 |  |  | 2 | 1 | 0 | 0 | 0 | 0 | 0 | 0 | 1 | 0 | 0 | 148 | 157 |  |
| K.SLFHYR.Q | Y | 27.24 | 821.4184 | 6 | 6.7 | 411.7192 | 2 | 37.68 | 9 | 10243 | Ana\_08.raw |  | 4.32E6 |  |  |  |  |  |  | 8.02E5 |  | 2 | 0 | 1 | 0 | 0 | 0 | 0 | 0 | 0 | 1 | 0 | 1487 | 1492 |  |
| R.LTSIDKWFLYK.M | Y | 25.41 | 1412.7704 | 11 | 3.7 | 471.9325 | 3 | 103.07 | 5 | 28822 | Ana\_04.raw |  |  |  |  | 2.25E5 |  |  |  |  |  | 1 | 0 | 0 | 0 | 0 | 1 | 0 | 0 | 0 | 0 | 0 | 870 | 880 |  |
| R.RTAVDSGIALLTNFQVTK.L | Y | 24.49 | 1933.0632 | 18 | 2.0 | 645.3630 | 3 | 116.96 | 6 | 32683 | Ana\_05.raw |  |  |  |  |  | 2.33E5 |  |  |  |  | 1 | 0 | 0 | 0 | 0 | 0 | 1 | 0 | 0 | 0 | 0 | 1454 | 1471 |  |
| R.IYAIAK.A | Y | 24.47 | 677.4112 | 6 | 2.7 | 339.7138 | 2 | 33.42 | 6 | 9153 | Ana\_05.raw | 1.79E6 |  |  | 2.13E6 |  | 4.03E6 |  |  |  |  | 3 | 1 | 0 | 0 | 1 | 0 | 1 | 0 | 0 | 0 | 0 | 851 | 856 |  |
| K.TSAC(+57.02)FEPSLDYM(+15.99)VTK.I | Y | 23.04 | 1763.7747 | 15 | 3.4 | 882.8976 | 2 | 82.49 | 4 | 22885 | Ana\_03.raw |  |  |  | 3.6E5 |  |  |  |  |  |  | 1 | 0 | 0 | 0 | 1 | 0 | 0 | 0 | 0 | 0 | 0 | 758 | 772 | Carbamidomethylation; Oxidation (M) |
| total 47 peptides |  |  |  |  |  |  |  |  |  |  |  |  |  |  |  |  |  |  |  |  |  |  |  |  |  |  |  |  |  |  |  |  |  |  |  |
| --- | --- | --- | --- | --- | --- | --- | --- | --- | --- | --- | --- | --- | --- | --- | --- | --- | --- | --- | --- | --- | --- | --- | --- | --- | --- | --- | --- | --- | --- | --- | --- | --- | --- | --- | --- |

Best Unique PSM (Scan F2:34421, m/z=1129.8750, z=3, RT=124.14, ppm=-1.8):

sp|P20029|BIP\_MOUSE

back to list

  

| Protein Coverage
| Supporting Peptides
| Best Unique PSM
|

Protein Coverage:

Supporting Peptides:

| Peptide | Uniq | -10lgP | Mass | Length | ppm | m/z | z | RT | Fraction | Scan | Source File | Area 10 | Area 01 | Area 02 | Area 03 | Area 04 | Area 05 | Area 06 | Area 07 | Area 08 | Area 09 | #Spec | #Spec 10 | #Spec 01 | #Spec 02 | #Spec 03 | #Spec 04 | #Spec 05 | #Spec 06 | #Spec 07 | #Spec 08 | #Spec 09 | Start | End | PTM |
| --- | --- | --- | --- | --- | --- | --- | --- | --- | --- | --- | --- | --- | --- | --- | --- | --- | --- | --- | --- | --- | --- | --- | --- | --- | --- | --- | --- | --- | --- | --- | --- | --- | --- | --- | --- |
| K.VTHAVVTVPAYFNDAQR.Q | Y | 105.13 | 1886.9639 | 17 | 4.9 | 629.9983 | 3 | 74.75 | 9 | 21299 | Ana\_08.raw | 1.28E7 | 1.73E7 | 1.49E7 | 1.24E7 | 1.81E7 |  | 1.06E7 | 1.48E7 | 2.93E7 | 1.52E7 | 17 | 2 | 2 | 2 | 2 | 1 | 0 | 2 | 2 | 2 | 2 | 166 | 182 |  |
| K.DNHLLGTFDLTGIPPAPR.G | Y | 100.51 | 1933.0057 | 18 | 4.5 | 645.3454 | 3 | 111.92 | 1 | 31027 | ANa\_10.raw | 1.59E7 | 1.19E7 | 4.45E7 | 2.47E7 | 1.13E7 | 7.89E6 | 4.51E7 | 1.75E7 | 7.05E7 | 5.9E6 | 85 | 9 | 10 | 10 | 9 | 6 | 6 | 10 | 9 | 8 | 8 | 476 | 493 |  |
| R.IINEPTAAAIAYGLDK.R | N | 98.74 | 1658.8879 | 16 | 2.1 | 830.4530 | 2 | 98.36 | 6 | 27817 | Ana\_05.raw | 1.5E8 | 1.38E8 | 1.16E8 | 1.71E7 | 1.84E7 | 6.76E7 | 5.7E7 | 5.77E7 | 1.85E8 | 9.55E7 | 49 | 5 | 6 | 6 | 2 | 5 | 4 | 3 | 6 | 7 | 5 | 199 | 214 |  |
| R.IEIESFFEGEDFSETLTR.A | Y | 95.63 | 2147.9897 | 18 | 0.8 | 1075.0031 | 2 | 130.82 | 7 | 36007 | Ana\_06.raw |  |  |  |  |  | 2.08E6 | 1.66E6 |  |  |  | 2 | 0 | 0 | 0 | 0 | 0 | 1 | 1 | 0 | 0 | 0 | 308 | 325 |  |
| R.IINEPTAAAIAYGLDKR.E | Y | 94.28 | 1814.9890 | 17 | 4.2 | 606.0062 | 3 | 87.41 | 9 | 24834 | Ana\_08.raw | 1.92E7 | 1.97E7 | 1.36E7 | 5.53E6 | 1.2E7 | 1.52E7 | 8.43E6 | 8.36E6 | 3.84E7 | 1.7E7 | 11 | 1 | 1 | 1 | 1 | 1 | 1 | 2 | 1 | 1 | 1 | 199 | 215 |  |
| R.ITPSYVAFTPEGER.L | Y | 93.90 | 1565.7725 | 14 | 3.3 | 783.8961 | 2 | 77.72 | 3 | 22004 | Ana\_02.raw | 6.38E7 | 6.85E7 | 6.14E7 | 3.63E7 | 6.07E7 | 5.29E7 | 3.65E7 | 4.29E7 | 1.14E8 | 4.53E7 | 41 | 5 | 5 | 4 | 3 | 5 | 3 | 3 | 5 | 4 | 4 | 62 | 75 |  |
| K.NQLTSNPENTVFDAK.R | Y | 92.64 | 1676.8005 | 15 | 1.1 | 839.4085 | 2 | 67.59 | 3 | 19220 | Ana\_02.raw | 2.82E7 | 2.72E7 | 2.09E7 | 1.04E7 | 2.39E7 | 1.63E7 | 1.37E7 | 1.61E7 | 4.78E7 | 2.74E7 | 18 | 1 | 1 | 2 | 1 | 2 | 1 | 1 | 3 | 3 | 3 | 83 | 97 |  |
| R.VEIIANDQGNR.I | N | 92.33 | 1227.6207 | 11 | 5.5 | 614.8210 | 2 | 37.88 | 1 | 10363 | ANa\_10.raw | 1.27E8 | 1.28E8 | 1.27E8 | 5.59E7 | 1.2E8 | 1.04E8 | 8.8E7 | 8.5E7 | 1.27E8 | 1.01E8 | 31 | 3 | 3 | 3 | 3 | 3 | 3 | 3 | 4 | 3 | 3 | 51 | 61 |  |
| K.LYGSGGPPPTGEEDTSEKDEL | Y | 90.64 | 2176.9648 | 21 | 1.8 | 1089.4917 | 2 | 64.46 | 3 | 18360 | Ana\_02.raw | 1.84E7 | 2.75E7 | 6.96E6 |  | 1.55E7 | 1.32E7 | 1.05E7 | 1.29E7 | 2.89E7 | 1.45E7 | 15 | 1 | 3 | 1 | 0 | 2 | 2 | 1 | 2 | 2 | 1 | 635 | 655 |  |
| K.ELEEIVQPIISK.L | Y | 90.06 | 1396.7812 | 12 | 1.8 | 699.3992 | 2 | 88.13 | 3 | 24773 | Ana\_02.raw | 2.83E7 | 4.06E7 | 2.82E7 | 8.19E6 | 1.44E7 | 1.85E7 | 1.47E7 | 1.23E7 | 6.51E7 | 2.94E7 | 15 | 2 | 1 | 2 | 1 | 2 | 2 | 1 | 2 | 1 | 1 | 623 | 634 |  |
| K.KSDIDEIVLVGGSTR.I | Y | 87.26 | 1587.8468 | 15 | 0.7 | 794.9312 | 2 | 74.97 | 3 | 21256 | Ana\_02.raw | 1.09E7 | 1.32E7 | 6.33E6 | 1.98E6 | 8.21E6 | 7.64E6 | 7.16E6 | 7.66E6 | 6.44E6 | 7.99E6 | 16 | 2 | 2 | 2 | 1 | 1 | 1 | 1 | 1 | 3 | 2 | 354 | 368 |  |
| K.IEWLESHQDADIEDFK.A | Y | 87.05 | 1973.9006 | 16 | -0.3 | 658.9739 | 3 | 93.62 | 3 | 26217 | Ana\_02.raw | 1.36E6 | 1.42E7 | 4.27E6 | 1.01E6 |  | 8.88E5 | 6.49E6 |  | 3.5E7 | 1.42E6 | 8 | 1 | 1 | 1 | 1 | 0 | 1 | 1 | 0 | 1 | 1 | 603 | 618 |  |
| K.SDIDEIVLVGGSTR.I | Y | 86.88 | 1459.7518 | 14 | 7.9 | 730.8890 | 2 | 91.12 | 7 | 25594 | Ana\_06.raw | 3.91E6 | 1.77E6 |  |  | 2.6E6 |  | 2.02E6 | 1.28E6 | 1.01E7 |  | 6 | 1 | 1 | 0 | 0 | 1 | 0 | 1 | 1 | 1 | 0 | 355 | 368 |  |
| K.TFAPEEISAMVLTK.M | Y | 82.81 | 1535.7905 | 14 | 6.0 | 768.9072 | 2 | 115.86 | 1 | 32031 | ANa\_10.raw | 1.46E6 |  |  | 1.78E5 |  | 1.6E6 |  |  |  | 7.9E6 | 4 | 1 | 0 | 0 | 1 | 0 | 1 | 0 | 0 | 0 | 1 | 140 | 153 |  |
| K.SQIFSTASDNQPTVTIK.V | Y | 80.49 | 1835.9265 | 17 | 5.3 | 918.9754 | 2 | 74.26 | 1 | 21030 | ANa\_10.raw | 8.57E6 | 8.8E5 | 2.76E6 | 4.09E6 | 9.51E6 | 2.25E6 | 6.34E6 | 5.51E6 | 2.63E7 | 7.98E6 | 13 | 1 | 1 | 1 | 1 | 1 | 1 | 2 | 2 | 2 | 1 | 449 | 465 |  |
| K.TFAPEEISAM(+15.99)VLTK.M | Y | 79.59 | 1551.7854 | 14 | 2.7 | 776.9021 | 2 | 96.83 | 1 | 27120 | ANa\_10.raw | 2E7 | 1.76E7 | 1.01E7 | 4.32E6 | 6.54E6 | 9.01E6 | 5.16E6 | 3.08E6 | 3.09E7 | 5.42E6 | 27 | 4 | 3 | 3 | 2 | 2 | 2 | 2 | 3 | 4 | 2 | 140 | 153 | Oxidation (M) |
| R.TWNDPSVQQDIK.F | Y | 79.15 | 1429.6837 | 12 | 1.9 | 715.8505 | 2 | 58.16 | 9 | 16508 | Ana\_08.raw | 7.59E6 | 8.34E6 | 6.93E6 | 2.69E6 | 5.48E6 | 5.94E6 | 4.77E6 | 5.58E6 | 1.49E7 |  | 9 | 1 | 1 | 1 | 1 | 1 | 1 | 1 | 1 | 1 | 0 | 103 | 114 |  |
| R.AKFEELNM(+15.99)DLFR.S | Y | 67.71 | 1527.7391 | 12 | 0.9 | 764.8775 | 2 | 85.68 | 6 | 24408 | Ana\_05.raw | 1.02E7 | 7.89E6 | 1.24E7 | 6.59E6 | 1.16E7 | 9.81E6 | 9.53E6 | 8.36E6 | 2.6E7 | 2.99E6 | 20 | 2 | 2 | 2 | 2 | 2 | 2 | 2 | 2 | 2 | 2 | 326 | 337 | Oxidation (M) |
| R.NELESYAYSLK.N | Y | 66.08 | 1315.6295 | 11 | 2.2 | 658.8235 | 2 | 84.35 | 4 | 23387 | Ana\_03.raw | 1.52E7 | 1.05E7 | 1.07E7 | 5.55E6 | 8.53E6 | 1.21E7 | 6.99E6 | 6.72E6 | 3.22E7 | 1.61E7 | 10 | 1 | 1 | 1 | 1 | 1 | 1 | 1 | 1 | 1 | 1 | 564 | 574 |  |
| K.ITITNDQNR.L | Y | 65.25 | 1073.5465 | 9 | 1.9 | 537.7816 | 2 | 26.89 | 3 | 7164 | Ana\_02.raw | 4.27E7 | 5.05E7 | 3.94E7 | 2.17E7 | 2.8E7 | 3.23E7 | 3.07E7 | 2.56E7 | 3.21E7 | 4.04E7 | 13 | 2 | 1 | 1 | 1 | 1 | 1 | 1 | 2 | 2 | 1 | 525 | 533 |  |
| K.FEELNM(+15.99)DLFR.S | Y | 64.65 | 1328.6071 | 10 | -0.8 | 665.3103 | 2 | 89.93 | 3 | 25247 | Ana\_02.raw | 1.17E7 | 1.24E7 | 5.2E6 | 5.03E6 | 9.17E6 | 7.85E6 | 6.75E6 | 6.61E6 | 2.11E7 | 9E6 | 10 | 1 | 1 | 1 | 1 | 1 | 1 | 1 | 1 | 1 | 1 | 328 | 337 | Oxidation (M) |
| R.LTPEEIER.M | Y | 64.11 | 985.5080 | 8 | 2.0 | 493.7622 | 2 | 44.87 | 6 | 12654 | Ana\_05.raw | 8.69E7 | 7.88E7 | 1.11E8 | 4.04E7 | 7.43E7 | 6.48E7 | 6.44E7 | 6.51E7 | 7.02E7 | 7.01E7 | 20 | 3 | 2 | 2 | 2 | 1 | 1 | 2 | 2 | 3 | 2 | 534 | 541 |  |
| K.VYEGERPLTK.D | Y | 60.67 | 1190.6295 | 10 | 8.2 | 596.3269 | 2 | 28.27 | 2 | 7478 | Ana\_01.raw | 7E7 | 3.55E7 | 7.42E7 | 1.05E7 | 5E7 | 2E7 | 5.28E7 | 1.48E7 | 3.7E7 | 6.03E7 | 18 | 2 | 1 | 2 | 1 | 2 | 1 | 2 | 1 | 3 | 3 | 466 | 475 |  |
| K.M(+15.99)KETAEAYLGK.K | Y | 60.26 | 1255.6118 | 11 | 5.3 | 628.8165 | 2 | 35.81 | 1 | 9723 | ANa\_10.raw | 4.86E6 | 6.01E6 | 1.16E7 | 3.98E6 | 1.1E7 | 8.52E6 | 9.47E6 | 5.54E6 |  | 3.83E6 | 13 | 1 | 1 | 2 | 1 | 2 | 2 | 2 | 1 | 0 | 1 | 154 | 164 | Oxidation (M) |
| K.ETAEAYLGK.K | Y | 48.86 | 980.4814 | 9 | 7.1 | 491.2515 | 2 | 42.92 | 5 | 11996 | Ana\_04.raw | 5.29E7 |  | 3.72E7 | 2.17E7 | 4.34E7 |  |  |  | 5.91E6 | 4.28E7 | 6 | 1 | 0 | 1 | 1 | 1 | 0 | 0 | 0 | 1 | 1 | 156 | 164 |  |
| K.VLEDSDLK.K | Y | 45.74 | 917.4705 | 8 | 3.4 | 459.7441 | 2 | 34.17 | 1 | 9236 | ANa\_10.raw | 3.63E7 | 3.33E7 | 3.58E7 | 1.62E7 |  |  | 2.65E7 |  |  |  | 5 | 1 | 1 | 1 | 1 | 0 | 0 | 1 | 0 | 0 | 0 | 346 | 353 |  |
| K.VLEDSDLKK.S | Y | 44.36 | 1045.5656 | 9 | 8.5 | 523.7945 | 2 | 27.98 | 7 | 7352 | Ana\_06.raw |  |  | 3.65E6 | 2.1E6 |  | 2.11E6 | 2.07E6 |  | 3.92E6 |  | 5 | 0 | 0 | 1 | 1 | 0 | 1 | 1 | 0 | 1 | 0 | 346 | 354 |  |
| K.DAGTIAGLNVM(+15.99)R.I | Y | 43.59 | 1232.6183 | 12 | 0.3 | 617.3166 | 2 | 60.58 | 3 | 17244 | Ana\_02.raw |  |  | 1.38E7 | 4.5E6 |  |  |  |  |  |  | 2 | 0 | 0 | 1 | 1 | 0 | 0 | 0 | 0 | 0 | 0 | 187 | 198 | Oxidation (M) |
| R.VM(+15.99)EHFIK.L | Y | 41.34 | 918.4633 | 7 | 5.0 | 460.2412 | 2 | 29.61 | 6 | 8008 | Ana\_05.raw |  |  | 1.54E7 | 5.44E6 | 1.08E7 | 6.85E6 | 6.02E6 | 7.96E6 |  |  | 7 | 0 | 0 | 2 | 1 | 1 | 1 | 1 | 1 | 0 | 0 | 263 | 269 | Oxidation (M) |
| K.TKPYIQVDIGGGQTK.T | Y | 32.31 | 1603.8569 | 15 | 4.2 | 535.6285 | 3 | 62.05 | 7 | 17572 | Ana\_06.raw |  |  |  |  | 2.64E6 |  | 3.21E6 | 2.87E6 |  |  | 3 | 0 | 0 | 0 | 0 | 1 | 0 | 1 | 1 | 0 | 0 | 125 | 139 |  |
| R.LIGDAAK.N | N | 28.78 | 686.3962 | 7 | 1.5 | 344.2059 | 2 | 21.83 | 4 | 5454 | Ana\_03.raw |  |  |  | 3.57E7 |  |  |  |  |  |  | 1 | 0 | 0 | 0 | 1 | 0 | 0 | 0 | 0 | 0 | 0 | 76 | 82 |  |
| K.EFFNGKEPSR.G | Y | 27.65 | 1209.5778 | 10 | -0.8 | 605.7957 | 2 | 41.38 | 3 | 11568 | Ana\_02.raw |  |  | 1.85E7 |  |  |  |  |  |  |  | 1 | 0 | 0 | 1 | 0 | 0 | 0 | 0 | 0 | 0 | 0 | 378 | 387 |  |
| K.IQQLVK.E | Y | 27.07 | 727.4592 | 6 | 6.3 | 364.7392 | 2 | 25.30 | 9 | 6409 | Ana\_08.raw | 1.51E7 | 2.02E7 | 2.2E7 | 1.04E7 | 1.76E7 |  | 1.52E7 | 1.21E7 | 1.48E7 | 2.59E6 | 9 | 1 | 1 | 1 | 1 | 1 | 0 | 1 | 1 | 1 | 1 | 372 | 377 |  |
| K.RLIGR.T | N | 26.60 | 613.4023 | 5 | 0.6 | 307.7086 | 2 | 18.67 | 4 | 4488 | Ana\_03.raw |  |  |  | 4.8E6 |  |  |  |  |  |  | 1 | 0 | 0 | 0 | 1 | 0 | 0 | 0 | 0 | 0 | 0 | 98 | 102 |  |
| total 34 peptides |  |  |  |  |  |  |  |  |  |  |  |  |  |  |  |  |  |  |  |  |  |  |  |  |  |  |  |  |  |  |  |  |  |  |  |
| --- | --- | --- | --- | --- | --- | --- | --- | --- | --- | --- | --- | --- | --- | --- | --- | --- | --- | --- | --- | --- | --- | --- | --- | --- | --- | --- | --- | --- | --- | --- | --- | --- | --- | --- | --- |

Best Unique PSM (Scan F9:21299, m/z=629.9983, z=3, RT=74.75, ppm=4.9):

sp|P52480|KPYM\_MOUSE

back to list

  

| Protein Coverage
| Supporting Peptides
| Best Unique PSM
|

Protein Coverage:

Supporting Peptides:

| Peptide | Uniq | -10lgP | Mass | Length | ppm | m/z | z | RT | Fraction | Scan | Source File | Area 10 | Area 01 | Area 02 | Area 03 | Area 04 | Area 05 | Area 06 | Area 07 | Area 08 | Area 09 | #Spec | #Spec 10 | #Spec 01 | #Spec 02 | #Spec 03 | #Spec 04 | #Spec 05 | #Spec 06 | #Spec 07 | #Spec 08 | #Spec 09 | Start | End | PTM |
| --- | --- | --- | --- | --- | --- | --- | --- | --- | --- | --- | --- | --- | --- | --- | --- | --- | --- | --- | --- | --- | --- | --- | --- | --- | --- | --- | --- | --- | --- | --- | --- | --- | --- | --- | --- |
| R.LAPITSDPTEAAAVGAVEASFK.C | Y | 122.54 | 2144.1001 | 22 | 3.6 | 1073.0612 | 2 | 120.74 | 3 | 33228 | Ana\_02.raw | 2.13E7 | 3.62E7 | 5.86E7 | 1.12E7 | 2.46E7 | 2.12E7 | 1.82E7 | 1.97E7 | 1.5E8 | 9.17E7 | 32 | 3 | 4 | 3 | 2 | 3 | 2 | 2 | 2 | 6 | 5 | 401 | 422 |  |
| R.EATESFASDPILYRPVAVALDTK.G | Y | 119.90 | 2492.2798 | 23 | 4.6 | 1247.1530 | 2 | 115.72 | 2 | 32215 | Ana\_01.raw | 6.35E7 | 2.04E7 | 1.33E7 | 2.36E6 | 4.66E6 | 1.21E7 | 1.25E7 | 2.03E6 | 7.65E7 | 3.48E7 | 12 | 1 | 2 | 1 | 1 | 1 | 1 | 1 | 1 | 2 | 1 | 93 | 115 |  |
| K.FGVEQDVDM(+15.99)VFASFIR.K | Y | 102.48 | 1874.8872 | 16 | 3.9 | 625.9721 | 3 | 135.09 | 6 | 37558 | Ana\_05.raw | 8.33E6 | 4.75E6 | 1.06E7 | 4.45E5 | 7.99E6 | 8.5E6 | 2.67E6 | 1.03E7 | 7.75E6 | 9.05E6 | 21 | 3 | 2 | 2 | 2 | 2 | 2 | 2 | 2 | 2 | 2 | 231 | 246 | Oxidation (M) |
| R.LNFSHGTHEYHAETIK.N | Y | 100.00 | 1882.8962 | 16 | 1.3 | 628.6402 | 3 | 41.44 | 3 | 11588 | Ana\_02.raw | 2.43E7 |  | 2.79E7 |  |  |  |  |  | 1.26E7 | 2.4E7 | 6 | 1 | 0 | 2 | 0 | 0 | 0 | 0 | 0 | 2 | 1 | 74 | 89 |  |
| K.KGVNLPGAAVDLPAVSEK.D | Y | 86.04 | 1763.9780 | 18 | 6.9 | 589.0040 | 3 | 81.55 | 1 | 23013 | ANa\_10.raw | 7.4E6 | 4.39E6 | 6.44E5 |  | 1.05E6 | 1.02E6 | 1.98E5 |  | 1.82E7 | 9.94E5 | 9 | 1 | 1 | 1 | 0 | 1 | 1 | 1 | 0 | 2 | 1 | 207 | 224 |  |
| K.GVNLPGAAVDLPAVSEK.D | Y | 83.86 | 1635.8832 | 17 | 0.0 | 818.9489 | 2 | 91.48 | 3 | 25659 | Ana\_02.raw | 1.8E7 | 3.28E6 | 3.04E6 | 2.91E5 |  | 2.94E6 | 2.2E5 | 8.11E5 | 2.58E7 | 1.75E7 | 14 | 2 | 1 | 2 | 1 | 0 | 2 | 1 | 1 | 2 | 2 | 208 | 224 |  |
| K.DAVLNAWAEDVDLR.V | Y | 77.80 | 1585.7736 | 14 | 5.2 | 793.8982 | 2 | 113.99 | 2 | 31753 | Ana\_01.raw |  | 9.88E6 |  | 5.4E6 | 2.01E6 |  |  |  | 5.5E5 | 1.04E7 | 6 | 0 | 1 | 0 | 1 | 1 | 0 | 0 | 0 | 2 | 1 | 476 | 489 |  |
| R.GDLGIEIPAEK.V | N | 74.84 | 1140.6025 | 11 | 4.3 | 571.3110 | 2 | 73.16 | 2 | 20840 | Ana\_01.raw | 4.84E7 | 2.82E7 | 2.57E7 | 1.13E7 | 2.2E7 | 2.42E6 | 1.26E7 | 1.57E7 | 7.59E7 | 3.85E7 | 16 | 2 | 1 | 2 | 1 | 1 | 1 | 1 | 2 | 3 | 2 | 295 | 305 |  |
| R.NTGIIC(+57.02)TIGPASR.S | Y | 74.46 | 1358.6976 | 13 | 3.8 | 680.3586 | 2 | 64.05 | 6 | 18386 | Ana\_05.raw | 3.34E7 | 2.38E7 | 2.21E7 | 8.11E6 | 1.54E7 | 1.47E7 | 9.31E6 | 1.1E7 | 6.51E7 | 3.08E7 | 15 | 2 | 2 | 1 | 1 | 2 | 1 | 1 | 1 | 3 | 1 | 44 | 56 | Carbamidomethylation |
| K.FGVEQDVDMVFASFIR.K | Y | 72.08 | 1858.8923 | 16 | -2.2 | 930.4514 | 2 | 137.11 | 6 | 38093 | Ana\_05.raw |  |  |  |  |  | 1.77E5 |  |  |  | 1.03E6 | 3 | 0 | 0 | 0 | 0 | 0 | 1 | 0 | 0 | 0 | 2 | 231 | 246 |  |
| R.LDIDSAPITAR.N | Y | 68.80 | 1170.6244 | 11 | 5.4 | 586.3226 | 2 | 66.31 | 1 | 18805 | ANa\_10.raw | 5.27E7 | 4.79E7 | 4.34E7 | 1.71E7 | 2.5E7 | 3.04E7 | 2.02E7 | 2.24E7 | 1.05E8 | 5.99E7 | 31 | 3 | 3 | 2 | 2 | 2 | 3 | 2 | 4 | 5 | 5 | 33 | 43 |  |
| R.KAADVHEVR.K | Y | 68.04 | 1023.5461 | 9 | 3.9 | 342.1906 | 3 | 16.49 | 5 | 3812 | Ana\_04.raw |  |  |  | 5.08E6 | 1.15E7 |  | 7.58E6 | 6.25E6 |  |  | 8 | 0 | 0 | 0 | 2 | 2 | 0 | 2 | 2 | 0 | 0 | 247 | 255 |  |
| K.IYVDDGLISLQVK.E | Y | 65.89 | 1461.8079 | 13 | 5.4 | 731.9152 | 2 | 104.90 | 6 | 29551 | Ana\_05.raw |  | 1.44E6 |  |  |  | 7.93E5 | 6.87E5 |  | 2.99E6 | 2.49E6 | 5 | 0 | 1 | 0 | 0 | 0 | 1 | 1 | 0 | 1 | 1 | 174 | 186 |  |
| R.APIIAVTR.N | Y | 62.83 | 839.5228 | 8 | 1.4 | 420.7693 | 2 | 44.17 | 3 | 12409 | Ana\_02.raw | 5.31E7 | 3.54E7 | 4.32E7 | 1.53E7 | 2.81E7 | 3.01E7 | 2.13E7 | 2.04E7 | 4.53E7 | 4.75E7 | 20 | 2 | 2 | 2 | 2 | 2 | 2 | 2 | 2 | 2 | 2 | 448 | 455 |  |
| K.GDYPLEAVR.M | Y | 62.54 | 1018.5083 | 9 | 4.3 | 510.2636 | 2 | 57.10 | 1 | 16129 | ANa\_10.raw | 3.63E7 | 2.37E7 | 2.6E7 | 1.18E7 | 1.48E7 | 2.11E7 | 1.63E7 | 1.42E7 |  | 3.52E7 | 17 | 2 | 2 | 2 | 2 | 2 | 2 | 2 | 1 | 0 | 2 | 368 | 376 |  |
| K.AADVHEVR.K | Y | 61.94 | 895.4512 | 8 | 2.6 | 448.7340 | 2 | 17.74 | 5 | 4227 | Ana\_04.raw |  |  |  | 6.96E5 | 4.8E6 |  |  | 5.34E5 |  |  | 3 | 0 | 0 | 0 | 1 | 1 | 0 | 0 | 1 | 0 | 0 | 248 | 255 |  |
| R.EAEAAIYHLQLFEELR.R | Y | 61.69 | 1930.9788 | 16 | 7.5 | 644.6717 | 3 | 119.75 | 4 | 32635 | Ana\_03.raw |  | 2.3E6 | 8.16E5 | 6.15E5 |  | 1.42E6 | 1.3E6 | 9.54E4 |  | 4.73E5 | 7 | 0 | 1 | 1 | 1 | 0 | 1 | 1 | 1 | 0 | 1 | 384 | 399 |  |
| R.VNLAM(+15.99)DVGK.A | Y | 53.35 | 961.4902 | 9 | 5.6 | 481.7551 | 2 | 39.74 | 1 | 10937 | ANa\_10.raw | 2.83E7 | 1.7E7 | 2.53E7 | 3.16E6 | 1.87E7 | 1.87E7 | 1.44E7 | 1.59E7 | 7.82E6 | 2.63E7 | 17 | 1 | 1 | 2 | 1 | 2 | 2 | 1 | 2 | 3 | 2 | 490 | 498 | Oxidation (M) |
| K.GSGTAEVELK.K | Y | 53.18 | 989.5029 | 10 | 6.1 | 495.7617 | 2 | 30.29 | 10 | 8162 | Ana\_09.raw | 4.48E7 |  | 3.3E7 | 1.8E7 | 2.37E7 |  |  | 2.02E7 |  | 5.52E7 | 6 | 1 | 0 | 1 | 1 | 1 | 0 | 0 | 1 | 0 | 1 | 126 | 135 |  |
| R.GIFPVLC(+57.02)K.D | Y | 51.62 | 932.5153 | 8 | 4.8 | 467.2672 | 2 | 85.24 | 1 | 24002 | ANa\_10.raw | 9.4E6 | 6.92E6 |  | 3.28E6 | 3.95E6 | 6.49E6 | 4.02E6 | 2.46E6 | 2.45E7 | 1.08E7 | 18 | 2 | 2 | 0 | 2 | 2 | 2 | 2 | 2 | 3 | 1 | 468 | 475 | Carbamidomethylation |
| K.ITLDNAYM(+15.99)EK.C | Y | 50.57 | 1212.5696 | 10 | 6.3 | 607.2959 | 2 | 52.77 | 1 | 14840 | ANa\_10.raw | 1.32E7 | 6.76E6 | 1.49E6 | 3.17E6 | 5.01E6 | 4.75E6 | 4.6E6 | 4.65E6 | 1.43E7 | 1.02E7 | 10 | 1 | 1 | 1 | 1 | 1 | 1 | 1 | 1 | 1 | 1 | 142 | 151 | Oxidation (M) |
| K.GADFLVTEVENGGSLGSK.K | Y | 48.38 | 1778.8687 | 18 | 1.4 | 890.4429 | 2 | 102.32 | 1 | 28573 | ANa\_10.raw | 8.94E5 |  |  |  |  |  |  |  |  |  | 1 | 1 | 0 | 0 | 0 | 0 | 0 | 0 | 0 | 0 | 0 | 189 | 206 |  |
| R.M(+15.99)QHLIAR.E | Y | 43.73 | 883.4698 | 7 | 2.4 | 442.7432 | 2 | 20.14 | 3 | 4981 | Ana\_02.raw |  | 0 | 1.31E7 |  | 8.04E6 |  |  |  | 2.05E6 | 1.3E7 | 5 | 0 | 1 | 1 | 0 | 1 | 0 | 0 | 0 | 1 | 1 | 377 | 383 | Oxidation (M) |
| K.GSGTAEVELKK.G | Y | 43.59 | 1117.5979 | 11 | 2.7 | 559.8077 | 2 | 25.40 | 4 | 6507 | Ana\_03.raw |  |  |  | 7.98E5 |  |  |  |  | 9.17E6 |  | 2 | 0 | 0 | 0 | 1 | 0 | 0 | 0 | 0 | 1 | 0 | 126 | 136 |  |
| R.QAHLYR.G | Y | 41.71 | 786.4136 | 6 | 2.5 | 394.2151 | 2 | 19.30 | 6 | 4700 | Ana\_05.raw |  |  | 2.39E6 | 1.95E6 |  | 4.74E6 | 2.33E6 |  |  |  | 4 | 0 | 0 | 1 | 1 | 0 | 1 | 1 | 0 | 0 | 0 | 462 | 467 |  |
| R.RFDEILEASDGIM(+15.99)VAR.G | Y | 38.85 | 1836.9039 | 16 | 0.1 | 613.3087 | 3 | 85.44 | 9 | 24301 | Ana\_08.raw |  |  |  |  |  |  |  |  | 2.64E6 |  | 1 | 0 | 0 | 0 | 0 | 0 | 0 | 0 | 0 | 1 | 0 | 279 | 294 | Oxidation (M) |
| K.DIQDLK.F | Y | 34.04 | 730.3861 | 6 | 3.0 | 366.2014 | 2 | 32.88 | 7 | 8862 | Ana\_06.raw | 1.39E7 |  |  | 6.31E6 | 8.85E6 | 1.23E7 | 7.64E6 | 7.15E6 |  |  | 6 | 1 | 0 | 0 | 1 | 1 | 1 | 1 | 1 | 0 | 0 | 225 | 230 |  |
| K.VFLAQK.M | N | 32.38 | 704.4221 | 6 | 4.7 | 353.2200 | 2 | 28.06 | 9 | 7299 | Ana\_08.raw |  |  |  |  |  |  |  |  | 2.79E7 | 2.04E7 | 2 | 0 | 0 | 0 | 0 | 0 | 0 | 0 | 0 | 1 | 1 | 306 | 311 |  |
| K.IENHEGVR.R | Y | 30.20 | 952.4726 | 8 | 2.1 | 318.4988 | 3 | 16.65 | 4 | 3852 | Ana\_03.raw |  |  |  | 1.77E5 | 2.03E6 |  |  |  |  |  | 2 | 0 | 0 | 0 | 1 | 1 | 0 | 0 | 0 | 0 | 0 | 271 | 278 |  |
| R.SVEM(+15.99)LK.E | Y | 23.29 | 721.3680 | 6 | 1.8 | 361.6920 | 2 | 20.78 | 5 | 5231 | Ana\_04.raw |  |  |  |  | 6.72E6 |  |  |  |  |  | 1 | 0 | 0 | 0 | 0 | 1 | 0 | 0 | 0 | 0 | 0 | 57 | 62 | Oxidation (M) |
| total 30 peptides |  |  |  |  |  |  |  |  |  |  |  |  |  |  |  |  |  |  |  |  |  |  |  |  |  |  |  |  |  |  |  |  |  |  |  |
| --- | --- | --- | --- | --- | --- | --- | --- | --- | --- | --- | --- | --- | --- | --- | --- | --- | --- | --- | --- | --- | --- | --- | --- | --- | --- | --- | --- | --- | --- | --- | --- | --- | --- | --- | --- |

Best Unique PSM (Scan F3:33228, m/z=1073.0612, z=2, RT=120.74, ppm=3.6):

sp|Q04447|KCRB\_MOUSE

back to list

  

| Protein Coverage
| Supporting Peptides
| Best Unique PSM
|

Protein Coverage:

Supporting Peptides:

| Peptide | Uniq | -10lgP | Mass | Length | ppm | m/z | z | RT | Fraction | Scan | Source File | Area 10 | Area 01 | Area 02 | Area 03 | Area 04 | Area 05 | Area 06 | Area 07 | Area 08 | Area 09 | #Spec | #Spec 10 | #Spec 01 | #Spec 02 | #Spec 03 | #Spec 04 | #Spec 05 | #Spec 06 | #Spec 07 | #Spec 08 | #Spec 09 | Start | End | PTM |
| --- | --- | --- | --- | --- | --- | --- | --- | --- | --- | --- | --- | --- | --- | --- | --- | --- | --- | --- | --- | --- | --- | --- | --- | --- | --- | --- | --- | --- | --- | --- | --- | --- | --- | --- | --- |
| K.NYEFM(+15.99)WNPHLGYILTC(+57.02)PSNLGTGLR.A | Y | 127.88 | 2968.3999 | 25 | -1.6 | 990.4723 | 3 | 127.28 | 3 | 34899 | Ana\_02.raw | 5.04E6 | 1.02E7 | 7.31E6 |  | 5.72E6 | 3.79E6 | 5.46E6 | 1.03E7 | 1.58E7 |  | 8 | 1 | 1 | 1 | 0 | 1 | 1 | 1 | 1 | 1 | 0 | 268 | 292 | Oxidation (M); Carbamidomethylation |
| K.TDLNPDNLQGGDDLDPNYVLSSR.V | Y | 117.13 | 2517.1619 | 23 | 4.1 | 1259.5934 | 2 | 100.62 | 9 | 28411 | Ana\_08.raw | 1.25E6 | 6.59E6 |  |  | 1.4E6 | 4.46E6 | 2.2E5 |  | 6.13E6 |  | 8 | 1 | 2 | 0 | 0 | 1 | 1 | 1 | 0 | 2 | 0 | 108 | 130 |  |
| R.GTGGVDTAAVGGVFDVSNADR.L | Y | 113.91 | 1963.9235 | 21 | 0.5 | 982.9695 | 2 | 89.92 | 6 | 25565 | Ana\_05.raw | 1.99E7 | 4.24E7 | 2.65E7 | 2.81E6 | 2.16E7 | 2.24E7 | 1.06E7 | 1.01E7 | 4.53E7 | 1.84E7 | 23 | 3 | 4 | 1 | 1 | 2 | 3 | 1 | 2 | 4 | 2 | 321 | 341 |  |
| R.HGGYQPSDEHK.T | Y | 99.91 | 1253.5425 | 11 | 2.7 | 627.7802 | 2 | 16.67 | 6 | 3828 | Ana\_05.raw |  |  |  | 1.29E7 | 2.45E7 | 3.2E7 | 1.85E7 | 1.2E7 |  |  | 10 | 0 | 0 | 0 | 2 | 2 | 2 | 2 | 2 | 0 | 0 | 97 | 107 |  |
| R.LGFSEVELVQM(+15.99)VVDGVK.L | Y | 99.42 | 1863.9652 | 17 | 3.1 | 932.9928 | 2 | 132.19 | 6 | 36746 | Ana\_05.raw | 4.66E7 | 3.61E7 | 3.76E7 | 2.56E6 | 4.03E7 | 5.68E7 | 3.39E7 | 2.23E7 | 5.62E7 | 7.05E6 | 45 | 5 | 6 | 5 | 3 | 4 | 6 | 4 | 5 | 4 | 3 | 342 | 358 | Oxidation (M) |
| R.LGFSEVELVQMVVDGVK.L | Y | 97.60 | 1847.9702 | 17 | 1.7 | 924.9940 | 2 | 135.22 | 6 | 37592 | Ana\_05.raw | 1.41E6 | 3.99E6 | 2.91E6 | 1.15E6 | 4.08E5 | 5.19E6 | 7.23E5 | 1.79E6 | 2.09E6 | 1.23E6 | 14 | 1 | 2 | 2 | 2 | 1 | 2 | 1 | 1 | 1 | 1 | 342 | 358 |  |
| K.LAVEALSSLDGDLSGR.Y | Y | 97.47 | 1601.8260 | 16 | 3.9 | 801.9234 | 2 | 106.97 | 7 | 29765 | Ana\_06.raw | 6.98E6 | 2.3E7 | 2.11E7 | 2.95E6 | 2.28E7 | 4.75E7 | 1.98E7 | 6.45E6 | 6.73E7 | 2.14E7 | 26 | 2 | 4 | 3 | 2 | 2 | 4 | 3 | 1 | 3 | 2 | 157 | 172 |  |
| R.LEQGQAIDDLM(+15.99)PAQK | Y | 93.42 | 1671.8137 | 15 | 1.7 | 836.9155 | 2 | 58.72 | 3 | 16694 | Ana\_02.raw | 2.26E7 | 2.7E7 | 2.43E7 | 6.89E6 | 1.66E7 | 1.95E7 | 1.1E7 | 1.23E7 | 3.67E7 | 1.09E7 | 24 | 3 | 2 | 2 | 2 | 3 | 3 | 2 | 2 | 3 | 2 | 367 | 381 | Oxidation (M) |
| R.FC(+57.02)TGLTQIETLFK.S | Y | 90.74 | 1556.7909 | 13 | 1.5 | 779.4039 | 2 | 127.31 | 3 | 34907 | Ana\_02.raw | 2.98E6 | 1.67E7 | 5.06E7 | 1.19E7 | 1.87E7 | 5.07E7 | 1.99E7 | 7.4E6 | 6.85E7 | 3.72E7 | 11 | 1 | 1 | 1 | 1 | 2 | 1 | 1 | 1 | 1 | 1 | 253 | 265 | Carbamidomethylation |
| K.DLFDPIIEER.H | Y | 83.50 | 1245.6240 | 10 | 1.5 | 623.8203 | 2 | 103.36 | 5 | 28897 | Ana\_04.raw | 6.11E7 | 9.75E7 | 1.07E8 | 2.91E7 | 7.4E7 | 8.51E7 | 3.4E7 | 3.99E7 | 1.15E8 | 6.39E7 | 57 | 7 | 6 | 6 | 6 | 6 | 5 | 2 | 7 | 7 | 5 | 87 | 96 |  |
| K.VLTPELYAELR.A | Y | 78.24 | 1302.7183 | 11 | 3.0 | 652.3683 | 2 | 94.41 | 6 | 26765 | Ana\_05.raw | 2.33E7 | 2.27E7 | 2.39E7 | 6.64E6 | 1.52E7 | 1.5E7 | 7.23E6 |  | 3.43E7 | 0 | 11 | 1 | 1 | 1 | 1 | 1 | 2 | 2 | 0 | 1 | 1 | 33 | 43 |  |
| K.SM(+15.99)TEAEQQQLIDDHFLFDKPVSPLLLASGM(+15.99)AR.D | Y | 76.43 | 3618.7698 | 32 | 1.1 | 905.7007 | 4 | 124.76 | 6 | 34724 | Ana\_05.raw | 1.26E6 | 4.86E6 | 1.13E7 | 4.21E6 | 7.42E6 | 8.26E7 | 1.37E7 | 2.81E7 | 3.43E7 | 7.96E6 | 21 | 2 | 1 | 3 | 2 | 2 | 3 | 1 | 3 | 2 | 2 | 178 | 209 | Oxidation (M) |
| K.TFLVWINEEDHLR.V | Y | 76.10 | 1670.8417 | 13 | 2.5 | 557.9559 | 3 | 111.35 | 5 | 30955 | Ana\_04.raw |  | 4.09E5 | 5.58E5 | 1.45E6 | 2.73E6 | 1.67E6 | 8.21E5 | 8.59E5 |  |  | 7 | 0 | 1 | 1 | 1 | 1 | 1 | 1 | 1 | 0 | 0 | 224 | 236 |  |
| K.RGTGGVDTAAVGGVFDVSNADR.L | Y | 67.69 | 2120.0247 | 22 | 0.2 | 707.6823 | 3 | 80.22 | 5 | 22761 | Ana\_04.raw |  |  |  |  | 7.26E5 |  |  |  |  |  | 1 | 0 | 0 | 0 | 0 | 1 | 0 | 0 | 0 | 0 | 0 | 320 | 341 |  |
| K.SMTEAEQQQLIDDHFLFDKPVSPLLLASGM(+15.99)AR.D | Y | 55.92 | 3602.7749 | 32 | 4.3 | 901.7048 | 4 | 126.23 | 2 | 34978 | Ana\_01.raw | 2.71E6 | 4.19E6 |  |  |  |  |  | 1.46E6 |  | 3.8E6 | 4 | 1 | 1 | 0 | 0 | 0 | 0 | 0 | 1 | 0 | 1 | 178 | 209 | Oxidation (M) |
| K.LLIEM(+15.99)EQR.L | Y | 42.43 | 1046.5430 | 8 | 0.9 | 524.2792 | 2 | 46.55 | 7 | 12986 | Ana\_06.raw |  | 1.39E7 | 2.27E7 | 7.84E6 | 1.77E7 | 1.96E7 | 1.2E7 | 1.41E7 | 1.26E7 |  | 9 | 0 | 1 | 1 | 1 | 1 | 2 | 1 | 1 | 1 | 0 | 359 | 366 | Oxidation (M) |
| R.FPAEDEFPDLSSHNNHM(+15.99)AK.V | Y | 40.53 | 2200.9482 | 19 | 1.8 | 551.2453 | 4 | 70.58 | 6 | 20253 | Ana\_05.raw |  |  |  |  | 9.35E5 | 2.86E6 |  |  |  |  | 2 | 0 | 0 | 0 | 0 | 1 | 1 | 0 | 0 | 0 | 0 | 14 | 32 | Oxidation (M) |
| K.FSEVLKR.L | Y | 35.51 | 877.5021 | 7 | 1.6 | 439.7591 | 2 | 32.44 | 6 | 8874 | Ana\_05.raw |  |  | 6E6 |  | 4.02E6 | 7.6E6 |  |  |  |  | 3 | 0 | 0 | 1 | 0 | 1 | 1 | 0 | 0 | 0 | 0 | 308 | 314 |  |
| K.LPHLGK.H | Y | 34.70 | 663.4067 | 6 | 7.4 | 332.7131 | 2 | 27.34 | 10 | 7245 | Ana\_09.raw | 2.93E6 |  |  |  | 3.18E6 | 4.36E6 | 1.22E6 |  | 1.45E6 | 2.6E6 | 6 | 1 | 0 | 0 | 0 | 1 | 1 | 1 | 0 | 1 | 1 | 299 | 304 |  |
| R.DWPDAR.G | N | 32.95 | 758.3347 | 6 | 5.0 | 380.1765 | 2 | 37.22 | 1 | 10158 | ANa\_10.raw | 1.23E7 |  | 2.1E7 | 7.65E6 |  |  | 1.18E7 | 1.1E7 | 5.75E6 |  | 6 | 1 | 0 | 1 | 1 | 0 | 0 | 1 | 1 | 1 | 0 | 210 | 215 |  |
| K.EVFTR.F | Y | 32.77 | 650.3387 | 5 | 4.4 | 326.1781 | 2 | 25.09 | 7 | 6472 | Ana\_06.raw |  |  | 1.26E7 |  | 1.11E7 | 1.33E7 | 8.82E6 |  |  |  | 4 | 0 | 0 | 1 | 0 | 1 | 1 | 1 | 0 | 0 | 0 | 248 | 252 |  |
| K.FSEVLK.R | Y | 29.46 | 721.4010 | 6 | 4.8 | 361.7095 | 2 | 41.11 | 8 | 11160 | Ana\_07.raw |  |  |  |  |  | 2.43E7 |  | 1.29E7 |  |  | 2 | 0 | 0 | 0 | 0 | 0 | 1 | 0 | 1 | 0 | 0 | 308 | 313 |  |
| R.YYALK.S | Y | 23.38 | 656.3533 | 5 | 4.4 | 329.1854 | 2 | 35.31 | 8 | 9451 | Ana\_07.raw |  |  |  |  |  |  |  | 1.03E7 |  | 1.03E7 | 2 | 0 | 0 | 0 | 0 | 0 | 0 | 0 | 1 | 0 | 1 | 173 | 177 |  |
| total 23 peptides |  |  |  |  |  |  |  |  |  |  |  |  |  |  |  |  |  |  |  |  |  |  |  |  |  |  |  |  |  |  |  |  |  |  |  |
| --- | --- | --- | --- | --- | --- | --- | --- | --- | --- | --- | --- | --- | --- | --- | --- | --- | --- | --- | --- | --- | --- | --- | --- | --- | --- | --- | --- | --- | --- | --- | --- | --- | --- | --- | --- |

Best Unique PSM (Scan F3:34899, m/z=990.4723, z=3, RT=127.28, ppm=-1.6):

sp|P09103|PDIA1\_MOUSE

back to list

  

| Protein Coverage
| Supporting Peptides
| Best Unique PSM
|

Protein Coverage:

Supporting Peptides:

| Peptide | Uniq | -10lgP | Mass | Length | ppm | m/z | z | RT | Fraction | Scan | Source File | Area 10 | Area 01 | Area 02 | Area 03 | Area 04 | Area 05 | Area 06 | Area 07 | Area 08 | Area 09 | #Spec | #Spec 10 | #Spec 01 | #Spec 02 | #Spec 03 | #Spec 04 | #Spec 05 | #Spec 06 | #Spec 07 | #Spec 08 | #Spec 09 | Start | End | PTM |
| --- | --- | --- | --- | --- | --- | --- | --- | --- | --- | --- | --- | --- | --- | --- | --- | --- | --- | --- | --- | --- | --- | --- | --- | --- | --- | --- | --- | --- | --- | --- | --- | --- | --- | --- | --- |
| R.TGPAATTLSDTAAAESLVDSSEVTVIGFFK.D | Y | 133.37 | 2984.4866 | 30 | 6.5 | 1493.2603 | 2 | 136.68 | 4 | 37080 | Ana\_03.raw | 1.11E7 | 5.18E6 | 4.81E6 | 0 | 1.29E7 | 1.16E6 | 4.29E6 |  | 8.69E6 | 6.94E6 | 14 | 1 | 2 | 1 | 1 | 1 | 1 | 2 | 0 | 3 | 2 | 135 | 164 |  |
| K.VDATEESDLAQQYGVR.G | Y | 112.64 | 1779.8275 | 16 | 3.3 | 890.9240 | 2 | 79.14 | 7 | 22383 | Ana\_06.raw | 4.6E7 | 5.19E7 | 3.58E7 | 1.89E7 | 2.27E7 | 2.97E7 | 2.99E7 | 2.76E7 | 5.17E7 | 2.38E7 | 34 | 4 | 4 | 4 | 3 | 2 | 3 | 4 | 4 | 3 | 3 | 84 | 99 |  |
| K.HNQLPLVIEFTEQTAPK.I | Y | 103.11 | 1964.0366 | 17 | 3.8 | 983.0294 | 2 | 102.74 | 9 | 28970 | Ana\_08.raw | 2.5E6 | 9.1E6 | 8.62E6 | 5.03E6 | 8.49E6 | 7.16E6 | 6.85E6 | 9.29E6 | 4.21E7 | 8.42E6 | 30 | 2 | 4 | 3 | 3 | 2 | 2 | 3 | 3 | 5 | 3 | 233 | 249 |  |
| K.VLVGANFEEVAFDEK.K | Y | 83.61 | 1665.8250 | 15 | -0.2 | 833.9196 | 2 | 105.37 | 4 | 28887 | Ana\_03.raw | 4.61E6 | 1.78E6 |  | 4.96E5 | 7.48E6 | 9.77E6 | 7.9E6 | 4.45E6 | 1.82E7 | 8.75E6 | 10 | 1 | 2 | 0 | 1 | 1 | 1 | 1 | 1 | 1 | 1 | 373 | 387 |  |
| R.EADDIVNWLK.K | Y | 76.65 | 1201.5979 | 10 | 3.0 | 601.8080 | 2 | 103.59 | 5 | 28960 | Ana\_04.raw | 1.52E6 | 2.52E7 | 2.79E7 |  | 2.18E7 | 2.2E7 | 9.61E6 | 6.66E6 | 3.84E7 | 2.13E7 | 17 | 1 | 3 | 3 | 0 | 1 | 1 | 1 | 1 | 5 | 1 | 123 | 132 |  |
| K.SNFEEALAAHK.Y | Y | 73.05 | 1215.5884 | 11 | 2.7 | 608.8031 | 2 | 51.56 | 3 | 14597 | Ana\_02.raw | 3.87E7 | 5.51E7 | 5.46E7 | 2.62E7 | 4.06E7 | 3.97E7 | 3.6E7 | 2.77E7 | 5.18E7 | 3.58E7 | 28 | 3 | 2 | 2 | 4 | 3 | 2 | 3 | 4 | 2 | 3 | 34 | 44 |  |
| K.M(+15.99)DSTANEVEAVK.V | Y | 71.91 | 1308.5867 | 12 | 1.8 | 655.3018 | 2 | 36.97 | 4 | 9925 | Ana\_03.raw | 9.35E6 | 1.01E7 |  | 1.98E7 |  | 3.37E6 |  |  | 8.88E5 |  | 6 | 1 | 2 | 0 | 1 | 0 | 1 | 0 | 0 | 1 | 0 | 427 | 438 | Oxidation (M) |
| K.YKPESDELTAEK.I | Y | 67.91 | 1408.6721 | 12 | 4.3 | 705.3464 | 2 | 37.65 | 7 | 10282 | Ana\_06.raw | 9.96E7 | 1.28E8 | 1.28E8 | 5.1E7 | 8.85E7 | 8.69E7 | 5.03E7 | 6.44E7 | 1.15E8 | 8.44E7 | 27 | 3 | 3 | 4 | 2 | 3 | 2 | 2 | 2 | 3 | 3 | 329 | 340 |  |
| K.DHENIIIAK.M | Y | 63.93 | 1051.5662 | 9 | 0.7 | 526.7907 | 2 | 34.54 | 3 | 9477 | Ana\_02.raw |  | 1.38E7 | 1.81E7 | 7.33E6 | 1.19E7 | 1.1E7 | 1.08E7 | 7.42E6 | 1.33E7 |  | 8 | 0 | 1 | 1 | 1 | 1 | 1 | 1 | 1 | 1 | 0 | 418 | 426 |  |
| K.LGETYKDHENIIIAK.M | Y | 62.73 | 1742.9203 | 15 | 4.4 | 581.9833 | 3 | 57.53 | 7 | 16245 | Ana\_06.raw | 9.52E6 | 7.39E5 | 2.65E7 | 1.17E7 | 1.88E7 | 8.09E6 | 1.41E7 | 5.7E6 | 2.53E7 | 1.1E7 | 17 | 1 | 1 | 2 | 2 | 2 | 1 | 2 | 1 | 3 | 2 | 412 | 426 |  |
| K.THILLFLPK.S | Y | 59.22 | 1080.6696 | 9 | 3.9 | 541.3442 | 2 | 85.96 | 2 | 24353 | Ana\_01.raw | 1.26E7 | 1.78E7 | 1.34E7 | 1.84E6 | 1.54E7 | 1.16E7 | 9.97E6 | 2.64E6 | 1.76E7 | 1.12E7 | 20 | 1 | 2 | 3 | 2 | 3 | 3 | 2 | 2 | 1 | 1 | 257 | 265 |  |
| K.LKAEGSEIR.L | Y | 58.96 | 1001.5505 | 9 | 4.8 | 501.7849 | 2 | 22.55 | 2 | 5595 | Ana\_01.raw | 3.28E7 | 3.79E7 | 3.1E7 | 1.33E7 | 5.26E6 | 2.63E7 | 1.27E7 | 5.28E6 | 4E7 | 2.33E7 | 20 | 2 | 2 | 2 | 2 | 2 | 2 | 2 | 2 | 2 | 2 | 72 | 80 |  |
| K.ALAPEYAK.A | Y | 58.23 | 861.4596 | 8 | 1.7 | 431.7378 | 2 | 33.96 | 4 | 9050 | Ana\_03.raw | 5.8E7 | 7.76E7 | 7.7E7 | 3.59E7 | 5.52E7 | 5.81E7 | 5.39E7 | 4.03E7 | 5.71E7 | 5.8E7 | 16 | 1 | 1 | 2 | 2 | 2 | 2 | 2 | 2 | 1 | 1 | 60 | 67 |  |
| R.NNFEGEITK.E | Y | 58.04 | 1050.4982 | 9 | 4.7 | 526.2589 | 2 | 43.99 | 6 | 12385 | Ana\_05.raw | 3.48E7 | 4.48E7 | 4.27E7 | 3E7 |  | 3.9E7 | 3.76E7 | 4.27E7 |  | 3.57E7 | 9 | 1 | 1 | 1 | 1 | 0 | 1 | 1 | 2 | 0 | 1 | 216 | 224 |  |
| K.VHSFPTLK.F | Y | 57.29 | 927.5178 | 8 | 2.3 | 464.7672 | 2 | 40.75 | 3 | 11376 | Ana\_02.raw |  |  | 2.74E7 | 1.2E7 | 2.06E7 | 1.86E7 | 1.79E7 | 1.45E7 | 3.65E6 | 1.36E7 | 13 | 0 | 0 | 2 | 2 | 2 | 1 | 2 | 2 | 1 | 1 | 439 | 446 |  |
| K.FFPASADR.T | Y | 57.25 | 909.4344 | 8 | 4.4 | 455.7265 | 2 | 44.99 | 2 | 12594 | Ana\_01.raw | 6.43E7 | 1.22E8 | 1.39E8 | 3.33E7 | 5.86E7 | 4.25E7 | 5.67E7 | 4.7E7 | 2.74E7 | 6.85E7 | 45 | 5 | 5 | 5 | 5 | 2 | 4 | 5 | 4 | 5 | 5 | 447 | 454 |  |
| K.ITEFC(+57.02)HR.F | Y | 56.62 | 961.4440 | 7 | 4.2 | 481.7313 | 2 | 24.86 | 10 | 6451 | Ana\_09.raw | 1.12E7 | 3.42E7 | 3.78E7 | 4.38E6 | 1.11E7 | 2.88E7 | 6.01E6 | 1.3E7 | 2.54E7 | 9.66E6 | 16 | 1 | 1 | 2 | 1 | 2 | 2 | 1 | 2 | 2 | 2 | 341 | 347 | Carbamidomethylation |
| R.TVIDYNGER.T | Y | 54.84 | 1065.5090 | 9 | 1.5 | 533.7626 | 2 | 43.23 | 7 | 11973 | Ana\_06.raw | 9.37E6 | 7.87E6 | 1.48E7 | 6.38E6 | 1.02E7 | 1.21E7 | 1.22E7 | 2.84E6 |  | 6.27E6 | 9 | 1 | 1 | 1 | 1 | 1 | 1 | 1 | 1 | 0 | 1 | 455 | 463 |  |
| R.LITLEEEM(+15.99)TK.Y | Y | 54.13 | 1221.6162 | 10 | 3.5 | 611.8175 | 2 | 56.09 | 7 | 15820 | Ana\_06.raw | 2.65E7 | 3.49E7 | 4.01E7 | 1.79E7 | 2.88E7 | 2.44E7 | 2.58E7 | 2.15E7 |  | 2.02E7 | 14 | 1 | 1 | 1 | 2 | 2 | 2 | 2 | 2 | 0 | 1 | 319 | 328 | Oxidation (M) |
| R.ILEFFGLK.K | Y | 52.89 | 965.5585 | 8 | 1.0 | 483.7870 | 2 | 109.62 | 3 | 30373 | Ana\_02.raw | 6.95E7 | 9.07E7 | 1.1E8 | 6.27E7 | 7.89E7 | 6.12E7 | 6.93E7 | 5.63E7 | 9.01E7 | 6.88E7 | 65 | 6 | 7 | 8 | 8 | 7 | 5 | 6 | 8 | 5 | 5 | 303 | 310 |  |
| K.KEEC(+57.02)PAVR.L | Y | 50.24 | 987.4807 | 8 | 3.8 | 330.1688 | 3 | 17.76 | 5 | 4235 | Ana\_04.raw |  |  |  | 2.62E6 | 1.01E7 | 4.99E6 | 4.41E6 | 6.21E6 |  |  | 9 | 0 | 0 | 0 | 2 | 2 | 2 | 2 | 1 | 0 | 0 | 311 | 318 | Carbamidomethylation |
| R.TLDGFKK.F | Y | 44.19 | 807.4490 | 7 | 6.3 | 404.7343 | 2 | 24.14 | 2 | 6142 | Ana\_01.raw |  | 2.43E6 |  | 3.46E6 |  |  | 4.72E6 | 3.11E6 | 7.83E6 |  | 5 | 0 | 1 | 0 | 1 | 0 | 0 | 1 | 1 | 1 | 0 | 464 | 470 |  |
| K.DGVVLFK.K | Y | 41.77 | 776.4432 | 7 | 4.1 | 389.2305 | 2 | 63.41 | 2 | 18065 | Ana\_01.raw | 3.44E6 | 7.35E6 | 1.06E7 | 4.08E6 | 6.85E6 | 3.52E6 |  |  | 2.67E6 | 5.82E6 | 8 | 1 | 1 | 1 | 1 | 1 | 1 | 0 | 0 | 1 | 1 | 203 | 209 |  |
| R.TLDGFK.K | Y | 39.96 | 679.3541 | 6 | -1.4 | 340.6838 | 2 | 31.98 | 3 | 8720 | Ana\_02.raw | 5.64E6 | 1.22E7 | 1.76E7 | 9.88E6 |  | 1.05E7 | 1.02E7 | 9.53E6 |  | 1.08E7 | 10 | 1 | 1 | 1 | 2 | 0 | 1 | 1 | 2 | 0 | 1 | 464 | 469 |  |
| K.IKPHLM(+15.99)SQEVPEDWDKQPVK.V | Y | 34.03 | 2419.2205 | 20 | 4.0 | 605.8148 | 4 | 59.00 | 2 | 16756 | Ana\_01.raw |  | 4.5E6 |  |  |  |  |  |  |  |  | 1 | 0 | 1 | 0 | 0 | 0 | 0 | 0 | 0 | 0 | 0 | 353 | 372 | Oxidation (M) |
| K.IFGGEIK.T | Y | 33.45 | 762.4276 | 7 | 4.0 | 382.2226 | 2 | 49.66 | 7 | 13928 | Ana\_06.raw | 2.33E7 |  | 4.05E7 | 1.75E7 | 2.66E7 | 2.76E7 | 2.79E7 | 2.11E7 | 2.89E7 | 2.56E7 | 11 | 1 | 0 | 1 | 1 | 1 | 2 | 1 | 2 | 1 | 1 | 250 | 256 |  |
| K.LLDFIK.H | N | 31.46 | 747.4531 | 6 | 3.0 | 374.7349 | 2 | 82.29 | 6 | 23493 | Ana\_05.raw | 2.28E7 | 3.08E7 |  | 1.44E7 | 2.03E7 | 2.29E7 | 1.66E7 | 1.65E7 | 4.21E7 | 2.27E7 | 18 | 1 | 2 | 2 | 2 | 2 | 2 | 1 | 2 | 3 | 1 | 227 | 232 |  |
| R.GYPTIK.F | N | 27.28 | 677.3748 | 6 | 0.7 | 339.6949 | 2 | 34.51 | 4 | 9212 | Ana\_03.raw |  |  |  | 5.61E6 |  |  | 3.07E6 | 4.42E6 |  |  | 3 | 0 | 0 | 0 | 1 | 0 | 0 | 1 | 1 | 0 | 0 | 100 | 105 |  |
| K.AVKDEL | Y | 27.06 | 673.3646 | 6 | 2.9 | 337.6906 | 2 | 27.26 | 6 | 7266 | Ana\_05.raw |  |  |  |  |  | 1.22E7 |  |  |  |  | 1 | 0 | 0 | 0 | 0 | 0 | 1 | 0 | 0 | 0 | 0 | 504 | 509 |  |
| K.YQLDK.D | Y | 25.55 | 665.3384 | 5 | 0.1 | 333.6765 | 2 | 24.96 | 4 | 6372 | Ana\_03.raw |  |  |  | 1.2E7 | 9.15E6 |  |  |  |  |  | 2 | 0 | 0 | 0 | 1 | 1 | 0 | 0 | 0 | 0 | 0 | 198 | 202 |  |
| K.AEGSEIR.L | Y | 22.72 | 760.3715 | 7 | 1.6 | 381.1936 | 2 | 34.17 | 5 | 9344 | Ana\_04.raw |  |  |  |  | 1.52E6 |  |  |  |  |  | 1 | 0 | 0 | 0 | 0 | 1 | 0 | 0 | 0 | 0 | 0 | 74 | 80 |  |
| total 31 peptides |  |  |  |  |  |  |  |  |  |  |  |  |  |  |  |  |  |  |  |  |  |  |  |  |  |  |  |  |  |  |  |  |  |  |  |
| --- | --- | --- | --- | --- | --- | --- | --- | --- | --- | --- | --- | --- | --- | --- | --- | --- | --- | --- | --- | --- | --- | --- | --- | --- | --- | --- | --- | --- | --- | --- | --- | --- | --- | --- | --- |

Best Unique PSM (Scan F4:37080, m/z=1493.2603, z=2, RT=136.68, ppm=6.5):

sp|P20152|VIME\_MOUSE

back to list

  

| Protein Coverage
| Supporting Peptides
| Best Unique PSM
|

Protein Coverage:

Supporting Peptides:

| Peptide | Uniq | -10lgP | Mass | Length | ppm | m/z | z | RT | Fraction | Scan | Source File | Area 10 | Area 01 | Area 02 | Area 03 | Area 04 | Area 05 | Area 06 | Area 07 | Area 08 | Area 09 | #Spec | #Spec 10 | #Spec 01 | #Spec 02 | #Spec 03 | #Spec 04 | #Spec 05 | #Spec 06 | #Spec 07 | #Spec 08 | #Spec 09 | Start | End | PTM |
| --- | --- | --- | --- | --- | --- | --- | --- | --- | --- | --- | --- | --- | --- | --- | --- | --- | --- | --- | --- | --- | --- | --- | --- | --- | --- | --- | --- | --- | --- | --- | --- | --- | --- | --- | --- |
| R.LLQDSVDFSLADAINTEFK.N | Y | 112.37 | 2125.0579 | 19 | 2.8 | 1063.5392 | 2 | 130.41 | 3 | 35722 | Ana\_02.raw | 2.33E7 |  | 5.49E6 | 1.6E6 | 4.57E6 | 9.03E6 | 4.39E6 | 3.02E6 | 8.7E6 | 1.46E7 | 12 | 2 | 0 | 1 | 1 | 1 | 1 | 1 | 1 | 3 | 1 | 79 | 97 |  |
| R.EM(+15.99)EENFALEAANYQDTIGR.L | Y | 107.24 | 2215.9692 | 19 | 1.4 | 1108.9934 | 2 | 95.47 | 3 | 26708 | Ana\_02.raw | 1.19E7 |  | 0 |  |  | 1.43E6 |  | 2.53E6 | 1.46E7 | 4.47E6 | 6 | 1 | 0 | 1 | 0 | 0 | 1 | 0 | 1 | 1 | 1 | 346 | 364 | Oxidation (M) |
| R.SLYSSSPGGAYVTR.S | Y | 92.66 | 1443.6993 | 14 | 2.4 | 722.8586 | 2 | 60.52 | 6 | 17327 | Ana\_05.raw | 1.25E8 | 6.42E7 | 5.13E7 | 2.34E7 | 4.55E7 | 4.89E7 | 3.51E7 | 3.75E7 | 1.86E8 | 8.21E7 | 34 | 6 | 3 | 2 | 2 | 2 | 3 | 3 | 4 | 5 | 4 | 51 | 64 |  |
| R.ISLPLPTFSSLNLR.E | Y | 89.93 | 1556.8926 | 14 | 4.8 | 779.4573 | 2 | 129.50 | 1 | 35595 | ANa\_10.raw | 5.92E7 | 5.94E7 | 9.77E7 | 1.06E7 | 1.45E7 | 7.36E7 | 6.89E7 | 6.49E6 | 3.65E8 | 2.44E8 | 67 | 13 | 7 | 5 | 3 | 6 | 3 | 2 | 6 | 10 | 12 | 411 | 424 |  |
| R.EEAESTLQSFR.Q | Y | 86.97 | 1295.5994 | 11 | 2.2 | 648.8084 | 2 | 61.11 | 6 | 17505 | Ana\_05.raw | 1.05E8 | 6.83E7 | 5.84E7 | 2.69E7 | 4.72E7 | 5.05E7 | 3.59E7 | 3.98E7 | 1.66E8 | 8.99E7 | 31 | 6 | 3 | 3 | 2 | 3 | 2 | 2 | 2 | 4 | 4 | 197 | 207 |  |
| R.KVESLQEEIAFLK.K | Y | 83.27 | 1532.8450 | 13 | 2.9 | 511.9571 | 3 | 95.10 | 3 | 26611 | Ana\_02.raw | 5.82E7 | 9.3E6 | 2.06E6 | 3.09E6 | 1.94E7 | 1.54E7 | 6.29E5 | 7.59E6 | 8.53E7 | 3.98E7 | 31 | 4 | 2 | 2 | 2 | 3 | 3 | 2 | 3 | 6 | 4 | 223 | 235 |  |
| R.KLLEGEESR.I | N | 81.04 | 1059.5560 | 9 | 8.7 | 354.1957 | 3 | 23.79 | 6 | 6172 | Ana\_05.raw | 1.35E8 | 7.36E7 | 8.47E7 | 3.98E7 | 3.65E7 | 5.87E7 | 4.47E7 | 2.25E7 | 1.49E8 | 2.57E7 | 25 | 5 | 2 | 1 | 1 | 2 | 2 | 2 | 2 | 5 | 3 | 402 | 410 |  |
| R.ETNLESLPLVDTHSK.R | Y | 80.01 | 1681.8523 | 15 | 2.0 | 561.6259 | 3 | 73.87 | 3 | 20957 | Ana\_02.raw | 2.26E7 | 1.49E7 | 1.67E7 | 1.51E6 | 1.13E7 | 1.1E7 | 1.12E7 | 1.33E7 | 2.65E6 | 2.11E7 | 14 | 1 | 1 | 2 | 1 | 1 | 1 | 1 | 2 | 1 | 3 | 425 | 439 |  |
| R.DGQVINETSQHHDDLE | Y | 78.20 | 1835.7922 | 16 | 1.8 | 918.9050 | 2 | 48.78 | 1 | 13669 | ANa\_10.raw | 2.41E7 | 2.61E6 |  |  | 2.47E7 | 1.7E7 | 1.14E7 |  | 6.43E7 | 3.93E7 | 14 | 2 | 1 | 0 | 0 | 3 | 1 | 1 | 0 | 3 | 3 | 451 | 466 |  |
| K.FADLSEAANR.N | Y | 73.99 | 1092.5199 | 10 | 2.4 | 547.2686 | 2 | 50.02 | 6 | 14230 | Ana\_05.raw | 6.63E7 | 4.03E7 | 3.73E7 |  | 3.4E7 | 3.94E7 | 2.86E7 | 2.53E7 | 1.11E8 | 5.86E7 | 23 | 2 | 2 | 2 | 2 | 2 | 1 | 2 | 3 | 4 | 3 | 295 | 304 |  |
| R.TNEKVELQELNDR.F | N | 73.46 | 1586.7900 | 13 | 7.9 | 794.4086 | 2 | 48.77 | 9 | 13667 | Ana\_08.raw | 1.95E7 | 6.68E6 | 4.32E6 |  | 2.98E6 | 7.99E6 | 2.95E6 |  | 3.01E7 | 1.28E7 | 10 | 1 | 1 | 2 | 0 | 1 | 1 | 1 | 0 | 2 | 1 | 101 | 113 |  |
| K.LLEGEESR.I | N | 70.89 | 931.4611 | 8 | 2.9 | 466.7392 | 2 | 26.90 | 6 | 7157 | Ana\_05.raw | 1.04E8 | 5.04E8 | 4.04E8 | 2.11E8 | 3.45E8 | 4.18E8 | 2.77E8 | 2.42E8 | 3.67E8 | 3.25E8 | 45 | 6 | 4 | 4 | 4 | 4 | 4 | 4 | 6 | 5 | 4 | 403 | 410 |  |
| K.VELQELNDR.F | N | 68.56 | 1114.5618 | 9 | 1.7 | 558.2891 | 2 | 53.93 | 6 | 15380 | Ana\_05.raw | 1.87E8 | 1.42E8 | 1.14E8 | 4.2E7 | 9.84E7 | 1.18E8 | 6.37E7 | 8.36E7 | 2.32E8 | 1.36E8 | 49 | 5 | 5 | 5 | 3 | 6 | 3 | 3 | 5 | 10 | 4 | 105 | 113 |  |
| K.VESLQEEIAFLK.K | Y | 66.70 | 1404.7500 | 12 | 2.7 | 703.3842 | 2 | 110.29 | 6 | 30954 | Ana\_05.raw |  |  |  | 5.42E6 | 5.27E6 | 1.12E7 | 5.8E6 |  |  |  | 4 | 0 | 0 | 0 | 1 | 1 | 1 | 1 | 0 | 0 | 0 | 224 | 235 |  |
| R.EYQDLLNVK.M | N | 65.67 | 1120.5764 | 9 | 3.7 | 561.2975 | 2 | 72.28 | 6 | 20741 | Ana\_05.raw | 1.18E8 | 9.27E7 | 7.55E7 | 3.42E7 | 5.46E7 | 8.33E7 | 3.64E7 | 6.08E7 | 2.11E8 | 9.03E7 | 38 | 6 | 4 | 3 | 3 | 2 | 4 | 1 | 6 | 4 | 5 | 382 | 390 |  |
| R.RQVDQLTNDK.A | Y | 65.29 | 1215.6207 | 10 | 3.8 | 608.8199 | 2 | 23.11 | 6 | 5951 | Ana\_05.raw | 7.85E6 |  | 2.78E6 | 8.62E5 |  | 3.25E6 | 1.72E6 | 8.99E5 | 8.72E6 | 6.17E6 | 8 | 1 | 0 | 1 | 1 | 0 | 1 | 1 | 1 | 1 | 1 | 159 | 168 |  |
| R.TYSLGSALRPSTSR.S | Y | 64.60 | 1494.7791 | 14 | 4.2 | 499.2690 | 3 | 59.68 | 6 | 17069 | Ana\_05.raw | 5.23E7 | 2.56E7 | 1.82E7 | 1.11E7 | 1.76E7 | 2.11E7 | 1.52E7 | 1.38E7 | 9.74E7 | 2.92E7 | 17 | 3 | 2 | 1 | 1 | 1 | 2 | 1 | 1 | 2 | 3 | 37 | 50 |  |
| R.QVQSLTC(+57.02)EVDALK.G | Y | 63.42 | 1489.7446 | 13 | 6.5 | 745.8844 | 2 | 79.25 | 1 | 22377 | ANa\_10.raw | 1.4E6 |  |  |  |  |  |  |  | 2.64E6 |  | 2 | 1 | 0 | 0 | 0 | 0 | 0 | 0 | 0 | 1 | 0 | 322 | 334 | Carbamidomethylation |
| R.LGDLYEEEM(+15.99)R.E | Y | 60.54 | 1269.5547 | 10 | 5.5 | 635.7881 | 2 | 53.13 | 1 | 14948 | ANa\_10.raw | 4.2E7 | 2.42E7 | 1.78E7 | 7.54E6 | 4.76E6 |  | 1.39E7 | 1.49E7 | 5.82E7 | 3.09E7 | 15 | 2 | 2 | 1 | 1 | 2 | 0 | 2 | 1 | 2 | 2 | 146 | 155 | Oxidation (M) |
| K.ILLAELEQLK.G | Y | 59.72 | 1168.7067 | 10 | 3.3 | 585.3625 | 2 | 107.93 | 6 | 30346 | Ana\_05.raw | 1.18E8 | 9.03E7 | 4.97E7 | 1.46E7 | 6.53E7 | 4.46E7 | 4.32E7 | 5.02E7 | 1.67E8 | 8.94E7 | 45 | 6 | 5 | 4 | 4 | 4 | 3 | 4 | 4 | 6 | 5 | 130 | 139 |  |
| K.NLQEAEEWYK.S | N | 58.98 | 1308.5986 | 10 | 2.0 | 655.3079 | 2 | 73.52 | 5 | 20937 | Ana\_04.raw | 2.36E7 | 1.34E7 | 8.6E6 | 4.03E6 | 1.09E7 |  | 5.99E6 | 8.4E6 | 9.23E6 | 8.81E6 | 9 | 1 | 1 | 1 | 1 | 1 | 0 | 1 | 1 | 1 | 1 | 283 | 292 |  |
| R.SYVTTSTR.T | Y | 58.12 | 913.4505 | 8 | 1.1 | 457.7330 | 2 | 23.24 | 3 | 6003 | Ana\_02.raw | 8.95E7 | 5.6E7 | 5.62E7 | 2.9E7 | 3.79E7 | 6.64E7 | 3.41E7 | 3.01E7 |  | 9.25E6 | 9 | 1 | 1 | 1 | 1 | 1 | 1 | 1 | 1 | 0 | 1 | 29 | 36 |  |
| R.QVDQLTNDK.A | Y | 53.64 | 1059.5197 | 9 | 5.3 | 530.7699 | 2 | 24.54 | 2 | 6279 | Ana\_01.raw | 6.02E6 | 8E6 | 1.49E7 | 2.89E6 |  | 8.78E6 | 4.07E6 | 6.9E6 |  | 3.86E6 | 9 | 1 | 1 | 1 | 1 | 0 | 1 | 2 | 1 | 0 | 1 | 160 | 168 |  |
| R.DNLAEDIM(+15.99)R.L | Y | 53.01 | 1091.4917 | 9 | 6.5 | 546.7567 | 2 | 52.38 | 9 | 14777 | Ana\_08.raw | 3.67E7 | 2.46E7 | 2.45E7 | 5.83E6 | 8.24E6 | 2.11E7 | 4.04E6 | 1.88E7 | 0 | 3.48E7 | 19 | 3 | 2 | 2 | 1 | 2 | 2 | 1 | 2 | 1 | 3 | 176 | 184 | Oxidation (M) |
| R.QDVDNASLAR.L | Y | 50.86 | 1087.5258 | 10 | 5.4 | 544.7731 | 2 | 29.97 | 6 | 8119 | Ana\_05.raw | 3.44E8 |  | 0 | 5.75E6 | 1.09E7 | 8.01E6 | 1.46E8 | 1.34E7 | 3.65E6 | 2.59E8 | 9 | 1 | 0 | 1 | 1 | 1 | 1 | 1 | 1 | 1 | 1 | 208 | 217 |  |
| R.QQYESVAAK.N | Y | 49.40 | 1022.5032 | 9 | 2.7 | 512.2603 | 2 | 25.16 | 4 | 6431 | Ana\_03.raw |  |  |  | 2.64E6 |  |  |  | 8.02E6 |  |  | 2 | 0 | 0 | 0 | 1 | 0 | 0 | 0 | 1 | 0 | 0 | 274 | 282 |  |
| R.LQDEIQNM(+15.99)KEEM(+15.99)AR.H | Y | 45.17 | 1765.7975 | 14 | -0.2 | 883.9059 | 2 | 29.64 | 9 | 7772 | Ana\_08.raw | 3.79E7 | 1.4E7 | 3.61E5 |  | 7.73E5 |  | 2.12E6 | 1.68E6 | 7.15E6 | 2.3E6 | 9 | 2 | 1 | 1 | 0 | 1 | 0 | 1 | 1 | 1 | 1 | 365 | 378 | Oxidation (M) |
| R.FANYIDK.V | Y | 44.13 | 869.4283 | 7 | 1.9 | 435.7223 | 2 | 46.17 | 6 | 13045 | Ana\_05.raw | 3.81E7 | 3.07E7 | 3.14E7 | 1.47E7 | 2.72E7 | 3.08E7 | 2.09E7 | 2.12E7 | 5.31E7 | 3.86E7 | 11 | 1 | 1 | 1 | 1 | 1 | 2 | 1 | 1 | 1 | 1 | 114 | 120 |  |
| R.FLEQQNK.I | N | 42.78 | 905.4606 | 7 | 6.4 | 453.7405 | 2 | 25.02 | 7 | 6450 | Ana\_06.raw | 7.66E6 | 2.16E7 | 2.19E8 | 1.48E8 | 2.21E8 | 2.67E8 | 2.03E8 | 8.66E7 |  | 9.67E7 | 14 | 1 | 1 | 1 | 2 | 2 | 2 | 2 | 2 | 0 | 1 | 123 | 129 |  |
| K.GTNESLER.Q | Y | 42.51 | 904.4250 | 8 | 3.3 | 453.2213 | 2 | 19.57 | 5 | 4841 | Ana\_04.raw |  |  |  |  | 3.27E6 |  |  |  |  |  | 1 | 0 | 0 | 0 | 0 | 1 | 0 | 0 | 0 | 0 | 0 | 335 | 342 |  |
| K.LQEEM(+15.99)LQR.E | Y | 40.78 | 1061.5176 | 8 | 2.0 | 531.7672 | 2 | 29.92 | 9 | 7849 | Ana\_08.raw |  |  |  | 7.19E6 | 1.18E7 | 1.13E7 | 1.65E7 |  | 9.89E6 | 1.83E7 | 6 | 0 | 0 | 0 | 1 | 1 | 1 | 1 | 0 | 1 | 1 | 189 | 196 | Oxidation (M) |
| R.LDLER.K | N | 35.02 | 644.3493 | 5 | 4.6 | 323.1834 | 2 | 33.12 | 7 | 8934 | Ana\_06.raw | 2.18E7 | 4.86E7 | 1.51E7 | 2.77E7 | 3.2E7 | 1.31E7 | 3.61E7 | 8.73E6 | 2.45E7 | 2.04E7 | 14 | 1 | 2 | 1 | 2 | 2 | 1 | 2 | 1 | 1 | 1 | 218 | 222 |  |
| R.SSVPGVR.L | Y | 34.71 | 700.3868 | 7 | 1.3 | 351.2011 | 2 | 22.58 | 3 | 5788 | Ana\_02.raw | 6.12E6 |  | 2.45E7 | 1.18E7 |  | 7.91E6 |  |  |  | 7.59E6 | 5 | 1 | 0 | 1 | 1 | 0 | 1 | 0 | 0 | 0 | 1 | 72 | 78 |  |
| K.M(+15.99)ALDIEIATYR.K | N | 27.59 | 1310.6541 | 11 | 3.9 | 656.3369 | 2 | 86.90 | 5 | 24570 | Ana\_04.raw |  |  |  |  | 3.45E5 |  |  |  |  |  | 1 | 0 | 0 | 0 | 0 | 1 | 0 | 0 | 0 | 0 | 0 | 391 | 401 | Oxidation (M) |
| R.SVSSSSYR.R | Y | 24.97 | 871.4036 | 8 | 0.5 | 436.7093 | 2 | 19.30 | 5 | 4751 | Ana\_04.raw |  |  |  |  | 9.65E6 |  |  |  |  |  | 1 | 0 | 0 | 0 | 0 | 1 | 0 | 0 | 0 | 0 | 0 | 5 | 12 |  |
| total 35 peptides |  |  |  |  |  |  |  |  |  |  |  |  |  |  |  |  |  |  |  |  |  |  |  |  |  |  |  |  |  |  |  |  |  |  |  |
| --- | --- | --- | --- | --- | --- | --- | --- | --- | --- | --- | --- | --- | --- | --- | --- | --- | --- | --- | --- | --- | --- | --- | --- | --- | --- | --- | --- | --- | --- | --- | --- | --- | --- | --- | --- |

Best Unique PSM (Scan F3:35722, m/z=1063.5392, z=2, RT=130.41, ppm=2.8):

sp|P08113|ENPL\_MOUSE

back to list

  

| Protein Coverage
| Supporting Peptides
| Best Unique PSM
|

Protein Coverage:

Supporting Peptides:

| Peptide | Uniq | -10lgP | Mass | Length | ppm | m/z | z | RT | Fraction | Scan | Source File | Area 10 | Area 01 | Area 02 | Area 03 | Area 04 | Area 05 | Area 06 | Area 07 | Area 08 | Area 09 | #Spec | #Spec 10 | #Spec 01 | #Spec 02 | #Spec 03 | #Spec 04 | #Spec 05 | #Spec 06 | #Spec 07 | #Spec 08 | #Spec 09 | Start | End | PTM |
| --- | --- | --- | --- | --- | --- | --- | --- | --- | --- | --- | --- | --- | --- | --- | --- | --- | --- | --- | --- | --- | --- | --- | --- | --- | --- | --- | --- | --- | --- | --- | --- | --- | --- | --- | --- |
| R.TDDEVVQREEEAIQLDGLNASQIR.E | Y | 123.96 | 2727.3311 | 24 | 2.6 | 910.1200 | 3 | 106.60 | 6 | 30001 | Ana\_05.raw |  | 1.25E7 |  |  | 5.91E6 | 7.38E6 | 1.19E6 | 5.01E5 | 7.05E6 |  | 6 | 0 | 1 | 0 | 0 | 1 | 1 | 1 | 1 | 1 | 0 | 44 | 67 |  |
| K.YSQFINFPIYVWSSK.T | Y | 97.62 | 1877.9352 | 15 | 1.9 | 939.9766 | 2 | 130.97 | 3 | 35877 | Ana\_02.raw |  | 3.56E6 | 1.08E6 | 4.09E5 | 1.79E6 | 2.2E6 | 1.39E6 | 1.59E6 | 3.79E6 | 7.54E5 | 9 | 0 | 1 | 1 | 1 | 1 | 1 | 1 | 1 | 1 | 1 | 271 | 285 |  |
| R.FQSSHHSTDITSLDQYVER.M | Y | 92.61 | 2249.0349 | 19 | 3.7 | 750.6884 | 3 | 72.97 | 1 | 20679 | ANa\_10.raw | 9.69E6 | 7.84E6 | 7.39E6 |  | 9.17E6 |  |  |  |  | 4.69E5 | 7 | 2 | 1 | 1 | 0 | 2 | 0 | 0 | 0 | 0 | 1 | 512 | 530 |  |
| K.GVVDSDDLPLNVSR.E | Y | 87.53 | 1484.7471 | 14 | 2.4 | 743.3826 | 2 | 78.25 | 3 | 22150 | Ana\_02.raw | 2.1E7 | 2.65E7 | 1.82E7 | 5.73E6 | 1.29E7 | 1.26E7 | 7.94E6 | 7.87E6 | 4.9E7 | 2.04E7 | 16 | 3 | 2 | 1 | 1 | 1 | 1 | 1 | 1 | 2 | 3 | 435 | 448 |  |
| K.EEASDYLELDTIK.N | Y | 85.12 | 1524.7195 | 13 | 0.5 | 763.3674 | 2 | 88.36 | 3 | 24833 | Ana\_02.raw | 7.04E6 | 1.12E7 | 1.12E7 | 4.74E5 | 5.98E6 | 5.51E5 | 4.84E6 | 4.35E6 | 1.95E7 | 8.63E6 | 10 | 1 | 1 | 1 | 1 | 1 | 1 | 1 | 1 | 1 | 1 | 253 | 265 |  |
| R.KYSQFINFPIYVWSSK.T | Y | 83.66 | 2006.0302 | 16 | 2.2 | 669.6855 | 3 | 127.49 | 7 | 35086 | Ana\_06.raw |  | 1.13E6 |  |  | 1.65E5 | 8.41E6 | 2.73E6 | 6.5E6 | 9.73E6 |  | 6 | 0 | 1 | 0 | 0 | 1 | 1 | 1 | 1 | 1 | 0 | 270 | 285 |  |
| R.IKEDEDDKTVM(+15.99)DLAVVLFETATLR.S | Y | 82.58 | 2766.3997 | 24 | 1.0 | 692.6079 | 4 | 134.69 | 4 | 36580 | Ana\_03.raw |  | 2.83E6 | 4.38E6 | 6.16E5 | 3.03E6 | 3.64E6 | 1.33E6 | 3.98E6 | 1.79E6 |  | 12 | 0 | 1 | 2 | 1 | 1 | 2 | 2 | 2 | 1 | 0 | 701 | 724 | Oxidation (M) |
| K.LGVIEDHSNR.T | Y | 77.87 | 1138.5730 | 10 | 4.9 | 570.2966 | 2 | 29.49 | 6 | 7969 | Ana\_05.raw |  | 2.56E7 | 1.59E7 | 2.09E7 | 3.57E7 | 2.45E7 | 3.29E7 | 2.26E7 |  | 1.94E7 | 15 | 0 | 1 | 1 | 3 | 2 | 2 | 2 | 2 | 0 | 2 | 494 | 503 |  |
| K.FAFQAEVNR.M | Y | 75.26 | 1080.5352 | 9 | 7.3 | 541.2788 | 2 | 63.77 | 1 | 18076 | ANa\_10.raw | 1.71E7 | 1.52E7 | 2.08E7 | 9.12E6 | 1.62E7 | 1.2E7 | 1.33E7 | 1.35E7 | 2.28E7 | 1.48E7 | 16 | 2 | 2 | 2 | 1 | 2 | 1 | 2 | 1 | 2 | 1 | 76 | 84 |  |
| K.SILFVPTSAPR.G | Y | 73.14 | 1186.6710 | 11 | 5.0 | 594.3458 | 2 | 83.70 | 1 | 23591 | ANa\_10.raw | 5.28E7 | 5.93E7 | 5.2E7 | 2.57E7 | 4.32E7 | 4.86E7 | 3.97E7 | 3.47E7 | 1.02E8 | 5.13E7 | 24 | 1 | 3 | 2 | 3 | 2 | 2 | 3 | 4 | 2 | 2 | 385 | 395 |  |
| R.ELISNASDALDK.I | N | 71.73 | 1274.6354 | 12 | 0.7 | 638.3254 | 2 | 63.17 | 7 | 17908 | Ana\_06.raw | 5.3E7 | 6.22E7 | 6.1E7 | 2.33E7 | 3.85E7 | 3.66E7 | 3.47E7 | 3.57E7 | 8.97E7 | 4.28E7 | 22 | 3 | 2 | 2 | 2 | 2 | 1 | 3 | 3 | 3 | 1 | 103 | 114 |  |
| K.EAESSPFVER.L | Y | 66.30 | 1149.5302 | 10 | 2.3 | 575.7737 | 2 | 44.98 | 7 | 12506 | Ana\_06.raw | 3.78E7 | 4.02E7 | 3.93E7 | 1.89E7 | 3.54E7 | 3.9E7 | 3.25E7 | 2.66E7 | 6E6 | 3.09E7 | 14 | 2 | 2 | 2 | 1 | 2 | 1 | 1 | 1 | 1 | 1 | 548 | 557 |  |
| K.DISTNYYASQK.K | Y | 65.23 | 1288.5935 | 11 | 1.8 | 645.3052 | 2 | 48.15 | 6 | 13646 | Ana\_05.raw | 2.95E7 | 2.72E7 | 2.71E7 | 1.47E7 | 2.48E7 | 2.42E6 | 2.01E7 | 1.86E7 | 3.55E7 | 2.5E7 | 13 | 1 | 1 | 2 | 1 | 1 | 1 | 2 | 1 | 2 | 1 | 672 | 682 |  |
| K.EFEPLLNWMK.D | Y | 60.70 | 1305.6427 | 10 | 3.6 | 653.8310 | 2 | 123.20 | 9 | 34257 | Ana\_08.raw | 4.84E5 | 2.71E6 |  |  |  | 1.63E5 |  |  | 5.44E6 |  | 4 | 1 | 1 | 0 | 0 | 0 | 1 | 0 | 0 | 1 | 0 | 614 | 623 |  |
| R.TDDEVVQR.E | Y | 60.55 | 960.4512 | 8 | -0.4 | 481.2327 | 2 | 21.61 | 5 | 5503 | Ana\_04.raw |  |  |  |  | 1.48E7 | 1.32E7 | 1.42E7 | 5.13E6 |  |  | 4 | 0 | 0 | 0 | 0 | 1 | 1 | 1 | 1 | 0 | 0 | 44 | 51 |  |
| K.SGTSEFLNK.M | Y | 60.18 | 981.4767 | 9 | 3.0 | 491.7471 | 2 | 39.40 | 6 | 10966 | Ana\_05.raw | 2.74E7 | 3.15E7 | 3.12E7 | 1.56E7 | 2.81E7 | 2.73E7 | 2.48E7 | 2.31E7 | 6.41E6 | 3.53E7 | 10 | 1 | 1 | 1 | 1 | 1 | 1 | 1 | 1 | 1 | 1 | 169 | 177 |  |
| R.SGYLLPDTK.A | Y | 60.00 | 992.5178 | 9 | 2.9 | 497.2676 | 2 | 57.17 | 7 | 16140 | Ana\_06.raw | 3.92E7 | 4.23E7 | 4.84E7 | 1.07E7 | 4.22E7 | 3.47E7 | 3.23E7 | 2.89E7 |  | 3.92E7 | 13 | 1 | 1 | 1 | 2 | 2 | 2 | 1 | 2 | 0 | 1 | 725 | 733 |  |
| R.GLFDEYGSK.K | Y | 58.98 | 1014.4658 | 9 | 4.4 | 508.2424 | 2 | 68.76 | 7 | 19507 | Ana\_06.raw | 1.97E7 | 2.18E7 | 2.52E7 | 1.37E7 | 1.89E7 | 2.1E7 | 2.09E7 | 1.79E7 | 2.84E7 | 1.59E7 | 15 | 2 | 1 | 2 | 2 | 2 | 2 | 1 | 1 | 1 | 1 | 396 | 404 |  |
| R.ELISNASDALDKIR.L | N | 58.86 | 1543.8206 | 14 | 5.5 | 515.6169 | 3 | 80.38 | 9 | 22886 | Ana\_08.raw |  |  |  |  |  |  |  |  | 2.43E6 |  | 1 | 0 | 0 | 0 | 0 | 0 | 0 | 0 | 0 | 1 | 0 | 103 | 116 |  |
| K.TFEINPR.H | Y | 58.22 | 875.4501 | 7 | 5.4 | 438.7347 | 2 | 46.51 | 1 | 12997 | ANa\_10.raw | 3.1E7 | 3.1E7 | 3.36E7 | 1.62E7 | 2.86E7 |  | 2.66E7 | 9.43E6 | 3.3E7 | 2.95E7 | 10 | 1 | 2 | 1 | 1 | 1 | 0 | 1 | 1 | 1 | 1 | 684 | 690 |  |
| R.KEAESSPFVER.L | Y | 57.97 | 1277.6251 | 11 | 4.8 | 639.8229 | 2 | 29.24 | 9 | 7659 | Ana\_08.raw | 6.75E6 | 1.95E7 | 1.06E7 | 1.24E7 | 0 | 8.85E6 | 2.48E7 | 6.81E6 | 7.75E6 | 5.73E6 | 14 | 1 | 2 | 1 | 2 | 1 | 1 | 2 | 1 | 2 | 1 | 547 | 557 |  |
| K.EFEPLLNWM(+15.99)K.D | Y | 56.54 | 1321.6376 | 10 | 4.4 | 661.8290 | 2 | 101.33 | 9 | 28598 | Ana\_08.raw | 1.21E6 | 1.21E6 | 6.09E6 | 3.23E6 | 5.68E6 | 6.12E6 | 1.04E6 |  | 8.21E6 |  | 9 | 1 | 1 | 1 | 1 | 1 | 1 | 1 | 0 | 2 | 0 | 614 | 623 | Oxidation (M) |
| K.TETVEEPLEEDEAAKEEK.E | Y | 53.18 | 2074.9429 | 18 | 2.9 | 692.6569 | 3 | 54.97 | 7 | 15489 | Ana\_06.raw |  |  |  |  |  |  | 3.69E6 |  |  |  | 1 | 0 | 0 | 0 | 0 | 0 | 0 | 1 | 0 | 0 | 0 | 286 | 303 |  |
| K.IYFM(+15.99)AGSSR.K | Y | 50.23 | 1046.4855 | 9 | 5.9 | 524.2531 | 2 | 46.26 | 6 | 13072 | Ana\_05.raw | 2.55E7 | 2.45E7 | 3.3E7 | 1.46E7 | 2.58E7 | 2.3E7 | 2.23E7 | 2.37E7 |  | 1.84E7 | 11 | 1 | 1 | 1 | 2 | 2 | 1 | 1 | 1 | 0 | 1 | 538 | 546 | Oxidation (M) |
| K.NLLHVTDTGVGM(+15.99)TR.E | Y | 48.91 | 1528.7667 | 14 | 2.8 | 510.5976 | 3 | 55.12 | 3 | 15642 | Ana\_02.raw | 7.38E6 | 4.4E6 | 5.35E6 |  |  | 4.56E6 |  |  | 2.13E7 | 3.59E6 | 8 | 2 | 1 | 1 | 0 | 0 | 1 | 0 | 0 | 2 | 1 | 143 | 156 | Oxidation (M) |
| K.EVEEDEYK.A | Y | 44.79 | 1039.4346 | 8 | 3.6 | 520.7264 | 2 | 25.30 | 5 | 6646 | Ana\_04.raw | 3.98E6 | 1.7E7 | 3.56E7 | 1.76E7 | 1.15E7 | 3.21E7 | 2.58E7 | 2.07E7 |  | 2.65E7 | 9 | 1 | 1 | 1 | 1 | 1 | 1 | 1 | 1 | 0 | 1 | 349 | 356 |  |
| K.LIINSLYK.N | Y | 39.72 | 962.5800 | 8 | 4.0 | 482.2992 | 2 | 77.72 | 7 | 22000 | Ana\_06.raw |  |  |  | 4.83E6 | 8.46E6 |  | 5.89E6 |  |  |  | 3 | 0 | 0 | 0 | 1 | 1 | 0 | 1 | 0 | 0 | 0 | 88 | 95 |  |
| K.YNDTFWK.E | Y | 36.23 | 972.4341 | 7 | 6.3 | 487.2274 | 2 | 69.33 | 7 | 19662 | Ana\_06.raw |  |  | 2.72E6 |  |  | 2.77E6 | 4.18E6 | 3.71E6 |  |  | 4 | 0 | 0 | 1 | 0 | 0 | 1 | 1 | 1 | 0 | 0 | 480 | 486 |  |
| K.YLNFVK.G | Y | 35.01 | 782.4326 | 6 | 3.7 | 392.2250 | 2 | 60.79 | 8 | 16986 | Ana\_07.raw |  | 1.37E7 | 1.48E7 | 7.49E6 | 1.29E7 | 1.37E7 | 1.2E7 | 8.57E6 | 1.81E7 | 1.24E7 | 9 | 0 | 1 | 1 | 1 | 1 | 1 | 1 | 1 | 1 | 1 | 429 | 434 |  |
| K.NLGTIAK.S | Y | 33.69 | 715.4229 | 7 | 7.0 | 358.7212 | 2 | 25.57 | 2 | 6613 | Ana\_01.raw |  | 9.02E6 |  |  | 1.06E7 | 9.48E6 |  |  |  |  | 3 | 0 | 1 | 0 | 0 | 1 | 1 | 0 | 0 | 0 | 0 | 162 | 168 |  |
| K.EFGTNIK.L | Y | 32.91 | 807.4127 | 7 | 3.1 | 404.7148 | 2 | 35.94 | 5 | 9877 | Ana\_04.raw |  | 3.31E7 | 2.39E7 | 1.33E7 | 1.9E7 | 1.66E7 | 1.65E7 | 1.36E7 |  | 1.18E7 | 8 | 0 | 1 | 1 | 1 | 1 | 1 | 1 | 1 | 0 | 1 | 487 | 493 |  |
| R.HPLIR.D | Y | 32.11 | 634.3915 | 5 | 2.2 | 318.2037 | 2 | 18.92 | 6 | 4580 | Ana\_05.raw |  |  |  |  |  | 2.66E7 | 1.96E7 |  |  |  | 2 | 0 | 0 | 0 | 0 | 0 | 1 | 1 | 0 | 0 | 0 | 691 | 695 |  |
| K.EIFLR.E | N | 28.74 | 676.3907 | 5 | 1.2 | 339.2031 | 2 | 46.69 | 3 | 13179 | Ana\_02.raw |  |  | 1.52E7 |  | 1.07E7 |  |  |  | 6.65E6 |  | 3 | 0 | 0 | 1 | 0 | 1 | 0 | 0 | 0 | 1 | 0 | 98 | 102 |  |
| R.KTLDM(+15.99)IK.K | Y | 27.37 | 863.4786 | 7 | 2.3 | 432.7476 | 2 | 20.32 | 3 | 5044 | Ana\_02.raw |  |  | 6.96E6 |  |  |  |  |  |  |  | 1 | 0 | 0 | 1 | 0 | 0 | 0 | 0 | 0 | 0 | 0 | 467 | 473 | Oxidation (M) |
| R.VFITDDFHDM(+15.99)M(+15.99)PK.Y | Y | 23.36 | 1626.7058 | 13 | 5.5 | 543.2455 | 3 | 71.04 | 7 | 20142 | Ana\_06.raw |  |  |  |  |  |  | 1.22E7 |  |  |  | 1 | 0 | 0 | 0 | 0 | 0 | 0 | 1 | 0 | 0 | 0 | 416 | 428 | Oxidation (M) |
| K.TLDM(+15.99)IK.K | Y | 22.97 | 735.3837 | 6 | 2.4 | 368.7000 | 2 | 24.72 | 3 | 6473 | Ana\_02.raw |  | 4.13E6 | 6.77E6 |  |  | 7.15E6 | 5.3E6 |  |  |  | 4 | 0 | 1 | 1 | 0 | 0 | 1 | 1 | 0 | 0 | 0 | 468 | 473 | Oxidation (M) |
| total 36 peptides |  |  |  |  |  |  |  |  |  |  |  |  |  |  |  |  |  |  |  |  |  |  |  |  |  |  |  |  |  |  |  |  |  |  |  |
| --- | --- | --- | --- | --- | --- | --- | --- | --- | --- | --- | --- | --- | --- | --- | --- | --- | --- | --- | --- | --- | --- | --- | --- | --- | --- | --- | --- | --- | --- | --- | --- | --- | --- | --- | --- |

Best Unique PSM (Scan F6:30001, m/z=910.1200, z=3, RT=106.60, ppm=2.6):

sp|Q9CZS1|AL1B1\_MOUSE

back to list

  

| Protein Coverage
| Supporting Peptides
| Best Unique PSM
|

Protein Coverage:

Supporting Peptides:

| Peptide | Uniq | -10lgP | Mass | Length | ppm | m/z | z | RT | Fraction | Scan | Source File | Area 10 | Area 01 | Area 02 | Area 03 | Area 04 | Area 05 | Area 06 | Area 07 | Area 08 | Area 09 | #Spec | #Spec 10 | #Spec 01 | #Spec 02 | #Spec 03 | #Spec 04 | #Spec 05 | #Spec 06 | #Spec 07 | #Spec 08 | #Spec 09 | Start | End | PTM |
| --- | --- | --- | --- | --- | --- | --- | --- | --- | --- | --- | --- | --- | --- | --- | --- | --- | --- | --- | --- | --- | --- | --- | --- | --- | --- | --- | --- | --- | --- | --- | --- | --- | --- | --- | --- |
| K.VGNPFELDTQQGPQVDKEQFER.I | Y | 124.59 | 2560.2192 | 22 | 3.9 | 854.4171 | 3 | 90.29 | 7 | 25368 | Ana\_06.raw | 2.16E7 | 3.05E7 |  | 1.01E7 | 1.92E7 | 8.14E6 | 1.43E7 | 6.52E6 | 2.08E7 | 1.6E7 | 10 | 1 | 2 | 0 | 1 | 1 | 1 | 1 | 1 | 1 | 1 | 350 | 371 |  |
| R.HEPVGVC(+57.02)GQIIPWNFPLVM(+15.99)QGWK.L | Y | 111.85 | 2707.3403 | 23 | 3.2 | 903.4570 | 3 | 130.08 | 6 | 36139 | Ana\_05.raw | 8.84E6 | 1.2E7 |  |  | 8.93E6 | 1.16E7 |  | 1.69E6 |  |  | 5 | 1 | 1 | 0 | 0 | 1 | 1 | 0 | 1 | 0 | 0 | 175 | 197 | Carbamidomethylation; Oxidation (M) |
| K.EAGFPPGVVNIITGYGPTAGAAIAQHMDVDK.V | Y | 105.26 | 3095.5386 | 31 | 3.2 | 1032.8568 | 3 | 131.44 | 1 | 36127 | ANa\_10.raw | 8.19E6 |  |  |  |  |  |  |  | 1.25E7 |  | 2 | 1 | 0 | 0 | 0 | 0 | 0 | 0 | 0 | 1 | 0 | 229 | 259 |  |
| K.TFPTVNPTTGEVIGHVAEGDR.A | Y | 88.64 | 2196.0811 | 21 | 3.2 | 733.0366 | 3 | 88.74 | 1 | 24955 | ANa\_10.raw | 6.51E6 | 1.54E7 | 2.03E7 | 7.47E6 | 3.43E6 | 1.75E6 | 1.05E7 | 8.76E6 | 3.54E7 |  | 9 | 1 | 1 | 1 | 1 | 1 | 1 | 1 | 1 | 1 | 0 | 55 | 75 |  |
| R.GFFIKPTVFGDVQDGM(+15.99)R.I | Y | 88.03 | 1928.9454 | 17 | -0.1 | 643.9890 | 3 | 97.32 | 3 | 27192 | Ana\_02.raw | 2.86E7 | 2.97E7 | 3.83E7 | 1.51E7 | 1.66E7 | 2.59E7 | 1.13E7 | 1.22E7 | 4.31E7 | 1.5E7 | 19 | 3 | 3 | 2 | 2 | 1 | 1 | 2 | 1 | 2 | 2 | 397 | 413 | Oxidation (M) |
| K.VAEQTPLSALYLASLIK.E | Y | 86.72 | 1816.0345 | 17 | 3.5 | 909.0277 | 2 | 131.59 | 3 | 36049 | Ana\_02.raw | 3.69E7 | 6.24E7 | 4.41E7 | 1.48E7 | 3.75E6 | 5.21E7 | 3.88E7 | 1.62E7 | 7.33E7 | 4.76E6 | 37 | 3 | 5 | 6 | 2 | 3 | 2 | 2 | 3 | 7 | 4 | 212 | 228 |  |
| K.VAFTGSTEVGHLIQK.A | Y | 85.02 | 1585.8464 | 15 | 3.2 | 793.9330 | 2 | 70.48 | 9 | 20121 | Ana\_08.raw | 1.75E6 | 1.17E7 | 1.36E7 | 7.14E6 | 1.74E6 | 1.29E7 | 7.86E6 | 1.43E6 | 1.96E7 | 1.76E6 | 12 | 1 | 2 | 1 | 1 | 1 | 1 | 1 | 1 | 2 | 1 | 260 | 274 |  |
| K.EEIFGPVQPLFK.F | Y | 83.42 | 1402.7496 | 12 | 4.2 | 702.3851 | 2 | 112.07 | 6 | 31411 | Ana\_05.raw | 4.71E7 | 5.67E7 | 5.17E7 | 2.66E7 | 3.9E7 | 4.3E7 | 2.88E7 | 2.68E7 | 7.73E7 | 3.96E7 | 30 | 4 | 3 | 4 | 2 | 3 | 2 | 2 | 3 | 4 | 3 | 417 | 428 |  |
| K.LAPALATGNTVVM(+15.99)K.V | Y | 74.15 | 1400.7698 | 14 | 0.1 | 701.3922 | 2 | 55.02 | 3 | 15613 | Ana\_02.raw | 2.14E6 |  | 8.12E6 |  |  | 7.9E6 |  |  |  |  | 4 | 1 | 0 | 1 | 0 | 0 | 2 | 0 | 0 | 0 | 0 | 198 | 211 | Oxidation (M) |
| R.TFVEESIYR.E | Y | 71.66 | 1142.5608 | 9 | 3.8 | 572.2899 | 2 | 66.07 | 2 | 18833 | Ana\_01.raw | 3.5E7 | 5.86E7 | 4.53E7 | 2.24E7 | 3.43E7 | 4.66E7 | 3.2E7 | 2.61E7 | 5.87E7 | 3.75E7 | 22 | 3 | 3 | 2 | 2 | 2 | 2 | 2 | 2 | 2 | 2 | 327 | 335 |  |
| K.EAGFPPGVVNIITGYGPTAGAAIAQHM(+15.99)DVDK.V | Y | 67.62 | 3111.5334 | 31 | 2.4 | 1038.1876 | 3 | 123.93 | 9 | 34447 | Ana\_08.raw | 2.57E7 | 5.8E6 | 2.31E6 |  | 1.46E6 |  | 6.05E6 | 5.51E6 | 9.37E6 |  | 7 | 1 | 1 | 1 | 0 | 1 | 0 | 1 | 1 | 1 | 0 | 229 | 259 | Oxidation (M) |
| R.KVGNPFELDTQQGPQVDKEQFER.I | Y | 66.01 | 2688.3142 | 23 | 6.2 | 673.0900 | 4 | 80.46 | 2 | 22836 | Ana\_01.raw |  | 3.71E6 |  | 1.25E6 |  |  |  |  |  |  | 2 | 0 | 1 | 0 | 1 | 0 | 0 | 0 | 0 | 0 | 0 | 349 | 371 |  |
| R.YGLAAAVFTR.D | Y | 64.47 | 1067.5763 | 10 | 3.2 | 534.7971 | 2 | 81.19 | 2 | 23038 | Ana\_01.raw | 5.82E6 | 0 | 7.64E6 | 4.39E6 | 6.56E6 | 1.25E7 | 6.13E6 | 4.89E6 |  | 6.32E6 | 9 | 1 | 1 | 1 | 1 | 1 | 1 | 1 | 1 | 0 | 1 | 444 | 453 |  |
| R.IAKEEIFGPVQPLFK.F | Y | 62.66 | 1714.9657 | 15 | 3.7 | 572.6646 | 3 | 102.44 | 6 | 28899 | Ana\_05.raw | 7.65E5 | 8.01E5 |  | 5.86E5 | 2.66E6 | 2.47E6 | 6.66E5 | 6.05E5 | 1.65E7 | 2.34E6 | 9 | 1 | 1 | 0 | 1 | 1 | 1 | 1 | 1 | 1 | 1 | 414 | 428 |  |
| K.LFINNEWHDAVSK.K | Y | 57.80 | 1571.7732 | 13 | 4.1 | 524.9338 | 3 | 79.65 | 6 | 22776 | Ana\_05.raw | 2.18E6 | 3.44E6 | 1.49E6 | 1.17E6 | 4.35E6 | 4.52E6 |  |  | 5.96E6 | 1.05E6 | 8 | 1 | 1 | 1 | 1 | 1 | 1 | 0 | 0 | 1 | 1 | 41 | 53 |  |
| R.YFAGWADK.W | N | 51.44 | 956.4392 | 8 | 4.4 | 479.2290 | 2 | 61.64 | 2 | 17544 | Ana\_01.raw | 4.65E6 | 8.73E6 |  | 3.34E6 |  | 4.88E6 | 2.83E6 |  | 6.68E6 | 1.87E6 | 7 | 1 | 1 | 0 | 1 | 0 | 1 | 1 | 0 | 1 | 1 | 150 | 157 |  |
| K.LLC(+57.02)GGER.L | Y | 51.35 | 803.3959 | 7 | 4.9 | 402.7072 | 2 | 23.27 | 2 | 5846 | Ana\_01.raw | 1.39E7 | 2.17E7 | 1.36E7 | 9.84E6 | 5.61E6 | 1.5E7 | 2.97E6 | 5.37E6 |  |  | 8 | 1 | 1 | 1 | 1 | 1 | 1 | 1 | 1 | 0 | 0 | 386 | 392 | Carbamidomethylation |
| R.LADLVER.D | N | 48.11 | 814.4548 | 7 | 4.7 | 408.2366 | 2 | 41.32 | 1 | 11418 | ANa\_10.raw | 3.78E7 | 4.92E7 | 5.17E7 | 1.9E7 | 3.96E7 | 4.43E7 | 2.92E7 | 3.15E7 | 2.71E7 | 3.07E7 | 16 | 1 | 1 | 2 | 1 | 2 | 1 | 2 | 2 | 3 | 1 | 110 | 116 |  |
| R.ADVDLAVK.A | Y | 47.96 | 829.4545 | 8 | 1.3 | 415.7350 | 2 | 38.37 | 3 | 10653 | Ana\_02.raw |  |  | 2.46E7 | 2.37E7 |  |  |  | 3.26E7 |  |  | 3 | 0 | 0 | 1 | 1 | 0 | 0 | 0 | 1 | 0 | 0 | 76 | 83 |  |
| R.VTLELGGK.S | N | 46.93 | 815.4752 | 8 | 4.9 | 408.7469 | 2 | 50.47 | 8 | 13979 | Ana\_07.raw | 2.75E7 | 3.49E7 | 3.43E7 | 1.61E7 | 2.77E7 | 3.58E7 | 2.3E7 | 1.7E7 | 1.6E7 | 2.95E7 | 11 | 1 | 1 | 1 | 1 | 1 | 1 | 1 | 1 | 2 | 1 | 284 | 291 |  |
| R.ELGEDGLR.A | Y | 45.82 | 887.4348 | 8 | 1.6 | 444.7254 | 2 | 35.22 | 3 | 9678 | Ana\_02.raw | 4.62E7 | 6.37E7 | 1.77E7 | 1.22E7 | 5.4E7 | 6.04E7 |  |  | 3E7 | 3.99E7 | 13 | 2 | 2 | 1 | 1 | 2 | 2 | 0 | 0 | 1 | 2 | 495 | 502 |  |
| K.IEEVIQR.A | Y | 44.82 | 885.4919 | 7 | 5.5 | 443.7557 | 2 | 35.27 | 2 | 9592 | Ana\_01.raw |  | 2.87E7 | 2.66E7 | 1.24E7 | 2.28E7 | 2.72E7 | 2.32E7 | 1.33E7 | 2.57E7 | 1.76E7 | 9 | 0 | 1 | 1 | 1 | 1 | 1 | 1 | 1 | 1 | 1 | 432 | 438 |  |
| K.KIEEVIQR.A | Y | 43.07 | 1013.5869 | 8 | 3.7 | 507.8026 | 2 | 31.16 | 6 | 8480 | Ana\_05.raw | 1.46E7 | 2.26E7 | 2.15E7 | 8.88E6 | 1.43E7 | 6.12E6 | 1.26E7 | 8.46E6 | 1.72E7 | 1.42E7 | 15 | 1 | 1 | 2 | 2 | 2 | 2 | 2 | 1 | 1 | 1 | 431 | 438 |  |
| R.LGSPWR.R | Y | 40.13 | 714.3813 | 6 | 2.9 | 358.1989 | 2 | 46.47 | 8 | 12776 | Ana\_07.raw | 2.28E6 | 5.39E6 | 5.68E6 | 2.73E6 | 5.53E6 | 7.15E6 | 4.17E6 | 3.46E6 | 5.23E6 |  | 9 | 1 | 1 | 1 | 1 | 1 | 1 | 1 | 1 | 1 | 0 | 91 | 96 |  |
| R.ILGYIR.L | Y | 33.86 | 733.4486 | 6 | 3.3 | 367.7328 | 2 | 55.27 | 6 | 15779 | Ana\_05.raw |  | 9.32E6 | 9.38E6 |  | 7.36E6 | 8.2E6 | 6.29E6 |  |  |  | 5 | 0 | 1 | 1 | 0 | 1 | 1 | 1 | 0 | 0 | 0 | 372 | 377 |  |
| K.TIPM(+15.99)DGEHFC(+57.02)FTR.H | Y | 33.41 | 1625.6967 | 13 | 4.4 | 542.9086 | 3 | 70.18 | 8 | 19706 | Ana\_07.raw |  |  |  | 4.4E5 |  |  | 3.74E6 | 2.18E6 |  |  | 3 | 0 | 0 | 0 | 1 | 0 | 0 | 1 | 1 | 0 | 0 | 162 | 174 | Oxidation (M); Carbamidomethylation |
| R.EFLER.T | N | 30.39 | 692.3493 | 5 | 2.6 | 347.1828 | 2 | 36.61 | 4 | 9820 | Ana\_03.raw |  |  |  | 9.92E6 |  |  |  |  |  | 1.45E6 | 2 | 0 | 0 | 0 | 1 | 0 | 0 | 0 | 0 | 0 | 1 | 336 | 340 |  |
| K.LAPALATGNTVVMK.V | Y | 24.96 | 1384.7748 | 14 | 5.3 | 693.3983 | 2 | 68.28 | 2 | 19463 | Ana\_01.raw | 6.76E5 | 6.97E5 |  |  |  |  |  |  |  |  | 2 | 1 | 1 | 0 | 0 | 0 | 0 | 0 | 0 | 0 | 0 | 198 | 211 |  |
| total 28 peptides |  |  |  |  |  |  |  |  |  |  |  |  |  |  |  |  |  |  |  |  |  |  |  |  |  |  |  |  |  |  |  |  |  |  |  |
| --- | --- | --- | --- | --- | --- | --- | --- | --- | --- | --- | --- | --- | --- | --- | --- | --- | --- | --- | --- | --- | --- | --- | --- | --- | --- | --- | --- | --- | --- | --- | --- | --- | --- | --- | --- |

Best Unique PSM (Scan F7:25368, m/z=854.4171, z=3, RT=90.29, ppm=3.9):

sp|Q921I1|TRFE\_MOUSE

back to list

  

| Protein Coverage
| Supporting Peptides
| Best Unique PSM
|

Protein Coverage:

Supporting Peptides:

| Peptide | Uniq | -10lgP | Mass | Length | ppm | m/z | z | RT | Fraction | Scan | Source File | Area 10 | Area 01 | Area 02 | Area 03 | Area 04 | Area 05 | Area 06 | Area 07 | Area 08 | Area 09 | #Spec | #Spec 10 | #Spec 01 | #Spec 02 | #Spec 03 | #Spec 04 | #Spec 05 | #Spec 06 | #Spec 07 | #Spec 08 | #Spec 09 | Start | End | PTM |
| --- | --- | --- | --- | --- | --- | --- | --- | --- | --- | --- | --- | --- | --- | --- | --- | --- | --- | --- | --- | --- | --- | --- | --- | --- | --- | --- | --- | --- | --- | --- | --- | --- | --- | --- | --- |
| K.AVLTSQETLFGGSDC(+57.02)TGNFC(+57.02)LFK.S | Y | 105.91 | 2551.1724 | 23 | 6.8 | 1276.6022 | 2 | 126.80 | 1 | 34876 | ANa\_10.raw | 7.94E6 | 8.52E5 | 2.32E6 | 1.36E6 | 1.11E7 | 4.13E6 | 7.41E6 | 1.65E7 | 8.9E6 | 9.83E6 | 16 | 2 | 1 | 1 | 1 | 2 | 2 | 2 | 2 | 1 | 2 | 619 | 641 | Carbamidomethylation |
| K.AVSSFFSGSC(+57.02)VPC(+57.02)ADPVAFPK.L | Y | 100.09 | 2229.0234 | 21 | 0.6 | 1115.5197 | 2 | 113.55 | 6 | 31793 | Ana\_05.raw | 2.6E7 | 1.92E7 | 2.23E7 | 3.69E6 | 5.98E6 | 1.98E7 | 1.17E7 | 2.86E6 | 4.58E7 | 3.59E7 | 19 | 2 | 2 | 2 | 1 | 1 | 2 | 2 | 1 | 3 | 3 | 168 | 188 | Carbamidomethylation |
| R.NQQEGVC(+57.02)PEGSIDNSPVK.W | Y | 98.39 | 1956.8846 | 18 | 0.4 | 979.4500 | 2 | 55.89 | 6 | 15956 | Ana\_05.raw | 1.41E7 | 8.08E6 | 8.03E6 | 1.9E6 | 5.31E6 | 1.07E7 | 5.73E6 | 7.22E6 | 1.54E7 | 1.05E7 | 11 | 2 | 1 | 1 | 1 | 1 | 1 | 1 | 1 | 1 | 1 | 344 | 361 | Carbamidomethylation |
| R.KPVDQYEDC(+57.02)YLAR.I | Y | 87.27 | 1655.7614 | 13 | 3.6 | 828.8909 | 2 | 54.24 | 9 | 15351 | Ana\_08.raw | 3.57E7 | 2.81E7 | 2.87E7 | 7.79E6 | 1.1E7 | 2.24E7 | 1.32E7 | 1.75E7 | 2.29E7 | 4.06E7 | 18 | 3 | 2 | 2 | 2 | 1 | 1 | 1 | 2 | 2 | 2 | 252 | 264 | Carbamidomethylation |
| K.DFQLFSSPLGK.D | Y | 86.93 | 1237.6343 | 11 | 6.2 | 619.8282 | 2 | 109.27 | 1 | 30349 | ANa\_10.raw | 1.62E7 | 1.03E7 | 1.22E7 | 4.51E6 | 5.72E6 | 1.04E7 | 6.74E6 | 6.19E6 | 2.02E7 | 1.49E7 | 21 | 3 | 2 | 2 | 1 | 2 | 2 | 2 | 2 | 3 | 2 | 300 | 310 |  |
| K.HQTVLDNTEGK.N | N | 86.72 | 1240.6047 | 11 | 7.1 | 621.3140 | 2 | 22.48 | 10 | 5654 | Ana\_09.raw | 1.77E7 |  | 1.1E7 | 3.29E6 | 7.69E5 |  | 2.72E5 | 2.55E6 |  | 1.78E7 | 9 | 1 | 0 | 1 | 1 | 1 | 0 | 1 | 1 | 0 | 3 | 555 | 565 |  |
| K.FDEFFSQGC(+57.02)APGYEK.N | N | 86.54 | 1780.7402 | 15 | -0.2 | 891.3773 | 2 | 83.89 | 3 | 23657 | Ana\_02.raw | 1.35E7 | 9.12E6 | 1.06E7 | 4.93E6 | 5.57E6 | 1.2E7 | 7.87E6 | 1.04E7 | 1.91E7 | 1.45E7 | 11 | 1 | 2 | 1 | 1 | 1 | 1 | 1 | 1 | 1 | 1 | 498 | 512 | Carbamidomethylation |
| R.SAGWVIPIGLLFC(+57.02)K.L | Y | 81.45 | 1559.8534 | 14 | 2.7 | 780.9361 | 2 | 135.34 | 6 | 37622 | Ana\_05.raw | 9.13E6 | 1.43E7 | 9.64E5 | 9.46E6 | 8.38E6 | 4.18E6 | 5.08E6 | 5.26E4 | 2.62E6 | 1.45E7 | 10 | 1 | 1 | 1 | 1 | 1 | 1 | 1 | 1 | 1 | 1 | 144 | 157 | Carbamidomethylation |
| K.DGGGDVAFVK.H | Y | 77.47 | 963.4661 | 10 | 3.3 | 482.7419 | 2 | 45.51 | 6 | 12851 | Ana\_05.raw | 2.52E7 | 3.23E6 | 1.67E7 | 5.51E6 | 1.01E7 | 2.08E7 | 8.4E6 | 9.86E6 | 6.27E5 | 8.14E6 | 17 | 2 | 1 | 2 | 2 | 2 | 2 | 1 | 2 | 1 | 2 | 216 | 225 |  |
| K.SKDFQLFSSPLGK.D | Y | 76.71 | 1452.7612 | 13 | 2.0 | 485.2620 | 3 | 88.14 | 6 | 25092 | Ana\_05.raw | 1.38E7 | 2.56E6 | 5.58E6 | 1.8E5 | 3.21E6 | 1.05E7 | 5.8E6 | 5.01E6 | 3.6E6 | 8.13E6 | 15 | 2 | 1 | 1 | 1 | 1 | 2 | 2 | 1 | 1 | 3 | 298 | 310 |  |
| K.ADRDQYELLC(+57.02)LDNTR.K | Y | 76.18 | 1880.8687 | 15 | 4.7 | 627.9664 | 3 | 80.36 | 6 | 22972 | Ana\_05.raw |  |  | 3.13E6 | 2.17E6 | 2.09E6 | 4.62E6 | 2.01E6 | 3.84E6 | 1.63E6 | 4.47E6 | 8 | 0 | 0 | 1 | 1 | 1 | 1 | 1 | 1 | 1 | 1 | 237 | 251 | Carbamidomethylation |
| K.IEC(+57.02)ESAETTEDC(+57.02)IEK.I | N | 75.00 | 1812.7394 | 15 | 5.0 | 907.3815 | 2 | 48.83 | 1 | 13681 | ANa\_10.raw | 1.96E7 | 1.29E7 | 1.47E7 | 5.97E6 | 7.32E6 | 1.58E7 | 9.6E6 | 1.08E7 | 2.62E7 | 2.2E7 | 10 | 1 | 1 | 1 | 1 | 1 | 1 | 1 | 1 | 1 | 1 | 384 | 398 | Carbamidomethylation |
| K.WC(+57.02)AVSEHENTK.C | Y | 71.11 | 1359.5878 | 11 | 2.5 | 680.8029 | 2 | 27.79 | 3 | 7448 | Ana\_02.raw | 6.19E6 |  | 9.91E6 |  |  | 1.35E7 |  | 7.2E6 | 1E7 | 4.8E6 | 10 | 1 | 0 | 2 | 0 | 0 | 2 | 0 | 2 | 2 | 1 | 27 | 37 | Carbamidomethylation |
| K.KGTDFQLNQLEGK.K | Y | 70.24 | 1476.7572 | 13 | 5.0 | 493.2621 | 3 | 64.52 | 6 | 18530 | Ana\_05.raw |  |  |  |  |  | 2.93E6 |  | 1.44E6 |  |  | 2 | 0 | 0 | 0 | 0 | 0 | 1 | 0 | 1 | 0 | 0 | 122 | 134 |  |
| K.LC(+57.02)QLC(+57.02)PGC(+57.02)GC(+57.02)SSTQPFFGYVGAFK.C | Y | 69.20 | 2740.1907 | 24 | 0.9 | 914.4050 | 3 | 121.16 | 6 | 33791 | Ana\_05.raw | 2.29E6 | 2.65E6 | 3.42E6 |  | 2.93E6 | 7.13E6 | 1.28E6 | 1.54E5 |  | 3.65E6 | 8 | 1 | 1 | 1 | 0 | 1 | 1 | 1 | 1 | 0 | 1 | 189 | 212 | Carbamidomethylation |
| K.DSAFGLLR.V | Y | 67.22 | 877.4657 | 8 | 3.1 | 439.7415 | 2 | 79.81 | 1 | 22531 | ANa\_10.raw | 1.72E7 | 9.53E6 | 1.38E7 | 4.87E6 | 7.46E6 | 1.73E7 | 7.16E6 | 1.03E7 | 2.06E6 | 1.84E7 | 21 | 2 | 1 | 2 | 2 | 2 | 3 | 2 | 3 | 1 | 3 | 316 | 323 |  |
| K.HTTIFEVLPEK.A | Y | 65.03 | 1312.7026 | 11 | 1.8 | 438.5756 | 3 | 73.15 | 3 | 20767 | Ana\_02.raw | 3.05E6 |  | 1.35E6 | 1.24E7 |  | 1.32E6 | 3.1E6 | 4.43E5 | 4.94E6 | 1.75E6 | 9 | 1 | 0 | 1 | 1 | 0 | 1 | 2 | 1 | 1 | 1 | 226 | 236 |  |
| R.LYLGHNYVTAIR.N | Y | 64.96 | 1418.7670 | 12 | 2.6 | 710.3926 | 2 | 68.10 | 9 | 19436 | Ana\_08.raw | 1.02E6 |  |  |  |  |  |  |  | 4.18E6 | 1.12E7 | 4 | 1 | 0 | 0 | 0 | 0 | 0 | 0 | 0 | 1 | 2 | 332 | 343 |  |
| R.DQYELLC(+57.02)LDNTR.K | Y | 61.71 | 1538.7035 | 12 | 1.5 | 770.3602 | 2 | 92.67 | 7 | 26005 | Ana\_06.raw | 2.13E6 |  |  |  |  |  | 0 |  |  |  | 2 | 1 | 0 | 0 | 0 | 0 | 0 | 1 | 0 | 0 | 0 | 240 | 251 | Carbamidomethylation |
| K.QEDFELLC(+57.02)PDGTR.K | N | 59.14 | 1578.6984 | 13 | -6.1 | 790.3516 | 2 | 104.11 | 9 | 29332 | Ana\_08.raw |  |  |  |  |  | 0 |  |  | 3.59E5 |  | 2 | 0 | 0 | 0 | 0 | 0 | 1 | 0 | 0 | 1 | 0 | 576 | 588 | Carbamidomethylation |
| K.GYYAVAVVK.A | Y | 53.91 | 968.5331 | 9 | 2.7 | 485.2751 | 2 | 57.46 | 6 | 16422 | Ana\_05.raw | 1.26E7 | 8.39E6 | 8.78E6 | 3.94E6 | 5.16E6 | 1.11E7 | 7.58E6 | 7.78E6 | 1.52E7 |  | 9 | 1 | 1 | 1 | 1 | 1 | 1 | 1 | 1 | 1 | 0 | 447 | 455 |  |
| K.EDLIWEILK.V | Y | 53.15 | 1157.6332 | 9 | 3.0 | 579.8256 | 2 | 129.93 | 6 | 36094 | Ana\_05.raw | 3.28E5 |  |  |  |  | 2.72E5 |  | 6.08E5 |  | 5.29E5 | 4 | 1 | 0 | 0 | 0 | 0 | 1 | 0 | 1 | 0 | 1 | 279 | 287 |  |
| K.LPEGTTPEK.Y | Y | 52.11 | 970.4971 | 9 | 2.2 | 486.2569 | 2 | 26.16 | 4 | 6741 | Ana\_03.raw |  |  |  | 1.46E7 | 1.44E7 | 4.67E7 | 2.71E7 | 2.72E7 |  |  | 5 | 0 | 0 | 0 | 1 | 1 | 1 | 1 | 1 | 0 | 0 | 659 | 667 |  |
| K.YLGAEYM(+15.99)QSVGNM(+15.99)R.K | Y | 51.71 | 1649.7178 | 14 | 3.2 | 825.8688 | 2 | 56.98 | 6 | 16281 | Ana\_05.raw | 1.6E7 |  | 2.74E6 |  | 1.76E6 | 8.74E6 | 4.87E6 | 7.73E6 | 9.22E6 | 2.47E6 | 9 | 2 | 0 | 1 | 0 | 1 | 1 | 1 | 1 | 1 | 1 | 668 | 681 | Oxidation (M) |
| K.TVLPPDGPR.L | Y | 51.21 | 950.5185 | 9 | 7.6 | 476.2701 | 2 | 43.12 | 2 | 12022 | Ana\_01.raw | 5.53E7 | 2.15E6 | 2.27E7 |  | 1.54E6 | 2.66E7 | 1.34E6 | 4.52E6 | 1.87E7 | 4.82E7 | 27 | 6 | 3 | 3 | 0 | 1 | 2 | 2 | 2 | 3 | 5 | 47 | 55 |  |
| K.EEYNGYTGAFR.C | N | 50.22 | 1305.5625 | 11 | 0.1 | 653.7886 | 2 | 61.80 | 6 | 17710 | Ana\_05.raw |  |  |  |  |  | 7.8E5 |  |  |  |  | 1 | 0 | 0 | 0 | 0 | 0 | 1 | 0 | 0 | 0 | 0 | 532 | 542 |  |
| R.IPSHAVVAR.K | Y | 49.36 | 948.5505 | 9 | 4.9 | 475.2849 | 2 | 24.02 | 2 | 6099 | Ana\_01.raw | 1.38E7 | 9.24E6 | 3.21E7 | 6.33E6 | 1.79E7 | 3.6E7 | 2.53E7 | 2.16E7 | 4.1E7 | 1.96E7 | 17 | 1 | 1 | 2 | 1 | 2 | 2 | 2 | 2 | 2 | 2 | 265 | 273 |  |
| K.VAQEHFGK.G | Y | 48.97 | 914.4610 | 8 | 1.8 | 458.2386 | 2 | 19.58 | 3 | 4789 | Ana\_02.raw |  | 1.14E7 | 1.76E7 | 5.02E6 |  | 2.01E7 | 1.94E6 | 9.52E6 |  | 3.24E7 | 7 | 0 | 1 | 1 | 1 | 0 | 1 | 1 | 1 | 0 | 1 | 288 | 295 |  |
| K.TSYPDC(+57.02)IK.A | Y | 48.33 | 982.4430 | 8 | 5.3 | 492.2314 | 2 | 37.54 | 1 | 10255 | ANa\_10.raw | 1.98E7 |  |  |  |  |  |  |  |  | 1.73E7 | 2 | 1 | 0 | 0 | 0 | 0 | 0 | 0 | 0 | 0 | 1 | 62 | 69 | Carbamidomethylation |
| K.GDVAFVK.H | N | 42.56 | 734.3962 | 7 | 4.2 | 368.2069 | 2 | 35.46 | 1 | 9613 | ANa\_10.raw | 1.29E7 |  | 1.12E7 |  |  |  |  |  |  | 7.32E6 | 4 | 1 | 0 | 1 | 0 | 0 | 0 | 0 | 0 | 0 | 2 | 548 | 554 |  |
| R.LLEAC(+57.02)TFHK.H | Y | 33.82 | 1117.5590 | 9 | -1.8 | 559.7858 | 2 | 45.24 | 6 | 12768 | Ana\_05.raw |  |  |  |  |  | 9.7E6 | 1.5E6 | 6.28E6 |  | 5.13E6 | 5 | 0 | 0 | 0 | 0 | 0 | 2 | 1 | 1 | 0 | 1 | 688 | 696 | Carbamidomethylation |
| K.NPAEWAK.N | N | 33.47 | 814.3973 | 7 | 4.4 | 408.2077 | 2 | 27.43 | 9 | 7102 | Ana\_08.raw | 6.86E6 |  |  |  |  |  |  |  | 4.21E6 |  | 2 | 1 | 0 | 0 | 0 | 0 | 0 | 0 | 0 | 1 | 0 | 566 | 572 |  |
| K.ASDTSITWNNLK.G | N | 30.66 | 1348.6622 | 12 | 3.1 | 675.3405 | 2 | 73.97 | 7 | 20970 | Ana\_06.raw |  |  |  |  |  |  | 1.46E6 |  |  |  | 1 | 0 | 0 | 0 | 0 | 0 | 0 | 1 | 0 | 0 | 0 | 456 | 467 |  |
| K.WC(+57.02)ALSHLER.T | N | 26.46 | 1170.5604 | 9 | 3.6 | 391.1955 | 3 | 62.06 | 7 | 17573 | Ana\_06.raw |  |  |  |  |  |  | 1.64E6 |  |  |  | 1 | 0 | 0 | 0 | 0 | 0 | 0 | 1 | 0 | 0 | 0 | 362 | 370 | Carbamidomethylation |
| R.TAGWNIPM(+15.99)GM(+15.99)LYNR.I | N | 23.52 | 1654.7595 | 14 | 2.7 | 828.3893 | 2 | 82.65 | 9 | 23513 | Ana\_08.raw |  |  |  |  |  |  |  |  | 7.72E5 |  | 1 | 0 | 0 | 0 | 0 | 0 | 0 | 0 | 0 | 1 | 0 | 479 | 492 | Oxidation (M) |
| total 35 peptides |  |  |  |  |  |  |  |  |  |  |  |  |  |  |  |  |  |  |  |  |  |  |  |  |  |  |  |  |  |  |  |  |  |  |  |
| --- | --- | --- | --- | --- | --- | --- | --- | --- | --- | --- | --- | --- | --- | --- | --- | --- | --- | --- | --- | --- | --- | --- | --- | --- | --- | --- | --- | --- | --- | --- | --- | --- | --- | --- | --- |

Best Unique PSM (Scan F1:34876, m/z=1276.6022, z=2, RT=126.80, ppm=6.8):

sp|P58252|EF2\_MOUSE

back to list

  

| Protein Coverage
| Supporting Peptides
| Best Unique PSM
|

Protein Coverage:

Supporting Peptides:

| Peptide | Uniq | -10lgP | Mass | Length | ppm | m/z | z | RT | Fraction | Scan | Source File | Area 10 | Area 01 | Area 02 | Area 03 | Area 04 | Area 05 | Area 06 | Area 07 | Area 08 | Area 09 | #Spec | #Spec 10 | #Spec 01 | #Spec 02 | #Spec 03 | #Spec 04 | #Spec 05 | #Spec 06 | #Spec 07 | #Spec 08 | #Spec 09 | Start | End | PTM |
| --- | --- | --- | --- | --- | --- | --- | --- | --- | --- | --- | --- | --- | --- | --- | --- | --- | --- | --- | --- | --- | --- | --- | --- | --- | --- | --- | --- | --- | --- | --- | --- | --- | --- | --- | --- |
| R.ALLELQLEPEELYQTFQR.I | Y | 104.48 | 2219.1472 | 18 | 3.7 | 1110.5850 | 2 | 131.99 | 6 | 36690 | Ana\_05.raw |  |  |  |  |  | 5.89E6 | 1.68E6 |  | 4.72E6 |  | 4 | 0 | 0 | 0 | 0 | 0 | 2 | 1 | 0 | 1 | 0 | 163 | 180 |  |
| K.IWC(+57.02)FGPDGTGPNILTDITK.G | Y | 97.47 | 2104.0298 | 19 | 2.7 | 1053.0250 | 2 | 130.20 | 9 | 36048 | Ana\_08.raw | 6.51E5 |  |  |  |  |  | 5.02E5 | 6.57E5 | 1.69E6 |  | 4 | 1 | 0 | 0 | 0 | 0 | 0 | 1 | 1 | 1 | 0 | 649 | 667 | Carbamidomethylation |
| K.AYLPVNESFGFTADLR.S | Y | 96.57 | 1798.8889 | 16 | -4.2 | 900.4479 | 2 | 114.91 | 3 | 31745 | Ana\_02.raw | 9.37E6 | 7.2E6 | 5.01E6 |  | 2.72E6 | 4.22E6 | 4.98E5 |  | 8.76E6 | 3.48E6 | 9 | 1 | 2 | 1 | 0 | 1 | 1 | 1 | 0 | 1 | 1 | 786 | 801 |  |
| R.WLPAGDALLQMITIHLPSPVTAQK.Y | Y | 90.88 | 2599.4197 | 24 | 2.2 | 867.4824 | 3 | 136.44 | 6 | 37920 | Ana\_05.raw |  | 1.11E6 |  |  |  | 2.09E6 |  |  | 2.33E6 | 2.91E6 | 4 | 0 | 1 | 0 | 0 | 0 | 1 | 0 | 0 | 1 | 1 | 343 | 366 |  |
| R.WLPAGDALLQM(+15.99)ITIHLPSPVTAQK.Y | Y | 88.04 | 2615.4146 | 24 | 3.9 | 872.8156 | 3 | 132.66 | 6 | 36877 | Ana\_05.raw | 2.33E7 | 6.96E6 | 1.4E7 | 5.18E6 | 1.26E7 | 1.43E7 | 2.49E6 | 1.27E7 | 3.03E7 | 1.33E7 | 10 | 1 | 1 | 1 | 1 | 1 | 1 | 1 | 1 | 1 | 1 | 343 | 366 | Oxidation (M) |
| R.YVEPIEDVPC(+57.02)GNIVGLVGVDQFLVK.T | Y | 87.42 | 2758.4250 | 25 | 7.7 | 920.4894 | 3 | 133.07 | 9 | 36814 | Ana\_08.raw | 1.27E6 | 2.66E6 |  | 1.26E6 |  |  |  |  | 6.79E6 |  | 4 | 1 | 1 | 0 | 1 | 0 | 0 | 0 | 0 | 1 | 0 | 457 | 481 | Carbamidomethylation |
| K.GVQYLNEIKDSVVAGFQWATK.E | Y | 86.72 | 2352.2114 | 21 | 6.2 | 785.0826 | 3 | 132.75 | 6 | 36902 | Ana\_05.raw | 2.27E6 | 1.14E6 | 6.26E5 |  | 1.31E6 | 2.05E6 | 6.08E5 | 9.92E5 | 2.76E6 |  | 8 | 1 | 1 | 1 | 0 | 1 | 1 | 1 | 1 | 1 | 0 | 668 | 688 |  |
| K.EGIPALDNFLDK.L | Y | 82.32 | 1330.6769 | 12 | 2.3 | 666.3472 | 2 | 114.82 | 6 | 32123 | Ana\_05.raw | 3.26E6 | 6.81E6 | 3.9E6 | 3.34E6 | 1.33E6 | 5.05E6 | 1.84E6 | 3.19E5 | 1.43E7 | 3.31E6 | 13 | 1 | 1 | 2 | 1 | 1 | 1 | 2 | 1 | 2 | 1 | 846 | 857 |  |
| R.ETVSEESNVLC(+57.02)LSK.S | Y | 80.94 | 1593.7556 | 14 | 5.3 | 797.8893 | 2 | 71.43 | 6 | 20499 | Ana\_05.raw | 7.61E6 | 1.24E7 | 6.86E6 | 4.07E6 | 5.16E6 | 4.75E6 | 1.44E5 | 3.68E6 | 2.31E7 | 8.57E6 | 11 | 1 | 1 | 1 | 1 | 1 | 1 | 1 | 1 | 2 | 1 | 581 | 594 | Carbamidomethylation |
| R.TFC(+57.02)QLILDPIFK.V | Y | 79.30 | 1493.7952 | 12 | 4.5 | 747.9082 | 2 | 130.21 | 1 | 35787 | ANa\_10.raw | 5.89E6 | 3.38E6 | 6.46E6 |  | 4.21E6 | 4.65E6 | 3.27E6 | 3.58E6 | 1.08E7 | 6.5E6 | 9 | 1 | 1 | 1 | 0 | 1 | 1 | 1 | 1 | 1 | 1 | 288 | 299 | Carbamidomethylation |
| R.LM(+15.99)EPIYLVEIQC(+57.02)PEQVVGGIYGVLNR.K | Y | 79.24 | 3004.5400 | 26 | 5.2 | 1002.5259 | 3 | 135.49 | 6 | 37660 | Ana\_05.raw |  |  |  |  |  | 1.14E6 |  | 5.89E5 |  |  | 2 | 0 | 0 | 0 | 0 | 0 | 1 | 0 | 1 | 0 | 0 | 740 | 765 | Oxidation (M); Carbamidomethylation |
| K.STAISLFYELSENDLNFIK.Q | Y | 78.92 | 2203.1047 | 19 | 0.9 | 1102.5607 | 2 | 135.97 | 6 | 37788 | Ana\_05.raw |  |  |  |  |  | 1.28E6 |  |  |  |  | 2 | 0 | 0 | 0 | 0 | 0 | 2 | 0 | 0 | 0 | 0 | 72 | 90 |  |
| K.EGIPALDNFLDKL | Y | 76.46 | 1443.7609 | 13 | 1.2 | 722.8885 | 2 | 133.59 | 3 | 36574 | Ana\_02.raw | 1.62E7 | 8.64E6 | 7.98E6 | 3.67E6 | 7.47E6 | 1.87E7 | 3.16E7 | 6.67E6 | 2.25E7 | 3.37E7 | 39 | 8 | 3 | 2 | 2 | 3 | 3 | 3 | 2 | 7 | 6 | 846 | 858 |  |
| R.NM(+15.99)SVIAHVDHGK.S | Y | 75.28 | 1322.6401 | 12 | 6.2 | 662.3314 | 2 | 22.84 | 9 | 5579 | Ana\_08.raw | 2.38E7 | 8.16E6 | 1.76E7 | 5.87E6 | 1.23E7 | 6.76E6 | 1.68E6 | 7.83E6 | 2.04E7 | 1.95E7 | 21 | 2 | 1 | 2 | 2 | 3 | 2 | 2 | 2 | 2 | 3 | 21 | 32 | Oxidation (M) |
| K.KEDLYLKPIQR.T | Y | 68.92 | 1401.7980 | 11 | 3.9 | 468.2751 | 3 | 45.55 | 3 | 12835 | Ana\_02.raw | 2.73E6 | 3.1E6 | 4.51E6 |  | 3.49E6 | 3.58E6 |  | 2.52E6 | 4.66E6 |  | 7 | 1 | 1 | 1 | 0 | 1 | 1 | 0 | 1 | 1 | 0 | 439 | 449 |  |
| K.ARPFPDGLAEDIDKGEVSAR.Q | Y | 68.50 | 2142.0706 | 20 | 2.4 | 536.5262 | 4 | 76.41 | 3 | 21641 | Ana\_02.raw |  |  | 3.57E6 |  |  |  |  |  | 3.88E6 |  | 2 | 0 | 0 | 1 | 0 | 0 | 0 | 0 | 0 | 1 | 0 | 606 | 625 |  |
| K.SDPVVSYR.E | Y | 67.64 | 921.4556 | 8 | 4.7 | 461.7372 | 2 | 39.35 | 7 | 10789 | Ana\_06.raw | 4.22E7 | 3.3E7 | 3.75E7 | 2.03E7 | 3.39E7 | 2.81E7 | 2.51E7 | 2.36E7 | 4.91E7 | 3E7 | 16 | 1 | 2 | 1 | 2 | 2 | 2 | 2 | 2 | 1 | 1 | 573 | 580 |  |
| K.EDLYLKPIQR.T | Y | 65.04 | 1273.7030 | 10 | 5.3 | 637.8622 | 2 | 56.99 | 2 | 16183 | Ana\_01.raw | 4.4E6 | 6.63E6 | 9.7E6 | 4.86E6 | 4.86E6 | 3.06E6 | 6.47E6 | 9.98E5 | 1.14E7 | 1.64E6 | 18 | 2 | 2 | 2 | 2 | 2 | 1 | 2 | 1 | 2 | 2 | 440 | 449 |  |
| R.VFSGVVSTGLK.V | Y | 61.66 | 1092.6179 | 11 | 1.2 | 547.3169 | 2 | 62.02 | 3 | 17674 | Ana\_02.raw | 3.22E7 | 3.03E7 | 3.51E7 | 1.78E7 | 2.72E7 | 1.96E7 | 1.8E7 | 1.97E7 | 5.38E7 | 3.58E7 | 18 | 2 | 2 | 2 | 1 | 2 | 1 | 2 | 2 | 2 | 2 | 416 | 426 |  |
| M.VNFTVDQIR.A | Y | 59.25 | 1090.5771 | 9 | 4.6 | 546.2983 | 2 | 71.48 | 8 | 20060 | Ana\_07.raw | 1.58E7 | 1.69E7 | 1.85E7 | 9.8E6 | 1.76E7 | 1.26E7 | 1.14E7 | 1.11E7 | 1.99E7 | 1.66E7 | 12 | 1 | 1 | 2 | 1 | 2 | 1 | 1 | 1 | 1 | 1 | 2 | 10 |  |
| K.GEGQLSAAER.A | Y | 58.58 | 1016.4886 | 10 | 4.2 | 509.2537 | 2 | 25.10 | 2 | 6460 | Ana\_01.raw | 2.83E7 | 4.71E7 | 6.12E7 | 3.34E7 | 4.55E7 | 5.27E7 | 4.01E7 | 3.44E7 | 1.52E7 | 3.93E7 | 16 | 2 | 1 | 2 | 1 | 2 | 1 | 1 | 3 | 1 | 2 | 240 | 249 |  |
| R.GGGQIIPTAR.R | N | 57.43 | 968.5403 | 10 | 4.1 | 485.2794 | 2 | 41.19 | 6 | 11522 | Ana\_05.raw | 5.66E7 | 4.77E7 | 5.67E7 | 2.34E7 | 4.63E7 | 3.96E7 | 3.42E7 | 3.28E7 | 2.32E7 | 5.23E7 | 19 | 2 | 2 | 2 | 2 | 2 | 2 | 2 | 2 | 2 | 1 | 717 | 726 |  |
| K.YEWDVAEAR.K | Y | 55.91 | 1137.5090 | 9 | -2.1 | 569.7606 | 2 | 66.54 | 3 | 18923 | Ana\_02.raw |  | 1.38E7 | 1.12E7 | 7.95E6 | 1.47E7 | 1.08E7 | 1.03E7 | 8.38E6 | 2.08E7 | 1.49E7 | 13 | 0 | 1 | 1 | 2 | 1 | 1 | 2 | 2 | 2 | 1 | 639 | 647 |  |
| K.VFDAIM(+15.99)NFR.K | Y | 55.34 | 1127.5433 | 9 | 4.4 | 564.7814 | 2 | 83.10 | 8 | 23287 | Ana\_07.raw | 1.15E7 | 9.62E6 | 1.76E7 | 8.61E6 | 1.34E7 | 1.03E7 | 7.96E6 | 1.12E7 | 1.96E7 | 1.14E7 | 17 | 1 | 1 | 2 | 2 | 2 | 2 | 2 | 1 | 2 | 2 | 300 | 308 | Oxidation (M) |
| K.EGALC(+57.02)EENM(+15.99)R.G | Y | 54.36 | 1223.4911 | 10 | 4.1 | 612.7553 | 2 | 26.57 | 7 | 6926 | Ana\_06.raw | 5.48E6 | 1.17E7 | 1.28E7 | 6.4E6 | 8.52E6 | 1.12E7 | 7.97E6 | 6.39E6 | 4.32E6 | 1.82E6 | 10 | 1 | 1 | 1 | 1 | 1 | 1 | 1 | 1 | 1 | 1 | 689 | 698 | Carbamidomethylation; Oxidation (M) |
| K.STLTDSLVC(+57.02)K.A | Y | 54.29 | 1122.5591 | 10 | 2.0 | 562.2880 | 2 | 59.21 | 7 | 16743 | Ana\_06.raw | 7.78E6 | 8.05E6 | 6.23E6 | 4.37E6 | 5E6 |  | 3.44E6 |  |  | 6.65E6 | 7 | 1 | 1 | 1 | 1 | 1 | 0 | 1 | 0 | 0 | 1 | 33 | 42 | Carbamidomethylation |
| R.KIWC(+57.02)FGPDGTGPNILTDITK.G | Y | 52.96 | 2232.1248 | 20 | 4.6 | 745.0523 | 3 | 121.39 | 2 | 33700 | Ana\_01.raw | 2.07E6 | 1.01E6 |  |  |  |  |  | 6.55E5 | 1.67E6 |  | 5 | 2 | 1 | 0 | 0 | 0 | 0 | 0 | 1 | 1 | 0 | 648 | 667 | Carbamidomethylation |
| K.TGTITTFEHAHNM(+15.99)R.V | Y | 52.76 | 1630.7522 | 14 | 4.6 | 816.3871 | 2 | 30.35 | 9 | 7988 | Ana\_08.raw | 2.1E7 |  |  | 4.75E6 | 7.74E6 | 1.65E7 |  |  | 1.87E6 | 1.39E7 | 6 | 1 | 0 | 0 | 1 | 1 | 1 | 0 | 0 | 1 | 1 | 482 | 495 | Oxidation (M) |
| K.FSVSPVVR.V | Y | 51.25 | 889.5021 | 8 | 4.2 | 445.7602 | 2 | 53.72 | 9 | 15186 | Ana\_08.raw | 3.5E7 | 3.34E7 | 3.52E7 | 1.69E7 | 2.52E7 | 2.62E7 | 2.26E7 | 2.13E7 | 4.89E7 | 3.11E7 | 13 | 2 | 1 | 1 | 1 | 1 | 1 | 1 | 1 | 2 | 2 | 499 | 506 |  |
| R.IM(+15.99)GPNYTPGK.K | Y | 49.66 | 1092.5273 | 10 | 5.8 | 547.2741 | 2 | 39.71 | 7 | 10899 | Ana\_06.raw |  |  | 3.75E7 | 1.85E7 |  |  | 2.71E7 |  | 1.83E7 |  | 4 | 0 | 0 | 1 | 1 | 0 | 0 | 1 | 0 | 1 | 0 | 429 | 438 | Oxidation (M) |
| K.AGIIASAR.A | Y | 49.63 | 757.4446 | 8 | 6.0 | 379.7318 | 2 | 25.56 | 9 | 6495 | Ana\_08.raw | 1.87E7 | 1.84E7 | 2.75E7 | 1.59E7 | 2.34E7 | 2.06E7 | 2E7 | 1.37E7 | 2.09E7 | 1.68E7 | 10 | 1 | 1 | 1 | 1 | 1 | 1 | 1 | 1 | 1 | 1 | 43 | 50 |  |
| R.FYAFGR.V | Y | 48.73 | 759.3704 | 6 | 3.7 | 380.6938 | 2 | 59.92 | 6 | 17137 | Ana\_05.raw | 2.35E7 | 2.14E7 | 2.5E7 | 1.3E7 | 2.06E7 | 1.83E7 | 1.63E7 | 1.28E7 | 3.06E7 | 2.4E7 | 18 | 2 | 2 | 2 | 2 | 1 | 2 | 2 | 1 | 2 | 2 | 410 | 415 |  |
| K.EGKPLLK.A | Y | 33.44 | 783.4854 | 7 | 2.9 | 392.7511 | 2 | 19.65 | 6 | 4813 | Ana\_05.raw |  |  | 6.02E6 | 6.09E6 | 1.09E7 | 9.72E6 |  |  |  |  | 4 | 0 | 0 | 1 | 1 | 1 | 1 | 0 | 0 | 0 | 0 | 331 | 337 |  |
| K.DLEEDHAC(+57.02)IPIK.K | Y | 24.96 | 1438.6761 | 12 | 2.0 | 720.3468 | 2 | 61.21 | 10 | 17341 | Ana\_09.raw |  |  |  |  |  |  |  |  |  | 6.31E5 | 1 | 0 | 0 | 0 | 0 | 0 | 0 | 0 | 0 | 0 | 1 | 560 | 571 | Carbamidomethylation |
| K.GVQYLNEIK.D | Y | 23.39 | 1062.5709 | 9 | 5.3 | 532.2955 | 2 | 62.14 | 1 | 17606 | ANa\_10.raw | 2.31E6 |  |  |  |  |  |  |  |  |  | 1 | 1 | 0 | 0 | 0 | 0 | 0 | 0 | 0 | 0 | 0 | 668 | 676 |  |
| total 35 peptides |  |  |  |  |  |  |  |  |  |  |  |  |  |  |  |  |  |  |  |  |  |  |  |  |  |  |  |  |  |  |  |  |  |  |  |
| --- | --- | --- | --- | --- | --- | --- | --- | --- | --- | --- | --- | --- | --- | --- | --- | --- | --- | --- | --- | --- | --- | --- | --- | --- | --- | --- | --- | --- | --- | --- | --- | --- | --- | --- | --- |

Best Unique PSM (Scan F6:36690, m/z=1110.5850, z=2, RT=131.99, ppm=3.7):

sp|P08249|MDHM\_MOUSE

back to list

  

| Protein Coverage
| Supporting Peptides
| Best Unique PSM
|

Protein Coverage:

Supporting Peptides:

| Peptide | Uniq | -10lgP | Mass | Length | ppm | m/z | z | RT | Fraction | Scan | Source File | Area 10 | Area 01 | Area 02 | Area 03 | Area 04 | Area 05 | Area 06 | Area 07 | Area 08 | Area 09 | #Spec | #Spec 10 | #Spec 01 | #Spec 02 | #Spec 03 | #Spec 04 | #Spec 05 | #Spec 06 | #Spec 07 | #Spec 08 | #Spec 09 | Start | End | PTM |
| --- | --- | --- | --- | --- | --- | --- | --- | --- | --- | --- | --- | --- | --- | --- | --- | --- | --- | --- | --- | --- | --- | --- | --- | --- | --- | --- | --- | --- | --- | --- | --- | --- | --- | --- | --- |
| R.LTLYDIAHTPGVAADLSHIETR.A | Y | 135.41 | 2392.2385 | 22 | 0.2 | 798.4203 | 3 | 114.83 | 3 | 31724 | Ana\_02.raw | 2.1E8 | 2.62E8 | 4.36E7 | 9.45E7 | 1.17E8 | 1.51E8 | 9.17E7 | 1.11E8 | 3.05E8 | 4.78E7 | 59 | 6 | 7 | 5 | 6 | 7 | 5 | 4 | 7 | 7 | 5 | 53 | 74 |  |
| K.VDFPQDQLATLTGR.I | Y | 102.28 | 1559.7943 | 14 | 1.7 | 780.9058 | 2 | 96.98 | 3 | 27100 | Ana\_02.raw | 5.32E7 | 8.77E7 | 8.85E7 | 2.48E7 | 9.31E6 | 2.37E7 | 1.73E7 | 1.57E7 | 1.51E8 | 6.09E7 | 43 | 4 | 5 | 5 | 5 | 2 | 4 | 4 | 4 | 6 | 4 | 216 | 229 |  |
| K.GC(+57.02)DVVVIPAGVPR.K | Y | 97.18 | 1337.7125 | 13 | 1.3 | 669.8644 | 2 | 78.05 | 3 | 22092 | Ana\_02.raw | 1.55E7 | 2.24E7 | 2.25E7 | 7.62E6 | 1.37E7 | 1.48E7 | 7.11E6 |  | 4.2E7 | 1.67E7 | 9 | 1 | 1 | 1 | 1 | 1 | 1 | 1 | 0 | 1 | 1 | 92 | 104 | Carbamidomethylation |
| K.ETEC(+57.02)TYFSTPLLLGK.K | Y | 94.41 | 1757.8546 | 15 | 0.7 | 879.9352 | 2 | 107.77 | 3 | 29897 | Ana\_02.raw | 3.26E7 | 4.27E7 | 3.48E7 | 1.98E7 | 1.19E7 | 2.68E7 | 1.61E7 | 1.61E7 | 6.2E7 | 2.92E7 | 11 | 1 | 2 | 1 | 1 | 1 | 1 | 1 | 1 | 1 | 1 | 282 | 296 | Carbamidomethylation |
| K.GYLGPEQLPDC(+57.02)LK.G | Y | 92.01 | 1488.7283 | 13 | 7.8 | 745.3772 | 2 | 88.88 | 6 | 25288 | Ana\_05.raw | 5.85E6 |  | 7.75E6 | 2.59E6 |  | 5.47E6 | 3.59E6 | 3.01E6 | 8.98E6 |  | 7 | 1 | 0 | 1 | 1 | 0 | 1 | 1 | 1 | 1 | 0 | 79 | 91 | Carbamidomethylation |
| K.VAVLGASGGIGQPLSLLLK.N | Y | 89.13 | 1792.0822 | 19 | 0.7 | 897.0490 | 2 | 129.72 | 3 | 35541 | Ana\_02.raw | 5.92E7 | 1.03E7 | 1.88E6 | 2.49E7 | 2.98E7 | 5.17E7 | 3.29E7 | 2.63E7 | 7.57E7 | 4.63E7 | 21 | 2 | 3 | 2 | 1 | 2 | 2 | 2 | 4 | 2 | 1 | 27 | 45 |  |
| K.AGAGSATLSM(+15.99)AYAGAR.F | Y | 83.05 | 1469.6932 | 16 | 1.3 | 735.8549 | 2 | 49.79 | 3 | 14085 | Ana\_02.raw | 2.82E7 | 2.76E7 | 3.3E7 | 1.03E7 | 1.84E7 | 2.29E7 |  | 1.52E7 | 3.97E7 | 1.67E7 | 12 | 2 | 2 | 1 | 1 | 1 | 1 | 0 | 1 | 2 | 1 | 242 | 257 | Oxidation (M) |
| K.EGVVEC(+57.02)SFVQSK.E | Y | 80.90 | 1367.6390 | 12 | -1.8 | 684.8256 | 2 | 57.76 | 6 | 16512 | Ana\_05.raw | 1.52E7 | 2.1E7 | 1.98E7 | 8.72E6 | 9.67E6 | 1.49E7 | 1.01E7 | 1.05E7 | 2.43E7 |  | 9 | 1 | 1 | 1 | 1 | 1 | 1 | 1 | 1 | 1 | 0 | 270 | 281 | Carbamidomethylation |
| R.VNVPVIGGHAGK.T | Y | 77.34 | 1146.6509 | 12 | 0.3 | 383.2244 | 3 | 47.38 | 3 | 13385 | Ana\_02.raw | 1.13E7 | 1.6E7 | 2.25E7 | 9.39E6 | 1.26E7 | 1.69E7 | 1.2E7 | 8.62E6 | 2.33E7 | 1.38E7 | 20 | 1 | 2 | 2 | 3 | 2 | 1 | 2 | 2 | 3 | 2 | 192 | 203 |  |
| K.TIIPLISQC(+57.02)TPK.V | Y | 76.30 | 1369.7639 | 12 | 2.3 | 685.8908 | 2 | 86.83 | 6 | 24732 | Ana\_05.raw | 6.22E6 |  | 5.11E6 | 2.32E5 | 1.68E6 | 5.54E6 | 3.67E6 | 2.96E6 | 1.51E7 | 9.93E5 | 9 | 1 | 0 | 1 | 1 | 1 | 1 | 1 | 1 | 1 | 1 | 204 | 215 | Carbamidomethylation |
| K.IFGVTTLDIVR.A | Y | 69.11 | 1232.7129 | 11 | 3.7 | 617.3660 | 2 | 109.58 | 6 | 30770 | Ana\_05.raw | 3.39E7 | 5.89E7 | 5.61E7 | 3.4E7 | 3.94E7 | 5.26E7 | 4E7 | 2.97E7 | 1.06E8 | 4.41E7 | 34 | 4 | 5 | 3 | 2 | 4 | 2 | 3 | 3 | 5 | 3 | 166 | 176 |  |
| K.HGVYNPNK.I | Y | 62.38 | 927.4562 | 8 | 2.3 | 464.7365 | 2 | 17.41 | 5 | 4115 | Ana\_04.raw |  |  |  | 1.11E7 | 0 | 2.28E6 | 1.62E7 | 1.12E7 |  |  | 6 | 0 | 0 | 0 | 1 | 1 | 1 | 1 | 2 | 0 | 0 | 158 | 165 |  |
| R.ANTFVAELK.G | Y | 59.22 | 991.5338 | 9 | 1.4 | 496.7749 | 2 | 59.61 | 3 | 16958 | Ana\_02.raw | 2.82E7 | 3.77E7 | 4.54E7 | 1.9E7 | 2.58E7 | 3.24E7 | 2.43E7 | 2.39E7 | 4.73E7 | 2.44E7 | 23 | 3 | 2 | 3 | 2 | 2 | 2 | 2 | 3 | 2 | 2 | 177 | 185 |  |
| K.M(+15.99)IAEAIPELK.A | Y | 58.00 | 1129.6052 | 10 | 0.6 | 565.8102 | 2 | 71.33 | 5 | 20330 | Ana\_04.raw | 2.25E7 | 1.14E7 | 0 | 5.47E6 | 7.02E6 | 1.23E7 | 1.73E7 | 1.46E7 | 3.01E7 | 1.38E7 | 16 | 3 | 2 | 1 | 1 | 1 | 1 | 2 | 1 | 2 | 2 | 315 | 324 | Oxidation (M) |
| R.IQEAGTEVVK.A | Y | 52.26 | 1072.5764 | 10 | -0.1 | 537.2954 | 2 | 30.01 | 3 | 8129 | Ana\_02.raw | 7.69E7 | 9.64E7 | 8.49E7 | 3.72E7 | 5.72E7 | 7.11E7 | 4.89E7 | 4.4E7 | 8.24E7 | 7.09E7 | 19 | 2 | 2 | 2 | 2 | 2 | 2 | 2 | 2 | 2 | 1 | 230 | 239 |  |
| K.ITPFEEK.M | Y | 50.44 | 862.4436 | 7 | 3.8 | 432.2307 | 2 | 41.28 | 6 | 11547 | Ana\_05.raw |  | 5.26E7 | 6.99E7 |  | 4.14E7 | 5.72E7 | 3.78E7 | 3.68E7 | 5.43E7 | 0 | 9 | 0 | 1 | 1 | 0 | 1 | 1 | 1 | 1 | 2 | 1 | 308 | 314 |  |
| K.AGAGSATLSMAYAGAR.F | Y | 48.10 | 1453.6984 | 16 | 5.8 | 727.8607 | 2 | 61.76 | 9 | 17572 | Ana\_08.raw |  |  |  |  |  |  |  |  | 5.92E6 |  | 1 | 0 | 0 | 0 | 0 | 0 | 0 | 0 | 0 | 1 | 0 | 242 | 257 |  |
| K.KGEDFVK.N | Y | 45.72 | 821.4283 | 7 | 1.6 | 411.7221 | 2 | 20.39 | 3 | 5068 | Ana\_02.raw |  |  | 4.14E7 | 1.32E7 | 1.64E6 | 2.24E7 | 2.14E7 | 1.64E7 |  |  | 6 | 0 | 0 | 1 | 1 | 1 | 1 | 1 | 1 | 0 | 0 | 329 | 335 |  |
| K.KHGVYNPNK.I | Y | 43.38 | 1055.5511 | 9 | 2.4 | 352.8585 | 3 | 16.30 | 6 | 3715 | Ana\_05.raw |  |  |  | 2.71E5 | 6.08E5 | 8.2E5 |  | 2.37E5 |  |  | 4 | 0 | 0 | 0 | 1 | 1 | 1 | 0 | 1 | 0 | 0 | 157 | 165 |  |
| K.NSPLVSR.L | Y | 39.83 | 771.4239 | 7 | 2.3 | 386.7201 | 2 | 23.32 | 5 | 6024 | Ana\_04.raw |  |  |  | 2.39E7 | 1.91E7 |  | 1.35E7 | 7.89E5 |  | 9.57E6 | 5 | 0 | 0 | 0 | 1 | 1 | 0 | 1 | 1 | 0 | 1 | 46 | 52 |  |
| K.GEDFVK.N | Y | 24.72 | 693.3333 | 6 | 3.0 | 347.6750 | 2 | 25.15 | 3 | 6611 | Ana\_02.raw |  |  | 4.23E6 |  |  |  |  |  |  |  | 1 | 0 | 0 | 1 | 0 | 0 | 0 | 0 | 0 | 0 | 0 | 330 | 335 |  |
| K.NLGIGK.I | Y | 24.45 | 600.3595 | 6 | 2.7 | 301.1878 | 2 | 26.40 | 7 | 6868 | Ana\_06.raw |  |  |  |  |  |  | 3.58E6 |  |  |  | 1 | 0 | 0 | 0 | 0 | 0 | 0 | 1 | 0 | 0 | 0 | 302 | 307 |  |
| total 22 peptides |  |  |  |  |  |  |  |  |  |  |  |  |  |  |  |  |  |  |  |  |  |  |  |  |  |  |  |  |  |  |  |  |  |  |  |
| --- | --- | --- | --- | --- | --- | --- | --- | --- | --- | --- | --- | --- | --- | --- | --- | --- | --- | --- | --- | --- | --- | --- | --- | --- | --- | --- | --- | --- | --- | --- | --- | --- | --- | --- | --- |

Best Unique PSM (Scan F3:31724, m/z=798.4203, z=3, RT=114.83, ppm=0.2):

A8DUK4|A8DUK4\_MOUSE

back to list

  

| Protein Coverage
| Supporting Peptides
| Best Unique PSM
|

Protein Coverage:

Supporting Peptides:

| Peptide | Uniq | -10lgP | Mass | Length | ppm | m/z | z | RT | Fraction | Scan | Source File | Area 10 | Area 01 | Area 02 | Area 03 | Area 04 | Area 05 | Area 06 | Area 07 | Area 08 | Area 09 | #Spec | #Spec 10 | #Spec 01 | #Spec 02 | #Spec 03 | #Spec 04 | #Spec 05 | #Spec 06 | #Spec 07 | #Spec 08 | #Spec 09 | Start | End | PTM |
| --- | --- | --- | --- | --- | --- | --- | --- | --- | --- | --- | --- | --- | --- | --- | --- | --- | --- | --- | --- | --- | --- | --- | --- | --- | --- | --- | --- | --- | --- | --- | --- | --- | --- | --- | --- |
| K.DFTPAAQAAFQKVVAGVAAALAHKYH | Y | 123.03 | 2681.4077 | 26 | 3.2 | 894.8127 | 3 | 134.25 | 3 | 36749 | Ana\_02.raw | 1.5E7 | 2.57E7 | 8.81E6 | 2.52E6 | 1.88E7 | 4.78E7 | 1.54E7 | 7.69E6 | 2.4E6 | 1.91E7 | 25 | 2 | 5 | 3 | 2 | 3 | 3 | 2 | 2 | 1 | 2 | 122 | 147 |  |
| R.YFDSFGDLSSASAIM(+15.99)GNAK.V | N | 118.64 | 1995.8883 | 19 | 0.2 | 998.9517 | 2 | 96.28 | 3 | 26921 | Ana\_02.raw | 2.76E8 | 4.65E8 | 6.29E8 | 8.1E7 | 2.76E8 | 2.77E8 | 1.35E8 | 3.23E8 | 2.53E8 | 1.02E8 | 111 | 11 | 18 | 21 | 6 | 10 | 10 | 5 | 14 | 7 | 9 | 42 | 60 | Oxidation (M) |
| R.YFDSFGDLSSASAIMGNAK.V | N | 109.65 | 1979.8934 | 19 | 3.7 | 990.9577 | 2 | 117.42 | 1 | 32434 | ANa\_10.raw | 7.66E7 | 4.55E7 |  |  | 1E6 | 1.7E7 | 4.83E5 | 1.79E7 | 4.73E7 | 1.94E7 | 29 | 6 | 5 | 0 | 0 | 1 | 2 | 1 | 4 | 4 | 6 | 42 | 60 |  |
| K.VITAFNDGLNHLDSLK.G | N | 107.13 | 1755.9155 | 16 | -0.9 | 878.9642 | 2 | 93.75 | 3 | 26254 | Ana\_02.raw | 3.11E8 | 5.66E8 | 7.11E8 | 3.69E7 | 2.66E8 | 2.99E8 | 1.72E8 | 3.31E8 | 2.34E8 | 1.57E8 | 130 | 14 | 16 | 19 | 9 | 11 | 10 | 9 | 18 | 12 | 12 | 68 | 83 |  |
| K.DFTPAAQAAFQKVVAGVAAALAHK.Y | Y | 104.86 | 2381.2854 | 24 | 3.5 | 596.3307 | 4 | 135.69 | 3 | 37120 | Ana\_02.raw | 1.43E6 | 2.96E6 | 2.64E6 | 7.24E5 | 9.54E5 | 3.02E6 | 1.58E6 | 2.81E6 | 1.28E6 |  | 13 | 1 | 2 | 2 | 1 | 1 | 1 | 2 | 2 | 1 | 0 | 122 | 145 |  |
| K.VNADEVGGEALGR.L | Y | 103.63 | 1285.6262 | 13 | 3.0 | 643.8223 | 2 | 53.86 | 6 | 15359 | Ana\_05.raw | 9.13E8 | 1.78E9 | 2.25E9 | 5.04E8 | 1.28E9 | 1.33E9 | 7.79E8 | 1.49E9 | 8.26E8 | 5.77E8 | 180 | 14 | 23 | 30 | 12 | 25 | 16 | 12 | 29 | 9 | 10 | 19 | 31 |  |
| R.LLVVYPWTQR.Y | N | 97.82 | 1273.7183 | 10 | 3.1 | 637.8684 | 2 | 102.18 | 6 | 28828 | Ana\_05.raw | 4.86E8 | 9.19E8 | 1.17E9 | 3.07E8 | 5.83E8 | 7.99E8 | 3.19E8 | 5.92E8 | 8.02E8 | 3.19E8 | 260 | 20 | 27 | 40 | 25 | 32 | 29 | 19 | 42 | 15 | 11 | 32 | 41 |  |
| K.KVITAFNDGLNHLDSLK.G | N | 97.49 | 1884.0105 | 17 | 1.2 | 629.0115 | 3 | 83.71 | 3 | 23607 | Ana\_02.raw | 1.12E7 | 3.81E7 | 3.09E7 | 4.66E6 | 1.16E7 | 1.96E7 | 8.98E6 | 1.67E7 | 1.67E7 | 1.03E6 | 20 | 2 | 3 | 2 | 2 | 2 | 2 | 2 | 2 | 2 | 1 | 67 | 83 |  |
| K.LHVDPENFR.L | N | 97.26 | 1125.5566 | 9 | 3.6 | 563.7876 | 2 | 55.51 | 6 | 15845 | Ana\_05.raw | 1.19E8 | 2.67E8 | 4.01E8 | 9.71E7 | 1.98E8 | 1.94E8 | 1.27E8 | 2.18E8 | 1.43E8 | 7.93E7 | 69 | 6 | 9 | 10 | 6 | 7 | 5 | 5 | 10 | 7 | 4 | 97 | 105 |  |
| K.GTFASLSELHC(+57.02)DK.L | N | 94.23 | 1463.6715 | 13 | 1.3 | 732.8440 | 2 | 63.48 | 3 | 18077 | Ana\_02.raw | 7.95E7 | 1.55E8 | 1.63E8 | 6.14E7 | 8.27E7 | 8.69E7 | 5.77E7 | 1.03E8 | 1.04E8 | 5.64E7 | 42 | 4 | 5 | 6 | 3 | 4 | 4 | 4 | 7 | 3 | 2 | 84 | 96 | Carbamidomethylation |
| R.LLGNM(+15.99)IVIVLGHHLGK.D | N | 84.05 | 1729.0072 | 16 | -1.2 | 433.2585 | 4 | 101.36 | 3 | 28253 | Ana\_02.raw |  | 4.98E5 | 1.64E7 | 2.5E6 | 3.74E6 | 1.69E5 | 2.94E6 | 3.52E6 |  |  | 21 | 0 | 1 | 5 | 4 | 3 | 1 | 2 | 5 | 0 | 0 | 106 | 121 | Oxidation (M) |
| K.DFTPAAQAAFQK.V | N | 76.41 | 1293.6353 | 12 | 0.9 | 647.8255 | 2 | 63.54 | 3 | 18097 | Ana\_02.raw | 2.73E7 | 1.02E8 | 1.47E8 |  | 7.19E7 | 4.43E7 | 3.16E7 | 6.02E7 | 3.65E7 | 2.35E7 | 24 | 2 | 4 | 4 | 0 | 3 | 2 | 2 | 4 | 2 | 1 | 122 | 133 |  |
| R.LLGNMIVIVLGHHLGK.D | N | 72.25 | 1713.0123 | 16 | 4.5 | 429.2623 | 4 | 127.85 | 8 | 35068 | Ana\_07.raw | 6.42E5 | 6.18E5 | 1.05E6 | 1.09E6 | 3E6 | 5.24E6 | 1.35E6 | 2.37E6 |  |  | 10 | 1 | 2 | 1 | 1 | 1 | 2 | 1 | 1 | 0 | 0 | 106 | 121 |  |
| K.VVAGVAAALAHK.Y | Y | 65.32 | 1105.6608 | 12 | 2.7 | 553.8392 | 2 | 49.03 | 3 | 13862 | Ana\_02.raw |  |  | 8.08E6 |  |  | 4.05E6 |  | 3.15E6 |  |  | 3 | 0 | 0 | 1 | 0 | 0 | 1 | 0 | 1 | 0 | 0 | 134 | 145 |  |
| K.AAVSGLWGK.V | Y | 63.08 | 887.4865 | 9 | 2.6 | 444.7516 | 2 | 59.75 | 6 | 17088 | Ana\_05.raw | 5.39E7 | 9.49E7 | 1.04E8 | 2.91E7 | 5.64E7 | 8.21E7 | 4.62E7 | 8.33E7 | 5.88E7 | 2.04E7 | 19 | 3 | 3 | 2 | 1 | 2 | 2 | 1 | 3 | 1 | 1 | 10 | 18 |  |
| M.VHLTDAEK.A | N | 61.87 | 911.4712 | 8 | 5.7 | 456.7455 | 2 | 21.62 | 6 | 5462 | Ana\_05.raw | 3.24E8 | 5.17E8 | 7.06E8 | 1.33E8 | 1E7 | 3.48E8 | 2.15E8 | 3.12E8 |  | 1.31E8 | 40 | 5 | 3 | 7 | 3 | 3 | 5 | 3 | 7 | 0 | 4 | 2 | 9 |  |
| total 16 peptides |  |  |  |  |  |  |  |  |  |  |  |  |  |  |  |  |  |  |  |  |  |  |  |  |  |  |  |  |  |  |  |  |  |  |  |
| --- | --- | --- | --- | --- | --- | --- | --- | --- | --- | --- | --- | --- | --- | --- | --- | --- | --- | --- | --- | --- | --- | --- | --- | --- | --- | --- | --- | --- | --- | --- | --- | --- | --- | --- | --- |

Best Unique PSM (Scan F3:36749, m/z=894.8127, z=3, RT=134.25, ppm=3.2):

sp|Q8VHX6|FLNC\_MOUSE

back to list

  

| Protein Coverage
| Supporting Peptides
| Best Unique PSM
|

Protein Coverage:

Supporting Peptides:

| Peptide | Uniq | -10lgP | Mass | Length | ppm | m/z | z | RT | Fraction | Scan | Source File | Area 10 | Area 01 | Area 02 | Area 03 | Area 04 | Area 05 | Area 06 | Area 07 | Area 08 | Area 09 | #Spec | #Spec 10 | #Spec 01 | #Spec 02 | #Spec 03 | #Spec 04 | #Spec 05 | #Spec 06 | #Spec 07 | #Spec 08 | #Spec 09 | Start | End | PTM |
| --- | --- | --- | --- | --- | --- | --- | --- | --- | --- | --- | --- | --- | --- | --- | --- | --- | --- | --- | --- | --- | --- | --- | --- | --- | --- | --- | --- | --- | --- | --- | --- | --- | --- | --- | --- |
| R.GQHVPGSPFQFTVGPLGEGGAHK.V | N | 114.28 | 2303.1445 | 23 | 3.8 | 576.7956 | 4 | 90.06 | 1 | 25304 | ANa\_10.raw | 1.86E7 | 5.87E6 | 2.37E7 | 6.44E6 | 1.54E7 | 1.46E7 |  | 1.38E7 | 3.15E7 | 4.2E6 | 18 | 3 | 2 | 2 | 1 | 2 | 1 | 0 | 3 | 2 | 2 | 2295 | 2317 |  |
| R.VEESTQVGGDPFPAVFGDFLGR.E | Y | 102.50 | 2323.1121 | 22 | 3.8 | 1162.5677 | 2 | 133.50 | 1 | 36703 | ANa\_10.raw | 1.63E6 |  |  |  |  |  |  |  |  |  | 1 | 1 | 0 | 0 | 0 | 0 | 0 | 0 | 0 | 0 | 0 | 2208 | 2229 |  |
| K.FNDEHIPDSPFVVPVASLSDDAR.R | Y | 93.29 | 2526.2026 | 23 | 5.5 | 843.0795 | 3 | 114.35 | 1 | 31642 | ANa\_10.raw | 2.36E6 | 1.21E6 |  | 9.44E5 | 1.11E6 | 5.05E6 | 4.86E5 | 6.25E5 | 7.88E6 |  | 8 | 1 | 1 | 0 | 1 | 1 | 1 | 1 | 1 | 1 | 0 | 2388 | 2410 |  |
| K.IEC(+57.02)DDKGDGSC(+57.02)DVR.Y | N | 89.31 | 1624.6458 | 14 | 0.4 | 813.3305 | 2 | 21.87 | 3 | 5563 | Ana\_02.raw | 1.78E7 | 2.73E7 | 2.13E7 | 1.72E6 | 7E6 | 2.34E7 | 5.65E6 | 1.04E7 | 2.12E7 | 1.83E7 | 19 | 2 | 2 | 2 | 1 | 2 | 2 | 2 | 2 | 2 | 2 | 617 | 630 | Carbamidomethylation |
| K.SPFVVNVAPPLDLSK.V | Y | 83.86 | 1581.8766 | 15 | 3.5 | 791.9484 | 2 | 106.95 | 9 | 30083 | Ana\_08.raw | 2.1E6 | 1.6E6 |  |  | 9.46E5 | 9.55E5 | 5.45E5 | 2.97E5 | 1.08E7 | 8.89E5 | 8 | 1 | 1 | 0 | 0 | 1 | 1 | 1 | 1 | 1 | 1 | 955 | 969 |  |
| R.SLTATGGNHVTAR.V | Y | 81.88 | 1283.6582 | 13 | 3.8 | 642.8388 | 2 | 24.28 | 1 | 6195 | ANa\_10.raw | 2.2E6 | 1.21E6 |  |  |  |  |  |  | 2.13E6 | 1.04E6 | 4 | 1 | 1 | 0 | 0 | 0 | 0 | 0 | 0 | 1 | 1 | 1280 | 1292 |  |
| R.VNVGEGSHPER.V | Y | 75.27 | 1179.5632 | 11 | 2.8 | 590.7905 | 2 | 21.58 | 9 | 5151 | Ana\_08.raw | 2.75E6 | 1.83E6 | 6.67E6 | 1.37E6 |  |  |  |  | 3.5E6 |  | 7 | 1 | 1 | 2 | 2 | 0 | 0 | 0 | 0 | 1 | 0 | 757 | 767 |  |
| K.TEAAEIVEGEDSAYSVR.F | Y | 75.10 | 1824.8376 | 17 | 3.3 | 913.4291 | 2 | 75.37 | 3 | 21366 | Ana\_02.raw | 1.46E6 | 7.46E5 | 3.98E5 |  |  |  |  |  |  |  | 3 | 1 | 1 | 1 | 0 | 0 | 0 | 0 | 0 | 0 | 0 | 2262 | 2278 |  |
| R.LIALLEVLSQK.R | N | 69.25 | 1225.7645 | 11 | 3.9 | 613.8919 | 2 | 129.98 | 7 | 35763 | Ana\_06.raw | 4.91E6 | 1.99E6 | 1.02E7 | 4.83E6 | 8.34E6 | 9.14E6 | 6.96E6 | 2.18E6 | 1.08E7 | 5.95E6 | 10 | 1 | 1 | 1 | 1 | 1 | 1 | 1 | 1 | 1 | 1 | 71 | 81 |  |
| R.GVAGVPAEFSIWTR.E | Y | 68.04 | 1488.7725 | 14 | 6.3 | 745.3982 | 2 | 113.33 | 1 | 31386 | ANa\_10.raw | 1.75E6 |  |  |  |  |  |  |  | 3.05E6 |  | 2 | 1 | 0 | 0 | 0 | 0 | 0 | 0 | 0 | 1 | 0 | 2328 | 2341 |  |
| K.DLAEDAPWK.K | N | 65.01 | 1043.4923 | 9 | 3.5 | 522.7552 | 2 | 72.72 | 6 | 20864 | Ana\_05.raw | 1.24E7 | 7.6E6 | 1.07E7 | 6.87E6 | 8.48E6 | 1.44E7 | 7.45E6 |  | 1.14E7 | 5.52E6 | 13 | 1 | 1 | 2 | 2 | 1 | 2 | 2 | 0 | 1 | 1 | 28 | 36 |  |
| K.AFGPGLEPTGC(+57.02)IVDRPAEFTIDAR.A | Y | 63.17 | 2588.2693 | 24 | 7.5 | 863.7702 | 3 | 103.67 | 9 | 29213 | Ana\_08.raw |  |  |  |  |  |  |  |  | 7.62E6 |  | 1 | 0 | 0 | 0 | 0 | 0 | 0 | 0 | 0 | 1 | 0 | 673 | 696 | Carbamidomethylation |
| K.LEPGGGAEAQAVR.Y | Y | 61.79 | 1253.6364 | 13 | 4.3 | 627.8281 | 2 | 34.01 | 9 | 9099 | Ana\_08.raw | 7.14E6 | 5.42E6 | 5.13E6 | 1.3E6 | 3.13E6 | 5.5E6 | 9.37E5 | 2.28E6 | 8.39E6 | 5.85E6 | 10 | 1 | 1 | 1 | 1 | 1 | 1 | 1 | 1 | 1 | 1 | 1016 | 1028 |  |
| K.YGGDEIPYSPFR.I | Y | 61.79 | 1399.6407 | 12 | 4.3 | 700.8306 | 2 | 91.01 | 6 | 25857 | Ana\_05.raw | 7.67E6 |  |  | 1.18E6 | 3.78E6 | 6.42E6 | 2.13E7 |  |  |  | 6 | 1 | 0 | 0 | 1 | 1 | 1 | 2 | 0 | 0 | 0 | 1617 | 1628 |  |
| K.HVTNSPFK.I | Y | 56.45 | 928.4766 | 8 | 1.0 | 465.2461 | 2 | 22.71 | 9 | 5536 | Ana\_08.raw | 1.2E7 |  |  |  | 1.8E6 |  |  |  | 9.18E6 |  | 3 | 1 | 0 | 0 | 0 | 1 | 0 | 0 | 0 | 1 | 0 | 2024 | 2031 |  |
| K.VAVGQEQAFSVNTR.G | Y | 55.71 | 1504.7634 | 14 | 5.3 | 753.3930 | 2 | 60.30 | 10 | 17067 | Ana\_09.raw |  |  |  |  |  |  |  |  |  | 5.8E5 | 1 | 0 | 0 | 0 | 0 | 0 | 0 | 0 | 0 | 0 | 1 | 979 | 992 |  |
| R.LTVTSLQETGLK.V | Y | 55.11 | 1288.7239 | 12 | 4.1 | 645.3719 | 2 | 69.30 | 9 | 19771 | Ana\_08.raw |  |  |  |  |  |  |  |  | 6.29E5 |  | 1 | 0 | 0 | 0 | 0 | 0 | 0 | 0 | 0 | 1 | 0 | 2412 | 2423 |  |
| K.NDNDTFTVK.Y | N | 54.27 | 1052.4774 | 9 | 5.4 | 527.2488 | 2 | 34.22 | 2 | 9284 | Ana\_01.raw | 2.84E7 | 2.74E7 |  |  |  |  |  |  | 8.6E6 | 1.49E7 | 5 | 1 | 1 | 0 | 0 | 0 | 0 | 0 | 0 | 2 | 1 | 825 | 833 |  |
| K.IQQNTFTR.W | N | 53.76 | 1006.5196 | 8 | 2.0 | 504.2681 | 2 | 28.64 | 3 | 7713 | Ana\_02.raw |  |  | 4.24E7 | 1.74E7 |  |  |  | 2.58E7 | 5.6E7 | 4.3E7 | 6 | 0 | 0 | 1 | 1 | 0 | 0 | 0 | 1 | 1 | 2 | 38 | 45 |  |
| K.YGGHPIPK.F | Y | 50.87 | 867.4603 | 8 | 3.3 | 434.7389 | 2 | 22.12 | 9 | 5329 | Ana\_08.raw | 2.7E6 |  |  |  |  |  |  |  | 3.8E6 | 4.51E6 | 3 | 1 | 0 | 0 | 0 | 0 | 0 | 0 | 0 | 1 | 1 | 1231 | 1238 |  |
| R.EVTTEFTVDAR.S | Y | 46.76 | 1266.6091 | 11 | 2.2 | 634.3132 | 2 | 61.26 | 5 | 17438 | Ana\_04.raw |  |  |  |  | 2.81E5 |  |  | 6.84E5 |  |  | 2 | 0 | 0 | 0 | 0 | 1 | 0 | 0 | 1 | 0 | 0 | 1269 | 1279 |  |
| R.FTVETR.G | N | 45.35 | 751.3864 | 6 | 2.0 | 376.7012 | 2 | 29.33 | 3 | 7925 | Ana\_02.raw | 4.96E7 | 4.71E7 | 4.24E7 | 1.96E7 | 3.51E7 | 4.5E7 | 2.76E7 | 2.49E7 | 4.87E7 | 3.52E7 | 15 | 2 | 1 | 1 | 2 | 1 | 2 | 2 | 2 | 1 | 1 | 1372 | 1377 |  |
| K.ILVGPSEIGDASK.V | Y | 44.90 | 1284.6925 | 13 | 3.1 | 643.3555 | 2 | 66.16 | 6 | 18994 | Ana\_05.raw |  | 7.17E6 | 7.63E6 |  | 4.04E6 | 5.42E6 | 7.96E5 | 1.1E6 |  | 7.66E6 | 7 | 0 | 1 | 1 | 0 | 1 | 1 | 1 | 1 | 0 | 1 | 2032 | 2044 |  |
| K.ATIQPVFDPSK.V | Y | 44.24 | 1201.6343 | 11 | 4.6 | 601.8271 | 2 | 65.37 | 7 | 18547 | Ana\_06.raw | 5.15E6 |  |  | 8.88E5 | 1.44E6 |  | 1.56E6 | 3.47E5 |  |  | 5 | 1 | 0 | 0 | 1 | 1 | 0 | 1 | 1 | 0 | 0 | 1148 | 1158 |  |
| R.GAGGQGQLDVR.M | Y | 44.04 | 1056.5312 | 11 | 6.7 | 529.2764 | 2 | 28.00 | 10 | 7450 | Ana\_09.raw |  |  |  |  |  | 8.99E6 |  |  | 1.99E6 | 8.12E6 | 3 | 0 | 0 | 0 | 0 | 0 | 1 | 0 | 0 | 1 | 1 | 993 | 1003 |  |
| R.APLQVAVLGPTGVAEPVEVR.D | Y | 42.56 | 2001.1259 | 20 | 6.0 | 1001.5762 | 2 | 102.66 | 10 | 28531 | Ana\_09.raw |  |  |  |  |  |  |  |  |  | 9.15E5 | 1 | 0 | 0 | 0 | 0 | 0 | 0 | 0 | 0 | 0 | 1 | 1476 | 1495 |  |
| K.LVSIDSK.A | N | 40.94 | 760.4330 | 7 | 5.1 | 381.2257 | 2 | 29.51 | 1 | 7861 | ANa\_10.raw | 6.01E7 | 6.64E7 | 5.26E7 |  |  | 4.66E7 | 2.86E7 | 3.95E7 | 4.72E7 | 3.77E7 | 8 | 1 | 1 | 1 | 0 | 0 | 1 | 1 | 1 | 1 | 1 | 115 | 121 |  |
| K.AEIAFEDR.K | Y | 40.72 | 949.4504 | 8 | 5.0 | 475.7349 | 2 | 47.26 | 10 | 13257 | Ana\_09.raw |  | 5.9E6 |  | 9.55E5 | 4.1E6 | 4.94E6 | 1.14E6 | 4.61E6 |  | 3.66E6 | 7 | 0 | 1 | 0 | 1 | 1 | 1 | 1 | 1 | 0 | 1 | 2358 | 2365 |  |
| K.VSGPGVEPHGVLR.E | Y | 39.73 | 1302.7043 | 13 | 9.7 | 435.2463 | 3 | 41.23 | 9 | 11364 | Ana\_08.raw |  |  |  |  |  |  |  |  | 7.45E5 |  | 1 | 0 | 0 | 0 | 0 | 0 | 0 | 0 | 0 | 1 | 0 | 1256 | 1268 |  |
| R.HIGISFTPK.E | Y | 39.53 | 998.5549 | 9 | 5.5 | 500.2874 | 2 | 52.34 | 1 | 14716 | ANa\_10.raw | 9.94E5 | 1.26E6 |  |  |  |  |  |  |  |  | 2 | 1 | 1 | 0 | 0 | 0 | 0 | 0 | 0 | 0 | 0 | 2001 | 2009 |  |
| K.GELTGEVR.M | N | 38.99 | 859.4399 | 8 | 4.0 | 430.7290 | 2 | 26.51 | 2 | 6920 | Ana\_01.raw |  | 1.32E7 |  | 5.95E6 |  | 6.47E6 | 1.14E7 |  |  |  | 6 | 0 | 1 | 0 | 1 | 0 | 1 | 3 | 0 | 0 | 0 | 1797 | 1804 |  |
| R.WC(+57.02)NEHLK.C | N | 37.58 | 985.4440 | 7 | 3.6 | 329.4898 | 3 | 24.09 | 9 | 6000 | Ana\_08.raw |  |  |  |  |  |  |  |  | 6.53E6 |  | 2 | 0 | 0 | 0 | 0 | 0 | 0 | 0 | 0 | 2 | 0 | 46 | 52 | Carbamidomethylation |
| K.AEGPGLSR.T | Y | 34.47 | 785.4031 | 8 | 2.5 | 393.7098 | 2 | 23.49 | 8 | 5898 | Ana\_07.raw |  |  |  | 5.13E6 |  |  |  | 1.39E6 |  |  | 3 | 0 | 0 | 0 | 2 | 0 | 0 | 0 | 1 | 0 | 0 | 873 | 880 |  |
| K.LKPGAPVR.S | Y | 29.40 | 836.5232 | 8 | 2.9 | 419.2701 | 2 | 21.73 | 9 | 5200 | Ana\_08.raw |  |  |  |  |  |  |  |  | 4.13E6 |  | 1 | 0 | 0 | 0 | 0 | 0 | 0 | 0 | 0 | 1 | 0 | 263 | 270 |  |
| K.TPC(+57.02)EEVYVK.H | Y | 22.54 | 1123.5220 | 9 | 1.6 | 562.7692 | 2 | 40.64 | 1 | 11213 | ANa\_10.raw | 1.34E6 |  |  |  |  |  |  |  |  |  | 1 | 1 | 0 | 0 | 0 | 0 | 0 | 0 | 0 | 0 | 0 | 2678 | 2686 | Carbamidomethylation |
| total 35 peptides |  |  |  |  |  |  |  |  |  |  |  |  |  |  |  |  |  |  |  |  |  |  |  |  |  |  |  |  |  |  |  |  |  |  |  |
| --- | --- | --- | --- | --- | --- | --- | --- | --- | --- | --- | --- | --- | --- | --- | --- | --- | --- | --- | --- | --- | --- | --- | --- | --- | --- | --- | --- | --- | --- | --- | --- | --- | --- | --- | --- |

Best Unique PSM (Scan F1:36703, m/z=1162.5677, z=2, RT=133.50, ppm=3.8):

sp|Q01853|TERA\_MOUSE

back to list

  

| Protein Coverage
| Supporting Peptides
| Best Unique PSM
|

Protein Coverage:

Supporting Peptides:

| Peptide | Uniq | -10lgP | Mass | Length | ppm | m/z | z | RT | Fraction | Scan | Source File | Area 10 | Area 01 | Area 02 | Area 03 | Area 04 | Area 05 | Area 06 | Area 07 | Area 08 | Area 09 | #Spec | #Spec 10 | #Spec 01 | #Spec 02 | #Spec 03 | #Spec 04 | #Spec 05 | #Spec 06 | #Spec 07 | #Spec 08 | #Spec 09 | Start | End | PTM |
| --- | --- | --- | --- | --- | --- | --- | --- | --- | --- | --- | --- | --- | --- | --- | --- | --- | --- | --- | --- | --- | --- | --- | --- | --- | --- | --- | --- | --- | --- | --- | --- | --- | --- | --- | --- |
| K.LADDVDLEQVANETHGHVGADLAALC(+57.02)SEAALQAIR.K | Y | 114.56 | 3671.7849 | 35 | 4.0 | 918.9572 | 4 | 128.46 | 9 | 35589 | Ana\_08.raw |  | 6.78E6 |  | 5.23E6 | 7.97E6 |  | 4.47E6 | 4.75E6 | 2.25E7 |  | 6 | 0 | 1 | 0 | 1 | 1 | 0 | 1 | 1 | 1 | 0 | 390 | 424 | Carbamidomethylation |
| R.ETVVEVPQVTWEDIGGLEDVKR.E | Y | 112.52 | 2497.2700 | 22 | 1.9 | 833.4322 | 3 | 119.23 | 3 | 32847 | Ana\_02.raw | 1.6E1 | 7.34E6 | 1.58E7 | 1.06E6 | 7.19E6 | 1.34E7 | 4.62E6 | 2.06E6 | 7.85E6 | 7.24E6 | 14 | 1 | 2 | 1 | 1 | 2 | 2 | 1 | 1 | 2 | 1 | 466 | 487 |  |
| K.NAPAIIFIDELDAIAPK.R | Y | 93.29 | 1809.9875 | 17 | 2.6 | 906.0034 | 2 | 135.56 | 7 | 37342 | Ana\_06.raw | 7.08E5 | 1.84E6 | 1.55E6 |  | 1.85E6 | 1.26E6 | 7.5E5 |  | 3.74E6 | 1.2E7 | 23 | 3 | 3 | 2 | 0 | 2 | 2 | 1 | 0 | 8 | 2 | 296 | 312 |  |
| R.ELQELVQYPVEHPDK.F | Y | 79.98 | 1822.9100 | 15 | 5.1 | 608.6470 | 3 | 71.31 | 9 | 20349 | Ana\_08.raw |  |  |  |  |  |  |  |  | 1.58E7 |  | 1 | 0 | 0 | 0 | 0 | 0 | 0 | 0 | 0 | 1 | 0 | 488 | 502 |  |
| R.LDQLIYIPLPDEK.S | Y | 79.78 | 1555.8497 | 13 | 1.1 | 778.9330 | 2 | 113.82 | 3 | 31460 | Ana\_02.raw | 2.47E7 | 3.08E7 | 2.3E7 | 1.32E7 | 1.65E7 | 2.28E7 | 1.26E7 | 1.12E7 | 4.85E7 | 2.43E7 | 20 | 2 | 2 | 2 | 2 | 2 | 2 | 2 | 2 | 2 | 2 | 639 | 651 |  |
| R.QAAPC(+57.02)VLFFDELDSIAK.A | Y | 79.59 | 1922.9447 | 17 | 4.8 | 962.4843 | 2 | 139.58 | 10 | 37981 | Ana\_09.raw | 2.08E5 | 1.91E5 |  |  | 1.97E5 | 4.94E5 |  |  | 1.1E6 | 6.49E5 | 6 | 1 | 1 | 0 | 0 | 1 | 1 | 0 | 0 | 1 | 1 | 568 | 584 | Carbamidomethylation |
| R.EVDIGIPDATGR.L | Y | 76.87 | 1241.6251 | 12 | 2.6 | 621.8215 | 2 | 69.37 | 3 | 19720 | Ana\_02.raw | 8.62E6 | 8.91E6 | 7.67E6 | 4.1E6 | 6.92E6 | 6.84E6 |  | 3.37E6 | 3.19E6 | 6.71E6 | 9 | 1 | 1 | 1 | 1 | 1 | 1 | 0 | 1 | 1 | 1 | 366 | 377 |  |
| R.GGNIGDGGGAADR.V | Y | 72.58 | 1115.4955 | 13 | 0.3 | 558.7552 | 2 | 20.90 | 7 | 5177 | Ana\_06.raw |  |  |  | 4.77E6 | 9.72E6 | 2.34E6 | 7.04E6 | 4.5E5 |  |  | 5 | 0 | 0 | 0 | 1 | 1 | 1 | 1 | 1 | 0 | 0 | 587 | 599 |  |
| R.GILLYGPPGTGK.T | Y | 69.84 | 1171.6600 | 12 | 6.9 | 586.8413 | 2 | 73.98 | 1 | 20954 | ANa\_10.raw | 6.75E6 | 2.09E6 | 8.2E6 | 4.46E6 | 3.66E6 | 6.98E6 | 5.96E6 | 5.28E6 | 1.67E7 | 5.78E6 | 14 | 1 | 1 | 2 | 2 | 1 | 2 | 1 | 2 | 1 | 1 | 240 | 251 |  |
| R.KYEM(+15.99)FAQTLQQSR.G | Y | 67.51 | 1644.7930 | 13 | 4.5 | 549.2740 | 3 | 52.48 | 9 | 14809 | Ana\_08.raw | 1.05E6 | 2.76E6 | 2E6 | 2.36E6 |  | 3.69E6 | 3.86E6 | 2.97E6 | 5.33E6 |  | 8 | 1 | 1 | 1 | 1 | 0 | 1 | 1 | 1 | 1 | 0 | 754 | 766 | Oxidation (M) |
| R.IVSQLLTLMDGLK.Q | Y | 65.85 | 1429.8214 | 13 | 3.1 | 715.9202 | 2 | 130.61 | 9 | 36161 | Ana\_08.raw |  |  |  |  |  |  |  |  | 1.02E6 |  | 1 | 0 | 0 | 0 | 0 | 0 | 0 | 0 | 0 | 1 | 0 | 324 | 336 |  |
| K.LAGESESNLR.K | Y | 64.23 | 1074.5305 | 10 | 3.3 | 538.2743 | 2 | 31.65 | 6 | 8637 | Ana\_05.raw | 3.2E7 | 3.65E7 | 3.21E7 | 1.43E7 | 2.44E7 | 2.25E7 |  |  | 4.16E7 |  | 7 | 1 | 1 | 1 | 1 | 1 | 1 | 0 | 0 | 1 | 0 | 278 | 287 |  |
| R.IVSQLLTLM(+15.99)DGLK.Q | Y | 62.47 | 1445.8163 | 13 | 2.9 | 723.9175 | 2 | 99.89 | 3 | 27869 | Ana\_02.raw | 4.06E5 |  | 1.44E6 |  | 7.09E5 | 1E6 |  |  | 2.99E6 | 8.04E5 | 7 | 1 | 0 | 2 | 0 | 1 | 1 | 0 | 0 | 1 | 1 | 324 | 336 | Oxidation (M) |
| R.WALSQSNPSALR.E | Y | 62.35 | 1328.6837 | 12 | 2.7 | 665.3510 | 2 | 73.57 | 7 | 20857 | Ana\_06.raw | 6.67E6 | 1.11E7 | 7.53E6 | 1.6E6 | 3.06E6 | 4.08E6 | 2.15E6 | 3.58E6 | 9.53E6 | 7.34E6 | 10 | 1 | 1 | 1 | 1 | 1 | 1 | 1 | 1 | 1 | 1 | 454 | 465 |  |
| K.M(+15.99)DELQLFR.G | Y | 60.05 | 1066.5117 | 8 | 0.5 | 534.2634 | 2 | 78.68 | 3 | 22261 | Ana\_02.raw | 4.97E6 |  | 5.09E6 | 2.74E6 |  | 3.96E6 | 1.69E6 |  |  |  | 5 | 1 | 0 | 1 | 1 | 0 | 1 | 1 | 0 | 0 | 0 | 46 | 53 | Oxidation (M) |
| K.DVDLEFLAK.M | Y | 59.17 | 1048.5441 | 9 | 1.6 | 525.2802 | 2 | 95.77 | 1 | 26833 | ANa\_10.raw | 2.72E6 | 1.51E6 | 1.81E6 | 1.9E5 | 8.35E5 | 1.85E6 | 1.46E6 | 9.71E5 | 1.62E6 | 2.47E6 | 10 | 1 | 1 | 1 | 1 | 1 | 1 | 1 | 1 | 1 | 1 | 669 | 677 |  |
| K.NVFIIGATNRPDIIDPAILRPGR.L | Y | 56.86 | 2517.4180 | 23 | 3.3 | 630.3638 | 4 | 112.90 | 5 | 31355 | Ana\_04.raw |  |  |  | 6.8E5 | 2.98E6 | 1.58E6 | 3.8E6 | 2.12E5 |  |  | 5 | 0 | 0 | 0 | 1 | 1 | 1 | 1 | 1 | 0 | 0 | 616 | 638 |  |
| R.KAFEEAEK.N | Y | 55.72 | 950.4708 | 8 | 4.3 | 476.2448 | 2 | 18.78 | 6 | 4529 | Ana\_05.raw |  |  |  | 2.15E6 | 2.77E6 | 4.56E6 | 3.27E6 | 2.55E6 |  |  | 5 | 0 | 0 | 0 | 1 | 1 | 1 | 1 | 1 | 0 | 0 | 288 | 295 |  |
| R.ESIESEIR.R | Y | 54.25 | 961.4716 | 8 | 2.1 | 481.7441 | 2 | 43.49 | 9 | 12056 | Ana\_08.raw | 1.23E7 | 1.1E7 | 1.27E7 | 6.33E6 | 1.17E7 | 1.09E7 | 8.37E6 | 8.18E6 | 1.59E7 | 1.34E7 | 10 | 1 | 1 | 1 | 1 | 1 | 1 | 1 | 1 | 1 | 1 | 701 | 708 |  |
| K.AIANEC(+57.02)QANFISIK.G | Y | 51.53 | 1577.7871 | 14 | 4.8 | 789.9046 | 2 | 74.57 | 9 | 21252 | Ana\_08.raw |  |  |  |  |  |  |  |  | 1.05E6 |  | 1 | 0 | 0 | 0 | 0 | 0 | 0 | 0 | 0 | 1 | 0 | 530 | 543 | Carbamidomethylation |
| R.DHFEEAM(+15.99)R.F | Y | 50.59 | 1049.4236 | 8 | 2.5 | 525.7204 | 2 | 20.71 | 3 | 5174 | Ana\_02.raw | 1.65E6 | 2.58E6 | 7.71E6 | 2.75E6 | 5.01E6 | 3.62E6 | 3.94E6 | 3.51E6 | 3.04E6 | 5.65E6 | 10 | 1 | 1 | 1 | 1 | 1 | 1 | 1 | 1 | 1 | 1 | 734 | 741 | Oxidation (M) |
| K.GVLFYGPPGC(+57.02)GK.T | Y | 49.61 | 1250.6117 | 12 | 2.5 | 626.3147 | 2 | 75.25 | 7 | 21321 | Ana\_06.raw | 1.22E6 |  |  | 1.34E6 | 1.15E6 | 9.27E5 | 0 |  |  |  | 5 | 1 | 0 | 0 | 1 | 1 | 1 | 1 | 0 | 0 | 0 | 513 | 524 | Carbamidomethylation |
| K.GDDLSTAILK.Q | Y | 47.18 | 1031.5498 | 10 | 2.9 | 516.7837 | 2 | 68.58 | 4 | 19087 | Ana\_03.raw |  |  |  | 2.57E6 |  |  |  |  |  |  | 1 | 0 | 0 | 0 | 1 | 0 | 0 | 0 | 0 | 0 | 0 | 9 | 18 |  |
| R.LEILQIHTK.N | Y | 47.00 | 1093.6495 | 9 | 4.9 | 547.8347 | 2 | 62.54 | 1 | 17727 | ANa\_10.raw | 1.44E6 | 9.06E5 | 1.07E6 |  | 4.52E5 |  |  |  | 1.29E6 |  | 6 | 1 | 1 | 1 | 0 | 1 | 0 | 0 | 0 | 2 | 0 | 378 | 386 |  |
| K.AIGVKPPR.G | Y | 45.05 | 836.5232 | 8 | 5.7 | 419.2712 | 2 | 25.56 | 2 | 6610 | Ana\_01.raw | 1.29E6 | 5.29E6 | 6.56E6 | 2.49E6 | 4.11E6 | 5E6 | 3.26E6 |  | 4.16E6 | 5.78E6 | 10 | 1 | 1 | 1 | 1 | 1 | 1 | 1 | 0 | 1 | 2 | 232 | 239 |  |
| K.EM(+15.99)VELPLR.H | Y | 44.28 | 1001.5215 | 8 | 4.3 | 501.7702 | 2 | 66.16 | 4 | 18435 | Ana\_03.raw |  |  |  | 3.04E6 |  |  |  |  |  |  | 1 | 0 | 0 | 0 | 1 | 0 | 0 | 0 | 0 | 0 | 0 | 218 | 225 | Oxidation (M) |
| R.RSVSDNDIR.K | Y | 41.48 | 1060.5261 | 9 | 5.7 | 531.2734 | 2 | 21.12 | 1 | 5140 | ANa\_10.raw | 0 |  |  |  |  |  |  |  |  |  | 1 | 1 | 0 | 0 | 0 | 0 | 0 | 0 | 0 | 0 | 0 | 745 | 753 |  |
| R.KGDIFLVR.G | Y | 39.86 | 946.5599 | 8 | 6.1 | 474.2901 | 2 | 57.76 | 7 | 16315 | Ana\_06.raw |  |  |  | 2.32E6 | 3.82E6 | 3.66E6 | 3.16E6 |  |  |  | 4 | 0 | 0 | 0 | 1 | 1 | 1 | 1 | 0 | 0 | 0 | 148 | 155 |  |
| K.FGM(+15.99)TPSK.G | Y | 38.97 | 782.3633 | 7 | 0.4 | 392.1891 | 2 | 20.89 | 7 | 5173 | Ana\_06.raw |  |  |  |  | 7.54E6 | 4.42E6 | 5.59E6 | 1.52E6 |  |  | 4 | 0 | 0 | 0 | 0 | 1 | 1 | 1 | 1 | 0 | 0 | 506 | 512 | Oxidation (M) |
| K.AFEEAEK.N | Y | 37.26 | 822.3759 | 7 | 2.0 | 412.1961 | 2 | 21.20 | 5 | 5365 | Ana\_04.raw |  |  |  |  | 4.47E6 |  |  |  |  |  | 1 | 0 | 0 | 0 | 0 | 1 | 0 | 0 | 0 | 0 | 0 | 289 | 295 |  |
| R.HPALFK.A | Y | 37.19 | 711.4067 | 6 | 4.9 | 356.7124 | 2 | 25.51 | 10 | 6651 | Ana\_09.raw |  |  |  |  |  |  |  |  | 1.21E7 | 4.42E6 | 2 | 0 | 0 | 0 | 0 | 0 | 0 | 0 | 0 | 1 | 1 | 226 | 231 |  |
| R.GFGSFR.F | Y | 35.96 | 669.3234 | 6 | 2.9 | 335.6700 | 2 | 44.33 | 5 | 12429 | Ana\_04.raw |  |  |  | 1.46E6 | 2.43E6 |  |  |  |  |  | 2 | 0 | 0 | 0 | 1 | 1 | 0 | 0 | 0 | 0 | 0 | 767 | 772 |  |
| K.GDIFLVR.G | Y | 35.65 | 818.4650 | 7 | 3.5 | 410.2412 | 2 | 70.15 | 2 | 19984 | Ana\_01.raw |  | 1.41E6 | 2.91E6 | 7.42E5 | 2.35E6 | 3.24E6 | 2.15E6 |  | 3.92E6 | 2.21E6 | 8 | 0 | 1 | 1 | 1 | 1 | 1 | 1 | 0 | 1 | 1 | 149 | 155 |  |
| R.SVSDNDIR.K | Y | 31.48 | 904.4250 | 8 | 0.3 | 453.2199 | 2 | 21.39 | 3 | 5403 | Ana\_02.raw |  |  | 2.96E6 | 5.95E6 |  |  |  |  |  |  | 2 | 0 | 0 | 1 | 1 | 0 | 0 | 0 | 0 | 0 | 0 | 746 | 753 |  |
| total 34 peptides |  |  |  |  |  |  |  |  |  |  |  |  |  |  |  |  |  |  |  |  |  |  |  |  |  |  |  |  |  |  |  |  |  |  |  |
| --- | --- | --- | --- | --- | --- | --- | --- | --- | --- | --- | --- | --- | --- | --- | --- | --- | --- | --- | --- | --- | --- | --- | --- | --- | --- | --- | --- | --- | --- | --- | --- | --- | --- | --- | --- |

Best Unique PSM (Scan F9:35589, m/z=918.9572, z=4, RT=128.46, ppm=4.0):

sp|P17182|ENOA\_MOUSE

back to list

  

| Protein Coverage
| Supporting Peptides
| Best Unique PSM
|

Protein Coverage:

Supporting Peptides:

| Peptide | Uniq | -10lgP | Mass | Length | ppm | m/z | z | RT | Fraction | Scan | Source File | Area 10 | Area 01 | Area 02 | Area 03 | Area 04 | Area 05 | Area 06 | Area 07 | Area 08 | Area 09 | #Spec | #Spec 10 | #Spec 01 | #Spec 02 | #Spec 03 | #Spec 04 | #Spec 05 | #Spec 06 | #Spec 07 | #Spec 08 | #Spec 09 | Start | End | PTM |
| --- | --- | --- | --- | --- | --- | --- | --- | --- | --- | --- | --- | --- | --- | --- | --- | --- | --- | --- | --- | --- | --- | --- | --- | --- | --- | --- | --- | --- | --- | --- | --- | --- | --- | --- | --- |
| R.SGETEDTFIADLVVGLC(+57.02)TGQIK.T | N | 107.52 | 2352.1519 | 22 | -0.4 | 1177.0828 | 2 | 135.91 | 9 | 37568 | Ana\_08.raw |  |  |  |  |  |  |  |  | 3.38E6 |  | 2 | 0 | 0 | 0 | 0 | 0 | 0 | 0 | 0 | 2 | 0 | 373 | 394 | Carbamidomethylation |
| K.FTASAGIQVVGDDLTVTNPK.R | Y | 106.83 | 2032.0476 | 20 | -3.6 | 1017.0274 | 2 | 97.23 | 3 | 27168 | Ana\_02.raw | 7.32E5 |  | 5.62E5 |  |  |  | 3.23E5 |  | 4.24E6 | 2.37E6 | 5 | 1 | 0 | 1 | 0 | 0 | 0 | 1 | 0 | 1 | 1 | 307 | 326 |  |
| K.LAM(+15.99)QEFM(+15.99)ILPVGASSFR.E | Y | 106.05 | 1927.9536 | 17 | 4.0 | 964.9880 | 2 | 110.11 | 3 | 30499 | Ana\_02.raw | 3.93E7 | 2.41E7 | 2.92E7 | 2.07E6 | 1.47E7 | 9.07E6 | 5.68E6 | 1.41E7 | 6.85E7 | 6.23E6 | 28 | 3 | 3 | 3 | 2 | 3 | 2 | 3 | 3 | 4 | 2 | 163 | 179 | Oxidation (M) |
| R.AAVPSGASTGIYEALELR.D | N | 105.40 | 1803.9366 | 18 | 2.6 | 902.9780 | 2 | 106.65 | 3 | 29607 | Ana\_02.raw | 6.13E7 | 4.7E7 | 3.13E7 | 8.56E6 | 3.96E7 | 2.32E7 | 3.05E7 | 7.51E6 | 2.34E8 | 5.23E7 | 45 | 5 | 4 | 3 | 3 | 4 | 4 | 3 | 4 | 9 | 6 | 33 | 50 |  |
| R.YITPDQLADLYK.S | Y | 99.60 | 1438.7344 | 12 | 1.3 | 720.3754 | 2 | 102.34 | 3 | 28510 | Ana\_02.raw | 1.04E8 | 7.94E7 | 7.77E7 | 2.54E7 | 4.65E7 | 5.98E7 | 2.65E7 | 2.13E7 | 2.11E8 | 1.36E8 | 31 | 4 | 4 | 3 | 2 | 3 | 2 | 2 | 3 | 4 | 4 | 270 | 281 |  |
| K.AGYTDQVVIGM(+15.99)DVAASEFYR.S | Y | 92.65 | 2207.0205 | 20 | 4.8 | 1104.5228 | 2 | 106.81 | 9 | 30043 | Ana\_08.raw |  |  |  |  |  |  |  |  | 2.12E6 |  | 1 | 0 | 0 | 0 | 0 | 0 | 0 | 0 | 0 | 1 | 0 | 234 | 253 | Oxidation (M) |
| K.DATNVGDEGGFAPNILENK.E | Y | 91.62 | 1959.9174 | 19 | 5.5 | 980.9714 | 2 | 92.13 | 1 | 25858 | ANa\_10.raw | 2.59E7 |  | 4.32E6 | 8.77E5 |  | 7.71E6 | 3.1E6 | 4.26E6 | 4.64E7 | 1.74E7 | 12 | 2 | 0 | 1 | 1 | 0 | 1 | 1 | 2 | 2 | 2 | 203 | 221 |  |
| K.SFVQNYPVVSIEDPFDQDDWGAWQK.F | Y | 81.84 | 2969.3508 | 25 | 3.5 | 990.7943 | 3 | 132.18 | 9 | 36578 | Ana\_08.raw | 1.15E7 | 8.9E6 |  |  | 4.22E6 | 6.69E6 | 5.54E6 |  | 1.55E7 | 4.46E6 | 7 | 1 | 1 | 0 | 0 | 1 | 1 | 1 | 0 | 1 | 1 | 282 | 306 |  |
| R.HIADLAGNPEVILPVPAFNVINGGSHAGNK.L | Y | 79.63 | 3020.5830 | 30 | -3.6 | 756.1503 | 4 | 116.24 | 9 | 32487 | Ana\_08.raw |  |  |  |  |  |  |  | 7.96E5 | 0 |  | 2 | 0 | 0 | 0 | 0 | 0 | 0 | 0 | 1 | 1 | 0 | 133 | 162 |  |
| K.VNQIGSVTESLQAC(+57.02)K.L | N | 78.29 | 1632.8141 | 15 | 2.6 | 817.4164 | 2 | 70.54 | 5 | 20099 | Ana\_04.raw |  | 3.84E6 | 3.12E6 | 9.38E5 | 2.54E6 |  | 9.35E5 | 1.67E6 | 1.88E7 |  | 7 | 0 | 1 | 1 | 1 | 1 | 0 | 1 | 1 | 1 | 0 | 344 | 358 | Carbamidomethylation |
| R.IGAEVYHNLK.N | Y | 71.12 | 1142.6084 | 10 | 4.9 | 572.3143 | 2 | 43.44 | 6 | 12219 | Ana\_05.raw |  | 1.36E7 | 2.1E7 | 3.54E6 | 1.76E7 | 2.16E7 | 1.61E7 | 1.37E7 | 2.49E7 | 3.14E7 | 20 | 0 | 2 | 2 | 2 | 3 | 2 | 2 | 3 | 1 | 3 | 184 | 193 |  |
| K.LAMQEFM(+15.99)ILPVGASSFR.E | Y | 70.60 | 1911.9586 | 17 | 2.0 | 956.9885 | 2 | 123.77 | 9 | 34402 | Ana\_08.raw | 4.84E6 |  |  |  |  |  |  | 4.8E5 | 6.36E6 | 3.04E6 | 7 | 2 | 0 | 0 | 0 | 0 | 0 | 0 | 1 | 2 | 2 | 163 | 179 | Oxidation (M) |
| K.LAM(+15.99)QEFMILPVGASSFR.E | Y | 69.68 | 1911.9586 | 17 | 4.5 | 956.9909 | 2 | 119.39 | 9 | 33290 | Ana\_08.raw | 3.49E6 |  |  |  |  |  |  |  | 9.15E6 | 2.08E6 | 3 | 1 | 0 | 0 | 0 | 0 | 0 | 0 | 0 | 1 | 1 | 163 | 179 | Oxidation (M) |
| K.KVNVVEQEK.I | Y | 68.50 | 1071.5924 | 9 | 2.7 | 536.8049 | 2 | 19.83 | 6 | 4869 | Ana\_05.raw | 6.76E7 | 3.53E7 | 3.17E7 | 1.27E7 | 2.16E7 | 2.46E7 | 1.87E7 | 1.38E7 |  | 6.19E7 | 18 | 3 | 1 | 2 | 2 | 2 | 2 | 2 | 2 | 0 | 2 | 81 | 89 |  |
| K.GVSQAVEHINK.T | Y | 65.19 | 1180.6200 | 11 | 0.6 | 591.3176 | 2 | 30.84 | 3 | 8369 | Ana\_02.raw | 2.69E7 |  | 2.77E7 | 9.62E6 | 2.47E7 | 1.36E7 | 8.88E6 | 1.01E7 |  | 2.6E7 | 11 | 1 | 0 | 2 | 2 | 2 | 1 | 1 | 1 | 0 | 1 | 61 | 71 |  |
| R.GNPTVEVDLYTAK.G | N | 60.02 | 1405.7089 | 13 | 7.8 | 703.8672 | 2 | 70.32 | 1 | 19928 | ANa\_10.raw | 2.28E6 |  | 4.63E6 |  |  |  | 1.36E6 |  | 0 | 8.25E6 | 5 | 1 | 0 | 1 | 0 | 0 | 0 | 1 | 0 | 1 | 1 | 16 | 28 |  |
| K.VNVVEQEK.I | Y | 57.99 | 943.4974 | 8 | 1.7 | 472.7568 | 2 | 22.92 | 3 | 5895 | Ana\_02.raw | 8.53E6 |  | 2.63E7 | 1.36E7 | 1.88E7 | 2.4E7 | 1.8E7 | 1.21E7 |  | 1.53E7 | 8 | 1 | 0 | 1 | 1 | 1 | 1 | 1 | 1 | 0 | 1 | 82 | 89 |  |
| R.IEEELGSK.A | Y | 56.38 | 903.4549 | 8 | 2.3 | 452.7357 | 2 | 27.24 | 6 | 7262 | Ana\_05.raw | 2.68E7 | 7.48E7 | 7.24E7 | 3.67E7 | 2.86E6 | 6.59E7 | 4.82E7 | 3.89E7 | 4.03E7 | 6.97E7 | 11 | 1 | 1 | 1 | 1 | 1 | 1 | 1 | 2 | 1 | 1 | 413 | 420 |  |
| K.LM(+15.99)IEM(+15.99)DGTENK.S | Y | 50.84 | 1311.5686 | 11 | 2.1 | 656.7930 | 2 | 33.66 | 3 | 9214 | Ana\_02.raw | 2.5E7 | 1.89E7 | 6.49E6 |  | 7.04E6 | 5.33E6 | 8.73E6 | 4.07E6 | 5.71E6 |  | 10 | 2 | 1 | 1 | 0 | 1 | 1 | 2 | 1 | 1 | 0 | 93 | 103 | Oxidation (M) |
| R.EIFDSR.G | Y | 47.44 | 765.3657 | 6 | 3.3 | 383.6914 | 2 | 35.43 | 6 | 9749 | Ana\_05.raw | 3.64E7 | 2.4E7 | 3.23E7 | 1.23E7 | 2.23E7 | 2.57E7 | 1.89E7 | 1.77E7 | 4.47E7 | 3.74E7 | 19 | 2 | 1 | 3 | 1 | 3 | 2 | 2 | 2 | 2 | 1 | 10 | 15 |  |
| K.TIAPALVSK.K | Y | 46.62 | 898.5487 | 9 | 4.1 | 450.2835 | 2 | 47.97 | 7 | 13411 | Ana\_06.raw | 5.57E7 | 3.06E7 | 3.65E7 | 1.41E7 | 2.58E7 | 3.05E7 | 2.16E7 | 2.04E7 | 1.04E8 | 4.87E7 | 18 | 2 | 2 | 1 | 2 | 2 | 1 | 2 | 2 | 2 | 2 | 72 | 80 |  |
| K.YNQILR.I | N | 46.49 | 805.4446 | 6 | 4.3 | 403.7313 | 2 | 31.44 | 9 | 8315 | Ana\_08.raw |  |  | 3.66E7 | 1.41E7 | 2.54E7 |  | 2.17E7 | 1.99E7 | 3.56E7 |  | 6 | 0 | 0 | 1 | 1 | 1 | 0 | 1 | 1 | 1 | 0 | 407 | 412 |  |
| K.SC(+57.02)NC(+57.02)LLLK.V | Y | 46.47 | 1006.4940 | 8 | 5.2 | 504.2569 | 2 | 48.18 | 10 | 13531 | Ana\_09.raw |  |  | 4.64E6 | 2.02E6 | 3.3E6 | 4.91E6 | 3.91E6 | 1.99E6 | 1.3E7 | 7.25E6 | 8 | 0 | 0 | 1 | 1 | 1 | 1 | 1 | 1 | 1 | 1 | 336 | 343 | Carbamidomethylation |
| K.EALELLK.T | Y | 43.89 | 814.4800 | 7 | 6.3 | 408.2498 | 2 | 62.18 | 1 | 17620 | ANa\_10.raw | 2.99E7 | 1.83E7 | 2.07E7 | 7.71E6 | 1.3E7 | 1.39E7 | 9.91E6 | 9.89E6 | 5.15E7 | 2.84E7 | 19 | 2 | 1 | 2 | 1 | 2 | 2 | 2 | 2 | 3 | 2 | 222 | 228 |  |
| K.YDLDFK.S | N | 41.22 | 799.3752 | 6 | 4.3 | 400.6966 | 2 | 62.17 | 1 | 17616 | ANa\_10.raw | 2.5E7 |  | 1.86E7 | 7.81E6 | 1.29E7 | 1.43E7 | 1.04E7 | 9.42E6 |  | 2.54E7 | 9 | 1 | 0 | 1 | 1 | 1 | 1 | 1 | 2 | 0 | 1 | 257 | 262 |  |
| K.GVPLYR.H | N | 36.82 | 703.4017 | 6 | 6.1 | 352.7103 | 2 | 37.00 | 10 | 10140 | Ana\_09.raw |  |  | 1.32E7 | 2.22E6 | 8.75E6 | 1.03E7 | 7.22E6 | 7.22E6 |  | 1.31E7 | 7 | 0 | 0 | 1 | 1 | 1 | 1 | 1 | 1 | 0 | 1 | 127 | 132 |  |
| R.SGKYDLDFK.S | Y | 36.59 | 1071.5237 | 9 | 6.9 | 536.7728 | 2 | 45.97 | 9 | 12825 | Ana\_08.raw |  |  |  |  |  |  |  |  | 2.67E6 |  | 1 | 0 | 0 | 0 | 0 | 0 | 0 | 0 | 0 | 1 | 0 | 254 | 262 |  |
| total 27 peptides |  |  |  |  |  |  |  |  |  |  |  |  |  |  |  |  |  |  |  |  |  |  |  |  |  |  |  |  |  |  |  |  |  |  |  |
| --- | --- | --- | --- | --- | --- | --- | --- | --- | --- | --- | --- | --- | --- | --- | --- | --- | --- | --- | --- | --- | --- | --- | --- | --- | --- | --- | --- | --- | --- | --- | --- | --- | --- | --- | --- |

Best Unique PSM (Scan F3:27168, m/z=1017.0274, z=2, RT=97.23, ppm=-3.6):

sp|Q62468|VILI\_MOUSE

back to list

  

| Protein Coverage
| Supporting Peptides
| Best Unique PSM
|

Protein Coverage:

Supporting Peptides:

| Peptide | Uniq | -10lgP | Mass | Length | ppm | m/z | z | RT | Fraction | Scan | Source File | Area 10 | Area 01 | Area 02 | Area 03 | Area 04 | Area 05 | Area 06 | Area 07 | Area 08 | Area 09 | #Spec | #Spec 10 | #Spec 01 | #Spec 02 | #Spec 03 | #Spec 04 | #Spec 05 | #Spec 06 | #Spec 07 | #Spec 08 | #Spec 09 | Start | End | PTM |
| --- | --- | --- | --- | --- | --- | --- | --- | --- | --- | --- | --- | --- | --- | --- | --- | --- | --- | --- | --- | --- | --- | --- | --- | --- | --- | --- | --- | --- | --- | --- | --- | --- | --- | --- | --- |
| K.VDVFTANTSLSSGPLPTFPLEQLVNK.S | Y | 101.10 | 2773.4539 | 26 | 0.3 | 1387.7346 | 2 | 133.34 | 9 | 36884 | Ana\_08.raw | 5.96E6 | 8.9E6 | 2.27E6 | 2.01E6 | 5.3E6 | 3.66E6 | 5.34E6 | 2.02E6 | 1.64E7 | 7.61E6 | 14 | 1 | 2 | 1 | 1 | 1 | 2 | 2 | 1 | 2 | 1 | 750 | 775 |  |
| R.KEEHLSTEDFTR.A | Y | 100.09 | 1490.7001 | 12 | -0.9 | 746.3566 | 2 | 37.87 | 3 | 10502 | Ana\_02.raw | 4.27E7 | 6.47E7 | 5.71E6 | 2.18E7 | 3.56E7 |  | 3.63E7 | 1.51E6 | 2.61E7 | 9.12E6 | 14 | 2 | 2 | 1 | 2 | 1 | 0 | 2 | 1 | 2 | 1 | 789 | 800 |  |
| K.HVETNSC(+57.02)DVQR.L | Y | 95.36 | 1343.5889 | 11 | 1.7 | 672.8029 | 2 | 18.21 | 3 | 4337 | Ana\_02.raw | 6.32E6 | 1.32E7 | 8E6 |  | 3.84E6 | 7.8E6 | 3.06E6 |  |  | 4.43E6 | 9 | 1 | 2 | 2 | 0 | 1 | 1 | 1 | 0 | 0 | 1 | 128 | 138 | Carbamidomethylation |
| R.IEDLELVPVESK.W | Y | 88.92 | 1369.7340 | 12 | 3.0 | 685.8763 | 2 | 84.82 | 6 | 24178 | Ana\_05.raw | 3.4E7 | 4.82E7 | 3.68E7 | 1.42E7 | 2.42E7 | 2.86E7 | 2.09E7 | 1.4E7 | 6.31E7 | 2.95E7 | 15 | 1 | 3 | 1 | 1 | 1 | 1 | 1 | 1 | 2 | 3 | 405 | 416 |  |
| K.SVEDLPEGVDPSR.K | Y | 81.19 | 1398.6626 | 13 | 4.3 | 700.3416 | 2 | 62.33 | 6 | 17872 | Ana\_05.raw | 3.22E7 | 5.01E7 | 3.74E7 | 1.51E7 | 2.5E7 | 3.34E7 | 2.35E7 | 1.82E7 | 4.95E7 | 2.7E7 | 20 | 2 | 2 | 2 | 1 | 2 | 2 | 2 | 2 | 3 | 2 | 776 | 788 |  |
| K.GSLNITTPGIQIWR.I | Y | 78.22 | 1554.8518 | 14 | 3.2 | 778.4357 | 2 | 107.83 | 1 | 29983 | ANa\_10.raw | 3.38E6 | 1.83E6 | 6.76E5 |  | 3.71E6 |  | 3.25E6 | 1.22E6 | 7.73E6 |  | 9 | 1 | 1 | 1 | 1 | 1 | 0 | 1 | 1 | 2 | 0 | 10 | 23 |  |
| R.EVQGNESETFR.S | Y | 77.85 | 1294.5790 | 11 | 2.4 | 648.2983 | 2 | 34.40 | 6 | 9451 | Ana\_05.raw | 4.99E7 | 8.18E7 | 5.16E7 | 2.52E7 | 4.19E7 | 5.6E7 | 3.86E7 | 2.65E7 | 5.75E7 | 4.2E7 | 26 | 2 | 3 | 3 | 2 | 3 | 3 | 2 | 3 | 3 | 2 | 98 | 108 |  |
| K.M(+15.99)VDDGSGEVQVWR.I | Y | 76.49 | 1492.6616 | 13 | 4.7 | 747.3416 | 2 | 61.88 | 9 | 17608 | Ana\_08.raw |  |  |  |  |  | 1.23E6 |  |  | 7.43E6 |  | 3 | 0 | 0 | 0 | 0 | 0 | 2 | 0 | 0 | 1 | 0 | 392 | 404 | Oxidation (M) |
| R.LQEENQVITPR.L | Y | 75.14 | 1325.6938 | 11 | 1.8 | 663.8554 | 2 | 43.23 | 3 | 12130 | Ana\_02.raw | 3.2E7 | 5.03E7 | 4.01E7 | 1.68E7 | 3.09E7 | 4.04E7 | 2.82E7 | 1.96E7 | 5.26E7 | 2.71E7 | 20 | 2 | 2 | 2 | 2 | 2 | 2 | 2 | 2 | 2 | 2 | 610 | 620 |  |
| R.GDVFLLDLGK.L | Y | 73.33 | 1075.5913 | 10 | 4.2 | 538.8052 | 2 | 109.36 | 7 | 30372 | Ana\_06.raw | 7.77E6 | 1.4E7 | 8.72E6 | 5.73E6 | 1.09E7 | 6.94E6 | 9.52E6 | 5.72E6 | 1.58E7 | 8.16E6 | 25 | 1 | 3 | 3 | 3 | 2 | 3 | 2 | 3 | 3 | 2 | 163 | 172 |  |
| R.ALGM(+15.99)TPAAFSALPR.W | Y | 71.80 | 1417.7388 | 14 | 6.6 | 709.8813 | 2 | 84.48 | 2 | 23943 | Ana\_01.raw | 1.2E7 | 1.3E7 | 9.12E6 | 2E6 | 6.54E6 |  | 6.8E6 |  | 2.53E7 |  | 7 | 1 | 1 | 1 | 1 | 1 | 0 | 1 | 0 | 1 | 0 | 801 | 814 | Oxidation (M) |
| K.AELGNSGDWSQIADEVM(+15.99)SPK.V | Y | 71.07 | 2148.9634 | 20 | 9.6 | 1075.4993 | 2 | 103.05 | 5 | 28816 | Ana\_04.raw |  |  |  |  | 1.76E6 | 2.15E6 |  |  | 3.21E6 |  | 3 | 0 | 0 | 0 | 0 | 1 | 1 | 0 | 0 | 1 | 0 | 730 | 749 | Oxidation (M) |
| R.SGAM(+15.99)SQALNFIK.A | Y | 69.16 | 1281.6387 | 12 | 4.2 | 641.8293 | 2 | 72.21 | 6 | 20721 | Ana\_05.raw | 1.23E7 | 1.83E7 | 1.63E7 | 7.01E6 | 1.16E7 | 1.96E7 | 1.15E7 | 1.07E7 | 3.36E6 | 9.47E6 | 11 | 1 | 1 | 2 | 1 | 1 | 1 | 1 | 1 | 1 | 1 | 309 | 320 | Oxidation (M) |
| K.AAISDSVVEPAAK.A | Y | 67.19 | 1256.6611 | 13 | 4.2 | 629.3405 | 2 | 48.68 | 6 | 13810 | Ana\_05.raw | 3.66E7 | 5.65E7 | 4.52E7 | 1.36E7 | 2.72E7 | 4.12E7 | 2.44E7 | 2.33E7 | 2.05E7 | 3.38E7 | 11 | 1 | 1 | 2 | 1 | 1 | 1 | 1 | 1 | 1 | 1 | 238 | 250 |  |
| R.ATSLNSNDVFILK.T | Y | 66.49 | 1420.7561 | 13 | 7.8 | 711.3909 | 2 | 92.22 | 7 | 25881 | Ana\_06.raw | 1.24E6 | 1.84E7 | 1.06E7 | 5.87E6 | 7.97E6 | 1.16E7 | 8.78E6 | 5.94E6 | 2.11E7 | 9.79E6 | 12 | 1 | 2 | 1 | 1 | 1 | 1 | 1 | 1 | 2 | 1 | 537 | 549 |  |
| R.KNNLEPVPSTR.L | Y | 65.30 | 1253.6727 | 11 | 2.8 | 418.8994 | 3 | 30.01 | 6 | 8131 | Ana\_05.raw | 1.79E7 | 3.06E7 | 1.18E7 | 7.2E6 | 7.4E6 | 1.21E7 | 8.39E6 | 6.58E6 | 2.01E7 | 1.47E7 | 15 | 2 | 2 | 1 | 1 | 1 | 1 | 1 | 1 | 2 | 3 | 506 | 516 |  |
| K.HEDC(+57.02)YILDQGGLK.I | Y | 64.67 | 1546.7086 | 13 | -3.6 | 774.3588 | 2 | 66.56 | 7 | 18889 | Ana\_06.raw |  |  |  | 4.67E6 |  |  | 4.17E6 |  |  |  | 3 | 0 | 0 | 0 | 1 | 0 | 0 | 2 | 0 | 0 | 0 | 282 | 294 | Carbamidomethylation |
| R.TYVGVVDGEK.E | Y | 63.19 | 1065.5342 | 10 | 1.7 | 533.7753 | 2 | 37.82 | 3 | 10487 | Ana\_02.raw |  | 1.47E7 | 4.36E7 | 6.27E6 | 3.29E7 | 1.47E7 | 2.89E7 | 2.02E7 | 1.04E7 | 2.76E7 | 17 | 0 | 1 | 2 | 1 | 3 | 2 | 2 | 2 | 2 | 2 | 206 | 215 |  |
| R.MVVYQGGTSR.K | Y | 62.82 | 1096.5336 | 10 | 2.6 | 549.2755 | 2 | 36.32 | 6 | 10018 | Ana\_05.raw | 1.43E7 |  | 6.61E6 | 2.42E6 | 8.34E6 | 1.49E7 | 3.64E6 | 6.75E6 | 5.95E6 | 1.24E7 | 9 | 1 | 0 | 1 | 1 | 1 | 1 | 1 | 1 | 1 | 1 | 496 | 505 |  |
| K.YNDEPVQIR.V | Y | 60.63 | 1132.5513 | 9 | 6.2 | 567.2864 | 2 | 47.15 | 10 | 13224 | Ana\_09.raw | 2.93E7 | 3.07E7 | 2.92E7 | 1.16E7 | 1.93E7 |  | 1.83E7 | 1.18E7 | 5.71E6 | 2.22E7 | 9 | 1 | 1 | 1 | 1 | 1 | 0 | 1 | 1 | 1 | 1 | 470 | 478 |  |
| K.AAATTVQEYLK.T | Y | 59.61 | 1193.6292 | 11 | 5.6 | 597.8252 | 2 | 64.62 | 10 | 18310 | Ana\_09.raw | 1.31E7 | 1.95E7 |  |  |  |  |  |  |  | 1.56E7 | 3 | 1 | 1 | 0 | 0 | 0 | 0 | 0 | 0 | 0 | 1 | 673 | 683 |  |
| K.RLQEENQVITPR.L | Y | 58.26 | 1481.7950 | 12 | 3.9 | 741.9077 | 2 | 38.59 | 2 | 10620 | Ana\_01.raw | 1.13E6 | 2.59E6 |  | 2.59E6 |  |  | 4.56E6 |  | 1.39E6 |  | 5 | 1 | 1 | 0 | 1 | 0 | 0 | 1 | 0 | 1 | 0 | 609 | 620 |  |
| R.DLETPIIVVK.Q | Y | 57.29 | 1125.6646 | 10 | -0.5 | 563.8393 | 2 | 85.61 | 3 | 24112 | Ana\_02.raw | 1.47E7 | 2.14E7 | 1.3E7 | 1.88E6 | 5.21E6 | 1.33E7 | 8.97E6 | 5.31E6 | 1.61E7 | 9.85E6 | 19 | 2 | 3 | 2 | 2 | 2 | 2 | 2 | 1 | 1 | 2 | 690 | 699 |  |
| K.NNLEPVPSTR.L | Y | 56.45 | 1125.5778 | 10 | 0.7 | 563.7966 | 2 | 36.10 | 3 | 9949 | Ana\_02.raw |  | 8.82E6 | 7.87E6 | 2.52E6 | 6.23E6 | 7.29E6 | 5.38E6 | 3.47E6 | 9.85E6 |  | 8 | 0 | 1 | 1 | 1 | 1 | 1 | 1 | 1 | 1 | 0 | 507 | 516 |  |
| K.LIIQWNGPESNR.M | Y | 55.71 | 1425.7365 | 12 | 5.0 | 713.8791 | 2 | 84.67 | 6 | 24137 | Ana\_05.raw | 1.75E6 |  |  |  |  | 1.29E6 | 2.64E5 |  |  |  | 3 | 1 | 0 | 0 | 0 | 0 | 1 | 1 | 0 | 0 | 0 | 173 | 184 |  |
| R.NVLAGEVEM(+15.99)SWK.S | Y | 54.69 | 1377.6598 | 12 | 0.7 | 689.8376 | 2 | 76.42 | 9 | 21774 | Ana\_08.raw |  |  | 2.88E6 |  |  |  |  | 1.79E6 | 7.91E6 |  | 3 | 0 | 0 | 1 | 0 | 0 | 0 | 0 | 1 | 1 | 0 | 147 | 158 | Oxidation (M) |
| R.LFEC(+57.02)SNQTGR.F | Y | 53.19 | 1210.5400 | 10 | 5.2 | 606.2805 | 2 | 34.98 | 1 | 9473 | ANa\_10.raw | 2.38E7 | 3.55E7 | 1.78E7 | 5.83E5 | 1.64E7 | 2.48E7 | 1.51E7 |  |  |  | 7 | 1 | 1 | 1 | 1 | 1 | 1 | 1 | 0 | 0 | 0 | 621 | 630 | Carbamidomethylation |
| R.M(+15.99)VVYQGGTSR.K | Y | 51.81 | 1112.5284 | 10 | 3.5 | 557.2734 | 2 | 36.49 | 6 | 10077 | Ana\_05.raw | 1.95E7 |  |  | 0 | 1.15E7 | 4E6 | 8.53E6 |  | 8.49E6 | 6.71E6 | 8 | 1 | 0 | 0 | 1 | 1 | 1 | 2 | 0 | 1 | 1 | 496 | 505 | Oxidation (M) |
| R.EVATRPLTQDLLK.H | Y | 51.50 | 1482.8406 | 13 | 2.2 | 495.2885 | 3 | 65.89 | 2 | 18783 | Ana\_01.raw | 3.86E6 | 6.15E6 |  |  |  | 1.63E6 | 3.28E6 |  | 3.37E6 |  | 6 | 1 | 1 | 0 | 0 | 0 | 1 | 1 | 0 | 2 | 0 | 269 | 281 |  |
| K.QVVVEGQEPANFWM(+15.99)ALGGK.A | Y | 50.39 | 2075.0146 | 19 | 4.5 | 1038.5193 | 2 | 130.50 | 7 | 35913 | Ana\_06.raw |  |  |  |  |  | 5.33E5 | 1.26E6 |  |  |  | 2 | 0 | 0 | 0 | 0 | 0 | 1 | 1 | 0 | 0 | 0 | 583 | 601 | Oxidation (M) |
| K.M(+15.99)VADTISR.T | Y | 44.83 | 907.4433 | 8 | 4.1 | 454.7308 | 2 | 26.08 | 6 | 6897 | Ana\_05.raw |  | 1.98E7 | 6.14E7 |  | 1.3E7 | 6.32E7 | 2.6E7 |  | 5.96E6 |  | 9 | 0 | 1 | 2 | 0 | 1 | 3 | 1 | 0 | 1 | 0 | 572 | 579 | Oxidation (M) |
| K.WTVPNR.T | Y | 44.44 | 771.4028 | 6 | 4.2 | 386.7103 | 2 | 44.66 | 2 | 12494 | Ana\_01.raw | 9.53E6 | 1.01E7 | 7.5E6 | 4.36E6 | 1.14E7 | 1.33E7 | 7.15E6 | 4.96E6 | 1.65E7 | 6.35E6 | 15 | 1 | 2 | 1 | 1 | 2 | 2 | 2 | 1 | 2 | 1 | 349 | 354 |  |
| K.AFEVTAR.A | Y | 43.12 | 792.4130 | 7 | 5.4 | 397.2159 | 2 | 37.97 | 7 | 10380 | Ana\_06.raw |  |  |  | 1.76E7 |  | 3.98E7 | 3.22E7 |  |  |  | 3 | 0 | 0 | 0 | 1 | 0 | 1 | 1 | 0 | 0 | 0 | 530 | 536 |  |
| K.LYHVSDSEGK.L | Y | 40.52 | 1133.5353 | 10 | 3.7 | 378.8538 | 3 | 26.86 | 6 | 7142 | Ana\_05.raw |  |  |  |  |  | 7.43E7 |  |  |  | 3.91E7 | 2 | 0 | 0 | 0 | 0 | 0 | 1 | 0 | 0 | 0 | 1 | 255 | 264 |  |
| K.EEHLSTEDFTR.A | Y | 38.91 | 1362.6051 | 11 | 8.8 | 682.3158 | 2 | 51.04 | 6 | 14521 | Ana\_05.raw |  |  | 4.72E6 |  |  | 3.76E6 |  |  |  | 3.25E6 | 3 | 0 | 0 | 1 | 0 | 0 | 1 | 0 | 0 | 0 | 1 | 790 | 800 |  |
| K.IFVWK.G | N | 31.25 | 691.4057 | 5 | 1.7 | 346.7107 | 2 | 66.89 | 3 | 19021 | Ana\_02.raw |  |  | 7.7E5 | 1.46E6 |  |  |  |  | 2.09E6 |  | 3 | 0 | 0 | 1 | 1 | 0 | 0 | 0 | 0 | 1 | 0 | 295 | 299 |  |
| K.THTVGSVAK.V | Y | 26.33 | 898.4872 | 9 | 2.6 | 450.2521 | 2 | 17.04 | 7 | 3952 | Ana\_06.raw |  |  |  |  |  |  | 1.81E6 |  |  |  | 1 | 0 | 0 | 0 | 0 | 0 | 0 | 1 | 0 | 0 | 0 | 361 | 369 |  |
| K.QGLVIR.K | Y | 22.30 | 684.4282 | 6 | 2.0 | 343.2221 | 2 | 36.75 | 4 | 9863 | Ana\_03.raw |  |  |  | 0 |  |  |  |  |  |  | 1 | 0 | 0 | 0 | 1 | 0 | 0 | 0 | 0 | 0 | 0 | 113 | 118 |  |
| total 38 peptides |  |  |  |  |  |  |  |  |  |  |  |  |  |  |  |  |  |  |  |  |  |  |  |  |  |  |  |  |  |  |  |  |  |  |  |
| --- | --- | --- | --- | --- | --- | --- | --- | --- | --- | --- | --- | --- | --- | --- | --- | --- | --- | --- | --- | --- | --- | --- | --- | --- | --- | --- | --- | --- | --- | --- | --- | --- | --- | --- | --- |

Best Unique PSM (Scan F9:36884, m/z=1387.7346, z=2, RT=133.34, ppm=0.3):

B1B0C7|B1B0C7\_MOUSE

back to list

  

| Protein Coverage
| Supporting Peptides
| Best Unique PSM
|

Protein Coverage:

Supporting Peptides:

| Peptide | Uniq | -10lgP | Mass | Length | ppm | m/z | z | RT | Fraction | Scan | Source File | Area 10 | Area 01 | Area 02 | Area 03 | Area 04 | Area 05 | Area 06 | Area 07 | Area 08 | Area 09 | #Spec | #Spec 10 | #Spec 01 | #Spec 02 | #Spec 03 | #Spec 04 | #Spec 05 | #Spec 06 | #Spec 07 | #Spec 08 | #Spec 09 | Start | End | PTM |
| --- | --- | --- | --- | --- | --- | --- | --- | --- | --- | --- | --- | --- | --- | --- | --- | --- | --- | --- | --- | --- | --- | --- | --- | --- | --- | --- | --- | --- | --- | --- | --- | --- | --- | --- | --- |
| R.GGSLPTHHQTHGSR.L | Y | 99.99 | 1470.7076 | 14 | 1.4 | 736.3621 | 2 | 15.74 | 3 | 3555 | Ana\_02.raw | 1.91E6 | 1.98E6 | 2.43E6 | 5.61E5 | 1.35E6 | 1.93E6 | 7.99E5 | 7.97E5 |  | 1.2E6 | 12 | 2 | 1 | 2 | 1 | 1 | 2 | 1 | 1 | 0 | 1 | 2756 | 2769 |  |
| R.EHLLM(+15.99)ALADLDELLVR.A | Y | 99.85 | 1865.9921 | 16 | 7.2 | 623.0091 | 3 | 134.91 | 6 | 37507 | Ana\_05.raw |  |  |  |  | 1.46E5 | 8.06E5 |  |  |  |  | 2 | 0 | 0 | 0 | 0 | 1 | 1 | 0 | 0 | 0 | 0 | 1479 | 1494 | Oxidation (M) |
| R.GHTPTHPGTLNQR.Q | Y | 97.14 | 1414.7065 | 13 | 2.5 | 708.3623 | 2 | 21.20 | 9 | 5022 | Ana\_08.raw | 5.52E6 | 6.78E6 | 5.02E6 | 1.79E6 | 7.18E5 | 4.03E6 |  |  | 6.35E6 | 4.59E6 | 12 | 2 | 2 | 1 | 1 | 1 | 1 | 0 | 0 | 3 | 1 | 648 | 660 |  |
| R.VVSGSGTQEASILVTIQQTLSPSHSQSVVHPVR.I | Y | 85.83 | 3427.8059 | 33 | 2.3 | 857.9608 | 4 | 124.34 | 6 | 34618 | Ana\_05.raw |  | 1.86E6 |  |  |  | 1.61E6 |  | 1.43E6 |  |  | 3 | 0 | 1 | 0 | 0 | 0 | 1 | 0 | 1 | 0 | 0 | 2497 | 2529 |  |
| R.SIEYSPQLEDASAK.E | Y | 83.97 | 1536.7307 | 14 | 2.6 | 769.3746 | 2 | 60.99 | 1 | 17277 | ANa\_10.raw | 5.98E6 |  |  |  |  |  |  |  |  |  | 1 | 1 | 0 | 0 | 0 | 0 | 0 | 0 | 0 | 0 | 0 | 93 | 106 |  |
| R.LPAIEPSDQGQYLC(+57.02)R.A | Y | 82.09 | 1745.8406 | 15 | 2.8 | 873.9300 | 2 | 77.53 | 1 | 21909 | ANa\_10.raw | 9.1E5 | 6.14E5 |  |  |  |  |  |  | 6.65E5 |  | 3 | 1 | 1 | 0 | 0 | 0 | 0 | 0 | 0 | 1 | 0 | 1919 | 1933 | Carbamidomethylation |
| R.GM(+15.99)VFGIPDGVLELVPQR.G | Y | 81.47 | 1841.9709 | 17 | 4.4 | 921.9968 | 2 | 130.88 | 1 | 35977 | ANa\_10.raw | 1.84E6 |  |  |  | 1.21E6 |  |  | 1.11E6 |  | 1.03E6 | 4 | 1 | 0 | 0 | 0 | 1 | 0 | 0 | 1 | 0 | 1 | 486 | 502 | Oxidation (M) |
| R.VIPYFTQTPYSFLPLPTIK.D | Y | 81.35 | 2224.2183 | 19 | 7.8 | 1113.1251 | 2 | 133.98 | 10 | 36553 | Ana\_09.raw | 2.87E6 | 2.48E6 | 1.36E6 | 1.35E6 | 1.27E6 | 2.67E6 | 1.06E6 | 8.24E5 | 4.99E6 | 2.34E6 | 17 | 2 | 1 | 2 | 1 | 1 | 2 | 2 | 2 | 2 | 2 | 3650 | 3668 |  |
| R.HQTHGSLLR.L | Y | 81.27 | 1047.5574 | 9 | 0.4 | 524.7862 | 2 | 18.37 | 3 | 4391 | Ana\_02.raw | 1.72E7 | 1.61E7 | 3.97E6 | 1.16E6 | 2.23E6 | 4.28E6 | 2.28E6 | 2.33E6 | 1.1E7 | 4.57E6 | 11 | 1 | 1 | 1 | 1 | 1 | 1 | 1 | 1 | 1 | 2 | 2187 | 2195 |  |
| R.LLQVTPTDSGEYVC(+57.02)R.V | Y | 75.46 | 1736.8403 | 15 | 0.2 | 869.4276 | 2 | 73.45 | 6 | 21069 | Ana\_05.raw | 3.01E5 | 1.87E6 |  |  |  | 1.06E6 |  | 6.49E5 | 0 |  | 5 | 1 | 1 | 0 | 0 | 0 | 1 | 0 | 1 | 1 | 0 | 2482 | 2496 | Carbamidomethylation |
| R.LPQVSPADSGDYVC(+57.02)R.V | Y | 74.77 | 1662.7671 | 15 | 5.5 | 832.3954 | 2 | 65.54 | 9 | 18692 | Ana\_08.raw |  |  |  |  |  |  |  |  | 2.77E6 |  | 1 | 0 | 0 | 0 | 0 | 0 | 0 | 0 | 0 | 1 | 0 | 2097 | 2111 | Carbamidomethylation |
| R.YELGSGLAVLR.S | Y | 72.58 | 1176.6503 | 11 | 0.5 | 589.3327 | 2 | 90.53 | 3 | 25405 | Ana\_02.raw | 4.61E5 | 2.32E6 | 7.67E5 | 8.43E5 |  | 1.31E6 | 9.75E5 |  |  | 1.07E6 | 7 | 1 | 1 | 1 | 1 | 0 | 1 | 1 | 0 | 0 | 1 | 3978 | 3988 |  |
| R.LHQVSPVDSGEYVC(+57.02)R.V | Y | 71.54 | 1744.8202 | 15 | 2.6 | 582.6155 | 3 | 54.46 | 6 | 15532 | Ana\_05.raw | 3.1E6 | 3.99E6 |  |  |  | 3.64E6 |  | 1.77E6 | 4.96E6 | 2.61E6 | 7 | 1 | 1 | 0 | 0 | 0 | 1 | 0 | 1 | 2 | 1 | 2388 | 2402 | Carbamidomethylation |
| R.ALSSAGQHVAR.A | Y | 66.23 | 1095.5785 | 11 | 5.8 | 366.2022 | 3 | 21.31 | 2 | 5177 | Ana\_01.raw | 6.77E6 | 6.91E6 | 4E6 | 1.19E6 |  |  | 2.37E6 | 4.44E6 | 9.5E6 | 3.54E6 | 12 | 2 | 2 | 1 | 1 | 0 | 0 | 1 | 2 | 2 | 1 | 1934 | 1944 |  |
| K.AFAYLQVPER.V | Y | 63.95 | 1192.6240 | 10 | 3.0 | 597.3211 | 2 | 81.33 | 1 | 22948 | ANa\_10.raw | 2.54E6 | 2.67E6 |  | 8.78E5 |  | 2.68E6 |  | 1.4E6 | 5.74E6 | 2.44E6 | 7 | 1 | 1 | 0 | 1 | 0 | 1 | 0 | 1 | 1 | 1 | 3640 | 3649 |  |
| K.SPAYTLVWTR.L | Y | 62.52 | 1192.6240 | 10 | 0.9 | 597.3198 | 2 | 82.74 | 9 | 23540 | Ana\_08.raw |  |  |  |  | 1.93E5 | 9.35E5 | 2.72E5 | 5.39E5 | 2.41E6 |  | 5 | 0 | 0 | 0 | 0 | 1 | 1 | 1 | 1 | 1 | 0 | 1798 | 1807 |  |
| R.GSLGTSGETC(+57.02)R.C | Y | 60.65 | 1123.4928 | 11 | 4.4 | 562.7562 | 2 | 22.45 | 1 | 5584 | ANa\_10.raw | 3.61E6 | 4.07E6 |  |  |  |  |  |  |  |  | 2 | 1 | 1 | 0 | 0 | 0 | 0 | 0 | 0 | 0 | 0 | 883 | 893 | Carbamidomethylation |
| R.HPTPLALGQFHTVTLLR.S | Y | 58.79 | 1900.0682 | 17 | 2.2 | 634.3647 | 3 | 95.17 | 1 | 26672 | ANa\_10.raw | 2.04E5 |  |  |  |  |  |  |  | 6.22E5 |  | 2 | 1 | 0 | 0 | 0 | 0 | 0 | 0 | 0 | 1 | 0 | 3733 | 3749 |  |
| R.IAHVELADAGQYR.C | Y | 56.57 | 1441.7313 | 13 | 0.8 | 481.5848 | 3 | 58.23 | 6 | 16650 | Ana\_05.raw |  | 1.39E6 | 6.59E5 |  | 1.46E6 | 3.04E6 |  |  | 3.84E6 | 1.15E6 | 6 | 0 | 1 | 1 | 0 | 1 | 1 | 0 | 0 | 1 | 1 | 3530 | 3542 |  |
| K.AGLSSGFVGC(+57.02)VR.E | Y | 55.95 | 1208.5972 | 12 | 7.6 | 605.3104 | 2 | 67.63 | 6 | 19428 | Ana\_05.raw |  |  |  |  |  | 2.73E6 |  |  |  |  | 1 | 0 | 0 | 0 | 0 | 0 | 1 | 0 | 0 | 0 | 0 | 3795 | 3806 | Carbamidomethylation |
| K.VDSYGGFLR.Y | Y | 55.43 | 1012.4977 | 9 | 2.9 | 507.2576 | 2 | 70.23 | 5 | 20010 | Ana\_04.raw | 1.57E6 | 2.43E6 |  |  | 2.56E6 | 2.24E6 | 1.12E6 | 1.97E6 |  | 1.07E6 | 7 | 1 | 1 | 0 | 0 | 1 | 1 | 1 | 1 | 0 | 1 | 607 | 615 |  |
| R.IEGNTLVIPR.V | Y | 55.33 | 1110.6396 | 10 | 3.4 | 556.3290 | 2 | 65.92 | 4 | 18372 | Ana\_03.raw | 3.38E6 | 5.04E5 | 4.04E6 | 1.58E6 | 3.49E6 | 3.48E6 |  | 2.25E6 | 3.04E6 | 2.77E6 | 9 | 1 | 1 | 1 | 1 | 1 | 1 | 0 | 1 | 1 | 1 | 3246 | 3255 |  |
| R.EVEQC(+57.02)TC(+57.02)PPGYR.G | Y | 52.90 | 1494.6232 | 12 | 7.1 | 748.3242 | 2 | 38.25 | 10 | 10520 | Ana\_09.raw |  |  |  |  |  |  |  |  |  | 6.86E6 | 1 | 0 | 0 | 0 | 0 | 0 | 0 | 0 | 0 | 0 | 1 | 1122 | 1133 | Carbamidomethylation |
| K.VTSYGGELR.F | Y | 52.65 | 980.4927 | 9 | 5.6 | 491.2564 | 2 | 36.75 | 10 | 10062 | Ana\_09.raw | 2.16E7 | 8.57E6 | 9.91E6 | 4.01E6 | 7.53E6 | 8.38E6 | 5.94E6 | 5.12E6 |  | 8.65E6 | 11 | 2 | 1 | 1 | 1 | 1 | 1 | 1 | 1 | 0 | 2 | 1002 | 1010 |  |
| R.SYEIIFR.E | Y | 51.68 | 926.4861 | 7 | 5.1 | 464.2527 | 2 | 78.78 | 8 | 22098 | Ana\_07.raw | 1.36E6 | 1.8E6 | 2.04E6 | 8.93E5 | 9.81E5 | 1.36E6 | 1.21E6 | 1.26E6 | 3.44E6 | 9.04E5 | 10 | 1 | 1 | 1 | 1 | 1 | 1 | 1 | 1 | 1 | 1 | 1458 | 1464 |  |
| K.ESLEVQIHPSR.S | Y | 50.91 | 1293.6677 | 11 | 2.5 | 647.8428 | 2 | 49.24 | 6 | 13987 | Ana\_05.raw |  | 3.15E5 |  |  |  | 4.84E5 |  |  |  |  | 2 | 0 | 1 | 0 | 0 | 0 | 1 | 0 | 0 | 0 | 0 | 1674 | 1684 |  |
| R.SPTNLANR.Q | Y | 48.13 | 871.4512 | 8 | 0.7 | 436.7332 | 2 | 20.98 | 3 | 5266 | Ana\_02.raw |  |  | 1.08E7 |  |  | 1.43E7 |  | 1.05E7 |  |  | 3 | 0 | 0 | 1 | 0 | 0 | 1 | 0 | 1 | 0 | 0 | 3697 | 3704 |  |
| R.NLIHTSNSR.A | Y | 47.08 | 1040.5363 | 9 | 6.2 | 347.8549 | 3 | 21.59 | 9 | 5156 | Ana\_08.raw | 1.93E6 | 9.63E5 | 9.97E5 |  | 1.7E6 |  |  |  | 2.97E6 | 1.18E6 | 7 | 1 | 1 | 1 | 0 | 1 | 0 | 0 | 0 | 2 | 1 | 1749 | 1757 |  |
| K.RGGSLPAR.H | Y | 45.88 | 812.4617 | 8 | 3.9 | 407.2397 | 2 | 18.28 | 6 | 4360 | Ana\_05.raw |  |  |  | 2.04E6 | 3.81E6 | 4.87E6 |  | 2.56E6 |  |  | 4 | 0 | 0 | 0 | 1 | 1 | 1 | 0 | 1 | 0 | 0 | 2179 | 2186 |  |
| R.ALEVEEC(+57.02)R.C | Y | 45.83 | 1004.4597 | 8 | 5.2 | 503.2397 | 2 | 35.25 | 6 | 9694 | Ana\_05.raw |  | 6.48E6 | 4.48E6 | 1.53E6 |  | 1.17E7 | 2.76E6 | 3.16E6 |  | 3.79E6 | 7 | 0 | 1 | 1 | 1 | 0 | 1 | 1 | 1 | 0 | 1 | 1524 | 1531 | Carbamidomethylation |
| R.ATFSSVPR.A | Y | 45.03 | 863.4501 | 8 | 4.9 | 432.7344 | 2 | 40.65 | 7 | 11185 | Ana\_06.raw | 9.27E6 | 1.13E7 | 1.31E7 | 4.51E6 | 9.16E6 | 1.15E7 | 6.91E6 | 7.14E6 | 4.4E6 | 9.34E6 | 10 | 1 | 1 | 1 | 1 | 1 | 1 | 1 | 1 | 1 | 1 | 1495 | 1502 |  |
| R.EVSEAVVEK.L | Y | 40.88 | 988.5076 | 9 | 7.6 | 495.2648 | 2 | 29.44 | 6 | 7954 | Ana\_05.raw |  |  |  |  |  | 1.72E7 |  |  |  |  | 1 | 0 | 0 | 0 | 0 | 0 | 1 | 0 | 0 | 0 | 0 | 110 | 118 |  |
| R.LEPTVPEDSGR.Y | Y | 39.69 | 1198.5829 | 11 | 6.4 | 600.3026 | 2 | 41.33 | 2 | 11468 | Ana\_01.raw | 8.29E6 | 1.14E7 | 4.85E6 |  |  |  |  |  | 3.5E6 | 7.07E6 | 5 | 1 | 1 | 1 | 0 | 0 | 0 | 0 | 0 | 1 | 1 | 3340 | 3350 |  |
| R.FDAGSGM(+15.99)ATIR.H | Y | 37.14 | 1140.5233 | 11 | 3.1 | 571.2707 | 2 | 42.64 | 8 | 11626 | Ana\_07.raw | 3.16E6 | 4.42E6 | 5.65E6 |  | 3.24E6 |  |  | 3.15E6 | 4.32E6 | 1.79E6 | 7 | 1 | 1 | 1 | 0 | 1 | 0 | 0 | 1 | 1 | 1 | 3722 | 3732 | Oxidation (M) |
| R.VQVSPER.T | Y | 36.46 | 813.4344 | 7 | 6.3 | 407.7271 | 2 | 22.68 | 8 | 5652 | Ana\_07.raw |  |  |  |  |  | 6.8E6 | 3.04E6 | 2.59E6 |  |  | 3 | 0 | 0 | 0 | 0 | 0 | 1 | 1 | 1 | 0 | 0 | 1957 | 1963 |  |
| R.LSFDQPNDFK.G | Y | 34.81 | 1209.5665 | 10 | 1.2 | 605.7913 | 2 | 73.22 | 4 | 20369 | Ana\_03.raw |  |  |  | 1.35E6 |  |  |  |  |  |  | 1 | 0 | 0 | 0 | 1 | 0 | 0 | 0 | 0 | 0 | 0 | 542 | 551 |  |
| R.VDGGRPVLR.S | Y | 27.17 | 967.5563 | 9 | 7.3 | 484.7889 | 2 | 23.75 | 10 | 6082 | Ana\_09.raw | 1.24E6 |  |  |  |  |  | 9.74E5 |  |  | 1.18E6 | 3 | 1 | 0 | 0 | 0 | 0 | 0 | 1 | 0 | 0 | 1 | 4014 | 4022 |  |
| total 37 peptides |  |  |  |  |  |  |  |  |  |  |  |  |  |  |  |  |  |  |  |  |  |  |  |  |  |  |  |  |  |  |  |  |  |  |  |
| --- | --- | --- | --- | --- | --- | --- | --- | --- | --- | --- | --- | --- | --- | --- | --- | --- | --- | --- | --- | --- | --- | --- | --- | --- | --- | --- | --- | --- | --- | --- | --- | --- | --- | --- | --- |

Best Unique PSM (Scan F3:3555, m/z=736.3621, z=2, RT=15.74, ppm=1.4):

E9PZ16|E9PZ16\_MOUSE

back to list

  

| Protein Coverage
| Supporting Peptides
| Best Unique PSM
|

Protein Coverage:

Supporting Peptides:

| Peptide | Uniq | -10lgP | Mass | Length | ppm | m/z | z | RT | Fraction | Scan | Source File | Area 10 | Area 01 | Area 02 | Area 03 | Area 04 | Area 05 | Area 06 | Area 07 | Area 08 | Area 09 | #Spec | #Spec 10 | #Spec 01 | #Spec 02 | #Spec 03 | #Spec 04 | #Spec 05 | #Spec 06 | #Spec 07 | #Spec 08 | #Spec 09 | Start | End | PTM |
| --- | --- | --- | --- | --- | --- | --- | --- | --- | --- | --- | --- | --- | --- | --- | --- | --- | --- | --- | --- | --- | --- | --- | --- | --- | --- | --- | --- | --- | --- | --- | --- | --- | --- | --- | --- |
| R.GGSLPTHHQTHGSR.L | Y | 99.99 | 1470.7076 | 14 | 1.4 | 736.3621 | 2 | 15.74 | 3 | 3555 | Ana\_02.raw | 1.91E6 | 1.98E6 | 2.43E6 | 5.61E5 | 1.35E6 | 1.93E6 | 7.99E5 | 7.97E5 |  | 1.2E6 | 12 | 2 | 1 | 2 | 1 | 1 | 2 | 1 | 1 | 0 | 1 | 2762 | 2775 |  |
| R.EHLLM(+15.99)ALADLDELLVR.A | Y | 99.85 | 1865.9921 | 16 | 7.2 | 623.0091 | 3 | 134.91 | 6 | 37507 | Ana\_05.raw |  |  |  |  | 1.46E5 | 8.06E5 |  |  |  |  | 2 | 0 | 0 | 0 | 0 | 1 | 1 | 0 | 0 | 0 | 0 | 1479 | 1494 | Oxidation (M) |
| R.GHTPTHPGTLNQR.Q | Y | 97.14 | 1414.7065 | 13 | 2.5 | 708.3623 | 2 | 21.20 | 9 | 5022 | Ana\_08.raw | 5.52E6 | 6.78E6 | 5.02E6 | 1.79E6 | 7.18E5 | 4.03E6 |  |  | 6.35E6 | 4.59E6 | 12 | 2 | 2 | 1 | 1 | 1 | 1 | 0 | 0 | 3 | 1 | 648 | 660 |  |
| R.VVSGSGTQEASILVTIQQTLSPSHSQSVVHPVR.I | Y | 85.83 | 3427.8059 | 33 | 2.3 | 857.9608 | 4 | 124.34 | 6 | 34618 | Ana\_05.raw |  | 1.86E6 |  |  |  | 1.61E6 |  | 1.43E6 |  |  | 3 | 0 | 1 | 0 | 0 | 0 | 1 | 0 | 1 | 0 | 0 | 2503 | 2535 |  |
| R.SIEYSPQLEDASAK.E | Y | 83.97 | 1536.7307 | 14 | 2.6 | 769.3746 | 2 | 60.99 | 1 | 17277 | ANa\_10.raw | 5.98E6 |  |  |  |  |  |  |  |  |  | 1 | 1 | 0 | 0 | 0 | 0 | 0 | 0 | 0 | 0 | 0 | 93 | 106 |  |
| R.LPAIEPSDQGQYLC(+57.02)R.A | Y | 82.09 | 1745.8406 | 15 | 2.8 | 873.9300 | 2 | 77.53 | 1 | 21909 | ANa\_10.raw | 9.1E5 | 6.14E5 |  |  |  |  |  |  | 6.65E5 |  | 3 | 1 | 1 | 0 | 0 | 0 | 0 | 0 | 0 | 1 | 0 | 1919 | 1933 | Carbamidomethylation |
| R.GM(+15.99)VFGIPDGVLELVPQR.G | Y | 81.47 | 1841.9709 | 17 | 4.4 | 921.9968 | 2 | 130.88 | 1 | 35977 | ANa\_10.raw | 1.84E6 |  |  |  | 1.21E6 |  |  | 1.11E6 |  | 1.03E6 | 4 | 1 | 0 | 0 | 0 | 1 | 0 | 0 | 1 | 0 | 1 | 486 | 502 | Oxidation (M) |
| R.VIPYFTQTPYSFLPLPTIK.D | Y | 81.35 | 2224.2183 | 19 | 7.8 | 1113.1251 | 2 | 133.98 | 10 | 36553 | Ana\_09.raw | 2.87E6 | 2.48E6 | 1.36E6 | 1.35E6 | 1.27E6 | 2.67E6 | 1.06E6 | 8.24E5 | 4.99E6 | 2.34E6 | 17 | 2 | 1 | 2 | 1 | 1 | 2 | 2 | 2 | 2 | 2 | 3658 | 3676 |  |
| R.HQTHGSLLR.L | Y | 81.27 | 1047.5574 | 9 | 0.4 | 524.7862 | 2 | 18.37 | 3 | 4391 | Ana\_02.raw | 1.72E7 | 1.61E7 | 3.97E6 | 1.16E6 | 2.23E6 | 4.28E6 | 2.28E6 | 2.33E6 | 1.1E7 | 4.57E6 | 11 | 1 | 1 | 1 | 1 | 1 | 1 | 1 | 1 | 1 | 2 | 2193 | 2201 |  |
| R.LLQVTPTDSGEYVC(+57.02)R.V | Y | 75.46 | 1736.8403 | 15 | 0.2 | 869.4276 | 2 | 73.45 | 6 | 21069 | Ana\_05.raw | 3.01E5 | 1.87E6 |  |  |  | 1.06E6 |  | 6.49E5 | 0 |  | 5 | 1 | 1 | 0 | 0 | 0 | 1 | 0 | 1 | 1 | 0 | 2488 | 2502 | Carbamidomethylation |
| R.LPQVSPADSGDYVC(+57.02)R.V | Y | 74.77 | 1662.7671 | 15 | 5.5 | 832.3954 | 2 | 65.54 | 9 | 18692 | Ana\_08.raw |  |  |  |  |  |  |  |  | 2.77E6 |  | 1 | 0 | 0 | 0 | 0 | 0 | 0 | 0 | 0 | 1 | 0 | 2103 | 2117 | Carbamidomethylation |
| R.YELGSGLAVLR.S | Y | 72.58 | 1176.6503 | 11 | 0.5 | 589.3327 | 2 | 90.53 | 3 | 25405 | Ana\_02.raw | 4.61E5 | 2.32E6 | 7.67E5 | 8.43E5 |  | 1.31E6 | 9.75E5 |  |  | 1.07E6 | 7 | 1 | 1 | 1 | 1 | 0 | 1 | 1 | 0 | 0 | 1 | 3986 | 3996 |  |
| R.LHQVSPVDSGEYVC(+57.02)R.V | Y | 71.54 | 1744.8202 | 15 | 2.6 | 582.6155 | 3 | 54.46 | 6 | 15532 | Ana\_05.raw | 3.1E6 | 3.99E6 |  |  |  | 3.64E6 |  | 1.77E6 | 4.96E6 | 2.61E6 | 7 | 1 | 1 | 0 | 0 | 0 | 1 | 0 | 1 | 2 | 1 | 2394 | 2408 | Carbamidomethylation |
| R.ALSSAGQHVAR.A | Y | 66.23 | 1095.5785 | 11 | 5.8 | 366.2022 | 3 | 21.31 | 2 | 5177 | Ana\_01.raw | 6.77E6 | 6.91E6 | 4E6 | 1.19E6 |  |  | 2.37E6 | 4.44E6 | 9.5E6 | 3.54E6 | 12 | 2 | 2 | 1 | 1 | 0 | 0 | 1 | 2 | 2 | 1 | 1934 | 1944 |  |
| K.AFAYLQVPER.V | Y | 63.95 | 1192.6240 | 10 | 3.0 | 597.3211 | 2 | 81.33 | 1 | 22948 | ANa\_10.raw | 2.54E6 | 2.67E6 |  | 8.78E5 |  | 2.68E6 |  | 1.4E6 | 5.74E6 | 2.44E6 | 7 | 1 | 1 | 0 | 1 | 0 | 1 | 0 | 1 | 1 | 1 | 3648 | 3657 |  |
| K.SPAYTLVWTR.L | Y | 62.52 | 1192.6240 | 10 | 0.9 | 597.3198 | 2 | 82.74 | 9 | 23540 | Ana\_08.raw |  |  |  |  | 1.93E5 | 9.35E5 | 2.72E5 | 5.39E5 | 2.41E6 |  | 5 | 0 | 0 | 0 | 0 | 1 | 1 | 1 | 1 | 1 | 0 | 1798 | 1807 |  |
| R.GSLGTSGETC(+57.02)R.C | Y | 60.65 | 1123.4928 | 11 | 4.4 | 562.7562 | 2 | 22.45 | 1 | 5584 | ANa\_10.raw | 3.61E6 | 4.07E6 |  |  |  |  |  |  |  |  | 2 | 1 | 1 | 0 | 0 | 0 | 0 | 0 | 0 | 0 | 0 | 883 | 893 | Carbamidomethylation |
| R.HPTPLALGQFHTVTLLR.S | Y | 58.79 | 1900.0682 | 17 | 2.2 | 634.3647 | 3 | 95.17 | 1 | 26672 | ANa\_10.raw | 2.04E5 |  |  |  |  |  |  |  | 6.22E5 |  | 2 | 1 | 0 | 0 | 0 | 0 | 0 | 0 | 0 | 1 | 0 | 3741 | 3757 |  |
| R.IAHVELADAGQYR.C | Y | 56.57 | 1441.7313 | 13 | 0.8 | 481.5848 | 3 | 58.23 | 6 | 16650 | Ana\_05.raw |  | 1.39E6 | 6.59E5 |  | 1.46E6 | 3.04E6 |  |  | 3.84E6 | 1.15E6 | 6 | 0 | 1 | 1 | 0 | 1 | 1 | 0 | 0 | 1 | 1 | 3538 | 3550 |  |
| K.AGLSSGFVGC(+57.02)VR.E | Y | 55.95 | 1208.5972 | 12 | 7.6 | 605.3104 | 2 | 67.63 | 6 | 19428 | Ana\_05.raw |  |  |  |  |  | 2.73E6 |  |  |  |  | 1 | 0 | 0 | 0 | 0 | 0 | 1 | 0 | 0 | 0 | 0 | 3803 | 3814 | Carbamidomethylation |
| K.VDSYGGFLR.Y | Y | 55.43 | 1012.4977 | 9 | 2.9 | 507.2576 | 2 | 70.23 | 5 | 20010 | Ana\_04.raw | 1.57E6 | 2.43E6 |  |  | 2.56E6 | 2.24E6 | 1.12E6 | 1.97E6 |  | 1.07E6 | 7 | 1 | 1 | 0 | 0 | 1 | 1 | 1 | 1 | 0 | 1 | 607 | 615 |  |
| R.IEGNTLVIPR.V | Y | 55.33 | 1110.6396 | 10 | 3.4 | 556.3290 | 2 | 65.92 | 4 | 18372 | Ana\_03.raw | 3.38E6 | 5.04E5 | 4.04E6 | 1.58E6 | 3.49E6 | 3.48E6 |  | 2.25E6 | 3.04E6 | 2.77E6 | 9 | 1 | 1 | 1 | 1 | 1 | 1 | 0 | 1 | 1 | 1 | 3254 | 3263 |  |
| R.EVEQC(+57.02)TC(+57.02)PPGYR.G | Y | 52.90 | 1494.6232 | 12 | 7.1 | 748.3242 | 2 | 38.25 | 10 | 10520 | Ana\_09.raw |  |  |  |  |  |  |  |  |  | 6.86E6 | 1 | 0 | 0 | 0 | 0 | 0 | 0 | 0 | 0 | 0 | 1 | 1122 | 1133 | Carbamidomethylation |
| K.VTSYGGELR.F | Y | 52.65 | 980.4927 | 9 | 5.6 | 491.2564 | 2 | 36.75 | 10 | 10062 | Ana\_09.raw | 2.16E7 | 8.57E6 | 9.91E6 | 4.01E6 | 7.53E6 | 8.38E6 | 5.94E6 | 5.12E6 |  | 8.65E6 | 11 | 2 | 1 | 1 | 1 | 1 | 1 | 1 | 1 | 0 | 2 | 1002 | 1010 |  |
| R.SYEIIFR.E | Y | 51.68 | 926.4861 | 7 | 5.1 | 464.2527 | 2 | 78.78 | 8 | 22098 | Ana\_07.raw | 1.36E6 | 1.8E6 | 2.04E6 | 8.93E5 | 9.81E5 | 1.36E6 | 1.21E6 | 1.26E6 | 3.44E6 | 9.04E5 | 10 | 1 | 1 | 1 | 1 | 1 | 1 | 1 | 1 | 1 | 1 | 1458 | 1464 |  |
| K.ESLEVQIHPSR.S | Y | 50.91 | 1293.6677 | 11 | 2.5 | 647.8428 | 2 | 49.24 | 6 | 13987 | Ana\_05.raw |  | 3.15E5 |  |  |  | 4.84E5 |  |  |  |  | 2 | 0 | 1 | 0 | 0 | 0 | 1 | 0 | 0 | 0 | 0 | 1674 | 1684 |  |
| R.SPTNLANR.Q | Y | 48.13 | 871.4512 | 8 | 0.7 | 436.7332 | 2 | 20.98 | 3 | 5266 | Ana\_02.raw |  |  | 1.08E7 |  |  | 1.43E7 |  | 1.05E7 |  |  | 3 | 0 | 0 | 1 | 0 | 0 | 1 | 0 | 1 | 0 | 0 | 3705 | 3712 |  |
| R.NLIHTSNSR.A | Y | 47.08 | 1040.5363 | 9 | 6.2 | 347.8549 | 3 | 21.59 | 9 | 5156 | Ana\_08.raw | 1.93E6 | 9.63E5 | 9.97E5 |  | 1.7E6 |  |  |  | 2.97E6 | 1.18E6 | 7 | 1 | 1 | 1 | 0 | 1 | 0 | 0 | 0 | 2 | 1 | 1749 | 1757 |  |
| K.RGGSLPAR.H | Y | 45.88 | 812.4617 | 8 | 3.9 | 407.2397 | 2 | 18.28 | 6 | 4360 | Ana\_05.raw |  |  |  | 2.04E6 | 3.81E6 | 4.87E6 |  | 2.56E6 |  |  | 4 | 0 | 0 | 0 | 1 | 1 | 1 | 0 | 1 | 0 | 0 | 2185 | 2192 |  |
| R.ALEVEEC(+57.02)R.C | Y | 45.83 | 1004.4597 | 8 | 5.2 | 503.2397 | 2 | 35.25 | 6 | 9694 | Ana\_05.raw |  | 6.48E6 | 4.48E6 | 1.53E6 |  | 1.17E7 | 2.76E6 | 3.16E6 |  | 3.79E6 | 7 | 0 | 1 | 1 | 1 | 0 | 1 | 1 | 1 | 0 | 1 | 1524 | 1531 | Carbamidomethylation |
| R.ATFSSVPR.A | Y | 45.03 | 863.4501 | 8 | 4.9 | 432.7344 | 2 | 40.65 | 7 | 11185 | Ana\_06.raw | 9.27E6 | 1.13E7 | 1.31E7 | 4.51E6 | 9.16E6 | 1.15E7 | 6.91E6 | 7.14E6 | 4.4E6 | 9.34E6 | 10 | 1 | 1 | 1 | 1 | 1 | 1 | 1 | 1 | 1 | 1 | 1495 | 1502 |  |
| R.EVSEAVVEK.L | Y | 40.88 | 988.5076 | 9 | 7.6 | 495.2648 | 2 | 29.44 | 6 | 7954 | Ana\_05.raw |  |  |  |  |  | 1.72E7 |  |  |  |  | 1 | 0 | 0 | 0 | 0 | 0 | 1 | 0 | 0 | 0 | 0 | 110 | 118 |  |
| R.LEPTVPEDSGR.Y | Y | 39.69 | 1198.5829 | 11 | 6.4 | 600.3026 | 2 | 41.33 | 2 | 11468 | Ana\_01.raw | 8.29E6 | 1.14E7 | 4.85E6 |  |  |  |  |  | 3.5E6 | 7.07E6 | 5 | 1 | 1 | 1 | 0 | 0 | 0 | 0 | 0 | 1 | 1 | 3348 | 3358 |  |
| R.FDAGSGM(+15.99)ATIR.H | Y | 37.14 | 1140.5233 | 11 | 3.1 | 571.2707 | 2 | 42.64 | 8 | 11626 | Ana\_07.raw | 3.16E6 | 4.42E6 | 5.65E6 |  | 3.24E6 |  |  | 3.15E6 | 4.32E6 | 1.79E6 | 7 | 1 | 1 | 1 | 0 | 1 | 0 | 0 | 1 | 1 | 1 | 3730 | 3740 | Oxidation (M) |
| R.VQVSPER.T | Y | 36.46 | 813.4344 | 7 | 6.3 | 407.7271 | 2 | 22.68 | 8 | 5652 | Ana\_07.raw |  |  |  |  |  | 6.8E6 | 3.04E6 | 2.59E6 |  |  | 3 | 0 | 0 | 0 | 0 | 0 | 1 | 1 | 1 | 0 | 0 | 1957 | 1963 |  |
| R.LSFDQPNDFK.G | Y | 34.81 | 1209.5665 | 10 | 1.2 | 605.7913 | 2 | 73.22 | 4 | 20369 | Ana\_03.raw |  |  |  | 1.35E6 |  |  |  |  |  |  | 1 | 0 | 0 | 0 | 1 | 0 | 0 | 0 | 0 | 0 | 0 | 542 | 551 |  |
| R.VDGGRPVLR.S | Y | 27.17 | 967.5563 | 9 | 7.3 | 484.7889 | 2 | 23.75 | 10 | 6082 | Ana\_09.raw | 1.24E6 |  |  |  |  |  | 9.74E5 |  |  | 1.18E6 | 3 | 1 | 0 | 0 | 0 | 0 | 0 | 1 | 0 | 0 | 1 | 4022 | 4030 |  |
| total 37 peptides |  |  |  |  |  |  |  |  |  |  |  |  |  |  |  |  |  |  |  |  |  |  |  |  |  |  |  |  |  |  |  |  |  |  |  |
| --- | --- | --- | --- | --- | --- | --- | --- | --- | --- | --- | --- | --- | --- | --- | --- | --- | --- | --- | --- | --- | --- | --- | --- | --- | --- | --- | --- | --- | --- | --- | --- | --- | --- | --- | --- |

Best Unique PSM (Scan F3:3555, m/z=736.3621, z=2, RT=15.74, ppm=1.4):

sp|Q8BMS1|ECHA\_MOUSE

back to list

  

| Protein Coverage
| Supporting Peptides
| Best Unique PSM
|

Protein Coverage:

Supporting Peptides:

| Peptide | Uniq | -10lgP | Mass | Length | ppm | m/z | z | RT | Fraction | Scan | Source File | Area 10 | Area 01 | Area 02 | Area 03 | Area 04 | Area 05 | Area 06 | Area 07 | Area 08 | Area 09 | #Spec | #Spec 10 | #Spec 01 | #Spec 02 | #Spec 03 | #Spec 04 | #Spec 05 | #Spec 06 | #Spec 07 | #Spec 08 | #Spec 09 | Start | End | PTM |
| --- | --- | --- | --- | --- | --- | --- | --- | --- | --- | --- | --- | --- | --- | --- | --- | --- | --- | --- | --- | --- | --- | --- | --- | --- | --- | --- | --- | --- | --- | --- | --- | --- | --- | --- | --- |
| K.KLDALTTGFGFPVGAATLADEVGVDVAQHVAEDLGK.A | Y | 133.13 | 3610.8518 | 36 | 2.7 | 903.7227 | 4 | 139.27 | 9 | 38434 | Ana\_08.raw | 3.72E6 | 2.07E6 |  |  |  | 1.35E6 |  |  | 8.13E6 | 3.27E6 | 6 | 1 | 1 | 0 | 0 | 0 | 1 | 0 | 0 | 1 | 2 | 570 | 605 |  |
| R.DSIFSNLIGQLDYK.G | Y | 99.94 | 1611.8145 | 14 | 2.0 | 806.9161 | 2 | 131.78 | 6 | 36622 | Ana\_05.raw | 8.28E6 | 7.54E6 |  | 1.86E6 | 4.45E6 | 9.27E6 | 5.11E6 | 3.98E6 |  | 6.66E6 | 9 | 1 | 1 | 0 | 2 | 1 | 1 | 1 | 1 | 0 | 1 | 423 | 436 |  |
| R.TIEYLEEVAVNFAK.G | Y | 95.57 | 1624.8348 | 14 | 4.5 | 813.4283 | 2 | 129.66 | 6 | 36021 | Ana\_05.raw | 3.78E6 |  | 2.84E6 | 1.08E6 | 2.99E6 | 6.59E6 | 6.76E5 | 2.71E6 | 6.5E6 | 4.88E6 | 12 | 1 | 0 | 1 | 1 | 2 | 2 | 1 | 1 | 2 | 1 | 236 | 249 |  |
| R.LPAKPEVSSDEDVQYR.V | Y | 93.22 | 1831.8951 | 16 | 0.9 | 611.6395 | 3 | 50.95 | 3 | 14419 | Ana\_02.raw |  | 3.07E6 | 2.87E6 | 1.58E6 | 3.28E6 | 4.83E6 | 1.57E6 |  | 9.57E6 | 4.72E6 | 8 | 0 | 1 | 1 | 1 | 1 | 1 | 1 | 0 | 1 | 1 | 661 | 676 |  |
| K.AGLEQGSDAGYLAESQK.F | Y | 92.40 | 1722.8060 | 17 | 5.1 | 862.4147 | 2 | 61.86 | 7 | 17510 | Ana\_06.raw | 5.27E6 | 5.25E6 | 3.61E6 | 2.07E6 | 1.93E6 | 3.46E6 | 2.43E6 | 2.23E6 | 9.32E6 | 4.08E6 | 10 | 1 | 1 | 1 | 1 | 1 | 1 | 1 | 1 | 1 | 1 | 310 | 326 |  |
| K.ADM(+15.99)VIEAVFEDLGVK.H | Y | 86.96 | 1650.8174 | 15 | 0.8 | 826.4166 | 2 | 132.34 | 3 | 36248 | Ana\_02.raw | 5.39E6 | 2.88E6 | 3.19E6 | 1.49E6 |  | 3.73E6 |  | 5.9E5 | 8.11E6 |  | 8 | 1 | 1 | 1 | 2 | 0 | 1 | 0 | 1 | 1 | 0 | 441 | 455 | Oxidation (M) |
| K.TVLGVPEVLLGILPGAGGTQR.L | Y | 78.40 | 2046.1837 | 21 | 3.4 | 683.0709 | 3 | 134.54 | 1 | 36982 | ANa\_10.raw | 2.05E7 | 3.62E6 | 4.67E6 | 1.83E6 | 5.19E6 | 9.95E6 | 1.18E7 | 2.45E6 | 8.91E6 | 1.34E7 | 21 | 2 | 2 | 3 | 1 | 2 | 2 | 2 | 2 | 2 | 3 | 167 | 187 |  |
| K.M(+15.99)GLVDQLVEPLGPGIK.S | Y | 73.23 | 1680.9120 | 16 | -2.0 | 841.4615 | 2 | 106.40 | 3 | 29545 | Ana\_02.raw | 6.58E5 | 5.96E6 | 2.44E6 | 1.8E5 | 2.94E6 | 6.8E5 | 2.47E6 | 8.62E5 | 1.41E7 |  | 9 | 1 | 1 | 1 | 1 | 1 | 1 | 1 | 1 | 1 | 0 | 215 | 230 | Oxidation (M) |
| K.DTTASAVAVGLR.Q | Y | 64.97 | 1159.6196 | 12 | 3.6 | 580.8192 | 2 | 58.56 | 4 | 16269 | Ana\_03.raw |  |  | 6.59E6 | 2.95E6 | 4.87E6 | 7.36E6 | 4.16E6 | 4.12E6 | 8.7E6 | 0 | 8 | 0 | 0 | 1 | 1 | 1 | 1 | 1 | 1 | 1 | 1 | 520 | 531 |  |
| K.SLNSEM(+15.99)DNILANLR.L | Y | 64.45 | 1604.7828 | 14 | -8.2 | 803.3922 | 2 | 96.65 | 1 | 27071 | ANa\_10.raw | 1.18E6 |  |  |  | 8.24E5 |  |  |  | 2.75E6 | 0 | 5 | 1 | 0 | 0 | 0 | 1 | 0 | 0 | 0 | 2 | 1 | 647 | 660 | Oxidation (M) |
| K.EVESVTPEHC(+57.02)IFASNTSALPINQIAAVSK.R | Y | 55.95 | 3111.5547 | 29 | 2.1 | 1038.1943 | 3 | 111.93 | 6 | 31377 | Ana\_05.raw |  |  |  |  |  | 7.59E5 |  |  |  |  | 1 | 0 | 0 | 0 | 0 | 0 | 1 | 0 | 0 | 0 | 0 | 461 | 489 | Carbamidomethylation |
| R.THINYGVK.G | Y | 55.71 | 930.4923 | 8 | 7.3 | 466.2568 | 2 | 23.47 | 9 | 5794 | Ana\_08.raw |  |  |  |  |  |  |  |  | 6.7E6 |  | 1 | 0 | 0 | 0 | 0 | 0 | 0 | 0 | 0 | 1 | 0 | 39 | 46 |  |
| K.DTTVTGLGR.G | Y | 52.64 | 918.4771 | 9 | -1.4 | 460.2451 | 2 | 35.64 | 5 | 9782 | Ana\_04.raw | 9.15E6 | 0 | 1.49E7 |  | 8.75E6 | 1.59E6 |  |  | 1.01E7 |  | 6 | 1 | 1 | 1 | 0 | 1 | 1 | 0 | 0 | 1 | 0 | 391 | 399 |  |
| K.DGPGFYTTR.C | Y | 50.18 | 1012.4614 | 9 | 2.0 | 507.2390 | 2 | 47.33 | 4 | 12994 | Ana\_03.raw |  |  | 8.97E6 | 1.6E6 | 3.66E6 | 5.07E6 |  |  |  |  | 4 | 0 | 0 | 1 | 1 | 1 | 1 | 0 | 0 | 0 | 0 | 541 | 549 |  |
| R.ILQEGVDPK.K | Y | 47.14 | 997.5444 | 9 | 3.9 | 499.7814 | 2 | 35.96 | 6 | 9910 | Ana\_05.raw | 9.8E6 | 3.63E6 |  | 6.49E5 | 8.58E6 | 1.05E7 | 7.03E6 | 6.34E6 | 0 | 1.01E7 | 9 | 1 | 1 | 0 | 1 | 1 | 1 | 1 | 1 | 1 | 1 | 561 | 569 |  |
| K.LTTYAM(+15.99)TVPFVR.Q | Y | 46.77 | 1413.7327 | 12 | 3.1 | 707.8758 | 2 | 80.74 | 9 | 22988 | Ana\_08.raw | 1.24E6 | 4.14E3 |  |  | 1.13E6 | 1.1E6 | 6.37E5 |  | 2.22E6 |  | 6 | 1 | 1 | 0 | 0 | 1 | 1 | 1 | 0 | 1 | 0 | 268 | 279 | Oxidation (M) |
| R.FGGGSVELLK.Q | Y | 46.31 | 1005.5494 | 10 | 7.0 | 503.7855 | 2 | 65.31 | 9 | 18624 | Ana\_08.raw |  |  |  |  |  |  |  |  | 8.31E6 |  | 1 | 0 | 0 | 0 | 0 | 0 | 0 | 0 | 0 | 1 | 0 | 611 | 620 |  |
| K.FGELALTK.E | Y | 43.06 | 877.4909 | 8 | 3.2 | 439.7541 | 2 | 62.93 | 6 | 18048 | Ana\_05.raw | 3.96E6 | 2.99E6 | 3.94E6 | 1.31E6 | 1.29E6 | 3.07E6 | 2.07E6 | 2.14E6 | 8.14E6 | 2.71E6 | 10 | 1 | 1 | 1 | 1 | 1 | 1 | 1 | 1 | 1 | 1 | 327 | 334 |  |
| K.ALTSFER.D | Y | 40.00 | 822.4235 | 7 | 1.1 | 412.2195 | 2 | 40.15 | 3 | 11193 | Ana\_02.raw |  |  | 7.56E6 |  |  |  |  |  | 5.33E6 |  | 2 | 0 | 0 | 1 | 0 | 0 | 0 | 0 | 0 | 1 | 0 | 416 | 422 |  |
| K.GLYPAPLK.I | Y | 32.86 | 857.5010 | 8 | 1.3 | 429.7584 | 2 | 55.70 | 2 | 15800 | Ana\_01.raw | 2.35E6 | 4.1E6 | 3.1E6 |  |  | 3.4E6 |  | 1.47E6 |  | 2.75E6 | 6 | 1 | 1 | 1 | 0 | 0 | 1 | 0 | 1 | 0 | 1 | 296 | 303 |  |
| K.VIGM(+15.99)HYFSPVDK.M | Y | 32.84 | 1407.6857 | 12 | 5.2 | 470.2383 | 3 | 53.83 | 9 | 15221 | Ana\_08.raw |  |  |  |  |  |  |  |  | 2.48E6 | 1.06E6 | 2 | 0 | 0 | 0 | 0 | 0 | 0 | 0 | 0 | 1 | 1 | 494 | 505 | Oxidation (M) |
| K.GDVAVIR.I | Y | 31.18 | 728.4181 | 7 | 3.4 | 365.2176 | 2 | 28.67 | 2 | 7601 | Ana\_01.raw |  | 8.37E6 | 3.26E6 |  | 3.73E6 | 6.41E6 | 5.15E6 |  | 8.7E6 | 3.56E6 | 7 | 0 | 1 | 1 | 0 | 1 | 1 | 1 | 0 | 1 | 1 | 47 | 53 |  |
| R.GQQQVFK.G | Y | 30.19 | 833.4395 | 7 | 0.0 | 417.7271 | 2 | 22.73 | 5 | 5842 | Ana\_04.raw |  |  |  |  | 2.5E6 | 6.33E6 |  |  |  |  | 2 | 0 | 0 | 0 | 0 | 1 | 1 | 0 | 0 | 0 | 0 | 400 | 406 |  |
| R.INSPNSKVNTLNK.E | Y | 29.87 | 1427.7732 | 13 | 1.3 | 714.8948 | 2 | 107.49 | 2 | 30079 | Ana\_01.raw |  | 5.53E4 |  |  |  |  |  |  |  |  | 1 | 0 | 1 | 0 | 0 | 0 | 0 | 0 | 0 | 0 | 0 | 54 | 66 |  |
| K.M(+15.99)QLLEIITTDK.T | Y | 25.91 | 1319.7007 | 11 | 3.8 | 660.8601 | 2 | 87.53 | 9 | 24869 | Ana\_08.raw |  |  |  |  |  |  |  |  | 2E6 |  | 1 | 0 | 0 | 0 | 0 | 0 | 0 | 0 | 0 | 1 | 0 | 506 | 516 | Oxidation (M) |
| total 25 peptides |  |  |  |  |  |  |  |  |  |  |  |  |  |  |  |  |  |  |  |  |  |  |  |  |  |  |  |  |  |  |  |  |  |  |  |
| --- | --- | --- | --- | --- | --- | --- | --- | --- | --- | --- | --- | --- | --- | --- | --- | --- | --- | --- | --- | --- | --- | --- | --- | --- | --- | --- | --- | --- | --- | --- | --- | --- | --- | --- | --- |

Best Unique PSM (Scan F9:38434, m/z=903.7227, z=4, RT=139.27, ppm=2.7):

sp|P68369|TBA1A\_MOUSE

back to list

  

| Protein Coverage
| Supporting Peptides
| Best Unique PSM
|

Protein Coverage:

Supporting Peptides:

| Peptide | Uniq | -10lgP | Mass | Length | ppm | m/z | z | RT | Fraction | Scan | Source File | Area 10 | Area 01 | Area 02 | Area 03 | Area 04 | Area 05 | Area 06 | Area 07 | Area 08 | Area 09 | #Spec | #Spec 10 | #Spec 01 | #Spec 02 | #Spec 03 | #Spec 04 | #Spec 05 | #Spec 06 | #Spec 07 | #Spec 08 | #Spec 09 | Start | End | PTM |
| --- | --- | --- | --- | --- | --- | --- | --- | --- | --- | --- | --- | --- | --- | --- | --- | --- | --- | --- | --- | --- | --- | --- | --- | --- | --- | --- | --- | --- | --- | --- | --- | --- | --- | --- | --- |
| R.FDGALNVDLTEFQTNLVPYPR.I | N | 117.80 | 2408.2012 | 21 | 4.2 | 1205.1129 | 2 | 130.54 | 9 | 36141 | Ana\_08.raw | 2.24E6 |  |  | 7.33E5 | 1.14E6 |  | 9.17E5 |  | 3.51E6 | 1.14E6 | 8 | 1 | 0 | 0 | 2 | 1 | 0 | 2 | 0 | 1 | 1 | 244 | 264 |  |
| R.AVFVDLEPTVIDEVR.T | N | 110.83 | 1700.8984 | 15 | 1.1 | 851.4575 | 2 | 114.52 | 3 | 31642 | Ana\_02.raw | 2.19E8 | 1.64E8 | 9.42E7 | 6.97E7 | 8.84E7 | 1.23E8 | 6.61E7 | 7.37E7 | 3.08E8 | 1.69E8 | 63 | 7 | 5 | 6 | 6 | 5 | 4 | 5 | 7 | 10 | 8 | 65 | 79 |  |
| K.TIGGGDDSFNTFFSETGAGK.H | N | 106.28 | 2006.8857 | 20 | 2.8 | 1004.4530 | 2 | 107.23 | 7 | 29832 | Ana\_06.raw | 1.21E8 | 1.32E8 | 9.97E7 | 4.88E7 | 5.95E7 | 8.51E7 | 4.25E7 | 5.38E7 | 2.49E8 | 1.17E8 | 54 | 6 | 7 | 5 | 6 | 6 | 4 | 3 | 7 | 5 | 5 | 41 | 60 |  |
| R.IHFPLATYAPVISAEK.A | N | 101.55 | 1755.9559 | 16 | 1.2 | 878.9863 | 2 | 104.62 | 6 | 29472 | Ana\_05.raw | 8.28E7 | 9.05E7 | 9.31E7 | 5.04E7 | 9.02E7 | 1.11E8 | 4.49E7 | 3.3E7 | 2.83E8 | 1.42E8 | 85 | 11 | 9 | 6 | 8 | 8 | 8 | 8 | 7 | 11 | 9 | 265 | 280 |  |
| R.AVC(+57.02)M(+15.99)LSNTTAIAEAWAR.L | N | 100.31 | 1879.8921 | 17 | -1.1 | 940.9523 | 2 | 100.08 | 1 | 27997 | ANa\_10.raw | 9.41E6 | 1.3E7 | 8.38E6 | 1.22E6 | 1.18E6 | 1.01E7 | 6.35E6 |  | 1.37E7 | 2.31E6 | 14 | 2 | 2 | 2 | 1 | 1 | 2 | 2 | 0 | 1 | 1 | 374 | 390 | Carbamidomethylation; Oxidation (M) |
| R.AVC(+57.02)MLSNTTAIAEAWAR.L | N | 97.01 | 1863.8971 | 17 | 2.5 | 932.9582 | 2 | 118.70 | 1 | 32766 | ANa\_10.raw | 4.22E6 | 1.14E6 |  | 9.92E5 |  | 1.45E6 |  |  | 6.04E6 | 5.52E6 | 9 | 2 | 1 | 0 | 1 | 0 | 2 | 0 | 0 | 1 | 2 | 374 | 390 | Carbamidomethylation |
| R.AFVHWYVGEGM(+15.99)EEGEFSEAR.E | N | 92.50 | 2345.0059 | 20 | 3.4 | 782.6785 | 3 | 94.03 | 6 | 26666 | Ana\_05.raw | 1.18E6 | 3.45E6 | 2.64E6 | 4.66E5 | 3.25E6 | 5.36E6 | 3.55E6 |  | 2.72E6 | 3.51E6 | 9 | 1 | 1 | 1 | 1 | 1 | 1 | 1 | 0 | 1 | 1 | 403 | 422 | Oxidation (M) |
| R.TIQFVDWC(+57.02)PTGFK.V | N | 92.37 | 1597.7599 | 13 | -2.2 | 799.8854 | 2 | 112.71 | 3 | 31165 | Ana\_02.raw |  | 3.05E7 | 2.58E7 | 1.34E7 | 1.16E7 | 1.82E7 | 1.25E7 | 9.58E6 |  |  | 8 | 0 | 1 | 2 | 1 | 1 | 1 | 1 | 1 | 0 | 0 | 340 | 352 | Carbamidomethylation |
| K.VGINYQPPTVVPGGDLAK.V | N | 78.26 | 1823.9781 | 18 | 1.3 | 912.9976 | 2 | 87.23 | 3 | 24540 | Ana\_02.raw | 2.69E7 | 6.86E7 | 4.84E7 | 1.55E7 | 3.76E7 | 3.39E7 | 2.24E7 | 2.24E7 | 1.81E8 | 7.09E7 | 26 | 3 | 3 | 2 | 2 | 1 | 3 | 2 | 1 | 5 | 4 | 353 | 370 |  |
| R.QLFHPEQLITGK.E | N | 73.59 | 1409.7666 | 12 | 4.3 | 705.8936 | 2 | 84.37 | 8 | 23640 | Ana\_07.raw | 1.33E7 | 8.46E6 | 9.75E6 | 7.29E6 | 9.99E6 | 1.49E7 | 4.79E6 | 1.32E7 | 3.21E7 | 3.98E6 | 20 | 2 | 2 | 2 | 2 | 2 | 2 | 1 | 2 | 3 | 2 | 85 | 96 |  |
| R.LIGQIVSSITASLR.F | Y | 72.26 | 1456.8613 | 14 | 3.7 | 729.4406 | 2 | 130.02 | 9 | 36001 | Ana\_08.raw |  |  |  |  |  |  |  |  | 4.81E6 | 7.09E5 | 2 | 0 | 0 | 0 | 0 | 0 | 0 | 0 | 0 | 1 | 1 | 230 | 243 |  |
| R.NLDIERPTYTNLNR.L | N | 68.75 | 1717.8748 | 14 | 4.1 | 859.9482 | 2 | 64.89 | 9 | 18502 | Ana\_08.raw | 1.14E7 | 8.44E6 |  |  |  |  |  |  | 1.47E7 | 5.59E6 | 6 | 2 | 1 | 0 | 0 | 0 | 0 | 0 | 0 | 2 | 1 | 216 | 229 |  |
| K.DVNAAIATIK.T | N | 66.51 | 1014.5709 | 10 | 2.0 | 508.2938 | 2 | 60.74 | 3 | 17289 | Ana\_02.raw | 6.9E7 | 6.27E7 | 6.56E7 | 2.23E7 | 4.02E7 | 4.48E7 | 3.11E7 | 3.01E7 | 1.15E8 | 6.17E7 | 22 | 2 | 2 | 3 | 2 | 1 | 2 | 2 | 4 | 3 | 1 | 327 | 336 |  |
| R.GHYTIGK.E | N | 56.84 | 774.4024 | 7 | 3.9 | 388.2100 | 2 | 21.28 | 7 | 5302 | Ana\_06.raw |  |  |  |  | 8.67E6 |  | 9.09E6 | 6.3E6 |  | 6.29E6 | 4 | 0 | 0 | 0 | 0 | 1 | 0 | 1 | 1 | 0 | 1 | 106 | 112 |  |
| K.EIIDLVLDR.I | N | 55.23 | 1084.6128 | 9 | 5.0 | 543.3164 | 2 | 104.20 | 6 | 29366 | Ana\_05.raw |  |  | 2.86E6 | 5.73E5 | 6.85E5 | 2E6 | 6.98E5 | 4.89E5 |  | 1.65E7 | 7 | 0 | 0 | 1 | 1 | 1 | 1 | 1 | 1 | 0 | 1 | 113 | 121 |  |
| K.EDAANNYAR.G | N | 51.28 | 1022.4417 | 9 | 2.5 | 512.2294 | 2 | 20.32 | 5 | 5091 | Ana\_04.raw |  |  |  |  | 1.64E7 |  |  |  |  |  | 1 | 0 | 0 | 0 | 0 | 1 | 0 | 0 | 0 | 0 | 0 | 97 | 105 |  |
| R.LSVDYGK.K | N | 49.40 | 780.4017 | 7 | 5.0 | 391.2101 | 2 | 36.09 | 10 | 9863 | Ana\_09.raw | 4.18E7 | 1.14E5 | 0 | 2.01E7 | 6.17E5 |  |  |  | 5.95E7 | 4.31E7 | 9 | 2 | 2 | 1 | 1 | 1 | 0 | 0 | 0 | 1 | 1 | 157 | 163 |  |
| K.YM(+15.99)AC(+57.02)C(+57.02)LLYR.G | N | 48.26 | 1264.5403 | 9 | 3.4 | 633.2795 | 2 | 70.56 | 4 | 19630 | Ana\_03.raw |  | 2.9E6 |  | 6.52E5 | 3.43E6 | 2.96E6 | 1.84E6 | 1.41E6 |  | 2.38E6 | 7 | 0 | 1 | 0 | 1 | 1 | 1 | 1 | 1 | 0 | 1 | 312 | 320 | Oxidation (M); Carbamidomethylation |
| K.FDLM(+15.99)YAK.R | N | 48.10 | 902.4208 | 7 | 5.6 | 452.2202 | 2 | 58.43 | 1 | 16509 | ANa\_10.raw | 3.91E7 | 3.27E7 | 3.75E7 | 1.76E7 | 3.22E7 | 3.02E7 | 2.45E7 |  | 5.73E7 | 3.33E7 | 14 | 2 | 2 | 1 | 1 | 1 | 1 | 2 | 0 | 2 | 2 | 395 | 401 | Oxidation (M) |
| R.EDMAALEK.D | N | 43.53 | 905.4164 | 8 | 6.1 | 453.7182 | 2 | 34.03 | 9 | 9103 | Ana\_08.raw | 2.12E6 |  |  |  |  |  |  |  | 5.61E6 |  | 2 | 1 | 0 | 0 | 0 | 0 | 0 | 0 | 0 | 1 | 0 | 423 | 430 |  |
| R.EDM(+15.99)AALEK.D | N | 42.79 | 921.4113 | 8 | 2.4 | 461.7140 | 2 | 20.33 | 5 | 5094 | Ana\_04.raw |  |  |  | 5.31E6 | 3.63E7 |  | 3.53E6 | 3.07E6 |  |  | 4 | 0 | 0 | 0 | 1 | 1 | 0 | 1 | 1 | 0 | 0 | 423 | 430 | Oxidation (M) |
| R.LSVDYGKK.S | N | 36.03 | 908.4967 | 8 | 3.4 | 455.2572 | 2 | 26.90 | 10 | 7103 | Ana\_09.raw | 3.62E6 |  |  |  |  |  |  |  |  | 3.48E6 | 2 | 1 | 0 | 0 | 0 | 0 | 0 | 0 | 0 | 0 | 1 | 157 | 164 |  |
| K.FDLMYAK.R | N | 31.78 | 886.4258 | 7 | 4.7 | 444.2223 | 2 | 67.31 | 9 | 19210 | Ana\_08.raw |  |  |  |  |  |  |  |  | 1.44E6 |  | 1 | 0 | 0 | 0 | 0 | 0 | 0 | 0 | 0 | 1 | 0 | 395 | 401 |  |
| total 23 peptides |  |  |  |  |  |  |  |  |  |  |  |  |  |  |  |  |  |  |  |  |  |  |  |  |  |  |  |  |  |  |  |  |  |  |  |
| --- | --- | --- | --- | --- | --- | --- | --- | --- | --- | --- | --- | --- | --- | --- | --- | --- | --- | --- | --- | --- | --- | --- | --- | --- | --- | --- | --- | --- | --- | --- | --- | --- | --- | --- | --- |

Best Unique PSM (Scan F9:36001, m/z=729.4406, z=2, RT=130.02, ppm=3.7):

A2ACG7|A2ACG7\_MOUSE

back to list

  

| Protein Coverage
| Supporting Peptides
| Best Unique PSM
|

Protein Coverage:

Supporting Peptides:

| Peptide | Uniq | -10lgP | Mass | Length | ppm | m/z | z | RT | Fraction | Scan | Source File | Area 10 | Area 01 | Area 02 | Area 03 | Area 04 | Area 05 | Area 06 | Area 07 | Area 08 | Area 09 | #Spec | #Spec 10 | #Spec 01 | #Spec 02 | #Spec 03 | #Spec 04 | #Spec 05 | #Spec 06 | #Spec 07 | #Spec 08 | #Spec 09 | Start | End | PTM |
| --- | --- | --- | --- | --- | --- | --- | --- | --- | --- | --- | --- | --- | --- | --- | --- | --- | --- | --- | --- | --- | --- | --- | --- | --- | --- | --- | --- | --- | --- | --- | --- | --- | --- | --- | --- |
| K.EETVLATVQALQTASHLSQQADLR.N | Y | 123.22 | 2608.3457 | 24 | 5.8 | 870.4609 | 3 | 123.58 | 3 | 33949 | Ana\_02.raw |  | 1.43E6 | 2.87E6 | 5.87E5 | 2.04E6 | 1.25E6 |  | 2.3E5 | 3.27E6 |  | 7 | 0 | 1 | 1 | 1 | 1 | 1 | 0 | 1 | 1 | 0 | 123 | 146 |  |
| R.LGKEETVLATVQALQTASHLSQQADLR.N | Y | 112.02 | 2906.5461 | 27 | 5.2 | 727.6476 | 4 | 123.15 | 1 | 33919 | ANa\_10.raw | 6.02E6 |  | 5.02E6 | 1.77E6 |  | 1.91E6 |  | 6.7E5 |  | 5.67E6 | 6 | 1 | 0 | 1 | 1 | 0 | 1 | 0 | 1 | 0 | 1 | 120 | 146 |  |
| K.NFESLSEAFSVASAAAALSQNR.Y | Y | 110.21 | 2269.0974 | 22 | 2.4 | 1135.5587 | 2 | 132.30 | 6 | 36775 | Ana\_05.raw |  | 2.99E6 |  | 1.55E6 |  | 1.87E6 | 5.46E6 |  | 8.97E6 |  | 7 | 0 | 1 | 0 | 1 | 0 | 1 | 2 | 0 | 2 | 0 | 213 | 234 |  |
| K.TPFSLVGNVFELNFK.N | Y | 107.14 | 1710.8981 | 15 | 6.6 | 856.4620 | 2 | 133.31 | 6 | 37060 | Ana\_05.raw |  | 1.31E6 | 4.71E5 | 4.92E5 | 3.33E4 | 1.89E6 |  | 5.81E5 | 0 |  | 8 | 0 | 1 | 1 | 1 | 1 | 2 | 0 | 1 | 1 | 0 | 291 | 305 |  |
| R.YHVPVVVVPEGSTSDTQEQAILR.L | Y | 106.14 | 2523.2969 | 23 | 4.8 | 842.1102 | 3 | 92.84 | 1 | 26051 | ANa\_10.raw | 3.94E6 | 2.71E6 |  | 0 |  |  |  |  | 1.06E7 |  | 4 | 1 | 1 | 0 | 1 | 0 | 0 | 0 | 0 | 1 | 0 | 235 | 257 |  |
| K.KNFESLSEAFSVASAAAALSQNR.Y | Y | 99.41 | 2397.1924 | 23 | 5.8 | 800.0760 | 3 | 125.79 | 9 | 34918 | Ana\_08.raw |  |  |  |  |  | 8.88E6 |  |  | 8.1E6 |  | 2 | 0 | 0 | 0 | 0 | 0 | 1 | 0 | 0 | 1 | 0 | 212 | 234 |  |
| R.NIVEEIEDLVAR.L | Y | 84.69 | 1398.7354 | 12 | 2.9 | 700.3770 | 2 | 134.41 | 3 | 36787 | Ana\_02.raw | 5.53E6 | 9.58E6 | 6.56E6 | 4.55E5 | 3.97E6 | 1.48E7 | 1.08E6 | 1.32E6 | 1.08E7 | 7.44E6 | 12 | 1 | 2 | 1 | 1 | 1 | 2 | 1 | 1 | 1 | 1 | 147 | 158 |  |
| K.TGQEVVFVAEPDNK.N | Y | 81.52 | 1531.7518 | 14 | 7.9 | 766.8892 | 2 | 61.74 | 1 | 17490 | ANa\_10.raw | 6.27E6 | 7.58E6 |  | 3.38E6 | 5.63E6 | 6.71E6 | 5.11E6 | 4.73E6 | 6.45E6 | 5.51E6 | 9 | 1 | 1 | 0 | 1 | 1 | 1 | 1 | 1 | 1 | 1 | 411 | 424 |  |
| K.NPILWNVADVVIK.F | Y | 80.86 | 1479.8450 | 13 | 4.0 | 740.9327 | 2 | 127.91 | 7 | 35194 | Ana\_06.raw | 1E6 | 4.37E5 | 1.49E6 | 1.02E6 | 5.32E5 | 1.16E6 | 2.06E6 | 1.22E6 | 5.65E5 |  | 9 | 1 | 1 | 1 | 1 | 1 | 1 | 1 | 1 | 1 | 0 | 460 | 472 |  |
| R.LQVSNVLSQPLAQAAVK.L | Y | 77.84 | 1765.0098 | 17 | 2.3 | 883.5142 | 2 | 97.28 | 1 | 27245 | ANa\_10.raw | 3.67E6 | 1.84E6 | 0 |  | 5.55E5 | 5.43E5 |  | 4.23E5 | 1.6E6 | 5.66E5 | 8 | 1 | 1 | 1 | 0 | 1 | 1 | 0 | 1 | 1 | 1 | 258 | 274 |  |
| K.LSSGYYDFSVR.V | Y | 66.56 | 1292.6036 | 11 | 1.1 | 647.3098 | 2 | 78.78 | 6 | 22534 | Ana\_05.raw | 4.37E6 | 3.77E6 |  | 4.09E6 | 2.61E6 | 6.92E6 | 2.82E6 | 4.82E6 | 6.89E6 | 4.26E6 | 9 | 1 | 1 | 0 | 1 | 1 | 1 | 1 | 1 | 1 | 1 | 309 | 319 |  |
| K.FPEEEAPSTVLSQSLFTPK.Q | Y | 50.39 | 2106.0520 | 19 | 6.2 | 1054.0398 | 2 | 115.73 | 2 | 32218 | Ana\_01.raw |  | 2.38E6 |  |  |  |  |  |  |  |  | 1 | 0 | 1 | 0 | 0 | 0 | 0 | 0 | 0 | 0 | 0 | 473 | 491 |  |
| K.FELDTSER.K | Y | 49.26 | 995.4559 | 8 | 5.2 | 498.7379 | 2 | 50.13 | 1 | 14069 | ANa\_10.raw | 6.11E6 | 8.57E6 |  | 4.78E6 |  |  | 6.7E6 | 5.14E6 | 1.03E7 | 8.35E6 | 7 | 1 | 1 | 0 | 1 | 0 | 0 | 1 | 1 | 1 | 1 | 429 | 436 |  |
| R.YIANTVELR.V | Y | 48.86 | 1077.5818 | 9 | 4.6 | 539.8007 | 2 | 54.73 | 1 | 15428 | ANa\_10.raw | 4.54E5 | 1.04E7 | 4.94E6 | 2.33E6 | 4.25E6 | 5.16E6 | 4.83E6 | 2.43E6 |  | 9E6 | 9 | 1 | 1 | 1 | 1 | 1 | 1 | 1 | 1 | 0 | 1 | 326 | 334 |  |
| R.M(+15.99)LAQHAVK.R | Y | 40.92 | 912.4851 | 8 | 0.8 | 457.2502 | 2 | 17.67 | 7 | 4147 | Ana\_06.raw |  |  |  | 7.94E5 | 2.91E6 | 2.07E6 | 2.6E6 | 1.33E6 |  |  | 5 | 0 | 0 | 0 | 1 | 1 | 1 | 1 | 1 | 0 | 0 | 588 | 595 | Oxidation (M) |
| R.VTYPAK.A | Y | 35.71 | 677.3748 | 6 | 2.5 | 339.6955 | 2 | 21.38 | 4 | 5317 | Ana\_03.raw |  |  |  | 1.09E6 | 1.01E6 |  | 1.12E6 |  |  |  | 3 | 0 | 0 | 0 | 1 | 1 | 0 | 1 | 0 | 0 | 0 | 364 | 369 |  |
| total 16 peptides |  |  |  |  |  |  |  |  |  |  |  |  |  |  |  |  |  |  |  |  |  |  |  |  |  |  |  |  |  |  |  |  |  |  |  |
| --- | --- | --- | --- | --- | --- | --- | --- | --- | --- | --- | --- | --- | --- | --- | --- | --- | --- | --- | --- | --- | --- | --- | --- | --- | --- | --- | --- | --- | --- | --- | --- | --- | --- | --- | --- |

Best Unique PSM (Scan F3:33949, m/z=870.4609, z=3, RT=123.58, ppm=5.8):

sp|P07901|HS90A\_MOUSE

back to list

  

| Protein Coverage
| Supporting Peptides
| Best Unique PSM
|

Protein Coverage:

Supporting Peptides:

| Peptide | Uniq | -10lgP | Mass | Length | ppm | m/z | z | RT | Fraction | Scan | Source File | Area 10 | Area 01 | Area 02 | Area 03 | Area 04 | Area 05 | Area 06 | Area 07 | Area 08 | Area 09 | #Spec | #Spec 10 | #Spec 01 | #Spec 02 | #Spec 03 | #Spec 04 | #Spec 05 | #Spec 06 | #Spec 07 | #Spec 08 | #Spec 09 | Start | End | PTM |
| --- | --- | --- | --- | --- | --- | --- | --- | --- | --- | --- | --- | --- | --- | --- | --- | --- | --- | --- | --- | --- | --- | --- | --- | --- | --- | --- | --- | --- | --- | --- | --- | --- | --- | --- | --- |
| R.GVVDSEDLPLNISR.E | N | 102.97 | 1512.7783 | 14 | 0.9 | 757.3971 | 2 | 86.49 | 3 | 24346 | Ana\_02.raw | 6.13E7 | 7.03E7 | 3.65E6 | 3.61E6 | 5.27E7 | 4.2E7 | 2.46E7 | 2.66E7 | 1.07E8 | 7.09E7 | 35 | 3 | 5 | 4 | 2 | 4 | 2 | 2 | 4 | 4 | 5 | 388 | 401 |  |
| K.HSQFIGYPITLFVEK.E | Y | 102.09 | 1777.9403 | 15 | -1.4 | 593.6532 | 3 | 116.76 | 3 | 32223 | Ana\_02.raw |  | 7.55E6 | 1.51E7 | 3.33E6 | 2.95E6 | 1.19E7 | 3.98E6 | 1.72E6 | 1.25E6 | 8.95E5 | 15 | 0 | 2 | 3 | 2 | 1 | 2 | 2 | 1 | 1 | 1 | 210 | 224 |  |
| R.VFIM(+15.99)DNC(+57.02)EELIPEYLNFIR.G | Y | 100.52 | 2430.1599 | 19 | 7.9 | 1216.0968 | 2 | 133.47 | 2 | 36952 | Ana\_01.raw | 1.05E6 | 3.88E6 | 1.85E6 | 1.59E6 |  | 4.48E6 |  |  |  | 2.84E6 | 7 | 1 | 2 | 1 | 1 | 0 | 1 | 0 | 0 | 0 | 1 | 369 | 387 | Oxidation (M); Carbamidomethylation |
| K.HLEINPDHSIIETLR.Q | Y | 93.25 | 1785.9373 | 15 | 4.8 | 596.3226 | 3 | 82.34 | 6 | 23506 | Ana\_05.raw | 1.01E6 |  | 5.8E5 |  | 1E6 | 1.4E6 |  |  |  | 1.67E6 | 5 | 1 | 0 | 1 | 0 | 1 | 1 | 0 | 0 | 0 | 1 | 634 | 648 |  |
| R.NPDDITNEEYGEFYK.S | Y | 86.66 | 1832.7740 | 15 | -0.1 | 917.3942 | 2 | 79.60 | 3 | 22508 | Ana\_02.raw | 1.02E7 | 1.61E7 | 1.28E7 | 7.19E6 | 1.13E7 | 1.39E7 |  | 1.02E7 | 1.49E7 | 9.55E6 | 11 | 1 | 2 | 1 | 1 | 2 | 1 | 0 | 1 | 1 | 1 | 301 | 315 |  |
| R.TLTIVDTGIGM(+15.99)TK.A | N | 76.46 | 1364.7222 | 13 | 2.8 | 683.3703 | 2 | 75.39 | 6 | 21614 | Ana\_05.raw | 1.7E7 | 2.94E7 | 3.4E7 | 1.31E7 | 2.19E7 | 1.58E7 | 1.83E7 | 1.87E7 | 6.4E6 | 1.47E7 | 22 | 2 | 2 | 3 | 3 | 2 | 1 | 2 | 2 | 1 | 4 | 88 | 100 | Oxidation (M) |
| K.EGLELPEDEEEK.K | N | 76.27 | 1415.6304 | 12 | 4.3 | 708.8255 | 2 | 63.16 | 6 | 18113 | Ana\_05.raw | 1.47E7 | 2.12E7 | 2.24E7 | 9.03E6 |  | 1.3E7 | 1.23E7 | 9.36E6 | 1.85E7 |  | 9 | 1 | 1 | 2 | 1 | 0 | 1 | 1 | 1 | 1 | 0 | 548 | 559 |  |
| K.EDQTEYLEER.R | N | 73.56 | 1310.5626 | 10 | 2.1 | 656.2900 | 2 | 46.56 | 3 | 13135 | Ana\_02.raw | 8.17E7 | 1.16E8 | 9.68E7 | 5.07E7 | 8.64E7 | 7.77E7 | 6.62E7 | 7.11E7 | 9.8E7 | 7.47E7 | 27 | 3 | 3 | 2 | 4 | 3 | 1 | 3 | 3 | 2 | 3 | 192 | 201 |  |
| R.YYTSASGDEM(+15.99)VSLK.D | Y | 73.25 | 1565.6919 | 14 | 0.6 | 783.8537 | 2 | 53.16 | 3 | 15067 | Ana\_02.raw | 3.47E6 | 5.4E6 | 6.41E6 | 3.1E6 |  |  | 3.64E6 |  |  | 2.12E6 | 6 | 1 | 1 | 1 | 1 | 0 | 0 | 1 | 0 | 0 | 1 | 466 | 479 | Oxidation (M) |
| R.RAPFDLFENR.K | Y | 72.08 | 1263.6360 | 10 | -3.6 | 422.2178 | 3 | 83.40 | 3 | 23524 | Ana\_02.raw | 1.07E6 | 7.69E5 | 2.36E6 | 5.04E6 | 4.9E6 | 3.63E6 | 8.32E6 | 1.1E6 | 2.42E5 | 2.13E6 | 23 | 1 | 1 | 3 | 4 | 3 | 3 | 4 | 2 | 1 | 1 | 347 | 356 |  |
| K.ADLINNLGTIAK.S | N | 71.56 | 1241.6979 | 12 | 2.5 | 621.8578 | 2 | 85.58 | 6 | 24379 | Ana\_05.raw | 9.14E6 | 4.85E7 | 4.8E7 | 1.77E7 | 3.55E7 | 3.13E7 | 2.49E7 | 2.43E7 | 6.64E7 | 2.83E7 | 23 | 2 | 3 | 3 | 1 | 3 | 1 | 1 | 3 | 4 | 2 | 101 | 112 |  |
| K.HNDDEQYAWESSAGGSFTVR.T | N | 71.42 | 2254.9514 | 20 | 1.3 | 752.6588 | 3 | 83.49 | 4 | 23153 | Ana\_03.raw |  |  |  | 5.26E5 |  |  |  |  |  |  | 1 | 0 | 0 | 0 | 1 | 0 | 0 | 0 | 0 | 0 | 0 | 154 | 173 |  |
| K.DQVANSAFVER.L | Y | 70.76 | 1234.5942 | 11 | 3.6 | 618.3066 | 2 | 47.84 | 5 | 13492 | Ana\_04.raw | 1.97E7 | 2.16E7 | 2.6E7 | 1.04E7 | 1.99E7 | 2.04E7 | 1.49E7 | 1.41E7 | 3.13E7 | 1.83E7 | 16 | 1 | 2 | 2 | 2 | 1 | 2 | 1 | 2 | 2 | 1 | 501 | 511 |  |
| R.APFDLFENR.K | Y | 68.73 | 1107.5349 | 9 | 1.4 | 554.7755 | 2 | 93.49 | 5 | 26307 | Ana\_04.raw | 6.5E6 | 4.17E6 | 6.66E6 | 1.49E6 | 4.34E6 | 4.44E6 | 3.33E6 | 2.15E6 | 1.01E7 | 4.84E6 | 11 | 2 | 1 | 1 | 1 | 1 | 1 | 1 | 1 | 1 | 1 | 348 | 356 |  |
| R.YESLTDPSK.L | N | 68.33 | 1038.4869 | 9 | 5.5 | 520.2536 | 2 | 35.77 | 1 | 9709 | ANa\_10.raw | 4.69E7 | 6.87E7 | 7.75E7 | 3.49E7 | 5.34E7 | 5.02E7 | 4.55E7 | 4.71E7 | 6.1E7 | 4.21E7 | 21 | 2 | 2 | 3 | 2 | 2 | 2 | 1 | 3 | 2 | 2 | 61 | 69 |  |
| R.ELISNSSDALDK.I | Y | 67.73 | 1290.6302 | 12 | 2.2 | 646.3238 | 2 | 52.16 | 6 | 14851 | Ana\_05.raw | 1.23E7 |  | 1.45E7 |  | 1.86E7 | 1.66E7 | 9.19E6 | 1.43E7 | 3.09E7 | 1.68E7 | 13 | 1 | 0 | 1 | 0 | 2 | 2 | 1 | 2 | 2 | 2 | 47 | 58 |  |
| K.YIDQEELNK.T | N | 66.73 | 1150.5505 | 9 | 4.3 | 576.2850 | 2 | 41.44 | 6 | 11597 | Ana\_05.raw | 7.59E7 | 8.68E7 | 1.08E8 | 3.52E7 | 7.65E7 | 5.89E7 | 4.76E7 | 3.83E7 | 4.98E7 | 6.56E7 | 10 | 1 | 1 | 1 | 1 | 1 | 1 | 1 | 1 | 1 | 1 | 285 | 293 |  |
| K.LGIHEDSQNR.K | Y | 66.64 | 1167.5632 | 10 | 0.8 | 584.7894 | 2 | 23.44 | 2 | 5903 | Ana\_01.raw | 1.21E7 | 9.81E6 | 5.82E6 | 3.27E6 | 4.02E6 | 5.52E6 | 5.48E5 | 1.17E6 | 6.99E6 | 8.59E6 | 10 | 1 | 1 | 1 | 1 | 1 | 1 | 1 | 1 | 1 | 1 | 448 | 457 |  |
| K.HIYFITGETK.D | Y | 66.13 | 1207.6237 | 10 | 1.0 | 604.8197 | 2 | 54.35 | 3 | 15418 | Ana\_02.raw | 3.34E6 | 8.73E6 | 9.48E6 | 4.18E6 | 7.08E6 | 7.74E6 | 6.07E6 | 5.87E6 |  | 4.71E6 | 9 | 1 | 1 | 1 | 1 | 1 | 1 | 1 | 1 | 0 | 1 | 491 | 500 |  |
| K.EGLELPEDEEEKKK.Q | N | 65.05 | 1671.8202 | 14 | 7.1 | 558.2846 | 3 | 42.33 | 3 | 11863 | Ana\_02.raw | 0 | 9.31E6 | 1.33E7 | 1.55E6 | 7.96E6 |  |  |  |  |  | 5 | 1 | 1 | 1 | 1 | 1 | 0 | 0 | 0 | 0 | 0 | 548 | 561 |  |
| R.ALLFVPR.R | Y | 57.34 | 814.5065 | 7 | 3.9 | 408.2621 | 2 | 80.01 | 8 | 22432 | Ana\_07.raw |  | 1.57E7 | 1.75E7 |  | 1.35E7 | 1.54E7 | 1.33E7 | 1.23E7 | 2.06E7 | 1.04E7 | 13 | 0 | 1 | 3 | 0 | 1 | 2 | 1 | 1 | 2 | 2 | 340 | 346 |  |
| K.LGIHEDSQNRK.K | Y | 51.39 | 1295.6582 | 11 | 4.3 | 648.8392 | 2 | 20.85 | 9 | 4901 | Ana\_08.raw |  | 7.85E5 |  |  |  |  |  |  | 4.78E5 |  | 2 | 0 | 1 | 0 | 0 | 0 | 0 | 0 | 0 | 1 | 0 | 448 | 458 |  |
| K.ELHINLIPSK.Q | Y | 51.38 | 1162.6710 | 10 | 1.1 | 388.5647 | 3 | 75.91 | 4 | 21118 | Ana\_03.raw |  | 1.19E6 |  | 1.05E6 | 1.91E6 | 1.67E6 | 6.2E5 | 1.18E6 | 4.39E5 | 9.15E5 | 9 | 0 | 1 | 0 | 1 | 1 | 2 | 1 | 1 | 1 | 1 | 75 | 84 |  |
| K.TKPIWTR.N | N | 48.99 | 900.5181 | 7 | 0.7 | 301.1802 | 3 | 33.07 | 3 | 9038 | Ana\_02.raw | 1.67E7 | 2.38E7 | 5.34E5 | 5.35E6 |  |  |  |  |  | 1.51E7 | 5 | 1 | 1 | 1 | 1 | 0 | 0 | 0 | 0 | 0 | 1 | 294 | 300 |  |
| K.EGLELPEDEEEKK.K | N | 44.93 | 1543.7252 | 13 | 0.3 | 772.8701 | 2 | 51.90 | 2 | 14657 | Ana\_01.raw |  | 1.38E7 |  | 0 |  |  | 2.61E6 |  | 7.3E6 |  | 5 | 0 | 2 | 0 | 1 | 0 | 0 | 1 | 0 | 1 | 0 | 548 | 560 |  |
| K.FYEQFSK.N | Y | 44.89 | 947.4388 | 7 | -0.1 | 474.7267 | 2 | 46.34 | 3 | 13069 | Ana\_02.raw | 1.09E7 | 1.32E7 | 1.48E7 | 7.22E6 | 1.11E7 | 1.18E7 | 1.01E7 | 9.08E6 | 1.53E7 | 1.11E7 | 10 | 1 | 1 | 1 | 1 | 1 | 1 | 1 | 1 | 1 | 1 | 438 | 444 |  |
| R.DNSTM(+15.99)GYM(+15.99)AAK.K | Y | 43.67 | 1219.4849 | 11 | 3.4 | 610.7518 | 2 | 21.28 | 4 | 5287 | Ana\_03.raw |  |  |  | 1.22E6 |  |  | 2.16E6 | 1.69E6 |  |  | 3 | 0 | 0 | 0 | 1 | 0 | 0 | 1 | 1 | 0 | 0 | 622 | 632 | Oxidation (M) |
| K.FENLC(+57.02)K.I | N | 42.97 | 809.3741 | 6 | 0.8 | 405.6947 | 2 | 28.27 | 3 | 7600 | Ana\_02.raw | 8.65E6 | 1.22E7 | 1.26E7 | 7.61E6 | 9.52E6 | 1.1E7 | 8.16E6 | 7.56E6 | 9.31E6 | 9.82E6 | 10 | 1 | 1 | 1 | 1 | 1 | 1 | 1 | 1 | 1 | 1 | 569 | 574 | Carbamidomethylation |
| K.LSELLR.Y | N | 42.95 | 729.4385 | 6 | 5.6 | 365.7285 | 2 | 43.93 | 2 | 12274 | Ana\_01.raw | 2.33E7 | 2.55E7 | 3.68E7 | 1.67E7 | 3.13E7 | 2.93E7 | 2.32E7 | 2.44E7 | 6.01E6 | 2.41E7 | 21 | 2 | 2 | 2 | 2 | 2 | 2 | 2 | 3 | 2 | 2 | 460 | 465 |  |
| K.EIFLR.E | N | 28.74 | 676.3907 | 5 | 1.2 | 339.2031 | 2 | 46.69 | 3 | 13179 | Ana\_02.raw |  |  | 1.52E7 |  | 1.07E7 |  |  |  | 6.65E6 |  | 3 | 0 | 0 | 1 | 0 | 1 | 0 | 0 | 0 | 1 | 0 | 42 | 46 |  |
| K.TLVSVTK.E | Y | 27.83 | 746.4538 | 7 | 0.9 | 374.2345 | 2 | 29.63 | 2 | 7903 | Ana\_01.raw |  | 8.17E6 | 1.07E7 |  |  |  |  |  |  |  | 2 | 0 | 1 | 1 | 0 | 0 | 0 | 0 | 0 | 0 | 0 | 541 | 547 |  |
| total 31 peptides |  |  |  |  |  |  |  |  |  |  |  |  |  |  |  |  |  |  |  |  |  |  |  |  |  |  |  |  |  |  |  |  |  |  |  |
| --- | --- | --- | --- | --- | --- | --- | --- | --- | --- | --- | --- | --- | --- | --- | --- | --- | --- | --- | --- | --- | --- | --- | --- | --- | --- | --- | --- | --- | --- | --- | --- | --- | --- | --- | --- |

Best Unique PSM (Scan F3:32223, m/z=593.6532, z=3, RT=116.76, ppm=-1.4):

sp|Q99KI0|ACON\_MOUSE

back to list

  

| Protein Coverage
| Supporting Peptides
| Best Unique PSM
|

Protein Coverage:

Supporting Peptides:

| Peptide | Uniq | -10lgP | Mass | Length | ppm | m/z | z | RT | Fraction | Scan | Source File | Area 10 | Area 01 | Area 02 | Area 03 | Area 04 | Area 05 | Area 06 | Area 07 | Area 08 | Area 09 | #Spec | #Spec 10 | #Spec 01 | #Spec 02 | #Spec 03 | #Spec 04 | #Spec 05 | #Spec 06 | #Spec 07 | #Spec 08 | #Spec 09 | Start | End | PTM |
| --- | --- | --- | --- | --- | --- | --- | --- | --- | --- | --- | --- | --- | --- | --- | --- | --- | --- | --- | --- | --- | --- | --- | --- | --- | --- | --- | --- | --- | --- | --- | --- | --- | --- | --- | --- |
| R.NDANPETHAFVTSPEIVTALAIAGTLK.F | Y | 129.54 | 2779.4392 | 27 | 2.0 | 927.4888 | 3 | 132.55 | 3 | 36304 | Ana\_02.raw | 1.01E7 | 2.02E7 | 9.74E6 | 5.09E6 | 1.93E6 | 1.55E7 | 5.51E6 | 2.63E6 | 9.25E6 | 3.75E6 | 10 | 1 | 1 | 1 | 1 | 1 | 1 | 1 | 1 | 1 | 1 | 480 | 506 |  |
| R.NAVTQEFGPVPDTAR.Y | Y | 100.75 | 1600.7845 | 15 | -0.6 | 801.3990 | 2 | 70.21 | 3 | 19954 | Ana\_02.raw | 1.74E7 | 2.96E7 | 1.53E7 | 8.31E6 | 1.33E7 | 1.84E7 | 9.8E6 | 1.17E7 | 2.11E7 | 9.79E6 | 18 | 2 | 2 | 1 | 1 | 2 | 3 | 1 | 3 | 2 | 1 | 634 | 648 |  |
| K.DINQEVYNFLATAGAK.Y | Y | 96.95 | 1752.8682 | 16 | 3.7 | 877.4446 | 2 | 131.32 | 6 | 36488 | Ana\_05.raw | 1.32E7 | 2.14E7 | 1.67E7 | 3.56E6 | 1.4E7 | 2.04E7 | 1.21E7 | 1.14E7 | 1.81E7 | 9.09E6 | 21 | 2 | 3 | 2 | 2 | 2 | 2 | 2 | 2 | 2 | 2 | 145 | 160 |  |
| K.IVYGHLDDPANQEIER.G | Y | 81.63 | 1867.9064 | 16 | 5.5 | 934.9656 | 2 | 62.29 | 9 | 17737 | Ana\_08.raw | 8.99E6 | 1.28E7 |  | 5.78E6 |  | 6.65E6 |  | 7.61E6 | 1.19E7 | 8.15E6 | 8 | 1 | 1 | 0 | 1 | 0 | 1 | 0 | 1 | 2 | 1 | 69 | 84 |  |
| R.EHAALEPR.H | Y | 80.16 | 921.4668 | 8 | 1.2 | 461.7412 | 2 | 19.50 | 3 | 4759 | Ana\_02.raw | 1.62E7 | 1.35E7 | 2.52E7 | 8.33E6 | 1.28E7 | 2.27E7 |  | 8.62E6 |  | 1.66E7 | 10 | 1 | 1 | 2 | 1 | 1 | 2 | 0 | 1 | 0 | 1 | 672 | 679 |  |
| K.QGLLPLTFADPSDYNK.I | Y | 76.13 | 1777.8887 | 16 | 4.9 | 889.9559 | 2 | 130.71 | 7 | 35974 | Ana\_06.raw |  |  |  |  |  |  | 1.04E6 |  |  |  | 1 | 0 | 0 | 0 | 0 | 0 | 0 | 1 | 0 | 0 | 0 | 702 | 717 |  |
| R.SDFDPGQDTYQHPPK.D | Y | 74.11 | 1730.7535 | 15 | 3.1 | 866.3867 | 2 | 48.86 | 2 | 13758 | Ana\_01.raw | 1.19E7 | 2E6 | 2.62E6 | 5.56E6 | 7.51E6 | 1.83E7 | 6.74E6 | 5.82E6 | 0 | 4.29E6 | 11 | 1 | 1 | 1 | 1 | 1 | 2 | 1 | 1 | 1 | 1 | 535 | 549 |  |
| R.TDIANLAEEFK.D | Y | 73.89 | 1249.6190 | 11 | 4.8 | 625.8198 | 2 | 97.44 | 2 | 27457 | Ana\_01.raw | 4.32E6 | 5.92E6 | 8.36E6 |  | 6.36E6 | 5.92E6 | 4.43E6 | 3.21E6 | 5.48E6 | 2.6E6 | 9 | 1 | 1 | 1 | 0 | 1 | 1 | 1 | 1 | 1 | 1 | 313 | 323 |  |
| K.SQFTITPGSEQIR.A | Y | 69.85 | 1462.7416 | 13 | 2.4 | 732.3798 | 2 | 66.56 | 3 | 18931 | Ana\_02.raw | 1.88E7 | 2.54E7 | 1.62E7 | 7.56E6 | 1.62E7 | 1.6E7 | 1.09E7 | 1.05E7 | 2.14E7 | 1.34E7 | 13 | 1 | 3 | 1 | 1 | 1 | 1 | 1 | 1 | 2 | 1 | 412 | 424 |  |
| R.WVVIGDENYGEGSSR.E | Y | 66.37 | 1666.7587 | 15 | 1.0 | 834.3875 | 2 | 81.28 | 4 | 22564 | Ana\_03.raw |  |  | 1.56E6 | 3.47E5 | 1.78E6 | 8.29E5 | 5.27E5 | 3.05E6 |  | 1.11E6 | 7 | 0 | 0 | 1 | 1 | 1 | 1 | 1 | 1 | 0 | 1 | 657 | 671 |  |
| K.FNPETDFLTGK.D | Y | 65.21 | 1267.6084 | 11 | 6.4 | 634.8156 | 2 | 86.83 | 1 | 24447 | ANa\_10.raw | 1.63E7 | 2.45E7 | 2.4E7 |  | 1.2E7 | 2.35E7 |  | 1.38E7 | 2.19E7 | 4.93E6 | 10 | 2 | 2 | 1 | 0 | 1 | 1 | 0 | 1 | 1 | 1 | 507 | 517 |  |
| K.FKLEAPDADELPR.S | Y | 64.99 | 1499.7620 | 13 | 0.8 | 500.9283 | 3 | 79.45 | 3 | 22469 | Ana\_02.raw | 4.37E6 | 6.61E6 | 1.11E7 | 1.18E6 | 1.63E5 | 5.91E6 | 9.56E5 | 3.39E6 | 4.54E6 | 2.68E5 | 10 | 1 | 1 | 1 | 1 | 1 | 1 | 1 | 1 | 1 | 1 | 522 | 534 |  |
| R.LQLLEPFDK.W | Y | 59.62 | 1101.6069 | 9 | 2.6 | 551.8122 | 2 | 92.95 | 3 | 26045 | Ana\_02.raw | 1.22E6 | 5.48E6 | 5.48E6 | 2.7E6 | 2.22E6 | 3.97E6 | 2.91E6 | 2.8E6 | 3.3E6 | 1.58E6 | 10 | 1 | 1 | 1 | 1 | 1 | 1 | 1 | 1 | 1 | 1 | 565 | 573 |  |
| R.VGLIGSC(+57.02)TNSSYEDM(+15.99)GR.S | Y | 58.48 | 1860.7982 | 17 | 6.4 | 931.4124 | 2 | 61.51 | 6 | 17622 | Ana\_05.raw | 1.45E6 | 2.87E6 | 1.76E6 |  | 1.39E6 | 1.93E6 |  |  |  |  | 5 | 1 | 1 | 1 | 0 | 1 | 1 | 0 | 0 | 0 | 0 | 379 | 395 | Carbamidomethylation; Oxidation (M) |
| K.NTIVTSYNR.N | Y | 57.77 | 1066.5408 | 9 | 4.0 | 534.2798 | 2 | 36.40 | 2 | 9943 | Ana\_01.raw |  | 2.04E7 |  |  | 1.22E7 | 1.42E7 | 9.91E6 | 7.76E6 |  |  | 5 | 0 | 1 | 0 | 0 | 1 | 1 | 1 | 1 | 0 | 0 | 466 | 474 |  |
| K.VAM(+15.99)SHFEPSEYIR.Y | Y | 55.72 | 1580.7292 | 13 | 6.1 | 527.9202 | 3 | 64.56 | 1 | 18300 | ANa\_10.raw | 5.75E6 | 8.72E6 | 9.6E5 | 4.74E6 | 6.09E6 | 5.37E6 | 5.04E6 | 5.74E6 | 6.92E6 | 4.11E6 | 10 | 1 | 1 | 1 | 1 | 1 | 1 | 1 | 1 | 1 | 1 | 32 | 44 | Oxidation (M) |
| R.DGYAQILR.D | Y | 54.60 | 934.4872 | 8 | 3.2 | 468.2524 | 2 | 62.27 | 4 | 17325 | Ana\_03.raw |  |  |  | 2.37E6 | 3.29E6 | 3.92E6 | 3.16E6 | 1.79E6 | 6.96E6 | 5.59E6 | 7 | 0 | 0 | 0 | 1 | 1 | 1 | 1 | 1 | 1 | 1 | 430 | 437 |  |
| R.AKDINQEVYNFLATAGAK.Y | Y | 51.34 | 1952.0002 | 18 | -1.2 | 651.6732 | 3 | 116.61 | 5 | 32316 | Ana\_04.raw |  |  |  |  | 5.56E5 |  |  | 2.15E5 |  |  | 2 | 0 | 0 | 0 | 0 | 1 | 0 | 0 | 1 | 0 | 0 | 143 | 160 |  |
| R.IHETNLK.K | Y | 48.57 | 853.4658 | 7 | 2.5 | 427.7412 | 2 | 18.08 | 3 | 4293 | Ana\_02.raw |  |  | 2.58E6 | 3.96E6 | 6.56E6 | 1.13E7 | 6.74E6 |  |  |  | 5 | 0 | 0 | 1 | 1 | 1 | 1 | 1 | 0 | 0 | 0 | 694 | 700 |  |
| R.VDVSPTSQR.L | Y | 48.30 | 987.4985 | 9 | 3.7 | 494.7584 | 2 | 25.49 | 6 | 6718 | Ana\_05.raw | 1.64E7 | 2.56E7 | 3.44E7 |  | 2.23E7 | 3.25E7 | 2.11E7 | 1.54E7 | 1.04E7 |  | 8 | 1 | 1 | 1 | 0 | 1 | 1 | 1 | 1 | 1 | 0 | 556 | 564 |  |
| R.LNRPLTLSEK.I | Y | 42.32 | 1169.6768 | 10 | 4.0 | 585.8480 | 2 | 35.43 | 9 | 9545 | Ana\_08.raw | 8.33E5 | 2.89E6 |  | 2.94E6 | 5.64E6 | 3.43E6 | 1.64E6 |  | 1.36E6 | 1.4E6 | 8 | 1 | 1 | 0 | 1 | 1 | 1 | 1 | 0 | 1 | 1 | 59 | 68 |  |
| K.LTIQGLK.D | Y | 39.93 | 771.4854 | 7 | 4.8 | 386.7518 | 2 | 52.44 | 1 | 14743 | ANa\_10.raw | 6.74E5 | 3.86E6 | 3.24E6 |  | 2.09E6 |  | 1.78E6 | 1.08E6 | 3.89E6 |  | 7 | 1 | 1 | 1 | 0 | 1 | 0 | 1 | 1 | 1 | 0 | 724 | 730 |  |
| K.VAGILTVK.G | Y | 36.37 | 799.5167 | 8 | 3.6 | 400.7671 | 2 | 49.43 | 2 | 13933 | Ana\_01.raw |  | 2.98E6 | 3.02E6 | 7.57E5 |  | 1.88E6 | 1.04E6 |  |  | 1.4E6 | 6 | 0 | 1 | 1 | 1 | 0 | 1 | 1 | 0 | 0 | 1 | 251 | 258 |  |
| R.YDLLEK.N | Y | 33.73 | 779.4065 | 6 | 1.7 | 390.7112 | 2 | 51.16 | 4 | 14122 | Ana\_03.raw | 1.7E6 | 4.62E6 | 5.72E6 | 2.54E6 | 3.92E6 | 6.51E6 | 4.25E6 | 2.69E6 | 2.3E6 | 2.18E6 | 10 | 1 | 1 | 1 | 1 | 1 | 1 | 1 | 1 | 1 | 1 | 45 | 50 |  |
| K.LTGSLSGWTSPK.D | Y | 27.04 | 1232.6401 | 12 | 3.9 | 617.3298 | 2 | 89.60 | 10 | 25102 | Ana\_09.raw |  |  |  |  |  |  |  |  |  | 6.28E4 | 1 | 0 | 0 | 0 | 0 | 0 | 0 | 0 | 0 | 0 | 1 | 234 | 245 |  |
| K.NINIVR.K | Y | 22.36 | 727.4341 | 6 | 3.9 | 364.7257 | 2 | 31.02 | 9 | 8184 | Ana\_08.raw |  |  |  |  |  |  |  |  | 3.04E6 |  | 1 | 0 | 0 | 0 | 0 | 0 | 0 | 0 | 0 | 1 | 0 | 51 | 56 |  |
| total 26 peptides |  |  |  |  |  |  |  |  |  |  |  |  |  |  |  |  |  |  |  |  |  |  |  |  |  |  |  |  |  |  |  |  |  |  |  |
| --- | --- | --- | --- | --- | --- | --- | --- | --- | --- | --- | --- | --- | --- | --- | --- | --- | --- | --- | --- | --- | --- | --- | --- | --- | --- | --- | --- | --- | --- | --- | --- | --- | --- | --- | --- |

Best Unique PSM (Scan F3:36304, m/z=927.4888, z=3, RT=132.55, ppm=2.0):

sp|P97449|AMPN\_MOUSE

back to list

  

| Protein Coverage
| Supporting Peptides
| Best Unique PSM
|

Protein Coverage:

Supporting Peptides:

| Peptide | Uniq | -10lgP | Mass | Length | ppm | m/z | z | RT | Fraction | Scan | Source File | Area 10 | Area 01 | Area 02 | Area 03 | Area 04 | Area 05 | Area 06 | Area 07 | Area 08 | Area 09 | #Spec | #Spec 10 | #Spec 01 | #Spec 02 | #Spec 03 | #Spec 04 | #Spec 05 | #Spec 06 | #Spec 07 | #Spec 08 | #Spec 09 | Start | End | PTM |
| --- | --- | --- | --- | --- | --- | --- | --- | --- | --- | --- | --- | --- | --- | --- | --- | --- | --- | --- | --- | --- | --- | --- | --- | --- | --- | --- | --- | --- | --- | --- | --- | --- | --- | --- | --- |
| K.TPDQIM(+15.99)ELFDSITYSK.G | Y | 110.04 | 1902.8921 | 16 | 2.6 | 952.4558 | 2 | 129.94 | 3 | 35602 | Ana\_02.raw | 1.28E7 | 1.02E7 | 1.47E7 | 7.09E6 | 1.38E7 | 2.4E7 | 2.2E7 | 1.16E7 | 1.35E7 | 3.43E6 | 12 | 1 | 1 | 1 | 1 | 1 | 2 | 2 | 1 | 1 | 1 | 463 | 478 | Oxidation (M) |
| K.LQNQLQTDLSVIPVINR.A | Y | 96.12 | 1950.0898 | 17 | 1.5 | 976.0536 | 2 | 116.35 | 6 | 32526 | Ana\_05.raw | 1.4E7 | 2.12E7 | 6.79E6 | 1.05E7 | 1.33E7 | 1.72E7 | 1.37E7 | 8.5E6 | 2.16E7 | 6.01E6 | 27 | 2 | 3 | 2 | 4 | 2 | 4 | 4 | 2 | 2 | 2 | 641 | 657 |  |
| K.AVNQQTAVQPPATVR.T | Y | 93.43 | 1578.8478 | 15 | 4.2 | 790.4345 | 2 | 48.00 | 7 | 13422 | Ana\_06.raw | 9.32E6 | 2.39E7 | 1.46E7 | 5.49E6 | 1.09E7 | 1.99E7 | 1.01E7 | 7.89E6 | 3.12E6 | 5.34E6 | 17 | 1 | 3 | 1 | 2 | 2 | 3 | 2 | 1 | 1 | 1 | 523 | 537 |  |
| K.SDQIALPDFNAGAM(+15.99)ENWGLVTYR.E | Y | 90.17 | 2583.2063 | 23 | 1.8 | 1292.6128 | 2 | 127.76 | 6 | 35501 | Ana\_05.raw |  | 2.02E6 | 6.19E5 |  |  | 3.63E6 | 1.87E6 | 9.36E5 |  |  | 5 | 0 | 1 | 1 | 0 | 0 | 1 | 1 | 1 | 0 | 0 | 340 | 362 | Oxidation (M) |
| R.ESSLVFDSQSSSISNK.E | Y | 90.10 | 1713.8057 | 16 | 1.7 | 857.9116 | 2 | 64.81 | 6 | 18611 | Ana\_05.raw | 1.21E7 | 2.95E7 | 1.58E7 | 7.62E6 | 1.26E7 | 1.8E7 | 1.21E7 | 8.97E6 | 1.92E7 | 1.13E7 | 16 | 1 | 3 | 2 | 1 | 1 | 1 | 2 | 2 | 2 | 1 | 363 | 378 |  |
| R.YLSYTLNPDYIR.K | Y | 88.34 | 1516.7561 | 12 | 2.3 | 759.3871 | 2 | 91.62 | 3 | 25696 | Ana\_02.raw | 1.25E7 | 4.38E6 | 1.61E7 | 4.89E6 | 1.02E7 | 1.27E7 | 7.32E6 | 5.75E6 | 1.75E7 |  | 12 | 1 | 2 | 2 | 1 | 1 | 2 | 1 | 1 | 1 | 0 | 843 | 854 |  |
| K.TPDQIMELFDSITYSK.G | Y | 86.38 | 1886.8971 | 16 | 1.3 | 944.4571 | 2 | 134.08 | 9 | 37083 | Ana\_08.raw |  | 2.91E6 |  | 2.89E5 |  | 1.85E6 |  |  | 2.64E6 | 9.95E5 | 5 | 0 | 1 | 0 | 1 | 0 | 1 | 0 | 0 | 1 | 1 | 463 | 478 |  |
| R.AQIIHDSFNLASAK.M | Y | 85.71 | 1513.7888 | 14 | 4.2 | 757.9048 | 2 | 67.53 | 2 | 19240 | Ana\_01.raw | 2.54E6 | 2E7 | 1.04E7 | 6.38E6 | 1.06E7 | 1.56E7 | 8.84E6 | 4.74E6 |  | 2.37E6 | 12 | 1 | 2 | 1 | 1 | 1 | 2 | 2 | 1 | 0 | 1 | 658 | 671 |  |
| R.M(+15.99)LSSFLTEDLFK.K | Y | 83.30 | 1445.7112 | 12 | 2.7 | 723.8648 | 2 | 123.73 | 6 | 34454 | Ana\_05.raw | 4.25E6 | 2.94E7 | 3.58E7 | 1.22E7 | 2.18E7 | 4.22E7 | 2.63E7 | 2.18E7 | 3.31E7 | 1.16E7 | 17 | 1 | 3 | 2 | 1 | 2 | 2 | 1 | 3 | 1 | 1 | 485 | 496 | Oxidation (M) |
| R.FSSEFELQQLEQFK.A | Y | 77.68 | 1758.8464 | 14 | 3.2 | 880.4333 | 2 | 115.90 | 6 | 32409 | Ana\_05.raw | 1.79E7 | 3.53E7 | 1.97E7 | 1.36E7 | 5.46E6 | 1.09E7 | 1.69E7 | 1.32E7 | 2.84E7 | 1.18E7 | 15 | 1 | 2 | 1 | 1 | 1 | 2 | 2 | 2 | 2 | 1 | 908 | 921 |  |
| K.VVATTQM(+15.99)QAADAR.K | Y | 75.81 | 1376.6718 | 13 | 4.1 | 689.3459 | 2 | 24.32 | 1 | 6207 | ANa\_10.raw | 1.89E6 |  | 1.78E7 | 9.36E6 | 1.31E7 | 2.03E7 | 1.68E7 | 9.95E6 |  | 6.69E6 | 8 | 1 | 0 | 1 | 1 | 1 | 1 | 1 | 1 | 0 | 1 | 206 | 218 | Oxidation (M) |
| K.VVATTQMQAADAR.K | Y | 75.66 | 1360.6769 | 13 | 7.4 | 681.3508 | 2 | 37.55 | 2 | 10299 | Ana\_01.raw |  | 2.71E6 |  |  |  |  |  |  | 2.84E6 | 2.55E6 | 3 | 0 | 1 | 0 | 0 | 0 | 0 | 0 | 0 | 1 | 1 | 206 | 218 |  |
| K.M(+15.99)IPITLALDNTLFLVK.E | Y | 71.43 | 1817.0372 | 16 | 4.3 | 606.6890 | 3 | 133.83 | 6 | 37208 | Ana\_05.raw |  | 1.44E6 | 9.81E5 | 2.46E6 | 7.49E5 | 5.25E6 | 2.74E6 |  | 1.49E6 | 6.8E5 | 11 | 0 | 1 | 1 | 2 | 1 | 2 | 2 | 0 | 1 | 1 | 672 | 687 | Oxidation (M) |
| K.KLQNQLQTDLSVIPVINR.A | Y | 69.25 | 2078.1848 | 18 | 1.7 | 693.7367 | 3 | 106.93 | 6 | 30088 | Ana\_05.raw |  |  |  |  | 1.86E5 | 8.6E5 |  |  |  |  | 2 | 0 | 0 | 0 | 0 | 1 | 1 | 0 | 0 | 0 | 0 | 640 | 657 |  |
| R.TLDGTPAPNIDK.T | Y | 69.16 | 1240.6299 | 12 | 1.4 | 621.3231 | 2 | 43.67 | 3 | 12261 | Ana\_02.raw | 4E6 | 2.73E7 | 2.38E7 | 7.63E6 | 1.92E7 | 3.04E7 | 1.89E7 | 3.38E7 | 1.08E7 |  | 18 | 1 | 2 | 2 | 1 | 2 | 2 | 3 | 3 | 2 | 0 | 141 | 152 |  |
| K.ADNSATGFGTGTR.A | Y | 67.02 | 1253.5636 | 13 | 4.9 | 627.7922 | 2 | 33.51 | 7 | 9052 | Ana\_06.raw | 1.8E7 | 3.83E7 | 1.97E7 | 1.3E7 | 1.54E7 |  | 2.39E7 | 1.17E7 | 1.69E7 | 8.37E6 | 11 | 1 | 2 | 1 | 1 | 1 | 0 | 2 | 1 | 1 | 1 | 922 | 934 |  |
| K.TLIPDSYR.V | Y | 64.13 | 963.5025 | 8 | 3.7 | 482.7603 | 2 | 61.16 | 7 | 17300 | Ana\_06.raw | 9.17E6 | 3E7 | 2.21E7 | 1.03E7 | 1.71E7 | 2.36E7 | 1.86E7 | 1.03E7 | 1.9E7 | 1.24E7 | 16 | 1 | 3 | 1 | 2 | 2 | 1 | 1 | 1 | 2 | 2 | 79 | 86 |  |
| R.VM(+15.99)AVDALASSHPLSSPADEIK.T | Y | 56.71 | 2153.0674 | 21 | 2.5 | 718.6982 | 3 | 82.96 | 7 | 23415 | Ana\_06.raw |  | 1.89E6 | 1.58E6 |  |  |  | 1.16E6 |  |  |  | 3 | 0 | 1 | 1 | 0 | 0 | 0 | 1 | 0 | 0 | 0 | 442 | 462 | Oxidation (M) |
| K.HFLLDSK.S | Y | 52.41 | 858.4599 | 7 | 2.4 | 430.2383 | 2 | 39.60 | 3 | 11034 | Ana\_02.raw |  |  | 1.47E7 | 6.58E6 | 1.01E7 | 1.52E7 |  |  |  | 9.04E6 | 5 | 0 | 0 | 1 | 1 | 1 | 1 | 0 | 0 | 0 | 1 | 565 | 571 |  |
| K.DLM(+15.99)VLNDVYR.V | Y | 52.30 | 1252.6122 | 10 | 5.4 | 627.3168 | 2 | 85.06 | 9 | 24200 | Ana\_08.raw | 1.72E6 | 2.24E6 |  |  |  |  |  |  | 4.38E6 |  | 3 | 1 | 1 | 0 | 0 | 0 | 0 | 0 | 0 | 1 | 0 | 432 | 441 | Oxidation (M) |
| R.ALEQALEK.T | Y | 50.54 | 900.4916 | 8 | 4.9 | 451.2553 | 2 | 36.50 | 2 | 9976 | Ana\_01.raw |  | 3.03E7 |  |  |  |  |  |  |  |  | 1 | 0 | 1 | 0 | 0 | 0 | 0 | 0 | 0 | 0 | 0 | 935 | 942 |  |
| R.SEVYGPM(+15.99)KR.Y | Y | 50.07 | 1081.5226 | 9 | 9.8 | 541.7739 | 2 | 21.12 | 2 | 5111 | Ana\_01.raw | 2.86E6 | 1.02E7 | 5.35E6 |  |  |  |  |  |  | 2.29E6 | 5 | 1 | 2 | 1 | 0 | 0 | 0 | 0 | 0 | 0 | 1 | 711 | 719 | Oxidation (M) |
| R.TLDGTPAPNIDKTELVER.T | Y | 49.29 | 1968.0164 | 18 | 1.2 | 657.0135 | 3 | 71.13 | 4 | 19789 | Ana\_03.raw |  |  |  | 4.89E5 |  |  |  | 1.08E6 |  |  | 2 | 0 | 0 | 0 | 1 | 0 | 0 | 0 | 1 | 0 | 0 | 141 | 158 |  |
| R.SEVYGPM(+15.99)K.R | Y | 48.75 | 925.4215 | 8 | 3.7 | 463.7197 | 2 | 23.53 | 6 | 6088 | Ana\_05.raw |  |  | 1.23E7 |  | 2.39E6 | 8.61E6 | 4.07E6 | 4.03E6 |  |  | 5 | 0 | 0 | 1 | 0 | 1 | 1 | 1 | 1 | 0 | 0 | 711 | 718 | Oxidation (M) |
| R.SEYM(+15.99)EGDVKK.V | Y | 47.39 | 1200.5332 | 10 | 3.2 | 601.2758 | 2 | 21.02 | 2 | 5077 | Ana\_01.raw |  | 1.79E7 | 6.08E6 |  | 6.75E6 | 7.92E6 |  |  |  |  | 6 | 0 | 2 | 1 | 0 | 1 | 2 | 0 | 0 | 0 | 0 | 196 | 205 | Oxidation (M) |
| R.SEYM(+15.99)EGDVK.K | Y | 44.64 | 1072.4382 | 9 | 2.6 | 537.2278 | 2 | 25.83 | 8 | 6591 | Ana\_07.raw |  | 8.08E6 | 1.13E7 | 6.31E6 | 8.81E6 | 1.24E7 | 1.01E7 | 2.01E6 |  | 3.73E6 | 8 | 0 | 1 | 1 | 1 | 1 | 1 | 1 | 1 | 0 | 1 | 196 | 204 | Oxidation (M) |
| K.DVWILNR.Y | Y | 42.49 | 914.4974 | 7 | -0.4 | 458.2558 | 2 | 85.35 | 8 | 23906 | Ana\_07.raw | 2.29E5 | 0 | 8.46E5 |  | 5.22E5 | 1.66E6 |  | 3.45E5 | 4.57E6 |  | 7 | 1 | 1 | 1 | 0 | 1 | 1 | 0 | 1 | 1 | 0 | 836 | 842 |  |
| R.ANIDWVK.E | Y | 37.96 | 844.4443 | 7 | 5.2 | 423.2316 | 2 | 52.83 | 2 | 14934 | Ana\_01.raw |  | 2.61E6 | 1.83E6 |  | 7.32E5 |  |  |  |  |  | 3 | 0 | 1 | 1 | 0 | 1 | 0 | 0 | 0 | 0 | 0 | 945 | 951 |  |
| R.QYEMDSQFQGELADDLAGFYR.S | Y | 35.59 | 2482.0747 | 21 | -6.8 | 828.3599 | 3 | 132.77 | 7 | 36576 | Ana\_06.raw |  |  |  |  |  |  | 2.58E4 |  |  |  | 1 | 0 | 0 | 0 | 0 | 0 | 0 | 1 | 0 | 0 | 0 | 175 | 195 |  |
| K.TELVER.T | Y | 33.81 | 745.3970 | 6 | 3.8 | 373.7072 | 2 | 23.71 | 6 | 6148 | Ana\_05.raw |  |  | 3.32E7 | 1.68E7 | 1.95E7 | 3.35E7 | 6.3E6 | 1.62E7 |  |  | 6 | 0 | 0 | 1 | 1 | 1 | 1 | 1 | 1 | 0 | 0 | 153 | 158 |  |
| K.GFYISK.T | Y | 27.43 | 713.3748 | 6 | 2.4 | 357.6956 | 2 | 44.50 | 4 | 12172 | Ana\_03.raw |  |  |  | 1.74E6 |  |  |  |  |  |  | 1 | 0 | 0 | 0 | 1 | 0 | 0 | 0 | 0 | 0 | 0 | 4 | 9 |  |
| total 31 peptides |  |  |  |  |  |  |  |  |  |  |  |  |  |  |  |  |  |  |  |  |  |  |  |  |  |  |  |  |  |  |  |  |  |  |  |
| --- | --- | --- | --- | --- | --- | --- | --- | --- | --- | --- | --- | --- | --- | --- | --- | --- | --- | --- | --- | --- | --- | --- | --- | --- | --- | --- | --- | --- | --- | --- | --- | --- | --- | --- | --- |

Best Unique PSM (Scan F3:35602, m/z=952.4558, z=2, RT=129.94, ppm=2.6):

sp|P07356|ANXA2\_MOUSE

back to list

  

| Protein Coverage
| Supporting Peptides
| Best Unique PSM
|

Protein Coverage:

Supporting Peptides:

| Peptide | Uniq | -10lgP | Mass | Length | ppm | m/z | z | RT | Fraction | Scan | Source File | Area 10 | Area 01 | Area 02 | Area 03 | Area 04 | Area 05 | Area 06 | Area 07 | Area 08 | Area 09 | #Spec | #Spec 10 | #Spec 01 | #Spec 02 | #Spec 03 | #Spec 04 | #Spec 05 | #Spec 06 | #Spec 07 | #Spec 08 | #Spec 09 | Start | End | PTM |
| --- | --- | --- | --- | --- | --- | --- | --- | --- | --- | --- | --- | --- | --- | --- | --- | --- | --- | --- | --- | --- | --- | --- | --- | --- | --- | --- | --- | --- | --- | --- | --- | --- | --- | --- | --- |
| K.SALSGHLETVILGLLK.T | Y | 107.26 | 1649.9716 | 16 | -0.2 | 825.9929 | 2 | 132.40 | 3 | 36263 | Ana\_02.raw | 2.96E7 | 3.79E7 | 1.97E7 | 9.4E6 | 3.21E7 | 4.31E7 | 1.99E7 | 1.76E7 | 5.58E7 | 5.85E7 | 43 | 3 | 5 | 3 | 2 | 6 | 2 | 2 | 4 | 9 | 7 | 89 | 104 |  |
| R.AEDGSVIDYELIDQDAR.E | Y | 99.29 | 1907.8748 | 17 | -0.5 | 954.9442 | 2 | 95.93 | 7 | 26869 | Ana\_06.raw | 2.23E6 | 0 |  |  |  | 2.43E6 | 9.08E5 |  | 1.02E7 | 4.52E6 | 8 | 1 | 2 | 0 | 0 | 0 | 1 | 1 | 0 | 2 | 1 | 180 | 196 |  |
| R.RAEDGSVIDYELIDQDAR.E | Y | 99.01 | 2063.9758 | 18 | 6.0 | 689.0034 | 3 | 83.02 | 9 | 23622 | Ana\_08.raw | 2.28E5 | 1.62E6 | 8.43E5 | 1.74E6 | 3.16E6 | 3.15E6 |  | 2.1E6 | 1.96E7 | 2.04E6 | 13 | 1 | 1 | 1 | 1 | 1 | 1 | 0 | 1 | 4 | 2 | 179 | 196 |  |
| K.GVDEVTIVNILTNR.S | Y | 98.61 | 1541.8413 | 14 | 3.6 | 771.9307 | 2 | 123.64 | 6 | 34431 | Ana\_05.raw | 4.02E7 | 3.71E7 | 3.74E7 | 1.31E7 | 3.86E7 | 4.84E7 | 2.87E7 | 1.47E7 | 6.66E7 | 3.97E7 | 40 | 4 | 5 | 3 | 3 | 5 | 4 | 3 | 4 | 6 | 3 | 50 | 63 |  |
| K.GLGTDEDSLIEIIC(+57.02)SR.T | Y | 98.52 | 1776.8563 | 16 | 4.2 | 889.4391 | 2 | 125.74 | 4 | 34181 | Ana\_03.raw | 6.9E6 | 7.11E6 | 2.59E6 | 4.89E6 | 6.71E6 | 1.44E7 | 1.61E7 | 1.43E6 | 7.55E7 | 1.8E6 | 18 | 1 | 3 | 1 | 2 | 1 | 1 | 2 | 1 | 5 | 1 | 120 | 135 | Carbamidomethylation |
| K.GDLENAFLNLVQC(+57.02)IQNKPLYFADR.L | Y | 91.44 | 2837.4170 | 24 | 3.0 | 946.8157 | 3 | 136.61 | 6 | 37965 | Ana\_05.raw |  | 3.36E5 |  |  |  | 1.17E6 |  |  | 8.73E5 | 2.41E6 | 4 | 0 | 1 | 0 | 0 | 0 | 1 | 0 | 0 | 1 | 1 | 250 | 273 | Carbamidomethylation |
| K.TPAQYDASELK.A | Y | 90.48 | 1221.5876 | 11 | 5.9 | 611.8047 | 2 | 44.47 | 1 | 12389 | ANa\_10.raw | 8.03E7 | 9.32E7 | 6.37E7 | 3.39E7 | 5.22E7 | 8.06E7 | 5.3E7 | 4.59E7 | 1.21E8 | 4.91E7 | 14 | 1 | 1 | 2 | 2 | 1 | 2 | 1 | 1 | 2 | 1 | 105 | 115 |  |
| K.TDLEKDIISDTSGDFR.K | Y | 86.84 | 1810.8584 | 16 | 7.2 | 906.4430 | 2 | 91.87 | 9 | 26055 | Ana\_08.raw |  |  | 7.95E6 | 3.94E6 | 7.32E6 | 5.36E6 | 1.87E6 | 5.25E5 | 3.26E7 |  | 12 | 0 | 0 | 1 | 2 | 2 | 2 | 2 | 1 | 2 | 0 | 153 | 168 |  |
| K.TDLEKDIISDTSGDFRK.L | Y | 80.38 | 1938.9534 | 17 | 6.0 | 647.3289 | 3 | 84.61 | 1 | 23833 | ANa\_10.raw | 2.3E6 |  |  |  |  |  |  |  | 5.24E6 |  | 2 | 1 | 0 | 0 | 0 | 0 | 0 | 0 | 0 | 1 | 0 | 153 | 169 |  |
| R.TNQELQEINR.V | Y | 77.83 | 1243.6157 | 10 | 3.4 | 622.8173 | 2 | 41.01 | 6 | 11469 | Ana\_05.raw | 6.22E7 | 6.33E7 | 5.55E7 | 2.39E7 | 3.87E7 | 4.1E7 | 3.64E7 | 3.61E7 | 6.91E7 | 5.42E7 | 14 | 1 | 1 | 1 | 1 | 1 | 2 | 2 | 2 | 2 | 1 | 136 | 145 |  |
| K.SLYYYIQQDTK.G | Y | 73.02 | 1420.6874 | 11 | 0.9 | 711.3516 | 2 | 80.41 | 10 | 22652 | Ana\_09.raw | 5.57E6 | 1.14E7 |  | 1.48E6 | 7.03E6 | 1.14E7 | 4.64E6 | 5.79E6 | 2.56E7 | 7.44E6 | 11 | 1 | 2 | 0 | 1 | 1 | 1 | 1 | 1 | 2 | 1 | 314 | 324 |  |
| K.SYSPYDM(+15.99)LESIK.K | Y | 71.15 | 1447.6541 | 12 | 2.7 | 724.8362 | 2 | 83.61 | 6 | 23853 | Ana\_05.raw |  |  | 1.41E6 | 2.25E6 |  | 2.73E6 | 2.56E6 |  |  | 1.48E6 | 5 | 0 | 0 | 1 | 1 | 0 | 1 | 1 | 0 | 0 | 1 | 234 | 245 | Oxidation (M) |
| K.ELPSALKSALSGHLETVILGLLK.T | Y | 69.01 | 2388.3992 | 23 | 5.2 | 598.1102 | 4 | 135.98 | 6 | 37791 | Ana\_05.raw |  |  |  |  | 9.26E5 | 2.05E6 | 5.11E5 |  |  | 4.61E5 | 6 | 0 | 0 | 0 | 0 | 2 | 2 | 1 | 0 | 0 | 1 | 82 | 104 |  |
| R.DALNIETAVK.T | Y | 67.44 | 1072.5764 | 10 | 1.9 | 537.2965 | 2 | 65.00 | 5 | 18534 | Ana\_04.raw | 1.98E7 | 2.09E7 | 1.82E7 | 1.09E7 | 1.59E7 | 1.92E7 | 1.16E7 | 1.01E7 | 3.49E7 | 2.18E7 | 17 | 1 | 1 | 2 | 2 | 2 | 2 | 2 | 2 | 2 | 1 | 38 | 47 |  |
| K.DIISDTSGDFR.K | Y | 63.37 | 1224.5623 | 11 | 5.5 | 613.2917 | 2 | 76.51 | 6 | 21914 | Ana\_05.raw | 5.87E6 | 2.03E7 | 1.6E7 | 9.54E6 | 1.26E7 | 1.69E7 | 1.21E7 | 1.08E7 | 4.51E6 | 1.03E7 | 15 | 1 | 2 | 1 | 2 | 1 | 1 | 2 | 1 | 2 | 2 | 158 | 168 |  |
| R.QDIAFAYQR.R | Y | 58.75 | 1110.5458 | 9 | -0.1 | 556.2801 | 2 | 61.07 | 6 | 17493 | Ana\_05.raw | 4.24E6 | 4.98E6 | 2.66E6 | 3.23E6 | 6.67E6 | 9.39E6 | 4.03E6 | 5.62E6 | 1.09E7 |  | 11 | 1 | 1 | 1 | 1 | 1 | 3 | 1 | 1 | 1 | 0 | 69 | 77 |  |
| K.DIISDTSGDFRK.L | Y | 50.17 | 1352.6572 | 12 | 6.6 | 677.3403 | 2 | 65.09 | 10 | 18449 | Ana\_09.raw | 1.8E6 | 4.16E5 |  |  |  |  |  |  |  | 4.49E6 | 4 | 1 | 1 | 0 | 0 | 0 | 0 | 0 | 0 | 0 | 2 | 158 | 169 |  |
| R.ELYDAGVK.R | Y | 46.42 | 893.4494 | 8 | 5.0 | 447.7342 | 2 | 38.98 | 1 | 10699 | ANa\_10.raw | 5.02E7 | 5.18E7 | 5.45E7 | 2.36E7 | 4.29E7 | 5.44E7 | 3.47E7 | 2.98E7 | 5.6E7 | 3.79E7 | 12 | 1 | 1 | 2 | 1 | 1 | 1 | 1 | 1 | 1 | 2 | 197 | 204 |  |
| R.SVC(+57.02)HLQK.V | Y | 43.53 | 870.4382 | 7 | 1.7 | 436.2271 | 2 | 17.22 | 5 | 4052 | Ana\_04.raw |  |  |  |  | 7.71E6 |  | 4.72E6 | 2.92E6 |  |  | 3 | 0 | 0 | 0 | 0 | 1 | 0 | 1 | 1 | 0 | 0 | 221 | 227 | Carbamidomethylation |
| R.SEVDM(+15.99)LK.I | Y | 39.53 | 836.3950 | 7 | 5.0 | 419.2068 | 2 | 27.32 | 2 | 7183 | Ana\_01.raw | 2.45E7 | 2.94E7 | 2.79E7 |  | 1.95E7 |  |  |  |  |  | 4 | 1 | 1 | 1 | 0 | 1 | 0 | 0 | 0 | 0 | 0 | 296 | 302 | Oxidation (M) |
| K.WISIM(+15.99)TER.S | Y | 38.13 | 1050.5168 | 8 | 4.5 | 526.2681 | 2 | 64.54 | 6 | 18535 | Ana\_05.raw |  |  | 0 |  | 5.83E6 | 7.07E6 |  | 5.44E6 |  |  | 4 | 0 | 0 | 1 | 0 | 1 | 1 | 0 | 1 | 0 | 0 | 213 | 220 | Oxidation (M) |
| R.VYKEM(+15.99)YK.T | Y | 37.49 | 975.4735 | 7 | 3.7 | 488.7458 | 2 | 20.03 | 10 | 4825 | Ana\_09.raw | 2.23E6 |  |  |  |  | 7.35E5 |  |  |  | 2.12E6 | 3 | 1 | 0 | 0 | 0 | 0 | 1 | 0 | 0 | 0 | 1 | 146 | 152 | Oxidation (M) |
| K.SYSPYDMLESIK.K | Y | 35.50 | 1431.6592 | 12 | 5.3 | 716.8406 | 2 | 108.89 | 9 | 30594 | Ana\_08.raw |  |  |  |  |  |  |  |  | 0 |  | 1 | 0 | 0 | 0 | 0 | 0 | 0 | 0 | 0 | 1 | 0 | 234 | 245 |  |
| K.LM(+15.99)VALAK.G | Y | 35.42 | 760.4517 | 7 | 4.3 | 381.2347 | 2 | 41.92 | 5 | 11697 | Ana\_04.raw |  | 1.29E6 |  |  | 2.78E6 | 3.41E6 |  |  | 6.24E6 |  | 4 | 0 | 1 | 0 | 0 | 1 | 1 | 0 | 0 | 1 | 0 | 170 | 176 | Oxidation (M) |
| K.ELPSALK.S | Y | 30.89 | 756.4381 | 7 | 3.9 | 379.2278 | 2 | 35.76 | 9 | 9651 | Ana\_08.raw |  | 1.41E7 |  |  |  |  | 8.1E6 | 7.04E6 | 9.2E6 | 1.42E7 | 5 | 0 | 1 | 0 | 0 | 0 | 0 | 1 | 1 | 1 | 1 | 82 | 88 |  |
| K.SYSPYDM(+15.99)LESIKK.E | Y | 22.32 | 1575.7490 | 13 | 6.5 | 526.2604 | 3 | 68.00 | 9 | 19407 | Ana\_08.raw |  |  |  |  |  |  |  |  | 1.67E6 |  | 1 | 0 | 0 | 0 | 0 | 0 | 0 | 0 | 0 | 1 | 0 | 234 | 246 | Oxidation (M) |
| total 26 peptides |  |  |  |  |  |  |  |  |  |  |  |  |  |  |  |  |  |  |  |  |  |  |  |  |  |  |  |  |  |  |  |  |  |  |  |
| --- | --- | --- | --- | --- | --- | --- | --- | --- | --- | --- | --- | --- | --- | --- | --- | --- | --- | --- | --- | --- | --- | --- | --- | --- | --- | --- | --- | --- | --- | --- | --- | --- | --- | --- | --- |

Best Unique PSM (Scan F3:36263, m/z=825.9929, z=2, RT=132.40, ppm=-0.2):

sp|Q9R100|CAD17\_MOUSE

back to list

  

| Protein Coverage
| Supporting Peptides
| Best Unique PSM
|

Protein Coverage:

Supporting Peptides:

| Peptide | Uniq | -10lgP | Mass | Length | ppm | m/z | z | RT | Fraction | Scan | Source File | Area 10 | Area 01 | Area 02 | Area 03 | Area 04 | Area 05 | Area 06 | Area 07 | Area 08 | Area 09 | #Spec | #Spec 10 | #Spec 01 | #Spec 02 | #Spec 03 | #Spec 04 | #Spec 05 | #Spec 06 | #Spec 07 | #Spec 08 | #Spec 09 | Start | End | PTM |
| --- | --- | --- | --- | --- | --- | --- | --- | --- | --- | --- | --- | --- | --- | --- | --- | --- | --- | --- | --- | --- | --- | --- | --- | --- | --- | --- | --- | --- | --- | --- | --- | --- | --- | --- | --- |
| K.KPLDFETQPVSSIVFQAENPEPLVK.G | Y | 137.21 | 2811.4695 | 25 | -1.1 | 938.1627 | 3 | 119.33 | 3 | 32872 | Ana\_02.raw | 1.53E7 | 4.88E7 | 4.04E7 | 3.46E6 | 2.04E7 | 1.45E7 | 1.17E7 | 1.37E7 | 1.18E7 | 1.08E7 | 23 | 2 | 3 | 2 | 2 | 2 | 2 | 3 | 2 | 2 | 3 | 516 | 540 |  |
| K.IDSVTGEIFSAAPLDR.E | Y | 104.18 | 1689.8573 | 16 | 2.9 | 845.9384 | 2 | 106.58 | 6 | 29996 | Ana\_05.raw | 3.09E7 | 3.84E7 | 3.37E7 | 1.78E7 | 1.2E7 | 2.89E7 | 1.8E7 | 8.2E6 | 5.99E7 | 2.21E7 | 28 | 2 | 4 | 5 | 2 | 3 | 2 | 3 | 2 | 2 | 3 | 611 | 626 |  |
| K.VPSDGLFLIGEYEGK.V | Y | 92.32 | 1622.8191 | 15 | 4.2 | 812.4202 | 2 | 110.70 | 3 | 30645 | Ana\_02.raw | 1.72E7 | 2.85E7 | 2.1E7 | 1.5E7 | 1.18E7 | 9.85E5 | 1.56E7 | 4.21E5 | 5.5E7 | 2.27E7 | 32 | 3 | 4 | 3 | 3 | 4 | 2 | 4 | 2 | 4 | 3 | 387 | 401 |  |
| K.TNPPAVTFELTGETDGIFK.I | Y | 87.07 | 2036.0101 | 19 | 5.4 | 1019.0178 | 2 | 117.54 | 6 | 32838 | Ana\_05.raw | 2.7E6 | 4.34E6 |  | 1.3E6 | 8.22E5 | 6.62E6 | 2.45E6 |  | 1.54E6 | 3.18E6 | 8 | 1 | 1 | 0 | 1 | 1 | 1 | 1 | 0 | 1 | 1 | 52 | 70 |  |
| R.LEVVTDPTTNAGYVK.I | Y | 86.51 | 1605.8250 | 15 | -3.4 | 803.9170 | 2 | 65.91 | 3 | 18769 | Ana\_02.raw | 1.23E7 | 1.61E7 | 3.33E6 | 6.18E6 | 7.81E6 | 1.58E6 | 1.78E6 | 5.74E6 | 1.6E7 | 6.84E6 | 10 | 1 | 1 | 1 | 1 | 1 | 1 | 1 | 1 | 1 | 1 | 499 | 513 |  |
| R.FEEQVYNIPIR.I | Y | 79.75 | 1406.7194 | 11 | 1.8 | 704.3682 | 2 | 83.39 | 3 | 23520 | Ana\_02.raw | 2.33E7 | 3.19E7 | 2.77E7 | 1.2E7 | 1.99E7 | 2.03E7 | 1.7E7 | 1.28E7 | 6.18E7 | 1.86E7 | 29 | 5 | 2 | 4 | 2 | 2 | 2 | 1 | 3 | 4 | 4 | 734 | 744 |  |
| K.DKVENPQSPENKPLR.S | Y | 77.40 | 1749.9009 | 15 | 6.9 | 584.3116 | 3 | 25.44 | 3 | 6699 | Ana\_02.raw |  | 2.23E7 | 1.4E7 | 7.37E6 | 1.09E7 | 1.28E7 | 9.77E6 | 2.12E6 | 1.68E6 | 1.51E6 | 11 | 0 | 2 | 1 | 1 | 1 | 1 | 1 | 1 | 1 | 2 | 812 | 826 |  |
| K.TGAISLTPEGSQELDPVK.N | Y | 73.53 | 1840.9418 | 18 | -1.9 | 921.4764 | 2 | 83.30 | 3 | 23496 | Ana\_02.raw | 3.74E6 | 8.07E6 | 7.36E6 | 3.2E6 | 5.2E6 | 5.54E6 | 3.93E6 | 2.45E6 | 9.61E6 |  | 9 | 1 | 1 | 1 | 1 | 1 | 1 | 1 | 1 | 1 | 0 | 185 | 202 |  |
| K.APEPVEIR.E | Y | 71.84 | 909.4919 | 8 | 3.3 | 455.7548 | 2 | 40.31 | 6 | 11254 | Ana\_05.raw | 2.81E7 | 3.83E7 | 3.5E7 | 1.79E7 | 2.94E7 | 3.36E7 | 2.5E7 | 1.73E7 | 0 | 2.85E7 | 14 | 2 | 2 | 1 | 1 | 1 | 2 | 1 | 1 | 1 | 2 | 240 | 247 |  |
| K.NSHVFFATAK.D | Y | 71.47 | 1120.5665 | 10 | 3.7 | 561.2926 | 2 | 44.34 | 7 | 12310 | Ana\_06.raw | 9.85E6 | 8.2E6 | 1.39E7 | 6.97E6 | 1.36E7 | 1.33E7 | 3.6E6 | 7.57E6 | 5.05E6 | 6.72E6 | 16 | 1 | 1 | 2 | 2 | 2 | 2 | 2 | 2 | 1 | 1 | 303 | 312 |  |
| K.DINDNRPTFLQSK.Y | Y | 66.89 | 1546.7739 | 13 | 0.9 | 774.3949 | 2 | 59.69 | 5 | 16964 | Ana\_04.raw |  | 4.61E6 |  | 1.23E6 | 2.8E6 | 3.25E6 | 2.45E6 |  | 2.2E7 |  | 7 | 0 | 1 | 0 | 1 | 1 | 1 | 1 | 0 | 2 | 0 | 117 | 129 |  |
| K.FTFALGR.E | Y | 57.37 | 810.4388 | 7 | 3.2 | 406.2280 | 2 | 77.78 | 5 | 22097 | Ana\_04.raw | 4.18E6 | 9.99E6 | 1E7 | 5.45E6 | 7.18E6 | 1.09E7 | 8.4E6 | 5.66E6 | 1.99E7 | 6.69E6 | 14 | 1 | 2 | 1 | 1 | 2 | 2 | 1 | 1 | 2 | 1 | 702 | 708 |  |
| K.VENPQSPENKPLR.S | Y | 57.24 | 1506.7791 | 13 | -0.2 | 503.2668 | 3 | 28.32 | 5 | 7579 | Ana\_04.raw |  |  |  |  | 6.21E6 | 5.2E6 | 4.09E6 | 3.36E6 |  |  | 4 | 0 | 0 | 0 | 0 | 1 | 1 | 1 | 1 | 0 | 0 | 814 | 826 |  |
| R.EGLQSDWEVSK.I | Y | 55.04 | 1276.5935 | 11 | 5.4 | 639.3075 | 2 | 62.71 | 2 | 17859 | Ana\_01.raw | 2.51E6 | 6.35E6 | 7.26E6 | 3.87E6 | 5.41E6 |  |  | 3.72E6 |  | 2.42E6 | 7 | 1 | 1 | 1 | 1 | 1 | 0 | 0 | 1 | 0 | 1 | 709 | 719 |  |
| K.DGLLYHTR.A | Y | 53.57 | 973.4981 | 8 | 2.1 | 487.7574 | 2 | 36.87 | 3 | 10185 | Ana\_02.raw | 7.82E6 | 7.9E6 | 1.11E7 | 7.52E6 | 7.73E6 | 1.34E7 | 7.3E6 |  |  |  | 7 | 1 | 1 | 1 | 1 | 1 | 1 | 1 | 0 | 0 | 0 | 74 | 81 |  |
| K.LVDQTPK.V | Y | 46.99 | 799.4440 | 7 | 2.9 | 400.7304 | 2 | 21.14 | 7 | 5255 | Ana\_06.raw |  |  |  | 1.64E7 | 2.59E7 |  | 2.2E7 | 0 |  |  | 4 | 0 | 0 | 0 | 1 | 1 | 0 | 1 | 1 | 0 | 0 | 380 | 386 |  |
| R.ETESVYR.V | Y | 44.68 | 882.4083 | 7 | 2.3 | 442.2124 | 2 | 23.71 | 4 | 6012 | Ana\_03.raw |  |  | 1.5E7 | 1.53E7 | 7.4E6 | 2.29E7 | 8.83E6 | 9.88E6 |  |  | 6 | 0 | 0 | 1 | 1 | 1 | 1 | 1 | 1 | 0 | 0 | 627 | 633 |  |
| total 17 peptides |  |  |  |  |  |  |  |  |  |  |  |  |  |  |  |  |  |  |  |  |  |  |  |  |  |  |  |  |  |  |  |  |  |  |  |
| --- | --- | --- | --- | --- | --- | --- | --- | --- | --- | --- | --- | --- | --- | --- | --- | --- | --- | --- | --- | --- | --- | --- | --- | --- | --- | --- | --- | --- | --- | --- | --- | --- | --- | --- | --- |

Best Unique PSM (Scan F3:32872, m/z=938.1627, z=3, RT=119.33, ppm=-1.1):

sp|Q61879|MYH10\_MOUSE

back to list

  

| Protein Coverage
| Supporting Peptides
| Best Unique PSM
|

Protein Coverage:

Supporting Peptides:

| Peptide | Uniq | -10lgP | Mass | Length | ppm | m/z | z | RT | Fraction | Scan | Source File | Area 10 | Area 01 | Area 02 | Area 03 | Area 04 | Area 05 | Area 06 | Area 07 | Area 08 | Area 09 | #Spec | #Spec 10 | #Spec 01 | #Spec 02 | #Spec 03 | #Spec 04 | #Spec 05 | #Spec 06 | #Spec 07 | #Spec 08 | #Spec 09 | Start | End | PTM |
| --- | --- | --- | --- | --- | --- | --- | --- | --- | --- | --- | --- | --- | --- | --- | --- | --- | --- | --- | --- | --- | --- | --- | --- | --- | --- | --- | --- | --- | --- | --- | --- | --- | --- | --- | --- |
| R.TQLEELEDELQATEDAK.L | N | 116.11 | 1960.9113 | 17 | 1.2 | 981.4641 | 2 | 112.32 | 3 | 31061 | Ana\_02.raw | 1.36E8 | 2.22E8 | 1.17E8 | 7.57E7 | 1.05E8 | 1.7E8 | 8.31E7 | 4.83E7 | 2.7E8 | 1.45E8 | 68 | 10 | 10 | 5 | 5 | 5 | 4 | 5 | 9 | 8 | 7 | 1546 | 1562 |  |
| K.LQQLFNHTMFILEQEEYQR.E | N | 102.05 | 2466.2002 | 19 | 4.9 | 823.0780 | 3 | 118.36 | 6 | 33058 | Ana\_05.raw |  |  |  |  |  | 4.77E6 |  |  |  |  | 1 | 0 | 0 | 0 | 0 | 0 | 1 | 0 | 0 | 0 | 0 | 483 | 501 |  |
| K.LQQLFNHTM(+15.99)FILEQEEYQR.E | N | 96.51 | 2482.1951 | 19 | 2.7 | 828.4079 | 3 | 103.10 | 5 | 28829 | Ana\_04.raw | 6.34E6 |  | 2.18E7 |  | 2.15E7 | 2.26E7 | 1.24E6 | 8.44E4 | 2.31E7 |  | 8 | 1 | 0 | 1 | 0 | 1 | 1 | 1 | 1 | 2 | 0 | 483 | 501 | Oxidation (M) |
| R.EDQSILC(+57.02)TGESGAGK.T | N | 94.65 | 1550.6882 | 15 | 0.4 | 776.3517 | 2 | 46.91 | 3 | 13245 | Ana\_02.raw | 6.73E7 | 9.75E7 | 5.91E7 | 2.44E7 | 4.17E7 | 6.67E7 | 3.22E7 | 4.18E7 | 1.11E8 | 4.88E7 | 27 | 4 | 5 | 2 | 2 | 3 | 2 | 1 | 3 | 3 | 2 | 170 | 184 | Carbamidomethylation |
| K.LDPHLVLDQLR.C | N | 84.87 | 1317.7405 | 11 | 1.6 | 440.2548 | 3 | 92.61 | 3 | 25955 | Ana\_02.raw | 2.14E6 | 4.21E6 | 2.49E6 | 1.52E6 | 7.25E6 | 1.77E6 | 4.2E6 | 3.71E6 | 3.64E7 | 1.88E7 | 23 | 2 | 3 | 3 | 1 | 2 | 2 | 2 | 2 | 3 | 3 | 690 | 700 |  |
| R.ALELDPNLYR.I | N | 81.07 | 1202.6295 | 10 | 2.4 | 602.3235 | 2 | 85.18 | 6 | 24273 | Ana\_05.raw | 9.75E7 | 1.34E8 | 1.19E8 | 4.21E7 | 8.95E7 | 1.03E8 | 5.04E7 | 5.85E7 | 2.02E8 | 6.89E7 | 54 | 5 | 7 | 7 | 5 | 5 | 5 | 4 | 6 | 6 | 4 | 753 | 762 |  |
| R.HEM(+15.99)PPHIYAISESAYR.C | Y | 77.78 | 1915.8887 | 16 | 2.9 | 639.6387 | 3 | 65.47 | 6 | 18797 | Ana\_05.raw | 1.34E7 | 1.75E7 | 1.81E7 | 3.34E6 | 1.49E7 | 1.71E7 | 1.01E7 | 1.27E7 |  |  | 11 | 1 | 1 | 1 | 1 | 1 | 2 | 2 | 2 | 0 | 0 | 148 | 163 | Oxidation (M) |
| R.AGVLAHLEEER.D | N | 75.88 | 1222.6306 | 11 | 4.9 | 612.3256 | 2 | 56.76 | 1 | 16028 | ANa\_10.raw | 1.1E7 |  | 8.62E6 | 8.53E6 | 4.85E6 | 1.4E7 | 3.79E6 | 9.81E6 | 2.4E7 | 1.23E7 | 14 | 1 | 0 | 2 | 2 | 1 | 2 | 1 | 2 | 1 | 2 | 772 | 782 |  |
| K.KFDQLLAEEK.G | N | 75.17 | 1219.6448 | 10 | 2.0 | 610.8309 | 2 | 53.25 | 3 | 15096 | Ana\_02.raw | 7E7 | 8.1E7 | 7.96E7 | 3.21E7 | 6.01E7 | 7.54E7 | 3.96E7 | 3.99E7 | 1.26E8 | 5.97E7 | 37 | 4 | 4 | 3 | 3 | 4 | 4 | 4 | 3 | 4 | 4 | 1452 | 1461 |  |
| K.VEDM(+15.99)AELTC(+57.02)LNEASVLHNLK.D | N | 73.50 | 2301.0981 | 20 | -0.6 | 768.0396 | 3 | 95.93 | 9 | 27155 | Ana\_08.raw |  |  |  |  |  |  |  |  | 7.55E6 |  | 1 | 0 | 0 | 0 | 0 | 0 | 0 | 0 | 0 | 1 | 0 | 87 | 106 | Oxidation (M); Carbamidomethylation |
| K.FDQLLAEEK.G | N | 71.45 | 1091.5498 | 9 | 2.9 | 546.7838 | 2 | 58.18 | 6 | 16636 | Ana\_05.raw | 3.53E7 | 3.9E7 | 4.13E7 | 1.48E7 | 2.94E7 | 4.16E7 | 2.35E7 | 2.34E7 | 5.36E7 | 2.71E7 | 11 | 1 | 1 | 1 | 1 | 1 | 1 | 1 | 1 | 2 | 1 | 1453 | 1461 |  |
| R.ALEEALEAK.E | N | 68.87 | 972.5127 | 9 | 1.7 | 487.2645 | 2 | 44.90 | 3 | 12627 | Ana\_02.raw | 1.13E8 | 1.41E8 | 1.47E8 | 5.37E7 | 1.14E8 | 1.46E8 | 7.47E7 | 8.94E7 | 1.33E8 | 9.07E7 | 38 | 4 | 4 | 5 | 4 | 4 | 3 | 2 | 5 | 3 | 4 | 1491 | 1499 |  |
| R.IVFQEFR.Q | N | 60.19 | 937.5021 | 7 | 2.1 | 469.7593 | 2 | 70.64 | 3 | 20071 | Ana\_02.raw | 1.78E8 | 2.55E8 | 2.31E8 | 9.57E7 | 1.71E8 | 2.24E8 | 1.16E8 | 1.52E8 | 2.58E8 | 1.28E8 | 82 | 7 | 9 | 10 | 6 | 9 | 8 | 8 | 10 | 8 | 7 | 719 | 725 |  |
| R.NC(+57.02)AAYLK.L | N | 56.53 | 838.4007 | 7 | 3.5 | 420.2091 | 2 | 28.49 | 6 | 7650 | Ana\_05.raw | 3.89E7 |  | 4.95E7 | 2.34E7 | 4.81E7 | 7.1E7 | 3.25E7 | 2.9E7 | 3.32E7 | 3.28E7 | 11 | 1 | 0 | 1 | 2 | 1 | 1 | 1 | 2 | 1 | 1 | 822 | 828 | Carbamidomethylation |
| R.C(+57.02)IIPNHEK.R | N | 51.44 | 1009.5015 | 8 | 3.8 | 505.7599 | 2 | 28.00 | 5 | 7477 | Ana\_04.raw | 3.6E6 | 5.27E6 | 2.95E6 |  | 3.18E6 | 3.44E6 | 0 | 1.91E6 |  |  | 7 | 1 | 1 | 1 | 0 | 1 | 1 | 1 | 1 | 0 | 0 | 678 | 685 | Carbamidomethylation |
| K.VIQYLAHVASSHK.G | N | 49.69 | 1451.7885 | 13 | 1.2 | 484.9373 | 3 | 48.09 | 3 | 13589 | Ana\_02.raw | 2.84E6 |  | 3.39E6 |  |  |  |  |  |  | 4.06E6 | 3 | 1 | 0 | 1 | 0 | 0 | 0 | 0 | 0 | 0 | 1 | 191 | 203 |  |
| K.NVHELEK.S | N | 46.85 | 867.4450 | 7 | 2.4 | 434.7308 | 2 | 18.44 | 6 | 4415 | Ana\_05.raw |  |  |  | 4.05E7 | 8.23E7 | 8.16E7 | 6.58E7 | 1.74E8 |  |  | 6 | 0 | 0 | 0 | 1 | 1 | 1 | 1 | 2 | 0 | 0 | 1526 | 1532 |  |
| K.VKPLLQVTR.Q | N | 43.60 | 1052.6705 | 9 | 4.3 | 351.8990 | 3 | 47.58 | 8 | 13112 | Ana\_07.raw | 5.04E7 | 3.17E7 | 7.22E7 | 2.28E7 | 5.31E7 | 7.4E7 | 3.29E7 | 3.78E7 | 6.72E7 | 4.05E7 | 26 | 2 | 1 | 3 | 3 | 4 | 3 | 3 | 3 | 2 | 2 | 841 | 849 |  |
| R.QGFPNR.I | N | 43.46 | 717.3558 | 6 | 0.9 | 359.6855 | 2 | 22.62 | 3 | 5802 | Ana\_02.raw |  | 4.83E6 | 1.13E7 | 7.76E6 |  | 1.82E7 | 7.99E6 | 1.36E7 |  |  | 6 | 0 | 1 | 1 | 1 | 0 | 1 | 1 | 1 | 0 | 0 | 713 | 718 |  |
| R.NTNPNFVR.C | N | 43.08 | 960.4777 | 8 | 5.1 | 481.2486 | 2 | 29.96 | 10 | 8058 | Ana\_09.raw | 4.19E7 |  | 2.88E7 | 1.24E7 |  |  | 1.83E7 | 1.6E7 | 4.87E7 | 3.61E7 | 8 | 1 | 0 | 1 | 1 | 0 | 0 | 1 | 2 | 1 | 1 | 670 | 677 |  |
| K.ADFC(+57.02)IIHYAGK.V | N | 42.52 | 1293.6176 | 11 | 0.7 | 432.2134 | 3 | 70.59 | 3 | 20057 | Ana\_02.raw | 5.04E5 | 1.28E6 | 6.1E5 | 8.67E5 | 3.82E5 | 9.18E5 | 4.85E5 |  | 0 | 1.79E6 | 9 | 1 | 1 | 1 | 1 | 1 | 1 | 1 | 0 | 1 | 1 | 573 | 583 | Carbamidomethylation |
| R.KLQAQMK.D | N | 41.48 | 845.4793 | 7 | 4.2 | 423.7487 | 2 | 18.45 | 8 | 4358 | Ana\_07.raw |  |  |  | 1.72E5 |  |  |  | 5.1E5 |  |  | 2 | 0 | 0 | 0 | 1 | 0 | 0 | 0 | 1 | 0 | 0 | 1649 | 1655 |  |
| R.TVGQLYK.E | N | 38.67 | 807.4490 | 7 | 2.6 | 404.7328 | 2 | 33.40 | 4 | 8890 | Ana\_03.raw | 3.54E6 | 1.11E8 | 1.2E8 | 4.55E7 | 8.98E7 | 1.13E8 | 7.15E7 | 6.55E7 | 1.02E8 | 6.97E7 | 17 | 1 | 1 | 2 | 1 | 3 | 2 | 2 | 3 | 1 | 1 | 652 | 658 |  |
| R.ELDDATEANEGLSR.E | Y | 37.46 | 1518.6797 | 14 | 10.0 | 760.3547 | 2 | 47.29 | 9 | 13228 | Ana\_08.raw |  |  |  |  |  |  |  |  | 3.6E6 |  | 1 | 0 | 0 | 0 | 0 | 0 | 0 | 0 | 0 | 1 | 0 | 1906 | 1919 |  |
| K.ALSLAR.A | N | 35.77 | 629.3860 | 6 | 2.7 | 315.7011 | 2 | 31.28 | 6 | 8518 | Ana\_05.raw |  |  |  |  |  | 3.8E7 | 2.13E7 | 1.28E7 |  | 1.54E7 | 4 | 0 | 0 | 0 | 0 | 0 | 1 | 1 | 1 | 0 | 1 | 1485 | 1490 |  |
| R.QIVSNLEK.K | N | 33.88 | 929.5182 | 8 | 5.3 | 465.7688 | 2 | 35.36 | 9 | 9523 | Ana\_08.raw | 1.19E7 |  |  |  |  |  |  |  | 7.42E6 |  | 2 | 1 | 0 | 0 | 0 | 0 | 0 | 0 | 0 | 1 | 0 | 1441 | 1448 |  |
| K.ATDKTFVEK.L | Y | 31.34 | 1037.5393 | 9 | 5.0 | 519.7795 | 2 | 22.47 | 2 | 5566 | Ana\_01.raw |  | 4.26E6 |  |  |  | 2.86E6 |  |  |  |  | 2 | 0 | 1 | 0 | 0 | 0 | 1 | 0 | 0 | 0 | 0 | 544 | 552 |  |
| K.LM(+15.99)ATLR.N | N | 29.79 | 719.4000 | 6 | 2.6 | 360.7082 | 2 | 25.75 | 9 | 6555 | Ana\_08.raw |  |  |  |  |  |  |  |  | 6.95E6 |  | 1 | 0 | 0 | 0 | 0 | 0 | 0 | 0 | 0 | 1 | 0 | 664 | 669 | Oxidation (M) |
| R.AILTPR.I | N | 29.60 | 669.4174 | 6 | 3.2 | 335.7170 | 2 | 26.50 | 9 | 6805 | Ana\_08.raw | 3.95E7 | 5.44E7 | 6.82E7 | 2.88E7 | 4.05E7 | 7.21E7 | 3.34E7 | 3.05E7 | 6.5E6 | 2.28E7 | 14 | 1 | 2 | 1 | 2 | 1 | 2 | 2 | 1 | 1 | 1 | 395 | 400 |  |
| R.EQEVAELK.K | Y | 27.78 | 944.4814 | 8 | -6.2 | 473.2451 | 2 | 38.40 | 5 | 10610 | Ana\_04.raw |  |  | 1.44E6 | 5.08E6 | 5.41E6 |  |  |  |  |  | 3 | 0 | 0 | 1 | 1 | 1 | 0 | 0 | 0 | 0 | 0 | 1173 | 1180 |  |
| K.LQLEK.V | N | 26.40 | 629.3748 | 5 | 5.2 | 315.6963 | 2 | 24.82 | 10 | 6436 | Ana\_09.raw |  |  | 5.13E7 |  |  |  |  |  |  | 3.11E7 | 2 | 0 | 0 | 1 | 0 | 0 | 0 | 0 | 0 | 0 | 1 | 969 | 973 |  |
| total 31 peptides |  |  |  |  |  |  |  |  |  |  |  |  |  |  |  |  |  |  |  |  |  |  |  |  |  |  |  |  |  |  |  |  |  |  |  |
| --- | --- | --- | --- | --- | --- | --- | --- | --- | --- | --- | --- | --- | --- | --- | --- | --- | --- | --- | --- | --- | --- | --- | --- | --- | --- | --- | --- | --- | --- | --- | --- | --- | --- | --- | --- |

Best Unique PSM (Scan F6:18797, m/z=639.6387, z=3, RT=65.47, ppm=2.9):

Q5SV64|Q5SV64\_MOUSE

back to list

  

| Protein Coverage
| Supporting Peptides
| Best Unique PSM
|

Protein Coverage:

Supporting Peptides:

| Peptide | Uniq | -10lgP | Mass | Length | ppm | m/z | z | RT | Fraction | Scan | Source File | Area 10 | Area 01 | Area 02 | Area 03 | Area 04 | Area 05 | Area 06 | Area 07 | Area 08 | Area 09 | #Spec | #Spec 10 | #Spec 01 | #Spec 02 | #Spec 03 | #Spec 04 | #Spec 05 | #Spec 06 | #Spec 07 | #Spec 08 | #Spec 09 | Start | End | PTM |
| --- | --- | --- | --- | --- | --- | --- | --- | --- | --- | --- | --- | --- | --- | --- | --- | --- | --- | --- | --- | --- | --- | --- | --- | --- | --- | --- | --- | --- | --- | --- | --- | --- | --- | --- | --- |
| R.TQLEELEDELQATEDAK.L | N | 116.11 | 1960.9113 | 17 | 1.2 | 981.4641 | 2 | 112.32 | 3 | 31061 | Ana\_02.raw | 1.36E8 | 2.22E8 | 1.17E8 | 7.57E7 | 1.05E8 | 1.7E8 | 8.31E7 | 4.83E7 | 2.7E8 | 1.45E8 | 68 | 10 | 10 | 5 | 5 | 5 | 4 | 5 | 9 | 8 | 7 | 1577 | 1593 |  |
| K.LQQLFNHTMFILEQEEYQR.E | N | 102.05 | 2466.2002 | 19 | 4.9 | 823.0780 | 3 | 118.36 | 6 | 33058 | Ana\_05.raw |  |  |  |  |  | 4.77E6 |  |  |  |  | 1 | 0 | 0 | 0 | 0 | 0 | 1 | 0 | 0 | 0 | 0 | 493 | 511 |  |
| K.LQQLFNHTM(+15.99)FILEQEEYQR.E | N | 96.51 | 2482.1951 | 19 | 2.7 | 828.4079 | 3 | 103.10 | 5 | 28829 | Ana\_04.raw | 6.34E6 |  | 2.18E7 |  | 2.15E7 | 2.26E7 | 1.24E6 | 8.44E4 | 2.31E7 |  | 8 | 1 | 0 | 1 | 0 | 1 | 1 | 1 | 1 | 2 | 0 | 493 | 511 | Oxidation (M) |
| R.EDQSILC(+57.02)TGESGAGK.T | N | 94.65 | 1550.6882 | 15 | 0.4 | 776.3517 | 2 | 46.91 | 3 | 13245 | Ana\_02.raw | 6.73E7 | 9.75E7 | 5.91E7 | 2.44E7 | 4.17E7 | 6.67E7 | 3.22E7 | 4.18E7 | 1.11E8 | 4.88E7 | 27 | 4 | 5 | 2 | 2 | 3 | 2 | 1 | 3 | 3 | 2 | 170 | 184 | Carbamidomethylation |
| K.LDPHLVLDQLR.C | N | 84.87 | 1317.7405 | 11 | 1.6 | 440.2548 | 3 | 92.61 | 3 | 25955 | Ana\_02.raw | 2.14E6 | 4.21E6 | 2.49E6 | 1.52E6 | 7.25E6 | 1.77E6 | 4.2E6 | 3.71E6 | 3.64E7 | 1.88E7 | 23 | 2 | 3 | 3 | 1 | 2 | 2 | 2 | 2 | 3 | 3 | 721 | 731 |  |
| R.ALELDPNLYR.I | N | 81.07 | 1202.6295 | 10 | 2.4 | 602.3235 | 2 | 85.18 | 6 | 24273 | Ana\_05.raw | 9.75E7 | 1.34E8 | 1.19E8 | 4.21E7 | 8.95E7 | 1.03E8 | 5.04E7 | 5.85E7 | 2.02E8 | 6.89E7 | 54 | 5 | 7 | 7 | 5 | 5 | 5 | 4 | 6 | 6 | 4 | 784 | 793 |  |
| R.HEM(+15.99)PPHIYAISESAYR.C | Y | 77.78 | 1915.8887 | 16 | 2.9 | 639.6387 | 3 | 65.47 | 6 | 18797 | Ana\_05.raw | 1.34E7 | 1.75E7 | 1.81E7 | 3.34E6 | 1.49E7 | 1.71E7 | 1.01E7 | 1.27E7 |  |  | 11 | 1 | 1 | 1 | 1 | 1 | 2 | 2 | 2 | 0 | 0 | 148 | 163 | Oxidation (M) |
| R.AGVLAHLEEER.D | N | 75.88 | 1222.6306 | 11 | 4.9 | 612.3256 | 2 | 56.76 | 1 | 16028 | ANa\_10.raw | 1.1E7 |  | 8.62E6 | 8.53E6 | 4.85E6 | 1.4E7 | 3.79E6 | 9.81E6 | 2.4E7 | 1.23E7 | 14 | 1 | 0 | 2 | 2 | 1 | 2 | 1 | 2 | 1 | 2 | 803 | 813 |  |
| K.KFDQLLAEEK.G | N | 75.17 | 1219.6448 | 10 | 2.0 | 610.8309 | 2 | 53.25 | 3 | 15096 | Ana\_02.raw | 7E7 | 8.1E7 | 7.96E7 | 3.21E7 | 6.01E7 | 7.54E7 | 3.96E7 | 3.99E7 | 1.26E8 | 5.97E7 | 37 | 4 | 4 | 3 | 3 | 4 | 4 | 4 | 3 | 4 | 4 | 1483 | 1492 |  |
| K.VEDM(+15.99)AELTC(+57.02)LNEASVLHNLK.D | N | 73.50 | 2301.0981 | 20 | -0.6 | 768.0396 | 3 | 95.93 | 9 | 27155 | Ana\_08.raw |  |  |  |  |  |  |  |  | 7.55E6 |  | 1 | 0 | 0 | 0 | 0 | 0 | 0 | 0 | 0 | 1 | 0 | 87 | 106 | Oxidation (M); Carbamidomethylation |
| K.FDQLLAEEK.G | N | 71.45 | 1091.5498 | 9 | 2.9 | 546.7838 | 2 | 58.18 | 6 | 16636 | Ana\_05.raw | 3.53E7 | 3.9E7 | 4.13E7 | 1.48E7 | 2.94E7 | 4.16E7 | 2.35E7 | 2.34E7 | 5.36E7 | 2.71E7 | 11 | 1 | 1 | 1 | 1 | 1 | 1 | 1 | 1 | 2 | 1 | 1484 | 1492 |  |
| R.ALEEALEAK.E | N | 68.87 | 972.5127 | 9 | 1.7 | 487.2645 | 2 | 44.90 | 3 | 12627 | Ana\_02.raw | 1.13E8 | 1.41E8 | 1.47E8 | 5.37E7 | 1.14E8 | 1.46E8 | 7.47E7 | 8.94E7 | 1.33E8 | 9.07E7 | 38 | 4 | 4 | 5 | 4 | 4 | 3 | 2 | 5 | 3 | 4 | 1522 | 1530 |  |
| R.IVFQEFR.Q | N | 60.19 | 937.5021 | 7 | 2.1 | 469.7593 | 2 | 70.64 | 3 | 20071 | Ana\_02.raw | 1.78E8 | 2.55E8 | 2.31E8 | 9.57E7 | 1.71E8 | 2.24E8 | 1.16E8 | 1.52E8 | 2.58E8 | 1.28E8 | 82 | 7 | 9 | 10 | 6 | 9 | 8 | 8 | 10 | 8 | 7 | 750 | 756 |  |
| R.NC(+57.02)AAYLK.L | N | 56.53 | 838.4007 | 7 | 3.5 | 420.2091 | 2 | 28.49 | 6 | 7650 | Ana\_05.raw | 3.89E7 |  | 4.95E7 | 2.34E7 | 4.81E7 | 7.1E7 | 3.25E7 | 2.9E7 | 3.32E7 | 3.28E7 | 11 | 1 | 0 | 1 | 2 | 1 | 1 | 1 | 2 | 1 | 1 | 853 | 859 | Carbamidomethylation |
| R.C(+57.02)IIPNHEK.R | N | 51.44 | 1009.5015 | 8 | 3.8 | 505.7599 | 2 | 28.00 | 5 | 7477 | Ana\_04.raw | 3.6E6 | 5.27E6 | 2.95E6 |  | 3.18E6 | 3.44E6 | 0 | 1.91E6 |  |  | 7 | 1 | 1 | 1 | 0 | 1 | 1 | 1 | 1 | 0 | 0 | 709 | 716 | Carbamidomethylation |
| K.VIQYLAHVASSHK.G | N | 49.69 | 1451.7885 | 13 | 1.2 | 484.9373 | 3 | 48.09 | 3 | 13589 | Ana\_02.raw | 2.84E6 |  | 3.39E6 |  |  |  |  |  |  | 4.06E6 | 3 | 1 | 0 | 1 | 0 | 0 | 0 | 0 | 0 | 0 | 1 | 191 | 203 |  |
| K.NVHELEK.S | N | 46.85 | 867.4450 | 7 | 2.4 | 434.7308 | 2 | 18.44 | 6 | 4415 | Ana\_05.raw |  |  |  | 4.05E7 | 8.23E7 | 8.16E7 | 6.58E7 | 1.74E8 |  |  | 6 | 0 | 0 | 0 | 1 | 1 | 1 | 1 | 2 | 0 | 0 | 1557 | 1563 |  |
| K.VKPLLQVTR.Q | N | 43.60 | 1052.6705 | 9 | 4.3 | 351.8990 | 3 | 47.58 | 8 | 13112 | Ana\_07.raw | 5.04E7 | 3.17E7 | 7.22E7 | 2.28E7 | 5.31E7 | 7.4E7 | 3.29E7 | 3.78E7 | 6.72E7 | 4.05E7 | 26 | 2 | 1 | 3 | 3 | 4 | 3 | 3 | 3 | 2 | 2 | 872 | 880 |  |
| R.QGFPNR.I | N | 43.46 | 717.3558 | 6 | 0.9 | 359.6855 | 2 | 22.62 | 3 | 5802 | Ana\_02.raw |  | 4.83E6 | 1.13E7 | 7.76E6 |  | 1.82E7 | 7.99E6 | 1.36E7 |  |  | 6 | 0 | 1 | 1 | 1 | 0 | 1 | 1 | 1 | 0 | 0 | 744 | 749 |  |
| R.NTNPNFVR.C | N | 43.08 | 960.4777 | 8 | 5.1 | 481.2486 | 2 | 29.96 | 10 | 8058 | Ana\_09.raw | 4.19E7 |  | 2.88E7 | 1.24E7 |  |  | 1.83E7 | 1.6E7 | 4.87E7 | 3.61E7 | 8 | 1 | 0 | 1 | 1 | 0 | 0 | 1 | 2 | 1 | 1 | 701 | 708 |  |
| K.ADFC(+57.02)IIHYAGK.V | N | 42.52 | 1293.6176 | 11 | 0.7 | 432.2134 | 3 | 70.59 | 3 | 20057 | Ana\_02.raw | 5.04E5 | 1.28E6 | 6.1E5 | 8.67E5 | 3.82E5 | 9.18E5 | 4.85E5 |  | 0 | 1.79E6 | 9 | 1 | 1 | 1 | 1 | 1 | 1 | 1 | 0 | 1 | 1 | 583 | 593 | Carbamidomethylation |
| R.KLQAQMK.D | N | 41.48 | 845.4793 | 7 | 4.2 | 423.7487 | 2 | 18.45 | 8 | 4358 | Ana\_07.raw |  |  |  | 1.72E5 |  |  |  | 5.1E5 |  |  | 2 | 0 | 0 | 0 | 1 | 0 | 0 | 0 | 1 | 0 | 0 | 1680 | 1686 |  |
| R.TVGQLYK.E | N | 38.67 | 807.4490 | 7 | 2.6 | 404.7328 | 2 | 33.40 | 4 | 8890 | Ana\_03.raw | 3.54E6 | 1.11E8 | 1.2E8 | 4.55E7 | 8.98E7 | 1.13E8 | 7.15E7 | 6.55E7 | 1.02E8 | 6.97E7 | 17 | 1 | 1 | 2 | 1 | 3 | 2 | 2 | 3 | 1 | 1 | 683 | 689 |  |
| R.ELDDATEANEGLSR.E | Y | 37.46 | 1518.6797 | 14 | 10.0 | 760.3547 | 2 | 47.29 | 9 | 13228 | Ana\_08.raw |  |  |  |  |  |  |  |  | 3.6E6 |  | 1 | 0 | 0 | 0 | 0 | 0 | 0 | 0 | 0 | 1 | 0 | 1937 | 1950 |  |
| K.ALSLAR.A | N | 35.77 | 629.3860 | 6 | 2.7 | 315.7011 | 2 | 31.28 | 6 | 8518 | Ana\_05.raw |  |  |  |  |  | 3.8E7 | 2.13E7 | 1.28E7 |  | 1.54E7 | 4 | 0 | 0 | 0 | 0 | 0 | 1 | 1 | 1 | 0 | 1 | 1516 | 1521 |  |
| R.QIVSNLEK.K | N | 33.88 | 929.5182 | 8 | 5.3 | 465.7688 | 2 | 35.36 | 9 | 9523 | Ana\_08.raw | 1.19E7 |  |  |  |  |  |  |  | 7.42E6 |  | 2 | 1 | 0 | 0 | 0 | 0 | 0 | 0 | 0 | 1 | 0 | 1472 | 1479 |  |
| K.ATDKTFVEK.L | Y | 31.34 | 1037.5393 | 9 | 5.0 | 519.7795 | 2 | 22.47 | 2 | 5566 | Ana\_01.raw |  | 4.26E6 |  |  |  | 2.86E6 |  |  |  |  | 2 | 0 | 1 | 0 | 0 | 0 | 1 | 0 | 0 | 0 | 0 | 554 | 562 |  |
| K.LM(+15.99)ATLR.N | N | 29.79 | 719.4000 | 6 | 2.6 | 360.7082 | 2 | 25.75 | 9 | 6555 | Ana\_08.raw |  |  |  |  |  |  |  |  | 6.95E6 |  | 1 | 0 | 0 | 0 | 0 | 0 | 0 | 0 | 0 | 1 | 0 | 695 | 700 | Oxidation (M) |
| R.AILTPR.I | N | 29.60 | 669.4174 | 6 | 3.2 | 335.7170 | 2 | 26.50 | 9 | 6805 | Ana\_08.raw | 3.95E7 | 5.44E7 | 6.82E7 | 2.88E7 | 4.05E7 | 7.21E7 | 3.34E7 | 3.05E7 | 6.5E6 | 2.28E7 | 14 | 1 | 2 | 1 | 2 | 1 | 2 | 2 | 1 | 1 | 1 | 405 | 410 |  |
| R.EQEVAELK.K | Y | 27.78 | 944.4814 | 8 | -6.2 | 473.2451 | 2 | 38.40 | 5 | 10610 | Ana\_04.raw |  |  | 1.44E6 | 5.08E6 | 5.41E6 |  |  |  |  |  | 3 | 0 | 0 | 1 | 1 | 1 | 0 | 0 | 0 | 0 | 0 | 1204 | 1211 |  |
| K.LQLEK.V | N | 26.40 | 629.3748 | 5 | 5.2 | 315.6963 | 2 | 24.82 | 10 | 6436 | Ana\_09.raw |  |  | 5.13E7 |  |  |  |  |  |  | 3.11E7 | 2 | 0 | 0 | 1 | 0 | 0 | 0 | 0 | 0 | 0 | 1 | 1000 | 1004 |  |
| total 31 peptides |  |  |  |  |  |  |  |  |  |  |  |  |  |  |  |  |  |  |  |  |  |  |  |  |  |  |  |  |  |  |  |  |  |  |  |
| --- | --- | --- | --- | --- | --- | --- | --- | --- | --- | --- | --- | --- | --- | --- | --- | --- | --- | --- | --- | --- | --- | --- | --- | --- | --- | --- | --- | --- | --- | --- | --- | --- | --- | --- | --- |

Best Unique PSM (Scan F6:18797, m/z=639.6387, z=3, RT=65.47, ppm=2.9):

Q3UH59|Q3UH59\_MOUSE

back to list

  

| Protein Coverage
| Supporting Peptides
| Best Unique PSM
|

Protein Coverage:

Supporting Peptides:

| Peptide | Uniq | -10lgP | Mass | Length | ppm | m/z | z | RT | Fraction | Scan | Source File | Area 10 | Area 01 | Area 02 | Area 03 | Area 04 | Area 05 | Area 06 | Area 07 | Area 08 | Area 09 | #Spec | #Spec 10 | #Spec 01 | #Spec 02 | #Spec 03 | #Spec 04 | #Spec 05 | #Spec 06 | #Spec 07 | #Spec 08 | #Spec 09 | Start | End | PTM |
| --- | --- | --- | --- | --- | --- | --- | --- | --- | --- | --- | --- | --- | --- | --- | --- | --- | --- | --- | --- | --- | --- | --- | --- | --- | --- | --- | --- | --- | --- | --- | --- | --- | --- | --- | --- |
| R.TQLEELEDELQATEDAK.L | N | 116.11 | 1960.9113 | 17 | 1.2 | 981.4641 | 2 | 112.32 | 3 | 31061 | Ana\_02.raw | 1.36E8 | 2.22E8 | 1.17E8 | 7.57E7 | 1.05E8 | 1.7E8 | 8.31E7 | 4.83E7 | 2.7E8 | 1.45E8 | 68 | 10 | 10 | 5 | 5 | 5 | 4 | 5 | 9 | 8 | 7 | 1583 | 1599 |  |
| K.LQQLFNHTMFILEQEEYQR.E | N | 102.05 | 2466.2002 | 19 | 4.9 | 823.0780 | 3 | 118.36 | 6 | 33058 | Ana\_05.raw |  |  |  |  |  | 4.77E6 |  |  |  |  | 1 | 0 | 0 | 0 | 0 | 0 | 1 | 0 | 0 | 0 | 0 | 520 | 538 |  |
| K.LQQLFNHTM(+15.99)FILEQEEYQR.E | N | 96.51 | 2482.1951 | 19 | 2.7 | 828.4079 | 3 | 103.10 | 5 | 28829 | Ana\_04.raw | 6.34E6 |  | 2.18E7 |  | 2.15E7 | 2.26E7 | 1.24E6 | 8.44E4 | 2.31E7 |  | 8 | 1 | 0 | 1 | 0 | 1 | 1 | 1 | 1 | 2 | 0 | 520 | 538 | Oxidation (M) |
| R.EDQSILC(+57.02)TGESGAGK.T | N | 94.65 | 1550.6882 | 15 | 0.4 | 776.3517 | 2 | 46.91 | 3 | 13245 | Ana\_02.raw | 6.73E7 | 9.75E7 | 5.91E7 | 2.44E7 | 4.17E7 | 6.67E7 | 3.22E7 | 4.18E7 | 1.11E8 | 4.88E7 | 27 | 4 | 5 | 2 | 2 | 3 | 2 | 1 | 3 | 3 | 2 | 207 | 221 | Carbamidomethylation |
| K.LDPHLVLDQLR.C | N | 84.87 | 1317.7405 | 11 | 1.6 | 440.2548 | 3 | 92.61 | 3 | 25955 | Ana\_02.raw | 2.14E6 | 4.21E6 | 2.49E6 | 1.52E6 | 7.25E6 | 1.77E6 | 4.2E6 | 3.71E6 | 3.64E7 | 1.88E7 | 23 | 2 | 3 | 3 | 1 | 2 | 2 | 2 | 2 | 3 | 3 | 727 | 737 |  |
| R.ALELDPNLYR.I | N | 81.07 | 1202.6295 | 10 | 2.4 | 602.3235 | 2 | 85.18 | 6 | 24273 | Ana\_05.raw | 9.75E7 | 1.34E8 | 1.19E8 | 4.21E7 | 8.95E7 | 1.03E8 | 5.04E7 | 5.85E7 | 2.02E8 | 6.89E7 | 54 | 5 | 7 | 7 | 5 | 5 | 5 | 4 | 6 | 6 | 4 | 790 | 799 |  |
| R.HEM(+15.99)PPHIYAISESAYR.C | Y | 77.78 | 1915.8887 | 16 | 2.9 | 639.6387 | 3 | 65.47 | 6 | 18797 | Ana\_05.raw | 1.34E7 | 1.75E7 | 1.81E7 | 3.34E6 | 1.49E7 | 1.71E7 | 1.01E7 | 1.27E7 |  |  | 11 | 1 | 1 | 1 | 1 | 1 | 2 | 2 | 2 | 0 | 0 | 185 | 200 | Oxidation (M) |
| R.AGVLAHLEEER.D | N | 75.88 | 1222.6306 | 11 | 4.9 | 612.3256 | 2 | 56.76 | 1 | 16028 | ANa\_10.raw | 1.1E7 |  | 8.62E6 | 8.53E6 | 4.85E6 | 1.4E7 | 3.79E6 | 9.81E6 | 2.4E7 | 1.23E7 | 14 | 1 | 0 | 2 | 2 | 1 | 2 | 1 | 2 | 1 | 2 | 809 | 819 |  |
| K.KFDQLLAEEK.G | N | 75.17 | 1219.6448 | 10 | 2.0 | 610.8309 | 2 | 53.25 | 3 | 15096 | Ana\_02.raw | 7E7 | 8.1E7 | 7.96E7 | 3.21E7 | 6.01E7 | 7.54E7 | 3.96E7 | 3.99E7 | 1.26E8 | 5.97E7 | 37 | 4 | 4 | 3 | 3 | 4 | 4 | 4 | 3 | 4 | 4 | 1489 | 1498 |  |
| K.VEDM(+15.99)AELTC(+57.02)LNEASVLHNLK.D | N | 73.50 | 2301.0981 | 20 | -0.6 | 768.0396 | 3 | 95.93 | 9 | 27155 | Ana\_08.raw |  |  |  |  |  |  |  |  | 7.55E6 |  | 1 | 0 | 0 | 0 | 0 | 0 | 0 | 0 | 0 | 1 | 0 | 124 | 143 | Oxidation (M); Carbamidomethylation |
| K.FDQLLAEEK.G | N | 71.45 | 1091.5498 | 9 | 2.9 | 546.7838 | 2 | 58.18 | 6 | 16636 | Ana\_05.raw | 3.53E7 | 3.9E7 | 4.13E7 | 1.48E7 | 2.94E7 | 4.16E7 | 2.35E7 | 2.34E7 | 5.36E7 | 2.71E7 | 11 | 1 | 1 | 1 | 1 | 1 | 1 | 1 | 1 | 2 | 1 | 1490 | 1498 |  |
| R.ALEEALEAK.E | N | 68.87 | 972.5127 | 9 | 1.7 | 487.2645 | 2 | 44.90 | 3 | 12627 | Ana\_02.raw | 1.13E8 | 1.41E8 | 1.47E8 | 5.37E7 | 1.14E8 | 1.46E8 | 7.47E7 | 8.94E7 | 1.33E8 | 9.07E7 | 38 | 4 | 4 | 5 | 4 | 4 | 3 | 2 | 5 | 3 | 4 | 1528 | 1536 |  |
| R.IVFQEFR.Q | N | 60.19 | 937.5021 | 7 | 2.1 | 469.7593 | 2 | 70.64 | 3 | 20071 | Ana\_02.raw | 1.78E8 | 2.55E8 | 2.31E8 | 9.57E7 | 1.71E8 | 2.24E8 | 1.16E8 | 1.52E8 | 2.58E8 | 1.28E8 | 82 | 7 | 9 | 10 | 6 | 9 | 8 | 8 | 10 | 8 | 7 | 756 | 762 |  |
| R.NC(+57.02)AAYLK.L | N | 56.53 | 838.4007 | 7 | 3.5 | 420.2091 | 2 | 28.49 | 6 | 7650 | Ana\_05.raw | 3.89E7 |  | 4.95E7 | 2.34E7 | 4.81E7 | 7.1E7 | 3.25E7 | 2.9E7 | 3.32E7 | 3.28E7 | 11 | 1 | 0 | 1 | 2 | 1 | 1 | 1 | 2 | 1 | 1 | 859 | 865 | Carbamidomethylation |
| R.C(+57.02)IIPNHEK.R | N | 51.44 | 1009.5015 | 8 | 3.8 | 505.7599 | 2 | 28.00 | 5 | 7477 | Ana\_04.raw | 3.6E6 | 5.27E6 | 2.95E6 |  | 3.18E6 | 3.44E6 | 0 | 1.91E6 |  |  | 7 | 1 | 1 | 1 | 0 | 1 | 1 | 1 | 1 | 0 | 0 | 715 | 722 | Carbamidomethylation |
| K.VIQYLAHVASSHK.G | N | 49.69 | 1451.7885 | 13 | 1.2 | 484.9373 | 3 | 48.09 | 3 | 13589 | Ana\_02.raw | 2.84E6 |  | 3.39E6 |  |  |  |  |  |  | 4.06E6 | 3 | 1 | 0 | 1 | 0 | 0 | 0 | 0 | 0 | 0 | 1 | 228 | 240 |  |
| K.NVHELEK.S | N | 46.85 | 867.4450 | 7 | 2.4 | 434.7308 | 2 | 18.44 | 6 | 4415 | Ana\_05.raw |  |  |  | 4.05E7 | 8.23E7 | 8.16E7 | 6.58E7 | 1.74E8 |  |  | 6 | 0 | 0 | 0 | 1 | 1 | 1 | 1 | 2 | 0 | 0 | 1563 | 1569 |  |
| K.VKPLLQVTR.Q | N | 43.60 | 1052.6705 | 9 | 4.3 | 351.8990 | 3 | 47.58 | 8 | 13112 | Ana\_07.raw | 5.04E7 | 3.17E7 | 7.22E7 | 2.28E7 | 5.31E7 | 7.4E7 | 3.29E7 | 3.78E7 | 6.72E7 | 4.05E7 | 26 | 2 | 1 | 3 | 3 | 4 | 3 | 3 | 3 | 2 | 2 | 878 | 886 |  |
| R.QGFPNR.I | N | 43.46 | 717.3558 | 6 | 0.9 | 359.6855 | 2 | 22.62 | 3 | 5802 | Ana\_02.raw |  | 4.83E6 | 1.13E7 | 7.76E6 |  | 1.82E7 | 7.99E6 | 1.36E7 |  |  | 6 | 0 | 1 | 1 | 1 | 0 | 1 | 1 | 1 | 0 | 0 | 750 | 755 |  |
| R.NTNPNFVR.C | N | 43.08 | 960.4777 | 8 | 5.1 | 481.2486 | 2 | 29.96 | 10 | 8058 | Ana\_09.raw | 4.19E7 |  | 2.88E7 | 1.24E7 |  |  | 1.83E7 | 1.6E7 | 4.87E7 | 3.61E7 | 8 | 1 | 0 | 1 | 1 | 0 | 0 | 1 | 2 | 1 | 1 | 707 | 714 |  |
| K.ADFC(+57.02)IIHYAGK.V | N | 42.52 | 1293.6176 | 11 | 0.7 | 432.2134 | 3 | 70.59 | 3 | 20057 | Ana\_02.raw | 5.04E5 | 1.28E6 | 6.1E5 | 8.67E5 | 3.82E5 | 9.18E5 | 4.85E5 |  | 0 | 1.79E6 | 9 | 1 | 1 | 1 | 1 | 1 | 1 | 1 | 0 | 1 | 1 | 610 | 620 | Carbamidomethylation |
| R.KLQAQMK.D | N | 41.48 | 845.4793 | 7 | 4.2 | 423.7487 | 2 | 18.45 | 8 | 4358 | Ana\_07.raw |  |  |  | 1.72E5 |  |  |  | 5.1E5 |  |  | 2 | 0 | 0 | 0 | 1 | 0 | 0 | 0 | 1 | 0 | 0 | 1686 | 1692 |  |
| R.TVGQLYK.E | N | 38.67 | 807.4490 | 7 | 2.6 | 404.7328 | 2 | 33.40 | 4 | 8890 | Ana\_03.raw | 3.54E6 | 1.11E8 | 1.2E8 | 4.55E7 | 8.98E7 | 1.13E8 | 7.15E7 | 6.55E7 | 1.02E8 | 6.97E7 | 17 | 1 | 1 | 2 | 1 | 3 | 2 | 2 | 3 | 1 | 1 | 689 | 695 |  |
| R.ELDDATEANEGLSR.E | Y | 37.46 | 1518.6797 | 14 | 10.0 | 760.3547 | 2 | 47.29 | 9 | 13228 | Ana\_08.raw |  |  |  |  |  |  |  |  | 3.6E6 |  | 1 | 0 | 0 | 0 | 0 | 0 | 0 | 0 | 0 | 1 | 0 | 1943 | 1956 |  |
| K.ALSLAR.A | N | 35.77 | 629.3860 | 6 | 2.7 | 315.7011 | 2 | 31.28 | 6 | 8518 | Ana\_05.raw |  |  |  |  |  | 3.8E7 | 2.13E7 | 1.28E7 |  | 1.54E7 | 4 | 0 | 0 | 0 | 0 | 0 | 1 | 1 | 1 | 0 | 1 | 1522 | 1527 |  |
| R.QIVSNLEK.K | N | 33.88 | 929.5182 | 8 | 5.3 | 465.7688 | 2 | 35.36 | 9 | 9523 | Ana\_08.raw | 1.19E7 |  |  |  |  |  |  |  | 7.42E6 |  | 2 | 1 | 0 | 0 | 0 | 0 | 0 | 0 | 0 | 1 | 0 | 1478 | 1485 |  |
| K.ATDKTFVEK.L | Y | 31.34 | 1037.5393 | 9 | 5.0 | 519.7795 | 2 | 22.47 | 2 | 5566 | Ana\_01.raw |  | 4.26E6 |  |  |  | 2.86E6 |  |  |  |  | 2 | 0 | 1 | 0 | 0 | 0 | 1 | 0 | 0 | 0 | 0 | 581 | 589 |  |
| K.LM(+15.99)ATLR.N | N | 29.79 | 719.4000 | 6 | 2.6 | 360.7082 | 2 | 25.75 | 9 | 6555 | Ana\_08.raw |  |  |  |  |  |  |  |  | 6.95E6 |  | 1 | 0 | 0 | 0 | 0 | 0 | 0 | 0 | 0 | 1 | 0 | 701 | 706 | Oxidation (M) |
| R.AILTPR.I | N | 29.60 | 669.4174 | 6 | 3.2 | 335.7170 | 2 | 26.50 | 9 | 6805 | Ana\_08.raw | 3.95E7 | 5.44E7 | 6.82E7 | 2.88E7 | 4.05E7 | 7.21E7 | 3.34E7 | 3.05E7 | 6.5E6 | 2.28E7 | 14 | 1 | 2 | 1 | 2 | 1 | 2 | 2 | 1 | 1 | 1 | 432 | 437 |  |
| R.EQEVAELK.K | Y | 27.78 | 944.4814 | 8 | -6.2 | 473.2451 | 2 | 38.40 | 5 | 10610 | Ana\_04.raw |  |  | 1.44E6 | 5.08E6 | 5.41E6 |  |  |  |  |  | 3 | 0 | 0 | 1 | 1 | 1 | 0 | 0 | 0 | 0 | 0 | 1210 | 1217 |  |
| K.LQLEK.V | N | 26.40 | 629.3748 | 5 | 5.2 | 315.6963 | 2 | 24.82 | 10 | 6436 | Ana\_09.raw |  |  | 5.13E7 |  |  |  |  |  |  | 3.11E7 | 2 | 0 | 0 | 1 | 0 | 0 | 0 | 0 | 0 | 0 | 1 | 1006 | 1010 |  |
| total 31 peptides |  |  |  |  |  |  |  |  |  |  |  |  |  |  |  |  |  |  |  |  |  |  |  |  |  |  |  |  |  |  |  |  |  |  |  |
| --- | --- | --- | --- | --- | --- | --- | --- | --- | --- | --- | --- | --- | --- | --- | --- | --- | --- | --- | --- | --- | --- | --- | --- | --- | --- | --- | --- | --- | --- | --- | --- | --- | --- | --- | --- |

Best Unique PSM (Scan F6:18797, m/z=639.6387, z=3, RT=65.47, ppm=2.9):

sp|P21981|TGM2\_MOUSE

back to list

  

| Protein Coverage
| Supporting Peptides
| Best Unique PSM
|

Protein Coverage:

Supporting Peptides:

| Peptide | Uniq | -10lgP | Mass | Length | ppm | m/z | z | RT | Fraction | Scan | Source File | Area 10 | Area 01 | Area 02 | Area 03 | Area 04 | Area 05 | Area 06 | Area 07 | Area 08 | Area 09 | #Spec | #Spec 10 | #Spec 01 | #Spec 02 | #Spec 03 | #Spec 04 | #Spec 05 | #Spec 06 | #Spec 07 | #Spec 08 | #Spec 09 | Start | End | PTM |
| --- | --- | --- | --- | --- | --- | --- | --- | --- | --- | --- | --- | --- | --- | --- | --- | --- | --- | --- | --- | --- | --- | --- | --- | --- | --- | --- | --- | --- | --- | --- | --- | --- | --- | --- | --- |
| K.NPLSDPLYDC(+57.02)IFTVEGAGLTK.E | Y | 129.43 | 2309.1250 | 21 | 2.5 | 1155.5726 | 2 | 130.47 | 7 | 35904 | Ana\_06.raw | 5.81E6 | 1.14E7 | 1.22E7 | 4.24E6 | 1.12E7 | 1.25E7 | 8.7E6 | 7.54E6 | 1.72E7 | 2.25E7 | 19 | 1 | 2 | 2 | 2 | 2 | 2 | 2 | 2 | 1 | 3 | 610 | 630 | Carbamidomethylation |
| R.VVTNYNSAHDQNSNLLIEYFR.N | Y | 106.53 | 2496.2034 | 21 | 4.3 | 833.0787 | 3 | 101.62 | 9 | 28676 | Ana\_08.raw |  |  |  | 1.49E6 | 1.82E6 | 5.06E6 | 1.05E6 | 1.34E5 | 2.3E6 | 0 | 7 | 0 | 0 | 0 | 1 | 1 | 1 | 1 | 1 | 1 | 1 | 297 | 317 |  |
| R.GLLIEPAANSYLLAER.D | Y | 94.00 | 1728.9409 | 16 | 1.8 | 865.4793 | 2 | 109.55 | 6 | 30761 | Ana\_05.raw | 1.94E7 | 1.14E7 | 1.08E7 |  | 5.59E6 | 8.82E6 | 6.38E6 | 7.34E5 | 5.27E7 | 2.12E7 | 13 | 2 | 2 | 1 | 0 | 1 | 1 | 1 | 1 | 2 | 2 | 564 | 579 |  |
| R.DHHTADLC(+57.02)QEK.L | Y | 87.43 | 1352.5779 | 11 | 3.6 | 677.2986 | 2 | 20.79 | 9 | 4882 | Ana\_08.raw |  | 2.51E6 | 5.24E6 | 2.33E6 | 3.84E6 |  | 2.83E6 |  | 4.73E6 | 9.13E6 | 9 | 0 | 1 | 1 | 1 | 1 | 0 | 1 | 0 | 2 | 2 | 20 | 30 | Carbamidomethylation |
| R.DDREDITHTYK.Y | Y | 84.51 | 1391.6317 | 11 | 5.5 | 696.8270 | 2 | 30.41 | 2 | 8152 | Ana\_01.raw | 2.18E7 | 1.52E7 | 2.95E6 | 6.73E6 | 1.21E7 | 1.41E6 | 1.07E7 | 7.04E6 | 3.18E7 | 2.37E7 | 16 | 2 | 2 | 1 | 1 | 2 | 1 | 2 | 1 | 2 | 2 | 434 | 444 |  |
| R.EYVLTQQGFIYQGSVK.F | Y | 80.14 | 1858.9465 | 16 | 2.0 | 930.4824 | 2 | 93.73 | 1 | 26291 | ANa\_10.raw | 7.47E5 |  |  |  |  |  |  |  |  |  | 1 | 1 | 0 | 0 | 0 | 0 | 0 | 0 | 0 | 0 | 0 | 158 | 173 |  |
| K.YSGC(+57.02)LTESNLIK.V | Y | 78.44 | 1383.6704 | 12 | 5.5 | 692.8463 | 2 | 67.62 | 1 | 19165 | ANa\_10.raw | 2.18E6 | 2.88E6 |  |  | 5.25E5 |  |  |  | 8.74E6 | 3.54E6 | 5 | 1 | 1 | 0 | 0 | 1 | 0 | 0 | 0 | 1 | 1 | 550 | 561 | Carbamidomethylation |
| R.VDLFPTDIGLHK.L | Y | 74.21 | 1353.7292 | 12 | 3.5 | 452.2520 | 3 | 92.81 | 1 | 26043 | ANa\_10.raw | 2.77E7 | 7.26E6 | 1.75E7 | 5.26E6 | 1.31E7 | 1.01E7 | 8.46E6 | 6.79E6 | 5.02E7 | 2.24E7 | 22 | 3 | 2 | 2 | 1 | 2 | 2 | 2 | 2 | 3 | 3 | 651 | 662 |  |
| K.SEGTYC(+57.02)C(+57.02)GPVSVR.A | Y | 73.25 | 1470.6232 | 13 | 2.9 | 736.3210 | 2 | 47.24 | 9 | 13213 | Ana\_08.raw |  |  |  |  |  |  |  |  | 1.56E7 |  | 1 | 0 | 0 | 0 | 0 | 0 | 0 | 0 | 0 | 1 | 0 | 365 | 377 | Carbamidomethylation |
| R.DLYLENPEIK.I | Y | 62.05 | 1232.6288 | 10 | 7.2 | 617.3261 | 2 | 77.02 | 6 | 22057 | Ana\_05.raw | 1.29E7 |  | 7.27E6 | 1.03E6 | 5.09E6 | 5.71E6 | 4.97E6 | 4.24E6 | 2.74E7 | 1.37E7 | 10 | 1 | 0 | 1 | 1 | 1 | 1 | 1 | 1 | 2 | 1 | 580 | 589 |  |
| R.LTLYFEGR.G | Y | 60.51 | 997.5233 | 8 | 3.0 | 499.7704 | 2 | 76.40 | 9 | 21766 | Ana\_08.raw | 9.59E5 |  |  | 8.12E5 | 1.63E6 | 1.02E6 |  | 7.43E5 | 1.81E6 | 2.61E6 | 7 | 1 | 0 | 0 | 1 | 1 | 1 | 0 | 1 | 1 | 1 | 41 | 48 |  |
| K.SVEVSDPVPAGDLVK.A | Y | 60.14 | 1510.7878 | 15 | 5.9 | 756.4056 | 2 | 76.20 | 1 | 21551 | ANa\_10.raw | 1.54E7 | 6.93E6 | 2.54E6 | 2.74E6 | 7.11E5 | 5.87E6 | 4.31E6 | 3.93E6 | 2.31E7 | 1.3E7 | 10 | 1 | 1 | 1 | 1 | 1 | 1 | 1 | 1 | 1 | 1 | 634 | 648 |  |
| R.NEFGELESNK.S | Y | 58.96 | 1165.5251 | 10 | 2.4 | 583.7712 | 2 | 48.65 | 9 | 13630 | Ana\_08.raw | 2.18E7 | 1.54E7 | 1.73E7 |  | 1.32E7 | 1.28E7 | 9.56E6 | 1.07E7 | 3.96E7 | 2.07E7 | 12 | 2 | 1 | 1 | 0 | 2 | 1 | 1 | 1 | 2 | 1 | 318 | 327 |  |
| K.YPEGSPEER.E | Y | 52.35 | 1062.4618 | 9 | 4.0 | 532.2403 | 2 | 24.92 | 9 | 6276 | Ana\_08.raw | 0 |  |  | 8.95E6 | 1.5E7 | 1.83E7 | 1.1E7 | 1.11E7 | 3.17E7 | 8.98E6 | 8 | 1 | 0 | 0 | 1 | 1 | 1 | 1 | 1 | 1 | 1 | 445 | 453 |  |
| R.WKEHGC(+57.02)QQVK.Y | Y | 44.62 | 1298.6190 | 10 | 1.3 | 433.8808 | 3 | 20.25 | 9 | 4707 | Ana\_08.raw |  |  |  |  |  |  |  |  | 3.13E5 |  | 1 | 0 | 0 | 0 | 0 | 0 | 0 | 0 | 0 | 1 | 0 | 264 | 273 | Carbamidomethylation |
| R.EDITHTYK.Y | Y | 35.55 | 1005.4767 | 8 | 5.1 | 503.7482 | 2 | 24.71 | 2 | 6336 | Ana\_01.raw |  | 3.55E6 |  |  |  |  |  |  |  |  | 1 | 0 | 1 | 0 | 0 | 0 | 0 | 0 | 0 | 0 | 0 | 437 | 444 |  |
| K.LAEKEETGVAM(+15.99)R.I | Y | 29.77 | 1348.6656 | 12 | 4.0 | 675.3428 | 2 | 24.11 | 1 | 6138 | ANa\_10.raw | 3.31E6 |  |  |  |  |  |  |  |  | 2.62E6 | 2 | 1 | 0 | 0 | 0 | 0 | 0 | 0 | 0 | 0 | 1 | 465 | 476 | Oxidation (M) |
| R.LLLC(+57.02)AR.T | Y | 27.07 | 744.4316 | 6 | 5.5 | 373.2251 | 2 | 40.52 | 9 | 11136 | Ana\_08.raw |  |  |  |  |  |  |  |  | 5.98E6 |  | 1 | 0 | 0 | 0 | 0 | 0 | 0 | 0 | 0 | 1 | 0 | 507 | 512 | Carbamidomethylation |
| R.KLVAEVSLK.N | Y | 26.00 | 985.6171 | 9 | 3.4 | 493.8175 | 2 | 40.40 | 9 | 11099 | Ana\_08.raw |  |  |  |  |  |  |  |  | 2.25E6 |  | 1 | 0 | 0 | 0 | 0 | 0 | 0 | 0 | 0 | 1 | 0 | 601 | 609 |  |
| R.SSPIYVGR.V | Y | 25.41 | 877.4658 | 8 | 5.0 | 439.7423 | 2 | 37.33 | 1 | 10191 | ANa\_10.raw | 1.87E7 |  |  |  |  |  |  |  | 2.72E7 | 2.02E7 | 4 | 1 | 0 | 0 | 0 | 0 | 0 | 0 | 0 | 2 | 1 | 215 | 222 |  |
| K.LVAEVSLK.N | Y | 23.05 | 857.5222 | 8 | 5.7 | 429.7708 | 2 | 51.81 | 10 | 14585 | Ana\_09.raw |  |  |  |  |  |  |  |  |  | 8.31E6 | 1 | 0 | 0 | 0 | 0 | 0 | 0 | 0 | 0 | 0 | 1 | 602 | 609 |  |
| R.ILYEK.Y | N | 22.26 | 664.3795 | 5 | 0.9 | 333.1973 | 2 | 25.57 | 3 | 6743 | Ana\_02.raw |  |  | 1.69E7 |  |  |  |  |  |  |  | 1 | 0 | 0 | 1 | 0 | 0 | 0 | 0 | 0 | 0 | 0 | 545 | 549 |  |
| total 22 peptides |  |  |  |  |  |  |  |  |  |  |  |  |  |  |  |  |  |  |  |  |  |  |  |  |  |  |  |  |  |  |  |  |  |  |  |
| --- | --- | --- | --- | --- | --- | --- | --- | --- | --- | --- | --- | --- | --- | --- | --- | --- | --- | --- | --- | --- | --- | --- | --- | --- | --- | --- | --- | --- | --- | --- | --- | --- | --- | --- | --- |

Best Unique PSM (Scan F7:35904, m/z=1155.5726, z=2, RT=130.47, ppm=2.5):

sp|Q62261|SPTB2\_MOUSE

back to list

  

| Protein Coverage
| Supporting Peptides
| Best Unique PSM
|

Protein Coverage:

Supporting Peptides:

| Peptide | Uniq | -10lgP | Mass | Length | ppm | m/z | z | RT | Fraction | Scan | Source File | Area 10 | Area 01 | Area 02 | Area 03 | Area 04 | Area 05 | Area 06 | Area 07 | Area 08 | Area 09 | #Spec | #Spec 10 | #Spec 01 | #Spec 02 | #Spec 03 | #Spec 04 | #Spec 05 | #Spec 06 | #Spec 07 | #Spec 08 | #Spec 09 | Start | End | PTM |
| --- | --- | --- | --- | --- | --- | --- | --- | --- | --- | --- | --- | --- | --- | --- | --- | --- | --- | --- | --- | --- | --- | --- | --- | --- | --- | --- | --- | --- | --- | --- | --- | --- | --- | --- | --- |
| K.HQILEQAVEDYAETVHQLSK.T | Y | 114.57 | 2337.1602 | 20 | -0.8 | 780.0601 | 3 | 118.45 | 3 | 32653 | Ana\_02.raw | 3.22E6 | 4.02E6 | 1.31E6 | 6.89E5 | 1.35E6 | 3.94E6 | 9.7E5 | 1.86E6 | 3.73E6 | 1.3E6 | 10 | 1 | 1 | 1 | 1 | 1 | 1 | 1 | 1 | 1 | 1 | 1634 | 1653 |  |
| R.EVDDLEQWIAER.E | Y | 90.51 | 1501.7048 | 12 | 0.5 | 751.8600 | 2 | 111.41 | 6 | 31242 | Ana\_05.raw | 7.65E5 | 9.18E5 |  |  | 3.85E5 | 1.01E6 | 4.34E5 | 8.69E5 | 1.71E6 | 1.84E5 | 8 | 1 | 1 | 0 | 0 | 1 | 1 | 1 | 1 | 1 | 1 | 1706 | 1717 |  |
| R.SQNIITDSSSLNAEAIR.Q | Y | 89.69 | 1817.9119 | 17 | 2.1 | 909.9651 | 2 | 81.23 | 6 | 23209 | Ana\_05.raw |  | 7.58E5 |  |  |  | 2.71E5 |  |  | 2.68E6 |  | 3 | 0 | 1 | 0 | 0 | 0 | 1 | 0 | 0 | 1 | 0 | 1548 | 1564 |  |
| R.ALVADSHPESER.I | Y | 82.79 | 1309.6262 | 12 | 4.5 | 655.8234 | 2 | 26.62 | 1 | 6946 | ANa\_10.raw | 3.24E6 | 8.68E6 | 3.34E6 |  |  | 3.96E6 |  |  | 6.77E6 | 2.05E6 | 6 | 1 | 1 | 1 | 0 | 0 | 1 | 0 | 0 | 1 | 1 | 1657 | 1668 |  |
| R.DVEDEILWVGER.M | Y | 80.33 | 1458.6990 | 12 | 6.5 | 730.3615 | 2 | 111.54 | 6 | 31278 | Ana\_05.raw |  |  |  | 8.04E5 | 1.63E5 | 8.21E5 | 2.44E5 | 7.95E5 |  |  | 5 | 0 | 0 | 0 | 1 | 1 | 1 | 1 | 1 | 0 | 0 | 1493 | 1504 |  |
| R.DLDDFQSWLSR.T | Y | 76.19 | 1380.6310 | 11 | -0.7 | 691.3223 | 2 | 117.50 | 3 | 32411 | Ana\_02.raw | 4.28E6 | 5.35E6 | 4.77E6 |  | 3.13E6 | 5.36E6 | 2.44E6 | 1.49E6 | 1.64E6 | 1.35E6 | 9 | 1 | 1 | 1 | 0 | 1 | 1 | 1 | 1 | 1 | 1 | 1070 | 1080 |  |
| R.DASVAEAWLLGQEPYLSSR.E | Y | 73.95 | 2091.0273 | 19 | 4.7 | 1046.5259 | 2 | 128.60 | 1 | 35358 | ANa\_10.raw | 1.34E6 |  |  |  |  |  |  |  |  |  | 1 | 1 | 0 | 0 | 0 | 0 | 0 | 0 | 0 | 0 | 0 | 2025 | 2043 |  |
| R.LVSQDNFGFDLPAVEAATK.K | Y | 71.97 | 2021.0105 | 19 | 2.0 | 1011.5146 | 2 | 117.77 | 2 | 32766 | Ana\_01.raw | 1.28E6 | 1.89E6 |  |  |  |  | 3.51E5 | 1.66E5 |  |  | 4 | 1 | 1 | 0 | 0 | 0 | 0 | 1 | 1 | 0 | 0 | 444 | 462 |  |
| K.VLLLSQDYGK.H | Y | 68.57 | 1134.6284 | 10 | 6.4 | 568.3251 | 2 | 71.13 | 6 | 20414 | Ana\_05.raw |  |  | 1.12E6 | 5.9E5 | 1.42E6 | 1.58E6 | 2.49E5 | 6.25E5 | 1.53E6 | 1.2E6 | 8 | 0 | 0 | 1 | 1 | 1 | 1 | 1 | 1 | 1 | 1 | 549 | 558 |  |
| R.KHYASEEIK.E | Y | 67.11 | 1103.5610 | 9 | 2.0 | 552.7889 | 2 | 18.15 | 3 | 4315 | Ana\_02.raw | 2.77E6 | 3.74E6 | 8.09E5 | 6.93E5 | 1.64E6 |  | 1.39E6 | 7.42E5 | 2.75E6 | 2.99E6 | 16 | 2 | 2 | 1 | 1 | 2 | 0 | 2 | 2 | 2 | 2 | 1981 | 1989 |  |
| K.FANSLVGVQQQLQAFNTYR.T | Y | 61.39 | 2183.1123 | 19 | 1.4 | 728.7124 | 3 | 122.13 | 6 | 34044 | Ana\_05.raw |  | 8.57E5 | 1.05E6 | 3.96E5 | 1.11E6 | 2.18E6 | 5.91E5 |  |  |  | 6 | 0 | 1 | 1 | 1 | 1 | 1 | 1 | 0 | 0 | 0 | 330 | 348 |  |
| K.HDTSASTQSTPASSR.A | Y | 59.08 | 1531.6863 | 15 | 3.3 | 511.5711 | 3 | 17.48 | 6 | 4091 | Ana\_05.raw |  |  |  |  |  | 9.18E5 |  |  |  |  | 1 | 0 | 0 | 0 | 0 | 0 | 1 | 0 | 0 | 0 | 0 | 2310 | 2324 |  |
| K.VLDNAIETEK.M | Y | 58.14 | 1130.5819 | 10 | 1.1 | 566.2988 | 2 | 43.45 | 4 | 11859 | Ana\_03.raw | 2.82E6 |  | 3E6 | 6.23E5 | 2.51E6 | 3.33E6 | 2.23E6 | 1.19E6 |  | 2.88E6 | 8 | 1 | 0 | 1 | 1 | 1 | 1 | 1 | 1 | 0 | 1 | 293 | 302 |  |
| R.LTTLELLEVR.R | Y | 55.37 | 1185.6969 | 10 | 2.6 | 593.8572 | 2 | 105.69 | 7 | 29437 | Ana\_06.raw | 4.95E5 | 1.05E6 |  |  |  | 6.44E5 | 1.64E6 |  | 1.95E6 |  | 5 | 1 | 1 | 0 | 0 | 0 | 1 | 1 | 0 | 1 | 0 | 2079 | 2088 |  |
| R.VAVVNQIAR.Q | Y | 54.24 | 968.5767 | 9 | 0.2 | 485.2957 | 2 | 42.29 | 3 | 11851 | Ana\_02.raw |  | 3.12E6 | 3.75E6 | 1.49E6 | 4.09E6 | 3.69E6 | 2.42E6 | 1.83E6 |  |  | 8 | 1 | 1 | 1 | 1 | 1 | 1 | 1 | 1 | 0 | 0 | 905 | 913 |  |
| K.FM(+15.99)ELLEPLSER.K | Y | 52.04 | 1378.6802 | 11 | -4.8 | 690.3441 | 2 | 89.50 | 6 | 25455 | Ana\_05.raw | 8.86E5 | 1.12E6 | 1.81E6 | 5.29E5 | 1.83E6 | 5.47E5 | 1.11E6 | 8.49E5 |  |  | 8 | 1 | 1 | 1 | 1 | 1 | 1 | 1 | 1 | 0 | 0 | 1467 | 1477 | Oxidation (M) |
| R.DQNTVETLQR.M | Y | 50.75 | 1202.5891 | 10 | 5.4 | 602.3051 | 2 | 37.84 | 6 | 10487 | Ana\_05.raw |  |  |  |  | 2.64E7 | 1.69E6 |  | 2.12E6 |  |  | 4 | 0 | 0 | 0 | 0 | 1 | 2 | 0 | 1 | 0 | 0 | 1835 | 1844 |  |
| R.IDDIFER.S | Y | 50.32 | 906.4446 | 7 | 2.8 | 454.2309 | 2 | 63.88 | 2 | 18195 | Ana\_01.raw | 2.15E6 | 2.78E6 |  |  | 3.62E6 | 2.06E6 | 2.88E6 |  | 3.42E6 |  | 6 | 1 | 1 | 0 | 0 | 1 | 1 | 1 | 0 | 1 | 0 | 1541 | 1547 |  |
| R.LQAAYAGDK.A | Y | 49.52 | 935.4712 | 9 | 2.8 | 468.7442 | 2 | 27.34 | 7 | 7159 | Ana\_06.raw |  |  |  | 1.09E6 |  | 3.82E6 | 2.45E6 | 1.54E6 |  |  | 4 | 0 | 0 | 0 | 1 | 0 | 1 | 1 | 1 | 0 | 0 | 1870 | 1878 |  |
| K.HLLGVEDLLQK.H | Y | 45.74 | 1263.7186 | 11 | 2.7 | 632.8683 | 2 | 91.19 | 6 | 25907 | Ana\_05.raw | 1.14E5 |  |  |  |  | 2.14E5 |  |  |  | 2.09E5 | 3 | 1 | 0 | 0 | 0 | 0 | 1 | 0 | 0 | 0 | 1 | 559 | 569 |  |
| K.LLEVLSGER.L | Y | 45.21 | 1014.5709 | 9 | 7.6 | 508.2966 | 2 | 68.27 | 2 | 19458 | Ana\_01.raw | 2.23E6 | 2.36E6 |  |  |  |  |  |  | 4.49E6 | 2.16E6 | 4 | 1 | 1 | 0 | 0 | 0 | 0 | 0 | 0 | 1 | 1 | 91 | 99 |  |
| R.KHNLLASK.E | Y | 44.82 | 909.5396 | 8 | 3.1 | 455.7785 | 2 | 18.91 | 2 | 4383 | Ana\_01.raw | 1.21E6 | 1.43E6 |  |  |  |  |  |  |  | 8.14E5 | 3 | 1 | 1 | 0 | 0 | 0 | 0 | 0 | 0 | 0 | 1 | 1478 | 1485 |  |
| K.RHEAFEK.S | N | 43.82 | 915.4562 | 7 | 2.4 | 306.1601 | 3 | 16.80 | 6 | 3871 | Ana\_05.raw |  |  |  | 3.45E5 | 2.07E6 | 1.59E6 |  |  |  |  | 5 | 0 | 0 | 0 | 1 | 2 | 2 | 0 | 0 | 0 | 0 | 2058 | 2064 |  |
| R.FESLEPEM(+15.99)NNQASR.V | Y | 43.38 | 1666.7257 | 14 | 2.5 | 834.3722 | 2 | 46.61 | 3 | 13152 | Ana\_02.raw |  |  | 8.88E5 |  |  |  | 7.45E5 | 3.76E5 |  |  | 3 | 0 | 0 | 1 | 0 | 0 | 0 | 1 | 1 | 0 | 0 | 891 | 904 | Oxidation (M) |
| K.INAVVETGR.R | Y | 42.41 | 957.5243 | 9 | 3.1 | 479.7709 | 2 | 33.41 | 6 | 9149 | Ana\_05.raw |  |  |  | 4.91E6 | 8.75E6 | 8.86E6 | 7.69E6 |  |  |  | 4 | 0 | 0 | 0 | 1 | 1 | 1 | 1 | 0 | 0 | 0 | 1225 | 1233 |  |
| R.AFEDEM(+15.99)SGR.S | Y | 41.57 | 1056.4182 | 9 | 4.9 | 529.2190 | 2 | 25.76 | 7 | 6667 | Ana\_06.raw |  |  | 3.79E6 |  | 3.58E6 | 4E6 | 2.92E6 | 1.7E6 |  |  | 5 | 0 | 0 | 1 | 0 | 1 | 1 | 1 | 1 | 0 | 0 | 682 | 690 | Oxidation (M) |
| R.LVSDGNINSDR.I | Y | 39.98 | 1188.5735 | 11 | 6.7 | 595.2980 | 2 | 33.69 | 6 | 9233 | Ana\_05.raw |  |  | 2.21E6 |  |  | 2.73E6 |  |  |  |  | 2 | 0 | 0 | 1 | 0 | 0 | 1 | 0 | 0 | 0 | 0 | 1235 | 1245 |  |
| K.LLDPEDISVDHPDEK.S | Y | 36.54 | 1720.8156 | 15 | 0.0 | 574.6125 | 3 | 72.81 | 6 | 20890 | Ana\_05.raw |  |  |  |  |  | 4.28E6 |  |  |  |  | 1 | 0 | 0 | 0 | 0 | 0 | 1 | 0 | 0 | 0 | 0 | 250 | 264 |  |
| R.KHYASEEIKEK.L | Y | 36.45 | 1360.6986 | 11 | 2.9 | 454.5748 | 3 | 19.71 | 9 | 4537 | Ana\_08.raw |  |  |  |  |  |  |  |  | 5.83E5 |  | 1 | 0 | 0 | 0 | 0 | 0 | 0 | 0 | 0 | 1 | 0 | 1981 | 1991 |  |
| R.VQAVVAVAR.E | Y | 35.34 | 911.5552 | 9 | 5.0 | 456.7872 | 2 | 36.62 | 2 | 10014 | Ana\_01.raw | 4.97E6 | 6.64E6 |  |  |  |  |  |  | 4.26E6 |  | 3 | 1 | 1 | 0 | 0 | 0 | 0 | 0 | 0 | 1 | 0 | 478 | 486 |  |
| R.FSALER.L | Y | 35.03 | 721.3759 | 6 | 7.0 | 361.6977 | 2 | 36.95 | 7 | 10068 | Ana\_06.raw |  |  |  | 1.34E6 |  |  | 1.71E6 |  |  |  | 2 | 0 | 0 | 0 | 1 | 0 | 0 | 1 | 0 | 0 | 0 | 2073 | 2078 |  |
| K.HRPDLIDFDK.L | N | 33.90 | 1254.6356 | 10 | 1.1 | 419.2196 | 3 | 54.51 | 5 | 15458 | Ana\_04.raw |  |  |  |  | 4.77E5 |  |  |  |  |  | 1 | 0 | 0 | 0 | 0 | 1 | 0 | 0 | 0 | 0 | 0 | 215 | 224 |  |
| R.DLVAIEAK.L | Y | 32.92 | 857.4858 | 8 | -1.6 | 429.7495 | 2 | 53.11 | 4 | 14681 | Ana\_03.raw |  |  |  | 1.33E6 | 2.46E6 |  | 2.69E6 |  |  |  | 3 | 0 | 0 | 0 | 1 | 1 | 0 | 1 | 0 | 0 | 0 | 1004 | 1011 |  |
| R.LAGIEER.C | Y | 29.40 | 786.4235 | 7 | 1.1 | 394.2195 | 2 | 28.52 | 9 | 7448 | Ana\_08.raw |  | 1.18E7 |  |  |  |  |  |  | 1.25E7 |  | 2 | 0 | 1 | 0 | 0 | 0 | 0 | 0 | 0 | 1 | 0 | 824 | 830 |  |
| R.LFQLNR.E | Y | 26.27 | 789.4497 | 6 | 1.2 | 395.7326 | 2 | 54.53 | 5 | 15463 | Ana\_04.raw |  |  |  |  | 1.14E6 |  |  |  |  |  | 1 | 0 | 0 | 0 | 0 | 1 | 0 | 0 | 0 | 0 | 0 | 1700 | 1705 |  |
| K.LQQFLR.D | Y | 24.45 | 803.4653 | 6 | 1.5 | 402.7405 | 2 | 50.77 | 4 | 14009 | Ana\_03.raw |  |  |  | 0 |  |  | 1.29E6 |  | 3E6 |  | 3 | 0 | 0 | 0 | 1 | 0 | 0 | 1 | 0 | 1 | 0 | 1064 | 1069 |  |
| K.ADDIQKR.E | Y | 22.32 | 844.4402 | 7 | -9.0 | 423.2236 | 2 | 45.38 | 4 | 12427 | Ana\_03.raw |  |  |  | 6.27E6 |  |  |  |  |  |  | 1 | 0 | 0 | 0 | 1 | 0 | 0 | 0 | 0 | 0 | 0 | 1879 | 1885 |  |
| total 37 peptides |  |  |  |  |  |  |  |  |  |  |  |  |  |  |  |  |  |  |  |  |  |  |  |  |  |  |  |  |  |  |  |  |  |  |  |
| --- | --- | --- | --- | --- | --- | --- | --- | --- | --- | --- | --- | --- | --- | --- | --- | --- | --- | --- | --- | --- | --- | --- | --- | --- | --- | --- | --- | --- | --- | --- | --- | --- | --- | --- | --- |

Best Unique PSM (Scan F3:32653, m/z=780.0601, z=3, RT=118.45, ppm=-0.8):

sp|P51881|ADT2\_MOUSE

back to list

  

| Protein Coverage
| Supporting Peptides
| Best Unique PSM
|

Protein Coverage:

Supporting Peptides:

| Peptide | Uniq | -10lgP | Mass | Length | ppm | m/z | z | RT | Fraction | Scan | Source File | Area 10 | Area 01 | Area 02 | Area 03 | Area 04 | Area 05 | Area 06 | Area 07 | Area 08 | Area 09 | #Spec | #Spec 10 | #Spec 01 | #Spec 02 | #Spec 03 | #Spec 04 | #Spec 05 | #Spec 06 | #Spec 07 | #Spec 08 | #Spec 09 | Start | End | PTM |
| --- | --- | --- | --- | --- | --- | --- | --- | --- | --- | --- | --- | --- | --- | --- | --- | --- | --- | --- | --- | --- | --- | --- | --- | --- | --- | --- | --- | --- | --- | --- | --- | --- | --- | --- | --- |
| R.YFAGNLASGGAAGATSLC(+57.02)FVYPLDFAR.T | N | 157.78 | 2795.3376 | 27 | 3.3 | 1398.6807 | 2 | 131.60 | 3 | 36052 | Ana\_02.raw | 1.29E7 | 2.53E7 | 1.79E7 | 1.03E7 | 1.42E7 | 2.86E7 | 1.66E7 | 8.85E6 | 1.93E7 | 7.28E6 | 20 | 2 | 2 | 2 | 2 | 2 | 2 | 2 | 2 | 2 | 2 | 112 | 138 | Carbamidomethylation |
| R.YFPTQALNFAFK.D | N | 89.35 | 1445.7343 | 12 | 3.5 | 723.8770 | 2 | 119.53 | 6 | 33368 | Ana\_05.raw | 6.46E7 | 7.52E7 | 7.98E7 | 2.05E7 | 3.74E7 | 4.27E7 | 3.04E7 | 2.87E7 | 1.26E8 | 5.56E7 | 37 | 4 | 5 | 5 | 3 | 2 | 2 | 2 | 4 | 4 | 6 | 81 | 92 |  |
| R.AAYFGIYDTAK.G | Y | 83.35 | 1218.5920 | 11 | 4.4 | 610.3060 | 2 | 78.83 | 6 | 22549 | Ana\_05.raw | 2.06E7 | 4.72E7 | 4.08E7 | 2.78E7 | 3.4E7 | 4.16E7 | 3.11E7 | 2.46E7 | 3.98E7 | 2.2E7 | 20 | 2 | 2 | 2 | 2 | 2 | 2 | 2 | 2 | 2 | 2 | 189 | 199 |  |
| R.GNLANVIRYFPTQALNFAFK.D | N | 79.39 | 2283.2163 | 20 | 3.8 | 762.0823 | 3 | 132.85 | 6 | 36931 | Ana\_05.raw | 9.41E5 | 4.21E6 | 2.14E6 | 1.68E6 | 2.26E6 | 3.07E6 | 7.45E5 |  |  | 3.85E6 | 8 | 1 | 1 | 1 | 1 | 1 | 1 | 1 | 0 | 0 | 1 | 73 | 92 |  |
| R.GNLANVIRYFPTQALNFAFKDK.Y | N | 74.22 | 2526.3381 | 22 | 3.9 | 632.5943 | 4 | 131.11 | 6 | 36429 | Ana\_05.raw |  |  |  |  |  | 3.07E6 |  |  |  | 1.85E6 | 2 | 0 | 0 | 0 | 0 | 0 | 1 | 0 | 0 | 0 | 1 | 73 | 94 |  |
| K.EQGVLSFWR.G | Y | 68.04 | 1120.5665 | 9 | 2.3 | 561.2918 | 2 | 98.57 | 3 | 27525 | Ana\_02.raw | 1.46E7 | 2.12E7 | 2.98E7 | 1.09E7 | 1.55E7 | 2.21E7 | 1.19E7 | 1.54E7 | 3.26E7 | 1.27E7 | 20 | 2 | 3 | 2 | 2 | 2 | 2 | 2 | 1 | 2 | 2 | 64 | 72 |  |
| K.DFLAGGVAAAISK.T | Y | 67.82 | 1218.6608 | 13 | 6.3 | 610.3415 | 2 | 95.65 | 1 | 26803 | ANa\_10.raw | 3.9E7 | 5.84E7 | 3.33E7 | 1.16E7 | 2.38E7 | 2.96E7 | 1.62E7 | 1.4E7 | 1.02E8 | 5.13E7 | 31 | 3 | 5 | 3 | 2 | 3 | 2 | 2 | 2 | 5 | 4 | 11 | 23 |  |
| R.GM(+15.99)GGAFVLVLYDEIKK.Y | N | 63.07 | 1754.9276 | 16 | 3.4 | 585.9851 | 3 | 124.04 | 6 | 34538 | Ana\_05.raw |  | 7.37E5 | 3.63E6 | 1.16E6 | 0 | 2.44E6 | 1.3E6 | 3.79E5 |  | 8.26E5 | 10 | 0 | 1 | 2 | 2 | 1 | 1 | 1 | 1 | 0 | 1 | 281 | 296 | Oxidation (M) |
| K.TAVAPIER.V | N | 59.52 | 855.4814 | 8 | 3.6 | 428.7495 | 2 | 30.28 | 6 | 8211 | Ana\_05.raw | 1.75E8 | 2.05E8 | 2.08E8 | 9.71E7 | 1.41E8 | 1.92E8 | 1.3E8 | 1.07E8 | 1.7E7 | 1.71E8 | 27 | 3 | 3 | 3 | 3 | 3 | 2 | 2 | 4 | 3 | 1 | 24 | 31 |  |
| M.TDAAVSFAK.D | Y | 48.50 | 908.4603 | 9 | 6.8 | 455.2405 | 2 | 36.66 | 10 | 10035 | Ana\_09.raw |  | 4.65E6 |  |  |  |  |  |  | 1.11E7 | 2.45E7 | 3 | 0 | 1 | 0 | 0 | 0 | 0 | 0 | 0 | 1 | 1 | 2 | 10 |  |
| R.GNLANVIR.Y | N | 48.16 | 855.4926 | 8 | 2.8 | 428.7548 | 2 | 49.59 | 7 | 13906 | Ana\_06.raw | 2.89E7 | 3.37E7 | 4.18E7 | 1.46E7 | 2.59E7 | 2.78E7 | 2.19E7 | 1.67E7 | 5.58E7 | 2.48E7 | 15 | 1 | 2 | 2 | 1 | 2 | 1 | 1 | 1 | 3 | 1 | 73 | 80 |  |
| K.LLLQVQHASK.Q | N | 47.15 | 1135.6713 | 10 | 3.2 | 568.8447 | 2 | 40.37 | 2 | 11173 | Ana\_01.raw | 3.4E7 | 4.34E7 | 4.63E7 | 7.09E6 | 3.58E7 | 4.38E7 | 2.89E7 | 1.02E7 | 8.93E6 | 3.76E7 | 20 | 2 | 2 | 3 | 1 | 3 | 2 | 2 | 1 | 2 | 2 | 34 | 43 |  |
| R.GM(+15.99)GGAFVLVLYDEIK.K | N | 42.54 | 1626.8326 | 15 | 5.4 | 814.4280 | 2 | 130.53 | 5 | 35925 | Ana\_04.raw |  |  |  |  | 9.28E5 |  |  |  |  |  | 1 | 0 | 0 | 0 | 0 | 1 | 0 | 0 | 0 | 0 | 0 | 281 | 295 | Oxidation (M) |
| K.GAWSNVLR.G | N | 42.38 | 901.4770 | 8 | 3.8 | 451.7475 | 2 | 63.04 | 2 | 17954 | Ana\_01.raw | 6.61E6 | 9.17E6 | 1.15E7 |  | 7.43E6 | 6.82E6 | 5.84E6 | 6.65E6 | 4.72E6 | 5.97E6 | 11 | 1 | 1 | 2 | 0 | 1 | 1 | 1 | 2 | 1 | 1 | 273 | 280 |  |
| K.GM(+15.99)LPDPK.N | N | 40.23 | 772.3789 | 7 | 0.9 | 387.1971 | 2 | 25.25 | 3 | 6640 | Ana\_02.raw |  | 7.82E6 | 4.79E7 | 2.45E7 | 2.25E7 | 3.19E7 | 1.57E7 | 2.38E7 | 1.52E7 |  | 9 | 0 | 1 | 1 | 1 | 1 | 2 | 1 | 1 | 1 | 0 | 200 | 206 | Oxidation (M) |
| R.LAADVGK.A | N | 40.15 | 672.3806 | 7 | 0.8 | 337.1978 | 2 | 20.12 | 5 | 5026 | Ana\_04.raw |  |  |  |  | 1.41E7 |  |  |  |  |  | 1 | 0 | 0 | 0 | 0 | 1 | 0 | 0 | 0 | 0 | 0 | 141 | 147 |  |
| K.GLGDC(+57.02)LVK.I | Y | 37.18 | 860.4426 | 8 | 3.8 | 431.2302 | 2 | 47.40 | 9 | 13258 | Ana\_08.raw | 4.53E6 | 7.93E6 |  |  |  |  |  |  | 1.82E7 |  | 3 | 1 | 1 | 0 | 0 | 0 | 0 | 0 | 0 | 1 | 0 | 156 | 163 | Carbamidomethylation |
| K.QIFLGGVDK.R | Y | 34.56 | 975.5389 | 9 | 3.4 | 488.7784 | 2 | 64.02 | 8 | 17943 | Ana\_07.raw |  | 3.92E6 |  |  | 1.11E6 | 2.97E6 | 2.07E6 | 2.97E6 | 5.55E6 |  | 6 | 0 | 1 | 0 | 0 | 1 | 1 | 1 | 1 | 1 | 0 | 97 | 105 |  |
| R.TQFWR.Y | Y | 32.34 | 736.3657 | 5 | 8.3 | 369.1932 | 2 | 43.45 | 9 | 12042 | Ana\_08.raw |  |  |  |  |  |  |  |  | 1.95E7 |  | 1 | 0 | 0 | 0 | 0 | 0 | 0 | 0 | 0 | 1 | 0 | 107 | 111 |  |
| K.QIFLGGVDKR.T | Y | 26.84 | 1131.6400 | 10 | 3.4 | 378.2219 | 3 | 51.22 | 5 | 14488 | Ana\_04.raw |  |  |  | 1.44E6 | 1.4E6 |  |  |  |  |  | 2 | 0 | 0 | 0 | 1 | 1 | 0 | 0 | 0 | 0 | 0 | 97 | 106 |  |
| total 20 peptides |  |  |  |  |  |  |  |  |  |  |  |  |  |  |  |  |  |  |  |  |  |  |  |  |  |  |  |  |  |  |  |  |  |  |  |
| --- | --- | --- | --- | --- | --- | --- | --- | --- | --- | --- | --- | --- | --- | --- | --- | --- | --- | --- | --- | --- | --- | --- | --- | --- | --- | --- | --- | --- | --- | --- | --- | --- | --- | --- | --- |

Best Unique PSM (Scan F6:22549, m/z=610.3060, z=2, RT=78.83, ppm=4.4):

E9Q9C6|E9Q9C6\_MOUSE

back to list

  

| Protein Coverage
| Supporting Peptides
| Best Unique PSM
|

Protein Coverage:

Supporting Peptides:

| Peptide | Uniq | -10lgP | Mass | Length | ppm | m/z | z | RT | Fraction | Scan | Source File | Area 10 | Area 01 | Area 02 | Area 03 | Area 04 | Area 05 | Area 06 | Area 07 | Area 08 | Area 09 | #Spec | #Spec 10 | #Spec 01 | #Spec 02 | #Spec 03 | #Spec 04 | #Spec 05 | #Spec 06 | #Spec 07 | #Spec 08 | #Spec 09 | Start | End | PTM |
| --- | --- | --- | --- | --- | --- | --- | --- | --- | --- | --- | --- | --- | --- | --- | --- | --- | --- | --- | --- | --- | --- | --- | --- | --- | --- | --- | --- | --- | --- | --- | --- | --- | --- | --- | --- |
| R.YYVLGATFYPGPEC(+57.02)ER.L | Y | 102.87 | 1920.8716 | 16 | 0.5 | 961.4435 | 2 | 101.78 | 3 | 28363 | Ana\_02.raw | 0 |  | 1.01E6 |  | 1.66E6 | 4.27E6 | 7.83E5 | 7.89E5 | 1.29E7 |  | 7 | 1 | 0 | 1 | 0 | 1 | 1 | 1 | 1 | 1 | 0 | 1978 | 1993 | Carbamidomethylation |
| R.VLIDGVLQYLPFQAAGGK.I | Y | 99.01 | 1888.0458 | 18 | 4.0 | 945.0339 | 2 | 131.73 | 1 | 36212 | ANa\_10.raw | 2.01E7 | 1.35E7 | 9.5E6 | 2.42E6 | 8.36E6 | 1.79E7 | 1.16E7 | 5.82E6 | 8.7E6 | 1.29E7 | 22 | 3 | 2 | 2 | 2 | 2 | 2 | 2 | 2 | 2 | 3 | 526 | 543 |  |
| K.DILC(+57.02)QALAAYAAAC(+57.02)QAAGIK.I | Y | 97.89 | 2078.0288 | 20 | 4.6 | 1040.0265 | 2 | 134.90 | 9 | 37300 | Ana\_08.raw |  | 3.9E6 | 3.55E6 |  |  | 1.36E7 | 5.18E5 |  | 2.55E7 | 7.64E6 | 10 | 0 | 1 | 2 | 0 | 0 | 2 | 1 | 0 | 2 | 2 | 1497 | 1516 | Carbamidomethylation |
| R.LLAPGQEVFDDDR.C | Y | 96.91 | 1473.7100 | 13 | 5.4 | 737.8663 | 2 | 76.04 | 9 | 21663 | Ana\_08.raw | 2.29E7 | 2.15E7 | 1.52E7 | 1.11E6 | 1.15E7 | 1.72E7 | 1.18E7 | 1.06E7 | 5.46E7 | 2.18E7 | 18 | 2 | 2 | 1 | 1 | 2 | 2 | 2 | 1 | 3 | 2 | 391 | 403 |  |
| R.VPAAYAGTLC(+57.02)GLC(+57.02)GNYNK.N | Y | 81.73 | 1927.8921 | 18 | 2.1 | 964.9554 | 2 | 81.22 | 9 | 23126 | Ana\_08.raw |  |  |  |  |  |  |  |  | 1.28E6 |  | 1 | 0 | 0 | 0 | 0 | 0 | 0 | 0 | 0 | 1 | 0 | 1779 | 1796 | Carbamidomethylation |
| R.SLAAYTAAC(+57.02)QAAGAAVKPWR.T | Y | 80.80 | 2062.0417 | 20 | 2.6 | 688.3563 | 3 | 95.19 | 9 | 26953 | Ana\_08.raw |  |  |  |  |  |  | 7.31E5 | 5.48E5 | 1.01E7 |  | 3 | 0 | 0 | 0 | 0 | 0 | 0 | 1 | 1 | 1 | 0 | 2271 | 2290 | Carbamidomethylation |
| K.VLSPLEYFR.Q | Y | 76.92 | 1122.6073 | 9 | 4.3 | 562.3134 | 2 | 108.18 | 6 | 30409 | Ana\_05.raw | 2.91E7 | 2.72E7 | 2.49E7 | 9.62E6 | 1.76E7 | 1.91E7 | 1.5E7 | 1.18E7 | 6.8E7 | 3.01E7 | 23 | 2 | 4 | 2 | 2 | 2 | 2 | 2 | 3 | 2 | 2 | 2244 | 2252 |  |
| R.VDLPATVLTSVSVR.R | Y | 73.69 | 1455.8297 | 14 | 4.8 | 486.2862 | 3 | 103.63 | 9 | 29200 | Ana\_08.raw | 3.63E6 | 2.56E6 |  |  | 1E6 | 2.61E6 |  |  | 2.48E6 | 3.13E6 | 7 | 1 | 1 | 0 | 0 | 1 | 1 | 0 | 0 | 2 | 1 | 2496 | 2509 |  |
| R.SC(+57.02)QGSC(+57.02)AALSGLTGC(+57.02)TTR.C | Y | 71.29 | 1885.8081 | 18 | 3.9 | 943.9150 | 2 | 62.25 | 9 | 17726 | Ana\_08.raw |  |  |  |  |  |  |  |  | 1.16E6 |  | 1 | 0 | 0 | 0 | 0 | 0 | 0 | 0 | 0 | 1 | 0 | 2311 | 2328 | Carbamidomethylation |
| K.EC(+57.02)GILADPEGPFR.E | Y | 70.92 | 1459.6765 | 13 | 5.2 | 730.8493 | 2 | 85.50 | 9 | 24319 | Ana\_08.raw | 5.37E6 | 5.36E6 |  | 5.68E5 | 3.59E6 | 1E6 | 9.53E5 | 3.59E5 | 1.6E7 | 6.91E6 | 9 | 1 | 1 | 0 | 1 | 1 | 1 | 1 | 1 | 1 | 1 | 648 | 660 | Carbamidomethylation |
| R.VAYDLVYYVR.V | Y | 66.57 | 1259.6550 | 10 | 3.2 | 630.8368 | 2 | 96.16 | 6 | 27234 | Ana\_05.raw |  |  | 3.03E6 |  | 1.62E5 | 8.7E6 |  |  | 6.5E6 |  | 5 | 0 | 0 | 1 | 0 | 1 | 2 | 0 | 0 | 1 | 0 | 950 | 959 |  |
| R.VVAHGVEVAVR.R | Y | 66.03 | 1134.6509 | 11 | 4.5 | 568.3353 | 2 | 37.79 | 10 | 10381 | Ana\_09.raw | 1.28E7 | 1.31E7 | 1.04E7 | 3.56E6 | 8.06E6 | 1.35E7 | 9.39E6 | 7.01E6 | 1.77E7 | 1.55E7 | 19 | 2 | 2 | 1 | 2 | 2 | 2 | 2 | 2 | 2 | 2 | 509 | 519 |  |
| R.GSQAVSYTR.S | Y | 65.58 | 967.4723 | 9 | 4.2 | 484.7455 | 2 | 23.62 | 6 | 6117 | Ana\_05.raw |  |  |  | 6.46E6 | 7.86E6 | 1.01E7 |  | 4.2E6 |  | 1.36E7 | 5 | 0 | 0 | 0 | 1 | 1 | 1 | 0 | 1 | 0 | 1 | 1702 | 1710 |  |
| R.GSPAVSYVR.Q | Y | 62.31 | 934.4872 | 9 | 3.3 | 468.2524 | 2 | 35.97 | 1 | 9773 | ANa\_10.raw | 2.45E7 | 2.12E7 | 2.2E7 |  |  | 1.61E7 | 1.03E7 | 4.04E6 | 3.16E7 | 2.57E7 | 8 | 1 | 1 | 1 | 0 | 0 | 1 | 1 | 1 | 1 | 1 | 1305 | 1313 |  |
| R.VVVTVAGQVVGLAR.G | Y | 62.01 | 1366.8296 | 14 | 3.5 | 684.4244 | 2 | 87.28 | 9 | 24800 | Ana\_08.raw | 3.3E5 |  |  |  |  | 4.67E5 |  |  | 1.19E6 | 2.77E6 | 4 | 1 | 0 | 0 | 0 | 0 | 1 | 0 | 0 | 1 | 1 | 2088 | 2101 |  |
| R.VLADVQPC(+57.02)HNNDK.I | Y | 61.97 | 1508.7041 | 13 | 1.9 | 755.3608 | 2 | 30.03 | 6 | 8138 | Ana\_05.raw |  | 9.91E5 |  |  |  | 1.07E6 | 2.5E5 |  |  |  | 3 | 0 | 1 | 0 | 0 | 0 | 1 | 1 | 0 | 0 | 0 | 2455 | 2467 | Carbamidomethylation |
| K.IRDPHGPFAAC(+57.02)HK.V | Y | 60.29 | 1504.7357 | 13 | 5.7 | 502.5887 | 3 | 24.04 | 9 | 5986 | Ana\_08.raw |  |  |  |  |  |  |  |  | 4.4E6 |  | 1 | 0 | 0 | 0 | 0 | 0 | 0 | 0 | 0 | 1 | 0 | 2231 | 2243 | Carbamidomethylation |
| R.VTIPGNYYK.Q | Y | 58.57 | 1053.5494 | 9 | 3.9 | 527.7841 | 2 | 56.64 | 9 | 16052 | Ana\_08.raw | 9.37E6 | 8.13E6 |  |  |  |  | 5.63E6 |  | 2.39E7 | 9.97E6 | 7 | 1 | 1 | 0 | 0 | 0 | 0 | 1 | 0 | 3 | 1 | 960 | 968 |  |
| K.DPC(+57.02)HGITC(+57.02)RPQETC(+57.02)K.V | Y | 52.67 | 1857.7920 | 15 | 0.7 | 620.2717 | 3 | 27.17 | 9 | 7022 | Ana\_08.raw |  |  |  |  |  |  |  |  | 3.82E6 |  | 1 | 0 | 0 | 0 | 0 | 0 | 0 | 0 | 0 | 1 | 0 | 1223 | 1237 | Carbamidomethylation |
| K.VLVENEHR.G | Y | 49.07 | 994.5196 | 8 | 1.1 | 498.2676 | 2 | 20.14 | 3 | 4982 | Ana\_02.raw | 1.85E7 |  | 1.09E7 | 2.76E6 |  | 1.13E7 | 6.21E6 |  |  | 2.69E7 | 9 | 2 | 0 | 1 | 1 | 1 | 1 | 1 | 0 | 0 | 2 | 489 | 496 |  |
| R.NIVLQTNK.G | Y | 48.38 | 928.5342 | 8 | 3.3 | 465.2759 | 2 | 33.32 | 6 | 9123 | Ana\_05.raw | 1.36E7 | 1.25E7 | 1.25E7 | 3.68E6 | 9.02E6 | 3.4E6 | 8.69E6 | 8.28E5 |  | 1.3E7 | 9 | 1 | 1 | 1 | 1 | 1 | 1 | 1 | 1 | 0 | 1 | 2129 | 2136 |  |
| R.LPASLSEGR.L | Y | 46.98 | 928.4977 | 9 | 3.4 | 465.2577 | 2 | 39.56 | 2 | 10921 | Ana\_01.raw |  | 1.72E7 |  |  |  |  |  |  |  |  | 1 | 0 | 1 | 0 | 0 | 0 | 0 | 0 | 0 | 0 | 0 | 143 | 151 |  |
| R.GSQTVSYTR.A | Y | 46.06 | 997.4828 | 9 | 2.5 | 499.7500 | 2 | 23.60 | 1 | 5974 | ANa\_10.raw | 5.86E6 | 7.18E6 |  | 4.95E6 |  |  | 8.43E6 |  |  | 1.53E7 | 5 | 1 | 1 | 0 | 1 | 0 | 0 | 1 | 0 | 0 | 1 | 497 | 505 |  |
| K.LLNPQGAIR.D | Y | 43.95 | 980.5767 | 9 | 5.8 | 491.2985 | 2 | 50.43 | 2 | 14220 | Ana\_01.raw | 1.21E7 | 1.01E7 | 7.13E6 |  |  |  | 4.95E6 | 3.61E6 | 2.98E7 | 1.46E7 | 10 | 2 | 1 | 1 | 0 | 0 | 0 | 1 | 1 | 2 | 2 | 665 | 673 |  |
| R.GEIGFVR.I | Y | 43.76 | 776.4180 | 7 | 4.9 | 389.2182 | 2 | 47.01 | 1 | 13155 | ANa\_10.raw | 5.58E6 | 4.84E6 | 6.97E6 | 3.67E6 | 5.67E6 | 8.57E6 | 7.1E6 | 5.45E6 | 3.55E6 |  | 9 | 1 | 1 | 1 | 1 | 1 | 1 | 1 | 1 | 1 | 0 | 129 | 135 |  |
| K.NVPWYR.V | Y | 43.28 | 833.4184 | 6 | 5.3 | 417.7187 | 2 | 52.74 | 9 | 14893 | Ana\_08.raw | 8.58E5 |  | 1.14E6 |  | 8.9E5 |  | 1.02E6 |  | 3.31E6 |  | 5 | 1 | 0 | 1 | 0 | 1 | 0 | 1 | 0 | 1 | 0 | 2449 | 2454 |  |
| K.AYLC(+57.02)R.S | N | 33.21 | 681.3268 | 5 | 3.0 | 341.6717 | 2 | 24.86 | 6 | 6506 | Ana\_05.raw | 2.58E6 |  | 3.85E6 | 1.62E6 |  | 4.11E6 |  |  |  | 3.17E6 | 5 | 1 | 0 | 1 | 1 | 0 | 1 | 0 | 0 | 0 | 1 | 2266 | 2270 | Carbamidomethylation |
| K.IQVYR.Q | Y | 29.44 | 677.3860 | 5 | 6.1 | 339.7024 | 2 | 27.53 | 2 | 7252 | Ana\_01.raw | 7.85E6 | 7.36E6 | 1.06E7 |  |  | 8.65E6 | 6.79E6 |  | 8.88E6 | 1.28E7 | 7 | 1 | 1 | 1 | 0 | 0 | 1 | 1 | 0 | 1 | 1 | 544 | 548 |  |
| total 28 peptides |  |  |  |  |  |  |  |  |  |  |  |  |  |  |  |  |  |  |  |  |  |  |  |  |  |  |  |  |  |  |  |  |  |  |  |
| --- | --- | --- | --- | --- | --- | --- | --- | --- | --- | --- | --- | --- | --- | --- | --- | --- | --- | --- | --- | --- | --- | --- | --- | --- | --- | --- | --- | --- | --- | --- | --- | --- | --- | --- | --- |

Best Unique PSM (Scan F3:28363, m/z=961.4435, z=2, RT=101.78, ppm=0.5):

sp|P68368|TBA4A\_MOUSE

back to list

  

| Protein Coverage
| Supporting Peptides
| Best Unique PSM
|

Protein Coverage:

Supporting Peptides:

| Peptide | Uniq | -10lgP | Mass | Length | ppm | m/z | z | RT | Fraction | Scan | Source File | Area 10 | Area 01 | Area 02 | Area 03 | Area 04 | Area 05 | Area 06 | Area 07 | Area 08 | Area 09 | #Spec | #Spec 10 | #Spec 01 | #Spec 02 | #Spec 03 | #Spec 04 | #Spec 05 | #Spec 06 | #Spec 07 | #Spec 08 | #Spec 09 | Start | End | PTM |
| --- | --- | --- | --- | --- | --- | --- | --- | --- | --- | --- | --- | --- | --- | --- | --- | --- | --- | --- | --- | --- | --- | --- | --- | --- | --- | --- | --- | --- | --- | --- | --- | --- | --- | --- | --- |
| R.FDGALNVDLTEFQTNLVPYPR.I | N | 117.80 | 2408.2012 | 21 | 4.2 | 1205.1129 | 2 | 130.54 | 9 | 36141 | Ana\_08.raw | 2.24E6 |  |  | 7.33E5 | 1.14E6 |  | 9.17E5 |  | 3.51E6 | 1.14E6 | 8 | 1 | 0 | 0 | 2 | 1 | 0 | 2 | 0 | 1 | 1 | 244 | 264 |  |
| R.IHFPLATYAPVISAEK.A | N | 101.55 | 1755.9559 | 16 | 1.2 | 878.9863 | 2 | 104.62 | 6 | 29472 | Ana\_05.raw | 8.28E7 | 9.05E7 | 9.31E7 | 5.04E7 | 9.02E7 | 1.11E8 | 4.49E7 | 3.3E7 | 2.83E8 | 1.42E8 | 85 | 11 | 9 | 6 | 8 | 8 | 8 | 8 | 7 | 11 | 9 | 265 | 280 |  |
| R.AVC(+57.02)M(+15.99)LSNTTAIAEAWAR.L | N | 100.31 | 1879.8921 | 17 | -1.1 | 940.9523 | 2 | 100.08 | 1 | 27997 | ANa\_10.raw | 9.41E6 | 1.3E7 | 8.38E6 | 1.22E6 | 1.18E6 | 1.01E7 | 6.35E6 |  | 1.37E7 | 2.31E6 | 14 | 2 | 2 | 2 | 1 | 1 | 2 | 2 | 0 | 1 | 1 | 374 | 390 | Carbamidomethylation; Oxidation (M) |
| R.AVC(+57.02)MLSNTTAIAEAWAR.L | N | 97.01 | 1863.8971 | 17 | 2.5 | 932.9582 | 2 | 118.70 | 1 | 32766 | ANa\_10.raw | 4.22E6 | 1.14E6 |  | 9.92E5 |  | 1.45E6 |  |  | 6.04E6 | 5.52E6 | 9 | 2 | 1 | 0 | 1 | 0 | 2 | 0 | 0 | 1 | 2 | 374 | 390 | Carbamidomethylation |
| R.AFVHWYVGEGM(+15.99)EEGEFSEAR.E | N | 92.50 | 2345.0059 | 20 | 3.4 | 782.6785 | 3 | 94.03 | 6 | 26666 | Ana\_05.raw | 1.18E6 | 3.45E6 | 2.64E6 | 4.66E5 | 3.25E6 | 5.36E6 | 3.55E6 |  | 2.72E6 | 3.51E6 | 9 | 1 | 1 | 1 | 1 | 1 | 1 | 1 | 0 | 1 | 1 | 403 | 422 | Oxidation (M) |
| R.SIQFVDWC(+57.02)PTGFK.V | N | 90.27 | 1583.7443 | 13 | -0.4 | 792.8791 | 2 | 111.78 | 3 | 30920 | Ana\_02.raw | 2.9E7 | 4.15E7 | 3.5E7 | 1.83E6 | 2.48E7 | 2.5E7 | 1.98E7 | 1.83E7 | 6.88E7 | 4.04E7 | 19 | 3 | 2 | 2 | 1 | 1 | 1 | 1 | 3 | 2 | 3 | 340 | 352 | Carbamidomethylation |
| K.TIGGGDDSFTTFFC(+57.02)ETGAGK.H | Y | 83.48 | 2066.8892 | 20 | 5.0 | 1034.4570 | 2 | 112.42 | 6 | 31503 | Ana\_05.raw | 5.96E5 | 1.97E6 | 6.58E5 | 7.31E5 | 5.43E5 | 1.06E6 | 7.68E5 | 7.3E5 | 1.82E6 | 1.66E6 | 10 | 1 | 1 | 1 | 1 | 1 | 1 | 1 | 1 | 1 | 1 | 41 | 60 | Carbamidomethylation |
| R.LISQIVSSITASLR.F | N | 82.49 | 1486.8718 | 14 | 5.8 | 744.4475 | 2 | 132.23 | 1 | 36352 | ANa\_10.raw | 5.92E6 | 5.49E6 | 3.88E6 |  | 2.45E6 | 3.71E6 | 3.27E6 | 8.77E5 | 1.58E7 | 5.67E6 | 14 | 1 | 2 | 1 | 0 | 2 | 2 | 2 | 1 | 2 | 1 | 230 | 243 |  |
| K.VGINYQPPTVVPGGDLAK.V | N | 78.26 | 1823.9781 | 18 | 1.3 | 912.9976 | 2 | 87.23 | 3 | 24540 | Ana\_02.raw | 2.69E7 | 6.86E7 | 4.84E7 | 1.55E7 | 3.76E7 | 3.39E7 | 2.24E7 | 2.24E7 | 1.81E8 | 7.09E7 | 26 | 3 | 3 | 2 | 2 | 1 | 3 | 2 | 1 | 5 | 4 | 353 | 370 |  |
| R.AVFVDLEPTVIDEIR.N | Y | 77.94 | 1714.9141 | 15 | -1.9 | 858.4627 | 2 | 123.53 | 6 | 34402 | Ana\_05.raw | 1.12E6 | 1.58E5 | 2.43E6 | 1.07E6 |  | 2.47E6 |  | 9.29E5 | 4.22E6 | 7.44E5 | 8 | 1 | 1 | 1 | 1 | 0 | 1 | 0 | 1 | 1 | 1 | 65 | 79 |  |
| R.QLFHPEQLITGK.E | N | 73.59 | 1409.7666 | 12 | 4.3 | 705.8936 | 2 | 84.37 | 8 | 23640 | Ana\_07.raw | 1.33E7 | 8.46E6 | 9.75E6 | 7.29E6 | 9.99E6 | 1.49E7 | 4.79E6 | 1.32E7 | 3.21E7 | 3.98E6 | 20 | 2 | 2 | 2 | 2 | 2 | 2 | 1 | 2 | 3 | 2 | 85 | 96 |  |
| R.NLDIERPTYTNLNR.L | N | 68.75 | 1717.8748 | 14 | 4.1 | 859.9482 | 2 | 64.89 | 9 | 18502 | Ana\_08.raw | 1.14E7 | 8.44E6 |  |  |  |  |  |  | 1.47E7 | 5.59E6 | 6 | 2 | 1 | 0 | 0 | 0 | 0 | 0 | 0 | 2 | 1 | 216 | 229 |  |
| R.GHYTIGK.E | N | 56.84 | 774.4024 | 7 | 3.9 | 388.2100 | 2 | 21.28 | 7 | 5302 | Ana\_06.raw |  |  |  |  | 8.67E6 |  | 9.09E6 | 6.3E6 |  | 6.29E6 | 4 | 0 | 0 | 0 | 0 | 1 | 0 | 1 | 1 | 0 | 1 | 106 | 112 |  |
| K.DVNAAIAAIK.T | Y | 54.19 | 984.5604 | 10 | 3.9 | 493.2894 | 2 | 66.66 | 9 | 19016 | Ana\_08.raw | 1.76E6 | 2.92E6 | 1.88E6 | 1.41E6 | 3.23E6 | 2.44E6 | 1.82E6 | 1.44E6 | 5.76E6 |  | 9 | 1 | 1 | 1 | 1 | 1 | 1 | 1 | 1 | 1 | 0 | 327 | 336 |  |
| K.EDAANNYAR.G | N | 51.28 | 1022.4417 | 9 | 2.5 | 512.2294 | 2 | 20.32 | 5 | 5091 | Ana\_04.raw |  |  |  |  | 1.64E7 |  |  |  |  |  | 1 | 0 | 0 | 0 | 0 | 1 | 0 | 0 | 0 | 0 | 0 | 97 | 105 |  |
| R.LSVDYGK.K | N | 49.40 | 780.4017 | 7 | 5.0 | 391.2101 | 2 | 36.09 | 10 | 9863 | Ana\_09.raw | 4.18E7 | 1.14E5 | 0 | 2.01E7 | 6.17E5 |  |  |  | 5.95E7 | 4.31E7 | 9 | 2 | 2 | 1 | 1 | 1 | 0 | 0 | 0 | 1 | 1 | 157 | 163 |  |
| K.YM(+15.99)AC(+57.02)C(+57.02)LLYR.G | N | 48.26 | 1264.5403 | 9 | 3.4 | 633.2795 | 2 | 70.56 | 4 | 19630 | Ana\_03.raw |  | 2.9E6 |  | 6.52E5 | 3.43E6 | 2.96E6 | 1.84E6 | 1.41E6 |  | 2.38E6 | 7 | 0 | 1 | 0 | 1 | 1 | 1 | 1 | 1 | 0 | 1 | 312 | 320 | Oxidation (M); Carbamidomethylation |
| K.FDLM(+15.99)YAK.R | N | 48.10 | 902.4208 | 7 | 5.6 | 452.2202 | 2 | 58.43 | 1 | 16509 | ANa\_10.raw | 3.91E7 | 3.27E7 | 3.75E7 | 1.76E7 | 3.22E7 | 3.02E7 | 2.45E7 |  | 5.73E7 | 3.33E7 | 14 | 2 | 2 | 1 | 1 | 1 | 1 | 2 | 0 | 2 | 2 | 395 | 401 | Oxidation (M) |
| K.EIIDPVLDR.I | Y | 47.90 | 1068.5815 | 9 | 1.9 | 535.2991 | 2 | 69.84 | 3 | 19851 | Ana\_02.raw | 3.63E6 |  | 4.92E6 | 1.14E6 | 4.84E6 | 6.9E6 | 3.25E6 | 3.46E6 |  | 3.66E6 | 8 | 1 | 0 | 1 | 1 | 1 | 1 | 1 | 1 | 0 | 1 | 113 | 121 |  |
| R.EDMAALEK.D | N | 43.53 | 905.4164 | 8 | 6.1 | 453.7182 | 2 | 34.03 | 9 | 9103 | Ana\_08.raw | 2.12E6 |  |  |  |  |  |  |  | 5.61E6 |  | 2 | 1 | 0 | 0 | 0 | 0 | 0 | 0 | 0 | 1 | 0 | 423 | 430 |  |
| R.EDM(+15.99)AALEK.D | N | 42.79 | 921.4113 | 8 | 2.4 | 461.7140 | 2 | 20.33 | 5 | 5094 | Ana\_04.raw |  |  |  | 5.31E6 | 3.63E7 |  | 3.53E6 | 3.07E6 |  |  | 4 | 0 | 0 | 0 | 1 | 1 | 0 | 1 | 1 | 0 | 0 | 423 | 430 | Oxidation (M) |
| R.LSVDYGKK.S | N | 36.03 | 908.4967 | 8 | 3.4 | 455.2572 | 2 | 26.90 | 10 | 7103 | Ana\_09.raw | 3.62E6 |  |  |  |  |  |  |  |  | 3.48E6 | 2 | 1 | 0 | 0 | 0 | 0 | 0 | 0 | 0 | 0 | 1 | 157 | 164 |  |
| K.FDLMYAK.R | N | 31.78 | 886.4258 | 7 | 4.7 | 444.2223 | 2 | 67.31 | 9 | 19210 | Ana\_08.raw |  |  |  |  |  |  |  |  | 1.44E6 |  | 1 | 0 | 0 | 0 | 0 | 0 | 0 | 0 | 0 | 1 | 0 | 395 | 401 |  |
| total 23 peptides |  |  |  |  |  |  |  |  |  |  |  |  |  |  |  |  |  |  |  |  |  |  |  |  |  |  |  |  |  |  |  |  |  |  |  |
| --- | --- | --- | --- | --- | --- | --- | --- | --- | --- | --- | --- | --- | --- | --- | --- | --- | --- | --- | --- | --- | --- | --- | --- | --- | --- | --- | --- | --- | --- | --- | --- | --- | --- | --- | --- |

Best Unique PSM (Scan F6:31503, m/z=1034.4570, z=2, RT=112.42, ppm=5.0):

A0A0A0MQA5|A0A0A0MQA5\_MOUSE

back to list

  

| Protein Coverage
| Supporting Peptides
| Best Unique PSM
|

Protein Coverage:

Supporting Peptides:

| Peptide | Uniq | -10lgP | Mass | Length | ppm | m/z | z | RT | Fraction | Scan | Source File | Area 10 | Area 01 | Area 02 | Area 03 | Area 04 | Area 05 | Area 06 | Area 07 | Area 08 | Area 09 | #Spec | #Spec 10 | #Spec 01 | #Spec 02 | #Spec 03 | #Spec 04 | #Spec 05 | #Spec 06 | #Spec 07 | #Spec 08 | #Spec 09 | Start | End | PTM |
| --- | --- | --- | --- | --- | --- | --- | --- | --- | --- | --- | --- | --- | --- | --- | --- | --- | --- | --- | --- | --- | --- | --- | --- | --- | --- | --- | --- | --- | --- | --- | --- | --- | --- | --- | --- |
| R.FDGALNVDLTEFQTNLVPYPR.I | N | 117.80 | 2408.2012 | 21 | 4.2 | 1205.1129 | 2 | 130.54 | 9 | 36141 | Ana\_08.raw | 2.24E6 |  |  | 7.33E5 | 1.14E6 |  | 9.17E5 |  | 3.51E6 | 1.14E6 | 8 | 1 | 0 | 0 | 2 | 1 | 0 | 2 | 0 | 1 | 1 | 273 | 293 |  |
| R.IHFPLATYAPVISAEK.A | N | 101.55 | 1755.9559 | 16 | 1.2 | 878.9863 | 2 | 104.62 | 6 | 29472 | Ana\_05.raw | 8.28E7 | 9.05E7 | 9.31E7 | 5.04E7 | 9.02E7 | 1.11E8 | 4.49E7 | 3.3E7 | 2.83E8 | 1.42E8 | 85 | 11 | 9 | 6 | 8 | 8 | 8 | 8 | 7 | 11 | 9 | 294 | 309 |  |
| R.AVC(+57.02)M(+15.99)LSNTTAIAEAWAR.L | N | 100.31 | 1879.8921 | 17 | -1.1 | 940.9523 | 2 | 100.08 | 1 | 27997 | ANa\_10.raw | 9.41E6 | 1.3E7 | 8.38E6 | 1.22E6 | 1.18E6 | 1.01E7 | 6.35E6 |  | 1.37E7 | 2.31E6 | 14 | 2 | 2 | 2 | 1 | 1 | 2 | 2 | 0 | 1 | 1 | 403 | 419 | Carbamidomethylation; Oxidation (M) |
| R.AVC(+57.02)MLSNTTAIAEAWAR.L | N | 97.01 | 1863.8971 | 17 | 2.5 | 932.9582 | 2 | 118.70 | 1 | 32766 | ANa\_10.raw | 4.22E6 | 1.14E6 |  | 9.92E5 |  | 1.45E6 |  |  | 6.04E6 | 5.52E6 | 9 | 2 | 1 | 0 | 1 | 0 | 2 | 0 | 0 | 1 | 2 | 403 | 419 | Carbamidomethylation |
| R.AFVHWYVGEGM(+15.99)EEGEFSEAR.E | N | 92.50 | 2345.0059 | 20 | 3.4 | 782.6785 | 3 | 94.03 | 6 | 26666 | Ana\_05.raw | 1.18E6 | 3.45E6 | 2.64E6 | 4.66E5 | 3.25E6 | 5.36E6 | 3.55E6 |  | 2.72E6 | 3.51E6 | 9 | 1 | 1 | 1 | 1 | 1 | 1 | 1 | 0 | 1 | 1 | 432 | 451 | Oxidation (M) |
| R.SIQFVDWC(+57.02)PTGFK.V | N | 90.27 | 1583.7443 | 13 | -0.4 | 792.8791 | 2 | 111.78 | 3 | 30920 | Ana\_02.raw | 2.9E7 | 4.15E7 | 3.5E7 | 1.83E6 | 2.48E7 | 2.5E7 | 1.98E7 | 1.83E7 | 6.88E7 | 4.04E7 | 19 | 3 | 2 | 2 | 1 | 1 | 1 | 1 | 3 | 2 | 3 | 369 | 381 | Carbamidomethylation |
| K.TIGGGDDSFTTFFC(+57.02)ETGAGK.H | Y | 83.48 | 2066.8892 | 20 | 5.0 | 1034.4570 | 2 | 112.42 | 6 | 31503 | Ana\_05.raw | 5.96E5 | 1.97E6 | 6.58E5 | 7.31E5 | 5.43E5 | 1.06E6 | 7.68E5 | 7.3E5 | 1.82E6 | 1.66E6 | 10 | 1 | 1 | 1 | 1 | 1 | 1 | 1 | 1 | 1 | 1 | 70 | 89 | Carbamidomethylation |
| R.LISQIVSSITASLR.F | N | 82.49 | 1486.8718 | 14 | 5.8 | 744.4475 | 2 | 132.23 | 1 | 36352 | ANa\_10.raw | 5.92E6 | 5.49E6 | 3.88E6 |  | 2.45E6 | 3.71E6 | 3.27E6 | 8.77E5 | 1.58E7 | 5.67E6 | 14 | 1 | 2 | 1 | 0 | 2 | 2 | 2 | 1 | 2 | 1 | 259 | 272 |  |
| K.VGINYQPPTVVPGGDLAK.V | N | 78.26 | 1823.9781 | 18 | 1.3 | 912.9976 | 2 | 87.23 | 3 | 24540 | Ana\_02.raw | 2.69E7 | 6.86E7 | 4.84E7 | 1.55E7 | 3.76E7 | 3.39E7 | 2.24E7 | 2.24E7 | 1.81E8 | 7.09E7 | 26 | 3 | 3 | 2 | 2 | 1 | 3 | 2 | 1 | 5 | 4 | 382 | 399 |  |
| R.AVFVDLEPTVIDEIR.N | Y | 77.94 | 1714.9141 | 15 | -1.9 | 858.4627 | 2 | 123.53 | 6 | 34402 | Ana\_05.raw | 1.12E6 | 1.58E5 | 2.43E6 | 1.07E6 |  | 2.47E6 |  | 9.29E5 | 4.22E6 | 7.44E5 | 8 | 1 | 1 | 1 | 1 | 0 | 1 | 0 | 1 | 1 | 1 | 94 | 108 |  |
| R.QLFHPEQLITGK.E | N | 73.59 | 1409.7666 | 12 | 4.3 | 705.8936 | 2 | 84.37 | 8 | 23640 | Ana\_07.raw | 1.33E7 | 8.46E6 | 9.75E6 | 7.29E6 | 9.99E6 | 1.49E7 | 4.79E6 | 1.32E7 | 3.21E7 | 3.98E6 | 20 | 2 | 2 | 2 | 2 | 2 | 2 | 1 | 2 | 3 | 2 | 114 | 125 |  |
| R.NLDIERPTYTNLNR.L | N | 68.75 | 1717.8748 | 14 | 4.1 | 859.9482 | 2 | 64.89 | 9 | 18502 | Ana\_08.raw | 1.14E7 | 8.44E6 |  |  |  |  |  |  | 1.47E7 | 5.59E6 | 6 | 2 | 1 | 0 | 0 | 0 | 0 | 0 | 0 | 2 | 1 | 245 | 258 |  |
| R.GHYTIGK.E | N | 56.84 | 774.4024 | 7 | 3.9 | 388.2100 | 2 | 21.28 | 7 | 5302 | Ana\_06.raw |  |  |  |  | 8.67E6 |  | 9.09E6 | 6.3E6 |  | 6.29E6 | 4 | 0 | 0 | 0 | 0 | 1 | 0 | 1 | 1 | 0 | 1 | 135 | 141 |  |
| K.DVNAAIAAIK.T | Y | 54.19 | 984.5604 | 10 | 3.9 | 493.2894 | 2 | 66.66 | 9 | 19016 | Ana\_08.raw | 1.76E6 | 2.92E6 | 1.88E6 | 1.41E6 | 3.23E6 | 2.44E6 | 1.82E6 | 1.44E6 | 5.76E6 |  | 9 | 1 | 1 | 1 | 1 | 1 | 1 | 1 | 1 | 1 | 0 | 356 | 365 |  |
| K.EDAANNYAR.G | N | 51.28 | 1022.4417 | 9 | 2.5 | 512.2294 | 2 | 20.32 | 5 | 5091 | Ana\_04.raw |  |  |  |  | 1.64E7 |  |  |  |  |  | 1 | 0 | 0 | 0 | 0 | 1 | 0 | 0 | 0 | 0 | 0 | 126 | 134 |  |
| R.LSVDYGK.K | N | 49.40 | 780.4017 | 7 | 5.0 | 391.2101 | 2 | 36.09 | 10 | 9863 | Ana\_09.raw | 4.18E7 | 1.14E5 | 0 | 2.01E7 | 6.17E5 |  |  |  | 5.95E7 | 4.31E7 | 9 | 2 | 2 | 1 | 1 | 1 | 0 | 0 | 0 | 1 | 1 | 186 | 192 |  |
| K.YM(+15.99)AC(+57.02)C(+57.02)LLYR.G | N | 48.26 | 1264.5403 | 9 | 3.4 | 633.2795 | 2 | 70.56 | 4 | 19630 | Ana\_03.raw |  | 2.9E6 |  | 6.52E5 | 3.43E6 | 2.96E6 | 1.84E6 | 1.41E6 |  | 2.38E6 | 7 | 0 | 1 | 0 | 1 | 1 | 1 | 1 | 1 | 0 | 1 | 341 | 349 | Oxidation (M); Carbamidomethylation |
| K.FDLM(+15.99)YAK.R | N | 48.10 | 902.4208 | 7 | 5.6 | 452.2202 | 2 | 58.43 | 1 | 16509 | ANa\_10.raw | 3.91E7 | 3.27E7 | 3.75E7 | 1.76E7 | 3.22E7 | 3.02E7 | 2.45E7 |  | 5.73E7 | 3.33E7 | 14 | 2 | 2 | 1 | 1 | 1 | 1 | 2 | 0 | 2 | 2 | 424 | 430 | Oxidation (M) |
| K.EIIDPVLDR.I | Y | 47.90 | 1068.5815 | 9 | 1.9 | 535.2991 | 2 | 69.84 | 3 | 19851 | Ana\_02.raw | 3.63E6 |  | 4.92E6 | 1.14E6 | 4.84E6 | 6.9E6 | 3.25E6 | 3.46E6 |  | 3.66E6 | 8 | 1 | 0 | 1 | 1 | 1 | 1 | 1 | 1 | 0 | 1 | 142 | 150 |  |
| R.EDMAALEK.D | N | 43.53 | 905.4164 | 8 | 6.1 | 453.7182 | 2 | 34.03 | 9 | 9103 | Ana\_08.raw | 2.12E6 |  |  |  |  |  |  |  | 5.61E6 |  | 2 | 1 | 0 | 0 | 0 | 0 | 0 | 0 | 0 | 1 | 0 | 452 | 459 |  |
| R.EDM(+15.99)AALEK.D | N | 42.79 | 921.4113 | 8 | 2.4 | 461.7140 | 2 | 20.33 | 5 | 5094 | Ana\_04.raw |  |  |  | 5.31E6 | 3.63E7 |  | 3.53E6 | 3.07E6 |  |  | 4 | 0 | 0 | 0 | 1 | 1 | 0 | 1 | 1 | 0 | 0 | 452 | 459 | Oxidation (M) |
| R.LSVDYGKK.S | N | 36.03 | 908.4967 | 8 | 3.4 | 455.2572 | 2 | 26.90 | 10 | 7103 | Ana\_09.raw | 3.62E6 |  |  |  |  |  |  |  |  | 3.48E6 | 2 | 1 | 0 | 0 | 0 | 0 | 0 | 0 | 0 | 0 | 1 | 186 | 193 |  |
| K.FDLMYAK.R | N | 31.78 | 886.4258 | 7 | 4.7 | 444.2223 | 2 | 67.31 | 9 | 19210 | Ana\_08.raw |  |  |  |  |  |  |  |  | 1.44E6 |  | 1 | 0 | 0 | 0 | 0 | 0 | 0 | 0 | 0 | 1 | 0 | 424 | 430 |  |
| total 23 peptides |  |  |  |  |  |  |  |  |  |  |  |  |  |  |  |  |  |  |  |  |  |  |  |  |  |  |  |  |  |  |  |  |  |  |  |
| --- | --- | --- | --- | --- | --- | --- | --- | --- | --- | --- | --- | --- | --- | --- | --- | --- | --- | --- | --- | --- | --- | --- | --- | --- | --- | --- | --- | --- | --- | --- | --- | --- | --- | --- | --- |

Best Unique PSM (Scan F6:31503, m/z=1034.4570, z=2, RT=112.42, ppm=5.0):

sp|Q70IV5|SYNEM\_MOUSE

back to list

  

| Protein Coverage
| Supporting Peptides
| Best Unique PSM
|

Protein Coverage:

Supporting Peptides:

| Peptide | Uniq | -10lgP | Mass | Length | ppm | m/z | z | RT | Fraction | Scan | Source File | Area 10 | Area 01 | Area 02 | Area 03 | Area 04 | Area 05 | Area 06 | Area 07 | Area 08 | Area 09 | #Spec | #Spec 10 | #Spec 01 | #Spec 02 | #Spec 03 | #Spec 04 | #Spec 05 | #Spec 06 | #Spec 07 | #Spec 08 | #Spec 09 | Start | End | PTM |
| --- | --- | --- | --- | --- | --- | --- | --- | --- | --- | --- | --- | --- | --- | --- | --- | --- | --- | --- | --- | --- | --- | --- | --- | --- | --- | --- | --- | --- | --- | --- | --- | --- | --- | --- | --- |
| R.HQLVEVIGQLEETLPER.M | Y | 98.18 | 1989.0531 | 17 | 2.3 | 664.0265 | 3 | 115.14 | 3 | 31800 | Ana\_02.raw | 3.31E6 | 1.8E5 | 1.16E6 | 2.25E5 | 2.81E6 | 4.7E6 | 2.26E6 | 1.38E6 |  |  | 8 | 1 | 1 | 1 | 1 | 1 | 1 | 1 | 1 | 0 | 0 | 947 | 963 |  |
| R.HSSSATTSGQQKPLEK.T | Y | 93.73 | 1684.8380 | 16 | 2.8 | 843.4286 | 2 | 18.55 | 2 | 4269 | Ana\_01.raw | 3.87E6 | 4.53E6 |  |  |  |  |  |  |  | 1.81E6 | 4 | 1 | 2 | 0 | 0 | 0 | 0 | 0 | 0 | 0 | 1 | 403 | 418 |  |
| R.GHQGHVAAGAVSSTPSNER.T | Y | 90.69 | 1860.8827 | 19 | 0.1 | 621.3016 | 3 | 21.89 | 9 | 5252 | Ana\_08.raw |  |  |  |  |  |  | 7.17E5 |  | 4.05E6 |  | 2 | 0 | 0 | 0 | 0 | 0 | 0 | 1 | 0 | 1 | 0 | 475 | 493 |  |
| R.DTGSEVEAHGVSHR.G | Y | 86.16 | 1479.6702 | 14 | 5.9 | 740.8467 | 2 | 23.61 | 9 | 5842 | Ana\_08.raw | 9.87E5 | 4.39E5 | 9.18E6 | 3.71E6 | 7.43E5 | 1.44E7 |  | 5E5 | 1.68E7 | 5.68E5 | 12 | 1 | 1 | 1 | 2 | 1 | 2 | 0 | 1 | 2 | 1 | 1468 | 1481 |  |
| K.LTDVVDVSDEAGLDYLLSK.D | Y | 83.32 | 2051.0310 | 19 | 1.0 | 1026.5238 | 2 | 126.51 | 6 | 35179 | Ana\_05.raw |  |  |  |  |  | 1.65E6 | 3.81E5 |  |  |  | 2 | 0 | 0 | 0 | 0 | 0 | 1 | 1 | 0 | 0 | 0 | 694 | 712 |  |
| R.LDTQDTASSLQSDSTTETIAESIVTTILK.Q | Y | 82.75 | 3067.5295 | 29 | 7.5 | 1023.5248 | 3 | 138.39 | 1 | 37964 | ANa\_10.raw | 1.07E6 |  |  |  |  | 1.92E6 |  |  |  |  | 2 | 1 | 0 | 0 | 0 | 0 | 1 | 0 | 0 | 0 | 0 | 622 | 650 |  |
| K.VLEVPALSLGGAIGSAHLK.E | Y | 82.47 | 1831.0566 | 19 | 4.5 | 611.3622 | 3 | 112.07 | 6 | 31412 | Ana\_05.raw | 1.32E6 | 2.28E6 |  | 1.89E6 | 2.24E6 | 3.92E5 | 1.5E6 | 1.05E6 | 0 |  | 8 | 1 | 1 | 0 | 1 | 1 | 1 | 1 | 1 | 1 | 0 | 891 | 909 |  |
| R.WAEDQALYAEEAR.S | Y | 81.12 | 1550.7001 | 13 | 4.5 | 776.3608 | 2 | 73.11 | 6 | 20973 | Ana\_05.raw | 1.95E6 |  | 1.34E6 | 0 | 6.3E5 | 3.56E6 | 2.35E5 | 1.42E6 | 2.3E6 | 3.08E5 | 9 | 1 | 0 | 1 | 1 | 1 | 1 | 1 | 1 | 1 | 1 | 53 | 65 |  |
| R.QQLDELNWSTALAEGER.D | Y | 68.54 | 1958.9333 | 17 | -3.7 | 980.4703 | 2 | 124.29 | 6 | 34605 | Ana\_05.raw |  |  |  |  |  | 8.93E5 |  |  |  |  | 1 | 0 | 0 | 0 | 0 | 0 | 1 | 0 | 0 | 0 | 0 | 69 | 85 |  |
| R.ESVQLYEDEVR.E | Y | 67.85 | 1365.6412 | 11 | -2.7 | 683.8260 | 2 | 62.00 | 3 | 17667 | Ana\_02.raw |  |  | 8.89E5 | 1.66E6 | 1.84E6 | 3.74E6 | 1.71E6 | 1.87E6 | 1.6E6 |  | 7 | 0 | 0 | 1 | 1 | 1 | 1 | 1 | 1 | 1 | 0 | 197 | 207 |  |
| R.AALEALLGR.L | Y | 65.33 | 912.5392 | 9 | 3.9 | 457.2787 | 2 | 83.12 | 6 | 23721 | Ana\_05.raw | 5.98E6 | 5.83E6 | 5.12E6 | 2.27E6 | 3.93E6 | 6.8E6 | 2.45E6 | 2.89E6 | 7.99E6 | 4.02E6 | 10 | 1 | 1 | 1 | 1 | 1 | 1 | 1 | 1 | 1 | 1 | 128 | 136 |  |
| R.ATSPAAPPPR.L | Y | 65.01 | 963.5137 | 10 | 4.9 | 482.7665 | 2 | 21.86 | 1 | 5388 | ANa\_10.raw | 6.86E6 |  | 7.22E6 | 1.97E6 |  |  |  | 2.22E6 |  |  | 5 | 1 | 1 | 1 | 1 | 0 | 0 | 0 | 1 | 0 | 0 | 170 | 179 |  |
| R.VASTGSGASPGDAHQAPGEK.G | Y | 63.76 | 1822.8445 | 20 | 2.5 | 608.6236 | 3 | 21.32 | 3 | 5378 | Ana\_02.raw |  |  | 2.41E6 | 1.79E6 |  |  |  | 2.39E6 |  |  | 3 | 0 | 0 | 1 | 1 | 0 | 0 | 0 | 1 | 0 | 0 | 1494 | 1513 |  |
| R.ENLLLEEELR.S | Y | 60.34 | 1256.6611 | 10 | 3.1 | 629.3398 | 2 | 89.50 | 6 | 25457 | Ana\_05.raw |  |  |  |  |  | 2.19E5 |  |  |  |  | 1 | 0 | 0 | 0 | 0 | 0 | 1 | 0 | 0 | 0 | 0 | 35 | 44 |  |
| R.DYQELLQVK.T | Y | 58.95 | 1134.5920 | 9 | 0.2 | 568.3034 | 2 | 79.19 | 3 | 22402 | Ana\_02.raw |  | 5.64E5 | 5.36E5 |  |  | 8.42E5 |  |  |  |  | 3 | 0 | 1 | 1 | 0 | 0 | 1 | 0 | 0 | 0 | 0 | 293 | 301 |  |
| R.SPDREDGEEVPAGGILFK.R | Y | 57.36 | 1914.9323 | 18 | 1.9 | 639.3192 | 3 | 80.32 | 6 | 22959 | Ana\_05.raw |  |  |  |  |  | 1.1E6 |  | 3E5 |  |  | 2 | 0 | 0 | 0 | 0 | 0 | 1 | 0 | 1 | 0 | 0 | 1049 | 1066 |  |
| R.AEPTVIEK.E | Y | 56.25 | 885.4807 | 8 | 2.2 | 443.7486 | 2 | 28.42 | 6 | 7626 | Ana\_05.raw | 1.09E7 |  |  | 2.56E6 | 5.83E6 | 8.79E6 |  |  | 4.33E6 | 8.37E6 | 6 | 1 | 0 | 0 | 1 | 1 | 1 | 0 | 0 | 1 | 1 | 918 | 925 |  |
| R.NQALELEQLR.A | Y | 56.12 | 1212.6462 | 10 | 2.7 | 607.3320 | 2 | 69.80 | 1 | 19777 | ANa\_10.raw | 3.2E6 | 1.75E6 | 7.09E6 | 1.27E6 | 4.14E6 | 5.52E6 |  | 3.37E6 | 9.73E6 |  | 8 | 1 | 1 | 1 | 1 | 1 | 1 | 0 | 1 | 1 | 0 | 239 | 248 |  |
| R.AEVSTIHLQSPGR.K | Y | 50.84 | 1393.7313 | 13 | 5.6 | 465.5870 | 3 | 47.78 | 2 | 13452 | Ana\_01.raw | 2.26E6 | 2.86E6 | 2.83E6 |  | 2.32E6 | 2.99E6 |  |  | 3.66E6 |  | 6 | 1 | 1 | 1 | 0 | 1 | 1 | 0 | 0 | 1 | 0 | 590 | 602 |  |
| R.RATESVITR.E | Y | 50.61 | 1031.5724 | 9 | 3.9 | 516.7955 | 2 | 22.23 | 9 | 5365 | Ana\_08.raw | 4.37E6 | 5.46E6 | 4.13E6 |  |  |  | 3.29E6 |  | 4.2E6 | 1.87E6 | 6 | 1 | 1 | 1 | 0 | 0 | 0 | 1 | 0 | 1 | 1 | 462 | 470 |  |
| K.GTEQAGFDK.T | Y | 50.33 | 951.4297 | 9 | 4.7 | 476.7244 | 2 | 23.16 | 8 | 5799 | Ana\_07.raw |  |  |  | 2.04E6 |  |  | 9.05E5 | 9.7E5 |  |  | 3 | 0 | 0 | 0 | 1 | 0 | 0 | 1 | 1 | 0 | 0 | 1514 | 1522 |  |
| R.DLDAAHER.Q | Y | 49.58 | 925.4253 | 8 | 0.6 | 463.7202 | 2 | 19.21 | 5 | 4723 | Ana\_04.raw |  |  |  |  | 3.32E6 |  |  |  |  |  | 1 | 0 | 0 | 0 | 0 | 1 | 0 | 0 | 0 | 0 | 0 | 143 | 150 |  |
| R.ANLRPVTPTHGFLR.N | Y | 48.71 | 1577.8790 | 14 | 4.9 | 526.9695 | 3 | 52.42 | 9 | 14792 | Ana\_08.raw |  |  |  |  |  |  |  |  | 1.7E6 |  | 1 | 0 | 0 | 0 | 0 | 0 | 0 | 0 | 0 | 1 | 0 | 424 | 437 |  |
| R.ELEEALGAR.A | Y | 45.34 | 986.5032 | 9 | 3.7 | 494.2607 | 2 | 46.34 | 6 | 13096 | Ana\_05.raw | 6.81E6 |  |  | 2.3E6 | 8.15E3 | 8.8E6 | 3.74E6 | 3.75E6 |  | 3.33E6 | 7 | 1 | 0 | 0 | 1 | 1 | 1 | 1 | 1 | 0 | 1 | 119 | 127 |  |
| K.M(+15.99)RGDVGGEKPVK.V | Y | 44.31 | 1287.6605 | 12 | 4.0 | 430.2292 | 3 | 18.66 | 2 | 4305 | Ana\_01.raw | 3.07E5 | 1.41E6 | 1.24E6 |  |  | 1.34E6 |  |  |  |  | 6 | 1 | 2 | 2 | 0 | 0 | 1 | 0 | 0 | 0 | 0 | 879 | 890 | Oxidation (M) |
| R.YTNSVLQR.K | Y | 40.61 | 979.5087 | 8 | 6.1 | 490.7646 | 2 | 32.99 | 9 | 8778 | Ana\_08.raw |  |  |  |  |  |  |  |  | 3.82E6 |  | 1 | 0 | 0 | 0 | 0 | 0 | 0 | 0 | 0 | 1 | 0 | 344 | 351 |  |
| R.LQAEDEAR.L | Y | 39.83 | 930.4406 | 8 | 5.2 | 466.2300 | 2 | 21.37 | 7 | 5333 | Ana\_06.raw |  |  |  |  | 9.37E6 |  | 7.37E6 | 5.33E6 |  |  | 3 | 0 | 0 | 0 | 0 | 1 | 0 | 1 | 1 | 0 | 0 | 221 | 228 |  |
| R.YSWQDEIAQGTWR.R | Y | 37.45 | 1638.7427 | 13 | 2.9 | 820.3810 | 2 | 103.08 | 6 | 29069 | Ana\_05.raw |  |  |  |  |  | 9.69E5 |  |  |  |  | 1 | 0 | 0 | 0 | 0 | 0 | 1 | 0 | 0 | 0 | 0 | 864 | 876 |  |
| R.DEAGEIER.A | Y | 36.71 | 917.4090 | 8 | 1.0 | 459.7122 | 2 | 25.09 | 5 | 6582 | Ana\_04.raw |  |  |  |  | 3.56E6 | 7.79E6 |  | 3.36E6 |  |  | 3 | 0 | 0 | 0 | 0 | 1 | 1 | 0 | 1 | 0 | 0 | 1022 | 1029 |  |
| R.ELLELQR.E | Y | 36.16 | 899.5076 | 7 | 5.1 | 450.7634 | 2 | 60.32 | 6 | 17259 | Ana\_05.raw |  |  |  |  |  | 3.49E6 |  |  |  |  | 1 | 0 | 0 | 0 | 0 | 0 | 1 | 0 | 0 | 0 | 0 | 91 | 97 |  |
| R.AVESVVR.E | Y | 33.60 | 758.4286 | 7 | 3.5 | 380.2229 | 2 | 24.60 | 2 | 6296 | Ana\_01.raw |  | 7.85E5 |  |  | 9.99E5 |  |  |  |  |  | 2 | 0 | 1 | 0 | 0 | 1 | 0 | 0 | 0 | 0 | 0 | 1030 | 1036 |  |
| K.LEAQATK.E | Y | 33.28 | 759.4127 | 7 | 4.0 | 380.7151 | 2 | 29.41 | 6 | 7944 | Ana\_05.raw | 4.46E7 |  |  |  |  | 2.86E7 |  |  |  |  | 2 | 1 | 0 | 0 | 0 | 0 | 1 | 0 | 0 | 0 | 0 | 501 | 507 |  |
| K.EEASGELR.A | Y | 32.81 | 889.4141 | 8 | 0.9 | 445.7147 | 2 | 20.29 | 5 | 5081 | Ana\_04.raw |  |  |  |  | 7.07E6 |  |  |  |  |  | 1 | 0 | 0 | 0 | 0 | 1 | 0 | 0 | 0 | 0 | 0 | 910 | 917 |  |
| R.ELYSPSGER.D | Y | 32.78 | 1036.4825 | 9 | 7.7 | 519.2526 | 2 | 35.26 | 6 | 9696 | Ana\_05.raw |  |  |  |  |  | 1.66E6 |  |  |  |  | 1 | 0 | 0 | 0 | 0 | 0 | 1 | 0 | 0 | 0 | 0 | 1072 | 1080 |  |
| K.VTYVDR.K | Y | 32.53 | 751.3864 | 6 | 0.2 | 376.7006 | 2 | 22.48 | 3 | 5759 | Ana\_02.raw |  |  | 5.31E6 |  | 3.77E6 | 2.63E6 |  |  |  |  | 3 | 0 | 0 | 1 | 0 | 1 | 1 | 0 | 0 | 0 | 0 | 670 | 675 |  |
| R.ELEQALR.R | Y | 31.96 | 857.4606 | 7 | 3.5 | 429.7391 | 2 | 34.72 | 1 | 9392 | ANa\_10.raw | 1.59E7 |  |  |  |  |  |  |  |  |  | 1 | 1 | 0 | 0 | 0 | 0 | 0 | 0 | 0 | 0 | 0 | 208 | 214 |  |
| R.LDAELGAQR.R | Y | 30.91 | 971.5035 | 9 | 2.4 | 486.7602 | 2 | 39.30 | 7 | 10774 | Ana\_06.raw |  |  |  |  |  |  | 3.8E6 |  |  |  | 1 | 0 | 0 | 0 | 0 | 0 | 0 | 1 | 0 | 0 | 0 | 109 | 117 |  |
| R.LYDYVC(+57.02)R.V | Y | 29.65 | 987.4484 | 7 | 5.8 | 494.7343 | 2 | 49.96 | 10 | 14053 | Ana\_09.raw |  |  |  | 9.96E5 |  |  |  |  |  | 1.29E6 | 2 | 0 | 0 | 0 | 1 | 0 | 0 | 0 | 0 | 0 | 1 | 22 | 28 | Carbamidomethylation |
| K.GVFSSEPR.H | Y | 26.14 | 877.4293 | 8 | 1.4 | 439.7225 | 2 | 37.45 | 6 | 10364 | Ana\_05.raw |  |  |  |  |  | 4.23E6 |  |  |  |  | 1 | 0 | 0 | 0 | 0 | 0 | 1 | 0 | 0 | 0 | 0 | 939 | 946 |  |
| total 39 peptides |  |  |  |  |  |  |  |  |  |  |  |  |  |  |  |  |  |  |  |  |  |  |  |  |  |  |  |  |  |  |  |  |  |  |  |
| --- | --- | --- | --- | --- | --- | --- | --- | --- | --- | --- | --- | --- | --- | --- | --- | --- | --- | --- | --- | --- | --- | --- | --- | --- | --- | --- | --- | --- | --- | --- | --- | --- | --- | --- | --- |

Best Unique PSM (Scan F3:31800, m/z=664.0265, z=3, RT=115.14, ppm=2.3):

sp|P97429|ANXA4\_MOUSE

back to list

  

| Protein Coverage
| Supporting Peptides
| Best Unique PSM
|

Protein Coverage:

Supporting Peptides:

| Peptide | Uniq | -10lgP | Mass | Length | ppm | m/z | z | RT | Fraction | Scan | Source File | Area 10 | Area 01 | Area 02 | Area 03 | Area 04 | Area 05 | Area 06 | Area 07 | Area 08 | Area 09 | #Spec | #Spec 10 | #Spec 01 | #Spec 02 | #Spec 03 | #Spec 04 | #Spec 05 | #Spec 06 | #Spec 07 | #Spec 08 | #Spec 09 | Start | End | PTM |
| --- | --- | --- | --- | --- | --- | --- | --- | --- | --- | --- | --- | --- | --- | --- | --- | --- | --- | --- | --- | --- | --- | --- | --- | --- | --- | --- | --- | --- | --- | --- | --- | --- | --- | --- | --- |
| K.GLGTDEDAIIGILAYR.N | Y | 119.03 | 1675.8781 | 16 | 3.4 | 838.9492 | 2 | 130.46 | 7 | 35900 | Ana\_06.raw | 1.07E7 | 4.15E7 | 2.95E7 | 8.48E6 | 2.2E7 | 3.02E7 | 2.04E7 | 1.12E7 | 2.13E7 | 2.31E6 | 22 | 1 | 4 | 3 | 2 | 3 | 2 | 2 | 2 | 2 | 1 | 29 | 44 |  |
| K.AASGFNATEDAQTLR.K | Y | 110.72 | 1550.7324 | 15 | 2.2 | 776.3752 | 2 | 59.80 | 6 | 17104 | Ana\_05.raw | 3.64E7 | 5.34E7 | 4.46E7 | 1.94E7 | 2.36E7 | 2.85E7 | 2.65E7 | 2.09E7 | 5.01E7 | 3.32E7 | 17 | 3 | 2 | 2 | 1 | 2 | 1 | 2 | 1 | 2 | 1 | 10 | 24 |  |
| K.GAGTDEGC(+57.02)LIEILASR.T | Y | 104.85 | 1660.8090 | 16 | 0.4 | 831.4121 | 2 | 114.52 | 3 | 31643 | Ana\_02.raw | 2.24E7 | 3.79E7 | 1.7E7 | 7.99E6 | 1.81E7 | 2.43E7 | 2.74E6 | 1.18E7 | 4.42E7 | 1.45E7 | 21 | 2 | 3 | 3 | 2 | 2 | 2 | 2 | 2 | 2 | 1 | 101 | 116 | Carbamidomethylation |
| K.SETSGSFEDALLAIVK.C | Y | 92.90 | 1665.8461 | 16 | -1.0 | 833.9295 | 2 | 130.39 | 3 | 35716 | Ana\_02.raw | 3.12E7 | 4.78E7 | 6.19E7 | 1.75E7 | 2.92E7 | 4.11E7 | 3.29E7 | 2.13E7 | 4.83E7 | 3.43E7 | 17 | 1 | 2 | 2 | 2 | 1 | 2 | 2 | 2 | 2 | 1 | 226 | 241 |  |
| R.INQTYQQQYGR.S | Y | 90.93 | 1397.6687 | 11 | 2.4 | 699.8433 | 2 | 38.89 | 7 | 10651 | Ana\_06.raw |  | 4.16E7 | 4.31E7 | 1.94E7 | 2.44E7 |  | 2.86E7 |  | 3.06E7 | 2.45E7 | 8 | 0 | 1 | 1 | 1 | 1 | 0 | 1 | 0 | 2 | 1 | 124 | 134 |  |
| R.NHLLHVFDEYKR.I | Y | 82.18 | 1569.8052 | 12 | 0.1 | 393.4586 | 4 | 59.63 | 3 | 16964 | Ana\_02.raw | 3.95E6 | 7.53E6 | 9.51E6 | 5.44E6 | 4.52E6 | 5.64E6 | 5.53E6 | 3.85E6 | 2.42E6 | 5.47E6 | 12 | 1 | 1 | 1 | 2 | 1 | 1 | 1 | 1 | 1 | 2 | 203 | 214 |  |
| K.GLGTDDNTLIR.V | Y | 76.27 | 1173.5990 | 11 | 3.0 | 587.8085 | 2 | 59.75 | 7 | 16898 | Ana\_06.raw | 2.59E7 | 3.78E7 | 5.37E7 | 1.72E7 | 1.92E7 | 2.98E7 | 2.66E7 | 1.95E7 | 4.8E7 | 2.01E7 | 19 | 1 | 1 | 2 | 2 | 2 | 2 | 3 | 2 | 3 | 1 | 260 | 270 |  |
| R.DEGNYLDDALM(+15.99)K.Q | Y | 69.69 | 1398.5973 | 12 | 0.0 | 700.3059 | 2 | 78.22 | 3 | 22141 | Ana\_02.raw | 6.81E6 | 1.17E7 | 1.8E7 | 7.03E6 | 7.52E6 | 8.43E6 | 6.66E6 |  | 1.78E7 | 2.2E6 | 10 | 1 | 1 | 2 | 1 | 1 | 1 | 1 | 0 | 1 | 1 | 161 | 172 | Oxidation (M) |
| K.QDAQELYEAGEK.R | Y | 65.26 | 1379.6205 | 12 | 1.6 | 690.8186 | 2 | 44.98 | 3 | 12654 | Ana\_02.raw |  |  | 2.72E6 | 1.38E6 |  |  | 3.47E6 | 2.28E6 |  |  | 7 | 0 | 0 | 1 | 2 | 0 | 0 | 3 | 1 | 0 | 0 | 173 | 184 |  |
| R.TPEEIRR.I | Y | 62.11 | 899.4824 | 7 | 1.0 | 300.8351 | 3 | 18.80 | 3 | 4534 | Ana\_02.raw | 1.63E7 | 1.22E7 | 4.04E6 | 7.77E6 | 1.02E7 |  | 1.3E7 | 9.18E6 |  | 2.37E7 | 16 | 2 | 2 | 1 | 2 | 2 | 1 | 2 | 2 | 0 | 2 | 117 | 123 |  |
| R.SKPSYFAER.L | Y | 58.71 | 1083.5349 | 9 | 0.6 | 362.1858 | 3 | 29.04 | 3 | 7835 | Ana\_02.raw | 1.13E7 | 1.3E7 | 7.16E7 |  | 2.46E7 | 3.6E7 | 2.01E7 | 1.12E7 | 3.32E7 |  | 12 | 1 | 1 | 2 | 0 | 2 | 2 | 1 | 1 | 2 | 0 | 245 | 253 |  |
| K.SLYSFIK.G | Y | 55.68 | 856.4694 | 7 | 1.5 | 429.2426 | 2 | 78.15 | 3 | 22120 | Ana\_02.raw | 7.69E6 | 1.02E7 | 1.64E7 | 6.85E6 | 6.98E6 | 8.61E6 | 6.71E6 | 6.67E6 | 1.52E7 | 7.67E6 | 14 | 1 | 2 | 2 | 1 | 1 | 1 | 2 | 1 | 2 | 1 | 294 | 300 |  |
| K.FLSILC(+57.02)SR.N | Y | 52.14 | 994.5270 | 8 | 3.4 | 498.2725 | 2 | 85.18 | 8 | 23859 | Ana\_07.raw | 5.65E6 | 7.34E6 | 7.88E6 | 4.54E6 | 3.56E6 | 7.44E6 | 4.25E6 | 3.86E6 | 1.33E7 | 5.91E6 | 10 | 1 | 1 | 1 | 1 | 1 | 1 | 1 | 1 | 1 | 1 | 193 | 200 | Carbamidomethylation |
| R.VLVSLSAAGR.D | Y | 50.11 | 971.5764 | 10 | 2.7 | 486.7968 | 2 | 58.69 | 6 | 16786 | Ana\_05.raw | 1.64E7 | 2.45E7 | 2.54E7 | 1.09E7 | 1.32E7 | 1.75E7 | 1.54E7 | 7.89E6 | 1.77E7 | 1.54E7 | 15 | 2 | 2 | 2 | 1 | 1 | 1 | 1 | 1 | 2 | 2 | 151 | 160 |  |
| R.DLIEDLK.S | N | 49.65 | 844.4542 | 7 | 1.3 | 423.2349 | 2 | 75.59 | 4 | 21028 | Ana\_03.raw | 8.45E6 | 1.3E7 | 1.69E7 | 7.59E6 | 8.85E6 | 8.73E6 | 7.03E6 |  | 2.59E6 |  | 9 | 1 | 1 | 2 | 1 | 1 | 1 | 1 | 0 | 1 | 0 | 63 | 69 |  |
| R.AEIDM(+15.99)LDIR.A | Y | 47.46 | 1090.5328 | 9 | 5.0 | 546.2764 | 2 | 69.39 | 7 | 19680 | Ana\_06.raw | 3.07E6 | 2.33E7 |  |  | 2.72E6 | 1.98E7 | 1.72E7 | 1.2E7 | 1.25E7 | 3.4E6 | 12 | 1 | 2 | 0 | 0 | 1 | 2 | 1 | 2 | 2 | 1 | 276 | 284 | Oxidation (M) |
| R.WGTDEVK.F | Y | 40.24 | 833.3919 | 7 | 4.4 | 417.7051 | 2 | 37.97 | 7 | 10381 | Ana\_06.raw | 1.12E7 | 1.48E7 | 1.92E7 | 7.54E6 | 9.93E6 |  | 1.22E7 | 8.05E6 | 1.58E7 |  | 10 | 1 | 1 | 2 | 1 | 1 | 0 | 2 | 1 | 1 | 0 | 186 | 192 |  |
| R.TPEEIR.R | N | 39.89 | 743.3813 | 6 | 9.6 | 372.7015 | 2 | 22.17 | 7 | 5591 | Ana\_06.raw |  |  |  |  |  |  | 9.31E6 |  |  |  | 1 | 0 | 0 | 0 | 0 | 0 | 0 | 1 | 0 | 0 | 0 | 117 | 122 |  |
| K.DIEQSIK.S | Y | 37.04 | 831.4338 | 7 | 4.3 | 416.7260 | 2 | 36.57 | 7 | 9952 | Ana\_06.raw |  | 0 |  | 1.54E7 |  | 2.41E7 | 2.16E7 | 1.73E7 |  |  | 5 | 0 | 1 | 0 | 1 | 0 | 1 | 1 | 1 | 0 | 0 | 219 | 225 |  |
| total 19 peptides |  |  |  |  |  |  |  |  |  |  |  |  |  |  |  |  |  |  |  |  |  |  |  |  |  |  |  |  |  |  |  |  |  |  |  |
| --- | --- | --- | --- | --- | --- | --- | --- | --- | --- | --- | --- | --- | --- | --- | --- | --- | --- | --- | --- | --- | --- | --- | --- | --- | --- | --- | --- | --- | --- | --- | --- | --- | --- | --- | --- |

Best Unique PSM (Scan F7:35900, m/z=838.9492, z=2, RT=130.46, ppm=3.4):

sp|P16858|G3P\_MOUSE

back to list

  

| Protein Coverage
| Supporting Peptides
| Best Unique PSM
|

Protein Coverage:

Supporting Peptides:

| Peptide | Uniq | -10lgP | Mass | Length | ppm | m/z | z | RT | Fraction | Scan | Source File | Area 10 | Area 01 | Area 02 | Area 03 | Area 04 | Area 05 | Area 06 | Area 07 | Area 08 | Area 09 | #Spec | #Spec 10 | #Spec 01 | #Spec 02 | #Spec 03 | #Spec 04 | #Spec 05 | #Spec 06 | #Spec 07 | #Spec 08 | #Spec 09 | Start | End | PTM |
| --- | --- | --- | --- | --- | --- | --- | --- | --- | --- | --- | --- | --- | --- | --- | --- | --- | --- | --- | --- | --- | --- | --- | --- | --- | --- | --- | --- | --- | --- | --- | --- | --- | --- | --- | --- |
| K.VIHDNFGIVEGLM(+15.99)TTVHAITATQK.T | N | 130.51 | 2610.3477 | 24 | 5.5 | 871.1279 | 3 | 128.30 | 7 | 35304 | Ana\_06.raw | 7.83E7 | 9.81E6 | 2.26E7 | 1.72E7 | 9.1E7 | 9.02E7 | 4.8E7 | 2.11E7 | 2.46E8 | 7.79E7 | 49 | 7 | 4 | 6 | 3 | 6 | 5 | 3 | 4 | 7 | 4 | 161 | 184 | Oxidation (M) |
| R.VPTPNVSVVDLTC(+57.02)R.L | N | 122.91 | 1555.8029 | 14 | 4.5 | 778.9122 | 2 | 86.42 | 1 | 24334 | ANa\_10.raw | 5.36E7 | 4.48E7 | 2.7E7 | 7.86E6 | 2.03E7 | 2.56E7 | 1.32E7 | 1.69E7 | 1.11E8 | 3.78E7 | 34 | 4 | 5 | 3 | 2 | 2 | 3 | 2 | 2 | 6 | 5 | 233 | 246 | Carbamidomethylation |
| K.VIHDNFGIVEGLMTTVHAITATQK.T | N | 118.44 | 2594.3525 | 24 | 3.9 | 865.7948 | 3 | 133.03 | 6 | 36982 | Ana\_05.raw | 4.14E7 | 3.6E6 |  | 1.32E6 |  | 5.78E6 | 1.27E6 | 8.41E6 | 6.48E7 | 3.99E7 | 17 | 3 | 2 | 0 | 1 | 0 | 2 | 1 | 2 | 4 | 2 | 161 | 184 |  |
| K.LISWYDNEYGYSNR.V | N | 103.38 | 1778.7900 | 14 | 2.5 | 890.4045 | 2 | 90.86 | 6 | 25815 | Ana\_05.raw | 3.08E7 | 4.93E7 | 3.07E7 | 1.57E7 | 2.41E7 | 2.7E7 | 2.26E7 | 3.11E7 | 9.11E7 | 3.17E7 | 30 | 3 | 3 | 2 | 2 | 2 | 3 | 3 | 4 | 5 | 3 | 308 | 321 |  |
| K.WGEAGAEYVVESTGVFTTM(+15.99)EK.A | N | 101.11 | 2306.0413 | 21 | 4.8 | 1154.0334 | 2 | 114.99 | 1 | 31808 | ANa\_10.raw | 2.87E7 | 3.59E7 | 1.74E7 | 1.69E7 | 4.13E6 | 2.94E7 | 7.24E6 | 9.28E6 | 6.16E7 | 3.24E7 | 28 | 4 | 3 | 3 | 2 | 2 | 2 | 2 | 2 | 4 | 4 | 85 | 105 | Oxidation (M) |
| R.GAAQNIIPASTGAAK.A | N | 87.32 | 1368.7361 | 15 | -0.2 | 685.3752 | 2 | 47.05 | 3 | 13285 | Ana\_02.raw | 2.87E8 | 2.17E8 | 1.86E8 | 7.31E7 | 1.39E8 | 1.59E8 | 1.09E8 | 1.2E8 | 4.34E8 | 2.43E8 | 43 | 6 | 5 | 4 | 2 | 3 | 3 | 3 | 5 | 6 | 6 | 199 | 213 |  |
| K.IVSNASC(+57.02)TTNC(+57.02)LAPLAK.V | Y | 86.78 | 1818.8969 | 17 | 6.6 | 910.4617 | 2 | 64.02 | 1 | 18145 | ANa\_10.raw | 7.61E6 | 5.24E6 |  |  | 1.5E6 |  |  | 1.69E5 | 2.67E7 | 1.04E7 | 6 | 1 | 1 | 0 | 0 | 1 | 0 | 0 | 1 | 1 | 1 | 144 | 160 | Carbamidomethylation |
| K.LVINGKPITIFQER.D | N | 70.49 | 1626.9457 | 14 | 4.3 | 814.4836 | 2 | 87.99 | 9 | 24994 | Ana\_08.raw | 3.37E7 | 2.26E7 | 6.01E6 | 1.57E6 | 1.44E7 | 8.29E6 | 3.75E6 | 6.37E6 | 6.56E7 | 6.25E6 | 20 | 2 | 2 | 2 | 1 | 3 | 2 | 1 | 2 | 2 | 3 | 65 | 78 |  |
| R.VVDLM(+15.99)AYM(+15.99)ASK.E | N | 61.41 | 1258.5938 | 11 | 2.0 | 630.3054 | 2 | 57.57 | 6 | 16456 | Ana\_05.raw | 4.11E7 | 3.6E7 | 3.25E7 | 1.65E7 | 2.52E7 | 2.89E7 | 2.22E7 | 2.6E7 | 7.52E7 | 2.17E7 | 18 | 1 | 2 | 2 | 1 | 2 | 2 | 2 | 2 | 2 | 2 | 322 | 332 | Oxidation (M) |
| R.VIISAPSADAPM(+15.99)FVM(+15.99)GVNHEK.Y | N | 53.82 | 2244.0918 | 21 | 4.8 | 749.0414 | 3 | 78.99 | 9 | 22506 | Ana\_08.raw | 4.5E7 | 2.46E6 | 3.47E7 | 1.02E7 |  | 2.48E7 |  | 2.15E7 | 1.07E8 | 2.54E7 | 16 | 3 | 1 | 2 | 1 | 0 | 2 | 0 | 1 | 4 | 2 | 117 | 137 | Oxidation (M) |
| R.VVDLM(+15.99)AYM(+15.99)ASKE | N | 50.11 | 1387.6364 | 12 | 4.1 | 694.8283 | 2 | 59.10 | 1 | 16701 | ANa\_10.raw | 1.85E7 |  | 1.02E7 |  |  |  |  |  | 3.21E7 | 1.86E6 | 5 | 2 | 0 | 1 | 0 | 0 | 0 | 0 | 0 | 1 | 1 | 322 | 333 | Oxidation (M) |
| K.VGVNGFGR.I | N | 47.39 | 804.4242 | 8 | 4.7 | 403.2213 | 2 | 40.41 | 10 | 11185 | Ana\_09.raw | 1.1E7 | 6.3E6 |  |  | 5.54E6 |  | 6.05E6 | 4.29E6 | 1.08E7 | 8.79E6 | 7 | 1 | 1 | 0 | 0 | 1 | 0 | 1 | 1 | 1 | 1 | 4 | 11 |  |
| K.VIPELNGK.L | N | 46.26 | 868.5018 | 8 | 4.4 | 435.2601 | 2 | 43.55 | 1 | 12110 | ANa\_10.raw | 1.23E7 | 2.38E7 |  |  |  |  | 2.67E7 |  | 1.75E7 | 2.2E7 | 5 | 1 | 1 | 0 | 0 | 0 | 0 | 1 | 0 | 1 | 1 | 218 | 225 |  |
| K.LTGM(+15.99)AFR.V | N | 43.94 | 810.4058 | 7 | 3.5 | 406.2116 | 2 | 51.11 | 6 | 14544 | Ana\_05.raw | 1.75E8 | 1.23E8 | 1.4E8 | 1E6 | 9.21E7 | 5.4E7 | 8.67E7 | 9.07E7 | 1.83E8 | 1.49E8 | 19 | 2 | 2 | 2 | 1 | 1 | 2 | 1 | 3 | 2 | 3 | 226 | 232 | Oxidation (M) |
| K.LTGMAFR.V | N | 38.72 | 794.4109 | 7 | 2.7 | 398.2138 | 2 | 49.30 | 10 | 13852 | Ana\_09.raw | 4.37E6 |  |  |  |  |  |  |  |  | 3.88E6 | 2 | 1 | 0 | 0 | 0 | 0 | 0 | 0 | 0 | 0 | 1 | 226 | 232 |  |
| K.LVINGKPITIFQERDPTNIK.W | N | 36.92 | 2295.2949 | 20 | 6.9 | 574.8350 | 4 | 90.48 | 1 | 25421 | ANa\_10.raw | 2.56E6 |  |  |  |  |  |  |  |  |  | 1 | 1 | 0 | 0 | 0 | 0 | 0 | 0 | 0 | 0 | 0 | 65 | 84 |  |
| total 16 peptides |  |  |  |  |  |  |  |  |  |  |  |  |  |  |  |  |  |  |  |  |  |  |  |  |  |  |  |  |  |  |  |  |  |  |  |
| --- | --- | --- | --- | --- | --- | --- | --- | --- | --- | --- | --- | --- | --- | --- | --- | --- | --- | --- | --- | --- | --- | --- | --- | --- | --- | --- | --- | --- | --- | --- | --- | --- | --- | --- | --- |

Best Unique PSM (Scan F1:18145, m/z=910.4617, z=2, RT=64.02, ppm=6.6):

A0A0A0MQF6|A0A0A0MQF6\_MOUSE

back to list

  

| Protein Coverage
| Supporting Peptides
| Best Unique PSM
|

Protein Coverage:

Supporting Peptides:

| Peptide | Uniq | -10lgP | Mass | Length | ppm | m/z | z | RT | Fraction | Scan | Source File | Area 10 | Area 01 | Area 02 | Area 03 | Area 04 | Area 05 | Area 06 | Area 07 | Area 08 | Area 09 | #Spec | #Spec 10 | #Spec 01 | #Spec 02 | #Spec 03 | #Spec 04 | #Spec 05 | #Spec 06 | #Spec 07 | #Spec 08 | #Spec 09 | Start | End | PTM |
| --- | --- | --- | --- | --- | --- | --- | --- | --- | --- | --- | --- | --- | --- | --- | --- | --- | --- | --- | --- | --- | --- | --- | --- | --- | --- | --- | --- | --- | --- | --- | --- | --- | --- | --- | --- |
| K.VIHDNFGIVEGLM(+15.99)TTVHAITATQK.T | N | 130.51 | 2610.3477 | 24 | 5.5 | 871.1279 | 3 | 128.30 | 7 | 35304 | Ana\_06.raw | 7.83E7 | 9.81E6 | 2.26E7 | 1.72E7 | 9.1E7 | 9.02E7 | 4.8E7 | 2.11E7 | 2.46E8 | 7.79E7 | 49 | 7 | 4 | 6 | 3 | 6 | 5 | 3 | 4 | 7 | 4 | 187 | 210 | Oxidation (M) |
| R.VPTPNVSVVDLTC(+57.02)R.L | N | 122.91 | 1555.8029 | 14 | 4.5 | 778.9122 | 2 | 86.42 | 1 | 24334 | ANa\_10.raw | 5.36E7 | 4.48E7 | 2.7E7 | 7.86E6 | 2.03E7 | 2.56E7 | 1.32E7 | 1.69E7 | 1.11E8 | 3.78E7 | 34 | 4 | 5 | 3 | 2 | 2 | 3 | 2 | 2 | 6 | 5 | 259 | 272 | Carbamidomethylation |
| K.VIHDNFGIVEGLMTTVHAITATQK.T | N | 118.44 | 2594.3525 | 24 | 3.9 | 865.7948 | 3 | 133.03 | 6 | 36982 | Ana\_05.raw | 4.14E7 | 3.6E6 |  | 1.32E6 |  | 5.78E6 | 1.27E6 | 8.41E6 | 6.48E7 | 3.99E7 | 17 | 3 | 2 | 0 | 1 | 0 | 2 | 1 | 2 | 4 | 2 | 187 | 210 |  |
| K.LISWYDNEYGYSNR.V | N | 103.38 | 1778.7900 | 14 | 2.5 | 890.4045 | 2 | 90.86 | 6 | 25815 | Ana\_05.raw | 3.08E7 | 4.93E7 | 3.07E7 | 1.57E7 | 2.41E7 | 2.7E7 | 2.26E7 | 3.11E7 | 9.11E7 | 3.17E7 | 30 | 3 | 3 | 2 | 2 | 2 | 3 | 3 | 4 | 5 | 3 | 334 | 347 |  |
| K.WGEAGAEYVVESTGVFTTM(+15.99)EK.A | N | 101.11 | 2306.0413 | 21 | 4.8 | 1154.0334 | 2 | 114.99 | 1 | 31808 | ANa\_10.raw | 2.87E7 | 3.59E7 | 1.74E7 | 1.69E7 | 4.13E6 | 2.94E7 | 7.24E6 | 9.28E6 | 6.16E7 | 3.24E7 | 28 | 4 | 3 | 3 | 2 | 2 | 2 | 2 | 2 | 4 | 4 | 111 | 131 | Oxidation (M) |
| R.GAAQNIIPASTGAAK.A | N | 87.32 | 1368.7361 | 15 | -0.2 | 685.3752 | 2 | 47.05 | 3 | 13285 | Ana\_02.raw | 2.87E8 | 2.17E8 | 1.86E8 | 7.31E7 | 1.39E8 | 1.59E8 | 1.09E8 | 1.2E8 | 4.34E8 | 2.43E8 | 43 | 6 | 5 | 4 | 2 | 3 | 3 | 3 | 5 | 6 | 6 | 225 | 239 |  |
| K.IVSNASC(+57.02)TTNC(+57.02)LAPLAK.V | Y | 86.78 | 1818.8969 | 17 | 6.6 | 910.4617 | 2 | 64.02 | 1 | 18145 | ANa\_10.raw | 7.61E6 | 5.24E6 |  |  | 1.5E6 |  |  | 1.69E5 | 2.67E7 | 1.04E7 | 6 | 1 | 1 | 0 | 0 | 1 | 0 | 0 | 1 | 1 | 1 | 170 | 186 | Carbamidomethylation |
| K.LVINGKPITIFQER.D | N | 70.49 | 1626.9457 | 14 | 4.3 | 814.4836 | 2 | 87.99 | 9 | 24994 | Ana\_08.raw | 3.37E7 | 2.26E7 | 6.01E6 | 1.57E6 | 1.44E7 | 8.29E6 | 3.75E6 | 6.37E6 | 6.56E7 | 6.25E6 | 20 | 2 | 2 | 2 | 1 | 3 | 2 | 1 | 2 | 2 | 3 | 91 | 104 |  |
| R.VVDLM(+15.99)AYM(+15.99)ASK.E | N | 61.41 | 1258.5938 | 11 | 2.0 | 630.3054 | 2 | 57.57 | 6 | 16456 | Ana\_05.raw | 4.11E7 | 3.6E7 | 3.25E7 | 1.65E7 | 2.52E7 | 2.89E7 | 2.22E7 | 2.6E7 | 7.52E7 | 2.17E7 | 18 | 1 | 2 | 2 | 1 | 2 | 2 | 2 | 2 | 2 | 2 | 348 | 358 | Oxidation (M) |
| R.VIISAPSADAPM(+15.99)FVM(+15.99)GVNHEK.Y | N | 53.82 | 2244.0918 | 21 | 4.8 | 749.0414 | 3 | 78.99 | 9 | 22506 | Ana\_08.raw | 4.5E7 | 2.46E6 | 3.47E7 | 1.02E7 |  | 2.48E7 |  | 2.15E7 | 1.07E8 | 2.54E7 | 16 | 3 | 1 | 2 | 1 | 0 | 2 | 0 | 1 | 4 | 2 | 143 | 163 | Oxidation (M) |
| R.VVDLM(+15.99)AYM(+15.99)ASKE | N | 50.11 | 1387.6364 | 12 | 4.1 | 694.8283 | 2 | 59.10 | 1 | 16701 | ANa\_10.raw | 1.85E7 |  | 1.02E7 |  |  |  |  |  | 3.21E7 | 1.86E6 | 5 | 2 | 0 | 1 | 0 | 0 | 0 | 0 | 0 | 1 | 1 | 348 | 359 | Oxidation (M) |
| K.VGVNGFGR.I | N | 47.39 | 804.4242 | 8 | 4.7 | 403.2213 | 2 | 40.41 | 10 | 11185 | Ana\_09.raw | 1.1E7 | 6.3E6 |  |  | 5.54E6 |  | 6.05E6 | 4.29E6 | 1.08E7 | 8.79E6 | 7 | 1 | 1 | 0 | 0 | 1 | 0 | 1 | 1 | 1 | 1 | 30 | 37 |  |
| K.VIPELNGK.L | N | 46.26 | 868.5018 | 8 | 4.4 | 435.2601 | 2 | 43.55 | 1 | 12110 | ANa\_10.raw | 1.23E7 | 2.38E7 |  |  |  |  | 2.67E7 |  | 1.75E7 | 2.2E7 | 5 | 1 | 1 | 0 | 0 | 0 | 0 | 1 | 0 | 1 | 1 | 244 | 251 |  |
| K.LTGM(+15.99)AFR.V | N | 43.94 | 810.4058 | 7 | 3.5 | 406.2116 | 2 | 51.11 | 6 | 14544 | Ana\_05.raw | 1.75E8 | 1.23E8 | 1.4E8 | 1E6 | 9.21E7 | 5.4E7 | 8.67E7 | 9.07E7 | 1.83E8 | 1.49E8 | 19 | 2 | 2 | 2 | 1 | 1 | 2 | 1 | 3 | 2 | 3 | 252 | 258 | Oxidation (M) |
| K.LTGMAFR.V | N | 38.72 | 794.4109 | 7 | 2.7 | 398.2138 | 2 | 49.30 | 10 | 13852 | Ana\_09.raw | 4.37E6 |  |  |  |  |  |  |  |  | 3.88E6 | 2 | 1 | 0 | 0 | 0 | 0 | 0 | 0 | 0 | 0 | 1 | 252 | 258 |  |
| K.LVINGKPITIFQERDPTNIK.W | N | 36.92 | 2295.2949 | 20 | 6.9 | 574.8350 | 4 | 90.48 | 1 | 25421 | ANa\_10.raw | 2.56E6 |  |  |  |  |  |  |  |  |  | 1 | 1 | 0 | 0 | 0 | 0 | 0 | 0 | 0 | 0 | 0 | 91 | 110 |  |
| total 16 peptides |  |  |  |  |  |  |  |  |  |  |  |  |  |  |  |  |  |  |  |  |  |  |  |  |  |  |  |  |  |  |  |  |  |  |  |
| --- | --- | --- | --- | --- | --- | --- | --- | --- | --- | --- | --- | --- | --- | --- | --- | --- | --- | --- | --- | --- | --- | --- | --- | --- | --- | --- | --- | --- | --- | --- | --- | --- | --- | --- | --- |

Best Unique PSM (Scan F1:18145, m/z=910.4617, z=2, RT=64.02, ppm=6.6):

sp|P29758|OAT\_MOUSE

back to list

  

| Protein Coverage
| Supporting Peptides
| Best Unique PSM
|

Protein Coverage:

Supporting Peptides:

| Peptide | Uniq | -10lgP | Mass | Length | ppm | m/z | z | RT | Fraction | Scan | Source File | Area 10 | Area 01 | Area 02 | Area 03 | Area 04 | Area 05 | Area 06 | Area 07 | Area 08 | Area 09 | #Spec | #Spec 10 | #Spec 01 | #Spec 02 | #Spec 03 | #Spec 04 | #Spec 05 | #Spec 06 | #Spec 07 | #Spec 08 | #Spec 09 | Start | End | PTM |
| --- | --- | --- | --- | --- | --- | --- | --- | --- | --- | --- | --- | --- | --- | --- | --- | --- | --- | --- | --- | --- | --- | --- | --- | --- | --- | --- | --- | --- | --- | --- | --- | --- | --- | --- | --- |
| R.IAIAALEVLEEENLAENADK.M | Y | 130.26 | 2154.1055 | 20 | -0.1 | 1078.0599 | 2 | 125.18 | 3 | 34368 | Ana\_02.raw |  |  | 1.43E7 | 7.65E6 | 7.08E6 | 3.47E7 | 1.35E7 | 1.03E7 | 6.54E6 | 3.03E6 | 17 | 0 | 0 | 2 | 2 | 3 | 3 | 2 | 1 | 2 | 2 | 332 | 351 |  |
| R.AFYNNVLGEYEEYITK.L | Y | 98.71 | 1951.9203 | 16 | -0.1 | 976.9673 | 2 | 127.65 | 3 | 34992 | Ana\_02.raw |  | 2.02E7 | 4.86E7 | 9.85E6 | 7.05E6 | 2.43E7 | 3.2E6 | 1.43E7 | 3.82E7 | 1.93E7 | 10 | 0 | 2 | 1 | 1 | 1 | 1 | 1 | 1 | 1 | 1 | 114 | 129 |  |
| K.KTEQGPPSSEYIFER.E | Y | 97.16 | 1766.8474 | 15 | 3.3 | 589.9584 | 3 | 60.63 | 3 | 17259 | Ana\_02.raw | 1.5E7 | 3.25E7 | 3.06E7 | 1.19E7 | 1.11E7 | 2.15E7 | 1.39E7 | 1.3E7 | 5.84E6 | 1.04E7 | 19 | 2 | 2 | 2 | 1 | 2 | 2 | 2 | 2 | 2 | 2 | 32 | 46 |  |
| R.ALQDPNVAAFM(+15.99)VEPIQGEAGVIVPDPGYLTGVR.E | Y | 94.70 | 3438.7493 | 33 | 2.6 | 1147.2600 | 3 | 129.58 | 3 | 35500 | Ana\_02.raw |  |  | 6.59E6 |  | 1.97E6 |  |  |  |  |  | 2 | 0 | 0 | 1 | 0 | 1 | 0 | 0 | 0 | 0 | 0 | 218 | 250 | Oxidation (M) |
| K.IVFADGNFWGR.T | Y | 87.78 | 1280.6301 | 11 | 2.6 | 641.3240 | 2 | 103.86 | 6 | 29275 | Ana\_05.raw | 8.6E5 | 2.21E7 | 2.35E7 | 7.68E6 | 1.44E7 | 1.8E7 | 6.51E6 | 5.07E6 | 2.02E7 | 1.17E7 | 25 | 1 | 3 | 4 | 3 | 3 | 2 | 2 | 2 | 3 | 2 | 170 | 180 |  |
| K.TEQGPPSSEYIFER.E | Y | 83.40 | 1638.7526 | 14 | 2.1 | 820.3853 | 2 | 73.68 | 5 | 20985 | Ana\_04.raw | 9.14E6 | 1.74E7 | 1.34E7 | 6.27E6 | 1.06E7 | 1.14E7 | 7.71E6 | 8.12E6 | 4.09E6 | 6.07E6 | 11 | 1 | 2 | 1 | 1 | 1 | 1 | 1 | 1 | 1 | 1 | 33 | 46 |  |
| K.VLPM(+15.99)NTGVEAGETAC(+57.02)K.L | Y | 82.75 | 1691.7859 | 16 | 3.7 | 846.9034 | 2 | 46.94 | 6 | 13275 | Ana\_05.raw | 3.41E6 | 1.15E7 | 1.28E7 | 3.27E6 | 4.61E6 | 9.01E6 | 4.22E6 | 4.67E6 |  | 2.93E6 | 9 | 1 | 1 | 1 | 1 | 1 | 1 | 1 | 1 | 0 | 1 | 136 | 151 | Oxidation (M); Carbamidomethylation |
| R.LAPPLVIKEDEIR.E | Y | 70.46 | 1491.8660 | 13 | 4.8 | 498.2983 | 3 | 74.83 | 9 | 21323 | Ana\_08.raw |  | 5.61E6 | 0 |  | 3.73E6 | 6.02E6 |  | 2.63E6 | 5.96E6 | 4.34E5 | 7 | 0 | 1 | 1 | 0 | 1 | 1 | 0 | 1 | 1 | 1 | 414 | 426 |  |
| K.YGAHNYHPLPVALER.G | Y | 68.52 | 1735.8794 | 15 | 2.0 | 579.6349 | 3 | 61.10 | 6 | 17503 | Ana\_05.raw | 6.09E6 | 1.64E7 | 1.25E7 | 4.18E6 | 4.09E6 | 1.15E7 | 2.65E6 | 6.42E6 | 3.16E6 | 5.65E6 | 18 | 2 | 2 | 2 | 1 | 2 | 2 | 2 | 2 | 1 | 2 | 50 | 64 |  |
| K.LPSDVVTSVR.G | Y | 67.66 | 1071.5924 | 10 | 1.1 | 536.8041 | 2 | 55.04 | 3 | 15617 | Ana\_02.raw | 2.22E7 | 4.6E7 | 4.78E7 | 1.48E7 | 1.94E7 | 3.28E7 | 2.1E7 | 2.12E7 | 4.72E7 | 1.84E7 | 16 | 1 | 2 | 2 | 2 | 2 | 1 | 2 | 2 | 1 | 1 | 363 | 372 |  |
| K.GLLNAIVIR.E | Y | 54.03 | 967.6178 | 9 | 2.6 | 484.8174 | 2 | 97.55 | 4 | 26868 | Ana\_03.raw | 1.3E7 | 2.01E7 | 3.58E7 | 9.3E6 | 1.34E7 | 2.26E7 | 1.66E7 | 1.4E7 | 2.34E7 | 1.41E7 | 22 | 3 | 2 | 3 | 2 | 2 | 2 | 2 | 3 | 1 | 2 | 375 | 383 |  |
| R.ESVEIINK.T | Y | 47.71 | 930.5022 | 8 | 2.7 | 466.2596 | 2 | 39.21 | 6 | 10905 | Ana\_05.raw | 1.65E7 | 3.6E7 | 4.52E7 | 0 | 2.5E7 | 3.03E7 |  | 9.32E6 |  | 1.98E7 | 8 | 1 | 1 | 1 | 1 | 1 | 1 | 0 | 1 | 0 | 1 | 427 | 434 |  |
| K.GIYM(+15.99)WDVEGR.Q | Y | 45.24 | 1240.5547 | 10 | 4.5 | 621.2874 | 2 | 76.75 | 2 | 21808 | Ana\_01.raw |  | 1.3E7 |  | 8.34E5 | 3.41E6 | 6.87E6 |  | 5.11E6 | 4.76E6 |  | 6 | 0 | 1 | 0 | 1 | 1 | 1 | 0 | 1 | 1 | 0 | 67 | 76 | Oxidation (M) |
| K.GIYMWDVEGR.Q | Y | 42.31 | 1224.5597 | 10 | 8.5 | 613.2923 | 2 | 90.86 | 10 | 25435 | Ana\_09.raw |  |  |  |  |  |  |  |  |  | 0 | 1 | 0 | 0 | 0 | 0 | 0 | 0 | 0 | 0 | 0 | 1 | 67 | 76 |  |
| K.M(+15.99)GAILR.K | Y | 42.11 | 675.3738 | 6 | 3.7 | 338.6954 | 2 | 24.04 | 6 | 6245 | Ana\_05.raw |  |  | 1.33E7 |  | 6.89E6 | 7.5E3 |  |  |  |  | 3 | 0 | 0 | 1 | 0 | 1 | 1 | 0 | 0 | 0 | 0 | 352 | 357 | Oxidation (M) |
| K.LFNYNK.V | Y | 39.65 | 797.4072 | 6 | 4.9 | 399.7128 | 2 | 39.33 | 2 | 10849 | Ana\_01.raw |  | 9.97E6 |  | 8.63E6 |  |  | 1.13E7 |  |  |  | 3 | 0 | 1 | 0 | 1 | 0 | 0 | 1 | 0 | 0 | 0 | 130 | 135 |  |
| K.LTLTSR.A | N | 35.71 | 689.4072 | 6 | 3.2 | 345.7120 | 2 | 31.98 | 8 | 8464 | Ana\_07.raw | 1.23E7 | 2.82E7 | 3.57E7 | 1.36E7 | 9.62E6 | 2.57E7 | 1.92E7 | 1.31E7 | 9.52E6 | 1.04E7 | 16 | 1 | 2 | 2 | 2 | 1 | 2 | 2 | 2 | 1 | 1 | 108 | 113 |  |
| R.LAPPLVIK.E | Y | 33.07 | 849.5687 | 8 | 4.9 | 425.7937 | 2 | 69.89 | 8 | 19622 | Ana\_07.raw | 1.05E7 | 2.69E7 | 2.71E7 | 1.19E7 | 1.57E7 | 1.96E7 | 1.52E7 | 1.11E7 | 2.29E7 | 1.16E7 | 17 | 1 | 2 | 2 | 2 | 1 | 1 | 2 | 2 | 2 | 2 | 414 | 421 |  |
| R.WGYTVK.G | Y | 31.64 | 752.3857 | 6 | 9.3 | 377.2036 | 2 | 44.61 | 9 | 12409 | Ana\_08.raw |  | 3.28E6 |  |  |  |  |  |  | 1.85E6 |  | 2 | 0 | 1 | 0 | 0 | 0 | 0 | 0 | 0 | 1 | 0 | 156 | 161 |  |
| total 19 peptides |  |  |  |  |  |  |  |  |  |  |  |  |  |  |  |  |  |  |  |  |  |  |  |  |  |  |  |  |  |  |  |  |  |  |  |
| --- | --- | --- | --- | --- | --- | --- | --- | --- | --- | --- | --- | --- | --- | --- | --- | --- | --- | --- | --- | --- | --- | --- | --- | --- | --- | --- | --- | --- | --- | --- | --- | --- | --- | --- | --- |

Best Unique PSM (Scan F3:34368, m/z=1078.0599, z=2, RT=125.18, ppm=-0.1):

sp|P48678|LMNA\_MOUSE

back to list

  

| Protein Coverage
| Supporting Peptides
| Best Unique PSM
|

Protein Coverage:

Supporting Peptides:

| Peptide | Uniq | -10lgP | Mass | Length | ppm | m/z | z | RT | Fraction | Scan | Source File | Area 10 | Area 01 | Area 02 | Area 03 | Area 04 | Area 05 | Area 06 | Area 07 | Area 08 | Area 09 | #Spec | #Spec 10 | #Spec 01 | #Spec 02 | #Spec 03 | #Spec 04 | #Spec 05 | #Spec 06 | #Spec 07 | #Spec 08 | #Spec 09 | Start | End | PTM |
| --- | --- | --- | --- | --- | --- | --- | --- | --- | --- | --- | --- | --- | --- | --- | --- | --- | --- | --- | --- | --- | --- | --- | --- | --- | --- | --- | --- | --- | --- | --- | --- | --- | --- | --- | --- |
| R.AQHEDQVEQYKK.E | Y | 106.64 | 1501.7161 | 12 | 2.7 | 751.8673 | 2 | 21.29 | 9 | 5054 | Ana\_08.raw | 7.18E6 | 5.14E6 |  | 2.1E6 | 4.76E6 | 6.77E6 | 3.16E6 | 2.85E6 | 1.25E7 | 8.94E6 | 13 | 2 | 1 | 0 | 1 | 1 | 2 | 1 | 1 | 2 | 2 | 250 | 261 |  |
| R.SVGGSGGGSFGDNLVTR.S | Y | 91.06 | 1565.7434 | 17 | 0.3 | 783.8792 | 2 | 61.56 | 3 | 17538 | Ana\_02.raw | 2.5E6 | 5.02E6 | 6.34E6 | 2.45E6 | 5.61E6 | 5.62E6 | 3.05E6 | 3.9E6 | 1.07E7 | 3.11E6 | 10 | 1 | 1 | 1 | 1 | 1 | 1 | 1 | 1 | 1 | 1 | 629 | 645 |  |
| R.NSNLVGAAHEELQQSR.I | Y | 86.92 | 1751.8550 | 16 | 4.4 | 876.9386 | 2 | 55.56 | 9 | 15731 | Ana\_08.raw |  |  |  | 1.58E6 |  | 3.31E6 | 2.73E6 |  | 1.48E7 |  | 5 | 0 | 0 | 0 | 1 | 0 | 1 | 1 | 0 | 2 | 0 | 281 | 296 |  |
| R.M(+15.99)QQQLDEYQELLDIK.L | Y | 86.73 | 1908.9138 | 15 | 5.3 | 955.4692 | 2 | 109.02 | 6 | 30623 | Ana\_05.raw | 1.16E6 | 5.72E5 | 1.21E6 |  | 1.09E6 | 1.74E6 | 2.22E4 |  | 3.37E6 |  | 8 | 1 | 1 | 1 | 0 | 1 | 1 | 1 | 0 | 2 | 0 | 352 | 366 | Oxidation (M) |
| K.AAYEAELGDAR.K | Y | 76.41 | 1164.5410 | 11 | 3.0 | 583.2795 | 2 | 49.11 | 6 | 13947 | Ana\_05.raw |  | 1.28E7 | 2.03E7 | 7.79E6 | 1.31E7 | 1.88E7 | 1.3E7 | 8.16E6 |  |  | 7 | 0 | 1 | 1 | 1 | 1 | 1 | 1 | 1 | 0 | 0 | 79 | 89 |  |
| R.SGAQASSTPLSPTR.I | Y | 76.23 | 1358.6790 | 14 | 6.2 | 680.3510 | 2 | 34.39 | 8 | 9173 | Ana\_07.raw | 2.7E7 | 3.46E7 | 2.28E7 | 1.03E7 | 2E7 |  | 1.57E7 | 1.39E7 | 2.74E7 | 1.84E7 | 9 | 1 | 1 | 1 | 1 | 1 | 0 | 1 | 1 | 1 | 1 | 12 | 25 |  |
| R.AQHEDQVEQYK.K | Y | 74.26 | 1373.6211 | 11 | 7.4 | 687.8229 | 2 | 23.44 | 9 | 5781 | Ana\_08.raw |  |  |  |  |  |  |  |  | 1.37E6 |  | 1 | 0 | 0 | 0 | 0 | 0 | 0 | 0 | 0 | 1 | 0 | 250 | 260 |  |
| R.TALINSTGEEVAM(+15.99)R.K | Y | 72.71 | 1506.7347 | 14 | 2.7 | 754.3766 | 2 | 56.00 | 6 | 15990 | Ana\_05.raw | 6.11E6 | 6.12E6 | 6.16E6 | 1.83E6 | 4.39E6 | 4.8E6 | 3.03E6 | 9.21E5 | 1.24E7 | 4.34E6 | 10 | 1 | 1 | 1 | 1 | 1 | 1 | 1 | 1 | 1 | 1 | 528 | 541 | Oxidation (M) |
| R.IDSLSAQLSQLQK.Q | Y | 71.18 | 1429.7776 | 13 | -2.4 | 715.8943 | 2 | 83.46 | 3 | 23537 | Ana\_02.raw | 3.84E6 | 5.9E6 | 2.25E6 | 9.73E5 | 2.32E6 | 3.24E6 | 2.85E5 | 1.73E6 | 1.19E7 | 1.06E6 | 10 | 1 | 1 | 1 | 1 | 1 | 1 | 1 | 1 | 1 | 1 | 299 | 311 |  |
| R.ITESEEVVSR.E | Y | 71.04 | 1147.5720 | 10 | 4.1 | 574.7957 | 2 | 36.22 | 6 | 9985 | Ana\_05.raw |  |  |  |  |  | 3.04E7 | 5.99E7 |  |  |  | 2 | 0 | 0 | 0 | 0 | 0 | 1 | 1 | 0 | 0 | 0 | 63 | 72 |  |
| R.VAVEEVDEEGK.F | Y | 68.03 | 1202.5667 | 11 | 1.8 | 602.2917 | 2 | 37.30 | 1 | 10181 | ANa\_10.raw | 2.86E7 | 3.5E7 | 3.16E7 | 1.3E7 |  |  | 1.93E7 |  |  |  | 6 | 1 | 1 | 1 | 2 | 0 | 0 | 1 | 0 | 0 | 0 | 440 | 450 |  |
| K.EAALSTALSEK.R | Y | 64.96 | 1118.5819 | 11 | 2.8 | 560.2998 | 2 | 50.33 | 3 | 14241 | Ana\_02.raw | 1.49E7 | 1.65E7 | 1.46E7 |  |  |  |  |  | 2.48E7 | 1.27E7 | 5 | 1 | 1 | 1 | 0 | 0 | 0 | 0 | 0 | 1 | 1 | 145 | 155 |  |
| R.LSPSPTSQR.S | Y | 62.42 | 971.5036 | 9 | 4.9 | 486.7614 | 2 | 22.60 | 2 | 5614 | Ana\_01.raw | 2.55E7 | 2.32E7 |  | 1.05E7 |  | 2.84E7 |  |  |  |  | 4 | 1 | 1 | 0 | 1 | 0 | 1 | 0 | 0 | 0 | 0 | 389 | 397 |  |
| K.EGDLLAAQAR.L | Y | 59.83 | 1042.5406 | 10 | 3.5 | 522.2794 | 2 | 44.16 | 6 | 12435 | Ana\_05.raw | 2.56E7 | 2.62E7 | 2.59E7 | 1.03E7 | 2E7 | 2.58E7 | 1.5E7 | 1.55E7 | 3.12E7 | 1.94E7 | 10 | 1 | 1 | 1 | 1 | 1 | 1 | 1 | 1 | 1 | 1 | 124 | 133 |  |
| R.TLEGELHDLR.G | Y | 58.35 | 1181.6040 | 10 | 0.8 | 394.8756 | 3 | 62.99 | 3 | 17941 | Ana\_02.raw |  | 5.95E5 | 6.52E6 | 1.15E6 |  | 4.15E6 |  | 2.69E6 | 1.35E7 | 3.74E6 | 8 | 0 | 1 | 1 | 1 | 0 | 1 | 0 | 1 | 2 | 1 | 157 | 166 |  |
| K.NIYSEELR.E | Y | 56.09 | 1022.5032 | 8 | 2.5 | 512.2602 | 2 | 53.06 | 6 | 15127 | Ana\_05.raw |  | 1.58E7 |  | 6.79E6 | 1.16E7 | 1.64E7 | 1.15E7 |  | 2.33E7 | 1.3E7 | 7 | 0 | 1 | 0 | 1 | 1 | 1 | 1 | 0 | 1 | 1 | 209 | 216 |  |
| R.LADALQELR.A | Y | 55.81 | 1027.5662 | 9 | -0.9 | 514.7899 | 2 | 67.34 | 3 | 19148 | Ana\_02.raw |  |  | 1.03E7 | 5.9E6 | 1E7 |  | 5.87E6 | 6.15E6 |  |  | 5 | 0 | 0 | 1 | 1 | 1 | 0 | 1 | 1 | 0 | 0 | 241 | 249 |  |
| K.DLEALLNSK.E | Y | 55.32 | 1001.5393 | 9 | 2.2 | 501.7780 | 2 | 90.83 | 7 | 25516 | Ana\_06.raw |  | 1.44E6 |  | 1.67E5 | 1.29E6 | 1.03E6 | 2.44E5 | 7.28E4 | 1.68E6 |  | 7 | 0 | 1 | 0 | 1 | 1 | 1 | 1 | 1 | 1 | 0 | 136 | 144 |  |
| K.LLEGEEER.L | N | 52.51 | 973.4716 | 8 | 0.3 | 487.7432 | 2 | 27.67 | 3 | 7406 | Ana\_02.raw | 3.73E7 | 4.95E7 | 4.14E7 | 1.83E7 | 2.72E7 | 3.89E7 | 2.44E7 | 2.27E7 |  | 3.91E7 | 9 | 1 | 1 | 1 | 1 | 1 | 1 | 1 | 1 | 0 | 1 | 379 | 386 |  |
| K.EDLQELNDR.L | Y | 50.69 | 1130.5204 | 9 | 1.3 | 566.2682 | 2 | 45.52 | 4 | 12468 | Ana\_03.raw |  |  |  | 2.17E6 | 3.95E6 |  |  | 1.94E6 |  |  | 3 | 0 | 0 | 0 | 1 | 1 | 0 | 0 | 1 | 0 | 0 | 33 | 41 |  |
| R.SLETENAGLR.L | Y | 48.97 | 1088.5461 | 10 | 5.9 | 545.2836 | 2 | 38.62 | 2 | 10631 | Ana\_01.raw | 2.52E7 | 2.82E7 |  |  |  |  | 1.46E7 |  |  | 2.67E7 | 4 | 1 | 1 | 0 | 0 | 0 | 0 | 1 | 0 | 0 | 1 | 51 | 60 |  |
| K.LEAALGEAK.K | Y | 48.77 | 900.4916 | 9 | 5.4 | 451.2555 | 2 | 34.04 | 1 | 9198 | ANa\_10.raw | 1.74E7 | 2.15E7 | 1.74E7 | 6.92E6 | 1.29E7 | 1.59E7 | 1.07E7 | 1.09E7 | 2.92E7 | 2.01E7 | 10 | 1 | 1 | 1 | 1 | 1 | 1 | 1 | 1 | 1 | 1 | 172 | 180 |  |
| R.LKDLEALLNSK.E | Y | 47.03 | 1242.7183 | 11 | 3.9 | 622.3688 | 2 | 78.92 | 9 | 22485 | Ana\_08.raw | 1.15E6 | 3.29E5 |  |  |  | 4.19E5 |  |  | 8.16E6 |  | 5 | 1 | 1 | 0 | 0 | 0 | 1 | 0 | 0 | 2 | 0 | 134 | 144 |  |
| K.LALDM(+15.99)EIHAYR.K | Y | 45.78 | 1346.6653 | 11 | 4.6 | 449.8978 | 3 | 64.33 | 9 | 18340 | Ana\_08.raw |  |  |  |  |  | 2.57E6 |  |  | 1.12E6 |  | 2 | 0 | 0 | 0 | 0 | 0 | 1 | 0 | 0 | 1 | 0 | 367 | 377 | Oxidation (M) |
| R.DLEDSLAR.E | Y | 42.60 | 917.4454 | 8 | 6.1 | 459.7328 | 2 | 53.94 | 8 | 14994 | Ana\_07.raw |  | 1.29E7 | 8.28E6 | 4.32E6 | 6.41E6 | 9.01E6 | 6.02E6 | 5.26E6 | 1.02E7 |  | 8 | 0 | 1 | 1 | 1 | 1 | 1 | 1 | 1 | 1 | 0 | 322 | 329 |  |
| R.LAVYIDR.V | Y | 41.17 | 848.4756 | 7 | 3.2 | 425.2464 | 2 | 52.68 | 1 | 14813 | ANa\_10.raw | 3.57E6 | 3.3E6 | 2.16E6 |  |  | 1.61E6 | 1.65E6 | 9.7E5 |  | 3.45E6 | 7 | 1 | 1 | 1 | 0 | 0 | 1 | 1 | 1 | 0 | 1 | 42 | 48 |  |
| K.AQNTWGC(+57.02)GSSLR.T | Y | 35.82 | 1335.5990 | 12 | 0.7 | 668.8073 | 2 | 45.46 | 3 | 12806 | Ana\_02.raw |  |  | 1.47E6 |  | 1.55E6 | 1.14E6 |  |  |  |  | 3 | 0 | 0 | 1 | 0 | 1 | 1 | 0 | 0 | 0 | 0 | 516 | 527 | Carbamidomethylation |
| K.EELDFQK.N | Y | 35.44 | 907.4286 | 7 | 4.3 | 454.7236 | 2 | 42.86 | 2 | 11943 | Ana\_01.raw |  | 5.99E6 |  |  |  |  |  |  |  |  | 1 | 0 | 1 | 0 | 0 | 0 | 0 | 0 | 0 | 0 | 0 | 202 | 208 |  |
| R.LQEKEDLQELNDR.L | Y | 34.38 | 1628.8005 | 13 | -5.0 | 815.4035 | 2 | 47.13 | 9 | 13180 | Ana\_08.raw |  |  |  |  |  |  |  |  | 0 |  | 1 | 0 | 0 | 0 | 0 | 0 | 0 | 0 | 0 | 1 | 0 | 29 | 41 |  |
| K.TLDSVAK.E | Y | 32.39 | 732.4017 | 7 | 0.8 | 367.2084 | 2 | 20.96 | 5 | 5286 | Ana\_04.raw |  |  |  |  | 4.91E6 |  |  |  |  |  | 1 | 0 | 0 | 0 | 0 | 1 | 0 | 0 | 0 | 0 | 0 | 91 | 97 |  |
| R.LQLELSK.V | Y | 31.90 | 829.4909 | 7 | 5.5 | 415.7550 | 2 | 54.09 | 2 | 15313 | Ana\_01.raw |  | 0 |  |  | 3.72E6 |  |  |  |  | 2.94E6 | 4 | 0 | 1 | 0 | 0 | 1 | 0 | 1 | 0 | 0 | 1 | 102 | 108 |  |
| K.VREEFK.E | Y | 22.81 | 806.4286 | 6 | 1.0 | 404.2220 | 2 | 19.18 | 3 | 4664 | Ana\_02.raw |  |  | 1.68E7 |  |  |  |  |  |  |  | 1 | 0 | 0 | 1 | 0 | 0 | 0 | 0 | 0 | 0 | 0 | 109 | 114 |  |
| total 32 peptides |  |  |  |  |  |  |  |  |  |  |  |  |  |  |  |  |  |  |  |  |  |  |  |  |  |  |  |  |  |  |  |  |  |  |  |
| --- | --- | --- | --- | --- | --- | --- | --- | --- | --- | --- | --- | --- | --- | --- | --- | --- | --- | --- | --- | --- | --- | --- | --- | --- | --- | --- | --- | --- | --- | --- | --- | --- | --- | --- | --- |

Best Unique PSM (Scan F9:5054, m/z=751.8673, z=2, RT=21.29, ppm=2.7):

sp|Q64GA5|PA24C\_MOUSE

back to list

  

| Protein Coverage
| Supporting Peptides
| Best Unique PSM
|

Protein Coverage:

Supporting Peptides:

| Peptide | Uniq | -10lgP | Mass | Length | ppm | m/z | z | RT | Fraction | Scan | Source File | Area 10 | Area 01 | Area 02 | Area 03 | Area 04 | Area 05 | Area 06 | Area 07 | Area 08 | Area 09 | #Spec | #Spec 10 | #Spec 01 | #Spec 02 | #Spec 03 | #Spec 04 | #Spec 05 | #Spec 06 | #Spec 07 | #Spec 08 | #Spec 09 | Start | End | PTM |
| --- | --- | --- | --- | --- | --- | --- | --- | --- | --- | --- | --- | --- | --- | --- | --- | --- | --- | --- | --- | --- | --- | --- | --- | --- | --- | --- | --- | --- | --- | --- | --- | --- | --- | --- | --- |
| R.ELLHLVDAGFAINTPYPLVLPPVR.E | Y | 113.44 | 2643.4788 | 24 | 2.0 | 882.1686 | 3 | 132.58 | 1 | 36443 | ANa\_10.raw | 1.03E7 |  |  |  |  |  |  |  | 1E7 | 3.94E6 | 3 | 1 | 0 | 0 | 0 | 0 | 0 | 0 | 0 | 1 | 1 | 411 | 434 |  |
| K.LNIQADQAPVIAVLGSGGGLR.A | Y | 109.74 | 2048.1377 | 21 | 4.3 | 1025.0806 | 2 | 120.58 | 1 | 33243 | ANa\_10.raw | 4.87E7 |  |  |  |  |  |  |  | 5.62E7 | 1.81E7 | 10 | 4 | 0 | 0 | 0 | 0 | 0 | 0 | 0 | 3 | 3 | 35 | 55 |  |
| R.HEIPFPEVSEDQLK.E | Y | 100.79 | 1666.8202 | 14 | 4.4 | 556.6165 | 3 | 80.45 | 1 | 22706 | ANa\_10.raw | 5.42E7 |  |  |  |  |  |  |  | 7.63E7 | 1.15E7 | 12 | 6 | 0 | 0 | 0 | 0 | 0 | 0 | 0 | 4 | 2 | 463 | 476 |  |
| R.KLNIQADQAPVIAVLGSGGGLR.A | Y | 86.93 | 2176.2327 | 22 | 8.9 | 726.4246 | 3 | 107.81 | 1 | 29976 | ANa\_10.raw | 1.39E7 |  |  |  |  |  |  |  |  |  | 1 | 1 | 0 | 0 | 0 | 0 | 0 | 0 | 0 | 0 | 0 | 34 | 55 |  |
| K.LSDSYTPDLVR.D | Y | 81.25 | 1264.6299 | 11 | 4.8 | 633.3253 | 2 | 67.61 | 1 | 19163 | ANa\_10.raw | 5.83E7 |  |  |  |  |  |  |  | 7.73E7 | 1.89E7 | 10 | 4 | 0 | 0 | 0 | 0 | 0 | 0 | 0 | 4 | 2 | 523 | 533 |  |
| K.FIEGPVTYSEAPR.M | Y | 79.20 | 1464.7249 | 13 | 2.5 | 733.3715 | 2 | 67.45 | 9 | 19251 | Ana\_08.raw | 1.01E8 |  |  |  |  |  |  |  | 1.22E8 | 4.77E7 | 11 | 3 | 0 | 0 | 0 | 0 | 0 | 0 | 0 | 5 | 3 | 267 | 279 |  |
| K.TQNSWFEFTPHHAGYPALGAYVPITEFGSR.F | Y | 69.86 | 3379.6050 | 30 | 7.4 | 845.9148 | 4 | 128.25 | 1 | 35263 | ANa\_10.raw | 6.87E6 |  |  |  |  |  |  |  |  |  | 1 | 1 | 0 | 0 | 0 | 0 | 0 | 0 | 0 | 0 | 0 | 189 | 218 |  |
| K.WQWGTVYNFLYK.H | Y | 69.82 | 1603.7823 | 12 | 6.1 | 802.9033 | 2 | 130.78 | 10 | 35689 | Ana\_09.raw |  |  |  |  |  |  |  |  |  | 3.53E6 | 1 | 0 | 0 | 0 | 0 | 0 | 0 | 0 | 0 | 0 | 1 | 387 | 398 |  |
| R.ELQDSNLSSLK.K | Y | 66.55 | 1232.6248 | 11 | 8.9 | 617.3251 | 2 | 55.19 | 1 | 15561 | ANa\_10.raw | 2.16E7 |  |  |  |  |  |  | 5.37E5 | 2.39E7 | 1.01E7 | 8 | 3 | 0 | 0 | 0 | 0 | 0 | 0 | 1 | 2 | 2 | 150 | 160 |  |
| K.EQFLDHLLDR.F | Y | 64.73 | 1284.6462 | 10 | 4.9 | 429.2248 | 3 | 95.52 | 1 | 26769 | ANa\_10.raw | 1.09E7 |  |  |  |  |  |  |  | 1.19E7 | 2.23E7 | 7 | 2 | 0 | 0 | 0 | 0 | 0 | 0 | 0 | 3 | 2 | 340 | 349 |  |
| K.APASC(+57.02)YVLR.G | Y | 63.67 | 1035.5171 | 9 | 7.8 | 518.7698 | 2 | 46.25 | 1 | 12921 | ANa\_10.raw | 1.97E7 |  |  |  |  |  |  |  |  |  | 1 | 1 | 0 | 0 | 0 | 0 | 0 | 0 | 0 | 0 | 0 | 481 | 489 | Carbamidomethylation |
| R.ELQDSNLSSLKK.Q | Y | 61.36 | 1360.7197 | 12 | 7.0 | 681.3719 | 2 | 43.03 | 1 | 11947 | ANa\_10.raw | 5.65E6 |  |  |  |  |  |  |  | 1E7 |  | 2 | 1 | 0 | 0 | 0 | 0 | 0 | 0 | 0 | 1 | 0 | 150 | 161 |  |
| K.VAGNPGNIPR.V | Y | 60.61 | 993.5355 | 10 | 4.3 | 497.7772 | 2 | 32.01 | 1 | 8597 | ANa\_10.raw | 6.86E7 |  |  |  |  |  |  |  | 3.24E7 | 2.16E7 | 3 | 1 | 0 | 0 | 0 | 0 | 0 | 0 | 0 | 1 | 1 | 557 | 566 |  |
| K.DNC(+57.02)GDDIETWR.K | Y | 58.26 | 1379.5411 | 11 | 4.4 | 690.7809 | 2 | 61.62 | 9 | 17530 | Ana\_08.raw |  |  |  |  |  |  |  |  | 2.25E6 |  | 1 | 0 | 0 | 0 | 0 | 0 | 0 | 0 | 0 | 1 | 0 | 505 | 515 | Carbamidomethylation |
| K.NEWDFHESLEK.A | Y | 57.48 | 1432.6259 | 11 | 5.4 | 717.3241 | 2 | 69.62 | 1 | 19725 | ANa\_10.raw | 7.95E6 |  |  |  |  |  |  |  | 1.1E7 | 2.71E6 | 5 | 2 | 0 | 0 | 0 | 0 | 0 | 0 | 0 | 2 | 1 | 113 | 123 |  |
| R.VKDPQGSQTVEFK.K | Y | 57.22 | 1461.7463 | 13 | 6.6 | 731.8853 | 2 | 36.69 | 1 | 9996 | ANa\_10.raw | 1.75E7 |  |  |  |  |  |  |  |  | 7.21E6 | 2 | 1 | 0 | 0 | 0 | 0 | 0 | 0 | 0 | 0 | 1 | 577 | 589 |  |
| K.INILSEM(+15.99)R.K | Y | 42.75 | 990.5168 | 8 | 5.8 | 496.2686 | 2 | 78.98 | 1 | 22303 | ANa\_10.raw | 1.14E7 |  |  |  |  |  |  |  | 1.2E7 |  | 3 | 2 | 0 | 0 | 0 | 0 | 0 | 0 | 0 | 1 | 0 | 548 | 555 | Oxidation (M) |
| R.DLTFLR.G | Y | 40.53 | 763.4228 | 6 | 5.6 | 382.7208 | 2 | 73.70 | 1 | 20878 | ANa\_10.raw | 1.17E7 |  |  |  |  |  |  |  | 1.29E7 | 4.6E6 | 4 | 1 | 0 | 0 | 0 | 0 | 0 | 0 | 0 | 2 | 1 | 232 | 237 |  |
| R.KVAGNPGNIPR.V | Y | 39.08 | 1121.6305 | 11 | 3.6 | 561.8245 | 2 | 24.19 | 9 | 6032 | Ana\_08.raw | 1.35E6 |  |  |  |  |  |  |  | 0 | 1.95E6 | 3 | 1 | 0 | 0 | 0 | 0 | 0 | 0 | 0 | 1 | 1 | 556 | 566 |  |
| K.VLEALR.K | Y | 37.75 | 699.4279 | 6 | 5.4 | 350.7231 | 2 | 38.07 | 1 | 10422 | ANa\_10.raw | 2.28E7 |  |  |  |  |  |  |  | 2.08E7 | 1.22E7 | 5 | 2 | 0 | 0 | 0 | 0 | 0 | 0 | 0 | 2 | 1 | 28 | 33 |  |
| K.DPQGSQTVEFK.K | Y | 26.69 | 1234.5830 | 11 | -5.8 | 618.2952 | 2 | 40.99 | 1 | 11319 | ANa\_10.raw | 6.2E7 |  |  |  |  |  |  |  |  |  | 1 | 1 | 0 | 0 | 0 | 0 | 0 | 0 | 0 | 0 | 0 | 579 | 589 |  |
| R.YEKNEWDFHESLEK.A | Y | 24.77 | 1852.8268 | 14 | 4.0 | 618.6187 | 3 | 61.46 | 9 | 17485 | Ana\_08.raw |  |  |  |  |  |  |  |  | 1.37E6 |  | 1 | 0 | 0 | 0 | 0 | 0 | 0 | 0 | 0 | 1 | 0 | 110 | 123 |  |
| total 22 peptides |  |  |  |  |  |  |  |  |  |  |  |  |  |  |  |  |  |  |  |  |  |  |  |  |  |  |  |  |  |  |  |  |  |  |  |
| --- | --- | --- | --- | --- | --- | --- | --- | --- | --- | --- | --- | --- | --- | --- | --- | --- | --- | --- | --- | --- | --- | --- | --- | --- | --- | --- | --- | --- | --- | --- | --- | --- | --- | --- | --- |

Best Unique PSM (Scan F1:36443, m/z=882.1686, z=3, RT=132.58, ppm=2.0):

sp|P09411|PGK1\_MOUSE

back to list

  

| Protein Coverage
| Supporting Peptides
| Best Unique PSM
|

Protein Coverage:

Supporting Peptides:

| Peptide | Uniq | -10lgP | Mass | Length | ppm | m/z | z | RT | Fraction | Scan | Source File | Area 10 | Area 01 | Area 02 | Area 03 | Area 04 | Area 05 | Area 06 | Area 07 | Area 08 | Area 09 | #Spec | #Spec 10 | #Spec 01 | #Spec 02 | #Spec 03 | #Spec 04 | #Spec 05 | #Spec 06 | #Spec 07 | #Spec 08 | #Spec 09 | Start | End | PTM |
| --- | --- | --- | --- | --- | --- | --- | --- | --- | --- | --- | --- | --- | --- | --- | --- | --- | --- | --- | --- | --- | --- | --- | --- | --- | --- | --- | --- | --- | --- | --- | --- | --- | --- | --- | --- |
| K.DC(+57.02)VGPEVENAC(+57.02)ANPAAGTVILLENLR.F | Y | 125.32 | 2781.3425 | 26 | 2.0 | 928.1234 | 3 | 131.39 | 6 | 36509 | Ana\_05.raw | 2.17E6 | 8.31E6 | 6.99E6 | 2.64E6 |  | 9.78E6 | 6.55E6 | 5.59E6 | 1.96E7 | 1.05E7 | 9 | 1 | 1 | 1 | 1 | 0 | 1 | 1 | 1 | 1 | 1 | 98 | 123 | Carbamidomethylation |
| K.ITLPVDFVTADKFDENAK.T | Y | 101.94 | 2022.0309 | 18 | 4.5 | 675.0206 | 3 | 107.23 | 9 | 30153 | Ana\_08.raw | 2.91E7 | 2.18E7 | 2.34E7 | 1.04E7 | 1.7E7 | 2.19E7 | 1.56E7 | 1.16E7 | 9.55E7 | 3.19E7 | 26 | 2 | 2 | 2 | 2 | 3 | 3 | 2 | 2 | 5 | 3 | 280 | 297 |  |
| K.VSHVSTGGGASLELLEGK.V | N | 94.87 | 1739.9053 | 18 | 4.5 | 580.9783 | 3 | 70.41 | 1 | 19953 | ANa\_10.raw | 4.32E6 | 3.1E6 | 7.23E5 | 3.16E6 | 4.33E6 | 4.71E6 |  | 2.7E5 | 1.33E7 | 7.33E6 | 10 | 1 | 1 | 1 | 1 | 1 | 1 | 0 | 1 | 2 | 1 | 389 | 406 |  |
| K.ALESPERPFLAILGGAK.V | Y | 93.45 | 1767.9883 | 17 | 3.7 | 590.3389 | 3 | 112.64 | 6 | 31560 | Ana\_05.raw | 8.56E7 | 4.88E7 | 5.27E7 | 3.3E7 | 4.79E7 | 2.26E7 | 4.1E7 | 2.86E7 | 1.53E8 | 5.88E7 | 39 | 5 | 3 | 4 | 2 | 3 | 2 | 4 | 5 | 6 | 5 | 200 | 216 |  |
| K.LGDVYVNDAFGTAHR.A | N | 86.25 | 1633.7848 | 15 | 5.5 | 817.9042 | 2 | 71.26 | 9 | 20336 | Ana\_08.raw | 1.69E7 | 1.2E7 | 1.02E7 | 7.13E6 | 5.6E6 | 9.75E6 | 8.32E5 | 3.29E6 | 4.29E7 | 1.57E7 | 14 | 1 | 1 | 1 | 2 | 2 | 1 | 1 | 2 | 2 | 1 | 157 | 171 |  |
| K.VLNNM(+15.99)EIGTSLYDEEGAK.I | Y | 78.91 | 1997.9252 | 18 | 3.0 | 999.9729 | 2 | 78.13 | 7 | 22108 | Ana\_06.raw | 3.38E6 |  |  |  | 1.31E6 | 4.2E5 | 2.11E6 | 2E6 | 2.42E6 |  | 6 | 1 | 0 | 0 | 0 | 1 | 1 | 1 | 1 | 1 | 0 | 247 | 264 | Oxidation (M) |
| K.ITLPVDFVTADK.F | Y | 72.77 | 1317.7180 | 12 | -0.6 | 659.8659 | 2 | 102.14 | 3 | 28459 | Ana\_02.raw | 4.78E6 | 2.19E6 | 7.2E6 |  | 4.85E6 | 6.46E5 | 1.96E6 | 1.31E6 | 7.09E6 | 8.73E6 | 11 | 1 | 1 | 1 | 0 | 1 | 1 | 1 | 1 | 2 | 2 | 280 | 291 |  |
| K.YSLEPVAAELK.S | Y | 72.71 | 1218.6495 | 11 | 4.4 | 610.3347 | 2 | 80.59 | 3 | 22773 | Ana\_02.raw | 1.39E7 | 2.22E6 | 9.38E6 | 1.54E6 | 8.03E6 | 9.45E6 |  |  | 3.19E7 | 1.38E7 | 9 | 2 | 1 | 1 | 1 | 1 | 1 | 0 | 0 | 1 | 1 | 76 | 86 |  |
| R.FHVEEEGK.G | N | 67.73 | 973.4504 | 8 | 3.1 | 487.7340 | 2 | 23.14 | 3 | 5970 | Ana\_02.raw | 3.01E7 | 2.43E7 | 2.82E7 | 1.21E7 | 1.75E7 | 2.03E7 | 1.39E7 | 1.8E7 | 3.18E7 | 2.75E7 | 15 | 2 | 1 | 2 | 2 | 2 | 1 | 1 | 1 | 2 | 1 | 124 | 131 |  |
| R.FHVEEEGKGK.D | N | 65.88 | 1158.5669 | 10 | 4.6 | 580.2934 | 2 | 21.06 | 6 | 5279 | Ana\_05.raw | 6.62E6 | 2.7E6 |  |  | 9.92E5 | 1.76E6 |  |  |  | 6.94E6 | 5 | 1 | 1 | 0 | 0 | 1 | 1 | 0 | 0 | 0 | 1 | 124 | 133 |  |
| R.GC(+57.02)ITIIGGGDTATC(+57.02)C(+57.02)AK.W | Y | 63.47 | 1753.7798 | 17 | 3.6 | 877.9003 | 2 | 62.07 | 1 | 17588 | ANa\_10.raw | 1.18E6 |  |  |  |  | 4.06E5 |  |  |  |  | 2 | 1 | 0 | 0 | 0 | 0 | 1 | 0 | 0 | 0 | 0 | 366 | 382 | Carbamidomethylation |
| K.SLM(+15.99)DEVVK.A | Y | 51.43 | 935.4634 | 8 | 6.6 | 468.7420 | 2 | 41.35 | 6 | 11567 | Ana\_05.raw | 1.1E7 | 1.05E7 | 1.36E7 | 6.48E6 | 1.8E7 | 1.95E7 | 1.21E7 | 5.4E6 | 8.8E6 | 1.03E7 | 14 | 1 | 1 | 1 | 2 | 2 | 2 | 2 | 1 | 1 | 1 | 354 | 361 | Oxidation (M) |
| K.YAEAVGR.A | Y | 41.59 | 764.3817 | 7 | 5.3 | 383.2001 | 2 | 22.19 | 6 | 5659 | Ana\_05.raw |  |  |  | 9.55E6 |  | 0 | 5.29E6 | 5.15E6 |  |  | 4 | 0 | 0 | 0 | 1 | 0 | 1 | 1 | 1 | 0 | 0 | 324 | 330 |  |
| R.VDFNVPM(+15.99)K.N | N | 40.47 | 964.4688 | 8 | 2.4 | 483.2428 | 2 | 54.49 | 4 | 15085 | Ana\_03.raw | 2.14E7 | 6.7E6 | 9.05E6 | 4.48E6 | 4.19E6 | 7.5E6 |  | 1.28E7 |  | 1.87E7 | 11 | 2 | 1 | 1 | 1 | 1 | 1 | 0 | 2 | 0 | 2 | 23 | 30 | Oxidation (M) |
| K.AAVPSIK.F | Y | 37.35 | 684.4170 | 7 | 3.4 | 343.2169 | 2 | 28.57 | 9 | 7461 | Ana\_08.raw |  |  | 3.85E6 |  |  |  |  | 5.36E5 | 8.71E6 | 6.43E6 | 5 | 1 | 0 | 1 | 0 | 0 | 0 | 0 | 1 | 1 | 1 | 42 | 48 |  |
| R.AHSSM(+15.99)VGVNLPQK.A | Y | 37.25 | 1382.6976 | 13 | 3.0 | 461.9079 | 3 | 38.41 | 6 | 10663 | Ana\_05.raw | 0 |  |  |  |  | 4.99E6 |  |  |  | 5.23E6 | 4 | 1 | 1 | 0 | 0 | 0 | 1 | 0 | 0 | 0 | 1 | 172 | 184 | Oxidation (M) |
| K.ELNYFAK.A | Y | 36.65 | 883.4439 | 7 | 2.2 | 442.7302 | 2 | 55.22 | 4 | 15290 | Ana\_03.raw |  |  |  | 6.53E6 |  |  |  |  |  |  | 1 | 0 | 0 | 0 | 1 | 0 | 0 | 0 | 0 | 0 | 0 | 193 | 199 |  |
| K.DVLFLK.D | N | 30.36 | 733.4374 | 6 | 3.0 | 367.7271 | 2 | 76.93 | 6 | 22034 | Ana\_05.raw |  |  | 3.51E6 |  |  | 3.14E6 |  |  | 9.21E6 | 4.62E6 | 5 | 0 | 0 | 1 | 0 | 0 | 1 | 0 | 0 | 2 | 1 | 92 | 97 |  |
| K.IDAFR.A | Y | 29.76 | 620.3282 | 5 | 5.2 | 311.1730 | 2 | 31.77 | 8 | 8402 | Ana\_07.raw |  |  | 7.89E6 | 3.29E6 |  |  |  | 3.83E6 |  | 9.24E6 | 4 | 0 | 0 | 1 | 1 | 0 | 0 | 0 | 1 | 0 | 1 | 147 | 151 |  |
| K.IQLINNM(+15.99)LDK.V | Y | 25.35 | 1216.6486 | 10 | 2.8 | 609.3333 | 2 | 64.51 | 10 | 18280 | Ana\_09.raw |  |  |  |  |  |  |  |  |  | 1.38E6 | 1 | 0 | 0 | 0 | 0 | 0 | 0 | 0 | 0 | 0 | 1 | 221 | 230 | Oxidation (M) |
| total 20 peptides |  |  |  |  |  |  |  |  |  |  |  |  |  |  |  |  |  |  |  |  |  |  |  |  |  |  |  |  |  |  |  |  |  |  |  |
| --- | --- | --- | --- | --- | --- | --- | --- | --- | --- | --- | --- | --- | --- | --- | --- | --- | --- | --- | --- | --- | --- | --- | --- | --- | --- | --- | --- | --- | --- | --- | --- | --- | --- | --- | --- |

Best Unique PSM (Scan F6:36509, m/z=928.1234, z=3, RT=131.39, ppm=2.0):

sp|P49290|PERE\_MOUSE

back to list

  

| Protein Coverage
| Supporting Peptides
| Best Unique PSM
|

Protein Coverage:

Supporting Peptides:

| Peptide | Uniq | -10lgP | Mass | Length | ppm | m/z | z | RT | Fraction | Scan | Source File | Area 10 | Area 01 | Area 02 | Area 03 | Area 04 | Area 05 | Area 06 | Area 07 | Area 08 | Area 09 | #Spec | #Spec 10 | #Spec 01 | #Spec 02 | #Spec 03 | #Spec 04 | #Spec 05 | #Spec 06 | #Spec 07 | #Spec 08 | #Spec 09 | Start | End | PTM |
| --- | --- | --- | --- | --- | --- | --- | --- | --- | --- | --- | --- | --- | --- | --- | --- | --- | --- | --- | --- | --- | --- | --- | --- | --- | --- | --- | --- | --- | --- | --- | --- | --- | --- | --- | --- |
| R.VGPLLAC(+57.02)LFENQFR.R | Y | 107.94 | 1662.8551 | 14 | 3.5 | 832.4377 | 2 | 131.62 | 9 | 36433 | Ana\_08.raw | 1.85E7 | 4.89E6 |  | 2.1E6 |  | 3.69E6 |  | 2.07E6 | 6.65E7 | 9.28E7 | 12 | 1 | 1 | 0 | 1 | 0 | 1 | 0 | 1 | 4 | 3 | 630 | 643 | Carbamidomethylation |
| K.TPDNIDIWVGAIAEPLLPGAR.V | Y | 101.13 | 2217.1792 | 21 | 6.8 | 1109.6044 | 2 | 133.82 | 9 | 37011 | Ana\_08.raw |  |  |  |  |  |  |  |  | 2.87E6 |  | 1 | 0 | 0 | 0 | 0 | 0 | 0 | 0 | 0 | 1 | 0 | 609 | 629 |  |
| R.ALM(+15.99)FM(+15.99)QWGQFIDHDLDFSPESPAR.V | Y | 100.05 | 2869.2839 | 24 | 4.6 | 957.4397 | 3 | 130.34 | 9 | 36086 | Ana\_08.raw | 1.92E6 |  |  |  |  |  |  |  | 4.66E6 |  | 2 | 1 | 0 | 0 | 0 | 0 | 0 | 0 | 0 | 1 | 0 | 222 | 245 | Oxidation (M) |
| R.TNFLGLLATNQR.F | Y | 88.29 | 1346.7306 | 12 | 4.0 | 674.3752 | 2 | 102.75 | 9 | 28973 | Ana\_08.raw | 1.67E7 |  | 0 | 6.9E4 |  |  |  | 1.27E6 | 5.54E7 | 3.01E7 | 12 | 2 | 0 | 1 | 1 | 0 | 0 | 0 | 1 | 4 | 3 | 330 | 341 |  |
| R.VGPLLAC(+57.02)LFENQFRR.A | Y | 77.30 | 1818.9563 | 15 | 4.9 | 607.3290 | 3 | 126.44 | 1 | 34778 | ANa\_10.raw | 3.85E6 |  |  |  |  |  |  |  | 8.45E6 | 2.46E6 | 3 | 1 | 0 | 0 | 0 | 0 | 0 | 0 | 0 | 1 | 1 | 630 | 644 | Carbamidomethylation |
| R.ANIYPQGFVSC(+57.02)SR.I | Y | 76.75 | 1497.7034 | 13 | 5.7 | 749.8632 | 2 | 70.78 | 1 | 20058 | ANa\_10.raw | 3.49E6 |  |  |  |  |  |  |  | 6.84E6 | 7.01E6 | 3 | 1 | 0 | 0 | 0 | 0 | 0 | 0 | 0 | 1 | 1 | 692 | 704 | Carbamidomethylation |
| R.ASAPNSHVPLSSVFFASWR.I | Y | 74.21 | 2059.0276 | 19 | 3.3 | 687.3521 | 3 | 116.32 | 10 | 31993 | Ana\_09.raw |  |  |  |  |  |  |  |  | 1.42E7 | 1.2E6 | 2 | 0 | 0 | 0 | 0 | 0 | 0 | 0 | 0 | 1 | 1 | 491 | 509 |  |
| R.VANVFTLAFR.F | Y | 71.92 | 1136.6342 | 10 | 4.0 | 569.3266 | 2 | 110.11 | 1 | 30568 | ANa\_10.raw | 8.39E6 |  |  |  |  |  |  |  | 3.39E7 | 1.35E7 | 6 | 2 | 0 | 0 | 0 | 0 | 0 | 0 | 0 | 2 | 2 | 463 | 472 |  |
| R.IVC(+57.02)DNTGITTVSR.D | Y | 71.37 | 1434.7136 | 13 | 5.4 | 718.3680 | 2 | 48.63 | 1 | 13625 | ANa\_10.raw | 1.72E7 |  |  |  |  |  |  |  | 5.25E7 | 2.45E7 | 5 | 1 | 0 | 0 | 0 | 0 | 0 | 0 | 0 | 2 | 2 | 675 | 687 | Carbamidomethylation |
| R.IPC(+57.02)FLAGDTR.S | Y | 65.73 | 1148.5648 | 10 | 5.8 | 575.2930 | 2 | 73.27 | 1 | 20764 | ANa\_10.raw | 1.09E7 |  |  |  |  |  |  |  | 3.43E7 | 1.34E7 | 5 | 2 | 0 | 0 | 0 | 0 | 0 | 0 | 0 | 2 | 1 | 369 | 378 | Carbamidomethylation |
| R.SLPFGWTPGK.R | Y | 64.40 | 1088.5654 | 10 | 2.7 | 545.2914 | 2 | 89.40 | 10 | 25051 | Ana\_09.raw | 1.39E6 |  |  |  |  |  |  |  | 1.09E7 | 1.79E6 | 4 | 1 | 0 | 0 | 0 | 0 | 0 | 0 | 0 | 2 | 1 | 181 | 190 |  |
| R.DFLPLVLGR.A | Y | 62.17 | 1028.6018 | 9 | 6.6 | 515.3116 | 2 | 116.39 | 1 | 32167 | ANa\_10.raw | 4.37E6 |  |  |  |  |  |  |  | 1.29E7 | 1.89E6 | 5 | 2 | 0 | 0 | 0 | 0 | 0 | 0 | 0 | 2 | 1 | 434 | 442 |  |
| R.NGFLLPLVR.D | Y | 61.78 | 1027.6178 | 9 | 4.0 | 514.8182 | 2 | 102.72 | 9 | 28965 | Ana\_08.raw | 3.22E6 |  |  |  |  |  |  |  | 6.19E6 | 3.89E6 | 4 | 1 | 0 | 0 | 0 | 0 | 0 | 0 | 0 | 2 | 1 | 193 | 201 |  |
| R.IIHEGGIDPILR.G | Y | 61.38 | 1331.7561 | 12 | 5.3 | 444.9283 | 3 | 65.19 | 10 | 18475 | Ana\_09.raw | 1.29E7 |  |  |  |  |  |  |  | 2.96E7 | 2.2E7 | 5 | 2 | 0 | 0 | 0 | 0 | 0 | 0 | 0 | 1 | 2 | 510 | 521 |  |
| R.DHGLPGYNAWR.R | Y | 60.25 | 1284.6000 | 11 | 6.7 | 429.2101 | 3 | 63.54 | 9 | 18109 | Ana\_08.raw |  |  |  |  |  |  |  |  | 1.53E6 |  | 1 | 0 | 0 | 0 | 0 | 0 | 0 | 0 | 0 | 1 | 0 | 566 | 576 |  |
| K.LTALHTLFVR.E | Y | 55.52 | 1169.6920 | 10 | 3.5 | 390.9060 | 3 | 70.41 | 9 | 20102 | Ana\_08.raw |  |  |  |  |  |  |  |  | 2.92E6 |  | 1 | 0 | 0 | 0 | 0 | 0 | 0 | 0 | 0 | 1 | 0 | 385 | 394 |  |
| R.DVSNQIVR.F | Y | 51.25 | 929.4930 | 8 | 3.5 | 465.7554 | 2 | 34.86 | 1 | 9436 | ANa\_10.raw | 2.41E7 |  | 1.93E6 |  |  | 1.34E6 |  |  | 5.56E5 | 3.18E7 | 6 | 1 | 0 | 1 | 0 | 0 | 1 | 0 | 0 | 1 | 2 | 202 | 209 |  |
| R.DC(+57.02)IPFFR.S | Y | 47.93 | 953.4429 | 7 | 4.6 | 477.7309 | 2 | 87.23 | 9 | 24786 | Ana\_08.raw | 7.48E6 |  |  |  |  |  |  |  | 2.4E7 | 1.06E7 | 3 | 1 | 0 | 0 | 0 | 0 | 0 | 0 | 0 | 1 | 1 | 281 | 287 | Carbamidomethylation |
| R.WLPAEYEDHR.S | Y | 43.98 | 1314.5992 | 10 | 5.7 | 658.3107 | 2 | 54.61 | 9 | 15453 | Ana\_08.raw |  |  |  |  |  |  |  |  | 1.11E6 |  | 1 | 0 | 0 | 0 | 0 | 0 | 0 | 0 | 0 | 1 | 0 | 171 | 180 |  |
| R.NLAQLSR.V | Y | 43.34 | 800.4504 | 7 | 3.7 | 401.2340 | 2 | 33.06 | 10 | 8981 | Ana\_09.raw |  |  |  |  |  |  |  |  | 1.84E7 | 1.76E7 | 2 | 0 | 0 | 0 | 0 | 0 | 0 | 0 | 0 | 1 | 1 | 586 | 592 |  |
| R.GYC(+57.02)SNVDPR.V | Y | 39.68 | 1066.4502 | 9 | 4.4 | 534.2347 | 2 | 30.01 | 10 | 8076 | Ana\_09.raw | 6.53E6 |  |  |  |  |  |  |  |  | 7.83E6 | 2 | 1 | 0 | 0 | 0 | 0 | 0 | 0 | 0 | 0 | 1 | 454 | 462 | Carbamidomethylation |
| R.IGLDLAALNM(+15.99)QR.S | Y | 38.18 | 1329.7074 | 12 | 4.8 | 665.8641 | 2 | 92.15 | 10 | 25783 | Ana\_09.raw | 8.58E6 |  |  |  |  |  |  |  | 8.79E5 | 6.77E6 | 3 | 1 | 0 | 0 | 0 | 0 | 0 | 0 | 0 | 1 | 1 | 552 | 563 | Oxidation (M) |
| R.LAAELR.R | N | 36.27 | 671.3966 | 6 | 3.8 | 336.7068 | 2 | 26.28 | 9 | 6730 | Ana\_08.raw | 1.49E7 |  |  |  |  |  |  |  | 4.73E6 | 1.71E7 | 3 | 1 | 0 | 0 | 0 | 0 | 0 | 0 | 0 | 1 | 1 | 399 | 404 |  |
| R.TLGPYR.G | Y | 36.06 | 705.3810 | 6 | 3.3 | 353.6989 | 2 | 27.38 | 9 | 7089 | Ana\_08.raw |  |  |  |  |  |  |  |  | 1.37E7 |  | 1 | 0 | 0 | 0 | 0 | 0 | 0 | 0 | 0 | 1 | 0 | 448 | 453 |  |
| K.LNLSAWR.G | Y | 33.77 | 858.4712 | 7 | 3.0 | 430.2442 | 2 | 68.27 | 9 | 19482 | Ana\_08.raw |  |  |  |  |  |  |  |  | 2.64E6 |  | 1 | 0 | 0 | 0 | 0 | 0 | 0 | 0 | 0 | 1 | 0 | 708 | 714 |  |
| R.RFC(+57.02)GLSQPR.N | Y | 31.27 | 1119.5607 | 9 | 0.8 | 560.7881 | 2 | 32.69 | 10 | 8869 | Ana\_09.raw |  |  |  |  |  |  |  |  |  | 6.81E6 | 1 | 0 | 0 | 0 | 0 | 0 | 0 | 0 | 0 | 0 | 1 | 577 | 585 | Carbamidomethylation |
| K.LFQQVR.R | Y | 27.54 | 789.4497 | 6 | 4.7 | 395.7340 | 2 | 32.70 | 9 | 8691 | Ana\_08.raw |  |  |  |  |  |  |  |  | 5.94E6 | 1.47E7 | 2 | 0 | 0 | 0 | 0 | 0 | 0 | 0 | 0 | 1 | 1 | 545 | 550 |  |
| total 27 peptides |  |  |  |  |  |  |  |  |  |  |  |  |  |  |  |  |  |  |  |  |  |  |  |  |  |  |  |  |  |  |  |  |  |  |  |
| --- | --- | --- | --- | --- | --- | --- | --- | --- | --- | --- | --- | --- | --- | --- | --- | --- | --- | --- | --- | --- | --- | --- | --- | --- | --- | --- | --- | --- | --- | --- | --- | --- | --- | --- | --- |

Best Unique PSM (Scan F9:36433, m/z=832.4377, z=2, RT=131.62, ppm=3.5):

sp|P48962|ADT1\_MOUSE

back to list

  

| Protein Coverage
| Supporting Peptides
| Best Unique PSM
|

Protein Coverage:

Supporting Peptides:

| Peptide | Uniq | -10lgP | Mass | Length | ppm | m/z | z | RT | Fraction | Scan | Source File | Area 10 | Area 01 | Area 02 | Area 03 | Area 04 | Area 05 | Area 06 | Area 07 | Area 08 | Area 09 | #Spec | #Spec 10 | #Spec 01 | #Spec 02 | #Spec 03 | #Spec 04 | #Spec 05 | #Spec 06 | #Spec 07 | #Spec 08 | #Spec 09 | Start | End | PTM |
| --- | --- | --- | --- | --- | --- | --- | --- | --- | --- | --- | --- | --- | --- | --- | --- | --- | --- | --- | --- | --- | --- | --- | --- | --- | --- | --- | --- | --- | --- | --- | --- | --- | --- | --- | --- |
| R.YFAGNLASGGAAGATSLC(+57.02)FVYPLDFAR.T | N | 157.78 | 2795.3376 | 27 | 3.3 | 1398.6807 | 2 | 131.60 | 3 | 36052 | Ana\_02.raw | 1.29E7 | 2.53E7 | 1.79E7 | 1.03E7 | 1.42E7 | 2.86E7 | 1.66E7 | 8.85E6 | 1.93E7 | 7.28E6 | 20 | 2 | 2 | 2 | 2 | 2 | 2 | 2 | 2 | 2 | 2 | 112 | 138 | Carbamidomethylation |
| R.YFPTQALNFAFK.D | N | 89.35 | 1445.7343 | 12 | 3.5 | 723.8770 | 2 | 119.53 | 6 | 33368 | Ana\_05.raw | 6.46E7 | 7.52E7 | 7.98E7 | 2.05E7 | 3.74E7 | 4.27E7 | 3.04E7 | 2.87E7 | 1.26E8 | 5.56E7 | 37 | 4 | 5 | 5 | 3 | 2 | 2 | 2 | 4 | 4 | 6 | 81 | 92 |  |
| R.GNLANVIRYFPTQALNFAFK.D | N | 79.39 | 2283.2163 | 20 | 3.8 | 762.0823 | 3 | 132.85 | 6 | 36931 | Ana\_05.raw | 9.41E5 | 4.21E6 | 2.14E6 | 1.68E6 | 2.26E6 | 3.07E6 | 7.45E5 |  |  | 3.85E6 | 8 | 1 | 1 | 1 | 1 | 1 | 1 | 1 | 0 | 0 | 1 | 73 | 92 |  |
| R.GNLANVIRYFPTQALNFAFKDK.Y | N | 74.22 | 2526.3381 | 22 | 3.9 | 632.5943 | 4 | 131.11 | 6 | 36429 | Ana\_05.raw |  |  |  |  |  | 3.07E6 |  |  |  | 1.85E6 | 2 | 0 | 0 | 0 | 0 | 0 | 1 | 0 | 0 | 0 | 1 | 73 | 94 |  |
| R.AAYFGVYDTAK.G | Y | 72.82 | 1204.5764 | 11 | 5.8 | 603.2990 | 2 | 71.87 | 7 | 20378 | Ana\_06.raw | 4.97E6 | 1.19E7 | 1.6E7 | 8.14E6 | 1.39E7 | 1.31E7 | 8.32E6 | 7.99E6 | 8.12E6 | 4.85E6 | 11 | 1 | 1 | 2 | 1 | 1 | 1 | 1 | 1 | 1 | 1 | 189 | 199 |  |
| R.GM(+15.99)GGAFVLVLYDEIKK.Y | N | 63.07 | 1754.9276 | 16 | 3.4 | 585.9851 | 3 | 124.04 | 6 | 34538 | Ana\_05.raw |  | 7.37E5 | 3.63E6 | 1.16E6 | 0 | 2.44E6 | 1.3E6 | 3.79E5 |  | 8.26E5 | 10 | 0 | 1 | 2 | 2 | 1 | 1 | 1 | 1 | 0 | 1 | 281 | 296 | Oxidation (M) |
| K.TAVAPIER.V | N | 59.52 | 855.4814 | 8 | 3.6 | 428.7495 | 2 | 30.28 | 6 | 8211 | Ana\_05.raw | 1.75E8 | 2.05E8 | 2.08E8 | 9.71E7 | 1.41E8 | 1.92E8 | 1.3E8 | 1.07E8 | 1.7E7 | 1.71E8 | 27 | 3 | 3 | 3 | 3 | 3 | 2 | 2 | 4 | 3 | 1 | 24 | 31 |  |
| K.EQGFLSFWR.G | Y | 57.73 | 1168.5665 | 9 | 0.2 | 585.2906 | 2 | 117.61 | 6 | 32858 | Ana\_05.raw | 3.67E6 | 1.09E7 | 2.27E6 | 4.47E6 | 7.66E6 | 8.45E6 | 4.16E6 | 5.42E6 | 6.11E6 | 3.01E6 | 14 | 1 | 2 | 1 | 1 | 2 | 2 | 1 | 2 | 1 | 1 | 64 | 72 |  |
| K.DEGANAFFK.G | Y | 56.71 | 997.4504 | 9 | 6.3 | 499.7356 | 2 | 61.46 | 2 | 17490 | Ana\_01.raw | 1.28E7 | 2E7 | 3.81E7 | 1.71E7 | 1.5E7 | 3.49E7 | 2.23E7 | 2.3E7 |  | 1.12E7 | 9 | 1 | 1 | 1 | 1 | 1 | 1 | 1 | 1 | 0 | 1 | 264 | 272 |  |
| K.DFLAGGIAAAVSK.T | Y | 49.81 | 1218.6608 | 13 | 4.3 | 610.3403 | 2 | 97.04 | 1 | 27181 | ANa\_10.raw | 4.35E6 |  |  |  |  |  |  |  |  |  | 1 | 1 | 0 | 0 | 0 | 0 | 0 | 0 | 0 | 0 | 0 | 11 | 23 |  |
| R.GNLANVIR.Y | N | 48.16 | 855.4926 | 8 | 2.8 | 428.7548 | 2 | 49.59 | 7 | 13906 | Ana\_06.raw | 2.89E7 | 3.37E7 | 4.18E7 | 1.46E7 | 2.59E7 | 2.78E7 | 2.19E7 | 1.67E7 | 5.58E7 | 2.48E7 | 15 | 1 | 2 | 2 | 1 | 2 | 1 | 1 | 1 | 3 | 1 | 73 | 80 |  |
| K.LLLQVQHASK.Q | N | 47.15 | 1135.6713 | 10 | 3.2 | 568.8447 | 2 | 40.37 | 2 | 11173 | Ana\_01.raw | 3.4E7 | 4.34E7 | 4.63E7 | 7.09E6 | 3.58E7 | 4.38E7 | 2.89E7 | 1.02E7 | 8.93E6 | 3.76E7 | 20 | 2 | 2 | 3 | 1 | 3 | 2 | 2 | 1 | 2 | 2 | 34 | 43 |  |
| R.GM(+15.99)GGAFVLVLYDEIK.K | N | 42.54 | 1626.8326 | 15 | 5.4 | 814.4280 | 2 | 130.53 | 5 | 35925 | Ana\_04.raw |  |  |  |  | 9.28E5 |  |  |  |  |  | 1 | 0 | 0 | 0 | 0 | 1 | 0 | 0 | 0 | 0 | 0 | 281 | 295 | Oxidation (M) |
| K.GAWSNVLR.G | N | 42.38 | 901.4770 | 8 | 3.8 | 451.7475 | 2 | 63.04 | 2 | 17954 | Ana\_01.raw | 6.61E6 | 9.17E6 | 1.15E7 |  | 7.43E6 | 6.82E6 | 5.84E6 | 6.65E6 | 4.72E6 | 5.97E6 | 11 | 1 | 1 | 2 | 0 | 1 | 1 | 1 | 2 | 1 | 1 | 273 | 280 |  |
| K.GM(+15.99)LPDPK.N | N | 40.23 | 772.3789 | 7 | 0.9 | 387.1971 | 2 | 25.25 | 3 | 6640 | Ana\_02.raw |  | 7.82E6 | 4.79E7 | 2.45E7 | 2.25E7 | 3.19E7 | 1.57E7 | 2.38E7 | 1.52E7 |  | 9 | 0 | 1 | 1 | 1 | 1 | 2 | 1 | 1 | 1 | 0 | 200 | 206 | Oxidation (M) |
| R.LAADVGK.G | N | 40.15 | 672.3806 | 7 | 0.8 | 337.1978 | 2 | 20.12 | 5 | 5026 | Ana\_04.raw |  |  |  |  | 1.41E7 |  |  |  |  |  | 1 | 0 | 0 | 0 | 0 | 1 | 0 | 0 | 0 | 0 | 0 | 141 | 147 |  |
| K.QIFLGGVDR.H | Y | 37.21 | 1003.5450 | 9 | 0.6 | 502.7801 | 2 | 62.18 | 3 | 17718 | Ana\_02.raw |  |  | 3.91E6 |  | 3.46E6 |  | 1.58E6 |  |  |  | 3 | 0 | 0 | 1 | 0 | 1 | 0 | 1 | 0 | 0 | 0 | 97 | 105 |  |
| total 17 peptides |  |  |  |  |  |  |  |  |  |  |  |  |  |  |  |  |  |  |  |  |  |  |  |  |  |  |  |  |  |  |  |  |  |  |  |
| --- | --- | --- | --- | --- | --- | --- | --- | --- | --- | --- | --- | --- | --- | --- | --- | --- | --- | --- | --- | --- | --- | --- | --- | --- | --- | --- | --- | --- | --- | --- | --- | --- | --- | --- | --- |

Best Unique PSM (Scan F7:20378, m/z=603.2990, z=2, RT=71.87, ppm=5.8):

sp|Q00896|A1AT3\_MOUSE

back to list

  

| Protein Coverage
| Supporting Peptides
| Best Unique PSM
|

Protein Coverage:

Supporting Peptides:

| Peptide | Uniq | -10lgP | Mass | Length | ppm | m/z | z | RT | Fraction | Scan | Source File | Area 10 | Area 01 | Area 02 | Area 03 | Area 04 | Area 05 | Area 06 | Area 07 | Area 08 | Area 09 | #Spec | #Spec 10 | #Spec 01 | #Spec 02 | #Spec 03 | #Spec 04 | #Spec 05 | #Spec 06 | #Spec 07 | #Spec 08 | #Spec 09 | Start | End | PTM |
| --- | --- | --- | --- | --- | --- | --- | --- | --- | --- | --- | --- | --- | --- | --- | --- | --- | --- | --- | --- | --- | --- | --- | --- | --- | --- | --- | --- | --- | --- | --- | --- | --- | --- | --- | --- |
| K.DQSPASHEIATNLGDFAISLYR.E | N | 133.04 | 2404.1658 | 22 | 2.8 | 802.3981 | 3 | 126.74 | 3 | 34760 | Ana\_02.raw | 1.72E7 | 2.56E6 | 3.11E7 | 6.37E6 | 1.68E7 | 3.02E7 | 1.97E7 | 9.58E6 | 0 | 1.26E7 | 15 | 2 | 2 | 1 | 2 | 1 | 1 | 2 | 2 | 1 | 1 | 36 | 57 |  |
| K.SFQHLLQTLNRPDSELQLSTGNGLFVNNDLK.L | N | 129.80 | 3497.7903 | 31 | -1.3 | 875.4537 | 4 | 124.34 | 3 | 34149 | Ana\_02.raw |  |  | 1.92E7 | 7.14E6 |  | 8.97E6 | 1.67E7 | 1.48E7 |  |  | 7 | 0 | 0 | 1 | 2 | 0 | 1 | 1 | 2 | 0 | 0 | 113 | 143 |  |
| K.KLDQDTVFALANYILFK.G | N | 120.74 | 1998.0825 | 17 | -0.1 | 1000.0484 | 2 | 135.43 | 3 | 37052 | Ana\_02.raw | 6.31E6 | 3.74E6 | 5.83E6 | 1.95E7 | 1.37E7 | 1.27E7 | 9.22E6 | 2.6E6 |  | 1.09E7 | 18 | 2 | 2 | 3 | 2 | 2 | 1 | 2 | 2 | 0 | 2 | 193 | 209 |  |
| R.FDHPFLFIIFEEHTQSPLFVGK.V | N | 106.71 | 2647.3474 | 22 | 2.8 | 662.8460 | 4 | 132.43 | 6 | 36814 | Ana\_05.raw |  |  | 4.89E6 | 4.4E6 | 1.19E6 | 2.36E6 | 2.12E6 |  |  |  | 7 | 0 | 0 | 2 | 2 | 1 | 1 | 1 | 0 | 0 | 0 | 385 | 406 |  |
| K.M(+15.99)QHLEQTLSK.E | Y | 62.97 | 1229.6074 | 10 | 2.3 | 615.8124 | 2 | 27.28 | 6 | 7273 | Ana\_05.raw |  |  |  |  | 8.2E5 | 2.84E6 | 2.66E6 | 2.36E6 |  |  | 4 | 0 | 0 | 0 | 0 | 1 | 1 | 1 | 1 | 0 | 0 | 278 | 287 | Oxidation (M) |
| K.TLM(+15.99)SPLGITR.I | N | 50.49 | 1103.6008 | 10 | 4.5 | 552.8102 | 2 | 67.36 | 8 | 18917 | Ana\_07.raw |  |  | 1.72E7 | 3.32E6 | 3.23E6 | 1.2E6 | 4.92E6 | 7.58E6 |  |  | 6 | 0 | 0 | 1 | 1 | 1 | 1 | 1 | 1 | 0 | 0 | 319 | 328 | Oxidation (M) |
| R.LSISGEYNLK.T | Y | 50.18 | 1122.5920 | 10 | 4.1 | 562.3056 | 2 | 67.45 | 1 | 19120 | ANa\_10.raw | 1.46E6 | 1.57E6 |  | 1.28E6 | 3.64E5 | 1.16E6 | 1.98E6 | 9.87E5 |  |  | 7 | 1 | 1 | 0 | 1 | 1 | 1 | 1 | 1 | 0 | 0 | 309 | 318 |  |
| R.LAQIHFPR.L | Y | 49.23 | 980.5555 | 8 | 1.2 | 491.2856 | 2 | 56.05 | 3 | 15911 | Ana\_02.raw | 2.09E6 |  | 4.07E6 | 7.14E5 | 7.36E5 |  |  |  |  |  | 5 | 1 | 0 | 2 | 1 | 1 | 0 | 0 | 0 | 0 | 0 | 301 | 308 |  |
| K.KVINDFVEK.G | N | 48.83 | 1090.6022 | 9 | 4.5 | 546.3108 | 2 | 41.87 | 6 | 11729 | Ana\_05.raw |  |  | 6.07E6 |  |  | 4.04E6 |  | 2.35E6 |  |  | 3 | 0 | 0 | 1 | 0 | 0 | 1 | 0 | 1 | 0 | 0 | 173 | 181 |  |
| K.VINDFVEK.G | N | 48.25 | 962.5073 | 8 | 6.2 | 482.2639 | 2 | 46.91 | 2 | 13186 | Ana\_01.raw | 2.13E7 | 2.23E7 | 3.02E7 | 9.36E6 | 1.79E7 | 2.5E7 | 1.8E7 | 1.48E7 | 9.49E6 | 1.4E7 | 11 | 1 | 1 | 1 | 2 | 1 | 1 | 1 | 1 | 1 | 1 | 174 | 181 |  |
| K.LDQDTVFALANYILFK.G | N | 36.93 | 1869.9875 | 16 | 4.5 | 936.0052 | 2 | 138.35 | 4 | 37487 | Ana\_03.raw |  |  |  | 2.83E5 |  |  |  |  |  |  | 1 | 0 | 0 | 0 | 1 | 0 | 0 | 0 | 0 | 0 | 0 | 194 | 209 |  |
| K.FLEEAK.N | N | 33.46 | 735.3802 | 6 | 3.6 | 368.6987 | 2 | 27.77 | 2 | 7326 | Ana\_01.raw | 2.24E7 | 2.72E7 |  | 9.67E6 | 1.71E7 | 2.36E7 | 1.33E7 |  | 8.14E6 | 1.4E7 | 8 | 1 | 1 | 0 | 1 | 1 | 1 | 1 | 0 | 1 | 1 | 148 | 153 |  |
| K.IVEAVK.K | N | 30.08 | 657.4061 | 6 | 2.4 | 329.7111 | 2 | 20.74 | 5 | 5220 | Ana\_04.raw |  |  |  | 2.57E6 | 5.68E6 |  | 2.93E6 |  |  |  | 3 | 0 | 0 | 0 | 1 | 1 | 0 | 1 | 0 | 0 | 0 | 187 | 192 |  |
| total 13 peptides |  |  |  |  |  |  |  |  |  |  |  |  |  |  |  |  |  |  |  |  |  |  |  |  |  |  |  |  |  |  |  |  |  |  |  |
| --- | --- | --- | --- | --- | --- | --- | --- | --- | --- | --- | --- | --- | --- | --- | --- | --- | --- | --- | --- | --- | --- | --- | --- | --- | --- | --- | --- | --- | --- | --- | --- | --- | --- | --- | --- |

Best Unique PSM (Scan F6:7273, m/z=615.8124, z=2, RT=27.28, ppm=2.3):

sp|Q3V0K9|PLSI\_MOUSE

back to list

  

| Protein Coverage
| Supporting Peptides
| Best Unique PSM
|

Protein Coverage:

Supporting Peptides:

| Peptide | Uniq | -10lgP | Mass | Length | ppm | m/z | z | RT | Fraction | Scan | Source File | Area 10 | Area 01 | Area 02 | Area 03 | Area 04 | Area 05 | Area 06 | Area 07 | Area 08 | Area 09 | #Spec | #Spec 10 | #Spec 01 | #Spec 02 | #Spec 03 | #Spec 04 | #Spec 05 | #Spec 06 | #Spec 07 | #Spec 08 | #Spec 09 | Start | End | PTM |
| --- | --- | --- | --- | --- | --- | --- | --- | --- | --- | --- | --- | --- | --- | --- | --- | --- | --- | --- | --- | --- | --- | --- | --- | --- | --- | --- | --- | --- | --- | --- | --- | --- | --- | --- | --- |
| K.IDIDNSGYVSDYELQDLFK.E | Y | 108.61 | 2233.0425 | 19 | -1.4 | 1117.5270 | 2 | 129.14 | 6 | 35869 | Ana\_05.raw | 2.32E6 | 4.64E6 | 6.34E6 | 2.03E6 | 4.3E6 | 7.01E6 | 5.34E6 | 1.13E6 | 1.05E7 | 4.31E6 | 10 | 1 | 1 | 1 | 1 | 1 | 1 | 1 | 1 | 1 | 1 | 23 | 41 |  |
| R.EGITAIGGTSSISSEGTQHSYSEEEK.V | Y | 104.43 | 2683.2097 | 26 | 6.1 | 895.4160 | 3 | 66.47 | 6 | 19084 | Ana\_05.raw |  | 1.51E7 | 3.19E6 |  | 6.47E6 | 9.75E6 | 7.42E6 |  | 1.36E7 | 5.33E6 | 7 | 0 | 1 | 1 | 0 | 1 | 1 | 1 | 0 | 1 | 1 | 100 | 125 |  |
| K.SISTSLPVLDLIDAIAPNAVR.Q | Y | 99.27 | 2164.2102 | 21 | 3.5 | 1083.1162 | 2 | 138.54 | 7 | 38090 | Ana\_06.raw | 3.85E6 | 2.61E6 | 1.48E6 |  | 1.21E6 | 1.29E7 | 4.73E6 | 1.12E6 | 3.69E6 | 6.8E5 | 19 | 2 | 3 | 2 | 0 | 2 | 3 | 2 | 2 | 2 | 1 | 547 | 567 |  |
| R.EHLTDEDKLNNAK.Y | Y | 83.97 | 1525.7372 | 13 | 4.6 | 763.8794 | 2 | 27.39 | 2 | 7206 | Ana\_01.raw | 4.87E6 | 1.78E7 | 1.03E7 |  | 8.91E6 | 1.17E7 | 6.9E6 | 3.73E6 | 4.16E6 | 5.12E6 | 10 | 1 | 2 | 1 | 0 | 1 | 1 | 1 | 1 | 1 | 1 | 574 | 586 |  |
| R.EELEELQEAFNK.I | Y | 75.12 | 1477.6936 | 12 | 6.5 | 739.8589 | 2 | 93.18 | 2 | 26327 | Ana\_01.raw | 6.75E6 | 1.32E7 | 8.96E5 | 4.04E6 | 8.2E6 | 9.09E6 | 1.74E6 | 3E6 | 2.58E6 | 7.97E6 | 11 | 1 | 1 | 1 | 1 | 1 | 1 | 1 | 1 | 2 | 1 | 11 | 22 |  |
| K.ISFEEFVSLM(+15.99)QELK.S | Y | 71.85 | 1714.8488 | 14 | 1.1 | 858.4326 | 2 | 129.65 | 6 | 36018 | Ana\_05.raw |  | 3E6 |  |  |  | 4.1E6 | 4.01E6 |  |  |  | 3 | 0 | 1 | 0 | 0 | 0 | 1 | 1 | 0 | 0 | 0 | 71 | 84 | Oxidation (M) |
| K.VLSDLGEGEK.V | Y | 71.20 | 1045.5291 | 10 | 4.1 | 523.7739 | 2 | 40.60 | 6 | 11342 | Ana\_05.raw |  | 3.69E7 | 1.4E7 | 7.02E6 | 1.27E7 | 1.7E7 | 1.15E7 | 7.47E6 | 3.43E6 |  | 8 | 0 | 1 | 1 | 1 | 1 | 1 | 1 | 1 | 1 | 0 | 508 | 517 |  |
| K.VAFVNWINK.A | Y | 69.32 | 1089.5970 | 9 | 3.7 | 545.8078 | 2 | 92.62 | 6 | 26285 | Ana\_05.raw | 1.57E6 | 6.9E6 | 1.92E6 | 2.27E6 | 3.91E6 | 5.78E6 | 3.62E6 | 2.05E6 | 6.76E6 | 1.67E6 | 10 | 1 | 1 | 1 | 1 | 1 | 1 | 1 | 1 | 1 | 1 | 126 | 134 |  |
| K.EASLPLPGYK.V | Y | 68.34 | 1073.5757 | 10 | 2.3 | 537.7963 | 2 | 68.22 | 3 | 19398 | Ana\_02.raw |  |  | 3.4E6 | 2.79E6 | 4.91E6 | 4.46E6 | 3.67E6 |  | 2.47E6 | 9.09E4 | 8 | 0 | 0 | 1 | 1 | 2 | 1 | 1 | 0 | 1 | 1 | 42 | 51 |  |
| R.IYALPDDLVEVKPK.M | Y | 65.89 | 1598.8918 | 14 | 4.7 | 533.9738 | 3 | 90.55 | 6 | 25730 | Ana\_05.raw | 6.98E5 | 8.3E5 | 7.82E5 | 7.6E5 | 4.47E6 | 4.06E6 | 4.29E6 | 2.55E6 | 9.95E6 | 7.38E5 | 10 | 1 | 1 | 1 | 1 | 1 | 1 | 1 | 1 | 1 | 1 | 599 | 612 |  |
| R.TINNFSQDIK.D | Y | 65.77 | 1178.5931 | 10 | 1.4 | 590.3047 | 2 | 57.07 | 6 | 16307 | Ana\_05.raw |  | 1.49E7 | 1.11E7 | 6.31E6 | 9.55E6 | 1.18E7 | 9.3E6 | 0 | 1.12E7 |  | 9 | 0 | 1 | 1 | 1 | 1 | 1 | 2 | 1 | 1 | 0 | 287 | 296 |  |
| K.LSPEELLLR.W | N | 65.62 | 1068.6179 | 9 | 0.1 | 535.3163 | 2 | 96.12 | 3 | 26879 | Ana\_02.raw | 4.02E7 | 3.83E7 | 4.76E7 | 1.8E7 | 3.44E7 | 2.99E7 | 2.46E7 | 2.06E7 | 5.59E7 | 3.12E7 | 28 | 3 | 4 | 3 | 2 | 3 | 2 | 2 | 3 | 4 | 2 | 266 | 274 |  |
| K.M(+15.99)INLSEPDTIDER.A | Y | 62.27 | 1547.7137 | 13 | 5.5 | 774.8684 | 2 | 69.50 | 6 | 19939 | Ana\_05.raw |  | 1.92E6 | 2.79E6 |  | 4.48E6 | 4.42E6 | 3.41E6 | 9.66E5 |  |  | 6 | 0 | 1 | 1 | 0 | 1 | 1 | 1 | 1 | 0 | 0 | 168 | 180 | Oxidation (M) |
| K.IENC(+57.02)NYAVELGK.N | N | 57.83 | 1408.6656 | 12 | 3.3 | 705.3424 | 2 | 57.88 | 9 | 16420 | Ana\_08.raw |  |  |  |  |  |  |  |  | 1.71E6 |  | 1 | 0 | 0 | 0 | 0 | 0 | 0 | 0 | 0 | 1 | 0 | 459 | 470 | Carbamidomethylation |
| K.AYFHLLNQIAPK.G | N | 55.23 | 1413.7769 | 12 | 2.2 | 472.2673 | 3 | 91.09 | 7 | 25586 | Ana\_06.raw | 1.74E5 |  | 5.06E5 | 9.16E5 |  | 1.72E6 | 3.56E6 |  |  |  | 5 | 1 | 0 | 1 | 1 | 0 | 1 | 1 | 0 | 0 | 0 | 300 | 311 |  |
| K.ILVVADNNK.D | Y | 53.47 | 984.5604 | 9 | 3.1 | 493.2890 | 2 | 36.54 | 6 | 10092 | Ana\_05.raw |  | 4.62E6 | 5.54E6 | 2.16E6 | 3.9E6 | 5.43E6 |  | 2.13E6 |  |  | 6 | 0 | 1 | 1 | 1 | 1 | 1 | 0 | 1 | 0 | 0 | 59 | 67 |  |
| R.NEALIALLK.D | Y | 52.44 | 983.6015 | 9 | 4.7 | 492.8103 | 2 | 96.05 | 6 | 27204 | Ana\_05.raw | 2.28E6 | 8.57E6 | 1E7 | 4.06E6 | 4.88E6 | 7.48E6 | 6.37E6 | 3.65E6 | 4.97E6 | 3.04E6 | 12 | 1 | 1 | 2 | 2 | 1 | 1 | 1 | 1 | 1 | 1 | 247 | 255 |  |
| K.SLADGILLC(+57.02)K.M | Y | 49.05 | 1088.5900 | 10 | 0.8 | 545.3027 | 2 | 83.10 | 6 | 23714 | Ana\_05.raw |  | 2.6E6 | 1.46E6 | 4.25E5 |  | 3.46E6 | 1.44E6 | 3.4E5 | 1.8E6 |  | 7 | 0 | 1 | 1 | 1 | 0 | 1 | 1 | 1 | 1 | 0 | 158 | 167 | Carbamidomethylation |
| R.AGFM(+15.99)LQEADK.L | Y | 48.00 | 1124.5172 | 10 | 5.7 | 563.2691 | 2 | 52.75 | 8 | 14653 | Ana\_07.raw | 4.29E6 | 9.65E6 | 9.17E6 | 4.42E6 | 8.34E6 | 9.33E6 | 7.2E6 | 3.59E6 | 6.87E6 |  | 11 | 1 | 1 | 1 | 1 | 1 | 2 | 2 | 1 | 1 | 0 | 336 | 345 | Oxidation (M) |
| K.VTDDIIIK.W | Y | 45.78 | 915.5276 | 8 | 2.0 | 458.7720 | 2 | 55.20 | 4 | 15284 | Ana\_03.raw |  | 1.24E7 | 8.71E6 | 5.91E6 |  |  |  |  | 1.26E7 | 5.35E6 | 5 | 0 | 1 | 1 | 1 | 0 | 0 | 0 | 0 | 1 | 1 | 518 | 525 |  |
| K.YAISVAR.K | Y | 44.85 | 778.4337 | 7 | 4.7 | 390.2260 | 2 | 42.48 | 2 | 11820 | Ana\_01.raw |  | 8.93E6 | 1.12E6 | 5.07E6 | 7.17E6 | 1.09E7 | 7.64E6 |  | 6.33E6 | 2.78E6 | 8 | 0 | 1 | 1 | 1 | 1 | 1 | 1 | 0 | 1 | 1 | 587 | 593 |  |
| K.STSISSFK.D | Y | 39.84 | 855.4338 | 8 | 6.9 | 428.7271 | 2 | 34.34 | 2 | 9317 | Ana\_01.raw | 1.72E7 | 1.51E7 | 1.1E7 |  | 1.11E7 | 1.44E7 | 1.19E7 | 6.78E6 |  | 1.04E7 | 8 | 1 | 1 | 1 | 0 | 1 | 1 | 1 | 1 | 0 | 1 | 537 | 544 |  |
| K.WVNQTLK.S | Y | 23.17 | 887.4865 | 7 | 5.5 | 444.7530 | 2 | 38.83 | 5 | 10742 | Ana\_04.raw |  |  |  |  | 8.27E6 |  |  |  |  |  | 1 | 0 | 0 | 0 | 0 | 1 | 0 | 0 | 0 | 0 | 0 | 526 | 532 |  |
| R.NEALIALLKDGEDLEELM(+15.99)K.L | Y | 22.76 | 2159.1030 | 19 | 4.3 | 720.7114 | 3 | 121.14 | 6 | 33784 | Ana\_05.raw |  |  |  |  |  | 4.62E5 |  |  |  |  | 1 | 0 | 0 | 0 | 0 | 0 | 1 | 0 | 0 | 0 | 0 | 247 | 265 | Oxidation (M) |
| total 24 peptides |  |  |  |  |  |  |  |  |  |  |  |  |  |  |  |  |  |  |  |  |  |  |  |  |  |  |  |  |  |  |  |  |  |  |  |
| --- | --- | --- | --- | --- | --- | --- | --- | --- | --- | --- | --- | --- | --- | --- | --- | --- | --- | --- | --- | --- | --- | --- | --- | --- | --- | --- | --- | --- | --- | --- | --- | --- | --- | --- | --- |

Best Unique PSM (Scan F6:35869, m/z=1117.5270, z=2, RT=129.14, ppm=-1.4):

E9QA15|E9QA15\_MOUSE

back to list

  

| Protein Coverage
| Supporting Peptides
| Best Unique PSM
|

Protein Coverage:

Supporting Peptides:

| Peptide | Uniq | -10lgP | Mass | Length | ppm | m/z | z | RT | Fraction | Scan | Source File | Area 10 | Area 01 | Area 02 | Area 03 | Area 04 | Area 05 | Area 06 | Area 07 | Area 08 | Area 09 | #Spec | #Spec 10 | #Spec 01 | #Spec 02 | #Spec 03 | #Spec 04 | #Spec 05 | #Spec 06 | #Spec 07 | #Spec 08 | #Spec 09 | Start | End | PTM |
| --- | --- | --- | --- | --- | --- | --- | --- | --- | --- | --- | --- | --- | --- | --- | --- | --- | --- | --- | --- | --- | --- | --- | --- | --- | --- | --- | --- | --- | --- | --- | --- | --- | --- | --- | --- |
| R.ASGDKEAEGAPQVEAGK.R | Y | 94.44 | 1642.7798 | 17 | 6.9 | 822.4028 | 2 | 24.49 | 9 | 6129 | Ana\_08.raw |  | 4.59E6 | 3.97E6 |  |  | 4.38E6 | 1.15E6 | 1.24E6 | 1.63E7 | 8.37E6 | 7 | 0 | 1 | 1 | 0 | 0 | 1 | 1 | 1 | 1 | 1 | 494 | 510 |  |
| R.M(+15.99)QNDSAENETAEGEEKR.E | Y | 94.29 | 1952.8018 | 17 | 0.0 | 651.9412 | 3 | 21.54 | 3 | 5454 | Ana\_02.raw | 2.12E7 | 1.55E7 | 9.49E6 | 3.94E6 | 4.45E6 | 7.52E6 | 5.32E6 | 4.89E6 | 2.14E7 | 1.22E7 | 16 | 2 | 2 | 1 | 1 | 2 | 1 | 1 | 1 | 3 | 2 | 137 | 153 | Oxidation (M) |
| K.EFDPTITDGSLSGPSR.R | Y | 90.60 | 1677.7845 | 16 | 1.8 | 839.9011 | 2 | 74.19 | 6 | 21288 | Ana\_05.raw | 2.33E7 | 2.15E7 | 5.87E6 | 8.04E6 | 1.1E7 | 1.56E7 | 4.23E6 | 1.29E7 | 3.92E7 | 1.33E7 | 20 | 2 | 2 | 2 | 2 | 2 | 2 | 1 | 2 | 3 | 2 | 120 | 135 |  |
| R.ASGDKEAEGAPQVEAGKR.L | Y | 86.02 | 1798.8809 | 18 | 7.2 | 900.4542 | 2 | 24.04 | 1 | 6114 | ANa\_10.raw | 1.78E6 |  |  |  |  |  |  |  | 7.53E6 |  | 2 | 1 | 0 | 0 | 0 | 0 | 0 | 0 | 0 | 1 | 0 | 494 | 511 |  |
| K.GSVFSAPSASGTPNK.E | Y | 76.72 | 1405.6837 | 15 | 2.5 | 703.8509 | 2 | 49.92 | 6 | 14201 | Ana\_05.raw | 3.73E7 | 3.09E7 | 2.57E7 | 1.12E7 | 2.22E7 | 2.85E7 | 1.46E7 | 1.68E7 | 5.72E7 | 2.18E7 | 17 | 2 | 1 | 1 | 2 | 2 | 2 | 1 | 2 | 3 | 1 | 694 | 708 |  |
| R.LEQYTNAIEGTK.A | Y | 75.43 | 1365.6776 | 12 | 1.1 | 683.8469 | 2 | 52.33 | 3 | 14821 | Ana\_02.raw | 1.93E7 | 1.65E7 | 1.37E7 | 2.18E6 | 7.05E6 | 1.33E7 | 8.51E6 | 8.28E6 | 4.07E7 | 1.88E7 | 11 | 1 | 1 | 1 | 1 | 1 | 1 | 1 | 1 | 2 | 1 | 654 | 665 |  |
| K.IEGDGEQDM(+15.99)EEIAR.R | Y | 73.91 | 1606.6780 | 14 | 2.8 | 804.3485 | 2 | 42.27 | 6 | 11854 | Ana\_05.raw | 2.32E7 | 2.11E7 | 1.86E7 | 4.04E6 | 7.23E6 | 7.65E6 | 4.86E6 | 6.46E6 | 1.95E7 | 1.9E7 | 16 | 2 | 2 | 2 | 2 | 1 | 1 | 1 | 1 | 2 | 2 | 238 | 251 | Oxidation (M) |
| K.ASKPM(+15.99)KPAASDLPVPAEGVR.N | Y | 69.68 | 2036.0724 | 20 | 4.6 | 679.7012 | 3 | 51.02 | 1 | 14319 | ANa\_10.raw | 1.68E7 |  |  |  | 5.59E6 | 2E7 | 3.39E6 | 4.01E6 | 2.78E7 | 2.34E7 | 9 | 1 | 0 | 0 | 0 | 1 | 2 | 1 | 1 | 1 | 2 | 666 | 685 | Oxidation (M) |
| R.RGETENEEFEK.L | Y | 69.52 | 1366.6001 | 11 | 6.9 | 456.5438 | 3 | 23.73 | 1 | 6013 | ANa\_10.raw | 1.17E7 | 8.56E6 | 2.1E7 | 7.76E6 |  | 2.13E7 | 6.31E6 | 1.35E6 | 1.47E7 | 2.49E7 | 13 | 1 | 1 | 2 | 2 | 0 | 2 | 1 | 1 | 1 | 2 | 519 | 529 |  |
| K.EAEGAPQVEAGK.R | Y | 69.17 | 1184.5673 | 12 | 9.0 | 593.2963 | 2 | 25.12 | 9 | 6346 | Ana\_08.raw | 5E6 | 1.38E7 | 8.67E6 |  | 9.35E6 | 1.35E7 | 6.43E6 | 3.16E6 | 1.69E7 | 1.54E6 | 9 | 1 | 1 | 1 | 0 | 1 | 1 | 1 | 1 | 1 | 1 | 499 | 510 |  |
| R.YEVEETEVVIK.S | Y | 66.47 | 1336.6761 | 11 | 4.5 | 669.3483 | 2 | 70.07 | 6 | 20106 | Ana\_05.raw | 5.03E6 | 5.06E5 |  | 9.79E5 | 1.08E6 | 3.84E6 |  |  |  |  | 5 | 1 | 1 | 0 | 1 | 1 | 1 | 0 | 0 | 0 | 0 | 160 | 170 |  |
| R.LEVEGQEQER.I | Y | 64.47 | 1215.5731 | 10 | 1.1 | 608.7945 | 2 | 28.15 | 3 | 7563 | Ana\_02.raw | 3.82E7 | 3.3E7 | 1.38E7 | 1.23E7 | 2.14E7 | 3.05E7 | 1.39E7 | 2.21E7 |  | 3.62E7 | 9 | 1 | 1 | 1 | 1 | 1 | 1 | 1 | 1 | 0 | 1 | 270 | 279 |  |
| K.VLEEEEQR.R | Y | 63.77 | 1030.4930 | 8 | 5.5 | 516.2567 | 2 | 24.42 | 1 | 6240 | ANa\_10.raw | 4.55E7 |  | 2.62E7 | 1.4E7 | 2.36E7 | 3.3E7 | 1.88E7 | 1.47E7 | 6.16E6 | 3.88E7 | 9 | 1 | 0 | 1 | 1 | 1 | 1 | 1 | 1 | 1 | 1 | 554 | 561 |  |
| R.KVLEEEEQR.R | Y | 60.11 | 1158.5880 | 9 | 1.1 | 580.3019 | 2 | 20.28 | 3 | 5031 | Ana\_02.raw | 4.71E6 | 4.13E6 | 2.66E6 | 6.22E5 |  | 1.98E6 | 1.29E6 | 1.63E6 | 5.74E6 | 4.16E6 | 9 | 1 | 1 | 1 | 1 | 0 | 1 | 1 | 1 | 1 | 1 | 553 | 561 |  |
| K.EAEGAPQVEAGKR.L | Y | 57.78 | 1340.6683 | 13 | 8.4 | 671.3471 | 2 | 23.59 | 10 | 6029 | Ana\_09.raw | 4.77E5 |  |  |  |  |  |  |  | 3.19E6 | 8.71E5 | 3 | 1 | 0 | 0 | 0 | 0 | 0 | 0 | 0 | 1 | 1 | 499 | 511 |  |
| K.GGSLGENQVEK.M | Y | 57.43 | 1116.5411 | 11 | 2.5 | 559.2792 | 2 | 25.25 | 5 | 6630 | Ana\_04.raw | 1.64E7 |  |  | 9.5E6 | 1.41E7 |  |  | 1.36E7 | 2.86E7 | 2.46E7 | 6 | 1 | 0 | 0 | 1 | 1 | 0 | 0 | 1 | 1 | 1 | 198 | 208 |  |
| R.RM(+15.99)QNDSAENETAEGEEKR.E | Y | 56.63 | 2108.9028 | 18 | 0.9 | 703.9755 | 3 | 20.62 | 10 | 5027 | Ana\_09.raw |  |  |  |  |  |  |  |  |  | 9.49E5 | 1 | 0 | 0 | 0 | 0 | 0 | 0 | 0 | 0 | 0 | 1 | 136 | 153 | Oxidation (M) |
| R.LRLEVEGQEQER.I | Y | 53.36 | 1484.7583 | 12 | 3.8 | 495.9286 | 3 | 47.69 | 6 | 13505 | Ana\_05.raw |  |  |  |  |  | 3.78E6 |  | 2.15E6 |  |  | 2 | 0 | 0 | 0 | 0 | 0 | 1 | 0 | 1 | 0 | 0 | 268 | 279 |  |
| R.LKEEIER.R | Y | 48.93 | 915.5025 | 7 | 4.1 | 306.1760 | 3 | 22.64 | 9 | 5512 | Ana\_08.raw | 3.39E7 | 1.33E7 | 2.04E7 | 7.47E6 | 7.23E6 | 2.26E7 | 3.07E6 | 1.27E6 | 1.43E7 | 3.49E7 | 17 | 2 | 2 | 2 | 2 | 1 | 2 | 1 | 1 | 2 | 2 | 580 | 586 |  |
| K.VLEEEEQRR.K | Y | 48.77 | 1186.5941 | 9 | 4.3 | 594.3069 | 2 | 22.43 | 9 | 5435 | Ana\_08.raw | 1.07E7 |  |  |  |  |  |  |  | 1.71E7 | 4.59E6 | 5 | 2 | 0 | 0 | 0 | 0 | 0 | 0 | 0 | 2 | 1 | 554 | 562 |  |
| R.EEM(+15.99)RLEAER.I | Y | 46.91 | 1177.5397 | 9 | 3.5 | 589.7792 | 2 | 21.88 | 9 | 5247 | Ana\_08.raw | 7.05E6 | 4.4E6 |  |  | 2.95E6 | 2.8E6 |  |  | 1.46E7 |  | 6 | 1 | 1 | 0 | 0 | 1 | 1 | 0 | 0 | 2 | 0 | 16 | 24 | Oxidation (M) |
| R.INEWLTK.S | Y | 45.01 | 902.4861 | 7 | 4.6 | 452.2524 | 2 | 51.14 | 9 | 14402 | Ana\_08.raw |  | 2.81E6 | 2.24E6 | 1.62E6 |  |  |  |  | 9.27E6 | 3.27E6 | 5 | 0 | 1 | 1 | 1 | 0 | 0 | 0 | 0 | 1 | 1 | 721 | 727 |  |
| R.DDKPAFR.K | Y | 44.05 | 847.4188 | 7 | 4.7 | 424.7187 | 2 | 22.52 | 1 | 5605 | ANa\_10.raw | 9.47E6 |  | 2.16E7 | 6.43E6 |  |  |  | 5.6E6 |  | 1.59E7 | 5 | 1 | 0 | 1 | 1 | 0 | 0 | 0 | 1 | 0 | 1 | 430 | 436 |  |
| K.RLQEALER.Q | Y | 43.91 | 1013.5618 | 8 | 9.1 | 507.7928 | 2 | 26.82 | 2 | 7020 | Ana\_01.raw | 5.42E6 | 3.71E6 | 2.22E6 |  |  |  |  |  | 4.83E6 | 4.37E6 | 5 | 1 | 1 | 1 | 0 | 0 | 0 | 0 | 0 | 1 | 1 | 110 | 117 |  |
| R.GETENEEFEK.L | Y | 43.75 | 1210.4989 | 10 | 3.1 | 606.2586 | 2 | 29.97 | 4 | 7874 | Ana\_03.raw |  |  |  | 5.43E6 |  |  |  |  |  |  | 1 | 0 | 0 | 0 | 1 | 0 | 0 | 0 | 0 | 0 | 0 | 520 | 529 |  |
| R.LQEALER.Q | Y | 41.69 | 857.4606 | 7 | 3.5 | 429.7391 | 2 | 30.70 | 6 | 8345 | Ana\_05.raw |  | 3.87E7 |  | 1.32E7 |  | 3.67E7 | 1.78E7 | 1.9E7 |  | 4.85E7 | 6 | 0 | 1 | 0 | 1 | 0 | 1 | 1 | 1 | 0 | 1 | 111 | 117 |  |
| K.VTSPTKV | Y | 39.77 | 730.4225 | 7 | 5.3 | 366.2205 | 2 | 22.97 | 1 | 5757 | ANa\_10.raw | 6.12E6 |  | 8.04E6 |  |  |  | 4.33E6 | 5.98E5 |  | 8.88E6 | 5 | 1 | 0 | 1 | 0 | 0 | 0 | 1 | 1 | 0 | 1 | 762 | 768 |  |
| K.KGFTEVK.A | Y | 39.51 | 807.4490 | 7 | 2.8 | 404.7329 | 2 | 20.98 | 6 | 5250 | Ana\_05.raw |  |  | 4.45E6 |  |  | 4E6 |  |  |  | 5.69E6 | 3 | 0 | 0 | 1 | 0 | 0 | 1 | 0 | 0 | 0 | 1 | 463 | 469 |  |
| R.AEFLNK.S | Y | 39.13 | 720.3806 | 6 | 3.8 | 361.1989 | 2 | 27.16 | 9 | 7018 | Ana\_08.raw | 1.99E7 |  | 1.78E7 | 7.97E6 | 1.47E7 | 1.9E7 | 8.15E6 |  | 8.01E5 | 1.8E7 | 8 | 1 | 0 | 1 | 1 | 1 | 1 | 1 | 0 | 1 | 1 | 626 | 631 |  |
| K.RLEELR.R | N | 37.48 | 814.4661 | 6 | 4.2 | 408.2420 | 2 | 23.06 | 9 | 5653 | Ana\_08.raw | 2.19E7 |  |  |  |  |  |  |  | 1.74E7 |  | 2 | 1 | 0 | 0 | 0 | 0 | 0 | 0 | 0 | 1 | 0 | 511 | 516 |  |
| R.RRGETENEEFEK.L | Y | 37.27 | 1522.7012 | 12 | 5.8 | 508.5773 | 3 | 21.35 | 9 | 5073 | Ana\_08.raw | 1.13E7 |  | 1.5E7 |  |  |  |  |  | 1.09E7 | 1.1E7 | 6 | 2 | 0 | 1 | 0 | 0 | 0 | 0 | 0 | 1 | 2 | 518 | 529 |  |
| K.GFTEVK.A | Y | 35.09 | 679.3541 | 6 | 1.9 | 340.6850 | 2 | 25.44 | 5 | 6688 | Ana\_04.raw | 6.14E5 |  | 5.01E6 | 2.64E6 | 1.02E6 | 5.81E6 | 2.73E6 |  |  |  | 6 | 1 | 0 | 1 | 1 | 1 | 1 | 1 | 0 | 0 | 0 | 464 | 469 |  |
| R.IAYQR.N | Y | 32.99 | 649.3547 | 5 | 2.4 | 325.6854 | 2 | 20.43 | 5 | 5128 | Ana\_04.raw |  |  |  |  | 3.01E6 |  |  |  |  |  | 1 | 0 | 0 | 0 | 0 | 1 | 0 | 0 | 0 | 0 | 0 | 25 | 29 |  |
| K.SFLDR.K | N | 28.97 | 636.3231 | 5 | 4.4 | 319.1702 | 2 | 39.70 | 4 | 10747 | Ana\_03.raw |  |  |  | 1.68E6 |  |  |  |  |  |  | 1 | 0 | 0 | 0 | 1 | 0 | 0 | 0 | 0 | 0 | 0 | 457 | 461 |  |
| R.LEELR.R | N | 27.25 | 658.3650 | 5 | 2.0 | 330.1904 | 2 | 26.84 | 4 | 6940 | Ana\_03.raw |  |  | 1.02E7 | 5.2E6 | 5.96E6 |  | 5.97E6 |  |  |  | 4 | 0 | 0 | 1 | 1 | 1 | 0 | 1 | 0 | 0 | 0 | 512 | 516 |  |
| total 35 peptides |  |  |  |  |  |  |  |  |  |  |  |  |  |  |  |  |  |  |  |  |  |  |  |  |  |  |  |  |  |  |  |  |  |  |  |
| --- | --- | --- | --- | --- | --- | --- | --- | --- | --- | --- | --- | --- | --- | --- | --- | --- | --- | --- | --- | --- | --- | --- | --- | --- | --- | --- | --- | --- | --- | --- | --- | --- | --- | --- | --- |

Best Unique PSM (Scan F9:6129, m/z=822.4028, z=2, RT=24.49, ppm=6.9):

sp|P17156|HSP72\_MOUSE

back to list

  

| Protein Coverage
| Supporting Peptides
| Best Unique PSM
|

Protein Coverage:

Supporting Peptides:

| Peptide | Uniq | -10lgP | Mass | Length | ppm | m/z | z | RT | Fraction | Scan | Source File | Area 10 | Area 01 | Area 02 | Area 03 | Area 04 | Area 05 | Area 06 | Area 07 | Area 08 | Area 09 | #Spec | #Spec 10 | #Spec 01 | #Spec 02 | #Spec 03 | #Spec 04 | #Spec 05 | #Spec 06 | #Spec 07 | #Spec 08 | #Spec 09 | Start | End | PTM |
| --- | --- | --- | --- | --- | --- | --- | --- | --- | --- | --- | --- | --- | --- | --- | --- | --- | --- | --- | --- | --- | --- | --- | --- | --- | --- | --- | --- | --- | --- | --- | --- | --- | --- | --- | --- |
| K.STAGDTHLGGEDFDNR.M | N | 112.04 | 1690.7183 | 16 | 4.9 | 846.3705 | 2 | 45.17 | 2 | 12649 | Ana\_01.raw | 1.15E8 | 1.44E8 | 1.35E8 | 6.12E7 | 1.07E8 | 1E8 | 7.94E7 | 8.64E7 | 1.4E8 | 9.77E7 | 40 | 4 | 6 | 4 | 3 | 4 | 3 | 3 | 4 | 6 | 3 | 224 | 239 |  |
| R.TTPSYVAFTDTER.L | N | 99.69 | 1486.6940 | 13 | 5.3 | 744.3582 | 2 | 69.89 | 1 | 19802 | ANa\_10.raw | 1.62E8 | 1.76E8 | 1.5E8 | 6.46E7 | 1.23E8 | 1.06E8 | 7.24E7 | 8.59E7 | 2.27E8 | 1.42E8 | 51 | 5 | 5 | 6 | 3 | 7 | 6 | 2 | 6 | 6 | 5 | 38 | 50 |  |
| R.IINEPTAAAIAYGLDK.K | N | 98.74 | 1658.8879 | 16 | 2.1 | 830.4530 | 2 | 98.36 | 6 | 27817 | Ana\_05.raw | 1.5E8 | 1.38E8 | 1.16E8 | 1.71E7 | 1.84E7 | 6.76E7 | 5.7E7 | 5.77E7 | 1.85E8 | 9.55E7 | 49 | 5 | 6 | 6 | 2 | 5 | 4 | 3 | 6 | 7 | 5 | 173 | 188 |  |
| R.IINEPTAAAIAYGLDKK.G | N | 95.42 | 1786.9828 | 17 | 0.5 | 596.6685 | 3 | 86.45 | 3 | 24333 | Ana\_02.raw | 2.7E7 | 7.31E5 | 1.63E7 | 5.34E6 | 1.79E7 | 1.63E7 | 1.26E7 | 8.11E6 | 5.92E7 | 2.04E7 | 17 | 2 | 1 | 1 | 1 | 1 | 2 | 2 | 2 | 3 | 2 | 173 | 189 |  |
| K.VEIIANDQGNR.T | N | 92.33 | 1227.6207 | 11 | 5.5 | 614.8210 | 2 | 37.88 | 1 | 10363 | ANa\_10.raw | 1.27E8 | 1.28E8 | 1.27E8 | 5.59E7 | 1.2E8 | 1.04E8 | 8.8E7 | 8.5E7 | 1.27E8 | 1.01E8 | 31 | 3 | 3 | 3 | 3 | 3 | 3 | 3 | 4 | 3 | 3 | 27 | 37 |  |
| R.FEELNADLFR.G | N | 87.12 | 1252.6088 | 10 | 2.3 | 627.3131 | 2 | 97.91 | 6 | 27699 | Ana\_05.raw | 5.57E7 | 4.44E7 | 6.22E7 | 2.2E7 | 4.03E7 | 3.82E7 | 2.75E7 | 2.99E7 | 7.88E7 | 4.35E7 | 39 | 5 | 4 | 5 | 2 | 4 | 2 | 3 | 5 | 4 | 5 | 305 | 314 |  |
| R.ARFEELNADLFR.G | N | 78.55 | 1479.7469 | 12 | 0.6 | 494.2566 | 3 | 86.00 | 9 | 24462 | Ana\_08.raw | 3.8E5 |  |  | 4.24E5 |  | 2.1E6 | 1.72E6 | 1.02E6 | 1.11E7 | 1.35E6 | 8 | 1 | 0 | 0 | 1 | 0 | 1 | 1 | 1 | 2 | 1 | 303 | 314 |  |
| R.NQM(+15.99)AEKDEYEHK.Q | Y | 64.63 | 1536.6515 | 12 | -1.8 | 769.3316 | 2 | 17.37 | 3 | 4056 | Ana\_02.raw | 7.59E5 |  | 1.91E6 |  |  | 3.25E6 | 8.44E5 |  |  |  | 6 | 1 | 0 | 2 | 0 | 0 | 2 | 1 | 0 | 0 | 0 | 587 | 598 | Oxidation (M) |
| K.ITITNDK.G | N | 55.47 | 803.4388 | 7 | 4.4 | 402.7285 | 2 | 24.39 | 1 | 6231 | ANa\_10.raw | 1.08E7 |  |  | 2.48E7 | 4.24E7 | 4.79E7 | 3.85E7 | 2.79E7 |  | 3.03E7 | 7 | 1 | 0 | 0 | 1 | 1 | 1 | 1 | 1 | 0 | 1 | 504 | 510 |  |
| K.NQVAM(+15.99)NPTNTIFDAK.R | Y | 55.27 | 1678.7985 | 15 | 1.8 | 840.4080 | 2 | 69.17 | 6 | 19847 | Ana\_05.raw |  |  |  |  | 9.67E5 | 1.19E6 |  |  |  |  | 2 | 0 | 0 | 0 | 0 | 1 | 1 | 0 | 0 | 0 | 0 | 58 | 72 | Oxidation (M) |
| K.NAVESYTYNIK.Q | Y | 52.39 | 1300.6299 | 11 | 2.4 | 651.3238 | 2 | 62.02 | 6 | 17776 | Ana\_05.raw |  |  |  |  |  | 3.28E6 |  | 1.27E6 | 4.64E6 | 2.14E6 | 4 | 0 | 0 | 0 | 0 | 0 | 1 | 0 | 1 | 1 | 1 | 543 | 553 |  |
| K.LLQDFFNGK.E | N | 50.24 | 1080.5603 | 9 | 2.1 | 541.2886 | 2 | 91.11 | 8 | 25457 | Ana\_07.raw |  | 8.07E6 | 9.57E6 | 3.38E6 | 6.29E6 | 1.15E6 | 5.38E6 | 3.94E6 |  | 5.69E6 | 8 | 0 | 1 | 1 | 1 | 1 | 1 | 1 | 1 | 0 | 1 | 352 | 360 |  |
| K.DAGTITGLNVLR.I | Y | 49.98 | 1228.6775 | 12 | 1.2 | 615.3467 | 2 | 86.13 | 6 | 24535 | Ana\_05.raw |  |  |  |  |  | 4.27E6 | 1.8E6 |  |  |  | 2 | 0 | 0 | 0 | 0 | 0 | 1 | 1 | 0 | 0 | 0 | 161 | 172 |  |
| R.KFEDATVQSDM(+15.99)K.H | Y | 45.51 | 1413.6445 | 12 | 2.5 | 707.8313 | 2 | 27.23 | 6 | 7258 | Ana\_05.raw |  |  |  |  |  | 8.01E5 |  | 5.27E5 |  |  | 2 | 0 | 0 | 0 | 0 | 0 | 1 | 0 | 1 | 0 | 0 | 78 | 89 | Oxidation (M) |
| K.VQVEYK.G | N | 40.81 | 764.4069 | 6 | 1.4 | 383.2112 | 2 | 24.93 | 3 | 6541 | Ana\_02.raw | 3.23E7 | 4.68E7 | 5.56E7 | 2.79E7 | 4.3E7 | 5.09E7 | 3.53E7 | 2.75E7 |  | 3.07E7 | 10 | 1 | 1 | 1 | 1 | 1 | 1 | 1 | 2 | 0 | 1 | 104 | 109 |  |
| K.RNTTIPTK.Q | N | 40.62 | 929.5294 | 8 | 1.0 | 465.7724 | 2 | 18.51 | 3 | 4438 | Ana\_02.raw |  |  | 3.47E6 | 1.52E6 | 2.62E6 | 3.09E6 | 2.5E6 | 1.67E6 |  | 1.8E6 | 7 | 0 | 0 | 1 | 1 | 1 | 1 | 1 | 1 | 0 | 1 | 419 | 426 |  |
| K.EIAEAYLGGK.V | Y | 38.94 | 1049.5393 | 10 | 5.6 | 525.7798 | 2 | 54.40 | 3 | 15434 | Ana\_02.raw |  |  | 9.43E5 |  |  |  |  |  |  |  | 1 | 0 | 0 | 1 | 0 | 0 | 0 | 0 | 0 | 0 | 0 | 130 | 139 |  |
| K.DNNLLGK.F | N | 35.26 | 772.4079 | 7 | 3.2 | 387.2125 | 2 | 30.19 | 5 | 8155 | Ana\_04.raw |  |  | 3.03E7 |  | 2.51E7 |  | 1.95E7 |  |  |  | 3 | 0 | 0 | 1 | 0 | 1 | 0 | 1 | 0 | 0 | 0 | 455 | 461 |  |
| R.GTLEPVEK.A | N | 34.42 | 871.4651 | 8 | 4.6 | 436.7418 | 2 | 27.03 | 10 | 7143 | Ana\_09.raw | 1.19E7 |  | 2.4E7 |  |  |  |  |  |  | 1.34E7 | 3 | 1 | 0 | 1 | 0 | 0 | 0 | 0 | 0 | 0 | 1 | 315 | 322 |  |
| R.LIGDAAK.N | N | 28.78 | 686.3962 | 7 | 1.5 | 344.2059 | 2 | 21.83 | 4 | 5454 | Ana\_03.raw |  |  |  | 3.57E7 |  |  |  |  |  |  | 1 | 0 | 0 | 0 | 1 | 0 | 0 | 0 | 0 | 0 | 0 | 51 | 57 |  |
| K.RLIGR.K | N | 26.60 | 613.4023 | 5 | 0.6 | 307.7086 | 2 | 18.67 | 4 | 4488 | Ana\_03.raw |  |  |  | 4.8E6 |  |  |  |  |  |  | 1 | 0 | 0 | 0 | 1 | 0 | 0 | 0 | 0 | 0 | 0 | 73 | 77 |  |
| R.NTTIPTK.Q | N | 23.26 | 773.4283 | 7 | 0.8 | 387.7218 | 2 | 21.30 | 4 | 5291 | Ana\_03.raw |  |  |  | 3.64E7 |  |  |  |  |  |  | 1 | 0 | 0 | 0 | 1 | 0 | 0 | 0 | 0 | 0 | 0 | 420 | 426 |  |
| total 22 peptides |  |  |  |  |  |  |  |  |  |  |  |  |  |  |  |  |  |  |  |  |  |  |  |  |  |  |  |  |  |  |  |  |  |  |  |
| --- | --- | --- | --- | --- | --- | --- | --- | --- | --- | --- | --- | --- | --- | --- | --- | --- | --- | --- | --- | --- | --- | --- | --- | --- | --- | --- | --- | --- | --- | --- | --- | --- | --- | --- | --- |

Best Unique PSM (Scan F3:4056, m/z=769.3316, z=2, RT=17.37, ppm=-1.8):

sp|Q9ERD7|TBB3\_MOUSE

back to list

  

| Protein Coverage
| Supporting Peptides
| Best Unique PSM
|

Protein Coverage:

Supporting Peptides:

| Peptide | Uniq | -10lgP | Mass | Length | ppm | m/z | z | RT | Fraction | Scan | Source File | Area 10 | Area 01 | Area 02 | Area 03 | Area 04 | Area 05 | Area 06 | Area 07 | Area 08 | Area 09 | #Spec | #Spec 10 | #Spec 01 | #Spec 02 | #Spec 03 | #Spec 04 | #Spec 05 | #Spec 06 | #Spec 07 | #Spec 08 | #Spec 09 | Start | End | PTM |
| --- | --- | --- | --- | --- | --- | --- | --- | --- | --- | --- | --- | --- | --- | --- | --- | --- | --- | --- | --- | --- | --- | --- | --- | --- | --- | --- | --- | --- | --- | --- | --- | --- | --- | --- | --- |
| K.GHYTEGAELVDSVLDVVR.K | N | 135.92 | 1957.9745 | 18 | 3.6 | 979.9980 | 2 | 127.77 | 6 | 35505 | Ana\_05.raw | 2.36E7 | 5.44E7 | 1.6E8 | 4.71E7 | 1.26E8 | 1.66E8 | 6.78E7 | 6.96E7 | 3.65E8 | 1.97E8 | 32 | 2 | 4 | 5 | 2 | 4 | 2 | 1 | 5 | 4 | 3 | 104 | 121 |  |
| K.NSSYFVEWIPNNVK.V | N | 110.81 | 1695.8257 | 14 | 0.6 | 848.9207 | 2 | 109.87 | 3 | 30437 | Ana\_02.raw | 3.45E7 | 3.29E7 | 2.2E7 | 1.6E7 | 2.71E7 | 1.96E7 | 1.72E7 | 1.87E7 | 6.7E7 | 2.73E7 | 21 | 2 | 3 | 2 | 1 | 4 | 1 | 3 | 1 | 3 | 1 | 337 | 350 |  |
| R.LHFFM(+15.99)PGFAPLTAR.G | N | 85.95 | 1619.8282 | 14 | 5.4 | 540.9529 | 3 | 102.58 | 1 | 28641 | ANa\_10.raw | 4.18E6 |  | 8.63E5 | 9.96E4 | 1.8E6 | 1.13E6 | 3.47E5 |  |  | 2.45E6 | 7 | 1 | 0 | 1 | 1 | 1 | 1 | 1 | 0 | 0 | 1 | 263 | 276 | Oxidation (M) |
| R.FPGQLNADLR.K | N | 78.02 | 1129.5880 | 10 | 3.0 | 565.8030 | 2 | 69.05 | 6 | 19811 | Ana\_05.raw | 7.68E7 | 5.71E7 | 5.55E7 | 2.67E7 | 6.02E7 | 4.21E7 | 2.93E7 | 4.94E7 | 1.57E8 | 8.76E7 | 45 | 4 | 4 | 4 | 4 | 4 | 5 | 3 | 5 | 6 | 6 | 242 | 251 |  |
| K.LAVNM(+15.99)VPFPR.L | N | 76.83 | 1158.6219 | 10 | 4.1 | 580.3206 | 2 | 79.35 | 6 | 22695 | Ana\_05.raw | 8.22E7 | 5.68E7 | 5.35E7 | 2.41E7 | 4.94E7 | 5.03E7 | 3.23E7 | 3.95E7 | 1.48E8 | 5.62E7 | 48 | 6 | 5 | 4 | 4 | 4 | 4 | 4 | 6 | 7 | 4 | 253 | 262 | Oxidation (M) |
| R.AILVDLEPGTM(+15.99)DSVR.S | N | 73.85 | 1630.8236 | 15 | 3.2 | 816.4217 | 2 | 86.14 | 1 | 24257 | ANa\_10.raw | 1.6E7 | 1.38E7 | 6.82E5 | 2.16E6 | 7.97E6 | 2.12E6 | 5.93E6 | 7.31E6 | 3.21E7 | 1.06E7 | 13 | 2 | 1 | 1 | 1 | 1 | 1 | 1 | 1 | 2 | 2 | 63 | 77 | Oxidation (M) |
| R.IM(+15.99)NTFSVVPSPK.V | N | 72.89 | 1334.6904 | 12 | 2.0 | 668.3538 | 2 | 67.16 | 3 | 19097 | Ana\_02.raw | 4.81E7 | 3.48E7 | 2.62E7 | 1.36E7 | 3.4E7 | 2.79E7 | 2.17E7 | 2.24E7 | 6.59E7 | 3.08E7 | 21 | 2 | 3 | 2 | 2 | 2 | 2 | 2 | 2 | 3 | 1 | 163 | 174 | Oxidation (M) |
| R.ALTVPELTQQM(+15.99)FDAK.N | N | 71.14 | 1706.8549 | 15 | 4.9 | 854.4389 | 2 | 89.92 | 3 | 25244 | Ana\_02.raw | 3.6E7 | 2.33E7 | 1.45E7 | 4.92E6 | 1.46E7 | 1.85E7 | 9.93E6 | 1.33E7 | 7.58E7 | 9.88E6 | 26 | 4 | 2 | 3 | 1 | 2 | 3 | 3 | 1 | 5 | 2 | 283 | 297 | Oxidation (M) |
| K.MSSTFIGNSTAIQELFK.R | Y | 69.76 | 1872.9291 | 17 | 3.1 | 937.4747 | 2 | 121.60 | 9 | 33856 | Ana\_08.raw |  |  |  |  |  |  |  | 1.83E6 | 2.21E7 |  | 2 | 0 | 0 | 0 | 0 | 0 | 0 | 0 | 1 | 1 | 0 | 363 | 379 |  |
| K.LAVNMVPFPR.L | N | 69.21 | 1142.6270 | 10 | 5.4 | 572.3239 | 2 | 92.17 | 1 | 25869 | ANa\_10.raw | 6.59E6 |  |  |  |  | 1.83E5 |  |  | 1.45E6 |  | 3 | 1 | 0 | 0 | 0 | 0 | 1 | 0 | 0 | 1 | 0 | 253 | 262 |  |
| R.ISEQFTAM(+15.99)FR.R | N | 65.28 | 1244.5859 | 10 | 1.5 | 623.3012 | 2 | 70.89 | 3 | 20141 | Ana\_02.raw | 2.85E7 | 2.58E7 | 1.92E7 | 1E7 | 8.26E6 | 1.54E7 | 6.67E6 | 1.69E7 | 2.69E7 | 1.4E7 | 10 | 1 | 1 | 1 | 1 | 1 | 1 | 1 | 1 | 1 | 1 | 381 | 390 | Oxidation (M) |
| R.EIVHIQAGQC(+57.02)GNQIGAK.F | N | 61.23 | 1821.9155 | 17 | 6.6 | 911.9710 | 2 | 49.98 | 9 | 14043 | Ana\_08.raw |  |  |  |  |  |  |  |  | 4.97E6 |  | 2 | 0 | 0 | 0 | 0 | 0 | 0 | 0 | 0 | 2 | 0 | 3 | 19 | Carbamidomethylation |
| R.ALTVPELTQQMFDAK.N | N | 51.60 | 1690.8600 | 15 | 9.7 | 846.4455 | 2 | 117.55 | 10 | 32318 | Ana\_09.raw |  | 3.09E6 |  |  |  | 1.51E6 |  | 3.44E5 |  | 2.19E6 | 4 | 0 | 1 | 0 | 0 | 0 | 1 | 0 | 1 | 0 | 1 | 283 | 297 |  |
| R.KLAVNM(+15.99)VPFPR.L | N | 49.45 | 1286.7169 | 11 | 2.9 | 429.9142 | 3 | 66.65 | 5 | 19005 | Ana\_04.raw |  |  |  | 2.22E6 | 4.19E6 | 8.25E6 |  | 2.66E6 | 3.2E6 |  | 5 | 0 | 0 | 0 | 1 | 1 | 1 | 0 | 1 | 1 | 0 | 252 | 262 | Oxidation (M) |
| R.YLTVATVFR.G | N | 35.17 | 1068.5968 | 9 | 2.3 | 535.3069 | 2 | 89.29 | 1 | 25099 | ANa\_10.raw | 1.14E6 |  |  |  |  |  |  |  |  |  | 1 | 1 | 0 | 0 | 0 | 0 | 0 | 0 | 0 | 0 | 0 | 310 | 318 |  |
| total 15 peptides |  |  |  |  |  |  |  |  |  |  |  |  |  |  |  |  |  |  |  |  |  |  |  |  |  |  |  |  |  |  |  |  |  |  |  |
| --- | --- | --- | --- | --- | --- | --- | --- | --- | --- | --- | --- | --- | --- | --- | --- | --- | --- | --- | --- | --- | --- | --- | --- | --- | --- | --- | --- | --- | --- | --- | --- | --- | --- | --- | --- |

Best Unique PSM (Scan F9:33856, m/z=937.4747, z=2, RT=121.60, ppm=3.1):

sp|Q8BFZ3|ACTBL\_MOUSE

back to list

  

| Protein Coverage
| Supporting Peptides
| Best Unique PSM
|

Protein Coverage:

Supporting Peptides:

| Peptide | Uniq | -10lgP | Mass | Length | ppm | m/z | z | RT | Fraction | Scan | Source File | Area 10 | Area 01 | Area 02 | Area 03 | Area 04 | Area 05 | Area 06 | Area 07 | Area 08 | Area 09 | #Spec | #Spec 10 | #Spec 01 | #Spec 02 | #Spec 03 | #Spec 04 | #Spec 05 | #Spec 06 | #Spec 07 | #Spec 08 | #Spec 09 | Start | End | PTM |
| --- | --- | --- | --- | --- | --- | --- | --- | --- | --- | --- | --- | --- | --- | --- | --- | --- | --- | --- | --- | --- | --- | --- | --- | --- | --- | --- | --- | --- | --- | --- | --- | --- | --- | --- | --- |
| R.SYELPDGQVITIGNER.F | N | 136.16 | 1789.8846 | 16 | 0.5 | 895.9501 | 2 | 101.19 | 3 | 28209 | Ana\_02.raw | 2.56E9 | 2.49E9 | 2.2E9 | 5.02E8 | 1.2E9 | 1.61E9 | 8.03E8 | 1.08E9 | 4.17E9 | 2.27E9 | 445 | 51 | 48 | 45 | 42 | 44 | 40 | 40 | 47 | 46 | 42 | 240 | 255 |  |
| R.TTGIVM(+15.99)DSGDGVTHTVPIYEGYALPHAILR.L | N | 134.68 | 3198.6018 | 30 | 0.6 | 1067.2085 | 3 | 109.44 | 6 | 30732 | Ana\_05.raw | 5.15E8 | 1.48E8 | 1.08E8 | 2.77E7 | 1.97E8 | 1.61E8 | 1.12E8 | 4.1E7 | 1.33E9 | 1.87E8 | 94 | 17 | 6 | 5 | 8 | 10 | 7 | 7 | 11 | 15 | 8 | 149 | 178 | Oxidation (M) |
| R.TTGIVMDSGDGVTHTVPIYEGYALPHAILR.L | N | 130.29 | 3182.6069 | 30 | 5.3 | 1061.8818 | 3 | 113.09 | 9 | 31668 | Ana\_08.raw | 1.04E8 | 4.47E7 | 3.97E6 | 1.57E7 | 0 | 4.85E7 | 5.93E6 | 1.32E7 | 2.9E8 | 1.9E8 | 37 | 6 | 3 | 1 | 3 | 1 | 3 | 1 | 3 | 8 | 8 | 149 | 178 |  |
| R.DLTDYLM(+15.99)KILTER.G | N | 84.48 | 1625.8334 | 13 | -0.5 | 813.9235 | 2 | 131.53 | 3 | 36031 | Ana\_02.raw | 2.95E6 | 3.96E6 | 7.7E6 | 1.52E6 | 5.67E6 | 8.87E6 | 6.41E6 | 2.09E6 | 2.69E6 | 6.16E6 | 17 | 2 | 1 | 2 | 1 | 2 | 2 | 2 | 2 | 1 | 2 | 185 | 197 | Oxidation (M) |
| R.HQGVMVGM(+15.99)GQK.D | N | 84.12 | 1186.5587 | 11 | 1.7 | 594.2877 | 2 | 16.20 | 3 | 3687 | Ana\_02.raw | 8.78E6 | 2.67E7 | 5.55E6 | 4.81E6 | 4.46E6 | 2.99E7 | 5.97E6 | 1.31E7 | 1.79E7 | 2.08E7 | 21 | 2 | 2 | 2 | 2 | 1 | 3 | 3 | 2 | 1 | 3 | 41 | 51 | Oxidation (M) |
| R.HQGVM(+15.99)VGMGQK.D | N | 69.21 | 1186.5587 | 11 | 1.9 | 594.2878 | 2 | 15.71 | 5 | 3571 | Ana\_04.raw | 1.62E7 | 9.07E6 |  |  | 1.39E6 | 1.35E7 | 8.82E5 | 7.09E6 | 1.92E6 | 1.76E7 | 12 | 1 | 1 | 0 | 0 | 1 | 2 | 2 | 2 | 1 | 2 | 41 | 51 | Oxidation (M) |
| R.HQGVM(+15.99)VGM(+15.99)GQK.D | N | 65.50 | 1202.5536 | 11 | 7.8 | 602.2888 | 2 | 21.12 | 1 | 5142 | ANa\_10.raw | 3.51E8 | 1.68E8 | 6.85E8 | 2.25E8 | 4.15E8 | 7.33E8 | 4.21E8 | 3.56E8 | 9.25E7 | 3.14E8 | 59 | 8 | 4 | 5 | 7 | 6 | 4 | 5 | 8 | 6 | 6 | 41 | 51 | Oxidation (M) |
| R.DLTDYLM(+15.99)K.I | N | 58.74 | 1013.4739 | 8 | 3.9 | 507.7462 | 2 | 92.37 | 6 | 26220 | Ana\_05.raw | 8.4E8 | 6.93E8 | 6.22E8 | 2.72E8 | 4.42E8 | 4.88E8 | 3.63E8 | 5.27E8 | 1.32E9 | 5.32E8 | 291 | 38 | 34 | 28 | 21 | 30 | 22 | 17 | 33 | 35 | 33 | 185 | 192 | Oxidation (M) |
| R.DLTDYLMK.I | N | 58.29 | 997.4790 | 8 | 4.0 | 499.7487 | 2 | 92.24 | 1 | 25887 | ANa\_10.raw | 3.85E7 | 6.67E6 | 1.26E5 | 5.05E5 |  | 6.74E6 | 2.41E5 | 3.81E6 | 2.57E7 | 2.99E6 | 21 | 5 | 2 | 1 | 1 | 0 | 2 | 1 | 1 | 5 | 3 | 185 | 192 |  |
| K.IIAPPER.K | N | 57.46 | 794.4650 | 7 | 1.7 | 398.2404 | 2 | 33.34 | 3 | 9119 | Ana\_02.raw | 3.97E9 | 4.12E9 | 3.95E9 | 1.66E9 | 3.05E9 | 3.75E9 | 2.4E9 | 2.4E9 | 4.68E9 | 3.63E9 | 283 | 36 | 29 | 33 | 22 | 29 | 22 | 16 | 34 | 31 | 31 | 330 | 336 |  |
| R.LDLAGRDLTDYLM(+15.99)K.I | N | 51.33 | 1638.8286 | 14 | 3.2 | 820.4242 | 2 | 95.36 | 9 | 26994 | Ana\_08.raw | 9.63E6 |  |  |  |  |  |  |  | 1.04E7 |  | 3 | 1 | 0 | 0 | 0 | 0 | 0 | 0 | 0 | 2 | 0 | 179 | 192 | Oxidation (M) |
| K.IIAPPERK.Y | N | 46.97 | 922.5599 | 8 | 3.9 | 462.2891 | 2 | 23.58 | 9 | 5830 | Ana\_08.raw | 8.34E7 | 2.85E7 | 2.04E7 |  | 1.06E7 | 3.16E7 | 5.24E6 | 4.71E6 | 8.32E7 | 2.96E7 | 18 | 3 | 2 | 2 | 0 | 2 | 2 | 1 | 1 | 3 | 2 | 330 | 337 |  |
| R.VAPDEHPILLTEAPLNPK.I | Y | 46.91 | 1953.0570 | 18 | 9.9 | 652.0327 | 3 | 94.50 | 2 | 26684 | Ana\_01.raw |  | 2.08E6 |  | 5.77E6 |  |  | 3.17E4 |  |  |  | 3 | 0 | 1 | 0 | 1 | 0 | 0 | 1 | 0 | 0 | 0 | 97 | 114 |  |
| K.C(+57.02)DVDIR.K | N | 43.98 | 776.3487 | 6 | 4.4 | 389.1833 | 2 | 28.80 | 8 | 7498 | Ana\_07.raw |  | 4.69E7 | 4.42E7 | 2.63E7 | 5.45E7 | 4.38E7 | 3.39E7 | 3.7E7 |  | 9.42E6 | 9 | 0 | 1 | 1 | 1 | 1 | 1 | 1 | 2 | 0 | 1 | 286 | 291 | Carbamidomethylation |
| K.IKIIAPPER.K | N | 42.43 | 1035.6440 | 9 | 0.5 | 346.2221 | 3 | 44.96 | 3 | 12647 | Ana\_02.raw | 3.02E6 |  | 7.18E6 | 2.01E6 | 2.08E6 | 3.03E6 | 1.58E6 |  | 4.82E6 | 2.45E6 | 12 | 1 | 0 | 2 | 2 | 1 | 2 | 1 | 0 | 2 | 1 | 328 | 336 |  |
| K.ILTER.G | N | 39.01 | 630.3701 | 5 | 3.4 | 316.1934 | 2 | 20.56 | 6 | 5102 | Ana\_05.raw | 2.01E7 |  |  | 2.76E6 | 1.13E7 | 9.27E6 | 2.65E8 | 1.7E8 | 7.79E6 |  | 14 | 1 | 0 | 0 | 3 | 3 | 1 | 4 | 1 | 1 | 0 | 193 | 197 |  |
| R.LDLAGR.D | N | 35.64 | 643.3653 | 6 | -0.6 | 322.6897 | 2 | 30.23 | 3 | 8192 | Ana\_02.raw | 7.61E8 | 7.49E8 | 8.47E8 | 3.81E8 | 6.81E8 | 6.9E7 | 4E5 | 1.11E2 | 9.23E8 | 6.75E8 | 38 | 3 | 3 | 4 | 6 | 6 | 3 | 1 | 1 | 9 | 2 | 179 | 184 |  |
| K.RGILTLK.Y | N | 29.35 | 799.5280 | 7 | 4.0 | 400.7729 | 2 | 41.36 | 6 | 11572 | Ana\_05.raw |  |  |  |  |  | 1.41E8 |  | 9.14E7 |  |  | 2 | 0 | 0 | 0 | 0 | 0 | 1 | 0 | 1 | 0 | 0 | 63 | 69 |  |
| R.GIHETTFNSIM(+15.99)K.C | Y | 26.14 | 1392.6708 | 12 | 3.3 | 465.2324 | 3 | 46.97 | 6 | 13285 | Ana\_05.raw |  |  |  |  | 2.57E6 | 2.83E6 |  |  |  |  | 2 | 0 | 0 | 0 | 0 | 1 | 1 | 0 | 0 | 0 | 0 | 274 | 285 | Oxidation (M) |
| R.GILTLK.Y | N | 25.30 | 643.4268 | 6 | 5.7 | 322.7225 | 2 | 51.01 | 9 | 14360 | Ana\_08.raw |  | 1.76E8 | 1.95E8 |  |  | 1.81E8 | 1.2E8 |  | 2.57E2 |  | 6 | 0 | 2 | 1 | 0 | 0 | 1 | 1 | 0 | 1 | 0 | 64 | 69 |  |
| total 20 peptides |  |  |  |  |  |  |  |  |  |  |  |  |  |  |  |  |  |  |  |  |  |  |  |  |  |  |  |  |  |  |  |  |  |  |  |
| --- | --- | --- | --- | --- | --- | --- | --- | --- | --- | --- | --- | --- | --- | --- | --- | --- | --- | --- | --- | --- | --- | --- | --- | --- | --- | --- | --- | --- | --- | --- | --- | --- | --- | --- | --- |

Best Unique PSM (Scan F2:26684, m/z=652.0327, z=3, RT=94.50, ppm=9.9):

sp|P22599|A1AT2\_MOUSE

back to list

  

| Protein Coverage
| Supporting Peptides
| Best Unique PSM
|

Protein Coverage:

Supporting Peptides:

| Peptide | Uniq | -10lgP | Mass | Length | ppm | m/z | z | RT | Fraction | Scan | Source File | Area 10 | Area 01 | Area 02 | Area 03 | Area 04 | Area 05 | Area 06 | Area 07 | Area 08 | Area 09 | #Spec | #Spec 10 | #Spec 01 | #Spec 02 | #Spec 03 | #Spec 04 | #Spec 05 | #Spec 06 | #Spec 07 | #Spec 08 | #Spec 09 | Start | End | PTM |
| --- | --- | --- | --- | --- | --- | --- | --- | --- | --- | --- | --- | --- | --- | --- | --- | --- | --- | --- | --- | --- | --- | --- | --- | --- | --- | --- | --- | --- | --- | --- | --- | --- | --- | --- | --- |
| K.DQSPASHEIATNLGDFAISLYR.E | N | 133.04 | 2404.1658 | 22 | 2.8 | 802.3981 | 3 | 126.74 | 3 | 34760 | Ana\_02.raw | 1.72E7 | 2.56E6 | 3.11E7 | 6.37E6 | 1.68E7 | 3.02E7 | 1.97E7 | 9.58E6 | 0 | 1.26E7 | 15 | 2 | 2 | 1 | 2 | 1 | 1 | 2 | 2 | 1 | 1 | 36 | 57 |  |
| K.SFQHLLQTLNRPDSELQLSTGNGLFVNNDLK.L | N | 129.80 | 3497.7903 | 31 | -1.3 | 875.4537 | 4 | 124.34 | 3 | 34149 | Ana\_02.raw |  |  | 1.92E7 | 7.14E6 |  | 8.97E6 | 1.67E7 | 1.48E7 |  |  | 7 | 0 | 0 | 1 | 2 | 0 | 1 | 1 | 2 | 0 | 0 | 113 | 143 |  |
| R.FDHPFLFIIFEEHTQSPIFVGK.V | N | 106.71 | 2647.3474 | 22 | 2.8 | 662.8460 | 4 | 132.43 | 6 | 36814 | Ana\_05.raw |  |  | 4.89E6 | 4.4E6 | 1.19E6 | 2.36E6 | 2.12E6 |  |  |  | 7 | 0 | 0 | 2 | 2 | 1 | 1 | 1 | 0 | 0 | 0 | 385 | 406 |  |
| K.ELDQDTVFALANYILFK.G | Y | 94.81 | 1999.0302 | 17 | 3.2 | 1000.5255 | 2 | 139.17 | 6 | 38625 | Ana\_05.raw | 3.25E5 | 0 | 8.5E5 | 4.71E5 | 2.82E6 | 3.37E6 |  | 4.04E5 | 6.11E5 | 7.11E5 | 12 | 1 | 1 | 1 | 1 | 2 | 2 | 0 | 2 | 1 | 1 | 193 | 209 |  |
| K.KPFDPENTEEAEFHVDK.S | Y | 77.48 | 2030.9221 | 17 | 1.6 | 677.9824 | 3 | 66.36 | 6 | 19053 | Ana\_05.raw |  |  | 2.23E6 | 2.96E5 | 2.25E6 | 1.21E6 | 1.56E6 | 1.71E6 | 3.41E6 |  | 7 | 0 | 0 | 1 | 1 | 1 | 1 | 1 | 1 | 1 | 0 | 214 | 230 |  |
| K.M(+15.99)QHLEQTLNK.E | N | 68.21 | 1256.6183 | 10 | 3.4 | 629.3185 | 2 | 26.47 | 6 | 7020 | Ana\_05.raw |  | 3.75E6 |  |  | 3.18E6 | 3.98E6 | 1.46E6 | 2.28E6 | 2.26E6 |  | 6 | 0 | 1 | 0 | 0 | 1 | 1 | 1 | 1 | 1 | 0 | 278 | 287 | Oxidation (M) |
| K.TLM(+15.99)SPLGITR.I | N | 50.49 | 1103.6008 | 10 | 4.5 | 552.8102 | 2 | 67.36 | 8 | 18917 | Ana\_07.raw |  |  | 1.72E7 | 3.32E6 | 3.23E6 | 1.2E6 | 4.92E6 | 7.58E6 |  |  | 6 | 0 | 0 | 1 | 1 | 1 | 1 | 1 | 1 | 0 | 0 | 319 | 328 | Oxidation (M) |
| K.KVINDFVEK.G | N | 48.83 | 1090.6022 | 9 | 4.5 | 546.3108 | 2 | 41.87 | 6 | 11729 | Ana\_05.raw |  |  | 6.07E6 |  |  | 4.04E6 |  | 2.35E6 |  |  | 3 | 0 | 0 | 1 | 0 | 0 | 1 | 0 | 1 | 0 | 0 | 173 | 181 |  |
| K.VINDFVEK.G | N | 48.25 | 962.5073 | 8 | 6.2 | 482.2639 | 2 | 46.91 | 2 | 13186 | Ana\_01.raw | 2.13E7 | 2.23E7 | 3.02E7 | 9.36E6 | 1.79E7 | 2.5E7 | 1.8E7 | 1.48E7 | 9.49E6 | 1.4E7 | 11 | 1 | 1 | 1 | 2 | 1 | 1 | 1 | 1 | 1 | 1 | 174 | 181 |  |
| K.FLEEAK.N | N | 33.46 | 735.3802 | 6 | 3.6 | 368.6987 | 2 | 27.77 | 2 | 7326 | Ana\_01.raw | 2.24E7 | 2.72E7 |  | 9.67E6 | 1.71E7 | 2.36E7 | 1.33E7 |  | 8.14E6 | 1.4E7 | 8 | 1 | 1 | 0 | 1 | 1 | 1 | 1 | 0 | 1 | 1 | 148 | 153 |  |
| K.IVEAVK.E | N | 30.08 | 657.4061 | 6 | 2.4 | 329.7111 | 2 | 20.74 | 5 | 5220 | Ana\_04.raw |  |  |  | 2.57E6 | 5.68E6 |  | 2.93E6 |  |  |  | 3 | 0 | 0 | 0 | 1 | 1 | 0 | 1 | 0 | 0 | 0 | 187 | 192 |  |
| R.LSISGDYNLK.T | Y | 26.81 | 1108.5764 | 10 | -1.7 | 555.2946 | 2 | 65.72 | 3 | 18722 | Ana\_02.raw |  |  | 1.56E5 |  |  |  |  |  |  |  | 1 | 0 | 0 | 1 | 0 | 0 | 0 | 0 | 0 | 0 | 0 | 309 | 318 |  |
| total 12 peptides |  |  |  |  |  |  |  |  |  |  |  |  |  |  |  |  |  |  |  |  |  |  |  |  |  |  |  |  |  |  |  |  |  |  |  |
| --- | --- | --- | --- | --- | --- | --- | --- | --- | --- | --- | --- | --- | --- | --- | --- | --- | --- | --- | --- | --- | --- | --- | --- | --- | --- | --- | --- | --- | --- | --- | --- | --- | --- | --- | --- |

Best Unique PSM (Scan F6:38625, m/z=1000.5255, z=2, RT=139.17, ppm=3.2):

sp|O88329|MYO1A\_MOUSE

back to list

  

| Protein Coverage
| Supporting Peptides
| Best Unique PSM
|

Protein Coverage:

Supporting Peptides:

| Peptide | Uniq | -10lgP | Mass | Length | ppm | m/z | z | RT | Fraction | Scan | Source File | Area 10 | Area 01 | Area 02 | Area 03 | Area 04 | Area 05 | Area 06 | Area 07 | Area 08 | Area 09 | #Spec | #Spec 10 | #Spec 01 | #Spec 02 | #Spec 03 | #Spec 04 | #Spec 05 | #Spec 06 | #Spec 07 | #Spec 08 | #Spec 09 | Start | End | PTM |
| --- | --- | --- | --- | --- | --- | --- | --- | --- | --- | --- | --- | --- | --- | --- | --- | --- | --- | --- | --- | --- | --- | --- | --- | --- | --- | --- | --- | --- | --- | --- | --- | --- | --- | --- | --- |
| K.GSPLGGVITNYLLEK.S | Y | 99.72 | 1559.8558 | 15 | 2.2 | 780.9369 | 2 | 121.91 | 6 | 33985 | Ana\_05.raw | 5.56E6 | 2.69E7 | 1.55E7 | 4.54E6 | 1.02E7 | 1.26E7 | 8.83E6 | 6.87E6 | 3.7E7 | 1.39E7 | 20 | 2 | 2 | 2 | 2 | 2 | 2 | 2 | 2 | 2 | 2 | 171 | 185 |  |
| K.EQLLQSNPVLEAFGNAK.T | N | 89.06 | 1856.9631 | 17 | 4.9 | 929.4933 | 2 | 118.25 | 6 | 33029 | Ana\_05.raw | 3.57E6 | 4.38E6 | 6.64E6 | 3.2E6 | 3.35E6 | 8.57E6 | 1.27E6 | 9.64E5 | 9.53E6 | 1.37E6 | 17 | 2 | 1 | 2 | 1 | 2 | 3 | 1 | 2 | 2 | 1 | 133 | 149 |  |
| R.NFHIFYQLLAGADAQLLK.A | Y | 87.57 | 2061.1047 | 18 | 3.9 | 688.0449 | 3 | 133.60 | 6 | 37142 | Ana\_05.raw |  | 7.09E5 |  | 3.23E5 |  | 1.36E6 |  |  |  | 8.74E5 | 4 | 0 | 1 | 0 | 1 | 0 | 1 | 0 | 0 | 0 | 1 | 197 | 214 |  |
| K.VVTVLNVTQAQYAR.D | Y | 86.94 | 1560.8624 | 14 | 4.4 | 781.4419 | 2 | 80.51 | 6 | 23013 | Ana\_05.raw |  | 3.43E6 |  | 9.84E5 |  | 3.03E6 |  |  | 7E6 | 2.66E6 | 5 | 0 | 1 | 0 | 1 | 0 | 1 | 0 | 0 | 1 | 1 | 329 | 342 |  |
| K.GDILLVSDHVVELLTK.M | Y | 83.08 | 1749.9875 | 16 | 4.7 | 584.3392 | 3 | 130.08 | 7 | 35794 | Ana\_06.raw |  | 2.37E6 |  | 1.33E6 | 1.67E6 | 1.62E6 | 2.26E6 |  |  |  | 5 | 0 | 1 | 0 | 1 | 1 | 1 | 1 | 0 | 0 | 0 | 967 | 982 |  |
| K.VLGSLTLSSEELAYGK.T | Y | 77.95 | 1665.8824 | 16 | 4.1 | 833.9519 | 2 | 94.24 | 3 | 26383 | Ana\_02.raw |  |  | 4.15E5 |  |  | 3.22E5 |  |  | 1.79E6 |  | 3 | 0 | 0 | 1 | 0 | 0 | 1 | 0 | 0 | 1 | 0 | 660 | 675 |  |
| R.AVQHAM(+15.99)SVIGFSEEEIR.Q | Y | 74.76 | 1917.9254 | 17 | 1.0 | 640.3164 | 3 | 71.78 | 9 | 20486 | Ana\_08.raw |  |  |  |  |  |  |  |  | 1.18E6 |  | 1 | 0 | 0 | 0 | 0 | 0 | 0 | 0 | 0 | 1 | 0 | 246 | 262 | Oxidation (M) |
| K.VTYNVTGFIDK.N | Y | 65.42 | 1255.6448 | 11 | 3.1 | 628.8316 | 2 | 84.76 | 6 | 24161 | Ana\_05.raw | 9.55E5 | 3.77E6 |  | 5.07E5 | 4.72E5 | 3.1E6 | 1.6E6 |  | 6.52E6 | 1.55E6 | 8 | 1 | 1 | 0 | 1 | 1 | 1 | 1 | 0 | 1 | 1 | 512 | 522 |  |
| R.DQC(+57.02)ILITGESGAGK.T | N | 63.82 | 1447.6976 | 14 | 4.0 | 724.8590 | 2 | 57.28 | 3 | 16260 | Ana\_02.raw |  |  | 2.51E5 |  | 6.11E5 | 1.58E6 |  |  |  |  | 3 | 0 | 0 | 1 | 0 | 1 | 1 | 0 | 0 | 0 | 0 | 94 | 107 | Carbamidomethylation |
| R.EEGPVLVADTVK.K | Y | 60.48 | 1255.6659 | 12 | 3.6 | 628.8425 | 2 | 63.62 | 5 | 18135 | Ana\_04.raw | 5.43E6 |  | 7.64E6 | 2.73E6 | 6.13E6 | 7.2E6 | 4.23E6 | 2.16E6 |  | 2.07E6 | 8 | 1 | 0 | 1 | 1 | 1 | 1 | 1 | 1 | 0 | 1 | 889 | 900 |  |
| K.GHVILTDAK.K | Y | 59.77 | 952.5342 | 9 | 0.7 | 477.2747 | 2 | 23.37 | 3 | 6042 | Ana\_02.raw |  |  | 7.66E6 |  | 8.97E6 | 1.24E7 |  |  |  |  | 3 | 0 | 0 | 1 | 0 | 1 | 1 | 0 | 0 | 0 | 0 | 919 | 927 |  |
| K.VEYFDNGIIC(+57.02)NLIEHSQR.G | Y | 59.22 | 2206.0476 | 18 | 2.4 | 736.3583 | 3 | 124.47 | 2 | 34503 | Ana\_01.raw |  | 1.88E6 |  |  |  |  |  |  |  | 4.73E5 | 2 | 0 | 1 | 0 | 0 | 0 | 0 | 0 | 0 | 0 | 1 | 430 | 447 | Carbamidomethylation |
| K.VLDNTWPAAPYR.C | Y | 57.06 | 1401.7041 | 12 | 4.4 | 701.8624 | 2 | 80.07 | 6 | 22891 | Ana\_05.raw | 5.28E5 | 6.59E5 |  |  |  | 1.36E6 |  |  |  |  | 3 | 1 | 1 | 0 | 0 | 0 | 1 | 0 | 0 | 0 | 0 | 800 | 811 |  |
| R.YLGLLENVR.V | N | 56.80 | 1075.6025 | 9 | 3.7 | 538.8105 | 2 | 86.42 | 2 | 24481 | Ana\_01.raw | 6.48E6 | 8.49E6 | 6.45E6 | 2.58E6 | 3.07E6 | 6.17E6 | 4.11E6 | 2.56E6 | 1.54E7 | 4.08E6 | 12 | 2 | 2 | 1 | 1 | 1 | 1 | 1 | 1 | 1 | 1 | 611 | 619 |  |
| K.SLFPEGNPK.E | Y | 56.20 | 987.5025 | 9 | 4.5 | 494.7608 | 2 | 54.69 | 7 | 15410 | Ana\_06.raw | 7.2E6 | 1.12E7 | 6.61E5 | 3.62E6 | 5.99E6 | 8.41E6 | 6.45E6 | 2.88E6 | 1.59E7 | 5.14E6 | 12 | 1 | 2 | 1 | 1 | 1 | 1 | 1 | 1 | 2 | 1 | 545 | 553 |  |
| R.GILAM(+15.99)LDEEC(+57.02)LRPGVVSDSTFLAK.L | Y | 54.91 | 2636.3188 | 24 | 5.1 | 879.7847 | 3 | 118.64 | 2 | 32993 | Ana\_01.raw |  | 1.77E6 |  |  |  | 1.37E6 |  |  |  |  | 2 | 0 | 1 | 0 | 0 | 0 | 1 | 0 | 0 | 0 | 0 | 448 | 471 | Oxidation (M); Carbamidomethylation |
| R.GDYIGLQGNPK.L | Y | 54.87 | 1160.5825 | 11 | 7.3 | 581.3028 | 2 | 46.69 | 9 | 13042 | Ana\_08.raw |  |  |  |  |  |  |  |  | 1.11E7 |  | 1 | 0 | 0 | 0 | 0 | 0 | 0 | 0 | 0 | 1 | 0 | 871 | 881 |  |
| K.ASYPQSVPIPFR.G | Y | 52.51 | 1360.7139 | 12 | 4.2 | 681.3671 | 2 | 85.71 | 9 | 24378 | Ana\_08.raw | 6.49E6 |  | 6.11E6 | 3.79E6 | 6.57E6 | 6.78E6 | 6.15E6 |  | 1.35E7 | 4.82E6 | 8 | 1 | 0 | 1 | 1 | 1 | 1 | 1 | 0 | 1 | 1 | 859 | 870 |  |
| K.RPPTAGTQFK.N | Y | 50.21 | 1101.5930 | 10 | 5.5 | 551.8068 | 2 | 23.07 | 9 | 5654 | Ana\_08.raw |  | 1.16E7 |  |  |  | 9.65E6 | 6.74E6 | 4.85E6 | 1.71E7 | 4.86E6 | 9 | 0 | 2 | 0 | 0 | 0 | 1 | 1 | 1 | 3 | 1 | 559 | 568 |  |
| K.VIQGPEGGGNRK.L | Y | 50.06 | 1210.6418 | 12 | 5.5 | 606.3315 | 2 | 20.15 | 2 | 4789 | Ana\_01.raw |  | 9.62E5 |  |  |  |  |  |  |  | 9.19E5 | 2 | 0 | 1 | 0 | 0 | 0 | 0 | 0 | 0 | 0 | 1 | 1016 | 1027 |  |
| K.TLFYLEEQR.R | Y | 49.09 | 1197.6029 | 9 | 0.7 | 599.8091 | 2 | 82.39 | 4 | 22857 | Ana\_03.raw |  |  |  | 1.8E6 |  |  | 3.19E6 |  |  |  | 2 | 0 | 0 | 0 | 1 | 0 | 0 | 1 | 0 | 0 | 0 | 685 | 693 |  |
| K.VIQGPEGGGNR.K | Y | 49.00 | 1082.5469 | 11 | -4.0 | 542.2786 | 2 | 22.01 | 3 | 5608 | Ana\_02.raw | 1.24E7 |  | 1.4E7 |  | 2.29E6 | 5.38E6 | 3.7E6 | 2.4E6 |  | 1.16E7 | 7 | 1 | 0 | 1 | 0 | 1 | 1 | 1 | 1 | 0 | 1 | 1016 | 1026 |  |
| R.SGAALTLANFIYQSM(+15.99)AQK.F | Y | 46.63 | 1928.9666 | 18 | 5.6 | 965.4960 | 2 | 124.22 | 7 | 34238 | Ana\_06.raw |  | 1.54E6 | 1.31E6 |  |  | 1.91E6 | 2.21E6 | 1.14E6 | 1.06E6 |  | 8 | 0 | 1 | 1 | 0 | 0 | 1 | 2 | 1 | 2 | 0 | 769 | 786 | Oxidation (M) |
| K.GSNAM(+15.99)EVTVR | Y | 45.79 | 1078.5077 | 10 | 4.6 | 540.2636 | 2 | 24.79 | 6 | 6482 | Ana\_05.raw |  |  | 4.05E6 |  |  | 5.1E6 |  |  |  |  | 2 | 0 | 0 | 1 | 0 | 0 | 1 | 0 | 0 | 0 | 0 | 1034 | 1043 | Oxidation (M) |
| R.LFYQWK.C | Y | 41.03 | 883.4592 | 6 | 4.3 | 442.7388 | 2 | 71.74 | 9 | 20475 | Ana\_08.raw |  |  |  |  |  | 6.58E5 |  | 5.71E5 | 8.15E5 |  | 3 | 0 | 0 | 0 | 0 | 0 | 1 | 0 | 1 | 1 | 0 | 823 | 828 |  |
| R.AGYAFR.Q | N | 39.23 | 683.3391 | 6 | 4.8 | 342.6784 | 2 | 32.87 | 10 | 8925 | Ana\_09.raw |  |  |  |  |  |  |  |  | 4.23E6 | 2.43E6 | 2 | 0 | 0 | 0 | 0 | 0 | 0 | 0 | 0 | 1 | 1 | 623 | 628 |  |
| K.YM(+15.99)DIEFDFK.G | N | 30.07 | 1222.5216 | 9 | 4.5 | 612.2708 | 2 | 86.63 | 9 | 24625 | Ana\_08.raw |  |  |  |  |  |  |  |  | 0 |  | 1 | 0 | 0 | 0 | 0 | 0 | 0 | 0 | 0 | 1 | 0 | 162 | 170 | Oxidation (M) |
| total 27 peptides |  |  |  |  |  |  |  |  |  |  |  |  |  |  |  |  |  |  |  |  |  |  |  |  |  |  |  |  |  |  |  |  |  |  |  |
| --- | --- | --- | --- | --- | --- | --- | --- | --- | --- | --- | --- | --- | --- | --- | --- | --- | --- | --- | --- | --- | --- | --- | --- | --- | --- | --- | --- | --- | --- | --- | --- | --- | --- | --- | --- |

Best Unique PSM (Scan F6:33985, m/z=780.9369, z=2, RT=121.91, ppm=2.2):

sp|P14206|RSSA\_MOUSE

back to list

  

| Protein Coverage
| Supporting Peptides
| Best Unique PSM
|

Protein Coverage:

Supporting Peptides:

| Peptide | Uniq | -10lgP | Mass | Length | ppm | m/z | z | RT | Fraction | Scan | Source File | Area 10 | Area 01 | Area 02 | Area 03 | Area 04 | Area 05 | Area 06 | Area 07 | Area 08 | Area 09 | #Spec | #Spec 10 | #Spec 01 | #Spec 02 | #Spec 03 | #Spec 04 | #Spec 05 | #Spec 06 | #Spec 07 | #Spec 08 | #Spec 09 | Start | End | PTM |
| --- | --- | --- | --- | --- | --- | --- | --- | --- | --- | --- | --- | --- | --- | --- | --- | --- | --- | --- | --- | --- | --- | --- | --- | --- | --- | --- | --- | --- | --- | --- | --- | --- | --- | --- | --- |
| R.ADHQPLTEASYVNLPTIALC(+57.02)NTDSPLR.Y | Y | 139.78 | 2995.4709 | 27 | 3.3 | 999.5009 | 3 | 117.34 | 6 | 32783 | Ana\_05.raw | 1.62E7 | 1.88E7 | 2.6E6 | 1.22E6 | 7.56E6 | 3.5E6 | 4.38E6 | 1.97E6 | 1E7 | 5.78E6 | 11 | 1 | 1 | 1 | 1 | 1 | 1 | 1 | 1 | 2 | 1 | 129 | 155 | Carbamidomethylation |
| R.FTPGTFTNQIQAAFR.E | Y | 114.02 | 1697.8525 | 15 | 1.8 | 849.9351 | 2 | 113.53 | 3 | 31381 | Ana\_02.raw | 3.15E7 | 3.36E7 | 3.66E7 | 2.04E7 | 3.16E7 | 3.61E7 | 1.43E7 | 2.61E7 | 4.37E7 | 2.5E7 | 26 | 2 | 3 | 3 | 2 | 3 | 3 | 2 | 2 | 3 | 3 | 103 | 117 |  |
| R.AIVAIENPADVSVISSR.N | Y | 96.37 | 1739.9417 | 17 | 4.3 | 870.9818 | 2 | 93.98 | 6 | 26653 | Ana\_05.raw | 1.37E7 | 9.77E6 |  | 1.34E6 |  | 2E6 |  | 5.81E5 | 3.93E7 | 1.97E7 | 11 | 2 | 1 | 0 | 2 | 0 | 1 | 0 | 1 | 2 | 2 | 64 | 80 |  |
| R.DPEEIEKEEQAAAEK.A | Y | 96.01 | 1714.7897 | 15 | 5.3 | 858.4067 | 2 | 57.21 | 6 | 16349 | Ana\_05.raw | 3.93E6 |  |  | 2.24E6 | 2.76E6 | 7.5E6 | 1.21E6 |  | 4.06E6 | 1.81E6 | 9 | 1 | 0 | 0 | 2 | 1 | 2 | 1 | 0 | 1 | 1 | 206 | 220 |  |
| K.FAAATGATPIAGR.F | Y | 91.88 | 1202.6407 | 13 | 0.3 | 602.3278 | 2 | 46.88 | 3 | 13236 | Ana\_02.raw | 4.59E7 | 4.81E7 | 4.67E7 | 2.22E7 | 3.65E7 | 3.37E7 | 2.91E7 | 2.81E7 | 5.01E5 | 3.46E7 | 16 | 1 | 2 | 1 | 2 | 1 | 1 | 2 | 2 | 2 | 2 | 90 | 102 |  |
| R.EHPWEVM(+15.99)PDLYFYRDPEEIEKEEQAAAEK.A | Y | 73.06 | 3593.6296 | 29 | 5.9 | 899.4200 | 4 | 108.33 | 9 | 30444 | Ana\_08.raw |  |  |  |  |  |  |  |  | 1.21E7 |  | 1 | 0 | 0 | 0 | 0 | 0 | 0 | 0 | 0 | 1 | 0 | 192 | 220 | Oxidation (M) |
| R.EHPWEVM(+15.99)PDLYFYR.D | Y | 63.47 | 1896.8505 | 14 | 6.1 | 633.2946 | 3 | 105.37 | 3 | 29289 | Ana\_02.raw |  |  | 1.51E6 | 8.1E5 | 4.37E5 |  | 8.49E5 | 5.64E5 |  |  | 5 | 0 | 0 | 1 | 1 | 1 | 0 | 1 | 1 | 0 | 0 | 192 | 205 | Oxidation (M) |
| K.SDGIYIINLK.R | Y | 51.93 | 1134.6284 | 10 | 2.1 | 568.3227 | 2 | 92.74 | 4 | 25610 | Ana\_03.raw | 9.56E5 | 1.88E6 | 1.08E6 | 6.78E5 | 1.23E6 | 5.23E5 | 7.83E5 | 7.5E5 | 3.51E6 | 1.22E6 | 10 | 1 | 1 | 1 | 1 | 1 | 1 | 1 | 1 | 1 | 1 | 43 | 52 |  |
| R.LLVVTDPR.A | Y | 47.01 | 911.5440 | 8 | 1.4 | 456.7799 | 2 | 52.80 | 3 | 14960 | Ana\_02.raw | 2.04E7 | 1.76E7 | 2.49E7 | 1.14E7 | 2.02E7 | 1.68E7 | 1.53E7 | 1.37E7 | 3.13E7 | 2.08E7 | 15 | 1 | 1 | 2 | 2 | 1 | 2 | 2 | 2 | 1 | 1 | 121 | 128 |  |
| R.YVDIAIPC(+57.02)NNK.G | Y | 43.95 | 1305.6387 | 11 | 4.7 | 653.8297 | 2 | 66.76 | 6 | 19171 | Ana\_05.raw | 2.44E6 | 4.59E5 |  | 7.55E5 |  | 5.38E5 | 8.32E5 |  |  |  | 5 | 1 | 1 | 0 | 1 | 0 | 1 | 1 | 0 | 0 | 0 | 156 | 166 | Carbamidomethylation |
| total 10 peptides |  |  |  |  |  |  |  |  |  |  |  |  |  |  |  |  |  |  |  |  |  |  |  |  |  |  |  |  |  |  |  |  |  |  |  |
| --- | --- | --- | --- | --- | --- | --- | --- | --- | --- | --- | --- | --- | --- | --- | --- | --- | --- | --- | --- | --- | --- | --- | --- | --- | --- | --- | --- | --- | --- | --- | --- | --- | --- | --- | --- |

Best Unique PSM (Scan F6:32783, m/z=999.5009, z=3, RT=117.34, ppm=3.3):

sp|Q61598|GDIB\_MOUSE

back to list

  

| Protein Coverage
| Supporting Peptides
| Best Unique PSM
|

Protein Coverage:

Supporting Peptides:

| Peptide | Uniq | -10lgP | Mass | Length | ppm | m/z | z | RT | Fraction | Scan | Source File | Area 10 | Area 01 | Area 02 | Area 03 | Area 04 | Area 05 | Area 06 | Area 07 | Area 08 | Area 09 | #Spec | #Spec 10 | #Spec 01 | #Spec 02 | #Spec 03 | #Spec 04 | #Spec 05 | #Spec 06 | #Spec 07 | #Spec 08 | #Spec 09 | Start | End | PTM |
| --- | --- | --- | --- | --- | --- | --- | --- | --- | --- | --- | --- | --- | --- | --- | --- | --- | --- | --- | --- | --- | --- | --- | --- | --- | --- | --- | --- | --- | --- | --- | --- | --- | --- | --- | --- |
| K.SPYLYPLYGLGELPQGFAR.L | Y | 115.64 | 2140.0991 | 19 | 3.8 | 714.3763 | 3 | 131.11 | 6 | 36428 | Ana\_05.raw | 6.42E6 | 4.97E6 | 5.56E6 | 1.85E6 | 3.72E6 | 1.51E7 | 1.04E7 | 4.91E6 | 3.35E7 | 2.05E7 | 14 | 1 | 1 | 1 | 1 | 1 | 2 | 2 | 1 | 2 | 2 | 222 | 240 |  |
| K.FDLGQDVIDFTGHSLALYR.T | Y | 112.74 | 2166.0745 | 19 | -1.5 | 723.0310 | 3 | 131.58 | 3 | 36045 | Ana\_02.raw |  | 9.77E6 | 5.27E6 | 2.32E6 | 6.06E6 | 9.89E6 | 3.55E6 | 3.76E6 | 8.62E6 | 1.08E6 | 9 | 0 | 1 | 1 | 1 | 1 | 1 | 1 | 1 | 1 | 1 | 175 | 193 |  |
| K.VPSTEAEALASSLM(+15.99)GLFEK.R | Y | 91.21 | 1994.9871 | 19 | 4.7 | 998.5055 | 2 | 133.21 | 5 | 36643 | Ana\_04.raw | 1.12E7 | 7.66E6 | 1.32E7 | 1.34E6 | 7.49E6 | 1.59E7 | 1.03E7 | 4.3E6 | 1.57E7 | 1.61E7 | 21 | 2 | 2 | 2 | 2 | 2 | 2 | 2 | 2 | 3 | 2 | 119 | 137 | Oxidation (M) |
| K.VPSTEAEALASSLMGLFEK.R | Y | 90.02 | 1978.9921 | 19 | 5.7 | 990.5090 | 2 | 137.40 | 9 | 37962 | Ana\_08.raw | 4.92E5 |  |  |  |  |  |  |  | 3.26E6 | 4.8E5 | 4 | 1 | 0 | 0 | 0 | 0 | 0 | 0 | 0 | 2 | 1 | 119 | 137 |  |
| K.EIRPALELLEPIEQK.F | Y | 88.50 | 1776.9985 | 15 | 4.6 | 593.3428 | 3 | 105.08 | 7 | 29279 | Ana\_06.raw | 1.23E6 | 5.98E5 | 1.22E7 | 5.06E5 | 2.23E6 | 1.2E7 | 5.02E6 | 2.58E6 | 2.01E7 | 2.51E6 | 16 | 3 | 1 | 2 | 1 | 1 | 2 | 2 | 1 | 2 | 1 | 365 | 379 |  |
| K.DLGTDSQIFISR.A | Y | 86.31 | 1350.6779 | 12 | 5.7 | 676.3500 | 2 | 85.60 | 1 | 24105 | ANa\_10.raw | 4.97E6 | 4.06E6 | 4.58E6 |  | 3.64E6 | 3.34E6 |  |  | 9.84E6 | 4.1E6 | 7 | 1 | 1 | 1 | 0 | 1 | 1 | 0 | 0 | 1 | 1 | 391 | 402 |  |
| R.TDDYLDQPC(+57.02)C(+57.02)ETINR.I | Y | 84.88 | 1898.7775 | 15 | 1.3 | 950.3973 | 2 | 63.81 | 9 | 18194 | Ana\_08.raw |  | 7.49E5 |  |  |  |  |  |  | 8.94E5 |  | 2 | 0 | 1 | 0 | 0 | 0 | 0 | 0 | 0 | 1 | 0 | 194 | 208 | Carbamidomethylation |
| K.FVSISDLFVPK.D | Y | 84.17 | 1250.6910 | 11 | 2.8 | 626.3546 | 2 | 122.10 | 6 | 34034 | Ana\_05.raw | 2.58E7 | 4.19E7 | 4.38E7 | 9.21E6 | 2.88E7 | 3.57E7 | 2.5E7 | 2.3E7 | 6.83E7 | 4.29E7 | 29 | 4 | 2 | 4 | 2 | 3 | 2 | 2 | 4 | 2 | 4 | 380 | 390 |  |
| K.VLHM(+15.99)DQNPYYGGESASITPLEDLYK.R | Y | 77.95 | 2855.3323 | 25 | 3.6 | 952.7881 | 3 | 104.61 | 1 | 29163 | ANa\_10.raw | 1.96E6 | 2.83E6 |  |  |  |  |  | 5.31E5 |  | 2.4E6 | 4 | 1 | 1 | 0 | 0 | 0 | 0 | 0 | 1 | 0 | 1 | 30 | 54 | Oxidation (M) |
| K.MLLFTEVTR.Y | Y | 58.20 | 1108.5951 | 9 | 5.7 | 555.3080 | 2 | 94.27 | 6 | 26730 | Ana\_05.raw |  |  |  |  |  | 3.64E5 |  |  |  |  | 1 | 0 | 0 | 0 | 0 | 0 | 1 | 0 | 0 | 0 | 0 | 90 | 98 |  |
| R.DWNVDLIPK.F | Y | 50.54 | 1098.5709 | 9 | 6.3 | 550.2962 | 2 | 96.83 | 9 | 27405 | Ana\_08.raw | 3.42E5 | 1.96E6 | 2.55E6 | 1.05E6 | 2.85E5 | 8.46E5 | 1.11E6 | 6.51E5 | 2.74E6 |  | 9 | 1 | 1 | 1 | 1 | 1 | 1 | 1 | 1 | 1 | 0 | 71 | 79 |  |
| K.M(+15.99)LLFTEVTR.Y | Y | 49.13 | 1124.5900 | 9 | 4.9 | 563.3050 | 2 | 92.79 | 2 | 26215 | Ana\_01.raw | 3.06E5 | 7.29E5 | 7.88E5 | 7.46E5 | 0 | 3.68E6 | 6.38E5 | 3.55E6 | 6.67E5 | 2.33E5 | 12 | 1 | 1 | 1 | 1 | 1 | 2 | 1 | 2 | 1 | 1 | 90 | 98 | Oxidation (M) |
| R.TFEGVDPK.K | Y | 42.79 | 891.4338 | 8 | 3.0 | 446.7255 | 2 | 37.39 | 4 | 10051 | Ana\_03.raw | 1.26E7 | 1.54E7 | 2.05E7 | 9.91E6 |  | 1.84E7 |  | 1.13E7 | 7.34E6 | 1.76E7 | 9 | 1 | 1 | 1 | 2 | 0 | 1 | 0 | 1 | 1 | 1 | 157 | 164 |  |
| K.VGQVIR.V | Y | 37.39 | 670.4126 | 6 | 4.5 | 336.2151 | 2 | 22.79 | 8 | 5687 | Ana\_07.raw |  |  | 4.96E6 | 4.34E6 | 7.87E6 | 9.17E6 |  | 3.49E6 |  |  | 5 | 0 | 0 | 1 | 1 | 1 | 1 | 0 | 1 | 0 | 0 | 294 | 299 |  |
| K.LYSESLAR.Y | Y | 37.34 | 937.4869 | 8 | 3.2 | 469.7522 | 2 | 38.91 | 3 | 10816 | Ana\_02.raw |  |  | 2.52E7 | 1.08E7 | 1.75E7 | 2.1E7 |  | 1.4E7 | 1.63E7 | 2.23E7 | 7 | 0 | 0 | 1 | 1 | 1 | 1 | 0 | 1 | 1 | 1 | 211 | 218 |  |
| R.FKLPGQPPASM(+15.99)GR.G | Y | 35.07 | 1400.7234 | 13 | 3.0 | 467.9165 | 3 | 53.95 | 5 | 15289 | Ana\_04.raw |  | 1.3E6 |  |  | 3.4E6 |  |  |  |  |  | 2 | 0 | 1 | 0 | 0 | 1 | 0 | 0 | 0 | 0 | 0 | 56 | 68 | Oxidation (M) |
| K.LPGQPPASM(+15.99)GR.G | Y | 34.26 | 1125.5601 | 11 | 5.6 | 563.7905 | 2 | 30.07 | 6 | 8151 | Ana\_05.raw | 3.92E6 | 3.7E6 |  |  |  | 2.14E6 | 1.42E6 | 1.35E6 |  |  | 5 | 1 | 1 | 0 | 0 | 0 | 1 | 1 | 1 | 0 | 0 | 58 | 68 | Oxidation (M) |
| K.VIEGSFVYK.G | Y | 28.78 | 1040.5542 | 9 | 6.0 | 521.2875 | 2 | 56.75 | 9 | 16088 | Ana\_08.raw | 1.03E7 |  |  |  |  |  |  |  | 2E7 |  | 2 | 1 | 0 | 0 | 0 | 0 | 0 | 0 | 0 | 1 | 0 | 104 | 112 |  |
| R.M(+15.99)TGSEFDFEEM(+15.99)KR.K | Y | 24.30 | 1637.6702 | 13 | 5.5 | 819.8469 | 2 | 56.17 | 7 | 15843 | Ana\_06.raw |  |  |  |  |  |  | 8.38E5 |  |  |  | 1 | 0 | 0 | 0 | 0 | 0 | 0 | 1 | 0 | 0 | 0 | 424 | 436 | Oxidation (M) |
| total 19 peptides |  |  |  |  |  |  |  |  |  |  |  |  |  |  |  |  |  |  |  |  |  |  |  |  |  |  |  |  |  |  |  |  |  |  |  |
| --- | --- | --- | --- | --- | --- | --- | --- | --- | --- | --- | --- | --- | --- | --- | --- | --- | --- | --- | --- | --- | --- | --- | --- | --- | --- | --- | --- | --- | --- | --- | --- | --- | --- | --- | --- |

Best Unique PSM (Scan F6:36428, m/z=714.3763, z=3, RT=131.11, ppm=3.8):

A0A7N9VRC4|A0A7N9VRC4\_MOUSE

back to list

  

| Protein Coverage
| Supporting Peptides
| Best Unique PSM
|

Protein Coverage:

Supporting Peptides:

| Peptide | Uniq | -10lgP | Mass | Length | ppm | m/z | z | RT | Fraction | Scan | Source File | Area 10 | Area 01 | Area 02 | Area 03 | Area 04 | Area 05 | Area 06 | Area 07 | Area 08 | Area 09 | #Spec | #Spec 10 | #Spec 01 | #Spec 02 | #Spec 03 | #Spec 04 | #Spec 05 | #Spec 06 | #Spec 07 | #Spec 08 | #Spec 09 | Start | End | PTM |
| --- | --- | --- | --- | --- | --- | --- | --- | --- | --- | --- | --- | --- | --- | --- | --- | --- | --- | --- | --- | --- | --- | --- | --- | --- | --- | --- | --- | --- | --- | --- | --- | --- | --- | --- | --- |
| K.GFFDPNTHENLTYLQLLER.C | N | 112.87 | 2306.1331 | 19 | 4.9 | 769.7220 | 3 | 126.30 | 2 | 34999 | Ana\_01.raw | 2.27E6 | 6.61E6 | 4.35E6 |  |  | 1.49E7 |  | 1.3E6 | 1.31E7 | 9.32E6 | 7 | 1 | 1 | 1 | 0 | 0 | 1 | 0 | 1 | 1 | 1 | 774 | 792 |  |
| R.GSGTSPDEGDAQDSSESAR.Q | Y | 96.10 | 1851.7355 | 19 | 1.5 | 926.8764 | 2 | 24.49 | 6 | 6385 | Ana\_05.raw | 1.61E6 | 2.67E6 | 1.98E6 | 1.15E6 | 2.26E6 | 2.91E6 | 1.6E6 | 7.65E5 |  | 7.58E5 | 9 | 1 | 1 | 1 | 1 | 1 | 1 | 1 | 1 | 0 | 1 | 2602 | 2620 |  |
| R.GHVVPVWDILTSNYVSR.D | Y | 95.87 | 1941.0109 | 17 | 4.4 | 648.0137 | 3 | 122.37 | 6 | 34105 | Ana\_05.raw | 9.77E5 |  |  | 2.9E5 | 1.93E6 | 3.64E6 |  | 4.31E5 | 3.13E6 | 1.33E6 | 7 | 1 | 0 | 0 | 1 | 1 | 1 | 0 | 1 | 1 | 1 | 3046 | 3062 |  |
| R.YQAGGLTVHDVTTTLTSLLAR.A | Y | 87.12 | 2216.1802 | 21 | 4.5 | 739.7373 | 3 | 132.88 | 1 | 36531 | ANa\_10.raw | 1.16E7 | 1.25E7 | 7.96E6 | 4.09E6 | 8.71E6 | 9.25E6 |  | 5.8E6 | 1.66E7 | 1.13E7 | 10 | 1 | 1 | 1 | 1 | 1 | 1 | 0 | 1 | 1 | 2 | 2998 | 3018 |  |
| R.GHEVPVWDILTSNYVSR.D | Y | 86.88 | 1970.9850 | 17 | 0.4 | 658.0026 | 3 | 120.07 | 6 | 33510 | Ana\_05.raw |  | 2.93E5 |  | 1.61E6 | 1.53E6 | 3.23E6 | 6.09E5 | 5.63E5 | 2.82E5 |  | 7 | 0 | 1 | 0 | 1 | 1 | 1 | 1 | 1 | 1 | 0 | 2531 | 2547 |  |
| R.RYQAGGLTVHDVTTTLTSLLAR.A | Y | 86.73 | 2372.2812 | 22 | 5.6 | 594.0809 | 4 | 130.04 | 6 | 36126 | Ana\_05.raw |  |  |  | 6.46E5 |  | 1.54E6 |  |  |  |  | 2 | 0 | 0 | 0 | 1 | 0 | 1 | 0 | 0 | 0 | 0 | 2997 | 3018 |  |
| K.GFFDPNTHENLTYLQLLR.R | Y | 86.26 | 2177.0906 | 18 | 1.1 | 726.7050 | 3 | 125.08 | 5 | 34494 | Ana\_04.raw | 1.66E6 | 5.73E6 | 5.4E6 | 6.25E5 | 9.18E6 | 4.52E6 | 7.27E6 | 5.9E6 | 8.46E6 | 2.54E6 | 10 | 1 | 1 | 1 | 1 | 1 | 1 | 1 | 1 | 1 | 1 | 2393 | 2410 |  |
| K.VLFSSYLSETR.R | Y | 64.62 | 1300.6663 | 11 | 1.4 | 651.3413 | 2 | 92.95 | 6 | 26375 | Ana\_05.raw |  |  |  | 1.15E6 | 3.57E6 | 3.5E6 | 1.81E6 |  |  |  | 4 | 0 | 0 | 0 | 1 | 1 | 1 | 1 | 0 | 0 | 0 | 2649 | 2659 |  |
| K.VSFPGLR.R | Y | 60.60 | 774.4388 | 7 | 2.7 | 388.2277 | 2 | 66.50 | 6 | 19093 | Ana\_05.raw | 8.67E5 | 3.89E6 |  |  | 2.76E6 | 2.54E6 | 7.1E5 | 1.6E6 | 6.37E6 | 3.96E6 | 8 | 1 | 1 | 0 | 0 | 1 | 1 | 1 | 1 | 1 | 1 | 2695 | 2701 |  |
| R.VPVDVAYQR.G | Y | 60.32 | 1045.5557 | 9 | 3.7 | 523.7870 | 2 | 47.83 | 4 | 13140 | Ana\_03.raw | 1.53E7 | 1.46E7 |  | 7.37E6 | 1.13E7 | 5.75E6 | 9.37E6 |  | 4.65E6 | 1.42E7 | 8 | 1 | 1 | 0 | 1 | 1 | 1 | 1 | 0 | 1 | 1 | 746 | 754 |  |
| R.ILSDPSDDTK.G | N | 56.91 | 1089.5189 | 10 | 5.1 | 545.7695 | 2 | 28.12 | 3 | 7553 | Ana\_02.raw | 7.89E6 | 1.53E7 | 5.42E6 |  |  |  | 2.54E6 |  | 4.9E6 |  | 5 | 1 | 1 | 1 | 0 | 0 | 0 | 1 | 0 | 1 | 0 | 1725 | 1734 |  |
| K.ELLAQFSSGSLTLPM(+15.99)LK.R | Y | 52.59 | 1849.9858 | 17 | -1.2 | 925.9991 | 2 | 121.88 | 3 | 33518 | Ana\_02.raw |  | 5.92E5 | 2.56E5 |  |  | 7.41E5 |  |  |  |  | 3 | 0 | 1 | 1 | 0 | 0 | 1 | 0 | 0 | 0 | 0 | 2552 | 2568 | Oxidation (M) |
| R.EDPQGALGK.A | Y | 52.52 | 913.4504 | 9 | 5.7 | 457.7351 | 2 | 25.95 | 2 | 6737 | Ana\_01.raw | 7.16E6 | 6.91E6 |  |  |  |  |  |  |  |  | 2 | 1 | 1 | 0 | 0 | 0 | 0 | 0 | 0 | 0 | 0 | 3026 | 3034 |  |
| R.EVPESLR.Q | Y | 32.66 | 828.4341 | 7 | 5.3 | 415.2265 | 2 | 39.01 | 7 | 10688 | Ana\_06.raw |  | 3.56E6 |  |  |  |  | 0 |  |  |  | 2 | 0 | 1 | 0 | 0 | 0 | 0 | 1 | 0 | 0 | 0 | 2470 | 2476 |  |
| K.GSSVHHLSEELR.R | Y | 31.66 | 1349.6687 | 12 | 5.0 | 450.8991 | 3 | 27.54 | 9 | 7142 | Ana\_08.raw |  |  |  |  |  |  |  |  | 3.41E6 |  | 1 | 0 | 0 | 0 | 0 | 0 | 0 | 0 | 0 | 1 | 0 | 2429 | 2440 |  |
| K.LLSAER.A | N | 30.63 | 687.3915 | 6 | 4.5 | 344.7046 | 2 | 23.39 | 1 | 5905 | ANa\_10.raw | 1.15E7 | 1.14E7 | 2.09E7 |  |  |  | 1.01E6 |  |  |  | 4 | 1 | 1 | 1 | 0 | 0 | 0 | 1 | 0 | 0 | 0 | 1650 | 1655 |  |
| K.VTFAGLR.D | Y | 27.94 | 762.4388 | 7 | 3.7 | 382.2281 | 2 | 34.75 | 2 | 9437 | Ana\_01.raw |  | 2.98E5 |  |  |  |  |  |  |  |  | 1 | 0 | 1 | 0 | 0 | 0 | 0 | 0 | 0 | 0 | 0 | 566 | 572 |  |
| K.TLDALR.S | Y | 25.94 | 687.3915 | 6 | 4.2 | 344.7045 | 2 | 23.90 | 6 | 6203 | Ana\_05.raw |  |  |  |  | 2.21E6 | 5.57E6 |  |  |  |  | 2 | 0 | 0 | 0 | 0 | 1 | 1 | 0 | 0 | 0 | 0 | 1877 | 1882 |  |
| K.LLAAER.A | N | 25.36 | 671.3966 | 6 | 3.7 | 336.7068 | 2 | 23.66 | 2 | 5979 | Ana\_01.raw |  | 1.33E6 |  |  |  |  |  |  |  |  | 1 | 0 | 1 | 0 | 0 | 0 | 0 | 0 | 0 | 0 | 0 | 119 | 124 |  |
| total 19 peptides |  |  |  |  |  |  |  |  |  |  |  |  |  |  |  |  |  |  |  |  |  |  |  |  |  |  |  |  |  |  |  |  |  |  |  |
| --- | --- | --- | --- | --- | --- | --- | --- | --- | --- | --- | --- | --- | --- | --- | --- | --- | --- | --- | --- | --- | --- | --- | --- | --- | --- | --- | --- | --- | --- | --- | --- | --- | --- | --- | --- |

Best Unique PSM (Scan F6:6385, m/z=926.8764, z=2, RT=24.49, ppm=1.5):

sp|Q8R0W0|EPIPL\_MOUSE

back to list

  

| Protein Coverage
| Supporting Peptides
| Best Unique PSM
|

Protein Coverage:

Supporting Peptides:

| Peptide | Uniq | -10lgP | Mass | Length | ppm | m/z | z | RT | Fraction | Scan | Source File | Area 10 | Area 01 | Area 02 | Area 03 | Area 04 | Area 05 | Area 06 | Area 07 | Area 08 | Area 09 | #Spec | #Spec 10 | #Spec 01 | #Spec 02 | #Spec 03 | #Spec 04 | #Spec 05 | #Spec 06 | #Spec 07 | #Spec 08 | #Spec 09 | Start | End | PTM |
| --- | --- | --- | --- | --- | --- | --- | --- | --- | --- | --- | --- | --- | --- | --- | --- | --- | --- | --- | --- | --- | --- | --- | --- | --- | --- | --- | --- | --- | --- | --- | --- | --- | --- | --- | --- |
| K.GFFDPNTHENLTYLQLLER.C | N | 112.87 | 2306.1331 | 19 | 4.9 | 769.7220 | 3 | 126.30 | 2 | 34999 | Ana\_01.raw | 2.27E6 | 6.61E6 | 4.35E6 |  |  | 1.49E7 |  | 1.3E6 | 1.31E7 | 9.32E6 | 7 | 1 | 1 | 1 | 0 | 0 | 1 | 0 | 1 | 1 | 1 | 774 | 792 |  |
| R.GSGTSPDEGDAQDSSESAR.Q | Y | 96.10 | 1851.7355 | 19 | 1.5 | 926.8764 | 2 | 24.49 | 6 | 6385 | Ana\_05.raw | 1.61E6 | 2.67E6 | 1.98E6 | 1.15E6 | 2.26E6 | 2.91E6 | 1.6E6 | 7.65E5 |  | 7.58E5 | 9 | 1 | 1 | 1 | 1 | 1 | 1 | 1 | 1 | 0 | 1 | 2602 | 2620 |  |
| R.GHVVPVWDILTSNYVSR.D | Y | 95.87 | 1941.0109 | 17 | 4.4 | 648.0137 | 3 | 122.37 | 6 | 34105 | Ana\_05.raw | 9.77E5 |  |  | 2.9E5 | 1.93E6 | 3.64E6 |  | 4.31E5 | 3.13E6 | 1.33E6 | 7 | 1 | 0 | 0 | 1 | 1 | 1 | 0 | 1 | 1 | 1 | 6136 | 6152 |  |
| R.YQAGGLTVHDVTTTLTSLLAR.A | Y | 87.12 | 2216.1802 | 21 | 4.5 | 739.7373 | 3 | 132.88 | 1 | 36531 | ANa\_10.raw | 1.16E7 | 1.25E7 | 7.96E6 | 4.09E6 | 8.71E6 | 9.25E6 |  | 5.8E6 | 1.66E7 | 1.13E7 | 10 | 1 | 1 | 1 | 1 | 1 | 1 | 0 | 1 | 1 | 2 | 2998 | 3018 |  |
| R.GHEVPVWDILTSNYVSR.D | Y | 86.88 | 1970.9850 | 17 | 0.4 | 658.0026 | 3 | 120.07 | 6 | 33510 | Ana\_05.raw |  | 2.93E5 |  | 1.61E6 | 1.53E6 | 3.23E6 | 6.09E5 | 5.63E5 | 2.82E5 |  | 7 | 0 | 1 | 0 | 1 | 1 | 1 | 1 | 1 | 1 | 0 | 2531 | 2547 |  |
| R.RYQAGGLTVHDVTTTLTSLLAR.A | Y | 86.73 | 2372.2812 | 22 | 5.6 | 594.0809 | 4 | 130.04 | 6 | 36126 | Ana\_05.raw |  |  |  | 6.46E5 |  | 1.54E6 |  |  |  |  | 2 | 0 | 0 | 0 | 1 | 0 | 1 | 0 | 0 | 0 | 0 | 2997 | 3018 |  |
| K.GFFDPNTHENLTYLQLLR.R | Y | 86.26 | 2177.0906 | 18 | 1.1 | 726.7050 | 3 | 125.08 | 5 | 34494 | Ana\_04.raw | 1.66E6 | 5.73E6 | 5.4E6 | 6.25E5 | 9.18E6 | 4.52E6 | 7.27E6 | 5.9E6 | 8.46E6 | 2.54E6 | 10 | 1 | 1 | 1 | 1 | 1 | 1 | 1 | 1 | 1 | 1 | 2393 | 2410 |  |
| K.VLFSSYLSETR.R | Y | 64.62 | 1300.6663 | 11 | 1.4 | 651.3413 | 2 | 92.95 | 6 | 26375 | Ana\_05.raw |  |  |  | 1.15E6 | 3.57E6 | 3.5E6 | 1.81E6 |  |  |  | 4 | 0 | 0 | 0 | 1 | 1 | 1 | 1 | 0 | 0 | 0 | 2649 | 2659 |  |
| K.VSFPGLR.R | Y | 60.60 | 774.4388 | 7 | 2.7 | 388.2277 | 2 | 66.50 | 6 | 19093 | Ana\_05.raw | 8.67E5 | 3.89E6 |  |  | 2.76E6 | 2.54E6 | 7.1E5 | 1.6E6 | 6.37E6 | 3.96E6 | 8 | 1 | 1 | 0 | 0 | 1 | 1 | 1 | 1 | 1 | 1 | 2695 | 2701 |  |
| R.VPVDVAYQR.G | Y | 60.32 | 1045.5557 | 9 | 3.7 | 523.7870 | 2 | 47.83 | 4 | 13140 | Ana\_03.raw | 1.53E7 | 1.46E7 |  | 7.37E6 | 1.13E7 | 5.75E6 | 9.37E6 |  | 4.65E6 | 1.42E7 | 8 | 1 | 1 | 0 | 1 | 1 | 1 | 1 | 0 | 1 | 1 | 746 | 754 |  |
| R.ILSDPSDDTK.G | N | 56.91 | 1089.5189 | 10 | 5.1 | 545.7695 | 2 | 28.12 | 3 | 7553 | Ana\_02.raw | 7.89E6 | 1.53E7 | 5.42E6 |  |  |  | 2.54E6 |  | 4.9E6 |  | 5 | 1 | 1 | 1 | 0 | 0 | 0 | 1 | 0 | 1 | 0 | 1725 | 1734 |  |
| K.ELLAQFSSGSLTLPM(+15.99)LK.R | Y | 52.59 | 1849.9858 | 17 | -1.2 | 925.9991 | 2 | 121.88 | 3 | 33518 | Ana\_02.raw |  | 5.92E5 | 2.56E5 |  |  | 7.41E5 |  |  |  |  | 3 | 0 | 1 | 1 | 0 | 0 | 1 | 0 | 0 | 0 | 0 | 2552 | 2568 | Oxidation (M) |
| R.EDPQGALGK.A | Y | 52.52 | 913.4504 | 9 | 5.7 | 457.7351 | 2 | 25.95 | 2 | 6737 | Ana\_01.raw | 7.16E6 | 6.91E6 |  |  |  |  |  |  |  |  | 2 | 1 | 1 | 0 | 0 | 0 | 0 | 0 | 0 | 0 | 0 | 6116 | 6124 |  |
| R.EVPESLR.Q | Y | 32.66 | 828.4341 | 7 | 5.3 | 415.2265 | 2 | 39.01 | 7 | 10688 | Ana\_06.raw |  | 3.56E6 |  |  |  |  | 0 |  |  |  | 2 | 0 | 1 | 0 | 0 | 0 | 0 | 1 | 0 | 0 | 0 | 2470 | 2476 |  |
| K.GSSVHHLSEELR.R | Y | 31.66 | 1349.6687 | 12 | 5.0 | 450.8991 | 3 | 27.54 | 9 | 7142 | Ana\_08.raw |  |  |  |  |  |  |  |  | 3.41E6 |  | 1 | 0 | 0 | 0 | 0 | 0 | 0 | 0 | 0 | 1 | 0 | 2429 | 2440 |  |
| K.LLSAER.A | N | 30.63 | 687.3915 | 6 | 4.5 | 344.7046 | 2 | 23.39 | 1 | 5905 | ANa\_10.raw | 1.15E7 | 1.14E7 | 2.09E7 |  |  |  | 1.01E6 |  |  |  | 4 | 1 | 1 | 1 | 0 | 0 | 0 | 1 | 0 | 0 | 0 | 1650 | 1655 |  |
| K.VTFAGLR.D | Y | 27.94 | 762.4388 | 7 | 3.7 | 382.2281 | 2 | 34.75 | 2 | 9437 | Ana\_01.raw |  | 2.98E5 |  |  |  |  |  |  |  |  | 1 | 0 | 1 | 0 | 0 | 0 | 0 | 0 | 0 | 0 | 0 | 566 | 572 |  |
| K.TLDALR.S | Y | 25.94 | 687.3915 | 6 | 4.2 | 344.7045 | 2 | 23.90 | 6 | 6203 | Ana\_05.raw |  |  |  |  | 2.21E6 | 5.57E6 |  |  |  |  | 2 | 0 | 0 | 0 | 0 | 1 | 1 | 0 | 0 | 0 | 0 | 1877 | 1882 |  |
| K.LLAAER.A | N | 25.36 | 671.3966 | 6 | 3.7 | 336.7068 | 2 | 23.66 | 2 | 5979 | Ana\_01.raw |  | 1.33E6 |  |  |  |  |  |  |  |  | 1 | 0 | 1 | 0 | 0 | 0 | 0 | 0 | 0 | 0 | 0 | 119 | 124 |  |
| total 19 peptides |  |  |  |  |  |  |  |  |  |  |  |  |  |  |  |  |  |  |  |  |  |  |  |  |  |  |  |  |  |  |  |  |  |  |  |
| --- | --- | --- | --- | --- | --- | --- | --- | --- | --- | --- | --- | --- | --- | --- | --- | --- | --- | --- | --- | --- | --- | --- | --- | --- | --- | --- | --- | --- | --- | --- | --- | --- | --- | --- | --- |

Best Unique PSM (Scan F6:6385, m/z=926.8764, z=2, RT=24.49, ppm=1.5):

sp|P63101|1433Z\_MOUSE

back to list

  

| Protein Coverage
| Supporting Peptides
| Best Unique PSM
|

Protein Coverage:

Supporting Peptides:

| Peptide | Uniq | -10lgP | Mass | Length | ppm | m/z | z | RT | Fraction | Scan | Source File | Area 10 | Area 01 | Area 02 | Area 03 | Area 04 | Area 05 | Area 06 | Area 07 | Area 08 | Area 09 | #Spec | #Spec 10 | #Spec 01 | #Spec 02 | #Spec 03 | #Spec 04 | #Spec 05 | #Spec 06 | #Spec 07 | #Spec 08 | #Spec 09 | Start | End | PTM |
| --- | --- | --- | --- | --- | --- | --- | --- | --- | --- | --- | --- | --- | --- | --- | --- | --- | --- | --- | --- | --- | --- | --- | --- | --- | --- | --- | --- | --- | --- | --- | --- | --- | --- | --- | --- |
| K.GIVDQSQQAYQEAFEISK.K | Y | 114.45 | 2039.9800 | 18 | 2.8 | 1021.0001 | 2 | 102.54 | 9 | 28918 | Ana\_08.raw | 2.44E7 | 3.62E7 | 2.44E7 |  | 2.33E7 | 2.84E7 | 4.1E6 | 5.7E5 | 7.65E7 | 3.8E7 | 25 | 3 | 4 | 2 | 0 | 3 | 2 | 1 | 2 | 5 | 3 | 140 | 157 |  |
| K.TAFDEAIAELDTLSEESYK.D | Y | 113.65 | 2130.9844 | 19 | 1.4 | 1066.5010 | 2 | 133.18 | 3 | 36469 | Ana\_02.raw | 3.21E7 | 8.66E7 | 3.78E7 | 2.89E7 | 2.12E7 | 5.73E7 | 2.64E7 | 3.05E7 | 1.41E8 | 4.65E7 | 54 | 4 | 10 | 6 | 3 | 4 | 2 | 2 | 3 | 16 | 4 | 194 | 212 |  |
| K.SVTEQGAELSNEER.N | Y | 94.98 | 1547.7063 | 14 | 1.2 | 774.8614 | 2 | 40.66 | 3 | 11348 | Ana\_02.raw | 1.01E8 | 1.01E8 | 8.4E7 | 3.4E7 | 8.21E7 | 7.83E7 | 5.55E7 | 6.4E7 | 1.45E8 | 8.78E7 | 27 | 3 | 2 | 1 | 3 | 3 | 1 | 2 | 4 | 5 | 3 | 28 | 41 |  |
| K.DSTLIMQLLR.D | N | 86.96 | 1188.6537 | 10 | 3.2 | 595.3360 | 2 | 124.83 | 6 | 34743 | Ana\_05.raw | 2.39E7 | 6.87E5 | 1.3E7 | 1.26E7 | 1.88E7 | 3.66E7 | 1.08E7 | 1.91E7 | 7.38E7 | 4.73E7 | 34 | 4 | 5 | 2 | 2 | 2 | 2 | 2 | 4 | 6 | 5 | 213 | 222 |  |
| R.YLAEVAAGDDKK.G | Y | 86.25 | 1278.6455 | 12 | 2.0 | 640.3313 | 2 | 32.35 | 3 | 8823 | Ana\_02.raw | 1.12E8 | 1.14E8 | 9.06E7 | 3.28E7 | 5.22E7 | 8.47E7 | 5.77E7 | 5.05E7 | 1.4E8 | 1.08E8 | 34 | 5 | 3 | 4 | 2 | 3 | 4 | 2 | 3 | 4 | 4 | 128 | 139 |  |
| K.KEM(+15.99)QPTHPIR.L | N | 74.15 | 1251.6394 | 10 | 1.2 | 626.8278 | 2 | 17.18 | 3 | 3993 | Ana\_02.raw | 5.44E6 | 7.42E6 | 8.01E6 | 8.48E6 | 5.39E6 | 2.87E7 | 1.6E7 | 1.39E7 |  | 3.87E6 | 17 | 1 | 2 | 1 | 3 | 1 | 3 | 3 | 2 | 0 | 1 | 158 | 167 | Oxidation (M) |
| K.FLIPNASQPESK.V | Y | 74.06 | 1329.6929 | 12 | 5.4 | 665.8573 | 2 | 66.41 | 6 | 19066 | Ana\_05.raw | 4.02E7 | 4.08E7 | 3.35E7 | 1.94E7 | 2.88E7 | 3.03E7 | 2.39E7 | 1.96E7 | 5.85E7 | 3.49E7 | 16 | 1 | 1 | 2 | 1 | 2 | 2 | 2 | 2 | 2 | 1 | 104 | 115 |  |
| K.DSTLIM(+15.99)QLLR.D | N | 72.98 | 1204.6486 | 10 | 0.9 | 603.3321 | 2 | 111.49 | 3 | 30844 | Ana\_02.raw | 1.53E8 | 1.71E8 | 1.8E8 | 9.51E7 | 1.58E8 | 1.44E8 | 1.08E8 | 1.2E8 | 2.28E8 | 1.14E8 | 72 | 9 | 8 | 7 | 4 | 8 | 6 | 6 | 8 | 7 | 9 | 213 | 222 | Oxidation (M) |
| R.DIC(+57.02)NDVLSLLEK.F | Y | 72.14 | 1417.7123 | 12 | 3.4 | 709.8658 | 2 | 131.24 | 3 | 35952 | Ana\_02.raw | 9.1E6 | 5.48E7 | 4.45E7 | 1.32E7 | 3.86E7 | 5.8E7 | 3.21E7 | 2.4E7 | 1.2E8 | 6.36E7 | 37 | 7 | 2 | 5 | 1 | 3 | 2 | 2 | 2 | 7 | 6 | 92 | 103 | Carbamidomethylation |
| K.EM(+15.99)QPTHPIR.L | N | 62.40 | 1123.5444 | 9 | 1.2 | 562.7802 | 2 | 18.72 | 3 | 4509 | Ana\_02.raw | 4.53E7 | 9.05E7 | 7.1E7 | 2.25E7 | 6.13E7 | 6.14E7 | 4.07E7 | 2.22E7 |  | 7.8E7 | 18 | 3 | 2 | 2 | 1 | 2 | 2 | 2 | 1 | 0 | 3 | 159 | 167 | Oxidation (M) |
| R.NLLSVAYK.N | N | 49.16 | 906.5175 | 8 | 4.4 | 454.2680 | 2 | 66.05 | 2 | 18826 | Ana\_01.raw | 5.03E7 | 6.33E7 | 4.71E7 |  |  | 4.5E7 |  |  | 1.12E8 | 5.55E7 | 15 | 3 | 3 | 2 | 0 | 0 | 1 | 0 | 0 | 4 | 2 | 42 | 49 |  |
| R.VVSSIEQK.T | Y | 48.91 | 888.4916 | 8 | 4.0 | 445.2549 | 2 | 22.56 | 2 | 5596 | Ana\_01.raw | 3.26E7 | 2.03E7 | 7.34E7 | 4.3E7 | 4.15E7 | 1.02E8 | 5.9E6 | 1.56E6 |  | 4.8E7 | 10 | 1 | 1 | 1 | 1 | 1 | 1 | 1 | 2 | 0 | 1 | 61 | 68 |  |
| K.M(+15.99)KGDYYR.Y | N | 48.58 | 947.4171 | 7 | 2.1 | 474.7168 | 2 | 18.80 | 5 | 4584 | Ana\_04.raw |  |  |  | 8.41E6 | 1.76E7 | 5.44E6 | 1.17E7 | 8.68E6 |  |  | 9 | 0 | 0 | 0 | 2 | 2 | 2 | 2 | 1 | 0 | 0 | 121 | 127 | Oxidation (M) |
| R.EKIETELR.D | Y | 47.31 | 1016.5502 | 8 | -1.5 | 339.8568 | 3 | 28.44 | 3 | 7650 | Ana\_02.raw | 3.24E6 | 3.74E6 | 3.32E6 | 8.96E6 | 2.25E6 | 7.07E5 | 7.84E6 |  | 1.52E7 | 3.81E6 | 11 | 1 | 1 | 1 | 2 | 1 | 1 | 1 | 0 | 2 | 1 | 84 | 91 |  |
| R.YLAEVAAGDDK.K | Y | 39.06 | 1150.5505 | 11 | 8.3 | 576.2873 | 2 | 51.94 | 3 | 14710 | Ana\_02.raw |  |  | 1.03E7 | 6E6 | 7.65E7 | 1.49E5 |  |  |  |  | 8 | 0 | 0 | 2 | 3 | 1 | 2 | 0 | 0 | 0 | 0 | 128 | 138 |  |
| R.YDDM(+15.99)AAC(+57.02)M(+15.99)K.S | Y | 37.62 | 1135.3984 | 9 | 4.2 | 568.7089 | 2 | 19.52 | 5 | 4822 | Ana\_04.raw |  |  |  | 5.63E5 | 4.48E6 |  | 1.34E6 | 9.13E5 |  |  | 4 | 0 | 0 | 0 | 1 | 1 | 0 | 1 | 1 | 0 | 0 | 19 | 27 | Oxidation (M); Carbamidomethylation |
| K.VFYLK.M | N | 26.21 | 668.3897 | 5 | 5.0 | 335.2038 | 2 | 46.61 | 9 | 13016 | Ana\_08.raw |  |  | 2.77E7 |  |  |  |  | 1.58E7 | 3.93E7 |  | 4 | 0 | 0 | 1 | 0 | 0 | 0 | 0 | 1 | 2 | 0 | 116 | 120 |  |
| total 17 peptides |  |  |  |  |  |  |  |  |  |  |  |  |  |  |  |  |  |  |  |  |  |  |  |  |  |  |  |  |  |  |  |  |  |  |  |
| --- | --- | --- | --- | --- | --- | --- | --- | --- | --- | --- | --- | --- | --- | --- | --- | --- | --- | --- | --- | --- | --- | --- | --- | --- | --- | --- | --- | --- | --- | --- | --- | --- | --- | --- | --- |

Best Unique PSM (Scan F9:28918, m/z=1021.0001, z=2, RT=102.54, ppm=2.8):

sp|P17751|TPIS\_MOUSE

back to list

  

| Protein Coverage
| Supporting Peptides
| Best Unique PSM
|

Protein Coverage:

Supporting Peptides:

| Peptide | Uniq | -10lgP | Mass | Length | ppm | m/z | z | RT | Fraction | Scan | Source File | Area 10 | Area 01 | Area 02 | Area 03 | Area 04 | Area 05 | Area 06 | Area 07 | Area 08 | Area 09 | #Spec | #Spec 10 | #Spec 01 | #Spec 02 | #Spec 03 | #Spec 04 | #Spec 05 | #Spec 06 | #Spec 07 | #Spec 08 | #Spec 09 | Start | End | PTM |
| --- | --- | --- | --- | --- | --- | --- | --- | --- | --- | --- | --- | --- | --- | --- | --- | --- | --- | --- | --- | --- | --- | --- | --- | --- | --- | --- | --- | --- | --- | --- | --- | --- | --- | --- | --- |
| K.ELASQPDVDGFLVGGASLKPEFVDIINAK.Q | Y | 116.49 | 3028.5757 | 29 | -8.6 | 1010.5239 | 3 | 130.39 | 3 | 35715 | Ana\_02.raw | 1.67E7 | 3.57E7 | 2.77E7 | 1.04E7 | 2.53E7 | 2.9E7 | 1.76E7 | 1.27E7 | 5.96E7 | 1.08E7 | 18 | 1 | 2 | 2 | 2 | 2 | 2 | 2 | 2 | 2 | 1 | 220 | 248 |  |
| R.HVFGESDELIGQK.V | Y | 104.29 | 1457.7150 | 13 | 6.0 | 729.8691 | 2 | 60.37 | 6 | 17276 | Ana\_05.raw | 1.23E7 | 1.07E7 | 2.2E7 | 1.15E7 | 2.14E7 | 2.15E7 | 1.4E7 | 1.35E7 | 1.9E7 | 1.3E7 | 16 | 1 | 1 | 2 | 2 | 2 | 2 | 2 | 2 | 1 | 1 | 101 | 113 |  |
| K.VVLAYEPVWAIGTGK.T | Y | 100.49 | 1601.8817 | 15 | 0.7 | 801.9487 | 2 | 111.48 | 3 | 30840 | Ana\_02.raw | 5.31E7 | 5.26E7 | 4.78E7 | 2.66E7 | 2.45E7 | 3.24E7 | 3.05E7 | 7.95E6 | 9.42E7 | 5.22E7 | 32 | 3 | 4 | 4 | 3 | 2 | 3 | 3 | 2 | 5 | 3 | 161 | 175 |  |
| K.VSHALAEGLGVIAC(+57.02)IGEK.L | Y | 100.25 | 1822.9611 | 18 | 4.6 | 608.6638 | 3 | 106.10 | 2 | 29719 | Ana\_01.raw | 5.93E5 | 1.41E6 |  |  | 8.27E5 |  | 5.93E5 | 8.63E5 | 1.08E6 | 6.29E5 | 8 | 1 | 1 | 0 | 0 | 1 | 0 | 1 | 1 | 2 | 1 | 114 | 131 | Carbamidomethylation |
| K.TATPQQAQEVHEK.L | Y | 95.41 | 1465.7161 | 13 | 1.7 | 733.8666 | 2 | 20.92 | 3 | 5247 | Ana\_02.raw | 6.95E7 | 6.37E7 | 5.27E7 | 2.37E7 | 4.77E6 | 5.99E7 | 2.5E7 | 1.09E7 | 9.53E7 | 4.39E6 | 26 | 4 | 3 | 2 | 2 | 1 | 2 | 2 | 2 | 4 | 4 | 176 | 188 |  |
| K.SNVNDGVAQSTR.I | Y | 89.40 | 1246.5902 | 12 | 2.2 | 624.3038 | 2 | 23.38 | 4 | 5915 | Ana\_03.raw | 3.06E7 |  | 2.8E7 | 1.81E7 | 2.9E7 | 3.29E7 | 1.91E7 | 1.06E7 |  | 2.92E7 | 20 | 1 | 0 | 1 | 3 | 4 | 3 | 3 | 4 | 0 | 1 | 195 | 206 |  |
| R.IIYGGSVTGATC(+57.02)K.E | Y | 80.06 | 1325.6649 | 13 | 4.6 | 663.8428 | 2 | 49.00 | 1 | 13732 | ANa\_10.raw | 2.1E7 | 2.24E7 | 1.87E7 | 8.1E6 | 1.58E7 | 1.72E7 | 7.92E6 | 8.34E6 | 4.27E7 | 2.25E7 | 10 | 1 | 1 | 1 | 1 | 1 | 1 | 1 | 1 | 1 | 1 | 207 | 219 | Carbamidomethylation |
| K.IAVAAQNC(+57.02)YK.V | Y | 72.29 | 1136.5648 | 10 | 2.7 | 569.2912 | 2 | 35.17 | 3 | 9664 | Ana\_02.raw |  |  | 0 | 6.55E6 | 1.24E7 |  |  | 6.55E6 |  |  | 5 | 1 | 0 | 1 | 1 | 1 | 0 | 0 | 1 | 0 | 0 | 60 | 69 | Carbamidomethylation |
| R.RHVFGESDELIGQK.V | Y | 66.20 | 1613.8162 | 14 | 6.4 | 538.9495 | 3 | 47.10 | 9 | 13171 | Ana\_08.raw |  |  |  | 4.1E5 |  |  | 2.94E6 |  | 4.36E6 |  | 3 | 0 | 0 | 0 | 1 | 0 | 0 | 1 | 0 | 1 | 0 | 100 | 113 |  |
| K.DLGATWVVLGHSER.R | Y | 66.15 | 1538.7841 | 14 | 1.6 | 770.4005 | 2 | 88.67 | 9 | 25182 | Ana\_08.raw |  |  |  | 2.6E5 | 1.26E6 |  | 5E5 | 9.09E5 | 1.45E6 | 4.53E5 | 6 | 0 | 0 | 0 | 1 | 1 | 0 | 1 | 1 | 1 | 1 | 86 | 99 |  |
| K.VVFEQTK.V | Y | 51.85 | 849.4596 | 7 | 5.1 | 425.7392 | 2 | 28.24 | 2 | 7468 | Ana\_01.raw | 4.38E7 | 4.41E7 | 3.96E7 | 1.24E7 | 1.87E7 | 3.19E7 | 2.33E7 | 1.85E7 | 4.22E7 | 4.4E7 | 10 | 1 | 1 | 1 | 1 | 1 | 1 | 1 | 1 | 1 | 1 | 143 | 149 |  |
| K.VIADNVK.D | Y | 34.16 | 757.4333 | 7 | 3.8 | 379.7254 | 2 | 21.87 | 6 | 5549 | Ana\_05.raw |  |  |  | 1.43E7 | 1.75E7 | 1.03E7 | 1.44E7 | 1.21E7 |  |  | 5 | 0 | 0 | 0 | 1 | 1 | 1 | 1 | 1 | 0 | 0 | 150 | 156 |  |
| total 12 peptides |  |  |  |  |  |  |  |  |  |  |  |  |  |  |  |  |  |  |  |  |  |  |  |  |  |  |  |  |  |  |  |  |  |  |  |
| --- | --- | --- | --- | --- | --- | --- | --- | --- | --- | --- | --- | --- | --- | --- | --- | --- | --- | --- | --- | --- | --- | --- | --- | --- | --- | --- | --- | --- | --- | --- | --- | --- | --- | --- | --- |

Best Unique PSM (Scan F3:35715, m/z=1010.5239, z=3, RT=130.39, ppm=-8.6):

A2AIM4|A2AIM4\_MOUSE

back to list

  

| Protein Coverage
| Supporting Peptides
| Best Unique PSM
|

Protein Coverage:

Supporting Peptides:

| Peptide | Uniq | -10lgP | Mass | Length | ppm | m/z | z | RT | Fraction | Scan | Source File | Area 10 | Area 01 | Area 02 | Area 03 | Area 04 | Area 05 | Area 06 | Area 07 | Area 08 | Area 09 | #Spec | #Spec 10 | #Spec 01 | #Spec 02 | #Spec 03 | #Spec 04 | #Spec 05 | #Spec 06 | #Spec 07 | #Spec 08 | #Spec 09 | Start | End | PTM |
| --- | --- | --- | --- | --- | --- | --- | --- | --- | --- | --- | --- | --- | --- | --- | --- | --- | --- | --- | --- | --- | --- | --- | --- | --- | --- | --- | --- | --- | --- | --- | --- | --- | --- | --- | --- |
| K.ATDAEADVASLNR.R | N | 104.41 | 1331.6317 | 13 | 1.9 | 666.8244 | 2 | 50.96 | 3 | 14424 | Ana\_02.raw | 1.77E8 | 1.79E8 | 1.44E8 | 4.85E7 | 1.11E8 | 1.34E8 | 6.86E7 | 9.21E7 | 2.34E8 | 1.17E8 | 42 | 6 | 5 | 4 | 3 | 5 | 2 | 2 | 6 | 6 | 3 | 78 | 90 |  |
| K.KATDAEADVASLNR.R | N | 95.49 | 1459.7267 | 14 | 1.5 | 730.8717 | 2 | 44.60 | 3 | 12539 | Ana\_02.raw | 1.62E7 | 1.34E7 | 9.9E6 | 2.4E6 | 6.87E6 | 1.07E7 | 4.07E6 | 5.16E6 | 8.73E6 | 1.13E7 | 10 | 1 | 1 | 1 | 1 | 1 | 1 | 1 | 1 | 1 | 1 | 77 | 90 |  |
| R.RIQLVEEELDR.A | N | 84.32 | 1398.7466 | 11 | 2.6 | 467.2574 | 3 | 65.03 | 3 | 18527 | Ana\_02.raw | 9.3E6 | 1.67E7 | 8.94E6 | 1.65E6 | 1.81E6 | 5.12E6 | 2.92E6 | 2.99E6 | 2.48E7 | 6.83E6 | 14 | 2 | 2 | 2 | 1 | 1 | 1 | 1 | 1 | 2 | 1 | 91 | 101 |  |
| R.IQLVEEELDR.A | N | 82.25 | 1242.6455 | 10 | 1.3 | 622.3308 | 2 | 73.05 | 3 | 20740 | Ana\_02.raw | 3.57E8 | 3.31E8 | 2.77E8 | 9.71E7 | 2.29E8 | 2.25E8 | 1.22E8 | 1.62E8 | 5.58E8 | 2.86E8 | 75 | 11 | 8 | 8 | 5 | 8 | 6 | 5 | 8 | 9 | 7 | 92 | 101 |  |
| K.SLIASEEEYSTK.E | Y | 81.64 | 1355.6456 | 12 | 3.0 | 678.8321 | 2 | 57.22 | 6 | 16351 | Ana\_05.raw | 9.72E7 | 1.39E8 | 1.1E8 | 4.4E7 | 7.8E7 | 1.03E8 | 5.42E7 | 6.26E7 | 1.6E8 | 8.66E7 | 24 | 2 | 3 | 2 | 2 | 2 | 2 | 2 | 2 | 4 | 3 | 206 | 217 |  |
| K.QLEEEQQALQK.K | Y | 78.14 | 1342.6729 | 11 | 3.3 | 672.3459 | 2 | 40.74 | 6 | 11388 | Ana\_05.raw | 1.52E7 | 1.18E7 | 1.23E7 | 5.3E6 | 1.23E7 | 1.95E7 | 8.26E6 | 1.36E7 | 7.71E6 | 5.08E6 | 10 | 1 | 1 | 1 | 1 | 1 | 1 | 1 | 1 | 1 | 1 | 38 | 48 |  |
| K.YSESVKDAQEK.L | Y | 76.12 | 1282.6040 | 11 | 1.0 | 642.3099 | 2 | 22.13 | 9 | 5330 | Ana\_08.raw | 6.95E6 | 4.25E6 | 3.19E6 |  |  | 8.78E6 | 1.6E6 |  | 5.78E6 | 2.03E6 | 11 | 2 | 1 | 1 | 0 | 0 | 3 | 1 | 0 | 2 | 1 | 60 | 70 |  |
| R.KLVILEGELER.S | Y | 72.08 | 1297.7605 | 11 | 1.9 | 433.5949 | 3 | 74.53 | 6 | 21387 | Ana\_05.raw | 1.69E7 | 1.98E7 | 1.39E7 | 3.11E6 | 1.44E7 | 1.6E7 | 4.59E6 | 4.25E6 | 1.18E7 | 1.61E7 | 17 | 2 | 2 | 2 | 1 | 2 | 2 | 1 | 1 | 2 | 2 | 168 | 178 |  |
| K.EDKYEEEIK.L | N | 72.04 | 1181.5452 | 9 | 2.1 | 591.7811 | 2 | 32.97 | 6 | 9023 | Ana\_05.raw | 1.95E8 | 2.31E8 | 2.88E8 | 1.16E8 | 2.33E8 | 2.95E8 | 1.67E8 | 1.68E8 | 2.25E8 | 2.57E8 | 32 | 4 | 3 | 4 | 2 | 3 | 3 | 3 | 3 | 4 | 3 | 218 | 226 |  |
| K.LDKENAIDR.A | N | 67.85 | 1072.5513 | 9 | 4.1 | 537.2851 | 2 | 24.94 | 6 | 6533 | Ana\_05.raw | 2.16E8 | 1.3E8 | 2.74E8 | 1.07E8 | 1.45E8 | 3.41E8 | 1.07E8 | 1.24E8 | 2.05E8 | 2.3E8 | 43 | 4 | 2 | 5 | 4 | 5 | 5 | 4 | 6 | 2 | 6 | 13 | 21 |  |
| K.SLIASEEEYSTKEDKYEEEIK.L | Y | 64.74 | 2519.1802 | 21 | 1.6 | 840.7354 | 3 | 71.94 | 9 | 20532 | Ana\_08.raw |  |  |  |  |  |  |  |  | 9.75E6 |  | 1 | 0 | 0 | 0 | 0 | 0 | 0 | 0 | 0 | 1 | 0 | 206 | 226 |  |
| K.LVILEGELER.S | Y | 62.58 | 1169.6655 | 10 | 3.7 | 585.8422 | 2 | 84.97 | 6 | 24217 | Ana\_05.raw | 2.39E7 | 2.63E7 | 1.89E7 | 6.01E6 | 1.75E7 | 1.83E7 | 4.09E6 | 7.8E6 | 4.62E7 | 2.14E7 | 25 | 3 | 3 | 2 | 2 | 2 | 2 | 2 | 3 | 3 | 3 | 169 | 178 |  |
| K.LKGTEDEVEK.Y | Y | 57.43 | 1146.5768 | 10 | 2.2 | 574.2969 | 2 | 20.73 | 7 | 5123 | Ana\_06.raw | 1.07E8 | 9.8E7 | 4.64E7 | 1.57E7 | 6.21E7 | 1.07E8 | 3.22E7 | 2.91E7 | 8.49E6 | 8.41E7 | 29 | 2 | 3 | 4 | 2 | 3 | 4 | 4 | 3 | 2 | 2 | 50 | 59 |  |
| K.M(+15.99)ELQEM(+15.99)QLK.E | Y | 53.27 | 1180.5468 | 9 | 3.3 | 591.2826 | 2 | 35.67 | 6 | 9825 | Ana\_05.raw |  |  |  | 8.57E6 | 3.05E7 | 2.43E7 | 1.3E7 | 4.04E7 |  | 3.62E6 | 6 | 0 | 0 | 0 | 1 | 1 | 1 | 1 | 1 | 0 | 1 | 141 | 149 | Oxidation (M) |
| R.KYEEVAR.K | N | 49.33 | 893.4606 | 7 | 0.3 | 447.7377 | 2 | 18.38 | 3 | 4393 | Ana\_02.raw |  |  | 8.04E7 | 1.25E8 | 2.72E8 | 3.48E8 | 1.53E8 | 1.89E8 |  |  | 16 | 0 | 0 | 1 | 3 | 3 | 3 | 3 | 3 | 0 | 0 | 161 | 167 |  |
| K.YEEVAR.K | N | 47.64 | 765.3657 | 6 | 2.4 | 383.6910 | 2 | 20.43 | 5 | 5125 | Ana\_04.raw |  |  |  |  | 1.16E7 | 4.79E6 | 2.17E7 | 1.63E7 |  |  | 4 | 0 | 0 | 0 | 0 | 1 | 1 | 1 | 1 | 0 | 0 | 162 | 167 |  |
| R.LATALQK.L | N | 46.81 | 743.4541 | 7 | 3.3 | 372.7356 | 2 | 25.37 | 6 | 6681 | Ana\_05.raw | 1.38E8 | 0 | 2.34E8 |  | 1.67E8 | 2.57E8 | 1.38E8 | 1.32E8 | 3.55E7 |  | 8 | 1 | 1 | 1 | 0 | 1 | 1 | 1 | 1 | 1 | 0 | 106 | 112 |  |
| K.YEEEIK.L | N | 45.29 | 809.3807 | 6 | 2.5 | 405.6986 | 2 | 25.49 | 6 | 6720 | Ana\_05.raw | 8.07E6 | 9.98E6 | 1.32E7 | 5.51E6 | 8.47E6 | 1.49E7 | 7.89E6 | 6.66E6 |  | 2.44E6 | 9 | 1 | 1 | 1 | 1 | 1 | 1 | 1 | 1 | 0 | 1 | 221 | 226 |  |
| R.QLEEELR.T | Y | 41.84 | 915.4661 | 7 | 5.6 | 458.7429 | 2 | 37.50 | 1 | 10241 | ANa\_10.raw | 2.81E7 |  |  | 8.28E6 |  |  |  | 2.44E7 | 2.21E7 |  | 4 | 1 | 0 | 0 | 1 | 0 | 0 | 0 | 1 | 1 | 0 | 192 | 198 |  |
| R.AEFAER.S | N | 40.91 | 721.3395 | 6 | 2.7 | 361.6780 | 2 | 22.34 | 6 | 5706 | Ana\_05.raw |  |  |  | 1.21E8 | 2.46E7 | 1.7E8 | 1.42E8 | 1.07E8 |  |  | 16 | 0 | 0 | 0 | 3 | 3 | 3 | 3 | 4 | 0 | 0 | 239 | 244 |  |
| R.AM(+15.99)KDEEKM(+15.99)ELQEM(+15.99)QLK.E | Y | 29.84 | 2027.9214 | 16 | 2.0 | 676.9824 | 3 | 31.96 | 3 | 8713 | Ana\_02.raw |  |  | 1.14E7 |  |  | 4.81E7 |  |  |  |  | 3 | 0 | 0 | 1 | 0 | 0 | 2 | 0 | 0 | 0 | 0 | 134 | 149 | Oxidation (M) |
| K.EAKHIAEDSDR.K | Y | 27.62 | 1269.5948 | 11 | 2.4 | 424.2066 | 3 | 16.26 | 7 | 3715 | Ana\_06.raw |  |  |  | 1.07E5 |  |  | 1.97E5 |  |  |  | 2 | 0 | 0 | 0 | 1 | 0 | 0 | 1 | 0 | 0 | 0 | 150 | 160 |  |
| K.EAETRAEFAER.S | N | 24.38 | 1307.6106 | 11 | -1.4 | 654.8116 | 2 | 65.68 | 1 | 18622 | ANa\_10.raw | 0 |  |  |  |  |  |  |  |  |  | 1 | 1 | 0 | 0 | 0 | 0 | 0 | 0 | 0 | 0 | 0 | 234 | 244 |  |
| total 23 peptides |  |  |  |  |  |  |  |  |  |  |  |  |  |  |  |  |  |  |  |  |  |  |  |  |  |  |  |  |  |  |  |  |  |  |  |
| --- | --- | --- | --- | --- | --- | --- | --- | --- | --- | --- | --- | --- | --- | --- | --- | --- | --- | --- | --- | --- | --- | --- | --- | --- | --- | --- | --- | --- | --- | --- | --- | --- | --- | --- | --- |

Best Unique PSM (Scan F6:16351, m/z=678.8321, z=2, RT=57.22, ppm=3.0):

sp|Q9CWJ9|PUR9\_MOUSE

back to list

  

| Protein Coverage
| Supporting Peptides
| Best Unique PSM
|

Protein Coverage:

Supporting Peptides:

| Peptide | Uniq | -10lgP | Mass | Length | ppm | m/z | z | RT | Fraction | Scan | Source File | Area 10 | Area 01 | Area 02 | Area 03 | Area 04 | Area 05 | Area 06 | Area 07 | Area 08 | Area 09 | #Spec | #Spec 10 | #Spec 01 | #Spec 02 | #Spec 03 | #Spec 04 | #Spec 05 | #Spec 06 | #Spec 07 | #Spec 08 | #Spec 09 | Start | End | PTM |
| --- | --- | --- | --- | --- | --- | --- | --- | --- | --- | --- | --- | --- | --- | --- | --- | --- | --- | --- | --- | --- | --- | --- | --- | --- | --- | --- | --- | --- | --- | --- | --- | --- | --- | --- | --- |
| K.RAEISNAIDQYVTGTIGEGEDLVK.W | Y | 113.95 | 2577.2922 | 24 | 5.4 | 860.1093 | 3 | 128.73 | 1 | 35396 | ANa\_10.raw | 3.26E6 | 3.09E6 | 1.31E6 | 2.83E5 |  | 2.47E6 | 1.44E6 | 1.12E6 | 2.6E6 | 1.16E6 | 9 | 1 | 1 | 1 | 1 | 0 | 1 | 1 | 1 | 1 | 1 | 484 | 507 |  |
| K.WEALFEEVPELLTEAEK.K | Y | 98.18 | 2032.0040 | 17 | -1.8 | 1017.0075 | 2 | 134.99 | 3 | 36943 | Ana\_02.raw |  | 1.14E6 | 1.36E6 |  |  | 2.53E5 |  | 1.71E6 | 2.16E6 |  | 5 | 0 | 1 | 1 | 0 | 0 | 1 | 0 | 1 | 1 | 0 | 508 | 524 |  |
| K.WEALFEEVPELLTEAEKK.E | Y | 93.44 | 2160.0989 | 18 | 2.2 | 721.0418 | 3 | 133.24 | 6 | 37040 | Ana\_05.raw |  | 2.35E6 | 1.21E6 |  | 1.32E6 | 2.54E6 |  |  |  | 4.76E4 | 5 | 0 | 1 | 1 | 0 | 1 | 1 | 0 | 0 | 0 | 1 | 508 | 525 |  |
| K.AFTHTAQYDEAISDYFR.K | Y | 92.87 | 2033.9119 | 17 | 3.3 | 678.9802 | 3 | 97.33 | 1 | 27261 | ANa\_10.raw | 4.26E6 | 1.54E6 |  | 1.78E6 | 3.18E6 | 2.6E6 |  | 8.07E5 | 2.26E6 |  | 7 | 1 | 1 | 0 | 1 | 1 | 1 | 0 | 1 | 1 | 0 | 178 | 194 |  |
| R.SGVAYIVAPSGSTADK.V | Y | 87.23 | 1521.7675 | 16 | 2.7 | 761.8931 | 2 | 60.06 | 6 | 17181 | Ana\_05.raw |  |  |  |  |  | 1.75E6 |  | 7.21E5 | 2.26E6 |  | 3 | 0 | 0 | 0 | 0 | 0 | 1 | 0 | 1 | 1 | 0 | 554 | 569 |  |
| K.LSGVSVSSDAFFPFR.D | Y | 84.71 | 1614.8042 | 15 | 6.5 | 808.4146 | 2 | 119.24 | 6 | 33291 | Ana\_05.raw | 1.98E6 | 1.02E6 | 5.97E6 | 1.96E5 | 1.49E6 | 2.32E6 | 2.44E5 | 7.98E5 | 4.59E6 | 8.57E5 | 11 | 1 | 1 | 1 | 1 | 1 | 1 | 1 | 1 | 1 | 2 | 531 | 545 |  |
| R.AEISNAIDQYVTGTIGEGEDLVK.W | Y | 84.66 | 2421.1912 | 23 | 4.2 | 1211.6079 | 2 | 131.70 | 7 | 36257 | Ana\_06.raw |  |  |  |  |  |  | 5.3E5 |  |  |  | 1 | 0 | 0 | 0 | 0 | 0 | 0 | 1 | 0 | 0 | 0 | 485 | 507 |  |
| R.GAVDIPAAASFK.H | Y | 66.41 | 1145.6080 | 12 | 5.2 | 573.8143 | 2 | 74.42 | 6 | 21354 | Ana\_05.raw | 5.04E6 | 0 | 4.44E6 | 3.06E6 | 4.98E6 | 3.54E6 | 2.51E6 | 3.07E6 |  | 3.74E6 | 9 | 1 | 1 | 1 | 1 | 1 | 1 | 1 | 1 | 0 | 1 | 255 | 266 |  |
| K.DGQVIGIGAGQQSR.I | Y | 62.53 | 1384.7058 | 14 | 8.7 | 693.3662 | 2 | 48.88 | 6 | 13876 | Ana\_05.raw |  |  | 1.56E7 | 2.98E5 |  | 1.67E6 | 1.18E6 |  |  |  | 4 | 0 | 0 | 1 | 1 | 0 | 1 | 1 | 0 | 0 | 0 | 438 | 451 |  |
| R.VVVC(+57.02)NLYPFVK.T | Y | 60.39 | 1336.7213 | 11 | 2.6 | 669.3697 | 2 | 94.44 | 9 | 26752 | Ana\_08.raw |  |  |  |  |  |  | 3.89E5 |  | 2.75E6 | 1.07E6 | 3 | 0 | 0 | 0 | 0 | 0 | 0 | 1 | 0 | 1 | 1 | 98 | 108 | Carbamidomethylation |
| R.EVSDGIVAPGYEEEALK.I | Y | 49.91 | 1804.8730 | 17 | 2.2 | 903.4458 | 2 | 79.47 | 3 | 22474 | Ana\_02.raw |  |  | 9.07E5 |  | 8.16E5 | 3.75E5 | 3.87E5 | 1.18E6 |  |  | 5 | 0 | 0 | 1 | 0 | 1 | 1 | 1 | 1 | 0 | 0 | 336 | 352 |  |
| R.DVSELTGFPEM(+15.99)LGGR.V | Y | 49.23 | 1622.7610 | 15 | 1.6 | 812.3890 | 2 | 99.33 | 2 | 27951 | Ana\_01.raw |  | 5.78E5 |  |  | 6.45E5 | 6.77E5 | 5.25E5 | 3.97E5 |  |  | 5 | 0 | 1 | 0 | 0 | 1 | 1 | 1 | 1 | 0 | 0 | 50 | 64 | Oxidation (M) |
| R.LDFNLVR.V | Y | 43.05 | 875.4865 | 7 | 0.9 | 438.7509 | 2 | 81.93 | 4 | 22739 | Ana\_03.raw |  |  |  | 2.27E6 |  |  |  | 2.76E6 | 6.96E6 |  | 3 | 0 | 0 | 0 | 1 | 0 | 0 | 0 | 1 | 1 | 0 | 91 | 97 |  |
| K.SLFSNIVTK.N | Y | 41.96 | 1007.5651 | 9 | 6.8 | 504.7933 | 2 | 77.78 | 9 | 22158 | Ana\_08.raw | 2.89E6 | 3.07E6 |  |  |  |  |  |  | 4.53E6 |  | 3 | 1 | 1 | 0 | 0 | 0 | 0 | 0 | 0 | 1 | 0 | 398 | 406 |  |
| R.NIPEDAADM(+15.99)AR.L | Y | 34.22 | 1217.5347 | 11 | 1.0 | 609.7752 | 2 | 38.93 | 4 | 10510 | Ana\_03.raw |  |  |  | 2E6 |  |  |  |  |  |  | 1 | 0 | 0 | 0 | 1 | 0 | 0 | 0 | 0 | 0 | 0 | 80 | 90 | Oxidation (M) |
| R.DAGLAVR.D | Y | 32.92 | 700.3868 | 7 | 4.6 | 351.2023 | 2 | 26.52 | 1 | 6916 | ANa\_10.raw | 7.8E5 |  | 6.15E6 |  |  |  |  |  |  | 8.5E5 | 3 | 1 | 0 | 1 | 0 | 0 | 0 | 0 | 0 | 0 | 1 | 43 | 49 |  |
| K.DLPESALR.D | Y | 31.70 | 899.4712 | 8 | 8.7 | 450.7468 | 2 | 50.74 | 2 | 14311 | Ana\_01.raw |  | 5.73E5 |  |  |  |  |  |  |  |  | 1 | 0 | 1 | 0 | 0 | 0 | 0 | 0 | 0 | 0 | 0 | 409 | 416 |  |
| total 17 peptides |  |  |  |  |  |  |  |  |  |  |  |  |  |  |  |  |  |  |  |  |  |  |  |  |  |  |  |  |  |  |  |  |  |  |  |
| --- | --- | --- | --- | --- | --- | --- | --- | --- | --- | --- | --- | --- | --- | --- | --- | --- | --- | --- | --- | --- | --- | --- | --- | --- | --- | --- | --- | --- | --- | --- | --- | --- | --- | --- | --- |

Best Unique PSM (Scan F1:35396, m/z=860.1093, z=3, RT=128.73, ppm=5.4):

sp|P02088|HBB1\_MOUSE

back to list

  

| Protein Coverage
| Supporting Peptides
| Best Unique PSM
|

Protein Coverage:

Supporting Peptides:

| Peptide | Uniq | -10lgP | Mass | Length | ppm | m/z | z | RT | Fraction | Scan | Source File | Area 10 | Area 01 | Area 02 | Area 03 | Area 04 | Area 05 | Area 06 | Area 07 | Area 08 | Area 09 | #Spec | #Spec 10 | #Spec 01 | #Spec 02 | #Spec 03 | #Spec 04 | #Spec 05 | #Spec 06 | #Spec 07 | #Spec 08 | #Spec 09 | Start | End | PTM |
| --- | --- | --- | --- | --- | --- | --- | --- | --- | --- | --- | --- | --- | --- | --- | --- | --- | --- | --- | --- | --- | --- | --- | --- | --- | --- | --- | --- | --- | --- | --- | --- | --- | --- | --- | --- |
| R.YFDSFGDLSSASAIM(+15.99)GNAK.V | N | 118.64 | 1995.8883 | 19 | 0.2 | 998.9517 | 2 | 96.28 | 3 | 26921 | Ana\_02.raw | 2.76E8 | 4.65E8 | 6.29E8 | 8.1E7 | 2.76E8 | 2.77E8 | 1.35E8 | 3.23E8 | 2.53E8 | 1.02E8 | 111 | 11 | 18 | 21 | 6 | 10 | 10 | 5 | 14 | 7 | 9 | 42 | 60 | Oxidation (M) |
| R.YFDSFGDLSSASAIMGNAK.V | N | 109.65 | 1979.8934 | 19 | 3.7 | 990.9577 | 2 | 117.42 | 1 | 32434 | ANa\_10.raw | 7.66E7 | 4.55E7 |  |  | 1E6 | 1.7E7 | 4.83E5 | 1.79E7 | 4.73E7 | 1.94E7 | 29 | 6 | 5 | 0 | 0 | 1 | 2 | 1 | 4 | 4 | 6 | 42 | 60 |  |
| K.VITAFNDGLNHLDSLK.G | N | 107.13 | 1755.9155 | 16 | -0.9 | 878.9642 | 2 | 93.75 | 3 | 26254 | Ana\_02.raw | 3.11E8 | 5.66E8 | 7.11E8 | 3.69E7 | 2.66E8 | 2.99E8 | 1.72E8 | 3.31E8 | 2.34E8 | 1.57E8 | 130 | 14 | 16 | 19 | 9 | 11 | 10 | 9 | 18 | 12 | 12 | 68 | 83 |  |
| R.LLVVYPWTQR.Y | N | 97.82 | 1273.7183 | 10 | 3.1 | 637.8684 | 2 | 102.18 | 6 | 28828 | Ana\_05.raw | 4.86E8 | 9.19E8 | 1.17E9 | 3.07E8 | 5.83E8 | 7.99E8 | 3.19E8 | 5.92E8 | 8.02E8 | 3.19E8 | 260 | 20 | 27 | 40 | 25 | 32 | 29 | 19 | 42 | 15 | 11 | 32 | 41 |  |
| K.KVITAFNDGLNHLDSLK.G | N | 97.49 | 1884.0105 | 17 | 1.2 | 629.0115 | 3 | 83.71 | 3 | 23607 | Ana\_02.raw | 1.12E7 | 3.81E7 | 3.09E7 | 4.66E6 | 1.16E7 | 1.96E7 | 8.98E6 | 1.67E7 | 1.67E7 | 1.03E6 | 20 | 2 | 3 | 2 | 2 | 2 | 2 | 2 | 2 | 2 | 1 | 67 | 83 |  |
| K.LHVDPENFR.L | N | 97.26 | 1125.5566 | 9 | 3.6 | 563.7876 | 2 | 55.51 | 6 | 15845 | Ana\_05.raw | 1.19E8 | 2.67E8 | 4.01E8 | 9.71E7 | 1.98E8 | 1.94E8 | 1.27E8 | 2.18E8 | 1.43E8 | 7.93E7 | 69 | 6 | 9 | 10 | 6 | 7 | 5 | 5 | 10 | 7 | 4 | 97 | 105 |  |
| K.GTFASLSELHC(+57.02)DK.L | N | 94.23 | 1463.6715 | 13 | 1.3 | 732.8440 | 2 | 63.48 | 3 | 18077 | Ana\_02.raw | 7.95E7 | 1.55E8 | 1.63E8 | 6.14E7 | 8.27E7 | 8.69E7 | 5.77E7 | 1.03E8 | 1.04E8 | 5.64E7 | 42 | 4 | 5 | 6 | 3 | 4 | 4 | 4 | 7 | 3 | 2 | 84 | 96 | Carbamidomethylation |
| R.LLGNM(+15.99)IVIVLGHHLGK.D | N | 84.05 | 1729.0072 | 16 | -1.2 | 433.2585 | 4 | 101.36 | 3 | 28253 | Ana\_02.raw |  | 4.98E5 | 1.64E7 | 2.5E6 | 3.74E6 | 1.69E5 | 2.94E6 | 3.52E6 |  |  | 21 | 0 | 1 | 5 | 4 | 3 | 1 | 2 | 5 | 0 | 0 | 106 | 121 | Oxidation (M) |
| K.DFTPAAQAAFQK.V | N | 76.41 | 1293.6353 | 12 | 0.9 | 647.8255 | 2 | 63.54 | 3 | 18097 | Ana\_02.raw | 2.73E7 | 1.02E8 | 1.47E8 |  | 7.19E7 | 4.43E7 | 3.16E7 | 6.02E7 | 3.65E7 | 2.35E7 | 24 | 2 | 4 | 4 | 0 | 3 | 2 | 2 | 4 | 2 | 1 | 122 | 133 |  |
| R.LLGNMIVIVLGHHLGK.D | N | 72.25 | 1713.0123 | 16 | 4.5 | 429.2623 | 4 | 127.85 | 8 | 35068 | Ana\_07.raw | 6.42E5 | 6.18E5 | 1.05E6 | 1.09E6 | 3E6 | 5.24E6 | 1.35E6 | 2.37E6 |  |  | 10 | 1 | 2 | 1 | 1 | 1 | 2 | 1 | 1 | 0 | 0 | 106 | 121 |  |
| K.DFTPAAQAAFQKVVAGVATALAHKYH | Y | 62.31 | 2711.4182 | 26 | 3.7 | 904.8167 | 3 | 134.20 | 7 | 36974 | Ana\_06.raw |  |  | 8.73E5 |  | 2.78E6 |  | 4.11E5 | 3.38E6 |  |  | 4 | 0 | 0 | 1 | 0 | 1 | 0 | 1 | 1 | 0 | 0 | 122 | 147 |  |
| M.VHLTDAEK.A | N | 61.87 | 911.4712 | 8 | 5.7 | 456.7455 | 2 | 21.62 | 6 | 5462 | Ana\_05.raw | 3.24E8 | 5.17E8 | 7.06E8 | 1.33E8 | 1E7 | 3.48E8 | 2.15E8 | 3.12E8 |  | 1.31E8 | 40 | 5 | 3 | 7 | 3 | 3 | 5 | 3 | 7 | 0 | 4 | 2 | 9 |  |
| K.VNSDEVGGEALGR.L | Y | 45.63 | 1301.6211 | 13 | 0.4 | 651.8181 | 2 | 53.82 | 6 | 15348 | Ana\_05.raw | 5.16E7 | 1.02E8 | 1.32E8 | 3.66E7 | 7.3E7 | 8.22E7 | 2.84E7 | 7.86E7 |  | 3.5E7 | 13 | 1 | 1 | 2 | 2 | 1 | 2 | 1 | 2 | 0 | 1 | 19 | 31 |  |
| total 13 peptides |  |  |  |  |  |  |  |  |  |  |  |  |  |  |  |  |  |  |  |  |  |  |  |  |  |  |  |  |  |  |  |  |  |  |  |
| --- | --- | --- | --- | --- | --- | --- | --- | --- | --- | --- | --- | --- | --- | --- | --- | --- | --- | --- | --- | --- | --- | --- | --- | --- | --- | --- | --- | --- | --- | --- | --- | --- | --- | --- | --- |

Best Unique PSM (Scan F7:36974, m/z=904.8167, z=3, RT=134.20, ppm=3.7):

sp|Q04857|CO6A1\_MOUSE

back to list

  

| Protein Coverage
| Supporting Peptides
| Best Unique PSM
|

Protein Coverage:

Supporting Peptides:

| Peptide | Uniq | -10lgP | Mass | Length | ppm | m/z | z | RT | Fraction | Scan | Source File | Area 10 | Area 01 | Area 02 | Area 03 | Area 04 | Area 05 | Area 06 | Area 07 | Area 08 | Area 09 | #Spec | #Spec 10 | #Spec 01 | #Spec 02 | #Spec 03 | #Spec 04 | #Spec 05 | #Spec 06 | #Spec 07 | #Spec 08 | #Spec 09 | Start | End | PTM |
| --- | --- | --- | --- | --- | --- | --- | --- | --- | --- | --- | --- | --- | --- | --- | --- | --- | --- | --- | --- | --- | --- | --- | --- | --- | --- | --- | --- | --- | --- | --- | --- | --- | --- | --- | --- |
| R.VAVVQYSGQGQQQPGR.A | Y | 107.05 | 1700.8594 | 16 | 3.4 | 851.4398 | 2 | 46.14 | 6 | 13038 | Ana\_05.raw | 1.6E7 | 1.78E7 |  | 6.32E6 | 1.35E7 | 1.62E7 | 9.71E6 | 1.22E7 | 3.19E6 | 9.47E6 | 10 | 1 | 1 | 1 | 1 | 1 | 1 | 1 | 1 | 1 | 1 | 870 | 885 |  |
| R.DAEEVISQTIDTIVDM(+15.99)IK.N | Y | 103.07 | 2035.0031 | 18 | 2.9 | 1018.5117 | 2 | 141.77 | 6 | 39252 | Ana\_05.raw | 4.23E6 | 1.6E6 | 1.19E6 |  |  | 4.8E6 | 9.13E5 |  | 1.67E6 | 1.14E6 | 15 | 3 | 2 | 3 | 0 | 0 | 2 | 1 | 0 | 2 | 2 | 222 | 239 | Oxidation (M) |
| R.VLLFSDGNSQGATAEAIEK.A | Y | 86.19 | 1948.9741 | 19 | 3.6 | 975.4978 | 2 | 87.34 | 6 | 24871 | Ana\_05.raw | 3.25E6 | 3.79E6 | 2.57E6 |  | 4.9E5 | 2.95E6 | 1.76E6 |  | 1.56E6 |  | 8 | 2 | 1 | 1 | 0 | 1 | 1 | 1 | 0 | 1 | 0 | 936 | 954 |  |
| K.ENYAELLDDGFLK.N | Y | 86.13 | 1525.7300 | 13 | 0.2 | 763.8724 | 2 | 117.02 | 3 | 32290 | Ana\_02.raw | 1.33E6 | 1.09E7 | 1.23E7 | 2.79E6 | 0 | 8.42E6 | 2.55E6 | 2.79E6 | 1.31E7 | 6.69E6 | 13 | 1 | 2 | 2 | 1 | 1 | 1 | 1 | 1 | 2 | 1 | 788 | 800 |  |
| K.TAEYDVAFGER.H | Y | 85.24 | 1256.5673 | 11 | 1.2 | 629.2917 | 2 | 65.96 | 6 | 18939 | Ana\_05.raw | 3.25E7 | 2.89E7 | 4.12E7 | 1.95E7 | 2.94E7 | 3.19E7 | 2.42E7 | 2.45E7 | 3.61E7 | 2.91E7 | 19 | 2 | 2 | 2 | 1 | 2 | 2 | 2 | 3 | 1 | 2 | 988 | 998 |  |
| K.GLEELLIGGSHLK.E | Y | 84.43 | 1364.7664 | 13 | -0.4 | 683.3902 | 2 | 98.34 | 3 | 27463 | Ana\_02.raw | 1.07E7 | 1.47E7 | 2.89E7 | 4.73E6 | 1.46E7 | 2.7E7 | 4.81E6 | 3.67E6 | 3.02E7 | 1.64E7 | 21 | 2 | 2 | 2 | 2 | 2 | 2 | 2 | 2 | 3 | 2 | 135 | 147 |  |
| R.GDEGPPGPEGLR.G | Y | 79.22 | 1179.5520 | 12 | 3.7 | 590.7855 | 2 | 45.81 | 7 | 12756 | Ana\_06.raw |  |  |  |  |  |  | 1.23E7 |  | 4.13E6 |  | 2 | 0 | 0 | 0 | 0 | 0 | 0 | 1 | 0 | 1 | 0 | 478 | 489 |  |
| R.VPNYQALLR.G | Y | 72.90 | 1072.6029 | 9 | 4.6 | 537.3112 | 2 | 76.49 | 9 | 21793 | Ana\_08.raw | 1.28E7 | 1.75E7 | 1.88E7 | 8.62E6 | 1.44E7 | 1.56E7 | 8.17E6 | 1.38E7 | 2.44E7 | 1.08E7 | 21 | 2 | 2 | 2 | 2 | 2 | 2 | 2 | 3 | 2 | 2 | 1003 | 1011 |  |
| R.DTTPLSVLC(+57.02)GADIQVVSVGIK.D | Y | 71.20 | 2171.1506 | 21 | 7.2 | 1086.5905 | 2 | 130.33 | 2 | 36090 | Ana\_01.raw |  | 3.01E6 | 1.82E6 |  |  |  |  |  |  |  | 2 | 0 | 1 | 1 | 0 | 0 | 0 | 0 | 0 | 0 | 0 | 736 | 756 | Carbamidomethylation |
| R.LSIIATDHTYR.R | Y | 64.71 | 1288.6775 | 11 | 0.8 | 430.5668 | 3 | 53.80 | 3 | 15255 | Ana\_02.raw | 5.83E6 | 8.86E6 | 1.07E7 | 4.02E6 | 7.47E6 | 8.92E6 | 6.25E6 | 5.98E6 | 8.96E6 |  | 9 | 1 | 1 | 1 | 1 | 1 | 1 | 1 | 1 | 1 | 0 | 199 | 209 |  |
| K.VFSVAITPDHLEPR.L | Y | 63.71 | 1579.8358 | 14 | 2.1 | 527.6203 | 3 | 83.36 | 6 | 23786 | Ana\_05.raw |  | 9.14E5 |  | 1.66E6 |  | 1.12E7 | 1.39E6 |  | 1.13E7 |  | 7 | 0 | 1 | 0 | 1 | 0 | 1 | 1 | 0 | 3 | 0 | 185 | 198 |  |
| K.GTYTDC(+57.02)AIK.K | Y | 55.99 | 1027.4645 | 9 | 2.6 | 514.7408 | 2 | 31.03 | 3 | 8426 | Ana\_02.raw | 8.17E6 | 1.46E7 | 1.45E7 |  | 9.68E6 |  |  | 7.49E6 | 1.77E6 | 7.44E6 | 7 | 1 | 1 | 1 | 0 | 1 | 0 | 0 | 1 | 1 | 1 | 125 | 133 | Carbamidomethylation |
| R.LLPPTQNNR.I | Y | 54.17 | 1051.5774 | 9 | 5.2 | 526.7987 | 2 | 29.87 | 2 | 7977 | Ana\_01.raw | 2.16E7 | 3E7 | 2.58E7 | 9.12E6 | 1.2E7 | 2.41E7 | 1.02E7 | 1.18E7 | 1.99E7 | 1.01E7 | 24 | 3 | 3 | 3 | 2 | 2 | 3 | 2 | 2 | 2 | 2 | 713 | 721 |  |
| R.GVLYQTVSR.K | Y | 52.79 | 1021.5556 | 9 | 3.9 | 511.7870 | 2 | 45.57 | 1 | 12715 | ANa\_10.raw | 1.34E7 | 1.54E7 | 2.05E7 | 7.63E6 | 1.43E7 | 1.62E7 | 1.11E7 | 1.24E7 |  | 1.1E7 | 9 | 1 | 1 | 1 | 1 | 1 | 1 | 1 | 1 | 0 | 1 | 1012 | 1020 |  |
| R.IALVITDGR.S | Y | 48.12 | 956.5654 | 9 | 2.3 | 479.2911 | 2 | 67.07 | 7 | 19032 | Ana\_06.raw |  | 1.17E6 |  |  |  |  | 1.26E6 | 9.37E5 | 1.23E6 |  | 4 | 0 | 1 | 0 | 0 | 0 | 0 | 1 | 1 | 1 | 0 | 722 | 730 |  |
| R.LSKDELVK.F | Y | 42.26 | 930.5386 | 8 | 0.7 | 311.1870 | 3 | 26.60 | 3 | 7069 | Ana\_02.raw |  |  | 2.02E6 |  | 1.34E6 |  |  |  |  |  | 2 | 0 | 0 | 1 | 0 | 1 | 0 | 0 | 0 | 0 | 0 | 644 | 651 |  |
| R.FLSAGR.A | Y | 35.44 | 649.3547 | 6 | 4.2 | 325.6860 | 2 | 26.64 | 6 | 7072 | Ana\_05.raw |  |  |  | 4.4E6 |  | 9.53E6 |  |  |  |  | 2 | 0 | 0 | 0 | 1 | 0 | 1 | 0 | 0 | 0 | 0 | 856 | 861 |  |
| total 17 peptides |  |  |  |  |  |  |  |  |  |  |  |  |  |  |  |  |  |  |  |  |  |  |  |  |  |  |  |  |  |  |  |  |  |  |  |
| --- | --- | --- | --- | --- | --- | --- | --- | --- | --- | --- | --- | --- | --- | --- | --- | --- | --- | --- | --- | --- | --- | --- | --- | --- | --- | --- | --- | --- | --- | --- | --- | --- | --- | --- | --- |

Best Unique PSM (Scan F6:13038, m/z=851.4398, z=2, RT=46.14, ppm=3.4):

sp|Q9D1A2|CNDP2\_MOUSE

back to list

  

| Protein Coverage
| Supporting Peptides
| Best Unique PSM
|

Protein Coverage:

Supporting Peptides:

| Peptide | Uniq | -10lgP | Mass | Length | ppm | m/z | z | RT | Fraction | Scan | Source File | Area 10 | Area 01 | Area 02 | Area 03 | Area 04 | Area 05 | Area 06 | Area 07 | Area 08 | Area 09 | #Spec | #Spec 10 | #Spec 01 | #Spec 02 | #Spec 03 | #Spec 04 | #Spec 05 | #Spec 06 | #Spec 07 | #Spec 08 | #Spec 09 | Start | End | PTM |
| --- | --- | --- | --- | --- | --- | --- | --- | --- | --- | --- | --- | --- | --- | --- | --- | --- | --- | --- | --- | --- | --- | --- | --- | --- | --- | --- | --- | --- | --- | --- | --- | --- | --- | --- | --- |
| K.LPDGSEIPLPPILLGK.L | Y | 109.91 | 1657.9653 | 16 | 1.8 | 829.9914 | 2 | 129.49 | 3 | 35477 | Ana\_02.raw | 2.07E7 | 1.39E7 | 2.11E7 | 6.34E5 | 5.72E6 | 2.4E7 | 7.46E6 | 4.15E5 | 3.4E7 | 1.69E7 | 17 | 2 | 2 | 2 | 1 | 2 | 2 | 1 | 1 | 2 | 2 | 69 | 84 |  |
| R.LVPDM(+15.99)IPEVVSEQVSSYLSK.K | Y | 103.67 | 2235.1345 | 20 | 0.8 | 1118.5754 | 2 | 133.25 | 5 | 36653 | Ana\_04.raw | 6.23E5 | 8.55E6 | 4.9E6 | 8.38E5 | 5.7E6 | 9.71E6 | 3.28E6 | 2.19E6 | 5.38E5 | 1.94E6 | 17 | 1 | 2 | 2 | 1 | 2 | 3 | 2 | 1 | 1 | 2 | 344 | 363 | Oxidation (M) |
| R.WRYPSLSLHGIEGAFSGSGAK.T | Y | 90.13 | 2219.1123 | 21 | 3.2 | 555.7871 | 4 | 107.97 | 9 | 30351 | Ana\_08.raw |  |  |  |  |  | 3.61E5 | 7.24E5 |  | 2.79E6 |  | 3 | 0 | 0 | 0 | 0 | 0 | 1 | 1 | 0 | 1 | 0 | 309 | 329 |  |
| R.EGGSIPVTLTFQEATGK.N | Y | 89.43 | 1733.8835 | 17 | 1.5 | 867.9503 | 2 | 92.34 | 3 | 25884 | Ana\_02.raw | 1.93E6 | 2.13E6 | 6.46E6 | 1.92E6 | 3.6E6 | 2.38E6 | 3.13E6 | 7.17E5 | 1.66E7 | 1.41E6 | 10 | 1 | 1 | 1 | 1 | 1 | 1 | 1 | 1 | 1 | 1 | 414 | 430 |  |
| K.AVFQYIDENQDR.Y | Y | 84.29 | 1496.6896 | 12 | 0.6 | 749.3525 | 2 | 69.90 | 3 | 19869 | Ana\_02.raw | 9.75E6 | 1.98E7 | 9.96E6 | 2.84E6 | 6.19E6 | 1.04E7 | 5.56E6 | 4.72E6 | 1.68E7 | 7.11E6 | 10 | 1 | 1 | 1 | 1 | 1 | 1 | 1 | 1 | 1 | 1 | 6 | 17 |  |
| K.M(+15.99)LAAYLYEVSQLK.N | Y | 83.98 | 1543.7955 | 13 | 3.1 | 772.9074 | 2 | 111.89 | 3 | 30950 | Ana\_02.raw |  | 8.15E5 | 1.69E6 | 2.95E6 |  | 6.05E5 |  | 3.44E5 |  |  | 7 | 0 | 1 | 1 | 2 | 0 | 2 | 0 | 1 | 0 | 0 | 462 | 474 | Oxidation (M) |
| K.TVFGVEPDLTR.E | Y | 80.19 | 1232.6400 | 11 | 4.5 | 617.3301 | 2 | 80.36 | 6 | 22971 | Ana\_05.raw | 1.95E7 | 3.83E7 | 2.93E7 | 1.44E7 | 2.38E7 | 2.84E7 | 1.42E7 | 1.23E7 | 4.86E7 | 1.89E7 | 24 | 2 | 4 | 2 | 2 | 3 | 2 | 2 | 2 | 2 | 3 | 403 | 413 |  |
| R.YPSLSLHGIEGAFSGSGAK.T | Y | 72.34 | 1876.9319 | 19 | 3.9 | 626.6537 | 3 | 106.79 | 6 | 30051 | Ana\_05.raw |  | 1.02E6 |  | 3.27E5 |  | 1.46E6 |  | 9.67E4 | 3.96E6 |  | 7 | 0 | 1 | 0 | 1 | 0 | 2 | 0 | 1 | 2 | 0 | 311 | 329 |  |
| K.NVM(+15.99)LLPVGSADDGAHSQNEK.L | Y | 72.23 | 2096.9797 | 20 | 6.0 | 700.0047 | 3 | 59.38 | 2 | 16872 | Ana\_01.raw |  | 5.07E6 |  | 1.88E6 | 3.43E5 | 3.86E6 | 3.45E6 | 1.84E6 | 1.88E6 |  | 7 | 0 | 1 | 0 | 1 | 1 | 1 | 1 | 1 | 1 | 0 | 431 | 450 | Oxidation (M) |
| K.ILIPGINDAVAPVTDEEHALYDHIDFDM(+15.99)EEFAK.D | Y | 68.14 | 3742.7712 | 33 | 2.7 | 936.7026 | 4 | 130.68 | 9 | 36178 | Ana\_08.raw | 1.19E7 | 2.35E6 | 1.4E7 |  | 7.32E6 | 1.86E7 | 9.75E6 | 4.41E6 | 2.28E7 | 9.32E6 | 9 | 1 | 1 | 1 | 0 | 1 | 1 | 1 | 1 | 1 | 1 | 257 | 289 | Oxidation (M) |
| R.LVPDMIPEVVSEQVSSYLSK.K | Y | 63.27 | 2219.1394 | 20 | 4.3 | 740.7236 | 3 | 135.47 | 9 | 37453 | Ana\_08.raw | 1E6 |  |  |  |  |  |  |  | 2.86E6 |  | 3 | 1 | 0 | 0 | 0 | 0 | 0 | 0 | 0 | 2 | 0 | 344 | 363 |  |
| K.KFAELQSPNK.F | Y | 57.92 | 1160.6189 | 10 | 4.4 | 581.3193 | 2 | 31.55 | 3 | 8594 | Ana\_02.raw |  |  | 1.45E6 |  |  |  |  |  |  |  | 1 | 0 | 0 | 1 | 0 | 0 | 0 | 0 | 0 | 0 | 0 | 364 | 373 |  |
| R.LGGSVELVDIGK.Q | Y | 57.24 | 1185.6604 | 12 | 6.3 | 593.8412 | 2 | 78.17 | 1 | 22081 | ANa\_10.raw | 5.02E6 |  |  | 5.62E6 |  |  |  |  | 2.81E6 | 2.75E6 | 4 | 1 | 0 | 0 | 1 | 0 | 0 | 0 | 0 | 1 | 1 | 55 | 66 |  |
| K.QKLPDGSEIPLPPILLGK.L | Y | 53.20 | 1914.1189 | 18 | 3.8 | 639.0493 | 3 | 122.05 | 5 | 33714 | Ana\_04.raw |  | 9.95E5 |  |  | 9.16E5 | 1.5E6 | 3.96E5 |  |  |  | 4 | 0 | 1 | 0 | 0 | 1 | 1 | 1 | 0 | 0 | 0 | 67 | 84 |  |
| R.LNYIEGTK.M | Y | 49.29 | 936.4916 | 8 | 0.5 | 469.2533 | 2 | 41.53 | 4 | 11290 | Ana\_03.raw |  |  |  | 8.05E6 |  | 1.95E7 | 1.36E7 |  |  |  | 3 | 0 | 0 | 0 | 1 | 0 | 1 | 1 | 0 | 0 | 0 | 454 | 461 |  |
| K.DVGAETLLHSC(+57.02)K.K | Y | 42.50 | 1328.6394 | 12 | 2.1 | 665.3284 | 2 | 55.23 | 3 | 15675 | Ana\_02.raw |  |  | 1.76E6 |  |  |  |  |  |  | 1.33E6 | 2 | 0 | 0 | 1 | 0 | 0 | 0 | 0 | 0 | 0 | 1 | 290 | 301 | Carbamidomethylation |
| total 16 peptides |  |  |  |  |  |  |  |  |  |  |  |  |  |  |  |  |  |  |  |  |  |  |  |  |  |  |  |  |  |  |  |  |  |  |  |
| --- | --- | --- | --- | --- | --- | --- | --- | --- | --- | --- | --- | --- | --- | --- | --- | --- | --- | --- | --- | --- | --- | --- | --- | --- | --- | --- | --- | --- | --- | --- | --- | --- | --- | --- | --- |

Best Unique PSM (Scan F3:35477, m/z=829.9914, z=2, RT=129.49, ppm=1.8):

sp|Q02788|CO6A2\_MOUSE

back to list

  

| Protein Coverage
| Supporting Peptides
| Best Unique PSM
|

Protein Coverage:

Supporting Peptides:

| Peptide | Uniq | -10lgP | Mass | Length | ppm | m/z | z | RT | Fraction | Scan | Source File | Area 10 | Area 01 | Area 02 | Area 03 | Area 04 | Area 05 | Area 06 | Area 07 | Area 08 | Area 09 | #Spec | #Spec 10 | #Spec 01 | #Spec 02 | #Spec 03 | #Spec 04 | #Spec 05 | #Spec 06 | #Spec 07 | #Spec 08 | #Spec 09 | Start | End | PTM |
| --- | --- | --- | --- | --- | --- | --- | --- | --- | --- | --- | --- | --- | --- | --- | --- | --- | --- | --- | --- | --- | --- | --- | --- | --- | --- | --- | --- | --- | --- | --- | --- | --- | --- | --- | --- |
| R.ATYLNSFSHVGTGIVHAINNVVR.G | Y | 117.66 | 2468.2925 | 23 | -0.9 | 823.7707 | 3 | 122.83 | 3 | 33759 | Ana\_02.raw | 5.74E6 | 6.59E6 | 1.04E7 | 3.75E6 | 2.6E7 | 5.61E7 | 4.44E6 | 2.85E7 | 4.05E7 | 5.93E6 | 29 | 3 | 2 | 3 | 3 | 4 | 4 | 3 | 3 | 2 | 2 | 923 | 945 |  |
| R.EKDFDSLAQPSFFDR.F | Y | 86.67 | 1800.8318 | 15 | -0.1 | 901.4230 | 2 | 92.14 | 3 | 25832 | Ana\_02.raw | 2.09E7 | 3.2E7 | 2.8E7 | 1.07E7 | 1.93E7 | 2.84E7 | 6.65E5 | 1.41E7 | 3E7 | 1.76E7 | 20 | 2 | 2 | 2 | 2 | 3 | 2 | 2 | 1 | 2 | 2 | 1014 | 1028 |  |
| K.NLEWIAGGTWTPSALK.F | Y | 84.31 | 1742.8992 | 16 | 6.6 | 872.4626 | 2 | 119.10 | 2 | 33113 | Ana\_01.raw | 2.46E6 | 1.39E6 | 2.57E6 |  | 2.58E5 | 1.96E6 |  | 8.82E5 | 5.4E6 | 8.45E5 | 8 | 1 | 1 | 1 | 0 | 1 | 1 | 0 | 1 | 1 | 1 | 708 | 723 |  |
| K.DFDSLAQPSFFDR.F | Y | 81.65 | 1543.6942 | 13 | -0.4 | 772.8541 | 2 | 107.84 | 3 | 29913 | Ana\_02.raw | 1.04E6 | 4.01E6 | 4.7E6 |  | 9.93E5 | 3.05E6 | 7.15E5 |  | 2.86E6 | 9.53E5 | 8 | 1 | 1 | 1 | 0 | 1 | 1 | 1 | 0 | 1 | 1 | 1016 | 1028 |  |
| R.DIANSPHELYR.N | Y | 77.33 | 1313.6364 | 11 | 7.7 | 657.8305 | 2 | 46.75 | 1 | 13073 | ANa\_10.raw | 2.46E7 | 2.69E7 | 2.79E7 | 6.6E6 | 7.06E6 | 2.43E7 | 4.83E6 | 4.19E6 | 2.04E7 | 1.73E7 | 17 | 2 | 2 | 2 | 1 | 1 | 2 | 1 | 1 | 3 | 2 | 218 | 228 |  |
| R.VGVVQYSHEGTFEAIR.L | Y | 77.00 | 1790.8951 | 16 | 4.4 | 597.9750 | 3 | 73.87 | 7 | 20941 | Ana\_06.raw | 1.04E7 | 2.18E7 | 7.37E6 | 7.15E6 |  | 1.64E7 | 7.5E6 | 8.66E6 | 1.14E7 | 8.98E6 | 10 | 1 | 2 | 1 | 1 | 0 | 1 | 1 | 1 | 1 | 1 | 675 | 690 |  |
| K.HEAYGEC(+57.02)YK.V | Y | 73.89 | 1155.4655 | 9 | 8.5 | 578.7449 | 2 | 24.16 | 1 | 6154 | ANa\_10.raw | 2.94E6 | 5.95E6 | 1.11E7 | 4.9E6 | 9.61E6 | 1.12E7 | 6.99E6 | 6.33E6 | 5.43E6 |  | 9 | 1 | 1 | 1 | 1 | 1 | 1 | 1 | 1 | 1 | 0 | 255 | 263 | Carbamidomethylation |
| R.RDDDPLNAR.M | Y | 68.33 | 1070.5105 | 9 | 1.8 | 536.2635 | 2 | 26.21 | 3 | 6946 | Ana\_02.raw | 2.04E7 | 1.92E7 | 1.78E7 | 9.02E6 | 1.78E7 | 1.63E7 | 1.02E7 |  | 1.05E7 | 1.72E7 | 10 | 1 | 1 | 1 | 2 | 1 | 1 | 1 | 0 | 1 | 1 | 883 | 891 |  |
| R.LFAVAPNR.N | Y | 66.13 | 886.5024 | 8 | 5.1 | 444.2608 | 2 | 48.65 | 2 | 13697 | Ana\_01.raw | 2.04E7 | 2.91E7 | 3.08E7 | 1.7E7 | 3.07E7 | 3.11E7 | 2.18E7 | 1.63E7 | 2.54E7 | 1.76E7 | 10 | 1 | 1 | 1 | 1 | 1 | 1 | 1 | 1 | 1 | 1 | 202 | 209 |  |
| R.VFAVVITDGR.H | Y | 62.18 | 1075.6025 | 10 | 2.8 | 538.8101 | 2 | 72.18 | 6 | 20713 | Ana\_05.raw |  |  |  | 4.76E5 | 3.78E5 | 1.49E6 | 7.95E5 | 1.47E6 |  |  | 6 | 0 | 0 | 0 | 1 | 1 | 1 | 1 | 2 | 0 | 0 | 740 | 749 |  |
| K.NFVINVVNR.L | Y | 55.17 | 1073.5981 | 9 | 5.2 | 537.8091 | 2 | 73.68 | 1 | 20873 | ANa\_10.raw | 9.43E6 | 1.62E6 | 2.45E6 | 5.68E6 | 1E7 | 9.72E6 |  |  | 2.01E6 | 6.97E6 | 8 | 1 | 1 | 1 | 1 | 1 | 1 | 0 | 0 | 1 | 1 | 651 | 659 |  |
| R.DDDLNLR.A | Y | 53.00 | 859.4036 | 7 | 3.7 | 430.7107 | 2 | 37.80 | 9 | 10280 | Ana\_08.raw | 2.15E7 |  | 2.67E7 | 2.06E5 | 1.89E7 | 2.26E7 | 2.08E7 | 1.4E7 | 5.81E6 | 2.05E7 | 11 | 1 | 0 | 1 | 1 | 1 | 1 | 2 | 2 | 1 | 1 | 754 | 760 |  |
| R.RFVEDVSR.R | Y | 50.09 | 1006.5195 | 8 | 5.2 | 504.2697 | 2 | 29.34 | 2 | 7813 | Ana\_01.raw | 5.69E7 | 4.97E7 |  |  | 3.42E7 | 5.17E7 | 2.53E7 | 2.58E7 | 5.6E7 | 4.14E7 | 8 | 1 | 1 | 0 | 0 | 1 | 1 | 1 | 1 | 1 | 1 | 869 | 876 |  |
| R.LGEQNFHK.V | Y | 49.82 | 971.4824 | 8 | -1.5 | 486.7478 | 2 | 26.94 | 4 | 6970 | Ana\_03.raw | 5.27E6 | 4.76E6 | 6.57E6 | 6.56E6 |  | 5.64E6 | 6E6 | 4.57E6 | 6.96E6 | 1.05E6 | 13 | 1 | 2 | 1 | 2 | 1 | 1 | 1 | 1 | 2 | 1 | 859 | 866 |  |
| R.NLNEQGLR.D | Y | 45.52 | 942.4883 | 8 | 1.7 | 472.2522 | 2 | 31.70 | 4 | 8386 | Ana\_03.raw |  |  |  | 1.16E7 | 2.29E7 |  | 1.73E7 | 1.77E7 | 2.87E7 |  | 5 | 0 | 0 | 0 | 1 | 1 | 0 | 1 | 1 | 1 | 0 | 210 | 217 |  |
| R.FVEDVSR.R | Y | 44.20 | 850.4185 | 7 | 4.1 | 426.2182 | 2 | 32.94 | 5 | 8982 | Ana\_04.raw |  | 3.93E7 | 3.61E7 | 5.65E6 | 1.84E7 | 2.32E7 | 1.24E7 |  |  | 2.06E7 | 7 | 0 | 1 | 1 | 1 | 1 | 1 | 1 | 0 | 0 | 1 | 870 | 876 |  |
| K.FAYNQLIK.E | Y | 37.65 | 995.5440 | 8 | 1.6 | 498.7801 | 2 | 74.25 | 5 | 21150 | Ana\_04.raw | 2.77E6 |  | 1.43E6 |  | 4E6 | 1.31E6 |  |  |  |  | 4 | 1 | 0 | 1 | 0 | 1 | 1 | 0 | 0 | 0 | 0 | 724 | 731 |  |
| K.ISLGDR.A | Y | 35.26 | 659.3602 | 6 | 4.2 | 330.6888 | 2 | 29.47 | 10 | 7915 | Ana\_09.raw | 6.3E6 | 9.14E6 | 9.79E6 | 3.44E6 | 5.82E6 |  |  | 3.54E6 | 7.31E6 | 6.45E6 | 8 | 1 | 1 | 1 | 1 | 1 | 0 | 0 | 1 | 1 | 1 | 1003 | 1008 |  |
| R.VNSLSSFK.E | Y | 30.13 | 880.4654 | 8 | 5.2 | 441.2423 | 2 | 44.17 | 8 | 12085 | Ana\_07.raw |  |  |  |  |  |  |  | 3.97E6 |  |  | 1 | 0 | 0 | 0 | 0 | 0 | 0 | 0 | 1 | 0 | 0 | 696 | 703 |  |
| K.SLQGIR.S | Y | 25.64 | 672.3918 | 6 | 1.5 | 337.2037 | 2 | 23.98 | 3 | 6244 | Ana\_02.raw | 3.56E6 | 5.95E6 | 1.18E7 |  |  |  |  |  |  |  | 3 | 1 | 1 | 1 | 0 | 0 | 0 | 0 | 0 | 0 | 0 | 136 | 141 |  |
| total 20 peptides |  |  |  |  |  |  |  |  |  |  |  |  |  |  |  |  |  |  |  |  |  |  |  |  |  |  |  |  |  |  |  |  |  |  |  |
| --- | --- | --- | --- | --- | --- | --- | --- | --- | --- | --- | --- | --- | --- | --- | --- | --- | --- | --- | --- | --- | --- | --- | --- | --- | --- | --- | --- | --- | --- | --- | --- | --- | --- | --- | --- |

Best Unique PSM (Scan F3:33759, m/z=823.7707, z=3, RT=122.83, ppm=-0.9):

sp|Q8BK48|EST2E\_MOUSE

back to list

  

| Protein Coverage
| Supporting Peptides
| Best Unique PSM
|

Protein Coverage:

Supporting Peptides:

| Peptide | Uniq | -10lgP | Mass | Length | ppm | m/z | z | RT | Fraction | Scan | Source File | Area 10 | Area 01 | Area 02 | Area 03 | Area 04 | Area 05 | Area 06 | Area 07 | Area 08 | Area 09 | #Spec | #Spec 10 | #Spec 01 | #Spec 02 | #Spec 03 | #Spec 04 | #Spec 05 | #Spec 06 | #Spec 07 | #Spec 08 | #Spec 09 | Start | End | PTM |
| --- | --- | --- | --- | --- | --- | --- | --- | --- | --- | --- | --- | --- | --- | --- | --- | --- | --- | --- | --- | --- | --- | --- | --- | --- | --- | --- | --- | --- | --- | --- | --- | --- | --- | --- | --- |
| K.DFHPVPSIIGINNDEYGWILPTIM(+15.99)DPAQK.I | Y | 120.96 | 3295.6223 | 29 | 3.2 | 1099.5515 | 3 | 132.31 | 6 | 36778 | Ana\_05.raw |  |  |  |  |  | 4.97E6 | 2.9E6 |  |  |  | 2 | 0 | 0 | 0 | 0 | 0 | 1 | 1 | 0 | 0 | 0 | 331 | 359 | Oxidation (M) |
| K.GDFM(+15.99)FVIPALQVAHFQR.S | Y | 103.16 | 1991.0087 | 17 | 3.0 | 664.6788 | 3 | 126.88 | 6 | 35274 | Ana\_05.raw | 7.84E6 | 5.12E5 | 7.73E6 |  | 1.01E6 | 3.56E6 | 9.04E6 | 5.2E6 | 1.51E6 |  | 12 | 2 | 2 | 2 | 0 | 1 | 1 | 1 | 1 | 2 | 0 | 412 | 428 | Oxidation (M) |
| R.SHAPVYFYEFQHRPSFFK.D | Y | 92.46 | 2286.1011 | 18 | 3.6 | 572.5346 | 4 | 94.05 | 6 | 26673 | Ana\_05.raw |  |  |  | 1.62E6 |  | 1.57E6 |  | 7.45E5 | 4.48E6 |  | 4 | 0 | 0 | 0 | 1 | 0 | 1 | 0 | 1 | 1 | 0 | 429 | 446 |  |
| R.FAPPEAPEPWSGVR.D | N | 86.12 | 1538.7517 | 14 | 5.9 | 770.3876 | 2 | 86.16 | 9 | 24504 | Ana\_08.raw | 1.81E7 | 2.23E7 | 1.18E7 | 6.41E5 | 1.13E7 | 1.22E7 | 9.26E6 |  | 5.15E7 | 1.25E7 | 18 | 3 | 2 | 2 | 1 | 2 | 2 | 2 | 0 | 2 | 2 | 73 | 86 |  |
| R.LGVLGFFSTGDQHAK.G | Y | 81.60 | 1575.8044 | 15 | 4.6 | 526.2778 | 3 | 95.63 | 9 | 27074 | Ana\_08.raw | 1.57E6 | 1.74E6 | 2.81E6 | 7.15E5 | 3.31E6 |  | 3.95E6 | 2.06E6 | 2.26E6 | 1.16E6 | 9 | 1 | 1 | 1 | 1 | 1 | 0 | 1 | 1 | 1 | 1 | 179 | 193 |  |
| K.GNWGYLDQVAALR.W | N | 77.33 | 1461.7365 | 13 | 2.1 | 731.8770 | 2 | 109.87 | 5 | 30585 | Ana\_04.raw | 3.63E6 | 6.43E6 | 5.15E6 | 1.69E6 | 5.89E5 | 8.04E6 | 2.05E6 | 1.04E6 | 3.39E7 | 3.58E6 | 11 | 1 | 1 | 1 | 1 | 1 | 1 | 1 | 1 | 2 | 1 | 194 | 206 |  |
| K.IIPGVVDGEFLPK.H | Y | 71.40 | 1382.7809 | 13 | 5.0 | 692.4012 | 2 | 102.26 | 2 | 28721 | Ana\_01.raw | 1.94E7 | 2.97E7 | 2.21E7 | 9.76E5 | 1.18E7 | 1.09E7 | 6.25E6 | 2.74E6 | 7.09E7 | 1.62E7 | 30 | 4 | 4 | 3 | 1 | 3 | 3 | 3 | 2 | 4 | 3 | 309 | 321 |  |
| K.LPYTEEEEQLSR.R | Y | 71.26 | 1492.7045 | 12 | 5.0 | 747.3632 | 2 | 63.97 | 9 | 18241 | Ana\_08.raw | 1.07E7 | 1.94E7 | 1.35E7 | 5.94E6 | 9.75E6 | 1.53E7 | 3.67E6 | 3.2E6 | 1.64E7 | 1.01E7 | 11 | 1 | 2 | 1 | 1 | 1 | 1 | 1 | 1 | 1 | 1 | 474 | 485 |  |
| K.HPQELM(+15.99)ASK.D | Y | 65.00 | 1055.5070 | 9 | 0.3 | 528.7609 | 2 | 17.67 | 3 | 4156 | Ana\_02.raw |  |  | 5.09E6 | 1.41E6 | 7.57E6 | 1.85E7 | 8.01E6 | 4.74E6 |  |  | 6 | 0 | 0 | 1 | 1 | 1 | 1 | 1 | 1 | 0 | 0 | 322 | 330 | Oxidation (M) |
| K.DFRPPYVK.A | Y | 57.59 | 1020.5392 | 8 | 0.5 | 341.1872 | 3 | 54.20 | 3 | 15375 | Ana\_02.raw |  |  | 6.82E6 |  |  |  |  |  | 2.59E6 |  | 3 | 0 | 0 | 2 | 0 | 0 | 0 | 0 | 0 | 1 | 0 | 447 | 454 |  |
| R.LQFWTK.T | N | 45.17 | 821.4435 | 6 | 4.6 | 411.7309 | 2 | 68.62 | 6 | 19692 | Ana\_05.raw |  |  |  |  | 8.96E5 | 9.97E5 |  |  | 5.19E6 |  | 3 | 0 | 0 | 0 | 0 | 1 | 1 | 0 | 0 | 1 | 0 | 535 | 540 |  |
| R.GKNEAEM(+15.99)LAINK.V | Y | 39.37 | 1332.6708 | 12 | 0.6 | 667.3431 | 2 | 33.74 | 3 | 9241 | Ana\_02.raw |  |  | 4.7E5 |  |  | 1.67E6 |  |  | 1.83E6 |  | 3 | 0 | 0 | 1 | 0 | 0 | 1 | 0 | 0 | 1 | 0 | 294 | 305 | Oxidation (M) |
| K.IEEITR.K | Y | 36.31 | 759.4127 | 6 | 5.8 | 380.7158 | 2 | 25.70 | 2 | 6657 | Ana\_01.raw |  | 8.97E6 | 1.55E7 |  |  | 1.01E7 | 1.99E7 |  |  |  | 4 | 0 | 1 | 1 | 0 | 0 | 1 | 1 | 0 | 0 | 0 | 360 | 365 |  |
| K.YWANFAR.H | N | 27.37 | 926.4399 | 7 | 5.5 | 464.2298 | 2 | 61.88 | 9 | 17609 | Ana\_08.raw |  | 5.32E6 |  |  |  | 3.96E6 |  |  | 9.53E6 |  | 3 | 0 | 1 | 0 | 0 | 0 | 1 | 0 | 0 | 1 | 0 | 490 | 496 |  |
| K.IQELK.G | N | 25.42 | 629.3748 | 5 | 3.5 | 315.6958 | 2 | 26.96 | 7 | 7048 | Ana\_06.raw |  |  |  | 2.03E7 |  | 5.22E7 | 3.21E7 |  |  |  | 3 | 0 | 0 | 0 | 1 | 0 | 1 | 1 | 0 | 0 | 0 | 546 | 550 |  |
| total 15 peptides |  |  |  |  |  |  |  |  |  |  |  |  |  |  |  |  |  |  |  |  |  |  |  |  |  |  |  |  |  |  |  |  |  |  |  |
| --- | --- | --- | --- | --- | --- | --- | --- | --- | --- | --- | --- | --- | --- | --- | --- | --- | --- | --- | --- | --- | --- | --- | --- | --- | --- | --- | --- | --- | --- | --- | --- | --- | --- | --- | --- |

Best Unique PSM (Scan F6:36778, m/z=1099.5515, z=3, RT=132.31, ppm=3.2):

sp|P63038|CH60\_MOUSE

back to list

  

| Protein Coverage
| Supporting Peptides
| Best Unique PSM
|

Protein Coverage:

Supporting Peptides:

| Peptide | Uniq | -10lgP | Mass | Length | ppm | m/z | z | RT | Fraction | Scan | Source File | Area 10 | Area 01 | Area 02 | Area 03 | Area 04 | Area 05 | Area 06 | Area 07 | Area 08 | Area 09 | #Spec | #Spec 10 | #Spec 01 | #Spec 02 | #Spec 03 | #Spec 04 | #Spec 05 | #Spec 06 | #Spec 07 | #Spec 08 | #Spec 09 | Start | End | PTM |
| --- | --- | --- | --- | --- | --- | --- | --- | --- | --- | --- | --- | --- | --- | --- | --- | --- | --- | --- | --- | --- | --- | --- | --- | --- | --- | --- | --- | --- | --- | --- | --- | --- | --- | --- | --- |
| R.ALM(+15.99)LQGVDLLADAVAVTM(+15.99)GPK.G | Y | 107.26 | 2144.1221 | 21 | 5.2 | 715.7184 | 3 | 132.88 | 6 | 36940 | Ana\_05.raw | 1.57E7 | 1.08E7 | 1.68E7 | 8.53E6 | 1.04E7 | 1.66E7 | 1.14E7 | 4.99E6 | 2.82E7 | 1.08E7 | 19 | 2 | 2 | 2 | 2 | 2 | 2 | 2 | 1 | 2 | 2 | 38 | 58 | Oxidation (M) |
| R.TALLDAAGVASLLTTAEAVVTEIPKEEK.D | Y | 95.46 | 2839.5430 | 28 | 1.6 | 947.5231 | 3 | 139.64 | 3 | 38090 | Ana\_02.raw | 1.82E6 | 2.14E7 | 4.03E6 | 2.34E6 | 4.58E6 | 7.12E6 | 2.73E6 |  | 8.88E6 | 1.36E6 | 13 | 1 | 3 | 2 | 1 | 1 | 1 | 1 | 0 | 2 | 1 | 527 | 554 |  |
| R.GVM(+15.99)LAVDAVIAELKK.Q | Y | 77.51 | 1571.8956 | 15 | 2.1 | 524.9736 | 3 | 124.37 | 6 | 34626 | Ana\_05.raw | 1.83E6 | 9.23E5 | 9.86E5 | 1.04E6 | 8.16E5 | 3.37E6 | 1.12E6 | 1.18E6 | 1.1E6 | 6.92E5 | 17 | 2 | 2 | 2 | 1 | 2 | 2 | 1 | 2 | 2 | 1 | 143 | 157 | Oxidation (M) |
| R.GYISPYFINTSK.G | Y | 76.33 | 1388.6976 | 12 | 5.5 | 695.3599 | 2 | 82.73 | 9 | 23536 | Ana\_08.raw | 3.36E6 | 3.64E6 | 1.78E6 | 8.81E5 | 1.47E6 | 3.62E6 | 2.56E6 | 1.34E6 | 9.66E6 | 1.83E6 | 10 | 1 | 1 | 1 | 1 | 1 | 1 | 1 | 1 | 1 | 1 | 222 | 233 |  |
| K.DM(+15.99)AIATGGAVFGEEGLNLNLEDVQAHDLGK.V | Y | 74.83 | 3099.4819 | 30 | 2.4 | 1034.1704 | 3 | 119.98 | 4 | 32695 | Ana\_03.raw |  |  |  | 1.4E6 |  | 3.06E6 |  | 2.52E6 |  |  | 3 | 0 | 0 | 0 | 1 | 0 | 1 | 0 | 1 | 0 | 0 | 315 | 344 | Oxidation (M) |
| R.TVIIEQSWGSPK.V | Y | 73.53 | 1343.7085 | 12 | 5.9 | 672.8655 | 2 | 73.63 | 7 | 20873 | Ana\_06.raw |  |  |  |  |  |  | 3.01E6 |  |  |  | 1 | 0 | 0 | 0 | 0 | 0 | 0 | 1 | 0 | 0 | 0 | 61 | 72 |  |
| K.ISSVQSIVPALEIANAHR.K | Y | 72.98 | 1904.0479 | 18 | 3.9 | 635.6924 | 3 | 103.95 | 8 | 28857 | Ana\_07.raw |  | 8.28E6 |  | 1.77E6 |  |  | 1.2E6 | 2.72E6 |  |  | 5 | 0 | 1 | 0 | 2 | 0 | 0 | 1 | 1 | 0 | 0 | 251 | 268 |  |
| K.TLNDELEIIEGM(+15.99)K.F | Y | 70.10 | 1519.7439 | 13 | 1.0 | 760.8800 | 2 | 97.98 | 3 | 27367 | Ana\_02.raw | 2.11E6 | 3.08E6 | 6.94E6 | 6.44E5 | 1.61E6 | 1.87E6 | 1.83E6 |  | 6.68E6 | 1.9E6 | 11 | 1 | 1 | 2 | 1 | 2 | 1 | 1 | 0 | 1 | 1 | 206 | 218 | Oxidation (M) |
| K.VGGTSDVEVNEK.K | Y | 70.09 | 1232.5884 | 12 | 5.7 | 617.3050 | 2 | 29.08 | 2 | 7728 | Ana\_01.raw | 2.98E7 | 4.01E7 |  | 1.68E7 | 2.89E7 | 3.59E7 | 2.45E7 |  | 3.26E7 |  | 8 | 1 | 2 | 0 | 1 | 1 | 1 | 1 | 0 | 1 | 0 | 406 | 417 |  |
| K.TLNDELEIIEGMK.F | Y | 69.82 | 1503.7490 | 13 | 0.3 | 752.8820 | 2 | 114.17 | 9 | 31951 | Ana\_08.raw | 6.93E5 |  |  |  |  |  |  |  | 2.34E6 | 1.69E6 | 3 | 1 | 0 | 0 | 0 | 0 | 0 | 0 | 0 | 1 | 1 | 206 | 218 |  |
| R.TALLDAAGVASLLTTAEAVVTEIPK.E | Y | 68.29 | 2453.3628 | 25 | 2.5 | 1227.6918 | 2 | 144.01 | 9 | 39570 | Ana\_08.raw | 1.17E5 |  | 4.19E5 |  |  |  |  |  | 2.55E5 | 3.37E5 | 4 | 1 | 0 | 1 | 0 | 0 | 0 | 0 | 0 | 1 | 1 | 527 | 551 |  |
| R.VTDALNATR.A | Y | 63.06 | 959.5036 | 9 | 4.2 | 480.7611 | 2 | 31.15 | 7 | 8323 | Ana\_06.raw | 3.19E7 | 3.87E7 | 4.44E7 | 2.02E7 | 2.7E7 | 3.55E7 | 2.73E7 | 2.61E7 | 1.23E7 | 2.64E7 | 11 | 1 | 1 | 1 | 1 | 1 | 1 | 1 | 1 | 1 | 2 | 421 | 429 |  |
| K.APGFGDNR.K | Y | 58.63 | 832.3828 | 8 | 4.0 | 417.2003 | 2 | 24.96 | 1 | 6421 | ANa\_10.raw | 8.6E6 | 1.94E7 | 2.12E7 | 1.12E7 | 1.4E7 |  | 1.48E7 |  |  | 4.92E6 | 7 | 1 | 1 | 1 | 1 | 1 | 0 | 1 | 0 | 0 | 1 | 302 | 309 |  |
| R.ALMLQGVDLLADAVAVTM(+15.99)GPK.G | Y | 58.59 | 2128.1272 | 21 | -0.1 | 710.3829 | 3 | 135.28 | 6 | 37606 | Ana\_05.raw | 9.67E5 | 1.28E6 |  |  |  | 7.1E5 |  |  | 3.9E6 | 1.15E6 | 5 | 1 | 1 | 0 | 0 | 0 | 1 | 0 | 0 | 1 | 1 | 38 | 58 | Oxidation (M) |
| K.NAGVEGSLIVEK.I | Y | 58.07 | 1214.6506 | 12 | 0.4 | 608.3328 | 2 | 58.46 | 4 | 16237 | Ana\_03.raw | 9.43E6 | 5.69E6 | 1.41E7 | 6.65E6 | 8.8E6 | 1.16E7 |  | 6.49E6 | 1.29E7 | 5.68E6 | 11 | 1 | 1 | 2 | 1 | 1 | 1 | 0 | 1 | 2 | 1 | 482 | 493 |  |
| K.APGFGDNRK.N | Y | 53.70 | 960.4777 | 9 | 3.9 | 481.2480 | 2 | 20.40 | 6 | 5047 | Ana\_05.raw |  | 2.52E6 |  | 2.13E6 | 8E5 | 4.21E6 | 3.3E6 |  |  | 4.49E6 | 10 | 0 | 1 | 0 | 2 | 1 | 2 | 2 | 0 | 0 | 2 | 302 | 310 |  |
| K.GANPVEIR.R | Y | 51.01 | 854.4610 | 8 | 2.3 | 428.2388 | 2 | 33.75 | 6 | 9255 | Ana\_05.raw | 2.51E7 | 3.27E7 |  |  | 2.52E7 | 2.54E7 | 2.04E7 | 1.96E7 | 4.74E6 | 2.46E7 | 8 | 1 | 1 | 0 | 0 | 1 | 1 | 1 | 1 | 1 | 1 | 134 | 141 |  |
| K.VGLQVVAVK.A | Y | 44.93 | 911.5804 | 9 | 5.3 | 456.7999 | 2 | 60.57 | 8 | 16924 | Ana\_07.raw | 7.09E6 | 1.26E7 | 1.33E7 | 6.44E6 | 9.94E6 | 9.77E6 | 7.61E6 | 6.3E6 | 1.33E7 | 9.43E6 | 10 | 1 | 1 | 1 | 1 | 1 | 1 | 1 | 1 | 1 | 1 | 293 | 301 |  |
| K.VGEVIVTK.D | Y | 43.05 | 843.5065 | 8 | 2.6 | 422.7617 | 2 | 33.29 | 5 | 9091 | Ana\_04.raw | 2.47E7 | 3.46E7 | 3.26E7 | 1.47E7 | 2.69E7 | 2.71E7 | 2.22E7 | 2.04E7 | 3.03E7 | 2.66E7 | 10 | 1 | 1 | 1 | 1 | 1 | 1 | 1 | 1 | 1 | 1 | 345 | 352 |  |
| K.TLNDELEIIEGM(+15.99)KFDR.G | Y | 39.33 | 1937.9404 | 16 | 8.0 | 646.9926 | 3 | 105.48 | 2 | 29555 | Ana\_01.raw |  | 6.13E5 |  |  |  | 5.53E5 |  |  | 1.67E6 |  | 3 | 0 | 1 | 0 | 0 | 0 | 1 | 0 | 0 | 1 | 0 | 206 | 221 | Oxidation (M) |
| K.LSDGVAVLK.V | Y | 36.88 | 900.5280 | 9 | 3.1 | 451.2727 | 2 | 48.06 | 3 | 13579 | Ana\_02.raw | 8.11E6 | 1.3E7 | 1.68E7 |  | 1.48E7 | 1.42E7 | 1.2E7 |  | 1.48E7 | 1.02E7 | 9 | 1 | 1 | 1 | 0 | 1 | 1 | 1 | 0 | 2 | 1 | 397 | 405 |  |
| K.IPAM(+15.99)TIAK.N | Y | 34.60 | 859.4837 | 8 | 2.2 | 430.7501 | 2 | 29.21 | 4 | 7649 | Ana\_03.raw |  |  |  | 9.53E6 |  | 2.51E7 |  | 1.66E7 |  |  | 3 | 0 | 0 | 0 | 1 | 0 | 1 | 0 | 1 | 0 | 0 | 474 | 481 | Oxidation (M) |
| K.DDAM(+15.99)LLK.G | Y | 33.58 | 820.4000 | 7 | 4.3 | 411.2090 | 2 | 35.02 | 5 | 9599 | Ana\_04.raw |  |  | 1.16E7 | 4.13E6 | 0 | 8.98E6 | 9.2E6 |  | 7.76E6 |  | 6 | 0 | 0 | 1 | 1 | 1 | 1 | 1 | 0 | 1 | 0 | 353 | 359 | Oxidation (M) |
| K.IGIEIIK.R | N | 29.98 | 784.5058 | 7 | 3.4 | 393.2615 | 2 | 68.89 | 10 | 19518 | Ana\_09.raw |  |  | 8.19E6 | 4.07E6 | 6.24E6 | 4.79E6 | 4.31E6 |  | 3.9E6 | 6.23E6 | 8 | 0 | 0 | 2 | 1 | 1 | 1 | 1 | 0 | 1 | 1 | 463 | 469 |  |
| K.EKLNER.L | Y | 23.18 | 787.4188 | 6 | 1.6 | 394.7173 | 2 | 33.09 | 8 | 8799 | Ana\_07.raw |  |  |  |  |  |  |  | 6.91E5 |  |  | 1 | 0 | 0 | 0 | 0 | 0 | 0 | 0 | 1 | 0 | 0 | 388 | 393 |  |
| total 25 peptides |  |  |  |  |  |  |  |  |  |  |  |  |  |  |  |  |  |  |  |  |  |  |  |  |  |  |  |  |  |  |  |  |  |  |  |
| --- | --- | --- | --- | --- | --- | --- | --- | --- | --- | --- | --- | --- | --- | --- | --- | --- | --- | --- | --- | --- | --- | --- | --- | --- | --- | --- | --- | --- | --- | --- | --- | --- | --- | --- | --- |

Best Unique PSM (Scan F6:36940, m/z=715.7184, z=3, RT=132.88, ppm=5.2):

sp|O08601|MTP\_MOUSE

back to list

  

| Protein Coverage
| Supporting Peptides
| Best Unique PSM
|

Protein Coverage:

Supporting Peptides:

| Peptide | Uniq | -10lgP | Mass | Length | ppm | m/z | z | RT | Fraction | Scan | Source File | Area 10 | Area 01 | Area 02 | Area 03 | Area 04 | Area 05 | Area 06 | Area 07 | Area 08 | Area 09 | #Spec | #Spec 10 | #Spec 01 | #Spec 02 | #Spec 03 | #Spec 04 | #Spec 05 | #Spec 06 | #Spec 07 | #Spec 08 | #Spec 09 | Start | End | PTM |
| --- | --- | --- | --- | --- | --- | --- | --- | --- | --- | --- | --- | --- | --- | --- | --- | --- | --- | --- | --- | --- | --- | --- | --- | --- | --- | --- | --- | --- | --- | --- | --- | --- | --- | --- | --- |
| K.AEAVQSFLAFIQHLR.T | Y | 97.62 | 1728.9310 | 15 | 0.6 | 577.3180 | 3 | 134.05 | 3 | 36693 | Ana\_02.raw |  | 7.39E6 | 5.36E5 | 1.39E6 | 3.03E6 | 7.57E6 | 7E6 | 1.51E6 | 2.31E6 | 5.24E5 | 10 | 0 | 2 | 1 | 1 | 1 | 1 | 1 | 1 | 1 | 1 | 323 | 337 |  |
| K.EFYSYENEPVGIENLK.R | Y | 95.10 | 1929.8995 | 16 | 3.4 | 965.9603 | 2 | 96.88 | 6 | 27425 | Ana\_05.raw | 6.07E6 | 8.4E6 | 8.8E5 | 2.19E6 |  | 4.82E6 | 3.92E6 | 1.05E6 | 7.35E6 | 4.38E5 | 9 | 1 | 1 | 1 | 1 | 0 | 1 | 1 | 1 | 1 | 1 | 134 | 149 |  |
| K.IEDSFVTAVLAEETR.A | Y | 88.20 | 1678.8413 | 15 | 2.9 | 840.4304 | 2 | 120.45 | 4 | 32816 | Ana\_03.raw | 2.07E6 | 1.43E7 | 1.43E7 | 1.22E6 | 1.57E6 | 1.25E7 | 9.55E6 |  | 2.83E6 | 1.34E6 | 22 | 2 | 4 | 2 | 2 | 2 | 3 | 4 | 0 | 2 | 1 | 222 | 236 |  |
| R.SGFTTANQVLGVSSK.A | Y | 86.80 | 1494.7678 | 15 | 0.6 | 748.3917 | 2 | 74.60 | 7 | 21143 | Ana\_06.raw | 6.09E6 | 1.15E7 | 7.57E6 | 3.37E6 | 1.59E6 | 4.47E6 | 4.38E6 | 1.01E6 | 3.17E6 | 4.35E6 | 10 | 1 | 1 | 1 | 1 | 1 | 1 | 1 | 1 | 1 | 1 | 199 | 213 |  |
| R.SNLNIFQYIGK.A | Y | 81.60 | 1295.6874 | 11 | 1.7 | 648.8521 | 2 | 112.62 | 3 | 31144 | Ana\_02.raw | 6.78E6 | 1.59E7 | 2.01E7 | 9.59E6 | 2.91E6 | 1.14E7 | 1.02E7 | 5.68E6 | 1.25E7 | 1.12E7 | 17 | 1 | 1 | 3 | 2 | 2 | 1 | 2 | 2 | 2 | 1 | 645 | 655 |  |
| K.AIPIVGQVLER.V | Y | 74.27 | 1193.7131 | 11 | 0.9 | 597.8644 | 2 | 97.29 | 3 | 27184 | Ana\_02.raw | 8.37E6 | 4.81E6 | 1.68E7 | 5.21E6 | 3.46E6 | 6.59E6 | 2.94E6 | 1.67E6 | 1.48E7 | 9.7E6 | 19 | 2 | 2 | 2 | 2 | 2 | 2 | 2 | 1 | 2 | 2 | 286 | 296 |  |
| K.YAEAGEGPVSHLATTVLQR.Y | Y | 69.92 | 1998.0170 | 19 | 5.6 | 667.0167 | 3 | 89.59 | 2 | 25346 | Ana\_01.raw |  | 1.06E6 | 9.42E5 |  |  |  |  |  | 1.8E6 |  | 3 | 0 | 1 | 1 | 0 | 0 | 0 | 0 | 0 | 1 | 0 | 492 | 510 |  |
| K.SGSSSAYTGYVER.S | Y | 68.28 | 1362.6051 | 13 | 4.6 | 682.3129 | 2 | 48.37 | 6 | 13715 | Ana\_05.raw | 1.23E7 | 2.09E7 | 2.02E7 | 7.41E6 | 7.26E6 | 1.28E7 | 1.11E7 | 6.62E6 | 1.52E7 | 1.4E7 | 10 | 1 | 1 | 1 | 1 | 1 | 1 | 1 | 1 | 1 | 1 | 610 | 622 |  |
| R.YDVSFITDEVK.K | Y | 66.53 | 1314.6343 | 11 | 1.7 | 658.3256 | 2 | 94.12 | 3 | 26350 | Ana\_02.raw |  |  | 7.71E5 |  |  |  |  |  |  |  | 1 | 0 | 0 | 1 | 0 | 0 | 0 | 0 | 0 | 0 | 0 | 511 | 521 |  |
| R.AFALNFQQTIAGK.I | Y | 65.97 | 1407.7510 | 13 | 1.0 | 704.8835 | 2 | 98.01 | 6 | 27726 | Ana\_05.raw | 8.83E5 | 6.92E5 | 3.94E6 | 6.8E5 | 5E5 | 1.58E6 | 1.91E6 | 8.04E5 |  | 1.86E6 | 9 | 1 | 1 | 1 | 1 | 1 | 1 | 1 | 1 | 0 | 1 | 237 | 249 |  |
| K.SDSSIILQER.F | Y | 60.84 | 1146.5880 | 10 | 2.4 | 574.3027 | 2 | 53.81 | 6 | 15343 | Ana\_05.raw |  | 2.8E7 | 6.45E4 | 5.06E6 | 5.34E6 | 7.14E6 | 8.4E6 | 4.45E6 |  |  | 7 | 0 | 1 | 1 | 1 | 1 | 1 | 1 | 1 | 0 | 0 | 383 | 392 |  |
| K.NALLPEGIPLLLK.Y | Y | 60.57 | 1389.8595 | 13 | 3.5 | 695.9395 | 2 | 128.35 | 6 | 35659 | Ana\_05.raw | 1.09E6 | 1.37E7 | 1.83E7 | 7.72E6 | 9.71E6 | 8.28E6 | 3.99E6 | 9.48E6 | 2.52E7 | 1.63E7 | 10 | 1 | 1 | 1 | 1 | 1 | 1 | 1 | 1 | 1 | 1 | 479 | 491 |  |
| R.YDVSFITDEVKK.T | Y | 59.86 | 1442.7292 | 12 | 5.3 | 481.9196 | 3 | 82.68 | 9 | 23523 | Ana\_08.raw | 9.65E5 | 1.77E6 | 7.22E5 |  |  | 1.08E6 | 6.2E5 |  | 2.16E6 | 1.85E5 | 7 | 1 | 1 | 1 | 0 | 0 | 1 | 1 | 0 | 1 | 1 | 511 | 522 |  |
| K.VTYQAQQDK.V | Y | 59.85 | 1079.5247 | 9 | 3.5 | 540.7715 | 2 | 20.99 | 7 | 5206 | Ana\_06.raw |  |  |  | 3.46E6 |  |  | 4.6E6 | 0 |  |  | 3 | 0 | 0 | 0 | 1 | 0 | 0 | 1 | 1 | 0 | 0 | 176 | 184 |  |
| K.EM(+15.99)AVHNYDR.F | Y | 58.70 | 1149.4873 | 9 | -0.4 | 575.7507 | 2 | 18.95 | 3 | 4589 | Ana\_02.raw | 6.72E5 |  | 6.72E6 | 2.87E6 | 1.53E6 | 5.04E6 | 4.5E6 | 9.7E5 |  | 1.85E6 | 13 | 1 | 0 | 2 | 2 | 1 | 2 | 2 | 2 | 0 | 1 | 598 | 606 | Oxidation (M) |
| K.GKPQDSVGYK.I | Y | 58.32 | 1077.5454 | 10 | 4.8 | 539.7826 | 2 | 21.35 | 10 | 5276 | Ana\_09.raw | 4.52E6 | 1.02E7 | 9.58E6 | 2.5E6 | 2.98E6 | 4.83E6 | 1.18E6 | 1.4E6 |  | 4E6 | 9 | 1 | 1 | 1 | 1 | 1 | 1 | 1 | 1 | 0 | 1 | 49 | 58 |  |
| K.NILLSIGELPK.E | Y | 58.04 | 1195.7175 | 11 | 4.8 | 598.8689 | 2 | 109.81 | 8 | 30369 | Ana\_07.raw | 1.12E6 | 1.9E6 | 2.15E6 | 1.47E5 | 8.21E5 | 1.52E6 | 3.09E6 | 6.92E5 | 6.71E5 | 5.59E5 | 11 | 1 | 1 | 1 | 1 | 1 | 1 | 2 | 1 | 1 | 1 | 558 | 568 |  |
| K.NLEPENLSK.A | Y | 53.25 | 1042.5294 | 9 | -1.3 | 522.2713 | 2 | 38.73 | 3 | 10760 | Ana\_02.raw |  | 7.98E6 | 1.27E7 | 4.18E6 |  |  |  |  |  | 5.37E6 | 4 | 0 | 1 | 1 | 1 | 0 | 0 | 0 | 0 | 0 | 1 | 314 | 322 |  |
| R.TTAAAVILK.N | Y | 41.66 | 886.5488 | 9 | 1.3 | 444.2823 | 2 | 47.38 | 6 | 13410 | Ana\_05.raw |  |  | 1.1E7 |  | 4.9E6 | 8.03E6 | 6.12E6 |  |  | 4.46E6 | 5 | 0 | 0 | 1 | 0 | 1 | 1 | 1 | 0 | 0 | 1 | 541 | 549 |  |
| K.SIFQGK.S | Y | 29.60 | 678.3701 | 6 | 2.9 | 340.1933 | 2 | 28.02 | 3 | 7524 | Ana\_02.raw |  |  | 4.53E6 |  |  |  |  |  |  |  | 1 | 0 | 0 | 1 | 0 | 0 | 0 | 0 | 0 | 0 | 0 | 100 | 105 |  |
| total 20 peptides |  |  |  |  |  |  |  |  |  |  |  |  |  |  |  |  |  |  |  |  |  |  |  |  |  |  |  |  |  |  |  |  |  |  |  |
| --- | --- | --- | --- | --- | --- | --- | --- | --- | --- | --- | --- | --- | --- | --- | --- | --- | --- | --- | --- | --- | --- | --- | --- | --- | --- | --- | --- | --- | --- | --- | --- | --- | --- | --- | --- |

Best Unique PSM (Scan F3:36693, m/z=577.3180, z=3, RT=134.05, ppm=0.6):

sp|Q00897|A1AT4\_MOUSE

back to list

  

| Protein Coverage
| Supporting Peptides
| Best Unique PSM
|

Protein Coverage:

Supporting Peptides:

| Peptide | Uniq | -10lgP | Mass | Length | ppm | m/z | z | RT | Fraction | Scan | Source File | Area 10 | Area 01 | Area 02 | Area 03 | Area 04 | Area 05 | Area 06 | Area 07 | Area 08 | Area 09 | #Spec | #Spec 10 | #Spec 01 | #Spec 02 | #Spec 03 | #Spec 04 | #Spec 05 | #Spec 06 | #Spec 07 | #Spec 08 | #Spec 09 | Start | End | PTM |
| --- | --- | --- | --- | --- | --- | --- | --- | --- | --- | --- | --- | --- | --- | --- | --- | --- | --- | --- | --- | --- | --- | --- | --- | --- | --- | --- | --- | --- | --- | --- | --- | --- | --- | --- | --- |
| K.SFQHLLQTLNRPDSELQLSTGNGLFVNNDLK.L | N | 129.80 | 3497.7903 | 31 | -1.3 | 875.4537 | 4 | 124.34 | 3 | 34149 | Ana\_02.raw |  |  | 1.92E7 | 7.14E6 |  | 8.97E6 | 1.67E7 | 1.48E7 |  |  | 7 | 0 | 0 | 1 | 2 | 0 | 1 | 1 | 2 | 0 | 0 | 113 | 143 |  |
| K.KLDQDTVFALANYILFK.G | N | 120.74 | 1998.0825 | 17 | -0.1 | 1000.0484 | 2 | 135.43 | 3 | 37052 | Ana\_02.raw | 6.31E6 | 3.74E6 | 5.83E6 | 1.95E7 | 1.37E7 | 1.27E7 | 9.22E6 | 2.6E6 |  | 1.09E7 | 18 | 2 | 2 | 3 | 2 | 2 | 1 | 2 | 2 | 0 | 2 | 193 | 209 |  |
| R.FDHPFLFIIFEEHTQSPIFVGK.V | N | 106.71 | 2647.3474 | 22 | 2.8 | 662.8460 | 4 | 132.43 | 6 | 36814 | Ana\_05.raw |  |  | 4.89E6 | 4.4E6 | 1.19E6 | 2.36E6 | 2.12E6 |  |  |  | 7 | 0 | 0 | 2 | 2 | 1 | 1 | 1 | 0 | 0 | 0 | 385 | 406 |  |
| K.ELISQFLLNR.R | Y | 70.71 | 1231.6924 | 10 | 4.7 | 616.8564 | 2 | 111.73 | 6 | 31325 | Ana\_05.raw | 2.93E6 | 9.46E6 | 9.66E6 | 2.64E6 | 6.58E6 | 8.33E6 | 3.17E6 | 6.69E6 |  |  | 12 | 1 | 1 | 2 | 1 | 2 | 2 | 1 | 2 | 0 | 0 | 288 | 297 |  |
| K.M(+15.99)QHLEQTLNK.E | N | 68.21 | 1256.6183 | 10 | 3.4 | 629.3185 | 2 | 26.47 | 6 | 7020 | Ana\_05.raw |  | 3.75E6 |  |  | 3.18E6 | 3.98E6 | 1.46E6 | 2.28E6 | 2.26E6 |  | 6 | 0 | 1 | 0 | 0 | 1 | 1 | 1 | 1 | 1 | 0 | 278 | 287 | Oxidation (M) |
| K.DQSPASHEIATNLGDFALR.L | Y | 66.04 | 2040.9865 | 19 | 3.4 | 681.3384 | 3 | 99.00 | 2 | 27863 | Ana\_01.raw |  | 1.79E6 | 1.23E6 |  |  |  | 1.13E6 |  |  |  | 3 | 0 | 1 | 1 | 0 | 0 | 0 | 1 | 0 | 0 | 0 | 36 | 54 |  |
| K.TLM(+15.99)SPLGITR.I | N | 50.49 | 1103.6008 | 10 | 4.5 | 552.8102 | 2 | 67.36 | 8 | 18917 | Ana\_07.raw |  |  | 1.72E7 | 3.32E6 | 3.23E6 | 1.2E6 | 4.92E6 | 7.58E6 |  |  | 6 | 0 | 0 | 1 | 1 | 1 | 1 | 1 | 1 | 0 | 0 | 319 | 328 | Oxidation (M) |
| K.KVINDFVEK.G | N | 48.83 | 1090.6022 | 9 | 4.5 | 546.3108 | 2 | 41.87 | 6 | 11729 | Ana\_05.raw |  |  | 6.07E6 |  |  | 4.04E6 |  | 2.35E6 |  |  | 3 | 0 | 0 | 1 | 0 | 0 | 1 | 0 | 1 | 0 | 0 | 173 | 181 |  |
| K.VINDFVEK.G | N | 48.25 | 962.5073 | 8 | 6.2 | 482.2639 | 2 | 46.91 | 2 | 13186 | Ana\_01.raw | 2.13E7 | 2.23E7 | 3.02E7 | 9.36E6 | 1.79E7 | 2.5E7 | 1.8E7 | 1.48E7 | 9.49E6 | 1.4E7 | 11 | 1 | 1 | 1 | 2 | 1 | 1 | 1 | 1 | 1 | 1 | 174 | 181 |  |
| K.LDQDTVFALANYILFK.G | N | 36.93 | 1869.9875 | 16 | 4.5 | 936.0052 | 2 | 138.35 | 4 | 37487 | Ana\_03.raw |  |  |  | 2.83E5 |  |  |  |  |  |  | 1 | 0 | 0 | 0 | 1 | 0 | 0 | 0 | 0 | 0 | 0 | 194 | 209 |  |
| K.FLEEAK.N | N | 33.46 | 735.3802 | 6 | 3.6 | 368.6987 | 2 | 27.77 | 2 | 7326 | Ana\_01.raw | 2.24E7 | 2.72E7 |  | 9.67E6 | 1.71E7 | 2.36E7 | 1.33E7 |  | 8.14E6 | 1.4E7 | 8 | 1 | 1 | 0 | 1 | 1 | 1 | 1 | 0 | 1 | 1 | 148 | 153 |  |
| K.IVEAVK.K | N | 30.08 | 657.4061 | 6 | 2.4 | 329.7111 | 2 | 20.74 | 5 | 5220 | Ana\_04.raw |  |  |  | 2.57E6 | 5.68E6 |  | 2.93E6 |  |  |  | 3 | 0 | 0 | 0 | 1 | 1 | 0 | 1 | 0 | 0 | 0 | 187 | 192 |  |
| total 12 peptides |  |  |  |  |  |  |  |  |  |  |  |  |  |  |  |  |  |  |  |  |  |  |  |  |  |  |  |  |  |  |  |  |  |  |  |
| --- | --- | --- | --- | --- | --- | --- | --- | --- | --- | --- | --- | --- | --- | --- | --- | --- | --- | --- | --- | --- | --- | --- | --- | --- | --- | --- | --- | --- | --- | --- | --- | --- | --- | --- | --- |

Best Unique PSM (Scan F6:31325, m/z=616.8564, z=2, RT=111.73, ppm=4.7):

sp|Q02053|UBA1\_MOUSE

back to list

  

| Protein Coverage
| Supporting Peptides
| Best Unique PSM
|

Protein Coverage:

Supporting Peptides:

| Peptide | Uniq | -10lgP | Mass | Length | ppm | m/z | z | RT | Fraction | Scan | Source File | Area 10 | Area 01 | Area 02 | Area 03 | Area 04 | Area 05 | Area 06 | Area 07 | Area 08 | Area 09 | #Spec | #Spec 10 | #Spec 01 | #Spec 02 | #Spec 03 | #Spec 04 | #Spec 05 | #Spec 06 | #Spec 07 | #Spec 08 | #Spec 09 | Start | End | PTM |
| --- | --- | --- | --- | --- | --- | --- | --- | --- | --- | --- | --- | --- | --- | --- | --- | --- | --- | --- | --- | --- | --- | --- | --- | --- | --- | --- | --- | --- | --- | --- | --- | --- | --- | --- | --- |
| R.NEEDATELVGLAQAVNAR.S | Y | 124.54 | 1898.9333 | 18 | 1.1 | 950.4750 | 2 | 105.55 | 6 | 29724 | Ana\_05.raw | 2.46E6 | 2.38E6 |  | 4.89E5 | 1.37E6 | 2.38E6 | 2.72E6 | 1.04E6 | 1.52E7 | 1.09E6 | 10 | 2 | 1 | 0 | 1 | 1 | 1 | 1 | 1 | 1 | 1 | 351 | 368 |  |
| R.LAGTQPLEVLEAVQR.S | Y | 83.36 | 1622.8992 | 15 | 3.8 | 541.9757 | 3 | 107.38 | 1 | 29866 | ANa\_10.raw | 1.7E6 | 1.55E6 | 1.33E6 |  | 3.04E6 | 7.25E5 | 1.43E6 | 1.94E6 | 8.07E5 | 2.24E6 | 9 | 1 | 1 | 1 | 0 | 1 | 1 | 1 | 1 | 1 | 1 | 679 | 693 |  |
| R.AAVASLLQSVQVPEFTPK.S | Y | 82.36 | 1884.0356 | 18 | -2.7 | 943.0226 | 2 | 121.41 | 3 | 33397 | Ana\_02.raw | 1.59E7 | 4.38E6 | 5.99E6 | 3.53E6 | 8.96E6 | 1.97E7 | 3.47E6 | 9.77E6 | 2.29E7 | 9.21E6 | 29 | 4 | 2 | 2 | 2 | 3 | 3 | 2 | 4 | 3 | 4 | 785 | 802 |  |
| K.DNPGVVTC(+57.02)LDEAR.H | Y | 79.96 | 1444.6616 | 13 | 3.8 | 723.3408 | 2 | 63.24 | 9 | 18017 | Ana\_08.raw |  | 1.91E6 |  |  |  |  |  |  | 5.28E6 |  | 2 | 0 | 1 | 0 | 0 | 0 | 0 | 0 | 0 | 1 | 0 | 227 | 239 | Carbamidomethylation |
| K.SLPASLVEPDFVM(+15.99)TDFAK.Y | Y | 76.20 | 1981.9706 | 18 | 1.8 | 991.9943 | 2 | 115.61 | 3 | 31929 | Ana\_02.raw | 1.44E6 | 2.08E6 | 2.57E6 |  | 2.23E6 | 4.02E6 | 4.08E5 | 5.43E5 |  |  | 9 | 1 | 2 | 1 | 0 | 2 | 1 | 1 | 1 | 0 | 0 | 305 | 322 | Oxidation (M) |
| K.YFLVGAGAIGC(+57.02)ELLK.N | Y | 72.57 | 1609.8538 | 15 | 2.0 | 805.9358 | 2 | 124.80 | 6 | 34733 | Ana\_05.raw |  |  |  |  |  | 1.02E6 |  |  | 7.22E5 | 1.34E6 | 3 | 0 | 0 | 0 | 0 | 0 | 1 | 0 | 0 | 1 | 1 | 471 | 485 | Carbamidomethylation |
| R.IYDDDFFQNLDGVANALDNIDAR.M | Y | 63.79 | 2613.1982 | 23 | -1.6 | 1307.6044 | 2 | 134.62 | 3 | 36845 | Ana\_02.raw |  |  | 3.08E5 |  |  |  |  |  |  | 4.06E5 | 2 | 0 | 0 | 1 | 0 | 0 | 0 | 0 | 0 | 0 | 1 | 559 | 581 |  |
| R.DEFEGLFK.Q | Y | 60.66 | 983.4600 | 8 | 0.7 | 492.7376 | 2 | 92.17 | 6 | 26166 | Ana\_05.raw | 6.78E6 | 5.84E6 | 6.57E6 | 2.89E6 | 5.42E6 | 6.03E6 | 3.96E6 | 3.92E6 | 1.19E7 | 6.35E6 | 13 | 1 | 1 | 1 | 2 | 1 | 1 | 2 | 1 | 2 | 1 | 650 | 657 |  |
| R.YDGQVAVFGSDFQEK.L | Y | 60.55 | 1688.7682 | 15 | 9.2 | 845.3991 | 2 | 84.75 | 9 | 24117 | Ana\_08.raw | 1.47E6 | 9.22E5 |  |  |  | 6.9E5 |  |  | 4.04E6 | 7.41E5 | 6 | 1 | 1 | 0 | 0 | 0 | 1 | 0 | 0 | 2 | 1 | 451 | 465 |  |
| R.AENYDISPADR.H | Y | 58.95 | 1249.5575 | 11 | 0.2 | 625.7861 | 2 | 46.35 | 4 | 12706 | Ana\_03.raw |  | 2.4E7 | 3.17E7 | 1.04E7 | 2.52E7 | 2.65E7 | 1.27E7 | 1.2E7 |  | 2.68E7 | 8 | 0 | 1 | 1 | 1 | 1 | 1 | 1 | 1 | 0 | 1 | 870 | 880 |  |
| K.LKSDTAAAAVR.Q | Y | 55.51 | 1101.6141 | 11 | 4.7 | 551.8170 | 2 | 22.41 | 9 | 5427 | Ana\_08.raw |  |  |  |  |  |  |  |  | 3.3E6 |  | 1 | 0 | 0 | 0 | 0 | 0 | 0 | 0 | 0 | 1 | 0 | 527 | 537 |  |
| R.LDQPM(+15.99)TEIVSR.V | Y | 48.36 | 1303.6442 | 11 | 2.3 | 652.8309 | 2 | 45.70 | 3 | 12876 | Ana\_02.raw |  |  | 8.55E6 | 4.17E6 |  |  | 6.23E6 |  |  |  | 3 | 0 | 0 | 1 | 1 | 0 | 0 | 1 | 0 | 0 | 0 | 1011 | 1021 | Oxidation (M) |
| K.ATLPSPDKLPGFK.M | Y | 47.82 | 1369.7605 | 13 | 4.4 | 457.5961 | 3 | 79.70 | 8 | 22348 | Ana\_07.raw | 2.61E5 | 2.1E6 |  |  |  | 6.44E5 | 9.92E5 | 1.49E6 | 1.17E6 | 4.95E5 | 8 | 1 | 1 | 0 | 0 | 0 | 2 | 1 | 1 | 1 | 1 | 831 | 843 |  |
| R.GLGVEIAK.N | Y | 45.08 | 785.4647 | 8 | 5.6 | 393.7418 | 2 | 45.81 | 2 | 12843 | Ana\_01.raw | 2.19E6 | 7.34E5 | 2.94E6 |  | 2.23E6 | 2.45E6 | 1.63E6 | 2.18E6 | 4.15E6 | 2.85E6 | 9 | 1 | 1 | 1 | 0 | 1 | 1 | 1 | 1 | 1 | 1 | 82 | 89 |  |
| K.LVVADTR.G | Y | 39.70 | 772.4443 | 7 | 4.0 | 387.2310 | 2 | 29.34 | 7 | 7766 | Ana\_06.raw |  |  |  | 5.26E6 | 6.63E6 | 4.65E6 | 5.7E6 | 9.38E5 | 7.01E6 | 7.7E6 | 7 | 0 | 0 | 0 | 1 | 1 | 1 | 1 | 1 | 1 | 1 | 186 | 192 |  |
| K.NIILGGVK.A | Y | 32.50 | 812.5120 | 8 | 3.9 | 407.2648 | 2 | 56.03 | 7 | 15802 | Ana\_06.raw |  |  |  |  |  |  | 2.15E6 |  |  |  | 1 | 0 | 0 | 0 | 0 | 0 | 0 | 1 | 0 | 0 | 0 | 90 | 97 |  |
| R.VGEFC(+57.02)HSR.G | Y | 29.83 | 990.4341 | 8 | 4.0 | 331.1533 | 3 | 21.87 | 10 | 5450 | Ana\_09.raw |  |  |  |  |  |  |  |  |  | 1.3E6 | 1 | 0 | 0 | 0 | 0 | 0 | 0 | 0 | 0 | 0 | 1 | 175 | 182 | Carbamidomethylation |
| R.GGIVSQVK.V | Y | 28.30 | 786.4600 | 8 | 3.9 | 394.2388 | 2 | 27.59 | 5 | 7356 | Ana\_04.raw |  | 5.96E6 | 4.56E6 | 2.54E6 | 3.86E6 | 4E6 |  | 2.59E6 |  | 3.82E6 | 7 | 0 | 1 | 1 | 1 | 1 | 1 | 0 | 1 | 0 | 1 | 289 | 296 |  |
| R.LEELK.A | N | 25.94 | 630.3588 | 5 | -1.9 | 316.1861 | 2 | 28.29 | 4 | 7373 | Ana\_03.raw |  |  |  | 2.65E6 |  |  |  |  |  |  | 1 | 0 | 0 | 0 | 1 | 0 | 0 | 0 | 0 | 0 | 0 | 826 | 830 |  |
| R.AEVSQPR.L | Y | 25.04 | 785.4031 | 7 | 3.8 | 393.7103 | 2 | 33.24 | 10 | 9030 | Ana\_09.raw |  | 1.55E7 |  |  |  |  |  |  |  | 1.44E7 | 3 | 0 | 2 | 0 | 0 | 0 | 0 | 0 | 0 | 0 | 1 | 128 | 134 |  |
| total 20 peptides |  |  |  |  |  |  |  |  |  |  |  |  |  |  |  |  |  |  |  |  |  |  |  |  |  |  |  |  |  |  |  |  |  |  |  |
| --- | --- | --- | --- | --- | --- | --- | --- | --- | --- | --- | --- | --- | --- | --- | --- | --- | --- | --- | --- | --- | --- | --- | --- | --- | --- | --- | --- | --- | --- | --- | --- | --- | --- | --- | --- |

Best Unique PSM (Scan F6:29724, m/z=950.4750, z=2, RT=105.55, ppm=1.1):

sp|P26040|EZRI\_MOUSE

back to list

  

| Protein Coverage
| Supporting Peptides
| Best Unique PSM
|

Protein Coverage:

Supporting Peptides:

| Peptide | Uniq | -10lgP | Mass | Length | ppm | m/z | z | RT | Fraction | Scan | Source File | Area 10 | Area 01 | Area 02 | Area 03 | Area 04 | Area 05 | Area 06 | Area 07 | Area 08 | Area 09 | #Spec | #Spec 10 | #Spec 01 | #Spec 02 | #Spec 03 | #Spec 04 | #Spec 05 | #Spec 06 | #Spec 07 | #Spec 08 | #Spec 09 | Start | End | PTM |
| --- | --- | --- | --- | --- | --- | --- | --- | --- | --- | --- | --- | --- | --- | --- | --- | --- | --- | --- | --- | --- | --- | --- | --- | --- | --- | --- | --- | --- | --- | --- | --- | --- | --- | --- | --- |
| K.SQEQLAAELAEYTAK.I | Y | 96.16 | 1650.8101 | 15 | 6.4 | 826.4176 | 2 | 94.96 | 3 | 26571 | Ana\_02.raw | 1.27E7 | 8.06E6 | 5.45E6 | 2.01E6 | 1.37E6 | 1.2E7 | 1.97E6 | 3.78E6 | 1.84E7 | 6.91E6 | 18 | 2 | 2 | 1 | 2 | 2 | 2 | 1 | 2 | 2 | 2 | 413 | 427 |  |
| K.APDFVFYAPR.L | N | 92.42 | 1181.5869 | 10 | 0.6 | 591.8011 | 2 | 90.23 | 3 | 25325 | Ana\_02.raw | 2.64E7 | 2.83E7 | 3.11E7 | 1.23E7 | 2.32E7 | 2.65E7 | 1.73E7 | 1.56E7 | 6.81E7 | 2.59E7 | 19 | 2 | 2 | 2 | 2 | 3 | 1 | 2 | 2 | 2 | 1 | 264 | 273 |  |
| K.FYPEDVAEELIQDITQK.L | Y | 90.07 | 2036.9941 | 17 | 7.2 | 1019.5117 | 2 | 137.44 | 6 | 38176 | Ana\_05.raw | 8.77E5 | 2.33E6 | 3.61E5 |  | 1.41E6 | 6.58E5 |  |  | 1.66E6 | 2.28E6 | 11 | 1 | 2 | 1 | 0 | 2 | 1 | 0 | 0 | 2 | 2 | 84 | 100 |  |
| R.QLLTLSNELSQAR.D | Y | 89.19 | 1471.7994 | 13 | 2.8 | 736.9091 | 2 | 119.28 | 6 | 33300 | Ana\_05.raw |  |  | 1.12E6 | 6.87E5 | 6.04E5 | 2.15E6 | 3.52E5 |  |  |  | 5 | 0 | 0 | 1 | 1 | 1 | 1 | 1 | 0 | 0 | 0 | 530 | 542 |  |
| K.IGFPWSEIR.N | N | 79.81 | 1103.5763 | 9 | 1.5 | 552.7963 | 2 | 106.42 | 3 | 29550 | Ana\_02.raw | 4.77E7 | 3.97E7 | 3.6E7 | 2.4E7 | 2.68E7 | 2.31E7 | 2.06E7 | 1.59E7 | 7.65E7 | 3.15E7 | 42 | 6 | 5 | 5 | 3 | 4 | 2 | 3 | 4 | 6 | 4 | 238 | 246 |  |
| K.IAQDLEM(+15.99)YGINYFEIK.N | Y | 74.03 | 1961.9445 | 16 | 4.0 | 981.9834 | 2 | 119.16 | 6 | 33269 | Ana\_05.raw | 1.91E6 |  | 6.49E6 |  | 4.22E6 | 1.27E7 | 1.74E6 | 4.05E5 | 1.29E7 | 4.29E6 | 10 | 1 | 0 | 1 | 0 | 2 | 1 | 2 | 1 | 1 | 1 | 194 | 209 | Oxidation (M) |
| R.RKPDTIEVQQM(+15.99)K.A | N | 73.07 | 1487.7766 | 12 | 3.2 | 496.9344 | 3 | 22.01 | 9 | 5292 | Ana\_08.raw | 2.16E7 | 2.51E7 | 2.92E6 |  | 1.68E7 | 2.83E6 | 1.34E7 | 1.31E6 | 3.44E7 | 2E7 | 15 | 2 | 2 | 1 | 0 | 2 | 1 | 2 | 1 | 2 | 2 | 295 | 306 | Oxidation (M) |
| R.KPDTIEVQQM(+15.99)K.A | N | 65.95 | 1331.6755 | 11 | 8.0 | 666.8503 | 2 | 23.40 | 9 | 5768 | Ana\_08.raw |  |  |  |  |  |  |  |  | 1.07E6 |  | 1 | 0 | 0 | 0 | 0 | 0 | 0 | 0 | 0 | 1 | 0 | 296 | 306 | Oxidation (M) |
| K.KAPDFVFYAPR.L | N | 63.38 | 1309.6819 | 11 | 6.5 | 437.5707 | 3 | 76.87 | 1 | 21734 | ANa\_10.raw | 9.37E5 | 2.73E6 | 6.46E5 | 4.16E6 | 5.64E6 | 5.79E6 | 1.76E6 | 1.44E6 | 3.11E6 | 1.53E6 | 13 | 1 | 1 | 1 | 2 | 2 | 1 | 1 | 1 | 1 | 2 | 263 | 273 |  |
| K.SGYLSSER.L | Y | 60.57 | 897.4192 | 8 | 2.0 | 449.7178 | 2 | 28.33 | 6 | 7599 | Ana\_05.raw |  |  | 1.48E7 | 8.27E6 | 1.34E7 | 1.61E7 | 1.19E7 | 5.88E6 | 9.62E6 | 9.72E6 | 8 | 0 | 0 | 1 | 1 | 1 | 1 | 1 | 1 | 1 | 1 | 144 | 151 |  |
| R.KENPVQFK.F | Y | 57.29 | 988.5341 | 8 | 4.9 | 495.2768 | 2 | 24.74 | 2 | 6345 | Ana\_01.raw | 9.43E6 | 1.44E7 |  | 4.41E6 | 5.9E6 | 1.12E7 | 4.3E6 | 2.69E6 | 8.66E6 | 7.62E6 | 10 | 1 | 1 | 0 | 1 | 1 | 1 | 1 | 1 | 2 | 1 | 72 | 79 |  |
| R.NISFNDKK.F | N | 53.33 | 964.4977 | 8 | 3.7 | 483.2579 | 2 | 24.54 | 1 | 6278 | ANa\_10.raw | 8.5E6 | 8.21E6 | 2.97E6 |  | 3.22E6 |  |  |  | 1.13E7 | 8.28E6 | 7 | 1 | 1 | 1 | 0 | 1 | 0 | 0 | 0 | 2 | 1 | 247 | 254 |  |
| K.ALQLEEER.R | Y | 52.35 | 986.5032 | 8 | 3.9 | 494.2608 | 2 | 42.49 | 6 | 11925 | Ana\_05.raw |  |  |  | 3.07E6 | 1.85E7 | 1.84E7 | 1.33E7 | 1.33E7 | 1.28E6 |  | 6 | 0 | 0 | 0 | 1 | 1 | 1 | 1 | 1 | 1 | 0 | 372 | 379 |  |
| K.LFFLQVK.D | N | 52.26 | 893.5374 | 7 | 5.5 | 447.7784 | 2 | 97.47 | 1 | 27302 | ANa\_10.raw | 4.42E6 | 1.25E6 | 5.07E6 | 3.12E6 | 4.39E6 | 1.18E6 | 4.28E6 | 2.58E6 | 6.28E6 | 4.57E6 | 11 | 1 | 1 | 1 | 1 | 2 | 1 | 1 | 1 | 1 | 1 | 101 | 107 |  |
| R.AKEAQDDLVK.T | Y | 51.28 | 1115.5822 | 10 | 7.2 | 558.8024 | 2 | 22.32 | 2 | 5519 | Ana\_01.raw |  | 3.31E6 |  |  |  | 2.5E6 |  |  |  | 2.03E6 | 3 | 0 | 1 | 0 | 0 | 0 | 1 | 0 | 0 | 0 | 1 | 449 | 458 |  |
| K.IALLEEAR.R | Y | 49.82 | 913.5233 | 8 | 5.8 | 457.7715 | 2 | 60.13 | 2 | 17094 | Ana\_01.raw | 1.25E7 | 1.89E7 | 1.35E7 | 7.21E6 | 1.31E7 | 1.48E7 | 1.14E7 | 8.33E6 | 2.08E7 | 1.14E7 | 17 | 2 | 1 | 2 | 2 | 2 | 2 | 2 | 1 | 1 | 2 | 428 | 435 |  |
| K.VSAQEVR.K | Y | 47.53 | 787.4188 | 7 | 1.8 | 394.7174 | 2 | 20.46 | 7 | 5042 | Ana\_06.raw |  |  |  | 4.68E6 | 1.8E7 |  | 6.89E6 | 2.4E6 |  |  | 4 | 0 | 0 | 0 | 1 | 1 | 0 | 1 | 1 | 0 | 0 | 65 | 71 |  |
| K.EAQDDLVK.T | Y | 44.68 | 916.4501 | 8 | 1.6 | 459.2331 | 2 | 26.96 | 4 | 6976 | Ana\_03.raw |  |  |  | 7.55E6 | 1.42E7 | 1.54E7 |  | 4.13E6 |  |  | 6 | 0 | 0 | 0 | 2 | 2 | 1 | 0 | 1 | 0 | 0 | 451 | 458 |  |
| K.FVIKPIDK.K | N | 43.65 | 958.5851 | 8 | 2.6 | 480.3011 | 2 | 51.47 | 8 | 14282 | Ana\_07.raw | 4.59E6 | 5.85E6 | 9.31E6 | 3.11E6 | 4.09E6 | 4.78E6 | 3.21E6 | 1.94E6 |  | 5.26E6 | 11 | 1 | 1 | 2 | 2 | 1 | 1 | 1 | 1 | 0 | 1 | 255 | 262 |  |
| R.NISFNDK.K | N | 41.03 | 836.4028 | 7 | 4.1 | 419.2104 | 2 | 31.34 | 9 | 8280 | Ana\_08.raw | 5.22E6 |  |  |  |  |  |  |  | 9.89E6 |  | 2 | 1 | 0 | 0 | 0 | 0 | 0 | 0 | 0 | 1 | 0 | 247 | 253 |  |
| K.ENPVQFK.F | Y | 38.09 | 860.4392 | 7 | 0.1 | 431.2269 | 2 | 36.14 | 5 | 9936 | Ana\_04.raw |  | 4.44E6 |  |  | 2.69E6 | 3.98E6 |  |  |  | 3.11E6 | 4 | 0 | 1 | 0 | 0 | 1 | 1 | 0 | 0 | 0 | 1 | 73 | 79 |  |
| K.DSAM(+15.99)LEYLK.I | Y | 27.32 | 1084.5110 | 9 | 6.4 | 543.2662 | 2 | 61.69 | 7 | 17458 | Ana\_06.raw |  |  |  |  | 1.64E6 |  | 1.65E6 |  |  |  | 2 | 0 | 0 | 0 | 0 | 1 | 0 | 1 | 0 | 0 | 0 | 185 | 193 | Oxidation (M) |
| K.KVSAQEVR.K | Y | 26.40 | 915.5137 | 8 | -1.3 | 458.7635 | 2 | 17.63 | 5 | 4190 | Ana\_04.raw |  |  |  |  | 1.76E6 |  |  |  |  |  | 1 | 0 | 0 | 0 | 0 | 1 | 0 | 0 | 0 | 0 | 0 | 64 | 71 |  |
| K.ALQLEEERR.R | Y | 25.93 | 1142.6044 | 9 | 1.6 | 381.8760 | 3 | 33.27 | 5 | 9086 | Ana\_04.raw |  |  |  |  | 2.13E6 |  |  |  |  |  | 1 | 0 | 0 | 0 | 0 | 1 | 0 | 0 | 0 | 0 | 0 | 372 | 380 |  |
| R.LIPQR.V | N | 22.99 | 625.3911 | 5 | 2.7 | 313.7037 | 2 | 25.29 | 4 | 6473 | Ana\_03.raw |  |  |  | 1.38E6 |  |  |  |  |  |  | 1 | 0 | 0 | 0 | 1 | 0 | 0 | 0 | 0 | 0 | 0 | 152 | 156 |  |
| R.IQVWHAEHR.G | Y | 22.87 | 1174.5996 | 9 | 4.1 | 392.5421 | 3 | 33.82 | 7 | 9144 | Ana\_06.raw |  |  |  |  |  |  | 2.65E6 |  |  |  | 1 | 0 | 0 | 0 | 0 | 0 | 0 | 1 | 0 | 0 | 0 | 172 | 180 |  |
| total 26 peptides |  |  |  |  |  |  |  |  |  |  |  |  |  |  |  |  |  |  |  |  |  |  |  |  |  |  |  |  |  |  |  |  |  |  |  |
| --- | --- | --- | --- | --- | --- | --- | --- | --- | --- | --- | --- | --- | --- | --- | --- | --- | --- | --- | --- | --- | --- | --- | --- | --- | --- | --- | --- | --- | --- | --- | --- | --- | --- | --- | --- |

Best Unique PSM (Scan F3:26571, m/z=826.4176, z=2, RT=94.96, ppm=6.4):

A0A0A6YXW6|A0A0A6YXW6\_MOUSE

back to list

  

| Protein Coverage
| Supporting Peptides
| Best Unique PSM
|

Protein Coverage:

Supporting Peptides:

| Peptide | Uniq | -10lgP | Mass | Length | ppm | m/z | z | RT | Fraction | Scan | Source File | Area 10 | Area 01 | Area 02 | Area 03 | Area 04 | Area 05 | Area 06 | Area 07 | Area 08 | Area 09 | #Spec | #Spec 10 | #Spec 01 | #Spec 02 | #Spec 03 | #Spec 04 | #Spec 05 | #Spec 06 | #Spec 07 | #Spec 08 | #Spec 09 | Start | End | PTM |
| --- | --- | --- | --- | --- | --- | --- | --- | --- | --- | --- | --- | --- | --- | --- | --- | --- | --- | --- | --- | --- | --- | --- | --- | --- | --- | --- | --- | --- | --- | --- | --- | --- | --- | --- | --- |
| R.WLHGNEELSPESYLVFEPLKEPGEGATTYLVTSVLR.V | N | 142.88 | 4060.0469 | 36 | 7.1 | 1016.0262 | 4 | 132.66 | 6 | 36876 | Ana\_05.raw |  | 7.57E6 | 4.15E6 | 1.89E6 | 1.01E7 | 7.26E6 | 4.18E6 | 3.8E6 | 9.92E6 |  | 8 | 0 | 1 | 1 | 1 | 1 | 1 | 1 | 1 | 1 | 0 | 255 | 290 |  |
| K.AVQNSC(+57.02)GC(+57.02)YSVSSVLPGC(+57.02)AER.W | N | 98.45 | 2299.9983 | 21 | 2.8 | 1151.0096 | 2 | 77.54 | 9 | 22093 | Ana\_08.raw |  |  |  |  |  |  |  |  | 6.18E5 |  | 1 | 0 | 0 | 0 | 0 | 0 | 0 | 0 | 0 | 1 | 0 | 166 | 186 | Carbamidomethylation |
| R.NPEGAVFTWEPSTGK.D | N | 95.62 | 1618.7627 | 15 | 3.5 | 810.3914 | 2 | 86.65 | 6 | 24681 | Ana\_05.raw | 2.03E7 | 2.17E7 | 1.84E7 | 5.23E6 | 1.51E7 | 1.85E7 | 1.88E7 | 2.28E7 | 5.17E7 | 1.07E7 | 16 | 1 | 2 | 2 | 1 | 2 | 1 | 2 | 2 | 1 | 2 | 145 | 159 |  |
| R.WLHGNEELSPESYLVFEPLK.E | N | 91.10 | 2386.1843 | 20 | 0.7 | 796.4026 | 3 | 126.02 | 6 | 35045 | Ana\_05.raw |  | 1.53E6 |  |  | 1.1E6 | 2.14E6 | 4.94E6 |  |  |  | 4 | 0 | 1 | 0 | 0 | 1 | 1 | 1 | 0 | 0 | 0 | 255 | 274 |  |
| R.NPTIYPLTLPR.A | Y | 90.95 | 1283.7238 | 11 | 4.4 | 642.8720 | 2 | 91.62 | 1 | 25728 | ANa\_10.raw | 6.33E7 | 5.93E7 | 5.14E7 | 1.99E7 | 4.05E7 | 4.16E7 | 3.52E7 | 3.81E7 | 1.48E8 | 4.66E7 | 39 | 5 | 5 | 3 | 3 | 3 | 2 | 3 | 4 | 6 | 5 | 5 | 15 |  |
| K.EPGEGATTYLVTSVLR.V | N | 80.70 | 1691.8729 | 16 | 4.0 | 846.9471 | 2 | 106.72 | 9 | 30020 | Ana\_08.raw |  |  |  | 2.81E5 | 2.89E5 | 2.29E5 | 1.75E6 | 2.65E5 | 1.46E6 |  | 6 | 0 | 0 | 0 | 1 | 1 | 1 | 1 | 1 | 1 | 0 | 275 | 290 |  |
| R.WNSGASFK.C | N | 45.95 | 895.4188 | 8 | 4.8 | 448.7188 | 2 | 42.14 | 8 | 11475 | Ana\_07.raw |  |  |  | 3.67E6 |  | 1.24E7 | 1.2E7 | 1.04E7 | 1.79E7 |  | 5 | 0 | 0 | 0 | 1 | 0 | 1 | 1 | 1 | 1 | 0 | 187 | 194 |  |
| R.VSAELWK.Q | Y | 45.04 | 831.4490 | 7 | 4.2 | 416.7336 | 2 | 54.54 | 1 | 15369 | ANa\_10.raw | 3.61E6 |  |  |  | 3.77E6 |  | 3.52E6 |  | 1.52E7 | 2.45E6 | 5 | 1 | 0 | 0 | 0 | 1 | 0 | 1 | 0 | 1 | 1 | 291 | 297 |  |
| K.C(+57.02)TVTHPESDTLTGTIAK.I | Y | 44.86 | 1829.8829 | 17 | 6.4 | 610.9722 | 3 | 55.38 | 8 | 15416 | Ana\_07.raw |  |  |  |  |  |  |  | 3.46E6 |  |  | 1 | 0 | 0 | 0 | 0 | 0 | 0 | 0 | 1 | 0 | 0 | 195 | 211 | Carbamidomethylation |
| total 9 peptides |  |  |  |  |  |  |  |  |  |  |  |  |  |  |  |  |  |  |  |  |  |  |  |  |  |  |  |  |  |  |  |  |  |  |  |
| --- | --- | --- | --- | --- | --- | --- | --- | --- | --- | --- | --- | --- | --- | --- | --- | --- | --- | --- | --- | --- | --- | --- | --- | --- | --- | --- | --- | --- | --- | --- | --- | --- | --- | --- | --- |

Best Unique PSM (Scan F1:25728, m/z=642.8720, z=2, RT=91.62, ppm=4.4):

sp|Q9WUM4|COR1C\_MOUSE

back to list

  

| Protein Coverage
| Supporting Peptides
| Best Unique PSM
|

Protein Coverage:

Supporting Peptides:

| Peptide | Uniq | -10lgP | Mass | Length | ppm | m/z | z | RT | Fraction | Scan | Source File | Area 10 | Area 01 | Area 02 | Area 03 | Area 04 | Area 05 | Area 06 | Area 07 | Area 08 | Area 09 | #Spec | #Spec 10 | #Spec 01 | #Spec 02 | #Spec 03 | #Spec 04 | #Spec 05 | #Spec 06 | #Spec 07 | #Spec 08 | #Spec 09 | Start | End | PTM |
| --- | --- | --- | --- | --- | --- | --- | --- | --- | --- | --- | --- | --- | --- | --- | --- | --- | --- | --- | --- | --- | --- | --- | --- | --- | --- | --- | --- | --- | --- | --- | --- | --- | --- | --- | --- |
| R.KSDLFQDDLYPDTAGPEAALEAEEWFEGK.N | Y | 119.74 | 3270.4880 | 29 | 4.8 | 1091.1752 | 3 | 132.27 | 6 | 36767 | Ana\_05.raw |  | 7.29E6 |  |  |  | 7.66E6 | 1.96E6 |  | 8.19E6 |  | 4 | 0 | 1 | 0 | 0 | 0 | 1 | 1 | 0 | 1 | 0 | 353 | 381 |  |
| K.KPTDTASVQNEAK.L | Y | 113.44 | 1387.6943 | 13 | 3.1 | 694.8566 | 2 | 19.90 | 3 | 4897 | Ana\_02.raw | 1.02E7 |  | 1.28E7 | 1.97E6 | 5.39E6 | 1.62E7 | 3.5E6 | 2.25E6 | 1.23E7 | 1.04E7 | 14 | 2 | 0 | 2 | 1 | 1 | 2 | 1 | 1 | 2 | 2 | 428 | 440 |  |
| R.YFEITDESPYVHYLNTFSSK.E | Y | 93.39 | 2439.1270 | 20 | 7.8 | 814.0559 | 3 | 121.41 | 1 | 33462 | ANa\_10.raw | 6.34E6 | 1.44E7 |  |  | 2.79E6 | 1.14E7 | 6.09E6 | 7.18E6 | 4.39E6 |  | 7 | 1 | 1 | 0 | 0 | 1 | 1 | 1 | 1 | 1 | 0 | 292 | 311 |  |
| R.AIFLADGNVFTTGFSR.M | Y | 91.67 | 1714.8678 | 16 | 3.8 | 858.4444 | 2 | 121.13 | 7 | 33444 | Ana\_06.raw | 3.08E6 | 4.89E6 | 6.45E6 | 2.17E5 | 8.62E5 | 7.5E6 | 4.74E6 | 3.83E5 | 1.26E7 | 5.29E6 | 18 | 1 | 2 | 2 | 1 | 2 | 3 | 2 | 2 | 1 | 2 | 224 | 239 |  |
| R.VTWDSSFC(+57.02)AVNPR.F | Y | 68.89 | 1537.6984 | 13 | -1.2 | 769.8555 | 2 | 84.46 | 5 | 23908 | Ana\_04.raw |  | 1.04E6 | 4.6E5 | 4.93E5 | 1.69E6 | 2.14E6 |  |  | 2.28E6 |  | 6 | 0 | 1 | 1 | 1 | 1 | 1 | 0 | 0 | 1 | 0 | 32 | 44 | Carbamidomethylation |
| K.ETIC(+57.02)SQDER.I | Y | 67.20 | 1136.4768 | 9 | 0.7 | 569.2461 | 2 | 21.00 | 3 | 5271 | Ana\_02.raw |  |  | 4.71E6 | 2.89E6 | 4.04E6 | 5.86E6 | 1.78E6 | 3.51E6 |  | 0 | 7 | 0 | 0 | 1 | 1 | 1 | 1 | 1 | 1 | 0 | 1 | 453 | 461 | Carbamidomethylation |
| R.HVFGQAVK.N | Y | 59.66 | 884.4868 | 8 | -0.2 | 443.2506 | 2 | 21.88 | 3 | 5567 | Ana\_02.raw | 1.98E7 | 2.06E7 | 2.47E7 |  | 2.28E6 | 1.65E7 | 6.74E6 | 7.24E6 | 1.82E7 |  | 8 | 1 | 1 | 1 | 0 | 1 | 1 | 1 | 1 | 1 | 0 | 12 | 19 |  |
| K.HGYIPGK.N | Y | 54.11 | 770.4075 | 7 | 3.5 | 386.2124 | 2 | 24.42 | 6 | 6365 | Ana\_05.raw | 9.96E6 | 5.11E6 |  | 7.74E6 | 1.12E7 | 1.58E7 | 7.22E6 | 1.36E6 |  |  | 7 | 1 | 1 | 0 | 1 | 1 | 1 | 1 | 1 | 0 | 0 | 392 | 398 |  |
| R.KQEIVAEK.E | Y | 48.35 | 943.5338 | 8 | 2.4 | 472.7753 | 2 | 18.17 | 4 | 4332 | Ana\_03.raw |  |  |  | 5.05E6 | 8.42E6 |  | 8.49E6 | 6.95E6 |  |  | 4 | 0 | 0 | 0 | 1 | 1 | 0 | 1 | 1 | 0 | 0 | 205 | 212 |  |
| K.SELSC(+57.02)APK.K | Y | 48.09 | 890.4167 | 8 | 4.0 | 446.2174 | 2 | 23.95 | 2 | 6072 | Ana\_01.raw | 5.32E6 | 8.8E6 |  |  | 8.13E6 | 1.14E7 |  | 5.94E6 |  | 2.26E6 | 6 | 1 | 1 | 0 | 0 | 1 | 1 | 0 | 1 | 0 | 1 | 420 | 427 | Carbamidomethylation |
| K.NADPILISLK.H | Y | 46.14 | 1082.6335 | 10 | 4.0 | 542.3262 | 2 | 85.50 | 7 | 24081 | Ana\_06.raw | 3.55E6 | 4.14E6 | 9.63E5 | 8.2E5 | 3.3E6 |  | 2.01E6 |  | 7.51E6 | 1.31E6 | 8 | 1 | 1 | 1 | 1 | 1 | 0 | 1 | 0 | 1 | 1 | 382 | 391 |  |
| K.LEQQLAK.M | Y | 37.73 | 828.4705 | 7 | 6.2 | 415.2451 | 2 | 22.77 | 1 | 5694 | ANa\_10.raw | 1.06E6 |  |  |  |  |  |  |  |  | 5.33E6 | 2 | 1 | 0 | 0 | 0 | 0 | 0 | 0 | 0 | 0 | 1 | 465 | 471 |  |
| R.VGIVAWHPTAR.N | Y | 33.79 | 1205.6669 | 11 | 3.4 | 402.8976 | 3 | 61.20 | 5 | 17422 | Ana\_04.raw |  |  |  |  | 5.33E6 | 3.56E6 |  |  | 3.09E6 |  | 3 | 0 | 0 | 0 | 0 | 1 | 1 | 0 | 0 | 1 | 0 | 133 | 143 |  |
| K.LDEILK.E | Y | 27.43 | 729.4272 | 6 | 2.4 | 365.7218 | 2 | 43.35 | 5 | 12127 | Ana\_04.raw |  | 4.11E6 |  | 2.05E6 | 3.75E6 | 4.94E6 | 3.21E6 |  |  |  | 5 | 0 | 1 | 0 | 1 | 1 | 1 | 1 | 0 | 0 | 0 | 441 | 446 |  |
| total 14 peptides |  |  |  |  |  |  |  |  |  |  |  |  |  |  |  |  |  |  |  |  |  |  |  |  |  |  |  |  |  |  |  |  |  |  |  |
| --- | --- | --- | --- | --- | --- | --- | --- | --- | --- | --- | --- | --- | --- | --- | --- | --- | --- | --- | --- | --- | --- | --- | --- | --- | --- | --- | --- | --- | --- | --- | --- | --- | --- | --- | --- |

Best Unique PSM (Scan F6:36767, m/z=1091.1752, z=3, RT=132.27, ppm=4.8):

sp|Q9DBJ1|PGAM1\_MOUSE

back to list

  

| Protein Coverage
| Supporting Peptides
| Best Unique PSM
|

Protein Coverage:

Supporting Peptides:

| Peptide | Uniq | -10lgP | Mass | Length | ppm | m/z | z | RT | Fraction | Scan | Source File | Area 10 | Area 01 | Area 02 | Area 03 | Area 04 | Area 05 | Area 06 | Area 07 | Area 08 | Area 09 | #Spec | #Spec 10 | #Spec 01 | #Spec 02 | #Spec 03 | #Spec 04 | #Spec 05 | #Spec 06 | #Spec 07 | #Spec 08 | #Spec 09 | Start | End | PTM |
| --- | --- | --- | --- | --- | --- | --- | --- | --- | --- | --- | --- | --- | --- | --- | --- | --- | --- | --- | --- | --- | --- | --- | --- | --- | --- | --- | --- | --- | --- | --- | --- | --- | --- | --- | --- |
| K.HLEGLSEEAIM(+15.99)ELNLPTGIPIVYELDK.N | Y | 144.30 | 3038.5522 | 27 | 0.1 | 1013.8582 | 3 | 132.48 | 3 | 36284 | Ana\_02.raw | 7.51E6 | 1.02E7 | 8.51E6 | 1.93E6 | 5.1E6 | 1.07E7 | 4.99E6 | 5.15E6 | 2.42E7 | 1.13E7 | 10 | 1 | 1 | 1 | 1 | 1 | 1 | 1 | 1 | 1 | 1 | 196 | 222 | Oxidation (M) |
| R.SYDVPPPPM(+15.99)EPDHPFYSNISK.D | Y | 99.21 | 2432.0994 | 21 | 1.9 | 811.7086 | 3 | 78.59 | 9 | 22389 | Ana\_08.raw | 2.25E7 | 1.82E7 | 1.86E7 | 8.71E6 | 1.42E7 | 1.68E7 | 9.17E6 | 1.39E7 | 4.9E7 | 1.72E7 | 11 | 1 | 1 | 1 | 1 | 1 | 1 | 1 | 1 | 2 | 1 | 118 | 138 | Oxidation (M) |
| R.HGESAWNLENR.F | Y | 91.54 | 1311.5956 | 11 | 6.2 | 656.8091 | 2 | 43.92 | 1 | 12225 | ANa\_10.raw | 5.56E6 | 3.31E6 | 1.85E6 |  | 2.68E6 |  | 3.46E6 |  | 1.33E7 | 2.85E6 | 9 | 1 | 1 | 1 | 0 | 1 | 0 | 2 | 0 | 2 | 1 | 11 | 21 |  |
| R.ALPFWNEEIVPQIK.E | Y | 86.05 | 1682.9031 | 14 | 2.7 | 842.4611 | 2 | 126.37 | 6 | 35138 | Ana\_05.raw | 5.52E5 | 7.94E6 | 5.3E7 | 2.61E6 | 3.52E7 | 9.37E6 | 2.89E7 | 2.94E6 | 1.22E8 | 5.07E7 | 29 | 3 | 4 | 4 | 2 | 2 | 2 | 2 | 2 | 4 | 4 | 163 | 176 |  |
| R.HYGGLTGLNK.A | Y | 75.01 | 1058.5509 | 10 | 3.2 | 530.2844 | 2 | 36.64 | 6 | 10125 | Ana\_05.raw |  |  | 1.61E7 |  | 1.38E7 | 1.38E7 | 1.15E7 |  |  |  | 4 | 0 | 0 | 1 | 0 | 1 | 1 | 1 | 0 | 0 | 0 | 91 | 100 |  |
| R.YADLTEDQLPSC(+57.02)ESLK.D | Y | 72.62 | 1867.8510 | 16 | 6.1 | 934.9384 | 2 | 80.84 | 2 | 22943 | Ana\_01.raw |  | 8.51E5 |  |  |  |  |  |  | 1.84E6 |  | 2 | 0 | 1 | 0 | 0 | 0 | 0 | 0 | 0 | 1 | 0 | 142 | 157 | Carbamidomethylation |
| R.VLIAAHGNSLR.G | Y | 54.79 | 1149.6619 | 11 | -0.2 | 384.2278 | 3 | 37.00 | 3 | 10226 | Ana\_02.raw | 8.15E6 |  | 7.49E6 | 3E6 | 6E6 | 7.53E6 | 5.17E6 | 3.9E6 | 3.19E6 | 9.4E6 | 9 | 1 | 0 | 1 | 1 | 1 | 1 | 1 | 1 | 1 | 1 | 181 | 191 |  |
| R.KAMEAVAAQGK.V | Y | 52.87 | 1102.5804 | 11 | 7.1 | 552.3014 | 2 | 20.56 | 10 | 5004 | Ana\_09.raw |  |  |  |  |  |  |  |  |  | 1.61E6 | 1 | 0 | 0 | 0 | 0 | 0 | 0 | 0 | 0 | 0 | 1 | 241 | 251 |  |
| R.FSGWYDADLSPAGHEEAK.R | Y | 50.98 | 1978.8696 | 18 | 3.7 | 660.6329 | 3 | 79.99 | 3 | 22613 | Ana\_02.raw |  | 3.98E6 | 2.8E6 | 8.81E3 | 1.47E6 |  | 1.28E6 |  |  |  | 5 | 0 | 1 | 1 | 1 | 1 | 0 | 1 | 0 | 0 | 0 | 22 | 39 |  |
| K.AMEAVAAQGK.V | Y | 28.07 | 974.4855 | 10 | 5.6 | 488.2527 | 2 | 24.00 | 1 | 6104 | ANa\_10.raw | 5.51E6 |  |  |  |  |  |  |  |  | 5.69E6 | 2 | 1 | 0 | 0 | 0 | 0 | 0 | 0 | 0 | 0 | 1 | 242 | 251 |  |
| total 10 peptides |  |  |  |  |  |  |  |  |  |  |  |  |  |  |  |  |  |  |  |  |  |  |  |  |  |  |  |  |  |  |  |  |  |  |  |
| --- | --- | --- | --- | --- | --- | --- | --- | --- | --- | --- | --- | --- | --- | --- | --- | --- | --- | --- | --- | --- | --- | --- | --- | --- | --- | --- | --- | --- | --- | --- | --- | --- | --- | --- | --- |

Best Unique PSM (Scan F3:36284, m/z=1013.8582, z=3, RT=132.48, ppm=0.1):

sp|Q922F4|TBB6\_MOUSE

back to list

  

| Protein Coverage
| Supporting Peptides
| Best Unique PSM
|

Protein Coverage:

Supporting Peptides:

| Peptide | Uniq | -10lgP | Mass | Length | ppm | m/z | z | RT | Fraction | Scan | Source File | Area 10 | Area 01 | Area 02 | Area 03 | Area 04 | Area 05 | Area 06 | Area 07 | Area 08 | Area 09 | #Spec | #Spec 10 | #Spec 01 | #Spec 02 | #Spec 03 | #Spec 04 | #Spec 05 | #Spec 06 | #Spec 07 | #Spec 08 | #Spec 09 | Start | End | PTM |
| --- | --- | --- | --- | --- | --- | --- | --- | --- | --- | --- | --- | --- | --- | --- | --- | --- | --- | --- | --- | --- | --- | --- | --- | --- | --- | --- | --- | --- | --- | --- | --- | --- | --- | --- | --- |
| K.GHYTEGAELVDSVLDVVR.K | N | 135.92 | 1957.9745 | 18 | 3.6 | 979.9980 | 2 | 127.77 | 6 | 35505 | Ana\_05.raw | 2.36E7 | 5.44E7 | 1.6E8 | 4.71E7 | 1.26E8 | 1.66E8 | 6.78E7 | 6.96E7 | 3.65E8 | 1.97E8 | 32 | 2 | 4 | 5 | 2 | 4 | 2 | 1 | 5 | 4 | 3 | 104 | 121 |  |
| K.NSSYFVEWIPNNVK.V | N | 110.81 | 1695.8257 | 14 | 0.6 | 848.9207 | 2 | 109.87 | 3 | 30437 | Ana\_02.raw | 3.45E7 | 3.29E7 | 2.2E7 | 1.6E7 | 2.71E7 | 1.96E7 | 1.72E7 | 1.87E7 | 6.7E7 | 2.73E7 | 21 | 2 | 3 | 2 | 1 | 4 | 1 | 3 | 1 | 3 | 1 | 337 | 350 |  |
| R.LHFFM(+15.99)PGFAPLTAR.G | N | 85.95 | 1619.8282 | 14 | 5.4 | 540.9529 | 3 | 102.58 | 1 | 28641 | ANa\_10.raw | 4.18E6 |  | 8.63E5 | 9.96E4 | 1.8E6 | 1.13E6 | 3.47E5 |  |  | 2.45E6 | 7 | 1 | 0 | 1 | 1 | 1 | 1 | 1 | 0 | 0 | 1 | 263 | 276 | Oxidation (M) |
| R.FPGQLNADLR.K | N | 78.02 | 1129.5880 | 10 | 3.0 | 565.8030 | 2 | 69.05 | 6 | 19811 | Ana\_05.raw | 7.68E7 | 5.71E7 | 5.55E7 | 2.67E7 | 6.02E7 | 4.21E7 | 2.93E7 | 4.94E7 | 1.57E8 | 8.76E7 | 45 | 4 | 4 | 4 | 4 | 4 | 5 | 3 | 5 | 6 | 6 | 242 | 251 |  |
| K.LAVNM(+15.99)VPFPR.L | N | 76.83 | 1158.6219 | 10 | 4.1 | 580.3206 | 2 | 79.35 | 6 | 22695 | Ana\_05.raw | 8.22E7 | 5.68E7 | 5.35E7 | 2.41E7 | 4.94E7 | 5.03E7 | 3.23E7 | 3.95E7 | 1.48E8 | 5.62E7 | 48 | 6 | 5 | 4 | 4 | 4 | 4 | 4 | 6 | 7 | 4 | 253 | 262 | Oxidation (M) |
| R.ALTVPELTQQM(+15.99)FDAK.N | N | 71.14 | 1706.8549 | 15 | 4.9 | 854.4389 | 2 | 89.92 | 3 | 25244 | Ana\_02.raw | 3.6E7 | 2.33E7 | 1.45E7 | 4.92E6 | 1.46E7 | 1.85E7 | 9.93E6 | 1.33E7 | 7.58E7 | 9.88E6 | 26 | 4 | 2 | 3 | 1 | 2 | 3 | 3 | 1 | 5 | 2 | 283 | 297 | Oxidation (M) |
| K.LAVNMVPFPR.L | N | 69.21 | 1142.6270 | 10 | 5.4 | 572.3239 | 2 | 92.17 | 1 | 25869 | ANa\_10.raw | 6.59E6 |  |  |  |  | 1.83E5 |  |  | 1.45E6 |  | 3 | 1 | 0 | 0 | 0 | 0 | 1 | 0 | 0 | 1 | 0 | 253 | 262 |  |
| K.IREEYPDR.I | N | 65.00 | 1076.5250 | 8 | 2.5 | 539.2711 | 2 | 25.17 | 6 | 6612 | Ana\_05.raw | 8.6E7 | 1.16E8 | 1.13E8 | 5.23E7 | 7.83E7 | 1.03E8 | 7.1E7 | 6.1E7 | 5.8E7 | 1.33E8 | 27 | 3 | 2 | 2 | 3 | 2 | 2 | 3 | 3 | 4 | 3 | 155 | 162 |  |
| R.ISVYYNESSSK.K | Y | 59.38 | 1275.5983 | 11 | -2.7 | 638.8047 | 2 | 43.52 | 9 | 12067 | Ana\_08.raw | 2.96E6 |  |  |  |  |  |  | 8.5E5 | 2.43E6 | 2.28E6 | 4 | 1 | 0 | 0 | 0 | 0 | 0 | 0 | 1 | 1 | 1 | 47 | 57 |  |
| R.ALTVPELTQQMFDAK.N | N | 51.60 | 1690.8600 | 15 | 9.7 | 846.4455 | 2 | 117.55 | 10 | 32318 | Ana\_09.raw |  | 3.09E6 |  |  |  | 1.51E6 |  | 3.44E5 |  | 2.19E6 | 4 | 0 | 1 | 0 | 0 | 0 | 1 | 0 | 1 | 0 | 1 | 283 | 297 |  |
| R.KLAVNM(+15.99)VPFPR.L | N | 49.45 | 1286.7169 | 11 | 2.9 | 429.9142 | 3 | 66.65 | 5 | 19005 | Ana\_04.raw |  |  |  | 2.22E6 | 4.19E6 | 8.25E6 |  | 2.66E6 | 3.2E6 |  | 5 | 0 | 0 | 0 | 1 | 1 | 1 | 0 | 1 | 1 | 0 | 252 | 262 | Oxidation (M) |
| R.YLTVATVFR.G | N | 35.17 | 1068.5968 | 9 | 2.3 | 535.3069 | 2 | 89.29 | 1 | 25099 | ANa\_10.raw | 1.14E6 |  |  |  |  |  |  |  |  |  | 1 | 1 | 0 | 0 | 0 | 0 | 0 | 0 | 0 | 0 | 0 | 310 | 318 |  |
| R.AALVDLEPGTM(+15.99)DSVR.S | Y | 33.34 | 1588.7766 | 15 | 3.1 | 795.3981 | 2 | 71.52 | 9 | 20412 | Ana\_08.raw |  |  |  |  |  |  |  |  | 1.84E6 |  | 1 | 0 | 0 | 0 | 0 | 0 | 0 | 0 | 0 | 1 | 0 | 63 | 77 | Oxidation (M) |
| total 13 peptides |  |  |  |  |  |  |  |  |  |  |  |  |  |  |  |  |  |  |  |  |  |  |  |  |  |  |  |  |  |  |  |  |  |  |  |
| --- | --- | --- | --- | --- | --- | --- | --- | --- | --- | --- | --- | --- | --- | --- | --- | --- | --- | --- | --- | --- | --- | --- | --- | --- | --- | --- | --- | --- | --- | --- | --- | --- | --- | --- | --- |

Best Unique PSM (Scan F9:12067, m/z=638.8047, z=2, RT=43.52, ppm=-2.7):

sp|P16406|AMPE\_MOUSE

back to list

  

| Protein Coverage
| Supporting Peptides
| Best Unique PSM
|

Protein Coverage:

Supporting Peptides:

| Peptide | Uniq | -10lgP | Mass | Length | ppm | m/z | z | RT | Fraction | Scan | Source File | Area 10 | Area 01 | Area 02 | Area 03 | Area 04 | Area 05 | Area 06 | Area 07 | Area 08 | Area 09 | #Spec | #Spec 10 | #Spec 01 | #Spec 02 | #Spec 03 | #Spec 04 | #Spec 05 | #Spec 06 | #Spec 07 | #Spec 08 | #Spec 09 | Start | End | PTM |
| --- | --- | --- | --- | --- | --- | --- | --- | --- | --- | --- | --- | --- | --- | --- | --- | --- | --- | --- | --- | --- | --- | --- | --- | --- | --- | --- | --- | --- | --- | --- | --- | --- | --- | --- | --- |
| K.IAIPDFGTGAM(+15.99)ENWGLVTYR.E | Y | 104.97 | 2226.0779 | 20 | 6.5 | 1114.0535 | 2 | 127.37 | 6 | 35399 | Ana\_05.raw | 1.21E6 | 2.11E6 |  |  |  | 3.1E6 | 2.92E6 | 9.63E5 | 8.64E5 |  | 7 | 1 | 1 | 0 | 0 | 0 | 1 | 2 | 1 | 1 | 0 | 341 | 360 | Oxidation (M) |
| K.TSDFWDSLQEASNLPVK.E | Y | 101.89 | 1935.9214 | 17 | 4.0 | 968.9718 | 2 | 123.28 | 6 | 34337 | Ana\_05.raw | 2.17E6 | 2.93E6 | 3.79E6 | 3.92E5 | 1.97E6 | 1.12E7 | 2.6E6 | 1.13E6 | 1.66E7 | 1.01E6 | 12 | 1 | 2 | 1 | 1 | 1 | 1 | 2 | 1 | 1 | 1 | 508 | 524 |  |
| R.ETNLLYDPLLSASSNQQR.V | Y | 99.50 | 2048.0173 | 18 | 0.6 | 1025.0166 | 2 | 102.90 | 3 | 28657 | Ana\_02.raw | 5.32E5 | 4.54E6 | 7.73E5 | 5.08E5 | 1.23E6 |  |  |  | 5.26E6 |  | 6 | 1 | 1 | 1 | 1 | 1 | 0 | 0 | 0 | 1 | 0 | 361 | 378 |  |
| R.SIAATDHEPTDAR.K | Y | 82.96 | 1382.6426 | 13 | 1.8 | 692.3298 | 2 | 27.21 | 5 | 7232 | Ana\_04.raw | 4.74E6 | 2.71E7 | 5.09E6 |  | 1.35E7 | 1.15E7 | 9.51E6 | 3.31E6 | 5.6E6 | 2.76E6 | 13 | 1 | 2 | 1 | 0 | 2 | 2 | 2 | 1 | 1 | 1 | 208 | 220 |  |
| R.SSFIDDAFALAR.A | Y | 72.27 | 1311.6459 | 12 | 1.3 | 656.8311 | 2 | 121.87 | 6 | 33975 | Ana\_05.raw | 2.96E6 | 2.45E7 | 2.22E7 | 4.18E6 | 2.17E6 | 2.4E7 | 1.14E7 | 3.33E6 | 2.67E7 | 1.29E7 | 16 | 2 | 2 | 2 | 2 | 1 | 1 | 1 | 1 | 2 | 2 | 647 | 658 |  |
| R.RPSGEQVPIR.R | Y | 61.28 | 1137.6254 | 10 | 4.9 | 569.8228 | 2 | 26.64 | 2 | 6960 | Ana\_01.raw | 1.81E6 | 8.31E6 |  | 1.77E6 |  | 4.83E6 |  |  | 7.36E6 | 1.79E6 | 6 | 1 | 1 | 0 | 1 | 0 | 1 | 0 | 0 | 1 | 1 | 141 | 150 |  |
| R.LPDFINPVHYDLEVK.A | Y | 60.45 | 1797.9301 | 15 | -5.6 | 600.3140 | 3 | 107.97 | 9 | 30348 | Ana\_08.raw |  |  |  | 2.01E5 |  | 6.48E5 | 4.39E5 |  | 3.06E6 |  | 4 | 0 | 0 | 0 | 1 | 0 | 1 | 1 | 0 | 1 | 0 | 85 | 99 |  |
| K.INPDHIGFYR.V | Y | 58.30 | 1230.6145 | 10 | 1.4 | 411.2127 | 3 | 58.96 | 3 | 16768 | Ana\_02.raw | 1.33E6 | 8.01E6 | 3.42E6 | 2.58E6 | 4.59E6 | 3.74E6 | 6.85E5 |  | 6.39E5 | 3.19E6 | 11 | 1 | 2 | 1 | 1 | 1 | 1 | 1 | 0 | 1 | 2 | 610 | 619 |  |
| K.SEEDFLPWER.V | Y | 56.80 | 1306.5829 | 10 | 4.4 | 654.3016 | 2 | 96.87 | 9 | 27415 | Ana\_08.raw | 1.73E6 | 1.69E6 |  | 3.99E5 | 2.36E6 | 3.1E6 |  | 3.73E5 | 3.04E6 | 5.93E5 | 8 | 1 | 1 | 0 | 1 | 1 | 1 | 0 | 1 | 1 | 1 | 676 | 685 |  |
| K.EYSALSNM(+15.99)PEEK.S | Y | 56.50 | 1412.6129 | 12 | 0.7 | 707.3142 | 2 | 42.15 | 6 | 11815 | Ana\_05.raw |  | 2.5E6 |  | 6.52E5 |  | 2.38E6 |  |  | 2.19E6 |  | 4 | 0 | 1 | 0 | 1 | 0 | 1 | 0 | 0 | 1 | 0 | 244 | 255 | Oxidation (M) |
| K.TQDVFTVIR.Y | Y | 51.11 | 1077.5818 | 9 | 2.2 | 539.7994 | 2 | 73.36 | 3 | 20819 | Ana\_02.raw |  | 3.7E6 | 3.41E6 | 2.76E6 |  |  |  |  | 1.37E6 | 2.04E6 | 5 | 0 | 1 | 1 | 1 | 0 | 0 | 0 | 0 | 1 | 1 | 838 | 846 |  |
| K.GSASIPVNLR.L | Y | 48.47 | 1012.5665 | 10 | 0.0 | 507.2906 | 2 | 58.17 | 4 | 16154 | Ana\_03.raw |  |  | 3.75E6 | 2.81E6 | 2.77E6 | 5.02E6 | 2.79E6 | 1.89E6 | 7E6 |  | 7 | 0 | 0 | 1 | 1 | 1 | 1 | 1 | 1 | 1 | 0 | 765 | 774 |  |
| K.NNIEWLNVNR.Q | Y | 47.19 | 1270.6418 | 10 | 4.1 | 636.3308 | 2 | 81.26 | 6 | 23216 | Ana\_05.raw |  |  |  |  |  | 5.81E5 |  |  |  |  | 1 | 0 | 0 | 0 | 0 | 0 | 1 | 0 | 0 | 0 | 0 | 925 | 934 |  |
| R.YISYNSYGK.T | Y | 46.23 | 1093.5081 | 9 | 6.6 | 547.7649 | 2 | 46.45 | 2 | 13043 | Ana\_01.raw |  | 7.34E6 | 5.09E6 |  |  |  |  |  | 4.39E6 | 5.44E6 | 4 | 0 | 1 | 1 | 0 | 0 | 0 | 0 | 0 | 1 | 1 | 847 | 855 |  |
| K.LLYGLASVK.D | Y | 42.54 | 962.5800 | 9 | 6.1 | 482.3002 | 2 | 71.95 | 9 | 20535 | Ana\_08.raw |  |  |  |  |  |  |  |  | 2.58E6 |  | 1 | 0 | 0 | 0 | 0 | 0 | 0 | 0 | 0 | 1 | 0 | 810 | 818 |  |
| K.TTYM(+15.99)EDGQIR.S | Y | 37.73 | 1228.5394 | 10 | 2.9 | 615.2787 | 2 | 33.60 | 6 | 9207 | Ana\_05.raw |  |  |  |  |  | 5.46E6 |  |  |  |  | 1 | 0 | 0 | 0 | 0 | 0 | 1 | 0 | 0 | 0 | 0 | 198 | 207 | Oxidation (M) |
| K.STYSISIIHPK.E | Y | 34.25 | 1244.6765 | 11 | 3.4 | 623.3477 | 2 | 58.04 | 9 | 16472 | Ana\_08.raw |  |  |  |  |  |  |  |  | 2.05E6 |  | 1 | 0 | 0 | 0 | 0 | 0 | 0 | 0 | 0 | 1 | 0 | 233 | 243 |  |
| R.FTAIER.K | Y | 30.89 | 735.3915 | 6 | 4.3 | 368.7046 | 2 | 31.81 | 6 | 8686 | Ana\_05.raw |  |  |  |  |  | 1.44E7 | 1.14E7 |  |  |  | 2 | 0 | 0 | 0 | 0 | 0 | 1 | 1 | 0 | 0 | 0 | 286 | 291 |  |
| R.FLLDSK.A | Y | 30.33 | 721.4010 | 6 | 5.0 | 361.7096 | 2 | 46.52 | 9 | 12989 | Ana\_08.raw |  | 1.89E6 |  |  |  |  |  |  | 2.77E6 |  | 2 | 0 | 1 | 0 | 0 | 0 | 0 | 0 | 0 | 1 | 0 | 552 | 557 |  |
| R.EQVLETVK.N | Y | 28.94 | 944.5178 | 8 | 2.5 | 473.2674 | 2 | 35.07 | 3 | 9634 | Ana\_02.raw |  |  | 1.44E8 |  |  |  |  |  |  |  | 1 | 0 | 0 | 1 | 0 | 0 | 0 | 0 | 0 | 0 | 0 | 917 | 924 |  |
| R.FTINDR.Y | Y | 23.21 | 764.3817 | 6 | 4.9 | 383.2000 | 2 | 39.30 | 8 | 10625 | Ana\_07.raw |  |  |  |  |  |  | 2.03E6 | 1.02E6 |  |  | 2 | 0 | 0 | 0 | 0 | 0 | 0 | 1 | 1 | 0 | 0 | 873 | 878 |  |
| total 21 peptides |  |  |  |  |  |  |  |  |  |  |  |  |  |  |  |  |  |  |  |  |  |  |  |  |  |  |  |  |  |  |  |  |  |  |  |
| --- | --- | --- | --- | --- | --- | --- | --- | --- | --- | --- | --- | --- | --- | --- | --- | --- | --- | --- | --- | --- | --- | --- | --- | --- | --- | --- | --- | --- | --- | --- | --- | --- | --- | --- | --- |

Best Unique PSM (Scan F6:35399, m/z=1114.0535, z=2, RT=127.37, ppm=6.5):

sp|O88569|ROA2\_MOUSE

back to list

  

| Protein Coverage
| Supporting Peptides
| Best Unique PSM
|

Protein Coverage:

Supporting Peptides:

| Peptide | Uniq | -10lgP | Mass | Length | ppm | m/z | z | RT | Fraction | Scan | Source File | Area 10 | Area 01 | Area 02 | Area 03 | Area 04 | Area 05 | Area 06 | Area 07 | Area 08 | Area 09 | #Spec | #Spec 10 | #Spec 01 | #Spec 02 | #Spec 03 | #Spec 04 | #Spec 05 | #Spec 06 | #Spec 07 | #Spec 08 | #Spec 09 | Start | End | PTM |
| --- | --- | --- | --- | --- | --- | --- | --- | --- | --- | --- | --- | --- | --- | --- | --- | --- | --- | --- | --- | --- | --- | --- | --- | --- | --- | --- | --- | --- | --- | --- | --- | --- | --- | --- | --- |
| R.NM(+15.99)GGPYGGGNYGPGGSGGSGGYGGR.S | Y | 115.60 | 2204.8928 | 25 | 4.8 | 1103.4590 | 2 | 51.55 | 1 | 14478 | ANa\_10.raw | 1.34E7 | 1.32E7 | 8.19E6 | 5.04E6 | 1.63E7 | 6.95E6 | 1.15E7 | 1.06E7 | 1.66E7 | 5.26E6 | 15 | 1 | 1 | 1 | 2 | 2 | 1 | 2 | 2 | 2 | 1 | 326 | 350 | Oxidation (M) |
| K.LFIGGLSFETTEESLR.N | Y | 97.66 | 1797.9148 | 16 | 0.7 | 899.9653 | 2 | 119.08 | 3 | 32809 | Ana\_02.raw | 1.6E6 | 6.96E5 | 1.09E7 | 1.43E6 | 6.9E6 | 7.9E6 | 4.99E6 | 7.36E6 | 3.74E6 | 2.25E6 | 14 | 1 | 2 | 2 | 1 | 2 | 1 | 2 | 1 | 1 | 1 | 23 | 38 |  |
| R.EESGKPGAHVTVK.K | Y | 93.35 | 1337.6938 | 13 | 0.6 | 669.8546 | 2 | 18.25 | 3 | 4350 | Ana\_02.raw | 8.11E7 | 7.53E7 | 7.08E7 | 1.14E7 | 2.15E7 | 3.28E7 | 1.53E7 | 1.05E7 | 9.03E7 | 7.72E7 | 26 | 3 | 3 | 3 | 2 | 2 | 2 | 2 | 2 | 3 | 4 | 100 | 112 |  |
| R.GGGGNFGPGPGSNFR.G | Y | 91.02 | 1376.6221 | 15 | 1.8 | 689.3195 | 2 | 55.12 | 6 | 15733 | Ana\_05.raw | 8.39E7 | 9.41E7 | 7.8E7 | 3.19E7 | 6.69E7 | 6.53E7 | 5.24E7 | 5.4E7 | 1.24E8 | 6.69E7 | 27 | 4 | 2 | 3 | 2 | 3 | 1 | 2 | 3 | 4 | 3 | 214 | 228 |  |
| R.GFGFVTFDDHDPVDK.I | Y | 85.88 | 1694.7576 | 15 | -1.2 | 848.3850 | 2 | 92.59 | 3 | 25947 | Ana\_02.raw | 4.59E7 | 5.31E7 | 5.39E7 | 2.31E7 | 3.33E7 | 4.4E7 | 3.33E7 | 3.09E7 | 8.2E7 | 5.25E7 | 45 | 6 | 5 | 4 | 3 | 2 | 4 | 4 | 6 | 5 | 6 | 154 | 168 |  |
| K.EDTEEHHLR.D | N | 83.32 | 1164.5160 | 9 | 3.3 | 583.2672 | 2 | 17.28 | 6 | 4025 | Ana\_05.raw |  |  | 1.9E7 | 3.48E7 | 6.33E7 | 9.35E7 | 4.88E7 | 5.08E7 |  |  | 13 | 0 | 0 | 2 | 2 | 2 | 2 | 3 | 2 | 0 | 0 | 121 | 129 |  |
| K.IDTIEIITDR.Q | Y | 69.92 | 1187.6398 | 10 | 3.4 | 594.8292 | 2 | 84.71 | 1 | 23862 | ANa\_10.raw | 2.5E7 | 3E7 | 2.48E7 | 7.37E5 |  | 1.98E7 |  | 1.11E7 | 5.44E7 | 2.9E7 | 16 | 1 | 3 | 1 | 1 | 0 | 1 | 0 | 3 | 4 | 2 | 138 | 147 |  |
| R.DYFEEYGK.I | Y | 64.39 | 1049.4341 | 8 | 4.9 | 525.7269 | 2 | 62.93 | 2 | 17921 | Ana\_01.raw | 4.04E7 | 4.55E7 | 5.08E7 | 2.68E7 | 4.02E7 | 3.98E7 | 3.36E7 | 3.24E7 | 5.4E7 | 3.91E7 | 20 | 2 | 2 | 2 | 2 | 2 | 2 | 2 | 2 | 2 | 2 | 130 | 137 |  |
| R.GGNFGFGDSR.G | Y | 62.08 | 1012.4362 | 10 | 3.0 | 507.2269 | 2 | 49.90 | 6 | 14195 | Ana\_05.raw | 5.45E7 | 5.06E7 | 5.81E7 | 2.68E7 | 5.37E7 | 5.12E7 | 4.17E7 | 3.34E7 | 6.17E7 | 4.23E7 | 23 | 2 | 2 | 2 | 3 | 2 | 2 | 2 | 3 | 3 | 2 | 204 | 213 |  |
| K.TLETVPLER.K | Y | 55.19 | 1056.5815 | 9 | 5.1 | 529.3007 | 2 | 58.49 | 7 | 16537 | Ana\_06.raw | 4.55E6 |  |  |  | 6.37E6 |  | 5.11E6 | 5E6 | 3.73E6 | 5.44E6 | 6 | 1 | 0 | 0 | 0 | 1 | 0 | 1 | 1 | 1 | 1 | 4 | 12 |  |
| R.NYYEQWGK.L | Y | 46.88 | 1086.4771 | 8 | 3.8 | 544.2479 | 2 | 58.91 | 8 | 16452 | Ana\_07.raw |  |  | 1.01E7 | 5.79E6 | 1.03E7 |  | 5.42E6 | 9.13E6 | 1.47E7 |  | 6 | 0 | 0 | 1 | 1 | 1 | 0 | 1 | 1 | 1 | 0 | 39 | 46 |  |
| K.LFVGGIK.E | N | 39.49 | 732.4534 | 7 | 3.0 | 367.2350 | 2 | 54.54 | 7 | 15367 | Ana\_06.raw | 2.73E7 | 3.67E7 | 3.65E7 | 1.76E7 | 2.9E7 | 2.64E7 | 2.21E7 | 2.26E7 | 6.24E7 | 3.64E7 | 40 | 4 | 4 | 4 | 4 | 5 | 3 | 4 | 4 | 4 | 4 | 114 | 120 |  |
| K.LTDC(+57.02)VVM(+15.99)R.D | Y | 37.86 | 1008.4733 | 8 | 0.0 | 505.2439 | 2 | 34.33 | 6 | 9431 | Ana\_05.raw |  | 6.54E6 |  |  |  | 4.2E6 | 0 | 7.72E6 |  |  | 4 | 0 | 1 | 0 | 0 | 0 | 1 | 1 | 1 | 0 | 0 | 47 | 54 | Carbamidomethylation; Oxidation (M) |
| K.KLFVGGIK.E | N | 36.91 | 860.5483 | 8 | 1.7 | 431.2822 | 2 | 45.34 | 6 | 12802 | Ana\_05.raw |  |  | 2.12E6 | 7.05E5 | 1.67E6 | 1.76E6 | 1.1E6 |  |  |  | 5 | 0 | 0 | 1 | 1 | 1 | 1 | 1 | 0 | 0 | 0 | 113 | 120 |  |
| total 14 peptides |  |  |  |  |  |  |  |  |  |  |  |  |  |  |  |  |  |  |  |  |  |  |  |  |  |  |  |  |  |  |  |  |  |  |  |
| --- | --- | --- | --- | --- | --- | --- | --- | --- | --- | --- | --- | --- | --- | --- | --- | --- | --- | --- | --- | --- | --- | --- | --- | --- | --- | --- | --- | --- | --- | --- | --- | --- | --- | --- | --- |

Best Unique PSM (Scan F1:14478, m/z=1103.4590, z=2, RT=51.55, ppm=4.8):

sp|P14211|CALR\_MOUSE

back to list

  

| Protein Coverage
| Supporting Peptides
| Best Unique PSM
|

Protein Coverage:

Supporting Peptides:

| Peptide | Uniq | -10lgP | Mass | Length | ppm | m/z | z | RT | Fraction | Scan | Source File | Area 10 | Area 01 | Area 02 | Area 03 | Area 04 | Area 05 | Area 06 | Area 07 | Area 08 | Area 09 | #Spec | #Spec 10 | #Spec 01 | #Spec 02 | #Spec 03 | #Spec 04 | #Spec 05 | #Spec 06 | #Spec 07 | #Spec 08 | #Spec 09 | Start | End | PTM |
| --- | --- | --- | --- | --- | --- | --- | --- | --- | --- | --- | --- | --- | --- | --- | --- | --- | --- | --- | --- | --- | --- | --- | --- | --- | --- | --- | --- | --- | --- | --- | --- | --- | --- | --- | --- |
| K.HEQNIDC(+57.02)GGGYVK.L | Y | 106.31 | 1475.6464 | 13 | 1.7 | 738.8317 | 2 | 33.98 | 6 | 9328 | Ana\_05.raw | 3.93E7 | 5.34E7 | 4.25E7 | 2.17E7 | 3.54E7 | 3.61E7 | 3E7 | 1.22E7 | 4.51E7 | 3.98E7 | 20 | 2 | 2 | 2 | 2 | 2 | 2 | 2 | 2 | 2 | 2 | 99 | 111 | Carbamidomethylation |
| K.KPEDWDEEM(+15.99)DGEWEPPVIQNPEYK.G | Y | 96.22 | 2975.2808 | 24 | 4.7 | 992.7722 | 3 | 97.10 | 1 | 27197 | ANa\_10.raw | 8.46E6 |  |  |  | 4.06E6 | 3.49E5 | 1.21E6 | 5.04E6 | 1.71E7 |  | 6 | 1 | 0 | 0 | 0 | 1 | 1 | 1 | 1 | 1 | 0 | 249 | 272 | Oxidation (M) |
| K.DM(+15.99)HGDSEYNIM(+15.99)FGPDIC(+57.02)GPGTK.K | Y | 87.61 | 2472.0032 | 22 | 4.3 | 825.0118 | 3 | 88.81 | 7 | 24973 | Ana\_06.raw | 5.94E6 | 9.92E6 | 1.02E7 | 5.2E6 | 3.22E6 | 1E7 | 8.29E6 | 1.09E7 | 1.48E7 |  | 10 | 1 | 1 | 1 | 1 | 1 | 1 | 1 | 2 | 1 | 0 | 121 | 142 | Oxidation (M); Carbamidomethylation |
| K.IKDPDAAKPEDWDER.A | Y | 75.68 | 1783.8376 | 15 | 0.5 | 446.9669 | 4 | 45.72 | 3 | 12882 | Ana\_02.raw | 2.97E7 | 2.25E7 | 2.07E7 |  | 2.27E7 | 1.61E7 | 2.11E7 | 9.52E6 | 6.04E6 | 2.12E7 | 14 | 2 | 1 | 2 | 0 | 2 | 1 | 2 | 1 | 1 | 2 | 208 | 222 |  |
| K.DKGLQTSQDAR.F | Y | 75.32 | 1217.6000 | 11 | 5.1 | 609.8104 | 2 | 18.80 | 6 | 4536 | Ana\_05.raw |  |  |  | 6.67E5 | 1.65E6 | 1.61E6 | 2.37E6 | 1.2E6 |  |  | 6 | 0 | 0 | 0 | 1 | 1 | 1 | 2 | 1 | 0 | 0 | 63 | 73 |  |
| K.EQFLDGDAWTNR.W | Y | 71.70 | 1450.6477 | 12 | 3.7 | 726.3338 | 2 | 83.02 | 6 | 23692 | Ana\_05.raw | 1.08E7 | 4.13E6 | 9.03E5 | 6.86E6 | 1.1E7 | 1.19E7 | 8.07E6 | 1.28E7 | 7.3E6 | 5.82E6 | 17 | 2 | 2 | 1 | 2 | 2 | 2 | 2 | 2 | 1 | 1 | 25 | 36 |  |
| K.SGTIFDNFLITNDEAYAEEFGNETWGVTK.A | Y | 67.02 | 3267.4883 | 29 | 4.4 | 1090.1748 | 3 | 134.89 | 6 | 37501 | Ana\_05.raw |  |  |  |  |  | 2.31E6 |  |  |  |  | 2 | 0 | 0 | 0 | 0 | 0 | 2 | 0 | 0 | 0 | 0 | 323 | 351 |  |
| K.IDNSQVESGSLEDDWDFLPPKK.I | Y | 66.64 | 2518.1863 | 22 | 8.4 | 840.4097 | 3 | 102.42 | 9 | 28886 | Ana\_08.raw |  | 7.36E6 |  |  |  |  |  |  | 4.4E6 |  | 2 | 0 | 1 | 0 | 0 | 0 | 0 | 0 | 0 | 1 | 0 | 186 | 207 |  |
| K.GLQTSQDAR.F | Y | 64.14 | 974.4781 | 9 | 2.3 | 488.2474 | 2 | 18.84 | 5 | 4598 | Ana\_04.raw |  |  |  |  | 4.89E6 |  |  |  |  |  | 1 | 0 | 0 | 0 | 0 | 1 | 0 | 0 | 0 | 0 | 0 | 65 | 73 |  |
| K.KVHVIFNYK.G | Y | 55.93 | 1146.6549 | 9 | 0.6 | 383.2258 | 3 | 42.19 | 3 | 11822 | Ana\_02.raw | 1.2E7 | 1.23E7 | 1.74E7 | 8.24E6 | 1.51E7 | 1.45E7 | 1.1E7 | 9.53E6 |  |  | 10 | 1 | 1 | 2 | 2 | 1 | 1 | 1 | 1 | 0 | 0 | 143 | 151 |  |
| K.LFPSGLDQK.D | Y | 55.51 | 1003.5338 | 9 | 4.8 | 502.7766 | 2 | 59.12 | 1 | 16707 | ANa\_10.raw | 4.02E7 | 4.54E7 | 5.04E7 | 2.56E7 | 4.07E7 | 3.62E7 | 3.23E7 | 3.46E7 | 6.34E7 | 4.09E7 | 25 | 3 | 3 | 2 | 2 | 2 | 2 | 3 | 3 | 3 | 2 | 112 | 120 |  |
| K.VHVIFNYK.G | Y | 55.06 | 1018.5600 | 8 | 8.1 | 510.2914 | 2 | 53.97 | 1 | 15201 | ANa\_10.raw | 4.02E6 |  |  | 2.02E6 |  |  | 2.86E6 |  |  | 4.97E6 | 4 | 1 | 0 | 0 | 1 | 0 | 0 | 1 | 0 | 0 | 1 | 144 | 151 |  |
| R.QIDNPDYK.G | Y | 53.92 | 991.4611 | 8 | 3.9 | 496.7397 | 2 | 26.84 | 1 | 7019 | ANa\_10.raw | 8.08E6 | 9.85E6 |  |  |  |  | 8.3E6 | 7.69E6 |  |  | 4 | 1 | 1 | 0 | 0 | 0 | 0 | 1 | 1 | 0 | 0 | 279 | 286 |  |
| K.FYGDLEKDK.G | Y | 51.49 | 1113.5342 | 9 | 4.9 | 557.7771 | 2 | 41.57 | 5 | 11586 | Ana\_04.raw | 8.48E6 | 1.19E7 | 7.39E6 |  | 6.66E6 | 8.91E6 |  | 3.93E6 | 2.89E6 | 7.82E6 | 8 | 1 | 1 | 1 | 0 | 1 | 1 | 0 | 1 | 1 | 1 | 56 | 64 |  |
| K.IDDPTDSKPEDWDKPEHIPDPDAK.K | Y | 49.01 | 2759.2561 | 24 | 0.2 | 690.8214 | 4 | 68.21 | 3 | 19394 | Ana\_02.raw | 4.7E6 |  | 5.76E6 | 3.81E6 | 5.88E6 |  | 5.79E6 |  |  | 1.43E6 | 6 | 1 | 0 | 1 | 1 | 1 | 0 | 1 | 0 | 0 | 1 | 225 | 248 |  |
| R.FYALSAK.F | Y | 47.91 | 798.4276 | 7 | 3.0 | 400.2223 | 2 | 51.28 | 7 | 14422 | Ana\_06.raw |  |  | 3.62E7 | 1.86E7 | 2.93E7 | 2.94E7 | 2.87E7 | 2.17E7 | 2.82E7 | 3.03E7 | 11 | 0 | 0 | 1 | 1 | 2 | 1 | 2 | 2 | 1 | 1 | 74 | 80 |  |
| K.FEPFSNK.G | Y | 46.98 | 867.4126 | 7 | 2.6 | 434.7147 | 2 | 49.19 | 5 | 13905 | Ana\_04.raw | 4.52E7 | 4.07E7 | 4.3E7 | 2.32E7 | 3.78E7 | 3.89E7 | 3.62E7 | 3.22E7 | 3E7 | 3.61E7 | 20 | 2 | 1 | 2 | 2 | 2 | 2 | 2 | 3 | 2 | 2 | 81 | 87 |  |
| K.NVLINK.D | Y | 39.11 | 699.4279 | 6 | 3.4 | 350.7224 | 2 | 26.66 | 6 | 7079 | Ana\_05.raw |  |  | 2.16E7 | 1.07E7 |  | 1.72E7 |  |  |  |  | 3 | 0 | 0 | 1 | 1 | 0 | 1 | 0 | 0 | 0 | 0 | 154 | 159 |  |
| K.IDNSQVESGSLEDDWDFLPPK.K | Y | 38.28 | 2390.0913 | 21 | 4.6 | 1196.0585 | 2 | 117.19 | 8 | 32281 | Ana\_07.raw |  |  |  |  |  |  |  | 2.54E5 |  |  | 1 | 0 | 0 | 0 | 0 | 0 | 0 | 0 | 1 | 0 | 0 | 186 | 206 |  |
| K.GEWKPR.Q | Y | 33.69 | 771.4027 | 6 | 3.0 | 386.7098 | 2 | 20.69 | 4 | 5103 | Ana\_03.raw |  |  | 1.17E7 | 5.11E6 |  |  | 8.69E6 | 5.7E6 |  |  | 4 | 0 | 0 | 1 | 1 | 0 | 0 | 1 | 1 | 0 | 0 | 273 | 278 |  |
| K.FYGDLEK.D | Y | 30.51 | 870.4123 | 7 | 2.3 | 436.2144 | 2 | 43.83 | 9 | 12159 | Ana\_08.raw |  | 3.24E7 | 3.77E7 | 1.57E7 | 2.66E7 |  |  | 2.17E7 | 2.9E7 |  | 6 | 0 | 1 | 1 | 1 | 1 | 0 | 0 | 1 | 1 | 0 | 56 | 62 |  |
| total 21 peptides |  |  |  |  |  |  |  |  |  |  |  |  |  |  |  |  |  |  |  |  |  |  |  |  |  |  |  |  |  |  |  |  |  |  |  |
| --- | --- | --- | --- | --- | --- | --- | --- | --- | --- | --- | --- | --- | --- | --- | --- | --- | --- | --- | --- | --- | --- | --- | --- | --- | --- | --- | --- | --- | --- | --- | --- | --- | --- | --- | --- |

Best Unique PSM (Scan F6:9328, m/z=738.8317, z=2, RT=33.98, ppm=1.7):

A0A0U1RNJ1|A0A0U1RNJ1\_MOUSE

back to list

  

| Protein Coverage
| Supporting Peptides
| Best Unique PSM
|

Protein Coverage:

Supporting Peptides:

| Peptide | Uniq | -10lgP | Mass | Length | ppm | m/z | z | RT | Fraction | Scan | Source File | Area 10 | Area 01 | Area 02 | Area 03 | Area 04 | Area 05 | Area 06 | Area 07 | Area 08 | Area 09 | #Spec | #Spec 10 | #Spec 01 | #Spec 02 | #Spec 03 | #Spec 04 | #Spec 05 | #Spec 06 | #Spec 07 | #Spec 08 | #Spec 09 | Start | End | PTM |
| --- | --- | --- | --- | --- | --- | --- | --- | --- | --- | --- | --- | --- | --- | --- | --- | --- | --- | --- | --- | --- | --- | --- | --- | --- | --- | --- | --- | --- | --- | --- | --- | --- | --- | --- | --- |
| K.VHLTGINVNPNALFPPVEFPAPR.G | Y | 108.31 | 2498.3433 | 23 | 1.5 | 833.7896 | 3 | 127.03 | 6 | 35311 | Ana\_05.raw | 2.51E6 | 0 |  |  |  | 2.23E6 |  | 2.33E6 |  |  | 4 | 1 | 1 | 0 | 0 | 0 | 1 | 0 | 1 | 0 | 0 | 803 | 825 |  |
| R.TLLEGSGLESIINIIHSSLAEPR.V | Y | 88.44 | 2448.3223 | 23 | 3.5 | 817.1176 | 3 | 137.24 | 6 | 38125 | Ana\_05.raw | 1.24E6 | 2.52E6 |  |  | 2.19E6 | 2.46E6 |  |  |  |  | 4 | 1 | 1 | 0 | 0 | 1 | 1 | 0 | 0 | 0 | 0 | 2474 | 2496 |  |
| R.TLEAVQDLLEQGR.Q | Y | 83.14 | 1470.7678 | 13 | -2.1 | 736.3896 | 2 | 105.70 | 6 | 29766 | Ana\_05.raw |  |  |  | 5.6E6 |  | 5.06E6 | 3.05E6 | 4.54E5 |  |  | 4 | 0 | 0 | 0 | 1 | 0 | 1 | 1 | 1 | 0 | 0 | 430 | 442 |  |
| K.LGPVGGVFNLAM(+15.99)VLR.D | Y | 81.07 | 1557.8701 | 15 | 2.5 | 779.9443 | 2 | 129.86 | 6 | 36076 | Ana\_05.raw |  |  | 1.77E6 |  | 3.64E6 | 6.15E6 | 8.54E6 |  |  |  | 4 | 0 | 0 | 1 | 0 | 1 | 1 | 1 | 0 | 0 | 0 | 1953 | 1967 | Oxidation (M) |
| R.LLLPEDPLISGLLNSQALK.A | Y | 81.06 | 2033.1771 | 19 | 5.1 | 1017.6010 | 2 | 134.22 | 6 | 37314 | Ana\_05.raw | 1.64E6 | 1.94E6 |  | 4.35E5 |  | 1.11E6 | 7.92E5 | 2.97E5 | 2.01E6 |  | 9 | 1 | 2 | 0 | 1 | 0 | 1 | 1 | 1 | 2 | 0 | 1202 | 1220 |  |
| K.DNLEFFLTNLGK.V | Y | 76.49 | 1409.7190 | 12 | 0.6 | 705.8672 | 2 | 129.11 | 4 | 35066 | Ana\_03.raw |  |  |  | 0 |  |  |  |  |  |  | 1 | 0 | 0 | 0 | 1 | 0 | 0 | 0 | 0 | 0 | 0 | 791 | 802 |  |
| K.VSDLLLSTDER.T | Y | 63.82 | 1246.6405 | 11 | 8.2 | 624.3326 | 2 | 66.31 | 2 | 18906 | Ana\_01.raw |  | 2.75E6 |  |  |  |  |  |  |  | 4.87E5 | 2 | 0 | 1 | 0 | 0 | 0 | 0 | 0 | 0 | 0 | 1 | 534 | 544 |  |
| R.SDEAVKPLGVK.V | Y | 62.88 | 1141.6343 | 11 | 5.4 | 571.8275 | 2 | 30.43 | 2 | 8157 | Ana\_01.raw | 5.12E5 | 1.72E6 |  |  |  |  |  |  | 1.6E6 |  | 3 | 1 | 1 | 0 | 0 | 0 | 0 | 0 | 0 | 1 | 0 | 523 | 533 |  |
| R.GYTVLGVEGR.V | Y | 55.22 | 1049.5505 | 10 | 8.0 | 525.7867 | 2 | 60.52 | 2 | 17214 | Ana\_01.raw |  | 4.6E6 | 6.57E5 |  | 2.73E6 |  |  |  |  |  | 3 | 0 | 1 | 1 | 0 | 1 | 0 | 0 | 0 | 0 | 0 | 469 | 478 |  |
| K.TGTVALEVR.L | Y | 51.10 | 944.5291 | 9 | 4.2 | 473.2738 | 2 | 42.66 | 2 | 11880 | Ana\_01.raw |  | 5.94E6 |  |  |  |  |  |  |  |  | 1 | 0 | 1 | 0 | 0 | 0 | 0 | 0 | 0 | 0 | 0 | 928 | 936 |  |
| R.VFTTVGSAEK.R | Y | 50.98 | 1037.5393 | 10 | 5.5 | 519.7798 | 2 | 36.10 | 10 | 9867 | Ana\_09.raw | 2.73E6 | 5.28E6 |  | 1.51E6 |  | 1.81E6 |  |  |  | 1.45E6 | 5 | 1 | 1 | 0 | 1 | 0 | 1 | 0 | 0 | 0 | 1 | 1686 | 1695 |  |
| R.VQEVQQVSTNK.R | Y | 50.72 | 1258.6517 | 11 | 0.9 | 630.3337 | 2 | 26.60 | 4 | 6874 | Ana\_03.raw |  |  |  | 1.15E6 |  |  |  |  | 3.26E6 |  | 2 | 0 | 0 | 0 | 1 | 0 | 0 | 0 | 0 | 1 | 0 | 479 | 489 |  |
| R.DTSFEQHVLLHTGGK.G | Y | 50.58 | 1667.8267 | 15 | 1.8 | 556.9505 | 3 | 60.79 | 2 | 17299 | Ana\_01.raw |  | 2.37E6 |  |  |  | 2.46E6 |  |  |  |  | 2 | 0 | 1 | 0 | 0 | 0 | 1 | 0 | 0 | 0 | 0 | 1716 | 1730 |  |
| K.FDASFFGVHPK.Q | Y | 48.99 | 1250.6084 | 11 | 5.0 | 417.8788 | 3 | 81.26 | 9 | 23137 | Ana\_08.raw |  |  |  | 1.03E6 |  | 1.34E6 |  | 6.15E5 | 1.22E6 |  | 4 | 0 | 0 | 0 | 1 | 0 | 1 | 0 | 1 | 1 | 0 | 60 | 70 |  |
| K.VLEALLPLK.S | Y | 48.50 | 994.6426 | 9 | 3.7 | 498.3304 | 2 | 97.83 | 8 | 27233 | Ana\_07.raw | 2.92E5 | 2.61E5 |  | 4.12E5 | 4.02E5 | 2.32E6 | 1.94E5 | 3.38E5 | 3.23E6 | 1.55E6 | 9 | 1 | 1 | 0 | 1 | 1 | 1 | 1 | 1 | 1 | 1 | 2374 | 2382 |  |
| R.ALIAEATK.L | Y | 40.86 | 815.4752 | 8 | 5.1 | 408.7469 | 2 | 30.52 | 2 | 8183 | Ana\_01.raw |  | 2.68E6 |  |  |  |  |  |  |  |  | 1 | 0 | 1 | 0 | 0 | 0 | 0 | 0 | 0 | 0 | 0 | 1945 | 1952 |  |
| K.AQVEDAFR.Y | Y | 38.71 | 934.4508 | 8 | 5.3 | 468.2352 | 2 | 41.70 | 2 | 11581 | Ana\_01.raw | 3.59E6 | 5.49E6 |  |  |  |  | 2.73E6 |  |  |  | 3 | 1 | 1 | 0 | 0 | 0 | 0 | 1 | 0 | 0 | 0 | 1825 | 1832 |  |
| K.LQASVR.C | N | 33.78 | 672.3918 | 6 | 3.0 | 337.2042 | 2 | 19.76 | 5 | 4904 | Ana\_04.raw |  |  |  |  | 4.25E6 |  |  |  |  |  | 1 | 0 | 0 | 0 | 0 | 1 | 0 | 0 | 0 | 0 | 0 | 1744 | 1749 |  |
| R.LTQGEVYK.E | Y | 29.72 | 936.4916 | 8 | 2.7 | 469.2544 | 2 | 27.62 | 2 | 7281 | Ana\_01.raw |  | 7.84E6 |  |  |  |  |  |  |  |  | 1 | 0 | 1 | 0 | 0 | 0 | 0 | 0 | 0 | 0 | 0 | 986 | 993 |  |
| R.VTAIYIDPATHR.Q | Y | 26.58 | 1355.7197 | 12 | 4.5 | 452.9159 | 3 | 55.07 | 2 | 15613 | Ana\_01.raw |  | 1.86E6 |  |  |  |  |  |  |  |  | 1 | 0 | 1 | 0 | 0 | 0 | 0 | 0 | 0 | 0 | 0 | 1053 | 1064 |  |
| R.LQVVDRPLPVR.G | Y | 25.94 | 1290.7771 | 11 | 5.1 | 431.2685 | 3 | 53.95 | 9 | 15261 | Ana\_08.raw |  |  |  |  |  |  |  |  | 3.02E6 |  | 1 | 0 | 0 | 0 | 0 | 0 | 0 | 0 | 0 | 1 | 0 | 374 | 384 |  |
| R.SFDDSGSGYC(+57.02)R.S | Y | 22.53 | 1249.4669 | 11 | 2.5 | 625.7423 | 2 | 39.11 | 4 | 10567 | Ana\_03.raw |  |  |  | 7.21E5 |  |  |  |  |  |  | 1 | 0 | 0 | 0 | 1 | 0 | 0 | 0 | 0 | 0 | 0 | 214 | 224 | Carbamidomethylation |
| total 22 peptides |  |  |  |  |  |  |  |  |  |  |  |  |  |  |  |  |  |  |  |  |  |  |  |  |  |  |  |  |  |  |  |  |  |  |  |
| --- | --- | --- | --- | --- | --- | --- | --- | --- | --- | --- | --- | --- | --- | --- | --- | --- | --- | --- | --- | --- | --- | --- | --- | --- | --- | --- | --- | --- | --- | --- | --- | --- | --- | --- | --- |

Best Unique PSM (Scan F6:35311, m/z=833.7896, z=3, RT=127.03, ppm=1.5):

sp|P19096|FAS\_MOUSE

back to list

  

| Protein Coverage
| Supporting Peptides
| Best Unique PSM
|

Protein Coverage:

Supporting Peptides:

| Peptide | Uniq | -10lgP | Mass | Length | ppm | m/z | z | RT | Fraction | Scan | Source File | Area 10 | Area 01 | Area 02 | Area 03 | Area 04 | Area 05 | Area 06 | Area 07 | Area 08 | Area 09 | #Spec | #Spec 10 | #Spec 01 | #Spec 02 | #Spec 03 | #Spec 04 | #Spec 05 | #Spec 06 | #Spec 07 | #Spec 08 | #Spec 09 | Start | End | PTM |
| --- | --- | --- | --- | --- | --- | --- | --- | --- | --- | --- | --- | --- | --- | --- | --- | --- | --- | --- | --- | --- | --- | --- | --- | --- | --- | --- | --- | --- | --- | --- | --- | --- | --- | --- | --- |
| K.VHLTGINVNPNALFPPVEFPAPR.G | Y | 108.31 | 2498.3433 | 23 | 1.5 | 833.7896 | 3 | 127.03 | 6 | 35311 | Ana\_05.raw | 2.51E6 | 0 |  |  |  | 2.23E6 |  | 2.33E6 |  |  | 4 | 1 | 1 | 0 | 0 | 0 | 1 | 0 | 1 | 0 | 0 | 803 | 825 |  |
| R.TLLEGSGLESIINIIHSSLAEPR.V | Y | 88.44 | 2448.3223 | 23 | 3.5 | 817.1176 | 3 | 137.24 | 6 | 38125 | Ana\_05.raw | 1.24E6 | 2.52E6 |  |  | 2.19E6 | 2.46E6 |  |  |  |  | 4 | 1 | 1 | 0 | 0 | 1 | 1 | 0 | 0 | 0 | 0 | 2476 | 2498 |  |
| R.TLEAVQDLLEQGR.Q | Y | 83.14 | 1470.7678 | 13 | -2.1 | 736.3896 | 2 | 105.70 | 6 | 29766 | Ana\_05.raw |  |  |  | 5.6E6 |  | 5.06E6 | 3.05E6 | 4.54E5 |  |  | 4 | 0 | 0 | 0 | 1 | 0 | 1 | 1 | 1 | 0 | 0 | 430 | 442 |  |
| K.LGPVGGVFNLAM(+15.99)VLR.D | Y | 81.07 | 1557.8701 | 15 | 2.5 | 779.9443 | 2 | 129.86 | 6 | 36076 | Ana\_05.raw |  |  | 1.77E6 |  | 3.64E6 | 6.15E6 | 8.54E6 |  |  |  | 4 | 0 | 0 | 1 | 0 | 1 | 1 | 1 | 0 | 0 | 0 | 1955 | 1969 | Oxidation (M) |
| R.LLLPEDPLISGLLNSQALK.A | Y | 81.06 | 2033.1771 | 19 | 5.1 | 1017.6010 | 2 | 134.22 | 6 | 37314 | Ana\_05.raw | 1.64E6 | 1.94E6 |  | 4.35E5 |  | 1.11E6 | 7.92E5 | 2.97E5 | 2.01E6 |  | 9 | 1 | 2 | 0 | 1 | 0 | 1 | 1 | 1 | 2 | 0 | 1202 | 1220 |  |
| K.DNLEFFLTNLGK.V | Y | 76.49 | 1409.7190 | 12 | 0.6 | 705.8672 | 2 | 129.11 | 4 | 35066 | Ana\_03.raw |  |  |  | 0 |  |  |  |  |  |  | 1 | 0 | 0 | 0 | 1 | 0 | 0 | 0 | 0 | 0 | 0 | 791 | 802 |  |
| K.VSDLLLSTDER.T | Y | 63.82 | 1246.6405 | 11 | 8.2 | 624.3326 | 2 | 66.31 | 2 | 18906 | Ana\_01.raw |  | 2.75E6 |  |  |  |  |  |  |  | 4.87E5 | 2 | 0 | 1 | 0 | 0 | 0 | 0 | 0 | 0 | 0 | 1 | 534 | 544 |  |
| R.SDEAVKPLGVK.V | Y | 62.88 | 1141.6343 | 11 | 5.4 | 571.8275 | 2 | 30.43 | 2 | 8157 | Ana\_01.raw | 5.12E5 | 1.72E6 |  |  |  |  |  |  | 1.6E6 |  | 3 | 1 | 1 | 0 | 0 | 0 | 0 | 0 | 0 | 1 | 0 | 523 | 533 |  |
| R.GYTVLGVEGR.V | Y | 55.22 | 1049.5505 | 10 | 8.0 | 525.7867 | 2 | 60.52 | 2 | 17214 | Ana\_01.raw |  | 4.6E6 | 6.57E5 |  | 2.73E6 |  |  |  |  |  | 3 | 0 | 1 | 1 | 0 | 1 | 0 | 0 | 0 | 0 | 0 | 469 | 478 |  |
| K.TGTVALEVR.L | Y | 51.10 | 944.5291 | 9 | 4.2 | 473.2738 | 2 | 42.66 | 2 | 11880 | Ana\_01.raw |  | 5.94E6 |  |  |  |  |  |  |  |  | 1 | 0 | 1 | 0 | 0 | 0 | 0 | 0 | 0 | 0 | 0 | 928 | 936 |  |
| R.VFTTVGSAEK.R | Y | 50.98 | 1037.5393 | 10 | 5.5 | 519.7798 | 2 | 36.10 | 10 | 9867 | Ana\_09.raw | 2.73E6 | 5.28E6 |  | 1.51E6 |  | 1.81E6 |  |  |  | 1.45E6 | 5 | 1 | 1 | 0 | 1 | 0 | 1 | 0 | 0 | 0 | 1 | 1688 | 1697 |  |
| R.VQEVQQVSTNK.R | Y | 50.72 | 1258.6517 | 11 | 0.9 | 630.3337 | 2 | 26.60 | 4 | 6874 | Ana\_03.raw |  |  |  | 1.15E6 |  |  |  |  | 3.26E6 |  | 2 | 0 | 0 | 0 | 1 | 0 | 0 | 0 | 0 | 1 | 0 | 479 | 489 |  |
| R.DTSFEQHVLLHTGGK.G | Y | 50.58 | 1667.8267 | 15 | 1.8 | 556.9505 | 3 | 60.79 | 2 | 17299 | Ana\_01.raw |  | 2.37E6 |  |  |  | 2.46E6 |  |  |  |  | 2 | 0 | 1 | 0 | 0 | 0 | 1 | 0 | 0 | 0 | 0 | 1718 | 1732 |  |
| K.FDASFFGVHPK.Q | Y | 48.99 | 1250.6084 | 11 | 5.0 | 417.8788 | 3 | 81.26 | 9 | 23137 | Ana\_08.raw |  |  |  | 1.03E6 |  | 1.34E6 |  | 6.15E5 | 1.22E6 |  | 4 | 0 | 0 | 0 | 1 | 0 | 1 | 0 | 1 | 1 | 0 | 60 | 70 |  |
| K.VLEALLPLK.S | Y | 48.50 | 994.6426 | 9 | 3.7 | 498.3304 | 2 | 97.83 | 8 | 27233 | Ana\_07.raw | 2.92E5 | 2.61E5 |  | 4.12E5 | 4.02E5 | 2.32E6 | 1.94E5 | 3.38E5 | 3.23E6 | 1.55E6 | 9 | 1 | 1 | 0 | 1 | 1 | 1 | 1 | 1 | 1 | 1 | 2376 | 2384 |  |
| R.ALIAEATK.L | Y | 40.86 | 815.4752 | 8 | 5.1 | 408.7469 | 2 | 30.52 | 2 | 8183 | Ana\_01.raw |  | 2.68E6 |  |  |  |  |  |  |  |  | 1 | 0 | 1 | 0 | 0 | 0 | 0 | 0 | 0 | 0 | 0 | 1947 | 1954 |  |
| K.AQVEDAFR.Y | Y | 38.71 | 934.4508 | 8 | 5.3 | 468.2352 | 2 | 41.70 | 2 | 11581 | Ana\_01.raw | 3.59E6 | 5.49E6 |  |  |  |  | 2.73E6 |  |  |  | 3 | 1 | 1 | 0 | 0 | 0 | 0 | 1 | 0 | 0 | 0 | 1827 | 1834 |  |
| K.LQASVR.C | N | 33.78 | 672.3918 | 6 | 3.0 | 337.2042 | 2 | 19.76 | 5 | 4904 | Ana\_04.raw |  |  |  |  | 4.25E6 |  |  |  |  |  | 1 | 0 | 0 | 0 | 0 | 1 | 0 | 0 | 0 | 0 | 0 | 1746 | 1751 |  |
| R.LTQGEVYK.E | Y | 29.72 | 936.4916 | 8 | 2.7 | 469.2544 | 2 | 27.62 | 2 | 7281 | Ana\_01.raw |  | 7.84E6 |  |  |  |  |  |  |  |  | 1 | 0 | 1 | 0 | 0 | 0 | 0 | 0 | 0 | 0 | 0 | 986 | 993 |  |
| R.VTAIYIDPATHR.Q | Y | 26.58 | 1355.7197 | 12 | 4.5 | 452.9159 | 3 | 55.07 | 2 | 15613 | Ana\_01.raw |  | 1.86E6 |  |  |  |  |  |  |  |  | 1 | 0 | 1 | 0 | 0 | 0 | 0 | 0 | 0 | 0 | 0 | 1053 | 1064 |  |
| R.LQVVDRPLPVR.G | Y | 25.94 | 1290.7771 | 11 | 5.1 | 431.2685 | 3 | 53.95 | 9 | 15261 | Ana\_08.raw |  |  |  |  |  |  |  |  | 3.02E6 |  | 1 | 0 | 0 | 0 | 0 | 0 | 0 | 0 | 0 | 1 | 0 | 374 | 384 |  |
| R.SFDDSGSGYC(+57.02)R.S | Y | 22.53 | 1249.4669 | 11 | 2.5 | 625.7423 | 2 | 39.11 | 4 | 10567 | Ana\_03.raw |  |  |  | 7.21E5 |  |  |  |  |  |  | 1 | 0 | 0 | 0 | 1 | 0 | 0 | 0 | 0 | 0 | 0 | 214 | 224 | Carbamidomethylation |
| total 22 peptides |  |  |  |  |  |  |  |  |  |  |  |  |  |  |  |  |  |  |  |  |  |  |  |  |  |  |  |  |  |  |  |  |  |  |  |
| --- | --- | --- | --- | --- | --- | --- | --- | --- | --- | --- | --- | --- | --- | --- | --- | --- | --- | --- | --- | --- | --- | --- | --- | --- | --- | --- | --- | --- | --- | --- | --- | --- | --- | --- | --- |

Best Unique PSM (Scan F6:35311, m/z=833.7896, z=3, RT=127.03, ppm=1.5):

sp|Q62167|DDX3X\_MOUSE

back to list

  

| Protein Coverage
| Supporting Peptides
| Best Unique PSM
|

Protein Coverage:

Supporting Peptides:

| Peptide | Uniq | -10lgP | Mass | Length | ppm | m/z | z | RT | Fraction | Scan | Source File | Area 10 | Area 01 | Area 02 | Area 03 | Area 04 | Area 05 | Area 06 | Area 07 | Area 08 | Area 09 | #Spec | #Spec 10 | #Spec 01 | #Spec 02 | #Spec 03 | #Spec 04 | #Spec 05 | #Spec 06 | #Spec 07 | #Spec 08 | #Spec 09 | Start | End | PTM |
| --- | --- | --- | --- | --- | --- | --- | --- | --- | --- | --- | --- | --- | --- | --- | --- | --- | --- | --- | --- | --- | --- | --- | --- | --- | --- | --- | --- | --- | --- | --- | --- | --- | --- | --- | --- |
| K.HVINFDLPSDIEEYVHR.I | Y | 118.82 | 2082.0171 | 17 | 4.4 | 521.5139 | 4 | 116.86 | 4 | 31880 | Ana\_03.raw | 3.22E6 | 2.14E6 | 7.24E6 | 1.45E6 | 3.25E6 | 8.94E6 | 4.28E6 | 4.56E6 | 2.52E7 | 5.82E6 | 17 | 1 | 2 | 2 | 2 | 2 | 1 | 2 | 1 | 2 | 2 | 512 | 528 |  |
| R.VGNLGLATSFFNER.N | Y | 92.66 | 1523.7732 | 14 | 2.9 | 762.8961 | 2 | 110.40 | 6 | 30981 | Ana\_05.raw | 1.07E7 | 9.62E6 | 9.33E6 | 5.29E6 | 5.81E6 | 8.54E6 | 5.39E6 | 5.34E6 | 2.53E7 | 1.05E7 | 13 | 1 | 1 | 1 | 1 | 1 | 1 | 1 | 1 | 3 | 2 | 535 | 548 |  |
| R.SFLLDLLNATGK.D | Y | 90.50 | 1290.7183 | 12 | 0.9 | 646.3670 | 2 | 130.59 | 3 | 35770 | Ana\_02.raw | 6.76E6 | 7.73E6 | 9.14E6 | 5.45E6 | 7.1E6 | 8.02E6 | 3.05E6 |  | 1.03E7 | 8.97E6 | 9 | 1 | 1 | 1 | 1 | 1 | 1 | 1 | 0 | 1 | 1 | 429 | 440 |  |
| K.TAAFLLPILSQIYADGPGEALR.A | Y | 83.67 | 2315.2524 | 22 | 4.5 | 772.7615 | 3 | 136.24 | 9 | 37656 | Ana\_08.raw | 2.28E5 |  |  |  |  | 4.92E5 |  |  | 1.2E6 |  | 3 | 1 | 0 | 0 | 0 | 0 | 1 | 0 | 0 | 1 | 0 | 231 | 252 |  |
| R.LEQELFSGGNTGINFEK.Y | Y | 76.82 | 1881.9108 | 17 | 3.7 | 941.9661 | 2 | 93.58 | 9 | 26518 | Ana\_08.raw |  |  |  |  |  |  |  |  | 6.93E5 |  | 1 | 0 | 0 | 0 | 0 | 0 | 0 | 0 | 0 | 1 | 0 | 146 | 162 |  |
| K.DLLDLLVEAK.Q | Y | 69.76 | 1127.6438 | 10 | 2.0 | 564.8303 | 2 | 131.05 | 7 | 36072 | Ana\_06.raw | 1.18E6 | 4.83E5 |  | 2.33E5 | 8.76E5 | 1.52E6 | 7.15E5 |  | 2.83E6 | 1.62E6 | 8 | 1 | 1 | 0 | 1 | 1 | 1 | 1 | 0 | 1 | 1 | 555 | 564 |  |
| R.DREEALHQFR.S | Y | 69.33 | 1299.6320 | 10 | 2.8 | 650.8251 | 2 | 32.73 | 9 | 8699 | Ana\_08.raw | 7.62E6 |  |  |  |  |  |  |  | 9.67E5 |  | 3 | 2 | 0 | 0 | 0 | 0 | 0 | 0 | 0 | 1 | 0 | 479 | 488 |  |
| R.VGSTSENITQK.V | Y | 64.84 | 1162.5830 | 11 | 0.5 | 582.2991 | 2 | 24.23 | 4 | 6165 | Ana\_03.raw | 8.02E6 | 7.19E6 | 1.01E7 | 4.44E6 | 1.73E6 | 1.45E6 | 3.18E6 | 2.46E6 | 7.52E6 | 8.35E6 | 10 | 1 | 1 | 1 | 1 | 1 | 1 | 1 | 1 | 1 | 1 | 408 | 418 |  |
| K.SPILVATAVAAR.G | Y | 63.78 | 1167.6975 | 12 | 3.6 | 584.8582 | 2 | 72.31 | 6 | 20751 | Ana\_05.raw | 4.95E6 | 6.3E6 | 3.95E6 | 2.35E6 | 4.13E6 | 4.63E6 | 2.84E6 | 2.65E6 | 1.01E7 | 5.24E6 | 10 | 1 | 1 | 1 | 1 | 1 | 1 | 1 | 1 | 1 | 1 | 492 | 503 |  |
| R.M(+15.99)LDM(+15.99)GFEPQIR.R | N | 53.09 | 1367.6213 | 11 | 2.3 | 684.8195 | 2 | 67.10 | 3 | 19082 | Ana\_02.raw |  | 4.78E6 | 8.23E6 | 3.92E6 | 9.64E6 | 5.84E6 | 6.13E6 | 6.13E6 | 1.59E7 | 8.18E6 | 9 | 0 | 1 | 1 | 1 | 1 | 1 | 1 | 1 | 1 | 1 | 352 | 362 | Oxidation (M) |
| R.YTRPTPVQK.H | Y | 46.84 | 1088.5978 | 9 | 1.7 | 363.8738 | 3 | 20.85 | 3 | 5220 | Ana\_02.raw | 7.8E6 | 1.06E7 | 8.44E6 | 4.02E6 |  |  |  |  | 7.95E6 | 4.47E6 | 10 | 2 | 2 | 2 | 2 | 0 | 0 | 0 | 0 | 1 | 1 | 200 | 208 |  |
| K.YLVLDEADR.M | Y | 41.99 | 1092.5450 | 9 | -1.6 | 547.2789 | 2 | 61.85 | 3 | 17624 | Ana\_02.raw |  |  | 3.12E6 |  |  |  |  |  |  |  | 1 | 0 | 0 | 1 | 0 | 0 | 0 | 0 | 0 | 0 | 0 | 343 | 351 |  |
| K.DKDAYSSFGSR.G | Y | 40.48 | 1231.5469 | 11 | 4.2 | 616.7833 | 2 | 33.51 | 9 | 8943 | Ana\_08.raw |  | 1.79E6 |  |  |  |  |  |  | 2.75E6 | 1.51E6 | 3 | 0 | 1 | 0 | 0 | 0 | 0 | 0 | 0 | 1 | 1 | 65 | 75 |  |
| R.IVEQDTM(+15.99)PPK.G | Y | 33.54 | 1172.5747 | 10 | 7.1 | 587.2988 | 2 | 28.55 | 7 | 7521 | Ana\_06.raw |  |  |  |  |  |  | 6.56E5 |  |  |  | 1 | 0 | 0 | 0 | 0 | 0 | 0 | 1 | 0 | 0 | 0 | 364 | 373 | Oxidation (M) |
| K.SSFFGDR.G | Y | 32.15 | 814.3610 | 7 | 5.1 | 408.1898 | 2 | 46.45 | 10 | 13004 | Ana\_09.raw |  |  |  |  |  |  |  |  | 1.09E6 | 1.91E6 | 2 | 0 | 0 | 0 | 0 | 0 | 0 | 0 | 0 | 1 | 1 | 82 | 88 |  |
| R.FSGGFGAR.D | Y | 28.64 | 797.3820 | 8 | 1.0 | 399.6987 | 2 | 39.51 | 4 | 10692 | Ana\_03.raw |  |  |  | 3.91E6 |  |  | 6.2E6 |  |  |  | 2 | 0 | 0 | 0 | 1 | 0 | 0 | 1 | 0 | 0 | 0 | 593 | 600 |  |
| K.VVWVEEIDKR.S | Y | 27.54 | 1271.6874 | 10 | 4.3 | 424.9049 | 3 | 65.96 | 9 | 18815 | Ana\_08.raw |  |  |  |  |  |  |  |  | 2.78E5 |  | 1 | 0 | 0 | 0 | 0 | 0 | 0 | 0 | 0 | 1 | 0 | 419 | 428 |  |
| total 17 peptides |  |  |  |  |  |  |  |  |  |  |  |  |  |  |  |  |  |  |  |  |  |  |  |  |  |  |  |  |  |  |  |  |  |  |  |
| --- | --- | --- | --- | --- | --- | --- | --- | --- | --- | --- | --- | --- | --- | --- | --- | --- | --- | --- | --- | --- | --- | --- | --- | --- | --- | --- | --- | --- | --- | --- | --- | --- | --- | --- | --- |

Best Unique PSM (Scan F4:31880, m/z=521.5139, z=4, RT=116.86, ppm=4.4):

sp|Q8BZF8|PGM5\_MOUSE

back to list

  

| Protein Coverage
| Supporting Peptides
| Best Unique PSM
|

Protein Coverage:

Supporting Peptides:

| Peptide | Uniq | -10lgP | Mass | Length | ppm | m/z | z | RT | Fraction | Scan | Source File | Area 10 | Area 01 | Area 02 | Area 03 | Area 04 | Area 05 | Area 06 | Area 07 | Area 08 | Area 09 | #Spec | #Spec 10 | #Spec 01 | #Spec 02 | #Spec 03 | #Spec 04 | #Spec 05 | #Spec 06 | #Spec 07 | #Spec 08 | #Spec 09 | Start | End | PTM |
| --- | --- | --- | --- | --- | --- | --- | --- | --- | --- | --- | --- | --- | --- | --- | --- | --- | --- | --- | --- | --- | --- | --- | --- | --- | --- | --- | --- | --- | --- | --- | --- | --- | --- | --- | --- |
| R.DPSGHDQEPQAVLSPLIAIALK.I | Y | 110.56 | 2298.2219 | 22 | 1.7 | 767.0826 | 3 | 130.99 | 6 | 36395 | Ana\_05.raw | 4.85E6 | 2.93E6 |  | 1.44E6 |  | 5.21E6 | 5.52E6 | 2.83E6 |  |  | 6 | 1 | 1 | 0 | 1 | 0 | 1 | 1 | 1 | 0 | 0 | 529 | 550 |  |
| K.TDSFEYVDPVDGTVTK.K | Y | 89.12 | 1771.8152 | 16 | 4.1 | 886.9185 | 2 | 81.82 | 3 | 23096 | Ana\_02.raw | 4.77E6 | 7.13E6 | 5.41E6 | 2.48E6 | 4.6E6 | 7.12E6 | 1.11E6 |  | 7.14E6 | 1.17E6 | 9 | 1 | 1 | 1 | 1 | 1 | 1 | 1 | 0 | 1 | 1 | 476 | 491 |  |
| K.TIEEYAIC(+57.02)PDLR.I | Y | 84.93 | 1478.7075 | 12 | 3.0 | 740.3632 | 2 | 84.32 | 6 | 24042 | Ana\_05.raw | 3.65E6 |  | 4.08E6 | 1.69E5 | 1.88E6 | 4.24E6 | 9.28E5 | 6.6E5 | 5.48E6 | 3.62E6 | 9 | 1 | 0 | 1 | 1 | 1 | 1 | 1 | 1 | 1 | 1 | 158 | 169 | Carbamidomethylation |
| R.NYLPNFIQSVLSSIDLR.D | Y | 79.20 | 1978.0524 | 17 | 1.3 | 990.0348 | 2 | 140.11 | 6 | 38852 | Ana\_05.raw |  |  |  |  |  | 4.38E5 |  |  |  |  | 1 | 0 | 0 | 0 | 0 | 0 | 1 | 0 | 0 | 0 | 0 | 39 | 55 |  |
| R.VEIVDPVDIYLNLLR.N | Y | 75.33 | 1769.9927 | 15 | 3.7 | 886.0069 | 2 | 137.20 | 7 | 37763 | Ana\_06.raw |  |  |  |  |  | 4.41E5 | 1.2E5 |  | 1.44E6 |  | 3 | 0 | 0 | 0 | 0 | 0 | 1 | 1 | 0 | 1 | 0 | 191 | 205 |  |
| R.RPTGLFEGQR.N | Y | 74.00 | 1159.6097 | 10 | 6.3 | 580.8158 | 2 | 36.05 | 1 | 9798 | ANa\_10.raw | 1.03E7 | 1.41E7 |  | 1.62E6 | 9.23E6 | 1.08E7 | 6.61E6 |  | 1.59E7 | 7.54E6 | 8 | 1 | 1 | 0 | 1 | 1 | 1 | 1 | 0 | 1 | 1 | 29 | 38 |  |
| K.GGEYGFGAAFDADGDR.Y | Y | 73.48 | 1603.6538 | 16 | 2.0 | 802.8358 | 2 | 84.36 | 6 | 24054 | Ana\_05.raw |  | 8.98E5 |  |  | 2.71E6 | 1.38E6 |  |  |  |  | 3 | 0 | 1 | 0 | 0 | 1 | 1 | 0 | 0 | 0 | 0 | 283 | 298 |  |
| R.FDYEGLEPK.A | Y | 56.03 | 1096.5077 | 9 | -0.4 | 549.2609 | 2 | 70.87 | 3 | 20136 | Ana\_02.raw | 1.03E7 | 1.04E6 | 9.77E6 | 4.52E6 | 9.21E6 | 1.05E7 | 5.38E6 | 5.67E6 | 4.58E6 | 5.31E6 | 17 | 1 | 1 | 2 | 2 | 2 | 2 | 2 | 2 | 2 | 1 | 433 | 441 |  |
| R.LYAESYER.D | Y | 55.74 | 1029.4767 | 8 | 5.9 | 515.7487 | 2 | 39.85 | 2 | 11014 | Ana\_01.raw |  | 1.72E7 |  | 7.93E6 |  |  | 1.16E7 | 1.02E7 |  |  | 4 | 0 | 1 | 0 | 1 | 0 | 0 | 1 | 1 | 0 | 0 | 521 | 528 |  |
| R.NIFDFNAIK.S | N | 53.89 | 1080.5603 | 9 | 2.7 | 541.2889 | 2 | 101.32 | 6 | 28604 | Ana\_05.raw | 8.19E6 | 1.24E7 | 1.49E7 |  | 7.16E6 | 9.52E6 |  | 4.93E6 | 1.63E7 | 8.14E6 | 9 | 1 | 1 | 1 | 1 | 1 | 1 | 0 | 1 | 1 | 1 | 206 | 214 |  |
| R.IIFSDASR.L | Y | 53.12 | 907.4763 | 8 | -0.1 | 454.7454 | 2 | 45.30 | 3 | 12754 | Ana\_02.raw | 1.35E7 | 1.8E7 | 2.37E7 | 8.88E6 | 1.72E7 | 2.14E7 | 1.37E7 | 1.3E7 | 5.75E6 | 1.15E7 | 10 | 1 | 1 | 1 | 1 | 1 | 1 | 1 | 1 | 1 | 1 | 497 | 504 |  |
| R.DLEALVTDK.S | Y | 53.07 | 1002.5233 | 9 | 5.6 | 502.2718 | 2 | 76.18 | 1 | 21546 | ANa\_10.raw | 6.3E6 | 1.02E4 | 1.12E7 | 5.08E6 | 9.17E6 | 1.27E7 | 7.64E6 | 7.46E6 | 1.12E7 | 5.83E6 | 10 | 1 | 1 | 1 | 1 | 1 | 1 | 1 | 1 | 1 | 1 | 449 | 457 |  |
| K.SFIGQQFAVGSHIYSIAK.T | Y | 51.53 | 1952.0155 | 18 | 3.0 | 651.6810 | 3 | 105.59 | 6 | 29736 | Ana\_05.raw |  | 1.11E6 |  |  |  | 1.59E6 |  |  |  |  | 2 | 0 | 1 | 0 | 0 | 0 | 1 | 0 | 0 | 0 | 0 | 458 | 475 |  |
| K.FNVANGGPAPDVVSDK.I | Y | 47.43 | 1585.7736 | 16 | -9.9 | 793.8862 | 2 | 70.46 | 8 | 19779 | Ana\_07.raw |  |  |  |  |  |  |  | 8.36E5 |  |  | 1 | 0 | 0 | 0 | 0 | 0 | 0 | 0 | 1 | 0 | 0 | 136 | 151 |  |
| K.ISQIHER.T | Y | 45.74 | 881.4719 | 7 | 3.2 | 441.7446 | 2 | 19.12 | 8 | 4560 | Ana\_07.raw |  |  | 1E7 |  | 9.06E6 | 1.61E7 | 6.21E6 | 1.36E6 |  |  | 5 | 0 | 0 | 1 | 0 | 1 | 1 | 1 | 1 | 0 | 0 | 551 | 557 |  |
| R.SM(+15.99)PTSTALDR.V | Y | 45.61 | 1093.5073 | 10 | 3.7 | 547.7629 | 2 | 28.79 | 8 | 7493 | Ana\_07.raw | 9.01E6 | 2.54E7 | 2.58E7 | 9.03E6 | 2.06E7 | 1.43E7 | 1.25E7 | 1.44E7 | 6.78E6 | 4.95E6 | 11 | 1 | 1 | 1 | 1 | 1 | 1 | 2 | 1 | 1 | 1 | 339 | 348 | Oxidation (M) |
| R.KQSVEEIVR.D | Y | 43.06 | 1086.6033 | 9 | 2.1 | 544.3101 | 2 | 31.81 | 6 | 8688 | Ana\_05.raw |  | 5.6E6 | 4.81E6 |  | 3.78E6 | 4.46E6 |  |  |  | 2.73E6 | 5 | 0 | 1 | 1 | 0 | 1 | 1 | 0 | 0 | 0 | 1 | 411 | 419 |  |
| K.VPVYETPAGWR.F | Y | 40.31 | 1273.6455 | 11 | 2.2 | 637.8314 | 2 | 76.36 | 8 | 21437 | Ana\_07.raw |  |  |  |  |  |  |  | 4.06E6 |  |  | 1 | 0 | 0 | 0 | 0 | 0 | 0 | 0 | 1 | 0 | 0 | 355 | 365 |  |
| K.SLLTGPSQLK.I | Y | 28.17 | 1042.6023 | 10 | -4.9 | 522.3058 | 2 | 75.48 | 4 | 20997 | Ana\_03.raw |  |  |  | 2.49E4 |  |  |  |  |  |  | 1 | 0 | 0 | 0 | 1 | 0 | 0 | 0 | 0 | 0 | 0 | 215 | 224 |  |
| K.FKPFR.V | Y | 23.74 | 693.3962 | 5 | -0.7 | 347.7051 | 2 | 28.48 | 3 | 7664 | Ana\_02.raw |  | 4.13E6 | 3.57E6 |  |  |  |  |  |  |  | 2 | 0 | 1 | 1 | 0 | 0 | 0 | 0 | 0 | 0 | 0 | 186 | 190 |  |
| K.IYQISK.T | N | 22.72 | 750.4276 | 6 | 3.8 | 376.2225 | 2 | 33.82 | 7 | 9143 | Ana\_06.raw |  |  |  |  |  |  | 4.54E6 |  |  |  | 1 | 0 | 0 | 0 | 0 | 0 | 0 | 1 | 0 | 0 | 0 | 152 | 157 |  |
| total 21 peptides |  |  |  |  |  |  |  |  |  |  |  |  |  |  |  |  |  |  |  |  |  |  |  |  |  |  |  |  |  |  |  |  |  |  |  |
| --- | --- | --- | --- | --- | --- | --- | --- | --- | --- | --- | --- | --- | --- | --- | --- | --- | --- | --- | --- | --- | --- | --- | --- | --- | --- | --- | --- | --- | --- | --- | --- | --- | --- | --- | --- |

Best Unique PSM (Scan F6:36395, m/z=767.0826, z=3, RT=130.99, ppm=1.7):

sp|P18760|COF1\_MOUSE

back to list

  

| Protein Coverage
| Supporting Peptides
| Best Unique PSM
|

Protein Coverage:

Supporting Peptides:

| Peptide | Uniq | -10lgP | Mass | Length | ppm | m/z | z | RT | Fraction | Scan | Source File | Area 10 | Area 01 | Area 02 | Area 03 | Area 04 | Area 05 | Area 06 | Area 07 | Area 08 | Area 09 | #Spec | #Spec 10 | #Spec 01 | #Spec 02 | #Spec 03 | #Spec 04 | #Spec 05 | #Spec 06 | #Spec 07 | #Spec 08 | #Spec 09 | Start | End | PTM |
| --- | --- | --- | --- | --- | --- | --- | --- | --- | --- | --- | --- | --- | --- | --- | --- | --- | --- | --- | --- | --- | --- | --- | --- | --- | --- | --- | --- | --- | --- | --- | --- | --- | --- | --- | --- |
| K.NIILEEGKEILVGDVGQTVDDPYTTFVK.M | Y | 116.24 | 3091.5964 | 28 | -0.1 | 1031.5393 | 3 | 129.25 | 3 | 35411 | Ana\_02.raw | 2.31E7 | 1.77E7 | 1.25E7 | 7.33E6 | 1.17E7 | 2.12E7 | 1.29E7 | 9.63E6 | 3.71E7 | 1.69E7 | 12 | 1 | 1 | 1 | 1 | 1 | 1 | 2 | 1 | 2 | 1 | 46 | 73 |  |
| K.EILVGDVGQTVDDPYTTFVK.M | Y | 110.31 | 2195.0996 | 20 | 1.8 | 1098.5591 | 2 | 111.68 | 3 | 30894 | Ana\_02.raw | 3.29E7 | 5.97E7 | 4.02E7 | 2.76E7 | 2.61E7 | 4.08E7 | 9.75E6 | 2.17E7 | 1.17E8 | 5.73E7 | 28 | 3 | 5 | 2 | 3 | 3 | 2 | 1 | 3 | 3 | 3 | 54 | 73 |  |
| K.HELQANC(+57.02)YEEVKDR.C | Y | 100.21 | 1789.8053 | 14 | 4.2 | 597.6116 | 3 | 39.59 | 6 | 11026 | Ana\_05.raw | 2.83E7 | 2.3E7 | 1.78E7 | 8.19E6 | 1.52E7 | 1.7E7 | 1.11E7 | 1.13E7 | 3.1E7 | 2.81E7 | 14 | 2 | 2 | 1 | 1 | 1 | 1 | 1 | 1 | 3 | 1 | 133 | 146 | Carbamidomethylation |
| K.KEDLVFIFWAPENAPLK.S | Y | 98.43 | 2016.0720 | 17 | 4.0 | 673.0340 | 3 | 129.43 | 6 | 35955 | Ana\_05.raw |  |  |  | 2.45E6 |  | 2.84E6 | 2.82E6 |  |  |  | 3 | 0 | 0 | 0 | 1 | 0 | 1 | 1 | 0 | 0 | 0 | 96 | 112 |  |
| K.HELQANC(+57.02)YEEVK.D | Y | 83.34 | 1518.6772 | 12 | 3.9 | 760.3489 | 2 | 36.92 | 9 | 10012 | Ana\_08.raw | 5.06E6 | 5.15E5 | 4.32E6 | 7.23E5 | 5.66E6 | 2.82E6 | 1.25E6 | 4.7E6 | 9.52E6 | 5.22E6 | 13 | 1 | 1 | 1 | 1 | 2 | 1 | 1 | 2 | 2 | 1 | 133 | 144 | Carbamidomethylation |
| R.YALYDATYETK.E | N | 83.02 | 1336.6187 | 11 | 4.5 | 669.3196 | 2 | 67.48 | 6 | 19384 | Ana\_05.raw | 1.01E8 | 8.25E7 | 6.75E7 | 3.81E7 | 5.44E7 | 6.22E7 | 4.74E7 | 5.21E7 | 1.48E8 | 1.01E8 | 32 | 5 | 4 | 2 | 2 | 2 | 2 | 2 | 4 | 4 | 5 | 82 | 92 |  |
| K.AVLFC(+57.02)LSEDKK.N | Y | 58.22 | 1308.6747 | 11 | 9.9 | 655.3511 | 2 | 59.79 | 9 | 16993 | Ana\_08.raw | 7.69E6 | 6.26E6 | 2.29E6 | 2.67E6 |  |  | 1.08E6 |  | 2.41E7 | 1.19E7 | 12 | 2 | 2 | 1 | 2 | 0 | 0 | 1 | 0 | 2 | 2 | 35 | 45 | Carbamidomethylation |
| K.M(+15.99)LPDKDC(+57.02)R.Y | Y | 53.88 | 1049.4634 | 8 | 2.8 | 525.7404 | 2 | 19.42 | 6 | 4742 | Ana\_05.raw |  |  | 2.02E7 | 1.15E7 | 2.26E7 | 1.31E7 | 9.91E6 | 1.4E7 |  | 6.26E6 | 13 | 0 | 0 | 2 | 2 | 2 | 2 | 2 | 2 | 0 | 1 | 74 | 81 | Oxidation (M); Carbamidomethylation |
| K.LGGSAVISLEGKPL | Y | 53.73 | 1339.7711 | 14 | 3.0 | 670.8948 | 2 | 90.62 | 6 | 25752 | Ana\_05.raw | 3.5E4 | 8.75E6 | 8.46E6 | 2.69E6 | 4.02E6 | 6.23E6 | 1.7E6 |  | 3.37E7 | 1.6E7 | 11 | 1 | 1 | 1 | 1 | 1 | 1 | 1 | 0 | 2 | 2 | 153 | 166 |  |
| K.NIILEEGK.E | Y | 49.19 | 914.5073 | 8 | 4.0 | 458.2627 | 2 | 45.58 | 5 | 12798 | Ana\_04.raw | 3.92E7 | 3.64E7 | 4.72E7 | 2.02E7 | 4.18E7 | 3.67E7 | 2.84E7 | 3.01E7 | 5.81E7 | 4.07E7 | 13 | 1 | 1 | 2 | 1 | 1 | 1 | 1 | 2 | 1 | 2 | 46 | 53 |  |
| K.MLPDKDC(+57.02)R.Y | Y | 44.57 | 1033.4685 | 8 | 5.5 | 517.7444 | 2 | 22.08 | 9 | 5315 | Ana\_08.raw |  |  |  |  |  |  |  |  | 5.03E6 |  | 1 | 0 | 0 | 0 | 0 | 0 | 0 | 0 | 0 | 1 | 0 | 74 | 81 | Carbamidomethylation |
| K.MIYASSK.D | N | 36.27 | 798.3945 | 7 | 8.1 | 400.2078 | 2 | 23.99 | 10 | 6161 | Ana\_09.raw |  |  |  | 5.13E6 | 7.27E6 |  |  |  |  | 5.64E6 | 3 | 0 | 0 | 0 | 1 | 1 | 0 | 0 | 0 | 0 | 1 | 115 | 121 |  |
| total 12 peptides |  |  |  |  |  |  |  |  |  |  |  |  |  |  |  |  |  |  |  |  |  |  |  |  |  |  |  |  |  |  |  |  |  |  |  |
| --- | --- | --- | --- | --- | --- | --- | --- | --- | --- | --- | --- | --- | --- | --- | --- | --- | --- | --- | --- | --- | --- | --- | --- | --- | --- | --- | --- | --- | --- | --- | --- | --- | --- | --- | --- |

Best Unique PSM (Scan F3:35411, m/z=1031.5393, z=3, RT=129.25, ppm=-0.1):

Z4YJV4|Z4YJV4\_MOUSE

back to list

  

| Protein Coverage
| Supporting Peptides
| Best Unique PSM
|

Protein Coverage:

Supporting Peptides:

| Peptide | Uniq | -10lgP | Mass | Length | ppm | m/z | z | RT | Fraction | Scan | Source File | Area 10 | Area 01 | Area 02 | Area 03 | Area 04 | Area 05 | Area 06 | Area 07 | Area 08 | Area 09 | #Spec | #Spec 10 | #Spec 01 | #Spec 02 | #Spec 03 | #Spec 04 | #Spec 05 | #Spec 06 | #Spec 07 | #Spec 08 | #Spec 09 | Start | End | PTM |
| --- | --- | --- | --- | --- | --- | --- | --- | --- | --- | --- | --- | --- | --- | --- | --- | --- | --- | --- | --- | --- | --- | --- | --- | --- | --- | --- | --- | --- | --- | --- | --- | --- | --- | --- | --- |
| K.HWLDSPWPGFFTLDGQPR.S | Y | 104.97 | 2155.0276 | 18 | 5.4 | 719.3537 | 3 | 130.59 | 6 | 36286 | Ana\_05.raw |  |  |  |  |  | 3.13E6 |  |  |  |  | 1 | 0 | 0 | 0 | 0 | 0 | 1 | 0 | 0 | 0 | 0 | 579 | 596 |  |
| R.HHVLHDQNVDKR.T | Y | 98.53 | 1496.7596 | 12 | 1.5 | 499.9279 | 3 | 16.55 | 3 | 3791 | Ana\_02.raw | 1.31E6 | 2.58E6 | 1.77E6 | 4.86E5 | 1.35E6 | 1.75E6 |  | 7.52E5 | 2.01E6 |  | 9 | 1 | 1 | 2 | 1 | 1 | 1 | 0 | 1 | 1 | 0 | 683 | 694 |  |
| K.VFHLPTTTFIGGQEPALPLR.E | Y | 88.69 | 2193.1946 | 20 | 6.6 | 732.0770 | 3 | 117.90 | 1 | 32558 | ANa\_10.raw | 1.46E7 | 2.13E7 | 1.74E7 | 3.26E6 | 6.53E6 | 1.45E7 | 9.83E6 | 8.12E6 | 7.74E5 | 1.16E7 | 16 | 1 | 3 | 3 | 1 | 1 | 1 | 1 | 2 | 2 | 1 | 181 | 200 |  |
| R.HHVLHDQNVDK.R | Y | 77.95 | 1340.6586 | 11 | 4.0 | 671.3392 | 2 | 18.26 | 2 | 4176 | Ana\_01.raw |  | 2.47E5 |  |  |  |  |  |  |  |  | 1 | 0 | 1 | 0 | 0 | 0 | 0 | 0 | 0 | 0 | 0 | 683 | 693 |  |
| R.FGLEGC(+57.02)EVLIPALK.T | Y | 77.20 | 1544.8271 | 14 | 4.1 | 773.4240 | 2 | 119.82 | 6 | 33444 | Ana\_05.raw | 5.15E6 | 5.34E6 | 8.39E6 | 1.75E6 | 2.72E6 | 7.67E6 | 5.32E5 | 1.16E6 | 1.29E7 | 7.82E6 | 19 | 2 | 2 | 2 | 2 | 2 | 2 | 1 | 2 | 2 | 2 | 274 | 287 | Carbamidomethylation |
| R.FLDTAFDLDAFK.K | Y | 75.70 | 1401.6815 | 12 | 2.0 | 701.8494 | 2 | 123.13 | 6 | 34297 | Ana\_05.raw |  | 8.28E5 | 4.03E6 | 1.71E6 | 1.05E6 | 3.89E6 | 6.36E5 | 7.85E5 | 1.25E6 |  | 8 | 0 | 1 | 1 | 1 | 1 | 1 | 1 | 1 | 1 | 0 | 1005 | 1016 |  |
| R.IEQLSPFPFDLLLK.E | Y | 64.79 | 1658.9282 | 14 | -1.5 | 830.4702 | 2 | 134.96 | 6 | 37521 | Ana\_05.raw | 6.59E5 |  |  | 3.85E5 |  | 1.6E6 | 2.24E5 |  | 2.57E6 | 1.46E6 | 8 | 1 | 0 | 0 | 1 | 0 | 2 | 2 | 0 | 1 | 1 | 926 | 939 |  |
| K.LEAADEGSGDM(+15.99)K.Y | Y | 63.45 | 1237.5132 | 12 | 0.3 | 619.7640 | 2 | 20.17 | 4 | 4943 | Ana\_03.raw |  |  |  | 2.24E6 | 4.08E6 | 1.81E6 | 2.93E6 | 6.71E5 |  |  | 5 | 0 | 0 | 0 | 1 | 1 | 1 | 1 | 1 | 0 | 0 | 333 | 344 | Oxidation (M) |
| R.LSGQDVER.G | Y | 59.04 | 902.4457 | 8 | 2.9 | 452.2315 | 2 | 24.34 | 7 | 6249 | Ana\_06.raw | 0 | 4.84E6 | 2.14E7 | 8E6 |  |  | 4.53E6 | 8.34E6 |  | 1.16E7 | 7 | 1 | 1 | 1 | 1 | 0 | 0 | 1 | 1 | 0 | 1 | 669 | 676 |  |
| R.FLDTAFDLDAFKK.F | Y | 58.59 | 1529.7765 | 13 | 1.5 | 510.9335 | 3 | 108.19 | 6 | 30411 | Ana\_05.raw |  |  |  |  | 6.33E5 | 5.45E5 | 4.66E5 |  |  |  | 3 | 0 | 0 | 0 | 0 | 1 | 1 | 1 | 0 | 0 | 0 | 1005 | 1017 |  |
| K.SWDIFFR.N | Y | 56.16 | 969.4708 | 7 | 2.9 | 485.7441 | 2 | 112.51 | 3 | 31112 | Ana\_02.raw |  | 3.92E6 | 1.54E6 | 2.56E6 | 1.62E6 | 3.26E6 | 1.31E6 | 1.43E6 |  |  | 7 | 0 | 1 | 1 | 1 | 1 | 1 | 1 | 1 | 0 | 0 | 75 | 81 |  |
| R.RELVTNR.T | Y | 50.46 | 886.4984 | 7 | 2.9 | 444.2578 | 2 | 18.85 | 6 | 4557 | Ana\_05.raw |  |  |  |  |  | 2.12E6 |  |  |  |  | 1 | 0 | 0 | 0 | 0 | 0 | 1 | 0 | 0 | 0 | 0 | 639 | 645 |  |
| R.FEEFLQR.K | Y | 48.16 | 967.4763 | 7 | 2.5 | 484.7466 | 2 | 66.25 | 7 | 18803 | Ana\_06.raw |  | 5.72E6 | 8.56E6 | 3.92E6 | 6.71E6 | 6.98E6 | 4.72E6 | 3.68E6 | 7.63E6 | 4.09E6 | 9 | 0 | 1 | 1 | 1 | 1 | 1 | 1 | 1 | 1 | 1 | 260 | 266 |  |
| R.NM(+15.99)EEEVAITR.I | Y | 44.39 | 1206.5551 | 10 | 5.1 | 604.2879 | 2 | 41.08 | 7 | 11318 | Ana\_06.raw |  |  | 8.99E6 | 4.41E6 |  |  | 6.76E6 | 2.66E6 |  |  | 4 | 0 | 0 | 1 | 1 | 0 | 0 | 1 | 1 | 0 | 0 | 916 | 925 | Oxidation (M) |
| K.THLTELQR.F | Y | 41.93 | 996.5352 | 8 | 4.7 | 499.2772 | 2 | 29.92 | 8 | 7844 | Ana\_07.raw |  |  |  | 1.17E6 |  |  | 1.94E6 | 1.61E6 |  |  | 3 | 0 | 0 | 0 | 1 | 0 | 0 | 1 | 1 | 0 | 0 | 997 | 1004 |  |
| K.LVEDHLAVQSLIR.A | Y | 39.18 | 1491.8409 | 13 | 3.3 | 498.2892 | 3 | 80.07 | 9 | 22801 | Ana\_08.raw |  |  |  |  |  |  |  |  | 7.17E5 |  | 1 | 0 | 0 | 0 | 0 | 0 | 0 | 0 | 0 | 1 | 0 | 123 | 135 |  |
| K.VYYDLTR.E | Y | 38.94 | 928.4654 | 7 | 2.7 | 465.2412 | 2 | 54.61 | 7 | 15389 | Ana\_06.raw |  |  |  | 2.28E6 |  |  | 2.26E6 | 2.23E6 | 7.71E6 |  | 4 | 0 | 0 | 0 | 1 | 0 | 0 | 1 | 1 | 1 | 0 | 904 | 910 |  |
| R.SSPYPTDVAR.V | Y | 37.04 | 1091.5247 | 10 | 5.2 | 546.7725 | 2 | 39.85 | 10 | 11011 | Ana\_09.raw |  | 2.56E7 | 2.47E7 |  | 2.66E7 |  | 1.77E7 |  | 1.93E6 | 1.71E7 | 6 | 0 | 1 | 1 | 0 | 1 | 0 | 1 | 0 | 1 | 1 | 453 | 462 |  |
| R.TSFDEM(+15.99)LPGTHFQR.V | Y | 34.05 | 1680.7566 | 14 | 3.4 | 561.2614 | 3 | 72.83 | 7 | 20649 | Ana\_06.raw |  |  |  | 2.39E6 | 2.94E5 | 3.83E6 | 2.86E6 |  |  |  | 4 | 0 | 0 | 0 | 1 | 1 | 1 | 1 | 0 | 0 | 0 | 866 | 879 | Oxidation (M) |
| K.IC(+57.02)EEAFTR.S | Y | 33.27 | 1024.4647 | 8 | 4.4 | 513.2419 | 2 | 43.88 | 2 | 12260 | Ana\_01.raw |  | 8.68E6 |  |  |  |  |  |  |  |  | 1 | 0 | 1 | 0 | 0 | 0 | 0 | 0 | 0 | 0 | 0 | 561 | 568 | Carbamidomethylation |
| total 20 peptides |  |  |  |  |  |  |  |  |  |  |  |  |  |  |  |  |  |  |  |  |  |  |  |  |  |  |  |  |  |  |  |  |  |  |  |
| --- | --- | --- | --- | --- | --- | --- | --- | --- | --- | --- | --- | --- | --- | --- | --- | --- | --- | --- | --- | --- | --- | --- | --- | --- | --- | --- | --- | --- | --- | --- | --- | --- | --- | --- | --- |

Best Unique PSM (Scan F6:36286, m/z=719.3537, z=3, RT=130.59, ppm=5.4):

sp|Q60597|ODO1\_MOUSE

back to list

  

| Protein Coverage
| Supporting Peptides
| Best Unique PSM
|

Protein Coverage:

Supporting Peptides:

| Peptide | Uniq | -10lgP | Mass | Length | ppm | m/z | z | RT | Fraction | Scan | Source File | Area 10 | Area 01 | Area 02 | Area 03 | Area 04 | Area 05 | Area 06 | Area 07 | Area 08 | Area 09 | #Spec | #Spec 10 | #Spec 01 | #Spec 02 | #Spec 03 | #Spec 04 | #Spec 05 | #Spec 06 | #Spec 07 | #Spec 08 | #Spec 09 | Start | End | PTM |
| --- | --- | --- | --- | --- | --- | --- | --- | --- | --- | --- | --- | --- | --- | --- | --- | --- | --- | --- | --- | --- | --- | --- | --- | --- | --- | --- | --- | --- | --- | --- | --- | --- | --- | --- | --- |
| K.HWLDSPWPGFFTLDGQPR.S | Y | 104.97 | 2155.0276 | 18 | 5.4 | 719.3537 | 3 | 130.59 | 6 | 36286 | Ana\_05.raw |  |  |  |  |  | 3.13E6 |  |  |  |  | 1 | 0 | 0 | 0 | 0 | 0 | 1 | 0 | 0 | 0 | 0 | 583 | 600 |  |
| R.HHVLHDQNVDKR.T | Y | 98.53 | 1496.7596 | 12 | 1.5 | 499.9279 | 3 | 16.55 | 3 | 3791 | Ana\_02.raw | 1.31E6 | 2.58E6 | 1.77E6 | 4.86E5 | 1.35E6 | 1.75E6 |  | 7.52E5 | 2.01E6 |  | 9 | 1 | 1 | 2 | 1 | 1 | 1 | 0 | 1 | 1 | 0 | 687 | 698 |  |
| K.VFHLPTTTFIGGQEPALPLR.E | Y | 88.69 | 2193.1946 | 20 | 6.6 | 732.0770 | 3 | 117.90 | 1 | 32558 | ANa\_10.raw | 1.46E7 | 2.13E7 | 1.74E7 | 3.26E6 | 6.53E6 | 1.45E7 | 9.83E6 | 8.12E6 | 7.74E5 | 1.16E7 | 16 | 1 | 3 | 3 | 1 | 1 | 1 | 1 | 2 | 2 | 1 | 185 | 204 |  |
| R.HHVLHDQNVDK.R | Y | 77.95 | 1340.6586 | 11 | 4.0 | 671.3392 | 2 | 18.26 | 2 | 4176 | Ana\_01.raw |  | 2.47E5 |  |  |  |  |  |  |  |  | 1 | 0 | 1 | 0 | 0 | 0 | 0 | 0 | 0 | 0 | 0 | 687 | 697 |  |
| R.FGLEGC(+57.02)EVLIPALK.T | Y | 77.20 | 1544.8271 | 14 | 4.1 | 773.4240 | 2 | 119.82 | 6 | 33444 | Ana\_05.raw | 5.15E6 | 5.34E6 | 8.39E6 | 1.75E6 | 2.72E6 | 7.67E6 | 5.32E5 | 1.16E6 | 1.29E7 | 7.82E6 | 19 | 2 | 2 | 2 | 2 | 2 | 2 | 1 | 2 | 2 | 2 | 278 | 291 | Carbamidomethylation |
| R.FLDTAFDLDAFK.K | Y | 75.70 | 1401.6815 | 12 | 2.0 | 701.8494 | 2 | 123.13 | 6 | 34297 | Ana\_05.raw |  | 8.28E5 | 4.03E6 | 1.71E6 | 1.05E6 | 3.89E6 | 6.36E5 | 7.85E5 | 1.25E6 |  | 8 | 0 | 1 | 1 | 1 | 1 | 1 | 1 | 1 | 1 | 0 | 1009 | 1020 |  |
| R.IEQLSPFPFDLLLK.E | Y | 64.79 | 1658.9282 | 14 | -1.5 | 830.4702 | 2 | 134.96 | 6 | 37521 | Ana\_05.raw | 6.59E5 |  |  | 3.85E5 |  | 1.6E6 | 2.24E5 |  | 2.57E6 | 1.46E6 | 8 | 1 | 0 | 0 | 1 | 0 | 2 | 2 | 0 | 1 | 1 | 930 | 943 |  |
| K.LEAADEGSGDM(+15.99)K.Y | Y | 63.45 | 1237.5132 | 12 | 0.3 | 619.7640 | 2 | 20.17 | 4 | 4943 | Ana\_03.raw |  |  |  | 2.24E6 | 4.08E6 | 1.81E6 | 2.93E6 | 6.71E5 |  |  | 5 | 0 | 0 | 0 | 1 | 1 | 1 | 1 | 1 | 0 | 0 | 337 | 348 | Oxidation (M) |
| R.LSGQDVER.G | Y | 59.04 | 902.4457 | 8 | 2.9 | 452.2315 | 2 | 24.34 | 7 | 6249 | Ana\_06.raw | 0 | 4.84E6 | 2.14E7 | 8E6 |  |  | 4.53E6 | 8.34E6 |  | 1.16E7 | 7 | 1 | 1 | 1 | 1 | 0 | 0 | 1 | 1 | 0 | 1 | 673 | 680 |  |
| R.FLDTAFDLDAFKK.F | Y | 58.59 | 1529.7765 | 13 | 1.5 | 510.9335 | 3 | 108.19 | 6 | 30411 | Ana\_05.raw |  |  |  |  | 6.33E5 | 5.45E5 | 4.66E5 |  |  |  | 3 | 0 | 0 | 0 | 0 | 1 | 1 | 1 | 0 | 0 | 0 | 1009 | 1021 |  |
| K.SWDIFFR.N | Y | 56.16 | 969.4708 | 7 | 2.9 | 485.7441 | 2 | 112.51 | 3 | 31112 | Ana\_02.raw |  | 3.92E6 | 1.54E6 | 2.56E6 | 1.62E6 | 3.26E6 | 1.31E6 | 1.43E6 |  |  | 7 | 0 | 1 | 1 | 1 | 1 | 1 | 1 | 1 | 0 | 0 | 75 | 81 |  |
| R.RELVTNR.T | Y | 50.46 | 886.4984 | 7 | 2.9 | 444.2578 | 2 | 18.85 | 6 | 4557 | Ana\_05.raw |  |  |  |  |  | 2.12E6 |  |  |  |  | 1 | 0 | 0 | 0 | 0 | 0 | 1 | 0 | 0 | 0 | 0 | 643 | 649 |  |
| R.FEEFLQR.K | Y | 48.16 | 967.4763 | 7 | 2.5 | 484.7466 | 2 | 66.25 | 7 | 18803 | Ana\_06.raw |  | 5.72E6 | 8.56E6 | 3.92E6 | 6.71E6 | 6.98E6 | 4.72E6 | 3.68E6 | 7.63E6 | 4.09E6 | 9 | 0 | 1 | 1 | 1 | 1 | 1 | 1 | 1 | 1 | 1 | 264 | 270 |  |
| R.NM(+15.99)EEEVAITR.I | Y | 44.39 | 1206.5551 | 10 | 5.1 | 604.2879 | 2 | 41.08 | 7 | 11318 | Ana\_06.raw |  |  | 8.99E6 | 4.41E6 |  |  | 6.76E6 | 2.66E6 |  |  | 4 | 0 | 0 | 1 | 1 | 0 | 0 | 1 | 1 | 0 | 0 | 920 | 929 | Oxidation (M) |
| K.THLTELQR.F | Y | 41.93 | 996.5352 | 8 | 4.7 | 499.2772 | 2 | 29.92 | 8 | 7844 | Ana\_07.raw |  |  |  | 1.17E6 |  |  | 1.94E6 | 1.61E6 |  |  | 3 | 0 | 0 | 0 | 1 | 0 | 0 | 1 | 1 | 0 | 0 | 1001 | 1008 |  |
| K.LVEDHLAVQSLIR.A | Y | 39.18 | 1491.8409 | 13 | 3.3 | 498.2892 | 3 | 80.07 | 9 | 22801 | Ana\_08.raw |  |  |  |  |  |  |  |  | 7.17E5 |  | 1 | 0 | 0 | 0 | 0 | 0 | 0 | 0 | 0 | 1 | 0 | 123 | 135 |  |
| K.VYYDLTR.E | Y | 38.94 | 928.4654 | 7 | 2.7 | 465.2412 | 2 | 54.61 | 7 | 15389 | Ana\_06.raw |  |  |  | 2.28E6 |  |  | 2.26E6 | 2.23E6 | 7.71E6 |  | 4 | 0 | 0 | 0 | 1 | 0 | 0 | 1 | 1 | 1 | 0 | 908 | 914 |  |
| R.SSPYPTDVAR.V | Y | 37.04 | 1091.5247 | 10 | 5.2 | 546.7725 | 2 | 39.85 | 10 | 11011 | Ana\_09.raw |  | 2.56E7 | 2.47E7 |  | 2.66E7 |  | 1.77E7 |  | 1.93E6 | 1.71E7 | 6 | 0 | 1 | 1 | 0 | 1 | 0 | 1 | 0 | 1 | 1 | 457 | 466 |  |
| R.TSFDEM(+15.99)LPGTHFQR.V | Y | 34.05 | 1680.7566 | 14 | 3.4 | 561.2614 | 3 | 72.83 | 7 | 20649 | Ana\_06.raw |  |  |  | 2.39E6 | 2.94E5 | 3.83E6 | 2.86E6 |  |  |  | 4 | 0 | 0 | 0 | 1 | 1 | 1 | 1 | 0 | 0 | 0 | 870 | 883 | Oxidation (M) |
| K.IC(+57.02)EEAFTR.S | Y | 33.27 | 1024.4647 | 8 | 4.4 | 513.2419 | 2 | 43.88 | 2 | 12260 | Ana\_01.raw |  | 8.68E6 |  |  |  |  |  |  |  |  | 1 | 0 | 1 | 0 | 0 | 0 | 0 | 0 | 0 | 0 | 0 | 565 | 572 | Carbamidomethylation |
| total 20 peptides |  |  |  |  |  |  |  |  |  |  |  |  |  |  |  |  |  |  |  |  |  |  |  |  |  |  |  |  |  |  |  |  |  |  |  |
| --- | --- | --- | --- | --- | --- | --- | --- | --- | --- | --- | --- | --- | --- | --- | --- | --- | --- | --- | --- | --- | --- | --- | --- | --- | --- | --- | --- | --- | --- | --- | --- | --- | --- | --- | --- |

Best Unique PSM (Scan F6:36286, m/z=719.3537, z=3, RT=130.59, ppm=5.4):

A0A140LHI8|A0A140LHI8\_MOUSE

back to list

  

| Protein Coverage
| Supporting Peptides
| Best Unique PSM
|

Protein Coverage:

Supporting Peptides:

| Peptide | Uniq | -10lgP | Mass | Length | ppm | m/z | z | RT | Fraction | Scan | Source File | Area 10 | Area 01 | Area 02 | Area 03 | Area 04 | Area 05 | Area 06 | Area 07 | Area 08 | Area 09 | #Spec | #Spec 10 | #Spec 01 | #Spec 02 | #Spec 03 | #Spec 04 | #Spec 05 | #Spec 06 | #Spec 07 | #Spec 08 | #Spec 09 | Start | End | PTM |
| --- | --- | --- | --- | --- | --- | --- | --- | --- | --- | --- | --- | --- | --- | --- | --- | --- | --- | --- | --- | --- | --- | --- | --- | --- | --- | --- | --- | --- | --- | --- | --- | --- | --- | --- | --- |
| R.KVAYNPFGPGQFFDLSIR.C | Y | 117.19 | 2055.0576 | 18 | 1.7 | 686.0276 | 3 | 125.61 | 6 | 34941 | Ana\_05.raw |  | 7.7E6 | 1.18E7 | 4.19E6 | 1.37E7 | 1.72E7 | 1.76E7 | 6.49E6 | 2.84E6 | 5.45E6 | 14 | 0 | 2 | 1 | 1 | 2 | 2 | 2 | 1 | 1 | 2 | 241 | 258 |  |
| K.VAYNPFGPGQFFDLSIR.C | Y | 113.64 | 1926.9628 | 17 | 1.0 | 964.4896 | 2 | 131.01 | 3 | 35887 | Ana\_02.raw |  | 8.8E7 | 5.22E7 | 2.05E7 | 3.47E7 | 7.9E7 | 4.64E7 | 2.46E7 | 9.41E7 | 5.35E7 | 14 | 0 | 2 | 2 | 2 | 1 | 2 | 2 | 1 | 1 | 1 | 242 | 258 |  |
| R.VPYVGALQGGLTVR.R | Y | 91.53 | 1428.8088 | 14 | 3.6 | 715.4142 | 2 | 92.12 | 6 | 26154 | Ana\_05.raw | 3.26E7 | 4.35E7 | 1.65E7 | 6.41E6 | 1.69E7 | 2.43E7 | 1.28E7 | 2.58E6 | 9.47E7 | 2.39E7 | 27 | 2 | 3 | 3 | 2 | 2 | 2 | 2 | 3 | 5 | 3 | 172 | 185 |  |
| K.VGSSGDIALHLNPR.I | Y | 83.46 | 1434.7579 | 14 | 7.9 | 718.3919 | 2 | 60.28 | 6 | 17248 | Ana\_05.raw | 5.3E7 | 7.92E7 | 9.84E7 | 4.51E7 | 8.32E7 | 6.58E7 | 5.46E7 | 4.59E7 | 1.25E8 | 5.28E7 | 35 | 4 | 6 | 3 | 2 | 5 | 2 | 2 | 4 | 5 | 2 | 207 | 220 |  |
| K.VFANGQHLFDFSHR.F | Y | 79.46 | 1673.8063 | 14 | 4.2 | 558.9451 | 3 | 82.46 | 7 | 23281 | Ana\_06.raw | 5.47E6 | 5.82E6 | 1.21E7 | 5.71E6 | 6.35E6 | 1.3E7 | 7.81E6 | 3.43E6 | 8.54E6 | 4.93E6 | 16 | 1 | 1 | 2 | 2 | 1 | 2 | 2 | 2 | 2 | 1 | 266 | 279 |  |
| K.GYVLPTAR.N | Y | 77.90 | 875.4865 | 8 | 5.1 | 438.7527 | 2 | 46.73 | 9 | 13055 | Ana\_08.raw | 6.89E7 | 8.55E7 | 1.08E8 | 5.26E7 | 8.93E7 | 7.68E7 | 7.12E7 | 6.57E7 | 1.22E8 | 7.24E7 | 34 | 4 | 3 | 4 | 4 | 3 | 2 | 4 | 3 | 4 | 3 | 192 | 199 |  |
| K.VVFNTM(+15.99)QSGQWGK.E | Y | 74.23 | 1496.7083 | 13 | 4.6 | 749.3648 | 2 | 64.20 | 6 | 18431 | Ana\_05.raw | 1.68E7 | 1.94E7 | 2.17E7 | 1.02E7 | 2.01E7 | 1.79E7 | 1.32E7 | 1.48E7 | 3.19E7 | 5.36E6 | 10 | 1 | 1 | 1 | 1 | 1 | 1 | 1 | 1 | 1 | 1 | 74 | 86 | Oxidation (M) |
| K.HFELVFM(+15.99)VM(+15.99)PEHYK.V | N | 66.60 | 1837.8531 | 14 | 0.9 | 613.6255 | 3 | 87.26 | 6 | 24849 | Ana\_05.raw |  | 4.64E6 |  |  |  | 2.36E6 |  | 3.27E6 | 5.31E6 |  | 4 | 0 | 1 | 0 | 0 | 0 | 1 | 0 | 1 | 1 | 0 | 100 | 113 | Oxidation (M) |
| R.NFVINFK.V | Y | 62.14 | 880.4807 | 7 | 1.6 | 441.2483 | 2 | 82.51 | 6 | 23554 | Ana\_05.raw | 4.46E7 | 6.46E7 | 7.51E7 | 4.35E7 | 5.55E7 | 7.11E7 | 4.49E7 | 5.02E7 | 1.08E8 | 5.45E7 | 41 | 5 | 4 | 6 | 5 | 5 | 2 | 3 | 3 | 3 | 5 | 200 | 206 |  |
| R.IGDSVVR.N | Y | 51.24 | 744.4130 | 7 | 4.1 | 373.2153 | 2 | 27.99 | 8 | 7250 | Ana\_07.raw | 1.13E8 | 1.71E8 | 1.68E8 | 9.17E7 | 1.28E8 | 1.64E8 | 1.28E8 | 7.85E7 | 9.31E7 | 1.22E8 | 15 | 1 | 1 | 1 | 1 | 1 | 3 | 2 | 2 | 1 | 2 | 221 | 227 |  |
| R.FDGWDK.V | N | 39.80 | 766.3286 | 6 | 1.2 | 384.1720 | 2 | 45.07 | 6 | 12714 | Ana\_05.raw | 1.3E7 | 2.21E7 | 1.84E7 | 5.62E6 | 1.81E7 | 2.2E7 | 1.55E7 | 1.41E7 | 2.87E7 | 1.03E7 | 16 | 1 | 2 | 2 | 2 | 1 | 2 | 1 | 2 | 2 | 1 | 68 | 73 |  |
| K.VVVNGNSFYEYGHR.L | Y | 35.33 | 1639.7743 | 14 | 3.6 | 547.6006 | 3 | 67.22 | 7 | 19075 | Ana\_06.raw |  |  |  |  |  |  | 3.84E6 |  |  |  | 1 | 0 | 0 | 0 | 0 | 0 | 0 | 1 | 0 | 0 | 0 | 114 | 127 |  |
| total 12 peptides |  |  |  |  |  |  |  |  |  |  |  |  |  |  |  |  |  |  |  |  |  |  |  |  |  |  |  |  |  |  |  |  |  |  |  |
| --- | --- | --- | --- | --- | --- | --- | --- | --- | --- | --- | --- | --- | --- | --- | --- | --- | --- | --- | --- | --- | --- | --- | --- | --- | --- | --- | --- | --- | --- | --- | --- | --- | --- | --- | --- |

Best Unique PSM (Scan F6:34941, m/z=686.0276, z=3, RT=125.61, ppm=1.7):

sp|Q8K419|LEG4\_MOUSE

back to list

  

| Protein Coverage
| Supporting Peptides
| Best Unique PSM
|

Protein Coverage:

Supporting Peptides:

| Peptide | Uniq | -10lgP | Mass | Length | ppm | m/z | z | RT | Fraction | Scan | Source File | Area 10 | Area 01 | Area 02 | Area 03 | Area 04 | Area 05 | Area 06 | Area 07 | Area 08 | Area 09 | #Spec | #Spec 10 | #Spec 01 | #Spec 02 | #Spec 03 | #Spec 04 | #Spec 05 | #Spec 06 | #Spec 07 | #Spec 08 | #Spec 09 | Start | End | PTM |
| --- | --- | --- | --- | --- | --- | --- | --- | --- | --- | --- | --- | --- | --- | --- | --- | --- | --- | --- | --- | --- | --- | --- | --- | --- | --- | --- | --- | --- | --- | --- | --- | --- | --- | --- | --- |
| R.KVAYNPFGPGQFFDLSIR.C | Y | 117.19 | 2055.0576 | 18 | 1.7 | 686.0276 | 3 | 125.61 | 6 | 34941 | Ana\_05.raw |  | 7.7E6 | 1.18E7 | 4.19E6 | 1.37E7 | 1.72E7 | 1.76E7 | 6.49E6 | 2.84E6 | 5.45E6 | 14 | 0 | 2 | 1 | 1 | 2 | 2 | 2 | 1 | 1 | 2 | 265 | 282 |  |
| K.VAYNPFGPGQFFDLSIR.C | Y | 113.64 | 1926.9628 | 17 | 1.0 | 964.4896 | 2 | 131.01 | 3 | 35887 | Ana\_02.raw |  | 8.8E7 | 5.22E7 | 2.05E7 | 3.47E7 | 7.9E7 | 4.64E7 | 2.46E7 | 9.41E7 | 5.35E7 | 14 | 0 | 2 | 2 | 2 | 1 | 2 | 2 | 1 | 1 | 1 | 266 | 282 |  |
| R.VPYVGALQGGLTVR.R | Y | 91.53 | 1428.8088 | 14 | 3.6 | 715.4142 | 2 | 92.12 | 6 | 26154 | Ana\_05.raw | 3.26E7 | 4.35E7 | 1.65E7 | 6.41E6 | 1.69E7 | 2.43E7 | 1.28E7 | 2.58E6 | 9.47E7 | 2.39E7 | 27 | 2 | 3 | 3 | 2 | 2 | 2 | 2 | 3 | 5 | 3 | 196 | 209 |  |
| K.VGSSGDIALHLNPR.I | Y | 83.46 | 1434.7579 | 14 | 7.9 | 718.3919 | 2 | 60.28 | 6 | 17248 | Ana\_05.raw | 5.3E7 | 7.92E7 | 9.84E7 | 4.51E7 | 8.32E7 | 6.58E7 | 5.46E7 | 4.59E7 | 1.25E8 | 5.28E7 | 35 | 4 | 6 | 3 | 2 | 5 | 2 | 2 | 4 | 5 | 2 | 231 | 244 |  |
| K.VFANGQHLFDFSHR.F | Y | 79.46 | 1673.8063 | 14 | 4.2 | 558.9451 | 3 | 82.46 | 7 | 23281 | Ana\_06.raw | 5.47E6 | 5.82E6 | 1.21E7 | 5.71E6 | 6.35E6 | 1.3E7 | 7.81E6 | 3.43E6 | 8.54E6 | 4.93E6 | 16 | 1 | 1 | 2 | 2 | 1 | 2 | 2 | 2 | 2 | 1 | 290 | 303 |  |
| K.GYVLPTAR.N | Y | 77.90 | 875.4865 | 8 | 5.1 | 438.7527 | 2 | 46.73 | 9 | 13055 | Ana\_08.raw | 6.89E7 | 8.55E7 | 1.08E8 | 5.26E7 | 8.93E7 | 7.68E7 | 7.12E7 | 6.57E7 | 1.22E8 | 7.24E7 | 34 | 4 | 3 | 4 | 4 | 3 | 2 | 4 | 3 | 4 | 3 | 216 | 223 |  |
| K.VVFNTM(+15.99)QSGQWGK.E | Y | 74.23 | 1496.7083 | 13 | 4.6 | 749.3648 | 2 | 64.20 | 6 | 18431 | Ana\_05.raw | 1.68E7 | 1.94E7 | 2.17E7 | 1.02E7 | 2.01E7 | 1.79E7 | 1.32E7 | 1.48E7 | 3.19E7 | 5.36E6 | 10 | 1 | 1 | 1 | 1 | 1 | 1 | 1 | 1 | 1 | 1 | 74 | 86 | Oxidation (M) |
| K.HFELVFM(+15.99)VM(+15.99)PEHYK.V | N | 66.60 | 1837.8531 | 14 | 0.9 | 613.6255 | 3 | 87.26 | 6 | 24849 | Ana\_05.raw |  | 4.64E6 |  |  |  | 2.36E6 |  | 3.27E6 | 5.31E6 |  | 4 | 0 | 1 | 0 | 0 | 0 | 1 | 0 | 1 | 1 | 0 | 100 | 113 | Oxidation (M) |
| R.NFVINFK.V | Y | 62.14 | 880.4807 | 7 | 1.6 | 441.2483 | 2 | 82.51 | 6 | 23554 | Ana\_05.raw | 4.46E7 | 6.46E7 | 7.51E7 | 4.35E7 | 5.55E7 | 7.11E7 | 4.49E7 | 5.02E7 | 1.08E8 | 5.45E7 | 41 | 5 | 4 | 6 | 5 | 5 | 2 | 3 | 3 | 3 | 5 | 224 | 230 |  |
| R.IGDSVVR.N | Y | 51.24 | 744.4130 | 7 | 4.1 | 373.2153 | 2 | 27.99 | 8 | 7250 | Ana\_07.raw | 1.13E8 | 1.71E8 | 1.68E8 | 9.17E7 | 1.28E8 | 1.64E8 | 1.28E8 | 7.85E7 | 9.31E7 | 1.22E8 | 15 | 1 | 1 | 1 | 1 | 1 | 3 | 2 | 2 | 1 | 2 | 245 | 251 |  |
| R.FDGWDK.V | N | 39.80 | 766.3286 | 6 | 1.2 | 384.1720 | 2 | 45.07 | 6 | 12714 | Ana\_05.raw | 1.3E7 | 2.21E7 | 1.84E7 | 5.62E6 | 1.81E7 | 2.2E7 | 1.55E7 | 1.41E7 | 2.87E7 | 1.03E7 | 16 | 1 | 2 | 2 | 2 | 1 | 2 | 1 | 2 | 2 | 1 | 68 | 73 |  |
| K.VVVNGNSFYEYGHR.L | Y | 35.33 | 1639.7743 | 14 | 3.6 | 547.6006 | 3 | 67.22 | 7 | 19075 | Ana\_06.raw |  |  |  |  |  |  | 3.84E6 |  |  |  | 1 | 0 | 0 | 0 | 0 | 0 | 0 | 1 | 0 | 0 | 0 | 114 | 127 |  |
| total 12 peptides |  |  |  |  |  |  |  |  |  |  |  |  |  |  |  |  |  |  |  |  |  |  |  |  |  |  |  |  |  |  |  |  |  |  |  |
| --- | --- | --- | --- | --- | --- | --- | --- | --- | --- | --- | --- | --- | --- | --- | --- | --- | --- | --- | --- | --- | --- | --- | --- | --- | --- | --- | --- | --- | --- | --- | --- | --- | --- | --- | --- |

Best Unique PSM (Scan F6:34941, m/z=686.0276, z=3, RT=125.61, ppm=1.7):

sp|P01872|IGHM\_MOUSE

back to list

  

| Protein Coverage
| Supporting Peptides
| Best Unique PSM
|

Protein Coverage:

Supporting Peptides:

| Peptide | Uniq | -10lgP | Mass | Length | ppm | m/z | z | RT | Fraction | Scan | Source File | Area 10 | Area 01 | Area 02 | Area 03 | Area 04 | Area 05 | Area 06 | Area 07 | Area 08 | Area 09 | #Spec | #Spec 10 | #Spec 01 | #Spec 02 | #Spec 03 | #Spec 04 | #Spec 05 | #Spec 06 | #Spec 07 | #Spec 08 | #Spec 09 | Start | End | PTM |
| --- | --- | --- | --- | --- | --- | --- | --- | --- | --- | --- | --- | --- | --- | --- | --- | --- | --- | --- | --- | --- | --- | --- | --- | --- | --- | --- | --- | --- | --- | --- | --- | --- | --- | --- | --- |
| R.DLHVPIPAVAEM(+15.99)NPNVNVFVPPR.D | Y | 120.07 | 2540.3208 | 23 | 1.5 | 847.7821 | 3 | 116.91 | 1 | 32301 | ANa\_10.raw | 6.58E7 |  |  |  |  |  |  |  | 1.38E8 | 3.28E7 | 10 | 3 | 0 | 0 | 0 | 0 | 0 | 0 | 0 | 4 | 3 | 98 | 120 | Oxidation (M) |
| K.GFSPADISVQWLQR.G | Y | 95.62 | 1602.8154 | 14 | 3.9 | 802.4182 | 2 | 118.35 | 9 | 33028 | Ana\_08.raw | 3.81E7 |  |  | 6.8E5 |  | 7.2E5 | 3.12E5 |  | 6.65E7 | 2.35E7 | 14 | 5 | 0 | 0 | 1 | 0 | 1 | 1 | 0 | 3 | 3 | 356 | 369 |  |
| K.LVESGFTTDPVTIENK.G | Y | 81.44 | 1748.8832 | 16 | 4.0 | 875.4523 | 2 | 78.47 | 9 | 22357 | Ana\_08.raw | 1.61E7 |  |  |  |  |  |  | 8.63E5 | 5.82E7 | 1.1E7 | 6 | 1 | 0 | 0 | 0 | 0 | 0 | 0 | 1 | 3 | 1 | 155 | 170 |  |
| K.GVASVC(+57.02)VEDWNNR.K | Y | 76.48 | 1504.6729 | 13 | 4.6 | 753.3472 | 2 | 67.50 | 9 | 19266 | Ana\_08.raw |  |  |  |  |  |  |  |  | 7.57E6 |  | 1 | 0 | 0 | 0 | 0 | 0 | 0 | 0 | 0 | 1 | 0 | 287 | 299 | Carbamidomethylation |
| K.LIC(+57.02)EATNFTPKPITVSWLK.D | Y | 74.98 | 2217.1868 | 19 | 2.1 | 740.0711 | 3 | 109.47 | 9 | 30744 | Ana\_08.raw | 4.62E6 |  |  |  |  |  |  |  | 1.18E7 |  | 2 | 1 | 0 | 0 | 0 | 0 | 0 | 0 | 0 | 1 | 0 | 133 | 151 | Carbamidomethylation |
| K.YLATSQVLLSPK.S | Y | 65.83 | 1318.7496 | 12 | 6.1 | 660.3861 | 2 | 72.85 | 1 | 20646 | ANa\_10.raw | 2.89E7 |  |  |  |  |  | 6.38E5 | 1.44E6 | 7.18E7 | 1.62E7 | 7 | 1 | 0 | 0 | 0 | 0 | 0 | 1 | 1 | 3 | 1 | 65 | 76 |  |
| K.SILEGSDEYLVC(+57.02)K.I | Y | 65.31 | 1511.7178 | 13 | 1.6 | 756.8674 | 2 | 79.27 | 9 | 22582 | Ana\_08.raw | 8.75E6 |  |  |  |  |  |  | 7.06E5 | 3.46E7 | 5.99E6 | 5 | 1 | 0 | 0 | 0 | 0 | 0 | 0 | 1 | 2 | 1 | 77 | 89 | Carbamidomethylation |
| R.KEFVC(+57.02)TVTHR.D | Y | 62.00 | 1275.6394 | 10 | 5.8 | 426.2229 | 3 | 27.47 | 10 | 7288 | Ana\_09.raw | 1.08E7 |  |  |  |  |  |  |  | 1.56E7 | 1.02E7 | 6 | 2 | 0 | 0 | 0 | 0 | 0 | 0 | 0 | 2 | 2 | 300 | 309 | Carbamidomethylation |
| R.DGFSGPAPR.K | Y | 59.34 | 902.4246 | 9 | 4.4 | 452.2215 | 2 | 32.00 | 9 | 8483 | Ana\_08.raw | 6.94E7 |  |  |  |  |  |  |  | 6.49E7 | 2.49E7 | 6 | 2 | 0 | 0 | 0 | 0 | 0 | 0 | 0 | 3 | 1 | 121 | 129 |  |
| K.EFVC(+57.02)TVTHR.D | Y | 55.46 | 1147.5444 | 9 | 3.8 | 383.5235 | 3 | 32.91 | 9 | 8754 | Ana\_08.raw | 5.67E6 |  |  |  |  |  |  |  | 5.86E6 | 5.26E6 | 3 | 1 | 0 | 0 | 0 | 0 | 0 | 0 | 0 | 1 | 1 | 301 | 309 | Carbamidomethylation |
| K.FISKPNEVHK.H | Y | 52.55 | 1197.6505 | 10 | 0.3 | 599.8327 | 2 | 23.08 | 9 | 5659 | Ana\_08.raw |  |  |  |  |  |  |  |  | 4.79E7 |  | 3 | 0 | 0 | 0 | 0 | 0 | 0 | 0 | 0 | 3 | 0 | 318 | 327 |  |
| R.ESATVTC(+57.02)LVK.G | Y | 51.99 | 1106.5642 | 10 | 9.7 | 554.2947 | 2 | 44.50 | 9 | 12374 | Ana\_08.raw |  |  |  |  |  |  |  |  | 1.69E7 |  | 1 | 0 | 0 | 0 | 0 | 0 | 0 | 0 | 0 | 1 | 0 | 346 | 355 | Carbamidomethylation |
| K.HPPAVYLLPPAR.E | Y | 48.85 | 1329.7557 | 12 | 1.9 | 444.2600 | 3 | 65.57 | 1 | 18591 | ANa\_10.raw | 2.16E6 |  |  |  |  |  |  |  | 6.37E6 | 1.68E6 | 4 | 1 | 0 | 0 | 0 | 0 | 0 | 0 | 0 | 2 | 1 | 328 | 339 |  |
| R.GQLLPQEK.Y | Y | 43.34 | 911.5076 | 8 | 4.6 | 456.7632 | 2 | 35.83 | 10 | 9783 | Ana\_09.raw | 1.85E7 |  |  |  |  |  |  |  | 4.3E7 | 1.38E7 | 4 | 1 | 0 | 0 | 0 | 0 | 0 | 0 | 0 | 2 | 1 | 370 | 377 |  |
| R.DLPSPQK.K | Y | 36.08 | 783.4127 | 7 | 3.4 | 392.7149 | 2 | 26.17 | 1 | 6802 | ANa\_10.raw | 2.93E6 |  |  |  |  |  |  |  |  | 4.65E6 | 2 | 1 | 0 | 0 | 0 | 0 | 0 | 0 | 0 | 0 | 1 | 310 | 316 |  |
| K.NLVAM(+15.99)GC(+57.02)LAR.D | Y | 29.49 | 1119.5529 | 10 | 6.8 | 560.7875 | 2 | 44.45 | 9 | 12357 | Ana\_08.raw |  |  |  |  |  |  |  |  | 1.01E7 |  | 1 | 0 | 0 | 0 | 0 | 0 | 0 | 0 | 0 | 1 | 0 | 21 | 30 | Oxidation (M); Carbamidomethylation |
| R.EQLNLR.E | Y | 27.10 | 771.4239 | 6 | 4.3 | 386.7209 | 2 | 30.73 | 1 | 8235 | ANa\_10.raw | 2.6E7 |  |  |  |  |  |  |  |  |  | 1 | 1 | 0 | 0 | 0 | 0 | 0 | 0 | 0 | 0 | 0 | 340 | 345 |  |
| total 17 peptides |  |  |  |  |  |  |  |  |  |  |  |  |  |  |  |  |  |  |  |  |  |  |  |  |  |  |  |  |  |  |  |  |  |  |  |
| --- | --- | --- | --- | --- | --- | --- | --- | --- | --- | --- | --- | --- | --- | --- | --- | --- | --- | --- | --- | --- | --- | --- | --- | --- | --- | --- | --- | --- | --- | --- | --- | --- | --- | --- | --- |

Best Unique PSM (Scan F1:32301, m/z=847.7821, z=3, RT=116.91, ppm=1.5):

A0A075B5P6|A0A075B5P6\_MOUSE

back to list

  

| Protein Coverage
| Supporting Peptides
| Best Unique PSM
|

Protein Coverage:

Supporting Peptides:

| Peptide | Uniq | -10lgP | Mass | Length | ppm | m/z | z | RT | Fraction | Scan | Source File | Area 10 | Area 01 | Area 02 | Area 03 | Area 04 | Area 05 | Area 06 | Area 07 | Area 08 | Area 09 | #Spec | #Spec 10 | #Spec 01 | #Spec 02 | #Spec 03 | #Spec 04 | #Spec 05 | #Spec 06 | #Spec 07 | #Spec 08 | #Spec 09 | Start | End | PTM |
| --- | --- | --- | --- | --- | --- | --- | --- | --- | --- | --- | --- | --- | --- | --- | --- | --- | --- | --- | --- | --- | --- | --- | --- | --- | --- | --- | --- | --- | --- | --- | --- | --- | --- | --- | --- |
| K.DLHVPIPAVAEM(+15.99)NPNVNVFVPPR.D | Y | 120.07 | 2540.3208 | 23 | 1.5 | 847.7821 | 3 | 116.91 | 1 | 32301 | ANa\_10.raw | 6.58E7 |  |  |  |  |  |  |  | 1.38E8 | 3.28E7 | 10 | 3 | 0 | 0 | 0 | 0 | 0 | 0 | 0 | 4 | 3 | 99 | 121 | Oxidation (M) |
| K.GFSPADISVQWLQR.G | Y | 95.62 | 1602.8154 | 14 | 3.9 | 802.4182 | 2 | 118.35 | 9 | 33028 | Ana\_08.raw | 3.81E7 |  |  | 6.8E5 |  | 7.2E5 | 3.12E5 |  | 6.65E7 | 2.35E7 | 14 | 5 | 0 | 0 | 1 | 0 | 1 | 1 | 0 | 3 | 3 | 357 | 370 |  |
| K.LVESGFTTDPVTIENK.G | Y | 81.44 | 1748.8832 | 16 | 4.0 | 875.4523 | 2 | 78.47 | 9 | 22357 | Ana\_08.raw | 1.61E7 |  |  |  |  |  |  | 8.63E5 | 5.82E7 | 1.1E7 | 6 | 1 | 0 | 0 | 0 | 0 | 0 | 0 | 1 | 3 | 1 | 156 | 171 |  |
| K.GVASVC(+57.02)VEDWNNR.K | Y | 76.48 | 1504.6729 | 13 | 4.6 | 753.3472 | 2 | 67.50 | 9 | 19266 | Ana\_08.raw |  |  |  |  |  |  |  |  | 7.57E6 |  | 1 | 0 | 0 | 0 | 0 | 0 | 0 | 0 | 0 | 1 | 0 | 288 | 300 | Carbamidomethylation |
| K.LIC(+57.02)EATNFTPKPITVSWLK.D | Y | 74.98 | 2217.1868 | 19 | 2.1 | 740.0711 | 3 | 109.47 | 9 | 30744 | Ana\_08.raw | 4.62E6 |  |  |  |  |  |  |  | 1.18E7 |  | 2 | 1 | 0 | 0 | 0 | 0 | 0 | 0 | 0 | 1 | 0 | 134 | 152 | Carbamidomethylation |
| K.YLATSQVLLSPK.S | Y | 65.83 | 1318.7496 | 12 | 6.1 | 660.3861 | 2 | 72.85 | 1 | 20646 | ANa\_10.raw | 2.89E7 |  |  |  |  |  | 6.38E5 | 1.44E6 | 7.18E7 | 1.62E7 | 7 | 1 | 0 | 0 | 0 | 0 | 0 | 1 | 1 | 3 | 1 | 66 | 77 |  |
| K.SILEGSDEYLVC(+57.02)K.I | Y | 65.31 | 1511.7178 | 13 | 1.6 | 756.8674 | 2 | 79.27 | 9 | 22582 | Ana\_08.raw | 8.75E6 |  |  |  |  |  |  | 7.06E5 | 3.46E7 | 5.99E6 | 5 | 1 | 0 | 0 | 0 | 0 | 0 | 0 | 1 | 2 | 1 | 78 | 90 | Carbamidomethylation |
| R.KEFVC(+57.02)TVTHR.D | Y | 62.00 | 1275.6394 | 10 | 5.8 | 426.2229 | 3 | 27.47 | 10 | 7288 | Ana\_09.raw | 1.08E7 |  |  |  |  |  |  |  | 1.56E7 | 1.02E7 | 6 | 2 | 0 | 0 | 0 | 0 | 0 | 0 | 0 | 2 | 2 | 301 | 310 | Carbamidomethylation |
| R.DGFSGPAPR.K | Y | 59.34 | 902.4246 | 9 | 4.4 | 452.2215 | 2 | 32.00 | 9 | 8483 | Ana\_08.raw | 6.94E7 |  |  |  |  |  |  |  | 6.49E7 | 2.49E7 | 6 | 2 | 0 | 0 | 0 | 0 | 0 | 0 | 0 | 3 | 1 | 122 | 130 |  |
| K.EFVC(+57.02)TVTHR.D | Y | 55.46 | 1147.5444 | 9 | 3.8 | 383.5235 | 3 | 32.91 | 9 | 8754 | Ana\_08.raw | 5.67E6 |  |  |  |  |  |  |  | 5.86E6 | 5.26E6 | 3 | 1 | 0 | 0 | 0 | 0 | 0 | 0 | 0 | 1 | 1 | 302 | 310 | Carbamidomethylation |
| K.FISKPNEVHK.H | Y | 52.55 | 1197.6505 | 10 | 0.3 | 599.8327 | 2 | 23.08 | 9 | 5659 | Ana\_08.raw |  |  |  |  |  |  |  |  | 4.79E7 |  | 3 | 0 | 0 | 0 | 0 | 0 | 0 | 0 | 0 | 3 | 0 | 319 | 328 |  |
| R.ESATVTC(+57.02)LVK.G | Y | 51.99 | 1106.5642 | 10 | 9.7 | 554.2947 | 2 | 44.50 | 9 | 12374 | Ana\_08.raw |  |  |  |  |  |  |  |  | 1.69E7 |  | 1 | 0 | 0 | 0 | 0 | 0 | 0 | 0 | 0 | 1 | 0 | 347 | 356 | Carbamidomethylation |
| K.HPPAVYLLPPAR.E | Y | 48.85 | 1329.7557 | 12 | 1.9 | 444.2600 | 3 | 65.57 | 1 | 18591 | ANa\_10.raw | 2.16E6 |  |  |  |  |  |  |  | 6.37E6 | 1.68E6 | 4 | 1 | 0 | 0 | 0 | 0 | 0 | 0 | 0 | 2 | 1 | 329 | 340 |  |
| R.GQLLPQEK.Y | Y | 43.34 | 911.5076 | 8 | 4.6 | 456.7632 | 2 | 35.83 | 10 | 9783 | Ana\_09.raw | 1.85E7 |  |  |  |  |  |  |  | 4.3E7 | 1.38E7 | 4 | 1 | 0 | 0 | 0 | 0 | 0 | 0 | 0 | 2 | 1 | 371 | 378 |  |
| R.DLPSPQK.K | Y | 36.08 | 783.4127 | 7 | 3.4 | 392.7149 | 2 | 26.17 | 1 | 6802 | ANa\_10.raw | 2.93E6 |  |  |  |  |  |  |  |  | 4.65E6 | 2 | 1 | 0 | 0 | 0 | 0 | 0 | 0 | 0 | 0 | 1 | 311 | 317 |  |
| K.NLVAM(+15.99)GC(+57.02)LAR.D | Y | 29.49 | 1119.5529 | 10 | 6.8 | 560.7875 | 2 | 44.45 | 9 | 12357 | Ana\_08.raw |  |  |  |  |  |  |  |  | 1.01E7 |  | 1 | 0 | 0 | 0 | 0 | 0 | 0 | 0 | 0 | 1 | 0 | 22 | 31 | Oxidation (M); Carbamidomethylation |
| R.EQLNLR.E | Y | 27.10 | 771.4239 | 6 | 4.3 | 386.7209 | 2 | 30.73 | 1 | 8235 | ANa\_10.raw | 2.6E7 |  |  |  |  |  |  |  |  |  | 1 | 1 | 0 | 0 | 0 | 0 | 0 | 0 | 0 | 0 | 0 | 341 | 346 |  |
| total 17 peptides |  |  |  |  |  |  |  |  |  |  |  |  |  |  |  |  |  |  |  |  |  |  |  |  |  |  |  |  |  |  |  |  |  |  |  |
| --- | --- | --- | --- | --- | --- | --- | --- | --- | --- | --- | --- | --- | --- | --- | --- | --- | --- | --- | --- | --- | --- | --- | --- | --- | --- | --- | --- | --- | --- | --- | --- | --- | --- | --- | --- |

Best Unique PSM (Scan F1:32301, m/z=847.7821, z=3, RT=116.91, ppm=1.5):

A0A075B6A0|A0A075B6A0\_MOUSE

back to list

  

| Protein Coverage
| Supporting Peptides
| Best Unique PSM
|

Protein Coverage:

Supporting Peptides:

| Peptide | Uniq | -10lgP | Mass | Length | ppm | m/z | z | RT | Fraction | Scan | Source File | Area 10 | Area 01 | Area 02 | Area 03 | Area 04 | Area 05 | Area 06 | Area 07 | Area 08 | Area 09 | #Spec | #Spec 10 | #Spec 01 | #Spec 02 | #Spec 03 | #Spec 04 | #Spec 05 | #Spec 06 | #Spec 07 | #Spec 08 | #Spec 09 | Start | End | PTM |
| --- | --- | --- | --- | --- | --- | --- | --- | --- | --- | --- | --- | --- | --- | --- | --- | --- | --- | --- | --- | --- | --- | --- | --- | --- | --- | --- | --- | --- | --- | --- | --- | --- | --- | --- | --- |
| K.DLHVPIPAVAEM(+15.99)NPNVNVFVPPR.D | Y | 120.07 | 2540.3208 | 23 | 1.5 | 847.7821 | 3 | 116.91 | 1 | 32301 | ANa\_10.raw | 6.58E7 |  |  |  |  |  |  |  | 1.38E8 | 3.28E7 | 10 | 3 | 0 | 0 | 0 | 0 | 0 | 0 | 0 | 4 | 3 | 99 | 121 | Oxidation (M) |
| K.GFSPADISVQWLQR.G | Y | 95.62 | 1602.8154 | 14 | 3.9 | 802.4182 | 2 | 118.35 | 9 | 33028 | Ana\_08.raw | 3.81E7 |  |  | 6.8E5 |  | 7.2E5 | 3.12E5 |  | 6.65E7 | 2.35E7 | 14 | 5 | 0 | 0 | 1 | 0 | 1 | 1 | 0 | 3 | 3 | 357 | 370 |  |
| K.LVESGFTTDPVTIENK.G | Y | 81.44 | 1748.8832 | 16 | 4.0 | 875.4523 | 2 | 78.47 | 9 | 22357 | Ana\_08.raw | 1.61E7 |  |  |  |  |  |  | 8.63E5 | 5.82E7 | 1.1E7 | 6 | 1 | 0 | 0 | 0 | 0 | 0 | 0 | 1 | 3 | 1 | 156 | 171 |  |
| K.GVASVC(+57.02)VEDWNNR.K | Y | 76.48 | 1504.6729 | 13 | 4.6 | 753.3472 | 2 | 67.50 | 9 | 19266 | Ana\_08.raw |  |  |  |  |  |  |  |  | 7.57E6 |  | 1 | 0 | 0 | 0 | 0 | 0 | 0 | 0 | 0 | 1 | 0 | 288 | 300 | Carbamidomethylation |
| K.LIC(+57.02)EATNFTPKPITVSWLK.D | Y | 74.98 | 2217.1868 | 19 | 2.1 | 740.0711 | 3 | 109.47 | 9 | 30744 | Ana\_08.raw | 4.62E6 |  |  |  |  |  |  |  | 1.18E7 |  | 2 | 1 | 0 | 0 | 0 | 0 | 0 | 0 | 0 | 1 | 0 | 134 | 152 | Carbamidomethylation |
| K.YLATSQVLLSPK.S | Y | 65.83 | 1318.7496 | 12 | 6.1 | 660.3861 | 2 | 72.85 | 1 | 20646 | ANa\_10.raw | 2.89E7 |  |  |  |  |  | 6.38E5 | 1.44E6 | 7.18E7 | 1.62E7 | 7 | 1 | 0 | 0 | 0 | 0 | 0 | 1 | 1 | 3 | 1 | 66 | 77 |  |
| K.SILEGSDEYLVC(+57.02)K.I | Y | 65.31 | 1511.7178 | 13 | 1.6 | 756.8674 | 2 | 79.27 | 9 | 22582 | Ana\_08.raw | 8.75E6 |  |  |  |  |  |  | 7.06E5 | 3.46E7 | 5.99E6 | 5 | 1 | 0 | 0 | 0 | 0 | 0 | 0 | 1 | 2 | 1 | 78 | 90 | Carbamidomethylation |
| R.KEFVC(+57.02)TVTHR.D | Y | 62.00 | 1275.6394 | 10 | 5.8 | 426.2229 | 3 | 27.47 | 10 | 7288 | Ana\_09.raw | 1.08E7 |  |  |  |  |  |  |  | 1.56E7 | 1.02E7 | 6 | 2 | 0 | 0 | 0 | 0 | 0 | 0 | 0 | 2 | 2 | 301 | 310 | Carbamidomethylation |
| R.DGFSGPAPR.K | Y | 59.34 | 902.4246 | 9 | 4.4 | 452.2215 | 2 | 32.00 | 9 | 8483 | Ana\_08.raw | 6.94E7 |  |  |  |  |  |  |  | 6.49E7 | 2.49E7 | 6 | 2 | 0 | 0 | 0 | 0 | 0 | 0 | 0 | 3 | 1 | 122 | 130 |  |
| K.EFVC(+57.02)TVTHR.D | Y | 55.46 | 1147.5444 | 9 | 3.8 | 383.5235 | 3 | 32.91 | 9 | 8754 | Ana\_08.raw | 5.67E6 |  |  |  |  |  |  |  | 5.86E6 | 5.26E6 | 3 | 1 | 0 | 0 | 0 | 0 | 0 | 0 | 0 | 1 | 1 | 302 | 310 | Carbamidomethylation |
| K.FISKPNEVHK.H | Y | 52.55 | 1197.6505 | 10 | 0.3 | 599.8327 | 2 | 23.08 | 9 | 5659 | Ana\_08.raw |  |  |  |  |  |  |  |  | 4.79E7 |  | 3 | 0 | 0 | 0 | 0 | 0 | 0 | 0 | 0 | 3 | 0 | 319 | 328 |  |
| R.ESATVTC(+57.02)LVK.G | Y | 51.99 | 1106.5642 | 10 | 9.7 | 554.2947 | 2 | 44.50 | 9 | 12374 | Ana\_08.raw |  |  |  |  |  |  |  |  | 1.69E7 |  | 1 | 0 | 0 | 0 | 0 | 0 | 0 | 0 | 0 | 1 | 0 | 347 | 356 | Carbamidomethylation |
| K.HPPAVYLLPPAR.E | Y | 48.85 | 1329.7557 | 12 | 1.9 | 444.2600 | 3 | 65.57 | 1 | 18591 | ANa\_10.raw | 2.16E6 |  |  |  |  |  |  |  | 6.37E6 | 1.68E6 | 4 | 1 | 0 | 0 | 0 | 0 | 0 | 0 | 0 | 2 | 1 | 329 | 340 |  |
| R.GQLLPQEK.Y | Y | 43.34 | 911.5076 | 8 | 4.6 | 456.7632 | 2 | 35.83 | 10 | 9783 | Ana\_09.raw | 1.85E7 |  |  |  |  |  |  |  | 4.3E7 | 1.38E7 | 4 | 1 | 0 | 0 | 0 | 0 | 0 | 0 | 0 | 2 | 1 | 371 | 378 |  |
| R.DLPSPQK.K | Y | 36.08 | 783.4127 | 7 | 3.4 | 392.7149 | 2 | 26.17 | 1 | 6802 | ANa\_10.raw | 2.93E6 |  |  |  |  |  |  |  |  | 4.65E6 | 2 | 1 | 0 | 0 | 0 | 0 | 0 | 0 | 0 | 0 | 1 | 311 | 317 |  |
| K.NLVAM(+15.99)GC(+57.02)LAR.D | Y | 29.49 | 1119.5529 | 10 | 6.8 | 560.7875 | 2 | 44.45 | 9 | 12357 | Ana\_08.raw |  |  |  |  |  |  |  |  | 1.01E7 |  | 1 | 0 | 0 | 0 | 0 | 0 | 0 | 0 | 0 | 1 | 0 | 22 | 31 | Oxidation (M); Carbamidomethylation |
| R.EQLNLR.E | Y | 27.10 | 771.4239 | 6 | 4.3 | 386.7209 | 2 | 30.73 | 1 | 8235 | ANa\_10.raw | 2.6E7 |  |  |  |  |  |  |  |  |  | 1 | 1 | 0 | 0 | 0 | 0 | 0 | 0 | 0 | 0 | 0 | 341 | 346 |  |
| total 17 peptides |  |  |  |  |  |  |  |  |  |  |  |  |  |  |  |  |  |  |  |  |  |  |  |  |  |  |  |  |  |  |  |  |  |  |  |
| --- | --- | --- | --- | --- | --- | --- | --- | --- | --- | --- | --- | --- | --- | --- | --- | --- | --- | --- | --- | --- | --- | --- | --- | --- | --- | --- | --- | --- | --- | --- | --- | --- | --- | --- | --- |

Best Unique PSM (Scan F1:32301, m/z=847.7821, z=3, RT=116.91, ppm=1.5):

sp|Q9JKF1|IQGA1\_MOUSE

back to list

  

| Protein Coverage
| Supporting Peptides
| Best Unique PSM
|

Protein Coverage:

Supporting Peptides:

| Peptide | Uniq | -10lgP | Mass | Length | ppm | m/z | z | RT | Fraction | Scan | Source File | Area 10 | Area 01 | Area 02 | Area 03 | Area 04 | Area 05 | Area 06 | Area 07 | Area 08 | Area 09 | #Spec | #Spec 10 | #Spec 01 | #Spec 02 | #Spec 03 | #Spec 04 | #Spec 05 | #Spec 06 | #Spec 07 | #Spec 08 | #Spec 09 | Start | End | PTM |
| --- | --- | --- | --- | --- | --- | --- | --- | --- | --- | --- | --- | --- | --- | --- | --- | --- | --- | --- | --- | --- | --- | --- | --- | --- | --- | --- | --- | --- | --- | --- | --- | --- | --- | --- | --- |
| R.ILAIGLINEALDEGDAQK.T | Y | 104.24 | 1882.0046 | 18 | 2.8 | 942.0122 | 2 | 129.74 | 6 | 36042 | Ana\_05.raw | 6.63E6 |  | 6.2E5 | 4.41E5 | 2.47E6 | 4.43E6 | 1.38E6 | 2.85E6 | 1.29E7 | 6.62E6 | 16 | 2 | 0 | 1 | 1 | 2 | 2 | 2 | 2 | 2 | 2 | 539 | 556 |  |
| K.LEGVLAEVAQHYQDTLIR.A | Y | 103.73 | 2054.0796 | 18 | 1.3 | 685.7014 | 3 | 123.23 | 3 | 33859 | Ana\_02.raw | 5.87E6 |  | 5.16E6 | 9.13E5 | 1.03E7 | 1.02E7 | 6.55E6 | 1.02E6 | 1.82E7 | 1.05E7 | 10 | 2 | 0 | 1 | 1 | 1 | 1 | 1 | 1 | 1 | 1 | 568 | 585 |  |
| R.FALGISAINEAVDSGDVGR.T | Y | 90.25 | 1889.9482 | 19 | 6.8 | 945.9878 | 2 | 115.59 | 6 | 32328 | Ana\_05.raw | 1.55E6 | 3.35E6 | 7.24E5 | 8.85E5 | 6.12E5 | 3.91E6 | 9.03E5 | 1.14E6 | 6.63E6 |  | 9 | 1 | 1 | 1 | 1 | 1 | 1 | 1 | 1 | 1 | 0 | 623 | 641 |  |
| K.NVIFEIGPTEEVGDFEVK.A | Y | 78.82 | 2020.9993 | 18 | 5.1 | 1011.5121 | 2 | 121.49 | 1 | 33482 | ANa\_10.raw | 2.19E6 | 0 | 4.85E5 |  |  |  | 1.36E5 |  | 1.12E6 |  | 5 | 1 | 1 | 1 | 0 | 0 | 0 | 1 | 0 | 1 | 0 | 1587 | 1604 |  |
| R.FQPGETLTEILETPATNEQEAEHQR.A | Y | 74.27 | 2867.3572 | 25 | 1.5 | 956.7944 | 3 | 112.00 | 9 | 31386 | Ana\_08.raw | 8.8E5 |  | 4.47E6 | 3.36E6 | 2.04E6 | 3.21E6 | 9.17E5 |  | 4.54E6 | 2.23E6 | 8 | 1 | 0 | 1 | 1 | 1 | 1 | 1 | 0 | 1 | 1 | 1398 | 1422 |  |
| K.LGLAPQIQDLYGK.V | Y | 66.48 | 1414.7820 | 13 | 1.9 | 708.3996 | 2 | 94.79 | 9 | 26848 | Ana\_08.raw |  |  | 1.01E6 |  |  |  |  |  | 9.86E5 |  | 2 | 0 | 0 | 1 | 0 | 0 | 0 | 0 | 0 | 1 | 0 | 162 | 174 |  |
| K.IFYPETTDIYDR.K | Y | 63.83 | 1531.7195 | 12 | 5.5 | 766.8712 | 2 | 84.64 | 10 | 23784 | Ana\_09.raw | 3.73E6 | 3.71E6 |  | 1.36E6 | 1.71E6 | 1.11E6 |  | 1.25E6 | 9.63E6 | 2.8E6 | 8 | 1 | 1 | 0 | 1 | 1 | 1 | 0 | 1 | 1 | 1 | 131 | 142 |  |
| R.EEYLLLR.L | Y | 49.73 | 934.5123 | 7 | 3.8 | 468.2652 | 2 | 70.68 | 9 | 20175 | Ana\_08.raw |  |  |  |  |  |  |  |  | 1.56E6 |  | 1 | 0 | 0 | 0 | 0 | 0 | 0 | 0 | 0 | 1 | 0 | 1018 | 1024 |  |
| K.TLQALQIPAAK.L | Y | 47.99 | 1152.6866 | 11 | 5.4 | 577.3537 | 2 | 66.80 | 9 | 19056 | Ana\_08.raw |  |  |  |  |  |  |  |  | 4.13E6 | 2.34E6 | 2 | 0 | 0 | 0 | 0 | 0 | 0 | 0 | 0 | 1 | 1 | 557 | 567 |  |
| R.VAADTFTALK.N | Y | 46.69 | 1035.5601 | 10 | 4.6 | 518.7897 | 2 | 56.95 | 9 | 16141 | Ana\_08.raw | 6.39E6 | 6.84E6 | 3.88E6 |  | 4.61E6 |  |  |  | 9.42E6 | 3.45E6 | 6 | 1 | 1 | 1 | 0 | 1 | 0 | 0 | 0 | 1 | 1 | 232 | 241 |  |
| R.EEIQSSISGVTAAYNR.E | Y | 45.88 | 1723.8376 | 16 | 9.0 | 862.9338 | 2 | 77.27 | 2 | 21948 | Ana\_01.raw |  | 4.09E6 | 5.9E5 |  | 9.84E5 |  | 0 |  |  |  | 4 | 0 | 1 | 1 | 0 | 1 | 0 | 1 | 0 | 0 | 0 | 723 | 738 |  |
| K.TDPVDIYK.S | Y | 43.71 | 949.4756 | 8 | 3.2 | 475.7466 | 2 | 51.49 | 7 | 14484 | Ana\_06.raw |  |  |  |  |  |  | 3.53E6 | 1.76E6 |  | 5.37E6 | 3 | 0 | 0 | 0 | 0 | 0 | 0 | 1 | 1 | 0 | 1 | 1089 | 1096 |  |
| R.YQELINDIAK.D | Y | 41.09 | 1205.6292 | 10 | 7.1 | 603.8261 | 2 | 71.27 | 10 | 20180 | Ana\_09.raw |  | 3.77E6 |  |  |  |  |  |  |  | 2.47E6 | 2 | 0 | 1 | 0 | 0 | 0 | 0 | 0 | 0 | 0 | 1 | 1478 | 1487 |  |
| K.VDQIQEIVTGNPTVIK.M | Y | 40.65 | 1752.9622 | 16 | 6.3 | 877.4939 | 2 | 93.11 | 10 | 26041 | Ana\_09.raw |  |  |  |  |  |  |  |  |  | 0 | 1 | 0 | 0 | 0 | 0 | 0 | 0 | 0 | 0 | 0 | 1 | 1038 | 1053 |  |
| K.GVLLEIEDLQANQFK.N | Y | 40.43 | 1715.9093 | 15 | -0.7 | 858.9613 | 2 | 120.74 | 4 | 32892 | Ana\_03.raw |  |  |  | 3.02E5 |  |  |  | 4.41E4 |  |  | 2 | 0 | 0 | 0 | 1 | 0 | 0 | 0 | 1 | 0 | 0 | 1572 | 1586 |  |
| K.FDVPGDENAEM(+15.99)DAR.T | Y | 40.17 | 1580.6412 | 14 | 4.2 | 791.3312 | 2 | 59.03 | 6 | 16880 | Ana\_05.raw | 1.34E6 |  |  |  | 3.69E6 | 6.06E6 |  |  |  |  | 3 | 1 | 0 | 0 | 0 | 1 | 1 | 0 | 0 | 0 | 0 | 1369 | 1382 | Oxidation (M) |
| K.ITLQDVVSHSK.K | Y | 39.11 | 1225.6666 | 11 | 0.6 | 409.5630 | 3 | 64.28 | 9 | 18326 | Ana\_08.raw |  |  |  |  |  |  |  |  | 5.29E5 |  | 1 | 0 | 0 | 0 | 0 | 0 | 0 | 0 | 0 | 1 | 0 | 925 | 935 |  |
| K.ATFYGEQVDYYK.S | Y | 39.04 | 1482.6666 | 12 | 3.4 | 742.3431 | 2 | 77.70 | 4 | 21597 | Ana\_03.raw |  |  |  | 9.64E5 |  |  |  | 1.57E6 |  |  | 2 | 0 | 0 | 0 | 1 | 0 | 0 | 0 | 1 | 0 | 0 | 1517 | 1528 |  |
| R.EEVITLIR.S | Y | 38.13 | 971.5651 | 8 | 4.4 | 486.7920 | 2 | 73.10 | 10 | 20690 | Ana\_09.raw |  |  |  |  |  |  |  |  |  | 2.43E7 | 1 | 0 | 0 | 0 | 0 | 0 | 0 | 0 | 0 | 0 | 1 | 894 | 901 |  |
| R.LIVDVIR.F | Y | 36.12 | 826.5276 | 7 | 1.2 | 414.2716 | 2 | 73.95 | 4 | 20571 | Ana\_03.raw |  | 1.86E6 | 1.17E6 | 7.99E5 | 1.16E6 | 1.52E6 | 9.09E5 | 7.6E5 |  | 1.61E6 | 8 | 0 | 1 | 1 | 1 | 1 | 1 | 1 | 1 | 0 | 1 | 1391 | 1397 |  |
| R.HTDNVIQWLNAM(+15.99)DEIGLPK.I | Y | 31.51 | 2209.0837 | 19 | 5.7 | 737.3727 | 3 | 122.63 | 8 | 33713 | Ana\_07.raw |  |  |  |  | 0 |  |  | 6.97E5 |  |  | 2 | 0 | 0 | 0 | 0 | 1 | 0 | 0 | 1 | 0 | 0 | 112 | 130 | Oxidation (M) |
| K.IELEK.Y | N | 28.53 | 630.3588 | 5 | 2.4 | 316.1874 | 2 | 27.29 | 6 | 7277 | Ana\_05.raw |  |  | 5.73E6 |  | 3.47E6 | 5.37E6 | 3.66E6 |  |  |  | 4 | 0 | 0 | 1 | 0 | 1 | 1 | 1 | 0 | 0 | 0 | 187 | 191 |  |
| R.TLSALR.S | Y | 26.67 | 659.3966 | 6 | 1.4 | 330.7060 | 2 | 27.01 | 3 | 7200 | Ana\_02.raw |  |  | 4.99E6 | 1.78E6 |  |  |  |  |  |  | 2 | 0 | 0 | 1 | 1 | 0 | 0 | 0 | 0 | 0 | 0 | 642 | 647 |  |
| K.LTELGTVDPK.N | Y | 25.95 | 1071.5812 | 10 | 7.8 | 536.8021 | 2 | 46.50 | 3 | 13118 | Ana\_02.raw |  |  | 2.95E6 |  |  |  |  |  |  |  | 1 | 0 | 0 | 1 | 0 | 0 | 0 | 0 | 0 | 0 | 0 | 1466 | 1475 |  |
| K.IQSLAR.M | Y | 22.26 | 686.4075 | 6 | -4.0 | 344.2096 | 2 | 21.80 | 3 | 5538 | Ana\_02.raw |  |  | 7.32E5 |  |  |  |  |  |  |  | 1 | 0 | 0 | 1 | 0 | 0 | 0 | 0 | 0 | 0 | 0 | 812 | 817 |  |
| total 25 peptides |  |  |  |  |  |  |  |  |  |  |  |  |  |  |  |  |  |  |  |  |  |  |  |  |  |  |  |  |  |  |  |  |  |  |  |
| --- | --- | --- | --- | --- | --- | --- | --- | --- | --- | --- | --- | --- | --- | --- | --- | --- | --- | --- | --- | --- | --- | --- | --- | --- | --- | --- | --- | --- | --- | --- | --- | --- | --- | --- | --- |

Best Unique PSM (Scan F6:36042, m/z=942.0122, z=2, RT=129.74, ppm=2.8):

sp|P05784|K1C18\_MOUSE

back to list

  

| Protein Coverage
| Supporting Peptides
| Best Unique PSM
|

Protein Coverage:

Supporting Peptides:

| Peptide | Uniq | -10lgP | Mass | Length | ppm | m/z | z | RT | Fraction | Scan | Source File | Area 10 | Area 01 | Area 02 | Area 03 | Area 04 | Area 05 | Area 06 | Area 07 | Area 08 | Area 09 | #Spec | #Spec 10 | #Spec 01 | #Spec 02 | #Spec 03 | #Spec 04 | #Spec 05 | #Spec 06 | #Spec 07 | #Spec 08 | #Spec 09 | Start | End | PTM |
| --- | --- | --- | --- | --- | --- | --- | --- | --- | --- | --- | --- | --- | --- | --- | --- | --- | --- | --- | --- | --- | --- | --- | --- | --- | --- | --- | --- | --- | --- | --- | --- | --- | --- | --- | --- |
| R.VRPASSAASVYAGAGGSGSR.I | Y | 103.38 | 1806.8972 | 20 | 3.9 | 603.3087 | 3 | 36.92 | 9 | 10011 | Ana\_08.raw | 5.2E7 | 3.61E7 | 2.07E7 |  | 3.6E6 | 4.56E6 |  |  | 5.04E7 | 3.15E7 | 12 | 2 | 2 | 2 | 0 | 1 | 1 | 0 | 0 | 2 | 2 | 27 | 46 |  |
| K.NQNINLENSLGDVEAR.Y | Y | 90.45 | 1784.8652 | 16 | 2.4 | 893.4420 | 2 | 84.03 | 6 | 23965 | Ana\_05.raw |  |  | 1.91E6 |  | 9.06E5 | 4.02E6 |  | 7.69E5 | 9.6E6 | 5.91E6 | 6 | 0 | 0 | 1 | 0 | 1 | 1 | 0 | 1 | 1 | 1 | 308 | 323 |  |
| K.AQM(+15.99)EQLNGVLLHLESELAQTR.A | Y | 87.82 | 2395.2166 | 21 | 5.3 | 799.4171 | 3 | 130.27 | 2 | 36074 | Ana\_01.raw | 0 | 2.48E6 | 1.73E6 |  | 4.03E6 |  | 3.49E6 | 2.45E6 | 6.98E6 |  | 7 | 1 | 1 | 1 | 0 | 1 | 0 | 1 | 1 | 1 | 0 | 326 | 346 | Oxidation (M) |
| R.LQLETEIEALKEELLFM(+15.99)K.K | Y | 78.64 | 2192.1650 | 18 | 3.5 | 731.7315 | 3 | 132.91 | 6 | 36949 | Ana\_05.raw | 7.22E4 | 2.66E6 | 2.96E6 | 1.27E6 |  | 7.97E6 |  |  | 8.84E6 | 5.42E6 | 7 | 1 | 1 | 1 | 1 | 0 | 1 | 0 | 0 | 1 | 1 | 190 | 207 | Oxidation (M) |
| R.AQIFANSVDNAR.I | Y | 70.91 | 1304.6472 | 12 | 4.1 | 653.3336 | 2 | 51.92 | 1 | 14588 | ANa\_10.raw | 7.96E6 | 7.47E6 | 5.5E6 |  |  |  |  |  |  | 6.37E6 | 4 | 1 | 1 | 1 | 0 | 0 | 0 | 0 | 0 | 0 | 1 | 131 | 142 |  |
| K.LEAEIATYR.R | Y | 70.55 | 1064.5502 | 9 | 4.4 | 533.2847 | 2 | 55.19 | 5 | 15652 | Ana\_04.raw | 5.83E6 | 7.74E6 | 8.13E6 | 3.58E6 | 7.08E6 | 6.87E6 | 4.3E6 | 3.93E6 |  | 5.99E6 | 9 | 1 | 1 | 1 | 1 | 1 | 1 | 1 | 1 | 0 | 1 | 366 | 374 |  |
| R.KVVDDTNITR.L | Y | 64.95 | 1159.6196 | 10 | 4.6 | 580.8198 | 2 | 26.51 | 1 | 6911 | ANa\_10.raw | 3.09E6 | 7.69E6 | 4.62E6 | 2.15E6 | 3.8E6 |  |  |  | 1.37E7 | 3.59E6 | 7 | 1 | 1 | 1 | 1 | 1 | 0 | 0 | 0 | 1 | 1 | 180 | 189 |  |
| R.IVLQIDNAR.L | N | 63.88 | 1040.5978 | 9 | 4.1 | 521.3083 | 2 | 60.33 | 6 | 17263 | Ana\_05.raw | 1.45E8 | 1.35E8 | 6.92E6 | 5.18E7 | 1.12E8 | 9.67E7 | 6E7 |  | 9.07E7 | 9.46E7 | 17 | 2 | 1 | 1 | 2 | 1 | 1 | 2 | 0 | 3 | 4 | 143 | 151 |  |
| R.DAETTLTELR.R | Y | 61.07 | 1147.5720 | 10 | 2.9 | 574.7949 | 2 | 70.78 | 4 | 19692 | Ana\_03.raw |  |  | 2.06E7 | 1.07E7 | 2.2E7 | 1.93E7 | 1.21E7 | 1.14E7 | 5.18E7 | 2.84E7 | 11 | 0 | 0 | 2 | 1 | 1 | 2 | 1 | 1 | 2 | 1 | 284 | 293 |  |
| R.AQYEALAQK.N | Y | 58.78 | 1020.5240 | 9 | 3.8 | 511.2712 | 2 | 35.05 | 6 | 9635 | Ana\_05.raw | 2.88E7 | 2.86E7 | 1.88E7 | 8.21E6 | 1.85E7 | 2.3E7 | 1.39E7 | 1.23E7 | 3.78E7 | 2.56E7 | 10 | 1 | 1 | 1 | 1 | 1 | 1 | 1 | 1 | 1 | 1 | 247 | 255 |  |
| K.ETM(+15.99)QDLNDR.L | Y | 58.10 | 1136.4768 | 9 | 3.7 | 569.2478 | 2 | 21.35 | 7 | 5327 | Ana\_06.raw |  |  |  | 4.68E6 | 1.99E6 |  | 8.47E6 | 6.31E6 |  |  | 4 | 0 | 0 | 0 | 1 | 1 | 0 | 1 | 1 | 0 | 0 | 74 | 82 | Oxidation (M) |
| R.LAADDFR.V | N | 57.33 | 806.3922 | 7 | 4.2 | 404.2051 | 2 | 42.34 | 2 | 11777 | Ana\_01.raw | 2.24E8 | 2.78E8 | 2.22E8 | 1.32E8 | 2.61E8 | 2.64E8 | 1.84E8 | 1.78E8 | 2.86E8 | 1.96E8 | 49 | 5 | 5 | 6 | 5 | 6 | 2 | 5 | 6 | 5 | 4 | 152 | 158 |  |
| R.STTFSTNYR.S | Y | 56.08 | 1075.4934 | 9 | 5.0 | 538.7567 | 2 | 40.84 | 1 | 11275 | ANa\_10.raw | 3.17E7 |  | 2.1E7 | 9.69E6 | 2.39E7 | 2.67E7 | 1.67E7 | 1.41E7 | 4.29E7 |  | 8 | 1 | 0 | 1 | 1 | 1 | 1 | 1 | 1 | 1 | 0 | 7 | 15 |  |
| K.VVDDTNITR.L | Y | 51.59 | 1031.5248 | 9 | 2.5 | 516.7709 | 2 | 30.03 | 5 | 8107 | Ana\_04.raw | 3.69E7 | 4.09E7 | 2.62E7 | 1.39E7 | 2.52E7 | 3.29E7 | 1.89E7 | 1.68E7 | 5.4E7 | 3.49E7 | 10 | 1 | 1 | 1 | 1 | 1 | 1 | 1 | 1 | 1 | 1 | 181 | 189 |  |
| R.DAETTLTELRR.T | Y | 50.91 | 1303.6732 | 11 | 5.9 | 435.5676 | 3 | 57.75 | 9 | 16382 | Ana\_08.raw | 7.49E6 |  |  |  |  |  |  |  | 1.26E7 |  | 3 | 1 | 0 | 0 | 0 | 0 | 0 | 0 | 0 | 2 | 0 | 284 | 294 |  |
| R.LASYLDK.V | N | 49.65 | 808.4330 | 7 | 0.3 | 405.2239 | 2 | 36.28 | 3 | 10002 | Ana\_02.raw | 1.07E8 | 1.23E8 | 1.07E8 | 4.67E7 | 1.05E8 | 1.26E8 | 7.91E7 | 7.01E7 | 1.06E8 | 8.02E7 | 17 | 3 | 2 | 1 | 2 | 1 | 1 | 2 | 2 | 2 | 1 | 83 | 89 |  |
| R.QAQEYEALLNIK.V | Y | 46.50 | 1418.7405 | 12 | 4.9 | 710.3810 | 2 | 119.37 | 9 | 33285 | Ana\_08.raw |  |  | 9.9E5 |  |  |  | 3.15E5 |  | 4.69E6 | 2.81E6 | 4 | 0 | 0 | 1 | 0 | 0 | 0 | 1 | 0 | 1 | 1 | 352 | 363 |  |
| K.IREHLEK.K | Y | 35.23 | 923.5188 | 7 | 1.1 | 308.8472 | 3 | 17.55 | 3 | 4117 | Ana\_02.raw |  | 6.33E6 | 4.65E6 |  | 4.39E6 | 5.91E6 |  |  |  | 5.27E6 | 6 | 0 | 1 | 2 | 0 | 1 | 1 | 0 | 0 | 0 | 1 | 104 | 110 |  |
| K.IIEDLR.A | Y | 33.40 | 757.4333 | 6 | 5.2 | 379.7259 | 2 | 39.93 | 1 | 10996 | ANa\_10.raw | 7.93E6 |  | 8.16E6 | 2.35E6 | 6.5E6 |  | 4.12E6 |  |  |  | 5 | 1 | 0 | 1 | 1 | 1 | 0 | 1 | 0 | 0 | 0 | 125 | 130 |  |
| R.VKYETELAM(+15.99)R.Q | Y | 27.57 | 1254.6278 | 10 | 7.6 | 628.3259 | 2 | 39.26 | 1 | 10786 | ANa\_10.raw | 7.5E6 |  |  |  |  |  |  |  |  |  | 1 | 1 | 0 | 0 | 0 | 0 | 0 | 0 | 0 | 0 | 0 | 159 | 168 | Oxidation (M) |
| K.SLETENRR.L | Y | 22.43 | 1003.5046 | 8 | -0.3 | 502.7595 | 2 | 17.37 | 5 | 4103 | Ana\_04.raw |  |  |  |  | 7.43E5 |  |  |  |  |  | 1 | 0 | 0 | 0 | 0 | 1 | 0 | 0 | 0 | 0 | 0 | 92 | 99 |  |
| total 21 peptides |  |  |  |  |  |  |  |  |  |  |  |  |  |  |  |  |  |  |  |  |  |  |  |  |  |  |  |  |  |  |  |  |  |  |  |
| --- | --- | --- | --- | --- | --- | --- | --- | --- | --- | --- | --- | --- | --- | --- | --- | --- | --- | --- | --- | --- | --- | --- | --- | --- | --- | --- | --- | --- | --- | --- | --- | --- | --- | --- | --- |

Best Unique PSM (Scan F9:10011, m/z=603.3087, z=3, RT=36.92, ppm=3.9):

sp|Q9JKR6|HYOU1\_MOUSE

back to list

  

| Protein Coverage
| Supporting Peptides
| Best Unique PSM
|

Protein Coverage:

Supporting Peptides:

| Peptide | Uniq | -10lgP | Mass | Length | ppm | m/z | z | RT | Fraction | Scan | Source File | Area 10 | Area 01 | Area 02 | Area 03 | Area 04 | Area 05 | Area 06 | Area 07 | Area 08 | Area 09 | #Spec | #Spec 10 | #Spec 01 | #Spec 02 | #Spec 03 | #Spec 04 | #Spec 05 | #Spec 06 | #Spec 07 | #Spec 08 | #Spec 09 | Start | End | PTM |
| --- | --- | --- | --- | --- | --- | --- | --- | --- | --- | --- | --- | --- | --- | --- | --- | --- | --- | --- | --- | --- | --- | --- | --- | --- | --- | --- | --- | --- | --- | --- | --- | --- | --- | --- | --- |
| R.VESVFETLVEDSPEEESTLTK.L | Y | 117.37 | 2367.1216 | 21 | 3.1 | 1184.5718 | 2 | 122.77 | 3 | 33744 | Ana\_02.raw | 1.55E6 | 1.85E6 | 3.88E6 | 1.87E6 | 1.11E6 | 2.54E6 | 5.38E6 | 1.53E6 | 3.26E6 |  | 14 | 2 | 1 | 2 | 2 | 1 | 1 | 2 | 2 | 1 | 0 | 556 | 576 |  |
| R.SLAEDFAEQPIKDAVITVPAFFNQAER.R | Y | 89.49 | 3005.5134 | 27 | 0.7 | 1002.8458 | 3 | 132.41 | 7 | 36473 | Ana\_06.raw |  |  |  |  |  |  | 1.72E6 |  | 2.15E6 |  | 2 | 0 | 0 | 0 | 0 | 0 | 0 | 1 | 0 | 1 | 0 | 159 | 185 |  |
| R.DAVIYPILVEFTR.E | Y | 87.29 | 1534.8395 | 13 | 3.4 | 768.4296 | 2 | 132.55 | 3 | 36305 | Ana\_02.raw | 1.44E5 | 2.31E6 | 1.96E6 | 1.87E6 | 3.38E6 | 2.06E6 | 1.62E6 | 1.93E6 | 2.4E6 | 7.2E5 | 12 | 1 | 1 | 1 | 2 | 1 | 1 | 2 | 1 | 1 | 1 | 439 | 451 |  |
| R.VEFEELC(+57.02)ADLFDR.V | Y | 80.60 | 1641.7344 | 13 | 0.2 | 821.8746 | 2 | 122.54 | 3 | 33688 | Ana\_02.raw |  |  | 1.27E6 | 2.44E5 | 8.97E5 | 7.95E5 | 4.07E5 | 2.12E5 |  | 1.16E6 | 7 | 0 | 0 | 1 | 1 | 1 | 1 | 1 | 1 | 0 | 1 | 346 | 358 | Carbamidomethylation |
| K.VLQLINDNTATALSYGVFR.R | Y | 78.32 | 2094.1108 | 19 | 3.2 | 1048.0660 | 2 | 127.91 | 7 | 35195 | Ana\_06.raw |  |  |  |  |  |  | 2.07E6 | 1.27E6 |  |  | 2 | 0 | 0 | 0 | 0 | 0 | 0 | 1 | 1 | 0 | 0 | 199 | 217 |  |
| K.DAVITVPAFFNQAER.R | Y | 73.49 | 1676.8522 | 15 | -0.5 | 839.4329 | 2 | 113.43 | 3 | 31357 | Ana\_02.raw | 4.84E5 | 2.55E6 | 6.33E5 |  |  | 1E6 |  | 1.52E6 |  |  | 5 | 1 | 1 | 1 | 0 | 0 | 1 | 0 | 1 | 0 | 0 | 171 | 185 |  |
| K.AANSLEAFIFETQDK.L | Y | 66.66 | 1682.8151 | 15 | 5.9 | 842.4198 | 2 | 119.30 | 7 | 32960 | Ana\_06.raw |  |  |  |  |  |  | 4.48E5 |  |  |  | 1 | 0 | 0 | 0 | 0 | 0 | 0 | 1 | 0 | 0 | 0 | 739 | 753 |  |
| R.EVEEEPGLR.S | Y | 58.04 | 1056.5087 | 9 | 5.4 | 529.2645 | 2 | 34.89 | 2 | 9479 | Ana\_01.raw | 1.33E7 | 5.44E6 |  | 2.51E6 | 1.19E7 | 1.34E7 | 1.25E7 | 9.96E6 | 9.93E6 | 1.65E7 | 9 | 1 | 1 | 0 | 1 | 1 | 1 | 1 | 1 | 1 | 1 | 452 | 460 |  |
| K.LGNTISSLFGGGTSSDAK.E | Y | 49.49 | 1710.8424 | 18 | 3.6 | 856.4316 | 2 | 92.78 | 2 | 26213 | Ana\_01.raw |  | 2E5 |  |  |  |  |  |  |  |  | 1 | 0 | 1 | 0 | 0 | 0 | 0 | 0 | 0 | 0 | 0 | 577 | 594 |  |
| K.AVGKEELGK.N | Y | 46.56 | 929.5181 | 9 | 4.4 | 465.7684 | 2 | 20.21 | 8 | 4895 | Ana\_07.raw |  |  |  | 1.47E6 |  | 2.88E6 | 8.87E5 | 1.06E6 |  |  | 4 | 0 | 0 | 0 | 1 | 0 | 1 | 1 | 1 | 0 | 0 | 399 | 407 |  |
| R.M(+15.99)GPYPQR.K | Y | 44.38 | 863.3959 | 7 | 3.9 | 432.7069 | 2 | 21.49 | 5 | 5458 | Ana\_04.raw |  |  |  |  | 6.17E6 |  | 4.93E6 | 3.06E6 |  |  | 3 | 0 | 0 | 0 | 0 | 1 | 0 | 1 | 1 | 0 | 0 | 473 | 479 | Oxidation (M) |
| K.LEELTLR.D | Y | 44.22 | 872.4967 | 7 | 4.2 | 437.2575 | 2 | 54.05 | 9 | 15291 | Ana\_08.raw | 3.68E6 | 4.62E6 |  | 2.62E6 |  |  | 4.54E6 |  | 6.25E6 |  | 5 | 1 | 1 | 0 | 1 | 0 | 0 | 1 | 0 | 1 | 0 | 723 | 729 |  |
| R.TLGGLEM(+15.99)ELR.L | Y | 38.84 | 1133.5751 | 10 | 4.9 | 567.7976 | 2 | 75.19 | 7 | 21302 | Ana\_06.raw | 2.8E6 |  |  | 2.03E5 | 6.64E5 | 1.4E6 | 9.95E5 |  | 1.3E6 |  | 6 | 1 | 0 | 0 | 1 | 1 | 1 | 1 | 0 | 1 | 0 | 269 | 278 | Oxidation (M) |
| K.TPVTVTLK.E | Y | 35.97 | 857.5222 | 8 | 4.0 | 429.7701 | 2 | 40.53 | 9 | 11140 | Ana\_08.raw |  |  |  |  |  |  |  |  | 6.28E5 |  | 1 | 0 | 0 | 0 | 0 | 0 | 0 | 0 | 0 | 1 | 0 | 68 | 75 |  |
| K.VITFNR.Y | Y | 35.46 | 748.4232 | 6 | 5.2 | 375.2208 | 2 | 42.05 | 7 | 11624 | Ana\_06.raw |  |  |  |  |  | 1.79E6 | 1.72E6 |  |  |  | 2 | 0 | 0 | 0 | 0 | 0 | 1 | 1 | 0 | 0 | 0 | 481 | 486 |  |
| K.VQEVLLK.A | Y | 32.15 | 827.5116 | 7 | 5.1 | 414.7652 | 2 | 41.91 | 1 | 11605 | ANa\_10.raw | 2.18E6 | 2.39E6 | 3.03E6 |  |  |  |  |  |  | 2.56E6 | 4 | 1 | 1 | 1 | 0 | 0 | 0 | 0 | 0 | 0 | 1 | 392 | 398 |  |
| R.FLGDSAAGM(+15.99)AIK.N | Y | 26.61 | 1195.5907 | 12 | -0.2 | 598.8025 | 2 | 59.49 | 4 | 16525 | Ana\_03.raw |  |  |  | 1.38E6 |  |  | 3.73E6 |  |  |  | 2 | 0 | 0 | 0 | 1 | 0 | 0 | 1 | 0 | 0 | 0 | 80 | 91 | Oxidation (M) |
| K.NATLAEQAK.L | Y | 23.51 | 944.4927 | 9 | 5.9 | 473.2564 | 2 | 34.32 | 7 | 9289 | Ana\_06.raw |  |  |  |  |  |  | 2.83E7 |  |  |  | 1 | 0 | 0 | 0 | 0 | 0 | 0 | 1 | 0 | 0 | 0 | 869 | 877 |  |
| total 18 peptides |  |  |  |  |  |  |  |  |  |  |  |  |  |  |  |  |  |  |  |  |  |  |  |  |  |  |  |  |  |  |  |  |  |  |  |
| --- | --- | --- | --- | --- | --- | --- | --- | --- | --- | --- | --- | --- | --- | --- | --- | --- | --- | --- | --- | --- | --- | --- | --- | --- | --- | --- | --- | --- | --- | --- | --- | --- | --- | --- | --- |

Best Unique PSM (Scan F3:33744, m/z=1184.5718, z=2, RT=122.77, ppm=3.1):

sp|P47738|ALDH2\_MOUSE

back to list

  

| Protein Coverage
| Supporting Peptides
| Best Unique PSM
|

Protein Coverage:

Supporting Peptides:

| Peptide | Uniq | -10lgP | Mass | Length | ppm | m/z | z | RT | Fraction | Scan | Source File | Area 10 | Area 01 | Area 02 | Area 03 | Area 04 | Area 05 | Area 06 | Area 07 | Area 08 | Area 09 | #Spec | #Spec 10 | #Spec 01 | #Spec 02 | #Spec 03 | #Spec 04 | #Spec 05 | #Spec 06 | #Spec 07 | #Spec 08 | #Spec 09 | Start | End | PTM |
| --- | --- | --- | --- | --- | --- | --- | --- | --- | --- | --- | --- | --- | --- | --- | --- | --- | --- | --- | --- | --- | --- | --- | --- | --- | --- | --- | --- | --- | --- | --- | --- | --- | --- | --- | --- |
| K.EAGFPPGVVNIVPGFGPTAGAAIASHEGVDK.V | Y | 99.34 | 2960.5032 | 31 | 0.2 | 987.8419 | 3 | 121.01 | 6 | 33752 | Ana\_05.raw | 1.27E7 | 4.33E6 | 1.1E7 | 1.29E6 | 5.98E6 | 8.45E6 | 2.25E6 | 2.86E6 | 1.01E7 | 1.8E2 | 26 | 5 | 2 | 2 | 2 | 3 | 3 | 1 | 3 | 4 | 1 | 229 | 259 |  |
| K.TIPIDGDFFSYTR.H | Y | 97.76 | 1530.7354 | 13 | 1.1 | 766.3758 | 2 | 107.98 | 3 | 29951 | Ana\_02.raw | 1E7 | 1.92E7 | 1.61E7 | 1.09E7 | 5.11E6 | 1.22E7 | 8.24E6 | 6.98E6 | 4.01E7 | 1.85E7 | 28 | 2 | 4 | 3 | 4 | 1 | 3 | 2 | 3 | 3 | 3 | 162 | 174 |  |
| R.TFVQENVYDEFVER.S | Y | 91.86 | 1773.8209 | 14 | -1.7 | 887.9163 | 2 | 102.49 | 3 | 28550 | Ana\_02.raw | 9.21E6 | 9.9E6 | 1.13E7 | 6.69E6 | 7.13E6 | 1.28E7 | 5.49E6 | 4.6E6 | 3.35E7 |  | 11 | 1 | 1 | 1 | 1 | 2 | 1 | 1 | 1 | 2 | 0 | 327 | 340 |  |
| K.VAFTGSTEVGHLIQVAAGSSNLK.R | Y | 87.50 | 2285.2014 | 23 | -1.9 | 762.7396 | 3 | 119.39 | 1 | 32942 | ANa\_10.raw | 2.62E6 |  | 3.89E6 | 2.06E6 | 1.32E6 | 7.51E6 |  | 1.48E6 | 7.35E6 |  | 7 | 1 | 0 | 1 | 1 | 1 | 1 | 0 | 1 | 1 | 0 | 260 | 282 |  |
| R.GYFIQPTVFGDVK.D | Y | 82.39 | 1469.7554 | 13 | 5.5 | 735.8890 | 2 | 107.41 | 7 | 29880 | Ana\_06.raw | 2.03E7 | 2.69E7 | 2.1E7 | 7.4E6 | 1.13E7 | 1.69E7 | 5.52E6 | 8E6 | 3.95E7 | 2.15E7 | 15 | 2 | 2 | 2 | 1 | 1 | 1 | 1 | 1 | 2 | 2 | 397 | 409 |  |
| R.ELGEYGLQAYTEVK.T | Y | 75.88 | 1598.7827 | 14 | 3.4 | 800.4014 | 2 | 89.56 | 6 | 25473 | Ana\_05.raw |  |  | 4.97E5 | 7.31E5 | 1.04E6 | 6.73E5 |  | 5.33E5 | 3.59E6 |  | 6 | 0 | 0 | 1 | 1 | 1 | 1 | 0 | 1 | 1 | 0 | 495 | 508 |  |
| R.TEQGPQVDETQFK.K | Y | 75.48 | 1505.6997 | 13 | 6.2 | 753.8618 | 2 | 50.29 | 2 | 14180 | Ana\_01.raw | 2.28E7 | 2.6E7 | 1.73E7 | 6.59E6 | 1.35E7 | 1.68E7 | 8.91E6 | 1.13E7 |  | 1.44E7 | 10 | 1 | 2 | 1 | 1 | 1 | 1 | 1 | 1 | 0 | 1 | 358 | 370 |  |
| K.VAEQTPLTALYVANLIK.E | Y | 74.62 | 1843.0454 | 17 | 3.0 | 922.5328 | 2 | 128.61 | 9 | 35628 | Ana\_08.raw | 3.31E6 | 1.18E7 | 3.12E6 | 4.58E6 | 2.18E6 | 1.05E7 | 4.18E6 | 3.26E6 | 1.48E7 | 5.51E6 | 10 | 1 | 1 | 1 | 1 | 1 | 1 | 1 | 1 | 1 | 1 | 212 | 228 |  |
| R.VVGNPFDSR.T | Y | 73.68 | 989.4930 | 9 | 2.3 | 495.7549 | 2 | 54.61 | 5 | 15484 | Ana\_04.raw | 2.03E7 | 6.29E5 | 2.31E7 | 9.93E6 | 2.15E7 | 2.15E7 | 1.42E7 | 1.38E7 |  | 1.96E7 | 9 | 1 | 1 | 1 | 1 | 1 | 1 | 1 | 1 | 0 | 1 | 349 | 357 |  |
| K.EEIFGPVM(+15.99)QILK.F | Y | 59.12 | 1418.7479 | 12 | 3.3 | 710.3835 | 2 | 111.58 | 6 | 31288 | Ana\_05.raw | 4.63E6 | 2.38E6 | 3.38E6 | 2.44E6 | 2.71E6 | 3.63E6 | 1.03E6 | 2.97E6 | 1.73E7 | 6.32E5 | 17 | 1 | 1 | 3 | 2 | 2 | 2 | 1 | 2 | 2 | 1 | 417 | 428 | Oxidation (M) |
| K.YGLAAAVFTK.D | Y | 57.37 | 1039.5702 | 10 | 0.1 | 520.7924 | 2 | 78.89 | 5 | 22395 | Ana\_04.raw |  |  |  |  | 1.48E6 | 2.09E6 | 4.81E5 | 3.3E5 |  |  | 4 | 0 | 0 | 0 | 0 | 1 | 1 | 1 | 1 | 0 | 0 | 444 | 453 |  |
| R.AAFQLGSPWR.R | Y | 56.69 | 1131.5825 | 10 | 4.1 | 566.8008 | 2 | 85.53 | 9 | 24327 | Ana\_08.raw |  |  |  | 3.43E5 |  |  | 3.97E5 |  | 1.55E6 |  | 3 | 0 | 0 | 0 | 1 | 0 | 0 | 1 | 0 | 1 | 0 | 87 | 96 |  |
| K.TIEEVVGR.A | Y | 53.31 | 901.4869 | 8 | 0.8 | 451.7511 | 2 | 43.70 | 4 | 11934 | Ana\_03.raw | 1.58E7 |  | 1.69E7 | 6.66E6 | 1.53E7 |  |  | 7.05E6 | 4.81E6 | 8.79E6 | 7 | 1 | 0 | 1 | 1 | 1 | 0 | 0 | 1 | 1 | 1 | 431 | 438 |  |
| R.VTLELGGK.S | N | 46.93 | 815.4752 | 8 | 4.9 | 408.7469 | 2 | 50.47 | 8 | 13979 | Ana\_07.raw | 2.75E7 | 3.49E7 | 3.43E7 | 1.61E7 | 2.77E7 | 3.58E7 | 2.3E7 | 1.7E7 | 1.6E7 | 2.95E7 | 11 | 1 | 1 | 1 | 1 | 1 | 1 | 1 | 1 | 2 | 1 | 284 | 291 |  |
| K.LLC(+57.02)GGGAAADR.G | Y | 45.83 | 1059.5131 | 11 | 1.5 | 530.7646 | 2 | 29.55 | 5 | 7963 | Ana\_04.raw |  |  | 1.21E7 |  | 8.82E6 |  |  |  | 8.5E5 |  | 3 | 0 | 0 | 1 | 0 | 1 | 0 | 0 | 0 | 1 | 0 | 386 | 396 | Carbamidomethylation |
| K.VAFTGSTEVGHLIQVAAGSSNLKR.V | Y | 37.95 | 2441.3027 | 24 | 5.0 | 611.3360 | 4 | 110.93 | 6 | 31117 | Ana\_05.raw |  |  |  | 9.58E5 |  | 2.13E6 |  |  |  |  | 2 | 0 | 0 | 0 | 1 | 0 | 1 | 0 | 0 | 0 | 0 | 260 | 283 |  |
| K.LGPALATGNVVVM(+15.99)K.V | Y | 30.31 | 1384.7748 | 14 | 2.5 | 693.3964 | 2 | 65.36 | 9 | 18639 | Ana\_08.raw |  |  |  |  |  |  |  |  | 1.97E6 |  | 1 | 0 | 0 | 0 | 0 | 0 | 0 | 0 | 0 | 1 | 0 | 198 | 211 | Oxidation (M) |
| K.EEIFGPVMQILK.F | Y | 23.76 | 1402.7529 | 12 | 9.8 | 702.3906 | 2 | 128.34 | 1 | 35287 | ANa\_10.raw | 3.52E6 |  |  |  |  |  |  |  |  |  | 1 | 1 | 0 | 0 | 0 | 0 | 0 | 0 | 0 | 0 | 0 | 417 | 428 |  |
| total 18 peptides |  |  |  |  |  |  |  |  |  |  |  |  |  |  |  |  |  |  |  |  |  |  |  |  |  |  |  |  |  |  |  |  |  |  |  |
| --- | --- | --- | --- | --- | --- | --- | --- | --- | --- | --- | --- | --- | --- | --- | --- | --- | --- | --- | --- | --- | --- | --- | --- | --- | --- | --- | --- | --- | --- | --- | --- | --- | --- | --- | --- |

Best Unique PSM (Scan F6:33752, m/z=987.8419, z=3, RT=121.01, ppm=0.2):

sp|Q9EQK5|MVP\_MOUSE

back to list

  

| Protein Coverage
| Supporting Peptides
| Best Unique PSM
|

Protein Coverage:

Supporting Peptides:

| Peptide | Uniq | -10lgP | Mass | Length | ppm | m/z | z | RT | Fraction | Scan | Source File | Area 10 | Area 01 | Area 02 | Area 03 | Area 04 | Area 05 | Area 06 | Area 07 | Area 08 | Area 09 | #Spec | #Spec 10 | #Spec 01 | #Spec 02 | #Spec 03 | #Spec 04 | #Spec 05 | #Spec 06 | #Spec 07 | #Spec 08 | #Spec 09 | Start | End | PTM |
| --- | --- | --- | --- | --- | --- | --- | --- | --- | --- | --- | --- | --- | --- | --- | --- | --- | --- | --- | --- | --- | --- | --- | --- | --- | --- | --- | --- | --- | --- | --- | --- | --- | --- | --- | --- |
| K.ELPSGVEELLNLGHDPLADR.G | Y | 109.12 | 2173.1016 | 20 | -0.4 | 725.3741 | 3 | 129.30 | 3 | 35424 | Ana\_02.raw | 2.06E7 | 8.92E5 | 1.27E7 | 7.07E6 |  | 1.5E7 | 6.55E6 | 7.61E6 | 2.62E7 |  | 9 | 2 | 1 | 1 | 1 | 0 | 1 | 1 | 1 | 1 | 0 | 418 | 437 |  |
| R.LAQDPFPLYPGELLEK.D | Y | 86.24 | 1828.9611 | 16 | 1.7 | 915.4894 | 2 | 124.49 | 3 | 34186 | Ana\_02.raw | 2.68E7 | 1.1E7 | 1.11E7 | 3.95E6 | 1.68E7 | 3.5E6 | 1.33E7 |  | 3.77E7 | 7.24E6 | 16 | 2 | 2 | 2 | 2 | 2 | 2 | 1 | 0 | 2 | 1 | 92 | 107 |  |
| R.GIQDVYVLSEQQGLLLK.A | Y | 79.46 | 1902.0461 | 17 | 4.0 | 952.0342 | 2 | 117.88 | 2 | 32796 | Ana\_01.raw | 8.82E5 | 1.7E6 |  | 4.14E5 |  | 5.92E5 | 6.19E5 | 8.01E5 | 7.93E4 | 1.64E6 | 8 | 1 | 1 | 0 | 1 | 0 | 1 | 1 | 1 | 1 | 1 | 319 | 335 |  |
| K.ALQPLEEGEGEEK.V | Y | 71.85 | 1427.6780 | 13 | 2.5 | 714.8480 | 2 | 50.42 | 4 | 13905 | Ana\_03.raw | 1.04E7 | 1.05E7 | 7.54E6 | 3.66E6 | 6.44E6 | 7.77E6 | 5.85E6 | 4.86E6 | 1.37E7 | 9.28E6 | 10 | 1 | 1 | 1 | 1 | 1 | 1 | 1 | 1 | 1 | 1 | 336 | 348 |  |
| K.SFFLQPGER.L | Y | 70.13 | 1079.5399 | 9 | 2.4 | 540.7785 | 2 | 72.75 | 6 | 20873 | Ana\_05.raw | 8.31E6 | 1.18E7 | 6.32E6 | 1.1E6 | 5.95E6 | 7.41E6 | 5.62E6 | 5.1E6 | 1.07E7 | 7.16E6 | 13 | 1 | 2 | 1 | 1 | 1 | 1 | 1 | 1 | 2 | 2 | 307 | 315 |  |
| K.DITPLQVVLPNTALHLK.A | Y | 68.89 | 1871.0880 | 17 | 5.0 | 624.7064 | 3 | 118.22 | 6 | 33021 | Ana\_05.raw | 3.01E6 | 7.95E6 | 5.74E6 | 1.11E6 | 1.01E6 | 6.85E6 | 4.87E6 | 3.3E6 | 1.24E7 | 2.21E6 | 13 | 2 | 1 | 1 | 1 | 1 | 1 | 1 | 1 | 1 | 3 | 108 | 124 |  |
| K.LFSVPDFVGDAC(+57.02)K.A | Y | 64.60 | 1453.6912 | 13 | 3.8 | 727.8557 | 2 | 108.53 | 5 | 30242 | Ana\_04.raw | 1.43E6 |  |  | 3.67E5 | 6.32E5 |  |  |  |  | 2.01E6 | 4 | 1 | 0 | 0 | 1 | 1 | 0 | 0 | 0 | 0 | 1 | 561 | 573 | Carbamidomethylation |
| R.GPLEYVPSAK.V | Y | 62.81 | 1059.5601 | 10 | 5.6 | 530.7903 | 2 | 65.44 | 8 | 18361 | Ana\_07.raw |  |  | 6.56E6 |  | 4.14E6 |  | 3.51E6 | 6.32E6 |  | 1.76E6 | 5 | 0 | 0 | 1 | 0 | 1 | 0 | 1 | 1 | 0 | 1 | 361 | 370 |  |
| K.EVEVVEIIQATVIK.Q | Y | 59.87 | 1568.9025 | 14 | 3.4 | 785.4612 | 2 | 130.84 | 7 | 36011 | Ana\_06.raw |  | 8.12E5 |  |  |  |  | 7.76E5 |  |  |  | 2 | 0 | 1 | 0 | 0 | 0 | 0 | 1 | 0 | 0 | 0 | 156 | 169 |  |
| R.IEGEGSVLQAK.L | Y | 58.13 | 1129.5979 | 11 | 5.2 | 565.8091 | 2 | 45.84 | 7 | 12766 | Ana\_06.raw | 7.17E6 | 8.84E6 | 7.24E6 | 4.54E6 | 7.56E6 | 8.94E6 | 8.26E6 | 4.49E6 | 1.57E7 | 8.37E6 | 10 | 1 | 1 | 1 | 1 | 1 | 1 | 1 | 1 | 1 | 1 | 735 | 745 |  |
| K.AQQLADVEAK.K | Y | 55.90 | 1071.5560 | 10 | 4.3 | 536.7876 | 2 | 34.77 | 7 | 9424 | Ana\_06.raw | 8.92E6 |  |  | 3.36E6 |  | 2.78E6 | 4.53E6 |  |  | 7.59E6 | 5 | 1 | 0 | 0 | 1 | 0 | 1 | 1 | 0 | 0 | 1 | 784 | 793 |  |
| K.ILDQSEAEK.A | Y | 54.03 | 1031.5134 | 9 | 3.5 | 516.7658 | 2 | 27.06 | 6 | 7205 | Ana\_05.raw |  |  |  |  | 8.77E6 | 1.46E7 | 6.49E6 |  |  |  | 3 | 0 | 0 | 0 | 0 | 1 | 1 | 1 | 0 | 0 | 0 | 693 | 701 |  |
| R.DLAVAGPEM(+15.99)QVK.L | Y | 50.03 | 1272.6383 | 12 | 1.1 | 637.3271 | 2 | 52.15 | 7 | 14676 | Ana\_06.raw |  | 5.7E6 | 0 | 1.45E6 |  |  | 3.15E6 | 2.65E6 | 1.27E7 | 8.91E5 | 7 | 0 | 1 | 1 | 1 | 0 | 0 | 1 | 1 | 1 | 1 | 809 | 820 | Oxidation (M) |
| K.EM(+15.99)TEALGPGTIR.D | Y | 48.54 | 1289.6285 | 12 | 7.6 | 645.8264 | 2 | 52.63 | 7 | 14814 | Ana\_06.raw |  |  | 6.72E5 | 1.12E6 | 2.34E6 | 1.09E6 | 1.92E6 | 3.31E6 |  |  | 6 | 0 | 0 | 1 | 1 | 1 | 1 | 1 | 1 | 0 | 0 | 797 | 808 | Oxidation (M) |
| K.VLQPSAAR.N | Y | 48.46 | 840.4817 | 8 | 5.1 | 421.2503 | 2 | 23.44 | 2 | 5904 | Ana\_01.raw |  | 1.48E7 |  | 3.62E6 | 7.06E5 |  | 4.4E6 |  |  | 2.09E6 | 5 | 0 | 1 | 0 | 1 | 1 | 0 | 1 | 0 | 0 | 1 | 445 | 452 |  |
| R.VTGEEWLVR.S | Y | 47.63 | 1087.5662 | 9 | 3.5 | 544.7922 | 2 | 73.34 | 6 | 21038 | Ana\_05.raw |  |  |  |  |  | 9.57E5 |  |  |  |  | 1 | 0 | 0 | 0 | 0 | 0 | 1 | 0 | 0 | 0 | 0 | 191 | 199 |  |
| K.AQALAIETEAELER.V | Y | 47.09 | 1542.7889 | 14 | -4.9 | 772.3979 | 2 | 80.64 | 1 | 22758 | ANa\_10.raw | 3.26E2 | 0 |  |  |  |  |  |  |  |  | 2 | 1 | 1 | 0 | 0 | 0 | 0 | 0 | 0 | 0 | 0 | 748 | 761 |  |
| R.VLFAPVR.M | Y | 46.66 | 800.4908 | 7 | 4.5 | 401.2545 | 2 | 61.16 | 6 | 17522 | Ana\_05.raw | 5.63E6 | 5.36E6 | 6.18E6 | 2.61E6 |  | 4.69E6 | 4.17E6 | 3.63E6 | 9.12E6 | 5.39E6 | 9 | 1 | 1 | 1 | 1 | 0 | 1 | 1 | 1 | 1 | 1 | 43 | 49 |  |
| R.GAVASVTFDDFHK.N | Y | 43.48 | 1392.6674 | 13 | 5.1 | 465.2321 | 3 | 65.63 | 9 | 18718 | Ana\_08.raw | 1.41E6 | 4.34E4 |  |  | 8.54E5 | 0 |  |  | 2.44E6 | 2.92E6 | 7 | 1 | 2 | 0 | 0 | 1 | 1 | 0 | 0 | 1 | 1 | 581 | 593 |  |
| K.VEVVEER.Q | Y | 39.22 | 858.4446 | 7 | 5.5 | 430.2320 | 2 | 26.35 | 10 | 6918 | Ana\_09.raw |  |  | 2.73E7 |  |  |  |  |  |  | 5.16E6 | 2 | 0 | 0 | 1 | 0 | 0 | 0 | 0 | 0 | 0 | 1 | 371 | 377 |  |
[truncated: 4,936,491 more chars]
