# Supplementary material for: Proteomic Analysis of Paraffin-Embedded Intestines from Schistosoma mansoni Infection in Mice: Highlighting Molecular Players During Acute Schistosomiasis
Source: Proteomes. 2026 Jul 29;14(3):39. doi: 10.3390/proteomes14030039 (PMC13510969; doi:10.3390/proteomes14030039)
Supplement: Supplementary file 1 [file proteomes-14-00039-s001.zip › Supplementary Figure S1 - Evaluation of spleen weight ratio to body weight at weeks 5 and 7.pdf]

Supplementary Figure S1

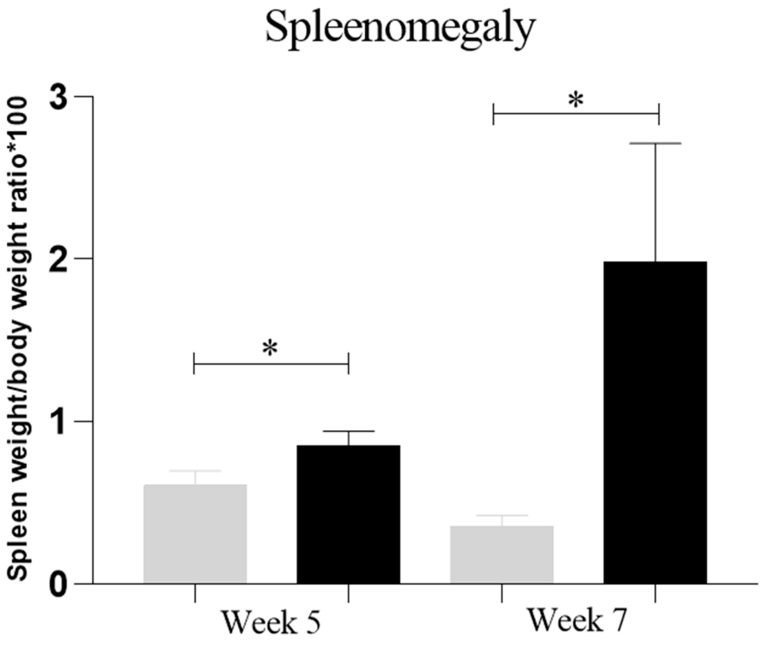

Supplementary Figure S1. Evaluation of spleen weight / body weight ratios for animals at weeks 5 and 7. Significant increase in spleen weight/body weight ratio was observed for infected animals (black bars) compared to control animals (grey bars) at both time points. Data are expressed as mean ± standard deviation. Data normality was assessed using Mann-Whitney Test and *t* Test. Asterisk (*) indicates $p < 0.05$.
