# Supplementary material for: Proteomic Analysis of Paraffin-Embedded Intestines from Schistosoma mansoni Infection in Mice: Highlighting Molecular Players During Acute Schistosomiasis
Source: Proteomes. 2026 Jul 29;14(3):39. doi: 10.3390/proteomes14030039 (PMC13510969; doi:10.3390/proteomes14030039)
Supplement: Supplementary file 1 [file proteomes-14-00039-s001.zip › Supplementary Figure S2 - Overview of Intestinal tissue histology from S. mansoni infected animals.pdf]

Supplementary Figure S2

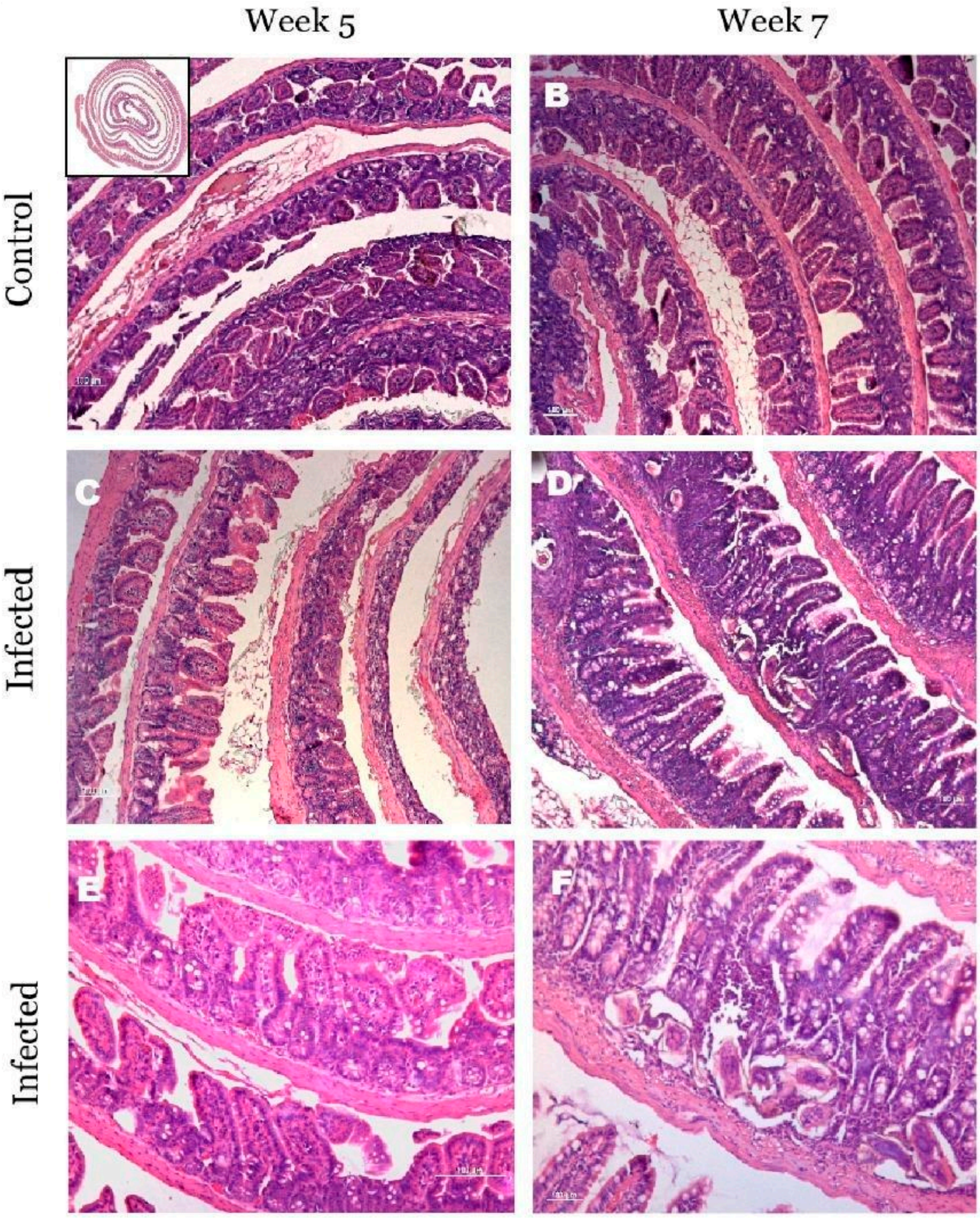

Supplementary Figure S2. Overview of Intestinal tissue histology from *S. mansoni* infected animals. Hematoxylin-eosin staining of a 5 µm longitudinal section of the small intestine. Prior to paraffin-embedment the small intestine was concentrically arranged to provide a panoramic view of its entire length (**A**). Inflammatory infiltrate and Goblet cell hyperplasia were more evident at week 7 post-infection at 20x magnification. (**A-D**) show sections of the intestine at 10x magnification, while E and F show them at 20x magnification.
