# Supplementary material for: Proteomic Analysis of Paraffin-Embedded Intestines from Schistosoma mansoni Infection in Mice: Highlighting Molecular Players During Acute Schistosomiasis
Source: Proteomes. 2026 Jul 29;14(3):39. doi: 10.3390/proteomes14030039 (PMC13510969; doi:10.3390/proteomes14030039)
Supplement: Supplementary file 1 [file proteomes-14-00039-s001.zip › Supplementary Figure S3A,B - Cumulative abundance of intestinal proteome.pdf]

Supplementary Figure S3

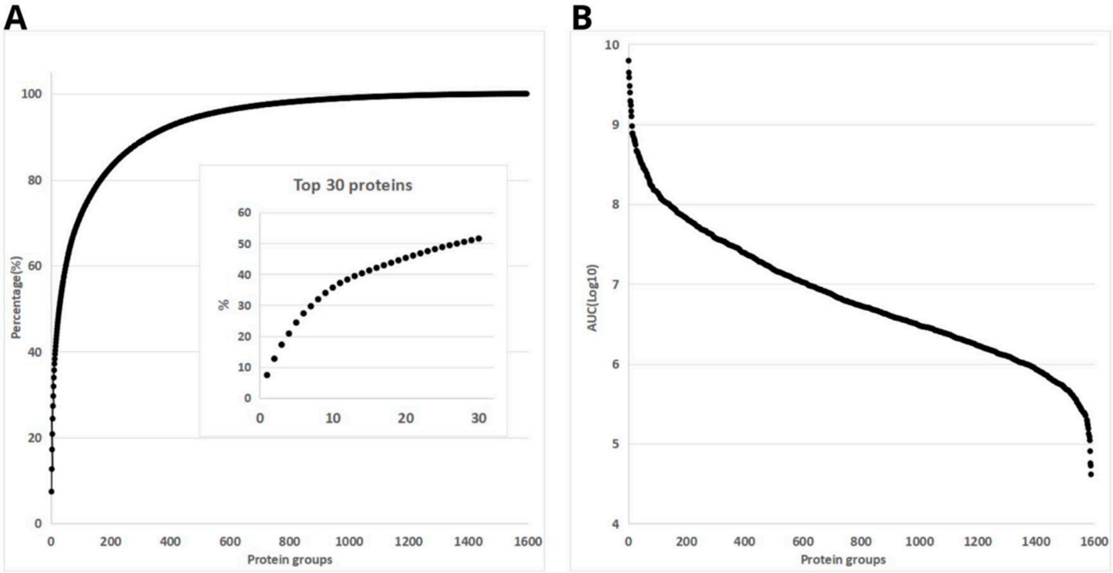

Supplementary Figure S3. Cumulative abundance of intestinal proteome. (**A**) Proteomic analysis of intestinal tissue revealed that 30 proteins account for approximately 60% of the cumulative protein abundance. (**B**) The dynamic range analysis indicated a difference of five orders of magnitude between the most abundant and the least abundant protein components.
