# Supplementary material for: Proteomic Analysis of Paraffin-Embedded Intestines from Schistosoma mansoni Infection in Mice: Highlighting Molecular Players During Acute Schistosomiasis
Source: Proteomes. 2026 Jul 29;14(3):39. doi: 10.3390/proteomes14030039 (PMC13510969; doi:10.3390/proteomes14030039)
Supplement: Supplementary file 1 [file proteomes-14-00039-s001.zip › Supplementary Figure S4 - STRING access.pdf]

Supplementary Figure S4

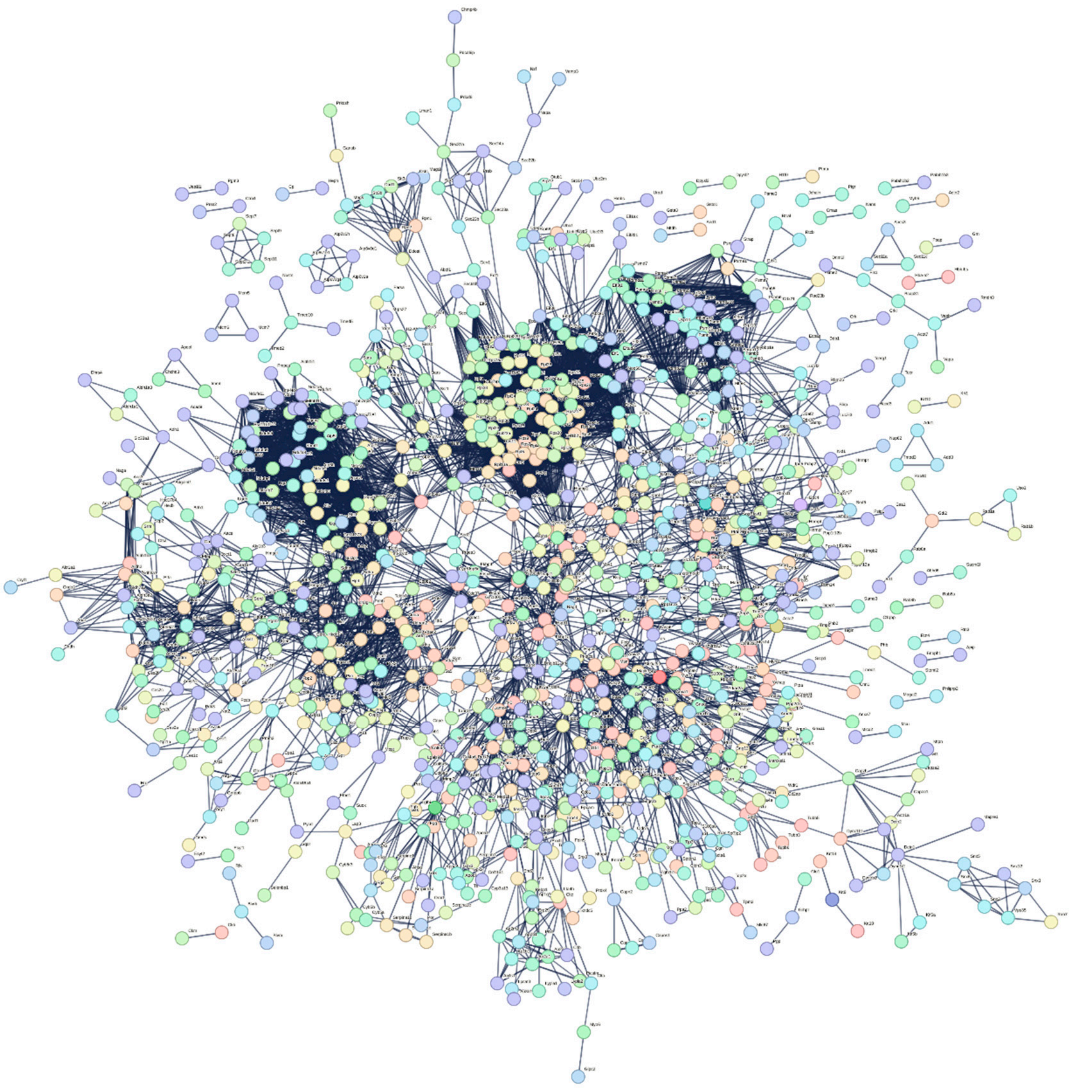

Supplementary Figure S4. STRING protein–protein interaction network of the compositional data. A minimum required interaction score of 0.900 (highest confidence) was applied, and disconnected proteins were not shown. Following the enrichment analysis, the proteins associated with the enriched Reactome term - Immune System were used to construct a second protein–protein interaction network (Figure 5 main text) using the same parameters.

The complete protein-protein interaction networks are available at:

https://string-db.org/cgi/network?taskId=bjmq4HEmnIdc&sessionId=bUkNfgqzQZzp

https://string-db.org/cgi/network?taskId=brkO9sFCTB0H&sessionId=bDZNfzCaexpw
