# Supplementary material for: Proteomic Analysis of Paraffin-Embedded Intestines from Schistosoma mansoni Infection in Mice: Highlighting Molecular Players During Acute Schistosomiasis
Source: Proteomes. 2026 Jul 29;14(3):39. doi: 10.3390/proteomes14030039 (PMC13510969; doi:10.3390/proteomes14030039)
Supplement: Supplementary file 1 [file proteomes-14-00039-s001.zip › Supplementary Table S1 - Compositional analysis of the intestinal proteomes during S. mansoni infection and Reactome-based pathway enrichment.pdf]

Supplementary Table S1

Table S1: Compositional analysis of the intestinal proteomes during *S. mansoni* infection and Reactome-based pathway enrichment.

**Top leading enriched pathways**		
Reactome Pathways	Count in network	FDR value
Metabolism	388 of 1754	$2.47e^{-78}$
Metabolism of RNA	183 of 599	$2.46e^{-50}$
L13a-mediated translational silencing of Ceruloplasmin expression	90 of 109	$1.01e^{-49}$
GTP hydrolysis and joining of the 60S ribosomal subunit	90 of 110	$1.01e^{-49}$
Cap-dependent Translation Initiation	92 of 117	$1.01e^{-49}$
Innate Immune System	228 of 945	$5.14e^{-49}$
Metabolism of proteins	307 of 1616	$4.23e^{-48}$
Formation of a pool of free 40S subunits	83 of 99	$1.53e^{-46}$
Translation	108 of 225	$1.42e^{-43}$
Immune System	296 of 1615	$1.51e^{-43}$
Neutrophil degranulation	158 of 526	$5.58e^{-43}$
Nonsense Mediated Decay (NMD) independent of the Exon Junction Complex (EJC)	75 of 92	$1.68e^{-41}$
**Other relevant enriched pathways**		
**Reactome Pathways**	**Count in network**	**FDR value**
Cellular response to stress	122 of 448	$2.04e^{-29}$
Adaptive immune system	125 of 687	$3.75e^{-17}$
GLI3 is processed into GLI3R by the proteasome	34 of 57	$7.44e^{-16}$
Ubiquitin-dependent degradation of Cyclin D	32 of 50	$1.31e^{-15}$
Programmed Cellular Death	48 of 132	$1.54e^{-15}$
Degradation of GLI1 by the proteasome	33 of 55	$1.69e^{-15}$
Autodegradation of E3 ubiquitin ligase COP1	32 of 51	$1.89e^{-15}$
Ubiquitin-mediated degradation of phosphorylated Cdc25A	32 of 51	$1.89e^{-15}$
Apoptosis	41 of 101	$9.83e^{-15}$
Degradation of beta-catenin by the destruction complex	36 of 78	$2.53e^{-14}$
Activation of NF-kappaB in B cells	33 of 64	$3.23e^{-14}$
Response to hypoxia	33 of 65	$4.46e^{-14}$
Autodegradation of Cdh1 by Cdh1: APC/C	32 of 63	$1.07e^{-13}$
Fc epsilon receptor signaling (FCERI)	42 of 121	$2.75e^{-13}$
NF-kB activation mediated by FCERI	34 of 77	$3.46e^{-13}$
APC/C: Cdc20 mediated degradation of Securin	60 of 243	$3.62e^{-13}$
Presentation of MHC class II antigen	42 of 126	$7.74e^{-13}$
UCH proteinases	35 of 86	$8.76e^{-13}$
Cell cycle - mitosis	91 of 482	$1.59e^{-13}$
Interleukin-1 signaling	37 of 102	$2.70e^{-12}$
T cell receptor (TCR) Signaling	36 of 103	$1.26e^{-11}$
Apoptotic execution phase	26 of 49	$1.19e^{-11}$
Platelet degranulation	37 of 121	$1.51e^{-10}$
Biological oxidations	49 of 207	$2.22e^{-10}$
DNA replication	37 of 126	$3.89e^{-10}$
TCF-dependent signaling in response to WNT	40 of 154	$1.42e^{-09}$
Interleukin-mediated signaling	54 of 262	$1.80e^{-09}$

Cytokine Signaling in the Immune System	67 of 397	2.00e-08
Detoxification of reactive oxygen species	18 of 33	2.10e-08
Processing and presentation of antigens by MHC class I	61 of 354	5.13e-08
Apoptotic cleavage of cellular proteins	17 of 37	3.81e-07
Intrinsic pathway for apoptosis	16 of 37	1.78e-06
Apoptosis induced by DNA fragmentation	9 of 12	2.92e-05
Collagen degradation	16 of 55	0.00011
Collagen formation	18 of 77	0.00034
Necrosis regulation	13 of 51	0.0015
Fibronectin matrix formation	4 of 4	0.0056
Apoptotic cleavage of cell adhesion proteins	7 of 11	0.00062
Apoptotic factor-mediated response	8 of 16	0.00069
Iron uptake and transport	14 of 56	0.0011
Collagen biosynthesis and modifying enzymes	15 of 63	0.0011
Collagen chain trimerization	9 of 36	0.0121
Elastic fibre formation	9 of 40	0.0206
Heme group degradation	6 of 19	0.0224
Molecules associated with elastic fibers	8 of 33	0.0224
Degradation of keratan sulfate	5 of 13	0.0231
Regulation of apoptosome activity	4 of 9	0.0355
Toll-like Receptor Cascades	26 of 143	0.00038
Cross-linking of collagen fibrils	5 of 16	0.0437
Heme signaling	4 of 10	0.0456

To explore the STRING enrichment data in Figure 5 in more detail, please access the following links:

https://string-db.org/cgi/network?taskId=bjmq4HEmnIdc&sessionId=bUkNfgqzQZzp
https://string-db.org/cgi/network?taskId=bElSISIgMylQ&sessionId=bFoN4JUnDSyV
